# Deep sequencing analysis of the heterogeneity of seed and commercial lots of the bacillus Calmette-Guérin (BCG) tuberculosis vaccine substrain Tokyo-172

**DOI:** 10.1038/srep17827

**Published:** 2015-12-04

**Authors:** Takayuki Wada, Fumito Maruyama, Tomotada Iwamoto, Shinji Maeda, Taro Yamamoto, Ichiro Nakagawa, Saburo Yamamoto, Naoya Ohara

**Affiliations:** 1 Department of International Health, Institute of Tropical Medicine, Nagasaki University, Nagasaki, 852-8523, Japan; 2 Department of Microbiology, Kyoto University Graduate School of Medicine, Kyoto, 606-8501, Japan; 3 Department of Infectious Diseases, Kobe Institute of Health, Kobe, 650-0046, Japan; 4 Department of Mycobacterium Reference and Research, The Research Institute of Tuberculosis, Tokyo, 204-0022, Japan; 5 Japan BCG Laboratory, Tokyo, 204-0022, Japan; 6 Department of Oral Microbiology, Graduate School of Medicine, Dentistry and Pharmaceutical Sciences, Okayama University, Okayama, 700-8558, Japan; 7 The Advanced Research Center for Oral and Craniofacial Sciences, Dental School, Okayama University, Okayama, 700-8558, Japan

## Abstract

BCG, only vaccine available to prevent tuberculosis, was established in the early 20th century by prolonged passaging of a virulent clinical strain of *Mycobacterium bovis*. BCG Tokyo-172, originally distributed within Japan in 1924, is one of the currently used reference substrains for the vaccine. Recently, this substrain was reported to contain two spontaneously arising, heterogeneous subpopulations (Types I and II). The proportions of the subpopulations changed over time in both distributed seed lots and commercial lots. To maintain the homogeneity of live vaccines, such variations and subpopulational mutations in lots should be restrained and monitored. We incorporated deep sequencing techniques to validate such heterogeneity in lots of the BCG Tokyo-172 substrain without cloning. By bioinformatics analysis, we not only detected the two subpopulations but also detected two intrinsic variations within these populations. The intrinsic variants could be isolated from respective lots as colonies cultured on plate media, suggesting analyses incorporating deep sequencing techniques are powerful, valid tools to detect mutations in live bacterial vaccine lots. Our data showed that spontaneous mutations in BCG vaccines could be easily monitored by deep sequencing without direct isolation of variants, revealing the complex heterogeneity of BCG Tokyo-172 and its daughter lots currently in use.

*Mycobacterium bovis* bacillus Calmette-Guérin (BCG) was established in 1921 after 13 years and 230 subcultures of its virulent parental Nocard strain on a glycerinated beef-bile-potato medium^1^. BCG has been the only officially registered live vaccine against tuberculosis (TB) for almost a century, and effective BCG mass vaccination campaigns against TB have been conducted worldwide. Since its development, the original strain of BCG, maintained at the Pasteur Institute, has been distributed and used to produce substrains^2^,^3^. These substrains have been used for vaccination and for culture with appropriate passaging to maintain the strains; this has caused accumulation of genetic difference among the substrains. Recent studies have reported the genetic diversity among these substrains^4^,^5^,^6^; this variation has affected the respective substrains phenotypically, modifying the lipid components of the cell wall and affecting carbon metabolism and antigenicity, for example^5^,^7^,^8^,^9^,^10^,^11^. Moreover, such genetic diversity has the potential to affect the stability of the vaccine during medium- and long-term storage. Recently, the World Health Organization (WHO) declared that BCG vaccine substrains should be evaluated for appropriate approval and application worldwide^10^,^12^.

In addition to difference among substrains, variations within each substrain have arisen by spontaneous mutations. A well-studied example is BCG Tokyo (BCG Japan), one of the ancestral substrains, which was initially transferred from the Pasteur Institute to Japan in 1924^13^. A freeze-dried seed lot, Tokyo-172, was found to contain at least two subpopulations that exhibited different colony morphologies (Types I and II)^14^,^15^. Consecutive seed lots of the substrain were propagated and subcultured to mass-produce the final bulk stock prior to the improvement of freeze-drying techniques, which may have been involved in the generation of mutations and the accumulation of genetic variants within the substrain. As a concrete example, the Type I subpopulation of Tokyo-172, which exhibits smooth colonies, is genetically characterized by a unique 22-bp deletion, which has been named RD16^15^, in the JTY3475 gene (an ortholog of Rv3405 of *Mycobacterium tuberculosis* H37Rv) compared with reference sequences of clinical strains, such as *M. bovis* AF2122/97 and *M. tuberculosis* H37Rv. In contrast to the thorough description of the Type I genomic sequence^16^, the Type II subpopulation, which can be distinguished from Type I organisms by its rough-shaped colonies, has remained genetically uncharacterized. Recently, a frameshift missense error of *pps*A, found exclusively in the Type II subpopulation, was reported to affect the cell wall components^17^. Therefore, a complicated heterogeneous mixture of the two subpopulations exists in the BCG Tokyo-172 substrain. Such natural diversification, e.g., heterogeneity on duplication of the DU2 region in the BCG Danish substrain^5^, commonly occurs in BCG substrains and can be exacerbated by continuous passage without appropriate cloning due to natural mutations and selective pressure in culture media.

Deep sequencing using next-generation sequencing (NGS) technologies has been developing rapidly and has enabled researchers to analyse variations in nucleotides in genetically distinct populations of various species^18^,^19^,^20^,^21^. Deep sequencing may unveil not only *a priori* differences between substrains but also *de novo* heterogeneity within a substrain without requiring the cloning of variants. As an example, the intra-lot variation in the oral poliovirus vaccine has already been successfully evaluated by NGS techniques^22^,^23^, providing important information for maintenance of the homogeneity of live vaccines. Such an approach allows us to observe, characterize, and examine undesirable heterogeneity in microbial resources caused by spontaneous genetic mutations.

In this study, we used NGS techniques to describe the heterogeneity in separate lots of the BCG Tokyo-172 substrain, including two commercial lots from overseas distributees. This approach can be used to detect genetic variations in live resources and contributes to the management of heterogeneity in cell seed lot systems.

## Results

### Genomic characterization of two subpopulations in the BCG Tokyo-172 substrain

In a prior investigation of the heterogeneity of seed and commercial lots, the genomic variation between Type I and II subpopulations of the BCG Tokyo-172 substrain was verified by mapping analysis using NGS short reads. In addition to an already-known difference (a frameshift mutation in the *pps*A gene)^17^, six nucleotide substitutions were newly identified (Table 1). In comparison with outgroup substrains, these nucleotide changes were assigned to both Type I and II subpopulations, indicating that genetic diversification occurred not in one of the two subpopulations unilaterally, but in both subpopulations after their divergence.

### Subpopulations in seed/commercial lots of BCG Tokyo-172

**Figure 1:**
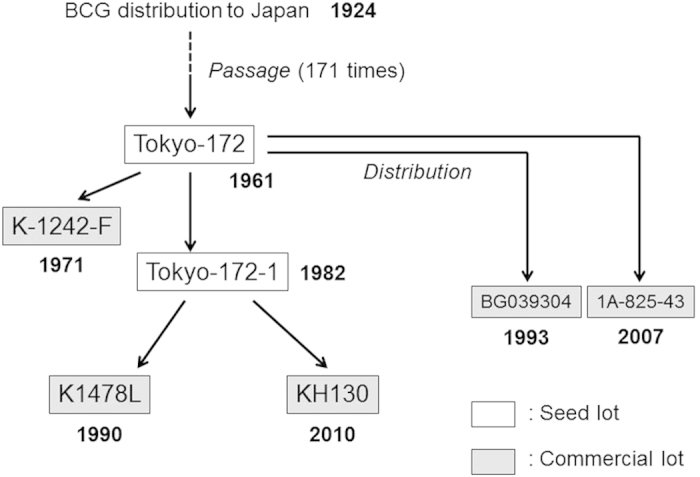
A historical record of recent lots of BCG Tokyo analysed in this study. Seven rectangle boxes correspond to BCG lots that were subjected to deep sequencing. Arrows indicate culturing processes to establish or produce descendant lots from each seed lot.

Next, to assess the heterogeneity in lots of BCG Tokyo-172, five domestic seed and commercial lots and two foreign commercial lots were sampled and characterized genetically (Fig. 1). The domestic lots included two sequential seeds, Tokyo-172 and its daughter seed lot, Tokyo-172-1. Three remaining lots (K-1242-F, K1478L, and KH130) were commercially available; these had been propagated and freeze-dried from Tokyo-172 or Tokyo-172-1. The two foreign lots, BG039304 and 1A-825-43, were commercial lots received from Taiwan and Thailand, respectively. BCG substrains in current use in both areas had been obtained from Japan after the establishment of Tokyo-172^2^,^24^,^25^ and were then produced independently as applied vaccines in the respective countries.

**Figure 2:**
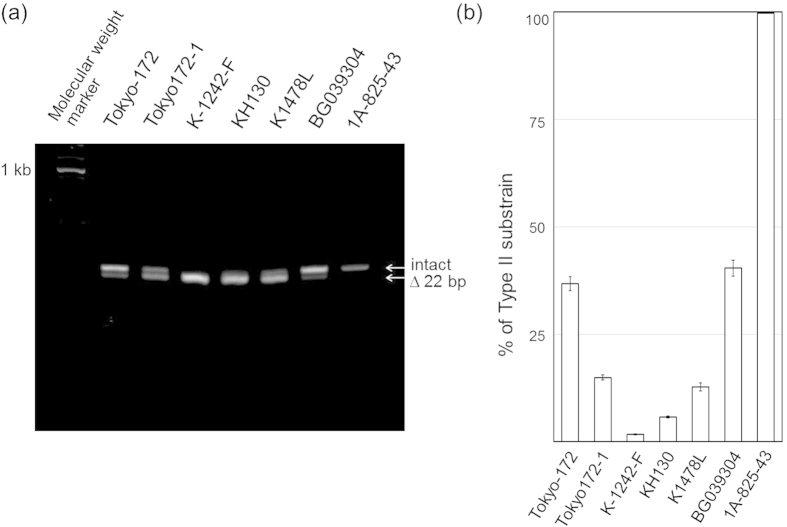
Heterogeneity of Type I and II subpopulations in each BCG lot was monitored based on the RD16 region. (**A**) Agarose gel electrophoresis of PCR products of the RD16 region. (**B**) Ratios of intact/deleted RD16 in each lot were calculated from the copy numbers derived from quantitative real-time PCR. The Y-axis shows the percentage of respective lots that were of the Type II subpopulation. The error bars indicate the propagated standard deviation (±2σ) of respective ratios.

First, from each lot, the two subpopulations, Types I and II, were quantified according to the RD16 genomic deletion. Two seed lots, Tokyo-172 and Tokyo-172-1, contained 36.8% ± 1.6% and 14.9% ± 0.6% of the Type II subpopulation, respectively (Fig. 2). The Type II subpopulation was predominated in domestic commercial lots (K-1242-F: 1.7% ± 0.1%, K1478L: 12.8% ± 0.9%, and KH130: 5.8% ± 0.2%). Overall, the proportion of the Type II subpopulation decreased with increasing passages. These results correspond with those of a previous report^14^. A commercial lot from Taiwan (BG039304) contained 40.4% ± 1.9% of the Type II subpopulation, showing genetic similarity to the original seed lot, Tokyo-172. In contrast, the RD16 deletion genotype was not detected by polymerase chain reaction (PCR) in a commercial lot from Thailand 1A-825-43.

### Deep sequencing analysis

Deep sequencing based on an Illumina platform provides massive sequence data at each nucleotide position in a genome; this analysis can capture genetic fluctuations quantitatively in a population of bacilli. With this perspective, the collected BCG lots were subjected to deep sequencing to evaluate the potential for use of this technique to measure the proportion of subpopulations and to detect spontaneous inherent variations.

**Figure 3:**
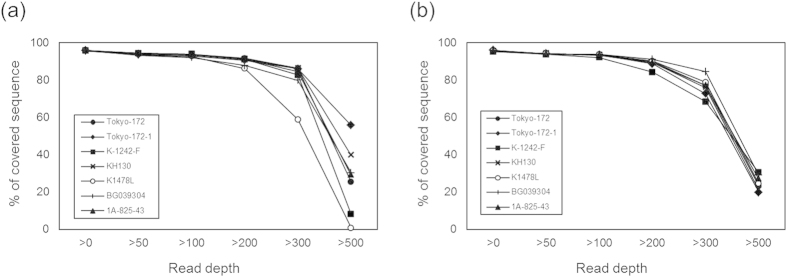
Percentage of covered nucleotides of the reference genome sequence (NC_012207, 4,171,711 bp) mapped with short reads from seven seed/commercial lots of Tokyo-172 by Bowtie2 (incorporated in Breseq v0.24rc6). The X-axis shows the depth of reads covering the reference genome, and the Y-axis shows the % of the reference sequence mapped with the depth. Short reads were obtained twice (**A,B**) from the same lots and used for subsequent analysis.

To monitor genomic regions as broadly as possible, the read data from each BCG lot were mapped to reference sequences (BCG Tokyo-172 substrain Type I). The average depths mapped to the references ranged from 299.07 to 484.65 (average: 400.05, by a mapping tool Bowtie 2; Supplementary Table 1). With the read quantity, about 90% of nucleotide sequences of the reference genomes were mapped by the reads with a read depth of more than 200 (Fig. 3), which guaranteed subsequent detection of heterogeneity in a broad range with high accuracy. Indeed, the seven polymorphisms found between the two subpopulations (Table 1) were successfully detected, and the proportions of Type I and II subpopulations in each lot were determined for all lots, except for K-1242-F, the commercial lot having the lowest proportion of the Type II subpopulation in our samples (Table 2).

We incorporated three bioinformatics tools to detect heterogeneity of BCG lots. LoFreq called the variation most consistently, although this tool cannot detect indel variations, such as the insertion variant at 3,192,638 (*pps*A). SNVer and Breseq can detect both substitution and indel variations in their separate, respective files. However, the proportion of variants with this insertion, as determined by SNVer, deviated from that of the other variant calls inconsistently. In contrast, the insertion could be detected by Breseq, consistent with other substitutions. Breseq could also detect the 22-bp deletion of RD16, although its sensitivity for detection of substitutive variants seemed lower than those of LoFreq and SNVer (Table 2). For example, Breseq failed to consistently detect variations at positions 644,562 (JTY_0567) and 3,606,131 (JTY_3278), although these variations were detected by LoFreq and SNVer.

To apply this method to other substrains, a reference sequence must be applied by integration of several genomes to account for the deleted regions of each substrain. For this purpose, we supplemented the entire sequence of BCG Tokyo-172 with other genome sequences (BCG Moreau RDJ, another candidate of the reference substrain of BCG, and *M. bovis* AL2122/97; Supplementary GenBank File 1). When it was used to replace the original sequence, the variation could be detected exactly as described in Table 2.

### Monitoring intrinsic heterogeneity in BCG lots

In addition to the complexity of Type I and II substrains, spontaneous heterogeneity in the BCG lots was also determined by variant calls. From monitoring of NGS data, we estimated that only two lots may have contained minor heterogeneity (Table 3) in over 5% of the total population.

One of the two intrinsic variants (Tokyo-172) was detected in the most ancestral seed lot in this study (Table 3). The variant was not found in descendant lots, indicating that the variant had not been positively selected in subsequent passages. The other heterogeneous lot was 1A-825-43; this was a commercial lot, and the variant would not be inherited in subsequent propagation, although the variant may be contaminated from its seed lot. To verify this bioinformatic estimation, we cultured the BCG lots on agar plates and tried to isolate variant clones directly from the colonies grown. As a result, mutated colonies were indeed isolated from the two BCG lots (Table 3).

## Discussion

BCG Tokyo-172 is one of four candidate WHO reference strains^26^,^27^. Two types of colonies showing different morphologies were found when Tokyo-172 was cultured on agar plates^15^; and these variants were thought to be caused by complex heterogeneity^16^,^17^. In this study, to monitor hidden heterogeneity in lot stocks of BCG Tokyo-172, deep sequencing analysis was performed. We successfully identified and characterized two subpopulations (Types I and II) of this strain, providing insights into the acquisition of heterogeneity in vaccine strains.

BCG has been used in vaccine production for almost a century. This long period of maintenance and worldwide distribution has resulted in wide genetic diversity among substrains^5^,^15^. This could also cause genetic heterogeneity within respective substrains, such as Type I and II subpopulations in BCG Tokyo-172. Although there is no evidence showing that heterogeneity affects the efficacy or antigenicity of the BCG vaccine directly, this feature should be controlled by certain procedures in order to maintain the stability of the vaccine. Monitoring of unpredictable variation across the entire genome was difficult in the past; however, with major advancements in deep sequencing analysis based on NGS technologies, such monitoring has become possible. Indeed, deep sequencing is now suitable for effective detection of spontaneous variation within a biotic resource and may become the new standard for quality control of new lots.

In this study, we examined seven derivative lots of Tokyo-172 to investigate their heterogeneity. The substrain BCG Tokyo-172 has been reported to include two subpopulations, identifiable by their colony morphology and genetic differences^15^,^17^. This readily detectable diversity provides a good indicator to evaluate the use of NGS techniques to identify heterogeneity within BCG lots. In our study, seven nucleotide polymorphisms were identified between Type I and II subpopulations and could be representative of heterogeneous mutations in each lot. The results showed that deep sequencing analysis could be applies as a standard method to test the heterogeneity of BCG vaccines quantitatively.

If we are to assume that there was a historical process of subdivision of the BCG Tokyo-172 substrain, it is important that both Type I and II subpopulations possess their own unique polymorphisms. Other substrains (BCG Russia and Moreau) that were distributed from the Pasteur Institute during the same period as BCG Tokyo do not share the seven polymorphisms of the subpopulations. Thus, these polymorphisms may have accumulated separately during passages in Japan. However, it is not clear why such heterogeneity has occurred, particularly because the cloning processes of the substrain over this period have not been recorded.

The heterogeneity in each lot of BCG Tokyo-172 can be interpreted as a mixture of the major polymorphisms between the two subpopulations; in contrast, the number of spontaneous variants was low. Respective subpopulations were highly clonal, suggesting that they may have been generated via cloning steps just before the establishment of Tokyo-172. After establishment of Tokyo-172, the proportion of the Type II subpopulation may have decreased during passaging. The most dramatic decrease was found in K-1242-F, a commercial lot propagated directly from Tokyo-172. However, it is not clear why this lot had such a lower proportion of the Type II subpopulation than the other lots derived from Tokyo-172-1. It is possible that some conditions of lot preparation, such as nutrient components and temperature in culture, may affect the proportions of certain subpopulations. Further *in vitro* experiments are needed to check this hypothesis. In contrast to the heterogeneity in domestic lots, the Type I subpopulation could not be detected in a commercial lot from Thailand. Therefore, some type of cloning process may have been used to purify the Type II subpopulation in this country.

In contrast to the distinct colony morphology of the subpopulations^15^, it is generally more difficult to identify heterogeneity because there are no obvious clues to isolate distinct variants. From this background, deep sequencing analyses based on NGS techniques can be used to survey unavoidable spontaneous variations without colony isolation. This strategy has been already reported antecedently for the monitoring of live attenuated poliovirus vaccines^21^,^22^,^23^. If genome-wide screening of heterogeneity can be incorporated into production of the BCG vaccine, it would be possible to assess the genetic uniformity of the vaccine, which could provide a more sustainable supply of stable vaccine lots.

Our approach revealed the complex heterogeneity of a substrain of BCG Tokyo-172. It is important to establish a corresponding reference genome sequence for other substrains in order to maintain high mapping quality. In this study, we constructed an artificial reference genome from three complete genome sequences of *M. bovis* and BCG substrains; however, further studies are require in order to verify the usefulness of this reference sequence for other substrains. Currently, the entire genome sequences of two of four candidate WHO reference substrains (i.e., BCG Danish 1331 and Russia I) have not been completed. Accumulation of such basic data may improve a variety of analytical methods for evaluation of various BCG substrains in the future.

## Methods

### Culture of BCG lots and DNA preparation

Cloned subpopulations (Types I and II) and seed/commercial lots of BCG Tokyo-172 and were cultured in 40 mL of Middlebrook 7H9 liquid broth with gentle shaking at 37 °C. The culture time was restricted to 2 days (about two generations of the substrain) to restrain heterogeneity in the culture as much as possible. Genomic DNAs were prepared from cultured bacilli according to a previous report^28^. Briefly, centrifuged bacilli were delipidated using chloroform and methanol and then lysed using egg-white lysozyme and proteinase K. Genomic DNA was purified by phenol/chloroform extraction from the lysates. The quality and quantity of purified genomic DNA were evaluated based on the OD_260_ and agarose electrophoresis.

### Determination of nucleotide variations between BCG Tokyo-172 subpopulations

Purified DNAs from two substrains were sequenced on an Illumina MiSeq platform to obtain short reads (DRA: DRR029468 and DRR029469), and which were mapped to the complete genome sequence of BCG Tokyo-172 Type I (GenBank: NC_012207) to identify nucleotide variations that were distinct between the two subpopulations using CLC Genomics Workbench (Qiagen Science Inc.). To determine genetic differences between the Type I and II subpopulations, PCR fragments with the respective variant positions were verified by conventional Sanger sequencing. Primer sequences are listed in Supplementary Table 2.

### Quantification of the Type I subpopulation in BCG lots

The proportion of subpopulations Types I and II in respective BCG lots was determined by the ratio of the genome region RD16 deleted genotype, a unique genetic feature of the Type I subpopulation, to the intact genotype, corresponding to the Type II subpopulation, using specific real-time PCR probes (Supplementary Table 3). The DNA concentration of both types was analysed in triplicate in three independent experiments, using a modified protocol of a previous report^14^. Subsequently, the ratios of the subpopulations were calculated, taking into account the propagation of errors^29^.

### Deep sequencing

Purified DNAs from BCG lots were subsequently analysed using an Illumina Genome Analyzer IIx (GAIIx; Illumina, Inc.). Libraries were constructed using a Paired-End DNA Sample Prep Kit (Illumina, Inc.), and each library was assigned to one lane per lot to obtain sufficient data quantity to detect minor heterogenic mutations. To avoid variants that arose during library construction as much as possible, libraries were constructed twice independently from the same purified DNAs. In this study, 75 bp of both strands were read by GAIIx, but only the forward strands were used for analysis owing to the low quality of the reverse strands. The quality score (QV) of the read data was checked by FastQC (ver. 0.10.1; Supplementary Figure S1).

### Detection of heterogenic variation

To find heterogenic variation in the reads, three bioinformatics tools, Breseq (ver. 0.24rc6)^30^, LoFreq (ver. 0.6.1)^31^, and SNVer (ver. 0.5.2)^32^ were used. All three tools are based on mapping to a reference genome sequence, finding subpopulational reads possessing nucleotide variants among the total mapped reads, and applying the statistical criteria of strand bias, base call quality, and mapping quality to remove pseudo-positive heterogeneity. Before analysis, the original read data were trimmed by SolexaQA (ver. 2.2)^33^ to remove low-quality base calls; from this analysis, base calls with a quality score (QV) lower than 20 were excluded, and reads shorter than 25 bp were removed. As a condition of Breseq, to filter variant calls, base calls with a QV lower than 20 were excluded from the calculation. For LoFreq and SNVer calculations, Burrows-Wheeler Alignment (ver 0.7.7)^34^ was used as the mapping tool. BCG Tokyo-172 Type I (RefSeq: NC_012207) was used as the reference genome sequence. Mapping results are summarized in Supplementary Table S1.

### Construction of a supplemented reference sequence

To supplement unique deletions in the genome sequence of BCG Tokyo-172, other entire genome sequences, i.e., those of BCG Moreau RDJ (GenBank: AM412059.2) and *M. bovis* AL2122/97 (RefSeq: NC_002945.3), were used. In brief, the three sequences were aligned using mauveAligner (ver 2.4.0)^35^, and we verified that these sequences could be regarded as entirely syntenic. Deleted regions longer than 20 bp in BCG Tokyo-172 were determined by MAFFT (ver. 7)^36^. Finally, the regions were supplemented manually to construct an intentional genomic sequence (4,419,199 bp, 47,788 bp larger than the original sequence). This sequence was automatically annotated by MiGAP^37^ and edited using In silico Molecular Cloning (ver. 5.3; In Silico Biology, Inc.) and Artemis (ver. 16.0.0)^38^.

### Sequencing of variant positions

Putative intrinsic variations emerging in BCG lots were identified from the variation calls described above as variants representing over 5% of the read data, in duplicate, of each lot called by more than two of the three tools. Unreliable variants (located in paralogous or highly polymorphic genes, transposons, and repetitive unit regions) were excluded to avoid false-positive or -negative calls. To verify the filtered variants in the two BCG lots (Tokyo-172 and 1A-825-43), bacilli were cultured directly on 7H11 agar plates, and colonies were isolated. After the respective colonies were suspended in DNA-free water and heat killed, the supernatants were used as PCR templates to check the putative variant nucleotides by sequencing. PCR and sequencing primers are listed in Supplementary Table S4.

## Additional Information

**How to cite this article**: Wada, T. *et al.* Deep sequencing analysis of the heterogeneity of seed and commercial lots of the bacillus Calmette-Guérin (BCG) tuberculosis vaccine substrain Tokyo-172. *Sci. Rep.* **5**, 17827; doi: 10.1038/srep17827 (2015).

## Supplementary Material

Supplementary Materials

## Figures and Tables

**Table 1:** Single nucleotide polymorphisms between subpopulations of *M. bovis* BCG Tokyo.

Position*	BCG Tokyo		Early distributed strains		*M. bovis* AF2122/97	Gene	Annotation
	I	II	BCG Russia	BCG Moreau			
253,186§	A	G	A	A	A	*pck*A	Y390C
644,562§	T	C	T	T	T	JTY_0567	I144T
765,342§	C	T	T	T	T	*rpo*C	A68V
2,717,585§	C	T	T	T	T	intergenic†	–
3,192,638	.	A	.	.	.	*pps*A	frameshift
3,606,131§	G	A	A	A	A	JTY_3278	T54I
4,087,391§	C	T	T	T	T	JTY_3735	A184V

^*^Correspondent to nucleotide positions of genome sequence of *M. bovis* BCG Tokyo Type I (Accesion No. NC012207).

^†^Left gene: *clp*X, right gene: *mmu*M.

^§^SNVs identified in this study.

**Table 2:** Detection of heterogeneity of type I/II variants in seed and commercial lots of *M. bovis* BCG Tokyo.

Position* (gene)	BCG Tokyo		Frequency of nucleotide variants (=Type II) estimated by each package†						1A-825-43
	I	II	Tokyo-172	Tokyo-172-1	K-1242-F	KH130	K1478L	BG039304	
253,186 (*pck*A)	A	G	Breseq 35.8/35.7	16.5/17.5	n.d./n.d.	2.4/4.4	13.0/9.0	40.5/39.1	100/100
			LoFreq 35.1/35.6	16/17.9	n.d./n.d.	2.4/4.6	12.4/8.8	40.0/39.1	100/100
			SNVer 36.8/36.4	17.3/17.9	n.d./n.d.	n.d./4.6	13.5/9.0	41.2/40.0	100/100
644,562 (JTY_0567)	T	C	Breseq 35.6/n.d§	19.0/n.d.	n.d./n.d.	2.6/3.4	9.4/9.6	40.0/n.d.	100/100
			LoFreq 32.8/34.8	17.3/17.0	n.d./n.d.	2.4/3.3	9.3/9.3	38.3/47.2	99.8/99.6
			SNVer 35.0/36.1	18.3/18.0	n.d./n.d.	2.5/3.7	10.1/9.3	40.9/48.5	99.8/99.6
765,342 (*rpo*C)	C	T	Breseq 42.2/33.3	16.7/18.8	n.d./n.d.	2.7/n.d.	10.8/11.7	40.4/40.9	100/100
			LoFreq 40.5/34.4	15.2/19.1	n.d./n.d.	2.6/3.5	10.8/11.3	39.0/41.2	99.7/99.6
			SNVer 43.8/35.0	18.0/19.1	n.d./n.d.	n.d./n.d.	11.9/11.6	41.8/42.0	99.7/99.6
2,717,585 (intergenic†)	C	T	Breseq 40.0/38.5	18.0/20.9	1.4/n.d.	7.2/6.8	10.9/12.4	43.2/47.7	100/100
			LoFreq 39.3/38.5	16.6/21.4	n.d./n.d.	6.8/6.6	10.4/12.4	41.9/47.7	100/99.8
			SNVer 41.9/39.8	19.0/22.5	n.d./n.d.	7.0/6.8	11.3/12.8	44.7/48.5	100/99.8
3,192,638 (*pps*A)	.	A	Breseq 34.3/35.2	17.1/19.8	n.d./n.d.	4.3/2.9	10.2/9.8	38.1/45.3	100/n.d.
			*LoFreq –*	–	–	–	–	–	–
			SNVer 23.7/47.2	n.d./15.3	n.d./n.d.	n.d./n.d.	n.d./8.6	26.4/29.3	46.6/47.2
3,606,131 (JTY_3278)	G	A	Breseq n.d./40.6	n.d./19.6	n.d./n.d.	4.7/5.0	n.d./10.2	n.d./n.d.	100/100
			LoFreq 43.2/39.9	22.5/19.2	3.1/n.d.	4.7/4.6	12.1/10.1	37.3/41.6	100/100
			SNVer 44.9/41.4	23.7/20.1	n.d./n.d.	5.1/n.d.	13.1/10.4	39.1/43.3	100/100
4,087,391 (JTY_3735)	C	T	Breseq 38.8/39.1	17.1/18.3	n.d./n.d.	3.4/3.3	9.0/13.8	43.0/42.7	100/100
			LoFreq 38.8/40.2	17.0/18.8	n.d./n.d.	3.3/3.7	9.0/14.2	42.3/43.8	100/99.7
			SNVer 39.7/40.9	17.5/19.5	n.d./n.d.	3.5/3.7	9.9/14.7	43.9/44.6	100/99.7

^*^Correspondent to nucleotide positions of genome sequence of *M. bovis* BCG Tokyo Type I (Accesion No. NC012207).

^†^Two frequency scores estimated from two read data of each BCG lot are tandemly indicated respectively.

^§^n.d. not detected.

^‡^Left gene: *clp*X, right gene: *mmu*M.

**Table 3:** Variant calls of the intrinsic heterogeneity in the respective lots.

Heterogenic lot	Position*	Reference nt	Variant nt	Gene	Amino acid change	Proportion of variants in each lot estimated *in silico* (%)†			No of variant colonies
						Breseq	LoFreq	SNVer	
Tokyo172	2,655,559	A	G	*sub*I	M1T	11.3/9.60	10.9/9.54	11.6/10.4	2/189
1A-825-43	4,369,503	G	A	*yid*C	P249L	7.60/4.00	6.69/4.18	7.47/4.55	5/216

^*^Correspondent to nucleotide positions of genome sequence of *M. bovis* BCG Tokyo Type I (Accesion No. NC012207).

^*^These variants were also called when our artificial reference (Tokyo_suppl.gbk) was used as the reference for analysis.

^†^Two frequency scores estimated from two read data of each BCG lot are tandemly indicated respectively.
