# Supplementary material for: Deep sequencing analysis of the heterogeneity of seed and commercial lots of the bacillus Calmette-Guérin (BCG) tuberculosis vaccine substrain Tokyo-172
Source: Sci Rep. 2015 Dec 4;5:17827. doi: 10.1038/srep17827 (PMC4669467; doi:10.1038/srep17827)
Supplement: Supplementary Materials [file srep17827-s1.pdf]

Deep sequencing analysis of the heterogeneity of seed and commercial lots of the bacillus Calmette-Guérin (BCG) tuberculosis vaccine substrain Tokyo-172

Takayuki Wada[1+], Fumito Maruyama[2+], Tomotada Iwamoto[3], Shinji Maeda[4], Taro Yamamoto[1], Ichiro Nakagawa[2], Saburo Yamamoto[5], and Naoya Ohara[6, 7*]

[1] Department of International Health, Institute of Tropical Medicine, Nagasaki University, Nagasaki, 852-8523, Japan.
[2] Department of Microbiology, Kyoto University Graduate School of Medicine, Kyoto, 606-8501, Japan
[3] Department of Infectious Diseases, Kobe Institute of Health, Kobe, 650-0046, Japan.
[4] Department of Mycobacterium Reference and Research, The Research Institute of Tuberculosis, Tokyo, 204-0022, Japan.
[5] Japan BCG Laboratory, Tokyo, 204-0022, Japan.
[6] Department of Oral Microbiology, Graduate School of Medicine, Dentistry and Pharmaceutical Sciences, Okayama University, Okayama, 700-8558, Japan.
[7] The Advanced Research Center for Oral and Craniofacial Sciences, Dental School, Okayama University, Okayama, 700-8558, Japan

*To whom correspondence should be addressed: oharan@md.okayama-u.ac.jp
[+] these authors contributed equally to this work

Supplementary Table 1: Summary of short reads of BCG lots and their mapping results to the entire genome sequence of BCG Tokyo-172 Type I.

Supplementary Table 2: Primers used to analyze sequences of polymorphisms between two subpopulations of *M. bovis* BCG Tokyo.

Supplementary Table 3: Primers and probes used to calculate proportion of the subpopulations in lots of *M. bovis* BCG Tokyo.

Supplementary Table 4: Primers used to analyze sequences of intrinsic variation found in seed/lot of *M. bovis* BCG Tokyo.

Supplementary Figure 1: Base quality of the original short read data from seven lots of BCG Tokyo-172 in this study.

Supplementary GenBank file 1:

Supplementary Table 1. Summary of short reads of BCG lots and their mapping results to the entire genome sequence of BCG Tokyo-172 Type I.

Lot name	No. of original reads	Accession number	Mapping results (Bowtie2/Breseq)*												Mapping results (BWA/LoFreq, SNVer)*											
			Read count mapped	nt count mapped (bp)	Depth			Fraction of reference sequence covered with depth (%)						Read count mapped	nt count mapped (bp)	Depth			Fraction of reference sequence covered with depth (%)					
					Average	Max	Min	>0	>50	>100	>200	>300	>500			Average	Max	Min	>0	>50	>100	>200	>300	>500
Tokyo-172	27,896,099	DRR029466	26,887,482	1,816,543,346	415.52	1,098	0	4199340 (96.1%)	4128080 (94.4%)	4106693 (93.9%)	4008267 (91.7%)	3771251 (86.3%)	1110751 (25.4%)	26,879,918	1,882,150,460	430.53	1,098	0	4367595 (99.9%)	4283981 (98.0%)	4259832 (97.4%)	4153200 (95.0%)	3901699 (89.2%)	1149872 (26.3%)
	26,471,891	DRR029475	26,437,301	1,720,176,730	393.48	1,515	0	4194484 (95.9%)	4124602 (94.3%)	4098342 (93.7%)	3940240 (90.1%)	3373267 (77.2%)	1013464 (23.2%)	26,432,568	1,785,089,054	408.33	1,515	0	4366674 (99.9%)	4282806 (98.0%)	4254098 (97.3%)	4085260 (93.4%)	3497009 (80.0%)	1052599 (24.1%)
Tokyo-172-1	32,628,931	DRR029467	31,654,813	2,118,730,530	484.65	1,170	0	4186678 (95.8%)	4098053 (93.7%)	4067634 (93.0%)	3963623 (90.7%)	3762164 (86.1%)	2435411 (55.7%)	31,651,771	2,197,179,819	502.59	1,170	0	4352288 (99.6%)	4253770 (97.3%)	4221341 (96.6%)	4109768 (94.0%)	3897673 (89.2%)	2526247 (57.8%)
	25,297,882	DRR029476	24,588,663	1,647,856,916	376.94	1,297	0	4194698 (96.0%)	4120558 (94.3%)	4085529 (93.5%)	3878084 (88.7%)	3184348 (72.8%)	863908 (19.8%)	24,584,910	1,709,840,798	391.11	1,601	0	4361215 (99.8%)	4275920 (97.8%)	4237894 (96.9%)	4018396 (91.9%)	3301903 (75.5%)	899070 (20.6%)
K-1242-F	24,483,377	DRR029463	24,056,335	1,608,914,827	368.03	1,025	0	4193791 (95.9%)	4127534 (94.4%)	4107188 (93.9%)	3980979 (91.1%)	3616378 (82.7%)	352597 (8.1%)	24,052,795	1,664,925,387	380.84	1,025	0	4356387 (99.6%)	4280034 (97.9%)	4257477 (97.4%)	4121928 (94.3%)	3737416 (85.5%)	360964 (8.3%)
	27,513,538	DRR029472	26,676,449	1,768,525,061	404.54	2,246	0	4165431 (95.3%)	4099522 (93.8%)	4032037 (92.2%)	3679009 (84.2%)	2989421 (68.4%)	1330116 (30.4%)	26,674,916	1,834,563,988	419.64	2,334	0	4325541 (98.9%)	4253953 (97.3%)	4182746 (95.7%)	3813381 (87.2%)	3101007 (70.9%)	1380522 (31.6%)
KH130	29,545,752	DRR029465	28,830,464	1,946,297,278	445.20	1,270	0	4179980 (95.6%)	4118281 (94.2%)	4094044 (93.6%)	3992588 (91.3%)	3775848 (86.4%)	1734696 (39.7%)	28,828,339	2,016,182,587	461.19	1,270	0	4342989 (99.3%)	4273576 (97.8%)	4246848 (97.1%)	4136826 (94.6%)	3907890 (89.4%)	1792636 (41.0%)
	25,743,281	DRR029474	25,084,853	1,683,174,834	385.02	1,361	0	4179857 (95.6%)	4117682 (94.2%)	4086261 (93.5%)	3908412 (89.4%)	3328609 (76.1%)	928323 (21.2%)	25,083,466	1,744,433,750	399.03	1,370	0	4342810 (99.3%)	4272406 (97.7%)	4238288 (96.9%)	4048655 (92.6%)	3445974 (78.8%)	962569 (22.0%)
K1478L	19,206,854	DRR029464	18,899,887	1,307,450,819	299.07	836	0	4191260 (95.9%)	4103857 (93.9%)	4057747 (92.8%)	3769495 (86.2%)	2575612 (58.9%)	34654 (0.8%)	18,897,900	1,353,042,840	309.50	836	0	4349113 (99.5%)	4252079 (97.3%)	4202638 (96.1%)	3897551 (89.2%)	2663644 (60.9%)	35847 (0.8%)
	26,856,614	DRR029473	26,129,235	1,739,432,764	397.88	1,544	0	4186834 (95.8%)	4119794 (94.2%)	4092749 (93.6%)	3944211 (90.2%)	3450143 (78.9%)	1067518 (24.4%)	26,127,804	1,801,848,096	412.16	1,544	0	4351060 (99.5%)	4274902 (97.8%)	4245158 (97.1%)	4086281 (93.5%)	3569942 (81.7%)	1103412 (25.2%)
BG039304	27,623,419	DRR029462	26,734,412	1,789,860,639	409.42	1,102	0	4195801 (96.0%)	4076730 (93.3%)	4024940 (92.1%)	3841626 (87.9%)	3492113 (79.9%)	1325660 (30.3%)	26,728,938	1,854,708,755	424.25	1,103	0	4360280 (99.7%)	4229448 (96.7%)	4174163 (95.5%)	3979892 (91.0%)	3617602 (82.8%)	1371372 (31.4%)
	27,325,895	DRR029471	26,469,690	1,781,586,909	407.53	1,273	0	4192264 (95.9%)	4106074 (93.9%)	4069221 (93.1%)	3916732 (89.6%)	3525586 (80.6%)	1187896 (27.2%)	26,465,616	1,848,055,454	422.73	1,286	0	4357351 (99.7%)	4260137 (97.4%)	4220707 (96.5%)	4058610 (92.8%)	3653987 (83.6%)	1233830 (28.2%)
1A-825-43	28,456,604	DRR029461	27,599,796	1,827,622,378	418.06	1,241	0	4183518 (95.7%)	4125510 (94.4%)	4100984 (93.8%)	3982555 (91.1%)	3697873 (84.6%)	1261961 (28.9%)	27,597,251	1,892,629,082	432.93	1,051	0	4348243 (99.5%)	4281373 (97.9%)	4253727 (97.3%)	4125887 (94.4%)	3825790 (87.5%)	1302518 (29.8%)
	27,315,549	DRR029470	25,830,633	1,752,840,906	400.95	1,424	0	4176951 (95.5%)	4113479 (94.1%)	4079886 (93.3%)	3903007 (89.3%)	3370236 (77.1%)	1170101 (26.8%)	25,828,929	1,818,585,219	415.99	1,424	0	4337241 (99.2%)	4266924 (97.6%)	4230545 (96.8%)	4043642 (92.5%)	3493326 (79.9%)	1218311 (27.9%)

* While Breseq is an all-in-one tool incorporating a mapping tool (Bowtie2), it is necessary to execute mapping analysis as a separated process to use LoFreq and SNVer. For the purpose, BWA, a very common tool for mapping analysis of short reads was utilized in this study.

Supplementary Table 2. Primers used to analyze sequences of polymorphisms between two subpopulations of *M. bovis* BCG Tokyo.

Target position	Primer sequence	Tm (C°)	Amplicon size (bp)
Amplification			
253186	TGGATGCGATTTGGCAAGGACGGTC	60	557
	AGGTTGATCCAGTGCTGGAAGTAGTC		
644562	CCAACACCCGGGTCATCGATCTGG	60	549
	AAGCCATCTCCGAGCATGTGGTCAG		
765342	TCCCGCAACGAATCCGCAAGTGTC	60	547
	ATTTCGGCCTCGAGCGTGGAGAGCTC		
2717585	ATCTCGTTACACAGGTCGATGCACTC	60	554
	TGATCGCGTGCGGAGCGTCCACCAG		
3192638	AGGTGGACCCCGATCTGTCGCTTG	60	569
	TCCTGTTGCCGAGCGGCGCAGCGTG		
3606131	TGTAGAACTCGAGCGGATCTTGTGAC	60	539
	TCTGGACGTCGGCGGTCGAACGGTAG		
4087391	TCCAGCGCGACCACGATCACCGAG	60	564
	AATGCGGCCGAATGGTCGGTGCAG		
Sequencing			
253186	GTCAACCCGGAATTCGGCTTCTTCG		
644562	ATGCCAGGATTTGTTGAGGCACAC		
765342	TCGAGGATCTTGCGTAAAGCTGTC		
2717585	TTGGCTCTTCCCGCAGAACGAGCAC		
3192638	TCGTACTGTCCGGCGAACTGTCAG		
3606131	AGCGGATCTTGTGACGCGGACTC		
4087391	TGTCGATGGACCGCGACGCGCTG		

Supplementary Table 3. Primers and probes used to calculate proportion of the subpopulations in lots of *M. bovis* BCG Tokyo.

Target position	Primer sequence	Tm (C°)	Reference
Primers			
Type II specific forward	CGAAGCTGACCAGACTGTTGC	60	Shibayama et al. [14]
Type I specific forward	CTCGATCACCTGGGCACTC	62	This study
Reverse (common)	GGTCGAGAGCCCGTTCG		Shibayama et al. [14]
Probes (common)	FAM-TGATGTCAGCGGGCGCCT-TAMRA		Shibayama et al. [14]

The PCR condition was followed with Sibayama et al. [14], except for annealing temperature (Tm).

Supplementary Table 4. Primers used to analyze sequences of intrinsic variation found in seed/lot of *M. bovis* BCG Tokyo.

Target position	Primer sequence	Tm (C°)	Amplicon size (bp)
2,655,559	ACGCGCCATATGAGGTAATC*	60	351
	TGAGTCCTGCAATGTTCAGC		
4,369,503	CTCCTCCTCCTTTTCGATCA	60	368
	GCCACTTCCTAGATGCCAAC*		

* The primers were also used as sequencing primers.

Supplementary figure 1. Base quality of the original short read data from seven lots of BCG Tokyo-172 in this study. The data were acquired from the same lots twice (A and B, respectively). The X-axis corresponds to the position of bases from 5’ to 3’ in reads, and Y-axis shows the phred scores (QV). The red line indicates the median value; the blue line indicates the mean quality; the yellow box shows 25-75% range of the total reads; the whiskers show the 10-90% range of the total reads. The graphs were generated by a free tool FastQC (http://www.bioinformatics.babraham.ac.uk/projects/fastqc/).

(A) 1st data collection

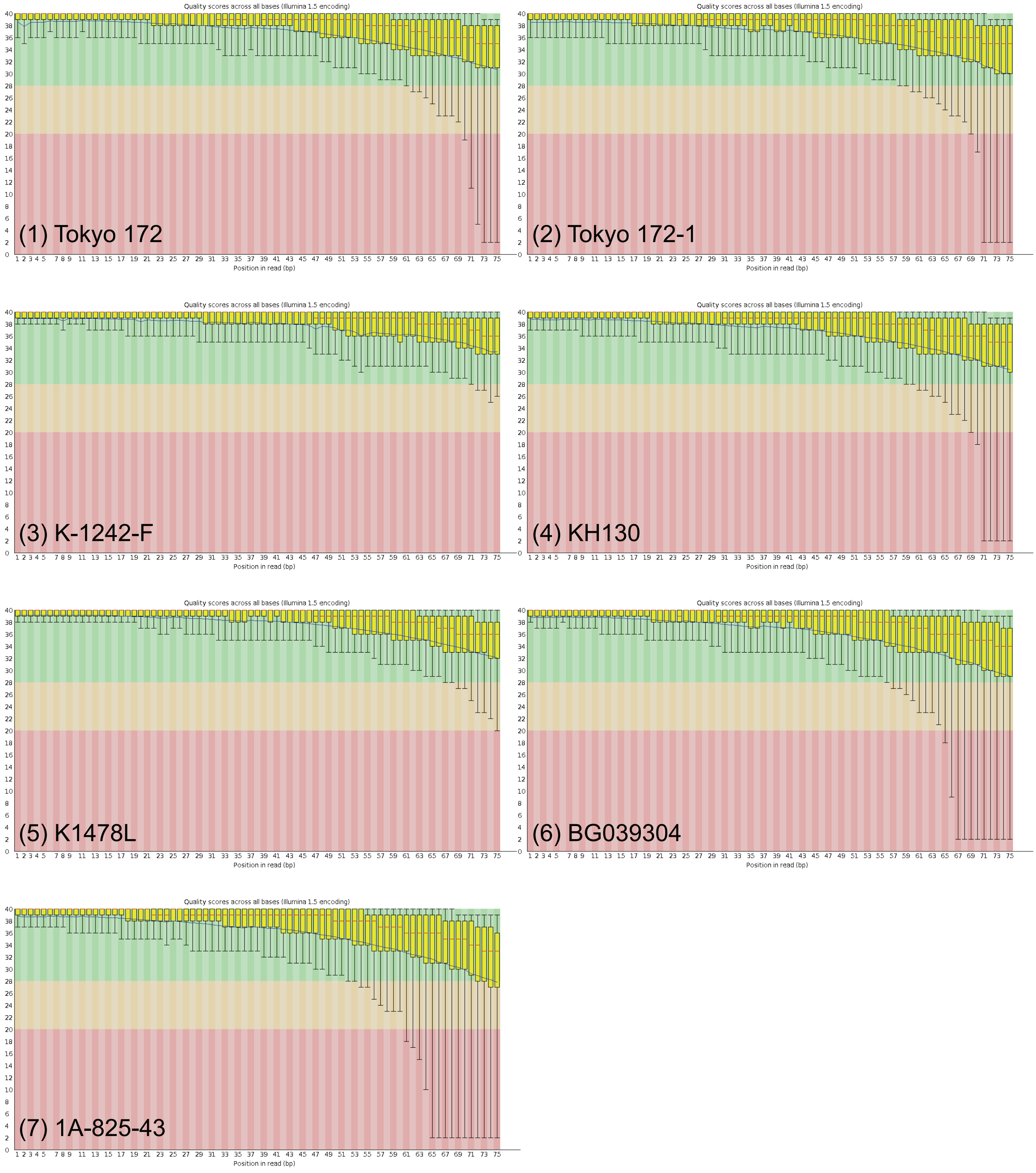

(A) 2nd data collection

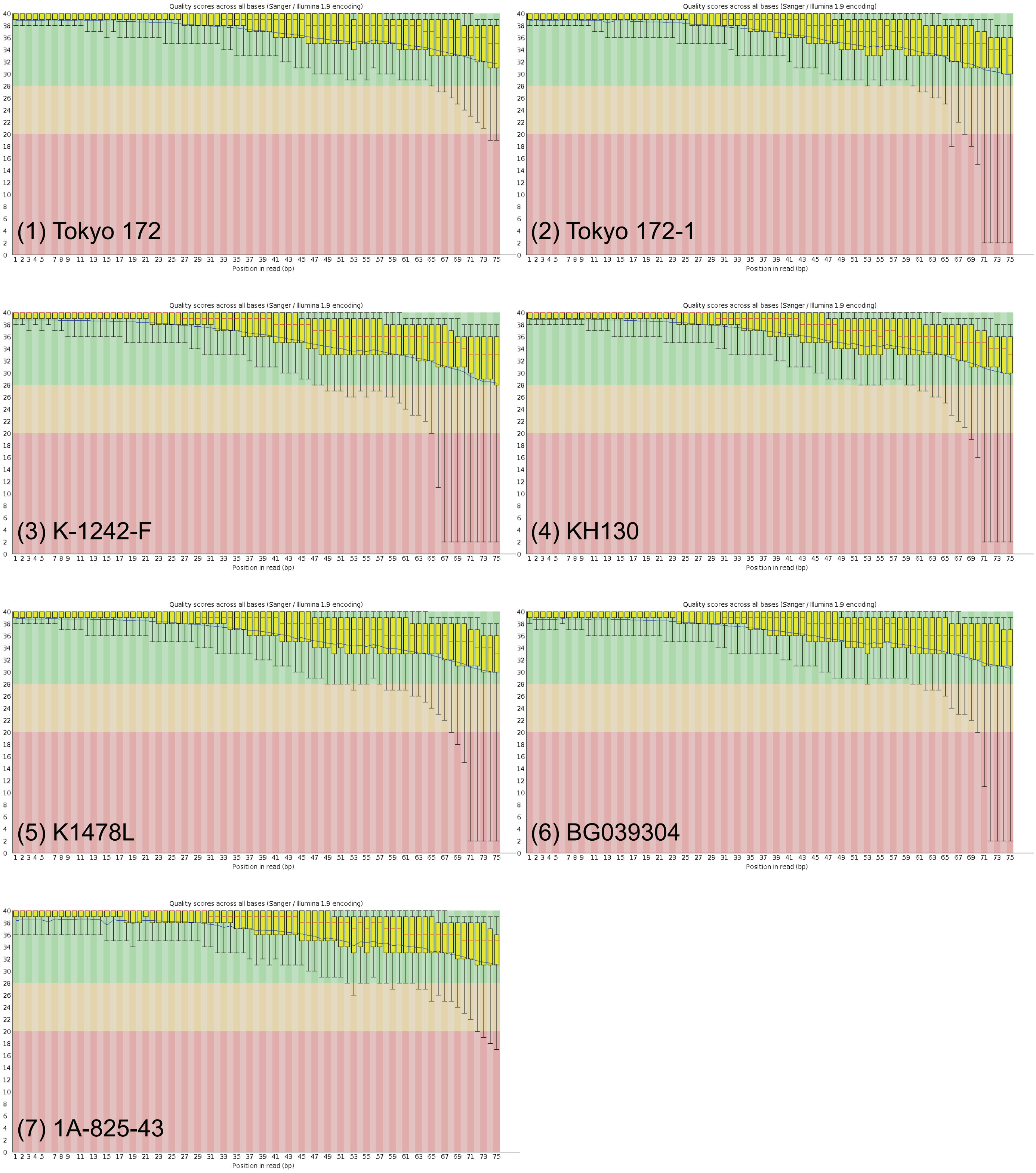

Supplementary GenBank file 1.

```
LOCUS       BCG_Tokyo_suppl_4419199 4419199 bp    DNA              12-9-2015
DEFINITION  BCG_Tokyo_suppl_4419199
FEATURES             Location/Qualifiers
     source          1..4419199
                     /note="molecular_form:circular"
                     /migap_code="248136284"
     CDS             1..1524
                     /codon_start=1
                     /gene="dnaA"
                     /note="gene_1"
                     /transl_table=11
                     /translation="LTDDPGSGFTTVWNAVVSELNGDPKVDDGPSSDANLSAPLTPQQ
                     RAWLNLVQPLTIVEGFALLSVPSSFVQNEIERHLRAPITDALSRRLGHQIQLGVRIAP
                     PATDEADDTTVPPSENPATTSPDTTTDNDEIDDSAAARGDNQHSWPSYFTERPRNTDS
                     ATAGVTSLNRRYTFDTFVIGASNRFAHAAALAIAEAPARAYNPLFIWGESGLGKTHLL
                     HAAGNYAQRLFPGMRVKYVSTEEFTNDFINSLRDDRKVAFKRSYRDVDVLLVDDIQFI
                     EGKEGIQEEFFHTFNTLHNANKQIVISSDRPPKQLATLEDRLRTRFEWGLITDVQPPE
                     LETRIAILRKKAQMERLAVPDDVLELIASSIERNIRELEGALIRVTAFASLNKTPIDK
                     ALAEIVLRDLIADANTMQISAATIMAATAEYFDTTVEELRGPGKTRALAQSRQIAMYL
                     CRELTDLSLPKIGQAFGRDHTTVMYAQRKILSEMAERREVFDHVKELTTRIRQRSKR"
     CDS             2052..3260
                     /codon_start=1
                     /gene="dnaN_1"
                     /note="gene_2"
                     /transl_table=11
                     /translation="MDAATTRVGLTDLTFRLLRESFADAVSWVAKNLPARPAVPVLSG
                     VLLTGSDNGLTISGFDYEVSAEAQVGAEIVSPGSVLVSGRLLSDITRALPNKPVGVHV
                     EGNRVALTCGNARFSLPTMPVEDYPTLPTLPEETGLLPAELFAEAISQVAIAAGRDDT
                     LPMLTGIRVEILGETVVLAATDRFRLAVRELKWSASSPDIEAAVLVPAKTLAEAAKAG
                     IGGSDVRLSLGTGPGVGKDGLLGISGNGKRSTTRLLDAEFPKFRQLLPTEHTAVATMD
                     VAELIEAIKLVALVADRGAQVRMEFADGSVRLSAGADDVGRAEEDLVVDYAGEPLTIA
                     FNPTYLTDGLSSLRSERVSFGFTTAGKPALLRPVSGDDRPVAGLNGNGPFPAVSTDYV
                     YLLMPVRLPG"
     CDS             3280..4437
                     /codon_start=1
                     /gene="recF"
                     /note="gene_3"
                     /transl_table=11
                     /translation="VYVRHLGLRDFRSWACVDLELHPGRTVFVGPNGYGKTNLIEALW
                     YSTTLGSHRVSADLPLIRVGTDRAVISTIVVNDGRECAVDLEIATGRVNKARLNRSSV
                     RSTRDVVGVLRAVLFAPEDLGLVRGDPADRRRYLDDLAIVRRPAIAAVRAEYERVVRQ
                     RTALLKSVPGARYRGDRGVFDTLEVWDSRLAEHGAELVAARIDLVNQLAPEVKKAYQL
                     LAPESRSASIGYRASMDVTGPSEQSDTDRQLLAARLLAALAARRDAELERGVCLVGPH
                     RDDLILRLGDQPAKGFASHGEAWSLAVALRLAAYQLLRVDGGEPVLLLDDVFAELDVM
                     RRRALATAAESAEQVLVTAAVLEDIPAGWDARRVHIDVRADDTGSMSVVLP"
     CDS             4704..4997
                     /codon_start=1
```

/note="gene_4"
/transl_table=11
/translation="VRVAEGMVLGQWSAVVGHQIAEHARPTALNDGVLSVIAESTAWA
TQLRIMQAQLLAKIAAAVGNDVVRSLKITGPAAPSWRKGPRHIAGRGPRDTYG"
CDS 5240..7267
/codon_start=1
/gene="gyrB"
/note="gene_5"
/transl_table=11
/translation="VAAQKKKAQDEYGAASITILEGLEAVRKRPGMYIGSTGERGLHH
LIWEVVDNAVDEAMAGYATTVNVVLLEDGGVEVADDGRGIPVATHASGIPTVDVVMTQ
LHAGGKFDSDAYAISGGLHGVGVSVVNALSTRLEVEIKRDGYEWSQVYEKSEPLGLKQ
GAPTKKTGSTVRFWADPAVFETTEYDFETVARRLQEMAFLNKGLTINLTDERVTQDEV
VDEVVSDVAEAPKSASERAAESTAPHKVKSRTFHYPGGLVDFVKHINRTKNAIHSSIV
DFSGKGTGHEVEIAMQWNAGYSESVHTFANTINTHEGGTHEEGFRSALTSVVNKYAKD
RKLLKDKDPNLTGDDIREGLAAVISVKVSEPQFEGQTKTKLGNTEVKSFVQKVCNEQL
THWFEANPTDSKVVVNKAVSSAQARIAARKARELVRRKSATDIGGLPGKLADCRSTDP
RKSELYVVEGDSAGGSAKSGRDSMFQAILPLRGKIINVEKARIDRVLKNTEVQAIITA
LGTGIHDEFDIGKLRYHKIVLMADADVDGQHISTLLLTLLFRFMRPLIENGHVFLAQP
PLYKLKWQRSDPEFAYSDRERDGLLEAGLKAGKKINKEDGIQRYKGLGEMDAKELWET
TMDPSVRVLRQVTLDDAAAADELFSILMGEDVDARRSFITRNAKDVRFLDV"
CDS 7302..9818
/codon_start=1
/note="gene_6"
/transl_table=11
/translation="MTDTTLPPDDSLDRIEPVDIQQEMQRSYIDYAMSVIVGRALPEV
RDGLKPVHRRVLYAMFDSGFRPDRSHAKSARSVAETMGNYHPHGDASIYDTLVRMAQP
WSLRYPLVDGQGNFGSPGNDPPAAMRYTEARLTPLAMEMLREIDEETVDFIPNYDGRV
QEPTVLPSRFPNLLANGSGGIAVGMATNIPPHNLRELADAVFWALENHDADEEETLAA
VMGRVKGPDFPTAGLIVGSQGTADAYKTGRGSIRMRGVVEVEEDSRGRTSLVITELPY
QVNHDNFITSIAEQVRDGKLAGISNIEDQSSDRVGLRIVIEIKRDAVAKVVINNLYKH
TQLQTSFGANMLAIVDGVPRTLRLDQLIRYYVDHQLDVIVRRTTYRLRKANERAHILR
GLVKALDALDEVIALIRASETVDIARAGLIELLDIDEIQAQAILDMQLRRLAALERQR
IIDDLAKIEAEIADLEDILAKPERQRGIVRDELAEIVDRHGDDRRTRIIAADGDVSDE
DLIAREDVVVTITETGYAKRTKTDLYRSQKRGGKGVQGAGLKQDDIVAHFFVCSTHDL
ILFFTTQGRVYRAKAYDLPEASRTARGQHVANLLAFQPEERIAQVIQIRGYTDAPYLV
LATRNGLVKKSKLTDFDSNRSGGIVAVNLRDNDELVGAVLCSADDDLLLVSANGQSIR
FSATDEALRPMGRATSGVQGMRFNIDDRLLSLNVVREGTYLLVATSGGYAKRTAIEEY
PVQGRGGKGVLTVMYDRRRGRLVGALIVDDDSELYAVTSGGGVIRTAARQVRKAGRQT
KGVRLMNLGEGDTLLAIARNAEESGDDNAVDANGADQTGN"
CDS 9963..10160
/codon_start=1
/gene="NCGM2209_0410"
/note="gene_7"
/transl_table=11
/translation="MRMAWSTVGAHIGQRPGQAAYQMLETRRRGSVLRLGNPKRGIVS
RRRYHTLRGARPTRPPPPMLG"
CDS 10190..10828
/codon_start=1
/note="gene_8"
/transl_table=11

```
                     /translation="VTGPAAAVRTPQPDPDASLGCGDGSPAEAYASELPDLSGPTPRA
                     PQRNPAPARPAEGGAGSRGDSAAGSSGGRSITAESRDARVQLSARRSRGPVRASMQIR
                     RIDPWSTLKVSLLLSVALFFVWMITVAFLYLVLGGMGVWAKLNSNVGDLLNNASGSSA
                     ELVSSGTIFGGAFLIGLVNIVLMTALATIGAFVYNLITDLIGGIEVTLADRD"
     tRNA            10887..10963
                     /note="identified by tRNAscan-SE; putative"
     tRNA            11112..11187
                     /note="identified by tRNAscan-SE; putative"
     CDS             complement(11123..11341)
                     /codon_start=1
                     /note="gene_9"
                     /transl_table=11
                     /translation="MLEHDSAGLTLDRYRHLYGSGVEAVGVAINALFTRDCDQTWSFE
                     VSELRFSLVEPRGLEPLTPALQRQCSTN"
     CDS             complement(11874..12311)
                     /codon_start=1
                     /note="gene_10"
                     /transl_table=11
                     /translation="MSEQVETRLTPRERLTRGLAYSAVGPVDVTRGLLELGVGLGLQS
                     ARSTAAGLRRRYREGRLAREVAAAQETLAQELTAAQDVVANLPQALQDARTQRRSKHH
                     LWIFAGIAAAILAGGAVAFSIVRRSSRPEPSPRPPSVEVQPRP"
     CDS             12486..13016
                     /codon_start=1
                     /note="gene_11"
                     /transl_table=11
                     /translation="VTNSPLATATATLHTNRGDIKIALFGNHAPKTVANFVGLAQGTK
                     DYSTQNASGGPSGPFYDGAVFHRVIQGFMIQGGDPTGTGRGGPGYKFADEFHPELQFD
                     KPYLLAMANAGPGTNGSQFFITVGKTPHLNRRHTIFGEVIDAESQRVVEAISKTATDG
                     NDRPTDPVVIESITIS"
     CDS             complement(13024..13176)
                     /codon_start=1
                     /note="gene_12"
                     /transl_table=11
                     /translation="MRLPLPVTPVAAKGERTWREGVRLNGPNGVSVYRHVPWRVHKVY
                     SSDEPT"
     CDS             complement(13222..13491)
                     /codon_start=1
                     /note="gene_13"
                     /transl_table=11
                     /translation="MAIASVTLVTDTPGRVLTGVAALGLILFASATWRARPRLAITPD
                     GLAIRGWFRTQLLRHSNIKIIRIDEFRRYGRLVRLLEIETVSGGC"
     CDS             complement(13713..13994)
                     /codon_start=1
                     /note="gene_14"
                     /transl_table=11
                     /translation="MPKSKVRKKNDFTVSAVSRTPMKVKVGPSSVWFVSLFIGLMLIG
                     LIWLMVFQLAAIGSQAPTALNWMAQLGPWNYAIAFAFMITGLLLTMRWH"
     CDS             14133..14876
                     /codon_start=1
                     /note="gene_15"
                     /transl_table=11
```

```
                     /translation="MIDRRRSAWRFSVPLVCLLAGLLLAATHGVSGGTEIRRSDAPRL
                     VDLVRRAQASVNRLATEREALTTRIDSVHGRSVDTALAAMQRRSAELAGVAAMNPVHG
                     PGLVVTLQDAQRDANGRFPRDASPDDLVVHQQDIEAVLNALWNAGAEAIQMQDQRIIA
                     MSIARCVGNTLLLNGRTYSPPYTIAAIGDAAAMQAALAAAPLVTLYKQYVVRFGLGYR
                     EEVHPDLQIVGYADPVRMHFAQPAGPLDY"
     CDS             14913..15611
                     /codon_start=1
                     /gene="trpG_1"
                     /note="gene_16"
                     /transl_table=11
                     /translation="MRILVVDNYDSFVFNLVQYLGQLGIEAEVWRNDDHRLSDEAAVA
                     GQFDGVLLSPGPGTPERAGASVSMVHACAAAHTPLLGVCLGHQAIGVAFGATVDRAPE
                     LLHGKTSSVFHTNVGVLQGLPDPFTATRYHSLTILPKSLPAVLRVTARTSSGVIMAVQ
                     HTGLPIHGVQFHPESILTEGGHRILANWLTCCGWTQDDTLVRRLENEVLTAISPHFPT
                     STASAGEATGRTSA"
     CDS             complement(15589..17469)
                     /codon_start=1
                     /note="gene_17"
                     /transl_table=11
                     /translation="MTTPSHLSDRYELGEILGFGGMSEVHLARDLRLHRDVAVKVLRA
                     DLARDPSFYLRFRREAQNAAALNHPAIVAVYDTGEAETPAGPLPYIVMEYVDGVTLRD
                     IVHTEGPMTPKRAIEVIADACQALNFSHQNGIIHRDVKPANIMISATNAVKVMDFGIA
                     RAIADSGNSVTQTAAVIGTAQYLSPEQARGDSVDARSDVYSLGCVLYEVLTGEPPFTG
                     DSPVSVAYQHVREDPIPPSARHEGLSADLDAVVLKALAKNPENRYQTAAEMRADLVRV
                     HNGEPPEAPKVLTDAERTSLLSSAAGNLSGPRTDPLPRQDLDDTDRDRSIGSVGRWVA
                     VVAVLAVLTVVVTIAINTFGGITRDVQVPDVRGQSSADAIATLQNRGFKIRTLQKPDS
                     TIPPDHVIGTDPAANTSVSAGDEITVNVSTGPEQREIPDVSTLTYAEAVKKLTAAGFG
                     RFKQANSPSTPELVGKVIGTNPPANQTSAITNVVIIIVGSGPATKDIPDVAGQTVDVA
                     QKNLNVYGFTKFSQASVDSPRPAGEVTGTNPPAGTTVPVDSVIELQVSKGNQFVMPDL
                     SGMFWVDAEPRLRALGWTGMLDKGADVDAGGSQHNRVVYQNPPAGTGVNRDGIITLRF
                     GQ"
     CDS             complement(17466..18761)
                     /codon_start=1
                     /note="gene_18"
                     /transl_table=11
                     /translation="MSPRVGVTLSGRYRLQRLIATGGMGQVWEAVDNRLGRRVAVKVL
                     KSEFSSDPEFIERFRAEARTTAMLNHPGIASVHDYGESQMNGEGRTAYLVMELVNGEP
                     LNSVLKRTGRLSLRHALDMLEQTGRALQIAHAAGLVHRDVKPGNILITPTGQVKITDF
                     GIAKAVDAAPVTQTGMVMGTAQYIAPEQALGHDASPASDVYSLGVVGYEAVSGKRPFA
                     GDGALTVAMKHIKEPPPPLPPDLPPNVRELIEITLVKNPAMRYRSGGPFADAVAAVRA
                     GRRPPRPSQTPPPGRAAPAAIPSGTTARVAANSAGRTAASRRSRPATGGHRPPRRTFS
                     SGQRALLWAAGVLGALAIIIAVLLVIKAPGDNSPQQAPTPTVTTTGNPPASNTGGTDA
                     SPRLNWTERGETRHSGLQSWVVPPTPHSRASLARYEIAQ"
     CDS             complement(18758..20197)
                     /codon_start=1
                     /note="gene_19"
                     /transl_table=11
                     /translation="MALIVLLLLNATMTQVFTADGLRADPRNQRVLLDEYSRQRGQIT
                     AGGQLLAYSVATDGRFRFLRVYPNPEVYAPVTGFYSLRYSSTALERAEDPILNGSDRR
                     LFGRRLADFFTGRDPRGGNVDTTINPRIQQAGWDAMQQGCYGPCKGAVVALEPSTGKI
                     LALVSSPSYDPNLLASHNPEVQAQAWQRLGDNPASPLTNRAISETYPPGSTFKVITTA
```

```
                     AALAAGATETEQLTAAPTIPLPGSTAQLENYGGAPCGDEPTVSLREAFVKSCNTAFVQ
                     LGIRTGADALRSMARAFGLDSPPRPTPLQVAESTVGPIPDSAALGMTSIGQKDVALTP
                     LANAEIAATIANGGITMRPYLVGSLKGPDLANISTTVGYQQRRAVSPQVAAKLTELMV
                     GAEKVAQQKGAIPGVQIASKTGTAEHGTDPRHTPPHAWYIAFAPAQAPKVAVAVLVEN
                     GADRLSATGGALAAPIGRAVIEAALQGEP"
     CDS             complement(20230..21639)
                     /codon_start=1
                     /note="gene_20"
                     /transl_table=11
                     /translation="MTTRLQAPVAVTPPLPTRRNAELLLLCFAAVITFAALLVVQANQ
                     DQGVPWDLTSYGLAFLTLFGSAHLAIRRFAPYTDPLLLPVVALLNGLGLVMIHRLDLV
                     DNEIGEHRHPSANQQMLWTLVGVAAFALVVTFLKDHRQLARYGYICGLAGLVFLAVPA
                     LLPAALSEQNGAKIWIRLPGFSIQPAEFSKILLLIFFSAVLVAKRGLFTSAGKHLLGM
                     TLPRPRDLAPLLAAWVISVGVMVFEKDLGASLLLYTSFLVVVYLATQRFSWVVIGLTL
                     FAAGTLVAYFIFEHVRLRVQTWLDPFADPDGTGYQIVQSLFSFATGGIFGTGLGNGQP
                     DTVPAASTDFIIAAFGEELGLVGLTAILMLYTIVIIRGLRTAIATRDSFGKLLAAGLS
                     STLAIQLFIVVGGVTRLIPLTGLTTPWMSYGGSSLLANYILLAILARISHGARRPLRT
                     RPRNKSPITAAGTEVIERV"
     CDS             complement(21636..23171)
                     /codon_start=1
                     /note="gene_21"
                     /transl_table=11
                     /translation="VTLVLRYAARSDRGLVRANNEDSVYAGARLLALADGMGGHAAGE
                     VASQLVIAALAHLDDDEPGGDLLAKLDAAVRAGNSAIAAQVEMEPDLEGMGTTLTAIL
                     FAGNRLGLVHIGDSRGYLLRDGELTQITKDDTFVQTLVDEGRITPEEAHSHPQRSLIM
                     RALTGHEVEPTLTMREARAGDRYLLCSDGLSDPVSDETILEALQIPEVAESAHRLIEL
                     ALRGGGPDNVTVVVADVVDYDYGQTQPILAGAVSGDDDQLTLPNTAAGRASAISQRKE
                     IVKRVPPQADTFSRPRWSGRRLAFVVALVTVLMTAGLLIGRAIIRSNYYVADYAGSVS
                     IMRGIQGSLLGMSLHQPYLMGCLSPRNELSQISYGQSGGPLDCHLMKLEDLRPPERAQ
                     VRAGLPAGTLDDAIGQLRELAANSLLPPCPAPRATSPPGRPAPPTTSETTEPNVTSSP
                     AAPSPTTSASAPTGTTPAIPTSASPAAPASPPTPWPVTSSPTMAALPPPPPQPGIDCR
                     AAA"
     CDS             complement(23269..23736)
                     /codon_start=1
                     /note="gene_22"
                     /transl_table=11
                     /translation="MQGLVLQLTRAGFLMLLWVFIWSVLRILKTDIYAPTGAVMMRRG
                     LALRGTLLGARQRRHAARYLVVTEGALTGARITLSEQPVLIGRADDSTLVLTDDYAST
                     RHARLSMRGSEWYVEDLGSTNGTYLDRAKVTTAVRVPIGTPVRIGKTAIELRP"
     CDS             complement(23860..25425)
                     /codon_start=1
                     /note="gene_23"
                     /transl_table=11
                     /translation="MGSQKRLVQRVERKLEQTVGDAFARIFGGSIVPQEVEALLRREA
                     ADGIQSLQGNRLLAPNEYIITLGVHDFEKLGADPELKSTGFARDLADYIQEQGWQTYG
                     DVVVRFEQSSNLHTGQFRARGTVNPDVETHPPVIDCARPQSNHAFGAEPGVAPMSDNS
                     SYRGGQGQGRPDEYYDDRYARPQEDPRGGPDPQGGSDPRGGYPPETGGYPPQPGYPRP
                     RHPGQGDYPEQIGYPDQGGYPEQRGYPDQRGYQDQGRGYPDQGQGGYPPPYEQRPPVS
                     PGPAAGYGAPGYDQGYRQSGGCGPSPGGGQPGYGGYGEYGRGPARHEEGSYVPSGPPG
                     PPEQRPAYPDQGGYDQGYQQGATTYGRQDYGGGADYTRYTESPQVPGYAPQGGGYAEP
                     AGRDYDYGQSGAPDYGQPAPGGYSGYGQGGYGSAGTSVTLQLDDGSGRTYQLREGSNI
```

```
                     IGRGQDAQFRLPDTGVSRRHLEIRWDGQVALLADLNSTNGTTVNNAPVQEWQLADGDV
                     IRLGHSEIIVRMH"
     tRNA            25625..25710
                     /note="identified by tRNAscan-SE; putative"
     CDS             25631..25996
                     /codon_start=1
                     /gene="EUTSA_v10022600mg"
                     /note="gene_24"
                     /transl_table=11
                     /translation="VAEWQTRWLQVPVSFGTWGFKSPFAHRTVRRVVTDIVVETAAMG
                     VVGTASARPRPAQGNPGIDVTIAGAVRFSSVAAAAGWRGASVPFYTARIAVSVLAACC
                     AIPVITSAAGRTALTRPAA"
     CDS             complement(25894..26862)
                     /codon_start=1
                     /note="gene_25"
                     /transl_table=11
                     /translation="VVLSTAFSQMFGIDYPIVSAPMDLIAGGELAAAVSGAGGLGLIG
                     GGYGDRDWLARQFDLAAGAPVGCGFITWSLARQPQLLDLALQYEPVAVMLSFGDPAVF
                     ADAIKSAGTRLVCQIQNRTQAERALQVGADVLVAQGTEAGGHGHGPRSTLTLVPEIVD
                     LVTARGTDIPVIAAGGIADGRGLAAALMLGAAGVLVGTRFYATVEALSTPQARDPLLA
                     ATGDDMCRTTIYDQLRRYPWPQGHTMSVLSNALTDQFEDTELDILHREEAMARYWRAV
                     PARDYSIANVTAGQAAGLVNAVLPAADVITGMAQQAARTLTAMRAV"
     CDS             complement(27004..27423)
                     /codon_start=1
                     /note="gene_26"
                     /transl_table=11
                     /translation="MAHPCATDPELWFGYPDDDGSDGAAKARAYERSATQARIQCLRR
                     CPLLQQRRCAQHAVEHRVEYGVWAGIKLPGGQYRKREQLAAAHDVLRRIAGGEINSRQ
                     LPDNAALLARNEGLEVTPVPGVVVHLPIAQVGPQPAA"
     CDS             27576..28346
                     /codon_start=1
                     /note="gene_27"
                     /transl_table=11
                     /translation="VSRESAGAAIRALRESRDWSLADLAAATGVSTMGLSYLERGARK
                     PHKSTVQKVENGLGLPPGTYSRLLVAADPDAELARLIAAQPSNPTAVRRAGAVVVDRH
                     SDTDVLEGYAEAQLDAIKSVIDRLPATTSNEYETYILSVIAQCVKAEMLAASSWRVAV
                     NAGADSTGRLMEHLRALEATRGALLERMPTSLSARFDRACAQSSLPEAVVAALIGVGA
                     DEMWDIRNRGVIPAGALPRVRAFVDAIEASHDADEGQQ"
     CDS             28939..29187
                     /codon_start=1
                     /note="gene_28"
                     /transl_table=11
                     /translation="LVQWAYAQAGVHLDRTTYQQINEGIPVPRSQVRPGDLVFPHPGH
                     VQLAIGNNLVVEAPHAGASVRVSSLGNNVQIRRPLSGR"
     CDS             29225..29587
                     /codon_start=1
                     /note="gene_29"
                     /transl_table=11
                     /translation="MSEQAGSSVAVIQERQALLARQHDAVAEADRELADVLASAHAAM
                     RESVRRLDAIAAELDRAVPDQDQLAVDTLMGAREFQTFLVAKQREIVAVVAAAHELDR
                     AKSAVLKRLRAQYTEPAR"
```

```
CDS             29702..31135
                /codon_start=1
                /note="gene_30"
                /transl_table=11
                /translation="VAFDAAMSTHEDLLATIRYVRDRTGDPNAWQTGLTPTEVTAVVT
                STTRSEQLDAILRKIRQRHSNLYYPAPPDREQGDAARAIADAEAALAHPNSATAQLDL
                QVVSAILNAHLKTVEGGESLHELQQEIEAAVRIRSDLDTPAGARDFQRFLIGKLKDIR
                EVVATASLDAASKSALMAAWTSLYDASKGDRGDADDRGPASVGSGGAPARGAGQQPEL
                PTRAEPDCLLDSLLLEDPGLLADDLQVPGGTSAAIPSASSTPSLPNLGGATMPGGGAT
                PALVPGVSAPGGLPLSGLLRGVGDEPELTDFDERGQEVRDPADYEHSNEPDERRADDR
                EGADEDAGLGKSESPPQAPTTVTLPNGETVTAASPQLAAAIKAAASGTPIADAFQQQG
                IAIPLPGTAVANPVDPARISAGDVGVFTDRHALALGPSKALLDGQIQHISAVRGRNFL
                GWIHPAATATAPARTEAPTPTRPAAAR"
CDS             31173..31490
                /codon_start=1
                /note="gene_31"
                /transl_table=11
                /translation="MTDRIHVQPAHLRQAAAHHQQTADYLRTVPSSHDAIRESLDSLG
                PIFSELRDTGRELLELRKQCYQQQADNHADIAQNLRTSAAMWEQHERAASRSLGNIID
                GSR"
CDS             31498..31803
                /codon_start=1
                /note="gene_32"
                /transl_table=11
                /translation="MTDANPAFDTVHPSGHILVRSCRGGYMHSVSLSEAAMETDAETL
                AEAILLTADVSCLKALLEVRNEIVAAGHTPSAQVPTTDDLNVAIEKLLAHQLRRRNR"
CDS             complement(31808..31951)
                /codon_start=1
                /note="gene_33"
                /transl_table=11
                /translation="MADSALQQQLDEVRALLTRARELFGPNPIEPPTDIAPDPDSTKT
                WLI"
CDS             32320..33138
                /codon_start=1
                /note="gene_34"
                /transl_table=11
                /translation="VIDRARASAMRRVESAMRELRQITGRVPVEIPRMRGAGGSDLDT
                TRLMPAVTVVQPADQACTDWPVAAAEDDEARLQRLLAFVARQEPRLNWAVGVNADGTT
                VLVTDVAHGWIPPGIALPEGVRLLAPARRAGRAPELVGITTCCKTYTPGDSLRRAVDS
                TAPTSSVQPRALPAIAGLSVELGIATQRHDGLPKIVHAMATAAGNGAAAEEVDLLRVH
                VDTALHHVLAQYPRVDPALLLNCMLLAATERSVTGDPIAANYHFAWFRELDSRR"
CDS             33208..33537
                /codon_start=1
                /note="gene_35"
                /transl_table=11
                /translation="MVSGSDSRSEPSQLSDRDLVESVLRDLSEAADKWEALVTQAETV
                TYSVDLGDVRAVANSDGRLLELTLHPGVMTGYAHGELADRVNLAITALRDEVEAENRA
                RYGGRLQ"
CDS             34279..36594
                /codon_start=1
                /note="gene_36"
```

/transl_table=11
/translation="MPTGLGYDFLRPVEDSGINDLKHYYFMADLADGQPLGRANLYSV
CFDLATTDRKLTPAWRTTIKRWFPGFMTFRFLECGLLTMVSNPLALRSDTDLERVLPV
LAGQMDQLAHDDGSDFLMIRDVDPEHYQRYLDILRPLGFRPALGFSRVDTTISWSSVE
EALGCLSHKRRLPLKTSLEFRERFGIEVEELDEYAEHAPVLARLWRNVKTEAKDYQRE
DLNPEFFAACSRHLHGRSRLWLFRYQGTPIAFFLNVWGADENYILLEWGIDRDFEHYR
KANLYRAALMLSLKDAISRDKRRMEMGITNYFTKLRIPGARVIPTIYFLRHSTDPVHT
ATLARMMMHNIQRPTLPDDMSEEFCRWEERIRLDQDGLPEHDIFRKIDRQHKYTGLKL
GGVYGFYPRFTGPQRSTVKAAELGEIVLLGTNSYLGLATHPEVVEASAEATRRYGTGC
SGSPLLNGTLDLHVSLEQELACFLGKPAAVLCSTGYQSNLAAISALCESGDMIIQDAL
NHRSLFDAARLSGADFTLYRHNDMDHLARVLRRTEGRRRIIVVDAVFSMEGTVADLAT
IAELADRHGCRVYVDESHALGVLGPDGRGASAALGVLARMDVVMGTFSKSFASVGGFI
AGDRPVVDYIRHNGSGHVFSASLPPAAAAATHAALRVSRREPDRRARVLAAAEYMATG
LARQGYQAEYHGTAIVPVILGNPTVAHAGYLRLMRSGVYVNPVAPPAVPEERSGFRTS
YLADHRQSDLDRALHVFAGLAEDLTPQGAAL"
CDS 36591..36854
/codon_start=1
/note="gene_37"
/transl_table=11
/translation="MKEAINATIQRILRTDRGITANQVLVDDLGFDSLKLFQLITELE
DEFDIAISFRDAQNIKTVGDVYTSVAVWFPETAKPAPLGKGTA"
CDS 36851..37246
/codon_start=1
/note="gene_38"
/transl_table=11
/translation="MTDDADLDLVRRTFAAFARGDLAELTQCFAPDVEQFVPGKHALA
GVFRGVDNVVACLGDTAAAADGTMTVTLEDVLSNTDGQVIAVYRLRASRAGKVLDQRE
AILVTVAGGRITRLSEFYADPAATESFWA"
CDS 37243..38931
/codon_start=1
/note="gene_39"
/transl_table=11
/translation="MTAALLSPAIAWQQISACTDRTLTITCEDSEVISYQDLIARAAA
CIPPLRRLDLKRGEPVLITAHTNLEFLSCFLGLMLHGAVPVPIPPREALKTTERFMTR
LGPLLRHHRVLICTPAEHDEIRAAASTDCQISRFTALAEAGDEQFGRATAQQLADTAT
ADWPLCTLDDDAYVQYTSGSTAAPRGVVITYRNLLSNMRAMAVGSQFQHGDVMGSWLP
LHHDMGLVGSLFAALFNSVSAVFTTPHRFLYDPLGFLRLLTSSGATHTFMPNFALEWL
INAYHRRGADIEGIDLHKMRRLIIASEPVHAEGMRRFAATFAGVGLAPTALGSGYGLA
EATVAVSMSAPNTGFRTETHAAAEVVTGGRVLPGYEVRIDAAPGARAGTIKLRGDSVA
AKAYVGGKKLDALDEEGFCDTHDLGFLVDDEIVILGRQDEVFIVHGENRFPYDIEFII
RGESEQHRTKVACFGVNERVVVVLESPLDSIIDKAEADRLRCQVVAATGLQLDELITV
RRGAIPTTTSGKLKRRAVAQAYRDGTLPRLATHAWTADPDSAPKTTRSSLEGAH"
CDS complement(39132..39812)
/codon_start=1
/note="gene_40"
/transl_table=11
/translation="MADPGPFVADLRAESDDLDALVAHLPADRWADPTPAPGWTIAHQ
IGHLLWTDRVALTAVTDEAGFAELMTAAAANPAGFVDDAATELAAVSPAELLTDWRVT
RGRLHEELLAVPDGRKLAWFGPPMSAASMATARLMETWAHGLDVADALGVIRPATQRL
RSIAHLGVRTRDYAFIVNNLTPPAEPFLVELRGPSGDTWSWGHPMRPSGSPGPPRTSA
SWSPSGAR"

```
CDS             complement(39860..41161)
                /codon_start=1
                /note="gene_41"
                /transl_table=11
                /translation="VIHSRMHARAPVDVWRSVRSLPDFWRLLQVRVASQFGDGLFQAG
                LAGALLFNPDRAADPMAIAGAFAVLFLPYSLLGPFAGALMDRWDRRWVLVGANTGRLA
                LIAGVGTILAVGAGDVPLLVGALVANGLARFVASGLSAALPHVVPREQVVTMNSVAIA
                SGAVSAFLGANFMLLPRWLLGSGDEGASAIVFLVAIPVSIALLWSLRFGPRVLGPDDT
                ERAIHGSAVYAVVTGWLHGARTVVQLPTVAAGLSGLAAHRMVVGINSLLILLLVRHVT
                ARAVGGLGTALLFFAATGLGAFLANVLTPTAIRRWGRYATANGALAAAATIQVAAAGL
                LVPVMVVCGFLLGVAGQVVKLCADSAMQMDVDDALRGHVFAVQDALFWVSYILSITVA
                AALIPEHGHAPVFVLFGSAIYLAGLVVHTIVGRRGQPVIGR"
CDS             41290..41895
                /codon_start=1
                /note="gene_42"
                /transl_table=11
                /translation="VAPHEDPEDHVAPAAQRVRAGTLLLANTDLLEPTFRRSVIYIVE
                HNDGGTLGVVLNRPSETAVYNVLPQWAKLAAKPKTMFIGGPVKRDAALCLAVLRVGAD
                PEGVPGLRHVAGRLVMVDLDADPEVLAAAVEGVRIYAGYSGWTIGQLEGEIERDDWIV
                LSALPSDVLVGPRADLWGQVLRRQPLPLSLLATHPIDLSRN"
CDS             complement(41987..42334)
                /codon_start=1
                /note="gene_43"
                /transl_table=11
                /translation="MFLAGVLCMCAAAASALFGSWSLFHTPTADPTALALRAMAPTQL
                AAAVMLAAGGVVAVAAPGHTALMVVIVCIAGAVGTLAAGSWQSAQYALRRETASPTAN
                CVGSCAVCTQACH"
CDS             complement(42416..43348)
                /codon_start=1
                /note="gene_44"
                /transl_table=11
                /translation="MIQIARTWRVFAGGMATGFIGVVLVTAGKASADPLLPPPPIPAP
                VSAPATVPPVQNLTALPGGSSNRFSPAPAPAPIASPIPVGAPGSTAVPPLPPPVTPAI
                SGTLRDHLREKGVKLEAQRPHGFKALDITLPMPPRWTQVPDPNVPDAFVVIADRLGNS
                VYTSNAQLVVYRLIGDFDPAEAITHGYIDSQKLLAWQTTNASMANFDGFPSSIIEGTY
                RENDMTLNTSRRHVIATSGADKYLVSLSVTTALSQAVTDGPATDAIVNGFQVVAHAAP
                AQAPAPAPGSAPVGLPGQAPGYPPAGTLTPVPPR"
CDS             43578..46454
                /codon_start=1
                /gene="leuS"
                /note="gene_45"
                /transl_table=11
                /translation="VPRADDADSDVPRYRYTAELAARLERTWQENWARLGTFNVPNPV
                GSLAPPDGAAVPDDKLFVQDMFPYPSGEGLHVGHPLGYIATDVYARYFRMVGRNVLHA
                LGFDAFGLPAEQYAVQTGTHPRTRTEANVVNFRRQLGRLGFGHDSRRSFSTTDVDFYR
                WTQWIFLQIYNAWFDTTANKARPISELVAEFESGARCLDGGRDWAKLTAGERADVIDE
                YRLVYRADSLVNWCPGLGTVLANEEVTADGRSDRGNFPVFRKRLRQWMMRITAYADRL
                LDDLDVLDWPEQVKTMQRNWIGRSTGAVALFSARAASDDGFEVDIEVFTTRPDTLFGA
                TYLVLAPEHDLVDELVAASWPAGVNPLWTYGGGTPGEAIAAYRRAIAAKSDLERQESR
                EKTGVFLGSYAINPANGEPVPIFIADYVLAGYGTGAIMAVPGHDQRDWDFARAFGLPI
                VEVIAGGNISESAYTGDGILVNSDYLNGMSVPAAKRAIVDRLESAGRGRARIEFKLRD
```

WLFARQRYWGEPFPIVYDSDGRPHALDEAALPVELPDVPDYSPVLFDPDDADSEPSPP
LAKATEWVHVDLDLGDGLKPYSRDTNVMPQWAGSSWYELRYTDPHNSERFCAKENEAY
WMGPRPAEHGPDDPGGVDLYVGGAEHAVLHLLYSRFWHKVLYDLGHVSSREPYRRLVN
QGYIQAYAYTDARGSYVPAEQVIERGDRFVYPGPDGEVEVFQEFGKIGKSLKNSVSPD
EICDAYGADTLRVYEMSMGPLEASRPWATKDVVGAYRFLQRVWRLVVDEHTGETRVAD
GVELDIDTLRALHRTIVGVSEDFAALRNNTATAKLIEYTNHLTKKHRDAVPRAAVEPL
VQMLAPLAPHIAEELWLRLGNTTSLAHGPFPKADAAYLVDETVEYPVQVNGKVRGRVV
VAADTDEETLKAAVLTDEKVQAFLAGATPRKVIVVAGRLVNLVI"

CDS complement(46564..47190)
/codon_start=1
/note="gene_46"
/transl_table=11
/translation="MSVVRSIGKKMQRISGPNALAVKGRPTQVYGHTHVRLDCRFMAD
SEFTAPEVTQLAEGLHRALSKLISMLRRGDPNGAAAGDLTLAQLSILVTLLDQGPIRM
TDLAAHERVRTPTTTVAIRRLEKIGLVKRSRDPSDLRAVLVDITPQGRAVHGESLANR
RAALAALLSQLPRSDLETLRKALAPLERLASGEPASGPASNSPARKRA"

CDS complement(47349..48083)
/codon_start=1
/note="gene_47"
/transl_table=11
/translation="MPKKYGVKEKDQVVAHILNLLLTGKLRSGDRVDRNEIAHGLGVS
RVPIQEALVQLEHDGIVSTRYHRGAFIERFDVATILEHHELDGLLNGIASARAAANPT
PRILGQLDAVMRSLRNSKESRAFAECVWEYRRTVNDEYAGPRLHATIRASQNLIPRVF
WMTYQNSRDDVLPFYEEENAAIHRREPEAARAACIGRSELMAQTMLAELFRRRVLVPP
EGACPGPFGAPIPGFARSYQPSSPVP"

CDS complement(48216..48893)
/codon_start=1
/note="gene_48"
/transl_table=11
/translation="VDIAFTWDHFFPLYGDPDGPHFECWTVLGAWAEQTSHIEIGALV
TCNSYRNPELLADMARTVDHISGGRLILGIGSGWKQKDYDEYGYRFGTAGSRLDDLAA
ALPRIKARLGKLNPPPTRDIPVLIGGGGERKTLRLVAEYADIWHSFTAGDSYLAKSAV
LSTHCSTVGRNPATIERSAAVDGGGLIASAEALAGLGVTLLTVGCDGPDYDLSAAAAL
CRWRDGR"

CDS 48831..49004
/codon_start=1
/note="gene_49"
/transl_table=11
/translation="VWAVRVAVERKEVVPGKSDVHTDVLAPADGVSDGAVMGGVLRLQ
LYADTNGEIGTHE"

CDS complement(49026..49922)
/codon_start=1
/note="gene_50"
/transl_table=11
/translation="VLSDDELTGLDEFALLAENAEQAGVNGPLPEVERVQAGAISALR
WGGSAPRVIFLHGGGQNAHTWDTVIVGLGEPALAVDLPGHGHSAWREDGNYSPQLNSE
TLAPVLRELAPGAEFVVGMSLGGLTAIRLAAMAPDLVGELVLVDVTPSALQRHAELTA
EQRGTVALMHGEREFPSFQAMLDLTIAAAPHRDVKSLRRGVFHNSRRLDNGNWVWRYD
AIRTFGDFAGLWDDVDALSAPITLVRGGSSGFVTDQDTAELHRRATHFRGVHIVEKSG
HSVQSDQPRALIEIVRGVLDTR"

CDS complement(50004..51107)

```
                     /codon_start=1
                     /note="gene_51"
                     /transl_table=11
                     /translation="MSEHQSLPAPEASTEVRVAIVGVGNCASSLVQGVEYYYNADDTS
                     TVPGLMHVRFGPYHVRDVKFVAAFDVDAKKVGFDLSDAIFASENNTIKIADVAPTNVI
                     VQRGPTLDGIGKYYADTTELSDAEPVDVVQALKEAKVDVLVSYLPVGSEEADKFYAQC
                     AIDAGVAFVNALPVFIASDPVWAKKFTDAGVPIVGDDIKSQVGATITHRVLAKLFEDR
                     GVQLDRTMQLNVGGNMDFLNMLERERLESKKISKTQAVTSNLKREFKTKDVHIGPSDH
                     VGWLDDRKWAYVRLEGRAFGDVPLNLEYKLEVWDSPNSAGVIIDAVRAAKIAKDRGIG
                     GPVIPASAYLMKSPPEQLPDDIARAQLEEFIIG"
     CDS             complement(51168..51710)
                     /codon_start=1
                     /note="gene_52"
                     /transl_table=11
                     /translation="MLELAILGLLIESPMHGYELRKRLTGLLGAFRAFSYGSLYPALR
                     RMQADGLIAENAAPAGTPVRRARRVYQLTDKGRRRFGELVADTGPHNYTDDGFGVHLA
                     FFNRTPAEARMRILEGRRRQVEERREGLREAVARASSSFDRYTRQLHQLGLESSEREV
                     KWLNELIAAERAAPNPAEQT"
     CDS             complement(51811..52680)
                     /codon_start=1
                     /note="gene_53"
                     /transl_table=11
                     /translation="VAKWLGAPLARGVSTATRAKDSDRQDACRILDDALRDGELSMEE
                     HRERVSAATKAVTLGDLQRLVADLQVESAPAQMPALKSRAKRTELGLLAAAFVASVLL
                     GVGIGWGVYGNTRSPLDFTSDPGAKPDGIAPVVLTPPRQLHSLGGLTGLLEQTRKRFG
                     DTMGYRLVIYPEYASLDRVDPADDRRVLAYTYRGGWGDATSSAKSIADVSVVDLSKFD
                     AKTAVSIMRGAPETLGLKQSDVKSMYLIVEPAKDPTTPAALSLSLYVSSDYGGGYLVF
                     AGDGTIKHVSYPS"
     CDS             52793..53227
                     /codon_start=1
                     /note="gene_54"
                     /transl_table=11
                     /translation="VRLQRQVVDYTLRRRSLLAEVYSGRTGVSEVCDANPYLLRAAKF
                     HGKPSRVICPICRKEQLTLVSWVFGEHLGAVSGSARTAEELILLATRFSEFAVHVVEV
                     CRTCSWNHLVKSYVLGAARPARPPRGSGGTRTARNGARTASE"
     CDS             53220..55691
                     /codon_start=1
                     /note="gene_55"
                     /transl_table=11
                     /translation="VNSDGRHHQSSSGAPRGPANPGQRGQVPPDDRLTAILPPVTDDR
                     SAPHADSIEAVKAALDGAPPMPPPRDPLEEVTAALAAPPGKPPRGDQLGGRRRPPGPP
                     GPPGSSGQPAGRLPQPRVDLPRVGQINWKWIRRSLYLTAAVVILLLMVTFTMAYLIVD
                     VPKPGDIRTNQVSTILASDGSEIAKIVPPEGNRVDVNLSQVPMHVRQAVIAAEDRNFY
                     SNPGFSFTGFARAVKNNLFGGDLQGGSTITQQYVKNALVGSAQHGWSGLMRKAKELVI
                     ATKMSGEWSKDDVLQAYLNIIYFGRGAYGISAASKAYFDKPVEQLTVAEGALLAALIR
                     RPSTLDPAVDPEGAHARWNWVLDGMVETKALSPNDRAAQVFPETVPPDLARAENQTKG
                     PNGLIERQVTRELLELFNIDEQTLNTQGLVVTTTIDPQAQRAAEKAVAKYLDGQDPDM
                     RAAVVSIDPHNGAVRAYYGGDNANGFDFAQAGLQTGSSFKVFALVAALEQGIGLGYQV
                     DSSPLTVDGIKITNVEGEGCGTCNIAEALKMSLNTSYYRLMLKLNGGPQAVADAAHQA
                     GIASSFPGVAHTLSEDGKGGPPNNGIVLGQYQTRVIDMASAYATLAASGIYHPPHFVQ
                     KVVSANGQVLFDASTADNTGDQRIPKAVADNVTAAMEPIAGYSRGHNLAGGRDSAAKT
```

GTTQFGDTTANKDAWMVGYTPSLSTAVWVGTVKGDEPLVTASGAAIYGSGLPSDIWKA
TMDGALKGTSNETFPKPTEVGGYAGVPPPPPPPPPSEVPPSETVIQPTVEIAPGITIP
IGPPTTITLAPPPPAPPAATPTPPP"
CDS 55688..57370
/codon_start=1
/note="gene_56"
/transl_table=11
/translation="VTGALSQSSNISPLPLAADLRSADNRDCPSRTDVLGAALANVVG
GPVGRHALIGRTRLMTPLRVMFAIALVFLALGWSTKAACLQSTGTGPGDQRVANWDNQ
RAYYQLCYSDTVPLYGAELLSQGKFPYKSSWIETDSNGTPQLRYDGQIAVRYMEYPVL
TGIYQYLSMAIAKTYTALSKVAPLPVVAEVVMFFNVAAFGLALAWLTTVWATSGLAGR
RIWDAALVAASPLVIFQIFTNFDALATGLATSGLLAWARRRPVLAGVLIGLGSAAKLY
PLLFLYPLLLLGIRAGRLNALARTMAAAAATWLLVNLPVMLLFPRGWSEFFRLNTRRG
DDMDSLYNVVKSFTGWRGFDPTLGFWEPPLVLNTVVTLLFVLCCAAIAYIALTAPHRP
RVAQLTFLTVASFLLVNKVWSPQFSLWLVPLAVLALPHRRILLAWMTIDALVWVPRMY
YLYGNPSRSLPEQWFTTTVLLRDIAVMVLCGLVVWQIYRPGRDLVRTGGPGALPACGG
VDDPVGGVFANAADAPPGRLPSWLRPRLGDEHARERTPDAGRDRTFSGQHRA"
CDS 57327..57965
/codon_start=1
/note="gene_57"
/transl_table=11
/translation="MQVAIALFPGNTALDAVGPYEVLQRVPSFDVVFVGHRRGEVRSD
NAMLGLLCDAAFDELTRPDVVIFPGGIGTRTLIHDQTVLDWVREAHRHTLLTTSVCTG
GLVLAAAGLLNGLTATTHWRVQDLFNSLGARYVPQRVVEHLPERVITAAGVSSGIDMG
LRLVELLVSREAAEASQLMIEYDPQPPVDAGSLAKASPATHRLALEFYQHRL"
CDS 58184..58474
/codon_start=1
/gene="rpsF"
/note="gene_58"
/transl_table=11
/translation="MRPYEIMVILDPTLDERTVAPSLETFLNVVRKDGGKVEKVDIWG
KRRLAYEIAKHAEGIYVVIDVKAAPATVSELDRQLSLNESVLRTKVMRTDKH"
CDS 58578..59072
/codon_start=1
/note="gene_59"
/transl_table=11
/translation="VAGDTTITIVGNLTADPELRFTPSGAAVANFTVASTPRIYDRQT
GEWKDGEALFLRCNIWREAAENVAESLTRGARVIVSGRLKQRSFETREGEKRTVIEVE
VDEIGPSLRYATAKVNKASRSGGFGSGSRPAPAQTSSASGDDPWGSAPASGSFGGGDD
EPPF"
CDS 59114..59368
/codon_start=1
/gene="rpsR"
/note="gene_60"
/transl_table=11
/translation="MAKSSKRRPAPEKPVKTRKCVFCAKKDQAIDYKDTALLRTYISE
RGKIRARRVTGNCVQHQRDIALAVKNAREVALLPFTSSVR"
CDS 59401..59859
/codon_start=1
/gene="rplI"
/note="gene_61"

```
                     /transl_table=11
                     /translation="MKLILTADVDHLGSIGDTVEVKDGYGRNFLLPRGLAIVASRGAQ
                     KQADEIRRARETKSVRDLEHANEIKAAIEALGPIALPVKTSADSGKLFGSVTAADVVA
                     AIKKAGGPNLDKRIVRLPKTHIKAVGTHFVSVHLHPEIDVEVSLDVVAQS"
     CDS             60388..63012
                     /codon_start=1
                     /note="gene_62"
                     /transl_table=11
                     /translation="MAVVDDLAPGMDSSPPSEDYGRQPPQDLAAEQSVLGGMLLSKDA
                     IADVLERLRPGDFYRPAHQNVYDAILDLYGRGEPADAVTVAAELDRRGLLRRIGGAPY
                     LHTLISTVPTAANAGYYASIVAEKALLRRLVEAGTRVVQYGYAGAEGADVAEVVDRAQ
                     AEIYDVADRRLSEDFVALEDLLQPTMDEIDAIASSGGLARGVATGFTELDEVTNGLHP
                     GQMVIVAARPGVGKSTLGLDFMRSCSIRHRMASVIFSLEMSKSEIVMRLLSAEAKIKL
                     SDMRSGRMSDDDWTRLARRMSEISEAPLFIDDSPNLTMMEIRAKARRLRQKANLKLIV
                     VDYLQLMTSGKKYESRQVEVSEFSRHLKLLAKELEVPVVAISQLNRGPEQRTDKKPML
                     ADLRESGCLTASTRILRADTGAEVAFGELMRSGERPMVWSLDERLRMVARPMINVFPS
                     GRKEVFRLRLASGREVEATGSHPFMKFEGWTPLAQLKVGDRIAAPRRVPEPIDTQRMP
                     ESELISLARMIGDGSCLKNQPIRYEPVDEANLAAVTVSAAHSDGAAIRDDYLAARVPS
                     LRPARQRLPRGRCTPIAAWLAGLGLFTKRSHEKCVPEAVFRAPNDQVALFLRHLWSAG
                     GSVRWDPTNGQGRVYYGSTSRRLIDDVAQLLLRVGIFSWITHAPKLGGHDSWRLHIHG
                     AKDQVRFLRHVGVHGAEAVAAQEMLRQLKGPVRNPNLDSAPKKVWAQVRNRLSAKQMM
                     DIQLHEPTMWKHSPSRSRPHRAEARIEDRAIHELARGDAYWDTVVEITSIGDQHVFDG
                     TVSGTHNFVANGISLHNSLEQDADVVILLHRPDAFDRDDPRGGEADFILAKHRNGPTK
                     TVTVAHQLHLSRFANMAR"
     CDS             63192..63884
                     /codon_start=1
                     /note="gene_63"
                     /transl_table=11
                     /translation="MITRYKPESGFVARSGGPDRKRPHDWIVWHFTHADNLPGIITAG
                     RLLADSAVTPTTEVAYNPVKELRRHKVVAPDSRYPASMASDHVPFYIAARSPMLYVVC
                     KGHSGYSGGAGPLVHLGVALGDIIDADLTWCASDGNAAASYTKFSRQVDTLGTFVDFD
                     LLCQRQWHNTDDDPNRQSRRAAEILVYGHVPFELVSYVCCYNTETMTRVRTLLDPVGG
                     VRKYVIKPGMYY"
     CDS             63901..64959
                     /codon_start=1
                     /note="gene_64"
                     /transl_table=11
                     /translation="MITYGSGDLLRADTEALVNTVNCVGVMGKGIALQFKRRYPEMFT
                     AYEKACKRGEVTIGKMFVVDTGQLDGPKHIINFPTKKHWRAPSKLAYIDAGLIDLIRV
                     IRELNIASVAVPPLGVGNGGLDWEDVEQRLVSAFQQLPDVDAVIYPPSGGSRAIEGVE
                     GLRMTWGRAVILEAMRRYLQQRRAMEPWEDPAGISHLEIQKLMYFANEADPDLALDFT
                     PGRYGPYSERVRHLLQGMEGAFTVGLGDGTARVLANQPISLTTKGTDAITDYLATDAA
                     ADRVSAAVDTVLRVIEGFEGPYGVELLASTHWVATREGAKEPATAAAAVRKWTKRKGR
                     IYSDDRIGVALDRILMTA"
     CDS             complement(65004..65231)
                     /codon_start=1
                     /note="gene_65"
                     /transl_table=11
                     /translation="MTKGYCPGGRWGFGDLAVCDGEKYPDGSFWHQWMQTWFTGPQFY
                     FDCVSGGEPLPGPPPPGGCGGAIPSEQPNAP"
     CDS             complement(65392..65604)
```

```
                     /codon_start=1
                     /note="gene_66"
                     /transl_table=11
                     /translation="VSKAGARAESRAIAAQSDASYTIATAAVPSRVSASSWYVDAGTA
                     STEQDFRLFSGMSAHYDSLPRLIRKR"
CDS                  65763..66686
                     /codon_start=1
                     /note="gene_67"
                     /transl_table=11
                     /translation="VRVVRAPALILVDAANPLAGKPFYVDPASAAMVAARNANPPNAE
                     LTSVANTPQSYWLDQAFPPATVGGTVARYTGAAQAAGAMPVLTLYGIPHRDCGSYASG
                     GFATGTDYRGWIDAVASGLGSSPATIIVEPDALAMADCLSPDQRQERFDLVRYAVDTL
                     TRDPAAAVYVDAGHSRWLSAEAMAARLNDVGVGRARGFSLNVSNFYTTDEEIGYGEAI
                     SGLTNGSHYVIDTSRNGAGPAPDAPLNWCNPSGRALGAPPTTATAGAHADAYLWIKRP
                     GESDGTCGRGEPQAGRFVSQYAIDLAHNAGQ"
CDS                  66915..68354
                     /codon_start=1
                     /note="gene_68"
                     /transl_table=11
                     /translation="LAREISRQTFLRGAAGALAAGAVFGSVRATADPAASGWEALSSA
                     LGGKVLQPDDGPQFATAKQVFNTNYNGYTPAVIVTPTSQLDVQKAMAFAAANNLKVAP
                     RGGGHSYVGASTANGAMVLDLRQLPGDINYDATTGRVTVTPATGLYAMHQVLAAAGRG
                     IPTGTCPTVGVAGHALGGGLGANSRHAGLLCDQLTSASVVLPSGQAVTASATDHPDLF
                     WALRGGGGGNFGVTTSLTFATFPSGDLDVVNLNFPPQSFAQVLVGWQNWLRTADRGSW
                     ALADATVDPLGTHCRILATCPAGSGGSVAAAIVSAVGTQPTGTENHTFNYLDLVRYLA
                     VGNLNPSPLGYVGGSDVFTTITPATAQGIASAVDAFPRGAGRMLAIMHALDGALATVS
                     PGATAFPWRRQSALVQWYVETSGSPSEATSWLNTAHQAVRAYSVGGYVNYLEVNQPPA
                     RYFGPNLSRLSAVRQKYDPSRVMFSGLNF"
CDS                  68612..71551
                     /codon_start=1
                     /note="gene_69"
                     /transl_table=11
                     /translation="METGSPGKRPVLPKRARLLVTAGMGMLALLLFGPRLVDIYVDWL
                     WFGEVGFRSVWITVLLTRLAIVAAVALVVAGIVLAALLLAYRSRPFFVPDEPQRDPVA
                     PLRSAVMRRPRLFGWGIAVTLGVVCGLIASFDWVKVQLFVHGGTFGIVDPEFGYDIGF
                     FVFDLPFYRSVLNWLFVAVVLAFLASLLTHYLFGGLRLTTGRGMLTQAARVQLAVFAG
                     AVVLLKAVAYWLDRYELLSSGRKEPTFTGAGYTDIHAELPAKLVLVAIAVLCAVSFFT
                     AIFLRDLRIPAMAAALLVLSAILVGGLWPLLMEQFSVRPNAADVERPYIQRNIEATRE
                     AYRIGGDWVQYRSYPGIGTKQPRDVPVDVTTIAKVRLLDPHILSRTFTQQQQLKNFFS
                     FAEILDIDRYRIDGELQDYIVGVRELSPKSLTGNQTDWINKHTVYTHGNGFVAAPANR
                     VNAAARDAENISDSNSGYPIYAVSDIASLGSGRQVIPVEQPRVYYGEVIAQADPDYAI
                     VGGAPGSAPREYDTDTSKYTYTGAGGVSIGNWFNRTVFATKFAQHKFLFSREIGSESK
                     VLIHRDPKERVQRVAPWLTTDDNPYPVVVNGRIVWIVDAYTTLDTYPYAQRSSLEGPV
                     TSPTGIVRQGKQVSYVRNSVKATVDAYDGTVTLFQFDRDDPVLRTWMRAFPGTVKSED
                     QIPDELRAHFRYPEDLFEVQRSLLAKYHVDEPREFFTTNAFWSVPSDPTNDANATQPP
                     FYVLVGDQQSAQPSFRLASAMVGYNREFLSAYISAHSDPANYGKLTVLELPTDTLTQG
                     PQQIQNSMISDTRVASERTLLERSNRIHYGNLLSLPIADGGVLYVEPLYTERISTSPS
                     SSTFPQLSRVLVSVREPRTEGGVRVGYAPTLAESLDQVFGPGTGRVATAPGGDAASAP
                     PPGAGGPAPPQAVPPPRTTQPPAAPPRGPDVPPATVAELRETLADLRAVLDRLEKAID
                     AAETPGG"
CDS                  71618..71857
```

```
                     /codon_start=1
                     /note="gene_70"
                     /transl_table=11
                     /translation="MATIQVRDLPEDVAETYRRRATAAGQSLQTYMRTKLIEGVRGRD
                     KAEVIEILEQALASTASPGISRETIEASRRELRGG"
     CDS             71850..72251
                     /codon_start=1
                     /note="gene_71"
                     /transl_table=11
                     /translation="VDECVVDAAAVVDALAGKGASAIVLRGLLKESISNAPHLLDAEV
                     GHALRRAVLSDEISEEQARAALDALPYLIDNRYPHSPRLIEYTWQLRHNVTFYDALYV
                     ALATALDVPLLTGDSRLAAAPGLPCEIKLVR"
     CDS             complement(72303..74540)
                     /codon_start=1
                     /note="gene_72"
                     /transl_table=11
                     /translation="MSAEQPTIIYTLTDEAPLLATYAFLPIVRAFAEPAGIKIEASDI
                     SVAARILAEFPDYLTEEQRVPDNLAELGRLTQLPDTNIIKLPNISASVPQLVAAIKEL
                     QDKGYAVPDYPADPNTDQEKAIKERYARCLGSAVNPVLRQGNSDRRAPKAVKEYARKH
                     PHSMGEWSMASRTHVAHMRHGDFYAGEKSMTLDRARNVRMELLAKSGKTIVLKPEVPL
                     DDGDVIDSMFMSKKALCDFYEEQMQDAFETGVMFSLHVKATMMKVSHPIVFGHAVRIF
                     YKDAFAKHQELFDDLGVNVNNGLSDLYSKIESLPASQRDEIIEDLHRCHEHRPELAMV
                     DSARGISNFHSPSDVIVDASMPAMIRAGGKMYGADGKLKDTKAVNPESTFSRIYQEII
                     NFCKTNGQFDPTTMGTVPNVGLMAQQAEEYGSHDKTFEIPEDGVANIVDVATGEVLLT
                     ENVEAGDIWRMCIVKDAPIRDWVKLAVTRARISGMPVLFWLDPYRPHENELIKKVKTY
                     LKDHDTEGLDIQIMSQVRSMRYTCERLVRGLDTIAATGNILRDYLTDLFPILELGTSA
                     KMLSVVPLMAGGGMYETGAGGSAPKHVKQLVEENHLRWDSLGEFLALGAGFEDIGIKT
                     GNERAKLLGKTLDAAIGKLLDNDKSPSRKTGELDNRGSQFYLAMYWAQELAAQTDDQQ
                     LAEHFASLADVLTKNEDVIVRELTEVQGEPVDIGGYYAPDSDMTTAVMRPSKTFNAAL
                     EAVQG"
     CDS             complement(74658..75227)
                     /codon_start=1
                     /note="gene_73"
                     /transl_table=11
                     /translation="LAPTDRRVRADAARNRARVLEVAYQTFAADGLSVPVDEIARRAG
                     VGAGTVYRHFPTKEALFQAVIADRMHRIIDKGHALLKSKHPGDALFAFLRSMVLQWGA
                     TDRGLVEALAGVGIEISSAAPEAEADFLDLLTDLLRAAQRAGTVRPDVDVLEVKTLLV
                     GCQAMQSYNAELAAKVTDVALDGLRANRK"
     CDS             75330..76241
                     /codon_start=1
                     /note="gene_74"
                     /transl_table=11
                     /translation="MTKWTAADIPDQTGRTAVITGANTGLGFETAAALAAHGAHVVLA
                     VRNLDKGKQAAARITEATPGAEVELQELDLTSLASVRAAAAQLKSDHQRIDLLINNAG
                     VMYTPRQTTADGFEMQFGTNHLGHFALTGLLIDRLLPVAGSRVVTISSVGHRIRAAIH
                     FDDLQWERRYRRVAAYGQAKLANLLFTYELQRRLAPGGTTIAVASHPGVSNTELVRNM
                     PRPLVAVAAILAPLMQDAELGALPTLRAATDPAVRGGQYFGPDGFGEIRGYPKVVASS
                     AQSHDEQLQRRLWAVSEELTGVVYPVG"
     CDS             complement(76266..77651)
                     /codon_start=1
                     /note="gene_75"
```

```
                     /transl_table=11
                     /translation="MTISVFDLFTIGIGPSSSHTVGPMRAANQFVVALRRRGHLDDLE
                     AMRVDLFGSLAATGAGHGTMSAILLGLEGCQPETITTEHKERRLAEIAASGVTRIGGV
                     IPVPLTERDIDLHPDIVLPTHPNGMTFTAAGPHGRVLATETYFSVGGGFIVTEQTSGN
                     SGQHPCSVALPYVSAQELLDICDRLDVSISEAALRNETCCRTENEVRAALLHLRDVMV
                     ECEQRSIAREGLLPGGLRVRRRAKVWYDRLNAEDPTRKPEFAEDWVNLVALAVNEENA
                     SGGRVVTAPTNGAAGIVPAVLHYAIHYTSAGAGDPDDVTVRFLLTAGAIGSLFKERAS
                     ISGAEVGCQGEVGSAAAMAAAGLAEILGGTPRQVENAAEIAMEHSLGLTCDPIAGLVQ
                     IPCIERNAISAGKAINAARMALRGDGIHRVTLDQVIDTMRATGADMHTKYKETSAGGL
                     AINVAVNIVEC"
     CDS             complement(77648..78925)
                     /codon_start=1
                     /gene="glyA"
                     /note="gene_76"
                     /transl_table=11
                     /translation="MNTLNDSLTAFDPDIAALIDGELRRQESGLEMIASENYAPLAVM
                     QAQGSVLTNKYAEGYPGRRYYGGCEFVDGVEQLAIDRVKALFGAEYANVQPHSGATAN
                     AATMHALLNPGDTILGLSLAHGGHLTHGMRINFSGKLYHATAYEVSKEDYLVDMDAVA
                     EAARTHRPKMIIAGWSAYPRQLDFARFRAIADEVDAVLMVDMAHFAGLVAAGVHPSPV
                     PHAHVVTSTTHKTLGGPRGGIILCNDPAIAKKINSAVFPGQQGGPLGHVIAAKATAFK
                     MAAQPEFAQRQQRCLDGARILAGRLTQPDVAERGIAVLTGGTDVHLVLVDLRDAELDG
                     QQAEDRLAAVDITVNRNAVPFDPRPPMITSGLRIGTPALAARGFSHNDFRAVADLIAA
                     ALTATNDDQLGPLRAQVQRLAARYPLYPELHRT"
     CDS             79530..80267
                     /codon_start=1
                     /note="gene_77"
                     /transl_table=11
                     /translation="VSVDPVDPVDPVDPVDPVDPVDPVDPVDPVDPVDAVVAAGSDGL
                     TVARIESEIGALEFLNELRTELKSGQFRPQPVRERKIPKPGGLGKVRRLGIPTVADRV
                     VQAALKLVLEPIFETDFEPVSYGFRPARRAHDTIAEIHLFGTQEYRWVLDADIKACFD
                     RIDHADLMDRVRHRIKDKRVLRLVNWQRIRHRWNWTDVRRWLTDPTGRWHPISADGIT
                     LFNPAAVPIRRYRYRGNTIPTPWTQAV"
     CDS             80698..81747
                     /codon_start=1
                     /note="gene_78"
                     /transl_table=11
                     /translation="MLFAALRDMQWRKRRLVITIISTGLIFGMTLVLTGLANGFRVEA
                     RHTVDSMGVDVFVVRSGAAGPFLGSIPFPDVDLARVAAEPGVMAAAPLGSVGTIMKEG
                     TSTRNVTVFGAPEHGPGMPRVSEGRSPSKPDEVAASSTMGRHLGDTVEVGARRLRVVG
                     IVPNSTALAKIPNVFLTTEGLQKLAYNGQPNITSIGIIGMPRQLPEGYQTFDRVGAVN
                     DLVRPLKVAVNSISIVAVLLWIVAVLIVGSVVYLSALERLRDFAVFKAIGTPTRSIMA
                     GLALQALVIALLAAVVGVVLAQVLAPLFPMIVAVPVGAYLALPVAAIVIGLFASVAGL
                     KRVVTVDPAQAFGGP"
     CDS             81750..82742
                     /codon_start=1
                     /note="gene_79"
                     /transl_table=11
                     /translation="VGDLSIQNLVVEYYSGGYALRPINGLNLDVAAGSLVMLLGPSGC
                     GKTTLLSCLGGILRPKSGAIKFDEVDITTLQGAELANYRRNKVGIVFQAFNLVPSLTA
                     VENVMVPLRSAGMSRRASRRRAEELLARVNLAERMNHRPGDLSGGQQQRVAVARAIAL
                     DPPLILADEPTAHLDFIQVEEVLRLIRELADGERVVVVATHDSRMLPMADRVVELTPD
```

FAETNRPPETVHLQAGEVLFEQSTMGDLIYVVSEGEFEIVHELADGGEELVKVAGPGD
YFGEIGVLFHLPRSATVRARSDATAVGYTVQAFRERLGVGGLRDLIEHRALAND"
CDS 82822..84057
/codon_start=1
/note="gene_80"
/transl_table=11
/translation="MGDLSISQVSARPGRIGIRARQMFDGYRFQRGPVLVVVEDGRIS
AVDFAGSACPDMNLVDLGESTLLPGLVDAHAHLCWDPDGRPEDLAGDPHAVLVGRARR
HAAAALRSGITTIRDLGDRDYAALALREEYRQKTTVGPELVVSGPPLTRSGGHCWFLG
GVADSVEELVDAVQERAARGADWIKVMATGGFVTTASDPWQPQYGSGQLAAVVAAAEQ
VGLPVTAHAHATAGIAAAVAAGVDGIEHCTFLSEGSAAASPDVVEAIVAQGVWCGMTI
PRVYPEMPENLVAVVQDGWRNIRRLIDAGARVALSTDAGVAPGRRHDVLPDDLVYLSR
HGFTSTEVLTGATAAAAASCGLGHRKGRIAPGYDADLLAVAAGVDHDPAGLCDVKAVW
RSGTQVPLQASAVGYNTPS"
CDS 84070..85242
/codon_start=1
/note="gene_81"
/transl_table=11
/translation="MQDSIFNLLTEEQLRGRNTLKWNYFGPDVVPLWLAEMDFPTAPA
VLDGVRACVDNEEFGYPPLGEDSLPRATADWCRQRYGWCPRPDWVRVVPDVLKGMEVV
VEFLTRPESPVALPVPAYMPFFDVLHVTGRQRVEVPMVQQDSGRYLLDLDALQAAFVR
GAGSVIICNPNNPLGTAFTEAELRAIVDIAARHGARVIADEIWAPVVYGSRHVAAASV
SEAAAEVVVTLVSASKGWNLPGLMCAQVILSNRRDAHDWDRINMLHRMGASTVGIRAN
IAAYHHGESWLDELLPYLRANRDHLARALPELAPGVEVNAPDGTYLSWVGFRALALPS
EPAEYLLSKAKVALSPGIPFGAAVGSGFARLNFATTRAILDRAIEAIAAALRDIID"
CDS complement(85257..85646)
/codon_start=1
/note="gene_82"
/transl_table=11
/translation="MPAVTTPSNHWGDERRKLSHQPPVRGQILGRRQARRLSQHFARV
GVEAPPKRLQEMLLGAPAADEEWTDVKFALIVTQLNHEKRVAKFHRLQRRATHSLICL
GLVLVALNFLICLAYIFFSLTQHAAAL"
CDS complement(85710..86468)
/codon_start=1
/note="gene_83"
/transl_table=11
/translation="VFVHGYMMGGQLWRRVSERLAGRGLRCIAPTWPLGAHPKPLRPG
ADQTIGGVAGIVADVLAALELKDVVLVGNDTGGVVTQLVAVHYPERLGALVLTSCDAF
EHFPPPILKPVILAAKSATLFRAAIQVMRAPAARNRAYAGLSHHNIDHLTRAWVRPAL
SNPAIAEDLRQLSLSLRTEVTTAVAARLPEFDKPALIAWSADDVFFALENGQRLAATI
PRARFEVIEGARTFSMVDSPDRLADQLSTVAVRT"
CDS 86602..87207
/codon_start=1
/note="gene_84"
/transl_table=11
/translation="MEIKRRTQEERSAATREALITGARKLWGLRGYAEVGTPEIATEA
GVTRGAMYHQFADKAALFRDVVEVVEQDVMARMATLVAASGAATPADAIRAAVDAWLE
VSGDPEVRQLVLLDAPVVLGWAGFRDVAQRYSLGMTEQLITEAIRAGQLARQPVRPLA
QVLIGALDEAAMFIATADDPKRARRETRQVLRRLIDGMLNG"
CDS complement(87282..87875)
/codon_start=1

```
                     /note="gene_85"
                     /transl_table=11
                     /translation="MNAVESTLRRVAKDLTGLRQRWALVGGFAVSARSEPRFTRDVDI
                     VVAVANDDAAESLVRQLLTQQYHLLASVEQDAARRLAAVRLGATADTAANVVVDLLFA
                     SCGIEPEIAEAAEEIEILPDLVAPVATTAHLIAMKLLARDDDRRPQDRSDLRALVDAA
                     SPQDIQDARKAIELITLRGFHRDRDLAAEWTRLAAKW"
     CDS             complement(87872..88078)
                     /codon_start=1
                     /note="gene_86"
                     /transl_table=11
                     /translation="VAVSVAAQKLRLALDMYEVGEQMQRMRLGRERPNADVVEIEAAI
                     DAWRMTRPGAEEGDSAGPTSTRFT"
     CDS             88278..89099
                     /codon_start=1
                     /note="gene_87"
                     /transl_table=11
                     /translation="VEPKRSRLVVCAPEPSHAREFPDVAVFSGGRANASQAERLARAV
                     GRVLADRGVTGGARVRLTMANCADGPTLVQINLQVGDTPLRAQAATAGIDDLRPALIR
                     LDRQIVRASAQWCPRPWPDRPRRRLTTPAEALVTRRKPVVLRRATPLQAIAAMDAMDY
                     DVHLFTDAETGEDAVVYRAGPSGLRLARQHHVFPPGWSRCRAPAGPPVPLIVNSRPTP
                     VLTEAAAVDRAREHGLPFLFFTDQATGRGQLLYSRYDGNLGLITPTGDGVADGLA"
     CDS             89096..89554
                     /codon_start=1
                     /note="gene_88"
                     /transl_table=11
                     /translation="MSPGSRRASPQSAREVVELDRDEAMRLLASVDHGRVVFTRAALP
                     AIRPVNHLVVDGRVIVRTRLTAKVSVAVRSSADAGVVVAYEADDLDPRRRTGWSVVVT
                     GLATEVSDPEQVARYQRLLHPWVNMAMDTVVAIEPEIVTGIRIVADSRTP"
     CDS             89649..89993
                     /codon_start=1
                     /note="gene_89"
                     /transl_table=11
                     /translation="VESEPLYKLKAEFFKTLAHPARIRILELLVERDRSVGELLSSDV
                     GLESSNLSQQLGVLRRAGVVAARRDGNAMIYSIAAPDIAELLAVARKVLARVLSDRVA
                     VLEDLRAGGSAT"
     CDS             89998..90477
                     /codon_start=1
                     /note="gene_90"
                     /transl_table=11
                     /translation="MGWVAKIFRVGRVVEPAAPLPAAIAEPPAGVRGSLQIRHVDAGS
                     CNGCEVEISGAFGPVYDAERFGARLVASPRHADALLVTGVVTHNMAGPLRKTLEATPR
                     PRVVIACGDCALNRGVFADAYGVVGAVGEVVPVDVEIAGCPPTPAAIMAALRSVTGK"
     CDS             90474..92402
                     /codon_start=1
                     /note="gene_91"
                     /transl_table=11
                     /translation="MTAAPTAGGVVTSGVGVAGVGVGLLGMFGPVRVVHVGWLLPLSG
                     VHIELDRLGGFFMALTGAVAAPVGCYLIGYVRREHLGRVPMAVVPLFVAAMLLVPAAG
                     SVTTFLLAWELMAIASLILVLSEHARPQVRSAGLWYAVMTQLGFIAILVGLVVLAAAG
                     GSDRFAGLGAVCDGVRAAVFMLTLVGFGSKAGLVPLHAWLPRAHPEAPSPVSALMSAA
                     MVNLGIYGIVRFDLQLLGPGPRWWGLALLAVGGTSALYGVLQASVAADLKRLLAYSTT
```

ENMGLITLALGAATLFADTGAYGPASIAAAAAMLHMIAHAAFKSLAFMAAGSVLAATG
LRDLDLLGGLARRMPATTVFFGVAALGACGLPLGAGFVSEWLLVQSLIHAAPGHDPIV
ALTTPLAVGVVALATGLSVAAMTKAFGIGFLARPRSTQAEAAREAPASMRAGMAIAAG
ACLVLAVAPLLVAPMVRRAAATLPAAQAVKFTGLGAVVRLPAMSGSIAPGVIAAAVLA
AALAVAVLARWRFRRRPAPARLPLWACGAADLTVRMQYTATSFAEPLQRVFGDVLRPD
TDIEVTHTAESRYMAERITYRTAVADAIEQRLYTPVVGAVAAMAELLRRAHTGSVHRY
LAYGALGVLIVLVVARCT"

CDS 92402..93352
/codon_start=1
/note="gene_92"
/transl_table=11
/translation="MSYLAGAAQIGGVMVGAPLVIGMTRQVRARWEGRAGAGLLQPWR
DLLKQLGKQQITPAGTTIVFAAAPVIVAGTTLLIAAIAPLVATGSPLDPSADLFAVVG
LLFLGTVALTLAGIDTGTSFGGMGASREITIAALVEPTILLAVFALSIPAGSANLGAL
VASTIDHPGHVVSLAGVLAFVALVIVIVAETGRLPVDNPATHLELTMVHEAMVLEYAG
PRLALVEWAAGMRLTVLLALLANLFLPWGIAGAAPTALDVLTGVVAVAAKVAILAVLL
ATFEVFLAKLRLFRVPELLAGSFLLALLAVTAANFFTVGA"

CDS 93363..94025
/codon_start=1
/note="gene_93"
/transl_table=11
/translation="MSNANFSILVDFAAGGLVLASVLIVWRRDLRAIVRLLAWQGAAL
AAIPLLRGIRDNDRALIAVGIAVLALRALVLPWLLARAVGAEAAAQREATPLVNTASS
LLITAGLTLTAFAITQPVVNLEPGVTINAVPAAFAVVLIALFVMTTRLHAVSQAAGFL
MLDNGIAATAFLLTAGVPLIVELGASLDVLFAVIVIGVLTGRLRRIFGDADLDKLREL
RD"

CDS 94025..95491
/codon_start=1
/note="gene_94"
/transl_table=11
/translation="MTGLLLAAILAPLAASIASLITGWRRTTATLTALSATTVLACAV
AMGFWMGSGAQFGLGGLLRADALTVVMLVVIGIVGTLATAASIGYIDTELAHGHIDGR
SARLYGVLTPAFLCATVLAVCANNIGVIWVAIEATTVITAFLVGHRRTRTALEATWKY
VVICSVGIAVAFLGTVLLYFAARDSGAAAAGALNLDILAEHAAGLDPGVARLAGGLLL
IGYGAKAGLFPFHTWLADAHSQAPAPVSALMSGVLLAVAFSVLIRLRPILDAVSGPAY
LRNGLLVVGLATLLVAVLMLTVTGDVKRMLAYSSMEHMGLIAIAAAAGTTLAIAALLL
HVLAHGIGKTVLFLAGGQLQAAHDSTAIADITGVMRRSRLIGVSFAVGLIVLLGLPPF
AMFASELAIARSLANERLAWVLGAALLLIAIGFTALARNSGRMLLGTPAAGAPAITVP
ATAAAALMVGIVVSAALGITAGPLADLLGIAASNVGLP"

CDS 95491..96966
/codon_start=1
/note="gene_95"
/transl_table=11
/translation="MSASWLRHRVSERGLIATAEQLWADSFRLALVAAHDDGDSLRVV
YLFLAGYPDRRVELEYVVPADNPEIRSLAYLSFPAGRFEREMADLYGIRPVGHPKPRR
LVRHAHWPDWHPMRTDAGPAPEFTDTGAFPFLAVEGPGVYEIPVGPVHAGLIEPGHFR
FSVAGETIVRLKARLWFVHRGIEKLFHGRPATAAVDLAERISGDTSAAHALAHSLAIE
DALGIELPHEIHRLRALIVELERLYNHAADLGALANDVGYSLANAHAQRIRENLLRRN
AAVTGHRLLRGAIRAGGVALRALPDTDELAALAVDLAEVATLTLANSVVYDRFAGTAV
LHPDDASALGCLGYVARASGLRSDARVEHPTIVLPITEIGAPDGDVLARYTVRRDEFA
ASAALAQHIVESHTGPIEYAATLHPVGAPSSGIGIVEGWRGTIVHRVEIDVDGRITRA

```
                     KVVDPSWFNWPALPVAMADTIVPDFPLANKSFNQSYAGNDL"
CDS                  97058..97675
                     /codon_start=1
                     /note="gene_96"
                     /transl_table=11
                     /translation="VVKGRSGSLSWRRVRTGDLGLAVWGGREEYRAVKPGTPGIQPKG
                     DMMTVTVVDAGPGRVSRSVEVAAPAAELFAIVADPRRHRELDGSGTVRGNIKVPAKLV
                     VGSKFSTKMKLFGLPYRITSRVTALKPNELVEWSHPLGHRWRWEFESLSPTLTRVTET
                     FDYHAAGAIKNGLKFYEMTGFAKSNAAGIEATLAKLSDQYARGRA"
CDS                  97832..98425
                     /codon_start=1
                     /note="gene_97"
                     /transl_table=11
                     /translation="VDQPWNANIHYDALLDAMVPLGTQCVLDVGCGDGLLAARLARRI
                     PYVTAVDIDAPVLRRAQTRFANAPIRWLHADIMTAELPNAGFDAVVSNAALHHIEDTR
                     TALSRLGGLVTPGGTLAVVTFVTPSLRNGLWHLTSWVACGMANRVKGKWEHSAPIKWP
                     PPQTLHELRSHVRALLPGACIRRLLYGRVLVTWRAPV"
CDS                  98554..99324
                     /codon_start=1
                     /note="gene_98"
                     /transl_table=11
                     /translation="VAKNQNRIRNRWELITCGLGGHVTYAPDDAALAARLRASTGLGE
                     VWRCLRCGDFALGGPQGRGAPEDAPLIMRGKALRQAIIIRALGVERLVRALVLALAAW
                     AVWEFRGARGAIQATLDRDLPVLRAAGFKVDQMTVIHALEKALAAKPSTLALITGMLA
                     AYAVLQAVEGVGLWLLKRWGEYFAVVATSIFLPLEVHDLAKGITTTRVVTFSINVAAV
                     VYLLISKRLFGVRGGRKAYDVERRGEQLLDLERAAMLT"
CDS                  99758..100525
                     /codon_start=1
                     /note="gene_99"
                     /transl_table=11
                     /translation="MAVTVGVICAIPQELAYLRGVLVDAKRQQVAQILFDSGQLDAHR
                     VVLAAAGMGKVNTGLTATLLADRFGCRTIVFTGVAGGLDPELCIGDIVIADRVVQHDF
                     GLLTDERLRPYQPGHIPFIEPTERLGYPVDPAVIDRVKHRLDGFTLAPLSTAAGGGGR
                     QPRIYYGTILTGDQYLHCERTRNRLHHELGGMAVEMEGGAVAQICASFDIPWLVIRAL
                     SDLAGADSGVDFNRFVGEVAASSARVLLRLLPVLTAC"
CDS                  100657..102942
                     /codon_start=1
                     /gene="ctpA"
                     /note="gene_100"
                     /transl_table=11
                     /translation="MTAAVTGEHHASVQRIQLRISGMSCSACAHRVESTLNKLPGVRA
                     AVNFGTRVATIDTSEAVDAAALCQAVRRAGYQADLCTDDGRSASDPDADHARQLLIRL
                     AIAAVLFVPVADLSVMFGVVPATRFTGWQWVLSALALPVVTWAAWPFHRVAMRNARHH
                     AASMETLISVGITAATIWSLYTVFGNHSPIERSGIWQALLGSDAIYFEVAAGVTVFVL
                     VGRYFEARAKSQAGSALRALAALSAKEVAVLLPDGSEMVIPADELKEQQRFVVRPGQI
                     VAADGLAVDGSAAVDMSAMTGEAKPTRVRPGGQVIGGTTVLDGRLIVEAAAVGADTQF
                     AGMVRLVEQAQAQKADAQRLADRISSVFVPAVLVIAALTAAGWLIAGGQPDRAVSAAL
                     AVLVIACPCALGLATPTAMMVASGRGAQLGIFLKGYKSLEATRAVDTVVFDKTGTLTT
                     GRLQVSAVTAAPGWEADQVLALAATVEAASEHSVALAIAAATTRRDAVTDFRAIPGRG
                     VSGTVSGRAVRVGKPSWIGSSSCHPNMRAARRHAESLGETAVFVEVDGEPCGVIAVAD
                     AVKDSARDAVAALADRGLRTMLLTGDNPESAAAVATRVGIDEVIADILPEGKVDVIEQ
```

```
                     LRDRGHVVAMVGDGINDGPALARADLGMAIGRGTDVAIGAADIILVRDHLDVVPLALD
                     LARATMRTVKLNMVWAFGYNIAAIPVAAAGLLNPLVAGAAMAFSSFFVVSNSLRLRKF
                     GRYPLGCGTVGGPQMTAPSSA"
     CDS             complement(102889..103335)
                     /codon_start=1
                     /note="gene_101"
                     /transl_table=11
                     /translation="VGIAQIALGTVQGFGLDVGLTHQHPTGAGTHLLNESTSWSIALG
                     VIMVGAALWPSAAAGLAGVLTAFVAILTGYVIVDALSGAVSTTRILTHLPVVIGAVLA
                     IMVWRSASGPRPRPDAVAAEPDIVLPDNASRGRRRGHLWPTDGSAA"
     CDS             complement(103784..104743)
                     /codon_start=1
                     /note="gene_102"
                     /transl_table=11
                     /translation="VDVSTRQAAEADLAGKAAQYRPDELARYAQRVMDWLHPDGDLTD
                     TERARKRGITLSNQQYDGMSRLSGYLTPQARATFEAVLAKLAAPGATNPDDHTPVIDT
                     TPDAAAIDRDTRSQAQRNHDGLLAGLRALIASGELGQHNGLPVSIVVTTTLTDLQTGA
                     GKGFTGGGTLLPMADVIRMTSHAHHYSPASGRYPQAIFDHGTPLALYHTKRLASPAQR
                     IMLFANDRGCTKPGCDAPAYHSQAHHVTGWTSTGRTDITDLTLACDPDNRLAEKGWTT
                     RKNTHGHTEWLPPPHLDHGQPWTNTFHHPERFLHNQDDDDKPD"
     CDS             105399..106790
                     /codon_start=1
                     /note="gene_103"
                     /transl_table=11
                     /translation="MAIPPEVHSGLLSAGCGPGSLLVAAQQWQELSDQYALACAELGQ
                     LLGEVQASSWQGTAATQYVAAHGPYLAWLEQTAINSAVTAAQHVAAAAAYCSALAAMP
                     TPAELAANHAIHGVLIATNFFGINTVPIALNEADYVRMWLQAADTMAAYQAVADAATV
                     AVPSTQPAPPIRAPGGDAADTWLDVLSSIGQLIRDILDFIANPYKYFLEFFEQFGFSP
                     AVTVVLALVALQLYDFLWYPYYASYGLLLLPFFTPTLSALTALSALIHLLNLPPAGLL
                     PIAAALGPGDQWGANLAVAVTPATAAVPGGSPPTSNPAPAAPSSNSVGSASAAPGISY
                     AVPGLAPPGVSSGPKAGTKSPDTAADTLATAGAARPGLARAHRRKRSESGVGIRGYRD
                     EFLDATATVDAATDVPAPANAAGSQGAGTLGFAGTAPTTSGAAAGMVQLSSHSTSTTV
                     PLLPTTWTTDAEQ"
     CDS             106809..107678
                     /codon_start=1
                     /note="gene_104"
                     /transl_table=11
                     /translation="MTLKVKGEGLGAQVTGVDPKNLDDITTDEIRDIVYTNKLVVLKD
                     VHPSPREFIKLGRIIGQIVPYYEPMYHHEDHPEIFVSSTEEGQGVPKTGAFWHIDYMF
                     MPEPFAFSMVLPLAVPGHDRGTYFIDLARVWQSLPAAKRDPARGTVSTHDPRRHIKIR
                     PSDVYRPIGEVWDEINRTTPPIKWPTVIRHPKTGQEILYICATGTTKIEDKDGNPVDP
                     EVLQELMAATGQLDPEYQSPFIHTQHYQVGDIILWDNRVLMHRAKHGSAAGTLTTYRL
                     TMLDGLKTPGYAA"
     CDS             107675..108226
                     /codon_start=1
                     /note="gene_105"
                     /transl_table=11
                     /translation="MSHTDLTPCTRVLASSGTVPIAEELLARVLEPYSCKGCRYLIDA
                     QYSATEDSVLAYGNFTIGESAYIRSTGHFNAVELILCFNQLAYSAFAPAVLNEEIRVL
                     RGWSIDDYCQHQLSSMLIRKASSRFRKPLNPQKFSARLLCRDLQVIERTWRYLKVPCV
                     IEFWDENGGAASGEIELAALNIP"
```

```
CDS             108231..109853
                /codon_start=1
                /note="gene_106"
                /transl_table=11
                /translation="MGGKKFQAMPQLPSTVLDRVFEQARQQPEAIALRRCDGTSALRY
                RELVAEVGGLAADLRAQSVSRGSRVLVISDNGPETYLSVLACAKLGAIAVMADGNLPI
                AAIERFCQITDPAAALVAPGSKMASSAVPEALHSIPVIAVDIAAVTRESEHSLDAASL
                AGNADQGSEDPLAMIFTSGTTGEPKAVLLANRTFFAVPDILQKEGLNWVTWVVGETTY
                SPLPATHIGGLWWILTCLMHGGLCVTGGENTTSLLEILTTNAVATTCLVPTLLSKLVS
                ELKSANATVPSLRLVGYGGSRAIAADVRFIEATGVRTAQVYGLSETGCTALCLPTDDG
                SIVKIEAGAVGRPYPGVDVYLAATDGIGPTAPGAGPSASFGTLWIKSPANMLGYWNNP
                ERTAEVLIDGWVNTGDLLERREDGFFYIKGRSSEMIICGGVNIAPDEVDRIAEGVSGV
                REAACYEIPDEEFGALVGLAVVASAELDESAARALKHTIAARFRRESEPMARPSTIVI
                VTDIPRTQSGKVMRASLAAAATADKARVVVRG"
CDS             109846..110094
                /codon_start=1
                /note="gene_107"
                /transl_table=11
                /translation="VAEPVRDRILAAVCDVLYIDEADLIDGDETDLRDLGLDSVRFVL
                LMKQLGVNRQSELPSRLAANPSIAGWLRELEAVCTEFG"
CDS             110076..117614
                /codon_start=1
                /note="gene_108"
                /transl_table=11
                /translation="VHRVRLSRSQRNLYNGVRQDNNPALYLIGKSYRFRRLELARFLA
                ALHATVLDNPVQLCVLENSGADYPDLVPRLRFGDIVRVGSADEHLQSTWCSGILGKPL
                VRHTVHTDPNGYVTGLDVHTHHILLDGGATGTIEADLARYLTTDPAGETPSVGAGLAK
                LREAHRRETAKVEESRGRLSAVVQRELADEAYHGGHGHSVSDAPGTAAKGVLHESATI
                CGNAFDAILTLSEAQRVPLNVLVAAAAVAVDASLRQNTETLLVHTVDNRFGDSDLNVA
                TCLVNSVAQTVRFPPFASVSDVVRTLDRGYVKAVRRRWLREEHYRRMYLAINRTSHVE
                ALTLNFIREPCAPGLRPFLSEVPIATDIGPVEGMTVASVLDEEQRTLNLAIWNRADLP
                ACKTHPKVAERIAAALESMAAMWDRPIAMIVNDWFGIGPDGTRCQGDWPARQPSTPAW
                FLDSARGVHQFLGRRRFVYPWVAWLVQRGAAPGDVLVFTDDDTDKTIDLLIACHLAGC
                GYSVCDTADEISVRTNAITEHGDGILVTVVDVAATQLAVVGHDELRKVVDERVTQVTH
                DALLATKTAYIMPTSGTTGQPKLVRISHGSLAVFCDAISRAYGWGAHDTVLQCAPLTS
                DISVEEIFGGAACGARLVRSAAMKTGDLAALVDDLVARETTIVDLPTAVWQLLCADGD
                AIDAIGRSRLRQIVIGGEAIRCSAVDKWLESAASQGISLLSSYGPTEATVVATFLPIV
                CDQTTMDGALLRLGRPILPNTVFLAFGEVVIVGDLVADGYLGIDGDGFGTVTAADGSR
                RRAFATGDRVTVDAEGFPVFSGRKDAVVKISGKRVDIAEVTRRIAEDPAVSDVAVELH
                SGSLGVWFKSQRTREGEQDAAAATRIRLVLVSLGVSSFFVVGVPNIPRKPNGKIDSDN
                LPRLPQWSAAGLNTAETGQRAAGLSQIWSRQLGRAIGPDSSLLGEGIGSLDLIRILPE
                TRRYLGWRLSLLDLIGADTAANLADYAPTPDAPTGEDRFRPLVAAQRPAAIPLSFAQR
                RLWFLDQLQRPAPVYNMAVALRLRGYLDTEALGAAVADVVGRHESLRTVFPAVDGVPR
                QLVIEARRADLGCDIVDATAWPADRLQRAIEEAARHSFDLATEIPLRTWLFRIADDEH
                VLVAVAHHIAADGWSVAPLTADLSAAYASRCAGRAPDWAPLPVQYVDYTLWQREILGD
                LDDSDSPIAAQLAYWENALAGMPERLRLPTARPYPPVADQRGASLVVDWPASVQQQVR
                RIARQHNATSFMVVAAGLAVLLSKLSGSPDVAVGFPIAGRSDPALDNLVGFFVNTLVL
                RVNLAGDPSFAELLGQVRARSLAAYENQDVPFEVLVDRLKPTRAMTHHPLIQVMLAWQ
                DNPVGQLNLGDLQATPMPIDTRTARMDLVFSLAERFSEGSEPAGIGGAVEYRTDVFEA
                QAIDVLIERLRKVLVAVAAAPERTVSSIDALDGTERARLDEWGNRAVLTAPAPTPVSI
                PQMLAAQVARIPEAEAVCCGDASMTYRELDEASNRLAHRLAGCGAGPGECVALLFERC
```

APAVVAMVAVLKTGAAYLPIDPANPPPRVAFMLGDAVPVAAVTTAGLRSRLAGHDLPI
IDVVDALAAYPGTPPPMPAAVNLAYILYTSGTTGEPKGVGITHRNVTRLFASLPARLS
AAQVWSQCHSYGFDASAWEIWGALLGGGRLVIVPESVAASPNDFHGLLVAEHVSVLTQ
TPAAVAMLPTQGLESVALVVAGEACPAALVDRWAPGRVMLNAYGPTETTICAAISAPL
RPGSGMPPIGVPVSGAALFVLDSWLRPVPAGVAGELYIAGAGVGVGYWRRAGLTASRF
VACPFGGSGARMYRTGDLVCWRADGQLEFLGRTDDQVKIRGYRIELGEVATALAELAG
VGQAVVIAREDRPGDKRLVGYATEIAPGAVDPAGLRAQLAQRLPGYLVPAAVVVIDAL
PLTVNGKLDHRALPAPEYGDTNGYRAPAGPVEKTVAGIFARVLGLERVGVDDSFFELG
GDSLAAMRVIAAINTTLNADLPVRALLHASSTRGLSQLLGRDARPTSDPRLVSVHGDN
PTEVHASDLTLDRFIDADTLATAVNLPGPSPELRTVLLTGATGFLGRYLVLELLRRLD
VDGRLICLVRAESDEDARRRLEKTFDSGDPELLRHFKELAADRLEVVAGDKSEPDLGL
DQPMWRRLAETVDLIVDSAAMVNAFPYHELFGPNVAGTAELIRIALTTKLKPFTYVST
ADVGAAIEPSAFTEDADIRVISPTRTVDGGWAGGYGTSKWAGEVLLREANDLCALPVA
VFRCGMILADTSYAGQLNMSDWVTRMVLSLMATGIAPRSFYEPDSEGNRQRAHFDGLP
VTFVAEAIAVLGARVAGSSLAGFATYHVMNPHDDGIGLDEYVDWLIEAGYPIRRIDDF
AEWLQRFEASLGALPDRQRRHSVLPMLLASNSQRLQPLKPTRGCSAPTDRFRAAVRAA
KVGSDKDNPDIPHVSAPTIINYVTNLQLLGLL"

CDS 117789..119774
/codon_start=1
/note="gene_109"
/transl_table=11
/translation="MGTHGATKSATSAVPTPRSNSMAMVRLAIGLLGVCAVVAAFGLV
SGARRYAEAGNPYPGAFVSVAEPVGFFAASLAGALCLGALIHVVMTAKPEPDGLIDAA
AFRIHLLAERVSGLWLGLAATMVVIQAAHDTGVGPARLLASGALSDSVAASEMARGWI
VAAICALVVATALRLYTRWLGHVVLLVPTVLAVVATAVTGNPGQGPDHDYATSAAIVF
AVAFATLTGLKIAAALAGTTPSRAVLVTQVTCGALALAYGAMLLYLFIPGWAVDSDFA
RLGLLAGVILTSVWLFDCWRLLVRPPHAGRRRGGGSGAALAMMAAMASIAAMAVMTAP
RFLTHAFTAWDVFLGYELPQPPTIARVLTVWRFDSLIGAAGVVLAIGYAAGFAALRRR
GNSWPVGRLIAWLTGCAALVFTSGSGVRAYGSAMFSVHMAEHMTLNMFIPVLLVLGGP
VTLALRVLPVTGDGRPPGAREWLTWLLHSRVTTFLSHPITAFVLFVASPYIVYFTPLF
DTFVRYHWGHEFMAIHFLVVGYLFYWAIIGIDPGPRRLPYPGRIGLLFAVMPFHAFFG
IALMTMSSTVGATFYRSVNLPWLSSIIADQHLGGGIAWSLTELPVIMVIVALVTQWAR
QDRRVASREDRHADSDYADDELEAYNAMLRELSRMRR"

CDS complement(119990..122248)
/codon_start=1
/note="gene_110"
/transl_table=11
/translation="VAAPVVGDADLQSVRRIRLDVSGMSCAACASRVETKLNKIPGVR
ASVNFATRVATIDAVGMAADELCGVVEKAGYHAAPHTETTVLDKRTKDPDGAHARRLL
RRLLVAAVLFVPLADLSTLFAIVPSARVPGWGYILTALAAPVVTWAAWPFHSVALRNA
RHRTTSMETLISVGIVAATAWSLSSVFGDQPPREGSGIWRAILNSDSIYLEVAAGVTV
FVLAGRYFEARAKSKAGSALRALAELGAKNVAVLLPDGAELVIPASELKKRQRFVTRP
GETIAADGVVVDGSAAIDMSAMTGEAKPVRAYPAASVVGGTVVMDGRLVIEATAVGAD
TQFAAMVRLVEQAQTQKARAQRLADHIAGVFVPVVFVIAGLAGAAWLVSGAGADRAFS
VTLGVLVIACPCALGLATPTAMMVASGRGAQLGIFIKGYRALETIRSIDTVVFDKTGT
LTVGQLAVSTVTMAGSGTSERDREEVLGLAAAVESASEHAMAAAIVAASPDPGPVNGF
VAVAGCGVSGEVGGHHVEVGKPSWITRTTPCHDAALVSARLDGESRGETVVFVSVDGV
VRAALTIADTLKDSAAAAVAALRSRGLRTILLTGDNRAAADAVAAQVGIDSAVADMLP
EGKVDVIQRLREEGHTVAMVGDGINDGPALVGADLGLAIGRGTDVALGAADIILVRDD
LNTVPQALDLARATMRTIRMNMIWAFGYNVAAIPIAAAGLLNPLIAGAAMAFSSFFVV
SNSLRLRNFGAQ"

```
CDS             122392..123906
                /codon_start=1
                /note="gene_111"
                /transl_table=11
                /translation="VTPVTTFPLVDAILAGRDRNLDGVILIAAQHLLQTTHAMLRSLF
                RVGLDPRNVAVIGKCYSTHPGVVDAMRADGIYVDDCSDAYAPHESFDTQYTRHVEWFF
                AESWARLTAGRTARVVLLDDGGSLLAVAGAMLDASADVIGIEQTSAGYAKIVGCALGF
                PVINIARSSAKLLYESPIIAARVTQTAFERTAGIDSSAAILITGAGAIGTALADVLRP
                LHDRVDVYDTRSGCMTPIDLPNAIGGYDVIIGATGATSVPASMHELLRPGVLLMSASS
                SDREFDAVALRRRTTPNPDCHADLRVADGSVDATLLNSGFPVNFDGSPMCGDASMALT
                MALLAAAVLYASVAVADEMSSDHPHLGLIDQGDIVASFLNIDVPLQALSRLPLLSIDG
                YRRLQVRSGHTLFRQGERADHFFVIESGELEALVDGKVILRLGAGDHFGEACLLGGMR
                RIATVRACEPSVLWELDGKAFGDALHGDAAMREIAYGVARTRLMHAGASESLMV"
CDS             complement(124055..124339)
                /codon_start=1
                /gene="rpmB"
                /note="gene_112"
                /transl_table=11
                /translation="MSARCQITGRTVGFGKAVSHSHRRTRRRWPPNIQLKAYYLPSED
                RRIKVRVSAQGIKVIDRDGHRGRRRAARAGSAPAHFARQAGSSLRTAAIL"
CDS             124449..125645
                /codon_start=1
                /note="gene_113"
                /transl_table=11
                /translation="MRTPVILVAGQDHTDEVTGALLRRTGTVVVEHRFDGHVVRRMTA
                TLSRGELITTEDALEFAHGCVSCTIRDDLLVLLRRLHRRDNVGRIVVHLAPWLEPQPI
                CWAIDHVRVCVGHGYPDGPAALDVRVAAVVTCVDCVRWLPQSLGEDELPDGRTVAQVT
                VGQAEFADLLVLTHPEPVAVAVLRRLAPRARITGGVDRVELALAHLDDNSRRGRTDTP
                HTPLLAGLPPLAADGEVAIVEFSARRPFHPQRLHAAVDLLLDGVVRTRGRLWLANRPD
                QVMWLESAGGGLRVASAGKWLAAMAASEVAYVDLERRLFADLMWVYPFGDRHTAMTVL
                VCGADPTDIVNALNAALLSDDEMASPQRWQSYVDPFGDWHDDPCHEMPDAAGEFSAHR
                NSGESR"
CDS             complement(125740..130617)
                /codon_start=1
                /note="gene_114"
                /transl_table=11
                /translation="VKIPGVATVLGGVTNGVAQTVRAGARLPGSAAAAVQTLASPVLE
                LTGPVVQSVVQTTGRAIGVRGSHNESPDGMTPPVRWRSGRRVHFDLDPLLPFPRWHEH
                AAMVEEPVRRIPGVAEAHVEGSLGRLVVELEPDVDSDIAVDEVRDVVSAVAADIFLAG
                SVSSPNSAPFADPGNPLAILVPLTAAAMDLVAMGATVTGWVARLPAAPQTTRALAALI
                NHQPRMVSLMESRLGRVGTDIALAATTAAANGLTQSLGTPLLDLVQRSLQISEAAAHR
                RVWRDREPALASPRRPQAPVVPIISSAGAKSQEPRHSWAAAAAGEASHVVVGGSIDAA
                IDTAKGSRAGPVEQYVVNQAANGSLIAAASALVAGGGTEDAAGAILAGVPRAAHMGRQA
                FAAVLGRGLANTGQLVLDPGALRRLDRVRVVVIDGAALRGDNRAVLHAQGDEPGWDDD
                RVYEVADALLHGEQAPEPDPDELPATGARLRWAPAQGPSATPAQGLEHADLVVDGQCV
                GSVDVGWEVDPYAIPLLQTAHRTGARVVLRHVAGTEDLSASVGSTHPPGTPLLKLVRE
                LRADRGPVLLITAVHRDFASTDTLAALAIADVGVALDDPRGATPWTADLITGTDLAAA
                VRILSALPVARAASESAVHLAQGGTTLAGLLLVTGEQDKTTNPASFRRWLNPVNAAAA
                TALVSGMWSAAKVLRMPDPTPQPLTAWHALDPEIVYSRLAGGSRPLAVEPGIPAWRRI
                LDDLSYEPVMAPLRGPARTLAQLAVATRHELADPLTPILAVGAAASAIVGSNIDALLV
                AGVMTVNAITGGVQRLRAEAAAAELFAEQDQLVRRVVVPAVATTRRRLEAARHATRTA
```

TVSAKSLRVGDVIDLAAPEVVPADARLLVAEDLEVDESFLTGESLPVDKQVDPVAVND
PDRASMLFEGSTIVAGHARAIVVATGVGTAAHRAISAVADVETAAGVQARLRELTSKV
LPMTLAGGAAVTALALLRRASLRQAVADGVAIAVAAVPEGLPLVATLSQLAAAQRLTA
RGALVRSPRTIEALGRVDTICFDKTGTLTENRLRVVCALPSSTAAERDPLPQTTDAPS
AEVLRAAARASTQPHNGEGHAHATDEAILAAASALAGSLSSQGDSEWVVLAEVPFESS
RGYAAAIGRVGTDGIPMLMLKGAPETILPRCRLADPGVDHEHAESVVRHLAEQGLRVL
AVAQRTWDNGTTHDDETDADAVDAVAHDLELIGYVGLADTARPSSRPLIEALLDAERN
VVLITGDHPITARAIARQLGLPADARVVTGAELAVLDEEAHAKLAADMQVFARVSPEQ
KVQIVASLQRCGRVTAMVGDGANDAAAIRMADVGIGVSGRGSSAARGAADIVLTDDDL
GVLLDALVEGRSMWAGVRDAVTILVGGNVGEVLFTVIGTAFGAGRAPVGTRQLLLVNL
LTDMFPALAVAVTSQFAEPDDAEYPTDDAAERAQREHRRAVLIGPTPSLDAPLLRQIV
NRGVVTAAGATAAWAIGRWTPGTERRTATMGLTALVMTQLAQTLLTRRHSPLVIATAL
GSAGVLVGIIQTPVISHFFGCTPLGPVAWTGVFSATAGATAVSALAPKWLASTVGVVQ
PDERPDDAEDSDAGG"

CDS complement(130971..131213)
/codon_start=1
/note="gene_115"
/transl_table=11
/translation="VFRVESNGNPDLVPVETLHSGDPITDVNGGGQRYIVLESKTVGD
SCVVLELESRVNHQLQVIEKSFPAGYHVGRAHHRIL"

CDS 131459..132949
/codon_start=1
/note="gene_116"
/transl_table=11
/translation="MSLLITSPATVAAAATHLAGIGSALSTANAAAAAPTTALSVAGA
DEVSVLIAALFEAYAQEYQALSAQALAFHDQFVQALNMGAVCYAAAETANATPLQALQ
TVQQNVLTVVNAPTQALLGRPIIGNGANGLPNTGQDGGPGGLLFGNGGNGGSGGVDQA
GGNGGAAGLIGNGGSGGVGGPGIAGSAGGAGGAGGLLFGNGGPGGAGGIGTTGDGGPG
GAGGNAIGLFGSGGTGGMGGVGGMGGVGNGGNAGNGGTAGLFGHGGAGGAGGIGSADG
GLGGGGGNGRFMGNGGVGGAGGYGASGDGGNAGNGGLGGVFGDGGAGGTGGLGDVNGG
LAGIGGNAGFVGNGGAGGNGQLGSGAVSSAGGMGGNGGLVFGNGGPGGLGGPGTSAGN
GGMGGNAVGLFGQGGAGGAGGSGFGAGIPGGRGGDGGSGGLIGDGGTGGGAGAGDAAA
SAGGNGGNARLIGNGGDGGPGMFGGPGGAGGSGGTIFGFAGTPGPS"

CDS 133097..133846
/codon_start=1
/note="gene_117"
/transl_table=11
/translation="MRVGPVGHQCAECVREGARAVRQPRTPFGGRQRSATPVVTYTLI
SLNALVFVMQVTVMGLERQLALWPPAVASGQTYRLVTSAFLHYGAMHLLLNMWALYVV
GPPLEMWLGRLRFGALYAVSALGGSVLVYLIAPLNTATAGASGAVFGLFGATFMVARR
LHLDVRWVVALIVINLAFTFLAPAISWQGHVGGLVTGALVAATYVYAPRERRNLIQAT
VTITVLVAFVVLIGWRTVDLLALFGGRLNLS"

CDS 134069..136084
/codon_start=1
/note="gene_118"
/transl_table=11
/translation="VRRVGRLAVWDRPERRSGIPALDGLRAIAVALVLASHGGIPGMG
GGFIGVDAFFVLSGFLITSLLLDELGRTGRIDLSGFWIRRARRLLPALVLMVLTVSAA
RALFPDQALTGLRSDAIAAFLWTANWRFVAQNTDYFTQGAPPSPLQHTWSLGVEEQYY
VVWPLLLIGATLLLAARARRRCRRATVGGVRFAAFLIASLGTMASATAAVAFTSAATR
DRIYFGTDTRAQALLIGSAAAALLVRDWPSLNRGWCLIRTRWGRRIARLLPFVGLAGL

AVTTHVATGSVGEFRHGLLIVVAGAAVIVVASVAMEQRGAVARILAWRPLVWLGTISY
GVYLWHWPIFLALNGQRTGWSGPALFAARCAATVVLAGASWWLIEQPIRRWRPARVPL
LPLAAATVASAAAVTMLVVPVGAGPGLREIGLPPGVSAVAAVSPSPPEASQPAPGPRD
PNRPFTVSVFGDSIGWTLMHYLPPTPGFRFIDHTVIGCSLVRGTPYRYIGQTLEQRAE
CDGWPARWSAQVNRDQPDVALLIVGRWETVDRVNEGRWTHIGDPTFDAYLNAELQRAL
SIVGSTGVRVMVTTVPYSRGGEKPDGRLYPEDQPERVNKWNAMLHNAISQHSNVGMID
LNKKLCPDGVYTAKVDGIKVRSDGVHLTQEGVKWLIPWLEDSVRVAS"

CDS 136366..137322
/codon_start=1
/note="gene_119"
/transl_table=11
/translation="MKVWITGAGGMMGSHLAEMLLAAGHDVYATYCRPTIDPSDLQFN
GAEVDITDWCSVYDSIATFRPDAVFHLAAQSYPAVSWARPVETLTTNMVGTAIVFEAL
RRVRPHAKIIVAGSSAEYGFVDPSEVPINERRELRPLHPYGVSKAATDMLAYQYHKSY
GMHTVVARIFNCTGPRKVGDALSDFVRRCTWLEHHPEQSAIRVGNLKTKRTIVDVRDL
NRALMLMLDKGEAGADYNVGGSIAYEMGDVLKQVIAACKRDDIVPEVDPALLRPTDEK
IIYGDCSKLAAITGWQQEICLTQTIADMFDYWRSKSESALMV"

CDS 137363..137986
/codon_start=1
/note="gene_120"
/transl_table=11
/translation="LTVTDSQYRQKVCTARTAEEIFVETIAVKTRILNDRVLLEAARA
IGDRLIAGYRAGARVFMCGNGGSAADAQHFAAELTGHLIFDRPPLGAEALHANSSHLT
AVANDYDYDTVFARALEGSARPGDTLFAISTSGNSMSVLRAAKTARELGVTVVAMTGE
SGGQLAEFADFLINVPSRDTGRIQESHIVFIHAISEHVEHALFAPRQ"

CDS 138087..138590
/codon_start=1
/note="gene_121"
/transl_table=11
/translation="VVGDYVRNWRQFEWLPGAARALKKLRAWAPYIVVVTNQQGVGAG
LMSAVDVMVIHRHLQMQLASDGVLIDGFQVCPHHRSQRCGCRKPRPGLVLDWLRRHPD
SEPLLSIVVGDSLSDLELAHNVAAAAGACASVQIGGASSGGVADASFDSLWEFAVAVG
HARGERG"

CDS 138590..139657
/codon_start=1
/note="gene_122"
/transl_table=11
/translation="MAILRGRAPLRLGLGGGGTDVEPYSSQFGGRILSVTIDKYAYAF
AERGTGDEIAFRSPDRDRAGQASIDDLASLEEDFPLHVAVYRRVIAEFNGGTPFPLQL
ATQVDAPPGSGLGSSSALVVAMLLTTCALIGSSPGPYELARLAWEIERVDLGMAGGWQ
DHYAAAFGGFNFMESRPNGEVVVNPLRIRREVIAELEASLLLYFGGVSRLSSEVIADQ
QRNVVERDADALAATHSICAEALEMKDLLVVGDIPGFADSLLRGWQAKKRTSTRISNP
AIEHAYQVAQSSGMVAGKVSGAGGGGFLMMIVDPRRRIEVARSLERECGGSVAPCLFT
KGGAVTWHIPESTAPRKAWSC"

CDS 140034..140222
/codon_start=1
/note="gene_123"
/transl_table=11
/translation="MTTMIMTFVVPQRVTRATKGRARSLLRVSRRLTDTFRAPLAWTP
QERADRYVARMPIAVIAD"

CDS complement(140345..141100)

```
                     /codon_start=1
                     /note="gene_124"
                     /transl_table=11
                     /translation="MRRVVRYLSVVVAITLMLTAESVSIATAAVPPLQPIPGVASVSP
                     ANGAVVGVAHPVVVTFTTPVTDRRAVERSIRISTPHNTTGHFEWVASNVVRWVPHRYW
                     PPHTRVSVGVQELTEGFETGDALIGVASISAHTFTVSRNGEVLRTMPASLGKPSRPTP
                     IGSFHAMSKERTVVMDSRTIGIPLNSSDGYLLTAHYAVRVTWSGVYVHSAPWSVNSQG
                     YANVSHGCINLSPDNAAWYFDAVTVGDPIEVVG"
     CDS             141278..142222
                     /codon_start=1
                     /note="gene_125"
                     /transl_table=11
                     /translation="VLFRQLEYFVAVAQERHFARAAEKCYVSQPALSSAIAKLERELN
                     VTLINRGHSFEGLTREGERLVVWAKRILAEHAAFKAEVDAVRSGITGTLRLGTVPTAS
                     TTASLVLSAFCSAHPLAKVQVCSRLAATELYRRLREFELDAVIVHPETQDSDDVDLVP
                     LYEEQYVLLSPADMLPPGTSTLVWRDAAQLPLALLTADMRDRQVIDAAFADHAVSAIP
                     QVETDSVASLFAQVATGNWASIVPHTWLWAMPMSGPTGGEIRAVELVDPVLKAQIALA
                     TNALGPGSPVARALITCAQALALNEFFDTQLRGITRRR"
     CDS             complement(142206..143924)
                     /codon_start=1
                     /note="gene_126"
                     /transl_table=11
                     /translation="VLTDGCHLVVDALKANDVDTIYGVVGIPITDLARAAQASGIRYI
                     GFRHEASAGNAAAAAGFLTARPGVCLTTSGPGFLNGLPALANATTNCFPMIQISGSSS
                     RPMVDLQRGDYQDLDQLNAARPFVKAAYRIGQVQDIGRGVARAIRTATSGRPGGVYLD
                     IPGDVLGQAVEASAASGAIWRPVDPAPRLLPAPEAIDRALDVLAQAQRPLLVLGKGAA
                     YAQADNVIREFVEHTGIPFLPMSMAKGLLPDSHPQSAAAARSLAMARADVVLLVGARL
                     NWLLGNGESPQWSADAKFIQVDIEASEFDSNRPIVAPLTGDIGSVMSALLEAAADRSS
                     VASAAWTGELADRKARNSAKMRRRLADDHHPMRFYNALGAIRSVLQRNPDVYVVNEGA
                     NALDLARNIIDMHLPRHRLDSGTWGVMGIGMGYAIAAAVETGRPVVAIEGDSAFGFSG
                     MEFETICRYRLPVTVVILNNGGVYRGDEATIFRSAAPVWRHDPAPTVLNAHARHELIA
                     EAFGGKGYHVSTPTELESALTDALASNGPSLIDCELDPADGVESGHLAKLNTTSAATP
                     AISGDG"
     CDS             144076..145704
                     /codon_start=1
                     /note="gene_127"
                     /transl_table=11
                     /translation="VLIVPNPHTEHMEGAFAMASDFGPRIADLVEVAATRLPEAPALV
                     VTADRIAISHRDLARLVDELAGQLTRSGLLPGDRVALRMGSNAEFVVALLAASRADLV
                     VVPLDPALPITEQRVRSQAAGARVVLIDADGPHDRAEPTTRWWPLTVNVGGDSGPSGG
                     TLSVHLDAATEPNPATSTPEGLRPDDAMIMFTGGTTGLPKMVPWTHANIASSVRAIIT
                     GYRLSPRDATVAVMPLYHGHGLIASLLATLASGGAVSLPARGRFSAHTFWDDIKAVGA
                     TWYTAVPTIHQILLERSATEPSGRKPAALRFIRSCSAPLTAQAALALQTEFAAPVVCA
                     FGMTEATHQVTTTQIEGIDQTETPVVSTGLVGRSTGAQIRIVGSDGLPLPAGAVGEIW
                     LRGTTVVRGYLGDPTITAANFTDGWLRTGDLGSLSAAGDLSIRGRIKELINRGGEKIS
                     PERVEGVLASHPNVMEAAVFGVPHQLYGEAVAAVIVPRESAPPTREELVQFCRERLAA
                     FEIPASFQEASGLPHTAKGSLDRRAVAERFGHSV"
     CDS             complement(145705..147849)
                     /codon_start=1
                     /note="gene_128"
                     /transl_table=11
```

/translation="MADRVNASQGAAAAPTANGPGGVRNVVLVGPSGGGKTTLIEALL
VAAKVLSRPGSVTEGTTVCDFDEAEIRQQRSVGLAVASLAYDGIKVNLVDTPGYADFV
GELWAGLRAADCALFVIAANEGVDEPTKSLWQECSQVGMPRAVVITKLDHARANYREA
LTAAQDAFGDKVLPLYLPSGDGLIGLLSQALYEYADGKRTTRTPAESDTERIEEARGA
LIEGIIEESEDESLMERYLGGETIDESVLIQDLEKAVARGSFFPVIPVCSSTGVGTLE
LLEVATRGFPSPMEHPLPEVFTPQGVPHAELACDNDAPLLAEVVKTTSDPYVGRVSLV
RVFSGTIRPDTTVHVSGHFSSFFGGGTSNTHPDHDEDERIGVLSFPLGKQQRPAAAVV
AGDICAIGKLSRAETGDTLSDKAEPLVLKPWTMPEPLLPIAIAAHAKTDEDKLSVGLG
RLAAEDPTLRIEQNQETHQVVLWCMGEAHAGVVLDTLANRYGVSVDTIELRVPLRETF
AGNAKGHGRHIKQSGGHGQYGVCDIEVEPLPEGSGFEFLDKVVGGAVPRQFIPSVEKG
VRAQMDKGVHAGYPVVDIRVTLLDGKAHSVDSSDFAFQMAGALALREAAAATKVILLE
PIDEISVLVPDDFVGAVLGDLSSRRGRVLGTETAGHDRTVIKAEVPQVELTRYAIDLR
SLAHGAASFTRSFARYEPMPESAAARVKAGAG"

CDS complement(147985..148419)
/codon_start=1
/note="gene_129"
/transl_table=11
/translation="VGEFDPKLRFAQSPVARLATSTPDGTPHLVPVVFALGARRPAEA
TGADVIYTAVDAKRKTTQRLRRLANLEHNPRASVLVDSYADDWTQLWWVRADGVAAIH
RDGEVMRAAYRLLRAKYAQYQSVPLNGPVIAIAVQRWASWHA"

CDS 148754..148936
/codon_start=1
/note="gene_130"
/transl_table=11
/translation="VSKHRIVAAMRHCGVPVIQEDGSLYYQGRDTSGRLTEVVAVEAD
DGDLIITHAMPKEWKR"

CDS 148933..149301
/codon_start=1
/note="gene_131"
/transl_table=11
/translation="MTKKPRNPADYVIGDDVEVSDVDLKQEEVYVDGERLTDERVEQM
ASESLRLAREREANLIPGGKSLSGGSAHSPAVQVVVSKATHAKLKELARSRKMSVSKL
LRPVLDEFVQRETGRILPRR"

CDS 149610..151226
/codon_start=1
/note="gene_132"
/transl_table=11
/translation="MSFVSVAPEIVVAAATDLAGIGSAISAANAAAAAPTTAVLAAGA
DEVSAAIAALFSGHAQAYQALSAQAAAFHQQFVQTLAGGAGAYAAAEAQVEQQLLAAI
NAPTQALLGRPLIGNGADGAPGTGQAGGAGGILYGNGGNGGSGAAGQAGGAGGPAGLI
GHGGSGGAGGHGGWLWGNGGVGGSGGAGVGAGVAGGHGGAGGAAGLWGAGGGGGNGGN
GADANIVSGGDGGLGGAGGGGGWLYGDGGAGGHGGQGAIGLGGGAGGDGGQGGAGRGL
WGTGGAGGHGGQGGGTGGPPLPGQAGMGAAGGAGGLIGNGGAGGDGGVGASGGVAGVG
GAGGNAMLIGHGGAGGAGGDSSFANGAAGGAGGAGGHLFGNGGSGGHGGAVTAGNTGI
GGAGGVGGDARLIGHGGAGGAGGDRAGALVGRDGGPGGNGGAGGQLYGNGGDGGPGGQ
GGQAFGANNIGGTGGAGGNGGPAILSGNGGNGGAGGAGGAGGAGGGAGGVGGAGGAPG
TGGTLQAAVSGLVTALFGAPGQPGDTGQPG"

CDS 151378..152445
/codon_start=1
/note="gene_133"
/transl_table=11

/translation="MSNSRRRSLRWSWLLSVLAAVGLGLATAPAQAAPPALSQDRFAD
FPALPLDPSAMVAQVGPQVVNINTKLGYNNAVGAGTGIVIDPNGVVLTNNHVIAGATD
INAFSVGSGQTYGVDVVGYDRTQDVAVLQLRGAGGLPSAAIGGGVAVGEPVVAMGNSG
GQGGTPRAVPGRVVALGQTVQASDSLTGAEETLNGLIQFDAAIQPGDSGGPVVNGLGQ
VVGMNTAASDNFQLSQGGQGFAIPIGQAMAIAGQIRSGGGSPTVHIGPTAFLGLGVVD
NNGNGARVQRVVGSAPAASLGISTGDVITAVDGAPINSATAMADALNGHHPGDVISVT
WQTKSGGTRTGNVTLAEGPPA"

CDS 152554..154359
/codon_start=1
/note="gene_134"
/transl_table=11
/translation="MNEAEHSVEHPPVQGSHVEGGVVEHPDAKDFGSAAALPADPTWF
KHAVFYEVLVRAFFDASADGSGDLRGLIDRLDYLQWLGIDCIWLPPFYDSPLRDGGYD
IRDFYKVLPEFGTVDDFVALVDAAHRRGIRIITDLVMNHTSESHPWFQESRRDPDGPY
GDYYVWSDTSERYTDARIIFVDTEESNWSFDPVRRQFYWHRFFSHQPDLNYDNPAVQE
AMIDVIRFWLGLGIDGFRLDAVPYLFEREGTNCENLPETHAFLKRVRKVVDDEFPGRV
LLAEANQWPGDVVEYFGDPNTGGDECHMAFHFPLMPRIFMAVRRESRFPISEIIAQTP
PIPDMAQWGIFLRNHDELTLEMVTDEERDYMYAEYAKDPRMKANVGIRRRLAPLLDND
RNQIELFTALLLSLPGSPVLYYGDEIGMGDVIWLGDRDGVRIPMQWTPDRNAGFSTAN
PGRLYLPPSQDPVYGYQAVNVEAQRDTSTSLLNFTRTMLAVRRRHPAFAVGAFQELGG
SNPSVLAYVRQVAGDDGDTVLCVNNLSRFPQPIELDLQQWTNYTPVELTGHVEFPRIG
QVPYLLTLPGHGFYWFQLTTHEVGAPPTCGGERRL"

CDS 154462..155829
/codon_start=1
/note="gene_135"
/transl_table=11
/translation="MTRSDTLATKLPWSDWLPRQRWYAGRNRELATVKPGVVVALRHN
LDLVLVDVTYTDGATERYQVLVGWDFEPASEYGTKAAIGVADDRTGFDALYDVAGPQF
LLSLIVSSAVCGTSTGEVTFTREPDVELPFAAQPRVCDAEQSNTSVIFDRRAILKVFR
RVSSGINPDIELNRVLTRAGNPHVARLLGAYQFGRPNRSPTDALAYALGMVTEYEANA
AEGWAMATASVRDLFAEGDLYAHEVGGDFAGESYRLGEAVASVHATLADSLGTAQATF
PVDRMLARLSSTVAVVPELREYAPTIEQQFQKLAAEAITVQRVHGDLHLGQVLRTPES
WLLIDFEGEPGQPLDERRAPDSPLRDVAGVLRSFEYAAYGPLVDQATDKQLAARAREW
VERNRAAFCDGYAVASGIDPRDSALLLGAYELDKAVYETGYETRHRPGWLPIPLRSIA
RLTAS"

CDS 155897..156676
/codon_start=1
/note="gene_136"
/transl_table=11
/translation="VQREIYDGEARLSWVLAALAGILGATAFTHSAGYFVTFMTGNSQ
RAVLGLFGDDAWMSVTASLLILFFVAGVVIASVCRRHFWAAHPHGPTVLTTFSLIFAA
GVDIMLGGWHESMLDFVPILFVVFGIGALNTSFVKDGEVSVPLSYVTGTLVKMGQGIE
RHLAGGKVEDWLGYFLLHASFVLGAAAGGAISMVVTGPQMLAVAAVVCAATTGYTYLH
ADRRGLVNQKRPQPGKRLFRALRRGELDSGTSTPATNYGSS"

CDS complement(156808..157830)
/codon_start=1
/note="gene_137"
/transl_table=11
/translation="MTFFEQVRRLRSAATTLPRRLAIAAMGAVLVYGLVGTFGGPATA
GAFSRPGLPVEYLQVPSASMGRDIKVQFQGGGPHAVYLLDGLRAQDDYNGWDINTPAF
EEYYQSGLSVIMPVGGQSSFYTDWYQPSQSNGQNYTYKWETFLTREMPAWLQANKGVS

PTGNAAVGLSMSGGSALILAAYYPQQFPYAASLSGFLNPSEGWWPTLIGLAMNDSGGY
NANSMWGPSSDPAWKRNDPMVQIPRLVANNTRIWVYCGNGTPSDLGGDNIPAKFLEGL
TLRTNQTFRDTYAADGGRNGVFNFPPNGTHSWPYWNEQLVAMKADIQHVLNGATPPAA
PAAPAA"
CDS 158077..158532
/codon_start=1
/note="gene_138"
/transl_table=11
/translation="MRTFESVADLAAAAGEKVGQSDWVTITQEEVNLFADATGDHQWI
HVDPERAAAGPFGTTIAHGFMTLALLPRLQHQMYTVKGVKLAINYGLNKVRFPAPVPV
GSRVRATSSLVGVEDLGNGTVQATVSTTVEVEGSAKPACVAESIVRYVA"
CDS complement(158545..159888)
/codon_start=1
/note="gene_139"
/transl_table=11
/translation="MPVRRRAGERLPTVWDFETDPQYQSKLDWVEKFMAEELEPLDLV
ALDPYDKKNADTMAILRPLQRQVKDQGLWAAHLRPELGGQGFGQVKLALLNEIIGRSR
WAPSAFGCQAPDSGNAEILALFGTDEQKARYLRPLLDGEITSCYSMTEPQGGSDPGLF
VTAATRDAAGNGDWIINGEKWFSTNAKHASFFIVMAVTKPEARTYEKMSLFIVPADTP
GIEIVRNVGVGAESTRHASHGYIRYHDVRVPADHVLGGEGQAFMIAQTRLGGGRIHHA
MRTIALARRAFDMMCERALSRQTRHGRLADLQMTQEKIADSWIQIEQFRLLVLRTAWL
IDKHHDYQKVRRDIAAVKVAMPQVLHDVVQRAMHLHGALGVSDEMPFVKMMLAAESLG
IADGATELHKMTVARRTLREYQPVTTLFPSQHIPTRRAHAEAWLAQRLEHAIAEF"
CDS complement(159930..161012)
/codon_start=1
/note="gene_140"
/transl_table=11
/translation="MTGISRRTFGLAAGFGAIGAGGLGGGCSTRSGPTPTPEPASRGV
GVVLSHEQFRTDRLVAHAQAAEQAGFRYVWASDHLQPWQDNEGHSMFPWLTLALVGNS
TSSILFGTGVTCPIYRYHPATVAQAFASLAILNPGRVFLGLGTGERLNEQAATDTFGN
YRERHDRLIEAIVLIRQLWSGERISFTGHYFRTDELKLYDTPAMPPPIFVAASGPQSA
TLAGRYGDGWIAQARDINDAKLLAAFAAGAQAAGRDPTTLGKRAELFAVVGDDKAAAR
AADLWRFTAGAVDQPNPVEIQRAAESNPIEKVLANWAVGTDPGVHIGAVQAVLDAGAV
PFLHFPQDDPITAIDFYRTNVLPELR"
CDS 161099..161581
/codon_start=1
/note="gene_141"
/transl_table=11
/translation="MTPQARPARRADVRELSRTMARAFYDDPFMSWLLSNDNARTARL
TRLFATIVRHQHLAGGGVEVARGAAGIGGAALWDPPDRWRESRRQQLAMTPGFLRVFG
FRTAKARAALDVMMRVHPEEPHWYLAAIGSDPTVRGQGFGQVLMRSRLDRCDAEHCPA
"
CDS 162001..162393
/codon_start=1
/note="gene_142"
/transl_table=11
/translation="MIALPALEGVEHRHVDVAEGVRIHVADAGPADGPAVMLVHGFPQ
NWWEWRDLIGPLAADGNRVLCPDLRGAGWSSAPRSRYTKTEMADDLAAVLDGLGVAKV
KLVAHDWGGPVAFIMMLRHPEKVTGFSA"
CDS 162496..162903
/codon_start=1

/note="gene_143"
/transl_table=11
/translation="VISDPKGRYFRLLTGWVGGGFRVPGDDVRLYLDCMREPGHAEAG
SRWYRTFQTREMLRWLRGEYNDARVDVPVRWLHGTGDPVITPDLLDGYAERASDFEVE
LVDGVGHWIVEQRPELVLDRVRAFLAAGTEQRD"
CDS complement(162874..163479)
/codon_start=1
/note="gene_144"
/transl_table=11
/translation="VTAVAAGALVVETDSFRLRLLDGLVASIGERGYRATTVSDIVRH
ARTSKRTFYDRFTSKEQCFLELLLADNETLGNSIRAAVDPNADWHDQIRQAVEAYVTH
IESRPAVTLSWIREFPSLGAAAYPVQRRGMEQLTSLLIELSASPGFRRANLPPLNVPL
AVILLGGLRELTALTVEDGQPIRNIVEPAVDASIALLGPRS"
CDS 163596..164921
/codon_start=1
/note="gene_145"
/transl_table=11
/translation="MSEVVTAAPAPPVVRLPPAVRGPKLFQGLAFVVSRRRLLGRFVR
RYGKAFTANILMYGRVVVVADPQLARQVFTSSPEELGNIQPNLSRMFGSGSVFALDGD
DHRRRRRLLAPPFHGKSMKNYETIIEEETLRETANWPQGQAFATLPSMMHITLNAILR
AIFGAGGSELDELRRLIPPWVTLGSRLAALPKPKRDYGRLSPWGRLAEWRRQYDTVID
KLIEAERADPNFADRTDVLALMLRSTYDDGSIMSRKDIGDELLTLLAAGHETTAATLG
WAFERLSRHPDVLAALVEEVDNGGHELRQAAILEVQRARTVIDFAARRVNPPVYQLGE
WVIPRGYSIIINIAQIHGDPDVFPQPDRFDPQRYIGSKPSPFAWIPFGGGTRRCVGAA
FANMEMDVVLRTVLRHFTLETTTAAGERSHGRGVAFTPKDGGRVVMRRR"
CDS complement(164942..165490)
/codon_start=1
/note="gene_146"
/transl_table=11
/translation="MTSNQKAILAGGCFWGLQDLIRNQPGVVSTRVGYSGGNIPNATY
RNHGTHAEAVEIIFDPTVTDYRTLLEFFFQIHDPTTKDRQGNDRGTSYRSAIFYFDEQ
QKRIALDTIADVEASGLWPGKVVTEVSPAGDFWEAEPEHQDYLQRYPNGYTCHFVRPG
WRLPRRTAESALRASLSPELGT"
CDS 165553..166056
/codon_start=1
/note="gene_147"
/transl_table=11
/translation="VSASEFSRAELAAAFEKFEKTVARAAATRDWDCWVQHYTPDVEY
IEHAAGIMRGRQRVRAWIQETMTTFPGSHMVAFPSLWSVIDESTGRIICELDNPMLDP
GDGSVISATNISIITYAGNGQWCRQEDIYNPLRFLRAAMKWCRKAQELGTLDEDAARW
MRRHGGP"
CDS 166057..167079
/codon_start=1
/note="gene_148"
/transl_table=11
/translation="MNAPKLVIGANGFLGSHVTRQLVADCAPQKGEVRAMVRPAANTR
SIDDLPLTRFHGDVFDTATVAEAMAGCDDVYYCVVDTRAWLRDPSPLFRTNVAGLRNV
LDVATDASLRRFVFTSSYATVGRRRGHVATEEDRVDTRKVTPYVRSRVAAEDLVLQYA
HDAGLPAVAMCVSTTYGGGDWGRTPHGAFIAGAVFGRLPFTMRGIRLEAVGVDDAARA
LILAAERGRNGERYLISERMMPLQEVVRIAADEAGVPPPRWSISVPVLYALGALGSLR
ARLTGKDTELSLASVRMMRSEADVDHGKAVRELGWQPRPVEESIREAARFWAAMRTVG

KDPAAS"
CDS 167140..167520
/codon_start=1
/note="gene_149"
/transl_table=11
/translation="MSNRIVLEPSADHPITIEPTNRRVQVRVNGEVVADTAAALCLQE
ASYPAVQYIPLADVVQDRLIRTETSTYCPFKGEASYYSVTTDAGDIVDDVMWTYENPY
PAVAAIAGHVACYPDKAEISIFPG"
CDS complement(167501..167911)
/codon_start=1
/note="gene_150"
/transl_table=11
/translation="MTPFDDPQAELAWMFLQSLCEGGDLDEGFALLSNDFTYWSIVTR
TELDKKTFRRAVERRKQVFEVNIELIRCVNEGETVVVEGHCDGVSADRTRYDSPFVCI
FETRDGMIISLREYSDTQSLAEVYPVACATPGRC"
CDS 167956..168867
/codon_start=1
/note="gene_151"
/transl_table=11
/translation="VVVEAVVTFAGAAGFAHTLAPLRRGQQDPCFRVPGDGTIWRTSL
LPTGPVTARISRAGRDAARCVAWGSGAEEFVDMAPAMLGAADDASDFVPLHPAVAAAH
RRLPNLRLGRTGQVLEALIPAVIEQRVPGADAFRSWRLLVSKYGTQAPGPAPPGMRVP
PSAEVWRHIPSWEFHRANVDPGRARAVVGCAQRAASLERLVSLPAARAAEALTSLPGV
GVWTAAETTQRVFGDADAVSVGDYHIPKMIGWTLVGRPVDDAGMLELLEPMRPHRHRV
VRLLEASGLAREPRRGPRLPVQNIRAL"
CDS complement(168934..170412)
/codon_start=1
/note="gene_152"
/transl_table=11
/translation="MAPGDWSVFAWHAANLPTMPEAEDIGNEAAGGRLGVSIRSAGYL
RKWFLLGITIGVIAGLGAVVFYLALKYTSEFLLGYLADYQIPTPVGEGGHRGSTGFAR
PWAIPLVTTGGAVLSALIVAKLAPEATGHGTDEAIESVHGDPRAIRGRAVLVKMVASA
LTIGSGGSGGREGPTAQISAGFCSLLTRRLNLSNEDGRTAVALGIGAGIGAIFAAPLG
GAALGASIPYRDDFDYRNLLPGFIASGTAYAVLGAFLGFDPLFGYIDAEYRFEKAWPL
LWFVVIGLIAAAVGYLYARVFHASVAITRRLPGGPVLKPAIGGLLVGLLGLPIPQILS
SGYGWAQLAADRGTLLSIPLWIVIVLPIAKILATSLSIGTGGSGGLFGPGIVIGAFVG
AAIWRLGELTELPGVPHEPGIFVVVAMMACFGSVSRAPLAVMIMVAEMTGSFSVVPGA
IIAVGIAALLLSRTNVTIYETQRLNRQTAEAERGGSDRPTTA"
CDS 170811..171356
/codon_start=1
/note="gene_153"
/transl_table=11
/translation="VFSERGYDGATFQEIAVRADLTRPAINHYFANKRVLYQEVVEQT
HELVIVAGIERARREPTLMGRLAVVVDFAMEADAQYPASTAFLATTVLESQRHPELSR
TENDAVRATREFLVWAVNDAIERGELAADVDVSSLAETLLVVLCGVGFYIGFVGSYQR
MATITDSFQQLLAGTLWRPPT"
CDS 171445..172398
/codon_start=1
/note="gene_154"
/transl_table=11
/translation="VTELDDVSSLPSSRRTAGDTWAITESVGATALGVAAARAVETAA

TNPLIRDEFAKVLVSSAGTAWARLADADLAWLDGDQLGRRVHRVACDYQAVRTHFFDE
YFGAAVDAGVRQVVILAAGLDARAYRLNWPAGTVVYEIDQPSVLEYKAGILQSHGAVP
TARRHAVAVDLRDDWPAALIAAGFDGTQPTAWLAEGLLPYLPGDAADRLFDMVTALSA
PGSQVAVEAFTMNTKGNTQRWNRMRERLGLDIDVQALTYHEPDRSDAAQWLATHGWQV
HSVSNREEMARLGRAIPQDLVDETVRTTLLRGRLVTPAQPA"

CDS 172441..173373
/codon_start=1
/note="gene_155"
/transl_table=11
/translation="MRTHDDTWDIKTSVGATAVMVAAARAVETDRPDPLIRDPYARLL
VTNAGAGAIWEAMLDPTLVAKAAAIDAETAAIVAYLRSYQAVRTNFFDTYFASAVAAG
IRQVVILASGLDSRAYRLDWPAGTIVYEIDQPKVLSYKSTTLAENGVTPSAGRREVPA
DLRQDWPAALRDAGFDPTARTAWLAEGLLMYLPAEAQDRLFTQVGAVSVAGSRIAAET
APVHGEERRAEMRARFKKVADVLGIEQTIDVQELVYHDQDRASVADWLTDHGWRARSQ
RAPDEMRRVGRWVEGVPMADDPTAFAEFVTAERL"

CDS 173468..174988
/codon_start=1
/note="gene_156"
/transl_table=11
/translation="MSDRVKAVAPPDGRTMMTTESVARKTQKSETEAPREPAPVSDEK
QTDVAKTVARLRKTFASGRTRSVEWRKQQLRALQKLMDENEDAIAAALAEDLDRNPFE
AYLADIATTSAEAKYAAKRVRRWMRRRYLLLEVPQLPGRGWVEYEPYGTVLIIGAWNY
PFYLTLGPAVGAIAAGNAVVLKPSEIAAASAHLMTELVYRYLDTEAIAVVQGDGAVSQ
ELIAQGFDRVMFTGGTEIGRKVYEGAAPHLTPVTLELGGKSPVIVAADADVDVAAKRI
AWIKLLNAGQTCVAPDYVLADATVRDELVSKITAALTKFRSGAPQGMRIVNQRQFDRL
SGYLAAAKTDAAADGGGVVVGGDCDASNLRIQPTVVVDPDPDGPLMSNEIFGPILPVV
TVKSLDDAIRFVNSRPKPLSAYLFTKSRAVRERVIREVPAGGMMVNHLAFQVSTAKLP
FGGVGASGMGAYHGRWGFEEFSHRKSVLTKPTRPDLSSFIYPPYTERAIKVARRLF"

CDS 175063..175923
/codon_start=1
/note="gene_157"
/transl_table=11
/translation="MPGVQDRVIVVTGAGGGLGREYALTLAGEGASVVVNDLGGARDG
TGAGSAMADEVVAEIRDKGGRAVANYDSVATEDGAANIIKTALDEFGAVHGVVSNAGI
LRDGTFHKMSFENWDAVLKVHLYGGYHVLRAAWPHFREQSYGRVVVATSTSGLFGNFG
QTNYGAAKLGLVGLINTLALEGAKYNIHANALAPIAATRMTQDILPPEVLEKLTPEFV
APVVAYLCTEECADNASVYVVGGGKVQRVALFGNDGANFDKPPSVQDVAARWAEITDL
SGAKIAGFKL"

CDS 175930..176898
/codon_start=1
/note="gene_158"
/transl_table=11
/translation="MKACVVKELSGPSGMVYTDIDEVSGDGGKVVIDVRAAGVCFPDL
LLTKGEYQLKLTPPFVPGMETAGVVRSAPSDAGFHVGERVSAFGVLGGYAEQIAVPVA
NVVRSPVELDDAGAVSLLVNYNTMYFALARRAALRPGDTVLVLGAAGGVGTAAVQIAK
AMQAGKVIAMVHREGAIDYVASLGADVVLPLTEGWAQQVRDHTYGQGVDIVVDPIGGP
TFDDALGVLAIDGKLLLIGFAAGAVPTLKVNRLLVRNISVVGVGWGEYLNAVPGSAAL
FAWGLNQLVFLGLRPPPPQRYPLSEAQAALQSLDDGGVLGKVVLEP"

CDS complement(176895..177182)
/codon_start=1
/note="gene_159"

/transl_table=11
/translation="MLTLPDDRAPTGLPDPGIEALAHTKIASTISTVVADGYAVVLST
ADIANSLLANAIGYPIAASVALVTPAAGANSSCWPADPSQHHRIAESRACA"
CDS complement(177773..179539)
/codon_start=1
/note="gene_160"
/transl_table=11
/translation="MAPFGFTPKARHNRGVALRSTYRLDRWVMGPVDKEGWGLSYVFA
QPSVLAAAATDLAGIGSAINQATAAVAAPTTGLAAAAADEVSTALATLFGAYGQQFQA
ISAQVAAFHNEFTQRLAAAANAFVNAEATNTSALVQEATAGLFKPTSPPVLPPMFNQN
TAIIMGGTGSPIPTPSYVNAITTLFIDPVVSNPVVKALVTPEELYPITGVKSLPFQTS
VQLGLQILDGAIWEQINAGNHVTVFGYSQSAVIASLEMQHLISLGPNAPSPSQLNFIL
IGNEMNPNGGILARIPGLNVTTLGLPFYGATPDNPYPTTTYTLEYDGFADFPRYPLNV
LSDINAVFGILTVHTTYADLTPAQIASATQLPTQGTTSNTYYIIETEHLPLLAPLRAI
PVIGPPLAALVEPNLEVIVNLGYGDPRFGYSTSPANVPTPFGLFPDVPASVVADALVA
GTQQGVNDFMVELPAALNTLPQTPMPAFPPYVPTLLPPPPPPQPATLINIADTFASVV
STGYSILLPTADLGLAFVTILPAYDLTLFVNQLAAGNLRAAIELPLAATIGLAALGGM
IEFIAIVVTLADITQQLQSFSI"
CDS complement(179549..180688)
/codon_start=1
/note="gene_161"
/transl_table=11
/translation="MVELATTGNAVTVFGWSQSAIIASLEMQRFTAMGGAAPSASDLN
FVLVGNEMNPNGGMLARFPDLTLPTLDLTFYGATPSDTIYPTAIYTLEYDGFADFSRY
PLNFISDLNAVAGITFVHTKYLDLTPAQVEGATKLPTSPGYTGVTDYYIIRTENRPLL
QPLRAVPVIGDPLADLIQPNLKVIVNLGYGDPNYGYSTSYADVRTPFGLWPNVPPQVI
ADALAAGTQEGILDFTADLQALSAQPLTLPQIQLPQPADLVAAVAAAPTPAEVVNTLA
RIISTNYAVLLPTVDIALALVTTLPLYTTQLFVRQLAAGNLINAIGYPLAATVGLGTI
DSGRRGIAHPPRGGLGHRSKHRGPRHLTDSRRHRRPPTTVYRPRQ"
CDS 180627..180959
/codon_start=1
/gene="MRGA423_00980"
/note="gene_162"
/transl_table=11
/translation="MALCDQPKTVTAFPVVASSTMIASCKMVKPSSTDVLRTKLLTPV
SGYNSSGVKTACSARRTDLQRIGGVNIDGGGSGIPVGPKNKVVKFAGNGGIIAAAAGV
GAAAGTAS"
CDS complement(181035..181259)
/codon_start=1
/note="gene_163"
/transl_table=11
/translation="MSYLIAEPRIVAAAAAEVAGIGSAVSTAGAAAAGPTCALAAAAG
DEVSAAIAKPFGAYGQEYQAVLAQVEAFHS"
CDS complement(181385..182215)
/codon_start=1
/note="gene_164"
/transl_table=11
/translation="MAVRELPGAWNFRDVADTATALRPGRLFRSSELSRLDDAGRATL
RRLGITDVADLRSSREVARRGPGRVPDGIDVHLLPFPDLADDDADDSAPHETAFKRLL
TNGGSNGESGESSQSINDAATRYMTDEYRQFPTRNGAQRALHRVVTLLAAGRPVLTHC
FAGKDRTGFVVALVLEAVGLDRDVIVADYLRSNDSVPQLRARISEMIQQRFDTELAPE

VVTFTKARLSDGVLGVRAEYLAAARQTIDETYGSLGGYLRDAGISQATVNRMRGVLLG
"
CDS complement(182217..183440)
/codon_start=1
/gene="fadE2"
/note="gene_165"
/transl_table=11
/translation="MDFAMSAKAIDYRTRLSDFMTEHVFGAEADYDDYRRAAGPADHT
APPIIEELKTKAKDRGLWNLFLSAESGLTNLEYAPLAEMTGWSMEIAPEALNCAAPDT
GNMEILHMFGTEQQRAQWLRPLLDGKIRSAFSMTEPAVASSDARNIETTISRDGADYV
INGRKWWTSGAADPRCKILIVMGRTNPDAAAHQQQSMVLVPIDTPGVTIVRSTPVFGW
QDRHGHCEIDYHNVRVPATNLLGEEGSGFAIAQARLGPGRIHHCMRALGAAERALALM
VNRVRNRVAFGRPLAEQGVVQQAIAQSRNEIDQARLLCEKAAWTIDQHGNKEARHLVA
MIKAVAPRVACDVIDRAIQVHGAAGVSDDTPLARLYGWHRAMRIFDGPDEVHLRSIAR
AELSREKSTFAAAVT"
CDS 183852..184952
/codon_start=1
/note="gene_166"
/transl_table=11
/translation="MTDPQTQSTRVGVVAESGPDERRVALVPKAVASLVNRGVAVVVE
AGAGERALLPDELYTAVGASIGDAWAADVVVKVAPPTAAEVGRLRGGQTLIGFLAPRN
ADNSIGALTQAGVQAFALEAIPRISRAQVMDALSSQANVSGYKAVLLAASESTRFFPM
LTTAAGTVKPATVLVLGVGVAGLQALATAKRLGARTTGYDVRPEVADQVRSVGAQWLD
LGISASGEGGYARELTDDERAQQQKALEEAISGFDVVITTALVPGRPAPTLVTAAAVE
AMKPGSVVVDLAGETGGNCELTEPGRTVVKHDVTIAAPLNLPATMPEHASELYSKNIT
ALLDLLIKDGRLAPDFDDEVIAQSCVTRGKDS"
CDS 184953..185285
/codon_start=1
/note="gene_167"
/transl_table=11
/translation="MCNELLENLAILVLSGFVGFAVISKVPNTLHTPLMSGTNAIHGI
VVLGALVVFGEIEHPSLVLQVILFVAVVFGTLNVIGGFIVTDRMLGMFKAKKPAVPAK
PDRDEALR"
CDS 185282..186709
/codon_start=1
/note="gene_168"
/transl_table=11
/translation="MNLHYLVEILYIISFSLFIYGLMGLTGPKTAVRGNLIAAAGMTI
AVAATLVMIRHTSQWPLIIAGLVVGVVLGVPPARLTKMTAMPQLVAFFNGVGGGTVAL
IALSEFIDTTGFSAFQHGESPTVHIVVASLFAAIIGSISFWGSIVAFGKLQEIISGRP
IGLGKAQQPINLLLLAVAVAAAVVIGLHAHPGSGGVALWWMIGLLVAAGVLGLMVVLP
IGGADMPVVISMLNAMTGLSAAAAGLALNNTAMIVAGMIVGASGSILTNLMAKAMNRS
IPAIVAGGFGGGGVAPSGGGDDKHVKATSAADAAIQMAYANQVIVVPGYGLAVAQAQH
AVKDLATLLEDRGVPVKYAIHPVAGRMPGHMNVLLAEAEVDYDAMKDMDDINDEFART
DVTIVIGANDVTNPAARNETSSPIYGMPILNVDKSRSVIVLKRSMNSGFAGIDNPLFY
ADGTTMLFGDAKKSVTEVSEELKAL"
CDS 187015..187659
/codon_start=1
/note="gene_169"
/transl_table=11
/translation="MPSDTSPNGLSRREELLAVATKLFAARGYHGTRMDDVADVIGLN

KATVYHYYASKSLILFDIYRQAAEGTLAAVHDDPSWTAREALYQYTVRLLTAIASNPE
RAAVYFQEQPYITEWFTSEQVAEVREKEQQVYEHVHGLIDRGIASGEFYECDSHVVAL
GYIGMTLGSYRWLRPSGRRTAKEIAAEFSTALLRGLIRDESIRNQSPLGTRKET"

CDS complement(187663..189069)
/codon_start=1
/note="gene_170"
/transl_table=11
/translation="MSYVIAAPEMLATAAADVDGIGSAIRAASASAAGPTTGLLAAAA
DEVSSAAAALFSEYARECQEVLKQAAAFHGEFTRALAAAGAAYAQAEASNTAAMSGTA
GSSGALGSVGMLSGNPLTALMMGGTGEPILSDRVLAIIDSAYIRPIFGPNNPVAQYTP
EQWWPFIGNLSLDQSIAQGVTLLNNGINAELQNGHDVVVFGYSQSAAVATNEIRALMA
LPPGQAPDPSRLAFTLIGNINNPNGGVLERYVGLYLTFLDMSFNGATPPDSPYQTYMY
TGQYDGYAHNPQYPLNILSDLNAFMGIRWVHNAYPFTAAEVANAVPLPTSPGYTGNTH
YYMFLTQDLPLLQPIRAIPFVGTPIAELIQPDLRVLVDLGYGYGYADVPTPASLFAPI
NPIAVASALATGTVQGPQAALVSIGLLPQSALPNTYPYLPSANPGLMFNFGQSSVTEL
SVLSGALGSVARLIPPIA"

CDS complement(189161..190669)
/codon_start=1
/note="gene_171"
/transl_table=11
/translation="MSHLVTAPDMLATAAAHVDEIASTLRAANAAAAGPTCNLLAAAG
DEVSAATAALFSAYGREYQAVVKQAAAFHSEFTRTLEAAGNAYAHAEAANAARVSHAL
DTINAPIRTLLGRTPLSPNGSSGAGGLPAIAQLAAESPITALIMGGTNNPLPDPEYVT
DINKAFIQTLFPGAVSQGLFTPEQFWPVTPDLGNLTFNQSVTEGVALLNTTVNNQLAL
DNKVVAFGYSQSATIINNYINSLMAMGSPNPDDISFVMIGSGNNPVGGLLARFPGFYI
PFLDVPFNGATPANSPYPTHIYTAQYDGIAHAPQFPLRILSDINAFMGYFYVHNTYPE
LMATQVDNAVPLPTSPGYTGNTQYYMFLTQDLPLLQPIRDIPYAGPPIADLFQPQLRV
LVDLGYADYGPGGNYADIPTPAGLFSIPNPFAVTYYLIKGSLQAPYGAIVEIGVEAGL
IGPEWFPDSYPWVPSINPGLNFYFGQPQVTLLSLMSGGLGNILHLIPPPVFT"

CDS 190837..192186
/codon_start=1
/note="gene_172"
/transl_table=11
/translation="MLTSLVSAVGSHHVTTDPDVLAGRSVDHTGRYRGRASALVRPGS
AEEVAEVLRVCRDAGAYVTVQGGRTSLVAGTVPEHDDVLLSTERLCVVSDVDTVERRI
EIGAGVTLAAVQHAASTAGLVFGVDLSARDTATVGGMASTNAGGLRTVRYGNMGEQVV
GLDVALPDGTVLRRHSRVRRDNTGYDLPALFVGAEGTLGVITALDLRLHPTPSHRVTA
VCGFAELAALVDAGRMFRDVEGIAALELIDGRAAALTREHLGVRPPVEADWLLLVELA
ADHDQTDRLADLLGGARMCGEPAVGVDAAAQQRLWRTRESLAEVLGVYGPPLKFDVSL
PLSAISGFARDAVALVHRHVPDSPEALPLLFGHIGEGNLHLNVLRCPPDREPALYAKM
MGLIAECGGNVSSEHGVGSRKRAYLGMSRQANDVAAMRRVKAALDPTGYLNAAVLFD"

CDS complement(192214..193365)
/codon_start=1
/note="gene_173"
/transl_table=11
/translation="VPAVQPWLYSNMPAIRGAVLDQIGVPRPYWRSKPISVVELRLDP
PDRGEVLVRIEAAGVCHSDLSVVDGTRVRPVPILLGHEAAGIVEQVGDGVDGVAVGQR
VVLVFLPRCGQCAACATDGRTPCEPGSAANKAGTLLGGGIRLSRGGRPVYHHLGVSGF
ATHVVVNRASVVPVPHEVPPTVAALLGCAVLTGGGAVLNVGDPQPGQSVAVVGLGGVG
MAAVLTALTYTDVRVVAVDQLPEKLSAAKALGAHEIYTPQQATAGGVKAAVVVEAVGH
PAALHTAIGLTAPGGRTITVGLPPPDVRISLSPLDFVTEGRSLIGSYLGSAVPSHDIP

RFVSLWQSGRLPVESLVTSTIRLDDINEAMDHLADGIAVRQLISFTGDL"
CDS 193347..193802
/codon_start=1
/note="gene_174"
/transl_table=11
/translation="MAALPAPEKLLRSDFPVLWPVGTRWADNDMFGHLNNAVYYQLFD
TAINAWINTSTGVDPLAMPVLGIVAESGCRYFSELRFPESLMVGLAVTRLGRSSVTYR
LGVFKEPDDAGVITALGHWVHVYVDRTSRRPVPIPEAIRSLLSTACVSG"
CDS 193901..194341
/codon_start=1
/gene="TB18"
/note="gene_175"
/transl_table=11
/translation="MPVLSKTVEVTADAASIMAIVADIERYPEWNEGVKGAWVLARYD
DGRPSQVRLDTAVQGIEGTYIHAVYYPGENQIQTVMQQGELFAKQEQLFSVVATGAAS
LLTVDMDVQVTMPVPEPMVKMLLNNVLEHLAENLKQRAEQLAAS"
CDS complement(194355..195170)
/codon_start=1
/note="gene_176"
/transl_table=11
/translation="LIKHDVVWVTLWPERPNNKPPPSPRQVPGNPGPTLKVLASHVNA
PLSAKPRSQLPLRRAQLSDEVAGHLRAAIMSGALRSGTFIRLDETAAELGVSVTPVRE
ALLKLRGEGMVGLEPHRGHVVLPLTRQDIDDIFWLQATIAQELATSATAHITDVEIDE
LDRINNALAGAIGSGDAKTIASIEFAFHRVFNKASRRIKLAWFLLNAARYMPAQVFAA
DPRWGADAVNSHRQLIAALRRRDTAAVIEHTVWQFTDGARRLTEALAETEVFG"
CDS 195225..196889
/codon_start=1
/gene="fadD5"
/note="gene_177"
/transl_table=11
/translation="LTAQLASHLTRALTLAQQQPYLARRQNWVNQLERHAMMQPDAPA
LRFVGNTMTWADLRRRVAALAGALSGRGVGFGDRVMILMLNRTEFVESVLAANMIGAI
AVPLNFRLTPTEIAVLVEDCAAHVMLTEAALAPVAIGVRNIQPLLSVIVVAGGSSQDS
VFGYEDLLNEAGDVHEPVDIPNDSPALIMYTAGTTGRPKGAVLTHANLTGQAMTALYT
SGANINSDVGFVGVPLFHIAGIGNMLTGLLLGLPTVIYPLGAFDPGQLLDVLEAEKVT
GIFLVPAQWQAVCTEQQARPRDLRLRVLSWGAAPAPDALLRQMSATFPETQILAAFGQ
TEMSPVTCMLLGEDAIAKRGSVGRVIPTVAARVVDQNMNDVPVGEVGEIVYRAPTLMS
CYWNNPEATAEAFAGGWFHSGDLVRMDSDGYVWVVDRKKDMIISGGENIYCAELENVL
ASHPDIAEVAVIGRADEKWGEVPIAVAAVTNDDLRIEDLGEFLTDRLARYKHPKALEI
VDALPRNPAGKVLKTELRLRYGACVNVERRSASAGFTERRENRQKL"
CDS 197093..197890
/codon_start=1
/note="gene_178"
/transl_table=11
/translation="VTTSTTLGGYVRDQLQTPLTLVGGFFRMCVLTGKALFRWPFQWR
EFILQCWFIMRVGFLPTIMVSIPLTVLLIFTLNILLAQFGAADISGSGAAIGAVTQLG
PLTTVLVVAGAGSTAICADLGARTIREEIDAMEVLGIDPIHRLVVPRVLASMLVATLL
NGLVITVGLVGGFLFGVYLQNVSGGAYLATLTLITGLPEVVIATIKAATFGLIAGLVG
CYRGLTVRGGSKGLGTAVNETVVLCVIALFAVNVILTTIGVRFGTGR"
CDS 197892..198761
/codon_start=1

/note="gene_179"
/transl_table=11
/translation="MSTAAVLRARFPRAVANLRQYGGAAARGLDEAGQLTWFALTSIG
QIAHALRYYRKETLRLIAQIGMGTGAMAVVGGTVAIVGFVTLSGSSLVAIQGFASLGN
IGVEAFTGFFAALINVRIAGPVVTGVALAATVGAGATAELGAMRISEEIDALEVMGIK
SISFLASTRIMAGLVVIIPLYALAMIMSFLSPQITTTVLYGQSNGTYEHYFQTFLRPD
DVFWSFLEALIITAIVMVSHCYYGYAAGGGPVGVGEAVGRSMRFSLVSVQVVVLFAAL
ALYGVDPNFNLTV"

CDS 198766..200130
/codon_start=1
/note="gene_180"
/transl_table=11
/translation="MTTPGKLNKARVPPYKTAGLGLVLVFALVVALVYLQFRGEFTPK
TQLTMLSARAGLVMDPGSKVTYNGVEIGRVDTISEVTRDGESAAKFILDVDPRYIHLI
PANVNADIKATTVFGGKYVSLTTPKNPTKRRITPKDVIDVRSVTTEINTLFQTLTSIA
EKVDPVKLNLTLSAAAEALTGLGDKFGESIVNANTVLDDLNSRMPQSRHDIQQLAALG
DVYADAAPDLFDFLDSSVTTARTINAQQAELDSALLAAAGFGNTTADVFDRGGPYLQR
GVADLVPTATLLDTYSPELFCTIRNFYDADPLAKAAAGGGNGYSLRTNSEILSGIGIS
LLSPLALATNGAAIGIGLVAGLIASPLAVAANLAGALPGIVGGAPNPYTYPENLPRVN
ARGGPGGAPGCWQPITRDLWPAPYLVMDTGASLAPYNHMEVGSPYAVEYVWGRQVGDN
TINP"

CDS 200127..201167
/codon_start=1
/note="gene_181"
/transl_table=11
/translation="MKITGTVVKLGIVSVVLLFFTVMIIVIFGQMRFDRTNGYTAEFS
NVSGLRQGQFVRASGVEIGKVKALHLVDGGRRVRVEFNIDRSVPLYQSTTAQIRYSDL
IGNRYVELKRGEGKGANDLLPPGGLIPLSRTSPALDLDALIGGFKPVFRALDPAKVNN
IANALITVFQGQGGTINDTLDQTAQLTSQIAERDQAIGEVVKNLNIVLDTTVKHRKEF
DETVNNLENLITGLRNHSDQLAGGLAHISNGAGTVADLLAENRTLVRKAVSYLDAIQQ
PVIDQRVELDDLLHKTPTALTALGRANGTYGDFQNFYLCDLQIKWNGFQAGGPVRTVK
LFSQPTGRCTPQ"

CDS 201164..202711
/codon_start=1
/note="gene_182"
/transl_table=11
/translation="MRTLEPPNRMRIGLMGIVVALLVVAVGQSFTSVPMLFAKPSYYG
QFTDSGGLHKGDRVRIAGLGVGTVEGLKIDGDHIVVKFSIGTNTIGTESRLAIRTDTI
LGRKVLEIEPRGAQALPPGGVLPVGQSTTPYQIYDAFFDVTKAASGWDIETVKRSLNV
LSETVDQTYPHLSAALDGVAKFSDTIGKRDEQITHLLAQANQVASILGDRSDQVDRLL
VNAKTLIAAFNERGRAVDALLGNISAFSAQVQNLINDNPNLNHVLEQLRILTDLLVDR
KEDLAETLTILGRFSASFGETFASGPYFKVLLANLVPGQILQPFVDAAFKKRGISPED
FWRSAGLPAYRWPDPNGTRFPNGAPPPPPPVLEGTPEHPGPAVPPGSPCSYTPPADGL
PRPWDPLPCANLTQGPFGGPDFPAPLDVATSPPNPDGPPPAPGLPIAGRPGEVPPNVP
GTPVPIPQEAPPGARTLPLGPAPGPAPPPAAPGPPAPPGPGPQLPAPFINPGGTGGSG
VTGGSEN"

CDS 202708..204300
/codon_start=1
/note="gene_183"
/transl_table=11
/translation="LSTIFDIRNLRLPQLSRASVVIGSLVVVLALAAGIVGVRLYQKL

TNNTVVAYFTQANALYVGDKVQIMGLPVGSIDKIEPAGDKMKVTFHYQNKYKVPANAS
AVILNPTLVASRNIQLEPPYRGGPVLADNAVIPVERTQVPTEWDELRDSVSHIIDELG
PTPEQPKGPFGEVIEAFADGLAGKGKQINTTLNSLSQALNALNEGRGDFFAVVRSLAL
FVNALHQDDQQFVALNKNLAEFTDRLTHSDADLSNAIQQFDSLLAVARPFFAKNREVL
THDVNNLATVTTTLLQPDPLDGLETVLHIFPTLAANINQLYHPTHGGVVSLSAFTNFA
NPMEFICSSIQAGSRLGYQESAELCAQYLAPVLDAIKFNYFPFGLNVASTASTLPKEI
AYSEPRLQPPNGYKDTTVPGIWVPDTPLSHRNTQPGWVVAPGMQGVQVGPITQGLLTP
ESLAELMGGPDIAPPSSGLQTPPGPPNAYDEYPVLPPIGLQAPQVPIPPPPPGPDVIP
GPVPPTPAPVGAPLPAEAGGGQ"

CDS 204297..205469
/codon_start=1
/note="gene_184"
/transl_table=11
/translation="MMSVLARMRVMRHRAWQGLVLLVLALLLSSCGWRGISNVAIPGG
PGTGPGSYTIYVQMPDTLAINGNSRVMVADVWVGSIRAIKLKNWVATLTLSLKKDVTL
PKNATAKIGQTSLLGSQHVELAAPPDPSPVPLKDGDTIPLKRSSAYPTTEQTLASIAT
LLRGGGLVNLEGIQQEINAIVTGRADQIRAFLGKLDTFTDELNQQRDDITRAIDSTNR
LLAYVGGRSEVLNRVLTDLPPLIKHFADKQELLINASDAVGRLSQSADQYLSAARGDL
HQDLQALQCPLKELRRAAPYLVGALKLILTQPFDVDTVPQLVRGDYMNLSLTLDLTYS
AIDNAFLTGTGFSGALRALEQSFGRDPETMIPDIRYTPNPNDAPGGPLVERGNRQC"

CDS 205463..207010
/codon_start=1
/note="gene_185"
/transl_table=11
/translation="VLTRFIRRQLILFAIVSVVAIVVLGWYYLRIPSLVGIGQYTLKA
DLPASGGLYPTANVTYRGITIGKVTAVEPTDQGARVTMSIASNYKIPVDASANVHSVS
AVGEQYIDLVSTGAPGKYFSSGQTITKGTVPSEIGPALDNSNRGLAALPTEKIGLLLD
ETAQAVGGLGPALQRLVDSTQAIVGDFKTNIGDVNDIIENSGPILDSQVNTGDQIERW
ARKLNNLAAQTATRDQNVRSILSQAAPTADEVNAVFSGVRDSLPQTLANLEVVFDMLK
RYHAGVEQLLVFLPQGAAIAQTVLTPTPGAAQLPLAPAINYPPPCLTGFLPASEWRSP
ADTSPRPLPSGTYCKIPQDAQLQVRGARNIPCVDVPGKRAATPKECRSKDPYVPLGTN
PWFGDPNQILTCPAPGARCDQPVKPGLVIPAPSINTGLNPAPADQVQGTPPPVSDPLQ
RPGSGTVQCNGQQPNPCVYTPTSGPSAVYSPASGELVGPDGVKYAVANSSTTGDDGWK
EMLAPAS"

CDS 206980..207687
/codon_start=1
/note="gene_186"
/transl_table=11
/translation="MEGDAGAGQLNPADANKSSSTEVKAADSAESDAGADQTGPQVKA
ADSAESDAGELGEDACPEQALVERRPSRLRRGWLVGIAATLLALAGGLGAAGYFALRS
HQESQSIAREDLAAIEAAKDCVAATQAPDAGAMSASMQKIIECGTGDFGAQASLYTSM
LVEAYQAASVHVQVTDMRAAVERNNNDGSVDVLVALRVKVSNTDSDAHEVGYRLRVRM
ALDEGRYKIAKLDQVTK"

CDS 207684..208652
/codon_start=1
/note="gene_187"
/transl_table=11
/translation="VTVVVEKTPTTLPQATPNGAAPWHVRAGAFAIDVLPGLAVAATM
ALTALTVPPGSAWRWLCACLLGLTILLLAVNRLLLPTITGWSLGRALTGIRVVRRDGS
AIGPWRLLVRDLAHLVDTLSLFVGWLWPLWDSRRRTFADLLLRTEVRRVEPVQRPAVI
RRLTAAVALAAAGACASATAVGAAVVYVNEWQTDHTRAQLATRGPKLVVDVLSYDPET

VQRDFERARSLATDRYRPQLSIQQDSVRESGPVRNQYWVTDSAVLSATPAQATMLLFM
QGERGTPPSQRYISATVRAIFQKSRGQWRLDDLAVVMKPRQPTGEK"
CDS 208649..209203
/codon_start=1
/note="gene_188"
/transl_table=11
/translation="MSPRRKFEPGEGALLAPQSIEPSRRWGLPLALTASAVVMAAAIS
ACALMRISHESHQRAAHKDIVMLSDVRSFMTMFTSPDPFHANEYAERVLSHATGDFAK
QYHERANDILIRISGVEPTTGTVLDAGVQRWNEDGSANVLVVTQITSKSADGKRVVSN
ANRWLVTAKQEGNEWKISSLLPVI"
CDS 209170..209904
/codon_start=1
/note="gene_189"
/transl_table=11
/translation="VEDQQSASGDLTQKSVANGESTDTASAATEGHRGEIDAAGEPDE
RGAAVADSQADEDDSAATAARGGKTRARRSRGRRLAITVGVAAALFVGSAAFAGATVE
PYLSERAVVATKLMVARTAANAITTLWTYTPENMDTLADRAANYLSGDFAAQYRRFVD
QIAAANKQAKITNDTEVTGAAVESLSGRDAVAIVYTNTTTTSPVTKNIPALKYLSYRL
FMKRYDARWLVTRMTTITSLDLTPQV"
CDS complement(209935..211044)
/codon_start=1
/note="gene_190"
/transl_table=11
/translation="MWIRAERVAVLTPTASLRRLTACYAALAVCAALACTTGQPAARA
ADGREMLAQAIATTRGSYLVYNFGGGHPMPLLNAGGHWYEMNNGGHLMIIKNASQRLS
PHLLVDTHTGDQARCEHNPGAHTGEGLWQASEIYPPLKAWQRMGRPTIAVNANFFDVR
GQKGGSWRSTGCSSPLGAYVDNTRGQGRANQAVTGTVAYAGKQGLSGGNELWSSLTTM
ILPVGGAPYVLRPKSRQDYDLATPVIEDLLNKNARFVAVAGIGLLSPGNTGQLHDGGP
SAARTALAYAKQKDEMYIFQGGNYTPDNIQDLFRGLGSDTAILLDGGGSSAIVLRRDT
GGMWAGAGSPKGSCDTRQVLCDSHERALPSWLAFN"
CDS complement(211124..212482)
/codon_start=1
/note="gene_191"
/transl_table=11
/translation="MSQAQPRPAAPNPKRNVKAIRTVRFWMAPIATTLALMSALAALY
LGGILNPMTNLRHFPIALVNEDAGPAGQQIVDGLVSGLDKNKFDIRVVSPDEARRLLD
TAAVYGSALIPPTFSSQLRDFGASAVTPTRTDRPAITISTNPRAGTLAASIAGQTLTR
ALTVVNGKVGERLTAEVAAQTGGVALAGAAAAGLASPIDVKSTAYNPLPNGTGNGLSA
FYYALLLLLAGFTGSIVVSTLVDSMLGYVPAEFGPVYRFAEQVNISRFRTLLVKWAVM
VVLALLTSGVYLAIAHGLGMPIPLGWQVWLYGVFAIIAVGVTSSSLIAVLGSMGLLVS
MLIFVILGLPSAGATVPLEAVPAFFRWLAQFEPMHQVFLGVRSLLYLNGNADAGLSQA
LTMTSIGLIIGLLLGGFITHLYDRSSFHRIPGAVEMAIAVEHQAQYQARQSARESSSE
QP"
CDS complement(212509..213243)
/codon_start=1
/note="gene_192"
/transl_table=11
/translation="MTATVEIRRAADRAVTTTSWLKSRHSFSFGDHYDPDNTHHGLLL
VNNDDQMEPASGFDPHPHRDMEIVTWVLRGALRHQDSAGNSGVIYPGLAQRMSAGTGI
LHSEMNDSATEPVHFVQMWVIPDATGITASYQQQEIDDELLRAGLVTIASGIPGQDAA
LTLHNSSASLHGARLRPGATVSLPCAPFLHLFVAYGRLTLEGGGELADGDAVRFTDAD

ARGLTANEPSEVLIWEMHAKLGDSAT"
CDS complement(213260..214288)
/codon_start=1
/note="gene_193"
/transl_table=11
/translation="VSETLRRLIDVSVLAENSGREPADERRGDFSAHTEPYRRELLAH
CYRMTGSLHDAEDLVQETLLRAWKAYEGFAGKSSLRTWLHRIATNTCLTALEGRRRRP
LPTGLGRPSADPSGELVERREVSWLEPLPDVTDDPADPSTIVGNRESVRLAFVAALQH
LSPRQRAVLLLRDVLQWKSAEVADAIGTSTVAVNSLLQRARSQLQTVRPSAADRLSAP
DSPEAQDLLARYIAAFEAYDIDRLVELFTAEAIWEMPPYTGWYQGAQAIVTLIHQQCP
AYSPGDMRLISLIANGQPAAAMYMRAGDVHLPFQLHVLDMAADRVSHVVAFLDTTLFP
KFGLPDSL"
CDS 214320..215159
/codon_start=1
/note="gene_194"
/transl_table=11
/translation="MTTTRTERNFAGIGDVRIVYDVWTPDTAPQAVVVLAHGLGEHAR
RYDHVAQRLGAAGLVTYALDHRGHGRSGGKRVLVRDISEYTADFDTLVGIATREYPGC
KRIVLGHSMGGGIVFAYGVERPDNYDLMVLSAPAVAAQDLVSPVVAVAAKLLGVVVPG
LPVQELDFTAISRDPEVVQAYNTDPLVHHGRVPAGIGRALLQVGETMPRRAPALTAPL
LVLHGTDDRLIPIEGSRRLVECVGSADVQLKEYPGLYHEVFNEPERNQVLDDVVAWLT
ERL"
CDS 215201..215950
/codon_start=1
/note="gene_195"
/transl_table=11
/translation="MTNDKMLARIAALLRQAEGTDNPHEADAFMSTAQRLATAASIDL
AVARSHAGNRSPAQAPTQRTITIGAAGTRGLRTYVQLFVLIAAANDVRCDVASNSTFV
YAYGFAEDIDTSHALYASLVVQMVRASDAYLASGAHRPTPTITARLNFQLAFGARVGQ
RLADAREQTRQEATKDRDRPPGTAIALRDKDIELHEYYRRSSKARGAWRASRATAGYS
SAARRAGDRAGRQARLGNNPELPGARAALGR"
CDS 215947..216456
/codon_start=1
/note="gene_196"
/transl_table=11
/translation="VIGADVPRDSQRARVYAAEAFVRTLFDRVTAHGSPTVEFFGTQL
TLPPEGRFGSVASVQRYVDDVLALPAVGQNWPTVSPVRVRARRAATAAHYENHGGTGT
IAVPDRHTAGWAMRELVVLHEVAHHLCQVPPPHGPEFVATVCTLTELVMGPEVGHVFR
VVYAQEGVR"
CDS 216501..218576
/codon_start=1
/note="gene_197"
/transl_table=11
/translation="MTDDERFSLLVGLTGASDLWPVRDERIPQGVPMCAGYVPGIPRL
GVPALLMSDAGLGVTNPGYRPGDTATALPAGLALAASFNPVLARSSGKAIGREARSRG
FNVQLAGAINLARDPRNGRNFEYLSEDPLLSATMAAESIIGIQQQGVIATTKHFSLNC
NETNRHWLDAVIDPDAHRESDLLAFEIVIERSQPGAVMAAYNKVNGDYAAGNDHLLND
VLKGAWGYRGWVMSDWGGTPSWECALAGLDQECGAQIDAVLWQSEAFTDRLRAAYADG
NLPKGRLSDMVRRILRSMFAVGIDRWKPAPAPDMNAHNEIAAQMARQGIVLLQNRGLL
PLAPESAGRIAVIGGYAHLGVPAGYGSSAVTPPGGYAGVIPIGGSGLAAGLRNLYLLP
SSPLSELRKRLPNAQFEFDPGINPAEAVLAARRADIAIVFAIRAEGEGFDSADLSLPW

GQDALIAAVASANANTVVVLETGNPVTMPWRDSVNAIMQAWYPGQAGGQAVAESVTGQ
VNPSGRLPITFPVDLGQTPRSQPRELGAPWGTSTTIHYTEGADVGYRWFASTNQTPMF
AFGHGLSYTSFEYRDLVVTGGHTVHASFSVTNTGDRSGADVPQLYMIAAPGESRLRLL
GFERVELEPGQTRRVRIEADPRLLARYDGEARSWRIEPGGYTVAVGASAVALKLAAKV
KLAGRGFGR"

CDS 218943..219599
/codon_start=1
/note="gene_198"
/transl_table=11
/translation="MDQQPNPPDVDAFLDSTLVGDDPALAAALAASDAAELPRIAVSA
QQGKFLCLLAGAIQARRVLEIGTLGGFSTIWLARGAGPQGRVVTLEYQPKHAEVARVN
LQRAGVADRVEVVVGPALDTLPTLAGGPFDLVFIDADKENNVVYIQWAIRLARRGAVI
VVDNVIRGGGILAESDDADAVAARRTLQMMGEHPGLDATAIQTVGRKGWDGFALALVR
"

CDS 219757..220149
/codon_start=1
/note="gene_199"
/transl_table=11
/translation="LLALRASFDRAAESTIAHFTFGLALLAGLYVAASPWIVGFSATR
GLPTCDLIVGIAVAYLAYGFASALDRTHGMTWTLPVLGVWVIFSPWVLPGVAVTAGMM
WSHIIAGAVVAVLGFYFGMRTRAAANQG"

CDS complement(220228..221955)
/codon_start=1
/note="gene_200"
/transl_table=11
/translation="MPQTTDEAASVSTVADIKPRSRDVTDGLEKAAARGMLRAVGMDD
EDFAKPQIGVASSWNEITPCNLSLDRLANAVKEGVFSAGGYPLEFGTISVSDGISMGH
EGMHFSLVSREVIADSVEVVMQAERLDGSVLLAGCDKSLPGMLMAAARLDLAAVFLYA
GSILPGRAKLSDGSERDVTIIDAFEAVGACSRGLMSRADVDAIERAICPGEGACGGMY
TANTMASAAEALGMSLPGSAAPPATDRRRDGFARRSGQAVVELLRRGITARDILTKEA
FENAIAVVMAFGGSTNAVLHLLAIAHEANVALSLQDFSRIGSGVPHLADVKPFGRHVM
SDVDHIGGVPVVMKALLDAGLLHGDCLTVTGHTMAENLAAITPPDPDGKVLRALANPI
HPSGGITILHGSLAPEGAVVKTAGFDSDVFEGTARVFDGERAALDALEDGTITVGDAV
VIRYEGPKGGPGMREMLAITGAIKGAGLGKDVLLLTDGRFSGGTTGLCVGHIAPEAVD
GGPIALLRNGDRIRLDVAGRVLDVLADPAEFASRQQDFSPPPPRYTTGVLSKYVKLVS
SAAVGAVCG"

CDS 222103..222393
/codon_start=1
/note="gene_201"
/transl_table=11
/translation="MTAAHGYTQQKDNYAKRLRRVEGQVRGIARMIEEDKYCIDVLTQ
ISAVTSALRSVALNLLDEHLSHCVTRAVAEGGPGADGKLAEASAAIARLVRS"

CDS 222521..223762
/codon_start=1
/note="gene_202"
/transl_table=11
/translation="MTAPTGTSATTTRPWTPRIATQLSVLACAAFIYVTAEILPVGAL
SAIARNLRVSVVLVGTLLSWYALVAAVTTVPLVRWTAHWPRRRALVVSLVCLTVSQLV
SALAPNFAVLAAGRVLCAVTHGLLWAVIAPIATRLVPPSHAGRATTSIYIGTSLALVV
GSPLTAAMSLMWGWRLAAVCVTGAAAAVALAARLALPEMVLRADQLEHVGRRARHHRN
PRLVKVSVLTMIAVTGHFVSYTYIVVIIRDVVGVRGPNLAWLLAAYGVAGLVSVPLVA

RPLDRWPKGAVIVGMTGLTAAFTLLTALAFGERHTAATALLGTGAIVLWGALATAVSP
MLQSAAMRSGGDDPDGASGLYVTAFQIGIMAGALLGGLLYERSLAMMLTASAGLMGVA
LFGMTVSQHLFENPTLSPGDG"

CDS 223839..224897
/codon_start=1
/note="gene_203"
/transl_table=11
/translation="MSRWKQGWTRGSLFAALNIAAVVAVLMLGAGVAVADPDAAPGDP
GGPGGPGGTAGPVDPPAVDLLAPPPDPLALPPALDPLAPPPPDPLAPPPPDPLAVPVA
AGPVAGQDPTPFVGPPPFRPPTFNPVDGAMVGVAKPIVINFAVPIADRAMAESAIHIS
SIPPVPGKFYWMSPTQVRWRPFEFWPANTAVNIDAAGTKSSFRTGDSLVATADDATHQ
MTITRNGVVQKTFPMSMGMVSGGHQTPNGTYYVLEKFATVVMDSSTYGVPVNSAQGYK
LTASDAVRIDNSGNFVHSAPWSVADQGKRNVTHGCINLSPANAKWFYDNFGSGDPVVV
KNSVGTYNKNDGAQDWQI"

CDS complement(224957..226804)
/codon_start=1
/note="gene_204"
/transl_table=11
/translation="VIQISRDMSSLGQTATTQALPDNSDGIQLTKFAADDILPLEYAP
PIGPELVSQDQLPAAWAYKRFRDLDDKESYRRKLLQELTDALAAQGSEAAEIATAALR
DLIDQMAEQGAVVLADIVESDDFLELVKRYDELMAREGSRSFIHRFLDLRRSPGMLTD
PAVNGALVHPLMIALISYAVGGPIRMIDARGKDAEPLSVLAQDNMLHIDNTPFNDEYK
ILITWRRGTAQGPAGQNFTFLPGTHKLARTCFVNEDGVPWSSENASIFTTPDSIRKVF
DAQRQLGGQDHPTVIEVTDSERPLSSVFAAGSLVHHRFRTASGSARSCIILVFHRVAD
NPGRMVSDVEDSSDVSLSELLTRGVPDESYQQRFIATLCAAADEIAELLLKWKKTPQR
PVSLPLQTKQIDGARFEEWISAATEAPEVREIRNRELTIPYGEVLSAEEFFDLIWRLM
RFDKHGPLDLILYHDNREEPRKWARNLIREMSADRLYERLLGWLADIQQPRPADCLRP
LQIHALISEVLKTLPLDEDQDPPADWHFDLLGMSHAEAARSVKHLLEDVAEALLRCED
MAAYLSTSLFAFWAVDAAYSLDGRRNLVVKDCARRLLRHYTMLSLTCFQ"

CDS 227111..230695
/codon_start=1
/note="gene_205"
/transl_table=11
/translation="MRTNCWWRLSGYVMRHRRDLLLGFGAALAGTVIAVLVPLVTKRV
IDDAIAADHRPLAPWAVVLVAAAGATYLLTYVRRYYGGRIAHLVQHDLRMDAFQALLR
WDGRQQDRWSSGQLIVRTTNDLQLVQALLFDVPNVLRHVLTLLLGVAVMTWLSVPLAL
LAVLLVPVIGLIAHRSRRLLAAATHCAQEHKAAVTGVVDAAVCGIRVVKAFGQEERET
VKLVMASRALYAAQLRVARLNAHFGPLLQTLPALGQMAVFALGGWMAAQGSITVGTFV
AFWACLTLLARPACDLAGMLTIAQQARAGAVRVLELIDSRPTLVDGTKPLSLEARLSL
EFQRVSFGYVADRPVLREISLSVRAGETLAVVGAPGSGKSTLASLATRCYDVTQGAVR
IGGQDVRELTLDSLRSAIGLVPEDAVLFSGTIGANIAYGRPDATPEQIATAARAAHIE
EFVNTLPDGYQTAVGARGLTLSGGQRQRIALARALLHQPRLLIMDDPTSAVDAVIECG
IQEVLREAIADRTAVIFTRRRSMLTLADRVAVLDSGRLLDVGTPDEVWERCPRYRELL
SPAPDLADDLVVAERSPVCRPVAGLGTKAAQHTNVHNPGPHDHPPGPDPLRRLLREFR
GPLALSLLLVAVQTCAGLLPPLLIRHGIDVGIRRHVLSALWWAALAGTATVVIRWVVQ
WGSAMVAGYTGEQVLFRLRSVVFAHAQRLGLDAFEDDGDAQIVTAVTADVEAIVAFLR
TGLVVAVISVVTLVGILVALLAIRARLVLLIFTTMPVLALATWQFRRASNWTYRRARH
RLGTVTATLREYAAGLRIAQAFRAEYRGLQSYFAHSDDYRRLGVRGQRLLALYYPFVA
LLCSLATTLVLLDGAREVRAGVISVGALVTYLLYIELLYTPIGELAQMFDDYQRAAVA
AGRIRSLLSTRTPSSPAARPVGTLRGEVVFDAVHYSYRTREVPALAGINLRIPAGQTV
VFVGSTGSGKSTLIKLVARFYDPTHGTVRVDGCDLREFDVDGYRNRLGIVTQEQYVFA

GTVRDAIAYGRPDATDAQVERAAREVGAHPMITALDNGYLHQVTAGGRNLSAGQLQLL
ALARARLVDPDILLLDEATVALDPATEAVVQRATLTLAARRTTLIVAHGLAIAEHADR
IVVLEHGTVVEDGAHTELLAAGGHYSRLWAAHTRLCSPEITQLQCIDA"
CDS 231001..231768
/codon_start=1
/note="gene_206"
/transl_table=11
/translation="LSNQCLGLGAPGRNTTGGTDIPHVVKRSGAKFREEFILRPDRVQ
MAPVNVISVAVVASDPLTRDGALARLSSHRELDVRAWQAGCETSVLLVLATTITAPLL
CQIEDVQKDGPSHAPKLVVVADEFSAEQVFRMIKLGLTGLLYRSQSTFDCIVETIRLS
AEGRLRLPERVQRYLVGRIKSTPTAEPDTPCAAALAEREVAVLRLLADGLSTHQVAVQ
LNYCERTIKNIVHDIVTRLKLRNRTHAVAHALRAGLI"
CDS 231881..232465
/codon_start=1
/note="gene_207"
/transl_table=11
/translation="MQGPRERMVVSAALLIRERGAHATAISDVLQHSGAPRGSAYHYF
PGGRTQLLCEAVDYAGEHVAAMINEAEGGLELLDALIDKYRQQLLSTDFRAGCPIAAV
SVEAGDEQDRERMAPVIARAAAVFDRWSDLTAQRFIADGIPPDRAHELAVLATSTLEG
AILLARVRRDLTPLDLVHRQLRNLLLAELPERSR"
CDS 232465..234711
/codon_start=1
/note="gene_208"
/transl_table=11
/translation="MTSSDWLPTACILCECNCGIVVQVDDRRLARIRGDKAHPGSAGY
TCNKALRLDHYQNNRARLSSPMRRRADGTYEEIDWDTAIVEIAEGFKQIRDTHGGDKI
FYYGGGGQGNHLGGAYSGAFLKALGSRYRSNALAQEKTGEAWVDFQLYGGHTRGEFEN
AEVSVFVGKNPWMSQSFPRARVVLNEIAKDPGRSMIVIDPVVTDTAKMADFHLRVQPG
CDAWCLAALAAVLVQENLCNEAFLAAHVHGVDTVRAALQEVPVADYAQRCGVDEELLR
AAARRIGTAASVSVFEDLGIQQAPNSTVCSYLNKLLWILTGNFAKKGGQHLHSSFAPL
FSQVSGRTPVTGAPIIAGLIPGNVVPEEILTEHPDRFRAMIVESGNPAHSLADSAACR
AAFQALELMVVVDVAMTETARLAHYVLPAASQFEKPEATFFNFEFPRNGFQLRRPLFP
PLPGTLPEPEIWARLVRALGVVDEADLRPLREAAAQGRQAYTEAFLAAAATNPTVAKL
TAYVLYETLGPTLPDGLAGAAALWGLAQKTAMAYPDAVRRAGHADGNALFDAILERPS
GVTFTVHNYEDDFALISHPDHKIALEIPEMLAEIRSLTQTPSRLTTPQLPIVLSVGER
RAYTANDIFRDPSWRKRDANGALRVSVEDAQALGLADGCLARITTAAGSAEATVEVTE
TMLAGHAALPNGFGLDYTGDDGRTVVAGVAPNALTSTRWRDPYAGTPWHKHVPAAIRR
ADAESPIW"
CDS complement(234752..236743)
/codon_start=1
/note="gene_209"
/transl_table=11
/translation="VTLAIPSGIDLSHIDADARPQDDLFGHVNGRWLAEHEIPADRAT
DGAFRSLFDRAETQVRDLIIQASQAGAAVGTDAQRIGDLYASFLDEEAVERAGVQPLH
DELATIDSAADATELAAALGTLQRAGVGGGIGVYVDTDSKDSTRYLVHFTQSGIGLPD
ESYYRDEQHAAVLAAYPGHIARMFGLVYGGESRDHAKTADRIVALETKLADAHWDVVK
RRDADLGYNLRTFAQLQTEGAGFDWVSWVTALGSAPDAMTELVVRQPDYLVTFASLWA
SVNVEDWKCWARWRLIRARAPWLTRALVAEDFEFYGRTLTGAQQLRDRWKRGVSLVEN
LMGDAVGKLYVQRHFPPDAKSRIDTLVDNLQEAYRISISELDWMTPQTRQRALAKLNK
FTAKVGYPIKWRDYSKLAIDRDDLYGNVQRGYAVNHDRELAKLFGPVDRDEWFMTPQT
VNAYYNPGMNEIVFPAAILQPPFFDPQADEAANYGGIGAVIGHEIGHGFDDQGAKYDG

DGNLVDWWTDDDRTEFAARTKALIEQYHAYTPRDLVDHPGPPHVQGAFTIGENIGDLG
GLSIALLAYQLSLNGNPAPVIDGLTGMQRVFFGWAQIWRTKSRAAEAIRRLAVDPHSP
PEFRCNGVVRNVDAFYQAFDVTEDDALFLDPQRRVRIWN"
CDS 236786..237445
/codon_start=1
/note="gene_210"
/transl_table=11
/translation="MPDGEQSQPPAQEDAEDDSRPDAAEAAAAEPKSSAGPMFSTYGI
ASTLLGVLSVAAVVLGAMIWSAHRDDSGERTYLTRVMLTAAEWTAVLINMNADNIDAS
LQRLHDGTVGQLNTDFDAVVQPYRQVVEKLRTHSSGRIEAVAIDTVHRELDTQSGAAR
PVVTTKLPPFATRTDSVLLVATSVSENAGAKPQTVHWNLRLDVSDVDGKLMISRLESI
R"
CDS 237442..238131
/codon_start=1
/note="gene_211"
/transl_table=11
/translation="MRNAWRLVVFDVLAPLATIAALAAIGVLLGWPLWWVSTCSVLVL
LVVEGVAINFWLLRRDSVTVGTDDDAPGLRLAVVFLCAAAISAAVVTGYLRWTTPDRD
FNRDSREVVHLATGMAETVASFSPSAPAAAVDRAAAMMVPEHAGGFKEQYAKSSADLA
RRGVTAQAATLAAGVEAIGPSAASVAVILRVSQSIPGQPTSQAARALRVTLTKRGSGW
LVLDVTPINAR"
CDS complement(238128..238631)
/codon_start=1
/note="gene_212"
/transl_table=11
/translation="VTLAAEPHPAPPQQPTVAWSEPDVDRRVEFWPTVAIRSALESGD
IATWQRIAAALKRDPYGRTARQVEEVLEGIPATGIANAFWEVLDRARTHLDANERAEV
ARQVGLLLDRSGLQRQEFASRIGVTAQDLTAYLDGIVSPSASLMIRMRRLSDRFVRAK
SVRAADS"
CDS complement(238628..241525)
/codon_start=1
/note="gene_213"
/transl_table=11
/translation="MRLSRNLRRCRWLVFTGWLLALVPAVYLAMTQSGNLTGGGFEVA
GSQSLLVHDQLDAHYPDRGAPALALVAAPRPDASYQDIDNAVALLRQIASELPGVTEA
PNPTQRPPQPDRPYVVSLRLDARNAGTSDVAKKLRDRIGVKGDQSGQTANGKVRLYVI
GQGALSAAAAANTKHDIANAERWNLPIILMVLVAVFGSLAAAAIPLALAVCTVVITMG
LVFVLSMHTTMSVFVTSTVSMFGIALAVDYSLFILMRYREELRCGRRPPDAVDAAMAT
SGLAVVLSGMTVIASLTGIYLINTPALRSMATGAILAVAVAMLTSATLTPAVLATFAR
AAAKRSALVHWSRRPASTQSWFWSRWVGWVMRRPWITALAASTVLLVMAAPATLMVLG
NSLLRQFDSSHEIRTGAAAAAQALGPGALGPVQVLVRFDAGGASAPEHSQTIAAIRHR
IAQAPNVVSVAPPRFADDNGSALLSAVLSVDPEDLGARDTITWMRTQLPRVAGAAQVD
VGGPTALIKDFDDRVSATQPLVLVFVAVIAFLMLLISIRSVFLAFKGVLMTLLSVAAA
YGSLVMVFQWGWARGLGFPALHSIDSTVPPLVLAMTFGLSMDYEIFLLTRIRERFLQT
GQTRDAVAYGVRTSARTITSAALIMIAVFCGFAFAGMPLVAEIGVACAVAIAVDATVV
RLVLVPALMAMFDRWNWWLPRWLAHILPSVDFDRPLPKVDLGDVVVIPDDFAAAIPPS
ADVRMVLKSAAKLKRLAPDAICVTDPLAFTGCGCDGKALDQVQLAYRNGIARAISWGQ
RPVHPVTVWRKRLAVALDALQTTTWECGGVQTHRAGPGYRRRSPVETTNVALPTGDRL
QIPTGAETLRFKGYLIMSRNSSHDYADFADLVDTMAPETAAAVLAGMDRYYSCQAPGR
QWMATQLVGRLADPQPSDLGDQSPGADAQAKWEEVRRRCLSVAVAMLEEAR"
CDS 241750..242160

```
                     /codon_start=1
                     /note="gene_214"
                     /transl_table=11
                     /translation="MKTGTATTRRRLLAVLIALALPGAAVALLAEPSATGASDPCAAS
                     EVARTVGSVAKSMGDYLDSHPETNQVMTAVLQQQVGPGSVASLTAHFEANPKVASDLH
                     ALSQPLTDLSTRCSLPISGLQAIGLMQAVQGARR"
     CDS             complement(242212..243495)
                     /codon_start=1
                     /note="gene_215"
                     /transl_table=11
                     /translation="MYRDYGATCTVTLKHVSHDAPARNLRQRVGALPRTRVGAPPAEG
                     VPPRGKYWWLRWAVLAIVAIVLAIEVALGWDQLAKAWVSLYRAKWWWLLAAVAAAGAS
                     MHSFAQIQRTLLKSAGVHVKQWRSEAAFYAANSLSTTLPGGPVLSATFLLRQQRIWGA
                     STVVASWQLVMSGVLQAVGLALLGLGGAFFLGAKNNPFSLLFTLGGFVTLLLLAQAVA
                     SRPELIEGIGRRVLSWANSVRGRPADAGLPKWRETLMQLESVSLGRRDLGVAFGWSLF
                     NWIADVACLGFAAYAAGDHASVGGLAVAYAAARAVGTIPLMPGGLLVVEAVLVPGLVS
                     SGMPLPSAISAMLIYRLISWLLIAAIGWVVFFFMFRTESTADSDNDRDPPTDPNLRLV
                     IQPQGTPCDDPVETTPQGPAPTPDLRPEGGETPPR"
     CDS             243620..244723
                     /codon_start=1
                     /note="gene_216"
                     /transl_table=11
                     /translation="MSASLDDASVAPLVRKTAAWAWRFLVILAAMVALLWVLNKFEVI
                     VVPVLLALMLSALLVPPVDWLDSRGLPRAVAVTLVLLSGFAVLGGILTFVVSQFIAGL
                     PHLVTEVERSIDSARRWLIEGPAHLRGEQIDNAGNAAIEALRNNQAKLTSGALSTAAT
                     ITELVTAAVLVLFTLIFFLYGGRSIWQYVTKAFPASVRDRVRAAGRAGYASLIGYARA
                     TFLVALTDAAGVGAGLAVMGVPLALPLASLVFFGAFIPLIGAVVAGFLAVVVALLAKG
                     IGYALITVGLLIAVNQLEAHLLQPLVMGRAVSIHPLAVVLAIAAGGVLAGVVGALLAV
                     PTAAFFNNAVQVLLGGNPFADVADVSSDHLTEV"
     CDS             complement(244720..247554)
                     /codon_start=1
                     /note="gene_217"
                     /transl_table=11
                     /translation="VFAWWGRTVVYRYRFIVIGVMVALCLGGGVFGLSLGKHVTQSGFY
                     DDGSQSVQASVLGDQVYGRDRSGHIVAIFQAPAGKTVDDPAWSKKVVDELNRFQQDHP
                     DQVLGWAGYLRASQATGMATADKKYTFVSIPLKGDDDDTILNNYKAIAPDLQRLDGGT
                     VKLAGLQPVAEALTGTIATDQRRMEVLALPLVAVVLFFVFGGVIAAGLPVMVGGLCIA
                     GALGIMRFLAIFGPVHYFAQPVVSLIGLGIAIDYGLFIVSRFREEIAEGYDTETAVRR
                     TVITAGRTVTFSAVLIVASAIGLLLFPQGFLKSLTYATIASVMLSAILSITVLPACLG
                     ILGKHVDALGVRTLFRVPFLANWKISAAYLNWLADRLQRTKTREEVEAGIWGKLVNRV
                     MKRPVLFAAPIVIIMILLIIPVGKLSLGGISEKYLPPTNSVRQAQEEFDKLFPGYRTN
                     PLTLVIQTSNHQPVTEAQIADIRSKAMAIGGFIEPDNDPANMWQERAYAVGASKDPSV
                     RVLQNGLINPADASKKLTELRAITPPKGITVLVGGTPALELDSIHGLFAKMPLMVVIL
                     LTTTIVLMFLAFGSVVLPIKATLMSALTLGSTMGILTWIFVDGHFSKWLNFTPTPLTA
                     PVIGLIIALVFGLSTDYEVFLVSRMVEARERGMSTQEAIRIGTAATGRIITAAALIVA
                     VVAGAFVFSDLVMMKYLAFGLMAALLLDATVVRMFLVPSVMKLLGDDCWWAPRWARRL
                     QTRIGLGEIHLPDERKRPVSNGRPARPPVTAGLVAARAAGDPRPPHDPTHPLAESPRP
                     ARSSPASSPELTPALEATAAPAAPSGASTTRMQIGSSTEPPTTRLAAAGRSVQSPAST
                     PPPTPTPPSAPSAGQTRAMPLAANRSTDAAGDPAEPTAALPIIRSDGDDSEAATEQLN
                     ARGTSDKTRQRRRGGGALSAQDLLRREGRL"
     CDS             complement(247620..248354)
```

/codon_start=1
/note="gene_218"
/transl_table=11
/translation="MMMSLTEDVTSQTSESLARHSVLAEDLSQDGLTSLGAPGARVLL
VWDAPNLDMGLGSILGRRPTALERPRFDALGRWLLARTAEIVAGRPGISTEPEATVFT
NIAPGSAEVVRPWVDALRNVGFAVFAKPKVDEDSDVDRDMLAHIDERYREGLAALVVA
SADGQAFRQPLEAVARSGTPVQVLGFREHASWALASDTLEFVDLEDIAGVFREPLPRI
GLDSLPEQGAWLQPFRPLSSLLTSRV"
CDS complement(248351..249184)
/codon_start=1
/note="gene_219"
/transl_table=11
/translation="LGINLDQGRLRRALMVHHGQMHAQPGVGLRPDTPVASGQLPSTS
IRSRRSGISKAQRETWERLWPELGLLALPQSPRGTPVDTRAWFGRDAPVVLEIGSGSG
TSTLAMAKAEPHVDVIAVDVYRRGLAQLLCAIDKVGSDGINIRLILGNAVDVLQHLIA
PDSLCGVRVFFPDPWPKARHHKRRLLQPATMALIADRLVPSGVLHAATDHPGYAEHIA
AAGDAEPRLVRVDPDTELLPISVVRPATKYERKAQLGGGAVIELLWKKHGCSERDLKI
R"
CDS 249274..250359
/codon_start=1
/note="gene_220"
/transl_table=11
/translation="MRGQGHQIFVDELARFATSSADQRVVAIAQRAAEPLRVAVRGRP
GVGCRTVARALQGAGSSSGMTVTPQARAADSDVDLVVYVTVEVVKPEDREAIAATRRP
VVAVLNKADLAGPLSGAGPIVMAQARCAQFSTLLGVPMESMIGLLAVAALDDLDDTLR
AALRALAAHPDGFDALDRAVAGFLAAALPVPTEVRLRLLDTLDLFGIALGMAAFRPGR
PSRTPAQLRTLLRRVSGVDAVIDKVTAAGSEVRYRRLLDAVAELEALAAQAKEIGGPI
GEFLRDDDTVLARMAAAVDVALAVGLDVGPLDDPAAHLPRAVRWHRYSLDNGDMHRTC
GADIARGSLRLWSLAGGMPLHRYRKSS"
CDS 250356..251834
/codon_start=1
/note="gene_221"
/transl_table=11
/translation="VIRAASDDPAGVDELVAAIAPGLAGLGLPVINRREVVLVTGPWL
AGVSGVRAALAERLPQRRFVETAELGPGDAPVAVVFVVSAATALTESDCVLLDTAAEH
TDAVVAVVSKIDVHRGWRDVLTSNRDRLAARASRYARVPWVGAAAAPELGEPYLDDLV
AAIQKQLADPAVARRNMLRAWESRLLMVARRFDGDAQSAGRRARVDALRQQRRTVLRQ
GRQSKSEHTIALRAQIQHARVKLSYFARNRCSLLRVELQEHVAGLSRKDIARFAAYTR
GRVQEVVAEVGEGAVAHLADVAQLLGVPVQPPVLENLPAVLPTVVAPPLTSRRLEIRL
TTLLGAGFGLGIALTLSRLVAGLTPGLAASGMVAGVAIGLAVTAWVVNARALLHDRVV
VDRWTGEVTASLRSVVEQLVATRVVAVETLLSTAISERDDAENARVADQVSIIDGELR
EHAVAAARAAALRDREMPAVRAALEAVRAELGEPGTPTTGLF"
CDS 252018..253838
/codon_start=1
/note="gene_222"
/transl_table=11
/translation="MTSATIPGLDTAPTNHQGLLSWVEEVAELTQPDRVVFTDGSEEE
FQRLCDQLVEAGTFIRLNPEKHKNSYLALSDPSDVARVESRTYICSAKEIDAGPTNNW
MDPGEMRSIMKDLYRGCMRGRTMYVVPFCMGPLGAEDPKLGVEITDSEYVVVSMRTMT
RMGKAALEKMGDDGFFVKALHSVGAPLEPGQKDVAWPCSETKYITHFPETREIWSYGS
GYGGNALLGKKCYSLRIASAMAHDEGWLAEHMLILKLISPENKAYYFAAAFPSACGKT

NLAMLQPTIPGWRAETLGDDIAWMRFGKDGRLYAVNPEFGFFGVAPGTNWKSNPNAMR
TIAAGNTVFTNVALTDDGDVWWEGLEGDPQHLIDWKGNDWYFRETETNAAHPNSRYCT
PMSQCPILAPEWDDPQGVPISGILFGGRRKTTVPLVTEARDWQHGVFIGATLGSEQTA
AAEGKVGNVRRDPMAMLPFLGYNVGDYFQHWINLGKHADESKLPKVFFVNWFRRGDDG
RFLWPGFGENSRVLKWIVDRIEHKAGGATTPIGTVPAVEDLDLDGLDVDAADVAAALA
VDADEWRQELPLIEEWLQFVGEKLPTGVKDEFDALKERLG"

CDS complement(253905..254876)
/codon_start=1
/note="gene_223"
/transl_table=11
/translation="VTHGMVLGKFMPPHAGHVYLCEFARRWVDELTIVVGSTAAEPIP
GAQRVAWMRELFPFDRVVHLANENPQRPWEHPDFWDIWKASLQGVLATRPDFVFGAEP
YNADFAQVLGARFVAVDHGRTVVPVTATDIRADPLGHWQHIPRCVRPAFVKRVSIIGP
ESTGKTTLAQAVAEKLRTKWVPERAKMLRELNGGSLIGLEWAEIVRGQIASEEALARD
ADRVLICDTDPLATTVWAEFLAGGCPQELRDLARRPYDLTLLTTPDVPWDADDGRCVP
GARGTFFARCEQALRAAGRSFVVITGGWEERLSVSLRAVEELVRARR"

CDS complement(254873..256306)
/codon_start=1
/note="gene_224"
/transl_table=11
/translation="VATLLVNPPGVRQDGLSYVDGLKLGDPWLYCSMPMEHLGMMSIK
AYAKTQGIAVTSVNGLVAGHGSVQETWLAMQSAAALSGTPRLVGFSCIDTFPEVLWLA
QRARQAWDGVRIVIGNAMATLNYERILRQHDCFDYVVVGDGEVAFTKLALALANDAAV
DDVPGLARRSEQGQILRTPSSLVDLDELPRPARDELPTVLADGFAASVFSTRGCPYRC
TFCGTGAMSAMLGKDSYRAKSVDAVVDEIDYLVSDYDVNFLSITDDLFISKHPGSQQR
AADFANAVLRRGISVNFMVDIRLDSVVDLDLFKHLHRAGLRRVFIGVETGSYEQLRAY
RKQILTRGQDAADTINALQQLGIDVIPGTIMFHPTVQPDELRETVRLLRATKYTVGFK
FMSRIVPYPGTPLYQAYSDAGYLTAKWPLGQWEFVDPEASRVYADVVAKVAPDVGISF
DEAEAYFLSRLDEWENVIAGRIAEATS"

CDS 256396..257913
/codon_start=1
/gene="fadD4"
/note="gene_225"
/transl_table=11
/translation="MQIRPYIAADKPAVILYPSGTVISFDELEARANRLAHWFRQAGL
REDDVVAILMENNEHVHAVMWAARRSGLYYVPINTHLTASEAAYIVDNSGAKAIVGSA
ALRETCHGLAEHLPGGLPDLLMLAGGGLVGWMTYPECVADQPDTPIEDEREGDLLQYS
SGTTGRPKGIKRELPHVSPDAAPGMMPALLDFWMDADSVYLSPAPMYHTAPSVWTMSA
LAAGVTTVVMEKFDAEGALDAIQRYRVTHAQFVPAMFVRMLKLPEAVRNSYDMSSLRR
VIHAAAPCPVQIKEQMIHWWGPIIDEYYASSEASGSTLITAEDWLTHPGSVGKPIQGG
VHIVGADGSELPPNQPGEIYFEGGYPFEYLNDPAKTAASRNKHGWVTVGDVGYLDDDG
YLFLTGRRHHMIISGGVNIYPQEAENLLVAHPKVLDAAVFGVPDDEMGQRVMAAVQTV
DSADANDQFAGELLAWLRDRLSHFKCPRSIAFEPQLPRTDTGKLYKSGLVEKYSV"

CDS complement(257955..259121)
/codon_start=1
/note="gene_226"
/transl_table=11
/translation="VRNELNDDEAMLVATVRAFIDRDVKPTVREVEHANSYPEAWIEQ
MKHIGIYGLAIDEQYGGSPVSMPCYVQVTQELARGWMSLAGAMGGHTVVAKLLTLFGT
EEQRRTYLPPMASGELRATMALTEPGGGSDLQNMSTTALADGPEGSAGLLINGCKTWI
SNARRSGLFAVLCKTDPNATPRHQGMSIVLVEPGPGLTVSRDLPKLGYKGVESCELSF

DNLRVPVSAILGGAMGQGFSQMMKGLETGRIQVAARALGVATAALEDSLAYAQQRESF
GRPIWQHQAVGNYLADMATKLTAARQLTRYAAERYDSGQRCDMEAGMAKLFASEVAME
IALNAVRIHGGYGYSTEYDVERYFRDSPLMILGEGTNEIQRNVIAGQLVARGGI"
CDS 259178..260191
/codon_start=1
/note="gene_227"
/transl_table=11
/translation="VASGYGGIRVGGPYFDDLSKGQVFDWAPGVTLSLGLAAAHQSIV
GNRLRLALDSDLCAAVTGMPGPLAHPGLVCDVAIGQSTLATQRVKANLFYRGLRFHRF
PAVGDTLYTRTEVVGLRANSPKPGRAPTGLAGLRMTTIDRTDRLVLDFYRCAMLPASP
DWKPGAVPGDDLSRIGADAPAPAADPTAHWDGAVFRKRVPGPHFDAGIAGAVLHSTAD
LVSGAPELARLTLNIAATHHDWRVSGRRLVYGGHTIGLALAQATRLLPNLATVLDWES
CDHTAPVHEGDTLYSELHIESAQAHADGGVLGLRSLVYAVSDSASEPDRQVLDWRFSA
LQF"
CDS complement(260188..261096)
/codon_start=1
/note="gene_228"
/transl_table=11
/translation="VSGNEVHPDLRRIAVVTPRQLVGPRTLPVMRALIVVAGLRMSRT
PPDIEVLTLESGVGVRLYRPAGSNEPAPALLWIHAGGYVMGTAQQDDRLCLRFSSRLG
ITVASVDYRLAPENPYPAALGDCYSALTWLASLPAVDPARVAIGGASAGGGLAAALAL
LARDRGGITPAFQLLVYPMLDDRTSIAPANPHYRLWNGRANRFGWRAYLGDADARVAV
PGRRDDLGGLAPAWIGVGTHDLLHDEDLAYAERLTAAGVPCQVEVVEGAFHGFDRVAP
NVGVSQRFFTSQCNSLRAALALSNRT"
CDS 261189..262517
/codon_start=1
/note="gene_229"
/transl_table=11
/translation="MSDPARGAEAEDAYGFPAGLWRWLQRHPPPALHRLTRFRSPLRG
PWLTSVFGLVLLVALPFVIITGLLSYIAYAPQLGQAIPGDVGWLRLPAFTWPTRPSWL
YRLTQGLHVGLGLVIIPVVLAKLWSVIPRLFVWPPARSIAQVLERLSVLMLVGGILFQ
IVTGVLNIQYDYIFGFSFYTGHYFGAWVFIAGFLLHIVVKIPHMVTGLRSIPMREVLG
TNVADTRAQPCDPDGLVSVNPGEATLSRRGALGLVGAGVLLIGVLTVGQTLGGFTRKA
ALLLPRGRVVSPGDFPVNKTAAAAGITAEAIGPDWRLVLRGGPAEVVLDRATLAGLPQ
RTARLPLACVEGWSAVRTWSGVPLAELALLAGVPAARSARVTSLQRGGAFGEAKLAAN
QIADPDALLALRVDGADLSLNHGYPARIIVPALPGVHNTKWVAGIEFHKR"
CDS 262519..263067
/codon_start=1
/note="gene_230"
/transl_table=11
/translation="MFDIATRFKNSYGSGPLHLLAMVSGFALLGYIVATARPSALWNQ
ATWWQSIAVWFVAAVVAHDLLLYPLYALADRILARLVGRRDVSAPRRRPELPVRNYIR
IPALAAGLTLLVFLPGIIRQGAPTYLDATGQTQEPFLGRWLLLTAVAFGISAAAYAIR
LVVAHVRRRRAGCSRVDAIDEE"
CDS 263077..264318
/codon_start=1
/note="gene_231"
/transl_table=11
/translation="MNQRRAAGSTGVAYIRWLLRARPADYMLALSVAGGSLPVVGKHL
KPLGGVTAIGVWGARHASDFLSATAKDLLTPGINEVRRRDRASTQEVSVAALRGIVSP
DDLAVEWPAPERTPPVCGALRHRRYVHRRRVLYGDDPAQLLDVWRRKDMPTKPAPVLI

FVPGGAWVHGSRAIQGYAVLSRLAAQGWVCLSIDYRVAPHHRWPRHILDVKTAIAWAR
ANVDKFGGDRNFIAVAGCSAGGHLSALAGLTANDPQYQAELPEGSDTSVDAVVGIYGR
YDWEDRSTPERARFVDFLERVVVQRTIDRHPEVFRDASPIQRVTRNAPPFLVIHGSRD
CVIPVEQARSFVERLRAVSRSQVGYLELPGAGHGFDLLDGARTGPTAHAIALFLNQVH
RSRAQFAKEVIQTPANCMVAL"

CDS complement(264281..264748)
/codon_start=1
/gene="O216_01215"
/note="gene_232"
/transl_table=11
/translation="MVNFGNHRDPVVGKPLNEVHLPQRVVAVQRGAGDFSDRLVEFAP
TPRGAQPPRPDVVVEIDLAVLPPHRVMELQRDINQLIAQWLKLMQPASDHLAKRVDTE
FLSVRIQFDHGDLECVHVHIRGLAVKQYRVPAAEPFHRSPLLIGRPYNWPAFG"

CDS complement(264971..265780)
/codon_start=1
/note="gene_233"
/transl_table=11
/translation="MNYFAGAADKVTWTETRTGSYGQSIVSRESVGVVGAIVAWNVPL
FLAVNKIAPALLAGCTIVLKPAAETPLTANALAEVFAEVGLPEGVLSVVPGGIETGQA
LTSNPDIDMFTFTGSSAVGREVGRRAAEMLKPCTLELGGKSAAIILEDVDLAAAIPMM
VFSGVMNAGQGCVNQTRILAPRSRYDEIVAAVTNFVTALPVGPPSDPAAQIGPLISEK
QRTRVEALHRQGHRGGRSVGVRRRPSRGLGQRLLYPIHERRWRGKWHGQCG"

CDS complement(265777..266127)
/codon_start=1
/note="gene_234"
/transl_table=11
/translation="MSDSATEYDKLFIGGKWTKPSTSDVIEVRCPATGEYVGKVPMAA
AADVDAAVAAARAAFDNGPWPSTPPHERAAVIAAAVKMLAERKDLFTKLLAAETGQPP
TIIETMHWMGSMGR"

CDS complement(266226..266990)
/codon_start=1
/note="gene_235"
/transl_table=11
/translation="VAVTDVFARRATLRRSLRLLADFRYEQRDPARFYRTLAADTAAM
IGDLWLATHSEPPVGRTLLDVGGGPGYFATAFSDAGVGYIGVEPDPDEMHAAGPAFTG
RPGMFVRASGMALPLADDSVDICLSSNVAEHVPRPWQLGTEMLRVTKPGGLVVLSYTV
WLGPFGGHEMGLSHYLGGARAAARYVRKHGHPAKNNYGSSLFAVSAAEGLRWAAGTGA
ALAVFPRYHPRWAWWLTSVPVLREFLVSNLVLVLTP"

CDS 267026..268180
/codon_start=1
/note="gene_236"
/transl_table=11
/translation="MSALRSVLLLCWRDIGHPQGGGSEAYLQRIGAQLAASGIAVTLR
TARYPGAPRHELVDGVRISRAGGRYSVYLWALLAMAAARCGLGPLRRVRPDVVVDTQN
GWPFVARLLYGRRSLVLVHHCHREQWPVAGRMMGRLGWYVESMLSPRLHRRNQYVTVS
LPSARDLIALGVDSERIAVVRNGLDEAPSPTLSGPRAPTPRVVVLSRLVPHKQIEDAL
AAVAELQPRIPGLHLDIVGGGWWRQRLVDHVHRLDIADAVTFHGHVDDVTKHHVLQSS
WVHLLPSRKEGWGLAVIEAAQHGVPTIGYRSSGGLADSIVDGVTGILVDDRAELVAWL
EQLLSDSVLRDQLGAKAQARSGEFSWRQSAEALRSVLEAVQASRFVSGVV"

CDS complement(268197..269927)
/codon_start=1

/note="gene_237"
/transl_table=11
/translation="VRWFRPGYALVLVLLLAAPLLRPGYLLLRDAVSTPRSYVSANAL
GLTSAPRATPQDFAVALASHLVDGGVVVKALLLLGLWLAGWGAARLVATALPAAGAAG
QFVAITLAIWNPYVAERLLQGHWSLLVGYGCLPWVATAMLTMRTTVGAGWFGLFGLAF
WVALAGLTPSGLLLAATVAVVCVAMPGAGRPRWQCGVAALGSALVGALPWLTASALGS
SLTSHTAANQLGVTAFAPRAEPGLGTLGSLASLGGIWNGEAVPSSRTTLFAVASAVVL
LAMVAIGLPTVARRPVAVPLLTLAAVSVMVPAVLATGPGLHALRVVVDAAPGLGVLRD
GQKWVALAVPGYTLSGAGTVLTLRRWLRPATAAVVCCLALVLTLPDLAWGVWGKVAPV
HYPSGWAAVAAAINADPRTVAVLPAGTMRRFSWSGSAPVLDPLPRWVRADVLTTGDLV
ISGVTVPGEDAHARAVQELLLTGPHPSTLAAAGVGWLVVESDSAGDMGAAARTLGRLA
AAHRDDELALYRVGGQTSGASSARLKATMLAHWAWLSMLLVGGAGAAGYWVRRHLHHC
EDTPASRAQD"
CDS complement(269937..271202)
/codon_start=1
/note="gene_238"
/transl_table=11
/translation="MLRFAACGAIGLGAALLIAALLLSTYTTRRIAEIPLDIDATLIS
DGTGTALDSASLATEHIVVNQDVPLVSQQQVTVESPANADVVTLQVGSSLRRTDKQKD
SGLLLAIVDTVTLNRKTAMAVSDDTHTGGAVQKPRGLNDENPPTAIPLRHDGLSYRFP
FHTEKKTYPYFDPIAQKAFDANYEGEEDVNGLTTYRFTQNVGYTPEGKLVAPLKYPSL
YAGDEDGKVTTSAAMWGLPGDPNEQITMTRYYAAQRTFWVDPVSGTIVKETERANHYF
ARDPLKPEVTFADYQVTSTEETVESQVNAARDERDRLALWSRVLPITFTAAGLVALVG
GGLFASFSLRTEGALMAASGDRDDHDYRRGGFEEPVPGAEAETEKLPTQRPDFPREPS
GSDPPRLGSAQPPPPPDAGHPDPGPPERR"
CDS 271343..272641
/codon_start=1
/note="gene_239"
/transl_table=11
/translation="MDSSVGTRSDLTKRGKTRLTLTAERMGPADESGAPIRPQTPHRH
TVLVTNGQVVGGTRGFLPAVEGMRACAAVGVVVTHVAFQTGHSSGVGGRLFGRFDLAV
AVFFAVSGFLLWRGHAAAARDLRSHPRTGPYLRSRVARIMPAYVVAVVVILSLLPDAD
HASLTVWLANLTLTQIYVPLTLTGGLTQMWSLSVEVAFYAALPVLALLGRRIPVGARV
PAIAALAALSWAWGWLPLDAGSGINPLTWPPAFFSWFAAGMLLAEWAYSPVGLPHRWA
RRRVAMAVTALLGYLVAASPLAGPEGLVPGTAAQFAVKTAMGSLVAFALVAPLVLDRP
DTSHRLLGSPAMVTLGRWSYGLFIWHLAALAMVFPVIGAFPFTGRMPTVLVLTLIFGF
AIAAVSYALVESPCREALRRWERRNEPISVGELQADAIAP"
CDS complement(272669..273082)
/codon_start=1
/note="gene_240"
/transl_table=11
/translation="MALKYLLDTSVIKRLSRPAVRRAVEPLAEAGAVARTQITDLEVG
YSARNETEWQRLMVALSAFDLIESTASHHRRALGIQRLLAARSQRGRKIPDLLIAAAG
EEHGLVVLHYDADFDLIAAVTGQPCQWIVPAGTID"
CDS complement(273073..273267)
/codon_start=1
/note="gene_241"
/transl_table=11
/translation="MAKHLVDIDEQALNMARTELGTTTIKDTVNAALRQATSQRVQRV
AAALDTLAAAPPEDRAEAWR"
CDS complement(273346..274326)

```
                     /codon_start=1
                     /note="gene_242"
                     /transl_table=11
                     /translation="VPELNTARGPIDTADLGVTLMHEHVFIMTTEIAQNYPEAWGDED
                     KRVAGAIARLGELKARGVDTIVDLTVIGLGRYIPRIARVAAATELNIVVATGLYTYND
                     VPFYFHYLGPGAQLDGPEIMTDMFVRDIEHGIADTGIKAGILKCATDEPGLTPGVERV
                     LRAVAQAHKRTGAPISTHTHAGLRRGLDQQRIFAEEGVELSRVVIGHCGDSTDVGYLE
                     ELIAAGSYLGMDRFGVDVISPFQDRVNIVARMCERGHADKMVLSHDACCYFDALPEEL
                     VPVAMPNWHYLHIHNDVIPALKQHGVTDEQLHTMLVDNPRRIFERQGGYQ"
     CDS             274421..276127
                     /codon_start=1
                     /note="gene_243"
                     /transl_table=11
                     /translation="MLLNPNHLTRKYPDRRSGEIMAATVDFFESRGKARLKHDDHERI
                     WYSDFLDFVGRERIFASLLTPASYGADDCRWDTYRISEFAEIMGFYGLSYWYPFQVTA
                     LGLGPIWMSANEDAKRKAAAGLEAGEVFAFGLSEQTHGADVYQTDMILTPSDGGWTAN
                     GEKYYIGNANVARMVSTFGKIAGTPESQEYVFFVADSQHERYDLIKNVVNSQNYVANY
                     ALRDYPVTEADILHRGAEAFHAALNTVNVCKYNLGWGAIGMCTHALYESVTHAANRHL
                     YGTVVTDFSHVRRLLTDAYVRLIAMKLVASRASDYMRSASAADRRYLLYSPLTKAKVT
                     SEGERVITALWDVIAAKGVEKDTFFETVAREIGLLPRLEGTVHINIGLLGKFMPNYLF
                     APDSTLPVIPRRDDAADDAFLFAQGPTGGLGKVRFHDWRASFDTCAHLPNVALLREQV
                     DVFAELLASATPDAAQQKDIDFAFGVGQLFANVPYAQLILEEARLSGVDEALIDEIFG
                     VLVRDFNTHAVELHGRSATTAEQARFAMRMVRRPVHDPARYDQIWKDHVLALNGAYQM
                     AP"
     CDS             276262..276951
                     /codon_start=1
                     /note="gene_244"
                     /transl_table=11
                     /translation="MPTVTWARVDPARRAAVVEAAEAEFGAHGFSRGSLNVIARRAGV
                     AKGSLFQYFADKRDLYAFIADIASQRVRSYMEDLIRELDPNRPFFEFLTDLLDGWVAY
                     FAEHPRERALHAAATLEVDTDARISVRSVLHRHYLDVLRPLVRDAHARGDLRADSDTG
                     ALMSLLLLIFPHLALAPYMRGLDPILGLDEPTPEQPALAVRRLVAVLAAAFDAQHPAT
                     NSAQTRSEEIT"
     CDS             276948..277892
                     /codon_start=1
                     /note="gene_245"
                     /transl_table=11
                     /translation="MTRTRSGSLAAGGLNWASLPLKLFAGGNAKFWDPADIDFTRDRA
                     DWEKLSDDERDYATRLCTQFIAGEEAVTEDIQPFMSAMRAEGRLADEMYLTQFAFEEA
                     KHTQVFRMWLDAVGISEDLHRYLDDLPAYRQIFYAELPECLNALSADPSPAAQVRASV
                     TYNHIVEGMLALTGYYAWHKICVERAILPGMQELVRRIGDDERRHMAWGTFTCRRHVA
                     ADDANWTVFETRMNELIPLALRLIEEGFALYGDQPPFDLSKDDFLQYSTDKGMRRFGT
                     ISNARGRPVAEIDVDYSPAQLEDTFADEDRRTLAAASA"
     CDS             complement(277968..279341)
                     /codon_start=1
                     /gene="gabD1"
                     /note="gene_246"
                     /transl_table=11
                     /translation="VPIATINPATGETVKTFTAATDDEVDAAIARAHRRFADYRQTSF
                     AQRARWANATADLLEAEADQAAAMMTLEMGKTLAAAKAEALKCAKGFRYYAENAEALL
                     ADEPADAAKVGASAAYGRYQPLGVILAVMPWNFPLWQAVRFAAPALMAGNVGLLKHAS
```

```
                     NVPQCALYLADVIARGGFPDGCFQTLLVSSGAVEAILRDPRVAAATLTGSEPAGQSVG
                     AIAGNEIKPTVLELGGSDPFIVMPSADLDAAVSTAVTGRVQNNGQSCIAAKRFIVHAD
                     IYDDFVDKFVARMAALRVGDPTDPDTDVGPLATEQGRNEVAKQVEDAAAAGAVIRCGG
                     KRLDRPGWFYPPTVITDISKDMALYTEEVFGPVASVFRAANIDEAVEIANATTFGLGS
                     NAWTRDETEQRRFIDDIVAGQVFINGMTVSYPELPFGGVKRSGYGRELSAHGIREFCN
                     IKTVWIA"
     CDS             complement(279529..280977)
                     /codon_start=1
                     /note="gene_247"
                     /transl_table=11
                     /translation="MGWFSAPEYWLGRLALERGTAIIYLIAFVAAAQQFRPLIGEHGM
                     LPVPRYLAGQSFWRTPSIFHFRYSDRVFAGVCWLGAVLSAAVVAGAASFVPLWATMLI
                     WLTLWVLYLSIVNVGQAWYSFGWESLLLETGFLMIFLGNERTAPPILTLLLARWLLFR
                     VEFGAGLIKMRGDSCWRSLTCLYYHHETQPMPGPLSWFFHHLPKPLHRIEVAGNHFAQ
                     LVVPFGLFTPQPAASIAAAIIVVTQLWLVASGNFSWLNWLTILLACSAIDTSSAAALL
                     PMPAQPALSAPPQWFAGLVVVFTAAVLLLSYWPARNLLSSHQRMNMSFNPFHLVNTYG
                     AFGSICRTRREVVIEGTDESPITEQTVWKAYEFKGKPGDPRRLPRQWAPYHLRLDWLM
                     WFAAISPGYALPWMTPFLNRLLRNDPATLKLLRHNPFPQSPPRYVRAQLYQYRFTTVA
                     ELRRDRAWWHRTLIGRYVPPMSLRKVASPPAD"
     CDS             complement(281012..285205)
                     /codon_start=1
                     /note="gene_248"
                     /transl_table=11
                     /translation="LSRKWLPVVGAVALALTFAQSPGQVSPDTKLDLTANPLRFLARA
                     TNLWNSDLPFGQAQNQAYGYLFPHGTFFVIGHLLGVPGWVTQRLWWAVLLTVGFWGLL
                     RVAEALGVGGPSSRVVGAVAFALSPRVLTTLGSISSETLPMMLAPWVLLPTILALRGT
                     SGRSVRALAAQAGLAVALMGAVNAIATLAGCLPAVIWWACHRPNRLWWRYTAWWLLAM
                     ALATLWWVMALTQLHGVSPPFLDFIESSGVTTQWSSLVEVLRGTDSWTPFVAPNATAG
                     APLVTGSAAILGTCLVAAAGLAGLTSPAMPARGRLVTMLLVGVVLLAVGHRGGLASPV
                     AHPVQAFLDAAGTPLRNVHKVGPVIRLPLVLGLAQLLSRVPLPGSAPRPAWLRAFAHP
                     ERDKRVAVAVVALTALVVSTSLAWTGRVAPPGTFGALPQYWQEAADWLRTHHAATPTP
                     GRVLVVPGAPFATQVWGTSHDEPLQVLGDGPWGVRDSIPLTPPQTIRALDSVQRLFAA
                     GRPSAGLADTLARQGISYVLVRNDLDPETSRSARPILLHRSIAGSPGLAKLAEFGAPV
                     GPDPLAGFVNDSGLRPRYPAIEIYRVSAPANPGAPYFAATDQLARVDGGPEVLLRLDE
                     RRRLQGQPPLGPVLMTADARAAGLPVPQVAVTDTPVARETDYGRVDHHSSAIRAPGDA
                     RHTYNRVPDYPVPGAEPVVGGWTGGRITVSSSSADATAMPDVAPASAPAAAVDGDPAT
                     AWVSNALQAAVGQWLQVDFDRPVTNAVVTLTPSATAVGAQVRRILIETVNGSTTLRFD
                     EAGKPLTAALPYGETPWVRFTAAATDDGSAGVQFGITDLAITQYDASGFAHPVQLRHT
                     VLVPGPPPGSAIAGWDLGSELLGRPGCAPGPDGVRCAASMALAPEEPANLSRTLTVPR
                     PVSVTPMVWVRPRQGPKLADLIAAPSTTRASGDSDLVDILGSAYAAADGDPATAWTAP
                     QRVVQHKTPPTLTLALPRPTVVTGLRLAASRSMLPAHPTVVAINLGDGPQVRQLQVGE
                     LTTLWLHPRVTDTVSVSLLDWDDVIDRNALGFDQLKPPGLAEVVVLGAGGAPIAPADA
                     ARNRARALTVDCDHGPVVAVAGRFVHTSIRTTVGALLDGEPVAALPCEREPIALPAGQ
                     QELLISPGAAFVVDGAQLSTPGAGLSSATVTSAETGAWGPTHREVRVPESATSRVLVV
                     PESINSGWVARTSTGARLTPIAVNGWQQAWVVPAGNPGTITLTFAPNSLYRASLAIGL
                     ALLPLLALLAFWRTGRRQLADRPTPPWRPGAWAAAGVLAAGAVIASIAGVMVMGTALG
                     VRYALRRRERLRDRVTVGLAAGGLILAGAALSRHPWRSVDGYAGNWASVQLLALISVS
                     VVAASVVATSESRGQDRMQ"
     CDS             complement(285261..285434)
                     /codon_start=1
                     /note="gene_249"
```

/transl_table=11
/translation="MNRIVAPAAASVVVGLLLGAAAIFGVTLMVQQDKKPPLPGGDPS
SSVLNRVEYGNRS"
CDS 285681..286715
/codon_start=1
/note="gene_250"
/transl_table=11
/translation="VPRSCAEPAGIPALLSPRDKLAQLLVVGVRDAADAQAVVTNYHV
GGILIGSDTDLTIFDGALAEIVAGGGPLPLAVSVDEEGGRLSRLRSLIGGTGPSAREL
AQTRTVQQVRDLARDRGRQMRKLGITIDFAPVVDVTDAPDDTVIGDRSFGSDPATVTA
YAGAYAQGLRDAGVLPVLKHFPGHGRGSGDSHNGGVTTPPLDDLVGDDLVPYRTLVTQ
APVGVMVGHLQVPGLTGSEPASLSKAAVNLLRTGTGYGAPPFDGPVFSDDLSGMAAIS
DRFGVSEAVLRTLQAGADIALWVTTKEVPAVLDRLEQALRAGELPMSAVDRSVVRVAT
MKGPNPGCGR"
CDS 286791..287405
/codon_start=1
/note="gene_251"
/transl_table=11
/translation="MAGGTKRLPRAVREQQMLDAAVQMFSVNGYHETSMDAIAAEAQI
SKPMLYLYYGSKEDLFGACLNREMSRFIDALRSSINFDQSPKDLLRNTIVSFLRYIDA
NRASWIVMYTQATSSQAFAHTVREGREQIVQLVAELVRAGTRGPLTDAEIEMMAVALV
GAGEAVATRLGIGDTDVDEAAEMMINLFWLGLKGAPVDRLETGH"
CDS 287467..287700
/codon_start=1
/note="gene_252"
/transl_table=11
/translation="MIRTQVQLPDELYRDAKRVAHEHEMTLAEVVRRGLEHMVRIYPR
RDAASDTWQPPTPRRLGPFRASEETWRELANEA"
CDS 287708..288145
/codon_start=1
/note="gene_253"
/transl_table=11
/translation="VLSIDTNILLYAQNRDCPEHDAAAAFLVECSGRADVAVCELVLM
ELYQLLRNPTVVTRPLEGPEAAEVCQTFRRNRRWALLENAPVMNEVWVLAATPRIARR
RLFDARLALTLRHHGVDEFATRNINGFTDFGFSRVWDPITSDG"
CDS complement(288175..289017)
/codon_start=1
/note="gene_254"
/transl_table=11
/translation="VTQPSGLKNLLRAAAGALPVVPRTDQLPNRTVTVEELPIDPANV
AAYAAVTGLRYGNQVPLTYPFALTFPSVMSLVTGFDFPFAAMGAIHTENHITQYRPIA
VTDAVGVRVRAENLREHRRGLLVDLVTNVSVGNDVAWHQVTTFLHQQRTSLSGEPKPP
PQKKPKLPPPAAVLRITPAKIRRYAAVGGDHNPIHTNPIAAKLFGFPTVIAHGMFTAA
AVLANIEARFPDAVRYSVRFAKPVLLPATAGLYVAEGDGGWDLTLRNMAKGYPHLTAT
VRGL"
CDS complement(289028..290392)
/codon_start=1
/gene="fabG"
/note="gene_255"
/transl_table=11
/translation="VAPKRSSDLFSQVVNSGPGSFLARQLGVPQPETLRRYRAGEPPL

```
                     TGSLLIGGAGRVVEPLRAALEKDYDLVGNNLGGRWADSFGGLVFDATGITEPAGLKGL
                     HEFFTPVLRNLGRCGRVVVVGGTPEAAASTNERIAQRALEGFTRSLGKELRRGATTAL
                     VYLSPDAKPAATGLESTMRFLLSAKSAYVDGQVFSVGADDSTPPADWEKPLDGKVAIV
                     TGAARGIGATIAEVFARDGAHVVAIDVESAAENLAETASKVGGTALWLDVTADDAVDK
                     ISEHLRDHHGGKADILVNNAGITRDKLLANMDDARWDAVLAVNLLAPLRLTEGLVGNG
                     SIGEGGRVIGLSSIAGIAGNRGQTNYATTKAGMIGITQALAPGLAAKGITINAVAPGF
                     IETQMTAAIPLATREVGRRLNSLLQGGQPVDVAEAIAYFASPASNAVTGNVIRVCGQA
                     MIGA"
     CDS             290534..291856
                     /codon_start=1
                     /note="gene_256"
                     /transl_table=11
                     /translation="VAPAAKNTSQTRRRVAVLGGNRIPFARSDGAYADASNQDMFTAA
                     LSGLVDRFGLAGERLDMVVGGAVLKHSRDFNLMRECVLGSELSPYTPAFDLQQACGTG
                     LQAAIAAADGIAAGRYEVAAAGGVDTTSDPPIGLGDDLRRTLLKLRRSRSNVQRLKLV
                     GTLPASLGVEIPANSEPRTGLSMGEHAAVTAKQMGIKRVDQDELAAASHRNMADAYDR
                     GFFDDLVSPFLGLYRDDNLRPNSSVEKLATLRPVFGVKAGDATMTAGNSTPLTDGASV
                     ALLASEQWAEAHSLAPLAYLVDAETAAVDYVNGNDGLLMAPTYAVPRLLARNGLSLQD
                     FDFYEIHEAFASVVLAHLAAWESEEYCKRRLGLDAALGSIDRSKLNVNGSSLAAGHPF
                     AATGGRILAQTAKQLAEKKAAKKGGGPLRGLISICAAGGQGVAAILEA"
     CDS             complement(292162..293997)
                     /codon_start=1
                     /note="gene_257"
                     /transl_table=11
                     /translation="VSHYRSNVRDQVFNLFEVLGVDKALGHGEFSDVDVDTARDMLAE
                     VSRLAEGPVAESFVEGDRNPPVFDPKTHSVMLPESFKKSVNAMLEAGWDKVGIDEALG
                     GMPMPKAVVWALHEHILGANPAVWMYAGGAGFAQILYHLGTEEQKKWAVLAAERGWGS
                     TMVLTEPDAGSDVGAARTKAVQQADGSWHIDGVKRFITSGDSGDLFENIFHLVLARPE
                     GAGPGTKGLSLYFVPKFLFDVETGEPGERNGVFVTNVEHKMGLKVSATCELAFGQHGV
                     PAKGWLVGEVHNGIAQMFEVIEQARMMVGTKAIATLSTGYLNALQYAKSRVQGADLTQ
                     MTDKTAPRVTITHHPDVRRSLMTQKAYAEGLRALYLYTATFQDAAVAEVVHGVDAKLA
                     VRVNDLMLPVVKGVGSEQAYAKLTESLQTLGGSGFLQDYPIEQYIRDAKIDSLYEGTT
                     AIQAQDFFFRKIVRDKGVALAHVSGQIQAFVDSGAGNGRLKTERALLAKALTNVQGMA
                     AALTGYLMAAQQDVTSLYKVGLGSVRFLMSVGDLIIGWLLQRQAAVAVAALDAGATGD
                     ERSFYEGKVAVASFFAKNFLPLLTSTREVIETLDNDIMELDEAAF"
     CDS             294369..294857
                     /codon_start=1
                     /note="gene_258"
                     /transl_table=11
                     /translation="VNSTNNLTPSSLREAFGHFPTGVVAIAAEVDGVRQGLAASTFVP
                     VSLEPPLVSFCVQNTSTTWPKLTGVPMLGISVLGEAHDAAVRTLAAKTGDRFAGLETV
                     SNDAGAVFIKGTSVWLESAIEQLVPAGDHTIVVLRVNQVKVDPNVAPIVFHRSVLRRL
                     GV"
     CDS             295212..296483
                     /codon_start=1
                     /note="gene_259"
                     /transl_table=11
                     /translation="MSKRILRLIIAQSGFYSAALQLGNVSIVLPFVVAELDAELWIAA
                     LIFPAFTAGGAIGNVVAPPAVAAVPRRHRLFIIVSCLAVLAGVNALCATIGKGSVAGI
                     LLVVNVTLIGVVSAISFVAFADLVAAMPSGTARARILLTEVGVGAALTAVVAATLSFV
                     PDQHPLSRNIHLLWTAAVAMAISAAICRALPHRIVPRVHAAPGLHKLVYVGWTAIRTN
```

GWYRRYLLVQVLFGSVVLGSSFHSIRVAAVPGDQPDEVVAVVLFVCVGLLGGIALWNR
VRERFGLVGLFVGSALVSIAAAVLSIAFDLAGAWPNVVAIGLVIALVSIANQSVFTAG
QLWIARDAEPGLRTSLISFGQLVINAGLVGMGLALGLIAQDHDAVWPVMIVLLLNLTA
AYSATRFAPAKSVDVRGLPQVSRTSRPKTGG"

CDS complement(296480..297226)
/codon_start=1
/note="gene_260"
/transl_table=11
/translation="MTYSASMRVWRGDESCGELREFTVEVNEGEVVLDVILRLQQTQT
PDLAVRWNCKAGKCGSCSAEINGKPRLMCMTRMSTFDEDEIVTVTPMRTFPVIRDLVT
DVSFNYQKAREIPSFAPPKELQPSEYRMAQVDVARSQEFRKCIECFLCQNVCHVVRDH
EENKDAFAGPRFLMRIAELEMHPLDTRDRRSQAQEEHGLGYCNITKCCTEVCPENIKI
TDNALIPMKERVADRKYDPVVWLGSKLFRR"

CDS complement(297227..299167)
/codon_start=1
/gene="sdhA"
/note="gene_261"
/transl_table=11
/translation="MVEVERHSYDVVVIGAGGAGLRAVIEARERGLKVAVVCKSLFGK
AHTVMAEGGCAAAMGNANPKDNWKTHFGDTMRGGKFLNNWRMAELHAKEAPDRVWELE
TYGALFDRTDDGRISQRNFGGHTYPRLAHVGDRTGLELIRTLQQKVVSLQQEDHAELG
DYEARIKVFAECTITELLKDQGAIAGAFGYWRESGRFIVFEAPAVVLATGGIGKSFKV
TSNSWEYTGDGHALALRAGATLINMEFVQFHPTGMVWPPSVKGILVTEGVRGDGGVLK
NSENSRFMFDYIPPVFKGQYAETEEEADQWLKDNDSARRTPDLLPRDEVARAINSEVK
AGRGTPHGGVYLDIASRLTPAEIKRRLPSMYHQFKELAEVDITTQAMEVGPTCHYVMG
GVEVDADTGAATVPGLFAAGECAGGMHGSNRLGGNSLSDLLVFGRRAGLGAADYVRAL
SSRPAVSAEAIDAAAQQALSPFEGPKDGSAPENPYALHMDLQYVMNDLVGIIRNADEI
SRALTLLAELWSRYHNVLVEGHRQYNPGWNLSIDLRNMLLVSECVARAALQRTESRGG
HTRDDHPGMDPNWRRILLVCRATETMGTGGSGSGDSNCHINVTQQLQTPMRPDLLELF
EISELEKYYTDEELAEHPGRRG"

CDS complement(299198..300019)
/codon_start=1
/note="gene_262"
/transl_table=11
/translation="MSAPTANRPAIGVFTPTRAQIPERTLRTDLWWLPPLLTNLGLLA
FICYATTRAFWGSQYWVEKYHYLTPFYSPCVSASCQPGASHLGVWFGHFPGWIPLGAM
VLPFLLGFRLTCYYYRKAYYRSVWQSPTSCAVPEPRAHYTGETRLPLIVQNTHRYFFY
IAVVVSLINTYDAIAAFHSPSGFGFGLGNVILTINVVLLWAYTISCHSCRHATGGRLK
HFSKHPVRYWIWTQVSKLNTRHMQFAWITLGTLALTDFYIMLVASGSITDLRFIG"

CDS complement(300099..300392)
/codon_start=1
/note="gene_263"
/transl_table=11
/translation="LSTTAELAELHDLVGGLRRCVTALKARFGDNPATRRIVIDADRI
LTDIELLDTDVSELDLERAAVPQPSEKIAIPDTEYDREFWRDVDDEGVGGHRY"

CDS complement(300537..301016)
/codon_start=1
/note="gene_264"
/transl_table=11
/translation="MNNLALWSRPVWDVEPWDRWLRDFFGPAATTDWYRPVAGDFTPA
AEIVKDGDDAVVRLELPGIDVDKDVNVELDPGQPVSRLVIRGEHRDEHTQDAGDKDGR

```
                     TLREIRYGSFRRSFRLPAHVTSEAIAASYDAGVLTVRVAGAYKAPAETQAQRIAITK"
CDS                  301230..303791
                     /codon_start=1
                     /note="gene_265"
                     /transl_table=11
                     /translation="MPTAGSSRAPAAAREIVVVGHGMVGHRLVEAVRARDADGSLRIT
                     VLAEEGDAAYDRVGLTSYTESWDRALLALPGNDYAGDQRVRLLLNTRVTQIDRATKSV
                     VTAAGQRHRYDTLVLATGSYAFVPPVPGHDLPACHVYRTFDDLDAIRAGAQRTLDGGH
                     TDGGVVIGGGLLGLEAANALRQFGLQTHVVEMMPRLMAQQIDEAGGALLARMIADLGI
                     AVHVGTGTESIESVKHSDGSVWARVRLSDGEVIDAGVVIFAAGIRPRDELARAAGLAI
                     GDRGGVLTDLSCRTSDPDIYAVGEVAAIDGRCYGLVGPGYTSAEVVADRLLDGSAEFP
                     EADLSTKLKLLGVDVASFGDAMGATENCLEVVINDAVKRTYAKLVLSDDATTLLGGVL
                     VGDASSYGVLRPMVGAELPGDPLALIAPAGSGAGAGALGVGALPDSAQICSCNNVTKG
                     ELKCAIADGCGDVPALKSCTAAGTSCGSCVPLLKQLLEAEGVEQSKALCEHFSQSRAE
                     LFEIITATEVRTFSGLLDRFGRGKGCDICKPVVASILASTGSDHILDGEQASLQDSND
                     HFLANIQKNGSYSVVPRVPGGDIKPEHLILIGQIAQDFGLYTKITGGQRIDLFGARVD
                     QLPLIWQRLVDGGMESGHAYGKAVRTVKSCVGSDWCRYGQQDSVQLAIDLELRYRGLR
                     APHKIKLGVSGCARECAEARGKDVGVIATEKGWNLYVAGNGGMTPKHAQLLASDLDKE
                     TLIRYIDRFLIYYIRTADRLQRTAPWVESLGLDHVREVVCEDSLGLAEEFEAAMQRHV
                     ANYKCEWKGVLEDPDKLSRFVSFVNAPDAVDSTVTFTERAGRKVPVSIGIPRVRS"
CDS                  303817..304173
                     /codon_start=1
                     /note="gene_266"
                     /transl_table=11
                     /translation="VTLLNDIQVWTTACAYDHLIPGRGVGVLLDDGSQVALFRLDDGS
                     VHAVGNVDPFSGAAVMSRGIVGDRGGRAMVQSPILKQAFALDDGSCLDDPRVSVPVYP
                     ARVTPEGRIQVARVAV"
CDS                  complement(304189..304713)
                     /codon_start=1
                     /note="gene_267"
                     /transl_table=11
                     /translation="VRILVTGGVRSGKSTHAEALLGDAADVVYVAPGRPAAGSDPDWD
                     ARVALHRARRPPTWLTVETADVATALSEARSPVLVDCLGTWLTAIMDGEALWSAATAD
                     VYAVLEARLDGLCAALTGLPTAIVVTNEVGLGVVPSHSSGVLFRDLLGTINRRVAAVC
                     DEVHLVIAGRVLKL"
CDS                  complement(304738..306222)
                     /codon_start=1
                     /note="gene_268"
                     /transl_table=11
                     /translation="MSGLLVAGTTSDAGKSAVTAGLCRALARRGVRVAPFKAQNMSNN
                     SMVCRGPDGTGVEIGRAQWVQALAARTTPEAAMNPVLLKPASDHRSHVVLMGKPWGEV
                     ASSSWCAGRRALAEAACRAFDALAARYDVVVAEGAGSPAEINLRAGDYVNMGLARHAG
                     LPTIVVGDIDRGGVFAAFLGTVALLAAEDQALVAGFVVNKFRGDSDLLAPGLRDLERV
                     TGRRVYGTLPWHPDLWLDSEDALDLQGRRAAGTGARRVAVVRLPRISNFTDVDALGLE
                     PDLDVVFASDPRALDDADLIVLPGTRATIADLAWLRARDLDRALLVHVAAGKPLLGIC
                     GGFQMLGRVIRDPYGIEGPGGQVTEVEGLGLLDVETAFSPHKVLRLPRGEGLGVPASG
                     YEIHHGRITRGDTAEEFLGGARDGPVFGTMWHGSLEGDALREAFLRETLGLAPSGSCF
                     LAARERRLDLLGDLVERHLDVDALLNLARHGCPPTLPFLAPGAP"
CDS                  complement(306241..307911)
                     /codon_start=1
                     /note="gene_269"
```

```
                     /transl_table=11
                     /translation="MTAPIWMASPPEVHSALLSSGPGPGPLLVSAEGWHSLSIAYAET
                     ADELAALLAAVQAGTWDGPTAAVYVAAHTPYLAWLVQASANSAAMATRQETAATAYGT
                     ALAAMPTLAELGANHALHGVLMATNFFGINTIPIALNESDYARMWIQAATTMASYQAV
                     STAAVAAAPQTTPAPQIVKANAPTAASDEPNQVQEWLQWLQKIGYTDFYNNVIQPFIN
                     WLTNLPFLQAMFSGFDPWLPSLGNPLTFLSPANIAFALGYPMDIGSYVAFLSQTFAFI
                     GADLAAAFASGNPATIAFTLMFTTVEAIGTIITDTIALVKTLLEQTLALLPAALPLLA
                     APLAPLTLAPASAAGGFAGLSGLAGLVGIPPSAPPVIPPVAAIAPSIPTPTPTPAPAP
                     APTAVTAPTPPPGPPPPPVTAPPPVTGAGIQSFGYLVGDLNSAAQARKAVGTGVRKKT
                     PEPDSAEAPAAAAAPEEQVQPQRRRRPKIKQLGRGYEYLDLDPETGHDPTGSPQGAGT
                     LGFAGTTHKASPGQVAGLITLPNDAFGGSPRTPMMPGTWDTDSATRVE"
     CDS             complement(308658..309062)
                     /codon_start=1
                     /note="gene_270"
                     /transl_table=11
                     /translation="MLRLPFGTPDFIEKIVTGSVNQVGRRTLYVLITTWDAAGGGPFA
                     ASAIATTGLAKTAEIVQSMFIGPVFNPLLKMLGADKIAIRASLCAAQLVGLGIMRYGV
                     RSEPLHSMSVEMLVDAIGPTMQRYLVGDIGRG"
     CDS             complement(309138..309881)
                     /codon_start=1
                     /note="gene_271"
                     /transl_table=11
                     /translation="MNLILTAHGTRRPSGVAMIADIAAQVSALVDRTVQVAFVDVLGP
                     SPSEVLSALSCRPAIVVPAFLSRGYHVRTDLPAHVAASAHPHVTVTPALGPCREIAQI
                     VTQQLVESGWRPGDSVILAAAGASDRRARADLHTTRTLVSELTGSWVDMGFAGTGGPD
                     VRTAVQRARDRAEANRGARRVVVASFLLAEGLFQERLRASGADVVTRPLGTHPGLAQL
                     VANRFRSAVARQQRLHRWHGTPTPVTLDL"
     CDS             complement(309878..311023)
                     /codon_start=1
                     /note="gene_272"
                     /transl_table=11
                     /translation="MAQAHSAPLTGYRIAVTSARRAEELCALLRRQGAEVCSAPAIKM
                     IALPDDDELQNNTEALIADPPDILVAHTGIGFRGWLAAAEGWGLANELLESLSSARII
                     SRGPKATGALRAAGLREEWSPDSESSHEVLEYLLESGVSRTRIAVQLHGAADSWDPFP
                     EFLGGLRFAGAQVVPIRVYRWKPAPLGGVFDHLVTGIARRQFDAVTFTSAPAAAAVLE
                     RSRELDIEDQLLAALRTDVHAMCVGPVTSRPLIRKGVPTSAPERMRLGALARHIAEEL
                     PLLGSCTFKAAGHVIEIRGTSVLVDDSVKPLSPSGMAILRALVHRPGGVVSRGDLLRV
                     LPGDGSDTHAVDTAVLRLRTALGDKNIVATVVKRGYRLAVDSRHDDV"
     CDS             complement(311123..312532)
                     /codon_start=1
                     /gene="narK-1"
                     /note="gene_273"
                     /transl_table=11
                     /translation="MGRSHQISDWDPEDSVAWEAGNKFIARRNLIWSVAAEHVGFSVW
                     SLWSVMVLFMPTSVYGFSAGDKFLLGATATLVGACLRFPYTFATAKFGGRNWTIFSAL
                     VLLIPTVGSILLLANPGLPLWPYLVCGALAGLGGGNFAASMTNINAFFPQRLKGAALA
                     LNAGGGNLGVPMVQLVGLLVIATAGDREPYWVCAIYLVLLAVAGLGAALYMDNLTEYR
                     IELNTMRAVVSEPHTWVISLLYIGTFGSFIGFSFAFGQVLQINFIASGQSTAQASLHA
                     AQIAFLGPLLGSLSRIYGGKLADRIGGGRVTLAAFCAMLLATGILISASTFGDHLAGP
                     MPTATMVGYVIGFTALFILSGIGNGSVYKMIPSIFEARSHSLQISEAERRQWSRSMSG
                     ALIGLAGAVGALGGVGVNLALRESYLTSGTATSAFWAFGVFYLVASVLTWAIYVRRGL
```

```
                     KSAGELVPATTAPAGLAYV"
     CDS             complement(312673..313218)
                     /codon_start=1
                     /note="gene_274"
                     /transl_table=11
                     /translation="VHTQVHTARLVHTADLDSETRQDIRQMVTGAFAGDFTETDWEHT
                     LGGMHALIWHHGAIIAHAAVIQRRLIYRGNALRCGYVEGVAVRADWRGQRLVSALLDA
                     VEQVMRGAYQLGALSSSARARRLYASRGWLPWHGPTSVLAPTGPVRTPDDDGTVFVLP
                     IDISLDTSAELMCDWRAGDVW"
     CDS             complement(313228..314130)
                     /codon_start=1
                     /note="gene_275"
                     /transl_table=11
                     /translation="MTTLEILRSGPLALVEDLGRAGLAHLGVGRSGAADRRSHTLANR
                     LVANPDDWATVEVTFGGFSARVRGGDVDIAVTGADTDPTVNGIMVGTNSIHHVRDGQV
                     ISLGTPRAGLRTYLAVRGGVCVEPVLGSRSYDVMSAIGPSPLRAGDVLPVGEHTDDYP
                     ELDQAPVAAIEEHLVELRVVPGPRDDWLVDPDALVHTIWMASNRSDRVGMRLQGRPLQ
                     HRWPDRQLPGEGVTRGAIQVPPNGLPVILGPDHPITGSYPVVGVITDEDIDKVAQIRP
                     GQYVRLHWARPRSRLPGQGVTQAW"
     CDS             complement(314147..314791)
                     /codon_start=1
                     /note="gene_276"
                     /transl_table=11
                     /translation="MDVFMDAALACTVLDYGDHALMLQCDSTADAMAWTDALRAAALP
                     GVVDIVAASRTVLVKLDAPRYQGVTRQRLRRLRVTPEAVAAADHRCDLVIDVVYDGPD
                     LAEVARCTGLTTAAVINAHTATGWRAGFSGSAPGFAYLIDGDPSLRVPRRPERRTSMP
                     PGSVALADGFSAIYPSQAPSDWQIIGHTDAVLWDVDRPQPALLTPGMWVQFRAA"
     CDS             complement(314875..315837)
                     /codon_start=1
                     /note="gene_277"
                     /transl_table=11
                     /translation="LQVAEAAAATGLFAGCSSPKPPPGTPGGAAVTITHLFGQTVIKE
                     PPKRVVSAGYTEQDDLLAVDVVPIAVTDWFGDQPFAVWPWAAPKLGGARPAVLNLDNG
                     IQIDRIAALKPDLIVAINAGVDADTYQQLSAIAPTVAQSGGDAFFEPWKDQARSIGQA
                     VFAADRMRSLIEAVDQKFAAVAQRHPRWRGKKALLLQGRLWQGNVVATLAGWRTDFLN
                     DMGLVIADSIKPFAVDQRGVIPRDHIKAVLDAADVLIWMTESPEDEKALLADPEIAAS
                     QATAQRRHIFTSKEQAGAIAFSSVLSCPVVAEQLPPQISQILGA"
     CDS             complement(315889..319518)
                     /codon_start=1
                     /note="gene_278"
                     /transl_table=11
                     /translation="VVGAGWHFWVDRGGTFTDVVARRPDGRLLTHKLLSDNPARYRDA
                     AVAGIRALLANGEAGTRVDAVRMGTTVATNALLERTGERTLLVITRGFGDALRIAYQN
                     RPRIFDRRIVLPEMLYERVVEVDERVTADGRVLRAPDLEALGEKMRQAHADGIRAVAV
                     VCLHSYLYPGHEREIGTLAQRIGFAQISLSSEVSPLMKLVPRGDTTVVDAYLSPVLRR
                     YINQVADQMRGVRLMFMQSNGGLAQAGHFRGKDAILSGPAGGIVGMVRMSALAGFDHV
                     IGFDMGGTSTDVSHYAGEYERVFTTQVAGVRLRAPMLDIHTVAAGGGSILHFDGSRYR
                     VGPDSAGADPGPACYRGGGPLCVTDANVMLGRIQPTHFPSVFGPSGDQPLDAGTVRRG
                     FTDLAADIAARTGDDRSPEQVAEGYLRIAVANMANAVKKISVQKGHDVTRYALTTFGG
                     AGGQHACAVADALGIRTVLIPPMAGVLSALGIGLADTTAMREQSVEIPLGPAAPQRLA
                     SVAESLERAARAELLDEGVPGERIRVVRRVHLRYEGTDTAIPVQLAEIETMATAFESS
```

HRALYTFLLDRPLIAEAISVEATGLTDQPDLSQLGDQANDTTGSSETVRIYSNGLWRD
APLRRREAMRPGDVLTGPAIIAEANATTVVDDGWQATMTETGHLLAQRVVTPPRPDAA
TRAGFEAGFEADPVLLEIFNNLFMSIAEQMGFRLEATAQSVNIRERLDFSCALFDPDG
NLVANAPHIPVHLGSMGTTVKEVIRRRLSGMKPGDVYAVNDPYHGGTHLPDITVITPV
FNTGGEDVLFFVASRGHHAEIGGITPGSMPADSREIHEEGVLFDNWLLAENGRFREAE
TRRLLTEAPFGSRNPDTNLADLRAQIAANQKGVDEVGKMIDHFGRDVVAAYMRHVQDN
AEEAVRRVIDRLDNGAYRYRMDSGATIAVRITVDRAARSATIDFTGTSAQLDTNFNAP
TSVVNAAVLYVFRTLVADDIPLNDGCLRPLRIVVPEGSMLAPTHPAAVVAGNVETSQA
ITGALFAALGVQAEGSGTMNNVTFGNERHQYYETVGSGSGAGDGYHGASVVQTHMTNS
RLTDPEVLEWRYPVLLREFAVRQGSGGAGRWRGGDGAVRRLEFTEPMTVSTLSGHRRV
RPYGMAGGSPGELGRNRVERADGSTVELAGCGSTHVEPGDTLVIETPGGGGYGPASTS
ARRRR"

CDS 319695..321086
/codon_start=1
/note="gene_279"
/transl_table=11
/translation="MALTTAPAIDYALPRQQDEGDHWIDDWRPEDPVFWETIGRPIAR
RNLIFSIFAEHVGFSVWMLWSIVVVQMTAAAPGHPAASGWALSASQALCLVAVPSGVG
AFLRLPYTFAIPIFGGRNWTTVSAALLVIPCLLLAWAVSHPSLPFAVLVVIAATAGFG
GGNFASSMANISFFYPEKDKGWALGLNAAGGNIGVAVVQKIIPPIVVAGSGVALSRAG
LFFVPLAVAAAVCAFLFMNNLTEAKADVKPVWQSLRHADTWIMSLLYIGTFGSFIGYS
AAFPTLLKTVFGRGDIALGWAFLGAGIGSLVRPLGGKLADRIGGARITAASFVMLAAG
AAAALWSVQSVNLPVFFVSFMFLFVATGIGNGSSYRMISRIFQVKGEVAGGDPETMVN
MRRQAAGALGIISSIGAFGGFVVPLAYAWSKVHFGNIEPALHFYVAFFLALLVVTWYC
YLRRTTPMGQVGV"

CDS complement(321128..321385)
/codon_start=1
/note="gene_280"
/transl_table=11
/translation="MTSSLVPISEARAHLSRLVRESADDDVVLMNHGRPAAILISAER
YESLMEELEDLRDRLSVHEREHVTMPLDKLGAELGVDIGRV"

CDS complement(321702..322895)
/codon_start=1
/note="gene_281"
/transl_table=11
/translation="MSRMAAPVSLDVHGRQVIVTHPGRVVFPAHNDRKGYTKFDLVRY
YLAVAEGAMRGVAGRPMILKRFVKGISAEAVFQKRAPANRPDWVDVAELHYASGRSAA
EAVIHDAAGLAWVINLGCVDLNPHPVLAGDLDHPDELRVDLDPMPGVAWQRVVEVALV
VREVLEDYGLTAWPKTSGSRGFHVYARIAPCWSFPQVRLAAQTVAREVERRLPDAATS
RWWKEEREGVFVDFNQNAKDRTVASAYSVRATPDARVSTPLHWEEVPGCDPAVFTMAT
VPSRLADIGDPWAGMDDAVGRLDRLLMLAEELGPPQKAQSAKPLIEIARAKTRAEAMA
ALDIWRDRYPGAAALLRPADVLVDGMRGPSSIWYRIRINLQHVPADQRPPQEELIADY
SPWPR"

CDS 322931..324613
/codon_start=1
/note="gene_282"
/transl_table=11
/translation="MPNLTDLPGQAVSKLQKSIGQYVARGTAELHYLRKIIESGAIGL
EPPLNYAALAADIRKWGEVGMLPSHNARRAPNRAAVIDEEGTLTFSELDEAAHAVANG
LLAKGVRAGEGVAILARNHRWFVIANYGAARVGARIILLNSEFSGPQIKEVSDREGAK
VIIYDDEYTKAVSLAQPPLGKLRALGVNPDDDKPSGSSDETLAELIAHSSTAPAPKAS

RRASIIILTSGTTGTPKGANRNTPPTLAPIGGILSHVPFKAGEVTLLPSPMFHALGYM
HAALAMFLGSTLVLRRRFKPALVLEDIEKHKATSMVVVPVMLSRILDQLEKTEPKPDL
SSLKIVFVSGSQLGAELATRALGDLGPVIYNMYGSTEVAFATIAGPKDLQFNPSTVGP
VVKGVTVKILDENGNEVPQGAVGRIFVGNAFPFEGYTGGGGKQIIDGLLSSGDVGYFD
ERGLLYVSGRDDEMIVSGGENVFPAEVEDLISGHPDVVEAAAIGVDDKEFGARLRAFV
VKKPGADLDEDTIKQYVRDHLARYKVPREVIFLDELPRNPTGKVLKRELRKL"

CDS complement(324630..326825)
/codon_start=1
/note="gene_283"
/transl_table=11
/translation="MSIAITPEHYELADSVRSLVARVAPSEVLHAALESPVENPPPYW
QAAAEQGLQGVHLAESVGGQGFGILELAVVLAEFGYGAVPGPFVPSAIASALIAAHDP
QAKVLAELATGAAIAAYALDSGLTATRHGDVLVIRGEVRAVPAAAQASVLVLPVAIES
RDEWVVLRNDQLEIEAVKSLDPLRPIAHVRANAVDVSDDALLSNLTMTTAHALMSTLL
SAEAVGVARWATDTASAYAKIREQFGRPIGQFQAIKHKCAEMIADTERATAAVWDAAR
ALDDAGESSSDVEFAAAVAATLAPATAQRCTQDCIQVHGGIGFTWEHDTNVYYRRALM
LAACFGRGSEYPQRVVDTATTAGMRPVDIDLDPSTEKLRAQIRAEVAALKAMPREPRT
VAIAEGGWVLPYLPKPWGRAASPVEQIIIAQEFTAGRVKRPQIAIATWIVPSIVAFGT
DNQKQRLLPPTFRGDIFWCQLFSEPGAGSDLASLATKATRVDGGWRITGQKIWTTGAQ
YSQWGALLARTDPSAPKHNGITYFLLDMKSEGVQVKPLRELTGKEFFNTVYLDDVFVP
DELVLGEVNRGWEVSRNTLTAERVSIGGSDSTFLPTLGEFVDFVRDYRFEGQFDQVAR
HRAGQLIAEGHATKLLNLRSTLLTLAGGDPMAPAAISKLLSMRTGQGYAEFAVSSFGT
DAVIGDTERLPGKWGEYLLASRATTIYGGTSEVQLNIIAERLLGLPRDP"

CDS complement(326939..328072)
/codon_start=1
/note="gene_284"
/transl_table=11
/translation="LTGRAATPGVIREFVGLPSRTAGRAAAGGHPCQGLYHHSVGRKP
KVALIAAHYQIDFSEHYLAEYMAIRGIGFLGWNTRFRGFESSFLLDHALVDIGVGVRW
LREVQGVETVVLLGNSGGGSLMAAYQSQAVDPNVTPLDGMRPAAGVTELPAADAYVAA
AAHPGRPDVLTAWMDAAVIDENDPVATDPELDLFDERNGPPYSPEFISRYRSAQVKRN
HTITDWAESELKRVRAAGFSDRPFSVMRTWADPRMVDPSIEPTKRRPNQCYAGTPVKA
NRSAHGIAAACTLRGWLGMWSLRVAQTRAAPHLARITCPALVLNAEADTGIFPSDAQQ
IYDGLASSDKTQVSIDTDHYFTTPGARSEQADTIAKWIAKRWR"

CDS complement(328069..328689)
/codon_start=1
/note="gene_285"
/transl_table=11
/translation="VPDFPTQRGRRTQAAIDAAARTVVVRNGILATTVADITAEAGRS
AASFYNYYDSKEAMVRQWALRFRDDANQRALSVIRHGLSDRERAYEAAAAHWYTYRNR
LAEAISVSQLAMVSDDFAQYWSEICQIPISFITETVKRAQAHGYCVGDDPQLMAEAIV
AMFNQFCYLQLSGKRSRRGQPDDQACIQTLANIYYRAIYSKEDSSN"

CDS 328786..329367
/codon_start=1
/note="gene_286"
/transl_table=11
/translation="MIKPHNTNTEFELGGINHVALVCSDMARTVDFYSNILGMPLIKA
LDLPGGQGQHFFFDAGNGDCVAFFWFADAPDRVPGLSSPVAIPGIGDITSAVSTMNHL
AFHVPAERFDAYRQRLKDKGVRVGPVLNHDDSETQVSAVVHPGVYVRSFYFQDPDGIT
LEFACWTKEFTTSDAQAVPKTAADRRPPVAADR"

CDS complement(329297..330022)

/codon_start=1
/note="gene_287"
/transl_table=11
/translation="MTRSDRPYRGVEAAERLATRRRQLLSAGLDLLGSDQHDIAELTI
RTICRRAGLSVRYFYESFTDKDEFVGRVFDWVVAELVATTQAAVTAVPAREQTRAGMA
NIVRTITADARVGRLLFSTQLANAVITRKRAESSALFAMLSGQHAVDTLHAPANDHVK
AVAHFAVGGVGQTISAWLAGDVRLDPDQLVDQLAALLDELTDPNLSRPRVAATAAKSG
ANDPQPPEVAGQPPSSARPARRS"
CDS 330112..331032
/codon_start=1
/note="gene_288"
/transl_table=11
/translation="MAISLVAHQPIPHVERPMADPPRLQLARRRRSAAGPGGNEDSLM
GVALLAGPANVIMELAMPGVGYGVLESRVESGRLDRHPIKRARTTFTYVAVAVAGSDD
QKAAFRRAVNKVHAQVYSTPESPVSYHAFDPELQLWVAACLYKGGVDVYRTFVGEMDD
EEADHHYRAGMAMGTTLQVPPQMWPPDRAAFDRYWRQSLDRVHIDDVVRDYLYPIVAL
RIRGIALPGPLRRLSEGIALLITTGFLPQRFRDEMRLPWDATKQRRFDALMAVLRTVN
RLMPRFVREFPFNLMLWDLDRRMRRGRPLV"
CDS complement(331072..331500)
/codon_start=1
/note="gene_289"
/transl_table=11
/translation="MFLIDVNVLLAAHRGDHPNHRTVRPWFDRLLAADDPFTVPNLVW
ASFLRLTTNRRIFEIPSPRADAFAFVEAVNAQPHHLPTSPGPRHLVLLRKLCDEADAS
GDLIPDAVLGAIAVEHHCAVVSLDRDFARFASVRHIRPPI"
CDS complement(331524..331781)
/codon_start=1
/note="gene_290"
/transl_table=11
/translation="MRTTVSISDELLATAKRRARERGQSLGAVIEDALRRELAAARTG
GARPTVPVFDAGTGPRPGIDLTSNRVLSEVLDEGLELNSRK"
CDS complement(331866..334616)
/codon_start=1
/note="gene_291"
/transl_table=11
/translation="MSFVIAAPEVIAAAATDLASLESSIAAANAAAAANTTALLAAGA
DEVSTAVAALFGAHGQAYQALSAQAQAFHAQFTQALTSGGGAYAAAEAAATSPLLAPI
NEFFLANTGRPLIGNGANGAPGTGADGAPGGWLIGNGGAGGSGAANNAVGGTGGTGGA
GGASGLLGSGGAGGAGGVATNTGGIGGAGGTGGNAVLFGAGGAGGASTNTTGGAGGAG
GDGGNAGLLFGAAGVGGAGGFALATTASGGAGGAGGAGGMFTDGGVGGVGGKGGFGGA
GGAGGNGGLFGAGGTGGAGGTIGAGVAGMGGAGGAGGAGGLFGAGGTGGSGGGGATTG
GDGGAGGNAGTLSLGAAGGAGGAGGEGLASAGGDGGAGGDGGNAGMFFGSGGAGGAGG
FGRTTGGIGGTGGNAGLLNGSGGAGGAGGAAITGPGGTGGAGGIPGLIGNGGNGGDGG
ASVTGTGGNGGAGGNGVQIGNGGNGGSGGTGAAAGKAGLGGLGGQLIGLDGSNAPVST
SVHTLQQAALNVVNEPFQTLTGRPLIGNGANGTPGTGAAGGAGGWLFGNGGNGGHGAT
NTAATATGGAGGAGGILFGTGGNGGTGGIATGAGGIGGAGGAGGVSLLIGSGGTGGNG
GNSIGVAGIGGAGGRGGDAGLLFGAAGTGGHGAAGGVPAGVGGAGGNGGLFANGGAGG
AGGFNAAGGNGGNGGLFGTGGTGGAGTNFGAGGNGGNGGLFGAGGTGGAAGSGGSGIT
TGGGGHGGNAGLLSLGASGGAGGSGGASSLAGGAGGTGGNGALLFGFGGAGGAGGHGG
AALTSIQQGGAGGAGGNGGLLFGSAGAGGAGGSGANALGAGTGGTGGDGGHAGVFGNG
GDGGAGGFGAGTGGSGGVGGNAVLIGNGGNGGNAGKAGATPGAGGTGGLLLGENGLNG

LP"
CDS complement(334880..337579)
/codon_start=1
/note="gene_292"
/transl_table=11
/translation="MSFVIAAPEVIAAAATDLASLGSSISAANAAAAANTTALMAAGA
DEVSTAIAALFGAHGQAYQALSAQAQAFHAQFVQALTSGGGAYAAAEAAAVSPLLDPI
NEFFLANTGRPLIGNGANGAPGTGANGGDGGWLIGNGGAGGSGAAGVNGGAGGNGGAG
GLIGNGGAGGAGGVASSGIGGSGGAGGNAMLFGAGGAGGAGGGVVALTGGAGGAGGAG
GNAGLLFGAAGVGGAGGFTNGSALGGAGGAGGAGGLFATGGVGGSGGAGSSGGAGGAG
GAGGLFGAGGTGGHGGFADSSFGGVGGAGGAGGLFGAGGEGGSGGHSLVAGGDGGAGG
NAGMLALGAAGGAGGIGGDGGTLTAGGIGGAGGAGGNAGLLFGSGGSGGAGGFGFADG
GQGGPGGNAGTVFGSGGAGGNGGVGQGFAGGIGGAGGTPGLIGNGGNGGNGGASAVTG
GNGGIGGTGVLIGNGGNGGSGGIGAGKAGVGGVSGLLLGLDGFNAPASTSPLHTLQQN
VLNVVNEPFQTLTGRPLIGNGANGTPGTGADGGAGGWLFGNGGNGGSGATGTNGGDGG
DGGAGGIFFGTGGTGGAGGVGTTGTGGDGGAGGAAFLVGSGGNGGSGGAGLTAGGDGG
DGGNAGSFFGAAGTGGAGASTKAGGTGGTGGNGGLFANGGAGGSGGLGGDAGTGGAGG
NGGLFGAGGTGGAGGSLGPGAGGAGGNGGLFGAGGTGGSGGHGTPAAVPGGAGGAGGN
AGLFSLGASGGAGGSGGSSLTDSGGIGGVGGAGGLLFGYGGAGGAGGYSNIGAGGAGG
AGGNAGMLSGSGGSGGTGGASGAAKGGVGGNGGTAGVFGNGGDGGAGGFGAGTGGNGG
VGGNAVLIGNGGNGGNGGKAGGTPGAGGTSGLLIGENGLNGLP"
CDS complement(337829..340333)
/codon_start=1
/note="gene_293"
/transl_table=11
/translation="MSFVIAAPEVIAAAATDLASLESSIAAANAAAAANTTALLAAGA
DEVSTAVAALFGAHGQAYQALSAQAQAFHAQFVQALTSGGGAYAAAEAAATSPLLAPI
NEFFLANTGRPLIGNGANGAPGTGADGAPGGWLIGNGGAGGSGAAGVNGGAGGNGGAG
GLIGNGGAGGAGGRASTGTGGAGGAGGAAGMLFGAAGVGGPGGFAAAFGATGGAGGAG
GNGGLFADGGVGGAGGATDAGTGGAGGSGGNGGLFGAGGTGGPGGFGIFGGGAGGDGG
SGGLFGAGGTGGSGGTSIINVGGNGGAGGDAGMLSLGAAGGAGGSGGSSPDGGGGAGG
IGGDGGTLFGSGGVGGLGFDAGGAGGAGGKAGLLSGAGGAGGAGGGSFAGAGGTGGAG
GAPGLVGNAGNGGNGGASANGAGAAGGAGGSGVLIGNGGNGGSGGTGAPAGTAGAGGL
GGQLLGRDGFNAPASTPLHTLQQQILNAINEPTQALTGRPLIGNGANGTPGTGADGGA
GGWLFGNGGNGGHGATGADGGDGGSGGAGGILSGIGGTGGSGGIGTTGQGGTGGTGGA
ALLIGSGGTGGSGGFGKDTGGAGGRGGDAGLFLGAAGTGGQAALSQNFIGAGGTAGAG
GTGGLFANGGAGGAGGFGANGGTGGNGLLFGAGGTGGAGTLGADGGAGGHGGLFGAGG
TGGAGGSSGGTFGGNGGSGGNAGLLALGASGGAGGSGGSALNVGGTGGVGGNGGSGGS
LFGFGGAGGTGGSSGIGSSGGTGGDGGTAGVFGNGGDGGAGGFGTGAGGTGGTGGNAV
LIGNGGNGGNGGKAGGTPGAGGTSGLIIGENGLNGL"
CDS complement(340599..340763)
/codon_start=1
/gene="O976_22825"
/note="gene_294"
/transl_table=11
/translation="MSSSATAAYSALSDDHTPAADNTEPGPGPLLSNAECTSGGEAIH
SVIAVNTTED"
CDS 340714..342234
/codon_start=1
/note="gene_295"
/transl_table=11

```
                     /translation="VWSSLSAEYAAVADELIGLLGAVQTGAWQGPSAAAYVAAHAPYL
                     AWLMRASETSAEAAARHETVAAAYTTAVAAMPTLVELAANHTLHGVLVATNFFGINTI
                     PIALNEADYARMWTQAASTMATYQAVAEAAVASAPQTTPAPPILAAEAADDDHDHDHD
                     HGGEPTPLDYLVAEILRIISGGRLIWDPAEGTMNGIPFEDYTDAAQPIWWVVRAIEFS
                     KDFETFVQELFVNPVEAFQFYFELLLFDYPTHIVQIVEALSQSPQLLAVALGSVISNL
                     GAVTGFAGLSGLAGMQPAAIPALAPVAAAPPTLPAVAMAPTMAAPGAAVASAAAPASA
                     PAASTVASATPAPPPAPGAAGFGYPYAIAPPGIGFGSGMSASASAQRKAPQPDSAAAA
                     AAAAAVRDQARARRRRRVTRRGYGDEFMDMNIDVDPDWGPPPGEDPVTSTVASDRGAG
                     HLGFAGTARREAVADAAGMTTLAGDDFGDGPTTPMVPGSWDPDRDAPGSAEPGDRG"
     CDS             342258..343166
                     /codon_start=1
                     /note="gene_296"
                     /transl_table=11
                     /translation="VRTEGDSWDITTSVGSTALFVATARALEAQKSDPLVVDPYAEAF
                     CRAVGGSWADVLDGKLPDHKLKSTDFGEHFVNFQGARTKYFDEYFRRAAAAGARQVVI
                     LAAGLDSRAYRLPWPDGTTVFELDRPQVLDFKREVLASHGAQPRALRREIAVDLRDDW
                     PQALRDSGFDAAAPSAWIAEGLLIYLPATAQERLFTGIDALAGRRSHVAVEDGAPMGP
                     DEYAAKVEEERAAIAEGAEEHPFFQLVYNERCAPAAEWFGERGWTAVATLLNDYLEAV
                     GRPVPGPESEAGPMFARNTLVSAARV"
     CDS             343423..345285
                     /codon_start=1
                     /note="gene_297"
                     /transl_table=11
                     /translation="VERDDIGMVAASPVASRVNGKVDADVVGRFATCCRALGIAVYQR
                     KRPPDLAAARSGFAALTRVAHDQCDAWTGLAAAGDQSIGVLEAASRTATTAGVLQRQV
                     ELADNALGFLYDTGLYLRFRATGPDDFHLAYAAALASTGGPEEFAKANHVVSGITERR
                     AGWRAARWLAVVINYRAERWSDVVKLLTPMVNDPDLDEAFSHAAKITLGTALARLGMF
                     APALSYLEEPDGPVAVAAVDGALAKALVLRAHVDEESASEVLQDLYAAHPENEQVEQA
                     LSDTSFGIVTTTAGRIEARTDPWDPATEPGAEDFVDPAAHERKAALLHEAELQLAEFI
                     GLDEVKRQVSRLKSSVAMELVRKQRGLTVAQRTHHLVFAGPPGTGKTTIARVVAKIYC
                     GLGLLKRENIREVHRADLIGQHIGETEAKTNAIIDSALDGVLFLDEAYALVATGAKND
                     FGLVAIDTLLARMENDRDRLVVIIAGYRADLDKFLDTNEGLRSRFTRNIDFPSYTSHE
                     LVEIAHKMAEQRDSVFEQSALHDLEALFAKLAAESTPDTNGISRRSLDIAGNGRFVRN
                     IVERSEEEREFRLDHSEHAGSGEFSDEELMTITADDVGRSVEPLLRGLGLSVRA"
     CDS             345282..346898
                     /codon_start=1
                     /note="gene_298"
                     /transl_table=11
                     /translation="MTNQQHDHDFDHDRRSFASRTPVNNNPDKVVYRRGFVTRHQVTG
                     WRFVMRRIAAGIALHDTRMLVDPLRTQSRAVLMGVLIVITGLIGSFVFSLIRPNGQAG
                     SNAVLADRSTAALYVRVGEQLHPVLNLTSARLIVGRPVSPTTVKSTELDQFPRGNLIG
                     IPGAPERMVQNTSTDANWTVCDGLNAPSRGGADGVGVTVIAGPLEDTGARAAALGPGQ
                     AVLVDSGAGTWLLWDGKRSPIDLADHAVTSGLGLGADVPAPRIIASGLFNAIPEAPPL
                     TAPIIPDAGNPASFGVPAPIGAVVSSYALKDSGKTISDTVQYYAVLPDGLQQISPVLA
                     AILRNNNSYGLQQPPRLGADEVAKLPVSRVLDTRRYPSEPVSLVDVTRDPVTCAYWSK
                     PVGAATSSLTLLAGSALPVPDAVHTVELVGAGNGGVATRVALAAGTGYFTQTVGGGPD
                     APGAGSLFWVSDTGVRYGIDNEPQGVAGGGKAVEALGLNPPPVPIPWSVLSLFVPGPT
                     LSRADALLAHDTLVPDSRPARPVSAEGGYR"
     CDS             346895..350887
                     /codon_start=1
                     /note="gene_299"
```

/transl_table=11
/translation="VSRLIFEARRRLAPPSSHQGTIIIEAPPELPRVIPPSLLRRALP
YLIGILIVGMIVALVATGMRVISPQTLFFPFVLLLAATALYRGNDKKMRTEEVDAERA
DYLRYLSVVRDNIRAQAAEQRASALWSHPDPAALASVPGSRRQWERDPHDPDFLVLRA
GRHTVPLATTLRVNDTADEIDLEPVSHSALRSLLDTQRSIGDVPTGIDLTKVSRITVL
GERAQVRAVLRAWIAQAVTWHDPTVLGVALAARDLEGRDWNWLKWLPHVDIPGRLDAL
GPARNLSTDPDELIALLGPVLADRPAFTGQPTDALRHLLIVVDDPDYDLGASPLAVGR
AGVTVVHCSASAPHREQYSDPEKPILRVAHGAIERWQTGGWQPYIDAADQFSADEAAH
LARRLSRWDSNPTHAGLRSAATRGASFTTLLGIEDASRLDVPALWAPRRRDEELRVPI
GVTGTGEPLMFDLKDEAEGGMGPHGLMIGMTGSGKSQTLMSILLSLLTTHSAERLIVI
YADFKGEAGADSFRDFPQVVAVISNMAEKKSLADRFADTLRGEVARREMLLREAGRKV
QGSAFNSVLEYENAIAAGHSLPPIPTLFVVADEFTLMLADHPEYAELFDYVARKGRSF
RIHILFASQTLDVGKIKDIDKNTAYRIGLKVASPSVSRQIIGVEDAYHIESGKEHKGV
GFLVPAPGATPIRFRSTYVDGIYEPPQTAKAVVVQSVPEPKLFTAAAVEPDPGTVIAD
TDEQEPADPPRKLIATIGEQLARYGPRAPQLWLPPLDETIPLSAALARAGVGPRQWRW
PLGEIDRPFEMRRDPLVFDARSSAGNMVIHGGPKSGKSTALQTFILSAASLHSPHEVS
FYCLDYGGGQLRALQDLAHVGSVASALEPERIRRTFGELEQLLLSRQQREVFRDRGAN
GSTPDDGFGEVFLVIDNLYGFGRDNTDQFNTRNPLLARVTELVNVGLAYGIHVIITTP
SWLEVPLAMRDGLGLRLELRLHDARDSNVRVVGALRRPADAVPHDQPGRGLTMAAEHF
LFAAPELDAQTNPVAAINARYPGMAAPPVRLLPTNLAPHAVGELYRGPDQLVIGQREE
DLAPVILDLAANPLLMVFGDARSGKTTLLRHIIRTVREHSTADRVAFTVLDRRLHLVD
EPLFPDNEYTANIDRIIPAMLGLANLIEARRPPAGMSAAELSRWTFAGHTHYLIIDDV
DQVPDSPAMTGPYIGQRPWTPLIGLLAQAGDLGLRVIVTGRATGSAHLLMTSPLLRRF
NDLQATTLMLAGNPADSGKIRGERFARLPAGRAILLTDSDSPTYVQLINPLVDAAAVS
GETQQKGSQS"

CDS 350884..351192
/codon_start=1
/note="gene_300"
/transl_table=11
/translation="MTLRVVPEGLAAASAAVEALTARLAAAHASAAPVITAVVPPAAD
PVSLQTAAGFSAQGVEHAVVTAEGVEELGRAGVGVGESGASYLAGDAAAAATYGVVGG
"

CDS 351195..352736
/codon_start=1
/note="gene_301"
/transl_table=11
/translation="MAAPIWMASPPEVHSALLSNGPGPGSLVAAATAWSQLSAEYAST
AAELSGLLGAVPGWAWQGPSAEWYVAAHLPYVAWLTQASADAAGAAAQHEAAAAAYTT
ALAAMPTLAELAANHVIHTVLVATNFFGINTIPITLNEADYVRMWLQAAAVMGLYQAA
SGAALASAPRTVPAPTVMNPGGGAASTVGAVNPWQWLLALLQQLWNAYTGFYGWMLQL
IWQFLQDPIGNSIKIIIAFLTNPIQALITYGPLLFALGYQIFFNLVGWPTWGMILSSP
FLLPAGLGLGLAAIAFLPIVLAPAVIPPASTPLAAAAVAAGSVWPAVSMAVTGAGTAG
AATPAAGAAPSAGAAPAPAAPATASFAYAVGGSGDWGPSLGPTVGGRGGIKAPAATVP
AAAAAAATRGQSRARRRRRSELRDYGDEFLDMDSDSGFGPSTGDHGAQASERGAGTLG
FAGTATKERRVRAVGLTALAGDEFGNGPRMPMVPGTWEQGSNEPEAPDGSGRGGGDGL
PHDSK"

CDS 352785..353078
/codon_start=1
/note="gene_302"
/transl_table=11
/translation="MSLLDAHIPQLVASQSAFAAKAGLMRHTIGQAEQAAMSAQAFHQ

GESSAAFQAAHARFVAAAAKVNTLLDVAQANLGEAAGTYVAADAAAASTYTGF"
CDS 353108..353398
/codon_start=1
/note="gene_303"
/transl_table=11
/translation="MSQIMYNYPAMLGHAGDMAGYAGTLQSLGAEIAVEQAALQSAWQ
GDTGITYQAWQAQWNQAMEDLVRAYHAMSSTHEANTMAMMARDTAEAAKWGG"
CDS 353409..354296
/codon_start=1
/note="gene_304"
/transl_table=11
/translation="MDATPNAVELTVDNAWFIAETIGAGTFPWVLAITMPYSDAAQRG
AFVDRQRDELTRMGLLSPQGVINPAVADWIKVVCFPDRWLDLRYVGPASADGACELLR
GIVALRTGTGKTSNKTGNGVVALRNAQLVTFTAMDIDDPRALVPILGVGLAHRPPARF
DEFSLPTRVGARADERLRSGVPLGEVVDYLGIPASARPVVESVFSGPRSYVEIVAGCN
RDGRHTTTEVGLSIVDTSAGRVLVSPSRAFDGEWVSTFSPGTPFAIAVAIQTLTACLP
DGQWFPGQRVSRDFSTQSS"
CDS 354343..355761
/codon_start=1
/note="gene_305"
/transl_table=11
/translation="MSGTVMQIVRVAILADSRLTEMALPAELPLREILPAVQRLVVPS
AQNGDGGQADSGAAVQLSLAPVGGQPFSLDASLDTVGVVDGDLLVLQPVPAGPAAPGI
VEDIADAAMIFSTSRLKPWGIAHIQRGALAAVIAVALLATGLTVTYRVATGVLAGLLA
VAGIAVASALAGLLITIRSPRSGIALSIAALVPIGAALALAVPGKFGPAQVLLGAAGV
AAWSLIALMIPSAERERVVAFFTAAAVVGASVALAAGAQLLWQLPLLSIGCGLIVAAL
LVTIQAAQLSALWARFPLPVIPAPGDPTPSAPPLRLLEDLPRRVRVSDAHQSGFIAAA
VLLSVLGSVAIAVRPEALSVVGWYLVAATAAAATLRARVWDSAACKAWLLAQPYLVAG
VLLVFYTTTGRYVAAFGAVLVLAVLMLAWVVVALNPGIASPESYSLPLRRLLGLVAAG
LDVSLIPVMAYLVGLFAWVLNR"
CDS 355758..357143
/codon_start=1
/note="gene_306"
/transl_table=11
/translation="MIRAAFACLAATVVVAGWWTPPAWAIGPPVVDAAAQPPSGDPGP
VAPMEQRGACSVSGVIPGTDPGVPTPSQTMLNLPAAWQFSRGEGQLVAIIDTGVQPGP
RLPNVDAGGDFVESTDGLTDCDGHGTLVAGIVAGQPGNDGFSGVAPAARLLSIRAMST
KFSPRTSGGDPQLAQATLDVAVLAGAIVHAADLGAKVINVSTITCLPADRMVDQAALG
AAIRYAAVDKDAVIVAAAGNTGASGSVSASCDSNPLTDLSRPDDPRNWAGVTSVSIPS
WWQPYVLSVASLTSAGQPSKFSMPGPWVGIAAPGENIASVSNSGDGALANGLPDAHQK
LVALSGTSYAAGYVSGVAALVRSRYPGLNATEVVRRLTATAHRGARESSNIVGAGNLD
AVAALTWQLPAEPGGGAAPAKPVADPPVPAPKDTTPRNVAFAGAAALSVLVGLTAATV
AIARRRREPTE"
CDS 357140..358135
/codon_start=1
/note="gene_307"
/transl_table=11
/translation="MNPIPSWPGRGRVTLVLLAVVPVALAYPWQSTRDYVLLGVAAAV
VIGLFGFWRGLYFTTIARRGLAILRRRRRIAEPATCTRTTVLVWVGPPASDTNVLPLT
LIARYLDRYGIRADTIRITSRVTASGDCRTWVGLTVVADDNLAALQARSARIPLQETA
QVAARRLADHLREIGWEAGTAAPDEIPALVAADSRETWRGMRHTDSDYVAAYRVSADA

```
                     ELPDTLPAIRSRPAQETWIALEIAYAAGSSTRYTVAAACALRTDWRPGGTAPVAGLLP
                     QHGNHVPALTALDPRSTRRLDGHTDAPADLLTRLHWPTPTAGAHRAPLTNAVSRT"
CDS                  complement(358122..359333)
                     /codon_start=1
                     /note="gene_308"
                     /transl_table=11
                     /translation="MSEMSGTFTADAIGPPVPIPDVPGADAGAEGLPSRSVLSARQRI
                     LVESSAIADVALRTAVASVLSATVTPAVVANALRHVNEGSERSNLNFYAELAAAHDPA
                     KSFPAPTELPKVTSRPASPLTEWVARGTVDNIAFASGFRAINPTMRQRWSALTANNIV
                     HAQHWRHRDGPRPTLCVIHGFMGSSYLLNGLFFSLPWYYRSGYDVLLYTLPFHGQRAE
                     KFSPFSGFGYFTSGLSGFAEAMAQAVYDFRSIVDYLRHIGVDRIALTSISLGGYTSAL
                     LASVESRLEAVIPNCPVVMPAKLFDEWFPANKLVKLGLRLTNISRDELIAGLAYHGPL
                     NYRPLLPKDRRMIITGLGDRMAPPEHAVTLWKQWDRCALHWFPGSHLLHVSQLDYLRR
                     MTVFLQGLMFD"
CDS                  359431..360216
                     /codon_start=1
                     /note="gene_309"
                     /transl_table=11
                     /translation="MWDPDVYLAFSGHRNRPFYELVSRVGLERARRVVDLGCGPGHLT
                     RYLARRWPGAVIEALDSSPEMVAAAAERGIDATTGDLRDWKPKPDTDVVVSNAALHWV
                     PEHSDLLVRWVDELAPGSWIAVQIPGNFETPSHAAVRALARREPYAKLMRDIPFRVGA
                     VVQSPAYYAELLMDTGCKVDVWETTYLHQLTGEHPVLDWITGSALVPVRERLSDESWQ
                     QFRQELIPLLNDAYPPRADGSTIFPFRRLFMVAEVGGARRSGG"
CDS                  complement(360205..361008)
                     /codon_start=1
                     /note="gene_310"
                     /transl_table=11
                     /translation="MSRAVRPYLVLATQRSGSTLLVESLRATGCAGEPQEFFQYLPST
                     GMAPQPREWFAGVDDDTILQLLDPLDPGTPDTATPVAWREHVRTSGRTPNGVWGGKLM
                     WNQTALLQQRAAQLPDRSGDGLRAAIRDVIGNEPVFVHVHRPDVVSQAVSFWRAVQTQ
                     VWRGHPDPKRDSQAVYHAGAIAHIIRNLRDQENGWRAWFAEEGIDPIDIAYPVLWRNL
                     TAIVASVLDAIGQDPKLAPAPMLERQANQRSDEWVDRYRAEAPRLGLPT"
CDS                  complement(361018..362415)
                     /codon_start=1
                     /note="gene_311"
                     /transl_table=11
                     /translation="VTSERATGQRENLLIVHWHDLGRYLGVYHHPDVYSPRLDRLAAE
                     GILFTRAHATAPLCTPSRGSLFTGRYPQSNGLVGLAHHGWEYRTGVQTLPQLLSESGW
                     YSALFGMQHETSYPKRLGFDEFDVSNSYCEYVVAKAQDWLHNRVPALDGQRFLLTAGF
                     FETHRPYPHERYRPADSAAVELPDYLPDTPEVRQDVAEFYGSIATADEAVGRLLDTLA
                     DTGLDASTWVVFVTDHGPAFPRAKSTLYDAGTGIALIIRPPTRRAMAPRVYDELFSGV
                     DLVPTLLDLLRLEVPADVEGVSHAPALLAPDTENAAVRDHVYTAKTYHDSFDPIRAIR
                     TKEYSYIENYAPRPLLDLPWDIQESPAGMAVAPLVKAPRPQRELYDLRADPTETNNLL
                     AGDDSTQGVAAIAADLAVRLHDWRQRTADVIPSDFAGSRIAERYTETYLRIHRKTPTG
                     RSAIAADRGIDEHCS"
CDS                  362594..364414
                     /codon_start=1
                     /note="gene_312"
                     /transl_table=11
                     /translation="VSFVIAQPEMIAAAAGELASIRSAINAANAAAAAQTTGVMSAAA
                     DEVSTAVAALFSSHAQAYQAASAQAAAFHAQVVRTLTVDAGAYASAEAANAGPNMLAA
```

VNAPAQALLGRPLIGNGANGAPGTGQAGGDGGLLFGNGGNGGSGAPGQAGGAGGAAGF
FGNGGNGGDGGAGANGGAGGTAGWFFGFGGNGGAGGIGVAGINGGLGGAGGDGGNAGF
FGNGGNGGMGGAGAAGVNAVNPGLATPVTPAANGGNGLNLVGVPGTAGGGADGANGSA
IGQAGGAGGDGGNASTSGGIGIAQTGGAGGAGGAGGDGAPGGNGGNGGSVEHTGATGS
SASGGNGATGGNGGVGAPGGAGGNGGHVSGGSVNTAGAGGKGGNGGTGGAGGPGGHGG
SVLSGPVGDSGNGGAGGDGGAGVSATDIAGTGGRGGNGGHGGLWIGNGGDGGAGGVGG
VGGAGAAGAIGGHGGDGGSVNTPIGGSEAGDGGKGGLGGDGGDGAAGGDGGAGGDGGG
RGIFGQFGAGGAGGAGGVGGAGGAGGTGGGGGNGGAIFNAGTPGAAGTGGDGGVGGTG
AAGGKGGAGGSGGVNGATGADGAKGLDGATGGKGNNGNPG"

CDS 364557..364784
/codon_start=1
/note="gene_313"
/transl_table=11
/translation="MTKEKISVTVDAAVLAAIDADARAAGLNRSEMIEQALRNEHLRV
ALRDYTAKTVPALDIDAYAQRVYQANRAAGS"

CDS 364781..365083
/codon_start=1
/note="gene_314"
/transl_table=11
/translation="LIAPGDIAPRRDSEHELYVAVLSNALHRAADTGRVITCPFIPGR
VPEDLLAMVVAVEQPNGTLLPELVQWLHVAALGAPLGNAGVAALREAASVVTALLC"

CDS 365343..365774
/codon_start=1
/note="gene_315"
/transl_table=11
/translation="VEVTDQRWLIDKSALVRLTDSPDMEIWSNRIERGLVHITGVTRL
EVGFSAECGEIARREFREPPLSAMPVEYLTPRIEDRALEVQTLLADRGHHRGPSIPDL
LIAATAELSGLTVLHVDKDFDAIAALTGQKTERLTHRPPSA"

CDS 365911..366543
/codon_start=1
/note="gene_316"
/transl_table=11
/translation="VGVPAKKKQQQGERSRESILDATERLMATKGYAATSISDIRDAC
GLAPSSIYWHFGSKEGVLAAMMERGAQRFFAAIPTWDEAHGPVEQRSERQLTELVSLQ
SQHPDFLRLFYLLSMERSQDPAVAAVVRRVRNTAIARFRDSITHLLPSDIPPGKADLV
VAELTAFAVALSDGVYFAGHLEPDTTDVERMYRRLRQALEALIPVLLEET"

CDS 366540..367448
/codon_start=1
/note="gene_317"
/transl_table=11
/translation="MNTGTAVITGASSGLGLQCARALLRRDASWHVVLAVRDPARGRA
AMEELGEPNRCSVLEVDLASVRSVRSFVETVRTTPLPPIRALVCNAGLQVVSGIAFTD
DGVEMTFGVNHLGHFALVTGILDWLARPARIVVVSSGTHDPSKHTGMPDPRYTCAADL
AHPPTDQNTPAEGRRRYTTSKLCNVLFTYELDRRLDHGEQGVMVNAFDPGLMPGSGLA
RDYPPILRLAYRLLSPMLRVLPFVHSTRVSGEHLAALAVDPRFAGVTGQYFAGAKAIR
SSAESYDRAKALDLWETSERLLAQVT"

CDS complement(367456..370899)
/codon_start=1
/note="gene_318"
/transl_table=11
/translation="LFNSGDGNIGFFNSGTGNFGIGNTGTGNFGIGNSGSTSTGLFNS

GDGNTGGFNPGNFNTGNFNTGSFNTGGFNAGNTNTGHFNTGNYNTGIANTGDVSTGAF
ISGNYSNGILWRGDYQGLIGYSYALTIPEIPAHLDVNIPIDIPITGSFTDLVVDNFTI
PIIGFESFAFSFHIHTEPDIGPIIVPSFVLSVPTFAIAVGGPTTAINISATAGLGPIT
IPIIDIPAAPGIGNSTTSPSSGFFNTGAGTASGFGNVGGNTSGLWNLASAASGVSGLL
NVGALGSGVANVGNTISGIYNTSPLDLGTPAFGSGLANIAGLLQGGAGTTILDLAGLG
NLNVGLANLGGSNFGIGNTGIFNVGFANVGNHNIGLANLGNYSVGFANSGNYHIGIAN
TGSANIGFANTGSGNIGIGLTGTGQIGFGSFNSGSHNIGLFNSGDGNVGFFNSGTGNV
GIGNTGTANFGIANSGSFNTGLGNTGSTNTSLFNPGNVNTGVGNTGSINTGSFNTGST
NTGSFNLGDHNTGSFNSGDYNTGYFNAGDYNTGVANTGNVNTGAFISGNYSNGFFWRG
DYQGLIGLSTTITIPEIPYRYDLSVPIDIPITGTVVATTPNSFTIPGFQIRVLLGPAA
VLVNEMIGPITIDVNQVIAIDSPIQQTISMVGTGGFGPIPIGISIGGTPGFGNSTTGP
SSGFFHTGAGHVSGFGNFGAGNMSGFGNFGAGNSGFFNAGGLGNSGLLNFGALQSGLA
NLGNTISGVYNTSTLDLATPAFGSGIANIGANLAGLFLDNTGNLTLNFGVANQGGLNA
GIGNLGSVNIGFVNTGDSNLGIGNLGDLNFGGVNIGGNNIGIANTGIFDIGLANLGSY
NIGLANLGDDNLGFGNAGSYNIGFANFGSDNLGFANTGSYNIGFANTGNNNIGVGLTG
NGQIGIGSLNSGSNNIGLFNSGSGNIGFFNSGTGNVGIFNAGTGNFGLANSGGFNTGI
GNAGSTNTGVFNPGDLNTGSFNPGSFNTGGFNPGSGNTGYLNTGDYNTGVANTGDVDT
GAFITGSYSNGFLVSGDYQGLIGLPLLGIPVTPGYFNLTGGPSSGFFNSGAGSVSGFV
NSGAGLSGYLNTGALGSGVANVGNTISGWLNASALDLATPGFLSGIGNFGTNLAGFFR
G"

CDS 370784..371038
/codon_start=1
/note="gene_319"
/transl_table=11
/translation="VLVLPELPMPKFPVPVLPMPKFPVPELKKPMFPSPELNKPMFPL
PEFRAPIPIWPVPVSPMPILLLPVLAKPILLLPVLATPML"

CDS complement(371043..377000)
/codon_start=1
/note="gene_320"
/transl_table=11
/translation="MDFVVSAPEVNSLRMYLGAGSGPMLAAAAAWDGLADELAVAASW
FGSVTSGLADAAWRGPAAVAMARAVAPYLGWLISATAQAEQAAAQARVAVATFEAARA
ATVHPAIVAANRAVLVSLVSSNLLGFNAPAIAATEAAYERMWAQDVAAMVGYHAGASA
AVSALMPFTQQLKKLAGLSERLTSAAAAAAGPPSAAGFNLGLANVGANNVGNGNVGVF
NVGFGNLGSYNLGFANLGSDNLGLANLGGHNIGFANTGSNNVGFGNTGSNNVGIGLTG
NGQIGFGSFNSGSHNIGLFNSGSGNVGLFNSGTGNFGIGNSGTGNFGLGNTGSTNTGW
FNTGDVNTGGFNPGSYNTGNFNTGNYNTGSFNAGNYNTGYFNTGDYNTGVANTGNVNT
GAFIAGNYSNGVLWRGDYQGLIGADIALEIPAIPINAQLFSMPIHQVMVMPGSVMTIP
GMRLPFTSIVPFVVYYGPVELPQSTLTLPTVTITVGGPTTTIDGNLTGMVGGVSIPLI
KIPAAPGFGNSTTSPSSGFFNAGAGTASGFGNFGGGASGFWNLASATSGLSGFGNVGA
LGSGVANVGNTISGLYNTSTSNLATPAFNSGLLHHSVGTMTLNFGLANVGGNNVGGAN
AGIFNVGLANLGDYNIGFGNLGGDNLGFAHAGSYNIGFANTGSNNLGFANTGDNNIGF
ANIGSNNIGIGLTGSGQIGFGSLNSGSHNIGLFNSGDGNIGLFNSGSGNFGIGNAGTG
NWGIGNSGAGNFGIGNAGSTNTGLFNSGDLNTGSLNPGSYNTGSVNTGSVNTGGFNAG
NYNTGYFNTGDYNTGMANTGNINTGAFISGNHSNGLLWRGDNQGLIDLAIGVDIPEIP
IVSVDVNIPIHIPITASFTDIVYSGLDLPPNTAVTVIFFGPVDIDPFTVPVIRITGPT
PVVMVGGPTTAINIGATVGVDAINIPIIHIPATPGFGNSTGGLSSGFFNSGAGSASGF
GNFGGAASGFMNLVSTTSGMSGFLNVGALGSGVANVGNTISGIYNVGTSDLSTPAVNS
GLANIGTNIAGLLRDGAGTAAINLGLANHGNLNVGFASLGGFNFGGATIGHNNVGIGN
TGIFDVGLANLGSYNIGFGNLGDDNLGFGNFGSYNIGFGNVGNDNLGFANAGGGNIGF
ANTGSNNVGFGNTGSNNVGIGLTGNGQIGFGSFNSGSGNIGLFNSGSNNIGFFNSGSG

NFGIANSGSFNTGIGNTGNTNTGLFNSGDVNTGAFNPGSFNTGSFNTGSFNTGGFNPG
NTNTGYLNIGNYNTGIANTGDVDTGAFITGNYSNGLFLSGDYQGLVGLNLVIDMPLPI
SLGVNIPIDIPITASAGNITLMGVTIPPTGDIVLSSIAGQRAHFGPITIPNITVVGPT
TTVAIGGPNTAITITGGGAIRIPLISIPAAPGFGNSTTNPSSGFFNTGAGGASGFGNF
GGANSGFWNLASATSGASGLLNVGALGSGLANVGTTVSGFYNTSTSDLATPAFNSGLA
NISTSIAGLLRDSTGTMVLNLGLANHGTLNVGIANLGDYNIGFANLGSANFGSANIGG
NNIGGANTGIFDIGLANLGSYNIGFGNFGDDNLGFGNLGSYNVGFGNLGNDNLGFANT
GSNNIGFANTGSNNIGIGLTGDGQIGFGSLNSGSGNIGLFNSGSGNIGFFNSGNGNVG
IGNTGTANFGLGNTGSTNTGFFNSGDVNTGIGNTGSFNTGSFNPGDSNTGDFNPGSYN
TGLGNTGDVDTGAFISGSYSNGFLWSGNYQGLIGLHAALAIPEIALTFGVDIPIHIPI
NIDAGVVTLQGFSIVAAENNIDFTPIIIPTINITLPTAAITVGGPTTSIGITASAGIG
SITIPIIDIPATSGFGNSTTSPSSGFFNSGAGSASGFLNVVAGASGISGYLNVGALGS
GVTNVGHTVSGFYNASALDLVTPAFASGLMRDGMGTMTLNLGLANLGSNNAGFGNTGI
FDVGVANLGNYNIGFGNFGDDNLALPT"

variation 375942..375971
/note="supplemented from AL2122/97"

CDS 377230..377874
/codon_start=1
/note="gene_321"
/transl_table=11
/translation="VYRVIAERRDMRRFVPGGVVSEDVLARLLHAAHAAPSVGLMQPW
RFIRITDETLKRRIHALVDDERLLTAEALGAREEEFLALKVEGILDCAELLVVALCDR
RGSYIFGRRTLPQMDLASVSCAIQNLWLAARSEGLGMGWVSLFDPQRLAALLAMPADA
EPVAILCLGPVPEFPDRPALELDGWAYARPLAEFVSENRWSYPSALATDHHHGE"

CDS complement(377862..378344)
/codon_start=1
/note="gene_322"
/transl_table=11
/translation="MAVIVRKWFGLGRLPADLRCQVEAEGLIYLAEYVAVTRRFTGVI
PGLRASHSIASYVGALAFTEQRVLGTLSMVPKLAGRVVDARWDGPQAGAATAEISPTG
LQLDLDVADVDPKFSGQLALHFKATIGEDVLSRLPRRSLAFDVPAEYVNLAVGVTYSP
"

CDS 378402..379118
/codon_start=1
/note="gene_323"
/transl_table=11
/translation="MTRPQALLAVSLAFVATAVYAVMWVGHSQDWGWLHSFDWSLLNA
AHDIGIKNPAWVRFWDGVSLILGPVVLRPLGLLAAMVALAKRKIRIALLLLACLPLNA
IMTIAAKSVAHRPRPATALVSAHSTSFPSGHALEATASVLALLTVLLPMLHSRFTRHI
AITVGALCVLTVGVARVALNVHHPTDVVAGWALGYLYFLVCLCVFRPPSIFGAQRASH
ALSPPVEVSRQPEPEVDTAR"

CDS 379220..379876
/codon_start=1
/note="gene_324"
/transl_table=11
/translation="MSRLLALLCAAVCTGCVAVVLAPVSLAVVNPWFANSVGNATQVV
SVVGTGGSTAKMDVYQRTAAGWQPLKTGITTHIGSAGMAPEAKSGYPATPMGVYSLDS
AFGTAPNPGGGLPYTQVGPNHWWSGDDNSPTFNSMQVCQKSQCPFSTADSENLQIPQY
KHSVVMGVNKAKVPGKGSAFFFHTTDGGPTAGCVAIDDATLVQIIRWLRPGAVIAIAK
"

CDS complement(379946..380437)

```
                     /codon_start=1
                     /note="gene_325"
                     /transl_table=11
                     /translation="VCCNGVVTPGDPADIAAIKQLKYRYLRALDTKHWDDFTDTLAED
                     VTGDYGSSVGTELHFTNRADLVDYLRQALGPGVITEHRVTHPEITVTGDTATGIWYLQ
                     DRVIVAEFNFMLIGAAFYHDQYRRTTDGWRISATGYDRTYEATMSLAGLNFNIRPGRA
                     LAD"
     CDS             380461..381690
                     /codon_start=1
                     /note="gene_326"
                     /transl_table=11
                     /translation="MSQSRYAGLSRSELAVLLPELLLIGQLIDRSGMAWCIQAFGRQE
                     MLQIAIEEWAGASPIYTKRMQKALNFEGDDVPTIFKGLQLDIGAPPQFMDFRFTLHDR
                     WHGEFHLDHCGALLDVEPMGDDYVVGMCHTIEDPTFDATAIATNPRAQVRPIHRPPRK
                     PADRHPHCAWTVIIDESYPEAEGIPALDAVRETKAATWELDNVDASDDGLVDYSGPLV
                     SDLDFGAFSHSALVRMADEVCLQMHLLNLSFAIAVRKRAKADAQLAISVNTRQLIGVA
                     GLGAERIHRAMALPGGIEGALGVLELHPLLNPAGYVLAETSPDRLVVHNSPAHADGAW
                     ISLCTPASVQPLQAIATAVDPHLKVRISGTDTDWTAELIEADAPASELPEVLVAKVSR
                     GSVFQFEPRRSLPLTVK"
     CDS             381845..383707
                     /codon_start=1
                     /note="gene_327"
                     /transl_table=11
                     /translation="MYDPLGLSIGTTNLVAAGNGGPPVTRRAVLTLYPHCAPKIGVPS
                     QNPNLIEPGALMSGFVERIGDAVALVSPDGSVHDPDLLLVEALDAMVLTAGADASSSE
                     IAIAVPAHWKPGAVHALRNGLRTHVGFVRSGMAPRLVSDAIAALTAVNSELGLPHGGV
                     VGLLDFGGSATYVTLVETKSDSRTSDFQPVSATARYQDFSGSQIDQALLLRVIDQFGY
                     GDDVDPASTAAVGQLGQLREQCRAAKERLSTDVATELFAELAGCSSSIEMTREQLEDL
                     IQDPLTGFIYAFDDMLARHNASWADLAAVVTVGGGANIPLVTQRLSFHTRRPVLTASQ
                     PGCAAAMGALLLANRGGERDSRTRTSIGLATAAAAGTSVIELPAGDVMVIDHEALTDR
                     ELAWSQTDFPSEAPARFEGDSYNEGGPCWSMRLNAVEPPKGPAWRRIRVSQLLIGVSA
                     VVAMTAIGGVALTLTAIERRPSPLPTPIVPGLAPMPPGSVVPSSRAPTPPPPPSTVAP
                     LPSAAPAPTTVAPAPPPPTQVVTTTTAPPVTTTPRPSPTTTTTTAPPSTTTTTELPVT
                     TTSTIPTIPTTTTTTVKMTTEWLHVPFLPVPIPVPIPQNPGAGEPQNPFGSLGSG"
     CDS             383779..384165
                     /codon_start=1
                     /note="gene_328"
                     /transl_table=11
                     /translation="VGDYGPFGFDPDEFDRVIREGSEGLRDAFERIGRFLSSSGAGTG
                     WSAIFEDLSRRSRPAPETAGEAGDGVWAIYTVDADGGARVEQVYATELDALRANKDNT
                     DPKRKVRFLPYGIAVSVLDDPVDEAQ"
     CDS             complement(384168..384830)
                     /codon_start=1
                     /note="gene_329"
                     /transl_table=11
                     /translation="MIVVWEHLCMNPEDDPEARIRELERPLADVARASELGGSQSGGY
                     TYPPGPPPPPYSYGGPFGGPSPRSSSGNRAWWILAAVVVVGVLVLVGGIAAFSAQRLS
                     QGNFVVLSPTPSVSRAVPTPTAQPATTLPPAGASLSVSGVNVNRTIACNDSIVSVSGM
                     SNTVVITGHCTSLTVSGMRNSVTADSVDTIEAAGFNNEVTYHSGSPKISNAGGSNSVQ
                     QG"
     CDS             384930..385775
```

```
                     /codon_start=1
                     /note="gene_330"
                     /transl_table=11
                     /translation="MAGFGALAAALPAPTAWADPSRPAAPAGPTPAPAAPAAATGGLL
                     FHDEFDGPAGSVPDPSKWQVSNHRTPIKNPVGFDRPQFFGQYRDSRQNVFLDGNSNLV
                     LRATREGNRYFGGLVHGLWRGGIGTTWEARIKFNCLAPGMWPAWWLSNDDPGRSGEID
                     LIEWYGNGTWPSGTTVHANPDGTAFETCPIGVDGGWHNWRVTWNPSGMYFWLDYADGI
                     EPYFSVPATGIEDLNEPIREWPFNDPGYTVFPVLNLAVGGSGGGDPATGSYPQEMLVD
                     WVRVF"
     CDS             385824..386438
                     /codon_start=1
                     /note="gene_331"
                     /transl_table=11
                     /translation="MEFLVTMTTRVPDSMPADAVERVRAREAARSRELAAQGKLLRLW
                     RPPLRPGEWRTLGLFAADDNGELEQLLASMPPRSWRTDDVTPLGAHPNDPVGQGITIA
                     PGKGPEFLIATTIMVPPGTPAQVVDDTVAREARRAPELAGRGHLVRLWALPDGPDGQR
                     TLGLWRARDPGELMAILESLPLAGWMTIETTPLSPHPDDPIRMP"
     CDS             complement(386462..387154)
                     /codon_start=1
                     /note="gene_332"
                     /transl_table=11
                     /translation="MGAFQEAAKLGFRYIETDVRATRDGVAVILHDRRLAPGVGLSGA
                     VDRLDWRDVRKAQLGAGQSIPTLEDLLTALPDMRVNIDIKAASAIEPTVNVIERCNAH
                     NRVLIGSFSERRRRRALRLLTKRVASSAGTGALLAWLTARPLGSRAYAWRMMRDIDCV
                     QLPSRLGGVPVITPARVRGFHAAGRQVHAWTVDEPDVMHTLLDMDVDGIITDRADLLR
                     DVLIARGEWDGA"
     tRNA            complement(387490..387563)
                     /note="identified by tRNAscan-SE; putative"
     CDS             complement(387594..388370)
                     /codon_start=1
                     /note="gene_333"
                     /transl_table=11
                     /translation="MFKRARAEIFDRNREVGISNVTTAASLVTFPVLAGILGGVVPSV
                     RTPSAAMVSGVQHFAAGIVMAAVAGEVLPDLRSRGPLWLIVVGFSAGVAVLVALRRFD
                     GHGEHQDGDDVGELPVGFLTVVAVDLFIDGLLVATGATVSSRTAIIITIALTVEVLFL
                     GLAVALRLAGSGMPRIRAAATTSALSLVIAVGGVSGAVALGRAGNTVLTLVLAFAAAA
                     LLWLVVEELLVEAHETPERPWMAVMFFAGFLILYGLGVME"
     CDS             388437..389105
                     /codon_start=1
                     /note="gene_334"
                     /transl_table=11
                     /translation="MSKVLVTGFGPYGVTPVNPAQLTAEELDGRTIAGATVISRIVPN
                     TFFESIAAAQQAIAEIEPALVIMLGEYPGRSMITVERLAQNVNDCGRYGLADCAGRVL
                     VGEPTDPAGPVAYHATVPVRAMVLAMRKAGVPADVSDAAGTFVCNHLMYGVLHHLAQK
                     GLPVRAGWIHLPCLPSVAALDHNLGVPSMSVQTAVAGVTAGIEAAIRQSADIREPIPS
                     RLQI"
     CDS             389177..389839
                     /codon_start=1
                     /note="gene_335"
                     /transl_table=11
                     /translation="VGRHELARDRRKSSAVLAAVLAPAAVFFATGGDVSTLAARADAN
```

PVLGDDAPCCVQIVPVAPLAFSSQISGGEIGTGLAASQFASASRWRIVSRYLPVGVAP
EQGLQVKTVLTARSISAAFPEIREIGGVRPDALRWHPNGLALDVMVPNPGTAEGIALG
NEIVAFVLKNATRFGMQDVIWRGAYYTPNGARTTGAGHYDHIHITTVGGGYPTGEELY
IR"

CDS 389871..390443
/codon_start=1
/gene="dcd"
/note="gene_336"
/transl_table=11
/translation="VLLSDRDLRAEISSGRLGIDPFDDTLVQPSSIDVRLDCLFRVFN
NTRYTHIDPAKQQDELTSLVQPVDGEPFVLHPGEFVLGSTLELFTLPDNLAGRLEGKS
SLGRLGLLTHSTAGFIDPGFSGHITLELSNVANLPITLWPGMKIGQLCMLRLTSPSEH
PYGSSRAGSKYQGQRGPTPSRSCQNFIRST"

CDS 390603..391880
/codon_start=1
/note="gene_337"
/transl_table=11
/translation="MAQLGHEVVGVDIDPGKVAKLAGGDIPFYEPGLRKLLTDNLAAG
RLRFTTDYDMAADFADVHFLGVGTPQKIGEYGADLRHVHAVIDALVPRLVRASILVGK
STVPVGTAAELGHRAGALAPRGVDVEIAWNPEFLREGFAVHDTLNPDRIVLGVQDDST
RAEVAVRELYAPLLAAGVPFLVTDLQTAELVKVSANAFLATKISFINAISEVCEAAGA
DVSQLADALGYDPRIGRQCLNAGLGFGGGCLPKDIRAFMARAGELGADQALTFLREVD
SINMRRRTKMVELATTACGGSLLGANIAVLGAAFKPESDDVRDSPALNVAGQLQLNGA
TVHVYDPKALDNAHRLFPTLNYAVSVAEACERADAVLVLTEWREFIDLEPADLANRVR
ARVIVDGRNCLDVTRWRRAGWRVFRLGVPRLGH"

CDS complement(391869..392495)
/codon_start=1
/note="gene_338"
/transl_table=11
/translation="VFAHPDDESFGLGAVLGDFTAQGTRLRGLCFTHGEASTLGRTDR
NLGEVRREELAAAAQVLGVDHVQLLAYPDNGLAQIPLNELTQRVVDALAGADLLLVFD
DNGVTGHPDHRRATEAALAAASTPGIPVLAWALPQPIADRLNAEFSASFGGRGHGHLD
IMIEVDRSRQLAAIGCHFTQSADNPVLWRRLELLGDREYLRWLRRSVP"

CDS 392641..393321
/codon_start=1
/note="gene_339"
/transl_table=11
/translation="MAGQSDRKAALLDQVARVGKALANGRRLQILDLLAQGERAVEAI
ATATGMNLTTASANLQALKSGGLVEARREGTRQYYRIAGEDVARLFALVQVVADEHLA
DVAVAAADVLGSPEDAITRAELLRRREAGEVTLVDVRPHEEYQAGHIPGAINIPIAEL
ADRLAELAGDRDIVAYCRGAYCVMAPDAVRIARDAGREVKRLDDGMLEWRLAGLPVDE
GAPVGHGD"

CDS 393355..394017
/codon_start=1
/note="gene_340"
/transl_table=11
/translation="VKRQPELLVAQHEHWQDTYRAHPVLYGTRPSEPGVYAAEVFNAD
GVQRVLELAAGHGRDTLYFAGQGFTVVATDFSDVAVAQLRRSAQARGVSARVQPIVHD
LRQPLPVKTGSIDGAFAHMALCMALSTSEIHAVVAEVGRVLRPGGKFIYTVRHTGDAH
YGAGQAHGDDIFECAGFAVHFFRRELVARLATGWVLEEVHDFEEGELPRRLWRVTVTK
PA"

```
CDS             complement(393985..395334)
                /codon_start=1
                /note="gene_341"
                /transl_table=11
                /translation="MASTLTTGLPPGPRLPRYLQSVLYLRFREWFLPAMHRKYGDVFS
                LRVPPYADNLVVYTRPEHIKEIFAADPRSLHAGEGNHILGFVMGEHSVLMTDEAEYAR
                MRSLLMPAFTRAALRGYRDMIASVAREHITRWRPHATINSLDHMNALTLDIILRVVFG
                VTDPKVKAELTSRLQQIINIHPAILAGVPYPSLKRMNPWKRFFHNQTKIDEILYREIA
                SRRIDSDLTARTDVLSRLLQTKDTPTKPLTDAELRDQLITLLLAGHETTAAALSWTLW
                ELAHAPEIQSQVVWAAVGGDDGFLEAVLKEGMRRHTVIASTARKVTAPAEIGGWRLPA
                GTVVNTSILLAHASEVSHPKPTEFRPSRFLDGSVAPNTWLPFGGGVRRCLGFGFALTE
                GAVILQEIFRRFTITAAGPSKGETPLVRNITTVPKHGAHLRLIPQRRLGGLGDSDPP"
CDS             395400..396002
                /codon_start=1
                /note="gene_342"
                /transl_table=11
                /translation="VQQQRTNRDKLLDGALACLRERGYGNTSSRDIARAAGVNIASIN
                YHFGSKDALLDDALGRCFSTWNQRVQEAFDHSRAAGPAGQILAVLEATVDSFEQIRPA
                VYACVESYAPALRSEALRERLAAGYADVRQHSVDLAGAALAGTDIAPPENLSTIVSVL
                MAVIDGLMIQWIADPSATPRSTEVIRALASIGAVVTSQLR"
CDS             complement(395983..396609)
                /codon_start=1
                /note="gene_343"
                /transl_table=11
                /translation="MRLTHPARRYLSSQAARPTGAFGRLLGRIWRAETADVNRIAVEL
                LAPGPGERVCEIGFGPGRTLGLLAAAGAQVSGVEVSTTMIAIAAHHNAKAIAAGLISL
                YHGDGVTLPVADHSLDKVLGVHNFYFSPDPRASLCDIARALRPGGRLVLTSISDDQPL
                AARFDPAIYRVPPTLDTAAWLGAAGFIDVGIKRSADHPATVWFTATAT"
CDS             complement(396636..397376)
                /codon_start=1
                /note="gene_344"
                /transl_table=11
                /translation="MARSIPADRFSAIVAASARVFIAHGYQRTQVQDVADALALAKGT
                LYGYAQGKAALFAAAVRYGDAQEALPLASELPVAAPVAGEIAAVVSARLAGEVTDMRL
                THALRATLPPGATTGDARAELAGIVTDLYSRLARHRIALKLVDRCAPELPDLAEVWFG
                TGRNAQVDAVQAYLVHRERAGLLILPGPAPMVARTIVELCALWAVHLHFDPSPEPWSI
                VQPGVIDDDAIAATLAEFVVRATTASSD"
CDS             397490..398656
                /codon_start=1
                /note="gene_345"
                /transl_table=11
                /translation="MSKTVLILGAGVGGLTTADTLRQLLPPEDRIILVDRSFDGTLGL
                SLLWVLRGWRRPDDVRVRPTAASLPGVEMVTATVAHIDIAAQVVHTDNSVIGYDALVI
                ALGAALNTDAVPGLSDALDADVAGQFYTLDGAAELRAKVEALEHGRIAVAIAGVPFKC
                PAAPFEAAFLIAAQLGDRYATGTVQIDTFTPDPLPMPVAGPEVGEALVSMLKDHGVGF
                HPRKALARVDEAARTMHFGDGTSEPFDLLAVVPPHVPSAAARSAGLSESGWIPVDPRT
                LSTSADNVWAIGDATVLTLPNGKPLPKAAVFAEAQAAVVAHGVARHLGYDVAERHFTG
                TGACYVETGDHQAAKGDGDFFAPSAPSVTLYPPSREFHEEKVAQELAWLTRWKT"
CDS             398731..399516
                /codon_start=1
                /note="gene_346"
```

/transl_table=11
/translation="LRKPASSLAKVDYSSAYLEQTHAFGELIRNVDQSTPVPTCPGWS
LGQLFRHVGRGDRWAAQIVRDRLDHFLDPRSVEGGKPPPDPDDAISWLYGGARLLVDA
VEQTGVETPVWTFLGPRPAGWWVRRRLHEVAVHRADVAITVGGEFTLEPNVAADGISE
FLERIAVQAGSGGTPLPLEDDDTLHLHATDPGLLEAGGWTVRRDERGVTWSHRHGKGA
VALRGGATELLLAMVRRLSVADTGIELLGDAGVWQKWLDRTPL"
CDS 399492..399917
/codon_start=1
/note="gene_347"
/transl_table=11
/translation="MAGSHAAVAAAHGNFQTMTTSEIATVLAWHDALNAADIETLVAL
STDDIDIGDAHGAVQGHDALRGWASSLTTTAELGRMYVHHGVVVVEQKITSGEDPGIA
RTGAAAFRVVQDHVASVFRHEDLASALAATELTEDDLVD"
CDS 399947..400813
/codon_start=1
/note="gene_348"
/transl_table=11
/translation="MRGIILAGGSGTRLYPITMGISKQLLPVYDKPMIYYPLTTLMMA
GIRDIQLITTPHDAPGFHRLLGDGAHLGVNISYATQDQPDGLAQAFVIGANHIGADSV
ALVLGDNIFYGPGLGTSLKRFQSISGGAIFAYWVANPSAYGVVEFGAEGMALSLEEKP
VTPKSNYAVPGLYFYDNDVIEIARGLKKSARGEYEITEVNQVYLNQGRLAVEVLARGT
AWLDTGTFDSLLDAADFVRTLERRQGLKVSIPEEVAWRMGWIDDEQLVQRARALVKSG
YGNYLLELLERN"
CDS 401457..402128
/codon_start=1
/note="gene_349"
/transl_table=11
/translation="MFDQVRGRMPSPEAIAHFDERFECHAPRTTRVSAAFIDRICSAT
RAENRAAAAQLVALGELFAYRWSRCGGREEWVMDTMAAVAAEVAAALRISQGLAASRL
RYARAMRERLPKTAEVFSAGDIGYLMFATIVYRTDLIVDPDVLAAVDAQLAANVARWP
SMTKGRPGLGRSIRSWRVPMPMRCGGARSIRPSASSGSGKAKTVCARSVAACWPSTHT
PSMRG"
CDS 402068..402823
/codon_start=1
/note="gene_350"
/transl_table=11
/translation="VCQIGGSLLAVDAHALDARLSALAGTVCEHDPRSREQRRADALG
ALAGGADRLGCGCGRADCAAGKRPAAPPVVIHLIAEAATINGTGSAPASQMNADGLIT
AELVAELAKTATLVPLVHPGDAPPEPGYAPSKALADFVRCRDLTCRWPGCDEPATNCD
LDHTIPYAAGGPTHASNLKCYCRTHHLVKTFWGWRDQQLPDGTLILTSPSGHTYVSTP
GSALLFPACATSAAASRHRKPTHPTTIATSAQR"
CDS 402823..402993
/codon_start=1
/note="gene_351"
/transl_table=11
/translation="MPKRRRTRAQDRAYRIATERRQNHAARQRAQVLTQTAAATDTHG
PPPDHNDDPPPF"
CDS complement(403163..404431)
/codon_start=1
/note="gene_352"
/transl_table=11

/translation="VDVTTHQLPWHTASHQRQRAFAQSAKLQDVLYEIRGPVHQHAAR
LEAEGHRILKLNIGNPAPFGFEAPDVIMRDIIQALPYAQGYSDSQGILSARRAVVTRY
ELVPGFPRFDVDDVYLGNGVSELITMTLQALLDNGDQVLIPSPDYPLWTASTSLAGGT
PVHYLCDETQGWQPDIADLESKITERTKALVVINPNNPTGAVYSCEILTQMVDLARKH
QLLLLADEIYDKILYDDAKHISLASIAPDMLCLTFNGLSKAYRVAGYRAGWLAITGPK
EHASSFIEGIGLLANMRLCPNVPAQHAIQVALGGHQSIEDLVLPGGRLLEQRDIAWTK
LNEIPGVSCVKPAGALYAFPRLDPEVYDIDDDEQLVLDLLLSEKILVTQGTGFNWPAP
DHLRLVTLPWSRDLAAAIERLGNFLVSYRQ"

CDS complement(404483..407095)
/codon_start=1
/note="gene_353"
/transl_table=11
/translation="MSMTAVVGVFALRRVWWLYKLVMSGQPASGRTDNLGTRIWTQIS
EVLGQRRLLKWSIPGLAHFFTMWGFFILLTVYIEAYGLLFEERFHIPVIGRWDALGFL
QDFFATAVFLGITTFAIIRILRNPREIGRSSRFYGSHNGGAWLVLLMIFNVIWTYVLV
RGSAVNNGTLPYGNGAFLSQLFGAILRPLGQPANEIIETTALLLHIGVMLAFLILVLH
SKHLHIFLAPINVTFKRLPDGLGPLLPLEADGKPIDFENPSEDAVFGRGKIEDFTWKG
MLDFATCTECGRCQSQCPAWNTGKPLSPKLVIMDLRDHWMAKAPYILGQKDASAGGEA
GHQEHHHVPESGFGRVPGHGPEQATRPLVGTEEQGGVIDPDVLWSCVTCGACVEQCPV
DIEHVDHIVDMRRYQVMMESEFPSELSVLFKNLETKGNPWGQNASDRTNWIDEVDFDV
PVYGQDVDSFDGYEYLFWVGCAGAYDDKAKKTTKAVAELLAVAGVKYLVLGAGETCNG
DSARRSGNEFLFQQLAQQAVETLDGLFEGVETVDRKIVVTCPHCFNTIGKEYRQLGAN
YTVLHHTQLLNRLVRDKRLVPVTPVSQDITYHDPCYLGRHNKVYEAPRELIGAAGASL
TEMPRHADRSFCCGAGGARMWMEEHIGKRINHERVDEALATDATAIATACPFCRVMVT
DGVNDRQEEAGRSGVEVLDVAQVLLGSLDHDKAQLPAKGTAAKQAQERAPKAAPKAAA
PVTPVEAPAEAPQAPAPAAPAAPVKGLGMAAGAKRPGAKKAAPTPAAPAAPAAPVKGL
GIAAGAKRPGAKKTPPPAPGLAEPAAQPQPEAKPQPEPAAPPKPQTDGDPAAPAAPVK
GLGIARGARPPGKR"

CDS complement(407240..409690)
/codon_start=1
/note="gene_354"
/transl_table=11
/translation="VTDSLTEVPPAARRALLELANAPTVPVKVLITGGIGTGKTTVLA
AARDTLRRSGLTVLACPPPDGEPPETALVIDDAQLLTDTELLRLTERVADSRLTVVAA
AEAREHHRALRALTMALERDRPRISLGPLPVAEHLRDCTAGLPFLIHAVSARAQAPAQ
AAKVALIERLRRLDEPTLDTLLMMSLTHELGVSDVAAALGISVTDARGLVDRAHASGL
IESSHTAAFLQSVHDAIAQIVGNAHHHEVETSLLRSQLDISPVSAELALRLAEHGLRD
ERLADILTRYAADTRDASVRCARLYRAAVHAGAKGLTVRLADALARTGDCTAAATLAD
DLLSSPDATERAAAVRVAASVAVHDGNTGHAAELFGWLGPHPDTMVSSAATIVFAANG
DLATARATLRLKDAGPPTMAARCARNLAEGLLLTMDQPYPVAMAKLGQAIATEQSLSQ
VIPDSPAALVTLAAIHAGDPVRARSVIGRAVRAGADPLFQRRHLLLSGWIKMQEGQLP
SASADVAAASAGTHLHRRDALWAAALQTAISRRTGDIGALQQHWYAAMEALAEYSLDL
FALLPLGELWVAAARMRQVDQLQHTLDQALTLLDSLGNPALWSNSLHWAGVHAGILAN
SPESVAPHGQALGAMVAHSTLAQALSDAGRTWLRVLAENVDADEVTAAARSLSHVGLT
SDATRLAGQAALQTSDARVSGAMLQLARDLKLGNDFGEPPSGAGDTEPASGTPPAPRQ
PPAGSPLSDREREVAELLLLGMPYRDIGARLFISAKTVEHHVARIRQRLGAGSRSEML
SMLRAMLAPESLTADERR"

CDS 409924..410463
/codon_start=1
/note="gene_355"
/transl_table=11

/translation="VANSLLDFVISLVRDPEAAARYAANPERSIAEAHLTDVTRADVN
SLIPVVSDSLSMSEPIGAAGGAHAGDRGNVWASGAATAALDAFAPHADAGVVQQHGAV
GSVLNQPTPPGPGVTPTDPRPFRAGPHETSALLTSAEIPDTTSEDGGLPTDHPAVWNH
PVVDPHTVEPDHHGYDIHG"

CDS 410646..412091
/codon_start=1
/note="gene_356"
/transl_table=11
/translation="MKMTSLIDYILSLFRSEDAARSFVAAPGRAMTSAGLIDIAPHQI
SSVAANVVPGLNLGAGDPMSGLRQAVAARHGFAQDVANVGFAGDAGAGVASVITTDVG
AGLASGLGAGFLGQGGLALAASSGGFGGQVGLAAQVGLGFTAVIEAEVGAQVGAGLGI
GTGLGAQAGMGFGGGVGLGLGGQAGGVIGGSAAGAIGAGVGGRLGGNGQIGVAGQGAV
GAGVGAGVGGQAGIASQIGVSAGGGLGGVGNVSGLTGVSSNAVLASNASGQAGLIASE
GAALNGAAMPHLSGPLAGVGVGGQAGAAGGAGLGFGAVGHPTPQPAALGAAGVVAKTE
AAAGVVGGVGGATAAGVGGAHGDILGHEGAALGSVDTVNAGVTPVEHGLVLPSGPLIH
GGTGGYGGMNPPVTDAPAPQVPARAQPMTTAAEHTPAVTQPQHTPVEPPVHDKPPSHS
VFDVGHEPPVTHTPPAPIELPSYGLFGLPGF"

CDS 412197..414050
/codon_start=1
/note="gene_357"
/transl_table=11
/translation="VTQPDDPRRVGVIVELIDHTIAIAKLNERGDLVQRLTRARQRIT
DPQVRVVIAGLLKQGKSQLLSSLLNLPAARVGDDEATVVITVVSYSAQPSARLVLAAG
PDGTTAAVDIPVDDISTDVRRAPHAGGREVLRVEVGAPSPLLRGGLAFIDTPGVGGLG
QPHLSATLGLLPEADAVLVVSDTSQEFTEPEMWFVRQAHQICPVGAVVATKTDLYPRW
REIVNANAAHLQRARVPMPIIAVSSLLRSHAVTLNDKELNEESNFPAIVKFLSEQVLS
RATERVRAGVLGEIRSATEQLAVSLGSELSVVNDPNLRDRLASDLERRKREAQQAVQQ
TALWQQVLGDGFNDLTADVDHDLRTRFRTVTEDAERQIDSCDPTAHWAEIGNDVENAI
ATAVGDNFVWAYQRSEALADDVARSFADAGLDSVLSAELSPHVMGTDFGRLKALGRME
SKPLRRGQKMIIGMRGSYGGVVMIGMLSSVVGLGLFNPLSVGAGLILGRMAYKEDKQN
RLLRVRSEAKANVRRFVDDISFVVSKQSRDRLKMIQRLLRDHYREIAEEITRSLTESL
QATIAAAQVAETERDNRIRELQRQLGILSQVNDNLAGLEPTLTPRASLGRA"

CDS 414047..415528
/codon_start=1
/note="gene_358"
/transl_table=11
/translation="VSTSDRVRAILHATIQAYRGAPAYRQRGDVFCQLDRIGARLAEP
LRIALAGTLKAGKSTLVNALVGDDIAPTDATEATRIVTWFRHGPTPRVTANHRGGRRA
NVPITRRGGLSFDLRRINPAELIDLEVEWPAEELIDATIVDTPGTSSLACDASERTLR
LLVPADGVPRVDAVVFLLRTLNAADVALLKQIGGLVGGSVGALGIIGVASRADEIGAG
RIDAMLSANDVAKRFTRELNQMGICQAVVPVSGLLALTARTLRQTEFIALRKLAGAER
TELNRALLSVDRFVRRDSPLPVDAGIRAQLLERFGMFGIRMSIAVLAAGVTDSTGLAA
ELLERSGLVALRNVIDQQFAQRSDMLKAHTALVSLRRFVQTHPVPATPYVIADIDPLL
ADTHAFEELRMLSLLPSRATTLNDDEIASLRRIIGGSGTSAAARLGLDPANSREAPRA
ALAAAQHWRRRAAHPLNDPFTTRACRAAVRSAEAMVAEFSARR"

CDS complement(415671..416204)
/codon_start=1
/note="gene_359"
/transl_table=11
/translation="MAALLAATALVAGCNTTIDGRPVASPGSGPTEPTFPTPRPTTAP
PGTTAPTLPTTPVSPTAPAGAIPLPPDSNGYVFIETKSGMTRCQINRDSVGCEAPFTN

```
                     SPLRDGEHANGIHITAGGSVQWVLGNLGAIPTVSIDYRTYEAQGWTIDATTDGTRFTN
                     NRTGHGMFVSIEKVDTF"
     CDS             416334..416750
                     /codon_start=1
                     /note="gene_360"
                     /transl_table=11
                     /translation="MALLPSTVVGVLLAAGAGRWYGKPKVLVDGWLDTAVGALRDGGC
                     NDVILVLGAVEVSAPAGVTAITAPDWQQGLSASVRAGLAQADREHADYAVLHVIDTPD
                     VNAKVVARVLGRALVSRSGLAGRGRIPAHSARRRGC"
     CDS             complement(416792..418093)
                     /codon_start=1
                     /gene="ansP-1"
                     /note="gene_361"
                     /transl_table=11
                     /translation="LFLVYGICGIFVFLILRALGELVLHRPSSGSFVSYAREFYGEKV
                     AFVAGWMYFLNWAMTGIVDTTAIAHYCHYWRAFQPIPQWTLALIALLVVLSMNLISVR
                     LFGELEFWASLIKVIALVTFLIVGTVFLAGRYKIDGQETGVSLWSSHGGIVPTGLLPI
                     VLVTSGVVFAYAAIELVGIAAGETAEPAKIMPRAINSVVLRIACFYVGSTVLLALLLP
                     YTAYKEHVSPFVTFFSKIGIDAAGSVMNLVVLTAALSSLNAGLYSTGRILRSMAINGS
                     GPRFTAPMSKTGVPYGGILLTAGIGLLGIILNAIKPSQAFEIVLHIAATGVIAAWATI
                     VACQLRLHRMANAGQLQRPKFRMPLSPFSGYLTLAFLAGVLILMYFDEQHGPWMIAAT
                     VIGVPALIGGWYLVRNRVTAVAHHAIDHTKSVAVVHSADPI"
     CDS             418019..418246
                     /codon_start=1
                     /gene="I917_02460"
                     /note="gene_362"
                     /transl_table=11
                     /translation="VQHQFAERAQDQEHEDAADPIDQEQTGPRRCKAAAGAEKKAGTD
                     GTTESDHLQLPAMEAFVIARVFAREPLVGDV"
     CDS             418768..419580
                     /codon_start=1
                     /note="gene_363"
                     /transl_table=11
                     /translation="MNVVTAHPKYPNDPLALVLIELRHPRTEPPVPSAISILKEELAR
                     WTPILEQEEVRQVNLETGEHTAHSQKKLVARDRRTAITFRPDAMTLEVTDYPGWEEFR
                     SIVHAMVTARQDVAPVDGCIRIGLRYINEIRASLAEPSGWAYWVAESLLGPGTQLADL
                     KLTTTAQRHVIQCEGPEPGDSLTLRYAGARGAVIQSTPFLQRLKEPPAEGDFFLIDID
                     SAWSDPCKGIPALDAHLVDEVAERLHTPIGPLFESLITSELRTKVLQQPGQE"
     CDS             419583..420236
                     /codon_start=1
                     /note="gene_364"
                     /transl_table=11
                     /translation="MTISFSSSNLRDDATSGNGDYRLDKLPETTPSTSVFDRADVTYR
                     QFTELHGQARDTRREAHVVELESKTGERARCAPMHALEQLADYGFAWRDIARVVGVSV
                     PAITKWRKGAGVTGENRLKIARLLALIDMLSDRFIGEPASWLEMPIQAGVGITRMDLL
                     ERGRYDLVLALASTHTGDGTVEYVLNETDKDWRETVVDNAFESYTAEDGVISIRPKR"
     CDS             420239..420898
                     /codon_start=1
                     /note="gene_365"
                     /transl_table=11
                     /translation="VPELETPDDPESIYLARLEDVGEHRPTFTGDIYRLGDGRMVMIL
```

QHPCALRHGVDLHPRLLVAPVRPDSLRSNWARAPFGTMPLPKLIDGQDHSADFINLEL
IDSPTLPTCERIAVLSQSGVNLVMQRWVYHSTRLAVPTHTYSDSTVGPFDEADLIEEW
VTDRVDDGADPQAAEHECASWLDERISGRTRRALLSDRQHASSIRREARSHRKSVKLA
D"

```
CDS             421125..423002
                /codon_start=1
                /gene="dnaK"
                /note="gene_366"
                /transl_table=11
                /translation="MARAVGIDLGTTNSVVSVLEGGDPVVVANSEGSRTTPSIVAFAR
                NGEVLVGQPAKNQAVTNVDRTVRSVKRHMGSDWSIEIDGKKYTAPEISARILMKLKRD
                AEAYLGEDITDAVITTPAYFNDAQRQATKDAGQIAGLNVLRIVNEPTAAALAYGLDKG
                EKEQRILVFDLGGGTFDVSLLEIGEGVVEVRATSGDNHLGGDDWDQRVVDWLVDKFKG
                TSGIDLTKDKMAMQRLREAAEKAKIELSSSQSTSINLPYITVDADKNPLFLDEQLTRA
                EFQRITQDLLDRTRKPFQSVIADTGISVSEIDHVVLVGGSTRMPAVTDLVKELTGGKE
                PNKGVNPDEVVAVGAALQAGVLKGEVKDVLLLDVTPLSLGIETKGGVMTRLIERNTTI
                PTKRSETFTTADDNQPSVQIQVYQGEREIAAHNKLLGSFELTGIPPAPRGIPQIEVTF
                DIDANGIVHVTAKDKGTGKENTIRIQEGSGLSKEDIDRMIKDAEAHAEEDRKRREEAD
                VRNQAETLVYQTEKFVKEQREAEGGSKVPEDTLNKVDAAVAEAKAALGGSDISAIKSA
                MEKLGQESQALGQAIYEAAQAASQATGAAHPGGEPGGAHPGSADDVVDAEVVDDGREA
                K"
CDS             422999..423706
                /codon_start=1
                /note="gene_367"
                /transl_table=11
                /translation="VTDGNQKPDGNSGEQVTVTDKRRIDPETGEVRHVPPGDMPGGTA
                AADAAHTEDKVAELTADLQRVQADFANYRKRALRDQQAAADRAKASVVSQLLGVLDDL
                ERARKHGDLESGPLKSVADKLDSALTGLGLVAFGAEGEDFDPVLHEAVQHEGDGGQGS
                KPVIGTVMRQGYQLGEQVLRHALVGVVDTVVVDAAELESVDDGTAVADTAENDQADQG
                NSADTLGEQAESEPSGS"
CDS             423742..424929
                /codon_start=1
                /note="gene_368"
                /transl_table=11
                /translation="MAQREWVEKDFYQELGVSSDASPEEIKRAYRKLARDLHPDANPG
                NPAAGERFKAVSEAHNVLSDPAKRKEYDETRRLFAGGGFGGRRFDSGFGGGFGGFGVG
                GDGAEFNLNDLFDAASRTGGTTIGDLFGGLFGRGGSARPSRPRRGNDLETETELDFVE
                AAKGVAMPLRLTSPAPCTNCHGSGARPGTSPKVCPTCNGSGVINRNQGAFGFSEPCTD
                CRGSGSIIEHPCEECKGTGVTTRTRTINVRIPPGVEDGQRIRLAGQGEAGLRGAPSGD
                LYVAVHVRPDKIFGRDGDDLTVTVPVSFTELALGSTLSVPTLDGTVGVRVPKGTADGR
                ILRVRGRGVPKRSGGSGDLLVTVKVAVPPNLAGAAQEALEAYAAAERSSGFNPRAGWA
                GNR"
CDS             424929..425309
                /codon_start=1
                /note="gene_369"
                /transl_table=11
                /translation="MAKNPKDGESRTFLISVAAELAGMHAQTLRTYDRLGLVSPRRTS
                GGGRRYSLHDVELLRQVQHLSQDEGVNLAGIKRIIELTSQVEALQSRLQEMAEELAVL
                RANQRREVAVVPKSTALVVWKPRR"
variation        425301..425402
                /note="supplemented from AL2122/97"
```

CDS complement(425434..435957)
/codon_start=1
/note="gene_370"
/transl_table=11
/translation="MSFAVLPPEINSARLYVGAGLAPMLDAAAAWDGLADELGSAAAS
FSAVTAGLAGSSWLGAASTAMTGAAAPYLGWLSAAAAQAQQAATQTRLAAAAFEAALA
ATVHPAIISANRALFVSLVVSNLLGQNAPAIAATEAAYEQMWAQDVAAMFGYHAGASA
AVSALTPFGQALPTVAGGGALVSAAAAQVTTRVFRNLGLANVGEGNVGNGNVGNFNLG
SANIGNGNIGSGNIGSSNIGFGNVGPGLTAALNNIGFGNTGSNNIGFGNTGSNNIGFG
NTGDGNRGIGLTGSGLLGFGGLNSGTGNIGLFNSGTGNVGIGNSGTGNWGIGNSGNSY
NTGFGNSGDANTGFFNSGIANTGVGNAGNYNTGSYNPGNSNTGGFNMGQYNTGYLNSG
NYNTGLANSGNVNTGAFITGNFNNGFLWRGDHQGLIFGSPGFFNSTSAPSSGFFNSGA
GSASGFLNSGANNSGFFNSSSGAIGNSGLANAGVLVSGVINSGNTVSGLFNMSLVAIT
TPALISGFFNTGSNMSGFFGGPPVFNLGLANRGVVNILGNANIGNYNILGSGNVGDFN
ILGSGNLGSQNILGSGNVGSFNIGSGNIGVFNVGSGSLGNYNIGSGNLGIYNIGFGNV
GDYNVGFGNAGDFNQGFANTGNNNIGFANTGNNNIGIGLSGDNQQGFNIASGWNSGTG
NSGLFNSGTNNVGIFNAGTGNVGIANSGTGNWGIGNPGTDNTGILNAGSYNTGILNAG
DFNTGFYNTGSYNTGGFNVGNTNTGNFNVGDTNTGSYNPGDTNTGFFNPGNVNTGAFD
TGDFNNGFLVAGDNQGQIAIDLSVTTPFIPINEQMVIDVHNVMTFGGNMITVTEASTV
FPQTFYLSGLFFFGPVNLSASTLTVPTITLTIGGPTVTVPISIVGALESRTITFLKID
PAPGIGNSTTNPSSGFFNSGTGGTSGFQNVGGGSSGVWNSGLSSAIGNSGFQNLGSLQ
SGWANLGNSVSGFFNTSTVNLSTPANVSGLNNIGTNLSGVFRGPTGTIFNAGLANLGQ
LNIGSANLGDFNLGSGNVGSFNVFSGNQGSYNIGPANLGNYNIGFANLGNYNIGFGNA
GDFNQGFANTGNNNIGFANTGNNNIGIGLSGDNQQGFNFAGGWNSGTANIGLFNSGTN
NVGIGNSGTGNWGIGNSGSGNTGIGNTGSTNTGFFNTGIVNTGVANAGSYNTGWYNTG
DTNTGIANLGDFNTGFYNTGNFSTGFANQGDIATGAFITGDMGNGAFWRGDQQGLFSA
GYRVHVPEIPAHVTVEVPVNIPITASFTNTVYSGITLEQINFGFTIDIAGIPLLAGAI
SKAVLPPITGTGPAITVNIGDPGGSTAIRIPATASVGPFDVTFVNIAATTGFFNATTD
PSSGFFNGGPGTVSGIANIGANISGFQNVANSATSGFNNYGSLQSGLANLGDTVSGVF
NTGIGAPANVSGMFNIGSNLAGFFHDQATGMSMFNLGLGNIGQFNVGFSNVGDSNAGL
ANIGSFNLGSGNLGSFNVFGGNQGSYNIGPANLGNYNIGLGNLGSYNFGFGNAGDFNL
GFANTGNNNIGFANTGNNNIGIGLSGDNQQGFNFAGGWNSGSGNSGLFNSGTNNIGLF
NSGTGNIGIGNSGTGNWGIANTGDTNTGIFNTGDVNTGLLNAGNVNTGIFNTGHYNTG
SFNAGSFNTAGFNPGSYNTGYLNTGSYNTGLANSGDVNTGGFITGNYSNGFWWRGDYQ
GLAGISQTITVPDTAVPVKLHVPIFLDIPVTGTLGTFTVHGFRFPEITGDIFLIGIPF
NAATLDAFSFPNISIVLPNIGINLGSGPDPLIDIAGTGGLLPIKIPLIDIPAAPGFGN
STTTPSSGFFNAGTGTVSGVGNVGSNSSGFFNLTSGSSGISGVQNFGELISGGFNFGN
TVSGLVNASTLGLSMPANLSGGGNVGATVAGFVNNTQILNLGFGNVGSGNVGHGNIGD
SNVGLGNLGNANVGHGNIGSFNVFSGNRGSYNIGPANLGNYNIGLGNLGSYNFGFGNA
GDFNLGFANSGSNNIGFANTGNNNIGIGLSGHNQQGFGSWNSGTANTGLFNSGTNNIG
LFNSGTGNIGIGNSGIGNTGIGNPGVGNTGLGNSGTGNWGLWNPGTGNMGVANVGTYN
TGGYNVGSTNTGIANVGIANTGSYNTGSTNTGSFNDGDFNTGFYNTGDYNTGFYNTGD
VNTGAFIGGNFSNGAFWQSDHQGQWGAHYAITVPQIPLLNFSLNIPVNIPIHLDFGTL
AVNGFQIPAITLRALGVTHFSVGPIIVPRIAGTLPVIDINIGDPGGSSSIPITITSGA
GPVVIPLLDIPPAPGFGNSTTGPSSGFFNSGTGSSSGFGNVGANNSGFWNTAFAGIGN
SGLQNFGSLQSGWANLGNTVSGFYNTSAADFATPANLSGLSNVGADLTGVLRGPNGST
FNAGLANLGQFNVGSANLGSANLGSANLGNSNVGFGNIGNANIGGANIGDFNVGIANT
GPGLTAAVNNIGIGNTGNYNIGVGNTGNYNIGFGNTGNNNIGIGLSGDNQIGFGPLNA
GIANMGLFNLGDNNFGMANAGNFNQGIANTGNNNIGLFNTGNNNVGIGLTGDGLSGFS
SLNSGAGNTGFFNSGTANTGLFNSGTGNTGLFNSGTGNVGIGNMGTGGFGVGLSGDSQ
VGIGGTNSGSFNIGLFNSGTGNVGIGNSGTGNVGIGNTGTGNTGIGNSGNYNTGLLNA

GLVNTGIANPGNHNTGLFNIGTFNTGIANPGHYNTGSYNTGSYNTGMANAGDYGTGAF
ITGSMNNGLLWRADRQGLLAANYTITIERPAAFLNVDIPVNIPITGDITNVSIPAITF
PRIDASGSVDIGILSGTVLAPVGPITLHGGDASAPLDTPIEIDFGPSPAINLNIGKPD
GSTVINIVGGAGAGPISIPIIDLRPAPGFFNATTGPSSGFLNWGAGSASGLLNFGNNS
GLYNFATSSMGNSGFQNYGSLQSGWANLGNSISGIYNTGLGAPANVSGLLNIGTNLAG
WLQNGPTETTFSVGLANLGFWNLGSANIGNYNLGSANIGVYNLGSANIGDFNLGSANI
GDFNLGSANIGSSNIGFGNVGPGLTAAIGNIGFGNTGNGNIGIGNTGTGNIGFGNTGN
GNIGIGLTGDTMTGFGGWNSGTGNIGLFNSGTGNIGFGNSGTGNWGIGNSGDYNTGIG
NTGSTNSGFFNTGLVNTGIGNSGDYNTGLFNAGNTNTGSFNPGDYNTGGFNPGNYNTG
YFNPGNSNTGFANSGDVNTGAFNSGNYSNGFFWRGDYQGLGGFAYQSAVSEIPWSYDI
GSNIEIPIEGDINAITQDAFTIDEFEIPIKLRVSVCVIYIPFKGCVKHVSVTIPITTE
HLGPYEIDASTINPDQPIDTAFTQTLDFAGSGTVGAFPFGFGWQQSPGFFNSTTTPSS
GFFNSGAGGASGFLNDAAAAVSGLGNVFTETSGFFNAGGVGNSGFQNFGNLLSGWANL
GNTVSGFYNTSMLDLATQALISGFGNHGARLSGILNNGSGP"

CDS complement(436108..436752)
/codon_start=1
/note="gene_371"
/transl_table=11
/translation="VTDASVHPDELDPEYHHHGGFPEYGPASPGAGFGQFVATMRRLQ
DLAVAADPGDAVWDEAAERAAALVELLSPFEADEGKAPAGRTPGLPGMGSLLLPPWTV
TRYGTDGVEMRGSFSRFHVGGNSAVHGGVLPLLFDHMFGMISHAAGRPISRTAFLHVD
YRRITPIDVPLIVRGRVTNTEGRKAFVCAELFDSDETLLAEGNGLMVRLLPGQP"

CDS complement(436749..438047)
/codon_start=1
/note="gene_372"
/transl_table=11
/translation="MPAIVLIGAQWGDEGKGKATDLLGGRVQWVVRYQGGNNAGHTVV
LPTGENFALHLIPSGVLTPGVTNVIGNGVVIDPGVLLNELRGLQDRGVDTAKLLISAD
AHLLMPYHIAIDKVTERYMGSKKIGTTGRGIGPCYQDKIARIGIRVADVLDPEQLTHK
VEAACEFKNQVLVKIYNRKALDPAQVVDALLEQAEGFKHRIADTRLLLNAALEAGETV
LLEGSQGTLLDVDHGTYPYVTSSNPTAGGAAVGSGIGPTRIGTVLGILKAYTTRVGSG
PFPTELFDEHGEYLSKTGREFGVTTGRRRRCGWFDAVIARYAARVNGITDYFLTKLDV
LSSLESVPVCVGYEIDGRRTRDMPMTQRDLCRAKPVYEELPGWWEDISGAREFDDLPA
KARDYVLRLEQLAGAPVSCIGVGPGREQTIVRRDVLQDRP"

CDS 438138..438785
/codon_start=1
/note="gene_373"
/transl_table=11
/translation="MYTAENAPGVAVLLSGDADVPGPLTGLPTHQDNLDTVIGRYSRL
IVVGADADLGAVLTRLLRTDRLDVEVGYVPRRRSPATRAYRLPAGRRAARRARCGVAR
RVPLIRDETGSVIVGRAQWLPAEEQALIHGEAVVDDTVLFDGDVAGVCIEPTLTLPGL
RAAVDGAGKWRRWIGGRAAQLGTTGAAVLRDGVAAPRPVRRSTFYRNVEGWLLVR"

CDS 438796..439575
/codon_start=1
/note="gene_374"
/transl_table=11
/translation="VSETGQRESVRPSPIFLGLLGLTAVGGALAWLAGETVQPLAYAG
VFVMVIAGWLVSLCLHEFGHAFTAWRFGDHDVAVRGYLTLDPRRYSHPMLSLGLPMLF
IALGGIGLPGAAVYVHTWFMTTARRTLVSLAGPTVNLALAMLLLAATRLLFDPIHAVL
WAGVAFLAFLQLTALVLNLLPIPGLDGYAALEPHLRPETQRALAPAKQFALVFLLVLF
LAPTLNGWFFGVVYWLFDLSGVSHRLAAAGSVLTRFWSIWF"

```
CDS             complement(439580..439984)
                /codon_start=1
                /note="gene_375"
                /transl_table=11
                /translation="MGDLLGPEPILLPGDSDAEAELLANESPSIVAAAHPSASVAWAV
                LAEGALADDKTVTAYAYARTGYHRGLDQLRRHGWKGFGPVPYSHQPNRGFLRCVAALA
                RAAAAIGETDEYGRCLDLLDDCDPAARPALGL"
CDS             440100..440927
                /codon_start=1
                /note="gene_376"
                /transl_table=11
                /translation="MSNAPEPDRSAGESGSEPAGERSADPGEERTESYPLVPHDAETE
                TVVITTSDNDAAVTQPEAQRERRFTAPGFDAKETQVIVTAHEAATEVFQTNQAPTTPP
                RMPTGMPPKTAVPQSIPPRTEATSVRQRTWGWALAVVVIVLALAAIAILGTVLLTRGK
                HSKMSQEDQVRQAIQSLDIAIQTGDLTALRSLTCGSTRDGYVDYDERDWAETYRRVSA
                AKQYPVIASIDQVVVNGAHAEANVTTFMAFDPQVRSTRSLDLQFRDDQWKICQSSSN"
CDS             441149..442531
                /codon_start=1
                /note="gene_377"
                /transl_table=11
                /translation="VSIRPAENSTLDIRHVIGIGTPKAVDLWLDVVTELPDRARELGS
                LSKAELGKLGPLLDGTNAVELFESIDDKLAAEALHAMDPSLAATFLEALDSDHAANIL
                REFKEPKREALLTLLPLERAMVLRGLLSWPEDCAAAHMVPETLTVRPNMTVSQAVASV
                RERASGLRSDARTTAYVYVTDADSHLLGVIAFRALVLANPEQRVRELMGDDLIVVSPL
                TDKELAAQTIMGHNLMAVPVVDADNRLLGIIAEDEAIDIAEEEATEDAERQGGSAPLE
                VPYLRASPWLLWRKRVVWLLVLFAAEAYTGSVLRAFSDEMEAVIALAFFIPLLIGTGG
                NTGTQIATTLVRAMATGQVRFRDVPAVLAKELSTGVLVGLTMAAAAVVRAWTLGVGPQ
                VTLTVALTVAAIVVWSSLVAAVLPPLLKKLRIDPAIVSGPMIATIVDGTGLLIYFLVA
                HLTLTELHGL"
CDS             complement(442543..443577)
                /codon_start=1
                /note="gene_378"
                /transl_table=11
                /translation="MPIATPEVYAEMLGQAKQNSYAFPAINCTSSETVNAAIKGFADA
                GSDGIIQFSTGGAEFGSGLGVKDMVTGAVALAEFTHVIAAKYPVNVALHTDHCPKDKL
                DSYVRPLLAISAQRVSKGGNPLFQSHMWDGSAVPIDENLAIAQELLKAAAAAKIILEI
                EIGVVGGEEDGVANEINEKLYTSPEDFEKTIEALGAGEHGKYLLAATFGNVHGVYKPG
                NVKLRPDILAQGQQVAAAKLGLPADAKPFDFVFHGGSGSLKSEIEEALRYGVVKMNVD
                TDTQYAFTRPIAGHMFTNYDGVLKVDGEVGVKKVYDPRSYLKKAEASMSQRVVQACND
                LHCAGKSLTH"
CDS             443673..444356
                /codon_start=1
                /note="gene_379"
                /transl_table=11
                /translation="MSTAVTAMPDILDPMYWLGANGVFGSAVLPGILIIVFIETGLLF
                PLLPGESLLFTGGLLSASPAPPVTIGVLAPCVALVAVLGDQTAYFIGRRIGPALFKKE
                DSRFFKKHYVTESHAFFEKYGKWTIILARFVPIARTFVPVIAGVSYMRYPVFLGFDIV
                GGVAWGAGVTLAGYFLGSVPFVHMNFQLIILALVFVSLLPALVSAARVYRARRNAPQS
                DPDPLVLPE"
CDS             complement(444345..445475)
                /codon_start=1
```

```
                     /note="gene_380"
                     /transl_table=11
                     /translation="MNLANRAASAETAVTQRHLRRLWALPGTQLAVVAWPSTRRDRLF
                     GSWHYWWQAHLLDCLVDAQLRDPQPQRRARINRQVRSHRVRNNFSWLNSYYDDMAWLA
                     LALERADRVAGVRRRRALPKLTNQFVEAWVPEDGGGIPWRKQDQFFNAPANGPAGLFL
                     ARYPDQYGKRLKRAEQMADWIDRTLIDPETHLVFDGIKAGSLVRAQYTYCQGVVLGLE
                     TELAVRTGPAARARHCARVHRLVAAVNEHMAPLGVLRGAGGGDGGLFAGITARYLALV
                     ATTLPGDSADDAAARDTARAIVLASAQSAWDYRQTVDGLPVFGAFWDREAELPTAGGE
                     QARSVRGAVHSSAIAERDLSVQLSGWMLMEAAHSAAAVSSLG"
     CDS             complement(445500..446093)
                     /codon_start=1
                     /note="gene_381"
                     /transl_table=11
                     /translation="VKRLDLVAGPNGAGKSTFVALTLAPLLPGIVFVNADEIAKQRWP
                     DDPTSHAYQAAQVAADTRARLIDLGRPFIAETVFSHPSKLELIRTARTAGYTVVLHVL
                     VIPEGLAVERVRHRVAAGGHDVPETKIRERHRRLAELVAQAITLADGATVYDNSRLAG
                     PRIVAQFSGGGIIGRACWPSWTPPPLMSRWSNRPETA"
     CDS             complement(446122..446511)
                     /codon_start=1
                     /note="gene_382"
                     /transl_table=11
                     /translation="VPKAVDRVTRVAADLVDSAAAEGARQSRSAKQQLDHWARVGRAV
                     SNQHTASRRRVEAALAGHLPMTDLTLEEGVVFNAEISAAIEERLSRTNYGDVLAAQGI
                     TTVALNDAGDIVEHRPDGTSVVLAATP"
     CDS             complement(446592..447812)
                     /codon_start=1
                     /note="gene_383"
                     /transl_table=11
                     /translation="MNGMATPALLPGVDLAAFAAALAARLRDAGIPVSASGQASLVQA
                     LQQLVPRTPAALYWGARLTLVSRVDELATFDAVFASLFGVFGSAEPDGANRPPPPIAG
                     PRTPVAGVGHRAKRRSCAAQAQNLPWDTRSLTMASAGQGGPSRTLPDVLPSRIVARAD
                     EPFDQFDPDDLRLLGAWLEATMARWPRRRSMRFESSPHGKRIDLRATMNASRSTGWES
                     VLLARIRPRRRPRRVLLLCDVSRSMQPYAAIYLHLMRAAVLRRAGGHPEVFAFSTSLT
                     RLTSVLSHRSAEMALHRANARVTDRYGGTFIGRSVAALLAPPHGNALRGAVVIIASDG
                     WDSDPPDVLVHALTRVRRRAELLVWLNPRAAHPEFQPRAGSMAAALPYCDLFLPAHSL
                     AGLHQLLLALAGAR"
     CDS             complement(447809..448411)
                     /codon_start=1
                     /note="gene_384"
                     /transl_table=11
                     /translation="MKIANEFTVSAPIEQAWSRLCDLEQMIPLMPGAQLIGHEGDEYL
                     GKVKVKVGPVTSEFSGKVHFVEQDRNQHRAVFDAKGKEARGTGNAAATVAAQLHEVGE
                     RTRVTVDTDLKIVGKLAQFGSGMLQQVSEKLLGQFVDSLEAELAAQSSESPQGTPPAT
                     EAAPIDLLQLADGGQLKKYGSALLAALTVLLLIWVLRRRR"
     CDS             complement(448425..449321)
                     /codon_start=1
                     /note="gene_385"
                     /transl_table=11
                     /translation="VTFASPDDVIRRFDEQNYLLDTGTASAIYLAVTLGRPLLLEGEP
                     GVGKTTAAKTLAVVLDTTLIRLQCYEGLTANEALYDWNYQRQLLSIRLAEARGKGISD
                     ISEADLYTEAYLVDRPILRCVRHRGPTPPVLLIDEIDRADDEFEALLLEFLGESAVTV
```

PELGTFLAECPPIAVLTSNRSRDLHDALRRRCLYHWIDYPEPDRAAAIVRRTVPGATA
PLIENATQFVCTARDLDLDKPPGVAETIDWVAALVALGVADLTAADSSPALASLGALA
KTPDDRTQIRDAYQAFTECSHA"
CDS complement(449318..449911)
/codon_start=1
/note="gene_386"
/transl_table=11
/translation="MTATQITGVVLAAGRSNRLGTPKQLLPYRDTTVLGATLDVARQA
GFDQLILTLGGAASAVRAAMALDGTDVVVVEDVERGCAASLRVALARVHPRATGIVLM
LGDQPQVAPATLRRIIDVGPATEIMVCRYADGVGHPFWFSRTVFGELARLHGDKGVWK
LVHSGRHPVRELAVDGCVPLDVDTWDDYRRLLESVPS"
CDS complement(449908..450663)
/codon_start=1
/note="gene_387"
/transl_table=11
/translation="MSISDRAAQLVAARTPFVRATVVRAQQPTSARPGDEAILLADGT
IEGFVGGHCAQNSVRKAAMGVLQAGESVLLRVLPDGDVHFPEAPGACVVVNPCLAGGS
LEIFLTPQLPAPLIQIYGETPIADALIELCGLLGYDARRDTDPADTDALPTAIVIASH
GGPEAEIIRTALDNGVGYVGLVASTVRGASILDSLDLSDAERARVHTPVGLAIGAKTP
AEIAVSIAAELIATLRGGGPRGRKALADENGGA"
CDS complement(450682..453000)
/codon_start=1
/note="gene_388"
/transl_table=11
/translation="MRKEDPRFIRGRGTYVDDVALPGMLHLAILRSPYAHARIVRIDV
TAAQAHPKVKAVVTGADLAAKGLAWMPTLANDVQAVLATDKTRFQGQEVASVVAEDRY
SARDACELVDVDYEPRDPVVDARTALDPSAPVIRTDLEGKSDNHIFDWETGDAAATEA
VFAKADVVVQQEIVYPRVHPAPMETCGAVADLDPVTGKLTLWTTSQAPHAHRTLYALV
AGLPEHKIRVISPDIGGGFGNKVPIYPGYVCAIVASLLLDKPVKWMEDRSENLTSTGF
ARDYIMVGEIAANRDGKILAIRSNVLADHGAFNAQAAPAKYPAGFFGVFTGSYDIEAA
YCHMTAVYTNKAPGGVAYACSFRITEAVYFVERLVDCLAFELKMDPAELRLRNLLRPN
QFPYQSKTGWVYDSGDYETTMRKAMNMIGYEALRAEQKQRRARGELMGIGMSFFTEAV
GAGPRKDMDILGLGMADGCELRVHPTGKAVLRLSVQTQGQGHETTFAQIVAEELGIAP
DDIEVVHGDTDQTPFGLGTYGSRSTPVSGGAAALVARKVRDKAKIIASGMLEVSVADL
QWEKGKFHVKGDPSAAVTIADIAMRAHGAGDLPEGIEGGLDAEVCYNPSNLTYPYGAY
FCVVDIDPGTAVVKVRRFLAVDDCGTRINPMIIEGQVHGGIVDGIGMALMEMIAFDED
GNCLGGSLMDYLIPTALEVPHLETGHTVTPSPHHPIGAKGIGESATVGSPPAVVNAVV
DALAPFGVRHADMPLTPSRVWEAMQGRATPPI"
CDS complement(453078..453557)
/codon_start=1
/note="gene_389"
/transl_table=11
/translation="MQVNMTVNGEPVTAEVEPRMLLVHFLRDQLRLTGTHWGCDTSNC
GTCVVEVDGVPVKSCTMLAVMASGHSIRTVEGLAGPDGQLDPVQEGFMRCHGLQCGFC
TPGMLITARALLDRNPDPDEQTIREAISGQICRCTGYTTIVRSIQWAAAHQTVKAQS"
CDS complement(453572..454432)
/codon_start=1
/note="gene_390"
/transl_table=11
/translation="VDHAIGLLDRLGEGARVVAGGHSLLPMMKLRIANPEYLVDINDL
APELGYVVVGGINNPNLVRLGAMTRHREILDSDALAAVCPIFRDAERVIADPVVRNRG

TLGGSLCQADPAEDLSTVCTVLDAVCLAKGPSGEREIAIDDFLVGPYETALAHNEVLI
EVRIPLRHNTSSAYAKVERRVGDWAITAAGAAVTLDGQTILAARVGLTAVNPDPVALA
ELSAGLVGQPATEEVFAEAGRRAAQACTPVTDVRGTAEYKRHLAGELTVRTLRTAAGR
VLGAPAAPEA"
CDS complement(454508..455674)
/codon_start=1
/note="gene_391"
/transl_table=11
/translation="VREVLAELMAIWAAGDTAGVATVVRTLRSAPRPPGAAMVVAPDG
SVSGSVSGGCVEGAVYELAAEVAQTGIPRLEHYGVSDDTAFAVGLTCGGIIDVFVEPV
SRATFPELGELADDIGAQRPVAIATVIAHPDERRVGRRLVIRPDTKSPVTGSLGSARA
DAAVIDDARGLLAVGRSEILEYGPDGQRRGEGMEVFVSSHAPRPRMLVFGAIDFAAAL
ARQGSFLGYRVTVCDARAVFATPARFPTADDVVVAWPHRYLAAQAEAGGIDERTVICV
LTHDPKFDVPVLEVALRLGVGYVGAMGSRKTHDDRMDRLRAAGLTDAELSRLSSPIGL
DLGARTPEETAVSIAADIIARRWGGGGRPLADIAGRIHHDAQVAGEFKDYLTRH"
CDS 455699..456664
/codon_start=1
/note="gene_392"
/transl_table=11
/translation="MTPAQLRAYSAVVRLGSVRAAAAELGLSDAGVSMHVAALRKELD
DPLFTRTGAGLAFTPGGLRLASRAVEILGLQQQTAIEVTEAAHGRRLLRIAASSAFAE
HAAPGLIELFSSRADDLSVELSVHPTSRFRELICSRAVDIAIGPASESSIGSDGSIFL
RPFLKYQIITVVAPNSPLAAGIPMPALLRHQQWMLGPSAGSVDGEIATMLRGLAIPES
QQRIFQSDAAALEEVMRVGGATLAIGFAVAKDLAAGRLVHVTGPGLDRAGEWCVATLA
PSARQPAVSELVGFISTPRCIQAMIRGSGVGVTRFRPKVHVTLWS"
CDS 456909..457136
/codon_start=1
/note="gene_393"
/transl_table=11
/translation="VVVSGRWEAGNADGNGGSAGLIGSGGAGGDGGSGGATGAGGEGG
DAGASGSINGNAGDPGNSGERGAVGKPGAPG"
CDS 457255..457470
/codon_start=1
/note="gene_394"
/transl_table=11
/translation="MSVYKVIDIIGTSPTSWEQAAAEAVQRARDSVDDIRVARVIEQD
MAVDSAGKITYRIKLEVSFKMRPAQPR"
CDS complement(457546..458196)
/codon_start=1
/note="gene_395"
/transl_table=11
/translation="MSALGPGPTEWGAPTGGVGPWAGDLPDDPRYDPVLLRDGDARNV
VDAYRYWTREAIIADIDTRRHPLHVAIENFGHDANIGSVVRTANAFAVHTVHIVGRRR
WNRRGAMVTDRYQRLCHHDSTTGLLEFAAGAGLTVVAVDNVPGAARLEQTALPRECLL
LFGQEGPGITDDARAGAAVTVSIAQFGSTRSINAGVAAGIAMHAWIRQHADLGRAW"
CDS complement(458193..459101)
/codon_start=1
/note="gene_396"
/transl_table=11
/translation="VRILVAWATCGAVVLSGLTGCSGSSHSGRTYGAQSARTGESLAV
LGWNMSVSNLRWSGDYVLIDVDASPTDPHAPHAKPEDIRFGLYGALAHPMESAALGSC

GDAMAHVRDVVSPLSAPAGRLTGTVCLGPLKERSAVRGVYTYSPRDRIPGTAAAYPAA
FPVGMLPTNQNDAGLVVKTTSVSAWRADGMQLGKPQLGDPVAFTGNGYMLLGLEVDAV
PDRYRDDSAARGGPMMLLAAPTLPGRGLSPACATYGSSVLILPDALLDAVHISASLCT
QGEINEALLYATVATVGTHAALWTSR"

CDS complement(459119..459658)
/codon_start=1
/gene="pyrE"
/note="gene_397"
/transl_table=11
/translation="VAGPDRAELAELVRRLSVVHGRVTLSSGREADYYVDLRRATLHH
RASALIGRLMRELTADWDYSVVGGLTLGADPVATAIMHAPGRPIDAFVVRKSAKAHGM
QRLIEGSEVTGQRVLVVEDTSTTGNSALTAVHAVQDVGGEVVGVATVVDRATGAAEAI
EAEGLRYRSVLGLADLGLD"

CDS complement(459739..460593)
/codon_start=1
/note="gene_398"
/transl_table=11
/translation="MVPLWFTLSALCFVGAVVLLYVDIDRRRGRSRRRKSWARSHGFD
YERESTEILKRWTRGVMSTVGDVAAHNVVLGQIRGEAVYIFDLEEVATVIALHRKVGT
NVVVDLRLKGLKEPRESDIWLLGAIGPRMVYSTNLDAARRACDRRMVTFAHTAPDCAE
IMWNEQNWTLVSMPIASTRAQWDEGLRTVRQFNDLLRVLPPLPQEMPQQTGVGPRGAA
PGRPVAPGGPAELPPRRAQPDPATTVLPDPARRAPEPIRRDEGRSEGVRRPPPAGRNG
QQATNYQH"

CDS complement(460734..463280)
/codon_start=1
/note="gene_399"
/transl_table=11
/translation="VDSFNPTTKTQAALTAALQAASTAGNPEIRPAHLLMALLTQNDG
IAAPLLEAVGVEPATVRAETQRLLDRLPQATGASTQPQLSRESLAAITTAQQLATELD
DEYVSTEHVMVGLATGDSDVAKLLTGHGASPQALREAFVKVRGSARVTSPEPEATYQA
LQKYSTDLTARAREGKLDPVIGRDNEIRRVVQVLSRRTKNNPVLIGEPGVGKTAIVEG
LAQRIVAGDVPESLRDKTIVALDLGSMVAGSKYRGEFEERLKAVLDDIKNSAGQIITF
IDELHTIVGAGATGEGAMDAGNMIKPMLARGELRLVGATTLDEYRKHIEKDAALERRF
QQVYVGEPSVEDTIGILRGLKDRYEVHHGVRITDSALVAAATLSDRYITARFLPDKAI
DLVDEAASRLRMEIDSRPVEIDEVERLVRRLEIEEMALSKEEDEASAERLAKLRSELA
DQKEKLAELTTRWQNEKNAIEIVRDLKEQLEALRGESERAERDGDLAKAAELRYGRIP
EVEKKLDAALPQAQAREQVMLKEEVGPDDIADVVSAWTGIPAGRLLEGETAKLLRMED
ELGKRVIGQKAAVTAVSDAVRRSRAGVSDPNRPTGAFMFLGPTGVGKTELAKALADFL
FDDERAMVRIDMSEYGEKHTVARLIGAPPGYVGYEAGGQLTEAVRRRPYTVVLFDEIE
KAHPDVFDVLLQVLDEGRLTDGHGRTVDFRNTILILTSNLGSGGSAEQVLAAVRATFK
PEFINRLDDVLIFEGLNPEELVRIVDIQLAQLGKRLAQRRLQLQVSLPAKRWLAQRGF
DPVYGARPLRRLVQQAIGDQLAKMLLAGQVHDGDTVPVNVSPDADSLILG"

CDS 463308..464585
/codon_start=1
/note="gene_400"
/transl_table=11
/translation="MLVGLFNAVNVESVPLNFTHLCIRRRFAASLESRSVGLEDRDAL
RVLQNAFKLDDPELVRRFYAHWFALDASVRDLFPPDMGAQRAAFGQALHWVYGELVAQ
RAEEPVAFLAQLGRDHRKYGVLPTQYDTLRRALYTTLRDYLGHPSRGAWTDAVDEAAG
QSLNLIIGVMSGAADADDAPAWWDGTVVEHIRVSRDLAVARLQLDRPLHYYPGQYVNV
HVPQCPRRWRYLSPAIPADPNGRIEFHVRVVPGGLVSNAIVGETRPGDRWRLSGPHGA

FRVDRDGGDVLMVAGSTGLAPLRALIIDLSRFAVNPRVHLFFGARYACELYDLPTLWQ
IAAHNPWLSVSPVSEYNGDPAWAADYPDVSAPRGLHVRQTGRLPDVVSRYGGWGDRQI
LICGGPAMVRATKAALIAKGAPPERIQHDPLSR"
CDS 464689..467946
/codon_start=1
/note="gene_401"
/transl_table=11
/translation="MSKLLPRGTVTLLLADVEGSTWLWETHPDDMGAAVARLDKAVSG
VIAAHDGVRPVEQGEGDSFVLAFACASDAVAAALDLQRARLAPIRLRIGVHTGEVALR
DEGNYAGPTINRTARLRDLAHGGQTVLSGVTESLVIDRLPDKAWLVDLGTHALRDLSR
PERVMQLCHPELRIDFPPLRVANDDVAHGLPVHLTRFVGRGAQITEVHRLVTDNRLVT
LTGAGGVGKTRLAAQLAAQIAGEFGRAWFVDLAPITDPDLVPVTVAGALGLHDQPGRS
TTDTVLRFLGGRPALVVLDNCEHLLDATAALVLALVKACRGVRLLATCREPLRVEGEV
SYRVPSLSLSDEAVEMFCYRAQRVRPDFRLTDDNSAAVTEICKRLDGLPLAIELAAAR
LRSMTLDEIIDGLRDRFALLTGGARTAAHRQQTLWASVDWSYTLLTEPERTLFRRLAV
FVGCFFVDDAQAVACSGDVQRYQVLDEITLLVDKSLVMADDNSGRTCYRLCETMRHYA
LEKLSEAGEVDAVFARHRDYYTALAARVDNPGPSDYSHCLDQAETEIDNLRAAFVWNR
ENSDTEGALALASSLLRVWMTRGRIQEGRAWFDSILADENARHLEVAAAVRARALADK
ALLDIFVDAAAGMEQAQQALVIAREVDEPALLSRALTACGLIAVAVARADAAASYFAE
AIDLARAVDDRWRLAQILTFQAVDAVVAGDPVAARPAAQEARELAAAIGDHSNALWCR
WCLGYAQLMRGELAAAAAQFGEVVDEAEASQEVLHKANSLQGLAFALAYQGELSAARA
AADAALEAAELGEYFAGMGYSALTTAALAAGDVQTAQHASEAAWRNLSLALPLSAAVQ
RAFNAQAALAGGDLSAARRWCDDAVQSMTGHHLAMALATRARIAVAEGKREEAERDAH
KALACAAESGAHLDLPDVLECLAGLASDAGTHHAAARLFGAAEAIRQQIGSVRFAIYR
SDYVQSVTALRDAMGEKDFDAAWAEGAALSIKETIAYAQRGHSWRKRPATGWESLTPT
EIDVVRLVGEGLANKDIATRLFVSPRTVQTHLTHVYTKLGFTSRLQLAQAAARRT"
CDS complement(467950..469281)
/codon_start=1
/note="gene_402"
/transl_table=11
/translation="MDFGALPPEINSARIYSGPGSRPLMQAAAAWQRLANELTATAAS
YSSVISGLTGDDWLGPSALSMAAAAVPYVAWMRATAASAEQAAAQAVAAANAYESAYA
ATVPPTVIAANRRTMLSLVKTNVFGQNTPAIATSEAQYGAMWAQDIVAMEGYAGASAA
ASQLPPFTPPPATTSGAGSLSDAAATAAQAVVPAAAATDVSLLPTLQSFLPPPFDAIP
NPIEDLDVLVAAAVAVAAGSLGVSAAQLGEIYRHDVVDEAQKAPHCPAESDQTPAGAA
GDGDLPEVGGRVTSPPQPPVAALTGYSANIGGLSVPHSWNLPPAVRQVAAMFPGATPM
YMTGSSDGSYAGLAAAGLAGTGLAGLTARGGSAPTPAAAAPAGAGGAGPAATRPAAQQ
TPAVPAAAAGSAIPGLPPGLPPGVVANLAATLAAIPGATIIVVPPSPNANQ"
CDS 469699..470874
/codon_start=1
/gene="purT"
/note="gene_403"
/transl_table=11
/translation="MLLGSAEPSRELAIALQGLGAEVIAVDGYVGAPAHRIADQSVVV
TMTDAEELTAVIRRLQPDFLVTVTAAVSVDALDAVEQADGECTELVPNARAVRCTADR
EGLRRLAADQLGLPTAPFWFVGSLGELQAVAVHAGFPLLVSPVAGVAGQGSSVVAGPN
EVEPAWQRAAGHQVQPQTGGVSPRVCAESVVEIEFLVTMIVVCSQGPNGPLIEFCAPI
GHRDADAGELESWQPQKLSTAALDAAKSIAARIVKALGGRGVFGVELMINGDEVYFAD
VTVCPAGSAWVTVRSQRLSVFELQARATLGLAVDTLMISPGAARVINPDHTAGRAAVG
AAPPADALTGALGVPESDVVIFGRGLGVALATAPEVAIARERAREVASRLNVPDSRE"
CDS 470871..471293

```
                     /codon_start=1
                     /note="gene_404"
                     /transl_table=11
                     /translation="VSYAGDITPLQAWEMLSDNPRAVLVDVRCEAEWRFVGVPDLSSL
                     GREVVYVEWATSDGTHNDNFLAELRDRIPADADQHERPVIFLCRSGNRSIGAAEVATE
                     AGITPAYNVLDGFEGHLDAEGHRGATGWRAVGLPWRQG"
     CDS             471290..472510
                     /codon_start=1
                     /note="gene_405"
                     /transl_table=11
                     /translation="MTDESSVRTPKALPDGVSQATVGVRGGMLRSGFEETAEAMYLTS
                     GYVYGSAAVAEKSFAGELDHYVYSRYGNPTVSVFEERLRLIEGAPAAFATASGMAAVF
                     TSLGALLGAGDRLVAARSLFGSCFVVCSEILPRWGVQTVFVDGDDLSQWERALSVPTQ
                     AVFFETPSNPMQSLVDIAAVTELAHAAGAKVVLDNVFATPLLQQGFPLGVDVVVYSGT
                     KHIDGQGRVLGGAILGDREYIDGPVQKLMRHTGPAMSAFNAWVLLKGLETLAIRVQHS
                     NASAQRIAEFLNGHPSVRWVRYPYLPSHPQYDLAKRQMSGGGTVVTFALDCPEDVAKQ
                     RAFEVLDKMRLIDISNNLGDAKSLVTHPATTTHRAMGPEGRAAIGLGDGVVRISVGLE
                     DTDDLIADIDRALS"
     CDS             complement(472507..473919)
                     /codon_start=1
                     /gene="ndh-1"
                     /note="gene_406"
                     /transl_table=11
                     /translation="MTLSSGEPSAVGGRHRVVIIGSGFGGLNAAKALKRADVDITLIS
                     KTTTHLFQPLLYQVATGILSEGDIAPTTRLILRRQKNVRVLLGEVNAIDLKAQTVTSK
                     LMDMTTVTPYDSLIVAAGAQQSYFGNDEFATFAPGMKTIDDALELRGRILGAFEAAEV
                     STDHAERERRLTFVVVGAGPTGVEVAGQIVELAERTLAGAFRTITPSECRVILLDAAP
                     AVLPPMGPKLGLKAQRRLEKMDAEVQLNAMVTAVDYKGITIKEKDGGERRIECACKVW
                     AAGVAASPLGKMIAEGSDGTEIDRAGRVIVEPDLTVKGHPNVFVVGDLMFVPGVPGVA
                     QGAIQGARYATTVIKHMVKGNDDPANRKPFHYFNKGSMATISRHSAVAQVGKLEFAGY
                     FAWLAWLVLHLVYLVGYRNRIAALFAWGISFMGRARGQMAITSQMIYARLVMTLMEQQ
                     AQGALAAAEQAEHAEQEAAG"
     CDS             474194..474385
                     /codon_start=1
                     /note="gene_407"
                     /transl_table=11
                     /translation="MRERLPKVGQVFAAGEIDYHMFQTLVYRTDLITDPQVLARVDAE
                     LALRVRGWPSMTRGSWPPR"
     CDS             474394..475386
                     /codon_start=1
                     /note="gene_408"
                     /transl_table=11
                     /translation="VAVADPDAVRQVRERARDREVSIWNSADGMGEVYAQLYATDAQA
                     LDARLNALVATVCAGDPRSTDQRRADALGALAAGADRLACRCDNPDCAAEGRPVSAVV
                     IHVVAEQASVKGHGQAPAALLGGDGLIPAELVAELAKTAGLQPIPVPAGTEPGYRPSV
                     KLAAFVRARDLTCRAPGCDRPATQCDLDHTIAFADGGATHAANLKCLCRLHHLLATFC
                     GWRAQQLPDGTVIWTLPGNQTYVTTPGSALLFPALCTPTGDPPAPEPARADRRGQRTA
                     MMPRRASTRTQNRAHCIAAERHRNHQARRIAQAAVIATETHGPPPDPDDDPPPF"
     CDS             complement(475402..476061)
                     /codon_start=1
                     /note="gene_409"
```

/transl_table=11
/translation="MVGGPLQTVFDAIEERTRHRAETTTREICESVGGADTVLSRIDK
NPELEPLLSQAIEAATRTSMEAKRRLLAQAAAAALEDDQKVEPASLIVATLSQLEPVH
IHALVRLAKAAKSSPDQDEIQRREVMRAASKVEPVPVLAALIQTGVAIATTTVWHGNG
TGTPAEESGHILIHDVSDFGHRLLAYLRAADAGAELLILPSGGSAPTGDHPTPHPSTS
R"

CDS 476184..476624
/codon_start=1
/note="gene_410"
/transl_table=11
/translation="VVAGHEEADRGSMDWMPLGDYETFRHWSGKPRAWGPQESGWRAW
FGGKIVDGLCEVLDEHLAVRRRGVPAAIGCVPWLSSEAVAETLLALSAFCVVIDKGTS
FPSRLRNPDKGFPNVALLRLRDMAPSEHGSRCSSARGRLCLSMS"

CDS 476606..477022
/codon_start=1
/note="gene_411"
/transl_table=11
/translation="MPEHELGPVRALGWLREDRKPLLNAKLLVLGHLALNVYDPDNGY
GEEVLDFEPRTVWWGSANWTVRAGSHLEVGFACDDPTLVEEATAFVADVIAFSEPIDT
TCAGPEPNLVQVEFDDAAMAEAMEEMAEPDDDGEDW"

CDS 477096..477464
/codon_start=1
/note="gene_412"
/transl_table=11
/translation="LLATFWGWRAQQLPDGTVIWTLPGDQTYVTTPGSALLFPALCTP
TGDPPRPDPARADRRGQRTAMMPRRASTRAQNRAHYIAAERHRNHQARRIAHVVTQTA
TTAPETNGPPPDPDDDPPPF"

CDS complement(477959..478600)
/codon_start=1
/note="gene_413"
/transl_table=11
/translation="MGVIARVVGVAACGLSLAVLAAAPTAGAEPTGALPPMTSSGSGP
VIGDGDAALRQRISQQLFSFGDPTVQEVDGSDAAQFITAAAAVADRDVASVFLPLQRV
LGCQQNTAGSGAGFGARAYRRTDGQWGGAMLVVAKSTVSDVDALKACVKSGWRKATAG
TPTSMCNNGWTYPPFADTRRGEEGYFVLLAGTASDFCSAPNANYRTTASSWPG"

CDS complement(478607..479836)
/codon_start=1
/note="gene_414"
/transl_table=11
/translation="MPVLRRLGCSVLALGLLAGCAPPRTGPASSPTNNGAKADAVIRI
VRDFMTQAHLKAVLVRVTVAGKEVVTRAVGDSMTGVPATTAMHFRNGAVAISYVATLL
LKLVDEKKLRLDDKLSRWLPDFPHADRVTLGQLAQMTSGYPDYVLGNEAFDAELYANP
FRQWTTQELLDQISSRPLLYDPGTNWNYAHTNYLLLGLALEKAAGQDMPTLLQRKVLS
PLGLTATANSDTPAIPEPALHAFTSERRAALKIPAGVPFYEESTFWNPSWTITHGAIQ
TTTIYDMEATAVGIGSGRLLSADSYKKMVSTELRGKTRAQPGCPTCFEQNDGYSYGLG
IVISGHWLLQNPMFAGYAAVEAYLPSQRVAVAVAVTYAPEAFDDQGNYRNQADILFRK
IGAEVAPNDAPPMPPGR"

CDS complement(479846..481033)
/codon_start=1
/note="gene_415"
/transl_table=11

/translation="MSTPTPPALDRDDPLGLDASLSSDEIAVRDTVRRFCAEHVTPHV
AAWFEDGDLPVARDLAKQFGELGLLGMQLHGHGCGGASAVHYGLACRELEAADSGIRS
LVSVQGSLAMFAIASFGSDEQKRQWLPGMATGDLLGCFGLTEPDVGSDPAAMKTRARR
DGPDWVITGGKMWITNGSVADVAIVWAATDDGIRGFIVPTDTPGFTANTIGHKLSLRA
SITSELVLDNVRLPADAMLPGATGLRAPLACLSEARYGIVWGAMGAARSAWQCALDYA
RQRTQFGRPIAGFQLTQAKLVDMAVELHKGQLLSLHLGRLKDRVGLRPDQVSFGKLNN
TREALKICRTARTILGGNGISLEYPVIRHMVNLESVLTYEGTPEMHQLVLGQAFTGLA
AFR"

CDS 481069..481440
/codon_start=1
/note="gene_416"
/transl_table=11
/translation="MRPRRALAGLAADVVAVLVFCAVGRRSHAEGLSVTGLAATAWPF
LTGTGIGWVLARGWRRPTALAPTGVIVWLCTIVVGMVLRKVSSAGVAASFVVVASAVT
AVLLLGWRAAVALMAPHRADG"

CDS complement(481635..482729)
/codon_start=1
/gene="mmpL1"
/note="gene_417"
/transl_table=11
/translation="MDNVDKLTEDLANLTDDTERMDTTQRQLLAQLDPTIATMQTVKD
LAQTLTSAFSGLVTQMEDMTRNATVMGRTFDAANNDDSFYLPPEAFQNPDFQRGLKLF
LSPDGTCARFVITHRGDPASAEGISHIDPIMQAADEAVKGTPLQAASIYLAGTSSTYK
DIHEGTLYDVMIAVVASLCLIFIIMLGITRSVVASAVIVGTVALSLGSAFGLSVLIWQ
HILHMPLHWLVLPMAIIVMLAVGSDYNLLLIARFQEEIGAGLKTGMIRAMAGTGRVVT
IAGLVFAFTMGSMVASDLRVVGQIGTTIMIGLLFDTLVVRSYMTPALATLLGRWFWWP
RRVDRLARQPQVLGPRRTTALSAERAALLQ"

CDS complement(482735..484510)
/codon_start=1
/note="gene_418"
/transl_table=11
/translation="VRSQRLAGHLSAAARTIHALSLPIILFWVALTIVVNVVAPQLQS
VARTHSVALGPHDAPSLIAMKRIGKDFQQFDSDTTAMVLLEGQEKLGDEAHRFYDVLV
TKLSQDTTHVQHIENFWGDPLTAAGSQSADGKAAYVQLNLTGDQGGSQANESVAAVQR
IVDSVPPPPGIKAYVTGPGPLGADRVVYGDRSLHTITGISIAVIAIMLFIAYRSLSAA
LIMLLTVGLELLAVRGIISTFAVNDLMGLSTFTVNVLVALTIAASTDYIIFLVGRYQE
ARATGQNREAAYYTMFGGTAHVVLASGLTVAGAMYCLGFTRLPYFNTLASPCAIGLVT
VMLASLTLAPAIIAVASRFGLFDPKRATTKRRWRRIGTVVVRWPGPVLAATLLIALIG
LLALPKYQTNYNERYYIPSAAPSNIGYLASDRHFPQARMEPEVLMVEADHDLRNPTDM
PILDRIAKTVFHTPGIARVQSITRPLGAPIDHSSIPFQLGMQSTMTIENLQNLKDRVA
DLSTLTDQLQRMIDITQRTQELTRQLTDATHDMNAHTRQMRDNANELRDRIADFDDFW
RPSEVSRTGSATASTFPSAGRCAPC"

CDS complement(484507..484935)
/codon_start=1
/note="gene_419"
/transl_table=11
/translation="MFGVAKRFWIPMVIVIVVAVAAVTVSRLHSVFGSHQHAPDTGNL
DPIIAFYPKHVLYEVFGPPGTVASINYLDADAQPHEVVNAAVPWSFTIVTTLTAVVAN
VVARGDGASLGCRITVNEVIREERIVNAYHAHTSCLVKSA"

CDS 486018..487013
/codon_start=1

/note="gene_420"
/transl_table=11
/translation="MQLLAKHQAPFSAAPNFAFDLAVAKTSEEDMAGLDLGHVNTIIN
GSEQVQPNTITKFLRRFRPYNLMPAAVKPSYGMAEAVVYLATTKAGSPPTSTEFDADS
LARGHAELSTFETERATRLIRYHSDDKEPLLRIVDPDSNIELGPGRIGEIWIHGKNVS
TGYHNADDALNRDKFQASIREASAGTPRSPWLRTGDLGFIVGDEFYIVGRMKDLIIQD
GVNHYPDDIETTVKEFTGGRVAAFSVSDDGVEHLVIAAEVRTEHGPDKVTIMDFSTIK
RLVVSALSKLHGLHVTDFLLVPPGALPKTTSGKISRAACAKQYGANKLQRVATFP"

CDS 487010..488392
/codon_start=1
/note="gene_421"
/transl_table=11
/translation="MTDGSVTADKLQKWFREYLSTHIECHPNEVSLDVPIRDLGLKSI
DVLAIPGDLGDRFGFCIPDLAVWDNPSANDLIDSLLNQRSADSLRESHGHADRNTQGR
GSINEPVAVIGVGCRFPGDIDGPERLWDFLTEKKCAITAYPDRGFTNAGTFAESGGFL
KDVAGFDNRFFDIPPDEALRMDPQQRLLLEVSWEALEHAGIIPESLRLSRTGVFVGVS
STDYVRLVSASAQQKSTIWDNTGGSSSIIANRISYFLDIQGPSIVIDTACSSSLVAVH
LACRSLSTWDCDIALVGGTNVLISPEPWGGFREAGILSQTGCCHAFDKSADGMVRGEG
CGVIVLQRLSDARLEGRRILAILTGSAVNQDGKSNGIMAPNPSAQIGVLENACKSARV
DPLEIGYVEAHGTGTSLGDRIEAHALGMVFGRKRPGSGPLMIGSIKPNIGHLEGAAGI
AGLIKAGVDG"

CDS 488496..491219
/codon_start=1
/note="gene_422"
/transl_table=11
/translation="VVAGRPRRAGVSSFGFGGTNAHVIVEEAGSVGADTVSGRADVGG
SGGGVVAWVISGKTASALAAQAGRLGRYVRARPALDVVDVGYSLVSTRSVFDHRAVVV
GQTRDELLAGLAGVVAGRPEAGVVCGVGKPAGKTAFVFAGQGSQWLGMGSELYAAYPV
FAEALDAVVDELDRHLRYPLRDVIWGHDQDLLNTTEFAQPALFAVEVALYRLLMSWGV
RPGLVLGHSVGELAAAHVAGALCLPDAAMLVAARGRLMQALPAGGAMFAVQAREDEVA
PMLGHDVSIAAVNGPASVVISGAHDAVSAIADRLRGQGRRVHRLAVSHAFHSALMEPM
IAEFTAVAAELSVGLPTIPVISNVTGQLVADDFASADYWARHIRAVVRFGDSVRSAHC
AGASRFIEVGPGGGLTSLIEASLADAQIVSVPTLRKDRPEPVSVMTAAAQGFVSGMGL
DWASVFSGYRPKRVELPTYAFQHQKFWLAPAPSVSDPTAAGQIGASDGGAELLASSGF
AARLAGRSADEQLAAAIEVVCEHAAAVLGRDGAAGLDAGQAFADSGFNSLSAVELRNR
LTAVTAVTLPATAIFDHPTPTELAQYLITQIDGHGSSAAAAANPAERIDALTDLFLQA
CDAGRDADGWKMVALASNTRERMSSPVRNNVSKNVALLADGISDVVVICIPTLTVLSD
QREYRDIANAMTGRHSVYSLTLPGFDSSDALPQNADMIVETVSNAIIDVVGGSCRFVL
SGYSSGGVLAYALCSHLSVKHQRNPLGVALIDTYLPSQIANPSMNEGFSPNDTGKGLS
REVIRVARMLNRLTATRLTAAATYAAIFQAWEPGRSMAPVLNIVAKDRIATVENLREE
RINRWRTAAAEAAYSVAEVPGDHFGMMSTSSEAIATEIHDWISGLVRGPHP"

CDS complement(491167..491985)
/codon_start=1
/note="gene_423"
/transl_table=11
/translation="MVATRGTRLAALALAPRLAGMAELVQITDKVHLARGHAVNWVLV
TDDTGVLLIDAGYPGDRAEVLASLNKLGYTPGDVRAIVLTHAHIDHLGSAIWFAREHS
TPVYCHAEEVGHAKREYRENASVFDVALRSWRPRVAVWGIHLLRRGGLTGDGIPTAQP
LTAEAAAGLPGQPMAIFTPGHTSGHCSYVVDGVLASGDALITGHPMLRHRGPQLLPAV
FSHSQQNSIRSLAALALLETNILAPGHGELWHGPIRKATDEALERAQKSNHVFR"

CDS 492063..493073

/codon_start=1
/note="gene_424"
/transl_table=11
/translation="VAELKLGYKASAEQFAPRELVELAVAAEAHGMDSATVSDHFQPW
RHQGGHAPFSLSWMTAVGERTNRLLLGTSVLTPTFRYNPAVIAQAFATMGCLYPNRVF
LGVGTGEALNEIATGYEGAWPEFKERFARLRESVGLMRQLWSGDRVDFDGDYYRLKGA
SIYDVPDGGVPVYIAAGGPAVAKYAGRAGDGFICTSGKGEELYTEKLMPAVREGAAAA
DRSVDGIDKMIEIKISYDPDPELALNNTRFWAPLSLTAEQKHSIDDPIEMEKAADALP
IEQIAKRWIVASDPDEAVEKVGQYVTWGLNHLVFHAPGHDQRRFLELFQSDLAPRLRR
LG"

CDS 493066..495138
/codon_start=1
/note="gene_425"
/transl_table=11
/translation="LADSSAIYLAAPESQTGKSTIALGLLHRLTAMVAKVGVFRPITR
LSAERDYILELLLAHTSAGLPYERCVGVTYQQLHADRDDAIAEIVDSYHAMADECDAV
VVVGSDYTDVTSPTELSVNARIAVNLGAPVLLTVRAKDRTPDQVASVVEVCLAELDTQ
RAHTAAVVANRCELSAIPAVTDALRRFTPPSYVVPEEPLLSAPTVAELTQAVNGAVVS
GDVALREREVMGVLAAGMTADHVLERLTDGMAVITPGDRSDVVLAVASAHAAEGFPSL
SCIVLNGGFQLHPAIAALVSGLRLRLPVIATALGTYDTASAAASARGLVTATSQRKID
TALELMDRHVDVAGLLAQLTIPIPTVTTPQMFTYRLLQQARSDLMRIVLPEGDDDRIL
KSAGRLLQRGIVDLTILGDEAKVRLRAAELGVDLDGATVIEPCASELHDQFADQYAQL
RKAKGITVEHAREIMNDATYFGTMLVHNCHADGMVSGAAHTTAHTVRPALEIIKTVPG
ISTVSSIFLMCLPDRVLAYGDCAIIPNPTVEQLADIAICSARTAAQFGIEPRVAMLSY
STGDSGKGADVDKVRAATELVRAREPQLPVEGPIQYDAAVEPSVAATKLRDSPVAGRA
TVLIFPDLNTGNNTYKAVQRSAGAIAIGPVLQGLRKPVNDLSRGALVDDIVNTVAITA
IQAQGVHE"

CDS 495131..496288
/codon_start=1
/note="gene_426"
/transl_table=11
/translation="MSSTVLVINSGSSSLKFQLVEPVAGMSRAAGIVERIGERSSPVA
DHAQALHRAFKMLAEDGIDLQTCGLVAVGHRVVHGGTEFHQPTLLDDTVIGKLEELSA
LAPLHNPPAVLGIKVARRLLANVAHVAVFDTAFFHDLPPAAATYAIDRDVADRWHIRR
YGFHGTSHQYVSERAAAFLGRPLDGLNQIVLHLGNGASASAIARGRPVETSMGLTPLE
GLVMGTRSGDLDPGVISYLWRTARMGVEDIESMLNHRSGMLGLAGERDFRRLRLVIET
GDRSAQLAYEVFIHRLRKYLGAYLAVLGHTDVVSFTAGIGENDAAVRRDALAGLQGLG
IALDQDRNLGPGHGARRISSDDSPIAVLVVPTNEELAIARDCLRVLGGRRA"

CDS complement(496342..498594)
/codon_start=1
/note="gene_427"
/transl_table=11
/translation="MAKASETERSGPGTQPADAQTATSATVRPLSTQAVFRPDFGDED
NFPHPTLGPDTEPQDRMATTSRVRPPVRRLGGGLVEIPRAPDIDPLEALMTNPVVPES
KRFCWNCGRPVGRSDSETKGASEGWCPYCGSPYSFLPQLNPGDIVAGQYEVKGCIAHG
GLGWIYLALDRNVNGRPVVLKGLVHSGDAEAQAMAMAERQFLAEVVHPSIVQIFNFVE
HTDRHGDPVGYIVMEYVGGQSLKRSKGQKLPVAEAIAYLLEILPALSYLHSIGLVYND
LKPENIMLTEEQLKLIDLGAVSRINSFGYLYGTPGFQAPEIVRTGPTVATDIYTVGRT
LAALTLDLPTRNGRYVDGLPEDDPVLKTYDSYGRLLRRAIDPDPRQRFTTAEEMSAQL
TGVLREVVAQDTGVPRPGLSTIFSPSRSTFGVDLLVAHTDVYLDGQVHAEKLTANEIV
TALSVPLVDPTDVAASVLQATVLSQPVQTLDSLRAARHGALDADGVDFSESVELPLME

VRALLDLGDVAKATRKLDDLAERVGWRWRLVWYRAVAELLTGDYDSATKHFTEVLDTF
PGELAPKLALAATAELAGNTDEHKFYQTVWSTNDGVISAAFGLARARSAEGDRVGAVR
TLDEVPPTSRHFTTARLTSAVTLLSGRSTSEVTEEQIRDAARRVEALPPTEPRVLQIR
ALVLGGALDWLKDNKASTNHILGFPFTSHGLRLGVEASLRSLARVAPTQRHRYTLVDM
ANKVRPTSTF"

CDS complement(498594..499580)
/codon_start=1
/note="gene_428"
/transl_table=11
/translation="MTRRALLARAAAPLAPLALAMVLASCGHSETLGVEATPTLPLPT
PVGMEIMPPQPPLPPDSSSQDCDPTASLRPFATKAEADAAVADIRARGRLIVGLDIGS
NLFSFRDPITGEITGFDVDIAGEVARDIFGVPSHVEYRILSAAERVTALQKSQVDIVV
KTMSITCERRKLVNFSTVYLDANQRILAPRDSPITKVSDLSGKRVCVARGTTSLRRIR
EIAPPPVIVSVVNWADCLVALQQREIDAVSTDDTILAGLVEEDPYLHIVGPDMADQPY
GVGINLDNTGLVRFVNGTLERIRNDGTWNTLYRKWLTVLGPAPAPPTPRYVD"

CDS complement(499580..500791)
/codon_start=1
/note="gene_429"
/transl_table=11
/translation="MTIGIVLAALGVASAFATSTTIEHRQQVLTAVLDHTEPLSFAAG
RLYTTLSVADAAAATAFIAQAEPGGVRLRYEQAITDASVAVTRASSGLTDESLVQLLG
RINAELAVYTGLVEIARANNRAGNPVGSSYLSEASGLMQSTILPDAQRLYQATSARVD
RETTASTQIPAPVILVVATTVVFGAFAHRWLARRTRRRINPGLVVGALGILVMVVWVG
TALTISTTASRSAKDTAAESLKTITNLAITAQQARADETLSLIRRGDEEVRKQAFYQR
IDAMQRQLNDYMARRHAVDKPDLQGADQLLVRWRQANDRINSYISVGNYRAATQVALG
KGEDDATPAFDKLDEALTKAMGQSRTQLRHDILNAHRGLAGAQVGGVVLSLGAAIAVA
LGLWPRLKEYR"

CDS 501053..501646
/codon_start=1
/note="gene_430"
/transl_table=11
/translation="VSDSGVAYWGRYGAAGLLLRAPRPDGTPAVLLQHRALWSHQGGT
WGLPGGARDSHETPEQTAVRESSEEAGLSAERLEVRATVVTAEVCGVDDTHWTYTTVV
ADAGELLDTVPNRESAELRWVAENEVADLPLHPGFAASWQRLRTAPATVPLARCDERR
QRLPRTIQIEAGVFLWCTPGDADQAPSPLGRRISSLL"

CDS complement(501630..502298)
/codon_start=1
/gene="thiE"
/note="gene_431"
/transl_table=11
/translation="VHESRLASARLYLCTDARRERGDLAQFAEAALAGGVDIIQLRDK
GSPGELRFGPLQARDELAACEILADAAHRYGALFAVNDRADIARAAGADVLHLGQRDL
PVNVARQILAPDTLIGRSTHDPDQVAAAAAGDADYFCVGPCWPTPTKPGRAAPGLGLV
RVAAELGGDDKPWFAIGGINAQRLPAVLDAGARRIVVVRAITSADDPRAAAEQLRSAL
TAAN"

CDS 502428..503450
/codon_start=1
/note="gene_432"
/transl_table=11
/translation="MASDLHTGSLAVIGGGVIGLSVARRAAQAGWPVRVHRSDERGAS
WVAGGMLAPHSEGWPGEERLLRLGLQSLRLWREGSFLDGLGPQLVTAHESLVVAVDRA

DVADLRTVADWLSAQGHPVIWESAARDVEPLLAQGIRHGFRAPTELAVDNRALLDALC
RDCERLGVRWSSQVSSLSDVDAHTVVIANGIDAPALWPGLPIRPVKGEVLRLRWRPGC
MPLPQRVIRARVRGRQVYLVPRSDGVVVGATQYEHGRDTAPVVSGVRDLLDDACTVLP
ALGEYELAECEAGLRPMTPDNLPLVQRLDSRTLVAAGHGRSGFLLAPWTAEQIVSELV
SVGAAS"

CDS 503447..503653
/codon_start=1
/note="gene_433"
/transl_table=11
/translation="MIVVVNEQQVEVDEQTTIAALLDSLGFGDRGIAVALNFSVLPRS
DWATKICELRKPVRLEVVTAVQGG"

CDS 503646..504404
/codon_start=1
/gene="thiG"
/note="gene_434"
/transl_table=11
/translation="VAESKLVIGDRSFASRLIMGTGGATNLAVLEQALIASGTELTTV
AIRRVDADGGTGLLDLLNRLGITPLPNTAGCRSAAEAVLTAQLAREALNTNWVKLEVI
ADERTLWPDAVELVRAAEQLVDDGFVVLPYTTDDPVLARRLEDTGCAAVMPLGSPIGT
GLGIANPHNIEMIVAGARVPVVLDAGIGTASDAALAMELGCDAVLLASAVTRAADPPA
MAAAMAAAVTAGYLARCAGRIPKRFWAQASSPAR"

CDS 504806..506278
/codon_start=1
/note="gene_435"
/transl_table=11
/translation="VLAVAVVVAFLTTGCIRWSTQSRPVVNGPAAAEFAVALRNRVST
DAMMAHLSKLQDIANANDGTRAVGTPGYQASVDYVVNTLRNSGFDVQTPEFSARVFKA
EKGVVTLGGNTVEARALEYSLGTPPDGVTGPLVAAPADDSPGCSPSDYDRLPVSGAVV
LVDRGVCPFAQKEDAAAQRGAVALIIADNIDEQAMGGTLGANTDVKIPVVSVTKSVGF
QLRGQSGPTTVKLTASTQSFKARNVIAQTKTGSSANVVMAGAHLDSVPEGPGINDNGS
GVAAVLETAVQLGNSPHVSNAVRFAFWGAEEFGLIGSRNYVESLDIDALKGIALYLNF
DMLASPNPGYFTYDGDQSLPLDARGQPVVPEGSAGIERTFVAYLKMAGKTAQDTSFDG
RSDYDGFTLAGIPSGGLFSGAEVKKSAEQAELWGGTADEPFDPNYHQKTDTLDHIDRT
ALGINGAGVAYAVGLYAQDLGGPNGVPVMADRTRHLIAKP"

CDS 506420..507862
/codon_start=1
/note="gene_436"
/transl_table=11
/translation="VAASVRTPIACLAAVVVIAGCTTVVDGRALSILNDPFRVGGLPA
TNGPSGARPDTPAASGTVINTNNGAIDKLSLLSVNDIEDYWMAVYSESLKGTFRPVGK
LVSYDSNDPSSPIVCHIDTYQLVNAFFSSRCNLIAWDRGVFMAVAQEYFGDMSVNGVL
AHEFGHALQVMANLVTRKDPTIVREQQADCFAGVYLWWVAEGKSTRFTLSTADGLDHV
LAGIITTRDPVMEADAENDDEHGSALDRVSAFQLGFINGTPACAAIDEDEVERRRGDL
PTTLRVDASGNPETGEVGINEETLSTLMELMGKIFSPKNPPTLSYQPAGCPDAKPSPP
AAYCPATNTIVVDLPALARMGKVASAAEHSLPQGDDTSLSIVMSRYALAVQHERGLPM
QSPWTALRTACLTGVAHRKMAVPIDLPSGQQLVLTAGDLDEAVSGLLTNRMVASDADG
VSVPAGFTRIAAFRAGVGGDMDACYARYPG"

CDS complement(507841..508167)
/codon_start=1
/note="gene_437"
/transl_table=11

/translation="MFIGITGLLLAVIGLMALWFPVYLDQYDAYGIKVTCGSGWRSNL
TQALYADGNDNTQALVTRCDTALLVRRAWAIPSVALGWLLVTGFLVMWVHNDQHQGQS
YPGYRA"
CDS complement(508412..508891)
/codon_start=1
/note="gene_438"
/transl_table=11
/translation="VRYNLPYRRRRPTGPPSGSGSGDRAGIVEAIQLCRGLAEGPLIA
GGHSYGGRQTSMVVAAGQAPVDVLTLFSYPVHPPGKPERVRTEHLPGIAVPTVFTHGT
ADPFGTLAQVRSAAAMVSAPTEVVEITGARHDLGSKTLDVARLAVDAALRLSAGQIA"
CDS complement(509038..509865)
/codon_start=1
/note="gene_439"
/transl_table=11
/translation="VNYLPLAPPGMTPPRVLSIAGSDSGGGAGIQADMRTMALLGVHA
CVAVTAVTVQNTLGVKDIHEVPNDVVAGQIEAVVTDIGVQAAKTGMLASSRIVATVAA
TWRRLELSVPLVVDPVCASMHGDPLLAPSALDSLRGQLFPLATLLTPNLDEARLLVDI
EVVDAESQRAAAKALHALGPQWVLVKGGHLRSSDGSCDLLYDGVSCYQFDAQRLPTGD
DHGGGDTLATAIAAALAHGFTVPDAVDFGKRWVTECLRAAYPLGRGHGPVSPLFRLS"
CDS complement(509862..511505)
/codon_start=1
/note="gene_440"
/transl_table=11
/translation="MTITVEPSVTTGPIAGSAKAYREIEAPGSGATLQVPFRRVHLST
GDHFDLYDTSGPYTDTDTVIDLTAGLPHRPGVVRDRGTQLQRARAGEITAEMAFIAAR
EDMSAELVRDEVARGRAVIPANHHHPESEPMIIGKAFAVKVNANIGNSAVTSSIAEEV
DKMVWATRWGADTIMDLSTGKNIHETREWILRNSPVPVGTVPIYQALEKVKGDPTELT
WEIYRDTVIEQCEQGVDYMTVHAGVLLRYVPLTAKRVTGIVSRGGSIMAAWCLAHHRE
SFLYTNFEELCDIFARYDVTFSLGDGLRPGSIADANDAAQFAELRTLGELTKIAKAHG
AQVMIEGPGHIPMHKIVENVRLEEELCEEAPFYTLGPLATDIAPAYDHITSAIGAAII
AQAGTAMLCYVTPKEHLGLPDRKDVKDGVIAYKIAAHAADLAKGHPRAQERDDALSTA
RFEFRWNDQFALSLDPDTAREFHDETLPAEPAKTAHFCSMCGPKFCSMRITQDVREYA
AEHGLETEADIEAVLAAGMAEKSREFAEHGNRVYLPITQ"
CDS complement(511657..511932)
/codon_start=1
/note="gene_441"
/transl_table=11
/translation="MAEKNTRRATSQREAVAKIREAETIVMNLPICGQVKIPRPEHLA
YYGGLAALAALELIDWPVALVIATGHILANNHHNRVLEELGEAMEEA"
CDS complement(511982..516601)
/codon_start=1
/note="gene_442"
/transl_table=11
/translation="MPVRAVATGFRATATLTGASITAATAVSATLAKTGVGTGMKVAI
IPLRAGAKALSGELSRETLGRNCWRGERRAWIEVRGLRSGGDDELGRVVLNAIQAHPG
VGSASLNYPLSRVVVAIDDPDTSLRELCRIVDDAEKAERHRHPDQAADQLAQSPGSLP
GDGVLLAVRAVTVAATAAGLGLALGGRALRWPRFPLVIEAAVAAVDHQPLLRRLLEDR
IGTAATATVLELAMAAAHTVTLSPAALSVDLTIQALKAAECRAGARAWRRHEPQLALH
ADEPADQPQSLWPRPARSTQPVQRSVARFALIQALSAVLVGAGTRDADMAATATLVAT
PKASRTTPEAFAAALGQGLADQHAVLPLRPESLRRLDRVDAIVIDPRVLCTDDLRVAR
IRGCGADELSTAWNRAQLVLTESGLRPGWHRVPGVSASGSDSAVEALFRPMHDRLASA

VVAEAHRTGADLVSVDVDALGELRPVFDDIRPLDDGASGSLDEALARAVAELRQAGRT
VAVLSSVGKQALSAADVALGVLPPPGAGAPPWYADVLLPDLGAAWRVLHAIPAARAAR
QRGNEISGGASALGALLMLPGVRGLGPGPVTTGAAAGLLSGYLLARKVVDAQAPRPAP
AHEWHAMSVEQVRKALPSPDEQAPAKAPPSPYPARALAGGLHTAKRGAQITQAPLNAL
WQLTKAVRAELSDPLTPMLALGAMASAVLGSPVDAVMVGSVLTGNSILAASQRLRAES
RLNRLLAQQIPPARKVLAGADDQPRYIEVRAEELRPGDIIEVRTHEVVPADARVIEEV
DVEVDESALTGESLSVTKQVEPTPGVDLIERRCMLYAGTTVVSGTAVAVVTAVGPDTQ
ERRAAELVSGDLSSVGLQHQLSRLTNQAWPVSMTGGALVTGLGLLRRRGLRQAVASGI
AVTVAAVPEGMPLVATLAQQASARRLSHFGALVRIPRSVEALGRVDMVCFDKTGTLSE
NRLRVAQVRPVAGHSREEVLRCAAHAAPASNGPQVHATDVAIVQAAAAAAASGTDGAE
PGAAEPAAHLPFRSGRSFSASVSGTELTVKGAPEVVLAACEGIGSSMDDAVAELAANG
LRVIAVAHRQLTAQQAQSVVDDPDEIARLCRDELSLVGFLGLSDTPRAQAAALLADLH
EHDLDIRLITGDHPITAAAIAEELGMQVSPEQVISGAEWDALSRKDQERAVAERVIFA
RMTPENKVQIVQTLEHSGRVCAMVGDGSNDAAAIRAATVGIGVVAHGSDPARVAADLV
LVDGRIESLLPAILEGRQLWQRVQAAVSVLLGGNAGEVAFAIIGSAITGTSPLNTRQL
LLVNMLTDALPAAALAVSKPSDPVTPATRGPDQRELWRAVGIRGATTAAAATVAWVMA
GFTGLPRRASTVALVALVAAQLGQTLVDSHAWLVVLTALGSLAALATLISIPVVSQLL
GCTPLDPLGWAQATAAATAATVAVAVLNRVLTGRDKSGQPNPQPPETDALSRDASPGA
PPGPRRRRRATARRKAPVKAPSATRQTTKPKGPPAHRSSSTYPRR"
CDS complement(516653..517096)
/codon_start=1
/note="gene_443"
/transl_table=11
/translation="MSVVGGTVRTVGRTVSGAATATTAAAGAVGGAAVSGIVGGVTGA
AKGIQKGLSSGSKSTAAAALAIGAIGVAGLVDWPILLAVGGGALLLRKLNRTPEVAAP
PVKAKLAPVPDKPAAAKEAPAKASKTTARKTSGRRAGTAELRSTN"
CDS complement(517297..518172)
/codon_start=1
/note="gene_444"
/transl_table=11
/translation="MPDGTIDGGHPQRPASPRLRSPLLRLATWNVNSIRTRLDRVLDW
LGRADVDVLAMQETKCPDGQFPALPLFELGYDVAHVGFDQWNGVAIASRVGLDDVRVG
FDGQPSWSGKPEVAATTEARALGATCGGIRVWSLYVPNGRALDDPHYTYKLDWLAALR
DTAEGWLRDDPAAPIALMGDWNIAPTDDDVWSTEFFAGCTHVSEPERKAFNAIVDAQF
TDVVRPFTPGPGVYTYWDYTQLRFPKKQGMRIDFILGSPALAARVMDAQIVREERKGK
APSDHAPVLVDLHAG"
CDS complement(518175..519083)
/codon_start=1
/note="gene_445"
/transl_table=11
/translation="MVSWPGLGTRVTVRYRRPAGSMPPLTDAVGRLLAVDPTVRVQTK
TGTIVEFSPVDVVALRVLTDAPVRTAAIRALEHAAAAAWPGVERTWLDGWLLRAGHGA
VLAANSAVPLDISAHTNTITEISAWYASRDLQPWLAVPDRLLPLPAGLAGERREQVLV
RDVSTGEPDRSVTLLDHPDDTWLRLYHQRLPLDMATPVIDGELAFGSYLGVAVARAAV
TDAPDGTRWVGLSAMRAADEQSATGSAGRQLWEALLGWGAGRGATRGYVRVHDTATSV
LAESLGFRLHHHCRYLPAQSVGWDTF"
CDS complement(519083..519676)
/codon_start=1
/gene="def"
/note="gene_446"
/transl_table=11

```
                     /translation="MAVVPIRIVGDPVLHTATTPVTVAADGSLPADLAQLIATMYDTM
                     DAANGVGLAANQIGCSLRLFVYDCAADRAMTARRRGVVINPVLETSEIPETMPDPDTD
                     DEGCLSVPGESFPTGRAKWARVTGLDADGSPVSIEGTGLFARMLQHETGHLDGFLYLD
                     RLIGRYARNAKRAVKSHGWGVPGLSWLPGEDPDPFGH"
     CDS             520013..520321
                     /codon_start=1
                     /note="gene_447"
                     /transl_table=11
                     /translation="MDSAMARAIRSGDDAEVADGLTRREHDILAFERQWWKFAGVKEE
                     AIKELFSMSATRYYQVLNALVDRPEALAADPMLVKRLRRLRASRQKARAARRLGFEVT
                     "
     CDS             520353..520847
                     /codon_start=1
                     /note="gene_448"
                     /transl_table=11
                     /translation="VLVTVGSMNERVPDSSGLPLRAMVMVLLFLGVVFLLLGWQALGS
                     SPNSEDDSSAISTMTTTTAAPTSTSVKPAAPRAEVRVYNISGAEGAAARTADRLKAAG
                     FTVTDVGNLSLPDVAATTVYYTEVEGERATADAVGRTLGAAVELRLPELSDQPPGVIV
                     VVTG"
     CDS             520880..521602
                     /codon_start=1
                     /note="gene_449"
                     /transl_table=11
                     /translation="MPKPADHRNHAAVSTSVLSALFLGAGAALLSACSSPQHASTVPG
                     TTPSIWTGSPAPSGLSGHDEESPGAQSLTSTLTAPDGTKVATAKFEFANGYATVTIAT
                     TGVGKLTPGFHGLHIHQVGKCEPNSVAPTGGAPGNFLSAGGHYHVPGHTGTPASGDLA
                     SLQVRGDGSAMLVTTTDAFTMDDLLSGAKTAIIIHAGADNFANIPPERYVQVNGTPGP
                     DETTLTTGDAGKRVACGVIGSG"
     CDS             521604..522734
                     /codon_start=1
                     /note="gene_450"
                     /transl_table=11
                     /translation="LPARRSAARIDFAGSPRPTLGVEWEFALVDSQTRDLSNEATAVI
                     AEIGENPRVHKELLRNTVEIVSGICECTAEAMQDLRDTLGPARQIVRDRGMELFCAGT
                     HPFARWSAQKLTDAPRYAELIKRTQWWGRQMLIWGVHVHVGIRSAHKVMPIMTSLLNY
                     YPHLLALSASSPWWGGEDTGYASNRAMMFQQLPTAGLPFHFQRWAEFEGFVYDQKKTG
                     IIDHMDEIRWDIRPSPHLGTLEVRICDGVSNLRELGALVALTHCLIVDLDRRLDAGET
                     LPTMPPWHVQENKWRAARYGLDAVIILDADSNERLVTDDLADVLTRLEPVAKSLNCAD
                     ELAAVSDIYRDGASYQRQLRVAQQHDGDLRAVVDALVAELVI"
     CDS             522791..523447
                     /codon_start=1
                     /note="gene_451"
                     /transl_table=11
                     /translation="VMADFAPVELAMFPLESAPLPDEDLPLHIFEPRYAALVRDCMDT
                     ADPRFGVVLISRGREVGGGDTRCDVGTLARITECADAGSGRYMLRCRVGERIRVCDWL
                     PDDPYPRAKVRFWPDQPGHPVTAAQLLEVEDRVVALFERIAAARGVRLPAREVVLGYP
                     VVDPADTGQRLYALACRVPMGPADRYAVLAAPSAADRLVRLGDALDSVAAMVEFELST
                     "
     CDS             complement(523627..525813)
                     /codon_start=1
                     /note="gene_452"
```

/transl_table=11
/translation="VTHPDPARQLTLTARLNTSAVDSRRGVVRLHPNAIAALGIREWD
AVSLTGSRTTAAVAGLAAADTAVGTVLLDDVTLSNAGLREGTEVIVSPVTVYGARSVT
LSGSTLATQSVPPVTLRQALLGKVMTVGDAVSLLPRDLGPGTSTSAASRALAAAVGIS
WTSELLTVTGVDPDGPVSVQPNSLVTWGAGVPAAMGTSTAGQVSISSPEIQIEELKGA
QPQAAKLTEWLKLALDEPHLLQTLGAGTNLGVLVSGPAGVGKATLVRAVCDGRRLVTL
DGPEIGALAAGDRVKAVASAVQAVRHEGGVLLITDADALLPAAAEPVASLILSELRTA
VATAGVVLIATSARPDQLDARLRSPELCDRELGLPLPDAATRKSLLEALLNPVPTGDL
NLDEIASRTPGFVVADLAALVREAALRAASRASADGRPPMLHQDDLLGALTVIRPLSR
SASDEVTVGDVTLDDVGDMAAAKQALTEAVLWPLQHPDTFARLGVEPPRGVLLYGPPG
CGKTFVVRALASTGQLSVHAVKGSELMDKWVGSSEKAVRELFRRARDSAPSLVFLDEL
DALAPRRGQSFDSGVSDRVVAALLTELDGIDPLRDVVMLGATNRPDLIDPALLRPGRL
ERLVFVEPPDAAARREILRTAGKSIPLSSDVDLDEVAAGLDGYSAADCVALLREAALT
AMRRSIDAANVTAADLATARETVRASLDPLQVASLRKFGTKGDLRS"

CDS complement(525810..526613)
/codon_start=1
/note="gene_453"
/transl_table=11
/translation="MTVLSICAGLTAIKFALEHQPKAAMALIAAAAILDGLDGRVARI
LDAQSRMGAEIDSLADAVNFGVTPALVLYVSMLSKWPVGWVVVLLYAVCVVLRLARYN
ALQDDGTQPAYAHEFFVGMPAPAGAVSMIGLLALKMQFGEGWWTSVWFLSFWVTGTSI
LLVSGIPMKKMHAVSVPPNYAAALLAVLAICAAAAVLAPYLLIWVIIIAYMCHIPFAV
RSQRWLAQHPEVWDDKPKQRRAVRRASRRAHPYRPSMARLGLRKPGRRL"

CDS complement(526667..527362)
/codon_start=1
/note="gene_454"
/transl_table=11
/translation="VARRPRPDGPQHLLALVRSAVPPVHPAGRPFIAAGLAIAAVGHR
YRWLRGTGLLAAAACAGFFRHPQRVPPTRPAAIVAPADGVICAIDSAAPPAELSMGDT
PLPRVSIFLSILDAHVQRAPVSGEVIAVQHRPGRFGSADLPEASDDNERTSVRIRMPN
GAEVVAVQIAGLVARRIVCDAHVGDKLAIGDTYGLIRFGSRLDTYLPAGAEPIVNVGQ
RAVAGETVLAECR"

CDS complement(527423..528601)
/codon_start=1
/note="gene_455"
/transl_table=11
/translation="MIRACRPITVPLTQAQGLVLGGDVVAPLSLPVFDNSAMDGYAVR
AEDTSGATPQNPVMLPVAEDIPAGRADMLTLQPVTAHRIMTGAPVPTGATAIVPVEAT
DGGVDSVAIRQQATPGKHIRRSGEDVAAGTTVLHNGQIVTPAVLGLAAALGLAELPVL
PRQRVLVISTGSELASPGTPLQPGQIYESNSIMLAAAVRDAGAAVVATATAGDDVAQF
GAILDRYAVDADLIITSGGVSAGAYEVVKDAFGSADYRGGDHGVEFVKVAMQPGMPQG
VGRVAGTPIVTLPGNPVSALVSFEVFIRPPLRMAMGLPDPYRPHRSAVLTASLTSPRG
KRQFRRAILDHQAGTVISYGPPASHHLRWLASANGLLDIPEDVVEVAAGTQLQVWDLT
"

CDS complement(528659..529594)
/codon_start=1
/note="gene_456"
/transl_table=11
/translation="MTANDNKTRKWSAADVPDQSGRVVVVTGANTGIGYHTAAVFADR
GAHVVLAVRNLEKGNAARARIMAARPGAHVTLQQLDLCSLDSVRAAADALRTAYPRID
VLINNAGVMWTPKQVTKDGFELQFGTNHLGHFALTGLVLDHMLPVPGSRVVTVSSQGH

RIHAAIHFDDLQWERRYNRVAAYGQAKLANLLFTYELQRRLGEAGKSTIAVAAHPGGS
NTELTRNLPRLIRPVATVLGPLLFQSPEMGALPTLRAATDPTTQGGQYYGPDGFGEQR
GHPKVVQSSAQSHDKDLQRRLWTVSEELTGVSFGV"
CDS 529888..531510
/codon_start=1
/gene="groEL"
/note="gene_457"
/transl_table=11
/translation="MAKTIAYDEEARRGLERGLNALADAVKVTLGPKGRNVVLEKKWG
APTITNDGVSIAKEIELEDPYEKIGAELVKEVAKKTDDVAGDGTTTATVLAQALVREG
LRNVAAGANPLGLKRGIEKAVEKVTETLLKGAKEVETKEQIAATAAISAGDQSIGDLI
AEAMDKVGNEGVITVEESNTFGLQLELTEGMRFDKGYISGYFVTDPERQEAVLEDPYI
LLVSSKVSTVKDLLPLLEKVIGAGKPLLIIAEDVEGEALSTLVVNKIRGTFKSVAVKA
PGFGDRRKAMLQDMAILTGGQVISEEVGLTLENADLSLLGKARKVVVTKDETTIVEGA
GDTDAIAGRVAQIRQEIENSDSDYDREKLQERLAKLAGGVAVIKAGAATEVELKERKH
RIEDAVRNAKAAVEEGIVAGGGVTLLQAAPTLDELKLEGDEATGANIVKVALEAPLKQ
IAFNSGLEPGVVAEKVRNLPAGHGLNAQTGVYEDLLAAGVADPVKVTRSALQNAASIA
GLFLTTEAVVADKPEKEKASVPGGGDMGGMDF"
CDS complement(531576..532004)
/codon_start=1
/note="gene_458"
/transl_table=11
/translation="VGAKKVDLKRLAAALPDYPFAYLITVDDGHRVHTVAVEPVLREL
PDGPDGPRAVVDVGLIGGRTRQNLAHRSEVTLLWPPSDPSGYSLIVDGRAQASDAGPD
DDTARCGVVPIRALLHRDAAPDSPTAAKGCLHDCVVFSVP"
CDS complement(532031..533443)
/codon_start=1
/note="gene_459"
/transl_table=11
/translation="MFAGPGSGPLIAAATAWGELAEELLASIASLGSVTSELTSGAWL
GPSAAAMMAVATQYLAWLSTAAAQAEQAAAQAMAIATAFEAALAATVQPAVVAANRGL
MQLLAATNWFGQNAPALMDVEAAYEQMWALDVAAMAGYHFDASAAVAQLAPWQQVLRN
LGIDIGKNGQINLGFGNTGSGNIGNNNIGNNNIGSGNTGTGNIGTGNTGSGNLGLGNL
GDGNIGFGNTGSGNIGFGITGDHQMGFGGFNSGSGNIGFGNSGTGNVGLFNSGSGNIG
IGNSGSLNSGIGTSGTINAGLGSAGSLNTSFWNAGMQNAALGSAAGSEAALVSSAGYA
TGGMSTAALSSGILASALGSTGGLQHGLANVLNSGLTNTPVAAPASAPVGGLDSGNPN
PGSGSAAAGSGANPGLRSPGTSYPSFVNSGSNDSGLRNTAVREPSTPGSGIPKSNFYP
SPDRESAYASPRIGQPVGSE"
CDS 533634..534191
/codon_start=1
/note="gene_460"
/transl_table=11
/translation="MQLRVCHLPRNVAGMASTDAAAQELLRDAFTRLIEHVDELTDGL
TDQLACYRPTPSANSIAWLLWHSARVQDIQVAHVAGVEEVWTRDGWVDRFGLDLPRHD
TGYGHRPEDVAKVRAPADLLSGYYHAVHKLTLEYIAGMTADELSRVVDTSWNPPVTVS
ARLVSIVDDCAQHLGQAAYLRGIAR"
CDS complement(534371..535069)
/codon_start=1
/gene="rskA"
/note="gene_461"
/transl_table=11

```
                     /translation="MTEHTDFELLELATPYALNAVSDDERADIDRRVAAAPSPVAAAF
                     NDEVRAVRETMAVVSAATTAEPPAHLRTAILDATKPEVRRQSRWRTAAFASAAAIAVG
                     LGAFDLGVLTRPSPPPTVAEQVLTAPDVRTVSRPLGAGTATVVFSRDRNTGLLVMNNV
                     APPSRGTVYQMWLLGGAKGPRSAETMGTAAVTPSTTATLTDLGASTALAFTVEPGTGS
                     PQPTGTILAELPLG"
     CDS             complement(535113..535676)
                     /codon_start=1
                     /note="gene_462"
                     /transl_table=11
                     /translation="MTGPPRLSSDLDALLRRVAGHDQAAFAEFYDHTKSRVYGLVMRV
                     LRDTGYSEETTQEIYLEVWRNASEFDSAKGSALAWLLTMAHRRAVDRVRCEQAGNQRE
                     VRYGAANVDPASDVVADLAIAGDERRRVTECLKALTDTQRQCIELAYYGGLTYVEVSR
                     RLAANLSTIKSRMRDALRSLRNCLDVS"
     CDS             complement(535725..536507)
                     /codon_start=1
                     /note="gene_463"
                     /transl_table=11
                     /translation="VNIVVVTSVSALAVAVVHSVAFAIGRRIGRYNVVDVVWGLGFVA
                     VAVAAATLGHGDPVRRWLLLALVSTWGLRLSWHMYRKTAGQGEDPRYADLLRGATPVQ
                     ALRKVFGLQGLLTLFVSFPLQLSAVTGPTPKPLLAVGGVGLAVWLVGITFEAVGDWQL
                     WVFKSDPANRGVIMDRGLWAWTRHPNYFGDACVWWGLWLITINDWAPLATVGSPLLMT
                     YLLVDVSGARLTERYLKGRPGFAEYQRRTAYFVPRPPRSARR"
     CDS             complement(536504..537787)
                     /codon_start=1
                     /note="gene_464"
                     /transl_table=11
                     /translation="MTVETSQTPSAAIDSDRWPAVAKVPRGPLAAASAAIANRLLRRT
                     ATHLPLRLVYSDGTATGAADPRAPSLFIHRPDALARRIGRHGLIGFGESYMAGEWSSK
                     ELTRVLTVLAGSVDELVPRSLHWLRPITPTFRPSWPDHSRDQARRNIAVHYDLSNDLF
                     AAFLDETMTYSCAMFTDLLAQPTPAWTELAAAQRRKIDRLLDVAGVQQGSHVLEIGTG
                     WGELCIRAAARGAHIRSVTLSVEQQRLARQRVAAAGFGHRVEIDLCDYRDVDGQYDSV
                     VSVEMIEAVGYRSWPRYFAALEQLVRPGGPVAIQAITMPHHRMLATRHTQTWIQKYIF
                     PGGLLPSTQAIIDITGQHTGLRIVDAASLRPHYAETLRLWRERFMQRRDGLAHLGFDE
                     VFARMWELYLAYSEAGFRSGYLDVYQWTLIREGPP"
     CDS             complement(537784..538515)
                     /codon_start=1
                     /note="gene_465"
                     /transl_table=11
                     /translation="VLTQTLTPAIYRTTISHCRQVPVHHSFAYRSYSWYVDVDNLPQL
                     PWWLRPFARFHADDHFADPFSCPPHSSLRDRLDAFFAARGLAVPDGRITALLQARVLG
                     YVFNPLSIFWCHDRDGQLRHVIAEVHNTYGGRHAYLLPPADLPVVTAKNFYVSPFHQL
                     AGYYLIRAPRPDRELDVTVTLHRDRRQVCPEFTATLRGQRRPATTRQIAMMQIISPLA
                     PMVVAARIRIQGIRLWLRRVPVVPR"
     CDS             complement(538509..539804)
                     /codon_start=1
                     /note="gene_466"
                     /transl_table=11
                     /translation="VAVVGSGVAGLTAAYILSGRDRVTLYEADGRLGGHAHTHYLDNG
                     GGPRGTDVVGVDSAFLVHNDRTYPTLCRLFAELGVATQESEMSMSVRADDIGLEYAGA
                     LGARGLFACRQSLRPRYLCMLAEILRFHRAAARLLREETDNAEDKPETLEAFLSRHHF
                     SQYFVDYFITPLVAAVWSCGGADALRYPARYLFVFLDHHGMLSVFGSPTWRTVTGGSA
```

NYVQAIAAQLDEVSTRTPVHSLRRLPDGVLVGAGDGPSRRFDAAVVAVHPDQALLLLD
EPTPAERAVLGAIAYSTNSAQLHTDESVLPRHHRARASWNYLVTPGQHQVVVSYDISR
LMRLDGGRRYLVTLGGHDRVDPSSVIAEMTYSHPLYTPESVAAQRLLPTLGDNRVVFA
GAYHGWGFHEDGAASGLRAARRLGADWPAAIPQEAMVAC"
CDS complement(539868..542771)
/codon_start=1
/gene="mmpL5_2"
/note="gene_467"
/transl_table=11
/translation="VSTKFANDSNTNARPEKPFIARMIHAFAVPIILGWLAVCVVVTV
FVPSLEAVGQERSVSLSPKDAPSFEAMGRIGMVFKEGDSDSFAMVIIEGNQPLGDAAH
KYYDGLVAQLRADKKHVQSVQDLWGDPLTAAGVQSNDGKAAYVQLSLAGNQGTPLANE
SVEAVRSIVESTPAPPGIKAYVTGPSALAADMHHSGDRSMARITMVTVAVIFIMLLLV
YRSIITVVLLLITVGVELTAARGVVAVLGHSGAIGLTTFAVSLLTSLAIAAGTDYGIF
IIGRYQEARQAGEDKEAAYYTMYRGTAHVILGSGLTIAGATFCLSFARMPYFQTLGIP
CAVGMLVAVAVALTLGPAVLHVGSRFGLFDPKRLLKVRGWRRVGTVVVRWPLPVLVAT
CAIALVGLLALPGYKTSYNDRDYLPDFIPANQGYAAADRHFSQARMKPEILMIESDHD
MRNPADFLVLDKLAKGIFRVPGISRVQAITRPEGTTMDHTSIPFQISMQNAGQLQTIK
YQRDRANDMLKQADEMATTIAVLTRMHSLMAEMASTTHRMVGDTEEMKEITEELRDHV
ADFDDFWRPIRSYFYWEKHCYGIPICWSFRSIFDALDGIDKLSEQIGVLLGDLREMDR
LMPQMVAQIPPQIEAMENMRTMILTMHSTMTGIFDQMLEMSDNATAMGKAFDAAKNDD
SFYLPPEVFKNKDFQRAMKSFLSSDGHAARFIILHRGDPQSPEGIKSIDAIRTAAEES
LKGTPLEDAKIYLAGTAAVFHDISEGAQWDLLIAAISSLCLIFIIMLIITRAFIAAAV
IVGTVALSLGASFGLSVLLWQHILAIHLHWLVLAMSVIVLLAVGSDYNLLLVSRFKQE
IGAGLKTGIIRSMGGTGKVVTNAGLVFAVTMASMAVSDLRVIGQVGTTIGLGLLFDTL
IVRSFMTPSIAALLGRWFWWPLRVRSRPARTPTVPSETQPAGRPLAMSSDRLG"
CDS complement(542768..543190)
/codon_start=1
/note="gene_468"
/transl_table=11
/translation="VLMRTWIPLVILVVVIVGGFTVHRIRGFFGSENRPSYSDTNLEN
SKPFNPKHLTYEIFGPPGTVADISYFDVNSEPQRVDGAVLPWSLHITTNDAAVMGNIV
AQGNSDSIGCRITVDGKVRAERVSNEVNAYTYCLVKSA"
CDS 543371..544132
/codon_start=1
/note="gene_469"
/transl_table=11
/translation="VRNGTLRYRQPSYKSHGVRYPLAVAQLGFQRARTEENKRQRAAA
LVEAARSLALETGVASVTLTAVAGRAGIHYSAVRRYFTSHKEVLLHLAAEGWARWSGT
VCEQLGEPGPMSAPRVAEALANGLAADPLFCDLLANLHLHLEQEVDVDRVIEVKRTSI
AAVIALVDAIESALPALGRSGAFDILLAAYSLAATLWQIANPPERLTDAYAEEPELLP
PEWNLDFAAALTRLLTATLLGLLAGSPCECRSPTR"
CDS 544475..546010
/codon_start=1
/note="gene_470"
/transl_table=11
/translation="MASPPEVHSALLSSGPGPGPVLAAATGWSSLGREYAAVAEELGA
LLAAVQAGVWQGPSAESFAAACLPYLSWLTQASADCAAAAARLEAVTAAYAAALVAMP
TLAELAANHATHGAMVATNFFGINTIPIAVNEADYVRMWLQAATTMATYQAVADSAVR
SIPDSVPPPRILKSNAQSQHSSSNNSGGADPVDDFIAEILKIITGGRVIWDPEAGTVN
GLPYDAYTNPGTLMWWIARSLELLQDFQEFAKLLFTNPVKAFQFLVDLILFDWPTHML

QLATWLAENPQLLVAALTPAISGLGAVSGLAGLTGLVPQPPVVPAPAPDAVVPTVLPL
AGTATPTTAPASAPAAGAAPGPPAGTATATSASVPTSAGGFPPYLVGSGPGIDFDAGT
PAGSRRAQPAADNVTAVAAAQVSARHQARRRRRAAAKERGNADEFVDMDSGPAIPPSG
ERDAWASNSGVGGLGFAGTASNETVAAPAGLTTLADDEFQCGPRMPMLPGAWDLGTWD
RGD"

CDS complement(546016..546339)
/codon_start=1
/note="gene_471"
/transl_table=11
/translation="MSQAPGNEIPHTLAALDWGGITCQSGAGCTNRASYVVHLHAVDE
CNDPDLDPFGNTVEILCIACLWHVEAKVLLQVGRLTRSPGAFCLTCGAPVREPTDIMR
DVAAL"

CDS complement(546655..547101)
/codon_start=1
/note="gene_472"
/transl_table=11
/translation="MSRLSSILRAGAAFLVLGIAAATFPQSAAADSTEDFPIPRRMIA
TTCDAEQYLAAVRDTSPVYYQRYMIDFNNHANLQQATINKAHWFFSLSPAERRDYSEH
FYNGDPLTFAWVNHMKIFFNNKGVVAKGTEVCNGYPAGDMSVWNWA"

CDS complement(547169..548083)
/codon_start=1
/note="gene_473"
/transl_table=11
/translation="MPTPDFQTLLYTTAGPVATITLNRPEQLNTIVPPMPDEIEAAIG
LAERDQDIKVIVLRGAGRAFSGGYDFGGGFQHWGDAMMTDGRWDPGKDFAMVTARETG
PTQKFMAIWRASKPVIAQVHGWCVGGASDYALCADIVIASEDAVIGTPYSRMWGAYLT
GMWLYRLSLAKVKWHSLTGRPLTGVQAAEAELINEAVPFERLEARVAEIATELARIPL
SQLQAQKLIVNQAYENMGLASTQLLGGILDGLMRNTPDALEFIRTAQTQGVRAAVERR
DGPFGDYSQAPPELRPDPTHVITPDGSM"

CDS complement(548866..550887)
/codon_start=1
/note="gene_474"
/transl_table=11
/translation="MTFEPAPDGADPYLWLEDVTGAEALDWVRARNKPTTAAFCDAEF
ERMRVEALEVLDTDARIPYVNRRGNYLYNFWRDAANPRGLWRRTTLDSYRTDSPGWDV
LIDVDELGRADDQKWVWGGAGVIEPDYTRALIGLSPGGSDASIVREFDMLTREFVEDG
FQLPPAKSQITWEDPDTVLLGTDFGGDSLTTSGYPRVIKRWRRGKPLADAETIFEGAG
TDVRVNASADRTPGFERTLLGRALDFWNEEVYELRGSELIRIEAPTDASVSIHRDWLL
IELRTDWTVATTRYTAGSLLAAEYDEFLAGSAELQVVFEPDEHTALYQYAWTRDRLLI
VTLADVASRVEIATPGSWRREPLSGIPAATNTVIVSADSHGDEFFLDSSGFDTPSRLM
RGTDDGRLAEIKSAPAFFDAENMAVTQYFATSDDGTSIPYFVVRRTDADNPGPTLLNG
YGGFETSRTPTYDGVLGRLWLARGGTYALANIRGGGEYGPGWHTQAMREGRDKVAQDF
AAVATDLVTRGITTAEQLGARGGSNGGLLMGIMLTGYPEKFGALVCDVPLLDMKRYHL
LLAGASWMAEYGDPDNPDDWKFISEYSPYQNISANRKYPPVLMTTSTRDDRVHPGHAR
KMTAALQAAGHPVWYYENIEGGHAGAADNAQIAFKSALSFAFLWRMLAG"

CDS 550955..552478
/codon_start=1
/note="gene_475"
/transl_table=11
/translation="MTVFSRPGSAGALMSYESRYQNFIGGQWVAPVHGRYFENPTPVT
GQPFCEVPRSDAADIDKALDAAHAAAPGWGKTAPAERAAILNMIADRIDKNAAALAVA

EVWDNGKPVREALAADIPLAVDHFRYFAAAIRAQEGALSQIDEDTVAYHFHEPLGVVG
QIIPWNFPILMAAWKLAPALAAGNTAVLKPAEQTPASVLYLMSLIGDLLPPGVVNVVN
GFGAEAGKPLASSDRIAKVAFTGETTTGRLIMQYASHNLIPVTLELGGKSPNIFFADV
LAAHDDFCDKALEGFTMFALNQGEVCTCPSRSLIQADIYDEFLELAAIRTKAVRQGDP
LDTETMLGSQASNDQLEKVLSYIEIGKQEGAVIIAGGERAELGGDLSGGYYMQPTIFT
GTNNMRIFKEEIFGPVVAVTSFTDYDDAIGIANDTLYGLGAGVWSRDGNTAYRAGRDI
QAGRVWVNCYHLYPAHAAFGGYKQSGIGREGHQMMLQHYQHTKNLLVSYSDKALGFF"

CDS 552478..552969
/codon_start=1
/note="gene_476"
/transl_table=11
/translation="MNAPAGVLITAEAAALLAGLQDRHGPVMFHQSGGCCDGSAPMCY
PRADFLVGDRDILLGVLDVGEDGVPVWISGPQYQAWKHTQLIIDVVPGRGGGFSLEAP
EGVRFLSRGRVFSDAEKAMREAAPVITGAAYECGERPLVRGLVVDLDDPDATPGVCRA
SRR"

CDS 552990..553268
/codon_start=1
/note="gene_477"
/transl_table=11
/translation="VIPLPRSWQLTSAMLVGNAIGLLAGVACSVLVHARIRPDIVIAM
VVGIPSAIGLLVILFSGRRWVTMLGAFILALAPGWFGVLVAIQVASSG"

CDS 553261..553830
/codon_start=1
/note="gene_478"
/transl_table=11
/translation="VADNDYRSAPGTEPFVPDFDTGAHSQRFLSLAGQQDRAGKSWPG
STPKPQEDPVGVAPSASVEVLGSEPAATLAHSVTVPGRYTYLKWWKFVLVVLGVWIGA
GEVGLSLFYWWYHTLDKTAAVFVVLVYVVACTVGGLILALVPGRPLITALSLGVMSGP
FASVAAAAPLYGYYYCERMSHCLVGVIPY"

CDS 553894..555288
/codon_start=1
/note="gene_479"
/transl_table=11
/translation="VTHYDVVVLGAGPGGYVAAIRAAQLGLSTAIVEPKYWGGVCLNV
GCIPSKALLRNAELVHIFTKDAKAFGISGEVTFDYGIAYDRSRKVAEGRVAGVHFLMK
KNKITEIHGYGTFADANTLLVDLNDGGTESVTFDNAIIATGSSTRLVPGTSLSANVVT
YEEQILSRELPKSIIIAGAGAIGMEFGYVLKNYGVDVTIVEFLPRALPNEDADVSKEI
EKQFKKLGVTILTATKVESIADGGSQVTVTVTKDGVAQELKAEKVLQAIGFAPNVEGY
GLDKAGVALTDRKAIGVDDYMRTNVGHIYAIGDVNGLLQLAHVAEAQGVVAAETIAGA
ETLTLGDHRMLPRATFCQPNVASFGLTEQQARNEGYDVVVAKFPFTANAKAHGVGDPS
GFVKLVADAKHGELLGGHLVGHDVAELLPELTLAQRWDLTASELARNVHTHPTMSEAL
QECFHGLVGHMINF"

CDS 555296..555589
/codon_start=1
/note="gene_480"
/transl_table=11
/translation="MTRRASTDTPQIIMGAIGGVVTGYILWLAAISVGDGLTTVSQWS
RVVLLLSVLVAVCGAAGGLRLRSRGKLAWSAFAFSLPIPPVVLTVAVLADIYL"

CDS complement(555593..556165)
/codon_start=1
/note="gene_481"

/transl_table=11
/translation="MTGQNGQVARISPGKFRQLGPVNWLVAKLAARAVGAPQMHLFTT
LGYRQYLFWTFAIYTGRLLHGRLPGVDTELVILRVAHLRSCEYELQHHRRMARRRGLD
ANTQATIFAWPDVPDGDGPRKVLSARQQALLQATDELIKDRTITAGTWERLATHLDPR
LLIEFCLLATQYDAIAATITALAIPPDNPQ"
CDS complement(556162..557586)
/codon_start=1
/note="gene_482"
/transl_table=11
/translation="VSKTYVGSRVRQLRNERGFSQAALAQMLEISPSYLDQIEHDVRP
LTVAVLLRITEVFGVDATFFASQDDTRLVAELREVTLDRDLDIAIDPHEVAEMVSAHP
GLARAVVNLHRRYRITTAQLAAATEERFSDGSGRGSITMPHEEVRDYFYQRQNYLHAL
DTAAEDLTAQMRMHHGDLARELTRRLTEVHGVRINKRIDLGDTVLHRYDPATNTLEIS
SHLSPGQQVFKMAAELAYLEFGDLIDAMVTDGKFTSAESRTLARLGLANYFAAATVLP
YRQFHDVAENFRYDVERLSAFYSVSYETIAHRLSTLQRPSMRGVPFTFVRVDRAGNMS
KRQSATGFHFSSSGGTCPLWNVYETFANPGKILVQIAQMPDGRNYLWVARTVELRAAR
YGQPGKTFAIGLGCELRHAHRLVYSEGLDLSGDPNTAATPIGAGCRVCERDNCPQRAF
PALGRALDLDEHRSTVSPYLVKQL"
CDS 557738..558532
/codon_start=1
/note="gene_483"
/transl_table=11
/translation="VSLDKKLMPVPDGHPDVFDREWPLRVGDIDRAGRLRLDAACRHI
QDIGQDQLREMGFEETHPLWIVRRTMVDLIRPIEFGDMLRCRRWCSGTSNRWCEMRVR
VDGRKGGLIESEAFWIHVNRETEMPARIADDFLAGLHRTTSVDRLRWKGYLKPGSRDD
ASEIHEFPVRVTDIDLFDHMNNAVYWSVIEDYLASHAELLRGPLRVTIEHEAPVALGD
KLEIISHVHPAGSTEIFGPGLVDRAVTTLTYVVGDEPKAVASLFNL"
CDS 558807..560093
/codon_start=1
/note="gene_484"
/transl_table=11
/translation="MSVVGTPKSAEQIQQEWDTNPRWKDVTRTYSAEDVVALQGSVVE
EHTLARRGAEVLWEQLHDLEWVNALGALTGNMAVQQVRAGLKAIYLSGWQVAGDANLS
GHTYPDQSLYPANSVPQVVRRINNALQRADQIAKIEGDTSVENWLAPIVADGEAGFGG
ALNVYELQKALIAAGVAGSHWEDQLASEKKCGHLGGKVLIPTQQHIRTLTSARLAADV
ADVPTVVIARTDAEAATLITSDVDERDQPFITGERTREGFYRTKNGIEPCIARAKAYA
PFADLIWMETGTPDLEAARQFSEAVKAEYPDQMLAYNCSPSFNWKKHLDDATIAKFQK
ELAAMGFKFQFITLAGFHALNYSMFDLAYGYAQNQMSAYVELQEREFAAEERGYTATK
HQREVGAGYFDRIATTVDPNSSTTALTGSTEEGQFH"
CDS 560175..561035
/codon_start=1
/note="gene_485"
/transl_table=11
/translation="VSDAIQRVGVVGAGQMGSGIAEVSARAGVEVTVFEPAEALITAG
RNRIVKSLERAVSAGKVTERERDRALGLLTFTTDLNDLSDRQLVIEAVVEDEAVKSEI
FAELDRVVTDPDAVLASNTSSIPIMKVAAATKQPQRVLGLHFFNPVPVLPLVELVRTL
VTDEAAAARTEEFASTVLGKQVVRCSDRSGFVVNALLVPYLLSAIRMVEAGFATVEDV
DKAVVAGLSHPMGPLRLSDLVGLDTLKLIADKMFEEFKEPHYGPPPLLLRMVEAGQLG
KKSGRGFYTY"
CDS 561168..562028
/codon_start=1

/note="gene_486"
/transl_table=11
/translation="MTELRPFYEESQSIYDVSDEFFSLFLDPTMAYTCAYFEREDMTL
EEAQNAKFDLALDKLHLEPGMTLLDIGCGWGGGLQRAIENYDVNVIGITLSRNQFEYS
KAKLAKIPTERSVQVRLQGWDEFTDKVDRIVSIGAFEAFKMERYAAFFERSYDILPDD
GRMLLHTILTYTQKQMHEMGVKVTMSDVRFMKFIGEEIFPGGQLPAQEDIFKFAQAAD
FSVEKVQLLQQHYARTLNIWAANLEANKDRAIALQSEEIYNKYMHYLTGCEHFFRKGI
SNVGQFTLTK"
CDS complement(562128..562991)
/codon_start=1
/note="gene_487"
/transl_table=11
/translation="MSVQLTPHFGNVQAHYDLSDDFFRLFLDPTQTYSCAYFERDDMT
LQEAQIAKIDLALGKLNLEPGMTLLDIGCGWGATMRRAIEKYDVNVVGLTLSENQAGH
VQKMFDQMDTPRSRRVLLEGWEKFDEPVDRIVSIGAFEHFGHQRYHHFFEVPHRTLPA
DGKMLLHTIVRPTFKEGREKGLTLTHELVHFTKFILAEIFPGGWLPSIPTVHEYAEKV
GFRVTAVQSLQLHYARTLDMWATALEANKDQAIAIQSQTVYDRYMKYLTGCAKLFRQG
YTDVDQFTLEK"
CDS complement(563144..563338)
/codon_start=1
/note="gene_488"
/transl_table=11
/translation="LLVAGALPRLAKVWPYFLRRRPQQPPPAFPVWPLWYAALAWLHV
RQAGALLVVGLAIGAWRRAW"
CDS complement(563505..563993)
/codon_start=1
/note="gene_489"
/transl_table=11
/translation="MPDAGAGSRLRSWAYALRTTNPPADGPTDTVTRWLVVTRAAVLP
MTLVSGLVAGLLAIGEPGLDWRWLVLWWESHAPHIANNLMNDLYDTDVGTDSATYARA
RYAQHPAATGANRAAYTTPRRTTSCGSPERALEPTTPRWARAVGRSCWRRSPTGCCAP
RC"
CDS complement(564003..564773)
/codon_start=1
/note="gene_490"
/transl_table=11
/translation="MCQQDPQLAVVSGASSEWELATVAERIPAVTVKTDGRKRRWHQH
KVERRNELVDGTIEAIRRHGRFLSMDEIAAEIGVSKTVLYRYFVDKNDLTTAVMMRFT
QTTLIPNMIAALSADMDGFELTREIIRVYVETVAAQPEPYRFVMANSSASKSKVIADS
ERIIARMLAVMLRRRMQEAGMDTDGVEPWAYLIVGGVQLATHSWMSDPRMSSDELIDY
LTMLSWSALCGIVEAGGSLEKFREQPHPSPIVPAWGQV"
CDS complement(564813..565040)
/codon_start=1
/gene="J113_03350"
/note="gene_491"
/transl_table=11
/translation="VAPTKNPTATTFIRRSAIRRAASASGSAAAKDSAKEARTRRATV
GWVLKFSRGGGEPDTSALCATTPSSAITGTS"
CDS 564988..566214
/codon_start=1
/note="gene_492"

```
                     /transl_table=11
                     /translation="MALRRMKVVAVGFLVGATGVFLACRWAQADGADHAWLGYLGAAA
                     EAGMVGALADWFAVTALFKHPLGIPIPHTAIIKRKKDQLGEGLGTFVRENFLSPPVVE
                     TKLRDAQIPSRLGKWLSEATHAQRVAAETATVLRVLVELLRDEDIQQVIDRMIVRRIA
                     EPQWGPPAGRVLATLLAENRQEAFIQLLADRAFQWSLNAGVVIQRVVERDSPSWSPRF
                     IDHLVGDRIHRELMEFTDKVRRNPDHELRRSATRFLFDFADDLQHDPATVARADAIKE
                     ELMARDEIATAAAAAWKTLKRLVLEGVDDPSSALRTRITDAVIRIGESLRDDADLRDK
                     VDSWTVRAAQHLVSEYGVEITAIITETIERWDAEEASRRIELHVGRDLQFIRINGTVV
                     GAMAGLAIYAIAQLLF"
     CDS             566301..566723
                     /codon_start=1
                     /note="gene_493"
                     /transl_table=11
                     /translation="MSSEEKLAAKVSTKASDVASDIGSFIRSQRETAHVSMRQLAERS
                     GVSNPYLSQVERGLRKPSADVLSQIAKALRVSAEVLYVRAGILEPSETSQVRDAIITD
                     TAITERQKQILLDIYASFTHQNEATREECPSDPTPTDD"
     CDS             567077..567676
                     /codon_start=1
                     /note="gene_494"
                     /transl_table=11
                     /translation="MAENSNIDDIKAPLLAALGAADLALATVNELITNLRERAEETRT
                     DTRSRVEESRARLTKLQEDLPEQLTELREKFTAEELRKAAEGYLEAATSRYNELVERG
                     EAALERLRSQQSFEEVSARAEGYVDQAVELTQEALGTVASQTRAVGERAAKLVGIELP
                     KKAAPAKKAAPAKKAAPAKKAAAKKAPAKKAAAKKVTQK"
     CDS             567764..568051
                     /codon_start=1
                     /note="gene_495"
                     /transl_table=11
                     /translation="VSHLVGTVMLVLLVAVLVTAVYAFVHAALQRPDAYTAADKLTKP
                     VWLVILGAAVALASILYPVLGVLGMAMSACASGVYLVDVRPKLLEIQGKSR"
     CDS             568056..568502
                     /codon_start=1
                     /note="gene_496"
                     /transl_table=11
                     /translation="MKALVAVSAVAVVALLGVSSAQADPEADPGAGEANYGGPPSSPR
                     LVDHTEWAQWGSLPSLRVYPSQVGRTASRRLGMAAADAAWAEVLALSPEADTAGMRAQ
                     FICHWQYAEIRQPGKPSWNLEPWRPVVDDSEMLASGCNPGSPEESF"
     CDS             568502..569176
                     /codon_start=1
                     /note="gene_497"
                     /transl_table=11
                     /translation="VLGQPTRAQLAALVDHTLLKPETTRADVAALVAEAAELGVYAVC
                     VSPSMVPVAVQAGGVRVAAVTGFPSGKHVSSVKAHEAAAALASGASEIDMVIDIGAAL
                     CGDIDAVRSDIEAVRAAAAGAVLKVIVESAVLLGQSNAHTLVDACRAAEDAGADFVKT
                     STGCHPAGGATVRAVELMAETVGPRLGVKASGGIRTAADAVAMLNAGATRLGLSGTRA
                     VLDGLS"
     CDS             complement(569201..570247)
                     /codon_start=1
                     /note="gene_498"
                     /transl_table=11
                     /translation="VTNPQGPPNDPSPWARPGDQGPLARPPASSEASTGRLRPGEPAG
```

HIQEPVSPPTQPEQQPQTEHLAASHAHTRRSGRQAAHQAWDPTGLLAAQEEEPAAVKT
KRRARRDPLTVFLVLIIVFSLVLAGLIGGELYARHVANSKVAQAVACVVKDQATASFG
VAPLLLWQVATRHFTNISVETAGNQIRDAKGMQIKLTIQNVRLKNTPNSRGTIGALDA
TITWSSEGIKESVQNAIPILGAFVTSSVVTHPADGTVELKGLLNNITAKPIVAGKGLE
LQIINFNTLGFSLPKETVQSTLNEFTSSLTKNYPLGIHADSVQVTSTGVVSRFSTRDA
AIPTGIQNPCFSHI"
CDS complement(570244..571086)
/codon_start=1
/note="gene_499"
/transl_table=11
/translation="MRIALAQIRSGTDPAANLQLVGKYAGEAATAGAQLVVFPEATMC
RLGVPLRQVAEPVDGPWANGVRRIATEAGITVIAGMFTPTGDGRVTNTLIAAGPGTPN
QPDAHYHKIHLYDAFGFTESRTVAPGREPVVVVVDGVRVGLTVCYDIRFPALYTELAR
RGAQLIAVCASWGSGPGKLEQWTLLARARALDSMSYVAAAGQADPGDARTGVGASSAA
PTGVGGSLVASPLGEVVVSAGTQPQLLVADIDVDNVAAARDRIAVLRNQTDFVQIDKA
QSRG"
CDS complement(571212..571736)
/codon_start=1
/note="gene_500"
/transl_table=11
/translation="MPRSFDMSADYEGSVEEVHRAFYEADYWKARLAETPVDVATLES
IRVGGDSGDDGTIEVVTLQMVRSHNLPGLVTQLHRGDLSVRREETWGPVKEGIATASI
AGSIVDAPVNLWGTAVLSPIPESGGSRMTLQVTIQVRIPFIGGKLERLIGTQLSQLVT
IEQRFTTLWITNNV"
CDS 571763..572872
/codon_start=1
/gene="murB"
/note="gene_501"
/transl_table=11
/translation="MKRSGVGSLFAGAHIAEAVPLAPLTTLRVGPIARRVITCTSAEQ
VVAALRHLDSAAKTGADRPLVFAGGSNLVIAENLTDLTVVRLANSGITIDGNLVRAEA
GAVFDDVVVRAIEQGLGGLECLSGIPGSAGATPVQNVGAYGAEVSDTITRVRLLDRCT
GEVRWVSARDLRFGYRTSVLKHADGLAVPTVVLEVEFALDPSGRSAPLRYGELIAALN
ATSGERADPQAVREAVLALRARKGMVLDPTDHDTWSVGSFFTNPVVTQDVYERLAGDA
ATRKDGPVPHYPAPDGVKLAAGWLVERAGFGKGYPDAGAAPCRLSTKHALALTNRGGA
TAEDVVTLARAVRDGVHDVFGITLKPEPVLIGCML"
CDS 572934..574289
/codon_start=1
/note="gene_502"
/transl_table=11
/translation="VVIRVLFRPVSLIPVNNSSTPQSQGPISRRLALTALGFGVLAPN
VLVACAGKVTKLAEKRPPPAPRLTFRPADSAADVVPIAPISVEVGDGWFQRVALTNSA
GKVVAGAYSRDRTIYTITEPLGYDTTYTWSGSAVGHDGKAVPVAGKFTTVAPVKTINA
GFQLADGQTVGIAAPVIIQFDSPISDKAAVERALTVTTDPPVEGGWAWLPDEAQGARV
HWRPREYYPAGTTVDVDAKLYGLPFGDGAYGAQDMSLHFQIGRRQVVKAEVSSHRIQV
VTDAGVIMDFPCSYGEADLARNVTRNGIHVVTEKYSDFYMSNPAAGYSHIHERWAVRI
SNNGEFIHANPMSAGAQGNSNVTNGCINLSTENAEQYYRSAVYGDPVEVTGSSIQLSY
ADGDIWDWAVDWDTWVSMSALPPPAAKPAATQIPVTAPVTPSDAPTPSGTPTTTNGPG
G"
CDS complement(574270..575025)
/codon_start=1

```
                     /note="gene_503"
                     /transl_table=11
                     /translation="MTTIGTRKRVAVVTGASSGIGEATARTLAAQGFHVVAVARRADR
                     ITALANQIGGTAIVADVTDDAAVEALARALSRVDVLVNNAGGAKGLQFVADADLEHWR
                     WMWDTNVLGTLRVTRALLPKLIDSGDGLIVTVTSIAALEVYDGGAGYTAAKHAQGALH
                     RTLRGELLGKPVRLTEIAPGAVETEFSLVRFDGDQQRADAVYAGMTPLVAADVAEVIG
                     FVATRPSHVNLDQIVIRPRDQASASRRATHPVR"
     CDS             575208..576524
                     /codon_start=1
                     /note="gene_504"
                     /transl_table=11
                     /translation="VYSTNRTSQSLSRKPGRKHQLRSHRYVMPPSLHLSDSAAASVFR
                     AVRLRGPVGRDVIAGSTSLSIATVNRQVIALLEAGLLRERADLAVSGAIGRPRVPVEV
                     NHEPFVTLGIHIGARTTSIVATDLFGRTLDTVETPTPRNAAGAALTSLADSADRYLQR
                     WRRRRALWVGVTLGGAVDSATGHVDHPRLGWRQAPVGPVLADALGLPVSVASHVDAMA
                     GAELMLGMRRFAPSSSTSLYVYARETVGYALMIGGRVHCPASGPGTIAPLPVHSEMLG
                     GTGQLESTVSDEAVLAAARRLRIIPGIASRTRTGGSATAITDLLRVARAGNQQAKELL
                     AERARVLGGAVALLRDLLNPDEVVVGGQAFTEYPEAMEQVEAAFTAGSVLAPRDIRVT
                     VFGNRVQEAGAGIVSLSGLYADPLGALRRSGALDARLQDTAPEALA"
     CDS             576581..578014
                     /codon_start=1
                     /note="gene_505"
                     /transl_table=11
                     /translation="VRHDDGSGLIAQRRPVRGEGATRSRGPSGPSNRNVSAADDPRRV
                     ALLAVHTSPLAQPGTGDAGGMNVYMLQSALHLARRGIEVEIFTRATASADPPVVRVAP
                     GVLVRNVVAGPFEGLDKYDLPTQLCAFAAGVLRAEAVHEPGYYDIVHSHYWLSGQVGW
                     LARDRWAVPLVHTAHTLAAVKNAALADGDGPEPPLRTVGEQQVVDEADRLIVNTDDEA
                     RQVISLHGADPARIDVVHPGVDLDVFRPGDRRAARAALGLPVDERVVAFVGRIQPLKA
                     PDIVLRAAAKLPGVRIIVAGGPSGSGLASPDGLVRLADELGISARVTFLPPQSHTDLA
                     TLFRAADLVAVPSYSESFGLVAVEAQACGTPVVAAAVGGLPVAVRDGITGTLVSGHEV
                     GQWADAIDHLLRLCAGPRGRVMSRAAARHAATFSWENTTDALLASYRRAIGEYNAERQ
                     RRGGEVISDLVAVGKPRHWTPRRGVGA"
     CDS             578032..578562
                     /codon_start=1
                     /note="gene_506"
                     /transl_table=11
                     /translation="VQRVIQNALEVSQLKYSQHPRPGGAPPALIVELPGERKLKINTI
                     LSVGEHSVRVEAFVCRKPDENREDVYRFLLRRNRRLYGVAYTLDNVGDIYLVGQMALS
                     AVDADEVDRVLGQVLEVVDSDFNALLELGFRSSIQREWQWRLSRGESLQNLQAFAHLR
                     PTTMQSAQRDEKELGG"
     variation        578563..578620
                     /note="supplemented from BCG moreau"
     CDS             578971..579609
                     /codon_start=1
                     /note="gene_507"
                     /transl_table=11
                     /translation="VPLQVFVGFVAMMTLKVAIGPQNAFVLRQGIRREYVLVIVALCG
                     IADGALIAAGVGGFAALIHAHPNMTLVARFGGAAFLIGYALLAARNAWRPSGLVPSES
                     GPAALIGVVQMCLVVTFLNPHVYLDTVVLIGALANEESDLRWFFGAGAWAASVVWFAV
                     LGFSAGRLQPFFATPAAWRILDALVAVTMIGVAVVVLVTSPSVPTANVALII"
     CDS             579745..580515
```

```
                     /codon_start=1
                     /gene="gpmA"
                     /note="gene_508"
                     /transl_table=11
                     /translation="VRERLAGMANTGSLVLLRHGESDWNALNLFTGWVDVGLTDKGQA
                     EAVRSGELIAEHDLLPDVLYTSLLRRAITTAHLALDSADRLWIPVRRSWRLNERHYGA
                     LQGLDKAETKARYGEEQFMAWRRSYDTPPPPIERGSQFSQDADPRYADIGGGPLTECL
                     ADVVARFLPYFTDVIVGDLRVGKTVLIVAHGNSLRALVKHLDQMSDDEIVGLNIPTGI
                     PLRYDLDSAMRPLVRGGTYLDPEAAAAGAAAVAGQGRG"
     CDS             580689..581921
                     /codon_start=1
                     /note="gene_509"
                     /transl_table=11
                     /translation="VTVFSALLLAGVLSALALAVGGAVGMRLTSRVVEQRQRVATEWS
                     GITVSQMLQCIVTLMPLGAAVVDTHRDVVYLNERAKELGLVRDRQLDDQAWRAARQAL
                     GGEDVESDLSPRKRSATGRSGLSVHGHARLLSEEDRRFAVVFVHDQSDYARMEAARRD
                     FVANVSHELKTPVGAMALLAEALLASADDSETVRRFAEKVLIEANRLGDMVAELIELS
                     RLQGAERLPNMTDVDVDTIVSEAISRHKVAADNADIEVRTDAPSNLRVLGDQTLLVTA
                     LANLVSNAIAYSPRGSLVSISRRRRGANIEIAVTDRGIGIAPEDQERVFERFFRGDKA
                     RSRATGGSGLGLAIVKHVAANHDGTIRVWSKPGTGSTFTLALPALIEAYHDDERPEQA
                     REPELRSNRSQREEELSR"
     variation        582060..582189
                     /note="supplemented from AL2122/97"
     CDS             582270..582962
                     /codon_start=1
                     /note="gene_510"
                     /transl_table=11
                     /translation="VALMTSVLIVEDEESLADPLTFLLRKEGFEATVVTDGPAALAEF
                     DRAGADIVLLDLMLPGMSGTDVCKQLRARSSVPVIMVTARDSEIDKVVGLELGADDYV
                     TKPYSARELIARIRAVLRRGGDDDSEMSDGVLESGPVRMDVERHVVSVNGDTITLPLK
                     EFDLLEYLMRNSGRVLTRGQLIDRVWGADYVGDTKTLDVHVKRLRSKIEADPANPVHL
                     VTVCGLGYKLEG"
     CDS             complement(582959..584848)
                     /codon_start=1
                     /note="gene_511"
                     /transl_table=11
                     /translation="MSRLADRAKSYPLASFGAALLPPELGGPLPAQFVQRVDRYVTRL
                     PATSRFAVRAGLASLAAASYLTTGRSLPRLHPDERARVLHRIAALSPEVAAAVEGLKA
                     IVLLANGADTYAHELLARAQEHDAARPDAELTVILSADSPSVTRADAVVVGSGAGGAM
                     VARTLARAGLDVVVLEEGRRWTVEEFRSTHPVDRYAGLYRGAGATVALGRPAVVLPMG
                     RAVGGTTVVNSGTCFRPSLAVQRRWRDEFGLGLADPDQLGRRLDDAEQTLRVAPVPLE
                     IMGRNGRLLLQAAKSLGWRAAPIPRNAPGCRGCCQCAIGCPSNAKFGVHLNALPQACA
                     AGARIISWARVERILHRAGRAYGVRARRPDGTTLDVLADAVVVAAGATETPGLLRRSG
                     LGGHPRLGHNLALHPATMLAGLFDDDVFAWRGVLQSAAVHEFHESDGVLIEATSTPPG
                     MGSMVFPGYGAELLRWLDRAPQIATFGAMVADRGVGTVRSVRGETVVRYDIAPGEIAK
                     LRVALQAIGRLFFAAGAVEVLTGIPGAPPMRSLPELQDVLRRANPRSLHLAAFHPTGT
                     AAAGADEQLCPVDATGRLRGVEGVWVADASILPSCPEVNPQLSIMAMALAVADQTVAK
                     VVGVR"
     CDS             complement(584845..585174)
                     /codon_start=1
                     /note="gene_512"
```

```
                     /transl_table=11
                     /translation="MSFLLDPPLLFVCGVLIERRLPVDRRDAAEAAALGVFFGASFGL
                     YHNVPGLGMLWRPFRAQNGRDFMWNSGVFSVDVARAEWPLHAMAAAIFATYPFFIKLG
                     RRLGRRI"
     CDS             complement(585171..586121)
                     /codon_start=1
                     /note="gene_513"
                     /transl_table=11
                     /translation="VDDETRSAALPRWRGAAGRLEVWYATLSDPLTRTGLWVHCETVA
                     PTTGGPYAHGWVTWFPPDAPPGTERFGPQPAQPAAGPAWFDIAGVRMAPAELTGRTRS
                     LAWELSWKDTAAPLWTFPRVAWERELLPGAQVVIAPTAVFAGSLAVGETTHRVDSWRG
                     GVAHIYGHGNAKRWGWIHADLGDGDVLEVVTAVSHKPGLRRLAPLAFVRFRIDGKDWP
                     ASPLPSLRMRTTLGVRHWQLEGRIGGREALIRVDQPPERCVSLGYTDPDGAKAVCTNT
                     EQADIHIELGGRHWSVLGTGHAEVGLRGTAAPAIKEGTPA"
     CDS             586177..586893
                     /codon_start=1
                     /note="gene_514"
                     /transl_table=11
                     /translation="MNQSSVFQPPDRQRVDERIATTIADAILDGVFPPGSTLPPERDL
                     AERLGVNRTSLRQGLARLQQMGLIEVRHGSGSVVRDPEGLTHPAVVEALVRKLGPDFL
                     VELLEIRAALGPLIGRLAAARSTPEDAEALCAALEVVQQADTAAARQAADLAYFRVLI
                     HSTRNRALGLLYRWVEHAFGGREHALTGAYDDADPVLTDLRAINGAVLAGDPAAAAAT
                     VEAYLNASALRMVKSYRDRA"
     CDS             complement(586894..587664)
                     /codon_start=1
                     /note="gene_515"
                     /transl_table=11
                     /translation="VHPGQEVELDFAREWVEFYDPDNPEHLIAADLTWLLSRWACVFG
                     TPACQGTVAGRPNDGCCSHGAFLSDDDDRTRLADAVHKLTDDDWQFRAKGLRRKGYLE
                     LDEHDGQPQHRTRKHKGACIFLNRPGFAGGAGCALHSKALKLGVPPLTMKPDVCWQLP
                     IRRSQEWVTRPDGTEILKTTLTEYDRRGWGSGGADLHWYCTGDPAAHVGTKQVWQSLA
                     DELTELLGEKAYGELAAMCKRRSQLGLIAVHPATRAAQ"
     CDS             587816..588850
                     /codon_start=1
                     /note="gene_516"
                     /transl_table=11
                     /translation="VRLGVLDVGSNTVHLLVVDAHRGGHPTPMSSTKATLRLAEATDS
                     SGKITKRGADKLISTIDEFAKIAISSGCAELMAFATSAVRDAENSEDVLSRVRKETGV
                     ELQALRGEDESRLTFLAVRRWYGWSAGRILNLDIGGGSLEVSSGVDEEPEIALSLPLG
                     AGRLTREWLPDDPPGRRRVAMLRDWLDAELAEPSVTVLEAGSPDLAVATSKTFRSLAR
                     LTGAAPSMAGPRVKRTLTANGLRQLIAFISRMTAVDRAELEGVSADRAPQIVAGALVA
                     EASMRALSIEAVEICPWALREGLILRKLDSEADGTALIESSSVHTSVRAVGGQPADRN
                     AANRSRGSKP"
     CDS             588847..589779
                     /codon_start=1
                     /note="gene_517"
                     /transl_table=11
                     /translation="MTGPHPETESSGNRQISVAELLARQGVTGAPARRRRRRRGDSDA
                     ITVAELTGEIPIIRDDHHHAGPDAHASQSPAANGRVQVGEAAPQSPAEPVAEQVAEEP
                     TRTVYWSQPEPRWPKSPPQDRRESGPELSEYPRPLRHTHSDRAPAGPPSGAEHMSPDP
                     VEHYPDLWVDVLDTEVGEAEAETEVREAQPGRGERHAAAAAAGTDVEGDGAAEARVAR
```

RALDVVPTLWRGALVVLQSILAVAFGAGLFIAFDQLWRWNSIVALVLSVMVILGLVVS
VRAVRKTEDIASTLIAVAVGALITLGPLALLQSG"
CDS 589795..590637
/codon_start=1
/note="gene_518"
/transl_table=11
/translation="VRPAIKVGLSTASVYPLRAEAAFEYADRLGYDGVELMVWGESVS
QDIDAVRKLSRRYRVPVLSVHAPCLLISQRVWGANPILKLDRSVRAAEQLGAQTVVVH
PPFRWQRRYAEGFSDQVAALEAASTVMVAVENMFPFRADRFFGAGQSRERMRKRGGGP
GPAISAFAPSYDPLDGNHAHYTLDLSHTATAGTDSLDMARRMGPGLVHLHLCDGSGLP
ADEHLVPGRGTQPTAEVCQMLAGSGFVGHVVLEVSTSSARSANERESMLAESLQFART
HLLR"
CDS 590653..591528
/codon_start=1
/note="gene_519"
/transl_table=11
/translation="MNALFTTAMALRPLDSDPGNPACRVFEGELNEHWTIGPKVHGGA
MVALCANAARTAYGAAGQQPMRQPVAVSASFLWAPDPGTMRLVTSIRKRGRRISVADV
ELTQGGRTAVHAVVTLGEPEHFLPGVDGSGGASGTAPLLSANPVVELMAPEPPEGVVP
IGPGHQLAGLVHLGEGCDVRPVLSTLRSATDGRPPVIQLWARPRGVAPDALFALLCGD
LSAPVTFAVDRTGWAPTVALTAYLRALPADGWLRVLCTCVEIGQDWFDEDHIVVDRLG
RIVVQTRQLAMVPAQ"
CDS 591553..592440
/codon_start=1
/note="gene_520"
/transl_table=11
/translation="MLFGMARIAIIGGGSIGEALLSGLLRAGRQVKDLVVAERMPDRA
NYLAQTYSVLVTSAADAVENATFVVVAVKPADVEPVIADLANATAAAENDSAEQVFVT
VVAGITIAYFESKLPAGTPVVRAMPNAAALVGAGVTALAKGRFVTPQQLEEVSALFDA
VGGVLTVPESQLDAVTAVSGSGPAYFFLLVEALVDAGVGVGLSRQVATDLAAQTMAGS
AAMLLERMDQDQGGANGELMGLRVDLTASRLRAAVTSPGGTTAAALRELERGGFRMAV
DAAVQAAKSRSEQLRITPE"
CDS 592581..592817
/codon_start=1
/note="gene_521"
/transl_table=11
/translation="MTSTNGPSARDTGFVEGQQAKTQLLTVAEVAALMRVSKMTVYRL
VHNGELPAVRVGRSFRVHAKAVHDMLETSYFDAG"
CDS 593124..594254
/codon_start=1
/note="gene_522"
/transl_table=11
/translation="VSSSNGRGGAGGVGGSSEHPQYPKVVLVTGACRFLGGYLTARLA
QNPLINRVIAVDAIAPSKDMLRRMGRAEFVRADIRNPFIAKVIRNGEVDTVVHAAAAS
YAPRSGGSAALKELNVMGAMQLFAACQKAPSVRRVVLKSTSEVYGSSPHDPVMFTEDS
SSRRPFSQGFPKDSLDIEGYVRALGRRRPDIAVTILRLANMIGPAMDTTLSRYLAGPL
VPTIFGRDARLQLLHEQDALGALERAAMAGKAGTFNIGADGILMLSQAIRRAGRIPVP
VPGFGVWALDSLRRANHYTELNREQFAYLSYGRVMDTTRMRVELGYQPKWTTVEAFDD
YFRGRGLTPIIDPHRVRSWEGRAVGLAQRWGSRNPIPWSGLR"
CDS 594261..595337
/codon_start=1

/note="gene_523"
/transl_table=11
/translation="MGNVAGETRANVIPLHTNRSRVAARRRAGQRAESRQHPSLLSDP
NDRASAEQIAAVVREIDEHRRAAGATTSSTEATPNDLAQLVAAVAGFLRQRLTGDYSV
DEFGFDPHFNSAIVRPLLRFFFKSWFRVEVSGVENIPRDGAALVVANHAGVLPFDGLM
LSVAVHDEHPAHRDLRLLAADMVFDLPVIGEAARKAGHTMACTTDAHRLLASGELTAV
FPEGYKGLGKRFEDRYRLQRFGRGGFVSAALRTKAPIVPCSIIGSEEIYPMLTDVKLL
ARLFGLPYFPITPLFPLAGPVGLVPLPSKWRIAFGEPICTADYASTDADDPMVTFELT
DQVRETIQQTLYRLLAGRRNIFFG"
CDS complement(595341..596249)
/codon_start=1
/note="gene_524"
/transl_table=11
/translation="MTSQGDTTSGTQLKPPVEAVRSHYDKSNEFFKLWLDPSMTYSCA
YFERPDMTLEEAQYAKRKLALDKLNLEPGMTLLDIGCGWGSTMRHAVAEYDVNVIGLT
LSENQYAHDKAMFDEVDSPRRKEVRIQGWEEFDEPVDRIVSLGAFEHFADGAGDAGFE
RYDTFFKKFYNLTPDDGRMLLHTITIPDKEEAQELGLTSPMSLLRFIKFILTEIFPGG
RLPRISQVDYYSSNAGWKVERYHRIGANYVPTLNAWADALQAHKDEAIALKGQETYDI
YMHYLRGCSDLFRDKYTDVCQFTLVK"
CDS complement(596272..596772)
/codon_start=1
/note="gene_525"
/transl_table=11
/translation="MTVPEEAQTLIGKHYRAPDHFLVGREKIREFAVAVKDDHPTHYS
EPDAAAAGYPALVAPLTFLAIAGRRVQLEIFTKFNIPINIARVFHRDQKFRFHRPILA
NDKLYFDTYLDSVIESHGTVLAEIRSEVTDAEGKPVVTSVVTMLGEAAHHEADADATV
AAIASI"
CDS complement(596934..597857)
/codon_start=1
/note="gene_526"
/transl_table=11
/translation="MVSSHLGSPDQAGHVDLASPADPPPPDASASHSPVDMPAPVAAA
GSDRQPPIDLTAAAFFDVDNTLVQGSSAVHFGRGLAARHYFTYRDVLGFLYAQAKFQL
LGKENSNDVAAGRRKALAFIEGRSVAELVALGEEIYDEIIADKIWDGTRELTQMHLDA
GQQVWLITATPYELAATIARRLGLTGALGTVAESVDGIFTGRLVGEILHGTGKAHAVR
SLAIREGLNLKRCTAYSDSYNDVPMLSLVGTAVAINPDARLRSLARERGWEIRDFRIA
RKAARIGVPSALALGAAGGALAALASRRQSR"
CDS 598235..598672
/codon_start=1
/note="gene_527"
/transl_table=11
/translation="MISVSGAVKRMWLLLAIVVVAVVGGLGIYRLHSIFGVHEQPTVM
VKPDFDVPLFNPKRVTYEVFGPAKTAKIAYLDPDARVHRLDSVSLPWSVTVETTLPAV
SVNLMAQSNADVISCRIIVNGAVKDERSETSPRALTSCQVSSG"
CDS 598669..601575
/codon_start=1
/gene="mmpL2"
/note="gene_528"
/transl_table=11
/translation="MSERHAALTSLPPILPRLIRRFAVVIVLLWLGFTAFVNLAVPQL
EVVGKAHSVSMSPSDAASIQAIKRVGQVFGEFDSDNAVTIVLEGDQPLGGDAHRFYSD

LMRKLSADTRHVAHIQDFWGDPLTAAGSQSADDRAAYVVVYLVGNNETEAYDSVHAVR
HMVDTTPPPHGVKAYVTGPAALNADQAEAGDKSIAKVTAITSMVIAAMLLVIYRSVIT
AVLVLIMVGIDLGAIRGFIALLADHNIFSLSTFATNLLVLMAIAASTDYAIFMLGRYH
ESRYAGEDRETAFYTMFHGTAHVILGSGLTIAGAMYCLSFARLPYFETLGAPIAIGML
VAVLAALTLGPAVLTVGSFFKLFDPKRRMNTRRWRRVGTAIVRWPGPVLAATCLVASI
GLLALPSYRTTYDLRKFMPASMPSNVGDAAAGRHFSRARLNPEVLLIETDHDMRNPVD
MLVLDKVAKNIYHSPGIEQVKAITRPLGTTIKHTSIPFIISMQGVNSSEQMEFMKDRI
YDILVQVAAMNTSIETMHRMYALMGEVIDNTVDMDHLTHDMSDITATLRDHLADFEDF
FRPIRSYFYWEKHCFDVPLCWSIRSIFDMFDSVDQLSEKLEYLVKDMDILITLLPQMR
AQMPPMISAMTTMRDMMLIWHGTLGAFYKQQERNNKDPGAMGRVFDAAQIDDSFYLPQ
SAFENPDFKRGLKMFLSPDGKAARFVIALEGDPATPEGICRVEPIKREAREAIKGTPL
QGAAIYLGGTAATFKDIREGARYDLLIAGVAAISLILIIMMIITRSVVAAVVIVGTVV
LSMGASFGLSVLVWQDILGIELYWMVLAMSVILLLAVGSDYNLLLISRLKEEIGAGLN
TGIIRAMAGTGGVVTAAGMVFAVTMSLFVFSDLRIIGQIGTTIGLGLLFDTLVVRSFM
TPSIAALLGRWFWWPLRVRPRPASQMLRPFAPRRLVRALLLPSGQHPSATGAHE"

CDS 601583..601861
/codon_start=1
/note="gene_529"
/transl_table=11
/translation="VELLTRAGCAICVRVAEQLAELSSELGFDMMTIDVDVAASTGNP
GLRAEFGDRLPVVLLDGREHSYWEVDEHRLRADIARSTFGSPPDKRLP"

CDS 601911..603317
/codon_start=1
/gene="hemA"
/note="gene_530"
/transl_table=11
/translation="VSVLLFGVSHRSAPVVVLEQLSIDESDQVKIIDRVLASPLVTEA
MVLSTCNRVEVYAVVDAFHGGLSVIGQVLAEHSGMSMGELTKYAYVRYSEAAVEHLFA
VASGLDSAVIGEQQVLGQVRRAYAVAESNRTVGRVLHELAQRALSVGKRVHSETAIDA
AGASVVSVALGMAERKLGSLAGTTAVVIGAGAMGALSAVHLTRAGVGHIQVLNRSLSR
AQRLARRIRESGVPAEALALDRLANVLADADVVVSCTGAVRPVVSLADVHHALAAARR
DEATRPLVICDLGMPRDVDPAVARLPCVWVVDVDSVQHEPSAHAAAADVEAARHIVAA
EVASYLVGQRMAEVTPTVTALRQRAAEVVEAELLRLDNRLPGLQSVQREEVARTVRRV
VDKLLHAPTVRIKQLASAPGGDSYAEALRELFELDQTAVDAVATAGELPVVPSGFDAE
SRRGGGDMQSSPKRSPSN"

CDS 603354..604256
/codon_start=1
/gene="hemC"
/note="gene_531"
/transl_table=11
/translation="LLATTQAATVRDALIAGGHSAELVTISTEGDRSMAPIASLGVGV
FTTALREAMEAGLVDAAVHSYKDLPTAADPRFTVAAIPPRNDPRDAVVARDGLTLGEL
PVGSLVGTSSPRRAAQLRALGLGLEIRPLRGNLDTRLNKVSSGDLDAIVVARAGLARL
GRLDDVTETLEPVQMLPAPAQGALAVECRAGDSRLVAVLAELDDADTRAAVTAERALL
ADLEAGCSAPVGAIAEVVESIDEDGRVFEELSLRGCVAALDGSDVIRASGIGSCGRAR
ELGLSVAAELFELGARELMWGVRH"

CDS 604289..605986
/codon_start=1
/note="gene_532"
/transl_table=11
/translation="MTRGRKPRPGRIVFVGSGPGDPGLLTTRAAAVLANAALVFTDPD

VPEPVVALIGTDLPPVSGPAPAEPVAGNGDAAGGGSAQEHGRAASAVVSGGPDIRPAL
GDPADVAKTLTAEARSGVDVVRLVAGDPLTVDAVISEVNAVARTHLHIEIVPGLAASS
AVPTYAGLPLGSSHTVADVRIDPENTDWDALAAAPGPLILQATASHLAESARSLIDHQ
LAESTPCVVTAHGTTCQQRSVETTLQGLTDPAVLGATDPACSANGRDSQAGPLIVTIG
KTVTSRAKLNWWESRALYGWTVLVPRTKDQAGEMSERLTSYGALPVEVPTIAVEPPRS
PAQMERAVKGLVDGRFQWIVFTSTNAVRAVWEKFGEFGLDARAFSGVKIACVGESTAD
RVRAFGISPELVPSGEQSSLGLLDDFPPYDSVFDPVNRVLLPRADIATETLAEGLRER
GWEIEDVTAYRTVRAAPPPATTREMIKTGGFDAVCFTSSSTVRNLVGIAGKPHARTII
ACIGPKTAETAAEFGLRVDVQPDTAAIGPLVDALAEHAARLRAEGALPPPRKKSRRR"
CDS 606174..607061
/codon_start=1
/note="gene_533"
/transl_table=11
/translation="VLPMFVADGIDEPRPITSMPGVVQHTRDSLRRAAAAAVAAGVGG
LMLFGVPRDQDKDGVGSAGIDPDGILNVALRDLAKDLGEATVLMADTCLDEFTDHGHC
GVLDDRGRVDNDATVARYVELAVAQAESGAHVVGPSGMMDGQVAAIRDGLDAAGYIDV
VILAYAAKFASAFYGPFREAVSSSLSGDRRTYQQEPGNAAEALREIELDLDGGADIVM
VKPAMGYLDVVAAAADVSPVPVAAYQVSGEYAMIRAAAANNWIDERAAVLESLTGIRR
AGADIVLTYWAVDAAGWLT"
CDS 607074..607622
/codon_start=1
/note="gene_534"
/transl_table=11
/translation="MTPTGDTKPKLLFYEPGASWYWVLTGPLAAVSVLLLEISSGAGV
GLITPAIFLVMVSAFVALQVKAARIHTSVELTHDALRQGTETIRLAEIVKIYPEADGR
ETSGEEPAKWQSARTLGELVGVPRGRVGIGLKLTGGRTAQAWARRHQQLRAALTPLVQ
ERLGPVDSDVADVNGDDAGPAR"
CDS 607619..607918
/codon_start=1
/note="gene_535"
/transl_table=11
/translation="VIARYRAGAELFLACAALAGSAGSWSRTRSTVAVAPVIDGQPVT
LSVVYHPQPLVLTLLLATIAGVLSVVGTARLRRARAGLNAHPDGLNQRPPGGWCH"
CDS 608090..609532
/codon_start=1
/note="gene_536"
/transl_table=11
/translation="VSAAFIDRICSATRAENRAAAAQLVALGELFAYRWSRCGGREEW
VMDTMAAVAAEVAAALRISQGLAASRLRYARAMRERLPKTAEVFSAGDIGYLMFATIV
YRTDLIVDPDVLAAVDAQLAANVARWPSMTKARLAGQVDKIVARADADAVRRRKEYQA
QRQFWVGESQDGVCQIGGSLLAVDAHALDARLSALAGTVCEHDPRSREQRRADALGAL
AGGADRLGCGCGRADCAAGKRPAAPPVVIHLIAEAATINGTGSAPASQMNADGLITAE
LVAELAKTATLVPLVHPGDAPPEPGYAPSKALADFVRCRDLTCRWPGCDEPATNCDLD
HTIPYAAGGPTHASNLKCYCRTHHLVKTFWGWRDQQLPDGTLILTSPSGHTYVSTPGS
ALLFPSLCHFSGGIPAPEADPPYDHCDQRTAMMPKRRRTRAQDRAYRIATERRQNHAA
RQRAQVLTQTAAATDTHGPPPDHNDDPPPF"
CDS complement(609529..610005)
/codon_start=1
/note="gene_537"
/transl_table=11
/translation="MTTTIPTSKSACSVTTRPGNAAVDYGGAQIRAYLHHLATVVTIR

GEIDAANVEQISEHVRRFSLGTNPMVLDLSELSHFSGAGISLLCILDEDCRAAGVQWA
LVASPAVVEQLGGRCDQGEHESMFPMARSVHKALHDLADAIDRRRQLVLPLISRSA"
CDS 610294..611526
/codon_start=1
/note="gene_538"
/transl_table=11
/translation="VRAAEITGEIRALTGLRIVAAVWVVLFHFRPMLGDASPGFRDAL
APVLDCGAQGVDLFFILSGFVLTWNYLDRMGRSWSVRANLHFLWLRLARVWPVYLVTL
HLAAVWVIFTLHVGHVPSPEAGQLTAISYVRQILLVQLWFQPYFDGSSWDGPAWSISA
EWLAYLLFGLLILVIFRMKHATRARGLMWLAFAASLPPVVLLLASGQFYTPWSWLPRI
VTQFAAGALACAAVRRLRPTDRARRIAGYLSVLVGVAIVGILYLLHAHPLAGVEDSGG
VVDVLFVPLVISLAIGVGSLPALLSTRLMVFGGQISFCLYMVHELVHTAWGWAVQQYE
LALQDQPWKWNVVGLLAIALGAAILLYHFVEEPGRRWMRRMVDVKAASARSEPGEPVG
STRYQIDDALEGVSARAV"
CDS 611658..612353
/codon_start=1
/note="gene_539"
/transl_table=11
/translation="VSRPGTYVIGLTLLVGLVVGNPGCPRSYRPLTLDYRLNPVAVIG
DSYTTGTDEGGLGSKSWTARTWQMLAARGVRIAADVAAEGRAGYGVPGDHGNVFEDLT
ARAVQPDDALVVFFGSRNDQGMDPEDPEMLAEKVRDTFDLARHRAPSASLLVIAPPWP
TADVPGPMLRIRDVLGAQARAAGAVFVDPIADHWFVDRPELIGADGVHPNDAGHEYLA
DKIAPLISMELVG"
CDS complement(612642..613490)
/codon_start=1
/note="gene_540"
/transl_table=11
/translation="MADLTRRALLRWGAGAGAGAAGVWAFGALVDPLEPQAAPAPFEP
PTAGSSLPTRISGSFISAARGGIKTNWVISMPPGQSGQLRPVIALHGKDGNAGMMLDL
GVEQGLARLVKEGKPAFAVVGVDGGNTYWHRRSSGGDSGAMVLDELLPMLTSMGMDTS
RVGFLGWSMGGYGALLLGARLGPARTAGICAISPALFTSFTGSTPGAFDSYDDYVQHS
VLGLPALNSIPLRVDCGTSDRFYFATRQFVNQLHQPPAGSFSPGGHDASYWREQLPGE
LAWMAS"
CDS 613725..614075
/codon_start=1
/note="gene_541"
/transl_table=11
/translation="MGGCSITCLNISEVPNETNRKKNRQAGLDRSIRVIHGSFDDIPE
PDSGYDVVWSQDAILHAPDRRKVLEEAFRVLRPGGELIFTDPMQADDVPDGVLQPVYD
RLNLRDLGSMRFYA"
CDS 614068..614373
/codon_start=1
/note="gene_542"
/transl_table=11
/translation="MREAAQALGFEVLDQRDLVRNLRTHYSRVFEELEARRLELEGKS
SQEYLDKMRVGLKNWVEAADNGHSRVGHPTFPRTRLTPICQLPTAAIDSTAGRRRYR"
CDS 614508..615812
/codon_start=1
/note="gene_543"
/transl_table=11
/translation="MIAIGGVIGAGLFVGSGVVIRATGPAAFLTYALCGALIVLVMRM

LGEMAAANPSTGAFADYAAKALGGWAGFSVGWLYWYFWVIVVGFEAVAGGKVLTYWID
APLWLASLCLMMMMTATNLVSVSSFGEFEFWFAGVKVATIVGFLVLGTAFAFGLLPGH
GMDFSNLSAHGGFFPDGVGAVFAAIVVAIFSMTGTEVVTIAAAEAPDPQRAVQRAMST
VVARIVIFFVGSVFLLTVILPWNSLELGASPYVAALRHMGIGGADQIMNAVVLTAVLS
CLNSGLYTASRMLFVLAARQEAPAQLVKVNRRGVPTFAIMGSSVVGFLCVIMAWVSPA
TVFVFLLNSSGAVILFVYLLIALSQIVLRRQTSGQNLGVRMWLFPGLSIVTVTGIVAV
LARMAFDYAARSQLWLSLLSWAVVVGCYLVTTLVRRPLNRPW"
CDS complement(615796..616200)
/codon_start=1
/note="gene_544"
/transl_table=11
/translation="MVGMQLPQWLARFNRYVTNPIQRLWAGWLPAFAILEHVGRRSGK
PYRTPLNVFSADVDGRAGVAILLTYGPNRDWLKNITAAGGGRMRRYGKTFGVANPRRL
TKAEAAPYVSSRWRPVFARLPFDEAVLLTKAD"
CDS 616305..617693
/codon_start=1
/note="gene_545"
/transl_table=11
/translation="MGSTEQATSRVRGAARTSAQLFEAACSVIPGGVNSPVRAFTAVG
GTPRFITEAHGCWLIDADGNRYVDLVCSWGPMILGHAHPAVVEAVAKAAARGLSFGAP
TPAETQLAGEIIGRVAPVERIRLVNSGTEATMSAVRLARGFTGRAKIVKFSGCYHGHV
DALLADAGSGVATLGLCDDPQRPASPRSQSSRGLPSSPGVTGAAAADTIVLPYNDIDA
VQQTFARFGEQIAAVITEASPGNMGVVPPGPGFNAALRAITAEHGALLILDEVMTGFR
VSRSGWYGIDPVPADLFAFGKVMSGGMPAAAFGGRAEVMQRLAPLGPVYQAGTLSGNP
VAVAAGLATLRAADDAVYTALDANADRLAGLLSEALTDAVVPHQISRAGNMLSVFFGE
TPVTDFASARASQTWRYPAFFHAMLDAGVYPPCSAFEAWFVSAALDDAAFGRIANALP
AAARAAAQERPA"
CDS 617693..618301
/codon_start=1
/note="gene_546"
/transl_table=11
/translation="MPEETQVHVVRHGEVHNPTGILYGRLPGFHLSATGAAQAAAVAD
ALADRDIVAVIASPLQRAQETAAPIAARHDLAVETDPDLIESANFFEGRRVGPGDGAW
RDPRVWWQLRNPFTPSWGEPYVDIAARMTTAVDKARVRGAGHEVVCVSHQLPVWTLRL
YLTGKRLWHDPRRRDCALASVTSLIYDGDRLVDVVYSQPAAL"
CDS 618355..618966
/codon_start=1
/note="gene_547"
/transl_table=11
/translation="MRRLVIAAAVSALLLTGCSGRDAVAQGGTFEFVSPGGKTDIFYD
PPASRGRPGPLSGPELADPARSVSLDDFPGQVVVVNVWGQWCGPCRAEVSQLQRVYDA
TRGAGVSFLGIDVRDNNRQAPQDFINDRHVTYPSIYDPAMRTLIAFGGKYPTSVIPST
LVLDRQHRVAAVFLRELLAADLQPVVERVAEEEPSGRAPVGAQ"
CDS 618963..619742
/codon_start=1
/note="gene_548"
/transl_table=11
/translation="MTGFTEIAAVGPLLVAVGVCLLAGLVSFASPCVVPLVPGYLSYL
AAVVGVDEQLPAGVVKPPVAARWRVAGSAALFVAGFTTVFVLGTVAVLGMTTTLITNQ
LLLQRVGGVLIVVMGLVFVGFIGALQRQARFTPRQLTSVAGAPVLGAVFALGWTPCLG
PTLTGVITVASATEGASVARGIVLVIAYCLGLGIPFVLLAFGSAWAVAGLGWLRRHTR

AIQIFGGALLIAVGAALVTGVWNDVVSWLRDAFVSDVRLPI"
CDS 619796..621364
/codon_start=1
/note="gene_549"
/transl_table=11
/translation="MGTALVLLFLLALAAIPGALLPQRGLNAAKVDDYLAAHPLIGPW
LDELQAFDVFSSFWFTAIYVLLFVSLVGCLAPRTIEHARSLRATPVAAPRNLARLPKH
AHARLAGEPAALAATITGRLRGWRSITRQQGDSVEVSAEKGYLREFGNLVFHFALLGL
LVAVAVGKLFGYEGNVIVIADGGPGFCSASPAAFDSFRAGNTVDGTSLHPICVRVNNF
QAHYLPSGQATSFAADIDYQADPATADLIANSWRPYRLQVNHPLRVGGDRVYLQGHGY
APTFTVTFPDGQTRTSTVQWRPDNPQTLLSAGVVRIDPPAGSYPNPDERRKHQIAIQG
LLAPTEQLDGTLLSSRFPALNAPAVAIDIYRGDTGLDSGRPQSLFTLDHRLIEQGRLV
KEKRVNLRAGQQVRIDQGPAAGTVVRFDGAVPFVNLQVSHDPGQSWVLVFAITMMAGL
LVSLLVRRRRVWARITPTTAGTVNVELGGLTRTDNSGWGAEFERLTGRLLAGFEARSP
DMAEAAAGTGRDVD"
CDS 621361..622335
/codon_start=1
/note="gene_550"
/transl_table=11
/translation="LNTLHVNVGLARYSDWAFTSAVVALVVALLLLAFEFAQVRGRGL
APLAVPAGSVATDSATPGIVADQRHRPFDERVGRGGLAVAYLGIGLLLACVVLRGLAT
QRVPWGNMYEFINLTCLSGLIAGAVVLRRARYRPLWVFLLVPVLILLTVSGRWLYANA
APVMPALQSYWLPIHVSVVSLGSGVFLVAGVASILFLVRTSRLGEPTGEGALAGMVRR
LPDAQTLDGIAYRTTIFAFPVFGFGVIFGAIWAEEAWGRYWGWDPKETVSFVAWVVYA
AYLHARSTAGWRDRKAAWINVAGFVAMVFNLFFVNLVTVGLHSYAGVG"
CDS 622377..623594
/codon_start=1
/note="gene_551"
/transl_table=11
/translation="VLVTEHPRTGVGAPDSGNGGTDHPTVQLPPVPSVGAPPAAAGGE
TPTRSVAGFRTQRLDPTAYGAYYSGPDEGPASPAERPPYRLEPVPHTPYPELATTTLL
RPVKPPPSEGWRRLLYLLSGRLINAGEGPRAAHLNDLVAQVNRPLRGCYRIAVLSLKG
GVGKTTITATLGATFADLRGDRVVAVDANPDRGTLSQKVPLETPATVRHLLRDADGIE
RYSDVRGYTSKGPSGLEVLASDSDPASSDAFSADDYTRTLDILERFYGLVLTDCGTGL
LHSAMSAVLPRSDVLVVVSSGSIDGARSAAATLDWLQAHGHDDQVRNSIAVVNAVRPR
AGKVDVGKVVEHFSRRCRAVRVVPFDPHLEEGAEIALDRLRRETREALTELAAVVAAG
FPGDPRRCKPSFT"
CDS complement(623591..623752)
/codon_start=1
/note="gene_552"
/transl_table=11
/translation="VLYLLLVLILATLIYLGWRAARAQMNRPKTRVIGPDDDPEFLRR
LGHGDNNRS"
CDS 623799..624116
/codon_start=1
/note="gene_553"
/transl_table=11
/translation="VSEAPNDKTTRGVVDILVYATARLLLVVAVSAAIFGVARLIGLT
EFPVVVATLFGLIIAMPLGIWVFSPLRRRATAALAVAGERRRAERERLRARLRGESLP
EEQ"
CDS 624263..626008

/codon_start=1
/note="gene_554"
/transl_table=11
/translation="MSNLLVTPELVAAAAADLAGIGSAIGAANAAAGAPTMALLAAGA
DEVSAAVAAVFSSYAQQYQALSAAAAAFHDQFVRALAAGAGAYAGAEAANVEQQLLNA
INAPTLALLGRPLIGNGADGAAGTGQAGGAGGLLYGNGGNGGSGAAGQAGGAGGAAGL
IGHGGTGGVGGTGAAGGAGGTGGWLFGNGGAGGTGGAVTGVSTTGGPGGHGGDAGLYG
FGGAGGAGGFGQSGAAGGAGGAGGAGGWLYGDGGDGGAGGNGGNESGTGVSGVGGVGG
AGGAGGLLFGNGGDGGVGGDGGDGSSTQDSGGDGGAGGAGGAGGWLLGNGGAGGAGGA
ASIKVATGGLGGDGGDAGLFGFGGDGGWGGRGVDARFGAAGGAAGAGGAGGWLYGDGG
AGGVGGVGGAVFSLSSGDGGAGGAGGGGGWLFGNGGDGGAGGGGGGRFGSGSGAGGDG
AVGGAGGAGAWFGNGGAGGVGGGGGRGTTAIGGDGGAGGAGGAGGWLYGDGGAGGAGG
GGGRGGTGNDGGDGGDGGRGGDAQLLGNGGDGGAGGAGGPAGFGASPGAGAAGGGGGA
GGSLFGSPGTTGPHG"
CDS complement(626037..627044)
/codon_start=1
/note="gene_555"
/transl_table=11
/translation="MTEIATTSGARSVGLLSVGAYRPERVVTNDEICQHIDSSDEWIY
TRTGIKTRRFAADDESAASMATEACRRALSNAGLSAADIDGVIVTTNTHFLQTPPAAP
MVAASLGAKGILGFDLSAGCAGFGYALGAAADMIRGGGAATMLVVGTEKLSPTIDMYD
RGNCFIFADGAAAVVVGETPFQGIGPTVAGSDGEQADAIRQDIDWITFAQNPSGPRPF
VRLEGPAVFRWAAFKMGDVGRRAMDAAGVRPDQIDVFVPHQANSRINELLVKNLQLRP
DAVVANDIEHTGNTSAASIPLAMAELLTTGAAKPGDLALLIGYGAGLSYAAQVVRMPK
G"
CDS complement(627126..628004)
/codon_start=1
/note="gene_556"
/transl_table=11
/translation="VASFAQWVSGARPRTLPNAIAPVVAGTGAAAWLHAAVWWKALLA
LAVAVALVIGVNYANDYSDGIRGTDDDRVGPVRLVGSRLATPRSVLTAAMTSLALGAL
AGLVLALLSAPWLIAVGAICIAGAWLYTGGSKPYGYAGFGELAVFVFFGPVAVLGTQY
TQALRVDWVGLAQAVATGALSCSVLVANNLRDIPTDARADKITLAVRLGDARTRMLYQ
GLLAVAGVLTFVLMLATPWCVVGLVAAPLALRAAGPVRSGRGGRELIPVLRDTGLAML
VWALAVAGALAFGQLS"
CDS 628021..628815
/codon_start=1
/note="gene_557"
/transl_table=11
/translation="MHNNGRMLGVIGGSGFYTFFGSDTRTVNSDTPYGQPSAPITIGT
IGVHDVAFLPRHGAHHQYSAHAVPYRANMWALRALGVRRVFGPCAVGSLDPELEPGAV
VVPDQLVDRTSGRADTYFDFGGVHAAFADPYCPTLRAAVTGLPGVVDGGTMVVIQGPR
FSTRAESQWFAAAGCNLVNMTGYPEAVLARELELCYAAIALVTDVDAGVAAGDGVKAA
DVFAAFGENIELLKRLVRAAIDRVADERTCTHCQHHAGVPLPFELP"
CDS 628812..629852
/codon_start=1
/note="gene_558"
/transl_table=11
/translation="MRVLLTGAAGFIGSRVDAALRAAGHDVVGVDALLPAAHGPNPVL
PPGCQRVDVRDASALAPLLAGVDLVCHQAAMVGAGVNAADAPAYGGHNDFATTVLLAQ
MFAAGVRRLVLASSMVVYGQGRYDCPQHGPVDPLPRRRADLDNGVFEHRCPGCGEPVI

WQLVDEDAPLRPRSLYAASKTAQEHYALAWSEASGGSVVALRYHNVYGPGMPRDTPYS
GVAAIFRSAVEKGKPPKVFEDGGQMRDFVHVDDVAAANLAAVHLGEADRDGFTAVNVC
SGRPISILQVATAICDARGGSMSPAITGHYRSGDVRHIVADPARAARVLGFRAAVDPG
EGLREFAFAPLR"
CDS complement(629862..631295)
/codon_start=1
/note="gene_559"
/transl_table=11
/translation="VGLSSDDTRRREVVRDLAAGALLIGALFFPWNLYFGFRIPDSSK
TVFGLLLAVTSLSLASLAVTFAGRRSQLRLGLNVPYLLLVLAFVVFDAIQTIRLGGTV
HVPGGVGPGGWLGITGALLSAQPALTGATTDEGSHSRWLRATQFLGYASMLGAALSTG
FNLSWRVRYALEPAAGASGFGKQNLAVIDTAVVYGVVALAAVLVASRWLLRPTAAEAL
STVALGGSTLIAGSIVWSLPIGREIDAFHGIAQNTSTAGVGYEGYLVWAAAAAMCAPL
TLFRSPNAPPIDKTVWRAASRNGLLLIAVWCLGSVAMRLTDLVVAVLLNYPFSRYDSM
ALAAFDLATAVLAIWLRFNMATEALPARLISSLCGLLCTFTVSRVIVGVVLAPRFQAS
SGGSAHPVYGNDLAQQITSTFDVVLCGLALSILAAAIVIGRLRQLPQPPHTPALSRPA
GSPRIFRSAGSTHPVRPKIYRPPDHSS"
CDS 631604..633250
/codon_start=1
/note="gene_560"
/transl_table=11
/translation="MDVALGVAVTDRVARLALVDSAAPGTVIDQFVLDVAEHPVEVLT
ETVVGTDRSLAGENHRLVATRLCWPDQAKADELQHALQDSGVHDVAVISEAQAATALV
GAAHAGSAVLLVGDETATLSVVGDPDAPPTMVAVAPVAGADATSTVDTLMARLGDQAL
APGDVFLVGRSAEHTTVLADQLRAASTMRVQTPDDPTFALARGAAMAAGAATMAHPAL
VADATTSLPPAEAGQSGSEGEQLAYSQASDYELLPVDEYEEHDEYGAAADRSAPLSRR
SLLIGNAVVAFAVIGFASLAVAVAVTIRPTAASKPVEGHQNAQPGKFMPLLPTQQQAP
VPPPPPDDPTAGFQGGTIPAVQNVVPRPGTSPGVGGTPASPAPEAPAVPGVVPAPVPI
PVPIIIPPFPGWQPGMPTIPTAPPTTPVTTSATTPPTTPPTTPVTTPPTTPPTTPVTT
PPTTPPTTPVTTPPTTVAPTTVAPTTVAPTTVAPTTVAPATATPTTVAPQPTQQPTQQ
PTQQMPTQQQTVAPQTVAPAPQPPSGGRNGSGGGDLFGGF"
CDS 633283..633939
/codon_start=1
/note="gene_561"
/transl_table=11
/translation="MAGDAVTVVLPCLNEEESLPAVLAAIPAGYRALVVDNNSTDDTA
TVAARHGAQVVVEPRPGYGSAVHAGVLAATTPIVAVIDADGSMDAGDLPKLVAELDKG
ADLVTGRRRPVAGLHWPWVARVGTVVMSWRLRTRHRLPVHDIAPMRVARREALLDLGV
VDRRSGYPLELLVRAAAAGWRVVELDVSYGPRTGGKSKVSGSLRGSIIAILDFWKVIS
"
CDS 633936..634598
/codon_start=1
/note="gene_562"
/transl_table=11
/translation="VSCLPVSVLVVAKAPEPGRVKTRLAAAIGDKVAADIAAAALLDT
LDAVAAAPVTARAVALTGDLDSAADSAEIRRRLKSFTVFRQRGDAFADRLANAHVDAA
DGYPVLQIGMDTPQVTAELLADCARLLLQIPAVLGLAFDGGWWVLGIRTPTAAECLRA
VPMSQPDTGELTLKALRDNGIDVTLVQRLGDFDIVDDIALVRDCCAPGSRFAQATRAA
GL"
CDS complement(634619..635968)
/codon_start=1

/note="gene_563"
/transl_table=11
/translation="VRIGRREGLAVAIGFVLVGAAFVLPRLNLGIKPRSDIGLERFAT
RAGAAPIFGYWDAHVGWGTAPAVLTAVAVVAWGPVVAHRLPWRVLTLSTWATAAAWAF
SLAMIDGWQRGFAGRLTTRDEYLWQVPGIADIPATLRTFTSRILDFQPNSWVTHVSGH
PPGALLTFVWLDRIGLRGGGWAGLVCLLVGSSAAAAVLIAVRVLASEQMARRTAPFVA
VAPTAIWVAVSADGYFAGVAAWGIALLAVAVHGATRFPALVAAGAGLLLGWGVFLNYG
LVLIVLPGMAVLAAADWRPVLRALGPAVLAALVVAVSFAVAGFSWFDGYTLVQQRYWQ
GIAKDRPFGYWSWANLACVVCAIGLGSVAGLSRVFDRAAISRRSGCHLLLLAVLAAIA
LADLSMLSKAETERIWLPFTIWLTAAPALLPPRSHRLWLAVNAAGALLLNSIIFTNW"
CDS complement(635980..637068)
/codon_start=1
/note="gene_564"
/transl_table=11
/translation="VLGGSDPALVAVPTQHESLLGALRVGEQIDDDVALVVTTSGTTG
PPKGAMLTAAALTASASAAHDRLGGPGSWLLAVPPYHIAGLAVLVRSVIAGSVPVELN
VSAGFDVTELPNAIKRLGSGRRYTSLVAAQLAKALTDPAATAALAELDAVLIGGGPAP
RPILDAAAAAGITVVRTYGMSETSGGCVYDGVPLDGVRLRVLAGGRIAIGGATLAKGY
RNPVSPDPFAEPGWFHTDDLGALESGDSGVLTVLGRADEAISTGGFTVLPQPVEAALG
THPAVRDCAVFGLADDRLGQRVVAAIVVGDGCPPPTLEALRAHVARTLDVTAAPRELH
VVNVLPRRGIGKVDRAALVRRFAGEADQ"
CDS complement(637137..637439)
/codon_start=1
/note="gene_565"
/transl_table=11
/translation="VNRFLTSIVAWLRAGYPEGIPPTDSFAVLALLCRRLSHDEVKAV
ANELMRLGDFDQIDIGVVITHFTDELPSPEDVERVRARLAAQGWPLDDVRDREEHA"
CDS complement(637499..637777)
/codon_start=1
/note="gene_566"
/transl_table=11
/translation="VSAWFNYTATLKILIFSLLAGALLPGLFAVGVRLQAAGDGADAT
ARRRPLLVAVSWAIFALVLAVVIIGVLYIARDFIAHHTGWAFLGATPK"
CDS complement(637774..639027)
/codon_start=1
/note="gene_567"
/transl_table=11
/translation="VNLQLFLLLIVVVTALAFDFTNGFHDTGNAMATSIASGALAPRV
AVALSAVLNLIGAFLSTAVAATIAKGLIDANLVTLELVFAGLVGGIVWNLLTWLLGIP
SSSSHALIGGIVGATIAAVGLRGVIWSGVVSKVIVPAVVAALLATLVGAVGTWLVYRT
TRGVAEKRTERGFRRGQIGSASLVSLAHGTNDAQKTMGVIFLALMSYGAVSTTASVPP
LWVIVSCAVAMAAGTYLGGWRIIRTLGKGLVEIKPPQGMAAESSSAAVILLSAHFGYA
LSTTQVATGSVLGSGVGKPGAEVRWGVAGRMVVAWLVTLPLAGLVGAFTYGLVHFIGG
YPGAILGFALLWLTATAIWLRSRRAPIDHTNVNADWEGNLTAGLEAGAQPLADQRPPV
PAPPAPTPPPNHRAPQFGVTTRNAP"
CDS complement(639147..639533)
/codon_start=1
/note="gene_568"
/transl_table=11
/translation="MEILASRMLLRPADYQRSLSFYRDQIGLAIAREYGAGTVFFAGQ
SLLELAGYGEPDHSRGPFPGALWLQVRDLEATQTELVSRGVSIAREPRREPWGLHEMH

VTDPDGITLIFVEVPEGHPLRTDTRA"
CDS complement(639596..640429)
/codon_start=1
/note="gene_569"
/transl_table=11
/translation="MRPPISPQLLINRPAMQPVDLTGKRILLTGASSGIGAAATKQFG
LHRAVVVAVARRKDLLDAVADRITGDGGTAMSLPCDLSDMEAIDALVEDVEKRIGGID
ILINNAGRSIRRPLAESLERWHDVERTMVLNYYAPLRLIRGLAPGMLERGDGHIINVA
TWGVLSEASPLFSVYNASKAALSAVSRIIETEWGSQGVHSTTLYYPLVATPMIAPTKA
YDGLPALTAAEAAEWMVTAARTRPVRIAPRVAVAVNALDSIGPRWVNALMQRRNEQLN
P"
CDS complement(640576..641478)
/codon_start=1
/note="gene_570"
/transl_table=11
/translation="LSDNPFDAKAWRLVDGFDDLTDITYHRHVDDATVRVAFNRPEVR
NAFRPHTVDELYRVLDHARMSPDVGVVLLTGNGPSPKDGGWAFCSGGDQRIRGRSGYQ
YASGDTADTVDVARAGRLHILEVQRLIRFMPKVVICLVNGWAAGGGHSLHVVCDLTLA
SREYARFKQTDADVGSFDGGYGSAYLARQVGQKFAREIFFLGRTYTAEQMHQMGAVNA
VAEHAELETVGLQWAAEINAKSPQAQRMLKFAFNLLDDGLVGQQLFAGEATRLAYMTD
EAVEGRDAFLQKRPPDWSPFPRYF"
CDS complement(641896..642204)
/codon_start=1
/note="gene_571"
/transl_table=11
/translation="VRASPTSPPEQVVVDASAMVDLLARTSDRCSAVRARLARTAMHA
PAHFDAEVLSALGRMQRAGALTVAYVDAALEELRQVPVTRHGLSSLLAERGRAATPSA
"
CDS complement(642201..642419)
/codon_start=1
/note="gene_572"
/transl_table=11
/translation="MARLNVYVPDELAERARARGLNVSALTQAAISAELENSATDAWL
EGLEPRSTGARHDDVLGAIDAARDEFEA"
CDS complement(642659..644374)
/codon_start=1
/note="gene_573"
/transl_table=11
/translation="MSTAGDDAVGVPPACGGRSDAVGVPQLARESGAMRDQDCSGELL
RSPTHNGHLLVGALKRHQNKPVLFLGDTRLTGGQLADRISQYIQAFEALGAGTGVAVG
LLSLNRPEVLMIIGAGQARGYRRTALHPLGSLADHAYVLNDAGISSLIIDPNPMFVER
ALALLEQVDSLQQILTIGPVPDALKHVAVDLSAEAAKYQPQPLVAADLPPDQVIGLTY
TGGTTGKPKGVIGTAQSIATMTSIQLAEWEWPANPRFLMCTPLSHAGAAFFTPTVIKG
GEMIVLAKFDPAEVLRIIEEQRITATMLVPSMLYALLDHPDSHTRDLSSLETVYYGAS
AINPVRLAEAIRRFGPIFAQYYGQSEAPMVITYLAKGDHDEKRLTSCGRPTLFARVAL
LDEHGKPVKQGEVGEICVSGPLLAGGYWNLPDETSRTFKDGWLHTGDLAREDSDGFYY
IVDRVKDMIVTGGFNVFPREVEDVVAEHPAVAQVCVVGAPDEKWGEAVTAVVVLRSNA
ARDEPAIEAMTAEIQAAVKQRKGSVQAPKRVVVVDSLPLTGLGKPDKKAVRARFWEGA
GRAVG"
CDS 644452..646056
/codon_start=1

/note="gene_574"
/transl_table=11
/translation="MADADLVMTGTVLTVDDARPTAEAIAVADGRVIAVGDRSEVAGL
VGANTRVIDLGAGCVMPGFVEAHGHPLLEAVVLSDRFVDIRPVTMRDADDVVAAIRGE
VARRGPAGAYLVGWDPLLQSGLGEPTLTWLDSLAPNGPLVIIHNSGHKAYFNSHAAWL
NGLTRDTADPKGAKYGRDGNGELDGTAEEIGAILPLLAGVADPSNFGAMLRAECARLN
RAGLTTCSEMAFDPGYRPMVEAVRAELTVRLCTYEISNARMCTDATPGQGDDMLRQVG
IKIWVDGSPWVGNIDLTFPYLDTPATRAIGVPPGSRGCANYTREQLAEIVGAYFPRGW
QIACHVHGDGGVDTILDVYEEALRRNPRDDHRLRLEHVGAIRPDQLRRAAELGVTCSI
FVDQIHYWGDVIVDDLFGAQRGSRWMPAGSAVAAGMRISLHNDPPVTPEEPLRNISVA
ATRVAPSGRVLAPEERLTVEQAIRAQTIDAAWQLFAEDAIGSLQVGKYADMVVLSADP
RTVPPEQIADLAVRATFLAGRQVYRR"
CDS 646062..647033
/codon_start=1
/note="gene_575"
/transl_table=11
/translation="VLPPLEALLDRLYVVALPMRVRFRGITTREVALIEGPAGWGEFG
AFVEYQSAQACAWLASAIETAYCAPPPVRRDRVPINATVPAVAAAQVGEVLARFPGAR
TAKVKVAEPGQSLADDIERVNAVRELVPMVRVDANGGWGVAEAVAAAAALTADGPLEY
LEQPCATVAELAELRRRVDVPIAADESIRKAEDPLAVVRAQAADIAVLKVAPLGGISA
LLDIAARIAVPVVVSSALDSAVGIAAGLTAAAALPELDHACGLGTGGLFEEDVAEPAA
PVDGFLAVARTTPDPARLQALGAPPQRRQWWIDRVKACYSLLVPSFG"
CDS 647030..647818
/codon_start=1
/note="gene_576"
/transl_table=11
/translation="VINLAYDDNGTGDPVVFIAGRGGAGRTWHPHQVPAFLAAGYRCI
TFDNRGIGATENAEGFTTQTMVADTAALIETLDIAPARVVGVSMGAFIAQELMVVAPE
LVSSAVLMATRGRLDRARQFFNKAEAELYDSGVQLPPTYDARARLLENFSRKTLNDDV
AVGDWIAMFSMWPIKSTPGLRCQLDCAPQTNRLPAYRNIAAPVLVIGFADDVVTPPYL
GREVADALPNGRYLQIPDAGHLGFFERPEAVNTAMLKFFASVKA"
CDS 647861..649525
/codon_start=1
/note="gene_577"
/transl_table=11
/translation="VNPSTTQARVVVDELIRGGVRDVVLCPGSRNAPLAFALQDADRS
GRIRLHVRIDERTAGYLAIGLAIGAGAPVCVAMTSGTAVANLGPAVVEANYARVPLIV
LSANRPYELLGTGANQTMEQLGYFGTQVRASISLGLAEDAPERTSALNATWRSATCRV
LAAATGARTANAGPVHFDIPLREPLVPDPEPLGAVTPPGRPAGKPWTYTPPVTFDQPL
DIDLSVDTVVISGHGAGVHPNLAALPTVAEPTAPRSGDNPLHPLALPLLRPQQVIMLG
RPTLHRPVSVLLADAEVPVFALTTGPRWPDVSGNSQATGTRAVTTGAPRPAWLDRCAA
MNRHAIAAVREQLAAHPLTTGLHVAAAVSHALRPGDQLVLGASNPVRDVALAGLDTRG
IRVRSNRGVAGIDGTVSTAIGAALAYEGAHERTGSPDSPPRTIALIGDLTFVHDSSGL
LIGPTEPIPRSLTIVVSNDNGGGIFELLEQGDPRFSDVSSRIFGTPHDVDVGALCRAY
HVESRQIEVDELGPTLDQPGAGMRVLEVKADRSSLRQLHAAIKAAL"
CDS 649522..650037
/codon_start=1
/note="gene_578"
/transl_table=11
/translation="VISPKPLLHILIHGRSDELPDTRGRIVLRWLRIAVLIVTGLVTL
QSVLLVAGAWRNDIAIQRNMGVAQAEVLSAGPRRSTIEFVTPDRITYRPQLGVLYPSE

LSTGMRIYVEYNKRDPNLVRVQHRNAGLAIIPAGSIAVVAWLIAAAALVVLAVLDKRL
ERRENSASATG"
CDS 650108..651235
/codon_start=1
/note="gene_579"
/transl_table=11
/translation="VRVAIVAESFLPQVNGVSNSVVKVLEHLRRTGHEALVIAPDTPP
GEDRAERLHDGVRVHRVPSRMFPKVTTLPLGVPTFRMLRALRGFDPDVVHLASPALLG
YGGLHAARRLGVPTVAVYQTDVPGFASSYGIPMTARAAWAWFRHLHRLADRTLAPSTA
TMESLIAQGIPRVHRWARGVDVQRFAPSARNEVLRRRWSPDGKPIVGFVGRLAPEKHV
DRLTGLAASGAVRLVIVGDGIDRARLQSAMPTAVFTGARYGKELAEAYASMDVFVHSG
EHETFCQVVQEALASGLPVIAPDAGGPRDLITPHRTGLLLPVGEFEHRLPDAVAHLVH
ERQRYALAARRSVLGRSWPVVCDELLGHYEAVRGRRTTQAA"
CDS 651252..651956
/codon_start=1
/gene="ubiE"
/note="gene_580"
/transl_table=11
/translation="MSRAALDKDPRDVASMFDGVARKYDLTNTVLSLGQDRYWRRATR
SALRIGPGQKVLDLAAGTAVSTVELTKSGAWCVAADFSVGMLAAGAARKVPKVAGDAT
RLPFGDDVFDAVTISFGLRNVANQQAALREMARVTRPGGRLLVCEFSTPTNALFATAY
KEYLMRALPRVARAVSSNPEAYEYLAESIRAWPDQAVLAHQISRAGWSGVRWRNLTGG
IVALHAGYKPGKQTPQ"
CDS complement(651970..652308)
/codon_start=1
/note="gene_581"
/transl_table=11
/translation="MKGTKLAVVVGMTVAAVSLAAPAQADDYDAPFNNTIHRFGIYGP
QDYNAWLAKISCERLSRGVDGDAYKSATFLQRNLPRGTTQGQAFQFLGAAIDHYCPEH
VGVLQRAGTR"
CDS complement(652342..653019)
/codon_start=1
/note="gene_582"
/transl_table=11
/translation="MTESLDLEFESAYRGESVAFGEGVRPPWSIGEPQPELAALIVQG
KFRGDVLDVGCGEAAISLALAERGHTTVGLDLSPAAVELARHEAAKRGLANASFEVAD
ASSFTGYDGRFDTIVDSTLFHSMPVESREGYLQSIVRAAAPGASYFVLVFDRAAIPEG
PINAVTEDELRAAVSKYWIIDEIKPARLYARFPAGFAGMPALLDIREEPNGLQSIGGW
LLSAHLG"
CDS complement(653092..654426)
/codon_start=1
/note="gene_583"
/transl_table=11
/translation="LLDSRDTSTHGRGKDSVAQVNACHHRRRSPHSTNILVSVDDSAD
VVVVGAGPAGSAAAAWAARAGRDVLVIDTATFPRDKPCGDGLTPRAVAELHQLGLGKW
LADHIRHRGLRMSGFGGEVEVDWPGPSFPSYGSAVARLELDDRIRKVAEDTGARMLLG
AKAVAVHHDSSRRVVSLTLADGTEVGCRQLIVADGARSPLGRKLGRRWHRETVYGVAV
RGYLSTAYSDDPWLTSHLELRSPDGAVLPGYGWIFPLGNGEVNIGVGALSTSRRPADL
ALRPLISYYTDLRRDEWGFTGQPRAVSSALLPMGGAVSGVAGSNWMLIGDAAACVNPL
NGEGIDYGLETGRLAAELLDSRDLARLWPSLLADRYGRGFSVARRLALLLTFPRFLPT
TGPITMRSTALMNIAVRVMSNLVTDDDRDWVARVWRGGGQLSRLVDRRPPFS"

```
CDS             654352..655341
                /codon_start=1
                /note="gene_584"
                /transl_table=11
                /translation="VVAGVDLGDAVFAAAVRAGVARVEQLMDTELRQADEVMSDSLLH
                LFNAGGKRFRPLFTVLSAQIGPQPDAAAVTVAGAVIEMIHLATLYHDDVMDEAQVRRG
                APSANAQWGNNVAILAGDYLLATASRLVARLGPEAVRIIADTFAQLVTGQMRETRGTS
                ENVDSIEQYLKVVQEKTGSLIGAAGRLGGMFSGATDEQVERLSRLGGVVGTAFQIADD
                IIDIDSESDESGKLPGTDVREGVHTLPMLYALRESGPDCARLRALLNGPVDDDAEVRE
                ALTLLRASPGMARAKDVLAQYAAQARHELALLPDVPGRRALAALVDYTVSRHG"
CDS             655442..656302
                /codon_start=1
                /note="gene_585"
                /transl_table=11
                /translation="MTWHPHANRLKTFLLLVGMSALIVAVGALFGRTALMLAALFAVG
                MNVYVYFNSDKLALRAMHAQPVSELQAPAMYRIVRELATSAHQPMPRLYISDTAAPNA
                FATGRNPRNAAVCCTTGILRILNERELRAVLGHELSHVYNRDILISCVAGALAAVITA
                LANMAMWAGMFGGNRDNANPFALLLVALLGPIAATVIRMAVSRSREYQADESGAVLTG
                DPLALASALRKISGGVQAAPLPPEPQLASQAHLMIANPFRAGERIGSLFSTHPPIEDR
                IRRLEAMARG"
CDS             complement(656487..657572)
                /codon_start=1
                /note="gene_586"
                /transl_table=11
                /translation="LLLRFVDGYQMSGCRNTLNAMAANKREPKVVVLGGGSWGTTVAS
                ICARRGPTLQWVRSAVTAQDINDNHRNSRYLGNDVVLSDTLRATTDFTEAANCADVVV
                MGVPSHGFRGVLVELSKELRPWVPVVSLVKGLEQGTNMRMSQIIEEVLPGHPAGILAG
                PNIAREVAEGYAAAAVLAMPDQHLATRLSAMFRTRRFRVYTTDDVVGVETAGALKNVF
                AIAVGMGYSLGIGENTRALVIARALREMTKLGVAMGGKSETFPGLAGLGDLIVTCTSQ
                RSRNRHVGEQLGAGKPIDEIIASMSQVAEGVKAAGVVMEFANEFGLNMPIAREVDAVI
                NHGSTVEQAYRGLIAEVPGHEVHGSGF"
CDS             complement(657573..659033)
                /codon_start=1
                /note="gene_587"
                /transl_table=11
                /translation="MSVTPNAGCVDVVIVGAGISGLGAAYRIIERNPQLTYTILERRA
                RIGGTWDLFRYPGVRSDSSIFTLSFPYEPWTREEGIADGAHIREYLTDMAHKYGIDRH
                IEFNSYVRAADWDSSTDTWTVTFEQNGVHKHYRSRFVFFGSGYYNYDEGYTPDFGGIE
                KFGGAVVHPQHWPEDLDYTGKKIVVIGSGATAVTLIPSLTDRAEKVTMLQRSPTYLIS
                ASKYSTFAAVVRKALPPKTSHLIVRMYNALLEAVFWFLSRKTPVFVKWLLRRTAIKNL
                PEGYDIETHFTPRYNPWDQRLCLIPDADLYNAITSGRAEVVTDHIDHFDATGIALKSG
                GHLDADIIVTATGLQLQALGGAAISLDGVEIDPRDRFVYKAHMLEDVPNLFWCVGYTN
                ASWTLRADMTARATAKLLAHMAAHGHTRAAPHLGDEPMDEKPSWDIQAGYVKRAPYAL
                PKSGTKRPWNVRQNYLADAIDYRFDRIEEAMVFGAA"
CDS             complement(659111..659602)
                /codon_start=1
                /note="gene_588"
                /transl_table=11
                /translation="MADSSFDIVSKVDRQEVDNALNQAAKELATRFDFRGTDTKIAWK
                GDEAVELTSSTEERVKAAVDVFKEKLIRRDISLKAFEAGEPQASGKTYKVTGALKQGI
                SSENAKKITKLIRDAGPKNVKTQIQGDEVRVTSKKRDDLQAVIAMLKKADLDVALQFV
```

```
                     NYR"
tRNA                 659672..659755
                     /note="identified by tRNAscan-SE; putative"
CDS                  659881..660903
                     /codon_start=1
                     /note="gene_589"
                     /transl_table=11
                     /translation="VVELSPDRIMAIGGGYGPSKVLLTAVGLGLFTELGDEAMTAEAI
                     ADRLGLLKRPAIDFLDALVSLDLLARDGDGPGSHYRNTPETAHFLDEARPTYAGGLLK
                     IWNERNYRFWADLTEALKTGKAQSEVKQTGRPFFEALYADPRRLEAFMAAMDAASRRN
                     IELLAKRFPFERYRRLCDVGCADGLLSRIVAAAHPHLQCVSLDLPAVTEIARRKLTAE
                     GLGERVQACAGDFLADPLPAADVITMGQILHDWNLDRKQQLVAKAYEALSKEGAFIVI
                     ETLIDDARRENTTGLMMSLNMLIEFGDAFDYSAADFRGWCGEAGFRSFEVIPLAGGSS
                     AAVAYK"
CDS                  661082..662431
                     /codon_start=1
                     /note="gene_590"
                     /transl_table=11
                     /translation="MLRHGLRFLTACQRRYGSVFTLHVAGFGHMVYLSDPAAIKTVFA
                     GNPSVFHAGEANSMLAGLLGDSSLLLIDDDVHRDRRRLMSPPFHRDAVARQAEPIAEI
                     AAANIAGWPMAKAFAVAPKMSEITLEVILRTVIGASDPVRLAALRKVMPRLLNVGPWA
                     TLALANPSLLNNRLWSRLRRRIEEADALLYAEIADRRADPDLAARTDTLAMLVRAADE
                     DGRTMTERELRDQLITLLVAGHDTTATGLSWALERLTRHPVTLAKAVQAADASAAGDP
                     AGDEYLDAVAKETLRIRPVVYDVGRVLTEAVEVAGYRLPAGVMVVPAIGLVHASAQLY
                     PDPERFDPDRMVGATLSPTTWLPFGGGNRRCLGATFAMVEMRVVLREILRRVELSTTT
                     TSGERPKLKHVIMVPHRGARIRVRATRDVSATSQATAQGAGCPAARGGGPSRAVGSQ"
CDS                  662449..662832
                     /codon_start=1
                     /note="gene_591"
                     /transl_table=11
                     /translation="MGSPSGSRGDFWPPALVAYCPPAVAPGACTIEVPKEGTLMKAKV
                     GDWLVIKGATIDQPDHRGLIIEVRSSDGSPPYVVRWLETDHVATVIPGPDAVVVTAEE
                     QNAADERAQHRFGAVQSAILHARGT"
CDS                  662906..664990
                     /codon_start=1
                     /note="gene_592"
                     /transl_table=11
                     /translation="VPFDIARIEAAVTRAAREVACDDPDMPGTVAKAVADALGRGIAP
                     VEDIQDCVEARLGEAGLDDVARVYIIYRQRRAELRTAKALLGVRDELKLSLAAVTVLR
                     ERYLLHDEQGRPAESTGELMDRSARCVAAAEDQYEPGSSRRWAERFATLLRNLEFLPN
                     SPTLMNSGTDLGLLAGCFVLPIEDSLQSIFATLGQAAELQRAGGGTGYAFSHLRPAGD
                     RVASTGGTASGPVSFLRLYDSAAGVVSMGGRRRGACMAVLDVSHPDICDFVTAKAESP
                     SELPHFNLSVGVTDAFLRAVERNGLHRLVNPRTGKIVARMPAAELFDAICKAAHAGGD
                     PGLVFLDTINRANPVPGRGRIEATNPCGEVPLLPYESCNLGSINLARMLADGRVDWDR
                     LEEVAGVAVRFLDDVIDVSRYPFPELGEAARATRKIGLGVMGLAELLAALGIPYDSEE
                     AVRLATRLMRRIQQAAHTASRRLAEERGAFPAFTDSRFARSGPRRNAQVTSVAPTGTI
                     SLIAGTTAGIEPMFAIAFTRAIVGRHLLEVNPCFDRLARDRGFYRDELIAEIAQRGGV
                     RGYPRLPAEVRAAFPTAAEIAPQWHLRMQAAVQRHVEAAVSKTVNLPATGRSMTSAPS
                     MWPPGRQRSRASRCIATAAGKDRYCPTPRRNRYWRRLTRSSAAAVRAAPASSDGGSHG
                     ASRRRIAQNQRF"
CDS                  complement(665049..666341)
```

```
                     /codon_start=1
                     /note="gene_593"
                     /transl_table=11
                     /translation="LAQRLAQLSGKAVVVLGLPRGGVPVAFEVAKSLQAPLDVLVVRK
                     LGVPFQPELAFGAIGEDGVRVLNDDVVRGTHLDAAAMDAVERKQLIELQRRAERFRRG
                     RDRIPLTGRIAVIVDDGIATGATAKAACQVARAHGADKVVLAVPIGPDDIVARFAGYA
                     DEVVCLATPALFFAVGQGYRNFTQTSDDEVVAFLDRAHRDFAEAGAIDAAADPPLRDE
                     EVQVVAGPVPVAGHLTVPEKPRGIVVLAHGSGSSRHSIRNRYVAEVLTGAGFATLLFD
                     LLTPEEERNRANVFDIELLASRLIDVTGWLATQPDTASLPVGYFGASTGAGAALVAAA
                     DPRVNVRAVVSRGGRPDLAGDSLGSVVAPTLLIVGGRDQVVLELNQRAQAVIPGKCQL
                     TVVPGATHLFEEPGTLEQVAKLACDWFIDHLCGPGPSG"
     CDS             complement(666604..666945)
                     /codon_start=1
                     /note="gene_594"
                     /transl_table=11
                     /translation="MGEHAIKRHMRQRKPTKHPLAQKRGARILVLTDDPRRSVLIVPG
                     CHLDSMRREKNAYYFQDGNALVGMVVSGGTVEYDADDRTYVVQLTDGRHTTESSFEHS
                     SPSRSPQSDDL"
     CDS             complement(667413..668804)
                     /codon_start=1
                     /note="gene_595"
                     /transl_table=11
                     /translation="MAIRQHVGALFTDLYEVTMAQAYWAERMSGTAVFEIFFRKLPPG
                     RSYIMAAGLADVVEFLEAFRFDEQDLRYLRGLGQFSDEFLRWLAGVRFTGDVWAAPEG
                     TVIFPNEPAVQLIAPIIEAQLVETFVLNQIHLQSVLASKAARVVAAARGRPVVDFGAR
                     RAHGTDAACKVARTSYLAGAAGTSNLLAARQYGIPTFGTMAHSFVQAFDSEVAAFEAF
                     ARLYPATMLLVDTYDTLRGVDHVIELAKRLGNRFDVRAVRLDSGDLDELSKATRARLD
                     TAGLEQVEIFASSGLDENRIAALLAARCPIDGFGVGTQLVVAQDAPALDMAYKLVAYD
                     GSGRTKFSSGKVIYPGRKQVFRKLEHGVFCGDTLGEHGENLPGDPLLVPIMTNGRRIR
                     QHAPTLDGARDWARQQIDALPPELRSLEDTGYSYPVAVSDRIVGELARLRHADTAEAH
                     PGSNVVGAKAKRP"
     CDS             complement(668814..669956)
                     /codon_start=1
                     /note="gene_596"
                     /transl_table=11
                     /translation="VAGKPDVVTVLLGGDVMLGRGVDQILPHPGKPQLRERYMRDATG
                     YVRLAERVNGRIPLPVDWRWPWGEALAVLENTATDVCLINLETTITADGEFADRKPVC
                     YRMHPDNVPALTALRPHVCALANNHILDFGYQGLTDTVAALAGAGIQSVGAGADLLAA
                     RRSALVTVGHERRVIVGSVAAESSGVPESWAARRDRPGVWLIRDPAQRDVADDVAAQV
                     LADKRPGDIAIVSMHWGSNWGYATAPGDVAFAHRLIDAGIDMVHGHSSHHPRPIEIYR
                     GKPILYGCGDVVDDYEGIGGHESFRSELRLLYLTVTDPASGNLISLQMLPLRVSRMRL
                     QRASQTDTEWLRNTIERISRRFGIRVVTRPDNLLEVVPAANLTSKE"
     CDS             complement(670172..671308)
                     /codon_start=1
                     /note="gene_597"
                     /transl_table=11
                     /translation="VKVAISGAGVAGAALAHWLQRTGHTPTVIERAPKFRTGGYMIDF
                     WGVGYQVAKRMGITDQIAAAGYHMEHVRSVGPTGKVKADLGVDVFRRMVGDDFTSLPR
                     GDLAAAIYTTIEDQVETIFDDSIATIDEHRDGVRLTFERTAPRDFDLVIGADGLHSNV
                     RRLVFGPERDFEHYLGCKVAACVVDGYRPRDERSYVLYNTVDRQLARFALRGDRTMFL
                     FVFRAEHDNPGVAPKDELRDQFGDVGWESRDILAALDDVEDLYFDVVSQIRMDRWSRG
```

```
                     RVLLIGDAAGCISLLGGEGTGLAITEAYVLAGELARAGGDHRRAFDAYEKRLRPFIEG
                     KQASAAKFIWFFRHPNPIRPVVSQRCDAHDELRPAGDAVRRQRA"
     CDS             671402..672715
                     /codon_start=1
                     /note="gene_598"
                     /transl_table=11
                     /translation="VPDMLEVAAEPTRRRLLQLLAPGERTVTQLASQFTVTRSAISQH
                     LGMLAEAGLVTARKQGRERYYRLDERGVLRLRALMESFWSDELDRLVADAAHYPPSQG
                     DCAMPFEKAVVVPLDPTSTFALITQPDRLRRWMAVAARIELRTGGAYRWTVTPGHSAA
                     GTVIDVDPGKRVVFTWGWEDHGDPPPGGSTVTITLTPVDGGTEVRLVHDGLTAQQAAR
                     HAKGWNHFLDRLVVAGQHGDAGPDEWAAAPDPLDELSCAEATLAVLQHVLRGIGASDL
                     TRQTPCTEYDVSQLADHLLRSLAIIGAAAGAQLAPRDVDAPLETQVADAAQAVMEAWR
                     RRGLAGTVELNSNQVPATVPVGILCLEFLVHAWDFAIATGSQVIASEPVSEYVLAVAG
                     KVITPATRNSAGFAAPAAVGSFAPVLDRLIAFTGRQPTAGHVSAT"
     CDS             672729..673514
                     /codon_start=1
                     /note="gene_599"
                     /transl_table=11
                     /translation="MPKRSEYRQGTPNWVDLQTTDQSAAKKFYTSLFGWGYDDNPVPG
                     GGGVYSMATLNGEAVAAIAPMPPGAPEGMPPIWNTYIAVDDVDAVVDKVVPGGGQVMM
                     PAFDIGDAGRMSFITDPTGAAVGLWQANRHIGATLVNETGTLIWNELLTDKPDLALAF
                     YEAVVGLTHSSMEIAAGQNYRVLKAGDAEVGGCMEPPMPGVPNHWHVYFAVDDADATA
                     AKAAAAGGQVIAEPADIPSVGRFAVLSDPQGAIFSVLKPAPQQ"
     CDS             complement(673559..677479)
                     /codon_start=1
                     /note="gene_600"
                     /transl_table=11
                     /translation="MSFVIATPEMLTTAATDLAKIGSTITAANTAAAAVAKVLPASAD
                     EVSVAVAALFGTHAQEYQTVSAQVATFHDRFVQTLSAAASSYVAAEAVNVEQSLLAAV
                     NAPTQALFGRPLIGNGADGSPGTGQAGGPGGILYGNGGNGGSGAPGQRGGAGGAAGLI
                     GNGGNGGAGGVGTTGGAGGHGGAGGWLYGNGGAGGFGGAGAVGGNGGAGGTAGLFGVG
                     GAGGAGGNGIAGVTGTSASTPGGSGTAGGAGGIGGNGGAGGAGGVLMGNGGNGGAGGE
                     GGPGGAGGAGASGAHATNLGADGQAGGNGGNGGAGGTGGVGGPGGGHGLLGLGGSHGA
                     GGAGGSGGDGGAPGDGGNGATGTWGHNLGAGGTGGNGGNPGAGGAGGAGGASVGGSAH
                     GANGAPGTTSTSGGNGGDGGKGADAISSGQTGANGGRGGDGGQVGNGGAGGAGGRGGA
                     GGLGFGSEAPGRPGGAGGTGGAGGNGGTQAGDGGTGGAGGAGGDGGSGGAGSIGFNAS
                     APGAAGSPGGNGGNGGPGGAGGEGGAGGLALAASGQNGSQGAGGDGGAGGNGGTPGNG
                     GHGAAGALGVNGGVGGAGGHGGDPGVGGAGGQGGSGSTPGANGAPGNTPTSGGNGGNG
                     GRGADATGFGQTGASGGRGGDGGLVGNGGAGGAGGNGSKGLPGLGRLGNPGLDGGTGG
                     NGGAGGSGGAWAGNGGTGGAGGTGGVGGTGGSGSDGVNGSSAGADGHPGGTGGVGGTG
                     GKGGDGGDGGAAPNGVAGSQGPGGAGGDGGTGGVGGNGGRGIDGADGATAGARGQDGG
                     AGGAGGKGGRGGTGGPGGAGPAGTTGSQGAGGNGGSGGTGGDPGDGGNGANGSVFTNN
                     GIGGNGGNGGNAGPSGAGGSGGAGSTFGATGSSSSIHVNGGNGGNGGNGDHALSGNGA
                     AGGNGGNGGNGSLRGSGGAGGHGGNGGNASRGMGGDGGTGGAGGNAGQIGNGGAGGNG
                     GDGGTGSDGNPGAITGSGGRGGDGGVGGQGGSVAGDGADGGRGGAGGTGGTGLRGTTG
                     ATGATGTFDAGADGHGGNGGTGGVGGTGGAGGGGGNGGAGGKALSPTGNNGSQGAGGD
                     GGAGGAGGTGGTGGDGGRGAHGTLFSSLAGTGGTGGNGGTGGTGGTGGAGGAGGTGST
                     LGATGATGAAGRAGNGGVGGSGGLGSAFGPGGTGGMGGAGGTSTVSAGGDGGRGGFGG
                     DGLDASSGGNGGDGGHGGDGFRTAGAGGRGGDGGKGADPGGLFPIPGAGGKGGTGGTG
                     GTAHLGPLAIIGQSGQPGQFGSPGADGRGGAGGAGGGGGAGGSF"
     CDS             677801..678559
```

/codon_start=1
/note="gene_601"
/transl_table=11
/translation="MVGYVDVRAYAELNEFVELQARGLTVRRPFRSHQTVKDVLEAMG
IPHTEVDLILVNGDPADFSYRPVAGDRIAAYPMFEALDIGSTARLRPAPLRNPRFVVD
VNLGQLARLLRLLGFDTRWSSAADDPTLADISLGEQRILLTRDRGLLKRRAITHGLFV
HSQHPEEQALEVLRRLDLNGRLAPLSRCLRCNGELAAVSKDEVIGQLEPLTRRYYESF
SRCFGCGRIYWPGSHHARLVRLVERLRDQLTTST"

CDS complement(678688..679179)
/codon_start=1
/note="gene_602"
/transl_table=11
/translation="MTDQSYAVDIAHPPAALLRLVNPILRSLLHTPLAGPLRTQLMVV
SFTGRKTGRHFSIPLSAHVIDNDLYALTEAGWKHNFSDGAAAQVVYDGKTTAMRGELI
RDRAVVSELFLRAAQAYGVKRGQRMLGLSFRDRRIPTLEEFAEAVDRLKLVAIRLTPA
DNS"

CDS 679273..679488
/codon_start=1
/note="gene_603"
/transl_table=11
/translation="VDKTTVYLPDELKAAVKRAARQRGVSEAQVIRESIRAAVGGAKP
PPRGGLYAGSEPIARRVDELLAGFGER"

CDS 679485..679892
/codon_start=1
/note="gene_604"
/transl_table=11
/translation="VIIDTSALLAYFDAAEPDHAAVSECIDSSADALVVSPYVVAELD
YLVATRVGVDAELAVLRELAGGAWELANCGAAEIEQAARIVTKYQDQRIGIADAANVV
LADRYRTRTILTLDRRHFSALRPIGGGRFTVIP"

CDS complement(679952..680638)
/codon_start=1
/note="gene_605"
/transl_table=11
/translation="MKHFTAAVATVALSLALAGCSFNIKTDSAPTTSPTTTSPTTSTT
TTSATTSAQAAGPNYTIADYIRDNHIQETPVHHGDPGSPTIDLPVPDDWRLLPESSRA
PYGGIVYTQPADPNDPPTIVAILSKLTGDIDPAKVLQFAPGELKNLPGFQGSGDGSAA
TLGGFSAWQLGGSYSKNGKLRTVAQKTVVIPSQGAVFVLQLNADALDDETMTLMDAAN
VIDEQTTITP"

CDS 680792..683425
/codon_start=1
/note="gene_606"
/transl_table=11
/translation="MRARRLRRALAALLAVAGLFVPFIVGVPTAYDGEPVFVAIPVEH
VNTLIGTGTGAAIVGEINNFPGASVPFGMVQYSPDTVDNYAGYDYGNPHSTGFSMTHA
SVGCPAFGDISMLPTTTPLGSQPWSAWEEIAHDDTEVGVPGYYTVRFPGTGVIAELTA
TTRTGVGRFRYPRNGWPALFHVRSGASLAGNYAATLQIEDNTTITGSATSGGFCGKKN
LYTVYFAMKFSQPFSSYGTWDGYAVYPGSHSMNSSYSGGYVGFPAGSVLEVRTALSYV
SVDGARANLDAEGGASFDDIRAATSSEWNAALSRIAVAGRGPGDVDTFYTCLYRSLLH
PNTFNDVDGRYIGFDGVIHSVASGHTHYANFSDWDTYRSLAPLQGLLFPQRASDMIQS
LVTDAEQSGAYPRWALANSATGMMSGDSVVPLIVNLYAFGARDFDLKSALHYMVNAAT
QGGVGLDGFLERPGIAAYLRLGYGPQTAEFRANGRIAGASVTLEWSVDDFAISRFADS

LGDTATAAVFQNRSQYWQNLFNPTTGYISPRSAAGFFPDGPGFVAYPSGFGQDGYDEG
NAEQYLWWVPHNVAGLVTALGGRTAVVKRLDRFTKKLNVGPNEPYLWAGNEPGFGVPW
LYNYIGQPWKTQRTVDRVRGLFGPTPGGAPGNDDLGALSSWYVWAALGLYPSTPGTTI
LTVNTPLFDRAVIALPTGKSIQITAPGASGRNRLKYIDGLTIDRQPSNQTFLPESIVR
TGGDLTFSLAGTPNKVWGTAASAAPPSFGAGSSAVTVNIARPIIGIVPGATGTVTVDA
QRMIDGVDDYTVTPTSYVVGIAAEPLSGQFDDDGAVSASVAITVARSVPSGYYPIYVT
TSAGDSARTLIVLVVVAEAVE"

CDS complement(683448..685835)
/codon_start=1
/note="gene_607"
/transl_table=11
/translation="MRVDGRDIGVSGNLLQPLTRRTNDIIRAVLAAIYLVAVITSSLI
TRPQWVALEKSISEIVGVLSPSQSDLVYLGYGLAILALPFVILIGLIVSRQWKLLGAY
AAAGLMAVLPLSISSSRIAAPRWHFDLSDRLATLLAQFLDDPRWIAMLAAVLTVSGPW
LPARWRHWWWALLLAFVPIHLVVSAIVPARSLLGLAVGWLVGALVVLVVGTPALEVPL
DGAIRALAKRGFAVSGLAVVRPAGPGPLVLSAACEQPNAGACSEALIELYGPHQSGGG
ALRQLWLKLTLRGTETAPLQASMRRAVEHRALMAIAFGDLGMANTTVIAVSPLDRGWT
LYAHRPARGIGISECTKTTPTAHVWEALRTLHDQQISHGDLCSAEITVDNGAVLFGGF
GEAEYGATDAQLQSDLAQLLVTTSALYDAEAAVTAAIDTFGKQAVLAASRRLTKSAVP
KRIRESITDPNAVIASTRAEVMRQTGADQIKAETITRFSRGQLIQLVLIGALVYVAYP
FISTVPTFFSQLRTANWWWALLGLAVSALTYVGAAAALWACADGLVGFWKLSIMQVAN
TFAATTTPAGVGGLALSTRFLQKGGLTAVRATAAVALQQSVQVIVHLVLLILFSALAG
TSTDLSHFVPNATVLYLIAGVALGIVGTFLFVPKLRRWLATAVRPKLREVTNDLIALA
REPKRLALIVLGCAGTTLGAALALWASIEAFGGGTTFVTVTVVTMVGGTLASAAPTPG
GVGAVEAALIGGLAAFGVPAALGVPSVLLYRLLTCWLPVFAGWQVMHWLTRHEMI"

CDS 685973..686695
/codon_start=1
/note="gene_608"
/transl_table=11
/translation="MALQPVTRRSVPEEVFEQIATDVLTGEMPPGEALPSERRLAELL
GVSRPAVREALKRLSAAGLVEVRQGDVTTVRDFRRHAGLDLLPRLLFRNGELDISVVR
SILEARLRNFPKVAELAAERNEPELAELLQDSLRALDTEEDPIVWQRHTLDFWDHVVD
SAGSIVDRLMYNAFRAAYEPTLAALTTTMTAAAKRPSDYRKLADAICSGDPTGAKKAA
QDLLELANTSLMAVLVSQASRQ"

CDS 686692..687489
/codon_start=1
/note="gene_609"
/transl_table=11
/translation="MTTHAVIITYLRDQTQPAVDAIGGFYRTCVLTGKALVRRPFHWR
EAIEQGWFITSVSLLPTLAVSIPLTVLIIFTLNILLAEFGAADISGAGAALGAVTQLG
PLTTVLVIAGAGATAICADLGARTIREEIDAMEVLGIDPIHRLVVPRVVAATIVAALL
NGAVITIGLVGGFVFSVFIQHVSAGAYVGTLTLVTGLPEVIISVVKSATFGLIAGLVG
CYRGLTTKGGPKGVGTAVNETLVLCVIALFATNVVLTTIGVRFGTGH"

CDS 687491..688378
/codon_start=1
/note="gene_610"
/transl_table=11
/translation="MVESSTASAAAVLRARYPRTAASLDRYGGGTARRLERTGTFARF
TRISVVQIGWALRRYRRETLRLVAEIGMGTGAMAVVGGTVAIIGFVTLSGGSLIAIQG
FASLGNIGVEAFTGFFAALANTRVAAPIVSGVALAATVGAGATAQLGAMRISEEIDAL
EVMGIKSISFLVSTRILGGLVVIMPLYALALDMAFTSGQVVTTVFYGQSNGTYEHYFR

```
                     TFLRPEDVGWSVVEVVIIAVVVMITHCYYGYTASGGPVGVGQAVGRSMRFSLVSVVVV
                     VLLAELALYGVDPNFNLTV"
     CDS             688384..689598
                     /codon_start=1
                     /gene="mce2A"
                     /note="gene_611"
                     /transl_table=11
                     /translation="VPTLVTRKNRRAWLYVEGVVLLLVGALVLVLVYKQFRGEFTPKT
                     ELTMVASRAGLVMEAGSKVTYNGVEIGRVGSISEIERDGRPAAKLVLDVNPRYISLIP
                     VNVVADIEAATLFGNKYVALSAPKIPQQQRISSHDVIDVGSVTTEFNTLFETITSIAE
                     KVDPIELNATLSAVAQAPDGLGGKFGESIVNGNQILAQLNPRLPQLGYDVRRLADLGE
                     VYVDASPDLWSFLQNALTTARTLTSQQRDLDAALLAATGAGNTGEDVFARGGPYLARA
                     AADLVPTATLLDTYSPELFCMIRNFHDAAPKVADAVGGNGYSLAAAGTILGAPNPYVY
                     PDNLPRVNAHGGPGGRPGCWQTITRELWPAPYLVMDTGASLAPYNHVELGQPMFTEYV
                     WGRQYGENTINP"
     CDS             689595..690626
                     /codon_start=1
                     /gene="mce2B"
                     /note="gene_612"
                     /transl_table=11
                     /translation="MKTTGTTIKLGIVWLVLSVFTVMIIVVFGQVRFHHTTGYSAVFT
                     HVSGLRAGQFVRAAGVEVGKVAKVTLIDGDKQVLVDFTVDRSLSLDQATTASIRYLNL
                     IGDRYLELGRGHSGQRLAPGATIPLEHTHPALDLDALLGGFRPLFQTLDPDKVNSIAS
                     SIITVFQGQGATINDILDQTASLTATLADRDHAIGEVVNNLNTVLATTVKHQTEFDRT
                     VDKLEVLITGLKNRADPLAAAAAHISSAAGTLADLLGADRPLLHSSFGHLEGIQQPLI
                     DELAELDHVLGKLPDAYRIIGRAGGIYGDFFNFYLCDISLKVNGLQPGGPVRTVKLFG
                     QPTGRCTPQ"
     CDS             690623..692068
                     /codon_start=1
                     /gene="mce2C"
                     /note="gene_613"
                     /transl_table=11
                     /translation="MRTLTEFNRGRVGMMGAVVTVLVVGVAQSFTSVPMLFATPTYYA
                     QFADMGGINTGDKVEIAGVNVGLVRSLAIRGNRVLIGFSLPGKTIGMQSRAAIRTDTI
                     LGRKNLEIEPRGSEPLKPNGFLPLAQTTTPYQIYDAFVDVTKAATGWDIDAVKRSLNV
                     LSETFDQTAPHLSAALEGVKAFSDTVGRRGEQIEQLLANANRIARVLGDRSEQVNGLL
                     VNAKTLLAAFKQRSQALRILLTNVSEASAQVSGLITDNPNLNHVLAQLRTVSEELVKR
                     KNELADVAVLLGRYTAALTEAVGSGPFFKAMVVNLLPYQILQPWVDAAFKKRGIDPEN
                     FWRSAGLPEFRWPDPNGTRFPNGAPPAAPPVREGTPKHPGPAVPPGTPCSYTPAAGAL
                     PRPDNPLPCAGATVGPFGGPDFPAPLDVQPSPPNPDGPPPTPGILSAGRPGEPAPAVP
                     GIPMPLPPNAPPGARTQPLEPFPDGTGGSNQ"
     CDS             692065..693615
                     /codon_start=1
                     /gene="mce2Da"
                     /note="gene_614"
                     /transl_table=11
                     /translation="MSTIFDIRSLRLPKLSAKVVVVGGLVVVLAVVAAAAGARLYRKL
                     TTTTVVAYFSEALALYPGDKVQIMGVRVGSIDKIEPAGDKMRVTLHYSNKYQVPATAT
                     ASILNPSLVASRTIQLSPPYTGGPVLQDGAVIPIERTQVPVEWDQLRDSINGILRQLG
                     PTERQPKGPFGDLIESAADNLAGKGRQLNETLNSLSQALTALNEGRGDFVAITRSLAL
                     FVSALYQNDQQFVALNENLAEFTDWFTKSDHDLADTVERIDDVLGTVRKFVSDNRSVL
```

AADVNNLADATTTLVQPEPRDGLETALHVLPTYASNFNNLYYPLHSSLVGQFVFPNFA
NPIQLICSAIQAGSRLGYQESAELCAQYLAPVLDALKFNYLPFGSNPFSSAATLPKEV
AYSEERLRPPPGYKDTTVPGIFSRDTPFSHGNHEPGWVVAPGMQGMQVQPFTANMLTP
ESLAELLGGPDIAPPRRREPTCPDRRMRMTSPIRCRRRGTRSPRPSRLRAPQDSQARA
SEVRRERG"

CDS 693602..694798
/codon_start=1
/note="gene_615"
/transl_table=11
/translation="VSAGSANGKPNRWTLRCGVSAGHRGSVFLLAVLLAPVVLTSCTW
RGIANVPLPVGRGMGPDRMTIYVQMPDTLALNTNSRVRVADVWVGTVRDISLRNWIAT
LTLELEPTVRLPANATAKIGQTSLLGTQHVELAAPPIPSPQPLKSGDTIGLKNSSAYP
TVERTLASVALILTGGGIVNLDVIQTEILNILDGHAGQIREFLERLATFTAELNNQRG
DLTRAIDSTNQLLTIIANRNDTLDRVLTDVPPLIEHFADTGQLFADATESLGRFSEVA
NRALAATRPNLHQTLQSLQRPLRQLERASPYVVGALKLGLTAPFNIDEVPNVIRGDYV
NVSATFDVTLSALDNALLSGTGISGMLRALEQAWGRDPDTMIPDVRYTPNPNDAPGGP
LVERAE"

CDS 694803..696353
/codon_start=1
/gene="mce2F"
/note="gene_616"
/transl_table=11
/translation="MLTRAIKTQLVLLTVLAVIAVVVLGWYFLRIPSLVGIGRYTLYA
ELPRSGGLYRTANVTYRGITIGKVTGVEPTERGARATMSIDNGYQIPTDASANVHSVS
AVGEQFVDLVSTRTSGPYLRHGQTITTTTVPSQIGPALDAANRGLAVLPKDRVASVLH
EASEAVGGLGSSLNRLIEATQAIAHDVRGSLEDIDDIIERSAPIIDSQVNSGNEIARW
AANLNTLAAQTAQTDPAVRSILANAAPTADQVNATFSDVRESLPQTLANLEVVIDMLK
RYHNGVEQALVFLPQSGAIAQSVTTEFPGQAGLGVGGLALNQPPPCLTGFLPASEWRS
PADTSTAPLPKGTYCRIPMDASNVVRGARNNPCVDVPGKRAATPRECRSNEAYVPGGT
NPWYGDPNQMLSCPAPAARCDQPVKPGQVIPAPSVNNGINPLPADQLPGTPPPVNDPL
QRPGSGTVQCNGQQPNPCVYTPSTFPTTIYDVQSGKVVAPDGVVYSVEASTHAGADGW
KVMLAPTG"

CDS complement(696405..696800)
/codon_start=1
/note="gene_617"
/transl_table=11
/translation="VVNVRRALADTSVFIGIEATRFDPDRFAGYEWGVSVVTLGELRL
GVLQASGPEAAARRLSTYQLAQRFEPLGIDEAVSEAWALLVSKLRAAKLRVPINDSWI
AATAVAHGIAILTQDNDYAAMPDVEVITI"

CDS complement(696794..697051)
/codon_start=1
/note="gene_618"
/transl_table=11
/translation="MSATIPARDLRNHTAEVLRRVAAGEEIEVLKDNRPVARIVPLKR
RRQWLPAAEVIGELVRLGPDTTNLGEELRETLTQTTDDVRW"

CDS complement(697234..698469)
/codon_start=1
/note="gene_619"
/transl_table=11
/translation="MGVVERAIAPSVLAALADTPVVVVNGARQVGKTTLVARLDYPGS
SEVVSLDDVANRDAARDDPRAFVSRPVDTLVIDEAQLEPGLFRAIKAEVDRDRRPGRF

LLTGSARLLSAPDMADALVGRVEIIELWPFSQGERAGIADGFVDALFTAPRELIHGSD
MRRADLVDRIATGGFPDIVARSPSRRRAWFDNYLTTATQSVIREISPIERLAEMPRVL
RLCAARTGAELNVSALANDLSIPARTTAGYLALLEAAFLIHRVPAWSTNLSRKVIRRP
KLVVSDSGLACHLLGVTGATLDRPGRPLGPLLETFVANEIRKQLTWSTERPSLWHFRD
RGGAEVDLVLEHPDGRVCGIEVKATSTPRAEDLRGLRYLAERLDDRFQFGVLLTAAPE
ATPFGPTLAALPVSTLWAG"
CDS 698586..698759
/codon_start=1
/note="gene_620"
/transl_table=11
/translation="VADEERSEALLRERSLHYVASSRAGDMLVVTWSGQRSELLSQLK
IHAATTTWTPIFS"
CDS complement(698720..699133)
/codon_start=1
/note="gene_621"
/transl_table=11
/translation="VKPPLAVDTSVAIPLLVRTHTAHAAVVAWWAHREAALCGHALAE
TYSVLTRLPRDLRLAPMDAARLLTERFAAPLLLSSRTTEHLPRVLAQFEITGGAVYDA
LVALAAAEHRAELATRDARAKDTYEKIGVHVVVAA"
CDS complement(699130..699366)
/codon_start=1
/note="gene_622"
/transl_table=11
/translation="MKAVVDAAGRIVVPKPLREALGLQPGSTVEISRYGAGLHLIPTG
RTARLEEENGVLVATGETTIDDEVVFGLIDSGRK"
CDS complement(699470..699976)
/codon_start=1
/note="gene_623"
/transl_table=11
/translation="VPITPLLHESVARFAATGADITTRAEPDLFVSIDPDHLRRILTA
VLDNAITHGDGEIAVTAHARDGAVDIGVRDHGPGFADHFLPVAFDRFTRADTARGGRG
SGLGLAIVAALTTTHGGHANATNHPDGGAELRITLPTPRPPFHEELPRITSSDTKDPN
REHDTSDQ"
CDS complement(700090..700620)
/codon_start=1
/note="gene_624"
/transl_table=11
/translation="MRSPAEWPIRWRLTLVFSAAMALVLAAAGAVTVVQFRDAAHEAD
PDGALRGLTDDITADLVRELVTILPIVLVIAAVAAYLLSRAALRPVDRIRAAAQTLTT
TPHPDTDAPLPVPPTDDEIAWLATTLNTMLTRLQRALAHEQQFVADASHELRTPLALL
TTELELRCAGPDPPTS"
CDS complement(700604..701308)
/codon_start=1
/note="gene_625"
/transl_table=11
/translation="VSGVRILVIEDEPKMTALLARALTEEGHTVDTVADGRHAVAAVD
GGDYDAVVLDVMLPGIDGFEVCARLRRQRVWTPVLMLTARGAVTDRIAGLDGGADDYL
TKPFNLDELFARLRALSRRGPIPRPPTLEAGDLRLDPSEHRVWRADTEIRLSHKEFTL
LEALIRRPGIVHTRAQLLERCWDAAYEARSNIVDVYIRYLRDKIDRPFGVTSLETIRG
AGYRLRKDGGRHALPR"
CDS 701422..701733

```
                     /codon_start=1
                     /note="gene_626"
                     /transl_table=11
                     /translation="MNRIVQFGVSAVAAAAIGIGAGSGIAAAFDGEDEVTGPDADRAR
                     AAAVQAVPGGTAGEVETETGEGAAAYGVLVTRPDGTRVEVHLDRDFRVLDTEPADGDG
                     G"
     CDS             701829..702755
                     /codon_start=1
                     /note="gene_627"
                     /transl_table=11
                     /translation="MAALLAAAALALTACAGSDDKGEPDDGGDRGASLATTSDADWKP
                     VADILGRTGKLNDGSVYKIGFARSDLSVQTKGVTVAPALSLGSWVAFARTPDGQTMLM
                     GDLVVTEDELASVTDAVQAGGLQQTALHKHLLEQSPPIWWTHIAGHGDAADLARAVRS
                     ALDATDTPPPAPATSGQTSLDLDTAAIDEALGRSGTIAGGVYKFFIARRDPVTMSGML
                     IPPSMGLATALNFQPTGNGRAAINGDFVMTAAEVQDVVQALRGGGIDIVAIHNHGFDE
                     QPRLFYMHFWAENDAVALARTLRAAVDATAAR"
     CDS             702996..703580
                     /codon_start=1
                     /note="gene_628"
                     /transl_table=11
                     /translation="MNLAAWAERNGVARVTAYRWFHAGLLPVPARKVGRLILVDELAS
                     EAGAQPKTAVYARVSSADQKSDLDRQVARVTSWATAEQIPVDKVVTEVGSVLNGHRRK
                     FPAVLRDLSVTRIVVEHRDRFCRFGSEYVHAALAAQGRELVVVDSAEVDDDLVWDMTE
                     ILTSMCARLYGKRAAQNRAKRAVAAAAVDDHEAA"
     CDS             703582..704325
                     /codon_start=1
                     /note="gene_629"
                     /transl_table=11
                     /translation="MPRLEIPNGWCVQAFRFTLDPTAEQAHALARHFGARRKAYNWTV
                     AQLKADIQAWRATGAQTAKPSLRVLRKRWNTVKDEVCVNAETGTVWWPECSKEAYADG
                     IAGAVDAYWNWQQRRAGKRDGKRMGFPRFKKKGRDADRVSFTTGAMRVEPDRRHLTLP
                     VIGCVRTHENTRRIERLIAKDRARVLAITVRRNGTRLDASVRVLVQRPQQPNVELPES
                     RIGVDVGVRRLATVATADGACCPVLVPDG"
     CDS             704295..704765
                     /codon_start=1
                     /note="gene_630"
                     /transl_table=11
                     /translation="MLPGPGARRLTLGIIPEGGAHIDVPRKTVGAWQTADTMGIFQAL
                     PDVWGGWRTECWEDRFEEQLIRCNGALRLPELDLAAGMDSAREWLRDRIFQRFSDSPA
                     GQILKLSELLADVGPGLVVSDDAVTNGGARPNNEEWARFVAACDLVRGAHAESA"
     CDS             704810..705055
                     /codon_start=1
                     /note="gene_631"
                     /transl_table=11
                     /translation="MALNIKDPSVHQAVKQIAKITGESQARAVATAVNERLARLRSDD
                     LAARLLAIGHKTASRMSPEAKRLDHDALLYDERGLPA"
     CDS             705052..705453
                     /codon_start=1
                     /note="gene_632"
                     /transl_table=11
                     /translation="VIVDTSAIIAILRDEDDAAAYADALANADVRRLSAASYLECGIV
```

LDSQRDPVISRALDELIEEAEFVVEPVTERQARLARAAYADFGRGSGHPAGLNFGDCL
SYALAIDRREPLLWKGNDFGHTGVQRALDRR"
CDS complement(706041..706238)
/codon_start=1
/note="gene_633"
/transl_table=11
/translation="VTDEKCVRCGGDQLVEGAVVWNAPLRFKREGAGHFNRGTQVNAV
ACETCGHIDLYLESRARGSTK"
CDS complement(706318..707475)
/codon_start=1
/note="gene_634"
/transl_table=11
/translation="MDDELRGLLARYARGELSADDARRAILRYPKWRVAEIDGELETV
ALDDGTPMLIAESSASDGREYSGLELVRDIAPLVGGLSFDPDEPWGSAFRPGALPELQ
SWARTVELEDAVAKPGPGQRDLLYEGPWWVAVSPGTGRPAVHRADGLDVITIMTAPDA
AATFRRTERHRGLDVVRLGPALWGDLAKRSDFDGVRLNPLRPLAQLWPPHVPAMLVAG
CDPRPNAEPLPARTVAEIHLWLDQHGARQEKRELSNRATPVGEVTVARAWWNYDRREI
AFTRVAPASDTEGLGSVPSRILCAGKLRQSIQSKLAGLPRLTWRADAWHRQRAALAVG
WALELEKLVCGERVPFAALRTPEGAHLWHLEPQAFTARAIRKLRDRAASFR"
CDS complement(707527..707748)
/codon_start=1
/note="gene_635"
/transl_table=11
/translation="MYAEHDVVVLTRDVPDKSLIAGDVGAVVGRYAAGGYEVDFTAAN
GCTVAVVTLAGDDIRPRRRREIPHVREVA"
CDS 707947..708495
/codon_start=1
/note="gene_636"
/transl_table=11
/translation="MIAGVAAKRMNREQFFRAASGLDEDRLRKALWNLYWRGTANMRE
RIEAELASAGRARPARKIKPPADPDIVGWEVDEFVSLARSGAYLGGDRRVSPRERSRW
RFTFKRLAAEAQDALRAEDAEPAASALEQLIDLAREADGYDYFRSDDPVAAAGFVVSD
VAAAGHPHFREFAAEIGAAIPP"
CDS complement(708514..711081)
/codon_start=1
/note="gene_637"
/transl_table=11
/translation="VAEAFDATQAVARILAEHGPLSEDDIARRLLDSGVADPDAVLRA
LRLETEWPARQLVDDRWVWLPTLLAGRVFTHRLGADEAVHDMLGVTPDLDPITTLCEH
EEYGRLADGSAARIVLAGYDEELLERRGIPDEAIDPGGALLLEPGTLATLGAAAGDLV
GVRLTAAGLVLERIGTAGADTSVGARLAELVDPDEPAFFPAAVWTACVDDPAAFTEPV
APLREILDQHGLTHEDDWLAPGGFNFDAWRFENRCELLAFRHDLDPNDAVALYTLIKL
HETMSLLLEATDPDELPRDVLATAAETATETGSDSLVDLLGDIGAALADPLLAELLVA
ETVGTDSGGAAALGLLTEMLEPKVPRAARVAVRWLRAVALDRIGDVEAAERELLAAES
MDTEWPLPLLDLARIASDRGDAERGLALLRRAGTEPDHPLVRLLERHRAQPRRDLGRN
EACWCGSGRKYKKCHLGREALPLAERVDWLYAKASQHALSGDWTGLLAEVSYERFRYA
DSDDEDALAAALADPLVLDAVLFEGGAFAEFLEVRGSLLPDDERLLAEQWLLVERSVF
EVEHVQPGEGVIVRDVRTGDTHEVHERAASRQLRAGQLICARPVPAGDTMVFFGGIEP
VALHERAVLIELLDDEPDPVTLVAQLSRRFAPPTLVNTEGDSLAICEASVRVDDPAGI
QGALDGVYDRVDGEEPPRWIEHVTNDGMLRVRATLVLDGDTLRVETNSEPRMDRVLAT
LTRLDPAMTVLDDDRRPLRNTREAAALAEQMPVTGAGAPDPDSPELAAALEEFIRDYE

TSWLDQPIPALDGHTPRQAADDPTRRADLIKLLDTFPAGAGARGGMDADRLRTALGL"
CDS 711165..711914
/codon_start=1
/note="gene_638"
/transl_table=11
/translation="VTAPTRWTLADGRELLFFSLPGPRTSGTAAERVARHAQAQTFAG
DIRQRAIQLVVSEQEVASKITAATAGIATTTFPETPSIDDTIIGNDNRDTGVRLVDVK
QDGGTSPPPPFAPWDTPDGTPPPGTGLSPTLQQMILGGDPANLTGQGLADNVQRFVQS
LPANDPNTAWLRGQVADLQAHVADIEYARTHCSTNDWIDRTAQFASGAIVFSIGVLTA
ETGAGVVAAAAGGVGAATAGVSLLQCLVGSK"
CDS 711914..712153
/codon_start=1
/note="gene_639"
/transl_table=11
/translation="MDVLAAGIAAGALTLAAWGAWRPHYRAASYLVAGAVELALIGLL
VVTGQTLMAISVAFLVALGGPLVVVNHRRAERSRG"
CDS 712348..712575
/codon_start=1
/note="gene_640"
/transl_table=11
/translation="VRTTIDLPQDLHKQALAIARDTHRTLSETVADLMRRGLAANRPT
ALSSDPRTGLPLVSVGTVVTSEDVRSLEDEQ"
CDS 712572..712973
/codon_start=1
/note="gene_641"
/transl_table=11
/translation="VTVLLDANVLIALVVAEHVHHDAAADWLMASDTGFATCPMTQGS
LVRFLVRSGQSAAAARDVVSAVQCTSRHEFWPDALSFAGVEVAGVVGHRQVTDAYLAQ
LARSHDGQLATLDSGLAHLHGDVAVLIPTTT"
CDS 713102..714286
/codon_start=1
/gene="galTa"
/note="gene_642"
/transl_table=11
/translation="MSATPPPGGLDASVFIANERGRQLDEALPVGFCVVTAPTRWTLA
DGRDLLFFSLPGHVPAPVSDRRPLPERDPAPSRLRFDRATGQWVIVAAQRQDRTYKPP
AARCPLCPGPTGLSSEVPAPDYDVVVFENRFPSLAGAGIAPIGAPDGDGFVSAPGHGR
CEVICFSADHTGSFAGLDPAHARLVVHAWRHRTAELTALPGVAQVFCFENRGEEIGVT
LTHPHGQIYAYPYLTPRTAAMLRQARRHRKRHGDNLFASLLAREVADGSRIVVRGELF
TAFVPFAARWPVEVHIYPNRLVRNLTELNDGELDEFARIYLDVLQRFDRMYSSPLPYM
SALHQFSEVQRDGYFHVELMSIRRSATKLKYLAAAESAMDAFIADVIPESVAARLREL
GP"
CDS 714283..715374
/codon_start=1
/note="gene_643"
/transl_table=11
/translation="MTVSYGAPGRVNLIGEHTDYNLGFALPIALPRRTVVTFTPEHTG
AITARSDRADGSARIPLDTTPGQVTGWAAYAAGAIWALRGAGHPVPGGAMSITSDVEI
GSGLSSSAALIGAVLGAVGAATGTRIDRLERARLAQRAENDYVGAPTGLLDHLAALFG
APKTALLIDFRDITVRPVAFDPDACDVVLLLMDSRARHRHAGGEYALRRASCERAAAD
LGVSSLRAVQDRGLAALGAIADPIDARRARHVLTENQRVLDFAAALADSDFTAAGQLL

```
                     TASHESMREDFAITTERIDLIAESAVRAGALGARMTGGGFGGAVIALVPADRARDVAD
                     TVRRAAVTAGYDEPAVSRTYAAPGAAECC"
     CDS             complement(715496..715840)
                     /codon_start=1
                     /gene="CCDC5079_0579"
                     /note="gene_644"
                     /transl_table=11
                     /translation="MLCMVIRTRSHIRRTGVSASIACHRLHLEAKATPAVGQTQERSS
                     WGGIGSRAKPRSKAASSWPRDTAATTNTRAQDQSAILVTRLLERLGALAPGRMRQTDH
                     ALALVLGISEAG"
     CDS             715874..716926
                     /codon_start=1
                     /note="gene_645"
                     /transl_table=11
                     /translation="VNAWDYRFCERCGSPIGVVPWPSEESGTRQTAPARSFVPLVVLA
                     ATLLVVAVVVTAVGYAVTRPARNDREEPSSARGAATTGVPFAQAEAASCPDDPVLEAE
                     SIDLTSDGLAVSAAFMSACAGGDVESNSALEVTVADGRRDVAAGSFDFSADPLRIEPG
                     VPARRTLVFPPGMYWRTPDMLSGAPALAATRKGRSDRSAARGGSARTTMVAAASAAPA
                     YGSINAVAGAVLVELRDSDFPYVRVGIANRWVPQVSSKRVGLVAAGKTWTSADILRDH
                     LALRQRFGGARLVWSGHWTTFSGPDFWVTVVGPAQPTAAEANRWCDSNGFGADDCFAK
                     FISTLVGAKGTTVYRK"
     CDS             716937..717884
                     /codon_start=1
                     /note="gene_646"
                     /transl_table=11
                     /translation="MSFCVYCGAELADPTRCGACGAYKIGSTWHRTTTPTVGAATTAT
                     GWRPDPTGRHEGRYFVAGQPTDLVREGDAEAVDPLGQQQLDQSGAVGVSPSAVSGWVR
                     SGHRRLWWALAGVVAFLGLVGAGVVGTLFLNRDRESIDDKYLAALRRSGLTGEFNSDA
                     NAIARGKQVCRQLQDGGEQQGMPVDQVAVQYYCPQFSDGFHILETITVTGSFTLKDES
                     PNVYAPAITVSGSGCSGSAGYADIDRGTQVTVKNGQGDILATAFLQAGQGGRFLCTFP
                     FSFEITEGEDRYVVSVSRRGEMSYSFADLKANGLSLVLG"
     CDS             717977..718231
                     /codon_start=1
                     /note="gene_647"
                     /transl_table=11
                     /translation="MALSIKHPEADRLARALAARTGETLTEAVVTALRERLARETGRA
                     RVVPLRDELAAIRHRCAALPVVDNRSAEAILGYDERGLPA"
     CDS             718231..718626
                     /codon_start=1
                     /note="gene_648"
                     /transl_table=11
                     /translation="MVIDTSALVAMLSDEPDAERFEAAVEADHIRLMSTASYLETALV
                     IEARFGEPGGRELDLWLHRAAVDLVAVHADQADAARAAYRTYGKGRHRAGLNYGDCFS
                     YGLAKISGQPLLFKGEDFQHTDIATVALP"
     CDS             complement(718720..719376)
                     /codon_start=1
                     /note="gene_649"
                     /transl_table=11
                     /translation="MFYLVAATDVIDVAAVRGAVSATGPAAPLTYVVVSAVLGALFVP
                     GPILAASSGLLFGPLVGVFVTLGATVGTAVVASLVGRRAGRASARALLGGERADRTDA
                     LIERCGLWAVVGQRFVPGISDAFASYAFGTFGVPLWQMAVGAFIGSAPRAFAYTALGA
```

AIGDRSPLLASCAIAVWCVTAIIGAFAARHGYRQWRAHARGDGADGGVEDPDREVGAR
"
CDS 719592..719852
/codon_start=1
/note="gene_650"
/transl_table=11
/translation="MSEVASRELRNDTAGVLRRVRAGEDVTITVSGRPVAVLTPVRPR
RRRWLSKTEFLSRLRGAQADPGLRNDLAVLAGDTTEDLGPIR"
CDS 719849..720256
/codon_start=1
/note="gene_651"
/transl_table=11
/translation="VSTTPAAGVLDTSVFIATESGRQLDEALIPDRVATTVVTLAELR
VGVLAAATTDIRAQRLATLESVADMETLPVDDDAARMWARLRIHLAESGRRVRINDLW
IAAVAASRALPVITQDDDFAALDGAASVEIIRV"
CDS complement(720328..721479)
/codon_start=1
/note="gene_652"
/transl_table=11
/translation="VRIGVGVSTAPDVRRAAAEAAAHAREELAGGTPALAVLLGSRSH
TDQAVDLLAAVQASVEPAALIGCVAQGIVAGRHELENEPAVAVWLASGPPAETFHLDF
VRTGSGALITGYRFDRTAHDLHLLLPDPYSFPSNLLIEHLNTDLPGTTVVGGVVSGGR
RRGDTRLFRDRDVLTSGLVGVRLPGAHSVSVVSQGCRPIGEPYIVTGADGAVITELGG
RPPLHRLREIVLGMAPDEQELVSRGLQIGIVVDEHLAVPGQGDFLIRGLLGADPTTGA
IGIGEVVEVGATVQFQVRDAAAADKDLRLAVERAAAELPGPPVGGLLFTCNGRGRRMF
GVTDHDASTIEDLLGGIPLAGFFAAGEIGPVAGHNALHGFTASMALFVD"
CDS complement(721572..723299)
/codon_start=1
/note="gene_653"
/transl_table=11
/translation="VKLTDVDFAVEASGMVRAFNQAGVLDVSDVHVAQRLCALAGESD
ERVALAVAVAVRALRAGSVCVDLLSIARVAGHDDLPWPDPADWLAAVRASPLLADPPV
LHLYDDRLLYLDRYWREEEQVCADLLALLTSRRPAGVPDLRRLFPTGFDEQRRAAEIA
LSQGVTVLTGGPGTGKTTTVARLLALVAEQAELAGEPRPRIALAAPTGKAAARLAEAV
RREMAKLDATDRARLGDLHAVTLHRLLGAKPGARFRQDRQNRLPHNVIVVDETSMVSL
TLMARLAEAVRPGARLILVGDADQLASVEAGAVLADLVDGFSVRDDALVAQLRTSHRF
GKVIGTLAEAIRAGDGDAVLGLLRSGEERIEFVDDEDPAPRLRAVLVPHALRLREAAL
LGASDVALATLDEHRLLCAHRDGPTGVLHWNRRVQAWLAEETGQPPWTPWYAGRPLLV
TANDYGLRVYNGDTGVVLAGPTGLRAVISGASGPLDVATGRLGDVETMHAMTIHKSQG
SQVDEVTVLMPQEDSRLLTRELLYTAVARAKRKVRVVGSEASVRAAIARRAVRASGLR
MRLQSTGCG"
CDS complement(723296..723979)
/codon_start=1
/note="gene_654"
/transl_table=11
/translation="VDVDHAQLAPELARALLPMHDTPLGPAAAALTLRQIGVRDRLRE
LDFEMPLAGGDLRGRSPDVSLADVGELLASHLPGDDPLSPYADRLGSAGLGDQPLRGY
LAGSIDVVLRLPGQRYLVVDYKTNHLGDTAADYGFERLTEAMLHSDYPLQALLYVVVL
HRFLRWRQRDYAPARHLGGVLYLFVRGMCGAATPVTAGHPAGVFTWNPPTALVVALSD
LLDRGRLQS"
CDS complement(723886..726579)

/codon_start=1
/note="gene_655"
/transl_table=11
/translation="MDRFELLGPLPREGTTTVLEASAGTGKTFALAGLVTRYLAETAA
TLDEMLLITFNRAASRELRERVRGQIVEALGALQGDAPPSGELVEHLLRGSDAERAQK
RSRLRDALANFDAATIATTHEFCGSVLKSLGVAGDNAADVELKESLTDLVTEIVDDRY
LANFGRQETDPELTYAEALALALAVVDDPCAQLRPPDPEPGSKAAVRLRFAAEVLEEL
ERRKGRLRAQGFNDLLIRLATALEAADSPARDRMRERWRIVLVDEFQDTDPMQWRVLE
RAFSRHSALILIGDPKQAIYGFRGGDIHTYLKAAGTADARYTLGVNWRSDRALVESLQ
TVLRDATLGHADIVVRGTDAHHAGHRLASAPRPAPFRLRVVKRHTLGYDGTAHVPIEA
LRRHIPDDLAADVAALLASGATFAGRPVVAADIAVIVEHHKDARACRNALAEAGIPAI
YTGDTDVFASQAAKDWLCLLEAFDAPQRSGLVRAAACTMFFGETAESLAAEGDALTDR
VAGTLHEWADHARHRGVAAVFQAAQLAGMGRRVLSQRGGERDLTDLAHIAQLLHEAAH
RERLGLPGLRDWLRRQAKAGAGPPEHNRRLDSDAAAVQIMTVFVAKGLQFPIVYLPFA
FNRNVRSDDILLYHDDGTRCLYIGGKDGGAQRRTVEGLNRVEAAHDNLRLTYVALTRA
QSQVVAWWAPTFDEVNGGLSRLLRGRRPGQSQVPDRCTPRVTDEQAWAVFAQWEAAGG
PSVEESVIGARSSLEKPVPVPGFEVRHFHRRIDTTWRRTSYSDLVRGSEAVTVTSEPA
AGGRADEVEIAVFAAPGSGADLTSPLAALPSGASFGSLVHAVLETADPAAPDLAAELE
AQVPGTRRGGPWTSTTRSWLPNWPERCCRCTTRRWDPPPPH"

CDS complement(726579..729872)
/codon_start=1
/note="gene_656"
/transl_table=11
/translation="MALHLHRAERTDLLADGLGALLADPQPDPFAQELVLVAARGVER
WLSQRLSLVLGCGPGRADGVCAGIAFRNPQSLIAEITGTLDDDPWSPEALAWPLLAVI
DASLDEPWCRTLASHLGHFATTDAEAELRRGRRYSVARRLAGLFASYARQRPGLLAAW
LDGDLGELPGDLAWQPPLWRALVTTVGADPPHVRHDKTIARLRDGPADLPARLSLFGH
TRLACTDVQLLDALAVHHDLHLWLPHPSDELWRALAGFQGADGLLPRRQDTSRRAAQH
PLLETLGRDVRELQRALPAARATDEFLGATTKPDTLLGWLQADIAGNAPRPAERSLSD
ADRSVQVHACHGPARQIDVLREVLLGLLEDDPTLQPRDIVVMCPDIDTYAPLIVAGFG
LGEVAGDCHPAHRLRVRLADRALTQTNPLLSVAAELLTIAETRATASQLLNLAQAAPV
RAKFGFADDDLDTITTWVRESNIRWGFDPTHRRRYGLDTVVHNTWRFGLDRILTGVAM
SEDSQAWLDTALPLDDVGSNRVELAGRLAEFVERLHHVVGGLSGARPLVAWLDALATG
IDLLTACNDGWQRAQVQREFADVLARAGSRAAPLLRLPDVRALLDAQLAGRPTRANFR
TGTLTVCTMVPMRSVPHRVVCLVGLDDGVFPRLSHPDGDDVLAREPMTGERDIRSEDR
QLLLDAIGAATQTLVITYTGADERTGQPRPPAVPLAELLDALDQTTSAPVRERILVTH
PLQPFDRKNVTPGALLGAKPFTFDPAALAAAQAAAGKRCPPTAFISGRLPAPPAADVT
LADLLDFFKDPVKGFFRALDYTLPWDVDTVEDSIPVQVDALAEWTVGERMLRDMLRGL
HPDDAAHSEWRRGTLPPGRLGVRRAKEIRNRARDLAAAALAHRDGHGQAHDVDVDLGD
GRRLSGTVTPVFGGRTVSVTYSKLAPKHVLPAWIGLVTLAAQEPGREWSALCIGRSKT
RNHIARRLFVPPPDPVAVLRELVLLYDAGRREPLPLPLKTSCAWAQARRDGQDPYPPA
RECWQTNRFRPGDDDAPAHVRAWGPRAPFEVLLGKPRAGEEVAGEETRLGALAARLWL
PLLAAEGSV"

CDS complement(730149..730844)
/codon_start=1
/note="gene_657"
/transl_table=11
/translation="MSDPVSYTRKDSIAVISMDDGKVNALGPAMQQALNAAIDNADRD
DVGALVITGNGRVFSGGFDLKILTSGEVQPAIDMLRGGFELAYRLLSYPKPVVMACTG
HAIAMGAFLLSCGDHRVAAHAYNIQANEVAIGMTIPYAALEIMKLRLTRSAYQQATGL
AKTFFGETALAAGFIDEIALPEVVVSRAEEAAREFAGLNQHAHAATKLRSRADALTAI

```
                     RAGIDGIAAEFGL"
     CDS             complement(730893..731711)
                     /codon_start=1
                     /note="gene_658"
                     /transl_table=11
                     /translation="MGWVLSSWHEVTGVDSGTWLAWAALGLGVVALVVTKRQIQRNRR
                     LAAEQTRPYVAMFMEPHVADWHVIELVVRNFGRTAAYDVRFSFPNPPTVAQYENAANG
                     YADVVELRLPQELPMLAPGQEWRMVWDSALDRAEIGRGIESRFPGTVTYYDRPEQPRR
                     WRFWRRGRRPLETKVVLDWDALPPVARIELMTTHDLAKREKQKLELLRSLLTYFHYAS
                     KETRPDVFRSEIDRINRAAAETQDRWRARQVEVPTEVSQRSEGQGPQPTRIPAG"
     CDS             complement(731877..732590)
                     /codon_start=1
                     /note="gene_659"
                     /transl_table=11
                     /translation="VSKDRLYFRQLLSGRDFAVGDMFATQMRNFAYLIGDRTTGDCVV
                     VDPAYAAGDLLDALESDDMQLSGVLVTHHHPDHVGGSMMGFQLPGLAELLERASVPVH
                     VNTHEALWVSRVTGIPVGDLITHEHGDKVSVGDIDIELLHTPGHTPGSQCFLLDGRLV
                     AGDTLFLEGCGRTDFPGGDSDEMYRSLRQLAELPGDPTVFPGHWYSAEPSASLSEVKR
                     SNYVYRPASLDQWRMLMGG"
     CDS             732709..732921
                     /codon_start=1
                     /note="gene_660"
                     /transl_table=11
                     /translation="MLKRVEIEVDDDLIQKVIRRYRVKGAREAVNLALRTLLGEADTA
                     EHGHDDEYDEFSDPNAWVPRRSRDTG"
     tRNA            733051..733123
                     /note="identified by tRNAscan-SE; putative"
     tRNA            733160..733236
                     /note="identified by tRNAscan-SE; putative"
     CDS             733269..733436
                     /codon_start=1
                     /gene="rpmG"
                     /note="gene_661"
                     /transl_table=11
                     /translation="MASSTDVRPKITLACEVCKHRNYITKKNRRNDPDRLELKKFCPN
                     CGKHQAHRETR"
     CDS             733487..733963
                     /codon_start=1
                     /note="gene_662"
                     /transl_table=11
                     /translation="VALSADIVGMHYRYPDHYEVEREKIREYAVAVQNDDAWYFEEDG
                     AAELGYKGLLAPLTFICVFGYKAQAAFFKHANIATAEAQIVQVDQVLKFEKPIVAGDK
                     LYCDVYVDSVREAHGTQIIVTKNIVTNEEGDLVQETYTTLAGRAGEDGEGFSDGAA"
     CDS             733950..734378
                     /codon_start=1
                     /note="gene_663"
                     /transl_table=11
                     /translation="MALREFSSVKVGDQLPEKTYPLTRQDLVNYAGVSGDLNPIHWDD
                     EIAKVVGLDAAIAHGMLTMGIGGGYVTSWVGDPGAVTEYNVRFTAVVPVPNDGKGAEL
                     VFNGRVKSVDPESKSVTIALTATTGGKKIFGRAIASAKLA"
     CDS             734382..734882
```

```
                     /codon_start=1
                     /note="gene_664"
                     /transl_table=11
                     /translation="MALKTDIRGMIWRYPDYFIVGREQCREFARAVKCDHPAFFSEEA
                     AADLGYDALVAPLTFVTILAKYVQLDFFRHVDVGMETMQIVQVDQRFVFHKPVLAGDK
                     LWARMDIHSVDERFGADIVVTRNLCTNDDGELVMEAYTTLMGQQGDGSARLKWDKESG
                     QVIRTA"
     tRNA            735081..735153
                     /note="identified by tRNAscan-SE; putative"
     CDS             735294..735779
                     /codon_start=1
                     /gene="secE"
                     /note="gene_665"
                     /transl_table=11
                     /translation="VSDEGDVADEAVADGAENADSRGSGGRTALVTKPVVRPQRPTGK
                     RSRSRAAGADADVDVEEPSTAASEATGVAKDDSTTKAVSKAARAKKASKPKARSVNPI
                     AFVYNYLKQVVAEMRKVIWPNRKQMLTYTSVVLAFLAFMVALVAGADLGLTKLVMLVF
                     G"
     CDS             735811..736527
                     /codon_start=1
                     /gene="nusG"
                     /note="gene_666"
                     /transl_table=11
                     /translation="VTTFDGDTSAGEAVDLTEANAFQDAAAPAEEVDPAAALKAELRS
                     KPGDWYVVHSYAGYENKVKANLETRVQNLDVGDYIFQVEVPTEEVTEIKNGQRKQVNR
                     KVLPGYILVRMDLTDDSWAAVRNTPGVTGFVGATSRPSALALDDVVKFLLPRGSTRKA
                     AKGAASTAAAAEAGGLERPVVEVDYEVGESVTVMDGPFATLPATISEVNAEQQKLKVL
                     VSIFGRETPVELTFGQVSKI"
     CDS             736579..737007
                     /codon_start=1
                     /gene="rplK"
                     /note="gene_667"
                     /transl_table=11
                     /translation="MAPKKKVAGLIKLQIVAGQANPAPPVGPALGQHGVNIMEFCKAY
                     NAATENQRGNVIPVEITVYEDRSFTFTLKTPPAAKLLLKAAGVAKGSAEPHKTKVAKV
                     TWDQVREIAETKKTDLNANDVDAAAKIIAGTARSMGITVE"
     CDS             737074..737781
                     /codon_start=1
                     /gene="rplA"
                     /note="gene_668"
                     /transl_table=11
                     /translation="MSKTSKAYRAAAAKVDRTNLYTPLQAAKLAKETSSTKQDATVEV
                     AIRLGVDPRKADQMVRGTVNLPHGTGKTARVAVFAVGEKADAAVAAGADVVGSDDLIE
                     RIQGGWLEFDAAIAAPDQMAKVGRIARVLGPRGLMPNPKTGTVTADVAKAVADIKGGK
                     INFRVDKQANLHFVIGKASFDEKLLAENYGAAIDEVLRLKPSSSKGRYLKKITVSTTT
                     GPGIPVDPSITRNFAGE"
     CDS             complement(737855..738751)
                     /codon_start=1
                     /gene="mmaA4"
                     /note="gene_669"
                     /transl_table=11
```

```
                     /translation="MAEKPISPTKTRTRFEDIQAHYDVSDDFFALFQDPTRTYSCAYF
                     EPPELTLEEAQYAKVDLNLDKLDLKPGMTLLDIGCGWGTTMRRAVERLDVNVIGLTLS
                     KNQHARCEQVLASIDTNRSRQVLLQGWEDFAEPVDRIVSIEAFEHFGHENYDDFFKRC
                     FNIMPADGRMTVQSSVSYHPYEMAARGKKLSFETARFIKFIVTEIFPGGRLPSTEMMV
                     EHGEKAGFTVPEPLSLRPHYIKTLRIWGDTLQSNKDKAIEVTSEEVYNRYMKYLRGCE
                     HYFTDEMLDCSLVTYLKPGAAA"
     CDS             complement(738825..739706)
                     /codon_start=1
                     /note="gene_670"
                     /transl_table=11
                     /translation="MSDNSTGTTKSRSNVDDVQAHYDLSDAFFALFQDPTRTYSCAYF
                     ERDDMTLHEAQVAKLDLTLGKLGLEPGMTLLDVGCGWGSVMKRAVERYDVNVVGLTLS
                     KNQHAYCQQVLDKVDTNRSHRVLLSDWANFSEPVDRIVTIEAIEHFGFERYDDFFKFA
                     YNAMPADGVMLLHSITGLHVKQVIERGIPLTMEMAKFIRFIVTDIFPGGRLPTIETIE
                     EHVTKAGFTITDIQSLQPHFARTLDLWAEALQAHKDEAIEIQSAEVYERYMKYLTGCA
                     KAFRMGYIDCNQFTLAK"
     CDS             complement(739854..740717)
                     /codon_start=1
                     /note="gene_671"
                     /transl_table=11
                     /translation="MVNDLTPHFEDVQAHYDLSDDFFRLFLDPTQTYSCAHFEREDMT
                     LEEAQIAKIDLALGKLGLQPGMTLLDIGCGWGATMRRAIAQYDVNVVGLTLSKNQAAH
                     VQKSFDEMDTPLDRRVLLAGWEQFNEPVDRIVSIGAFEHFGHDRHADFFARAHKILPP
                     DGVLLLHTITGLTRQQMVDHGLPLTLWLARFLKFIATEIFPGGQPPTIEMVEEQSAKT
                     GFTLTRRQSLQPHYARTLDLWAEALQEHKSEAIAIQSEEVYERYMKYLTGCAKLFRVG
                     YIDVNQFTLAK"
     CDS             complement(740884..741744)
                     /codon_start=1
                     /note="gene_672"
                     /transl_table=11
                     /translation="MAKLRPYYEESQSAYDISDDFFALFLDPTWVYTCAYFERDDMTL
                     EEAQLAKVDLALDKLNLEPGMTLLDVGCGWGGALVRAVEKYDVNVIGLTLSRNHYERS
                     KDRLAAIGTQRRAEARLQGWEEFEENVDRIVSFEAFDAFKKERYLTFFERSYDILPDD
                     GRMLLHSLFTYDRRWLHEQGIALTMSDLRFLKFLRESIFPGGELPSEPDIVDNAQAAG
                     FTIEHVQLLQQHYARTLDAWAANLQAARERAIAVQSEEVYNNFMHYLTGCAERFRRGL
                     INVAQFTMTK"
     CDS             complement(741791..742696)
                     /codon_start=1
                     /note="gene_673"
                     /transl_table=11
                     /translation="VDIRSGTAVSGDVKLYYEDMGDLDHPPVLLIMGLGAQMLLWRTD
                     FCARLVAKGLRVIRYDNRDVGLSTKTERHRPGQPLATRLVRSWLGLPSQAAYTLEDMA
                     ADAAALLDHLDVKHAHVVGASMGGMIAQIFAARFAQRTKTLAVIFSSNNHRFLPPPAP
                     RALLALLTGPPPDSPRDVIVDNAVRVSKIIGSPAYPIPEDQVRAEAAESYDRNFHPWG
                     IAQQFSAILGSGSLLRYDRRIVAPTVVIHGRADKLMRPFGGRAVARAINGARLVLIDG
                     MGHDLPRQLWDRVIGELTRNFSEAG"
     CDS             complement(742708..744054)
                     /codon_start=1
                     /note="gene_674"
                     /transl_table=11
                     /translation="MGSTTHREVAKLDRVPLPVEAARVAATGWQVTRTAVRFIGRLPR
```

KGPWQQKVIKELPQTFADLGPTYVKFGQIIASSPGAFGESLSREFRGLLDRVPPAKTD
EVHKLFVEELGDEPARLFASFEEEPFASASIAQVHYATLRSGEEVVVKIQRPGIRRRV
AADLQILKRFAQTVELAKLGRRLSAQDVVADFADNLAEELDFRLEAQSMEAWVSHLHA
SPLGKNIRVPQVHWDFTTERVLTMERVHGIRIDNTAAIRKAGFDGVELVKALLFSVFE
GGLRHGLFHGDLHAGNLYVDEAGRIVFFDFGIMGRIDPRTRWLLRELVYALLVKKDHA
AAGKIVVLMGAVGTMKPETQAAKDLERFATPLTMQSLGDMSYADIGRQLSALADAYDV
KLPRELVLIGKQFLYVERYMKLLAPRWQMMSDPQLTGYFANFMVEVSREHQSDIEV"

CDS 744260..748456
/codon_start=1
/note="gene_675"
/transl_table=11
/translation="MRVVSAESTELFVGPSDAPLQLVRVAVTGCTEPPPIRIHGDGLA
GEAAARPGDDVIEVAVAVESPVVGERRTARVHTPDGPSLAFEFTVAEPGWTMFMISHF
HYDPVWWNTQGAYTSQWREDPPGRARQANGFELVRAHLEMARREPEYKFVLAEVDYLK
PYWDTHPQDRADLRRFLADGRIEVMGGTYNEPNTNLTSPETTIRNLVHGIGFQRDVLG
AEPATAWQLDVFGHDPQFPGLAADAGLTSSSWARGPHHQWGPAQGGVDRMQFCSEFEW
IAPSGRGLLTHYMPAHYSAGWSMDSSTSLADAEAATYALFDQLKKVALTRNVLLPVGT
DYTPPNKWVTAIHRDWGARYTWPRFVCALPKEFFAAVRAELAKRGWVPLPQTRDMNPI
YTGKDVSYIDTKQANRAAENAVLEAERFAVFAALLTGAEYPQAALAKAWVQLAYGAHH
DAITGSESDQVYLDLLTGWRDAWELGRAARDNSLRLLSGAVAASHDRVVVWNPLTQRR
TDIVTARVDPPLQAGVRVFDPDGAEVAALVEHDGRSVTWLACDVPSLGWRVYRLVPAD
EAPGWELVPGTDIANEHYRLAVDPERGGALSSLVQDGRQLIAAGRVANELALYEEYPS
HPTQGEGPWHLLPTGPVVCSSACPAQVQAYRGPLGQRLVVRGRIGTLLRYTQTLTLWD
GVDRVDCRTSIDEFTGEDRLLRLRWPCPVPGAMPISEVGDAVVGRGFALLHEGPESVD
TAQHPWTLDNPAYGWFGLSSAVRVRAGDGVRAVSVAEVVSPTETVSGPMARDLMVALV
RAGVTATCSGADKPRYGHLDVDSNLPDARIALGGPDRNTFTKAVLAEAAPAYTAELQR
QLAKTGTARVWVPAANPLARAWLPGADLRAPCALPVLVIDGRDEKHLRAAVASLADDL
ADAEIVVHQRAAPQMEPFEDRTVALLNRGVPSFAVDSEGTLHTALMRSCTGWPSGVWI
DQPRRTAPDGSNFQLQHWTHHFDYALVCGGGDWRRAGIPARSAQFSHPLLAVAPRRPQ
GELPAVGSLLHVEPADSVQLGALKAAGNRLAAGSARPVQPAAVALRLVQTTGADTPVT
IGCELGKVGALRPADLLETPLAMARARKSSIDLHGYQVATVLARLDVAADMANVLAAD
DVALAPHAETAQPQYARYWLHNRGPAPLGGLPAVAHLHPRRVRGQPGDDVVLRLTAAS
DCTDSVLGGVVDVVCPLGWPATPARLPFTLGAGAHLQADIALSIPAGAPPGPYPVRAQ
LRVVDTAVPAAWRQVVEDVCVVTVGADSDLEELVYLVDGPADIELAAGDRARLAVTIG
SRAHAELALDAHSISPWGTWEWIGPPALGAVLPARGMAKLAFDVTPPAWLEPGQWWAL
VRVGCAGQLVYSPAVKVSVT"

CDS 748453..749127
/codon_start=1
/note="gene_676"
/transl_table=11
/translation="MSGRSRLPGSSSRRDAARIVAERVVATVAGVAVAVDEVDAAEAR
LRDGPRAAALPASGTSEGRQLRRWLTQLIVTERVVAAEAAARGLTAAGAPAEADLLPD
ATARLEIGSVAAAVLADPLARALFAAVTARVAVTDDAVADYHARNPLRFAAPCPGQHG
WRAPAAAAPPLDQVRRAITEHLLGAARRRAFRVWLDARRNALVVLAPGYEHPGDPRQP
DNTRRH"

CDS 749127..750035
/codon_start=1
/note="gene_677"
/transl_table=11
/translation="MLTLCLDIGGTKIAAGLADPAGTLVHTAQRPTPAYGGAEQVWAA
VAEMIADALGVAGGAVGGVGIASAGPIDLHSGRVSPINIGSWGGFPLRDRVAAAVPGV

```
                     PVRLGGDGVCMALGEHWLGAGRGARFLLGLVVSTGVGGGLVLDGAPCLGRTGNAGHVG
                     HVVVDPDGSPCPCGGRGCVETIASGPSLARWARANGWSAPPGAGAKELAEAAGAGDPV
                     ALRAFRRGAAALAAMIASVGAVCDLDLAVIGGGVAKSGRLLFEPLRAALADHARLDFL
                     AGLRVVPAELGGAAGLVGAARLAAIA"
     CDS             750366..750902
                     /codon_start=1
                     /gene="rplJ"
                     /note="gene_678"
                     /transl_table=11
                     /translation="MARADKATAVADIAAQFKESTATLITEYRGLTVANLAELRRSLT
                     GSATYAVAKNTLIKRAASEAGIEGLDELFVGPTAIAFVTGEPVDAAKAIKTFAKEHKA
                     LVIKGGYMDGHPLTVAEVERIADLESREVLLAKLAGAMKGNLAKAAGLFNAPASQLAR
                     LAAALQEKKACPGPDSAE"
     CDS             750939..751331
                     /codon_start=1
                     /gene="rplL"
                     /note="gene_679"
                     /transl_table=11
                     /translation="MAKLSTDELLDAFKEMTLLELSDFVKKFEETFEVTAAAPVAVAA
                     AGAAPAGAAVEAAEEQSEFDVILEAAGDKKIGVIKVVREIVSGLGLKEAKDLVDGAPK
                     PLLEKVAKEAADEAKAKLEAAGATVTVK"
     CDS             complement(751324..752091)
                     /codon_start=1
                     /note="gene_680"
                     /transl_table=11
                     /translation="MPISYNIVISLILERYALGENMARMTSQTGVRDELLHAGVRLLD
                     DHGPDALQTRKVAAAAGTSTMAVYTHFGGMRGLIAAIAEEGLRQFDVALTVPQTADPV
                     ADLLAIGTAYRRYAIERPHMYRLMFGSTSAHGINVPARDVLTLKVAEIEHQHPSFAHV
                     VRAVHRCLLAGRFATALGADDDTAIVATAAQFWSQIHGFVMLELAGFYGDRGAAVEPV
                     LAAMTVNLLVALGDSPERAQCSLRAEQTQKNTLGRAT"
     CDS             752090..753595
                     /codon_start=1
                     /note="gene_681"
                     /transl_table=11
                     /translation="MTTAQAAESQNPYLEGFLAPVSTEVTATDLPVTGRIPEHLDGRY
                     LRNGPNPVAEVDPATYHWFTGDAMVHGVALRDGKARWYRNRWVRTPAVCAALGEPISA
                     RPHPRTGIIEGGPNTNVLTHAGRTLALVEAGVVNYELTDELDTVGPCDFDGTLHGGYT
                     AHPQRDPHTGELHAVSYSFARGHRVQYSVIGTDGHARRTVDIEVAGSPMMHSFSLTDN
                     YVVIYDLPVTFDPMQVVPASVPRWLQRPARLVIQSVLGRVRIPDPIAALGNRMQGHSD
                     RLPYAWNPSYPARVGVMPREGGNEDVRWFDIEPCYVYHPLNAYSECRNGAEVLVLDVV
                     RYSRMFDRDRRGPGGDSRPSLDRWTINLATGAVTAECRDDRAQEFPRINETLVGGPHR
                     FAYTVGIEGGFLVGAGAALSTPLYKQDCVTGSSTVASLDPDLLIGEMVFVPNPSARAE
                     DDGILMGYGWHRGRDEGQLLLLDAQTLESIATVHLPQRVPMGFHGNWAPTT"
     CDS             753676..754686
                     /codon_start=1
                     /note="gene_682"
                     /transl_table=11
                     /translation="MGVSIEVNGLTKSFGSSRIWEDVTLTIPAGEVSVLLGPSGTGKS
                     VFLKSLIGLLRPERGSIIIDGTDIIECSAKELYEIRTLFGVLFQDGALFGSMNLYDNT
                     AFPLREHTKKKESEIRDIVMEKLALVGLGGDEKKFPGEISGGMRKRAGLARALVLDPQ
                     IILCDEPDSGLDPVRTAYLSQLIMDINAQIDATILIVTHNINIARTVPDNMGMLFRKH
```

LVMFGPREVLLTSDEPVVRQFLNGRRIGPIGMSEEKDEATMAEEQALLDAGHHAGGVE
EIEGVPPQISATPGMPERKAVARRQARVREMLHTLPKKAQAAILDDLEGTHKYAVHEI
GQ"
CDS complement(755074..755325)
/codon_start=1
/note="gene_683"
/transl_table=11
/translation="MSRMFGDVYPDVPVPKSVWRWIDSAQHRLARAGAVGALSVVDLL
ICDTAAARGLVVLHDDADYELAERHLPDIRVRRVVSADD"
CDS complement(755552..755707)
/codon_start=1
/note="gene_684"
/transl_table=11
/translation="VSVTQIDLDDEALADVMRIAAVHTKKEAVNLAMRDYVERFRRIE
ALARSRE"
CDS complement(755783..756499)
/codon_start=1
/note="gene_685"
/transl_table=11
/translation="VEAGRADTVAPSHRWGLGAFLVVELVFLVASTSLAVVLTGHGPV
SAGVLALALAAPTVVAAGLAILITRLRGNGPRTDLRLRWSWRGLRLGLMFGFGGMLVT
IPASLVYTAIVGPEANSAVVRIFGGVRASWPWALVVFLVVVFVAPLCEEIIYRGLLWG
AVDRRWGRWAALVVTTVVFALAHLEFARAPLLVVVAIPIALARFYSGGLLASIVTHQV
TNLLPGIVLLLGLTGAISLP"
CDS complement(756775..757008)
/codon_start=1
/note="gene_686"
/transl_table=11
/translation="VADRIGAVVVVALTRTRRGLVSELELTAVENRVPSDCVVNFDNI
HTLPRTAFRRRITRLSPARLHEACQTLRASTGC"
CDS complement(757070..757321)
/codon_start=1
/gene="mazE2"
/note="gene_687"
/transl_table=11
/translation="MTMLSFRADDHDVDLADAWARRLHIGRSELLRDALRRHLAALAA
DQDVQAYTERPLTDDENALAEIADWGPAEDWADWADAAR"
CDS complement(757425..757862)
/codon_start=1
/note="gene_688"
/transl_table=11
/translation="MIVLDTTVLVYAKGAEHPLRDPCRDLVAAIADERIAATTTAEVI
QEFVHVRARRRDRSDAAALGRVTMPNCSRRYSPSIEATSKRGLTLFETTPGLEACDAV
LAAVAASAGATALVSADPAFADLSDVVHVIPDAAGMVSLLGDR"
CDS complement(757859..758113)
/codon_start=1
/note="gene_689"
/transl_table=11
/translation="MSMRLAHRLQILLDDECHRRITAVARERGVPVATVVREAIDRGL
VSPAGRRKSAGRRLLDAADMSVPEPRELKQELEALRARRG"
CDS 758227..760590

/codon_start=1
/note="gene_690"
/transl_table=11
/translation="MPQPRTHLPIPSAARTGLITYDAKDPDSTYPPIEQLRPPAGAPN
VLLILLDDVGFGASSAFGGPCRTSTAELLAGNGLRYNRFHTTALCSPTRQALLTGRNH
HSAGMGGITEIATGAPGYSSVLPNTMSPIARTLKLNGYNTAQFGKCHEVPVWQTSPVG
PFDAWPSGGGGFEYFYGFIGGEANQWYPSLYEGTTPVEVNRTPEEGYHFMADMTDKAL
GWIGQQKALAPDRPFFVYFAPGATHAPHHVPREWADKYRGRFDVGWDALREETFARQK
ELGVIPADCQLTARHAEIPAWDDMPEDLKPVLCRQMEVYAGFLEYTDHHVGRLVDGLQ
RLGVLDDTLVFYIIGDNGASAEGTINGTYNEMLNFNGLADIETPRFMTDRLDKFGGPE
SYNHYSVGWAHAMDTPYQWTKQVASHWGGTRNGTIVHWPNGIAAKGEMRWQFHHVIDV
APTILEAAGLPEPLFVNGVQQHPIEGVSMAYSFDDAQAPDRHETQYFEMFGNRGIYHK
GWTAVTKHKTPWILVGEQTVAFDDDVWELYDTTKDWSQAKDLAKEMPEKLHELQRLWL
IEATRYNVLPLDDDTASRINPDLAGRPVLIRGNTQVLFSNMGRLSENCVLNLKNKSHT
VTAEVEVPETGAEGVIVAQGASIGGWSLYANDGKLKYCYNLGGIKHFYAESADPLPAG
AHQVRMEFAYAGGGLGKGGEVTLYVDGQQVGEGHVEATLAIVFSADDGCDVGMDSGSP
VSPDYAPGSNAFNGRIKGVQLAIAEAAAAAGHLVDPEHAIRIALARQ"

CDS 760891..761229
/codon_start=1
/note="gene_691"
/transl_table=11
/translation="VTEGEVGVGLLDTSVFIARESGGAIADLPERVALSVMTIGELQL
GLLNAGDSATRSRRADTLALARTADQIPVSEAVMISLARLVADCRAAGVRRSVKLTDA
LIAATAEIKV"

CDS 761384..761614
/codon_start=1
/gene="TMLG_03155"
/note="gene_692"
/transl_table=11
/translation="LHGGLIDSTASIWSYCPRPDSQPWQAATRAFSPEATKGAMRALW
VSQREDLTADAKRVNLLGSMRRMWPKEVEIAS"

CDS 762125..765415
/codon_start=1
/gene="rpoB"
/note="gene_693"
/transl_table=11
/translation="VGGLEEVLYELSPIEDFSGSMSLSFSDPRFDDVKAPVDECKDKD
MTYAAPLFVTAEFINNNTGEIKSQTVFMGDFPMMTEKGTFIINGTERVVVSQLVRSPG
VYFDETIDKSTDKTLHSVKVIPSRGAWLEFDVDKRDTVGVRIDRKRRQPVTVLLKALG
WTSEQIVERFGFSEIMRSTLEKDNTVGTDEALLDIYRKLRPGEPPTKESAQTLLENLF
FKEKRYDLARVGRYKVNKKLGLHVGEPITSSTLTEEDVVATIEYLVRLHEGQTTMTVP
GGVEVPVETDDIDHFGNRRLRTVGELIQNQIRVGMSRMERVVRERMTTQDVEAITPQT
LINIRPVVAAIKEFFGTSQLSQFMDQNNPLSGLTHKRRLSALGPGGLSRERAGLEVRD
VHPSHYGRMCPIETPEGPNIGLIGSLSVYARVNPFGFIETPYRKVVDGVVSDEIVYLT
ADEEDRHVVAQANSPIDADGRFVEPRVLVRRKAGEVEYVPSSEVDYMDVSPRQMVSVA
TAMIPFLEHDDANRALMGANMQRQAVPLVRSEAPLVGTGMELRAAIDAGDVVVAEESG
VIEEVSADYITVMHDNGTRRTYRMRKFARSNHGTCANQCPIVDAGDRVEAGQVIADGP
CTDDGEMALGKNLLVAIMPWEGHNYEDAIILSNRLVEEDVLTSIHIEEHEIDARDTKL
GAEEITRDIPNISDEVLADLDERGIVRIGAEVRDGDILVGKVTPKGETELTPEERLLR
AIFGEKAREVRDTSLKVPHGESGKVIGIRVFSREDEDELPAGVNELVRVYVAQKRKIS
DGDKLAGRHGNKGVIGKILPVEDMPFLADGTPVDIILNTHGVPRRMNIGQILETHLGW

CAHSGWKVDAAKGVPDWAARLPDELLEAQPNAIVSTPVFDGAQEAELQGLLSCTLPNR
DGDVLVDADGKAMLFDGRSGEPFPYPVTVGYMYIMKLHHLVDDKIHARSTGPYSMITQ
QPLGGKAQFGGQRFGEMECWAMQAYGAAYTLQELLTIKSDDTVGRVKVYEAIVKGENI
PEPGIPESFKVLLKELQSLCLNVEVLSSDGAAIELREGEDEDLERAAANLGINLSRNE
SASVEDLA"

CDS 765460..769410
/codon_start=1
/note="gene_694"
/transl_table=11
/translation="VLDVNFFDELRIGLATAEDIRQWSYGEVKKPETINYRTLKPEKD
GLFCEKIFGPTRDWECYCGKYKRAPFKGIICERCGVEVTRAKVRRERMGHIELAAPVT
HIWYFKGVPSRLGYLLDLAPKDLEKIIYFAAYVITSVDEEMRHNELSTLEAEMAVERK
AVEDQRDGELEARAQKLEADLAELEAEGAKADARRKVRDGGEREMRQIRDRAQRELDR
LEDIWSTFTKLAPKQLIVDENLYRELVDRYGEYFTGAMGAESIQKLIENFDIDAEAES
LRDVIRNGKGQKKLRALKRLKVVAAFQQSGNSPMGMVLDAVPVIPPELRPMVQLDGGR
FATSDLNDLYRRVINRNNRLKRLIDLGAPEIIVNNEKRMLQESVDALFDNGRRGRPVT
GPGNRPLKSLSDLLKGKQGRFRQNLLGKRVDYSGRSVIVVGPQLKLHQCGLPKLMALE
LFKPFVMKRLVDLNHAQNIKSAKRMVERQRPQVWDVLEEVIAEHPVLLNRAPTLHRLG
IQAFEPMLVEGKAIQLHPLVCEAFNADFDGDQMAVHLPLSAEAQAEARILMLSSNNIL
SPASGRPLAMPRLDMVTGLYYLTTEVPGDTGEYQPASGDHPETGVYSSPAEAIMAADR
GVLSVRAKIKVRLTQLRPPVEIEAELFGHSGWQPGDAWMAETTLGRVMFNELLPLGYP
FVNKQMHKKVQAAIINDLAERYPMIVVAQTVDKLKDAGFYWATRSGVTVSMADVLVPP
RKKEILDHYEERADKVEKQFQRGALNHDERNEALVEIWKEATDEVGQALREHYPDDNP
IITIVDSGATGNFTQTRTLAGMKGLVTNPKGEFIPRPVKSSFREGLTVLEYFINTHGA
RKGLADTALRTADSGYLTRRLVDVSQDVIVREHDCQTERGIVVELAERAPDGTLIRDP
YIETSAYARTLGTDAVDEAGNVIVERGQDLGDPEIDALLAAGITQVKVRSVLTCATST
GVCATCYGRSMATGKLVDIGEAVGIVAAQSIGEPGTQLTMRTFHQGGVGEDITGGLPR
VQELFEARVPRGKAPIADVTGRVRLEDGERFYKITIVPDDGGEEVVYDKISKRQRLRV
FKHEDGSERVLSDGDHVEVGQQLMEGSADPHEVLRVQGPREVQIHLVREVQEVYRAQG
VSIHDKHIEVIVRQMLRRVTIIDSGSTEFLPGSLIDRAEFEAENRRVVAEGGEPAAGR
PVLMGITKASLATDSWLSAASFQETTRVLTDAAINCRSDKLNGLKENVIIGKLIPAGT
GINRYRNIAVQPTEEARAAAYTIPSYEDQYYSPDFGAATGAAVPLDDYGYSDYR"

CDS complement(769407..769781)
/codon_start=1
/gene="TBAG_03288"
/note="gene_695"
/transl_table=11
/translation="LFDSAAAITNPGHAWASAMERSGLLECVAGLDEQPFGEFTADKL
NPDRGSSRRVPRRQADGGIATHVERGGGQRQSGGQAGVVPQRMHGFPALAMQDRLIHH
GEQTQNRIAQAFRVRFCVCSPT"

CDS complement(769774..771687)
/codon_start=1
/note="gene_696"
/transl_table=11
/translation="MLSVGRGIADITGEAADCGMLGYGKSDQRTAGIHQRLRSRAFVF
RDDSQDGDARLLLIVAELPLPMQNVNEEVLRRLADLYGDTYSEQNTLITATHTHAGPG
GYCGYLLYNLTTSGFRPATFAAIVDGIVESVEHAHADVAPAEVPLSHGELYGASINRS
PSAFDRNPPADKAFFPKRVDPHTTLVRIDRGEATVGVIHFFATHGTSMTNRNHLISGD
NKGFAAYHWERTVGGADYLAGQPDFIAAFAQTNPGDMSPNVDGPLSPEAPPDREFDNT
RRTGLCQFEDAFTQLSGATPIGAGIDARFTYVDLGSVLVRGEYTPDGEERRTGRPMFG
AGAMAGTDEGPGFHGFRQGRNPFWDRLSRAMYRLARPTAAAQAPKGIVMPARLPNRIH

PFVQEIVPVQLVRIGRLYLIGIPGEPTIVAGLRLRRMVASIVGADLADVLCVGYTNAY
IHYVTTPEEYLEQRYEGGSTLFGRWELCALMQTVAELAEAMRDGRPVTLGRRPRPTRE
LSWVRGAPADAGSFGAVIAEPSATYRPGQAVEAVFVSALPNNDLRRGGTYLEVVRREG
ASWVRIADDGDWATSFRWQRQGRAGSHVSIRWDVPGDTTPGQYRIVHHGTARDRNGML
TAFSATTREFTVV"

CDS 771882..772640
/codon_start=1
/note="gene_697"
/transl_table=11
/translation="VLIGSHVSPTDPLAAAEAEGADVVQIFLGNPQSWKAPKPRDDAA
ALKAATLPIYVHAPYLINLASANNRVRIPSRKILQETCAAAADIGAAAVIVHGGHVAD
DNDIDKGFQRWRKALDRLETEVPVYLENTAGGDHAMARRFDTIARLWDVIGDTGIGFC
LDTCHTWAAGEALTDAVDRIKAITGRIDLVHCNDSRDEAGSGRDRHANLGSGQIDPDL
LVAAVKAAGAPVICETADQGRKDDIAFLRERTGS"

CDS 772672..773514
/codon_start=1
/note="gene_698"
/transl_table=11
/translation="MLRRVAILLAAVLAFAGCSGGTRLAAGFGNGNSVHTLDVDGAGR
SYRLYKPVGLPSSAPLVVMLHGGFGSAKQAERSYGWDELADSEKFLVAYPDGYHRAWN
ANGGGCCGRPAREGVDDIGFVRAVVADIANNVSIDPARVYVTGMSNGAIMSYTLACNT
SIFAAIGVVSGTQLDPCQSPRPVSVIHIHGTADPLVRYHGGPGAGFARIDGPPVPDLN
AFWREVNRCGALDTTTEGPVTTSGATCADNRRVVLLTVDDAGHRWPSFATQTLWRFFA
AHFR"

CDS 773574..775202
/codon_start=1
/note="gene_699"
/transl_table=11
/translation="MSDTHVVTNQVPPLENYNPASSPVLIEALIQEGGQWGLDEVNEV
GAISASCQAQRWGELADRNRPILHTHDAYGYRVDEVEYDPAYHELMRTAITHGMHAAP
WADDRPGAHVVRAAKTSVWTVEPGHICPISMTYAVVPALRYNSELAAVYEPLLTSREY
DPELKPATTKAGITAGMSMTEKQGGSDVRAGTTQATPNADGSYSLTGHKWFTSAPMCD
IFLVLAQAPDGLSCFLLPRVLPDGTRNRMFLQRLKDKLGNHANASSEVEYDGAVAWLV
GEEGRGVPTIIEMVNLTRLDCALGSATSMRTGLTRAVHHAQHRKAFGAYLIDQPLMRN
VLADLAVEAEAATIVAMRMAGATDNAVRGNETEALLRRIGLAAAKYWVCKRSTAHAAE
ALECLGGNGYVEDSGMPRLYREAPLMGIWEGSGNVSALDTLRAMATRPACVEVLFDEL
ARSAGQDPRLDGHVERLRPQLGDLDTIGYRARKIAEDICLALQGSLLVRHGHPAVAEA
FLATRLGGQWGGAYGTMPAGLDLAPILERALVKG"

CDS 775213..776151
/codon_start=1
/note="gene_700"
/transl_table=11
/translation="MTHAIRPVDFDNLKTMTYEVTGRIARITFNRPEKGNAIIADTPL
ELSALVERADLDPGVHVILVSGRGEGFCAGFDLSAYAEGSSSTGGGGAYQGTVLDGKT
QAVNHLPNQPWDPMIDYQMMSRFVRGFASLMHADKPTVVKIHGYCVAGGTDIALHADQ
VIAAADAKIGYPPTRVWGVPAAGLWAHRLGDQRAKRLLFTGDCITGAQAAEWGLAVEA
PEPADLDERTERLVARIAALPVNQLIMVKLALNSALLQQGVATSRMVSTVFDGAARHT
PEGHAFVADAVEHGFRDAVRRRDEPFGDYGRQASRV"

CDS 776154..776876
/codon_start=1
/note="gene_701"

```
                     /transl_table=11
                     /translation="MPAMTARSVVLSVLLGAHPAWATASELIQLTADFGIKETTLRVA
                     LTRMVGAGDLVRSADGYRLSDRLLARQRRQDEAMRPRTRAWHGNWHMLIVTSIGTDAR
                     TRAALRTCMHHKRFGELREGVWMRPDNLDLDLESDVAARVRMLTARDEAPADLAGQLW
                     DLSGWTEAGHRLLGDMAAATDMPGRFVVAAAMVRHLLTDPMLPAELLPADWPGAGLRA
                     AYHDFATAMAKRRDATQLLEVT"
     CDS             776873..777664
                     /codon_start=1
                     /note="gene_702"
                     /transl_table=11
                     /translation="MSDLVRVERKGRVTTVILNRPASRNAVNGPTAAALCAAFEQFDR
                     DDAASVAVLWGAGGTFCAGADLKAFGTPEANSVHRTGPGPMGPSRMMLSKPVIAAVSG
                     YAVAGGLELALWCDLRVAEEDAVFGVFCRRWGVPLIDGGTVRLPRLIGHSRAMDMILT
                     GRGVPADEALAMGLANRVVPKGQARQAAEELAAQLAALPQQCLRSDRLSALHQWGLPE
                     SAALDLEFASIARVAGEALEGARRFAAGAGRHGAPAPRAEQGDTL"
     CDS             complement(777676..780564)
                     /codon_start=1
                     /note="gene_703"
                     /transl_table=11
                     /translation="VQRTAAPTGSVPPDRHAARPFIPRMIRTFAVPIILGWLVTIAVL
                     NVTVPQLETVGQIQAVSMSPDAAPSMISMKHIGKVFEEGDSDSAAMIVLEGQRPLGDA
                     AHAFYDQMIGRLQADTTHVQSLQDFWGDPLTATGAQSSDGKAAYVQVKLAGNQGESLA
                     NESVEAVKTIVERLAPPPGVKVYVTGSAALVADQQQAGDRSLQVIEAVTFTVIIVMLL
                     LVYRSIITSAIMLTMVVLGLLATRGGVAFLGFHRIIGLSTFATNLLVVLAIAAATDYA
                     IFLIGRYQEARGLGQDRESAYYTMFGGTAHVVLGSGLTIAGATFCLSFTRLPYFQTLG
                     VPLAIGMVIVVAAALTLGPAIIAVTSRFGKLLEPKRMARVRGWRKVGAAIVRWPGPIL
                     VGAVALALVGLLTLPGYRTNYNDRNYLPADLPANEGYAAAERHFSQARMNPEVLMVES
                     DHDMRNSADFLVINKIAKAIFAVEGISRVQAITRPDGKPIEHTSIPFLISMQGTSQKL
                     TEKYNQDLTARMLEQVNDIQSNIDQMERMHSLTQQMADVTHEMVIQMTGMVVDVEELR
                     NHIADFDDFFRPIRSYFYWEKHCYDIPVCWSLRSVFDTLDGIDVMTEDINNLLPLMQR
                     LDTLMPQLTAMMPEMIQTMKSMKAQMLSMHSTQEGLQDQMAAMQEDSAAMGEAFDASR
                     NDDSFYLPPEVFDNPDFQRGLEQFLSPDGHAVRFIISHEGDPMSQAGIARIAKIKTAA
                     KEAIKGTPLEGSAIYLGGTAAMFKDLSDGNTYDLMIAGISALCLIFIIMLIITRSVVA
                     AAVIVGTVVLSLGASFGLSVLIWQHILGIELHWLVLAMAVIILLAVGADYNLLLVARL
                     KEEIHAGINTGIIRAMGGSGSVVTAAGLVFAFTMMSFAVSELTVMAQIGTTIGMGLLF
                     DTLIVRSFMTPSIAALLGKWFWWPQVVRQRPVPQPWPSPASARTFALV"
     CDS             complement(780567..780995)
                     /codon_start=1
                     /note="gene_704"
                     /transl_table=11
                     /translation="MIGTLKRAWIPLLILVVVAIAGFTVQRIRTFFGSEGILVTPKVF
                     ADDPEPFDPKVVEYEVSGSGSYVNINYLDLDAKPQRIDGAALPWSLTLKTTAPSAAPN
                     ILAQGDGTSITCRITVDGEVKDERTATGVDALTYCFVKSA"
     CDS             781080..781577
                     /codon_start=1
                     /note="gene_705"
                     /transl_table=11
                     /translation="VSVNDGVDQMGAEPDIMEFVEQMGGYFESRSLTRLAGRLLGWLL
                     VCDPERQSSEELATALAASSGGISTNARMLIQFGFIERLAVAGDRRTYFRLRPNAFAA
                     GERERIRAMAELQDLADVGLRALGDAPPQRSRRLREMRDLLAYMENVVSDALGRYSQR
                     TGEDD"
```

```
CDS             complement(781633..782130)
                /codon_start=1
                /note="gene_706"
                /transl_table=11
                /translation="VVEKPLRADRATHSRLATFALALAAAALPLAGCSSTANPPAATT
                TPATATTTTATSGPTAAPTVTTGESTTASIQIGDMLTYGSIGTTATLDCADGKSLNVA
                GSDNTLTVNGTCETVTVGGANNKIAFDRIDERLVVVGLDNTVTYKNGDPTIDNLGAGN
                RINKE"
CDS             complement(782132..782506)
                /codon_start=1
                /note="gene_707"
                /transl_table=11
                /translation="VKWNTVAASLAAGVITIAVALAAPPPAAHAKNGDTHVTGQGIER
                TLDCNESTLLVNGTQNIVTALGTCWAVTVMGSSNTVVADTIINDITVYGWDETVFFRN
                GDPFIWDRGRELGMVNRLQRVG"
CDS             782745..783401
                /codon_start=1
                /note="gene_708"
                /transl_table=11
                /translation="MAAQPQAPSAGGRPRAGKAVKSVARPAKLSRESIVEGALTFLDR
                EGWDSLTINALATQLGTKGPSLYNHVDSLEDLRRAVRIRVIDDIITMLNRVGAGRARD
                DAVLVMAGAYRSYAHHHPGRYSAFTRMPLGGDDPEYTAATRGAAAPVIAVLSSYGLDG
                EQAFYAALEFWSALHGFVLLEMTGVMDDIDTDAVFTDMVLRLAAGMERRTTHGGTAST
                "
CDS             783650..784024
                /codon_start=1
                /gene="rpsL"
                /note="gene_709"
                /transl_table=11
                /translation="MPTIQQLVRKGRRDKISKVKTAALKGSPQRRGVCTRVYTTTPKK
                PNSALRKVARVKLTSQVEVTAYIPGEGHNLQEHSMVLVRGGRVKDLPGVRYKIIRGSL
                DTQGVKNRKQARSRYGAKKEKG"
CDS             784024..784494
                /codon_start=1
                /note="gene_710"
                /transl_table=11
                /translation="MPRKGPAPKRPLVNDPVYGSQLVTQLVNKVLLKGKKSLAERIVY
                GALEQARDKTGTDPVITLKRALDNVKPALEVRSRRVGGATYQVPVEVRPDRSTTLALR
                WLVGYSRQRREKTMIERLANEILDASNGLGASVKRREDTHKMAEANRAFAHYRW"
CDS             784575..786680
                /codon_start=1
                /note="gene_711"
                /transl_table=11
                /translation="VAQKDVLTDLSRVRNFGIMAHIDAGKTTTTERILYYTGINYKIG
                EVHDGAATMDWMEQEQERGITITSAATTTFWKDNQLNIIDTPGHVDFTVEVERNLRVL
                DGAVAVFDGKEGVEPQSEQVWRQADKYDVPRICFVNKMDKIGADFYFSVRTMGERLGA
                NAVPIQLPVGAEADFEGVVDLVEMNAKVWRGETKLGETYDTVEIPADLAEQAEEYRTK
                LLEVVAESDEHLLEKYLGGEELTVDEIKGAIRKLTIASEIYPVLCGSAFKNKGVQPML
                DAVVDYLPSPLDVPPAIGHAPAKEDEEVVRKATTDEPFAALAFKIATHPFFGKLTYIR
                VYSGTVESGSQVINATKGKKERLGKLFQMHSNKENPVDRASAGHIYAVIGLKDTTTGD
                TLSDPNQQIVLESMTFPDPVIEVAIEPKTKSDQEKLSLSIQKLAEEDPTFKVHLDSET
```

GQTVIGGMGELHLDILVDRMRREFKVEANVGKPQVAYKETIKRLVQNVEYTHKKQTGG
SGQFAKVIINLEPFTGEEGATYEFESKVTGGRIPREYIPSVDAGAQDAMQYGVLAGYP
LVNLKVTLLDGAYHEVDSSEMAFKIAGSQVLKKAAALAQPVILEPIMAVEVTTPEDYM
GDVIGDLNSRRGQIQAMEERAGARVVRAHVPLSEMFGYVGDLRSKTQGRANYSMVFDS
YSEVPANVSKEIIAKATGE"

CDS 786911..788101
/codon_start=1
/note="gene_712"
/transl_table=11
/translation="VAKAKFQRTKPHVNIGTIGHVDHGKTTLTAAITKVLHDKFPDLN
ETKAFDQIDNAPEERQRGITINIAHVEYQTDKRHYAHVDAPGHADYIKNMITGAAQMD
GAILVVAATDGPMPQTREHVLLARQVGVPYILVALNKADAVDDEELLELVEMEVRELL
AAQEFDEDAPVVRVSALKALEGDAKWVASVEELMNAVDESIPDPVRETDKPFLMPVED
VFTITGRGTVVTGRVERGVINVNEEVEIVGIRPSTTKTTVTGVEMFRKLLDQGQAGDN
VGLLLRGVKREDVERGQVVTKPGTTTPHTEFEGQVYILSKDEGGRHTPFFNNYRPQFY
FRTTDVTGVVTLPEGTEMVMPGDNTNISVKLIQPVAMDEGLRFAIREGGRTVGAGRVT
KIIK"

CDS 788239..789036
/codon_start=1
/note="gene_713"
/transl_table=11
/translation="VLARYIKMQLLVLLCGGLVGPIFLVVYFTLGLGSLMSWMFYVGL
IITVADVLVALALTNYGAKTAAKTAALERSGVLALAQSTGLSETGTRINDQPLVKVHL
HISGPGITPFDTEDRVIASVTRLGNLTARKLVVLVNPATQQYLIDWERSALVNGLVPA
QFTVAEDNKTYDLSGQTGPLMEILQILKANNVPLNRMVDIRSNPALRQQVQAVVRRAA
ERQAPAAEPASQGSIAERLAELESLRASGAVNAAEYESKRAQIISEI"

CDS 789189..790016
/codon_start=1
/gene="fabG"
/note="gene_714"
/transl_table=11
/translation="VSARGGSLHGRVAFVTGAARAQGRSHAVRLAREGADIVALDICA
PVSGSVTYPPATSEDLGETVRAVEAEGRKVLAREVDIRDDAELRRLVADGVEQFGRLD
IVVANAGVLGWGRLWELTDEQWETVIGVNLTGTWRTLRATVPAMIDAGNGGSIVVVSS
SAGLKATPGNGHYAASKHALVALTNTLAIELGEFGIRVNSIHPYSVDTPMIEPEAMIQ
TFAKHPGYVHSFPPMPLQPKGFMTPDEISDVVVWLAGDGSGALSGNQIPVDKGALKY"

CDS 790030..791250
/codon_start=1
/note="gene_715"
/transl_table=11
/translation="MNAHVTSREGVNEFDDGIVIVGGGLAAARTAEQLRRAGYSGRLT
IVSDEVHLPYDRPPLSKEVLRSEVDDVALKPREFYDEKDIALRLGSAAVSLDTGEQTV
TLADGTVLGYDELVIATGLVPRRIPSLPDLDGIRVLRSFDESMALRKHASAARHAVVV
GAGFIGCEVAASLRGLGVDVVLVEPQPAPLASVLGEQIGQLVTRLHRDEGVDVRTGVT
VAEVRGKGHVDAVVLTDGTELPADLVVVGIGSTPATEWLEGSGVEVDNGVICDKAGRT
SAPNVWALGDVASWRDPMGHQARVEHWSNVADQARVVVPAMLGTDVPTGVVVPYFWSD
QYDVKIQCLGEPHATDVVHLVEDDGRKFLAYYERDGVLVGVVGGGMAGKVMKVRGKIA
AGAPIAEVLDQTQA"

CDS complement(791247..791501)
/codon_start=1
/note="gene_716"

```
                     /transl_table=11
                     /translation="VLGWTVKPGRVADGWQAPGVHLMARCSGPQPASERRADMDGGDI
                     DAAVARVRAAGALAEPSRQPDDMSAECADDQGARCHLGQL"
     CDS             complement(792114..793163)
                     /codon_start=1
                     /note="gene_717"
                     /transl_table=11
                     /translation="VTGTEHLVHTLRSQGRVCTSSGSPMYRELLELVAADVESGGVFA
                     SILADQKGAPEGQAVPLRLLGGLHRMVLDGRAPVLRRWYPSTGGTWQAEAAWPDIVRT
                     ATDQPESLRAALDRPPQTNEVGRSAALIGGLLIACLQFDLPIRLFEIGSSAGLNLRPD
                     RYRYRYLGGEWGLADSPVRIDNAWLGELPPTATVRIVERHGYDIAPIDVTSPDGELNA
                     LSYIWPDQTDRLERLRGAIAVARNIPADLHRQAAHAAVAGMTLTDDALTVLWHSITWQ
                     YLPADERAAIRAGIDALAAQADAHCPFVHLTLEPAHQRPGAQIKYLVRMRSWPGGHAR
                     VLGECHPHGPPVTWQ"
     CDS             complement(793160..793756)
                     /codon_start=1
                     /note="gene_718"
                     /transl_table=11
                     /translation="MPHESRVGRRRSTTPHHISDVAIELFAAHGFTDVSVDDIARAAG
                     IARRTLFRYYASKNAIPWGDFSTHLAQLQGLLDNIDSRIQLRDALRAALLAFNTFDES
                     ETIRHRKRMRVILQTPELQAYSMTMYAGWREVIAKFVARRSGGKTTDFMPQTVAWTML
                     GVALSAYEHWLRDESVSLTEALGAAFDVVGAGLDRLNQ"
     CDS             793921..794250
                     /codon_start=1
                     /note="gene_719"
                     /transl_table=11
                     /translation="VWGLLTVPAPAQARRADSSEFDPDRGWRLHPQVAVRPEPFGALL
                     YHFGTRKLSFLKNRTILAVVQTLADYPDIRSACRGAGVDDCDQDPYLHALSVLAGSNM
                     LVPRQTT"
     CDS             794247..795422
                     /codon_start=1
                     /note="gene_720"
                     /transl_table=11
                     /translation="MTSPVPRLIEQFERGLDAPICLTWELTYACNLACVHCLSSSGKR
                     DPGELSTRQCKDIIDELERMQVFYVNIGGGEPTVRPDFWELVDYATAHHVGVKFSTNG
                     VRITPEVATRLAATDYVDVQISLDGATAEVNDAIRGTGSFDMAVRALQNLAAAGFAGV
                     KISVVITRRNVAQLDEFATLASRYGATLRITRLRPSGRGTDVWADLHPTADQQVQLYD
                     WLVSKGERVLTGDSFFHLAPLGQSGALAGLNMCGAGRVVCLIDPVGDVYACPFAIHDH
                     FLAGNVLSDGGFQNVWKNSSLFRELREPQSAGACGSCGHYDSCRGGCMAAKFFTGLPL
                     DGPDPECVQGHSEPALARERHLPRPRADHSRGRRVSKPVPLTLSMRPPKRPCNESPV"
     CDS             795425..796615
                     /codon_start=1
                     /note="gene_721"
                     /transl_table=11
                     /translation="VAEAWFETVAIAQQRAKRRLPKSVYSSLIAASEKGITVADNVAA
                     FSELGFAPHVIGATDKRDLSTTVMGQEVSLPVIISPTGVQAVDPGGEVAVARAAAARG
                     TVMGLSSFASKPIEEVIAANPKTFFQVYWQGGRDALAERVERARQAGAVGLVVTTDWT
                     FSHGRDWGSPKIPEEMNLKTILRLSPEAITRPRWLWKFAKTLRPPDLRVPNQGRRGEP
                     GPPFFAAYGEWMATPPPTWEDIGWLRELWGGPFMLKGVMRVDDAKRAVDAGVSAISVS
                     NHGGNNLDGTPASIRALPAVSAAVGDQVEVLLDGGIRRGSDVVKAVALGARAVMIGRA
                     YLWGLAANGQAGVENVLDILRGGIDSALMGLGHASVHDLSPADILVPTGFIRDLGVPS
```

```
                     RRDV"
     CDS             796895..797560
                     /codon_start=1
                     /note="gene_722"
                     /transl_table=11
                     /translation="VIPLGSTEQHGPHLPLDTDTRIATAVARTVTARLHAEDLPIAQE
                     EWLMAPAIAYGASGEHQRFAGTISIGTEALTMLLVEYGRSAACWARRLVFVNGHGGNV
                     GALTRAVGLLRAEGRDAGWCPCTCPGGDPHAGHTETSVLLHLSPADVRTERWRAGNRA
                     PLPVLLPSMRRGGVAAVSETGVLGDPTTATAAEGRRIFAAMVDDCVRRVARWMPQPDG
                     MLT"
     CDS             797609..799021
                     /codon_start=1
                     /note="gene_723"
                     /transl_table=11
                     /translation="MTATRLPDGFAVQVDRRVRVLGDGSALLGGSPTRLLRLAPAARG
                     LLCDGRLKVRDEVSAELARILLDATVAHPRPPSGPSHRDVTVVIPVRNNASGLRRLVT
                     SLRGLRVIVVDDGSACPVESDDFVGAHCDIEVLHHPHSKGPAAARNTGLAACTTDFVA
                     FLDSDVTPRRGWLESLLGHFCDPTVALVAPRIVSLVEGENPVARYEALHSSLDLGQRE
                     APVLPHSTVSYVPSAAIVCRSSAIRDVGGFDETMHSGEDVDLCWRLIEAGARLRYEPI
                     ALVAHDHRTQLRDWIARKAFYGGSAAPLAVRHPDKTAPLVISGGALMAWILMSIGTGL
                     GRLASLVIAVLTGRRIARAMRCAETSFLDVLAVATRGLWAAALQLASAICRHYWPLAL
                     LAAILSRRCRRVVLIAAVVDGVVDWLRRREGADDDAEPIGPLTYLVLKRVDDLAYGAG
                     LWYGVVRERNIGALKPQIRT"
     CDS             799104..800462
                     /codon_start=1
                     /note="gene_724"
                     /transl_table=11
                     /translation="MDSSCVVTVLEAGPGLADPGLLAQTANGLQLPIGAGSPLVERYR
                     TRLTDRPVRHLPIVRGATVGGSGAINGGYFCRGLPSDFDRASIPGWAWSDVLEHFRAI
                     ETDLDFETPVHGRSGPIPVRRTHEMTGITESFMAAAEDAGFAWIADLNDVGPEMPSGV
                     GAVPLNIVNGVRTSSAVGYLMPALGRPNLTLLARTRAVRLRFSATTAVGVDAIGPGGP
                     VSLSADRIVLCAGAIQSAHLLMLSGVGEEEVLRSAGVKVLMALPVGMGCSDHPEWVMP
                     TNWAVAVDRPVLEVLLSTHDGIEIRPYTGGFVAMTGDGTAGHRDWPHIGVALMQPRAR
                     GRITLVSSDPQIPVRIEHRYDSEPADVAALRQGSALAHELCGAATRIGPAVWATSQHL
                     CGSAPMGTDDDPRAVVDPRCRVRGIENLWVIDGSVLPSITSRGPHATIVMLGHRAAEF
                     VQ"
     CDS             800947..801252
                     /codon_start=1
                     /note="gene_725"
                     /transl_table=11
                     /translation="VHVDRVRLTGTERELRAENQSPPIFRPQNTLGDGANGLPLAVCT
                     TTAHTCHTSHTHPSRWTPNPVPATKGVPAGLVQATFIIENLDPGNNDTPTPLHPNCD"
     CDS             802576..802881
                     /codon_start=1
                     /gene="rpsJ"
                     /note="gene_726"
                     /transl_table=11
                     /translation="VAGQKIRIRLKAYDHEAIDASARKIVETVVRTGASVVGPVPLPT
                     EKNVYCVIRSPHKYKDSREHFEMRTHKRLIDIIDPTPKTVDALMRIDLPASVDVNIQ"
     CDS             802898..803551
                     /codon_start=1
```

/gene="rplC"
/note="gene_727"
/transl_table=11
/translation="MARKGILGTKLGMTQVFDESNRVVPVTVVKAGPNVVTRIRTPER
DGYSAVQLAYGEISPRKVNKPLTGQYTAAGVNPRRYLAELRLDDSDAATEYQVGQELT
AEIFADGSYVDVTGTSKGKGFAGTMKRHGFRGQGASHGAQAVHRRPGSIGGCATPARV
FKGTRMAGRMGNDRVTVLNLLVHKVDAENGVLLIKGAVPGRTGGLVMVRSAIKRGEK"
CDS 803551..804222
/codon_start=1
/gene="rplD"
/note="gene_728"
/transl_table=11
/translation="MAAQEQKTLKIDVKTPAGKVDGAIELPAELFDVPANIALMHQVV
TAQRAAARQGTHSTKTRGEVSGGGRKPYRQKGTGRARQGSTRAPQFTGGGVVHGPKPR
DYSQRTPKKMIAAALRGALSDRARNGRIHAITELVEGQNPSTKSARAFLASLTERKQV
LVVIGRSDEAGAKSVRNLPGVHILAPDQLNTYDVLRADDVVFSVEALNAYIAANTTTS
EEVSA"
CDS 804222..804524
/codon_start=1
/gene="rplW"
/note="gene_729"
/transl_table=11
/translation="MATLADPRDIILAPVISEKSYGLLDDNVYTFLVRPDSNKTQIKI
AVEKIFAVKVASVNTANRQGKRKRTRTGYGKRKSTKRAIVTLAPGSRPIDLFGAPA"
CDS 804671..805513
/codon_start=1
/gene="rplB"
/note="gene_730"
/transl_table=11
/translation="MAIRKYKPTTPGRRGASVSDFAEITRSTPEKSLVRPLHGRGGRN
AHGRITTRHKGGGHKRAYRMIDFRRNDKDGVNAKVAHIEYDPNRTARIALLHYLDGEK
RYIIAPNGLSQGDVVESGANADIKPGNNLPLRNIPAGTLIHAVELRPGGGAKLARSAG
SSIQLLGKEASYASLRMPSGEIRRVDVRCRATVGEVGNAEQANINWGKAGRMRWKGKR
PSVRGVVMNPVDHPHGGGEGKTSGGRHPVSPWGKPEGRTRNANKSSNKFIVRRRRTGK
KHSR"
CDS 805554..805835
/codon_start=1
/gene="rpsS"
/note="gene_731"
/transl_table=11
/translation="MPRSLKKGPFVDEHLLKKVDVQNEKNTKQVIKTWSRRSTIIPDF
IGHTFAVHDGRKHVPVFVTESMVGHKLGEFAPTRTFKGHIKDDRKSKRR"
CDS 805832..806425
/codon_start=1
/gene="rplV"
/note="gene_732"
/transl_table=11
/translation="MTAATKATEYPSAVAKARFVRVSPRKARRVIDLVRGRSVSDALD
ILRWAPQAASGPVAKVIASAAANAQNNGGLDPATLVVATVYADQGPTAKRIRPRAQGR
AFRIRRRTSHITVVVESRPAKDQRSAKSSRARRTEASKAASKVGATAPAKKAAAKAPA
KKAPASSGVKKTPAKKAPAKKAPAKASETSAAKGGSD"

CDS 806425..807249
/codon_start=1
/gene="rpsC"
/note="gene_733"
/transl_table=11
/translation="VGQKINPHGFRLGITTDWKSRWYADKQYAEYVKEDVAIRRLLSS
GLERAGIADVEIERTRDRVRVDIHTARPGIVIGRRGTEADRIRADLEKLTGKQVQLNI
LEVKNPESQAQLVAQGVAEQLSNRVAFRRAMRKAIQSAMRQPNVKGIRVQCSGRLGGA
EMSRSEFYREGRVPLHTLRADIDYGLYEAKTTFGRIGVKVWIYKGDIVGGKRELAAAA
PAGADRPRRERPSGTRPRRSGASGTTATGTDAGRAAGGEEAAPDAAAPVEAQSTES"
CDS 807253..807669
/codon_start=1
/gene="rplP"
/note="gene_734"
/transl_table=11
/translation="MLIPRKVKHRKQHHPRQRGIASGGTTVNFGDYGIQALEHAYVTN
RQIESARIAINRHIKRGGKVWINIFPDRPLTKKPAETRMGSGKGSPEWWVANVKPGRV
LFELSYPNEGVARAALTRAIHKLPIKARIITREEQF"
CDS 807669..807902
/codon_start=1
/note="gene_735"
/transl_table=11
/translation="MAVGVSPGELRELTDEELAERLRESKEELFNLRFQMATGQLNNN
RRLRTVRQEIARIYTVLRERELGLATGPDGKES"
CDS 807902..808309
/codon_start=1
/gene="rpsQ"
/note="gene_736"
/transl_table=11
/translation="MAEAKTGAKAAPRVAKAAKAAPKKAAPNDAEAIGAANAANVKGP
KHTPRTPKPRGRRKTRIGYVVSDKMQKTIVVELEDRMRHPLYGKIIRTTKKVKAHDED
SVAGIGDRVSLMETRPLSATKRWRLVEILEKAK"
CDS 808478..810841
/codon_start=1
/note="gene_737"
/transl_table=11
/translation="MAPEATEAFNGTIELDIRDSEPDWGPYAAPVAPEHSPNILYLVW
DDVGIATWDCFGGLVEMPAMTRVAERGVRLSQFHTTALCSPTRASLLTGRNATTAGMA
TIEEFTDGFPNCNGRIPADTALLPEVLAEHGYNTYCVGKWHLTPLEESNMASTKRHWP
TSRGFERFYGFLGGETDQWYPDLVYDNHPVSPPGTPEGGYHLSKDIADKTIEFIRDAK
VIAPDKPWFSYVCPGAGHAPHHVFKEWADRYAGRFDMGYERYREIVLERQKALGIVPP
DTELSPINPYLDVPGPNGETWPLQDTVRPWDSLSDEEKKLFCRMAEVFAGFLSYTDAQ
IGRILDYLEESGQLDNTIIVVISDNGASGEGGPNGSVNEGKFFNGYIDTVAESMKLFD
HLGGPQTYNHYPIGWAMAFNTPYKLFKRYASHEGGIADPAIISWPNGIAAHGEIRDNY
VNVSDITPTVYDLLGMTPPGTVKGIPQKPMDGVSFIAALADPAADTGKTTQFYTMLGT
RGIWHEGWFANTIHAATPAGWSNFNADRWELFHIAADRSQCHDLAAEHPDKLEELKAL
WFSEAAKYNGLPLADLNLLETMTRSRPYLVSERASYVYYPDCADVGIGAAVEIRGRSF
AVLADVTIDTTGAEGVLFKHGGAHGGHVLFVRDGRLHYVYNFLGERQQLVSSSGPVPS
GRHLLGVRYLRTGTVPNSHTPVGDLELFFDENLVGALTNVLTHPGTFGLAGAAISVGR
NGGSAVSSHYEAPFAFTGGTITQVTVDVSGRPFEDVESDLALAFSRD"
CDS 810889..811788

/codon_start=1
/note="gene_738"
/transl_table=11
/translation="VLTELVDLPGGSFRMGSTRFYPEEAPIHTVTVRAFAVERHPVTN
AQFAEFVSATGYVTVAEQPLDPGLYPGVDAADLCPGAMVFCPTAGPVDLRDWRQWWDW
VPGACWRHPFGRDSDIADRAGHPVVQVAYPDAVAYARWAGRRLPTEAEWEYAARGGTT
ATYAWGDQEKPGGMLMANTWQGRFPYRNDGALGWVGTSPVGRFPANGFGLLDMIGNVW
EWTTTEFYPHHRIDPPSTACCAPVKLATAADPTISQTLKGGSHLCAPEYCHRYRPAAR
SPQSQDTATTHIGFRCVADPVSG"

CDS 812191..813030
/codon_start=1
/note="gene_739"
/transl_table=11
/translation="VLDVCIILGVLIAYVFSLLGYDWLAHTPGPLPQPDVGTTDDTVV
LIRFEELHTVANRLDVKVLVLPDDSMIDHRLQVLTTDTSVRLYPENELGDLQYPVGKL
PAQVATTIEAHGNPGAWPFDTYTTDTVQADVLVGAGDNRQYVPARVEVTGSLEGWDIS
AVRVGESSQTSDRPDNVIITLKRAKGPLVFDLGICLVLITLPTLALFVAIQMITGRRK
FQPPFGTWYAAMLFAVVPLRTILPGSPPAGAWIDRAVVIWVLIALAAAMVVYIVAWYR
ESD"

CDS 813516..813884
/codon_start=1
/gene="rplN"
/note="gene_740"
/transl_table=11
/translation="VIQQESRLKVADNTGAKEILCIRVLGGSSRRYAGIGDVIVATVK
DAIPGGNVKRGDVVKAVVVRTVKERRRPDGSYIKFDENAAVIIKPDNDPRGTRIFGPV
GRELREKRFMKIISLAPEVL"

CDS 813885..814202
/codon_start=1
/gene="rplX"
/note="gene_741"
/transl_table=11
/translation="MKVHKGDTVLVISGKDKGAKGKVLQAYPDRNRVLVEGVNRIKKH
TAISTTQRGARSGGIVTQEAPIHVSNVMVVDSDGKPTRIGYRVDEETGKRVRISKRNG
KDI"

CDS 814202..814765
/codon_start=1
/gene="rplE"
/note="gene_742"
/transl_table=11
/translation="MTTAQKVQPRLKERYRSEIRDALRKQFGYGNVMQIPTVTKVVVN
MGVGEAARDAKLINGAVNDLALITGQKPEVRRARKSIAQFKLREGMPVGVRVTLRGDR
MWEFLDRLTSIALPRIRDFRGLSPKQFDGVGSYTFGLAEQAVFHEVDVDKIDRVRGMD
INVVTSAATDDEGRALLRALGFPFKEN"

CDS 814770..814955
/codon_start=1
/gene="rpsN"
/note="gene_743"
/transl_table=11
/translation="MAKKALVNKAAGKPRFAVRAYTRCSKCGRPRAVYRKFGLCRICL
REMAHAGELPGVQKSSW"

```
CDS             815119..815517
                /codon_start=1
                /gene="rpsH"
                /note="gene_744"
                /transl_table=11
                /translation="MTMTDPIADFLTRLRNANSAYHDEVSLPHSKLKANIAQILKNEG
                YISDFRTEDARVGKSLVIQLKYGPSRERSIAGLRRVSKPGLRVYAKSTNLPRVLGGLG
                VAIISTSSGLLTDRQAARQGVGGEVLAYVW"
CDS             815541..816080
                /codon_start=1
                /gene="rplF"
                /note="gene_745"
                /transl_table=11
                /translation="MSRIGKQPIPVPAGVDVTIEGQSISVKGPKGTLGLTVAEPIKVA
                RNDDGAIVVTRPDDERRNRSLHGLSRTLVSNLVTGVTQGYTTKMEIFGVGYRVQLKGS
                NLEFALGYSHPVVIEAPEGITFAVQAPTKFTVSGIDKQKVGQIAANIRRLRRPDPYKG
                KGVRYEGEQIRRKVGKTGK"
CDS             816083..816451
                /codon_start=1
                /gene="rplR"
                /note="gene_746"
                /transl_table=11
                /translation="MAQSVSATRRISRLRRHTRLRKKLSGTAERPRLVVHRSARHIHV
                QLVNDLNGTTVAAASSIEADVRGVPGDKKARSVRVGQLIAERAKAAGIDTVVFDRGGY
                TYGGRIAALADAARENGLSF"
CDS             816471..817133
                /codon_start=1
                /note="gene_747"
                /transl_table=11
                /translation="MAEQPAGQAGTTDNRDARGDREGRRRDSGRGSRERDGEKSNYLE
                RVVAINRVSKVVKGGRRFSFTALVIVGDGNGMVGVGYGKAKEVPAAIAKGVEEARKSF
                FRVPLIGGTITHPVQGEAAAGVVLLRPASPGTGVIAGGAARAVLECAGVHDILAKSLG
                SDNAINVVHATVAALKLLQRPEEVAARRGLPIEDVAPAGMLKARRKSEALAASVLPDR
                TI"
CDS             817136..817333
                /codon_start=1
                /gene="rpmD"
                /note="gene_748"
                /transl_table=11
                /translation="MSQLKITQVRSTIGARWKQRESLRTLGLRRIRHSVIREDNAATR
                GLIAVVRHLVEVEPAQTGGKT"
CDS             817333..817773
                /codon_start=1
                /gene="rplO"
                /note="gene_749"
                /transl_table=11
                /translation="VTLKLHDLRPARGSKTARTRVGRGDGSKGKTAGRGTKGTRARKQ
                VPVTFEGGQMPIHMRLPKLKGFRNRFRTEYEIVNVGDINRLFPQGGAVGVDDLVAKGA
                VRKNALVKVLGDGKLTAKVDVSAHKFSGSARAKITAAGGSATEL"
CDS             817875..819677
                /codon_start=1
```

/note="gene_750"
/transl_table=11
/translation="MVAFLPSIPVVEDLRALVGRVDTARHHGVPNGCVLEFNLRSVPP
ETTGFDPLTVLTGGGRPMALRDAVAAIHRAAEDPRVAGLIARVQLPPSPAGAVQELRE
AIAAFSAVKPSLAWAETYPGTLSYYLASAFGEVWMQPSGSVGLVGFATNATFLRDALH
KAGIEAQFVARGEYKSAANLFTEDGFTDAHREAVTRMLDSLQDQVWQAVAKSRNIGVD
ALDELADRAPLLRDDAVTCGLIDRIGFRDQAYARMAELVGVEKGSPESSGSQTSPDEK
PPRMYLARYASSARPRLTPPVPSIPGRRSKPTIAVVTLEGPIVNGRGGPQFLPLGPSS
AGGDTIAAALREVAADDSVSAIVLRVDSPGGSVTASETIWREVARARDRGKPVVASMG
AVAASGGYYVSMGADAIVANPGTITGSIGVITGKLVVRDLKDRLGVGSDAVRTNANAD
AWSIDAPFTPDQQAHREAEADLFYSDFVERVAEGRKMTTDAVDVVARGRVWTGADALD
RGLVDELGGLRTAVRRAKVLAGLDEDTEVRIVSYPGSSLWDMVRPRPSSRPAAASLPD
AMGALLARSIVGIVEQVEQTLSGASVLWLGESRL"
CDS complement(819682..820587)
/codon_start=1
/note="gene_751"
/transl_table=11
/translation="MPRAHDDNWDLASSVGATATMVAAGRALATKDPRGLINDPFAEP
LVRAVGLDFFTKLIDGELDIATTGNLSPGRAQAMIDGIAVRTKYFDDYFRTATDGGVR
QVVILAAGLDARAYRLPWPAGTVVYEIDQPQVIDFKTTTLAGIGAKPTAIRRTVYIDL
RADWPAALQAAGLDSTAPTAWLAEGMLIYLPPDPRTGCSTTAPNSVLRAARSLPNLSR
ALWISTQAGYEKWRIRFASTAWTSTWRRWCIPANAATSSTTCAPRAGTLRAQCGPTYS
GAMVCPFPPHTTTIRSAKSSSSAVV"
CDS complement(820680..821783)
/codon_start=1
/note="gene_752"
/transl_table=11
/translation="MTYTGSIRCEGDTWDLASSVGATATMVAAARAMATRAANPLIND
QFAEPLVRAVGVDVLTRLASGELTASDIDDPERPNASMVRMAEHHAVRTKFFDEFFMD
ATRAGIRQVVILASGLDSRAYRLAWPAQTVVYEIDQPQVMEFKTRTLAELGATPTADR
RVVTADLRADWPTALGAAGFDPTQPTAWSAEGLLRYLPPEAQDRLLDNVTALSVPDSR
FATESIRNFKPHHEERMRERMTILANRWRAYGFDLDMNELVYFGDRNEPASYLSDNGW
LLTEIKSQDLLTANGFQPFEDEEVPLPDFFYVSARLQRKHRQYPAHRKPAPSWRHTAC
PVNELSKSAAYTMTRSDAHQASTTAPPPPGLTG"
CDS complement(821986..822642)
/codon_start=1
/note="gene_753"
/transl_table=11
/translation="MNFVDDPESAVLAAAKDMLRRGLVEGTAGNISARRSDGNVVITP
SSVDYAEMLLHDLVLVDAGGAVLHAKDGRSPSTELNLHLACYRAFDDIGSVIHSHPVW
ATMFAVAHEPIPACIDEFAIYCGGDVRCTEYAASGTPEVGRNAVRALEGRAAALIANH
GLVAVGPRPDQVLRVTALVERTAQIVWGARALGGPVPIPEDVCRNFTGVYGYLRANPL
"
CDS complement(822639..823583)
/codon_start=1
/note="gene_754"
/transl_table=11
/translation="LRGPGFAQLRRLADVVYDPWIDQRPLRIYSAEQLADRITAVAAD
VLVVESDSVGGPVFERGLRVVAATRGDPSNVDIPGATAAGIPVLHTPARNADAVAEMT
VALLLAVARHLIPADADVRSGNIFRDGTIPYQRFRGAEIAGLTAGLVGLGAVGRAVRW
RLSGLGLRVIAHDPYRDDAGHSLDELLAEADIVSMHAAVTDDTIGMIGAQQFAAMRDG

AVFLNTARSQLHDTDALVDALRGGKLAAAGLDHFTGEWLPTDHPLVSMPNVVLTPHIG
GATWNTEARQARMVADDLGALLSGNRPAHVVNPEVLGS"
CDS 823641..824996
/codon_start=1
/note="gene_755"
/transl_table=11
/translation="VAPVSRDDVTIGIDIGTTAVKAVAADDNGRVTARVRIGHQLAVP
APDRLEHDADEAWRRGPLAALDRLVGPDTRALAVAAMVPSLTAVDPAGRPITPGLLYG
DARGRVPNASVARAQSVPSVGETAEFLRWTAGQAPDASGYWPAPAVANYALSGEAVID
YATAVTTLPLFDGTGWNATACADCGVTVDRMPRVETFGVGVGQVRGTGAVLAVGAVDA
LCEQIVAGADRDGDVLVLCGATLIVWTTISAARQVPGLWTIPHTAPGKSQIGGASNAG
GLFLNWVDRVIGPGDPALADPRRVPVWLPYIRGERTPFHEPDRRAVLDGVDLSQDAAS
VRRAAYEASGFVVRQLIELSGAPVARIVAAGGGTRIQPWMQAIADATGRPVEVSRVAE
GAALGAAFLGRLAAGLESSIADAARWASTDRIVEPSADWAGPTKERYRRSLALSGSKL
A"
CDS 825030..825737
/codon_start=1
/note="gene_756"
/transl_table=11
/translation="VSFYAYAMTDHDQTAARREIADALLAALERRHEVADAIVEAANK
AAAVEAIVNLLGTSHLAAEAVMSMSFDQLTQDARTKIIAELDDLNKQLSFTVKERPAS
SGEGLELRPFSPDEDRDIFARRTEEMGAAGDGSGGPAGSVDDEIRAAQKRVDDEEAAW
FVAVDSGVKVGMVFGELVHGEVDVRIWIHPDHRKKGYGTAALRKSRSEMAWAFPAVPM
VARAPAAQPAQPGSAGR"
CDS complement(825826..826782)
/codon_start=1
/note="gene_757"
/transl_table=11
/translation="MTQTGSARFEGDSWDLASSVGLTATMVAAARAVAGRAPGALVND
QFAEPLVRAVGVDFFVRMASGELDPDELAEDEANGLRRFADAMAIRTHYFDNFFLDAT
RAGIRQAVILASGLDSRAYRLRWPAGTIVFEVDQPQVIDFKTTTLAGLGAAPTTDRRT
VAVDLRDDWPTALQKAGFDNAQRTAWIAEGLLGYLSAEAQDRLLDQITAQSVPGSQFA
TEVLRDINRLNEEELRGRMRRLAERFRRHGLDLDMSGLVYFGDRTDARTYLADHGWRT
ASASTTDLLAEHGLPPIDGDDAPFGEVIYVSAELKQKHQDTR"
CDS 826943..828268
/codon_start=1
/gene="secY"
/note="gene_758"
/transl_table=11
/translation="VLSAFISSLRTVDLRRKILFTLGIVILYRVGAALPSPGVNFPNV
QQCIKEASAGEAGQIYSLINLFSGGALLKLTVFAVGVMPYITASIIVQLLTVVIPRFE
ELRKEGQAGQSKMTQYTRYLAIALAILQATSIVALAANGGLLQGCSLDIIADQSIFTL
VVIVLVMTGGAALVMWMGELITERGIGNGMSLLIFVGIAARIPAEGQSILESRGGVVF
TAVCAAALIIIVGVVFVEQGQRRIPVQYAKRMVGRRMYGGTSTYLPLKVNQAGVIPVI
FASSLIYIPHLITQLIRSGSGVVGNSWWDKFVGTYLSDPSNLVYIGIYFGLIIFFTYF
YVSITFNPDERADEMKKFGGFIPGIRPGRPTADYLRYVLSRITLPGSIYLGVIAVLPN
LFLQIGAGGTVQNLPFGGTAVLIMIGVGLDTVKQIESQLMQRNYEGFLK"
CDS 828265..828810
/codon_start=1
/gene="adk"
/note="gene_759"

/transl_table=11
/translation="VRVLLLGPPGAGKGTQAVKLAEKLGIPQISTGELFRRNIEEGTK
LGVEAKRYLDAGDLVPSDLTNELVDDRLNNPDAANGFILDGYPRSVEQAKALHEMLER
RGTDIDAVLEFRVSEEVLLERLKGRGRADDTDDVILNRMKVYRDETAPLLEYYRDQLK
TVDAVGTMDEVFARALRALGK"
CDS 828813..829613
/codon_start=1
/note="gene_760"
/transl_table=11
/translation="MRPLARLRGRRVVPQRSAGELDAMAAAGAVVAAALRAIRAAAAP
GTSSLSLDEIAESVIRESGATPSFLGYHGYPASICASINDRVVHGIPSTAEVLAPGDL
VSIDCGAVLDGWHGDAAITFGVGALSDADEALSEATRESLQAGIAAMVVGNRLTDVAH
AIETGTRAAELRYGRSFGIVAGYGGHGIGRQMHMDPFLPNEGAPGRGPLLAAGSVLAI
EPMLTLGTTKTVVLDDKWTVTTADGSRAAHWEHTVAVTDDGPRILTLG"
CDS 829686..830219
/codon_start=1
/note="gene_761"
/transl_table=11
/translation="VARVSGAAAAEAALMRALYDEHAAVLWRYALRLTGDAAQAEDVV
QETLLRAWQHPEVIGDTARPARAWLFTVARNMIIDERRSARFRNVVGSTDQSGTPEQS
TPDEVNAALDRLLIADALAQLSAEHRAVIQRSYYRGWSTAQIATDLGIAEGTVKSRLH
YAVRALRLTLQELGVTR"
CDS 830283..831035
/codon_start=1
/note="gene_762"
/transl_table=11
/translation="MTMPLRGLGPPDDTGVREVSTGDDHHYAMWDAAYVLGALSAADR
REFEAHLAGCPECRGAVTELCGVPALLSQLDRDEVAAISESAPTVVASGLSPELLPSL
LAAVHRRRRRTRLITWVASSAAAAVLAIGVLVGVQGHSAAPQRAAVSALPMAQVGTQL
LASTVSISGEPWGTFINLRCVCLAPPYASHDTLAMVVVGRDGSQTRLATWLAEPGHTA
TPAGSISTPVDQIAAVQVVAADTGQVLLQRSL"
CDS 831350..831847
/codon_start=1
/note="gene_763"
/transl_table=11
/translation="VASDNRDPIAAARANWERSGWGDVSLGMVAVTSVMRAHQILLAR
VETALRPYDLSFSRFELLRLLAFSRIGALPITKASDRLQVHVTSVTHAIRRLEADGLV
RRVPHPTDGRTTLVQITELGRSTVEDATVTLNEQVFANVGMGAEESQALVSAVETLRR
NAGDF"
CDS 832205..832753
/codon_start=1
/note="gene_764"
/transl_table=11
/translation="VDPLMAHQRAQDAFAALLANVRADQLGGPTPCSEWTINDLIEHV
VGGNEQVGRWAASPIEPPARPDGLVAAHQAAAAVAHEIFAAPGGMSATFKLPLGEVPG
QVFIGLRTTDVLTHAWDLAAATGQSTDLDPELAVERLAAARALVGPQFRGPGKPFADE
KPCPRERPPADQLAAFLGRTVR"
CDS 832943..833806
/codon_start=1
/note="gene_765"
/transl_table=11

/translation="VLLAVMSDYSLGVPDETGLGADAARAREVALTQHIGVSAETDRA
VVPKLRQAYDSLVCGRRRLGAIGAEIENAVAHQRALGLDTPAGARNFSRFLATKAHDI
TRVLAATAAESQAGAARLRSLASSYQAVGFGPKPQEPPPDPVPFPPYQPKVWAACRAR
GQDPDKVVRTFHHAPMSARFRSLPAGDSVLYCGNDKYGLLHIQAKHGRQWHDIADARW
PSAGNWRYLADYAIGATLAYPERVEYNQDNDTFAVYRRVSLPDGRYVFTTRVIISARD
GKIITAFPQTT"

CDS 833840..834448
/codon_start=1
/note="gene_766"
/transl_table=11
/translation="VTGPPRSYTGRRDLIAEKLEPYFQISAMLPKNTRPTSETAEEFW
DNSLWCSWGDRETGYTRTVTVSICQVADGEREAEGVRDMMRLECPAGLDLRTPNPEAY
EITGQRPGEFVFVLGYLGHVRAIVGNCYIEIMPMGTRVELSKLADVALDIGRSVGCSA
YENDFTLPDIPTQWRNQPLGWYTQGLAPYLPGLSDPKDAAEG"

CDS 834679..834993
/codon_start=1
/note="gene_767"
/transl_table=11
/translation="VFSVKGEEGKQALDRWISWARRCRIPVFVELAGGIVRHRQAIDA
ALDHGLWQGLIESTNTKIRLLTRIAFGFRSPEALIALAMLALGGRRPALPGRTKHPRI
SQ"

CDS 835135..835653
/codon_start=1
/note="gene_768"
/transl_table=11
/translation="VIAAPEAIAAAATDLASIGSTIGAANAAAAANTTAVLAAGADQV
SVAIAAAFGAHGQAYQALSAQAATFHIQFVQALTAGAGSYAAAEAASAASITSPLLDA
INAPFLAALGRPLIGNGADGAPGTGAAGGAGGLLFGNGGAGGSGAPGGAGGLLFGNGG
AGGPGASGGALG"

CDS complement(836031..836588)
/codon_start=1
/note="gene_769"
/transl_table=11
/translation="MTRQQLAHLLRRACAVVGDVDVLVLGSQSILGSFDENELPPQAT
ASQEADIAFVNDPARDKADHVDVAIGEMSDFHRSNGVYAEGVHIDTAILPNGWRDRLV
SWTVESSRPAKPRFLEPHDLAVAKLAAGREKDKAFVAALIRSGLLDVGVIQARVLLLP
EETDPRIGQRIAAWLNYYGAGNHSS"

CDS complement(836585..837124)
/codon_start=1
/note="gene_770"
/transl_table=11
/translation="VFFGFTSQHWDMETLLKTSEAAQILGVSRQHVVNMCDRGEMVCV
HVGSHRRVPSSEVERVTSRRLTREEERSLWLHRALLSPLLTEPDTVVSAARENLRRWS
GMHRRDGMAGWYFTKWQRVLNDGLDAVMHVLTSPSEDAREMRQNSPFAGILPEATRVA
VLRSFKDHWDREHERAMTE"

CDS complement(837204..837350)
/codon_start=1
/note="gene_771"
/transl_table=11
/translation="MLGLAMEAVASTGAAAPLGSHAAQIYAKFAADHADLDFSAVIET
LRGS"

```
CDS             837846..840239
                /codon_start=1
                /note="gene_772"
                /transl_table=11
                /translation="MSFVLAMPEVLGSAATDLAALGSVLGAADAAAAATTTGIVAAVQ
                DEVSAAIAALFSAHGRAYQVASAQAAAVHAQFVEALSAGAGAYASAEAAGAAVLANPA
                QSVQQDLLAAVNAQSVALTGRPLIGNGANGAPGTGANGAPGGWLLGNGGAGGSAAAGS
                GLPGGAGGAAGLFGTGGAGGAGGSSTVGDGGAGGAGGSGGWLLGTGGVGGVGGLGAGA
                GGAGGVGGAGGLLGAGGHGGAGGLGAVTGGVGGAGGAGGLLAGLLAGPGGAGGTGGRG
                FLNDGGVGGAGGNAGLLFGAGGTGGSGGAGLGGDGGAGGAGGNAGVLFGNAGSGGTGG
                FGDTDGGAGGAGGDAGWLGSGGVGGAGGFGETGDGGVGGAGGKAGLLIGNGGAGGAGV
                LIGNGGNAGIGGTGPTAGDTGAGGISGLLLGADGFNAPASASPLHTLKQQALAAINAP
                TQTLTGRPLIGNGTPGAVGSGATGAPGGWLLGDGGAGGSGAAGSGAPGGAGGAAGLWG
                TGGAGGAGGWLLGDGGAGGIGGASTVLGGTGGGGGVGGLWGAGGAGGAGGTGLVGGDG
                GAGGAGGTGGLLAGLIGAGGGHGGTGGLSTNGDGGVGGAGGNAGMLAGPGGAGGAGGD
                GENLDTGGDGGAGGSAGLLFGSGGAGGAGGFGFLGGDGGAGGNAGLLLSSGGAGGFGG
                FGTAGGVGGAGGNAGWLGFGGAGGIGGIGGNANGGAGGNGGTGGQLWGSGGAGGEGGA
                ALSVGDTGGAGGVGGSAGLIGTGGNGGNGGTGANAGSPGTGGAGGLLLGQNGLNGLP"
CDS             840638..843367
                /codon_start=1
                /note="gene_773"
                /transl_table=11
                /translation="MSWVMVSPELVVAAAADLAGIGSAISSANAAAAVNTTGLLTAGA
                DEVSTAIAALFGAQGQAYQAASAQAAAFYAQFVQALSAGGGAYAAAEAAAVSPLLAPI
                NAQFVAATGRPLIGNGANGAPGTGANGGPGGWLIGNGGAGGSGAPGAGAGGNGGAGGL
                FGSGGAGGAGGNAFGAGEVGGAGGAGGNAMLFGAGGAGGAGGAGGNAGMLFGAAGVGG
                VGGFSNGGATGGAGGAGGAGGLFTTGGVGGAGGAGGDGGDGGAGGLFGAGGTGGAGGF
                GTAVLAGTGGAGGPGGAGGLFGAGGEGGSGGSGNLTGGAGGAGGNAGTLATGDGGAGG
                TGGASRSGGFGGAGGAGGDAGMFFGSGGSGGAGGAGGSGGFGLPSGGKGGAGGDAGML
                FGSGGSGGAGGISRSVGDGAAGGAGGAPGLIGNGGNGGNGGASTGGGDGGPGGAGGTG
                VLIGNGGSGGTGATLGKAGIGGTGGVLLGLDGFTAPASTSPLHTLQQDVINMVNDPFQ
                TLTGRPLIGNGANGTPGTGADGGAGGWLFGNGGNGGQGTIGGVNGGAGGAGGAGGILF
                GTGGTGGSGGPGATGLGGIGGAGGAALLFGSGGAGGSGGAGAVGGNGGAGGNAGALLG
                AAGAGGAGGAGAVGGNGGAGGNGGLFANGGAGGPGGFGSPAGAGGIGGAGGNGGLFGA
                GGTGGAGGGSTLAGGAGGAGGNGGLFGAGGTGGAGSHSTAAGVSGGAGGAGGDAGLLS
                LGASGGAGGSGGSSLTAAGVVGGIGGAGGLLFGSGGAGGSGGFSNSGNGGAGGAGGDA
                GLLVGSGGAGGAGASATGAATGGDGGAGGKSGAFGLGGDGGAGGATGLSGAFHIGGKG
                GVGGSAVLIGNGGNGGNGGNSGNAGKSGGAPGPSGAGGAGGLLLGENGLNGLM"
variation        842676..842723
                /note="supplemented from AL2122/97"
CDS             843458..843715
                /codon_start=1
                /note="gene_774"
                /transl_table=11
                /translation="MRTTVSISDEILAAAKRRARERGQSLGAVIEDALRREFAAAHVG
                GARPTVPVFDGGTGPRRGIDLTSNRALSEVLDEGLELNSRK"
CDS             843739..844167
                /codon_start=1
                /note="gene_775"
                /transl_table=11
                /translation="MFLLDANVLLAAHRGDHPNHRTVRPWFDRLLAADDPFTVPNLVW
```

ASFLRLATNRRIFEIPSPRAEAFAFVEAVTAQPHHLPTNPGPRHLVLLRKLCDEADAS
GDLIPDAVLAAIAVGHHCAVVSLDRDFARFASVRHIRPPL"
CDS complement(844248..844385)
/codon_start=1
/note="gene_776"
/transl_table=11
/translation="VVRKHAFHWRYDSTEELELLNQLWQLVSLRLNFFTPTKKALGFR
P"
CDS 844520..844789
/codon_start=1
/note="gene_777"
/transl_table=11
/translation="VGLNYLGQVRAIVGDCVIHIMPMGTGVELSKLADLALDIGRSVG
CSAYENDFTLPDIPTQWRNQPLGWYTQGLAPYLPGLSDPKDAAEG"
CDS complement(844858..845742)
/codon_start=1
/note="gene_778"
/transl_table=11
/translation="MTTIAFLGLGNMGAPMSANLVGAGHVVRGFDPAPTAASGAAAHG
VAVFRSAPEAVAEADVVITMLPTGEVVRRCYTDVLAAARPATLFIDSSTISVTDAREV
HALAESHGMLQLDAPVSGGVKGAAAATLAFMVGGDESTLRRARPVLEPMAGKIIHCGA
AGAGQAAKVCNNMVLAVQQIAIAEAFVLAEKLGLSAQSLFDVITGATGNCWAVHTNCP
VPGPVPTSPANNDFKPGFSTALMNKDLGLAMDAVAATGATAPRGSHAADIYAKFAADH
ADLDFSAVIHTLRARADA"
CDS complement(845753..846925)
/codon_start=1
/note="gene_779"
/transl_table=11
/translation="MFVLNDDERVIVETAAAFAGKRLAPHALEWDAAKHFPVDVLREA
AELGMAAIYCRDDVGGSGLRRLDGARIFEQLAIADPVTAAFLSIHNMCAWMIDSFGTD
EQRKDWIPRLATMGVIASYCLTEPGAGSDAGALSTRAVRHGSGKGGDYVLDGVKQFIS
GAAASDVYVVMARTGAEGPRGVSAFVVEKGTPGLSFGAPEAKMGWHAQPTAQVVLDGV
RVPAEAMLGGADGEGAGFGIAMSGLNGGRLNIAACSLGGAQAAFDKAGAYVRDRQAFG
GSLLDEPTVRFTLADMATGLQTSRMLLWRAASALDDDDADKVELCAMAKRYVTDTCFE
VADQALQLHGGYGYLREYGLEKIVRDLRVHRILEGTNEIMRLVIGRAEAARFRATV"
CDS complement(846932..848464)
/codon_start=1
/note="gene_780"
/transl_table=11
/translation="MTTQISHFIDGQRTAGQSTRSADVFDPNTGQIQAKVPMAGKSDI
DAAVASAVEAQKGWAAWNPQRRARVLMRFIELVNDTIDELAELLSREHGKTLADARGD
VQRGIEVIEFCLGIPHLLKGEYTEGAGPGIDVYSLRQPLGVVAGITPFNFPAMIPLWK
AGPALACGNAFVLKPSERDPSVPVRLAELFIEAGLPAGVFQVVHGDKEAVDAILHHPD
IKAVGFVGSSDIAQYIYAGAAATGKRAQCFGGAKNHMIVMPDADLDQAVDALIGAGYG
SAGERCMAISVAVPVGDQTAERLRARLIERINNLRVGHSLDPKADYGPLVTGAALARV
RDYIGQGVAAGAELVVDGRDRASDDLTFGLPEGDANLEGGFFIGPTLFDHVAAHMSIY
TDEIFGPVLCMVRARDYEEALRLPSEHEYGNGVAIFTRDGDAARDFVSRVQVGMVGVN
VPIPVPVAYHTFGGWKRSGFGDLNQHGPAAIQFYTKVKTVTSRWPSGIKDGAEFVIPT
MS"
CDS 848670..850424
/codon_start=1

```
                     /note="gene_781"
                     /transl_table=11
                     /translation="MSFVIVARDALAAAAADLAQIGSAVNAGNLAAANPTTAVAAAAA
                     DEVSAALAALFGAHAREYQAAAAQAAAYHEQFVHRLSAAATSYAVTEVTIATSLRGAL
                     GSAPASVSDGFQAFVYGPIHATGQQWINSPVGEALAPIVNAPTNVLLGRDLIGNGVTG
                     TAAAPNGGPGGLLFGDGGAGYTGGNGGSAGLIGNGGTGGAGFAGGVGGMGGTGGWLMG
                     NGGMGGAGGVGGNGGAGGQALLFGNGGLGGAGGAGGVDGAIGRGGWFIGTGGMATIGG
                     GGNGQSIVIDFVRHGQTPGNAAMLIDTAVPGPGLTALGQQQAQAIANALAAKGPYAGI
                     FDSQLIRTQQTAAPLANLLGMAPQVLPGLNEIHAGIFEDLPQISPAGLLYLVGPIAWT
                     LGFPIVPMLAPGSTDVNGIVFNRAFTGAVQTIYDASLANPVVAADGNITSVAYSSAFT
                     IGVGTMMNVDNPHPLLLLTHPVPNTGAVVVQGNPEGGWTLVSWDGIPVGPASLPTALF
                     VDVRELITAPQYAAYDIWESLFTGDPAAVINAVRDGADEVGAAVVQFPHAVADDVIDA
                     TGHPYLSGLPIGLPSLIP"
     CDS             complement(850614..852551)
                     /codon_start=1
                     /note="gene_782"
                     /transl_table=11
                     /translation="MVGFAWLPPETNSLRMYLGAGSRPLLAAAGAWDGLAEELHAAAS
                     SFGSVTSELAGGAWQGPASAAMANAAGPYASWLTAAGAQAELAARQARAAAGAFEEAL
                     AGVVHPAVVQANRVRTWLLAVSNVFGQNAPAIAAMESTYEQMWAQDVAVMAGYHAASS
                     AAAAQLASWQPALPNINLGVGNIGNLNVGNGNTGDYNLGNGNLGNANFGGGNGSAFHG
                     QISSFNVGSGNIGNFNLGSGNGNVGIGPSSFNVGSGNIGNANVGGGNSGDNNFGFGNF
                     GNANIGIGNAGPNMSSPAVPTPGNGNVGIGNGGNGNFGGGNTGNANIGLGNVGDGNVG
                     FGNSGSYNFGFGNTGNNNIGIGLTGSNQIGFGGLNSGSGNIGFGNSGTGNIGFFNSGS
                     GNFGVGNSGVTNTGVANSGNINTGFGNSGFINTGFGNALSVNTGFGNSGQANTGIGNA
                     GDFNTGNFNGGIINTGSFNSGAFNSGSFNGGDANSGFLNSGLTNTGFANSGNINTGGF
                     NAGNLNTGFGNTTDGLGENSGFGNAGSGNSGFNNSGKGNSGAQNVGNLQISGFANSGQ
                     SVTGYNNSVSVTSGFGNKGTGLFSGFMSGFGNTGFLQSGFGNLEANPDNNSATSGFGN
                     SGKQDSGGFNSIDFVSGFFHR"
     CDS             complement(852853..853023)
                     /codon_start=1
                     /note="gene_783"
                     /transl_table=11
                     /translation="VAEQRYQAVLAVISDGLSISQVAEKVGVSRQTLHTWLARYEAEG
                     LDGLRIGTGTAL"
     tRNA            complement(853150..853224)
                     /note="identified by tRNAscan-SE; putative"
     CDS             complement(853252..853977)
                     /codon_start=1
                     /note="gene_784"
                     /transl_table=11
                     /translation="MNLGQTLVGIATWPARAGLAAADTGLNMAGAAVDMAKQALGDAG
                     GASGSTSMANMLGIDDTIARANRLARLLDDDMPLGRAIAPNGPMDRMLRPGGVVDLLT
                     QPGGLLDRLTAEGGAMQRALQPGGLADQLLAEDGLIERVLSEDGLADRLLAEGGLIDK
                     ITAKDGPLEQLADVADTLARLTPGMEALEPAIATLQDAVIALTMVVNPLSSIAERIPL
                     PGRRPARRSSSRSVRSQRVVDSE"
     CDS             complement(854146..855030)
                     /codon_start=1
                     /note="gene_785"
                     /transl_table=11
                     /translation="LRWGVESICTQLTELGVPIAPSTYYDHINREPSRRELRDGELKE
```

```
                     HISRVHAANYGVYGARKVWLTLNREGIEVARCTVERLMTKLGLSGTTRGKARRTTIAD
                     PATARPADLVQRRFGPPAPNRLWVADLTYVSTWAGFAYVAFVTDAYARRILGWRVAST
                     MATSMVLDAIEQAIWTRQQEGVLDLKDVIHHTDRGSQYTSIRFSERLAEAGIQPSVGA
                     VGSSYDNALAETINGLYKTELIKPGKPWRSIEDVELATARWVDWFNHRRLYQYCGDVP
                     PVELEAAYYAQRQRPAAG"
CDS                  complement(855081..855350)
                     /codon_start=1
                     /note="gene_786"
                     /transl_table=11
                     /translation="MVAEIRGQHDSEWAAISEVARLLGVGCAETVRKWVRQAQVDAGA
                     RPGTTTEESAELKRLRRDNAELRRANAILKTASAFFAAELDRPAR"
CDS                  855477..856220
                     /codon_start=1
                     /note="gene_787"
                     /transl_table=11
                     /translation="MRKGVDLVTAGTPGENTTPEARVLVVDDEANIVELLSVSLKFQG
                     FEVYTATNGAQALDRARETRPDAVILDVMMPGMDGFGVLRRLRADGIDAPALFLTARD
                     SLQDKIAGLTLGGDDYVTKPFSLEEVVARLRVILRRAGKGNKEPRNVRLTFADIELDE
                     ETHEVWKAGQPVSLSPTEFTLLRYFVINAGTVLSKPKILDHVWRYDFGGDVNVVESYV
                     SYLRRKIDTGEKRLLHTLRGVGYVLREPR"
CDS                  856265..857722
                     /codon_start=1
                     /note="gene_788"
                     /transl_table=11
                     /translation="MARHLRGRLPLRVRLVAATLILVATGLVASGIAVTSMLQHRLTS
                     RIDRVLLEEAQIWAQITLPLAPDPYPIHNPDRPPSRFYVRVISPDGQSYTALNDNTAI
                     PAVPANNDVGRHPTTLPSIGGSKTLWRAVSVRASDGYLTTVAIDLADVRSTVRSLVLL
                     QVGIGSAVLVVLGVAGYAVVRRSLRPLAEFEQTAAAIGAGQLDRRVPQWHPRTEVGRL
                     SLALNGMLAQIQRAVASAESSAEKARDSEDRMRQFITDASHELRTPLTTIRGFAELYR
                     QGAARDVGMLLSRIESEASRMGLLVDDLLLLARLDAHRPLELCRVDLLALASDAAHDA
                     RAMDPKRRITLEVLDGPGTPEVLGDESRLRQVLRNLVANAIQHTPESADVTVRVGTEG
                     DDAILEVADDGPGMSQEDALRVFERFYRADSSRARASGGTGLGLSIVDSLVAAHGGAV
                     TVTTALGEGCCFRVSLPRVSDVDQLSLTPVVPGPP"
CDS                  complement(857694..858095)
                     /codon_start=1
                     /note="gene_789"
                     /transl_table=11
                     /translation="VSIFTKIINRELPGRFVYEDDDVVAFLTIEPMTQGHTLVVPRAE
                     IDHWQNVDPALFGRVMSVSQLIGKAVCRAFSTQRAGMIIAGLEVPHLHIHVFPTRSLS
                     DFGFANVDRNPSPGSLDEAQAKIRAALAQLA"
CDS                  complement(858135..858554)
                     /codon_start=1
                     /note="gene_790"
                     /transl_table=11
                     /translation="MTQTTQSPALIASQSSWRCVQAHDREGWLALMADDVVIEDPIGK
                     SVTNPDGSGIKGKEAVGAFFDTHIAANRLTVTCEETFPSSSPDEIAHILVLHSEFDGG
                     FTSEVRGVFTYRVNKAGLITNMRGYWNLDMMTFGNQE"
CDS                  complement(858567..859694)
                     /codon_start=1
                     /note="gene_791"
                     /transl_table=11
```

/translation="VKTKGALIWEFNQPWSVEEIEIGDPRKDEVKIQMEAAGMCRSDH
HLVTGDIPMAGFPVLGGHEGAGIVTEVGPGVDDFAPGDHVVLAFIPSCGKCPSCQAGM
RNLCDLGAGLLAGESVTDGSFRIQARGQNVYPMTLLGTFSPYMVVHRSSVVKIDPSVP
FEVACLVGCGVTTGYGSAVRTADVRPGDDVAIVGLGGVGMAALQGAVSAGARYVFAVE
PVEWKRDQALKFGATHVYPDINAALMGIAEVTYGLMAQKVIITVGKLDGADVDSYLTI
TAKGGTCVLTAIGSLVDTQVTLNLAMLTLLQKNIQGTIFGGGNPHYDIPKLLSMYKAG
KLNLDDMVTTAYKLEQINDGYQDMLNGKNIRGVIRYTDDDR"
CDS complement(859793..860338)
/codon_start=1
/note="gene_792"
/transl_table=11
/translation="VAGYPRDELEDVVHRWLQANRTAERRGDWTLLADFYTDDATYGW
NVGPNEDVMCVGIDEIRDIALGQEMDGLQGWRYPYQRVVIDEKQGEVVGFWKQVATDA
NGAEQEVYGIGGSWFRYAGGGKWNWQRDFFDFGHVSALYLELIKAGKLSPGMQKRIER
AVSGNKVPGYYPLGKTPVPLW"
CDS complement(860341..860547)
/codon_start=1
/note="gene_793"
/transl_table=11
/translation="MGYRVEADRDLCQGHAMCELEAPEYFRVPKRGQVEILDPEPPEE
ARGVIKHAVWACPTQALSIRETGE"
CDS complement(860550..861905)
/codon_start=1
/note="gene_794"
/transl_table=11
/translation="MSAVALPRVSGGHDEHGHLEEFRTDPIGLMQRVRDECGDVGTFQ
LAGKQVVLLSGSHANEFFFRAGDDDLDQAKAYPFMTPIFGEGVVFDASPERRKEMLHN
AALRGEQMKGHAATIEDQVRRMIADWGEAGEIDLLDFFAELTIYTSSACLIGKKFRDQ
LDGRFAKLYHELERGTDPLAYVDPYLPIESFRRRDEARNGLVALVADIMNGRIANPPT
DKSDRDMLDVLIAVKAETGTPRFSADEITGMFISMMFAGHHTSSGTASWTLIELMRHR
DAYAAVIDELDELYGDGRSVSFHALRQIPQLENVLKETLRLHPPLIILMRVAKGEFEV
QGHRIHEGDLVAASPAISNRIPEDFPDPHDFVPARYEQPRQEDLLNRWTWIPFGAGRH
RCVGAAFAIMQIKAIFSVLLREYEFEMAQPPESYRNDHSKMVVQLAQPACVRYRRRTG
V"
CDS complement(861905..862732)
/codon_start=1
/note="gene_795"
/transl_table=11
/translation="MPRFEPHPARRTTVVAGASSGIGAATATELAGRGFPVALGARRM
DKLAELVDKIRADGGEAVAFPLDVTDPESVKSFVAQTVEALGEVELLVSSAGDMLPGQ
LHEVSTEAFAEQVQIHLVGANRLATAVLPAMVARRRGDLIFVGSDVGLRQRPHMGAYG
AAKAGLAAMVTNLQMELEGTGVRASIVHPGPTLTGMGWQLSAEQVGPMLADWAKWGQA
RHNYFLRPSDLARAIAFVAETPRGCVVVNMEIQPEAPLRDAPAHRQKLVLGEEGMPG"
CDS complement(862732..862869)
/codon_start=1
/note="gene_796"
/transl_table=11
/translation="MEARVALGALLRRIRNYEVDDDNVVRVHSSNVRGFAHLPISVQA
R"
CDS complement(863936..864577)
/codon_start=1

/note="gene_797"
/transl_table=11
/translation="VSSDVLVTTPAQRQTEPHAEAVSRNRRQQATFRKVLAAAMATLR
EKSYADLTVRLVAARAKVAPATAYTYFSSKNHLIAEVYLDLVRQVPCVTDVNVPMPIR
VTSSLRHLALVVADEPEIGAACTAALLDGGADPAVRAVRDRIGAEIHRRITSAIGPGA
DPGTVFALEMAFFGALVQAGSGTFTYHEIADRLGYVVGLILAGANEPSTGGSE"
CDS 864779..866248
/codon_start=1
/note="gene_798"
/transl_table=11
/translation="MALWGDGISALLIDGKLSDGRAGTFPTVNPATEEVLGVAADADA
EDMGRAIEAARRAFDSTDWSRNTELRVRCVRQLRDAMQQHVEELRELTISEVGAPRML
TASAQLEGPVGDLSFAADTAESYPWKQDLGEASPLGIATRRTLAREAVGVVGAITPWN
FPHQINLAKLGPALAAGNTVVLKPAPDTPWCAAALGEIIVEHTDFPPGVVNIVTSSSH
ALGALLAKDPRVDMISFTGSTATGRAVMADAAATIKKVFLELGGKSAFVVLDDADLAA
ASAVSAFSACMHAGQGCAITTRLVVPRARYEEAVAIAAATMSSIRPGDPNDPGTVCGP
LISARQRDRVQGYLDLAVAEGGRFACGGARPADREVGFYIEPTVIAGLTNDARVAREE
IFGPVLTVIAHDGDDDAVRIANDSPYGLSGTVYGADPQRAARIASRLRVGTVNVNGGV
WYCADAPFGGYKQSGIGREMGLLGFEEYLEAKLIATAAN"
CDS 866279..867025
/codon_start=1
/note="gene_799"
/transl_table=11
/translation="MFDSKVAIVTGAAQGIGQAYAQALAREGASVVVADINADGAAAV
AKQIVADGGTVIHVPVDVSDEDSAKAMVDRAVGAFGGIDYLVNNAAIYGGMKLDLLLT
VPLDYYKKFMSVNHDGVLVCTRAVYKHMAKRGGGAIVNQSSTAAWLYSNFYGLAKVGV
NGLTQQLARELGGMKIRINAIAPGPIDTEATRTVTPAELVKNMVQTIPLSRMGTPEDL
VGMCLFLLSDSASWITGQIFNVDGGQIIRS"
CDS 867123..868010
/codon_start=1
/note="gene_800"
/transl_table=11
/translation="MTAHPETPRLGYIGLGNQGAPMAKRLLDWPGGLTVFDVRVEAMA
PFVEGGATAAASVSDVAEADIISITVFDDAQVSSVITADNGLATHAKPGTIVAIHSTI
ADTTAVDLAEKLKPQGIHIVDAPVSGGAAAAAKGELAVMVGADDEAFQRIKEPFSRWA
SLLIHAGEPGAGTRMKLARNMLTFVSYAAAAEAQRLAEACGLDLVALGKVVRHSDSFT
GGAGAIMFRNTTAPMEPADPLRPLLEHTRGLGEKDLSLALALGEVVSVDLPLAQLALQ
RLAAGLGVPHPDTEPAKET"
CDS 868010..868441
/codon_start=1
/note="gene_801"
/transl_table=11
/translation="MDELRRTGLDKMNEVYAWDMPDMPGEFFALTVDHLFGRIWTRPG
LSMRDRRMAVIAVLTAQGQSDLLEVQVNAVLHNDELTIDELRELAVFITHYVGFPLGS
RLNSAIERVAAKRKQAAENGSLPDTKANVAEVLAKESGKSS"
CDS 868453..869721
/codon_start=1
/note="gene_802"
/transl_table=11
/translation="VRVLVIGSGAREHALLLALGKDPQVSGLIVAPGNAGTARIAEQH
DVDITSAEAVVALAREVGADMVVIGPEVPLVLGVADAVRAAGIVCFGPGKDAARIEGS

KAFAKDVMAAAGVRTANSEIVDSPAHLDAALDRFGPPAGDPAWVVKDDRLAAGKGVVV
TADRDVARAHGAALLEAGHPVLLESYLDGPEVSLFCVVDRTVVVPLLPAQDFKRVGED
DTGLNTGGMGAYAPLPWLPDNIYREVVSRIVEPVAAELVRRGSSFCGLLYVGLAITAR
GPAVVEFNCRFGDPETQAVLALLESPLGQLLHAAATGKLADFGELRWRDGVAVTVVLA
AENYPGRPRVGDVVVGSEAEGVLHAGTTRRDDGAIVSSGGRVLSVVGTGADLSAARAH
AYEILSSIRLPGGHFRSDIGLRAAEGKISV"

CDS complement(869718..871301)
/codon_start=1
/note="gene_803"
/transl_table=11
/translation="VSTPFGWTFPYAWPRMPILATNVVCTSQPLAAQAGLRMLADGGN
AVDAAVATAITLTVVEPVSNGIGSDAFSIVWDGQKLHGLNASGRSPSAWTPEYFGGNA
VPVLGWNSVTVPGAVSAWVELHARFGRLPFETLFEPAISYGRNGFLVSPTVAAQWAAQ
VPLFASQPGFADAFMPGGRAPKPGELFTFPDHAATLEKIAATNGEEFYRGELAAKLEA
HSAANGGVMRADDLAAHRVDWVDTITGTYRGYTIHQIPPNGQGIVALIALGILEHFDM
SSWSVDSAESVHVQIEALKLAFADAQACVADIDYMPVHPKRLLDKEYLRQRATLIDPK
RAMPAATGIPRGGTVYLAAADAAGMMVSMIQSNYLGFGSGVVVPGTGISLHNRGSDFT
VVPRHPNRVGPRKRPYHTIIPGFVTRDGAPVMSFGVMGGMMQPQGHVQVLVRIADYGQ
NPQAACDGPRFRWVNGMRVSFENGFPDSTLDELRQRGHDLVAVADYSQFGSCQAIWRL
DDGYLAASDPRRDGQAAAC"

CDS complement(871307..872008)
/codon_start=1
/note="gene_804"
/transl_table=11
/translation="MSTGSFVSAARAGKMTNWAIARPPGQTQALRPVIALHGLGGSAS
AVMDGGVEQGLAQAVNAGLPPFAVVSVDGGSSYWHQRASGEDAGAMVLNELIPLLDTQ
RLDTSRVAFLGWSMGGYGALLLGSRLGPARTAAICAVSPALWLSAGAVAPGSFDGPDD
WSANSVFGLPALGSIPIRVDCGNSDPFYAATKQFVAQLPHPPAGGFSPGGHNGGFWSA
QLPAELTWFAPLLTG"

CDS 872325..872897
/codon_start=1
/note="gene_805"
/transl_table=11
/translation="LVSAAAELLGEGGFEAVRHRAVARRAGLPLASTTYYFSSLDDLI
ARAVEHIGMIEVAQLRARVSALSRRRRGPETTAVVLVDLLVGEMSSPGLAEQLISRYE
RHIACTRLPDLRESMRRSLRQRAEAVAEAIERSGRSAQIELVCTLICAVDGSVVSALV
EGRDPRAAALATVVDLIDVLAPVDQRPVPF"

CDS complement(872851..873630)
/codon_start=1
/note="gene_806"
/transl_table=11
/translation="MYFVGVDLAWAGRNPTGVAAVDADGCLVGVGAARDDASVLAALR
PYVVGDCLVAFDAPLVVANRTGQRPAEAALNRDFRQFEAGAYPANTEKPEFADVPRAA
RLARQLALDMDPLSSATRRAIEVYPHPATVALFRLPRALKYKAKPGRSVDLLKSELLR
LMDGVEGLAQAGVRMQVAGQPDWVSLRRQVTVAQRKSDLRAAEDPIDAVVCAYVALYA
QRRPADVTIYGDFTTGYIVTPSLPTDFRTAPDAGRRARARR"

CDS 873875..875293
/codon_start=1
/note="gene_807"
/transl_table=11
/translation="VSIPNVLATRYASAEMVAIWSPEAKVVSERRLWLAVLRAQAELG

VAVADSVLADYERVVDDVDLASISARERVLRHDVKARIEEFNALAGHEHVHKGMTSRD
LTENVEQLQIRRSLEVIFAHGVAAVARLAERAVSYRDLIMAGRSHNVAAQATTLGKRF
ASAAQEMMIALRRLRELIDRYPLRGIKGPMGTGQDMLDLLGGDRAALADLERRVADFL
GFATVFNSVGQVYPRSLDHDVVSALVQLGAGPSSLAHTIRLMAGHELATEGFAPGQVG
SSAMPHKMNTRSCERVNGLQVVLRGYASMVAELAGAQWNEGDVFCSVVRRVALPDSFF
AVDGQIETFLTVLDEFGAYPAVIGRELDRYLPFLATTKVLMAAVRAGMGRESAHRLIS
EHAVATALAMREHGAEPDLLDRLAADPRLPLGRDALEAALADKKAFAGAAGDQVDDVV
AMVDALVSRYPDAAKYTPGAIL"

CDS 875280..876542
/codon_start=1
/note="gene_808"
/transl_table=11
/translation="VQFFSVMTTAAGLSGIDLTDLDNFADGFPHHLFAIHRREAPVYW
HRPTEHTPDGEGFWSVATYAETLEVLRDPVTYSSVTGGQRRFGGTVLQDLPVAGQVLN
MMDDPRHTRIRRLVSSGLTPRMIRRVEDDLRRRARGLLDGVEPGAPFDFVVEIAAELP
MQMICILLGVPETDRHWLFEAVEPGFDFRGSRRATMPRLNVEDAGSRLYTYALELIAG
KRAEPADDMLSVVANATIDDPDAPALSDAELYLFFHLLFSAGAETTRNSIAGGLLALA
ENPDQLQTLRSDFELLPTAIEEIVRWTSPSPSKRRTASRAVSLGGQPIEAGQKVVVWE
GSANRGPSVFDRADEFDITRKPNPHLGFGQGVHYCLGANLARLELRVLFEELLSRFGS
VRVVEPAEWTRSNRHTGIRHLVVELRGG"

CDS complement(876539..877144)
/codon_start=1
/note="gene_809"
/transl_table=11
/translation="LPYATTPGRLLAQLISDITVAVWTTLWMLVGLAVHDAISIIGEA
GRQIEIGSHGIAGNLAAAGQDAQRIPVVGDALSNPITAASQAALDIAGAGHNLDTTAG
WLAVVLALAVAATPILAVAMPWLFLRLRFCRRKWTVTTLAATPAGRQLLALRALANRP
PGKLAAVSTDPVGAWRREDPATMRALAALELRAAGIPLRGD"

CDS 877210..878103
/codon_start=1
/note="gene_810"
/transl_table=11
/translation="MRPALSDYQHVASGKVREIYRVDDEHLLLVASDRISAYDYVLDS
TIPDKGRVLTAMSAFFFGLVDAPNHLAGPPDDPRIPDEVLGRALVVRRLEMLPVECVA
RGYLTGSGLLDYQATGKVCGIALPPGLVEASRFATPLFTPATKAALGDHDENISFDRV
VEMVGALRANQLRDRTLQTYVQAADHALTRGIIIADTKFEFGIDRHGNLLLADEIFTP
DSSRYWPADDYRAGVVQTSFDKQFVRSWLTGSESGWDRGSDRPPPPLPEHIVEATRAR
YINAYERISELKFDDWIGPGA"

CDS 878100..880259
/codon_start=1
/note="gene_811"
/transl_table=11
/translation="MMHRTALPSPPVAKRVQTRREHHGDVFVDPYEWLRDKDSPEVIA
YLEAENDYTERTTAHLEPLRQKIFHEIKARTKETDLSVPTRRGNWWYYARTFEGKQYG
VHCRCPVTDPDDWNPPEFDERTEIPGEQLLLDENVEADGHDFFALGAASVSLDDNLLA
YSVDVVGDERYTLRFKDLRTGEQYPDEIAGIGAGVTWAADNRTVYYTTVDAAWRPDTV
WRYRLGSGESSERVYHEADDRFWLAVGRTRSNAYLLIAAGSSITSEVRYAHAADPTAQ
FSVVLPRRDGVEYSVEHAVIAGQDRFLILHNDGAVNFTLVEAPVEDPARQRTLIAHRD
DVRLDAVDALAGHLVVSYRREALPRVQLWPIGPDGNYGEPEEISFDSELMSAGLGPNP
NWDSPKLRVGAGSFVTPVRIYDIDLVTGERTLLKEQPVLGGYRREDYVERRDWAYGDD
GTRIPVSIVHRADIEFPAPALIYGYGAYEICEDPRFSIARLSLLDRGMVFVVAHVRGG

GEMGRLWYENGKLLDKKNTFTDFIAVARHLVDTGLTSQQQLVALGGSAGGLLMGAVAN
MAPDLFAGILAQVPFVDPLTTILDPSLPLTVTEWDEWGNPLNDSDVYAYVKSYSPYEN
VTAQKYPAILAMTSLNDTRVYYVEPAKWVAALRHAKTDGNSVLLKTQMHAGHGGISGR
YERWKETAFQYGWLLATADSDRYGGGQGNDLDGAAPA"

CDS complement(880687..882324)
/codon_start=1
/note="gene_812"
/transl_table=11
/translation="VTGIDMLGNAMVEACPAEGDAPVPITPAGRPRSGQRSYPDRLDV
GLLRTAGVCVLASVMAHVDVTVVSVAQRTFVADFGSTQAVVAWTMTGYMLALATVIPT
AGWAADRFGTRRLFMGSVLAFTLGSLLCAVAPNILLLIIFRVVQGFGGGMLTPVSFAI
LAREAGPKRLGRVMAVVGIPMLLGPVGGPILGGWLIGAYGWRWIFLVNLPVGLSALVL
AAIVFPRDRPAASENFDYMGLLLLSPGLATFLFGVSSSPARGTMADRHVLIPAITGLA
LIAAFVAHSWYRTEHPLIDMRLFQNRAVAQANMTMTVLSLGLFGSFLLLPSYLQQVLH
QSPMQSGVHIIPQGLGAMLAMPIAGAMMDRRGPAKIVLVGIMLIAAGLGTFAFGVARQ
ADYLPILPTGLAIMGMGMGCSMMPLSGAAVQTLAPHQIARGSTLISVNQQVGGSIGTA
LMSVLLTYQFNHSEIIATAKKVALTPESGAGRGAAVDPSSLPRQTNFAAQLLHDLSHA
YAVVFVIATALVVSTLIPAAFLPKQQASHRRAPLLSA"

CDS 882507..883193
/codon_start=1
/note="gene_813"
/transl_table=11
/translation="VSVSGIGESTLADVDAFCAEMDARSVPVSLLVAPRMRDDYRLDR
DPRTVDWLTGRRAAGDALVLHGYDEAATKRRRGEFAMLRAHEANLRLMAADRVLEHLG
LRTRLFAAPGWLVSPGVRTALPANGFRLLADLHGITDLVRLTTVRARVLGIGEGFLAE
PWWCRMVVMSAERIARRGGVVRIAVAARHLRKSGPLQAMLDAVDLAMLQGCTPMVYRW
RADAAVLDAA"

CDS 883209..883784
/codon_start=1
/note="gene_814"
/transl_table=11
/translation="VALTCTDMSDAVAGSDAEGLTADAIVVGAGLAGLVAACELADRG
LRVLILDQENRANVGGQAFWSFGGLFLVNSPEQRRLGIRDSHELALQDWLGTAAFDRP
EDYWPEQWAHAYVDFAAGEKRSWLRARGLKIFRWWAGPSVVVTTRRGTATRCPVSTSP
GVLGRLWSTYSCVSCVIAPRCALRTATRSTN"

CDS 883667..884908
/codon_start=1
/note="gene_815"
/transl_table=11
/translation="VPRFHITWGTGPALVDIFVRQLRDRPTVRFAHRHQVDKLIVEGN
AVTGVRGTVLEPSDEPRGAPSSRKSVGKFEFRASAVIVASGGIGGNHELVRKNWPRRM
GRIPKQLLSGVPAHVDGRMIGIAQKAGAAVINPDRMWHYTEGITNYDPIWPRHGIRII
PGPSSLWLDAAGKRLPVPLFPGFDTLGTLEYITKSGHDYTWFVLNAKIIEKEFALSGQ
EQNPDLTGRRLGQLLRSRAHAGPPGPVQAFIDRGVDFVHANSLRELVAAMNELPDVVP
LDYETVAAAVTARDREVVNKYSKDGQITAIRAARRYRGDRFGRVVAPHRLTDPKAGPL
IAVKLHILTRKTLGGIETDLDARVLKADGTPLAGLYAAGEVAGFGGGGVHGYRALEGT
FLGGCIFSGRAAGRGAAEDIR"

CDS complement(884943..885581)
/codon_start=1
/note="gene_816"
/transl_table=11

```
                     /translation="MQLTHFGHSCLLAEFGQTRLLFDPGTFSHGFEGITGLSAILITH
                     QHPDHIDVTRLPTLLEDNPAAELYADPQTAAQLGEPWRAVHVGDELPLAELTVRAVGG
                     CHAVIHPEIPVIENISYLVGDSKHRARLMHPGDALFVPGEQVDVLATPAAAPWMKISE
                     AVDYLRAVAPARAVPIHQAIVAPDARGIYYGRLTEMTTTDFQVLPEESAVTF"
     CDS             complement(885584..885910)
                     /codon_start=1
                     /gene="MRGA327_04910"
                     /note="gene_817"
                     /transl_table=11
                     /translation="VTRPATAQSRLSCGNAGVAHTCSNPKRGVALVTATAAPAIAGQR
                     YCEFNGTAWVLIHAVAGIWSHIGGQVSGSAWALGAAMSVANKRPATSIVARINRIGNA
                     PIRAQS"
     CDS             885792..886286
                     /codon_start=1
                     /note="gene_818"
                     /transl_table=11
                     /translation="MAGAAVAVTRATPRFGFEQVCATPAFPHDSRDWAVAGRVTVVHP
                     DGQWQLQAQVLHWRGDTARGGQIAASVFGTAVAALRACQLGAPLQSPSVTDDEPTRMA
                     AVISGPVIMHTYLVAHVSSSTISELTLWSSGPPQVPWPTVADSAVLDALTAPLCEAYI
                     GSCP"
     CDS             886392..886631
                     /codon_start=1
                     /note="gene_819"
                     /transl_table=11
                     /translation="VARVVVHVMPKAEILDPQGQAIVGALGRLGHLGISDVRQGKRFE
                     LEVDDTVDDTTLAEIAESLLANTVIEDWTISRDPQ"
     CDS             886628..887302
                     /codon_start=1
                     /note="gene_820"
                     /transl_table=11
                     /translation="VTARIGVVTFPGTLDDVDAARAARQVGAEVVSLWHADADLKGVD
                     AVVVPGGFSYGDYLRAGAIARFAPVMDEVVAAADRGMPVLGICNGFQVLCEAGLLPGA
                     LTRNVGLHFICRDVWLRVASTSTAWTSRFEPDADLLVPLKSGEGRYVAPEKVLDELEG
                     EGRVVFRYHDNVNGSLRDIAGICSANGRVVGLMPHPEHAIEALTGPSDDGLGLFYSAL
                     DAVLTG"
     CDS             complement(887319..887792)
                     /codon_start=1
                     /note="gene_821"
                     /transl_table=11
                     /translation="MTESIGEPLSTNLIERYLRARGRRYFRGHHDAEFFFVANAHLRL
                     HVHLEISPAYRDVFTIRVSPAYFFPATDHTRLAEIVNAWNLQNHEVTAIVHGSSDPHR
                     IGVAAERSLIRDRIRFDDFATFVDNAVSAATELFGQLTAAGLPPTATPPLLRDAG"
     CDS             complement(887940..888668)
                     /codon_start=1
                     /note="gene_822"
                     /transl_table=11
                     /translation="MTLANNGTGMDHFLTPTEYLDAGHPLVRTTAATLIRDAVSDTER
                     VRRIYYYVRDVPYDVLASFRYLAQGHHRASDVIGHGVAFCMGKASSFVALCRAAGVPA
                     RIAFQTIDAPDKEFLSPQVRALWGGRTGRPFPWHSLGEAYLGRRWVKLDATIDAPTAA
                     RLGKPYRQEFDGATPIPTVEGTILRENGSYADYPSAVAQWYERIAQSVLKALQSTEVH
                     ALVAADEELWTGPPVELADATHRL"
```

```
CDS             complement(888665..889708)
                /codon_start=1
                /note="gene_823"
                /transl_table=11
                /translation="MNAKDDPHFGLMLAATVNGLAVGSYREMVVVSQTAEEYGFDSVW
                LCDHFLTISPGEYAKVAGIAADTGSATGTETGGAGQCAPSRSLPLLECWTALAALSRD
                TTKLRLGTSVLCNSYRHPSVLAKMAATLDVISQGRLDLGLGAGWFRRESQAYGIPFPP
                VGDRVSALAESLQVIKAVWTEPNPTYAGRFYTLDGATCDPPPVQRPHPPLWIGGEGDR
                VQRIAAKHAQGLNVRWWSPQQVTQRRGFLTQASEAAGRDPDTLRLSVTLLLAPTQSGE
                EEVRIREEFASIPEPGLIVGTPDRCVERIREYQDRGVGHFLFTIPHVVKSDYLHIIGS
                DIIPRVKTEVTIP"
CDS             complement(889705..890514)
                /codon_start=1
                /note="gene_824"
                /transl_table=11
                /translation="MTSVKLDLDAADLRISRGSVPASTQLAEALKAQIIQQRLPRGGR
                LPSERELIDRSGLSRVTVRAAVGMLQRQGWLVRRQGLGTFVADPVEQELSCGVRTITE
                VLLSCGVTPQVDVLSHQTGPAPQRISETLGLVEVLCIRRRIRTGDQPLALVTAYLPPG
                VGPAVEPLLSGSADTETTYAMWERRLGVRIAQATHEIHAAGASPDVADALGLAVGSPV
                LVVDRTSYTNDGKPLEVVVFHHRPERYQFSVTLPRTLPGSGAGIIEKRDFA"
CDS             890587..890892
                /codon_start=1
                /note="gene_825"
                /transl_table=11
                /translation="MTSPVAVIARFMPRPDARSALRALLDAMITPTRAEDGCRSYDLY
                ESADGGELVLFERYRSRIALDEHRGSPHYLNYRAQVGELLTRPVAVTVLAPLDEASA"
CDS             complement(891005..891706)
                /codon_start=1
                /note="gene_826"
                /transl_table=11
                /translation="VVLELDDGTELRVDEVLFATGRAPRTDDIGLETIGLTPGSWLDV
                DDTCRVRAVDDGWLYAAGDVNHRALLTHQGKYQARIAGTAIGARAAGRPLDTTSWGMH
                ATTADHHAVPQAFFTDPEAAAVGLTADQAAQAGHRIKAIDVEIGDVVMGAKLFADGYT
                GRARMVVDVDRGHLLGVTMVGPGAAELLHSATVAVAGQVPIDRLWHAVPCFPTISELW
                LRLLESYRDSFYLLV"
CDS             complement(891604..892503)
                /codon_start=1
                /note="gene_827"
                /transl_table=11
                /translation="MTAAQQDQAPMATPGCREGETYDVVVLGAGPVGQNVADRARAGG
                LRVAVVERELVGGECSYWACVPSKALLRPVIAISDARRVDGAREAVDGSINTAGVFGR
                RNRYVAHWDDTGQADWVSGIGATLIRGDGRLDGPRRVVVTKSSGESVALTARHAVVIC
                TGSRPALPDLPGITEARPWTNRQATDNSTVPDRLAIVGAGGVGVEMATAWQGLGASVT
                LLARGSGLLPRMEPFVGELIGRGLADAGVDVRVGVSVRALGRPNPLAQWSSSWTTVPS
                CGSTRYSSPPAEHREPTTSAWRQ"
CDS             892896..893591
                /codon_start=1
                /note="gene_828"
                /transl_table=11
                /translation="MVVVGTDAHKYSHTFVATDEVGRQLGEKTVKATTAGHATAIMWA
                REQFGLELIWGIEDCRNMSARLERDLLAAGQQVVRVPTKLMAQTRKSARSRGKSDPID
```

ALAVARAVLRETDLPLATHDETSRELKLLTDRRDVLVAQRTSAINRLRWLVHELDPER
APAARSLDAASTSRPCGPGWTPARIGRRTRARRADRHHPAHRRDQHPGPAHQRPSPPS
RPRTAGNPWLARS"
CDS 893624..893989
/codon_start=1
/note="gene_829"
/transl_table=11
/translation="VTRFKSEAAFACHAAVAPIPVWSGNTAGQMRLSRSGNRQLNAAL
HRIALTQIRMTDSRGQAYYQRLQDAGKTKRAALRCLKRRLARTVFQALRTVHQPSSEH
TQPAAACHRSYCSRSCLSG"
CDS complement(893979..894776)
/codon_start=1
/note="gene_830"
/transl_table=11
/translation="MNNLYRDLAPVTEAAWAEIELEAARTFKRHIAGRRVVDVSDPGG
PVTAAVSTGRLIDVKAPTNGVIAHLRASKPLVRLRVPFTLSRNEIDDVERGSKDSDWE
PVKEAAKKLAFVEDRTIFEGYSAASIEGIRSASSNPALTLPEDPREIPDVISQALSEL
RLAGVDGPYSVLLSADVYTKVSETSDHGYPIREHLNRLVDGDIIWAPAIDGAFVLTTR
GGDFDLQLGTDVAIGYASHDTDTVRLYLQETLTFLCYTAEASVALSH"
CDS complement(894773..895774)
/codon_start=1
/note="gene_831"
/transl_table=11
/translation="VPAVSPQPILAPLTPAAIFLVATIGADGEATVHDALSKISGLVR
AIGFRDPTKHLSVVVSIGSDAWDRLFAGPRPTELHPFVELTGPRHTAPATPGDLLFHI
RAETMDVCFELAGRILKSMGDAVTVVDEVHGFRFFDNRDLLGFVDGTENPSGPIAIKA
TTIGDEDRNFAGSCYVHVQKYVHDMASWESLSVTEQERVIGRTKLDDIELDDNAKPAN
SHVALNVITDDDGTERKIVRHNMPFGEVGKGEYGTYFIGYSRTPTVTEQMLRNMFLGD
PAGNTDRVLDFSTAVTGGLFFSPTIDFLDHPPPLPQAATPTLAAGSLSIGSLKGSPR"
CDS 895825..897126
/codon_start=1
/note="gene_832"
/transl_table=11
/translation="MAATAHGLCEFIDASPSPFHVCATVAGRLLGAGYRELREADRWP
DKPGRYFTVRAGSLVAWNAEQSGHTQVPFRIVGAHTDSPNLRVKQHPDRLVAGWHVVA
LQPYGGVWLHSWLDRDLGISGRLSVRDGTGVSHRLVRIDDPILRVPQLAIHLAEDRKS
LTLDPQRHINAVWGVGERVESFVGYVAQRAGVAAADVLAADLMTHDLTPSALIGASVN
GTASLLSAPRLDNQASCYAGMEALLAVDVDSASSGFVPVLAIFDHEEVGSASGHGAQS
DLLSSVLERIVLAAGGTREDFLRRLTTSMLASADMAHATHPNYPDRHEPSHPIEVNAG
PVLKVHPNLRYATDGRTAAAFALACQRAGVPMQRYEHRADLPCGSTIGPLAAARTGIP
TVDVGAAQLAMHSARELMGAHDVAAYSAALQAFLSAELSEA"
CDS 897138..897485
/codon_start=1
/note="gene_833"
/transl_table=11
/translation="MALKVEMVTFDCSDPAKLAGWWAEQFDGTTRELLPGEFVVVART
DGPRLGFQKVPDPAPGKNRVHLDFTTKDLDAEVLRLVAAGASEVGRHQVGESFRWVVL
ADPEGNAFCVAGQ"
CDS complement(897479..898135)
/codon_start=1
/note="gene_834"

/transl_table=11
/translation="MSRHWPLFDLRITTPRLQLQLPTEELCDQLIDTILEGVHDPDRM
PFSVPWTRASREDLPFNTLSHLWQQLAGFKRDDWSLPLAVLVDGRAVGVQALSSKDFP
ITRQVDSGSWLGLRYQGHGYGTEMRAAVLYFAFAELEAQVATSRSFVDNPASIAVSRR
NGYRDNGLDRVAREGAMAEALLFRLTRDDWQRHRTVEVRVDGFDRCRPLFGPLEPPRY
"

CDS 898291..900591
/codon_start=1
/note="gene_835"
/transl_table=11
/translation="MSPLARTPRKTSVLDTVEHAATTPDQPQPYGELGLKDDEYRRIR
QILGRRPTDTELAMYSVMWSEHCSYKSSKVHLRYFGETTSDEMRAAMLAGIGENAGVV
DIGDGWAVTFKVESHNHPSYVEPYQGAATGVGGIVRDIMAMGARPVAVMDQLRFGAAD
APDTRRVLDGVVRGIGGYGNSLGLPNIGGETVFDPCYAGNPLVNALCVGVLRQEDLHL
AFASGAGNKIILFGARTGLDGIGGVSVLASDTFDAEGSRKKLPSVQVGDPFMEKVLIE
CCLELYAGGLVIGIQDLGGAGLSCATSELASAGDGGMTIQLDSVPLRAKEMTPAEVLC
SESQERMCAVVSPKNVDAFLAVCRKWEVLATVIGEVTDGDRLQITWHGETVVDVPPRT
VAHEGPVYQRPVARPDTQDALNADRSAKLSRPVTGDELRATLLALLGSPHLCSRAFIT
EQYDRYVRGNTVLAEHADGGMLRIDESTGRGIAVSTDASGRYTLLDPYAGAQLALAEA
YRNVAVTGATPVAVTNCLNFGSPEDPGVMWQFTQAVRGLADGCADLGIPVTGGNVSFY
NQTGSAAILPTPVVGVLGVIDDVRRRIPTGLGAEPGETLMLLGDTRDEFDGSVWAQVT
ADHLGGLPPVVDLAREKLLAAVLSSASRDGLVSAAHDLSEGGLAQAIVESALAGETGC
RIVLPEGADPFVLLFSESAGRVLVAVPRTEESRFRGMCEARGLPAVRIGVVDQGSDAV
EVQGLFAVSLAELRATSEAVLPRYFG"

CDS 900588..901217
/codon_start=1
/note="gene_836"
/transl_table=11
/translation="MSRLRALSLAAGLVGWSLVSPRLPAPWRIPLQAGLGSVLVLVTR
ATMGLWPPRLWAGLRLGWAAGAAAATAIAATTPVPMVRLSMSARELPASVPVWLVWHI
PGGTVWAEEAAFRGALATIGARAFGRSGGRILQAGAFGLSHIADARATGEPLVLTVLA
TGIAGWMFGWLADRSGSLAAPLLTHLAINEAGAVAAVLVQRRSGISTRL"

CDS 901326..902294
/codon_start=1
/note="gene_837"
/transl_table=11
/translation="LAPQVHRLRAAEHPRPDYVLLHISDTHLIGGDRRLYGAVDADDR
LGELLEQLNQSGLRPDAIVFTGDLADKGEPAAYRKLRGLVEPFAAQLGAELVWVMGNH
DDRAELRKFLLDEAPSMAPLDRVCMIDGLRIIVLDTSVPGHHHGEIRASQLGWLAEEL
ATPAPDGTILALHHPPIPSVLDMAVTVELRDQAALGRVLRGTDVRAILAGHLHYSTNA
TFVGIPVSVASATCYTQDLTVAAGGTRGRDGAQGCNLVHVYPDTVVHSVIPLGGGETV
GTFVSPGQARRKIAESGIFIEPSRRDSLFKHPPMVLTSSAPRSPVD"

CDS complement(902239..903837)
/codon_start=1
/note="gene_838"
/transl_table=11
/translation="MPKISSRDGGRPAQRTVNPIIVTRRGKIARLESGLTPQEAQIED
LVFLRKVLNRADIPYLLIRNHKNRPVLAINIELRAGLERALAAACATEPMYAKTIDEP
GLSPVLVATDGLSQLVDPRVVRLYRRRIAPGGFRYGPAFGVELQFWVYEETVIRCPVE
NSLSRKVLPRNEITPTNVKLYGYKWPTLDGMFAPHASDVVFDIDMVFSWVDGSDPEFR
ARRMAQMSQYVVGEGDDAEARIRQIDELKYALRSVNMFAPWIRRIFIATDSTPPPWLA

EHPKITIVRAEDHFSDRSALPTYNSHAVESQLHHIPGLSEHFLYSNDDMFFGRPLKAS
MFFSPGGVTRFIEAKTRIGLGANNPARSGFENAARVNRQLLFDRFGQVITRHLEHTAV
PLRKSVLIEMEREFPEEFARTAASPFRSDTDISVTNSFYHYYALMTGRAVPQEKAKVL
YVDTTSYAGLRLLPKLRKHRGYDFFCLNDGSFPEVPAAQRAERVVSFLERYFPIPAPW
EKIAADVSRRDFAVPRTSAPSEGA"

CDS 904142..904531
/codon_start=1
/note="gene_839"
/transl_table=11
/translation="MSARDRVDPAKTRQVVLALADWLRDETLPAPDTDVLAAAVRLTA
RTLAALAPGASVEVRIPPFAAVQCISGPRHTRGTPPNVVQTDPRTWLLVATGLSGVAQ
ARGSGALQLSGSRAGEIEAWLPLVDLG"

CDS 904618..906201
/codon_start=1
/note="gene_840"
/transl_table=11
/translation="VAVDSDYVTDRAAGSRQTVTGQQPEQDLNSPREECGVFGVWAPG
EDVAKLTYYGLYALQHRGQEAAGIAVADGSQVLVFKDLGLVSQVFDEQTLAAMQGHVA
IGHCRYSTTGDTTWENAQPVFRNTAAGTGVALGHNGNLVNAAALAARARDAGLIATRC
PAPATTDSDILGALLAHGAADSTLEQAALDLLPTVRGAFCLTFMDENTLYACRDPYGV
RPLSLGRLDRGWVVASETAALDIVGASFVRDIEPGELLAIDADGVRSTRFANPTPKGC
VFEYVYLARPDSTIAGRSVHAARVEIGRRLARECPVEADLVIGVPESGTPAAVGYAQE
SGVPYGQGLMKNAYVGRTFIQPSQTIRQLGIRLKLNPLKEVIRGKRLIVVDDSIVRGN
TQRALVRMLREAGAVELHVRIASPPVKWPCFYGIDFPSPAELIANAVENEDEMLEAVR
HAIGADTLGYISLRGMVAASEQPTSRLCTACFDGKYPIELPRETALGKNVIEHMLANA
ARGAALGELAADDEVPVGR"

CDS 906232..907326
/codon_start=1
/note="gene_841"
/transl_table=11
/translation="MTDLAKGPGKDPGSRGITYASAGVDIEAGDRAIDLFKPLASKAT
RPEVRGGLGGFAGLFTLRGDYREPVLAASSDGVGTKLAIAQAMDKHDTVGLDLVAMVV
DDLVVCGAEPLFLLDYIAVGRIVPERLSAIVAGIADGCMRAGCALLGGETAEHPGLIE
PDHYDISATGVGVVEADNVLGPDRVKPGDVIIAMGSSGLHSNGYSLVRKVLLEIDRMN
LAGHVEEFGRTLGEELLEPTRIYAKDCLALAAETRVRTFCHVTGGGLAGNLQRVIPHG
LIAEVDRGTWTPAPVFTMIAQRGRVRRTEMEKTFNMGVGMIAVVAPEDTTRALAVLTA
RHLDCWVLGTVCKGGKQGPRAKLVGQHPRF"

CDS complement(907412..907594)
/codon_start=1
/note="gene_842"
/transl_table=11
/translation="MGRGRAKAKQTKVARELKYSSPQTDFQRLQRELSGTGTDRLDGD
GPSDDDSWNDEDDWRR"

CDS complement(907741..908847)
/codon_start=1
/note="gene_843"
/transl_table=11
/translation="VAAVPAPDPGPDAGAIWHYGDPLGEQRAGQADAVLVDRSHRAVL
TLDGGDRQTWLHSISTQHVSDLPEGASTQNLSLDGQGRVEDHWIQTELGGTTYLDTEP
WRGEPLLAYLRKMVFWSMVTPRAADMAVLSLLGPRLAEERVLDALGLDVLPAEWLAVP
LAGGGIVRRMPDGLAGQIELDVVVKRGDRADWQRRLTQAGVRPAGIWAYEAHRVAHRV

PARRPRLGVDTDERTIPHEVGWIGGPGAGAVHLNKGCYRGQETVARVHNLGRPPRMLV
LLHLDESVQRPSTGDAVLAGGRTVGRLGTVVEHVELGPVALALLKRGLPGDTALVTGP
EAEVAAVIDVDSLPPADDVGAGRRAVERLRGGIR"
CDS complement(908897..909088)
/codon_start=1
/gene="asnS"
/note="gene_844"
/transl_table=11
/translation="LGQPLQVRFNQTGSAVAHQQCLENAIPPHSGKIIGVQQRHTRLQ
DLTVKRDHHARLPQHVPAA"
CDS 909101..909799
/codon_start=1
/note="gene_845"
/transl_table=11
/translation="MDLPEPDLPRWRRAVEVATQRWVASTADEGALRLIYSRGREGGS
APTAYVMVSPVPARVIGARRDGVSAITLDRGLPADGGDAMPWLMASAKTLSYAVNMAV
LRHAARQGAGDVIFVSTDGYVLEGPRSTVVIATDGDQGGGNPCLLTPPPWYPILRGTT
QQALFEVARAKGYDCDYRALRVADLFDSQGIWLVSSMTLAARVHTLDGRRLPRTPIAE
VFAELVDAAIVSDR"
CDS complement(909845..910525)
/codon_start=1
/note="gene_846"
/transl_table=11
/translation="VSSGAGSDATGAGGVHAAGSGDRAVAAAVERAKATAARNIPAFD
DLPVPADTANLREGADLNNALLALLPLVGVWRGEGEGRGPDGDYRFGQQIVVSHDGGD
YLNWESRSWRLTATGDYQEPGLREAGFWRFVADPYDPSESQAIELLLAHSAGYVELFY
GRPRTQSSWELVTDALARSRSGVLVGGAKRLYGIVEGGDLAYVEERVDADGGLVPHLS
ARLSRFVG"
CDS complement(910688..910990)
/codon_start=1
/gene="sseC2"
/note="gene_847"
/transl_table=11
/translation="MCSGPKQGLTLPASVDLEKETVITGRVVDGDGQAVGGAFVRLLD
SSDEFTAEVVASATGDFRFFAAPGSWTLRALSAAGNGDAVVQPSGAGIHEVDVKIT"
CDS complement(910992..911825)
/codon_start=1
/note="gene_848"
/transl_table=11
/translation="MARCDVLVSADWAESNLHAPKVVFVEVDEDTSAYDRDHIAGAIK
LDWRTDLQDPVKRDFVDAQQFSKLLSERGIANEDTVILYGGNNNWFAAYAYWYFKLYG
HEKVKLLDGGRKKWELDGRPLSSDPVSRPVTSYTASPPDNTIRAFRDEVLAAINVKNL
IDVRSPDEFSGKILAPAHLPQEQSQRPGHIPGAINVPWSRAANEDGTFKSDEELAKLY
ADAGLDNSKETIAYCRIGERSSHTWFVLRELLGHQNVKNYDGSWTEYGSLVGAPIELG
S"
CDS complement(912118..912540)
/codon_start=1
/note="gene_849"
/transl_table=11
/translation="MTTMIVASVATGALATIARWLLTRRSVILREVGPETTPAAPART
AELGLSGAGPTVVHFRAPGCAPCDRVRRGVGDVCADLGDVAHIEVDLDSNPQAARRFS

VLSLPTTLIFDVDGRQRYRTSGVPKAADLRSALKPLLA"
CDS complement(912537..913349)
/codon_start=1
/note="gene_850"
/transl_table=11
/translation="MPMRKVLVGVTGAAIVVAVLIVGAVGADFGASIYAEYRLSTTVR
KAANLRSDPFVAILRFPFIPQAMREHYAELEIKAFAVEHAGSGTATLEATMHSIDLSY
ASWLIRPDAKLPVGELESRIIIDSMHLGRYLGISDLMVAAPRQESNDATGGTTESGIS
GSRGLVFSGTPISANFAHRVSVLVDLSVASDDRATLVITPTAVVTGPDTADQPVPDDK
RDAVLHAFASKLPNQKLPFGVVPNTVGARGSDVIIEGITRGVTISLDEFKQS"
CDS 913482..914246
/codon_start=1
/note="gene_851"
/transl_table=11
/translation="LELLLLTSELYPDPVLPALSLLPHTVRTAPAEASSLLEAGNADA
VLVDARNDLSSGRGLCRLLSSTGRSIPVLAVVSEGGLVAVSADWGLDEILLPSTGPAE
IDARLRLVVGRRGDLADQESLGKVSLGELVIDEGTYTARLRGRPLDLTYKEFELLKYL
AQHAGRVFTRAQLLHEVWGYDFFGGTRTVDVHVRRLRAKLGPEHEALIGTVRNVGYKA
VRPARGRPPAADPDDEDADPGRDGMQEPLVDPLRSQ"
CDS 914243..915190
/codon_start=1
/note="gene_852"
/transl_table=11
/translation="VTALDWRSALTADEQRSVRALVTATTAVDGVAPVGEQVLRELGQ
QRTEHLLVAGSRPGGPIIGYLNLSPPRGAGGAMAELVVHPQSRRRGIGTAMARAALAK
TAGRNQFWAHGTLDPARATASALGLVGVRELIQMRRPLRDIPEPTIPDGVVIRTYAGT
SDDAELLRVNNAAFAGHPEQGGWTAVQLAERRGEAWFDPDGLILAFGDSPRERPGRLL
GFHWTKVHPDHPGLGEVYVLGVDPAAQRRGLGQMLTSIGIVSLARRLGGRKTLDPAVE
PAVLLYVESDNVAAVRTYQSLGFTTYSVDTAYALAGTDN"
CDS 915233..915745
/codon_start=1
/gene="pstB"
/note="gene_853"
/transl_table=11
/translation="VAKRLDLTDVNIYYGSFHAVADVSLAILPRSVTALIGPSGCGKT
TVLRTLNRMHEVIPGARVEGAVLLDDQDIYAPGIDPVGVRRAIGMVFQRPNPFPAMSI
RNNVVAGLKLQGVRNRKVLDDTAESSLRGANLWDEVKDRLDKPGGGLSGGSSSGCASH
GQSPCNPTCC"
CDS 915700..916008
/codon_start=1
/gene="pstB"
/note="gene_854"
/transl_table=11
/translation="LCIARAIAVQPDVLLMDEPCSSLDPISTMAIEDLISELKQQYTI
VIVTHNMQQAARVSDQTAFFNLEAVGKPGRLVEIASTEKIFSNPNQKATEDYISGRFG
"
CDS complement(916064..916705)
/codon_start=1
/note="gene_855"
/transl_table=11
/translation="MRTAYHEQLSELSERLGEMCGLAGIAMERATQALLQADLVLAEQ

VISDHEKIATLSARAEESAFVLLALQAPVAGDLRAIVSAIQMVADIDRMGALALHVAK
IARRRHPQHALPEEVNGYFAEMGRVAVELGNSAQEVVLSHDPEKAAQIREEDDAMDDL
HRHLFTVLMDREWKHGVAAAVDVTLLSRFYERFADHAVEVARRVIFQATGAFP"

CDS complement(916763..918817)
/codon_start=1
/note="gene_856"
/transl_table=11
/translation="MSDGESAAPWARLSESAFPDGVDRWITVPPATWVAAQGPRDTQN
VGCHATGAVSVADLIARLGPAFPDLPTHRHVAPEPEPSGRGPKVPDDADDQQDTEAIA
IPAHSLEFLSELPDLRAANYPRADHARREPELPGKQLTGSARVRPLRIRRTSPAPAKP
APNSGRRPMVLAARSLAALFAALALALTGGAWQWSASKNSRLNMVSALDPHSGDIVNP
SGQHGDENFLLVGMDSRAGANANIGAGDAEDAGGARSDTVMLVNIPASRERVVAVSFP
RDLAITPIQCEAWNPETGKYGPIYDEKTGTMGPRLVYTETKLNSAFSFGGPKCLVKVI
QKLSGLSINRFIAIDFVGFARMVEALGGVEVCSTTPLRDYELGTVLEHAGRQVIDGPT
ALNYVRARQVTTESNGDYGRIKRQQLFLSSLLRSMISTDTLFNLSRLNNVVNMFIGNS
YVDNVKTKDLVELGRSLQHMAAGHVTFVTVPTGITDQNGDEPPRTSDMKALFTAIIDD
DPLPLENDHNAQRLGNTPSTPPTTTKKAPQAGLTNEIQHQQVTTTSPKEVTVQVSNST
GQAGLATTATDQLKRNGFNVMAPDDYPSSLLATTVFFSPGNEQAAATVAAVFGQSKIE
RVTGIGQLVQVVLGQDFSAVRAPLPSGSTVSVQISRNSSSPPTKLPEDLTVTNAADTT
CE"

CDS complement(918983..920116)
/codon_start=1
/note="gene_857"
/transl_table=11
/translation="LRIGPIELASPVVLAPMAGVTNVAFRALCRQLEQSKVGTVSGLY
VCEMVTARALIERHPVTMHMTTFSADESPRSLQLYTVDPDTTYAAARMIAGEGLADHI
DMNFGCPVPKVTKRGCGAALPFKRRLFGQIVAAAVRATEGTDIPVTVKFRIGIDDAHH
THLDAGRIAEAEGAAAVALHARTAAQRYSGTADWEQIARLKQHVRTIPVLGNGDIYDA
GDALAMMSTTGCDGVVIGRGCLGRPWLFAELSAAFTGSPAPTPPTLGEVADIIRRHGT
LLAAHFGEDKGMRDIRKHIAWYLHGFPAGSALRRALAMVKTLDELDCLLDRLDGTVPF
PDSATGARGRQGSPARVALPDGWLTDPDDCRVPEGADAMGSGG"

CDS complement(920240..921256)
/codon_start=1
/gene="desA1"
/note="gene_858"
/transl_table=11
/translation="MSAKLTDLQLLHELEPVVEKYLNRHLSMHKPWNPHDYIPWSDGK
NYYALGGQDWDPDQSKLSDVAQVAMVQNLVTEDNLPSYHREIAMNMGMDGAWGQWVNR
WTAEENRHGIALRDYLVVTRSVDPVELEKLRLEVVNRGFSPGQNHQGHYFAESLTDSV
LYVSFQELATRISHRNTGKACNDPVADQLMAKISADENLHMIFYRDVSEAAFDLVPNQ
AMKSLHLILSHFQMPGFQVPEFRRKAVVIAVGGVYDPRIHLDEVVMPVLKKWRIFERE
DFTGEGAKLRDELALVIKDLELACDKFEVSKQRQLDREARTGKKVSAHELHKTAGKLA
MSRR"

CDS complement(921418..922059)
/codon_start=1
/note="gene_859"
/transl_table=11
/translation="VQTGQNRGRWSGVPLESRHALRRDNLVAAGVQLLGGAGGPALTV
RAVCRHAGLTERYFYESFADREHFVRAVYDDVCTRAMATLTSAQTPREAVEQFVELMV
DDPVRGRVLLLAPAVEPALTRSGAEWMPNFIELLQRKLSRIVDPVLQKLVATSLIGAL
TGLFTAYLNGRLGATRKQFIDYCVNMLLSTAATYAPHRERGESEHSIPAGPHN"

CDS 922257..923195
/codon_start=1
/note="gene_860"
/transl_table=11
/translation="VSPLGYESPPLPLGPDSLTWRYFGDWRGMLQGPWAGSMQNMHPQ
LGAAVEDHSTFFRERWPRLLRSLYPIGGVVFDGDRAPVTGVQVRDYHITIKGVDGAGR
RYHALNPDVFYWAHATFFVGTLHVAERFCGGLTEAQRRQLFDEHVQWYRMYGMSMRPV
PATWEEFQDYWDHMCRNVLENNFAARAVLDLTELPKPPFAQRVPDWLWAAPRKLLARF
FVWLTVGLYDPPVRELMGYRWLRRDEWLHRRFGDIVQLVFALVPFRFRKHPRARAGWD
RATGRIPADAPLVQTPARNLPPPDERDNPTHYCPKV"
CDS complement(923247..923588)
/codon_start=1
/note="gene_861"
/transl_table=11
/translation="VVEVFRMLADATRVQVLWSLADREMSVNELAEQVGKPAPSVSQH
LAKLRMARLVRTRRDGTTIFYRLENEHVRQLVIDAVFNAEHAGPGIPRHHRAAGGLQS
VAKASATKDVG"
CDS complement(923697..924119)
/codon_start=1
/note="gene_862"
/transl_table=11
/translation="MPAGMAGFRRWAQTNDPTAHAESLAIRAACTKLGTEHLVGTTLN
VLAHPCPMCYGSLYYCSPDEVVFLTSRDAYEPHYVDDRRYFEPATFYDEFAKEWQDRR
LPMRQEHRPDIRAGAVDVYRFRQEPNGGERSAIAAPTG"
CDS 924009..924371
/codon_start=1
/note="gene_863"
/transl_table=11
/translation="VLSAQLGARGTDGKALGVGGRIIGLGPSSKTCHACRHVQDIGWD
EKWQCDGCSITHQRDDNAAINLARYEEPPSVVGPVGAAVKRGADRKTGPGPAGGREAR
KGTGHPAGEQPRDGVLVA"
CDS 924476..925381
/codon_start=1
/note="gene_864"
/transl_table=11
/translation="MVRADRDRWDLATSVGATATMVAAQRALAADPRYALIDDPYAAP
LVRAVGMDVYTRLVDWQIPVEGDSEFDPQRMATGMACRTRFFDQFFLDATHSGIGQFV
ILASGLDARAYRLAWPVGSIVYEVDMPEVIEFKTATLSDLGAEPATERRTVAVDLRDD
WATALQTAGFDPKVPAAWSAEGLLVYLPVEAQDALFDNITALSAPGSRLAFEFVPDTA
IFADERWRNYHNRMSELGFDIDLNELVYHGQRGHVLDYLTRDGWQTSALTVTQLYEAN
GFAYPDDELATAFADLTYSSATLMR"
CDS complement(925400..926215)
/codon_start=1
/note="gene_865"
/transl_table=11
/translation="MLPETNQDEVQPNAPVALVTVEIRHPTTDSLTESANRELKHLLI
NDLPIERQAQDVSWGMTAPGGAPTPVADRFVRYVNRDNTTAASLKNQAIVVETTAYRS
FEAFTDVVMRVVDARAQVSSIVGLERIGLRFVLEIRVPAGVDGRITWSNWIDEQLLGP
QRFTPGGLVLTEWQGAAVYRELQPGKSLIVRYGPGMGQALDPNYHLRRITPAQTGPFF
LLDIDSFWTPSGGSIPEYNRDALVSTFQDLYGPAQVVFQEMITSRLKDELLRQ"
tRNA complement(926306..926381)

```
                     /note="identified by tRNAscan-SE; putative"
     tRNA            926505..926581
                     /note="identified by tRNAscan-SE; putative"
     tRNA            926616..926692
                     /note="identified by tRNAscan-SE; putative"
     tRNA            926719..926795
                     /note="identified by tRNAscan-SE; putative"
     CDS             927457..930189
                     /codon_start=1
                     /note="gene_866"
                     /transl_table=11
                     /translation="VSYVSVLPATLATAATEVARIGSALSLASAVAAAQTSAVQAAAA
                     DEVSAAIAALFSAHGRDFQALSARAAAFHHEFVQALAAGAGSYAVAEIAAASPLQSLI
                     DVFNAPIQAATGRPLIGNGANGQPGTGAPGGPGGWLIGNGGAGGSGAPGAIGGAGGPA
                     GLIGVGGAGGAGGDSAVAGVIGGAGGAGGAALLFGAGGAGGAGGSGGSGAAGGAGGAG
                     GAGGLFASGGSGGFGGFASTGTGGAGGTGGAGGLFASGGVGGTGGGAGSGGTGGVGGT
                     GGAGGLFASGGAGGAGGSGGTGGAGGTGGAGGLFGAGGAGGLGGQGNHTGGHGGAGGS
                     AGLLALGDGGAGGAGGAATTGTGGAGGAGGKAGLLFGSGGAGGSGGAAGTFGDTGNSG
                     GAGGAGGKAGLLFGSGGAGGSGGAGGFANGSTGGAGGAGGGAGLIGNGGNGGSGGTSV
                     ATGGAGNGGAGGAGGGAGLIGNGGNGGSGGMGDAPGGTGVGGIGGLLLGLDGANAPAS
                     TNPLHTAQQQALAAVNAPIQAVTGRPLIGNGANGAPGSGAPGGHGGWLFGGGGTGGSG
                     VSGGAGGDGGAGGILFGAGGAGGAGGAVTGTGATGGSGGAGGGALLFGAGGAGGAGGS
                     SGIGGFAAGGAGGPGGAGGLFNGGGAGGAGGSGVSGGAGGEGGAGGAGGLFAGGGIGG
                     AGGFGGFRGGEGGAGGAGGLFAGGGAGGAGGSGNNVGGAGGAGGVGGLFGAGGAGGSG
                     GGGSVAGDGGAGGNAGLLAPGLAGGAGGGGGQGFDTGGAGGPGGDAGLLVGSGGVGGA
                     GGFGLTTGGPGAAGGDAGLLFGSGGAGGAGGSGRTDLGGAGGAGGKAGLIGNGGNGGA
                     GGAGGAGGPGGAAFGLGNGGNGGNGGTGTSAGSPGAGGAGGSLIGAEGLPGLLP"
     CDS             complement(930416..933064)
                     /codon_start=1
                     /note="gene_867"
                     /transl_table=11
                     /translation="MSFVIAAPDLVAMATEDLAGIGASLTAANAAAAVPTSGLLAAAG
                     DEVSAAIAALFSSHGQQYQAMSAQAAAFHARFVQALAGAMGAYAAAEAANASPLQTLE
                     QGLLGAINAPAAALSGRPFIGNGTNGAPGTGEAGGPGGWLLGNGGNGGSGAPGQTGGA
                     GGAAGLLGHGGTGGAGGTGASGGKGGTGGWLWGSGGAGGAGGSGGGSGGAGGNALMFG
                     IGGNGGAGGAASGVGNGGVGGAGGAGGALVAIGGAGGAGGAATTGTGGAGGAGSNALG
                     LFLGLGGSGGQGGDSAMGSGGAGGAGGSGGAASPFGIDIGIGGAGGHGGAGTNGGAGG
                     AGGAGGSSGTVFALDLSWGGAGGNGGAATTGTGGAGGTGGFAVAPDFIGFGAAYGGAG
                     GLGGAATGAGGTGGTGGVGAGGFAALGVGVGGAGGAGGAATETGGIGGAGGLGVGLLG
                     GAGGAGGPGGAASAGSGGHGGTGGDALGLIGAGIGGVGGVGGAATDTGGNGGAGGSGT
                     GLLGGVGGAGGHGGGASVGTGGSGGAGGDGFGFVGAGGNGGNAGTGVGVNGANGGNGG
                     SATGALAAVGGAGAAGGDATSGTGGFGGAGGSARGLIFALGGAGAAGGDASTGVGGPG
                     GPGGTGTASSPFGIAIAIGGAGAQGGAGTSGATGGAGGDGVFEGIAVLGLGFGGAAGA
                     GGAATGDGATGGAGGFGGAGAGIANFLGFSVLHGGAGGAGGTATGTGGNGGAGGGGGL
                     SSPVILGIGIGGAGGDGGGALGVLGGMGGDGGDGGEAVAVGIAVGGAGGAGGAAPTGN
                     GGAGGNGGDALGLVGVGGNGGNAGTGFGANTGGNGGDTTIVVNGMLAPSTLGYGGNGG
                     NGVNGGAGGTGGKAGVFGAPGQNGLP"
     CDS             933661..934176
                     /codon_start=1
                     /note="gene_868"
                     /transl_table=11
```

/translation="LIVSIEDVRRIANYEELAAHFQTDLREPPEADTNVPGPCRVVGS
SDRTFGTDWSEFRSAGYHGVTDDLRPGGPVMVETVSQAIALYPDPSTARGVFHRLESS
LAECAGLHDPYFDFILGRPDASTVRIGAAGWSHVYRLKSSVFISVGVLGIEPAEPIAN
VILQTISDRIQ"

```
CDS             complement(934157..934513)
                /codon_start=1
                /gene="TBCG_00826"
                /note="gene_869"
                /transl_table=11
                /translation="VISPARLTAWIAASALILLTGTELFGTALSWVKLSPIWHVFSFA
                VDTRPIATLLDYASHPIDPRSYSSVAVTAVGAEATLLAFFFATVGVVASTALNTLRSS
                PPLLLSSPRLTTGCDR"
CDS             complement(934789..935604)
                /codon_start=1
                /note="gene_870"
                /transl_table=11
                /translation="MSDSPDYDPVLLAWVTPIVTALADVVPAEQLMLVGAQCRDLLHW
                RFCRGVPPRATNDTDIAGTLNNWDHFEAIRATFRALGSTGHRFLIADRAVDALPFGEV
                ESPTGTTRHPPGNQLMNVHGCTDAYLRADVLPLPGGLTVHLPQPPNYAVLKLHAWLDR
                SADHDYKDGPDLALVVHWYAGDLDRLYAKPDQWALRRHDFDLRTAAAALLGHDMRASV
                SAPEAAVLATRATQADHDLLAQHFAVGRPGWPTTTASRRPLVEALLDQLTPGS"
CDS             complement(935582..936610)
                /codon_start=1
                /note="gene_871"
                /transl_table=11
                /translation="MDQIGADLAEAVERHLTEYGVRVLGGLSALNSAHPESLDLEIDA
                HPLTITALYLPHLSATAALQAWDTAGAGSPLLVVGPRLHPSSAETLRARGLWYIDGAG
                NAYLRHQGGLLIDVRGRRSAVSAQPGTLGDGLHSDGPRNPFTPKRAQVVCVLLDAPQL
                VDAPLRAIAASAGVSVGMAKETMDTLRTTGFFEHLGSRRRLVRTDELLDLWAAAYPGG
                LGRANKLLVASGDIHTWSAPDGLAVAVSGEQALPDEIRNPESLMLYVDTPAPGLPADL
                LIHNRWHRDPHGSIVIRKLFWRNLPDEQPGLAPTALIYADLLASREPRQVEVAHLMRR
                QDERLARL"
CDS             937458..938081
                /codon_start=1
                /note="gene_872"
                /transl_table=11
                /translation="VPPVSEAARAAGLVDVRGVVPDAAIDLRYATANNFTGTQLYPPG
                ARCLVHESMAEGLAAAAAVLRPHGQVLVFWDCYRPHDVQVRMFDVVPNPAWVARPGKY
                AHSHEAGRSVDVTFASAQRQCPSVRRSGELCLADMGTDFDDFSSRATAFATQGVSAEA
                QANRAHLRAAMQAGGLTVYSGEWWHFDGPGPGPGAGVDRPILEVPVD"
CDS             938168..938980
                /codon_start=1
                /note="gene_873"
                /transl_table=11
                /translation="VNDKRRAIYTHGYHESVLRSHRRRTAENSAGYLLPYLVPGLSVL
                DVGCGPGTITVDLAARVVPGSVTGVEPTDDALSLARAEAQLHRLSNISFTTSDVHKLD
                FPDDAFDVVHAHQVLQHVADPVRALQEMRRVCTPGGIVAARDADYSGFIWFPKLPALD
                RWLDLYERAARANGGEPDAGRRLLSWARAAGFDDVTPTASVWCFATASAREWWGLVWA
                DRILQSDLAHQLVDSGLATAAQLEEISTAWREWAAAPDGWLAIPHGEILCRA"
CDS             complement(939048..939680)
                /codon_start=1
```

/note="gene_874"
/transl_table=11
/translation="VAEMATVAEALALTRFHIFSHSWGGMLAQQYVLDKAPDAVSLTI
ANSTASIPEFSASLVSLKSCLDVATRSAIDRHEAAGTTHSAEYQAAIRTWNETYLCRT
RPWPRELTEAFANMGTEIFETMFGPSDFRIVGNVRDWDVVDRLADIAVPTLLVVGRFD
ECSPEHMREMQGRIAGSRLEFFESSSHMPFIEEPARFDRVMREFLRLHDI"
CDS 940667..941995
/codon_start=1
/note="gene_875"
/transl_table=11
/translation="VTTAAAIAREDRLRYTGPERCSGDGQVRAAGDRYSTVIWLLGGN
LLVRSAGFGYPFLAYHVAGRGHGAGAVGAVVAAYGLGWAVGQLLCGWLVDRVGARVTL
VSTMLVAAAVLVLMAGLHTVPGLLVGAMIAGLVCDAPRPVLGAVIAELVADPQRRAQL
DGWRYGWVLNIGAAITGGVGGVVAGWLDTPVLYWINGIGCAIFAGLAGRCIPADVCRR
TESGLRACTAMSKVGYRQALSDKRLVLLAVSGLATLTTLMGFFAAVPMLMSASGLGVG
AYGWVQLINALAVVAVTPLLTPWLSKQLALGPRPDILAGAGVWVTLCMAAAGLARTTV
GFSVAAAACSPGEIAWFVVAAGIVHRIAPPAHGGRYHGIWSMAVAASSVAAPILAAFN
LANGGRLVLAATTVTVGFFGAALCLPLARVLAAASCGPLSSKEPSRDSYQ"
CDS 941979..942983
/codon_start=1
/note="gene_876"
/transl_table=11
/translation="VTRTSEGLAAFVVDQLEELYRRMWVLRLLDMALEQLRIEGLING
PLQGGFGQEAVSVGAAAALGEGDVIITTHRPHAQHVGTDAPLGPVIADMLGATAGDLE
GADEDAHIADPRAGLPAAIRVVKQSPLLAIGHAYALWLRDTGRVTLCVTQDCDVDADA
FNEAADLAAVWQLPVVILVENIRGALSVHLGRYTHEPRVYRRAVAYGMPGVSVDGNDV
EAVRDCVANAVVRARAGGGPTLVQAITYRTTDFSGSDRGGYRDLAGSEQFLDPLIFAR
RRLIAAGTTRGRLDEQERAACQQVADAVAFAKARARPNGGGPISRPTSGWHQQPKTRF
"
CDS complement(943047..943697)
/codon_start=1
/note="gene_877"
/transl_table=11
/translation="MSNPQPEKVRVVVGDDHPLFREGVVRALSLSGSVNVVGEADDGA
AALELIKAHLPDVALLDYRMPGMDGAQVAAAVRSYELPTRVLLISAHDEPAIVYQALQ
QGAAGFLLKDSTRTEIVKAVLDCAKGRDVVAPSLVGGLAGEIRQRAAPVAPVLSARER
EVLNRIACGQSIPAIAAELYVAPSTVKTHVQRLYEKLGVSDRAAAVAEAMRQRLLD"
CDS 943769..945058
/codon_start=1
/note="gene_878"
/transl_table=11
/translation="LHSDVPSYGNLGRLGGRHEYGVLVAMTSSAELDRVRWAHQLRSY
RIASVLRIGVVGLMVAAMVVGTSRSEWPQQIVLIGVYAVAALWALLLAYSASRRFFAL
RRFRSMGRLEPFAFTAVDVLILTGFQLLSTDGIYPLLIMILLPVLVGLDVSTRRAAVV
LACTLVGFAVAVLGDPVMLRAIGWPETIFRFALYAFLCATALMVVRIEERHTRSVAGL
SALREELLAQTMTASEVLQRRIAEAIHDGPLQDVLAARQELIELDAVTPGDERVGRAL
AGLQSASERLRQATFELHPAVLEQVGLGPAVKQLAASTAQRSGIKISTDIDYPIRSGI
DPIVFGVVRELLSNVVRHSGATTASVRLGITDEKCVLDVADDGVGVTGDTMARRLGEG
HIGLASHRARVDAAGGVLVFLATPRGTHVCVELPLKR"
CDS complement(945271..946785)
/codon_start=1

/note="gene_879"
/transl_table=11
/translation="MPELATSGNAFDKRRFSRRGFLGAGIASGFALAACASKPTASGA
AGMTAAIDAAEAARPHSGRTVTATLTPQPARIDLGGPIVSTLTYGNTIPGPLIRATVG
DEIVVSVTNRLGDPTSVHWHGIALRNDMDGTEPATANIGPGGDFTYRFSVPDPGTYWA
HPHVGLQGDHGLYLPVVVDDPTEPGHYDAEWIIILDDWTDGIGKSPQQLYGELTDPNK
PTMQNTTGMPEGEGVDSNLLGGDGGDIAYPYYLINGRIPVAATSFKAKPGQRIRIRII
NSAADTAFRIALAGHSMTVTHTDGYPVIPTEVDALLIGMAERYDVMVTAAGGVFPLVA
LAEGKNALARALLSTGAGSPPDPQFRPDELNWRVGTVEMFTAATTANLGRPEPTHDLP
VTLGGTMAKYDWTINGEPYSTTNPLHVRLGQRPTLMFDNTTMMYHPIHLHGHTFQMIK
ADGSPGARKDTVIVLPKQKMRAVLVADNPGVWVMHCHNNYHQVAGMATRLDYIL"

CDS 946910..947326
/codon_start=1
/note="gene_880"
/transl_table=11
/translation="VGHVESGHVVWMRSAIVAVALGVTVAAVAAACWLPQLHRHVAHP
NHPLTTSVGSEFVINTDHGHLVDNSMPPCPERLATAVLPRSATPVLLPDVVAAAPGMT
AALTDPVAPAARGPPAAQGSVRTGQDLLTRFCLVRR"

CDS 947433..948647
/codon_start=1
/note="gene_881"
/transl_table=11
/translation="MNNNLPLANPVNPTSITSNPQILLANRAHRTLVRSRQTRDRYRL
LPEGYQVTPGRNRHPGTMVGNTPVLWIPELSGTSDPDRGFWAKLEGFNPGGMKDRPAL
YMVECARARGDIAPGAAIVESTSGTLGLGLALAGKVYRHPVTLVTDPGLEPIIARMLT
AYGAGVDMVTQPHPVGGWQQARKDRVAQLMAEYPGAWNPNQYGNPDNVGAYRSLALEL
VAQLGRIDVLVCSVGTGGHSAGVARVLREFNPDMRLIGVDTIGSTIFGQPASNRLMRG
LGSSIYPRNVDYRAFDEVHWVAPPEAVWACRSLAATHYASGGWSVGAVALVAGWAARN
LPADTTIAAVFPDGPQRYFDTIYNDAYCNEHELLGGQPPTEPDEIASPLDAVVTRWTR
STTVIDPTQVVS"

CDS 948647..949906
/codon_start=1
/note="gene_882"
/transl_table=11
/translation="MGARAIFRGFNRPSRVLMINQFGINIGFYMLMPYLADYLAGPLG
LAAWAVGLVMGVRNFSQQGMFFVGGTLADRFGYKPLIIAGCLIRTGGFALLVVAQSLP
SVLIAAAATGFAGALFNPAVRGYLAAEAGERKIEAFAMFNVFYQSGILLGPLVGLVLL
ALDFRITVLAAAGVFGLLTVAQLVALPQHRADSEREKTSILQDWRVVVRNRPFLTLAA
AMTGCYALSFQIYLALPMQASILMPRNQYLLIAAMFAVSGLVAVGGQLRITRWFAVRW
GAERSLVVGATILAASFIPVAVIPNGQRFGVAVAVMALVLSASLLAVASAALFPFEMR
AVVALSGDRLVATHYGFYSTIVGVGVLVGNLAIGSLMSAARRLNTDEIVWGGLILVGI
VAVAGLRRLDTFTSGSQNMTGRWAAPR"

CDS 949903..950235
/codon_start=1
/note="gene_883"
/transl_table=11
/translation="VTRDPHSPDCGREGSYRDTITRPLTDLPVAGYPLVPRVASPRYR
CTTPQCGRAVFNQDLANVDQYLVVNQLAHQLIDGSSLIPDADKRWDARRHADMTHHLT
SSLKENQS"

CDS complement(950232..951059)
/codon_start=1

/note="gene_884"
/transl_table=11
/translation="VDGFPGRGAVITGGASGIGLATGTEFARRGARVVLGDVDKPGLR
QAVNHLRAEGFDVHGVMCDVRHREEVTHLADEAFRLLGHVDVVFSNAGIVVGGPIVEM
THDDWRWVIDVDLWGSIHTVEAFLPRLLEQGTGGHVVFTASFAGLVPNAGLGAYGVAK
YGVVGLAETLAREVTADGIGVSVLCPMVVETNLVANSERIRGAACAQSSTTGSPGPLP
LQDDNLGVDDIAQLTADAILANRLYVLPHAASRASIRRRFERIDRTFDEQAAEGWRH"
CDS 951348..951986
/codon_start=1
/note="gene_885"
/transl_table=11
/translation="VSTGDIHSLVIASDYRVPDPGRVWPLLQRNKSALADIGAHHVLI
YASTHDSGRVLVMIGVRSREPIVELLRSRVFFDWFDAMGVDDIPAVFAGEIVDRFVAA
PTTTQSTPRVPGVVVAAFASVNNVSNLTAEVRSAIARFTAAGIRKTWVFQAFDDAHEV
LILQEFADEAGARQWIEHPDAAAEWMSGAGVGAYPPLFVGRFFDMMRIEALQ"
CDS complement(952027..953709)
/codon_start=1
/note="gene_886"
/transl_table=11
/translation="VTPQKSDACSDPVYTVGDYLLDRLAELGVSEIFGVPGDYNLQFL
DHIVAHPTIRWVGSANELNAGYAADGYGRLRGMSAVVTTFGVGELSVTNAIAGSYAEH
VPVVHIVGGPTKDAQGTRRALHHSLGDGDFEHFLRISREITCAQANLMPATAGREIDR
VLSEVREQKRPGYILLSSDVARFPTEPPAAPLPRYPGGTSPRALSLFTKAAIELIADH
QLTVLADLLVHRLQAVKELEALLAADVVPHATLMWGKSLLDESSPNFLGIYAGAASAE
RVRAAIEGAPVLVTAGVVFTDMVSGFFSQRIDPARTIDIGQYQSSVADQVFAPLEMSA
ALQALATILTGRGISSPPVVPPPAEPPPAMPARDEPLTQQMVWDRVCSALTPGNVVLA
DQGTSFYGMADHRLPQGVTFIGQPLWGSIGYTLPAAVGAAVAHPDRRTVLLIGDGAAQ
LTVQELGTFSREGLSPVIVVVNNDGYTVERAIHGETAPYNDIVSWNWTELPSALGVTN
HLAFRAQTYGQLDDALTVAAARRDRMVLVEVVLPRLEIPRLLGQLVGSMAPQ"
CDS 953774..954217
/codon_start=1
/note="gene_887"
/transl_table=11
/translation="MAIKESRDIVIEASPEEILDVIADFEAMTEWSPAHQSVEILETG
DDGRPSKVKMKVKTAGITDEQVVAYSWTDRSVRWTLVSSTQQRSQDGKYELTPKGDNT
LVQFEITVDPQVPLPGFVLKRAIKGTIDTATEALRSQVLKVKKGQ"
CDS 954223..955302
/codon_start=1
/note="gene_888"
/transl_table=11
/translation="VTTGGPLAGVKVIELGGIGPGPHAGMVLADLGADVVRVRRPGGL
TMPSEDRDLLHRGKRIVDLDVKTQPQAMLELAAKADVLLDCFRPGTCERLGIGPDDCA
SVNPRLIFARITGWGQDGPLASTAGHDINYLSQTGALAAFGYADRPPMPPLNLVADFG
GGSMLVLLGIVVALYERERSGVGQVVDAAMVDGVSVLAQMMWTMKGIGSLRDQRESFL
LDGGAPFYRCYETSDGKYMAVGAIEPQFFAALLSGLGLSAADVPTQLDVAGYPQMYDI
FAERFASRTRDEWTRVFAGTDACVTPVLAWSEAANNDHLKARSTVITAHGVQQAAPAP
RFSRTPAGPVRPPPAAATPIDEINW"
CDS 955415..955819
/codon_start=1
/note="gene_889"
/transl_table=11

```
                     /translation="MEALADVGVLASWSPLHKQVEVIDYYPDGRPHHVRATVKILGLV
                     DKEVLEYHWGPDWVCWDADQTFQQHGQHIEYTVKPEGVDRARVRFDITVEPAGPIPGF
                     IVKRASEHVLDAAAKGLQKLIAGAGDQGNAKS"
CDS                  955847..956320
                     /codon_start=1
                     /note="gene_890"
                     /transl_table=11
                     /translation="VIANLVAVAIRASREVVIEAPPEVIVEALADMDAVPSWSSVHKR
                     VEVVDTYSDGRPHHVKVTIKVAGIVDTELLEYHWGPDWVVWDAAKTAQQHGQHGEYNL
                     RREDNDKTRVRFTLTVEPSAPLPAFWVNIARKKILHAATEGLRKQVVGRRRFTSG"
CDS                  complement(956317..957510)
                     /codon_start=1
                     /note="gene_891"
                     /transl_table=11
                     /translation="MTVSRLRPYATTVFAEMSALATRIGAVNLGQGFPDEDGPPKMLQ
                     AAQDAIAGGVNQYPPGPGSAPLRRAIAAQRRRHFGVDYDPETEVLVTVGATEAIAAAV
                     LGLVEPGSEVLLIEPFYDSYSPVVAMAGAHRVTVPLVPDGRGFALDADALRRAVTPRT
                     RALIINSPHNPTGAVLSATELAAIAEIAVAANLVVITDEVYEHLVFDHARHLPLAGFD
                     GMAERTITISSAAKMFNCTGWKIGWACGPAELIAGVRAAKQYLSYVGGAPFQPAVALA
                     LDTEDAWVAALRNSLRARRDRLAAGLTEIGFAVHDSYGTYFLCADPRPLGYDDSTEFC
                     AALPEKVGVAAIPMSAFCDPAAGQASQQADVWNHLVRFTFCKRDDTLDEAIRRLSVLA
                     ERPAT"
CDS                  957640..958878
                     /codon_start=1
                     /note="gene_892"
                     /transl_table=11
                     /translation="VEFVEQEIDMSEEAFIYEAIRTPRGKQKNGSLHEVKPLSLVVGL
                     IDELRKRHPDLDENLISDVILGCVSPVGDQGGDIARAAVLASGMPVTSGGVQLNRFCA
                     SGLEAVNTAAQKVRSGWDDLVLAGGVESMSRVPMGSDGGAMGLDPATNYDVMFVPQGI
                     GADLIATIEGFSREDVDAYALRSQQKAAEAWSGGYFAKSVVPVRDQNGLLILDHDEHM
                     RPDTTKEGLAKLKPAFEGLAALGGFDDVALQKYHWVEKINHVHTGGNSSGIVDGAALV
                     MIGSAAAGKLQGLTPRARIVATATSGADPVIMLTGPTPATRKVLDRAGLTVDDIDLFE
                     LNEAFASVVLKFQKDLNIPDEKLNVNGGAIAMGHPLGATGAMILGTMVDELERRNARR
                     ALITLCIGGGMGVATIIERV"
CDS                  958883..961045
                     /codon_start=1
                     /note="gene_893"
                     /transl_table=11
                     /translation="MPDNTIQWDKDADGIVTLTMDDPSGSTNVMNEAYIESMGKAVDR
                     LVAEKDSITGVVVASAKKTFFAGGDVKTMIQARPEDAGDVFNTVETIKRQLRTLETLG
                     KPVVAAINGAALGGGLEIALACHHRIAADVKGSQLGLPEVTLGLLPGGGGVTRTVRMF
                     GIQNAFVSVLAQGTRFKPAKAKEIGLVDELVATVEELVPAAKAWIKEELKANPDGAGV
                     QPWDKKGYKMPGGTPSSPGLAAILPSFPSNLRKQLKGAPMPAPRAILAAVEGAQVDF
                     DTASRIESRYFASLVTGQVAKNMMQAFFFDLQAINAGGSRPEGIGKTPIKRIGVLGAG
                     MMGAGIAYVSAKAGYEVVLKDVSLEAAAKGKGYSEKLEAKALERGRTTQERSDALLAR
                     ITPTADAADFKGVDFVIEAVFENQELKHKVFGEIEDIVEPNAILGSNTSTLPITGLAT
                     GVKRQEDFIGIHFFSPVDKMPLVEIIKGEKTSDEALARVFDYTLAIGKTPIVVNDSRG
                     FFTSRVIGTFVNEALAMLGEGVEPASIEQAGSQAGYPAPPLQLSDELNLELMHKIAVA
                     TRKGVEDAGGTYQPHPAEAVVEKMIELGRSGRLKGAGFYEYADGKRSGLWPGLRETFK
                     SGSSQPPLQDMIDRMLFAEALETQKCLDEGVLTSTADANIGSIMGIGFPPWTGGSAQF
                     IVGYSGPAGTGKAAFVARTRELAAAYGDRFLPPESLLS"
```

```
CDS             complement(961113..962741)
                /codon_start=1
                /note="gene_894"
                /transl_table=11
                /translation="VQSDKTVLLEVDHELAGAARAAIAPFAELERAPEHVHTYRITPL
                ALWNARAAGHDAEQVVDALVSYSRYAVPQPLLVDIVDTMARYGRLQLVKNPAHGLTLV
                SLDRAVLEEVLRNKKIAPMLGARIDDDTVVVHPSERGRVKQLLLKIGWPAEDLAGYVD
                GEAHPISLHQEGWQLRDYQRLAADSFWAGGSGVVVLPCGAGKTLVGAAAMAKAGATTL
                ILVTNIVAARQWKRELVARTSLTENEIGEFSGERKEIRPATISTYQMITRRTKGEYRH
                LELFDSRDWGLIIYDEVHLLPAPVFRMTADLQSKRRLGLTATLIREDGREGDVFSLIG
                PKRYDAPWKDIEAQGWIAPAECVEVRVTMTDSERMMYATAEPEERYRICSTVHTKIAV
                VKSILAKHPDEQTLVIGAYLDQLDELGAELGAPVIQGSTRTSEREALFDAFRRGEVAT
                LVVSKVANFSIDLPEAAVAVQVSGTFGSRQEEAQRLGRILRPKADGGGAIFYSVVARD
                SLDAEYAAHRQRFLAEQGYGYIIRDADDLLGPAI"
CDS             complement(962879..965149)
                /codon_start=1
                /note="gene_895"
                /transl_table=11
                /translation="MTEHTPDIPLGSWLAALPDERLTQLLELRPDLAQPPPGSIAALA
                ARAQARQSVKAATDELDFLRLAVFDALLVLQADTAPVPIVRLLAVIGDRAAQADVLGA
                LADLKQRALAWGETAVRVATDAGTALPWHPGQVTLEGSSRSGDQLADLIAGLDPAQRD
                VLDKLLQGSPVGRTRDAAPGAPSDRPVPRLLAMGLLRRIDAETVILPRHVGQVLRGEQ
                PGPMEFTAPDPVVSTTTPDDADAAAAGAVIDLLREVDVLLENLGATPVAELRSGGLGV
                REFKRLAKATGIDEPRLGLILEIAAAAGLIASGMPDPEPPHSDGPFWAPTVAADRFAT
                MSPAERWHLLASAWLDLPGRPALIGTRGPDAKPYGALSDSLFSTAAPLDRRLLLGMLA
                ELPAGAGVDASRASATLIWRRPRWARRLQPAPIADLLTEGHALGLVGRGAISTPARAL
                LDEALEPATAPAAAVGVMARALPKPIDHFLVQADLTVVVPGPLQRELADDLTTVATVE
                SAGTAMVYRVSEQSIRHALDVGKSRDWLQEFFANRSKTPVPQGLTYLIDDVARRHGQL
                RIGMAASFVRCEDPTLLAQVVAAPEADGLALRALAPTVAVSPAPISEVLVTLRGAGFA
                PAAEDSTGAVVDVRTRGARVPTPQRRRPYRPPPRPNSEALKAVVAVLREVTAAPFANV
                RVDPAVTMSLLQRAAKDQATLVISYLDAAGVATQRVVAPITLRGGQLVAFDSSSGRLR
                DFAIHRITSVVSAHDR"
CDS             965217..965417
                /codon_start=1
                /note="gene_896"
                /transl_table=11
                /translation="VADIAEGKARKTRYVDHGWPTTDPDDHAVSELVTDRTGALSPFG
                ELTFPVPSDDLPYIHPVTVINR"
CDS             965501..965929
                /codon_start=1
                /gene="moaC"
                /note="gene_897"
                /transl_table=11
                /translation="MVDITEKATTKRTAVAAGILRTSAQVVALISTGGLPKGDALATA
                RVAGIMAAKRTSDLIPLCHQLALTGVDVDFTVGQLDIEITATVRSTDRTGVEMEALTA
                VSVAALTLYDMIKAVDPGALIDDIRVLHKEGGRRGTWTRR"
CDS             965926..966408
                /codon_start=1
                /note="gene_898"
                /transl_table=11
                /translation="MSTRSARIVVVSSRAAAGVYTDDCGPIIAGWLEQHGFSSVQPQV
```

VADGNPVGEALHDAVNAGVDVIITSGGTGISPTDTTPEHTVAVLDYVIPGLADAIRRS
GLPKVPTSVLSRGVCGVAGRTLIINLPGSPGGVRDGLGVLADVLDHALEQIAGGDHPR
"
CDS 966405..966830
/codon_start=1
/note="gene_899"
/transl_table=11
/translation="MTQVLRAALTDQPIFLAEHEELVSHRSAGAIVGFVGMIRDRDGG
RGVLRLEYSAHPSAAQVLADLVAEVAEESSGVRAVAASHRIGVLQVGEAALVAAVAAD
HRRAAFGTCAHLVETIKARLPVWKHQFFEDGTDEWVGSV"
CDS complement(966848..968071)
/codon_start=1
/note="gene_900"
/transl_table=11
/translation="MSGRHRKPTTSNVSVAKIAFTGAVLGGGGIAMAAQATAATDGEW
DQVARCESGGNWSINTGNGYLGGLQFTQSTWAAHGGGEFAPSAQLASREQQIAVGERV
LATQGRGAWPVCGRGLSNATPREVLPASAAMDAPLDAAAVNGEPAPLAPPPADPAPPV
ELAANDLPAPLGEPLPAAPADPAPPADLAPPAPADVAPPVELAVNDLPAPLGEPLPAA
PADPAPPADLAPPAPAELAPPAPADLAPPAPADLAPPVELAVNDLPAPLGEPLPAAPA
ELAPPADLAPASADLAPPAPADLAPPAPAELAPPAPADLAPPAAVNEQTAPGDQPATA
PGGPVGLATDLELPEPDPQPADAPPPGDVTEAPAETPQVSNIAYTKKLWQAIRAQDVC
GNDALDSLAQPYVIG"
CDS complement(968519..968797)
/codon_start=1
/note="gene_901"
/transl_table=11
/translation="VTQVSDESAGIQVTVRYFAAARAAAGAGSEKVTLRSGATVAELI
DGLSVRDVRLATVLSRCSYLRDGIVVRDDAVALSAGDTIDVLPPFAGG"
CDS complement(968801..969883)
/codon_start=1
/gene="moaA"
/note="gene_902"
/transl_table=11
/translation="MTLTALGMPALRSRTNGIADPRVVPTTGPLVDTFGRVANDLRVS
LTDRCNLRCSYCMPERGLRWLPGEQLLRPDELARLIHIAVTRLGVTSVRFTGGEPLLA
HHLDEVVAATARLRPRPEISLTTNGVGLARRAGALAEAGLDRVNVSLDSIDRAHFAAI
TRRDRLAHVLAGLAAAKAAGLTPVKVNAVLDPTTGREDVVDLLRFCLERGYQLRVIEQ
MPLDAGHSWRRNIALSADDVLAALRPHFRLRPDPAPRGSAPAELWLVDAGPNTPRGRF
GVIASVSHAFCSTCDRTRLTADGQIRSCLFSTEETDLRRLLRGGADDDAIEAAWRAAM
WSKPAGHGINAPDFIQPDRPMSAIGG"
CDS complement(969880..970269)
/codon_start=1
/note="gene_903"
/transl_table=11
/translation="MRLILNVIWLVFGGLWLALGYLLASLVCFLLIITIPFGFAALRI
ASYALWPFGRTIVEKPTAGTGALIGNVIWVLLFGIWLALGHLVSAAAMAVTIIGIPLA
LANLKLIPVSLVPLGKDIVGVNSQVPT"
CDS 970434..970841
/codon_start=1
/note="gene_904"
/transl_table=11

/translation="VPTGKVKWYDPDKGFGFLSQEGGEDVYVRSSALPTGVEALKAGQ
RVEFGIASGRRGPQALSLRLIEPPPSLSRPRREPAAEHKHSPDELHGMVEDMITLLES
TVQPELRKGRYPDRKTARRVAEVVRAVAREFES"
CDS complement(970960..972789)
/codon_start=1
/note="gene_905"
/transl_table=11
/translation="MSYVLATPEMVAAAANNLAQIGSTLSAANAAALAPTTGVLAAGA
DEVSAAVASLFSGHAQAYQTLGTQAAAFHERFIQALSTAAGAYGSAEAANASPLQQAL
NVINAPTQTLLGRPLIGNGTNGAPGTGQAGGPGGLLYGNGGNGGSGGVGQAGGAGGSA
GLIGIGGTGGAGGAGAVGGVGGNGGWLYGNGGAGGLGGTGVAGVNGGMGAAGGAGGNA
YLFGSGGAGGQGGMGAAGADGVNPTPTGTADAGSTGTDQTLGGNAIGGNGGPGDAGDA
MTSGGAGGSGGNAVSTVNGDAVGGEGGKGGEGAYGGAGGAGGSAASIGNAAIGGNGGA
GGNAQAPGGVGGAGGEGGDAQVGTNSPSNAEAGNGGSGGNGFDSFASGGTGGAGGTGG
AGGRGGLLIGDGGAGGAGGVGGTGGSGAPGGGGGAGGDGGAANTDSAGSSRKAFGGDG
GVGGDGASALGTGGEGGIGGQGGNGGAGGLLIGNGGAGGVGGTAGAGGTGGSGGAGGA
GGAGGGGTNSGPGAAFGGNGNTGGNGGNGGAPGALGGKGGSGGLIGRAGSDGGVGAGG
AGGAGGAGGTGGEGGTGGDGKTTDGNPGMGGSPGSAGQPGQPG"
CDS 973050..975002
/codon_start=1
/note="gene_906"
/transl_table=11
/translation="MAQQTQVTEEQARALAEESRESGWDKPSFAKELFLGRFPLGLIH
PFPKPSDAEEARTEAFLVKLREFLDTVDGSVIERAAQIPDEYVKGLAELGCFGLKIPS
EYGGLNMSQVAYNRVLMMVTTVHSSLGALLSAHQSIGVPEPLKLAGTAEQKRRFLPRC
AAGAISAFLLTEPDVGSDPARMASTATPIDDGQAYELEGVKLWTTNGVVADLLVVMAR
VPRSEGHRGGISAFVVEADSPGITVERRNKFMGLRGIENGVTRLHRVRVPKDNLIGRE
GDGLKIALTTLNAGRLSLPAIATGVAKQALKIAREWSVERVQWGKPVGQHEAVASKIS
FIAATNYALDAVVELSSQMADEGRNDIRIEAALAKLWSSEMACLVGDELLQIRGGRGY
ETAESLAARGERAVPVEQMVRDLRINRIFEGSSEIMRLLIAREAVDAHLTAAGDLANP
KADLRQKAAAAAGASGFYAKWLPKLVFGEGQLPTTYREFGALATHLRFVERSSRKLAR
NTFYGMARWQASLEKKQGFLGRIVDIGAELFAISAACVRAEAQRTADPVEGEQAYELA
EAFCQQATLRVEALFDALWSNTDSIDVRLANDVLEGRYTWLEQGILDQSEGTGPWIAS
WEPGPSTEANLARRFLTVSPSSEAKL"
CDS complement(975091..976251)
/codon_start=1
/note="gene_907"
/transl_table=11
/translation="VRIGVGVCTTPDARQAAVEAAGQARDELAGEAPSLAVLLGSRAH
TDRAADVLSAVLQMIDPPALVGCIAQAIVAGRHEIEDEPAVVVWLASGLAAETFQLDF
VRTGSGALITGYRFDRTARDLHLLLPDPYTFPSNLLIEHPNTDLPGTAVVGGVVSGGR
RRGDTRLFRDHDVLTSGVVGVRLPGMRGVPVVSQGCRPIGYPYIVTGADGILITELGG
RPPLQRLREIVEGLSPDERALVSHGLQIGIVVDEHLAAPGQGDFVIRGLLGADPSTGS
IEIDEVVQVGATMQFQVRDAAGADKDLRLTVERAAARLPGRAAGALLFTCNGRGRRMF
GVADHDASTIEELLGGIPLAGFFAAGEIGPIAGRNALHGFTASMALFVDDME"
CDS complement(976351..976839)
/codon_start=1
/note="gene_908"
/transl_table=11
/translation="VKRGVATLPVILVILLSVAAGAGAWLLVRGHGPQQPEISAYSHG
HLTRVGPYLYCNVVDLDDCQTPQAQGELPVSERYPVQLSVPEVISRAPWRLLQVYQDP

ANTTSTLFRPDTRLAVTIPTVDPQRGRLTGIVVQLLTLVVDHSGELRDVPHAEWSVRL
IF"
CDS complement(976836..978221)
/codon_start=1
/note="gene_909"
/transl_table=11
/translation="MYLLVHRAATADGADKSGLTALTWPVMANFAVDSAMAVALANTL
FFAAASGESKSRVALYLLITIAPFAVIAPLIGPALDRLQHGRRVALALSFGLRTALAV
VLIMNYDGATGSFPSWVLYPCALAMMVFSKSFSVLRSAVTPRVMPPTIDLVRVNSRLT
VFGLLGGTIAGGAIAAGVEFVCTHLFQLPGALFVVVAITIAGASLSMRIPRWVEVTSG
EVPATLSYHRDRGRLRRRWPEEVKNLGGTLRQPLGRNIITSLWGNCTIKVMVGFLFLY
PAFVAKAHEANGWVQLGMLGLIGAAAAVGNFAGNFTSARLQLGRPAVLVVRCTVLVTV
LAIAAAVAGSLAATAIATLITAGSSAIAKASLDASLQHDLPEESRASGFGRSESTLQL
AWVLGGAVGVLVYTELWVGFTAVSALLILGLAQTIVSFRGDSLIPGLGGNRPVMAEQE
TTRRGAAVAPQ"
CDS 978620..979408
/codon_start=1
/note="gene_910"
/transl_table=11
/translation="VTGPTEESAVATVADWPEGLAAVLRGAADQARAAVVEFSGPEAV
GDYLGVSYEDGNAATHRFIAHLPGYQGWQWAVVVASYSGADHATISEVVLVPGPTALL
APDWVPWEQRVRPGDLSPGDLLAPAKDDPRLVPGYTASGDAQVDETAAEIGLGRRWVM
SAWGRAQSAQRWHDGDYGPGSAMARSTKRVCRDCGFFLPLAGSLGAMFGVCGNELSAD
GHVVDRQYGCGAHSDTTAPAGGSTPIYEPYDDGVLDIIEKPAES"
CDS complement(979417..980748)
/codon_start=1
/note="gene_911"
/transl_table=11
/translation="VMNFMVLPPEVNSARIYAGAGPAPMLAAAVAWDGLAAELGMAAA
SFSLLISGLTAGPGSAWQGPAAAAMAAAAAPYLSWLNAATARAEGAAAGAKAAAAVYE
AARAATAHPALVAANRNQLLSLVLSNLFGQNLPAIAATEASYEQLWAQDVAAMVGYHG
GASTVASQLTPWQQLLSVLPPVVTAAPAGAVGVPAALAIPALGVENIGVGNFLGIGNI
GNNNVGSGNTGDYNFGIGNIGNANLGNGNIGNANLGSGNAGFFNFGNGNDGNTNFGSG
NAGFLNIGSGNEGSGNLGFGNAGDDNTGWGNSGDTNTGGFNSGDLNTGIGSPVTQGVA
NSGFGNTGTGHSGFFNSGNSGSGFQNLGNGSSGFGNASDTSSGFQNAGTALTRASSTW
ADSPRAWPIRAPSRLQVWRTRATTARECSIRVIISRVSSTGAPPKKVGNSG"
CDS complement(981023..981298)
/codon_start=1
/note="gene_912"
/transl_table=11
/translation="MSVENSQIREPPPLPPVLLEVWPVIAVGALAWLVAAVAAFVVPG
LASWRPVTVAGLATGLLGTTIFVWQLAAARRGARGAQAGLETYLDPK"
CDS complement(981295..981699)
/codon_start=1
/gene="MT0903"
/note="gene_913"
/transl_table=11
/translation="MSANEAITLVIDGGRTRSRIANAPGVIAPSLASVVNAESWDSET
GDDGFRNRSWRESRMTANDKSLASRASLSSTAEVTTEVQTRASETANDDFGSTHCRPT
THQQVVVPPAGAEATHPGPFADASSPRYVDSG"
CDS 981620..981907

/codon_start=1
/note="gene_914"
/transl_table=11
/translation="MTPGALAIRERVRPPSMTRVIASLADMGFVDRAPHPIDGRQVLV
SVSESGAELVKAARRARQEWLAERLATLNRSERDILRSAADLMLALVDESP"
CDS 981904..982770
/codon_start=1
/note="gene_915"
/transl_table=11
/translation="VTEGRCAQHPDGLDVQDVCDPDDPRLDDFRDLNSIDRRPDLPTG
KALVIAEGVLVVQRMLASRFTPLALFGTDRRLAELKDDLAGVGAPYYRASADVMARVI
GFHLNRGVLAAARRVPEPSVAQVVAGARTVAVLEGVNDHENLGSIFRNAAGLSVDAVV
FGTGCADPLYRRAVRVSMGHALLVPYARAADWPTELMTLKESGFRLLAMTPHGNACKL
PEAIAAVSHERIALLVGAEGPGLTAAALRISDVRVRIPMSRGTDSLNVATAAALAFYE
RTRSGHHIGPGT"
CDS 982767..983051
/codon_start=1
/note="gene_916"
/transl_table=11
/translation="VNDQRDQAVPWATGLAVAGFVAAVIAVAVVVLSLGLIRVHPLLA
VGLNIVAVSGLAPTLWGWRRTPVLRWFVLGAAVGVAGAWLALLALTLGDG"
CDS complement(983048..983809)
/codon_start=1
/note="gene_917"
/transl_table=11
/translation="MRELKVVGLDADGKNIICQGAIPSEQFKLPVDDRLRAALRDDSV
QPEQAQLDIEVTNVLSPKEIQARIRAGASVEQVAAASGSDIARIRRFAHPVLLERSRA
AELATAAHPVLADGPAVLTMQETVAAALVARGLNPDSLTWDAWRNEDSRWTVQLAWKA
GRSDNLAHFRFTPGAHGGTATAIDDTAHELINPTFNRPLRPLAPVAHLDFDEPEPAQP
TLTVPSAQPVSNRRGKPAIPAWEDVLLGVRSGGRR"
CDS complement(983966..985096)
/codon_start=1
/note="gene_918"
/transl_table=11
/translation="MADQLTPHLEIPTAIKPRDGRFGSGPSKVRLEQLQTLTTTAAAL
FGTSHRQAPVKNLVGRVRSGLAELFSLPDGYEVILGNGGATAFWDAAAFGLIDKRSLH
LTYGEFSAKFASAVSKNPFVGEPIIITSDPGSAPEPQTDPSVDVIAWAHNETSTGVAV
AVRRPEGSDDALVVIDATSGAGGLPVDIAETDAYYFAPQKNFASDGGLWLAIMSPAAL
SRIEAIAATGRWVPDFLSLPIAVENSLKNQTYNTPAIATLALLAEQIDWLVGNGGLDW
AVKRTADSSQRLYSWAQERPYTTPFVTDPGLRSQVVGTIDFVDDVDAGTVAKILRANG
IVDTEPYRKLGRNQLRVAMFPAVEPDDVSALTECVDWVVERL"
CDS 985343..986326
/codon_start=1
/note="gene_919"
/transl_table=11
/translation="MDVADDAEYVEMLATLSEGSVRRNFNPYTDIDWESPEFAVTDND
PRWILPATDPLGRHPWYQAQSRERQIEIGMWRQANVAKVGLHFESILIRGLMNYTFWM
PNGSPEYRYCLHESVEECNHTMMFQEMVNRVGADVPGLPRRLRWVSPLVPLVAGPLPV
AFFIGVLAGEEPIDHTQKNVLREGKSLHPIMERVMSIHVAEEARHISFAHEYLRKRLP
RLIRMQRFWISLYFPLTMRSLCNAIVVPPKAFWEEFDIPREVKKELFFGSPESRKWLC
DMFADARMLAHDTGLMNPIARLVWRLCKIDGKPSRYRSEPQRQHLAAAPAA"

```
CDS             986345..988072
                /codon_start=1
                /note="gene_920"
                /transl_table=11
                /translation="MPHVITQSCCNDASCVFACPVNCIHPTPDEPGFATSEMLYIDPV
                ACVDCGACVTACPVSAIAPNTRLDFEQLPFVEINASYYPKRPAGVKLAPTSKLAPVTP
                AAEVRVRRQPLTVAVVGSGPAAMYAADELLVQQGVQVNVFEKLPTPYGLVRSGVAPDH
                QNTKRVTRLFDRIAGHRRFRFYLNVEIGKHLGHAELLAHHHAVLYAVGAPDDRRLTID
                GMGLPGTGTATELVAWLNGHPDFNDLPVDLSHERVVIIGNGNVALDVARVLAADPHEL
                AATDIADHALSALRNSAVREVVVAARRGPAHSAFTLPELIGLTAGADVVLDPGDHQRV
                LDDLAIVADPLTRNKLEILSTLGDGSAPARRVGRPRIRLAYRLTPRRVLGQRRAGGVQ
                FSVTGTDELRQLDAGLVLTSIGYRGKPIPDLPFDEQAALVPNDGGRVIDPGTGEPVPG
                AYVAGWIKRGPTGFIGTNKSCSMQTVQALVADFNDGRLTDPVATPTALDQLVQARQPQ
                AIGCAGWRAIDAAEIARGSADGRVRNKFTDVAEMLAAATSAPKEPLRRRVLARLRDLG
                QPIVLTVPL"
CDS             complement(988055..988513)
                /codon_start=1
                /note="gene_921"
                /transl_table=11
                /translation="MAINVEPALSPHLVVDDAASAIDFYVKAFDAVELGRVPGPDGKL
                IHAALRINGFTVMLNDDVPQMCGGKSMTPTSLGGTPVTIHLTVTDVDAKFQRALNAGA
                TVVTALEDQLWGDRYGVVADPFGHHWSLGQPVREVNMDEIQAAMSSQGDG"
CDS             989216..989365
                /codon_start=1
                /gene="TMLG_01586"
                /note="gene_922"
                /transl_table=11
                /translation="VRLAGNVGNIPIPIDCTLRGLTQYSRTNNAEVVQSVETHHRPAN
                FDALT"
CDS             989775..991247
                /codon_start=1
                /note="gene_923"
                /transl_table=11
                /translation="MDYAKRIGQVGALAVVLGVGAAVTTHAIGSAAPTDPSSSSTDSP
                VDACSPLGGSASSLAAIPGASVPQVGVRQVDPGSIPDDLLNALIDFLAAVRNGLVPII
                ENRTPVANPQQVSVPEGGTVGPVRFDACDPDGNRMTFAVRERGAPGGPQHGIVTVDQR
                TASFIYTADPGFVGTDTFSVNVSDDTSLHVHGLAGYLGPFHGHDDVATVTVFVGNTPT
                DTISGDFSMLTYNIAGLPFPLSSAILPRFFYTKEIGKRLNAYYVANVQEDFAYHQFLI
                KKSKMPSQTPPEPPTLLWPIGVPFSDGLNTLSEFKVQRLDRQTWYECTSDNCLTLKGF
                TYSQMRLPGGDTVDVYNLHTNTGGGPTTNANLAQVANYIQQNSAGRAVIVTGDFNARY
                SDDQSALLQFAQVNGLTDAWVQVEHGPTTPPFAPTCMVGNECELLDKIFYRSGQGVTL
                QAVSYGNEAPKFFNSKGEPLSDHSPAVVGFHYVADNVAVR"
CDS             complement(991282..992403)
                /codon_start=1
                /gene="citA3"
                /note="gene_924"
                /transl_table=11
                /translation="MTVVPENFVPGLDGVVAFTTEIAEPDKDGGALRYRGVDIEDLVS
                QRVTFGDVWALLVDGNFGSGLPPAEPFPLPIHSGDVRVDVQAGLAMLAPIWGYAPLLD
                IDDATARQQLARASVMALSYVAQSARGIYQPAVPQRIIDECSTVTARFMTRWQGEPDP
                RHIEAIDAYWVSAAEHGMNASTFTARVIASTGADVAAALSGAIGAMSGPLHGGAPARV
```

LPMLDEVERAGDARSVVKGILDRGEKLMGFGHRVYRAEDPRARVLRAAAERLGAPRYE
VAVAVEQAALSELRERRPDRAIETNVEFWAAVVLDFARVPANMMPAMFTCGRTAGWCA
HILEQKRLGKLVRPSAIYVGPGPRSPESVDGWERVLTTA"

CDS complement(992490..995138)
/codon_start=1
/note="gene_925"
/transl_table=11
/translation="VRALLAQNRLVTLCGTGGVGKTRLAIQIASASELRDGLCFVDLA
PITESGIVAATAARAVGLPDQPGRSTMDSLRRFIGNRRMLMVLDNCEHLLDACAALVV
ELLGACPELTILATSREPIGMAGEITWRVPSMSITDEAVELFADRASRVQPGFTIANH
NAAAVGEICRRLDGIPLAIEFAAARVRSMSPLEIADGLDDCFRLLAGGVRGAVQRQQT
LRASIDWSHALLTETEQILFRRLAPFVGGFDLAAVRAVAAGSDLDPFSVLDQLTLLVD
KSLVVADDCQGRTRYRLLETVRRYALEKLGDSGEADVHARHRDYYTALAASLNTPADN
DHQRLVARAETEIDNLRAAFAWSRENGHITEALQLASSLQPIWFGRAHLREGLSWFNS
ILEDQRFHRLAVSTAVRARALADKAMLSTWLATSPVGATDIIAPAQQALAMAREVGDP
AALVRALTACGCSSGYNAEAAAPYFAEATDLARAIDDKWTLCQILYWRGVGTCISGDP
NALRAAAEECRDLADTIGDRFVSRHCSLWLSLAQMWAGNLTEALELSREITAEAEASN
DVPTKVLGLYTQAQVLAYCGASAAHAIAGACIAAATELGGVYQGIGYAAMTYAALAAG
DVTAALEASDAARPILRAQPDQVTMHQVLMAQLALAGGDAIAARQFANDAVDATNGWH
RMVALTIRARVATARGEPELARDDAHAALACGAELHIYQGMPDAMELLAGLAGEVGSH
SEGVRLLGAAAALRQQTRQVRFKIWDAGYQASVTALREAMGDEDFDRAWAEGAALSTD
EAIAYAQRGRGERKRPARGWGSLTPTERDVVRLVSEGLSNKDIAKRLFVSPRTVQTHL
THVYAKLGLASRVQLVDEAARRGSPS"

CDS complement(995140..995679)
/codon_start=1
/note="gene_926"
/transl_table=11
/translation="MCPEDMATAIAHLDHTVSEAITNHGGVQPVKRYEGDSFVAAFTR
ASDAAACALDLQRTSLAPIRLRIGLHTGEVQLRDELYVGPTINRTARLRDLAHGGQVV
LSAATGDLVTGRLPADAWLVDLGRHPLRGLPRPEWVMQLCHPDIREKFPPLRTAKSSP
TSILPAQFTTFVGRRAQIS"

CDS 995977..996294
/codon_start=1
/gene="O216_04955"
/note="gene_927"
/transl_table=11
/translation="MNCVEEHDTCLTTAITSSQHLRGAAKPISTLQFGEDTWPILETG
LSQRCSLPPKEIVFGAARWALAAARGMLPRPTTDSPPQRQRYPKRYRFLEHSCLEREM
RRL"

CDS 996341..997882
/codon_start=1
/note="gene_928"
/transl_table=11
/translation="LLNRMWEGDFGLFWGVGAMTGRCPTVAVVGAGMSGMCVAITLLS
AGITDVCIYEKADDVGGTWRDNTYPGLTCDVPSRLYQYSFAKNPNWTQMFSRGGEIQD
YLRGIAERYGLRHRIRFGATVVSARFDDGRWVLRTDSGTESTVDFLISATGVLHHPRI
PPIAGLDDFRGTVFHSARWDHTVPLLGRRIAVIGTGSTGVQLVCGLAGVAGKVTMFQR
TAQWVLPWPNPRYSKLARVFHRAFPCLGSLAYKAYSLSFETFAVALSNPGLHRKLVGA
VCRASLRRVRDPRLRRALTPDYEPMCKRLVMSGGFYRAIQRDDVELVTAGIDHVEHRG
IVTDDGVLHEVDVIVLATGFDSHAFFRPMQLTGRDGIRIDDVWQDGPHAHQTVAIPGF
PNFFMMLGPHSPVGNFPLTAVAESQAEHIVQWIKRWRHGEFDTMEPKSAATEAYNTVL

```
                     RAAMPNTVWTTGCDSWYLNKDGIPEVWPFAPAKHRAMLANLHPEEYDLRRYAAVRATS
                     RPQSA"
     CDS             complement(997860..998837)
                     /codon_start=1
                     /note="gene_929"
                     /transl_table=11
                     /translation="MRTEDDSWDVTTSVGSTGLLVAAARALETQKADPLAIDPYAEVF
                     CRAAGGEWADVLDGKLPDHYLTTGDFGEHFVNFQGARTRYFDEYFSRATAAGMKQVVI
                     LAAGLDSRAFRLQWPIGTTIFELDRPQVLDFKNAVLADYHIRPRAQRRSVAVDLRDEW
                     QIALCNNGFDANRPSAWIAEGLLVYLSAEAQQRLFIGIDTLASPGSHVAVEEATPLDP
                     CEFAAKLERERAANAQGDPRRFFQMVYNERWARATEWFDERGWRATATPLAEYLRRVG
                     RAVPEADTEAAPMVTAITFVSAVRTGLVADPARTSPSSTSIGFKRFEAD"
     CDS             999138..1000247
                     /codon_start=1
                     /note="gene_930"
                     /transl_table=11
                     /translation="MMPKIVSVQHSTRRHLTSFVGRKAELNDVRRLLSDKRLVTLTGP
                     DGMGKSRLALQIGAQIAHEFTYGRWDCDLATVTDRDCVSISMLNALGLPVQPGLSAID
                     TLVGVINDARVLLVLDHCEHLLDACAAIIDSLLRSCPRLTILTTSTEAIGLAGELTWR
                     VPPLSLTNDAIELFVDRARRVRSDFAINADTAVTVGEICRRLDGVPLAIELAAARTDT
                     LSPVEILAGLNDRFRLVAGAAGNAVRPEQTLCATVQWSHALLSGPERALLHRLAVFAG
                     GFDLDGAQAVGANDEDFEGYQTLGRFAELVDKAFVVVENNRGRAGYRLLYSVRQYALE
                     KLSESGEADAVLARYRKHLKQPNQVVRAGSGGVRY"
     CDS             1000405..1001841
                     /codon_start=1
                     /note="gene_931"
                     /transl_table=11
                     /translation="MDLPMAAADALFLWAETPTRPLHVGALAVLSQPDNGTGRYLRKV
                     FSAAVARQQVAPWWRRRPHRSLTSLGQWSWRTETEVDLDYHVRLSALPPRAGTAELWA
                     LVSELHAGMLDRSRPLWQVDLIEGLPGGRCAVYVKVHHALADGVSVMRLLQRIVTADP
                     HQRQMPTLWEVPAQASVAKHTAPRGSSRPLTLAKGVLGQARGVPGMVRVVADTTWRAA
                     QCRSGPLTLAAPHTPLNEPIAGARSVAGCSFPIERLRQVAEHADATINDVVLAMCGGA
                     LRAYLISRGALPGAPLIAMVPVSLRDTAVIDVFGQGPGNKIGTLMCSLATHLASPVER
                     LSAIRASMRDGKAAIAGRSRNQALAMSALGAAPLALAMALGRVPAPLRPPNVTISNVP
                     GPQGALYWNGARLDALYLLSAPVDGAALNITCSGTNEQITFGLTGCRRAVPALSILTD
                     QLAHELELLVGVSEAGPGTRLRRIAGRR"
     CDS             1002031..1003326
                     /codon_start=1
                     /gene="gltA"
                     /note="gene_932"
                     /transl_table=11
                     /translation="VADTDDTATLRYPGGEIDLQIVHATEGADGIALGPLLAKTGHTT
                     FDVGFANTAAAKSSITYIDGDAGILRYRGYPIDQLAEKSTFIEVCYLLIYGELPDTDQ
                     LAQFTGRIQRHTMLHEDLKRFFDGFPRNAHPMPVLSSVVNALSAYYQDALDPMDNGQV
                     ELSTIRLLAKLPTIAAYAYKKSVGQPFLYPDNSLTLVENFLRLTFGFPAEPYQADPEV
                     VRALDMLFILHADHEQNCSTSTVRLVGSSRANLFTSISGGINALWGPLHGGANQAVLE
                     MLEGIRDSGDDVSEFVRKVKNREAGVKLMGFGHRVYKNYDPRARIVKEQADKILAKLG
                     GDDSLLGIAKELEEAALTDDYFIERKLYPNVDFYTGLIYRALGFPTRMFTVLFALGRL
                     PGWIAHWREMHDEGDSKIGRPRQIYTGYAERDYVTIDAR"
     CDS             complement(1003367..1004974)
                     /codon_start=1
```

/note="gene_933"
/transl_table=11
/translation="MSDHDRDFDVVVVGGGHNGLVAAAYLARAGLRVRLLERLAQTGG
AAVSIQAFDGVEVALSRYSYLVSLLPSRIVADLGAPVRLARRPFSSYTPAPATAGRSG
LLIGPTGEPRAAHLAAIGAAPDAHGFAAFYRRCRLVTARLWPTLIEPLRTREQARRDI
VEYGGHEAAAAWQAMVDEPIGHAIAGAVANDLLRGVIATDALIGTFARMHEPSLMQNI
CFLYHLVGGGTGVWHVPIGGMGSVTSALATAAARHGAEIVTGADVFALDPDGTVRYHS
DGSDGAEHLVRGRFVLVGVTPAVLASLLGEPVAALAPGAQVKVNMVVRRLPRLRDDSV
TPQQAFAGTFHVNETWSQLDAAYSQAASGRLPDPLPCEAYCHSLTDPSILSARLRDAG
AQTLTVFGLHTPHSVFGDTEGLAERLTAAVLASLNSVLAEPIQDVLWTDAQSKPCIET
TTTLDLQRTLGMTGGNIFHGALSWPFADNDDPLDTPARQWGVATDHERIMLCGSGARR
GGAVSGIGGHNAAMAVLACLASRRKSP"

CDS complement(1005000..1005263)
/codon_start=1
/note="gene_934"
/transl_table=11
/translation="MGKGRKPTDSETLAHIRDLVAEEKALRAQLRHGGISESEEQQQL
RRIEIELDQCWDLLRQRRALRQTGGDPREAVVRPADQVEGYTG"

CDS 1005371..1006351
/codon_start=1
/note="gene_935"
/transl_table=11
/translation="VASKAGLGQTPATTDARRTQKFYRGSPGRPWLIGAVVIPLLIAA
IGYGAFERPQSVTGPTGVLPTLTPTSTRGASALSLSLLSISRSGNTVTLIGDFPDEAA
KAALMTALNGLLAPGVNVIDQIHVDPVVRSLDFSSAEPVFTASVPIPDFGLKVERDTV
TLTGTAPSSEHKDAVKRAATSTWPDMKIVNNIEVTGQAPPGPPASGPCADLQSAINAV
TGGPIAFGNDGASLIPADYEILNRVADKLKACPDARVTINGYTDNTGSEGINIPLSAQ
RAKIVADYLVARGVAGDHIATVGLGSVNPIASNATPEGRAKNRRVEIVVN"

CDS 1006364..1006516
/codon_start=1
/note="gene_936"
/transl_table=11
/translation="MDFVIQWSCYLLAFLGGSAVAWVVVTLSIKRASRDEGAAEAPSA
AETGAQ"

CDS 1006516..1007043
/codon_start=1
/note="gene_937"
/transl_table=11
/translation="MEHVHWWLAGLAFTLGMVLTSTLMVRPVEHQVLVKKSVRGSSAK
SKPPTARKPAVKSGTKREESPTAKTKVATESAAEQIPVAGEPAAEPIPVAGEPAARIP
VVPYAPYGPGSARAGADGSGPQGWLVKGRSDTRLYYTPEDPTYDPTVAQVWFQDEESA
ARAFFTPWRKSTRRT"

CDS complement(1007060..1008400)
/codon_start=1
/note="gene_938"
/transl_table=11
/translation="MNILSRIFARTPSLRTRVVVATAIGAAIPVLIVGTVVWVGITND
RKERLDRRLDEAAGFAIPFVPRGLDEIPRSPNDQDALITVRRGNVIKSNSDITLPKLQ
DDYADTYVRGVRYRVRTVEIPGPEPTSVAVGATYDATVAETNNLHRRVLLICTFAIGA
AAVFAWLLAAFAVRPFKQLAEQTRSIDAGDEAPRVEVHGASEAIEIAEAMRGMLQRIW
NEQNRTKEALASARDFAAVSSHELRTPLTAMRTNLEVLSTLDLPDDQRKEVLNDVIRT

QSRIEATLSALERLAQGELSTSDDHVPVDITDLLDRAAHDAARIYPDLDVSLVPSPTC
IIVGLPAGLRLAVDNAIANAVKHGGATLVQLSAVSSRAGVEIAIDDNGSGVPEGERQV
VFERFSRGSTASHSGSGLGLALVAQQAQLHGGTASLENSPLGGARLVLRLPGPS"
CDS complement(1008411..1009121)
/codon_start=1
/note="gene_939"
/transl_table=11
/translation="MGGMDTGVTSPRVLVVDDDSDVLASLERGLRLSGFEVATAVDGA
EALRSATENRPDAIVLDINMPVLDGVSVVTALRAMDNDVPVCVLSARSSVDDRVAGLE
AGADDYLVKPFVLAELVARVKALLRRRGSTATSSSETITVGPLEVDIPGRRARVNGVD
VDLTKREFDLLAVLAEHKTAVLSRAQLLELVWGYDFAADTNVVDVFIGYLRRKLEAGG
GPRLLHTVRGVGFVLRMQ"
CDS complement(1009252..1010739)
/codon_start=1
/note="gene_940"
/transl_table=11
/translation="VSRITTDQLRHAVLDRGSFVSWDSEPLAVPVADSYARELAAARA
ATGADESVQTGEGRVFGRRVAVVACEFDFLGGSIGVAAAERITAAVERATAERLPLLA
SPSSGGTRMQEGTVAFLQMVKIAAAIQLHNQARLPYLVYLRHPTTGGVFASWGSLGHL
TVAEPGALIGFLGPRVYELLYGDPFPSGVQTAENLRRHGIIDGVVALDRLRPMLDRAL
TVLIDAPEPLPAPQTPAPVPDVPTWDSVVASRRPDRPGVRQLLRHGATDRVLLSGTDQ
GEAATTLLALARFGGQPTVVLGQQRAVGGGGSTVGPAALREARRGMALAAELCLPLVL
VIDAAGPALSAAAEQGGLAGQIAHCLAELVTLDTPTVSILLGQGSGGPALAMLPADRV
LAALHGWLAPLPPEGASAIVFRDTAHAAELAAAQGIRSADLLKSGIVDTIVPEYPDAA
DEPIEFALRLSNAIAAEVHALRKIPAPERLATRLQRYRRIGLPRD"
CDS 1010766..1011497
/codon_start=1
/note="gene_941"
/transl_table=11
/translation="MIGITQAEAVLTIELQRPERRNALNSQLVEELTQAIRKAGDGSA
RAIVLTGQGTAFCAGADLSGDAFAADYPDRLIELHKAMDASPMPVVGAINGPAIGAGL
QLAMQCDLRVVAPDAFFQFPTSKYGLALDNWSIRRLSSLVGHGRARAMLLSAEKLTAE
IALHTGMANRIGTLADAQAWAAEIARLAPLAIQHAKRVLNDDGAIEEAWPAHKELFDK
AWGSQDVIEAQVARMEKRPPKFQGA"
CDS 1011503..1012621
/codon_start=1
/note="gene_942"
/transl_table=11
/translation="MVRRALRLAAGTASLAAGTWLLRALHGTPAALGADAASIRAVSE
QSPNYRDGAFVNLDPASMFTLDREELRLIVWELVARHSASRPAAPIPLASPNIYRGDA
SRLAVSWFGHSTALLEIDGYRVLTDPVWSDRCSPSDVVGPQRLHPPPVQLAALPAVDA
VVISHDHYDHLDIDTVVALVGMQRAPFLVPLGVGAHLRSWGVPQDRIVELDWNQSAQV
DELTVVCVPARHFSGRFLSRNTTLWASWAFVGPNHRAYFGGDTGYTKSFTQIGADHGP
FDLTLLPIGAYNTAWPDIHMNPEEAVRAHLDVTDSGSGMLVPVHWGTFRLAPHPWGEP
VERLLAAAEPEHVTVAVPLPGQRVDPTGPMRLHPWWRL"
CDS 1012741..1014294
/codon_start=1
/note="gene_943"
/transl_table=11
/translation="MVMLLTLTGCGSTHQALGPPSGLPDASPNERSAIQIPAGRIDDA
VAKVDGLVGELMQNTGIPGMAVAIVHGGKTLYAKGFGVRDVGKGGGPDNKVDADTVFQ

LASVSKSVGATVVAHAVTDNVVTWDTPVVSKLPWFALRDPYVTGQVTIADLYSHRSGL
PDHAGDLLEDLGYDRRQVLQRLKYLPLAPFRISYAYTNFGVTAAAEAVAAAAGQSWED
LSDEVLYRPLGMGSTSSRFTDFLARPNHAVNHVKVADRWEARYQRDPDAQSPAGGVSS
SLNDMTHWLAMVLADGVYNGRRITSPEALLLVYTPQVISRHPVSPRARASFYGYGFNV
GVTSSGRTEYSHSGAFGLGAAANFVVLPSEDLAIIALTNAGPIGVPETLTAEFMDLVQ
YGQVREDWAALYKKAFAPLNELAGSLVGKQSPANPAPSRPLNDYVGVYANDYWGPATV
TYHDGQLRLSLGPKNQTFDLTHWDGDTFTFTLSTENALPGSISKATFAGDTLNLEYYD
ADKLGTFTR"

CDS 1014291..1016684
/codon_start=1
/note="gene_944"
/transl_table=11
/translation="MTRSASATAGLTDAEVAQRVAEGKSNDIPERVTRTVGQIVRANV
FTRINAILGVLLLIVLATGSLINGMFGLLIIANSVIGMVQEIRAKQTLDKLAIIGQAK
PLVRRQSGTRTRSTNEVVLDDIIELGPGDQVVVDGEVVEEENLEIDESLLTGEADPIA
KDAGDTVMSGSFVVSGAGAYRATKVGSEAYAAKLAAEASKFTLVKSELRNGINRILQF
ITYLLVPAGLLTIYTQLFTTHVGWRESVLRMVGALVPMVPEGLVLMTSIAFAVGVVRL
GQRQCLVQELPAIEGLARVDVVCADKTGTLTESGMRVCEVEELDGAGRQESVADVLAA
LAAADARPNASMQAIAEAFHSPPGWVVAANAPFKSATKWSGVSFRDHGNWVIGAPDVL
LDPASVAARQAERIGAQGLRVLLLAAGSVAVDHAQAPGQVTPVALVVLEQKVRPDARE
TLDYFAVQNVSVKVISGDNAVSVGAVADRLGLHGEAMDARALPTGREELADTLDSYTS
FGRVRPDQKRAIVHALQSHGHTVAMTGDGVNDVLALKDADIGVAMGSGSPASRAVAQI
VLLNNRFATLPHVVGEGRRVIGNIERVANLFLTKTVYSVLLALLVGIECLIAIPLRRD
PLLFPFQPIHVTIAAWFTIGIPAFILSLAPNNERAYPGFVRRVMTSAVPFGLVIGVAT
FVTYLAAYQGRYASWQEQEQASTAALITLLMTALWVLAVIARPYQWWRLALVLASGLA
YVVIFSLPLAREKFLLDASNLATTSIALAVGVVGAATIEAMWWIRSRMLGVKPRVWR"

CDS 1016957..1017115
/codon_start=1
/note="gene_945"
/transl_table=11
/translation="VVHIVGLSIETTAPTDSAITPIMVREINIGEIPLGLRLGSDTTL
LDAALAGG"

CDS 1017241..1017420
/codon_start=1
/note="gene_946"
/transl_table=11
/translation="MGILDKVKNLLSQNADKVETVINKAGEFVDEQTQGNYSDAIHKL
QDAASNVVGMSDQQS"

CDS 1017426..1017860
/codon_start=1
/note="gene_947"
/transl_table=11
/translation="MAKLSGSIDVPLPPEEAWMHASDLTRYREWLTIHKVWRSKLPEV
LEKGTVVESYVEVKGMPNRIKWTIVRYKPPEGMTLNGDGVGGVKVKLIAKVAPKEHGS
VVSFDVHLGGPALLGPIGMIVAAALRADIRESLQNFVTVFAG"

CDS 1017958..1018731
/codon_start=1
/note="gene_948"
/transl_table=11
/translation="VPTRSSAPLGAPCWIDLTTSDVDRAQDFYGTVFGWAFESAGPDY
GGYINAAKGGHPVAGLMANRPEFQSPDGWATYFHTVDIGATVAKLAAAGGSSCLDPME

VPGKGFMSLAVDPSGAAFGLWQPLQHHGFEVIGEAGSPVWHQLTTRDYRSVIDFYRQV
FGWRTEQISDTDEFCYTTAWFDDQQLLGVMDGSSCLPEGVPSNWTIFFGAEDVDETLR
VICDNGGSVVRAAENTPYGRLAAAADPMGVVFNLSSLQA"

CDS 1018796..1019245
/codon_start=1
/note="gene_949"
/transl_table=11
/translation="MTRRLRPGWLVALSAAVIAASTWMPWLTTTVGGGGWVNAIGGTH
GSLELPHGFGPGQLIVLLSSTLLVVGAMAGRGLSVKLSSIAALVVSLLIVALTVWYYK
LNVNPPVSAEYGLYFGAAGGVCAVGCSLWAAVSAASPGRRRHREVVR"

CDS complement(1019380..1019610)
/codon_start=1
/gene="TBPG_02786"
/note="gene_950"
/transl_table=11
/translation="VRFYRDLVGMLDQTFAESYGSNGAIFGLPSSSLTLEIVETDHHE
QLCRYCSDKRGQQAALTGLQEVATQPVEQRPL"

CDS complement(1019777..1021285)
/codon_start=1
/note="gene_951"
/transl_table=11
/translation="MDITIVGKYLSTLPEDDDHPYRTGPWRPQTTEWDADDLTTVTGE
VPADLDGIYLRNTENPLHPAFATYHPFDGDGMIHVVGFRDGKAFYRNRFIRTDGFLAE
NEAGGPLWPGLAEPVQLAKREHGWGARGLMKDASSTDVIVHRGIALTSFYQCGDLYRI
DPYSANTLGKESWHGRFPFDWGVSAHPKVDNKTGELLFFNYSKQEPYMRYGVVDQNNE
LVHYVDVPLPGPRLPHDMAFTENYVILNDFPLFWDPRLLERDVHLPRFYPEIPSRFAV
VARRGNDIRWFEADPTFVLHFTNAYEQGDEIVLDGFYEGDPQPLDTGGTKWEKLFRFL
ALDRLQSRLHRWRLNMVTGAVHEEQLSESITEFGTINADYAASSYRYTYAATGKPSWF
LFDGLVKHDLLTGNHECYSFGDGVYGSETAMAPRVGSSAEDDGYLVTLTTDMNDDASY
CLVFDAARPGDGPICKLALPERISSGTHSAWVPGAELRRWDHAESPAAAVGL"

CDS complement(1021287..1022525)
/codon_start=1
/note="gene_952"
/transl_table=11
/translation="MDDGVWILGGYQSDFARNLSKENRDFADLTREVVDGTLTAAKVD
AADLAAAGVVHVANAFGEMFARQGHLGAMPATVCDDLWDTPATRHEAACASGSVATLA
AMADLRSGAYRVALVVGLELEKTVPGDTAAEHLSAAAWTGHEGAEARYLWPSMFAQVA
DEYDRRYGLDDTHLRAIAQLNFANARRNPNAQTRGWTIPDPITDDDATNPLTEGRLRR
FDCSQMTDGGAGLVLVSDAYLRDHRDARPIGRIDGWGHRTVGLGLRQKLDRVAQGDSA
PYLLPHVRATVLDALRRARVTLDDLDGIEVHDCFTPSEYLAIDHIGLTGPGESWKAIE
NGEIEIGGRLPINPSGGLIGGGHPVGASGVRMLLDAAKQVSGIAGDYQVENAEAFGTL
NFGGSTATTVSFVVSTTRGS"

CDS complement(1022618..1023889)
/codon_start=1
/note="gene_953"
/transl_table=11
/translation="MDFGLLPPEVNSSRMYSGPGPESMLAAAAAWDGVAAELTSAAVS
YGSVVSTLIVEPWMGPAAAAMAAAATPYVGWLAATAALAKETATQARAAAEAFGTAFA
MTVPPSLVAANRSRLMSLVAANILGQNSAAIAATQAEYAEMWAQDAAVMYSYEGASAA
ASALPPFTPPVQGTGPAGPAAAAAATQAAGAGAVADAQATLAQLPPGILSDILSALAA
NADPLTSGLLGIASTLNPQVGSAQPIVIPTPIGELDVIALYIASIATGSIALAITNTA

RPWHIGLYGNAGGLGPTQGHPLSSATDEPEPHWGPFGGAAPVSAGVGHAALVGALSVP
HSWTTAAPEIQLAVQATPTFSSSAGADPTALNGMPAGLLSGMALASLAARGTTGGGGT
RSGTSTDGQEDGRKPPVVVIREQPPPGNPPR"
CDS complement(1023904..1024203)
/codon_start=1
/note="gene_954"
/transl_table=11
/translation="MSFVTIQPVVLAAATGDLPTIGTAVSARNTAVCAPTTGVLPPAA
NDVSVLTAARFTAHTKHYRVVSKPAALVHGMFVALPAATADAYATTEAVNVVATG"
CDS 1024647..1026428
/codon_start=1
/note="gene_955"
/transl_table=11
/translation="MSAKERGDQNAVVDALRSIQPAVFIPASVVIVAMIVVSVVYSSV
AENAFVRLNSAITGGVGWWYILVATGFVVFALYCGISRIGTIRLGRDDELPEFSFWAW
LAMLFSAGMGIGLVFYGVAEPLSHYLRPPRSRGVPALTDAAANQAMALTVFHWGLHAW
AIYVVVGLGMAYMTYRRGRPLSVRWLLEPVVGRGRVEGALGHAVDVIAIVGTLFGVAT
SLGFGITQIASGLEYLGWIRVDNWWMVGMIAAITATATASVVSGVSKGLKWLSNINMA
LAAALALFVLLLGPTLFLLQSWVQNLGGYVQSLPQFMLRTAPFSHDGWLGDWTIFYWG
WWISWAPFVGMFIARISRGRTIREFIGAVLLVPTVIASLWFTIFGDSALLRQRNNGDM
LVNGAVDTNTSLFRLLDGLPIGAITSVLAVLVIVFFFVTSSDSGSLVIDILSAGGELD
PPKLTRVYWAVLEGVAAAVLLLIGGAGSLTALRTAAIATALPFSIVMVVACYAMTKAF
HFDLAATPRLLHVTVPDVVAAGNRRRHDISATLSGLIAVRDVDSGTYIVHPDTGALTV
TAPPDPLDDHVFESDRHVTRRNTTSSR"
CDS 1026960..1027247
/codon_start=1
/note="gene_956"
/transl_table=11
/translation="MATKPERKTERLAARLTPEQDALIRRAAEAEGTDLTNFTVTAAL
AHARDVLADRRLFVLTDAAWTEFLAALDRPVSHKPRLEKLFAARSIFDTEG"
CDS 1027244..1027744
/codon_start=1
/note="gene_957"
/transl_table=11
/translation="VSGYSAPRRISDADDVTSFSSGEPSLDDYLRKRALANHVQGGSR
CFVTCRDGRVVGFYALASGSVAHADAPGRVRRNMPDPVPVILLSRLAVDRKEQGRGLG
SHLLRDAIGRCVQAADSIGLRAILVHALHDEARAFYVHFDFEISPTDPLHLMLLMKDA
RALIGD"
tRNA complement(1027881..1027953)
/note="identified by tRNAscan-SE; putative"
CDS complement(1028057..1029376)
/codon_start=1
/note="gene_958"
/transl_table=11
/translation="MDAAQVIEPAHAGQDVDEAAVAARELSGAERALVGDLVRQARAE
GVALTGPDGLLKALTKTVLEAALQEEMTEHLGYDRHAAAGRGSGNSRNGSRNKKVITD
ACGQVEIAVPRDRNGTFEPVIVGKRKRRVTDVDRVVLSLYAKGLTTGEIAAHFADVYG
VSVSKDTISRITDRVIEEMQAWWSRPLEKVYAAVFIDAIMVKIRDGQVRNRPVYAAIG
VDLDGHKDILGMWAGEGDGESAKFWLAVLTELRNRGVKDIFFLVCDGLKGLPDSVSAA
FPLATVQTCIIHLIRNTFRYASRKYWDKISVDLKPIYTAASAAEARLRYEEFAEKWGK
PYPAITRLWDSAWEEFIPFLDYDVEIRRVPCSTNAIESLNARYRRAVRARGHFPNEQS

ALKTLYLVTRSLDPKGTGQTKWAVRWKPALNALAITFADRMPAAEER"
CDS 1029664..1030245
/codon_start=1
/note="gene_959"
/transl_table=11
/translation="VNLADWAESVGVNRHTAYRWFREGTLPVPAERVGRLILVKTAAS
ASAAAAGVVLYARVSSHDRRSDLDRQVARLTAWATERDLGVGQVVCEVGSGLNGKRPK
LRRILSDPDARVIVVEHRDRLARFGVEHLEAALSAQGRRIVVADPGETTDDLVCDMIE
VLTGMCARLYGRRGARNRAMRAVTEAKREPGAG"
CDS 1030257..1031897
/codon_start=1
/note="gene_960"
/transl_table=11
/translation="MRSCAQAAKVAEATGGVQLAGKPKPDGTPTFSRYVEIGVDFEAH
RPVVESVSVLFELYDGDANSYAATGGPGAQLPSGWMVTAAKFEVEWPADPQRAGLVRS
HFGARRKAFNWGLAQVKADLDAKAADPAHESVDWDLKSLRWAWNRAKDDVAPWWAENS
KECYSSGLADLAQGLANWKAGKNGTRKGRRVGFPRFKSGRRDPGRVRFTTGTMRIEDD
RRTITVPVIGPLRAKENTRRVQRHLVSGRAQILNMTLSQRWGRLFVAVCYALRTPTTR
SPLTQPTVRAGMDLGVRTLATVATLDTATGEQTIIEYPNPAPLKATLVARRRAGRELS
RRIPGSHGHRAVKAKLARLDRRCVHLRREAAHQLTTELAGTYGQVVIEDLDVAAMKRS
MRRRAFRRSVSDAAMGLVAPQLAYKTAKCSGVLTVADRWFASSQIHHGCTSPDGTPCR
LQGKGRIDKHLLCPVTGEVVDRDRNAALNLRDWPDNASRGPVGTTAPSAPGPTTTVGT
GHGADTGSSGAGGASVRPRPRRAGRGEAKTQTPQGDAA"
CDS complement(1032073..1033137)
/codon_start=1
/note="gene_961"
/transl_table=11
/translation="MPDRRHPYFAYGSNLCAHQMASRCPDAGAPRPAVLSDHNWLINQ
RGVATVEPFAGNKVHGVLWQLSERDLVRLDSAEGVPVRYRRERLTVHTDDTALPAWVY
IDHRVMPGRPRPGYLPRVIDGARHHGLPQRWIDYLHRWDPARWPLPVLPSSRSGPAPQ
SLSELLSQPGVIETSQLRSRFGFLAIHGGGLEQVTDLIAERSAEAAGASVYLLRHPDN
YPHHLPSARFDPAESARLAEFLDHVDVAVSLHGYDRIGRSTQLLAGGRNRALAAHLAR
HIQLPGYRVVTDLAAIPEELRGLHPDNPVNRVRDGGTQLELSIRVRGLGPRSTLPGVG
GMSPVTATLVQGLVTAARSW"
CDS complement(1033138..1034364)
/codon_start=1
/note="gene_962"
/transl_table=11
/translation="LAQDTRTSLKTSWYLLGPAFVAAIAYVDPGNVAANVSSGAQFGY
LLLWVIVAANVMAALVQYLSAKLGLVTGRSLPEAIGKRMGRPARLAYWAQAEIVAMAT
DVAEVIGGAIALRIMFNLPLPIGGIITGVVSLLLLTIQDRRGQRLFERVITALLLVIA
IGFTASFFVVTPPPNAVLGGLAPRFQGTESVLLAAPIMGATVMPHAVYLHSGLARDRH
GHPDPGPQRRRLLRVTRWDVGLAMLIAGGVNAAMLLVAALNMRGRGDTASIEGAYHAV
HDTLGATIAVLFAVGLLASGLASSSVGAYAGAMIMQGLLHWSVPMLVRRLITLGPALA
ILTLGFDPTRTLVLSQVVLSFGIPFAVLPLVKLTGSPAVMGGDTNHRATTWVGWVVAV
MVSLLNVMLIYLTVTG"
CDS complement(1034456..1035193)
/codon_start=1
/note="gene_963"
/transl_table=11
/translation="MTTTSDQNAAAPPRFDGLRALFINATLKRSPELSHTDGLIERSS

GIMREHGVQVDTLRAVDHDIATGVWPDMTEHGWATDEWPALYRRVLDAHILVLCGPIW
LGDNSSVMKRVIERLYACSSLLNEDGQYAYYGRAGGCLITGNEDGVKHCAMNVLYSLQ
HLGYTIPPQADAGWIGEAGPGPSYLDPGSGGPENDFTNRNTTFMTFNLMHIAQMLRVA
GGIPAYGNQRTKWDAGCRPDFANPDYR"
CDS complement(1035270..1036346)
/codon_start=1
/note="gene_964"
/transl_table=11
/translation="MAIPVVQLGTGNVGVHSLRALIADPEFELTGVWVSSDAKAGKDA
AELAGLADSTGVRASTDLNAVLATGPRCAVYNAMADNRLPEALEDYRRILAAGINIVG
SGPVFLQYPWQVIPDEIIKPLQDAARAGNSSLYVNGIDPGFANDLLPMALAGTCESIE
QIRCMEIVDYATYDSAVVMFDVMGFGKPMDQIPMLLQPGVLSLAWGSVVRQLAAGLGI
SLDGVEEMYVREPAPEAFNIASGHIPKGSAAALRFEVLGLVDGVPAVVLEHVTRLRAD
LCPEWPQPAQPGGSYRIEISGEPCYAMDICLSSRHGDHNHAGLVATAMRIVNAIPAVV
AAEPGIRTTLDLPLITGEGRYAAA"
CDS complement(1036400..1037191)
/codon_start=1
/note="gene_965"
/transl_table=11
/translation="MILDMFRLDDKVAVITGGGRGLGAAIALAFAQAGADVLIASRTS
SELDAVAEQIRAAGRRAHTVAADLAHPEVTAQLAGQAVGAFGKLDIVVNNVGGTMPNT
LLSTSTKDLADAFAFNVGTAHALTVAAVPLMLEHSGGGSVINISSTMGRLAARGFAAY
GTAKAALAHYTRLAALDLCPRVRVNAIAPGSILTSALEVVAANDELRAPMEQATPLRR
LGDPVDIAAAAVYLASPAGSFLTGKTLEVDGGLTFPNLDLPIPDL"
CDS 1037547..1038575
/codon_start=1
/gene="pstS-1"
/note="gene_966"
/transl_table=11
/translation="VTGGGATTGQASAKVDCGGKKTLKASGSTAQANAMTRFVNVFEQ
ACPGQTLNYTANGSGAGISEFNGNQTDFGGSDVPLSKDEAAAAQRRCGSPAWNLPVVF
GPIAVTYNLNSVSSLNLDGPTLAKIFNGSITQWNNPAIQALNRDFTLPGERIHVVFRS
DESGTTDNFQRYLQAASNGAWGKGAGKSFQGGVGEGARGNDGTSAAAKNTPGSITYNE
WSFAQAQHLTMANIVTSAGGDPVAITIDSVGQTIAGATISGVGNDLVLDTDSFYRPKR
PGSYPIVLATYEIVCSKYPDSQVGTAVKAFLQSTIGAGQSGLGDNGYIPIPDEFKSRL
STAVNAIA"
CDS 1038588..1039562
/codon_start=1
/note="gene_967"
/transl_table=11
/translation="VVTEPLTKPALVAVDMRPARRGERLFKLAASAAGSTIVIAILLI
AIFLLVRAVPSLRANHANFFTSTQFDTSDDEQLAFGVRDLFMVTALSSITALVLAVPV
AVGIAVFLTHYAPRRLSRPFGAMVDLLAAVPSIIFGLWGIFVLAPKLEPIARFLNRNL
GWLFLFKQGNVSLAGGGTIFTAGIVLSVMILPIVTSISREVFRQTPLIQIEAALALGA
TKWEVVRMTVLPYGRSGVVAASMLGLGRALGETVAVLVILRSAARPGTWSLFDGGYTF
ASKIASAASEFSEPLPTGAYISAGFALFVLTFLVNAAARAIAGGKVNG"
CDS 1039559..1040473
/codon_start=1
/note="gene_968"
/transl_table=11
/translation="VSPSTSIEALDQPVKPVVFRPLTLRRRIKNSVATTFFFTSFVVA

LIPLVWLLWVVIARGWFAVTRSGWWTHSLRGVLPEQFAGGVYHALYGTLVQAGVAAVL
AVPLGLMTAVYLVEYGTGRMSRVTTFTVDVLAGVPSIVAALFVFSLWIATLGFQQSAF
AVALALVLLMLPVVVRAGEEMLRLVPDELREASYALGVPKWKTIVRIVAPIAMPGIVS
GILLSIARVVGETAPVLVLVGYSHSINLDVFHGNMASLPLLIYTELTNPEHAGFLRVW
GAALTLIIVVATINLAAAMIRFVATRRR"

CDS complement(1040480..1041589)
/codon_start=1
/note="gene_969"
/transl_table=11
/translation="LLATPADTGLSQSESGIAGAGTGPPTPGAARWSPGDSATVAGPL
AADSRGGNWPSQTGHSPAVPNALQASLGHAVPPAGNKRKVWAVVGAAAIVLVAIVAAA
GYLVLRPSWSPTQASGQTVLPFTGIDFRLSPSGVAVDSAGNVYVTSEGMYGRVVKLAT
GSTGTTVLPFNGLYQPQGLAVDGAGTVYVTDFNNRVVTLAAGSNNQTVLPFDGLNYPE
GLAVDTQGAVYVADRGNNRVVKLAAGSKTQTVLPFTGLNDPDGVAVDNSGNVYVTDTD
NNRVVKLEAESNNQVVLPFTDITAPWGIAVDEAGTVYVTEHNTNQVVKLLAGSTTSTV
LPFTGLNTPLAVAVDSDRTVYVADRGNDRVVKLTS"

CDS complement(1041600..1042475)
/codon_start=1
/gene="pknD"
/note="gene_970"
/transl_table=11
/translation="VSDAVPQVGSQFGPYQLLRLLGRGGMGEVYEAEDTRKHRVVALK
LISPQYSDNAVFRARMQREADTAGRLTEPHIVPIHDYGEINGQFFVEMRMIDGTSLRA
LLKQYGPLTPARAVAIVRQIAAALDAAHANGVTHRDVKPENILVTASDFAYLVDFGIA
RAASDPGLTQTGTAVGTYNYMAPERFTGDEVTYRADIYALACVLGECLTGAPPYRADS
VERLIAAHLMDPAPQPSQLRPGRVPPALDQVIAKGMAKNPAERFMSAGDLAIAAHDAL
NHIRATPGHDDSAAR"

CDS complement(1042497..1043609)
/codon_start=1
/gene="pstS2"
/note="gene_971"
/transl_table=11
/translation="VKFARSGAAVSLLAAGTLVLTACGGGTNSSSSGAGGTSGSVHCG
GKKELHSSGSTAQENAMEQFVYAYVRSCPGYTLDYNANGSGAGVTQFLNNETDFAGSD
VPLNPSTGQPDRAAERCGSPAWDLPTVFGPIAITYNIKGVSTLNLDGPTTAKIFNGTI
TVWNDPQIQALNSGTDLPPTPISVIFRSDKSGTSDNFQKYLDGASNGAWGKGASETFN
GGVGVGASGNNGTSALLQTTDGSITYNEWSFAVGKQLNMAQIITSAGPDPVAITTESV
GKTIAGAKIMGQGNDLVLDTSSFYRPTQPGSYPIVLATYEIVCSKYPDATTGTAVRAF
MQAAIGPGQEGLDQYGSIPLPKSFQAKLAAAVNAIS"

CDS 1043825..1044040
/codon_start=1
/gene="pstBa"
/note="gene_972"
/transl_table=11
/translation="MACERLGGQSGAADVDAAAPAMAAVNLTLGFAGKTVLDQVSMGF
PARAVTSLMGPTGSGKTTFFAHPKPDE"

CDS 1044015..1044656
/codon_start=1
/gene="pstB-2"
/note="gene_973"
/transl_table=11

```
                     /translation="LRTLNRMNDKVSGYRYSGDVLLGGRSIFNYRDVLEFRRRVGMLF
                     QRPNPFPMSIMDNVLAGVRAHKLVPRKEFRGVAQARLTEVGLWDAVKDRLSDSPFRLS
                     GGQQQLLCLARTLAVNPEVLLLDEPTSALDPTTTEKIEEFIRSLADRLTVIIVTHNLA
                     QAARISDRAALFFDGRLVEEGPTEQLFSSPKHAETARYVAGLSGDVKDAKRGN"
CDS                  1044677..1045801
                     /codon_start=1
                     /note="gene_974"
                     /transl_table=11
                     /translation="VKIRLHTLLAVLTAAPLLLAAAGCGSKPPSGSPETGAGAGTVAT
                     TPASSPVTLAETGSTLLYPLFNLWGPAFHERYPNVTITAQGTGSGAGIAQAAAGTVNI
                     GASDAYLSEGDMAAHKGLMNIALAISAQQVNYNLPGVSEHLKLNGKVLAAMYQGTIKT
                     WDDPQIAALNPGVNLPGTAVVPLHRSDGSGDTFLFTQYLSKQDPEGWGKSPGFGTTVD
                     FPAVPGALGENGNGGMVTGCAETPGCVAYIGISFLDQASQRGLGEAQLGNSSGNFLLP
                     DAQSIQAAAAGFASKTPANQAISMIDGPAPDGYPIINYEYAIVNNRQKDAATAQTLQA
                     FLHWAITDGNKASFLDQAHFQPLPPAVVKLSDALIATISS"
CDS                  1045930..1046877
                     /codon_start=1
                     /note="gene_975"
                     /transl_table=11
                     /translation="VIPLLALVLVLVVLVIEAMGAIRLNGLHFFTATEWNPGNTYGET
                     VVTDGVAHPVGAYYGALPLIVGTLATSAIALIIAVPVSVGAALVIVERLPKRLAEAVG
                     IVLELLAGIPSVVVGLWGAMTFGPFIAHHIAPVIAHNAPDVPVLNYLRGDPGNGEGML
                     VSGLVLAVMVVPIIATTHDLFRQVPVLPREGAIALGMSNWECVRRVTLPWVSSGIVG
                     AVVLGLGRALGETMAVAMVSGAVLGAMPANIYATMTTIAATIVSQLDSAMTDSTNFAV
                     KTLAEVGLVLMVITLLTNVAARGMVRRVSRTALPVGRGI"
CDS                  1046879..1047784
                     /codon_start=1
                     /note="gene_976"
                     /transl_table=11
                     /translation="MGESAESGSRQLPAMSPPRRSVAYRRKIVDALWWAACVCCLAVV
                     ITPTLWMLIGVVSRAVPVFHWSVLVQDSQGNGGGLRNAIIGTAVLAIGVILVGGTVSV
                     LTGIYLSEFATGKTRSILRGAYEVLSGIPSIVLGYVGYLALVVYFDWGFSLAAGVLVL
                     SVMSIPYIAKATESALAQVPTSYREAAEALGLPAGWALRKIVLKTAMPGIVTGMLVAL
                     ALAIGETAPLLYTAGWSNSPPTGQLTDSPVGYLTYPIWTFYNQPSKSAQDLSYDAALL
                     LIVFLLLLIFIGRLINWLSRRRWDV"
CDS                  complement(1047761..1048636)
                     /codon_start=1
                     /note="gene_977"
                     /transl_table=11
                     /translation="VEFRRDVPLADNQGILTYMRAIWTGSIAFGLVNVPVKVYSATAD
                     HDIRFHQVHAKDNGRIRYKRVCEACGEVVDYRDLARAYESGDGQMVAITDDDIASLPE
                     ERSREIEVLEFVPAADVDPMMFDRSYFLEPDSKSSKSYVLLAKTLAETDRMAIVHFTL
                     RNKTRLAALRVKDFGKREVMMVHTLLWPDEIRDPDFPVLDQKVEIKPAELKMAGQVVD
                     SMADDFNPDRYHDTYQEQLQELIDTKLEGGQAFTAEDQPRLLDEPEDVSDLLAKLEAS
                     VKARSKANSNVPTPP"
CDS                  1048698..1050977
                     /codon_start=1
                     /note="gene_978"
                     /transl_table=11
                     /translation="MGSASEQRVTLTNADKVLYPATGTTKSDIFDYYAGVAEVMLGHI
                     AGRPATRKRWPNGVDQPAFFEKQLALSAPPWLSRATVAHRSGTTTYPIIDSATGLAWI
```

AQQAALEVHVPQWRFVAEPGSGELNPGPATRLVFDLDPGEGVMMAQLAEVARAVRDLL
ADIGLVTFPVTSGSKGLHLYTPLDEPVSSRGATVLAKRVAQRLEQAMPALVTSTMTKS
LRAGKVFVDWSQNSGSKTTIAPYSLRGRTHPTVAAPRTWAELDDPALRQLSYDEVLTR
IARDGDLLERLDADAPVADRLTRYRRMRDASKTPEPIPTAKPVTGDGNTFVIQEHHAR
RPHYDFRLERDGVLVSWAVPKNLPDNTSVNHLAIHTEDHPLEYATFEGAIPSGEYGAG
KVIIWDSGTYDTEKFHDDPHTGEVIVNLHGGRISGRYALIRTNGDRWLAHRLKNQKDQ
KVFEFDNLAPMLATHGTVAGLKASQWAFEGKWDGYRLLVEADHGAVRLRSRSGRDVTA
EYPQLRALAEDLADHHVVLDGEAVVLDSSGVPSFSQMQNRGRDTRVEFWAFDLLYLDG
RALLGTRYQDRRKLLETLANATSLTVPELLPGDGAQAFACSRKHGWEGVIAKRRDSRY
QPGRRCASWVKDKHWNTQEVVIGGWRAGEGGRSSGVGSLLMGIPGPGGLQFAGRVGTG
LSERELANLKEMLAPLHTDESPFDVPLPARDAKGITYVKPALVAEVRYSEWTPEGRLR
QSSWRGLRPDKKPSEVVRE"
CDS 1050974..1052908
/codon_start=1
/note="gene_979"
/transl_table=11
/translation="MKWVTYRSDHGERTGVLSGDAIYAMPPDVSLLDLVGRGADGLRT
AGERAVRSPAAVVALDEVTLAAPIPRPPSIRDSLCFLDHMRNCQEAMGGGRVLMDTWY
RIPAFYFACPSTVLGPYDDAPTAPGSAWQDFELEIAAVIGTSGKDLTVEQAERSIIGY
TIFNDWSARDLQMLEGQLRIGQAKGKDSGITLGPYLVTPDELEPYCRGGKLSLRVIAL
VNGTVIGSGSTAQMDWSFGEVIAYASRGVTLTPGDVFGSGTVPTCTLVEHLRPPESFP
GWLHDGDVVTLQVEGLGETRQTVRTSGTPFPLALRPNPDAEPDRRGVNPAPTRVPFTR
GLHEVADRVWAWTLPDGGYGFSNAGLVAGDGASLLVDTLFDLALTREMLAAMKPVTER
APITDALITHSNGDHTHGTQLLDRSVRIIAAKGTSEEIEHGPAPEMLARIQTADLGPV
ATRYLRDRFGHFDFSGIKLRNADLTFDRDLAIELGGRRVDLLNLGPAHTTADSVVHVA
DAGVLFAGDLLFIGCTPIVWAGPIANWVAACDAMIALDAPTVVPGHGPVTGPDGIRAV
RGYLAHIAEQAEAAYRKGLSLPEAVETIDLGEYASWLDSERVVVNVYQRYRELDPDTP
RQDLLALLVMQAEWAARHCT"
CDS complement(1053155..1054021)
/codon_start=1
/note="gene_980"
/transl_table=11
/translation="VRFSYAEAMTDFTFYIPLAKAAEAAGYSSMTIPDSIAYPFESDS
KYPYTPDGNREFMDGKPFIETFVLTAALGAVTTRLRFNFFVLKLPIRPPALVAKQAGS
LAALIGNRVGLGVGTSPWPEDYELMGVPFAKRGKRIDECIEIVRGLTTGDYFEFHGEF
YDIPKTKMTPAPTQPIPILVGGHADAALRRAARADGWMHGGGDPDELDRLIARVKRLR
EEAGKTSPFEIHVISLDGFTVDGVKRLEDKGVTDVIVGFRVPYTMGPDTEPLQTKIRN
LEMFAENVIAKV"
CDS complement(1054106..1054879)
/codon_start=1
/note="gene_981"
/transl_table=11
/translation="VVAVSTAAKSPTALAIAVRTQDSVVILTADGALDSSSSALLRDS
LTRATLEQPSAVIVNVTELQVAEESAWSVFISARWQADFRADVPVLLVCGHRAGRAAV
TRTGVARFMPVYPTEKAASKAIGRLARRNFKRSDAQLPANLNSLRESRQLVREWLTQW
SRPGLIPVALVVVNVFVENVLKHTGSDPVMRIESDGPTATIAVSDGSSAPAVRLASPP
KGIDVSGLAIVAALSRAWGSSPTSSGKTVWAIIGPENQL"
CDS complement(1055258..1056253)
/codon_start=1
/note="gene_982"
/transl_table=11

```
                     /translation="VRFQARRAIGPIRPTSAAWDRDFDPAGKRIAVVGTDAAAAHYIS
                     RLSESAASVTVFTQAPRRVVTGVPLWTTRAKRWLRRRTGAEHPAVAWATAAIDALTSS
                     GIRTSDGVEHPVDAIIYGTGFAIADQVGDQTLVGAGGVTIRQAWDDGMEPYLGVAVHG
                     FPNYFFITGPDTAAQARCVVECMKLMERTASRRIEVRRSSQQVFNERAQLKPAQPHRQ
                     TGGLEAFDLSSAATEDDQTYDGAATLTLAGARFRVRVRLTGHLDPIDGNYHWQGTVFD
                     SLPETSLTHARAATLTIGGRSAPARITEQTPWGTHSVAGVGPPPYARSGPASATT"
     CDS             1056327..1056803
                     /codon_start=1
                     /note="gene_983"
                     /transl_table=11
                     /translation="VAGTPQPRALGPDALDVSTDDLAGLLAGNTGRIKTVITDQKVIA
                     GIGNAYSDEILHVAKISPFATAGKLSGAQLTCLHEAMASVLSDAVRRSVGQGAAMLKG
                     EKRSGLRVHARTGLPCPVCGDTVREVSFADKSFQYCPTCQTGGKALADRRMSRLLK"
     CDS             1056809..1057570
                     /codon_start=1
                     /note="gene_984"
                     /transl_table=11
                     /translation="MLTGVTRQKILITGASSGLGAGMARSFAAQGRDLALCARRTDRL
                     TELKAELSQRYPDIKIAVAELDVNDHERVPKVFAELSDEIGGIDRVIVNAGIGKGARL
                     GSGKLWANKATIETNLVAALVQIETALDMFNQRGSGHLVLISSVLGVKGVPGVKAAYA
                     ASKAGVRSLGESLRAEYAQGPIRVTVLEPGYIESEMTAKSASTMLMVDNATGVKALVA
                     AIEREPGRAAVPWWPWAPLVRLMWVLPPRLTRRFA"
     CDS             complement(1057586..1059247)
                     /codon_start=1
                     /gene="pgi"
                     /note="gene_985"
                     /transl_table=11
                     /translation="MTSAPIPDITATPAWDALRRHHDQIGNTHLRQFFADDPGRGREL
                     TVSVGDLYIDYSKHRVTRETLALLIDLARTAHLEERRDQMFAGVHINTSEDRAVLHTA
                     LRLPRDAELVVDGQDVVTDVHAVLDAMGAFTDRLRSGEWTGATGKRISTVVNIGIGGS
                     DLGPVMVYQALRHYADAGISARFVSNVDPADLIATLADLDPATTLFIVASKTFSTLET
                     LTNATAARRWLTDALGDAAVSRHFVAVSTNKRLVDDFGINTDNMFGFWDWVGGRYSVD
                     SAIGLSLMTVIGRDAFADFLAGFHIIDRHFATAPLESNAPVLLGLIGLWYSNFFGAQS
                     RTVLPYSNDLSRFPAYLQQLTMESNGKSTRADGSPVSADTGEIFWGEPGTNGQHAFYQ
                     LLHQGTRLVPADFIGFAQPLDDLPTAEGTGSMHDLLMSNFFAQTQVLAFGKTAEEIAA
                     DGTPAHVVAHKVMPGNRPSTSILASRLTPSVLGQLIALYEHQVFTEGVVWGIDSFDQW
                     GVELGKTQAKALLPVITGAGSPPPQSDSSTDGLVRRYRTERGRAG"
     CDS             complement(1060208..1060480)
                     /codon_start=1
                     /note="gene_986"
                     /transl_table=11
                     /translation="MNLEMLESQPVPEIDTLREEIDRLDAEILALVKRRAEVSKAIGK
                     ARMASGGTRLVHSREMKVIERYSELGPDGKDLAILLLRLGRGRLGH"
     CDS             1060822..1063137
                     /codon_start=1
                     /note="gene_987"
                     /transl_table=11
                     /translation="MSVHATDAKPPGPSPADQLLDGLNPQQRQAVVHEGSPLLIVAGA
                     GSGKTAVLTRRIAYLMAARGVGVGQILAITFTNKAAAEMRERVVGLVGEKARYMWVST
                     FHSTCVRILRNQAALIEGLNSNFSIYDADDSRRLLQMVGRDLGLDIKRYSPRLLANAI
                     SNLKNELIDPHQALAGLTEDSDDLARAVASVYDEYQRRLRAANALDFDDLIGETVAVL
```

```
                     QAFPQIAQYYRRRFRHVLVDEYQDTNHAQYVLVRELVGRDSNDGIPPGELCVVGDADQ
                     SIYAFRGATIRNIEDFERDYPDTRTILLEQNYRSTQNILSAANSVIARNAGRREKRLW
                     TDAGAGELIVGYVADNEHDEARFVAEEIDALAEGSEITYNDVAVFYRTNNSSRSLEEV
                     LIRAGIPYKVVGGVRFYERKEIRDIVAYLRVLDNPGDAVSLRRILNTPRRGIGDRAEA
                     CVAVYAENTGVGFGDALVAAAQGKVPMLNTRAEKAIAGFVEMFDELRGRLDDDLGELV
                     EAVLERTGYRRELEASTDPQELARLDNLNELVSVAHEFSTDRENAAALGPDDEDVPDT
                     GVLADFLERVSLVADADEIPEHGAGVVTLMTLHTAKGLEFPVVFVTGWEDGMFPHMRA
                     LDNPTELSEERRLAYVGITRARQRLYVSRAIVRSSWGQPMLNPESRFLREIPQELIDW
                     RRTAPKPSFSAPVSGAGRFGSARPSPTRSGASRRPLLVLQVGDRVTHDKYGLGRVEEV
                     SGVGESAMSLIDFGSSGRVKLMHNHAPVTKL"
     CDS             complement(1063218..1064249)
                     /codon_start=1
                     /note="gene_988"
                     /transl_table=11
                     /translation="LSQHRIARSAAMAAIRTPRDRWPHHHRNEVTEIIPLDGFLDGLA
                     LYDELDFAELDDLDLGDDCVFDYEAQLLAAPELDDLDDADDLAPEWLVAPTVVLTPEV
                     TPVSRRVGQHRKQPIGAARGRLLISAMAAGAAAAAAHTAIQQSETPRTETVLTAHASA
                     LNEGSGSNPPRGVQVIAAQPAASAAVHNAEFARGVAFAEERAEREARLQRPLYVMPTK
                     GIFTSSFGYRWGVLHAGIDLANAIGTPIYAVSDGVVIDAGPTAGYGMWVKLLHADGTV
                     TLYGHVNTTLVSVGERVMAGDQIATMGSRGFSTGPHLHFEVLLGGTERVDPVPWLAKR
                     GLSVGNYTG"
     CDS             1064526..1065689
                     /codon_start=1
                     /gene="sucC"
                     /note="gene_989"
                     /transl_table=11
                     /translation="MDLFEYQAKELFAKHNVPSTPGRVTDTAEGAKAIATEIGRPVMV
                     KAQVKIGGRGKAGGVKYAATPQDAYEHAKNILGLDIKGHIVKKLLVAEASDIAEEYYL
                     SFLLDRANRTYLAMCSVEGGMEIEEVAATKPERLAKVPVNAVKGVDLDFARSIAEQGH
                     LPAEVLDTAAVTIAKLWELFVAEDATLVEVNPLVRTPDHKILALDAKITLDGNADFRQ
                     PGHAEFEDRAATDPLELKAKEHDLNYVKLDGQVGIIGNGAGLAMSTLDVVAYAGEKHG
                     GVKPANFLDIGGGASAEVMAAGLDVVLGDQQVKSVFVNVFGGITSCDAVATGIVKALG
                     MLGDEANKPLVVRLDGNNVEEGRRILTEANHPLVTLVATMDEAADKAAELASA"
     CDS             1065702..1066613
                     /codon_start=1
                     /note="gene_990"
                     /transl_table=11
                     /translation="MTHMSIFLSRDNKVIVQGITGSEATVHTARMLRAGTQIVGGVNA
                     RKAGTTVTHEDKGGRLIKLPVFGSVAEAMEKTGADVSIIFVPPTFAKDAIIEAIDAEI
                     PLLVVITEGIPVQDTAYAWAYNLEAGHKTRIIGPNCPGIISPGQSLAGITPANITGPG
                     PIGLVSKSGTLTYQMMFELRDLGFSTAIGIGGDPVIGTTHIDAIEAFEKDPDTKLIVM
                     IGEIGGDAEERAADFIKTNVSKPVVGYVAGFTAPEGKTMGHAGAIVSGSSGTAAAKQE
                     ALEAAGVKVGKTPSATAALAREILLSL"
     variation       1066622..1066677
                     /note="supplemented from BCG moreau"
     CDS             complement(1066675..1067523)
                     /codon_start=1
                     /note="gene_991"
                     /transl_table=11
                     /translation="MHYGLVLFTSDRGITPAAAARLAESHGFRTFYVPEHTHIPVKRQ
                     AAHPTAGDASLPDDRYMRTLDPWVSLGAASAVTSRIRLATAVALPVEHDPITLAKSIA
```

TLDHLSHGRVSVGVGFGWNTDELVDHGVPPGRRRTMLREYLEAMRALWTQEEACYDGE
FVKFGPSWAWPKPVQPHIPVLVGAAGTEKNFKWIARSADGWITTPRDVDIDEPVKLLQ
DIWAAAGRDGLPQIVALDVKPVPDKLARWAELGVTEVLFGMPDRSADDAAAYVERLAA
KLACCV"
CDS 1067688..1068599
/codon_start=1
/note="gene_992"
/transl_table=11
/translation="MTYSPGNPGYPQAQPAGSYGGVTPSFAHADEGASKLPMYLNIAV
AVLGLAAYFASFGPMFTLSTELGGGDGAVSGDTGLPVGVALLAALLAGVALVPKAKSH
VTVVAVLGVLGVFLMVSATFNKPSAYSTGWALWVVLAFIVFQAVAAVLALLVETGAIT
APAPRPKFDPYGQYGRYGQYGQYGVQPGGYYGQQGAQQAAGLQSPGPQQSPQPPGYGS
QYGGYSSSPSQSGSGYTAQPPAQPPAQSGSQQSHQGPSTPPTGFPSFSPPPPVSAGTG
SQAGSAPVNYSNPSGGEQSSSPGGAPV"
CDS 1068726..1070006
/codon_start=1
/note="gene_993"
/transl_table=11
/translation="VVALGIIAAVTLLQLLIANSDMTGAWGAIASMWLGVHLVPISIG
GRALGVMPLLPVLLMVWATARSTARATSPQSSGLVVRWVVASALGGPLLMAAIALAVI
HDASSVVTELQTPSALRAFTSVLVVHSVGAATGVWSRVGRRALAATALPDWLHDSMRA
AAAGVLALLGLSGVVTAGSLVVHWATMQELYGITDSIFGQFSLTVLSVLYAPNVIVGT
SAIAVGSSAHIGFATFSSFAVLGGDIPALPILAAAPTPPLGPAWVALLIVGASSGVAV
GQQCARRALPFVAAMAKLLVAAVAGALVMAVLGYGGGGRLGNFGDVGVDEGALVLGVL
FWFTFVGWVTVVIAGGISRRPKRLRPAPPVELDADESSPPVDMFDGAASEQPPASVAE
DVPPSHDDIANGLKAPTADDEALPLSDEPPPRAD"
CDS complement(1070206..1070385)
/codon_start=1
/gene="purM"
/note="gene_994"
/transl_table=11
/translation="MGCGGGGDGDVPGIATGVVGEPNGEHRHRCFRGDFGGPAFAIHT
NGDYPCRVVADSGIE"
CDS 1070302..1070769
/codon_start=1
/gene="purN"
/note="gene_995"
/transl_table=11
/translation="VPVFTVRLADHPSRDAWDVAITAATAAHEPDLVVSAGFMRILGP
QFLSRFYGRTLNTHPALLPAFPGTHGVADALAYGVKVTGATVHLVDAGTDTGPILAQQ
PVPVLDGDDEETLHERIKVTERRLLVAAVAALATHGVTVVGRTATMGRKVTIG"
CDS 1070766..1072337
/codon_start=1
/gene="purH"
/note="gene_996"
/transl_table=11
/translation="MSTDDGRRPIRRALISVYDKTGLVDLAQGLSAAGVEIISTGSTA
KTIADTGIPVTPVEQLTGFPEVLDGRVKTLHPRVHAGLLADLRKSEHAAALEQLGIEA
FELVVVNLYPFSQTVESGASVDDCVEQIDIGGPAMVRAAAKNHPSAAVVTDPLGYHGV
LAALRAGGFTLAERKRLASLAFQHIAEYDIAVASWMQQTLAPEHPVAAFPQWFGRSWR
RVAMLRYGENPHQQAALYGDPTAWPGLAQAEQLHGKDMSYNNFTDADAAWRAAFDHEQ

TCVAIIKHANPCGIAISSVSVADAHRKAHECDPLSAYGGVIAANTEVSVEMAEYVSTI
FTEVIVAPGYAPGALDVLARKKNIRVLVAAEPLAGGSELRPISGGLLIQQSDQLDAHG
DNPANWTLATGSPADPATLTDLVFAWRACRAVKSNAIVIAADGATVGVGMGQVNRVDA
ARLAVERGGERVRGAVAASDAFFPFPDGLETLAAAGVTAVVHPGGSVRDEEVTEAAAK
AGVTLYLTGARHFAH"

CDS 1072471..1073823
/codon_start=1
/note="gene_997"
/transl_table=11
/translation="VGELRAAGHRERGVKQEIRENLLTALADGDNVWPGILGFDDTVI
PQVERALIAGHDFVLLGERGQGKTRLLRALAGLLDEWTPVIAGAELGEHPYTPITPES
IRRAAQLGDDLPVAWKHRSERYTEKLATPDTSVADLVGDVDPIKVAEGRSLGDPETIA
YGLIPRAHRGIVAVNELPDLAERIQVSMLNVMEERDIQVRGYTLRLPLDVLVVASANP
EDYTNRGRIITPIKDRFGAEIRTHYPLELEAEMGVIVQEAHLSAQVPDYLMQVLARFA
RYLRESRSIDQRSGVSARFAIAAAETVAAAARHRGAVLGETDPVARVVDLGTVIDVLR
GKLEFESGEEGREQAVLEHLLRRATADTASRVLGGIDVGSLVTAVEGGSAVTTGERVS
AKDVLAAVPGLPVVDRIARKLGAESEGERAAALELALEALYLAKRVDKVCGEGQTVYG
"

CDS 1073816..1075834
/codon_start=1
/note="gene_998"
/transl_table=11
/translation="MAKSDGDDPLRPASPRLRSSRRHSLRYSAYTGGPDPLAPPVDLR
DALEQIGQDVMAGASPRRALSELLRRGTRNLTGADRLAAEVNRRRRELLRRNNLDGTL
QEIKKLLDEAVLAERKELARALDDDARFAELQLDALPASPAKAVQELAEYRWRSGQAR
EKYEQIKDLLGRELLDQRFAGMKQALAGATDDDRRRVTEMLDDLNDLLDKHARGEDTQ
RDFDEFMTKHGEFFPENPRNVEELLDSLAKRAAAAQRFRNSLSQEQRDELDALAQQAF
GSPALMRALDRLDAHLQAARPGEDWTGSQQFSGDNPFGMGEGTQALADIAELEQLAEQ
LSQSYPGASMDDVDLDALARQLGDQAAVDARTLAELERALVNQGFLDRGSDGQWRLSP
KAMRRLGETALRDVAQQLSGRHGERDHRRAGAAGELTGATRPWQFGDTEPWHVARTLT
NAVLRQAAAVHDRIRITVEDVEVAETETRTQAAVALLVDTSFSMVMENRWLPMKRTAL
ALHHLVCTRFRSDALQIIAFGRYARTVTAAELTGLAGVYEQGTNLHHALALAGRHLRR
HAGAQPVVLVVTDGEPTAHLEDFDGDGTSVFFDYPPHPRTIAHTVRGFDDMARLGAQV
TIFRLGSDPGLARFIDQVARRVQGRVVVPDLDGLGAAVVGDYLRFRRR"

CDS 1075888..1076109
/codon_start=1
/note="gene_999"
/transl_table=11
/translation="MKTLYLRNVPDDVVERLERLAELAKTSVSAVAVRELTEASRRAD
NPALLGDLPDIGIDTTELIGGIDAERAGR"

CDS 1076106..1076489
/codon_start=1
/note="gene_1000"
/transl_table=11
/translation="MIVVDASAALAALLNDGQARQLIAAERLHVPHLVDSEIASGLRR
LAQRDRLGAADGRRALQTWRRLAVTRYPVVGLFERIWEIRANLSAYDASYVALAEALN
CALVTADLRLSDTGQAQCPITVVPR"

CDS 1076635..1076982
/codon_start=1
/note="gene_1001"
/transl_table=11

/translation="VRVPSQWMISSRVTVAWNIVGYLVYAALAFVGGFAVWFSLFFAM
ATDGCHDSACDASYHVFPAMVTMWIGVGAVLLLTLVVMVRNSSRGNVVIGWPFVGLLA
LGLVYVAADAVLH"
CDS complement(1077001..1077675)
/codon_start=1
/note="gene_1002"
/transl_table=11
/translation="MKRTSRSLTAALLGIAALLAGCIKPNTFDPYANPGRGELDRRQK
IVNGRPDLETVQQQLANLDATIRAMIAKYSPQTRFSTGVTVSHLTNGCNDPFTRTIGR
QEASELFFGRPAPTPQQWLQIVTELAPVFKAAGFRPNNSVPGDPPQPLGAPNYSQIRD
DGVTINLVNGDNRGPLGYSYNTGCHLPAAWRTAPPPLNMRPANDPDVHYPYLYGSPGG
RTRDAY"
CDS complement(1077858..1078820)
/codon_start=1
/note="gene_1003"
/transl_table=11
/translation="MPQMPPPDSDPEEVKRWWHSLTPGQQDRVKQWFPNTLRNRDGIP
IAVRNELNLSVLQRELTRLQNGWLSRDGVWHTDTDKLADLRALRDTLAAHPGTSLILL
DTASDPRKVLAAVGVGDVDNAERVGVTMGGLNTRVSSSVGDMVKEAGIQRAKAAELRE
RAGWPNYDAVASIAWLGYDAPDGLKDVMHDWSARDAAGPLNRFDKGLAATTNVSDQHI
TAFGHSYGSLVTSLALQQGAPVSDVVLYGSPGTELTHASQLGVEPGHAFYMIGVNDHV
ANTIPEFGAFGSAPQDVPGMTQLSVNTGLAPGPLLSDGQLHERA"
CDS 1078728..1078979
/codon_start=1
/gene="GOARA_035_00080"
/note="gene_1004"
/transl_table=11
/translation="LLDPILLARGQRMPPPLDLLRVAVRRRHLRHCGWRMAELGIDLL
ARQVPGGASNRRGQILIGGLGVGQQTFDALRIAVLRMAC"
CDS complement(1079339..1079698)
/codon_start=1
/note="gene_1005"
/transl_table=11
/translation="VEASWYGRTLRAIPEVLSQVGYQQADHGESLLTSHHCCLGAAEG
ARPGWVGSSAGALSGLLDSWAEASTAHAAHIGDHSYGMHLAAVGFAEMEEHNAAALAA
VYPTGGGSARCDGVDVS"
CDS complement(1079794..1080396)
/codon_start=1
/note="gene_1006"
/transl_table=11
/translation="MSNSAQRDARNSRDESARASDTDRIQIAQLLAYAAEQGRLQLTD
YEDRLARAYAATTYQELDRLRADLPGAAIGPRRGGECNPAPSTLLLALLGGFERRGRW
NVPKKLTTFTLWGSGVLDLRYADFTSTEVDIRAYSIMGAQTILLPPEVNVEIHGHRVM
GGFDRKVVGEGTRGAPTVRIRGFSLGGDVGIKRKPRKPRK"
CDS 1080535..1080894
/codon_start=1
/note="gene_1007"
/transl_table=11
/translation="MSKELTAKKRAALNRLKTVRGHLDGIVRMLESDAYCVDVMKQIS
AVQSSLERANRVMLHNHLETCFSTAVLDGHGQAAIEELIDAVKFTPALTGPHARLGGA
AVGESATEEPMPDASNM"

CDS 1080951..1081247
/codon_start=1
/note="gene_1008"
/transl_table=11
/translation="MVWHGFLAKAVPTVVTGAVGVAAYEALRKMVVKAPLRAATVSVA
AWGIRLAREAERKAGESAEQARLMFADVLAEASERAGEEVPPLAVAGSDDGHDH"
CDS 1081237..1083615
/codon_start=1
/note="gene_1009"
/transl_table=11
/translation="MTTDVLSDTDVSLKVVSNASGRMRVCVTGFNVDAVRAVAIEETV
SQVTGVHAVHAYPRTASVVIWYSPELGDTAAVLSAITKAQHVPAELVPARAPHSAGVR
GVGVVRKITGGIRRMLSRPPGVDKPLKASRCGGRPRGPVRGSASWPGEQNRRERRTWL
PRVWLALPLGLLALGSSMFFGAYPWAGWLAFAATLPVQFVAGWPILRGAVQQARALTS
NMDTLIALGTLTAFVYSTYQLFAGGPLFFDTSALIIAFVVLGRHLEARATGKASEAIS
KLLELGAKEATLLVDGQELLVPVDQVQVGDLVRVRPGEKIPVDGEVTDGRAAVDESML
TGESVPVEKTAGDRVAGATVNLDGLLTVRATAVGADTALAQIVRLVEQAQGDKAPVQR
LADRVSAVFVPAVIGVAVATFAGWTLIAANPVAGMTAAVAVLIIACPCALGLATPTAI
MVGTGRGAELGILVKGGEVLEASKKIDTVVFDKTGTLTRARMRVTDVIAGQRRQPNQV
LRLAAAVESGSEHPIGAAIVAAAHERGLAIPAANAFTAVAGHGVRAQVNGGPVVVGRR
KLVDEQHLVLPDHLAAAAVEQEERGRTAVFVGQDGQVVGVLAVADTVKDDAADVVGRL
HAMGLQVAMITGDNARTAAAIAKQVGIEKVLAEVLPQDKVAEVRRLQDQGRVVAMVGD
GVNDAPALVQADLGIAIGTGTDVAIEASDITLMSGRLDGVVRAIELSRQTLRTIYQNL
GWAFGYNTAAIPLAALGALNPVVAGAAMGFSSVSVVTNSLRLRRFGRDGRTA"
CDS 1083612..1084244
/codon_start=1
/note="gene_1010"
/transl_table=11
/translation="MIHDLMLRWVVTGLFVLTAAECGLAIIAKRRPWTLIVNHGLHFA
MAVAMAVMAWPWGARVPTTGPAVFFLLAAVWFGATAVVAVRGTATRGLYGYHGLMMLA
TAWMYAAMNPRLLPVRSCTEYATEPDGSMPAMDMTAMNMPPNSGSPIWFSAVNWIGTV
GFAVAAVFWACRFVMERRQEATQSRLPGSIGQAMMAAGMAMLFFAMLFPV"
CDS complement(1084335..1085144)
/codon_start=1
/note="gene_1011"
/transl_table=11
/translation="MDSPVDYAGPAACGGPFARLTLNSPHNRNALSSTLVSQLHQGLS
AAEADPAVRLVVLGHTGGTFCAGADLSEAGGGGGDPYRMAVARAREMTALLRAIVESP
LPVVGAINGHVRAGGFGLVGACDMVVAGPESTFALTEARIGVAPAIISLTLLPKLSPR
AAARYYLTGEKFGAREAADIGLITMAADDVDAAVAALVADVGRGSPQGLAASKALTTA
AVLEGFDRDAERLTEESARLFVSDEAREGMLAFLQKRPPRWVQPATMRAAD"
CDS complement(1085144..1086310)
/codon_start=1
/note="gene_1012"
/transl_table=11
/translation="VTDTSFIESEERQALRKAVASWVANYGHEYYLDKARKHEHTSEL
WAEAGKLGFLGVNLPEEYGGGGAGMYELSLVMEEMAAAGSALLLMVVSPAINGTIIAK
FGTDDQKKRWLPGIADGSLTMAFAITEPDAGSNSHKITTTARRDGSDWIIKGQKVFIS
GIDQAQAVLVVGRSEEAKTGKLRPALFVVPTDAPGFSYTPIEMELVSPERQFQVFLDD
VRLPADALVGAEDAAIAHLFAGLNPERIMGAASAVGMGRFALGRAVDYVKTRKVWSTP
IGAHQGLAHPLAQCHIEVELAKLMTQKAATLYDHGDDFGAAEAANMAKYAAAEASSRA

VDQAVQSMGGNGLTKEYGVAAMMTSARLARIAPISREMVLNFVAQTSLGLPRSY"
CDS complement(1086307..1088316)
/codon_start=1
/note="gene_1013"
/transl_table=11
/translation="MLMGITRVLVANRGEIARRVFATCRRLGLGTVAVYTDPDAAAPH
VAEADARVRLPQTTDYLNAEAIIAAAQAAGADAVHPGYGFLSENAEFAAAVQEAGLTW
VGPPVDAVRAMGSKIESKKLMAAAGVPVLEELDPDAVTTAQLPVLVKASAGGGGRGMR
VVHELSALPAEVEAARREAQSAFGDPTVFCERYLPTGHHVEVQVMADTHGTVWAVGER
ECSFQRRHQKIIEEAPSPLVERVPGMRAKLFDAARLAASAIGYTGAGTVEFLADDSPG
REGEFYFLEMNTRLQVEHPVTEETTGLDLVELQLMIADCGRLDTEPPPAQGYSIEARL
YAEDPAHGWQPQAGVMHTIEVPGVRAQFDSLGQRTGIRLDSGIVDGSTVSIHYDPMLA
KVVSYGATRRQAALVLADALVRARLHGLRTNRELLVNVLRHPAFLDGATDTGFFDTHG
MAELSTPLADTATLRLSAIAAALADAEHNRASAGVFSSIPSGWRNLASGYQVKTYRDD
ADTEHRVEYRFTRTGLALPGDPVVQLVSADVDQVVLAQDGVAHGFTVARHGPDVYVDS
ARGPVHLVALSRFPEPSSAVEQGSLVAPMPGNVIRIGAEVGDTVTAGQPLIWLEAMKM
EHTIAAPADGVLTHVSVNTGQQVEVGAILARVEAPQNGPAEGDSP"
CDS complement(1088316..1089911)
/codon_start=1
/note="gene_1014"
/transl_table=11
/translation="VTVLQSTLDPNASAYDEAAATMSGKLDEINAELAKALAGGGPKY
VDRHHARGKLTPRERIELLVDPDSPFLELSPLAAYGSNFQIGASLVTGIGAVCGVECM
IVANDPTVKGGTSNPWTLRKILRANQIAFENRLPVISLVESGGADLPTQKEIFIPGGQ
MFRDLTRLSAAGIPTIALVFGNSTAGGAYVPGMSDHVVMIKERSKVFLAGPPLVKMAT
GEESDDESLGGAEMHARISGLADYFALDELDAIRIGRRIVARLNWIKQGPAPAPVTEP
LFDAEELIGIVPPDLRIPFDPREVIARIVDGSEFDEFKPLYGSSLVTGWARLHGYPLG
ILANARGVLFSEESQKATQFIQLANRADTPLLFLHNTTGYMVGKDYEEGGMIKHGSMM
INAVSNSTVPHISLLIGASYGAGHYGMCGRAYDPRFLFAWPSAKSAVMGGAQLSGVLS
IVARAAAEARGQQVDEAADAAMRAAVEGQIEAESLPLVLSGMLYDDGVIDPRDTRTVL
GMCLSAIANGPIKGTSNFGVFRM"
CDS complement(1089908..1091056)
/codon_start=1
/note="gene_1015"
/transl_table=11
/translation="VNIWTTPERQQLRKTVRAFAEREILPHVDEWERIGELPRGLHRL
AGAAGLLGAGFPEAVGGGGGDGADPVIICEEMHQAGAPGGVYASLFTCGIAVPHMVAS
GDERLIATYVRPTLAGEKIGALAITEPGGGSDVGHLRTSAVRDGDHYVINGAKTYITS
GVRADYVVTAVRTGGPGAAGVSLLVVEKDTPGFEVTRKLDKMGWRSSDTAELCYTDVA
VPATNLVGAENSGFTQIARAFVSERIGLAAQAYSSAQRCLDLTAQWCRDRETFGRPLI
SRQSVQNTLAEMARRIDVARVYAHHVVERQLAGETDLIAQVCFAKNTAVQAGEWVANQ
AVQLFGGMGYMAESEVERQYRDMRILGIGGGTTEILTALAAKTLGYQS"
CDS complement(1091053..1092756)
/codon_start=1
/note="gene_1016"
/transl_table=11
/translation="MAPDCGAVRIGNCSGFYGDRLSAMREMLTGGELDYLTGDYLAEL
TMLILGRDRMKNPDRGYAKTFLAQLEDCLGLAHDRGVRIVTNAGGLNPAGLANAVRAL
AARLGIPAQVAHVEGDDLQPRAAELGLGTPLTANAYLGAWGIVDCFERGADVVVTGRV
TDASVVVGAAAAHFGWGRTDYHRLAGAVVAGHVIECGVQATGGNYAFFTEIGDLTHAG
FPLAEIAADGSSVITKHHGTGGLVSVDTITAQLLYEITGARYANPDVTARMDSVELSP

DGPDRVRISGVIGEPPPPTYKVSLNSIGGFRNAMTFVLTGLDIDAKADLVRRQLEAAL
TVKPAELQWTLARTDHPDADTEETASALLTCVARDPDPANVGRQFSSAAVELALASYP
GFTATAPPGDGQVYGVFTPGYVDAGKVAHIAVHADGTRTEIPCATETLELAPAHPPAL
PDPLPAGPTRRVPLGLIAGARSGDKGGSANVGVWVRTDEQWRWLAHTLTVELLKELLP
ETAGLVVTRHVLPNLRALNFVIEAILGQGVAYQARFDPQAKGLGEWLRSRHVEIPETL
L"

CDS 1092934..1094427
/codon_start=1
/note="gene_1017"
/transl_table=11
/translation="MSFVVTAPPVLASAASDLGGIASMISEANAMAAVRTTALAPAAA
DEVSAAIAALFSSYARDYQTLSVQVTAFHVQFAQTLTNAGQLYAVVDVGNGVLLKTEQ
QVLGVINAPTQTLVGRPLIGDGTHGAPGTGQNGGAGGILWGNGGNGGSGAPGQPGGRG
GDAGLFGHGGHGGVGGPGIAGAAGTAGLPGGNGANGGSGGIGGAGGAGGNGGLLFGNG
GAGGQGGSGGLGGSGGTGGAGMAAGPAGGTGGIGGIGGIGGAGGVGGHGSALFGHGGI
NGDGGTGGMGGQGGAGGNGWAAEGITVGIGEQGGQGGDGGAGGAGGIGGSAGGIGGSQ
GAGGHGGDGGQGGAGGSGGVGGGGAGAGGDGGAGGIGGTGGNGSIGGAAGNGGNGGRG
GAGGMATAGSDGGNGGGGGNGGVGVGSAGGAGGTGGDGGAAGAGGAPGQCYFQQPAPQ
GLPIGTRGTGGEGGAGGAGGDGGQGDIGFDGARGVNGGPGRWRWRRR"

CDS 1094372..1095706
/codon_start=1
/note="gene_1018"
/transl_table=11
/translation="MAPGVSTAARGGGGGAGGDGSGTFNAQANKGGDGGAGGVGGAGG
TGGTGGVGADGGRGGDSGRGGDGGNAGHGGAAQFSGRGAYGGEGGSGGAGGNAGGAGT
GGTAGSGGAGGFGGNGADGGNGGNGGNGGFGGINGTFGTNGAGGTGGLGTLLGGHNGN
IGLNGATGGIGSTTLTNATVPLQLVNTTEPVVFISLNGGQMVPVLLDTGSTGLVMDSQ
FLTQNFGPVIGTGTAGYAGGLTYNYNTYSTTVDFGNGLLTLPTSVNVVTSSSPGTLGN
FLSRSGAVGVLGIGPNNGFPGTSSIVTAMPGLLNNGVLIDESAGILQFGPNTLTGGIT
ISGAPISTVAVQIDNGPLQQAPVMFDSGGINGTIPSALASLPSGGFVPAGTTISVYTS
DGQTLLYSYTTTATNTPFVTSGGVMNTGHVPFAQQPIYVSYSPTAIGTTTFN"

CDS 1095724..1095930
/codon_start=1
/gene="TBLG_03139"
/note="gene_1019"
/transl_table=11
/translation="VIGKGRLQRAFSIVAALICARKLIPKRKAHHGCAHAQKVAREHP
KPALAVEGPAGRVRAEHTGRAGVS"

CDS complement(1095923..1096930)
/codon_start=1
/note="gene_1020"
/transl_table=11
/translation="MSFVNVAPQLVSTAAADAARIGSAINTANTAAAATTQVLVAAQD
EVSTAIAALFGSHGQHYQAISAQVAAYQERFVLALSQAGSTYAVAEAASATPLQQIEQ
ALLGVINTPTEALVGRKLIGDGAHGAPGTGQAGGAGGILWGNGGNGGSGAPGQAGGAG
GAAGLIGNGGAGGTGGAVSLARAGTAGGAGRGPVGGIGGAGGVGGAGGAAGAVTTITH
ASFNDPHGVAVNPGGNVYVTNFGSGTVSVINPATNTVTGSPITIGNGPSGVAVSPVTG
LVFVTNFDSNTVSVIDPTTNTVTGSPITVGTAPTGVAVNPVTGEVYVTNFAGDTVSVI
S"

CDS 1097256..1097633
/codon_start=1

/note="gene_1021"
/transl_table=11
/translation="LPPWSPIPVPQPHHHRGDNRELTVTPGPNPGPELGRAVFNRYPP
RHCRRDCRRAHLRAEAHTKRGRPTMAVPKRRKSRSNTRSRRSQWKAAKTELVGVTVAG
HAHKVPRRLLKAARLGLIDFDKR"

CDS complement(1097652..1099025)
/codon_start=1
/note="gene_1022"
/transl_table=11
/translation="MSFVNVAPQLVSTAAADAARIGSAINTANTAAAATTQVLAAAQD
EVSTAIAALFGSHGQHYQAISAQVAAYQERFVLALSQASSTYAVAEAASATPLQNVLD
AINAPVQSLTGRPLIGDGANGIDGTGQAGGNGGWLWGNGGNGGSGAPGQAGGAGGAAG
LIGNGGAGGAGGQGLPFEAGANGGAGGAGGWLFGNGGAGGVGGAGGAGTTFGVAGGDG
GTGGVGGHGGLIGVGGHGGDGGTGGTGGAVSLARAGTAGGAGGGPAGGIGGAGGVGGA
GGAAGAVTTITHASFNDPHGVAVNPGGNIYVTNQGSNTVSVIDPVTNTVTGSITDGNG
PSGVAVSPVTGLVFVTNFDSNTVSVIDPNTNTVTGSIPVGTGAYGVAVNPGGNIYVTN
QFSNTVSVIDPATNTVTGSPIPVGLDPTGVAVNPVTGVVYVTNSLDDTVSVITGEPAR
SVCSAAI"

CDS 1099429..1100082
/codon_start=1
/note="gene_1023"
/transl_table=11
/translation="VRESLRRSLSFNGYSVELAHDGVEALDMIASDRPDALVLDVMMP
RLDGLEVCRQLRSTGDDLPILVLTARDSVSERVAGLDAGADDYLPKPFALEELLARMR
ALLRRTKPEDAAESMAMRFSDLTLDPVTREVNRGQRRISLTRTEFALLEMLIANPRRV
LTRSRILEEVWGFDFPTSGNALEVYVGYLRRKTEADGEPRLIHTVRGVGYVLRETPP"

CDS 1100082..1101596
/codon_start=1
/note="gene_1024"
/transl_table=11
/translation="MWWFRRRDRAPLRATSSLSLRWRVMLLAMSMVAMVVVLMSFAVY
AVISAALYSDIDNQLQSRAQLLIASGSLAADPGKAIEGTAYSDVNAMLVNPGQSIYTA
QQPGQTLPVGAAEKAVIRGELFMSRRTTADQRVLAIRLTNGSSLLISKSLKPTEAVMN
KLRWVLLIVGGIGVAVAAVAGGMVTRAGLRPVGRLTEAAERVARTDDLRPIPVFGSDE
LARLTEAFNLMLRALAESRERQARLVTDAGHELRTPLTSLRTNVELLMASMAPGAPRL
PKQEMVDLRADVLAQIEELSTLVGDLVDLSRGDAGEVVHEPVDMADVVDRSLERVRRR
RNDIHFDVEVIGWQVYGDTAGLSRMALNLMDNAAKWSPPGGHVGVRLSQLDASHAELV
VSDRGPGIPVQERRLVFERFYRSASARALPGSGLGLAIVKQVVLNHGGLLRIEDTDPG
GQPPGTSIYVLLPGRRMPIPQLPGATAGARSTDIENSRGSANVISVESQSTRAT"

CDS 1101973..1103034
/codon_start=1
/note="gene_1025"
/transl_table=11
/translation="VSAGIGGAAASLVGFNRAPAGPSGGPVAASAAPSIPAANMPPGS
VEQVAAKVVPSVVMLETDLGRQSEEGSGIILSAEGLILTNNHVIAAAAKPPLGSPPPK
TTVTFSDGRTAPFTVVGADPTSDIAVVRVQGVSGLTPISLGSSSDLRVGQPVLAIGSP
LGLEGTVTTGIVSALNRPVSTTGEAGNQNTVLDAIQTDAAINPGNSGGALVNMNAQLV
GVNSAIATLGADSADAQSGSIGLGFAIPVDQAKRIADELISTGKASHASLGVQVTNDK
DTPGAKIVEVVAGGAAANAGVPKGVVVTKVDDRPINSADALVAAVRSKAPGATVALTF
QDPSGGSRTVQVTLGKAEQ"

CDS 1103034..1103579

/codon_start=1
/note="gene_1026"
/transl_table=11
/translation="MKVAAQCSKLGYTVAPMEQRAELVVGRALVVVVDDRTAHGDEDH
SGPLVTELLTEAGFVVDGVVAVSADEVEIRNALNTAVIGGVDLVVSVGGTGVTPRDVT
PEATRDILDREILGIAEAIRASGLSAGIVDAGLSRGLAGVSGSTLVVNLAGSRYAVRD
GMATLNPLAAQIIGQLSSLEI"
CDS complement(1103599..1104054)
/codon_start=1
/note="gene_1027"
/transl_table=11
/translation="MLKGFKEFLARGNIVDLAVAVVIGTAFTALVTKFTDSIITPLIN
RIGVNAQSDVGILRIGIGGGQTIDLNVLLSAAINFFLIAFAVYFLVVLPYNTLRKKGE
VEQPGDTQVVLLTEIRDLLAQTNGDSPGRHGGRGTPSPTDGPLASTESQ"
CDS 1104377..1105123
/codon_start=1
/note="gene_1028"
/transl_table=11
/translation="MNRQPIVQLSNLSWTFREGETRRQVLDHITFDFEPGEFVALLGQ
SGSGKSTLLNLISGIEKPTTGDVTINGFAITQKTERDRTLFRRDQIGIVFQFFNLIPT
LTVLENITLPQELAGVSQRKAAVVARDLLEKVGMADRERTFPDKLSGGEQQRVAISRA
LAHNPMLVLADEPTGNLDSDTGDKVLDVLLDLTRQAGKTLIMATHSPSMTQHADRVVN
LQGGRLIPALNRENQTDQPASTILLPTSYE"
CDS 1105116..1107683
/codon_start=1
/note="gene_1029"
/transl_table=11
/translation="MNDQAPVAYAPLWRTAWRRLRQRPFQYILLVLGIALGVAMIVAI
DVSSNSAQRAFDLSAAAITGKSTHRLVSGPAGVDQQLYVDLRRHGYDFSAPVIEGYVL
ARGLGNRAMQFMGTDPFAESAFRSPLWSNQNIAELGGFLTRPNGVVLSRQVAQKYGLA
VGDRIALQVKGAPTTVTLVGLLTPADEVSNQKLSDLIIADISTAQELFHMPGRLSHID
LIIKDEATATRIQQRLPAGVRMETSDTQRDTVKQMTDAFTVNLTALSLIALLVGIFLI
YNTVTFNVVQRRPFFAILRCLGVTREQLFWLIMTESLVAGLIGTGLGLLIGIWLGEGL
IGLVTQTINDFYFVINVRNVSVSAESLLKGLIIGIFAAMLATLPPAIEAMRTVPASTL
RRSSLESKITKLMPWLWVAWFGLGSFGVLMLWLPGNNLVVAFVGLFSVLIALALIAPP
LTRFVMLRLAPGLGRLLGPIGRMAPRNIVRSLSRTSIAIAALMMAVSLMVGVSISVGS
FRQTLANWLEVTLKSDVYVSPPTLTSGRPSGNLPVDAVRNISKWPGVRDAVMARYSSV
FAPDWGREVELMAVSGDISDGKRPYRWIDGNKDTLWPRFLAGKGVMLSEPMVSRQHLQ
MPPRPITLMTDSGPQTFPVLAVFSDYTSDQGVILMDRASYRAHWQDDDVTTMFLFLAS
GANSGALIDQLQAAFAGREDIVIQSTHSVREASMVIFDRSFTITIALQLVATVVAFIG
VLSALMSLELDRAHELGVFRAIGMTTRQLWKLMFIETGLMGGMAGLMALPTGCILAWI
LVRIINVRSFGWTLQMHFESAHFLRALLVAVVAALAAGMYPAWRLGRMTIRTAIREE"
CDS 1107690..1108850
/codon_start=1
/note="gene_1030"
/transl_table=11
/translation="MRKAGLTGVVLVLTLTLVAFWWWQRPRTNAVAADSLVGVLVDEN
NAGYSLATVPGAIRFPRDLGPHYDYQTEWWYYTGNLETADGRLFGYQLTFFRRALAPP
GEGVAIADASSWRTTQVYMAHFAISDISNRGFDPAEKFSRQALGLAGASSEPYAVWLD
DWYARESNNNSVQLFARTQNTVLDLTLTQTLPPILQGNAGLSVKGAQPGNASNYYSLV
RQESRGTVSVNGDTFMVSGLSWKDHEYMTSALAPEDVGWDWFGLQFYNGTALMLFQIR

QADGSVTRFSSGTFVAGDGGVIPLESSDFRIKTTDRWTSDQSGATYPIAWEIEIERIG
LTLRGAALMANQELRLSRTYWEGAVALEGRYQGMPISGRGYVEMTGYVQRLS"
CDS complement(1108979..1109980)
/codon_start=1
/note="gene_1031"
/transl_table=11
/translation="VRMPTATSMIPAVSLGDPQFTANVHDGIARITELINSELSQADE
VMRDTVAHLVDAGGTPFRPLFTVLAAQLGSDPDGWEVTVAGAAIELMHLGTLCHDRVV
DESDMSRKTPSDNTRWTNNFAILAGDYRFATASQLASRLDPEAFAVVAEAFAELITGQ
MRATRGPASHIDTIEHYLRVVHEKTGSLIAASGQLGAALSGAAEEQIRRVARLGRMIG
AAFEISRDIIAISGDSATLSGADLGQAVHTLPMLYALREQTPDTSRLRELLAGPIHDD
HVAEALTLLRCSPGIGKAKNVVAAYAAQAREELPYLPDRQPRRALATLIDHAVSACD"
CDS complement(1110017..1110673)
/codon_start=1
/note="gene_1032"
/transl_table=11
/translation="VAESSLNPSLVSRISAFLRPDWTRTVRARRFAAAGLVMLAGVAA
LRSNPEDDRAEVVVAAHDLRPGTALTPGDVRLEKRSATTLPDGSQADLDAVVGSTLAS
PTRRGEVLTDVRLLGSRLAESTAGPDARIVPLHLADSALVDLVRVGDVVDVLAAPVTD
SPAALRLLATDAIVVLVSAQQKAQAADSDRVVLVALPARLANTVAGAALGQTVTLTLH
"
CDS complement(1110746..1111078)
/codon_start=1
/note="gene_1033"
/transl_table=11
/translation="VPTYSYECTQCANRFDVVQAFTDDALTTCERCSGRLRKLFNAVG
VVFKGTGFYRTDSRESGKKSKSQTNGSSTSESTKSSGSSGSSGSSESKASGSTEKSTS
STTAAAAV"
CDS complement(1111152..1111823)
/codon_start=1
/note="gene_1034"
/transl_table=11
/translation="VLIARLFVGAVPALARDGRGCVIVEDMAIASKSALRDQLLAARR
RVADDVRAAEARMLRGHLERMVTSDSTVCAYVPVGGEPGSIEMLDVLLRRAGRVLLPV
ARTAGGDLPLPLRWGEYRAGGLARARWGLLEPPEPWLPEAALAQASLVLVPALAVDRQ
SVRLGRGRGFYDRSLRCRDPHARLVAVVRTVELVDVLPSEPHDVPMTHALTPERGLIA
LPCGE"
CDS 1111846..1112766
/codon_start=1
/note="gene_1035"
/transl_table=11
/translation="MSRPEVLTPFTAIVPAAGLGTRFLPATKTVPKELLPVVDTPGIE
LVAAEAAAAGAERLVIVTSEGKDGVVAHFVEDLVLEGTLEARGKIAMLAKVRRAPALI
KVESVVQAEPLGLGHAIGCVEPTLSPDEDAVAVLLPDDLVLPTGVLETMSKVRASRGG
TVLCAIEVAREEISAYGVFDVEPVPDGDYTDDPNVLKVRGMVEKPKAETAPSRYAAAG
RYVLDRAIFDALRRIDRGAGGEVQLTDAIALLIAEGHPVHVVVHQGSRHDLGNPGGYL
KAAVDFALDRDDYGPDLRRWLVARLGLTEQ"
CDS 1112969..1114123
/codon_start=1
/note="gene_1036"
/transl_table=11

```
                     /translation="MPGFDQAAIDGYAVRSVDVAGVGDTGGVQVFADHGDLDGRDVLT
                     LPVMGTIEAGARTLSRLQPRQAVRVQTGAPLPTLADAVLPLRWTDGGMSRVRVLRGAP
                     SGAYVRRAGDDVQPGDVAVRAGTIIGAAQVGLLAAVGRERVLVHPRPRLSVMAVGGEL
                     VDISRTPGNGQVYDVNSYALAAAGRDAGAEVNRVGIVSNDPTELGEIVEGQLNRAEVV
                     VIAGGVGGAAAEAVRSVLSELGEMEVVRVAMHPGSVQGFGQLGRDGVPTFLLPANPVS
                     ALVVFEVMVRPLIRLSLGKRHPMRRIVSARTLSPITSVAGRKGYLRGQLMRDQDSGEY
                     LVQALGGAPGASSHLLATLAEANCLVVVPTGAEQIRTGEIVDVAFLAQHG"
     CDS             1114186..1114797
                     /codon_start=1
                     /note="gene_1037"
                     /transl_table=11
                     /translation="MAVGPLRVSAGVIRLRPVRMRDGVHWSRIRLADRAHLEPWEPSA
                     DGEWTVRHTVAAWPAVCSGLRSEARNGRMLPYVIELDGQFCGQLTIGNVTHGALRSAW
                     IGYWVPSAATGGGVATGALALGLDHCFGPVMLHRVEATVRPENAASRAVLAKVGFREE
                     GLLRRYLEVDRAWRDHLLMAITVEEVYGSVASTLVRAGHASWP"
     CDS             1114958..1116034
                     /codon_start=1
                     /note="gene_1038"
                     /transl_table=11
                     /translation="MPSIPQSLLWISLVVLWLFVLVPMLISKRDAVRRTSDVALATRV
                     LNGGAGARLLKRGGPAAGHRWGYLPPEGQGDDPDWKPEEDWRDDPVEDGFADVEHDID
                     EDQEADDARRRGAVVMKVAAPQTAGADEPDYLDVDVVEEDSEALPVGAGAAVGESADE
                     ADAEAADGVAGHADPEADPVEYEYEYEYVEDTCGLELEEDDQEAPPTVASGTSRRRRF
                     DTKTAAAVSARKYTFRKRALIVMAVILVGSAAAAFELTPVAWWICGSATGVTVLYLAY
                     LRRQTRIEEKVRRRRMQRIARARLGVQNTRDREYDVVPSRLRRPGAVVLEIDDEDPIF
                     THLESAAPIRNYGWPRDLPRAVGQ"
     tRNA            1116085..1116160
                     /note="identified by tRNAscan-SE; putative"
     CDS             complement(1116747..1116956)
                     /codon_start=1
                     /note="gene_1039"
                     /transl_table=11
                     /translation="MPEPFRIAREKVRVLRPAGRITIHTSYAGQCPPMRYAPNAAAGN
                     IGLTMFDRNAFVNLSRSAGLVDVEQ"
     CDS             1117322..1118323
                     /codon_start=1
                     /note="gene_1040"
                     /transl_table=11
                     /translation="LDGIAELTGARVEDLAGMDVFQGCPAEGLVSLAASVQPLRAAAG
                     QVLLRQGEPAVSFLLISSGSAEVSHVGDDGVAIIARALPGMIVGEIALLRDSPRSATV
                     TTIEPLTGWTGGRGAFATMVHIPGVGERLLRTARQRLAAFVSPIPVRLADGTQLMLRP
                     VLPGDRERTVHGHIQFSGETLYRRFMSARVPSPALMHYLSEVDYVDHFVWVVTDGSDP
                     VADARFVRDETDPTVAEIAFTVADAYQGRGIGSFLIGALSVAARVDGVERFAARMLSD
                     NVPMRTIMDRYGAVWQREDVGVITTMIDVPGPGELSLGREMVDQINRVARQVIEAVG"
     CDS             1118341..1119099
                     /codon_start=1
                     /note="gene_1041"
                     /transl_table=11
                     /translation="VRPPLAPQFAADLLVKTVSTLRSSGAALGRLTTMRKAVLAVGSV
                     CWLVGCSSGASSTTASTGDIAKVAEVKSGFGPEYTVTDVTPRAIDPGFFSARKLPDGL
                     SFDPANCAQVAAGPQLPTGLQGNMAAVSAEGNGNRFVVIAVETSQPLPAPSPGKDCSK
```

VTFSGTQLRGGIEVVDVPHIDGTQTLGVHRVLQAVVGGSARTGELYDYSARFGDYQVI
VIANPLVIPGRPVARVDTQRARDLLVQAVAAVRG"
CDS complement(1119105..1119722)
/codon_start=1
/note="gene_1042"
/transl_table=11
/translation="MCDKLGGVAIAVQGALFEHNERRQLGDGAFIDIRSGWLTGGEEL
LDALLSTVPWRAERRQMYDRVVDVPRLVSFHDLTIEDPPHPQLARMRRRLNDIYGGEL
GEPFTTAGLCYYRDGSDSVAWHGDTIGRGSTEDTMVAIVSLGATRVFALRPRGRGPSL
RLPLAHGDLLVMGGSCQRTFEHAVPKTSAPTGPRVSIQFRPRDVR"
CDS 1119759..1120967
/codon_start=1
/note="gene_1043"
/transl_table=11
/translation="VGVELGSNSEVGALRVVILHRPGAELRRLTPRNTDQLLFDGLPW
VSRAQDEHDEFAELLASRGAEVLLLSDLLTEALHHSGAARMQGIAAAVDAPRLGLPLA
QELSAYLRSLDPGRLAHVLTAGMTFNELPSDTRTDVSLVLRMHHGGDFVIEPLPNLVF
TRDSSIWIGPRVVIPSLALRARVREASLTDLIYAHHPRFTGVRRAYESRTAPVEGGDV
LLLAPGVVAVGVGERTTPAGAEALARSLFDDDLAHTVLAVPIAQQRAQMHLDTVCTMV
DTDTMVMYANVVDTLEAFTIQRTPDGVTIGDAAPFAEAAAKAMGIDKLRVIHTGMDPV
VAEREQWDDGNNTLALAPGVVVAYERNVQTNARLQDAGIEVLTIAGSELGTGRGGPRC
MSCPAARDPL"
CDS complement(1121002..1122570)
/codon_start=1
/note="gene_1044"
/transl_table=11
/translation="MTARPPESCVLAKDRPEEPVVPVVSPGPLVPVADFGPLDRLRGW
IVTGLITLLATVTRFLNLGSLTDAGTPIFDEKHYAPQAWQVLNNHGVEDNPGYGLVVH
PPVGKQLIAIGEAIFGYNGFGWRFTGALLGVLLVALVVRIVRRISRSTLVGAIAGVLL
ICDGVSFVTARTALLDGFLTFFVVAAFGALIVDRDQVRERMHIALLAGRSAATVWGPR
VGVRWWRFGAGVLLGLACATKWSGVYFVLFFGAMALAFDVAARRQYQVQRPWLGTVRR
DVLPSGYALGLIPFAVYLATYAPWFASETAIDRHAVGQAVGRNSVVPLPDAVRSLWHY
TAKAFHFHAGLTNSAGNYHPWESKPWTWPMSLRPVLYAIDQQDVAGCGAQSCVKAEML
VGTPAMWWLAVPVLAYAGWRMFVRRDWRYAVVLVGYCAGWLPWFADIDRQMYFFYAAT
MAPFLVMGISLVLGDILYHPGQGSERRTLGLIVVCCYVALVVTNFAWLYPVLTGLPIS
QQTWNLEIWLPSWR"
CDS 1122596..1123453
/codon_start=1
/note="gene_1045"
/transl_table=11
/translation="MSSGRLLLGATPLGQPSDASPRLAAALATADVVAAEDTRRVRKL
AKALDIRIGGRVVSLFDRVEALRVTALLDAINNGATVLVVSDAGTPVISDPGYRLVAA
CIDAGVSVTCLPGPSAVTTALVMSGLPAEKFCFEGFAPRKGAARRAWLAELAEERRTC
VFFESPRRLAACLNDAVEQLGGARPAAICRELTKVHEEVVRGSLDELAIWAAGGVLGE
ITVVVAGAAPHAELSSLIAQVEEFVAAGIRVKDACSEVAAAHPGVRTRQLYDAVLQSR
RETGGPAQP"
CDS complement(1123463..1124686)
/codon_start=1
/note="gene_1046"
/transl_table=11
/translation="MRLAIVGTAAAAAIGGTLAVAPLTLSTPERVAGGTCSAGQQCDR

LAAVLMPDTATPSGPAAAEHAVPAPFEPVADTIAPGLVPRPGVPAAAAVPRVGPPAVP
GLPNIPGAAGPALPPPPALPNLAAPSVPGVGIPGIGIPGIGIPGIGIPGVPDPITGVN
TAAAVVNGVLGVGGTAAGVVTASAVAVTYLVLAVNALESSGILPTARGTASTVASLLL
PGAQSAAAALPAVGLPALPGVTPASLLAMAAAAGLPGVGFPSLPGVSPTDLMAMAAAA
GLPTSLPGLAGMSPAELTALVAGGLPMLAAAGLPAGLAGVDPATLAAALPALAAGGLP
PGLPALPGVDPAALAAALPALAAGLPALPAGLPPLPAVPALPAPPPLPGPPPLPALPS
RLCTPGFGPIGVCIP"
CDS complement(1124796..1126040)
/codon_start=1
/note="gene_1047"
/transl_table=11
/translation="VLRAVGRATSRLDLPPPAALTGEWFGALAVIAPSVSIQPVSGDD
VFSGPPGTGGPDATGAVGGGWVGYLSYPDAGADGRPHRIPEAAGGWTDCVLRRDRDGQ
WWYESLSGAPIADWLASALATTRASVARPAPACRIDWEPADRAAHRDGVLACLEAIGA
GEVYQACVCTQFAGTVTGSPLDFFIDGFGRTAPSRSAFVAGPWGAVASLSPELFLRRR
GSVVTSSPIKGTLPLDAPPSALRASAKEVAENIMIVDLVRNDLGRVAVTGTVTVPELL
VVRPAPGVWHLVSTVSARVPLEEPMSALLDAAFPPASVTGTPKLRARQLISQWERYRR
GIYCGTVGLASPVAGCELNVAIRTVEFDTAGNAVLGVGGGITADSDPDAEWAECLHKA
APIVGLPAATRTTPARLASKVR"
CDS 1126453..1127991
/codon_start=1
/note="gene_1048"
/transl_table=11
/translation="LRGQYEDLMEPLFPQGNPAQQRYPPWPASYDASLRVSWRQLQPT
DPRTLPPDAPDDRKYDFSVIDNALTRLADRGMRLTLRVYAYSSCCKASYPDGTNIAIP
DWERAIASTNTSYPGPATDPSTGVVQVVPNFNDSTYLNDFAQLLAALGRRYDGDERLS
VFEFSGYGDFSENHVAYLRDTLGAPGPGPDESVATLGYYSQFRDQNITTASIKQLIAA
NVSAFPHTQLVTSPANPEIVRELFADEVTNKLAAPVGVRSDCLGVDAPLPAWAESSTS
HYVQTKDPVVAALRQRLATAPVITEWCELPTGSSPRAYYEKGLRDVIRYHVSMTSSVN
FPDQTATSPMDPALYLVWAQANAAAGYRYSVEAQPGSQALAGKVATISVTWTNYGAAA
ATEKWVPGYRLVDSTGQVVRTLPAAVDLKTLVSDQRGDRSSDQPTPASVAETVRVDLS
GLPAGHYTLRAAIDWQQHKPNGSHVVNYPSMLLSRDGRDDSGFYPVATLDIPRDAQTA
VNAS"
CDS complement(1128018..1129577)
/codon_start=1
/note="gene_1049"
/transl_table=11
/translation="MKPYYVTTAIAYPNAAPHVGHAYEYIATDAIARFKRLDGYDVRF
LTGTDEHGLKVAQAAAAAGVPTAALARRNSDVFQRMQEALNISFDRFIRTTDADHHEA
SKELWRRMSAAGDIYLDNYSGWYSVRDERFFVESETQLVDGTRLTVETGTPVTWTEEQ
TYFFRLSAYTDKLLAHYHANPDFIAPETRRNEVISFVSGGLDDLSISRTSFDWGVQVP
EHPDHVMYVWVDALTNYLTGAGFPDTDSELFRRYWPADLHMIGKDIIRFHAVYWPAFL
MSAGIELPRRIFAHGFLHNRGEKMSKSVGNIVDPVALAEALGVDQVRYFLLREVPFGQ
DGSYSDEAIVTRINTDLANELGNLAQRSLSMVAKNLDGRVPNPGEFADADAALLATAD
GLLERVRGHFDAQAMHLALEAIWLMLGDANKYFSVQQPWVLRKSESEADQARFRTTLY
VTCEVVRIAALLIQPVMPESAGKILDLLGQAPNQRSFAAVGVRLTPGTALPPPTGVFP
RYQPPQPPEGK"
CDS 1129717..1130457
/codon_start=1
/note="gene_1050"
/transl_table=11

/translation="VRSLVERAAAAGVTAVVTVADDLESARWVTRAAEWDRRVYAAVA
LHPTRADALTDAARAELERLVAHPRVVAVGETGIDMYWPGRLDGCAEPHVQREAFAWH
IDLAKRTGKPLMIHNRQADRDVLDVLRAEGAPDTVILHCFSSDAAMARTCVDAGWLLS
LSGTVSFRNARELREAVPLMPVEQLLVETDAPYLTPHPHRGLANEPYCLPYTVRALAE
LVNRRPEEVALITTSNARRAYGLGWMRQ"

CDS 1130665..1131753
/codon_start=1
/note="gene_1051"
/transl_table=11
/translation="MLRLVVGALLLVLAFAGGYAVAACKTVTLTVDGTAMRVTTMKSR
VIDIVEENGFSVDDRDDLYPAAGVQVHDADTIVLRRSRPLQISLDGHDAKQVWTTAST
VDEALAQLAMTDTAPAAASRASRVPLSGMALPVVSAKTVQLNDGGLVRTVHLPAPNVA
GLLSAAGVPLLQSDHVVPAATAPIVEGMQIQVTRNRIKKVTERLPLPPNARRVEDPEM
NMSREVVEDPGVPGTQDVTFAVAEVNGVETGRLPVANVVVTPAHEAVVRVGTKPGTEV
PPVIDESIWDAIAGCEAGGNWAINTGNGYYGGVQFDQGTWEANGGLRYAPRADLATRE
EQIAVAEVTRLRQGWGAWPVCAVRAGAR"

CDS 1131726..1132679
/codon_start=1
/gene="ksgA"
/note="gene_1052"
/transl_table=11
/translation="MCCTSGCALTIRLLGRTEIRRLAKELDFRPRKSLGQNFVHDANT
VRRVVAASGVSRSDLVLEVGPGLGSLTLALLDRGATVTAVEIDPLLASRLQQTVAEHS
HSEVHRLTVVNRDVLALRREDLAAAPTAVVANLPYNVAVPALLHLLVEFPSIRVVTVM
VQAEVAERLAAEPGSKEYGVPSVKLRFFGRVRRCGMVSPTVFWPIPRVYSGLVRIDRY
ETSPWPTDDAFRRRVFELVDIAFAQRRKTSRNAFVQWAGSGSESANRLLAASIDPARR
GETLSIDDFVRLLRRSGGSDEATSTGRDARAPDISGHASAS"

CDS 1132729..1133685
/codon_start=1
/note="gene_1053"
/transl_table=11
/translation="VSASDGNTAELWVPTGSVTVRVPGKVNLYLAVGDRREDGYHELT
TVFHAVSLVDEVTVRNADVLSLELVGEGADQLPTDERNLAWQAAELMAEHVGRAPDVS
IMIDKSIPVAGGMAGGSADAAAVLVAMNSLWELNVPRRDLRMLAARLGSDVPFALHGG
TALGTGRGEELATVLSRNTFHWVLAFADSGLLTSAVYNELDRLREVGDPPRLGEPGPV
LAALAAGDPDQLAPLLGNEMQAAAVSLDPALARALRAGVEAGALAGIVSGSGPTCAFL
CTSASSAIDVGAQLSGAGVCRTVRVATGPVPGARVVSAPTEV"

CDS 1134200..1135834
/codon_start=1
/note="gene_1054"
/transl_table=11
/translation="LSRFTEKMFHNARTATTGMVTGEPHMPVRHTWGEVHERARCIAG
GLAAAGVGLGDVVGVLAGFPVEIAPTAQALWMRGASLTMLHQPTPRTDLAVWAEDTMT
VIGMIEAKAVIVSEPFLVAIPILEQKGMQVLTVADLLASDPIGPIEVGEDDLALMQLT
SGSTGSPKAVQITHRNIYSNAEAMFVGAQYDVDKDVMVSWLPCFHDMGMVGFLTIPMF
FGAELVKVTPMDFLRDTLLWAKLIDKYQGTMTAAPNFAYALLAKRLRRQAKPGDFDLS
TLRFALSGAEPVEPADVEDLLDAGKPFGLRPSAILPAYGMAETTLAVSFSECNAGLVV
DEVDADLLAALRRAVPATKGNTRRLATLGPLLQDLEARIIDEQGDVMPARGVGVIELR
GESLTPGYLTMGGFIPAQDEHGWYDTGDLGYLTEEGHVVVCGRVKDVIIMAGRNIYPT
DIERAAGRVDGVRPGCAVAVRLDAGHSRESFAVAVESNAFEDPAEVRRIEHQVAHEVV
AEVDVRPRNVVVLGPGTIPKTPSGKLRRANSVTLVT"

```
CDS             complement(1135908..1136483)
                /codon_start=1
                /note="gene_1055"
                /transl_table=11
                /translation="MAEPLLVVGLGNPGANYARTRHNLGFVVADLLAARLGAKFKAHK
                RSGAEVATGRSAGRSLVLAKPRCYMNESGRQIGPLAKFYSVAPANIIVIHDDLDLEFG
                RIRLKIGGGEGGHNGLRSVVAALGTKDFQRVRIGIGRPPGRKDPAAFVLENFTPAERA
                EVPTICEQAADATELLIEQGMEPAQNRVHAW"
CDS             complement(1136496..1137143)
                /codon_start=1
                /note="gene_1056"
                /transl_table=11
                /translation="MAKSASNQLRVTVRTETGKGASRRARRAGKIPAVLYGHGAEPQH
                LELPGHDYAAVLRHSGTNAVLTLDIAGKEQLALTKALHIHPIRRTIQHADLLVVRRGE
                KVVVEVSVVVEGQAGPDTLVTQETNSIEIEAEALSIPEQLTVSIEGAEPGTQLTAGQI
                ALPAGVSLISDPDLLVVNVVKAPTAEELEGEVAGAEEAEEAAVEAGEAEAAGESE"
CDS             complement(1137360..1137533)
                /codon_start=1
                /note="gene_1057"
                /transl_table=11
                /translation="MTCTAGDCTPGTGSSSQPAPAKQRYLVQLTITSLANEAVKHASD
                IEAIIAGFVVAAK"
CDS             1137710..1138060
                /codon_start=1
                /gene="MRGA327_06380"
                /note="gene_1058"
                /transl_table=11
                /translation="LAIISVFGVMFGFFIGAQPGGVGIDTVRSVRLLGATGSPVTPTL
                SRYFHSGTGTSVVVETALVVGLVDKIDWKSGDFGPQATADIASVATATATAKGLTESC
                GQRRPAKPAGCPQG"
CDS             complement(1138071..1139051)
                /codon_start=1
                /note="gene_1059"
                /transl_table=11
                /translation="LSHDWTDNRKNLMLFAGRAHPELAEQVAKELDVHVTSQDAREFA
                NGEIFVRFHESVRGCDAFVLQSCPAPVNRWLMEQLIMIDALKRGSAKRITAVMPFYPY
                ARQDKKHRGREPISARLIADLLKTAGADRIVTVDLHTDQIQGFFDGPVDHMRGQNLLT
                GYIRDNYPDGNMVVVSPDSGRVRIAEKWADALGGVPLAFIHKTRDPRVPNQVVSNRVV
                GDVAGRTCVLIDDMIDTGGTIAGAVALLHNDGAGDVIIAATHGVLSDPAAQRLASCGA
                REVIVTNTLPIGEDKRFPQLTVLSIAPLLASTIRAVFENGSVTGLFDGDA"
CDS             complement(1139143..1140630)
                /codon_start=1
                /note="gene_1060"
                /transl_table=11
                /translation="MTFPGDTAVLVLAAGPGTRMRSDTPKVLHTLAGRSMLSHVLHAI
                AKLAPQRLIVVLGHDHQRIAPLVGELADTLGRTIDVALQDRPLGTGHAVLCGLSALPD
                DYAGNVVVTSGDTPLLDADTLADLIATHRAVSAAVTVLTTTLDDPFGYGRILRTQDHE
                VMAIVEQTDATPSQREIREVNAGVYAFDIAALRSALSRLSSNNAQQELYLTDVIAILR
                SDGQTVHASHVDDSALVAGVNNRVQLAELASELNRRVVAAHQLAGVTVVDPATTWIDV
                DVTIGRDTVIHPGTQLLGRTQIGGRCVVGPDTTLTDVAVGDGASVVRTHGSSSSIGDG
                AAVGPFTYLRPGTALGADGKLGAFVEVKNSTIGTGTKVPHLTYVGDADIGEYSNIGAS
```

```
                     SVFVNYDGTSKRRTTVGSHVRTGSDTMFVAPVTIGDGAYTGAGTVVREDVPPGALAVS
                     AGPQRNIENWVQRKRPGSPAAQASKRASEMACQQPTQPPDADQTP"
tRNA                 complement(1140646..1140717)
                     /note="identified by tRNAscan-SE; putative"
CDS                  1140819..1141478
                     /codon_start=1
                     /note="gene_1061"
                     /transl_table=11
                     /translation="VAGPDKGPDKAPENPTRVTRARMTGTERRHQLIGIARSLFAERG
                     YDGTSIEEIAQRANVSKPVVYEHFGGKEGLYAVVVDREMSALLDGITSSLTNNRSRVR
                     VERVALALLTYVEERTDGFRIMIRDSPASISSGTYSSLLNDAVSQVSSILAGDFARRG
                     LDPDLAPLYAQALVGSVSMTAQWWLDAREPKKEVVAAHLVNLVWNGLTHLEADPRLQD
                     E"
CDS                  1141537..1145241
                     /codon_start=1
                     /note="gene_1062"
                     /transl_table=11
                     /translation="MTAPGPACSDTPIAGLVELALSAPTFQQLMQRAGGRPDELTLIA
                     PASARLLVASALARQGPLLVVTATGREADDLAAELRGVFGDAVALLPSWETLPHERLS
                     PGVDTVGTRLMALRRLAHPDDAQLGPPLGVVVTSVRSLLQPMTPQLGMMEPLTLTVGD
                     ESPFDGVVARLVELAYTRVDMVGRRGEFAVRGGILDIFAPTAEHPVRVEFWGDEITEM
                     RMFSVADQRSIPEIDIHTLVAFACRELLLSEDVRARAAQLAARHPAAESTVTGSASDM
                     LAKLAEGIAVDGMEAVLPVLWSDGHALLTDQLPDGTPVLVCDPEKVRTRAADLIRTGR
                     EFLEASWSVAALGTAENQAPVDVEQLGGSGFVELDQVRAAAARTGHPWWTLSQLSDES
                     AIELDVRAAPSARGHQRDIDEIFAMLRAHIATGGYAALVAPGTGTAHRVVERLSESDT
                     PAGMLDPGQAPKPGVVGVLQGPLRDGVIIPGANLVVITETDLTGSRVSAAEGKRLAAK
                     RRNIVDPLALTAGDLVVHDQHGIGRFVEMVERTVGGARREYLVLEYASAKRGGGAKNT
                     DKLYVPMDSLDQLSRYVGGQAPALSRLGGSDWANTKTKARRAVREIAGELVSLYAKRQ
                     ASPGHAFSPDTPWQAELEDAFGFTETVDQLTAIEEVKADMEKPIPMDRVICGDVGYGK
                     TEIAVRAAFKAVQDGKQVAVLVPTTLLADQHLQTFGERMSGFPVTIKGLSRFTDAAES
                     RAVIDGLADGSVDIVIGTHRLLQTGVRWKDLGLVVVDEEQRFGVEHKEHIKSLRTHVD
                     VLTMSATPIPRTLEMSLAGIREMSTILTPPEERYPVLTYVGPHDDKQIAAALRRELLR
                     DGQAFYVHNRVSSIDAAAARVRELVPEARVVVAHGQMPEDLLETTVQRFWNREHDILV
                     CTTIVETGLDISNANTLIVERADTFGLSQLHQLRGRVGRSRERGYAYFLYPPQVPLTE
                     TAYDRLATIAQNNELGAGMAVALKDLEIRGAGNVLGIEQSGHVAGVGFDLYVRLVGEA
                     LETYRDAYRAAADGQTVRTAEEPKDVRIDLPVDAHLPPDYIASDRLRLEGYRRLAAAS
                     SDREVAAVVDELTDRYGALPEPARRLAAVARLRLLCRGSGITDVTAASAATVRLSPLT
                     LPDSAQVRLKRMYPGAHYRATTATVQVPIPRAGGLGAPRIRDVELVQMVADLITALAG
                     KPRQHIGITNPSPPGEDGRGRNTTIKERQP"
CDS                  1145241..1146218
                     /codon_start=1
                     /note="gene_1063"
                     /transl_table=11
                     /translation="MIVVLVDPRRPTLVPVEAIEFLRGEVQYTEEMPVAVPWSLPAAR
                     SAHAGNDAPVLLSSDPNHPAVITRLAAGARLISAPDSQRGERLVDAVAMMDKLRTAGP
                     WESEQTHDSLRRYLLEETYELLDAVRSGSVDQLREELGDLLLQVLFHARIAEDASQSP
                     FTIDDVADTLMRKLGNRAPGVLAGESISLEDQLAQWEAAKASEKARKSVADDVHTGQP
                     ALALAQKVIQRAQKAGLPAHLIPDEITSVSVSADVDAENTLRTAVLDFIDRLRCAERA
                     IAVARRGSNVAEQLDVTPLGVITEQEWLAHWPTAVNDSRGGSKKRKGMR"
CDS                  1146357..1147037
                     /codon_start=1
```

/note="gene_1064"
/transl_table=11
/translation="MLLASSCTWQLSLFIPDGVPPPPGDPVPPVDTHAGGRPADQLRE
WAEKRAAALGIPVIALEAYAYAARVAEVENPKCHLAWTTLAGIGRVESHHGTYRGATI
APNGDVSPPIRGVRLDGTGGTLRIVDRDGGGLDGDAAVERAMGPMQFISETWRLYGVA
ARNDGIANVDNIDDAALSAAGYLCWRGKDLATPRGWITALRAYNNSVIYARAVRDWAT
AYAAGHPL"

CDS 1147134..1148423
/codon_start=1
/gene="eno"
/note="gene_1065"
/transl_table=11
/translation="VPIIEQVGAREILDSRGNPTVEVEVALIDGTFARAAVPSGASTG
EHEAVELRDGGDRYGGKGVQKAVQAVLDEIGPAVIGLNADDQRLVDQALVDLDGTPDK
SRLGGNAILGVSLAVAKAAADSAELPLFRYVGGPNAHILPVPMMNILNGGAHADTAVD
IQEFMVAPIGAPSFVEALRWGAEVYHALKSVLKKEGLSTGLGDEGGFAPDVAGTTAAL
DLISRAIESAGLRPGADVALALDAAATEFFTDGTGYVFEGTTRTADQMTEFYAGLLGA
YPLVSIEDPLSEDDWDGWAALTASIGDRVQIVGDDIFVTNPERLEEGIERGVANALLV
KVNQIGTLTETLDAVTLAHHGGYRTMISHRSGETEDTMIADLAVAIGSGQIKTGAPAR
SERVAKYNQLLRIEEALGDAARYAGDLAFPRFACETK"

CDS 1148428..1149114
/codon_start=1
/note="gene_1066"
/transl_table=11
/translation="MPEAKRPESKRRSPASRPGKAGDSVRGGRATKPSAKPSTPAPHA
SRKTTRTPHEHIVEPIKRAITESVEKRSEQRLGFTARRAAILAAVVCVLTLTIARPVR
TYFAQRAEMEQLAATEAMLRRQIADLEEQQVKLADPAYIAAQARERLGFVMPGDIPFQ
VQLPSTPLAPPQPGSDAATATNNEPWYTALWHTIADDPHLPPAAPPAPEPGRPGPLPP
ASPNPEQPGG"

CDS 1149107..1149598
/codon_start=1
/note="gene_1067"
/transl_table=11
/translation="VVDRADLEVVTRQLGRAPRGVLAIAYRCPNGEPGVVKTAPRLPD
GTPFPTLYYLTHPVLTAAASRLETTGLMREMNRRLGQDAELAAAYRRAHESYLSERDA
LEPLGTTVSAGGMPDRVKCLHVLIAHSLAKGPGLNPFGDEALALLAAEPRTAATLVAG
QWR"

CDS 1149589..1150548
/codon_start=1
/note="gene_1068"
/transl_table=11
/translation="VALTRVAAIDCGTNSIRLLIADVGAGLARGELHDVHRETRIVRL
GQGVDATGRFAPEAIARTRTALTDYAELLTFHHAERVRMVATSAARDVVNRDVFFAMT
ADVLGAALPGSAAEVITGAEEAELSFRGAVGELGSAGAPFVVVDLGGGSTEIVLGEHE
VVASYSADIGCVRLTERCLHSDPPTLQEVSTARRLVRERLEPALRTVPLELARTWVGL
AGTMTTLSALAQSMTAYDAAAIHLSRVPGADLLEVCQRLIGMTRKQRAALAPMHPGRA
DVIGGGAIVVEELARELRERAGIDQLTVSEHDILDGIALSLAG"

CDS complement(1150997..1151677)
/codon_start=1
/note="gene_1069"
/transl_table=11

/translation="MTLVLVIDDEPQILRALRINLTVRGYQVITASTGAGALRAAAEH
PPDVVILDLGLPDMSGIDVLGGLRGWLTAPVIVLSARTDSSDKVQALDAGADDYVTKP
FGMDEFLARLRAAVRRNTAAAELEQPVIETDSFTVDLAGKKVIKDGAEVHLTPTEWGM
LEMLARNRGKLVGRGELLKEVWGPAYATETHYLRVYLAQLRRKLEDDPSHPKHLLTES
GMGYRFEA"
CDS complement(1151674..1154256)
/codon_start=1
/note="gene_1070"
/transl_table=11
/translation="VTLLFADLCAIFTPYRWMIEHVTTKRGQLRIYLGAAPGVGKTYA
MLGEAHRRLERGTDVVAAVVETHGRNKTAKLLEGIEMISPRYVEYRGARFPELDVEAV
LRRHPQVVLVDELAHTNTPGSKNPKRWQDVQEILDAGITVISTVNIQHLEGLNDVVEQ
ITGIEQKEKIPDEIVRAADQVELVDITPEALRRRLAHGNVYAAERVDAALSNYFRTGN
LTALREIALLWLADQVDAALEKYRADKKITATWEARERVVVAVTGGPESETLVRRASR
IASKSSAELMVVHVIRGDDLAGVSAPQLGRVRELATSLGATMHTVVGDDVPTALLDFA
REMNATQLVVGTSRRSRWARLFDEGIGARTVQESGGIDVHMVTHPAASRASGWSRVSP
RERHIASWLAALVVPSVICAITVAWLDRFMGIGGESALFFIGVLIVALLGGVAPAALS
ALLSGMLLNYFLTEPRYTWTIAEPDAAVTEFVLLAMAVAVAVLVDGAASRTREARRAS
QEAELLALFAGSVLRGADLATLLQRVRETYSQRAVTMLRVRQGASTGETVACVGTNPC
RDVDSADTAIEVGDDEFWMLMAGRKLAARDRRVLTAVATQAAGLVKQRELAEEAGQAE
AIARADELRRSLLSAVSHDLRTPLAAAKVAVSSLRTEDVAFSPEDTAELLATIEESID
QLTALVANLLDSSRLAAGVIRPQLRRAYLEEAVQRALVSIGKGATGFYRSGIDRVKVD
VGDAVAMADAGLLERVLANLIDNALRYAPDCVVRVDAGRVRERVLINVIDEGPGVPRG
TEEQLFAPFQRPGDHDNTTGVGLGMSVARGFVEAMGGTISATDTPGGGLTVVIDLAAP
EDRP"
CDS 1154582..1156297
/codon_start=1
/note="gene_1071"
/transl_table=11
/translation="MSGTSWLQFAALIAVLLLTAPALGGYLAKIYGDEAKKPGDRVFG
PIERVIYQVCRVDPGSEQRWSTYALSVLAFSVMSFLLLYGIARFQGVLPFNPTDKPAV
TDHVAFNAAVSFMTNTNWQSYSGEATMSHFTQMTGLAVQNFVSASAGMCVLAALIRGL
ARKRASTLGNFWVDLARTVLRIMFPLSFVVAILLVSQGVIQNLHGFIVANTLEGAPQL
IPGGPVASQVAIKQLGTNGGGFFNVNSAHPFENYTPIGNFVENWAILIIPFALCFAFG
KMVHDRRQGWAVLAIMGIIWIGMSVAAMSFEAKGNPRLDALGVTQQTTVDQSGGNLEG
KEVRFGVGASGLWAASTTGTSNGSVNSMHDSYTPLGGMVPLAHMMLGEVSPGGTGVGL
NGLLVMAILAVFIAGLMVGRTPEYLGKKIQATEMKLVTLYILAMPIALLSFAAASVLI
SSALASRNNPGPHGLSEILYAYTSGANNNGSAFAGLTASTWSYDTTIGVAMLIGRFFL
IIPVLAIAGSLARKGTTPVTAATFPTHKPLFVGLVIGVVLIVGGLTFFPALALGPIVE
QLSTQ"
CDS 1156297..1158423
/codon_start=1
/note="gene_1072"
/transl_table=11
/translation="MIARMETSATAAAATSAPRLRLAKRSLFDPMIVRSALPQSLRKL
APRVQARNPVMLVVLVGAVITTLAFLRDLASSTAQENVFNGLVAAFLWFTVLFANFAE
AMAEGRGKAQAAALRKVRSETMANRRTAAGNIESVPSSRLDLDDVVEVSAGETIPSDG
EIIEGIASVDESAITGESAPVIRESGGDRSAVTGGTVVLSDRIVVRITAKQGQTFIDR
MIALVEGAARQQTPNEIALNILLAGLTIIFLLAVVTLQPFAIYSGGGQRVVVLVALLV
CLIPTTIGALLSAIGIAGMDRLVQHNVLATSGRAVEAAGDVNTLLLDKTGTITLGNRQ
ATEFVPINGVSAEAVADAAQLSSLADETPEGRSIVVLAKDEFGLRARDEGVMSHARFV

PFTAETRMSGVDLAEVSGIRRIRKGAAAAVMKWVRDHGGHPTEEVGAIVDGISSGGGT
PLVVAEWTDNSSARAIGVVHLKDIVKVGIRERFDEMRRMSIRTVMITGDNPATAKAIA
QEAGVDDFLAEATPEDKLALIKREQQGGRLVAMTGDGTNDAPALAQADVGVAMNTGTQ
AAREAGNMVDLDSDPTKLIEVVEIGKQLLITRGALTTFSIANDVAKYFAIIPAMFVGL
YPVLDKLNVMALHSPRSAILSAVIFNALVIVALIPLALRGVRFRAESASAMLRRNLLI
YGLGGLVVPFIGIKLVDLVIVALGVS"

CDS 1158423..1158992
/codon_start=1
/gene="kdpC"
/note="gene_1073"
/transl_table=11
/translation="MRRQLLPALTMLLVFTVITGIVYPLAVTGVGQLFFGDQANGALL
ERDGQVIGSAHIGQQFTAAKYFHPRPSSAGDGYDAAASSGSNLGPTNEKLLAAVAERV
TAYRKENNLPADTLVPVDAVTGSGSGLDPAISVVNAKLQAPRVAQARNISIRQVERLI
EDHTDARGLGFLGERAVNVLRLNLALDRL"

CDS complement(1158996..1160456)
/codon_start=1
/note="gene_1074"
/transl_table=11
/translation="LLLGVLAVVTVVLVAVGVVSVLSLSGYVTAMNDAELVESLHALN
HSYTRYRDSAQTSTPTGNLPMSQAVLEFTGQTPGNLIAVLHDGVVIGSAVFSEDGARP
APPDVIRAIEAQVWDGGPPRVESLGSLGAYQVDSSAAGADRLFVGVSLSLANQIIARK
KVTTVALVGAALVVTAALTVWVVGYALRPLRRVAATAAEVATMPLTDDDHQISVRVRP
GDTDPDNEVGIVGHTLNRLLDNVDGALAHRVDSDLRMRQFITDASHELRTPLAAIQGY
AELTRQDSSDLPPTTEYALARIESEARRMTLLVDELLLLSRLSEGEDLETEDLDLTDL
VINAVNDAAVAAPTHRWVKNLPDEPVWVNGDHARLHQLVSNLLTNAWVHTQPGVTVTI
GITCHRTGPNAPCVELSVTDDGPDIDPEILPHLFDRFVRASKSRSNGSGHGLGLAIVS
SIVKAHRGSVTAESGNGQTVFRVRLPMIEQQIATTA"

CDS complement(1160533..1161306)
/codon_start=1
/note="gene_1075"
/transl_table=11
/translation="MTTMSGYTRSQRPRQAILGQLPRIHRADGSPIRVLLVDDEPALT
NLVKMALHYEGWDVEVAHDGQEAIAKFDKVGPDVLVLDIMLPDVDGLEILRRVRESDV
YTPTLFLTARDSVMDRVTGLTSGADDYMTKPFSLEELVARLRGLLRRSSHLERPADEA
LRVGDLTLDGASREVTRDGTPISLSSTEFELLRFLMRNPRRALSRTEILDRVWNYDFA
GRTSIVDLYISYLRKKIDSDREPMIHTVRGIGYMLRPPE"

CDS complement(1161488..1161979)
/codon_start=1
/note="gene_1076"
/transl_table=11
/translation="MPAPITKGRLGKPVEFEHKAQVVDNDDGIVLDHTVQQGNPPDAP
QLAPAVAWVKKRAGRTPRTVTADRGYGEAAVDQQLTEVGVKNVLIPRKGKPSQDRRAE
EHRKAFRRTIKWRTGCEGRISHLKRGYGWDRGRIGGLEGTRTWVGHGVFAHNLVTISA
LPA"

CDS complement(1161945..1162631)
/codon_start=1
/note="gene_1077"
/transl_table=11
/translation="VPHPTTLMKLTTRCGSAAIDGLNEALLAKAAEAKLLGTNRIRAD
TTVARANVSYPTDLGLLAKAMRRIAATGKRIQAAGGAVRTRVGDRSRAAGRRAHAVAA

KLRSRAELGRDEARAAVLRFTGELAELAQAAAQEAQQLLDNAKQAVLRAKAKAAALAA
RGERDAVAGRRCGGLVRAVNDLTELLNATRQIVAQTRQRVAGITSDGASRRVSLHDGD
ARPDHQGSAR"
CDS complement(1162665..1163003)
/codon_start=1
/note="gene_1078"
/transl_table=11
/translation="LIPGRMVLNWEDGLNALVAEGIEAIVFRTLGDQCWLWESLLPDE
VRRLPEELARVDALLDDPAFFAPFVPFFDPRRGRPSTPMEVYLQLMFVKFRYRLGYES
LCREVADSIT"
CDS complement(1163114..1163398)
/codon_start=1
/note="gene_1079"
/transl_table=11
/translation="MTINYQFGDVDAHGAMIRALAGSLEAEHQAIISDVLTASDFWGG
AGSAACQGFITQLGRNFQVIYEQANAHGQKVQAAGNNMAQTDSAVGSSWA"
CDS complement(1163425..1163721)
/codon_start=1
/note="gene_1080"
/transl_table=11
/translation="MASRFMTDPHAMRDMAGRFEVHAQTVEDEARRMWASAQNISGAG
WSGMAEATSLDTMTQMNQAFRNIVNMLHGVRDGLVRDANNYEQQEQASQQILSS"
CDS complement(1163867..1165042)
/codon_start=1
/note="gene_1081"
/transl_table=11
/translation="MDFGALPPEINSARMYAGAGAGPMMAAGAAWNGLAAELGTTAAS
YESVITRLTTESWMGPASMAMVAAAQPYLAWLTYTAEAAAHAGSQAMASAAAYEAAYA
MTVPPEVVAANRALLAALVATNVLGINTPAIMATEALYAEMWAQDALAMYGYAAASGA
AGMLQPLSPPSQTTKPGGLAAQSAAVGSAAATAAVNQVSVADLISSLPNAVSGLASPV
TSVLDSTGLSGIIADIDALLATPFVANIINSAVNTAAWYVNAAIPTAIFLANALNSGA
PVAIAEGAIEAAEGAASAAAAGLADSVTPAGLGASLGEATLVGRLSVPAAWSTAAPAT
TAGATALEGSGWTVAAEEEAGPVTGMMPGMASAAKGTGAYAGPRYGFKPTVMPKQVVV"
CDS complement(1165119..1165946)
/codon_start=1
/note="gene_1082"
/transl_table=11
/translation="MSFLKTVPEELTAAAAQLGTIGAAMAAQNAAAAAPTTAIAPAAL
DEVSALQAALFTAYGTFYQQVSAEAQAMHDMFVNTLGISAGTYGVTESLNSSAAASPL
SGITGEASAIIQATTGLFPPELSGGIGNILNIGAGNWASATSTLIGLAGGGLLPAEEA
AEAASALGGEAALGELGALGAAEAALGEAGIAAGLGSASAIGMLSVPPAWAGQATLVS
TTSTLPGAGWTAAAPQAAAGTFIPGMPGVASAARNSAGFGAPRYGVKPIVMPKPATV"
CDS complement(1166734..1166973)
/codon_start=1
/note="gene_1083"
/transl_table=11
/translation="LLGSQHLPTKRSYPRSALSTYLVFDALIAHGDRHDRNWAVHVPP
LETKYVEALCPSFDHAASLGFTLTDQTPAQHLHDG"
CDS complement(1167142..1167579)
/codon_start=1
/note="gene_1084"

/transl_table=11
/translation="VRIRLTAGQAGDNPQLLPLLDDYRHASTEYALGSTDFRLLADKA
YSHPSTRAALRSKKIKHTIPERQDQIDRRKAKGSAGGRPPAFDAALYGLRNTVERGFH
RLKQWRGIATRYDKYALTYLGGVLLACAVIHARVGTPKLGDTP"
CDS complement(1167662..1168069)
/codon_start=1
/note="gene_1085"
/transl_table=11
/translation="VTRVGVISDEFWAVVEPLMPSHEGKPGRRFSDHRLILEGIAWRF
RTGSPWRDLPAEFGPWQTVWKRHHRWSLDGTCDEVFAHVAAVFGVDAEVAEDIEKLLS
VDSTNVRAHQHSAGACSDTLATGGTVGLQEIRR"
CDS complement(1168352..1169377)
/codon_start=1
/note="gene_1086"
/transl_table=11
/translation="MCAHQFFGLVHNPVVAAAIGKPEPPPVDSDIGLPTTVPFEPWSV
ADFSRYLSTLGLPAAGDAVTLHRILSSMERAGLLLPLGWDPRLPVMGQKYISQGTISK
GQRGGNLWLSEVFGAELIIPSYNAVTVQLAGHDDAGNPVDSWGTGLVVDHNHVITNKH
VVTGLAGTSAGLSVYPSSNHAEAELVNFSGTAHPHPTLDVAVIKFEMPEGKYIPRLGG
MAFRDPDWADEVYVFGYPRVPMTAEMAITVQRGEVVNPAATTIPGRQKIFLYSAIARP
GNSGGPIVAQDGRVIGLVVEDSAEAPSTGTGPNAAPFYRGIPSSEVIRALDELDFGGI
VEMDTLP"
CDS 1169663..1170247
/codon_start=1
/note="gene_1087"
/transl_table=11
/translation="MAIWDRLVEVAAEQHGYVTTRDARDIGVDPVQLRLLAGRGRLER
VGRGVYRVPVLPRGEHDDLAAAVSWTLGRGVISHESALALHALADVNPSRIHLTVPRN
NHPRAAGGELYRVHRRDLQAAHVTSVDGIPVTTVARTIKDCVKTGTDPYQLRAAIERA
EAEGTLRRGSAAELRAALDETTAGLRARPKRASA"
CDS 1170358..1171125
/codon_start=1
/note="gene_1088"
/transl_table=11
/translation="MIVVAQFAATLTDDTGAPLLLVKGGSSLELRRGIPDSRTSKDFD
TVARRDIELIHEQLADAGETGWEGFTAIFTAPEEIDVPGMPVKPRRFTAKLSYRGRAF
ATVPIEVSSVEAGNADQFDTLTSDALGLVGVPAAVAVPCMTIPWQIAQKLHAVTAVLE
EPKVNDRAHDLVDLQLLEGLLLDADLMPTRSACIAIFEARAQHPWPPRVATLPHWPLI
YAGALEGLDHLELARTVDAAAQAVQRFVARIDRATKR"
CDS 1171995..1173242
/codon_start=1
/note="gene_1089"
/transl_table=11
/translation="MTSSHLIDTEQLLADQLAQASPDLLRGLLSTFIAALMGAEADAL
CGAGYRERSDERSNQRNGYRHRDFDTRAATIDVAIPKLRQGSYFPDWLLQRRKRAERA
LTSVVATCYLLGVSTRRMERLVETLGVTKLSKSQVSIMAKELDEAVEAFRTRPLDAGP
YTFLAADALVLKVREAGRVVGVHTLIATGVNAEGYREILGIQVTSAEDGAGWLAFFRD
LVARGLSGVALVTSDAHAGLVAAIGATLPAAAWQRCRTHYAANLMAATPKPSWPWVRT
LLHSIYDQPDAESVVAQYDRVLDALTDKLPAVAEHLDTARTDLLAFTAFPKQIWRQIW
SNNPQERLNREVRRRTDVVGIFPDRASIIRLVGAVLAEQHDEWIEGRRYLGLEVLTRA
RAALTSTEEPAKQQTTNTPALTT"

```
CDS             complement(1173610..1174626)
                /codon_start=1
                /note="gene_1090"
                /transl_table=11
                /translation="MSWAERTVSALEGAFRSEVRARRVNGPHRDVIVSLDGAEFLVRW
                LTTGWPRQVAEALHATSRPDILAAPTMSPGARKAAHDAGVGWVDESGAADIHYRNTST
                GTTLVIETKGAPPAPLDARIGWRRATLAVCEALLANITGPTVASVVEATGLSMGSSAQ
                ALKFLEKNGHLASATARGPKSARLIVDRDALLDAYAEAADKLRSPISISTGVLWRDPT
                AGVVKAGQLWDAAGIEWAATSALSASLLAPMQTEIAPMEIYVPGRSWSDLRRAAMAAG
                LQEIAGGRLILRFFPTPACARLTEQNLQGFRSMLWPRVYADLRTAGVRGEDAAEHLRE
                AMTK"
CDS             complement(1174682..1174864)
                /codon_start=1
                /gene="TMFG_02872"
                /note="gene_1091"
                /transl_table=11
                /translation="MLRDLVLLDPDGERLVFGMRAHPDSAALLTRADDLEELIGFAAR
                QRSASVGSHVAIELHP"
CDS             1174958..1175404
                /codon_start=1
                /note="gene_1092"
                /transl_table=11
                /translation="MGKGAAFDECACYTTRRAARQLGQAYDRALRPSGLTNTQFSTLA
                VISLSEGSAGIDLTMSELAARIGVERTTLTRNLEVMRRDGLVRVMAGADARCKRIELT
                AKGRAALQKAVPLWRGVQAEVTASVGDWPRVRRDIANLGQAAEACR"
CDS             1175449..1176357
                /codon_start=1
                /note="gene_1093"
                /transl_table=11
                /translation="MMARQRFRDQVVLITGASSGIGEATAKAFAREGAVVALAARREG
                ALRRVAREIEAAGGRAMVAPLDVSSSESVRAMVADVVGEFGRIDVVFNNAGVSLVGPV
                DAETFLDDTREMLEIDYLGTVRVVREVLPIMKQQRSGRIMNMSSVVGRKAFARFAGYS
                SAMHAIAGFSDALRQELRGSGIAVSVIHPALTQTPLLANVDPADMPPPFRSLTPIPVH
                WVAAAVLDGVARRRARVVVPFQPRLLMVGDAFSPRYGDRVVRLLESKIFGRLIGSYRG
                SVYRHQPTESAKAQAAQPERGYSSAR"
CDS             complement(1176516..1177271)
                /codon_start=1
                /note="gene_1094"
                /transl_table=11
                /translation="MRADVTAEHLTQVVRDIAVIDIDDGVAFNLDTSSVQEIRERADY
                PGLRVRVAMSVGPWQGIAAWDVSTGEPIAPWPTRVTIDRILGEPITLLGYAPETIIAE
                KGVTILERGITSTRWRDYVDIVQLDRRGIDDDELLRSARAVAQYRGATLEPVAPHLAG
                YGAVAQAKWATEHGRCQHCWRHWKPAHVGRRNMDLLDAKQVSEMIGVPVGTLRHWRHS
                DIGPASFTLGRRVVYRRDEVSRWISKRESATRR"
CDS             1178294..1178683
                /codon_start=1
                /note="gene_1095"
                /transl_table=11
                /translation="MDCCEERGVARHKGLSQVGTPGCPRWSQAVSCRCSAYREAAVTA
                VQMPLTPGYGETPLPHDELAALLPEVVEVLDKPITRADVYDLEQGLQDQVFDLLMPTA
                VEGSLSLDELLSDHFVRDLHARMFGPV"
```

```
CDS             complement(1178582..1178857)
                /codon_start=1
                /note="gene_1096"
                /transl_table=11
                /translation="MDSHKVCMNNNTQLPTGPIIGVHPAVRDGVERVAYLDGDLLRCN
                TDVEFTSSPPPGPVLYRTKHTRVEIADEMVTEKLIKRQRAFNSRRHQ"
CDS             1179137..1179475
                /codon_start=1
                /note="gene_1097"
                /transl_table=11
                /translation="VATRHQRAGIDDRWHKRVKGPDGNRRTVRSAVCGRVSRWRVRWV
                DGGGEEHSKSFQRKPDAQAPDPCRTVDARQGHGPVRNAHLGARLLRLTAVYGSVRLLP
                CGASMWGIAC"
CDS             1179469..1179813
                /codon_start=1
                /note="gene_1098"
                /transl_table=11
                /translation="VLTVRSSPTAVTGKGIVESTTKTKRDRHVPVPEPVWRRLHAELP
                TDPNALVFPGRKGGFLPLGEYRWAFDNAGDQVGIEGWYRTVWGTPRPRWRSAQALTSR
                SCNGSLDTQQRR"
tRNA            1179967..1180040
                /note="identified by tRNAscan-SE; putative"
CDS             1180199..1180963
                /codon_start=1
                /note="gene_1099"
                /transl_table=11
                /translation="MSVDYPQMAATRGRIEPAPRRVRGYLGHVLVFDTSAARYVWEVP
                YYPQYYIPLADVRMEFLRDENHPQRVQLGPSRLHSLVSAGQTHRSAARVFDVDGDSPV
                AGTVRFNWDPLRWFEEDEPIYGHPRNPYQRADALRSHRHVRVELDGIVLADTRSPVLL
                FETGIPTRYYIDPADIAFEHLEPTSTQTLCPYKGTTSGYWSVRVGDAVHRDLAWTYHY
                PLPAVAPIAGLVAFYNEKVDLTVDGVALPRPHTQFS"
CDS             1181916..1183148
                /codon_start=1
                /note="gene_1100"
                /transl_table=11
                /translation="MSRAGDDAERSDEEERRMSVMNGREVARESRDAQVFEFGTAPGS
                AVVKIPVQGGPIGGIAISRDGSLLVVTNNGTDTVSVVGTDTCRVTQTVTSVNEPFAIA
                MGNAEANRAYVSTVSSAYDAIAVIDVATNTVLGTHPLALSVSDLTLSPDDKYLYVSRN
                GTRGADVAVLDTTTGALIDVVDVSQAPGTTTQCVRMSPDGSVLYVGANGPSGGLLVVI
                TTRAQSDGGRIGSRSRSRQKSSKPRGNQAAAGLRVVATIDIGSSVRDVALSPDGAIAY
                VASCGSDFGAVVDVIDTRTHQITSSRAISEIGGLVTRVSVSGDADRAYLVSEDRVTVL
                CTRTHDVIGTIRTGQPSCVVESPDGKYLYIADYSGTITRTAVASTIVSGTEQLALQRR
                GSMQWFSPELQQYAPALA"
CDS             1183255..1184886
                /codon_start=1
                /note="gene_1101"
                /transl_table=11
                /translation="MYGTMQDFPLTITAIMRHGCGVHGRRTVTTATGEGYRHSSYRDV
                GQRAGQLANALRRLGVTGDQRVATFMWNNTEHLVTYFAVPSMGAVLHTLNIRLFPEQI
                AYVTNEAEDRVILVDLSLARLLAPVLPKLDTVHTVIAVGEGDTTPLREAGKTVLRFAE
                LIDAESPDFGWPQIDENSAAAMCYTSGTTGNPKGVVYSHRSSFLHTMAACTTNGIGVG
```

SSDKVLPIVPMFHANGWGLPYAALMAGADLVLPDRHLDARSLIHMVETLKPTLAGAVP
TIWNDVMHYLEKDPDHDMSSLRLVACGGSAVPESLMRTFEDKHDVQIRQLWGMTETSP
LATMAWPPPGTPDDQHWAFRITQGQPVCGVETRIVDDDGQVLPNDGNAVGEVEVRGPW
IAGSYYGGRDESKFDSGWLRTGDVGRIDEQGFITLTDRAKDVIKSGGEWISSVELENC
LIAHPDVLEAAVVGVPDERWQERPLAVVVVREGATVSAGDLRAFLADKVVRWWLPERW
AFVDEIPRTSVGKYDKKAIRSRYAEGAYQITEVHT"

CDS 1184968..1186026
/codon_start=1
/note="gene_1102"
/transl_table=11
/translation="MSLRVIQWATGSVGVAAIKGVLQHPELELVGCWVHSAAKSGKDV
GEIIGSPPLGVIATNSIDDVLALDADAVIYAPLLPSVDEVAALLRSGKNVVTPLGWFY
PSEKEAAPLEVAAQAGNATLHGAGIGPGAVTELFPLLLSVMSTGVTFVRSEEFSDLRS
YGAPDVLRYVMGFGGTPDSALTGPMQKILDGGFLQSVRLCVDRLGFAADPQIRTSQEV
AVATAPIDSPIGVIEPGQVAGRRFHWEALVEDTVVVQIAVNWLMGSENLDPPWSFGPA
GERYEIEVRGSPDTCVTIKGWQPQTVAAGLKSNPGIVATAAHCVNAIPATCAAPAGIQ
SFFDLPLITGRAAPGLAR"

CDS 1186079..1186552
/codon_start=1
/note="gene_1103"
/transl_table=11
/translation="MAKSVVVEQSRAIPVQSEDAFGGTLAAALPVICSHWYGLIPPIK
EVRDQTGAWDSVGQARVITMVGGGRVREELTSVDPPRSFGYTLTDIKGPLAPLVALVE
GKWSFAPADTGTTVTWQWTIHPRSALAAPVLPVFARMWRGYARGVLEKLSALLVG"

CDS 1186586..1187449
/codon_start=1
/note="gene_1104"
/transl_table=11
/translation="MCRLFGLHSGTDAVTATFWLLNASDSLAEQSRRNPDGTGLGVFD
EHHQPRLHKQPIAAWQDADFATEAHELTGTTFVAHVRYATTGSLDIRNTHPFLQDGRI
FAHNGVVEGLDVLDERLREVGADDLVLGQTDSERVFALITASIRARDGNESAGLIDAL
RWLAANVPIYAVNVLLSTATDVWALRYPESHELYILDRRGDGAPEFHLRSKRIRAHST
HLRERSSVVFATEPMDDNPRWRLLDAGELVHVDAALRVNRSLVLPDPPRHPIRREDLS
EPVLHAQHTSA"

CDS 1187523..1188311
/codon_start=1
/note="gene_1105"
/transl_table=11
/translation="LRGIADESPAAARLLLDSDVLVGTSAGATVAAQISSGCPLDTLY
ERQLAETSAEIDPGVDIDAITDLFLTAVTEPHISTRRRLQRIGAVALAVDTVPESVRR
QVIAQRLPSHDWPDRVLRVTAIDIATGELVVFHRESNVALVDAVAASCSVPGAWPPVT
IAGRRYMDGGVASSVNLGVADDCDAAVVLVPAGADAPSPFGGGAAAEIAAATGMVFAV
FADDDSLAAFGPNPLDPLCRVNSAMAGRQQGRREAQAVARLLGV"

CDS complement(1188312..1189394)
/codon_start=1
/note="gene_1106"
/transl_table=11
/translation="MPAPAALRVRGSSSPRVALALGSGGARGYAHIGVIQALRERGYD
IVGIAGSSMGAVVGGVHAAGRLDEFAHWAKSLTQRTILRLLDPSISAAGILRAEKILD
AVRDIVGPVAIEQLPIPYTAVATDLLAGKSVWFQRGPLDAAIRASIAIPGVIAPHEVD
GRLLADGGILDPLPMAPIAGVNADLTIAVSLNGSEAGPARDAEPNVTAEWLNRMVRST

SALFDVSAARSLLDRPTARAVLSRFGAAAAESDSWSQAPEIEQRPAGPPADREEAADT
PGLPKMGSFEVMNRTIDIAQSALARHTLAGYPADLLIEVPRSTCRSLEFHRAVEVIAV
GRALATQALEAFEIDDDESAAATIEG"

CDS complement(1189475..1189894)
/codon_start=1
/note="gene_1107"
/transl_table=11
/translation="VRPSRYAPLLCAMVLALAWLSAVAGCSRGGSSKAGRSSSVAGTL
PAGVVGVSPAGVTTRVDAPAESTEEEYYQACHAARLWMDAQPGSGESLIEPYLAVVQA
SPSGVAGSWHIRWAALTPARQAAVIVAARAAANAECG"

CDS 1190006..1190572
/codon_start=1
/note="gene_1108"
/transl_table=11
/translation="VVMPLVTPTTAVPSPGPTRLRVADLLRATDQAADDVLGGRCDHL
LPDGGVPQTQRWYTRIHGDEELDIWLISWVPGQPTELHDHGGSLGALTVLSGSLNEYR
WDGRRLRRRRLDAGDQAGFPLGWVHDVVWAPRPIGGPDAAGMAVAPTLSVHAYSPPLT
AMSYYEITERNTLRRQRTELTDQPEGSG"

CDS 1190569..1190964
/codon_start=1
/note="gene_1109"
/transl_table=11
/translation="MSRIDRVLEAARRRYRRLAADQVPEAARRGAVLVDIRPQAQRAR
EGEVPGALVIERNVLEWRCDPTSDARLPQAVDDDVEWVILCSEGYTSSLAAASLLDLG
LHRATDVVGGYRALAAGGVLAELGGAVGG"

CDS complement(1190992..1193007)
/codon_start=1
/note="gene_1110"
/transl_table=11
/translation="VSFVLVSPSQLMAAAADVAGIGSAISAANAAALAPTSVLAAAGA
DEVSAAVAALFSAHAGQYQQLGARAALFHEQFVQALTGAASAYASAEATNVEQQVLGL
INAPTQALWGRPLIGNGADGTAANPNGGAGGLLYGNGGNGFSQTTAGLTGGTGGSAGL
IGNGGNGGAGGAGANGGAGGNGGWLYGSGGNGGAGGAGPAGAIGAPGVAGGAGGAGGT
AGLFGNGGVGGVGGDGGQGGNGAGAGASGTKGGDAGAGGAGGAGGWIHGHGGAGGDGG
AGGAGGQASPGAPGPPSQPGGAGGAGGAGGRGGDGGSAGWLSGNGGDAGNGGGGGTAG
GAGNGGQFGGDGGTGGTGGTAGAGGNGGRGAVLFGHGGNAGHGGAGGNGAAAGAGGEH
VMATAGKGGTGGVGGDGGGGAGGGGGLLYGNGGAGGNGGAGGAGNSGGDGGTGLNAAL
GGNGGGGGVGGNAGAGGTGGSAGWLSGNGGAGGSGGSAGAGGAGGKGGDTPNGLAINP
GIGGNGGDTGNAGNGGNGGSAARLFGGGGAGGAGGTGSTAGSGGSGGTNPPTGLQAAG
GNGGSGHAGGHGGNGGGAGLLGGGTGGNGGGGGQGGLGAAAGGVDGNGGNGGNGGKGG
DAQLVGDGGNGGNGGKGGAGLIAGLDGAGGAGGTRGLIFGNAGTPGQ"

CDS complement(1193340..1194278)
/codon_start=1
/gene="HIRU_S771"
/note="gene_1111"
/transl_table=11
/translation="VNYGNGGNAGNGGNGGRGSVLFGHGGNAGHGGAGGNGAVSGNGG
SSITAVGGKGGTGGGGGGGAGGTGGDAGLLYGNGGAGGTGGSGGAGARGGDGGAGSGT
AQGGDGGAGGVGGNGGNGGNGGSAGWLSGNGGTGGGGGTAGAGGQGGNGNSGIDPGNG
GQGADTGNAGNGGHGGSAAKLFGDGGAGGAGGMGSTGGTGGGGGFGGGTGGNGGNGHA
GGAGGSGGTAGLLGSGGTGGDGGNGGLGAGSGAKGNGGNGGDGGKGGDAQLIGNGGNG

GNGGKGGTGLMPGINGTGGAGGSRGQISGNPGTPGQ"
variation 1193936..1194259
/note="supplemented from BCG moreau"
CDS complement(1194301..1195641)
/codon_start=1
/note="gene_1112"
/transl_table=11
/translation="MREMSYMIAVPDMLSSAAGDLASIGSSINASTRAAAAATTRLLP
AAADEVSAHIAALFSGHGEGYQAIARQMAAFHDQFTLALTSSAGAYASAEATNVEQQV
LGLINAPTQALWGRPLIGNGADGTAANPNGGAGGLLYGNGGNGFSQTTAGLTGGTGGS
AGLIGNGGNGGAGGAGANGGAGGNGGWLYGSGGNGGAGGAGPAGAIGAPGVAGGAGGA
GGSAGLFGNGGAGGAGGAGGQGGAGIGGADGTKGGDAGAGGAGGAGGWIHGHGGVGGD
GGTGGQGGDGVQGEPGDTGAAGGAGGAGGRGGDGGSAGWLSGNGGDAGTGGGGGNAGA
GGEGGIFGGNGGNGGTGWISGGGGDGGHGGGLFTHGGDGNHGRFGGNGGTGGNGGNTR
LGIYGKGGTVVFGRGVNAGHSGADGNLLYGSGGGEDDMGNGGNAGTRWYRCPRA"
variation 1194709..1194807
/note="supplemented from AL2122/97"
CDS complement(1195994..1197757)
/codon_start=1
/note="gene_1113"
/transl_table=11
/translation="MTEPAAATTTNASDEPATGAEQAVDTAATPQTPEPQPIRSTWWI
RHYTFTGTAMGLVFVWFSMTPSLLPRGPLFQGLVSGICGAFGYGLGVFAVWLVRYMRS
HNSSPPPPRWAWPPLIAVGAVGMVGMAVQFHVWQDDVRDLMGVEHLRWYDYPLAAALS
LVVLFTLVEIGQFIRWLFRFLVGQVDRIAPFRVSAAIVVVLLVVLTITLLNGVVLKFA
MNSMNSTFAAVNNEMNPDSAPPKTPLRSGGPGSLVSWESLGHQGRIFVHSGPTIADLT
AFNGTPAVEPIRTYAGLNSADGIMATAELAARELARTGGLRRAVVAVATSTGTGWINE
AEASALEYMYNGDTAIVSMQYSFLPSWLSFLVDKENARHAGEALFEAVDKLIRQLPES
QRPKLVVFGESLGSFGGEAPFMNLNNILARTDGALFSGPTFNNTVWNSLTANRDAGSP
QWLPIYDDGRNVRFVARARDLQRPDAPWGRLRVVYLQHASDPIAWWTPRLLFREPDWL
REQRGYDVLPQTRWIPVVTFVQVSADMAVATHVPDGHGHRYVATVADGWAAVLSPPGW
TQQKTERLQPLLHANAKPFGS"
CDS complement(1197754..1198527)
/codon_start=1
/note="gene_1114"
/transl_table=11
/translation="MTYETILVERDQRVGIITLNRPQALNALNSQVMNEVTSAATELD
DDPDIGAIIITGSAKAFAAGADIKEMADLTFADAFTADFFATWGKLAAVRTPTIAAVA
GYALGGGCELAMMCDVLIAADTAKFGQPEIKLGVLPGMGGSQRLTRAIGKAKAMDLIL
TGRTMDAAEAERSGLVSRVVPADDLLTEARATATTISQMSASAARMAKEAVNRAFESS
LSEGLLYERRLFHSAFATEDQSEGMAAFIEKRAPQFTHR"
CDS complement(1198539..1199576)
/codon_start=1
/note="gene_1115"
/transl_table=11
/translation="VTGESHEVLTNVEGGVGFVTLNRPKAINSLNQTMVDLLATVLMS
WEHEDAVHAVVLSGAGERGLCAGGDVVAVYHSARKDGVEARRFWRHEYLLNALIGRFA
KPYVALMDGIVMGGGVGVSAHANTRVVTDTSKVAMPEVGIGFIPDVGGVYLLSRAPGA
LGLHAALTGAPFSGADAIALGFADHFVPHGDLDAFTQKIVTGGVESALAAHAVEPPPS
TLAAQRDWIDECYAGDSVADIVAALRKQGGEPAVNASDLIASRSPIALSVTLQAVRRA
AKLDTLEDVLIQDYRVSSASLRSHDLVEGIRAQLIDKDRNPNWSPATLDAITAADIEA

```
                     YFEPVDDDLSF"
     CDS             1199763..1200599
                     /codon_start=1
                     /note="gene_1116"
                     /transl_table=11
                     /translation="VRETSNPVFRSLPKQRGGYAQFGTGTAQQGFPADPYLAPYREAK
                     ATRPLTIDDVVTKTGLTLAMLAGTAVVSYFLVVSNVALAMPLTLVGALGGLALVLVAT
                     FGRKQDNPAIVLSYAALEGLFLGAISFVLANFTVASANAGVLIGEAILGTMGVFFGML
                     VVYKTGAIRVTPKFTRMVVAALFGVLVLMLGNLVLAMFNVGGGEGLGLRSPGPLGIIF
                     SLVCIGIAAFSFLIDFDAADQMIRAGAPEKAAWGVALGLTVTLVWLYIEILRLLSYLQ
                     NE"
     CDS             1200706..1201566
                     /codon_start=1
                     /note="gene_1117"
                     /transl_table=11
                     /translation="MREMGAQPFIGSEALAAGLISWHELGKYYTAIMPNVYLDKRLKP
                     SLRQRVIAAWLWSGRKGVIAGASASALHGAKWVDDHALVELIWRNARAPNGVRTKDEL
                     LLDGEVQRLCGLTVTTVERTAFDLGRRPPLGQAITRLDALANATDFKINDVRELARKH
                     PHTRGLRQLDKALDLVDPGAQSPKETWLRLLLINAGFPRPSTQIPLLGVYGHPKYFLD
                     MGWEDIMLAVEYDGEQHRLSRDQFVKDVERLEYIRRAGWTHIRVLADHKGPDVVRRVR
                     QAWDTLTSRR"
     CDS             complement(1201640..1202857)
                     /codon_start=1
                     /note="gene_1118"
                     /transl_table=11
                     /translation="MPEAVIVSTARSPIGRAMKGSLVGMRPDDLAVQMVRAALDKVPA
                     LNPHQIDDLMMGCGLPGGESGFNIARVVAVALGYDFLPGTTVNRYCSSSLQTTRMAFH
                     AIKAGEGDAFISAGVETVSRFAKGNSDSWPDTKNPLFDGAQERSAAAAAGADEWHDPR
                     TDQKLPDIYIAMGQTAENVAIMTGISREEQDRWGVRSQNRAEEAIKNGFFEREITPVT
                     LPDGTTVSTDDGPRPGTTYEKVSELKPAFRPNGTVTAGNACPLNDGAAAVVITSDTKA
                     KELGLTPLARIVSTGVSGLSPEIMGLGPIEASKKALERAGMAITDIDLVEINEAFAVQ
                     VLGSARELGIDEDKLNISGGAIALGHPFGMTGARITTTLLNNLQTYDKTFGLETMCVG
                     GGQGMAMVIERLA"
     CDS             complement(1202910..1203860)
                     /codon_start=1
                     /note="gene_1119"
                     /transl_table=11
                     /translation="MRVPRRSTIALATAGALASTGTAYLGARNLLVGQATHARTVIPK
                     SFDAPPRADGVYTRGGGPVQRWRREVPFDVHLMIFGDSTATGYGCASAEEVPGVLIAR
                     GLAEQTGKRIRLSTKAIVGATSKGVCGQVDAMFVVGPPPDAAVIMIGANDITALNGIG
                     PSAQRLADCVRRLRTRGAVVVVGTCPDLGVITAIPQPLRALAHTRGVRLARAQTAAVK
                     AAGGVPVPLGHLLAPKFRAMPELMFSADRYHPSAPAYALAADLLFLALRDALTEKLDI
                     PIHETPSRPGTATLEPGHTRHSMMSRLRRPRPARAVPTGG"
     CDS             1204077..1205144
                     /codon_start=1
                     /note="gene_1120"
                     /transl_table=11
                     /translation="MTAPSKVSGSPRVVISPRDVLKARRLEARKFAISDGAPVEVVES
                     GPSLVARLAALASRVAVRPVLAVGSYLPHAPWPWGVIDQAARVLLPASTTVRAAVSLP
                     NASAQLVRASGVLPADGTRRAVLYLHGGAFLTCGANSHGRLVELLSKFADSPVLVVDY
                     RLIPKHSIGMALDDCHDGYRWLRLLGYEPEQIVLAGDSAGGYLALALAQRLQEVGEEP
```

AALVAISPLLQLAKEHKQAHPNIKTDAMFPARAFDALDALVASAAARNQVDGEPEELY
EPLEHITPGLPRTLIHVSGSEVLLHDAQLAAAKLAAAGVPAEVRVWPGQVHDFQVAAS
MLPEAIRSLRQIGEYIREATG"

CDS 1205201..1206595
/codon_start=1
/note="gene_1121"
/transl_table=11
/translation="MRIAQHISELIGGTPLVRLNSVVPDGAGTVAAKVEYLNPGGSSK
DRIAVKMIEAAEASGQLKPGGTIVEPTSGNTGVGLALVAQRRGYKCVFVCPDKVSEDK
RNVLIAYGAEVVVCPTAVPPHDPASYYSVSDRLVRDIDGAWKPDQYANPEGPASHYVT
TGPEIWADTEGKVTHFVAGIGTGGTITGAGRYLKEVSGGRVRIVGADPEGSVYSGGAG
RPYLVEGVGEDFWPAAYDPSVPDEIIAVSDSDSFDMTRRLAREEAMLVGGSCGMAVVA
ALKVAEEAGPDALIVVLLPDGGRGYMSKIFNDAWMSSYGFLRSRLDGSTEQSTVGDVL
RRKSGALPALVHTHPSETVRDAIGILREYGVSQMPVVGAEPPVMAGEVAGSVSERELL
SAVFEGRAKLADAVSAHMSPPLRMIGAGELVSAAGKALRDWDALMVVEEGKPVGVITR
YDLLGFLSEGAGRR"

CDS 1207043..1207519
/codon_start=1
/note="gene_1122"
/transl_table=11
/translation="MPTESYTPWITRVLAAFIDWAPYVVLVGIGWVIMLVTQTSSCVT
SISEYDVGQFCVSQPSMIGQLVQWLLSVGGLAYLVWNYGYRQGTTGSSIGKSVLKFKV
VSETTGQPIGFGMSVVRQLAHFIDAIICFVGFLFPLWDAKRQTLADKIMTTVCVPI"

CDS 1207551..1208717
/codon_start=1
/note="gene_1123"
/transl_table=11
/translation="MSEDRTGHQGISGPATRAIHAGYRPDPATGAVNVPIYASSTFAQ
DGVGGLRGGFEYARTGNPTRAALEASLAAVEEGAFARAFSSGMAATDCALRAMLRPGD
HVVIPDDAYGGTFRLIDKVFTRWDVQYTPVRLADLDAVGAAITPRTRLIWVETPTNPL
LSIADITAIAELGTDRSAKVLVDNTFASPALQQPLRLGADVVLHSTTKYIGGHSDVVG
GALVTNDEELDEEFAFLQNGAGAVPGPFDAYLTMRGLKTLVLRMQRHSENACAVAEFL
ADHPSVSSVLYPGLPSHPGHEIAARQMRGFGGMVSVRMRAGRRAAQDLCAKTRVFILA
ESLGGVESLIEHPSAMTHASTAGSQLEVPDDLVRLSVGIEDIADLLGDLEQALG"

CDS complement(1208788..1209282)
/codon_start=1
/gene="greA"
/note="gene_1124"
/transl_table=11
/translation="MTDTQVTWLTQESHDRLKAELDQLIANRPVIAAEINDRREEGDL
RENGGYHAAREEQGQQEARIRQLQDLLSNAKVGEAPKQSGVALPGSVVKVYYNGDKSD
SETFLIATRQEGVSDGKLEVYSPNSPLGGALIDAKVGETRSYTVPNGSTVSVTLVSAE
PYHS"

CDS complement(1209468..1209929)
/codon_start=1
/note="gene_1125"
/transl_table=11
/translation="LHPAGVNPGMTHTPIPRPDARYGRPRLSRRARRRVAIALGVLVA
AAGIVIAVIGYQRISTSAVTGSLVGYRLVDDETASVTISVTRSDPSRPVACIVRVRAT
NGSETGRRELLVPPSEATTVQVTTTVKSSQPPVMADVYGCGTEVPSYLRLP"

CDS 1210004..1210870

```
                     /codon_start=1
                     /note="gene_1126"
                     /transl_table=11
                     /translation="VSELRLMAVHAHPDDESSKGAATLARYADEGHRVLVVTLTGGER
                     GEILNPAMDLPDVHGRIAEIRRDEMTKAAEILGVEHTWLGFVDSGLPKGDLPPPLPDD
                     CFARVPLEVSTEALVRVVREFRPHVMTTYDENGGYPHPDHIRCHQVSVAAYEAAGDFC
                     RFPDAGEPWTVSKLYYVHGFLRERMQMLQDEFARHGQRGPFEQWLAYWDPDHDFLTSR
                     VTTRVECSKYFSQRDDALRAHATQIDPNAEFFAAPLAWQERLWPTEEFELARSRIPAR
                     PPETELFAGIEP"
CDS                  1210867..1211133
                     /codon_start=1
                     /note="gene_1127"
                     /transl_table=11
                     /translation="VNQILLSVIAEGGPGNTGPDFGKASPVGLLVIVLLVIATLFLVR
                     SMNQQLKKVPKSFDRDHPELDQAADEGTDRDGPARPPGPPHESG"
CDS                  1211120..1213141
                     /codon_start=1
                     /note="gene_1128"
                     /transl_table=11
                     /translation="MSPANPSGTNTLALATSPYLRQHADNPVHWQQWTPQALAEAAAR
                     AVPILLSVGYAACHWCHVMAHESFDDDEVAAAMNAGFVCIKVDREERPDIDAVYMNAT
                     VALTGQGGWPMTCFLTPNGRPFFCGTYYPKAAFLQLLSAISETWRERRAEVEQASDHI
                     AAELRSMASGLPGGGPEVAPELCDDAVAGVLREQDTAHGGFGGAPKFPPSALLEALMR
                     HYERTRSPAALEAVARTGNAMARGGIYDQLGGGFARYSVDGAWVVPHFEKMLYDNALL
                     LRAYAHWARRTGDPLARRVAAQTARFLLDELGSKAPADMFTSSLDADADGREGSTYVW
                     TPVQLTEVLGGDDGRWAAEVFGVTEAGTFEHGTSVLQLPADPDDAARLDRVRAALLVA
                     RLARAQPARDDKVVTSWNGLAITALAEASVALDDPALAHAARRCATRLLDLHVVDGRL
                     RRASLGGVVGDSAAILEDHAMLATGLLALYQLTSEGAWLTAATGLLDTAVAHFGDPQR
                     PGRWFDTADDAERLMLRPSDPLDGATPSGASSIAEALLTAGHVVDGARAERYWQLAAD
                     TLRAHAVLLARAPRSAGHWLAVAEAVVRGPLQIAVACDLPRSSLLADARRLAPGGAIV
                     VGGAAGSSALLVGRDRVAGADAAYVCRGRVCDLPVTSAAELATALGVPG"
CDS                  complement(1213240..1213968)
                     /codon_start=1
                     /note="gene_1129"
                     /transl_table=11
                     /translation="MSGQADTATTAEARTPAHAAHHLVEGVARVLTKPRFRGWIHVYS
                     AGTAVLAGASLVAVSWAVGSAKAGLTTLAYTAATITMFTVSATYHRVNWKSATARNWM
                     KRADHSMIFVFIAGSYTPFALLALPAHDGRVVLSIVWGGAIAGILLKMCWPAAPRSVG
                     VPLYLLLGWVAVWYTATILHNAGVTALVLLFVGGALYSIGGILYAVRWPDPWPTTFGY
                     HEFFHACTAVAAICHYIAMWFVVF"
CDS                  1214079..1214867
                     /codon_start=1
                     /note="gene_1130"
                     /transl_table=11
                     /translation="VEIIPPRLKEPLYRLYELRLRQGLAASKSDLPRHIAVLCDGNRR
                     WARSAGYDDVSYGYRMGAAKIAEMLRWCHEAGIELATVYLLSTENLQRDPDELAALIE
                     IITDVVEEICAPANHWSVRTVGDLGLIGEEPARRLRGAVESTPEVASFHVNVAVGYGG
                     RREIVDAVRALLSKELANGATAEELVDAVTVEGISENLYTSGQPDPDLVIRTSGEQRL
                     SGFLLWQSAYSEMWFTEAHWPAFRHVDFLRALRDYSARHRRYGR"
CDS                  1215044..1217515
                     /codon_start=1
```

/note="gene_1131"
/transl_table=11
/translation="MSFVVVAPEVLAAAASDLAGIGSTLAQANAAALAPTTAVLAAGA
DEVSAAIASLFGAHGQAYQAVSAQMSAFHAQFMQALTGAGGAYAAAEAVNVSAAQSVE
QDLLAAINARFERIFGRPLIGDGANGGPGQDGGPGGLLYGNGGNGGTSTTVGMAGGNG
GAAGLIGNGGFGGGGGPGAAGGNGGAGGWLFGNGGAGGAGGLGVAPGVPGGAGGAGGA
GGVGGPAGLWGHGGAGGAGGAGVAGAGGFEGTIGAGGAGGVGGAGGVGGAGGAGGWLY
GDAGAGGDGGVGGAGGTGGLGNRGGAGGAGGAGGVGGAGGAAGLWGGGGAGGVGGTGG
GAGLGAQSVTFSSSLSGLSGGDGGAGGAGGAGGAGGTGGWLYGGGGAAGSGGDGGTGG
QGGAGGAGVFSLFGSGGGPGGNGGVGGVGGVGGAGGRAGLFGVGGLGGAGGDAGDSGE
GGFGGPGLAGGLFGNPGNGGVGGIGGDAAAGGAGGAGGNGGWLFGNGGAGGSGGDGGA
AGRGGAGNLGSAGGINAPAGNPGSGSVGIGGAGGAGGTAGLFGDGGAGGAGAAGGFGG
ISAATPSAGSEGAMGGAGGVGGNARLLGTGGAGGVGGGGGVGGLGGLGTAINNVDAGS
GAGGGGGGAGGTGGTAGLLYGVGGAGGGGGAGGDGGRGGVATPGGQGGDAGDGGAGGA
GGNGGGGASGAGGWLLGTGGAGGAGGNGGNGGKAGFSPGPTNFGLNGAGGGGGGVGGN
GATGPWLFGDGGAGGGGGAGGIGGDGGPTPGSTGAGAAGGHGGDAQLIGNGGHGGAGG
TGVPNGSGGAGGLSGLLFGEPGANG"

variation 1217074..1217115
/note="supplemented from AL2122/97"

CDS 1217701..1218012
/codon_start=1
/note="gene_1132"
/transl_table=11
/translation="VGYGDRREFVDAVAVEAICENLNTSGQPDPDLVIRTSGEQRLSG
HRGPTGGVSRRRLLRALRDYSTPHASIPYVPPPYRSDGIHASRLAVESVFDALAGRVE
L"

CDS 1218478..1218783
/codon_start=1
/note="gene_1133"
/transl_table=11
/translation="VRGAVNAVAGQVTGNGGSGNSGTSAAAANPNSDNTASIADRGTS
AIMTTASATASSTGVDGGIAATYAVASQWDGGYVANYTINQFGRDFDDRLAVAIHFA"

CDS 1218877..1219059
/codon_start=1
/note="gene_1134"
/transl_table=11
/translation="VDSITVGYQAAQTGGYSPPTNLLINGQAVTIDQTPITSSPTTPP
PTTPPEIPTGGTVIST"

CDS 1219251..1219706
/codon_start=1
/note="gene_1135"
/transl_table=11
/translation="VGTNLPTEVGQILSAPTSIDYNYPTTGVWDASYDICLDSTPKTT
GVNQQEIMIWFNHQGSIQPVGSPVGNTTIEGKNFVVWDGSNGMNNAMAYVATEPIEVW
SFDVMSFVDHTATMEPITDSWYLTSIRAGLEPWSDGVGLGVDSFSAKVN"

CDS 1220121..1222673
/codon_start=1
/note="gene_1136"
/transl_table=11
/translation="MSFVIAAPEALVAVASDLAGIGSALAEANAAALAPTTALLAAGA
DEVSAAIAALFGAHGQAYQTVSAQASAFHAQFVQALTGGGGAYAAAEAANVSAAQSTD

QRLLDLINGPTQALLGRPLIGDGANGGPGQDGGPGGLLYGNGGNGGTSTTAGVAGGNG
GAAGLIGNGGAGGGGGAGAAGGNGGAGGWLYGNGGAGGAGGTSVIPGVAGGNGGAGGS
AGLWGTGGAGGDGGNGRSGPVNVAGSAGGNGGAGGAAGLFGDAGAGGNGGKGGAGGAA
FSINFTAGDGGAGGAGGSGGHALLWGAGGAGGNGGSGGTGGAGGSTAGAGGNGGAGGG
GGTGGLLFGNGGAGGHGAAAGNGLAAGNGVSSSGGGGAGGTGGAGGDGGAGGAGGNAR
LWGVGGAGGAGGDGGAGGAGGKGGSGLSGNANGGAGGDSGRGGTGGAGGEGGAAGLLV
GTGGHGGDGGAGGAAVKGGDGGAAAGTGIAGAGGRGGAGGSGGSGGDGGGGAAGPAGW
LFGDGGAGGNGGAAAAGGAGGQAGGGGGNGGNGGNGGNGGNGGNGATGGWLYGNGGAG
GQGATAGAGGAGANGVSSTNGGGNGGIGGTGGSGGAGGNAGLLGVGGAGGHGASGGAG
DRGGAGGTGFISSDGGAGGDGGDGGNGGAGGTGGLLFGAGGNGGPGGSGGAADIGGNG
GAGNGGGTDGNGGNGGSGGGAGSGGDGGGAGGNGAWLFGNGGAGGGGGKGGNGAGGGL
GGGSFGLPGLNGSGGDGGDGGNGAPGGVLYGNGGAGGQGSSGGIGGPGATGGAGGKGG
DGGDAQLIGDGGNGGNGGAGGTGGTPGPGGPGGSGGLGGLLFGQTGTAGVSP"

CDS complement(1222891..1223763)
/codon_start=1
/note="gene_1137"
/transl_table=11
/translation="MSTPLALTEEELVGLRGLGEQIDLLEVEEVYLPLARLIHLQVAA
RQRLFAATAEFLGEPQQNPDRPVPFIIGVAGSVAVGKSTTARVLQALLARWDHHPRVD
LVTTDGFLYPNAELQRRNLMHRKGFPESYNRRALMRFVTSVKSGSDYACAPVYSHLHY
DIIPGAEQVVRHPDILILEGLNVLQTGPTLMVSDLFDFSLYVDARIEDIEQWYVSRFL
AMRTTAFADPESHFHHYAAFSDSQAVVAAREIWRTINRPNLVENILPTRPRATLVLRK
DADHSINRLRLRKL"

CDS 1224217..1225497
/codon_start=1
/gene="glyA"
/note="gene_1138"
/transl_table=11
/translation="MSAPLAEVDPDIAELLAKELGRQRDTLEMIASENFAPRAVLQAQ
GSVLTNKYAEGLPGRRYYGGCEHVDVVENLARDRAKALFGAEFANVQPHSGAQANAAV
LHALMSPGERLLGLDLANGGHLTHGMRLNFSGKLYENGFYGVDPATHLIDMDAVRATA
LEFRPKVIIAGWSAYPRVLDFAAFRSIADEVGAKLLVDMAHFAGLVAAGLHPSPVPHA
DVVSTTVHKTLGGGRSGLIVGKQQYAKAINSAVFPGQQGGPLMHVIAGKAVALKIAAT
PEFADRQRRTLSGARIIADRLMAPDVAKAGVSVVSGGTDVHLVLVDLRDSPLDGQAAE
DLLHEVGITVNRNAVPNDPRPPMVTSGLRIGTPALATRGFGDTEFTEVADIIATALAT
GSSVDVSALKDRATRLARAFPLYDGLEEWSLVGR"

CDS 1225602..1226429
/codon_start=1
/note="gene_1139"
/transl_table=11
/translation="MAQKPVADALTLELEPVVEANMTRHLDTEDIWFAHDYVPFDQGE
NFAFLGGRDWDPSQSTLPRTITDACEILLILKDNLAGHHRELVEHFILEDWWGRWLGR
WTAEEHLHAIALREYLVVTREVDPVANEDVRVQHVMKGYRAEKYTQVETLVYMAFYER
CGAVFCRNLAAQIEEPILAGLIDRIARDEVRHEEFFANLVTHCLDYTRDETIAAIAAR
AADLDVLGADIEAYRDKLQNVADAGIFGKPQLRQLISDRITAWGLAGEPSLKQFVTG"

CDS 1226640..1227941
/codon_start=1
/note="gene_1140"
/transl_table=11
/translation="VTDTRTYVLDTSVLLSDPWACSRFAEHDVVVPLVVISELEAKRH
HHELGWFARQALRLFDDLRLEHGRLDQPIPVGTQGGTLHVELNHTDPAVLPAGFRTDS

```
                     NDSRILSCAANLAAEGKRVTLVSKDIPLRVKAAAVGLAADEYHAQDVVVSGWSGMHEL
                     ETASADIDALFADGEIDLVEARDLPCHTGIRLLGGGSHALGRVNAHKRVQLVRGDREA
                     FGLRGRSAEQRVALDLLLDESVGIVSLGGKAGTGKSALALCAGLEAVLERRTHRKVVV
                     FRPLYAVGGQELGYLPGSESEKMGPWAQAVFDTLEGLASPAVLEEVLSRGMLEVLPLT
                     HIRGRSLHDSFVIVDEAQSLERNVLLTVLSRLGTGSRVVLTHDIAQRDNLRVGRHDGV
                     AAVIEKLKGHPLFAHITLLRSERSPIAALVTEMLEEITGPR"
     CDS             1228028..1228903
                     /codon_start=1
                     /note="gene_1141"
                     /transl_table=11
                     /translation="VPKRPDNQTWRYWRTVTGVVVAGAVLVVGGLSGRVTRAENLSCS
                     VIKCVALTFDDGPGPYTDRLLHILTDNDAKATFFLIGNKVAANPAGARRIADAGMEIG
                     SHTWEHPNMTTIPPEDIPGQFSRANDVIAAATGRTPTLYRPAGGLSNDAVRQAAAKVG
                     QAEILWDVIPFDWINDSNTAATRHMLMTQIKPGSVVLFHDTYSSTVDVVYQFIPVLKA
                     NGYRLVTVSELLGPRAPGSSYGSRENGPPVNELRDIPASEIPPLPNTSSPKPMSNFPI
                     TDIAGQNSGGPNNGA"
     CDS             complement(1228906..1229787)
                     /codon_start=1
                     /note="gene_1142"
                     /transl_table=11
                     /translation="MTVPPAGPYGNYPYGPNTYGQDLYWGGQPQGGSYPPAYPPQQYP
                     PGWPAGPYPPGPPPPGPGSKTPWLILAGLAVLGVILLVVILVIGLRGDNKSTTATSPA
                     TSAPTSQPFSQQTATGCTPNVSGGVQPIGDSISAGKLSFPTSAAPGWSAFSDDQNPNL
                     IDAVGVGHEVAGADQWMMQAEVAITNFVTTMDVAAQASKLMQCVADGPGYAGSSPTLG
                     PTKTSSITVDGVRAARVDADITIADSSRNVKGDSVTIIAVDTKPVTVFLGATPIGDAT
                     SRATVERVIEALKVNKS"
     CDS             complement(1229784..1231208)
                     /codon_start=1
                     /gene="fumC"
                     /note="gene_1143"
                     /transl_table=11
                     /translation="MAVDADSANYRIEHDTMGEVRVPAKALWRAQTQRAVENFPISGR
                     GLERTQIRALGLLKGACAQVNSDLGLLAPEKADAIIAAAAEIADGQHDDQFPIDVFQT
                     GSGTSSNMNTNEVIASIAAKGGVTLHPNDDVNMSQSSNDTFPTATHIAATEAAVAHLI
                     PALQQLHDALAAKALDWHTVVKSGRTHLMDAVPVTLGQEFSGYARQIEAGIERVRACL
                     PRLGELAIGGTAVGTGLNAPDDFGVRVVAVLVAQTGLSELRTAANSFEAQAARDGLVE
                     ASGALRTIAVSLTKIANDIRWMGSGPLTGLAEIQLPDLQPGSSIMPGKVNPVLPEAVT
                     QVAAQVIGNDAAIAWGGANGAFELNVYIPMMARNILESFKLLTNVSRLFAQRCIAGLT
                     ANVEHLRRLAESSPSIVTPLNSAIGYEEAAAVAKQALKERKTIRQTVIDRGLIGDRLS
                     IEDLDRRLDVLAMAKAEQLDSDRL"
     CDS             complement(1231239..1232327)
                     /codon_start=1
                     /gene="glpX"
                     /note="gene_1144"
                     /transl_table=11
                     /translation="MTAEGSGSSTAAVASHDPSHTRPSRREAPDRNLAMELVRVTEAG
                     AMAAGRWVGRGDKEGGDGAAVDAMRELVNSVSMRGVVVIGEGEKDHAPMLYNGEEVGN
                     GDGPECDFAVDPIDGTTLMSKGMTNAISVLAVADRGTMFDPSAVFYMNKIAVGPDAAH
                     VLDITAPISENIRAVAKVKDLSVRDMTVCILDRPRHAQLIHDVRATGARIRLITDGDV
                     AGAISACRPHSGTDLLAGIGGTPEGIIAAAAIRCMGGAIQAQLAPRDDAERRKALEAG
                     YDLNQVLTTEDLVSGENVFFCATGVTDGDLLKGVRYYPGGCTTHSIVMRSKSGTVRMI
```

EAYHRLSKLNEYSAIDFTGDSSAVYPLP"
CDS 1232383..1233027
/codon_start=1
/note="gene_1145"
/transl_table=11
/translation="VPAEPQPTPRPAKPRLLQDGRDMFWSLAPLVVGCILLAGLVGMC
SFQLGGTKRGPIPSYDAAQALRADAKTLGFPIRLPQLPGGWTPNSGGRGGIENGRADP
ATGQRRNAATSIVGFISPTGRYLSLTQSNADEDKLVGSIHPSMYPTGTVDVGGTRWVV
YEGSDENGAVEPVWTTRLTGPGGATQLAITGAGSIDQFRTLASATQSQPPLPAR"
CDS complement(1233034..1234062)
/codon_start=1
/note="gene_1146"
/transl_table=11
/translation="LFTPLFKWFTKRFNTGLSAACTLLSALAAVVVPVGALVGLAIVQ
IARMVDSVADWVRTTDLSTLGDKILQFVNGLFDRVPFLHITVTADALRKAMISVAQNV
GEWLLHFLRDAAGSLAGVITSAIIFVYVFVALLVNREKLRTLIGQLNPLGEDVTDLYL
QKMGSMVRGTVNGQFVIAACQGVAGAASIYIAGFHHGFFIFAIVLTALSIIPLGGGIV
TIPFGIGMIFYGNIAGGIFVLLWHLLVVTNIDNVLRPILVPRDARLNSALMLLSVFAG
ITMFGPWGIIIGPVLMILIVTTIDVYLAVYKGVELEQFEAPPVRRRWLPRRGPATSRN
APPPSTAE"
CDS complement(1234302..1234613)
/codon_start=1
/note="gene_1147"
/transl_table=11
/translation="MRPIHIAQLDKARPVLILTREVVRPHLTNVTVAPITTTVRGLAT
EVPVDAVNGLNQPSVVSCDNIQTIPVCDLGRQIGYLLASQEPALAEAIGNAFDLDWVV
A"
CDS complement(1234613..1234933)
/codon_start=1
/note="gene_1148"
/transl_table=11
/translation="MYLPWGVVLAGGANGFGAGAYQTGTICEVSTQIAVRLPDEIVAF
IDDEVRGQHARSRAAVVLRALERERRRRLAERDAEILATNTSATGDLDTLAGHCARTA
LDID"
CDS 1234943..1235632
/codon_start=1
/note="gene_1149"
/transl_table=11
/translation="MVVDSCVAESRYGPVRGADDGRVKVWKGIRYAAPPLGDLRFRTP
EPPERWTEVADATTFGPACPQPAIPNMPLDLGASQSEDCWSLNIWAPADTEPGDGKPV
MVWLHGGAYILGSGSQPLYNGRRLAASGDVVVVTVNYRLGALGFLDLSSFNTSRRRFD
SNIGLRDVLAVLRWVADNIAVFGGDPEKVTLFGESARESSRPCSPPRRPRVCSRRRSP
RAHRRHRSTTR"
CDS 1235706..1236104
/codon_start=1
/note="gene_1150"
/transl_table=11
/translation="VPTAAILSASSEVFNEVPVRNPGTLAFVPIVDGDLLPDYPVKLA
QEGRSHPVPLIIGTNKHESALFRLMRSPLMPITPRDHVDVHPDCRRTARSASANRGAD
RLRVLAMAAQSTLIEYGYRRRLPDAVGVAR"
CDS 1236058..1236468

/codon_start=1
/note="gene_1151"
/transl_table=11
/translation="MATDVGFRMPSVWLAEGHSGVAPVYLYRFDYSTPLLKLLLVRAA
HATELPYVWGNLGGSQDPALKLGDAKAAIAVSRRVRTRWINFATRGKPTGPDGEPDWP
CYEEAHRACLIIGRRDAVVHDVDAHIRATWGSKW"
CDS complement(1236486..1237598)
/codon_start=1
/note="gene_1152"
/transl_table=11
/translation="MLRRMGDASLTTELGRVLVTGGAGFVGANLVTTLLDRGHWVRSF
DRAPSLLPAHPQLEVLQGDITDADVCAAAVDGIDTIFHTAAIIELMGGASVTDEYRQR
SFAVNVGGTENLLHAGQRAGVQRFVYTSSNSVVMGGQNIAGGDETLPYTDRFNDLYTE
TKVVAERFVLAQNGVDGMLTCAIRPSGIWGNGDQTMFRKLFESVLKGHVKVLVGRKSA
RLDNSYVHNLIHGFILAAAHLVPDGTAPGQAYFINDAEPINMFEFARPVLEACGQRWP
KMRISGPAVRWVMTGWQRLHFRFGFPAPLLEPLAVERLYLDNYFSIAKARRDLGYEPL
FTTQQALTECLPYYVSLFEQMKNEARAEKTAATVKP"
CDS complement(1237608..1237865)
/codon_start=1
/note="gene_1153"
/transl_table=11
/translation="MVCDPNGDDTGRTHATVPVSQLGYEACRDELMEVVRLLEQGGLD
LDASLRLWERGEQLAKRCEEHLAGARQRVSDVLAGDEAQNG"
CDS complement(1237855..1239102)
/codon_start=1
/gene="xseA"
/note="gene_1154"
/transl_table=11
/translation="VTQNSAENPFPVRAVAIRVAGWIDKLGAVWVEGQLAQITMRPDA
KTVFMVLRDPAADMSLTVTCSRDLVLSAPVKLAEGVQVVVCGKPSFYTGRGTFSLRLS
EIRAVGIGELLARIDRLRRLLDAEGLFDPRLKRPIPYLPNMIGLITGRASAAERDVTT
VASARWPAARFAVRNVAVQGPNAVGQIVEALRELDRDPDVDVIVLARGGGSVEDLLPF
SDETLCRAIAACRTPVVSAVGHEPDNPLCDLVVDLRAATPTDAAKKVVPDTAAEQRLI
DDLRRRSAQALRNWVSREQRAVAQLRSRPVLADPMTMVSVRAEEVHRARSTLRRNLTL
MVAAETERIGHLAARLATLGPAATLARGYAIVQTVAQTGPEGGSEPQVLRSVHDAPEG
TKLRVRVADGALAAVSEGQTNGL"
CDS complement(1239099..1239671)
/codon_start=1
/note="gene_1155"
/transl_table=11
/translation="MKLPRTILMYPMTLASQAAHVVMRFQQGLAELVIKGDNTLETLF
PPKDEKPEWATFDEDLPDALEGTSIPLLGLSDASEAKNDDRRSDGRFALYSVSDTPET
TTASRSADRSTNPKTAKHPKSATKPTVPTPAVAAELDYPALTLAQLRARLHTLDVPEL
EALLAYEQATKARAPFQTLLANRITRATAK"
CDS 1239800..1240834
/codon_start=1
/gene="ispH"
/note="gene_1156"
/transl_table=11
/translation="VSAQSTLNAMVPTVDMGIPGASVSSRSVADRPNRKRVLLAEPRG
YCAGVDRAVETVERALQKHGPPVYVRHEIVHNRHVVDTLAKAGAVFVEETEQVPEGAI

VVFSAHGVAPTVHVSASERNLQVIDATCPLVTKVHNEARRFARDDYDILLIGHEGHEE
VVGTAGEAPDHVQLVDGVDAVDQVTVRDEDKVVWLSQTTLSVDETMEIVGRLRRRFPK
LQDPPSDDICYATQNRQVAVKAMAPECELVIVVGSRNSSNSVRLVEVALGAGARAAHL
VDWADDIDSAWLDGVTTVGVTSGASVPEVLVRGVLERLAECGYDIVQPVTTANETLVF
ALPRELRSPR"
CDS complement(1240851..1241765)
/codon_start=1
/note="gene_1157"
/transl_table=11
/translation="VPWWAAILIAVTATAIGYAIDAGSGHKALTLVFTGCYIAGCVGA
VLAVRQSDLFTALVQPPLILFCAVPGAYWLFHGGTIGKFKDLLINCGYSLIERFPLML
GTAAGVLLIGLVRWYLGTALFDSIARKLSSLMTGDSDDDGGRRSAQRPARTRSRHARP
PSEDNREPIAERRSRRRPRPQNDPHPRRNAHERPAPRSSRFDSYRSYQPSEPSGPAEP
VNRYERRGARYQPYARYEPTYEPQRRRARPSEPTNPTHHPISQVRYRGSATRDARRDN
YREEQRFDRRDRSRAPRRPPAESWEYDV"
CDS 1241897..1242970
/codon_start=1
/note="gene_1158"
/transl_table=11
/translation="VSLSLGIVGLPNVGKSTLFNALTRNNVVAANYPFATIEPNEGVV
SLPDPRLDKLAELFGSQRVVPAPVTFVDIAGLIKGASEGAGLGNKFLAHIRECDAICQ
VVRVFVDDDVTHVTGRVDPQSDIEVVETELILADLQTLERATGRLEKEARTNKARKPV
YDAALRAQQVLDAGKTLFAAGVDAAALRELNLLTTKPFLYVFNADEAVLTDPARVGEL
RALVAPADAVFLDAAIESELTELDDESAAELLESIGQSERGLDALARAGFHTLKLQTF
LTAGPKEARAWTIHQGDTAPKAAGVIHSDFEKGFIKAEIVSYDDLVAAGSMAAAKAAG
KVRIEGKDYVMADGDVVEFRFNV"
CDS 1243070..1243255
/codon_start=1
/note="gene_1159"
/transl_table=11
/translation="VTVDDALLAKAAELTGVKEKSTLLREGLQTLVRVESARRLAALG
GTDPQATAAPRRRTSPR"
CDS 1243252..1243626
/codon_start=1
/note="gene_1160"
/transl_table=11
/translation="VILVDTSVWIEHLRAADARLVELLGDDEAGCHPLVIEELALGSI
KQRDVVLDLLANLYQFPVVTHDEVLRLVGRRRLWGRGLGAVDANLLGSVALVGGARLW
TRDKRLKAACAESGVALAEEVS"
CDS 1243886..1244527
/codon_start=1
/note="gene_1161"
/transl_table=11
/translation="MIALATLLTLINQVVGTPYIPGGDSPAGTDCSELASWVSNAATA
RPVFGDRFNTGNEEAALAARGFQQGTAPNALVIGWNGHHTAVTLPDGTPVSSGEGGGV
RVGGGGAYQPKFTHHMYLPMDVDAGEDQPPAPDEPVTAVDDVEPEMPAPCPTQRPPVT
PRHNLCNKLRTMPGALSAALAAAAPVWPAPISGCRGFSTSLLAKRNHPVIVGK"
CDS 1244657..1244830
/codon_start=1
/note="gene_1162"
/transl_table=11

/translation="MADEPRLEAGAHPFEEGRDKAPELRATQMDHVRFTEGRRERNRD
RLERSQQFRQPGR"
CDS complement(1244757..1245032)
/codon_start=1
/note="gene_1163"
/transl_table=11
/translation="MGALGTVRGLQDSNTAFVGALHSGNLLGATGAVLQAPGNAVNGF
LFGQTSISQSIDVSPEYGYELVAVSDPVGGTAGSARAGHGYVHADLR"
CDS 1245275..1245598
/codon_start=1
/note="gene_1164"
/transl_table=11
/translation="MIFIVVKFETKPEWTERWPDLVASFTAATRAEEGNLWFEWSRSL
DDPAEYVLVESFRDGEAGGVHVNSDHFRQAMRELPKALASTPKIISQTIDATGWSAMG
EMTVG"
CDS complement(1245613..1246323)
/codon_start=1
/note="gene_1165"
/transl_table=11
/translation="MVHGVDPSTLALDRALNETRTGDLWLFRGRSRPDRAIQTLTNAP
VNHVGMTVAIDDLPPLIWHAELGDKLLDVWTGTNHRGVQLNDARQVVQQWAGRYRQRC
WLRQLTPHANRDQEDKLLRVIARMNGTPFPTTARLTGRWLRGRLPTLNDWLRGIPVLD
RKVREQTQRRKQQQRTMGLATAYCAETVAITYEEMGLLVTDKDAHWFDPGKFWSGDSL
PLAPGYRLGHEIAVDVGG"
CDS complement(1246506..1246706)
/codon_start=1
/note="gene_1166"
/transl_table=11
/translation="MGRSVRHGDDLFGRNVAMTARVAGQAVGGQILVGEPVHDAVSDC
ADIRFGSYRLFSLDAAPGPDLD"
CDS complement(1246652..1247146)
/codon_start=1
/note="gene_1167"
/transl_table=11
/translation="MLSGGREAVKTVWQTANLVRKEGFGAAVRSSIEDPADWAEVERP
DLARVTPDGRVVILFSDIEESTALDERIGDRTWVKLIGAHDKLVHELVRRWSGHMVTS
QGDGFMIAFARAEQAVRCGIDIQDALRNSAKRKRNQGIRVRIGTTWGARCGTVTICSA
ATSQ"
CDS 1247349..1248749
/codon_start=1
/note="gene_1168"
/transl_table=11
/translation="LVDGGGGASDLLVIFGITGDLARKMTFRALYRLERHQLLDCPIL
GVASDDMSVGQLVKWARESIGRTEKIDDAVFDRLAGRLSYLHGDVTDSQLYDSLAELI
GSACRPLYYLEMPPALFAPIVENLANVRLLERARVAVEKPFGHDLASALELNARLRAV
LGEDQILRVDHFLGKQPVVELEYLRFANQALAELWDRNSISEIHITMAEDFGVEDRGK
FYDAVGALRDVVQNHLLQVLALVTMEPPVGSSADDLNDKKAEVFRAMAPLDPDRCVRG
QYLGYTEVAGVASDSATETYVALRTEIDNWRWAGVPIFVRAGKELPAKVTEVRLFLRR
VPALAFLPNRRPAEPNQIVLRIDPDPGMRLQISAHTDDSWRDIHLDSSFAVDLGEPIR
PYERLLYAGLVGDHQLFAREDSIEQTWRIVQPLLDNPGEIHRYDRGSWGPEAAQSLLR
GHRGWQSPWLPRGTDA"

```
CDS             1248771..1249793
                /codon_start=1
                /note="gene_1169"
                /transl_table=11
                /translation="MQLGMIGLGRMGANIVRRLAKGGHDCVVYDHDPDAVKAMAGEDR
                TTGVASLRELSQRLSAPRVVWVMVPAGNITTAVIEELANTLEAGDIVIDGGNTYYRDD
                LRHEKLLFKKGIHLLDCGTSGGVWGRERGYCLMIGGDGDAFARAEPIFATVAPGVAAA
                PRTPGRDGEVAPSEQGYLHCGPCGSGHFVKMVHNGIEYGMMASLAEGLNILRNADVGT
                RVQHGDAETAPLPNPECYQYDFDIPEVAEVWRRGSVIGSWLLDLTAIALRESPDLAEF
                SGRVSDSGEGRWTAIAAIDEGVPAPVLTTALQSRFASRDLDDFANKALSAMRKQFGGH
                AEKPAN"
CDS             complement(1249786..1250694)
                /codon_start=1
                /note="gene_1170"
                /transl_table=11
                /translation="MTIWRVPSKVTSGPVSAVSSSPQAVAFSGARGITLVADEWNRGA
                AAADRPTILMLHGGGQNRFSWKNTGQILADEGHHVVALDTRGPGDSDRAPGADYAVET
                PTTDVLHVVEAIGRRVVVVEASMGGLTGILVAERAGPQTVNGLVLVDVVPRYEKEGNA
                RIRDFMLGNIDGFGSLEEAADAVAEYLPHRDKPRSPEGLKRNLRLRDGRWHWHWDPAM
                MTAPGHDPQLRTENFERAAMGLTIPVLLIRGKLSDVVSSDGARDFLAKVPNAEFVELS
                NAGRTAAGDDNDAFTDVVVDFVRRLS"
CDS             1250814..1251719
                /codon_start=1
                /note="gene_1171"
                /transl_table=11
                /translation="MAEPHWIDVKGPNGDLKALTWGPAGAPVALCLHGFPDTAYGWRK
                VAPRLAESGWHVVAPFMRGYAPSSIPADGSYHVGALMHDALRVRSAAGGTERDVIIGH
                DWGAIAATGLAAMPDSPFAKAVIMSVPPSAAFRPLGRVPERGRLLRELPHQLLRSWYI
                LYFQLPWLPERSASWVVPLLWRRWSPGYHAEEDLRHVDAAIGTPEGRRAALGPYRATM
                RNTRAPADYADLNRLWTEAPKLPVLYLHGHDDGCATSAFTHWTARVLPAGSEVAVVEH
                AGHFLQLEQPDKIAELIVAFIGSPG"
CDS             1251739..1252968
                /codon_start=1
                /note="gene_1172"
                /transl_table=11
                /translation="MAAVDAQFYWMSAKVPNDQFLLYAFDGEPTDLERAVAQVYRRAR
                GCPGLGMRVQDRGALAYPQWVPTPVQRDQLVCHDLADRSWQGCLAAVVGLAGKQLDMR
                RMPWRLHVFTPVHDVPGVSGLGTVAVMQFAHALGDGARASAMAAWLFGRPAAVPEIAR
                SRAGFLPWRAAHAARAHLRLVRDTNAGLVAPGVGSRPPLSTNARPEGVRAVRTLLRRR
                SQLAGPTVTVTVLAAVSTGLLGLLGGDVDTLGAEVPMAKPGVPRSYNHFGNVVVGLYP
                RLEPDERVRRIATDLANARRRFEHPAMLSADRAFAAVPAALLRWGVSQFDAEVRPVRV
                AGNTVVSSVYRGAADLSFGDAPVVLTAGYPALSPAMGLTHGVHGIGDTVAISVHAAES
                AVSDIDAYMRLLDAALQ"
CDS             complement(1252972..1253577)
                /codon_start=1
                /note="gene_1173"
                /transl_table=11
                /translation="MSELTVLQAVRLKGRVITTDLAQTLGEDLADVAATVDRLTAAGL
                LVDATPLRISPSGRMRLDDLLAEERNRADSTVLAAAYRDFRSVNADFKRLVTDWQLKG
                EKPNTHDDAEYDAAVLSRLDGVHRRVGPIIGTVAMQLPRLSRYPVKLRAALDKVKAGD
                IAWLTRPLIDSYHTVWFELHEELIQAVGLTRDEAAKSGDAQ"
```

```
CDS             complement(1253574..1255046)
                /codon_start=1
                /gene="ppdK"
                /note="gene_1174"
                /transl_table=11
                /translation="MTRITRANGCPDGTLENAVVALDGGANYPREILGNKGHGIDMMR
                RHHLPVPPAFCITTEVGVRYLAAPESTIAAIWDDVLDRMSWLETETSCTFGRGPNPLL
                VSVRSGATQSMPGMMDTILDVGMTDAVERVLARPGAADFAHDTRRRFTSMYRRIVGSA
                GPITDDPYAQLRASIEAVFASWNSPRAVAYRDHHGLDDQGGTAVVVQAMVFGNLTANS
                GAGVLSSRNPITGANEPFGEWLPGGQGDDVVSGLVAVAPITALRDQQPAVYDQLMAAA
                RSLERMAGDVQEIEFTVEDSQLWLLQTRGAERSAQAAVRLALQLHHEGLIDDTETLRR
                VTPTHIETLLRPSLQPETRLAAPLLAKGLPACPGVVSGTAYTEVDEALDAADRGEPVI
                LVRDHTRPEDVMGMLAAQGIVTEVGGAASHAAVVSRELGRVAVVGCGPGVAAALAGKE
                ITVDGYEGEVRQGVLALSAWSESDTPELRELADIAQRISS"
CDS             complement(1255259..1256614)
                /codon_start=1
                /note="gene_1175"
                /transl_table=11
                /translation="MCSTREEITEAFASLATALSRVLGLTFDALTTPERLALLEHCET
                ARRQLPSVEHTLINQIGEQSTEEELGGKLGLTLADRLRITRSEAKRRVAEAADLGQRR
                ALTGEPLPPLLTATAKAQRHGLIGDGHVEVIRAFVHRLPSWVDLKTLEKAERDLAKQA
                TQYRPDQLAKLAARIMDCLNPDGDYTDEDRARRRGLTLGKQDVDGMSRLSGYVTPELR
                ATIEAVWAKLAAPGMCNPEQKAPCVNGAPSKEQARRDTRSCPQRNHDALNAGLRSLLT
                SGNLGQHNGLPASIIVTTTLKDLEAAAGAGLTGGGTILPISDVIRLARHANHYLAIFD
                RGKALALYHTKRLASPAQRIMLYAKDSGCSAPGCDVPGYYCEVHHVTPYAQCRNTDVN
                DLTLGCGGHHPLAERGWTTRKNAHGDTEWLPPPHLDHGQPRVNTFHHPEKLLADDEGD
                P"
CDS             complement(1256716..1258077)
                /codon_start=1
                /note="gene_1176"
                /transl_table=11
                /translation="VALAKALDLSTSYVNQLENDQRPITVPVLLLLTERFDLSAQYFS
                SDSDARLVADLSDVFTDIGVEHAVSGAQIEEFVARMPEVGHSLVAVHRRLRAATEELE
                GYRSRATAETELPPARPMPFEEVRDFFYDRNNYIHDLDMAAERMFTESGMRTGGLDIQ
                LAELMRDRFGISVVIDDNLPDTAKRRYHPDTKVLRVAHWLMPGQRAFQIATQLALVGQ
                SDLISSIVATDDQLSTEARGVARIGLANYFAGAFLLPYREFHRAAEQLRYDIDLLGRR
                FGVGFETVCHRLSTLQRPRQRGIPFIFVRTDKAGNISKRQSATAFHFSRVGGSCPLWV
                VHDAFAQPERIVRQVAQMPDGRSYFWVAKTTAADGLGYLGPHKNFAVGLGCDLAHAHK
                LVYSTGVVLDDPSTEVPIGAGCKICNRTSCAQRAFPYLGGRVAVDENAGSSLPYSSTE
                QSV"
CDS             1258272..1259777
                /codon_start=1
                /note="gene_1177"
                /transl_table=11
                /translation="MLMHAVRAWRSADDFPCTEHMAYKIAQVAADPVDVDPEVADMVC
                NRIIDNAAVSAASMVRRPVTVARHQALAHPVRHGAKVFGVEGSYSADWAAWANGVAAR
                ELDFHDTFLAADYSHPADNIPPLVAVAQQLGVCGAELIRGLVTAYEIHIDLTRGICLH
                EHKIDHVAHLGPAVAAGIGTMLRLDQETIYHAIGQALHLTTSTRQSRKGAISSWKAFA
                PAHAGKVGIEAVDRAMRGEGSPAPIWEGEDGVIAWLLAGPEHTYRVPLPAPGEPKRAI
                LDSYTKQHSAEYQSQAPIDLACRLRERIGDLDQIASIVLHTSHHTHVVIGTGSGDPQK
                FDPDASRETLDHSLPYIFAVALQDGCWHHERSYAPERARRSDTVALWHKISTVEDPEW
```

```
                     TRRYHCADPAKKAFGARAEVTLHSGEVIVDELAVADAHPLGTRPFERKQYVEKFTELA
                     DGVVEPVEQQRFLAVVESLADLESGAVGGLNVLVDPRVLDKAPVIPPGIFR"
CDS                  1259774..1260955
                     /codon_start=1
                     /note="gene_1178"
                     /transl_table=11
                     /translation="MTGPLAAARSVAATKSMTAPTVDERPDIKKGLAGVVVDTTAISK
                     VVPQTNSLTYRGYPVQDLAARCSFEQVAFLLWRGELPTDAELALFSQRERASRRVDRS
                     MLSLLAKLPDNCHPMDVVRTAISYLGAEDPDEDDAAANRAKAMRMMAVLPTIVAIDMR
                     RRRGLPPIAPHSGLGYAQNFLHMCFGEVPETAVVSAFEQSMILYAEHGFNASTFAARV
                     VTSTQSDIYSAVTGAIGALKGRLHGGANEAVMHDMIEIGDPANAREWLRAKLARKEKI
                     MGFGHRVYRHGDSRVPTMKRALERVGTVRDGQRWLDIYQVLAAEMASATGILPNLDFP
                     TGPAYYLMGFDIASFTPIFVMSRITGWTAHIMEQATANALIRPLSAYCGHEQRVLPGT
                     F"
CDS                  1260967..1262697
                     /codon_start=1
                     /note="gene_1179"
                     /transl_table=11
                     /translation="MGFLQPRLPDIDLAEWSQGSRSQKIRPMAQHWAEVGFGTPVLLH
                     LFYVAKILLYVLVGWLIVLTTKGIDGFTDAAAWYAEPIVFEKVVLYTMLFEVIGLGCG
                     FGPLNNRFFPPMGSILYWMRFGTIRLPPWPDRVPWTRGTKRKPVDVALYALLVMMLLS
                     ALFTDGAGPIPELGTTVGLLPAWQIVLILLLLGVLGLRDKVIFLAARGEVYATLTVTF
                     LFGRLNGIDMIVAAKLVFLVIWIGAATSKLNRHFPFVISTMMSNNPLFRPRFIKRMFF
                     KKFPGDLRPGLLSRIVAHVSTVIEMCVPVVLFVAHGGWPTVVAATIMVCFHLGILTAI
                     PMGVPLEWNVFMIFGVLSLFVGHACLGLADVKNPVPLAILIAVVAGIVIAGNVFPRKI
                     SFLAAMRYYAGNWDTTLWCIKPSAEDKINRGIVAIASMPAAQLERFYGKDRAQIPMYL
                     GYAFRAMNSHGRALFTLAHRAMAGHDEDDYVITDGERVCSTAVGWNFGDGHLHNEQLI
                     AAMQQRCGFQPGEVRVVLLDAQPIHRQTQEYRLVDAATGEFERGYVRVADMVNRQPWD
                     DDVPVHVLPG"
CDS                  complement(1262709..1264988)
                     /codon_start=1
                     /gene="metE"
                     /note="gene_1180"
                     /transl_table=11
                     /translation="VTQPVRRQPFTATITGSPRIGPRRELKRATEGYWAGRTSRSELE
                     AVAATLRRDTWSALAAAGLDSVPVNTFSYYDQMLDTAVLLGALPPRVSPVSDGLDRYF
                     AAARGTDQIAPLEMTKWFDTNYHYLVPEIGPSTTFTLHPGKVLAELKEALGQGIPARP
                     VIIGPITFLLLSKAVDGAGAPIERLEELVPVYSELLSLLADGGAQWVQFDEPALVTDL
                     SPDAPALAEAVYTALCSVSNRPAIYVATYFGDPGAALPALARTPVEAIGVDLVAGADT
                     SVAGVPELAGKTLVAGVVDGRNVWRTDLEAALGTLATLLGSAATVAVSTSCSTLHVPY
                     SLEPETDLDDALRSWLAFGAEKVREVVVLARALRDGHDAVADEIASSRAAIASRKRDP
                     RLHNGQIRARIEAIVASGAHRGNAAQRRASQDARLHLPPLPTTTIGSYPQTSAIRVAR
                     AALRAGEIDEAEYVRRMRQEITEVIALQERLGLDVLVHGEPERNDMVQYFAEQLAGFF
                     ATQNGWVQSYGSRCVRPPILYGDVSRPRAMTVEWITYAQSLTDKPVKGMLTGPVTILA
                     WSFVRDDQPLADTANQVALAIRDETVDLQSAGIAVIQVDEPALRELLPLRRADQAEYL
                     RWAVGAFRLATSGVSDATQIHTHLCYSEFGEVIGAIADLDADVTSIEAARSHMEVLDD
                     LNAIGFANGVGPGVYDIHSPRVPSAEEMADSLRAALRAVPAERLWVNPDCGLKTRNVD
                     EVTASLHNMVAAAREVRAG"
CDS                  1265564..1265800
                     /codon_start=1
                     /note="gene_1181"
```

/transl_table=11
/translation="MAAYQKFGQEHAAAIRGGAVLHPTATATTVRVTGARGGDVVTGD
GPYEAADLDEQGPFPMETVYLWEDGPNGTTRMTL"
CDS complement(1265914..1267770)
/codon_start=1
/note="gene_1182"
/transl_table=11
/translation="MSFLVLPPEVNSALMFAGAGSGPTLAAAAAWDGLAAELGQAANS
FSSATAALADTAWQGPAATAMAAAAAPYASWLSTAATRALSAAAQAKAAAAVYEAARA
ATVDPLLVAANRHQLVSLVLSNLFGQNAPAIAATEAAYEQLWAADVAAMVSYHSGASA
VAAQLAPWAQAVRALPNPTAPALASGPAALAIPALGIGNTGIGNIFSIGNIGDYNLGN
GNTGNANLGSGNTGQANLGSGNTGFFNFGSGNTANTNFGSGNLGNLNLGSGNDGNGNF
GLGNIGDGNRGSGNVGSFNFGTANAGSFNVGSANHGSPNVGFANLGNNNLGIANLGNN
NLGIANLGNNNIGIGLTGDNMIGIGALNSGIGNLGFGNSGNNNIGLFNSGNNNIGFFN
SGDSNFGFFNSGDTNTGFGNAGFTNTGFGNAGSGNFGFGNAGNNNFGFGNSGFENMGV
GNSGAYNTGSFNSGTLNTGDLNSGDFNTGWANSGDINTGGFHSGDLNTGFGSPVDQPV
MNSGFGNIGTGNSGFNNSGDANSGFQNTNTGAFFIGHSGLLNSGGGQHVGISNSGTGF
NTGLFNTGFNNTGIGNSATNAAFTTTSGVANSGDNSSGGFNAGNDQSGFFDG"
CDS 1267956..1268198
/codon_start=1
/note="gene_1183"
/transl_table=11
/translation="MQLGNQNTMRFAGRPQRFRQSAYPLFNPNSAIALGHPFGGSGAR
LMTTVLHHMPDKGIRYGLQTMCEGRGQANATIVELL"
CDS 1268248..1268589
/codon_start=1
/note="gene_1184"
/transl_table=11
/translation="MVITINRPEARNAVNGAVSIVVGDALEEAHDNPDVRAVVITGAG
DKSLCAGADLKAIARRENPYHPHHGEWGIAGYRHHFIDKPTSAAVSGTALDDGAEPAL
ASDLVVADEHT"
CDS complement(1269114..1270130)
/codon_start=1
/note="gene_1185"
/transl_table=11
/translation="MTSYDTDLLVVGGGPGGLATALHARARGLSVIVAEPRENPIDKA
CGEGLMPGGLAELTSLGVDPVGLPFHGIAYVGEHRRVQARFRTGPGRGVRRTTLHAAL
AARAKEQDTEWIRSRVATIQQDAHGVTAAGVRAKWLVAADGLHSAVRRAVGIKATAGT
PRRYGVRWHYRLPVWSDFVEVHWSRWGEAYVTPVEPDLVGVAILSRQRPELAWFPSLA
HHLQDASRGHARGCGPLRQVVSRRVAGRVLLVGDAAGYEDALTGEGISLAVKQAAAAV
SAIVDDTPASYEAAWHRITRDYRLVTRGLVLASTPRAARRAIVPLCALLPTAFRYGVN
ILAY"
CDS complement(1270127..1270627)
/codon_start=1
/note="gene_1186"
/transl_table=11
/translation="VYYLLILAVVFERLAELVVAQRNARWSFAQGGKEFGRPHYVVMV
ILHTALLLGCVVEPWALHRPFIPWLGWPMLAVVVASQGLRWWCVKSLGKRWNTRVIVL
PHATLVRRGPYRWMRHPNYVAVVAEGFALPLVHTAWLTALVFTLANATLLTVRLRVEN
SVLGYI"
CDS complement(1270632..1271081)

/codon_start=1
/note="gene_1187"
/transl_table=11
/translation="MCRLVRRVPAGGPGFTGPGVGPARGGVISRHPALPVLTQVYEPA
FLVALLTTIRTASNLARRRYGPWNGPRRSRAIQHSAATIRAVERPGNGGPTRQNLNPS
GASNVADNMESIAGGAIPPFGLGCIAGPARIVCIGPGFSSESVLLRW"
CDS 1271115..1271837
/codon_start=1
/note="gene_1188"
/transl_table=11
/translation="MSQSTTPYRSSVLAEFRRAITNVAVPHHEPPGIVRRRRVVVGVT
LVIGAVMLGFSLRRTPGESSFYWLTLALAAVWIAGALMSGPLHLGGICWRGRNQRPVI
TGTTVGLLLAGIFGVGAMIVRAIPGAAEPIARVLQFAHQGTLLPILLITLINGIAEEM
FFRGALYTALGRRYPVTISTVLYVGATMASANLMLGFAAIFVGTVCALERRASGGVLA
PILTHFVWGLIMVFALPPLFAV"
CDS complement(1271845..1272651)
/codon_start=1
/note="gene_1189"
/transl_table=11
/translation="MPDSGIAALTPVTGLNVTLTDRVLSVRINRPSSLNSLTVPILTG
IADTLERAAADPVVKVVRLGGVGRGFSSGVSMSVDDVWGGGPPTAIVEEANRAVRAVA
ALPHPVVAVVQGPAVGVAVSLALACDFILASDSAFFMLANTKVALMPDGGASALVAAA
TGRIRAMRLALLAEQLPAREALAWGLISAVYPDSDFEAEVDKVISRLLAGPALAFAQA
KNAINAAALTELEPTFARELDGQEVLLRTHDFAEGAAAFLQRRTPNFTGS"
CDS complement(1272794..1273600)
/codon_start=1
/note="gene_1190"
/transl_table=11
/translation="MSNYRIDTRTIVPGLAVTLADGVLSVTIDRPESLNSLTKPVLAG
MADAIEGAATDPRVKVVRLGGAGRGFSSGGAISVDDVWASGPPTDTVAEANRTVRAIV
ALPQPVVAVVQGPTVGCGVSLALACDLVLASDNAFFMLAHTNVGLMPDGGASALVQAA
IGRIRAMHMALLPDRVPAAEALSWGLVSAVYPAADFDAEVDKLISRLLAGPALAIAKT
KNAINAATLTELAPTLLRELDGQALLLRTDDFAEGATAFQQRRTPMFTGR"
CDS 1273704..1274786
/codon_start=1
/gene="mcr"
/note="gene_1191"
/transl_table=11
/translation="MAGPLSGLRVVELAGIGPGPHAAMILGDLGADVVRIDRPSSVDG
ISRDAMLRNRRIVTADLKSDQGLELALKLIAKADVLIEGYRPGVTERLGLGPEECAKV
NDRLIYARMTGWGQTGPRSQQAGHDINYISLNGILHAIGRGDERPVPPLNLVGDFGGG
SMFLLVGILAALWERQSSGKGQVVDAAMVDGSSVLIQMMWAMRATGMWTDTRGANMLD
GGAPYYDTYECADGRYVAVGAIEPQFYAAMLAGLGLDAAELPPQNDRARWPELRALLT
EAFASHDRDHWGAVFANSDACVTPVLAFGEVHNEPHIIERNTFYEANGGWQPMPAPRF
SRTASSQPRPPAATIDIEAVLTDWDG"
CDS 1274798..1275550
/codon_start=1
/note="gene_1192"
/transl_table=11
/translation="MKTKDAVAVVTGGASGLGLATTKRLLDAGAQVVVVDLRGDDVVG
GLGDRARFAQADVTDEAAVSNALELADSLGPVRVVVNCAGTGNAIRVLSRDGVFPLAA

FRKIVDINLVGTFNVLRLGAERIAKTEPIGEERGVIINTASVAAFDGQIGQAAYSASK
GGVVGMTLPIARDLASKLIRVVTIAPGLFDTPLLASLPAEAKASLGQQVPHPSRLGNP
DEYGALVLHIIENPMLNGEVIRLDGAIRMAPR"
CDS complement(1275678..1275833)
/codon_start=1
/note="gene_1193"
/transl_table=11
/translation="MAAFRLAVMWIDDSSADVIKADFEALYHGDMLVEGDTSEQLEDL
HPLPTAS"
CDS 1276065..1278410
/codon_start=1
/note="gene_1194"
/transl_table=11
/translation="MLQRIARLAIAAPRRIIGFAVFVFIAAAVFGVPVADSLSPGGFQ
DPRSESARAIEVLTDKFGQSGQKMLIVVTAAAGADSPPAREVGTDIVEVLRRSPLVYN
VTSPWTVPPTAAADLLSTDGKSGLIVVNVKGGENDAQNHAQTLSDEVAHDRDGVTVRA
GGSAMEYAQINRQNKDDLLVMELIAIPLSFLVLIWVFGGLLAAGLPMAQAVLAVVGSM
AVLRLVTFATEVSTFALNLSTALGLALAIDYTLLIVSRYRDELAEGSDRDEALIRTMA
TSGRTVLFSAVTVALSMSATALFPMYFLKSFAYAGVATVAFVATASIVITPAAIVLLG
PRLDALDVRRLVRRLLGRPDPVHKPVKQLFWYRSSKFVMRRWLPVGTAVVALLVLLGL
PFLSVKWGFPDDRVLPRSASARQVGDILRDDFGHDPATQIPIVVPDARGLGPVELDSY
AAELSRVPDVSAVAAPTGTFVDGSWVGTPRGATGLAEGSAFLTVSSTAPLFSRASDIQ
LKRLHQVAGPAGRSVVMAGVAQVNRDSVDAVTDRLPMVLGLIAAITYVLLFLLTGSVV
LPAKALVCNVLSLTAAFGALVWIFQEGHFGALGTTPSGTLVANMPVLLFCIAFGLSMD
YEVFLVSRIREYWLESGAARPARRSVAEVHAANDESVALGVARTGRVITAAALVMSMS
FAALIAAHVSFMRMFGLGLTLAVAADATLVRMVVVPAFMHVTGRWNWWAPRPLAWLHE
RFGVSEAAEPVSRRRSHAGGLGKIAGRSDGQTIPASLTRNG"
CDS 1278543..1279193
/codon_start=1
/note="gene_1195"
/transl_table=11
/translation="MTSGAAASASRVDHPLFARIWPVVAAHEAEAIRALRRENLAGLS
GRVLEVGAGVGTNFAYYPVAVEQVIAMEPEPRLAAKARIAAADAPVPIVVTDKTVEEF
RDTETFDAVVCSLVLCSVSDPGAVLAHLRSLLRRGGELRYLEHVASAGARGRVQRFVD
ATFWPRLAGNCHTHRHTERAILDAGFVVDSSRREWAFPAWVPLPVSELALGRAHRT"
CDS complement(1279671..1279946)
/codon_start=1
/gene="TMFG_02334"
/note="gene_1196"
/transl_table=11
/translation="LTPNNEDVAGRITESLKQMRTDPSAQPAPEQPIAAATSFYPSTN
MTPWSRVSTDLISSLLVGAVEAQHAADGRRVLAVLGLVERAARGRTS"
CDS complement(1279943..1281307)
/codon_start=1
/note="gene_1197"
/transl_table=11
/translation="MRSDTREEISAALDAYHASLSRVLDLKCDALTTPELLACLQRLE
VERRRQGAAEHALINQLAGQACEEELGGTLRTALANRLHITPGEASRRIAEAEDLGER
RALTGEPLPAQLTATAAAQREGKIGREHIKEIQAFFKELSAAVDLGIREAAEAQLAEL
ATSRRPDHLHGLATQLMDWLHPDGNFSDQERARKRGITMGKQEFDGMSRISGLLTPEL
RATIEAVLAKLAAPGACNPDDQTPLVDDTPDADAVRRDTRSQAQRNHDAFLAALRGLL

```
                     ASGELGQHKGLPVTIVVSTTLKELEAATGKGVTGGGSRVPMSDLIRMASNANHYLALF
                     DGAKPLALYHTKRLASPAQRIMLYAKDRGCSRPGCDAPAYHSEVHHVTPWTTTHRTDI
                     NDLTLACGPDNRLVEKGWKTRKNAHGDTEWLPPPHLDHGQPRINRYHHPAKILCEQDD
                     DEPH"
     CDS             1281536..1281943
                     /codon_start=1
                     /note="gene_1198"
                     /transl_table=11
                     /translation="VTRVGVISDEFWAVVEPLMPSHEGKPGRRFSDHRLILEGIAWRF
                     RTGSPWRDLPAEFGPWQTVWKRHHRWSLDGTCDEVFAHVAAVFGVDAEVAEDIEKLLS
                     VDSTNVRAHQHSAGACSDTLATGGTVGLQEIRR"
     CDS             1282026..1282463
                     /codon_start=1
                     /note="gene_1199"
                     /transl_table=11
                     /translation="VRIRLTAGQAGDNPQLLPLLDDYRHASTEYALGSTDFRLLADKA
                     YSHPSTRAALRSKKIKHTIPERQDQIDRRKAKGSAGGRPPAFDAALYGLRNTVERGFH
                     RLKQWRGIATRYDKYALTYLGGVLLACAVIHARVGTPKLGDTP"
     CDS             complement(1282547..1283260)
                     /codon_start=1
                     /note="gene_1200"
                     /transl_table=11
                     /translation="MRVAVLSGAGISAESGVPTFRDDKNGLWARFDPYELSSTQGWLR
                     NPERVWGWYLWRHYLVANVEPNDGHRAIAAWQDHAEVSVITQNVDDLHERAGSGAVHH
                     LHGSLFEFRCARCGVPYTDALPEMPEPAIEVEPPVCDCGGLIRPDIVWFGEPLPEEPW
                     RSAVEATGSADVMVVVGTSAIVYPAAGLPDLALARGTAVIEVNPEPTPLSGSATISIR
                     ESASQALPGLLERLPALLK"
     CDS             1283298..1283663
                     /codon_start=1
                     /note="gene_1201"
                     /transl_table=11
                     /translation="VELRDWLRVDVKAGKPLFDQLRTQVIDGVRAGALPPGTRLPTVR
                     DLAGQLGVAANTVARAYRELESAAIVETRGRFGTFISRFDPTDAAMAAAAKEYVGVAR
                     ALGLTKSDAMRYLTHVPDD"
     CDS             complement(1283641..1284489)
                     /codon_start=1
                     /note="gene_1202"
                     /transl_table=11
                     /translation="VSAHKPAKQRVALTGVSETALLTLNARAAEARRRDTIIDDPMAV
                     ALVESIDFGFAKFGPTGQGFALRARAFDMAAQHYLDQHPAATVVALAEGLQTSFWRLD
                     VAIPGGQFRWLTVDLPPIVDLRTRLLPSSPRVSVCAQSALDYSWMDSVDPAGGVFITA
                     EGLLMYLQPEQALGLIAQCAQTFPGGQMLFDLPPRWFAGWSRLGLRTSLRYKVPRMPF
                     SMSVAQAADLVNKVPGVVAVRDLRVPPGRGLWVNMALSTVYRLPVFDPLRPCLTLLEF
                     SRPARG"
     CDS             complement(1284486..1285073)
                     /codon_start=1
                     /note="gene_1203"
                     /transl_table=11
                     /translation="MPRAYVAVASFSGGLVQSGMAKFAAFLRGVNVGGVNLKMAEVAT
                     ALTDAGFCNVRTILASGNVLLESTCGAAEVREKTEATLRERFGYDAWALIYDVDTVRT
                     IVAAYPFECELEGYQSYVTFVADAAILDELSALADTAGPDENISRGPDPLGVLYWQVP
```

```
                     KGSTLDSTIGQTMGKKRYKSSTTTRNLRTLAKVLR"
CDS                  1285072..1285515
                     /codon_start=1
                     /note="gene_1204"
                     /transl_table=11
                     /translation="MARQVFDDKLLAVISGNSIGVLATIKHDGRPQLSNVQYHFDPRK
                     LLIQVSIAEPRAKTRNLRRDPRASILVDADDGWSYAVAEGTAQLTPPAAAPDDDTVEA
                     LIALYRNIAGEHPDWDDYRQAMVTDRRVLLTLPISHVYGLPPGMR"
CDS                  1285673..1285861
                     /codon_start=1
                     /note="gene_1205"
                     /transl_table=11
                     /translation="MGESKSPQESSSEGETKRKFREALDRKMAQSSSGSDHKDGGGKQ
                     SRAHGPVASRREFRRKSG"
CDS                  1285949..1286536
                     /codon_start=1
                     /note="gene_1206"
                     /transl_table=11
                     /translation="VPNLQLVQEPAADALLNANPFALLVGMLLDQQVPMETAFAGPKK
                     IADRMGSFDAGDIADYDPDKFVALCSERPAIHRFPGSMAKRIQALAQIIVDRYDGDAA
                     ALWTAGEPDGNELLRRLKGLPGFGEQKARIFLALLGKQYGVTPKGWQVAAGEFGQPGT
                     YLSVADIVDAGSLGQVRSHKRQRKAAAKAEGKAPT"
CDS                  1286533..1286679
                     /codon_start=1
                     /note="gene_1207"
                     /transl_table=11
                     /translation="VKTHLTCPCGEAITGKDEDELVELTQAHLASVHPGLEYDRDAIL
                     FMAY"
CDS                  complement(1286699..1287742)
                     /codon_start=1
                     /note="gene_1208"
                     /transl_table=11
                     /translation="LAAVVITSAAAFALCPNAAADPATPQPNPTQQLPGLPALAQLSP
                     IIQQAAMNPAQATQLLMAAASAFAGNPAVPTESKNVASSVNQFVAEPTNPDSAALGVP
                     APHGVALPEAIPVPHVPPLGAEPGVQAHLPTGIDPSHAAGPAPAVAPTVTPPVAAPPA
                     SAPAPAPDAAQPVAVPGPPPAPPAPRAAAPAPASAAPAPAAAPAPASGFGADAPPTQD
                     FMYPSIGPNCVADGSNSIATALSVAGPAKIPLPGPGPGQTAYVFTAVGTPGPADVQRL
                     PLNVTWVNLTTGKSGSATLRPRSDINPDGPTTLTVIADTGSGSIMSTIFGQVTTKDRQ
                     CQFMPTIGSTVVP"
variation            1287100..1287171
                     /note="supplemented from BCG moreau"
CDS                  complement(1287822..1288505)
                     /codon_start=1
                     /note="gene_1209"
                     /transl_table=11
                     /translation="MPTIWTFVRAAAVLVGSSAALLTGGIAHADPAPAPAPAPNIPQQ
                     LISSAANAPQILQNLATALGATPPLSAPKVAEPAPAAPGITATFPGLTPAAPAAAAAP
                     ALTPSIPGVNAPIPGITPAAPALPVTAPAAAPTIPGVNAPIPGITAPAPAAAAVPASV
                     PGVPSAKVDLPQLPYLPLQVPQQLSLPADLPALASGVIPAAPIAPTPPAPGAPALPPG
                     PPSLLAALP"
CDS                  1288635..1289930
```

```
                     /codon_start=1
                     /gene="pimE"
                     /note="gene_1210"
                     /transl_table=11
                     /translation="MCRTLIDGPVRSAIAKVRQIDTTSSTPAAARRVTSPPARETRAA
                     VLLLVLSVGARLAWTYLAPNGANFVDLHVYVSGAASLDHPGTLYGYVYADQTPDFPLP
                     FTYPPFAAVVFYPLHLVPFGLIALLWQVVTMAALYGAVRISQRLMGGTAETGHFAAML
                     WTAIAIWIEPLRSTFDYGQINVLLMLAALWAVYTPRWWLSGLLVGVASGVKLTPAITA
                     VYLVGVRRLHAAAFSVVVFLATVGVSLLVVGDEARYYFTDLLGDAGRVGPIATSFNQS
                     WRGAISRILGHDAGFGPLVLAAIASTAVLAILAWRALDRSDRLGKLLVVELFGLLLSP
                     ISWTHHWVWLVPLMIWLIDGPARERPGARILGWGWLVLTIVGVPWLLSFAQPSIWQIG
                     RPWYLAWAGLVYVVATLATLGWIAASERYVRIRPRRMAN"
     CDS             complement(1289927..1290211)
                     /codon_start=1
                     /gene="phhB"
                     /note="gene_1211"
                     /transl_table=11
                     /translation="MAVLTDEQVDAALHDLNGWQRAGGVLRRSIKFPTFMAGIDAVRR
                     VAERAEEVNHHPDIDIRWRTVTFALVTHAVGGITENDIAMAHDIDAMFGA"
     CDS             1290238..1290663
                     /codon_start=1
                     /note="gene_1212"
                     /transl_table=11
                     /translation="MLNQIVVAGAIVRGCTVLVAQRVRPPELAGRWELPGGKVAAGET
                     ERAALARELAEELGLEVADLAVGDRVGDDIALNGTTTLRAYRVHLLGGEPRARDHRAL
                     CWVTAAELHDVDWVPADRGWIADLARTLNGSAADVHRRC"
     CDS             1290971..1294669
                     /codon_start=1
                     /note="gene_1213"
                     /transl_table=11
                     /translation="VTVTPHVGGPLEELLERSGRFFTPGEFSADLRTVTRRGGREGDV
                     FYRDRWSHDKVVRSTHGVNCTGSCSWKIYVKDGIITWETQQTDYPSVGPDRPEYEPRG
                     CPRGASFSWYSYSPTRVRYPYARGVLVEMYREAKTRLGDPVLAWADIQADPERRRRYQ
                     QARGKGGLVRVSWAEASEMVAAAHVHTIKTYGPDRVAGFSPIPAMSMVSHAAGSRFVE
                     LIGGVMTSFYDWYADLPVASPQVFGDQTDVPESGDWWDASYLVMWGSNVPITRTPDAH
                     WMAEARYRGAKVVVVSPDYADNTKFADEWVRCAAGTDTALAMAMGHVILSECYVRNQV
                     PFFVDYVRRYTDLPFLIKLEKRGDLLVPGKFLTAADIGEESENAAFKPALLDELTNTV
                     VVPQGSLGFRFGEDGVGKWNLDLGSVVPALSVEMDKAVNGDRSAELVTLPSFDTIDGH
                     GETVSRGLPVRRAGKHLVCTVFDLMLAHYGVARAGLPGEWPTGYHDRTQQNTPAWQES
                     ITGVPAAQAIRFAKEFARNATESGGRSMIIMGGGICHWFHSDVMYRSVLALLMLTGSM
                     GRNGGGWAHYVGQEKVRPLTGWQTMAMATDWSRPPRQVPGASYWYAHTDQWRYDGYGA
                     DKLASPVGRGRFAGKHTMDLLTSATAMGWSPFYPQFDRSSLDVADEARAAGRDVGDYV
                     AEQLAQHKLKLSITDPDNPVNWPRVLTVWRANLIGSSGKGGEYFLRHLLGTDSNVQSD
                     PPTDGVHPRDVVWDSDIPEGKLDLIMSIDFRMTSTTLVSDVVLPAATWYEKSDLSSTD
                     MHPYVHSFSPAIDPPWETRSDFGAFAAIARAFSALAKRHLGTRTDVVLTALQHDTPDE
                     MAYPDGTERDWLATGEVPVPGRTMSKLTVVERDYTAIYDKWLTLGPLIDQFGMTTKGY
                     TVHPFREVSELAANFGVMNSGVAVGRPAITTAKRMADVILALSGTCNGRLAVEGFLEL
                     EKRTGQRLAHLAEGSEERRITYADTQARPVPVITSPEWSGSESGGRRYAPFTINIEHL
                     KPFHTLTGRMHFYLAHDWVEELGEQLPVYRPPLDMARLFNQPELGPTDDGLGLTVRYL
                     TPHSKWSFHSTYQDNLYMLSLSRGGPTMWMSPGDAAKINVRDNDWVEAVNANGIYVCR
                     AIVSHRMPEGVVFVYHVQERTVDTPRTETNGKRGGNHNALTRVRIKPSHLAGGYGQHA
```

FAFNYLGPTGNQRDEVTVVRRRSQEVRY"
CDS 1294708..1296384
/codon_start=1
/note="gene_1214"
/transl_table=11
/translation="MKVMAQMAMVMNLDKCIGCHTCSVTCKQAWTNRSGTEYVWFNNV
ETRPGVGYPRTYEDQERWRGGWVRDKKGRLRLRDGGRIHKLLRIFANPKLPTIGDYYE
PWTYDYENLTSAPAGDTFPTAAPRSLISGNPMKVSWGSNWDDNLAGSPEIVPNDPVLK
KVNQVNQEVKLKLEETFMFYLPRICEHCLNPSCVASCPSGAMYKRTEDGIVLVDQDRC
RGWRMCVSGCPYKKVYFNHKTGKAEKCTLCYPRIEVGLPTVCSETCVGRLRYLGLVLY
DVDQVLQAASVESDTDLYEAQRRILLDPHDPRVIAGARAEGIADEWIEAAQRSPVYAL
INTYRVALPLHPEYRTMPMVWYIPPLSPVVDAVSRDGHDGEDLGNLFGALDALRIPIA
YLAELFTAGDTEVVAGVLRRLAAMRCYMRDINLGRETQPHIPESVGMTEEQIYQMYRL
LAVAKYEERYVIPTSYAGELPAAAMTDDMGCSLSVDGGPGMYESGPFGQGSPTPVPIA
VESFHALQHAGSAATGGAGRSRVNLLNWDPNGAAAGLFPEPQPSKDVVQR"
CDS 1296441..1297046
/codon_start=1
/note="gene_1215"
/transl_table=11
/translation="VWQSASLLLAYPDDGLAERLHMVDALRAHQTGPAAALLGRTVAE
LRALAPMAAAAQYVETFDMRRRSTMYLTYWTAGDTRNRGREMLAFATAYRDAGVKPPR
TEAPDYLPVVLEFAATVDPEAGRRLLTEHRVPIDVLRGALADAKSPYEYTVAAICETL
PAATNQEVRRAQRLAQSGPPAEAVGLQPFTLTVPPKRAEGA"
CDS 1297049..1297789
/codon_start=1
/note="gene_1216"
/transl_table=11
/translation="LAVLDLVEIFWDAAPYVVVAIAVVGTWWRYRYDKFGWTTRSSQL
YESRLLSIGSPMFHFGSLLVIMGHVMGLFIPDSWTRAFGMSDHLYHLQALLLGAPAGF
ATLLGIGLLIYRRRIQTPVWLATTRNDKLMYLVLVCAIVAGLACTLMGATHEGDMHDY
RRSVSVWFRSIWMLAPRGDLMAQATLYYQVHVLIALALFVLWPFTRLVHAFSAPIAYL
FRPYIVYRSREVAAKHELIGSAPRRRGW"
CDS 1297811..1299697
/codon_start=1
/note="gene_1217"
/transl_table=11
/translation="VPFRNVAIVAHVDHGKTTLVDAMLRQSGALRERGELQERVMDTG
DLEREKGITILAKNTAVHRHHPDGTVTVINVIDTPGHADFGGEVERGLSMVDGLLLLV
DASEGPLPQTRFVLRKALAAHLPVILVVNKTDRPDARIAEVVDASHDLLLDVASDLDD
EAAAAAEHALGLPTLYASGRAGVASTTAPPDGQVPDGTNLDPLFEVLEKHVPPPKGEP
DAPLQALVTNLDASTFLGRLALIRIYNGRIRKGQQVAWIRQVDGQQTVTTAKITELLA
TEGVERKPTDAAVAGDIVAVAGLPEIMIGDTLAASANPVALPRITVDEPAISVTIGTN
TSPLAGKVGGHKLTARMVRSRLDAELVGNVSIRVVDIGAPDAWEVQGRGELALAVLVE
QMRREGFELTVGKPQVVTKTIDGTLHEPFESMTVDCPEEYIGAVTQLMAARKGRMVEM
ANHTTGWVRMDFVVPSRGLIGWRTDFLTETRGSGVGHAVFDGYRPWAGEIRARHTGSL
VSDRAGAITPFALLQLADRGQFFVEPGQQTYEGMVVGINPRPEDLDINVTREKKLTNM
RSSTADVIETLAKPLQLDLERAMELCAPDECVEVTPEIVRIRKVELAAAARARSRART
KARG"
CDS complement(1299581..1299928)
/codon_start=1
/gene="PPL_02504"

/note="gene_1218"
/transl_table=11
/translation="VLRGVSVLWGAGGGLTVHPANTIHASATNAPTVTQTRRTGLGTP
IRLSSTPTSVTRLRKAGLDFAVALRSRGQRAKLLATRLGARPAASPGGGGQLDFADPH
DLRGHLDAFVGRT"
CDS 1299963..1301702
/codon_start=1
/note="gene_1219"
/transl_table=11
/translation="MGIDWIGPGFNPHLLSDLSPVNAAISALVLPSAFRPIPDPNTPT
GSRWEMDPTLLVSADVTNNHPFTVTYKIRPEAQWTDNAPIAADDFWYLWQQMVTQPGV
VDPAGYHLITSVQSLEGGKQAVVTFAQPYPAWRELFTDILPAHIVKDIPGGFASGLAR
ALPVTGGQFRVENIDPQRDEILIARNDRYWGPPSKPGIILFRRAGAPAALADSVRNGD
TQVAQVHGGSAAFAQLSAIPDVRTARIVTPRVMQFTLRANVPKLADTQVRKAILGLLD
VDLLAAVGAGTDNTVTLDQAQIRSPSDPGYVPTAPPAMSSAAALGLLEASGFQVDTNT
SVSPAPSVPDSTTTSVSTGPPEVIRGRISKDGEQLTLVIGVAANDPTSVAVANTAADQ
LRDVGIAATVLALDPVTLYHDALNDNRVDAIVGWRQAGGNLATLLASRYGCPALQATT
VPAANAPTTAPSAPIGPTPSAAPDTATPPPTAPRRPSDPGALVKAPSNLTGICDRSIQ
SNIDAALNGTKNINDVITAVEPRLWNMSTVLPILQDTTIVAAGPSVQNVSLSGAVPVG
IVGDAGQWVKTGQ"
CDS complement(1301730..1302335)
/codon_start=1
/note="gene_1220"
/transl_table=11
/translation="MTVSAPAKANPYRRRGEVLERALYDATLAELESAGYGGLTMEGI
AARAQTGKAALYRRWAGKRELVLAAVQYALPPVPEPRADRSARENLLAVFTANCEILA
GKTALPSMEIVSQLLHEPELRAIFINSVWAPRLRIVESILQAGVRSGEIDPATLTPMT
ARIGPALIHQHVLFTGSPPDREQLTRIIDAMILTTGERRES"
CDS complement(1302407..1302892)
/codon_start=1
/note="gene_1221"
/transl_table=11
/translation="VSETVGSFAAPATKNLPSKLWTLLTKGTYPLTAARISSIPVEYV
LAFVEGSNMGQMMGNLAMRSLTPTLKGPLELLPNAVRPAVSATLGNADTIGGLSVPPS
WVADKSITPLAKAVPTSAPGGPSGTSWAQLGLASLAGGAVGAVAARTRSGVILRSPAA
G"
CDS complement(1302906..1303448)
/codon_start=1
/note="gene_1222"
/transl_table=11
/translation="MDFTIFPPEFNSLNIQGSARPFLVAANAWKNLSNELSYAASRFE
SEINGLITSWRGPSSTIMAAAVAPFRAWIVTTASLAELVADHISVVAGAYEAAHAAHV
PLPVIETNRLTRLALATTNIFGIHTPAIFALDALYAQYWSQDGEAMNLYATMAAAAAR
LTPFSPPGADRQPGRAGQTL"
CDS complement(1303466..1303768)
/codon_start=1
/note="gene_1223"
/transl_table=11
/translation="VSFVTTRPDSIGETAANLHEIGVTMSAHDDGVTPLITNVESPAH
DLVSIVTSMLFSMHGELYKAIARQAHVIHESFVQTLQTSKTSYWLTELANRAGTST"
CDS 1303948..1304859

```
                     /codon_start=1
                     /note="gene_1224"
                     /transl_table=11
                     /translation="MSETPRLLFVHAHPDDESLSNGATIAHYTSRGAQVHVVTCTLGE
                     EGEVIGDRWAQLTADHADQLGGYRIGELTAALRALGVSAPIYLGGAGRWRDSGMAGTD
                     QRSQRRFVDADPRQTVGALVAIIRELRPHVVVTYDPNGGYGHPDHVHTHTVTTAAVAA
                     AGVGSGTADHPGDPWTVPKFYWTVLGLSALISGARALVPDDLRPEWVLPRADEIAFGY
                     SDDGIDAVVEADEQARAAKVAALAAHATQVVVGPTGRAAALSNNLALPILADEHYVLA
                     GGSAGARDERGWETDLLAGLGFTASGT"
CDS                  1305029..1305391
                     /codon_start=1
                     /note="gene_1225"
                     /transl_table=11
                     /translation="LVVLALLTVDGVVSALAGALLMPWYIGSAPFPISALISGLVNAA
                     LVWAAARWTTSSRVAALPLWAWLLTVAAMSFGGPGDDVILGGQGLLVYGALVFVVAGA
                     VPPAWVLWRRRVQADGSG"
CDS                  complement(1305399..1306325)
                     /codon_start=1
                     /note="gene_1226"
                     /transl_table=11
                     /translation="MSFVFAAPEALAAAAADMAGIGSTLNAANVVAAVPTTGVLAAAA
                     DEVSTQVAALLSAHAQGYQQLSRQMMTAFHDQFVQALRASADAYATAEASAAQTMVNA
                     VNAPARALLGHPLISADASTGGGSNALSRVHSMFLGTGGSSALGGSAAANAAASGALQ
                     LQPTGGASGLSAVGALLPRAGAAAAAALPALAAESIGNAIKNLYNAVEPWVQYGFNLT
                     AWAVGWLPYIGILAPQINFFYYLGEPIVQAVLFNAIDFVDGTVTFSQALTNIETATAA
                     SINQFINTEINWIRGFLPPLPPISPPGFPSLP"
CDS                  1306716..1309145
                     /codon_start=1
                     /gene="fbiC"
                     /note="gene_1227"
                     /transl_table=11
                     /translation="MTARGDELADLCASAARVRDAGLVSAGRHGPSGRLAISYSRKVF
                     IPVTRLCRDNCHYCTFVTVPGKLRAQGSSTYMEPDEILDVARRGAEFGCKEALFTLGD
                     RPEARWRQAREWLGERGYDSTLSYVRAMAIRVLEQTGLLPHLNPGVMSWSEMSRLKPV
                     APSMGMMLETTSRRLFETKGLAHYGSPDKDPAVRLRVLTDAGRLSIPFTTGLLVGIGE
                     TLSERADTLHAIRKSHKEFGHIQEVIVQNFRAKEHTAMAAFPDAGIEDYLATVAVARL
                     VLGPGMRIQAPPNLVSGDECRALVGAGVDDWGGVSPLTPDHVNPERPWPALDELAAVT
                     AEAGYDMVQRLTAQPKYVQAGAAWIDPRVRGHVVALADPATGLARDVNPVGMPWQEPD
                     DVASWGRVDLGAAIDTQGRNTAVRSDLASAFGDWESIREQVHELAVRAPERIDTDVLA
                     ALRSAERAPAGCTDGEYLALATADGPALEAVAALADSLRRDVVGDEVTFVVNRNINFT
                     NICYTGCRFCAFAQRKGDADAYSLSVGEVADRAWEAHVAGATEVCMQGGIDPELPVTG
                     YADLVRAVKARVPSMHVHAFSPMEIANGVTKSGLSIREWLIGLREAGLDTIPGTAAEI
                     LDDEVRWVLTKGKLPTSLWIEIVTTAHEVGLRSSSTMMYGHVDSPRHWVAHLNVLRDI
                     QDRTGGFTEFVPLPFVHQNSPLYLAGAARPGPSHRDNRAVHALARIMLHGRISHIQTS
                     WVKLGVRRTQVMLEGGANDLGGTLMEETISRMAGSEHGSAKTVAELVAIAEGIGRPAR
                     QRTTTYALLAA"
CDS                  complement(1309251..1309535)
                     /codon_start=1
                     /note="gene_1228"
                     /transl_table=11
                     /translation="MSLTVGAGVASADPVDAVINTTCNYGQVVAALNATDPGAAAQFN
```

ASPVAQSYLRNFLAAPPPQRAAMAAQLQAVPGAAQYIGLVESVAGSCNNY"
CDS complement(1309784..1311808)
/codon_start=1
/note="gene_1229"
/transl_table=11
/translation="MTNPYPNLLSPLDLGFTTLRNRVVMGSMHTGLEDRARHIDRLAD
YFAERARGGVGLIITGGYAPNRTGWLLPFASELVTSAQARRHRRINRAVHDSGAKILL
QILHAGRYAYHPLAVSASPIKAPITPFRPRALSARGVEATIADFARCAQLARDAGYDG
VEIMGSEGYLLNQFLAPRTNKRTDSWGGTPANRRRFPVEIIRRSRAAVGSDFIICYRL
SMADYVAEGQSWDEIVALATEVEGAGATIINSGFGWHEARVPTIVTSVPGGAFVDISS
AVAEHVTIPVVASNRINMPQAAERILAETQVRLISMARPMLSDPDWVLKAQSNRVDEI
NTCISCNQACLDHAFARKTVSCLLNPRAGRETQLVLSPTRRARSVAVVGAGPAGLATA
ANAAQRGHRVTLFEANDFIGGQFDMARRIPGKEEFSETIRYFSTILAKHGVEVRLGTR
VAAQELTGYDEVVLATGVAPRIPAIPGIDHPMVLTYAEAITGVRPVGRTVAVVGAGGI
GFDVTELLVTDSSPTLNLKEWKAEWGVADPREARGALTTPLPAPPAREVYLLQRTKGP
QGKRLGKTTGWVHRASLKAKGVHQLSGVNYEQINDDGLHISFGPKRRRPQLLAVDNVV
VCAGQEPVRDLESELRRHGINPHISGGAAVAAELDAKRAIKQGTELAARL"
CDS complement(1311805..1312374)
/codon_start=1
/note="gene_1230"
/transl_table=11
/translation="MALPHAILVSLCEQASSGYELARRFDRSIGYFWTATHQQIYRTL
RVMENNNWVRATTVLQHGRPDKKVYAISDSGRAELARWIAEPLSPTRPGRGSALTDSS
TRDIAVKLRGAGYGDVAALYTQVTALRAERVKSLDTYRGIEKRTFADPSALDGAALHQ
YLVLRGGIRAEESAIDWLDEVAEALQEKR"
CDS 1312587..1312913
/codon_start=1
/note="gene_1231"
/transl_table=11
/translation="VTYTIAEPCVDIKDKACIEECPVDCIYEGARMLYIHPDECVDCG
ACEPVCPVEAIFYEDDVPEQWSHYTQINADFFAELGSPGGAAKVGMTENDPQAVKDLA
PQSEDA"
CDS 1312946..1314034
/codon_start=1
/note="gene_1232"
/transl_table=11
/translation="VSASLPVFPWDTLADAKALAGAHPDGIVDLSVGTPVDPVAPLIQ
EALAAASAAPGYPATAGTARLRESVVAALARRYGITRLTEAAVLPVIGTKELIAWLPT
LLGLGGADLVVVPELAYPTYDVGARLAGTRVLRADALTQLGPQSPALLYLNSPSNPTG
RVLGVDHLRKVVEWARGRGVLVVSDECYLGLGWDAEPVSVLHPSVCDGDHTGLLAVHS
LSKSSSLAGYRAGFVVGDLEIVAELLAVRKHAGMMVPAPVQAAMVAALDDDAHERQQR
ERYAQRRAALLPALGSAGFAVDYSDAGLYLWATRGEPCRDSVAWLAQRGILVAPGDFY
GPGGAQHVRVALTATDERVAAAVGRLTC"
CDS complement(1314062..1316881)
/codon_start=1
/note="gene_1233"
/transl_table=11
/translation="VDPHRDLESRAFAGNWRVYQQQALDAFDADVAAGDNRAYLVLPP
GAGKTMIGLEAARRLGRRSLVLVPNTAVQAQWAAAWDNSFPSSDRSASKCGTERGLAS
AMNVLTYQSLAVIDAETDSTVRREVLRNRDQQALLDLLHPNGRAVIERAATLGPWTLV
LDECHHLLATWGALVSALASVLGAQTALIGLTATPATELTAWQHTLHDELFGTADFVI

PTPALVREGDLAPYQELVYLTQPTPEEQAWIGTHRARFADLMLALIDQKVGSMSLAAW
LHTRIVDRATREGNQIAWSTFERAEPDLACSGLRFAYDGLIPLPDGVRLREQHRIAPD
AQDWVNVLTDFSVGHLQQSADPRDAHALTAIKRVLPGLGYRLTSRGVRVATSPVDRLC
ALSESKIAATAHILDTEDAVLGARLRALVLCDFESMTGALPTSLKGAPVSEQSGSAQL
VAAMLAASDHRRRTPLHALLVTGQTFACPAAIEDDLIAFCAERGALVTAEPLDAHPSL
RVMRGTGGFTPRTWVALATEYFLAGRARVLVGTRSLLGEGWDCAAVNVNIDLTSATTQ
AAITQMRGRAIRNDPSDGHKVADNWSVCCIATEHPRGDADYLRLVRKHDGYYAATPQG
LIESGVTHCDPSLSPYGPPVTDTHAITARALQRVAERAQARSWWRIGEPYEGVDVATI
RVRSRQPLGVAAPRIPASALTPPVPGQFSPVRLARGAVAAVSVVGASTATAVASANLG
MLAGAGTAGAIVAAGVGLVATAAAAESRRLDHAPNALEQLAAVVADALYAAGGAQRGS
AALRLASDPEGWIRCQLDGVPTEQSLRFTAALDELLAPLAEPRYLIGRKILTPPARPV
ARRLFAVRAVVGLSLPGTVAWHAVPRWFARNKDRRQHLAQAWRKHIGPPRQLPADSPQ
GQAILDLFRGDNPLSVTTQLRTTWR"

CDS 1317260..1323565
/codon_start=1
/gene="mas-1"
/note="gene_1234"
/transl_table=11
/translation="MGFGSIHPRLVQGDCVVRTATATSVAVIGMACRLPGGIDSPQRL
WEALLRGDDLVGEIPADRWDANVYYDPEPGVPGRSVSRWGAFLDDVGGFDCDFFGLTE
REATAIDPQHRLLLEVSWEAIEHAGVDPATLAESQTGVFVGLTHGDYELLSADCGAAE
GPYGFTGTSNSFASGRVAYTLGLHGPAVTVDTACSSGLTAVHQACRSLDDGESDLALA
GGVVVTLEPRKSVSGSLQGMLSPTGRCHAFDEAADGFVSGEGCVVLLLKRLPDAVRDG
DRVLAIVRGTAANQDGRTVNIAAPSAQAQIAVYQQALAAAGVEASTVGMVEAHGTGTP
VGDPVEYASLAAVYGTEGPCALTSVKTNFGHLQSASGPLGLMKTILALRHGVVPQNLH
FCRLPDQLAEIDTELFVPQANTSWPDNTGQPRRAAVSSYGMSGTNVHAILEQAPVSEP
AASGPELTPEAGGLALFPVSATSAEQLHVTAARLADWVDQNGNAGSRVSMRDLGYTLS
CRRAHRPVRTVVTASSFDELSAALRDVAGDQIPYQPAVGHDDRGPVWVFSGQGSQWPG
MGTELLVAEPVFAATVAAMEPVIARESGFSVTEAMSAPQTVSGIDRVQPTIFAVQVAL
AAALKSYGVRPGAIIGHSLGEAAAAVVAGALSLHDGLRVICRRSRLMSRIAGSGAMAS
VELPGQQVLSELAIRGISDVVLSVVASPTSTVVGGATQSIRDLVAAWEQQDVLAREVA
VDVASHTPQVDPILDELLEVLAEVDPTAPEIPYYSATLWDPRERPSFTGEYWVENLRY
TVRFAAAVQAALKDGYRVFGELAPHPLLTYAVEQNAASLDMPIATLAAMRRGEQLPFG
LRGFVADVHNAGAKVDFSVQYPDGRLVDAPLPSWTHRTLMLSREDSHRSHTGAVQAVH
PLLGAHVHLLEEPERHVWQAGVGTGAHPWLGDHRIHNVAAFPGAAYCEMALAAARTTL
GELSEVRDIKFEQTLLLDEQTVVSSAATIAAPGILQFAVESHQEGEPARRASAMLHAL
EEMPQPPGYDTNALTAAHESSMSGEELRKMFNSLGIQYGPAFSGLVAVHTARGAVTTV
LAEVALPGAIRSQQSAYASHPALLDACFQSVLVHPEVQKATVGGLMLPVGVRRLRNYH
STRSAHYCLARVTSSSRAGECEADLDVFDQAGTVLLTVEGLRLAAGISEHERANRVFD
ERLLTIEWERGELPEVPQIDAGSWLLLSASEADPLTAQLADALNAVGAQSTSVASASD
VAQLRSLLGGRLTGVVVVTGPPTGGLTQCGRDYVSQLVGIARELAELPGEPPRLFVVT
RSAASVLPSDLANLEQAGLRGLMRVIDSEHPHLGATAIDVDNDETVAALVASQLQSGS
QEDETAWRNGIWYTARLRPGPLRPAERRTAVVEYRRDGMRLQIRTPGDLESLEFVTFD
RVPPGPGEIEVAVTASSVNFADVLVAFGRYPTFEGYRQQLGIDFAGVVTAVGPDVTEH
RIGDHVGGMSANGCWSTFVRCDARLAVTLPPELPVAAAAAVPTASATAWYALHDLARI
CSDDKVLIHSGTGGVGQAAIAIARAAGCEIFATAGSAQRRQLLHDMGVEHVYDSRSTE
FAEQIRGDTDGYGVDVVLNSLPGAAQRAGIELLAFGGRFVEIGKRDIYGDTRLGLFPF
RRNLSLYAVDLALLTHSHPHTVRRLLKTVYQHTVEGTLPVPQTTHYPIHDAAVAIRLV
GGAGHTGKVVLDVPRTGEGVAVVPPEQVRTSRPDGAYLVTGGLGGLGLFLAGELAAAG
CGRIVLNSRSTPSPHATRVIERLRAAGADIQVECGDIADAATAHRVVAVATASGLPVR
GVLHAAAVVEDATLANVTDELIDRCWAPKVHGAWNIHRATAAQPLEWFCLFSSAAALV

GSPGQGAYAAANSWLDAFAHWRRAQGLPATSIAWGAWAEIGRATALAEGTGAAIAPAE
GARAFQTLLRYGRAYSGYAPIMGTPWLTAFAQRSRFAEAFHATGQNQPATGKFLAELG
SLPREEWPRTVRRLVSDQISLLLRRTIDPDRPLSDYGLDSLGNLELRTRIETETGIRV
SPTKITTVRGLAEHVCDELAAAQSAPV"

CDS 1323618..1325036
/codon_start=1
/note="gene_1235"
/transl_table=11
/translation="MLRVGPLTIGTLDDWAPSTGSTVSWRPSAVAHTKASQAPISDVP
VSYMQAQHIRGYCEQKAKGLDYSRLMVVSCQQPGQCDIRAANYVINAHLRRHDTYRSW
FQYNGNGQIIRRTIQDPADIEFVPVHHGELTLPQIREIVQNTPDPLQWGCFRFGIVQG
CDHFTFFASVDHVHVDAMIVGVTLMEFHLMYAALVGGHAPLELPPAGSYDDFCRRQHT
FSSTLTVESPQVRAWTKFAEGTNGSFPDFPLPLGDPSKPSDADIVTVMMLDEEQTAQF
ESVCTAAGARFIGGVLACCGLAEHELTGTTTYYGLTPRDTRRTPADAMTQGWFTGLIP
ITVPIAGSAFGDAARAAQTSFDSGVKLAEVPYDRVVELSSTLTMPRPNFPVVNFLDAG
AAPLSVLLTAELTGTNIGVYSDGRYSYQLSIYVIRVEQGTAVAVMFPDNPIARESVAR
YLATLKSVFQRVAESGQQQNVA"

CDS 1325103..1328111
/codon_start=1
/note="gene_1236"
/transl_table=11
/translation="VVGCWVALALVLPMAVPSLAEMAQRHPVAVLPADAPSSVAVRQM
AEAFHESGSENILVVLLTDEKGLGAADENVYHTLVDRLRNDAKDVVMLQDFLTTPPLR
EVLGSKDGKAWILPIGLAGDLGTPKSYHAYTDVERIVKRTVAGTTLTANVTGPAATVA
DLTDAGARDRASIELAIAVMLLVILMVIYRNPVTMLLPLVTIGASLMTAQALVAGVSL
VGGLAVSNQAIVLLSAMIAGAGTDYAVFLISRYHEYVRLGEHPERAVQRAMMSVGKVI
AASAATVGITFLGMRFAKLGVFSTVGPALAIGIAVSFLAAVTLLPAILVLASPRGWVA
PRGERMATFWRRAGTRIVRRPKAYLGASLIGLVALASCASLAHFNYDDRKQLPPSDPS
SVGYAAMEHHFSVNQTIPEYLIIHSAHDLRTPRGLADLEQLAQRVSQIPGVAMVRGVT
RPNGETLEQARATYQAGQVGNRLGGASRMIDERTGDLNRLASGANLLADNLGDVRGQV
SRAVAGVRSLVDALAYIQNQFGGNKTFNEIDNAARLVSNIHALGDALQVNFDGIANSF
DWLDSVVAALDTSPVCDSNPMCGNARVQFHKLQTARDNGTLDKVVGLARQLQSTRSPQ
TVSAVVNDLGRSLNSVVRSLKSLGLDNPDAARARLISMQNGANDLASAGRQVADGVQM
LVDQTKNMGIGLNQASAFLMAMGNDASQPSMAGFNVPPQVLKSEEFKKVAQAFISPDG
HTVRYFIQTDLNPFSTAAMDQVNTIIDTAKGAQPNTSLADASISMSGYPVMLRDIRDY
YERDMRLIVAVTVVVVILILMALLRAIVAPLYLVGSVVISYMSAIGLGVVVFQVFLGQ
ELHWSVPGLAFVVLVAVGADYNMLLASRLRDESALGVRSSVIRTVRCTGGVITAAGLI
FAASMSGLLFSSIGTVVQGGFIIGVGILIDTFVVRTITVPAMATLLGRASWWPGHPWQ
RCAPEEGQMSARMSARTKTVFQAVADGSKR"

CDS complement(1328115..1329230)
/codon_start=1
/note="gene_1237"
/transl_table=11
/translation="MLSRTRFSMQRQMKRVIAGAFAVWLVGWAGGFGTAIAASEPAYP
WAPGPPPSPSPVGDASTAKVVYALGGARMPGIPWYEYTNQAGSQYFPNAKHDLIDYPA
GAAFSWWPTMLLPPGSHQDNMTVGVAVKDGTNSLDNAIHHGTDPAAAVGLSQGSLVLD
QEQARLANDPTAPAPDKLQFTTFGDPTGRHAFGASFLARIFPPGSHIPIPFIEYTMPQ
QVDSQYDTNHVVTAYDGFSDFPDRPDNLLAVANAAIGAAIAHTPIGFTGPGDVPPQNI
RTTVNSRGATTTTYLVPVNHLPLTLPLRYLGMSDAEVDQIDSVLQPQIDAAYARNDNW
FTRPVSVDPVRGLDPLTAPGSIVEGARGLLGSPAFGG"

CDS complement(1329359..1331095)

/codon_start=1
/note="gene_1238"
/transl_table=11
/translation="MSDSSVLSLLRERAGLQPDDAAFTYIDYEQDWAGITETLTWSEV
FRRTRIVAHEVRRHCTTGDRAVILAPQGLAYIAAFLGSMQAGAIAVPLSVPQIGSHDE
RVSAVLADASPSVILTTSAVAEAVAEHIHRPNTNNVGPIIEIDSLDLTGNSPSFRVKD
LPSAAYLQYTSGSTRAPAGVMISHRNLQANFQQLMSNYFGDRNGVAPPDTTIVSWLPF
YHDMGLVLGIIAPILGGYRSELTSPLAFLQRPARWLHSLANGSPSWSAAPNFAFELAV
RKTTDADIEGLDLGNVLGITSGAERVHPNTLSRFCNRFAPYNFREDMIRPSYGLAEAT
LYVASRNSGDKPEVVYFEPDKLSTGSANRCEPKTGTPLLSYGMPTSPTVRIVDPDTCI
ECPAGTIGEIWVKGDNVAEGYWNKPDETRHTFGAMLVHPSAGTPDGSWLRTGDLGFLS
EDEMFIVGRMKDMLIVYGRNHYPEDIESTVQEITGGRVAAISVPVDHTEKLVTVIELK
LLGDSAGEAMDELDVIKNNVTAAISRSHGLNVADLVLVPPGSIPTTTSGKIRRAACVE
QYRLQQFTRLDG"
CDS complement(1331272..1332888)
/codon_start=1
/note="gene_1239"
/transl_table=11
/translation="MRIAGVGLGQLLLALDATVVSLVDAPRGLDLPVASTALIDSDDV
RLGLAAAAGSADVFFLIGVTDDEAVRWVDDQARQRAPVAIFVKHPSDSVVAGAVRAGS
AVVAVEPRARWERLYHLVNHVLEHHGDRADPTDDSGTDLFGLAQSLADRIHGMISIED
AQSHVLAYSASNDEADELRRLSILGRAGPPEHLQWIGQWGIFDALRAGREVVRVAERP
ELGLRPRLAIGIHQPGVGALRPPVFAGTIWVQQGSQPLADDAEEMLRGAAVLAARIMS
RLATQPNTHALRVQQLLGLAELNATTAPVDVSTIARELGVAAEGNATLIGFDTAENRD
TAVRHVRLVDVMALSASAFRHDAQVAANGSRIYVLLPQTTTGRAVTSWVRGTISALRA
ELGVALRAAIAGPVAGLAEVNPARVEVDRVLESAERHPILGQVTSLAEARTTVLLDEI
VTLVGTDQRLVDPRIRDLGAQDPVLAQTLRAYLDAFGDIGAAARSLQVHPNTVRYRIR
RIEQLLSTSLGDPDVRLLFSLGLRAMERTA"
CDS 1332961..1334604
/codon_start=1
/note="gene_1240"
/transl_table=11
/translation="MLAGMDAITQVPVPANEPVHDYAPKSPERTRLRTELASLADHPI
DLPHVIGGRHRMGDGERIDVVQPHRHAARLGTLTNATHADAAAAVEAAMSAKSDWAAL
PFDERAAVFLRAADLLAGPWREKIAAATMLGQSKSVYQAEIDAVCELIDFWRFNVAFA
RQILEQQPISGPGEWNRIDYRPLDGFVYAITPFNFTSIAGNLPTAPALMGNTVIWKPS
ITQTLAAYLTMQLLEAAGLPPGVINLVTGDGFAVSDVALADPRLAGIHFTGSTATFGH
LWQWVGTNIGRYHSYPRLVGETGGKDFVVAHASARPDVLRTALIRGAFDYQGQKCSAV
SRAFIAHSVWQRMGDELLAKAAELRYGDITDLSNYGGALIDQRAFVKNVDAIERAKGA
AAVTVAVGGEYDDSEGYFVRPTVLLSDDPTDESFVIEYFGPLLSVHVYPDERYEQILD
VIDTGSRYALTGAVIADDRQAVLTALDRLRFAAGNFYVNDKPTGAVVGRQPFGGARGS
GTNDKAGSPLNLLRWTSARSIKETFVAATDHIYPHMAVD"
CDS 1334604..1335593
/codon_start=1
/note="gene_1241"
/transl_table=11
/translation="MAGWFAHTLRPAMLAAGRSDRLGRIVERSPLTRGVVRRFVPGDT
LDDVVDIVTALRDSGRYLSIDYLGENVTDADDAAAAVRAYLGLLDVLGRRGDIACDGV
RPLEVSLKLSALGQALDRDGQKIALDNARAICERAERVGAWVTVDAEDHTTTDSTLSI
SGDLRVDFPWLGTVVQAYLRRTLADCAELAAVGARVRLCKGAYDEPASVAYRDAAQVT
DSYLRCLRVLTAGRGYPMVATHDPVIIAAVPGITRESGRSQGDFEYQMLYGVRDDEQR

RLTGAGNHVRVYVPFGTRWYGYFLRRLAERPANLAFFLRALTDRRRARGCAER"
CDS 1335675..1336547
/codon_start=1
/gene="sigI"
/note="gene_1242"
/transl_table=11
/translation="MSQHDPVSAAWRAHRAYLVDLAFRMVGDIGVAEDMVQEAFSRLL
RAPVGDIDDERGWLIVVTSRLCLDHIKSASTRRERPQDIAAWHDGDASVSSVDPADRV
TLDDEVRLALLIMLERLGPAERVVFVLHEIFGLPYQQIATTIGSQASTCRQLAHRARR
KINESRIAASVEPAQHRVVTRAFIEACSNGDLDTLLEVLDPGVAGEIDARKGVVVVGA
DRVGPTILRHWSHPATVLVAQPVCGQPAVLAFVNRALAGVLALSIEAGKITKIHVLVQ
PSTLDPLRAELGGG"
CDS 1336563..1337441
/codon_start=1
/note="gene_1243"
/transl_table=11
/translation="MTMKSLAALDRPSWLSSSAWPWQPYLLSHHQGGIAVTDIGDGPA
VLFVHVGSWSFVWRDVLLRLANDFRCVAIDAPGCGLSDRLSTPPTLAQAADAITSVID
ALQLRDLTLVAHDLGGPAGFLAAARRGDRVAALAAVNCFAWRPTGPLFRGMLAAMGSA
PVRELDAAINALARATSTRFGAGRHWSRADRAAFRAGIDAPARRAWHAYFRDARRAHA
LYTDVDAALRGGLADRPLLTIFGQFNDPLRFQPRWKELFPTARQLQVRRGNHFPMCDD
PDLVAGALTSFVQRST"
CDS 1337472..1338428
/codon_start=1
/note="gene_1244"
/transl_table=11
/translation="LRFAAAPPHAKLLAMAVAIARPKLEGNIAVGEDRRIGFAEFGAP
QGRAVFWLHGTPGARRQIPTEARVYAEHHNIRLIGVDRPGIGASTPHQYETILAFADD
LRTIADTLGIDKMAVVGLSGGGPYTLACAAGLPDRVVAAGVLGGVAPTRGPDAISGGL
MRLGSAVAPLLQVGGTPLRLGASLLIRAARPVASPALDLYGLLSPRADRHLLARPEFK
AMFLDDLLNGSRKQLAAPFADVIAFARDWGFRLDEVKVPVRWWHGDHDHIVPFSHGEH
VVSRLPDAKLLHLPGESHLAGLGRGEEILSTLMQIWDRDLRK"
CDS 1338510..1339337
/codon_start=1
/note="gene_1245"
/transl_table=11
/translation="MLLPVLEPADRPCDAPGWFLYLTDIPRAGVEYGQLLAVLPLQRM
LPAGDGHPVLVLPGLLAGDGSTWILRRILRRLGYAAYGWGLGRNIGPTAKAVSGMRDL
LDKLHSRYHTPVSLIGWSLGGIFARGLARDHPSAVRQVITLGSPFGMRDTCETRSAWS
FNRYAHLHTERHELPLEMESEPLPVPTTAIYSRCDGMVAWQTCMNSPSERAENIAVRS
SHIGYGHNPPVVWAIADRLAQPQGAWAPFRPPKVLSPLFPRPDTPAEAVSTPQTRPA"
CDS 1339377..1340798
/codon_start=1
/note="gene_1246"
/transl_table=11
/translation="VLLASLNPAVVSAADIADAVRIDGDVLSRSDLVGAATSVAERVA
GAHRVAVLATPTASTVLAITGCLIAGVPVVPVPADVGVTERRHMLTDSGVQAWLGPLP
DDPAGLPHIPVRTHARSWHRYPEPSPGAIAMVVYTSGTTGPPKGVQLSRRAIAADLDA
LAEAWQWTAEDVLVHGLPLYHVHGLVLGLLGSLRFGNRFVHTGKPTPAGYAQACYEAH
GTLFFGVPTVWSRVAADQAAAGALKPARLLVSGSAALPVPVFDKLVQLTGHRPVERYG
ASESLITLSTRADGERRPGWVGLPLAGVQTRLVDDDGGEVPHDGETVGKLQVRGPTLF

DGYLNQPDATAAAFDADSWYRTGDVAVVDGSGMHRIVGRESVDLIKSGGYRVGAGEIE
TVLLGHPDVAEAAVVGVPDDDLGQRIVAYVVGSANVDADGLINFVAQQLSVHKRPREV
RIVDALPRNALGKVLKKQLLSEG"
CDS complement(1340831..1342117)
/codon_start=1
/note="gene_1247"
/transl_table=11
/translation="VQIAPARVAWQQPSPRIRELIREGARIALNPSPEWIEELDRATI
AANPAIANDPVLAKVVQTANRANLVYWAAANLRDPGARVPANLGTEPLRMARDLVRRG
LDTVAFNIYRTGEHIGWRFWMGIAFELTSDPQELRELLDVSARSVNDFIEATLTGIAA
QVQSEHDELTRSTHAERLEVVGLILDGAPISPERAEAKLGYPLSRAHTAAIIWSDELD
GDHSYLDRAADLFCHAVGSTRPLTVVAGAASRWAWVTDADGLDIDTVQAAVDNAPGAR
IAIGTTANGVEGFRRSHLEALITQRTLSRLRSTQRVAFFADVKMVALISQNPDAASEF
ITSTLGDLESASPDLQTALLTFINEQCNASRAAKRLHTHRNTFLRRLESAQRLLPRPL
DHTSVHVAVALEALQWRGNKAHALSSPGRRSNSVPA"
CDS 1342566..1342871
/codon_start=1
/note="gene_1248"
/transl_table=11
/translation="VHVSFVMAYPEMLAAAADTLQSIGATTVASNAAAAAPTTGVVPP
AADEVSALTAAHFAAHAAMYQSVSARAAAIHDQFVATLASSASSYAATEVANAAAAS"
CDS 1342918..1344090
/codon_start=1
/note="gene_1249"
/transl_table=11
/translation="MVDFGALPPEINSARMYAGPGSASLVAAAKMWDSVASDLFSAAS
AFQSVVWGLTVGSWIGSSAGLMVAAASPYVAWMSVTAGQAELTAAQVRVAAAAYETAY
GLTVPPPVIAENRAELMILTATNLLGQNTPAIAVNEAEYGEMWAQDAAAMFGYAAATA
TATATLLPFEEAPEMTSAGGLLEQAVAVEEASDTAAANQLMNNVPQALQQLAQPTKSI
WPFDQLSELWKAISPHLSPLSNIVSMLNNHVSMTNSGVSMASTLSSMLKGFAPAAAQA
VETAAQNGVRAMSSLGSSLGSSGLGGGVAANLGRAASVGSLSVPPAWAAANQAVTPAA
RALPLTSLTSAAERGPGQMLGGLPVGQMGARAGGGLSGVLRVPPRPYVMPHSPAAG"
CDS 1344225..1344521
/codon_start=1
/note="gene_1250"
/transl_table=11
/translation="MASRFMTDPHAMRDMAGRFEVHAQTVEDEARRMWASAQNISGAG
WSGMAEATSLDTMAQMNQAFRNIVNMLHGVRDGLVRDANNYEQQEQASQQILSS"
CDS 1344572..1344856
/codon_start=1
/note="gene_1251"
/transl_table=11
/translation="MTINYQFGDVDDHGAMIRAQAGLLEAEHQAIIRDVLTASDFWGG
AGSAACQGFITQLGRNFQVIYEQANAHGQKVQAAGNNMAQTDSAVGSSWA"
CDS complement(1344924..1346171)
/codon_start=1
/note="gene_1252"
/transl_table=11
/translation="MTSSHLIDTEQLLADQLAQASPDLLRGLLSTFIAALMGAEADAL
CGAGYRERSDERSNQRNGYRHRDFDTRAATIDVAIPKLRQGSYFPGWLLQRRKRAERA
LTSVVATCYLLGVSTRRMERLVETLGVTKLSKSQVSIMAKELDEAVEAFRTRPLDAGP

YTFLAADALVLKVREAGRVVGVHTLIATGVNAEGYREILGIQVTSAEDGAGWLAFFRD
LVARGLSGVALVTSDAHAGLVAAIGATLPAAAWQRCRTHYAANLMAATPKPSWPWVRT
LLHSIYDQPDAESVVAQYDRVLDALTDKLPAVAEHLDTARTDLLAFTAFPKQIWRQIW
SNNPQERLNREVRRRTDVVGIFPDRASIIRLVGAVLAEQHDEWIEGRRYLGLEVLTRA
RAALTSTEEPAKQQTTNTPALTT"

CDS 1346508..1347785
/codon_start=1
/note="gene_1253"
/transl_table=11
/translation="MKRVALACLVGSAIEFYDFLIYGTAAALVFPTVFFPHLDPTVAA
VASMGTFAVAFLSRPFGAAVFGYFGDRLGRKKTLVATLLIMGLATVTVGLVPTTVAIG
AAAPLILTTMRLLQGFAVGGEWAGSALLSAEYAPASKRGWYGMFTVVGGGIALVLTSL
TFLGVNYTIGESSPTFMQWGWRIPFLVSAALIAVALYVRFNIDETPVFARERADEKTR
LGPAETPIAQVLRRQRREIVLAAGSAVCCFGFVYLASTYLASYAQTRLGYSRGSILFD
SVLGGLLCIVFTALSSALCDQLGRRRVLLAGWAVALPWSLLVMPLIDSGSPSLFAVAV
VGMYAIGGFGFGPTASFIPELFATSYRYTGSALAANLAGVAGGALPPVIAGALVATYG
SWAIGVMLAILALISLVCTYRLPETAGSALVSR"

CDS complement(1347782..1348726)
/codon_start=1
/note="gene_1254"
/transl_table=11
/translation="VTGAAGIGLATLAADGSVLDTWFPAPELTESGTSATSRLAVSDV
PVELAALIGRDDDRRTETIAVRTVIGSLDDVAADPYDAYLRLHLLSHRLVAPHGLNAG
GLFGVLTNVVWTNHGPCAIDGFEAVRARLRRRGPVTVYGVDKFPRMVDYVVPTGVRIA
DADRVRLGAHLAPGTTVMHEGFVNYNAGTLGASMVEGRISAGVVVGDGSDVGGGASIM
GTLSGGGTHVISIGKRCLLGANSGLGISLGDDCVVEAGLYVTAGTRVTMPDSNSVKAR
ELSGSSNLLFRRNSVSGAVEVLARDGQGIALNEDLHAN"

CDS 1348826..1349890
/codon_start=1
/gene="dapE"
/note="gene_1255"
/transl_table=11
/translation="VLDLRGDPIELTAALIDIPSESRKEARIADEVEAALRAQASGFE
IIRNGNAVLARTKLNRSSRVLLAGHLDTVPVAGNLPSRRENDQLHGCGAADMKSGDAV
FLHLAATLAEPTHDLTLVFYDCEEIDSAANGLGRIQRELPDWLSADVAILGEPTAGCI
EAGCQGTLRVVLSVTGTRAHSARSWLGDNAIHKLGAVLDRLAVYRARSVDIDGCTYRE
GLSAVRVAGGVAGNVIPDAASVTINYRFAPDRSVAAALQHVHDVFDGLDVQIEQTDAA
AGALPGLSEPAAKALVEAAGGQVRAKYGWTDVSRFAALGIPAVNYGPGDPNLAHCRDE
RVPVGNITAAVDLLRRYLGG"

CDS complement(1349887..1350471)
/codon_start=1
/note="gene_1256"
/transl_table=11
/translation="VLLAYVLITKGEFGAAASMLEPAAATLERTGYSWGPLSLMLLAT
AIAQQGHIAESAKTLQRAEARHGTKSALFAPELGLARAWTRAAAQDMTGAIAAAREAA
RTAERAGQAAVALCAWHNAVRLGDIRAVDPVTRLAAEIDCTVGNILVKHARGLADGDA
AELTAVAEELAGIGMAAAAADATKAAARLGPQQR"

CDS complement(1350502..1352190)
/codon_start=1
/note="gene_1257"
/transl_table=11

```
                     /translation="MRVWKHVEAAVDSPDRCGVVLVGPHGVGKTLLAQLAAEQVMSED
                     GRSGRARWVVGTAPGRAIPFGAFRHLISLPASGADIGRPAALLRAARSSLTGDAGDLL
                     LVVDDAHNLDPLSATLVYQLARAGAARLVVTVASEAEPPDAIAALWSDDLLTRVAIEP
                     LDRAQTAAFVESALDATLDVADADELFRRSLGNPLYLRHLIDGGGLEHVDGRWRCRDE
                     DRRPLSGVIDEYLCALPEPARAVVDYLAIAEPLARTDLVALVGGEQLDTLGQAEAAGA
                     VRVGPDSDTSEIFVGHPLYADRARAVLTAEHAHALRVSLVAQLAKHPSDHVSDQLRLS
                     SLAIDVPASATPAAVTDAATAAGQALRLGDVRLAERLARAALDRSDALAARLPLAYAL
                     GWQGRGREADAVLAAVNPAELTETELMAWAIPRAANRFWMLNEPERATAFLQTTRSRV
                     TEPTARSTLDALAATFAMNSGNLPRAITLATEVLSGPAADDMAVAWAASAAALSSARM
                     GRFGDVDRLAERASAAEHPGLLRFTVGLAQITSLLLAGDVAPAQELAKRFTDFA"
     CDS             1352285..1352848
                     /codon_start=1
                     /note="gene_1258"
                     /transl_table=11
                     /translation="MSAKIDITGDWTVAVYCAASPTHAELLELAAEVGAAIAGRGWTL
                     VWGGGHVSAMGAVASAARACGGWTVGVIPKMLVYRELADHDADELIVTDTMWERKQIM
                     EDRSDAFIVLPGGVGTLDELFDAWTDGYLGTHDKPIVMVDPWGHFDGLRAWLNGLLDT
                     GYVSPTAMERLVVVDNVKDALRACAPS"
     CDS             1352898..1354691
                     /codon_start=1
                     /gene="fadD6"
                     /note="gene_1259"
                     /transl_table=11
                     /translation="VSDYYGGAHTTVRLIDLATRMPRVLADTPVIVRGAMTGLLARPN
                     SKASIGTVFQDRAARYGDRVFLKFGDQQLTYRDANATANRYAAVLAARGVGPGDVVGI
                     MLRNSPSTVLAMLATVKCGAIAGMLNYHQRGEVLAHSLGLLDAKVLIAESDLVSAVAE
                     CGASRGRVAGDVLTVEDVERFATTAPATNPASASAVQAKDTAFYIFTSGTTGFPKASV
                     MTHHRWLRALAVFGGMGLRLKGSDTLYSCLPLYHNNALTVAVSSVINSGATLALGKSF
                     SASRFWDEVIANRATAFVYIGEICRYLLNQPAKPTDRAHQVRVICGNGLRPEIWDEFT
                     TRFGVARVCEFYAASEGNSAFINIFNVPRTAGVSPMPLAFVEYDLDTGDPLRDASGRV
                     RRVPDGEPGLLLSRVNRLQPFDGYTDPVASEKKLVRNAFRDGDCWFNTGDVMSPQGMG
                     HAAFVDRLGDTFRWKGENVATTQVEAALASDQTVEECTVYGVQIPRTGGRAGMAAITL
                     RAGAEFDGQALARTVYGHLPGYALPLFVRVVGSLAHTTTFKSRKVELRNQAYGADIED
                     PLYVLAGPDEGYVPYYAEYPEEVSLGRRPQG"
     CDS             1354889..1355713
                     /codon_start=1
                     /note="gene_1260"
                     /transl_table=11
                     /translation="MAIVNRTPDSFYDKGATFSDAAARDAVHRAVADGADVIDVGGVK
                     AGPGERVDVDTEITRLVPFIEWLRGAYPDQLISVDTWRAQVAKAACAAGADLINDTWG
                     GVDPAMPEVAAEFGAGLVCAHTGGALPRTRPFRVSYGTTTRGVVDAVISQVTAAAERA
                     VAAGVAREKVLIDPAHDFGKNTFHGLLLLRHVADLVMTGWPVLMALSNKDVVGETLGV
                     DLTERLEGTLAATALAAAAGARMFRVHEVAATRRVLEMVASIQGVRPPTRTVRGLA"
     CDS             1355710..1356684
                     /codon_start=1
                     /note="gene_1261"
                     /transl_table=11
                     /translation="MTASELVAGDLAGGRAPGALPLDTTWHRPGWTIGELEAAKAGRT
                     ISVVLPALNEEATIESVIDSISPLVDGLVDELIVLDSGSTDDTEIRAIASGARVVSRE
                     QALPEVPVRPGKGEALWRSLAATSGDIVVFIDSDLINPHPLFVPWLVGPLLTGEGIQL
                     VKSFYRRPLQVSDVTSGVCATGGGRVTELVARPLLAALRPELGCVLQPLSGEYAASRE
```

LLTSLPFAPGYGVEIGLLIDTFDRLGLDAIAQVNLGVRAHRNRPLDELGAMSRQVIAT
LLSRCGIPDSGVGLTQFLPGGPDDSDYTRHTWPVSLVDRPPMKVMRPR"
CDS 1356723..1357091
/codon_start=1
/note="gene_1262"
/transl_table=11
/translation="VALVLVYLVVLVLVAIVLFAAASLLFGRGEQLPPLPRATTATTL
PAFGVTRADVDAVKFTQVLRGYKTSEVDWVLERLGRELEALRSQLGAIHASSEDAEAE
SDASNPSRGETVVHYRSDPA"
CDS 1357088..1357702
/codon_start=1
/note="gene_1263"
/transl_table=11
/translation="VSGDGLVRCPWAEVRPGPDAQLYRDYHDNEWGRPLYGRVALFER
MSLEAFQSGLSWLIILRKRENFRRAFSGFDIDKIARYTDTDVRRLLADDGIVRNRAKI
EATIANARAAADLGSSEDLSELLWSFAPPPRPRPVDGSEIPSVSTESKAMSRELKRRG
FRFVGPTTAYALMQATGMVDDHIQACWVPTERPFDQPGCPMAAR"
CDS 1357869..1358036
/codon_start=1
/note="gene_1264"
/transl_table=11
/translation="MAAMKPRTGDGPLEATKEGRGIVMRVPLEGGGRLVVELTPDEAA
ALGDELKGVTS"
CDS complement(1358064..1359227)
/codon_start=1
/note="gene_1265"
/transl_table=11
/translation="MRVAMLTREYPPEVYGGAGVHVTELVAYLRRLCAVDVHCMGAPR
PGAFAYRPDPRLGSANAALSTLSADLVMANAASAATVVHSHTWYTALAGHLAAILYDI
PHILTAHSLEPLRPWKKEQLGGGYQVSTWVEQTAVLAANAVIAVSSAMRNDMLRVYPS
LDPNLVHVIRNGIDTETWYPAGPARTGSVLAELGVDPNRPMAVFVGRITRQKGVVHLV
TAAHRFRSDVQLVLCAGAADTPEVADEVRVAVAELARNRTGVFWIQDRLTIGQLREIL
SAATVFVCPSVYEPLGIVNLEAMACATAVVASDVGGIPEVVADGITGSLVHYDADDAT
GYQARWAEAVNALVADPATAERYGHAGRQRCIQEFSWAYIAEQTLDIYRKVCA"
CDS 1359402..1360616
/codon_start=1
/gene="glgC"
/note="gene_1266"
/transl_table=11
/translation="MREVPHVLGIVLAGGEGKRLYPLTADRAKPAVPFGGAYRLIDFV
LSNLVNARYLRICVLTQYKSHSLDRHISQNWRLSGLAGEYITPVPAQQRLGPRWYTGS
ADAIYQSLNLIYDEDPDYIVVFGADHVYRMDPEQMVRFHIDSGAGATVAGIRVPRENA
TAFGCIDADDSGRIRSFVEKPLEPPGTPDDPDTTFVSMGNYIFTTKVLIDAIRADADD
DHSDHDMGGDIVPRLVADGMAAVYDFSDNEVPGATDRDRAYWRDVGTLDAFYDAHMDL
VSVHPVFNLYNKRWPIRGESENLAPAKFVNGGSAQESVVGAGSIISAASVRNSVLSSN
VVVDDGAIVEGSVIMPGTRVGRGAVVRHAILDKNVVVGPGEMVGVDLEKDRERFAISA
GGVVAVGKGVWI"
CDS complement(1360859..1361191)
/codon_start=1
/note="gene_1267"
/transl_table=11

```
                     /translation="VLASAATDLAGIGSALSAANAAAAAPTTAMLAACADEVSAVVAS
                     LFARHAQAYQALSLQATAFHQQFVQALTGAGGAYAAAEAVNAAVAQSVQQDVLNVINA
                     PTQALFDR"
     CDS             complement(1361325..1362986)
                     /codon_start=1
                     /note="gene_1268"
                     /transl_table=11
                     /translation="LDRPWRRPGALRYALERVRGVAKPPITVTDPPADVVIERDVEVP
                     TRDGTLLRINVFRSAEGGARPVIASIHPYGKDALPRRRGNRWTFSPQYRMLRQPKPLT
                     FSALTGWEAPDPAWWTAQGFVVVNADSRGCGRSDGTGDLLSHQEAEDTYDLVGWLADQ
                     SWSDGRVVMLGVSYLAISQYAVAALQPPALRAICPWEGFTDAYRDLAFPGGIRESGFT
                     RLWSRGVRRRTRQTYDMEQMQEAHPLRDDFWRSRVPDLSAIKVPMLVCGSFSDNNLHS
                     RGSIRAFTRSGCGHARLYTHRGGKWETFYSATALSEQLKFLRDALAGSSGSRSVRLEV
                     REDRDTITAVREETQWPLAGTRWRPMYLAGPGLLATEPPPTAGSIRFQTRSRAAAFNW
                     TIPEDIELTGPMAARLWVQLDGCDDANLFVGVEKWRDGQFVAFEGSYGWGRDRVTTGW
                     QRVSLRELDPELSQPWEPVPACARPRPVTAGEVVAVDVALGPSATLFRAGEQLRLVVG
                     GRWLSPRNPLTGQFPAAYPRPPRGRVTLHWGPRYDAHLLIPEVPG"
     CDS             complement(1363038..1363712)
                     /codon_start=1
                     /note="gene_1269"
                     /transl_table=11
                     /translation="MHIGLKIFIWGVLGLVVFGALLFGPAGTFDYWQAWVFLAAFVST
                     TIGPTIYLARNDPAALQRRMRSGPLAEGRTIQKFIVIGAFLGFFAMMVLSACDHRYGW
                     SSVPAAVCVIGDVLVMTGLGIAMLVVIQNRYAASTVRVEAGQILASDGLYKIVRHPMY
                     AGNVVMMTGIPLALGSYWAMFILVPGTLVLVFRILDEEKLLTQELSGYREYRQLVRYR
                     LVPYVW"
     CDS             complement(1363721..1365367)
                     /codon_start=1
                     /note="gene_1270"
                     /transl_table=11
                     /translation="VSSTVIDRARPAGHRAPHRGSGFTGTLGLLRLYLRRDRVSLPLW
                     VLLLSVPLATVYIASVETVYPDRSARAAAAAAIMASPAQRALYGPVYNDSLGAVGIWK
                     AGMFHTLIAVAVILTVIRHTRADEESGRAELIDSTVVGRYANLTGALLLSFGASIATG
                     AIGALGLLATDVAPAGSVAFGVALAASGMVFTAVAAVAAQLSPSARFTRAVAFAVLGT
                     AFALRAIGDAGSGTLSWCSPLGWSLQVRPYAGERWWVLLLSLATAAVLTVLAYRLRAG
                     RDVGAGLIAERPGAGTAGPMLSEPFGLAWRLNRGSLLLWTVGLCLYGLVMGSVVHGIG
                     DQLGDNTAVRDIVTRMGGTGALEQAFLALAFTMIGMVAAAFAVSLTLRLHQEETGLRA
                     ETLLAGAVSRTHWLASHLAMALAGSAVATLISGVAAGLAYGMTVGDVGGKLPTVVGTA
                     AVQLPAVWLLSAVTVGLFGLAPRFTPVAWGVLVGFIALYLLGSLAGFPQMLLNLEPFA
                     HIPRVGGGDFTAVPLLWLLAIDAALITLGAMAFRRRDVRC"
     CDS             complement(1365364..1366299)
                     /codon_start=1
                     /note="gene_1271"
                     /transl_table=11
                     /translation="MSADNHQVPIEIRGLTKHFGSVRALDGLDLTVREGEVHGFLGPN
                     GAGKSTTLRILLGLVKADGGSVRLLGGDPWTDAVDLHRHIAYVPGDVTLWPSLTGGET
                     IDLLARMRGGIDNARRAELIERFGLDPTKKARTYSKGNRQKVSLISALSSHATLLLLD
                     EPSSGLDPLMENVFQQCIGEARQRGVTVLLSSHILAETEALCEKVTIIRAGKTVESGS
                     LDALRHLSRTSIKAEMIGDPGDLSRIKGVEDISIEGTTVRAQVDSESLRELIQVLGHA
                     GVRSLVSQPPTLEELFLRHYSLGPEVAAEQQVATP"
     CDS             complement(1366289..1366963)
```

```
                     /codon_start=1
                     /note="gene_1272"
                     /transl_table=11
                     /translation="LTGDERTFNILNMRSADLTAHARIREAAIEQFGRHGFGVGLRAI
                     AEAAGVSAALVIHHFGSKEGLRKACDDFVAEEIRSSKAAALKSNDPTTWLAQMAEIES
                     YAPLMAYLVRSMQSGGELAKMLCQKMIDNAEEYLDEGVRAGTVKPSRDPRARARFLAI
                     TGGGGFLLYLQMHENPTDLRAALRDYAHDMVLPSLEVYTEGLLADRAMYEAFLAEAQQ
                     GEAHVG"
CDS             complement(1367069..1367812)
                     /codon_start=1
                     /note="gene_1273"
                     /transl_table=11
                     /translation="VLGRRSNQARLPDGVANWTYAAGMDGTPGHDDMPGQPAPSRGES
                     LWAHAEGSISEDVILAGARERATDIGAGAVTPAVGALLCLLAKLSGGKAVAEVGTGAG
                     VSGLWLLSGMRDDGVLTTIDIEPEHLRLARQAFAEAGIGPSRTRLISGRAQEVLTRLA
                     DASYDLVFIDADPIDQPDYVAEGVRLLRSGGVIVVHRAALGGRAGDPGARDAEVIAVR
                     EAARLIAEDERLTPALVPLGDGVLAAVRD"
CDS             1368120..1368752
                     /codon_start=1
                     /note="gene_1274"
                     /transl_table=11
                     /translation="MSHPQQVRDDQWVEPSDQLQGTAVFDATGDKATMPSWDELVRQH
                     ADRVYRLAYRLSGNQHDAEDLTQETFIRVFRSVQNYQPGTFEGWLHRITTNLFLDMVR
                     RRARIRMEALPEDYDRVPADEPNPEQIYHDARLGPDLQAALASLPPEFRAAVVLCDIE
                     GLSYEEIGATLGVKLGTVRSRIHRGRQALRDYLAAHPEHGECAVHVNPVR"
CDS             1368910..1369374
                     /codon_start=1
                     /note="gene_1275"
                     /transl_table=11
                     /translation="MADPGSVGHVFRRAFSWLPAQFASQSDAPVGAPRQFRSTEHLSI
                     EAIAAFVDGELRMNAHLRAAHHLSLCAQCAAEVDDQSRARAALRDSHPIRIPSTLLGL
                     LSEIPRCPPEGPSKGSSGGSSQGPPDGAAAGFGDRFADGDGGNRGRQSRVRR"
CDS             1369455..1371029
                     /codon_start=1
                     /note="gene_1276"
                     /transl_table=11
                     /translation="VDTDNAMPARFSAQIQNEDEVTSDQGNNGGPNGGGRLAPRPVFR
                     PPVDPASRQAFGRPSGVQGSFVAERVRPQKYQDQSDFTPNDQLADPVLQEAFGRPFAG
                     AESLQRHPIDAGALAAEKDGAGPDEPDDPWRDPAAAAALGTPALAAPAPHGALAGSGK
                     LGVRDVLFGGKVSYLALGILVAIALVIGGIGGVIGRKTAEVVDAFTTSKVTLSTTGNA
                     QEPAGRFTKVAAAVADSVVTIESVSDQEGMQGSGVIVDGRGYIVTNNHVISEAANNPS
                     QFKTTVVLNDGKEVPANLVGRDPKTDLAVLKVDNVDNLTVARLGDSSKVRVGDEVLAV
                     GAPLGLRSTVTQGIVSALHRPVPLSGEGSDTDTVIDAIQTDASINHGNSGGPLIDMDA
                     QVIGINTAGKSLSDSASGLGFAIPVNEMKLVANSLIKDGKIVHPTLGINTRSVSNAIA
                     SGAQVANVKAGSPAQKGGILENDVIVKVGNRAVADSDEFVVAVRQLAIGQDAPIEVVR
                     EGRHVTLTVKPDPDST"
CDS             1371031..1371426
                     /codon_start=1
                     /gene="tatB"
                     /note="gene_1277"
                     /transl_table=11
```

/translation="VFANIGWGEMLVLVMVGLVVLGPERLPGAIRWAASALRQARDYL
SGVTSQLREDIGPEFDDLRGHLGELQKLRGMTPRAALTKHLLDGDDSLFTGDFDRPTP
KKPDAAGSAGPDATEQIGAGPIPFDSDAT"

CDS complement(1371459..1372289)
/codon_start=1
/note="gene_1278"
/transl_table=11
/translation="VDVAHLMAAAVLFDIDGVLVLSWRAIPGAAETVRQLTHRGIACA
YLTNTTTRTRRQIAEALGAAGIPVAADDVITAGVLTAEYLHGAYPGARCFLVNNGDIT
EDLPGIDVVLSTEIGPEDCPEAPDVVVLGSAGPQFDHRTLSRVYGWMLDGVPVVAMHR
NMTWNTTDGLRIDTGMYLTGMEQACGKTATAIGKPAAEGFLAAADRVGVDPQQMVMIG
DDLHNDVLAAQAVGMTGVLVRTGKFRQQTLDRWLAGASATRPHHVIDSVAGLPPLLGC
"

CDS complement(1372400..1373863)
/codon_start=1
/note="gene_1279"
/transl_table=11
/translation="MTDRPHDWHRLSPRMLLVHPVHEMLRQLPVLIGSVVLGSATGNP
VWPLAALGVTVVFGVLRWFFTTYRIDDENVSLRTGILSRRAVSVPRNRIRSVQTEARL
LHRLLGLTVLRVGTGQEARGEAAFELDAVDSARVPRLRALLLAESLAPVEPTGRVLAR
WQSSWLRYAPLSFSGLVMIGAVIGLGYQTGLAVRLPESGFARSAVDAAQRAGVVLVVA
VTVLLVVGVSALLAVLFSWLTYGNLLLRRGGSGQEGVLHLRHGLLRVREHTYDMRRLR
GATLREPLLVRLLRGARLDAVMTGVHGEGQSSMLLPPCPFETATAVLTDLIDNTDAAA
GPLRRHGPAAARRRWTRALLVPTLAGVALIAAAPILGVPGWAWTLWAVLTAGCAGLAV
DRVRSLGHRVADGWLVARAGSLQRRRDCIACTGIIGWTVRQTLFQRRAGVATLVAATA
AGRKGYQVLDVPAELAWSVAGAASPWVADSVWLRHGS"

CDS complement(1373860..1374294)
/codon_start=1
/note="gene_1280"
/transl_table=11
/translation="MWALSASLGWILPVIAQLVWWAVHPQPPWPHLAAAALTAVAMVV
HIGVVPLWRYRVHRWEISPQAVFTRTGWLVQERRITPISRVQTVDTYRGPMDRLFGLA
NVTVTTASSAGAVHIEALDTDVADRVVAQLTDIAALRGEDAT"

CDS 1374488..1375045
/codon_start=1
/note="gene_1281"
/transl_table=11
/translation="MSRQWHWLAATLLLITTAACSRPGTEEPDCPTKITLPPGATPTT
TLDPRCIVRATTTGTADGDAASRWTGTVRIAGFYASICNAVWDGNVSLAGKDELTGKA
TLILVETSCPGKVVAGELVLKGNVGSDSLAITWAHPELPQRAFDLGAGQGTIRRSGDR
AEGTFNSDMGGGTEFFLTWSLTMRN"

CDS complement(1375345..1376490)
/codon_start=1
/note="gene_1282"
/transl_table=11
/translation="MSGTRDGDLNAAIRTALGKVIDPELRRPITELGMVKSIDTGPDG
SVHVEIYLTIAGCPKKSEITERVTRAVADVPGTSAVRVSLDVMSDEQRTELRKQLRGD
TREPVIPFAQPDSLTRVYAVASGKGGVGKSTVTVNLAAAMAVRGLSIGVLDADIHGHS
IPRMMGTTDRPTQVESMILPPIAHQVKVISIAQFTQGNTPVVWRGPMLHRALQQFLAD
VYWGDLDVLLLDLPPGTGDVAISVAQLIPNAELLVVTTPQLAAAEVAERAGSIALQTR
QRIVGVVENMSGLTLPDGTTMQVFGEGGGRLVAERLSRAVGADVPLLGQIPLDPALVA

AGDSGVPLVLSSPDSAIGKELHSIADGLSTRRRGLAGMSLGLDPTRR"
CDS complement(1376530..1377597)
/codon_start=1
/note="gene_1283"
/transl_table=11
/translation="VHAVITPVAAVAASGIDLSGPVVIAMKRPPTSFRVAVATIPAPP
PPMIVNSPGALGIPAMALSAYRNAELKMAAAAPGCGVSWNLLAGIGRIESMHANGGAT
DARGTAIQPIYGPTLDGTLPGNEIIIQSSVGNRVTYARAMGPMQFLPGTWARYATDGD
DDGVADPQNLFDSTLAAARYLCSGGLNLRDPAQVMAALLRYNNSMPYAQNVLGWAAGY
ATGVFPVDLPPITGPPPPLGDAHLENPEGLGPGLPINVNGLTADGPMAHLPLIDLTPR
QAALNPPPMFPWMAPDPSAPMPGCTLICIGSHGPPVGAPPFPPTAPPPPFLPAAPPPP
DPLAGPPGDAGLAPPAPAPAG"
CDS complement(1377890..1378432)
/codon_start=1
/note="gene_1284"
/transl_table=11
/translation="VSKPFAPRRLYTPRTSRTLAPRLDPEAVGRTTESIARFFGTGRY
LLVQTLLVLTWIVLNLFAVGLRWDPYPFILLNLAFSTQASYAAPLILLAQNRQEKRDR
AVFEEDRRRAAQTKADTEYNARELAALRLAIGEVPTRDYLRHELDSLRALLAELQPTD
PDVAQPRVADEAEQHAKKSG"
CDS complement(1378429..1379736)
/codon_start=1
/note="gene_1285"
/transl_table=11
/translation="MGSVNRVYLARLSRMSVLGPLGESFGRVRDVVISISIVRQQPRV
LGLVVDLATRRKIFIPILRVAAIEPHAVTLSTGNVSLHRFEQRPGEALALGQVLDTLV
KVNDPALPELAGVDVVVTDLGVEQTRSRDWMVTRVAVRTQRRLRRRGPVHVVDWHNVA
GLTPSALAMPGQDVAQLLDQFEGWKAVDVADAIRGLPPKRRHEVFKALHDKRLADVLQ
ELPELDQAEVLSQLGTERAADVLEEMDPDDAADLLAVLNPTEAELLLTRMDPGDSGQV
RRLLTHSPDTAGGLMTSDPVVLTPDTSIAEALARVRDPDLTPALASMVFVARPPTATP
TGHYLGCVHLQRLLRDPPAELVGGVVDTDLLTLTPETPLAAVTRYFAAYNLVCGPVVD
DENHLLGAVTVDDLLDHLLPHDWRVDMPELDPSGAPDRPGGPR"
CDS complement(1379798..1380394)
/codon_start=1
/note="gene_1286"
/transl_table=11
/translation="MTAPSGSSGESAHDAAGGPPPVGERPPEQPIADAPWAPPASSPM
ADHPPPAYPPSGYPPAYQPGYPTDYPPPMPPGGYAPPGYPPPGTSSAGYGDIPYPPMP
PPYGGSPGGYYPEPGYLDGYGPSQPGMNTMALVSLISALVGVLCCIGSIVGIVFGAIA
INQIKQTREEGYGLAVAGIVIGIATLLVYMIAGIFAIP"
CDS 1380616..1381071
/codon_start=1
/note="gene_1287"
/transl_table=11
/translation="VPALPTPPKGWPVGSYPTYAEAQRAVDYLSEQQFPVQQVTIVGV
DLMQVERVTGRLTWPKVLGGGVLSGAWLGLFIGLVLGFFSPNPWSALVTGLVAGVFFG
LITSAVPYAMARGTRDFSSTMQLVAGRYDVLCDPQNAEKARDLLARLAI"
CDS 1381092..1382498
/codon_start=1
/note="gene_1288"
/transl_table=11

/translation="VVMSRGRIPRLGAAVLVALTTAAAACGADSQGLVVSFYTPATDG
ATFTAIAQRCNQQFGGRFTIAQVSLPRSPNEQRLQLARRLTGNDRTLDVMALDVVWTA
EFAEAGWALPLSDDPAGLAENDAVADTLPGPLATAGWNHKLYAAPVTTNTQLLWYRPD
LVNSPPTDWNAMIAEAARLHAAGEPSWIAVQANQGEGLVVWFNTLLVSAGGSVLSEDG
RHVTLTDTPAHRAATVSALQILKSVATTPGADPSITRTEEGSARLAFEQGKAALEVNW
PFVFASMLENAVKGGVPFLPLNRIPQLAGSINDIGTFTPSDEQFRIAYDASQQVFGFA
PYPAVAPGQPAKVTIGGLNLAVAKTTRHRAEAFEAVRCLRDQHNQRYVSLEGGLPAVR
ASLYSDPQFQAKYPMHAIIRQQLTDAAVRPATPVYQALSIRLAAVLSPITEIDPESTA
DELAAQAQKAIDGMGLLP"

CDS 1382495..1383418
/codon_start=1
/note="gene_1289"
/transl_table=11
/translation="VTSVEQRTATAVFSRTGSRMAERRLAFMLVAPAAMLMVAVTAYP
IGYALWLSLQRNNLATPNDTAFIGLGNYHTILIDRYWWTALAVTLAITAVSVTIEFVL
GLALALVMHRTLIGKGLVRTAVLIPYGIVTVVASYSWYYAWTPGTGYLANLLPYDSAP
LTQQIPSLGIVVIAEVWKTTPFMSLLLLAGLALVPEDLLRAAQVDGASAWRRLTKVIL
PMIKPAIVVALLFRTLDAFRIFDNIYVLTGGSNNTGSVSILGYDNLFKGFNVGLGSAI
SVLIFGCVAVIAFIFIKLFGAAAPGGEPSGR"

CDS 1383423..1384247
/codon_start=1
/note="gene_1290"
/transl_table=11
/translation="VGARRATYWAVLDTLVVGYALLPVLWIFSLSLKPTSTVKDGKLI
PSTVTFDNYRGIFRGDLFSSALINSIGIGLITTVIAVVLGAMAAYAVARLEFPGKRLL
IGAALLITMFPSISLVTPLFNIERAIGLFDTWPGLILPYITFALPLAIYTLSAFFREI
PWDLEKAAKMDGATPGQAFRKVIVPLAAPGLVTAAILVFIFAWNDLLLALSLTATKAA
ITAPVAIANFTGSSQFEEPTGSIAAGAIVITIPIIVFVLIFQRRIVAGLTSGAVKG"

CDS 1384252..1385433
/codon_start=1
/note="gene_1291"
/transl_table=11
/translation="MAEIVLDHVNKSYPDGHTAVRDLNLTIADGEFLILVGPSGCGKT
TTLNMIAGLEDISSGELRIAGERVNEKAPKDRDIAMVFQSYALYPHMTVRQNIAFPLT
LAKMRKADIAQKVSETAKILDLTNLLDRKPSQLSGGQRQRVAMGRAIVRHPKAFLMDE
PLSNLDAKLRVQMRGEIAQLQRRLGTTTVYVTHDQTEAMTLGDRVVVMYGGIAQQIGT
PEELYERPANLFVAGFIGSPAMNFFPARLTAIGLTLPFGEVTLAPEVQGVIAAHPKPE
NVIVGVRPEHIQDAALIDAYQRIRALTFQVKVNLVESLGADKYLYFTTESPAVHSVQL
DELAEVEGESALHENQFVARVPAESKVAIGQSVELAFDTARLAVFDADSGANLTIPHR
A"

CDS complement(1385510..1386610)
/codon_start=1
/note="gene_1292"
/transl_table=11
/translation="VFPGFDALPEVLRPVARPQPPNAHPVAQPPAQALVDCGVYVCGQ
RLPGKYTYAAALREVREIELTGQEAFVWIGLHEPDENQMQDVADVFGLHPLAVEDAVH
AHQRPKLERYDETLFLVLKTVNYVPHESVVLAREIVETGEIMIFVGKDFVVTVRHGEH
GGLSEVRKRMDADPEHLRLGPYAVMHAIADYVVDHYLEVTNLMETDIDSIEEVAFAPG
RKLDIEPIYLLKREVVELRRCVNPLSTAFQRMQTESKDLISKEVRRYLRDVADHQTEA
ADQIASYDDMLNSLVQAALARVGMQQNMDMRKISAWAGIIAVPTMIAGIYGMNFHFMP
ELDSRWGYPTVIGGMVLICLFLYHVFRNRNWL"

```
CDS             1386781..1387770
                /codon_start=1
                /note="gene_1293"
                /transl_table=11
                /translation="VSASPLKVAVTGAAGQIGYSLLFRLASGSLLGPDRPIELRLLEI
                EPALQALEGVVMELDDCAFPLLSGVEIGSDPQKIFDGVSLALLVGARPRGAGMERSDL
                LEANGAIFTAQGKALNAVAADDVRVGVTGNPANTNALIAMTNAPDIPRERFSALTRLD
                HNRAISQLAAKTGAAVTDIKKMTIWGNHSATQYPDLFHAEVAGKNAAEVVNDQAWIED
                EFIPTVAKRGAAIIDARGASSAASAASATIDAARDWLLGTPADDWVSMAVVSDGSYGV
                PEGLISSFPVTTKGGNWTIVSGLEIDEFSRGRIDKSTAELADERSAVTELGLI"
CDS             1387846..1388106
                /codon_start=1
                /note="gene_1294"
                /transl_table=11
                /translation="MRTTLTLDDDVVRLVEDAVHRERRPMKQVINDALRRALAPPVKR
                QEQYRLEPHESAVRSGLDLAGFNKLADELEDEALLDATRRAR"
CDS             1388103..1388534
                /codon_start=1
                /note="gene_1295"
                /transl_table=11
                /translation="VIIPDINLLLYAVITGFPQHRRAHAWWQDTVNGHTRIGLTYPAL
                FGFLRIATSARVLAAPLPTADAIAYVREWLSQPNVDLLTAGPRHLDIALGLLDKLGTA
                SHLTTDVQLAAYGIEYDAEIHSSDTDFARFADLKWTDPLRE"
CDS             complement(1388557..1390245)
                /codon_start=1
                /note="gene_1296"
                /transl_table=11
                /translation="MEYLIAAQDVLVAAAADLEGIGSALAAANRAAEAPTTGLLAAGA
                DEVSAAIASLFSGNAQAYQALSAQAAAFHQQFVRALSSAAGSYAAAEAANASPMQAVL
                DVVNGPTQLLLGRPLIGDGANGGPGQNGGDGGLLYGNGGNGGSSSTPGQPGGRGGAAG
                LIGNGGAGGAGGPGANGGAGGNGGWLYGNGGLGGNGGAATQIGGNGGNGGHGGNAGLW
                GNGGAGGAGAAGAAGANGQNPVSHQVTHATDGADGTTGPDGNGTDAGSGSNAVNPGVG
                GGAGGIGGDGTNLGQTDVSGGAGGDGGDGANFASGGAGGNGGAAQSGFGDAVGGNGGA
                GGNGGAGGGGGLGGAGGSANVANAGNSIGGNGGAGGNGGIGAPGGAGGAGGNANQDNP
                PGGNSTGGNGGAGGDGGVGASADVGGAGGFGGSGGRGGLLLGTGGAGGDGGVGGDGGI
                GAQGGSGGNGGNGGIGADGMANQDGDGGDGGNGGDGGAGGAGGVGGNGGTGGAGGLFG
                QSGSPGSGAAGGLGGAGGNGGAGGGGGTGFNPGAPGDPGTQGATGANGQHGLNG"
CDS             complement(1390233..1390517)
                /codon_start=1
                /note="gene_1297"
                /transl_table=11
                /translation="VAIAGVGRATSHARHCKQDGKQQREDPGDSHTEVSEPIVTATDA
                VGTGLRVLGPIHTVGAGIQANTQCTVKQRDTLRADPLVNLAAREAPRWSI"
CDS             1390527..1391285
                /codon_start=1
                /note="gene_1298"
                /transl_table=11
                /translation="VVGSTPDSEAMLLAAIYVAALRSYGFAAHAETAADPVAKLDSGA
                FTVVPAFTGQMLQTLQPDASVRSDAQVYRAIVSALPEGIAAGDYTTAAEDKPALVVTQ
                STAKAWGGGDLSELPSHCRGLLVGRVAGAHTPAAVGPCRLPAPREFRNDATMFAALRA
                GQLVAAWTTTADPDIPADLIMLTDGKPALIRAENIVPLYRRNALTERKLLAVNEVAGV
```

```
                     LDTTALIGMRRQVAAGADPAAVAAGWLAEHPLGR"
     CDS             complement(1391366..1392196)
                     /codon_start=1
                     /note="gene_1299"
                     /transl_table=11
                     /translation="MEGFAGKVAVVTGAGSGIGQALAIELARSGAKVAISDVDTDGLA
                     DTEHRLKAISTPVKTDRLDVTEREAFLAYADAVNEHFGTVNQIYNNAGIAFTGDIEVS
                     QFKDIERVMDVDFWGVVNGTKAFLPHLIASGDGHVINISSVFGLFSAPGQAAYNSAKF
                     AVRGFTEALRQEMALAGHPVKVTTVHPGGVKTAIARNATAAEGLDQAELAETFDKRVA
                     HLSPQRAAQIILTGVAKNKARVLVGVDAKVLDLVVRLTGSGYQRIFPIITGRLIPRPR
                     "
     CDS             complement(1392253..1392546)
                     /codon_start=1
                     /note="gene_1300"
                     /transl_table=11
                     /translation="VSDDHPYHVAITATAARDLQRLPEKIAAACVEFVFGPLLNNPHR
                     LGKPLRNDLEGLHSARRGDYRVVYAIDDGHHRVEIIHIARRSASYRMNPCRPR"
     CDS             complement(1392543..1392860)
                     /codon_start=1
                     /note="gene_1301"
                     /transl_table=11
                     /translation="MLGVQGLAILLATIAVMAVVPLGEVRNRLSEYVAEVELTHERIT
                     ITRHGHPAAVLISADDLASIEETLEVLRTPGASEAIREGLADVAAGRFVSNDEIRNRY
                     TAR"
     CDS             complement(1392925..1396620)
                     /codon_start=1
                     /gene="kgd"
                     /note="gene_1302"
                     /transl_table=11
                     /translation="VANISSPFGQNEWLVEAMYRKFRDDPSSVDPSWHEFLVDYSPEP
                     TSQPAAEPTRVTSPLVAERAAAAAPQAPPKPADTAAAGNGVVAALAAKTAVPPPAEGD
                     EVAVLRGAAAAVVKNMSASLEVPTATSVRAVPAKLLIDNRIVINNQLKRTRGGKISFT
                     HLLGYALVQAVKKFPNMNRHYTEVDGKPTAVTPAHTNLGLAIDLQGKDGKRSLVVAGI
                     KRCETMRFAQFVTAYEDIVRRARDGKLTTEDFAGVTISLTNPGTIGTVHSVPRLMPGQ
                     GAIIGVGAMEYPAEFQGASEERIAELGIGKLITLTSTYDHRIIQGAESGDFLRTIHEL
                     LLSDGFWDEVFRELSIPYLPVRWSTDNPDSIVDKNARVMNLIAAYRNRGHLMADTDPL
                     RLDKARFRSHPDLEVLTHGLTLWDLDRVFKVDGFAGAQYKKLRDVLGLLRDAYCRHIG
                     VEYAHILDPEQKEWLEQRVETKHVKPTVAQQKYILSKLNAAEAFETFLQTKYVGQKRF
                     SLEGAESVIPMMDAAIDQCAEHGLDEVVIGMPHRGRLNVLANIVGKPYSQIFTEFEGN
                     LNPSQAHGSGDVKYHLGATGLYLQMFGDNDIQVSLTANPSHLEAVDPVLEGLVRAKQD
                     LLDHGSIDSDGQRAFSVVPLMLHGDAAFAGQGVVAETLNLANLPGYRVGGTIHIIVNN
                     QIGFTTAPEYSRSSEYCTDVAKMIGAPIFHVNGDDPEACVWVARLAVDFRQRFKKDVV
                     IDMLCYRRRGHNEGDDPSMTNPYMYDVVDTKRGARKSYTEALIGRGDISMKEAEDALR
                     DYQGQLERVFNEVRELEKHGVQPSESVESDQMIPAGLATAVDKSLLARIGDAFLALPN
                     GFTAHPRVQPVLEKRREMAYEGKIDWAFGELLALGSLVAEGKLVRLSGQDSRRGTFSQ
                     RHSVLIDRHTGEEFTPLQLLATNSDGSPTGGKFLVYDSPLSEYAAVGFEYGYTVGNPD
                     AVVLWEAQFGDFVNGAQSIIDEFISSGEAKWGQLSNVVLLLPHGHEGQGPDHTSARIE
                     RFLQLWAEGSMTIAMPSTPSNYFHLLRRHALDGIQRPLIVFTPKSMLRHKAAVSEIKD
                     FTEIKFRSVLEEPTYEDGIGDRNKVSRILLTSGKLYYELAARKAKDNRNDLAIVRLEQ
                     LAPLPRRRLRETLDRYENVKEFFWVQEEPANQGAWPRFGLELPELLPDKLAGIKRISR
                     RAMSAPSSGSSKVHAVEQQEILDEAFG"
```

```
CDS             complement(1396762..1397550)
                /codon_start=1
                /note="gene_1303"
                /transl_table=11
                /translation="MSARRIRSWKRFDNRSANAAEPDPQLAGTGGRPKVSTRALAQVI
                ERSSRIQGPAAQAYVARLRRAHPGASPAKIVAKLEKRFLSVVTASGAAVGTAATLPGI
                GTLAAWFAAAGEVVVFLEATALFVLALASVHAIPLDHRERRRALVLAVLVGDNTTAVA
                DLLGPGRTSGGWVSETMASLPLPAISSLNSRMLKYVVKRFALKRGALMFGKLVPMGIG
                AIIGAIGNRLVGKKLVRNARSAFGTPPARWPVTLHVLPTVRDAS"
CDS             1397747..1399486
                /codon_start=1
                /note="gene_1304"
                /transl_table=11
                /translation="MTTAIRRAAGSSYFRNPWPALWAMMVGFFMIMLDSTVVAIANPT
                IMAQLRIGYATVVWVTSAYLLAYAVPMLVAGRLGDRFGPKNLYLIGLGVFTVASLGCG
                LSSGAGMLIAARVVQGVGAGLLTPQTLSTITRIFPAHRRGVALGAWGTVASVASLVGP
                LAGGALVDSMGWEWIFFVNVPVGVIGLILAAYLIPALPHHPHRFDWFGVGLSGAGMFL
                IVFGLQQGQSANWQPWIWAVIVGGIGFMSLFVYWQARNAREPLIPLEVFNDRNFSLSN
                LGIAIIAFAGTGMMLPVTFYAQAVCGLSPTHTAVLFAPTAIVGGVLAPFVGMIIDRSH
                PLCVLGFGFSVLAIAMTWLLCEMAPGTPIWRLVLPFIALGVAGAFVWSPLTVTATRNL
                RPHLAGASSGVFNAVRQLGAVLGSASMAAFMTSRIAAEMPGGVDALTGPAGQDATVLQ
                LPEFVREPFAAAMSQSMLLPAFVALFGIVAALFLVDFTGAAVAKEPLPESDGDADDDD
                YVEYILRREPEEDCDTQPLRASRPAAAAASRSGAGGPLAVSWSTSAQGMPPGPPGRRA
                WQADTESTAPSAL"
CDS             complement(1399389..1402808)
                /codon_start=1
                /note="gene_1305"
                /transl_table=11
                /translation="VFVTGDSIVYSASDLAAAARCQYALLREFDAKLGRGPAVAVDDE
                LMARAAVLGSAHEGRRLDQLRHEFGDAVAIIGRPAYTPAGLAAAADATRRAIANHAPV
                VYQAAMFDGRFVGFADFLIRDGHRYRVADTKLARSPTVTALLQLAAYADALVHSGVPV
                AADAELELGDGTIVRYRVGELIPVYRSQRALLQRLLDGHYTAGTAVRWDDERVQACFR
                CPQCTERLRASDDLLLVGGMRVRQRDKLLEAGITTIAELADHTAPVPGLTTNALGKLT
                AQAKLQIRQRDTGAPQFEIVDPRPLTLLPEPNPGDLFFDFEGDPLWTADGKQWGLEYL
                FGVLEAGRAGVFRPLWAHDRTAERQALTDFLAIVARRRRRHPNMHIYHYAPYEKTALL
                RLVGRYGIGEDDVDDLLRNGVLVDLYPLVRKSIRVGTDSFSLKALEPLYLGTQPRSGD
                VTTAADSINSYARYCELRAAGRIDEAATVLKEIEGYNHYDCRSTRALRDWLLMRAWEA
                GVTPIGAQPVPDADPIDDGDSLASVLSKFTGDAAAGERTPEQTAVALLAAARGYHRRE
                DKPFWWAHFDRLNYPVDEWSDSTDVFLASEASVTVDWHMPPRARKPQRRVRLTGELAR
                GDLNGNVFALYEPPAPPGMTDNPDRRAAGPAAVVETDDPTVPTEVVIVERTGSDGNTF
                QQLPFALAPGPPVPTTALRESIESTAAAVASGSPQLPSTALMDVLLRRPPRTRSGAAL
                PRSSDPVTDIAAAALDLDSSYLAVHGPPGTGKTYTAARVIAELVTEHAWRIGVVAQSH
                ATVENLLEGVISAGLDPGQVAKKPHDHTAGRWQSIDGSQYTEFIRDTAGCVIGGTAWD
                FANGNRVPKASLDLLVIDEAGQFCLANTIAVAPAATNLLLLGDPQQLPQVSQGTHPEP
                VDTSALSWLVDGQHTLPDERGYFLDRSYRMHPAVCAAVSALSYEGRLCSHTERTAVRR
                LDGYPPGVHTRGVHHKGNSIESPEEAEAILAELRQLLGSPWTDEHGTRPLAASDVLVL
                APYNAQVALVRRRLASAGLGGADGVRVGTVDKFQGGQAPVVFISMTASSADDVPRGIS
                FLLNRNRLNVAVSRAQYAAVIVRSELLTQYLPATPDGLVDLGAFLGLTSTS"
CDS             complement(1402864..1403472)
                /codon_start=1
                /note="gene_1306"
```

```
                     /transl_table=11
                     /translation="VPGVWSPPCPTTPRVGVVAALVAATLTGCGSGDSTVAKTPEATP
                     SLSTAHPAPPSSEPSPPSATAAPPSNHSAAPVDPCAVNLASPTIAKVVSELPRDPRSE
                     QPWNPEPLAGNYNECAQLSAVVIKANTNAGNPTTRAVMFHLGKYIPQGVPDTYGFTGI
                     DTSQCTGDTVALTYASGIGLNNVVKFRWNGGGVELIGNTTGG"
     CDS             1403451..1405229
                     /codon_start=1
                     /note="gene_1307"
                     /transl_table=11
                     /translation="VVTTHRAPYRHRARGCGRRGRVTTKLDGLMAFPEYSPAASAATF
                     ADLQIHPRVLRAIGDVGYESPTAIQAATIPALMAGSDVVGLAQTGTGKTAAFAIPMLS
                     KIDITSKVPQALVLVPTRELALQVAEAFGRYGAYLSQLNVLPIYGGSSYAVQLAGLRR
                     GAQVVVGTPGRVIDHLERATLDLSRVDFLVLDEADEMLTMGFADDVERILSETPEYKQ
                     VALFSATMPPAIRKLSAKYLHDPFEVTCKAKTAVAENISQSYIQVARKMDALTRVLEV
                     EPFEAMIVFVRTKQATEEIAEKLRARGFSAAAISGDVPQAQRERTITALRDGDIDILV
                     ATDVAARGLDVERISHVLNYDIPHDTESYVHRIGRTGRAGRSGAALIFVSPRELHLLK
                     AIEKATRQTLTEAQLPTVEDVNTQRVAKFADSITNALGGPGIELFRRLVEEYEREHDV
                     PMADIAAALAVQCRGGEAFLMAPDPPLSRRNRDQRRDRPQRPKRRPDLTTYRVAVGKR
                     HKIGPGAIVGAIANEGGLHRSDFGQIRIGPDFSLVELPAKLPRATLKKLAQTRISGVL
                     IDLRPYRPPDAARRHNGGKPRRKHVG"
     CDS             1405226..1406377
                     /codon_start=1
                     /note="gene_1308"
                     /transl_table=11
                     /translation="MTLPKERAAQGGLERIAHVDRVASLTGIRAVAALLVVGTHAAYT
                     TGKYTHGYWGLMSSRMEIGVPIFFVLSGFLLFRPWVKSAATGGPPPSLSRYAWHRVRR
                     IMPAYTVTVLLAYLVYHFRTAGPNPGHTWVGLFRNLTLTQIYTDGYLGAFLHQGLTQM
                     WSLAVEVAFYLALPALAYLLLVLVCRRRWQPRLLLATMAGLTMISPAWLILVHNTHWM
                     PDGARLWLPTYLAWFVGGMMLAVLAAMGVRCYAFVAIPLAVICYFIVSTPIAGAPTTS
                     PTALAEALVKTAFYAVIAVLAVAPLALGDQGWYAQLLASRPMVFLGEISYEIFLIHLV
                     TMEIAMVDVLGYRVYTSSMVNLCLVTLVLTIPLAWLLHRFTRVQGDRPS"
     CDS             complement(1406346..1406645)
                     /codon_start=1
                     /note="gene_1309"
                     /transl_table=11
                     /translation="VNTDVLAGLMAELPEGMVVTDPAVTDGYRQDRAFDPSAGKPLAI
                     IRPRRARWVVRMLTSLLMFPGRDEADERAMIAEFVVPIVTPASAAARKAGHPGPE"
     CDS             complement(1406642..1407901)
                     /codon_start=1
                     /note="gene_1310"
                     /transl_table=11
                     /translation="MRNSNRGPAFLILFATLMAAAGDGVSIVAFPWLVLQREGSAGQA
                     SIVASATMLPLLFATLVAGTAVDYFGRRRVSMVADALSGAAVAGVPLVAWGYGGDAVN
                     VLVLAVLAALAAAFGPAGMTARDSMLPEAAARAGWSLDRINGAYEAILNLAFIVGPAI
                     GGLMIATVGGITTMWITATAFGLSILAIAALQLEGAGKPHHTSRPQGLVSGIAEGLRF
                     VWNLRVLRTLGMIDLTVTALYLPMESVLFPKYFTDHQQPVQLGWALMAIAGGGLVGAL
                     GYAVLAIRVPRRVTMSTAVLTLGLASMVIAFLPPLPVIMVLCAVVGLVYGPIQPIYNY
                     VIQTRAAQHLRGRVVGVMTSLAYAAGPLGLLLAGPLTDAAGLHATFLALALPIVCTGL
                     VAIRLPALRELDLAPQADIDRPVGSAQ"
     CDS             1407993..1408799
                     /codon_start=1
```

/note="gene_1311"
/transl_table=11
/translation="MHPKTGRAFRSPVEPGSGWPGDPATPQTPVAADAAQVSALAGGA
GSICELNALISVCRACPRLVSWREEVAVVKRRAFADQPYWGRPVPGWGSKRPRLLILG
LAPAAHGANRTGRMFTGDRSGDQLYAALHRAGLVNSPVSVDAADGLRANRIRITAPVR
CAPPGNSPTPAERLTCSPWLNAEWRLVSDHIRAIVALGGFAWQVALRLAGASGTPKPR
FGHGVVTELGAGVRLLGCYHPSQQNMFTGRLTPTMLDDIFREAKKLAGIE"
CDS 1408792..1409625
/codon_start=1
/note="gene_1312"
/transl_table=11
/translation="LSDVKTVVVSGASVAGTAAAYWLGRHGYSVTMVERHPGLRPGGQ
AIDVRGPALDVLERMGLLAAAQEHKTRIRGASFVDRDGNELFRDTESTPTGGPVNSPD
IELLRDDLVELLYGATQPSVEYLFDDSISTLQDDGDSVRVTFERAAAREFDLVIGADG
LHSNVRRLVFGPEEQFVKRLGTHAAIFTVPNFLELDYWQTWHYGDSTMAGVYSARNNT
EARAALAFMDTELRIDYRDTEAQFAELQRRMAEDGWVRAQLLHYIAAHRISISTKCRR
S"
CDS 1409688..1409918
/codon_start=1
/note="gene_1313"
/transl_table=11
/translation="LSGQGTSVALLGAYILAGELKAAGDDYQLGFANYHAEFHGFVER
NQWLVSDNIPGGAPIPQEEFERIVHSITIKDY"
CDS complement(1410044..1410517)
/codon_start=1
/note="gene_1314"
/transl_table=11
/translation="MPLRKTDPMDISRWLERHVGVQLLRLHDAIYRGTNGRIGHRIPG
APPSLLLHTTGAKTSQPRTTSLTYARDGDAYLIVASKGGDPRSPGWYHNLKANPDVEI
NVGPKRFGVTAKPVQPHDPDYARLWQIVNENNANRYTNYQSRTSRPIPVVVLTRR"
CDS complement(1410498..1410932)
/codon_start=1
/note="gene_1315"
/transl_table=11
/translation="MPCVFCAIIAGEAPAIRIYEDGGYLAILDIRPFTRGHTLVLPKR
HTVDLTDTPPEALADMVAIGQRIARAARATKLADATHIAINDGRAAFQTVFHVHLHVL
PRRNGDKLSVAKGMMLRRDPDREATGRILREALAQQDAAAQD"
CDS 1410991..1412379
/codon_start=1
/note="gene_1316"
/transl_table=11
/translation="VDPTDLAFAGAAAQARMLADGALTAPMLLEVYLQRIERLDSHLR
AYRVVQFDRARAEAEAAQQRLDAGERLPLLGVPIAIKDDVDIAGEVTTYGSAGHGPAA
TSDAEVVRRLRAAGAVIIGKTNVPELMIMPFTESLAFGATRNPWCLNRTPGGSSGGSA
AAVAAGLAPVALGSDGGGSIRIPCTWCGLFGLKPQRDRISLEPHDGAWQGLSVNGPIA
RSVMDAALLLDATTTVPGPEGEFVAAAARQPGRLRIALSTRVPTPLPVRCGKQELAAV
HQAGALLRDLGHDVVVRDPDYPASTYANYLPRFFRGISDDADAQAHPDRLEARTRAIA
RLGSFFSDRRMAALRAAEVVLSSRIQSIFDDVDVVVTPGAATGPSRIGAYQRRGAVST
LLLVVQRVPYFQVWNLTGQPAAVVPWDFDGDGLPMSVQLVGRPYDEATLLALAAQIES
ARPWAHRRPSVS"
CDS 1412454..1413647

/codon_start=1
/note="gene_1317"
/transl_table=11
/translation="VTDHVREADDANIDDLLGDLGGTARAERAKLVEWLLEQGITPDE
IRATNPPLLLATRHLVGDDGTYVSAREISENYGVDLELLQRVQRAVGLARVDDPDAVV
HMRADGEAAARAQRFVELGLNPDQVVLVVRVLAEGLSHAAEAMRYTALEAIMRPGATE
LDIAKGSQALVSQIVPLLGPMIQDMLFMQLRHMMETEAVNAGECAAGKPLPGARQVTV
AFADLVGFTQLGEVVSAEELGHLAGRLAGLARDLTAPPVWFIKTIGDAVMLVCPDPAP
LLDTVLKLVEVVDTDNNFPRLRAGVASGMAVSRAGDWFGSPVNVASRVTGVARPGAVL
VADSVREALGDAPEADGFQWSFAGPRRLRGIRGDVRLFRVRRGATRTGSGGAAQDDDL
AGSSP"

CDS 1413946..1414500
/codon_start=1
/note="gene_1318"
/transl_table=11
/translation="MHGRRYGRPGGWQQAQQPDASGAAEWFAGRLPEDWFDGDPTVIV
DREEITVIGKLPGLESPEEESAARASGRVSRFRDETRPERMTIADEAQNRYGRKVSWG
VEVGGERILFTHIAVPVMTRLKQPERQVLDTLVDAGVARSRSDALAWSVKLVGEHTEE
WLAKLRTAMSAVDDLRAQGPDLPA"

CDS complement(1414520..1416310)
/codon_start=1
/gene="pknH"
/note="gene_1319"
/transl_table=11
/translation="MSDAQDSRVGSMFGPYHLKRLLGRGGMGEVYEAEHTVKEWTVAV
KLMTAEFSKDPVFRERMKREARIAGRLQEPHVVPIHDYGEVDGQMFLEMRLVEGTDLD
SVLKRFGPLTPPRAVAIITQIASALDAAHADGVMHRDVKPQNILITRDDFAYLVDFGI
ASATTDEKLTQLGTAVGTWKYMAPERFSNDEVTYRADIYALACVLHECLTGAPPYRAD
SAGTLVSSHLMGPIPQPSAIRPGIPKAFDAVVARGMAKKPEDRYASAGDLALAAHEAL
SDPDQDHAADILRRSQESTLPGTAAVTAQPPTMPTVTPPPIQAAPTGQPSWAPNSGPM
PASGPTPTPQYYQGGGWGAPPSGGPSPWAQTPRKTNPWPFVAGAAAVVLVLVLGAIGI
WIAIRPKPVQPPQPVAEERLSALLLNSSEVNAVMGSSSMQPGKPITSMDSSPVTVSLP
DCQGALYTSQDPVYAGTGYTAINGLISSEPGDNYEHWVNQAVVAFPTADKARAFVQTS
ADKWKNCAGKTVTVTNKAKTYRWTFADVKGSPPTITVIDTQEGAEGWECQRAMSVANN
VVVDVNACGYQITNQAGQIAAKIVDKVNKE"

CDS complement(1416651..1417817)
/codon_start=1
/note="gene_1320"
/transl_table=11
/translation="MAGSATVEKRLDFGLLGPLQMTIDGTPVPSGTPKQRAVLAMLVI
NRNRPVGVDALITALWEEWPPSGARASIHSYVSNLRKLLGGAGIDPRVVLAAAPPGYR
LSIPDNTCDLGRFVAEKTAGVHAAAAGRFEQASRHLSAALREWRGPVLDDLRDFQFVE
PFATALVEDKVLAHTAKAEAEIACGRASAVIAELEALTFEHPYREPLWTQLITAYYLS
DRQSDALGAYRRVKTTLADDLGIDPGPTLRALNERILRQQPLDAKKSAKTTAAGTVTV
LDQRTMASGQQAVAYLHDIASGRGYPLQAAATRIGRLHDNDIVLDSANVSRHHAVIVD
TGTNYVINDLRSSNGVHVQHERIRSAVTLNDGDHIRICDHEFTFQISAGTHGGT"

CDS complement(1418128..1418826)
/codon_start=1
/note="gene_1321"
/transl_table=11
/translation="MTTSKIATAFKTATFALAAGAVALGLASPADAAAGTMYGDPAAA

AKYWRQQTYDDCVLMSAADVIGQVTGREPSERAIIKVAQSTPSVVHPGSIYTKPADAE
HPNSGMGTSVADIPTLLAHYGVDAVITDEDHATATGVATGMAALEQYLGSGHAVIVSI
NAEMIWGQPVEETDSAGNPRSDHAVVVTGVDTENGIVHLNDSGTPTGRDEQIPMETFV
EAWATSHDFMAVTT"
CDS complement(1419049..1419423)
/codon_start=1
/note="gene_1322"
/transl_table=11
/translation="MTTMITLRRRFAVAVAGVATAAATTVTLAPAPANAADVYGAIAY
SGNGSWGRSWDYPTRAAAEATAVKSCGYSDCKVLTSFTACGAVAANDRAYQGGVGPTL
AAAMKDALTKLGGGYIDTWACN"
CDS complement(1419484..1420218)
/codon_start=1
/note="gene_1323"
/transl_table=11
/translation="MKHPPCSVVAAATAILAVVLAIGGCSTEGDAGKASDTAATASNG
DAAMLLKQATDAMRKVTGMHVRLAVTGDVPNLRVTKLEGDISNTPQTVATGSATLLVG
NKSEDAKFVYVDGHLYSDLGQPGTYTDFGNGASIYNVSVLLDPNKGLANLLANLKDAS
VAGSQQADGVATTKITGNSSADDIATLAGSRLTSEDVKTVPTTVWIASDGSSHLVQIQ
IAPTKDTSVTLTMSDWGKQVTATKPV"
CDS complement(1420431..1420772)
/codon_start=1
/note="gene_1324"
/transl_table=11
/translation="MLSPLSPRIIAAFTTAVGAAAIGLAVATAGTAGANTKDEAFIAQ
MESIGVTFSSPQVATQQAQLVCKKLASGETGTEIAEEVLSQTNLTTKQAAYFVVDATK
AYCPQYASQLT"
CDS complement(1420880..1422724)
/codon_start=1
/note="gene_1325"
/transl_table=11
/translation="MRSRDFWGSAARLVKRLAPQRRLSIAVITLGIAGTTIGVIVPRI
LGHATDLLFNGVIGRGLPGGITKAQAVASARARGDNTFADLLSGMNVVPGQGVDFAAV
ERTLALALALYLAAALMIWAQARLLNLTVQKTMVRLRTDVEDKVHRLPLSYFDGQQRG
ELLSRVTNDIDNLQSSLSMTISQLVTSILTMVAVLAMMVSISGLLALITLLTVPLSLL
VTRAITRRSQPLFVAHWTSTGRLNAHLEETYSGFTVVKTFGHQAAARERFHELNDDVY
QAGFGAQFLSGLVQPATAFIGNLGYVAVAVAGGLQVATGQITLGSIQAFIQYIRQFNM
PLSQLAGMYNALQSGVASAERVFDVLDEPEESPEPEPELPNLTGRVEFEHVNFAYLPG
TPVIRDLSLVAEPGSTVAIVGPTGAGKTTLVNLLMRFYEIGSGRILIDGVDIASVSRQ
SLRSRIGMVLQDTWLYDGTIAENIAYGRPEATTDEIVEAARAAHVDRFVNTLPAGYQT
RVSGDGGSISVGEKQLITIARAFLARPQLLILDEATSSVDTRTELLIQRAMRELRRDR
TSFIIAHRLSTIRDADHILVVQTGQIVERGNHAELLARRGVYYQMTRA"
CDS complement(1422772..1424538)
/codon_start=1
/note="gene_1326"
/transl_table=11
/translation="VCDSYPMLLALLRQHIRPYRRLVAMLMMLQLVSTLASLYLPTVN
AAIVDDGVAKGDTATIVRLGAVMLGVTGLQVLCAIGAVYLGSRTGAGFGRDLRSAMFE
HIITFSERETARFGAPTLLTRSTNDVRQILFLVQMTATVLVTAPIMCVGGIIMAIHQE
AALTWLLLVSVPILAVANYWIISHMLPLFRRMQSLIDGINRVMRDQLSGVRVVRAFTR
EGYERDKFAQANTALSNAALSAGNWQALMLPVTTLTINASSVALIWFGGLRIDSGQMQ

VGSLIAFLSYFAQILMAVLMATMTLAVLPRASVCAERITEVLSTPAALGNPDNPKFPT
DGVTGVVRLAGATFTYPGADCPVLQDISLTARPGTTTAIVGSTGSGKSTLVSLICRLY
DVTAGAVLVDGIDVREYHTERLWSAIGLVPQRSYLFSGTVADNLRYGGGPDQVVTEQE
MWEALRVAAADGFVQTDGLQTRVAQGGVNFSGGQRQRLAIARAVIRRPAIYVFDDAFS
ALDVHTDAKVHASLRQVSGDATIIVVTQRISNAAQADQVIVVDNGKIVGTGTHETLLA
DCPTYAEFAASQSLSATVGGVG"

CDS 1424667..1425224
/codon_start=1
/note="gene_1327"
/transl_table=11
/translation="VRRKVRRLTLAVSALVALFPAVAGCSDSGDNKPGATIPSTPANA
EGRHGPFFPQCGGVSDQTVTELTRVTGLVNTAKNSVGCQWLAGGGILGPHFSFSWYRG
SPIGRERKTEELSRASVEDINIDGHSGFIAIGNEPSLGDSLCEVGIQFSDDFIEWSVS
FSQKPFPPPCDIAKELTRQSIANSK"

CDS 1425221..1425763
/codon_start=1
/note="gene_1328"
/transl_table=11
/translation="MRRVLVGAAALITALLVLTGCTKSISGTAVKAGGAGVPRNNNSQ
ERYPNLLKECEVLTTDILAKTVGADPLDIQSTFVGAICRWQAANPAGLIDITRFWFEQ
GSLSNERKVAEGLKYQVETRAIQGVDSIVMRTGDPNGACGVASDAAGVVGWWVNPQAP
GIDACGQAIKLMELTLATNA"

CDS complement(1425908..1426414)
/codon_start=1
/note="gene_1329"
/transl_table=11
/translation="VNEQYRNLVLMRHAKSAYPDGIADHDRPLAPRGIREAGLAGGWL
RANLPAVDAVLCSTATRARQTLAHTGIDAPARYAERLYGAAPGTVIEEINRVGDNVTS
LLVVGHEPTTSALAIVLASISGTDAAVAERISEKFPTSGIAVLRVAGHWADVEPGCAA
LVGFHVPR"

CDS complement(1426844..1426996)
/codon_start=1
/gene="HMPREF1169_02723"
/note="gene_1330"
/transl_table=11
/translation="MVTRQQIDRKADHAHGFQGLTDYLWRELIVFEDVTGDYDEFGAH
IGGQRF"

CDS 1426952..1427887
/codon_start=1
/note="gene_1331"
/transl_table=11
/translation="MRVIGLPVYLLPGNHDPLDASSVYTSTLFRAERPDNVVVLDRAG
VHEVRPGVQIVAAPWRSKAPTTDPVAEVLAGLPTDAAIRLLVAHGGVDALDPDHDKPS
LIRLAALDDALTRQAIHYVALGDKHSLTQVGSSGRVWYSGAPEVTNFDDVEPDPGHVL
VVDIDESDPRHPVTVDARRIGRWRFVTLHHQVDTSRDIADLDLNLDLMTDKDRTVVRL
ALTGSLTVTDRAALDTCLDKYARLFAWLGLWERHTDLAVIPVDAEFTDLGIGGFAAAA
VDELVATARGGDDESAVDAQAALALLLRLADRGAA"

CDS 1427884..1430511
/codon_start=1
/note="gene_1332"
/transl_table=11

/translation="VKLHRLALTNYRGIAHRDVEFPDHGVVVVCGANEIGKSSMVEAL
DLLLEYKDRSTKKEVKQVKPTNADVGSEVIAEISSGPYRFVYRKRFHKRCETELTVLA
PRREQLTGDEAHERVRTMLAETVDTELWHAQRVLQAASTAAVDLSGCDALSRALDLAA
GDDAALSGTESLLIERIEAEYARYFTPTGRPTGEWSAAVSRLAAAEAAVADCAAAVAE
VDDGVRRHTELTEQVAELSQQLLAHQLRLEAARVAAEKIAAITDDAREAKLIATAAAA
TSGASTAAHAGRLGLLTEIDTRTAAVVAAEAKARQAADEQATARAEAEACDAALTEAT
QVLTAVRLRAESARRTLDQLADCEEADRLAARLARIDDIEGDRDRVCAELSAVTLTEE
LLSRIERAAAAVDRGGAQLASISAAVEFTAAVDIELGVGDQRVSLSAGQSWSVTATGP
TEVKVPGVLTARIVPGATALDFQAKYAAAQQELADALAAGEVADLAAARSADLCRREL
LSRRDQLTATLAGLCGDEQVDQLRSRLEQLCAGQPAELDLVSTDTATARAELDAVEAA
RIAAEKDCETRRQIAAGAARRLAETSTRATVLQNAAAAESAELGAAMTRLACERASVG
DDELAAKAEADLRVLQTAEQRVIDLADELAATAPDAVAAELAEAADAVELLRERHDEA
IRALHEVGVELSVFGTQGRKGKLDAAETEREHAASHHARVGRRARAARLLRSVMARHR
DTTRLRYVEPYRAELHRLGRPVFGPSFEVEVDTDLRIRSRTLDDRTVPYECLSGGAKE
QLGILARLAGAALVAKEDAVPVLIDDALGFTDPERLAKMGEVFDTIGADGQVIVLTCS
PTRYGGVKGAHRIDLDAIQ"

CDS 1430532..1432118
/codon_start=1
/note="gene_1333"
/transl_table=11
/translation="MDTQSDYVVVGTGSAGAVVASRLSTDPATTVVALEAGPRDKNRF
IGVPAAFSKLFRSEIDWDYLTEPQPELDGREIYWPRGKVLGGSSSMNAMMWVRGFASD
YDEWAARAGPRWSYADVLGYFRRIENVTAAWHFVSGDDSGVTGPLHISRQRSPRSVTA
AWLAAARECGFAAARPNSPRPEGFCETVVTQRRGARFSTADAYLKPAMRRKNLRVLTG
ATATRVVIDGDRAVGVEYQSDGQTRIVYARREVVLCAGAVNSPQLLMLSGIGDRDHLA
EHDIDTVYHAPEVGCNLLDHLVTVLGFDVEKDSLFAAEKPGQLISYLLRRRGMLTSNV
GEAYGFVRSRPELKLPDLELIFAPAPFYDEALVPPAGHGVVFGPILVAPQSRGQITLR
SADPHAKPVIEPRYLSDLGGVDRAAMMAGLRICARIAQARPLRDLLGSIARPRNSTEL
DEATLELALATCSHTLYHPMGTCRMGSDEASVVDPQLRVRGVDGLRVADASVMPSTVR
GHTHAPSVLIGEKAADLIRS"

CDS complement(1432135..1433772)
/codon_start=1
/note="gene_1334"
/transl_table=11
/translation="MVLTGCSASGTQLELAPTADRRAAVGTTSDINQQDPATLQDGGN
LRLSLTDFPPNFNILHIDGNNAEVAAMMKATLPRAFIIGPDGSTTVDTNYFTSIELTR
TAPQVVTYTINPEAVWSDGTPITWRDIASQIHAISGADKAFEIASSSGAERVASVTRG
VDDRQAVVTFAKPYAEWRGMFAGNGMLLPASMTATPEAFNKGQLDGPGPSAGPFVVSA
LDRTAQRIVLTRNPRWWGARPRLDSITYLVLDDAARLPALQNNTIDATGVGTLDQLTI
AARTKGISIRRAPGPSWYHFTLNGAPGSILADKALRLAIAKGIDRYTIARVAQYGLTS
DPVPLNNHVFVAGQDGYQDNSGVVAYNPEQAKRELDALGWRRSGAFREKDGRQLVIRD
LFYDAQSTRQFAQIAQHTLAQIGVKLELQAKSGSGFFSDYVVNVGAFDIAQFGWVGDAF
PLSSLTQIYASDGESNFGKIGSPQIDAAIERTLAELDPGKARALANQVDELIWAEGFS
LPLTQSPGTVAVRSTLANFGATGLADLDYTAIGFMRR"

CDS complement(1433903..1435741)
/codon_start=1
/note="gene_1335"
/transl_table=11
/translation="MSPLLEVTDLAVTFRTDGDPVTAVRGISYRVEPGEVVAMVGESG
SGKSAAAMAVVGLLPEYAQVRGSVRLQGTELLGLADNAMSRFRGKAIGTVFQDPMSAL
TPVYTVGDQIAEAIEVHQPRVGKKAARRRAVELLDLVGISQPQRRSRAFPHELSGGER

QRVVIAIAIANDPDLLICDEPTTALDVTVQAQILDVLKAARDVTGAGVLIITHDLGVV
AEFADRALVMYAGRVVESAGVNDLYRDRRMPYTVGLLGSVPRLDAAQGTRLVPIPGAP
PSLAGLAPGCPFAPRCPLVIDECLTAEPELLDVATDHRAACIRTELVTGRSAADIYRV
KTEARPAALGDASVVVRVRHLVKTYRLAKGVVLRRAIGEVRAVDGISLELRQGRTLGI
VGESGSGKSTTLHEILELAAPQSGSIEVLGTDVATLGTAERRSLRRDIQVVFQDPVAS
LDPRLPVFDLIAEPLQANGFGKNETHARVAELLDIVGLRHGDASRYPAEFSGGQKQRI
GIARALALQPKILALDEPVSALDVSIQAGIINLLLDLQEQFGLSYLFVSHDLSVVKHL
AHQVAVMLAGTVVEQGDSEEVFGNPKHEYTRRLLGAVPQPDPARRG"
CDS complement(1435738..1436613)
/codon_start=1
/note="gene_1336"
/transl_table=11
/translation="MTEFASRRTLVVRRFLRNRAAVASLAALLLLFVSAYALPPLLPY
SYDDLDFNALLQPPGTKHWLGTNALGQDLLAQTLRGMQKSMLIGVCVAVISTGIAATV
GAISGYFGGWRDRTLMWVVDLLLVVPSFILIAIVTPRTKNSANIMFLVLLLAGFGWMI
SSRMVRGMTMSLREREFIRAARYMGVSSRRIIVGHVVPNVASILIIDAALNVAAAILA
ETGLSFLGFGIQPPDVSLGTLIADGTASATAFPWVFLFPASILVLILVCANLTGDGLR
DALDPASRSLRRGVR"
CDS complement(1436610..1437587)
/codon_start=1
/note="gene_1337"
/transl_table=11
/translation="MTRYLARRLLNYLVLLALASFLTYCLTSLAFSPLESLMQRSPRP
PQAVIDAKAHDLGLDRPILARYANWVSHAVRGDFGTTITGQPVGTELGRRIGVSLRLL
VVGSVFGTVAGVVIGAWGAIRQYRLSDRVMTTLALLVLSTPTFVVANLLILGALRVNW
AVGIQLFDYTGETSPGVAGGVWDRLGDRLQHLILPSLTLALAAAAGFSRYQRNAMLDV
LGQDFIRTARAKGLTRRRALLKHGLRTALIPMATLFAYGVAGLVTGAVFVEKIFGWHG
MGEWMVRGISTQDTNIVAAITVFSGAVVLLAGLLSDVIYAALDPRVRVS"
CDS 1437794..1438285
/codon_start=1
/note="gene_1338"
/transl_table=11
/translation="VTVTDDYLANNVDYASGFKGPLPMPPSKHIAIVACMDARLDVYR
MLGIKEGEAHVIRNAGCVVTDDVIRSLAISQRLLGTREIILLHHTDCGMLTFTDDDFK
RAIQDETGIRPTWSPESYPDAVEDVRQSLRRIEVNPFVTKHTSLRGFVFDVATGKLNE
VTP"
CDS 1438379..1439377
/codon_start=1
/note="gene_1339"
/transl_table=11
/translation="MAITINMVNPTGFIRYEDVEQEAMTSDVTVGPAPGQYQLSHLRL
LEAEAIHVIREVAAEFERPVLLFSGGKDSIVMLHLALKAFRPGRLPFPVMHVDTGHNF
DEVIATRDELVAAAGVRLVVASVQDDIDAGRVVETIPSRNPIQTVTLLRAIRENQFDA
AFGGARRDEEKARAKERVFSFRDEFGQWDPKAQRPELWNLYNGRHHKGEHIRVFPLSN
WTEFDIWSYIGAEQVRLPSIYFAHRRKVFQRDGMLLAVHRHMQPRADEPVFEATVRFR
TVGDVTCTGCVESSASTVAEVIAETAVARLTERGATRADDRISEAGMEDRKRQGYF"
CDS 1439377..1441221
/codon_start=1
/note="gene_1340"
/transl_table=11
/translation="MTTLLRLATAGSVDDGKSTLIGRLLYDSKAVMEDQWASVEQTSK

```
                     DRGHDYTDLALVTDGLRAEREQGITIDVAYRYFATPKRKFIIADTPGHIQYTRNMVTG
                     ASTAQLVIVLVDARHGLLEQSRRHAFLASLLGIRHLVLAVNKMDLLGWDQEKFDAIRD
                     EFHAFAARLDVQDVTSIPISALHGDNVVTKSDQTPWYEGPSLLSHLEDVYIAGDRNMV
                     DVRFPVQYVIRPHTLEHQDHRSYAGTVASGVMRSGDEVVVLPIGKTTRITAIDGPNGP
                     VAEAFPPMAVSVRLADDIDISRGDMIARTHNQPRITQEFDATVCWMADNAVLEPGRDY
                     VVKHTTRTVRARIAGLDYRLDVNTLHRDKTATALKLNELGRVSLRTQVPLLLDEYTRN
                     ASTGSFILIDPDTNGTVAAGMVLRDVSARTPSPNTVRHRSLVTAQDRPPRGKTVWFTG
                     LSGSGKSSVAMLVERKLLEKGISAYVLDGDNLRHGLNADLGFSMADRAENLRRLSHVA
                     TLLADCGHLVLVPAISPLAEHRALARKVHADAGIDFFEVFCDTPLQDCERRDPKGLYA
                     KARAGEITHFTGIDSPYQRPKNPDLRLTPDRSIDEQAQEVIDLLESSS"
     CDS             1441275..1441760
                     /codon_start=1
                     /note="gene_1341"
                     /transl_table=11
                     /translation="MRMSAKAEYAVRAMVQLATAASGTVVKTDDLAAAQGIPPQFLVD
                     ILTNLRTDRLVRSHRGREGGYELARPGTEISIADVLRCIDGPLASVRDIGLGDLPYSG
                     PTTALTDVWRALRASMRSVLEETTLADVAGGALPEHVAQLADDYRAQESTRHGASRHG
                     D"
     CDS             1441818..1443035
                     /codon_start=1
                     /note="gene_1342"
                     /transl_table=11
                     /translation="MVSTHAVVAGETLSALALRFYGDAELYRLIAAASGIADPDVVNV
                     GQRLIMPDFTRYTVVAGDTLSALAARFYGDASLYPLIAAVNGIADPGVIDVGQVLVIF
                     IGRSDGFGLRIVDRNENDPRLWYYRFQTSAIGWNPGVNVLLPDDYRTSGRTYPVLYLF
                     HGGGTDQDFRTFDFLGIRDLTAGKPIIIVMPDGGHAGWYSNPVSSFVGPRNWETFHIA
                     QLLPWIEANFRTYAEYDGRAVAGFSMGGFGALKYAAKYYGHFASASSHSGPASLRRDF
                     GLVVHWANLSSAVLDLGGGTVYGAPLWDQARVSADNPVERIDSYRNKRIFLVAGTSPD
                     PANWFDSVNETQVLAGQREFRERLSNAGIPHESHEVPGGHVFRPDMFRLDLDGIVARL
                     RPASIGAAAERAD"
     CDS             1443084..1443716
                     /codon_start=1
                     /note="gene_1343"
                     /transl_table=11
                     /translation="MCVSVGESVAQSLQQWDRKLWDVAMLHACNAVDETGRKRYPTLG
                     VGTRFRTALRDSLDIYGVMATPGVDLEKTRFPVGVRSDLLPDKRPDIADVLYGIHRWL
                     HGHADESSVEFEVSPYVNASAALRIANDGKIQLPKSAILGLLAVAVFAPENKGEVIPP
                     DYQLSWYDHVFFISVWWGWQDHFREIVNVDRASLVALDFGDLWNGWTPVG"
     CDS             complement(1443855..1445258)
                     /codon_start=1
                     /note="gene_1344"
                     /transl_table=11
                     /translation="MLNRRKAILDYLQGAVWVLPTFGVAIGLGSGAVLSMIPVKSGTL
                     IDKLMFQGTPGDARGVLIVVSATMITTIGIVFSLTVLSLQIASSQFSVRLLRTFLRDV
                     PNQVVLAIFACTFAYSTGGLHTVGEHRDGGAFIPKVAVTGSLALAFVSIAALIYFLHH
                     LMHSIQIDTIMDKVRLRTLGLVDQLYPESDTADRQVETPPSPPADAVPLLAPHSGYLQ
                     TVDVDDIAELAAASRYTALLVTFVGDYVTAGGLLGWCWRRGTAPGAPGSDFPQRCLRH
                     VHIGFERTLQQDIRFGLRQMVDIALRALSPALNDPYTAIQVVHHLSAVESVLASRALP
                     DDVRRDRAGELLFWLPYPSFATYLHVGCAQIRRYGSREPLVLTALLQLLSAVAQNCVD
                     PSRRVAVQTQIALVVRAAQREFADESDRAMVLGAAARATEVVERPGTLAPPPSTFGQV
                     AAAQAAASTIRSADRDG"
```

```
CDS             1445590..1445745
                /codon_start=1
                /note="gene_1345"
                /transl_table=11
                /translation="VPVFDGYTRMVARLMGSLAVLRSVSIPKGYRDFGFGSLRAVAPK
                NCPDVSG"
CDS             complement(1445872..1446207)
                /codon_start=1
                /note="gene_1346"
                /transl_table=11
                /translation="MFTRRFAASMVGTTLTAATLGLAALGFAGTASASSTDEAFLAQL
                QADGITPPSAARAIKDAHAVCDALDEGHSAKAVIKAVAKATGLSAKGAKTFAVDAASA
                YCPQYVTSS"
tRNA            complement(1446563..1446638)
                /note="identified by tRNAscan-SE; putative"
CDS             1446752..1448404
                /codon_start=1
                /gene="argS"
                /note="gene_1347"
                /transl_table=11
                /translation="VTPADLAELLKATAAAVLAERGLDASALPQMVTVERPRIPEHGD
                YASNLAMQLAKKVGTNPRELAGWLAEALTKVDGIASAEVAGPGFINMRLETAAQAKVV
                TSVIDAGHSYGHSLLLAGRKVNLEFVSANPTGPIHIGGTRWAAVGDALGRLLTTQGAD
                VVREYYFNDHGAQIDRFANSLIAAAKGEPTPQDGYAGSYITNIAEQVLQKAPDALSLP
                DAELRETFRAIGVDLMFDHIKQSLHEFGTDFDVYTHEDSMHTGGRVENAIARLRETGN
                IYEKDGATWLRTSAFGDDKDRVVIKSDGKPAYIAGDLAYYLDKRQRGFDLCIYMLGAD
                HHGYIARLKAAAAAFGDDPATVEVLIGQMVNLVRDGQPVRMSKRAGTVLTLDDLVEAI
                GVDAARYSLIRSSVDTAIDIDLALWSSASNENPVYYVQYAHARLSALARNAAELALIP
                DTNHLELLNHDKEGTLLRTLGEFPRVLETAASLREPHRVCRYLEDLAGDYHRFYDSCR
                VLPQGDEQPTDLHTARLALCQATRQVIANGLAIIGVTAPERM"
CDS             1448401..1449744
                /codon_start=1
                /note="gene_1348"
                /transl_table=11
                /translation="VNELLHLAPNVWPRNTTRDEVGVVCIAGIPLTQLAQEYGTPLFV
                IDEDDFRSRCRETAAAFGSGANVHYAAKAFLCSEVARWISEEGLCLDVCTGGELAVAL
                HASFPPERITLHGNNKSVSELTAAVKAGVGHIVVDSMTEIERLDAIAGEAGIVQDVLV
                RLTVGVEAHTHEFISTAHEDQKFGLSVASGAAMAAVRRVFATDHLRLVGLHSHIGSQI
                FDVDGFELAAHRVIGLLRDVVGEFGPEKTAQIATVDLGGGLGISYLPSDDPPPIAELA
                AKLGTIVSDESTAVGLPTPKLVVEPGRAIAGPGTITLYEVGTVKDVDVSATAHRRYVS
                VDGGMSDNIRTALYGAQYDVRLVSRVSDAPPVPARLVGKHCESGDIIVRDTWVPDDIR
                PGDLVAVAATGAYCYSLSSRYNMVGRPAVVAVHAGNARLVLRRETVDDLLSLEVR"
CDS             1449748..1451073
                /codon_start=1
                /note="gene_1349"
                /transl_table=11
                /translation="VPGDEKPVGVAVLGLGNVGSEVVRIIENSAEDLAARVGAPLVLR
                GIGVRRVTTDRGVPIELLTDDIEELVAREDVDIVVEVMGPVEPSRKAILGALERGKSV
                VTANKALLATSTGELAQAAESAHVDLYFEAAVAGAIPVIRPLTQSLAGDTVLRVAGIV
                NGTTNYILSAMDSTGADYASALADASALGYAEADPTADVEGYDAAAKAAILASIAFHT
                RVTADDVYREGITKVTPADFGSAHALGCTIKLLSICERITTDEGSQRVSARVYPALVP
```

```
                     LSHPLAAVNGAFNAVVVEAEAAGRLMFYGQGAGGAPTASAVTGDLVMAARNRVLGSRG
                     PRESKYAQLPVAPMGFIETRYYVSMNVADKPGVLSAVAAEFAKREVSIAEVRQEGVVD
                     EGGRRVGARIVVVTHLATDAALSETVDALDDLDVVQGVSSVIRLEGTGL"
CDS                  1451070..1452152
                     /codon_start=1
                     /note="gene_1350"
                     /transl_table=11
                     /translation="MTVPPTATHQPWPGVIAAYRDRLPVGDDWTPVTLLEGGTPLIAA
                     TNLSKQTGCTIHLKVEGLNPTGSFKDRGMTMAVTDALAHGQRAVLCASTGNTSASAAA
                     YAARAGITCAVLIPQGKIAMGKLAQAVMHGAKIIQIDGNFDDCLELARKMAADFPTIS
                     LVNSVNPVRIEGQKTAAFEIVDVLGTAPDVHALPVGNAGNITAYWKGYTEYHQLGLID
                     KLPRMLGTQAAGAAPLVLGEPVSHPETIATAIRIGSPASWTSAVEAQQQSKGRFLAAS
                     DEEILAAYHLVARVEGVFVEPASAASIAGLLKAIDDGWVARGSTVVCTVTGNGLKDPD
                     TALKDMPSVSPVPVDPVAVVEKLGLA"
CDS                  1452370..1453320
                     /codon_start=1
                     /note="gene_1351"
                     /transl_table=11
                     /translation="MVTQALLPSGLVASAVVAASSANLGPGFDSVGLALSLYDEIIVE
                     TTDSGLTVTVDGEGGDQVPLGPEHLVVRAVQHGLQAAGVSAAGLAVRCRNAIPHSRGL
                     GSSAAAVVGGLAAVNGLVVQTDSSPSSDAELIQLASEFEGHPDNAAAAVLGGAVVSWT
                     DHSGDRPNYSAVSLRLHPDIRLFTAIPEQRSSTAETRVLLPAQVSHDDARFNVSRAAL
                     LVVALTERPDLLMAATEDLLHQPQRAAAMTASAEYLRLLRRHNVAAALSGAGPSLIAL
                     STDSELPTDAVEFGAAKGFAVTELTVGEAVRWSPTVRVPG"
CDS                  1453577..1455385
                     /codon_start=1
                     /note="gene_1352"
                     /transl_table=11
                     /translation="VTDTDLITAGESTDGKPSDAAATDPPDLNADEPAGSLATMVLPE
                     LRALANRAGVKGTSGMRKNELIAAIEEIRRQANGAPAVDRSAQEHDKGDRPPSSEAPA
                     TQGEQTPTEQIDSQSQQVRPERRSATREAGPSGSGERAGTAADDTDNRQGGQQDAKTE
                     ERGTDAGGDQGGDQQASGGQQARGDEDGEARQGRRGRRFRDRRRRGERSGDGAEAELR
                     EDDVVQPVAGILDVLDNYAFVRTSGYLPGPHDVYVSMNMVRKNGMRRGDAVTGAVRVP
                     KEGEQPNQRQKFNPLVRLDSINGGSVEDAKKRPEFGKLTPLYPNQRLRLETSTERLTT
                     RVIDLIMPIGKGQRALIVSPPKAGKTTILQDIANAITRNNPECHLMVVLVDERPEEVT
                     DMQRSVKGEVIASTFDRPPSDHTSVAELAIERAKRLVEQGKDVVVLLDSITRLGRAYN
                     NASPASGRILSGGVDSTALYPPKRFLGAARNIEEGGSLTIIATAMVETGSTGDTVIFE
                     EFKGTGNAELKLDRKIAERRVFPAVDVNPSGTRKDELLLSPDEFAIVHKLRRVLSGLD
                     SHQAIDLLMSQLRKTKNNYEFLVQVSKTTPGSMDSD"
CDS                  1455536..1455778
                     /codon_start=1
                     /gene="rpmE"
                     /note="gene_1353"
                     /transl_table=11
                     /translation="MKSDIHPAYEETTVVCGCGNTFQTRSTKPGGRIVVEVCSQCHPF
                     YTGKQKILDSGGRVARFEKRYGKRKVGADKAVSTGK"
CDS                  1455868..1456941
                     /codon_start=1
                     /gene="prfA"
                     /note="gene_1354"
                     /transl_table=11
```

/translation="MTQPVQTIDVLLAEHAELELALADPALHSNPAEARRVGRRFARL
APIVATHRKLTSARDDLETARELVASDESFAAEVAALEARVGELDAQLTDMLAPRDPH
DADDIVLEVKSGEGGEESALFAADLARMYIRYAERHGWAVTVLDETTSDLGGYKDATL
AIASKADTPDGVWSRMKFEGGVHRVQRVPVTESQGRVHTSAAGVLVYPEPEEVGQVQI
DESDLRIDVFRSSGKGGQGVNTTDSAVRITHLPTGIVVTCQNERSQLQNKTRALQVLA
ARLQAMAEEQALADASADRASQIRTVDRSERIRTYNFPENRITDHRIGYKSHNLDQVL
DGDLDALFDALSAADKQSRLRQS"

CDS 1456938..1457915
/codon_start=1
/note="gene_1355"
/transl_table=11
/translation="MTSAPATMRWGNLPLAGESGTMTLRQAIDLAAALLAEAGVDSAR
CDAEQLAAHLAGTDRGRLPLFEPPGDEFFGRYRDIVTARARRVPLQHLIGTVSFGPVV
LHVGPGVFVPRPETEAILGWATAQSLPARPLIVDACTGSGALAVALAQHRANLGLKAR
IIGIDDSDCALDYARRNAAGTPVELVRADVTTPCLLPELDGQVDLMVSNPPYIPDAAV
LEPEVAQHDPHHALFGGPDGMTVISAVVGLAGRWLRPGGLFAVEHDDTTSSSTVDLVS
STKLFVDVQARKDLAGRPRFVTAMRWGHLPLAGENGAIDPRQRRCRAKR"

CDS 1457931..1458584
/codon_start=1
/note="gene_1356"
/transl_table=11
/translation="LTETFDCADPEQRSRGIVSAVGAIKAGQLVVMPTDTVYGIGADA
FDSSAVAALLSAKGRGRDMPVGVLVGSWHTIEGLVYSMPDGARELIRAFWPGALSLVV
VQAPSLQWDLGDAHGTVMLRMPLHPVAIELLREVGPMAVSSANISGHPPPVDAEQARS
QLGDHVAVYLDAGPSEQQAGSTIVDLTGATPRVLRQGPVSTERIAEVLGVDAASLFG"

CDS 1458665..1459882
/codon_start=1
/note="gene_1357"
/transl_table=11
/translation="LVQYGLEVSSDVAGVAGGLLALSYRGAGVPLRELALVGLTAAII
TYFATGPVRMLASRLGAVAYPRERDVHVTPTPRMGGLAMFLGIVGAVFLASQLPALTR
GFVYSTGMPAVLVAGAVIMGIGLIDDRWGLDALTKFAGQITAASVLVTMGVAWSVLYI
PVGGVGTIVLDQASSILLTLALTVSIVNAMNFVDGLDGLAAGLGLITALAICMFSVGL
LRDHGGDVLYYPPAVISVVLAGACLGFLPHNFHRAKIFMGDSGSMLIGLMLAAASTTA
AGPISQNAYGARDVFALLSPFLLVVAVMFVPMLDLLLAIVRRTRAGRSAFSPDKMHLH
HRLLQIGHSHRRVVLIIYLWVGIGAFGAASSIFFNPRDTAAVMLGAIVVAGVATLIPL
LRRGDDYYDPDLD"

CDS 1460169..1460624
/codon_start=1
/note="gene_1358"
/transl_table=11
/translation="VFPSVAFRPVRLFFINVGLAAVAMLVAGVFGHLTVGMFLGLGLL
LGLLNALLVRRSAESITAKEHPLKRSMALNSASRLAIITILGLIIAYIFRPAGLGVVF
GLAFFQVLLVATTALPVLKKLRTATEEPVATYSSNGQTGGSEGRSASDD"

CDS 1460617..1461369
/codon_start=1
/note="gene_1359"
/transl_table=11
/translation="MTETILAAQIEVGEHHTATWLGMTVNTDTVLSTAIAGLIVIALA
FYLRAKVTSTDVPGGVQLFFEAITIQMRNQVESAIGMRIAPFVLPLAVTIFVFILISN
WLAVLPVQYTDKHGHTTELLKSAAADINYVLALALFVFVCYHTAGIWRRGIVGHPIKL

LKGHVTLLAPINLVEEVAKPISLSLRLFGNIFAGGILVALIALFPPYIMWAPNAIWKA
FDLFVGAIQAFIFALLTILYFSQAMELEEEHH"
CDS 1461418..1461663
/codon_start=1
/note="gene_1360"
/transl_table=11
/translation="MDPTIAAGALIGGGLIMAGGAIGAGIGDGVAGNALISGVARQPE
AQGRLFTPFFITVGLVEAAYFINLAFMALFVFATPVK"
CDS 1461694..1462209
/codon_start=1
/note="gene_1361"
/transl_table=11
/translation="MGEVSAIVLAASQAAEEGGESSNFLIPNGTFFVVLAIFLVVLAV
IGTFVVPPILKVLRERDAMVAKTLADNKKSDEQFAAAQADYDEAMTEARVQASSLRDN
ARADGRKVIEDARVRAEQQVASTLQTAHEQLKRERDAVELDLRAHVGTMSATLASRIL
GVDLTASAATR"
CDS 1462216..1463556
/codon_start=1
/note="gene_1362"
/transl_table=11
/translation="MSTFIGQLFGFAVIVYLVWRFIVPLVGRLMSARQDTVRQQLADA
AAAADRLAEASQAHTKALEDAKSEAHRVVEEARTDAERIAEQLEAQADVEAERIKMQG
ARQVDLIRAQLTRQLRLELGHESVRQARELVRNHVADQAQQSATVDRFLDQLDAMAPA
TADVDYPLLAKMRSASRRALTSLVDWFGTMAQDLDHQGLTTLAGELVSVARLLDREAV
VTRYLTVPAEDATPRIRLIERLVSGKVGAPTLEVLRTAVSKRWSANSDLIDAIEHVSR
QALLELAERAGQVDEVEDQLFRFSRILDVQPRLAILLGDCAVPAEGRVRLLRKVLERA
DSTVNPVVVALLSHTVELLRGQAVEEAVLFLAEVAVARRGEIVAQVGAAAELSDAQRT
RLTEVLSRIYGHPVTVQLHIDAALLGGLSIAVGDEVIDGTLSSRLAAAEARLPD"
CDS 1463601..1465250
/codon_start=1
/note="gene_1363"
/transl_table=11
/translation="MAELTIPADDIQSAIEEYVSSFTADTSREEVGTVVDAGDGIAHV
EGLPSVMTQELLEFPGGILGVALNLDEHSVGAVILGDFENIEEGQQVKRTGEVLSVPV
GDGFLGRVVNPLGQPIDGRGDVDSDTRRALELQAPSVVHRQGVKEPLQTGIKAIDAMT
PIGRGQRQLIIGDRKTGKTAVCVDTILNQRQNWESGDPKKQVRCVYVAIGQKGTTIAA
VRRTLEEGGAMDYTTIVAAAASESAGFKWLAPYTGSAIAQHWMYEGKHVLIIFDDLTK
QAEAYRAISLLLRRPPGREAYPGDVFYLHSRLLERCAKLSDDLGGGSLTGLPIIETKA
NDISAYIPTNVISITDGQCFLETDLFNQGVRPAINVGVSVSRVGGAAQIKAMKEVAGS
LRLDLSQYRELEAFAAFASDLDAASKAQLERGARLVELLKQPQSQPMPVEEQVVSIFL
GTGGHLDSVPVEDVRRFETELLDHMRASEEEILTEIRDSQKLTEEAADKLTEVIKNFK
KGFAATGGGSVVPDEHVEALDEDKLAKEAVKVKKPAPKKKK"
CDS 1465257..1466174
/codon_start=1
/note="gene_1364"
/transl_table=11
/translation="MAATLRELRGRIRSAGSIKKITKAQELIATSRIARAQARLESAR
PYAFEITRMLTTLAAEAALDHPLLVERPEPKRAGVLVVSSDRGLCGAYNANIFRRSEE
LFSLLREAGKQPVLYVVGRKAQNYYSFRNWNITESWMGFSEQPTYENAAEIASTLVDA
FLLGTDNGEDQRSDSGEGVDELHIVYTEFKSMLSQSAEAHRIAPMVVEYVEEDIGPRT
LYSFEPDATMLFESLLPRYLTTRVYAALLESAASELASRQRAMKSATDNADDLIKALT

LMANRERQAQITQEISEIVGGANALAEAR"
CDS 1466214..1467674
/codon_start=1
/note="gene_1365"
/transl_table=11
/translation="MTTTAEKTDRPGKPGSSDTSGRVVRVTGPVVDVEFPRGSIPELF
NALHAEITFESLAKTLTLEVAQHLGDNLVRTISLQPTDGLVRGVEVIDTGRSISVPVG
EGVKGHVFNALGDCLDEPGYGEKFEHWSIHRKPPAFEELEPRTEMLETGLKVVDLLTP
YVRGGKIALFGGAGVGKTVLIQEMINRIARNFGGTSVFAGVGERTREGNDLWVELAEA
NVLKDTALVFGQMDEPPGTRMRVALSALTMAEWFRDEQGQDVLLFIDNIFRFTQAGSE
VSTLLGRMPSAVGYQPTLADEMGELQERITSTRGRSITSMQAVYVPADDYTDPAPATT
FAHLDATTELSRAVFSKGIFPAVDPLASSSTILDPSVVGDEHYRVAQEVIRILQRYKD
LQDIIAILGIDELSEEDKQLVNRARRIERFLSQNMMAAEQFTGQPGSTVPVKETIEAF
DRLCKGDFDHVPEQAFFLIGGLDDLAKKAESLGAKL"
CDS 1467688..1468053
/codon_start=1
/note="gene_1366"
/transl_table=11
/translation="MAELNVEIVAVDRNIWSGTAKFLFTRTTVGEIGILPRHIPLVAQ
LVDDAMVRVEREGEKDLRIAVDGGFLSVTEEGVSILAESAEFESEIDEAAAKQDSESD
DPRIAARGRARLRAVGAID"
CDS 1468073..1468495
/codon_start=1
/note="gene_1367"
/transl_table=11
/translation="MIGMVVLVVVLGLAVLALSYRLWKLRQGGTAGIMRDIPAVGGHG
WRHGVIRYRGGEAAFYRLSSLRLWPDRRLSRRGVEIISRRAPRGDEFDIMTDEIVVVE
LCDSTQDRRVGYEIALDRGALTAFLSWLESRPSPRAPP"
CDS complement(1468542..1469876)
/codon_start=1
/note="gene_1368"
/transl_table=11
/translation="VRNVRLFRALLGVDKRTVIEDIEFEEDDAGDGARVIARVRPRSA
VLRRCGRCGRKASWYDRGAGLRQWRSLDWGTVEVFLEAEAPRVNCPTHGPTVVAVPWA
RHHAGHTYAFDDTVAWLAVACSKTAVCELMRIAWRTVGAIVARVWADTEKRIDRFANL
RRIGIDEISYKRHHRYLTVVVDHDSGRLVWAAPGHDKATLGLFFDALGAERAAQITHV
SADAADWIADVVTERCPDAIQCADPFHVVAWATEALDVERRRAWNDARAIARTEPKWG
RGRPGKNAAPRPGRERARRLKGARYALWKNPEDLTERQSAKLAWIAKTDPRLYRAYLL
KESLRHVFSVKGEEGKQALDRWISWAQRCRIPVFVELAARIKRHRVAIDAALDHGLSQ
GLIESTNTKIRLLTRIAFGFRSPQALIALAMLTLAGHRPTLPGRHNHPQISQ"
CDS complement(1470042..1470623)
/codon_start=1
/note="gene_1369"
/transl_table=11
/translation="MAVHLTRIYTRTGDDGTTGLSDMSRVAKTDARLVAYADCDEANA
AIGAALALGHPDTQITDVLRQIQNDLFDAGADLSTPIVENPKHPPLRIAQSYIDRLEG
WCDAYNAGLPALKSFVLPGGSPLSALLHVARTVVRRAERSAWAAVDAHPEGVSVLPAK
YLNRLSDLLFILSRVANPDGDVLWRPGGDRTAS"
CDS 1470692..1471948
/codon_start=1
/note="gene_1370"

```
                     /transl_table=11
                     /translation="VAERFVVTGGNRLSGEVAVGGAKNSVLKLMAATLLAEGTSTITN
                     CPDILDVPLMAEVLRGLGATVELDGDVARITAPDEPKYDADFAAVRQFRASVCVLGPL
                     VGRCKRARVALPGGDAIGSRPLDMHQAGLRQLGAHCNIEHGCVVARAETLRGAEIQLE
                     FPSVGATENILMAAVVAEGVTTIHNAAREPDVVDLCTMLNQMGAQVEGAGSPTMTITG
                     VPRLHPTEHRVIGDRIVAATWGIAAAMTRGDISVAGVDPAHLQLVLHKLHDAGATVTQ
                     TDASFRVTQYERPKAVNVATLPFPGFPTDLQPMAIALASIADGTSMITENVFEARFRF
                     VEEMIRLGADARTDGHHAVVRGLPQLSSAPVWCSDIRAGAGLVLAGLVADGDTEVHDV
                     FHIDRGYPLFVENLVSLGAEIERVCC"
rRNA                 1472217..1473751
rRNA                 1474031..1477166
                     /note="identified by RNAmmer; putative"
rRNA                 1477273..1477384
                     /note="identified by RNAmmer; putative"
CDS                  complement(1477505..1478002)
                     /codon_start=1
                     /note="gene_1371"
                     /transl_table=11
                     /translation="MIHYRTIDSPIGPLTLAGHGSVLTNLRMLEQTYEPSRTHWTPDP
                     GAFSGAVDQLNAYFAGELTEFDVELDLRGTDFQQRVWKALLTIPYGETRSYGEIADQI
                     GAPGAARAVGLANGHNPIAIIVPCHRVIGASGKLTGYGGGINRKRALLELEKSRAPAD
                     LTLFD"
CDS                  complement(1477999..1479489)
                     /codon_start=1
                     /note="gene_1372"
                     /transl_table=11
                     /translation="VHDDFERCYRAVQSKDARFDGWFVVAVLTTGVYCRPSCPVRPPF
                     ARNVRFLPTAAAAQGEGFRACKRCRPDASPGSPEWNVRSDVVARAMRLIADGTVDRDG
                     VSGLAAQLGYTIRQLERLLQAVVGAGPLALARAQRMQTARVLIETTNLPFGDVAFAAG
                     FSSIRQFNDTVRLACDGTPTALRARAAARFESATASAGTVSLRLPVRAPFAFEGVFGH
                     LAATAVPGCEEVRDGAYRRTLRLPWGNGIVSLTPAPDHVRCLLVLDDFRDLMTATARC
                     RRLLDLDADPEAIVEALGADPDLRAVVGKAPGQRIPRTVDEAEFAVRAVLAQQVSTKA
                     ASTHAGRLVAAYGRPVHDRHGALTHTFPSIEQLAEIDPGHLAVPKARQRTINALVASL
                     ADKSLVLDAGCDWQRARGQLLALPGVGPWTAEVIAMRGLGDPDAFPASDLGLRLAAKK
                     LGLPAQRRALTVHSARWRPWRSYATQHLWTTLEHPVNQWPPQEKIA"
CDS                  complement(1479570..1481195)
                     /codon_start=1
                     /note="gene_1373"
                     /transl_table=11
                     /translation="MSAKKSTAQRLGRVLETVTRQSGRLPETPAYGSWLLGRVSESQR
                     RRRVRIQVMLTALVVTANLLGIGVALLLVTIAIPEPSIVRDTPRWLTFGVVPGYVLLA
                     LALGSYALTRQTVQALRWAIEGRKPTREEERRTFLAPWRVAVGHLMFWGVGTALLTTL
                     YGLINNAFIPRFLFAVSFCGVLVATATYLHTEFALRPFAAQALEAGPPPRRLAPGILG
                     RTMVVWLLGSGVPVVGIALMAMFEMVLLNLTRMQFATGVLIISMVTLVFGFILMWILA
                     WLTATPVRVVRAALRRVERGELRTNLVVFDGTELGELQRGFNAMVAGLRERERVRDLF
                     GRHVGREVAAAAERERSKLGGEERHVAVVFIDIVGSTQLVTSRPPADVVKLLNKFFAI
                     VVDEVDRHHGLVNKFEGDASLTIFGAPNRLPCPEDKALAAARAIADRLVNEMPECQAG
                     IGVAAGQVIAGNVGARERFEYTVIGEPVNEAARLCELAKSRPGKLLASAQAVDAASEE
                     ERARWSLGRHVKLRGHDQPVRLAKPVGLTKPRR"
CDS                  complement(1481265..1482872)
                     /codon_start=1
```

/note="gene_1374"
/transl_table=11
/translation="MPAKKTMAQRLGQALETMTRQCGQLPETPAYGSWLLGRVSESPS
RRWVRIKRIVTVYIMTANLTGIVVALLVVTFAFPVPSIYTDAPWWVTFGVAPAYATLA
LAIGTYWITTRIVRASIRWAIEERAPSQADGRNTLLLPFRVAAVHLILWDIGGALLAT
LYGLANRVFVTIILFSVTICGVLVATNCYLFTEFALRPVAAKALEAGRPPRRFAPGIM
GRTMTVWSLGSGVPVTGIATTALYVLLVHNLTETQLASAVLILSITTLIFGFLVMWIL
AWLTAAPVRVVRAALKRVEQGDLRGDLVVFDGTELGELQRGFNAMVNGLRERERVRDL
FGRHVGREVAAAAERERPQLGGEDRHAAVVFVDIVGSTQLVDNQPAAHVVKLLNRFFA
IVVNEVDRHHGLINKFAGDAALAIFGAPNRLDRPEDAALAAARAIADRLANEMPEVQA
GIGVAAGQIVAGNVGAKQRFEYTVVGKPVNQAARLCELAKSHPARLLASSDTLHAASE
TERAHWSLGETVTLRGHEQPTRLAVPT"
CDS complement(1482885..1484456)
/codon_start=1
/note="gene_1375"
/transl_table=11
/translation="MRRVRIQGMLTVAILVTNVIGLIVGAMLLTVAFPKPSVILDAPH
WVSFGIVPGYCVLAFILGTYWLTRQTARALRWAIEERTPSHDEARSAFLVPLRVALAV
LFLWGAAAALWTIIYGLANRLFIPRFLFSMGVIGVVAATSCYLLTEFALRPMAAQALE
VGATPRSLVRGIVGRTMLVWLLCSGVPNVGVALTAIFDDTFWELSNDQFMITVLILWA
PLLIFGFILMWILAWLTATPVRVVREALNRVEQGDLSGDLVVFDGTELGELQRGFNRM
VEGLRERERVRDLFGRHVGREVAAAAERERPKLGGEERHVAVVFVDIVGSTQLVTSRP
AAEVVMLLNRFFTVIVDEVNHHRGLVNKFQGDASLAVFGAPNRLSHPEDAALATARAI
ADRLASEMPECQAGIGVAAGQVVAGNVGAHERFEYTVIGEPVNEAARLCELAKSYPSR
LLASSQTLRGASENECARWSLGETVTLRGHDQPIRLASPVQQLQMPAQSADIVGGALG
DHQTHTIYRGAHPTD"
CDS 1484659..1485330
/codon_start=1
/note="gene_1376"
/transl_table=11
/translation="VRLVIAQCTVDYIGRLTAHLPSARRLLLFKADGSVSVHADDRAY
KPLNWMSPPCWLTEESGGQAPVWVVENKAGEQLRITIEGIEHDSSHELGVDPGLVKDG
VEAHLQALLAEHIQLLGEGYTLVRREYMTAIGPVDLLCRDERGGSVAVEIKRRGEIDG
VEQLTRYLELLNRDSVLAPVKGVFAAQQIKPQARILATDRGIRCLTLDYDTMRGMDSG
EYRLF"
CDS complement(1485684..1486139)
/codon_start=1
/gene="mce"
/note="gene_1377"
/transl_table=11
/translation="MTTDQVHARHMLATSLVTGLDHVGIAVADLDVAIEWYHDHLGMI
LVHEEINDDQGIREALLAVPGSAAQIQLMAPLDESSVIAKFLDKRGPGIQQLACRVSD
LDAMCRRLRSQGVRLVYETARRGTANSRINFIHPKDAGGVLIELVEPAP"
CDS 1486233..1487402
/codon_start=1
/note="gene_1378"
/transl_table=11
/translation="VIVAGARTPIGKLMGSLKDFSASELGAIAIKGALEKANVPASLV
EYVIMGQVLTAGAGQMPARQAAVAAGIGWDVPALTINKMCLSGIDAIALADQLIRARE
FDVVVAGGQESMTKAPHLLMNSRSGYKYGDVTVLDHMAYDGLHDVFTDQPMGALTEQR
NDVDMFTRSEQDEYAAASHQKAAAAWKDGVFADEVIPVNIPQRTGDPLQFTEDEGIRA

NTTAAALAGLKPAFRGDGTITAGSASQISDGAAAVVVMNQEKAQELGLTWLAEIGAHG
VVAGPDSTLQSQPANAINKALDREGISVDQLDVVEINEAFAAVALASIRELGLNPQIV
NVNGGAIAVGHPLGMSGTRITLHAALQLARRGSGVGVAALCGAGGQGDALILRAG"
CDS 1487532..1488446
/codon_start=1
/note="gene_1379"
/transl_table=11
/translation="VTRPRPPLGPAMAGAVDLSGIKQRAQQNAAASTDADRALSTPSG
VTEITEANFEDEVIVRSDEVPVVVLLWSPRSEVCVDLLDTLSGLAAAAKGKWSLASVN
VDVAPRVAQIFGVQAVPTVVALAAGQPISSFQGLQPADQLSRWVDSLLSATAGKLKGA
ASSEESTEVDPAVAQARQQLEDGDFVAARKSYQAILDANPGSVEAKAAIRQIEFLIRA
TAQRPDAVSVADSLSDDIDAAFAAADVQVLNQDVSAAFERLIALVRRTSGEERTRVRT
RLIELFELFDPADPEVVAGRRNLANALY"
CDS complement(1488525..1490327)
/codon_start=1
/note="gene_1380"
/transl_table=11
/translation="MSFVIAAPETLVRAASDLANIGSTLGAANAAALGPTTELLAAGA
DEVSAAIASLFAAHGQAYQAVSAQMSAFHAQFVQTFTAGAGAYASAEAAAAAPLEGLL
NIVNTPTQLLLGRPLIGNGANGAPGTGQAGGAGGLLYGNGGAGGSGAPGQAGGPGGAA
GLFGNGGAGGAGGDGPGNGAAGGAGGAGGLLFGSGGAGGPGGVGNTGTGGLGGDGGAA
GLFGAGGIGGAGGPGFNGGAGGAGGRSGLFEVLAAGGAGGTGGLSVNGGTGGTGGTGG
GGGLFSNGGAGGAGGFGVSGSAGGNGGTGGDGGIFTGNGGTGGAGGTGTGNQLVGGEG
GAGGAGGNAGILFGAGGIGGTGGTGLGAPDPGGTGGKGGVGGIGGAGALFGPGGAGGT
GGFGASSADQMAGGIGGSGGSGGAAKLIGDGGAGGTGGDSVRGAAGSGGTGGTGGLIG
DGGAGGAGGTGIEFGSVGGAGGNAAGLSGAGGAGGAGGFGETAGDGGAGGNAGLLNGD
GGAGGAGGLGIAGDGGNGGKGGKAGMVGNGGDGGAGGASVVANGGVGGSGGNATLIGN
GGNGGNGGVGSAPGKGGAGGTAGLLGLNGSPGLS"
CDS complement(1490479..1492674)
/codon_start=1
/note="gene_1381"
/transl_table=11
/translation="MSRSEKLTGEHLAPEPAEMARLVAGTHHNPHGILGAHEYGDHTV
IRAFRPHAVEVVALVGKDRFSLQHLDSGLFAVALPFVDLIDYRLQVTYEGCEPHTVAD
AYRFLPTLGEVDLHLFAEGRHERLWEVLGAHPRSFTTADGVVSGVSFAVWAPNAKGVS
LIGEFNGWNGHEAPMRVLGPSGVWELFWPDFPCDGLYKFRVHGADGVVTDRADPFAFG
TEVPPQTASRVTSSDYTWGDDDWMAGRALRNPVNEAMSTYEVHLGSWRPGLSYRQLAR
ELTDYIVDQGFTHVELLPVAEHPFAGSWGYQVTSYYAPTSRFGTPDDFRALVDALHQA
GIGVIVDWVPAHFPKDAWALGRFDGTPLYEHSDPKRGEQLDWGTYVFDFGRPEVRNFL
VANALYWLQEFHIDGLRVDAVASMLYLDYSRPEGGWTPNVHGGRENLEAVQFLQEMNA
TAHKVAPGIVTIAEESTSWPGVTRPTNIGGLGFSMKWNMGWMHDTLDYVSRDPVYRSY
HHHEMTFSMLYAFSENYVLPLSHDEVVHGKGTLWGRMPGNNHVKAAGLRSLLAYQWAH
PGKQLLFMGQEFGQRAEWSEQRGLDWFQLDENGFSNGIQRLVRDINDIYRCHPALWSL
DTTPEGYSWIDANDSANNVLSFMRYGSDGSVLACVFNFAGAEHRDYRLGLPRAGRWRE
VLNTDATIYHGSGIGNLGGVDATDDPWHGRPASAVLVLPPTSALWLTPA"
CDS complement(1492682..1494748)
/codon_start=1
/note="gene_1382"
/transl_table=11
/translation="VPGRVEIDDVAPVVSCGVYPAKAVVGEVVPVSAAVWREGHEAVA
ATLVVRYLGVRYPHLTDRPRARVLPTPSEPQQRVKPLLIPMTSGQEPFVFHGQFTPDR

VGLWTFRVDGWGDPIHTWRHGLIAKLDAGQGETELSNDLLVGAVLLERAATGVPRGLR
DPLLAAAAALRTPGDPVTRTALALTPEIEELLADYPLRDLVTRGEQFGVWVDRPLARF
GAWYEMFPRSTGGWDDDGNPVHGTFATAAAELPRIAGMGFDVVYLPPIHPIGKVHRKG
RNNSPTAAPTDVGSPWAIGSDEGGHDTVHPSLGTIDDFDDFVSAARDLGMEVALDLAL
QCAPDHPWAREHRQWFTELPDGTIAYAENPPKKYQDIYPLNFDNDPEGLYDEVLRVVQ
HWVNHGVKFFRVDNPHTKPPNFWAWLIAQVKTVDPDVLFLSEAFTPPARQYGLAKLGF
TQSYSYFTWRTTKWELTEFGNQIAELADYRRPNLFVNTPDILHAVLQHNGPGMFAIRA
VLAATMSPAWGMYCGYELFEHRAVREGSEEYLDSEKYELRPRDFASALDQGRSLQPFI
TRLNIIRRLHPAFQQLRTIHFHHVDNDALLAYSKFDPATGDCVLVVVTLNAFGPEEAT
LWLDMAALGMEDYDRFWVRDEITGEEYQWGQANYIRIDPARAVAHIINMPAVPYESRN
TLLRRR"

CDS 1494926..1497517
/codon_start=1
/note="gene_1383"
/transl_table=11
/translation="VKALRRFTVRAHLPERLAALDQLSTNLRWSWDKPTQDLFAAIDP
ALWEQCGHDPVALLGAVNPARLDELALDAEFLGALDELAADLNDYLSRPLWYQEQQDA
GVAAQALPTGIAYFSLEFGVAEVLPNYSGGLGILAGDHLKSASDLGVPLIAVGLYYRS
GYFRQSLTADGWQHETYPSLDPQGLPLRLLTDANGDPVLVEVALGDNAVLRARIWVAQ
VGRVPLLLLDSDIPENEHDLRNVTDRLYGGDQEHRIKQEILAGIGGVRAIRAYTAVEK
LTPPEVFHMNEGHAGFLGIERIRELVTDAGLDFDTALTVVRSSTVFTTHTPVPAGIDR
FPLEMVQRYVNDQRGDGRSRLLPGLPADRIVALGAEDDPAKFNMAHMGLRLAQRANGV
SLLHGRVSRAMFNELWAGFDPDEVPIGSVTNGVHAPTWAAPQWLQLGRELAGSDSLRE
PVVWQRLHQVDPAHLWWIRSQLRSMLVEDVRARLRQSWLERGATDAELGWIATAFDPN
VLTVGFARRVPTYKRLTLMLRDPGRLEQLLLDEQRPIQLIVAGKSHPADDGGKALIQQ
VVRFADRPQFRHRIAFLPNYDMSMARLLYWGCDVWLNNPLRPLEACGTSGMKSALNGG
LNLSIRDGWWDEWYDGENGWEIPSADGVADENRRDDLEAGALYDLLAQAVAPKFYERD
ERGVPQRWVEMVRHTLQTLGPKVLASRMVRDYVEHYYAPAAQSFRRTAGAQFDAAREL
ADYRRRAEEAWPKIEIADVDSTGLPDTPLLGSQLTLTATVRLAGLRPNDVTVQGVLGR
VDSGDVLMDPVTVEMAHTGTGDGGYEIFSTTTPLPLAGPVGYTVRVLPRHPMLAASNE
LGLVTLA"

CDS complement(1497557..1499551)
/codon_start=1
/note="gene_1384"
/transl_table=11
/translation="VSESVSMSVPELLAIAVAALGGTRRRGQQEMAAAVAHAFETGEH
LVVQAGTGTGKSLAYLVPAIIRALCDDAPVVVSTATIALQRQLVDRDLPQLVDSLTNA
LPRRPKFALLKGRRNYLCLNKIHNSVTASDHDDERPQEELFDPVAVTALGRDVQRLTA
WASTTVSGDRDDLKPGVGDRSWSQVSVSARECLGVARCPFGSECFSERARGAAGLADV
VVTNHALLAIDAVAESAVLPEHRLLVVDEAHELADRVTSVAAAELTSATLGMAARRIT
RLVDPKVTQRLQAASATFSSAIHDARPGRIDCLDDEMATYLSALRDAASAARSAIDTG
SDTTTASVRAEAGAVLTEISDTASRILASFAPAIPDRSDVVWLEHEDNHESARAVLRV
APLSVAELLATQVFARATTVLTSATLTIGGSFDAMATAWGLTADTPWRGLDVGSPFQH
AKSGILYVAAHLPPPGRDGSGSAEQLTEIAELITAAGGRTLGLFSSMRAARAATEAMR
ERLSTPVLCQGDDSTSTLVEKFTADAATSLFGTLSLWQGVDVPGPSLSLVLIDRIPFP
RPDDPLLSARQRAVAARGGNGFMTVAASHAALLLAQGSGRLLRRVTDRGVVAVLDSRM
ATARYGEFLRASLPPFWQTTNATQVRAALRRLARADAKAH"

CDS complement(1499575..1500855)
/codon_start=1
/note="gene_1385"
/transl_table=11

/translation="LTDKYELTMLAAALRDGSANRPTTFEVFARRLPTGRRYGVVAGT
GRLLEALPQFRFDADACELLAQFLDPATVRYLREFRFRGDIDGYAEGELYFPGSPVLS
VRGSFAECVLLETLVLSIFNHDTAIASAAARMVSAAGGRPLIEMGSRRTHERAAVAAA
RAAYIAGFAASSNLAAQRRYGVPAHGTAAHAFTMLHAQHGGPTELAERAAFRAQVEAL
GPGTTLLVDTYDVTTGVANAVAAAGAELGAIRIDSGELGVLARQAREQLDRLGATRTR
IVVSGDLDEFSIAALRGEPVDSYGVGTSLVTGSGAPTANMVYKLVEVDGVPVQKRSSY
KESPGGRKEALRRSRATGTITEELVHPAGRPPVIVEPHRVLTLPLVRAGQPVADTSLA
AARQLVASGLRSLPADGLKLAPGEPAIPTRTIPA"
CDS 1501023..1501328
/codon_start=1
/gene="clpS"
/note="gene_1386"
/transl_table=11
/translation="MAVVSAPAKPGTTWQRESAPVDVTDRAWVTIVWDDPVNLMSYVT
YVFQKLFGYSEPHATKLMLQVHNEGKAVVSAGSRESMEVDVSKLHAAGLWATMQQDR"
CDS 1501354..1501944
/codon_start=1
/note="gene_1387"
/transl_table=11
/translation="VRRWKRVETRDGPRFRSSLAPHEAALLKNLAGAMIGLLDDRDSS
SPSDELEEITGIKTGHAQRPGDPTLRRLLPDFYRPDDLDDDDPTAVDGSESFNAALRS
LHEPEIIDAKRVAAQQLLDTVPDNGGRLELTESDANAWIAAVNDLRLALGVMLEIGPR
GPERLPGNHPLAAHFNVYQWLTVLQEYLVLVLMGSR"
CDS 1501961..1502995
/codon_start=1
/note="gene_1388"
/transl_table=11
/translation="MNSITDVGGIRVGHYQRLDPDASLGAGWACGVTVVLPPPGTVGA
VDCRGGAPGTRETDLLDPANSVRFVDALLLAGGSAYGLAAADGVMRWLEEHRRGVAMD
SGVVPIVPGAVIFDLPVGGWNCRPTADFGYSACAAAGVDVAVGTVGVGVGARAGALKG
GVGTASATLQSGVTVGVLAVVNAAGNVVDPATGLPWMADLVGEFALRAPPAEQIAALA
QLSSPLGAFNTPFNTTIGVIACDAALSPAACRRIAIAAHDGLARTIRPAHTPLDGDTV
FALATGAVAVPPEAGVPAALSPETQLVTAVGAAAADCLARAVLAGVLNAQPVAGIPTY
RDMFPGAFGS"
CDS 1503006..1503443
/codon_start=1
/note="gene_1389"
/transl_table=11
/translation="LLRKGTVYVLVIRADLVNAMVAHARRDHPDEACGVLAGPEGSDR
PERHIPMTNAERSPTFYRLDSGEQLKVWRAMEDADEVPVVIYHSHTATEAYPSRTDVK
LATEPDAHYVLVSTRDPHRHELRSYRIVDGAVTEEPVNVVEQY"
CDS 1503465..1503746
/codon_start=1
/note="gene_1390"
/transl_table=11
/translation="MNVTVSIPTILRPHTGGQKSVSASGDTLGAVISDLEANYSGISE
RLMDPSSPGKLHRFVNIYVNDEDVRFSGGLATAIADGDSVTILPAVAGG"
CDS 1503756..1504727
/codon_start=1
/note="gene_1391"
/transl_table=11

```
                     /translation="MTRYDSLLQALGNTPLVGLQRLSPRWDDGRDGPHVRLWAKLEDR
                     NPTGSIKDRPAVRMIEQAEADGLLRPGATILEPTSGNTGISLAMAARLKGYRLICVMP
                     ENTSVERRQLLELYGAQIIFSAAEGGSNTAVATAKELAATNPSWVMLYQYGNPANTDS
                     HYCGTGPELLADLPEITHFVAGLGTTGTLMGTGRFLREHVANVKIVAAEPRYGEGVYA
                     LRNMDEGFVPELYDPEILTARYSVGAVDAVRRTRELVHTEGIFAGISTGAVLHAALGV
                     GAGALAAGERADIALVVADAGWKYLSTGAYAGSLDDAETALEGQLWA"
     CDS             1504826..1505440
                     /codon_start=1
                     /note="gene_1392"
                     /transl_table=11
                     /translation="VVGGTTILTFVALLYLVELIDQLSGSRLDVNGIRPLKTDGLWGV
                     IFAPLLHANWHHLMANTIPLLVLGFLMTLAGLSRFVWATAIIWILGGLGTWLIGNVGS
                     SCGPTDHIGASGLIFGWLAFLLVFGLFVRKGWDIVIGLVVLFVYGGILLGAMPVLGQC
                     GGVSWQGHLSGAVAGVVAAYLLSAPERKARALKRAGARSGHPKL"
     CDS             1505437..1506252
                     /codon_start=1
                     /note="gene_1393"
                     /transl_table=11
                     /translation="MNSPLAPVGVFDSGVGGLTVARAIIDQLPDEDIVYVGDTGNGPY
                     GPLTIPEIRAHALAIGDDLVGRGVKALVIACNSASSACLRDARERYQVPVVEVILPAV
                     RRAVAATRNGRIGVIGTRATITSHAYQDAFAAARDTEITAVACPRFVDFVELGVTSGR
                     QVLGLAQGYLEPLQRAEVDTLVLGCTHYPLLSGLIQLAMGENVTLVSSAEETAKEVVR
                     VLTEIDLLRPHDAPPATRIFEATGDPEAFTKLAARFLGPVLGGVQPVHPSRIH"
     CDS             1506330..1507100
                     /codon_start=1
                     /note="gene_1394"
                     /transl_table=11
                     /translation="VRITVLGCSGSVVGPDSPASGYLLRAPHTPPLVIDFGGGVLGAL
                     QRHADPASVHVLLSHLHADHCLDLPGLFVWRRYHPSRPSGKALLYGPSDTWSRLGAAS
                     SPYGGEIDDCSDIFDVHHWADSEPVTLGALTIVPRLVAHPTESFGLRITDPSGASLAY
                     SGDTGICDQLVELARGVDVFLCEASWTHSPKHPPDLHLSGTEAGMVAAQAGVRELLLT
                     HIPPWTSREDVISEAKAEFDGPVHAVVCDETFEVRRAG"
     CDS             1507117..1507896
                     /codon_start=1
                     /gene="rph"
                     /note="gene_1395"
                     /transl_table=11
                     /translation="VSKREDGRLDHELRPVIITRGFTENPAGSVLIEFGHTKVLCTAS
                     VTEGVPRWRKATGLGWLTAEYAMLPSATHSRSDRESVRGRLSGRTQEISRLIGRSLRA
                     CIDLAALGENTIAIDCDVLQADGGTRTAAITGAYVALADAVTYLSAAGKLSDPRPLSC
                     AIAAVSVGVVDGRIRVDLPYEEDSRAEVDMNVVATDTGTLVEIQGTGEGATFARSTLD
                     KLLDMALGACDTLFAAQRDALALPYPGVLPQGPPPPKAFGT"
     CDS             1507944..1508549
                     /codon_start=1
                     /note="gene_1396"
                     /transl_table=11
                     /translation="VTKLLVASRNRKKLAELRRVLDGAGLSGLTLLSLGDVSPLPETP
                     ETGVTFEDNALAKARDAFSATGLASVADDSGLEVAALGGMPGVLSARWSGRYGDDAAN
                     TALLLAQLCDVPDERRGAAFVSACALVSGSGEVVVRGEWPGTIAREPRGDGGFGYDPV
                     FVPYGDDRTAAQLSPAEKDAVSHRGRALALLLPALRSLATG"
     CDS             complement(1508546..1508908)
```

/codon_start=1
/note="gene_1397"
/transl_table=11
/translation="MTAPETPAAQHAEPAIAVERIRTALLGYRIMAWTTGLWLIALCY
EIVVRYVVKVDNPPTWIGVVHGWVYFTYLLLTLNLAVKVRWPLGKTAGVLLAGTIPLL
GIVVEHFQTKEIKARFGL"
CDS complement(1508905..1509285)
/codon_start=1
/note="gene_1398"
/transl_table=11
/translation="VSTTRRRRPALIALVIIATCGCLALGWWQWTRFQSTSGTFQNLG
YALQWPLFAWFCVYAYRNFVRYEETPPQPPTGGAAAAIPAGLLPERPKPAQQPPDDPV
LREYNAYLAELAKDDARKQNRTTA"
CDS 1509396..1509650
/codon_start=1
/note="gene_1399"
/transl_table=11
/translation="MTSSPSTVSTTLLSILRDDLNIDLTRVTPDARLVDDVGLDSVAF
AVGMVAIEERLGVALSEEELLTCDTVGELEAAIAAKYRDE"
CDS 1509643..1511208
/codon_start=1
/note="gene_1400"
/transl_table=11
/translation="MSELAAVLTRSMQASAGDLMVLDRETSLWCRHPWPEVHGLAESV
AAWLLDHDRPAAVGLVGEPTVELVAAIQGAWLAGAAVSILPGPVRGANDQRWADATLT
RFLGIGVRTVLSQGSYLARLRSVDTAGVTIGDLSTAAHTNRSATPVASEGPAVLQGTA
GSTGAPRTAILSPGAVLSNLRGLNQRVGTDAATDVGCSWLPLYHDMGLAFVLSAALAG
APLWLAPTTAFTASPFRWLSWLSDSGATMTAAPNFAYNLIGKYARRVSEVDLGALRVT
LNGGEPVDCDGLTRFAEAMAPFGFDAGAVLPSYGLAESTCAVTVPVPGIGLLADRVID
GSGAHKHAVLGNPIPGMEVRISCGDQAAGNASREIGEIEIRGASMMAGYLGQQPIDPD
DWFATGDLGYLGAGGLVVCGRAKEVISIAGRNIFPTEVELVAAQVRGVREGAVVALGT
GDRSTRPGLVVAAEFRGPDEANARAELIQRVASECGIVPSDVVFVSPGSLPRTSSGKL
RRLAVRRSLEMAD"
CDS 1511208..1512368
/codon_start=1
/note="gene_1401"
/transl_table=11
/translation="MTAGSDLDDFRGLLAKAFDERVVAWTAEAEAQERFPRQLIEHLG
VCGVFDAKWATDARPDVGKLVELAFALGQLASAGIGVGVSLHDSAIAILRRFGKSDYL
RDICDQAIRGAAVLCIGASEESGGSDLQIVETEIRSRDGGFEVRGVKKFVSLSPIADH
IMVVARSVDHDPTSRHGNVAVVAVPAAQVSVQTPYRKVGAGPLDTAAVCIDTWVPADA
LVARAGTGLAAISWGLAHERMSIAGQIAASCQRAIGITLARMMSRRQFGQTLFEHQAL
RLRMADLQARVDLLRYALHGIAEQGRLELRTAAAVKVTAARLGEEVISECMHIFGGAG
YLVDETTLGKWWRDMKLARVGGGTDEVLWELVAAGMTPDHDGYAAVVGASKA"
CDS complement(1512335..1512967)
/codon_start=1
/note="gene_1402"
/transl_table=11
/translation="MTKPTSAGQADDALVRLARERFDLPDQVRRLARPPVPSLEPPYG
LRVAQLTDAEMLAEWMNRPHLAAAWEYDWPASRWRQHLNAQLEGTYSLPLIGSWHGTD
GGYLELYWAAKDLISHYYDADPYDLGLHAAIADLSKVNRGFGPLLLPRIVASVFANEP

RCRRIMFDPDHRNTATRRLCEWAGCKFLGEHDTTNRRMALYALEAPTTAA"
tRNA 1513090..1513173
/note="identified by tRNAscan-SE; putative"
CDS 1513409..1515988
/codon_start=1
/note="gene_1403"
/transl_table=11
/translation="MARGLQGVMLRSFGARDHTATVIETISIAPHFVRVRMVSPTLFQ
DAEAEPAAWLRFWFPDPNGSNTEFQRAYTISEADPAAGRFAVDVVLHDPAGPASSWAR
TVKPGATIAVMSLMGSSRFDVPEEQPAGYLLIGDSASIPGMNGIIETVPNDVPIEMYL
EQHDDNDTLIPLAKHPRLRVRWVMRRDEKSLAEAIENRDWSDWYAWATPEAAALKCVR
VRLRDEFGFPKSEIHAQAYWNAGRAMGTHRATEPAATEPEVGAAPQPESAVPAPARGS
WRAQAASRLLAPLKLPLVLSGVLAALVTLAQLAPFVLLVELSRLLVSGAGAHRLFTVG
FAAVGLLGTGALLAAALTLWLHVIDARFARALRLRLLSKLSRLPLGWFTSRGSGSIKK
LVTDDTLALHYLVTHAVPDAVAAVVAPVGVLVYLFVVDWRVALVLFGPVLVYLTITSS
LTIQSGPRIVQAQRWAEKMNGEAGSYLEGQPVIRVFGAASSSFRRRLDEYIGFLVAWQ
RPLAGKKTLMDLATRPATFLWLIAATGTLLVATHRMDPVNLLPFMFLGTTFGARLLGI
AYGLGGLRTGLLAARHLQVTLDETELAVREHPREPLDGEAPATVVFDHVTFGYRPGVP
VIQDVSLTLRPGTVTALVGPSGSGKSTLATLLARFHDVERGAIRVGGQDIRSLAADEL
YTRVGFVLQEAQLVHGTAAENIALAVPDAPAEQVQVAAREAQIHDRVLRLPDGYDTVL
GANSGLSGGERQRLTIARAILGDTPVLILDEATAFADPESEYLVQQALNRLTRDRTVL
VIAHRLHTITRADQIVVLDHGRIVERGTHEELLAAGGRYCRLWDTGQGSRVAVAAAQD
GTR"
CDS 1515985..1517724
/codon_start=1
/note="gene_1404"
/transl_table=11
/translation="MIRTWIALVPNDHRARLIGFALLAFCSVVARAVGTVLLVPLMAA
LFGEAPQRAWLWLGWLSAATVAGWVLDAVTARIGIELGFAVLNHTQHDVADRLPVVRL
DWFTAENTATARQAIAATGPELVGLVVNLVTPLTSAILLPAVIALALLPISWQLGVAA
LAGVPLLLGALWASAAFARRADTAADKANTALTERIIEFARTQQALRAARRVEPARSL
VGNALASQHTATMRLLGMQIPGQLLFSIASQLALIVLAGTTAALTITGTLTVPEAIAL
IVVMVRYLEPFTAVSELAPALESTRATLGRIGSVLTAPVMVAGSGTWRDGAVVPRIEF
DDVAFGYDGGSGPVLDGVSFCLQPGTTTAIVGPSGCGKSTILALIAGLHQPTRGRVLI
DGTDVATLDARAQQAVCSVVFQHPYLFHGTIRDNVFAADPGASDDQFAQAVRLARVDE
LIARLPDGANTIVGEAGSALSGGERQRVSIARALLKAAPVLLVDEATSALDAENEAAV
VDALAADPRSRTRVIVAHRLASIRHADRVLFVDDGRVVEDGSISELLTAGGRFSQFWR
QQHEAAEWQILAE"
CDS 1517853..1518596
/codon_start=1
/gene="fabG"
/note="gene_1405"
/transl_table=11
/translation="MASLLNARTAVITGGAQGLGLAIGQRFVAEGARVVLGDVNLEAT
EVAAKRLGGDDVALAVRCDVTQADDVDILIRTAVERFGGLDVMVNNAGITRDATMRTM
TEEQFDQVIAVHLKGTWNGTRLAAAIMRERKRGAIVNMSSVSGKVGMVGQTNYSAAKA
GIVGMTKAAAKELAHLGIRVNAIAPGLIRSAMTEAMPQRIWDQKLAEVPMGRAGEPSE
VASVAVFLASDLSSYMTGTVLDVTGGRFI"
CDS 1519125..1519496
/codon_start=1
/note="gene_1406"

/transl_table=11
/translation="MARTLALRASAGLVAGMAMAAITLAPGARAETGEQFPGDGVFLV
GTDIAPGTYRTEGPSNPLILVFGRVSELSTCSWSTHSAPEVSNENIVDTNTSMGPMSV
VIPPTVAAFQTHNCKLWMRIS"
CDS complement(1519562..1520347)
/codon_start=1
/note="gene_1407"
/transl_table=11
/translation="MQTTPGKRQRRQRGSINPEDIISGAFELAQQVSIDNLSMPLLGK
HLGVGVTSIYWYFRKKDDLLNAMTDRALSKYVFATPYIEAGDWRETLRNHARSMRKTF
ADNPVLCDLILIRAALSPKTARLGAQEMEKAIANLVTAGLSLEDAFDIYSAVSVHVRG
SVVLDRLSRKSQSAGSGPSAIEHPVAIDPATTPLLAHATGRGHRIGAPDETNFEYGLE
CILDHAGRLIEQSSKAAGEVAVRRPTATADAPTPGARAKAVAR"
CDS complement(1520367..1522238)
/codon_start=1
/note="gene_1408"
/transl_table=11
/translation="MCNDTATPQLEELVTTVANQLMTVDAATSAEVSQRVLAYLVEQL
GVDVSFLRHNDRDRRATRLVAEWPPRLNIPDPDPLRLIYFADADPVFALCEHAKEPLV
FRPEPATEDYQRLIEEARGVPVTSAAAVPLVSGEITTGLLGFIKFGDRKWHEAELNAL
MTIATLFAQVQARVAAEARLRYLADHDDLTGLHNRRALLQHLDQRLAPGQPGPVAALF
LDLDRLKAINDYLGHAAGDQFIHVFAQRIGDALVGESLIARLGGDEFVLIPASPMSAD
AAQPLAERLRDQLKDHVAIGGEVLTRTVSIGVASGTPGQHTPSDLLRRADQAALAAKH
AGGDSVAIFTADMSVSGELRNDIELHLRRGIESDALRLVYLPEVDLRTGDIVGTEALV
RWQHPTRGLLAPGCFIPVAESINLAGELDRWVLRRACNEFSEWQSAGLGHDALLRINV
SAGQLVTGGFVDFVADTIGQHGLDASSVCLEITENVVVQDLHTARATLARLKEVGVHI
AIDDFGTGYSAISLLQTLPIDTLKIDKTFVRQLGTNTSDLVIVRGIMTLAEGFQLDVV
AEGVETEAAARILLDQRCYRAQGFLFSRPVPGEAMRHMLSARRLPPTCIPATDPALS"
CDS complement(1522247..1524394)
/codon_start=1
/note="gene_1409"
/transl_table=11
/translation="VTIPHEGGSTGILVLRDDDHDDVLVLDRLRSDPSIEFVDRFAEQ
LAGVRRLLPQPDPDLLEEAKRWAYYPWRRMVVAILGPRGFRAVRLDRNRHLITAEEQR
ALHALRVGVVGLSAGHAIAYTLAAEGACGTLRLADFDKIELSNLNRVPVGVFDIGLNK
AMIAARRIAELDPYLAVDLVTSGLSPESVDEFLDGLDVVIEECDSLDIKVILRQAACA
RGVPVLMATSDRGLVDVERYDVEPGRPIFHGLLGDIDADKLCGLTTKDKVPHVLNILD
CQELSARCAASMIEVDQTLWGWPQLAGDIWVGAATVAEAVRRIGLGEPLESGRVRVDV
SAALDRLDQPPMPSRGNGWLLESVPPTAPAEPQPTSEIVAQAAIRAPSGGNVQPWHVV
AKQHSLTIRLAPEHTSAMDIAFRGSAVAVGAAMFNARVAAAAHRVLGSVEFDESQPDS
PLQATMHFGRGDDPSLAALYRPMLLRTTNRHHGMPGHVHPATVELLTNTAAAEGARLQ
LLLSRNEIDRAATILAAADRIRYLTPRLHEEMMSELRWPGDPSLDAGIDVRSLELDSG
ELRVLDILRRSDVVARLAQWDCGTALEDNTNERVSASSALAIVYVDGATLTDFARGGS
AMQAVWIVAQQHGLAVQPMSPIFLYARGRHDLDQASPHFAAQLHRLQLDFRELVKPGK
EGHEVLIFRLFHAPPPSVCSRRRVRHAIPEPHR"
CDS complement(1524391..1525146)
/codon_start=1
/note="gene_1410"
/transl_table=11
/translation="MTTAQLRPIAPQKLHFSENLSVWVSDAQCRLVVSQPALDPTLWN
TYLQGALRAYSKHGVECTLDLDAISDGSDTQLFFAAIDIGGDVVGGARVIGPLRSADD

SHAVVEWAGNPGLSAVRKMINDRAPFGVVEVKSGWVNSDAQRSDAIAAALARALPLSM
SLLGVQFVMGTAAAHALDRWRSSGGVIAARIPAAAYPDERYRTKMIWWDRRTLANHAE
PKQLSRMLVESRKLLRDVEALSATTAATAGAEQ"
CDS complement(1525655..1526521)
/codon_start=1
/note="gene_1411"
/transl_table=11
/translation="LIDYEEMFRGAMQARAMVANPDQWADSDRDQVNTRHYLSTSMRV
ALDRGEFFLVYQPIIRLADNRIIGAEALLRWEHPTLGTLLPGRFIDRAENNGLMVPLT
AFVLEQACRHVRSWRDHSTDPQPFVSVNVSASTICDPGFLVLVEGVLGETGLPAHALQ
LELAEDARLSRDEKAVTRLQELSALGVGIAIDDFGIGFSSLAYLPRLPVDVVKLGGKF
IECLDGDIQARLANEQITRAMIDLGDKLGITVTAKLVESPSQAARLRAFGCKAAQGWH
FAKALPVDFFRE"
CDS 1527199..1530453
/codon_start=1
/note="gene_1412"
/transl_table=11
/translation="VAALVEAVTLLTADVEGSTRLSQTRLNELAADYPTLDQNISEAV
AAHGGVTRPVDQEVGSGLVVAFLRAGDAIACALELQLSTLAPMRPRVGVHTGDVRLRG
DGTITGSAINESACLRDLAHEGQTLLSAATGDLVIDQLPANTWLTDVGKYPLRGLHRQ
ERVIQLCHRDLRNEFPPLRMSVGNRSSLPAQFTTFVGRDAQINEVQEVLTNYRLVTLR
GEGGVGKTRLAIQIAAASEFRDGLCFVDLAPIADPGMVSTTAAHALGLIDRPGSSTFD
TLSHAIGNCHMLMVLDNCEHVLDACAELVVELLGACPELSILATSRESIGVTGEVTWV
VPSLSPANEAIQLFTERARLVQPNFEIVADNFAAVSEICRRLDGMPLAIELAAARLRS
LSPNEIANSLDDRFRLLTGGARSTVQRQQTLRASMDWSYALLTDTERILFRRLAVFVG
GFDLTAASEVAAAGGDDFVERYSVLDQLTLLVDKSLVVAEESRGSTRYRLLETVRQYA
LEKLNESEEIDGVRARHRTHYATMAAGLNVPASTDYEQRLLQAEAEIDNLRAAFTWSR
GNGDIAAALQLASALQPLWSQGRMREGLAWLESILEREGDNHLVPAGVWARALAEKVI
LKAWPATSPMGAPDIVAQAHHALALARDAGDCAVLARALVACGCGSGCDTEAAQPYFA
EAIELARAINDEWTLSQIDYWQVVGIFISGQPIPLRAAAEQARELADSIGNRFVSRQC
RLFACLAQIWEGDANGALALSRDVTAEAEVANDVVTKVLGLYVEAMALSYIGDSAART
IAGAALEAATELGGIYQDLGYGAITRAALAAGDVAAIEASEASWDLRNQHNVVTAHHE
LMAQAALVRGDVTTARRFADEAVLASTGWHLMMALIARARVAIAQDELGKARDDAHAA
VACGVGVQTYLAMPDALELLAGLAGEAGNHGQAVRLFGAAAAQRQRTGEVRHKIWDAG
YEAATAALRDAMGDEDFTAAWAEGAAAPLDEAIAYAQRGRGERKRPSNGWDALTPAEH
KIVKLVTEGLVTKDIAARLFVSPRTVQTHLTHIYTKLDVTSRVQLVQEAAQHST"
CDS 1530682..1531287
/codon_start=1
/note="gene_1413"
/transl_table=11
/translation="MRLWGVQSEAITAAMVLSRKVSAIIAGHCGVRLVDQGVGDGFVA
AFAHASDAVACALELHQAPLSPIVLRIGIHTGEAQLVDERIYAGATMNLAAELRDLAH
GGQTVMSGATEDAVLGRLPMRAWLIGLRPMEGSPEGHNFPQSQRIAQLCHPNLRNTFP
PLRMRIADASGIPYVGRILVNVQVVPHWEGGCAAAGMVLAG"
CDS complement(1531335..1531571)
/codon_start=1
/gene="TBOG_01870"
/note="gene_1414"
/transl_table=11
/translation="MMALMNEIDLYEHKTPLPISPTSEGTPMTETHFRQTLYECAVKL
RELAYTLPQGVGEHALLRMSEQMIVTAGQVAPRV"

```
CDS             1531710..1532732
                /codon_start=1
                /note="gene_1415"
                /transl_table=11
                /translation="MGGARRLKLDGSIPNQLARAADAAVALERNGFDGGWTAEASHDP
                FLPLLLAAEHTSRLELGTNIAVAFARNPMIVANVGWDLQTYSKGRLILGLGTQIRPHI
                EKRFSMPWGHPARRMREFVAALRAIWLAWQDGTKLCFEGEFYTHKIMTPMFTPEPQPY
                PVPRVFIAAVGEAMTEMCGEVADGHLGHPMVSKRYLTEVSVPALLRGLARSGRDRSAF
                EVSCEVMVATGADDAELAAACTATRKQIAFYGSTPAYRKVLEQHGWGDLHPELHRLSK
                LGEWEAMGGLIDDEMLGAFAVVGPVDTIAGALRNRCEGVVDRVLPIFMAASQECINAA
                LQDFRR"
CDS             complement(1532805..1533995)
                /codon_start=1
                /note="gene_1416"
                /transl_table=11
                /translation="VVDFGALPPEINSARMYAGPGSASLVAAAQMWDSVASDLFSAAS
                AFQSVVWGLTVGSWIGSSAGLMVAAASPYVAWMSVTAGQAELTAAQVRVAAAAYETAY
                GLTVPPPVIAENRAELMILIATNLLGQNTPAIAVNEAEYGEMWAQDAAAMFGYAATAA
                TATEALLPFEDAPLITNPGGLLEQAVAVEEAIDTAAANQLMNNVPQALQQLAQPTKSI
                WPFDQLSELWKAISPHLSPLSNIVSMLNNHVSMTNSGVSMASTLHSMLKGFAPAAAQA
                VETAAQNGVQAMSSLGSQLGSSLGSSGLGAGVAANLGRAASVGSLSVPPAWAAANQAV
                TPAARALPLTSLTSAAQTAPGHMLGGLPLGQLTNSGGGFGGVSNALRMPPRAYVMPRV
                PAAG"
CDS             complement(1534310..1534972)
                /codon_start=1
                /note="gene_1417"
                /transl_table=11
                /translation="VTDDVRDVNTETTDATEVAEIDSAAGEAGDSATEAFDTDSATES
                TAQKGQRHRDLWRMQVTLKPVPVILILLMLISGGATGWLYLEQYRPDQQTDSGAARAA
                GAAASDGTIALLSYSPDTLDQDFATARSHLAGDFLSYYDQFTQQIVAPAAKQKSLKTT
                AKVVRAAVSELHPDSAVVLVFVDQSTTSKDSPNPSMAASSVMVTLAKVDGNWLITKFT
                PV"
CDS             complement(1534969..1535754)
                /codon_start=1
                /note="gene_1418"
                /transl_table=11
                /translation="MAETTEPPSDAGTSQADAMALAAEAEAAEAEALAAAARARARAA
                RLKREALAMAPAEDENVPEEYADWEDAEDYDDYDDYEAADQEAARSASWRRRLRVRLP
                RLSTIAMAAAVVIICGFTGLSGYIVWQHHEATERQQRAAAFAAGAKQGVINMTSLDFN
                KAKEDVARVIDSSTGEFRDDFQQRAADFTKVVEQSKVVTEGTVNATAVESMNEHSAVV
                LVAATSRVTNSAGAKDEPRAWRLKVTVTEEGGQYKMSKVEFVP"
CDS             complement(1536045..1538006)
                /codon_start=1
                /note="gene_1419"
                /transl_table=11
                /translation="MAAEMDWDKTVGAAEDVRRIFEHIPAILVGLEGPDHRFVAVNAA
                YRGFSPLLDTVGQPAREVYPELEGQQIYEMLDRVYQTGEPQSGSEWRLQTDYDGSGVE
                ERYFDFVVTPRRRADGSIEGVQLIVDDVTSRVRARQAAEARVEELSERYRNVRDSATV
                MQQALLAASVPVVPGADIAAEYLVAAEDTAAGGDWFDALALGDRLVLVVGDVVGHGVE
                AAAVMSQLRTALRMQISTGYTVVEALEAVDRFHKQVPGSKSATMCVGSLDFTSGEFQY
                CTAGHPPPLLVTADASARYVEPTGAGPLGSGTGFPVRSEVLNIGDAILFYTDGLIERP
```

GRPLEASTAEFADLAASIASGSGGFVLDAPARPIDRLCSDTLELLLRSTGYNDDVTLL
AMQRRAPTPPLHITLDATINAARTVRAQLREWLAEIGADHSDIADIVHAISEFVENAV
EHGYATDVSKGIVVEAALAGDGNVRASVIDRGQWKDHRDGARGRGRGLAMAEALVSEA
RIMHGAGGTTATLTHRLSRPARFVTDTMVRRAAFQQTIDSEFVSLVESGRIVVRGDVD
STTAATLDRQIAVESRSGIAPVTIDLSAVTHLGSAGVGALAAACDRARKQGTECVLVA
PPGSPAHHVLSLVQLPVVGADTEDIFAQE"

CDS complement(1538145..1538531)
/codon_start=1
/note="gene_1420"
/transl_table=11
/translation="MNPTQAGSFTTPVSNALKATIQHHDSAVIIHARGEIDAANEHTW
QDLVTKAAAATTAPEPLVVNLNGLDFMGCCAVAVLAHKAERCRRRGVDVRLVSRDRAV
ARIIHACGYGDVLPVHPTTESALSAT"

CDS 1538782..1539573
/codon_start=1
/note="gene_1421"
/transl_table=11
/translation="VDLHSRPPWSNNAVRRLGVALRDGVDPPVDCPSYAEVMLWHADL
AAEVQDRIEGRSWSASELLVTSRAKSQDTLLAKLRRRPYLQLNTIQDIAGVRIDADLL
LGEQTRLAREIADHFGADQPAIHDLRDHPHAGYRAVHVWLRLPAGRVEIQIRTILQSL
WANFYELLADAYGRGIRYDERPEQLAAGVVPAQLQELVGVMQDASADLAMHEAEWQHC
AEIEYPGQRAMALGEASKNKATVLATTKFRLERAINEAESAGGGG"

CDS 1539575..1539802
/codon_start=1
/note="gene_1422"
/transl_table=11
/translation="VAGYVVEYNRRTHVRRITEFATPQEAMEHRLKLEAERTDSNIEI
VALVSKSLGTLKQTHSRYFTGEELNVGNGAR"

CDS complement(1539874..1541079)
/codon_start=1
/note="gene_1423"
/transl_table=11
/translation="VNLDGNQASIREVCDAGLLSGAVTMVWQREKLLQVNEIGYRDID
AGVPMQRDTLFRIASMTKPVTVAAAMSLVDEGKLALRDPITRWAPELCKVAVLDDAAG
PLDRTHPARRAILIEDLLTHTSGLAYGFSVSGPISRAYQRLPFGQGPDVWLAALATLP
LVHQPGDRVTYSHAIDVLGVIVSRIEDAPLYQVIDERVLGPAGMTDTGFYVSADAQRR
AATMYRLDEQDRLRHDVMGPPHVTPPSFCNAGGGLWSTADDYLRFVRMLLGDGTVDGV
RVLSPESVRLMRTDRLTDEQKRHSFLGAPFWVGRGFGLNLSVVTDPAKSRPLFGPGGL
GTFSWPGAYGTWWQADPSADLILLYLIQHCPDLSVDAAAAVAGNPSLAKLRTAQPKFV
RRTYRALGL"

CDS 1541382..1542167
/codon_start=1
/note="gene_1424"
/transl_table=11
/translation="MNGLISQACGSHRPRRPSSLGAVAILIAATLFATVVAGCGKKPT
TASSPSPGSPSPEAQQILQDSSKATKGLHSVHVVVTVNNLSTLPFESVDADVTNQPQG
NGQAVGNAKVRMKPNTPVVATEFLVTNKTMYTKRGGDYVSVGPAEKIYDPGIILDKDR
GLGAVVGQVQNPTIQGRDAIDGLATVKVSGTIDAAVIDPIVPQLGKGGGRLPITLWIV
DTNASTPAPAANLVRMVIDKDQGNVDITLSNWGAPVTIPNPAG"

CDS 1542838..1543833
/codon_start=1

/note="gene_1425"
/transl_table=11
/translation="VGYFAVQGVRLVEPSWMPLWAFVIAMVLLRSSLAGFGHYALHRA
QRGLNRVFNNAFDLNYVALSLVTADGHTLLHHPYTQSEVDIKKNVFTMMMRLPWLYRV
PVHTIHKFGHMLSGMAIRIVDVFRITRKVGVEESYGSWRAALPHFLGSAGVRLLLVSE
LVVFAIAGDFWPWALQFVATLWVSTFLVVASHEFEDDTQGGAVNGEDWGVDQLEHAND
LTVIGNRYVDCFLSAGLSSHRVHHVLPFQRSGFANIVTEDVLREEAAKFGVEWLPAKG
FITDRLPRLCRKYLLTPSRQAKERHWGFVREHCSPAALKASASYVVAGFVGIGSV"
CDS 1543830..1545011
/codon_start=1
/note="gene_1426"
/transl_table=11
/translation="MNVSAESGAPRRAGQRHEVGLAQLPPAPPTTVAVIEGLATGTPR
RVVNQSDAADRVAELFLDPGQRERIPRVYQNSRITTRRMAVDPLDAKFDVFRREPAAI
RDRMHLFYEHAVPLAVDVSKRALAGLPYRAAEIGLLVLATSTGFIAPGVDVAIVKELG
LSPSISRVVVNFMGCAAAMNALGTATNYVRAHPAMKALVVCIELCSVNAVFADDINDV
VIHSLFGDGCAALVIGASQVQEKLEPGKVVVRSSFSQLLDNTEDGIVLGVNHNGITCE
LSENLPGYIFSGVAPVVTEMLWDNGLQISDIDLWAIHPGGPKIIEQSVRSLGISAELA
AQSWDVLARFGNMLSVSLIFVLETMVQQAESAKAISTGVAFAFGPGVTVEGMLFDIIR
R"
CDS 1545065..1545565
/codon_start=1
/note="gene_1427"
/transl_table=11
/translation="MADNLRWDALQLRDGDIIISAPSKSGLTWTQRLVSLLVFDGPDL
PGPLSTVSPWLDQTIRPIEEVVATLDAQQHRRFIKTHTPLDGLVLDDRVSYICVGRDP
RDAAVSMLYQSANMNEDRMRILHEAVVPFHERIAPPRLRNSVMRAARPRSSGIGWRGR
ISLPLA"
CDS 1545532..1545999
/codon_start=1
/note="gene_1428"
/transl_table=11
/translation="MEGPNQPPPGIGFTHLKGIGTLANILHQLGTVWVRRHLPNVALF
HYADYQADLAGELLRLARVLGIAATRDRARDLAQYATLDAMRSRASEIAPNTTDGIWH
SDERFFRRGGSGDWQQFFTEAEHLRYYHRINQLAPPDLLAWAHEGRRGYDPAN"
CDS 1546839..1548158
/codon_start=1
/note="gene_1429"
/transl_table=11
/translation="MTGRRLARFPAFRAGVAQDDDVGSTLSQGSTTGVLSGPNWSYWP
SRVLGSADPTTIAHRHGTHRITSPDETWLALQPFLAPAGITRVADVTWLDCLGIPTVQ
AVRPASLTLSVSQGKAASYRAAQVSAVMESLEGWHAENVTADLWSATARDLEADLTYD
PAQLRHRPGSLYHAGVKLDWMVATTLLTGRRTWVPWTAVLVNVATRDCWEPPMFEMDT
TGLASGNCYDEATLHALYEVMERHSVAAAVAGETMFEVPTDDVAGSDSAHLVEMIRDA
GDDVDLARIDVWDGYYCFAAELTSATLEVTFGGFGLHHDPNVALSRAITEAAQSRITA
ISGAREDLPSAIYHRFGRVHTYAKARKTSLRLNRARPTPWRVPDVDSLPELVASAATA
VANRSGTEPLAVVCDFADACVPVVKVLAPGLVLSSASPMRTPLQEAE"
CDS 1548155..1549648
/codon_start=1
/note="gene_1430"
/transl_table=11

```
                     /translation="MTACGRIVVTAGPTISAADIRSVVPDAEVAPPIAFGQALSYDLR
                     SGDTLLIVDGLFFQQPSVRHKELLTLMADGVRVVGSSSMGALRAAELHPFGMEGYGWV
                     FESYRDGVLEADDEVGVVHGDADDGYPVFVDALVNMRHTLARAVATGVVCSELAERII
                     ETARATPFTMRTWARLLSEVGAPDQRGLAAQLRSLRVDVKHADALLALRQLGQRPRVE
                     PLRPGPPPTVWSRRWRQPWAPPTSVAASADHGESFVDVTDLEVLSFLSVSSVDYWAYR
                     PALQQVAAWYWTLKHPEQSGSVGERAARAVAEVASEGYGRALEFIAYRYALATGIIDE
                     TGFPEAVAAHWLTTEERHGLGNDPISISARVITRTLFVVRLLPAIDHFLDLLRKDSRL
                     PRWRAMAAHALCKRDDLARQKPHLNLGRPDPTQLKRLFGARWGTQVNRIELARRGLMT
                     EDAFYAAATPFAVAAVDDQLPRIEVGTLGPAPLSADVPERHFDFGSV"
     CDS             complement(1549586..1550266)
                     /codon_start=1
                     /note="gene_1431"
                     /transl_table=11
                     /translation="VSPSPTYTPPKLASMPGIDFDALYRGESPGEGLPPITTPPWDTK
                     APKDNVIGWHTGGWVHGDVLDIGCGLGDNAIYLARNGYQVTGLDISPTALTTAKRRAS
                     DAGVDVKFAVGDATKLTGYTGAFDTVIDCGMFHCLDDDGKRSYAASVHRATRPGATLL
                     LSCFSNAMPPDEEWPRSTVSEQTLRDVLGGAGWDIESLEPATVRRELDGTEVEMAFWN
                     VRAQRRGS"
     CDS             complement(1550235..1551524)
                     /codon_start=1
                     /note="gene_1432"
                     /transl_table=11
                     /translation="MFDLVSLAGVDSRDDEASLTARIAELERVKSAAAAGQARAAAAL
                     DKLRRCNEADAGVPARRRGRGVASEVALARRDSPARGGRHLGFAKALVYEMPHTLAAL
                     EVGRLSEWRATLIVRESACLDVEDRRALDAELCADMSALDGMGDARIAAAARAIAYRL
                     DAQAVVERAARAETERTVTIRPAPDTMTWVTALLPVARGVSVYAALKRAADTTFDDRT
                     RGQVMADTLVERVTGQPAEAAQPVAVNLVLSDETLLAGDRAPAVVDGYGPIPAAVARN
                     LVRDAVADTRSRATLRRLYRHPRSGALVAMESRARRFPKGLAAFIGLRDQRCRMPYCD
                     APIRHRDHAQPHHRGGPTTATNGLGSCERCNYVKEAPGWRVSTDTDETGRHTAEFTTP
                     TGMYYHCTAPPLPGPLEIDVSQVEARIGVALTHLHAA"
     CDS             1551661..1552242
                     /codon_start=1
                     /note="gene_1433"
                     /transl_table=11
                     /translation="MGAAGDAAIGRESRELMSAADVGRTISRIAHQIIEKTALDDPVG
                     PDAPRVVLLGIPTRGVTLANRLAGNITEYSGIHVGHGALDITLYRDDLMIKPPRPLAS
                     TSIPAGGIDDALVILVDDVLYSGRSVRSALDALRDVGRPRAVQLAVLVDRGHRELPLR
                     ADYVGKNVPTSRSESVHVRLREHDGRDGVVISR"
     CDS             1552239..1553198
                     /codon_start=1
                     /gene="pyrB"
                     /note="gene_1434"
                     /transl_table=11
                     /translation="MTPRHLLTAADLSRDDATAILDDADRFAQALVGRDIKKLPTLRG
                     RTVVTMFYENSTRTRVSFEVAGKWMSADVINVSAAGSSVGKGESLRDTALTLRAAGAD
                     ALIIRHPASGAAHLLAQWTGAHNDGPAVINAGDGTHEHPTQALLDALTIRQRLGGIEG
                     RRIVIVGDILHSRVARSNVMLLDTLGAEVVLVAPPTLLPVGVTGWPATVSHDFDAELP
                     AADAVLMLRVQAERMNGGFFPSVREYSVRYGLTERRQAMLPGHAVVLHPGPMVRGMEI
                     TSSVADSSQSAVLQQVSNGVQVRMAVLFHVLVGAQDAGKEGAA"
     CDS             1553195..1554487
                     /codon_start=1
```

/gene="pyrC"
/note="gene_1435"
/transl_table=11
/translation="VSVLIRGVRPYGEGERVDVLVDDGQIAQIGPDLAIPDTADVIDA
TGHVLLPGFVDLHTHLREPGREYAEDIETGSAAAALGGYTAVFAMANTNPVADSPVVT
DHVWHRGQQVGLVDVHPVGAVTVGLAGAELTEMGMMNAGAAQVRMFSDDGVCVHDPLI
MRRALEYATGLGVLIAQHAEEPRLTVGAFAHEGPMAARLGLAGWPRAAEESIVARDAL
LARDAGARVHICHASAAGTVEILKWAKDQGISITAEVTPHHLLLDDARLASYDGVNRV
NPPLREASDAVALRQALADGIIDCVATDHAPHAEHEKCVEFAAARPGMLGLQTALSVV
VQTMVAPGLLSWRDIARVMSENPACIARLPDQGRPLEVGEPANLTVVDPDATWTVTGA
DLASRSANTPFESMSLPATVTATLLRGKVTARDGKIRA"

CDS 1554484..1554981
/codon_start=1
/note="gene_1436"
/transl_table=11
/translation="MNSGTLAGSLIFAAVLVMLIAVLARLMMRGWRRRSERQAELLGD
LPDVPEHVSSATVTTRGLYVGATLSPAWNERVTVGDLGYRSKAVLTRYPSGIMVERAR
AQPIWIPTESIAAIRMERGVAGKVVAGIGILAIRWRLPSGTEIDVGFRADNRDEYQEW
LEEPV"

CDS 1554978..1556108
/codon_start=1
/note="gene_1437"
/transl_table=11
/translation="LSKAVLVLEDGRVFTGRPFGATGQALGEAVFSTGMSGYQETLTD
PSYHRQIVVATAPQIGNTGWNGEDSESRGERIWVAGYAVRDPSPRASNWRATGTLEDE
LIRQRIVGIAGIDTRGVVRHLRSRGSMKAGVFSDGALAEPADLIARVRAQQSMLGADL
AGEVSTAEPYVVEPDGPPGVSRFTVAALDLGIKTNTPRNFARRGIRCHVLPASTTFEQ
IAELNPHGVFLSNGPGDPATADHVVALTREVLGAGIPLFGICFGNQILGRALGLSTYK
MVFGHRGINIPVVDHATGRVAVTAQNHGFALQGEAGQSFATPFGPAVVSHTCANDGVV
EGVKLVDGRAFSVQYHPEAAAGPHDAEYLFDQFVELMAGEGR"

CDS 1556108..1559455
/codon_start=1
/gene="carB"
/note="gene_1438"
/transl_table=11
/translation="VPRRTDLHHVLVIGSGPIVIGQACEFDYSGTQACRVLRAEGLQV
SLVNSNPATIMTDPEFADHTYVEPITPAFVERVIAQQAERGNKIDALLATLGGQTALN
TAVALYESGVLEKYGVELIGADFDAIQRGEDRQRFKDIVAKAGGESARSRVCFTMAEV
RETVAELGLPVVVRPSFTMGGLGSGIAYSTDEVDRMAGAGLAASPSANVLIEESIYGW
KEFELELMRDGHDNVVVVCSIENVDPMGVHTGDSVTVAPAMTLTDREYQRMRDLGIAI
LREVGVDTGGCNIQFAVNPRDGRLIVIEMNPRVSRSSALASKATGFPIAKIAAKLAIG
YTLDEIVNDITGETPACFEPTLDYVVVKAPRFAFEKFPGADPTLTTTMKSVGEAMSLG
RNFVEALGKVMRSLETTRAGFWTAPDPDGGIEEALTRLRTPAEGRLYDIELALRLGAT
VERVAEASGVDPWFIAQINELVNLRNELVAAPVLNAELLRRAKHSGLSDHQIASLRPE
LAGEAGVRSLRVRLGIHPVYKTVDTCAAEFEAQTPYHYSSYELDPAAETEVAPQTERP
KVLILGSGPNRIGQGIEFDYSCVHAATTLSQAGFETVMVNCNPETVSTDYDTADRLYF
EPLTFEDVLEVYHAEMESGSGGPGVAGVIVQLGGQTPLGLAHRLADAGVPIVGTPPEA
IDLAEDRGAFGDLLSAAGLPAPKYGTATTFAQARRIAEEIGYPVLVRPSYVLGGRGME
IVYDEETLQGYITRATQLSPEHPVLVDRFLEDAVEIDVDALCDGAEVYIGGIMEHIEE
AGIHSGDSACALPPVTLGRSDIEKVRKATEAIAHGIGVVGLLNVQYALKDDVLYVLEA
NPRASRTVPFVSKATAVPLAKACARIMLGATIAQLRAEGLLAVTGDGAHAARNAPIAV

KEAVLPFHRFRRADGAAIDSLLGPEMKSTGEVMGIDRDFGSAFAKSQTAAYGSLPAQG
TVFVSVANRDKRSLVFPVKRLADLGFRVLATEGTAEMLRRNGIPCDDVRKHFEPAQPG
RPTMSAVDAIRAGEVNMVINTPYGNSGPRIDGYEIRSAAVAGNIPCITTVQGASAAVQ
GIEAGIRGDIGVRSLQELHRVIGGVER"

CDS 1559452..1560276
/codon_start=1
/gene="pyrF"
/note="gene_1439"
/transl_table=11
/translation="VTGFGLRLAEAKARRGPLCLGIDPHPELLRGWDLATTADGLAAF
CDICVRAFADFAVVKPQVAFFESYGAAGFAVLERTIAELRAADVLVLADAKRGDIGAT
MSAYATAWVGDSPLAADAVTASPYLGFGSLRPLLEVAAAHGRGVFVLAATSNPEGAAV
QNAAADGRSVAQLVVDQVGAANEAAGPGPGSIGVVVGATAPQAPDLSAFTGPVLVPGV
GVQGGRPEALGGLGGAASSQLLPAVAREVLRAGPGVPELRAAGERMRDAVAYLAAV"

CDS 1560471..1560779
/codon_start=1
/note="gene_1440"
/transl_table=11
/translation="VTLRVVPESLAGASAAIEAVTARLAAAHAAAAPFIAAVIPPGSD
SVSVCNAVEFSVHGSQHVAMAAQGVEELGRSGVGVAESGASYAARDALAAASYLSGGL
"

CDS 1560776..1562395
/codon_start=1
/note="gene_1441"
/transl_table=11
/translation="MTEPWIAFPPEVHSAMLNYGAGVGPMLISATQNGELSAQYAEAA
SEVEELLGVVASEGWQGQAAEAFVAAYMPFLAWLIQASADCVEMAAQQHAVIEAYTAA
VELMPTQVELAANQIKLAVLVATNFFGINTIPIAINEAEYVEMWVRAATTMATYSTVS
RSALSAMPHTSPPPLILKSDELLPDTGEDSDEDGHNHGGHSHGGHARMIDNFFAEILR
GVSAGRIVWDPVNGTLNGLDYDDYVYPGHAIWWLARGLEFFQDGEQFGELLFTNPTGA
FQFLLYVVVVDLPTHIAQIATWLGQYPQLLSAALTGVIAHLGAITGLAGLSGLSAIPS
AAIPAVVPELTPVAAAPPMLAVAGVGPAVAAPGMLPASAPAPAAAAGATAAGPTPPAT
GFGGFPPYLVGGGGPGIGFGSGQSAHAKAAASDSAAAESAAQASARAQARAARRGRSA
AKARGHRDEFVTMDMGFDAAAPAPEHQPGARASDCGAGPIGFAGTVRKEAVVKAAGLT
TLAGDDFGGGPTMPMMPGTWTHDQGVFDEHR"

CDS 1562956..1563273
/codon_start=1
/note="gene_1442"
/transl_table=11
/translation="VALPQLTDEQRAAALEKAAAARRARAELKDRLKRGGTNLTQVLK
DAESDEVLGKMKVSALLEALPKVGKVKAQEIMTELEIAPTRRLRGLGDRQRKALLEKF
GSA"

CDS 1563408..1564034
/codon_start=1
/gene="gmk"
/note="gene_1443"
/transl_table=11
/translation="VSVGEGPDTKPTARGQPAAVGRVVVLSGPSAVGKSTVVRCLRER
IPNLHFSVSATTRAPRPGEVDGVDYHFIDPTRFQQLIDQGELLEWAEIHGGLHRSGTL
AQPVRAAAATGVPVLIEVDLAGARAIKKTMPEAVTVFLAPPSWQDLQARLIGRGTETA
DVIQRRLDTARIELAAQGDFDKVVVNRRLESACAELVSLLVGTAPGSP"

```
CDS             1564100..1564432
                /codon_start=1
                /note="gene_1444"
                /transl_table=11
                /translation="VSISQSDASLAAVPAVDQFDPSSGASGGYDTPLGITNPPIDELL
                DRVSSKYALVIYAAKRARQINDYYNQLGEGILEYVGPLVEPGLQEKPLSIALREIHAD
                LLEHTEGE"
CDS             1564448..1565704
                /codon_start=1
                /gene="dfp"
                /note="gene_1445"
                /transl_table=11
                /translation="VVDHKRIPKQVIVGVSGGIAAYKACTVVRQLTEASHRVRVIPTE
                SALRFVGAATFEALSGEPVCTDVFADVPAVPHVHLGQQADLVVVAPATADLLARAAAG
                RADDLLTATLLTARCPVLFAPAMHTEMWLHPATVDNVATLRRRGAVVLEPATGRLTGA
                DSGAGRLPEAEEITTLAQLLLERHDALPYDLAGRKLLVTAGGTREPIDPVRFIGNRSS
                GKQGYAVARVAAQRGADVTLIAGHTAGLVDPAGVEVVHVSSAQQLADAVSKHAPTADV
                LVMAAAVADFRPAQVATAKIKKGVEGPPTIELLRNDDVLAGVVRARAHGQLPNMRAIV
                GFAAETGDANGDVLFHARAKLRRKGCDLLVVNAVGEGRAFEVDSNDGWLLASDGTESA
                LQHGSKTLMASRIVDAIVTFLAGCSS"
CDS             1565832..1567043
                /codon_start=1
                /note="gene_1446"
                /transl_table=11
                /translation="VSEKGRLFTSESVTEGHPDKICDAISDSVLDALLAADPRSRVAV
                ETLVTTGQVHVVGEVTTSAKEAFADITNTVRARILEIGYDSSDKGFDGATCGVNIGIG
                AQSPDIAQGVDTAHEARVEGAADPLDSQGAGDQGLMFGYAINATPELMPLPIALAHRL
                SRRLTEVRKNGVLPYLRPDGKTQVTIAYEDNVPVQLDTVVISTQHAADIDLEKTLDPD
                IREKVLNTVLDDLAHETLDASTVRVLVNPTGKFVLGGPMGDAGLTGRKIIVDTYGGWA
                RHGGGAFSGKDPSKVDRSAAYAMRWVAKNVVAAGLAERVEVQVAYAIGKAAPVGLFVE
                TFGTETEDPVKIEKAIGEVFDLRPGAIIRDLNLLRPIYAPTAAYGHFGRTDVELPWEQ
                LDKVDDLKRAI"
CDS             complement(1567116..1568591)
                /codon_start=1
                /note="gene_1447"
                /transl_table=11
                /translation="MPDYHALIVGAGFSGIGAAIKLDRAGFSDYLVVEAGDGVGGTWH
                WNTYPGIAVDIPSFSYQFSFEQSRHWSRTYAPGHELKAYAEHCVDKYGIRSRIRLNTK
                VLAAEFDDEHSLWRVQTDPGGEITARFLISACGILTVPKLPDIDGVDSFEGVTMHTAR
                WDHTQDLTGKRVGIIGTGASAVQVIPEMAPIVSHLTVFQRTPIWCFPKFDVPLPTAVR
                WAMRIPGGKAVHRLLSQAFVEATFPIAAHYFAVFPLAKHMESAGRRYLRQQVHDPVVR
                EQLTPRYAVGCKRPGFHNTYLSTFNRDNVRLVTEPIDKITPTAVATTDGASHEIDVLV
                LATGFKVLDTDSIPTYAVTGTGGASLSRFWDEHRLQAYEGVSVPGYPNFFTVFGPYGY
                VGSSYFALIETQAHHIIRCLKRARRTGATRIEVTEEANARYFAEVMRRRHRQVFWQDS
                CRLANSYYFDKNGDVPLRPTTTVEAYWRSRRFDLGDYRISS"
CDS             complement(1568591..1569976)
                /codon_start=1
                /note="gene_1448"
                /transl_table=11
                /translation="MATATTQRPLKGPAKRMSTWTMTREAITIGFDAGDGFLGRLRGS
                DITRFRCAGRRFVSISHPDYVDHVLHEARLKYVKSDEYGPIRATAGLNLLTDEGDSWA
```

RHRGALNSTFARRHLRGLVGLMIDPIADVTAALVPGAQFDMHQSMVETTLRVVANALF
SQDFGPLVQSMHDLATRGLRRAEKLERLGLWGLMPRTVYDTLIWCIYSGVHLPPPLRE
MQEITLTLDRAINSVIDRRLAEPTNSADLLNVLLSADGGIWPRQRVRDEALTFMLAGH
ETTANAMSWFWYLMALNPQARDHMLTELDDVLGMRRPTADDLGKLAWTTACLQESQRY
FSSVWIIAREAVDDDIIDGHRIRRGTTVVIPIHHIHHDPRWWPDPDRFDPGRFLRCPT
DRPRCAYLPFGGGRRICIGQSFALMEMVLMAAIMSQHFTFDLAPGYHVELEATLTLRP
KHGVHVIGRRR"

CDS 1570093..1571088
/codon_start=1
/note="gene_1449"
/transl_table=11
/translation="VYATRVLCEVANERGVPTADVLAGTAIEPADLDDPDAVVGALDE
ITAVRRLLARLPDDAGIGIDVGSRFALTHFGLFGFAVMSCGTLRELLTIAMRYFALTT
MHVDITLFETADDCLVELDASHLPADVRGFFIERDIAGIIATTTSFALPLAAKYADQV
SAELAVDAELLRPLLELVPVHDVAFGRAHNRVHFPRAMFDEPLPQADRHTLEMCIAQC
DVLMQRNERRRGITALVRSKLFRDSGLFPTFTDVAGELDMHPRTLRRRLAEEGTSFRA
LLGEARSTVAVDLLRNVGLTVQQVSTRLGYTEVSTFSHAFKRWYGVAPSEYSRRG"

CDS complement(1571134..1572492)
/codon_start=1
/note="gene_1450"
/transl_table=11
/translation="MSFLFAQPEMLGAAATDLASIGSAISTANAAAAAATTRVLAAGA
DEVSAAVAALFSGHAQTYQALSTQAAAFHQQIVQTLTSTAGAYASAEAANVEQQLLGA
INAPTMALLGRPLIGHGADGAPGTGQAGGAGGILYGNGGNGGSGATGQAGGAGGAAGL
IGHGGAGGLGGTGASGGAGGAGGWLWGNGGAGGNGGVGVAGDPGGVGGAGGAGGAAGL
WGSGGSGGTGGQGGVGGGKSGDGGTGGIGGAGGGGGWLHGDGGAGGHGGQGGTGVSSG
GNGGAGGTGGDGRGLSGSGGAGGHGGQTGVGGKVGENNFGGAGGAGGTGGLIGNGGAG
GTGGKGGDGFGVFGKGGAGGTGGRGGAAGLIGDAGTGGTGGKGGTAGEDGTGGNGGTG
GNGGAAVLIGNGGGGGAGGNGGAGNDGTPGNGGGGGVGGTGGTLFGQPGQPGPPGQPG
PA"

CDS complement(1572747..1573148)
/codon_start=1
/note="gene_1451"
/transl_table=11
/translation="MILVDSDVLIAHLRGVVAARDWLVSARKDGPLAISVVSTAELIG
GMRTAERREVWRLLASFRVQPATEVIARRAGDMMRRYRRSHNRIGLGDYLIAATADVQ
GLQLATLNVWHFPMFEQLKPPFAVPGHRPRA"

CDS complement(1573145..1573402)
/codon_start=1
/note="gene_1452"
/transl_table=11
/translation="MKRTNIYLDEEQTASLDKLAAQEGVSRAELIRLLLNRALTTAGD
DLASDLQAINDSFGTLRHLDPPVRRSGGREQHLAQVWRATS"

CDS complement(1573485..1574444)
/codon_start=1
/note="gene_1453"
/transl_table=11
/translation="MTEPTVARPDIDPVLKMLLDTFPVTFTAADGVEVARARLRQLKT
PAELLPELRIEERTVGYDGLTDIPVRVYWPPVVRDNLPVVVYYHGGGWSLGGLDTHDP
VARAHAVGAQAIVVSVDYRLAPEHPYPAGIDDSWAALRWVGENAAELGGDPSRIAVAG
DSAGGNISAVMAQLARDVGGPPLVFQLLWYPTTMADLSLPSFTENADAPILDRDVIDA

FLAWYVPGLDISDHTMLPTTLAPGNADLSGLPPAFIGTAEHDPLRDDGACYAELLTAA
GVSVELSNEPTMVHGYVNFALVVPAAAEATGRGLAALKRALHA"
CDS complement(1574469..1575431)
/codon_start=1
/note="gene_1454"
/transl_table=11
/translation="MPSLDNTADEKPAIDPILLKVLDAVPFRLSIDDGIEAVRQRLRD
LPRQPVHPELRVVDLAIDGPAGPIGTRIYWPPTCPDQAEAPVVLYFHGGGFVMGDLDT
HDGTCRQHAVGADAIVVSVDYRLAPEHPYPAAIEDAWAATRWVAEHGRQVGADLGRIA
VAGDSAGGTIAAVIAQRARDMGGPPIVFQLLWYPSTLWDQSLPSLAENADAPILDVKA
IAAFSRWYAGEIDLHNPPAPMAPGRAENLADLPPAYIAVAGYDPLRDDGIRYGELLAA
AGVPVEVHNAQTLVHGYVGYAGVVPAATEATNRGLVALRVVLHG"
CDS complement(1575498..1575767)
/codon_start=1
/gene="J113_09755"
/note="gene_1455"
/transl_table=11
/translation="MKHKCANRNAPKPNTNAHVHQGIANNQSPVADNSSAGTNHRRSR
PMGWATAAAASSTAIDALNAGCNIACPVNSLPGGKRNAITVKYTP"
CDS 1575652..1576167
/codon_start=1
/note="gene_1456"
/transl_table=11
/translation="LVPALLLSATGDWLLAIPWWTWAFVFGLGAFLLAHLCFIGALLP
LARQAAPSRGRVAAVVAMCVASAGLLVWFWPHLGKDNLTIPVTVYIVALSAMVCTALL
ARLPTIWTAVGAVCFAASDSMIGIGRFILGNEALAVPIWWSYAAAEILITAGFFFGRE
VPDNAAAPTDS"
CDS 1576239..1578215
/codon_start=1
/note="gene_1457"
/transl_table=11
/translation="VLPMLSVPHLDRDFDYLVPAEHSDDAQPGVRVRVRFHGRLVDGF
VLERRSDSDHHGKLGWLDRVVSPEPVLTTEIRRLVDAVAARYAGTRQDVLRLAVPARH
ARVEREITTAPGRPVVAPVDPSGWAAYGRGRQFLAALADSRAARAVWQALPGELWADR
FAEAAAQTVRAGRTVLAIVPDQRDLDTLWQAATALVDEHSVVALSAGLGPEARYRRWL
AALRGSARLVIGTRSAVFAPLSELGLVMVWADADDSLAEPRAPYPHAREVAMLRAHQA
RCAALIGGYARTAEAHALVRSGWAHDVVAPRPEVRARSPRVVALDDSGYDDARDPAAR
TARLPSIALRAARSALQSGAPVLVQVPRRGYIPSLACGRCRAIARCRSCTGPLSLQGA
GSPGAVCRWCGRVDPTLRCVRCGSDVVRAVVVGARRTAEELGRAFPGTAVITSAGDTL
VPQLDAGPALVVATPGAEPRAPGGYGAALLLDSWALLGRQDLRAAEDALWRWMTAAAL
VRPRGAGGVVTVVAESSIPTVQSLIRWDPVGHAEAELAARTEVGLPPSVHIAALDGPA
GTVTALLEAARLPDPDRLQADLLGPVDLPPGVRRPAGIPADAPVIRMLLRVCREQGLE
LAASLRRGIGVLSARQTRQTRSLVRVQIDPLHIG"
CDS complement(1578233..1579057)
/codon_start=1
/note="gene_1458"
/transl_table=11
/translation="MTVYTPTSERQAPATTHRQMWALGDYAAIAEELLAPLGPILVST
SGIRRGDRVLDVAAGSGNVSIPAAMAGAHVTASDLTPELLRRAQARAAAAGLELGWRE
ANAEALPFSAGEFDAVLSTIGVMFAPRHQRTADELARVCRRGGKISTLNWTPEGFYGK
LLSTIRPYRPTLPAGAPHEVWWGSEDYVSGLFRDHVSDIRTRRGSLTVDRFGCPDECR

DYFKNFYGPAINAYRSIADSPECVATLDAEITELCREYLCDGVMQWEYLIFTARKC"
CDS 1579229..1579708
/codon_start=1
/note="gene_1459"
/transl_table=11
/translation="MPTEYPATAEESVDVITDALLTASRLLVAISAHSIAQVDENITI
PQFRTLVILSNHGPINLATLATLLGVQPSATGRMVDRLVGAELIDRLPHPTSRRELLA
ALTKRGRDVVRQVTEHRRTEIARIVEQMAPAERHGLVRALTAFTEAGGEPDARYEIE"
CDS complement(1579780..1580604)
/codon_start=1
/note="gene_1460"
/transl_table=11
/translation="MTIDTPAREDQTLAATHRAMWALGDYALMAEEVMAPLGPILVAA
AGIGPGVRVLDVAAGSGNISLPAAKTGATVISTDLTPELLQRSQARAAQQGLTLQYQE
ANAQALPFADDEFDTVISAIGVMFAPDHQAAADELVRVCRPGGTIGVISWTCEGFFGR
MLATIRPYRPSVSADLPPSALWGREAYVTGLLGDGVTGLKTARGLLEVKRFDTAQAVH
DYFKNNYGPTIEAYAHIGDNAVLAAELDRQLVELAAQYLSDGVMEWEYLLLTAEKR"
CDS 1580801..1581739
/codon_start=1
/gene="fmt"
/note="gene_1461"
/transl_table=11
/translation="VRLVFAGTPEPALASLRRLIESPSHDVIAVLTRPDAASGRRGKP
QPSPVAREAAERGIPVLRPSRPNSAEFVAELSDLAPECCAVVAYGALLGGPLLAVPPH
GWVNLHFSLLPAWRGAAPVQAAIAAGDTITGATTFQIEPSLDSGPIYGVVTEVIQPTD
TAGDLLKRLAVSGAALLSTTLDGIADQRLTPRPQPADGVSVAPKITVANARVRWDLPA
AVVERRIRAVTPNPGAWTLIGDLRVKLGPVHLDAAHRPSKPLPPGGIHVERTSVWIGT
GSEPVRLGQIQPPGKKLMNAADWARGARLDLAARAT"
CDS 1581736..1583109
/codon_start=1
/note="gene_1462"
/transl_table=11
/translation="MTPRSRGPRRRPLDPARRAAFETLRAVSARDAYANLVLPALLAQ
RGIGGRDAAFATELTYGTCRARGLLDAVIGAAAERSPQAIDPVLLDLLRLGTYQLLRT
RVDAHAAVSTTVEQAGIEFDSARAGFVNGVLRTIAGRDERSWVGELAPDAQNDPIGHA
AFVHAHPRWIAQAFADALGAAVGELEAVLASDDERPAVHLAARPGVLTAGELARAVRG
TVGRYSPFAVYLPRGDPGRLAPVRDGQALVQDEGSQLVARALTLAPVDGDTGRWLDLC
AGPGGKTALLAGLGLQCAARVTAVEPSPHRADLVAQNTRGLPVELLRVDGRHTDLDPG
FDRVLVDAPCTGLGALRRRPEARWRRQPADVAALAKLQRELLSAAIALTRPGGVVLYA
TCSPHLAETVGAVADALRRHPVHALDTRPLFEPVLAGLGEGPHVQLWPHRHGTDAMFA
AALRRLT"
CDS 1583143..1583832
/codon_start=1
/note="gene_1463"
/transl_table=11
/translation="MAGSTGGPLIAPSILAADFARLADEAAAVNGADWLHVDVMDGHF
VPNLTIGLPVVESLLAVTDIPMDCHLMIDNPDRWAPPYAEAGAYNVTFHAEATDNPVG
VARDIRAAGAKAGISVKPGTPLEPYLDILPHFDTLLVMSVEPGFGGQRFIPEVLSKVR
AVRKMVDAGELTILVEIDGGINDDTIEQAAEAGVDCFVAGSAVYGADDPAAAVAALRR
QAGAASLHLSL"
CDS 1583829..1584848

/codon_start=1
/note="gene_1464"
/transl_table=11
/translation="MNVEQVKSIDEAMGLAIEHSYQVKGTTYPNPPVGAVIVDPNGRI
VGAGGTEPAGGDHAEVVALRRAGGLATGAIVVVTMEPCNHYGKTPPCVNALIEARVGT
VVYAVADPNGIAGGGAGRLSAAGLQVRSGVLAEQVAAGPLREWLHKQRTGLPHVTWKY
ATSIDGRSAAADGSSQWISSEAARLDLHRRRAIADAILVGTGTVLADDPALTARLADG
SLAPQQPLRVVVGKRDIPPEARVLNDEARTMMIRTHEPMEVLRALSDRTDVLLEGGPT
LAGAFLRAGAINRILAYVAPILLGGPVTAVDDVGVSNITNALRWQFDSVEKVGPDLLL
SLVAR"

CDS complement(1584845..1586449)
/codon_start=1
/note="gene_1465"
/transl_table=11
/translation="MGREGPGHEAPGELISMRAGRRVAISAGSLAVLLGALDTYVVVT
IMRDIMNSVGIPINQLHRITWIVTMYLLGYIAAMPLLGRASDRFGRKLMLQVSLAGFI
IGSVVTALAGHFGDFHMLIAGRTIQGVASGALLPITLALGADLWSQRNRAGVLGGIGA
AQELGSVLGPLYGIFIVWLLHDWRDVFWINVPLTAIAMVMIHFSLPSHDRSTEPERVD
LVGGLLLALALGLAVIGLYNPNPDGKHVLPDYGAPLLVGALVAAVAFFGWERFARTRL
IDPAGVHFRPFLSALGASVAAGAALMVTLVDVELFGQGVLQMDQAQAAGMLLWFLIAL
PIGAVTGGWIATRAGDRAVAFAGLLIAAYGYWLISHWPVDLLADRHNILGLFTVPAMH
TDLVVAGLGLGLVIGPLSSATLRVVPSAQHGIASAAVVVARMTGMLIGVAALSAWGLY
RFNQILAGLSAAIPPNASLLERAAAIGARYQQAFALMYGEIFTITAIVCVFGAVLGLL
ISGRKEHADEPEVQEQPTLAPQVEPL"

CDS complement(1586407..1587117)
/codon_start=1
/note="gene_1466"
/transl_table=11
/translation="MRTPRRHCRRIAVLAAVSIAATVVAGCSSGSKPSGGPLPDAKPL
VEEATAQTKALKSAHMVLTVNGKIPGLSLKTLSGDLTTNPTAATGNVKLTLGGSDIDA
DFVVFDGILYATLTPNQWSDFGPAADIYDPAQVLNPDTGLANVLANFADAKAEGRDTI
NGQNTIRISGKVSAQAVNQIAPPFNATQPVPATVWIQETGDHQLAQAQLDRGSGNSVQ
MTLSKWGEKVQVTKPPVS"

CDS 1587202..1587807
/codon_start=1
/note="gene_1467"
/transl_table=11
/translation="MFTGIVEERGEVTGREALVDAARLTIRGPMVTADAGHGDSIAVN
GVCLTVVDVLPDGQFTADVMAETLNRSNLGELRPGSRVNLERAAALGSRLGGHIVQGH
VDATGEIVARCPSEHWEVVRIEMPASVARYVVEKGSITVDGISLTVSGLGAEQRDWFE
VSLIPTTRELTTLGSAAVGTRVNLEVDVVAKYVERLMRSAG"

CDS 1587834..1588136
/codon_start=1
/note="gene_1468"
/transl_table=11
/translation="MSCIPDEIDTPDVLIDRDILDRNIGRMSSAVAAKGIARRPHVKT
HKLPEIAHMQLRAGARPDGGHHRGSRGIRRPRRRRRIHHLPIVDRHTPSRPAPSAG"

CDS 1588021..1588536
/codon_start=1
/note="gene_1469"
/transl_table=11

/translation="VATIGEVEVFVDHGADDVFITYPLWIGTRQADRLRQLADRARIA
VGAGTAEGASNTGARLADAAGAIDVLIEIDSGHHRSGVRAEQVLEVAHAVGEAGLHLV
GVFTFPGHSYAPGKPGEAGEQERRALNDAANALVAVGFPISCRSGGSTPTALLTAADG
ASETSRRLCAR"
CDS 1588526..1588927
/codon_start=1
/note="gene_1470"
/transl_table=11
/translation="VLGDAQQLELGRCAPADIALTVAATVVSRQDCRSGLRRIVLDCG
SKILGSDRPAWATGFGRLIDHADARIAALSEHHATVVWPDDAPLPPVGTRLRVIPNHV
CLTTNLVDDVAVVRDATLIDRWKVAARGKNH"
CDS 1589032..1590309
/codon_start=1
/note="gene_1471"
/transl_table=11
/translation="MTRLDSVERAVADIAAGKAVIVIDDEDRENEGDLIFAAEKATPE
MVAFMVRYTSGYLCVPLDGAICDRLGLLPMYAVNQDKHGTAYTVTVDARNGIGTGISA
SDRATTMRLLADPTSVADDFTRPGHVVPLRAKDGGVLRRPGHTEAAVDLARMAGLQPA
GAICEIVSQKDEGSMAHTDELRVFADEHGLALITIADLIEWRRKHEKHIERVAEARIP
TRHGEFRAIGYTSIYEDVEHVALVRGEIAGPNADGDDVLVRVHSECLTGDVFGSRRCD
CGPQLDAALAMVAREGRGVVLYMRGHEGRGIGLMHKLQAYQLQDAGADTVDANLKLGL
PADARDYGIGAQILVDLGVRSMRLLTNNPAKRVGLDGYGLHIIERVPLPVRANAENIR
YLMTKRDKLGHDLAGLDDFHESVHLPGEFGGAL"
CDS 1590306..1590788
/codon_start=1
/note="gene_1472"
/transl_table=11
/translation="VKGGAGVPDLPSLDASGVRLAIVASSWHGKICDALLDGARKVAA
GCGLDDPTVVRVLGAIEIPVVAQELARNHDAVVALGVVIRGQTPHFDYVCDAVTQGLT
RVSLDSSTPIANGVLTTNTEEQALDRAGLPTSAEDKGAQATVAALATALTLRELRAHS
"
CDS 1590785..1591249
/codon_start=1
/note="gene_1473"
/transl_table=11
/translation="VTAAPNDWDVVLRPHWTPLFAYAAAFLIAVAHVAGGLLLKVGSS
GVVFQTADQVAMGALGLVLAGAVLLFARPRLRVGSAGLSVRNLLGDRIVGWSEVIGVS
FPGGSRWARIDLADDEYIPVMAIQAVDKDRAVAAMDTVRSLLARYRPDLCAR"
CDS 1591334..1591960
/codon_start=1
/note="gene_1474"
/transl_table=11
/translation="VVLALAACTESVAGRAMRATDRSSGLPTSAKPARARDLLLQDGD
RAPFGQVTQSRVGDSYFTSAVPPECSAALLFKGSPLRPDGSSDHAEAAYNVTGPLPYA
ESVDVYTNVLNVHDVVWNGFRDVSHCRGDAVGVSRAGRSTPMRLRYFATLSDGVLVWT
MSNPRWTCDYGLAVVPHAVLVLSACGFKPGFPMAEWASKRRAQLDSQV"
CDS 1592140..1592613
/codon_start=1
/note="gene_1475"
/transl_table=11
/translation="MGELRLVGGVLRVLVVVGAVFDVAVLNAGAASADGPVQLKSRLG

DVCLDAPSGSWFSPLVINPCNGTDFQRWNLTDDRQVESVAFPGECVNIGNALWARLQP
CVNWISQHWTVQPDGLVKSDLDACLTVLGGPDPGTWVSTRWCDPNAPDQQWDSVP"
CDS 1592677..1594617
/codon_start=1
/gene="uvrC"
/note="gene_1476"
/transl_table=11
/translation="VPDPATYRPAPGSIPVEPGVYRFRDQHGRVIYVGKAKSLRSRLT
SYFADVASLAPRTRQLVTTAAKVEWTVVGTEVEALQLEYTWIKEFDPRFNVRYRDDKS
YPVLAVTLGEEFPRLMVYRGPRRKGVRYFGPYSHAWAIRETLDLLTRVFPARTCSAGV
FKRHRQIDRPCLLGYIDKCSAPCIGRVDAAQHRQIVADFCDFLSGKTDRFARALEQQM
NAAAEQLDFERAARLRDDLSALKRAMEKQAVVLGDGTDADVVAFADDELEAAVQVFHV
RGGRVRGQRGWIVEKPGEPGDSGIQLVEQFLTQFYGDQAALDDAADESANPVPREVLV
PCLPSNAEELASWLSGLRGSRVVLRVPRRGDKRALAETVHRNAEDALQQHKLKRASDF
NARSAALQSIQDSLGLADAPLRIECVDVSHVQGTDVVGSLVVFEDGLPRKSDYRHFGI
REAAGQGRSDDVACIAEVTRRRFLRHLRDQSDPDLLSPERKSRRFAYPPNLYVVDGGA
PQVNAASAVIDELGVTDVAVIGLAKRLEEVWVPSEPDPIIMPRNSEGLYLLQRVRDEA
HRFAITYHRSKRSTRMTASALDSVPGLGEHRRKALVTHFGSIARLKEATVDEITAVPG
IGVATATAVHDALRPDSSGAAR"
CDS 1594614..1595519
/codon_start=1
/note="gene_1477"
/transl_table=11
/translation="MMNHARGVENRSEGGGIDVVLVTGLSGAGRGTAAKVLEDLGWYV
ADNLPPQLITRMVDFGLAAGSRITQLAVVMDVRSRGFTGDLDSVRNELATRAITPRVV
FMEASDDTLVRRYEQNRRSHPLQGEQTLAEGIAAERRMLAPVRATADLIIDTSTLSVG
GLRDSIERAFGGDGGATTSVTVESFGFKYGLPMDADMVMDVRFLPNPHWVDELRPLTG
QHPAVRDYVLHRPGAAEFLESYHRLLSLVVDGYRREGKRYMTIAIGCTGGKHRSVAIA
EALMGLLRSDQQLSVRALHRDLGRE"
CDS 1595516..1596544
/codon_start=1
/note="gene_1478"
/transl_table=11
/translation="MTDGIVALGGGHGLYATLSAARRLTPYVTAVVTVADDGGSSGRL
RSELDVVPPGDLRMALAALASDSPHGRLWATILQHRFGGSGALAGHPIGNLMLAGLSE
VLADPVAALDELGRILGVKGRVLPMCPVALQIEADVSGLEADPRMFRLIRGQVAIATT
PGKVRRVRLLPTDPPATRQAVDAIMAADLVVLGPGSWFTSVIPHVLVPGLAAALRATS
ARRALVLNLVAEPGETAGFSVERHLHVLAQHAPGFTVHDIIIDAERVPSEREREQLRR
TATMLQAEVHFADVARPGTPLHDPGKLAAVLDGVCARDVGASEPPVAATQEIPIDGGR
PRGDDAWR"
CDS 1596541..1597518
/codon_start=1
/note="gene_1479"
/transl_table=11
/translation="MTTDVKDELSRLVVKSVSARRAEVTSLLRFAGGLHIVGGRVVVE
AELDLGSIARRLRKEIFELYGYTAVVHVLSASGIRKSTRYVLRVANDGEALARQTGLL
DMRGRPVRGLPAQVVGGSIDDAEAAWRGAFLAHGSLTEPGRSSALEVSCPGPEAALAL
VGAARRLGVGAKAREVRGADRVVVRDGEAIGALLTRMGAQDTRLVWEERRLRREVRAT
ANRLANFDDANLRRSARAAVAAAARVERALEILGDTVPEHLASAGKLRVEHRQASLEE
LGRLADPPMTKDAVAGRIRRLLSMADRKAKVDGIPDTESVVTPDLLEDA"
CDS complement(1597528..1598136)

```
                     /codon_start=1
                     /note="gene_1480"
                     /transl_table=11
                     /translation="VLLGILTLLLSACQTASASGYNEPRGYDRATLKLVFSMDLGMCL
                     NRFTYDSKLAPSRPQVVACDSREARIRNDGFHANAPSCMRIDYELITQNHRAYYCLKY
                     LVRVGYCYPAVTTPGKPPSVLLYAPSACDESLPSPRVATALVPGTRSANREFSRFVVT
                     EIKSLGAGGRCDSASVSLQPPEEIEGPAIPPASSQLVCVAPK"
     CDS             1598353..1599672
                     /codon_start=1
                     /note="gene_1481"
                     /transl_table=11
                     /translation="MHVGALAICDPSDAPEYSFQRLRELIIERLPEIPQLRWRVTGAP
                     LGLDRPWFVEDEELDIDFHIRRIGVPAPGGRRELEELVGRLMSYKLDRSRPLWELWVI
                     EGVEGGRIATLTKMHHAIVDGVSGAGLGEILLDITPEPRPPQQETVGFVGFQIPGLER
                     RAIGALINVGIMTPFRIVRLLEQTVRQQIAALGVAGKPARYFEAPKTRFNAPVSPHRR
                     VTGTRVELARAKAVKDAFGVKLNDVVLALVAGAARQYLQKRDELPAKPLIAQIPVSTR
                     SEETKADVGNQVSSMTASLATHIEDPAKRLAAIHESTLSAKEMAKALSAHQIMGLTET
                     TPPGLLQLAARAYTASGLSHNLAPINLVVSNVPGPPFPLYMAGARLDSLVPLGPPVMD
                     VALNITCFSYQDYLDFGLVTTPEVANDIDEMADAIEPALAELERAAE"
     CDS             complement(1599694..1600908)
                     /codon_start=1
                     /note="gene_1482"
                     /transl_table=11
                     /translation="VELLNAANGLRPLSGSGYSTVLAFWLGWPTSEVPGVYLGASVLD
                     ALRRGRRGDFGGLKGKAALALTAAAWVILAVIRYRGATTPGPVLEAGLTEQLGPDYAK
                     ELATLPTEPMRSRGRNLPLRTAMARRRYVETTNVVCYGPYGRANLADIWRRRDLPRDA
                     KAPVLVQVPGGAWVLGWRRPQAYPLMSHLAARGWVCVSLNYRVSPRHTWPDHIVDVKR
                     ALAWVKENIAAYGGDPNFVAISGGSAGGHLCALAALTPNDPRFQPGFEQVDTSVAAAV
                     PVYGRYDWFTTDAPGRREFVGLLETFVVKRKFSTHRDIFVDASPIHHVRADAPPFFVL
                     HGRHDSLIPVAEAHAFVEELRAVSKSPVAYADLPHAQHAFDVFGSPRAHHTAEAVARF
                     LSWVYATNPPAT"
     CDS             complement(1600956..1602539)
                     /codon_start=1
                     /note="gene_1483"
                     /transl_table=11
                     /translation="LIATMRRAGLIAPLRPDRYLRIVAAMRREGMGFTAGFAGAARRC
                     PDRPGLIDELGTLTWRQLDERGNALAAALQALPAGPPRVVGIMCRNHRGFVDALLAVN
                     RIGAHILLLNTSFAGPALAEVVTREGVDTVVYDEEFSATVDRALAEKPQATRIVAWTD
                     EDHDLTVEKLVAAHAGRRPEHTGSHGKVILLTSGTTGTPKGARHSGGGIGTLKAILDR
                     TPWRAEEVTVIVAPMFHAWGFSQLVLASSLACTIVTRRRFDPEATLDLIDRHHATGLV
                     VVPVMFDRIMDLPAEIRNRYDGRSLRFAAASGSRMRPDVVIAFMDQFGDVIYNNYNAT
                     EAGMIATATPADLRTAPDTAGRPAEGTEIRILDQQFTEVPTGEVGTIYVRNDSQFDGY
                     TSGAAKDFHAGFMSSGDVGYLDENGRLFVVGRDDEMIVSGGENIYPIEVEKTLATHPD
                     VAEAAVIGVDDQQYGQRLAAFVVLKPGVSATPETLKQHVRDNLANYKVPRDIAVLDEL
                     PRGITGKILRTELQSRVGS"
     CDS             complement(1602567..1603394)
                     /codon_start=1
                     /note="gene_1484"
                     /transl_table=11
                     /translation="LSETDSPGNGDDAGIGDIGKFDPGLTQRLISVLRPVLKTYHRSQ
                     VHGLDSFPPGGALVVANHSGGMFPMDVPVFSVDFYDKFGYDRPVYTLSHDILFMGLTG
```

DLFRRTGYIRATRENAAKALRSGGVVVVFPGGDYDAYRPTFAENVIDFNGRKGYVSTA
VEAGVPIVPAVSIGGQESQLYLSRGTWLARRLGLKRLLRSDILPISFGFPFGFSAAIP
PNLPLPAKIVMQVLDPINLTKQFGEDPDVDAVDEHVRSVMQQALNDLAAKRRFPILG"
CDS 1603513..1604781
/codon_start=1
/note="gene_1485"
/transl_table=11
/translation="VAEAGGGPISVIARHMQLIRDDFISELFDKMKAEIRGLDYDARM
ADLWRASITENFVTAVHYLDRDTPQSLVEAPAAALAYARAAAQRDIPLSGLVRAHRLG
HARFLEVAMQYVSLLEPADRVSTIIELVNRSARLVDLVADQLIVAYEHEHDRWLSRRS
GLQQQWVSELLADTPVDVPRAERALGYRLDGVHIAAVVWVDSAVPIGDVVAQFDQVRC
LLAGELGPELGPVANSLMVPTDEREARLWFSPAPTRAFAPSRIRAAFESAGIRARLAC
GRVGDGLRGFRASLKQAERVKALALAGGARPGGRVMFYDDVAPVALLADDLEELRRFV
TDVLGDLSVDDERNSWLRETLREFLLRNRSYVATADAMILHRNTIQYRVIQAMELCGQ
NLDDPDAAFRVQMALEVCRWMAPAVLRAKQ"
CDS 1605021..1606607
/codon_start=1
/note="gene_1486"
/transl_table=11
/translation="MSFVFAVPEMVAATASDLASLGAALSEATAAAAIPTTQVLAAAA
DEVSAAIAELFGAHGQEFQALSAQASAFHDRFVRALSAAAGWYVDAEAANAALVDTAA
TGASELGSGGRTALILGSTGTPRPPFDYMQQVYDRYIAPHYLGYAFSGLYTPAQFQPW
TGIPSLTYDQSVAEGAGYLHTAIMQQVAAGNDVVVLGFSQGASVATLEMRHLASLPAG
VAPSPDQLSFVLLGNPNNPNGGILARFPGLYLQSLGLTFNGATPDTDYATTIYTTQYD
GFADFPKYPLNILADVNALLGIYYSHSLYYGLTPEQVASGIVLPVSSPDTNTTYILLP
NEDLPLLQPLRGIVPEPLLDLIEPDLRAIIELGYDRTGYADVPTPAALFPVHIDPIAV
PPQIGAAIGGPLTALDGLLDTVINDQLNPVVTSGIYQAGAELSVAAAGYGAPAGVTNA
IFIGQQVLPILVEGPGALVTADTHYLVDAIQDLAAGDLSGFNQNLQLIPATNIALLVF
AAGIPAVAAVAILTGQDFPV"
CDS 1606718..1608487
/codon_start=1
/note="gene_1487"
/transl_table=11
/translation="MGFLKPDLPDVDHDTWLTQPRRTRLQVVTRDWVEHGFGTPYAVY
LLYLTKIAVYVAAGAAIISLTPGLGGLSRIGDWWTQPIVYQKVIVFTLLFEVLGFGCG
SGPLTGRFWPPIGGFLYWLRPNTIRLPAWPDKVPFTQGDTRTVVDVALYAIVLIGGVW
ALLSPGSPGPGGTPVTAAGDVGLINPVLVVPTIVALGVLGLRDKTIFLAARGEHYWLK
LFVFFFPFTDQIAAFKIIMLCLWWGAATSKLNHHFPYVVAVMTSNNALLRSRVFNPIK
HLLYRDHANDLRPSWLPKLMAHGGGTTAEFLVPGILVLVADGHPWRWFLIGFMVLFHL
NILSNLPMGVPLEWNVFFIFSLCYLFGHYGAITATDLRSPLLLAIVIAVVAVVIMGNL
LPEKISFLPAMRYYAGNWATSIWCFRGDAEATMETSVVKSSALVVNQLAKLYDGATAE
IMTDQVAAFRAMHTHGRALNGLLPRALDDEAHYRIREGEIVAGPLVGWNFGEGHLHNE
QLVAAVQRRCNFADGDLRVIILEGQPIHVQKQWYRIVDAKTGLFEAGYVTVEDMLSRQ
PWPEPGDEFPVHVTTQRGTPSKP"
CDS 1608484..1609905
/codon_start=1
/note="gene_1488"
/transl_table=11
/translation="MTTAVVVGAGPNGLAAAIHLARHGVDVQVLEARDTIGGGARSGE
LTVPGVIHDHCSAFHPLGVGSPFWAAIDLQRYGLTWKWPDVDCAHPLDDGTAGVLYRS
IEATAAGLGPDGKRWQRAVGDLAAGFDELAEDLLRPVLNMPRHPIRLARFGPRAALPA

TAMARRFHTERARALFGGAAAHVYTRLDRPLTASLGLMILASGHRHGWPVARGGSGSI
TKALAAALDAYGGTVATGVTVTSRRDIPDADIVMLDLSPAAVLGIYGDVMPTRINRSY
RRYRAGSSAFKVDFAIEGDVGWTNPDCRRAGTVHLGGTFAEIADTERQRAQGTMVQRP
FVLVGQQYLADPSRSVGNINPIWAYAHVPFGYTGDATAAVIDQIERFAPGFRDRIVAT
VSTSTTELQTYNRNFIGGDIIGGANDRLQVIFRPRVAVDPYAIGVPGVYLCSQSAPPG
AGIHGLCGYHAAESALRWLRKRR"

CDS 1610069..1610890
/codon_start=1
/note="gene_1489"
/transl_table=11
/translation="MRAVFGCAIAVVGIAGSVVAGPADIHLVAAKQSYGFAVASVLPT
RGQVVGVAHPVVVTFSAPITNPANRHAAERAVEVKSTPAMTGKFEWLDNDVVQWVPDR
FWPAHSTVELSVGSLSSDFKTGPAVVGVASISQHTFTVSIDGVEEGPPPPLPAPHHRV
HFGEDGVMPASMGRPEYPTPVGSYTVLSKERSVIMDSSSVGIPVDDPDGYRLSVDYAV
RITSRGLYVHSAPWALPALGLENVSHGCISLSREDAEWYYNAVDMATRSLCRNSS"

CDS complement(1610976..1611596)
/codon_start=1
/note="gene_1490"
/transl_table=11
/translation="MAIVNRFNIKVIAGAGLFAAAIALSPDAAADPLMTGGYACIQGM
AGDAPVAAGDPVAAGGPAATGACSAALTDMAGVPFVAPGPVPAAAPVPIGAPVPIPGA
PVPIPGAPVPIPGAPVPIPGGPVPIPGAPVPVPAVPAPVIPVGTPLIALGPVLAGAPG
DGVVSAPIIGMSGVKDALTDPAPAGGPVPGQPVLPGPSASAPAGAR"

CDS 1611962..1612981
/codon_start=1
/note="gene_1491"
/transl_table=11
/translation="VTVRVGINGFGRIGRNFYRALLAQQEQGTADVEVVAANDITDNS
TLAHLLKFDSILGRLPCDVGLEGDDTIVVGRAKIKALAVREGPAALPWGDLGVDVVVE
STGLFTNAAKAKGHLDAGAKKVIISAPATDEDITIVLGVNDDKYDGSQNIISNASCTT
NCLAPLAKVLDDEFGIVKGLMTTIHAYTQDQNLQDGPHKDLRRARAAALNIVPTSTGA
AKAIGLVMPQLKGKLDGYALRVPIPTGSVTDLTVDLSTRASVDEINAAFKAAAEGRLK
GILKYYDAPIVSSDIVTDPHSSIFDSGLTKVIDDQAKVVSWYDNEWGYSNRLVDLVTL
VGKSL"

CDS 1612984..1614222
/codon_start=1
/gene="pgk"
/note="gene_1492"
/transl_table=11
/translation="MSVANLKDLLAEGVSGRGVLVRSDLNVPLDEDGTITDAGRIIAS
APTLKALLDADAKVVVAAHLGRPKDGPDPTLSLAPVAVALGEQLGRHVQLAGDVVGAD
ALARAEGLTGGDILLLENIRFDKRETSKNDDDRRALAKQLVELVGTGGVFVSDGFGVV
HRKQASVYDIATLLPHYAGTLVADEMRVLEQLTSSTQRPYAVVLGGSKVSDKLGVIES
LATKADSIVIGGGMCFTFLAAQGFSVGTSLLEDDMIEVCRGLLETYHDVLRLPVDLVV
TEKFAADSPPQTVDVGAVPNGLMGLDIGPGSIKRFSTLLSNAGTIFWNGPMGVFEFPA
YAAGTRGVAEAIVAATGKGAFSVVGGGDSAAAVRAMNIPEGAFSHISTGGGASLEYLE
GKTLPGIEVLSREQPTGGVL"

CDS 1614219..1615004
/codon_start=1
/gene="tpiA"
/note="gene_1493"

```
                     /transl_table=11
                     /translation="VSRKPLIAGNWKMNLNHYEAIALVQKIAFSLPDKYYDRVDVAVI
                     PPFTDLRSVQTLVDGDKLRLTYGAQDLSPHDSGAYTGDVSGAFLAKLGCSYVVVGHSE
                     RRTYHNEDDALVAAKAATALKHGLTPIVCIGEHLDVREAGNHVAHNIEQLRGSLAGLL
                     AEQIGSVVTAYEPVWAIGTGRVASAADAQEVCAAIRKELASLASPRIADTVRVLYGGS
                     VNAKNVGDIVAQDDVDGGLVGGASLDGEHFATLAAIAAGGPLP"
CDS                  complement(1615020..1615247)
                     /codon_start=1
                     /note="gene_1494"
                     /transl_table=11
                     /translation="MAPDRHALGLGLLVGALERGMAAGVIQRVPLPPLSHLLLAALTE
                     SALQIADATDKDRTRVEVERAFMALLEGLRV"
CDS                  complement(1615616..1616035)
                     /codon_start=1
                     /note="gene_1495"
                     /transl_table=11
                     /translation="MSASNAFVEGFADFWKAPSPDRLTDHLHPDVVLVRPLSPPRHGL
                     GAAQREFTRILGLLPDLHGEVDRWSQAGDVVFIEFRLIARLGSEVVEWPVVDRFLLRG
                     DKAVERVSYFDSLPLLIKVVKHPSAWRGWLTTMRSRA"
CDS                  1616372..1616725
                     /codon_start=1
                     /note="gene_1496"
                     /transl_table=11
                     /translation="VAGVTAAVSARLKADEARRPGFYAAGSGPLPQVRGSTLPVMELA
                     LQITLIVTSVLVVLLVLLHRAKGGGLSTLFGGGVQSSLSGSTVVEKNLDRLTLFVTGI
                     WLVSIIGVALLIKYR"
CDS                  complement(1616864..1618312)
                     /codon_start=1
                     /note="gene_1497"
                     /transl_table=11
                     /translation="MLSAAAADVASIGAALSAANGAAAPTTAGVLAAGADEVSAAIAS
                     LFSGYARDYQALSAQMARFHQQFVQALTASVGSYAAAEAANASPLQALEQQVLAAINA
                     PTQTLLGRPLIGNGADGLPGQNGGAGGLLWGNGGNGGAGDAAHPNGGNGGDAGMFGNG
                     GAGGAGYSPAAGTGAAGGAGGAGGAGGWLSGNGGAGGNGGTGASGADGGGGLPPVPAS
                     PGGNGGGGGAGGAAGMFGTGGAGGTGGDGGAGGAGDSPNSGANGARGGDGGNGAAGGA
                     GGRLFGNGGAGGNGGTAGQGGDGGTALGAGGIGGDGGTGGAGGTGGTAGIGGSSAGAG
                     GAGGDGGAGGTGGGSSMIGGKGGTGGNGGVGGTGGASALTIGNGSSAGAGGAGGAGGT
                     GGTGGYIESLDGKGQAGNGGNGGNGAAGGAGGGGTGAGGNGGAGGNGGDGGPSQGGGN
                     PGFGGDGGTGGPGGVGVPDGIGGANGAQGKHG"
CDS                  1618446..1620746
                     /codon_start=1
                     /note="gene_1498"
                     /transl_table=11
                     /translation="MQVVYTSATHWGVFTARVHGGDIAAVAALASDTNPAPQLQNLPGA
                     VRHRSRIANPAVRRGWLQHGPGPSSARGAEEFVEVSWDELIELLASELRRTVDRYGNE
                     AIYGSSYGWASAGRFHHAQSQVHRFLNMLGGYTASRHSYSAGASEVIFPHIVGAALFE
                     ALAETTTWDVIVDHTALLVAFGGLPVKNTAVMPGGTTAHPDRDYVGRYRARGGRLVSV
                     SPLRDDIAAIAGPLDDRCRWLAPVPGTDVAIMLGLAYVLATESLADRAFLGRYCTGYE
                     RFERYLLGLDDGIPKTPEWAAALSGLAAGDLRDLARRMAEHRTLITTSLSLQRIEHGE
                     QTVWMAATLAAMLGQIGLPGGGFGHGYSSNGVGNPPLACGLPALPQGNNPVSTFIPVA
                     AISELLQRPGQRLAYNGRLLELPDIKCVYWAGGNPFHHHQNLPRLRRALSRVDTIVVH
```

EQYWTAMAKHADIVVPTTTSFERDDFAASKTNPTLIAMPAMVPPYANARDDYHTFSAL
AHRLGFGKQFTEGRSAREWLEHMYDKWSAELDFPVPSFAEFWRTGRLELPTRTGLTWL
ADFRADPAAHPLGTPSGRIEIFSDTVDAFALPDCAGHPTWYEPSEWLGGPRAARYPLH
LIANQPRTRLHSQLDHGGASMASKIRGREPIRIHPDDAAARELTDGDIVRVFNDRGAC
LAGVVIDDGLRPKVVQLSTGAWFDPADPRDPDSMCVHGNPNALSNDSGTSSLAHGSTG
QHVLVQIERFTGELPPVRAHEPPRLA"

CDS complement(1620862..1621326)
/codon_start=1
/note="gene_1499"
/transl_table=11
/translation="VLIERQSVVAAPAEQVWQRVVTPEGINDELRPWMTMSVPRGAKG
MTVDTVPIGAPIGRAWLRLFGVLPFDYDRLSIAELEPGRRFREDSTMLSMRQWQHERT
VTPEGDTKTIVRDRITFQTRAGLRFAAPLIAAGLRALFGHRHRRLQRHFAQG"

CDS complement(1621954..1622364)
/codon_start=1
/note="gene_1500"
/transl_table=11
/translation="MTVMADRSGRPAPVRRRMKTLTQAALNADKTVEQVEDVLDGLGK
TMAELNSSLSQLNSTVERLEDGLDHLEGTLHSLDDLAKRLIVLVEPVEAIVDRIDYIV
SLGETVMSPLSVTEHAVRGVLDRLRNRTVHEPTN"

CDS complement(1622381..1623124)
/codon_start=1
/note="gene_1501"
/transl_table=11
/translation="VSSSIEIFPDSDILVAAAGKRLVGAIGAAVAARGQALIVLTGGG
NGIALLRYLSAQAQQIEWSKVHLFWGDERYVPEDDDERNLKQARRALLNHVDIPSNQV
HPMAASDGDFGGDLDAAALAYEQVLAASAAPGDPAPNFDVHLLGMGPEGHINSLFPHS
PAVLESTRMVVAVDDSPKPPPRRITLTLPAIQRSREVWLLVSGPGKADAVAAAIGGAD
PVSVPAAGAVGRQNTLWLLDRDAAAKLPS"

CDS complement(1623121..1624032)
/codon_start=1
/note="gene_1502"
/transl_table=11
/translation="MIVDLPDTTTTAVNKKLDELREKIGAVAMGRVLTLIIAPDSEAM
LEESIEAANDASHEHPSRIIVTMRGDPYADRPRLDAQLRVGADAGAGEFVVLRLSGPL
AGHADSVVIPFLLPDIPVVAWWPDIAPAVPAQDALGKLAIRRITDATNAIDPLSAIKS
RLAGYGAGDTDLAWSRITYWRALLTSAVDQPPHEPIESALVSGLKTEPALDVLAGWLA
SRIEGPVRRAVGELKVELVRNSETIVLSRPQEGITATLTRTGKPDALVPLARRVTGEC
LAEDLRRLDPDEIYCAALEGIKKVQYR"

CDS complement(1624085..1625629)
/codon_start=1
/note="gene_1503"
/transl_table=11
/translation="MKPAHAAASWRNPLRDKRDKRLPRIAGPCGMVIFGVTGDLARKK
VMPAVYDLANRGLLPPTFSLVGFARRDWSTQDFGQVVYNAVQEHCRTPFRQQNWDRLA
EGFRFVPGTFDDDDAFAQLAETLEKLDAERGTGGNHAFYLAIPPKSFPVVCEQLHKSG
LARPQGDRWSRVVIEKPFGHDLASARELNKAVNAVFPEEAVFRIDHYLGKETVQNILA
LRFANQLFDPIWNAHYVDHVQITMAEDIGLGGRAGYYDGIGAARDVIQNHLMQLLALT
AMEEPVSFHPAALQAEKIKVLSATRLAEPLDQTTSRGQYAAGWQGGEKVVGLLDEEGF
AEDSTTETFAAITLEVDTRRWAGVPFYLRTGKRLGRRVTEIALVFRRAPHLPFDATMT
DELGTNAMVIRVQPDEGVTLRFGSKVPGTAMEVRDVNMDFSYGSAFAEDSPEAYERLI

```
                     LDVLLGEPSLFPVNAEVELAWEILDPALEHWAAHGTPDAYEAGTWGPESSLEMLRRTG
                     REWRRP"
CDS                  complement(1625626..1626747)
                     /codon_start=1
                     /gene="tal"
                     /note="gene_1504"
                     /transl_table=11
                     /translation="MTAQNPNLAALSAAGVSVWLDDLSRDRLRSGNLQELIDTKSVVG
                     VTTNPSIFQKALSEGHTYDAQIAELAARGADVDATIRTVTTDDVRSACDVLVPQWEDS
                     DGVDGRVSIEVDPRLAHETEKTIQQAIELWKIVDRPNLFIKIPATKAGLPAISAVLAE
                     GISVNVTLIFSVQRYREVMDAYLTGMEKARQAGHSLSKIHSVASFFVSRVDTEIDKRL
                     DRIGSRQALELRGQAGVANARLAYAAYREVFEDSDRYRSLKVDGARVQRPLWASTGVK
                     NPDYSDTLYVTELVAPHTVNTMPEKTIDAVADHGVIQGDTVTGTASDAQAVFDQLGAI
                     GIDLTDVFAVLEEEGVRKFEASWNELLQETRAHLDTAAQ"
CDS                  complement(1626764..1628866)
                     /codon_start=1
                     /note="gene_1505"
                     /transl_table=11
                     /translation="VTTLEEISALTRPRHPDDWTEIDSAAVDTIRVLAADAVQKVGNG
                     HPGTAMSLAPLAYTLFQRTMRHDPSDTHWLGRDRFVLSAGHSSLTLYIQLYLGGFGLE
                     LSDIESLRTWGSKTPGHPEFRHTPGVEITTGPLGQGLASAVGMAMASRYERGLFDPDA
                     EPGASPFDHYIYVIASDGDIEEGVTSEASSLAAVQQLGNLIVFYDRNQISIEDDTNIA
                     LCEDTAARYRAYGWHVQEVEGGENVVGIEEAIANAQAVTDRPSFIALRTVIGYPAPNL
                     MDTGKAHGAALGDDEVAAVKKIVGFDPDKTFQVREDVLTHTRGLVARGKQAHERWQLE
                     FDAWARREPERKALLDRLLAQKLPDGWDADLPHWEPGSKALATRAASGAVLSALGPKL
                     PELWGGSADLAGSNNTTIKGADSFGPPSISTKEYTAHWYGRTLHFGVREHAMGAILSG
                     IVLHGPTRAYGGTFLQFSDYMRPAVRLAALMDIDTIYVWTHDSIGLGEDGPTHQPIEH
                     LSALRAIPRLSVVRPADANETAYAWRTILARRNGSGPVGLILTRQGVPVLDGTDAEGV
                     ARGGYVLSDAGGLQPGEEPDVILIATGSEVQLAVAAQTLLADNDILARVVSMPCLEWF
                     EAQPYEYRDAVLPPTVSARVAVEAGVAQCWHQLVGDTGEIVSIEHYGESADHKTLFRE
                     YGFTAEAVAAAAERALDN"
CDS                  complement(1629305..1633531)
                     /codon_start=1
                     /note="gene_1506"
                     /transl_table=11
                     /translation="MSLVIVAPETVAAAALDVARIGSSIGVANSAAAGSTTSVLAAGA
                     DEVSAAIATLFGSHAREYQAISTQVAAFHDRFAQTLSAAVGSYVSAEATNAAPLATLE
                     HNVLNALNAPTQALLGRPLIGDGAAGAPGTGQAGGAGGILWGNGGAGGSGAPGQVGGA
                     GGAAGLFGTGGAGGAGGAGAAGGAGGSGGWLLGNGGVGGAGGQSLLGGATGGAGGNAG
                     LFGVGGTGGPGGPGGPGGPGGPGGPGGVGGTGGAGGLGGTLYGAGGHGGAGGPGPIGG
                     VGGHGGVGGAAGLLGVGGHGGAGGHGAAGIAGAAGKGGIFPDGSNGGAGGDAGDGGTG
                     GRGGWLAGAGGAGGDGGIGGTGGAGGAGFSRALIVAGDNGGDGGNGGMGGAGGAGGPG
                     GAGGLISLLGGQGAGGDGGDGGAGGVGGDRGAGANGNQAVNAGAGGAGGHGGDPGAGG
                     AGGTGGAGSTIGAHGAAGASPTSGGNGGAGGNGAHFSSGGKAGGNGGAGGAGGLVGNG
                     GAGGAGGNGAPGAPPSGGDPNGGGGGAGGAGGKGGDGGAQAGDGGAGGAGGKGGNGGN
                     GATGATGLNGLGAGADGTDGGKGGNGGAGGGGGAGGQGGKALAATHQDGSMGAGGAGG
                     NGGAGGMGGDGGNGAKGTFDNGGDGVGGNGGNGGSRGIGGAGGIGGAGSTAGADGARG
                     ATPTSGGNGGTGGNGANATVAGGAGGAGGKGGNGGLVGNGGAGGKGGDGMAGVAGSSP
                     TTAGESGTSGQNGGAGGAGGAGGRGGDFGGDGGTGGAGGNGADGGAGGNGANGANATT
                     PGAKGGDGGHGGPGAQGGNGGQGGPGGLAGNLFGQNGIQGVGGSGGKGGAGGLAGDGG
                     NGANGNFAFGDGNGGHGGNGGNPGAGGQGGSGGAGSTPGAKGAHGFTPTSGGDGGDGG
```

NGGNSQVVGGNGGDGGNGGNGGSAGTGGNGGRGGDGAFGGMSANATNPGENGPNGNPG
GNGGAGGAGGAGLNGGNGGAGGNGGLGGFGGNGAAGANGVAVGAPGQPGGAGGHGGAG
GNGGAGGNGGQGVVSDGAGGAGGAGGDGGAPGDGANGGNGQGAGAFAGGGGGRGGDGG
NAGNAGAGGPAGTGSTAGKAGPAGSILHDGGNGGHGGHGAASGGNGGPGGHGGNGGNG
GTGANGGNGGIGGTGGAGSTGAKGVLGTNEGDGGDGGRGGNGGRGGNGGQGLTGAGGN
GGTGGTPGNGGNGGNGASGDLVTSPGDGGGGGRGGDAGRGGDAGLGGSSGPGGTPGDW
GTGGTGGTGGTGGQGANGGLTGGRGGTGGNGGAGGTGGTGHNGSQPGMGGNGGAGGFG
GNGFAGVGGRGGMGGSGGTGGTGDAGPFGTGTGGTGGHGGQGGGGGFSILLGLGGLGG
LGSPGSIATGTAGGAGGGGGFGGLGGGEFV"

variation 1632810..1632836
/note="supplemented from AL2122/97"

CDS 1633981..1634859
/codon_start=1
/note="gene_1507"
/transl_table=11
/translation="MSTLLAYLALTKPRVIELLLVTAIPAMLLADRGAIHPLLMLNTL
VGGMMAATGANTLNCVADADIDKVMKRTARRPLAREAVPTRNALALGLTLTVISFFWL
WCATNLLAGVLALVTVAFYVFVYTLWLKRRTSQNVVWGGAAGCMPVMIGWSAITGTIA
WPALAMFAIIFFWTPPHTWALAMRYKQDYQVAGVPMLPAVATERQVTKQILIYTWLTV
AATLVLALATSWLYGAVALVAGGWFLTMAHQLYAGVRAGEPVRPLRLFLQSNNYLAVV
FCALAVDSVIALPTLH"

CDS complement(1634908..1637337)
/codon_start=1
/note="gene_1508"
/transl_table=11
/translation="MSLVIVAPETVAAAALDVARIGSSIGVANSAAAGSTTSVLAAGA
DEVSAAIATLFGSHAREYQAISTQVAAFHDRFAQTLSAAVGSYVSAEATNAAPLATLE
HNVLNALNAPTQALLGRPLIGDGAAGAPGTGQAGGAGGILWGNGGAGGSGAPGQVGGA
GGAAGLFGTGGAGGAGGAGAAGGAGGSGGWLLGNGGVGGAGGQSLLGGATGGAGGNAG
LFGVGGTGGPGGPGGPGGVGGTGGAGGLGGTLYGAGGHGGAGGPGPIGGVGGHGGVGG
AAGLLGVGGHGGAGGHGAAGIAGAAGKGGIFPDGSNGGAGGDAGDGGAGGRGGWLAGA
GGAGGDGGIGGTGGAGGAGFSRALIVAGDNGGDGGNGGMGGAGGAGGPGGAGGLISLL
GGQGAGGDGGDGGAGGVGGDRGAGANGNQAVNGGAGGHGGNGGNPGTGGAGGTGGAGS
ITGAQGAIGATPTSGGNGGAGGNGANATTAGTNGANGGPGGHGGLVGNGGAGGAGGNG
ANGAAGTNASDSGAVGGKGNSGGNGGQGGAGGDGGTLAGNGGAGGTGGRGADGGLGGS
GAEGANATTAGERGQDGGKGGNGGVGGTGGNAVAPGANGGHGGNGGNPGFSGAGGLGG
LSGDGVTRAAQGATPDFADTGGKGGNGGNGANAVAPGGTGASGGAGGNAGAGGKGGEN
IIGDGGGGGNGGAGGQGGDGTAGAGGDGGAGGQGGDGTAGAGGDGGAGGKGGDGGDGG
SDPTEGRGFGGLGGAGGAGGKGGAGTLLGLTVFGDNGGAGVLGDSTDPDGSGGAGGAG
GAGGAGGDPTI"

variation 1636096..1636302
/note="supplemented from AL2122/97"

CDS 1637456..1638754
/codon_start=1
/note="gene_1509"
/transl_table=11
/translation="VHSAHSPYYRLVALRETSPRIHELIREAARIALNPTQEWLDEFD
RAILAANPSIAADPALATVVKRSNRAHLIHFAAANLRNPGAPVPANLGPEPLRMARDL
VRVGLDALALDIYRIGQNVAWRRWTDIAFGLTSDPDELHELLDVPFRTANEFVDTTLA
GITTEMQLERDKLTRDVPAERRKIVQLLIDGAPISREHAEARLGYPLDRSHTAAVIWG
DQAQGDHSHLDRVADAFGHAGGCPHPLVVVAGAATRWVWVKDAPGFDIDLIHEVLHDI

PDARIAIGATAPGIEGFRRSHRDALTTARMIIRLESPHRVAFFTDVEMVALLTENAEG
ADDFIQRTLGNLESASPALKTTLLTFINQQCNASRAARLLFTHRNTLMNRLETAQRLL
PRPLADTTIHVAVALEAQQWREKQTSDPPAKKESNGTKMR"

CDS complement(1638782..1639768)
/codon_start=1
/note="gene_1510"
/transl_table=11
/translation="MHAIEVTETGGPGVLRHVDQPQPQPGHGELLIKAEAIGVNFIDT
YFRSGQYPRELPFVIGSEVCGTVEAVGPGVTAADTAISVGDRVVSASANGAYAEFCTA
PASLTAKVPDDVTSEVAASALLKGLTAHYLLKSVYPVKRGDTVLVHAGAGGVGLILTQ
WATHLGVRVITTVSTAEKAKLSKDAGADVVLDYPEDAWQFAGRVRELTGGTGVQAVYD
GVGATTFDASLASLAVRGTLALFGAASGPVPPVDPQRLNAAGSVYLTRPSLFHFTRTG
EEFSWRAAELFDAIGSEAITVAVGGRYPLADALRAHQDLEARKTVGSVVLLP"

CDS 1639788..1640651
/codon_start=1
/note="gene_1511"
/transl_table=11
/translation="MKLARPDVFHPRVVLAGWPQQPAGDGDDAGLVAALRHRGLHAGW
LSWDDPEIVHADLVILRATRDYPARLDEFLAWTTRVANLLNSRPVVAWNVERRYLRDL
MDRGVPTVPGEVYVPGEPVRLPRKGQVFVGPTIGTGTRRCSARFAAEFVAQLHAAGQA
VLVQPGGSGDETVLVFLGGEPSHAFTKQADTWRQTEPDFEIWDVGAAAVAGAAAQVGV
DPGELLYARAHITGGSRDPRLLELQLVDPSLGWQWLDPDIRNLAQRDFALCVQSALER
LGLGPFSHRRP"

CDS complement(1640601..1641533)
/codon_start=1
/note="gene_1512"
/transl_table=11
/translation="VPYDRAVSPSLRVQRVIAAIVILTQGGIAVTGAIVRVTASGLGC
PTWPQCFPGSFTPVVVAEVPRVHQAVEFGNRMVTFAVVIAAALAVLVVTRARRRTEVL
AYAWLMPVSTVVQAMIGGITVRTGLLWWTVAIHLLASMTMVWLAVLLYVKIGQPDDGV
VHELVVSPLRALTALSALNLAAVLVTGTLVTAAGPHAGDRSPSRTVPRLKVEITTLVH
MHSSLLVAYLALLIGLGFGLLAVGATRAILVRLAVLLALVATQAAVGTTQYFTGVPAA
LVAIHVAGAAAVTAATAALWASMGERAQPQPLQR"

CDS complement(1641645..1642430)
/codon_start=1
/note="gene_1513"
/transl_table=11
/translation="VTQTNRPAFPAGTFSPDPRPNAVPLMLAAQFSLELKLLLRNGEQ
LLLTMFIPITLLVGLTLLPMGSFGHNRAATFVPVIMALAVISTAFTGQAIAVAFDRRY
GALKRLGATPLPVWGIIAGKSLAVVAVVFLQAIILGAIGFALGWRPALTALTLGAGII
ALGTAGFAALGLLLGGTLRAEIVLAVANLMWFVFAGFGALTLESNVIPTAFKWVARVT
PSGALTEALSQAMTVSVDWFGIVVLAVWGALAALAALRWFRFT"

CDS complement(1642427..1643368)
/codon_start=1
/note="gene_1514"
/transl_table=11
/translation="VNRAPDTPEVVLRLRGVCKRYGSITAVSNLDLDVHDAEVMALLG
PNGAGKTTTVEMCEGFVRPDAGSIEVLGLDPITDNARLRARIGVMLQGGGGYPAARAG
EMLDLVASYAANPLDPHWLLDTLGLTEAARTTYRRLSGGQQQRLALACALVGRPQLVF
LDEPTAGMDAHARVLVWELIDALRRDGVTVVLTTHHLKEAEELADRLVIIDHGVTVAA
GTPAELMRSGAKDQLRFTAPPRLDLSLLASALPEGYQATELTPGEYLVEGPVDPQVLA

```
                     TVTAWCAQIDVLATDMRVEQRSLEDVFLDLTGRKLRQ"
     CDS             complement(1643524..1645299)
                     /codon_start=1
                     /note="gene_1515"
                     /transl_table=11
                     /translation="MAARHHTLSWSIASLHGDEQAVGAPLTTTELTALARTRLFGATG
                     TVLMAIGALGAGARPVVQDPTFGVRLLNLPSRIQTVSLTMTTTGAVMMALAWLMLGRF
                     TLGRRRMSRGELDRTLLLWMLPLLIAPPMYSKDVYSYLAQSEIGRDGLDPYRVGPASG
                     LGLGHVFTLSVPSLWRETPAPYGPLFLWIGRGISSLTGENIVAAVLCHRLVVLIGVTL
                     IVWATPRLAQRCGVAEVSALWLGAANPLLIMHLVAGIHNEALMLGLMLTGVEFALRGL
                     DMANTPRPSPETWRLGPATIRASRRPELGASPRAGASRAVKPRPEWGPLAMLLAGSIL
                     ITLSSQVKLPSLLAMGFVTTVLAYRWGGNLRALLLAAAVMASLTLAIMAILGWASGLG
                     FGWINTLGTANVVRSWMSPPTLLALGTGHVGILLGLGDHTTAVLSLTRAIGVLIITVM
                     VCWLLLAVLRGRLHPIGGLGVALAVTVLLFPVVQPWYLLWAIIPLAAWATRPGFRVAA
                     ILATLIVGIFGPTANGDRFALFQIVDATAASAIIVILLIALTYTRLPWRPLAAEQVVT
                     AAESASKTPATRRPTAAPDAYADST"
     CDS             1645488..1646153
                     /codon_start=1
                     /note="gene_1516"
                     /transl_table=11
                     /translation="VRLLLESGSITAGEIGDRLGLSAAGVRRHLDALIEAGDAEASAA
                     APWQQVGRGRPAKRYRLTAAGRAKLDHSYDDLASAAMRQLREIGGEEAVRTFARRRID
                     AILADVAPADGPDDAALEAAAERIATALSKAGYVATTTRVGGPIHGVQFCQHHCPVSH
                     VAEEFPELCETEQQAMAEVLGTHVQRLATIVNGDCACTTHVPLSPAPSPRPPATSTEG
                     VSR"
     CDS             1646150..1648690
                     /codon_start=1
                     /note="gene_1517"
                     /transl_table=11
                     /translation="MTLTPEASKSVAQPPTQAPLTQEEAIASLGRYGYGWADSDVAGA
                     NAQRGLSEAVVRDISAKKNEPDWMLQSRLKALRIFDRKPIPKWGSNLDGIDFDNIKYF
                     VRSTEKQAASWDDLPEDIRNTYDRLGIPEAEKQRLVAGVAAQYESEVVYHQIREDLEA
                     QGVIFLDTDTGLREHPDIFKEYFGTVIPAGDNKFSALNTAVWSGGSFIYVPPGVHVDI
                     PLQAYFRINTENMGQFERTLIIADEGSYVHYVEGCLPAGELITTADGDLRPIESIRVG
                     DFVTGHDGRPHRVTAVQVRDLDGELFTFTPMSPANAFSVTAEHPLLAIPRDEVRVMRK
                     ERNGWKAEVNSTKLRSAEPRWIAAKDVAEGDFLIYPKPKPIPHRTVLPLEFARLAGYY
                     LAEGHACLTNGCESLIFSFHSDEFEYVEDVRQACKSLYEKSGSVLIEEHKHSARVTVY
                     TKAGYAAMRDNVGIGSSNKKLSDLLMRQDETFLRELVDAYVNGDGNVTRRNGAVWKRV
                     HTTSRLWAFQLQSILARLGHYATVELRRPGGPGVIMGRNVVRKDIYQVQWTEGGRGPK
                     QARDCGDYFAVPIKKRAVREAHEPVYNLDVENPDSYLAYGFAVHNCTAPIYKSDSLHS
                     AVVEIIVKPHARVRYTTIQNWSNNVYNLVTKRARAEAGATMEWIDGNIGSKVTMKYPA
                     VWMTGEHAKGEVLSVAFAGEDQHQDTGAKMLHLAPNTSSNIVSKSVARGGGRTSYRGL
                     VQVNKGAHGSRSSVKCDALLVDTVSRSDTYPYVDIREDDVTMGHEATVSKVSENQLFY
                     LMSRGLTEDEAMAMVVRGFVEPIAKELPMEYALELNRLIELQMEGAVG"
     CDS             1648687..1649880
                     /codon_start=1
                     /note="gene_1518"
                     /transl_table=11
                     /translation="MTAPGLTAAVEGIAHNKGELFASFDVDAFEVPHGRDEIWRFTPL
                     RRLRGLHDGSARATGSATITVSERPGVYTQTVRRGDPRLGEGGVPTDRVAAQAFSSFN
                     SATLVTVERDTQVVEPVGITVTGPGEGAVAYGHLQVRIEELGEAVVVIDHRGGGTYAD
```

NVEFVVDDAARLTAVWIADWADDTVHLSAHHARIGKDAVLRHVTVMLGGDVVRMSAGV
RFCGAGGDAELLGLYFADDGQHLESRLLVDHAHPDCKSNVLYKGALQGDPASSLPDAH
TVWVGDVLIRAQATGTDTFEVNRNLVLTDGARADSVPNLEIETGEIVGAGHASATGRF
DDEQLFYLRSRGIPEAQARRLVVRGFFGEIIAKIAVPEVRERLTAAIEHELEITESTE
KTTVS"
CDS 1649877..1650677
/codon_start=1
/note="gene_1519"
/transl_table=11
/translation="MTILEIKDLHVSVENPAEADHEIPILRGVDLTVKSGETHALMGP
NGSGKSTLSYAIAGHPKYHVTSGTITLDGADVLAMSIDERARAGLFLAMQYPVEVPGV
SMSNFLRSAATAIRGEPPKLRHWVKEVKAAMAALDIDPAFAERSVNEGFSGGEKKRHE
ILQLELLKPKIAILDETDSGLDVDALRVVSEGVNRYAESQHGGILLITHYTRILRYIH
PEYVHVFVGGRIVESGGSELADELDQNGYVRFSPASGRYPHQPAPTGA"
CDS 1650679..1651932
/codon_start=1
/note="gene_1520"
/transl_table=11
/translation="MTASVNSLDLAAIRADFPILKRIMRGGNPLAYLDSGATSQRPLQ
VLDAEREFLTASNGAVHRGAHQLMEEATDAYEQGRADIALFVGADTDELVFTKNATEA
LNLVSYVLGDSRFERAVGPGDVIVTTELEHHANLIPWQELARRTGATLRWYGVTDDGR
IDLDSLYLDDRVKVVAFTHHSNVTGVLTPVSELVSRAHQSGALTVLDACQSVPHQPVD
LHELGVDFAAFSGHKMLGPNGIGVLYGRRELLAQMPPFLTGGSMIETVTMEGATYAPA
PQRFEAGTPMTSQVVGLAAAARYLGAIGMAAVEAHERELVAAAIEGLSGIDGVRILGP
TSMRDRGSPVAFVVEGVHAHDVGQVLDDGGVAVRVGHHCALPLHRRFGLAATARASFA
VYNTADEVDRLVAGVRRSRHFFGRA"
CDS 1651929..1652417
/codon_start=1
/note="gene_1521"
/transl_table=11
/translation="VTLRLEQIYQDVILDHYKHPQHRGLREPFGAQVYHVNPICGDEV
TLRVALSEDGTRVTDVSYDGQGCSISQAATSVLTEQVIGQRVPRALNIVDAFTEMVSS
RGTVPGDEDVLGDGVAFAGVAKYPARVKCALLGWMAFKDALAQASEAFEEVTDERNQR
TG"
CDS 1652392..1652739
/codon_start=1
/note="gene_1522"
/transl_table=11
/translation="MSETSAPAEELLADVEEAMRDVVDPELGINVVDLGLVYGLDVQD
GDEGTVALIDMTLTSAACPLTDVIEDQSRSALVGSGLVDDIRINWVWNPPWGPDKITE
DGREQLRALGFTV"
CDS complement(1652834..1654663)
/codon_start=1
/note="gene_1523"
/transl_table=11
/translation="LGHYIANVRDLEFNLLEVLDIGAVLGTGRYSDLDVDTVRTILAE
AARLAEGPIAESFGYADRNPPVFDPNTHSISVPDELAKTVQAIKEAGWWRLGLAEEIG
GMPAPPPLAWAVNEMIYCANPSACFFNLGPVLAQSLYIEGNDEQRRWAAEGVQRGWQA
TMVLTEPDAGSDVGAGRTKAFEQPDGTWHIEGVKRFISGGDVGNTAENIFHLVLARPE
GAGPGTKGLSLFYVPNYLFDPDTFELGARNGVYVTGLEHKMGLKSSPTCELTFGGADV
PAVGYLVGGVHNGIAQMFTVIEHARMTIGVKSAGTLSTGYLNALAFAKERVQGADLTQ

MTDKTAPRVTIMHHPDVRRSLMTQKAYAEGLRALYLYAAAHQDDAVAQRVSGADHDMA
HRVDDLLLPIVKGVGSERAYEILTESLQTLGGSGFLVDYPLEQYIRDAKIDSLYEGTT
AIQALDFFFRKIVRDHGKALQFVLAQVTHTVENIDPSLKPQAELLRTALDDITAMTGA
LTGYLMSAAQHSSDIYKVGLGSVRYLLAVGDLLIGWRLLVLAGVAHAALADGPSQNDE
AFYRGKIAVAAFFAKNMLPKLTGVRSVIENIDDDIMRVPEDAF"

CDS complement(1654770..1655882)
/codon_start=1
/note="gene_1524"
/transl_table=11
/translation="MSFVVANTEFVSGAAGNLARLGSMISAANSAAAAQTTAVAAAGA
DEVSAAVAALFGAHGQTYQVLSAQAAAFHSQFVQALSGGAQAYAAAEATNFGPLQPLF
DVINAPTLALLNRPLIGNGADGTAANPNGQAGGLLIGNGGNGFSPAAGPGGNGGAAGL
LGHGGNGGVGALGANGGAGGTGGWLFGNGGAGGNSGGGGGAGGIGGSAVLFGAGGAGG
ISPNGMGAGGSGGNGGLFFGNGGAGASSFLGGGGAGGRAFLFGDGGAGGAALSAGSAG
RGGDAGFFYGNGGAGGSGAGGASSAHGGAGGQAGLFGNGGEGGDGGALGGNGGNGGNA
QLIGNGGDGGDGGGAGAPGLGGRGGLLLGLPGANGT"

CDS 1656124..1658097
/codon_start=1
/note="gene_1525"
/transl_table=11
/translation="MTLTACEVTAAEAPFDRVSKTIPHPLSWGAALWSVVSVRWATVA
LLLFLAGLVAQLNGAPEAMWWTLYLACYLAGGWGSAWAGAQALRNKALDVDLLMIAAA
VGAVAIGQIFDGALLIVIFATSGALDDIATRHTAESVKGLLDLAPDQAVVVQGDGSER
VVAASELVVGDRVVVRPGDRIPADGAVLSGASDVDQRSITGESMPVAKARGDEVFAGT
VNGSGVLHLVVTRDPSQTVVARIVELVADASATKAKTQLFIEKIEQRYSLGMVAATLA
LIVIPLMFGADLRPVLLRAMTFMIVASPCAVVLATMPPLLSAIANAGRHGVLVKSAVV
VERLADTSIVALDKTGTLTRGIPRLASVAPLDPNVVDARRLLQLAAAAEQSSEHPLGR
AIVAEARRRGIAIPPAKDFRAVPGCGVHALVGNDFVEIASPQSYRGAPLAELAPLLSA
GATAAIVLLDGVAIGVLGLTDQLRPDAVESVAAMAALTAAPPVLLTGDNGRAAWRVAR
NAGITDVRAALLPEQKVEVVRNLQAGGHQVLLVGDGVNDAPAMAAARAAVAMGAGADL
TLQTADGVTIRDELHTIPTIIGLARQARRVVTVNLAIAATFIAVLVLWDLFGQLPLPL
GVVGHEGSTVLVALNGMRLLTNRSWRAAASAAR"

CDS 1658141..1658515
/codon_start=1
/note="gene_1526"
/transl_table=11
/translation="VTTRDLTAAYFQQTISANSNVLVYFWAPLCAPCDLFTPTYEASS
RKHFDVVHGKVNIETEKDLASIAGVKLLPTLMAFKKGKLVFKQAGIANPAIMDNLVQQ
LRAYTFKSPAGEGIGPGTKTSS"

CDS 1658531..1658902
/codon_start=1
/note="gene_1527"
/transl_table=11
/translation="VTTRDLTAAQFNETIQSSDMVLVDYWASWCGPCRAFAPTFAESS
EKHPDVVHAKVDTEAERELAAAAQIRSIPTIMAFKNGKLLFNQAGALPPAALESLVQQ
LKAYEVEAGEATTQNGRAQQA"

CDS 1658972..1659781
/codon_start=1
/note="gene_1528"
/transl_table=11
/translation="VSLVLVEHPRPEIAQITLNRPERMNSMAFDVMVPLKEALAQVSY

DNSVRVVVLTGAGRGFSSGADHKSAGVVPHVENLTRPTYALRSMELLDDVILMLRRLH
QPVIAAVNGPAIGGGLCLALAADIRVASSSAYFRAAGINNGLTASELGLSYLLPRAIG
SSRAFEIMLTGRDVSAEEAERIGLVSRQVPDEQLLDACYAIAARMAGFSRPGIELTKR
TLWSGLDAASLEAHMQAEGLGQLFVRLLTANFEEAVAARAEQRAPVFTDDT"

CDS 1659817..1661445
/codon_start=1
/note="gene_1529"
/transl_table=11
/translation="VITATDLEVRAGARILLAPDGPDLRVQPGDRIGLVGRNGAGKTT
TLRILAGEVEPYAGSVTRAGEIGYLPQDPKVGDLDVLARDRVLSARGLDVLLTDLEKQ
QALMAEVADEDERDRAIRRYGQLEERFVALGGYGAESEAGRICASLGLPERVLTQRLR
TLSGGQRRRVELARILFAASESGAGNSTTLLLDEPTNHLDADSLGWLRDFLRLHTGGL
VVISHNVDLVADVVNKVWFLDAVRGQVDVYNMGWQRYVDARATDEQRRIRERANAERK
AAALRAQAAKLGAKATKAVAAQNMLRRADRMMAALDEERVADKVARIKFPTPAACGRT
PLVANGLGKTYGSLEVFTGVDLAIDRGSRVVILGLNGAGKTTLLRLLAGVEQPDTGVL
EPGYGLRIGYFAQEHDTLDNDATVWENVRHAAPDAGEQDLRGLLGAFMFTGPQLEQPA
GTLSGGEKTRLALAGLVASTANVLLLDEPTNNLDPASREQVLDALRSYRGAVVLVTHD
PGAAAALGPQRVVLLPDGTEDYWSDEYRDLIELA"

CDS 1661542..1661733
/codon_start=1
/note="gene_1530"
/transl_table=11
/translation="MRKSKKTRDQLLRELRNAYEGGASIRNLAATTGRSYGSIHSMLR
ESGTTMRGRGGPNRRPRPR"

CDS complement(1661802..1662365)
/codon_start=1
/note="gene_1531"
/transl_table=11
/translation="VPKVSEDHLAARRRQILDGARRCFAEYGYDKATVRRLEQAIGMS
RGAIFHHFRDKDALFFALAREDTERMAAVASREGLIGVMRDMLAAPDQFDWLATRLEI
ARKLRNDPDFSRGWAERSAELAAATTDRLRRQKQANRVRDDVPSDVLRCYLDLVLDGL
LARLASGEDPQRLAAVLDLVENSVRRS"

CDS complement(1662376..1665207)
/codon_start=1
/note="gene_1532"
/transl_table=11
/translation="VTSKSVNSFGAHDTLKVGEKSYQIYRLDAVPNTAKLPYSLKVLA
ENLLRNEDGSNITKDHIEAIANWDPKAEPSIEIQYTPARVVMQDFTGVPCIVDLATMR
EAIADLGGNPDKVNPLAPADLVIDHSVIADLFGRADAFERNVEIEYQRNGERYQFLRW
GQGAFDDFKVVPPGTGIVHQVNIEYLASVVMTRDGVAYPDTCVGTDSHTTMVNGLGVL
GWGVGGIEAEAAMLGQPVSMLIPRVVGFRLTGEIQPGVTATDVVLTVTEMLRQHGVVG
KFVEFYGEGVAEVPLANRATLGNMSPEFGSTAAIFPIDEETIKYLRFTGRTPEQVALV
EAYAKAQGMWHDPKHEPEFSEYLELNLSDVVPSIAGPKRPQDRIALAQAKSTFREQIY
HYVGNGSPDSPHDPHSKLDEVVEETFPASDPGQLTFANDDVATDETVHSAAAHADGRV
SNPVRVKSDELGEFVLDHGAVVIAAITSCTNTSNPEVMLGAALLARNAVEKGLTSKPW
VKTTIAPGSQVVNDYYDRSGLWPYLEKLGFYLVGYGCTTCIGNSGPLPEEISKAVNDN
DLSVTAVLSGNRNFEGRINPDVKMNYLASPPLVIAYALAGTMDFDFQTQPLGQDKDGK
NVFLRDIWPSQQDVSDTIAAAINQEMFTRNYADVFKGDDRWRNLPTPSGNTFEWDPNS
TYVRKPPYFEGMTAKPEPVGNISGARVLALLGDSVTTDHISPAGAIKPGTPAARYLDE
HGVDRKDYNSFGSRRGNHEVMIRGTFANIRLRNQLLDDVSGGYTRDFTQPGGPQAFIY
DAAQNYAAQHIPLVVFGGKEYGSGSSRDWAAKGTLLLGVRAVIAESFERIHRSNLIGM

GVIPLQFPEGKSASSLGLDGTEVFDITGIDVLNDGKTPKTVCVQATKGDGATIEFDAV
VRIDTPGEADYYRNGGILQYVLRNILKSG"
CDS 1665365..1665925
/codon_start=1
/note="gene_1533"
/transl_table=11
/translation="MTGPYFPQTIPFLPSYIPQDVDMTAVKAEVAALGVSAPPAATPG
LLEVVQHARDEGIDLKIVLLDHNPPNDTPLRDIATVVGADYSDATVLVLSPNYVGSYS
TQYPRVTLEAGEDHSKTGNPVQSAQNFVHELSTPEFPWSALTIVLLIGVLAAAVGARL
MQLRGRRSATSTDAAPGAGDDLNQGV"
CDS 1666151..1667569
/codon_start=1
/note="gene_1534"
/transl_table=11
/translation="MRRNRRGSPARPAARFVRPAIPSALSVALLVCTPGLATADPQTD
TIAALIADVAKANQRLQDLSDEVQAEQESVNKAMVDVETARDNAAAAEDDLEVSQRAV
KDANAAIAAAQHRFDTFAAATYMNGPSVSYLSASSPDEIIATVTAAKTLSASSQAVMA
NLQRARTERVNTESAARLAKQKADKAAADAKASQDAAVAALTETRRKFDEQREEVQRL
AAERDAAQARLQAARLVAWSSEGGQGAPPFRMWDPGSGPAGGRAWDGLWDPTLPMIPS
ANIPGDPIAVVNQVLGISATSAQVTANMGRKFLEQLGILQPTDTGITNAPAGSAQGRI
PRVYGRQASEYVIRRGMSQIGVPYSWGGGNAAGPSKGIDSGAGTVGFDCSGLVLYSFA
GVGIKLPHYSGSQYNLGRKIPSSQMRRGDVIFYGPNGSQHVTIYLGNGQMLEAPDVGL
KVRVAPVRTAGMTPYVVRYIEY"
CDS 1667580..1668305
/codon_start=1
/note="gene_1535"
/transl_table=11
/translation="MRHTRFHPIKLAWITAVVAGLMVGVATPADAEPGQWDPTLPALV
SAGAPGDPLAVANASLQATAQATQTTLDLGRQFLGGLGINLGGPAASAPSAATTGASR
IPRANARQAVEYVIRRAGSQMGVPYSWGGGSLQGPSKGVDSGANTVGFDCSGLVRYAF
AGVGVLIPRFSGDQYNAGRHVPPAEAKRGDLIFYGPGGGQHVTLYLGNGQMLEASGSA
GKVTVSPVRKAGMTPFVTRIIEY"
CDS 1668444..1669577
/codon_start=1
/note="gene_1536"
/transl_table=11
/translation="MTSAGGFPAGAGGYQTPGGHSASPAHEAPPGGAEGLAAEVHTLE
RAIFEVKRIIVGQDQLVERMLVGLLSKGHVLLEGVPGVAKTLAVETFARVVGGTFSRI
QFTPDLVPTDIIGTRIYRQGREEFDTELGPVVANFLLADEINRAPAKVQSALLEVMQE
RHVSIGGRTFPMPSPFLVMATQNPIEHEGVYPLPEAQRDRFLFKINVGYPSPEEEREI
IYRMGVTPPQAKQILSTGDLLRLQEIAANNFVHHALVDYVVRVVFATRKPEQLGMNDV
KSWVAFGASPRASLGIIAAARSLALVRGRDYVIPQDVIEVIPDVLRHRLVLTYDALAD
EISPEIVINRVLQTVALPQVNAVPQQGHSVPPVMQAAAAASGR"
CDS 1669574..1670527
/codon_start=1
/note="gene_1537"
/transl_table=11
/translation="VTESKAPAVVHPPSMLRGDIDDPKLAAALRTLELTVKQKLDGVL
HGDHLGLIPGPGSEPGESRLYQPGDDVRRMDWAVTARTTHPHVRQMIADRELETWLVV
DMSASLDFGTACCEKRDLAVAAAAAITFLNSGGGNRLGALIANGAAMTRVPARTGRQH
QHTMLRTIATMPQAPAGVRGDLAVAIDALRRPERRRGMAVIISDFLGPINWMRPLRAI

AARHEVLAIEVLDPRDVELPDVGDVVLQDAESGVVREFSIDPALRDDFARAAAAHRAD
VARTIRGCGAPLLSLRTDRDWLADIVRFVASRRRGALAGHQ"
CDS 1670538..1671545
/codon_start=1
/note="gene_1538"
/transl_table=11
/translation="MTLPLLGPMTLSGFAHSWFFLFLFVVAGLVALYILMQLARQRRM
LRFANMELLESVAPKRPSRWRHVPAILLVLSLLLFTIAMAGPTHDVRIPRNRAVVMLV
IDVSQSMRATDVEPSRMVAAQEAAKQFADELTPGINLGLIAYAGTATVLVSPTTNREA
TKNALDKLQFADRTATGEAIFTALQAIATVGAVIGGGDTPPPARIVLFSDGKETMPTN
PDNPKGAYTAARTAKDQGVPISTISFGTPYGFVEIDDQRQPVPVDDETMKKVAQLSGG
NSYNAATLAELRAVYSSLQQQIGYETIKGDASVGWLRLGALALALAALAALLINRRLP
T"
CDS complement(1671618..1672460)
/codon_start=1
/note="gene_1539"
/transl_table=11
/translation="MTDPFLGSEALAAGVLTPYELRSRYVALHKDVYVPQGVELTAQL
RAKALWLRSRRRGVLAGYSASAFHGAKWIDADLPAAIIDTNRRRAPGLQVWEERIEPD
EICVIEGMRVTTPERTALDLTSRFPLDPAVAAVDALIQATDLKVADVEPLIERYRGRR
GMKAARAALDLVDGGAQSPKETWLRLLLIRAGFPRPQTQIAVRNEWGWAEAHLDMGWQ
DIKVAAEYDGDHHLTSRYHYRKDILRHEKVQHRYGWIVVRVVAEDHPADIIRRVGEAR
AFRA"
CDS 1672601..1673344
/codon_start=1
/note="gene_1540"
/transl_table=11
/translation="VTATATEGAKPPFVSRSVLVTGGNRGIGLAIAQRLAADGHKVAV
THRGSGAPKGLFGVECDVTDSDAVDRAFTAVEEHQGPVEVLVSNAGLSADAFLMRMTE
EKFEKVINANLTGAFRVAQRASRSMQRNKFGRMIFIGSVSGSWGIGNQANYAASKAGV
IGMARSIARELSKANVTANVVAPGYIDTDMTRALDERIQQGALQFIPAKRVGTPAEVA
GVVSFLASEDASYISGAVIPVDGGMGMGH"
CDS 1673363..1674172
/codon_start=1
/note="gene_1541"
/transl_table=11
/translation="MTGLLDGKRILVSGIITDSSIAFHIARVAQEQGAQLVLTGFDRL
RLIQRITDRLPAKAPLLELDVQNEEHLASLAGRVTEAIGAGNKLDGVVHSIGFMPQTG
MGINPFFDAPYADVSKGIHISAYSYASMAKALLPIMNPGGSIVGMDFDPSRAMPAYNW
MTVAKSALESVNRFVAREAGKYGVRSNLVAAGPIRTLAMSAIVGGALGEEAGAQIQLL
EEGWDQRAPIGWNMKDATPVAKTVCALLSDWLPATTGDIIYADGGAHTQLL"
CDS 1674178..1675212
/codon_start=1
/gene="hemH"
/note="gene_1542"
/transl_table=11
/translation="MQFDAVLLLSFGGPEGPEQVRPFLENVTRGRGVPAERLDAVAEH
YLHFGGVSPINGINRTLIAELEAQQELPVYFGNRNWEPYVEDAVTAMRDNGVRRAAVF
ATSAWSGYSSCTQYVEDIARARRAAGRDAPELVKLRPYFDHPLFVEMFADAITAAAAT
VRGDARLVFTAHSIPTAADRRCGPNLYSRQVAYATRLVAAAAGYCDFDLAWQSRSGPP
QVPWLEPDVTDQLTGLAGAGINAVIVCPIGFVADHIEVVWDLDHELRLQAEAAGIAYA

RASTPNADPRFARLARGLIDELRYGRIPARVSGPDPVPGCLSSINGQPCRPPHCVASV
SPARPSAGSP"
CDS complement(1675178..1676086)
/codon_start=1
/note="gene_1543"
/transl_table=11
/translation="VIRVSRVEQLHCKIMWCPSVSLSIWANAWLAGKAAPDDVLDALS
LWAPTQSVAAYDAVAAGHTGLPWPDVHDAGTVSLLQTLRAAVGRRRLRGTINVVLPVP
GDVRGLAAGTQFEHDALAAGEAVIVANPEDPGSAVGLVPEFSYGDVDEAAQSEPLTPE
LCALSWMVYSLPGAPVLEHYELGDAEYALRSAVRSAAEALSTIGLGSSDVANPRGLVE
QLLESSRQHRVPDHAPSRALRVLENAAHVDAIIAVSAGLSRLPIGTQSLSDAQRATDA
LRPLTAVVRSARMSAVTAILHSAWPD"
CDS 1676102..1676536
/codon_start=1
/note="gene_1544"
/transl_table=11
/translation="MPVALIWLIAALVLVGAEALTGDMFLLMLGGGALAASVSSWLLA
WPMWADGAVFLLVSVLLLVLVRPAVRRRLTQTKGVQLGIEALEGKKAVVLGRVARDGG
QVKLDGQVWTARPLNDGDVFEPGDSVTVVQIDGATAVVFKDV"
CDS 1676558..1677703
/codon_start=1
/note="gene_1545"
/transl_table=11
/translation="VQGAVAGLVFLAVLVIFAIIVVAKSVALIPQAEAAVIERLGRYS
RTVSGQLTLLVPFIDRVRARVDLRERVVSFPPQPVITEDNLTLNIDTVVYFQVTVPQA
AVYEISNYIVGVEQLTTTTLRNVVGGMTLEQTLTSRDQINAQLRGVLDEATGRWGLRV
ARVELRSIDPPPSIQASMEKQMKADREKRAMILTAEGTREAAIKQAEGQKQAQILAAE
GAKQAAILAAEADRQSRMLRAQGERAAAYLQAQGQAKAIEKTFAAIKAGRPTPEMLAY
QYLQTLPEMARGDANKVWVVPSDFNAALQGFTRLLGKPGEDGVFRFEPSPVEDQPKHA
ADGDDAEVAGWFSTDTDPSIARAVATAEAIARKPVEGSLGTPPRLTQ"
CDS 1677713..1678069
/codon_start=1
/note="gene_1546"
/transl_table=11
/translation="MSGLTSPKTYAVLAALQAGDAVACAIPLPPIARLLDDLDVPVSV
RPVLPVVKAASAVGLLSVTRFPALARLTTAMLTLYFILAVGAHVRVRDRVVNAIPAAS
FLTLFALMTAKGPERT"
CDS 1678103..1678333
/codon_start=1
/note="gene_1547"
/transl_table=11
/translation="VSVGEVEVLKVENSRVRAEQLAKLYELRSSRDRVRVDAALAELS
RAAAARGCAGTSGLGNNLMAPGPPHSLLGRDR"
CDS 1678483..1679790
/codon_start=1
/note="gene_1548"
/transl_table=11
/translation="VSQCFAVKGIGGADQATLGSAEILVKYAQLADKRARVYVLVSTW
LVVWGIWHVYFVEAVFPNAILWLHYYAASYEFGFVRRGLGGELIRMLTGDHFFAGAYT
VLWTSITVWLIALAVVVWLILSTGNRSERRIMLALLVPVLPFAFSYAIYNPHPELFGM
TALVAFSIFLTRAHTSRTRVILSTLYGLTMAVLALIHEAIPLEFALGAVLAIIVLSKN

ATGATRRICTALAIGPGTVSVLLLAVVGRRDIADQLCAHIPHGMVENPWAVATTPQRV
LDYIFGRVESHADYHDWVCEHVTPWFNLDWITSAKLVAVVGFRALFGAFLLGLLFFVA
TTSMIRYVSAVPVRTFFAELRGNLALPVLASALLVPLFITAVDWTRWWVMITLDVAIV
YILYAIDRPEIEQPPSRRNVQVFVCVVLVLAVIPTGSANNIGR"

CDS complement(1680369..1681127)
/codon_start=1
/note="gene_1549"
/transl_table=11
/translation="VTAPAICNTTETVHGIATSLGAVARQASLPRIVGTVVGITVLVV
VALLVPVPTAVELRDWAKSLGAWFPLAFLLVHTVVTVPPFPRTAFTLAAGLLFGSVVG
VFIAVVGSTASAVIAMLLVRATGWQLNSLVRRRAINRLDERLRERGWLAILSLRLIPV
VPFAAINYAAGASGVRILSFAWATLAGLLPGTAAVVILGDAFAGSGSPLLILVSVCTG
ALGLTGLVYEIRNYRRQHRRMPGYDDPVREPALI"

CDS 1681318..1683165
/codon_start=1
/note="gene_1550"
/transl_table=11
/translation="VSIDVPERADLEQVRGRWRNAVAGVLSKSNRTDSAQLGDHPERL
LDTQTADGFAIRALYTAFDELPEPPLPGQWPFVRGGDPLRDVHSGWKVAEAFPANGAT
ADTNAAVLAALGEGVSALLIRVGESGVAPDRLTALLSGVYLNLAPVILDAGADYRPAC
DVMLALVAQLDPGQRDTLSIDLGADPLTASLRDRPAPPIEEVVAVASRAAGERGLRAI
TVDGPAFHNLGATAATELAATVAAAVAYLRVLTESGLVVSDALRQISFRLAADDDQFM
TLAKMRALRQLWARVAEVVGDPGGGAAVVHAETSLPMMTQRDPWVNMLRCTLAAFGAG
VGGADTVLVHPFDVAIPGGFPGTAAGFARRIARNTQLLLLEESHVGRVLDPAGGSWFV
EELTDRLARRAWQRFQAIEARGGFVEAHDFLAGQIAECAARRADDIAHRRLAITGVNE
YPNLGEPALPPGDPTSPVRRYAAGFEALRDRSDHHLARTGARPRVLLLPLGPLAEHNI
RTTFATNLLASGGIEAIDPGTVDAGTVGNAVADAGSPSVAVICGTDARYRDEVADIVQ
AARAAGVSRVYLAGPEKALGDAAHRPDEFLTAKINVVQALSNLLTRLGA"

CDS 1683166..1685418
/codon_start=1
/note="gene_1551"
/transl_table=11
/translation="MTTKTPVIGSFAGVPLHSERAAQSPTEAAVHTHVAAAAAAHGYT
PEQLVWHTPEGIDVTPVYIAADRAAAEAEGYPLHSFPGEPPFVRGPYPTMYVNQPWTI
RQYAGFSTAADSNAFYRRNLAAGQKGLSVAFDLATHRGYDSDHPRVQGDVGMAGVAID
SILDMRQLFDGIDLSTVSVSMTMNGAVLPILALYVVAAEEQGVAPEQLAGTIQNDILK
EFMVRNTYIYPPKPSMRIISDIFAYTSAKMPKFNSISISGYHIQEAGATADLELAYTL
ADGVDYIRAGLNAGLDIDSFAPRLSFFWGIGMNFFMEVAKLRAGRLLWSELVAQFAPK
SAKSLSLRTHSQTSGWSLTAQDVFNNVARTCIEAMAATQGHTQSLHTNALDEALALPT
DFSARIARNTQLVLQQESGTTRPIDPWGGSYYVEWLTHRLARRARAHIAEVAEHGGMA
QAISDGIPKLRIEEAAARTQARIDSGQQPVVGVNKYQVPEDHEIEVLKVENSRVRAEQ
LAKLQRLRAGRDEPAVRAALAELTRAAAEQGRAGADGLGNNLLALAIDAARAQATVGE
ISEALEKVYGRHRAEIRTISGVYRDEVGKAPNIAAATELVEKFAEADGRRPRILIAKM
GQDGHDRGQKVIATAFADIGFDVDVGSLFSTPEEVARQAADNDVHVIGVSSLAAGHLT
LVPALRDALAQVGRPDIMIVVGGVIPPGDFDELYAAGATAIFPPGTVIADAAIDLLHR
LAERLGYTLD"

CDS 1685486..1685734
/codon_start=1
/note="gene_1552"
/transl_table=11
/translation="MTVRLDQQTRQRLQDIVKGGYRSANAAIVDAINKRWEALHDEQL

DAAYAAAIHDNPAYPYESEAERSAARARRNARQQRSAQ"
CDS 1685731..1686048
/codon_start=1
/note="gene_1553"
/transl_table=11
/translation="VNAPLRGQVYRCDLGYGAKPWLIVSNNARNRHTADVVAVRLTTT
RRTIPTWVAMGPSDPLTGYVNADNIETLGKDELGDYLGEVTPATMNKINTALATALGL
PWP"
CDS 1686048..1687049
/codon_start=1
/note="gene_1554"
/transl_table=11
/translation="MAASHDDDTVDGLATAVRGGDRAALPRAITLVESTRPDHREQAQ
QLLLRLLPDSGNAHRVGITGVPGVGKSTAIEALGMHLIERGHRVAVLAVDPSSTRTGG
SILGDKTRMARLAVHPNAYIRPSPTSGTLGGVTRATRETVVLLEAAGFDVILIETVGV
GQSEVAVANMVDTFVLLTLARTGDQLQGIKKGVLELADIVVVNKADGEHHKEARLAAR
ELSAAIRLIYPREALWRPPVLTMSAVEGRGLAELWDTVERHRQVLTGAGEFDARRRDQ
QVDWTWQLVRDAVLDRVWSNPTVRKVRSELERRVRAGELTPALAAQQILEIANLTDR"
CDS 1687102..1688391
/codon_start=1
/note="gene_1555"
/transl_table=11
/translation="VMVDTGVDHRAVSSHDGPDAGRRVFGAADPRFACVVRAFASMFP
GRRFGGGALAVYLDGQPVVDVWKGWADRAGWVPWSADSAPMVFSATKGMTATVIHRLA
DRGLIDYEAPVAEYWPAFGANGKATLTVRDVMRHQAGLSGLRGATQQDLLDHVVMEER
LAAAVPGRLLGKSAYHALTFGWLMSGLARAVTGKDMRLLFREELAEPLDTDGLHLGRP
PADAPTRVAEIIMPQDIAANAVLTCAMRRLAHRFSGGFRSMYFPGAIAAVQGEAPLLD
AEIPAANGVATARALARMYGAIANGGEIDGIRFLSRELVTGLTRNRRQVLPDRNLLVP
LNFHLGYHGMPIGNVMPGFGHVGLGGSIGWTDPETGVAFALVHNRLLSPLVMTDHAGF
VGIYHLIRQAAAQARKRGYQPVTPFGAPYSEPGAAAG"
CDS complement(1688464..1689081)
/codon_start=1
/note="gene_1556"
/transl_table=11
/translation="VLDVGCGSGRMALPLTGYLNSEGRYAGFDISQKAIAWCQEHITS
AHPNFQFEVSDIYNSLYNPKGKYQSLDFRFPYPDASFDVVFLTSVFTHMFPPDVEHYL
DEISRVLKPGGRCLCTYFLLNDESLAHIAEGKSAHNFQHEGPGYRTIHKKRPEEAIGL
PETFVRDVYGKFGLAVHEPLHYGSWSGREPHLSFQDIVIATKTAS"
CDS complement(1689295..1689486)
/codon_start=1
/note="gene_1557"
/transl_table=11
/translation="VIEIVGTSPDGVDAAIQGGLARAAQTMRALDWFEVQSIRGHLVD
GAVAHFQVTMKVGFRLEDS"
CDS 1689568..1689966
/codon_start=1
/note="gene_1558"
/transl_table=11
/translation="VPSGEPSTAGHFEHLPRGSFGRILSVLNAAADHHPRELLVVGIA
TFDQKRPAVGVDEHDPGGAATPAVVINYESRSSAGGTIGHSTTSQVACCLYQQPKRPA
LRPTKAAATTAATTWIERVQNRRGRHSALV"

```
CDS             1690041..1691039
                /codon_start=1
                /note="gene_1559"
                /transl_table=11
                /translation="MSEPYVLEFYRRARAAADKITPDVEIIFVDDGSPEAALQQAVSL
                LDSDPCVRVIQLSRNFGHHKAMMTGLAHATGDLVFLIDSDLEEDPALLEPFYEKLIST
                GADVVFGCHARRPGGWLRNFGPKIHYRASALLCDPPLHENTLTVRLMTADYVRSLVQH
                QERELSIAGLWQITGFYQVPMSVNKAWKGTTTYTFRRKVATLVDNVTSFSNKPLVFIF
                YLGAAIFIISSSAAGYLIIDRIFFRALQAGWASVIVSIWMLGGVTIFCIGLVGIYVSK
                VFIETKQRPYTIIRRIYGSDLTTREPSSLKTAFPAAHLSNGKRVTSEPEGLATGNR"
CDS             1691051..1691872
                /codon_start=1
                /note="gene_1560"
                /transl_table=11
                /translation="MIPVKVENNTSLDQVQDALNCVGYAVVEDVLDEASLAATRDRMY
                RVQERILTEIGKERLARAGELGVLRLMMKYDPHFFTFLEIPEVLSIVDRVLSETAILH
                LQNGFILPSFPPFSTPDVFQNAFHQDFPRVLSGYIASVNIMFAIDPFTRDTGATLVVP
                GSHQRIEKPDHTYLARNAVPVQCAAGSLFVFDSTLWHAAGRNTSGKDRLAINHQFTRS
                FFKQQIDYVRALGDAVVLEQPARTQQLLGWYSRVVTNLDEYYQPPDKRLYRKGQG"
CDS             1692085..1692984
                /codon_start=1
                /note="gene_1561"
                /transl_table=11
                /translation="MAWRKLGRIFAPSGELDWSRSHAALPVPEWIEGDIFRIYFSGRD
                GQNRSSIGSVIVDLAVGGKILDIPAEPILRPGARGMFDDCGVSIGSIVRAGDTRLLYY
                TGWNLAVTVPWKNTIGVAISEAGAPFERWSTFPVVALDERDPFSLSYPWVIQDGGTYR
                MWYGSNLGWGEGTDEIPHVIRYAQSRDGVHWEKQDRVHIDTSGSDNSAACRPCVVRDA
                GVYRMWFCARGAKYRIYCATSEDGLTWRQLGKDEGIDVSPDSWDSDMIEYPCVFDHRG
                QRFMLYSGDGYGRTGFGLAVLEN"
CDS             complement(1693157..1694269)
                /codon_start=1
                /note="gene_1562"
                /transl_table=11
                /translation="MTGRELAYIAEAHSCGHLAGDGPFTRRSHAWLEQQTGCRKALLT
                PSCTAALEMMALLLDIEEGDEVILPSYTFVSTANAFVLRGGVPVFVDIRPDTLNIDET
                RIVDAITPRTKAIVPVHYAGVACEMDAIMKIATHHNLAVVEDAAQGAMASYRGRALGS
                IGDLGALSFHETKNVISGEGGALLVNSEDFLLRAEILREKGTNRSRFLRNEVDKYTWQ
                DKGSSYLPSELVAAFLWAQFEEAERITRIRLDLWNRYHESFESLEQRGLLRRPIIPQG
                CSHNAHMYYVLLAPSADREEVLARLTSKGIGAVFHYVPLHDSPAGRRYGRTNGNLTVT
                NDVASRLIRLPMWVGLQEVDQSRVVEALTRILTLRA"
CDS             complement(1694442..1695107)
                /codon_start=1
                /note="gene_1563"
                /transl_table=11
                /translation="MTKPLVIFGSGDIAQLAHYYFTRDSEYEVVAFTVDRDYASVSEF
                CGLPLVAFDEVAQRFPPESHAMFVALAYAKLNGVRKEKYLAAKALGYELASYVSSHAT
                VLNDGRIGENVFLLEDNTIQPFVSIGNNVTLWSGNHIGHHSTIHDHCFLASHIVVSGG
                VVIEEQSFIGVNATLRDHITIGSRCVVGAGALLLGDADADGVYIGTKTERRPVPSTEL
                RKI"
CDS             1695300..1696064
                /codon_start=1
```

/note="gene_1564"
/transl_table=11
/translation="MWTMVLLLGLGMAIDPARLGLAVVMLSRRRPMLNLFAFWVGGMV
AGVGIALAVLVFMRDVALAAIQGVVSAANEFREAVGILAGGRLHIVIGVIMLLLAARM
VARARAQVGVPVGPVGVADGGMSALALAQRPPGLVARLEVRTQQMLQGDVVWPAFVVG
VASSAPPFESVVALTVIMASGAEIGTQFGAFVVFTLLVLAVIEIPLVAYLAIPQQTQQ
VMLRFQDWVRSNRRQISLTILIGVGFLFLYQGVTSL"
CDS 1696097..1697032
/codon_start=1
/note="gene_1565"
/transl_table=11
/translation="VVSVNPAKPLISVCIPMYNNGATIERCLRSILEQEGVEFEIVVV
DDDSSDDCAAIAATMLRPGDRLLRNEPRLGLNRNHNKCLEVARGGLIQFVHGDDRLLP
GALQTLSRRFEDPSVGMAFAPRRVESDDIKWQQRYGRVHTRFRKLRDRNHGPSLVLQM
VLHGAKENWIGEPTAVMFRRQLALDAGGFRTDIYQLVDVDFWLRLMLRSAVCFVPHEL
SVRRHTAATETTRVMATRRNVLDRQRILTWLIVDPLSPNRVRSAAALWWIPAWLAMIV
EVAVLGPQRRTHLKALAPAPFREFAHARRQLPLAD"
CDS 1697424..1698497
/codon_start=1
/note="gene_1566"
/transl_table=11
/translation="VVDPTATDSPKVSIVSISYNQEEYIREALDGFAAQRTEFPVEVI
IADDASTDATPRIIGEYAARYPQLFRPILRQTNIGVHANFKDVLSAARGEYLALCEGD
DYWTDPLKLSKQVKYLDRHPETTVCFHPVRVIYEDGAKDSEFPPLSWRRDLSVDALLA
RNFIQTNSVVYRRQPSYDDIPANVMPIDWYLHVRHAVGGEIAMLPETMAVYRRHAHGI
WHSAYTDRRKFWETRGHGMAATLEAMLDLVHGHREREAIVGEVSAWVLREIGKTPGRQ
GRALLLKSIADHPRMTMLSLQHRWAQTPWRRFKRRLSTELSSLAALAYATRRRALEGR
DGGYRETTSPPTGRGRNVRGSHA"
CDS complement(1698486..1698686)
/codon_start=1
/gene="TBJG_03742"
/note="gene_1567"
/transl_table=11
/translation="MPSSGTALAHPDQSLEDIRTGGLTDLKDGPQGYLMALSVVESAA
TGPIPHAPSIEARRAIYQDLGM"
CDS 1698731..1700482
/codon_start=1
/note="gene_1568"
/transl_table=11
/translation="MSVVESSLPGVLRERASFQPNDKALTFIDYERSWDGVEETLTWS
QLYRRTLNLAAQLREHGSTGDRALILAPQILDYVVSFIASLQAGIVAVPLSIPQGGAH
DERTVSVFADTAPAIVLTASSVVDNVVEYVQPQPGQNAPAVIEVDRLDLDARPSSGSR
SAAHGHPDILYLQYTSGSTRTPAGVMVSNKNLFANFEQIMTSYYGVYGKVAPPGSTVV
SWLPFYHDMGFVLGLILPILAGIPAVLTSPIGFLQRPARWIQMLASNTLAFTAAPNFA
FDLASRKTKDEDMEGLDLGGVHGILNGSERVQPVTLKRFIDRFAPFNLDPKAIRPSYG
MAEATVYVATRKAGQPPKIVQFDPQKLPDGQAERTESDGGTPLVSYGIVDTQLVRIVD
PDTGIERPAGTIGEIWVHGDNVAIGYWQKPEATERTFSATIVNPSEGTPAGPWLRTGD
SGFLSEGELFIMGRIKDLLIVYGRNHSPDDIEATIQTISPGRCAAIAVSEHGAEKLVA
IIELKKKDESDDEAAERLGFVKREVTSAISKSHGLSVADLVLVSPGSIPITTSGKIRR
AQCVELYRQDEFTRLDA"
CDS complement(1700718..1704137)

/codon_start=1
/note="gene_1569"
/transl_table=11
/translation="VVKRRQSNGNVTVNAVIVISPRHRLPGLYGYRVARHDEAKAGGL
FDRIGNFVVRWPLIVIGCWIAVAAALTLLLPTLQAQAAKREQAPLPPGAPSMVLQKEM
SAAFQEKIETSALLLVLLTNENGLGPADEAVYRKLIENLRADTQDKISVQDFLAVPEM
KELLASKDNKAWNLPITFAGDAASPETQAAFKRVAAIVKQTVAGTSLTVHLSGPIATV
ADLTELGEKDVRIIEIGTAVSVLIILILVYRNLVTMLVPLATIGASVVTAQGTLSGLA
EFGLAVNMQAIVFMSAVMIGAGTDYAVFLISRYHDYVRHGEKSDMAVKKALMSIGKVI
TASAATVAVTFLAMVFTKLEVFSAVGPAIAVAITVSLLGAVTLLPAILTLTGRRGWIK
PRRDLTSRMWRRSGVRIVRRPTIHLVGSLIVLVALAGCTLLIRFNYDDLKTVPQHVES
VKGYEAMNRHFPMNAMTPMVLFIKSPRDLRTPGALADIEMMSREIAELPNIVMVRGLT
RPNGEPLKETKVSFQAGEVGGKLDEATTLLEEHGGELDQLTGGAHQLADALAQIRNEI
NGAVASSSGIVNTLQAMMDLMGGDKTIRQLENASQYVGRMRALGDNLSGTVTDAEQIA
TWASPMVNALNSSPVCNSDPACRTSRAQLAAIVQAQDDGLLRSIRALAVTLQQTQEYQ
TLARTVSTLDGQLKQVVSTLKAVDGLPTKLAQMQQGANALADGSAALAAGVQELVDQV
KKMGSGLNEAADFLLGIKRDADKPSMAGFNIPPQIFSRDEFKKGAQIFLSADGHAARY
FVQSALNPATTEAMDQVNDILRVADSARPNTELEDATIGLAGVPTALRDIRDYYNSDM
KFIVIATIVIVFLILVILLRALVAPIYLIGSVLISYLSALGIGTLVFQLILGQEMHWS
LPGLSFILLVAIGADYNMLLISRIRDESPHGIRIGVIRTVGSTGGVITSAGLIFAASM
FGLVGASINTMAQAGFTIGIGIVLDTFLVRTVTVPALTTMIGRANWWPSELGRDPSTP
PTKADRWLRRVKGHRRKAPIPAPKPPHTKVVRNTNGHASKAATKSVPNGKPADLAEGN
GEYLIDHLRRHSLPLFGYAAMPAYDVVDGVSKPNGDGAHIGKEPVDHLLGH"

CDS 1704340..1705125
/codon_start=1
/note="gene_1570"
/transl_table=11
/translation="VALRYRLQSNPLVGKLTTKYFLPLGTRQVGDHVVFFNFGYEEDP
PMALPLSESDEPNRYCIQLYHQTASQVDLTGKEVLEVSCGAGGGASYIARNLGPASYT
GLDLNPASIDLCRAKHRLPGLQFVQGDAQNLPFPDESFDAVVNVEASHQYPDFRGFLA
EVARVLRPGGHFLYTDSRRNPVVAEWEAALADAPLRTISQRDIGAQAKRGLDANTARS
QEAIGRRAPVLLAGLTRCAVRVLDWDLRRGGGFSYRIYLFAKD"

CDS 1705155..1706399
/codon_start=1
/note="gene_1571"
/transl_table=11
/translation="MKFVVASYGTRGDIEPCAAVGLELQRRGHDVCLAVPPNLIGFVE
TAGLSAVAYGSRDSQEQLDEQFLHNAWKLQNPIKLLREAMAPVTEGWAELSAMLTPVA
AGADLLLTGQIYQEVVANVAEHHGIPLAALHFYPVRANGEIAFPARLPAPLVRSTITA
IDWLYWRMTKGVEDAQRRELGLPKASTPAPRRMAVRGSLEIQAYDALCFPGLAAEWGG
RRPFVGALTMESATDADDEVASWIAADTPPIYFGFGSMPIGSLADRVAMISAACAELG
ERALICSGPSDATGIPQFDHVKVVRVVSHAAVFPTCRAVVHHGGAGTTAAGLRAGIPT
LILWVTSDQPIWAAQIKQLKVGRGRRFSSATKESLIADLRTILAPDYVTRAREIASRM
TKPAASVTATADLLEDAARRAR"

CDS 1706446..1707231
/codon_start=1
/note="gene_1572"
/transl_table=11
/translation="LYAPLVSLMITVPVFGQHEYTHALVADLEREGADYLIVDNRGDY
PRIGTERVSTPGENLGWAGGSELGFRLAFAEGYSHAMTLNNDTRVSKGFVAALLDSRL
PADAGMVGPMFDVGFPFAVADEKPDAESYVPRARYRKVPAVEGTALVMSRDCWDAVGG

MDLSTFGRYGWGLDLDLALRARKSGYGLYTTEMAYINHFGRKTANTHFGGHRYHWGAS
AAMIRGLRRTHGWPAAMGILREMGMAHHRKWHKSFPLTCPASC"
CDS complement(1707209..1708393)
/codon_start=1
/note="gene_1573"
/transl_table=11
/translation="MAVPPNLIEFVESAGLTGVAYGPDSDEQINTVAAFVRNLTRAQN
PLNLARAVKELFVEGWAEMGTTLTTLADGADLVMTGQTYHGVAANVAEYYDIPAAALH
HFPMQVNGQIAIPSIPTPATLVRATMKVSWRLYAYVSKDADRAQRRELGLPPAPAPAV
RRLAERGAPEIQAYDPVFFPGLAAEWSDRRPFVGPLTMELHSEPNEELESWIAAGTPP
IYFGFGSTPVQTPVQTLAMISDVCAQLGERALIYSPAANSTRIRHADHVKRVGLVNYS
TILPKCRAVVHHGGAGTTAAGLRAGMPTLILWDVADQPIWAGAVQRLKVGSAKRFTNI
TRGSLLKELRSILAPECAARAREISTRMTRPTAAVTAAADLLEATARQTPGSTPSSSP
GR"
CDS complement(1708512..1714775)
/codon_start=1
/gene="pks5"
/note="gene_1574"
/transl_table=11
/translation="MGCRLPGGIDSPDRLWEALLRGDDLVTEIPADRWDIDEYYDPEP
GVPGRTDCKWGAYLDNVGDFDPEFFGIGEKEAIAIDPQHRLLLETSWEAMEHGGLTPN
QMASRTGVFVGLVHTDYILVHADNQTFEGPYGNTGTNACFASGRVAYAMGLQGPAITV
DTACSSGLTAIHLACRSLHDGESDIALAGGVYVMLEPRRFASGSALGMLSATGRCHAF
DVSADGFVSGEGCVMLALKRLPDALADGDRILAVIRGTAANQDGHTVNIATPSRSAQV
AAYREALDVAGVDPATVGMVEAHGPGTPVGDPIEYASLAEVYGNDGPCALASVKTNFG
HTQSAAGALGLMKAVLALQHGVVPQNLHFTALPDKLAAIETNLFVPQEITPWPGADQE
TPRRAAVSSYGMTGTNVHAIVEQAPVPAPESGAPGDTPATPGIDGALLFALSASSQDA
LRQTAARLADWVDAQGPELAPADLAYTLARRRGHRPVRTAVLAATTAELTEALREVAT
GETPYPPAVGQDDRGPVWVFSGQGSQWAGMGADLLATEPVFAATIAAIEPLIAAESGF
SVTEAMTAPEVVTGIDRVQPTLFAMQVALAATMKSYGVAPGAVIGHSLGESAAAVVAG
ALCLEDGVRVICRRSALMTRIAGAGAMASVELPAQQVLSELMARGVNDAVVAVVASPQ
STVIGGATQTVRDLVAAWEQRDVLAREVAVDVASHSPQVDPILDELAEALAEISPLQP
EIPYYSATSFDPREEPYCDAYYWVDNLRHTVRFAAAVQAALEDGYRVFTELTPHPLLT
HAVDQTARSLDMSAAALAGMRREQPLPHGLRALAGDLYAAGAAVDFAVLYPTGRLINA
PLPTWNHRRLLLDDTTRRIAHANTVAVHPLLGSHVRLPEEPERHVWQGEVGTVTQPWL
ADHQIHGAAALPGAAYCEMALAAARAVLGEASEVRDIRFEQMLLLDDETPIGVTATVE
APGVVPLTVETSHDGRYTRQLAAVLHVVREADDAPDQPPQKNIAELLASHPHKVDGAE
VRQWLDKRGHRLGPAFAGLVDAYIAEGAGDTVLAEVNLPGPLRSQVKAYGVHPVLLDA
CFQSVAAHPAVQGMADGGLLLPLGVRRLRSYGSARHARYCCTTVTACGVGVEADLDVL
DEHGAVVLAVRGLQLGTGASQASERARVLGERLLSIEWHERELPENSHAEPGAWLLIS
TCDATDLVAAQLTDALKVHDAQCTTMSWPQRADHAAQAARLRDQLGTGGFTGVFVLTA
PQTGDPDAESPVRGGELVKHVVRIAREIPEITAQEPRLYVVLTHNAQAVLSGDRPNLEQ
GGMRGLLRVIGAEHPHLKASYVDVDEQTGAESVARQLLAASGEDETAWRNDQWYTARL
CPAPLRPEERQTTVVDHAEAGMRLQIRTPGDLQTLEFAALDRVPPGPGEIEVAVTASS
INFADVLVTFGRYQTLDGRQPQLGTDFAGVVSAVGPGVSELKVGDRVGGMSPNGCWAT
FVTCDARLATRLPEGLTDAQAAAVTTASATAWYGLQDLARIKAGDKVLIHSATGGVGQ
AAIAIARAAGAQIYATAGNEKRRDLLRDMGIEHVYDSRSVEFAEQIRRDTAGYGVDIV
LNSVTGAAQLAGLKLLALGGRFIEIGKRDIYSNTRLELLPFRRNLAFYGLDLGLMSVS
HPAAVRELLSTVYRLTVEGVLPMPQSTHYPLAEAATAIRVMGAAEHTGKLILDVPHAG
RSSVVLPPEQARVFRSDGSYIITGGLGGLGLFLAEKMANAGAGRIVLSSRSQPSQKAL
ETIELVRAIGSDVVVECGDIAQPDTADRLVTAATATGLPLRGVLHAAAVVEDATLANI

TDELIERDWAPKAYGAWQLHRATADQPLDWFCSFSSAAALVGSPGQGAYAAANSWLDT
FTHWRRAQDLPATSIAWGAWGQIGRAIAFAEQTGDAIAPEEGAYAFETLLRHNRAYSG
YAPVIGSPWLTAFAQHSPFAEKFQSLGQNRSGTSKFLAELVDLPREEWPDRLRRLLSK
QVGLILRRTIDTDRLLSEYGLDSLSSQELRARVEAETGIRISATEINTTVRGLADLMC
DKLAADRDAPAPA"

CDS complement(1715382..1715822)
/codon_start=1
/note="gene_1575"
/transl_table=11
/translation="VQSSHIDGEIVGALIPDLAVLHSEDASRAAVGREKHRCSLDPLG
GGFRSRRASMPAGALLLSAVIAIQLDRMNARVFGDGWIGAQACMWVNKFHEESTVTAL
SPSSPIAQGSIARHPETMQSAYVRIAEGGSRDVAPAAQLQRRRP"

CDS 1715931..1717685
/codon_start=1
/note="gene_1576"
/transl_table=11
/translation="VVASSIPTALRERASVHPNGAAITYIDYEQDWAGVAETLTWSQL
YRRMLNVAEPLRHVGATGDRAVILAPQGIEYVVGFLGALQAGRIAVPLPVPHAGAHDE
RTISVLSDTSPAVILTTSGAVDDVRECAQPQPGQSAPSIVELDLLDLDSRQRSRSPGA
RPTGRDTPETAYLQYTSGSTRTPAGVMVSNKNVFANFEQIVADFFAPEGGVVPPDLTV
VSWLPLYHDMGLLLGAIMPILAGVPTVLTSPVGFLQRPARWIQLLARNGRTISAGPNF
AFELAVRKTSDDGMDGLDLAGVHTILNGSERVHPATLKRFAERFGRFNFAAAALRPAY
GMAEATVYIATRNVNEPPEIVDFESEKLPAGQAIRCPSGSGTPLVSYGVPRSQLVRIV
DPDTCIECPQGSVGEIWVQGGNVASGYWHKPEESKRTFGARIVTPSAGTPEAPWLRTG
DSGFVSGGELFIIGRIKDLLIVYGRNHAPDDIEATIQEITSGRCAAIAVPDHGTEKLV
AIIELKKRGDSDEDVADRLRIVKRDVAAAIFDSHGLSVADLVLVSPGSIPITTSGKIR
RAQCVQLYRRREFTRLDA"

CDS 1717802..1718905
/codon_start=1
/note="gene_1577"
/transl_table=11
/translation="MSDGAVVRALVLEAPRRLVVRQYRLPRIGDDDALVRVEACGLCG
TDHEQYTGELAGGFAFVPGHETVGTIAAIGPRAEQRWGVSAGDRVAVEVFQSCRQCAN
CRGGEYRRCVRHGLADMYGFIPVDREPGLWGGYAEYQYLAPDSMVLRVAGDLSPEVAT
LFNPLGAGIRWGVTIPETKPGDVVAVLGPGIRGLCAAAAAKGAGAGFVMVTGLGPRDA
DRLALAAQFGADLAVDVAIDDPVAALTEQTGGLADVVVDVTAKAPAAFAQAIALARPA
GTVVVAGTRGVGSGAPGFSPDVVVFKELRVLGALGVDATAYRAALDLLVSGRYLFASL
PRRCVRLEGAEDLLATMAGERDGVPPIHGVLTP"

CDS 1718902..1719468
/codon_start=1
/note="gene_1578"
/transl_table=11
/translation="MTTSRVPLLPVDEAKAAADEAGVPDYMAELSIFQVLLNHPRLAR
TFNDLLATMLWHGTLDSRLRELVIMRIGWLTDCDYEWTQHWRVASGLGVSADDLLGVR
DWQGYNGFGPAEQAVLAATDDVVREGAVSAQSWSACERELHCDKVVLIELVTVISAWR
MVASILHSLEVPLEDGVSSWPPDGLSPR"

CDS complement(1719545..1719979)
/codon_start=1
/note="gene_1579"
/transl_table=11
/translation="MSDPLTAQEQHKRRQAVRELMPRTPFIGGLGIVFERYEPDDVVI

RLPFRTDLTNDGTYFHGGVIASVMDTAGAAAAWSNHDFDRGTRAATVAMSIQYTGAAK
RCDLLCHARTARRRKELTFTEITATDPDGNIVAHAVQTYRIV"
CDS 1720111..1721166
/codon_start=1
/note="gene_1580"
/transl_table=11
/translation="VAAVSNAGGFGILGAVAHSPKRLESELTWIEEHTGGKPYGVDVL
LPPKYIGAEQGGIDAQQARELIPEGHRTFVDDLLVRYGIPAVTDRQRSSSAGGLHISP
KGYQPLLDVAFAHDIRLIASALGPPPPDLVERAHNHDVLVAALAGTAQHARRHAAAGV
DLIVAQGTEAGGHTGEVATMVLVPEVVDAVSPTPVLAAGGIARGRQIAAALALGAEGV
WCGSVWLTTEEAETPPVVKDKFLAATSSDTVRSRSLTGKPARMLRTAWTDEWDRPDSP
DPLGMPLQSALVSDPQLRINQAAGQPGAKARELATYFVGQVVGSLDRVRSARSVVLDM
VEEFIDTVGQLQGLVQR"
CDS 1721163..1721840
/codon_start=1
/note="gene_1581"
/transl_table=11
/translation="VSRASARRRRAVSDEDKSQRRDEILAAAKIVFAHKGFHATTVAD
IAKQAGLAYGLIYWYFDSKDDLFHALMAGEEEALRAHVAAELARVGGSTEAPLRALLQ
AAVQATFEFFETDKATVKLLFRDAYALGGRFEEHLGGIYERFIDDIEAVVVAAQRRGE
VVEAPSRMAAYTLAALVGQLAHRRLNTDDNVTAAQVADFVVSLVLDGLRPRALAVGAR
GGRAART"
CDS 1722405..1722641
/codon_start=1
/note="gene_1582"
/transl_table=11
/translation="MTAALHNDVVTVASAPKLRVVRDVPPAPASKKVARRLDAQPFGT
GGDPLVDGAARLLSIPLRHLYAALWRVGLLEVQA"
CDS 1722948..1726073
/codon_start=1
/gene="ileS"
/note="gene_1583"
/transl_table=11
/translation="VTDNAYPKLAGGAPDLPALELEVLDYWSRDDTFRASIARRDGAP
EYVFYDGPPFANGLPHYGHLLTGYVKDIVPRYRTMRGYKVERRFGWDTHGLPAELEVE
RQLGITDKSQIEAMGIAAFNDACRASVLRYTDEWQAYVTRQARWVDFDNDYKTLDLAY
MESVIWAFKQLWDKGLAYEGYRVLPYCWRDETPLSNHELRMDDDVYQSRQDPAVTVGF
KVVGGQPDNGLDGAYLLVWTTTPWTLPSNLAVAVSPDITYVQVQAGDRRFVLAEARLA
AYARELGEEPVVLGTYRGAELLGTRYLPPFAYFMDWPNAFQVLAGDFVTTDDGTGIVH
MAPAYGEDDMVVAEAVGIAPVTPVDSKGRFDVTVADYQGQHVFDANAQIVRDLKTQSG
PAAVNGPVLIRHETYEHPYPHCWRCRNPLIYRSVSSWFVRVTDFRDRMVELNQQITWY
PEHVKDGQFGKWLQGARDWSISRNRYWGTPIPVWKSDDPAYPRIDVYGSLDELERDFG
VRPANLHRPYIDELTRPNPDDPTGRSTMRRIPDVLDVWFDSGSMPYAQVHYPFENLDW
FQGHYPGDFIVEYIGQTRGWFYTLHVLATALFDRPAFKTCVAHGIVLGFDGQKMSKSL
RNYPDVTEVFDRDGSDAMRWFLMASPILRGGNLIVTEQGIRDGVRQVLLPLWNTYSFL
ALYAPKVGTWRVDSVHVLDRYILAKLAVLRDDLSESMEVYDIPGACEHLRQFTEALTN
WYVRRSRSRFWAEDADAIDTLHTVLEVTTRLAAPLLPLITEIIWRGLTRERSVHLTDW
PAPDLLPSDADLVAAMDQVRDVCSAASSLRKAKKLRVRLPLPKLIVAVENPQLLRPFV
DLIGDELNVKQVELTDAIDTYGRFELTVNARVAGPRLGKDVQAAIKAVKAGDGVINPD
GTLLAGPAVLTADEYNSRLVAADPESTAALPDGAGLVVLDGTVTAELEAEGWAKDRIR
ELQELRKSTGLDVSDRIRVVMSVPAEREDWARTHRDLIAGEILATDFEFADLADGVAI

GDGVRVSIEKT"
CDS 1726270..1727676
/codon_start=1
/gene="dinX"
/note="gene_1584"
/transl_table=11
/translation="VESRWVLHLDMDAFFASVEQLTRPTLRGRPVLVGGLGGRGVVAG
ASYEARAYGARSAMPMHQARRLIGVTAVVLPPRGVVYGIASRRVFDTVRGLVPVVEQL
SFDEAFAEPPQLAGAVAEDVETFCERLRRRVRDETGLIASVGAGSGKQIAKIASGLAK
PDGIRVVRHAEEQALLSGLPVRRLWGIGPVAEEKLHRLGIETIGQLAALSDAEAANIL
GATIGPALHRLARGIDDRPVVERAEAKQISAESTFAVDLTTMEQLHEAIDSIAEHAHQ
RLLRDGRGARTITVKLKKSDMSTLTRSATMPYPTTDAGALFTVARRLLPDPLQIGPIR
LLGVGFSGLSDIRQESLFADSDLTQETAAAHYVETPGAVVPAAHDATMWRVGDDVAHP
ELGHGWVQGAGHGVVTVRFETRGSGPGSARTFPVDTGDISNASPLDSLDWPDYIGQLS
VEGSAGASAPTVDDVGDR"
CDS complement(1727641..1728621)
/codon_start=1
/note="gene_1585"
/transl_table=11
/translation="MGANHVRNDPIMARLTVITTGGTISTTAGPDGVLRPTHCGATLI
AGLDMDSDIEVVDLMALDSSKLTPADWDRIGAAVQEAFRGGADGVVITHGTDTLEETA
LWLDLTYAGSRPVVLTGAMLSADAPGADGPANLRDALAVAADPAARDLGVLVSFGGRV
LQPLGLHKVANPDLCGFAGESLGFTSGGVRLTRTKTRPYLGDLGAAVAPRVDIVAVYP
GSDAVAMDACVAAGARAVVLEALGSGNAGAAVIEGVRRHCRDGSDPVVIAVSTRVAGA
RVGAGYGPGHDLVEAGAVMVPRLPPSQARVLLMAALAANSPVADVIDRWG"
CDS 1728673..1729281
/codon_start=1
/gene="lspA"
/note="gene_1586"
/transl_table=11
/translation="VPDEPTGSADPLTSTEEAGGAGEPNAPAPPRRLRMLLSVAVVVL
TLDIVTKVVAVQLLPPGQPVSIIGDTVTWTLVRNSGAAFSMATGYTWVLTLIATGVVV
GIFWMGRRLVSPWWALGLGMILGGAMGNLVDRFFRAPGPLRGHVVDFLSVGWWPVFNV
ADPSVVGGAILLVILSIFGFDFDTVGRRHADGDTVGRRKADG"
CDS 1729316..1730200
/codon_start=1
/note="gene_1587"
/transl_table=11
/translation="MRVDTGLARLLGLSRTAAAALAEEGAVELNGVPAGKSDRLVSGA
LLQVRLPEAPAPLQNTPIDIEGMTILYSDDDIVAVDKPAAVAAHASVGWTGPTVLGGL
AAAGYRITTSGVHERQGIVHRLDVGTSGVMVVAISERAYTVLKRAFKYRTVDKRYHAL
VQGHPDPSSGTIDAPIGRHRGHEWKFAITKNGRHSLTHYDTLEAFVAASLLDVHLETG
RTHQIRVHFAALHHPCCGDLVYGADPKLAKRLGLDRQWLHARSLAFAHPADGRRVEIV
SPYPADLQHALKILRGEG"
CDS complement(1730207..1730800)
/codon_start=1
/note="gene_1588"
/transl_table=11
/translation="MRWIGVLVTALVLSACAANPPANTTSPTAGQSLDCTKPATIVQQ
LVCHDRQLTSLDHRLSTAYQQALAHRRSAALEAAQSSWTMLRDACAQDTDPRTCVQEA
YQTRLVQLAIADPATATPPVLTYRCPTQDGPLTAQFYNQFDPKTAVLNWKGDQVIVFV

ELSGSGARYGRQGIEYWEHQGEVRLDFHGATFVCRTS"
CDS complement(1730855..1731265)
/codon_start=1
/note="gene_1589"
/transl_table=11
/translation="MGLLSRLRKREPISIYDKIGGHEAIEVVVEDFYVRVLADDQLSA
FFSGTNMSRLKGKQVEFFAAALGGPEPYTGAPMKQVHQGRGITMHHFSLVAGHLADAL
TAAGVPSETITEILGVIAPLAVDVTSGESTTAPV"
CDS 1731493..1732518
/codon_start=1
/note="gene_1590"
/transl_table=11
/translation="MNLGDLTNFVEKPLAAVSNIVNTPNSAGRYRPFYLRNLLDAVQG
RNLNDAVKGKVVLITGGSSGIGAAAAKKIAEAGGTVVLVARTLENLENVANDIRAIRG
NGGTAHVYPCDLSDMDAIAVMADQVLGDLGGVDILINNAGRSIRRSLELSYDRIHDYQ
RTMQLNYLGAVQLILKFIPGMRERHFGHIVNVSSVGVQTRAPRFGAYIASKAALDSLC
DALQAETVHDNVRFTTVHMALVRTPMISPTTIYDKFPTLTPDQAAGVITDAIVHRPRR
ASSPFGQFAAVADAVNPAVMDRVRNRAFNMFGDSSAAKGSESQTDTSELDKRSETFVR
ATRGIHW"
CDS 1732523..1733326
/codon_start=1
/note="gene_1591"
/transl_table=11
/translation="MSLPKPNNQTTVVITGASSGIGVELARGLAGRGFPLMLVARRRE
RLDELADQLRQEHCVGVEVLPLDLADTQARAQLADRLRSDAIAGLCNSAGFGTSGRFW
ELPFARESEEVVLNALALMELTHAALPGMVKRGAGAVLNIASIAGFQPIPYMAVYSAT
KAFVLTFSEAVQEELHGTGVSVTALCPGPVPTEWAEIASAERFSIPLAQVSPHDVAEA
AIAGMLSGKRTVVPGIVPKFVSTSGRFAPRSLLLPAIRIGNRLRGGPSR"
CDS 1733624..1734055
/codon_start=1
/note="gene_1592"
/transl_table=11
/translation="MASVELSADVPISPQDTWDHVSELSELGEWLVIHEGWRSELPDQ
LGEGVQIVGVARAMGMRNRVTWRVTKWDPPHEVAMTGSGKGGTKYGVTLTVRPTKGGS
ALGLRLELGGRALFGPLGSAAARAVKGDVEKSLKQFAELYG"
CDS 1734123..1737677
/codon_start=1
/gene="dnaE"
/note="gene_1593"
/transl_table=11
/translation="MSGSSAGSSFVHLHNHTEYSMLDGAAKITPMLAEVERLGMPAVG
MTDHGNMFGASEFYNSATKAGIKPIIGVEAYIAPGSRFDTRRILWGDPSQKADDVSGS
GSYTHLTMMAENATGLRNLFKLSSHASFEGQLSKWSRMDAELIAEHAEGIIITTGCPS
GEVQTRLRLGQDREALEAAAKWREIVGPDNYFLELMDHGLTIERRVRDGLLEIGRALN
IPPLATNDCHYVTRDAAHNHEALLCVQTGKTLSDPNRFKFDGDGYYLKSAAEMRQIWD
DEVPGACDSTLLIAERVQSYADVWTPRDRMPVFPVPDGHDQASWLRHEVDAGLRRRFP
AGPPDGYRERAAYEIDVICSKGFPSYFLIVADLISYARSAGIRVGPGRGSAAGSLVAY
ALGITDIDPIPHGLLFERFLNPERTSMPDIDIDFDDRRRGEMVRYAADKWGHDRVAQV
ITFGTIKTKAALKDSARIHYGQPGFAIADRITKALPPAIMAKDIPLSGITDPSHERYK
EAAEVRGLIETDPDVRTIYQTARGLEGLIRNAGVHACAVIMSSEPLTEAIPLWKRPQD
GAIITGWDYPACEAIGLLKMDFLGLRNLTIIGDAIDNVRANRGIDLDLESVPLDDKAT

YELLGRGDTLGVFQLDGGPMRDLLRRMQPTGFEDVVAVIALYRPGPMGMNAHNDYADR
KNNRQAIKPIHPELEEPLREILAETYGLIVYQEQIMRIAQKVASYSLARADILRKAMG
KKKREVLEKEFEGFSDGMQANGFSPAAIKALWDTILPFADYAFNKSHAAGYGMVSYWT
AYLKANYPAEYMAGLLTSVGDDKDKAAVYLADCRKLGITVLPPDVNESGLNFASVGQD
IRYGLGAVRNVGANVVGSLLQTRNDKGKFTDFSDYLNKIDISACNKKVTESLIKAGAF
DSLGHARKGLFLVHSDAVDSVLGTKKAEALGQFDLFGSNDDGTGTADPVFTIKVPDDE
WEDKHKLALEREMLGLYVSGHPLNGVAHLLAAQVDTAIPAILDGDVPNDAQVRVGGIL
ASVNRRVNKNGMPWASAQLEDLTGGIEVMFFPHTYSSYGADIVDDAVVLVNAKVAVRD
DRIALIANDLTVPDFSNAEVERPLAVSLPTRQCTFDKVSALKQVLARHPGTSQVHLRL
ISGDRITTLALDQSLRVTPSPALMGDLKELLGPGCLGS"

```
CDS             complement(1737726..1739930)
                /codon_start=1
                /note="gene_1594"
                /transl_table=11
                /translation="VAAHGRKEARREIRHALPGFNLRPSEAPGFFVGFAAVGARQRRE
                ARWVWPADGMVVMNFSVLPPEINSALMFAGAGPGPMLAAASAWTGLAGDLGSAAASFS
                AVTSQLATGSWQGPASAAMTGVAASYARWLTTAAAQAEQAAGQAQAAVSAFEAALAAT
                VHPGAVSANRGRLRSLVASNLLGQNAPAIAAVEAVYEQMWAADVAAMLGYHGEASAVA
                LSLTPFTPSPSAAATPGGAVIIAGFPFLDLGNVTIGGFNLASGNLGLGNLGSFNPGSA
                NTGSVNLGNANIGDLNLGSGNIGSYNLGGGNTGDLNPGSGNTGTLNWGSGNIGSYNLG
                GGNLGSYNLGSGNTGDTNFGGGNTGNLNVGGGNTGNSNFGFGNTGNVNFGNGNTGDTN
                FGSGNLGSGNIGFGNKGSHNIGFGNSGNNNIGFGLTGDNQIGFGALNSGSGNLGFGNS
                GNGNIGFFNSGNNNIGMGNSGNGVGALSVEFGSSAERSSGFGNSGELSTGIGNSGQLS
                TGWFNSATTSTGWFNSGTTNTGWFNSGTTNTGIGNSGGNLVTGSMGLFNSGHTNTGSF
                NAGSMNTGDFNSGNVNTGYFNSGNINTGFFNSGDLNTGLFNSVNQPVQNSGWLHTGTN
                NSGYANAGTFNSGFDNNARDEHAEFVTGNSGLANVGNYNAGIINVGDHLSGFRNSVPT
                ITGTANISGFVNAGTSISGFFNFGSLMSGFANFDDEVSGYLNGDSRASGWIH"
CDS             1740036..1741862
                /codon_start=1
                /gene="fadD11"
                /note="gene_1595"
                /transl_table=11
                /translation="MTTTERPTTMCEAFQRTAVMDPDAVALRTPGGNQTMTWRDYAAQ
                VRRVAAGLAGLGVRRGDTVSLMMANRIEFYPLDVGAQHVGATSFSVYNTLPAEQLTYV
                FDNAGTKVVICEQQYVDRVRASGVPIEHIVCVDGAPPGTLSLTDLYAAASGDFFDFES
                TWRAVQPEDIVTLIYTSGTTGNPKGVEMTHANLLFEGYAIDEVLGIRFGDRVTSFLPS
                AHIADRMTGLYLQEMFGTQVTAVADARTIAAALPDVRPTVWGAVPRVWEKLKAGIEFT
                VARETDEMKRQALAWAMSVAGKRANALLAGESMSDQLVAEWAKADESVLSKLRERLGF
                GELRWALSGAAPIPKETLAFFAGIGIPIAEIWGMSELSCVATASHPRDGRLGTVGKLL
                PGLQGKIAEDGEYLVRGPLVMKGYRKEPAKTAEAIDSDGWLHTGDVFDIDSDGYLRVV
                DRKKELIINAAGKNMSPANIENTILAACPMVGVMMAIGDGRTYNTALLVFDADSLGPY
                AAQRGLDASPAALAADPEVIARIAAGVAEGNAKLSRVEQIKRFRILPTLWEPGGDEIT
                LTMKLKRRRIAAKYSAEIEELYASELRPQVYEPAAVPSTQPA"
CDS             1741876..1743741
                /codon_start=1
                /note="gene_1596"
                /transl_table=11
                /translation="VTAREVGRIGLRKLLQRIGIVAESMTPLATDPVEVTQLLDARWY
                DERLRALADELGRDPDSVRAEAAGYLREMAASLDERAVQAWRGFSRWLMRAYDVLVDE
                DQITQLRKLDRKATLAFAFSHRSYLDGMLLPEAILANRLSPALTFGGANLNFFPMGAW
                AKRTGAIFIRRQTKDIPVYRFVLRAYAAQLVQNHVNLTWSIEGGRTRTGKLRPPVFGI
```

LRYITDAVDEIDGPEVYLVPTSIVYDQLHEVEAMTTEAYGAVKRPEDLRFLVRLARQQ
GERLGRAYLDFGEPLPLRKRLQEMRADKSGTGSEIERIALDVEHRINRATPVTPTAVV
SLALLGADRSLSISEVLATVRPLASYIAARNWAVAGAADLTNRSTIRWTLHQMVASGV
VSVYDAGTEAVWGIGEDQHLVAAFYRNTAIHILVDRAVAELALLAAAETTTNGSVSPA
TVRDEALSLRDLLKFEFLFSGRAQFEKDLANEVLLIGSVVDTSKPAAAADVWRLLESA
DVLLAHLVLRPFLDAYHIVADRLAAHEDDSFDEEGFLAECLQVGKQWELQRNIASAES
RSMELFKTALRLARHRELVDGADATDIAKRRQQFADEIATATRRVNTIAELARRQ"

CDS 1743869..1744069
/codon_start=1
/gene="TMEG_01459"
/note="gene_1597"
/transl_table=11
/translation="MVATTTHFPKQKAPCGHMVDGDHHIERDDEGLAYGDLKFSCGCR
EIRLSTTTDPCGYARFDTTARC"

CDS 1744111..1745862
/codon_start=1
/note="gene_1598"
/transl_table=11
/translation="MTAQHNIVVIGGGGAGLRAAIAIAETNPHLDVAIVSKVYPMRSH
TVSAEGGAAAVTGDDDSLDEHAHDTVSGGDWLCDQDAVEAFVAEAPKELVQLEHWGCP
WSRKPDGRVAVRPFGGMKKLRTWFAADKTGFHLLHTLFQRLLTYSDVMRYDEWFATTL
LVDDGRVCGLVAIELATGRIETILADAVILCTGGCGRVFPFTTNANIKTGDGMALAFR
AGAPLKDMEFVQYHPTGLPFTGILITEAARAEGGWLLNKDGYRYLQDYDLGKPTPEPR
LRSMELGPRDRLSQAFVHEHNKGRTVDTPYGPVVYLDLRHLGADLIDAKLPFVRELCR
DYQHIDPVVELVPVRPVVHYMMGGVHTDINGATTLPGLYAAGETACVSINGANRLGSN
SLPELLVFGARAGRAAADYAARHQKSDRGPSSAVRAQARTEALRLERELSRHGQGGER
IADIRADMQATLESAAGIYRDGPTLTKAVEEIRVLQERFATAGIDDHSRTFNTELTAL
LELSGMLDVALAIVESGLRREESRGAHQRTDFPNRDDEHFLAHTLVHRESDGTLRVGY
LPVTITRWPPGERVYGR"

CDS 1745865..1746617
/codon_start=1
/note="gene_1599"
/transl_table=11
/translation="MMDRIVMEVSRYRPEIESAPTFQAYEVPLTREWAVLDGLTYIKD
HLDGTLSFRWSCRMGICGSSGMTINGDPKLACATFLADYLPGPVRVEPMRNFPVIRDL
VVDISDFMAKLPSVKPWLVRHDEPPVEDGEYRQTPAELDAFKQFSMCINCMLCYSACP
VYALDPDFLGPAAIALGQRYNLDSRDQGAADRRDVLAAADGAWACTLVGECSTACPKG
VDPAGAIQRYKLTAATHALKKLLFPWGGGGGG"

CDS 1746614..1746994
/codon_start=1
/note="gene_1600"
/transl_table=11
/translation="MSAYRQPVERYWWARRRSYLRFMLREISCIFVAWFVLYLVLVLR
AVGAGGNSYQRFLDFSANPVVVVLNVVALSFLLLHAVTWFGSAPRAMVIQVRGRRVPA
RAVLAGHYAAWLVVSVIVAWMVLS"

CDS 1747072..1747368
/codon_start=1
/note="gene_1601"
/transl_table=11
/translation="MVTALVAPVLLLLFGLAFPLGWLDAPDHGHLLAMVRNPITKLVV
LVLVVLALFHAAHRFRFVLDHGLQLGRFDRVIALWCYGMAVLGSATAGWMLLTM"

CDS 1747448..1748044
/codon_start=1
/note="gene_1602"
/transl_table=11
/translation="VTQIADRPTDPSPWSPRETELLAVTLRLLQEHGYDRLTVDAVAA
SARASKATVYRRWPSKAELVLAAFIEGIRQVAVPPNTGNLRDDLLRLGELICREVGQH
ASTIRAVLVEVSRNPALNDVLQHQFVDHRKALIQYILQQAVDRGEISSAAISDELWDL
LPGYLIFRSIIPNRPPTQDTVQALVDDVILPSLTRSTG"
CDS 1748183..1748629
/codon_start=1
/note="gene_1603"
/transl_table=11
/translation="VQGISVTGLVKRGWMVLVAVAVVAVAGFSVYRLHGIFGSHDTTS
TAGGVANDIKPFNPKQVTLEVFGAPGTVATINYLDVDATPRQVLDTTLPWSYTITTTL
PAVFANVVAQGDSNSIGCRITVNGVVKDERIVNEVRAYTFCLDKSS"
CDS 1748704..1751529
/codon_start=1
/note="gene_1604"
/transl_table=11
/translation="VGVAAITNAAVPQLEVVGEAHNVAQSSPDDPSLQAMKRIGKVFH
EFDSDSAAMIVLEGDKPLGNDAHRFYDTLLRNLSNDTKHVEHVQDFWGDPLTAAGSQS
TDGKAAYVQVYLAGNQGEALSIESVDAVRDIVAHTPPPAGVKAYVTGAAPLMADQFQV
GSKGTAKVTGITLVVIAVMLLFVYRSVVTMVLVLITVLIELAAARGIVAFLGNAGVIG
LSTYSTNLLTLLVIAAGTDYAIFVLGRYHEARYAAQDRETAFYTMYRGTAHVVLGSGL
TVAGAVYCLSFTRLPYFQSLGIPASIGVMIALAAALSLAPSVLILGSRFGCFEPKRRM
RTRGWRRIGTAIVRWPGPILAVACAIAVVGLLALPGYKTSYDARYYMPATAPANIGYM
AAERHFPQARLNPELLMIETDHDMRNPADMLILDRIAKAVFHLPGIGLVQAMTRPLGT
PIDHSSIPFQISMQSVGQIQNLKYQRDRAADLLKQAEELGKTIEILQRQYALQQELAA
ATHEQAESFHQTIATVKELRDRIANFDDFFRPIRSYFYWEKHCYDIPSCWALRSVFDT
IDGIDQLGEQLASVTVTLDKLAAIQPQLVALLPDEIASQQINRELALANYATMSGIYA
QTAALIENAAAMGQAFDAAKNDDSFYLPPEAFDNPDFQRGLKLFLSADGKAARMIISH
EGDPATPEGISHIDAIKQAAHEAVKGTPMAGAGIYLAGTAATFKDIQDGATYDLLIAG
IAALSLILLIMMIITRSLVAALVIVGTVALSLGASFGLSVLVWQHLLGIQLYWIVLAL
AVILLLAVGSDYNLLLISRFKEEIGAGLNTGIIRAMAGTGGVVTAAGLVFAATMSSFV
FSDLRVLGQIGTTIGLGLLFDTLVVRAFMTPSIAVLLGRWFWWPQRVRPRPASRMLRP
YGPRPVVRELLLREGNDDPRTQVATHR"
CDS 1751539..1751985
/codon_start=1
/note="gene_1605"
/transl_table=11
/translation="MPLSGEYAPSPLDWSREQADTYMKSGGTEGTQLQGKPVILLTTV
GAKTGKLRKTPLMRVEHDGQYAIVASLGGAPKNPVWYHNVVKNPRVELQDGTVTGDYD
AREVFGDEKAIWWQRAVAVWPDYASYQTKTDRQIPVFVLTPVRAGG"
CDS 1752020..1753309
/codon_start=1
/note="gene_1606"
/transl_table=11
/translation="VSAELSQSPSSSPLFSLSGADIDRAAKRIAPVVTPTPLQPSDRL
SAITGATVYLKREDLQTVRSYKLRGAYNLLVQLSDEELAAGVVCSSAGNHAQGFAYAC
RCLGVHGRVYVPAKTPKQKRDRIRYHGGEFIDLIVGGSTYDLAAAAALEDVERTGATL
VPPFDDLRTIAGQGTIAVEVLGQLEDEPDLVVVPVGGGGCIAGITTYLAERTTNTAVL

GVEPAGAAAMMAALAAGEPVTLDHVDQFVDGAAVNRAGTLTYAALAAAGDMVSLTTVD
EGAVCTAMLDLYQNEGIIAEPAGALSVAGLLEADIEPGSTVVCLISGGNNDVSRYGEV
LERSLVHLGLKHYFLVDFPQEPGALRRFLDDVLGPNDDITLFEYVKRNNRETGEALVG
IELGSAADLDGLLARMRATDIHVEALEPGSPAYRYLL"

CDS 1753362..1753565
/codon_start=1
/note="gene_1607"
/transl_table=11
/translation="MSRTNIDIDDELAAEVMRRFGLTTKRAAVDLALRRLVGSPLSRE
FLLGLEGVGWEGDLDDLRSDRPD"

CDS 1753571..1753975
/codon_start=1
/note="gene_1608"
/transl_table=11
/translation="MILIDTSAWVEYFRATGSIAAVEVRRLLSEEAARIAMCEPIAME
ILSGALDDNTHTTLERLVNGLPSLNVDDAIDFRAAAGIYRAARRAGETVRSINDCLIA
ALAIRHGARIVHRDADFDVIARITNLQAASFR"

CDS complement(1753992..1754651)
/codon_start=1
/note="gene_1609"
/transl_table=11
/translation="LDTSAIPATRLLAYTCTHDQVGNRALGDRPSQYLTGGQLAIKAA
LTLGSPYTAMLFMGEEWGASSPFFQFFCSHPEPELAHSTVAGRKEEFAEHGWAADDIPD
PQDPQTFQRCKLNWAEAGSGEHARLHRFYRDLIALRHNEADLADPWLDHLMVDYDEQQ
RWVVMRRGQLMIACNLGAEPTCVPVSGELVLAWESPIIGDNSTELAAYSLAILRAAEP
A"

CDS complement(1754549..1755733)
/codon_start=1
/note="gene_1610"
/transl_table=11
/translation="MPEFRVWAPKPALVRLDVNGAVHAMTRSADGWWHTTVAAPADAR
YGYLLDDDPTVLPDPRSARQPDGVHARSQRWEPPGQFGAARTDTGWPGRSVEGAVIYE
LHIGTFTTAGTFDAAIEKLDYLVDLGIDFVELMPVNSFAGTRGWGYDGVLWYSVHEPY
GGPDGLVRFIDACHTRRLGVLIDAVFNHLGPSGNYLPRFGPYLSSASNPWGDGINIAG
ADSDEVRHYIIDCALRWMRDFHADGLRLDAVHALVDTTAVHVLEELANATRWLSGQLG
RPLSLIAETDRNDPRLITRPSHGGYGIPPNGMTTSITPSIPRYPVSGKATMRISARWP
LSRTRCATATSTPARIRRSGVVGTGVHWTLLRSRPPGCSPTPAPTTKSATALSATAHR
NT"

CDS complement(1755726..1758023)
/codon_start=1
/note="gene_1611"
/transl_table=11
/translation="MAFPVISTYRVQMRGRSNGFGFTFADAENLLDYLDDLGVSHLYL
SPILTAVGGSTHGYDVTDPTTVSPELGGSDGLARLSAAARSRGMGLIVDIVPSHVGVG
KPEQNAWWWDVLKFGRSSAYAEFFDIDWELGDGRIILPLLGSDSDVANLRVDGDLLRL
GDLALPVAPGSGDGTGPAVHDRQHYRLVGWRHGLCGYRRFFSITSLAGLRQEDRAVFD
ASHAEVARWFTEGLVDGVRVDHLDGLSDPSGYLAQLRELLGPNAWIVVEKILAVDEAL
EPTLPVDGSTGYDVLREIGGVLVDPQGESPLTALVESAGVDYQEMPAMLADLKVHAAV
HTLASELRRLRRCIAAAAGADHPLLPAAVAALLRHIGRYRCDYPGQAAVLPCALAETH
STTPQLAPGLQLIAAAVARGGEPAVRLQQLCGAVSAKAVEDCMFYRDARLVSLNEVGG
EPRRFGVGAAEFHHRAATRARLWPRSMTTLSTHDTKRGEDVRARIGVLSQVPWLWAKF

IGHAQAIAPAPDAVTGQFLWQNVFGVWPVSGEVSAALRGRLHTYAEKAIREAAWHTSW
HNPNRAFEDDVHGWLDLVLDGPLASELTGLVAHLNSHAESDALAAKLLALTVPGVPDV
YQGSELWDDSLVDPDNRRPVDYGTRRVALKALQHPKIRVLAAALRLRRTHPESFLGGA
YHPVFAAGPAADHVVAFRRGDDILVAVTRWTVRLQQTGWDHTVLPLPDGSWTDALTGF
TASGHTPAVELFADLPVVLLVRDNA"

CDS complement(1758027..1760195)
/codon_start=1
/note="gene_1612"
/transl_table=11
/translation="VMSSNNAGESDGTGPALPTVWPGNAYPLGATYDGAGTNFSLFSE
IAEKVELCLIDEDGVESRIPLDEVDGYVWHAYLPNITPGQRYGFRVHGPFDPAAGHRC
DPSKLLLDPYGKSFHGDFTFGQALYSYDVNAVDPDSTPPMVDSLGHTMTSVVINPFFD
WAYDRSPRTPYHETVIYEAHVKGMTQTHPSIPPELRGTYAGLAHPVIIDHLNELNVTA
VELMPVHQFLHDSRLLDLGLRNYWGYNTFGFFAPHHQYASTRQAGSAVAEFKTMVRSL
HEAGIEVILDVVYNHTAEGNHLGPTINFRGIDNTAYYRLMDHDLRFYKDFTGTGNSLN
ARHPHTLQLIMDSLRYWVIEMHVDGFRFDLASTLARELHDVDRLSAFFDLVQQDPVVS
QVKLIAEPWDVGEGGYQVGNFPGLWTEWNGKYRDTVRDYWRGEPATLGEFASRLTGSS
DLYEATGRRPSASINFVTAHDGFTLNDLVSYNDKHNEANGENNRDGESYNRSWNCGVE
GPTDDPDILALRARQMRNMWATLMVSQGTPMIAHGDEIGRTQYGNNNVYCQDSELSWM
DWSLVDKNADLLAFARKATTLRKNHKVFRRRRFFEGEPIRSGDEVRDIAWLTPSGREM
THEDWGRGFDRCVAVFLNGEAITAPDARGERVVDDSFLLCFNAHDHDVEFVMPHDGYA
QQWTGELDTNDPVGDIDLTVTATDTFSVPARSLLVLRKTL"

CDS complement(1760231..1762420)
/codon_start=1
/note="gene_1613"
/transl_table=11
/translation="MLTLSPPRPPALTPEPALPPVTMGTRTTGFYRHDLDGLRGVAIA
LVAVFHVWFGRVSGGVDVFLALSGFFFGGKILRAALNPDLSLSPIAEVIRLIRRLLPA
LVVVLAGCALLTIAIQPQTRWEAFANQSLASLGYYQNWELASTVSNYLRAGEAVSPLQ
HIWSMSVQGQFYLAFLLLVAGCAYLLRRLFRGPRAPYLRTMFVVLLSTLTLASFIYAI
VAHHAYQATAYYNTFARAWELLAGALVGAVVPHVRWPMWLRTAVATAALAAILSCGAL
IDGVKEFPGPWALVPVGATMLMILAGANRQGHPGTRDRLPLPNRLLATAPLVALGAMA
YSWYLWHWPLLIFWLSYTGHRHANFVEGAAVLLVSGLLAYLTTRLVEDPLRYRAPAGV
RSPAAVPPIPWRLRLRRPTIVLGSVVALLGVALTATSFTWREHVIVQRAAGKELSGLS
SRDYPGARALIDHVRVPKLRMRPTVLEVRHDLPTSTKDGCISDFVNPAIINCTYGDVD
APRTIALAGGSHAEHWLTALDLLGRMHHFKVVTYLKMGCPLSTEEVPLIMGNNAPYPQ
CHQWVQAAMAKLVADHPDYVFTTSTRPWNIKPGDVMPATYVGIWQTFADNNIPVLAMR
DTPWLVKDGQPFIPADCLAKGGNPQSCGIARSKVLVDRNPTLDFVARFPLLKPLDMSD
AICRTDTCRAVEGNVLVVYRDSHHLTPTYMRTMTSELGRQIAANTDWW"

CDS complement(1762519..1763211)
/codon_start=1
/note="gene_1614"
/transl_table=11
/translation="MKRSMKSGSFAIGLAMMLAPMVAAPGLAAADPATRPVDYQQITD
VVIARGLSQRGVPFSWAGGGISGPTRGTGTGINTVGFDASGLIQYAYAGAGLKLPRSS
GQMYKVGQKVLPQQARKGDLIFYGPEGTQSVALYLGKGQMLEVGDVVQVSPVRTNGMT
PYLVRVLGTQPTPVQQAPVQPAPVQQAPVQQAPVQQAPVQQAPVQQAPVQQAPVQQAP
VQPPPFGTARSR"

CDS complement(1763451..1764023)
/codon_start=1
/note="gene_1615"

/transl_table=11
/translation="MICSGVNPPVAAAMEIEGNAATPRPIPVTVAKTSAIGEHACMAP
PNQPEDTQPDAGTESRSALDEPDERTPPRVLTLRIAPWDVVCTVVILAVFLVMVTMTS
WPSRLFAFTDNVCPPDACPLVPFGVNYYIYPVMWGGIGAAIATAVIGPFVSMLKGWYM
SFWPIISIAVITVTSIAGYAIAGFSERYWH"
CDS 1763983..1765296
/codon_start=1
/note="gene_1616"
/transl_table=11
/translation="MAAATGGLTPEQIIAVDGAHLWHPYSSIGREAVSPVVAVAAHGA
WLTLIRDGQPIEVLDAMSSWWTAIHGHGHPALDQALTTQLRVMNHVMFGGLTHEPAAR
LAKLLVDITPAGLDTVFFSDSGSVSVEVAAKMALQYWRGRGLPGKRRLMTWRGGYHGD
TFLAMSICDPHGGMHSLWTDVLAAQVFAPQVPRDYDPAYSAAFEAQLAQHAGELAAVV
VEPVVQGAGGMRFHDPRYLHDLRDICRRYEVLLIFDEIATGFGRTGALFAADHAGVSP
DIMCVGKALTGGYLSLAATLCTADVAHTISAGAAGALMHGPTFMANPLACAVSVASVE
LLLGQDWRTRITELAAGLTAGLDTARALPAVTDVRVCGAIGVIECDRPVDLAVATPAA
LDRGVWLRPFRNLVYAMPPYICTPAEITQITSAMVEVARLVGSLP"
CDS 1765293..1766453
/codon_start=1
/note="gene_1617"
/transl_table=11
/translation="MKAATQARIDDSPLAWLDAVQRQRHEAGLRRCLRPRPAVATELD
LASNDYLGLSRHPAVIDGGVQALRIWGAGATGSRLVTGDTKLHQQFEAELAEFVGAAA
GLLFSSGYTANLGAVVGLSGPGSLLVSDARSHASLVDACRLSRARVVVTPHRDVDAVD
AALRSRDEQRAVVVTDSVFSADGSLAPVRELLEVCRRHGALLLVDEAHGLGVRGGGRG
LLYELGLAGAPDVVMTTTLSKALGSQGGVVLGPTPVRAHLIDAARPFIFDTGLAPAAV
GAARAALRVLQAEPWRPQAVLNHAGELARMCGVAAVPDSAMVSVILGEPESAVAAAAA
CLDAGVKVGCFRPPTVPAGTSRLRLTARASLNAGELELARRVLTDVLAVARR"
CDS 1766450..1767130
/codon_start=1
/gene="bioD"
/note="gene_1618"
/transl_table=11
/translation="LTILVVTGTGTGVGKTVVCAALASAARQAGIDVAVCKPVQTGTA
RGDDDLAEVGRLAGVTQLAGLARYPQPMAPAAAAEHAGMALPARDQIVRLIADLDRPG
RLTLVEGAGGLLVELAEPGVTLRDVAVDVAAVALVVVTADLGTLNHTKLTLEALAAQQ
VSCAGLVIGSWPDPPGLVAASNRSALARIATVRAALPAGAASLDAGDFAAMSAAAFDR
NWVAGLVG"
CDS 1767130..1767639
/codon_start=1
/note="gene_1619"
/transl_table=11
/translation="MVHSIELVFDSDTEAAIRRIWAGLAAAGIPSQAPASRPHVSLAV
AERIAPEVDEPLGAVARRLPLDCVIGAPVLFGRANVVFTRLVVPTSELLALHAEVHRL
CGPHLAPAPMANSLPGQWTAHVTLARRVGGHQLGRALRIAGRPSRIDGRFAGLRRWDG
NTRAEYLLG"
variation 1767857..1777103
/note="supplemented from AL2122/97"
CDS 1767905..1768315
/codon_start=1
/note="gene_1620"

/transl_table=11
/translation="MTTTPARFNHLVTVTDLETGDRAVCDRDQVAETIRAWFPDAPLE
VREALVRLQAALNRHEHTGELEAFLRISVEHADAAGGDECGPAILAGRSGPEQAAINR
QLGLAGDDEPDGDDTPPWSRMIGLGGGSPAEDER"
CDS 1768281..1768832
/codon_start=1
/note="gene_1621"
/transl_table=11
/translation="LAAEAQRKTSADGEHRGNRTLGGPTGGAWIASGPWAGSYLGETN
GAAGRRIAELERPKAKQRQREGQDHGRQARYSGLGPMGYKPESERHSTKTDTAIGAAL
GISAGTYRRLKRIDNATHSDDKEIRRFAEKQMAPLVAGSPSWNARKPRSANARVVASV
HRSPMPALVPWNQSRLSATLTRR"
CDS 1768790..1769290
/codon_start=1
/note="gene_1622"
/transl_table=11
/translation="MEPKPSQRHTDKEVGAALGISAGTYKRLKRIDNATRSDDKEIRL
FAEKQMAPLAAGSPSWNGRKPSSGNRKAATMAARLDILAWGPWAQARIGASFDENRHC
YRRSPRHLRRHLPAAQTNRQRNPQRVGGVGGPAPLSRGRPRLALSYLRGSLHLQNSKR
VAHQHI"
CDS complement(1769234..1770655)
/codon_start=1
/note="gene_1623"
/transl_table=11
/translation="MTEFDDIKNLSLPETRDAAKQLLDSVAGDLTGEAAQRFQALTRH
AEELRAEQRRRGREAEEELRRYRAGELRVVPGAPTGGDDGDAPPGNSLRDTAFRTLDS
CVRDGLMSSRAAETAETLCRTGPPQSTSWAQRWLAATGSRDYLGAFVKRVSNPVAGHT
VWTDREAAAWREAAAVAAEQRAMGLVDTQGGFLIPAALDPAILLSGDGSTNPIRQVAR
VVQTTSEIWRGVTSEGAEARWYSEAQEVSDDSPALAQPAVPNYRGSCWIPFSIELEGD
AASFVGEIGKILADSVEQLQTAAFVNGSGNGEPTGFVSALTGTSDQVVVGAGSEAIVA
ADVYALQSALPPRFQASAAFAANLSTINTLRQAETSNGALKFPSLHDSPPMLAGKSVL
EVSHMDTVDSAVTATNHPLVLGDWKQFLIVDRVGSMVELVPHLFGPNRRPTGQRGFFA
WFRVGSDVLVRNAFRVLKVETTA"
CDS complement(1770663..1771196)
/codon_start=1
/note="gene_1624"
/transl_table=11
/translation="LSSILFRMAELRSGEGRTVHGTIVPYNEATTVRDFDGEFQEMFA
PGAFRRSIAERGHKLKLLVSHDARTRYPVGRAVELREEPHGLFGAFEIADTPDGDEAL
ANVKAGVVDSFSVGFRPIRDRREGDVLVRVEAALLEVSLTGVPAYSGAQIAGVRAESL
TVVSRSTAEAWLSLLDW"
CDS complement(1771349..1771819)
/codon_start=1
/note="gene_1625"
/transl_table=11
/translation="MPRPPKPARLKLVEGRSPGRDSGGRKVPESPKFIRQAPDAPDWL
DAEALAEWRRVAPTLERLDLLKPEDRALLSAYCETWSVYVAAVQRVRAEGLTITSPKS
GVVHRNPAVTVAETARMHLLRLASEFGLTPAAEQRLAVAPGDDGDGLNPFAPDR"
CDS complement(1771900..1772214)
/codon_start=1
/note="gene_1626"

/transl_table=11
/translation="VTPINRPLTNDERQLMHELAVQVVCSQTGCSPDAAVEALESFAK
DGTLILRGDTENAYLEAGGNVLVHADRDWLAFHASYPGNDPLRDARPIEQDDDQGAGS
PS"
CDS complement(1772211..1772483)
/codon_start=1
/note="gene_1627"
/transl_table=11
/translation="MAETPDHAELRRRIADMAFNADVGMATCKRCGDAVPYIILPNLQ
TGEPVMGVADNKWKRANCPVDVGKPCPFLIAEGVADSTDDTIEVDQ"
CDS complement(1772497..1772892)
/codon_start=1
/note="gene_1628"
/transl_table=11
/translation="MTAVAITPASGGRHSVRFAYDSAIVSLIKSTIPAYARSWSAHTR
CWFIDADWTPLLAAELRYHGHTVTGPADPAQQQCTDWAKALFRAVGPQRTPAVYRALS
KVLHPDAPTGCPILQQQLNAARTALTNPA"
CDS complement(1773088..1774503)
/codon_start=1
/note="gene_1629"
/transl_table=11
/translation="MADIPYGTDYPDAPWIDRDGHVLIDDGGKPTQVHRGQARIAYRL
AERYQDKLLHVAGIGWHSWDGRRWAADDRGEAKRAVLAELRQALSDSLNDKELRADVR
KCESASGVAGVLDLAAALVPFAATLADLDSDPHLLNVANGTLDLHTLKLRPHAPADRI
TKICRGAYQSDTESPLWQAFLTRVLPDEGVRGFVQRLAGVGLLGTVREHVLAILIGVG
ANGKSVFDKAIRYALGDYACTAEPDLFMHRENAHPTGEMDLRGVRWVAVSESEKDRRL
AESTIKRLTGGDTIRARKMRQDFVEFTPSHTPLLITNHLPRVPGDDTAIWRRIRVVPF
EVVIPADEQDRELDARLQLEADSILSWAVAGWSDYQRIGLSQPDAVLAATSNYREDSD
TIKRFIDDECVTSSPVLKATTTHLFEAWQRWRVQEGVPEISRKAFGQSLDTHGYPVTD
KARDGRWRAGIAVRGADDFDD"
CDS complement(1774503..1774901)
/codon_start=1
/note="gene_1630"
/transl_table=11
/translation="MTAGAGGSPPTRRCSATEDRAPATVATPSSADPTASRAVSWWSV
HEHVAPVLDAAGSWPMAGTPAWRQLDDADPRKWAAICDAARHWALRVETCQEAMAQAS
RDVSAAADWPGIAREIVRRRGVYIPRAGVA"
CDS complement(1774898..1775119)
/codon_start=1
/note="gene_1631"
/transl_table=11
/translation="VSTIYHHRGRVAALSRSRASDDPEFIAAKTDLVAANIADYLIRT
LAAAPPLTDEQRTRLAELLRPVRRSGGAR"
CDS complement(1775175..1775690)
/codon_start=1
/note="gene_1632"
/transl_table=11
/translation="MSRHHNIVIVCDHGRKGDGRIEHERCDLVAPIIWVDETQGWLPQ
APAVATLLDDDNQPRAVIGLPPNESRLRPEMRRDGWVRLHWEFACLRYGAAGVRTCEQ
RPVRVRNGDLQTLCENVPRLLTGLAGNPDYAPGFAVQSDAVVVAMWLWRTLCESDTPN
KLRATPTRGSC"

```
CDS             complement(1775687..1776865)
                /codon_start=1
                /note="gene_1633"
                /transl_table=11
                /translation="VVAWDLDRLHRRPIELEAFMSLADEKRLALATVAGDVDLATPQG
                RLVARLKGSVAAHETEHKKARQRRAARQKAERGHPNWSKAFGYLPGPNGPEPDPRTAP
                LVKQAYADILAGASLGDVCRQWNDAGAFTITGRPWTTTTLSKFLRKPRNAGLRAYKGA
                RYGPVDRDAIVGKAQWSPLVDEATFWAAQAVLDAPGRAPGRKSVRRHLLTGLAGCGKC
                GNHLAGSYRTDGQVVYVCKACHGVAILADNIEPILYHIVAERLAMPDAVDLLRREIHD
                AAEAETIRLELETLYGELDRLAVERAEGLLTARQVKISTDIVNAKITKLQARQQDQER
                LRVFDGIPLGTPQVAGMIAELSPDRFRAVLDVLAEVVVQPVGKSGRIFNPERVQVNWR
                "
CDS             complement(1776753..1777994)
                /codon_start=1
                /note="gene_1634"
                /transl_table=11
                /translation="VDVSTRQAAEADLAGKAAQYRPDELARYAQRVMDWLHPDGDLTD
                TERARKRGITLSNQQYDGMSRLSGYLTPQARATFEAVLAKLAAPGATNPDDHTPVIDT
                TPDAAAIDRDTRSQAQRNHDGLLAGLRALIASGELGQHNGLPVSIVVTTTLTDLQTGA
                GKGFTGGGTLLPMADVIRMTSHAHHYSPASGRYPQAIFDHGTPLALYHTKRLASPAQR
                IMLFANDRGCTKPGCDAPAYHSQAHHVTGWTSTGRTDITDLTLACDPDNRLAEKGWTT
                RKNTHGHTEWLPPPHLDHGQPWTCEIHYTCACCCLPPNLRRPLRRTARRGPPTRGLPK
                AVRAAKMGARRVPRQRRQRINRQAPPRLRADVGRHHRRQDRRRGGLGPGPAPSPSHRA
                GSLHVISRREAAGPGHRRRRR"
CDS             1778827..1779924
                /codon_start=1
                /note="gene_1635"
                /transl_table=11
                /translation="MGSARAEMTKDAGEYLVTQAATRPTNDAGQDGGNNSDILVVARQ
                QVLQRGEGLNQDQVLAVLQLPDDRLEELLALAHEVRMRWCGPEVEVEGIISLKTGGCP
                EDCHFCSQSGLFASPVRSAWLDIPSLVEAAKQTAKSGATEFCIVAAVRGPDERLMAQV
                AAGIEAIRNEVEINIACSLGMLTAEQVDQLAARGVHRYNHNLETARSFFANVVTTHTW
                EERWQTLSMVRDAGMEVCCGGILGMGETLQQRAEFAAELAELGPDEVPLNFLNPRPGT
                PFADLEVMPVGDALKAVAAFRLALPRTMLRFAGGREITLGDLGAKRGILGGINAVIVG
                NYLTTLGRPAEADLELLDELQMPLKALNASL"
CDS             1780245..1780826
                /codon_start=1
                /note="gene_1636"
                /transl_table=11
                /translation="MLGLSATGVLVGGLWAWIAPPIHAVVAITRAGERVHEYLGSESQ
                NFFIAPFMLLGLLSVLAVVASALMWQWREHRGPQMVAGLSIGLTTAAAIAAGVGALVV
                RLRYGALDFDTVPLSRGDHALTYVTQAPPVFFARRPLQIALTLMWPAGIASLVYALLA
                AGTARDDLGGYPAVDPSSNARTEALETPQAPVS"
CDS             complement(1780991..1782331)
                /codon_start=1
                /note="gene_1637"
                /transl_table=11
                /translation="MVEPGNLAGATGAEWIGRPPHEELQRKVRPLLPSDDPFYFPPAG
                YQHAVPGTVLRSRDVELAFMGLIPQPVTATQLLYRTTNMYGNPEATVTTVIVPAELAP
                GQTCPLLSYQCAIDAMSSRCFPSYALRRRAKALGSLTQMELLMISAALAEGWAVSVPD
                HEGPKGLWGSPYEPGYRVLDGIRAALNSERVGLSPATPIGLWGYSGGGLASAWAAEAC
```

GEYAPDLDIVGAVLGSPVGDLGHTFRRLNGTLLAGLPALVVAALQHSYPGLARVIKEH
ANDEGRQLLEQLTEMTTVDAVIRMAGRDMGDFLDEPLEDILSTPEVSHVFGDTKLGSA
VPTPPVLIVQAVHDYLIDVSDIDALADSYTAGGANVTYHRDLFSEHVSLHPLSAPMTL
RWLTDRFAGKPLTDHRVRTTWPTIFNPMTYAGMARLAVIAAKVITGRKLSRRPL"

CDS complement(1782588..1783298)
/codon_start=1
/note="gene_1638"
/transl_table=11
/translation="MAHGSTAHEVLAVVFQVRGVGMSRGAAKPQLNVLLWQRAKEPQR
GAWSLPGGRLRNDEDMTSSVRRQLAEKVDLRELAHLEQLAVFSDPHRLPGIRMIASTY
LGVVPSPATPELPADTRWHPVSSLPPMAFDHGPMVTHARTRLIAKMSYTNIGFALAPK
EFALSTLRDIYGAALGYQVDATNLQRVLARRRVITQTGTIAQSGRSGGRPAALYRFTD
SQLRVTDEFAALRPPGQL"

CDS 1783347..1784396
/codon_start=1
/note="gene_1639"
/transl_table=11
/translation="MTVLNRTDTLVDELTADITNTPLGYGGVDGDERWAAEIRRLAHL
RGATVLAHNYQLPAIQDVADHVGDSLALSRVAAEAPEDTIVFCGVHFMAETAKILSPH
KTVLIPDQRAGCSLADSITPDELRAWKDEHPGAVVVSYVNTTAAVKALTDICCTSSNA
VDVVASIDPDREVLFCPDQFLGAHVRRVTGRKNLHVWAGECHVHAGINGDELADQARA
HPDAELFVHPECGCATSALYLAGEGAFPAERVKILSTGGMLEAAHTTRARQVLVATEV
GMLHQLRRAAPEVDFRAVNDRASCKYMKMITPAALLRCLVEGADEVHVDPGIAASGRR
SVQRMIEIGHPGGGE"

CDS 1784396..1785979
/codon_start=1
/note="gene_1640"
/transl_table=11
/translation="MAGPAWRDAADVVVIGTGVAGLAAALAADRAGRSVVVLSKAAQT
HVTATHYAQGGIAVVLPDNDDSVDAHVADTLAAGAGLCDPDAVYSIVADGYRAVTDLV
GAGARLDESVPGRWALTREGGHSRRRIVHAGGDATGAEVQRALQDAAGMLDIRTGHVA
LRVLHDGTAVTGLLVVRPDGCGIISAPSVILATGGLGHLYSATTNPAGSTGDGIALGL
WAGVAVSDLEFIQFHPTMLFAGRAGGRRPLITEAIRGEGAILVDRQGNSITAGVHPMG
DLAPRDVVAAAIDARLKATGDPCVYLDARGIEGFASRFPTVTASCRAAGIDPVRQPIP
VVPGAHYSCGGIVTDVYGQTELLGLYAAGEVARTGLHGANRLASNSLLEGLVVGGRAG
KAAAAHAAAAGRSRATSSATWPEPISYTALDRGDLQRAMSRDASMYRAAAGLHRLCDS
LSGAQVRDVACRRDFEDVALTLVAQSVTAAALARTESRGCHHRAEYPCTVPEQARSIV
VRGADDANAVCVQALVAVC"

CDS 1785979..1786836
/codon_start=1
/note="gene_1641"
/transl_table=11
/translation="MGLSDWELAAARAAIARGLDEDLRYGPDVTTLATVPASATTTAS
LVTREAGVVAGLDVALLTLDEVLGTNGYRVLDRVEDGARVPPGEALMTLEAQTRGLLT
AERTMLNLVGHLSGIATATAAWVDAVRGTKAKIRDTRKTLPGLRALQKYAVRTGGGVN
HRLGLGDAALIKDNHVAAAGSVVDALRAVRNAAPDLPCEVEVDSLEQLDAVLPEKPEL
ILLDNFAVWQTQTAVQRRDSRAPTVMLESSGGLSLQTAATYAETGVDYLAVGALTHSV
RVLDIGLDM"

CDS 1786885..1787643
/codon_start=1
/note="gene_1642"

/transl_table=11
/translation="VARTFEDLVAEAASASVGGWDFSWLDGRATEERPSWGYQRQLSQ
RLANATAALDLETGGGEVLAGAGNFPPTMVATEAWPPNAAMATRRLHPMGAVVVITGD
KPPLPFADAAFDLVTSRHPSTRWWTEIARVLRAGGSYFAQHVGPATLWDLREHFLGPR
EHNGADQYAQVVRTCITDAGLEIVDLQMERLRVEFFDVGAVIYFLRKVIWFLPDFTVE
GYHDRLRALHERIQAEGPFVTYSTRALIEARKPS"
CDS complement(1787664..1788074)
/codon_start=1
/note="gene_1643"
/transl_table=11
/translation="MSAKDHPNNAPGVPMVFPLWLERLQVKYINRALKPIARYLPGTA
TIEHRGRKSGKPYQTIVTAYRKDGVLAIALAHGKTDWVKNVLAAGEADVHFARGVVHV
INPRIVPAGSDGQGLPRMARLQLRRIGVFVGDIA"
CDS 1788147..1789490
/codon_start=1
/note="gene_1644"
/transl_table=11
/translation="MVYVTAPPPVLTRIDLRGAELTAAELRAALPRGGADVEAVLPTV
RPIVAAVAERGAEAALDFGASFDGVRPHAIRVPDAALDAALAGLDCDVCEALQVMVER
TRAVHSGQRRTDVTTTLGPGATVTERWVPVERVGLYVPGGNAVYPSSVVMNVVPAQAA
GVDSLVVASPPQAQWDGMPHPTILAAARLLGVDEVWAVGGAQAVALLAYGGTDTDGAA
LTPVDMITGPGNIYVTAAKRLCRSRVGIDAEAGPTEIAILADHTADPVHVAADLISQA
EHDELAASVLVTPSEDLADATDAELAGQLQTTVHRERVTAALTGRQSAIVLVDDVDAA
VLVVNAYAAEHLEIQTADAPQVASRIRSAGAIFVGPWSPVSLGDYCAGSNHVLPTAGC
ARHSSGLSVQTFLRGIHVVEYTEAALKDVSGHVITLATAEDLPAHGEAVRRRFER"
CDS 1789487..1790629
/codon_start=1
/note="gene_1645"
/transl_table=11
/translation="MTRSGHPVTLDDLPLRADLRGKAPYGAPQLAVPVRLNTNENPHP
PTRALVDDVVRSVREAAIDLHRYPDRDAVALRADLAGYLTAQTGIQLGVENIWAANGS
NEILQQLLQAFGGPGRSAIGFVPSYSMHPIISDGTHTEWIEASRANDFGLDVDVAVAA
VVDRKPDVVFIASPNNPSGQSVSLPDLCKLLDVAPGIAIVDEAYGEFSSQPSAVSLVE
EYPSKLVVTRTMSKAFAFAGGRLGYLIATPAVIDAMLLVRLPYHLSSVTQAAARAALR
HSDDTLSSVAALIAERERVTTSLNDMGFRVIPSDANFVLFGEFADAPAAWRRYLEAGI
LIRDVGIPGYLRATTGLAEENDAFLRASARIATDLVPVTRSPVGAP"
CDS 1790626..1791258
/codon_start=1
/gene="hisB"
/note="gene_1646"
/transl_table=11
/translation="MTTTQTAKASRRARIERRTRESDIVIELDLDGTGQVAVDTGVPF
YDHMLTALGSHASFDLTVRATGDVEIEAHHTIEDTAIALGTALGQALGDKRGIRRFGD
AFIPMDETLAHAAVDLSGRPYCVHTGEPDHLQHTTIAGSSVPYHTVINRHVFESLAAN
ARIALHVRVLYGRDPHHITEAQYKAVARALRQAVEPDPRVSGVPSTKGAL"
CDS 1791255..1791875
/codon_start=1
/gene="hisH"
/note="gene_1647"
/transl_table=11
/translation="VTAKSVVVLDYGSGNLRSAQRALQRVGAEVEVTADTDAAMTADG

LVVPGVGAFAACMAGLRKISGERIIAERVAAGRPVLGVCVGMQILFACGVEFGVQTPG
CGHWPGAVIRLEAPVIPHMGWNVVDSAAGSALFKGLDVDARFYFVHSYAAQRWEGSPD
ALLTWATYRAPFLAAVEDGALAATQFHPEKSGDAGAAVLSNWVDGL"
CDS 1791888..1792622
/codon_start=1
/note="gene_1648"
/transl_table=11
/translation="MPLILLPAVDVVEGRAVRLVQGKAGSQTEYGSAVDAALGWQRDG
AEWIHLVDLDAAFGRGSNHELLAEVVGKLDVQVELSGGIRDDESLAAALATGCARVNV
GTAALENPQWCARVIGEHGDQVAVGLDVQIIDGEHRLRGRGWETDGGDLWDVLERLDS
EGCSRFVVTDITKDGTLGGPNLDLLAGVADRTDAPVIASGGVSSLDDLRAIATLTHRG
VEGAIVGKALYARRFTLPQALAAVRD"
CDS 1792630..1793442
/codon_start=1
/note="gene_1649"
/transl_table=11
/translation="MHLDSLVAPLVEQASAILDAATALFLVGHRADSAVRKKGNDFAT
EVDLAIERQVVAALVAATGIEVHGEEFGGPAVDSRWVWVLDPIDGTINHAAGSPLAAI
LLGLLHDGVPVAGLTWMPFTDQRYTAVAGGPLIKNGVPQPPLADAELANVLVGVGTFS
ADSRGQFPGRYRLAVLEKLSRVSSRLRMHGSTGIDLVFVADGILGGAISFGGHVWDHA
AGVALVRAAGGVVTDLAGQPWTPASRSALAGPLRVHAQILEILGSIGEPEDY"
CDS 1793444..1794247
/codon_start=1
/note="gene_1650"
/transl_table=11
/translation="MYADRDLPGAGGLAVRVIPCLDVDDGRVVKGVNFENLRDAGDPV
ELAAVYDAEGADELTFLDVTASSSGRATMLEVVRRTAEQVFIPLTVGGGVRTVADVDS
LLRAGADKVAVNTAAIACLDLLADMARQFGSQCIVLSVDARTVPVGSAPTPSGWEVTT
HGGRRGTGMDAVQWAARGADLGVGEILLNSMDADGTKAGFDLALLRAVRAAVTVPVIA
SGGAGAVEHFAPAVAAGADAVLAASVFHFRELTIGQVKAALAAEGITVR"
CDS 1794244..1794591
/codon_start=1
/gene="hisI"
/note="gene_1651"
/transl_table=11
/translation="MTLDPKIAARLKRNADGLVTAVVQERGSGDVLMVAWMNDEALAR
TLQTREATYYSRSRAEQWVKGATSGHTQHVHSVRLDCDGDAVLLTVDQVGGACHTGDH
SCFDAAVLLEPDD"
CDS 1794772..1795854
/codon_start=1
/note="gene_1652"
/transl_table=11
/translation="MLKRVPWTVVLPSLAFVALVLTWGKQIGPVVGLLAAVLLAGAVL
AAVNHAEVVAARVGEPFGSLVLAVAVTTIEVALIVALMVSGGDDAATLARDTVFAAVM
ITTNGIAGLSLLLGSLRYGVTLFNPHGSGAALATVTTLATLSLVLPTFTTSQSGPELS
PGQLIFAGAASLGLYVLFLFTQTVRHRDFFLPVAQKGAVEDDSHADPPSTRAALLSLG
LLLVALVAVVGLAKVESPVIEEVVSAAGFPQSFVGVVIATLVLLPETLAAARAARQGR
LQTSLNLAYGSAMASIGLTIPTIALASLWLSGPLQLGLGAIQLVLLVLTVVVSVLTVV
PGRATRLQGEVHLVLLAAYLFLAVVP"
CDS complement(1795889..1796353)
/codon_start=1

/note="gene_1653"
/transl_table=11
/translation="MKTGDTVADFELPDQTGTPRRLSVLLSDGPVVLFFYPAAMTPGC
TKEACHFRDLAKEFAEVRASRVGISTDPVRKQAKFAEVRRFDYPLLSDAQGTVAAQFG
VKRGLLGKLMPVKRTTFVIDTDRKVLDVISSEFSMDAHADKALATLRAIRSG"

CDS 1796494..1798044
/codon_start=1
/note="gene_1654"
/transl_table=11
/translation="VHADLAATTSREDFRLLAAEHRVVPVTRKVLADSETPLSAYRKL
AANRPGTFLLESAENGRSWSRWSFIGAGAPTALTVREGQAVWLGAVPKDAPTGGDPLR
ALQVTLELLATADRQSEPGLPPLSGGMVGFFAYDMVRRLERLPERAVDDLCLPDMLLL
LATDVAAVDHHEGTITLIANAVNWNGTDERVDWAYDDAVARLDVMTAALGQPLPSTVA
TFSRPEPRHRAQRTVEEYGAIVEYLVDQIAAGEAFQVVPSQRFEMDTDVDPIDVYRIL
RVTNPSPYMYLLQVPNSDGAVDFSIVGSSPEALVTVHEGWATTHPIAGTRWRGRTDDE
DVLLEKELLADDKERAEHLMLVDLGRNDLGRVCTPGTVRVEDYSHIERYSHVMHLVST
VTGKLGEGRTALDAVTACFPAGTLSGAPKVRAMELIEEVEKTRRGLYGGVVGYLDFAG
NADFAIAIRTALMRNGTAYVQAGGGVVADSNGSYEYNEARNKARAVLNAIAAAETLAA
PGANRSGC"

CDS 1798082..1798741
/codon_start=1
/note="gene_1655"
/transl_table=11
/translation="MIGIAQLLLVVAAGALWMAARLPWVVIGSFDELGPPKEVTLTGA
SWSTALLPLALLMLAAAVAALAVRGWPLRALAVLLAAASFAVGYLGISLWVVPDVAAR
GADLAHVPVVTLVGSARHYWGAVAAVLAAVCALLAAVFLMSSAAIRGSAGEDMARYAA
PRARRSIARRQHSNAAGRAAPQDDGPDMGPRMSERMIWEALDEGRDPTDREQESDTEG
R"

CDS 1798801..1799649
/codon_start=1
/note="gene_1656"
/transl_table=11
/translation="VGDSGKGTAGMSPATVLDSILEGVRADVAAREASVSLSEIKAAA
AAAPPPLDVMAALREPGIGVIAEVKRASPSAGALATIADPAKLAQAYQDGGARIVSVV
TEQRRFQGSLDDLDAVRASVSIPVLRKDFVVQPYQIHEARAHGADMLLLIVAALEQSV
LVSMLDRTESLGMTALVEVHTEQEADRALKAGAKVIGVNARDLMTLDVDRDCFARIAP
GLPSSVIRIAESGVRGTADLLAYAGAGADAVLVGEGLVTSGDPRAAVADLVTAGTHPS
CPKPAR"

CDS 1799718..1800950
/codon_start=1
/note="gene_1657"
/transl_table=11
/translation="MSAAIAEPTSHDPDSGGHFGGPSGWGGRYVPEALMAVIEEVTAA
YQKERVSQDFLDDLDRLQANYAGRPSPLYEATRLSQHAGSARIFLKREDLNHTGSHKI
NNVLGQALLARRMGKTRVIAETGAGQHGVATATACALLGLDCVIYMGGIDTARQALNV
ARMRLLGAEVVAVQTGSKTLKDAINEAFRDWVANADNTYYCFGTAAGPHPFPTMVRDF
QRIIGMEARVQIQGQAGRLPDAVVACVGGGSNAIGIFHAFLDDPGVRLVGFEAAGDGV
ETGRHAATFTAGSPGAFHGSFSYLLQDEDGQTIESHSISAGLDYPGVGPEHAWLKEAG
RVDYRPITDSEAMDAFGLLCRMEGIIPAIESAHAVAGALKLGVELGRGAVIVVNLSGR
GDKDVETAAKWFGLLGND"

CDS 1800950..1801762

```
                     /codon_start=1
                     /gene="trpA"
                     /note="gene_1658"
                     /transl_table=11
                     /translation="MVAVEQSEASRLGPVFDSCRANNRAALIGYLPTGYPDVPASVAA
                     MTALVESGCDIIEVGVPYSDPGMDGPTIARATEAALRGGVRVRDTLAAVEAISIAGGR
                     AVVMTYWNPVLRYGVDAFARDLAAAGGLGLITPDLIPDEAQQWLAASEEHRLDRIFLV
                     APSSTPERLAATVEASRGFVYAASTMGVTGARDAVSQAAPELVGRVKAVSDIPVGVGL
                     GVRSRAQAAQIAQYADGVIVGSALVTALTEGLPRLRALTGELAAGVRLGMSA"
CDS                  1801759..1803168
                     /codon_start=1
                     /gene="lgt"
                     /note="gene_1659"
                     /transl_table=11
                     /translation="MMRMLPSYIPSPPRGVWYLGPLPVRAYAVCVITGIIVALLIGDR
                     RLTARGGERGMTYDIALWAVPFGLIGGRLYHLATDWRTYFGDGGAGLAAALRIWDGGL
                     GIWGAVTLGVMGAWIGCRRCGIPLPVLLDAVAPGVVLAQAIGRLGNYFNQELYGRETT
                     MPWGLEIFYRRDPSGFDVPNSLDGVSTGQVAFVVQPTFLYELIWNVLVFVALIYIDRR
                     FIIGHGRLFGFYVAFYCAGRFCVELLRDDPATLIAGIRINSFTSTFVFIGAVVYIILA
                     PKGREAPGALRGSEYVVDEALEREPAELAAAVASAASAVGPVGPGEPNQPDDVAEAV
                     KAEVAEVTDEVAAESVVQVADRDGESTPAVEETSEADIEREQPGDLAGQAPAAHQVDA
                     EAASAAPEEPAALASEAHDETEPEVPEKAAPIPDPAKPDELAVAGPGDDPAEPDGIRR
                     QDDFSSRRRRWWRLRRRRQ"
CDS                  1803889..1804284
                     /codon_start=1
                     /note="gene_1660"
                     /transl_table=11
                     /translation="MLKGVTDPLQHGAFEPGWQSAPPGYPPPYPQYPGPGSYFDPFAP
                     YGRHPVTGQPFSDKSKTVAGLLQLLGLFGIAGIGRIYLGHTGLGIAQLLVGWVTCGLG
                     AVIWGVIDALLILTDKVGDPWGRPLRDGS"
CDS                  1804274..1804672
                     /codon_start=1
                     /note="gene_1661"
                     /transl_table=11
                     /translation="MEASGRQRRYAAAGSVVLLAGALGYIGLVDPHNSNSLYPPCLFK
                     LLTGWNCPACGGLRMIHDLLHGELAASINDNVFLLVGVPVLASWVLLRRRHGDLALPI
                     PVMIAVAVAVIAWTVLRNLPGFPLVPTISG"
CDS                  1804864..1806198
                     /codon_start=1
                     /gene="pyk3"
                     /note="gene_1662"
                     /transl_table=11
                     /translation="MDVARMNFSHGDYDDHKVAYERVRVASDATGRAVGVLADLQGPK
                     IRLGRFASGATHWAEGETVRITVGACEGSHDRVSTTYKRLAQDAVAGDRVLVDDGKVA
                     LVVDAVEGDDVVCTVVEGGPVSDNKGISLPGMNVTAPALSEKDIEDLTFALNLGVDMV
                     ALSFVRSPADVELVHEVMDRIGRRVPVIAKLEKPEAIDNLEAIVLAFDAVMVARGDLG
                     VELPLEEVPLVQKRAIQMARENAKPVIVATQMLDSMIENSRPTRAEASDVANAVLDGA
                     DALMLSGETSVGKYPLAAVRTMSRIICAVEENSTAAPPLTHIPRTKRGVISYAARDIG
                     ERLDAKALVAFTQSGDTVRRLARLHTPLPLLAFTAWPEVRSQLAMTWGTETFIVPKMQ
                     STDGMIRQVDKSLLELARYKRGDLVVIVAGAPPGTVGSTNLIHVHRIGEDDV"
CDS                  1806224..1807108
```

/codon_start=1
/note="gene_1663"
/transl_table=11
/translation="MSDFDELLAVLDLNAVASDLFTGSHPSKNPLRTFGGQLMAQSFV
ASSRTLTRHHLPPSAFSVHFINGGDTAKDIEFQVIRLRDERRFANRRVDAVQDGTLLS
SAMVSYMAGGRGLEHALDPPQVAEPHTRPPIGELLRGYEETVPHFVNALQPIEWRYAN
DPAWIMRDKGDRLAYNRVWVKALGEMPDDPVLHTATLLYSSDTTVLDSVITTHGLSWG
FDRIFAASANHSVWFHRQVNFDDWVLYSTSSPVAADSRGLGSGHFFDRSGKLIATVVQ
EGVLKYFPATPDSAAGRS"
CDS 1807166..1808620
/codon_start=1
/note="gene_1664"
/transl_table=11
/translation="MVAAAGEPLNCQRANPEVTVKLPSADVVPRLRGRQRVVVHVDSR
TARCVGALALVCAACWLIALLAGDYRHAQWAVAGRLGWSLTVLAAVAFIARGIFLGRP
VTAMHATAAGLFLLAGLAAHVLVADLLGEILIAGSGWALMWPTSAHPRPEDLPRVWAL
INATRADSLAPFAMQAGKSHHFSAAGTAALAYRTRIGYAVVSGDPIGDEAQFPQLVAD
FAAMCHMHGWRIVVVGCSERRLGLWSDPMVVGQSLRPIPIGRDVVIDVSNFEMTGRRF
RNLRQAVKRTHNFGVTTEIVAEQQLDDQRQAELAEVLAASPSGARTDRGFCMNLDGVL
EGRYPGIQLIIARDASGRVQGFHRYATAGGGSDMSLDVPWRRRGAPNGIDERLSADMI
AAAKDAGVQRLSLAFAAFPDLFGANQLGRLQRVCRALIHILDPLIALESLYRYLRKFH
ALDERRYVLISMTQVFALALVLLSLEFVPRRRHL"
CDS complement(1808554..1810284)
/codon_start=1
/note="gene_1665"
/transl_table=11
/translation="MNRPSAVSRRQRDLLAASGLLGPRLPRILAAVALGVLSLGSALA
LAGVSAWLITRAWQMPPVLDLSVAVVAVRAFAISRGVLHYCERLATHDTALRAAGRAR
TLIYHRLAHGPAAAAVGLHSGDLAARVGADVDELANMLVRALVPIAVAAVLAVAATAV
VAAVSVPAAVVLAVCLLVAGVVAPWLAGRTAAAQEAIARQHRGMRDTSAMIALEHAPE
LRVAGALRNVIADSQRRQHAWADALDAAARTGAIAEAMPTAAIGASLLGAVVAGIGMA
PTVAPTTLAILMLLPLSAFEATVALPAAAVQLTRSRIAAARLLDLTGSNRVRETESTV
SARLPVGTGVLAADVCCGHQEAQSIRVTIDLPPGARLAVTGASGAGKTTLLMTLAGLL
PPVHGRVLLDGTNLSDFDEDELRSAVSFFAEDAHIFATTVRDNLLTARGDCPDDELIE
ALDRVGLCGWLAGLPEGLSTVLIGGAQAVSAGQRRRLLLARAVLSPARIVLLDEPVEH
LDAANADLLRDLLAPNSGIMSAMRTVVVATHHLPNDIQCAELSIATDQRCRRRGTNSS
DNNTNASAKT"
CDS complement(1810281..1811864)
/codon_start=1
/note="gene_1666"
/transl_table=11
/translation="VACGVGISGCAIGSAIVLASIVAGVIDPANPGMAGLRRWLGPLS
ILLVLWGLRASIQWLQARLAQRGASAVIADLSGQVLTAVTARRPSQLAAQRDAAAVLI
TRGLDGLRPYFTGYLPTLLLAAILTPATVAVIGLYDLKSMAIVVITLPLIPIFMVLIG
LATTNPSAAALAAMTAVQARLLDLIAGIPTLRALGRASGPEQRIAELSADHRRSAMAT
LRIAFLSALVLELLATLGVALVAVGIGLRLVFGEMSLTAGLTVLLLAPEVYWPLRRVG
VQFHAAADGRTAADKAFALLGESPSPTPGRRTVTARGGVIRLERLSVRGRDGRAPYDL
TADIEPGRVTVLTGRNGAGKSTTLQAIAGLTAPSSGRITVAGVDVTNLAPAAWWRQLS
WLPQRPVLVPGTVRHNLVLLGPVDDLERACAAAGFDAVLDELPRGLDTVLGRGGVGLS
LGQRQRLGLARALGSPAAVLLLDEPTAHLDARTEQHVLGAIVERARAGATVLVVAHRQ
QVAAAGDRVVEVNSDGFRR"

```
CDS             complement(1811951..1812991)
                /codon_start=1
                /note="gene_1667"
                /transl_table=11
                /translation="VVLQELWFGVIAALFLGFFILEGFDFGVGMLMAPFAHVGMGDPE
                THRRTALNTIGPVWDGNEVWLITAGAAIFAAFPGWYATVFSALYLPLLAILFGMILRA
                VAIEWRGKIDDPKWRTGADFGIAAGSWLPALLWGVAFAILVRGLPVDANGHVALSIPD
                VLNAYTLLGGLATAGLFSLYGAVFIALKTSGPIRDDAYRFAVWLSLPVAGLVAGFGLW
                TQLAYGKDWTWLVLAVAGCAQAAATVLVWRRVSDGWAFMCTLIVVAAVVVLLFGALYP
                NLVPSTLNPQWSLTIHNASSTPYTLKIMTWVTAFFAPLTVAYQTWTYWVFRQRISAER
                IPPPTGLARRAP"
CDS             complement(1813021..1814478)
                /codon_start=1
                /note="gene_1668"
                /transl_table=11
                /translation="MNVVDISRWQFGITTVYHFIFVPLTIGLAPLIAVMQTLWVVTDN
                PAWYRLTKFFGKLFLINFAIGVATGIVQEFQFGMNWSEYSRFVGDVFGAPLAMEGLAA
                FFFESTFIGLWIFGWNRLPRLVHLACIWIVAIAVNVSAFFIIAANSFMQHPVGAHYNP
                TTGRAELSSIVVLLTNNTAQAAFTHTVSGALLTAGTFVAAVSAWWLVRSSTTHADSDT
                QAMYRPATILGCWVALAATAGLLFTGDHQGKLMFQQQPMKMASAESLCDTQTDPNFSV
                LTVGRQNNCDSLTRVIEVPYVLPFLAEGRISGVTLQGIRDLQQEYQQRFGPNDYRPNL
                FVTYWSFRMMIGLMAIPVLFALIALWLTRGGQIPNQRWFSWLALLTMPAPFLANSAGW
                VFTEMGRQPWVVVPNPTGDQLVRLTVKAGVSDHSATVVATSLLMFTLVYAVLAVIWCW
                LLKRYIVEGPLEHDAEPAAHGAPRDDEVAPLSFAY"
CDS             complement(1814589..1815122)
                /codon_start=1
                /note="gene_1669"
                /transl_table=11
                /translation="LLPHLWKSTLASGILSLILGVLVLAWPGISILVAAMAFGVYLLI
                TGVAQVAFAFSLHVSAGGRILLFISGAASLILAVLAFRHFGDAVLLLAIWIGIGFIFR
                GVATTVSAISDPMLPGRGWSIFVGVISLIAGIVVMASPFESIWILALVVGIWLVVIGA
                CEIASSFAIRKASQTLG"
CDS             complement(1815205..1816461)
                /codon_start=1
                /note="gene_1670"
                /transl_table=11
                /translation="VRTQARAPTQHYAESVARRQRVLTITAWLAVVVTGSFALMQLAT
                GAGGWYIALINVFTAVTFAIVPLLHRFGGLVAPLTFIGTAYVAIFAIGWDVGTDAGAQ
                FFFLVAAALVVLLVGIEHTALAVGLAAVAAGLVIALEFLVPPDTGLQPPWAMSVSFVL
                TTVSACGVAVATVWFALRDTARAEAVMEAEHDRSEALLANMLPASIAERLKEPERNII
                ADKYDEASVLFADIVGFTERASSTAPADLVRFLDRLYSAFDELVDQHGLEKIKVSGDS
                YMVVSGVPRPRPDHTQALADFALDMTNVAAQLKDPRGNPVPLRVGLATGPVVAGVVGS
                RRFFYDVWGDAVNVASRMESTDSVGQIQVPDEVYERLKDDFVLRERGHINVKGKGVMR
                TWYLIGRKVAADPGEVRGAEPRTAGV"
tRNA            complement(1816603..1816679)
                /note="identified by tRNAscan-SE; putative"
CDS             complement(1816693..1816941)
                /codon_start=1
                /gene="ADRA2C"
                /note="gene_1671"
                /transl_table=11
```

/translation="VQLGQLDGFLAVAGLADNFISLLSQHLGQVHADERFIFRDQDPT
WDSGIGVGGGAGHDGHCVVSGRDREPRGAVSRDVAPLE"
CDS 1816849..1817388
/codon_start=1
/note="gene_1672"
/transl_table=11
/translation="MDLAEMLREEGYEIVGEAGDGQEAVELAELHKPDLVIMDVKMPR
RDGIDAASEIASKRIAPIVVLTAFSQRDLVERARDAGAMAYLVKPFSISDLIPAIELA
VSRFREITALEGEVATLSERLETRKLVERAKGLLQTKHGMTEPDAFKWIQRAAMDRRT
TMKRVAEVVLETLGTPKDT"
CDS complement(1817456..1818658)
/codon_start=1
/note="gene_1673"
/transl_table=11
/translation="MSAPEPVYILGAGMHPWGKWGNDFTEYGVVAARAALRDAGVDWR
HVQLVAGADTIRNGYPGFVAGATFAQKLGWTGVPVSSSYAACASGSQALQSARAQILA
GFCDVALVIGADTTPKGFFAPVGGERKGDPDWQRFHLIGATNTVYFALLARRRMDLYG
ATVEDFAQVKVKNSRHGLDNPNARYRKENSIDDVLASPVVSDPLRLLDICATSDGAAA
LIVASKSFTEKHLGSVAGVPSVRAISTVTPKYPQHLPELPDIATDSTAAVPAPERVFK
DQILDAAYAEAGIGPEDLSLAEVYDLSTALELDWYEHLGLCPKGEAEALLRSGATTLG
GRVPVNPSGGLACFGEAIPAQAIAQVCELTWQLRGQATGRQVADAKVGVTANQGLFGH
GSSVIVAR"
CDS complement(1818661..1819152)
/codon_start=1
/note="gene_1674"
/transl_table=11
/translation="VPEVTREEPAIDGWFTTDKAGNPHLLGGKCPQCGTYVFPPRADN
CPNPACGSDTLESVGLSTRGKLWSYTENRYAPPPPYPAPDPFEPFAVAVVELADEGLI
VLGKVVDGTLAADLKVGMEMELTTMPLFADDDGVQRIVYAWRIPSRAGDDAERSDAEE
RRR"
CDS 1819310..1821970
/codon_start=1
/note="gene_1675"
/transl_table=11
/translation="LLDGNSLAFRAFYALPAENFKTRGGLTTNAVYGFTAMLINLLRD
EAPTHIAAAFDVSRQTFRLQRYPEYKANRSSTPDEFAGQIDITKEVLGALGITVLSEP
GFEADDLIATLATQAENEGYRVLVVTGDRDALQLVSDDVTVLYPRKGVSELTRFTPEA
VVEKYGLTPRQYPDFAALRGDPSDNLPGIPGVGEKTAAKWIAEYGSLRSLVDNVDAVR
GKVGDALRANLASVVRNRELTDLVRDVPLAQTPDTLRLQPWDRDHIHRLFDDLEFRVL
RDRLFDTLAAAGGPEVDEGFDVRGGALAPGTVRQWLAEHAGDGRRAGLTVVGTHLPHG
GDATAMAVAAADGEGAYLDTATLTPDDDAALAAWLADPAKPKALHEAKAAVHDLAGRG
WTLEGVTSDTALAAYLVRPGQRSFTLDDLSLRYLRRELRAETPQQQQLSLLDDDDTDA
ETIQTTILRARAVIDLADALDAELARIDSTALLGEMELPVQRVLAKMESAGIAVDLPM
LTELQSQFGDQIRDAAEAAYGVIGKQINLGSPKQLQVVLFDELGMPKTKRTKTGYTTD
ADALQSLFDKTGHPFLQHLLAHRDVTRLKVTVDGLLQAVAADGRIHTTFNQTIAATGR
LSSTEPNLQNIPIRTDAGRRIRDAFVVGDGYAELMTADYSQIEMRIMAHLSGDEGLIE
AFNTGEDLHSFVASRAFGVPIDEVTGELRRRVKAMSYGLAYGLSAYGLSQQLKISTEE
ANEQMDAYFARFGGVRDYLRAVVERARKDGYTSTVLGRRRYLPELDSSNRQVREAAER
AALNAPIQGSAADIIKVAMIQVDKALNEAQLASRMLLQVHDELLFEIAPGERERVEAL
VRDKMGGAYPLDVPLEVSVGYGRSWDAAAH"
CDS 1822133..1823578

```
                     /codon_start=1
                     /gene="rpsA"
                     /note="gene_1676"
                     /transl_table=11
                     /translation="MPSPTVTSPQVAVNDIGSSEDFLAAIDKTIKYFNDGDIVEGTIV
                     KVDRDEVLLDIGYKTEGVIPARELSIKHDVDPNEVVSVGDEVEALVLTKEDKEGRLIL
                     SKKRAQYERAWGTIEALKEKDEAVKGTVIEVVKGGLILDIGLRGFLPASLVEMRRVRD
                     LQPYIGKEIEAKIIELDKNRNNVVLSRRAWLEQTQSEVRSEFLNNLQKGTIRKGVVSS
                     IVNFGAFVDLGGVDGLVHVSELSWKHIDHPSEVVQVGDEVTVEVLDVDMDRERVSLSL
                     KATQEDPWRHFARTHAIGQIVPGKVTKLVPFGAFVRVEEGIEGLVHISELAERHVEVP
                     DQVVAVGDDAMVKVIDIDLERRRISLSLKQANEDYTEEFDPAKYGMADSYDEQGNYIF
                     PEGFDAETNEWLEGFEKQRAEWEARYAEAERRHKMHTAQMEKFAAAETAGRGADDQSS
                     ASSAPSEKTAGGSLASDAQLAALREKLAGSA"
     CDS             1823604..1824827
                     /codon_start=1
                     /note="gene_1677"
                     /transl_table=11
                     /translation="MLRIGLTGGIGAGKSLLSTTFSQCGGIVVDGDVLAREVVQPGTE
                     GLASLVDAFGRDILLADGALDRQALAAKAFRDDESRGVLNGIVHPLVARRRSEIIAAV
                     SGDAVVVEDIPLLVESGMAPLFPLVVVVHADVELRVRRLVEQRGMAEADARARIAAQA
                     SDQQRRAVADVWLDNSGSPEDLVRRARDVWNTRVQPFAHNLAQRQIARAPARLVPADP
                     SWPDQARRIVNRLKIACGHKALRVDHIGSTAVSGFPDFLAKDVIDIQVTVESLDVADE
                     LAEPLLAAGYPRLEHITQDTEKTDARSTVGRYDHTDSAALWHKRVHASADPGRPTNVH
                     LRVHGWPNQQFALLFVDWLAANPGAREDYLTVKCDADRRADGELARYVTAKEPWFLDA
                     YQRAWEWADAVHWRP"
     CDS             complement(1824978..1825421)
                     /codon_start=1
                     /note="gene_1678"
                     /transl_table=11
                     /translation="VRAVDEYTVHPWGLYLARPTPGRAQFHYLESWLLPSLGLRATVF
                     HFNPSHKRDHDYYLDVGEYTPGPSVWRSEDHYLDIEVRTGGGAELADVDELLDAVRHG
                     LLTPTVAEQAVRHAVDAVEGLARNGYDLTRWLATKGMELTWRSGS"
     CDS             1825486..1827762
                     /codon_start=1
                     /note="gene_1679"
                     /transl_table=11
                     /translation="MAHPIWSHEACRYIDTPVLVAFCARSPHGVSAGGSRLVGVAFAT
                     EHPVVAHSEYRAVEEIVRAGGHFEVVSPHAPAGDQPAAIDELERRINAGERDVVLLGA
                     TGTGKSATTAWLIERLQRPTLVMAPNKTLAAQLANELREMLPHNAVEYFVSYYDYYQP
                     EAYIAQTDTYIEKDSSINDDVERLRHSATSALLSRRDVVVVASVSCIYGLGTPQSYLD
                     RSVELKVGEEVPRDGLLRLLVDVQYTRNDMSFTRGSFRVRGDTVEIIPSYEELAVRIE
                     FFGDEIEALYYLHPLTGEVIRQVDSLRIFPATHYVAGPERMAHAVSAIEEELAERLAE
                     LESQGKLLEAQRLRMRTNYDIEMMRQVGFCSGIENYSRHIDGRGPGTPPATLLDYFPE
                     DFLLVIDESHVTVPQIGGMYEGDISRKRNLVEYGFRLPSACDNRPLTWEEFADRIGQT
                     VYLSATPGPYELSQSGGEFVEQVIRPTGLVDPKVVVKPTKGQIDDLIGEIRTRADADQ
                     RVLVTTLTKKMAEDLTDYLLEMGIRVRYLHSEVDTLRRVELLRQLRLGDYDVLVGINL
                     LREGLDLPEVSLVAILDADKEGFLRSSRSLIQTIGRAARNVSGEVHMYADKITDSMRE
                     AIDETERRRAKQIAYNEANGIDPQPLRKKIADILDQVYREADDTAVVEVGGSGRNASR
                     GRRAQGEPGRAVSAGVFEGRDTSAMPRAELADLIKDLTAQMMAAARDLQFELAARFRD
                     EIADLKRELRGMDAAGLK"
     CDS             1827759..1829174
```

```
                     /codon_start=1
                     /note="gene_1680"
                     /transl_table=11
                     /translation="VTETASETGSWRQLLSRYLGTSIVLAGGVALYATNEFLTISLLP
                     STIADIGGSRLYAWVTTLYLVGSVVAATTVNTMLLRVGARSSYLMGLAVFGLASLVCA
                     AAPSMQILVAGRTLQGIAGGLLAGLGYALINSTLPKSLWTRGSALVSAMWGVATLIGP
                     ATGGLFAQLGLWRWAFGVMTLLTALMAMLVPVALGAGRVGPGGETPVGSTHKVPVWSL
                     LLMGAAALAISVAALPNYLVQTAGLLAAAALLVAVFVVVDWRIHAAVLPPSVFGSGPL
                     KWIYLTMSVQMIAAMVDTYVPLFGQRLGHLTPVAAGFLGAALAVGWTVGEVASASLNS
                     ARVIGHVVAAAPLVMASGLALGAVTQRADAPVGIIALWALALLIIGTGIGIAWPHLTV
                     RAMDSVADPAESSAAAAAINVVQLISGAFGAGLAGVVVNTAKGGEVAAARGLYMAFTV
                     LAAAGVIASYQATHRDRRLPR"
CDS             complement(1829163..1830731)
                     /codon_start=1
                     /note="gene_1681"
                     /transl_table=11
                     /translation="MSASTLEQPAAAHVDELVARMRGRLLDPLAIAVLAAVISGAWAS
                     RPSLWFDEGATISASASRTLPELWSLLGHIDAVHGLYYLLMHGWFAIFPPTELWSRLP
                     SCLAIGAAAAGVVVFAKQFSGRTTAVCAGAVFAILPRVTWAGIEARSSALSVAAAVWL
                     TVLLVAAVRCNTQRRWLLYALVLMLSILVSINLALLVPAYATMVPLLASGKSRKSPVI
                     WWTVVTAAALGAMTPFILFAHGQVWQVGWIAGLNRNIILDVIHRQYFDHSVPFAILAG
                     LIVAAGIAAHLAGARGPGGDTHRLVLVSAAWIVVPTAVVLIYSATVEPIYYPRYLILT
                     APAAAVILAVCVVTIARKPWLIAGVVFLLAAAAFPNYFFTQRGPYAKEGWDYSQVADV
                     ISAHAKPGDCLLVDNTAGWRPGPIRALLATRPAAFRSLIDVERGTYGPKVGTLWDGHV
                     AVWLTTAKIDKCPTLWTIANRDKSLPDHQVGEMLSPGTGFGRTPVYRFPSYLGFRIVE
                     RWQFHYSQVVKSTR"
CDS             1831042..1831482
                     /codon_start=1
                     /note="gene_1682"
                     /transl_table=11
                     /translation="MSAYKTVVVGTDGSDSSMRAVDRAAQIAGADAKLIIASAYLPQH
                     EDARAADILKDESYKVTGTAPIYEILHDAKERAHNAGAKNVEERPIVGAPVDALVNLA
                     DEEKADLLVVGNVGLSTIAGRLLGSVPANVSRRAKVDVLIVHTT"
CDS             complement(1831489..1832283)
                     /codon_start=1
                     /note="gene_1683"
                     /transl_table=11
                     /translation="MLCARTDNHQGTGNVVTSAHMTRANDDDAGAAGIGAVAHMTTVD
                     DNYTGHVERGKAARRFLPGATILKASVGPMDNNAYLVTCSATGETLLIDAANDAEVLI
                     DLVRRYAPKLALIVTSHQHFDHWQALQAVAAATGAPTAAHPIDADPLPVKPDRLLTHG
                     DSVRIGELTFDVIHLRGHTPGSIALALGGPVTGGVTQLFTGDCLFPGGVGKTWQPADF
                     TQLLDDVTTRVFDVYADSTVIYPGHGDDTELGAERPSLSEWRARGW"
CDS             1832296..1835250
                     /codon_start=1
                     /note="gene_1684"
                     /transl_table=11
                     /translation="LAGEMSFSERDSVADRLIVKGAREHNLRSVDLDLPRDALIVFTG
                     LSGSGKSSLAFDTIFAEGQRRYVESLSAYARQFLGQMDKPDVDFIEGLSPAVSIDQKS
                     TNRNPRSTVGTITEVYDYLRLLYARAGTPHCPTCGERVARQTPQQIVDQVLAMPEGTR
                     FLVLAPVVRTRKGEFADLFDKLNAQGYSRVRVDGVVHPLTDPPKLKKQEKHDIEVVVD
                     RLTVKAAAKRRLTDSVETALNLADGIVVLEFVDHELGAPHREQRFSEKLACPNGHALA
```

VDDLEPRSFSFNSPYGACPECSGLGIRKEVDPELVVPDPDRTLAQGAVAPWSNGHTAE
YFTRMMAGLGEALGFDVDTPWRKLPAKARKAILEGADEQVHVRYRNRYGRTRSYYADF
EGVLAFLQRKMSQTESEQMKERYEGFMRDVPCPVCAGTRLKPEILAVTLAGESKGEHG
AKSIAEVCELSIADCADFLNALTLGPREQAIAGQVLKEIRSRLGFLLDVGLEYLSLSR
AAATLSGGEAQRIRLATQIGSGLVGVLYVLDEPSIGLHQRDNRRLIETLTRLRDLGNT
LIVVEHDEDTIEHADWIVDIGPGAGEHGGRIVHSGPYDELLRNKDSITGAYLSGRESI
EIPAIRRSVDPRRQLTVVGAREHNLRGIDVSFPLGVLTSVTGVSGSGKSTLVNDILAA
VLANRLNGARQVPGRHTRVTGLDYLDKLVRVDQSPIGRTPRSNPATYTGVFDKIRTLF
AATTEAKVRGYQPGRFSFNVKGGRCEACTGDGTIKIEMNFLPDVYVPCEVCQGARYNR
ETLEVHYKGKTVSEVLDMSIEEAAEFFEPIAGVHRYLRTLVDVGLGYVRLGQPAPTLS
GGEAQRVKLASELQKRSTGRTVYILDEPTTGLHFDDIRKLLNVINGLVDKGNTVIVIE
HNLDVIKTSDWIIDLGPEGGAGGGTVVAQGTPEDVAAVPASYTGKFLAEVVGGGASAA
TSRSNRRRNVSA"

CDS complement(1835307..1835564)
/codon_start=1
/note="gene_1685"
/transl_table=11
/translation="MPDEPTPPEATTPNSESDPRYDSAGVPTFESVREKIETRYGTAL
GATELDAESPQGRRLEDQYAQRQRAAAERLAQIRESMHTDE"

CDS complement(1835580..1836971)
/codon_start=1
/note="gene_1686"
/transl_table=11
/translation="MHGWVPLAVQVVTAVVLVLAAGWRSRHWQRRWLPTAAAIGATLA
WGTRWYVTGNGLANERPPSTLWIWVALTGAAATVLILGWRSARWWRRGASLLAVPLCL
LSATLTLNLWVGYFPTVQTAWNQLTSGPLPDQADQAAVAALAHSGVRPSHGTLLPVVI
PSDASHFKHRGELVYLPPAWFDREHRSENPPPPQLPTVMMIGGQFNTPADWARAGNAV
KTLDDFAAAHSGNAPVVVFVDSGGAFNNDTECVNGRRGNAADHLTKDVVPYMVSKFGV
SPEQTSWGIVGWSMGGTCAVDLTVMHPTLFSAFVDIAGDFYPNAGNKTQTIVRLFGGN
EDAWSAFDPTTVITRHGSYTGLSGWFAISSPGPPSPDNAVADTTTMRLAGRDAAANPG
NQAAAANALCALGRANGIYCAVVPQPGKHDWPFADRVFAAALPWLAGQLATPGVPKIP
LPGTTQQIAGTGR"

CDS complement(1837108..1840443)
/codon_start=1
/note="gene_1687"
/transl_table=11
/translation="VTLTKPRSATRGNSRYHWVPAAAGWTVGVIATLSLLASVSPLIR
WIIKVPREFINDYLFNFPDTNFAWSFVLALLAAALTARKRIAWLVLLANMVLAAVVNA
AEIAAGGNTAAESFGENLGFAVHVVAIVVLVLGYREFWAKVRRGALFRAAAVWLAGAV
VGIVASWGLVELFPGSLAPDERLGYAANRVVGFALADPDLFTGRPHVFLNAIFGLFGA
FALIGAAIVLFLSQRADNALTGEDESAIRGLLDLYGKDDSLGYFATRRDKSVVFASSG
RACITYRVEVGVCLASGDPVGDHRAWPQAVDAWLRLCQTYGWAPGVMGASSQGAQTYR
EAGLTALELGDEAILRPADFKLSGPEMRGVRQAVTRARRAGLTVRIRRHRDIAEDEMA
QTITRADSWRDTETERGFSMALGRLGDPADSDCLLVEAIDPHNQVLAMLSLVPWGTTG
VSLDLMRRSPQSPNGTIELMVSELALHAESLGITRISLNFAVFRAAFEQGAQLGAGPV
ARLWRGLLVFFSRWWQLETLYRSNMKYQPEWVPRYACYEDARVIPRVGVASVIAEGFL
VLPFSRRNRVHTGHHPAVPERLAATGLLHHDGSAPDVSGLRQVGLTNGDGVERRLPEQ
VRVRFDKLEKLRSSGIDAFPVGRPPSHTVAQALAADHQASVSVSGRIMRIRNYGGVLF
AQLRDWSGEMQVLLDNSRLDQGCAAEFNAATDLGDLVEMTGHMGASKTGTPSLIVSGW
RLIGKCLRPLPNKWKGLLDPEARVRTRYLDLAVNAESRALITARSSVLRAVRETLFAK
GFVEVETPILQQLHGGATARPFVTHINTYSMDLFLRIAPELYLKRLCVGGVERVFELG

RAFRNEGVDFSHNPEFTLLEAYQAHAGYLEWIDGCRELIQNAAQAANGAPIAMRPRTD
KGSDGTRHHLEPVDISGIWPVRTVHDAISEALGERIDADTGLTTLRKLCDAAGVPYRT
QWDAGAVVLELYEHLVECRTEQPTFYIDFPTSVSPLTRPHRSKRGVAERWDLVAWGIE
LGTAYSELTDPVEQRRRLQEQSLLAAGGDPEAMELDEDFLQAMEYAMPPTGGLGMGID
RVVMLITGRSIRETLPFPLAKPH"

CDS 1840947..1841468
/codon_start=1
/note="gene_1688"
/transl_table=11
/translation="VRIEDSLRVAADADLDLVEVAPNARPPVCKIMDYGKYKYEAAQK
ARESRRNQQQTVVKEQKLRPKIDDHDYETKKGHVVRFLEAGSKVKVTIMFRGREQSRP
ELGYRLLQRLGADVADYGFIETSAKQDGRNMTMVLAPHRGAKTRARARHPGEPAGGPP
PKPTAGDSKAAPN"

CDS 1841518..1841712
/codon_start=1
/gene="rpmI"
/note="gene_1689"
/transl_table=11
/translation="MPKAKTHSGASKRFRRTGTGKIVRQKANRRHLLEHKPSTRTRRL
DGRTVVAANDTKRVTSLLNG"

CDS 1841774..1842163
/codon_start=1
/gene="rplT"
/note="gene_1690"
/transl_table=11
/translation="MARVKRAVNAHKKRRSILKASRGYRGQRSRLYRKAKEQQLHSLN
YAYRDRRARKGEFRKLWIARINAAARLNDITYNRLIQGLKAAGVEVDRKNLADIAISD
PAAFTALVDVARAALPEDVNAPSGEAA"

CDS 1842196..1842978
/codon_start=1
/note="gene_1691"
/transl_table=11
/translation="VLTERSARVATAVKLHRHVGRRRAGRFLAEGPNLVAAALARGLV
REVFVTEVAARRHELLLAAHEASVHLVTERAAKALSDTVTPAGLVAVCDLPATRLEDV
LAGSPQLIAVTVEIREPGNAGTVIRIADAMGAAAVILAGRSVDPYNGKCLRASTGSIF
AIPVVVAPDVGAAIADLRAAGLQVLATAVDGEMALDDADRLLAEPTAWLFGPEAHGLS
AEIAALADHRVHIPMSGGAESLNVAAAAAICLYESARALGRR"

CDS complement(1842989..1844044)
/codon_start=1
/note="gene_1692"
/transl_table=11
/translation="MTVASRTSADPLGPDSLTWKYFGDLRTGMMGVWIGAIQNMYPEL
GAGVEEHSILLREPLQRVARSVYPIMGVVYDGDRAAQTGQQIKGYHRTIKGVDAEGRR
YHALNPDTFYWAHATFFMLVIKVAEYFCGGLTEAEKHQLFEEHVRWYRMYGMSMRPVP
KSWEDFQDYWDRVCRDKLEINQATVDILQMRIPKPRFVLMPTPIWDQLFKPLIAGQRW
IAAGLFDPAVREKAGMHWTPGDEVLLRVFGKVVELAFLAVPDEIRLHPRALAAYRRAA
GRTRHDAPLVQAPGFMAPPRDRQGLPMHYFPPRSHRFTRSALDPAKALMERAGALVHS
TLSLAGVRPARGPSRAA"

CDS 1844354..1845286
/codon_start=1
/note="gene_1693"

```
                     /transl_table=11
                     /translation="MSFLTVAPDMVTAAAGNLESVGSALNEAAAAAAPATVGLAAPAA
                     DRVSAVVAAMLGAYARDFQGISAQIAGFHNQFVGALRGGAAAYASAEAANVQQTVVNA
                     VNAPAQALLGHPLIGPETVGSSAAAVSFGFGPLLLAGSDPLLAVPFSYPASLPTPFGP
                     VTMTLNGSFDPLTQQVVFDSGSLTAPAPFVYGLGAVGPALTTMTALQNSGTAFSGAVQ
                     SGNLLGAAGALLQAPGNAVTGFLFGQTAISQSIPGPSNLGYESVGISVPVGGLLAPLQ
                     PVTVTLTPTSGMPTAIQLSGTQFGGLLPALLNGF"
     CDS             1845364..1846314
                     /codon_start=1
                     /note="gene_1694"
                     /transl_table=11
                     /translation="LPGSARTTYPCHVEVGPQDSESGAPDETATAMASPVPRQRSALR
                     WLRTVNRSPGLVSFIHRARRLLPGDPEFGDPLSTAGEGGPRAAARAADRLLRDRDAAS
                     REVGLSVLQVWQALTEAVSRRPANPEVTLVFTDLVGFSTWSLHAGDDATLTLLRQVAR
                     AVESPLLDAGGHIVKRLGDGIMAVFRNPTVALRAVLVAQDAVKSLEVQGYTPRMRIGI
                     HTGRPQRLAADWLGVDVNIAARVMERATKGGIMISQPTLDLIPQSELDALGVVARRVR
                     KPVFASKPTGIPPDLAIYRIKTVSESTAADNFDEMSPDAQ"
     CDS             1846321..1847127
                     /codon_start=1
                     /note="gene_1695"
                     /transl_table=11
                     /translation="MIYRVACLLARIRFTVGYVAALASVSTTILMHGPQVHAQVIRHA
                     STNLHNLAHGHLGTLWNSAFVIDEGPLYFWLPCLACLLAVAELQLRSLRLTVAFVVGH
                     IGATLLVAAVLAGAIEIGWLPWSISRVSDVGMSYGALAALGALTAAIPGRWRPAWIGW
                     WVSLGLATATIGGGFTDAGHTVALLLGMLVTACFTRPARWTLGRCALLAVASGFCLVL
                     LAHSWWSLVSGSALGLLGALGAAGFARWTRARATSLPPGALAIPQPALSR"
     CDS             1847290..1848348
                     /codon_start=1
                     /note="gene_1696"
                     /transl_table=11
                     /translation="VGDPPLESIVSMLSPEALTTAVDAAQQAIALADTLDVLARVKTE
                     HLGDRSPLALARQALAVLPKEQRAEAGKRVNAARNAAQRSYDERLATLRAERDAAVLV
                     AEGIDVTLPSTRVPAGARHPIIMLAEHVADTFIAMGWELAEGPEVETEQFNFDALNFP
                     ADHPARGEQDTFYIAPEDSRQLLRTHTSPVQIRTLLARELPVYIISIGRTFRTDELDA
                     THTPIFHQVEGLAVDRGLSMAHLRGTLDAFARAEFGPSARTRIRPHFFPFTEPSAEVD
                     VWFANKIGGADWVEWGGCGMVHPNVLRATGIDPDLYSGFAFGMGLERTLQFRNGIPDM
                     RDMVEGDVRFSLPFGVGA"
     CDS             1848348..1850843
                     /codon_start=1
                     /gene="pheT"
                     /note="gene_1697"
                     /transl_table=11
                     /translation="MRLPYSWLREVVAVGASGWDVTPGELEQTLLRIGHEVEEVIPLG
                     PVDGPVTVGRVADIEELTGYKKPIRACAVDIGDRQYREIICGATNFAVGDLVVVALPG
                     ATLPGGFTISARKAYGRNSDGMICSAAELNLGADHSGILVLPPGAAEPGADGAGVLGL
                     DDVVFHLAITPDRGYCMSVRGLARELACAYDLDFVDPASNSRVPPLPIEGPVWPLTVQ
                     PETGVRRFALRPVIGIDPAAVSPWWLQRRLLLCGIRATCPAVDVTNYVMLELGHPMHA
                     HDRNRISGTLGVRFARSGETAVTLDGIERKLDTADVLIVDDAATAAIGGVMGAASTEV
                     RADSTDVLLEAAIWDPAAVSRTQRRLHLPSEAARRYERTVDPAISVAALDRCARLLAD
                     IAGGEVSPTLTDWRGDPPCDDWSPPPIRMGVDVPDRIAGVAYPQGTTARRLAQIGAVV
                     THDGDTLTVTPPSWRPDLRQPADLVEEVLRLEGLEVIPSVLPPAPAGRGLTAGQQRRR
```

TIGRSLALSGYVEILPTPFLPAGVFDLWGLEADDSRRMTTRVLNPLEADRPQLATTLL
PALLEALVRNVSRGLVDVALFAIAQVVQPTEQTRGVGLIPVDRRPTDDEIAMLDASLP
RQPQHVAAVLAGLREPRGPWGPGRPVEAADAFEAVRIIARASRVDVTLRPAQYLPWHP
GRCAQVFVGESSVGHAGQLHPAVIERSGLPKGTCAVELNLDAIPCSAPLPAPRVSPYP
AVFQDVSLVVAADIPAQAVADAVRAGAGDLLEDIALFDVFTGPQIGEHRKSLTFALRF
RAPDRTLTEDDASAARDAAVQSAAERVGAVLRG"

CDS complement(1850937..1853993)
/codon_start=1
/note="gene_1698"
/transl_table=11
/translation="MSFLLVEPDLVTAAAANLAGIRSALSEAAAAASTPTTALASAGA
DEVSAAVSRLFGAYGQQFQALNARAATFHAEFVSLLNGGAAAYTGAEAASVSSMQALL
DAVNAPTQTLLGRPLIGNGADGVAGTGSNAGGNGGPGGILYGNGGNGGAGGPDGGAGG
NGGAAGLIGNGGAGGAGGVGGAGGAGGAGGTGGLLYGNGGAGGNGGSAAAAGGAGGNA
LLFGNGGNGGSGASGGAAGHAGTIFGNGGNAGAGSGLAGADGGLFGNGGDGGSSTSKA
GGAGGNALFGNGGDGGSSTVAAGGAGGNTLVGNGGAGGAGGTSGLTGSGVAGGAGGSV
GLWGSGGAGGDGGAATSLLGVGMNAGAGGAGGNAGLLYGNGGAGGAGGNGGDTTVPLF
DSGVGGAGGAGGNASLFGNGGTGGVGGKGGTSSDLASATSGAGGAGGAGGVGGLLYGN
GGNGGAGGIGGAAINILANAGAGGAGGAAGSSFIGNGGNGGAGGAGGAAALFSSGVGG
AGGSGGTALLLGSGGAGGNGGTGGANSGSLFASPGGTGGAGGHGGAGGLIWGNGGAGG
NGGNGGTTADGALEGGTGGIGGTGGSAIAFGNGGQGGAGGTGGDHSGGNGIGGKGGAS
GNGGNAGQVFGDGGTGGTGGAGGAGSGTKAGGTGSDGGHGGNATLIGNGGDGGAGGAG
GAGSPAGAPGNGGTGGTGGVLFGQSGSSGPPGAAALAFPSLSSSVPILGPYEDLIANT
VANLASIGNTWLADPAPFLQQYLANQFGYGQLTLTALTDATRDFAIGLAGIPPSLQSA
LQALAAGDVSGAVTDVLGAVVKVFVSGVDASDLSNILLLGPVGDLFPILSIPGAMSQN
FTNVVMTVTDTTIAFSIDTTNLTGVMTFGLPLAMTLNAVGSPITTAIAFAESTTAFVS
AVQAGNLQAAAAALVGAPANVANGFLNGEARLPLALPTSATGGIPVTVEVPVGGILAP
LQPFQATAVIPVIGPVTVTLEGTPAGGIVPALVNYAPTQLAQAIAP"

CDS 1854187..1855245
/codon_start=1
/gene="argC"
/note="gene_1699"
/transl_table=11
/translation="MQNRQVANATKVAVAGASGYAGGEILRLLLGHPAYADGRLRIGA
LTAATSAGSTLGEHHPHLTPLAHRVVEPTEAAVLGGHDAVFLALPHGHSAVLAQQLSP
ETLIIDCGADFRLTDAAVWERFYGSSHAGSWPYGLPELPGARDQLRGTRRIAVPGCYP
TAALLALFPALAADLIEPAVTVVAVSGTSGAGRAATTDLLGAEVIGSARAYNIAGVHR
HTPEIAQGLRAVTDRDVSVSFTPVLIPASRGILATCTARTRSPLSQLRAAYEKAYHAE
PFIYLMPEGQLPRTGAVIGSNAAHIAVAVDEDAQTFVAIAAIDNLVKGTAGAAVQSMN
LALGWPETDGLSVVGVAP"

CDS 1855242..1856456
/codon_start=1
/gene="argJ"
/note="gene_1700"
/transl_table=11
/translation="VTDLAGTTRLLRAQGVTAPAGFRAAGVAAGIKASGALDLALVFN
EGPDYAAAGVFTRNQVKAAPVLWTQQVLTTGRLRAVILNSGGANACTGPAGFADTHAT
AEAVAAALSDWGTETGAIEVAVCSTGLIGDRLPMDKLLAGVAHVVHEMHGGLVGGDEA
AHAIMTTDNVPKQVALHHHDNWTVGGMAKGAGMLAPSLATMLCVLTTDAAAEPAALER
ALRRAAAATFDRLDIDGSCSTNDTVLLLSSGASEIPPAQADLDEAVLRVCDDLCAQLQ
ADAEGVTKRVTVTVTGAATEDDALVAARQIARDSLVKTALFGSDPNWGRVLAAVGMAP

ITLDPDRISVSFNGAAVCVHGVGAPGAREVDLSDADIDITVDLGVGDGQARIRTTDLS
HAYVEENSAYSS"
CDS 1856453..1857337
/codon_start=1
/note="gene_1701"
/transl_table=11
/translation="MSRIEALPTHIKAQVLAEALPWLKQLHGKVVVVKYGGNAMTDDT
LRRAFAADMAFLRNCGIHPVVVHGGGPQITAMLRRLGIEGDFKGGFRVTTPEVLDVAR
MVLFGQVGRELVNLINAHGPYAVGITGEDAQLFTAVRRSVTVDGVATDIGLVGDVDQV
NTAAMLDLVAAGRIPVVSTLAPDADGVVHNINADTAAAAVAEALGAEKLLMLTDIDGL
YTRWPDRDSLVSEIDTGTLAQLLPTLESGMVPKVEACLRAVIGGVPSAHIIDGRVTHC
VLVELFTDAGTGTKVVRG"
CDS 1857334..1858536
/codon_start=1
/gene="argD"
/note="gene_1702"
/transl_table=11
/translation="MTGASTTTATMRQRWQAVMMNNYGTPPIALASGDGAVVTDVDGR
TYIDLLGGIAVNVLGHRHPAVIEAVTRQMSTLGHTSNLYATEPGIALAEELVALLGAD
QRTRVFFCNSGAEANEAAFKLSRLTGRTKLVAAHDAFHGRTMGSLALTGQPAKQTPFA
PLPGDVTHVGYGDVDALAAAVDDHTAAVFLEPIMGESGVVVPPAGYLAAARDITARRG
ALLVLDEVQTGMGRTGAFFAHQHDGITPDVVTLAKGLGGGLPIGACLAVGPAAELLTP
GLHGSTFGGNPVCAAAALAVLRVLASDGLVRRAEVLGKSLRHGIEALGHPLIDHVRGR
GLLLGIALTAPHAKDAEATARDAGYLVNAAAPDVIRLAPPLIIAEAQLDGFVAALPAI
LDRAVGAP"
CDS 1858533..1859456
/codon_start=1
/note="gene_1703"
/transl_table=11
/translation="VIRHFLRDDDLSPAEQAEVLELAAELKKDPVSRRPLQGPRGVAV
IFDKNSTRTRFSFELGIAQLGGHAVVVDSGSTQLGRDETLQDTAKVLSRYVDAIVWRT
FGQERLDAMASVATVPVINALSDEFHPCQVLADLQTIAERKGALRGLRLSYFGDGANN
MAHSLLLGGVTAGIHVTVAAPEGFLPDPSVRAAAERRAQDTGASVTVTADAHAAAAGA
DVLVTDTWTSMGQENDGLDRVKPFRPFQLNSRLLALADSDAIVLHCLPAHRGDEITDA
VMDGPASAVWDEAENRLHAQKALLVWLLERS"
CDS 1859453..1859965
/codon_start=1
/note="gene_1704"
/transl_table=11
/translation="MSRAKAAPVAGPEVAANRAGRQARIVAILSSAQVRSQNELAALL
AAEGIEVTQATLSRDLEELGAVKLRGADGGTGIYVVPEDGSPVRGVSGGTDRMARLLG
ELLVSTDDSGNLAVLRTPPGAAHYLASAIDRAALPQVVGTIAGDDTILVVAREPTTGA
QLAGMFENLR"
CDS 1859974..1861170
/codon_start=1
/note="gene_1705"
/transl_table=11
/translation="MSERVILAYSGGLDTSVAISWIGKETGREVVAVAIDLGQGGEHM
DVIRQRALDCGAVEAVVVDARDEFAEGYCLPTVLNNALYMDRYPLVSAISRPLIVKHL
VAAAREHGGGIVAHGCTGKGNDQVRFEVGFASLAPDLEVLAPVRDYAWTREKAIAFAE
ENAIPINVTKRSPFSIDQNVWGRAVETGFLEHLWNAPTKDIYAYTEDPTINWGVPDEV

IVGFERGVPVSVDGKPVSMLAAIEELNRRAGAQGVGRLDVVEDRLVGIKSREIYEAPG
AMVLITAHTELEHVTLERELGRFKRQTDQRWAELVYDGLWYSPLKAALEAFVAKTQEH
VSGEVRLVLHGGHIAVNGRRSAESLYDFNLATYDEGDSFDQSAARGFVYVHGLSSKLA
ARRDLR"
CDS 1861250..1862662
/codon_start=1
/note="gene_1706"
/transl_table=11
/translation="VSTNEGSLWGGRFAGGPSDALAALSKSTHFDWVLAPYDLTASRA
HTMVLFRAGLLTEEQRDGLLAGLDSLAQDVADGSFGPLVTDEDVHAALERGLIDRVGP
DLGGRLRAGRSRNDQVAALFRMWLRDAVRRVATGVLDVVGALAEQAAAHPSAIMPGKT
HLQSAQPILLAHHLLAHAHPLLRDLDRIVDFDKRAAVSPYGSGALAGSSLGLDPDAIA
ADLGFSAAADNSVDATAARDFAAEAAFVFAMIAVDLSRLAEDIIVWSSTEFGYVTLHD
SWSTGSSIMPQKKNPDIAELARGKSGRLIGNLAGLLATLKAQPLAYNRDLQEDKEPVF
DSVAQLELLLPAMAGLVASLTFNVQRMAELAPAGYTLATDLAEWLVRQGVPFRSAHEA
AGAAVRAAEQRGVGLQELTDDELAAISPELTPQVREVLTIEGSVSARDCRGGTAPGRV
AEQLNAIGEAAERLRRQLVR"
CDS 1862789..1863832
/codon_start=1
/note="gene_1707"
/transl_table=11
/translation="VFGALPPYRYSQRELTDSFVSIPDFEGYEDIVRQLHASAKVNSR
HLVLPLEKYPKLTDFGEANKIFIEKAVDLGVQALAGALDESGLRPEDLDVLITATVTG
LAVPSLDARIAGRLGLRADVRRVPLFGLGCVAGAAGVARLHDYLRGAPDGVAALVSVE
LCSLTYPGYKPTLPGLVGSALFADGAAAVVAAGVKRAQDIGADGPDILDSRSHLYPDS
LRTMGYDVGSAGFELVLSRDLAAVVEQYLGNDVTTFLASHGLSTTDVGAWVTHPGGPK
IINAITETLDLSPQALELTWRSLGEIGNLSSASVLHVLRDTIAKPPPSGSPGLMIAMG
PGFCSELVLLRWH"
CDS 1863915..1870295
/codon_start=1
/gene="pks8"
/note="gene_1708"
/transl_table=11
/translation="MNSTPEDLVKALRRSLKQNERLKRENRDLLARTTEPVAVVGMGC
RYPGGVDSPETLWELVAHGRDAVSEFPADRGWDVAGLFDPDPDAVGKSYTRCGGFLTD
VAGFDAEFFGIAPSEALAMDPQQRLLLEVSWEALERAGIDPITLRGSQTGVFAGVFHG
SYGGQGRVPGDLERYGLRGSTLSVASGRVAYVLGLQGPAVSVDTACSSSLVALHLAVQ
SLRLGECDLALVGGVTVMATPAMFIEFSRQRALSADGRCKAYAGAADGTAFAEGAGVL
VLARLADARRLGHPVLALVRGSAVNQDGASNGLATPNGPAQQRVITAALASARLGVAD
VDVVEGHGTGTTLGDPIEAQAILATYGQRPADRPLWLGSIKSNIGHTSAAAGVAGVIK
MVQAMRHGVLPKTLHVDVPTPHVDWSAGAVSLLTEPRPWHVPGRPRRAGVSSFGISGT
NAHVILEEAPAVEPVGAAHGNDPVAVPWVLSARSAQALTNQARRLLAWVGADENVRPL
DVGWSLVNTRSLFDHRAVVVGADRTQLMEGLTGLAAGVPGADVVAGRAQTVGKTAFVF
PGQGAQWLGMGAQLCATAPVFAEHIHRCERALREHVEWSLLDVLRGAPGAPGLDRVDV
VQPALWAVMVSLAELWRSVGVVPDAVIGHSQGEIAAAYVAGALSLWDAAAVVALRSRL
LVRLGGAGGMVSLACGQPQAEKLASQWGDRLNIAAVNGVSSVVLAGETDAVTELMQRC
EAEGIRARRIDVDYASHSAQVDAIREELIAALRGIEPRTSTVAFFSTVTGELMDTAGV
NAEYWYRSIRQPVQFERAVRNAFDGGYRVFVESSPHPVLIAGIEETLVDCDRGATGEP
IVIPTLGRDDGGVGRFWLSAGQAHVAGVGVDWRAAFADLGGRRVELPTYAFARQRFWL
DGLGAVGGDLGGVGLVGAEHGLLAAVVQRPDSGGVVLTGRISVVAAPWLADHAVGPVV
LFPGTGFVELALRAGDEVGCSVLQELTLQAPLVLPADGVRVQVVVGGVEQSGTRNVWV

YSAAGQADSSPGWTLHAQGVLGVGSVQPAAELSVWPPVGARAMDVADGYQVLAARGYG
YGPAFRGLQALWRRGAEVFADVTLPEGVPIRGFGIHPAVLDAALHAWGIVEGEQQTML
PFSWQGVCLHASGAARVRVRLAPVGRGAVPVELADPQGLPVLSVRQLMVRPVSAAALS
RSTAGDRGLLEMIWTPVPLEGGDIGDDAVVWELPPHAGAQAGGDVLAAVYRGVHEVLE
VLQSWLASDATGLGVVVTRGAVGPVDDDVTDLAGAAVWGLVRSAQAEHPGRVVLVDTD
GSVAVEDAVGFGARSGEPQLVVRRGRVYAARLAPVAAGLTLPSASAGGWRLVAGGGGT
LADVVVAPVAPVELATGQVRVAVGAVGVNFRDVLVALGMYPGGGELGVDGAGVVVEVG
PGVTGLAVGDRVMGLLGLVGSEAVVDARLVTMVPAGWSLVEAAAVPVAFLTAFYGLSV
LAEVAAGQKVLVHAGTGGVGMAAVSLARYWGAEVFVTASRAKWDTLRAMGFDDIHISD
SRSLEFEEAFLRATEGSGVDVVLNSLAGEFTDASLRLLPSGGRFIELGKTDIRDGQTV
AERHRGVRYRAFDLVEAGPDRIAAMLSEVVGLLAAGVLARLPVKTFDARCAPAAYRFV
SQARHIGKVVLTIPDGPGGQSGLAGGTVVVTGGTGMAGSAVATHLVRRHGVANLVLVS
RSGEQADRAAEVAALLREGGAQVAVVSCDVADRDALAALLAGLDPRYPLKGVFHAAGV
LDDAVITGLTPDRVDTVLRAKVDGAWNLHELTEDMDLSAFVVFSSMAGIVGTPAQGNY
AAANAFLDGLVAYRRSRGLAGLSVAWGLWEQASAMTRHLGERDRARMTQAGLAPLTTE
QALGFLDTALQADRAVVVAARLDRAALAGAGAALPALFSQLAAGPTRRRIDAADTAVS
MSGLVSRLHALTPERRQRELTDLVISNAAAVLGRSSSVDINAHKAFQDLGFDSLTAVE
LRNRLKTATGLTLSPTLIFDYPTPATLAEHLDSRLVTASGSDQQSLSDRVDDITRELV
VLLDQPDLSANVKAHLRTRLQTMLTSLTTEDDDIAAATESQLFAILDEELGS"

CDS 1870315..1875120
/codon_start=1
/gene="pks8"
/note="gene_1709"
/transl_table=11
/translation="MSGTTTHVDYLKRLTADLRRTRRRLSDLEAKLSEPVAVVGMGCR
YPGGVDSPETLWELVAQGRDAVSDFPADRGWDVYGLFDPDPDACGKMYTRRGTFLEHA
GDFDAGFFGIGPSEALAMDPQQRLLLEVSWEALERTGIDPTKLRGSATGVFAGVIHAG
YGGQLSGELEGYGLTGSTLSVASGRVAYVLGLEGPAVSVDTACSSSLVALHLAVQSLR
SGECDLALAGGVTVMATPAAFVEFSRQRALARDGRCKVYAGAADGTAWSEGAGVLVVE
RLVDARRLGHPVLALVRGSAVNQDGASNGLTAPNGPSQQRVIRAALASARLRAVEVDV
VEGHGTGTMLGDPIEAQALLATYGQDRVEPLWLGSIKSNIGHTSAAAGVAGVIKMVQA
MRHGVMPKTLHVDVPTPHVDWSVGAVSLLTQPRAWSVHGRPRRAGVSSFGISGTNAHV
ILEQAPVVESVVPEVASPTAASAVPWVLSARSEQALAGQAQRLLAFVAANPDLDPIDV
GWSLVKTRAMFEHRAVVVGADRGALLAGLAALAAGESGAGVAVGRARSVGKTVFVFPG
QGAQWVGMGAQLYAELPLFALAFDAVAEELDRHLRLPLRNVLWEGDEALLTSTEFAQP
ALFAIEVALATLLQHWGISPDFLIGHSVGEIAAAHLAGVLSLTDAAGLVAARGRLMAE
LPAGGVMVVAASEEEVLPVLVDGANLAAVNAPHSVVVSGCEAAVSDIADHFARRGRRV
HRLAVSHAFHSLLMEPMLAEFTRIAAGISVSKPRIPLVSNVTGQMAGAGYGDGQYWVE
HARRPVRFVEGVQLLNAVGATRFVEVGPGGGLTALVEQSLPLGEALSVAMMRREHPEV
SSVLGAVATLFTAGAQMDWPAVFGSPGRRIELPTYAFQRQRYWLPPTSAGSADISGVG
LLAARHGLLGAVVEQPDSDVVVLTGRLSVGEQRWLADHVIAGVVLLAGAAFVELALRA
ADQVDCGVVEELTVVTPLVLPTVGGVQLQVVVGVGEMGQRPVSIYSRNAESDSGWVLH
ARGVLGAKAVAPAADLSVWPPLGAAPVDVDGAYQRFAELGYEYGRAFQGLTAMWRRES
ELFADVAVPDDVDVTLSGFGIHPLVLDAALHAMGMVGEQAATMLPFSWQGVSLHAAGA
SRVRARIAPAGDGTVSVELADQAGLPVLSVQALVMRSVSSQLLSAAVAAADAAGRGLL
EVAWLPVELAHNDISADLVVWELESFQDGVGPVYSATHRVLVALQSWLAQERAGGLVV
LTQGSVGQDATNLAGAAVWGLVRSAQAEHPGRVMLVDSDGSMDVGDVIGCGEEQLMIR
NGTAYAARLAQLRPQPILQLPDTNSGWRLVAGGAGTLEDLTLASCPAKELAPGQVRIE
VRALGVNFRDVLVALGIYPGAAELGAEGAGVVTEVGPGVTGLAVGDPVMGLLGVAGSE
AVVDARLVVKLPNRWPLTDAAGVPVVFLTAYCALRVLAQVQPGESVLVHAAAGGVGMA
AVQLARLWGLEVFATASRGKWDTLHTMGCDNTHVADSRTLAFEETFWLTTEGRGVDVV

LNSLAGEFTDASLRLLPRGGRFIEMGKTEFGTPRSLPRTILGWPTGLST"
CDS 1875096..1876628
/codon_start=1
/note="gene_1710"
/transl_table=11
/translation="VAYRAFDLMEAGPQRIAQMLAELVELFKTEALHRLPVKSWDVRH
AREAYRFLSQARHVGKVVLTMPDAWAAGTVLITGGTGMAGSAVARHLVSRYGVRQVVL
ASRAGEHTESVAALVDELGSAGARVQVVSCDVADRDAVAGLVASQPDLTAVFHAAGVL
DDAVITGLTPERVDKVLRAKVDGAWNLHELTRHLDVSAFVLFSSMAGIVGAPGQANYA
AANAFLDGLAAYRRSRGLAALSVAWGLWEQASAMTEHLGERDRVRMSRVGLAPLPTNQ
AMGFLDAALLADRPVVVAARLDRAALAGAELPALFSQLVAGPIRRIIDGADEVSGSGL
ASRLHGLTPEQRHRELTELVCSNAAIVLGHSGTEIDAHKAFQDLGFDSLTAVELRNRL
KTATGLTLPPTLIFDYPTAAELAEHLDIQLANAPAVTVDQPNPSTRFNEVTRELQALL
DQPNWNPDDKTRLIKRLQAILTDCTAPPASSGPSTTHDDEDITTATESQLFAILDDEL
GP"
CDS 1876634..1879687
/codon_start=1
/note="gene_1711"
/transl_table=11
/translation="VQPTGIAIIGLACRFPTVVSPGDLWDLLRDGREATGSIDNVADF
DADFFNLSPREASAMDPRQRLALELTWELLEDAFVVPETLRGQPIAVYLGAMNDDYAV
LTLAADRVDHHAFAGTSRAIIANRVSFAFGLRGPSVTIDSGQSSSLVAVHLACESVRT
GEAPLAIAGGVHLNLARETAMLEQEFGAVSPSGHTYAFDERADGYVPGDGGGLVLLKP
VQAALDDGDRIHAIIRGSAVGNAGHSATGLTVPSVAGQVDVIRRAMSGAGVDCHQVHY
VEAHGTGTKIGDPIEARALGEIFAARQRRPVSVGSVKTNIGHTGGAAGIAGLLKAVLA
IENAVIPPSLNYVGAPIDLDSLGLRVDTALTPWPVADEPRRAGVSSFGMGGTNAHVIL
EQGPTQSPEIVESVAAAGSNAPVAVPWVLAARSPQALTNQAGRLLAHLTADDGLTALD
VGWSLVSTRSVFDHRAVVVGADRGRLMAGLAGLAAGEPGAGVVVGRARSVGKTVFVFP
GQGSQWLGMGRQLYGRYSVFARAFDEVVAVLDGQLRLSVRQVMWGADAGLLESTEFAQ
PALFVVQVALAALLQDWGVLPDLVMGHSVGEIAAAYVAGALSLVDAARVVAARGRLMQ
ALPAGGVMVAVAASEDEVAPLLTEGVCIAAVNAPESVVISGEQAAVGVVVDRLVGLGR
RVRRLAVSHAFHSVLMDPMVEEFSKVLADVCVRAPRIGLVSNVTGQLAGAGYGSPAYW
VEHVRKPVRFFDGVGLAESLGARVFVEVGPGAGLEASVALLARDRPEVESVLAGVGRL
FAEGVAVDWSSVFAGLGGRRVELPTYGFARQRFWLGDNGELSVDQTGKDAGAIARLQS
LAPPELQRQLVELVCFHAAIVLGRKSSHDIDPECAFQDLGFDSMSGVELRNRLQMAIG
LPGLSLPRTLIFDYPTASALAECLGQLLGGQHESSDDESIWQLLKNIPIHQLRRTGLL
DKLLLLAGQPEESLAGRTVSDEVIDSLSPEALIGLALDEDENDIR"
CDS 1879822..1880895
/codon_start=1
/note="gene_1712"
/transl_table=11
/translation="VGRGMSVIAGVFGALPPHRYSQSEITDSFVEFPGLKEHEEIIRR
LHAAAKVNGRHLVLPLQQYPSLTDFGDANEIFIEKAVDLGVEALLGALDDANLRPSDI
DMIATATVTGVAVPSLDARIAGRLGLRPDVRRMPLFGLGCVAGAAGVARLRDYLRGAP
DDVAVLVSVELCSLTYPAVKPTVSSLVGTALFGDGAAAVVAVGDRRAEQVRAGGPDIL
DSRSSLYPDSLHIMGWDVGSHGLRLRLSPDLTNLIERYLANDVTTFLDAHRLTKDDIG
AWVSHPGGPKVIDAVATSLALPPEALELTWRSLGEIGNLSSASILHILRDTIEKRPPS
GSAGLMLAMGPGFCTELVLLRWR"
CDS complement(1880878..1882170)
/codon_start=1
/note="gene_1713"

```
                     /transl_table=11
                     /translation="VRYPLGEALLALYRWRGPLINAGVGGHGYTYLLGAEANRFVFAN
                     ADAFSWSQTFESLVPVDGPTALIVSDGADHRRRRSVVAPGLRHHHVQRYVATMVSNID
                     TVIDGWQPGQRLDIYQELRSAVRRSTAESLFGQRLAVHSDFLGEQLQPLLDLTRRPPQ
                     VMRLQQRVNSPGWRRAMAARKRIDDLIDAQIADARTAPRPDDHMLTTLISGCSEEGTT
                     LSDNEIRDSIVSLITAGYETTSGALAWAIYALLTVPGTWESAASEVARVLGGRVPAAD
                     DLSALTYLNGVVHETLRLYSPGVISARRVLRDLWFDGHRIRAGRLLIFSAYVTHRLPE
                     IWPEPTEFRPLRWDPNAADYRKPAPHEFIPFSGGLHRCIGAVMATTEMTVILARLVAR
                     AMLQLPAQRTHRIRAANFAALRPWPGLTVEIRKSAPAQ"
CDS                  complement(1882185..1883960)
                     /codon_start=1
                     /note="gene_1714"
                     /transl_table=11
                     /translation="MAHLLGAEAVHLAYPTQVVFEAVTLGVNDGARIGIVGRNGDGKS
                     SLLGLLTGQLRPDSGRVTRRSGLRVNALSQTDTLDPNRTVGWTLIGDQPEHQWAGNPR
                     IRDVVAGLVSDIAWDTPVSTLSGGQRRRVQLASLLVGEWDVIALDEPTNHLDIQGITW
                     LADHLRRRWARNTGGLLVVTHDRWFLDEVATTTWEVHDGIVEPFEGGYAAYVLQRVER
                     DRLTAAAEAKRQNLLRKELAWLRRGAPARTCKPKFRIEAANQLIADVPPPRNTVELAK
                     LAAARLGKDVVDLLGVSVSYQPSGGRPVLRDIEWRIGPGERIGIVGANGAGKSTLLGL
                     IAGTVQPGVGRVKRGKTVRLAVLDQHGDDLAPFADDRIADVLGRLRGGYQVEGREVTP
                     TQLLERLGFRRDQLSARVDDLSGGQRRRLQLMLTLLSEPNVLLLDEPTNDVDTEMLTA
                     TEDLLDSWAGTLIVVSHDRYLLERVTDQQYAILDDRLRHLPGGIDEYLQLAARVSAPA
                     PAERPAPPAMSGAQRRATEKELAAVDRQLARLADRVAAKHTELAEHDQSDHVGITRLT
                     QQLRVLQDHVAAMENRWLELSEMLE"
variation             1883991..1884047
                     /note="supplemented from BCG moreau"
CDS                  1884795..1885142
                     /codon_start=1
                     /note="gene_1715"
                     /transl_table=11
                     /translation="MIRAVWNGTVLAEAPRTVRVEGNHYFPPESLHREHLIESPTTSI
                     CPWKGLAHYYNVVVDGPYGPVNPDAAWYYRRPSPLARRIKNHVAFWHGVTVEGESESR
                     HGLARRVVAWLGK"
CDS                  1885150..1885542
                     /codon_start=1
                     /note="gene_1716"
                     /transl_table=11
                     /translation="MPTVGPADHAAGLDRRATPDQLPIWRIGIISGLVGMLCCVGPTI
                     LALVGIISAATAFAWANDLYDNYAWWFRVSGLAVLAILVWWALRHRNRCSVNAIRRLR
                     WRLMAVLAIAVGTYGVLSAVTTWFGTFV"
CDS                  complement(1885551..1886882)
                     /codon_start=1
                     /note="gene_1717"
                     /transl_table=11
                     /translation="VATIAASPTHNALGKAARRLLPLLFVLYVINFVDRANISVAALA
                     MNADLRLSATAYGTAAGVFFLGYVLFQVPANAALARFGAGRTLTAVVLAWGVCSAATA
                     LVTSAHTLYLARFALGVAEGGFFPGVIAYLTVWFPCAQRARAVATFLLAIPVANTVGL
                     PLSGLIVGHVHMAGLPGWRAMFVIEALPALLLAPLLRRLLPDNPQRASWLTPEERAEL
                     SARLTEDTPAPTGRSSGAGWDLVLFAVVYGGLYFALYALQFFLPQLVASLAHGTATLT
                     AATLAALPYGVAALAMLAWSHRSIDRSGAQAGHITLPTTAAGSAALGAALSPMSPIVT
                     LSWLTIAVAGILAAMPAFWSRCTAALAGPRVAVAIATVNAVASLASFAGPYATGHLKD
```

ATGTYHLALLTVAAVLAAAAACSLLLRHAGRTVCANDSEIMLHPSPATPFV"
CDS complement(1886975..1887907)
/codon_start=1
/note="gene_1718"
/transl_table=11
/translation="MTITDPAVSAHADATIGLFEITDHITIDSTQGAHTVEMWCPVIG
DGAFQRVLDVEVTSEDPYDLTREPEFGNLMLYSRLRLATAASWSIRYVVERRAIGHAP
DPARARPLATAQLFSRALIPEAHVDVDERTRTLAQDVVGPETNPLEQARRIYDYVTGA
MDYDATKQSFLGSTEHALTCSVGNCNDIHALFVSLCRSVDIPARFVLGQALELPQPGA
QDCEVCGYHCWAEFFVAGLGWLPADASCATKYGTHGLFANLQANHIAWSIGRDILLAP
PQRAGRSLFFAGPYAEIDGETHPAQRQIRFTAMT"
CDS complement(1887935..1888591)
/codon_start=1
/note="gene_1719"
/transl_table=11
/translation="MSGAKKLIFEQFALVGQALSSGHRLELLDLLVQGERSVDALARA
SGLTFANASQHLLQLRRAGLVTSRRDGKRVIYALSDPQVWDVVRAVRAVAERNLASVG
SLVRQYYTDRDSLEPISRDELQARVAAGSVLVLDVRPAMEYAAGHLPGAVSIPLDELA
ERLDELPSGIDIVACCRGPYCVYAYDALELLRPNGFSARRLDGGFSEWLAADLPVVRT
"
CDS complement(1888916..1889650)
/codon_start=1
/note="gene_1720"
/transl_table=11
/translation="MADRSVRPLRHLVHAVTGGQPPSEAQVRQAAWIARCVGRGGSAP
LHRDDVSALAETLQAKEFAPGAVVFHADQTADGVWIVRHGLIELAVGSRRRRAVVNIL
HPGDVDGDIPLLLEMPMVYTGRALTQATCLFLDRQAFERLLATHPAIARRWLSSVAQR
VSTAQIRLMGMLGRPLPAQVAQLLLDEAIDARIELAQRTLAAMLGAQRPSINKILKEF
ERDRLITVGYAVIEITDQHGLRARAQ"
CDS 1889920..1890426
/codon_start=1
/note="gene_1721"
/transl_table=11
/translation="VDVPAATGRTHLQFRRFAACPICHLHLRSFANRHQEVADSGITE
VVFFHSAADALRGYQSLLPFAVIADPDRVQYREFGVEKSLGAITHPRALWAAVRGSAA
MLHRNDPERAGVGFGDGTTHLGLPADFLLDADGTVAAVHYGRHADDQWSVDQLIDINR
SLGGKGTQ"
CDS 1890423..1890971
/codon_start=1
/note="gene_1722"
/transl_table=11
/translation="VTHSRLIGALTVVAIIVTACGSQPKSQPAVAPTGDAAAATQVPA
GQTVPAQLQFSAKTLDGHDFHGESLLGKPAVLWFWAPWCPTCQGEAPVVGQVAASHPE
VTFVGVAGLDQVPAMQEFVNKYPVKTFTQLADTDGSVWANFGVTQQPAYAFVDPHGNV
DVVRGRMSQDELTRRVTALTSR"
CDS 1891066..1891974
/codon_start=1
/note="gene_1723"
/transl_table=11
/translation="MTMARVRRGTELLLSPQSPPATGGLIVLTGLRLLAGLIWLYNVV
WKVPPDFGERGRRDLYHFTHLAVEHPVFTPFSWVIEHAVLPYFTAFGWGVLFAESALA

VLLLTGTAVRLAALIGIGQSVAIGLSVAESPGEWPWAYAMLLGIHVVLLFTCSTRYAA
VDAVRAAATGSAARTAAQRLLAGWGIVLGLIGLVAVWRGLGDDRPAYVGIRALEFSLG
EYNLRGALALIAIALAMLAAAKRGWRTVALVAAVVAVAAAAAIYLQVGRTAVWLGGTN
TTAAVFVCAAVVSLATEFRIGRVEGA"

CDS 1891974..1893095
/codon_start=1
/note="gene_1724"
/transl_table=11
/translation="MATPGVVQEVVSVAAEHAERVDTDCAFPAEAVDALRKTGLLGLV
LPREIGGMGSGPVEFTEVVAQLSAACGSTAMIYLMHMAAAVTVAASPPPGLPDLLADM
ASGKQLGTLAFSEPGSRSHFWAPVSTASADGDGIAVRADKSWVTSAGFADVYVVSVGS
ADGAAGDVDLYAVPADTPGLRVAGTFTGMGLRGNASAPMAVDIRIPDSYRLGEAGGGF
GIMMQTVLPWFNLGNAAVSLGLATAATGAAVKHVGTARLEHLGGSLAELPTIRAQIAR
MGTTLAAQKAYLEVAANSVSSPDDTTLTHVLGVKASVNDAALTITESAMRVCGGAAFS
KHLPIERAFRDARAGSVMAPTADALYDFYGRAVTGLPLF"

CDS 1893104..1893928
/codon_start=1
/note="gene_1725"
/transl_table=11
/translation="MSTEPLVVGAVAYTPNVVPIWEGIRGYFQDSESPDTQMDFVLYS
NYARLVDSLIAGHIDIAWNTNLAYVRTVLQTGGRCTPLAQRDTDVDYTTVFVAHAGSD
LHGAKDIAGKRLALGSADSAHAAILPLYYLRRAGIAESDLQVIRFDTDIGKHGDTGRS
ELDAVDAVLAGEADVAAIGSSTWAAMGAAELMGESLTEVWRTDGYCHCMFTALDTLPA
ERYQPWLDRLLAMSWDDSEHRKILELEGLRRWVPPHLDGYKPLFEAVQEQGIDPRW"

CDS 1893922..1894917
/codon_start=1
/gene="moeX"
/note="gene_1726"
/transl_table=11
/translation="MVIIELMRRVVGLAQGATAEVAVYGDRDRDLAERWCANTGNTLV
RADVDQTGVGTLVVRRGHPPDPASVLGPDRLPGVRLWLYTNFHCNLCCDYCCVSSSPS
TPHRELGAERIGRIVGEAARWGVRELFLTGGEPFLLPDIDTIIATCVKQLPTTVLTNG
MVFKGRGRRALESLPRGLALQISLDSATPELHDAHRGAGTWVKAVAGIRLALSLGFRV
RVAATVASPAPGELTAFHDFLDGLGIAPGDQLVRPIALEGAASQGVALTRESLVPEVT
VTADGVYWHPVAATDERALVTRTVEPLTPALDMVSRLFAEQWTRAAEEAALFPCA"

CDS 1895147..1895995
/codon_start=1
/note="gene_1727"
/transl_table=11
/translation="VTTAHNGSAPRFQRTRSGYDPVAVNHYIAELVLRQQAQHCEIET
LKAEIASLKDENAALKDTSPSAQAVTDRMAKMLRLAVDEVFQMQSEARAEAATLVSAA
RDEAEAVRTQKREMLADMNARQRALESEHADVMRRAREEAEQLVAQATAEVERMRVID
ARRREKAEQELDAEIIRLRTDAQFQIDDQLQATQQECEKRLGEAKIEADRRLHVADEQ
IEHGLSEARRTLEEISQRRVGILEQLARIHAQLENIPALLESARHSETEPLQSINGAV
AELRAI"

CDS 1896216..1899212
/codon_start=1
/note="gene_1728"
/transl_table=11
/translation="VDLNFSMVTRPIERLVATAQNGLEVLRLGGLETGSVPSPSQIVE
SVPMYKLRRYFPPDNRPGQPPVGPPVLMVHPMMMSADMWDVTREDGAVGILHASGLDP

WVIDFGSPDEVEGGMRRNLADHIVALSEAVDTVKDATGHDVHFVGYSQGGMFCYQAAA
YRRSKDIASVVAFGSPVDTLAALPMGIPANMGAAVADFMADHVFNRLDIPSWMARMGF
QMMDPLKTAKARVDFVRQLHDREALLPREQQRRFLESEGWIAWSGPAISELLKQFIAH
NRMMTGGFAISGQMVTLTDITCPILAFVGEVDDIGQPASVRGIRRAAPNSEVYECLIR
AGHFGLVVGSRAAQQSWPTVADWVRWISGDGTKPENIHLMADQPAEHTDSGVAFSSRV
AHGIGEVSEAALALARGAADAVVAANRSVRTLAVETVRTLPRLARLGQLNDHTRISLG
RIIDEQAHDAPKGEFLLFDGRVHTYEAVNRRINNVVRGLIAVGVRQGDRVGVLMETRP
SALVAIAALSRLGAVAVVMRPDTDLSASVRLGRVTEILTDPTNLDAARQLPGQVLVLG
GGESRDLDLPADALEQGQVIDMEKIDPDAVELPAWYRPNPGLARDLAFIAFSSADGDL
VAKQITNYRWAVSAFGTASTAALGRRDTVYCLTPLHHESALLVSLGGAVVGGTRIALS
RGLRPDRFVAEVRQYGVTVVSYTWAMLRDVVDDPAFVLHGNHPVRLFIGSGMPTGLWE
RVVEAFAPAHVVEFFATTDGQAVLANVAGAKIGSKGRPLPGAGRVELGAYDAEHDLIL
ENDRGFVQVAGVNQVGVLLAQSRGPIDPTASVKRGVFAPADTWISTDYLFWRDDDGDY
WLAGGRGSVVRTARGMVYTEPVTNALGLITGVDLAVTYGVLVRGRHVAVSAVTLLPGA
TITAADLTEAVASMPVGLGPDIVHVVPQLTLSGTYRPTVSALRANGIPKAGRQAWYFN
SGGNEYRRLTPAVRTELTGQHRRGNA"

CDS 1899205..1899429
/codon_start=1
/note="gene_1729"
/transl_table=11
/translation="MLDEALLAILVCPADRGPLVLVEDGDIQVLYNPRLRRAYRIEDG
IPVLLVDEAREVDEDEHARLMARGRPAAPQ"

CDS complement(1899395..1900018)
/codon_start=1
/note="gene_1730"
/transl_table=11
/translation="VAAPDNSRRRPGRPAGSSDTRERILSSARELFAHNGIDRTSIRA
VAAKAGVDAALVHHYFGTKQQLFAAAIHIPIDPMVIIGPIREAPVEELGYKLPSLLLP
IWDSELGAGLIATLRSLISGSDVGLARSFLEEVVTVELGSRVDNPPGTGKIRTQFVAS
QLMGVVMARYIVRIEPFASLPAEQIVQTIAPNLQRYLTGELPDDLAP"

CDS complement(1900020..1900700)
/codon_start=1
/note="gene_1731"
/transl_table=11
/translation="MILLVPILIITLMYFMFENFPHRPGTPSGFNTACLVLLGLFPLF
VMFVITAITMQRERASGTLERILTTPLRRLDLLAGYGTAFSIAAAAQATLACIVAFWF
LGFDTAGSPVWVFAIAIVNAVLGVGLGLLCSAFARTEFQAVQFIPLVMVPQLLLAGII
VPRALMPTWLEWISNVMPASYALEALQQVGAHPELTGIAVRDVVVVLSFAVASLCLAA
VTLRRRTS"

CDS complement(1900772..1901539)
/codon_start=1
/note="gene_1732"
/transl_table=11
/translation="MMISSSDELLRDGADPAVIIDQLRVIRGKRLALQDVSVRVACGT
ITGLLGPSGSGKTTLIRCIVGSQIIASGSVSVLGQPAGSAELRHRVGYMPQDPTIYND
LRVIDNIRYFAELCGVDRQAADEVIEAVDLRDHRTARCANLSGGQRARVSLACALVGR
PDLLVLDEPTIGLDPVLRVELWDRFTALARRGTTLLVSSHVMDEADRCGDLLLLRQGQ
LLAHTTPHRLRKETGCTSLEEAFLSIVRRTTTVPAAG"

CDS 1901598..1902209
/codon_start=1
/note="gene_1733"

/transl_table=11
/translation="MNAEELAIDPVAAAHRLLGATIAGRGVRAMVVEVEAYGGVPDGP
WPDAAAHSYRGRNGRNDVMFGPPGRLYTYRSHGIHVCANVACGPDGTAAAVLLRAAAI
EDGAELATSRRGQTVRAVALARGPGNLCAALGITMADNGIDLFDPSSPVRLRLNDTHR
ARSGPRVGVSQAADRPWRLWLTGRPEVSAYRRSSRAPARGASD"
CDS 1902230..1903495
/codon_start=1
/note="gene_1734"
/transl_table=11
/translation="MILDELSWRGLIAQSTDLDTLAAEAQRGPMTVYAGFDPTAPSLH
AGHLVPLLTLRRFQRAGHRPIVLAGGATGMIGDPRDVGERSLNEADTVAEWTERIRGQ
LERFVDFDDSPMGAIVENNLEWTGSLSAIEFLRDIGKHFSVNVMLARDTIRRRLAGEG
ISYTEFSYLLLQANDYVELHRRHGCTLQIGGADQWGNIIAGVRLVRQKLGATVHALTV
PLVTAADGTKFGKSTGGGSLWLDPQMTSPYAWYQYFVNTADADVIRYLRWFTFLSADE
LAELEQATAQRPQQRAAQRRLASELTVLVHGEAATAAVEHASRALFGRGELARLDEAT
LAAALRETTVAELKPGSPDGIVDLLVASGLSASKGAARRTIHEGGVSVNNIRVDNEEW
VPQSSDFLHGRWLVLRRGKRSIAGVERIG"
CDS 1904146..1904529
/codon_start=1
/note="gene_1735"
/transl_table=11
/translation="MTAHTHDGTRTWRTGRQATTLLALLAGVFGGAASCAAPIQADMM
GNAFLTALTNAGIAYDQPATTVALGRSVCPMVVAPGGTFESITSRMAEINGMSRDMAS
TFTIVAIGTYCPAVIAPLMPNRLQA"
CDS 1904568..1905320
/codon_start=1
/note="gene_1736"
/transl_table=11
/translation="VVDDRQGRRGGRRPRSAAADNRPAFRDGPAIPPGIHARQLAPEI
RRELSTLDRATADAVACHLVAAGELIDDDPEAALRHARAARVRASRIAAVREAVGIAA
YRCGDWAQALAELRAARRMGSKSPLLALIADCERGLGRPQRAIELARGSEAVELSGDA
ADELRIVAAGARADLGQLEQALTVLSTPQLDPGRTGSTAARLFYAYAEILLALGRGDE
ALQWFLRSAAADIDGVTDAEDRVDELGAREQK"
CDS 1905317..1906378
/codon_start=1
/note="gene_1737"
/transl_table=11
/translation="MKSIAQEHDCLLIDLDGTVFCGRQPTGGAVQSLSQVRSRKLFVT
NNASRSADEVAAHLCELGFTATGEDVVTSAQSAAHLLAGQLAPGARVLIVGTEALANE
VAAVGLRPVRRFEDRPDAVVQGLSMTTGWSDLAEAALAIRAGALWVAANVDPTLPTER
GLLPGNGSMVAALRTATGMDPRVAGKPAPALMTEAVARGDFRAALVVGDRLDTDIEGA
NAAGLPSLMVLTGVNSAWDAVYAEPVRRPTYIGHDLRSLHQDSKLLAVAPQPGWQIDV
GGGAVTVCANGDVDDLEFIDDGLSIVRAVASAVWEARAADLHQRPLRIEAGDERARAA
LQRWSLMRSDHPVTSVGTQ"
CDS 1906375..1906551
/codon_start=1
/note="gene_1738"
/transl_table=11
/translation="MTIDPDQIRAEIDALLASLPDPADAENGPSLAELEGIARRLSEA
HEVLLAALESAEKG"
CDS 1906559..1907365

/codon_start=1
/note="gene_1739"
/transl_table=11
/translation="VARRARVDAELVRRGLARSRQQAAELIGAGKVRIDGLPAVKPAT
AVSDTTALTVVTDSERAWVSRGAHKLVGALEAFAIAVAGRRCLDAGASTGGFTEVLLD
RGAAHVVAADVGYGQLAWSLRNDPRVVVLERTNARGLTPEAIGGRVDLVVADLSFISL
ATVLPALVGCASRDADIVPLVKPQFEVGKGQVGPGGVVHDPQLRARSVLAVARRAQEL
GWHSVGVKASPLPGPSGNVEYFLWLRTQTDRALSAKGLEDAVHRAISEGP"

CDS 1907365..1908288
/codon_start=1
/gene="ppnK"
/note="gene_1740"
/transl_table=11
/translation="VTAHRSVLLVVHTGRDEATETARRVEKVLGDNKIALRVLSAEAV
DRGSLHLAPDDMRAMGVEIEVVDADQHAADGCELVLVLGGDGTFLRAAELARNASIPV
LGVNLGRIGFLAEAEAEAIDAVLEHVVAQDYRVEDRLTLDVVVRQGGRIVNRGWALNE
VSLEKGPRLGVLGVVVEIDGRPVSAFGCDGVLVSTPTGSTAYAFSAGGPVLWPDLEAI
LVVPNNAHALFGRPMVTSPEATIAIEIEADGHDALVFCDGRREMLIPAGSRLEVTRCV
TSVKWARLDSAPFTDRLVRKFRLPVTGWRGK"

CDS 1908302..1910065
/codon_start=1
/note="gene_1741"
/transl_table=11
/translation="VLTELRIESLGAISVATAEFDRGFTVLTGETGTGKTMVVTGLHL
LGGARADATRVRSGADRAVVEGRFTTTDLDDATVAGLQAVLDSSGAERDEDGSVIALR
SISRDGPSRAYLGGRGVPAKSLSGFTNELLTLHGQNDQLRLMRPDEQRGALDRFAAAG
EAVQRYRKLRDAWLTARRDLVDRRNRARELAQEADRLKFALNEIDTVDPQPGEDVALV
ADIARLSELDTLREAATTARATLCGTPDADAFDRGAVDSLGRARAALQSSDDAALRGL
AEQVGEALTVVVDAVAELGAYLDELPADASALDAKLARQAQLRTLTRKYAADIDGVLR
WADEARARLAQLDVSEEGLAALERRTGELAHELGQAAVDLSTIRRKAAKRLAKEVSAE
LSALAMADAEFTIGVTTELADHGDPVALALASGELARAGADGVDAVEFGFVAHRGMTV
LPLAKSASGGELSRVMLSLEVVLATSRKQAAGTTMVFDEIDAGVGGWAAVQIGRRLAR
LARTHQVIVVTHLPQVAAYADVHLMVQRTGRDGASGVRRLTSEDRVAELARMLAGLGD
SDSGRAHARELLETAQNDELT"

CDS 1910161..1911342
/codon_start=1
/note="gene_1742"
/transl_table=11
/translation="MRMSALLSRNTSRPGLIGIARVDRNIDRLLRRVCPGDIVVLDVL
DLDRITADALVEAEIAAVVNASSSVSGRYPNLGPEVLVTNGVTLIDETGPEIFKKVKD
GAKVRLYEGGVYAGDRRLIRGTERTDHDIADLMREAKSGLVAHLEAFAGNTIEFIRSE
SPLLIDGIGIPDVDVDLRRRHVVIVADEPSGPDDLKSLKPFIKEYQPVLVGVGTGADV
LRKAGYRPQLIVGDPDQISTEVLKCGAQVVLPADADGHAPGLERIQDLGVGAMTFPAA
GSATDLALLLADHHGAALLVTAGHAANIETFFDRTRVQSNPSTFLTRLRVGEKLVDAK
AVATLYRNHISGGAIALLALTMLIAIIVALWVSRTDGVVLHWIIDYWNRFSLWVQHLV
S"

CDS 1911364..1912308
/codon_start=1
/note="gene_1743"
/transl_table=11
/translation="MISLRQHAVSLAAVFLALAMGVVLGSGFFSDTLLSSLRSEKRDL

YTQIDRLTDQRDALREKLSAADNFDIQVGSRIVHDALVGKSVVIFRTPDAHDDDIAAV
SKIVGQAGGAVTATVSLTQEFVEANSAEKLRSVVNSSILPAGSQLSTKLVDQGSQAGD
LLGIALLSNADPAAPTVEQAQRDTVLAALRETGFITYQPRDRIGTANATVVVTGGALS
TDAGNQGVSVARFAAALAPRGSGTLLAGRDGSANRPAAVAVTRADADMAAEISTVDDI
DAEPGRITVILALHDLINGGHVGHYGTGHGAMSVTVSQ"

CDS 1912448..1914208
/codon_start=1
/gene="pyrG"
/note="gene_1744"
/transl_table=11
/translation="VRKHPQTATKHLFVSGGVASSLGKGLTASSLGQLLTARGLHVTM
QKLDPYLNVDPGTMNPFQHGEVFVTEDGAETDLDVGHYERFLDRNLPGSANVTTGQVY
STVIAKERRGEYLGDTVQVIPHITDEIKRRILAMAQPDADGNRPDVVITEIGGTVGDI
ESQPFLEAARQVRHYLGREDVFFLHVSLVPYLAPSGELKTKPTQHSVAALRSIGITPD
ALILRCDRDVPEALKNKIALMCDVDIDGVISTPDAPSIYDIPKVLHREELDAFVVRRL
NLPFRDVDWTEWDDLLRRVHEPHETVRIALVGKYVELSDAYLSVAEALRAGGFKHRAK
VEICWVASDGCETTSGAAAALGDVHGVLIPGGFGIRGIEGKIGAIAYARARGLPVLGL
CLGLQCIVIEAARSVGLTNANSAEFDPDTPDPVIATMPDQEEIVAGEADLGGTMRLGS
YPAVLEPDSVVAQAYQTTQVSERHRHRYEVNNAYRDKIAESGLRFSGTSPDGHLVEFV
EYPPDRHPFVVGTQAHPELKSRPTRPHPLFVAFVGAAIDYKAGELLPVEIPEIPEHTP
NGSSHRDGVGQPLPEPASRG"

CDS 1914303..1914824
/codon_start=1
/note="gene_1745"
/transl_table=11
/translation="VTREVVEHFGAVAIVAMDDNGNIPMVYQYRHTYGRRLWELPAGL
LDVAGEPPHLTAARELREEVGLQASTWQVLVDLDTAPGFSDESVRVYLATGLREVGRP
EAHHEEADMTMGWYPIAEAARRVLRGEIVNSIAIAGVLAVHAVTTGFAQPRPLDTEWI
DRPTAFAARRAER"

CDS 1914821..1915756
/codon_start=1
/gene="xerD"
/note="gene_1746"
/transl_table=11
/translation="MKTLALQLQGYLDHLTIERGVAANTLSSYRRDLRRYSKHLEERG
ITDLAKVGEHDVSEFLVALRRGDPDSGTAALSAVSAARALIAVRGLHRFAAAEGLAEL
DVARAVRPPTPSRRLPKSLTIDEVLSLLEGAGGDKPSDGPLTLRNRAVLELLYSTGAR
ISEAVGLDLDDIDTHARSVLLRGKGGKQRLVPVGRPAVHALDAYLVRGRPDLARRGRG
TAAIFLNARGGRLSRQSAWQVLQDAAERAGITAGVSPHMLRHSFATHLLEGGADVRVV
QELLGHASVTTTQIYTLVTVHALREVWAGAHPRAR"

CDS complement(1915830..1917194)
/codon_start=1
/note="gene_1747"
/transl_table=11
/translation="MYSSSREEAVAAFDNLDTALNRVLKVSPDDLTIPECLAMLQRCE
KIRRRLPAAEHPFINKLADQTDQTELGGKLPFALAERLHISRGEASRRIHEAADLGPR
RTLTGQPLPPLLTATAAAQRAGHLGPAHVQVIRCFLHQLPHHVDLPTREKAEAELATL
GGRFRPDQLHKLATKLADCLNPDGNYNDTDRARRRSIILGNQGPDGMSAISGYLTPEA
RATVDAVLAKLAAPGMANPADDTPCLAGTPSQAAIEADTRSAGQRHHDGLLAALRALL
CSGELGQHNGLPAAIIVSTSLTELQSRAGHALTGGGTLLPMSDVIRLASHANHYLRIF
DHGRELALYHTKRLASPGQRIVLYAKDRGCSFPNCDVPGYLTEVHHVTDFAQCQETDI

NELTQGCGPHHQLATTGGWITRKRKDGTTEWLPPAHLDHGQPRTNSYFHPEKLLHDSD
EDDP"
CDS complement(1917455..1917616)
/codon_start=1
/gene="BN45_40178"
/note="gene_1748"
/transl_table=11
/translation="VIQQFNERNAPVFLISLKPAASGWTSNLFASVVDDGNAFGSVPT
VDDIRGLLA"
CDS complement(1917749..1918498)
/codon_start=1
/note="gene_1749"
/transl_table=11
/translation="MIRKARIAPLRWSVWRMAAGIRNITTTGQIGDGREAAAVDYVLA
HAGAGNIDDVLATIDKFAYEKSMLINVGDEKGTLLDAAVRRADPALALELGTYLGYSA
LRIARAAPEARVYSVELAEANASNARRIWAHAGVDDRVVCVVGTIGDGGRTLDALTEH
GFATGTLDFVFLDHDKKAYLPDLQSILDRGWLHPGSIVVADNVRVPGAPKYRAYMRRQ
QGMSWNTIEHKTHLEYQTLVPDLVLESEYLG"
CDS complement(1918404..1920074)
/codon_start=1
/note="gene_1750"
/transl_table=11
/translation="MPDDIAAADPTDTQPHLRRDLANRHIQLIAIGGAIGTGLFMGSG
RTISLAGPAVMVVYGIIGFFVFFVLRAMGELLLSNLNYKSFVDFAADLLGPAAGFFVG
WSYWFAWVVTGIADLVAITSYARFWWPGLPIWVPALVTVALILAVNLFSVRHFGELEF
WFALIKVAAIVCLIAVGAILVATNFVSPHGVHATIENLWNDNGFFPTGFLGVVSGFQI
AFFAYIGVELVGTAAAETADPRRTLPRAINAVPLRVAVFYIGALLAILAVVPWRQFAS
GESPFVTMFSLAGLAAAASVVNFVVVTAAASSANSGFFSTGRMLFGLADEGHAPAAFH
QLNRGGVPAPALLLTAPLLLTSIPLLYAGRSVIGAFTLVTTVSSLLFMFVWAMIIISY
LVYRRRHPQRHTDSVYKMPGGVVMCWAVLVFFAFVIWTLTTETETATALAWFPLWFVL
LAVGWLVTQRRQSRRSFGFHCQVVGVRQQLGRGMARLAMKIHARPKLRSAVVVEPVSA
GEPGARRSAKSVRKLASDDSQSAHCPVAVVGLADGGRDPQYHHDGPDR"
CDS complement(1920115..1921272)
/codon_start=1
/note="gene_1751"
/transl_table=11
/translation="MDFGALPPEVNSGRMYCGPGSAPMVAAASAWNGLAAELSVAAVG
YERVITTLQTEEWLGPASTLMVEAVAPYVAWMRATAIQAEQAASQARAAAAAYETAFA
AIVPPPLIAANRARLTSLVTHNVFGQNTASIAATEAQYAEMWAQDAMAMYGYAGSSAT
ATKVTPFAPPPNTTSPSAAATQLSAVAKAAGTSAGAAQSAIAELIAHLPNTLLGLTSP
LSSALTAAATPGWLEWFINWYLPISQLFYNTVGLPYFAIGIGNSLITSWRALGWIGPE
AAEAAAAAPAAVGAAVGGTGPVSAGLGNAATIGKLSVPPNWAGASPSLAPTVGSASAP
LVSDIVEQPEAGAAGNLLGGMPLAGSGTGTGGAGPRYGFRVTVMSRPPFAG"
CDS complement(1921312..1922496)
/codon_start=1
/note="gene_1752"
/transl_table=11
/translation="MTLDVPVNQGHVPPGSVACCLVGVTAVADGIAGHSLSNFGALPP
EINSGRMYSGPGSGPLMAAAAAWDGLAAELSSAATGYGAAISELTNMRWWSGPASDSM
VAAVLPFVGWLSTTATLAEQAAMQARAAAAAFEAAFAMTVPPPAIAANRTLLMTLVDT
NWFGQNTPAIATTESQYAEMWAQDAAAMYGYASAAAPATVLTPFAPPPQTTNATGLVG

```
                     HATAVAALRGQHSWAAAIPWSDIQKYWMMFLGALATAEGFIYDSGGLTLNALQFVGGM
                     LWSTALAEAGAAEAAAGAGGAAGWSAWSQLGAGPVAASATLAAKIGPMSVPPGWSAPP
                     ATPQAQTVARSIPGIRSAAEAAETSVLLRGAPTPGRSRAAHMGRRYGRRLTVMADRPN
                     VG"
CDS                  1923500..1924960
                     /codon_start=1
                     /note="gene_1753"
                     /transl_table=11
                     /translation="VLQRIARELLSGVAVAIVALPLAIAFGITATGTSQGALIGLYGA
                     IFAGFFAAVFGGTPGQVTGPTGPITVVATATIAEHGLEGAFFAFILAGVFQILFGACR
                     LGSLIRYVPHPVISGFMGGIAILIIMTQLDQVRSSSLLVLVTVVLLLASGRFIKAIPP
                     SLLVLVLVSSVLPLAAPWLRDLRAGPVSINRTVDYIGEIPQAMPSFDFPQVANSTMLQ
                     VLLSAVAIALLGSLDSLLTSLVMDNIRGTRHRSNKELIGQGIGNIAAGLFGGLAGAGA
                     TVRSVVNVRNGGQTALSAATHSVVLFVFVAGLGAVVQYIPLAVLSGILILVAVGMFDW
                     HAMRKAHVSPRGDVIVMFTTMIITVVVDLTIAVMVGIALSLLVHRLRSRQRKAKVTQD
                     DTGTYRIDGPLSFLSVDGVFGSLRDGREDVSLDLQHVTYLDTSGAQALLYFIDHSEKD
                     GVAVSIKRIPPRLESQLTALADNEQRDKLRTVLESA"
CDS                  1925071..1925934
                     /codon_start=1
                     /note="gene_1754"
                     /transl_table=11
                     /translation="MTDHPDTGNGIGLTGRPPRAIPDPTPRSSHGPAKVIAMCNQKGG
                     VGKTTSTINLGAALGEYGRRVLLVDMDPQGALSAGLGVPHYELDKTIHNVLVEPRVSI
                     DDVLIHSRVKNMDLVPSNIDLSAAEIQLVNEVGREQTLARALYPVLDRYDYVLIDCQP
                     SLGLLTVNGLACTDGVIIPTECEFFSLRGLALLTDTVDKVRDRLNPKLDISGILITRY
                     DPRTVNSREVMARVVERFGDLVFDTVITRTVRFPETSVAGEPITTWAPKSAGALAYRA
                     LARELIDRFGM"
CDS                  1925931..1926767
                     /codon_start=1
                     /gene="scpA"
                     /note="gene_1755"
                     /transl_table=11
                     /translation="VNGLQNSLANGGTAPENGYSAGFRVRLTNFEGPFDLLLQLIFAH
                     QLDVTEVALHQVTDDFIAYTKAIGARLELEETTAFLVIAATLLDLKAARLLPAGQVDD
                     EEDLALLEVRDLLFARLLQYRAFKHVAEMFAELEATALRSYPRAVSLEDGFVGLLPEV
                     MLGVDAHRFAEIAAIALTPRPAPTVATEHLHELMVSVPEQAEHLLAMLKARGSGQWAS
                     FSELVADCTAPIEIVGRFLALLELYRTRAVAFEQSEPLGALQVSWTGDDAERSDEKER
                     RL"
CDS                  1926764..1927459
                     /codon_start=1
                     /gene="scpB"
                     /note="gene_1756"
                     /transl_table=11
                     /translation="VTEHMPEHDPSYGIPDIAEPAELDADELKRVLEALLLVIDTPVT
                     ADALAAATEQPVYRVAAKLQLMADELTGRDSGIDLRHTSEGWRMYTRARFAPYVEKLL
                     LDGARTKLTRAALETLAVVAYRQPVTRARVSAVRGVNVDAVMRTLLARGLITEVGTDA
                     DTGAVTFATTELFLERLGLTSLSELPDIAPLLPDVDTIDDLSESLDSEPRFIKLTGEL
                     ASEQTLSFDVDRD"
CDS                  1927459..1928220
                     /codon_start=1
                     /note="gene_1757"
```

```
                     /transl_table=11
                     /translation="MAEPEESREPRGIRLQKVLSQAGIASRRAAEKMIVDGRVEVDGH
                     VVTELGTRVDPQVAVVRVDGARVVLDDSLVYLALNKPRGMHSTMSDDRGRPCIGDLIE
                     RKVRGTKKLFHVGRLDADTEGLMLLTNDGELAHRLMHPSHEVPKTYLATVTGSVPRGL
                     GRTLRAGIELDDGPAFVDDFAVVDAIPGKTLVRVTLHEGRNRIVRRLLAAAGFPVEAL
                     VRTDIGAVSLGKQRPGSVRALRSNEIGQLYQAVGL"
     CDS             1928217..1928909
                     /codon_start=1
                     /gene="cmk"
                     /note="gene_1758"
                     /transl_table=11
                     /translation="VSRLSAAVVAIDGPAGTGKSSVSRRLARELGARFLDTGAMYRIV
                     TLAVLRAGADPSDIAAVETIASTVQMSLGYDPDGDSCYLAGEDVSVEIRGDAVTRAVS
                     AVSSVPAVRTRLVELQRTMAEGPGSIVVEGRDIGTVVFPDAPVKIFLTASAETRARRR
                     NAQNVAAGLADDYDGVLADVRRRDHLDSTRAVSPLQAAGDAVIVDTSDMTEAEVVAHL
                     LELVTRRSEAVR"
     CDS             1928906..1930297
                     /codon_start=1
                     /gene="engA"
                     /note="gene_1759"
                     /transl_table=11
                     /translation="VTQDGTWVDESDWQLDDSEIAESGAAPVVAVVGRPNVGKSTLVN
                     RILGRREAVVQDIPGVTRDRVCYDALWTGRRFVVQDTGGWEPNAKGLQRLVAEQASVA
                     MRTADAVILVVDAGVGATAADEAAARILLRSGKPVFLAANKVDSEKGESDAAALWSLG
                     LGEPHAISAMHGRGVADLLDGVLAALPEVGESASASGGPRRVALVGKPNVGKSSLLNK
                     LAGDQRSVVHEAAGTTVDPVDSLIELGGDVWRFVDTAGLRRKVGQASGHEFYASVRTH
                     AAIDSAEVAIVLIDASQPLTEQDLRVISMVIEAGRALVLAYNKWDLVDEDRRELLQRE
                     IDRELVQVRWAQRVNISAKTGRAVHKLVPAMEDALASWDTRIATGPLNTWLTEVTAAT
                     PPPVRGGKQPRILFATQATARPPTFVLFTTGFLEAGYRRFLERRLRETFGFDGSPIRV
                     NVRVREKRAGKRR"
     CDS             1930480..1931283
                     /codon_start=1
                     /note="gene_1760"
                     /transl_table=11
                     /translation="MALAQQVPNLGLARFSVQDKSILITGATGSLGRVAARALADAGA
                     RLTLAGGNSAGLAELVNGAGIDDAAVVTCRPDSLADAQQMVEAALGRYGRLDGVLVAS
                     GSNHVAPITEMAVEDFDAVMDANVRGAWLVCRAAGRVLLEQGQGGSVVLVSSVRGGLG
                     NAAGYSAYCPSKAGTDLLAKTLAAEWGGHGIRVNALAPTVFRSAVTEWMFTDDPKGRA
                     TREAMLARIPLRRFAEPEDFVGALIYLLSDASSFYTGQVMYLDGGYTAC"
     CDS             1931277..1932191
                     /codon_start=1
                     /note="gene_1761"
                     /transl_table=11
                     /translation="MLTSHGFSRAAVVGAGLMGRRIAGVLASAGLDVAITDTNAEILH
                     AAAVEAARVAGAGRGSVAAAADLAAAIPDADLVIEAVVENLAVKQELFERLATLAPDA
                     VLATNTSVLPIGAVTERVEDGSRVIGTHFWNPPDLIPVVEVVPSARTAPDTADRVVAL
                     LTQVGKLPVRVGRDVPGFIGNRLQHALWREAIALVAEGVCDPKTVDLVVRNTIGLRLA
                     TLGPLENADYIGLDLTLAIHDAVIPSLNHDPHPSPLLRELVAAGQLGARTGHGFLDWP
                     AGAREATTARLAQHIAAQLQANEKGRGT"
     CDS             1932194..1933024
                     /codon_start=1
```

/note="gene_1762"
/transl_table=11
/translation="MTFAWPLGAAESTLEFYDLSHPWGHGAPAWPYFEDVQIERLHGM
AKSRVLTQKITTVMHSGTHIDAPAHVVEGTPFLDEIPLSAFFGTGVVVSIPKGKWGMV
TAEDLQNATPDIRPGDIVVVNTGWHHKYADSAEYYAYSPGFDKKAGEWFAAKGVKAVG
TDTQALDHPLATAIAPHGPAEAQGGLLPWAVREYEAQTGRKVLDDFPDWEPCHRAILS
QGIYGFENVGGDLDKVTGKRVTFAAFPWRWVGGDGCIVRLVAIVDPTGSYRIETGKAA
"
CDS 1933024..1933374
/codon_start=1
/note="gene_1763"
/transl_table=11
/translation="MKLTRASQAPRYVAPAHHEVSTMRLQGREAGRTERFWVGLSVYR
PGGTAEPAPTREETVYVVLDGELVVTVDGAETVLGWLDSVHLAKGELRSIHNRTDRQA
LLLVTVAHPVAEVA"
CDS 1933427..1934050
/codon_start=1
/note="gene_1764"
/transl_table=11
/translation="MSIVITVAPTGPIATKADNPALPTSPEEIATAVEQAYHAGAAVA
HIHLRDENERPTADPNIARRAMDLIGERCPILIQLSTGVGLTVPFEQREQLVELRPRM
ATLNPCSMSFGAGEFRNPPQAVRRLAARMRELDIKPELEIYDTGHLEACLRLWAEDLL
AEPLQFSIVLGVRGGMAATADNLLTMVRRLPPGRSGKSSRSVRPTWN"
CDS 1934041..1934244
/codon_start=1
/note="gene_1765"
/transl_table=11
/translation="MELTAMGLALGGNARVGLEDTLYLRKGELAPSNLALVSRTIRLA
EALDLPIASVEEAEAALQLPGTS"
CDS 1934258..1935037
/codon_start=1
/note="gene_1766"
/transl_table=11
/translation="VSAEEQDTRSGGIQVIARAAELLRVLQAHPGGLSQAEIGERVGM
ARSTVSRILNALEDEGLVASRGARGPYRLGPEITRMATTVRLGVVTEMHPFLTELSRE
LDETVDLSILDGDRADVVDQVVPPQRLRAVSAVGESFPLYCCANGKALLAALPPERQA
RALPSRLAPLTANTITDRAALRDELNRIRVDGVAYDREEQTEGICAVGAVLRGVSVEL
VAVSVPVPAQRFYGREAELAGALLAWVSKVDAWFNGTEDRK"
tRNA 1935230..1935303
/note="identified by tRNAscan-SE; putative"
CDS complement(1935647..1936036)
/codon_start=1
/note="gene_1767"
/transl_table=11
/translation="VIVLDASAAVELMLTTPAGAAVARRLRGETVHAPAHFDVEVIGA
IRQAVVRQLISDHEGLVVVVNFLSLPVRRWPLKPFTQRAYQLRSTHTVADGAYVALAE
GLGVPLITCDGRLAQSHGHNAEIELVA"
CDS complement(1936033..1936260)
/codon_start=1
/note="gene_1768"
/transl_table=11

/translation="MSAMVQIRNVPDELLHELKARAAAQRMSLSDFLLARLAEIAEEP
ALDDVLDRLAALPRRDLGASAAELVDEARSE"
CDS 1936478..1937962
/codon_start=1
/note="gene_1769"
/transl_table=11
/translation="VIVPAREPEPQPRRVLNGLSDVRAFFHNNTMPLYFISPTPFNLL
GIYRWIRNFFYLTYYDSFEGEHSRVFVPRRRDRRDFDGMGDVCNHLLRDPETLEFIKN
RGPGGKACFVMLDEETQALARQAGLEVMHPPAELRHRLESKIVMTRLADEAGVPSVPH
VIGRVSSYDELSALAHGAGLGDDLVVEAAYGNAGSATFFVRGLRDWDQCAGGIVGQPE
IKVMKRIRNVEVCIEATVTRHGTVIGPAMTSLVGYPELTPYRGAWCGNDVWRGALPPA
QTRAAREMVAKLGDVLSREGYRGYFEVDLLHDLDADELYLGEVNPRLSGASPMTNLTT
EAYADMPLFLFHLLEYMDVDYELDIEAINSRWERGYGEDEVWGQLIMSETSPDLELFT
ATPRTGMWRLNHDGRVSFARQGNDWATMLDESEAFYMRVAAPGDLRCEGAQLGVLVTR
GHLQTDDYQLTERGRRWIDGLKAQFASTPLTPAAPIVSRLVARA"
CDS 1937959..1939206
/codon_start=1
/note="gene_1770"
/transl_table=11
/translation="VSGGVPAGLALDNWLSSPYSHWAFQHVEDFMPTTVIARGTEPVV
TLPADNAPIADIGLTSTDGIATTVGAVMAATATDGWAVAHRGALVAEQYLDGLGPRTR
HLLFSVSKSLVAAVVGALHGAGAIELDAPVTAYVPALADCGYAGATVRHLLDMRSGVA
FSENYDDPAAEIHVREQVIGWAPKRGPDLPATLRDYLLTLRRKSAHGGPFEYRSCETD
VLGWICEAAAGQPMPELMSELLWSRIGAQCDATIALDVAGAAGTGIFDGGISACLTDM
IRFGSLYLRDGVSLAGQQVVPAAWIADTFDGGPDSRQAFAASPDDNPMPGGMYRNQVW
FPYPGSNVALCVGMCGQLIYVNRAAEVVAAKLSTQPHSHEPHMLDTLRAFDAVAHELS
GIRSSSTNDPQRPSPPAQEASPG"
CDS complement(1939249..1939668)
/codon_start=1
/note="gene_1771"
/transl_table=11
/translation="MVGNEENELQDLRNLRRPCFSRAEAPIGVYNGEQAIIVYDLRPV
PHWPKYWIQALAKHFQRQLKPSPKIDISLLDDRIRFSVFVSTDVSAKDLCKLDDAVYN
AVRNAGRAIENEQAALDHKLAEVRKRRMDTWDESYFR"
CDS complement(1939658..1940368)
/codon_start=1
/note="gene_1772"
/transl_table=11
/translation="VQPYGQYCPVARAAELLGDRWTLLIVRELLFGPLRFTEIERGLP
GISRSVLAQRLRRLQHDRIIEAVPEHTGGGYRFTVAGEELRPVLQTLGDWVSRWLMAD
PTPAECDPELLTLWISRRVNTEALPGRRVVVEFRYHGERPLWAWLVLEPGDISVCLHD
PCLPVDLTVRGHPRDLYRVYSGRSTLAAEISAERIELDGLPAMRRAFPSWMAWSPFAP
AMRQAVVSVDQMPEAHGG"
CDS 1940469..1941854
/codon_start=1
/note="gene_1773"
/transl_table=11
/translation="MTATLTKTLGSLDDFRGTLCVPGDPDYPRVRAIWNGQVAREPAL
IATCHDACDVRTVLRRAVDAGMVTAVRGGGHNVAGTALCDGGVVIDLSAMRAVSLDPA
TGRVRVQGGATLADLDHATVPFARVAPAGIVTTTGVGGLTLGGGVGWTTRRFGLSCDN
LVAVRLVTAAGDYLSVDDERDPELMWGLRGGGGNFGIVTEFEFATHPFGPVAVAGFVV

```
                     YRLDDGPAVLRGYRQFAAAAPEEVTTIVVLRHAPPAPWIPVDQRGKPVVMIGAVHTGS
                     IQTGIEALRPVKSLARPVADTVWPTPFLAHQAVLDASNPAGHRYYWKSDYLAELNDEA
                     IDLLVEQTAQLSSPDSLIGIFQLGGAAARGGERSCFPSRHARFMVNYATHWTEAREDD
                     LHRQWTRDAIEALAPYGLGTAYVNFTADDAPMHVETLYSTTEFSRLVTLKNRLDPDNV
                     FRNNHNIRPSA"
CDS                  1941887..1942456
                     /codon_start=1
                     /note="gene_1774"
                     /transl_table=11
                     /translation="MDLYSNLVEAEQRLVALVSSIDADSYSSPTPCDRWDVRALLSHA
                     LASIDAFAAAVDGAPGPDMAQVFSGADIVGDDPLGATQRITRRSQAAWSTVRDLNAEL
                     STFIGVMPAGQALAIITFSTVVHGWDLAVATGQAGELPEHLAEAAQQVAAELVPVLRP
                     RGLFAHDVDLAGEATPTQRLVALTGRKPR"
CDS                  complement(1942481..1943251)
                     /codon_start=1
                     /note="gene_1775"
                     /transl_table=11
                     /translation="MSVNGLPGAHNAGLQPIDSKGCHTRRTRHTKVLFVSKGVLANGR
                     GRWLAIAASLVVSAAILYAQGAEHTCCRETPAAIPTGPDSAPANAPRIASPTEADLLA
                     ASAPVAAQQFQFALPAGVASEEGLQVKTIWVARAVSVLFPQITNIFGYRQDPLKWHPN
                     GLAIDVMIPNHHSDEGIQLGNQVAGLALANAKRWGVLHVIWRQGYYPGIGAPSWTADY
                     GSETLNHYDHVHIATDGGGYPTGRETYYVGSMSPTPPE"
CDS                  complement(1943248..1944186)
                     /codon_start=1
                     /note="gene_1776"
                     /transl_table=11
                     /translation="VARTDDDNWDLTSSVGVTATIVAVGRALATKDPRGLINDPFAEP
                     LVRAVGLDLFTKMMDGELDMSTIADVSPAVAQAMVYGNAVRTKYFDDYLLNATAGGIR
                     QVAILASGLDSRAYRLPWPTRTVVYEIDQPKVMEFKTTTLADLGAEPSAIRRAVPIDL
                     RADWPTALQAAGFDSAAPTAWLAEGLLIYLKPQTQDRLFDNITALSAPGSMVATEFVT
                     GIADFSAERARTISNPFRCHGVDVDLASLVYTGPRNHVLDYLAAKGWQPEGVSLAELF
                     RRSGLDVRAADDDTIFISGCLTDHSSISPPTAAGWR"
variation            1944211..1944324
                     /note="supplemented from AL2122/97"
CDS                  complement(1944366..1945832)
                     /codon_start=1
                     /note="gene_1777"
                     /transl_table=11
                     /translation="VRALDRLPHEIATAVVETVNCADPGAAFDELDAKINAGMKAYAI
                     PGVAVAVWAGGQEYVKGYGVTNVDHPMPVDGDTVFRIGSTTKTFTGTVMMRLVERGKV
                     DLDSPVRRYIPDFAVADESASATVTVRQLLNHTAGWDGRNGQDFGRGDDAVALYVKAM
                     TRLPQLTPPGTAFAYNNSGLVVAGRIIELVAGTTYESTVQRLLLDPLQLAHTRYFSDQ
                     IIGLNVAASHSVVDGKPIAVTDFWTFPRSCNPTGGLMSTARDQLRYAQFHLGDGRAPN
                     GEQILSRQSLKAMRSNPGAGGTLWVELTGMGVTWMLRPSAENVTIVEHGGTWKGQRSG
                     FVMVPDRNFAMTVLTNSDGGFHMINDLFASDWALQRFAGLSNLPATPQRLGAVDLAPY
                     EGRYIAKQVAQNGDLETTVIDFRARDGQLAGSMSTDDANPDGQNSANLGLAFYRPDYG
                     LDLGPDNKPTGSRSNFVRGPDGNIAWFCSQHGRLFRRQ"
CDS                  1946351..1947907
                     /codon_start=1
                     /gene="gabD2"
                     /note="gene_1778"
```

/transl_table=11
/translation="MPAPSAEVFDRLRNLAAIKDVAARPTRTIDEVFTGKPLTTIPVG
TAADVEAAFAEARAAQTDWAKRPVIERAAVIRRYRDLVIENREFLMDLLQAEAGKARW
AAQEEIVDLIANANYYARVCVDLLKPRKAQPLLPGIGKTTVCYQPKGVVGVISPWNYP
MTLTVSDSVPALVAGNAVVLKPDSQTPYCALACAELLYRAGLPRALYAIVPGPGSVVG
TAITDNCDYLMFTGSSATGSRLAEHAGRRLIGFSAELGGKNPMIVARGANLDKVAKAA
TRACFSNAGQLCISIERIYVEKDIAEEFTRKFGDAVRNMKLGTAYDFSVDMGSLISEA
QLKTVSGHVDDATAKGAKVIAGGKARPDIGPLFYEPTVLTNVAPEMECAANETFGPVV
SIYPVADVDEAVEKANDTDYGLNASVWAGSTAEGQRIAARLRSGTVNVDEGYAFAWGS
LSAPMGGMGLSGVGRRHGPEGLLKYTESQTIATARVFNLDPPFGIPATVWQKSLLPIV
RTVMKLPGRR"
CDS complement(1947917..1948465)
/codon_start=1
/note="gene_1779"
/transl_table=11
/translation="MAVESSMLALGTPAPSFTLPQPATGATVSLDELTGPALVVTFIC
NHCPYVQHVAAGLATLGRDLADQGVPMVGISSNDVVTYPQDGPDQMVAEARRHGWTFP
YLYDETQDVARAFSAACTPDTFVFDGQRRLVYRGQLDDSRPGNGRPVTAADVRAAVDA
LLAGRPVNPDQRPSIGCGIKWR"
CDS complement(1948529..1949125)
/codon_start=1
/note="gene_1780"
/transl_table=11
/translation="MITFRLRLPCRTILRVFSRNPLVRGTDRLEAVVMLLAVTVSLLT
IPFAAAAGTAVHDSRSHVYAHQAQTRHPATATVIDHEGVIDSNTTATSAPPRTKITVP
ARWVVNGIERSGEVNAKPGTKSGDRVGIWVDSAGQLVDEPAPPARAIADAALAALGLW
LSVAAVAGALLALTRAILIRVRNASWQHDIDSLFCTQR"
CDS complement(1949448..1949690)
/codon_start=1
/note="gene_1781"
/transl_table=11
/translation="MTNVGDQGVDAVFGVIYPPQVALVSFGKPAQRVCAVDGAIHVMT
TVLATLPADHGCSDDHRGALFFLSINELTRCAAVTG"
CDS complement(1949965..1950558)
/codon_start=1
/note="gene_1782"
/transl_table=11
/translation="VFLYVAVGSLVVARLLLYPLRPADLTPPYWVAMGATAITVLAGA
HIVEMADAPMAIVTSGLVAGASVVFWAFGPWLIPPLVAASIWKHVVHRVPLRYEATLW
SVVFPLGMYGVGAYRLGLAAHLPIVESIGEFEGWVALAVWTITFVAMLHHLAATIGRS
GRSSHAIGAADDTHAIICRPPRSFDHQVRAFRRNQPM"
CDS complement(1950561..1950803)
/codon_start=1
/gene="TBCG_01691"
/note="gene_1783"
/transl_table=11
/translation="MRDTTFGPVVTRLCGWTYALSVVLLWVTCTAYAVLIAVSTTRIV
IFRKEFADDLADPRRGFGMFTFVAASDVLGTRLVGQ"
CDS complement(1950902..1952860)
/codon_start=1
/note="gene_1784"

/transl_table=11
/translation="VTVTPRTGSRIEELLARSGRFFIPGEISADLRTVTRRGGRDGDV
FYRDRWSHHKVVRSTHGVNCTGSCSWKIYVKDGIITWETQETDYPSVGPDRPEYEPRG
CPRGAAFSWYTYSPTRVRHPYARGVLVEMYREAKARLGDPVAAWADIQADPRRRRRYQ
RARGKGGLVRVSWAEATEMIAAAHVHTISTYGPDRVAGFSPIPAMSMVSHAAGSRFVE
LIGGVMTSFYDWYADLPVASPQVFGDQTDVPESGDWWDVVWQCASVLLTYPNSRQLGT
AEELLAHIDGPAADLLGRTVSELRRADPLTAATRYVDTFDLRGRATLYLTYWTAGDTR
NRGREMLAFAQTYRSTDVAPPRGETPDFLPVVLEFAATVDPEAGRRLLSGYRVPIAAL
CNALTEAALPYAHTVAAVCRTGDMMGELFWTVVPYVTMTIVAVGSWWRYRYDKFGWTT
RSSQLYESRLLRIASPMFHFGILVVIVGHGIGLVIPQSWTQAAGLSEGAYHVQAVVLG
SIAGITTLAGVTLLIYRRRTRGPVFMATTVNDKVMYLVLVAAIVAGLGATALGSGVVG
EAYNYRETVSVWFRSVWVLQPRGDLMAEAPLYYQIHVLIGLALFALWPFTRLVHAFSA
PIGYLFRPYIIYRSREELVLTRPRRRGW"
CDS complement(1952857..1954044)
/codon_start=1
/note="gene_1785"
/transl_table=11
/translation="MRGQAANLVLATWISVVNFWAWNLIGPLSTSYARDMSLSSAEAS
LLVATPILVGALGRIVTGPLTDRFGGRAMLIAVTLASILPVLAVGVAATMGSYALLVF
FGLFLGVAGTIFAVGIPFANNWYQPARRGFSTGVFGMGMVGTALSAFFTPRFVRWFGL
FTTHAIVAAALASTAVVAMVVLRDAPYFRPNADPVLPRLKAAARLPVTWEMSFLYAIV
FGGFVAFSNYLPTYITTIYGFSTVDAGARTAGFALAAVLARPVGGWLSDRIAPRHVVL
ASLAGTALLAFAAALQPPPEVWSAATFITLAVCLGVGTGGVFAWVARRAPAASVGSVT
GIVAAAGGLGGYFPPLVMGATYDPVDNDYTVGLLLLVATALVACTYTALHAREPVSEE
ASR"
CDS 1954283..1954615
/codon_start=1
/note="gene_1786"
/transl_table=11
/translation="MTSELSLVATGKGSNIMCGDQSDHVLQHWTVDISIDEHEGLTRA
KARLRWREKELVGVGLARLNPADRNVPEIGDELSVARALSDLGKRMLKVSTHDIEAVT
HQPARLLY"
CDS complement(1954629..1956311)
/codon_start=1
/note="gene_1787"
/transl_table=11
/translation="MIPTMTSAGWAPGVVQFREYQRRWLRGDVLAGLTVAAYLIPQAM
AYATVAGLPPAAGLWASIAPLAIYALLGSSRQLSIGPESATALMTAAVLAPMAAGDLR
RYAVLAATLGLLVGLICLLAGTARLGFLASLLSRPVLVGYMAGIALVMISSQLGTITG
TSVEGNEFFSEVHSFATSVTRVHWPTFVLAMSVLALLTMLTRWAPRAPGPIIAVLAAT
MLVAVMSLDAKGIAIVGRIPSGLPTPGVPPVSVEDLRALIIPAAGIAIVTFTDGVLTA
RAFAARRGQEVNANAELRAVGACNIAAGLTHGFPVSSSSSRTALADVVGGRTQLYSLI
ALGLVVIVMVFASGLLAMFPIAALGALVVYAALRLIDLSEFRRLARFRRSELMLALAT
TAAVLGLGVFYGVLAAVALSILELLRRVAHPHDSVLGFVPGIAGMHDIDDYPQAKRVP
GLVVYRYDAPLCFANAEDFRRRALTVVDQDPGQVEWFVLNAESNVEVDLTALDALDQL
RTELLRRGIVFAMARVKQDLRESLRAASLLDKIGEDHIFMTLPTAVQAFRRR"
CDS 1956379..1956591
/codon_start=1
/note="gene_1788"
/transl_table=11
/translation="MELAARMGETLTQAVVVAVREQLARRTGRTRSISLREELAAIGR

RCAALPVLDTRAADTILGYDERGLPA"
CDS 1956591..1956839
/codon_start=1
/note="gene_1789"
/transl_table=11
/translation="MVIDTSALVAMLNDEPEAQRFEIAVAADHVWLMSTASYPEMATV
IETRFGEPGGREPKVSGQPLLYTGDDFACIDIRAVLAG"
CDS 1956920..1957585
/codon_start=1
/note="gene_1790"
/transl_table=11
/translation="MGRVRTGGLLLRTRVPGNRFADYRITVHVQQARTVLDPFPRDGY
RGVFESGQVRIESHDGAVISSRAHPRAAFFGRSGLRRNIRWDPLDSVYFAGYAMWNYL
TTPYLLTREGVAVEEGAPWQQEGETWRRLIVSFPPDIDTHSPRQTFYVDASGLLRRHD
YVPEVVGHWARAAHYCADPVDVDGFVFPTCRWVHPIGPGNRSLPFPTLVSILLTDIRV
ETD"
CDS 1957679..1959379
/codon_start=1
/note="gene_1791"
/transl_table=11
/translation="MDGTAESREGTQFGPYRLRRLVGRGGMGDVYEAEDTVRERIVAL
KLMSETLSSDPVFRTRMQREARTAGRLQEPHVVPIHDFGEIDGQLYVDMRLINGVDLA
AMLRRQGPLAPPRAVAIVRQIGSALDAAHAAGATHRDVKPENILVSADDFAYLVDFGI
ASATTDEKLTQLGNTVGTLYYMAPERFSESHATYRADIYALTCVLYECLTGSPPYQGD
QLSVMGAHINQAIPRPSTVRPGIPVAFDAVIARGMAKNPEDRYVTCGDLSAAAHAALA
TADQDRATDILRRSQVAKLPVPSTHPVSPGTRWPQPTPWAGGAPPWGPPSSPLPRSAR
QPWLWVGVAVAVVVALAGGLGIALAHPWRSSGPRTSAPPPPPPADAVELRVLNDGVFV
GSSVAPTTIDIFNEPICPPCGSFIRSYASDIDTAVADKQLAVRYHLLNFLDDQSHSKN
YSTRAVAASYCVAGQNDPKLYASFYSALFGSDFQPQENAASDRTDAELAHLAQTVGAE
PTAISCIKSGADLGTAQTKATNASETLAGFNASGTPFVWDGSMVVNYQDPSWLARLIG
"
CDS 1959767..1960081
/codon_start=1
/gene="MDA_GLEAN10011451"
/note="gene_1792"
/transl_table=11
/translation="VQRTQPIQATHRVQVMHARHATHCRTATHNTVSRLPIIPTLPAV
AAAPAIATLPAVATEPATATLPAVATEPATATAPVVAADPATAMLSMLAIERLITMCG
FR"
CDS complement(1960136..1960666)
/codon_start=1
/note="gene_1793"
/transl_table=11
/translation="MTRSYRPAPPIERVVLLNDRGDATGVADKATVHTGDTPLHLAFS
SYVFDLHDQLLITRRAATKRTWPAVWTNSCCGHPLPGESLPGAIRRRLAAELGLTPDR
VDLILPGFRYRAAMADGTVENEICPVYRVQVDQQPRPNSDEVDAIRWLSWEQFVRDVT
AGVIAPVSPWCRSQLG"
CDS 1960813..1962243
/codon_start=1
/note="gene_1794"
/transl_table=11

/translation="MPLAEGSTFAGFTIVRQLGSGGMGEVYLARHPRLPRQDALKVLR
ADVSADGEYRARFNREADAAASLWHPHIVAVHDRGEFDGQLWIDMDFVDGTDTVSLLR
DRYPNGMPGPEVTEIITAVAEALDYAHERRLLHRDVKPANILIANPDSPDRRIMLADF
GIAGWVDDPSGLTATNMTVGTVSYAAPEQLMGNELDGRADQYALAATAFHLLTGSPPF
QHANPAVVISQHLSASPPAIGDRVPELTPLDPVFAKALAKQPKDRYQRCVDFARALGH
RLGGAGDPDDTRVSQPVAVAAPAKRSLLRTAVIVPAVLAMLLVMAVAVTVREFQRADD
ERAAQPARTRTTTSAGTTTSVAPASTTRPAPTTPTTTGAADTATASPTAAVVAIGALC
FPLGSTGTTKTGATAYCSTLQGTNTTIWSLTEDTVASPTVTATADPTEAPLPIEQESP
IRVCMQQTGQTRRECREEIRRSNGWP"

CDS 1962311..1964902
/codon_start=1
/note="gene_1795"
/transl_table=11
/translation="MSQPAAPPVLTVRYEGSERTFAAGHDVVVGRDLRADVRVAHPLI
SRAHLLLRFDQGRWVAIDNGSLNGLYLNNRRVPVVDIYDAQRVHIGNPDGPALDFEVG
RHRGSAGRPPQTTSIRLPNLSAGAWPTDGPPQTGTLGSGQLQQLPPATTRIPAAPPSG
PQPRYPTGGQQLWPPSGPQRAPQIYRPPTAAPPPAGARGGTEAGNLATSMMKILRPGR
LTGELPPGAVRIGRANDNDIVIPEVLASRHHATLVPTPGGTEIRDNRSINGTFVNGAR
VDAALLHDGDVVTIGNIDLVFADGTLARREENLLETRVGGLDVRGVTWTIDGDKTLLD
GISLTARPGMLTAVIGPSGAGKSTLARLVAGYTHPTDGTVTFEGHNVHAEYASLRSRI
GMVPQDDVVHGQLTVKHALMYAAELRLPPDTTKDDRTQVVARVLEELEMSKHIDTRVD
KLSGGQRKRASVALELLTGPSLLILDEPTSGLDPALDRQVMTMLRQLADAGRVVLVVT
HSLTYLDVCDQVLLLAPGGKTAFCGPPTQIGPVMGTTNWADIFSTVADDPDAAKARYL
ARTGPTPPPPPVEQPAELGDPAHTSLFRQFSTIARRQLRLIVSDRGYFVFLALLPFIM
GALSMSVPGDVGFGFPNPMGDAPNEPGQILVLLNVGAVFMGTALTIRDLIGERAIFRR
EQAVGLSTTAYLIAKVCVYTVLAVVQSAIVTVIVLVGKGGPTQGAVALSKPDLELFVD
VAVTCVASAMLGLALSAIAKSNEQIMPLLVVAVMSQLVFSGGMIPVTGRVPLDQMSWV
TPARWGFAASAATVDLIKLVPGPLTPKDSHWHHTASAWWFDMAMLVALSVIYVGFVRW
KIRLKAC"

CDS 1965335..1966006
/codon_start=1
/note="gene_1796"
/transl_table=11
/translation="VGLLIRLAELLVVMLPLIGVLYVGIKALSSFTRRLGEASGDLAS
DSPAMPRPTTVENDAARWRAITRAVEAHERTDARWLEYELDAAKLLDFPVMTDMRDPL
TTAFHKAKLQADFHKPLRAEDLLDDPDAAGHYLDAVRDYVTAFDTAEAEAMRRRRTGF
SREEQQRLARAQSLLRVASDAGATAQERERAYRLARTELDGLIVLPDRTRAGIERGIA
GELDD"

CDS complement(1966003..1966494)
/codon_start=1
/note="gene_1797"
/transl_table=11
/translation="MFTIVGVIVALIGAFVQSRRHRHRPAADIHMLWWMVLIVGVVSI
IGAGYHVFDGERTAELIGYTRGDGGFQWENAMGDLAIGVVGLMAYRFRGHFWLATIVV
LTIQYVGDAAGHIYYWVVENNTNPYNIGVPLWTDILLPIVMWALYAWSWHSNGDAVPK
GQP"

CDS complement(1966644..1968242)
/codon_start=1
/note="gene_1798"
/transl_table=11
/translation="MTDTIQSLLRQHVSDPTIAVKYGGLQWTWSQYLAESAARAAALI

```
                     TIADPQRPTHIGSLLGNTPEMLAQLAAAGLGGYVLCGLNTTRRGDALAADVRRADCQI
                     VVTDADHRALLDGLDLAGARILDTSTPRWAELVAGDGAFVPYREVDTMDPFMMIFTSG
                     TSGNPKAVPVSHLMATFAGRSLTERFGLTEQDTCYVSMPLFHSNAVVAGWAPAVVSGA
                     AIAPATFSATGFLDDVRRYHATYMNYVGKPLAYILATPERDDDADNPLRVAFGNEAND
                     KDIEEFSRRFGVQVEDGFGSTENAVIVIREPGTPPGSIGRGAHGVAVYNGETVTECAV
                     ARFDAHGALTNADEAIGELVNTTGSGFFTGYYNDPEANAERMRHGMYWSGDLAYRDSE
                     GWIYLAGRTADWMRVDGENLTAAPIERILLRYKAINRVAVYAVPDEYVGDQVMAALVL
                     RAGDTFDPDAFEAFLDAQPDLSTKARPRYIRIAADLPSTATHKVLKRQLIDEGTAVGK
                     ADTLWVREPRGSAYHHASGPAKAI"
CDS                  1968440..1969678
                     /codon_start=1
                     /note="gene_1799"
                     /transl_table=11
                     /translation="MGETTTCAIIGGGPAGMVLGLLLARAGVQVTLLEKHGDFLRDFR
                     GDTVHPTTMRLLDELGLWERFAALPYSEVRTATLHSNGRAVTYIDFERLHQPYPYVAM
                     VPQWDLLNLLAEAAQAEPSFTLRMKTEVTGLLREGGKVTGVRYQGAEGPGELRAELTV
                     ACDGRWSIARHEAGLKAREFPVNFDVWWFKLPREGDAEFSFLPRFSPGKGLGVIPREG
                     YFQIAYLGPKGTDAQLRERGIEEFRRDVSELLPEATASVAALASTDEVKHLNVKVNRL
                     RRWHIDGLLCIGDAAHAMSPVAGVGINLAVQDAVAAATILAEPLREHRVSSRHLAAVR
                     RRRAFPTAVTQAVQRVLHRRLLGPLLQGRDPTPPAALLGLVERLPWLSAVPAYFVGLG
                     VRPEHAPAFARRGPGNRKGP"
CDS                  1970016..1970222
                     /codon_start=1
                     /note="gene_1800"
                     /transl_table=11
                     /translation="VASCACSIRLCPNSSTDCRPSHAYQLDAFAAAVLRGQAVKTTPK
                     DAVENMSAIHAIYRAAGLPSRNPS"
CDS                  complement(1970289..1973522)
                     /codon_start=1
                     /note="gene_1801"
                     /transl_table=11
                     /translation="MNFSVLPPEINSALIFAGAGPEPMAAAATAWDGLAMELASAAAS
                     FGSVTSGLVGGAWQGASSSAMAAAAAPYAAWLAAAAVQAEQTAAQAAAMIAEFEAVKT
                     AVVQPMLVAANRADLVSLVMSNLFGQNAPAIAAIEATYEQMWAADVSAMSAYHAGASA
                     IASALSPFSKPLQNLAGLPAWLASGAPAAAMTAAAGIPALAGGPTAINLGIANVGGGN
                     VGNANNGLANIGNANLGNYNFGSGNFGNSNIGSASLGNNNIGFGNLGSNNVGVGNLGN
                     LNTGFANTGLGNFGFGNTGNNNIGIGLTGNNQIGIGGLNSGTGNFGLFNSGSGNVGFF
                     NSGNGNFGIGNSGNFNTGGWNSGHGNTGFFNAGSFNTGMLDVGNANTGSLNTGSYNMG
                     DFNPGSSNTGTFNTGNANTGFLNAGNINTGVFNIGHMNNGLFNTGDMNNGVFYRGVGQ
                     GSLQFSITTPDLTLPPLQIPGISVPAFSLPAITLPSLTIPAATTPANITVGAFSLPGL
                     TLPSLNIPAATTPANITVGAFSLPGLTLPSLNIPAATTPANITVGAFSLPGLTLPSLN
                     IPAATTPANITVGAFSLPGLTLPSLNIPAATTPANITVSGFQLPPLSIPSVAIPPVTV
                     PPITVGAFNLPPLQIPEVTIPQLTIPAGITIGGFSLPAIHTQPITVGQIGVGQFGLPS
                     IGWDVFLSTPRITVPAFGIPFTLQFQTNVPALQPPGGGLSTFTNGALIFGEFDLPQLV
                     VHPYTLTGPIVIGSFFLPAFNIPGIDVPAINVDGFTLPQITTPAITTPEFAIPPIGVG
                     GFTLPQITTQEIITPELTINSIGVGGFTLPQITTPPITTPPLTIDPINLTGFTLPQIT
                     TPPITTPPLTIDPINLTGFTLPQITTPPITTPPLTIDPINLTGFTLPQITTPPITTPP
                     LTIDPINLTGFTLPQITTPPITTPPLTIEPIGVGGFTTPPLTVPGIHLPSTTIGAFAI
                     PGGPGYFNSSTAPSSGFFNSGAGGNSGFGNNGSGLSGWFNTNPAGLLGGSGYQNFGGL
                     SSGFSNLGSGVSGFANRGILPFSVASVVSGFANIGTNLAGFFQGTTS"
variation             1971007..1971081
```

```
                     /note="supplemented from AL2122/97"
variation             1971884..1971961
                     /note="supplemented from BCG moreau"
CDS                  complement(1973726..1974961)
                     /codon_start=1
                     /note="gene_1802"
                     /transl_table=11
                     /translation="LLNPGARNAAPLQQQALVPRANPGPNPAPNPPATGPQPPNATQL
                     TPNPAPAPDPAPAAAPDPGATLAGATTSLAEWVTGPDSPNKTLERFGISGTDLGIPWD
                     NGDPANRQVLMIFGDTFGYCAVDGHQWRYNTLFRSQDRDLGNGVHVTSGDASNRYSGS
                     PVRQPGFSKQLINSIKWARDETGIIPTAGIAVGKTQYVNFMSIRNWGRDGEWTTNYSG
                     IAVSKDNGQTWGVFPGTIRASGPDSGGKARFVPGNENFQMGAYLKSNDGYLYSFGTPP
                     GRGGSAYLARVPQRFVPDLTKYQYWNGDSNSWVPNKPDAATPVIPGPVGEMSVQYNTY
                     LKQYLALYTNGMNDVVARTAPAPQGPWSAEQMLVSSWQMPGGIYAPMMHPWSTGKDVY
                     FSLSLWSAYNVMLMHTVLP"
CDS                  1975092..1975292
                     /codon_start=1
                     /gene="NCGM2209_2107"
                     /note="gene_1803"
                     /transl_table=11
                     /translation="MALGGGGAFAASPLHGAAIAGGARPIGTMSPIAAAHASDTDTIR
                     GIGDMSHLPGLDVAIDVRSSLM"
CDS                  complement(1975601..1977145)
                     /codon_start=1
                     /gene="plcB_2"
                     /note="gene_1804"
                     /transl_table=11
                     /translation="VSQSHIGGVSRREFLAKVAAGGAGALMSFAGPVIEKAYGAGPCS
                     GHLTDIEHFVFFMQENRSFDHYFGTLSGTDGFNTVSPLFQQKGWNPMTQALDATGVTM
                     PYRFDTTRGPFLDGACVNDPDHSWVAMHESWNGGVNDNWLPAQAKTRSAAHTPTVMGY
                     YTRQDIPIHYLLADAFTVCDRYFCSVLGPTLPNRLYWLSATIDPDGQNGGPELQSPTF
                     QPVRRFGWRIMPQNLSDAGVSWKVYRNKTLGPISSVLTYGSLVTSFKQSADPRSDLVR
                     FGVAPSYPASFAADVLANRLPRVSWVIPNVLESEHPAVPAAAGAFAIVNILRILLANP
                     AVWEKTALIVSYDENGGFFDHVVPATAPAGTPGEYVTVPDIDQVPGSGGIRGPIGLGF
                     RVPCFVISPYSRGPQMVHDTFDHTSQLRLLETRFGVPVPNLTAWRRSVTGDMTSTFNF
                     AVPPNSSWPNLDYPGLHALSTVPQCVPNAALGTINRGIPYRVPDPQIMPTQETTPTRG
                     IPSGPC"
CDS                  complement(1977274..1978179)
                     /codon_start=1
                     /note="gene_1805"
                     /transl_table=11
                     /translation="LETLRPDTLEVSDRLTLRSLGRWGREHGVTTVMISHERLDRFAG
                     QLLPRRAAQKFADFANARTAANYDTVVCTTGFAREEFDRIGATNTVTVPLGVDLKTFH
                     PRRRCARVRQHWATPTQILLVHCGRLSVEKHADRSIDALAALCDAGVDARLVIAGEGP
                     LRARLERKATGLPIDFTGFISDRHAVAGLLASADVALAPGPHETFGLAALESLACGTP
                     AVVSRTSALTEIITADSGACADNRPEAIAHAVRTIVSRPERHRRRCARRRAEIFTWQR
                     AAASMLATLGAMAVSTRCGDTQDTA"
CDS                  1978644..1979747
                     /codon_start=1
                     /note="gene_1806"
                     /transl_table=11
```

/translation="MLETAGLWGKRADMIVRGCLPYNAEPPPAVLAGSDITPINAFYV
RNHGPVPDIAPQHWRLTVGGLVDNPLTVTYERLTTEFDQHCVVATLACAGNRRAELLR
VRQIPGKEPWAHGAISTAQWCGVRLADILQAADVHIDEGLHVAFDAPDVAEEARPIQP
YGSSIPLSKALSPEVLLAWQMNSEPLPRAHGGPVRVVVPGFIGARSVKWVTAITVQPG
ASQNYFQALDYRILPADADADIVGPGEGISLSSLALNCDILDPTDGDDVPAGALTIRG
YGMAGDGRSVERVDVSVDDGLTWQQADLHAAPSQWSWRPWSLTVDVEPGPLGITARAW
DDTGALQPESAVSLWNPRGYGNNAWARVALRVS"

CDS 1979948..1982785
/codon_start=1
/note="gene_1807"
/transl_table=11
/translation="VTAIGRLIHRYAIWIVGVWALAAIIGNNFAPPLEQVITAEDQPF
SPAGTATSRAVERSAAAFSQAPGDNIGYLVLERNGVLNDQDRAYYDALVVALRRDSRH
VIEVVDWWGTPAIAEVARSDDHHAVTAALRFGGMVGTSQAGESITAARSIVTQLHPPD
GLHVFVTGPGATIVDEFAAIDRQTQLITATTIVVLLILLLIVYRSAITATVPLLSVVV
SLAVAKPIVSVLVDRDFIGISLFSLGLSVAVVVGAGTGFAMFLIGRYHERRRQHIAPA
AALADAYRGVAPAIAGATFIVVTSLGAVGWLSLARIGMFATTGILCSIGVLAVGLAAL
TLTPALVALASRANLLKPPQHKRIQRQFRRLGTHVARWPAPILVASGVFVLIMMIALP
RVPIGWDEAAATPSAAESNRGYRAADRHFAPNQLLPTQVMIETDHDIRNPAGLTAIER
ITAAIMAIGGVRMVQSASHPNGMVSKQAALTASAGNLGDQLDEFSDQLTSRQATFTNL
EAAVRDVVSALDLVQAGIRQDGYGLGQVSLAVRLMQQAITKLQGSAGDVFDIFDPLRR
FVAAIPECRANPVCSVAQEVVQWANTVTESCAKLADAAGQLARGIADVASATSGVSGL
PNALDGIGGQLAQVRESAAGVQELLNNVGAAPLRELPDYLRELAAVSQSAPGVDLYAA
RRILTDPNMRAVLDYFVSPNGHATRLLVYGDGSEWGDDGAQRARAIVTAVAEETDEGT
LRPTAVELTGVGPATRDLQDLVGSDLTLLAVITLAVIFAIAALLLRSPLAGLVVVGTI
ATSYICALGASVVIWKHILGDNLHWSVLPIAFVLLISVGSAYNLLFALRIREESPAGP
RTSVIRAFAATGMVVTAAGIVFGTTMFALAASTSLSVAQIGVTVGMGLLLDALVIRGF
VLPALMVLLGRWLWWPRRSVSNRQVPEPSPA"

CDS 1983106..1983762
/codon_start=1
/note="gene_1808"
/transl_table=11
/translation="MVTTWALLFAPVPAASADPPDPTVSDGACPDVEVVFARGTGEPP
GVGGIGEDFIDALRSKIGEKSMGVYGVDYPATTDFPTAMAGIYDAGTHVEQTAANCPQ
SKLVLGGFSQGAAVMGFVTAAAIPDGAPLDAPRPMPPEVADHVAAVTLFGMPSVAFMH
SIGAPPIVIGPLYAEKTIQLCAPGDPVCSSGGNWAAHNGYADDGMVEQAAVFAAGRLG
"

CDS complement(1984029..1986476)
/codon_start=1
/note="gene_1809"
/transl_table=11
/translation="VTPLLNSINAPVLAATGRPLIGNGANGAPGTGANGGDAGWLIGN
GGAGGSGAKGANGGAGGPGGAAGLFGNGGAGGAGGTATANNGIGGAGGAGGSAMLFGA
GGAGGAGGAATSLVGGIGGTGGTGGNAGMLAGAAGAGGAGGFSFSTAGGAGGAGGAGG
LFTTGGVGGAGGQGHTGGAGGAGGAGGLFGAGGMGGAGGFGDHGTLGTGGAGGDGGGG
GLFGAGGDGGAGGSGLTTGGAAGNGGNAGTLSLGAAGGAGGTGGAGGTVFGGGKGGAG
GAGGNAGMLFGSGGGGGTGGFGFAAGGQGGVGGSAGMLSGSGGSGGAGGSGGPAGTAA
GGAGGAGGAPGLIGNGGNGGNGGESGGTGGVGGAGGNAVLIGNGGEGGIGALAGKSGF
GGFGGLLLGADGYNAPESTSPWHNLQQDILSFINEPTEALTGRPLIGNGDSGTPGTGD
DGGAGGWLFGNGGNGGAGAAGTNGSAGGAGGAGGILFGTGGAGGAGGVGTAGAGGAGG
AGGSAFLIGSGGTGGVGGAATTTGGVGGAGGNAGLLIGAAGLGGCGGGAFTAGVTTGG

```
                 AGGTGGAAGLFANGGAGGAGGTGSTAGGAGGAGGAGGLYAHGGTGGPGGNGGSTGAGG
                 TGGAGGPGGLYGAGGSGGAGGHGGMAGGGGGVGGNAGSLTLNASGGAGGSGGSSLSGK
                 AGAGGAGGSAGLFYGSGGAGGNGGYSLNGTGGDGGTGGAGQITGLRSGFGSAGGAGGA
                 GGNGGAGGKAGLYGNGGDGGAGGDGATSGKGGAGGNAVVIGNGGNGGNAGKAGGTAGA
                 GGAGGLVLGRDGQHGLT"
variation        1984113..1984148
                 /note="supplemented from AL2122/97"
CDS              complement(1986473..1986757)
                 /codon_start=1
                 /note="gene_1810"
                 /transl_table=11
                 /translation="MSFVIAVPETIAAAATDLADLGSTIAGANAAAAANTTSLLAAGA
                 DEISAAIAALFGAHGRAYQAASAEAAAFHGRFVQALTTGGAPMRPPRPPP"
CDS              1987435..1988862
                 /codon_start=1
                 /note="gene_1811"
                 /transl_table=11
                 /translation="MERLSGLDAFFLYMETPSQPLNVCCVLELDTSTMPGGYTYGRFH
                 AALEKYVKAAPEFRMKLADTELNLDHPVWVDDDNFQIRHHLRRVAMPAPGGRRELAEI
                 CGYIAGLPLDRDRPLWEMWVIEGGARSDTVAVMLKVHHAVVDGVAGANLLSHLCSLQP
                 DAPAPQPVRGTGGGNVLQIAASGLVGFASRPVRLATVVPATVLTLVRTLLRAREGRTM
                 AAPFSAPPTPFNGPLGRLRNIAYTQLDMRDVKRVKDRFGVTINDVVVALCAGALRRFL
                 LEHGVLPEAPLVATVPVSVHDKSDRPGRNQATWMFCRVPSQISDPAQRIRTIAAGNTV
                 AKDHAAAIGPTLLHDWIQFGGSTMFGAAMRILPHISITHSPAYNLILSNVPGPQAQLY
                 FLGCRMDSMFPLGPSLATRASTSPSCPSTGNWVSALSPAPTCCRTCGAWQTGFPRRSK
                 SCWSAVMTSRKAATTRTPESYVQNR"
CDS              complement(1988850..1989233)
                 /codon_start=1
                 /note="gene_1812"
                 /transl_table=11
                 /translation="MSDFDTERVSRAVAAALVGPGGVALVVKVFAGLPGVIHTPARRG
                 FFRSNPERIQIGDWRYEVAHDGRLLAAHMVNGIVIAEDALIAEAVGPHLARALGQIVS
                 RYGATVIPNINAAIEVLGTGTDYRF"
CDS              complement(1989233..1990021)
                 /codon_start=1
                 /note="gene_1813"
                 /transl_table=11
                 /translation="MQSSSLDPVASERLSHAEKSFTSDLSINEFALLHGAGFEPIELV
                 MGVSVYHVGFQFSGMRQQQELGVLTEATYRARWNAMARMQAEADALKADGIVGVRLNW
                 RHHGEGGEHLEFMAVGTAVRYTAKPGAFRRPNGQAFSSHLSGQDMVTLLRSGFAPVAF
                 VMGNCVFHIAVQGFMQTLRQIGRNMEMPQWTQGNYQARELAMSRMQSEAERDGATGVV
                 GVHFAISNYAWGVHTVEFYTAGTAVRRTGSGETITPSFVLPMDS"
CDS              complement(1990161..1990622)
                 /codon_start=1
                 /note="gene_1814"
                 /transl_table=11
                 /translation="VPGLGQIALNSAPIFGGAMLAIAAGQFKGPDFRALIRQDMDLLD
                 RLPADATKRRANLQRTIDARIDDLIDAADKSHALRKAAMSYRGNWRDIVLLVCVLLFT
                 IIWWNVNHGRANWLPTFVLLILLAAVTAVYALRGALRAATSLMRGRRGADR"
CDS              complement(1991039..1992295)
                 /codon_start=1
```

/note="gene_1815"
/transl_table=11
/translation="MSSTATSGAAVVSPAERVEVLFEELAELAGQRNAIDGRIVEIVA
ELDRDGLWGVTGARSVAGLVAWKMGCSSGNAHTIATVARRLPEFPRCARGMREGRLSL
DQVGVIAGRAGEGSDAHYAQLAGVATVNQLRTALKLEPRPEPEPDFRPEPRPSITRSA
DEQFSCWRIKLPHVEAAKFDAALQSHLDALIAEYKRDHDNSDGVSDQRPPLPGNVEAF
LRLVEAGWDAEVARRPHGQHTTVVMHLDVQERAAGLHLGPLLSESERRYLLCDATFEA
WFERDGQVIGCGRTTRQINRRLRRALEHRDRTCVVPGCGATRGLHAHHIRHWQDGGAT
ELANLVLVCPYHHRAHHRGLITITGPADNLTVADSAGRPLSAGSLARASTKPPPAVAP
WPGPTGERADWWWYEPFQPQPPPISN"

CDS complement(1992922..1993137)
/codon_start=1
/note="gene_1816"
/transl_table=11
/translation="LWVADITFVRTWQGFCYTAFVTDVCTRKIVVRAVSATMRTEDLP
VQVFNHAVWQSNSDLSELVHHSDPGSQ"

CDS 1993511..1993786
/codon_start=1
/note="gene_1817"
/transl_table=11
/translation="VHVIGDQDSIAAVLNRLRRAQGQLAGVISMIEQGRDCRDVVTQL
AAVSRALDRAGFKIVAAGLKECVSGATASGAAPLSAAELEKLFLALA"

CDS 1993854..1994213
/codon_start=1
/note="gene_1818"
/transl_table=11
/translation="MSDQPRHHQVLDDLLPQHRALRHQIPQVYQRFVALGDAALTDGA
LSRKVKELVALAIAVVQGCDGCVASHAQAAVRAGATAQEAAEAIGVTILMHGGPATIH
GARAYAAFCEFADTTPS"

CDS 1994394..1996268
/codon_start=1
/note="gene_1819"
/transl_table=11
/translation="MSYLVVVPELVAAAATDLANIGSSISAANAAAAAPTTALVAAGG
DEVSAAIAALFGAHARAYQALSAQAAMFHEQFVRALAAGGNSYAVAEAATAQSVQQDL
LNLINAPTQALLGRPLIGNGANGLPGTGQNGGDGGILYGNGGNGGSGGVNQAGGNGGN
AGLWGNGGSGGAGGNATTAGRNGFNGGAGGSGGLLWGNGGAGGAGGHGGPAPLVGGVG
TTGGAGGNGGGAGLFYGFGGAGGNGGMGGVAPSTGPSMGILPAGGVGGPGGSGGASAL
AFGSGGVGGAGGLGGPTDGTVQGVGGFGGQGGNGGQSGLLFGNAGAGGAGAAGGAGTG
DTESFGGHGGAGGDGGAVGLIGNGGGGGNGGAGGTGSPGAVVGGNGGVGGLGGAGSPG
GLLYGTGGAGGNGGPGGDGGTGATVGFAGSGGFGGAGGIAQLFGTGGMGGSGGGIGAG
TTTVVPPDVAPVGGTGGNGGRAGLLLGVGGMGGNGGATSVGGTLYAAGGNGGDGGLVW
GNGGTGGSGGAGGAGSVGNGGAGGNAALLFGNGGAGGAGGAGGIGAGGAGGFGAVLFG
NGGAGGSGAPGGIGAGGNGGNALLVGNGGNGGAGTGGAAGGAGGSGGLLFGQNGMPGP
"

CDS 1996424..1997668
/codon_start=1
/note="gene_1820"
/transl_table=11
/translation="VHEVAAREQRSDGPMRLDAQGRLQRYEEAFADYDAPFAFVDLDA
MWGNADQLLARAGDKPIRVASKSLRCRPLQREILDASERFDGLLTFTLTETLWLAGQG

FSNLLLAYPPTDRAALRALGELTAKDPDGAPIVMVDSVEHLDLIERTTDKPVRLCLDF
DAGYWRAGGRIKIGSKRSPLHTPEQARALAVEIARRPALTLAALMCYEAHIAGLGDNV
AGKRVHNAIIRRMQRMSFEELRERRARAVELVREVADIKIVNAGGTGDLQLVAQEPLI
TEATAGSGFYAPTLFDSYSTFTLQPAAMFALPVCRRPGAKTVTALGGGYLASGVGAKD
RMPTPYLPVGLKLNALEGTGEVQTPLSGDAARRLKLGDKVYFRHTKAGELCERFDHLH
LVRGAEVVDTVPTYRGEGRTFL"

CDS 1997757..1998962
/codon_start=1
/note="gene_1821"
/transl_table=11
/translation="MTPITALPTELAAMREVVETLAPIERAAGEPGEHKAAEWIVERL
RTAGAQDARIEEEQYLDGYPRLHLKLSVIGVAAGVAGLLSRRLRIPAALAGVGAGLAI
ADDCANGPRIVRKRTETPRTTWNAVAEAGDPAGQLTVVVCAHHDAAHSGKFFEAHIEE
VMVELFPGIVERIDTQLPNWWGPILAPALAGVGALRGSRPMMIAGTVGSALAAALFAD
IARSPVVPGANDNLSAVALLVALAERLRERPVKGVRVLLVSLGAEETLQGGIYGFLAR
HKPELDRDRTYFLNFDTIGSPELIMLEGEGPTVMEDYFYRPFRDLVIRAAERADAPLR
RGIRSRNSTDAVLMSRAGYPTACFVSINRHKSVANYHLMSDTPENLCYETVSHAVTVA
ESVIRELAR"

CDS 1998959..2000245
/codon_start=1
/note="gene_1822"
/transl_table=11
/translation="MSPIWSNWPGEQVCAPSAIVRPTSEAELADVIAQAAKRGERVRA
VGSGHSFTDIACTDGVMIDMTGLQRVLDVDQPTGLVTVEGGAKLRALGPQLAQRRLGL
ENQGDVDPQSITGATATATHGTGVRFQNLSARIVSLRLVTAGGEVLSLSEGDDYLAAR
VSLGALGVISQVTLQTVPLFTLHRHDQRRSLAQTLERLDEFVDGNDHFEFFVFPYADK
ALTRTMHRSDEQPKPTPGWQRMVGENFENGGLSLICQTGRRFPSVAPRLNRLMTNMMS
SSTVQDRAYKVFATQRKVRFTEMEYAIPRENGREALQRVIDLVRRRSLPIMFPIEVRF
SAPDDSFLSTAYGRDTCYIAVHQYAGMEFESYFRAVEEIMDDYAGRPHWGKRHYQTAA
TLRERYPQWDRFAAVRDRLDPDRVFLNDYTRRVLGP"

CDS complement(2000818..2001513)
/codon_start=1
/note="gene_1823"
/transl_table=11
/translation="VLRRAVAALDEIAAEPGHLRLVDLCERLGLAKSTTRRLLVGLVE
VGLVSVDSHGRFALGERLLGFGSVTGAHIAAAFRPTVERVARATDGETVDLSVLRGQR
MWFVDQIESSYRLRAVSAVGLRFPLNGTANGKAALAALDDADAEAALCRLDPMVAEGL
RREIVEIRRTGIAFDRNEHTPGISAAAIARRALGDNVIAISVPAPTARFLEKEQRIIA
ALRAAADSPDWTR"

CDS 2001630..2002970
/codon_start=1
/note="gene_1824"
/transl_table=11
/translation="MRALPAGRHFFRGSDGYEAARRGTVWHRRVPDRYPEVIVQAVSA
DDIVSAIRYATVNGHKVSVVSGGHSFAASHLRDGAVLLDVSRIDHASIDADKGRAVVG
PGKGGSVLMAELEAQGLFFPGGHCRGVCLGGYLLQGGYGWNSRIYGPACESVIGLDVI
TADGAQIHCDADNHADLYWAARGAGPGFFGVVTSFYLKLYPRPATCGTSVYVYPFDLA
DEVFTWARAVSAEVDPRVELQALASRGEPSMGIDVPVISLASPAFADSPEEAEQALAL
FGTCPVVEQALVKVPYMPTDLPAWYDVAMTHYLSDHHYAVDNMWTSASAEDLLPGIRS
ILDTLPPHPAHFLWLNWGPCPPRQDMAYSIEADIYLALYGSWKDPADEAKYADWARSH
MAAMSHLAVGIQLADENLGARPARFASDAAMAKLDRVRAEYDPDGLFNSWMGRI"

```
CDS             2002970..2003788
                /codon_start=1
                /note="gene_1825"
                /transl_table=11
                /translation="MASDLYLGYRNDDADTPFGKFFKPEMAPLPQHVVVALQHGPQAG
                MALLAFDDAASIVDEGYQQTENGYGILGDGSMQVSVRTDMPGVTPAMWAWWFGWHGSD
                TRRYKLWHPRAHLSARWKDGDQDSGAGRRGAQRYVGRWSMISEYIGSTKLGAAIQFVE
                PAAMGLPDDSDDTVSICARLGSADAPVDAGWFVHQVRSTPGGSEMRSRFWMGGPHIAV
                RKAPEVASKAVRPIASKLIGVSESTARNLLVYCAQEMNHLAGFLADLWESFGDE"
CDS             complement(2003793..2004353)
                /codon_start=1
                /note="gene_1826"
                /transl_table=11
                /translation="VPGNDWIVGGNRRTIAAERIYAAATDLITRYGLNALDIDKLARE
                VHCSRATIYRRAGGKAQIRDVVLTRAAARIADGVRSDVETLRGRERVVAAILLSLQRI
                RSDPLGKLMFGSIHGGAGELAWLTESPLLADFATELTGIAGGDPQGAKWVVRVVLSLM
                YWPAENDEAERRLVEKYVAPAFAEQS"
CDS             2004544..2005758
                /codon_start=1
                /note="gene_1827"
                /transl_table=11
                /translation="MTIAKDANTFFGAESVQDPYPLYERMRAAGSVHRIANSDFYAVC
                GWDAVNEAIGRPEDFSSNLTATMTYTAEGTAKPFEMDPLGGPTHVLATADDPAHAVHR
                KLVLRHLAAKRIRVMEQFTVQAADRLWVDGMQDGCIEWMGAMANRLPMMVVAELIGLP
                DPDIAQLVKWGYAATQLLEGLVENDQLVAAGVALMELSGYIFEQFDRAAADPRDNLLG
                ELATACASGELDTLTAQVMMVTLFAAGGESTAALLGSAVWILATRPDIQQQVRANPEL
                LGAFIEETLRYEPPFRGQYRHVRNATTLDGTELPADSHLLLLWGAANRDPAQFEAPGE
                FRLDRAGGKGHISFGKGAHFCVGAALARLEARIVLRLLLDRTSVIEAADVGGWLPSIL
                VRRIERLELAVQ"
CDS             complement(2005879..2006385)
                /codon_start=1
                /note="gene_1828"
                /transl_table=11
                /translation="LGKDRGGIGTREFDRSGGDMRVSLFLSDAAQADAQSGKVHALGL
                GWRQCQTPTPPFALVLFLDIDWDETNKQHQLKCQLLTADGDPVVVPGPHGPQRILFEA
                AAEAGRAPGAIHGTSVRMPLTLNIPAGIPLEPGIYEWRVEVEGYERATAVEAFIVAGG
                GHPPASCG"
CDS             complement(2006484..2008277)
                /codon_start=1
                /note="gene_1829"
                /transl_table=11
                /translation="MCAHEYAEQRSAVSGIEGLLTWLGGGHWRELGERHERSTHAVAG
                VIVAVGAALAGLLASLAVSEAAQGPISSPIGAASLALVLGLLVGAVTRGTASGPARGR
                AGVTGRASVAVAVGFVVGELAALVMFSGAIDRRLDEQAMHSADATPAAVQASASLQQA
                RNARTALDSAVERARGRLDDALVVARCEYHPTPACPQTRITGVPGRGPETRTANQLLA
                DAQRELDNALAARDHQAPALDAKMAHDEQALAEVRQAVVADAGRGLGSRWVAMNDLTL
                ASAGALTARMLAIAFFALLYLLPLILRLWRGDTTHDRHAAARAERERAELEADTAIAI
                KRAEVRRAAEIMWAEHQLTQTRLAIEAQAEIDREQQRRRVVEALEGPVRASSERTLQP
                VEDEVYLPIAAETEAASRTVAQLPAGAAHHRPGIAKNLPAQVQPEGAVEPREKRATPV
                IRSIPDATKAAARWIRPLVPPFVARMLDNTTAPLRTARQVFEEVEEIAFSFKRTHKVT
                VNAEGSDPNDQPPLESHSPAAPAESNPIASSDSARRSRLATNDDHPPLAQVPPRDLAS
```

```
                     LSVGSTGELTQREGPHELRSPDGPRQLPPPR"
     CDS             2008497..2009060
                     /codon_start=1
                     /note="gene_1830"
                     /transl_table=11
                     /translation="MQNHDYVTYEEFGRRFFEVAVTPDRVAAAFADIAGSEFAMEPIS
                     QGPGGIAKVSANVKIREPRVTRKLGDLITFVIHIPLSIDLLLDLRLDKQRFMVAGDIA
                     LRATARAAEPLLLIVDVAKPRPSDITVNVSSKSIRGEVLRILAGVDGEIRRFIAQYVS
                     AEIDSPKSQAAQVINVAEQLDSTWSGP"
     CDS             complement(2009100..2011283)
                     /codon_start=1
                     /note="gene_1831"
                     /transl_table=11
                     /translation="MGDMTELAPSLVELTRRFGIATEYTDWTGRQVLVSEATLVAALA
                     ALGVPAQTEQQRNDALAAQLRSYWARPLPATIVMRAGEQTQFRVHVTDGAPADVWLQL
                     EDGTTRAEVVQVDNFTPPFDLDGRWIGEASFVLPADLPLGYHRVNLRSGDSQASAAVV
                     VTPDWLGLPDKLAGRRAWGLAVQLYSVRSRQSWGIGDLTDLANLALWSASAHGAGYVL
                     VNPLHAATLPGPAGRSKPIEPSPYLPTSRRFVNPLYLRVEAIPELVDLPKRGRVQRLR
                     TNVQQHADQLDTIDRDSAWAAKRAALKLVHRVPRSAGRELAYAAFRTREGRALDDFAT
                     WCALAETYGDDWHRWPKSLRHPDASGVADFVDKHADAVDFHRWLQWQLDEQLASAQSQ
                     ALRAGMSLGIMADLAVGVHPNGADAWALQDVLAQGVTAGAPPDEFNQLGQDWSQPPWR
                     PDRLAEQEYRPFRALIQAALRHAGAVRIDHIIGLFRLWWIPDGAPPTQGTYVRYDHDA
                     MIGIVALEAHRAGAVVVGEDLGTVEPWVRDYLLLRGLLGTSILWFEQDRDCGPAGTPL
                     PAERWREYCLSSVTTHDLPPTAGYLAGDQVRLRESLGLLTNPVEAELESARADRAAWM
                     AELRRVGLLADGAEPDSEEAVLALYRYLGRTPSRLLAVALTDAVGDRRTQNQPGTTDE
                     YPNWRVPLTGPDGQPMLLEDIFTDRRAATLAEAVRAATTSPMSCW"
     CDS             2011538..2013058
                     /codon_start=1
                     /note="gene_1832"
                     /transl_table=11
                     /translation="VAEESRGQRGSGYGLGLSTRTQVTGYQFLARRTAMALTRWHVRM
                     EIEPGRRQTLAVVASVSAALVICLGALLWSFISPSGQLNESPIIADRDSGALYVRVGD
                     RLYPALNLASARLITGRPDNPHLVRSSQIATMPRGPLVGIPGAPSSFSPKSPPASSWL
                     VCDTVATSSSIGSLQGVTVTVIDGTPDLTGHRQILSGSDAVVLRYGGDAWVIREGRRS
                     RIEPTNRAVLLPLGLTPEQVSQARPMSRALFDALPVGPELLVPEVPNAGGPATFPGAP
                     GPIGTVIVTPQISGPQQYSLVLGDGVQTLPPLVAQILQNAGSAGNTKPLTVEPSTLAK
                     MPVVNRLDLSAYPDNPLEVVDIREHPSTCWWWERTAGENRARVRVVSGPTIPVAATEM
                     NKVVSLVKADTSGRQADQVYFGPDHANFVAVTGNNPGAQTSESLWWVTDAGARFGVED
                     SKEARDALGLTLTPSLAPWVALRLLPQGPTLSRADALVEHDTLPMDMTPAELVVPK"
     CDS             2013055..2017230
                     /codon_start=1
                     /note="gene_1833"
                     /transl_table=11
                     /translation="MKRGFARPTPEKPPVIKPENIVLSTPLSIPPPEGKPWWLIVVGV
                     VVVGLLGGMVAMVFASGSHVFGGIGSIFPLFMMVGIMMMMFRGMGGGQQQMSRPKLDA
                     MRAQFMLMLDMLRETAQESADSMDANYRWFHPAPNTLAAAVGSPRMWERKPDGKDLNF
                     GVVRVGVGMTRPEVTWGEPQNMPTDIELEPVTGKALQEFGRYQSVVYNLPKMVSLLVE
                     PWYALVGEREQVLGLMRAIICQLAFSHGPDHVQMIVVSSDLDQWDWVKWLPHFGDSRR
                     HDAAGNARMVYTSVREFAAEQAELFAGRGSFTPRHASSSAQTPTPHTVIIADVDDPQW
                     EYVISAEGVDGVTFFDLTGSSMWTDIPERKLQFDKTGVIEALPRDRDTWMVIDDKAWF
                     FALTDQVSIAEAEEFAQKLAQWRLAEAYEEIGQRVAHIGARDILSYYGIDDPGNIDFD
```

SLWASRTDTMGRSRLRAPFGNRSDNGELLFLDMKSLDEGGDGPHGVMSGTTGSGKSTL
VRTVIESLMLSHPPEELQFVLADLKGGSAVKPFAGVPHVSRIITDLEEDQALMERFLD
ALWGEIARRKAICDSAGVDDAKEYNSVRARMRARGQDMAPLPMLVVVIDEFYEWFRIM
PTAVDVLDSIGRQGRAYWIHLMIASQTIESRAEKLMENMGYRLVLKARTAGAAQAAGV
PNAVNLPAQAGLGYFRKSLEDIIRFQAEFLWRDYFQPGVSIDGEEAPALVHSIDYIRP
QLFTNSFTPLEVSVGGPDIEPVVAQPNGEMLESDDIEGGEDEDEEGVRTPKVGTVIID
QLRKIKFEPYRLWQPPLTQPVAIDDLVNRFLGRPWHKEYGSACNLVFPIGIIDRPYKH
DQPPWTVDTSGPGANVLILGAGGSGKTTALQTLICSAALTHTPQQVQFYCLAYSSTAL
TTVSRIPHVGEVAGPTDPYGVRRTVAELLALVRERKRSFLECGIASMEMFRRRKFGGE
AGPVPDDGFGDVYLVIDNYRALAEENEVLIEQVNVIINQGPSFGVHVVVTADRESELR
PPVRSGFGSRIELRLAAVEDAKLVRSRFAKDVPVKPGRGMVAVNYVRLDSDPQAGLHT
LVARPALGSTPDNVFECDSVVAAVSRLTSAQAPPVRRLPARFGVEQVRELASRDTRQG
VGAGGIAWAISELDLAPVYLNFAENSHLMVTGRRECGRTTTLATIMSEIGRLYAPGAS
SAPPPAPGRPSAQVWLVDPRRQLLTALGSDYVERFAYNLDGVVAMMGELAAALAGREP
PPGLSAEELLSRSWWSGPEIFLIVDDIQQLPPGFDSPLHKAVPFVNRAADVGLHVIVT
RTFGGWSSAGSDPMLRALHQANAPLLVMDADPDEGFIRGKMKGGPLPRGRGLLMAEDT
GVFVQVAATEVRR"

CDS complement(2017245..2018426)
/codon_start=1
/note="gene_1834"
/transl_table=11
/translation="MTTPGEDHAGSFYLPRLEYSTLPMAVDRGVGWKTLRDAGPVVFM
NGWYYLTRREDVLAALRNPKVFSSRKALQPPGNPLPVVPLAFDPPEHTRYRRILQPYF
SPAALSKALPSLRRHTVAMIDAIAGRGECEAMADLANLFPFQLFLVLYGLPLEDRDRL
IGWKDAVIAMSDRPHPTEADVAAARELLEYLTAMVAERRRNPGPDVLSQVQIGEDPLS
EIEVLGLSHLLILAGLDTVTAAVGFSLLELARRPQLRAMLRDNPKQIRVFIEEIVRLE
PSAPVAPRVTTEPVTVGGMTLPAGSPVRLCMAAVNRDGSDAMSTDELVMDGKVHRHWG
FGGGPHRCLGSHLARLELTLLVGEWLNQIPDFELAPDYAPEIRFPSKSFALKNLPLRW
S"

CDS 2018656..2018829
/codon_start=1
/note="gene_1835"
/transl_table=11
/translation="VGHAQCYAVDPDLFPIDDSGNSILAEHEVRPEDMQLTRDGVAAC
PEMALILEEDDAD"

CDS 2019081..2020193
/codon_start=1
/note="gene_1836"
/transl_table=11
/translation="VEREDWLDFGALPPEINSGRMYCGPGSGPMLAAAAAWDGVAVEL
GLAATGYASVIAELTGAPWVGAASLSMVAAATPYVAWLSQAAARAEQAGMQAAAAAAA
YEAAFVMTVPPPVITANRVLVMTLIATNFFGQNSAAIAVAEAQYAEMWAQDAVAMYGY
AAASASASRLIPFAAPPKTTNSAGVVAQVAAVAAMPGLLQRLSSAASVSWSNPNDWWL
VRLLGSITPTERTTIVRLLGQSYFATGMAQFFASIAQQLTFGPGGTTAGSGGAWYPTP
QFAGLGASRAVSASLARANKIGALSVPPSWVKTTALTEPGAHAVSANPTVGSSHGPHG
LLRGLPLGSRITRRSGAFAHRYGFRHSVVARPPSAG"

CDS 2020272..2020571
/codon_start=1
/note="gene_1837"
/transl_table=11
/translation="MSFVTTQPEALAAAAGSLQGIGSALNAQNAAAATPTTGVVPAAA

DEVSALTAAQFAAHAQIYQAVSAQAAAIHEMFVNTLQMSSGSYAATEAANAAAAG"
CDS 2020585..2021766
/codon_start=1
/note="gene_1838"
/transl_table=11
/translation="MDFGALPPEVNSVRMYAGPGSAPMVAAASAWNGLAAELSSAATG
YETVITQLSSEGWLGPASAAMAEAVAPYVAWMSAAAAQAEQAATQARAAAAAFEAAFA
ATVPPPLIAANRASLMQLISTNVFGQNTSAIAAAEAQYGEMWAQDSAAMYAYAGSSAS
ASAVTPFSTPPQIANPTAQGTQAAAVATAAGTAQSTLTEMITGLPNALQSLTSPLLQS
SNGPLSWLWQILFGTPNFPTSISALLTDLQPYASFFYNTEGLPYFSIGMGNNFIQSAK
TLGLIGSAAPAAVAAAGDAAKGLPGLGGMLGGGPVAAGLGNAASVGKLSVPPVWSGPL
PGSVTPGAAPLPVSTVSAAPEAAPGSLLGGLPLAGAGGAGAGPRYGFRPTVMARPPFA
G"
CDS 2022181..2023272
/codon_start=1
/note="gene_1839"
/transl_table=11
/translation="MSFATPQPEKGFGMDFGALPPEINSGRMYCGPGSGPMLAAAAAW
DGVAVELGLAATGYASVIAELTGAPWVGAASLSMVAAATPYVAWLSQAAARAEQAGMQ
AAAAAAAYEAAFVMTVPPPVITANRVLVMTLIATNFFGQNSAAIAVAEAQYAEMWAQD
AVAMYGYAAASASASRLIPFAAPPKTTNSAGVVAQAVASVSWPNPNDWWLVRLLGSIT
PTERTTIVRLLGQSYLATGMARFLTSIAQQLTFGPGGTTAGSGGAWYPTPQFAGLGAG
PAVSASLARAEPVGRLSVPPSWAVAAPAFAEKPEAGTPMSVIGEASSCGQGGLLRGIP
LARAGRRTGAFAHRYGFRHSVITRSPSAG"
CDS 2023699..2023998
/codon_start=1
/note="gene_1840"
/transl_table=11
/translation="MSFVTTQPEALAAAAANLQGIGTTMNAQNAAAAAPTTGVVPAAA
DEVSALTAAQFAAHAQMYQTVSAQAAAIHEMFVNTLVASSGSYAATEAANAAAAG"
CDS 2024142..2024438
/codon_start=1
/note="gene_1841"
/transl_table=11
/translation="MASRFMTDPHAMRDMAGRFEVHAQTVEDEARRMWASAQNISGAG
WSGQAEATSLDTMTQMNQAFRNIVNMLHGVRDGLVRDANNYEQQEQASQQILSS"
CDS 2024489..2024773
/codon_start=1
/note="gene_1842"
/transl_table=11
/translation="MTINYQFGDVDAHGAMIRAQAASLEAEHQAIVRDVLAAGDFWGG
AGSVACQEFITQLGRNFQVIYEQANAHGQKVQAAGNNMAQTDSAVGSSWA"
CDS 2024861..2025763
/codon_start=1
/note="gene_1843"
/transl_table=11
/translation="MDQQSTRTDITVNVDGFWMLQALLDIRHVAPELRCRPYVSTDSN
DWLNEHPGMAVMREQGIVVNDAVNEQVAARMKVLAAPDLEVVALLSRGKLLYGVIDDE
NQPPGSRDIPDNEFRVVLARRGQHWVSAVRVGNDITVDDVTVSDSASIAALVMDGLES
IHHADPAAINAVNVPMEEMLEATKSWQESGFNVFSGGDLRRMGISAATVAALGQALSD
PAAEVAVYARQYRDDAKGPSASVLSLKDGSGGRIALYQQARTAGSGEAWLAICPATPQ

LVQVGVKTVLDTLPYGEWKTHSRV"
CDS 2026035..2027546
/codon_start=1
/note="gene_1844"
/transl_table=11
/translation="MTAVADAPQADIEGVASPQAVVVGVMAGEGVQIGVLLDANAPVS
VMTDPLLKVVNSRLRELGEAPLEATGRGRWALCLVDGAPLRATQSLTEQDVYDGDRLW
IRFIADTERRSQVIEHISTAVASDLSKRFARIDPIVAVQVGASMVATGVVLATGVLGW
WRWHHNTWLTTIYTAVIGVLVLAVAMLLLMRAKTDADRRVADIMLMSAIMPVTVAAAA
APPGPVGSPQAVLGFGVLTVAAALALRFTGRRLGIYTTIVIIDALTMLAALARMVAAT
SAVTLLSSLLLICVVAYHAAPALSRRLAGIRLPVFPSATSRWVFEARPDLPTTVVVSG
GSAPVLEGPSSVRDVLLQAERARSFLSGLLTGLGVMVVVCMTSLCDPHTGQRWLPLIL
AGFTSGFLLLRGRSYVDRWQSITLAGTAVIIAAAVCVRYALELSSPLAVSIVAAILVL
LPAAGMAAAAHVPHTIYSPLFRKFVEWIEYLCLMPIFPLALWLMNVYAAIRYR"
CDS 2027524..2029281
/codon_start=1
/note="gene_1845"
/transl_table=11
/translation="MQRFGTGSSRSWCGRAGTATIAAVLLASGALTGLPPAYAISPPT
IDPGALPPDGPPGPLAPMKQNAYCTEVGVLPGTDFQLQPKYMEMLNLNEAWQFGRGDG
VKVAVIDTGVTPHPRLPRLIPGGDYVMAGGDGLSDCDAHGTLVASMIAAVPANGAVPL
PSVPRRPVTIPTTETPPPPQTVTLSPVPPQTVTVIPAPPPEEGVPPGAPVPGPEPPPA
PGPQPPAVDRGGGTVTVPSYSGGRKIAPIDNPRNPHPSAPSPALGPPPDAFSGIAPGV
EIISIRQSSQAFGLKDPYTGDEDPQTAQKIDNVETMARAIVHAANMGASVINISDVMC
MSARNVIDQRALGAAVHYAAVDKDAVIVAAAGDGSKKDCKQNPIFDPLQPDDPRAWNA
VTTVVTPSWFHDYVLTVGAVDANGQPLSKMSIAGPWVSISAPGTDVVGLSPRDDGLIN
AIDGPDNSLLVPAGTSFSAAIVSGVAALVRAKFPELSAYQIINRLIHTARPPARGVDN
QVGYGVVDPVAALTWDVPKGPAEPPKQLSAPLVVPQPPAPRDMVPIWVAAGGLAGALL
IGGAVFGTATLMRRSRKQQ"
CDS 2029278..2030498
/codon_start=1
/gene="eccE5"
/note="gene_1846"
/transl_table=11
/translation="MKAQRSFGLALSWPRVTAVFLVDVLILAVASHCPDSWQADHHVA
WWVGVGVAAVVTLLSVVSYHGITVISGLATWVRDWSADPGTTLGAGCTPAIDHQRRFG
RDTVGVREYNGRLVSVIEVTCGESGPSGRHWHRKSPVPMLPVVAVADGLRQFDIHLDG
IDIVSVLVRGGVDAAKASASLQEWEPQGWKSEERAGDRTVADRRRTWLVLRMNPQRNV
AAVACRDSLASTLVAATERLVQDLDGQSCAARPVTADELTEVDSAVLADLEPTWSRPG
WRHLKHFNGYATSFWVTPSDITSETLDELCLPDSPEVGTTVVTVRLTTRVGSPALSAW
VRYHSDTRLPKEVAAGLNRLTGRQLAAVRASLPAPTHRPLLVIPSRNLRDHDELVLPV
GQELEHATSSFVGQ"
CDS 2030495..2032327
/codon_start=1
/note="gene_1847"
/transl_table=11
/translation="MTRPQAAAEDARNAMVAGLLASGISVNGLQPSHNPQVAAQMFTT
ATRLDPKMCDAWLARLLAGDQSIEVLAGAWAAVRTFGWETRRLGVTDLQFRPEVSDGL
FLRLAITSVDSLACAYAAVLAEAKRYQEAAELLDATDPRHPFDAELVSYVRGVLYFRT
KRWPDVLAQFPEATQWRHPELKAAGAAMATTALASLGVFEEAFRRAQEAIEGDRVPGA
ANIALYTQGMCLRHVGREEEAVELLRRVYSRDAKFTPAREALDNPNFRLILTDPETIE

ARTDPWDPDSAPTRAQTEAARHAEMAAKYLAEGDAELNAMLGMEQAKKEIKLIKSTTK
VNLARAKMGLPVPVTSRHTLLLGPPGTGKTSVARAFTKQLCGLTVLRKPLVVETSRTK
LLGRYMADAEKNTEEMLEGALGGAVFFDEMHTLHEKGYSQGDPYGNAIINTLLLYMEN
HRDELVVFGAGYAKAMEKMLEVNQGLRRRFSTVIEFFSYTPQELIALTQLMGRENEDV
ITEEESQVLLPSYTKFYMEQSYSEDGDLIRGIDLLGNAGFVRNVVEKARDHRSFRLDD
EDLDAVLASDLTEFSEDQLRRFKELTREDLAEGLRAAVAEKKTK"

CDS 2032954..2033145
/codon_start=1
/note="gene_1848"
/transl_table=11
/translation="MSVKSKNGRLAARVLVALAALFAMIALTGSACLAEGPPLGRNPQ
GAPAPVGGTVIVAPMHSGV"

CDS 2033248..2035215
/codon_start=1
/note="gene_1849"
/transl_table=11
/translation="MLPNFAVLPPEVNSARVFAGAGSAPMLAAAAAWDDLASELHCAA
MSFGSVTSGLVVGWWQGSASAAMVDAAASYIGWLSTSAAHAEGAAGLARAAVSVFEEA
LAATVHPAMVAANRAQVASLVASNLFGQNAPAIAALESLYEWMWAQDAAAMAGYYVGA
SAVATQLASWLQRLQSIPGAASLDARLPSSAEAPMGVVRAVNSAIAANAAAAQTVGLV
MGGSGTPIPSARYVELANALYMSGSVPGVIAQALVTPQGLYPVVVIKNLTFDSSVAQG
AVILESAIRQQIAAGNNVTVFGYSQSATISSLVMANLAASADPPSPDELSFTLIGNPN
NPNGGVATRFPGISFPSLGVTATGATPHNLYPTKIYTIEYDGVADFPRYPLNFVSTLN
AIAGTYYVHSNYFILTPEQIDAAVPLTNTVGPTMTQYYIVRTENLPLLEPLRSVPIVG
NPLANLVQPNLKVIVNLGYGDPAYGYSTSPPNVATPFGLFPEVSPVVIADALAAGTQQ
GIGDFAYDVSHLELPLPADGSTMPSTAPGSGTPVPPLSIDSLIDDLQVANRNLANTIS
KVAATSYATVLPTADIANAALTIVPSYNIHLFLEGIQQALKGDPMGLVNAVGYPLAAD
VALFTAAGGLQLLIIISAGRTIANDISAIVP"

CDS 2035796..2037067
/codon_start=1
/note="gene_1850"
/transl_table=11
/translation="MDFGLLPPEINSGRMYTGPGPGPMLAAATAWDGLAVELHATAAG
YASELSALTGAWSGPSSTSMASAAAPYVAWMSATAVHAELAGAQARLAIAAYEAAFAA
TVPPPVIAANRAQLMVLIATNIFGQNTPAIMMTEAQYMEMWAQDAAAMYGYAGSSATA
SRMTAFTEPPQTTNHGQLGAQSSAVAQTAATAAGGNLQSAFPQLLSAVPRALQGLALP
TASQSASATPQWVTDLGNLSTFLGGAVTGPYTFPGVLPPSGVPYLLGIQSVLVTQNGQ
GVSALLGKIGGKPITGALAPLAEFALHTPILGSEGLGGGSVSAGIGRAGLVGKLSVPQ
GWTVAAPEIPSPAAALQATRLAAAPIAATDGAGALLGGMALSGLAGRAAAGSTGHPIG
SAAAPAVGAAAAAVEDLATEANIFVIPAMDD"

CDS 2037179..2038570
/codon_start=1
/note="gene_1851"
/transl_table=11
/translation="MDFGVLPPEINSGRMYAGPGSGPMLAAAAAWDGLATELQSTAAD
YGSVISVLTGVWSGQSSGTMAAAAAPYVAWMSATAALAREAAAQASAAAAAYEAAFAA
TVPPPVVAANRAELAVLAATNIFGQNTGAIAAAEARYAEMWAQDAAAMYGYAGSSSVA
TQVTPFAAPPPTTNAAGLATQGVAVAQAVGASAGNARSLVSEVLEFLATAGTNYNKTV
ASLMNAVTGVPYASSVYNSMLGLGFAESKMVLPANDTVISTIFGMVQFQKFFNPVTPF
NPDLIPKSALGAGLGLRSAISSGLGSTAPAISAGASQAGSVGGMSVPPSWAAATPAIR
TVAAVFSSTGLQAVPAAAISEGSLLSQMALASVAGGALGGAAARATGGFLGGGRVTAV

```
                     KKSLKDSDSPDKLRRVVAHMMEKPESVQHWHTDEDGLDDLLAELKKKPGIHAVHMAGG
                     NKAEIAPTISESG"
     CDS             complement(2038718..2040340)
                     /codon_start=1
                     /gene="PE_PGRS32b"
                     /note="gene_1852"
                     /transl_table=11
                     /translation="LQIVGQTALDAINSPVQTLTGRPLIGNGANGVAGTGQNGGDGGW
                     LYGNGGNGGSGGTGQNGGNGGSAGLWGSGGNGGQGGAGANGAAGQPGKAGGSGGNGGA
                     GGWIYGHGGHGGAGGNGGNATAPGGASAGFDGGAGGNGGSGGRGGLLFGNGGNGSVGG
                     MGGQGTNDTAGDSAGSGGLGGNGGNGAQGGWLIGNGGQGGDSGAGGGTDSTQTGVMNG
                     ASGGSAGIAGNGGDAGLVGNGGAGGNGGNGAAGSALGTTIFGGSGGVGGSGGDGGNGG
                     WLFGSGASGGNGGQGGDAGTNGFAGFGGSAGGGGWVGAVNFGPISVQGFGLFGHGGDG
                     GNGGDVGAGSLSIQFGASGGDGGQGGVLYGNGGNGGNAGSGGGTGFEGSAGQGGAAIL
                     IGNGGAGGNGATGGTGVGNIIQEAGGDGSDGGAGGSGGLLFGSGGAGGIGGAGGVGGS
                     GNDGGNGGDGGQGGASGLGIGNGGPGGSGGTGGAGGTGGSAGTGGAGGDGGNAALLIG
                     TGGDGGDGVPPAPGGQGGKGGLIGLPGQNGQP"
     CDS             complement(2040374..2040670)
                     /codon_start=1
                     /gene="PE_PGRS32"
                     /note="gene_1853"
                     /transl_table=11
                     /translation="MDSPCGDNGQEVWTSQMIVAPAFVDAAAKDLATIGSAISRANAE
                     ALVPITALLPAGADDVSAAIAALFATHGQAYQELSAHAVAFHEQFVQLMSAGAA"
     CDS             complement(2040818..2041135)
                     /codon_start=1
                     /note="gene_1854"
                     /transl_table=11
                     /translation="VSTLLSIPLMIGLAVPAHAGPSGDDAVFLASLERAGITYSHPDQ
                     AIASGKAVCALVESGESGLQVVNELRTRNPGFSMDGCCKFAAISAHVYCPHQITKTSV
                     SAK"
     CDS             2041867..2042166
                     /codon_start=1
                     /note="gene_1855"
                     /transl_table=11
                     /translation="MAFVLVCPDALAIAAGQLRHVGSVIAARNAVAAPATAELAPAAA
                     DEVSALTATQFNFHAAMYQAVGAQAIAMNEAFVAMLGASADSYAATEAANIIAVS"
     CDS             2042181..2043392
                     /codon_start=1
                     /note="gene_1856"
                     /transl_table=11
                     /translation="MTAALDFATLPPEINSARMYSGAGSAPMLAAASAWHGLSAELRA
                     SALSYSSVLSTLTGEEWHGPASASMTAAAAPYVAWMSVTAVRAEQAGAQAEAAAAAYE
                     AAFAATVPPPVIEANRAQLMALIATNVLGQNAPAIAATEAQYAEMWSQDAMAMYGYAG
                     ASAAATQLTPFTEPVQTTNASGLAAQSAAIAHATGASAGAQQTTLSQLIAAIPSVLQG
                     LSSSTAATSASGPSGLLGILGSGSSWLDKLWALLDPNSNFWNTIASSGLFLPSNTIAP
                     FLGLLGGVAAADAAGDVLGEATSGGLGGALVAPLGSAGGLGGTVAAGLGNAATVGTLS
                     VPPSWTAAAPLASPLGSALGGTPMVAPPPAVAAGMPGMPFGTMGGQGFGRAVPQYGFR
                     PNFVARPPAAG"
     CDS             2043713..2044945
                     /codon_start=1
```

/note="gene_1857"
/transl_table=11
/translation="MLDFGALPPEINSGRMYAGPGSGPLLAAAAAWDALAAELYSAAA
SYGSTIEGLTVAPWMGPSSITMAAAVAPYVAWISVTAGQAEQAGAQAKIAAGVYETAF
AATVPPPVIEANRALLMSLVATNIFGQNTPAIAATEAHYAEMWAQDAAAMYGYAGSSA
TASQLAPFSEPPQTTNPSATAAQSAVVAQAAGAAASSDITAQLSQLISLLPSTLQSLA
TTATATSASAGWDTVLQSITTILANLTGPYSIIGLGAIPGGWWLTFGQILGLAQNAPG
VAALLGPKAAAGALSPLAPLRGGYIADITPLGGGATGGIARAIYVGSLSVPQGWAEAA
PVMRAVASVLPGTGAAPALAAEAPGALFGEMALSSLAGRALAGTAVRSGAGAARVAGG
SVTEDVASTTTIIVIPAD"

CDS complement(2044929..2045198)
/codon_start=1
/gene="MGAST_03725"
/note="gene_1858"
/transl_table=11
/translation="MDCNSAANPSQATAAANIGPAPGPRYISPEVISGGCNPKSIATP
FLNRPLTGWPPATGHSGDDTVLLSSPVGANTQFKCHLESPVSPRV"

CDS 2045089..2045640
/codon_start=1
/note="gene_1859"
/transl_table=11
/translation="LQPPEITSGEMYLGPGAGPMLAAAVAWDGLAAELQSMAASYASI
VEGMASESWLGPSSAGMAAAAAPYVTWMSGTSAQAKAAADQARAAVVAYETAFAAVVP
PPQIAANRSQLISLVATNIFGQNTAAIAATEAEYGEMWAQDTMAMFGYASSSATASRL
TPFTAPPQTTNPSGLAGQAAATG"

CDS 2045671..2046489
/codon_start=1
/note="gene_1860"
/transl_table=11
/translation="VTTALSSAAAQFPFDIIPTLLQGLATLSTQYTQLMGQLINAIFG
PTGATTYQNLFVTAANVTKFSTWANDAMSAPNLGMTEFKVFWQPPPAPEIPKSSLGAG
LGLRSGLSAGLAHAASAGLGQANLVGDLSVPPSWASATPAVRLVANTLPATSLAAAPA
TQIPANLLGQMALGSMTGGALGAAAPAIYTGSGARARANGGTPSAEPVKLEAVIAQLQ
KQPDAVRHWNVDKADLDGLLDRLSKQPGIHAVHVSNGDKPKVALPDTQLGSHST"

CDS 2046770..2047084
/codon_start=1
/note="gene_1861"
/transl_table=11
/translation="MLVALLLSAATMIGLAAPGKADPTGDDAAFLAALDQAGITYADP
GHAITAAKAMCGLCANGVTGLQLVADLRDYNPGLTMDSAAKFAAIASGAYCPEHLEHH
PS"

CDS 2047238..2047942
/codon_start=1
/note="gene_1862"
/transl_table=11
/translation="MQTLTVADFALRLAVGVGCGAIIGLERQWRARMAGLRTNALVAT
GATLFVLYAVATEDSSPTRVASYVVSGIGFLGGGVILREGFNVRGLNTAATLWCSAAV
GVLAASGHLVFTLIGTGTIVAVHLLGRPLGRLVDRDNAVEDEGLQPYQVRVICRPKAE
TYVRAHIVQRTSSNDITLRGIRTGPAGDDNITLTAHLLMVGHTPAKLERLVAELSLQP
GVYAVHWYAGEHAQAE"

CDS complement(2047952..2049154)

```
                     /codon_start=1
                     /note="gene_1863"
                     /transl_table=11
                     /translation="LTRVVVIGSGFAGLWAALGAARRLDELAVPAGTVDVMVVSNKPF
                     HDIRVRNYEADLSACRIPLGDVLGPAGVAHVTAEVTAIDADGRRVTTSTGASYSYDRL
                     VLASGSHVVKPALPGLAEFGFDVDTYDGAVRLQQHLQGLAGGPLTSAAATVVVVGAGL
                     TGIETACELPGRLHALFARGDGVTPRVVLIDHNPFVGSDMGLSARPVIEQALLDNGVE
                     TRTGVSVAAVSPGGVTLSSGERLAAATVVWCAGMRASRLTEQLPVARDRLGRLQVDDY
                     LRVIGVPAMFAAGDVAAARMDDEHLSVMSCQHGRPMGRYAGCNVINDLFDQPLLALRI
                     PWYVTVLDLGSAGAVYTEGWERKVVSQGAPAKTTKQSINTRRIYPPLNGSRADLLAAA
                     APRVQPRP"
     CDS             complement(2049476..2049907)
                     /codon_start=1
                     /note="gene_1864"
                     /transl_table=11
                     /translation="MITNLRRRTAMAAAGLGAALGLGILLVPTVDAHLANGSMSEVMM
                     SEIAGLPIPPIIHYGAIAYAPSGASGKAWHQRTPARAEQVALEKCGDKTCKVVSRFTR
                     CGAVAYNGSKYQGGTGLTRRAAEDDAVNRLEGGRIVNWACN"
     CDS             2050274..2051218
                     /codon_start=1
                     /note="gene_1865"
                     /transl_table=11
                     /translation="VDAVAGFFSALPEEMRDPVLFAIPCFLLLLILEWTAARKLESIE
                     TAATGQPRPASGAYLTRDSVASISMGLVSIATTAGWKSLALLGYAAIYAYLAPWQLSA
                     HRWYTWVIAIVGVDLLYYSYHRIAHRVRLIWATHQAHHSSEYFNFATALRQKWNNSGE
                     ILMWVPLPLMGLPPWMVFCSWSLNLIYQFWVHTERIDRLPRWFEFVFNTPSHHRVHHG
                     MDPVYLDKNYGGILIIWDRLFGSFQPELFRPHYGLTKRVDTFNIWKLQTREYVAIVRD
                     WRSATRLRDRLGYVFGPPGWEPRTIDKSNAAASLVTSR"
     CDS             2051323..2051988
                     /codon_start=1
                     /note="gene_1866"
                     /transl_table=11
                     /translation="VVRLVPRAFAATVALLAAGFSPATASADPVLVFPGMEIRQDNHV
                     CTLGYVDPALKIAFTAGHCRGGGAVTSRDYKVIGHLRAFRDNTPSGSTVATHELIADY
                     EAIVLADDVTASNILPSGRALESRPGVVLHPGQAVCHFGVSTGETCGTVESVNNGWFT
                     MSHGVLSEKGDSGGPVYLAPDGGPAQIVGIFNSVWGGFPAAVSWRSTSEQVHADLGVT
                     PLA"
     CDS             2052069..2052755
                     /codon_start=1
                     /note="gene_1867"
                     /transl_table=11
                     /translation="VGKRRDAREQIEAKIVELGRRQLLDHGAAGLSLRAIARNLGMVS
                     SAVYRYVSSRDELLTLLLVDAYSDLADTVDRARDDTVADSWSDDVIAIARAVRGWAVT
                     NPARWALLYGSPVPGYHAPPDRTAGVATRVVGAFFDAIAAGIATGDIRLTDDVAPQPM
                     SSDFEKIRQEFGFPGDDRVVTKCFLLWAGVVGAISLEVFGQYGADMLTDPGVVFDAQT
                     RLLVAVLAEH"
     CDS             2053390..2054853
                     /codon_start=1
                     /note="gene_1868"
                     /transl_table=11
                     /translation="MSTDIPATVSAETVTSWSDDVDVTVIGFGIAGGCAAVSAAAAGA
```

RVLVLERAAAAGGTTALAGGHFYLGGGTTVQLATGHPDSPEEMYKYLVAVSREPDHDK
IRAYCDGSVEHFNWLEGLGFQFERSYFPGKAVIQPNTEGLMFTGNEKVWPFLELAVPA
PRGHKVPVPGDTGGAAMVIDLLLKRAASLGIQIRYETGATELIVDGTGKVTGVMWKRF
SETGAIKAKSVIIAAGGFVMNPDMVAKYTPKLAEKPFVLGNTYDDGLGIRLGVSAGGA
TQHMDQMFITAPPYPPSILLTGIIVNKLGQRFVAEDSYHSRTAGFIMEQPDSAAYLIV
DEAHLEHPKMPLVPLIDGWETVVEMEAALGIPPGNLAATLDRYNAYAARGADPDFHKQ
PEFLAAQDNGPWGAFDMSLGKAMYAGFTLGGLATSVDGQVLRDDGAVVAGLYAVGACA
SNIAQDGKGYASGTQLGEGSFFGRRAGAHAAARAQGM"

CDS complement(2054973..2056478)
/codon_start=1
/note="gene_1869"
/transl_table=11
/translation="MSFVVTIPEALAAVATDLAGIGSTIGTANAAAAVPTTTVLAAAA
DEVSAAMAALFSGHAQAYQALSAQAALFHEQFVRALTAGAGSYAAAEAASAAPLEGVL
DVINAPALALLGRPLIGNGANGAPGTGANGGDGGILIGNGGAGGSGAAGMPGGNGGAA
GLFGNGGAGGAGGNVASGTAGFGGAGGAGGLLYGAGGAGGAGGRAGGGVGGIGGAGGA
GGNGGLLFGAGGAGSVGGLAADAGDGGAGGDGGLFFGVGGAGGAGGTGTNVTGGAGGA
GGNGGLLFGAGGVGGVGGDGVAFLGTAPGGPGGAGGAGGLFGVGGAGGAGGIGLVGNG
GAGGSGGSALLWGDGGAGGAGGVGSTTGGAGGAGGNAGLLVGAGGAGGAGALGGGATG
VGGAGGNGGTAGLLFGAGGAGGAGGFGFGGAGGAGGLGGKAGLIGDGGDGGAGGNGTG
AKGGDGGAGGGAILVGNGGNGGNAGSGTPNGSAGTGGAGGLLGKNGMNGLP"

CDS complement(2056613..2058532)
/codon_start=1
/note="gene_1870"
/transl_table=11
/translation="LGPKLFKPSIDWSRAFPDSVYWVGKAWTISAICVLAILVLLRYL
TPWGRQFWRITRAYFVGPNSVRVWLMLGVLLLSVVLAVRLNVLFSYQGNDMYTALQKA
FEGIASGDGTVKRSGVRGFWMSIGVFSVMAVLHVTRVMADIYLTQRFIIAWRVWLTHH
LTQDWLDGRAYYRDLFIDETIDNPDQRIQQDVDIFTAGAGGTPNAPSNGTASTLLFGA
VQSIISVISFTAILWNLSGTLNIFGVSIPRAMFWTVLVYVFVATVISFIIGRPLIWLS
FRNEKLNAAFRYALVRLRDAAEAVGFYRGERVEGTQLQRRFTPVIDNYRRYVRRSIAF
NGWNLSVSQTIVPLPWVIQAPRLFAGQIDFGDVGQTATSFGNIHDSLSFFRNNYDAFA
SFRAAIIRLHGLVDANEKGRALPAVLTRPSDDESVELNDIEVRTPAGDRLIDPLDVRL
DRGGSLVITGRSGAGKTTLLRSLAELWPYASGTLHRPGGENETMFLSQLPYVPLGTLR
DVVCYPNSAAAIPDATLRDTLTKVALAPLCDRLDEERDWAKVLSPGEQQRVAFARILL
TKPKAVFLDESTSALDTGLEFALYQLLRSELPDCIVVSVSHRPALERLHENQLELLGG
GQWRLAPVEAAPAEV"

CDS 2058603..2060246
/codon_start=1
/note="gene_1871"
/transl_table=11
/translation="MSTDTAPAQTMHAGRLIARRLKASGIDTVFTLSGGHLFSIYDGC
REEGIRLIDTRHEQTAAFAAEGWSKVTRVPGVAALTAGPGITNGMSAMAAAQQNQSPL
VVLGGRAPALRWGMGSLQEIDHVPFVAPVARFAATAQSAENAGLLVDQALQAAVSAPS
GVAFVDFPMDHAFSMSSDNGRPGALTELPAGPTPAGDALDRAAGLLSTAQRPVIMAGT
NVWWGHAEAALLRLVEERHIPVLMNGMARGVVPADHRLAFSRARSKALGEADVALIVG
VPMDFRLGFGGVFGSTTQLIVADRVEPAREHPRPVAAGLYGDLTATLSALAGSGGTDH
QGWIEELATAETMARDLEKAELVDDRIPLHPMRVYAELAALLERDALVVIDAGDFGSY
AGRMIDSYLPGCWLDSGPFGCLGSGPGYALAAKLARPQRQVVLLQGDGAFGFSGMEWD
TLVRHNVAVVSVIGNNGIWGLEKHPMEALYGYSVVAELRPGTRYDEVVRALGGHGELV
SVPAELRPALERAFASGLPAVVNVLTDPSVAYPRRSNLA"

```
CDS             2060351..2062687
                /codon_start=1
                /note="gene_1872"
                /transl_table=11
                /translation="VPKTTRAQPGRLSSRFWRLLGASTEKNRSRSLADVTASAEYDKE
                AADLSDEKLRKAAGLLNLDDLAESADIPQFLAIAREAAERRTGLRPFDVQLLGALRML
                AGDVIEMATGEGKTLAGAIAAAGYALAGRHVHVVTINDYLARRDAEWMGPLLDAMGLT
                VGWITADSTPDERRTAYDRDVTYASVNEIGFDVLRDQLVTDVNDLVSPNPDVALIDEA
                DSVLVDEALVPLVLAGTTHRETPRLEIIRLVAELVGDKDADEYFATDSDNRNVHLTEH
                GARKVEKALGGIDLYSEEHVGTTLTEVNVALHAHVLLQRDVHYIVRDDAVHLINASRG
                RIAQLQRWPDGLQAAVEAKEGIETTETGEVLDTITVQALINRYATVCGMTGTALAAGE
                QLRQFYQLGVSPIPPNKPNIREDEADRVYITTAAKNDGIVEHITEVHQRGQPVLVGTR
                DVAESEELHERLVRRGVPAVVLNAKNDAEEARVIAEAGKYGAVTVSTQMAGRGTDIRL
                GGSDEADHDRVAELGGLHVVGTGRHHTERLDNQLRGRAGRQGDPGSSVFFSSWEDDVV
                AANLDHNKLPMATDENGRIVSPRTGSLLDHAQRVAEGRLLDVHANTWRYNQLIAQQRA
                IIVERRNTLLRTVTAREELAELAPKRYEELSDKVSEERLETICRQIMLYHLDRGWADH
                LAYLADIRESIHLRALGRQNPLDEFHRMAVDAFASLAADAIEAAQQTFETANVLDHEP
                GLDLSKLARPTSTWTYMVNDNPLSDDTLSALSLPGVFR"
CDS             2062884..2063513
                /codon_start=1
                /note="gene_1873"
                /transl_table=11
                /translation="MEPVLTQNRVLTVPNMLSVIRLALIPAFVYVVLSAHANGWGVAI
                LVFSGVSDWADGKIARLLNQSSRLGALLDPAVDRLYMVTVPIVFGLSGIVPWWFVLTL
                LTRDALLAGTLPLLWSRGLSALPVTYVGKAATFGFMVGFPTILLGQCDPLWSHVLLAC
                GWAFLIWGMYAYLWAFVLYAVQMTMVVRQMPKLKGRAHRPAAQNAGERG"
CDS             2063500..2064429
                /codon_start=1
                /note="gene_1874"
                /transl_table=11
                /translation="VNVAESDRLLGGYDPNAGYSAHAGAQPQRIPVPSLLRALLSEHL
                DAGYAAVAAERERAAAPRCWQARAVSWMWQALAATLVAAVFAAAVAQARSVAPGVRAA
                QQLLVASVRSTQAAATTLAQRRSTLSAKVDDVRRIVLADDAEGQRLLARLDVLSLAAA
                SAPVVGPGLTVTVTDPGASPNLSDVSKQRVSGSQQIILDRDLQLVVNSLWESGAEAIS
                IDGVRIGPNVTIRQAGGAILVDNNPTSSPYTILAVGPPHAMQDVFDRSAGLYRLRLLE
                TSYGVGVSVNVGDGLALPAGATRDVKFAKQIGP"
CDS             2064458..2064823
                /codon_start=1
                /note="gene_1875"
                /transl_table=11
                /translation="MGSDTAWSPARMIGIAALAVGIVLGLVFHPGVPEVIQPYLPIAV
                VAALDAVFGGLRAYLERIFDPKVFVVSFVFNVLVAALIVYVGDQLGVGTQLSTAIIVV
                LGIRIFGNTAALRRRLFGA"
CDS             2065005..2065718
                /codon_start=1
                /note="gene_1876"
                /transl_table=11
                /translation="LTEILRGGRSRLVFGTLAILLCLVLGVAIVTQVRQTDSGDSLET
                ARPADLLVLLDSLRQREATLNAEVIDLQNTLNALQASGNTDQAALESAQARLAALSIL
                VGAVGATGPGVMITIDDPGPGVAPEVMIDVINELRAAGAEAIQINDAHRSVRVGVDTW
                VVGVPGSLTVDTKVLSPPYSILAIGDPPTLAAAMNIPGGAQDGVKRVGGRMVVQQADR
```

VDVTALRQPKQHQYAQPVK"
CDS 2065756..2066160
/codon_start=1
/note="gene_1877"
/transl_table=11
/translation="VSDIPSDLHYTAEHEWIRRSGDDTVRVGITDYAQSALGDVVFVQ
LPVIGTAVTAGETFGEVESTKSVSDLYAPISGKVSAVNSDLDGTPQLVNSDPYGAGWL
LDIQVDSSDVAALESALTTLLDAEAYRGTLTE"
CDS 2066400..2066888
/codon_start=1
/note="gene_1878"
/transl_table=11
/translation="VTDMNPDIEKDQTSDEVTVETTSVFRADFLSELDAPAQAGTESA
VSGVEGLPPGSALLVVKRGPNAGSRFLLDQAITSAGRHPDSDIFLDDVTVSRRHAEFR
LENNEFNVVDVGSLNGTYVNREPVDSAVLANGDEVQIGKFRLVFLTGPKQGEDDGSTG
GP"
CDS 2066885..2067628
/codon_start=1
/note="gene_1879"
/transl_table=11
/translation="VSAPDSPALAGMSIGAVLDLLRPDFPDVTISKIRFLEAEGLVTP
RRASSGYRRFTAYDCARLRFILTAQRDHYLPLKVIRAQLDAQPDGELPPFGSPYVLPR
LVPVAGDSAGGVGSDTASVSLTGIRLSREDLLERSEVADELLTALLKAGVITTGPGGF
FDEHAVVILQCARALAEYGVEPRHLRAFRSAADRQSDLIAQIAGPLVKAGKAGARDRA
DDLAREVAALAITLHTSLIKSAVRDVLHR"
CDS 2067654..2068241
/codon_start=1
/note="gene_1880"
/transl_table=11
/translation="VFDVTVETWRPPRRRTERESDTGCMCGGQTQMGEVRVVGIRVEQ
PQNQPVLLLREANGDRYLPIWIGQSEAAAIALEQQGVEPPRPLTHDLIRDLIAALGHS
LKEVRIVDLQEGTFYADLIFDRNIKVSARPSDSVAIALRVGVPIYVEEAVLAQAGLLI
PDESDEEATTAVREDEVEKFKEFLDSVSPDDFKAT"
CDS 2068634..2069266
/codon_start=1
/note="gene_1881"
/transl_table=11
/translation="LGEQPRQDQLDFADHTGTAGDGNDGAAAASGPVQPGLFPDDSVP
DELVGYRGPSACQIAGITYRQLDYWARTSLVVPSIRSAAGSGSQRLYSFKDILVLKIV
KRLLDTGISLHNIRVAVDHLRQRGVQDLANITLFSDGTTVYECTSAEEVVDLLQGGQG
VFGIAVSGAMRELTGVIADFHGERADGGESIAAPEDELASRRKHRDRKIG"
CDS 2069625..2072450
/codon_start=1
/note="gene_1882"
/transl_table=11
/translation="VSDHSTFADRHIGLDSQAVATMLAVIGVDSLDDLAVKAVPAGIL
DTLTDTGAAPGLDSLPPAASEAEALAELRALADANTVAVSMIGQGYYDTHTPPVLLRN
IIENPAWYTAYTPYQPEISQGRLEALLNFQTLVTDLTGLEIANASMLDEGTAAAEAMT
LMHRAARGPVKRVVVDADVFTQTAAVLATRAKPLGIEIVTADLRAGLPDGEFFGAIAQ
LPGASGRITDWSALVQQAHDRGALVAVGADLLALTLIAPPGEIGADVAFGTTQRFGVP
MGFGGPHAGYLAVHAKHARQLPGRLVGVSVDSDGTPAYRLALQTREQHIRRDKATSNI

CTAQVLLAVLAAMYASYHGAGGLTAIARRVHAHAEAIAGALGDALVHDKYFDTVLARV
PGRADEVLARAKANGINLWRVDADHVSVACDEATTDTHVAVVLDAFGVAAAAPAHADI
ATRTSEFLTHPAFTQYRTETSMMRYLRALADKDIALDRSMIPLGSCTMKLNAAAEMES
ITWPEFGRQHPFAPASDTAGLRQLVADLQSWLVLITGYDAVSLQPNAGSQGEYAGLLA
IHEYHASRGEPHRDICLIPSSAHGTNAASAALAGMRVVVVDCHDNGDVDLDDLRAKVG
EHAERLSALMITYPSTHGVYEHDIAEICAAVHDAGGQVYVDGANLNALVGLARPGKFG
GDVSHLNLHKTFCIPHGGGGPGVGPVAVRAHLAPFLPGHPFAPELPKGYPVSSAPYGS
ASILPITWAYIRMMGAEGLRAASLTAITSANYIARRLDEYYPVLYTGENGMVAHECIL
DLRGITKLTGITVDDVAKRLADYGFHAPTMSFPVAGTLMVEPTESESLAEVDAFCEAM
IGIRAEIDKVGAGEWPVDDNPLRGAPHTAQCLLASDWDHPYTREQAAYPLGTAFRPKV
WPAVRRIDGAYGDRNLVCSCPPVEAFA"
CDS complement(2072677..2073537)
/codon_start=1
/note="gene_1883"
/transl_table=11
/translation="MSIDFTPDPQLYPFESRWFDSSRGRIHYVDEGTGPPILLCHGNP
TWSFLYRDIIVALRDRFRCVAPDYLGFGLSERPSGFGYQIDEHARVIGEFVDHLGLDR
YLSMGQDWGGPISMAVAVERADRVRGVVLGNTWFWPADTLAMKAFSRVMSSPPVQYAI
LRRNFFVERLIPAGTEHRPSSAVMAHYRAVQPNAAARRGVAEMPKQILAARPLLARLA
REVPATLGTKPTLLIWGMKDVAFRPKTIIPRLSATFPDHVLVELPNAKHFIQEDAPDR
IAAAIIERFG"
CDS 2073680..2074444
/codon_start=1
/note="gene_1884"
/transl_table=11
/translation="MLLLHGYPSSSFDFRAVIPHLTGQAWVTMDFLGFGLSDKPRPHR
YSLLEQAHLVETVVAHTVTGAVVVLAHDMGTSVTTELLARDLDGRLPFDLRRAVLSNG
SVILERASLRPIQKVLRSPLGPVAARLVSRGGFTRGFGRIFSPAHPLSAQEAQAQWEL
LCYNDGNRIPHLLISYLDERIRHAQRWHGAVRDWPKPLGFVWGLDDPVATTNVLNGLR
ELRPSAAVVELPGLGHYPQVEAPKAYAEAALSLLVD"
CDS complement(2074449..2076257)
/codon_start=1
/note="gene_1885"
/transl_table=11
/translation="MRYSSCYVTMRDGVRIAIDLYLPAGLTSAARLPAILHQTRYYRS
LQLRWPLRMLLGGKPLQHIAADKRRRRRFVASGYAWVDVDVRGSGASFGARVCEWSSD
EIRDGAEIVDWIVRQPWCNGTVAALGNSYDGTSAELLLVNQHPAVRVIAPCFSLFDVY
TDIAFPGGIHAAWFTDTWGRYNEALDRNALHEVVGWWAKLPVTGMQPVQEDRDRSLRD
GAIAAHRGNYDVHQIAGSLTFRDDVSASDPYRGQPDARLEPIGTPIESGSINLISPHN
YWRDVQASGAAIYSYSGWFDGGYAHAAIKRFLTVSTPGSHLILGPWNHTGGWRVDPLR
GLSRPDFDHDGELLRFIDHHVKGADTGIGSEPPVHYFTMVENRWKSADTWPPPATTQS
YYLSADRQLRPDAPDCDSGADEYVVDQTAGTGERSRWRSQVGIGGHVCYPDRKAQDAK
LLTYTSAPLDHPLEVTGHVVVTLFITSTSSDGTFFVYLEDVDPRGRVAYITEGQLRAI
HRRLSDGPPPYRQVVPYRTFASGDAWPLVPGEIARLTFDLLPTSYLFQPGHRIRIAIA
GADASHFAILPGCAPTVRVYRSRMHASRIDLPVIQP"
CDS complement(2076351..2078384)
/codon_start=1
/note="gene_1886"
/transl_table=11
/translation="MGRHSKPDPEDSVDDLSDGHAAEQQHWEDISGSYDYPGVDQPDD
GPLSSEGHYSAVGGYSASGSEDYPDIPPRPDWEPTGAEPIAAAPPPLFRFGHRGPGDW

QAGHRSADGRRGVSIGVIVALVAVVVMVAGVILWCFFGDALSNRSHTAAARCVGGKDT
VAVIADPSIADQVKESADSYNASAGPVGDRCVAVAVTSAGSDAVINGFIGKWPTELGG
QPGLWIPSSSISAARLTGAAGSQAISDSRSLVISPVLLAVRPELQQALANQNWAALPG
LQTNPNSLSGLDLPAWGSLRLAMPSSGNGDAAYLAGEAVAAASAPAGAPATAGIGAVR
TLMGARPKLADDSLTAAMDTLLKPGDVATAPVHAVVTTEQQLFQRGQSLSDAENTLGS
WLPPGPAAVADYPTVLLSGAWLSQEQTSAASAFARYLHKPEQLAKLARAGFRVSDVKP
PSSPVTSFPALPSTLSVGDDSMRATLADTMVTASAGVAATIMLDQSMPNDEGGNSRLS
NVVAALENRIKAMPPSSVVGLWTFDGREGRTEVPAGPLADPVNGQPRPAALTAALGKQ
YSSGGGAVSFTTLRLIYQEMLANYHVGQANSVLVITAGPHTDQTLDGPGLQDFIRKSA
DPAKPIAVNIIDFGADPDRATWEAVAQLSGGSYQNLETSASPDLATAVNIFLS"
CDS 2078307..2078507
/codon_start=1
/note="gene_1887"
/transl_table=11
/translation="VLLFRRMPVGQVVNGILGVGLAVPTHTGVCVLSVEGRRLSSTSY
APALPDGAGSLPQAGGAPTAAR"
CDS complement(2078504..2080729)
/codon_start=1
/note="gene_1888"
/transl_table=11
/translation="MTDRVSVGNLRIARVLYDFVNNEALPGTDIDPDSFWAGVDKVVA
DLTPQNQALLNARDELQAQIDKWHRRRVIEPIDMDAYRQFLTEIGYLLPEPDDFTITT
SGVDAEITTTAGPQLVVPVLNARFALNAANARWGSLYDALYGTDVIPETDGAEKGPTY
NKVRGDKVIAYARKFLDDSVPLSSGSFGDATGFTVQDGQLVVALPDKSTGLANPGQFA
GYTGAAESPTSVLLINHGLHIEILIDPESQVGTTDRAGVKDVILESAITTIMDFEDSV
AAVDAADKVLGYRNWLGLNKGDLAAAVDKDGTAFLRVLNRDRNYTAPGGGQFTLPGRS
LMFVRNVGHLMTNDAIVDTDGSEVFEGIMDALFTGLIAIHGLKASDVNGPLINSRTGS
IYIVKPKMHGPAEVAFTCELFSRVEDVLGLPQNTMKIGIMDEERRTTVNLKACIKAAA
DRVVFINTGFLDRTGDEIHTSMEAGPMVRKGTMKSQPWILAYEDHNVDAGLAAGFSGR
AQVGKGMWTMTELMADMVETKIAQPRAGASTAWVPSPTAATLHALHYHQVDVAAVQQG
LAGKRRATIEQLLTIPLAKELAWAPDEIREEVDNNCQSILGYVVRWVDQGVGCSKVPD
IHDVALMEDRATLRISSQLLANWLRHGVITSADVRASLERMAPLVDRQNAGDVAYRPM
APNFDDSIAFLAAQELILSGAQQPNGYTEPILHRRRREFKARAAEKPAPSDRAGDDAA
R"
CDS complement(2081005..2081400)
/codon_start=1
/note="gene_1889"
/transl_table=11
/translation="VILVDSNIPMYLVGASHPHKLDAQRLLESALSGGERLVTDAEVL
QEICHRYVAIKRREAIQPAFDAIIGVVDEVLPIERTDVEHARDALLRYQTLSARDALH
IAVMAHHDITRLMSFDRGFDSYPGIKRLA"
CDS complement(2081397..2081660)
/codon_start=1
/note="gene_1890"
/transl_table=11
/translation="MSKRLQVLLDPDEWEELREIARRHRTTVSEWVRRTLREAREREP
RGDLDMKLRSVRAAARHEFPTADVEQMLEEIERGRGAEREGSR"
CDS complement(2081719..2082288)
/codon_start=1
/note="gene_1891"
/transl_table=11

/translation="MFGDGGNGGFSFFDGNGGDGGTGGTLIGNGGDGGNSVQTDGFLR
GHGGDGGNAVGLIGNGGAGGAGSAGTGVFAPGGGSGGNGGNGALLVGNGGAGGSGGPT
QIPSVAVPVTGAGGTGGNGGTAGLIGNGGNGGAAGVSGDGTPGTGGNGGYAQLIGDGG
DGGPGDSGGPGGSGGTGGTLAGQNGSPGG"
CDS complement(2082447..2083265)
/codon_start=1
/note="gene_1892"
/transl_table=11
/translation="MSFVVAAPEVVVAAASDLAGIGSAIGAANAAAAVPTMGVLAAGA
DEVSAAVADLFGAHAQAYQALSAQAALFHEQFVHAMTAGAGAYAGAEAADAAALDVLN
GPFQALFGRPLIGDGANGAPGQPGGPGGLLYGNGGNGGNGGIGQPGGAGGDAGLIGNG
GNGGIGGPGRPALPVVPAGSADCSSATAATAVPADSVPDPSALPVGSAGPVEPPSGCS
AMAGPAGPAGSARPASRAGPAAPAAPADCCTATAATAAMSHREPPTAERAVTPG"
CDS complement(2083428..2084477)
/codon_start=1
/note="gene_1893"
/transl_table=11
/translation="MDVLSAVLLALLLIGANAFFVGAEFALISARRDRLEALAEQGKA
TAVTVIRAGEQLPAMLTGAQLGVTVSSILLGRVGEPAVVKLLQLSFGLSGVPPALLHT
LSLAVALAIVVALHVLLGEMVPKNIALAGPERTAMLLVPPYLVYVRLARPFIAFYNNC
ANAILRLVGVQPKDELDIAVSTAELSEMIAESLSEGLLDHEEHTRLTRALRIRTRLVA
DVAVPLVNIRAVQVSAVGSGPTIGGVEQALAQTGYSRFPVVDRGGRFIGYLHIKDVLT
LGDNPQTVIDLAVVRPLPRVPQSLPLADALSRMRRINSHLALVTADNGSVVGMVALED
VVEDLVGTMRDGTHR"
CDS complement(2084477..2085844)
/codon_start=1
/note="gene_1894"
/transl_table=11
/translation="VNLTDTVATILAILALTAGTGVFVAAEFSLTALDRSTVEANARG
GTSRDRFIQRAHHRLSFQLSGAQLGISITTLATGYLTEPLVAELPHPGLVAVGMSDRV
ADGLITFFALVIVTSLSMVFGELVPKYLAVARPLRTARSVVAGQVLFSLLLTPAIRLT
NGAANWIVRRLGIEPAEELRSARTPQELVSLVRSSARSGALDDATAWLMRRSLQFGAL
TAEELMTPRSKIVALQTDDTIADLVAAAAASGFSRFPVVEGDLDATVGIVHVKQVFEV
PPGDRAHTLLTTVAEPVAVVPSTLDGDAVMAQVRASALQTAMVVDEYGGTAGMVTLED
LIEEIVGDVRDEHDDATPDVVAAGNGWRVSGLLRIDEVASATGYRAPDGPYETIGGLV
LRELGHIPVAGETVELTALDQDGLPDDSMRWLATVIQMDGRRIDSLELIKMGGHADPG
SGRGR"
CDS complement(2086018..2087454)
/codon_start=1
/note="gene_1895"
/transl_table=11
/translation="MRFLDGHPPGYDLTYNDVFIVPNRSEVASRFDVDLSTADGSGTT
IPVVVANMTAVAGRRMAETVARRGGIVILPQDLPIPAVKQTVAFVKSRDLVLDTPVTL
APDDSVSDAMALIHKRAHGVAVVILEGRPIGLVRESSCLGVDRFTRVRDIAVTDYVTA
PAGTEPRKIFDLLEHAPVDVAVLTDADGTLAGVLSRTGAIRAGIYTPATDSAGRLRIG
AAVGINGDVGAKARALAEAGVDVLVIDTAHGHQVKTLDAIKAVSALDLGLPLAAGNVV
SAEGTRDLLKAGANVVKVGVGPGAMCTTRMMTGVGRPQFSAVLECASAARQLGGHIWA
DGGIRHPRDVALALAAGASNVMIGSWFAGTYESPGDLMRDRDDQPYKESYGMASKRAV
VARTGADNPFDRARKALFEEGISTSRMGLDPDRGGVEDLIDHITSGVRSTCTYVGASN
LAELHERAVVGVQSGAGFAEGHPLPAGW"
CDS complement(2087490..2087699)

```
                     /codon_start=1
                     /note="gene_1896"
                     /transl_table=11
                     /translation="VVSTAAQLGIPTPGFSSALSYYDALRTARLPAALTQAQRDFFGA
                     HTYGRIDEPGKFHTLWSSDRTEVPV"
     CDS             2087798..2088298
                     /codon_start=1
                     /gene="W823_12425"
                     /note="gene_1897"
                     /transl_table=11
                     /translation="VVEELRPDDAAATPDGGQVARRDVPAEFGAAGLDLVEALSVGHD
                     LGGVQRLADVFGERGGVGGLLAELARSQTAGGRALPHGSRESAGKHRFGDAGHRHTQV
                     QRGLDGPTAGAFLLGPVHDDVDERFAGLGIGLPQHLGGDLDQVAVQIAIVPLGEHIGD
                     RRRGQT"
     CDS             complement(2088970..2089920)
                     /codon_start=1
                     /note="gene_1898"
                     /transl_table=11
                     /translation="VSALAFTILAVLLAGPTPALLARATWPLRAPRAAMVLWQAIALA
                     AVLSSFSAGIAIASRLLMPGPDGRPTTSFVGAAGRLGWPLWAAYITVFALTVLVGARL
                     AVAVVRVATATRRRRAHHRMVVDLVGVGHNGALAQPCARARDLRVLDVAQPLAYCLPG
                     VRSRVVVSEGTLTALADAEVAAILTHERAHLRARHDLVLEAFTAVHAAFPRLVRSANA
                     LGAAQLLVELLADDAAVRAAGRTPLARALVACASGRAPSGALAVGGPSTVLRVRRLSG
                     RGNSAVLSAAAYLAAAAVLVVPTVALAVPWLTQLQRLFIA"
     CDS             complement(2089935..2090351)
                     /codon_start=1
                     /note="gene_1899"
                     /transl_table=11
                     /translation="MAKLTRLGDLERAVMDHLWSRTEPQTVRQVHEALSARRDLAYTT
                     VMTVLQRLAKKNLVLQIRDDRAHRYAPVHGRDELVAGLMVDALAQAEYSGSRQAALVH
                     FVERVGADEADALRRALAELEAGHGNRPPAGAATET"
     CDS             2090629..2091051
                     /codon_start=1
                     /note="gene_1900"
                     /transl_table=11
                     /translation="VQPSPDSPAPLNVTVPFDSELGLQFTELGPDGARAQLDVRPKLL
                     QLTGVVHGGVYCAMIESIASMAAFAWLNSHGEGGSVVGVNNNTDFLRSISSGMVYGTA
                     EPLHRGRRQQLWLVTITDDTDRVVARGQVRLQNLEARP"
     CDS             2091100..2091402
                     /codon_start=1
                     /gene="ureA"
                     /note="gene_1901"
                     /transl_table=11
                     /translation="MRLTPHEQERLLLSYAAELARRRRARGLRLNHPEAIAVIADHIL
                     EGARDGRTVAELMASGREVLGRDDVMEGVPEMLAEVQVEATFPDGTKLVTVHQPIA"
     CDS             2091399..2091713
                     /codon_start=1
                     /gene="ureB"
                     /note="gene_1902"
                     /transl_table=11
                     /translation="MIPGEIFYGSGDIEMNAAALSRLQMRIINAGDRPVQVGSHVHLP
```

QANRALSFDRATAHGYRLDIPAATAVRFEPGIPQIVGLVPLGGRREVPGLTLNPPGRL
DR"
CDS 2091713..2093446
/codon_start=1
/gene="ureC"
/note="gene_1903"
/transl_table=11
/translation="MARLSRERYAQLYGPTTGDRIRLADTNLLVEVTEDRCGGPGLAG
DEAVFGGGKVLRESMGQGRASRADGAPDTVITGAVIIDYWGIIKADIGIRDGRIVGIG
KAGNPDIMTGVHRDLVVGPSTEIISGNRRIVTAGTVDCHVHLICPQIIVEALAAGTTT
IIGGGTGPAEGTKATTVTPGEWHLARMLESLDGWPVNFALLGKGNTVNPDALWEQLRG
GASGFKLHEDWGSTPAAIDTCLAVADVAGVQVALHSDTLNETGFVEDTIGAIAGRSIH
AYHTEGAGGGHAPDIITVAAQPNVLPSSTNPTRPHTVNTLDEHLDMLMVCHHLNPRIP
EDLAFAESRIRPSTIAAEDVLHDMGAISMIGSDSQAMGRVGEVVLRTWQTAHVMKARR
GALEGDPSGSQAADNNRVRRYIAKYTICPAIAHGMDHLIGSVEVGKLADLVLWEPAFF
GVRPHVVLKGGAIAWAAMGDANASIPTPQPVLPRPMFGAAAATAAATSVHFVAPQSID
ARLADRLAVNRGLAPVADVRAVGKTDLPLNDALPSIEVDPDTFTVRIDGQVWQPQPAA
ELPMTQRYFLF"
CDS 2093446..2094081
/codon_start=1
/note="gene_1904"
/transl_table=11
/translation="MTSLAVLLTLADSRLPTGAHVHSGGIEEAIAAGLVTGLATLEAF
LKRRVRTHGLLTASIAAAVHRGELAVDDADRETDARTPAPAARHASRSQGRGLIRLAR
RVWPDSGWEELGPRPHLAVVAGRVGALSGLAPEHNALHLVYITMTGSAIAAQRLLALD
PAEVTVVTFQLSELCEQIAQEATAGLADLSDPLLDTLAQRHDERVRPLFVS"
CDS 2094092..2094766
/codon_start=1
/note="gene_1905"
/transl_table=11
/translation="MATHSHPHSHTVPARPRRVRKPGEPLRIGVGGPVGSGKTALVAA
LCRQLRGELSLAVLTNDIYTTEDADFLRTHAVLPDDRIAAVQTGGCPHTAIRDDITAN
LDAIDELMAAHDALDLILVESGGDNLTATFSSGLVDAQIFVIDVAGGDKVPRKGGPGV
TYSDLLVVNKTDLAALVGADLAVMARDADAVRDGRPTVLQSLTEDPAASDVVAWVRSQ
LAADGV"
CDS 2094756..2095400
/codon_start=1
/note="gene_1906"
/transl_table=11
/translation="MESSVLVVASPNRLPRIDCRGGVQARRTAPDTVHLVSAAATPLG
GDTMRIRVIVERGAQLRLRSAAATVALPGVDTLTSHAHWEIDVTGTLDVDLEPTVVAA
SARHLSHATLRLHDDGRVRLRERVQIGRCNEREGFWSSSLQADRHGRPLLRHRVELGA
GSLADDVIAAPRATISELRYPATAFTDAIDARSTVLALAGGGTLSTWQADRLPG"
CDS complement(2095403..2096743)
/codon_start=1
/gene="ndh-2"
/note="gene_1907"
/transl_table=11
/translation="VIIGSGFGGLNAAKKLKRADVDIKLIARTTHHLFQPLLYQVATG
IISEGEIAPPTRVVLRKQRNVQVLLGNVTHIDLAGQCVVSELLGHTYQTPYDSLIVAA
GAGQSYFGNDHFAEFAPGMKSIDDALELRGRILSAFEQAERSSDPERRAKLLTFTVVG

AGPTGVEMAGQIAELAEHTLKGAFRHIDSTKARVILLDAAPAVLPPMGAKLGQRAAAR
LQKLGVEIQLGAMVTDVDRNGITVKDSDGTVRRIESACKVWSAGVSASRLGRDLAEQS
RVELDRAGRVQVLPDLSIPRYPNVFVVGDMAAVEGVPGVAQGAIQGAKYVASTIKAEL
AGANPAEREPFQYFDKGSMATVSRFSAVAKIGPVEFSGFIAWLIWLVLHLAYLIGFKT
KITTLLSWTVTFLSTRRGQLTITDQQAFARTRLEQLAELAAEAQGSAASAKVAS"

CDS complement(2096935..2097858)
/codon_start=1
/note="gene_1908"
/transl_table=11
/translation="VTIRLGLQIPNFSYGTGVEKLFPSVIAQAREAEAAGYDSLFVMD
HFYQLPMLGTPDQPMLEAYTALGALATATERLQLGALVTGNTYRSPTLLAKIITTLDV
VSAGRAILGIGAGWFELEHRQLGFEFGTFSDRFNRLEEALQILEPMVKGERPTFFGDW
YTTESAMAEPRYRDRIPILIGGGGEKKTFAIAARFADHLNIVAAVDELPRKMRALAAR
CDEAGRDRSTLQTSLLLTVMIDETLSPDAIPAEMSGRVVVGSPAQIADQIQAKVLDAG
VDGLIINLAPHGYLPGVITTAAEALRPLLGV"

CDS complement(2097897..2098574)
/codon_start=1
/note="gene_1909"
/transl_table=11
/translation="MAVEVLVTGGDTDLGRTMAEGFRNDGHKVTLVGARRGDLEVAAK
ELDVDAVVCDTTDPTSLTEARGLFPRHLDTIVNVPAPSWDAGDPRAYSVSDTANAWRN
ALDATVLSVVLTVQSVGDHLRSGGSIVSVVAENPPAGGAESAIKAALSNWIAGQAAVF
GTRGITINTVACGRSVQTGYEGLSHTPAPVAAEIARLALFLTTPAARHITGQTLHVSH
GALAHFG"

CDS 2098736..2099521
/codon_start=1
/note="gene_1910"
/transl_table=11
/translation="VRWIGLSTGLVSAMLVAGLVACGSNSPASSPAGPTQGARSIVVF
AAASLQSAFTQIGEQFKAGNPGVNVNFAFAGSSELATQLTQGATADVFASADTAQMDS
VAKAGLLAGHPTNFATNTMVIVAAAGNPKKIRSFADLTRPGLNVVVCQPSVPCGSATR
RIEDATGIHLNPVSEELSVTDVLNKVITGQADAGLVYVSDALSVATKVTCVRFPEAAG
VVNVYAIAVLKRTSQPALARQFVAMVTAAAGRRILDQSGFAKP"

CDS 2099524..2100318
/codon_start=1
/note="gene_1911"
/transl_table=11
/translation="MHPPTDLPRWVYLPAIAGIVFVAMPLVAIAIRVDWPRFWALITT
PSSQTALLLSVKTAAASTVLCVLLGVPMALVLARSRGRLVRSLRPLILLPLVLPPVVG
GIALLYAFGRLGLIGRYLEAAGISIAFSTAAVVLAQTFVSLPYLVISLEGAARTAGAD
YEVVAATLGARPGTVWWRVTLPLLLPGVVSGSVLAFARSLGEFGATLTFAGSRQGVTR
TLPLEIYLQRVTDPDAAVALSLLLVVVAALVVLGVGARTPIGTDTR"

CDS 2100325..2101434
/codon_start=1
/note="gene_1912"
/transl_table=11
/translation="MSKLQLRAVVADRRLDVEFSVSAGEVLAVLGPNGAGKSTALHVI
AGLLRPDAGLVRLGDRVLTDTEAGVNVATHDRRVGLLLQDPLLFPHLSVAKNVAFGPQ
CRRGMFGSGRARTRASALRWLREVNAEQFADRKPRQLSGGQAQRVAIARALAAEPDVL
LLDEPLTGLDVAAAAGIRSVLRSVVARSGCAVVLTTHDLLDVFTLADRVLVLESGTIA
EIGPVADVLTAPRSRFGARIAGVNLVNGTIGPDGSLRTQSGAHWYGTPVQDLPTGHEA

IAVFPPTAVAVYPEPPHGSPRNIVGLTVAEVDTRGPMVLVRGHDQPGGAPGLAACITV
DAATELRVAPGSRVWFSVKAQEVALHPAPHQHASS"
CDS 2101487..2102464
/codon_start=1
/note="gene_1913"
/transl_table=11
/translation="MHQVDPNLTRRKGRLAALAIAAMASASLVTVAVPATANADPEPA
PPVPTTAASPPSTAAAPPAPATPVAPPPPAAANTPNAQPGDPNAAPPPADPNAPPPPV
IAPNAPQPVRIDNPVGGFSFALPAGWVESDAAHLDYGSALLSKTTGDPPFPGQPPPVA
NDTRIVLGRLDQKLYASAEATDSKAAARLGSDMGEFYMPYPGTRINQETVSLDANGVS
GSASYYEVKFSDPSKPNGQIWTGVIGSPAANAPDAGPPQRWFVVWLGTANNPVDKGAA
KALAESIRPLVAPPPAPAPAPAEPAPAPAPAGEVAPTPTTPTPQRTLPA"
CDS 2102916..2103221
/codon_start=1
/note="gene_1914"
/transl_table=11
/translation="MDITATTEFSAMNLDGKTGIGWLGYIVIGGIAGWLASKIVKGGG
SGILMNVVIGVVGAFGAGLVLNALGVDVNHGGYWFTFFVALGGAVVLLWIVGMVRKT"
CDS 2103296..2104336
/codon_start=1
/note="gene_1915"
/transl_table=11
/translation="MVSPATTATMSAWQVRRPGPMDTGPLERVTTRVPRPAPSELLVA
VHACGVCRTDLHVTEGDLPVHRERVIPGHEVVGEVIEVGSAVGAAAGGEFDRGDRVGI
AWLRHTCGVCKYCRRGSENLCPQSRYTGWDADGGYAEFTTVPAAFAHHLPSGYSDSEL
APLLCAGIIGYRSLLRTELPPGGRLGLYGFGGSAHITAQVALAQGAEIHVMTRGARAR
KLALQLGAASAQDAADRPPVPLDAAILFAPVGDLVLPALEALDRGGILAIAGIHLTDI
PDLNYQQHLFQERQIRSVTSNTRADARAFFDFAAQHHIEVTTPEYPLGQADRALGDLS
AGRIAGAAVLLI"
CDS complement(2104343..2105065)
/codon_start=1
/note="gene_1916"
/transl_table=11
/translation="VSHLSVMHRFRIYVDIAVVVLVLVLTNLIAHFTTPWASIATVPA
AAVGLVILVRSRGLGWAELGLSRQHWKSGLVYALAAVALVVAVISVGVLLPITRPMFM
NHHYATISGAVIASMVMIPLQTVIPEELAFRGVLHGALNRAWGFRGVAVAGSVLFGLW
HIATSLGLTSSNVGFTRLFGGGIIGLVAGVMLAVLATGVAGFVFSWLRRRSGSLIAPI
ALHWSLNGMGALAAALVWHLST"
CDS complement(2105106..2105861)
/codon_start=1
/note="gene_1917"
/transl_table=11
/translation="VTVAPRRLAWTNARQSYPVRVAHVLSVNLARVRANPDPRAQSKL
TGIDKVAASEAVMVRAPGSMHAGVGSGLVGDTVGNPKLHGGDDQAVYAYAREDLDAWE
TQLHRTLHNGMFGENLTTSGVDVTYARIGERWRIGSDGLVLEVSAPRIPCRTFAAFLD
LRYWIKTFTRAAKPGAYLRVIAPGTVRAGDTITVDYRPEHNVTVGLVFRARTSESELL
PQLLAADALAAELKAYARERTPSPPPVDSADDV"
CDS complement(2105858..2106718)
/codon_start=1
/note="gene_1918"
/transl_table=11

/translation="VPGRTSIGVKIRDKVQDKVIAITGGARGIGLATAAALHNLGAKV
AIGDIDEAMAKESGADLDLDMYGKLDVTDPDSFSGFLDAVERQLGPIDVLVNNAGIMP
VGRIVDEPDPVTRRILDINVYGVILGSKLAAQRMVPRGRGHVINVASLAGEIYAVGVA
TYCASKHAVVAFTDSARLEYRSAGVKFSMVLPSFVNTELIAGTGGIKGFKNAEPADIA
DAIVGLIVHPKPRVRVTKAAGSMIVAQRFMPRQVSEGLNRLLGGEHVFTDDVDMEKRR
TYEARARGEE"

CDS 2106814..2109228
/codon_start=1
/note="gene_1919"
/transl_table=11
/translation="VHVPERLLDAVRVLDLSDGCSAGGTDMVTRLLADLGADVLKVQP
PGGSPGRHVRPTLAGTSIGFAMHNANKRSAVLNPLDESDRRRFLDLAASADIVVDCGL
PGQAAAYGASCAELADRYRHLVALSITDFGAAGPRSSWRATDPVLYAMSGALSRSGPT
AGTPVLPPDGIASATAAVQAAWAVLVAYFNRLRCGTGDYIDFSRFDAVVMALDPPFGA
HGQVAAGIRSTGRWRGRPKNQDAYPIYPCRDGYVRFCVMAPRQWRGLRRWLGEPEDFQ
DPKYDVIGARLAAWPQISVLVAKLCAEKTMKELVAAGQALGVPITAVLTPSRILASEH
FQAVGAITDAELVPGVRTGVPTGYFVVDGKRAGFRTPAPAAGQDEPRWLADPAPVPPP
SGRVGGYPFEGLRILDLGIIVAGGELSRLFGDLGAEVIKVESADHPDGLRQTRVGDAM
SESFAWTHRNHLALGLDLRNSEGKAIFGRLVAESDAVFANFKPGTLTSLGFSYDVLHA
FNPRIVLAGSSAFGNRGPWSTRMGYGPLVRAATGVTRVWTSDEAQPDNSRHPFYDATT
IFPDHVVGRVGALLALAALIHRDRTGGGAHVHISQAEVVVNQLDTMFVAEAARATDVA
EIHPDTSVHAVYPCAGDDEWCVISIRSDDEWRRATSVFGQPELANDPRFGASRSRVAN
RSELVAAVSAWTSTRTPVQAAGALQAAGVAAGPMNRPSDILEDPQLIERNLFRDMVHP
LIARPLPAETGPAPFRHIPQAPQRPAPLPGQDSVQICRKLLGMTADETERLINERVMF
GPAVTA"

CDS 2109540..2111000
/codon_start=1
/note="gene_1920"
/transl_table=11
/translation="VLIGYGQVNHRGDIDAEKQSIEPVDLMAAAARKAADSTVLEAVD
SIRVVHMLSAHYRNPGQLLGERIKARTFTTGYSGVGGNMPQSLVNRACLDIQRGRAGV
VLLAGAETWRTRTGLRAKGSKLEWTVQDESVPLPDMAGDDVPMAGAAELRINLDRPAY
VYPIFEQALRIAYGESIENHRKRIGELWARFSAVAADNPHAWIRNPVTADEIWQPGPQ
NRMVSWPYTKLMNSNNMVDQGAALLLTSVERATRLRIPAERWVYPQAGTDAHDTPAVA
DRHRLHRSTAIRIAGARALELAGLGLDDIEYVDLYSCFPSAVQVAAIELGLDTDDPAR
PLTVTGGLTFAGGPWSNYVTHSIATMAELLAANPGRRGLITANGGYLTKHSFGVYGTE
PPSEFRWEDMQPAVDREPTGDGLVEWEGIGTVEAWTTPVNRDGQPEKAFLAVRTPDGS
RSLAVITDPASVQATVREDIAGVKVAVAPDGTATLR"

CDS 2111099..2113198
/codon_start=1
/note="gene_1921"
/transl_table=11
/translation="VQILVTDATGAVGRSVTRQLIAAGHTVSGIAQHPHDALDPRVDY
VCASLRNPVLQELAGEADAVIHLAPVDTSAPGGVGITGLAHVANAAARAGARLLFVSQ
AAGRPELYRQAETLVSTGWAPSLVIRIAPPVGRQLDWMVCRTVATLLRSKVSARPIRV
LHLDDLVRFLVLALNTDRNGVVDLATPDTTNVVTAWRLLRSVDPHLRTRRVRSWEQLI
PEVDIAAVQEDWNFEFGWQATEAIVDTGRGLVGRRLHPAGATNGSGQLALPVEAPPRS
VPSHGEPLGSAAPEGLEGEFDDRIDERFPVFSSASLAEALPGPLTPMTLDVQLSGLRA
AGRAMGRVLALGGVVADEWERRAIAVFGHRPYIGVSANIVAAAQLPGWDAQAVARRAL
GEQPQVTELLPFGRPQLAGGPLGSVAKVVVTARSLALLRHLRSDTHHYVAAADAEHLA
AGQLASLPDAGLEVRIRLLRDRIHQGWILTVLWVIDTGVTAATLEHTRAGSAVSGGGM

IMESGRIGAEIAPLAAVLRADPPLCALANDGNLASIRALSAPAAAAVDAVIARIGHRG
LGEAELANLTFADDPALLLKTAAEIAARPAGPAHPATLIQRLAAGTRSARELAHGTTI
RFTHELRMTLRELGSRRVAADVIDVVDDVFYLTCDELITTPADARLRIKRRRAERERL
QAQRPPDVIDHAWVPVE"
CDS complement(2113212..2114447)
/codon_start=1
/note="gene_1922"
/transl_table=11
/translation="MASPTTFVIVGGGLAGAKAVEALRRSDFGGRIILFGDEEHLPYD
RPPLSKEFLAGKKSLSDFTIQTSDWYRDHDVDVRLGVRVSSLDRSAHTVELPDGAAVR
YDKLLLATGSAPRRPPIPGSDAAGVHYLRSYNDAVALNSVLVQGSSLAVVGAGWIGLE
VAASARQRGVDVTVVETAIQPLLAALGEAVGKVFADLHRDQGVDLRLQTQLEEITAAD
GKATGLKMRDGSTVAADAVLVAVGAKPNVELAQQAGLAMGEGGVLVDASLRTSDPDIY
AVGDIAAAEHPLLGTRVRTEHWANALKQPAVAAAGMLGRPGEYAELPYLFTDQYDLGM
EYVGHAPSCDRVVFRGNVAGREFLSFWLDGDSRVLAGMNVNVWDVVDDVKGLIRSGNP
VDVDRLVDPQWPLADLTTN"
CDS complement(2114514..2115161)
/codon_start=1
/note="gene_1923"
/transl_table=11
/translation="MRLLVIKPEPLARRLLKLAGTTYAAEAGIRIRDKPMPLFQLLVL
CMLASKPIGAATAARAARELFCSGLRTPKAVLSAERQTMISAFGRAHYVRYDESSATR
LTAIAHRVRDEYSGDLRELAQRTRPDVSAAKRMLKTFNGIGDTGADIFLREVQDVWIW
VRPYFDDRATAAAKQLGLPTDPKKLASVAPSSNALLAAALVRVALDDELRLQVTG"
CDS complement(2115247..2115624)
/codon_start=1
/note="gene_1924"
/transl_table=11
/translation="MNLKREFVHRVQRFVVNPIGRQLPMTMLETIGRKTGQPRRTAVG
GRVVDNQFWMVSEHGEHSDYVYNIKANPAVRVRIGGRWRSGTAYLLPDDDPRQRLRGL
PRLNSAGVRAMGTDLLTIRVDLD"
CDS complement(2115659..2116903)
/codon_start=1
/note="gene_1925"
/transl_table=11
/translation="MAVNRRVPRVRDLAPLLQFNRPQFDTSKRRLGAALTIQDLRRIA
KRRTPRAAFDYADGAAEDELSIARARQGFRDIEFHPTILRDVTTVCAGWNVLGQPTVL
PFGIAPTGFTRLMHTEGEIAGARAAAAAGIPFSLSTLATCAIEDLVIAVPQGRKWFQL
YMWRDRDRSMALVRRAAAAGFDTMLVTVDVPVAGARLRDVRNGMSIPPALTLRTVLDA
MGHPRWWFDLLTTEPLAFASLDRWPGTVGEYLNTVFDPSLTFDDLAWIKSQWPGKLVV
KGIQTLDDARAVVDRGVDGIVLSNHGGRQLDRAPVPFHLLPHVARELGKHTEILVDTG
IMSGADIVAAIALGARCTLIGRAYLYGLMAGGEAGVNRAIEILQTGVIRTMRLLGVTC
LEELSPRHVTQLRRLGPIGAPT"
CDS 2116926..2117363
/codon_start=1
/note="gene_1926"
/transl_table=11
/translation="MKSASDPFDLKRFVYAQAPVYRSVVEELRAGRKRGHWMWFVFPQ
LRGLGSSPLAVRYGISSLEEAQAYLQHDLLGPRLHECTGLVNQVQGRSIEEIFGPPDD
LKLCSSMTLFARATDANQDFVALLAKYYGGGEDRRTVALLAVT"
CDS 2117418..2118122

/codon_start=1
/note="gene_1927"
/transl_table=11
/translation="MEKVIAVLMRPEPDDDWCARQRAQVADALLGLGVAGLSINVRDS
TVRDSLMTLTTLYPPVAAVVSLWTQQCYGEQVAAALRLLAQECDELGAYLVTESVPLT
FPSLVESGSRTPGLANIALLRRPDGLDQATWLTRWQRDHTQVAIEAQATFGYTQNWVV
RALTPEAPGIAGIVEELFPVAATTDLKAFFGAADDNDLRNRISRMVASTSAFGANQNI
DTVPTSRYVFRTPFKD"
CDS 2118133..2118576
/codon_start=1
/note="gene_1928"
/transl_table=11
/translation="MTTLNEAAALAAAERGLAVVSTVRADGTVQASLVNVGLLPHPVS
GEPSLGFTTYGKVKLGNLRARPQLAVTFRNGWQWATVEGRAQLVGPDDPRPWLVDGER
LRLLLREVFTAAGGTHDDWDEYDRVMAQEQRAVVLITPTRIYSNG"
CDS complement(2118582..2118794)
/codon_start=1
/note="gene_1929"
/transl_table=11
/translation="MYVCLCVGVTNQTVCDAVARGASTSKEVAAVCGAGGDCGRCRRT
LRAIIAAARLNPTQLDPAGRTSDAVC"
CDS 2119092..2119571
/codon_start=1
/note="gene_1930"
/transl_table=11
/translation="MQGDPDVLRLLNEQLTSELTAINQYFLHSKMQDNWGFTELAAHT
RAESFDEMRHAEEITDRILLLDGLPNYQRIGSLRIGQTLREQFEADLAIEYDVLNRLK
PGIVMCREKQDTTSAVLLEKIVADEEEHIDYLETQLELMDKLGEELYSAQCVSRPPT"
CDS complement(2119535..2119861)
/codon_start=1
/gene="Rcas_0453"
/note="gene_1931"
/transl_table=11
/translation="VTAHDWSTVLPSSATIVGNAGTTMVWPVAATSTPNTSAVKIMLR
RTGLSSGPPARIAVGAVVGAVGPAISALPSLFESMLELISGARSGIGESSSGHQVGGR
ETHCAE"
CDS 2120506..2121720
/codon_start=1
/note="gene_1932"
/transl_table=11
/translation="VCCVLSFVVGFAMLGALTFVPIYLGYVDGASATASGLRTLPMVI
GLLIASTGTGVLVGRTGRYKIFPVAGMALMAVAFLLMSQMDEWTPPLLQSLYLVVLGA
GIGLSMQVLVLIVQNTSSFEDLGVATSGVTFFRVVGASFGTATFGALFVNFLDRRLGS
ALTSGAVPVPAVPSPAVLHQLPQSMAAPIVRAYAESLTQVFLCAVSVTVVGFILALLL
REVPLTDIHDDADDLGDGFGVPRAESPEDVLEIAVRRMLPNGVRLRDIATQPGCGLGV
AELWALLRIYQYQRLFEAVRLTDIGRHLHVPYQVFEPVFDRLVQTGYAARDGDILTLT
PSGHRQVDSLAVLIRQWLLDHLAVAPGLKRQPDHQFEAALQHVTDAVLVQRDWYEDLG
DLSESRQLAATT"
CDS 2121775..2123127
/codon_start=1
/note="gene_1933"

/transl_table=11
/translation="MTATPLAAAAIAQLEAEGVDTVIGTVVNPAGLTQAKTVPIRRTN
TFANPGLGASPVWHTFCIDQCSIAFTADISVVGDQRLRIDLSALRIIGDGLAWAPAGF
FEQDGTPVPACSRGTLSRIEAALADAGIDAVIGHEVEFLLVDADGQRLPSTLWAQYGV
AGVLEHEAFVRDVNAAATAAGIAIEQFHPEYGANQFEISLAPQPPVAAADQLVLTRLI
IGRTARRHGLRVSLSPAPFAGSIGSGAHQHFSLTMSEGMLFSGGTGAAGMTSAGEAAV
AGVLRGLPDAQGILCGSIVSGLRMRPGNWAGIYACWGTENREAAVRFVKGGAGSAYGG
NVEVKVVDPSANPYLASAAILGLALDGMKTKAVLPSETTVDPTQLSDVDRDRAGILRL
AADQADAIAVLDSSKLLRCILGDPVVDAVVAVRQLEHERYGDLDPAQLADKFRMAWSV
"

CDS 2123130..2124266
/codon_start=1
/note="gene_1934"
/transl_table=11
/translation="MADSAGSDLTRHTAEVPLIDQHVHGCWLTEGNRRRFENALNEAN
TEPLADFDSGFDSQLGFAVRNHCAPILGLPRHVDPQTYWDRRSQFSEAELARRFLQAA
GVTDWLVETGIGYDVSGMASVAGLGELSGSHAHEVVRLEQVAEQAVQASGDYASAFNE
ILRRRAATAVATKSILAYRGGFDGDLTEPPAAQVAEAAKRWRDRGGVRLQDRVLLRFG
LHQALRLGKPLQFHVGFGDRDADLHKANPLYLLDFLRQSGNTPIVLLHCYPYEREAGY
LAQAFNNVYLDGGLSVHYLGARSPAFIGRLLELAPFRKIVYSSDGFGPAELHFLGATL
WRSGIQRVLRGFVERDDWCETDALRVVDLIAHGTAARIYRLGDR"

CDS complement(2124294..2125610)
/codon_start=1
/note="gene_1935"
/transl_table=11
/translation="VKDKLHWLAMHGVIRGIAAIGIRRGDLQARLIADPAVATDPVPF
YDEVRSHGALVRNRANYLTVDHRLAHDLLRSDDFRVVSFGENLPPPLRWLERRTRGDQ
LHPLREPSLLAVEPPDHTRYRKTVSAVFTSRAVSALRDLVEQTAINLLDRFAEQPGIV
DVVGRYCSQLPIVVISEILGVPEHDRPRVLEFGELAAPSLDIGIPWRQYLRVQQGIRG
FDCWLEGHLQQLRHAPGDDLMSQLIQIAESGDNETQLDETELRAIAGLVLVAGFETTV
NLLGNGIRMLLDTPEHLATLRQHPELWPNTVEEILRLDSPVQLTARVACRDVEVAGVR
IKRGEVVVIYLAAANRDPAVFPDPHRFDIERPNAGRHLAFSTGRHFCLGAALARAEGE
VGLRTFFDRFPDVRAAGAGSRRDTRVLRGWSTLPVTLGPARSMVSP"

CDS complement(2125660..2126046)
/codon_start=1
/note="gene_1936"
/transl_table=11
/translation="MVVAAGLSACGQAQTVPRKAARLTIDGVTHTTRPATCSQEHSYR
TIDIRNHDSTVQAVVLLSGDRVIPQWVKIRNVDGFNGSFWHGGVGNARADRARNTYTV
AGSAYGISSKKPNTVVSTDFNILAEC"

CDS complement(2126123..2126920)
/codon_start=1
/note="gene_1937"
/transl_table=11
/translation="MGREGATLFHANGWRVGAIDRNEDGLAALRVQLGAERLWARAVD
VTDKAALEGALADFCAGNVGGGLDMMWNNAGIGEGGWFEDVPYEAAVRVVDVNFKAVL
TGAYAALPYLKKAPGSLMFSTSSSSGTYGMPRIAVYSATKHAVKGLTEALSVEWQRHG
VRVADVLPGLIDTAILTSTRQHSDEGPYTISAEQIRAAAPKKGMFRLMPSSSVAEAAW
RAYQHPTRLHWYVPRSIRWIDRLKGVSPEFVRRHIAKSLATLEPKRK"

CDS complement(2126984..2127460)
/codon_start=1

```
                     /note="gene_1938"
                     /transl_table=11
                     /translation="MCLDQVMEGSATVHMAAPPDKIWTLIADVRNTGRFSPETFEAEW
                     LDGATGPALGARFRGHVRRNGIGPVYWTVCEVTACEPGREFGFAVLLGDRPVNNWHYR
                     LTPTADGTEVTESFRLPPSVLTTVYYRVFGGWLRQRRNIRDMTKTLQRIKDLVEAG"
     CDS             complement(2127499..2127921)
                     /codon_start=1
                     /note="gene_1939"
                     /transl_table=11
                     /translation="MTRIAKPLIKSAMAAGLVTASMSLSTAVAHAGPSPNWDAVAQCE
                     SGGNWAANTGNGKYGGLQFKPATWAAFGGVGNPAAASREQQIAVANRVLAEQGLDAWP
                     TCGAASGLPIALWSKPAQGIKQIINEIIWAGIQASIPR"
     CDS             complement(2128041..2128640)
                     /codon_start=1
                     /note="gene_1940"
                     /transl_table=11
                     /translation="LLTRPREIYLATAVSIGILLSLIAPLGPPLARADGTSQLAELVD
                     AAAERLEVADPVAAFKWRAQLPIEDSGRVEQQLAKLGEDARSQHIDPDYVTRVFDDQI
                     RATEAIEYSRFSDWKLNPASAPPEPPDLSASRSAIDSLNNRMLSQIWSHWSLLSAPSC
                     AAQLDRAKRDIVRSRHLDSLYQRALTTATQSYCQALPPA"
     CDS             complement(2128658..2129590)
                     /codon_start=1
                     /note="gene_1941"
                     /transl_table=11
                     /translation="MIGTAAAVVLPGLVGLAGGAATAGAFSRPGLPVEYLQVPSPSMG
                     RDIKVQFQSGGNNSPAVYLLDGLRAQDDYNGWDINTPAFEWYYQSGLSIVMPVGGQSS
                     FYSDWYSPACGKAGCQTYKWETLLTSELPQWLSANRAVKPTGSAAIGLSMAGSSAMIL
                     AAYHPQQFIYAGSLSALLDPSQGMGPSLIGLAMGDAGGYKAADMWGPSSDPAWERNDP
                     TQQIPKLVANNTRLWVYCGNGTPNELGGANIPAEFLENFVRSSNLKFQDAYNAAGGHN
                     AVFNFPPNGTHSWEYWGAQLNAMKGDLQSSLGAG"
     CDS             2130011..2131168
                     /codon_start=1
                     /note="gene_1942"
                     /transl_table=11
                     /translation="LRCENLDTVLGLSITPTTLGWVLAEGHGADGAILDRNELELHSG
                     RNAQAIHTAEQLAAEVLLAHEVAAAGDHRLRVIGVTWNAEASAQAALLVESLTGAGFD
                     NVVPVRRLRAIETLAQAIAPVIGCEQIAVCVLEHESATVVMVDTHDGKTQIAVKHVCR
                     GLSGLTSWLTGMFGRDAWRPAGVVVVGSDSEVSEFSWQLERVLPVPVFAQTMAQVTVA
                     RGAALAAAQSTEFTDAQLVADSVSQPTVAPRRSRHYAGAAAALAAAAVTFVASLSLAV
                     GIQLAPHNDTGTAKHGAHKPTPRIAKAVAPAVPPPPTVTPPVPARAPRPAAQHEPPAR
                     VTSGEALTEPNPPEEQPNASAPQQDRNDSQPITRVLEHIPGAYGDSAPPAE"
     CDS             complement(2131138..2131857)
                     /codon_start=1
                     /note="gene_1943"
                     /transl_table=11
                     /translation="MQPDAYPVRVRGDLDPALSRWQWLVKWFLAIPHYIVLFFLHVAA
                     VVVTVIAFFAILFTGRYPRTLFDFNVGVMRWRWRVAFYALSALGTDRYPPFSLQTKAE
                     YPADLEVDYPERLSRGLVLIKWWLLAIPHYLILAVFLSSGWRVFLIDPHDRVGIMWPS
                     LLVILLLVAVVALLFTGRYPIGLYNLVIGVNRWALRVRAYTTLMRDEYPPLRLDMGPR
                     EQVSQPATAASDYSAGGAESP"
     CDS             complement(2132222..2132395)
```

/codon_start=1
/note="gene_1944"
/transl_table=11
/translation="VVPVDLRRDWPTPLRQAGFDPNQPSAWLAEGLLAFLPPDAQDRL
LDNITALSAPGSR"

CDS complement(2132439..2132795)
/codon_start=1
/note="gene_1945"
/transl_table=11
/translation="MPRTNNDAWDLATSVGATATMVAAARAVATRADNPLIDDPFAEP
LVRAVGIDFFTRWAAGNIKATDVDDPDGTWGLQRLADLLAARTRYFDAFFRDATSAGI
RQAVILASGLDARAYR"

CDS complement(2132854..2133465)
/codon_start=1
/note="gene_1946"
/transl_table=11
/translation="VAHKTRREGRAGRSSEYSRGVSDAVWTLDASDGELVLRTGVVGR
AARLGHRLTIAMTRWQALVNWSGTDPVAGELVAEVDSFEVMRGEGGVKGLSEPEKALV
RANALKTLNASRFPHIRFTTEAIAQTGNGYRLTGKLHIRGKSREHVIDLHTEDLGAAW
RISADTTVRQSNYGVKPYSLLMGSIRVADEVSVAFTAVRAKDD"

CDS 2133519..2133926
/codon_start=1
/note="gene_1947"
/transl_table=11
/translation="MIRELVTTAAITGAAIGGAPVAGADPQRYDGDVPGMNYDASLGA
PCSSWERFIFGRGPSGQAEACHFPPPNQFPPAETGYWVISYPLYGVQQVGAPCPKPQA
AAQSPDGLPMLCLGARGWQPGWFTGAGFFPPEP"

CDS 2133943..2134254
/codon_start=1
/note="gene_1948"
/transl_table=11
/translation="MIMCEGRPTESPIPRWLRFVLTSDRAGSAWYIGAGFFFAPVLAV
LSPWPTITAVLWWIIGLAGLWLGLLGIAMAVGLARVLRSGAEIPEAYWRTLVDYRSAN
E"

CDS 2134264..2134482
/codon_start=1
/note="gene_1949"
/transl_table=11
/translation="MSFNPKDAVDAVRDIAANAVEKASDIVENAGHIIRGDIAGGASG
IVKDSIDIATHAVDRTKEVFTGKTDDEG"

CDS complement(2134517..2135647)
/codon_start=1
/note="gene_1950"
/transl_table=11
/translation="MHTAICDELGIEFPIFAFTHCRDVVVAVSKAGGFGVLGAVGFTP
EQLEIELNWIDEHIGDHPYGVDIVIPNKYEGMDSQLSADELAKTLRSMVPQEHLDFAR
KILADHGVPVEDADEDSLQLLGWTEATATPQVDAALKHPKMTMVANALGTPPADMIKH
IHDSGRKVAALCGSPSQARKHADAGVDIIIAQGGEAGGHCGEVGSIVLWPQVVKEVAP
VPVLAAGGIGSGQQIAAALALGTQGAWTGSQWLMVEEAANTAVQQAAYVKATSRDTVR
SRSFTGKPARMLRNDWTEAWEQPESPKPLGMPLQYMVSGMAVKATHKYPNETVDVAFN
PVGQVVGQFTKVEKTATVIERWVQEYLEATARLDALNAAASV"

```
CDS             2136299..2136622
                /codon_start=1
                /note="gene_1951"
                /transl_table=11
                /translation="MRAVVIDGAGSVRVNTQPDPALPGPDGVVVAVTAAGICGSDLHF
                YEGEYPFTEPVALGHEAVGTIVEAGPQVRTVGVGDLVMVSSVAGCGVCPGCEPMIQSC
                ASPAR"
CDS             2136619..2137323
                /codon_start=1
                /note="gene_1952"
                /transl_table=11
                /translation="MIFGAGVLGGAQADLLAVPAADFQVLKIPEGITTEQALLLTDNL
                ATGWAAAQRADISFGSAVAVIGLGAVGLCALRSAFIHGAATVFAVDRVKGRLQRAATW
                GATPIPSPAAETILAATRGRGADSVIDAVGTDASMSDALNAVRPGGTVSVVGVHDLQP
                FPLPALTCLLRSITLRMTMAPVQRTWPELIPLLQSGRLDVDGIFTTTLPLDEAAKGYA
                TARARSGEELKVLLTP"
CDS             complement(2137313..2138224)
                /codon_start=1
                /note="gene_1953"
                /transl_table=11
                /translation="MTTPEYGSLRSDDDHWDIVSNVGYTALLVAGWRALHTTGPKPLV
                QDEYAKHFITASADPYLEGLLANPRTSEDGTAFPRLYGVQTRFFDDFFNCADEAGIRQ
                AVIVAAGLDCRAYRLDWQPGTTVFEIDVPKVLEFKARVLSERGAVPKAHRVAVPADLR
                TDWPTPLTAAGFDPQRPSAWSVEGLLPYLTGDAQYALFARIDELCAPGSRVALGALGS
                RLDHEQLAALETAHPGVNMSGDVNFSALTYDDKTDPVEWLVEHGWAVDPVRSTLELQV
                GYGLTPPDVDVKIDSFMRSQYITAVRA"
CDS             complement(2138229..2138606)
                /codon_start=1
                /note="gene_1954"
                /transl_table=11
                /translation="VVGAIRPDGQGLVAFVGVTHGDDLDKARRLAEKLWNLRVLAGEK
                SASDMHAPILVISQFTLYADTAKGRRPSWNAAAPGAVAQPLIAAFAAALRQLGAHVEA
                GVFGAHMQVELVNDGPVTVMLEG"
CDS             2138718..2139026
                /codon_start=1
                /note="gene_1955"
                /transl_table=11
                /translation="VSVLVAFSVTPLGVGEGVGEIVTEAIRVVRDSGLPNQTDAMFTV
                IEGDTWAEVMAVVQRAVEAVAARAPRVSAVIKVDWRPGVTDAMTQKVATVERYLLRPE
                "
CDS             complement(2138992..2139507)
                /codon_start=1
                /note="gene_1956"
                /transl_table=11
                /translation="MIELEVHQADVTKLELDAITNAANTRLRHAGGVAAAIARAGGPE
                LQRESTEKAPIGLGEAVETTAGDMPARYVIHAATMELGGPTSGEIITAATAATLRKAD
                ELGCRSLALVAFGTGVGGFPLDDAARLMVGAVRRHRPGSLQRVVFAVHGDAAERAFSA
                AIQAGEDTARR"
CDS             2139785..2139934
                /codon_start=1
                /note="gene_1957"
```

```
                     /transl_table=11
                     /translation="MPAASVGRAEPEAPVAGVVPAVAAGRAVEAATAGKAVAAGRAVE
                     AEMAA"
     CDS             complement(2140068..2141405)
                     /codon_start=1
                     /note="gene_1958"
                     /transl_table=11
                     /translation="MDIAYQVLGDGPTDLLVLPGPFVPIDSIDDEPSLYRFHRRLASF
                     SRVIRLDHRGVGLSSRLAAITTLGPKFWAQDAIAVMDAVGCEQATIFAPSFHAMNGLV
                     LAADYPERVRSLIVVNGSARPLWAPDYPVGAQVRRADPFLTVALEPDAVERGFDVLSI
                     VAPTVAGDDVFRAWWDLAGNRAGPPSMARAVSKVIAEADVRDVLGHIEAPTLILHRVG
                     STYIPVGHGRYLAEHIAGSRLVELPGTDTLYWVGDTGPMLDEIEEFITGVRGGADAER
                     MLATIMFTDIVGSTQHAAALGDDRWRDLLDNHDTIVCHEIQRFGGREVNTAGDGFVAT
                     FTSPSAAIACADDIVDAVAALGIEVRIGIHAGEVEVRDASHGTDVAGVAVHIGARVCA
                     LAGPSEVLVSSTVRDIVAGSRHRFAERGEQELKGVPGRWRLCVLMRDDATRTR"
     CDS             2141485..2142777
                     /codon_start=1
                     /note="gene_1959"
                     /transl_table=11
                     /translation="MAVSARAGIVITGTEVLTGRVQDRNGPWIADRLLELGVELAHIT
                     ICGDRPADIEAQLRFMAEQGVDLIVTSGGLGPTADDMTVEVVARYCGRELVLDDELEN
                     RIANILKKLMGRNPAIEPANFDSIRAANRKQAMIPAGSQVIDPVGTAPGLVVPGRPAV
                     MVLPGPPRELQPIWSKAIQTAPVQDAIAGRTTYRQETIRIFGLPESSLADTLRDAEAA
                     IPGFDLVEITTCLRRGEIEMVTRFEPNAAQVYTQLARLLRDRHGHQVYSEDGASVDEL
                     VAKLLTGRRIATAESCTAGLLAARLTDRPGSSKYVAGAVVAYSNEAKAQLLGVDPALI
                     EAHGAVSEPVAQAMAAGALQGFGADTATAITGIAGPSGGTPEKPVGTVCFTVLLDDGR
                     TTTRTVRLPGNRSDIRERSTTVAMHLLRRTLSGIPGSP"
     CDS             complement(2142829..2144055)
                     /codon_start=1
                     /note="gene_1960"
                     /transl_table=11
                     /translation="MASFLGWTMDAFDYFLVVLVYADIATTFHHTKTDVAFLTTATLA
                     MRPVGALLFGLWADRVGRRVPLMVDVSFYSVIGFLCAFAPNFTVLVILRLLYGIGMGG
                     EWGLGAALSMEKVPAERRGVFSGLLQEGYAFGYLLASVAALVVMNWLGLSWRWLFGLS
                     IIPALISLIIRYRVKESEVWEAAQDRMRLTKTRIRDVLGNPAIVRRFVYLVLLMTAFN
                     WMSHGTQDVYPTFLTATTDHGAGLSSLTARWIVVIYNIGAIIGGLAFGTLSQRFSRRY
                     TIVFCAALGLPIVPLFAYSRTAAMLCLGSFLMQVFVQGAWGVIPAHLTEMSPDAIRGV
                     YPGVTYQLGNLLAAFNLPIQERLAESHGYPFALAATIVPVLLVVAVLTAIGKDATGIR
                     FGTTETAFLVRHRNRH"
     CDS             2144187..2144591
                     /codon_start=1
                     /note="gene_1961"
                     /transl_table=11
                     /translation="MVPFLMRAAVTGFALWVVTLFVPGMRFAGGDTTLQRVAIIFVVA
                     VIFGLVNAFIKPIVQILSIPLYILTLGLFHVVVNASMLWLTAWITEHTTHWGLQIDHF
                     WWTAIWAAILLSIVSWILSLLARDFRRVTRAH"
     CDS             2144903..2145208
                     /codon_start=1
                     /note="gene_1962"
                     /transl_table=11
                     /translation="VGGDIDASNEVAWQRLVSKSAAIAIAPGPFVIDIRDLDFMGSCA
```

YAVLAQESVRCRRRGVNMRLVSNQPIVARTIAACGLRRLIPLYAMVETALAPPPSAH"
CDS complement(2145256..2146218)
/codon_start=1
/note="gene_1963"
/transl_table=11
/translation="VAIGEQQVIVIGAGVSGLTSAICLAEAGWPVRVWAAALPQQTTS
AVAGAVWGPRPKEPVAKVRGWIEQSLHVFRDLAKDPATGVRMTPALSVGDRIETGAMP
PGLELIPDVRPADPADVPGGFRAGFHATLPMIDMPQYLDCLTQRLAATGCEIETRPLR
SLAEAAEAAPIVINCAGLGARELAGDATVWPRFGQHVVLTNPGLEQLFIERTGGSEWI
CYFAHPQRVVCGGISIPGRWDTTPEPEITERILQRCRRIQPRLAEAAVIETITGLRPD
RPSVRVEAEPIGRALCIHNYGHGGDGVTLSWGCAREVVNLVGGG"
CDS complement(2146248..2146718)
/codon_start=1
/note="gene_1964"
/transl_table=11
/translation="MRLKPAPSPAAAFAVAGLILAGWAGSVGLAGADPEPAPTPKTAI
DSDGTYAVGIDIAPGTYSSAGPVGDGTCYWKRMGNPDGALIDNALSKKPQVVTIEPTD
KAFKTHGCQPWQNTGSEGAAPAGVPGPEAGAQLQNQLGILNGLLGPTGGRVPQP"
CDS complement(2147058..2147552)
/codon_start=1
/note="gene_1965"
/transl_table=11
/translation="MRRKGAEMCWMCDHPEATAEEYLDEVYGIMLMHGWAVQHVECER
RPFAYTVGLTRRGLPELVVTGLSPRRGQRLLNIAARRALVGDLLTPGMQTTLPAGPLV
ETVQVTHPDAHLYCAIAIFGDKVTALQLVWADRRGRWPWAADFDEGRGTQPVLGMRAT
RRSA"
CDS complement(2147712..2149859)
/codon_start=1
/note="gene_1966"
/transl_table=11
/translation="MKYPVEGGGNQDWWPNRLNLKVLHQNPAVADPMGAAFDYAAEVA
TIDVDALTRDIEEVMTTSQPWWPADYGHYGPLFIRMAWHAAGTYRIHDGRGGAGGGMQ
RFAPLNSWPDNASLDKARRLLWPVKKKYGKKLSWADLIVFAGNCALESMGFKTFGFGF
GRVDQWEPDEVYWGKEATWLGDERYSGKRDLENPLAAVQMGLIYVNPEGPNGNPDPMA
AAVDIRETFRRMAMNDVETAALIVGGHTFGKTHGAGPADLVGPEPEAAPLEQMGLGWK
SSYGTGTGKDAITSGIEVVWTNTPTKWDNSFLEILYGYEWELTKSPAGAWQYTAKDGA
GAGTIPDPFGGPGRSPTMLATDLSLRVDPIYERITRRWLEHPEELADEFAKAWYKLIH
RDMGPVARYLGPLVPKQTLLWQDPVPAVSHDLVGEAEIASLKSQILASGLTVSQLVST
AWAAASSFRGSDKRGGANGGRIRLQPQVGWEVNDPDGDLRKVIRTLEEIQESFNSAAP
GNIKVSFADLVVLGGCAAIEKAAKAAGHNITVPFTPGRTDASQEQTDVESFAVLEPKA
DGFRNYLGKGNPLPAEYMLLDKANLLTLSAPEMTVLVGGLRVLGANYKRLPLGVFTEA
SESLTNDFFVNLLDMGITWEPSPADDGTYQGKDGSGKVKWTGSRVDLVFGSNSELRAL
VEVYGADDAQPKFVQDFVAAWDKVMNLDRFDVR"
CDS complement(2149972..2150424)
/codon_start=1
/note="gene_1967"
/transl_table=11
/translation="LSSVSSIPDYAEQLRTADLRVTRPRVAVLEAVNAHPHADTETIF
GVVRFALPDVSRQAVYDVLHALTAAGLVRKIQPSGSVARYESRVGDNHHHIVCRSCGV
IADVDCAVGEAPCLTASDHNGFLLDEAEVIYWGLCPDCSISDTSRSHP"
CDS complement(2150529..2151143)

```
                     /codon_start=1
                     /note="gene_1968"
                     /transl_table=11
                     /translation="MINMESTVAHAFHRFALAILGLALPVALVAYGGNGDSRKAAPLA
                     PKAAALGRSMPETPTGDVLTISSPAFADGAPIPEQYTCKGANIAPPLTWSAPFGGALV
                     VDDPDAPREPYVHWIVIGIAPGAGSTADGETPGGGISLPNSSGQPAYTGPCPPAGTGT
                     HHYRFTLYHLPAVPPLAGLAGTQAARVIAQAATMQARLIGTYEG"
     CDS             complement(2151205..2151810)
                     /codon_start=1
                     /note="gene_1969"
                     /transl_table=11
                     /translation="MTSTLHRTPLATAGLALVVALGGCGGGGGDSRETPPYVPKATTV
                     DATTPAPAAEPLTIASPMFADGAPIPVQFSCKGANVAPPLTWSSPAGAAELALVVDDP
                     DAVGGLYVHWIVTGIAPGSGSTADGQTPAGGHSVPNSGGRQGYFGPCPPAGTGTHHYR
                     FTLYHLPVALQLPPGATGVQAAQAIAQAASGQARLVGTFEG"
     CDS             complement(2151910..2153052)
                     /codon_start=1
                     /note="gene_1970"
                     /transl_table=11
                     /translation="MHAVGQALPVEVHNRKGNAPPNLLITRAITEGCSRVRPVKPDKE
                     NIMRAVVITKHGDPSVLQVRQRPDPPPPGPGQLRVAVRAAGVNFADHLARVGLYPDAP
                     KLPAVVGYEVAGTVEAVGDGVDPNRVGERVLAGTRFGGYCEIVNVAATDSVVLPDALS
                     FEQGAAVPVNYATAWAALHGYGSLRAGERVLIHAAAGGVGIAAVQFAKAAKAEVHGTA
                     SPQKHQKLAEFGVDRAIDYRRDGWWQGLGPYDVVLDALGGTSLRRSYTLLRPGGRLVG
                     YGISNMQHGEKRSMRRVAPHALSMLRGFNLMKQLEESKTVIGLNMLRLWDDRRTLEPW
                     IAPLTKALNDGTILPIVHAIVPFAEAPEAHRILAARENVGKVVLVP"
     CDS             2153014..2153766
                     /codon_start=1
                     /note="gene_1971"
                     /transl_table=11
                     /translation="VHFDWECLTDSVHRCRLPFCDVTVGLVRGRTGILLVDTGTTLGE
                     ATAIAADVKQIAGCQVTHVVLTHKHFDHVLGSSVFDQAEVFCAPEVVEYLRSATDRLR
                     EDALSYGADTAEVDRAIAALKPPQHGIYDAAVDLGDRTVTITHPGSGHTTADLVVVAP
                     ATGHADGPTVVFTGDLVEESADPDIDADSDLAAWPATLDRVLAIGGPDASYVPGHGKV
                     VDAQFVRRQRAWLRTRASRQPRETPATLPCKR"
     CDS             complement(2153744..2154112)
                     /codon_start=1
                     /note="gene_1972"
                     /transl_table=11
                     /translation="VPTQLRFDRWFLPLAVPLGLGPKNSELWVGAGSLHVKMGWAFAA
                     DIPLTSITKAEATNARVYAAGVHFGFGRWLVNGSRKGLVALTIDPPEQAKMWKKSMTV
                     RELWVSVTDPDALVTACTAK"
     CDS             2154286..2156586
                     /codon_start=1
                     /note="gene_1973"
                     /transl_table=11
                     /translation="MAIAETDTEVHTPFEQDFEKDVAATQRYFDSSRFAGIIRLYTAR
                     QVVEQRGTIPVDHIVAREAAGAFYERLRELFAARKSITTFGPYSPGQAVSMKRMGIEA
                     IYLGGWATSAKGSSTEDPGPDLASYPLSQVPDDAAVLVRALLTADRNQHYLRLQMSER
                     QRAATPAYDFRPFIIADADTGHGGDPHVRNLIRRFVEVGVPGYHIEDQRPGTKKCGHQ
                     GGKVLVPSDEQIKRLNAARFQLDIMRVPGIIVARTDAEAANLIDSRADERDQPFLLGA
```

TKLDVPSYKSCFLAMVRRFYELGVKELNGHLLYALGDSEYAAAGGWLERQGIFGLVSD
AVNAWREDGQQSIDGIFDQVESRFVAAWEDDAGLMTYGEAVADVLEFGQSEGEPIGMA
PEEWRAFAARASLHAARAKAKELGADPPWDCELAKTPEGYYQIRGGIPYAIAKSLAAA
PFADILWMETKTADLADARQFAEAIHAEFPDQMLAYNLSPSFNWDTTGMTDEEMRRFP
EELGKMGFVFNFITYGGHQIDGVAAEEFATALRQDGMLALARLQRKMRLVESPYRTPQ
TLVGGPRSDAALAASSGRTATTKAMGKGSTQHQHLVQTEVPRKLLEEWLAMWSGHYQL
KDKLRVQLRPQRAGSEVLELGIHGESDDKLANVIFQPIQDRRGRTILLVRDQNTFGAE
LRQKRLMTLIHLWLVHRFKAQAVHYVTPTDDNLYQTSKMKSHGIFTEVNQEVGEIIVA
EVNHPRIAELLTPDRVALRKLITKEA"
CDS 2156658..2156840
/codon_start=1
/gene="MCAN_19331"
/note="gene_1974"
/transl_table=11
/translation="MLSDGVCGGLSKIQRTGVHLRRPAVATTIAAPVYGPAPGEKKPR
SIWPMLLTPELNAAVR"
CDS complement(2156756..2163196)
/codon_start=1
/gene="PPE34"
/note="gene_1975"
/transl_table=11
/translation="MSAAAVAWDQLAMELASAAASFNSVTSGLVGESWLGPSSAAMAA
AVAPYLGWLAAAAAQAQRSATQAAALVAEFEAVRAAMVQPALVAANRSDLVSLVFSNF
FGQNAPAIAAIEAAYEQMWAIDVSVMSAYHAGASAVASALTPFTAPPQNLTDLPAQLA
AAPAAVVTAAITSSKGVLANLSLGLANSGFGQMGAANLGILNLGSLNPGGNNFGLGNV
GSNNVGLGNTGNGNIGFGNTGNGNIGFGLTGDNQQGFGGWNSGTGNIGLFNSGTGNIG
IGNTGTGNFGIGNSGTSYNTGIGNTGQANTGFFNAGIANTGIGNTGNYNTGSFNLGSF
NTGDFNTGSSNTGFFNPGNLNTGVGNTGNVNTGGFNSGNYSNGFFWRGDYQGLIGFSG
TLTIPAAGLDLNGLGSVGPITIPSITIPEIGLGINSSGALVGPINVPPITVPAIGLGV
SSSGALVGPINVPPITVPAIGLGVSSSGALVGPINVPPITVPAIGLGVSSSGALVGPI
NVPPITVPAIGLGVSSSGALVGPINVPPITVPAIGLGVSSSGALVGPINVPPITVPAI
GLGVSSSGALVGPINVPPITVPAIGLGVSSSGALVGPINVPPITVPAIGLGVSSSGAL
VGPINVPPITVPAIGLGVSSSGALVGPINVPPITVPAIGLGVSSSGALVGPINVPPIT
VPAIGLGVSSSGALVGPINVPPITVPAIGLGVSSSGALVGPINVPPITVPAIGLGVSS
SGALVGPINVPPITVPAIGLGVSSSGALVGPINVPPITVPAIGLGVSSSGALVGPINV
PPITLNSIGLELSAFQVINVGSISIPASPLAIGLFGVNPTVGSIGPGSISIQLGTPEI
PAIPPFFPGFPPDYVTVSGQIGPITFLSGGYSLPAIPLGIDVGGGLGPFTVFPDGYSL
PAIPLGIDVGGGLGPFTVFPDGYSLPAIPLGIDVGGGLGPFTVFPDGYSLPAIPLGID
VGGGLGPFTVFPDGYSLPAIPLGIDVGGGLGPFTVFPDGYSLPAIPLGIDVGGGLGPF
TVFPDGYSLPAIPLGIDVGGGLGPFTVFPDGYSLPAIPLGIDVGGGLGPFTVFPDGYS
LPAIPLGIDVGGAIGPLTTPPITIPAIPLGIDVSGSLGPINIPIEIAGTPGFGNSTTT
PSSGFFNSGTGGTSGFGNVGSGGSGFWNIAGNLGNSGFLNVGPLTSGILNFGNTVSGL
YNTSTLGLATSAFHSGVGNTDSQLAGFMRNAAGGTLFNFGFANDGTLNLGNANLGDYN
VGSGNVGSYNFGSGNIGNGSFGFGNIGSNNFGFGNVGSNNLGFANTGPGLTEALHNIG
FGNIGGNNYGFANIGNGNIGFGNTGTGNIGIGLTGDNQVGFGALNSGSGNIGFFNSGN
GNIGFFNSGNGNVGIGNSGNYNTGLGNVGNANTGLFNTGNVNTGIGNAGSYNTGSYNA
GDTNTGDLNPGNANTGYLNLGDLNTGWGNIGDLNTGALISGSYSNGILWRGDYQGLIG
YSDTLSIPAIPLSVEVNGGIGPIVVPDITIPGIPLSLNALGGVGPIVVPDITIPGIPL
SLNALGGVGPIVVPDITIPGIPLSLNALGGVGPIVVPDITIPGIPLSLNALGGVGPIT
VPGVPISRIPLTINIRIPVNITLNELPFNVAGIFTGYIGPIPLSTFVLGVTLAGGTLE
SGIQGFSVNPFGLNIPLSGATNAVTIPGFAINPFGLNVPLSGGTSPVTIPGFAINPFG

LDVPLSGGTNAVTIPGFAINPFGLDVPLSGGTNAVTIPGFAINPFGLDVPLSGGTNAV
TIPGFAINPFGLDVPLSGGTNAVTIPGFAINPFGLDVPLSGGTNAVTIPGFAINPFGL
DVPLSGGTNAVTIPGFAINPFGLDVPLSGGTSPVTIPGFAINPFGLDVPLSGGTSPVT
IPGFAINPFGLNVPLSGGTNAVTIPGFAINPFGLNVPLSGGTSPVTIPGFAINPFGLN
VPLSGGTNAVTIPGFAINPFGLNVPLSGGTSPVTIPGFAINPFGLNVPLSGGTSPVTI
PGFTIPGSPLNLTANGGLGPINITSAPGFGNSTTTPSSGFFNSGDGSASGFGNVGPGI
SGLWNQVPNALQGGVSGIYNVGQLASGVANLGNTVSGFNNTSTVGHLTAAFNSGVNNI
GQMLLGFFSPGAGP"

variation 2157389..2157526
/note="supplemented from BCG moreau"

variation 2157967..2158104
/note="supplemented from BCG moreau"

variation 2158407..2158475
/note="supplemented from AL2122/97"

variation 2159938..2160012
/note="supplemented from AL2122/97"

variation 2160438..2160587
/note="supplemented from BCG moreau"

variation 2160977..2161120
/note="supplemented from AL2122/97"

variation 2161172..2161243
/note="supplemented from BCG moreau"

variation 2161263..2161334
/note="supplemented from BCG moreau"

variation 2161847..2161990
/note="supplemented from AL2122/97"

CDS complement(2163603..2164607)
/codon_start=1
/note="gene_1976"
/transl_table=11
/translation="LVVFIPNNITALQTNMPGVFPQIGGFANTPPAFINTGTITVGGG
QINGVGFSIGAINVTPFTLPNVVIQPWSLGGISVDGFTLPEISTQEFTTPALTISPIG
VGALSLPDITTQQFTTPELTIDPITLGGFTLPQLSIPAITTPAFTIDPIALGGFTLPQ
IMTPEITTPPFAIDPIGLSGFTLPQVNIPEITTPEFTIQPVGLAAFTTPALTIARIHL
PSTTMGGFAIPAGPGYFNSSATPSSGFFNAGIGGNSGFGNSGSGLSGWFNTSPVGLLA
GSGYQNYGGLISGFSNLGSGISGFANTGTLPFAVTSLVSGLANIGNNLSGLFFQSTTP
"

CDS complement(2164690..2166375)
/codon_start=1
/note="gene_1977"
/transl_table=11
/translation="MAAAAAPYAGWLAAAATQAEQAATQAQVMVAEFEAVRLAMVQPA
LVAANRSGLISLVISNLFGQNAPAIAAAEAAYEEMWALDVSAMAAYHSGASAVAVALP
AFALPLRLPAGLAAGPAAVVTALTTAVGMPTFAGRAIAASLGLANVGGGNLGNANNGL
GNIGNANLGNNNLGSGNFGSFNIGSANLGGNNIGIGNAGANNFGLANLGNLNTGFANA
GIGNFGIANTGNNNIGNGLTGNNQIGIGGLNSGNGNVGLFNAGSANIGFFNSGNGNFG
IGNSGNFSTGLFNPGHGNTGFLNAGSFNTGMFDVGNANTGSFNVGHYNFGAFNPGPSN
TGTFNTGGANTGWFNTGSINTGAFNIGDMNNGLFNTGDMNNGVFYRGVGQGSLQFAIT
SPDLTLPSLEIPGISVPAFSLPAITLPSLTIPAVTTPANVTVGAFDLPGLTVPSLTIP
AAMTPANITVGAFDLPGLTVPSLTIPATTTPANITVGAFNLPQLSIPSVTFPPITIPA
GTALGAFNLPTLSIPSVTVPPITIPAGNHCRRIYATHDTHPVNKYTPNKYRRL"

CDS 2166305..2166562
/codon_start=1
/note="gene_1978"
/transl_table=11
/translation="VAACSAWVAAAASHPAYGAAAAAIATDDRPCHDPPTSPAVTEPK
ETAAEANSVASPSQADAAASIGPEPAPAKIKAELISGGNTE"
CDS complement(2167016..2167480)
/codon_start=1
/note="gene_1979"
/transl_table=11
/translation="MSGRKFSFEVTKTSSAPAATLFRLVTDGGNWATWAKPIVAQSSW
ARRGDPAPGGIGAIRKLGMWPVFVQEETVEYEQDRRHVYKLVGARTPVQDYFGEVVLT
PNASGGTDLRWSGSFTEKVRGTGPVMRAALGGAVRFFAGQLVKAAEREAVRR"
CDS 2167578..2168441
/codon_start=1
/note="gene_1980"
/transl_table=11
/translation="VFPRWPQQAHNHEVSRADTVSVPRAPTQAEVAAVLRIMTPLRKV
IKPKVYGIENVPTERALLVGNHNTLGLVDAPLLAAELWERGRIVRSLGDHAHFKIPGW
RDALTRTGVVEGTREITSELMRRGELVIVFPGGAREVNKRKNERYKLVWKNRLGFARL
AIQHGYPIVPFASVGAEHGIDIVLDNESPLLAPVQFLAEKLLGTKDGPALVRGVGLTP
VPRPERQYYWFGEPIDTTEFMGQQADDNAARRVRERAAAAIEHGIELMLAERAADPNR
SLVGRLLRSDA"
CDS complement(2168479..2169750)
/codon_start=1
/note="gene_1981"
/transl_table=11
/translation="MVRLIPSLLAMATVLGGVIGCSAHQPPTPASGCRQLDAFLKWHH
GVREFLQSAIDANSRCTGTADGSARKVAIFDWDNTVVKNDIGYATNYYMLQHSLVLQP
ANQDWHAASRYLTDAAANALSVACGKVVPAGKPLPTGSNALCANEILSLLDGETTTGQ
PAFVGNNVRRLAGPYAWSNALSAGYTAEELAGFADQAKKQNLAADVGATQQVGTQQVD
GYIRVYPQMKDLIGTLQAHGIDTWVVSASPEPIVKVWAGEVGLDDQHVVGVRSVADQS
GKLTAHLVGCGGVRDGDDSVMTYLDGKRCWANQVIFGVTGPQAFNQLAADRRQVLAAG
DSNSDATFVGDATVVSLVINRNQDDLMCRAYDGLFTRGGKWAINPMFIDPLPQHAPYV
CGEAFINPDGSKQPVLRNDGTPIPDQVDSVF"
CDS 2170022..2171137
/codon_start=1
/note="gene_1982"
/transl_table=11
/translation="MDSTVTASIRRMLGLLAATLLLGGCTGQHTTRTAASTTYTPHIK
ASSQDVLDGAINADEPGCSAAVGVEGKVIWSGVRGIADLASGAKITTDTVFDIASVSK
QFTATAILLLVEAGKLTLDDPISQYVPELPDWAQTVTVEQLMHQTSGIPDYVALLAAR
GYQVSDRTIEAEARQALAAAPELQFKPGTRFDYSNSNYLLLGEIVHRASGQPLPEFLS
AEIFQPLGLAMVVDPVGKVPNKAVSYEKGTGGNRSEYRVGNPAWEQIGDGGIQTTPSQ
LARWADNYRTGSVGGLKLLEAQLAGAVETEPGGGDRYGAGIVSRADGTLDHAGAWAGF
VTAFHISSDRRTSVAISCNTDKPDPVAMADALGRLWM"
CDS 2171152..2172468
/codon_start=1
/note="gene_1983"
/transl_table=11
/translation="LAAGTQAAIIHGMAQPPSLLTTDNGLPFGVQGACDSRFTGVIRA

FAGLYPGRKFGGGALSVYIDDRQVVDVWTGWSDRQGKVPWTADTGAMVFSATKGLAAT
VIHRLVDRGLLSYDAPVAEYWPEFGANGKSEVTVSDVLRHRSGLAHLKGVDKDEVMDH
LLMEQKLAAAPLNRQHGKLAYHAVTYGWLLSGLARAVTGKGMRELFREELARPLNTDG
IHLGRPPADSPTKAAQTLLPQAKVPTPLLDFIAPKVAGLSFSGLLGAVYFPGILSLLQ
DDMPFLDGEVPAVNGVVTARALAKTYGALANDGVIDGTRLLSSQAVRGLTGKSELWPD
LNLGLPFTYHQGYQSSPVPGLLEGYGHIGLGGTIGWADPETGSAFGYVHNRLLTLLLF
DIGSFAGLAALLNSAVVAARRDDPLEVPHFGAPYSEPRHEQAASGA"

CDS complement(2172505..2172885)
/codon_start=1
/note="gene_1984"
/transl_table=11
/translation="MDPADVINPTSTRDAALARVLAYRQRVRARPLLIRATLAVVGGG
LFVVSLPMIVLLPELGIPALLVAFRLLAVEAQWAVRAYAWTDWRFTQLREWFHRQVLV
TRAAILVGLFLAAVALVWLLVYEF"

CDS 2173042..2174904
/codon_start=1
/note="gene_1985"
/transl_table=11
/translation="MNDGSRQELRVRSGLLQIEDCLDADGGIALPAGTTLISLIERNI
KYVGDLVAYRYLDHARSAAGCALEVTWTQFGMRLAAIGAHVQRFAGPGDRVAILAPQG
IDYVCGFYAAIKAGTVAVPLFAPELPGHAERLDTALRDSEPAVILTTAAAKNAVEGFL
NNVPRLRKPTVLVIDQIPDREGELFVPVELDIDAVSHLQYTSGSTRPPVGVEITHRAV
GTNLVQMILSIDLLNRNTHGVSWLPLYHDMGLSMIGFPAVYGGHSTLMSPTAFVRRPL
RWIQALSEGSRTGRVVTAAPNFAYEWAAQRGLPAQGDDVDLSNVVLIIGSEPVSIDAV
TTFNKAFAPYGLPRTAFKPSYGIAEATLLVATIDHAAEPTVVYLDPEQLGAGHATRVA
PDAPNAVVHVSCGHVARSLWAVIVDPDTGPEAGAELPDGEIGEVWLQGDNVARGYWGR
PEETRMTFGARLQSPLAEGSHADGSAIDDTWLRTGDLGVYLDGELYITGRIADLLTID
GRNHYPQDIEATAAEASPMVRRGYITAFTVPASDGDDRNQRLVIIAERAAGTSRSDPR
PALDAIRAAVCNRHGLSVADLSFLPAGAIPRTTSGKLARQACRAQYLSGRLGVH"

CDS complement(2174912..2175391)
/codon_start=1
/note="gene_1986"
/transl_table=11
/translation="MKLTTMIKTAVAVVAMAAIATFAAPVALAAYPITGKLGSELTMT
DTVGQVVLGWKVSDLKSSTAVIPGYPVAGQVWEATATVNAIRGSVTPAVSQFNARTAD
GINYRVLWQAAGPDTISGATIPQGEQSTGKIYFDVTGPSPTIVAMNNGMEDLLIWEP"

CDS 2175625..2176401
/codon_start=1
/note="gene_1987"
/transl_table=11
/translation="VVTAIPGPSGAEPGESRALAGYPVTPPALPRPVIFDQRWTDLTF
IHWPVLPESVAGSYPPGTRPDVFADGMTYVGLVPFRMSSTKLGTALPIPYVGTFPETN
VRLYSIDNAGRHGVLFRSLETARLTVVPLTRIGLGIPYAWSRMRMMRSGKHITYHSVR
RWPRRGLRSLLTITIGDLVEPTPLEVWLTARWGAHTRKAGRTWWVPNEHKPWPLRAAE
IAELNDELIDASGVQPTGDRLRALFSPGVHARFGRPCVVQ"

CDS complement(2176405..2177172)
/codon_start=1
/note="gene_1988"
/transl_table=11
/translation="MSVLDLFDLHGKRALITGASTGIGKRVALAYVEAGAQVAIAARH
LDALEKLADEIGTSGGKVVPVCCDVSQHQQVTSMLDQVTAELGGIDIAVCNAGIITVT

PMLDMPLEEFQRLQNTNVTGVFLTAQAAAKAMVKQGQGGVIINTASMSGHIINVPQQV
SHYCASKAAVIHLTKAMAVELAPHKIRVNSVSPGYILTELVEPYTEYQPLWEPKIPLG
RLGRPEELAGLYLYLASEASSYMTGSDIVIDGGYTCP"
CDS complement(2177217..2177828)
/codon_start=1
/note="gene_1989"
/transl_table=11
/translation="LELCDLLEELGPAVATLIEGWTAHDLAAHIVLRERDLVAGLCIV
LPGPFQRFAERRRARLAQSKDFTWLVARIRSGPPMGFFRIGWVRTLANLNEFFVHHED
VRRASGRGPRSLTPEMDAALWRNVRRGSHFLSRRLHGCGLEIEWVGTGKRVRVRSGEP
TARLTGPPGELLLYVFGRRAVARVEVSGPLEAIAAVHRTHFGM"
CDS complement(2177873..2178397)
/codon_start=1
/note="gene_1990"
/transl_table=11
/translation="MTQIAFVAYPGVTALDVVGPYEVLRNLPHAQVRFVWLRGRRATS
HWLTLPALKAFGAIPVADERIVHQDNIVTSAGVSAGLDLALWLAGQLGGEARAKAIQL
AIEYDPQPPFDSGHMSKASPTTKAAATALLSKDSAKPANLTAATLLAWERALAAVQSR
RRKRQPVGAQARRP"
CDS complement(2178415..2178645)
/codon_start=1
/note="gene_1991"
/transl_table=11
/translation="MSPRHFTRVFSDEVGEAPGRYVERIRTEAARRQLEETHDTVVAI
AARCGFGTAETMRRSFIRRVGISPDQYRKAFA"
CDS complement(2178642..2178920)
/codon_start=1
/note="gene_1992"
/transl_table=11
/translation="MRSSQKVWTAAGVTAGIDLALALVEDDHGTEIAQTVARWLVLYL
RRPGGQTQFAAPVWMPRAKRTSIRRVQEAIEAEPGARTASANWLNVRP"
CDS 2179327..2179824
/codon_start=1
/gene="tpx"
/note="gene_1993"
/transl_table=11
/translation="MAQITLRGNAINTVGELPAVGSPAPAFTLTGGDLGVISSDQFRG
KSVLLNIFPSVDTPVCATSVRTFDERAAASGATVLCVSKDLPFAQKRFCGAEGTENVM
PASAFRDSFGEDYGVTIADGPMAGLLARAIVVIGADGNVAYTELVPEIAQEPNYEAAL
AALGA"
CDS complement(2179821..2180912)
/codon_start=1
/note="gene_1994"
/transl_table=11
/translation="VDFRYSTEQDDFRASLRGFLGRGAPVREMAAADGSDRRLWQRLC
TELELPALHVPPEHGGLGATLVETAIAFAELGRALTPIPFFAATVFAIEAILRMGDDEQ
RKRLLAGLLTGARIGTIAVSGHDVASATTVRAVRRDGRPALTGECTPVLHGHVADLFV
VPAVADGSIVLHVVAADAPGVTVTPLPSFDITRPVATLRLAGSPAEPLTAGTPDDMER
VLDVARVLLAAEMLGGAEACLDLAVQYAGRRTQFDRPIGSFQAVKHACADMMIEIDAT
RATVMFAAMSAANGDELQTVAPLAKAQTAETFVLCAGSALQIHGAIAFTWEHDLHLYY
RRAKTTEALFGSSARNRALLAERAGLVKA"

```
CDS             complement(2180914..2182143)
                /codon_start=1
                /note="gene_1995"
                /transl_table=11
                /translation="VDVSYPPEAEAFRDRIREFVAEHLPPGWPGPGALPPHEREEFAR
                HWRRALAGAGLVAVSWPTEYGGGGLSPMEQVVLAEEFARAGAPERAENDLLGIDLLGN
                TLIALGSEAQKRHFLPRILSGEHRWCQGFSEPEAGSDLASVRTRGVLDGDEWVINGHK
                IWTSAGTTANWIFLLARTDPSAAKHRGLSFLLVPMDQPGVVVRPIVNAAGHSSFSEVF
                LTDARTSAGNVVGRVGDGWSTAMTLLGFERGSHIATAAIDFERDLQRLCELARDRGLH
                TDPRVRDGLAWCYARVQIMRYRGYRDLTLALTGRPPGAEAAITKVIWSEYFRRYTDLA
                VEILGLEALGPRGPGNGGARLVPEAGTPNSPACWMDELLYARAATIYAGSSQIQRNVI
                GERLLGLPKEPRPEVLC"
CDS             complement(2182158..2183114)
                /codon_start=1
                /note="gene_1996"
                /transl_table=11
                /translation="MFVGRVGPVDRRSDGERSRRPREFEYIRYETIDDGRIAAITLDR
                PKQRNAQTRGMLVELGAAFELAEADDTVRVVILRAAGPAFSAGHDLGSADDIRERSPG
                PDQHPSYRCNGATFGGVESRNRQEWHYYFENTKRWRNLRKITIAQVHGAVLSAGLMLA
                WCCDLIVASEDTVFADVVGTRLGMCGVEYFGHPWEFGPRKTKELLLTGDCIGADEAHA
                LGMVSKVFPADELATSTIEFARRIAKVPTMAALLIKESVNQTVDAMGFSAALDGCFKI
                HQLNHAHWGEVTGGKLSYGTVEYGLEDWRAAPQIRPAIKQRP"
CDS             2183339..2184448
                /codon_start=1
                /note="gene_1997"
                /transl_table=11
                /translation="MEIGIFLMPAHPPERTLYDATRWDLDVIELADQLGYVEAWVGEH
                FTVPWEPICAPDLLLAQALLRTQQIKLAPGAHLLPYHHPVELAHRVAYFDHLAQGRFM
                LGVGASGIPGDWALYDVDGKNGEHREMTREALEIMLRIWTEDEPWEHRGKYWNANGIA
                PMFEGLMRRHIKPYQKPHPPIGVTGFSAGSETLKLAGERGYIPMSLDLNTEYVATHWD
                AVEEGALRSGRTPDRRDWRLVREVLVAETDEQAFRYAVDGTMGRAMREYVLPTFRMFG
                MTKFYKHNPSVPDDEVTPEYLAENTFVVGSVQTVVDKLEATYDQVGGFGHLLILGFDY
                SDNPGPWKESLRLLAHEVMPRLNARLATKPATAVV"
CDS             2184451..2186970
                /codon_start=1
                /note="gene_1998"
                /transl_table=11
                /translation="MAVRQVTVGYSDGTHKTMPVRCDQTVLDAAEEHGVAIVNECQSG
                ICGTCVATCTAGRYQMGRTEGLSDVERAARKILTCQTFVTSDCRIELQYPVDDNAALL
                VTGDGVVTAVELVSPSTAILRVDTSGMAGALRYRAGQFAQLQVPGTNVWRNYSYAHPA
                DGRGECEFIIRLLPDGVMSNYLRDRAQPGDHIALRCSKGSFYLRPIVRPVILVAGGTG
                LSAILAMAQSLDADVAHPVYLLYGVERTEDLCKLDELTELRRRVGRLEVHVVVARPDP
                DWDGRTGLVTDLLDERMLASGDADVYLCGPVAMVDAARTWLDHNGFHRVGLYYEKFVA
                SGAARRRTPARLDYAGVDIAEVCRRGRGTAVVIGGSIAGIAAAKMLSETFDRVIVLEK
                DGPHRRREGRPGAAQGWHLHHLLTAGQIELERIFPGIVDDMVREGAFKVDMAAQYRIR
                LGGTWKKPGTSDIEIVCAGRPLLEWCVRRRLDDEPRIDFRYESEVADLAFDRANNAIV
                GVAVDNGDADGGDGLQVVPAEFVVDASGKNTRVPEFLERLGVGAPEAEQDIINCFYST
                MQHRVPPERRWQDKVMVICYAYRPFEDTYAAQYYTDSSRTILSTSLVAYNCYSPPRTA
                REFRAFADLMPSPVIGENIDGLEPASPIYNFRYPNMLRLRYEKKRNLPRALLAVGDAY
                TSADPVSGLGMSLALKEVREMQALLAKYGAGHRDLPRRYYRAIAKMADTAWFVIREQN
                LRFDWMKDVDKKRPFYFGVLTWYMDRVLELVHDDLDAYREFLAVVHLVKPPSALMRPR
```

IASRVLGKWARTRLSGQKTLIARNYENHPIPAEPADQLVNA"
CDS 2186982..2188052
/codon_start=1
/note="gene_1999"
/transl_table=11
/translation="VSQVHRILNCRGTRIHAVADSPPDQQGPLVVLLHGFPESWYSWR
HQIPALAGAGYRVVAIDQRGYGRSSKYRVQKAYRIKELVGDVVGVLDSYGAEQAFVVG
HDWGAPVAWTFAWLHPDRCAGVVGISVPFAGRGVIGLPGSPFGERRPSDYHLELAGPG
RVWYQDYFAVQDGIITEIEEDLRGWLLGLTYTVSGEGMMAATKAAVDAGVDLESMDPI
DVIRAGPLCMAEGARLKDAFVYPETMPAWFTEADLDFYTGEFERSGFGGPLSFYHNID
NDWHDLADQQGKPLTPPALFIGGQYDVGTIWGAQAIERAHEVMPNYRGTHMIADVGHW
IQQEAPEETNRLLLDFLGGLRP"
CDS 2188049..2188564
/codon_start=1
/note="gene_2000"
/transl_table=11
/translation="VSCTFDMVPETVDHLDEVGLRRVFGCFPCGVIAVCAMVDDQPVG
MAASSFTSVSVDPPLVSICVQNCSTTWPKLRDRPRLGVSVLAEGHDAACMSLSRKEGN
RFAGVFWSELSSGGVVIAGAGAWLDCRPYAEIPAGDHLIALLEICAVRADPETPPLVF
HGSRFRRLESR"
CDS 2188561..2189622
/codon_start=1
/note="gene_2001"
/transl_table=11
/translation="MKTTDVRVRRAITAMAGGHAVVLTGDPNGDGYLVFAAQAATPRL
VAFAVRHTSGYLRVALPGAECERLHLPPMCDRDTTHCVSVDVRGTGTGISASDRAWTI
AALASATSVAADFQRPGHVVPVQAQADGVLGRRGPAEAAVDLARLAERRPAAALCEIV
SPDNPVQMAHHAESVEFAVEHGLAMVSIGELVAYRRRIEPQVVRFTAATLPTWAGASR
VIGFRDVYDLGEHLAVIVGAVGAGVPVPLHVHIECLTGDVFGSTACRCGEELNGALAR
MSAQGSGVVLYLRPPGPAQACGLFARGDAATDVMPETVTWILRDLGVYAIRLSDDVPG
FGLVMFGAIREASTLAAAG"
CDS 2189619..2190389
/codon_start=1
/note="gene_2002"
/transl_table=11
/translation="LNHPDLAGKVAIVTGAGAGIGLAVARRLADEGCHVLCADIDGDA
ADAAATKIGCGAAACRVDVSDEQQIIAMVDACVAAFGGVDKLVANAGVVHLASLIDTT
VEDFDRVIAINLRGAWLCTKHAAPRMIERGGGAIVNLSSLAGQVAVGGTGAYGMSKAG
IIQLSRITAAELRSSGIRSNTLLPAFVDTPMQQTAMAMFDGALGAGGARSMIARLQGR
MAAPEEMAGIVVFLLSDDASMITGTTQIADGGTIAALW"
CDS complement(2190599..2190928)
/codon_start=1
/note="gene_2003"
/transl_table=11
/translation="VTALPARGEVWWCEMAEIGRRPVVVLSRDAAIPRLRRALVAPCT
TTIRGLASEVVLEPGSDPIPRRSAVNLDSVESVSVAVLVNRLGRLADIRMRAICTALE
VAVDCSR"
CDS complement(2190925..2191302)
/codon_start=1
/note="gene_2004"
/transl_table=11

/translation="VKTARLQVTLRCAVDLINSSSDQCFARIEHVASDQADPRPGVWH
SSGMNRIRLSTTVDAALLTSARDMRAGITDAALIDEALAALLARHRSAEVDASYAAYD
KHPVDEPDEWGDLASWRRAAGDS"
CDS complement(2191299..2191784)
/codon_start=1
/note="gene_2005"
/transl_table=11
/translation="MSDNGASRVPPVDETPAAESAEPITAVSLAWLPAGDYERALDLW
PDFAGSDLVTGPDGPVAHPLYCRRMQQKLVEFAEAGFPGLAVAAIRVAPFAAWCAEQG
QEPDSPEARAEYAAYLTAHGDHDVMAWPPGRNQQCWCGSGHKYKKCCAAASFIDTEPA
P"
CDS 2191944..2193308
/codon_start=1
/note="gene_2006"
/transl_table=11
/translation="MRSDTREEISAALDAYHASLSRVLDLKCDALTTPELLACLQRLE
VERRRQGAAEHALINQLAGQACEEELGGTLRTALANRLHITPGEASRRIAEAEDLGER
RALTGEPLPAQLTATAAAQREGKIGREHIKEIQAFFKELSAAVDLGIREAAEAQLAEL
ATSRRPDHLHGLATQLMDWLHPDGNFSDQERARKRGITMGKQEFDGMSRISGLLTPEL
RATIEAVLAKLAAPGACNPDDQTPLVDDTPDADAVRRDTRSQAQRNHDAFLAALRGLL
ASGELGQHKGLPVTIVVSTTLKELEAATGKGVTGGGSRVPMSDLIRMASNANHYLALF
DGAKPLALYHTKRLASPAQRIMLYAKDRGCSRPGCDAPAYHSEVHHVTPWTTTHRTDI
NDLTLACGPDNRLVEKGWKTRKNAHGDTEWLPPPHLDHGQPRINRYHHPEKILCEPDD
DEPH"
CDS 2193568..2193804
/codon_start=1
/gene="MORY_10546"
/note="gene_2007"
/transl_table=11
/translation="VQPSTRMWTWTILSQRCDRTVEFAIVADDDVTGQDGSGDGTDPR
TMLGRFANQHNEWVRLSVRHVLDAGEALDAGQID"
CDS 2193979..2194380
/codon_start=1
/note="gene_2008"
/transl_table=11
/translation="VDRYNDQASGRALIEIRLCNERATPMPIPIGLWMFQTKLHVNAG
GADVFLPVCDVLEQDLAERDEEVRQLNLQYRNRLEYAIGRTCSAAWSVNGSRRPSAVW
TTWLPVAETPHTRARSVENALLSMDSRGGVT"
CDS complement(2194669..2195019)
/codon_start=1
/note="gene_2009"
/transl_table=11
/translation="MTVFRIKPDNYFGDVVLAAADRDGLRIFQYAVRSAHESGQATFD
IDGVQQRIVRESGTADMELGSQTVVWRFDDTKLVEILDKLSPLIDGEGPGHQYIDDLN
SPAPTLMISVDEYA"
CDS complement(2195030..2195533)
/codon_start=1
/note="gene_2010"
/transl_table=11
/translation="MPGDPKDMTAVQARAAWDAVNADIARYNARCGRTFVLPNEQAAY
DACIADKGSLLERQAAIRARLGELGVPVEGEPPPAPDPAGPQPNEGLPPPGVSPPAES

NLTVGPPSRPIQQARGGESLWDENGGEWRYFPGDNYRYPHWDYNPHDSPTARWQNIPI
GDLPTHK"
CDS 2195636..2195962
/codon_start=1
/gene="MGAST_28345"
/note="gene_2011"
/transl_table=11
/translation="VEADGVKGCGSGGYFGGHFVFDSDELCRALADIPGQCLCLGLAS
CLFGEGAAGSGVGDREVLFDVEARVVGILNGIDDPALGCADGSGAFTGKPTRLAQMLG
YATDYL"
CDS complement(2195953..2196144)
/codon_start=1
/note="gene_2012"
/transl_table=11
/translation="LLPTLSHIHAWDTEHLIEAAYYWTKVADQWEDVFLEMRNRSHFI
AWEGAGGDGCDSEPALTYR"
CDS complement(2196145..2196441)
/codon_start=1
/note="gene_2013"
/transl_table=11
/translation="MKAGELRVNIQQVAATASQWSGRSTELSVLAPPPLGQPFQPTTA
AVGGAHAAVGLAVAAFTARTHATASAVEAAAAEYANNEAAAAAEMAAVPQTRLV"
CDS 2196669..2196896
/codon_start=1
/note="gene_2014"
/transl_table=11
/translation="MEQIVIRNLPEGTKAALRVRAARHHHSVEAEARAILTAGLLGEE
VPMPVLLAADSGHDIDFEPERLGLIARTPQL"
CDS 2196893..2197204
/codon_start=1
/note="gene_2015"
/transl_table=11
/translation="VTYVLDTNVVSALRVPGRHPAVAAWADSVQVAEQFVVAITLAEI
ERGVIAKERTDPTQSEHLRRWFDDKVLRIFVFARRGTNLIMQPLAGHIGYSLYSGISW
F"
CDS 2197358..2197534
/codon_start=1
/note="gene_2016"
/transl_table=11
/translation="MARKATSPGKPAPTSGQYRPVGGGNEVTVPKGHRLPPSPKPGQK
WVNVDPTKNKSGRG"
CDS 2197674..2198051
/codon_start=1
/note="gene_2017"
/transl_table=11
/translation="VPPPDPAAMGTWKFFRASVDGRPVFKKEFDKLPDQARAALIVLM
QRYLVGDLAAGSIKPIRGDILELRWHEANNHFRVLFFRWGQHPVALTAFYKNQQKTPK
TKIETALDRQKIWKRAFGDTPPI"
CDS 2198093..2198542
/codon_start=1
/note="gene_2018"

/transl_table=11
/translation="MSIDFPLGDDLAGYIAEAIAADPSFKGTLEDAEEARRLVDALIA
LRKHCQLSQVEVAKRMGVRQPTVSGFEKEPSDPKLSTLQRYARALDARLRLVLEVPTL
REVPTWHRLSSYRGSARDHQVRVGADKEILMQTNWARHISVRQVEVA"
CDS 2198539..2199084
/codon_start=1
/note="gene_2019"
/transl_table=11
/translation="MTDRTDADDLDLQRVGARLAARAQIRDIRLLRTQAAVHRAPKPA
QGLTYDLEFEPAVDADPATISAFVVRISCHLRIQNQAADDDVKEGDTKDETQDVATAD
FEFAALFDYHLQEGEDDPTEEELTAYAATTGRFALYPYIREYVVYDLTGRLALPPLTLE
ILSRPMPVSPGAQWPATRGTP"
CDS 2199141..2199293
/codon_start=1
/note="gene_2020"
/transl_table=11
/translation="MQRWTVSPAARVEILGRYWWRIRRRATEGAKAKSKGKARRGSQF
KVLEHG"
CDS complement(2199636..2199932)
/codon_start=1
/note="gene_2021"
/transl_table=11
/translation="VSSRYLLSPAAQAHLEEIWDCTYDRWGVDQAEQYLRELQHAIDR
AAANPRIGRACDEIRPGYRKLSAGSHTLFYRVTGEGTIDVVRVLHQRMDVDRNL"
CDS complement(2199929..2200228)
/codon_start=1
/note="gene_2022"
/transl_table=11
/translation="VSQGLTPTTNNWYCAFVGKNTSFVLDEHYSAFIDGEIAAGRYRS
ASEVIRSALRLLEDRETQLRALREALEAGERSGSSTPFDFDGFLGRKRADASRGR"
CDS 2200320..2200661
/codon_start=1
/note="gene_2023"
/transl_table=11
/translation="MIDTTEAAYRLTYQPDGTSITVRENLVDILARELLGPIRGPQEV
LPFSPRSQYLVGHLAPVKLTGAALIDDNAVQARANAEALAEGGGVPAYAADETTPTPT
TTPKTAHPSRA"
CDS complement(2200821..2201228)
/codon_start=1
/note="gene_2024"
/transl_table=11
/translation="VIYLETSALVKLIRIEVESDALADWLDDRTELRWITSALTEVEL
SRAIRAVSPEGLPAVPSVLARLDRFEIDAVIRSTAAAYPNPALRSLDAIHLATAQTAG
SVAPLTALVTYDNRLKEAAEALSLAVVAPGQAR"
CDS complement(2201232..2201504)
/codon_start=1
/note="gene_2025"
/transl_table=11
/translation="VNEVSIRTLNQETSKVLARVKRGEEINLTERGKVIARIIPASAG
PLDSLISTGSVQPARVHGPAPRPTIPMRGGLDSGTLLERMRAEERY"
CDS complement(2201537..2202700)

/codon_start=1
/note="gene_2026"
/transl_table=11
/translation="LDQAVGLFIERGFHSVKLEDIAEAAGVTARALYRHYDNKQALLA
EAIRTGQDQYQSARRLTEGETEPTPRPLNADLEDLIAAAVASRALTVLWQREARYLNE
DDRTAVRRRINAIVAGMRDSVLLEVPDLSPQHSELRAWAVSSTLTSLGRHSLSLPGEE
LKKLLYQACMAAARTPPVCELPPLPAGDAARDEADVLFSRYETLLAAGARLFRAQGYP
AVNTSEIGKGAGIAGPGLYRSFSSKQAILDALIRRLDEWRCLECIRALRANQQAAQRL
RGLVQGHVRISLDAPDLVAVSVTELSHASVEVRDGYLRNQGDREAVWIDLIGKLVPAT
SVAQGRLLVAAAISFIEDVARTWHLTRYAGVADEISGLALAILTSGAGNLLRA"
CDS 2202905..2203162
/codon_start=1
/note="gene_2027"
/transl_table=11
/translation="VLLPRPQEVSDERLASLRGIGHPYHPCSILNIGLTKYIIAPVYA
ITLQFILITLLRVHISNRHIALLAPQEAVDEFAVRNDMSRR"
CDS 2203656..2204060
/codon_start=1
/note="gene_2028"
/transl_table=11
/translation="MVIVADKAAGRVADPVLRPVGALGDFFAMTLDTSVCMFKPPFAW
REYLLQCWFVARVSTLPGVLMTIPWAVISGFLFNVLLTDIGAADFSGTGCAIFTVNQS
RGTGSLERGRFIGPQDHRVAAALEVTAPLLRS"
CDS 2204145..2204993
/codon_start=1
/note="gene_2029"
/transl_table=11
/translation="MGEANIREQAIATMPRGGPDASWLDRRFQTDALEYLDRDDVPDE
VKQKIIGVLDRVGTLTNLHEKYARIALKLVSDIPNPRILELGAGHGKLSAKILELHPT
ATVTISDLDPTSVANIAAGELGTHPRARTQVIDATAIDGHDHSYDLAVFALAFHHLPP
TVACKAIAEATRVGKRFLIIDLKRQKPLSFTLSSVLLLPLHLLLLPWSSMRSSMHDGF
ISALRAYSPSALQTLARAADPGMQVEILPAPTRLFPPSLAVVFSRSSSAPTESSECSA
DRQPGE"
CDS complement(2204956..2206401)
/codon_start=1
/note="gene_2030"
/transl_table=11
/translation="VVGPRTRGYAIHKLGFCSVVMLGINSIIGAGIFLTPGEVIGLAG
PFAPMAYVLAGIFAGVVAIVFATAARYVRTNGASYAYTTAAFGRRIGIYVGVTHAITA
SIAWGVLASFFVSTLLRVAFPDKAWADAEQLFSVKTLTFLGFIGVLLAINLFGNRAIK
WANGTSTVGKAFALSAFIVGGLWIITTQHVNNYATAWSAYSATPYSLLGVAEIGKGTF
SSMALATIVALYAFTGFESIANAAEEMDAPDRNLPRAIPIAIFSVGAIYLLTLTVAML
LGSNKIAASGDTVKLAAAIGNATFRTIIVVGALISMFGINVAASFGAPRLWTALADSG
VLPTRLSRKNQYDVPMVSFAITASLALAFPLALRFDNLHLTGLAVIARFVQFIIVPIA
LIALARSQAVEHAAVRRNAFTDKVLPLVAIVVSVGLAVSYDYRCIFLVRGGPNYFSIA
LIVITFIVVPAMAYLHYYRIIRRVGDRPSTR"
CDS complement(2206580..2207266)
/codon_start=1
/note="gene_2031"
/transl_table=11
/translation="VRIKIFMLVTAVVLLCCSGVATAAPKTYCEELKGTDTGQACQIQ

MSDPAYNINISLPSYYPDQKSLENYIAQTRDKFLSAATSSTPREAPYELNITSATYQS
AIPPRGTQAVVLKVYQNAGGTHPTTTYKAFDWDQAYRKPITYDTLWQADTDPLPVVFP
IVQGELSKQTGQQVSIAPNAGLDPVNYQNFAVTNDGVIFFFNPGELLPEAAGPTQVLV
PRSAIDSMLA"
CDS complement(2207457..2208425)
/codon_start=1
/gene="nrdF1"
/note="gene_2032"
/transl_table=11
/translation="MTGKRVERVHAINWNRLLDAKDLQVWERLTGNFWLPEKIPLSND
LASWQTLSSTEQQTTIRVFTGLTLLDTAQATVGAVAMIDDAVTPHEEAVLTNMAFMES
VHAKSYSSIFSTLCSTKQIDDAFDWSEQNPYLQRKAQIIVDYYRGDDALKRKASSVML
ESFLFYSGFYLPMYWSSRGKLTNTADLIRLIIRDEAVHGYYIGYKCQRGLADLTDAER
ADHREYTCELLHTLYANEIDYAHDLYDELGWTDDVLPYMRYNANKALANLGYQPAFDR
DTCQVNPAVRAALDPGAGENHDFFSGSGSSYVMGTHQPTTDTDWDF"
CDS complement(2208650..2209069)
/codon_start=1
/note="gene_2033"
/transl_table=11
/translation="MIVDTSAVVALVQGERPHATLVAAALAGAHSPVMSAPTVAECLI
VLTARHGPVARTIFERLRSEIGLSVSSFTAEHAAATQRAFLRYGKGRHRAALNFGDCM
TYATAQLGHQPLLAVGNDFPQTDLEFRGVVGYWPGVA"
CDS complement(2209078..2209365)
/codon_start=1
/note="gene_2034"
/transl_table=11
/translation="VAICWYHLPVALNIKDPEVDRLAAELADRLHTSKTAAIRHALSA
QLAFLESRAGDREAQLLDILRTEIWPLLADRSPITKLEREQILGYDPATGV"
CDS 2209481..2211157
/codon_start=1
/note="gene_2035"
/transl_table=11
/translation="VSFLVVVPEFLTSAAADVENIGSTLRAANAAAAASTTALAAAGA
DEVSAAVAALFARFGQEYQAVSAQASAFHQQFVQTLNSASGSYAAAEATIASQLQTAQ
HDLLGAVNAPTETLLGRPLIGDGAPGTATSPNGGAGGLLYGNGGNGYSATASGVGGGA
GGSAGLIGNGGAGGAGGPNAPGGAGGNGGWLLGNGGIGGPGGASSIPGMSGGAGGTGG
ATGLLGWGANGGAGGLGDGVGVDRGTGGAGGRGGLLYGGYGVSGPGGDGRTVPLEIIH
VTEPTVHANVNGGPTSTILVDTGSAGLVVSPEDVGGILGVLHMGLPTGLSISGYSGGL
YYIFATYTTTVDFGNGIVTAPTAVNVVLLSIPTSPFAISTYFSALLADPTTTPFEAYF
GAVGVDGVLGVGPNAVGPGPSIPTMALPGDLNQGVLIDAPAGELVFGPNPLPAPNVEV
VGSPITTLYVKIDGGTPIPVPSIIDSGGVTGTIPSYVIGSGTLPANTNIEVYTSPGGD
RLYAFNTNDYRPTVISSGLMNTGFLPFRFQPVYIDYSPSGIGTTVFDHPA"
CDS complement(2211145..2211804)
/codon_start=1
/note="gene_2036"
/transl_table=11
/translation="VQMTPRSLVRIVGVVVATTLALVSAPAGGRAAHADPCSDIAVVF
ARGTHQASGLGDVGEAFVDSLTSQVGGRSIGVYAVNYPASDDYRASASNGSDDASAHI
QRTVASCPNTRIVLGGYSQGATVIDLSTSAMPPAVADHVAAVALFGEPSSGFSSMLWG
GGSLPTIGPLYSSKTINLCAPDDPICTGGGNIMAHVSYVQSGMTSQAATFAANRLDHA
G"

```
CDS             2211913..2212143
                /codon_start=1
                /note="gene_2037"
                /transl_table=11
                /translation="MTDSVVVRVKPGSHKGPLVEVGPNGELIIYVREPAIDGKANDAV
                TRLLAAHLQLPKSRVKLVSGATSRFKRFRLSR"
CDS             complement(2212228..2213139)
                /codon_start=1
                /note="gene_2038"
                /transl_table=11
                /translation="MVDPQLDGPQLAALAAVVELGSFDAAAERLHVTPSAVSQRIKSL
                EQQVGQVLVVREKPCRATTAGIPLLRLAAQTALLESEALAEMGGNASLKRTRITIAVN
                ADSMATWFSAVFDGLGDVLLDVRIEDQDHSARLLREGVAMGAVTTERNPVPGCRVHPL
                GEMRYLPVASRPFVQRHLSDGFTAAAAAKAPSLAWNRDDGLQDMLVRKAFRRAITRPT
                HFVPTTEGFTAAARAGLGWGMFPEKLAASPLADGSFVRVCDIHLDVPLYWQCWKLDSP
                IIARITDTVRAAASGLYRGQQRRRRPG"
CDS             2213248..2213847
                /codon_start=1
                /note="gene_2039"
                /transl_table=11
                /translation="VNSPLVVGFLACFTLIAAIGAQNAFVLRQGIQREHVLPVVALCT
                VSDIVLIAAGIAGFGALIGAHPRALNVVKFGGAAFLIGYGLLAARRAWRPVALIPSGA
                TPVRLAEVLVTCAAFTFLNPHVYLDTVVLLGALANEHSDQRWLFGLGAVTASAVWFAT
                LGFGAGRLRGLFTNPGSWRILDGLIAVMMVALGISLTVT"
CDS             complement(2214307..2214456)
                /codon_start=1
                /gene="FJ05194_2996"
                /note="gene_2040"
                /transl_table=11
                /translation="VLEFVMVKRAMKPVCHVDVTDNVALAVAALATGVMASPRMATIN
                ADTVA"
CDS             2215004..2215456
                /codon_start=1
                /note="gene_2041"
                /transl_table=11
                /translation="VRPGELVFDIGAGEGALTAHLVRAGARVVAVELHPRRVGVLRER
                FPGITVVHADAASIRLPGRPFRVVANPPYGISSRLLRTLLAPNSGLVAADLVLQRALV
                CKFASRNARRFTLTVGLMLPRRAFLPPPHVDSAVLVVRRRKCGDWQGR"
CDS             complement(2215976..2216536)
                /codon_start=1
                /note="gene_2042"
                /transl_table=11
                /translation="VSDALDEGLVQRIDARGTIEWSETCYRYTGAHRDALSGEGARRF
                GGRWNPPLLFPAIYLADSAQACMVEVERAAQAASTTAEKMLEAAYRLHTIDVTDLAVL
                DLTTPQAREAVGLENDDIYGDDWSGCQAVGHAAWFLHMQGVLVPAAGGVGLVVTAYEQ
                RTRPGQLQLRQSVDLTPALYQELRAT"
CDS             complement(2216533..2216874)
                /codon_start=1
                /note="gene_2043"
                /transl_table=11
                /translation="MGVNVLASTVSGAIERLGLTYEEVGDIVDASPRSVARWTAGQVV
```

PQRLNKQRLIELAYVADALAEVLPRDQANVWMFSPNRLLEHRKPADLVRDGEYQRVLA
LIDAMAEGVFV"
CDS 2216960..2217217
/codon_start=1
/gene="TMJG_00242"
/note="gene_2044"
/transl_table=11
/translation="MGIQFRLGPGDHKPVEDFLSRDHAGTTAITLDTNATRHQHDAAA
AAVDAGLDVYWEPAAERLAAHPASGSTSSLCETGSPTTRMP"
CDS complement(2217118..2217453)
/codon_start=1
/note="gene_2045"
/transl_table=11
/translation="VGRLEGKVAFITGVARGQGRSHAVRLADGQARALGKVDVEACGA
LVGEVEVWGRDVRDDRRVFVESPADEFGACRRVARQGIRVVGLPVSQRELVEPEAGCA
ARRSAAGSQ"
CDS complement(2217542..2217886)
/codon_start=1
/note="gene_2046"
/transl_table=11
/translation="VVISRAEIYWADLGPPSGSQPAKRRPVLVIQSDPYNASRLATVI
AAVITSNTALAAMPGNVFLPATTTRLPRDSVVNVTAIVTLNKTDLTDRVGEVPASLMH
EVDRGLRRVLDL"
CDS complement(2217880..2218128)
/codon_start=1
/note="gene_2047"
/transl_table=11
/translation="MKTAISLPDETFDRVSRRASELGMSRSEFFTKAAQRYLHELDAQ
LLTGQIDRALESIHGTDEAEALAVANAYRVLETMDDEW"
CDS complement(2218228..2220543)
/codon_start=1
/note="gene_2048"
/transl_table=11
/translation="VTTVVDAEVQLTVVSDAAGRMRVQATGFQFDAGRAVAIEDTVGK
VAGVQAVHAYPRTASIVIWYSRAICDTAAILSAIIDAETVPAAAVPAYASRSASNRKA
GVVQKIIDWSTRTLSGVRRDVAAQPSGETSDACCDGEDNEDREPEQLWQVAKLRRAAF
SGVLLTASLVAAWAYPLWPVVLGLKALALAVGASTFVPSSLKRLAEGRVGVGTLMTIA
ALGAVALGELGEAATLAFLFSISEGLEEYATARTRRGLRALLSLVPDQATVLREGTET
IVASTELHVGDQMIVKPGERLATDGIIRAGRTALDVSAITGESVPVEVGPGDEVFAGS
INGLGVLQVGVTATAANNSLARIVHIVEAEQVRKGASQRLADCIARPLVPSIMIAAAL
IAGTGSVLGNPLVWIERALVVLVAAAPCALAIAVPVTVVASIGAASRLGVLIKGGAAL
ETLGTIRAVALDKTGTLTANRPVVIDVATTNGATREEVLAVAAALEARSEHPLAVAVL
AATQATTAASDVQAVPGAGLIGRLDGRVVRLGRPGWLDAAELADHVACMQQAGATAVL
VERDQQLLGAIAVRDELRPEAAEVVAGLRTGGYQVTMLTGDNHATAAALAAQAGIEQV
HAELRPEDKAHLVAQLRARQPTAMVGDGVNDAPALAAADLGIAMGAMGTDVAIETADV
ALMGQDLRHLPQALDHARRSRQIMVQNVGLSLSIITVLMPLALFGILGLAAVVLVHEF
TEVIVIANGVRAGRIKPLAGPPKTPDRTIPG"
CDS complement(2220540..2220812)
/codon_start=1
/note="gene_2049"
/transl_table=11

```
                     /translation="VVTHELLVKAAGAVLTGLVGVSAYETLRKALGTAPIRRASVTVM
                     EWGLRGTRRAEAAAESARLTVADVVAEARGRIGEEAPLPAGARVDE"
     CDS             complement(2220865..2221221)
                     /codon_start=1
                     /note="gene_2050"
                     /transl_table=11
                     /translation="MLTCEMRESALARLGRALADPTRCRILVALLDGVCYPGQLAAHL
                     GLTRSNVSNHLSCLRGCGLVVATYEGRQVRYALADSHLARALGELVQVVLAVDTDQPC
                     VAERAASGEAVEMTGS"
     CDS             2221378..2222145
                     /codon_start=1
                     /note="gene_2051"
                     /transl_table=11
                     /translation="VVASGAATKGVTVMKQTPPAAVGRRHLLEISASAAGVIALSACS
                     GSPPEPGKGRPDTTPEQEVPVTAPEDLMREHGVLKRILLIYREGIRRLQADDQSPAPA
                     LNESAQIIRRFIEDYHGQLEEQYVFPKLEQAGKLTDITSVLRTQHQRGRVLTDRVLAA
                     TTAAAAFDQPARDTLAQDMAAYIRMFEPHEAREDTVVFPALRDVMSAVEFRDMAETFE
                     DEEHRRFGEAGFQSVVDKVADIEKSLGIYDLSQFTPS"
     CDS             2222241..2223194
                     /codon_start=1
                     /note="gene_2052"
                     /transl_table=11
                     /translation="MSAQQTNLGIVVGVDGSPCSHTAVEWAARDAQMRNVALRVVQVV
                     PPVITAPEGWAFEYSRFQEAQKREIVEHSYLVAQAHQIVEQAHKVALEASSSGRAAQI
                     TGEVLHGQIVPTLANISRQVAMVVLGYRGQGAVAGALLGSVSSSLVRHAHGPVAVIPE
                     EPRPARPPHAPVVVGIDGSPTSGLAAEIAFDEASRRGVDLVALHAWSDMGPLDFPRLN
                     WAPIEWRNLEDEQEKMLARRLSGWQDRYPDVVVHKVVVCDRPAPRLLELAQTAQLVVV
                     GSHGRGGFPGMHLGSVSRAVVNSGQAPVIVARIPQDPAVPA"
     CDS             2223453..2226113
                     /codon_start=1
                     /note="gene_2053"
                     /transl_table=11
                     /translation="VLLLESDPYHGLSDGEAAQRLERFGPNTLAVVTRASLLARILRQ
                     FHHPLIYVLLVAGTITAGLKEFVDAAVIFGVVVINAIVGFIQESKAEAALQGLRSMVH
                     THAKVVREGHEHTMPSDELVPGDLVLLAAGDKVPADLRLVRQTGLSVNESALTGESTP
                     VHKDEVALPEGTPVADRRNIAYSGTLVTAGHGAGIVVATGAETELGEIHRLVGAAEVV
                     ATPLTAKLAWFSKFLTIAILGLAALTFGVGLLRRQDAVETFTAAIALAVGAIPEGLPT
                     AVTITLAIGMARMAKRRAVIRRLPAVETLGSTTVICADKTGTLTENQMTVQSIWTPHG
                     EIRATGTGYAPDVLLCDTDDAPVPVNANAALRWSLLAGACSNDAALVRDGTRWQIVGD
                     PTEGAMLVVAAKAGFNPERLATTLPQVAAIPFSSERQYMATLHRDGTDHVVLAKGAVE
                     RMLDLCGTEMGADGALRPLDRATVLRATEMLTSRGLRVLATGMGAGAGTPDDFDENVI
                     PGSLALTGLQAMSDPPRAAAASAVAACHSAGIAVKMITGDHAGTATAIATEVGLLDNT
                     EPAAGSVLTGAELAALSADQYPEAVDTASVFARVSPEQKLRLVQALQARGHVVAMTGD
                     GVNDAPALRQANIGVAMGRGGTEVAKDAADMVLTDDDFATIEAAVEEGRGVFDNLTKF
                     ITWTLPTNLGEGLVILAAIAVGVALPILPTQILWINMTTAIALGLMLAFEPKEAGIMT
                     RPPRDPDQPLLTGWLVRRTLLVSTLLVASAWWLFAWELDNGAGLHEARTAALNLFVVV
                     EAFYLFSCRSLTRSAWRLGMFANRWIILGVSAQAIAQFAITYLPAMNMVFDTAPIDIG
                     VWVRIFAVATAITIVVATDTLLPRIRAQPP"
     CDS             complement(2226182..2226919)
                     /codon_start=1
                     /note="gene_2054"
```

/transl_table=11
/translation="VLPNAWDVPSALAYLAEGFTAIGTTSFGVSSSGGHPDGHRATRG
ANIALAAALAPLQCYVSVDIEDGYSDEPDAIADYVAQLSTAGINIEDSSAEKLIDPAL
AAAKIVAIKQRNPEVFVNARVDTYWLRQHADTTSTIQRALRYVDAGADGVFVPLANDP
DELAELTRNIPCPVNTLPVPGLTIADLGELGVARVSTGSVPYSAGLYAAAHAARAVRD
GEQLPRSVPYAELQARLVDYENRTSTT"
CDS complement(2227053..2228375)
/codon_start=1
/note="gene_2055"
/transl_table=11
/translation="MRRPLDPRDIPDELRRRLGLLDAVVIGLGSMIGAGIFAALAPAA
YAAGSGLLLGLAVAAVVAYCNAISSARLAARYPASGGTYVYGRMRLGDFWGYLAGWGF
VVGKTASCAAMALTVGFYVWPAQAHAVAVAVVVALTAVNYAGIQKSAWLTRSIVAVVL
VVLTAVVVAAYGSGAADPARLDIGVDAHVWGMLQAAGLLFFAFAGYARIATLGEEVRD
PARTIPRAIPLALGITLAVYALVAVAVIAVLGPQRLARAAAPLSEAMRVAGVNWLIPV
VQIGAAVAALGSLLALILGVSRTTLAMARDRHLPRWLAAVHPRFKVPFRAELVVGAVV
AALAATADIRGAIGFSSFGVLVYYAIANASALTLGLDEGRPRRLIPLVGLIGCVVLAF
ALPLSSVAAGAAVLGVGVAAYGVRRIITRRARQTDSGDTQRSGHPSAT"
CDS 2228617..2230059
/codon_start=1
/note="gene_2056"
/transl_table=11
/translation="MNDPWPRPTQGPAKTIETDYLVIGAGAMGMAFTDTLITESGARV
VMIDRACQPGGHWTTAYPFVRLHQPSAYYGVNSRALGNNTIDLVGWNQGLNELAPVGE
ICAYFDAVLQQQLLPTGRVDYFPMSEYLGDGRFRTLAGTEYVVTVNRRIVDATYLRAV
VPSMRPAPYSVAPGVDCVAPNELPKLGTRDRYVVVGAGKTGMDVCLWLLRNDVCPDKL
TWIMPRDSWLIDRATLQPGPTFVRQFRESYGATLEAIGAATSTDDLFDRLETAGTLLR
IDPSVRPSMYRCATVSHLELEQLRRIRDIVRMGHVQRIEPTTIVLDGGSVPATPTALY
IDCTADGAPQRPAKPVFDADHLTLQAVRGCQQVFSAAFIAHVEFAYEDDAVKNELCTP
IPHPDCDLDWMRLMHSDLGNFQRWLNDPDLTDWLSSARLNLLADLLPPLSHKPRVRER
VVSMFQKRLGTAGDQLAKLLDAATATTEQR"
CDS 2230069..2230821
/codon_start=1
/note="gene_2057"
/transl_table=11
/translation="VHHNRDVDLALVERPSSGYVYTTGWRLATTDIDEHQQLRLDGVA
RYIQEVGAEHLADAQLAEVHPHWIVLRTVIDVINPIELPSDITFHRWCAALSTRWCSM
RVQLQGSAGGHIETEGFWICVNKDTLTPSRLTDDCIARFGSTTENHRLKWRPWLTGPN
IDGTETPFPLRRTDIDPFEHVNNTIYWHGVHEILCQIPTLTAPYRAVLEYRSPIKSGE
PLTIRYEQHDDVVRMHFVVGDDVRAAALLRRL"
CDS 2230897..2231679
/codon_start=1
/note="gene_2058"
/transl_table=11
/translation="MSGRLIGKVALVSGGARGMGASHVRAMVAEGAKVVFGDILDEEG
KAVAAELADAARYVHLDVTQPAQWTAAVDTAVTAFGGLHVLVNNAGILNIGTIEDYAL
TEWQRILDVNLTGVFLGIRAVVKPMKEAGRGSIINISSIEGLAGTVACHGYTATKFAV
RGLTKSTALELGPSGIRVNSIHPGLVKTPMTDWVPEDIFQTALGRAAEPVEVSNLVVY
LASDESSYSTGAEFVVDGGTVAGLAHNDFGAVEVSSQPEWVT"
CDS complement(2231800..2232489)
/codon_start=1

/note="gene_2059"
/transl_table=11
/translation="MPASSAAGRDAAAYDAWYDSPTGRPILATEVAALRPLIEVFAQP
RLEIGVGTGRFADLLGVRFGLDPSRDALMFARRRGVLVANAVGEAVPFVSRHFGAVLM
AFTLCFVTDPAAIFRETRRLLADGGGLVIGFLPRGTPWADLYALRAARGQPGYRDARF
YTAAELEQLLADSGFRVIARRCTLHQPPGLARYDIEAAHDGIQAGAGFVAISAVDQAH
EPKDDHPLESE"
CDS complement(2232497..2232694)
/codon_start=1
/note="gene_2060"
/transl_table=11
/translation="MPTITVSSTSSLCGQALSGNPTFAEHLVRMGITSVSVHSGAIAA
TPGSVAAAERRLLLESARGDA"
CDS complement(2232715..2234211)
/codon_start=1
/note="gene_2061"
/transl_table=11
/translation="MDSPTNDGTCDAHPVTDEPFIDVRETHTAVVVLAGDRAFKAKKP
VVTDFCDFRTAEQRERACIREFELNSRLAAQSYLGIAHLSDPSGGHAEPVVVMRRYRD
KQRLASMVTAGLPVEGALDAIAEVLARFHQRAQRNRCIDTQGEVGAVARRWHENLAEL
RHHADKVVSGDVIRRIEHMVDEFVSGREVLFAGRIKEGCIVDGHADLLADDIFLVDGE
PALLDCLEFEDELRYLDRIDDAAFLAMDLEFLGRKDLGDYFLAGYAVRSGDTAPASLR
DFYIAYRAVVRAKVECVRFSQGKPEAAADAVRHLIIATQHLQHATVRLALVGGNPGTG
KSTLARGVAELVGAQVISTDDVRRRLRDCGVITGEPGVLDSGLYSRANVVAVYQEALR
KARLLLGSGHSVILDGTWGDPQMRACARRLAADTHSAIVEFRCSATVDVMADRIVARA
GGNSDATAEIAAALAARQADWDTGHRIDTAGPRERSVGQAYHIWRSAI"
CDS complement(2234233..2235120)
/codon_start=1
/note="gene_2062"
/transl_table=11
/translation="MSKPRKQHGVVVGVDGSLESDAAACWGATDAAMRNIPLTVVHVV
NADVATWPPMPYPETWGVWQEDEGRQIVANAVKLAKEAVGADRKLSVKSELVFSTPVP
TMVEISNEAEMVVLGSSGRGALARGLLGSVSSSLVRRAGCPVAVIHSDDAVIPDPQHA
PVLVGIDGSPVSELATAVAFDEASRRGVELIAVHAWSDVEVVELPGLDFSAVQQEAEL
SLAERLAGWQERYPDVPVSRVVVCDRPARKLVQKSASAQLVVVGSHGRGGLTGMLLGS
VSNAVLHAARVPVIVARQS"
CDS 2235239..2239222
/codon_start=1
/note="gene_2063"
/transl_table=11
/translation="VRCGIVVNVTGPPPTIDRRYHDAVIVGLDNVVDKATRVHAAAWT
KFLDDYLTRRPQRTGEDHCPLTHDDYRRFLAGKPDSVADFLAARGIRLPPGSPTDLTD
DTVYGLQNLERQTFLQLLNTGVPEGKSIASFARRLQVAGVRVAAHTSHRNYGHTLDAT
GLAEVFAVFVDGAVTAELGLPAEPNPAGLIETAKRLGANPGRCVVIDSCQTGLRAGRN
GGFALVIAVDAHGDAENLLSSGADAVVADLAAVTVGSGDAAISTIPDALQVYSQLKRL
LTGRRPAVFLDFDGTLSDIVERPEAATLVDGAAEALRALAAQCPVAVISGRDLADVRN
RVKVDGLWLAGSHGFELVAPDGSHHQNAAATAAIDGLAEAAAQLADALREIAGAVVEH
KRFAVAVHYRNVADDSVDNLIAAVRRLGHAAGLRVTTGRKVVELRPDIAWDKGKALDW
IGERLGPAEVGPDLRLPIYIGDDLTDEDAFDAVRFTGVGIVVRHNEHGDRRSAATFRL
ECPYTVCQFLSQLACDLQEAVQHDDPWTLVFHGYDPGQERLREALCAVGNGYLGSRGC
APESAESEAHYPGTYVAGVYNQLTDHIEGCTVDNESLVNLPNWLSLTFRIDGGAWFNV

DTVELLSYRQTFDLRRATLTRSLRFRDAGGRVTTMTQERFASMNRPNLVALQTRIESE
NWSGTVDFRSLVDGGVHNTLVDRYRQLSSQHLTTAEIEVLADSVLLRTQTSQSGIAIA
VAARSTLWRDGQRVDAQYRVARDTNRGGHDIQVTLSAGQSVTLEKVATIFTSRDAATL
TAAISAQRCLGEAGRYAELCQQHVRAWARLWERCAIDLTGNTEELRLVRLHLLHLLQT
ISPHTAELDAGVPARGLNGEAYRGHVFWDALFVAPVLSLRMPKVARSLLDYRYRRLPA
ARRAAHRAGHLGAMYPWQSGSDGSEVSQQLHLNPRSGRWTPDPSDRAHHVGLAVAYNA
WHYYQVTGDRQYLVDCGAELLVEIARFWVGLAKLDDSRGRYLIRGVIGPDEFHSGYPG
NEYDGIDNNAYTNVMAVWVILRAMEALDLLPLTDRRHLIEKLGLTTQERDQWDDVSRR
MFVPFHDGVISQFEGYSELAELDWDHYRHRYGNIQRLDRILEAEGDSVNNYQASKQAD
ALMLLYLLSSDELIGLLARLGYRFAPTQIPGTVDYYLARTSDGSTLSAVVHAWVLARA
NRSNAMEYFRQVLRSDIADVQGGTTQEGIHLAAMAGSIDLLQRCYSGLELRDDRLVLS
PQWPEALGPLEFPFVYRRHQLSLRISGRSATLTAESGDAEPIEVECRGHVQRLRCGHT
IEVGCSR"
CDS complement(2239321..2239665)
/codon_start=1
/gene="fdxA3"
/note="gene_2064"
/transl_table=11
/translation="VTYVIGSECVDVMDKSCVQECPVDCIYEGARMLYINPDECVDCG
ACKPACRVEAIYWEGDLPDDQHQHLGDNAAFFHQVLPGRVAPLGSPGGAAAVGPIGVD
TPLVAAIPVECP"
CDS complement(2239854..2241179)
/codon_start=1
/note="gene_2065"
/transl_table=11
/translation="MDEIESLIGLRPTPLTWPVVIAGDFLGVWDPPPSLPGAANHEIS
APTARISCMLIERRDAAARLRRALHRAPVVLLTGPRQAGKTTLSRLVGKSAPECTFDA
ENPVDATRLADPMLALSGLSGLITIDEAQRIPDLFPVLRVLVDRPVMPARFLILGSAS
PDLVGLASESLAGRVELVELSGLTVRDVGSSAADRLWLRGGLPPSFTARSNEDSAAWR
DGYITTFLERDLAQLGVRIPAATMRRAWTMLAHYHGQLFSGAELARSLDVAQTTARRY
LDALTDALVVRQLTPWFANIGKRQRRSPKIYIRDTGLLHRLLGIDDRLALERNPKLGA
SWEGFVLEQLAALLAPNPLYYWRTQQDAELDLYVELSGRPYGFEIKRTSTPSISRSMR
SALVDLQLARLAIVYPGEHRFPLSDTVVAVPADQILTTGSVDELLALLK"
CDS 2241282..2241509
/codon_start=1
/note="gene_2066"
/transl_table=11
/translation="VSRTNIEIDDELVAAAQRMYRLDSKRSAVDLALRRLVGEPLGRD
EALALQGSGFDFSDDEIESFSDTDRKLADES"
CDS 2241510..2241908
/codon_start=1
/note="gene_2067"
/transl_table=11
/translation="MIVDTSVWIAYLSTSESLASRWLADRIAADSTVIVPEVVMMELL
IGKTDEDTAALRRRLLQRFAIEPLAPVRDAEDAAAIHRRCRRGGDTVRSLIDCQVAAM
ALRIGVAVAHRDRDYEAIRTHCGLRTEPLF"
CDS complement(2242091..2242522)
/codon_start=1
/note="gene_2068"
/transl_table=11
/translation="MSDEIARLVADVFELAGLLRRSGEVVAAREGHTQARWQLLSVVS

DRALTVPQAARRLGVTRQGVQRVANDLVVCGLAELRHNPDHRTSPLLVLTENGRRVLQ
AITERAIVVNNRLADAVDPAALQATRDSLRRMIVALKAERP"
CDS 2242623..2243057
/codon_start=1
/note="gene_2069"
/transl_table=11
/translation="MSAPITNLQAAQRDAIMNRPAVNGFPHLAETLRRAGVRTNTWWL
PAMQSLYETDYGPVLDQGVPLIDGVAEVPAFDRTALVTALRADQAGQTSFREFAAAAW
RAGVLRYVVDLENRTCTYFGLHDQTYMEHYAAVEPSGGAPTS"
CDS 2243761..2244378
/codon_start=1
/note="gene_2070"
/transl_table=11
/translation="VSIVDARGREVRRATIEHNAAGLRELLELLSRAGAREVAIERPD
GPVVDTLLEAGITVVVISPNQLKNLRGRYGSAGNKDDRFDAFVLADTLRTDRSRLRPL
LPDTPATATLRRTCRPRKDLVAHRVALANQLRAHLRVVFPGVGLFADLDSPISLAFLT
FLPRFDCQDRADWLSVKRLAGWLAAAGYCGRAPRPAHRCPARRHR"
CDS 2244332..2244922
/codon_start=1
/note="gene_2071"
/transl_table=11
/translation="VLHDRLTGAPRGATGDEGAANAHITRAMVAALTSVATQIKTLDA
QIAEQLSLHADAHIFTSLPRSGTVRAARLLAEIGDCRARFPTPESLACLAGVAPSTRQ
SGKVKHVGFRWAADKQLRDAVCDFAGDSRRANLWAADRYNRAIARGHDHPHAVRILAR
AWLYAIWHCWQDGAAYHPANHRALQALLNQDQDRAA"
CDS complement(2245050..2246306)
/codon_start=1
/note="gene_2072"
/transl_table=11
/translation="MSSTATSGAAVVSPAERVEVLFEELAELAGQRNAIDGRIVEIVA
ELDRDGLWGVTGARSVAGLVAWKMGCSSGNAHTIATVARRLPEFPRCARGMREGRLSL
DQVGVIAGRAGEGSDAHYAQLAGVATVNQLRTALKLEPRPEPEPDFRPEPRPSITRSA
DEQFSCWRIKLPHVEAAKFDAALQSHLDALIAEYKRDHDNSDGVSDQRPPLPGNVEAF
LRLVEAGWDAEVARRPHGQHTTVVMHLDVQERAAGLHLGPLLSESERRYLLCDATFEA
WFERDGQVIGCGRTTRQINRRLRRALEHRDRTCVVPGCGATRGLHAHHIRHWQDGGAT
ELANLVLVCPYHHRAHHRGLITITGPADNLTVADSAGRPLSAGSLARASTKPPPAVAP
WPGPTGERADWWWYEPFQPQPPPISN"
CDS 2246660..2247235
/codon_start=1
/note="gene_2073"
/transl_table=11
/translation="MTELGDKFLAALVGTIRDTRFDIADMRNWRPGWFPTMHSRCLSN
LIHDRIWAHLVTLIASNPGTSIKDKGATREIVVGAHLRLRIKRHHAGDEISTYPTRTA
IEFWQQGSQPAFPGLEEVRIAVGYRWDPDTREIGAPLLSLRDGKDHVIWVVELDEPAA
GVKITWTPIEPTLPSIDFGDLGEDSGASGER"
CDS 2247232..2248272
/codon_start=1
/note="gene_2074"
/transl_table=11
/translation="MNGLGDVLAVARKARGLTQIELAELVGLTQPAINRYESGDRDPD
QHIVAKLAEILGVTDDLLIHGNRFRGALAVDAHMRRHKTTKASAWRQLEARLNLLRVH

ASFLFEEVAINSEQHVPAFDPEFTAAEDAARLVRAQWRMPMGPVVNLTRWMEAAGCLV
FEEDFATQRIDGLSQWVDDYPVMLINANAAPDRKRLTLAHELGHLVLHSTNPTENMET
EATAFAAEFLMPESEIRPELRRLDLGKLLELKREWGVSMQALLERAYRMGLVSAEART
KLYKAMNARGWKTKEPGIESIVREKPSLPAHIGMTLRSRGFTDQQAAAIAGYANPADN
PFRPEGGRLHAI"
CDS 2248514..2249233
/codon_start=1
/note="gene_2075"
/transl_table=11
/translation="MAGDQELELRFDVPLYTLAEASRYLVVPRATLATWADGYERRPA
NAPAVQGQPIITALPHPTGSHARLPFVGIAEAYVLNAFRRAGVPMQRIRPSLDWLIKN
VGPHALASQDLCTGGAEVLWRFAERSGEGSPDDLVVRGLIVPRSGQYVFKEIVEHYLQ
QISFADDNLASMIRLPQYGDANVVLDPRRGYGQPVFDGSGVRVADVLGPLRAGATFQA
VADDYGVTPDQLRDALDAIAA"
CDS 2249337..2249639
/codon_start=1
/note="gene_2076"
/transl_table=11
/translation="MREHYGETQAQSVSDHKWIAMTAECGWIGFHKDANIRRNAVERR
TVLDTGARLFCVPRADILAEQVAARYIASLAAIARAARFPGPFIYTVHPSKIVRVL"
CDS complement(2249655..2249942)
/codon_start=1
/note="gene_2077"
/transl_table=11
/translation="MKWAAKTDHLAIVLLPRHHRRHSRRGRALPARSRSALGWIIERY
RVTTDKASGIVNDPNDWCDEHDDPTYIVDLIKKVTTVSVETMKIVDGLAGG"
CDS complement(2250039..2250344)
/codon_start=1
/note="gene_2078"
/transl_table=11
/translation="MAMTLRDMDAVRPVNREAVDRHKARMRDEVRAFRLRELRAAQSL
TQVQVAALAHIRQSRVSSIENGDIGSAQVNTLRKYVSALGGELDITVRLGDETFTLA"
CDS complement(2250353..2250958)
/codon_start=1
/note="gene_2079"
/transl_table=11
/translation="VNVPWENAHGGALYCLIRGDEFSAWHRLLFQRPGCAESVLACRH
FLDGSPVARCSYPEEYHPCVISRIALLCDSVGWTADVERISAWLNGLDRETYELVFAA
IEVLEEEGPALGCPLVDTVRGSRHKNMKELRPGSQGRSEVRILFAFDPARQAIMLAAG
NKAGRWTQWYDEKIKAADEMFAEHLAQFEDTKPKRRKRKKG"
CDS complement(2250983..2251291)
/codon_start=1
/note="gene_2080"
/transl_table=11
/translation="MLGSGAVRYEVGANIDATGFGGIAAVHRLVTRLGLVTRLGLVER
VDAHSRFSSSNLPKSSRRISGRVSLSGMSNSAAKVVASTSSSPWGQPLSVGLRRRWRS
"
CDS complement(2251502..2252134)
/codon_start=1
/gene="K60_020990"
/note="gene_2081"

```
                     /transl_table=11
                     /translation="MTSVAQLEHYLEEHLTKELAWLLRAATEWHAQHCMNLGIDGYSM
                     QVYALDSTVLHARTLFEFFTQNTSVGQNANYYNCTVYKVPLIGSILYQFHWRRPIHSH
                     MMHAQDRRPVTQLPTYDDHAQTKPLNEMPVDFAKEIVRLWRVFVKDLNNHTNLQFRPI
                     GATAQTALASEINAAKRVRTNDVTQRQIAVGKETSRLEPNFSIPQIEWPA"
     CDS             complement(2252131..2252367)
                     /codon_start=1
                     /gene="TMCG_00113"
                     /note="gene_2082"
                     /transl_table=11
                     /translation="MRNVVPEVGNRDSAVPVVLAGPVSSVIDEIVREGAQQMLAGAQR
                     RCAASKLVRQRHETAILLGWFNAILLGWFNERQR"
     CDS             complement(2252574..2253470)
                     /codon_start=1
                     /note="gene_2083"
                     /transl_table=11
                     /translation="MTLIQTVTTDDLVIQVADRRLSRPDGSVFDDDYTKLVCWNTSFT
                     VGFTGLARIDPAQKKSTSEWLAETLCDYASFEDGVDALRYWASGQIGQLPTGKGWEDK
                     RLGIIIAGFDRRRIPLVAEISNFDPEAPIPANQNEFECYRIRRAPGHSASFRITGAAL
                     TEKMYANILLRRVPRMLKQQDGITRAARLMVALQRRISEDNPGVGRHAMAVAIPRERT
                     MPAVLSNLDAPSLNTMNSNFCYFDDAGFNYKQLGPHMAGGGWAWADFVAEADPSNPDM
                     QKVGGRVLKCPQPPPQAESTGC"
     CDS             complement(2253654..2258474)
                     /codon_start=1
                     /note="gene_2084"
                     /transl_table=11
                     /translation="MGSVHDVIEAFRKAPSNAERGTKFEQLMVRYFELDPTMAQQYDA
                     VWRWIDWPERRGRTDTGIDLVARERDTGNYTAIQCKFYEPTHTLAKGDIDSFFTASGK
                     TGFTNRVIISTTDRWGRNAEDALADQLVPVQRIGMAEIAESPIDWDIAWPAGDLQVNL
                     TPAKRHELRPHQQQAIDAVFRGFAVGNDRGKLIMACGTGKTFTALKIAERIAADNGGS
                     ARILLLVPSISLLSQTLREWTAQSELDVRAFAVCSDTKVSRSAEDYHVHDVPIPVTTD
                     ARVLLHEMAHRRCAQGLTVVFCTYQSLPTVAKAQRLGVDEFDLVMCDEAHRTTGVTLA
                     GDDESNFVRVHDGQYLKAARRLYMTATPRIFTESIKDRADQHSAELVSMDDELTFGPE
                     FHRLSFGEAVERGLLTDYKVMVLTVDQGVIAPRLQQELSGVSGELMLDDASKIVGCWN
                     GLAKRSGTGIVAGEPPMRRAVAFAKDIKTSKQVAELFPKVVEAYRELVDDGPGLACSV
                     RHVDGTFNALVRNEQLAWLKGVVAEDECRILSNARCLSEGVDVPALDAVLFLNPRNSI
                     VDVVQSVGRVMRKSPGKDYGYVILPVAVPEGVEPSAALADNKRFKVVWQVLNALRSHD
                     ERFDAMVNSIALNVKPTKTGEGSDKLLGGHIGPTSDEAGPAVAEQLAMFSLSQWQEAI
                     YARIVDKVGTRTYWEQWAADVADIAATLTTRIHALLGGADATAAAAFEQFLAGLRDNL
                     NDSITPDDAISMLSQHLITKPVFDALFAGHDFASHNPVSRAMQKMVDTVGGAGLEAET
                     ARLEGFYESVRRRAGEVTSAEGKQQVIAELYEKFFRIGFKKQAEALGIVYTPVEVVDF
                     IVRAADFVSRKHFGRGLTDEGVHILDGFAGTGTFITRLLQSDLITAADLTRKYSQELH
                     ANEIMLLAYYIAAVNIESTYHALAGKTADADAYEPFPGMALADTFQISEAGDSMDAIM
                     FPYNNARILRQLATPISVIIGNPPYSVGQSSANDLNANVKYPTLDGRIEQTYAKRSTA
                     QLKNSLYDSYIRAFRWATDRIGDNGVVGFVSNGGYIDGNTADGMRLSLADDYAAVYVY
                     NLRGNQRTAGELSRQEGGKVFGGGSRNTVAIFLGIKDPKHSGPCDVLYRDIGDYLSRE
                     EKLRIVGDGYLDTVEWQTVTPNLHGDWVNQRDDAFSAWPVIGDKKAALDVTRVFANYS
                     AGLKTSRDAWCYNFSRGALEANIGRTIDFYNSEVDRINEIRGRDAKTPPVDALITVDS
                     AKFSWDRINKRQVAQGIRIEFAPAGMRLGTYRPFTKEHAYLDPNQQLNNCTYQLPSMF
                     PTPEHGNVGYYVVGMGSDKPFSCLMLNAIPDLAFWGSSNGQFFPRWTYEKTEPRDGEL
                     DFESTTNAEVDDHGYRRVDNITGVILKLYRDTIGDQVTKDDIFYYVYGLLHDPAYRTK
```

YAADLKKMLPHIPTPETRERFDQLASAGRKLADLHVGYESVKPYPLDVQLKPGADPED
RETWRVEKMKWKSKQDHSTIIYNSRVTIAGIPDEAERYLLGSRSALGWIIDRYRVTTD
KASGIVNDPNDWCDEHANPTYIVDLIKKVTTVSVETMKIVDSIVALASAGSDST"
CDS complement(2258984..2259943)
/codon_start=1
/note="gene_2085"
/transl_table=11
/translation="MIKEIFAPHSHDAADSVDDTLESTAAGIRTVKISLLVLGLTALI
QIVIVVMSGSVALAADTIHNFADALTAVPLWIAFALGAKPATRRYTYGFGRVEDLAGS
FVVAMITMSAIIAGYEAIARLIHPQQIEHVGWVALAGLVGFIGNEWVALYRIRVGHRI
GSAALIADGLHARTDGFTSLAVLCSAGGVALGFPLADPIVGLLITAAILAVLRTAARD
VFRRLLDGVDPAMVDAAEQALAARPGVQAVRSVRMRWIGHRLHADAELDVDPALDLAQ
AHRIAHDAEHELTHTVPKLTTALIHAYPAEHGSSIPDRGRTVE"
CDS complement(2260097..2260981)
/codon_start=1
/note="gene_2086"
/transl_table=11
/translation="MSAATAKYGILVGVDGSAQSNAAVAWAAREAVMRQLPITLLHIV
APVVVGWPVGQLYANMTEWQKDNAQQVIEQAREALTNSLGESKPPQVHTELVFSNVVP
TLIDASQQAWLMVVGSQGMGALGRLLLGSISTALLHHARCPVAIIHSGNGATPDSDAP
VLVGIDGSPASEAATALAFDEASRRRVDLVALHAWTDLGMFPVLGMDWREREKREAEV
LAERLAGWQEQYPDVRVHRSLVCDKPARWLLEHSEQAQLVVVGSHGRGGFSGMLLGSV
SSAVAHSVRIPVIVVRPS"
CDS complement(2261021..2262697)
/codon_start=1
/note="gene_2087"
/transl_table=11
/translation="VRETLSQLRLRELLLEVQDRIEQIVEGRDRLDGLIDAILAITSG
LKLDATLRAIVHTAAELVDARYGALGVRGYDHRLVEFVYEGIDEETRHLIGSLPEGRG
VLGALIEEPKPIRLDDISRHPASVGFPLHHPPMRTFLGVPVRIRDEVFGNLYLTEKAD
GQPFSDDDEVLVQALAAAAGIAVDNARLFEESRTREAWIEATRDIGTQMLAGADPAMV
FRLIAEEALTLMAGAATLVAVPLDDEAPACEVDDLVIVEVAGEISPAVKQMTVAVSGT
SIGGVFHDRTPRRFDRLDLAVDGPVEPGPALVLPLRAADTVAGVLVALRSADEQPFSD
KQLDMMAAFADQAALAWRLATAQRQMREVEILTDRDRIARDLHDHVIQRLFAVGLTLQ
GAAPRARVPAVRESIYSSIDDLQEIIQEIRSAIFDLHAGPSRATGLRHRLDKVIDQLA
IPALHTTVQYTGPLSVVDTVLANHAEAVLREAVSNAVRHANATSLAINVSVEDDVRVE
VVDDGVGISGDITESGLRNLRQRADDAGGEFTVENMPTGGTLLRWSAPLR"
CDS complement(2262803..2263642)
/codon_start=1
/note="gene_2088"
/transl_table=11
/translation="MNQSHKPPSIVVGIDGSKPAVQAALWAVDEAASRDIPLRLLYAI
EPDDPGYAAHGAAARKLAAAENAVRYAFTAVEAADRPVKVEVEITQERPVTSLIRASA
AAALVCVGAIGVHHFRPERVGSTAAALALSAQCPVAIVRPHRVPIGRDAAWIVVEADG
SSDIGVLLGAVMAEARLRDSPVRVVTCRQSGVGDTGDDVRASLDRWLARWQPRYPDVR
VQSAAVHGELLDYLAGLGRSVHMVVLSASDQEHVEQLVGAPGNAVLQEAGCTLLVVGQ
QYL"
CDS complement(2263639..2264658)
/codon_start=1
/note="gene_2089"
/transl_table=11

```
                     /translation="MTEPAAWDEGKPRIITLTMNPALDITTSVDVVRPTEKMRCGAPR
                     YDPGGGGINVARIVHVLGGCSTALFPAGGSTGSLLMALLGDAGVPFRVIPIAASTRES
                     FTVNESRTAKQYRFVLPGPSLTVAEQEQCLDELRGAAASAAFVVASGSLPPGVAADYY
                     QRVADICRRSSTPLILDTSGGGLQHISSGVFLLKASVRELRECVGSELLTEPEQLAAA
                     HELIDRGRAEVVVVSLGSQGALLATRHASHRFSSIPMTAVSGVGAGDAMVAAITVGLS
                     RGWSLIKSVRLGNAAGAAMLLTPGTAACNRDDVERFFELAAEPTEVGQDQYVWHPIVN
                     PEASP"
     CDS             complement(2264675..2266720)
                     /codon_start=1
                     /note="gene_2090"
                     /transl_table=11
                     /translation="VLMTAAADVTRRSPRRVFRDRREAGRVLAELLAAYRDQPDVIVL
                     GLARGGLPVAWEVAAALHAPLDAFVVRKLGAPGHDEFAVGALASGGRVVVNDDVVRGL
                     RITPQQLRDIAEREGRELLRRESAYRGERPPTDITGKTVIVVDDGLATGASMFAAVQA
                     LRDAQPAQIVIAVPAAPESTCREFAGLVDDVVCATMPTPFLAVGESFWDFRQVTDEEV
                     RRLLATPTAGPSLRRPAASTAADVLRRVAIDAPGGVPTHEVLAELVGDARIVLIGESS
                     HGTHEFYQARAAMTQWLIEEKGFGAVAAEADWPDAYRVNRYVRGLGEDTNADEALSGF
                     ERFPAWMWRNTVVRDFVEWLRTRNQRYESGALRQAGFYGLDLYSLHRSIQEVISYLDK
                     VDPRAAARARARYACFDHACADDGQAYGFAAAFGAGPSCEREAVEQLVDVQRNALAYA
                     RQDGLLAEDELFYAQQNAQTVRDAEVYYRAMFSGRVTSWNLRDQHMAQTLGSLLTHLD
                     RHLDAPPARIVVWAHNSHVGDARATEVWADGQLTLGQIVRERYGDESRSIGFSTYTGT
                     VTAASEWGGIAQRKAVRPALHGSVEELFHQTADSFLVSARLSRDAEAPLDVVRLGRAI
                     GVVYLPATERQSHYLHVRPADQFDAMIHIDQTRALEPLEVTSRWIAGENPETYPTGL"
     CDS             complement(2266732..2267166)
                     /codon_start=1
                     /note="gene_2091"
                     /transl_table=11
                     /translation="MATTLPVQRHPRSLFPEFSELFAAFPSFAGLRPTFDTRLMRLED
                     EMKEGRYEVRAELPGVDPDKDVDIMVRDGQLTIKAERTEQKDFDGRSEFAYGSFVRTV
                     SLPVGADEDDIKATYDKGILTVSVAVSEGKPTEKHIQIRSTN"
     CDS             2267363..2268358
                     /codon_start=1
                     /gene="acg"
                     /note="gene_2092"
                     /transl_table=11
                     /translation="MPDTMVTTDVIKSAVQLACRAPSLHNSQPWRWIAEDHTVALFLD
                     KDRVLYATDHSGREALLGCGAVLDHFRVAMAAAGTTANVERFPNPNDPLHLASIDFSP
                     ADFVTEGHRLRADAILLRRTDRLPFAEPPDWDLVESQLRTTVTADTVRIDVIADDMRP
                     ELAAASKLTESLRLYDSSYHAELFWWTGAFETSEGIPHSSLVSAAESDRVTFGRDFPV
                     VANTDRRPEFGHDRSKVLVLSTYDNERASLLRCGEMLSAVLLDATMAGLATCTLTHIT
                     ELHASRDLVAALIGQPATPQALVRVGLAPEMEEPPPATPRRLIDEVFHVRAKDHR"
     CDS             complement(2268474..2269316)
                     /codon_start=1
                     /note="gene_2093"
                     /transl_table=11
                     /translation="VLDRYGTDVLAAGGRRRPRSVEHPVELGMVVEDAETGYVGAVVR
                     VEYGRIDLEDRYGKTRGFPLGPGYLLDGLPVILTAPRCAAAAGPRRTASGSVAVPGAR
                     ARVARASRIYVEGRHDAELIAAVWGADLRIEGVVVEHLGGVDDLVEIVAKFRPGPRRR
                     LGVLVDHLVAGSKEARIAEVVRRGPGGSDTLVVGHPYVDIWQAVKPQRVGLAAWPRVP
                     RHIEWKHGVCDALGWPHADQADIAAAWRRIRSQVRDWTDLEPALIGRVEELIDFVTQP
                     AGDE"
```

CDS 2269528..2269851
/codon_start=1
/note="gene_2094"
/transl_table=11
/translation="VSTYRSPDRAWQALADGTRRAIVERLAHGPLAVGELARDLPVSR
PAVSQHLKVLKTARLVCDRPAGTRRVYQLDPTGLAALRTDLDRFWTRALTGYAQLIDS
EGDDT"
CDS 2269848..2270336
/codon_start=1
/note="gene_2095"
/transl_table=11
/translation="MTRPRTDAIHHHVVVNAPIERAFAVFTTRFGDFKPREHNLLAIP
ITETVFECHAGGHIYDRGVDGSVCKWARVLVYEPPSRVLFTWDIGPTWRPETDLAKTS
EVEVRFTAQSAETTRVDLEHRHLDRHGPGWESVADGVDSEAGWPLYLRRYTDLLCIQV
QP"
CDS 2270333..2270974
/codon_start=1
/note="gene_2096"
/transl_table=11
/translation="MIAADDDTEKSMMDMARAERAELAAFLTTLTLQQWETPSLCAGW
SVKEVVAHMISYEDLGVFGLLKRFAKGRIVRANEVGVDEFAGLSPQELVDYVGRHLQP
RGLTAGFGGMIALVDGMIHHQDIRRPLGQPRTIPAQRLDRVLRLMPKNPRLRARPRIK
GLRLRATDLDWTIGTGPEVTGPGEALLMAMAGRPAAVSDLSGPGKPTLAGRLG"
CDS complement(2270981..2271955)
/codon_start=1
/note="gene_2097"
/transl_table=11
/translation="VALVSTARVDLVCEGGGVRGIGLVGAVDALADAGYRFPRVAGSS
AGAIVASLVAALQTAGEPVTRLAEMMRSIDYPKFLDRNLIGHVPLIGGGLSLLLSDGV
YRGAYLEQLLGGLLADLGVHTFGDLRTGEAPEQFAWSLVVTASDLSRRRLVRIPWDLD
SYGIHPDDFSVARAVHASSAIPFVFEPVRVRGATWVDGGLLSNFPVALFDRTDAEPRW
PTFGIRLSARPGIPPTRPVQGPVSLGIAAIETLVSNQDNAYIDDPCTVRRTIFVPAHD
VSPIDFDITAEQREALYQRGFQAGQKFLANWNYADYLADCGGPFTPSL"
CDS complement(2271957..2273030)
/codon_start=1
/note="gene_2098"
/transl_table=11
/translation="MASVSFEQATRRYPGTDRPALDRLDLIVGDGEFVVLVGPSGCGK
TTSLRMVAGLETLDCGRIRIGERDVTEVDPKDRDVAMVFQNYALYPHMTVAQNMGFAL
KVAKIGKAEIRERVLAAAKLLDLQSYLDRKPKDLSGGQRQRVAMGRAIVRRPQVFLMD
EPLSNLDAKLRGQTRNQIAALQRQLGTTTVYVTHDQVEAMTMGDRVAVLSDGVLQQCA
SPRELYRNPGNVFVAGFIGSPAMNLFRLSIADSTVSLGDWQILLPRAVVGTAAEVIIG
VRPEHLELGGAGIEMDVDMVEELGADAYLYGRIVSGGCEMDQSIVARVDGRGPPERGS
RVRLCPTPGHLHFFAVDGRRIPG"
CDS complement(2273033..2273875)
/codon_start=1
/note="gene_2099"
/transl_table=11
/translation="VGWADRIVHRHFIRGLALYAGLIGIAWCALFPIIWALSGSLKAD
GEVTEPTLFPSHPQWSNYREVFALMPFWRMFFNTVLYAGCVTAGQVFFCSLAGYAFAR
LQFRGRDTLFVLYLSTLMVPLTVTVIPQFILMRIVGWVDTPWAMIVPGLFGSAFGTYL

MRQFFRTLPTDLEEAAILDGCSPWQIYWRILLPHSRPAVLVLGVLTWVNVWNDFLWPL
LMIQRNSLATLTLGLVRLRGEYVARWPVLMAASMLMLVPLVILYAVAQRSFVRGIAVT
GLGG"
CDS complement(2273862..2274725)
/codon_start=1
/note="gene_2100"
/transl_table=11
/translation="MFVAPNLAAVVVFMLFPLGFSLYMSFQKWDLFTHATFVRLDNFR
NLFTSDPLFLIAVVNTAVYTVGTVVPTVIVSLVVAAFLNRKIKGISLFRTVVFLPLAI
SSVVMAVVWQFVFNTDNGLLNIMLGWLGIGPIPWLIEPRWAMVSLCLVSVWRSVPFAT
VVLLAAMQGVPETVYEAARIDGAGEIRQFVSITVPLIRGALSFVVVISIIHAFQAFDL
VYVLTGANGGPETATYVLGIMLFQHAFSFLEFGYASALAWVMFAILLVLTVLQLRITH
RRSWEASRGLG"
CDS complement(2274761..2276080)
/codon_start=1
/note="gene_2101"
/transl_table=11
/translation="MVNKPFERRSLLRGAGALTAASLAPWAAGCAADDDDALTFFFAA
NPDELRPRMRVVNEFQRRYPDIKVRALLSGPGVMQQLATFCAGGKCPDVLMAWELTYA
ELADRGVLLDLNTLLARDQAFAAELKSDSIGALYETFTFNGGQYAFPEQWSGNFLFYN
KQLFDDAGVPPPPGSWERPWSFAEFLDAAQALTKQGRSGRDRQWGFVNAWVSFYAAGL
FAMNNGVPWSVPRMNPTHLNFDHDGFLEAVQFYADLTNKHKVAPSAAEQQSMSTADLF
SVGKAGIALAGHWRYQTFDRADGLDFDVAPLPIGPRGRAACSDIGVTGLAIAATSRRK
DQAWEFVKFATGPVGQALIGESRLFVPVLRSAINSHGFANAHRRVGNLAVLSEGPAYS
EGLPVTPAWEKIAALMDRYFGPVLRGSRPATSLTGLSQAVDEVLRNP"
CDS complement(2276118..2276915)
/codon_start=1
/note="gene_2102"
/transl_table=11
/translation="MAPPNRDELLAAVERSPQAAAAHDRAGWVGLFTGDARVEDPVGS
QPQVGHEAIGRFYDTFIGPRDITFHRDLDIVSGTVVLRDLELEVAMDSAVTVFIPAFL
RYDLRPVTGEWQIAALRAYWELPAMMLQFLRTGSGATRPALQLSRALLGNQGLGGTAG
FLTGFRRAGRRHKKLVETFLNAASRADKSAAYHALSRTATMTLGEDELLDIVELFEQL
RGASWTKVTGAGSTVAVSLASDHRRGIMFADVPWRGNRINRIRYFPA"
CDS complement(2276915..2277475)
/codon_start=1
/note="gene_2103"
/transl_table=11
/translation="MRALIIVDVQNDFCEGGSLAVTGGAALARAISDYLAEAADYHHV
VATKDFHIDPGDDFSGTPDYSSSWPPHCVSGTPGADFHPSLDTSAIEAVFYKGAYTGA
YSGFEGVDENGTPLLNWLRQRGVDEVDVVGIATDHCVRQTAEDAVRNGLATRVLVDLT
AGVSADTTVAALEEMRTASVELVCSS"
CDS complement(2277516..2277866)
/codon_start=1
/note="gene_2104"
/transl_table=11
/translation="MYETVVVSTVVMHFAFIAYVLAGGFLALRWRRTMWLHVPAVIWG
IGIAAKRVDCPLTWVERWARTKAAMTPLSPDGFVAHYITGVIYPAGWVAAAQLVMFAI
VAASWTLYLWLPRR"
CDS complement(2277919..2279424)
/codon_start=1

/note="gene_2105"
/transl_table=11
/translation="MHERTVRARTATGIVEGFTRDGVHRWRSIPYARAPVGSLRFRAP
QPAQPWPGVRHCHTFANCAPQQRRYTVMGIGRYQTRSEDCLTLNVVTPEEPATQPLPV
MVFIHGGGYILGSSATPIYDGAALARRGCVYVSVNYRLGALGCLDLSSLSTPQITLDS
NVYLRDLVLALRWVHDNIAEFGGDPGNVTIFGESAGAHITATLLAVPAAKGLFARAIS
ESPAAGMVRSREVAAEFAARFANLIGARTQDAANALMQASPAQLVEAQHHLIRQGMRK
RLGAFPIGPVFGDDYLPMDPVEAMRSGRVHAVPLIVGTNAEEGRLFTRFLGMLPTNEP
MVEELLSGMKPADRERITAAYPNYPAPSACIQLGGDFAFSSAAWQIAEAHGANAPTYL
YRYDYAPRTLRWSGFGATHATELFAVFDIYRTRFGALLTAAADRRAALRVSNEVQRRW
RCFSQIGVPGDDWPAYTQDDRAVLVFDRRCRIEFDPHQHRRIAWDGFSLAN"
CDS 2279503..2280159
/codon_start=1
/note="gene_2106"
/transl_table=11
/translation="MRIAALVAVSLLIAGCPREVGGDVGQSQTIAPPAPAPSAAPSTP
PAAGAPITTIVSWIEAGHPVDPAAYHVATRDGVTTQLGDDVAFSASSGTVACMTDARH
TSGTLACLVRLANPPPRPETAYGEWKGGWVDFDGIHLQVGSARADPGPFVYGNGPELA
NGDTLSIGDYRCRSYQAGLFCVNYAHQSAVRFASAGIEPFGCLKPAPPPDGVGVAFGC
"
CDS complement(2280196..2282550)
/codon_start=1
/note="gene_2107"
/transl_table=11
/translation="VRGRNDHINIDGTANVLKAMAETGTGRIVFTSSGHQPRVEQMLA
DCGLEWVAVRCALIFGRNVDNWVQRLFALPVLPAGYADRVVQVVHSDDAQRLLVRALL
DTVIDSGPVNLAAPGELTFRRIAAALGRPMVPIGSPVLRRVTSFAELELLHSAPLMDV
TLLRDRWGFQPAWNAEECLEDFTLAVRGRIGLGKRTFSLPWRLANIQDLPAVDSPADD
GVAPRLAGPEGANGEFDTPIDPRFPTYLATNLSEALPGPFSPSSASVTVRGLRAGGVG
IAERLRPSGVIQREIAMRTVAVFAHRLYGAITSAHFMAATVPFAKPATIVSNSGFFGP
SMASLPIFGAQRPPSESSRARRWLRTLRNIGVFGVNLVGLSAGSPRDTDAYVADVDRL
ERLAFDNLATHDDRRLLSLILLARDHVVHGWVLASGSFMLCAAFNVLLRGLCGRDTAP
AAGPELVSARSVEAVQRLVAAARRDPVVIRLLAEPGERLDKLAVEAPEFHSAVLAELT
LIGHRGPAEVEMAATSYADNPELLVRMVAKTLRAVPAPQPPTPVIPLRAKPVALLAAR
QLRDREVRRDRMVRAIWVLRALLREYGRRLTEAGVFDTPDDVFYLLVDEIDALPADVS
GLVARRRAEQRRLAGIVPPTVFSGSWEPSPSSAAALAAGDTLRGVGVCGGRVRGRVRI
VRPETIDDLQPGEILVAEVTDVGYTAAFCYAAAVVTELGGPMSHAAVVAREFGFPCVV
DAQGATRFLPPGALVEVDGATGEIHVVELASEDGPALPGSDLSR"
CDS 2282446..2282640
/codon_start=1
/gene="TCDM_00422"
/note="gene_2108"
/transl_table=11
/translation="VTAGRENDAPGSGLGHRLEDIGGAVDIDVVVSATHPRTMRDHIR
TCHSTFDGGGIPDIGRNEIR"
CDS complement(2282765..2295166)
/codon_start=1
/note="gene_2109"
/transl_table=11
/translation="VERLKRTNRALLERSSEPIAIVGMSCRFPGGVDSPEGLWQMVAD
ARDVMSEFPTDRGWDLAGLFDPDPDVRHKSYARTGGFVDGVADFDPAFFGISPSEALA

MDPQHRMLLELSWEALERAGIDPTGLRGSATGVFAGLIVGGYGMLAEEIEGYRLTGMT
SSVASGRVAYVLGLEGPAVSVDTACSSSLVALHMAVGSLRSGECDLALAGGVTVNATP
TVFVEFSRHRGLAPDGRCKPYAGRADGVGWSEGGGMLVLQRLSDARRLGHPVLAVVVG
SAVNQDGASNGLTAPNGPSQQRVVRAALANAGLSAAEVDVVEGHGTGTTLGDPIEAQA
LLATYGQDRGEPGEPLWLGSVKSNMGHTQAAAGVAGVIKMVLAMRHELLPATLHVDVP
SPHVDWSAGAVELLTAPRVWPAGARTRRAGVSSFGISGTNAHVIIEAVPVVPRREAGW
AGPVVPWVVSAKSESALRGQAARLAAYVRGDDGLDVADVGWSLAGRSVFEHRAVVVGG
DRDRLLAGLDELAGDQLGGSVVRGTATAAGKTVFVFPGQGSQWLGMGIELLDTAPAFA
QQIDACAEAFAEFVDWSLVDVLRGAPGAPGLDRVDVVQPVLFAVMVSLAELWKSVAVH
PDAVIGHSQGEIAAAYVAGALSLRDAARVVTLRSKLLAGLAGPGGMVSIACGADQARD
LLAPFGDRVSIAVVNGPSAVVVSGEVGALEELIAVCSTKELRTRRIEVDYASHSVEVE
AIRGPLAEALSGIEPRSTRTVFFSTVTGNRLDTAGLDADYWYRNVRQTVLFDQAVRNA
CEQGYRTFIESSPHPALITGVEETFAACTDGDSEAIVVPTLGRGDGGLHRFLLSAASA
FVAGVAVNWRGTLDGAGYVELPTYAFDKRRFWLSAEGSGADVSGLGLGASEHPLLGAV
VDLPASGGVVLTGRLSPNVQPWLADHAVSDVVLFPGTGFVELAIRAGDEVGCSVLDEL
TLAAPLLLPATGSVAVQVVVDAGRDSNSRGVSIFSRADAQAGWLLHAEGILRPGSVEP
GADLSVWPPAGAVTVDVADGYERLATRGYRYGPAFRGLTAMWARGEEIFAEVRLPEAA
GGVGGFGVHPALLDAVLHAVVIAGDPDELALPFAWQGVSLHATGASAVRARIAPAGPS
AVSVELADGLGLPVLSVASMVARPVTERQLLAAVSGSGPDRLFEVIWSPASAATSPGP
TPAYQIFESVAADQDPVAGSYVRSHQALAAVQSWLTDHESGVLVVATRGAMALPREDV
ADLAGAAVWGLVRSAQTEHPGRIVLVDSDAATDDAAIAMALATGEPQVVLRGGQVYTA
RVRGSRAADAILVPPGDGPWRLGLGSAGTFENLRLEPVPNADAPLGPGQVRVAMRAIA
ANFRDIMITLGMFTHDALLGGEGAGVVVEVGPGVTEFSVGDSVFGFFPDGSGTLVAGD
VRLLLPMPADWSYAEAAAISAVFTTAYYAFIHLADVQPGQRVLIHAGTGGVGMAAVQL
ARHLGLEVFATASKGKWDTLRAMGFDDDHISDSRSLEFEDKFRAATGGRGFDVVLDSL
AGEFVDASLRLVAPGGVFLEMGKTDIRDPGVIAQQYPGVRYRAFDLFEAGPDRIAQIL
AELATLFGDGVLRLLPVTTFDVRRAPAALRYLSQARHTGKVVMLMPGSWAAGTVLITG
GTGMAGSAVARHVVARHGVRNLVLVSRRGPDAPGAAELVAELAAAGAQVQVVACDAAD
RAALAKVIADIPVQHPLSGVIHTAGALDDAVVMSLTPDRVDVVLRSKVDAAWHLHELT
RDLDVSAFVMFSSMAGLVGSSGQANYAAANSFLDALAAHRRAHGLPAISLGWGLWDQA
SAMTSGLDAADLARLGREGVLALSTAEALELFDTAMIVDEPFLAPARIDLTALRAHAV
AVPPMFSDLASAPTRRQVDDSVAAAKSKSALAHRLHGLPEAEQHAVLLGLVRLHIATV
LGNITPEAIDPDKAFQDLGFDSLTAVEMRNRLKSATGLSLSPTLIFDYPTPNRLASYI
RTELAGLPQEIKHTPAVRTTSEDPIAIVGMACRYPGGVNSPDDMWDMLIQGRDVLSEF
PADRGWDLAGLYNPDPDAAGACYTRTGGFVDGVGDFDPAFFGVGPSEALAMDPQQRML
LELSWEALERAGIDPTGLRGSATGVFAGVMTQGYGMFAAEPVEGFRLTGQLSSVASGR
VAYVLGLEGPAVSVDTACSSSLVALHMAVGSLRSGECDLALAGGVTVNATPDIFVEFS
RWRGLSPDGRCKAFAAAADGTGFSEGGGMLVLQRLSDARRLGHPVLAVVVGSAVNQDG
ASNGLTAPNGPSQQRVVRAALANAGLSAAEVDVVEGHGTGTTLGDPIEAQALLATYGQ
DRGEPGEPLWLGSVKSNMGHTQAAAGVAGVIKMVLAMRHELLPATLHVDVPSPHVDWS
AGAVELLTAPRVWPAGARTRRAGVSSFGISGTNAHVIIEAVPVVPRREAGWAGPVVPW
VVSAKSESALRGQAARLAAYVRGDDGLDVADVGWSLAGRSVFEHRAVVVGGDRDRLLA
GLDELAGDQLGGSVVRGTATAAGKTVFVFPGQGSQWLGMGMGLHAGYPVFAEAFNTVV
GELDRHLLRPLREVMWGHDENLLNSTEFAQPALFAVEVALFRLLGSWGVRPDFVMGHS
IGELSAAHVAGVLSLENAAVLVAARGRLMQALPAGGAMVAVQAAEEEVRPLLSAEVDI
AAVNGPASLVISGAQNAVAAVADQLRADGRRVHQLAVSHAFHSPLMDPMIDEFAAVAA
GIAIGRPTIGVISNVTGQLAGDDFGSAAYWRRHIRQAVRFADSVRFAQAAGGSRFLEV
GPSGGLVASIEESLPDVAVTTMSALRKDRPEPATLTNAVAQGFVTGMDLDWRAVVGEA
QFVELPTYAFQRRRFWLSGDGVAADAAGLGLAASEHALLGAVIDLPASGGVVLTGRLS
PRVQGWLADHSVAGVTIFPGAGFVELAIRAGDEVGCGVVDELTLAAPLVLPASGSVAV
QVVVNGPDESGVRGVSVYSRGDVGTGWVLHAEGALRAGSAEPTADLAMWPPAGAVPVE

VADGYQQLAERGYGYGLAFRGLTAMWRRGDEVFAEVALPADAGVSVTGFGVHPVLLDA
ALHAVVLSAESAERGQGSVLVPFSWQGVSLHAAGASAVRARIAPVGPSAVSIELADGL
GLPVLSVASMLARPVTDQQLRAAVSSSGPDRLFEVTWSPQPSAAVEPLPVCAWGTTED
SAAVVFESVPLAGDVVAGVYAATSSVLDVLQSWLTRDGAGVLVVMTRGAVALPGEDVT
DLAGAAVWGLVRSAQTEHPGRIVLVDSDAPLDDSALAAVVTTGEPQVLWRRGEVYTAR
VHGSRAVGGLLVPPSDRPWRLAMSTAGTFENLRLELIPDADAPLGPGQVRVAVSAIAA
NFRDVMIALGLYPDPDAVMGVEACGVVIETSLNKGSFAVGDRVMGLFPEGTGTVASTD
QRLLVKVPAGWSHTAAATTSVVFATAHYALVDLAAARSGQRVLIHAGTGGVGMAAVQL
ARHLGLEVFATASKGKWDTLRAMGFDDDHISDSRSLEFEDKFRAATGGRGFDVVLDSL
AGEFVDASLRLVAPGGVFLEMGKTDIRDPGVIAQQYPGVRYRAFDLFEAGPDRIAQIL
AELATLFGDGVLRPLPVTTFDVRRAPAALRYLSQARHTGKVVMLMPGSWAAGTVLITG
GTGMAGSAVARHVVARHGVRNLVLVSRRGPDAPGAAELVAELAAAGAQVQVVACDAAD
RAALAKVIADIPVQHPLSGVIHTAGALDDAVVMSLTPDRVDVVLRSKVDAAWHLHELT
RDLDVSAFVMFSSMAGLVGSSGQANYAAANSFLDALAAHRRAHGLPAISLGWGLWDQA
SAMTSGLATVDFKRFARDGIVAMSSADALQLFDTAMIVDEPFMLPAHIDFAALKVKFD
GGTLPPMFVDLINAPTRRQVDDSLAAAKSKSALLQRLEGLPEDEQHAVLLDLVRSHIA
TVLGSASPEAIDPDRAFQELGFDSLTAVEMRNRLKSATGLSLSPTLIFDYPNSAALAG
YMRRELLGSSPQDTSAVAAGEAELQRIVASIPVKRLRQAGVLDLLLALANETETSGQD
PALAPTAEQEIADMDLDDLVNAAFRNDDE"

CDS complement(2295527..2295751)
/codon_start=1
/note="gene_2110"
/transl_table=11
/translation="MLTRGEVRALPADAVVLSADDAADLSDRVYQVRCAAEDVVTALD
EGAAATELRDLCDELIRAARAADGWRRAGA"

CDS 2296055..2296390
/codon_start=1
/note="gene_2111"
/transl_table=11
/translation="MADRVLRGSRLGAVSYETDRNHDLAPRQIARYRTDNGEEFEVPF
ADDAEIPGTWLCRNGMEGTLIEGDLPEPKKVKPPRTHWDMLLERRSIEELEELLKERL
ELIRSRRRG"

CDS complement(2296365..2298989)
/codon_start=1
/note="gene_2112"
/transl_table=11
/translation="VKLGAWVAAQLPTTRTAVRTRLTRLVVSIVAGLLLYASFPPRNC
WWAAVVALALLAWVLTHRATTPVGGLGYGLLFGLVFYVSLLPWIGELVGPGPWLALAT
TCALFPGIFGLFAVVVRLLPGWPIWFAVGWAAQEWLKSILPFGGFPWGSVAFGQAEGP
LLPLVQLGGVALLSTGVALVGCGLTAIALEIEKWWRTGGQGDAPPAVVLPAACICLVL
FAAIVVWPQVRHAGSGSGGEPTVTVAVVQGNVPRLGLDFNAQRRAVLDNHVEETLRLA
ADVHAGLAQQPQFVIWPENSSDIDPFVNPDAGQRISAAAEAIGAPILIGTLMDVPGRP
RENPEWTNTAIVWNPGTGPADRHDKAIVQPFGEYLPMPWLFRHLSGYADRAGHFVPGN
GTGVVRIAGVPVGVATCWEVIFDRAPRKSILGGAQLLTVPSNNATFNKTMSEQQLAFA
KVRAVEHDRYVVVAGTTGISAVIAPDGGELIRTDFFQPAYLDSQVRLKTRLTPATRWG
PILQWILVGAAAAVVLVAMRQNGWFPRPRRSEPKGENDDSDAPPGRSEASGPPALSES
DDELIQPEQGGRHSSGFGRHRATSRSYMTTGQPAPPAPGNRPSQRVLVIIPTFNEREN
LPVIHRRLTQACPAVHVLVVDDSSPDGTGQLADELAQADPGRTHVMHRTAKNGLGAAY
LAGFAWGLSREYSVLVEMDADGSHAPEQLQRLLDAVDAGADLAIGSRYVAGGTVRNWP
WRRLVLSKTANTYSRLALGIGIHDITAGYRAYRREALEAIDLDGVDSKGYCFQIDLTW
RTVSNGFVVTEVPITFTERELGVSKMSGSNIREALVKVARWGIEGRLSRSDHARARPD

IARPGAGGSRVSRADVTE"
CDS complement(2299147..2300751)
/codon_start=1
/note="gene_2113"
/transl_table=11
/translation="VSQIPVKLLVNGRVYSPTHPEATAMAVRGDVVAWLGSDDVGRDQ
FPDADVQDLDGRFVAPGFVDSHIHLTATGLMLSGLDLRPATSRAQCLRMVADYAADHP
GQPLWGHGWDESAWPENAAPSTADLDAVLGDCPAYLARIDSHSALVSSGLRRLVPELA
AATGYTAQRPLTGDAHHLARAAARYLLTDVQLADARAVALQAIAAAGVVAVHECAGPE
IGGLDDWLRLRALEHGVEVIGYWGEAVATPAQARDLVTETGARGLAGDLFVDGALGSR
TAWLHEPYADAPDCIGTCHLDVDGIEAHVRACTKAEVTAGFHVIGDAAVSAAVAAFER
VVADLGVVAVARCGHRLEHVEMVTADQAAKLGAWGVIASVQPNFDELWGGGDGMYARR
LGAQRGSELNPLALLASQGVPLALGSDAPVTGFDPWASVRAAVNHRTPGSGVSARAAF
AAATRGGWRAGGVRDGRIGTLVPGAPASYAIWDAGDFDVDAPRDAVQRWSTDPRSRVP
ALPRLGPTDALPRCRQTVHRGAVIYG"
CDS complement(2300756..2301286)
/codon_start=1
/gene="fxsA"
/note="gene_2114"
/transl_table=11
/translation="VVSRLLLSYAVVELAVVFALAATIGFGWTLLVLLATFVLGFGLL
APLGGWQLGRRLLWLRSGLAEPRSALSDGALVTVASVLVLVPGLVTTTMGLLLLVPPI
RALARPGLTAIAVRGFLRNVPLTADAAANMAGAFGESGTDPDFIDGEVIDVIDVEPLT
LQPPRVAAEPPSPGSN"
CDS 2301359..2302072
/codon_start=1
/note="gene_2115"
/transl_table=11
/translation="MTTIEIDAPAGPIDALLGLPPGQGPWPGVVVVHDAVGYVPDNKL
ISERIARAGYVVLTPNMYARGGRARCITRVFRELLTKRGRALDDILAARDHLLAMPEC
SGRVGIVGFCMGGQFALVLSPRGFGATAPFYGTPLPRHLSETLNGACPIVASFGTRDP
LGIGAANRLRKVTAAKNIPADIKSYPGAGHSFANKLPGQPLVRIAGFGYNEAATEDAW
RRVFEFFGQHLRAGSPGEP"
CDS complement(2302321..2302587)
/codon_start=1
/note="gene_2116"
/transl_table=11
/translation="MAAKSARKGPTKAKKNLLDSLGVESVDYKDTATLRVFISDRGKI
RSRGVTGLTVQQQRQVAQAIKNAREMALLPYPGQDRQRRAALCP"
CDS complement(2302588..2302893)
/codon_start=1
/gene="rpsN"
/note="gene_2117"
/transl_table=11
/translation="VAKKSKIVKNQRRAATVARYASRRTALKDIIRSPSSAPEQRSTA
QRALARQPRDASPVRLRNRDAIDGRPRGHLRKFGLSRVRVRQLAHDGHLPGVRKASW"
CDS complement(2302895..2303059)
/codon_start=1
/gene="rpmG"
/note="gene_2118"
/transl_table=11

/translation="MARTDIRPIVKLRSTAGTGYTYTTRKNRRNDPDRLILRKYDPIL
RRHVDFREER"
CDS complement(2303059..2303295)
/codon_start=1
/gene="rpmB"
/note="gene_2119"
/transl_table=11
/translation="LSAHCQVTGRKPGFGNTVSHSHRRSRRRWSPNIQQRTYYLPSEG
RRIRLRVSTKGIKVIDRDGIEAVVARLRRQGQRI"
CDS 2304513..2304914
/codon_start=1
/note="gene_2120"
/transl_table=11
/translation="MLTVVCLLVVTVLAICYRPLLFATVDPEVAAARGVPVRALGIVF
AALMGVVAAQAVQIVGALLVMSLLITPAAAAARVVVAPVAAIATSVVFAEVSAVGGIL
LSLAPGVPVSVFVATISFVIYLICWLLRRRR"
CDS complement(2304915..2305319)
/codon_start=1
/note="gene_2121"
/transl_table=11
/translation="VTPTFSDLAEAQYLLLTTFTKDGRPKPVPIWAALDTDRGDRLLV
ITEKKSWKVKRIRNTPRVTLATCTLRGRPTSEAVEATAAILDESQTGAVYDAIVKRYG
IQGKLFTFVSKLRGGMRNNIGLELKVAESETG"
CDS complement(2305403..2308987)
/codon_start=1
/note="gene_2122"
/transl_table=11
/translation="VPEPTVLLLSTSDTDLISARSSGKNYRWANPSRLSDLELTDLLA
EASIVVIRILGGYRAWQSGIDTVIAGGVPAALVSGEQAADAELTDRSTVAAGTALQAH
IYLAHGGVDNLRELHAFLCDTVLMTGFGFTPPVATPTWGVLERPDAGKTGPTIAVLYY
RAQHLAGNTGYVEALCRAIEDAGGRPLPLYCASLRTAEPRLLERLGGADAMVVTVLAA
GGVKPAAASAGGDDDSWNVEHLAALDIPILQGLCLTSPRDQWCANDDGLSPLDVASQV
AVPEFDGRIITVPFSFKEIDDDGLISYVADPERCARVAGLAVRHARLRQVAPADKRVA
LVFSAYPTKHARIGNAVGLDTPASAVALLQAMRQRGYRVGDLPGVESNDGDALIHALI
ECGGHDPDWLTEGQLAGNPIRVSAKEYRDWFATLPAELTDVVTAYWGPPPGELFVDRS
HDPDGEIVIAALRAGNLVLMVQPPRGFGENPVAIYHDPDLPPSHHYLAAYRWLDTGFS
NGFGAHAVVHLGKHGNLEWLPGKTLGMSASCGPDAALGDLPLIYPFLVNDPGEGTQAK
RRAHAVLVDHLIPPMARAETYGDIARLEQLLDEHASVAALDPGKLPAIRQQIWTLIRA
AKMDHDLGLTERPEEDSFDDMLLHVDGWLCEIKDVQIRDGLHILGQNPTGEQELDLVL
AILRACQLFGGAHAIPGLRQALGLAEDGTDERATVDQTEAKARELVAALQATGWDPSA
ADRLTGNADAAAVLRFAATEVIPRLAGTATEIEQVLRALDGRFIPAGPSGSPLRGLVN
VLPTGRNFYSVDPKAVPSRLAWEAGVALADSLLARYRDEHGRWPRSVGLSVWGTSAMR
TAGDDIAEVLALLGVRPVWDDASRRVIDLAPMQPAELGRPRIDVTVRISGFFRDAFPH
VVTMLDDAVRLVADLDEAAEDNYVRAHAQADLAHHGDQRRATTRIFGSKPGTYGAGLL
QLIDSRSWRDDADLAQVYTAWGGFAYGRDLDGREAIDDMNRQYRRIAVAAKNTDTREH
DIADSDDYFQYHGGMVATVRALTGQAPAAYIGDNTRPDAIRTRTLSEETTRVFRARVV
NPRWMAAMRRHGYKGAFEMAATVDYLFGYDATAGVMADWMYEQLTQRYVLDAQNRTFM
TESNPWALHGMAERLLEAAGRGLWAQPAPETLDGLRQVLLETEGDLEA"
CDS 2309146..2309298
/codon_start=1
/note="gene_2123"

```
                     /transl_table=11
                     /translation="MSALLTELAAQAERQAIFRAEREASHAETTTQAVRDEDREWEGT
                     VGDGLG"
     CDS             2309291..2309701
                     /codon_start=1
                     /note="gene_2124"
                     /transl_table=11
                     /translation="LAEPRRGDLWLVSLGAARAGEPGKHRPAVVVSVDELLTGIDDEL
                     VVVVPVSSSRSRTPLRPPVAPSEGVAADSVAVCRGVRAVARARLVERLGALKPATMRA
                     IENALTLILGLPTGPERGEAATHSPVRWTGGRDP"
     CDS             2309685..2310776
                     /codon_start=1
                     /note="gene_2125"
                     /transl_table=11
                     /translation="VAGTRDADACPGALRPHQAADGALARIRLPGGMITAAQLATLAS
                     VASDFGSATLELTARGNVQLRGIRDVAAVADAVAKAGLLPSATHERVRNIVASPLSGR
                     AGGLADVRAWVGELDAAIRAEPRLAELGGRFWFGLDDGRADVSGLGADVGVQVFPDGP
                     RLLLTGRDTGVRVADVAETLIEVALRFVKIRETAWRVTELADIGELQSGVELGPSVRP
                     VTKTPVGWIPQDDSRVTLGAAVPLGVLPARVAECLAAIEAPLVITPWRSVLICDLDDA
                     TADAALRVLAPLGLVFDENSPWLNISACTGSPGCAHSAADVRADAARSLNVESAGHRH
                     FVGCERACGSPPAGEVLVATGGGYRRLRP"
     CDS             2310786..2311412
                     /codon_start=1
                     /gene="cobH"
                     /note="gene_2126"
                     /transl_table=11
                     /translation="VLDYLRDAAEIYRRSFAVIRAEADLARFPADVARVVVRLIHTCG
                     QVDVAEHVAYTDDVVARAGAALAAGAPVLCDSSMVAAGITTSRLPADNQIVSLVADPR
                     ATELAARRQTTRSAAGVELCAERLPGAVLAIGNAPTALFRLLELVDEGAPPPAAVLGG
                     PVGFVGSAQAKEELIERPRGMSYLVVRGRRGGSAMAAAAVNAIASDRE"
     CDS             2311409..2312935
                     /codon_start=1
                     /note="gene_2127"
                     /transl_table=11
                     /translation="MSARGTLWGVGLGPGDPELVTVKAARVIGEADVVAYHSAPHGHS
                     IARGIAEPYLRPGQLEEHLVYPVTTEATNHPGGYAGALEDFYADATERIATHLDAGRN
                     VALLAEGDPLFYSSYMHLHTRLTRRFNAVIVPGVTSVSAASAAVATPLVAGDQVLSVL
                     PGTLPVGELTRRLADADAAVVVKLGRSYHNVREALSASGLLGDAFYVERASTAGQRVL
                     PAADVDETSVPYFSLAMLPGGRRRALLTGTVAVVGLGPGDSDWMTPQSRRELAAATDL
                     IGYRGYLDRVEVRDGQRRHPSDNTDEPARARLACSLADQGRAVAVVSSGDPGVFAMAT
                     AVLEEAEQWPGVRVRVIPAMTAAQAVASRVGAPLGHDYAVISLSDRLKPWDVIAARLT
                     AAAAADLVLAIYNPASVTRTWQVGAMRELLLAHRDPGIPVVIGRNVSGPVSGPNEDVR
                     VVKLADLNPAEIDMRCLLIVGSSQTRWYSVDSQDRVFTPRRYPEAGRATATKSSRHSD
                     "
     CDS             complement(2312881..2314104)
                     /codon_start=1
                     /note="gene_2128"
                     /transl_table=11
                     /translation="VTDDHPRADIVSRQYHRWLYPHPIADLEAWTTANWEWFDPVHSH
                     RILWPDREYRPDLDILIAGCGTNQAAIFAFTNRAAKVVAIDISRPALDHQQYLKDKHG
                     LANLELHLLPIEELATLGRDFDLVVSTGVLHHLADPRAGMKELAHCLRRDGVVAAMLY
```

GKYGRIGVELLGSVFRDLGLGQDDASIKLAKEAISLLPTYHPLRNYLTKARDLLSDSA
LVDTFLHGRQRSYTVEECVDLVTSAGLVFQGWFHKAPYYPHDFFVPNSEFYAAVNTLP
EVKAWSVMERLETLNATHLFMACRRDRPKEQYTIDFSTVAALDYVPLMRTRCGVSGTD
MFWPGWRMAPSPAQLAFLQQVDGRRTIREIAGCVARTGEPSGGSLADLEEFGRKLFQS
LWRLDFVAVALPASG"

CDS complement(2314120..2314932)
/codon_start=1
/note="gene_2129"
/transl_table=11
/translation="LPAGADLADRFAELERRYDARLGVYVPATGTTAAIEYRADERFA
FCSTFKAPLVAAVLHQNPLTHLDKLITYTSDDIRSISPVAQQHVQTGMTIGQLCDAAI
RYSDGTAANLLLADLGGPGGGTAAFTGYLRSLGDTVSRLDAEEPELNRDPPGDERDTT
TPHAIALVLQQLVLGNALPPDKRALLTDWMARNTTGAKRIRAGFPADWKVIDKTGTGD
YGRANDIAVVWSPTGVPYVVAVMSDRAGGGYDAEPREALLAEAATCVAGVLA"

CDS 2315178..2315735
/codon_start=1
/note="gene_2130"
/transl_table=11
/translation="MTATASDDEAVTALALSAAKGNGRALEAFIKATQQDVWRFVAYL
SDVGSADDLTQETFLRAIGAIPRFSARSSARTWLLAIARHVVADHIRHVRSRPRTTRG
ARPEHLIDGDRHARGFEDLVEVTTMIADLTTDQREALLLTQLLGLSYADAAAVCGCPV
GTIRSRVARARDALLADAEPDDLTG"

CDS complement(2315725..2316420)
/codon_start=1
/note="gene_2131"
/transl_table=11
/translation="VRWRKSCNPHVEIVSSLAGRVPNPALPIGPVRIGGFGGVEGLRG
WLREERIDAVVDATHPFAVTITAHAAQVCGELGLPYLVLARPPWDPGTAIIAVSDIEA
ADVVAEQGYSRVFLTTGRSGIAAFANSDAWFLIRVVTAPDGTALPRRHKLVLSRGPYG
YHDEFALLREQRIDALVTKNSGGKMTRAKLDAAAALGISVVMIARPLLPAGVAAVDSV
HRAAMWVAGLPSR"

CDS complement(2316457..2317212)
/codon_start=1
/note="gene_2132"
/transl_table=11
/translation="MTVYFIGAGPGAADLITVRGQRLLQRCPVCLYAGSIMPDDLLAQ
CPPGATIVDTGPLTLEQIVRKLADADADGRDVARLHSGDPSLYSALAEQCRELDALGI
GYEIVPGVPAFAAAAAALKRELTVPGVAQTVTLTRVATLSTPMPPGEDLAALARSRAT
LVLHLAAAQIDAIVPRLLDGGYRPETPVAVVAFASWPQQRTLRGTLADIAARMHDAKI
TRTAVIVVGDVLTAEGFTDSYLYSVARHGRYAQ"

CDS complement(2317209..2318054)
/codon_start=1
/note="gene_2133"
/transl_table=11
/translation="MGWNVYDTEVISLVTAQPHTAVRRGGRAIVLSGDRSTPQALAVL
LTEHGRGDSKFSVLEQLGGPAERRRDGTARAWACDPPLDVDELNVIAVRYLPDERTSW
APDEAFAHDGQITKHPIRVLTLAALAPRPGQRLWDVGAGSGAIAVQWCRSWPGCTAVA
FERDERRRRNIGFNAAAFGVSVDVRGDAPDAFDDAARPSVIFLGGGVTQPGLLEACLD
SLPAGGNLVANAVTVESEAALAHAYSRLGGELRRFQHYLGEPLGGFTGWRPQLPVTQW
SVTKR"

CDS complement(2318035..2318223)

```
                     /codon_start=1
                     /note="gene_2134"
                     /transl_table=11
                     /translation="MLPAVQGLSPDGADLHVVASGDPLLHGIGSTLIRLFGHDNVTVF
                     AARVRGDVGVRPDGLERV"
     CDS             complement(2318298..2319086)
                     /codon_start=1
                     /note="gene_2135"
                     /transl_table=11
                     /translation="MPRARWLQSAALMGALAVVLITAAPVAADAYQVPAPPSPTASCD
                     VISPVAIPCVALGKFADAVAAECRRVGVPDARCVLPLAHRVTQAARDAYLQSWVHRTA
                     RFQDALQDPVPLRETQWLGTHNSFNSLSDSFTVSHADSNQQLSLAQQLDIDVRALELD
                     LHYLPRLEGHGAPGVTVCHGLGPKNANLGCTVEPLLATVLPQIANWLNAPGHTEEVIL
                     LYLEDQLKNASAYESVVATLDQVLRRADGTSLIYRPNPARRGPQ"
     CDS             complement(2319244..2319543)
                     /codon_start=1
                     /note="gene_2136"
                     /transl_table=11
                     /translation="VPPSSATPVVVFSVAVVVVCLIGGVAGSLWPRPAGRLRGGCYFA
                     FMGVAWVLLAISAIANAVKGSLWWDIWSLGLLVLIPAVVYGKMRRSRRISSDQDR"
     CDS             complement(2319530..2320501)
                     /codon_start=1
                     /note="gene_2137"
                     /transl_table=11
                     /translation="MLATLSQIRAWSTEHLIDAAGYWTETADRWEDVFLQMRNQAHAI
                     AWNGAGGDGLRQRTRADFSTVSGIADQLRRAATIARNGAGTIDAAQRRVMYAVEDAQD
                     AGFNVGEDLSVTDTKTTQPAAVQAARLAQAQALAGDIRLRVGQLVAAENEVSGQLAAT
                     TGDVGNVRFAGAPVVAHSAVQLVDFFKQDGPTPPPPGAPHPSGGADGPYSDPITSMML
                     PPAGTEAPVSDATKRWVDNMVNELAARPPDDPIAVEARRLAFQALHRPCNSAEWTAAV
                     AGFAGSSAGVVGTALAIPAGPADWALLGAALLGVGGSGAAVVNCATK"
     CDS             2320559..2320786
                     /codon_start=1
                     /gene="Si000203m.g"
                     /note="gene_2138"
                     /transl_table=11
                     /translation="MGVSCGRGSHPGSRSLRPGGEFRCRAADCCVDTTYRHRRGLEWL
                     AGFWRRCRGGELGAKALRLTGRNLKLTKYNL"
     CDS             2321266..2321580
                     /codon_start=1
                     /note="gene_2139"
                     /transl_table=11
                     /translation="VFVDVGLLHSGANESHYAGEHAHGGADQLSRGPLLSGMFGTFPV
                     AQTFHDAVGAAHAQQMRNLHAHRQALITVGEKARHAATGFTDMDDGNAAELKAVVCSC
                     AT"
     CDS             2321562..2323532
                     /codon_start=1
                     /note="gene_2140"
                     /transl_table=11
                     /translation="MQLRHINIRALIAEAGGDPWAIEHSLHAGRPAQIAELAEAFHAA
                     GRCTAEANAAFEEARRRFEASWNRENGEHPINDSAEVQRVTAALGVQSLQLPKIGVDL
                     ENIAADLAEAQRAAAGRIATLESQLQRIDDQLDQALELEHDPRLAAAERSELDALITC
```

LEQDAIDDTASALGQLQSIRAGYSDHLQQSLAMLRADGYDGAGLQGLDAPQSPVKLEE
PIQIPPPGTGAPEVHRWWTSLTSEERQRLIAEHPEQIGNLNGVPVSARSDANIAVMTR
DLNRVRDIATRYRTSVDDVLGDPAKYGLSAGDITRYRNADETKKGLDHNARNDPRNPS
PVYLFAYDPMAFGGKGRAAIAIGNPDTAKHTAVIVPGTSSSVKGGWLHDNHDDALNLF
NQAKAADPNNPTAVIAWMGYDAPNDFTDPRIATPMLARIGGAALAEDVNGLWVTHLGV
GQNVTVLGHSYGSTTVADAFALGGMHANDAVLLGCPGTDLAHSAASFHLDGGRVYVGA
ASTDPISMLGQLDSLSQYVNRGNLAGQLQGLAVGLGTDPAGDGFGSVRFRAEVPNSDG
INPHDHSYYYHRGSEALRSMADIASGHGDALASDGMLAQPRHQPGVEIDIPGLGSVEI
DIPGTPASIDPEWSRPPGSITDDHVFDAPLHR"

CDS 2323609..2324076
/codon_start=1
/note="gene_2141"
/transl_table=11
/translation="MHKPHSAGSSNPWDDSAHPLTDDQAMAQVVEPAKQIVAAADLQA
VRAGFSFTSCNDQGDPPYQGTVRMAFLLQGDHDAYFQHVRAAMLSHGWIDGPPPGQYF
HGITLHKNGVTANMSLALDHSYGEMILDGECRNTTDHHHDDETTNITNQLVQP"

CDS 2324129..2324395
/codon_start=1
/note="gene_2142"
/transl_table=11
/translation="VWHVATGDRRARIAPGIEELGPTLVETVRRRDALPRIAQAVVVA
ATRNYGVPDNETDLLRSPRPRWPPNAAGMQVECTDTAAAIALRG"

CDS 2324651..2324932
/codon_start=1
/note="gene_2143"
/transl_table=11
/translation="VYKLAARAVQIATNGDNPAFANIATVNGAVMLQQTLNTTPALVP
GERTDALALAEAYGQATAFAMEQDHPAWQSAANDVNAKDARMKAICGGG"

CDS complement(2325147..2325452)
/codon_start=1
/note="gene_2144"
/transl_table=11
/translation="VVTQPGVGVTTDGLVEHSLRRVDNVGHRGCSHMVGHGIRLLDDG
VHRGNFGLYRPFAGQDLVDLLIAGLCDLGQPIPQIVHRIRDLLALVAFDLALMRDGG"

variation 2325584..2325661
/note="supplemented from AL2122/97"

CDS 2325861..2327087
/codon_start=1
/note="gene_2145"
/transl_table=11
/translation="LGQSFTTGMTTGTPAAAGAQALSAGALHAATEPLPPPAPPPTTP
TVTTPTVATATTAGIPHIPDSAPTPSPAPIAPPTTDNASAMTPIAPMVANGPPASPAP
PAAAPAGPLPAYGADLRPPVTTPPATPPTPTGPISGAAVTPSSPAAGGSLMSPVVNKS
TAPATTQAQPSNPTPPLASATAAATTGAAAGDTSRRAAEQQRLRRILDTVARQEPGLS
WAAGLRDNGQTTLLVTDLASGWIPPHIRLPAHITLLEPAPRRRHATVTDLLGTTTVAA
AHHPHGYLSQPDPDTPALTGDRTARIAPTIDELGPTLVETVRRHDTLPRIAQAVVVAA
TRNYGVPDNETDLLHHKTTEIHQAVLTTYPNHDIATVVDWMLLAAINALIAGDQSGAN
YHLAWAIAAISTRRSR"

CDS 2327084..2328028
/codon_start=1
/note="gene_2146"

```
                     /transl_table=11
                     /translation="MTSIESHPEQYWAAAGRPGPVPLALGPVHPGGPTLIDLLMALFG
                     LSTNADLGGTNADIEGDDTDRRAHAADAARKFSANEANAAEQMQGVGAQGMAQMASGI
                     GGALSGALGGVMGPLTQLPQQAMQAGQGAMQPLMSAMQQAQGADGLAAVDGARLLDSI
                     GGEPGLGSGAGGGDVGGGGAGGTTPTGYLGPPPVPTSSPPTTPAGAPTKSATMPPPGG
                     ASPASAHMGAAGMPMVPPGAMGARGEGSGQEKPVEKRVTAPAVPNGQPVKGRLTVPPS
                     APTTKPTDGKPVVRRRILLPEHKDFGRIAPDEKTDAGE"
     CDS             2328015..2329016
                     /codon_start=1
                     /note="gene_2147"
                     /transl_table=11
                     /translation="MPVSDDSSSAFDLICAEIERQLRGGELLMDAAAASELLLTVRYQ
                     LDTQPRPLVIVHGPLFQAVKAARAQVYGRLIQLRHARCEVLDERWQLRPTGQRDVRAL
                     LIDVLNVLLAAITAAGVERAYACAERRAMAAAVVAKNYRDALGVELQCNSVCRAAAEA
                     IHALAHRTGATEDADCLPPVDVIHADVTRRMHGEVATDVVAAGELVIAARHLLDPMPR
                     GELSYGPLHEGGNAARKSVYRRLVQLWQARRAVTDGDVDLRDARTLLTDLDSILREMR
                     TAATIQQAYTRAERRAMAAAVVAKIRGDAMGLDAQRDAVHRAAADALHALQSVGIHQ"
     CDS             2329251..2329556
                     /codon_start=1
                     /note="gene_2148"
                     /transl_table=11
                     /translation="VSDMCDVVSFVGAAERVLRARFRPSPESGPPVHARRCGWSLGIS
                     AETLRRWAGQAEVDSGVVAGVSASRSGSVKTSELEQTIEILKVATSFFARKCDPRHR"
     CDS             2329535..2330140
                     /codon_start=1
                     /note="gene_2149"
                     /transl_table=11
                     /translation="VRPATPLICAFGDKHKHTYGVTPICRALAVHGVQIASRTYFADR
                     AAAPSKRALWDTTITEILAGYYEPDAEGKRPPECLYGSLKMWAHLQRQGFRWPSATVK
                     TIMRANGWRGVPLAAHITHHRTRPGRGPGPRPGGSAMAGFSNEPAGSGRLHLRADDVE
                     FRLHRVRGRRLRRCDRGLGMLADQRRSVRRTRITPRPSRLT"
     CDS             2330635..2332404
                     /codon_start=1
                     /note="gene_2150"
                     /transl_table=11
                     /translation="VAHELSAGSVFAGYRIERMLGAGGMGTVYLARNPDLPRSEALKV
                     LAAELSRDLDFRARFVREADVAAGLDHPNIVAVHQRGQFEGRLWIAMQFVDGGNAEDA
                     LRAATMTTARAVYVIGEVAKALDYAHQQGVIHRDIKPANFLLSRAAGGDERVLLSDFG
                     IARALGDTGLTSTGSVLATLAYAAPEVLAGQGFDGRADLYSLGCALFRLLTGEAPFAA
                     GAGAAVAVVAGHLHQPPPTVSDRVPGLSAAMDAVIATAMAKDPMRRFTSAGEFAHAAA
                     AALYGGATDGWVPPSPAPHVISQGAVPGSPWWQHPVGSVTALATPPGHGWPPGLPPLP
                     RRPRRYRRGVAAVAAVMVVAAAAVTAVTMTSHQPRTATPPSAAALSPTSSSTTPPQPP
                     IVTRSRLPGLLPPLDDVKNFVGIQNLVAHEPMLQPQTPNGSINPAECWPAVGGGVPSA
                     YDLGTVIGFYGLTIDEPPTGTAPNQVGQLIVAFRDAATAQRHLADLASIWRRCGGRTV
                     TLFRSEWRRPVELSTSVPEVVDGITTMVLTAQGPVLRVREDHAIAAKNNVLVDVDIMT
                     PDTSRGQQAVIGITNYILAKIPG"
     CDS             complement(2332421..2333635)
                     /codon_start=1
                     /note="gene_2151"
                     /transl_table=11
                     /translation="VRRVATHRIGCGHAGYLASARERAVNLADMGSRRFDAEVYARRL
```

ALAAAATADAGLAGLVITPGYDLCYLIGSRAETFERLTALVLPAAGAPAVVLPRLELA
ALKQSAAAELGLRVCDWVDGDDPYGLVSAVLGGAPVATAVTDSMPALHMLPLADALGV
LPVLATDVLRRLRMVKEETEIDALRKAGAAIDRVHARVPEFLVPGRTEADVAADIAEA
IVAEGHSEVAFVIVGSGPHGADPHHGYSDRELREGDIVVVDIGGTYGPGYHSDSTRTY
SIGEPDSDVAQSYSMLQRAQRAAFEAIRPGVTAEQVDAAARDVLAEAGLAEYFVHRTG
HGIGLCVHEEPYIVAGNDLVLVPGMAFSIEPGIYFPGRWGARIEDIVIVTEDGAVSVN
NCPHELIVVPVS"

variation 2333660..2333716
/note="supplemented from AL2122/97"

CDS 2333801..2334721
/codon_start=1
/note="gene_2152"
/transl_table=11
/translation="MWFRSFFGVPSSITAPDGRPVNAVRGFIDSMAVVITQQRPNRLA
VCLDLDWRPQFRVDLIPSYKAHRVAEPEPNGQPDVEEVPDELTPQVDMIMELLDAFGI
AMAGAPGFEADDVLGTLATRERRDPVIVVSGDRDLLQVVADDPVPVRVLYLGRGLAKA
TLFGPAEVAERYGLPAHRAGAAYAELALLRGDPSDGLPGVPGVGEKTAATLLARHGSL
DQIMAAADDRKTTMAKGLRTKLLAASAYIKAADRVVRVATDAPVTLSTPTDRLPLVAA
DPERTAELATRFGVESSIARLQKALDTLPG"

CDS complement(2334725..2335459)
/codon_start=1
/note="gene_2153"
/transl_table=11
/translation="MSGPQGSDPRQPWQPPGQGADHSSDPTVAAGYPWQQQPTQEATW
QAPAYTPQYQQPADPAYPQQYPQPTPGYAQPEQFGAQPTQLGVPGQYGQYQQPGQYGQ
PGQYGQPGQYAPPGQYPGQYGPYGQSGQGSKRSVAVIGGVIAVMAVLFIGAVLILGFW
APGFFVTTKLDVIKAQAGVQQVLTDETTGYGAKNVKDVKCNNGSDPTVKKGATFECTV
SIDGTSKRVTVTFQDNKGTYEVGRPQ"

CDS complement(2335501..2338221)
/codon_start=1
/note="gene_2154"
/transl_table=11
/translation="VTELAELDRFTAELPFSLDDFQQRACSALERGHGVLVCAPTGAG
KTVVGEFAVHLALAAGSKCFYTTPLKALSNQKHTDLTARYGRDQIGLLTGDLSVNGNA
PVVVMTTEVLRNMLYADSPALQGLSYVVMDEVHFLADRMRGPVWEEVILQLPDDVRVV
SLSATVSNAEEFGGWIQMVRGDTTVVVDEHRPVPLWQHVLVGKRMFDLFDYRIGEAEG
QPQVNRELLRHIAHRREADRMADWQPRRRGSGRPGFYRPPGRPEVIAKLDAEGLLPAI
TFVFSRAGCDAAVTQCLRSPLRLTSEEERARIAEVIDHRCGDLADSDLAVLGYYEWRE
GLLRGLAAHHAGMLPAFRHTVEELFTAGLVKAVFATETLALGINMPARTVVLERLVKF
NGEQHMPLTPGEYTQLTGRAGRRGIDVEGHAVVIWHPEIEPSEVAGLASTRTFPLRSS
FAPSYNMTINLVHRMGPQQAHRLLEQSFAQYQADRSVVGLVRGIERGNRILGEIAAEL
GGSDAPILEYARLRARVSELERAQARASRLQRRQAATDALAALRRGDIITITHGRRGG
LAVVLESARDRDDPRPLVLTEHRWAGRISSADYSGTTPVGSMTLPKRVEHRQPRVRRD
LASALRSAAAGLVIPAARRVSEAGGFHDPELESSREQLRRHPVHTSPGLEDQIRQAER
YLRIERDNAQLERKVAAATNSLARTFDRFVGLLTEREFIDGPATDPVVTDDGRLLARI
YSESDLLVAECLRTGAWEGLKPAELAGVVSAVVYETRGGDGQGAPFGADVPTPRLRQA
LTQTSRLSTTLRADEQAHRITPSREPDDGFVRVIYRWSRTGDLAAALAAADVNGSGSP
LLAGDFVRWCRQVLDLLDQVRNAAPNPELRATAKRAIGDIRRGVVAVDAG"

CDS complement(2338270..2339196)
/codon_start=1
/note="gene_2155"

/transl_table=11
/translation="VRAAGLLKRLNPRNRRSRVNPDATMSLVDHLTELRTRLLISLAA
ILVTTIFGFVWYSHSIFGLDSLGEWLRHPYCALPQSARADISADGECRLLATAPFDQF
MLRLKVGMAAGIVLACPVWFYQLWAFITPGLYQRERRFAVAFVIPAAVLFVAGAVLAY
LVLSKALGFLLTVGSDVQVTALSGDRYFGFLLNLLVVFGVSFEFPLLIVMLNLAGLLT
YERLKSWRRGLIFAMFVFAAIFTPGSDPFSMTALGAALTVLLELAIQIARVHDKRKAK
REAAIPDDEASVIDPPSPVPAPSVIGSHDDVT"
CDS complement(2339213..2339560)
/codon_start=1
/gene="tatA"
/note="gene_2156"
/transl_table=11
/translation="MHTRQLRRRDGAKSPGAAAAAVASGQRLEVIKVGSLSPWHWAIL
AVVVIVLFGAKKLPDAARSLGKSLRIFKSEVRELQNENKAEASIETPTPVQSQRVDPS
AASGQDSTEARPA"
CDS complement(2339532..2340482)
/codon_start=1
/note="gene_2157"
/transl_table=11
/translation="MSALSTRLVRLLNMVPYFQANPRITRAEAAAELGVTAKQLEEDL
NQLWMCGLPGYSPGDLIDFEFCGDTIEVTFSAGIDRPLKLTSPEATGLLVALRALADI
PGVVDPQAARSAIAKIAAAAGAVAAVAEQAPTESPAAAAVRAAVRNSRALTIDYYAAS
HDTLTTRIVDPIRVLLIGGHSYLEAWSREAEGVRLFRFDRIVDAAELGEPAVPPESAR
QAPPDTSLFDGDLSLPSATLRVAPSASWMLEYYPIRELRQLPDGSCEVAMTYASEDWM
TRLLLGFGSDVRVLAPESLAQRVRDAATAALDAYQAAAPP"
CDS complement(2340479..2341477)
/codon_start=1
/note="gene_2158"
/transl_table=11
/translation="MATSKVERLVNLVIALLSTRGYITAEKIRSSVAGYSDSPSVEAF
SRMFERDKNELRDLGIPLEVGRVSALEPTEGYRINRDAYALSPVELTPDEAAAVAVAT
QLWESPELITATQGALLKLRAAGVDVDPLDTGAPVAIASAAAVSGLRGSEDVLGILLS
AIDSGQVVQFSHRSSRAEPYTVRTVEPWGVVTEKGRWYLVGHDRDRDATRVFRLSRIG
AQVTPIGPAGATTVPAGVDLRSIVAQKVTEVPTGEQATVWVAEGRATALRRAGRSAGP
RQLGGRDGEVIELEIRSSDRLAREITGYGADAIVLQPGSLRDDVLARLRAQAGALA"
CDS complement(2341486..2342829)
/codon_start=1
/gene="pafA"
/note="gene_2159"
/transl_table=11
/translation="MGIETEFGVTCTFHGHRRLSPDEVARYLFRRVVSWGRSSNVFLR
NGARLYLDVGSHPEYATAECDSLVQLVTHDRAGEWVLEDLLVDAEQRLADEGIGGDIY
LFKNNTDSAGNSYGCHENYLIVRAGEFSRISDVLLPFLVTRQLICGAGKVLQTPKAAT
YCLSQRAEHIWEGVSSATTRSRPIINTRDEPHADAEKYRRLHVIVGDSNMSETTTMLK
VGTAALVLEMIESGVAFRDFSLDNPIRAIREVSHDVTGRRPVRLAGGRQASALDIQRE
YYTRAVEHLQTREPNAQIEQVVDLWGRQLDAVESQDFAKVDTEIDWVIKRKLFQRYQD
RYDMELSHPKIAQLDLAYHDIKRGRGIFDLLQRKGLAARVTTDEEIAEAVDQPPQTTR
ARLRGEFISAAQEAGRDFTVDWVHLKLNDQAQRTVLCKDPFRAVDERVKRLIASM"
CDS complement(2342896..2344371)
/codon_start=1
/note="gene_2160"

/transl_table=11
/translation="MSFVIASPEALLAAATDLAAIRSTIRAANAAAAVPTTGALAPAA
DEVSAGIAALFGAQAQSYQAVSAQAAAFHDRFVQLLNAGGGSYASAEIANAQQNLLNA
VNAPTQTLLGRPLVGDGADGASGPVGQPGGDGGILWGNGGNGGDSTSPGVAGGAGGSA
GLIGNGGRGGNGAPGGAGGNGGLGGLLLGNGGAGGVGGTGDNGVGDLGAGGGGGDGGL
GGRAGLIGHGGAGGNGGDGGHGGSGKAGGSGGSGGFGQFGGAGGLLYGNGGAAGSGGN
GGDAGTGVSSDGFAGLGGSGGRGGDAGLIGVGGGGGNGGDPGLGARLFQVGSRGGDGG
VGGWLYGDGGGGGDGGNGGLPFIGSTNAGNGGSARLIGNGGAGGSGGSGAPGSVSSGG
VGGAGNPGGSGGNGGVWYGNGGAGGAAGQGGPGMNTTSPGGPGGVGGHGGTAILFGDG
GAGGAGAAGGPGTPDGAAGPGGSGGTGGLLFGVPGPSGPDG"
CDS 2344554..2346206
/codon_start=1
/note="gene_2161"
/transl_table=11
/translation="MAGALFEPSFAAAHPAGLLRRPVTRTVVLSVAATSIAHMFEISL
PDPTELCRSDDGALVAAIEDCARVEAAASARRLSAIAELTGRRTGADQRADWACDFWD
CAAAEVAAALTISHGKASGQMHLSLALNRLPQVAALFLAGHLGARLFSIIAWRTYLVR
DPHALSLLDAALAEHAGAWGPLSAPKLEKAIDSWIDRYDPGALRRSRISARTRDLCIG
DPDEDAGTAALWGRLYATDAAMLDRRLTEMAHGVCEDDPRTLAQRRADALGALAAGAD
HLACGCGKPDCPSGAGNDERAAGVVIHVVADASALDAQPDPHLSGDEPPSRPLTPETT
LFEALTPDPEPDPPATHAPAELITTGGGVVPAPLLAELIRGGATISQVRHPGDLAAEP
HYRPSAKLAEFVRMRDLTCRFPGCDVPAEFCDIDHSAPWPLGPTHPSNLKCACRKHHL
LKTFWTGWRDVQLPDGTVIWTAPNGHTYTTHPGSRIFFPTWHTTTAELPQTSTAAVNV
DARGLMMPRRRRTRAAELAHRINAERALNDAYMAERNKPPSF"
CDS 2346405..2349446
/codon_start=1
/note="gene_2162"
/transl_table=11
/translation="MLVLHGFWSNSGGMRLWAEDSDLLVKSPSQALRSARPHPFAAPA
DLIAGIHPGKPATAVLLLPSLRSAPLDSPELIRLAPRPAARTDPMLLAWTVPVVDLDP
TAALAAFDQPAPDVRYGASVDYLAELAVFARELVERGRVLPQLRRDTHGAAACWRPVL
QGRDVVAMTSLVSAMPPVCRAEVGGHDPHELATSALDAMVDAAVRAALSPMDLLPPRR
GRSKRHRAVEAWLTALTCPDGRFDAEPDELDALAEALRPWDDVGIGTVGPARATFRLS
EVETENEETPAGSLWRLEFLLQSTQDPSLLVPAEQAWNDDGSLRRWLDRPQELLLTEL
GRASRIFPELVPALRTACPSGLELDADGAYRFLSGTAAVLDEAGFGVLLPSWWDRRRK
LGLVLSAYTPVDGVVGKASKFGREQLVEFRWELAVGDDPLSEEEIAALTETKSPLIRL
RGQWVALDTEQLRRGLEFLERKPTGRKTTAEILALAASHPDDVDTPLEVTAVRADGWL
GDLLAGAAAASLQPLDPPDGFTATLRPYQQRGLAWLAFLSSLGLGSCLADDMGLGKTV
QLLALETLESVQRHQDRGVGPTLLLCPMSLVGNWQQEAARFAPNLRVYAHHGGARLHG
EALRDHLERTDLVVSTYTTATRDIDELSEYEWNRVVLDEAQAVKNSLSRAAKAVRRLR
AAHRVALTGTPMENRLAELWSIMDFLNPGLLGSSERFRTRYAIPIERHGHTEPAERLR
ASTRPYILRRLKTDPAIIDDLPEKIEIKQYCQLTTEQASLYQAVVADMMEKIENTEGI
ERRGNVLAAMAKLKQVCNHPAQLLHDRSPVGRRSGKVIRLEEILEEILAEGDRVLCFT
QFTEFAELLVPHLAARFGRAARDIAYLHGGTPRKRRDEMVARFQSGDGPPIFLLSLKA
GGTGLNLTAANHVVHLDRWWNPAVENQATDRAFRIGQRRTVQVRKFICTGTLEEKIDE
MIEEKKALADLVVTDGEGWLTELSTRDLREVFALSEGAVGE"
CDS 2349439..2350272
/codon_start=1
/note="gene_2163"
/transl_table=11
/translation="VSSTWYPPPSRPRPVEGGIKARSTRGAIAQTWWSERFIAVLEDI

GLGNRLQRGRSYARKGQVISLQVDAGLVTALVQGSRARPYRIRIGIPAFGKSQWAHVE
RTLAENAWYAAKLLSGEMPEDIEDVFAGLGLSLFPGTARELSLDCSCPDYAVPCKHLA
ATFYLLAESFDEDPFAILAWRGRERE DLLANLAAARADGAAPAADHAEQVAQPLTDCL
DRYYARQADINVPSPPATPSTALLDQLPDTGLSARGRPLTELLRPAYHALTHHHNSAG
G"

CDS complement(2350251..2350685)
/codon_start=1
/note="gene_2164"
/transl_table=11
/translation="MKIVDANVLLYAVNTTSEHHKPSLRWLDGALSGADRVGFAWVPL
LAFVRLATKVGLFPRPLPREAAITQVADWLAAPSAVLVNPTVRHADILARMLTYVGTG
ANLVNDAHLAALAVEHRASIVSYDSDFGRFEGVRWDQPPALL"

CDS complement(2350692..2350946)
/codon_start=1
/note="gene_2165"
/transl_table=11
/translation="MRTTVTLDDDVEQLVRRRMAERQVSFKKALNDAIRDGASGRPAP
SHFSTRTADLGVPAVNLDRALQLAADLEDEELVRRQRRGS"

CDS 2352166..2352462
/codon_start=1
/note="gene_2166"
/transl_table=11
/translation="MSFVNVDPFGMLAAAATLESLGSHMAVSNAAVASVTTKVPPPAA
DYVSKKLSLFFSSHGQQYQVQAARGTAFHRKLVRTLANGALAYEEVEIANNEGF"

CDS 2352518..2353249
/codon_start=1
/note="gene_2167"
/transl_table=11
/translation="MPNFWALPPEINSTRIYLGPGSGPILAAAQGWNALASELEKTKV
GLQSALDTLLESYRGQSSQALIQQTLPYVQWLTTTAEHAHKTAIQLTAAANAYEQARA
AMVPPAMVRANRVQTTVLKAINWFGQFSTRIADKEADYEQMWFQDALVMENYWEAVQE
AIQSTSHFEDPPEMADDYDEAWMLNTVFDYHNENAKEEVIHLVPDVNKERGPIELVTK
VDKEGTIRLVYDGEPTFSYKEHPKF"

CDS complement(2353789..2354535)
/codon_start=1
/note="gene_2168"
/transl_table=11
/translation="VSFPYFISPEQAMRERSELARKGIARAKSVVALAYAGGVLFVAE
NPSRSLQKISELYDRVGFAAAGKFNEFDNLRRGGIQFADTRGYAYDRRDVTGRQLANV
YAQTLGTIFTEQAKPYEVELCVAEVAHYGETKPPELYRITYDGSIADEPHFVVMGGTT
EPIANALKESYAENASLTDALGIAVAALRAGSADTSGGDQPTLGVASLEVAVLDANRP
RRAFRRITGSALQALLVDQESPQSDGESSG"

CDS complement(2354532..2355407)
/codon_start=1
/note="gene_2169"
/transl_table=11
/translation="VTWPLPDRLSINSLSGTPAVDLSSFTDFLRRQAPELLPASISGG
APLAGGDAQLPHGTTIVALKYPGGVVMAGDRRSTQGNMISGRDVRKVYITDDYTATGI
AGTAAVAVEFARLYAVELEHYEKLEGVPLTFAGKINRLAIMVRGNLAAAMQGLLALPL
LAGYDIHASDPQSAGRIVSFDAAGGWNIEEEGYQAVGSGSLFAKSSMKKLYSQVTDGD
SGLRVAVEALYDAADDDSATGGPDLVRGIFPTAVIIDADGAVDVPESRIAELARAIIE

SRSGADTFGSDGGEK"
CDS complement(2355404..2355598)
/codon_start=1
/note="gene_2170"
/transl_table=11
/translation="MAQEQTKRGGGGGDDDDIAGSTAAGQERREKLTEETDDLLDEID
DVLEENAEDFVRAYVQKGGQ"
CDS complement(2355711..2357291)
/codon_start=1
/note="gene_2171"
/transl_table=11
/translation="MPASSAARCAARIVGGPRLYGMQRIIGTEVEYGISSPSDPTANP
ILTSTQAVLAYAAAAGIQRAKRTRWDYEVESPLRDARGFDLSRSAGPPPVVDADEVGA
ANMILTNGARLYVDHAHPEYSAPECTDPLDAVIWDKAGERVMEAAARHVASVPGAAKL
QLYKNNVDGKGASYGSHENYLMSRQTPFSAIITGLTPFLVSRQVVTGSGRVGIGPSGD
EPGFQLSQRSDYIEVEVGLETTLKRGIINTRDEPHADADRYRRLHVIIGDANLAETST
YLKLGTTALVLDLIEEGPAHAIDLTDLALARPVHAVHAISRDPSLRATVALADGRELT
GLALQRIYLDRVAKLVDSRDPDPRAADIVETWAHVLDQLERDPMDCAELLDWPAKLRL
LDGFRQRENLSWSAPRLHLVDLQYSDVRLDKGLYNRLVARGSMKRLVTEHQVLSAVEN
PPTDTRAYFRGECLRRFGADIAAASWDSVIFDLGGDSLVRIPTLEPLRGSKAHVGALL
DSVDSAVELVEQLTAEPR"
CDS 2357379..2358572
/codon_start=1
/note="gene_2172"
/transl_table=11
/translation="MSLSVRRPPAARAAAIVEAESWFLKRGLPSVLTMRGRCRRLWPR
SAPMLAAWAVVEGCLMAVFFVTDGGEVFISATPTTAQWVILALLAVALPLASLVGWLV
SQISSGRGQAAVATMAVAFAAASDVIESGPIQLLRTAVVVGLVLLQTGCGVGSVLGWA
VRMTLEHLATVGTLAVRALPIVLLTALVFFNTYVWLMAANINGERLPLAMVFLLAIAG
AFVVSKTVERVRPLLRSTTVMPQGSQSLAGTPFATMGDPSPGFPLTRAERLNVVFLLA
ASQLVEILVVASVGAAIYLVLGMIILTPPLLREWTHYDSMTTTVLGMTFPAPDSLIRM
CLFLGALTFMYISARAVDDAEYRAMFLDPLIDDLHTALLARNRYRNNVVTAPCAGVDA
GHVDD"
CDS 2358583..2359206
/codon_start=1
/note="gene_2173"
/transl_table=11
/translation="MSAPERVTGLSGQRYGEVLLVTPGEAGPQATVYNSFPLNDCPAE
LWSALDPQALATEHKAATALLNGPRYWLMNAIEKAPQGPPVTKTFGGIEMLQQATVLL
SSMNPAPYTVSQVSRNTVFVFNAGEEVYELQDPKGQRWVMQTWSQVVDPNLSRADLPK
LGERLNLPAGWSYHTRVLTSELRVDTTNREARVLQDDLTNSYSLVTA"
CDS complement(2359210..2361039)
/codon_start=1
/note="gene_2174"
/transl_table=11
/translation="MGESERSEAFGIPRDSPLSSGDAAELEQLRREAAVLREQLENAV
GSHAPTRSARDIHQLEARIDSLAARNSKLMETLKEARQQLLALREEVDRLGQPPSGYG
VLLATHDDDTVDVFTSGRKMRLTCSPNIDAASLKKGQTVRLNEALTVVEAGTFEAVGE
ISTLREILADGHRALVVGHADEERVVWLADPLIAEDLPDGLPEALNDDTRPRKLRPGD
SLLVDTKAGYAFERIPKAEVEDLVLEEVPDVSYADIGGLSRQIEQIRDAVELPFLHKE
LYREYSLRPPKGVLLYGPPGCGKTLIAKAVANSLAKKMAEVRGDDAHEAKSYFLNIKG

PELLNKFVGETERHIRLIFQRAREKASEGTPVIVFFDEMDSIFRTRGTGVSSDVETTV
VPQLLSEIDGVEGLENVIVIGASNREDMIDPAILRPGRLDVKIKIERPDAEAAQDIYS
KYLTEFLPVHADDLAEFDGDRSACIKAMIEKVVDRMYAEIDDNRFLEVTYANGDKEVM
YFKDFNSGAMIQNVVDRAKKNAIKSVLETGQPGLRIQHLLDSIVDEFAENEDLPNTTN
PDDWARISGKKGERIVYIRTLVTGKSSSASRAIDTESNLGQYL"

CDS 2361320..2361889
/codon_start=1
/note="gene_2175"
/transl_table=11
/translation="MRRNIRVTLGAATIVAALGLSGCSHPEFKRSSPPAPSLPPVTSS
PLEAAPITPLPAPEALIDVLSRLADPAVPGTNKVQLIEGATPENAAALDRFTTALRDG
SYLPMTFAANDIAWSDNKPSDVMATVVVTTAHPDNREFTFPMEFVSFKGGWQLSRQTA
EMLLAMGNSPDSTPSATSPAPAPSPTPPG"

CDS 2361897..2362190
/codon_start=1
/note="gene_2176"
/transl_table=11
/translation="MWIGWLEFDVLLGDVRSLKQKRSVTRPLVAELQRKFSVSAAETG
SHDLYRRAGIGVAVVSGDRSHAVDVLDNAERLVAAHPEFELLSVRRGLHRTDD"

CDS complement(2362219..2363061)
/codon_start=1
/note="gene_2177"
/transl_table=11
/translation="VSATGPFSIGERVQLTDAKGRRYTMSLTPGAEFHTHRGSIAHDA
VIGLEQGSVVKSSNGALFLVLRPLLVDYVMSMPRGPQVIYPKDAAQIVHEGDIFPGAR
VLEAGAGSGALTLSLLRAVGPAGQVISYEQRADHAEHARRNVSGCYGQPPDNWRLVVS
DLADSELPDGSVDRAVLDMLAPWEVLDAVSRLLVAGGVLMVYVATVTQLSRIVEALRA
KQCWTEPRAWETLQRGWNVVGLAVRPQHSMRGHTAFLVATRRLAPGAVAPAPLGRKRE
GRDG"

CDS 2363135..2363971
/codon_start=1
/note="gene_2178"
/transl_table=11
/translation="MADQPDPPTPRPALSPSRATDFKQCPLLYRFRAIDRLPEATSAA
QLRGSVVHAALEQLYGLPAGLRSPDTARSLVQRAWDQMVAAEPELAGELDPGQPTQLL
EDARALVSGYYRLEDPTRFDPQCCEQRVEVELADGTLLRGYIDRIDVAATGELRVVDY
KTGKAPPAARALAEFKAMFQMKFYAVALFRSRGVPPTRLRLIYLADGQLLDYSPDRDE
LLRFEKTLMAIWRAIQSAGETGDFRPNPSRLCDWCPHQQRCPAFGGTPPPYPGWPTEP
AA"

CDS complement(2363994..2364476)
/codon_start=1
/note="gene_2179"
/transl_table=11
/translation="VTHVLVLLLALLIGVVAGLRSLTAPAVVSWAAFLGWINLHGTWA
SWMGNFVTVVIVSVLAVAELVNDKRPKTPPRTVTPVFAVRIILGAFAGAVIGTAWGYR
WGGLGAGVIGAVLGTMGGYQARTRLVAARGGHDLPIALLEDSVAVLGGFAIVAAAAAL
"

CDS complement(2364555..2365409)
/codon_start=1
/gene="hisG"
/note="gene_2180"

```
                     /transl_table=11
                     /translation="MLRVAVPNKGALSEPATEILAEAGYRRRTDSKDLTVIDPVNNVE
                     FFFLRPKDIAIYVGSGELDFGITGRDLVCDSGAQVRERLALGFGSSSFRYAAPAGRNW
                     TTADLAGMRIATAYPNLVRKDLATKGIEATVIRLDGAVEISVQLGVADAIADVVGSGR
                     TLSQHDLVAFGEPLCDSEAVLIERAGTDGQDQTEARDQLVARVQGVVFGQQYLMLDYD
                     CPRSALKKATAITPGLESPTIAPLADPDWVAIRALVPRRDVNGIMDELAAIGAKAILA
                     SDIRFCRF"
     CDS             complement(2365412..2365693)
                     /codon_start=1
                     /note="gene_2181"
                     /transl_table=11
                     /translation="VQQSLAVKTFEDLFAELGDRARTRPADSTTVAALDGGVHALGKK
                     LLEEAGEVWLAAEHESNDALAEEISQLLYWTQVLMISRGLSLDDVYRKL"
     CDS             2365820..2367214
                     /codon_start=1
                     /note="gene_2182"
                     /transl_table=11
                     /translation="VTFPMWFAVPPEVPSAWLSTGMGPGPLLAAQYTEIATELASVLA
                     AVQASSWQGPSADRFVVAHQPFRYWLTHAATVATAAAAAHETAAAGYTSALGGMPTLA
                     ELAANHAMHGALVTTNFFGVNTIPIALNEADYLRMWIQAATVMSHYQAVAHESVAATP
                     STPPAPQIVTSAASSAASISFPDPTKLILQLLKDFLELLRYLAVELLPGPLGDLIAQV
                     LDWFISFVSGPVFTFLAYLVLDPLIYFGPFAPLTSPVLLPAGLTGLAGLGAVSGPAGP
                     MVERVHSDGPSRQSWPAATGVTLVGTNPAALVTTPAPAPTTSAAPTAPSTPGSSAAQG
                     LYAVGGPDGEGFNPIAKTTALAGVTTDAAAPAAKLPGDQAQSSASKATRLRRRLRQHR
                     FEFLADDGRLTMPNTPEMADVAAGNRGLDALGFAGTIPKSAPGSATGLTHLGGGFADV
                     LSQPMLPHTWDGSD"
     CDS             complement(2367211..2370789)
                     /codon_start=1
                     /note="gene_2183"
                     /transl_table=11
                     /translation="VTAADKHLYDTDLLDVLSQRVMVGDGAMGTQLQAADLTLDDFRG
                     LEGCNEILNETRPDVLETIHRNYFEAGADAVETNTFGCNLSNLGDYDIADRIRDLSQK
                     GTAIARRVADELGSPDRKRYVLGSMGPGTKLPTLGHTEYAVIRDAYTEAALGMLDGGA
                     DAILVETCQDLLQLKAAVLGSRRAMTRAGRHIPVFAHVTVETTGTMLLGSEIGAALTA
                     VEPLGVDMIGLNCATGPAEMSEHLRHLSRHARIPVSVMPNAGLPVLGAKGAEYPLLPD
                     ELAEALAGFIAEFGLSLVGGCCGTTPAHIREVAAAVANIKRPERQVSYEPSVSSLYTA
                     IPFAQDASVLVIGERTNANGSKGFREAMIAEDYQKCLDIAKDQTRDGAHLLDLCVDYV
                     GRDGVADMKALASRLATSSTLPIMLDSTETAVLQAGLEHLGGRCAINSVNYEDGDGPE
                     SRFAKTMALVAEHGAAVVALTIDEEGQARTAQKKVEIAERLINDITGNWGVDESSILI
                     DTLTFTIATGQEESRRDGIETIEAIRELKKRHPDVQTTLGLSNISFGLNPAARQVLNS
                     VFLHECQEAGLDSAIVHASKILPMNRIPEEQRNVALDLVYDRRREDYDPLQELMRLFE
                     GVSAASSKEDRLAELAGLPLFERLAQRIVDGERNGLDADLDEAMTQKPPLQIINEHLL
                     AGMKTVGELFGSGQMQLPFVLQSAEVMKAAVAYLEPHMERSDDDSGKGRIVLATVKGD
                     VHDIGKNLVDIVLSNNGYEVVNIGIKQPIATILEVAEDKSADVVGMSGLLVKSTVVMK
                     ENLEEMNTRGVAEKFPVLLGGAALTRSYVENDLAEIYQGEVHYARDAFEGLKLMDTIM
                     SAKRGEAPDENSPEAIKAREKEAERKARHQRSKRIAAQRKAAEEPVEVPERSDVAADI
                     EVPAPPFWGSRIVKGLAVADYTGLLDERALFLGQWGLRGQRGGEGPSYEDLVETEGRP
                     RLRYWLDRLSTDGILAHAAVVYGYFPAVSEGNDIVVLTEPKPDAPVRYRFHFPRQQRG
                     RFLCIADFIRSRELAAERGEVDVLPFQLVTMGQPIADFANELFASNAYRDYLEVHGIG
                     VQLTEALAEYWHRRIREELKFSGDRAMAAEDPEAKEDYFKLGYRGARFAFGYGACPDL
                     EDRAKMMALLEPERIGVTLSEELQLHPEQSTDAFVLHHPEAKYFNV"
```

CDS 2371015..2371893
/codon_start=1
/note="gene_2184"
/transl_table=11
/translation="VTPSEGNAPLPELHNTVVVAAFEGWNDAGDAASDAVAHLAASWQ
ALPIVEIDDEAYYDYQVNRPVIRQVDGVTRELQWPAMRISHCRPPGSDRDVVLMCGVE
PNMRWRTFCDELLAVIDKLNVDTVVILGALLADTPHTRPVPVSGAAYSAASARQFGLQ
ETRYEGPTGIAGVFQSACVGAGIPAVTFWAAVPHYVSHPPNPKATIALLRRVEDVLDV
EVPLADLPAQAEAWEREITETIAEDHELAEYVQTLEQHGDAAVDMNEALGNIDGDALA
AEFERYLRRRRPGFGR"
CDS complement(2371924..2372694)
/codon_start=1
/gene="PE_PGRS37"
/note="gene_2185"
/transl_table=11
/translation="LIGDGANGGPGQPGGPGGLLYGNGGHGGAGAAGQDRGAGNSAGL
IGNGGAGGAGGNGGIGGAGAPGGLGGDGGKGGFADEFTGGFAQGGRGGFGGNGNTGAS
GGMGGAGGAGGAGGAGGLLIGDGGAGGAGGIGGAGGVGGGGGAGGTGGGGVASAFGGG
NAFGGRGGDGGDGGDGGTGGAGGARGAGGAGGAGGWLSGHSGAHGAMGSGGEGGAGGG
GGARGEAGAGGGTSTGTNPGKAGAPGTQGDSGDPGPPG"
CDS complement(2372691..2372897)
/codon_start=1
/note="gene_2186"
/transl_table=11
/translation="MAIAALFGANAQEYQQISAQAAAYLAQFVAALTSGGGWYAAAET
ANATLAHEVLNAVNAPTQALLAGR"
CDS 2373338..2374807
/codon_start=1
/gene="ansP1"
/note="gene_2187"
/transl_table=11
/translation="LSAASQRVDAFGEEAGYHKGLKPRQLQMIGIGGAIGTGLFLGAS
GRLAKAGPGLFLVYGVCGVFVFLILRALGELVLHRPSSGSFVSYAREFFGEKAAYAVG
WMYFLHWAMTSIVDTTAIATYLQRWTIFTVVPQWILALIALTVVLSMNLISVEWFGEL
EFWAALIKVLALMAFLVVGTVFLAGRYPVDGHSTGLSLWNNHGGLFPTSWLPLLIVTS
GVVFAYSAVELVGTAAGETAEPEKIMPRAINSVVARIAIFYVGSVALLALLLPYTAYK
AGESPFVTFFSKIGFHGAGDLMNIVVLTAALSSLNAGLYSTGRVMHSIAMSGSAPRFT
ARMSKSGVPYGGIVLTAVITLFGVALNAFKPGEAFEIVLNMSALGIIAGWATIVLCQL
RLHKLANAGIMQRPRFRMPFSPYSGYLTLLFLLVVLVTMASDKPIGTWTVATLIIVIP
ALTAGWYLVRKRVMAVARERLGHTGPFPAVANPPVRSRD"
CDS 2374807..2375010
/codon_start=1
/note="gene_2188"
/transl_table=11
/translation="MLRRGESIIRNRYASKPPLYGMAMVFLAMAVVAVTAYFRMGWWS
IIGYAAAAIIGVIGFALAFRDLS"
CDS complement(2375030..2375911)
/codon_start=1
/note="gene_2189"
/transl_table=11
/translation="MTSLQGKVVFITGAARGIGAEVARRLHNKGAKLVLTDLSKSELA

VMGAELGGDDRLLTVVADVRDLPAMQAAAETAVERFGGIDVVVANAGIASYGSVLKVD
PQAFRRVLDVNLLGNFHTVRATLPALIDRRGYVLIVSSLAAFAAPPGMAPYNMSKAGN
EHFANALRLEVAHLGVSVGSAHMSWIDTALVRDTKADLPAFAELLARLPWPLNKTTSV
NKCAAAFVNGIEGRKDRVYCPGWVALFRWLKPLLSTRVGQRPIRNTVAKLMPQMDAEV
AALGRFASAYTESLENS"
CDS complement(2375937..2377181)
/codon_start=1
/note="gene_2190"
/transl_table=11
/translation="MQSWYCPPVPVLPGRGPQLRLYDSADRQVRPVAPGSKATMYVCG
ITPYDATHLGHAATYVTFDLIHRLWLDLGHELHYVQNITDIDDPLFERADRDGVDWRD
LAQAEVALFCEDMAALRVLPPQDYVGATEAIAEMVELIEKMLACGAAYVIDREMGEYQ
DIYFRADATLQFGYESGYDRDTMLRLCEERGGDPRRPGKSDELDALLWRAARPGEPSW
PSPFGPGRPGWHVECAAIALSRIGSGLDIQGGGSDLIFPHHEFTAAHAECVSGERRFA
RHYVHAGMIGWDGHKMSKSRGNLVLVSALRAQDVEPSAVRLGLLAGHYRADRFWSQQV
LDEATARLHRWRTATALPAGPAAVDVVARVRRYLADDLDTPKAIAALDGWVTDAVEYG
GHDAGAPKLVATAIDALLGVDL"
CDS complement(2377239..2378042)
/codon_start=1
/note="gene_2191"
/transl_table=11
/translation="VVSPAAPDLTDDLTDAELAADLAADAGKLLLQVRAEIGFDQPWT
LGEAGDRQANSLLLRRLQAERPGDAVLSEEAHDDLARLKSDRVWIIDPLDGTREFSTP
GRDDWAVHIALWRRSSNGQPEITDAAVALPARGNVVYRTDTVTSGAAPAGVPGTLRIA
VSATRPPAVLHRIRQTLAIQPVSIGSAGAKAMAVIDGYVDAYLHAGGQWEWDSAAPAG
VMLAAGMHASRLDGSPLRYNQLDPYLPDLLMCRAEVAPILLGAIADAWR"
CDS 2378133..2378363
/codon_start=1
/note="gene_2192"
/transl_table=11
/translation="MRTTVSLADDVAAAVQRLRKERSIGLSEAVNELIRAGLTKRQVA
NRFQQQTYDMGEGIDYSNIGDAIETLDGPASG"
CDS complement(2378573..2379388)
/codon_start=1
/note="gene_2193"
/transl_table=11
/translation="MTLRDDEHAVLADGELTVLGRIRSASNATFLCESTLGLRSLHCV
YKPVSGERPLWDFPDGTLAGRELSAYLVSTQLGWNLVPHTIIRDGPAGIGMLQLWVQQ
PGDAVDSDPLPGPDLVDLFPAHRPRPGYLPVLRAYDYAGDEVVLMHADDIRLRRMAVF
DVLINNADRKGGHILCGIDGQVYGVDHGLCLHVENKLRTVLWGWAGKPIDDQILQAVA
GLADALGGPLAEALAGRIAAAEIGALRRRAQSLLDQPVMPGPNGHRPIPWPAF"
CDS complement(2379372..2379959)
/codon_start=1
/note="gene_2194"
/transl_table=11
/translation="MARAIHVFRTPDRFVAGTVGQPGNRTFYLQAVHDSRVVSVVLEK
QQVAVLAERIGALLFEVNRRFGTPVPPEPTEIDDLSPLIMPVDAEFRVGTMGLGWDSE
AQSVVVELLAVTDAEFDASVVLDDTEEGPDAVRVFLTPESARQFATRSYRVISAGRPP
CPLCDEPLDPEGHICARTNGYRRDVLLGSGDDPAG"
CDS complement(2380023..2380733)
/codon_start=1

/note="gene_2195"
/transl_table=11
/translation="MTVILLRHARSTSNTAGVLAGRSGVDLDEKGREQATGLIDRIGD
LPIRAVASSPMLRCQRTVEPLAEALCLEPLIDDRFSEVDYGEWTGRKIGDLVDEPLWR
VVQAHPSAAVFPGGEGLAQVQTRAVAAVREHDRRLADQHGHDVLWLACTHGDVIKAVI
ADAFGMHLDSFQRITADPGSVSVVRYTQLRPFVLHVNHTGARLAPALQAAASAQGASP
EPNAAVPPGDAVIGGSTD"
CDS complement(2380730..2381473)
/codon_start=1
/note="gene_2196"
/transl_table=11
/translation="VSRIFFSGDAGASFTAVSQLGTEAAVVIYFARDIVRILSAWLHG
LVVKAHRNTDYRLGWYVIIGTIPICILGLFFKDDIRSGVRNLWVVVTALVVFSGVIAL
AEYVGRQSRHIERLTWRDAVVVGIAQTLALVPGVSRSGSTISAGLFLGLDRELAARFG
FLLAIPAVFASGLFSLPDAFHPVTEGMSATGPQLLVATLIAFVLGLTAVAWLLRFLVR
HNMYWFVGYRVLVGTGMLVLLATGTVAAT"
CDS 2381409..2381627
/codon_start=1
/gene="bthur0005_12730"
/note="gene_2197"
/transl_table=11
/translation="VPNWLTAVNEAPASPLKKIRDTIARCPEDDTGRNSVKPWAAANT
MTCHQDIAGAAVTTTTVPGYRRPVDGCA"
CDS complement(2381624..2381932)
/codon_start=1
/note="gene_2198"
/transl_table=11
/translation="MTVTADRHLADKREEFAVEDISTGIFASGYGQVGDGRSFSFHIE
HRSLVVEIYRPRVAGPVPQAEDVVAMAVRGLVDIDLTDERSLAAAVRDSVASAAPVSR
"
CDS 2382232..2383128
/codon_start=1
/note="gene_2199"
/transl_table=11
/translation="VRPLSGHPRAALFDNGTRQLVALRPGADSAAPASIMVFDDVHVA
PRVIFLPGPAAALTSDDHGTAFLAARGGYFVADLSSGHTARVNVADAAHTDFTAIARR
SDGKLVLGSADGAVYTLAKNPAVDPASGAATVASRTKIFARVDALVTQGNTTVVLDRG
QTSVTTIGADGHAQQALRAGQGATTMAADPLGRVLIADTRGGQLLVYGVDPLILRQAY
PVRQAPYGLAGSRELAWVSQTASNTVIGYDLTTGIPVEKVRYPTVQQPNSLAFDETSD
TLYVVSGSGAGVQVIEHAAGTR"
CDS 2383125..2383406
/codon_start=1
/note="gene_2200"
/transl_table=11
/translation="MSSRPAARRTWLPTGWGSEMSDEYEWAPLRLPPEVTRVSASTRL
SIEAEYRGWELARVRLYTDGSRRVLLRRKKSRWADAEANRRPDQPQLWL"
CDS 2383397..2384515
/codon_start=1
/note="gene_2201"
/transl_table=11
/translation="VALKAGASPRADRYRMYPLVRRLLFLIPPEHAHKLVFAVLRGVA

```
                     AVAPVCRLLRRLLGPTDPVLASTVFGVRFPAPLGLAAGFDKDGTALSSWGAMGFGYAE
                     IGTVTAHPQPGNPAPRLFRLADDRALLNRMGFNNHGARALAIRLARHRPEIPIGVNIG
                     KTKKTPAGDAVNDYRASARMVGPLASYLVVNVSSPNTPGLRDLQAVESLRPILSAVRA
                     ETSTPVLVKIAPDLSDSDLDDIADLAVELDLAGIVATNTTVSRDGLTTPGVDRLGPGG
                     ISGPPLAQRAVQVLRRLYDRVGDRLALISVGGIETADDAWERITAGASLLQGYTGFIY
                     GGERWAKDIHEGIARRLHDGGFGSLHEAVGSARRRQPS"
CDS                  complement(2384520..2385050)
                     /codon_start=1
                     /note="gene_2202"
                     /transl_table=11
                     /translation="MTTSPDPYAALPKLPSFSLTSTSITDGQPLATPQVSGIMGAGGA
                     DASPQLRWSGFPSETRSFAVTVYDPDAPTLSGFWHWAVANLPANVTELPEGVGDGREL
                     PGGALTLVNDAGMRRYVGAAPPPGHGVHRYYVAVHAVKVEKLDLPEDASPAYLGFNLF
                     QHAIARAVIFGTYEQR"
CDS                  complement(2385098..2386444)
                     /codon_start=1
                     /note="gene_2203"
                     /transl_table=11
                     /translation="VTDETGASSDHSDDVAQVVSRLIRFDTTNSGEPGTTKGEAECAR
                     WVAEQLAEVGYQPEYVESGAPGRGNVFARLAGADSSRGALLIHGHLDVVPAEPAEWSV
                     HPFSGAIEDGYVWGRGAVDMKDMVGMMIVVARHLRQAAIVPPRDLVFAFVADEEHGGK
                     YGSHWLVDNRPDLFDGITEAIGEVGGFSLTVPRHDGGERRLYLIETAEKGIQWMRLTA
                     RGRAGHGSMVHDQNAVTAVCEAVARLGRHQFPLVCTDTVAQFLAVVGEETGLAFDLDS
                     PDLAGTIDKLGPMARMLKAVLHDTANPTMLKAGYKANVVPATAEAVVDCRVLPGRRAA
                     FEAEVDALIGPDVTREWVSDLPSYETTFDGDLVAAMNAAVLAVDPDGRTVPYMLSGGT
                     DAKAFARLGIRCFGFSPLRLPPDLDFTSLFHGVDERVPIDGLRFGTEVLTHLLTHC"
variation            2386557..2386672
                     /note="supplemented from AL2122/97"
tRNA                 2386825..2386913
                     /note="identified by tRNAscan-SE; putative"
CDS                  complement(2387031..2387348)
                     /codon_start=1
                     /note="gene_2204"
                     /transl_table=11
                     /translation="MTRRLRVHNGVEDDLFEAFSYYADAAPDQIDRLYNLFVDAVTKR
                     IPQAPNAFAPLFKHYRHIYLRPFRYYVAYRTTDEAIDILAVRHGMENPNAVEAEISGR
                     TFE"
CDS                  complement(2387345..2387560)
                     /codon_start=1
                     /note="gene_2205"
                     /transl_table=11
                     /translation="VVVNRALLASVDALSRDEQIELVEHINGNLAEGMHISEANQALI
                     EARANDTDDAHWSTIDDFDKRIRARLG"
CDS                  complement(2387884..2388069)
                     /codon_start=1
                     /gene="DOK_13684"
                     /note="gene_2206"
                     /transl_table=11
                     /translation="VPPVRRALGNLIRIGEWDPTDHARYPIMVARSGQQRLILGRIVR
                     RLDSDCVMHSCRLQFRQ"
CDS                  2388259..2388873
```

                     /codon_start=1
                     /note="gene_2207"
                     /transl_table=11
                     /translation="LADELASYRGRDRLLVLGLARGGVPVGWEVASALGAELDVFLVR
                     KLGVPQWRELAMGALASGGGVVMNDDVVSSLRITDQQVRAAIDSETAELQRRELAYRG
                     GRPVVDPRARIVILVDDGIATGASMLAAVRTIRATGPESIVVAVPVGPATACRELAAE
                     ADDVVCATMPAAFEAVGQVYNDFHQVTDDEVRELLATPTTGAAT"
     CDS   complement(2389003..2389359)
                     /codon_start=1
                     /note="gene_2208"
                     /transl_table=11
                     /translation="MLIIALVLALIGLLALVFAVVTSNQLVAWVCIGASVLGVALLIV
                     DALRERQQGGADEADGAGETGVAEEADVDYPEEAPEESQAVDAGVIGSEEPSEEASEA
                     TEESAVSADRSDDSAK"
     CDS   complement(2389454..2390236)
                     /codon_start=1
                     /note="gene_2209"
                     /transl_table=11
                     /translation="MPLTPADVHNVAFSKPPIGKRGYNEDEVDAFLDLVENELTRLIE
                     ENSDLRQRINELDQELAAGGGAGVTPQATQAIPAYEPEPGKPAPAAVSAGMNEEQALK
                     AARVLSLAQDTADRLTNTAKAESDKMLADARANAEQILGEARHTADATVAEARQRADA
                     MLADAQSRSEAQLRQAQEKADALQADAERKHSEIMGTINQQRAVLEGRLEQLRTFERE
                     YRTRLKTYLESQLEELGQRGSAAPVDSNADAGGFDQFNRGKN"
     CDS   complement(2390504..2390794)
                     /codon_start=1
                     /note="gene_2210"
                     /transl_table=11
                     /translation="LVVFFQILGFALFIFWLLLIARVVVEFIRSFSRDWRPTGVTVVI
                     LEIIMSITDPPVKVLRRLIPQLTIGAVRFDLSIMVLLLVAFIGMQLAFGAAA"
     CDS   complement(2390956..2391612)
                     /codon_start=1
                     /note="gene_2211"
                     /transl_table=11
                     /translation="MSTLHKVKAYFGMAPMEDYDDEYYDDRAPSRGYARPRFDDDYGR
                     YDGRDYDDARSDSRGDLRGEPADYPPPGYRGGYADEPRFRPREFDRAEMTRPRFGSWL
                     RNSTRGALAMDPRRMAMMFEDGHPLSKITTLRPKDYSEARTIGERFRDGSPVIMDLVS
                     MDNADAKRLVDFAAGLAFALRGSFDKVATKVFLLSPADVDVSPEERRRIAETGFYAYQ
                     "
     CDS   complement(2391678..2392226)
                     /codon_start=1
                     /note="gene_2212"
                     /transl_table=11
                     /translation="MAELNRLLAAAELGHSGGVHWHMVGRIQRNKAGSLARWAHTAHS
                     VDSSRLVTALDRAVVAALAEHRRGERLRVYVQVSLDGDGSRGGVDSTTPGAVDRICAQ
                     VQESEGLELVGLMGIPPLDWDPDEAFDRLQSEHNRVRAMFPHAIGLSAGMSNDLEVAV
                     KHGSTCVRVGTALLGPRRLRSP"
     CDS   complement(2392460..2393122)
                     /codon_start=1
                     /gene="yfiH"
                     /note="gene_2213"
                     /transl_table=11

/translation="VSAPPFDTFNLGDHVGDDPAAVAANRARLAAAIGLPGNRVVWMN
QVHGDRVELVDQPRNTALDDTDGLVTATPRLALAVVTADCVPVLMADARAGIAAAVHA
GRAGAQRGVVVRALEVMLSLGAQVRDISALLGPAVSGRNYEVPAAMADEVEAALPGSR
TTTAAGTPGVDLRAGIACQLRDLGVESIDVDPRCTVADPTLFSHRRDAPTGRFASLVW
ME"
CDS complement(2393223..2394362)
/codon_start=1
/note="gene_2214"
/transl_table=11
/translation="MTPPHNYLAVIKVVGIGGGGVNAVNRMIEQGLKGVEFIAINTDA
QALLMSDADVKLDVGRDSTRGLGAGADPEVGRKAAEDAKDEIEELLRGADMVFVTAGE
GGGTGTGGAPVVASIARKLGALTVGVVTRPFSFEGKRRSNQAENGIAALRESCDTLIV
IPNDRLLQMGDAAVSLMDAFRSADEVLLNGVQGITDLITTPGLINVDFADVKGIMSGA
GTALMGIGSARGEGRSLKAAEIAINSPLLEASMEGAQGVLMSIAGGSDLGLFEINEAA
SLVQDAAHPDANIIFGTVIDDSLGDEVRVTVIAAGFDVSGPGRKPVMGETGGAHRIES
AKAGKLTSTLFEPVDAVSVPLHTNGATLSIGGDDDDVDVPPFMRR"
CDS complement(2394535..2395479)
/codon_start=1
/note="gene_2215"
/transl_table=11
/translation="MTEHNEDPQIERVADDAADEEAVTEPLATESKDEPAEHPEFEGP
RRRARRERAERRAAQARATAIEQARRAAKRRARGQIVSEQNPAKPAARGVVRGLKALL
ATVVLAVVGIGLGLALYFTPAMSAREIVIIGIGAVSREEVLDAARVRPATPLLQIDTQ
QVADRVATIRRVASARVQRQYPSALRITIVERVPVVVKDFSDGPHLFDRDGVDFATDP
PPPALPYFDVDNPGPSDPTTKAALQVLTALHPEVASQVGRIAAPSVASITLTLADGRV
VIWGTTDRCEEKAEKLAALLTQPGRTYDVSSPDLPTVK"
CDS complement(2395476..2396960)
/codon_start=1
/gene="murC"
/note="gene_2216"
/transl_table=11
/translation="VSTEQLPPDLRRVHMVGIGGAGMSGIARILLDRGGLVSGSDAKE
SRGVHALRARGALIRIGHDASSLDLLPGGATAVVTTHAAIPKTNPELVEARRRGIPVV
LRPAVLAKLMAGRTTLMVTGTHGKTTTTSMLIVALQHCGLDPSFAVGGELGEAGTNAH
HGSGDCFVAEADESDGSLLQYTPHVAVITNIESDHLDFYGSVEAYVAVFDSFVERIVP
GGALVVCTDDPGGAALAQRATELGIRVLRYGSVPGETMAATLVSWQQQGVGAVAHIRL
ASELATAQGPRVMRLSVPGRHMALNALGALLAAVQIGAPADEVLDGLAGFEGVRRRFE
LVGTCGVGKASVRVFDDYAHHPTEISATLAAARMVLEQGDGGRCMVVFQPHLYSRTKA
FAAEFGRALNAADEVFVLDVYGAREQPLAGVSGASVAEHVTVPMRYVPDFSAVAQQVA
AAASPGDVIVTMGAGDVTLLGPEILTALRVRANRSAPGRPGVLG"
CDS complement(2396957..2398033)
/codon_start=1
/note="gene_2217"
/transl_table=11
/translation="MAVADALVALDPRVRITALGTPRGLETRLVPQRGYHLELITAVP
MPRKPGGDLARLPSRVWRAVREARDVLDDVDADVVVGFGGYVALPAYLAARGLPLPPR
RRRRIPVVIHEANARAGLANRVGAHTADRVLSAVPDSGLRRAEVVGVPVRASIAALDR
AVLRAEARAHFGFPDDARVLLVFGGSQGAVSLNRAVSGAAADLAAAGVCVLHAHGPQN
VLELRRRAQGDPPYVAVPYLDRMELAYAAADLVICRAGAMTVAEVSAVGLPAIYVPLP
IGNGEQRLNALPVVNAGGGMVVADAALTPELVARQVAGLLTDPARLAAMTAAAARVGH
RDAAGQVARAALAVATGAGARTTT"

```
CDS             complement(2398089..2399759)
                /codon_start=1
                /note="gene_2218"
                /transl_table=11
                /translation="VLTRLLRRGTSDTDGSQTRGAEPVEGQRTGPEEASNPGSARPRT
                RFGAWLGRPMTSFHLIIAVAALLTTLGLIMVLSASAVRSYDDDGSAWVIFGKQVLWTL
                VGLIGGYVCLRMSVRFMRRIAFSGFAITIVMLVLVLVPGIGKEANGSRGWFVVAGFSM
                QPSELAKMAFAIWGAHLLAARRMERASLREMLIPLVPAAVVALALIVAQPDLGQTVSM
                GIILLGLLWYAGLPLRVFLSSLAAVVVSAAILAVSAGYRSDRVRSWLNPENDPQDSGY
                QARQAKFALAQGGIFGDGLGQGVAKWNYLPNAHNDFIFAIIGEELGLVGALGLLGLFG
                LFAYTGMRIASRSADPFLRLLTATTTLWVLGQAFINIGYVIGLLPVTGLQLPLISAGG
                TSTAATLSLIGIIANAARHEPEAVAALRAGRDDKVNRLLRLPLPEPYLPPRLEAFRDR
                KRANPQPAQTQPARKTPARRPDSLPGRWACPRDPARPARPIRRFVDQCIMELASGTRA
                SVAHGAFGHWKVSVTGEGHGQPAGRRARGNGAPARRCRLAVLWFLAVC"
CDS             complement(2399771..2401240)
                /codon_start=1
                /note="gene_2219"
                /transl_table=11
                /translation="VPDVLDPLGPGAPVLVAGGRVTGQAVAAVLTRFGATPTVCDDDP
                VMLRPHAERGLPTVSSSDAVQQITGYALVVASPGFSPATPLLAAAAAAGVPIWGDVEL
                AWRLDAAGCYGPPRSWLVVTGTNGKTTTTSMLHAMLIAGGRRAVLCGNIGSAVLDVLD
                EPAELLAVELSSFQLHWAPSLRPEAGAVLNIAEDHLDWHATMAEYTAAKARVLTGGVA
                VAGLDDSRAAALLDGSPAQVRVGFRLGEPAAGELGVRDAHLVDRAFSDDLTLLPVASI
                PVPGPVGVLDALAAAALARSVGVPAGAIADAVTSFRVGRHRAEVVAVADGITYVDDSK
                ATNPHAARASVLAYPRVVWIAGGLLKGASLHAEVAAMASRLVGAVLIGRDRAAVAEAL
                SRHAPDVPVVQVVAGEDTGMPATVEVPVACVLDVAKDDKAGETVGAAVMTAAVAAARR
                MAQPGDTVLLAPAGASFDQFTGYADRGEAFATAVRAVIR"
CDS             complement(2401233..2402330)
                /codon_start=1
                /note="gene_2220"
                /transl_table=11
                /translation="MRECAPMRQILIAVAVAVTVSILLTPVLIRLFTKQGFGHQIRED
                GPPSHHTKRGTPSMGGVAILAGIWAGYLGAHLAGLAFDGEGIGASGLLVLGLATALGG
                VGFIDDLIKIRRSRNLGLNKTAKTVGQITSAVLFGVLVLQFRNAAGLTPGSADLSYVR
                EIATVTLAPVLFVLFCVVIVSAWSNAVNFTDGLDGLAAGTMAMVTAAYVLITFWQYRN
                ACVTAPGLGCYNVRDPLDLALIAAATAGACIGFLWWNAAPAKIFMGDTGSLALGGVIA
                GLSVTSRTEILAVVLGALFVAEITSVVLQILTFRTTGRRMFRMAPFHHHFELVGWAET
                TVIIRFWLLTAITCGLGVALFYGEWLAAVGA"
CDS             complement(2402309..2403841)
                /codon_start=1
                /note="gene_2221"
                /transl_table=11
                /translation="MIELTVAQIAEIVGGAVADISPQDAAHRRVTGTVEFDSRAIGPG
                GLFLALPGARADGHDHAASAVAAGAAVVLAARPVGVPAIVVPPVAAPNVLAGVLEHDN
                DGSGAAVLAALAKLATAVAAQLVAGGLTIIGITGSSGKTSTKDLMAAVLAPLGEVVAP
                PGSFNNELGHPWTVLRATRRTDYLILEMAARHHGNIAALAEIAPPSIGVVLNVGTAHL
                GEFGSREVIAQTKAELPQAVPHSGAVVLNADDPAVAAMAKLTAARVVRVSRDNTGDVW
                AGPVSLDELARPRFTLHAHDAQAEVRLGVCGDHQVTNALCAAAVALECGASVEQVAAA
                LTAAPPVSRHRMQVTTRGDGVTVIDDAYNANPDSMRAGLQALAWIAHQPEATRRSWAV
                LGEMAELGEDAIAEHDRIGRLAVRLDVSRLVVVGTGRSISAMHHGAVLEGAWGSGEAT
                ADHGADRTAVNVADGDAALALLRAELRPGDVVLVKASNAAGLGAVADALVADDTCGSV
```

```
                     RP"
CDS                  complement(2403838..2405391)
                     /codon_start=1
                     /gene="murE"
                     /note="gene_2222"
                     /transl_table=11
                     /translation="VEAAPTGLRPNAVVGVRLAALADQVGAALAEGPAQRAVTEDRTV
                     TGVTLRAQDVSPGDLFAALTGSTTHGARHVGDAIARGAVAVLTDPAGVAEIAGRAAVP
                     VLVHPAPRGVLGGLAATVYGHPSERLTVIGITGTSGKTTTTYLVEAGLRAAGRVAGLI
                     GTIGIRVGGADLPSALTTPEAPTLQAMLAAMVERGVDTVVMEVSSHALALGRVDGTRF
                     AVGAFTNLSRDHLDFHPSMADYFEAKASLFDPDSALRARTAVVCIDDDAGRAMAARAA
                     DAITVSAADRPAHWRATDVAPTDAGGQQFTAIDPAGVGHHIGIRLPGRYNVANCLVAL
                     AILDTVGVSPEQAVPGLREIRVPGRLEQIDRGQGFLALVDYAHKPEALRSVLTTLAHP
                     DRRLAVVFGAGGDRDPGKRAPMGRIAAQLADLVVVTDDNPRDEDPTAIRREILAGAAE
                     VGGDAQVVEIADRRDAIRHAVAWARPGDVVLIAGKGHETGQRGGGRVRPFDDRVELAA
                     ALEALERRA"
CDS                  complement(2405468..2406388)
                     /codon_start=1
                     /note="gene_2223"
                     /transl_table=11
                     /translation="MLSPDEGLLTAGWATLRETLLVGQVPRGRKEAVAAAVAASLRCP
                     WCVDAHTTMLYAAGQTDTAAAILAGTAPAAGDPNAPYVAWAAGTGTPAGPPAPFGPDV
                     AAEYLGTAVQFHFIARLVLVLLDETFLPGGPRAQQLMRRAGGLVFARKVRAEHRPGRS
                     TRRLEPRTLPDDLAWATPSEPIATAFAALSHHLDTAPHLPPPTRQVVRRVVGSWHGEP
                     MPMSSRWTNEHTAELPADLHAPTRLALLTGLAPHQVTDDDVAAARSLLDTDAALVGAL
                     AWAAFTAARRIGTWIGAAAEGQVSRQNPTG"
CDS                  complement(2406499..2407119)
                     /codon_start=1
                     /note="gene_2224"
                     /transl_table=11
                     /translation="MPSADVGRQTRAQILRAAMDIASVKGLSGLSIGELAGRLGMSKS
                     GLFRHFGAKEQLQLATVEAAVSVFEAEVVAPAMAAPPGVDRVRALMHAWVGYLERDVF
                     PGGCFFAAAAADVDSQPGPVRDRIAATGRAGIAAITADVETAQRRGEIRADIEARQLA
                     FELHAYAMEANWALLLLDDDGAGERARTAIDAALARVGTTQEGVES"
CDS                  complement(2407112..2407978)
                     /codon_start=1
                     /note="gene_2225"
                     /transl_table=11
                     /translation="MLVSLMQFVTDLTPPPQLVAVWAEERGFAGLYVPEKTHVPISRS
                     TPWPGGELPDWYRRCYDPVVALAAAAAVTTRLRVGTGACLVAVHDPILLAKQIASLCA
                     MSGERFVLGVGFGWNVEELADHGVPFADRIAVTVDKLAAMRALWAAEPVHYEGTHASV
                     PPSWAWPKPAVAPPVLFGCRPSARAFEVIARHGDGWQPIEGYGELLGALPMLHAAFER
                     AGRDPATAQVCVYSSAGDPATLHEYRRAGVAEVALALPSAGRDQVLAALDRSAPLVDA
                     FAGDDREVKSHA"
CDS                  complement(2408081..2409553)
                     /codon_start=1
                     /note="gene_2226"
                     /transl_table=11
                     /translation="MSFVIAAPEVMAAAATDLANIGSSISAASAAAAGPTMGILAAGA
                     DEVSVAISALFGSHAQGYQTLSAQLAAYHNQFVRALNAGAGSYASAEAANVQQTLLNA
                     INAPTQTLLGRPLIGNGADGGPGQNGGPGGLLYGNGGNGGAGDTANPNGGNGGSAGLI
```

GNGGAGGAGAATGAGGAGGNGGWLYGNGGPGGAAGLGTAGGVSPAGGAGGAAGLWGHG
GAGGAGGSASGAPGAGGAGGDGGRGGLLYGDGGAGGAGGNGSNGVTGVHGGNGGAGGA
AGLIGNGGAGGDGGNGGLSNTGASGGAGGAGGAALIGNGGDGGHGGNGGHGNSGGAGG
AGGAGGHVGLIGNGGNGGAGGNGGNDNSSTLADAGSGGAGAAGGNGGLFYGNGGVGGR
GGNGGQAPTPGNAGDGGAGGNARLIGDGGRGGNGGEGGDGPPGVKGDGGNGGNGGNAV
VIGNGGNGGAGGFGIPVGSGGAGGSRGVLFGTPGANGADG"

CDS complement(2409763..2411508)
/codon_start=1
/note="gene_2227"
/transl_table=11
/translation="MLVAATQLFFLQVSHAAGLRAQAAGQLKVTDVQPAARGSIVDRN
NDRLAFTIEARALTFQPKRIRRQLEEARKKTSAAPDPQQRLRDIAQEVAGKLNNKPDA
AAVLKKLQSDETFVYLARAVDPAVASAICAKYPEVGAERQDLRQYPGGSLAANVVGGI
DWDGHGLLGLEDSLDAVLAGTDGSVTYDRGSDGVVIPGSYRNRHKAVHGSTVVLTLDN
DIQFYVQQQVQQAKNLSGAHNVSAVVLDAKTGEVLAMANDNTFDPSQDIGRQGDKQLG
NPAVSSPFEPGSVNKIVAASAVIEHGLSSPDEVLQVPGSIQMGGVTVHDAWEHGVMPY
TTTGVFGKSSNVGTLMLSQRVGPERYYDMLRKFGLGQRTGVGLPGESAGLVPPIDQWS
GSTFANLPIGQGLSMTLLQMTGMYQAIANDGVRVPPRIIKATVAPDGSRTEEPRPDDI
RVVSAQTAQTVRQMLRAVVQRDPMGYQQGTGPTAGVPGYQMAGKTGTAQQINPGCGCY
FDDVYWITFAGIATADNPRYVIGIMLDNPARNSDGAPGHSAAPLFHNIAGWLMQRENV
PLSPDPGPPLVLQAT"

CDS complement(2411799..2412761)
/codon_start=1
/note="gene_2228"
/transl_table=11
/translation="MLSPFDRPAPAKNTSQAKARAKARKAKAPKLVRPTPMERLAARL
TSIDLRPRTLANKVPFVVLVIGSLGVGLGLTLWLSTDAAERSYQLSNARERTRMLQQH
KEALERDVREAASAPALAEAARRQGMIPTRDTAHLVQDPDGNWVVVGTPKPADGVPPP
PLNTKLPEDPPPPPKPAAVPLEVPVRVTPGPDDPAPPARSGPEVLVRTPDGTATLGGA
THLPTQAGPQLPGPVPIPGAPGPMPAPPLGAVPSPAPAENPVPLQVGAAPPAGLPGPA
PVAATPGLSGGSQPMVAPPAPVPANGEQFGPVTAPVPTAPGAPR"

CDS complement(2412950..2413984)
/codon_start=1
/gene="mraW"
/note="gene_2229"
/transl_table=11
/translation="VADPGSGPTGFGHVPVLAQRCFELLTPALTRYYPDGSQAVLLDA
TIGAGGHAERFLEGLPGLRLIGLDRDPTALDVARSRLVRFADRLTLVHTRYDCLGAAL
AESGYAAVGSVDGILFDLGVSSMQLDRAERGFAYATDAPLDMRMDPTTPLTAADIVNT
YDEAALADILRRYGEERFARRIAAGIVRRRAKTPFTSTAELVALLYQAIPAPARRVGG
HPAKRTFQALRIAVNDELESLRTAVPAALDALAIGGRIAVLAYQSLEDRIVKRVFAEA
VASATPAGLPVELPGHEPRFRSLTHGAERASVAEIERNPRSTPVRLRALQRVEHRAQS
QQWATEKGDS"

CDS complement(2414142..2414573)
/codon_start=1
/note="gene_2230"
/transl_table=11
/translation="MFLGTYTPKLDDKGRLTLPAKFRDALAGGLMVTKSQDHSLAVYP
RAAFEQLARRASKAPRSNPEARAFLRNLAAGTDEQHPDSQGRITLSADHRRYASLSKD
CVVIGAVDYLEIWDAQAWQNYQQIHEENFSAASDEALGDIF"

CDS complement(2414922..2415326)

```
                     /codon_start=1
                     /note="gene_2231"
                     /transl_table=11
                     /translation="MPLSDHEQRMLDQIESALYAEDPKFASSVRGGGFRAPTARRRLQ
                     GAALFIIGLGMLVSGVAFKETMIGSFPILSVFGFVVMFGGVVYAITGPRLSGRMDRGG
                     SAAGASRQRRTKGAGGSFTSRMEDRFRRRFDE"
     CDS             2415592..2416212
                     /codon_start=1
                     /note="gene_2232"
                     /transl_table=11
                     /translation="LAIFLIDLPPSDMERRLGDALTVYVDAMRYPRGTETLRAPMWLE
                     HIRRRGWQAVAAVEVTAAEQAEAADTTALPSAAELSNAPMLGVAYGYPGAPGQWWQQQ
                     VVLGLQRSGFPRLAIARLMTSYFELTELHILPRAQGRGLGEALARRLLAGRDEDNVLL
                     STPETNGEDNRAWRLYRRLGFTDIIRGYHFAGDPRAFAILGRTLPL"
     CDS             2416332..2416991
                     /codon_start=1
                     /note="gene_2233"
                     /transl_table=11
                     /translation="MLAIAMLLMLVPLATGCLRVRASITISPDDLVSGEIIAAAKPKN
                     SKDTGPALDGDVPFSQKVAVSNYDSDGYVGSQAVFSDLTFAELPQLANMNSDAAGVNL
                     SLRRNGNIVILEGRADLTSVSDPDADVELTVAFPAAVTSTNGDRIEPEVVQWKLKPGV
                     VSTMSAQARYTDPNTRSFTGAGIWLGIAAFAAAGVVAVLAWIDRDRSPRLTASGDPPT
                     S"
     CDS             complement(2416988..2417893)
                     /codon_start=1
                     /note="gene_2234"
                     /transl_table=11
                     /translation="VTLNTIALELVPPNLEGGKERAIEDARKVVQYSAASGLDGRIRH
                     VMMPGMIAEDDDRPIPMQPKLDVLDFWSIIKPELAGVHGLCTQVTAFMDEPSLHRRLV
                     DLSDAGMEGIVFVGVPRTMQDGEGSGVAPTDALSLYRQLVANRGVIVIPTRDGEQGRL
                     NFKCSRGATYGMTQLLYSDAIVGFLREFARTTEHRPEILLSFGFVPKVETRIGLINWL
                     IQDPGNAAVADEQAFVQKLAGSEPARRRRLMVDLYKRVLDGVADLGFPLSIHLEATYG
                     VSAAAFETFAEMLAYWSPAEPGKPD"
     CDS             2418240..2419262
                     /codon_start=1
                     /note="gene_2235"
                     /transl_table=11
                     /translation="LHGRRRAAAHMGSDYDGLIADLEDFVLGGGKRLRPLFAYWGWHA
                     VASREPDPDVLLLFSALELLHAWALVHDDLIDRSATRRGRPTAQLRYAALHRDRDWRG
                     SPDQFGMSAAILLGDLAQVWADDIVSKVCQSALAPDAQRRVHRVWADIRNEVLGGQYL
                     DIVAEASAAESIESAMNVATLKTACYTVSRPLQLGTAAAADRSDVAAIFEHFGADLGV
                     AFQLRDDVLGVFGDPAVTGKPSGDDLKSGKRTVLVAEAVELADRSDPLAAKLLRTSIG
                     TRLTDAQVRELRTVIEAVGARAAAESRIAALTQRALATLASAPINATAKAGLSELAMM
                     AANRSA"
     CDS             2419299..2420969
                     /codon_start=1
                     /note="gene_2236"
                     /transl_table=11
                     /translation="LATAKDAVVQHLSRLFEFTTGPQGGPARLGFAGAVLITAGGLGA
                     GSVRQHDPLLESIHMSWLRFGHGLVLSSILLWTGVGVMLLAWLGLGRRVLAGEATEFT
                     MRATTVIWLAPLLLSVPVFSRDTYSYLAQGALLRDGLDPYAVGPVGNPNALLDDVSPI
```

```
                     WTITTAPYGPAFILVAKFVTVIVGNNVVAGTMLLRLCMLPGLALLVWATPRLASHLGT
                     HGPTALWICVLNPLVLIHLMGGVHNEMLMVGLMTAGIALTVQGRNVAGIILITVAIAV
                     KATAGIALPFLVWVWLRHLRERRGYRPVQAFLAAAAISLLIFVAVFAVLSAVAGVGLG
                     WLTALAGSVKIINWLTVPTGAANVIHALGRGLFTVDFYTLLRITRLIGIVIIAVSLPL
                     LWWRFRRDDRAALTGVAWSMLIVVLFVPAALPWYYSWPLAVAAPLAQSRRAIAAIAGL
                     STWVMVIFKPDGSHGMYSWLHFWIATACALTAWYVLYSVTGPARSAGCNPGGQYAIAW
                     ARRTTSRAWWACNASTGRALLTASREPSRVMVSDGSGVNSQRIISVSR"
     CDS             2420855..2421253
                     /codon_start=1
                     /gene="AMIS_14900"
                     /note="gene_2237"
                     /transl_table=11
                     /translation="MQCIDGTGVADGVTRAVAGDGQRRIGGKQPAHNLGVAIAPVVQD
                     GQQPRQALDHLTGVGEEDLRDHHATSAPHGHQMTFPQLLGHLADRNAEQLGDPGQVVR
                     RFVRVQNVIASENAAHPRKCRAWCAARHAR"
     CDS             2421326..2422498
                     /codon_start=1
                     /note="gene_2238"
                     /transl_table=11
                     /translation="LESALLDSRYLVQAKIASGGTSTVYRGLDVRLDRPVALKVMDAR
                     YAGDEQFLTRFRLEARAVARLNNRALVAVYDQGKDGRHPFLVMELIEGGTLRELLIER
                     GPMPPHAVVAVLRPVLGGLAAAHRAGLVHRDVKPENILISDDGDVKLADFGLVRAVAA
                     ASITSTGVILGTAAYLSPEQVRDGNADPRSDVYSVGVLVYELLTGHTPFTGDSALSIA
                     YQRLDADVPRASAVIDGVPPQFDELVACATARNPADRYADAIAMGADLEAIAEELALP
                     EFRVPAPRNSAQHRSAALYRSRITQQGQLGAKPVHHPTRQLTRQPGDCSEPASGSEPE
                     HEPITGQFAGIAIEEFIWARQHARRMVLVWVSVVLAITGLVASAAWTIGSNLSGLL"
     CDS             complement(2422640..2423305)
                     /codon_start=1
                     /note="gene_2239"
                     /transl_table=11
                     /translation="MRSKIPDLQRALEGRFDDHHALMCRLHLAHLDQLDAMIGALDEQ
                     IEQLMHPFCARRELIASIPGIGVGASATVISEIGADPAAWFPSAEHLASWVRLCPGNH
                     ESAGKRHHGARRKGNQHLQPVLVECAWAAVRTDGYLREYYRRQVRKFGGFRSPAANKK
                     AIIAVAHKLIVIIWHVLATGRPYQDLGADYFTTRMDPDKERRRLVAKLEAQGLGVTLE
                     PAA"
     CDS             complement(2423239..2423619)
                     /codon_start=1
                     /note="gene_2240"
                     /transl_table=11
                     /translation="VRCGVDVVGAWLCVAVQETDVGAPPLSGVSRLLLRARVPVSLVH
                     VWGLPSQRALLVATLSRKGAGGMHILYERCAFVDVGQDIIAVAVWVPGEGPDGRKTIK
                     RTLCARRSPICSGRWKGASMITMP"
     CDS             complement(2423690..2425078)
                     /codon_start=1
                     /note="gene_2241"
                     /transl_table=11
                     /translation="MNWTVDIPIDQLPSLPPLPTDLRTRLDAALAKPAAQQPTWPADQ
                     ALAMRTVLESVPPVTVPSEIVRLQEQLAQVAKGEAFLLQGGDCAETFMDNTEPHIRGN
                     VRALLQMAVVLTYGASMPVVKVARIAGQYAKPRSADIDALGLRSYRGDMINGFAPDAA
                     AREHDPSRLVRAYANASAAMNLVRALTSSGLASLHLVHDWNREFVRTSPAGARYEALA
                     TEIDRGLRFMSACGVADRNLQTAEIYASHEALVLDYERAMLRLSDGEDGEPQLFDLSA
```

HTVWIGERTRQIDGAHIAFAQVIANPVGVKLGPNMTPELAVEYVERLDPHNKPGRLTL
VSRMGNHKVRDLLPPIVEKVQATGHQVIWQCDPMHGNTHESSTGFKTRHFDRIVDEVQ
GFFEVHRALGTHPGGIHVEITGENVTECLGGAQDISETDLAGRYETACDPRLNTQQSL
ELAFLVAEMLRD"
CDS complement(2425169..2425675)
/codon_start=1
/note="gene_2242"
/transl_table=11
/translation="VRYFYDTEFIEDGHTIELISIGVVAEDGREYYAVSTEFDPERAG
SWVRTHVLPKLPPPASQLWRSRQQIRLDLEEFLRIDGTDSIELWAWVGAYDHVALCQL
WGPMTALPPTVPRFTRELRQLWEDRGCPRMPPRPRDVHDALVDARDQLRRFRLITSTD
DAGRGAAR"
CDS complement(2425685..2426623)
/codon_start=1
/note="gene_2243"
/transl_table=11
/translation="MVALYGACICSQGGRSFLEVFHWLQHDIVDRGRLPLLCCLVAFV
LTFLVTRSFVRFIHRRAADGRPARWWQPRNVHIGSVHIHHVAFGVVLVMISGLTLVTL
SVDGREPEFTIAASIFGVGAALVLDEYALILHLSDVYWEEDGRTSVDAVFAAVAVAGL
LIMGLHPLIFFLTVRQGANWVVLQTTLIAGLVLTLPLAVVVLLKGKVWTGLLGMFVVV
LLVVGAVRLSRPHAPWARWRYTRHPEKMRRALQRERTWRRPVVRIKLWLQYVIAGTPR
MPDERAVDAQLDQDVRPAPPPERTAPILISGSVWSD"
CDS 2426660..2427943
/codon_start=1
/note="gene_2244"
/transl_table=11
/translation="MSAWRAPEVGSRLGRRVLWCLLWLLAGVALGYVAWRLFGHTPYR
IDIDIYQMGARAWLDGRPLYGGGVLFHTPIGLNLPFTYPPLAAVLFSPFAWLQMPAAS
VAITVLTLVLLIASTAIVLTGLDAWPTSRLVPAPARLRRLWLAVLIVAPATIWLEPIS
SNFAFGQINVVLMTLVIVDCFPRRTPWPRGLMLGLGIALKLTPAVFLLYFLLRRDGRA
ALTALASFAVATLLGFVLAWRDSWEYWTHTLHHTDRIGAAALNTDQNIAGALARLTIG
DDERFALWVAGSLLVLAATIWAMRRVLRAGEPTLAVICVALFGLVVSPVSWSHHWVWM
LPAVLVIGLLGWRRRNVALAMLSLAGVVLMRWTPIDLLPQHRETTAVWWRQLAGMSYV
WWALAVIVVAGLTVTARMTPQRSLTRGLTPAPTAS"
CDS complement(2427944..2428687)
/codon_start=1
/note="gene_2245"
/transl_table=11
/translation="MWYYLFKYIFMGPLFTLLGRPKVEGLEYIPSSGPAILASNHLAV
ADSFYLPLVVRRRIWFLAKSEYFTGTGLKGWINRWFYSVSGQVPIDRTNADSAQGALQ
TAVVLLGQGKLLGMYPEGTRSPDGRLYKGKTGLARLALHTGVPVIPVAMIGTNVVNPP
GRKMLRFGRVTVRFGKPMDFSRFEGLAGNHFIERAVTDEVIYELMGLSGQEYVDIYAA
SVKDGRNAGGAGANPNSTDAARIPETAAG"
CDS complement(2428773..2429168)
/codon_start=1
/note="gene_2246"
/transl_table=11
/translation="VSGAHTDVRPELRKLAQAILDGIDPAVRVAAAMASGGGPGTGKC
QQVWCPLCALAALVTGEQHPLLTVIADHSLALLEVIRAIVDDIDRSAKPPPEGPPGGG
QTGASGGENTNGEGSMKSHYQAIPVTIEE"
CDS complement(2429165..2430307)

```
                     /codon_start=1
                     /note="gene_2247"
                     /transl_table=11
                     /translation="VLVVSTDQAHSLGDVLGIAVPPTGQGDPVRVLAYDPEAGGGFLD
                     ALALDTLALLEGRWLHVVETLDRRFPGSELSSIAPEELCALPGIQEVLGLHAVGELAA
                     ARRWDRIVVDCASTADALRMLTLPATFGLYVERAWPRHRRLSIGADDGRSAVLAELLE
                     RIRASVERLSTLLTDGALVSAHLVLTPERVVAAEAVRTLGSLALMGVRVEELLVNQLL
                     VQDENYEYRSLPDHPAFHWYAERIGEQRAVLDDLDATIGDVALVLVPHLAGEPIGPKA
                     LGGLLDSARRRQGSAPPGPLQPIVDLESGSGLASIYRLRLALPQLDPGTLTLGRADDD
                     LIVSAGGMRRRVRLASVLRRCTVLDAHLRGGELTVRFRPNPEVWPT"
     CDS             complement(2430424..2430858)
                     /codon_start=1
                     /gene="TB16"
                     /note="gene_2248"
                     /transl_table=11
                     /translation="VADKTTQTIYIDADPGEVMKAIADIEAYPQWISEYKEVEILEAD
                     DEGYPKRARMLMDAAIFKDTLIMSYEWPEDRQSLSWTLESSSLLKSLEGTYRLAPKGS
                     GTEVTYELAVDLAVPMIGMLKRKAERRLIDGALKDLKKRVEG"
     CDS             complement(2430963..2431352)
                     /codon_start=1
                     /note="gene_2249"
                     /transl_table=11
                     /translation="MNSIQIADETYVAADAARVSAAVADRCSWRRWWPDLRLQVTEDR
                     ADKGIRWTVTGALTGTMEIWLEPSMDGVLLHYFLHAEPTGVAAWQLARMNLARMTHHR
                     RVAGKKMAFEVKTVLERSRPIGVSPVT"
     CDS             2431793..2433319
                     /codon_start=1
                     /note="gene_2250"
                     /transl_table=11
                     /translation="VGAVTVPIYETSSAEQVRWVLQDSEAVVLFAETDSHATMVAELS
                     GSVPALREVLQIAGSGPNALDRLTEAGASVDPAELTARLAALRSTDPATLIYTSGTTG
                     RPKGCQLTQSNLVHEIKGARAYHPTLLRKGERLLVFLPLAHVLARAISMAAFHSKVTV
                     GFTSDIKNLLPMLAVFKPTVVVSVPRVFEKVYNTAEQNAANAGKGRIFAIAAQTAVDW
                     SEACDRGGPGLLLRAKHAVFDRLVYRKLRAALGGNCRAAVSGGAPLGARLGHFYRGAG
                     LTIYEGYGLSETSGGVAISQFNDLKIGTVGKPVPGNSLRIADDGELLVRGGVVFSGYW
                     RNEQATTEAFTDGWFKTGDLGAVDEDGFLTITGRKKEIIVTAGGKNVAPAVLEDQLRA
                     HPLISQAVVVGDAKPFIGALITIDPEAFEGWKQRNSKTAGASVGDLATDPDLIAEIDA
                     AVKQANLAVSHAESIRKFRILPVDFTEDTGELTPTMKVKRKVVAEKFASDIEAIYNKE
                     "
     CDS             complement(2433350..2434549)
                     /codon_start=1
                     /note="gene_2251"
                     /transl_table=11
                     /translation="LRLLTQGGPASLTQVSRVLLVTNDFPPRRGGIQSYLGEFVGRLV
                     GSRAHAMTVYAPQWKGADAFDDAARAAGYRVVRHPSTVMLPGPTVDVRMRRLIAEHDI
                     ETVWFGAAAPLALLAPRARLAGASRVLASTHGHEVGWSMLPVARSVLRRIGDGTDVVT
                     FVSSYTRSRFASAFGPAASLEYLPPGVDTDRFRPDPAARAELRKRYRLGERPTVVCLS
                     RLVPRKGQDTLVTALPSIRRRVDGAALVIVGGGPYLETLRKLAHDCGVADHVTFTGGV
                     ATDELPAHHALADVFAMPCRTRGAGMDVEGLGIVFLEASAAGVPVIAGNSGGAPETVQ
                     HNKTGLVVDGRSVDRVADAVAELLIDRDRAVAMGAAGREWVTAQWRWDTLAAKLADFL
                     RGDDAAR"
```

```
CDS             complement(2434604..2435437)
                /codon_start=1
                /note="gene_2252"
                /transl_table=11
                /translation="LLACLFVAELIGAAALSGNPVRDGPAAPAQVVAPADGFVALRVA
                DDRTVRLLSLGGAATDRLLSRIAAGIDAAVDEVVAFWGTDWSHDIFVVAAGSDEQFHA
                AAGGGLASQWADIAAITVVDRVDPARRTVVGQRIVFAPGAAHMSPAALRIVLGHELFH
                YAARADTALDAPRWLAEGVADFVARPKTPPPADAVSVALSLPSDTDLDTPGPQRSLAY
                DRAWWFARFVAAAYGTAKLRELYLATCGVGHFDLATAAHDVLGIDAAGLLARWQRWLM
                G"
CDS             complement(2435472..2436590)
                /codon_start=1
                /note="gene_2253"
                /transl_table=11
                /translation="MRSAIGFFASFTVSSGVLAANVLADPADDALAKLNELSRQAEQT
                TEALHSAQLDLNEKLAAQRAADQKLADNRTALDAARARLATFQTAVNKVAAATYMGGR
                THGMDAILTAESPQLLIDRLSVQRVMAHQMSTQMARFKAAGEQAVKAEQAAAKSAADA
                RSAAEQAAAVRANLQHKQSQLQVQIAVVKSQYVALTPEERTALADPGPVPAVAAIAPG
                APPAALPPGAPPGDGPAPGVAPPPGGMPGLPFVQPDGAGGDRTAVVQAALTQVGAPYA
                WGGAAPGGFDCSGLVMWAFQQAGIALPHSSQALAHGGQPVALSDLQPGDVLTFYSDAS
                HAGIYIGDGLMVHSSTYGVPVRVVPMDSSGPIYDARRY"
CDS             complement(2436815..2437066)
                /codon_start=1
                /note="gene_2254"
                /transl_table=11
                /translation="MARSGPDTSGGTAPGPGAPVVIDCDDCAARGSGCRDCVVSVLIG
                VPESLSHDERAALEVLADVGLAPRLRLVPVRRQRGSGVA"
CDS             2437176..2439113
                /codon_start=1
                /note="gene_2255"
                /transl_table=11
                /translation="VQGPNVAAMGATGGTQLSFADLAHAQGAAWTPADEMSLRETTFV
                VVDLETTGGRTTGNDATPPDAITEIGAVKVCGGAVLGEFATLVNPQHSIPPQIVRLTG
                ITTAMVGNAPTIDAVLPMFFEFAGDSVLVAHNAGFDIGFLRAAARRCDITWPQPQVLC
                TMRLARRVLSRDEAPSVRLAALARLFAVASNPTHRALDDARATVDVLHALIERVGNQG
                VHTYAELRSYLPNVTQAQRCKRVLAETLPHRPGVYLFRGPSGEVLYVGTAADLRRRVS
                QYFNGTDRRKRMTEMVMLASSIDHVECAHPLEAGVRELRMLSTHAPPYNRRSKFPYRW
                WWVALTDEAFPRLSVIRAPRHDRVVGPFRSRSKAAETTALLARCTGLRTCTTRLTRSA
                RHGPACPELEVSACPAARDVTAAQYAEAVLRAAALIGGLDNAALAAAVQQVTELAERR
                RYESAARLRDHLATAIEALWHGQRLRALAALPELIAAKPDGPREGGYQLAVIRHGQLA
                AAGRAPRGVPPMPVVDAIRRGAQAILPTPAPLGGALVEEIALIARWLAEPGVRIVGVS
                NDAAGLASPVRSAGPWAAWAATARSAQLAGEQLSRGWQSDLPTEPHPSREQLFGRTGV
                DCRTGPPQPLLPGRQPFSTAG"
CDS             2439120..2439749
                /codon_start=1
                /gene="O216_11990"
                /note="gene_2256"
                /transl_table=11
                /translation="VGDDRTGGVEHHSVPDRAPGTAQHRAHRGRVGLRIASSQLVKLG
                ARKPESGGIKRQLIHAARRNAPDRARGGGGQLVQPIVAVYHQHTGPARSKHPGHHFGE
                VGERASDQSSPGPGRIGQRPEKIEHGGHTDLAAYRGRVPVGGMEPRREAEPDPNLREA
```

ARDQVGSQVDAHPQRLQRVGATGQRGRRSVAVLDHRHTRSRHHNRRHGG"
CDS 2440327..2440869
/codon_start=1
/note="gene_2257"
/transl_table=11
/translation="MVSVGTIVWLSSELMFFAGLFAFYFSARAQAGGNWPPPPTELNL
YQAVPVTLVLIASSFTCQMGVFAAERGDIFGLRRWYVITFLMGLFFVLGQAYEYRNLM
SHGTSIPSSAYGSVFYLATGFHGLHVTGGLIAFIFLLVRTGMSKFTPAQATASIVVSY
YWHFVDIVWIALFTVIYFIR"
CDS 2440910..2441752
/codon_start=1
/note="gene_2258"
/transl_table=11
/translation="LTKLGFTRSGGSKSGRTRRRLRRRLSGGVLLLIALTIAGGLAAV
LTPTPQVAVADESSSALLRTGKQLFDTSCVSCHGANLQGVPDHGPSLIGVGEAAVYFQ
VSTGRMPAMRGEAQAPRKDPIFDEAQIDAIGAYVQANGGGPTVVRNPDGSIATQSLRG
NDLGRGGDLFRLNCASCHNFTGKGGALSSGKYAPDLAPANEQQILTAMLTGPQNMPKF
SNRQLSFEAKKDIIAYVKVATEARQPGGYLLGGFGPAPEGMAMWIIGMVAAIGLALWI
GARS"
CDS 2441827..2443038
/codon_start=1
/note="gene_2259"
/transl_table=11
/translation="VPGPNPQDGAKDGAKATAVPREPDEAALAAMSNQELLALGGKLD
GVRIAYKEPRWPVEGTKAEKRAERSVAVWLLLGGVFGLALLLIFLFWPWEFKAADGES
DFIYSLTTPLYGLTFGLSILSIAIGAVLYQKRFIPEEISIQERHDGASREIDRKTVVA
NLTDAFEGSTIRRRKLIGLSFGVGMGAFGLGTLVAFAGGLIKNPWKPVVPTAEGKKAV
LWTSGWTPRYQGETIYLARATGTEDGPPFIKMRPEDIDAGGMETVFPWRESDGDGTTV
ESHHKLQEIAMGIRNPVMLIRIKPSDLGRVVKRKGQESFNFGEFFAFTKVCSHLGCPS
SLYEQQSYRILCPCHQSQFDALHFAKPIFGPAARALAQLPITIDTDGYLVANGDFVEP
VGPAFWERTTT"
CDS 2443035..2444684
/codon_start=1
/note="gene_2260"
/transl_table=11
/translation="MSPKLSPPNIGEVLARQAEDIDTRYHPSAALRRQLNKVFPTHWS
FLLGEIALYSFVVLLITGVYLTLFFDPSMVDVTYNGVYQPLRGVEMSRAYQSALDISF
EVRGGLFVRQIHHWAALMFAAAIMVHLARIFFTGAFRRPRETNWVIGSLLLILAMFEG
YFGYSLPDDLLSGLGLRAALSSITLGMPVIGTWLHWALFGGDFPGTILIPRLYALHIL
LLPGIILALIGLHLALVWFQKHTQFPGPGRTEHNVVGVRVMPVFAFKSGAFFAAIVGV
LGLMGGLLQINPIWNLGPYKPSQVSAGSQPDFYMMWTEGLARIWPPWEFYFWHHTIPA
PVWVAVIMGLVFVLLPAYPFLEKRFTGDYAHHNLLQRPRDVPVRTAIGAMAIAFYMVL
TLAAMNDIIALKFHISLNATTWIGRIGMVILPPFVYFITYRWCIGLQRSDRSVLEHGV
ETGIIKRLPHGAYIELHQPLGPVDEHGHPIPLQYQGAPLPKRMNKLGSAGSPGSGSFL
FADSAAEDAALREAGHAAEQRALAALREHQDSIMGSPDGEH"
CDS complement(2444975..2445619)
/codon_start=1
/note="gene_2261"
/transl_table=11
/translation="MVSRYSAYRRGPDVISPDVIDRILVGACAAVWLVFTGVSVAAAV
ALMDLGRGFHEMAGNPHTTWVLYAVIVVSALVIVGAIPVLLRARRMAEAEPATRPTGA

SVRGGRSIGSGHPAKRAVAESAPVQHADAFEVAAEWSSEAVDRIWLRGTVVLTSAIGI
ALIAVAAATYLMAVGHDGPSWISYGLAGVVTAGMPVIEWLYSRQLRRVVAPQSS"
CDS complement(2445619..2446518)
/codon_start=1
/note="gene_2262"
/transl_table=11
/translation="MSGPNPPGREPDEPESEPVSDTGDERASGNHLPPVAGGGDKLPS
DQTGETDAYSRAYSAPESEHVTGGPYVPADLRLYDYDDYEESSDLDDELAAPRWPWVV
GVAAIIAAVALVVSVSLLVTRPHTSKLATGDTTSSAPPVQDEITTTKPAPPPPPPAPP
PTTEIPTATETQTVTVTPPPPPPPATTTAPPPATTTTAAAPPPTTTTPTGPRQVTYSV
TGTKAPGDIISVTYVDAAGRRRTQHNVYIPWSMTVTPISQSDVGSVEASSLFRVSKLN
CSITTSDGTVLSSNSNDGPQTSC"
CDS complement(2446704..2447123)
/codon_start=1
/note="gene_2263"
/transl_table=11
/translation="MHIEARLFEFVAAFFVVTAVLYGVLTSMFATGGVEWAGTTALAL
TGGMALIVATFFRFVARRLDSRPEDYEGAEISDGAGELGFFSPHSWWPIMVALSGSVA
AVGIALWLPWLIAAGVAFILASAAGLVFEYYVGPEKH"
CDS complement(2447131..2448117)
/codon_start=1
/note="gene_2264"
/transl_table=11
/translation="MLGALAVTVSGCSWSEALGIGWPEGITPEAHLNRELWIGAVIAS
LAVGVIVWGLIFWSAVFHRKKNTDTELPRQFGYNMPLELVLTVIPFLIISVLFYFTVV
VQEKMLQIAKDPEVVIDITSFQWNWKFGYQRVNFKDGTLTYDGADPERKRAMVSKPEG
KDKYGEELVGPVRGLNTEDRTYLNFDKVETLGTSTEIPVLVLPSGKRIEFQMASADVI
HAFWVPEFLFKRDVMPNPVANNSVNVFQIEEITKTGAFVGHCAEMCGTYHSMMNFEVR
VVTPNDFKAYLQQRIDGKTNAEALRAINQPPLAVTTHPFDTRRGELAPQPVG"
CDS 2448468..2450426
/codon_start=1
/note="gene_2265"
/transl_table=11
/translation="VCGLLAFVAAPAGAAGPEGADAASAIARASHLMRHRGPDESGTW
HAVDGASGGVVFGFNRLSIIDIAHSHQPLRWGPPEAPDRYVLVFNGEIYNYLELRDEL
RTQHGAVFATDGDGEAILAGYHHWGTEVLQRLRGMFAFALWDTVTRELFCARDPFGIK
PLFIATGAGGTAVASEKKCLLDLVELVGFDTEIDHRALQHYTVLQYVPEPETLHRGVR
RLESGCFARIRADQLAPVITRYFVPRFAASPITNDNDQARYDEITAVLEDSVAKHMRA
DVTVGAFLSGGIDSTAIAALAIRHNPRLITFTTGFEREGFSEIDVAVASAEAIGARHI
AKVVSADEFVAALPEIVWYLDEPVADPALVPLFFVAREARKHVKVVLSGEGADELFGG
YTIYREPLSLRPFDYLPKPLRRSMGKVSKPLPEGMRGKSLLHRGSLTLEERYYGNARS
FSGAQLREVLPGFRPDWTHTDVTAPVYAESAGWDPVARMQHIDLFTWLRGDILVKADK
ITMANSLELRVPFLDPEVFAVASRLPAGAKITRTTTKYALRRALEPIVPAHVLHRPKL
GFPVPIRHWLRAGELLEWAYATVGSSQAGHLVDIAAVYRMLDEHRCGSSDHSRRLWTM
LIFMLWHAIFVEHSVVPQISEPQYPVQL"
CDS complement(2450524..2451498)
/codon_start=1
/note="gene_2266"
/transl_table=11
/translation="VTIAVTGSIATDHLMRFPGRFSEQLLPEHLHKVSLSFLVDDLVM
HRGGVAGNMAFAIGVLGGEVALVGAAGADFADYRDWLKARGVNCDHVLISETAHTARF

TCTTDVDMAQIASFYPGAMSEARNIKLADVVSAIGKPELVIIGANDPEAMFLHTEECR
KLGLAFAADPSQQLARLSGEEIRRLVNGAAYLFTNDYEWDLLLSKTGWSEADVMAQID
LRVTTLGPKGVDLVEPDGTTIHVGVVPETSQTDPTGVGDAFRAGFLTGRSAGLGLERS
AQLGSLVAVLVLESTGTQEWQWDYEAAASRLAGAYGEHAAAEIVAVLA"
CDS 2451702..2452394
/codon_start=1
/note="gene_2267"
/transl_table=11
/translation="MPGPHSPNPGVGTNGPAPYPEPSSHEPQALDYPHDLGAAEPAFA
PGPADDAALPPAAYPGVPPQVSYPKRRHKRLLIGIVVALALVSAMTAAIIYGVRTNGA
NTAGTFSEGPAKTAIQGYLNALENRDVDTIVRNALCGIHDGVRDKRSDQALAKLSSDA
FRKQFSQVEVTSIDKIVYWSQYQAQVLFTMQVTPAAGGPPRGQVQGIAQLLFQRGQVL
VCSYVLRTAGSY"
CDS complement(2452402..2452758)
/codon_start=1
/note="gene_2268"
/transl_table=11
/translation="MTVQNEPSAKTHGVILTEAAAAKAKSLLDQEGRDDLALRIAVQP
GGCAGLRYNLFFDDRTLDGDQTAEFGGVRLIVDRMSAPYVEGASIDFVDTIEKQGFTI
DNPNATGSCACGDSFN"
CDS complement(2452858..2454003)
/codon_start=1
/note="gene_2269"
/transl_table=11
/translation="MKGSQASDDATGSLGPGRLQLPAMRVLVAPDCYGDSLSAVEAAA
AIATGWTRSRPGDSFIVAPQSDGGPGFVEVLGSRLGETRRLRVCGPLNTVVNAAWVFD
PGSATAYLECAQACGLGLLGGPPTPETALAAHSKGVGQLIAAALRAGAARIVVGLGGS
ACTDGGKGMIAELGGLDAARRQLADVEVIAASDVEYPLLGPWGTARVFAPQKGADMAT
VAVLEGRLAAWAIELDAAAGRGVSAEPGAGAAGGIGAGLLAVGGRYQSGAAIIAEHTH
FADDLADAELIVTGEGRFDEQSLHGKVVGAIAAAARPLAIPVIVLAGQVSLDKSALRS
AGIMAALSIAEYAGSVRLALADAANQLMGLASQVAARLGNSGPSGYR"
CDS 2454093..2454803
/codon_start=1
/note="gene_2270"
/transl_table=11
/translation="VKLLGHRKSHGHQRADASPDAGSKDGCRPDSGRTSGSDTSRGSQ
TTGPKGRPTPKRNQSRRHTKKGPVAPAPMTAAQARARRKSLAGPKLSREERRAEKAAN
RARMTERRERMMAGEEAYLLPRDRGPVRRYVRDVVDSRRNLLGLFMPSALTLLFVMFA
VPQVQFYLSPAMLILLALMTIDAIILGRKVGRLVDTKFPSNTESRWRLGLYAAGRASQ
IRRLRAPRPQVERGGDVG"
CDS 2454882..2455967
/codon_start=1
/gene="cobT"
/note="gene_2271"
/transl_table=11
/translation="MIGFAPVSTPDAAAEAAARARQDSLTKPRGALGSLEDLSVWVAS
CQQRCPPRQFERARVVVFAGDHGVARSGVSAYPPEVTAQMVANIDAGGAAINALADVA
GATVRVADLAVDADPLSERIGAHKVRRGSGNIATEDALTNDETAAAITAGQQIADEEV
DAGADLLIAGDMGIGNTTAAAVLVAALTDAEPVAVVGFGTGIDDAGWARKTAAVRDAL
FRVRPVLPDPVGLLRCAGGADLAAIAGFCAQAAVRRTPLLLDGVAVTAAALVAERLAP
GAHRWWQAGHRSSEPGHGLALAALGLDPIVDLHMRLGEGTGAAVALMVLRAAVAALSS

MATFTEAGVSTRSVDGVDRTAPPAVSP"
CDS 2456051..2456713
/codon_start=1
/note="gene_2272"
/transl_table=11
/translation="MTALPVVGAALGALAAAIAWAGAQVFGPSSPLSGMLTVAVLLVV
TRGLHIDGVADTADGLGCYGPPQRALAVMRDGSTGPFGVAAVVLVIALQGLAFATLTT
VGIAGITLAVLSGRVTAVLVCRRSVPAAHGSTLGSRVAGTQPAPVVAAWLAVLLAVSV
PAGPRPWQGPIAVLVAVTAGAALAAHCVHRFGGVTGDVLGSAIELSTTVSAVTLAGLA
RL"
CDS 2456871..2458409
/codon_start=1
/note="gene_2273"
/transl_table=11
/translation="MPASRLVRQVSAPRNLFGRLVAQGGFYTAGLQLGSGAVVLPVIC
AHQGLTWAAGLLYPAFCIGAILGNSLSPLILQRAGQLRHLLMAAISATAAALVVCNAA
VPWTGVGVAAVFLATTGAGGVVTGVSSVAYTDMISSMLPAVRRGELLLTQGAAGSVLA
TGVTLVIVPMLAHGNEMARYHDLLWLGAAGLVCSGIAALFVGPMRSVSVTTATRMPLR
EIYWMGFAIARSQPWFRRYMTTYLLFVPISLGTTFFSLRAAQSNGSLHVLVILSSIGL
VVGSMLWRQINRLFGVRGLLLGSALLNAAAALLCMVAESCGQWVHAWAYGTAFLLATV
AAQTVVAASISWISVLAPERYRATLICVGSTLAAVEATVLGVALGGIAQKHATIWPVV
VVLTLAVIAAVASLRAPTRIGVTADTSPQAATLQAYRPATPNPIHSDERSTPPDHLSV
RRGQLRHVWDSRRPAPPLNRPSCRRAARRPAPGKPAAALPQPRHPAVGVREGAPLDAG
QRIA"
CDS complement(2458335..2459441)
/codon_start=1
/note="gene_2274"
/transl_table=11
/translation="MTSGSLQFTVLRAVNPATDAQRESMLRAPGFGKYHTDHMVSIDY
AEGRGWHNARVIPYGPIELDPSAIVLHYAQEVFEGLKAYRWADGSIVSFRADANAARL
RSSARRLAIPELPDAVFIESLRQLIAVDKAWVPGAGGEEALYLRPFIFATEPGLGVRP
ATQYRYLLIASPAGAYFKGGIAPVSVWVSTEYVRACPGGTGAAKFGGNYAASLLAQAE
AAENGCDQVVWLDAVERRYIEEMGGMNIFFVLGSGGSARLVTPELSGSLLPGITRDSL
LQLAIDAGFAVEERRIDIDEWQKKAAAGEITEVFACGTAAVITPVARVRHGASEFRIA
DGQPGEVTMALRDTLTGIQRGTFADTHGWMARLG"
CDS complement(2459513..2460616)
/codon_start=1
/gene="gcvT"
/note="gene_2275"
/transl_table=11
/translation="VSDVPELIHGPLEDRHRELGASFAEFGGWLMPVSYAGTVSEHNA
TRTAVGLFDVSHLGKALVRGPGAAQFVNSALTNDLGRIGPGKAQYTLCCTESGGVIDD
LIAYYVSDDEIFLVPNAANTAAVVGALQAAAPGGLSITNLHRSYAVLAVQGPCSTDVL
TALGLPTEMDYMGYADASYSGVPVRVCRTGYTGEHGYELLPPWESAGVVFDALLAAVS
AAGGEPAGLGARDTLRTEMGYPLHGHELSLDISPLQARCGWAVGWRKDAFFGRAALLA
EKAAGPRRLLRGLRMVGRGVLRPGLAVLVGDETVGVTTSGTFSPTLQVGIGLALIDSD
AGIEDGQQINVDVRGRAVECQVVCPPFVAVKTR"
CDS 2460631..2461797
/codon_start=1
/note="gene_2276"
/transl_table=11

/translation="MGVPAGTLRQVYDSLDFDALEAAGIANPRERAGLLTYLDELGFT
VEEMVQAERRGRLFGLAGDVLLWSGPPIYTLATAADELGLSADDVARAWSLLGLTVAG
PDVPTLSQADVDALATWVALKALVGEDGAFGLLRVLGTAMARLAEAESTMIRAGSPNI
QMTHTHDELATARAYRAAAEFVPRIGALIDTVHRHHLASARTYFEGVIGDTSASVTCG
IGFADLSSFTALTQALTPAQLQDLLTEFDAAVTDVVHADGGRLVKFIGDAVMWVSSSP
ERLVRAAVDLVDHPGARAAELQVRAGLAYGTVLALNGDYFGNPVNLAARLVAAAAPGQ
ILAAAQLRDMLPDWPALAHGPLTLKGFDAPVMAFELHDNPRARDADTPSPAASD"

CDS 2461803..2463356
/codon_start=1
/note="gene_2277"
/transl_table=11
/translation="LPVTTEPGYLSPSVAVATSMPKRGVGAAVLIVPVVSTGEEDRPG
AVVASAEPFLRADTVAEIEAGLRALDATGASDQVHRLAVPSLPVGSVLTVGLGKPRRE
WPADTIRCAAGVAARALNSSEAVITTLAELPGDGICSATVEGLILGSYRFSAFRSDKT
APKDAGLRKITVLCCAKDAKKRALHGAAVATAVATARDLVNTPPSHLFPAELAKRAKT
LSESVGLDVEVIDEKALKKAGYGGVIGVGQGSSRPPRLVRLIHRGSRLAKNPQKAKKV
ALVGKGITFDTGGISIKPAASMHHMTSDMGGAAAVIATVTLAARLRLPIDVIATVPMA
ENMPSATAQRPGDVLTQYGGTTVEVLNTDAEGRLILADAIVRACEDKPDYLIETSTLT
GAQTVALGTRIPGVMGSDEFRDRVAAISQRVGENGWPMPLPDDLKDDLKSTVADLANV
SGQRFAGMLVAGVFLREFVAESVDWAHIDVAGPAYNTGSAWGYTPKGATGVPTRTMFA
VLEDIAKNG"

CDS complement(2463394..2465172)
/codon_start=1
/note="gene_2278"
/transl_table=11
/translation="MPATQQMSRLVDSPDGVRIAVYHEGNPDGPTVVLVHGFPDSHVL
WDGVVPLLAERFRIVRYDNRGVGRSSVPKPISAYTMAHFADDFDAVIGELSPGEPVHV
LAHDWGSVGVWEYLRRPGASDRVASFTSVSGPSQDHLVNYVYGGLRRPWRPRTFLRAI
SQTLRLSYMALFSVPVVAPLLLRVALSSAAVRRNMVGDIPVDQIHHSETLARDAAHSV
KTYPANYFRSFSSSRRGRAIPIVDVPVQLIVNSQDPYVRPYGYDQTARWVPRLWRRDI
KAGHFSPMSHPQVMAAAVHDFADLADGKQPSRALLRAQVGRPRGYFGDTLVSVTGAGS
GIGRETALAFAREGAEIVISDIDEATVKDTAAEIAARGGIAYPYVLDVSDAEAVEAFA
ERVSAEHGVPDIVVNNAGIGQAGRFLDTPAEQFDRVLAVNLGGVVNGCRAFGQRLVER
GTGGHIVNVSSMAAYAPLQSLSAYCTSKAATYMFSDCLRAELDAAGVGLTTICPGVID
TNIVATTGFHAPGTDEEKIDGRRGQIDKMFALRSYGPDKVADAIVSAVKKKKPIRPVA
PEAYALYGISRVLPQALRSTARLRVI"

CDS 2465436..2467097
/codon_start=1
/note="gene_2279"
/transl_table=11
/translation="MAFSVQMPALGESVTEGTVTRWLKQEGDTVELDEPLVEVSTDKV
DTEIPSPAAGVLTKIIAQEDDTVEVGGELAVIGDAKDAGEAAAPAPEKVPAAQPESKP
APEPPPVQPTSGAPAGGDAKPVLMPELGESVTEGTVIRWLKKIGDSVQVDEPLVEVST
DKVDTEIPSPVAGVLVSISADEDATVPVGGELARIGVAADIGAAPAPKPAPKPVPEPA
PTPKAEPAPSPPAAQPAGAAEGAPYVTPLVRKLASENNIDLAGVTGTGVGGRIRKQDV
LAAAEQKKRAKAPAPAAQAAAAPAPKAPPAPAPALAHLRGTTQKASRIRQITANKTRE
SLQATAQLTQTHEVDMTKIVGLRARAKAAFAEREGVNLTFLPFFAKAVIDALKIHPNI
NASYNEDTKEITYYDAEHLGFAVDTEQGLLSPVIHDAGDLSLAGLARAIADIAARARS
GNLKPDELSGGTFTITNIGSQGALFDTPILVPPQAAMLGTGAIVKRPRVVVDASGNES
IGVRSVCYVPLTYDHRLIDGADAGRFLTTIKHRLEEGAFEADLGL"

CDS 2467097..2468002

/codon_start=1
/note="gene_2280"
/transl_table=11
/translation="MANAVVAIAGSSGLIGSALTAALRAADHTVLRIVRRAPANSEEL
HWNPESGEFDPHALTDVDAVVNLCGVNIAQRRWSGAFKQSLRDSRITPTEVLSAAVAD
AGVATLINASAVGYYGNTKDRVVDENDSAGTGFLAQLCVDWETATRPAQQSGARVVLA
RTGVVLSPAGGMLRRMRPLFSVGLGARLGSGRQYMSWISLEDEVRALQFAIAQPNLSG
PVNLTGPAPVTNAEFTTAFGRAVNRPTPLMLPSVAVRAAFGEFADEGLLIGQRAIPSA
LERAGFQFHHNTIGEALGYATTRPG"
CDS 2468055..2468747
/codon_start=1
/note="gene_2281"
/transl_table=11
/translation="VTGSIRSKLSAIDVRQLGTVDYRTAWQLQRELADARVAGGADTL
LLLEHPAVYTAGRRTETHERPIDGTPVVGTDRGGKITWHGPGQLVGYPIIGLAEPLDV
VNYVRRLEESLIQVCADLGLHAGRVDGRSGVWLPGRPARKVAAIGVRVSRATTLHGFA
LNCDCDLAAFTAIVPCGISDAAVTSLSAELGRTVTVDEVRATVAAAVCAALDGVLPVG
DRVPSHAVPSPL"
CDS 2468744..2469679
/codon_start=1
/gene="lipA"
/note="gene_2282"
/transl_table=11
/translation="MSVAAEGRRLLRLEVRNAQTPIERKPPWIKTRARIGPEYTELKN
LVRREGLHTVCEEAGCPNIFECWEDREATFLIGGDQCTRRCDFCQIDTGKPAELDRDE
PRRVADSVRTMGLRYATVTGVARDDLPDGGAWLYAATVRAIKELNPSTGVELLIPDFN
GEPTRLAEVFESGPEVLAHNVETVPRIFKRIRPAFTYRRSLGVLTAARDAGLVTKSNL
ILGLGETSDEVRTALGDLRDAGCDIVTITQYLRPSARHHPVERWVKPEEFVQFARFAE
GLGFAGVLAGPLVRSSYRAGRLYEQARNSRALASR"
CDS 2469706..2470458
/codon_start=1
/note="gene_2283"
/transl_table=11
/translation="MAKPRNAAESKAAKAQANAARKAAARQRRAQLWQAFTLQRKEDK
RLLPYMIGAFLLIVGASVGVGVWAGGFTMLTMIPLGVLLGALVAFVIFGRRAQRTVYR
KAEGQTGAAAWALDNLRGKWRVTPGVAATGNLDAVHRVIGRPGVIFVGEGSAARVKPL
LAQEKKRTARLVGDVPIYDIIVGNGDGEVPLAKLERHLTRLPANITVKQMDTVESRLA
ALGSRAGAGVMPKGPLPTTAKMRSVQRTVRRK"
CDS complement(2470465..2470887)
/codon_start=1
/note="gene_2284"
/transl_table=11
/translation="MTAKSPPDYPGKTLGLPDTGPGSLAPMGRRLAALLIDWLIAYGL
ALLGVEFGVWSTPMLSTVVLVIWLLLGVAAVRLFGFTPGQLMLGLVVVAVGGRRPVGI
GRLVVRGLLIGLVVPPLFTDSDGRGLHDRLTATAVVRR"
CDS 2471086..2472522
/codon_start=1
/note="gene_2285"
/transl_table=11
/translation="VTEKTPDDVFKLAKDEKVEYVDVRFCDLPGIMQHFTIPASAFDK
SVFDDGLAFDGSSIRGFQSIHESDMLLLPDPETARIDPFRAAKTLNINFFVHDPFTLE

PYSRDPRNIARKAENYLISTGIADTAYFGAEAEFYIFDSVSFDSRANGSFYEVDAISG
WWNTGAATEADGSPNRGYKVRHKGGYFPVAPNDQYVDLRDKMLTNLINSGFILEKGHH
EVGSGGQAEINYQFNSLLHAADDMQLYKYIIKNTAWQNGKTVTFMPKPLFGDNGSGMH
CHQSLWKDGAPLMYDETGYAGLSDTARHYIGGLLHHAPSLLAFTNPTVNSYKRLVPGY
EAPINLVYSQRNRSACVRIPITGSNPKAKRLEFRSPDSSGNPYLAFSAMLMAGLDGIK
NKIEPQAPVDKDLYELPPEEAASIPQTPTQLSDVIDRLEADHEYLTEGGVFTNDLIET
WISFKRENEIEPVNIRPHPYEFALYYDV"

CDS complement(2472840..2475824)
/codon_start=1
/note="gene_2286"
/transl_table=11
/translation="VVVTKLATQRPKLPSVGRLGLVDPPAGERLAQLGWDRHEDQAHV
DLLWSLSRAPDADAALRALIRLSENPDTGWDELNAALLRERSLRGRLFSVLGSSLALG
DHLVAHPQSWKLLRGKVTLPSHDQLQRSFVECVEESEGMPGSLVHRLRTQYRDYVLML
AALDLAATVEDEPVLPFTVVAARLADAADAALAAALRVAEASVCGEHPPPRLAVIAMG
KCGARELNYVSDVDVIFVAERSDPRNARVASEMMRVASAAFFEVDAALRPEGRNGELV
RTLESHIAYYQRWAKTWEFQALLKARPVVGDAELGERYLTALMPMVWRACEREDFVVE
VQAMRRRVEQLVPADVRGRELKLGSGGLRDVEFAVQLLQLVHARSDESLRVASTVDAL
AALGEGGYIGREDAANMTASYEFLRLLEHRLQLQRLKRTHLLPDPEDEEAVRWLARAA
HIRPDGRNDAAGVLREELKKQNVRVSKLHTKLFYQPLLESIGPTGLEIAHGMTLEAAG
RRLAALGYEGPQTALKHMSALVNQSGRRGRVQSVLLPRLLDWMSYAPDPDGGLLAYRR
LSEALATESWYLATLRDKPAVAKRLMHVLGTSAYVPDLLMRAPRVIQQYEDGPAGPKL
LETEPAAVARALIASASRYPDPERAIAGARTLRRRELARIGSADLLGLLEVTEVCRAL
TSVWVAVLQAALDVMIRASLPDDDRAPAAIAVIGMGRLGGAELGYGSDADVMFVCEPA
TGVDDARAVKWSTSIAERVRALLGTPSVDPPLELDANLRPEGRNGPLVRTLGSYAAYY
EQWAQPWEIQALLRAHAVAGDAELGQRFLRMVDKTRYPPDGVSADSVREIRRIKARIE
SERLPRGADPNTHTKLGRGGLADIEWTVQLLQLQHAHQVPALHNTSTLQSLDVIAAAD
LVPAADVELLRQAWLTATRARNALVLVRGKPTDQLPGPGRQLNAVAVAAGWRNDDGGE
FLDNYLRVTRRAKAVVRKVFGS"

CDS complement(2475873..2477213)
/codon_start=1
/note="gene_2287"
/transl_table=11
/translation="MDRQKEFVLRTLEERDIRFVRLWFTDVLGFLKSVAIAPAELEGA
FEEGIGFDGSSIEGFARVSESDTVAHPDPSTFQVLPWATSSGHHHSARMFCDITMPDG
SPSWADPRHVLRRQLTKAGELGFSCYVHPEIEFFLLKPGPEDGSVPVPVDNAGYFDQA
VHDSALNFRRHAIDALEFMGISVEFSHHEGAPGQQEIDLRFADALSMADNVMTFRYVI
KEVALEEGARASFMPKPFGQHPGSAMHTHMSLFEGDVNAFHSADDPLQLSEVGKSFIA
GILEHACEISAVTNQWVNSYKRLVQGGEAPTAASWGAANRSALVRVPMYTPHKTSSRR
VEVRSPDSACNPYLTFAVLLAAGLRGVEKGYVLGPQAEDNVWDLTPEERRAMGYRELP
SSLDSALRAMEASELVAEALGEHVFDFFLRNKRTEWANYRSHVTPYELRTYLSL"

CDS complement(2477308..2478870)
/codon_start=1
/note="gene_2288"
/transl_table=11
/translation="MAAMWRRRPLSSALLSFGLLLGGLPLAAPPLAGATEEPGAGQTP
GAPVVAPQQSWNSCREFIADTSEIRTARCATVSVPVDYDQPGGTQAKLAVIRVPATGQ
RFGALLVNPGGPGASAVDMVAAMAPAIADTDILRHFDLVGFDPRGVGHSTPALRCRTD
AEFDAYRRDPMADYSPAGVTHVEQVYRQLAQDCVDRMGFSFLANIGTASVARDMDMVR
QALGDDQINYLGYSYGTELGTAYLERFGTHVRAMVLDGAIDPAVSPIEESISQMAGFQ
TAFNDYAADCARSPACPLGTDSAQWVNRYHSLVDPLVQKPGKTSDPRGLSYADATTGT

INALYSPQRWKYLTSGLLGLQRGSDAGDLLVLADDYDGRDADGHYSNDQDAFNAVRCV
DAPTPADPAAWVAADQRIRQVAPFLSYGQFTGSAPRDLCALWPVPATSTPHPAAPAGA
GKVVVVSTTHDPATPYQSGVDLARQLGAPLITFDGTQHTAVFDGNQCVDSAVMHYFLD
GTLPPTSLRCAP"
CDS complement(2478932..2480494)
/codon_start=1
/note="gene_2289"
/transl_table=11
/translation="VGMRLSRRDKIARMLLIWAALAAVALVLVGCIRVVGGRARMAEP
KLGQPVEWTPCRSSNPQVKIPGGALCGKLAVPVDYDRPDGDVAALALIRFPATGDKIG
SLVINPGGPGESGIEAALGVFQTLPKRVHERFDLVGFDPRGVASSRPAIWCNSDADND
RLRAEPQVDYSREGVAHIENETKQFVGRCVDKMGKNFLAHVGTVNVAKDLDAIRAALG
DDKLTYLGYSYGTRIGSAYAEEFPQRVRAMILDGAVDPNADPIEAELRQAKGFQDAFN
NYAADCAKNAGCPLGADPAKAVEVYHSLVDPLVDPDNPRISRPARTKDPRGLSYSDAI
VGTIMALYSPNLWQHLTDGLSELVDNRGDTLLALADMYMRRDSHGRYNNSGDARVAIN
CVDQPPVTDRDKVIDEDRRAREIAPFMSYGKFTGDAPLGTCAFWPVPPTSQPHAVSAP
GLVPTVVVSTTHDPATPYKAGVDLANQLRGSLLTFDGTQHTVVFQGDSCIDEYVTAYL
IGGTTPPSGAKC"
CDS 2481213..2482058
/codon_start=1
/gene="panB"
/note="gene_2290"
/transl_table=11
/translation="MSEQTIYGANTPGGSGPRTKIRTHHLQRWKADGHKWAMLTAYDY
STARIFDEAGIPVLLVGDSAANVVYGYDTTVPISIDELIPLVRGVVRGAPHALVVADL
PFGSYEAGPTAALAAATRFLKDGGAHAVKLEGGERVAEQIACLTAAGIPVMAHIGFTP
QSVNTLGGFRVQGRGDAAEQTIADAIAVAEAGAFAVVMEMVPAELATQITGKLTIPTV
GIGAGPNCDGQVLVWQDMAGFSGAKTARFVKRYADVGGELRRAAMQYAQEVAGGVFPA
DEHSF"
CDS 2482303..2483844
/codon_start=1
/note="gene_2291"
/transl_table=11
/translation="MPVEAPRPARHLEVERKFDVIESTVSPSFEGIAAVVRVEQSPTQ
QLDAVYFDTPSHDLARNQITLRRRTGGADAGWHLKLPAGPDKRTEMRAPLSASGDAVP
AELLDVVLAIVRDQPVQPVARISTHRESQILYGAGGDALAEFCNDDVTAWSAGAFHAA
GAADNGPAEQQWREWELELVTTDGTADTKLLDRLANRLLDAGAAPAGHGSKLARVLGA
TSPGELPNGPQPPADPVHRAVSEQVEQLLLWDRAVRADAYDAVHQMRVTTRKIRSLLT
DSQESFGLKESAWVIDELRELANVLGVARDAEVLGDRYQRELDALAPELVRGRVRERL
VDGARRRYQTGLRRSLIALRSQRYFRLLDALDALVSERAHATSGEESAPVTIDAAYRR
VRKAAKAAKTAGDQAGDHHRDEALHLIRKRAKRLRYTAAATGADNVSQEAKVIQTLLG
DHQDSVVSREHLIQQAIAANTAGEDTFTYGLLYQQEADLAERCREQLEAALRKLDKAV
RKARD"
CDS 2484394..2485104
/codon_start=1
/note="gene_2292"
/transl_table=11
/translation="VTRWDKRVDSGDWDAIAAEVSEYGGALLPRLITPGEAARLRKLY
ADDGLFRSTVDMASKRYGAGQYRYFHAPYPEPIERLKQALYPKLLPIARNWWAKLGRE
APWPDSLDDWLASCHAAGQTRSTALMLKYGTNDWNALHQDLYGELVFPLQVVINLSDP
ETDYTGGEFLLVEQRPRAQSRGTAMQLPQGHGYVLTTRDRPVRTSRGWSASPVRHGLS

TIRSGERYAMGLIFHDAA"
CDS complement(2485116..2486210)
/codon_start=1
/note="gene_2293"
/transl_table=11
/translation="VKVVIEADGGSRGNPGPAGYGAVVWTADHSTVLAESKQAIGRAT
NNVAEYRGLIAGLDDAVKLGATEAAVLMDSKLVVEQMSGRWKVKHPDLLKLYVQAQAL
ASQFRRINYEWVPRARNTYADRLANDAMDAAAQSAAADADPAKIVATESPTSPGWTGA
RGTPTRLLLLRHGQTELSEQRRYSGRGNPGLNEVGWRQVGAAAGYLARRGGIAAVVSS
PLQRAYDTAVTAARALALDVVVDDDLVETDFGAWEGLTFAEAAERDPELHRRWLQDTS
ITPPGGESFDDVLRRVRRGRDRIIVGYEGATVLVVSHVTPIKMLLRLALDAGSGVLYR
LHLDLASLSIAEFYADGASSVRLVNQTGYL"
CDS complement(2486207..2486944)
/codon_start=1
/note="gene_2294"
/transl_table=11
/translation="MKAGVAQQRSLLELAKLDAELTRIAHRATHLPQRAAYQQVQAEH
NAANDRMAALRIAAEDLDGQVSRFESEIDAVRKRGDRDRSLLTSGATDAKQLADLQHE
LDSLQRRQASLEDALLEVLERREELQAQQTAESRALQALRADLAAAQQALDEALAEID
QARHQHSSQRDMLTATLDPELAGLYERQRAGGGPGAGRLQGHRCGACRIEIGRGELAQ
ISAAAEDEVVRCPECGAILLQLEGFEE"
CDS complement(2486941..2488080)
/codon_start=1
/note="gene_2295"
/transl_table=11
/translation="MSVRLADVIDVLDQAYPPRLAQSWDSVGLVCGDPDDVVDSVTVA
VDATPAVVDQVPQAGLLLVHHPLLLRGVDTVAANTPKGVLVHRLIRTGRSLFTAHTNA
DSASPGVSDALAHAVGLTVDAVLDPVPGAADLDKWVIYVPRENSEAVRAAVFEAGAGH
IGDYSHCSWSVAGTGQFLAHDGASPAIGSVGTVERVAEDRVEVVAPARARAEVLAAMR
AAHPYEEPAFDIFALVPPPVGSGLGRIGRLPKPEPLRTFVARLEAALPPTATGVRAAG
DPDLLVSRVAVCGGAGDSLLATVAAADVQAYVTADLRHHPADEHCRASQVALIDVAHW
ASEFPWCGQAAEVLRSHFGASLPVRVCTICTDPWNLDHETGRDQA"
CDS complement(2488077..2489171)
/codon_start=1
/note="gene_2296"
/transl_table=11
/translation="VLWILGPHTGPLLFDAVASLDTSPLAAARYHGDQDVAPGVLDFA
VNVRHDRPPEWLVRQLAALLPELARYPSTDDVHRAQDAVAERHGRTRDEVLPLVGAAE
GFALLHNLSPVRAAIVVPAFTEPAIALSAAGITAHHVVLKPPFVLDTAHVPDDADLVV
VGNPTNPTSVLHLREQLLELRRPGRILVVDEAFADWVPGEPQSLADDSLPDVLVLRSL
TKTWSLAGLRVGYALGSPDVLARLTVQRAHWPLGTLQLTAIAACCAPRAVAAAAADAV
RLTALRAEMVAGLRSVGAEVVDGAAPFVLFNIADADGLRNYLQSKGIAVRRGDTFVGL
DARYLRAAVRPEWPVLVAAIAEWAKRGGRR"
CDS complement(2489679..2489855)
/codon_start=1
/note="gene_2297"
/transl_table=11
/translation="LALWYQAMIAKFGEQVVDAKVWAPAKRVGVHEAKTRLSELLRLV
YGGQRLRLPAAASR"
CDS 2489867..2490625
/codon_start=1

/note="gene_2298"
/transl_table=11
/translation="LSNRFVNDNGIVTDTTASGTNCPPPPRAAARRASSPGESPQLVI
FDLDGTLTDSARGIVSSFRHALNHIGAPVPEGDLATHIVGPPMHETLRAMGLGESAEE
AIVAYRADYSARGWAMNSLFDGIGPLLADLRTAGVRLAVATSKAEPTARRILRHFGIE
QHFEVIAGASTDGSRGSKVDVLAHALAQLRPLPERLVMVGDRSHDVDGAAAHGIDTVV
VGWGYGRADFIDKTSTTVVTHAATIDELREALGV"
CDS 2490618..2491109
/codon_start=1
/note="gene_2299"
/transl_table=11
/translation="VSDPLHVTFVCTGNICRSPMAEKMFAQQLRHRGLGDAVRVTSAG
TGNWHVGSCADERAAGVLRAHGYPTDHRAAQVGTEHLAADLLVALDRNHARLLRQLGV
EAARVRMLRSFDPRSGTHALDVEDPYYGDHSDFEEVFAVIESALPGLHDWVDERLARN
GPS"
CDS 2491163..2491924
/codon_start=1
/note="gene_2300"
/transl_table=11
/translation="VVAFTYLCFTVLAPWQLGKNAKTSRENQQIRYSLDTPPVPLKTL
LPQQDSSAPDAQWRRVTATGQYLPDVQVLARLRVVEGDQAFEVLAPFVVDGGPTVLVD
RGYVRPQVGSHVPPIPRLPVQTVTITARLRDSEPSVAGKDPFVRDGFQQVYSINTGQV
AALTGVQLAGSYLQLIEDQPGGLGVLGVPHLDPGPFLSYGIQWISFGILAPIGLGYFA
YAEIRARRREKAGSPPPDKPMTVEQKLADRYGRRR"
CDS complement(2491906..2492754)
/codon_start=1
/gene="cobD"
/note="gene_2301"
/transl_table=11
/translation="VALFGRAAAKLEQITYRDGRVAGAVHVGLLVGAVGLLGAALQRL
PGRCWPVAATATATWAALGGTSLARTGRQISDLLERDDVEAARRLLPSLCGRDPAQLG
GPGLTRAALESVAENTADAQVVPLLWAASSGVPAVLGYRAINTLDSMIGYRSPRYLRF
GWAAARLDDWANYVGARATAVLVVICAPVVGGSPRGAVRAWRRDAARHPSPNAGVVEA
AFAGALDVRLGGPTRYHHELQIRPTLGDGRSPKVADLRRAVVLSRVVQAGAAVLAVML
VYRRRP"
CDS 2492677..2492916
/codon_start=1
/gene="PRUPE_ppa007687mg"
/note="gene_2302"
/transl_table=11
/translation="VYRTGDPAVTVGDLLQLGGRPTEQGHRMTSFGVAEHDVEQAADQ
HADGPGLPDRCKHSGSVAHVVYAQLFMTSYGSYPR"
CDS 2492913..2493728
/codon_start=1
/note="gene_2303"
/transl_table=11
/translation="MKRPATRVADLLNPAAMLLPAANVIMQLAVPGVGYGVLESPVDS
GNVYKHPFKRARTTGTYLAVATIGTESDRALIRGAVDVAHRQVRSTASSPVSYNAFDP
KLQLWVAACLYRYFVDQHEFLYGPLEDATADAVYQDAKRLGTTLQVPEGMWPPDRVAF
DEYWKRSLDGLQIDAPVREHLRGVASVAFLPWPLRAVAGPFNLFATTGFLAPEFRAMM
QLEWSQAQQRRFEWLLSVLRLADRLIPHRAWIFVYQLYLWDMRFRARHGRRIV"

```
CDS             complement(2493823..2493948)
                /codon_start=1
                /note="gene_2304"
                /transl_table=11
                /translation="MTSDQLPATKADLYAAVDAMRADMRELLEQISTLIREATQK"
tRNA            complement(2494067..2494141)
                /note="identified by tRNAscan-SE; putative"
CDS             complement(2494187..2494648)
                /codon_start=1
                /note="gene_2305"
                /transl_table=11
                /translation="MLNVGATAPDFTLRDQNQQLVTLRGYRGAKNVLLVFFPLAFTGI
                CQGELDQLRDHLPEFENDDSAALAISVGPPPTHKIWATQSGFTFPLLSDFWPHGAVSQ
                AYGVFNEQAGIANRGTFVVDRSGIIRFAEMKQPGEVRDQRLWTDALAALTA"
CDS             complement(2494648..2495070)
                /codon_start=1
                /note="gene_2306"
                /transl_table=11
                /translation="VVAADHASNYARKLGIQRDQLIQEWGWDEDTDDDIRAAIEEACG
                GELLDEDTDEVIDVVLLWWRDGDGDLVDTLMDAIGPLAEDGVIWVVTPKTGQPGHVLP
                AEIAEAAPTAGLMPTSSVNLGNWSASRLVQPKSRAGKR"
CDS             complement(2495162..2495752)
                /codon_start=1
                /note="gene_2307"
                /transl_table=11
                /translation="VLIGWRAVPRRHGGELPRRGALALGCIALLLMGIVGCTTVTDGT
                AMPDTNVAPAYRSSVSASVSASAATSSIRESQRQQSLTTKAIRTSCDALAATSKDAID
                KVNAYVAAFNQGRNTGPTEGPAIDALNNSASTVSGSLSAALSAQLGDALNAYVDAARA
                VANAIGAHASTAEFNRRVDRLNDTKTKALKMCVAAF"
CDS             2495924..2498716
                /codon_start=1
                /gene="aceE"
                /note="gene_2308"
                /transl_table=11
                /translation="LTTDFARHDLAQNSNSASEPDRVRVIREGVASYLPDIDPEETSE
                WLESFDTLLQRCGPSRARYLMLRLLERAGEQRVAIPALTSTDYVNTIPTELEPWFPGD
                EDVERRYRAWIRWNAAIMVHRAQRPGVGVGGHISTYASSAALYEVGFNHFFRGKSHPG
                GGDQVFIQGHASPGIYARAFLEGRLTAEQLDGFRQEHSHVGGGLPSYPHPRLMPDFWE
                FPTVSMGLGPLNAIYQARFNHYLHDRGIKDTSDQHVWCFLGDGEMDEPESRGLAHVGA
                LEGLDNLTFVINCNLQRLDGPVRGNGKIIQELESFFRGAGWNVIKVVWGREWDALLHA
                DRDGALVNLMNTTPDGDYQTYKANDGGYVRDHFFGRDPRTKALVENMSDQDIWNLKRG
                GHDYRKVYAAYRAAVDHKGQPTVILAKTIKGYALGKHFEGRNATHQMKKLTLEDLKEF
                RDTQRIPVSDAQLEENPYLPPYYHPGLNAPEIRYMLDRRRALGGFVPERRTKSKALTL
                PGRDIYAPLKKGSGHQEVATTMATVRTFKEVLRDKQIGPRIVPIIPDEARTFGMDSWF
                PSLKIYNRNGQLYTAVDADLMLAYKESEVGQILHEGINEAGSVGSFIAAGTSYATHNE
                PMIPIYIFYSMFGFQRTGDSFWAAADQMARGFVLGATAGRTTLTGEGLQHADGHSLLL
                AATNPAVVAYDPAFAYEIAYIVESGLARMCGENPENIFFYITVYNEPYVQPPEPENFD
                PEGVLRGIYRYHAATEQRTNKAQILASGVAMPAALRAAQMLAAEWDVAADVWSVTSWG
                ELNRDGVAIETEKLRHPDRPAGVPYVTRALENARGPVIAVSDWMRAVPEQIRPWVPGT
                YLTLGTDGFGFSDTRPAARRYFNTDAESQVVAVLEALAGDGEIDPSVPVAAARQYRID
                DVAAAPEQTTDPGPGA"
```

CDS 2498776..2500020
/codon_start=1
/note="gene_2309"
/transl_table=11
/translation="VNDNQLAPVARPRSPLELLDTVPDSLLRRLKQYSGRLATEAVSA
MQERLPFFADLEASQRASVALVVQTAVVNFVEWMHDPHSDVGYTAQAFELVPQDLTRR
IALRQTVDMVRVTMEFFEEVVPLLARSEEQLTALTVGILKYSRDLAFTAATAYADAAE
ARGTWDSRMEASVVDAVVRGDTGPELLSRAAALNWDTTAPATVLVGTPAPGPNGSNSD
GDSERASQDVRDTAARHGRAALTDVHGTWLVAIVSGQLSPTEKFLKDLLAAFADAPVV
IGPTAPMLTAAHRSASEAISGMNAVAGWRGAPRPVLARELLPERALMGDASAIVALHT
DVMRPLADAGPTLIETLDAYLDCGGAIETCARKLFVHPNTVRYRLKRITDFTGRDPTQ
PRDAYVLRVAATVGQLNYPTPH"
CDS 2500259..2501167
/codon_start=1
/note="gene_2310"
/transl_table=11
/translation="VIALLAPGQGSQTEGMLSPWLQLPGAADQIAAWSKAADLDLARL
GTTASTEEITDTAVAQPLIVAATLLAHQELARRCVLAGKDVIVAGHSVGEIAAYAIAG
VIAADDAVALAATRGAEMAKACATEPTGMSAVLGGDETEVLSRLEQLDLVPANRNAAG
QIVAAGRLTALEKLAEDPPAKARVRALGVAGAFHTEFMAPALDGFAAAAANIATADPT
ATLLSNRDGKPVTSAAAAMDTLVSQLTQPVRWDLCTATLREHTVTAIVEFPPAGTLSG
IAKRELRGVPARAVKSPADLDELANL"
CDS 2501243..2501590
/codon_start=1
/gene="acpP"
/note="gene_2311"
/transl_table=11
/translation="VPVTQEEIIAGIAEIIEEVTGIEPSEITPEKSFVDDLDIDSLSM
VEIAVQTEDKYGVKIPDEDLAGLRTVGDVVAYIQKLEEENPEAAQALRAKIESENPDA
VANVQARLEAESK"
CDS 2501587..2502837
/codon_start=1
/note="gene_2312"
/transl_table=11
/translation="VSQPSTANGGFPSVVVTAVTATTSISPDIESTWKGLLAGESGIH
ALEDEFVTKWDLAVKIGGHLKDPVDSHMGRLDMRRMSYVQRMGKLLGGQLWESAGSPE
VDPDRFAVVVGTGLGGAERIVESYDLMNAGGPRKVSPLAVQMIMPNGAAAVIGLQLGA
RAGVMTPVSACSSGSEAIAHAWRQIVMGDADVAVCGGVEGPIEALPIAAFSMMRAMST
RNDEPERASRPFDKDRDGFVFGEAGALMLIETEEHAKARGAKPLARLLGAGITSDAFH
MVAPAADGVRAGRAMTRSLELAGLSPADIDHVNAHGTATPIGDAAEANAIRVAGCDQA
AVYAPKSALGHSIGAVGALESVLTVLTLRDGVIPPTLNYETPDPEIDLDVVAGEPRYG
DYRYAVNNSFGFGGHNVALAFGRY"
CDS 2502931..2504184
/codon_start=1
/note="gene_2313"
/transl_table=11
/translation="MTELVTGKAFPYVVVTGIAMTTALATDAETTWKLLLDRQSGIRT
LDDPFVEEFDLPVRIGGHLLEEFDHQLTRIELRRMGYLQRMSTVLSRRLWENAGSPEV
DTNRLMVSIGTGLGSAEELVFSYDDMRARGMKAVSPLTVQKYMPNGAAAAVGLERHAK
AGVMTPVSACASGAEAIARAWQQIVLGEADAAICGGVETRIEAVPIAGFAQMRIVMST
NNDDPAGACRPFDRDRDGFVFGEGGALLLIETEEHAKARGANILARIMGASITSDGFH

MVAPDPNGERAGHAITRAIQLAGLAPGDIDHVNAHATGTQVGDLAEGRAINNALGGNR
PAVYAPKSALGHSVGAVGAVESILTVLALRDQVIPPTLNLVNLDPEIDLDVVAGEPRP
GNYRYAINNSFGFGGHNVAIAFGRY"
CDS 2504215..2505636
/codon_start=1
/note="gene_2314"
/transl_table=11
/translation="MTIMAPEAVGESLDPRDPLLRLSNFFDDGSVELLHERDRSGVLA
AAGTVNGVRTIAFCTDGTVMGGAMGVEGCTHIVNAYDTAIEDQSPIVGIWHSGGARLA
EGVRALHAVGQVFEAMIRASGYIPQISVVVGFAAGGAAYGPALTDVVVMAPESRVFVT
GPDVVRSVTGEDVDMASLGGPETHHKKSGVCHIVADDELDAYDRGRRLVGLFCQQGHF
DRSKAEAGDTDIHALLPESSRRAYDVRPIVTAILDADTPFDEFQANWAPSMVVGLGRL
SGRTVGVLANNPLRLGGCLNSESAEKAARFVRLCDAFGIPLVVVVDVPGYLPGVDQEW
GGVVRRGAKLLHAFGECTVPRVTLVTRKTYGGAYIAMNSRSLNATKVFAWPDAEVAVM
GAKAAVGILHKKKLAAAPEHEREALHDQLAAEHERIAGGVDSALDIGVVDEKIDPAHT
RSKLTEALAQAPARRGRHKNIPL"
CDS 2505856..2506560
/codon_start=1
/note="gene_2315"
/transl_table=11
/translation="MCSWGGHAVACLGTAAALYGFDTENTVAIHMLDPGVRMRPTVGL
MVHQRVGARLQRVSGRLATAPAWTAVEVARQLRRPRALATLDAALRSMRCARSEIENA
VAEQRGRRGIVAARELLPFADGRAESAMESEARLVMIDHGLPLPELQYPIHGHGGEMW
RVDFAWPDMRLAAEYESIEWHAGPAEMLRDKTRWAKLQELGWTIVPIVVDDVRREPGR
LAARIARHLDRARMAG"
CDS complement(2506629..2508179)
/codon_start=1
/note="gene_2316"
/transl_table=11
/translation="VLMPHSAALNAARRSADLTALADGGALDVIVIGGGITGVGIALD
AATRGLTVALVEKHDLAFGTSRWSSKLVHGGLRYLASGNVGIARRSAVERGILMTRNA
PHLVHAMPQLVPLLPSMGHTKRALVRAGFLAGDALRVLAGTPAATLPRSRRIPASRVV
EIAPTVRRDGLDGGLLAYDGQLIDDARLVMAVARTAAQHGARILTYVGASNVTGTSVE
LTDRRTRQSFALSARAVINAAGVWAGEIDPSLRLRPSRGTHLVFDAKSFANPTAALTI
PIPGELNRFVFAMPEQLGRIYLGLTDEDAPGPIPDVPQPSSEEITFLLDTVNTALGTA
VGTKDVIGAYAGLRPLIDTGGAGVQGRTADVSRDHAVFESPSGVISVVGGKLTEYRYM
AEDVLNRAITLRHLRAAKCRTRNLPLIGAPANPGPAPGSGAGLPESLVARYGAEAANV
AAAATCERPTEPVADGIDVTRAEFEYAVTHEGALDVDDILDRRTRIGLVPRDRERVVA
VAKEFLSR"
CDS complement(2508173..2508742)
/codon_start=1
/note="gene_2317"
/transl_table=11
/translation="MLSMSNDRADTGGRILRAAASCVVDYGVDRVTLAEIARRAGVSR
PTVYRRWPDTRSIMASMLTSHIADVLREVPLDGDDREALVKQIVAVADRLRGDDLIMS
VMHSELARVYITERLGTSQQVLIEGLAARLTVAQRSGSVRSGDARRLATMVLLIAQST
IQSADIVDSILDSAALATELTHALNGYLC"
CDS 2508790..2510379
/codon_start=1
/note="gene_2318"
/transl_table=11

/translation="MKWDAWGDPAAAKPLSDGVRSLLKQVVGLADSEQPELDPAQVQL
RPSALSGADHDALARIVGTEYFRTADRDRLLHAGGKSTPDLLRRKDTGVQDAPDAVLL
PGGPNGEDAVADILHYCSDHGIAVVPFGGGTSVVGGLDPVRNDFRAVISLDMRRFDRL
HRIDEVSGEAELEAGVTGPEAERLLGEHGFSLGHFPQSFEFATIGGFAATRSSGQDSA
GYGRFNDMILGLRMITPVGVLDLGRVPASAAGPDLRQLAIGSEGVFGVITRVRLRVHR
IPESTRYEAWSFPDFATGVAALRTITQTGTGPTVVRLSDEAETGVNLATTEAIGETQI
TGGCLGITVFEGTQEHTESRHAETRALLAARGGTSLGEGPARAWERGRFAAPYLRDSL
LAAGALCETLETATVWSNTPVLKAAVTEALTTSLAASGTPALVMCHVSHVYPTGASLY
FTVVAGQRGDPIEQWLAAKKAASDAIMATGGTITHHHAVGSDHRPWMRAEVGDLGVTL
LRTIKATLDPAGILNPGKLIP"

CDS 2510376..2511305
/codon_start=1
/note="gene_2319"
/transl_table=11
/translation="MSAGQLRRHEIGKVTALTNPLSGHGAAVKAAHGAIARLKHRGVD
VVEIVGGDAHDARHLLAAAVAKGTDAVMVTGGDGVVSNALQVLAGTDIPLGIIPAGTG
NDHAREFGLPTKNPKAAADIVVDGWTETIDLGRIQDDNGIEKWFGTVAATGFDSLVND
RANRMRWPHGRMRYYIAMLAELSRLRPLPFRLVLDGTEEIVADLTLADFGNTRSYGGG
LLICPNADHSDGLLDITMAQSDSRTKLLRLFPTIFKGAHVELDEVSTTRAKTVHVECP
GINVYADGDFACPLPAEISAVPAALQVLRPRHG"

CDS 2511371..2511874
/codon_start=1
/note="gene_2320"
/transl_table=11
/translation="MSGHRKKAMLALAAASLAATLAPNAVAAAEPSWNGQYLVTLSAN
AKTGTSMAANRPEYPHKANYTFSSRCASDVCIATVVDAPPPKNEFIPRPIEYTWNGTQ
WVREISWQWDCLLPDGTIEYAPAKSITAYTPGQYGILTGVFHTDIASGTCKGNVDMPV
SAKPIVG"

CDS complement(2511907..2512362)
/codon_start=1
/note="gene_2321"
/transl_table=11
/translation="LRYRDLETVAAPTINVLRVWPEIVGAIVLLVIAAMGIGHGLRPS
PEPVPAPQKQLGCVRFALIFGLTVINPATFVYFTAVAVTLARALRATTAIAVVVGVAL
ASLLWQLLLVSAGAFLRSRATARVRRMTVLAGNAVIAAFGAVLVVHAFA"

CDS 2512481..2512699
/codon_start=1
/note="gene_2322"
/transl_table=11
/translation="VRLRPAEDSDDFLAWSSTDTTIDDAVHVTGPYDYLLHIRVCDTA
DLDRLLRRLKTSAEAAQTQTRIALRSRR"

CDS complement(2512728..2513240)
/codon_start=1
/note="gene_2323"
/transl_table=11
/translation="MRASNQFADVTSGVVYIHGSPAAVCPHVEWALSSTLQAKANLVW
TPQPALPPQLRAVTNWVGPVGTGARLANALRSWSVLRFEVTEDPSPGVDGQRFSHTPQ
LGLWSGAMSANGDIMVGEMRLRAMMAQGADTLAAELDSVLGTAWDQALEVYRDGGDAG
EVTWLSRGVG"

CDS complement(2513391..2514209)
/codon_start=1

```
                     /note="gene_2324"
                     /transl_table=11
                     /translation="MTALEVLGGWPVPAAAAAVIGPAGVLATHGDTARVFALASVTKP
                     LVARAAQVAVEEGVVNLDTPAGPPGSTVRHLLAHTSGLAMHSDQALARPGTRRMYSNY
                     GFTVLAESVQRESGIEFGRYLTEAVCEPLGMVTTRLDGGPAAAGFGATSTVADLAVFA
                     GDLLRPSTVSAQMHADATTVQFPGLDGVLPGYGVQRPNDWGLGFEIRNSKSPHWTGEC
                     NSTRTFGHFGQSGGFIWVDPKADLALVVLTARDFGDWALDLWPAISDAVLAEYT"
     CDS             complement(2514223..2515284)
                     /codon_start=1
                     /note="gene_2325"
                     /transl_table=11
                     /translation="MSGALETTEEFGNRFVAAIDSAGLAILVSVGHQTGLLDTMAGLP
                     PATSMEIAEAAGLEERYVREWLGGMTTGQIVEYDAGSSTYSLPAHRAGMLTRAAGPDN
                     LAVIAQFVSLLGEVEQKVIRCFREGGGVPYSEYPRFHKLMAEMSGMVFDAALIDVVLP
                     LVDGLPDRLRSGADVADFGCGSGRAVKLMAQAFGASRFTGIDFSDEAVAAGTEEAARL
                     GLANATFERHDLAELDKVGAYDVITVFDAIHDQAQPARVLQNIYRALRPGGVLLMVDI
                     KASSQLEDNVGVPLSTYLYTTSLMHCMTVSLALDGAGLGTVWGRQLATSMLADAGFTD
                     VTVAEIESDVLNNYYIARK"
     variation       2515298..2515350
                     /note="supplemented from AL2122/97"
     CDS             2515579..2516664
                     /codon_start=1
                     /note="gene_2326"
                     /transl_table=11
                     /translation="MSQTVRGVIARQKGEPVELVNIVVPDPGPGEAVVDVTACGVCHT
                     DLTYREGGINDEYPFLLGHEAAGIIEAVGPGVTAVEPGDFVILNWRAVCGQCRACKRG
                     RPRYCFDTFNAEQKMTLTDGTELTAALGIGAFADKTLVHSGQCTKVDPAADPAVAGLL
                     GCGVMAGLGAAINTGGVTRDDTVAVIGCGGVGDAAIAGAALVGAKRIIAVDTDDTKLD
                     WARTFGATHTVNAREVDVVQAIGGLTDGFGADVVIDAVGRPETYQQAFYARDLAGTVV
                     LVGVPTPDMRLDMPLVDFFSHGGALKSSWYGDCLPESDFPTLIDLYLQGRLPLQRFVS
                     ERIGLEDVEEAFHKMHGGKVLRSVVML"
     CDS             2516664..2517299
                     /codon_start=1
                     /note="gene_2327"
                     /transl_table=11
                     /translation="MAAIERVITHGTFELDGGSWEVDNNIWLVGDDSEVVVFDAAHHA
                     APIIDAVGGRKVVAVICTHGHNDHVTVAPELGTALDAPVLMHPGDAVLWRMTHPDKSF
                     RAVSDGDAVRVGGTELRALHTPGHSPGSVCWYAPELGPGTGTVFSGDTLFAGGPGATG
                     RSYSDFPTILRSISGRLGALPGDTVVHTGHGDSTTIGDEIVHYEEWVARGH"
     CDS             complement(2517376..2518974)
                     /codon_start=1
                     /note="gene_2328"
                     /transl_table=11
                     /translation="MQITPFLAGTAGLSLLFSSTQANVAGMLIGMALRAGARRQPVIG
                     CAAALVFGGLPALAFPAPSWWWLAWFGLVPLLLVVRAAPTSWEGALRAWTGMGGFVLA
                     TQYWLVTSAGPMLVLLAAGLGVLWLPAGWLAHRLLSVPVTTCRVGAALVVVPSAWVAA
                     EAVRSWQSLGGPWALLGASQWSQPVTLASASLGGVWLTSFLLVATNTAIASVLVCRAT
                     GGRLVALGCVIGCAGLGPASYLLGSVPVGGPTVRVALVQAGDIADAAARLAAGEEFTA
                     AVADQRPDLVVWGESSVGQDLTRHPDVLARLAELSQRVGADLLVNVDAPAPDGGIYKS
                     AVLVGAHEAVGSYRKTRLVPFGEYVPLRPLFGWITRYSKAAAKDRQRGAGPVVLAVNS
                     LHIAPLISYEMTFSDLTRHAARLGAALLVYQSSTSTFQGSWAQPQLAAQPAVRAVEAG
```

IPAVHASLSGDSSAFDTRGRRLAWCSAEFNGAIVVNVPLASNVTLYLRLGDWVPVTAF
VVMGAGFAVFLRRSLARVSDCADK"
CDS 2518973..2519926
/codon_start=1
/note="gene_2329"
/transl_table=11
/translation="MAKDLVATVPDLSGKLAIITGANSGLGFGLARRLSAAGADVIMA
IRNRAKGEAAVEEIRTAVPDAKLTIKALDLSSLASVAALGEQLMADGRPIDLLINNAG
VMTPPERVTTADGFELQFGSNHLGHFALTAHLLPLLRAAQRARVVSLSSLAARRGRIH
FDDLQFERSYAPMTAYGQSKLAVLMFARELDRRSRAAGWGIISNAAHPGLTKTNLQIA
GPSHGRDKPALMERLYKTSWRFAPFLWQEIEEGILPALYAAATPQADGGAFYGPRGRY
EVAGGGVREAKVPAAARNDADSKRLWEVSEQLTGVSYPKSR"
CDS complement(2519904..2521652)
/codon_start=1
/note="gene_2330"
/transl_table=11
/translation="LSIGVTNLAAVAADHSITRKPVLTLYRQRPPEVGVPSENPRLDE
PGLVITDFVDRVGDSVGIVAADGSVYRSEALVADALLALAYTATGGRALPGSVTVTYP
AHWGPAAVAALDSALRRASEWSHGTSSTAQPLSLLPDAAAALYAIRADPGIPARGIVA
VCDFGGSGTGITLVDAADEYRPVAATVRHQAFSGDLIDQSLLSYVMSELPGTGAFDPA
GTSAIGSLTKLRIECRKAKERLSSSTVTTLTDALGGDIRLTRNELEDTIRDSLDSVGR
ALEQTLARSGIRTAELVAIVSVGGGANIPAVTTTLSGRFCVPVVRTPRPQLTAAFGGA
LWAARRPGDTSATVLTAVTSATATAPADAPASVLQPALAWSEADEDSHIGPAPGYTAA
RPSLSFDHDAHAEPEPKSPPIPWYRLPAVIITGTTVAVLLVGAAVAIGLSTGDQPTAP
GTPQRPGVTTTAAPPPSPAPASDGPTTEPAPPVQAPATGGPAPPLQQPLPPPPTTTNT
QPAVTTDVITPAPTTPASAPPATTQPPATTQPPATTSPSPPPIPPIPPIPEIPQLPPG
IPQVPGIGQFSAISGS"
CDS 2522110..2523261
/codon_start=1
/note="gene_2331"
/transl_table=11
/translation="VFGVVGLLADVVYEGARSITGPLLASLGATGLVVGVVTGVGEAA
ALGLRLVSGPLADRSRRFWAWTIAGYTLTVVTVPLLGIAGALWVACALVIAERVGKAV
RGPAKDTLLSHAASVTGRGRGFAVHEALDQVGAMIGPLTVAGMLAITGNAYAPALGVL
TLPGGAALALLLWLQRRVPRPESYEDCPVVLGNPSAPRPWALPAQFWLYCGFTAITML
GFGTFGLLSFHMVSHGVLAAAMVPVVYAAAMAADALTALASGFSYDRYGAKTLAVLPI
LSILVVLFAFTDNVTMVVIGTLVWGAAVGIQESTLRGVVADLVASPRRASAYGVFAAG
LGAATAGGGALIGWLYDISIGTLVVVVIALELMALVMMFAIRLPRVAPS"
CDS 2523436..2524722
/codon_start=1
/note="gene_2332"
/transl_table=11
/translation="MGLNTAIATRVNGTPPPEVPIADIELGSLDFWALDDDVRDGAFA
TLRREAPISFWPTIELPGFVAGNGHWALTKYDDVFYASRHPDIFSSYPNITINDQTPE
LAEYFGSMIVLDDPRHQRLRSIVSRAFTPKVVARIEAAVRDRAHRLVSSMIANNPDRQ
ADLVSELAGPLPLQIICDMMGIPKADHQRIFHWTNVILGFGDPDLATDFDEFMQVSAD
IGAYATALAEDRRVNHHDDLTSSLVEAEVDGERLSSREIASFFILLVVAGNETTRNAI
THGVLALSRYPEQRDRWWSDFDGLAPTAVEEIVRWASPVVYMRRTLTQDIELRGTKMA
AGDKVSLWYCSANRDESKFADPWTFDLARNPNPHLGFGGGGAHFCLGANLARREIRVA
FDELRRQMPDVVATEEPARLLSQFIHGIKTLPVTWS"
CDS complement(2524976..2526046)

/codon_start=1
/note="gene_2333"
/transl_table=11
/translation="LIRNRFAVHHSRWHFAVLYTFLSMVNSCLGLWQKIVFGRRVAET
VIADPPIFIVGHWRTGTTLLHELLVVDDRHTGPTGYECLAPHHFLLTEWFAPYVEFLV
SKHRAMDNMDLSLHHPQEDEFVWCMQGLPSPYLTIAFPNRPPQYEEYLDLEQVAPREL
EIWKRTLFRFVQQVYFRRRKTVILKNPTHSFRIKVLLEVFPQAKFIHIVRDPYVVYPS
TIHLHKALYRIHGLQQPTFDGLDDKVVSTYVDLYRKLDEGRELVDPTRFYELRYEDLI
GDPEGQLRRLYQHLGLGDFECYLPRLRQYLADHADYKTNSYQLTVEQRAIVDEHWGEI
IDRYGYDRHTPEPARLRPAVGG"

CDS complement(2526139..2527608)
/codon_start=1
/note="gene_2334"
/transl_table=11
/translation="MTATQSPPEPAPDRVRLAGCPLAGTPDVGLTAQDATTALGVPTR
RRASSGGIPVATSMWRDAQTVRTYGPAVAKALALRVAGKARSRLTGRHCRKFMQLTDF
DPFDPAIAADPYPHYRELLAGERVQYNPKRDVYILSRYADVREAARNHDTLSSARGVT
FSRGWLPFLPTSDPPAHTRMRKQLAPGMARGALETWRPMVDQFARELVGGLLTQTPAD
VVSTVAAPMPMRAITSVLGVDGPDEAAFCRLSNQAVRITDVALSASGLISLVQGFAGF
RRLRALFTHRRDNGLLRECTVLGKLATHAEQGRLSDDELFFFAVLLLVAGYESTAHMI
STLFLTLADYPDQLTLLAQQPDLIPSAIEEHLRFISPIQNICRTTRVDYSVGQAVIPA
GSLVLLAWGAANRDPRQYEDPDVFRADRNPVGHLAFGSGIHLCPGTQLARMEGQAILR
EIVANIDRIEVVEPPTWTTNANLRGLTRLRVAVTPRVAP"

CDS 2528030..2528557
/codon_start=1
/note="gene_2335"
/transl_table=11
/translation="MRLPGRHVLYALSAVTMLAACSSNGARGGIASTNMNPTNPPATA
ETATVSPTPAPQSARTETWINLQVGDCLADLPPADLSRITVTIVDCATAHSAEVYLRA
PVAVDAAVVSMANRDCAAGFAPYTGQSVDTSPYSVAYLIDSHQDRTGADPTPSTVICL
LQPANGQLLTGSARR"

CDS 2528664..2528963
/codon_start=1
/note="gene_2336"
/transl_table=11
/translation="MTTPPDKARRRFLRDAYKNAERVARTALLTIDQDQLEQLLDYVD
ERLGEQPCDHTARHAQRWAQSHRIEWETLAEGLQEFGGYCDCEIVMNVEPEAIFG"

CDS 2529069..2529437
/codon_start=1
/note="gene_2337"
/transl_table=11
/translation="VADDSNDTATDVEPDYRFTLANERTFLAWQRTALGLLAAAVALV
QLVPELTIPGARQVLGVVLAILAILTSGMGLLRWQQADRAMRRHLPLPRHPTPGYLAV
GLCVVGVVALALVVAKAITG"

CDS 2529434..2529763
/codon_start=1
/note="gene_2338"
/transl_table=11
/translation="VNRHSTAASDRGLQAERTTLAWTRTAFALLVNGVLLTLKDTQGA
DGPAGLIPAGLAGAAASCCYVIALQRQRALSHRPLPARITPRGQVHILATAVLVLMVV
TAFAQLL"

```
CDS             2530215..2531084
                /codon_start=1
                /note="gene_2339"
                /transl_table=11
                /translation="MSYVAAEPGVLISPTDDLQSPRSAPAAHDENADGITGGTRDDSA
                PNSRFQLGRRIPEATAQEGFLVRPFTQQCQIIHTEGDHAVIGVSPGNSYFSRQRLRDL
                GLWGLTNFDRVDFVYTDVHVAESYEALGDSAIEARRKAVKNIRGVRAKITTTVNELDP
                AGARLCVRPMSEFQSNEAYRELHADLLTRLKDDEDLRAVCQDLVRRFLSTKVGPRQGA
                TATQEQVCMDYICAEAPLFLDTPAILGVPSSLNCYHQSLPLAAMLYARGSGLRASRNQ
                GHAIVTPDGSPAE"
CDS             2531081..2532271
                /codon_start=1
                /note="gene_2340"
                /transl_table=11
                /translation="MTATVLLEVPFSARGDRIPDAVAELRTREPIRKVRTITGAEAWL
                VSSYALCTQVLEDRRFSMKETAAAGAPRLNALTVPPEVVNNMGNIADAGLRKAVMKAI
                TPKAPGLEQFLRDTANSLLDNLITEGAPADLRNDFADPLATALHCKVLGIPQEDGPKL
                FRSLSIAFMSSADPIPAAKINWDRDIEYMAGILENPNITTGLMGELSRLRKDPAYSHV
                SDELFATIGVTFFGAGVISTGSFLTTALISLIQRPQLRNLLHEKPELIPAGVEELLRI
                NLSFADGLPRLATADIQVGDVLVRKGELVLVLLEGANFDPEHFPNPGSIELDRPNPTS
                HLAFGRGQHFCPGSALGRRHAQIGIEALLKKMPGVDLAVPIDQLVWRTRFQRRIPERL
                PVLW"
CDS             complement(2532456..2533370)
                /codon_start=1
                /note="gene_2341"
                /transl_table=11
                /translation="MVKTAMLGAVALVIALGGTCGVADALPLGQTDDPMIVAHRAGTR
                DFPENTVLAITNAVAAGVDGMWLTVQVSSDGVPVLYRPSDLATLTDGAGPVNSKTVQQ
                LQQLNAGWNFTTPGVEGHPYRQRATPIPTLEQAIGATPPDMTLFLDLKQTPPQPLVSA
                VAQVLTRTGAAGRSIVYSTNADITAAASRQEGLQVAESRDVTRQRLFNMALNHHCDPQ
                PDPGKWAGFELHRDVTVTEEFTLGSGISAVNAELWDEASVDCFRSQSGMKVMGFAVKT
                VDDYRLAHKIGLDAVLVDSPLAAQQWRH"
CDS             2533534..2534913
                /codon_start=1
                /note="gene_2342"
                /transl_table=11
                /translation="MSEMTARFSEIVGNANLLTGDAIPEDYAHDEELTGPPQKPAYAA
                KPATPEEVAQLLKAASENGVPVTARGSGCGLSGAARPVEGGLLISFDRMNKVLEVDTA
                NQVAVVQPGVALTDLDAATADTGLRYTVYPGELSSSVGGNVGTNAGGMRAVKYGVARH
                NVLGLQAVLPTGEIIRTGGRMAKVSTGYDLTQLIIGSEGTLALVTEVIVKLHPRLDHN
                ASVLAPFADFDQVMAAVPKILASGLAPDILEYIDNTSMAALISTQNLELGIPDQIRDS
                CEAYLLVALENRIADRLFEDIQTVGEMLMELGAVDAYVLEGGSARKLIEAREKAFWAA
                KALGADDIIDTVVPRASMPKFLSTARGLAAAADGAAVGCGHAGDGNVHMAIACKDPEK
                KKKLMTDIFALAMELGGAISGEHGVGRAKTGYFLELEDPVKISLMRRIKQSFDPAGIL
                NPGVVFGDT"
CDS             2535147..2536805
                /codon_start=1
                /note="gene_2343"
                /transl_table=11
                /translation="MSDNAKHHRDGHLVASGLQDRAARTPQHEGFLGPDRPWHLSFSL
                LLAGSFVLFSWWAFDYAGSGANKVILVLATVVGMFMAFNVGGNDVANSFGTSVGAGTL
```

```
                     TMKQALLVAAIFEVSGAVIAGGDVTETIRSGIVDLSGVSVDPRDFMNIMLSALSAAAL
                     WLLFANRMGYPVSTTHSIIGGIVGAAIALGMVSGQGGAALRMVQWDQIGQIVVSWVLS
                     PVLGGLVSYLLYGVIKRHILLYNEQAERRLTEIKKERIAHRERHKAAFDRLTEIQQIA
                     YTGALARDAVAANRKDFDPDELESDYYRELHEIDAKTSSVDAFRALQNWVPLVAAAGS
                     MIIVAMLLFKGFKHMHLGLTTMNNYFIIAMVGAAVWMATFIFAKTLRGESLSRSTFLM
                     FSWMQVFTASGFAFSHGSNDIANAIGPFAAILDVLRTGAIEGNAAVPAAAMVTFGVAL
                     CAGLWFIGRRVIATVGHNLTTMHPASGFAAELSAAGVVMGATVLGLPVSSTHILIGAV
                     LGVGIVNRSTNWGLMKPIVLAWVITLPSAAILASVGLVALRAIF"
     CDS             complement(2536912..2537832)
                     /codon_start=1
                     /note="gene_2344"
                     /transl_table=11
                     /translation="MPGLTCFEIFLAIAEAGSLGGAARELGLTQQAVSRRLASMEAQI
                     GVRLAIRTTRGSQLTPAGIVVAEWAARLLEVADEIDAGLGSLRTEGRQRIRVVASQTI
                     AEQLMPHWMLSLRAADMRRGGTVPEVILTATNSEHAIAAVRDGIADLGFIENPCPPTG
                     LGSVVVARDELVVVVPPGHKWARRSRVVSARELAQTPLVTREPNSGIRDSLTAALRDT
                     LGEDMQQAPPVLELSSAAAVRAAVLAGAGPAAMSRLAIADDLAFGRLLAVDIPALNLR
                     RQLRAIWVGGRTPPAGAIRDLLSHITSRST"
     CDS             complement(2537899..2538120)
                     /codon_start=1
                     /note="gene_2345"
                     /transl_table=11
                     /translation="MWLPYEATPPYERWPARDVDPKGPAACSIGGLRIVLASLRLVAV
                     AGSLSVMPIFEAPQSTLAWGHFSSMGRRH"
     CDS             2538119..2539414
                     /codon_start=1
                     /note="gene_2346"
                     /transl_table=11
                     /translation="MGAPRLIHVIRQIGALVVAAVTAAATINAYRPLARNGFASLWSW
                     FIGLVVTEFPLPTLASQLGGLVLTAQRLTRPVRAVSWLVAAFSALGLLNLSRAGRQAD
                     AQLTAALDSGLGPDRRTASAGLWRRPAGGGTAKTPGPLRMLRIYRDYAHDGDISYGEY
                     GRANHLDIWRRPDLDLTGTAPVLFQIPGGAWTTGNKRGQAHPLMSHLAELGWICVAIN
                     YRHSPRNTWPDHIIDVKRALAWVKAHISEYGGDPDFIAITGGSAGGHLSSLAALTPND
                     PRFQPGFEEADTRVQAAVPFYGVYDFTRLQDAMHPMMLPLLERMVVKQPRTANMQSYL
                     DASPVTHISADAPPFFVLHGRNDSLVPVQQARGFVDQLRQVSKQPVVYAELPFTQHAF
                     DLLGSARAAHTAIAVEQFLAEVYATQHAGSEPGPAVAIP"
     CDS             2539447..2540784
                     /codon_start=1
                     /note="gene_2347"
                     /transl_table=11
                     /translation="VKLLSPLDQMFARMEAPRTPMHIGAFAVFDLPKGAPRRFIRDLY
                     EAISQLAFLPFPFDSVIAGGASMAYWRQVQPDPSYHVRLSALPYPGTGRDLGALVERL
                     HSTPLDMAKPLWELHLIEGLTGRQFAMYFKAHHCAVDGLGGVNLIKSWLTTDPEAPPG
                     SGKPEPFGDDYDLASVLAAATTKRAVEGVSAVSELAGRLSSMVLGANSSVRAALTTPR
                     TPFNTRVNRHRRLAVQVLKLPRLKAVAHATDCTVNDVILASVGGACRRYLQELGDLPT
                     NTLTASVPVGFERDADTVNAASGFVAPLGTSIEDPVARLTTISASTTRGKAELLAMSP
                     NALQHYSVFGLLPIAVGQKTGALGVIPPLFNFTVSNVVLSKDPLYLSGAKLDVIVPMS
                     FLCDGYGLNVTLVGYTDKVVLGFLGCRDTLPHLQRLAQYTGAAFEELETAALP"
     CDS             complement(2540851..2541114)
                     /codon_start=1
                     /note="gene_2348"
```

```
                     /transl_table=11
                     /translation="VPTLFLDGQCLFGPVLVDPPAGPAALNLWSVVTGMAGLPHVYEL
                     QRPKSPADVELIAQQLRPYLDGRDWVSINRGEIVDIDRLAGRS"
     CDS             complement(2541142..2541543)
                     /codon_start=1
                     /note="gene_2349"
                     /transl_table=11
                     /translation="MTTVDFHFDPLCPFAYQTSVWIRDVRAQLGITINWRFFSLEEIN
                     LVAGKKHPWERDWSYGWSLMRIGALLRRTNMSLLDRWYAAIGHELHTLGGKPHDPAVA
                     RRLLCDVGVNAAILDAALDDPTTHDDVRADH"
     CDS             2541713..2543305
                     /codon_start=1
                     /note="gene_2350"
                     /transl_table=11
                     /translation="VFGLVVIVALVAAVVVGTVLGHRYRVGPPVLLILSGSLLGLIPR
                     FGDVQIDGEVVLLLFLPAILYWESMNTSFREIRWNLRVIVMFSIGLVIATAVAVSWTA
                     RALGMESHAAAVLGAVLSPTDAAAVAGLAKRLPRRALTVLRGESLINDGTALVLFAVT
                     VAVAEGAAGIGPAALVGRFVVSYLGGIMAGLLVGGLVTLLRRRIDAPLEEGALSLLTP
                     FAAFLLAQSLKCSGVVAVLVSALVLTYVGPTVIRARSRLQAHAFWDIATFLINGSLWV
                     FVGVQIPGAIDHIAGEDGGLPRATVLALAVTGVVIATRIAWVQATTVLGHTVDRVLKK
                     PTRHVGFRQRCVTSWAGFRGAVSLAAALAVPMTTNSGAPFPDRNLIIFVVSVVILVTV
                     LVQGTSLPTVVRWARMPEDVAHANELQLARTRSAQAALDALPTVADELGVAPDLVKHL
                     EKEYEERAVLVMADGADSATSDLAERNDLVRRVRLGVLQHQRQAVTTLRNQNLIDDIV
                     LRELQAAMDLEEVQLLDPADAE"
     CDS             2543697..2544431
                     /codon_start=1
                     /note="gene_2351"
                     /transl_table=11
                     /translation="VIAALAGALIAVTVPARPNRPEADREALWKIVHDRCEFGYRRTG
                     AYAPCTFVDEQSGTALYKADFDPYQFLLIPLARITGIEDPALRESAGRNYLYDAWAAR
                     FLVTARLNNSLPESDVVLTINPKNARTQDQLHIHISCSSPTTSAALRNVDTSEYVGWK
                     QLPIDLGGRRFQGLAVDTKAFESRNLFRDIYLKVTADGKKMENASIAVANVAQDQFLL
                     LLAEGTEDQPVAAETLQDHDCSITKS"
     CDS             2544854..2545087
                     /codon_start=1
                     /note="gene_2352"
                     /transl_table=11
                     /translation="VTLSLSDSTPPDVNGFGISLKIGSVDYQMPYQPVQSPTQVEATR
                     QGKSYTLTGTGHAVIPGQTGMRELPFGVHVTCP"
     CDS             2545147..2546001
                     /codon_start=1
                     /note="gene_2353"
                     /transl_table=11
                     /translation="VQARGQVLITAAELAGMIQAGDPVSILDVRWRLDEPDGHAAYLQ
                     GHLPGAVFVSLEDELSDHTIAGRGRHPLPSGASLQATVRRCGIRHDVPVVVYDDWNRA
                     GSARAWWVLTAAGIANVRILDGGLPAWRSAGGSIETGQVSPQLGNVTVLHDDLYAGQR
                     LTLTAQQAGAGGVTLLDARVPERFRGDVEPVDAVAGHIPGAINVPSGSVLADDGTFLG
                     NGALNALLSDHGIDHGGRVGVYCGSGVSAAVIVAALAVIGQDAALFPGSWSEWSSDPT
                     RPVGRGTA"
     CDS             complement(2546002..2547006)
                     /codon_start=1
```

/note="gene_2354"
/transl_table=11
/translation="VGAPLRHCLLVAAALSLGCGVAAADPGYVANVIPCEQRTLVLSA
FPAEADAVLAHTALDANPVVVADRRRYYLGSISGKKVIVAMTGIGLVNATNTTETAFA
RFTCASSIAIAAVMFSGVAGGAGRTSIGDVAIPARWTLDNGATFRGVDPGMLATAQTL
SVVLDNINTLGNPVCLCRNVPVVRLNHLGRQPQLFVGGDGSSSDKNNGQAFPCIPNGG
SVFGCQPCSAPDRSLGYTGNFFQAAGPWLKNALISNLNIVSTVNPGFDAVDQETAAAQ
AVADAHGVPFLGIRGMSDGPGDPLHLPGFPVQFFVYKQIAANNAARVTEAFLQNWAGV
"
CDS 2547301..2548524
/codon_start=1
/note="gene_2355"
/transl_table=11
/translation="VIPNPLEELTLEQLRSQRTSMKWRAHPADVLPLWVAEMDVKLPP
TVADALRRAIDDGDTGYPYGTEYAEAVREFACQRWQWHDLEVSRTAIVPDVMLGIVEV
LRLITDRGDPVIVNSPVYAPFYAFVSHDGRRVIPAPLRGDGRIDLDALQEAFSSARAS
SGSSGNVAYLLCNPHNPTGSVHTADELRGIAERAQRFGVRVVSDEIHAPLIPSGARFT
PYLSVPGAENAFALMSASKAWNLGGLKAALAIAGREAAADLARMPEEVGHGPSHLGVI
AHTAAFRTGGNWLDALLRGLDHNRTLLGALVDEHLPGVQYRWPQGTYLAWLDCRELGF
DDAASDEMTEGLAVVSDLSGPARWFLDHARVALSSGHVFGIGGAGHVRINFATSRAIL
IEAVSRMSRSLLERR"
CDS 2548747..2549385
/codon_start=1
/note="gene_2356"
/transl_table=11
/translation="MDQSANHACLPTPLASTTGRGQDHEMPVEETSTPQKLPQFRYHP
DPVGTGSIVADEVSCVSCEQRRPYTYTGPVYAEEELNEAICPWCIADGSAASRFDATF
TDAMWAVPDDVPEDVTEEVLCRTPGFTGWLQEEWLHHCGDAAAFLGPVGASEVADLPD
ALDALRNEYRGYDWPADKIEEFILTLDRNGLATAYLFRCLSCGVHLAYADFA"
CDS 2549431..2550381
/codon_start=1
/note="gene_2357"
/transl_table=11
/translation="LHNNGSDVAQLREEQIMDVLRTPDSRFEHLVGYPFAPHYVDVTA
GDTQPLRMHYVDEGPGDGPPIVLLHGEPTWSYLYRTMIPPLSAAGHRVLAPDLIGFGR
SDKPTRIEDYTYLRHVEWVTSWFENLDLHDVTLFVQDWGSLIGLRIAAEHGDRIARLV
VANGFLPAAQGRTPLPFYVWRAFARYSPVLPAGRLVNFGTVHRVPAGVRAGYDAPFPD
KTYQAGARAFPRLVPTSPDDPAVPANRAAWEALGRWDKPFLAIFGYRDPILGQADGPL
IKHIPGAAGQPHARIKASHFIQEDSGTELAERMLSWQQAT"
CDS 2550413..2550865
/codon_start=1
/note="gene_2358"
/transl_table=11
/translation="MAMEMAMMGLLGTVVGASAMGIGGIAKSIAEAYVPGVAAAKDRR
QQMNVDLQARRYEAVRVWRSGLCSASNAYRQWEAGSRDTHAPNVVGDEWFEGLRPHLP
TTGEAAKFRTAYEVRCDNPTLMVLSLEIGRIEKEWMVEASGRTPKHRG"
CDS 2550961..2552028
/codon_start=1
/note="gene_2359"
/transl_table=11
/translation="VTNGLRNYRLGVGYTVERRGRRRAEPLGTVADMKYLDVDGIGQV

SRIGLGTWQFGSREWGYGDRYATGAARDIVKRARALGVTLFDTAEIYGLGKSERILGE
ALGDDRTEVVVASKVFPVAPFPAVIKNRERASARRLQLNRIPLYQIHQPNPVVPDSVI
MPGMRDLLDSGDIGAAGVSNYSLARWRKADAALGRPVVSNQVHFSLAHPDALEDLVPF
AELENRIVIAYSPLAQGLLGGKYGLENRPGGVRALNPLFGTENLRRIEPLLATLRAIA
VDVDAKPAQVALAWLISLPGVVAIPGASSVEQLEFNVAAADIELSAQSRDALTDAARA
FRPVSTGRFLTDMVREKVSRR"

CDS complement(2552034..2553977)
/codon_start=1
/note="gene_2360"
/transl_table=11
/translation="MNAHVEQLEFQAEARQLLDLMVHSVYSNKDAFLRELISNASDAL
DKLRIEALRNKDLEVDTSDLHIEIDADKAARTLTVRDNGIGMAREEVVDLIGTLAKSG
TAELRAQLREAKNAAASEELIGQFGIGFYSSFMVADKVQLLTRKAGESAATRWESSGE
GTYTIESVEDAPQGTSVTLHLKPEDAEDDLHDYTSEWKIRNLVKKYSDFIAWPIRMDV
ERRTPASQEEGGEGGEETVTIETETLNSMKALWARPKEEVSEQEYKEFYKHVAHAWDD
PLEIIAMKAEGTFEYQALLFIPSHAPFDLFDRDAHVGIQLYVKRVFIMGDCDQLMPEY
LRFVKGVVDAQDMSLNVSREILQQDRQIKAIRRRLTKKVLSTIKDVQSSRPEDYRTFW
TQFGRVLKEGLLSDIDNRETLLGISSFVSTYSEEEPTTLAEYVERMKDGQQQIFYATG
ETRQQLLKSPHLEAFKAKGYEVLLLTDPVDEVWVGMVPEFDGKPLQSVAKGEVDLSSE
EDTSEAEREERQKEFADLLTWLQETLSDHVKEVRLSTRLTESPACLITDAFGMTPALA
RIYRASGQEVPVGKRILELNPSHPLVTGLRQAHQDRADDAEKSLAETAELLYGTALLA
EGGALEDPARFAELLAERLARTL"

CDS complement(2554051..2554869)
/codon_start=1
/note="gene_2361"
/transl_table=11
/translation="MLGVAPEAKGPPVKVHHLNCGTMNAFGIALLCHVLLVETDDGLV
LVDTGFGIQDCLDPGRVGLFRHVLRPAFLQAETAARQIEQLGYRTSDVRHIVLTHFDF
DHIGGIADFPEAHLHVTAAEARGAIHAPSLRERLRYRRGQWAHGPKLVEHGPDGEPWR
GFASAKPLDSIGTGVVLVPMPGHTRGHAAVAVDAGHRWVLHCGDAFYHRGTLDGRFRV
PFVMRAEEKLLSYNRNQLRDNQARIVELHRRHDPDLLIVCAHDPDLYQLARDTA"

CDS 2554990..2555682
/codon_start=1
/note="gene_2362"
/transl_table=11
/translation="VNDLLTRRLLTMGAAAAMLAAVLLLTPITVPAGYPGAVAPATAA
CPDAEVVFARGRFEPPGIGTVGNAFVSALRSKVNKNVGVYAVKYPADNQIDVGANDMS
AHIQSMANSCPNTRLVPGGYSLGAAVTDVVLAVPTQMWGFTNPLPPGSDEHIAAVALF
GNGSQWVGPITNFSPAYNDRTIELCHGDDPVCHPADPNTWEANWPQHLAGAYVSSGMV
NQAADFVAGKLQ"

CDS 2555788..2556030
/codon_start=1
/note="gene_2363"
/transl_table=11
/translation="MHAKVGDYLVVKGTTTERHDQHAEIIEVRSADGSPPYVVRWLVN
GHETTVYPGSDAVVVTATEHAEAEKRAAARAGHAAT"

CDS complement(2556071..2556994)
/codon_start=1
/note="gene_2364"
/transl_table=11
/translation="MTAPEPRVPVIDMWAPFVPSAEVIDDLREGFPVELLSYFEVFTK

TTISAEQFGAYAESLRRTDDQILDSLDDAGITRSLITGFDERSTCGVTFVHNASVAAV
AARYPDRFLPFAGADILAGDSAVDEFERWVVEHGFRGLSLRPFMIGRPASDPAYFPCY
AKCVELGVPVSIHTSADWTRTRLSDLGHPRHIDDVACRFPELTILMSHGGYPWVLQAC
LIAWKHPNVYLELAAHRPKYFASPGAGWEPLMRFGQTTIRNKIVYGTGGFLINRPYLQ
LCDEMRALPVPREVLEDWLWRNATRVLRLDT"

CDS complement(2556991..2557200)
/codon_start=1
/note="gene_2365"
/transl_table=11
/translation="MSHDIATEEADDGALDRCVLCDLTGKRVDVKEATCTGRPATTFE
QAFAVERDAGFDDFLHGPVGPRSTP"

CDS 2557784..2559073
/codon_start=1
/note="gene_2366"
/transl_table=11
/translation="MTQTLRLTALDEMFITDDIDIVPSVQIEARVSGRFDLDRLAAAL
RAAVAKHALARARLGRASLTARTLYWEVPDRADHLAVEITDEPVGEVRSRFYARAPEL
HRSPVFAVAVVRETVGDRLLLNFHHAAFDGMGGLRLLLSLARAYADEPDEVGGPPIEE
ARNLKGVAGSRDLFDVLIRARGLAKPAIDRKRTTRVAPDGGSPDGPRFVFAPLTIESD
EMATAVARRPEGATVNDLAMAALALTILQWNRTHDVPAADSVSVNMPVNFRPTAWSTE
VISNFASYLAIVLRVDEVTDLEKATAIVAGITGPLKQSGAAGWVVDLLEGGKVLPAML
KRQLQLLLPLVEDRFVESVCLSNLGRVDVPAFGGEAGDTTEVWFSPTAAMSVMPIGVG
LVGFGGTLRAMFRGDGRTIGGEALGRFAALYRDTLLT"

CDS complement(2559169..2559432)
/codon_start=1
/gene="TBGG_01523"
/note="gene_2367"
/transl_table=11
/translation="MGAIDTQQRLGVIPACAPAEKAGRVTKVSSSGPNSTPLPAARIG
PGTNNAPSAADTTTYARGAIGPVATTTALRTPSTWMTRATATR"

CDS 2559472..2559897
/codon_start=1
/note="gene_2368"
/transl_table=11
/translation="VVGQRFVPGISDALASYTFGAFGVPLWQMVVGSFIGSAPRVFVY
TALGASITNLSSPLVYSAIAVWCVTAIIGAFAARRWYRKWRARPRRRCGLAQLTTGSQ
QRHTSHRTPAGVVMPGSLSEHRRLRQEAPDRIEHHPPIE"

CDS complement(2559826..2560647)
/codon_start=1
/note="gene_2369"
/transl_table=11
/translation="LPVVAIVALVASGVITFIWSQQRRLIYFPSAGPVPSASSVLPAG
RDVVVETQDGMRLGGWYFPHTSGGSGPAVLVCNGNAGDRSMRAELAVALHGLGLSVLL
FDYRGYGGNPGRPSEQGLAADARAAQEWLSGQSDVDPARIAYFGESLGAAVAVGLAVQ
RPPAALVLRSPFTSLAEVGAVHYPWLPLRRLLLDHYPSIERIASVHAPVLVIAGGSDD
IVPATLSEWLVAAAAEPKRYVVVPGVGHNDPELLDGRVMLDAIRRFLTETAVLGQ"

CDS 2562394..2563110
/codon_start=1
/note="gene_2370"
/transl_table=11
/translation="MRADMSVTSMLDREVYVYAEVDKLIGLPAGTAKRWINGYERGVK

DHPPILRVTPGATPWVTWGEFVETRMLAEYRDRRKVPIVRQRAAIEELRARFNLRYPL
AHLRPFLSTHERDLTMGGEEIGLPDAEVTIRTGQALLGDARWLASIATPGRDEVGEAV
IVELPVDKAFPEIVINPSRYSGQPTFVGRRVSPVTIAQMVDGGEEREDLAADYGLSLK
QIQDAIDYTKKYRLARLVAA"

```
CDS             2563120..2563452
                /codon_start=1
                /note="gene_2371"
                /transl_table=11
                /translation="MLEVDKVTHVVDENLLRLGVALSPSEKTRPGLAARPSTTCYRKA
                SSTPTGSPSSGVGWVVISNDRHLRTRPVEAELAVAHKLKVVHLHGRVGGLVRVGTADA
                AGCAVAGH"
tRNA            complement(2563739..2563812)
                /note="identified by tRNAscan-SE; putative"
CDS             complement(2563818..2564273)
                /codon_start=1
                /note="gene_2372"
                /transl_table=11
                /translation="VTGAGIVETTTNRVRHVPVPEPVSERLRDELPTEPNALVFPSYR
                GGHLPIEEYRRAFDKGCKAVGIADLVPHGLRHTTASLAISAGANVKVVQRLLGHATAA
                MTLDRHGHLLSDDLAGVAGLLVQAIKSAAASLRYSDPDSVAVENISAAS"
CDS             2565410..2565754
                /codon_start=1
                /note="gene_2373"
                /transl_table=11
                /translation="VVAALHAGKAVTIAPQSMTLTTQQAADLLGVSRPTVVRLIKSGE
                LAAERIGNRHRLVLDDVLAYREARRQRQYDALAESAMDIDADEDPEVICEQLREARRV
                VAARRRTERRRA"
CDS             2565787..2566383
                /codon_start=1
                /note="gene_2374"
                /transl_table=11
                /translation="VVKPAARLSVVVGDVAANYDGRVVVAPTGQAVDVAVREGAGDVG
                YSVERENLPADDPVRNGNRWRVIAVDTEHHRIAARRLGDGARAAFSGDYLHEHITHGY
                AITVHASQGTTAHSTHAVLGDNTSRATLYVAMTPARESNTAYLCERTAGEGARVDLAG
                WDLWVSGKAEAMSDEKSASPVWCRVGARCDHRGKRSCW"
CDS             2566461..2566730
                /codon_start=1
                /note="gene_2375"
                /transl_table=11
                /translation="MMKEIELHLVDAAAPSGEIAIKDLAALATALQELTTRISRDPIN
                TPGPGRTKQFMEELSQLASAPGPDIDGGIDLTDDEFQAFLQAARS"
CDS             complement(2567027..2567881)
                /codon_start=1
                /note="gene_2376"
                /transl_table=11
                /translation="VPAPVSVRDDLCRLVALSPGDGRIAGLVRQVCARALSLPSLPCE
                VAVNEPESPAEAVVAEFAEQFSVDVSAITGEQRSLLWTHLGEDAFGAVVAMYIADFVP
                RVRAGLEALGVGKEYLGWVTGPISWDHNTDLSAAVFNGFLPAVARMRALDPVTSELVR
                LRGAAQHNCRVCKSLREVSALDAGGSETLYGEIERFDTSVLLDVRAKAALRYADALIW
                TPAHLAVDVAVEVRSRFSDDEAVELTFDIMRNASNKVAVSLGADAPRVQQGTERYRIG
                LDGQTVFG"
```

```
CDS             complement(2567892..2569265)
                /codon_start=1
                /note="gene_2377"
                /transl_table=11
                /translation="MIEPQHAVNIVLKEAARSGRADETMVLVTEKVEATLRWAGNSMT
                TNGVSHSRNVTVISIVRRGDSAFVGSVVSAEVDPSVLPGLVVSSQDAARSAPEAGDAA
                PLLADTGEPDDWDAPVPGTGAGVFTGIAGSLSRGFRGADRLYGYAHRSVSTTFLASST
                GLRRRYTQPTGAIEINAKRGDASAWVGIGTPDFVEVPIDLMLERLSTRLRWAQRTVEL
                PAGRYQTIMPPSTVADMMIYLGWSMAGRGAQEGRTAFSAPGGGTRVGERLTELPLTLF
                TDPAAPGLACTPFVAVSNSSETQSVFDNGMEISQVDWIRSGVINALAYPRATAAKFDA
                PVAVAADNLIMTGGSADLADMIAGTERGLLLTTLWYIREVDPTTLLLTGLTRDGVYLV
                EDGEVSAAVNNFRFNESPLDLLRRATEAGVSEPTLPREWSDWVTRTAMPPLRIPDFHM
                SSVSQAQ"
CDS             complement(2569262..2570779)
                /codon_start=1
                /note="gene_2378"
                /transl_table=11
                /translation="VTPNRGIDEDFLDLPRQQLADAALSAAATAGASHADLRVHRIST
                EIIQLRDGELETAVISRELGLAVRVIVAGTWGFASHAELAPDVAAATARHAVHVATVL
                AALNTERVRLAPEPVYTDAEWVSNYRIDPFGVPASEKIAVLRDYSGRLLDADGIDHVS
                ASLNAVKEQTFYADTFGSSITQQRVRLLPCLDAVAVDSAAGNFESMRTLAPPTARGWE
                VVAGDEIWNWTDELAQLPSLLAEKVRAPSVMPGPTDLVIDPTNLWLTIHESIGHATEY
                DRAIGYEAAYAGTSFATPDKLGTLRYGSPVMNVTADRTAEFGLATVGYDDEGVAAQSW
                DLVRDGVFVGYQLDRAFAPRLGEPRSNGCSYADSPHHVPIQRMANISLQPGIEDLSTA
                DLIGRVDDGIYIVGDKSWSIDMQRYNFQFTGQRFFRIRGGQLYGQLRDVAYQSSTTDF
                WNAMEAVGGPSTWRMGGAINCGKAQPGQVAAVSHGCPSALFRGVNVLNTRTEGGR"
CDS             2570813..2571685
                /codon_start=1
                /note="gene_2379"
                /transl_table=11
                /translation="MRDAPRRRTALAYALLAPSLVGVVAFLLLPILVVVWLSLHRWDL
                LGPLRYVGLTNWRSVLTDSGFADSLVVTAVFVAIVVPAQTVLGLLAASLLARRLPGTG
                LFRTLYVLPWICAPLAIAVMWRWILAPTDGAISTVLGHRIEWLTDPGLALPVVSAVVV
                WTNVGYVSLFFLAGLMAIPQDIHNAARTDGASAWQRFWRITLPMLRPTMFFVLVTGII
                SAAQVFDTVYALTGGGPQGSTDLVAHRIYAEAFGAAAIGRASVMAVVLFVILVGATVV
                QHLYFRRRISYELT"
CDS             2571672..2572496
                /codon_start=1
                /note="gene_2380"
                /transl_table=11
                /translation="MSSPSRVSNTAVYAVLTIGAVITLSPFLLGLLTSFTSAHQFATG
                TPLQLPRPPTLANYADIADAGFRRAAVVTALMTAVILLGQLTFSVLAAYAFARLQFRG
                RDALFWVYVATLMVPGTVTVVPLYLMMAQLGLRNTFWALVLPFMFGSPYAIFLLREHF
                RLIPDDLINAARLDGANTLDVIVHVVIPSSRPVLAALAMITVVSQWNNFMWPLVITSG
                HKWRVLTVATADLQSRFNDQWTLVMAATTVAIVPLIALFVTFQRHIVASIVVSGLK"
CDS             2572493..2573815
                /codon_start=1
                /note="gene_2381"
                /transl_table=11
                /translation="VTRPRQSTLVATALVLVAILLGVTAVLLGLSAEPRGGKIVVTVR
                LWDEPIAAAYRQSFAAFTRSHPDIEVRTNLVAYSTYFETLRTDVAGGSADDIFWLSNA
```

YFAAYADSGRLMKIQTDAADWEPAVVDQFTRSGVLWGVPQLTDAGIAVFYNADLLAAA
GVDPTQVDNLRWSRGDDDTLRPMLARLTVDADGRTANTPGFDARRVRQWGYNAANDPQ
AIYLNYIGSAGGVFQRDGKFAFDNPGAIEAFRYLVGLINDDHVAPPASDTNDNGDFSR
NQFLAGKMALFQSGTYSLAPVARDALFHWGVAMLPAGPAGRVSVTNGIAAAGNSASKH
PDAVRQVLAWMGSTEGNSYVGRHGAAIPAVLSAQPVYFDYWSARGVDVTPFFAVLNGP
RIAAPGGAGFAAGQQALEPYFDEMFLGRGDVTTTLRQAQAAANAATQR"

CDS complement(2573823..2574701)
/codon_start=1
/note="gene_2382"
/transl_table=11
/translation="VTIVVGYLAGKVGPSALHLAVRVARMHKTSLTVATIVRRHWPTP
SLARVDAEYELWSEQLAAASAREAQRYLRRLADGIEVSYHHRAHRSVSAGLLDVVEEL
EAGVLVLGSFPSGRRARVLIGSTADRLLHSSPVPVAITPRRYRCYTDRLTRLSCGYSA
TSGSVDVVRRCGHLASRYGVPMRVITFAVRGRTMYPPEVGLHAEASVLEAWAAQAREL
LEKLRINGVVSEDVVLQVVTGNGWAQALDAADWQDGEILALGTSPFGDVARVFLGSWS
GKIIRYSPVPVLVLPG"

CDS complement(2574698..2576095)
/codon_start=1
/note="gene_2383"
/transl_table=11
/translation="MLRRRPVSGAPVASGASGNLKRSFGTFQLTMFGVGATIGTGIFF
VLAQAVPEAGPGVIVSFIIAGIAAGLAAICYAELASAVPISGSAYSYAYTTLGEAVAM
VVAACLLLEYGVATAAVAVGWSGYVNKLLSNLFGFQMPHVLSAAPWDTHPGWVNLPAV
ILIGLCALLLIRGASESARVNAIMVLIKLGVLGMFMIIAFSAYSADHLKDFVPFGVAG
IGSAAGTIFFSYIGLDAVSTAGDEVKDPQKTMPRALIAALVVVTGVYVLVALAALGTQ
PWQDFAEQETAGLAIILDNVTHGEWASTILAAGAVVSIFTVTLVTMYGQTRILFAMGR
DGLLPARFAKVNPRTMTPVHNTVIVAIFASTLAAFIPLDSLADMVSIGTLTAFSVVAV
GVIVLRVREPDLPRGFKVPGYPVTPVLSVLACGYILASLHWYTWLAFSGWVAVAVIFY
LMWGRHHSALNEEVP"

CDS complement(2576129..2576680)
/codon_start=1
/note="gene_2384"
/transl_table=11
/translation="VLMIADEIQSGLACTGYPFFACDHGGVLPDIYLLGKTLGGGAVPL
SAMVADREIFGVVHPGEHGSTFGGNPLAAAIGTPVVSMVVWGECQARSAKLGAHLHQR
LADLIGDGAVALRGLGWWADVDIERALAIGTDMSMRLADRGVLLKDTYGAALRFAPPL
VITAQEIDCAVRRFADALWEAGS"

CDS complement(2576674..2577339)
/codon_start=1
/note="gene_2385"
/transl_table=11
/translation="MTNLADATQATMALVERHAAHNYSPLPVVAASAEGAWIADIDGL
RYLDWLAAYSAVNLGHRNPASTATAHAQVDTVTLLNRALHADRLGPLGAALAQLCGKD
VVLPMNSDAEAVESGLRVARKWGADVNGLPAGRHDIILANNNFHGHTSSVVSFSSDPA
AGSGVEPSTPGLRSVPFGDAAAPAQTIDDNTVADLLEPIPGQAGIIVPADDYLPAASS
TTC"

CDS complement(2577336..2578109)
/codon_start=1
/note="gene_2386"
/transl_table=11
/translation="MTPPAFFAVAYAINPWMDVTAPVDVQVAQAQWEHLHQTYLRLGH

SVDLIEPISGLPDMVYTANGGFITHDIAVVARFRFPERAGESRAYASWMSSVGYRPVT
TRHVNEGQGDLLMVGERVLAGYGFRTDQRAHAEIAAVLGLPVVSLELVDPRFYHLDTA
LAVLDDHTIAYYPPAFSTAAQEQLSALFPDAIVVGSADAFVFGLNAVSDGLNVVLPVA
AMGFAAQLRAAGFEPVGVDLSELLKGGGSVKCCTLEIHP"

CDS 2578309..2578755
/codon_start=1
/note="gene_2387"
/transl_table=11
/translation="MDRLDDTDERILAELAEHARATFAEIGHKVSLSAPAVKRRVDRM
LESGVIKGFTTVVDRNALGWNTEAYVQIFCHGRIAPDQLRAAWVNIPEVVSAATVTGT
SDAILHVLAHDMRHLEAALERIRSSADVERSESTVVLSNLIDRMPP"

CDS complement(2578984..2579832)
/codon_start=1
/note="gene_2388"
/transl_table=11
/translation="MTTTSAPARNGTRRPSRPIVLLIPVPGSSVIHDLWAGTKLLVVF
GISVLLTFYPGWVTIGMMAALVLAAARIAHIPRGALPSVPRWLWIVLAIGFLTAALAG
GTPVVAVGGVQLGLGGALHFLRITALSVVLLALGAMVSWTTNVAEISPAVATLGRPFR
VLRIPVDEWAVALALALRAFPMLIDEFQVLYAARRLRPKRMPPSRKARRQRHARELID
LLAAAITVTLRRADEMGDAITARGGTGQLSAHPGRPKLADWVTLAITAMASGTAVAIE
SLILHS"

CDS complement(2579829..2581799)
/codon_start=1
/note="gene_2389"
/transl_table=11
/translation="MAALCAVTAIISVVVPFAAGLALLGTVPTGLLAYRYRLRVLAAA
TVAAGMIAFLIAGLGGFMGVVHSAYIGGLTGIVKRRGRGTPTVVVSSLIGGFVFGAAM
VGMLAAMVRLRHLIFKVMTANVDGIAATLARMHMQGAAADVKRYFAEGLQYWPWVLLG
YFNIGIMIVSLIGWWALSRLLERMRGIPDVHKLDPPPGDDVDALIGPVPVRLDKVRFR
YPRAGQDALREVSLDVRAGEHLAIIGANGSGKTTLMLILAGRAPTSGTVDRPGTVGLG
KLGGTAVVLQHPESQVLGTRVADDVVWGLPLGTTADVGRLLSEVGLEALAERDTGSLS
GGELQRLALAAALAREPAMLIADEVTTMVDQQGRDALLAVLSGLTQRHRTALVHITHY
DNEADSADRTLSLSDSPDNTDMVHTAAMPAPVIGVDQPQHAPALELVGVGHEYASGTP
WAKTALRDINFVVEQGDGVLIHGGNGSGKSTLAWIMAGLTIPTTGACLLDGRPTHEQV
GAVALSFQAARLQLMRSRVDLEVASAAGFSASEQDRVAAALTVVGLDPALGARRIDQL
SGGQMRRVVLAGLLARAPRALILDEPLAGLDAASQRGLLRLLEDLRRARGLTVVVVSH
DFAGMEELCPRTLHLRDGVLESAAASEAGGMS"

CDS 2581963..2582454
/codon_start=1
/note="gene_2390"
/transl_table=11
/translation="MSPSPAAANRSEVGGPLPGLGADLLAVVARLNRLATQRIQMPLP
AAQARLLATIEAQGEARIGDLAAVDHCSQPTMTTQVRRLEDAGLVTRTADPGDARAVR
IRITPEGIRTLTAVRADRAAAIEPQLALLPPADRRVLADAVDVLRRLLDHAATTPGRA
TRQ"

CDS 2582706..2583854
/codon_start=1
/note="gene_2391"
/transl_table=11
/translation="MQFLSVIPEQVESAAQDLAGIRSALSASYAAAAGPTTAVVSAAE
DEVSTAIASIFGAYGRQCQVLSAQASAFHDEFVNLLKTGATAYRNTEFANAQSNVLNA

VNAPARSLLGHPSAAESVQNSAPTLGGGHSTVTAGLAAQAGRAVATVEQQAAAAVAPL
PSAGAGLAQVVNGVVTAGQGSAAKLATALQSAAPWLAKSGGEFIVAGQSALTGVALLQ
PAVVGVVQAGGTFLTAGTSAATGLGLLTLAGVEFSQGVGNLALASGTAATGLGLLGSA
GVQLFSPAFLLAVPTALGGVGSLAIAVVQLVQGVQHLSLVVPNVVAGIAALQTAGAQF
AQGVNHTMLAAQLGAPGIAVLQTAGGHFAQGIGHLTTAGNAAVTVLIS"

CDS complement(2583889..2585436)
/codon_start=1
/gene="narK-4"
/note="gene_2392"
/transl_table=11
/translation="MEQHTLLQREESPRSPAAPSLRRLGGSRHITHWDPEDLGAWEAG
NKGIARRNLLWSVVTVHLGYSVWTLWPVLELLMPQDVYGFSTSDKFLLGTIATLFGAF
LRMPYALASAIFGGRNWATFSAIVLLIPAIGTTVLLTHPGLPLWPYLVCAALTGLGGG
NFASSMSNANAFYPHRLKGSALGIAGGVGNLGVPAIQLVGLLAIATVGERKPYLVCAL
YVVLVAIAVIGVSLFMNNVEQHRVQVNRLRPIVSAVLSTRDTWLLSLLYLGTFGSFIG
FSFVFGQVLQTNFLACGQSPARATLHAVELAFVGPLLAAVARIYGGRLADRVGGSRLT
LIVFVAMTLAAGLLISASTLEGRHVGQHRGATMVGYFVCFVALFVLSGLGNGSVYKMI
PTIFEACSRSLDLSEAERRDWSRIISGVVIGFVAAFGALGGVGINMALRESYLSTGSG
TDAFWIFMMCYAAAAVLTWKVYDRRTVTDMGMLQAALVRQPASTPAELIGPRTQSDRF
SGCSISA"

CDS complement(2585670..2586197)
/codon_start=1
/note="gene_2393"
/transl_table=11
/translation="VRRQRSAVPILALLALLALLALIVGLGASGCAWKPPTTRPSPPN
TCKDSDGPTADTVRQAIAAVPIVVPGSKWVEITRGHTRNCRLHWVQIIPTIAAQSTPQ
QLLFFDRNIPLGSPTRNPKPYITVLPAGDDTVTVQYQWQIGSDQECCPTGIGTVRFHI
GSDGKLEALGSIPHQ"

CDS 2586161..2586658
/codon_start=1
/note="gene_2394"
/transl_table=11
/translation="VPESGRRYADDARHELSENWEFPLRFINASGVDPKARVPPVFLP
QIGRLTPDAVGEAIGIAADDIPMAARWIGSRPCSLIGQPNTMGDEMGYLGPGLAGQRC
VDRLVMGASRSTCSRLPVIASVDERLSVLKPVRPRLHSISFIFKGRPGEVYLTVTGYN
FRGVP"

CDS 2587083..2588729
/codon_start=1
/note="gene_2395"
/transl_table=11
/translation="MSDARVPRIPAALSAPSLNRGVGFTHAQRRRLGLTGRLPSAVLT
LDQQAERVWHQLQSLATDLGRNLLLEQLHYRHEVLYFKVLADHLPELMPVVYTPTVGE
AIQRFSDEYRGQRGLFLSIDEPDEIEEAFNTLGLGPEDVDLIVCTDAEAILGIGDWGV
GGIQIAVGKLALYTAGGGVDPRRCLAVSLDVGTDNEQLLADPFYLGNRHARRRGREYD
EFVSRYIETAQRLFPRAILHFEDFGPANARKILDTYGTDYCVFNDDMQGTGAVVLAAV
YSGLKVTGIPLRDQTIVVFGAGTAGMGIADQIRDAMVADGATLEQAVSQIWPLDRPGL
LFDDMDDLRDFQVPYAKNRHQLGVAVGDRVGLSDAIKIASPTILLGCSTVYGAFTKEV
VEAMTASCKHPMIFPLSNPTSRMEAIPADVLAWSNGRALLATGSPVAPVEFDETTYVI
GQANNVLAFPGIGLGVIVAGARLITRRMLHAAAKAIAHQANPTNPGDSLLPDVQNLRA
ISTTVAEAVYRAAVQDGVASRTHDDVRQAIVDTMWLPAYD"

CDS complement(2588726..2590297)

```
                     /codon_start=1
                     /note="gene_2396"
                     /transl_table=11
                     /translation="MNRTQLLTLIATGLGLFMIFLDALIVNVALPDIQRSFAVGEDGL
                     QWVVASYSLGMAVFIMSAATLADLYGRRRWYLIGVSLFTLGSIACGLAPSIAVLTTAR
                     GAQGLGAAAVSVTSLALVSAAFPEAKEKARAIGIWTAIASIGTTTGPTLGGLLVDQWG
                     WRSIFYVNLPMGALVLFLTLCYVEESCNERARRFDLSGQLLFIVAVGALVYAVIEGPQ
                     IGWTSVQTIVMLWTAAVGCALFVWLERRSSNPMMDLTLFRDTSYALAIATICTVFFAV
                     YGMLLLTTQFLQNVRGYTPSVTGLMILPFSAAVAIVSPLVGHLVGRIGARVPILAGLC
                     MLMLGLLMLIFSEHRSSALVLVGLGLCGSGVALCLTPITTVAMTAVPAERAGMASGIM
                     SAQRAIGSTIGFAVLGSVLAAWLSATLEPHLERAVPDPVQRHVLAEIIIDSANPRAHV
                     GGIVPRRHIEHRDPVAIAEEDFIEGIRVALLVATATLAVVFLAGWRWFPRDVQTAGSD
                     AKREAAYSDDRRVRG"
     CDS             2590772..2591704
                     /codon_start=1
                     /note="gene_2397"
                     /transl_table=11
                     /translation="MSIAEDITQLIGRTPLVRLRRVTDGAVADIVAKLEFFNPANSVK
                     DRIGVAMLQAAEQAGLIKPDTIILEPTSGNTGIALAMVCAARGYRCVLTMPETMSLER
                     RMLLRAYGAELILTPGADGMSGAIAKAEELAKTDQRYFVPQQFENPANPAIHRVTTAE
                     EVWRDTDGKVDIVVAGVGTGGTITGVAQVIKERKPSARFVAVEPAASPVLSGGQKGPH
                     PIQGIGAGFVPPVLDQDLVDEIITVGNEDALNVARRLAREEGLLVGISSGAATVAALQ
                     VARRPENAGKLIVVVLPDFGERYLSTPLFADVAD"
     CDS             2591708..2592397
                     /codon_start=1
                     /note="gene_2398"
                     /transl_table=11
                     /translation="MLTAMRGDIRAARERDPAAPTALEVIFCYPGVHAVWGHRLAHWL
                     WQRGARLLARAAAEFTRILTGVDIHPGAVIGARVFIDHATGVVIGETAEVGDDVTIYH
                     GVTLGGSGMVGGKRHPTVGDRVIIGAGAKVLGPIKIGEDSRIGANAVVVKPVPPSAVV
                     VGVPGQVIGQSQPSPGGPFDWRLPDLVGASLDSLLTRVARLEALGGGPQAAGVIRPPE
                     AGIWHGEDFSI"
     CDS             2592891..2593781
                     /codon_start=1
                     /note="gene_2399"
                     /transl_table=11
                     /translation="VGTTTVERLEPRLSPASRHITEAKAFGTECHVSSFTREQDPDRA
                     VRVEQIHGEAYVAAGHVYESALDELGRLDNSNAEFILDKARGSTRETEVIYLHAVPAE
                     PLSGSQGEGGLRIVGISAVGSIDDLSAFKAAKPSMGLAHQRKLYDAIEDLGHGGVKEI
                     AALSVTADAPPTVSYSLIREVLRLYHRTGEKLIITFAMPAYAKMVMNFGRFAMPQVGE
                     PFYAHRNNDPRTSNDLLLVPSIVEPSNFLENISRGVVTADDGPTARRRFATLCYMTDG
                     LDDYFMPLTRQVLSEGIQDI"
     CDS             complement(2593845..2594963)
                     /codon_start=1
                     /note="gene_2400"
                     /transl_table=11
                     /translation="MRAGRWGPGMTGLDPAEFLSLVEAAALAPSADNRREVQLEHAGR
                     RVRLWGDQTWRSAPEHRRIMSLVAIGAAVENVKLRAGRLGFETKVCWFPDSGNPGLVA
                     EIDVDRLPQTRVDPIEVAIERRRTNRRVRFRGPPLSQGELGALSAEATGIDGIQLHWF
                     DSPETRKQILRLVRLAETERFRSRELHEELFSAVRFDIGWTASSDDGLPPGSLEVEAW
                     MRPMFRGLRHWRVLRLLRTVGMHHALGLRAAYLPCRLAPHVGALTTSLDLASGALTAG
```

AVFERIWLRTTLLGAELQPFAASAVLSLPACEWVAPHVRAALVGGWNLLAPGHWPMMV
FRIGHARAPSVRTMRQSVEAYCYAPAERSGSDSESRFA"

CDS complement(2595083..2595892)
/codon_start=1
/note="gene_2401"
/transl_table=11
/translation="MGGVGGIDMVALARMGIGKFTIADPDVFEIRNSNRQYGAMRSTN
GQAKAEVMRNIVHDINPEAEIRAFCEPIGKENAATFLEGADVLVDGIDAFEIDLRRLL
YREAQQRGIYALGAGPLGFSTAWVVFDPKGMTFDRYFDLSDAMNTVDKFVAFIAGIAP
SATHRRSIDLSYVDIENRTGPSVGLACHLASGVVAAEVLKILLGHGRVYAAPYFHQFD
AYRSIYVRKRLRCGNRHPLQRVKRRLLARYINRRSAGVIPGLRYHRTEPSY"

CDS 2596670..2599150
/codon_start=1
/note="gene_2402"
/transl_table=11
/translation="MVPGEVHMSDTPSGPHPIIPRTIRLAAIPILLCWLGFTVFVSVV
VPPLEAIGETRAVAVAPDDAQSMRAMRRAGKVFNEFDSNSIAMVVLESDQPLGEKAHR
YYDHLVDTLVLDQSHIQHIQDFWRDPLTAAGAVSADGKAAYVQLYLAGNMGEALANES
VEAVRKIVANSTPPEGIRTYVTGPAALFADQIAAGDRSMKLITGLTFAVITVLLLLVY
RSIATTLLILPMVFIGLGATRGTIAFLGYHGMVGLSTFVVNILTALAIAAGTDYAIFL
VGRYQEARHIGQNREASFYTMYRGTANVILGSGLTIAGATYCLSFARLTLFHTMGPPL
AIGMLVSVAAALTLAPAIIAIAGRFGLLDPKRRLKTRGWRRVGTAVVRWPGPILATSV
ALALVGLLALPGYRPGYNDRYYLRAGTPVNRGYAAADRHFGPARMNPEMLLVESDQDM
RNPAGMLVIDKIAKEVLHVSGVERVQAITRPQGVPLEHASIPFQISMMGATQTMSLPY
MRERMADMLTMSDEMLVAINSMEQMLDLVQQLNDVTHEMAATTREIKATTSELRDHLA
DIDDFVRPLRSYFYWEHHCFDIPLCSATRSLFDTLDGVDTLTDQLRALTDDMNKMEAL
TPQFLALLPPMITTMKTMRTMMLTMRSTISGVQDQMADMQDHATAMGQAFDTAKSGDS
FYLPPEAFDNAEFQQGMKLFLSPNGKAVRFVISHESDPASTEGIDRIEAIRAATKDAI
KATPLQGAKIYIGGTAATYQDIRDGTKYDILIVGIAAVCLVFIVMLMITQSLIASLVI
VGTVLLSLGTAFGLSVLIWQHFVGLQVH"

CDS 2599163..2599558
/codon_start=1
/gene="mmpL5_2"
/note="gene_2403"
/transl_table=11
/translation="MSVIVLLAVGSDYNLLLVSRFKEEVGAGLKTGIIRAMAGTGAVV
TSAGLVFAFTMASMAVSELRVIGQVGTTIGLGLLFDTLVVRSFMTPSIAALLGRWFWW
PNMIHSRPTVPEAHTRQGARRIQPHLHRG"

CDS complement(2599644..2600885)
/codon_start=1
/note="gene_2404"
/transl_table=11
/translation="MSHVTAAPNVLAASAGELAAIGSTMRAANAAAAAPTAGVLAAGG
DDVSAGIAALFGARAQAYQAISAQAALFHDRFVQILQEGAAAYAMAEAANALPLQKAQ
GVVSELAQDRTGGTGTGQSRGAGGFGGVGQAGGKGWDGGPIGNGQVGEQHGAGQLGST
DGNPGVAGAAHGSGVSASHGSGATGAAGVADPGGSGAGVGSAAGNGTGAGSADAVGGA
GTGTDIVGSVRGDGGVGMASGDGGLSTGAAGASAEGGLMPGFGGAPWVGGHWGLGGEG
HSGAIGGVGEQVAPAVATAPAVSPATTSAVAAESGSTPATKAQAMHATTNPGNAAHQG
NPADPGNSARRADGGRDEQLLLLPLTSLRGLRHTLKKLSGLRARNGLLTASGDNASGS
GRPWDRDQLLRALGLRPPGHE"

tRNA complement(2601384..2601456)

/note="identified by tRNAscan-SE; putative"
CDS 2601574..2601993
/codon_start=1
/note="gene_2405"
/transl_table=11
/translation="MPVGGRQHVFEKLASILGLVAAPLMLLGLSACGRSAGKTSEPTC
PTEPIDAADSSTTPDPSCVVRATEINGNGSRIQTWTGSYDAAATQSGGVCGGTCNFHA
TVRFTVDEGQISGSVDQVYQAAMVAIATRPTSPSLAP"
CDS 2602213..2602506
/codon_start=1
/note="gene_2406"
/transl_table=11
/translation="MDATAPLVGGTALIGYVAVLGLGYVLGAKAGRRRYEQIASTYRA
LTGSPVARSMIEGGRRKIANRISPDAGFVTLAEIDNQTAVVQRGVERQPKTAR"
CDS complement(2602510..2604429)
/codon_start=1
/gene="dnaG"
/note="gene_2407"
/transl_table=11
/translation="MSGRISDRDIAAIREGARIEDVVGDYVQLRRAGADSLKGLCPFH
NEKSPSFHVRPNHGHFHCFGCGEGGDVYAFIQKIEHVSFVEAVELLADRIGHTISYTG
AATSVQRDRGSRSRLLAANAAAAAFYAQALQSDEAAPARQYLTERSFDAAAARKFGCG
FAPSGWDSLTKHLQRKGFEFEELEAAGLSRQGRHGPMDRFHRRLLWPIRTSAGEVVGF
GARRLFDDDAMEAKYVNTPETLLYKKSSVMFGIDLAKRDIAKGHQAVVVEGYTDVMAM
HLAGVTTAVASCGTAFGGEHLAMLRRLMMDDSFFRGELIYVFDGDEAGRAAALKAFDG
EQKLAGQSFVAVAPDGMDPCDLRLKCGDAALRDLVARRTPLFEFAIRAAIAEMDLDSA
EGRVAALRRCVPMVGQIKDPTLRDEYARQLAGWVGWADVAQVIGRVRGEAKRTKHPRL
GRLGSTTIARAAQRPTAGPPTELAVRPDPRDPTLWPQREALKSALQYPALAGPVFDAL
TVEGFTHPEYAAVRAAIDTAGGTSAGLSGAQWLDMVRQQTTSTVTSALISELGVEAIQ
VDDDKLPRYIAGVLARLQEVWLRRQIAEVKSKLQRMSPIEQGDEYHALFGDLVAMEAY
RRSLLEQASGDDLTA"
CDS complement(2604434..2605729)
/codon_start=1
/note="gene_2408"
/transl_table=11
/translation="VSASEHDPYDDFDRQRRVAEAPKTAGLPGTEGQYRSDFARDRAR
VLHSAALRRLADKTQVVGPREGDTPRTRLTHSLEVAQIGRGMAIGLGCDLDLVELAGL
AHDIGHPPYGHNGERALDEVAASHGGFEGNAQNFRILTSLEPKVVDAQGLSAGLNLTR
ASLDAVTKYPWMRGDGLGSQRRKFGFYDDDRESAVWVRQGAPPERACLEAQVMDWADD
VAYSVHDVEDGVVSERIDLRVLAAEEDAAALARLGEREFSRVSADELMAAARRLSRLP
VVAAVGKYDATLSASVALKRLTSELVGRFASAAIATTRAAAGPGPLVRFRADLQVPDL
VRAEVAVLKILALQFIMSDPRHLETQARQRERIHRVAHRLYSGAPQTLDPVYAAAFNT
AADDAARLRVVVDQIASYTEGRLERIDADQLGVSRNALD"
CDS 2605798..2607780
/codon_start=1
/note="gene_2409"
/transl_table=11
/translation="MRLVRLLGMVLTILAAGLLLGPPAGAQPPFRLSNYVTDNAGVLT
SSGRTAVTAAVDRLYADRRIRLWVVYVENFSGQSALNWAQRTTRTSELGNYDALLAVA
TTGREYAFLVPSAMPGVSEGQVDNVRRYQIEPALHDGDYSGAAVAAANGLNRSPSSSS
RVVLLVTVGIIVIVVAVLLVVMRHRNRRRRADELAAARRVDPTNVMALAAVPLQALDD

```
                     LSRSMVVDVDNAVRTSTNELALAIEEFGERRTAPFTQAVNNAKAALSQAFTVRQQLDD
                     NTPETPAQRRELLTRVIVSAAHADRELASQTEAFEKLRDLVINAPARLDLLTQQYVEL
                     TTRIGPTQQRLAELHTEFDAAAMTSIAGNVTTATERLAFADRNISAARDLADQAVSGR
                     QAGLVDAVRAAESALGQARALLDAVDSAATDIRHAVASLPAVVADIQTGIKRANQHLQ
                     QAQQPQTGRTGDLIAARDAAARALDRARGAADPLTAFDQLTKVDADLDRLLATLAEEQ
                     ATADRLNRSLEQALFTAESRVRAVSEYIDTRRGSIGPEARTRLAEAKRQLEAAHDRKS
                     SNPTEAIAYANAASTLAAHAQSLANADVQSAQRAYTRRGGNNAGAILGGIIIGDLLSG
                     GTRGGLGGWIPTSFGGSSNAPGSSPDGGFLGGGGRF"
     CDS             complement(2607865..2608047)
                     /codon_start=1
                     /note="gene_2410"
                     /transl_table=11
                     /translation="LLAAGDFWGGAGSVACQEFITQLGRNFQVIYEQANAHGQKVQAA
                     GNNMAQTDSAVGSSWA"
     CDS             complement(2608031..2609434)
                     /codon_start=1
                     /note="gene_2411"
                     /transl_table=11
                     /translation="VVNFSVLPPEINSGRMFFGAGSGPMLAAAAAWDGLAAELGLAAE
                     SFGLVTSGLAGGSGQAWQGAAAAMVVAAAPYAGWLAAAAARAGGAAVQAKAVAGAFEA
                     ARAAMVDPVVVAANRSAFVQLVLSNVFGQNAPAIAAAEATYEQMWAADVAAMVGYHGG
                     ASAAAALAPWQQAVPGLLGLLDSAQSSAQAVTAQAVGSTVPGPLQGINFGFGNIGSLN
                     LGSGNTGDTNVGSGNIGNTNLGGGNIGSFNLGSGNQGDINLGIGNVGNLNLGSGNFGS
                     QNLGSGNIGSTNVGSGNIGSTNVGSGNIGSTNVGSGNIGDTNFGNGNNGNFNFGSGNT
                     GSNNIGFGNTGSGNFGFGNTGNNNIGIGLTGDGQIGIGGLNSGSGNIGFGNSGTGNVG
                     LFNSGTGNVGFGNSGTANTGFGNAGNVNTGFWNGGSTNTGLANAGAGNTGFFDAGNYN
                     FGSLNAGNINSSFVGRG"
     variation        2608884..2611462
                     /note="supplemented from AL2122/97"
     CDS             complement(2610172..2612016)
                     /codon_start=1
                     /note="gene_2412"
                     /transl_table=11
                     /translation="VVNFSVLPPEINSGRMFFGAGSGPMLAAAAAWDGLAAELGLAAE
                     SFGLVTSGLAGGSGQAWQGAAAAAMVVAAAPYAGWLAAAAARAGGAAVQAKAVAGAFE
                     AARAAMVDPVVVAANRSAFVQLVLSNVFGQNAPAIAAAEATYEQMWAADVAAMVGYHG
                     GASAAAALAPWQQAVPGLSGLLGGAANAPAAAAQGAAQGLAELTLNLGVGNIGSLNLG
                     SGNIGGTNVGSGNVGGTNLGSGNYGSLNWGSGNTGTGNAGSGNTGDYNPGSGNFGSGN
                     FGSGNIGSLNVGSGNFGTLNLANGNNGDVNFGGGNTGDFNFGGGNNGTLNFGFGNTGS
                     GNFGFGNTGNNNIGIGLTGDGQIGIGGLNSGTGNIGFGNSGNNNIGFFNSGDGNIGFF
                     NSGDGNTGFGNAGNINTGFWNAGNLNTGFGSAGNGNVGIFDGGNSNSGSFNVGFQNTG
                     FGNSGAGNTGFFNAGDSNTGFANAGNVNTGFFNGGDINTGGFNGGNVNTGFGSALTQA
                     GANSGFGNLGTGNSGWGNSDPSGTGNSGFFNTGNGNSGFSNAGPAMLPGFNSGFANIG
                     SFNAGIANSGNNLAGISNSGDDSSGAVNSGSQNSGAFNAGVGLSGFFR"
     CDS             complement(2612154..2613533)
                     /codon_start=1
                     /note="gene_2413"
                     /transl_table=11
                     /translation="VAPVIDTVVNLAKRRGFVYPSGEIYGGTKSAWDYGPLGVELKEN
                     IKRQWWRSVVTGRDDVVGIDSSIILPREVWVASGHVDVFHDPLVESLITHKRYRADHL
                     IEAYEAKHGHPPPNGLADIRDPETGEPGQWTQPREFNMMLKTYLGPIETEEGLHYLRP
```

ETAQGIFVNFANVVTTARKKPPFGIGQIGKSFRNEITPGNFIFRTREFEQMEMEFFVE
PATAKEWHQYWIDNRLQWYIDLGIRRENLRLWEHPKDKLSHYSDRTVDIEYKFGFMGN
PWGELEGVANRTDFDLSTHARHSGVDLSFYDQINDVRYTPYVIEPAAGLTRSFMAFLI
DAYTEDEAPNTKGGMDKRTVLRLDPRLAPVKAAVLPLSRHADLSPKARDLGAELRKCW
NIDFDDAGAIGRRYRRQDEVGTPFCVTVDFDSLQDNAVTVRERDAMTQDRVAMSSVAD
YLAVRLKGS"

CDS 2613727..2614134
/codon_start=1
/note="gene_2414"
/transl_table=11
/translation="MVTSPSTPTAAHEDVGADEVGGHQHPADRFAECPTFPAPPPREI
LDAAGELLRALAAPVRIAIVLQLRESQRCVHELVDALHVPQPLVSQHLKILKAAGVVT
GERSGREVLYRLADHHLAHIVLDAVAHAGEDAI"

CDS 2614131..2614523
/codon_start=1
/note="gene_2415"
/transl_table=11
/translation="MSAAGVRSTRQRAAISTLLETLDDFRSAQELHDELRRRGENIGL
TTVYRTLQSMASSGLVDTLRTDTGESVYRRCSEHHHHHLVCRSCGSTIEVGDHEVEAW
AAEVATKHGFSDVSHTIEIFGTCSDCRS"

CDS complement(2614631..2615059)
/codon_start=1
/note="gene_2416"
/transl_table=11
/translation="VPSLPDRLASILRDVLPAEEEPDGALTVRHDGTFASLRVVSIAE
DLELVSLTQILAWDLPLTKRLTEQVAKQARDINFGSVSLREKVSEKAARRSSGRPASN
TADVMLRYNFPGTGLTDDALRTLILLVLETGATIRSALVG"

CDS complement(2615059..2615949)
/codon_start=1
/note="gene_2417"
/transl_table=11
/translation="VARDARKRTSSNFPQLPPAPDDYPTFPDTSTWPVVFPELPAAPY
GGPCRPPQHTSKAAAPRIPADRLPNHVAIVMDGNGRWATQRGLARTEGHKMGEAVVID
IACGAIELGIKWLSLYAFSTENWKRSPEEVRFLMGFNRDVVRRRRDTLKKLGVRIRWV
GSRPRLWRSVINELAVAEEMTKSNDVITINYCVNYGGRTEITEATREIAREVAAGRLN
PERITESTIARHLQRPDIPDVDLFLRTSGEQRSSNFMLWQAAYAEYIFQDKLWPDYDR
RDLWAACEEYASRTRRFGSA"

CDS complement(2615942..2616739)
/codon_start=1
/gene="recO"
/note="gene_2418"
/transl_table=11
/translation="MRLYRDRAVVLRQHKLGEADRIVTLLTRDHGLVRAVAKGVRRTR
SKFGARLEPFAHIEVQLHPGRNLDIVTQVVSVDAFATDIVADYGRYTCGCAILETAER
LAGEERAPAPALHRLTVGALRAVADGQRPRDLLLDAYLLRAMGIAGWAPALTECARCA
TPGPHRAFHIATGGSVCAHCRPAGSTTPPLGVVDLMSALYDGDWEAAEAAPQSARSHV
SGLVAAHLQWHLERQLKTLPLVERFYQADRSVAERRAALIGQDIAGG"

CDS 2616801..2618255
/codon_start=1
/note="gene_2419"
/transl_table=11

```
                     /translation="VVGASGSDAGAISGSGNQRLPTLTDLLYQLATRAVTSEELVRRS
                     LRAIDVSQPTLNAFRVVLTESALADAAAADKRRAAGDTAPLLGIPIAVKDDVDVAGVP
                     TAFGTQGYVAPATDDCEVVRRLKAAGAVIVGKTNTCELGQWPFTSGPGFGHTRNPWSR
                     RHTPGGSSGGSAAAVAAGLVTAAIGSDGAGSIRIPAAWTHLVGIKPQRGRISTWPLPE
                     AFNGVTVNGVLARTVEDAALVLDAASGNVEGDRHQPPPVTVSDFVGIAPGPLKIALST
                     HFPYTGFRAKLHPEILAATQRVGDQLELLGHTVVKGNPDYGLRLSWNFLARSTAGLWE
                     WAERLGDEVTLDRRTVSNLRMGHVLSQAILRSARRHEAADQRRVGSIFDIVDVVLAPT
                     TAQPPPMARAFDRLGSFGTDRAIIAACPSTWPWNLLGWPSINVPAGFTSDGLPIGVQL
                     MGPANSEGMLISLAAELEAVSGWATKQPQVWWTS"
     CDS             complement(2618252..2619181)
                     /codon_start=1
                     /note="gene_2420"
                     /transl_table=11
                     /translation="VFTFGEETGMTEFHSGFVCLVGRPNTGKSTLTNALVGAKVAITS
                     TRPQTTRHAIRGIVHSDDFQIILVDTPGLHRPRTLLGKRLNDLVRETYAAVDVIGLCI
                     PADEAIGPGDRWIVEQLRSTGPANTTLVVIVTKIDKVPKEKVVAQLVAVSELVTNAAE
                     IVPVSAMTGDRVDLLIDVLAAALPAGPAYYPDGELTDEPEEVLMAELIREAALQGVRD
                     ELPHSLAVVIDEVSPREGRDDLIDVHAALYVERDSQKGIVIGKGGARLREVGTAARSQ
                     IENLLGTKVYLDLRVKVAKNWQRDPKQLGRLGF"
     CDS             complement(2619228..2619569)
                     /codon_start=1
                     /note="gene_2421"
                     /transl_table=11
                     /translation="MMRRPITLAEQLDAEDAKLVVLARAAMARAEAGAGAAVRDVDGR
                     TYAAAPVALSALELTGLQAAVAAAVSSGATGLQAAVLVAGSVDDPGIAAVRELAPTAA
                     IIVTDRAGNPL"
     CDS             complement(2619541..2620848)
                     /codon_start=1
                     /note="gene_2422"
                     /transl_table=11
                     /translation="LTGYYQLLGSIVLIGLGGLFAAIDAAISTVSPARVDELVRDQRP
                     GAGSLRKVMADRPRYVNLVVLLRTSCEITATALLVVFIRYHFSMVWGLYLAAGIMVLA
                     SFVVVGVGPRTLGRQNAYSISLATALPLRLISWLLMPISRLLVLLGNALTPGRGFRNG
                     PFASEIELREVVDLAQQRGVVAADERRMIESVFELGDTPAREVMVPRTEMIWIESDKT
                     AGQAMTLAVRSGHSRIPVIGENVDDIVGVVYLKDLVEQTFCSTNGGRETTVARVMRPA
                     VFVPDSKPLDALLREMQRDRNHMALLVDEYGAIAGLVSIEDVLEEIVGEIADEYDQAE
                     TAPVEDLGDKRFRVSARLPIEDVGELYGVEFDDDLDVDTVGGLLALELGRVPLPGAEV
                     ISHGLRLHAEGGTDHRGRVRIGTVLLSPAEPDGADDEEADHPG"
     CDS             complement(2620845..2621393)
                     /codon_start=1
                     /note="gene_2423"
                     /transl_table=11
                     /translation="VREHLMSIEVANESGIDVSEAELVSVARFVIAKMDVNPCAELSM
                     LLLDTAAMADLHMRWMDLPGPTDVMSFPMDELEPGGRPDAPEPGPSMLGDIVLCPEFA
                     AEQAAAAGHSLGHELALLTIHGVLHLLGYDHAEPDEEKEMFALQDRLLEEWVADQVEA
                     YQHDRQDEKDRRLLDKSRYFDL"
     CDS             complement(2621397..2622455)
                     /codon_start=1
                     /note="gene_2424"
                     /transl_table=11
                     /translation="VTSRETRAADAAGARQADAQVRSSIDVPPDLVVGLLGSADENLR
```

ALERTLSADLHVRGNAVTLCGEPADVALAERVISELIAIVASGQSLTPEVVRHSVAML
VGTGNESPAEVLTLDILSRRGKTIRPKTLNQKRYVDAIDANTIVFGIGPAGTGKTYLA
MAKAVHALQTKQVTRIILTRPAVEAGERLGFLPGTLSEKIDPYLRPLYDALYDMMDPE
LIPKLMSAGVIEVAPLAYMRGRTLNDAFIVLDEAQNTTAEQMKMFLTRLGFGSKVVVT
GDVTQIDLPGGARSGLRAAVDILEDIDDIHIAELTSVDVVRHRLVSEIVDAYARYEEP
GSGLNRAARRASGARGRR"

CDS complement(2622726..2624039)
/codon_start=1
/note="gene_2425"
/transl_table=11
/translation="VVLPKPTPRGRELIRQAAKVALHPTPEWLDELDRATLAAHPSIA
ADPALATVVSRANRSHLIHFATANLRKPGQPVPANLGPDPLRMARDLVRRGLDASALD
VYRVGQNVAWQRWTEIAFGLTTDPQELHELLTLPFRSASEFIDATLAGLAAQMQLEYD
ELTRDVHAEHRRIVELILDGAPISRQSAEAKLGYPLDRSHTAAIIWYDDPDDNQNHLD
HTARAFGRALGCPQPLIAVASAATRWVWVSDAATLDTDRIHQVLDHAPHARIAVGTTA
RGIDGFRRSHRDALATQRMLARLRSQQRLAFFADIHMIAVLTENPDSAADFITSTLGD
LESASPQLLTTVLTYINEQCNASRAAHVLHTHRNTLLRRLETAQRLLPRPLDHTIIQV
AVAISALQWRGSQTSDPVETPVEGITSPPPESLGRRRSRLAQLER"

CDS 2624234..2624419
/codon_start=1
/note="gene_2426"
/transl_table=11
/translation="MSLVSVAPELVVTAVPDVARIGSSIGAPDTAAAARPTTSVLAAG
ADEVSADVVALFGWVAR"

CDS complement(2624518..2625306)
/codon_start=1
/note="gene_2427"
/transl_table=11
/translation="MVAMLFYVDTLPDTGAVAVVDGDEGFHAATVRRIRPGEQLVLGD
GVGRLARCVVEQAGRGGLRARVLRRWSVPPVRPPVTVVQALPKSERSELAIELATEAG
ADAFLAWQAARCVANWDGARVDKGLRRWRAVVRSAARQSRRARIPPVDGVLSTPMLVQ
RVREEVAAGAAVLVLHEEATERIVDIAAAQAGSLMLVVGPEGGIAPDELAALTDAGAV
AVRLGPTVLRTSTAAAVALGAVGVLTSRWDASASDCEYCDVTRR"

CDS complement(2625320..2626468)
/codon_start=1
/note="gene_2428"
/transl_table=11
/translation="VARDYYGLLGVSKNASDADIKRAYRKLARELHPDVNPDEAAQAK
FKEISVAYEVLSDPDKRRIVDLGGDPLESAAAGGNGFGGFGGLGDVFEAFFGGGFGGG
AASRGPIGRVRPGSDSLLRMRLDLEECATGVTKQVTVDTAVLCDRCQGKGTNGDSVPI
PCDTCGGRGEVQTVQRSLLGQMLTSRPCPTCRGVGVVIPDPCQQCMGDGRIRARREIS
VKIPAGVGDGMRVRLAAQGEVGPGGGPAGDLYVEVHEQAHDVFVREGDHLHCTVSVPM
VDAALGVTVTVDAILDGLSEITIPPGTQPGSVITLRGRGMPHLRSNTRGDLHVHVEVV
VPTRLDHQDIELLRELKGRRDREVAEVRSTHAAAGGLFSRLRETFTGR"

CDS complement(2626543..2627574)
/codon_start=1
/note="gene_2429"
/transl_table=11
/translation="MGSADERRFEVLRAIVADFVATQEPIGSKSLVERHNLGVSSATV
RNDMAVLEAEGYITQPHTSSGRVPTEKGYREFVDRLEDVKPLSSAERRAIQSFLESGV
DLDDVLRRAVRLLAQLTRQVAVVQYPTLSTSTVRHLEVIALTPARLLMVVITDSGRVD

QRIVELGDVIDDHQLAQLREILGQALEGKKLSAASVAVADLASQLGGAGGLGDAVGRA
ATVLLESLVEHTEERLLLGGTANLTRNAADFGGSLRSILEALEEQVVVLRLLAAQQEA
GKVTVRIGHETASEQMVGTSMVSTAYGTAHTVYGGMGVVGPTRMDYPGTIASVAAVAL
YIGDVLGAR"

CDS 2627746..2628063
/codon_start=1
/note="gene_2430"
/transl_table=11
/translation="MIFKGVREGKPYPEHGLSYRDWSQIPPQQIRLDELVTTTTVLAL
DRLLSEDSTFYGDLFPHAVKWRGTTYLEDGLHRAVRAALRNRTVLHARVFDMDASPGG
RRS"

CDS complement(2628090..2628596)
/codon_start=1
/note="gene_2431"
/transl_table=11
/translation="MKMVKSIAAGLTAAAAIGAAAAGVTSIMAGGPVVYQMQPVVFGA
PLPLDPASAPDVPTAAQLTSLLNSLADPNVSFANKGSLVEGGIGGTEARIADHKLKKA
AEHGDLPLSFSVTNIQPAAAGSATADVSVSGPKLSSPVTQNVTFVNQGGWMLSRASAM
ELLQAAGN"

CDS complement(2628696..2628911)
/codon_start=1
/note="gene_2432"
/transl_table=11
/translation="VSTNPFDDDNGAFFVLVNDEDQHSLWPVFADIPAGWRVVHGEAS
RAACLDYVEKNWTDLRPKSLRDAMAED"

CDS complement(2628889..2630184)
/codon_start=1
/note="gene_2433"
/transl_table=11
/translation="MNPTLAVLGAGAKAVAVAAKASVLRDMGVDVPDVIAVERIGVGA
NWQASGGWTDGAHRLGTSPEKDVGFPYRSALVPRRNAELDERMTRYSWQSYLIATASF
AEWIDRGRPAPTHRRWSQYLAWVADHIGLKVIHGEVERLAVTGDRWALCTHETTVQAD
ALMITGPGQAEKSLLPGNPRVLSIAQFWDRAAGHDRINAERVAVIGGGETAASMLNEL
FRHRVSTITVISPQVTLFTRGEGFFENSLFSDPTDWAALTFDERRDALARTDRGVFSA
TVQEALLADDRIHHLRGRVAHAVGRQGQIRLTLSTNRGSENFETVHGFDLVIDGSGAD
PLWFTSLFSQHTLDLLELGLGGPLTADRLQEAIGYDLAVTDVTPKLFLPTLSGLTQGP
GFPNLSCLGLLSDRVLGAGIFTPTKHNDTRRSGEHQSFR"

CDS complement(2630181..2634566)
/codon_start=1
/note="gene_2434"
/transl_table=11
/translation="MGPVAVTRADARGAIDDVMALSPLQQGLFSRATLVAAESGSEAA
EADPYVIAMAADAAGPLDIALLRDCAAAMLTRHPNLRASFLHGNLSRPVQVIPSSAEV
LWRHVRAHPSEVGALAAEERRRRFDVGRGPLIRFLLIELPDECWHLVIVAHHIVIDGW
SLPLFVSELLALYRAGGHVAALPAAPRPYRDYIGWLAGRDQTASRAMWADHLNGLDGP
TLLSPALADTPVQPGIPGRTEVRLDREATAELADAARTRGVTISTLVQMAWATTLSAF
TGRGDVTFGVTVSGRPSELSGVETMIGLFINTVPLRVRLDARATVGGQCAVLQRQFAM
LRDHSYLGFNEFRAIAGIGEMFDTLLVYENFPPGEVVGTAEFVANGVTFRPVALESLS
HFPVTVAAHRSTGELTLLVEVLDGALGTMAPESLGRRVLAVLQRLVSRWDRPLRDVDI
LLDGEHDPTAPGLPDVTTSAPAVHTRFAEIAAAQPDSVAVSWADGQLTYRELDALADR
LATGLRRADVSRETPVAVALSRGPRYVAAMLAVLKAGGMIVPLDPAMPGERVAEILRQ

TSAPVVIDEGVFAASVGADILEDDRAITVPVDQAAYVIFTSGTTGTPKGVIGTHRALS
AYADDHIERVLRPAAQRLGRPLRIAHAWSFTFDAAWQPLVALLDGHAVHIVDDHRQRD
AGALVEAIDRFGLDMIDTTPSMFAQLHNAGLLDRAPLAVLALGGEALGAATWRMIQQN
CARTAMTAFNCYGPTETTVEAVVAAVAEHARPVIGRPTCTTRAYVMDSWLRPVPDGVA
GELYLAGAQLTRGYLGRPAETAARFVAEPNGRGSRMYRTGDVVRRLPDGGLEFLGRSD
DQVKIRGFRVEPGEIAAVLNGHHAVHGCHVTARGHASGPRLTAYVAGGPQPPPVAELR
AMLLERLPRYLVPHHIVVLDELPLTPHGKIDENALAAINVTEGPATPPQTPTELVLAE
AFADVMETSNVDVTAGFLQMGLDSIVALSVVQAARRRGIALRARLMVECDTIRELAAA
IDSDAAWQAPANDAGEPIPVLPNTHWLYEYGDPRRLAQTEVIRLPDRITRERLDAVLA
AVVDGHEVLRCRFDRDAMALVAQPKTDILSEVWVSGELVTAVAEQTLGVLASLDPQAG
RLLSAVWLREPDGPGVLVLTAHVLAMDPASWRIVLGELDAGLHALAAGRAPSPARENT
SYRQWSRLLAQRAKALDSVDFWVAELEGADPPLGARRVAPQTDRVGELAITMSISDAD
LTARLLSTGRSMTDLLATAAARMVTAWRRQRGQQTPAPLLALETHGRADVHVDKTADT
SDTVGLLSAIYPLRIHCDGATDFARIPGSGIDYGLLRYLRADTAERLRAHREPQLLLN
YLGSLHVGVGDLAVDRALLADVGQLPEPEQPVRHELTVLAALLGPADAPVLATRWRTL
PDILSADDVATLQSLWQGALAEITA"

CDS complement(2634548..2639743)
/codon_start=1
/note="gene_2435"
/transl_table=11
/translation="VTNTADIGARLDEARLELLRRRLADRGLSSAAQDIGPHTDDRLS
DGQARMWFVQMADPSGALLNICVSYRITGDIDLARLRDAVNAVARRHRILRTTYPVGD
DGVAQPTVHADLRPGWTQYDLTDLSQRAQRLRLEVLAQREFCAPFELSRDAPLRITVV
RTAADEHVLLLVAHHIAWDDGSWRVFFTDLTQAYSRADLGADLGPEHRPSAASGPDTT
EADLNYWRAIMADPPEPLELPGPAGTCVPTSWRAARATLRLPADTAARVATMAKNTGC
TPYMVLLAAFGALVHRYTHSDDFLVAAPVLNRGAGTEDAIGYFGNTVAMRLRPQSAMS
FRELLTATRDIASGAFAHQRINLDRVVRELNPDRRHGAERMTRVSFGFREPDGGGFNP
PGIECERYDLRSNITQLPLGFMVEFDRAGVLVEAEHLVEILEPALAKQMLRHFGVLLD
NALAAPDNTLSGLALMDERDAARLREVSRGERFDTPVKTLVDLVNEQTTRTPDATAVV
YEGQHFTYHDLNEASNRLGHWLIEQGIGSEDRVAVLLDKSPDLIVTALGVVKSGAVYV
PVDPSYPQDRLDFILADCDAKLVLRTPVRELAGYRSDDPTDADRIRPLRPDNTAYLIY
TSGTTGLPKGVAVPHRPVAEYFVWFKGEYDVDDTDRLLQVASPSFDVSIAEIFGTLAC
GARMVIPRPGGLTDIGYLTALLRDEGITAMHFVPSLLGLFLSLPGVSQWRTLQRVPIG
GEPLPGEVADKFHATFDALLHNFYGPTETVINASRFKVVGPQGTRIVPIGRPKINTTM
HLLDDSLQPVPTGVIGEIYIGGTHVAYGYHRRAGLTAERFVADPFNPGSRMYRSGDLA
RRNADGDIEFVGRADEQVKIRGFRIELGDVAAAIAVDPTVGQAVVVVSDLPRLGKSLV
GYVTPAAGGDGPADVGVDLDRIRARVAAALPEYMLPAAYVVLDEIPITAHGKIDRAAL
PEPQIASDTEFRAPQTATERRLAQLFGELLGRDRVGADDSFFDLGGHSLLATKLVAAV
RNAFGVDVGVREIFEFATVTALAGHIDTLDSDSARPRLTRVDHDGPVRLSSSQMRSWF
NYRFDGPNAVNNIPFAAALHGPCDTNAFAAAITDVVARHEILRTVYREIGGVPHQIIQ
PPAEVPVRCAAGSDAAWLRAELNNERGYVFDLETDWPIRAALLSTPEQTVLSLVVHHI
AGDHWSAGVLFTDLLTAYRARSTGQRPSWAPLPVQYADYSVWQSALLDDGAGIVGPQR
DYWIRQLGGLAGETGLRPDFPRPALLSGAGDAVEFRLGAAIRDKLAAVSRDLGVTEFM
LLQAAVAVVLHKAGGGVDVPIGAPVAGRSEANLDQLIGFFINIVVLRNDLRGNPTLRE
VLQRTRQMALAAYAHQDLPFDQVVEAVNPQRSLSRNPLFDIVVHVREQMPQDHVIDTG
PDGDTTLRVLEPTFDAAQADLSVNFFACGDEYRGHVIYRTELYERATAQRFADWLVRV
VEAFADRPDQPLREVEMVSAQARRRILDRSNAGAGTARVYLLDDALKPVPVGVVGDVY
YGGGPAVGARLARPSETATRFVADPFAAQPGSRLYRNGERGVWKADGQLELLAEIERL
PTAQAAPVPAEPADTETERALAAILADVLEVGEVGRYDDFFNLGGDSILATQVAARAR
DGGIPLTARMVFEHPVLCELAAAVDAKPHVEAEPDDKHHAPMSTSGLSPDELSALTAS
WDQWP"

```
CDS             complement(2639736..2642750)
                /codon_start=1
                /note="gene_2436"
                /transl_table=11
                /translation="MAPKQLPDGRVAVLLSAHAEELIGPDARAIADYLERFPATTVTE
                VARQLRKTRRVRRHRAVLRAADRLELAEGLRALAAGREHPLIARSSLGSAPRQAFVFP
                GQGGHWPGMGAVAYRELPTYRTATDTCAAAFAAAGVDSPLPYLIAPPGTDERQAFCEI
                EIEGAQFVHAVALAEVWRSCGVLPDLTVGHSLGEVAAAYLAGSITLSDAVAVVAARAN
                VVGRLPGRYAVAALGIGEQDASALIATTGGWLELSVVNASSTVAVSGERQAVAAIVDT
                VRSSGHFARGITVGFPVHTSVLESLRDELCEQLPDSEFMEAPVQFIGGTTGDVVAPGT
                TFGDYWYANLRHTVRFDRAVESAIRCGARAFIEISAHPALLFAIGQNCEGAANLPDGP
                AVLVGSARRGERFVDALSANIVSAAVADPGYPWGDLGGDPLDGDVDLSGFPNAPMRAV
                PMWAHPEPLPPVSGLTIAVERWERMVPSTPVAGRHRHLAVLDLGAHRALAQTLCAAID
                SHPDTELSAARDAELILVIAPDFEHTDAVRAAGALADLVGAGLLDYPMHIGARCQSVC
                LVTVGAEQVDAADAVPSAGQAALAAMHRSIGFEHPEQTFSHLDLPSWDLDPVLGVSVI
                TAVLRGFGETALRGSVNGYTLFERTLADAPAVPNWSLDSGVLDDVVVTGGAGAIGMHY
                ARYLAEHGARRIVLLSRRAADQATVAMLRKQHGTVIVSPPCDITDPTQLSAIAAEYGG
                VGASLIVHAAGSVISGTAPGVTSAAVVDNFAAKVLGLAQMIELWPLRPDVRTLLCSSV
                MGVWGGHGVVAYSAANRLLDVMAAQLRAQGRHCVAVKWGLWQAPKAGEPARGIADAVT
                IARVERSGLRQMAPQQAIEASLHEFTVDPLVFAADAARLQMLLDSRQFERYEGPTDPN
                LTIVDAVRTQLAAVLGIPQAGEVNLQESLFDLGVDSMLALDLRNRLKRSIGATVSLAT
                LMGDITGDGLVAKLEDADERSHTAQKVDISRD"
CDS             complement(2642750..2644084)
                /codon_start=1
                /note="gene_2437"
                /transl_table=11
                /translation="MSDNDPVVIVGLAIEAPGGVETADDYWTLLSEQREGLGPFPTDR
                GWALRELFDGSRRNGFKPIHNLGGFLSSATTFDPEFFRISPREATAMDPQQRVGLRVA
                WRTLENSGINPDDLAGHDVGCYVGASALEYGPALTEFSHHSGHLITGTSLGVISGRIA
                YTLDLAGPALTVDTSCSSALAAFHTAVQAIRAGDCDLALAGGVCVMGTPGYFVEFSKQ
                HALSDDGHCRPYSAHASGTAWAEGAAMFLLQRRSRATADRRRVLAEVRASCLNSDGLS
                DGLTAPSGDAQTRLLRRAIAQAAVVPADVGMVEGHGTATRLGDRTELRSLAASYGTAP
                AGRGPLLGSVKSNIGHAQAAAGGLGLVKVILAAQHAAIPPTLHVDEPSREIDWEKQGL
                RLADKLTPWRAVDGWRTAAVSAFGMSGTNSHVIVSMPDTVSAPERGPECGEV"
CDS             complement(2644074..2648318)
                /codon_start=1
                /note="gene_2438"
                /transl_table=11
                /translation="VVHATACSEIIRAEVAELLGVRADALHPGANLVGQGLDSIRMMS
                LVGRWRRKGIAVDFATLAATPTIEAWSQLVSAGTGVAPTAVAAPGDAGLSQEGEPFPL
                APMQHAMWVGRHDHQQLGGVAGHLYVEFDGARVDPDRLRAAATRLALRHPMLRVQFLP
                DGTQRIPPAAGSRDFPISVADLRHVAPDVVDQRLAGIRDAKSHQQLDGAVFELALTLL
                PGERTRLHVDLDMQAADAMSYRILLADLAALYDGREPPALGYTYQEYRQAIEAEETLP
                QPVRDADRDWWAQRIPQLPDPPALPTRAGGERDRRRSTRRWHWLDPQTRDALFARARA
                RGITPAMTLAAAFANVLARWSASSRFLLNLPLFSRQALHPDVDLLVGDFTSSLLLDVD
                LTGARTAAARAQAVQEALRSAAGHSAYPGLSVLRDLSRHRGTQVLAPVVFTSALGLGD
                LFCPDVTEQFGTPGWIISQGPQVLLDAQVTEFDGGVLVNWDVREGVFAPGVIDAMFTH
                QVDELLRLAAGDDAWDAPSPSALPAAQRAVRAALNGRTAAPSTEALHDGFFRQAQQQP
                DAPAVFASSGDLSYAQLRDQASAVAAALRAAGLRVGDTVAVLGPKTGEQVAAVLGILA
                AGGVYLPIGVDQPRDRAERILATGSVNLALVCGPPCQVRVPVPTLLLADVLAAAPAEF
                VPGPSDPTALAYVLFTSGSTGEPKGVEVAHDAAMNTVETFIRHFELGAADRWLALATL
```

ECDMSVLDIFAALRSGGAIVVVDEAQRRDPDAWARLIDTYEVTALNFMPGWLDMLLEV
GGGRLSSLRAVAVGGDWVRPDLARRLQVQAPSARFAGLGGATETAVHATIFEVQDAAN
LPPDWASVPYGVPFPNNACRVVADSGDDCPDWVAGELWVSGRGIARGYRGRPELTAER
FVEHDGRTWYRTGDLARYWHDGTLEFVGRADHRVKISGYRVELGEIEAALQRLPGVHA
AAATVLPGGSDVLAAAVCVDDAGVTAESIRQQLADLVPAHMIPRHVTLLDRIPFTDSG
KIDRAEVGALLAAEVERSGDRSAPYAAPRTVLQRALRRIVADILGRANDAVGVHDDFF
ALGGDSVLATQVVAGIRRWLDSPSLMVADMFAARTIAALAQLLTGREANADRLELVAE
VYLEIANMTSADVMAALDPIEQPAQPAFKPWVKRFTGTDKPGAVLVFPHAGGAAAAYR
WLAKSLVANDVDTFVVQYPQRADRRSHPAADSIEALALELFEAGDWHLPAPLTLFGHC
MGAIVAFEFARLAERNGVPVRALWASSGQAPSTVAASGPLPTADRDVLADMVDLGGTD
PVLLEDEEFVELLVPAVKADYRALSGYSCPPDVRIRANIHAVGGNRDHRISREMLTSW
ETHTSGRFTLSHFDGGHFYLNDHLDAVARMVSADVR"

CDS 2648417..2650114
/codon_start=1
/note="gene_2439"
/transl_table=11
/translation="MPPKAADGRRPSPDGGLGGFVPFPADRAASYRAAGYWSGRTLDT
VLSDAARRWPDRLAVADAGDRPGHGGLSYAELDQRADRAAAALHGLGITPGDRVLLQL
PNGCQFAVALFALLRAGAIPVMCLPGHRAAELGHFAAVSAATGLVVADVASGFDYRPM
ARELVADHPTLRHVIVDGDPGPFVSWAQLCAQAGTGSPAPPADPGSPALLLVSGGTTG
MPKLIPRTHDDYVFNATASAALCRLSADDVYLVVLAAGHNFPLACPGLLGAMTVGATA
VFAPDPSPEAAFAAIERHGVTVTALVPALAKLWAQSCEWEPVTPKSLRLLQVGGSKLE
PEDARRVRTALTPGLQQVFGMAEGLLNFTRIGDPPEVVEHTQGRPLCPADELRIVNAD
GEPVGPGEEGELLVRGPYTLNGYFAAERDNERCFDPDGFYRSGDLVRRRDDGNLVVTG
RVKDVICRAGETIAASDLEEQLLSHPAIFSAAAVGLPDQYLGEKICAAVVFAGAPITL
AELNGYLDRRGVAAHTRPDQLVAMPALPTTPIGKIDKRAIVRQLGIATGPVTTQRCH"

CDS 2650210..2651130
/codon_start=1
/note="gene_2440"
/transl_table=11
/translation="MVLRPITGAIPPDGPWGIWASRRIIAGLMGTFGPSLAGTRVEQV
NSVLPDGRRVVGEWVYGPHNNAINAGPGGGAIYYVHGSGYTMCSPRTHRRLTSWLSSL
TGLPVFSVDYRLAPRYRFPTAATDVRAAWDWLAHVCGLAAEHMVIAADSAGGHLTVDM
LLQPEVAARPPAAVVLFSPLIDLTFRLGASRELQRPDPVVRADRAARSVALYYTGVDP
AHHRLALDVAGGPPLPPTLIQVGGAEILEADARQLDADIRAAGGICELQVWPDQMHVF
QALPRMTPEAAKAMTYVAQFIRSTTARGDL"

CDS complement(2651134..2652429)
/codon_start=1
/note="gene_2441"
/transl_table=11
/translation="MPAGVNPADLAAELAAVVTESVDEDYLLYECDGQWVLAAGVQAM
VELDSDELRVIRDGVTRRQQWSGRPGAALGEAVDRLLLETDQAFGWVAFEFGVHRYGL
QQRLAPHTPLARVFSPRTRIMVSEKEIRLFDAGIRHREAIDRLLATGVREVPQSRSVD
VSDDPSGFRRRVAVAVDEIAAGRYHKVILSRCVEVPFAIDFPLTYRLGRRHNTPVRSF
LLQLGGIRALGYSPELVTAVRADGVVITEPLAGTRALGRGPAIDRLARDDLESNSKEI
VEHAISVRSSLEEITDIAEPGSAAVIDFMTVRERGSVQHLGSTIRARLDPSSDRMAAL
EALFPAVTASGIPKAAGVEAIFRLDECPRGLYSGAVVMLSADGGLDAALTLRAAYQVG
GRTWLRAGAGIIEESEPEREFEETCEKLSTLTPYLVARQ"

CDS 2652939..2653148
/codon_start=1
/gene="TBCG_02329"

/note="gene_2442"
/transl_table=11
/translation="MFVIRLADGEEVHGECDELTINPATGVLTVCRVDGFEETTTHYS
PSAWRSVTHRKRGVGVRPSLVSTAQ"
CDS 2653246..2654499
/codon_start=1
/note="gene_2443"
/transl_table=11
/translation="MLHEFWVNFTHNLFKPLLLFFYFGFLIPIFKVRFEFPYVLYQGL
TLYLLLAIGWHGGEELAKIKPSNVGAIVGFMVVGFALNFVIGTLAYFLLSKLTAMRRV
DRATVAGYYGSDSAGTFATCVAVLTSVGMAFDAYMPVMLAVMEIPGCLVALYLVARLR
HRGMNEAGYMADEPGYTTAAMIGAGPGTPARPAHSDSLTAQAERGIEEELELSLEKRE
HPNWDEDGVKDSGTNASIFSRELLQEVFLNPGLVLLFGGIVIGLISGLQGQKVLHDDD
NFFVAAFQGVLCLFLLEMGMTASRKLKDLASAGSGFVFFGLLAPNLFATLGIIVAHGY
AYVTNNDFAPGTYVLFAVLCGAASYIAVPAVQRLAIPEASPTLPLAASLGLTFSYNVT
IGIPLYIEIARIVGQWFPATGASIG"
CDS complement(2654496..2655668)
/codon_start=1
/note="gene_2444"
/transl_table=11
/translation="MVFRQAPVELPGLAPMPGQPFGVYLHVPFCLTRCGYCDFNTYTP
AQLGGVSPDRWLLALRAELELAAAKLDAPTVHTVYVGGGTPSLLGGERLATLLDMVRD
HFVLAPDAEVSTEANPESTWPEFFATIRAAGYTRVSLGMQSVAPRVLATLDRVHSPGR
AAAAATEAIAEGFTHVNLDLIYGTPGESDDDLVRSVDATVQAGVDHVSAYALVVEHGT
ALARRVRRGELAAPDDDVLAHRYELVDARLSAAGFAWYEVSNWCRPGGECRHNLGYWD
GGQWWGAGPGAHGYIGVTRWWNVKHPNTYAEILAGATLPVAGFEQLGADALHTEDVLL
KVRLRQGLPLARLGAAERERAEAVLADGLLDYHGDRLVLTGRGRLLADAVVRTLLG"
CDS complement(2655729..2656193)
/codon_start=1
/note="gene_2445"
/transl_table=11
/translation="MTPGLLTTAGAGRPRDRCARIVCTVFIETAVVATMFVALLGLST
ISSKADDIDWDAIAQCESGGNWAANTGNGLYGGLQISQATWDSNGGVGSPAAASPQQQ
IEVADNIMKTQGPGAWPKCSSCSQGDAPLGSLTHILTFLAAETGGCSGSRDD"
CDS complement(2656190..2656756)
/codon_start=1
/note="gene_2446"
/transl_table=11
/translation="VRRMAIFGRGHGASEPGGTGEPAETPGRGRLTRSVIGWVGAVAV
VVSLAGSGWCGWVLFEKHQTDVAAGQALQAARSYVVKLATMDCERIDHNMRDILEGST
GEFKDKYGKSSAHLRQLLADNRVATHGTVVAASVKSATTNKVVVLMFIDQSVSNRNSP
TPQIDRSRIKVIMDKVNGRWLASKVELL"
CDS 2657184..2658851
/codon_start=1
/note="gene_2447"
/transl_table=11
/translation="MTTARPAKARNEGQWALGHREPLNANEELKKAGNPLDVRERIEN
IYAKQGFDSIDKTDLRGRFRWWGLYTQREQGYDGTWTGDDNIDKLEAKYFMMRVRCDG
GALSAAALRTLGQISTEFARDTADISDRQNVQYHWIEVENVPEIWRRLDDVGLQTTEA
CGDCPRVVLGSPLAGESLDEVLDPTWAIEEIVRRYIGKPDFADLPRKYKTAISGLQDV
AHEINDVAFIGVNHPEHGPGLDLWVGGGLSTNPMLAQRVGAWVPLGEVPEVWAAVTSV

FRDYGYRRLRAKARLKFLIKDWGIAKFREVLETEYLKRPLIDGPAPEPVKHPIDHVGV
QRLKNGLNAVGVAPIAGRVSGTILTAVADLMARAGSDRIRFTPYQKLVILDIPDALLD
DLIAGLDALGLQSRPSHWRRNLMACSGIEFCKLSFAETRVRAQHLVPELERRLEDINS
QLDVPITVNINGCPNSCARIQIADIGFKGQMIDDGHGGSVEGFQVHLGGHLGLDAGFG
RKLRQHKVTSDELGDYIDRVVRNFVKHRSEGERFAQWVIRAEEDDLR"

CDS 2658848..2659612
/codon_start=1
/note="gene_2448"
/transl_table=11
/translation="MSGETTRLTEPQLRELAARGAAELDGATATDMLRWTDETFGDIG
GAGGGVSGHRGWTTCNYVVASNMADAVLVDLAAKVRPGVPVIFLDTGYHFVETIGTRD
AIESVYDVRVLNVTPEHTVAEQDELLGKDLFARNPHECCRLRKVVPLGKTLRGYSAWV
TGLRRVDAPTRANAPLVSFDETFKLVKVNPLAAWTDQDVQEYIADNDVLVNPLVREGY
PSIGCAPCTAKPAEGADPRSGRWQGLAKTECGLHAS"

CDS 2659627..2660508
/codon_start=1
/note="gene_2449"
/transl_table=11
/translation="MQSAAMLRSGAIEAPPATMQSAAMLRSGAIEAPPATMQSAAMRW
GHLPLAEESGTIAPQLVLTAHGSKDPRSAANARAIAGRLARMRPGLDVRVAFCELNSP
NLVDVLNRCRGAAVVTPLLLADAYHARVDIPAQIASCRVGHRVRQASVLGEDIRLVSA
LHERLTELGVSPFDHTLGVVVLAIGSSHPAANARTSTVASRLAEGTQWAAVTTAFITR
PEASLADATDRLRRHGARRMVIAPWLLAPGILSDRVRGYAREAGIAMAQPLGAHPMVA
ATMWDRYRQAVAGRIAA"

CDS 2660587..2662476
/codon_start=1
/note="gene_2450"
/transl_table=11
/translation="MLSLSGCGGFHAGAPSTAGPCEIVPNGTPAPKTPPATVPSSRNL
ATNPEIATGYRRDMTVVRTAHYAAATANPLATQVACRVLRDGGTAADAVVAAQAVLGL
VEPQSSGIGGGGYLVYFDARTGSVQAYDGREVAPAAATENYLRWVSDVDRSAPRPNAR
ASGRSIGVPGILRMLEMVHNEHGRTPWRDLFGPAVTLADGGFDISARMGAAISDAAPQ
LRDDPEARKYFLNPDGSPKPAGTRLTNPAYSKTLSAIASAGANAFYSGDIAHDIVAAA
SDTSNGRTPGLLTIEDLAGYLAKRRQPLCTTYRGREICGMPSSGGVAVAATLGILEHF
PMSDYAPSKVDLNGGRPTVMGVHLIAEAERLAYADRDQYIADVDFVQLPGGSLTTLVD
PGYLAARAALISPQHSMGSARPGDFGAPTAVAPPVPEHGTSHLSVVDSYGNAATLTTT
VESSFGSYHLVDGFILNNQLSDFSAEPHATDGSPVANRVEPGKRPRSSMAPTLVFDHS
SAGRGALYAVLGSPGGSMIIQFVVKTLVAMLDWGLNPQQAVSLVDFGAANSPHTNLGG
ENPEINTSDDGDHDPLVQGLRALGHRVNLAEQSSGLSAITRSEAGWAGGADPRREGAV
MGDDA"

CDS 2662761..2664611
/codon_start=1
/note="gene_2451"
/transl_table=11
/translation="MGVLRLFANHSVVENNIVQTIASAAGTLSSIIFVLPALLMIGWW
SGFPYWTTAAVCALGGILGVMYSIPLRRALVTGSDLPYPEGVAGAEVLKIGDSAREME
HNRRGIGVIALGAAAAAGYALLASLRVINNSLSATFRVGSGATMIGASLSLALIGVGH
LVGVTVGVAMIVGLAIAFGVMLPIRTAGQLPPDGDYAVAVARIFSTDVRFIGAGAIAV
AAAWTFLKILGPILRGIADAAVSARTRRRGQAVGQTERDIPIHIVAMVVLLSLIPIGW
LLADFTDGTPLDDRRPGAIAAGVLLVLVIGLMVAAVCGYMAGLIGSSNSPISGVGILV
VVLAGLLIKTAYGPATGSQIPALVAYTVFTAALVFGVATISNDNLQDLKTGQLVGATP

WKQQVALIIGVLVGSVVMAPILQLMQAGFGFQGAPGATANALAAPQAALMSALAKGVF
GGSLNWSLVGVGALTGVIAVALDETLAKTTTNLRLPPLAVGMGMYLPAALTLMIPIGA
FLGRIYDSWARWSGDDDERKKRLGVMLATGLIVGESLYGVLFAVIVATTGKEEPLAMV
GDGFRFASQPLGAIVFAGLLAWLYQRTRVTASYRLAAPAGSSKPLPDLPG"
CDS 2665335..2666417
/codon_start=1
/note="gene_2452"
/transl_table=11
/translation="MSFLIASPEALAATATYLTGIGSAINAANAVAAAPTTEILAAGT
DEVSTAISALFGAHAQAYQALSAHVAAFHDQFVHTLTAGAGSYMAAEAAASPLQALQL
ELLNAINAPTLALLGRPLIGDGTDAAPGSGGAGGAGGILIGNGGTGGASDLAGTGRGG
VGGAGGAGGLFGIGGAGGGCGSAVAIGGDGGAGGAGGVFSGGGAGGAGDAIGGSGGAC
GTGGLLGGGGGAGGAGGAGGNGGGASNSASIGGDGGSGGAGGMLYGAGGVGGNGGAAV
AIGGDGGAGGRAGAIGNGGDGGNGGTSNTPGGSGGDGGNGGNAGLIGSGGNGGNAEIV
ISGGSVAGTGGNGGLLLGFNGTNGLP"
CDS complement(2666442..2667497)
/codon_start=1
/note="gene_2453"
/transl_table=11
/translation="MTYAIVVADATKRYGDFVALDHVDFVVPTGSLTALLGPSGSGKS
TLLRTIAGLDQPDTGTITINGRDVTRVPPQRRGIGFVFQHYAAFKHLTVRDNVAFGLK
IRKRPKAEIKAKVDNLLQVVGLSGFQSRYPNQLSGGQRQRMALARALAVDPEVLLLDE
PFGALDAKVREELRAWLRRLHDEVHVTTVLVTHDQAEALDVADRIAVLHKGRIEQVGS
PTDVYDAPANAFVMSFLGAVSTLNGSLVRPHDIRVGRTPNMAVAAADGTAGSTGVLRA
VVDRVVVLGFEVRVELTSAATGGAFTAQITRGDAEALALREGDTVYVRATRVPPIAGG
VSGVDDAGVERVKVTST"
CDS complement(2667514..2668332)
/codon_start=1
/note="gene_2454"
/transl_table=11
/translation="MTSLPAARYLVRSVALGYVFVLLIVPVALILWRTFEPGFGQFYA
WISTPAAISALNLSLLVVAIVVPLNVIFGVTTALVLARNRFRGKGVLQAIIDLPFAVS
PVIVGVSLILLWGSAGALGFVEQDLGFKIIFGLPGIVLASMFVTCPFVVREVEPVLHE
LGTDQEQAAATLGSGWWQTFWRITLPSIRWGLTYGIVLTVARTLGEYGAVIIVSSNLP
GTSQTLTLLVSDRYHRGAEYGAYALSTLLMAVSVVVLIVQMVLDARRARAVSEG"
CDS complement(2668329..2669180)
/codon_start=1
/note="gene_2455"
/transl_table=11
/translation="MTESLVGERRAPQFRARLSGPAGPPSVRVGMAVVWLSVIVLLPL
AAIVWQAAGGGWRAFWLAVSSHAAMESFRVTLTISTAVTVINLVFGLLIAWVLVRDDF
AGKRIVDAIIDLPFALPTIVASLVMLALYGNNSPVGLHFQHTATGVGVALAFVTLPFV
VRAVQPVLLEIDRETEEAAASLGANGAKIFTSVVLPSLTPALLSGAGLAFSRAIGEFG
SVVLIGGAVPGKTEVSSQWIRTLIENDDRTGAAAISVVLLSISFIVLLILRVVGARAA
KREEMAA"
CDS complement(2669177..2670247)
/codon_start=1
/note="gene_2456"
/transl_table=11
/translation="MLSLTLSEASCIASASRWRHIIPAGVVCALIAGIGVGCHGGPSD
VVGRAGPDRAHTSITLVAYAVPEPGWSAVIPAFNASEQGRGVQVITSYGASADQSRGV

ADGKPADLVNFSVEPDIARLVKAGKVDKDWDADATKGIPFGSVVTFVVRAGNPKNIRD
WDDLLRPGIEVITPSPLSSGSAKWNLLAPYAAKSDGGRNNQAGIDFVNTLVNEHVKLR
PGSGREATDVFVQGSGDVLISYENEAIATERAGKPVQHVTPPQTFKIENPLAVVATST
HLGAATAFRNFQYTVQAQKLWAQAGFRPVDPAVAADFADLFPVPAKLWTIADLGGWGS
VDPQLFDKATGSITKIYLRATG"

CDS 2670186..2670590
/codon_start=1
/note="gene_2457"
/transl_table=11
/translation="MCRQRDALAMQLASESVKESIGDLPVRDFGQRSRSGGKAIAEHC
RTHELHIRPRTGGESATTVQVGRSAANERADIAPRKTRCCVHVAKPNRIRLADQLARS
SMGEKPGHDHQRNQRDQNQRDVRPRHPGYLGA"

CDS complement(2670575..2670769)
/codon_start=1
/note="gene_2458"
/transl_table=11
/translation="MNGFLSWWDGVELWLSGLPFALQALAVMPVVLALAYFTAALLDA
LLGRVIQLIRRARRPDQAPR"

CDS 2670948..2672990
/codon_start=1
/note="gene_2459"
/transl_table=11
/translation="MVLHAQPPDQSTETAREAKALAGATDGATATSADLHAPMALSSS
SPLRNPFPPIADYAFLSDWETTCLISPAGSVEWLCVPRPDSPSVFGAILDRSAGHFRL
GPYGVSVPSARRYLPGSLIMETTWQTHTGWLIVRDALVMGKWHDIERRSRTHRRTPMD
WDAEHILLRTVRCVSGTVELMMSCEPAFDYHRLGATWEYSAEAYGEAIARANTEPDAH
PTLRLTTNLRIGLEGREARARTRMKEGDDVFVALSWTKHPPPQTYDEAADKMWQTTEC
WRQWINIGNFPDHPWRAYLQRSALTLKGLTYSPTGALLAASTTSLPETPRGERNWDYR
YAWIRDSTFALWGLYTLGLDREADDFFAFIADVSGANNNERHPLQVMYGVGGERSLVE
AELHHLSGYDHARPVRIGNGAYNQRQHDIWGSILDSFYLHAKSREQVPENLWPVLKRQ
VEEAIKHWREPDRGIWEVRGEPQHFTSSKVMCWVALDRGAKLAERQGEKSYAQQWRAI
ADEIKADILEHGVDSRGVFTQRYGDEALDASLLLVVLTRFLPPDDPRVRNTVLAIADE
LTEDGLVLRYRVHETDDGLSGEEGTFTICSFWLVSALVEIGEVGRAKRLCERLLSFAS
PLLLYAEEIEPRSGRHLGNFPQAFTHLALINAVVHVIRAEEEADSSGMFQPANAPM"

CDS complement(2673068..2673823)
/codon_start=1
/note="gene_2460"
/transl_table=11
/translation="VTNRWRWVVPLFAVFLAAGCTTTTTGKAGLAPNAVPRPLMGSLI
QRVPLDGAALSTLLNQPFQALPPFPPVFGGSDSLGDSDVSARPADCVGVGYLTQRNVY
RSVEVKSVARVSWRHDGSSVKVDDLDEGVVALPSAAAADDLFARFSAQWKECDGTTLT
VPASAFGQRSITDVRVADSVVAATVSLRRGTHSILASVPQARAVGVRGNCVVEVAVTF
FGITHPSDQGSADISTSAVDIAHAMMDRISELS"

CDS complement(2673820..2675790)
/codon_start=1
/note="gene_2461"
/transl_table=11
/translation="VTSVRTPCSQHRRDRPSAIGSQLPDADTLDTRQPPLQEIPISSF
ADKTFTAPAQIRNFCIIAHIDHGKSTLADRMLQLTGVVDERSMRAQYLDRMDIERERG
ITIKAQNVRLPWRVDKTDYVLHLIDTPGHVDFTYEVSRALEACEGAVLLVDAAQGIEA
QTLANLYLALDRDLHIIPVLNKIDLPAADPDRYAAEMAHIIGCEPAEVLRVSGKTGEG

VSDLLDEVVRQVPPPQGDAEAPTRAMIFDSVYDIYRGVVTYVRVVDGKISPRERIMMM
STGATHELLEVGIVSPEPKPCEGLGVGEVGYLITGVKDVRQSKVGDTVTSLSRARGAA
AEALTGYREPKPMVYSGLYPVDGSDYPNLRDALDKLQLNDAALTYEPETSVALGFGFR
CGFLGLLHMEITRERLEREFGLDLISTSPNVVYRVHKDDGTEIRVTNPSDWPEGKIRT
VYEPVVKTTIIAPSEFIGTIMELCQSRRGELGGMDYLSPERVELRYTMPLGEIIFDFF
DALKSRTRGYASLDYEEAGEQEAALVKVDILLQGEAVDAFSAIVHKDTAYAYGNKMTT
KLKELIPRQQFEVPVQAAIGSKIIARENIRAIRKDVLSKCYGGDITRKRKLLEKQKEG
KKRMKTIGRVEVPQEAFVAALSTDAAGDKGKK"

CDS 2675769..2676371
/codon_start=1
/note="gene_2462"
/transl_table=11
/translation="MASARKSQWKTLQRFAENLVFTEAPKLVRHLQNTQETLRTIRQA
VKITANIMTTAVPSPPAEIAAGRPVTSTSCPTAARARRLVYAPDLDGRADPGEIVWTW
VAYEQDPTRGKDRPVLVVGRDRSVLLGLLVSSQERHAADRDWVGIGSGAWDYEGRESW
VRLDRVLDVPEESIRREGAILEREVFDVVAARLRADYAWR"

CDS complement(2676542..2676970)
/codon_start=1
/note="gene_2463"
/transl_table=11
/translation="MRIADVLRNKGAAVVTINPDATVGELLAGLAEQNIGAMVVVGAE
GVVGIVSERDVVRQLHTYGASVLSRPVAKIMSTTVATCTKSDTVDKISVLMTENRVRH
VPVLDGKKLIGIVSIGDVVKSRMGELEAEQQQLQSYITQG"

CDS 2677230..2678072
/codon_start=1
/note="gene_2464"
/transl_table=11
/translation="MLEITLLGTGSPIPDPDRAGPSTLVRAGAQAFLVDCGRGVLQRA
AAVGVGAAGLSAVLLTHLHSDHIAELGDVLITSWVTNFAADPAPLPIIGPPGTAEVVE
ATLKAFGHDIGYRIAHHADLTTPPPIEVHEYTAGPAWDRDGVTIRVAPTDHRPVTPTI
GFRIESDGASVVLAGDTVPCDSLDQLAAGADALVHTVIRKDIVTQIPQQRVKDICDYH
SSVREAAATANRAGVGTLVMTHYVPAIGPGQEEQWRALAATEFSGRIEVGNDLHRVEV
HPRR"

CDS complement(2679142..2679981)
/codon_start=1
/note="gene_2465"
/transl_table=11
/translation="MWRTRVVHTTGYVYQSPVTASYNEARLTPRSSSRQNLVLNRVET
IPATRSYRYIDYWGTAVTAFDLHAPHTELTVTSSSVVETERPEPLAAKATWADLQSTA
VIDRFDEVLRPTPHTPASARVDAVGRRIRKCHEPSEAVVAAARWARSELDYIPGTTSV
HSSGLDALEQGKGVCQDFVHLSLMVLRSMGIPCRYVSGYLHPKRDAVVGKTVDGRSHA
WVQAWTGGWWHYDPTNDNEITEQYISVGVGRDYTDVSPLKGIYSGEGVTDLDVVVEIT
RLA"

CDS complement(2679981..2680958)
/codon_start=1
/note="gene_2466"
/transl_table=11
/translation="MLARNAEALYWIGRYVERADDTARILDVAVHQLLEDSSVDPDQA
SRLLLRVLGIEPPDHELDVWSLTDLVAFSTNSQGGSSIVDAISAARENAKSAREVTSS
ETWECLNTTYNALPERERAAKRLGPHEFLSFIEGRAAMFAGLADSTLLRDDGYRFMLL
GRAIERVDMTVRLLLSRVGDSASSPAWVTLLRSAGAHDTYLRTYRGVLDAGRVVEFMM

LDRLFPRSVFHSLKLAEHNLAELMHNPHSRIGATTEAQRLLGQARSELEFVQPGVLLE
TLESRLAGLQTTCRDVGDALALQYFHAAPWVAWSDAGQRGQLVGSQEES"
CDS complement(2680958..2682613)
/codon_start=1
/note="gene_2467"
/transl_table=11
/translation="MRRVSLPNQLNETRRRSPTRGERIFGGYNTSDVYAMAFDEMFDA
QGIVRGPYKGIYAELAPSDASELKARADALGRAFIDQGITFSLSGQERPFPLDLVPRV
ISAPEWTRLERGITQRVKALECYLDDIYGDQEILRDGVIPRRLVTSCEHFHRQAVGIV
PPNGVRIHVAGIDLIRDHRGDFRVLEDNLRSPSGVSYVMENRRTMARVFPNLFATHRV
RAVDDYASHLLRALRNSAATNEADPTVVVLTPGVYNSAYFEHSLLARQMGVELVEGRD
LFCRDNQVYMRTTEGERQVDVIYRRIDDAFLDPLQFRADSVLGVAGLVNAARAGNVVL
SSAIGNGVGDDKLVYTYVPTMIEYYLHEKPLLANVETLRCWLDDEREEVLDRIRELVL
KPVEGSGGYGIVFGPEASQAELAAVSQKIRDDPRSWIAQPMMELSTVPTRIEGTLAPR
YVDLRPFAVNDGNEVWVLPGGLTRVALVEGSRVVNSSQGGGSKDTWVLAPRASAAARE
LGAAQIVRSLPQPLCDPTVDASGYEPHDQQPQQQQQQQQQAFH"
CDS 2682723..2682983
/codon_start=1
/gene="rpsT"
/note="gene_2468"
/transl_table=11
/translation="VANIKSQQKRNRTNERARLRNKAVKSSLRTAVRAFREAAHAGDK
AKAAELLASTNRKLDKAASKGVIHKNQAANKKSALAQALNKL"
CDS complement(2682999..2683967)
/codon_start=1
/note="gene_2469"
/transl_table=11
/translation="VSEAKPLHLVLGDEELLVERAVADVLRSARQRAGTADVPVSRMR
AGDVGAYELAELLSPSLFAEERIVVLGAAAEAGKDAAAVIESAAADLPAGTVLVVVHS
GGGRAKSLANQLRSMGAQVHPCARITKVSERADFIRSEFASLRVKVDDETVTALLDAV
GSDVRELASACSQLVADTGGAVDAAAVRRYHSGKAEVRGFDIADKAVAGDVAGAAEAL
RWAMMRGEPLVVLADALAEAVHTIGRVGPQSGDPYRLAAQLGMPPWRVQKAQKQARRW
SRDTVATAMRLVAELNANVKGAVADADYALESAVRQVAELVADRGR"
CDS complement(2683980..2685524)
/codon_start=1
/note="gene_2470"
/transl_table=11
/translation="MGFGASRLDVRLVPAALVSWIVTAAGIVWPIGNVCALCCVVVAL
GGGALWWCVARRSWHAPRLGSISAGLVAVGMVGAGYGLAVALRSEAVDRHPITVAFGT
SALVTVTPSESPVSLGRGRLMFRATVQRLRDDETSGRVVVFARALDFGELMVGQPVQF
RARISRPARHDLTVAVFNATGRPTVGRAGPVHRAAHIVRHRFAAAVREVLPADQATML
PALVLGDTSTVTALTSREFRAAGLTHLTAVSGANVTIVCAAALVSARLIGPRAAVVCA
AVALVAFVILVQPTASVLRAAVMGAIALVGMLSARRRQAIPALSGSVLVLLAAAPHLA
VDIGFALSVAATGALVVIAPVWSRRLVDRGCPKVLADALAVAAAAQLVTAPLVAAISG
RVSLVAVVANLAVAAVIAPITVLGSVAAVLVVPWPAGAQVLIRFTGPEVWWVLRVAHW
ASGVPAATVPVAAGLPGVLLVGGATVFTVAQWRWRWFRAAMCKTMAVAVICLLAWSLS
GLVGPS"
CDS complement(2685539..2686432)
/codon_start=1
/note="gene_2471"
/transl_table=11

```
                     /translation="MRTELPAERLQRRLGAVPDIDSHAASAHLDPEPHDPTDDGPDHD
                     EPRDDPNSLLPRWLPDTSRGQGWADRIRADPGRAGAVALAVIAALAVLVTVFTLIRDR
                     TEPVMSAKLPPVEPVSPTNPRSSASPGSPDRSGLPVVVSVVGLVHTPGLVTLAPGARI
                     ADALQAAGGAVDGADTVGLNMARQLGDGEQIVVGLAPPSGQPRVLGSSVGAGTPGPAG
                     TSGTATTGPKTAPKTAEVLDLNTATVEQLDALPGIGPVTAAAIVAWRQRNGRFTSVDQ
                     LADVDGIGPARLDKLRNLVRV"
     CDS             complement(2686772..2687935)
                     /codon_start=1
                     /note="gene_2472"
                     /transl_table=11
                     /translation="MFLLAAASFTDFIGPESATAWRTLVPTDGAVVVRDGAGPGSEVV
                     GMALYMDLRLTVPGEVVLPTAGLSFVAVAPTHRRRGLLRAMCAELHRRIADSGYPVAA
                     LHASEGGIYGRFGYGPATTLHELTVDRRFARFHADAPGGGLGGSSVRLVRPTEHRGEF
                     EAIYERWRQQVPGGLLRPQVLWDELLAECKAAPGGDRESFALLHPDGYALYRVDRTDL
                     KLARVSELRAVTADAHCALWRALIGLDSMERISIITHPQDPLPHLLTDTRLARTTWRQ
                     DGLWLRIMNVPAALEARGYAHEVGEFSTVLEVSDGGRFALKIGDGRARCTPTDAAAEI
                     EMDRDVLGSLYLGAHRASTLAAANRLRTKDSQLLRRLDAAFASDVPVQTAFEF"
     CDS             complement(2688120..2688962)
                     /codon_start=1
                     /note="gene_2473"
                     /transl_table=11
                     /translation="VTVVVVTDTSCRLPADLREQWSIRQVPLHILLDGLDLRDGVDEI
                     PDDIHKRHATTAGATPVELSAAYQRALADSGGDGVVAVHISSALSGTFRAAELTAAEL
                     GPAVRVIDSRSAAMGVGFAALAAGRAAAAGDELDTVARAAAAAVSRIHAFVAVARLDN
                     LRRSGRISGAKAWLGTALALKPLLSVDDGKLVLVQRVRTVSNATAVMIDRVCQLVGDR
                     PAALAVHHVADPAAANDVAAALAERLPACEPAMVTAMGPVLALHVGAGAVGVCVDVGA
                     SPPA"
     CDS             complement(2689043..2689756)
                     /codon_start=1
                     /note="gene_2474"
                     /transl_table=11
                     /translation="LLVFADSLAYYGPTGGLPADDPRIWPNIVASQLDWDLELIGRIG
                     WTCRDVWWAATQDPRAWAALPRAGAVIFATGGMDSLPSVLPTALRELIRYVRPSWLRR
                     WVRDGYAWVQPRLSPVARAALPPHLTAEYLEKTRGAIDFNRPGIPIIASLPSVHIAET
                     YGKAHHGRAGTVAAITEWAQHHDIPLVDLKAAVAEQILSGYGNRDGIHWNFEAHQAVA
                     ELMLKALAEAGVPNEKSRG"
     CDS             complement(2689776..2690429)
                     /codon_start=1
                     /note="gene_2475"
                     /transl_table=11
                     /translation="VMLRHGQTDYNVGSRMQGQLDTELSELGRTQAVAAAEVLGKRQP
                     LLIVSSDLRRAYDTAVKLGERTGLVVRVDTRLRETHLGDWQGLTHAQIDADAPGARLA
                     WREDATWAPHGGESRVDVAARSRPLVAELVASEPEWGGADEPDRPVVLVAHGGLIAAL
                     SAALLKLPVANWPALGGMGNASWTQLSGHWAPGSDFESIRWRLDVWNASAQVSSDVL"
     CDS             complement(2690444..2690827)
                     /codon_start=1
                     /note="gene_2476"
                     /transl_table=11
                     /translation="MTANREAIDMARVAAGAAAAKLADDVVVIDVSGQLVITDCFVIA
                     SGSNERQVNAIVDEVEEKMRQAGYRPARREGAREGRWTLLDYRDIVVHIQHQDDRNFY
                     ALDRLWGDCPVVPVDLSANSAGAPQ"
```

```
CDS             complement(2690824..2691459)
                /codon_start=1
                /gene="nadD"
                /note="gene_2477"
                /transl_table=11
                /translation="MGGTFDPIHYGHLVAASEVADLFDLDEVVFVPSGQPWQKGRQVS
                AAEHRYLMTVIATASNPRFSVSRVDIDRGGPTYTKDTLADLHALHPDSELYFTTGADA
                LASIMSWQGWEELFELARFVGVSRPGYELRNEHITSLLGQLAKDALTLVEIPALAISS
                TDCRQRAEQSRPLWYLMPDSVVQYVSKCRLYCGACDAGARSTTSLAAGNGL"
CDS             2691734..2692006
                /codon_start=1
                /note="gene_2478"
                /transl_table=11
                /translation="MPASVSTVLVDTSVAVAPVVADHDHHEDTFQALRGRTLGLAGHA
                AFERRTLATVAKLLAHTFPATRFLGAGAAMSLLPELAPAEIAGGAV"
CDS             2692248..2693294
                /codon_start=1
                /note="gene_2479"
                /transl_table=11
                /translation="VDNLPIESAESTRLAKAAMTRRFYTRSVVKGEITLPAVPSMIDE
                YVTMCAGLFAGVGRKFSDEELAHLRAVLQGQLAEAYAASQRSTIVISYNAPMGPTLHY
                QVRAQWRTVAQEYENWIATREPPLFGTEPDARVWALANEAADPTTHRVLEIGAGTGRN
                ALALARRGHPVDVVEMTPKFADIIRSDAERDSLDVRVIMRDVFSTMDDLRQDYQLMVL
                SEVVPDFRTTQQLRNLFELAAQCLAPGARLVFNAFLANGDYAPDQAAREFGQQMYTGM
                CTRAEMSAAAAGLPLELVADDSVYDYEKTHLPPGAWPPTSWYADWIRGLDVFTTNVES
                CPIEMRWLVFQRRR"
CDS             complement(2693427..2694227)
                /codon_start=1
                /note="gene_2480"
                /transl_table=11
                /translation="MLQDAGIKADSVASSVTPKSVRAMVEALIDGERRPAVLADLARG
                SMRSKIPDLQRALEGRFDDHHALMCRLHLAHLDQLDAMIGALDEQIEQLMHPFCARRE
                LIASIPGIGVGASATVISEIGADPAAWFPSAEHLASWVRLCPGNHESAGKRHHGARRT
                GNQHLQPVLVECAWAAVRTDGYLREYYRRQVRKFGGFRSPAANKKAIIAVAHKLIVII
                WHVLATGRPYQDLGADYFTTRMDPDKERRRLVAKLEAQGLGVTLEPAA"
CDS             complement(2694515..2695957)
                /codon_start=1
                /note="gene_2481"
                /transl_table=11
                /translation="VAARRIRAARPLAPHGLPGHLVGFVEALRGSGISVGPSETVDAG
                RVMATLGLGDREVLREGIACAVLRRPDHRDTYDAMFDLWFPAALGARAVITTEDESAG
                SGGLPPDDVEAMRQLLLDLLANNQDLAGKDERLVEMIARIVEAYGKYSSSRGPSFSSY
                QALKAMALDELEGKLLAGLLAPYGDEPTATQEQIAKALAAQKIAQLRRMVDAETKRRT
                AEQLGREHVQMYGIPQLSENVEFLRASGEQLRQMRRVVAPLARTLATRLAARRRRARA
                GSIDLRKTLRKSMSTGGVPIDLVLHKPRPARPELVVLCDVSGSVAGFSHFTLLLVHAL
                RQQFSRVRVFAFIDSTDEVTHMFGPESDLAIAIQRITREAGVYARDGHSDYGNAFVSF
                MQGFPNVLSPRSSLLVLGDGRTNYRNPATDVLADMVTASRHAHWLNPEPKHLWGSGDS
                AVPRYQEVITMHECRSAKQLATVIDQLLPV"
CDS             complement(2695957..2696832)
                /codon_start=1
                /note="gene_2482"
```

```
                     /transl_table=11
                     /translation="VTVPARPTPLFADIADVSRRLAETGYLPDTATATAVFLADRLGK
                     PLLVEGPAGVGKTELARAVAQATGSGLVRLQCYEGVDEARALYEWNHAKQILRIQAGS
                     GDWEATKTDVLSEEFLLQRPLLTAIRRTEPTVLLIDETDKADIEIEGLLLEVLSDFAV
                     TVPELGTLTATRAPFVLLTSNATRELSEALKRRCLYLHIDFPTPELERRILLSRVPEL
                     PEHFAEELVRIIGVLRGMQLKKVPSIAETIDWGRTVLALGLDTIDDAVVAATLGVVLK
                     HQSDQQRATGELRLN"
     CDS             complement(2696859..2698106)
                     /codon_start=1
                     /gene="proA"
                     /note="gene_2483"
                     /transl_table=11
                     /translation="MTVPAPSQLDLRQEVHDAARRARVAARRLASLPTTVKDRALHAA
                     ADELLAHRDQILAANAEDLNAAREADTPAAMLDRLSLNPQRVDGIAAGLRQVAGLRDP
                     VGEVLRGYTLPNGLQLRQQRVPLGVVGMIYEGRPNVTVDAFGLTLKSGNAALLRGSSS
                     AAKSNEALVAVLRTALVGLELPADAVQLLSAADRATVTHLIQARGLVDVVIPRGGAGL
                     IEAVVRDAQVPTIETGVGNCHVYVHQAADLDVAERILLNSKTRRPSVCNAAETLLVDA
                     AIAETALPRLLAALQHAGVTVHLDPDEADLRREYLSLDIAVAVVDGVDAAIAHINEYG
                     TGHTEAIVTTNLDAAQRFTEQIDAAAVMVNASTAFTDGEQFGFGAEIGISTQKLHARG
                     PMGLPELTSTKWIAWGAGHTRPA"
     CDS             2698822..2699409
                     /codon_start=1
                     /note="gene_2484"
                     /transl_table=11
                     /translation="MPLLTIGDQFPAYQLTALIGGDLSKVDAKQPGDYFTTITSDEHP
                     GKWRVVFFWPKDFTFVCPTEIAAFSKLNDEFEDRDAQILGVSIDSEFAHFQWRAQHND
                     LKTLPFPMLSDIKRELSQAAGVLNADGVADRVTFIVDPNNEIQFVSATAGSVGRNVDE
                     VLRVLDALQSDELCACNWRKGDPTLDAGELLKASA"
     CDS             2699435..2699968
                     /codon_start=1
                     /note="gene_2485"
                     /transl_table=11
                     /translation="MSIEKLKAALPEYAKDIKLNLSSITRSSVLDQEQLWGTLLASAA
                     ATRNPQVLADIGAEATDHLSAAARHAALGAAAIMGMNNVFYRGRGFLEGRYDDLRPGL
                     RMNIIANPGIPKANFELWSFAVSAINGCSHCLVAHEHTLRTVGVDREAIFEALKAAAI
                     VSGVAQALATIEALSPS"
     CDS             complement(2699965..2700549)
                     /codon_start=1
                     /note="gene_2486"
                     /transl_table=11
                     /translation="MHFEAYPPEVNSANIYAGPGPDSMLAAARAWRSLDVEMTAVQRS
                     FNRTLLSLMDAWAGPVVMQLMEAAKPFVRWLTDLCVQLSEVERQIHEIVRAYEWAHHD
                     MVPLAQIYNNRAERQILIDNNALGQFTAQIADLDQEYDDFWDEDGEVMRDYRLRVSDA
                     LSKLTPWKAPPPIAHSTVLVAPVSPSTASSRTDT"
     CDS             complement(2700596..2700895)
                     /codon_start=1
                     /note="gene_2487"
                     /transl_table=11
                     /translation="MSFVITNPEALTVAATEVRRIRDRAIQSDAQVAPMTTAVRPPAA
                     DLVSEKAATFLVEYARKYRQTIAAAAVVLEEFAHALTTGADKYATAEADNIKTFS"
     CDS             complement(2701473..2701763)
```

/codon_start=1
/note="gene_2488"
/transl_table=11
/translation="VGLRDADERWDTVGQAIGLFLRGHTLRTAAPTALIVGTVLCAVN
QGATLAEGAATIGTWVRMVINYLVPFLVASVGYLGARRGVRRASGRSDPSAQ"
CDS complement(2701744..2703189)
/codon_start=1
/note="gene_2489"
/transl_table=11
/translation="MNLLDSTWFYWAVGIAIGLPAGLIVLTELHNILVRRNSHLARQA
SLLRNYLLPLGAVLLLLVKASEVPAEDPTVRVLTTAFGFLVLVLLLSLLNATLFQGAP
QQSWRKRLPAIFVDVARFALIGIGLAVILSYIWGVRVGGLFAALGVTSVVIGLMLQNS
VGQIVSGLFMLFEQPFRIDDWLETPTARGRVVEVNWRAVHIDTGSGLQIMPNSMLATT
AFTNLSRPAGAHECSITTTFSTSDPPDKVCAMLNRAASALPHVKPGVVPATIARGAAE
YRTTVRLTSPADEGPTQATFLRWVWYAARREGLHLDEADDEFSTAERVESALRTVVGP
ELRLSSSDQQSLARYARLVRYGTDEIVQHAGVVPMGITFVIAGSVRLTVTTDDGSVVA
IATLKKGTFLGLTALTRQPDPAGAVALEEVTALQIGREHLEQVVMNKPMLLQELGRVI
DERQRKAQQAIRRDLHQSPAAAGEHRGPARR"
CDS complement(2703186..2705378)
/codon_start=1
/note="gene_2490"
/transl_table=11
/translation="LTSGEALDSVAESESTPAKKRHKNVLRRRPRFRASIQSKLMVLL
LLTSIVSVAAIAAIVYQSGRTSLRAAAYERLTQLRESQKRAVETLFSDLTNSLVIYER
GLTVVDAVVRFTAGFDQLADATISPAQQQAIVNYYNNEFITPVERTTGDKLDITALLP
TSPAQRYLQAYYTAPFTSDQDAMRLDDAGDGSAWSAANAQFNSYFREIVTRFDYDDAV
LLDTRGNIVYTLSKDPDLGTNILTGPYRESNLRDAYLKALGANAVDFTWITDFKPYQP
QLGVPTAWLVAPVEAGGKTQGVLALPLPIDKINKIMTADRQWQAAGMGSGTETYLAGP
DSLMRSDSRLFLQDPEEYRKQVVAAGTSLDVVNRAIQFGGTTLLQPVATEGLRAAQRG
QTGTVTSTDYTGSRELEAYAPLNVPDSDLHWSILATRNDSEAFAAVASFSRALVLVTV
GIIVVICVASMLIAHAMVRPIRRLEVGTQKISAGDYEVNIPVKSRDEIGDLTAAFNEM
SRNLQTKEELLNEQRKENDRLLLSMMPEPVVERYRLGEQTIAQEHQDVTVLFADILGV
DEISSGLSGNELVKIVDELVRQFDSAAEHLGVERIRTLHNGYLAGCGVTTPRLDNIPR
TVDFALEMRRIVDRFNCQTGNDLHLRVGINTGDVISGLVGRSSVVYDMWGAAVSLAYQ
MHSGSPQPGIYVTSQVYEAMRDVWQFTAAGTISVGGLEEPIYRLSERS"
CDS 2705859..2706773
/codon_start=1
/note="gene_2491"
/transl_table=11
/translation="MANASETNVGPMAPRVCVVGSVNMDLTFVVDALPRPGETVLAAS
LTRTPGGKGANQAVAAARAGAQVQFSGAFGDDPAAAQLRAHLRANAVGLDRTVTVPGP
SGTAIIVVDASAENTVLVAPGANAHLTPVPSAVANCDVLLTQLEIPVATALAAARAAQ
SADAVVMVNASPAGQDRSSLQDLAAIADVVIANEHEANDWPSPPTHFVITLGVRGARY
VGADGVFEVPAPTVTPVDTAGAGDVFAGVLAANWPRNPGSPAERLRALRRACAAGVLA
TLVSGAGDCAPAAAAIDAALRANRHNGS"
CDS 2706987..2707424
/codon_start=1
/note="gene_2492"
/transl_table=11
/translation="VTVVAAVLQRTNVVQPLNTLRMVWIQVAGIIPATAGIAATVYAQ
LAMGDSWRIGVDEQENTTLVRTGPFKWVRHPIYTAMMAFGLGLLLVTPNLVALAGFIL

```
                     LVATLEVHVRRVEEPYLLRTHSAVYRGYTASVGRFVPGVGLIR"
     CDS             complement(2707421..2709460)
                     /codon_start=1
                     /gene="nadE"
                     /note="gene_2493"
                     /transl_table=11
                     /translation="MNFYSAYQHGFVRVAACTHHTTIGDPAANAASVLDMARACHDDG
                     AALAVFPELTLSGYSIEDVLLQDSLLDAVEDALLDLVTESADLLPVLVVGAPLRHRHR
                     IYNTAVVIHRGAVLGVVPKSYLPTYREFYERRQMAPGDGERGTIRIGGADVAFGTDLL
                     FAASDLPGFVLHVEICEDMFVPMPPSAEAALAGATVLANLSGSPITIGRAEDRRLLAR
                     SASARCLAAYVYAAAGEGESTTDLAWDGQTMIWENGALLAESERFPKGVRRSVADVDT
                     ELLRSERLRMGTFDDNRRHHRELTESFRRIDFALDPPAGDIGLLREVERFPFVPADPQ
                     RLQQDCYEAYNIQVSGLEQRLRALDYPKVVIGVSGGLDSTHALIVATHAMDREGRPRS
                     DILAFALPGFATGEHTKNNAIKLARALGVTFSEIDIGDTARLMLHTIGHPYSVGEKVY
                     DVTFENVQAGLRTDYLFRIANQRGGIVLGTGDLSELALGWSTYGVGDQMSHYNVNAGV
                     PKTLIQHLIRWVISAGEFGEKVGEVLQSVLDTEITPELIPTGEEELQSSEAKVGPFAL
                     QDFSLFQVLRYGFRPSKIAFLAWHAWNDAERGNWPPGFPKSERPSYSLAEIRHWLQIF
                     VQRFYSFSQFKRSALPNGPKVSHGGALSPRGDWRAPSDMSARIWLDQIDREVPKG"
     CDS             complement(2709746..2710876)
                     /codon_start=1
                     /gene="proB"
                     /note="gene_2494"
                     /transl_table=11
                     /translation="MRSPHRDAIRTARGLVVKVGTTALTTPSGMFDAGRLAGLAEAVE
                     RRMKAGSDVVIVSSGAIAAGIEPLGLSRRPKDLATKQAAASVGQVALVNSWSAAFARY
                     GRTVGQVLLTAHDISMRVQHTNAQRTLDRLRALHAVAIVNENDTVATNEIRFGDNDRL
                     SALVAHLVGADALVLLSDIDGLYDCDPRKTADATFIPEVSGPADLDGVVAGRSSHLGT
                     GGMASKVSAALLAADAGVPVLLAPAADAATALADASVGTVFAARPARLSARRFWVRYA
                     AEATGALTLDAGAVRAVVRQRRSLLAAGITAVSGRFCGGDVVELRAPDAAMVARGVVA
                     YDASELATMVGRSTSELPGELRRPVVHADDLVAVSAKQAKQV"
     CDS             complement(2710876..2712315)
                     /codon_start=1
                     /gene="obgE"
                     /note="gene_2495"
                     /transl_table=11
                     /translation="VPRFVDRVVIHTRAGSGGNGCASVHREKFKPLGGPDGGNGGRGG
                     SIVFVVDPQVHTLLDFHFRPHLTAASGKHGMGNNRDGAAGADLEVKVPEGTVVLDENG
                     RLLADLVGAGTRFEAAAGGRGGLGNAALASRVRKAPGFALLGEKGQSRDLTLELKTVA
                     DVGLVGFPSAGKSSLVSAISAAKPKIADYPFTTLVPNLGVVSAGEHAFTVADVPGLIP
                     GASRGRGLGLDFLRHIERCAVLVHVVDCATAEPGRDPISDIDALETELACYTPTLQGD
                     AALGDLAARPRAVVLNKIDVPEARELAEFVRDDIAQRGWPVFCVSTATRENLQPLIFG
                     LSQMISDYNAARPVAVPRRPVIRPIPVDDSGFTVEPDGHGGFVVSGARPERWIDQTNF
                     DNDEAVGYLADRLARLGVEEELLRLGARSGCAVTIGEMTFDWEPQTPAGEPVAMSGRG
                     TDPRLDSNKRVGAAERKAARSRRREHGDG"
     CDS             complement(2712401..2712661)
                     /codon_start=1
                     /gene="rpmA"
                     /note="gene_2496"
                     /transl_table=11
                     /translation="MAHKKGASSSRNGRDSAAQRLGVKRYGGQVVKAGEILVRQRGTK
                     FHPGVNVGRGGDDTLFAKTAGAVEFGIKRGRKTVSIVGSTTA"
```

CDS complement(2712676..2712987)
/codon_start=1
/gene="rplU"
/note="gene_2497"
/transl_table=11
/translation="MATYAIVKTGGKQYKVAVGDVVKVEKLESEQGEKVSLPVALVVD
GATVTTDAKALAKVAVTGEVLGHTKGPKIRIHKFKNKTGYHKRQGHRQQLTVLKVTGI
A"
CDS 2713338..2714813
/codon_start=1
/note="gene_2498"
/transl_table=11
/translation="MTAPLDRAPVTDLPANNKGRDRTHWLYLAVIFAVIAGVIVGLTA
PSTGKSLTVLGTVFVNLIKMMIAPVIFCTIVLGIGSVRKAAAVGKVGGLALAYFLTMS
SVALGIGLIVGNLLSPGRDLHLRPGAVGSGAALAGQAAESHGIAGFIQQIIPRSLPSA
LTEGNVLQVLLVALLVGFAVQGLGPAGESILRAVENLQKLVFKVLVMVLWLAPIGAFG
AIANIVATTGFNAVTNLLLLMAGFYLTCVVFVFGVLGVLLRIVSGLSIFRLLRYLARE
YLLIFATSSSEVVLPRLITKMKHLGVQSSTVGVVVPTGYSFNLDGTAIYLTMASLFIA
DAMGHRLTWGEQIALLAFMIIASKGAAGVSGAGLATLAGGLQAHRPELLDGVGLIVGI
DRFMSEARSLTNFSGNAVATILVASWTKTIDLSKADEVLRGRDPFDESTMVDPHDEEP
PAATPHGGGVPTNPALCDFEQVSLGGLVGRPAGPQRADVDG"
CDS complement(2714752..2717499)
/codon_start=1
/note="gene_2499"
/transl_table=11
/translation="VLDALTALDGRVRSAHSTVDRVDAVRVRDLLATHLETAGVLAAS
VHAPEASEEPESRLMLETQETRNADVERPHYMPLFVAPQPIPEPLADDEDVDDGPDYV
ADDSDADDEGQLDRPANRRRRRGRRGRGRGRGEQGGSDGDPVDQQSEPRAQQFTSADA
AETDDGDDRDSEDTEAGDNGEDENGSLEAGNRRRRRRRRRKSASGDDNDAALEGPLPD
DPPNTVVHERVPRAGDKAGNSQDGGSGSTEIKGIDGSTRLEAKRQRRRDGRDAGRRRP
PVLSEAEFLARREAVERVMVVRDRVRTEPPLPGTRYTQIAVLEDGIVVEHFVTSAASA
SLVGNIYLGIVQNVLPSMEAAFVDIGRGRNGVLYAGEVNWDAAGLGGADRKIEQALKP
GDYVVVQVSKDPVGHKGARLTTQVSLAGRFLVYVPGASSTGISRKLPDTERQRLKEIL
REVVPSDAGVIIRTASEGVKEDDIRADVARLRERWEQIEAKAQETKEKAAGAAVALYE
EPDVLVKVIRDLFNEDFVGLIVSGDEAWNTINEYVNSVAPELVSKLTKYESADGPDGQ
SAPDVFTVHRIDEQLAKAMDRKVWLPSGGTLVIDRTEAMTVIDVNTGKFTGAGGNLEQ
TVTKNNLEAAEEIVRQLRLRDIGGIVVIDFIDMVLESNRDLVLRRLTESLARDRTRHQ
VSEVTSLGLVQLTRKRLGTGLIEAFSTSCPNCSGRGILLHADPVDSAAATGRKSEPGA
RRGKRSKKSRSEESSDRSMVAKVPVHAPGEHPMFKAMAAGLSSLAGRGDEESGEPAAE
LAEQAGDQPPTDLDDTAQADFEDTEDTDEDEDELDADEDLEDLDDEDLDEDLDVEDSD
SDDEDSDEDAADADVDEEDAAGLDGSPGEVDVPGVTELAPTRPRRRVAGRPAGPPIRL
D"
CDS complement(2717943..2718377)
/codon_start=1
/gene="ndk"
/note="gene_2500"
/transl_table=11
/translation="LRHLVLGTVTERTLVLIKPDGIERQLIGEIISRIERKGLTIAAL
QLRTVSAELASQHYAEHEGKPFFGSLLEFITSGPVVAAIVEGTRAIAAVRQLAGGTDP
VQAAAPGTIRGDFALETQFNLVHGSDSAESAQREIALWFPGA"
CDS complement(2718396..2718767)

/codon_start=1
/note="gene_2501"
/transl_table=11
/translation="MTDRSREPADPWKGFSAVMAATLILEAIVVLLAIPVVDAVGGGL
RPASLGYLVGLAVLLILLTGLQRRPWAIWVNLGAQPVLVAGFAVYPGVGFIGVLFAAL
WVLIAYLRAEVRRRRDYRVSQ"
CDS complement(2718764..2720227)
/codon_start=1
/note="gene_2502"
/transl_table=11
/translation="MNSTNSGPPDSGSATGVVPTPDEIASLLQVEHLLDQRWPETRID
PSLTRISALMDLLGSPQRSYPSIHIAGTNGKTSVARMVDALVTALHRRTGRTTSPHLQ
SPVERISIDGKPISPAQYVATYREIEPLVALIDQQSQASAGKGGPAMSKFEVLTAMAF
AAFADAPVDVAVVEVGMGGRWDATNVINAPVAVITPISIDHVDYLGADIAGIAGEKAG
IITRAPDGSPDTVAVIGRQVPKVMEVLLAESVRADASVAREDSEFAVLRRQIAVGGQV
LQLQGLGGVYSDIYLPLHGEHQAHNAVLALASVEAFFGAGAQRQLDGDAVRAGFAAVT
SPGRLERMRSAPTVFIDAAHNPAGASALAQTLAHEFDFRFLVGVLSVLGDKDVDGILA
ALEPVFDSVVVTHNGSPRALDVEALALAAGERFGPDRVRTAENLRDAIDVATSLVDDA
AADPDVAGDAFSRTGIVITGSVVTAGAARTLFGRDPQ"
CDS complement(2720224..2722884)
/codon_start=1
/gene="valS"
/note="gene_2503"
/transl_table=11
/translation="VTASPHPAADMLPKSWDPAAMESAIYQKWLDAGYFTADPTSTKP
AYSIVLPPPNVTGSLHMGHALEHTMMDALTRRKRMQGYEVLWQPGTDHAGIATQSVVE
QQLAVDGKTKEDLGRELFVDKVWDWKRESGGAIGGQMRRLGDGVDWSRDRFTMDEGLS
RAVRTIFKRLYDAGLIYRAERLVNWSPVLQTAISDLEVNYRDVEGELVSFRYGSLDDS
QPHIVVATTRVETMLGDTAIAVHPDDERYRHLVGTSLAHPFVDRELAIVADEHVDPEF
GTGAVKVTPAHDPNDFEIGVRHQLPMPSILDTKGRIVDTGTRFDGMDRFEARVAVRQA
LAAQGRVVEEKRPYLHSVGHSERSGEPIEPRLSLQWWVRVESLAKAAGDAVRNGDTVI
HPASMEPRWFSWVDDMHDWCISRQLWWGHRIPIWYGPDGEQVCVGPDETPPQGWEQDP
DVLDTWFSSALWPFSTLGWPDKTAELEKFYPTSVLVTGYDILFFWVARMMMFGTFVGD
DAAITLDGRRGPQVPFTDVFLHGLIRDESGRKMSKSKGNVIDPLDWVEMFGADALRFT
LARGASPGGDLAVSEDAVRASRNFGTKLFNATRYALLNGAAPAPLPSPNELTDADRWI
LGRLEEVRAEVDSAFDGYEFSRACESLYHFAWDEFCDWYLELAKTQLAQGLTHTTAVL
AAGLDTLLRLLHPVIPFLTEALWLALTGRESLVSADWPEPSGISVDLVAAQRINDMQK
LVTEVRRFRSDQGLADRQKVPARMHGVRDSDLSNQVAAVTSLAWLTEPGPDFEPSVSL
EVRLGPEMNRTVVVELDTSGTIDVAAERRRLEKELAGAQKELASTAAKLANADFLAKA
PDAVIAKIRDRQRVAQQETERITTRLAALQ"
CDS complement(2722942..2724201)
/codon_start=1
/note="gene_2504"
/transl_table=11
/translation="VTATPREFDIVLYGATGFVGKLTAEYLARAGGDARIALAGRSTQ
RVLAVREALGESAQTWPILTADASLPSTLQAMAARAQVVVTTVGPYTRYGLPLVAACA
AAGTDYADLTGEPMFMRNSIDLYHKQAADTGARIVHACGFDSVPSDLSVYALYHAARE
DGAGELTDTNCVVRSFKGGFSGGTIASMLEVLSTASNDPDARRQLSDPYMLSPDRGAE
PELGPQPDLPSRRGRRLAPELAGVWTAGFIMAPTNTRIVRRSNALLDWAYGRRFCYSE
TMSVGSTVLAPVVSVVGGGVGNAMFGLASRYIRLLPRGLVKRVVPKPGTGPSAAARER
GYYRIETYTTTTTGARYLARMAQDGDPGYKATSVLLGECGLALALDRDKLSDMRGVLT

PAAAMGDALLERLPAAGVSLQTTRLAS"
CDS complement(2724291..2724491)
/codon_start=1
/note="gene_2505"
/transl_table=11
/translation="VRVRWKLVDHTGNGYYGGLQFTAGTWRANGGSGSAANASREEQI
RVAENVLRSQGIRAWPVCGRRG"
CDS complement(2725646..2726182)
/codon_start=1
/note="gene_2506"
/transl_table=11
/translation="MGRDKATLPLPGGTTTLVEHMVGILGQRCAPVFVMAAPGQPLPT
LPVPVLRDELPGLGPLPATGRGLRAAAEAGVRLAFVCAVDMPYLTVELIEDLARRAVQ
TDAEVVLPWDGRNHYLAAVYRTDLADRVDTLVGAGERKMSALVDASDALRIVMADSRP
LTNVNSAAGLHAPMQPGR"
CDS complement(2726253..2727374)
/codon_start=1
/note="gene_2507"
/transl_table=11
/translation="MTRSGDEAQLMTGVTGDLAGTELGLTPSLTKNAGVPTTDQPQKG
KDFTSDQEVRWCPGCGDYVILNTIRNFLPELGLRRENIVFISGIGCSSRFPYYLETYG
FHSIHGRAPAIATGLALAREDLSVWVVTGDGDALSIGGNHLIHALRRNINVTILLFNN
RIYGLTKGQYSPTSEVGKVTKSTPMGSLDHPFNPVSLALGAEATFVGRALDSDRNGLT
EVLRAAAQHRGAALVEILQDCPIFNDGSFDALRKEGAEERVIKVRHGEPIVFGANGEY
CVVKSGFGLEVAKTADVAIDEIIVHDAQVDDPAYAFALSRLSDQNLDHTVLGIFRHIS
RPTYDDAARSQVVAARNAAPSGTAALQSLLHGRDTWTVD"
CDS complement(2727371..2729347)
/codon_start=1
/note="gene_2508"
/transl_table=11
/translation="MEGGDVDPNGSGAGPESHDAAFHAAPDRQRLENVVIRFAGDSGD
GMQLTGDRFTSEAALFGNDLATQPNYPAEIRAPAGTLPGVSSFQIQIADYDILTAGDR
PDVLVAMNPAALKANIGDLPLGGMVIVNSDEFTKRNLTKVGYVTNPLESGELSDYVVH
TVAMTTLTLGAVEAIGASKKDGQRAKNMFALGLLSWMYGRELEHSEAFIREKFARKPE
IAEANVLALKAGWNYGETTEAFGTTYEIPPATLPPGEYRQISGNTALAYGIVVAGQLA
GLPVVLGSYPITPASDILHELSKHKNFNVVTFQAEDEIGGICAALGAAYGGALGVTST
SGPGISLKSEALGLGVMTELPLLVIDVQRGGPSTGLPTKTEQADLLQALYGRNGESPV
AVLAPRSPADCFETALEAVRIAVSYHTPVILLSDGAIANGSEPWRIPDVNALPPIKHT
FAKPGEPFQPYARDRETLARQFAIPGTPGLEHRIGGLEAANGSGDISYEPTNHDLMVR
LRQAKIDGIHVPDLEVDDPTGDAELLLIGWGSSYGPIGEACRRARRRGTKVAHAHLRY
LNPFPANLGEVLRRYPKVVAPELNLGQLAQVLRGKYLVDVQSVTKVKGVSFLADEIGR
FIRAALAGRLAELEQDKTLVARLSAATAGAGANG"
CDS complement(2729564..2730820)
/codon_start=1
/note="gene_2509"
/transl_table=11
/translation="MSGTVVAVPPRVARALDLLNFSLADVRDGLGPYLSIYLLLIHDW
DQASIGFVMAVGGIAAIVAQTPIGALVDRTTAKRALVVAGAVLVTAAAVAMPLFAGLY
SISVLQAVTGIASSVFAPALAAITLGAVGPQFFARRIGRNEAFNHAGNASAAGATGAL
AYFFGPVVVFWVLAGMALISVLATLRIPPDAVDHDLARGMDHAPGEPHPQPSRFTVLA
HNRELVIFGAAVVAFHFANAAMLPLVGELLALHNRDEGTALMSSCIVAAQVVMVPVAY

VVGTRADAWGRKPIFLVGFAVLTARGFLYTLSDNSYWLVGVQLLDGIGAGIFGALFPL
VVQDVTHGTGHFNISLGAVTTATGIGAALSNLVAGWIVVVAGYDAAFMSLGALAGAGF
LLYLVAMPETVDSDVRVRSRPTLGGK"

CDS complement(2730836..2732116)
/codon_start=1
/note="gene_2510"
/transl_table=11
/translation="MARIGDGGDLLKCSFCGKSQKQVKKLIAGPGVYICDECIDLCNE
IIEEELADADDVKLDELPKPAEIREFLEGYVIGQDTAKRTLAVAVYNHYKRIQAGEKG
RDSRCEPVELTKSNILMLGPTGCGKTYLAQTLAKMLNVPFAIADATALTEAGYVGEDV
ENILLKLIQAADYDVKRAETGIIYIDEVDKIARKSENPSITRDVSGEGVQQALLKILE
GTQASVPPQGARKHPHQEFIQIDTTNVLFIVAGAFAGLEKIIYERVGKRGLGFGAEVR
SKAEIDTTDHFADVMPEDLIKFGLIPEFIGRLPVVASVTNLDKESLVKILSEPKNALV
KQYIRLFEMDGVELEFTDDALEAIADQAIHRGTGARGLRAIMEEVLLPVMYDIPSRDD
VAKVVVTKETVQDNVLPTIVPRKPSRSERRDKSA"

CDS complement(2732245..2732478)
/codon_start=1
/gene="IMCC9480_1896"
/note="gene_2511"
/transl_table=11
/translation="VTARLKLGGQAAIADQHGIADQFHNATVGWSAGGRAAAGSATRG
PRWGYDRDTPETRIARLGEWGAVGLYVSFRREA"

CDS 2732407..2733315
/codon_start=1
/gene="mmuM"
/note="gene_2512"
/transl_table=11
/translation="VELVSDSVLISDGGLATELEARGHDLSDPLWSARLLVDAPHAIT
AVHTAYFRAGAQIATTASYQASFEGFAARGIGHDDATVLLRRSVELAQAARDEVGVGG
LSVAASVGPYGAALADGSEYRGCYGLSVAALMKWHLPRLEVLVDAGADMLALETIPDI
DEAEALVNLVRRLATPAWLSYTINGTRTRAGQPLTDAFAVAAGVPEIVAVGVNCCAPD
DVLPAIAFAVAHTGKPVIVYPNSGEGWDGRRRAWVGPRRFSGSSGQLAREWVAAGARI
VGGCCRVRPIDIAEIGRALTTAPPRG"

CDS 2733410..2735008
/codon_start=1
/note="gene_2513"
/transl_table=11
/translation="MGNQPEKSDVTAAPDTVEGDSHTAMTPRQRLTVLATGLGIFMVF
VDVNIVNVALPSIQKVFHTGEQGLQWAVAGYSLGMAAVLMSCALLGDRYGRRRSFVFG
VTLFVVSSIVCVLPVSLAVFTVARVIQGLGAAFISVLSLALLSHSFPNPRMKARAISN
WMAIGMVGAASAPALGGLMVDGLGWRSVFLVNVPLGAIVWLLTLVGVDESQDPEPTQL
DWVGQLTLIPAVALIAYTIIEAPRFDRQSAGFVAALLLAAGVLLWLFVRHEHRAAFPL
VDLKLFAEPLYRSVLIVYFVVMSCFFGTLMVITQHFQNVRDLSPLHAGLMMLPVPAGF
GVASLLAGRAVNKWGPQLPVLTCLAAMFIGLAIFAISMDHAHPVALVGLTIFGAGAGG
CATPLLHLGMTKVDDGRAGMAAGMLNLQRSLGGIFGVAFLGTIVAAWLGAALPNTMAD
EIPDPIARAIVVDVIVDSANPHAHAAFIGPGHRITAAQEDEIVLAADAVFVSGIKLAL
GGAAVLLTGAFVLGWTRFPRTPAS"

CDS complement(2735159..2735803)
/codon_start=1
/note="gene_2514"
/transl_table=11

/translation="VNSQNSQIQPQARYILPSFIEHSSFGVKESNPYNKLFEERIIFL
GVQVDDASANDIMAQLLVLESLDPDRDITMYINSPGGGFTSLMAIYDTMQYVRADIQT
VCLGQAASASAAVLLAAGTPGKRMALPNARVLIHQPSLSGVIQGQFSDLEIQAAEIERM
RTLMETTLARHTGKDAGVIRKDTDRDKILTAEEAKDYGIIDTVLEYRKLSAQTA"
CDS complement(2735800..2736402)
/codon_start=1
/gene="clpP"
/note="gene_2515"
/transl_table=11
/translation="VSQVTDMRSNSQGLSLTDSVYERLLSERIIFLGSEVNDEIANRL
CAQILLLAAEDASKDISLYINSPGGSISAGMAIYDTMVLAPCDIATYAMGMAASMGEF
LLAAGTKGKRYALPHARILMHQPLGGVTGSAADIAIQAEQFAVIKKEMFRLNAEFTGQ
PIERIEADSDRDRWFTAAEALEYGFVDHIITRAHVNGEAQ"
CDS complement(2736519..2737919)
/codon_start=1
/gene="tig"
/note="gene_2516"
/transl_table=11
/translation="VKSTVEQLSPTRVRINVEVPFAELEPDFQRAYKELAKQVRLPGF
RPGKAPAKLLEARIGREAMLDQIVNDALPSRYGQAVAESDVQPLGRPNIEVTKKEYGQ
DLQFTAEVDIRPKISLPDLSALTVSVDPIEIGEDDVDAELQSLRTRFGTLTAVDRPVA
VGDVVSIDLSATVDGEDIPNAAAEGLSHEVGSGRLIAGLDDAVVGLSADESRVFTAKL
AAGEHAGQEAQVTVTVRSVKERELPEPDDEFAQLASEFDSIDELRASLSDQVRQAKRA
QQAEQIRNATIDALLEQVDVPLPESYVQAQFDSVLHSALSGLNHDEARFNELLVEQGS
SRAAFDAEARTASEKDVKRQLLLDALADELQVQVGQDDLTERLVTTSRQYGIEPQQLF
GYLQERNQLPTMFADVRRELAIRAAVEAATVTDSDGNTIDTSEFFGKRVSAGEAEEAE
PADEGAARAASDEATT"
tRNA complement(2737956..2738032)
/note="identified by tRNAscan-SE; putative"
tRNA 2738169..2738239
/note="identified by tRNAscan-SE; putative"
CDS 2738283..2739467
/codon_start=1
/note="gene_2517"
/transl_table=11
/translation="MNQPDIKGSCASEFTKVRDAFERNFVLRNEVGAAVAVWVDGDLV
VNLWGGSADAGGTRPWQHDTLATVLSGTKALTATCVHQLVDRGELDLHAPVARYWPEF
GQAGKQAITLAMVMSHRSGAIGPRGRLGWEQVADWDFVCEQLAAAEPWWQPGAAQGYH
MTTFGFILGEVFRRVTGRTVGQYLRTEIAEPLGADVHIGLHPGEQLRCADLVDKPHIR
QLLADVQAPGYPTSLNEHPKAALSVSMGFAPDDELGSNDLQLWRQIEFPGTNGQVSAL
GLATFYNGLAQEKLLSREHMELVRVSQGGFDTDLVLGPRVADHGWGLGYMLNQRGVNG
PNPRIFGHGGLGGSFGFVDLEHRIGYAYVMNRFDATKANADPRSVVLSNEVYAALGVN
RS"
CDS complement(2739487..2740293)
/codon_start=1
/note="gene_2518"
/transl_table=11
/translation="VPEGHTLHRLARLHQRRFAGAPVSVSSPQGRFADSASALNGRVL
RRASAWGKHLFHHYVGGPVVHVHLGLYGTFTEWARPTDGWLPEPAGQVRMRMVGAEFG
TDLRGPTVCESIDDGEVADVVARLGPDPLRSDANPSSAWSRITKSRRPIGALLMDQTV
IAGVGNVYRNELLFRHRIDPQRPGRGIGEPEFDAAWNDLVSLMKVGLRRGKIIVVRPE

HDHGLPSYLPDRPRTYVYRRAGEPCRVCGGVIRTALLEGRNVFWCPVCQT"
CDS complement(2740299..2740787)
/codon_start=1
/note="gene_2519"
/transl_table=11
/translation="MSGMRVYLGADHAGYELKQRIIEHLKQTGHEPIDCGALRYDADD
DYPAFCIAAATRTVADPGSLGIVLGGSGNGEQIAANKVPGARCALAWSVQTAALAREH
NNAQLIGIGGRMHTVAEALAIVDAFVTTPWSKAQRHQRRIDILAEYERTHEAPPVPGA
PA"
CDS complement(2740889..2741512)
/codon_start=1
/note="gene_2520"
/transl_table=11
/translation="MLEKAPQKSVADFWFDPLCPWCWITSRWILEVAKVRDIEVNFHV
MSLAILNENRDDLPEQYREGMARAWGPVRVAIAAEQAHGAKVLDPLYTAMGNRIHNQG
NHELDEVITQSLADAGLPAELAKAATSDAYDNALRKSHHAGMDAVGEDVGTPTIHVNG
VAFFGPVLSKIPRGEEAGKLWDASVTFASYPHFFELKRTRTEPPQFD"
CDS 2741614..2744199
/codon_start=1
/note="gene_2521"
/transl_table=11
/translation="VALPNLTRDQAVERAALITVDSYQIILDVTDGNGAPGERTFRST
TTVVFDALPGADTVIDISAHTVRRASLNDQDLDVSGYDEAAGIPLRGLAQRNVVVVDA
DCHYSNTGEGLHRFVDPVDGETYLYSQFETADAKRMFACFDQPDLKATFDVRVTAPAH
WKVISNGAPLAAANGVHTFATTPRMSTYLVALIAGPYAAWTDTYIDDHGEIPLGIYCR
ASLAEYMDAERLFTQTKQGFGFYHKHFGLPYAFGKYDQLFVPEFNAGAMENAGAVTFL
EDYVFRSKVTRASYERRAETVLHEMAHMWFGDLVTMTWWDDLWLNESFATFASVLCQS
EATEFTEAWTTFATVEKSWAYRQDQLPSTHPIAADIPDLAAVEVNFDGITYAKGASVL
KQLVAYVGLERFLAGLRDYFRTHAFGNASFDDLLAALEKASGRDLSNWGEQWLKTTGL
NTLRPDFEVDAEGRFTRFAVTQSGAAPGAGETRVHRLAVGIYDDDGSKSSGKLVRVHR
EELDVSGPITNVPALVGVSRGKLILVNDDDLTYCSLRLDERSLQTALDRIADIAEPLP
RTLVWSAAWEMTREAELRARDFVSLVSGGVHAETEVGVAQRLLLQAQTALGCYAEPGW
ARERGWPQFADRLLELAREAEPGSDHQLAYINSLCSSVLSPRHVQTLGALLEGEPAAC
GLAGLAVDTDLRWRIVTALATAGAIDADGPETPRIDAEVQRDPTAAGKRHAAQARAAR
PQFVVKDEAFTTVVEDDTLANATGRAMIAGIAAPGQGELLKPFARRYFQAIPGVWARR
SSEVAQSVVIGLYPHWDISEQGITAAEEFLSDPEVPPALRRLVLEGQAAVQRSLRARN
FDADG"
CDS complement(2744272..2744739)
/codon_start=1
/note="gene_2522"
/transl_table=11
/translation="VARGDVATIEHAELPPGWVLTTSGRISGVTEPGELSVHYPFPIA
DLVALDDALTYSSRACQVRFAIYLGDLGRDTAARAREILGKVPTPDNAVLLAVSPNQC
AIEVVYGSQVRGRGAESAAPLGVAAASSAFEQGELVDGLISAIRVLSAGIAPG"
CDS complement(2744726..2744968)
/codon_start=1
/note="gene_2523"
/transl_table=11
/translation="VSAMEIHLFFVGIPLLLVVVLSVLIWSRKGPHPATYKLSEPWTH
PPILWAATDEVVGSAHGGHGHDASEFTVGGGASGTW"
CDS complement(2744995..2745663)

/codon_start=1
/note="gene_2524"
/transl_table=11
/translation="MAHGKKRRGHRSSGVAAGVTGPASCLHGVHSHRLASGVETHPPN
RHESASIWNRRRVLLLNSTYEPLTALSMRRAIVMVICGKADVVHEDPSGPVIHSSTRS
ILVPSVIQLRSYVRVPYRARVPMTRAALMHRDRFCCAYCGGKADTVDHVVPRSRGGAH
SWENCVACCSPCNHRKGDRLLTELGWALRRAPLPPTGPHWRLLSAVKELDPSWARYLG
EGAA"
CDS 2745788..2746192
/codon_start=1
/note="gene_2525"
/transl_table=11
/translation="MEGMDQMPKSFYDAVGGAKTFDAIVSRFYAQVAEDEVLRRVYPE
DDLAGAEERLRMFLEQYWGGPRTYSEQRGHPRLRMRHAPFRISLIERDAWLRCMHTAV
ASIDSETLDDEHRRELLDYLEMAAHSLVNSPF"
CDS 2746270..2747832
/codon_start=1
/note="gene_2526"
/transl_table=11
/translation="MGEPWWSRAVFYQVYPRSFADSNGDGVGDLDGLASRLDHLQQLG
VDAIWINPVTVSPMADHGYDVADPRDIDPLFGGMPAFERLVAAAHRQGIKVTMDVVPN
HTSSAHPWFQAALADLPGSPARDRYFFRDGRGPDGSLPPNNWESVFGGPAWTRVREPD
GNPGQWYLHLFDTEQPDLNSDNPEILDDFEKTLRFWLDRGVDGFRIDVAHGMAKPPGL
PDSPDLGIEVLHHRDDDPRFNHPNVHAIHRDIRTVIDEYPGAVTVGEVWVHDNARWAE
YLRPDELHLGFNFRLARTEFDAAEIRDAVANSLAAAALQNATPTWTLANHDVGREVSR
YGGGEIGLRRAKAMAVVMLALPGVVFLYNGQELGLPDVDLPDEVLQDPTWERSGRTER
GRDGCRVPIPWSGNIPPFGFSTCPDTWLPMPPEWAALTAEKQRADAGSTLSFFRLALR
LRRERNEFDGDVDWLAAPDDALIFRRHGGGLVCALNAAERPLALPAGEPILASAPLTD
ATLPPNAAAWLV"
CDS 2748448..2748912
/codon_start=1
/note="gene_2527"
/transl_table=11
/translation="VAALALAVVSLGVGIVGWFHRQPHDKPSPAPSAPTFTSQQISDA
KENVCAAHRIVRQAAVLNTNQANPVPGDPTGDLAVAANARLALYSGGDYLLRRLTAEP
ATPAELRDAVRSLANALQELAVNYLAGAPDSVVTPLRLALERDTRAVDPLCV"
CDS complement(2748944..2749597)
/codon_start=1
/note="gene_2528"
/transl_table=11
/translation="VVERGLWLPDPAHRADLATFVDHALRLDDAAVIRIRARSTGLLS
AWVATGFDVLASRVVAGKVRPDDLSVAARSLAHGLATTDASGYVDPGYSMDSAWRGGL
PPESGFTYLDDVPARVMLDLAHRGARLAKEHGSSAGPPVSLLDQEVIQVSSADVVVGL
PMRCVFALTAMGFLPQSAETISADELIRVRISPAWLRLDARFGSVYRHRGHAALVLR"
CDS complement(2749603..2750019)
/codon_start=1
/note="gene_2529"
/transl_table=11
/translation="VSVGFVTPVGVRWSDIDMYQHVNHATMVTILEEARVPFLKDAFG
ADITSTGLLIADVRVTYKGQLRLSDSPLQVTIWTKRLRAVDFTLGYEVRSVNAEPDSR
PAVIAESQLAAFHIEEQRLVRLSPHHREYLQRWFRG"

```
CDS             complement(2750016..2754887)
                /codon_start=1
                /note="gene_2530"
                /transl_table=11
                /translation="MTIDPGAKQDVEAWTTFTASADIPDWISKAYIDSYRGPRDDSSE
                ATKAAEASWLPASLLTPAMLGAHYRLGRHRAAGESCVAVYRADDPAGFGPALQVVAEH
                GGMLMDSVTVLLHRLGIAYAAILTPVFDVHRSPTGELLRIEPKAEGTSPHLGEAWMHV
                ALSPAVDHKGLAEVERLLPKVLADVQRVATDATALIATLSELAGEVESNAGGRFSAPD
                RQDVGELLRWLGDGNFLLLGYQRCRVADGMVYGEGSSGMGVLRGRTGSRPRLTDDDKL
                LVLAQARVGSYLRYGAYPYAIAVREYVDGSVVEHRFVGLFSVAAMNADVLEIPTISRR
                VREALAMAESDPSHPGQLLLDVIQTVPRPELFTLSAQRLLTMARAVVDLGSQRQALLF
                LRADRLQYFVSCLVYMPRDRYTTAVRMQFEDILVREFGGTRLEFTARVSESPWALMHF
                MVRLPEVGVAGEGAAAPPVDVSEANRIRIQGLLTEAARTWADRLIGAAAAGSVGQADA
                MHYAAAFSEAYKQAVTPADAIGDIAVITELTDDSVKLVFSERDEQGVAQLTWFLGGRT
                ASLSQLLPMLQSMGVVVLEERPFSVTRPDGLPVWIYQFKISPHPTIPLAPTVAERAAT
                AHRFAEAVTAIWHGRVEIDRFNELVMRAGLTWQQVVLLRAYAKYLRQAGFPYSQSYIE
                SVLNEHPATVRSLVDLFEALFVPVPSGSASNRDAQAAAAAVAADIDALVSLDTDRILR
                AFASLVQATLRTNYFVTRQGSARCRDVLALKLNAQLIDELPLPRPRYEIFVYSPRVEG
                VHLRFGPVARGGLRWSDRRDDFRTEILGLVKAQAVKNAVIVPVGAKGGFVVKRPPLPT
                GDPAADRDATRAEGVACYQLFISGLLDVTDNVDHATASVNPPPEVVRRDGDDAYLVVA
                ADKGTATFSDIANDVAKSYGFWLGDAFASGGSVGYDHKAMGITARGAWEAVKRHFREI
                GIDTQTQDFTVVGIGDMSGDVFGNGMLLSKHIRLIAAFDHRHIFLDPNPDAAVSWAER
                RRMFELPRSSWGDYDRSLISEGGGVYSREQKAIPLSAQVRAVLGIDGSVDGGAAEMAP
                PNLIRAILRAPVDLLFNGGIGTYIKAESESDADVGDRANDPVRVNANQVRAKVIGEGG
                NLGVTALGRVEFDLSGGRINTDALDNSAGVDCSDHEVNIKILIDSLVSAGTVKADERT
                QLLESMTDEVAQLVLADNEDQNDLMGTSRANAASLLPVHAMQIKYLVAERGVNRELEA
                LPSEKEIARRSEAGIGLTSPELATLMAHVKLGLKEEVLATELPDQDVFASRLPRYFPT
                ALRERFTPEIRSHQLRREIVTTMLINDLVDTAGITYAFRIAEDVGVTPIDAVRTYVAT
                DAIFGVGHIWRRIRAANLPIALSDRLTLDTRRLIDRAGRWLLNYRPQPLAVGAEINRF
                AAMVKALTPRMSEWLRGDDKAIVEKTAAEFASQGVPEDLAYRVSTGLYRYSLLDIIDI
                ADIADIDAAEVADTYFALMDRLGTDGLLTAVSQLPRHDRWHSLARLAIRDDIYGALRS
                LCFDVLAVGEPGESSEQKIAEWEHLSASRVARARRTLDDIRASGQKDLATLSVAARQI
                RRMTRTSGRGISG"
CDS             complement(2754991..2756667)
                /codon_start=1
                /note="gene_2531"
                /transl_table=11
                /translation="MAEFIYTMKKVRKAHGDKVILDDVTLSFYPGAKIGVVGPNGAGK
                SSVLRIMAGLDKPNNGDAFLATGATVGILQQEPPLNEDKTVRGNVEEGMGDIKIKLDR
                FNEVAELMATDYTDELMEEMGRLQEELDHADAWDLDAQLEQAMDALRCPPADEPVTNL
                SGGERRRVALCKLLLSKPDLLLLDEPTNHLDAESVQWLEQHLASYPGAILAVTHDRYF
                LDNVAEWILELDRGRAYPYEGNYSTYLEKKAERLAVQGRKDAKLQKRLTEELAWVRSG
                AKARQAKSKARLQRYEEMAAEAEKTRKLDFEEIQIPVGPRLGNVVVEVDHLDKGYDGR
                ALIKDLSFSLPRNGIVGVIGPNGVGKTTLFKTIVGLEKPDSGSVKVGETVKLSYVDQA
                RAGIDPRKTVWEVVSDGLDYIQVGQTEVPSRAYVSAFGFKGPDQQKPAGVLSGGERNR
                LNLALTLKQGGNLILLDEPTNDLDVETLGSLENALLNFPGCAVVISHDRWFLDRTCTH
                ILAWEGDDDNEAKWFWFEGNFGAYEENKVERLGVDAARPHRVTHRKLTRG"
CDS             complement(2756748..2757254)
                /codon_start=1
                /note="gene_2532"
                /transl_table=11
```

/translation="MFETPLTVVGHIVNDLQRRKVGDQEVVKFRVASNSRRRTSDGGW
EPGNSLFITVNCWGRLVTGVGAALGKGAPVIVVGHVYTSEYEDRDGIRRSSLEMRATS
VGPDLSRVIVRIEKPAYTGPSAGDLPAATGTGAAGAADAPASAADSVSDVVVDDAITG
HNPLPISA"
CDS complement(2758181..2760550)
/codon_start=1
/note="gene_2533"
/transl_table=11
/translation="VTKPAADASAVLTAEDTLVLASTATPVEMELIMGWLGQQRARHP
DSKFDILKLPPRNAPPAALTALVEQLEPGFASSPQSGEDRSIVPVRVIWLPPADRSRA
GKVAALLPGRDPYHPSQRQQRRILRTDPRRARVVAGESAKVSELRQQWRDTTVAEHKR
DFAQFVSRRALLALARAEYRILGPQYKSPRLVKPEMLASARFRAGLDRIPGATVEDAG
KMLDELSTGWSQVSVDLVSVLGRLASRGFDPEFDYDEYQVAAMRAALEAHPAVLLFSH
RSYIDGVVVPVAMQDNRLPPVHMFGGINLSFGLMGPLMRRSGMIFIRRNIGNDPLYKY
VLKEYVGYVVEKRFNLSWSIEGTRSRTGKMLPPKLGLMSYVADAYLDGRSDDILLQGV
SICFDQLHEITEYAAYARGAEKTPEGLRWLYNFIKAQGERNFGKIYVRFPEAVSMRQY
LGAPHGELTQDPAAKRLALQKMSFEVAWRILQATPVTATGLVSALLLTTRGTALTLDQ
LHHTLQDSLDYLERKQSPVSTSALRLRSREGVRAAADALSNGHPVTRVDSGREPVWYI
APDDEHAAAFYRNSVIHAFLETSIVELALAHAKHAEGDRVAAFWAQAMRLRDLLKFDF
YFADSTAFRANIAQEMAWHQDWEDHLGVGGNEIDAMLYAKRPLMSDAMLRVFFEAYEI
VADVLRDAPPDIGPEELTELALGLGRQFVAQGRVRSSEPVSTLLFATARQVAVDQELI
APAADLAERRVAFRRELRNILRDFDYVEQIARNQFVAREFKARQGRDRI"
CDS complement(2760547..2762289)
/codon_start=1
/note="gene_2534"
/transl_table=11
/translation="MSAADEQGEERATRKSAPDLRLPGSVAEILASPAGPKVGAFFDL
DGTLVAGFTAVILTQERLRRRDMGVGELLGMVQAGLNHTLGRIEFEDLIGKAAAALAG
RLLTDLEEIGERLFAQRIESRIYPEMRELVRAHVARGHTVVLSSSALTIQVGPVARFL
GINNMLTNKFETNEDGILTGGVLKPILWGPGKATAVQRFAAEHDIDLKDSYFYADGDE
DVALMYLVGNPRPTNPEGKMAAVAKRRGWPILKFNSRGGVGIRRQLRTLAGLSTIVPV
AAGAVGIGVLTGSRRRGVNFFTSTFSQLLLATSGVHLNVIGKENLTAQRPAVFIFNHR
NQVDPVIAGALVRDNWVGVGKKELASDPIMGTLGKLLDGVFIDRDDPVAAVETLHTVE
ERARNGLSIVIAPEGTRLDTTEVGSFKKGPFRIAMAAKIPIVPIVIRNAEIVASRNST
TINPGTVDVAVFPPIPVDDWTLDALPDRIAEVRQLYLDTLADWPVDGLPAVDLYAEQK
AARKARAQVAKATAKRVPAKKAPAKSAANKGAAATKAATKKASPKAKPSESKIAGKDG
EASASPSSSAKGRS"
CDS complement(2762286..2763800)
/codon_start=1
/note="gene_2535"
/transl_table=11
/translation="LCVELPNPRGVVAMAESGESPRLSDELGPVDYLMHRGEANPRTR
SGIMALELLDGTPDWDRFRTRFENASRRVLRLRQKVVVPTLPTAAPRWVVDPDFNLDF
HVRRVRVSGPATLREVLDLAEVILQSPLDISRPLWTATLVEGMADGRAAMLLHVSHAV
TDGVGGVEMFAQIYDLERDPPPRSTPPQPIPEDLSPNDLMRRGINHLPIAVVGGVLDA
LSGAVSMAGRAVLEPVSTVSGILGYARSGIRVLNRAAEPSPLLRRRSLTTRTEAIDIR
LADLHKAAKAGGGSINDAYLAGLCGALRRYHEALGVPISTLPMAVPVNLRAEGDAAGG
NQFTGVNLAAPVGTIDPVARMKKIRAQMTQRRDEPAMNIIGSIAPVLSVLPTAVLEGI
TGSVIGSDVQASNVPVYPGDTYLAGAKILRQYGIGPLPGVAMMVVLISRGGWCTVTVR
YDRASVRNDELFAQCLQAGFDEILALAGDPAPRVLPASFDTQGAGSVPRSVSGS"
CDS complement(2763990..2765255)

/codon_start=1
/note="gene_2536"
/transl_table=11
/translation="MHIASVTSRCSRAGAEALRQGAQLAADARDTCRAGALLLRGSPC
AIGWVAGWLSAEFPARVVTGHALSRISPRSIGRFGTSWAAQRADQILHAALVDAFGPD
FRDLVWHPTGEQSEAARRSGLLNLPHIPGPHRRYAAQTSDIPYGPGGRENLLDIWRRP
DLAPGRRAPVLIQVPGGAWTINGKRPQAYPLMSRMVELGWICVSINYSKSPRCTWPAH
IVDVKRAIAWVRENIADYGGDPDFITITGGSAGAHLAALAALSANDPALQPGFESADT
AVQAAAPYYGVYDLTNAENMHEMMMPFLEHFVMRSRYVDNPGLFKAASPISYVHSEAP
PFFVLHGEKDPMVPSAQSRAFSAALRDAGAATVSYAELPNAHHAFDLAATVRSRMVAE
AVSDFLGVIYGRRMGARKGSLALSSPPAS"
tRNA 2765443..2765516
/note="identified by tRNAscan-SE; putative"
CDS 2765617..2766387
/codon_start=1
/note="gene_2537"
/transl_table=11
/translation="MAQYDPVLLSVDKHVALITVNDPDRRNAVTDEMSAQLRAAIQRA
EGDPDVHAVVVTGAGKAFCAGADLSALGAGVGDPAEPRLLRLYDGFMAVSSCNLPTIA
AVNGAAVGAGLNLALAADVRIAGPAALFDARFQKLGLHPGGGATWMLQRAVGPQVARA
ALLFGMCFDAESAVRHGLALMVADDPVTAALELAAGPAAAPREVVLASKATMRATASP
GSLDLEQHELAKRLELGPQAKSVQSPEFAARLAAAQHR"
CDS complement(2766567..2767004)
/codon_start=1
/note="gene_2538"
/transl_table=11
/translation="VGGKGGASGMLIGNGGNGGSGGVGGAGGVGGAGGDGGNGGSGGN
ASTFGDENSIGGAGGTGGNGGNGANGGNGGAGGIAGGAGGSGGFLSGAAGVSGADGIG
GAGGAGGAGGAGGSGGEAGAGGLTNGPGSPGVSGTEGMAGAPG"
CDS complement(2767067..2768650)
/codon_start=1
/note="gene_2539"
/transl_table=11
/translation="VSLVIATPQLLATAALDLASIGSQVSAANAAAAMPTTEVVAAAA
DEVSAAIAGLFGAHARQYQALSVQVAAFHEQFVQALTAAAGRYASTEAAVERSLLGAV
NAPTEALLGRPLIGNGADGTAPGQPGAAGGLLFGNGGNGAAGGFGQTGGSGGAAGLIG
NGGNGGAGGTGAAGGAGGNGGWLWGNGGNGGVGGTSVAAGIGGAGGNGGNAGLFGHGG
AGGTGGAGLAGANGVNPTPGPAASTGDSPADVSGIGDQTGGDGGTGGHGTAGTPTGGT
GGDGATATAGSGKATGGAGGDGGTAAAGGGGGNGGDGGVAQGDIASAFGGDGGNGSDG
VAAGSGGGSGGAGGGAFVHIATATSTGGSGGFGGNGAASAASGADGGAGGAGGNGGAG
GLLFGDGGNGGAGGAGGIGGDGATGGPGEAAATLASRGLTAQTPRQNPMWSAARVVMA
ARAAAALASAAPAGPAARAATAAPAGCCSATAATAATPGPAGMAAPALPVGLAVTAAV
VAPRRFTKTRSLVSGRSVA"
CDS complement(2768732..2772073)
/codon_start=1
/note="gene_2540"
/transl_table=11
/translation="MLASMSKIHPGVDVVPVDWSADGVSELVPTGTVTLLLADIEGAT
HLPGSQLDTTAIAKLDRTLTELVREHRGVCPVEQGEGDSFLVAFARASDAVACALGLQ
RAPLAPIRLRIGMHTGEVSSPDEGNCVGPTIDRTARLRELAHGGQTVLSGTTSDLVAD
LLPKDAWLNDLGTYRLDDLPRPERVVQLCHPDLHNAFPPLRTRKVVGAHCLPAQLTRL

VGRVDEVAQVRGLLDVKRWVTLTGVGGVGKTRLATQVASAVADGYPDGVWYVNLAPIT
DPALVPIAAARVLGLPDQPGRSTVDTIVRRIGDRRMLVVLDNCEHLLDGCAALIVALL
GACPALRVLATSREPIAVAGEQIWRVPPLGHGEAIELFTDRAREARPELEITADNLAL
VTEICHRLDGIPLAIELAASRVRALALTEIVDSLHDRFRLLTGGSRIAVRRQQTMRAS
VDWSHALLTGPEQVLFRRLAVFPSGFDLDGAQAAAAGGDVQRYEVVDLLSLLADKSLV
VTEDSDGRTRYRLLETVRQYALEKLRESGDADAVRARHRDHYAAVAAGLDAPSVAGHE
RRLNQAELEIDNLRAAFAFSRENGDTGHALLLASCLQPLWRARGRLQEGLAWFAAALA
DHDAHPAGADPGLYARALADRALIDAVAGITDRLDDAQKALAIARDIEDPALLARALT
ACGGVAAYNADLARPWLAEAVGLARAVGDKWRLAEVLAWQAYVGFAGEGDPGATRAAG
EEARSLADEIGDAFLSRSCRWALAAANLWQGNLEAAVGLSREVIGESDAAHDMVSSCA
GQACLAHALAHRGDTEAAAAAQASIDTAVGLSPVLSGSACSALVFATLAAGDVAAAEH
ARESATRFFGASAAAIINDPTSSAQISCARGDLNAAHRLADGAASITRGVHRARALTT
RCRIEIAQGDRHRAERDAHDALGVAASIGAYLWVPDILECLASVMADAGSNREAVRLF
GAADAARGRMGAVRFGIYQAGCNSSLATLRKSMGDSEFDDAWAEGTALSIDEAIAYAQ
RGRGARKRPTSGWGALTPTELEVALLVGEGLSNKEIGVRLFISPRTVHSHLTHVYTKL
GLSSRLQLAQQAARRGESERGPSRP"

CDS complement(2772647..2774731)
/codon_start=1
/note="gene_2541"
/transl_table=11
/translation="LGGDSGNGTRAASGVDASDHGPGSGGNGGNGGNGAQASVAGGAG
GNGGDGGNAGRVGDGGAGGNGGDGAAGANGANSGAPGSDALALGQPGGNGGQGDAGQA
GGAGGAGGAGGSVSGDGGAGGNGGAGGNGGVGASGGAGARGANGIDSIGGTGGAGGGG
GDGGAGGVGGHGGDGGVGGAAPSGTVGSHGTGGVGGDGGLGGAGGVGGAGGNGGIGIT
VGGAGGAGGNGGDPGAGGRGGLGGDSGNGTSAANGVDASKHGPLTGGDGGVGGNGAKA
AAAGGDGGQGGDGGNAGLFGDGGAGGDGADGTAAEALGGDGGAGGAGGKGGDAGDIGD
GGDGGKGGDGAHGALGGLTVAGGNGGAGGAGGAGGAGGAFLGDGGNGGAGGQGGAGRG
GSPGGGGGVGGHGGAGGDAGMNGGGGTGGQGGNGAAGGAGWSPDSDLKGFDGFDGGSG
GAGGDGGAGGAGGTQTGDGGDGGAGGLGGAGGVGGNGVDGFDINETTGRDGGDGGDGG
YGGWGGAGGNGGAGGSAPAGEVGNRGVGGDGGDGGSGGDAGNGGLGGDGFTYLADLTE
SLAATEVTAATAGGAAPADKAVSVPQVARTARPASAPPAVTEATAGTAVTVGTATAAL
LMQATAGRVATAATAAWVGPGETVAPPVVTEATAGPAAPAASAPLRLAASAAVTAGTA
GAEVTADAVPVA"

CDS 2774624..2775184
/codon_start=1
/note="gene_2542"
/transl_table=11
/translation="LRTVATVTTVAAAAGPVVAGVHTAGRAGAVAAVAAQAAEASRAG
VTAVTAVPAITAMTAVTAVAAGSAEPAVTANAADTVAADLTRGCGPAGAAITAGPAVT
ANAADTAVAAFPAGASNGLSAVGAGGALAAAATTSSGTTRTAGPALATGSTVARHRPT
GATGSAGTARAAGLPRIASTAVAATT"

CDS 2775248..2775952
/codon_start=1
/gene="O217_13285"
/note="gene_2543"
/transl_table=11
/translation="LTAVGLASFARLPREGAVGAVLTYVRAIRTGRAATTVAAGPAVT
DQPAVTTGTALAAVRASHGGVGAVTALAAVATTVHGSAGRAGHTLGGAGAAGATLPTG
AWVTTIAAGPTVTILESHVAAATAAPVTGRPTSPAGTTVPANTLAARPTRRCVTASPT
GPTVTAATAGSAITTVAAVRYEHSIETVSSVTTGPAGPTVAAGTAGPAIAGVGPTRAA
GPTGPAVTSILTGGTG"

CDS complement(2776065..2777492)
/codon_start=1
/note="gene_2544"
/transl_table=11
/translation="MSYVIATPEMMATAAFDLARIGSQVSAASAVAAMPTTEVVAAGA
DEVSAGIAALFSAHAQEYQALSAQAAAFHDQFVHTLTAAARWYTATEIANAAAMRVVL
GAVNAPTQTLLGRPLIGDGAHGTAPGQPGGAGGLLFGNGGNGAAGAVGQVGGAGGAAG
LFGIGGAGGAGGAGAPGGTGGTGGWLAGGGGVGGMGGAGGGAGGAGGNAGLFGNGGAG
GAGGAGGGAGGAGGNAGWFGHGGAGGVGGVGAAGANGATPGQDGAAGVAGSDDGAGGD
GLAGSDGGDGGAGGVGGNGGRGGWLLGNGGAGGVGGVGGAGGAGAAGGAGGAGATGIN
GPAGISAAGGDGGAGGNGGAGGNGGVGGAGGAGGSAGLLGYVGRAGDGGAGGGGGLGG
APGDGGAGGNGGSWLAAGDGGAGGHGGDPGLGGAGGAGGGVGRRRCSRGGQWSGGRQR
RAGQRRQRRQRWQWRPRIGRRRSWR"
CDS complement(2777579..2777770)
/codon_start=1
/gene="TBCG_02430"
/note="gene_2545"
/transl_table=11
/translation="MLAGMREIDPGADVAPLDCSKVSKDDVGNPVAAGSVALLLADRV
GSTHLGGRRGKSEQGLSRR"
CDS 2777921..2778544
/codon_start=1
/note="gene_2546"
/transl_table=11
/translation="MVDTSAPASRLDTDPRRAHVSLSKHPYQIGVFGSGTIGPRVYEL
AYQVGAEIAKQGHILISGGMTGTMEASSRGASDADGLVVGVLPGDKFTDGNAYSTIKI
LSGMQFARNYITGLSCHGAIVVGGSSGAYEEARRVWEGRGPVVVLANSGSPTGASAQM
LSMQEIFGVAFPEDKPKPWRVFSAATPAESVSLVIGLIRKGYAQHEP"
CDS 2778534..2779286
/codon_start=1
/note="gene_2547"
/transl_table=11
/translation="MSRRIINEFGVQIYGATIGDTWAGLVRAVLDLGSQCFDEDRERI
ALSNVRIKSSVQNYPDLTIEEHCNSDQLKAMLDFMFNTDTMEDIDVVKSFSRGAKSYH
RRIKEGRMIEFVIERLSLIPESKKAVVVFPTYEDYAAVMRNHRDDYLPCLVSIQFRLL
PDGKDYVFHTTFYSRSMDAWQKGHGNLLSIAKLSDWVRENVSARIGRKIMLGPLDGMI
CDVHIYKETYAEACKRLANLDLRRTQFDAVRN"
CDS 2779339..2779560
/codon_start=1
/note="gene_2548"
/transl_table=11
/translation="VRTTLDLDDDVIAAARELASSQRRSLGSVISELARRGLMPGRVE
ADDGLPVIRVPAGTPPITPEMVRRALDED"
CDS 2779566..2779991
/codon_start=1
/note="gene_2549"
/transl_table=11
/translation="VALLDVNALVALAWDSHIHHARIREWFTANATLGWATCPLTEAG
FVRVSTNPKVLPSAIGIADARRVLVALRAVGGHRFLADDVSLVDDDVPLIVGYRQVTD
AHLLTLARRRGVRLVTFDAGVFTLAQQRPKTPVELLTIL"
CDS complement(2780014..2781195)

/codon_start=1
/note="gene_2550"
/transl_table=11
/translation="MSGEDSIRSFPVPDLGEGLQEVTVTCWSVAVGDDVEINQTLCSV
ETAKAEVEIPSPYAGRIVELGGAEGDVLKVGAELVRIDTGPTAVAQPNGEGAVPTLVG
YGADAAIETSRRTSRPLAAPVVRKLAKELAVDLAALQRGSGAGGVITRADVLAAARGG
VGAGPDVRPVHGVHARMAEKMTLSHKEIPTAKASVEVICAELLRLRDWFVSAAPEITP
FALTLRLLVIALKHNVILNSTWVDSGEGPQVHVHRGVHLGFGAATERGLLVPVVTDAQ
DKNTRELASRVAELITGAREGTLTPAELRGSTFTVSNFGALGVDDGVPVINHPEAAIL
GLGAIKPRPVVVGGEVVARPTMTLTCVFDHRVVDGAQVAQFMCELRDLIESPETALLD
L"

CDS complement(2781192..2782238)
/codon_start=1
/note="gene_2551"
/transl_table=11
/translation="MTQIADRPARPDETLAVAVSDITQSLTMVQAINRALYDAMAADE
RVLVFGEDVAVEGGVFRVTEGLADTFGADRCFDTPLAESAIIGIAVGLALRGFVPVPE
IQFDGFSYPAFDQVVSHLAKYRTRTRGEVDMPVTVRIPSFGGIGAAEHHSDSTESYWV
HTAGLKVVVPSTPGDAYWLLRHAIACPDPVMYLEPKRRYHSRGMVDTSRPEPPIGHAM
VRRSGTDVTVVTYGNLVSTALSSADTAEQQHDWSLEVIDLRSLAPLDFDTIAASIQRT
GRCVVMHEGPRSLGYGAGLAARIQEEMFYQLEAPVLRACGFDTPYPPARLEKLWLPGP
DRLLDCVERVLRQP"

CDS complement(2782249..2783322)
/codon_start=1
/note="gene_2552"
/transl_table=11
/translation="MLMSVDLEPVQLVGPDGTPTAERRYHRDLPEETLRWLYEMMVVT
RELDTEFVNLQRQGELALYTPCRGQEAAQVGAAACLRKTDWLFPQYRELGVYLVRGIP
PGHVGVAWRGTWHGGLQFTTKCCAPMSVPIGTQTLHAVGAAMAAQRLDEDSVTVAFLG
DGATSEGDVHEALNFAAVFTTPCVFYVQNNQWAISMPVSRQTAAPSIAHKAIGYGMPG
IRVDGNDVLACYAVMAEAAARARAGDGPTLIEAVTYRLGPHTTADDPTRYRSQEEVDR
WATLDPIPRYRTYLQDQGLWSQRLEEQVTARAKHVRSELRDAVFDAPDFDVDEVFTTV
YAEITPGLQAQREQLRAELARTD"

CDS complement(2783611..2784432)
/codon_start=1
/note="gene_2553"
/transl_table=11
/translation="MNLRAAGPGWLFCPADRPERFAKAAAAADVVILDLEDGVAEAQK
PAARNALRDTPLDPERTVVRINAGGTADQARDLEALAGTAYTTVMLPKAESAAQVIEL
APRDVIALVETARGAVCAAEIAAADPTVGMMWGAEDLIATLGGSSSRRADGAYRDVAR
HVRSTILLAASAFGRLALDAVHLDILDVEGLQEEARDAAAVGFDVTVCIHPSQIPVVR
KAYRPSHEKLAWARRVLAASRSERGAFAFEGQMVDSPVLTHAETMLRRAGEATSE"

CDS complement(2784429..2784890)
/codon_start=1
/note="gene_2554"
/transl_table=11
/translation="LWFEEFQIGTTYLHRPGRTVTEADNVLFTTLTMNTQSLHLDAAW
AGQQPGFRGERLVNSMFTLSTMVGLSVAQLTLGTIVANLGFSEVSFPKPVFHGDTLYA
ETVCTGKRESKSRPGEGIVTLEHIARNQHGEVVARAVRTTLVQKQSIKEAQ"

CDS complement(2784983..2786167)
/codon_start=1

/note="gene_2555"
/transl_table=11
/translation="MTTTTTTISGGILPKEYQDLRDTVADFARTVVAPVSAKHDAEHS
FPYEIVAKMGEMGLFGLPFPEEYGGMGGDYFALSLVLEELGKVDQSVAITLEAAVGLG
AMPIYRFGTEEQKQKWLPDLTSGRALAGFGLTEPGAGSDAGSTRTTARLEGDEWIING
SKQFITNSGTDITSLVTVTAVTGTTGTAADAKKEISTIIVPSGTPGFTVEPVYNKVGW
NASDTHPLTFADARVPRENLLGARGSGYANFLSILDEGRIAIAALATGAAQGCVDESV
KYANQRQSFGQPIGAYQAIGFKIARMEARAHVARTAYYDAAAKMLAGKPFKKEAAIAK
MISSEAAMDNSRDATQIHGGYGFMNEYPVARHYRDSKVLEIGEGTTEVQLMLIARSLG
LQ"
CDS complement(2786172..2788136)
/codon_start=1
/note="gene_2556"
/transl_table=11
/translation="MFDTVLVANRGEIAVRVIRTLRRLGIRSVAVYSDPDVDARHVLE
ADAAVRLGPAPARESYLDIGKVLDAAARTGAQAIHPGYGFLAENADFAAACERARVVF
LGPPARAIEVMGDKIAAKNAVAAFDVPVVPGVARAGLTDDALVTAAAEVGYPVLIKPS
AGGGGKGMRLVQDPARLPEALVSARREAMSSFGDDTLFLERFVLRPRHIEVQVLADAH
GNVVHLGERECSLQRRHQKVIEEAPSPLLDPQTRERIGVAACNTARCVDYVGAGTVEF
IVSAQRPDEFFFMEMNTRLQVEHPVTEAITGLDLVEWQLRVGAGEKLGFAQNDIELRG
HAIEARVYAEDPAREFLPTGGRVLAVFEPAGPGVRVDSSLLGGTVVGSDYDPLLTKVI
AHGADREEALDRLDQALARTAVLGVQTNVEFLRFLLADERVRVGDLDTAVLDERSADF
TARPAPDDVLAAGGLYRQWALARRAQGDLWAAPSGWRGGGHMAPVRTAMRTPLRSETV
SVWGPPESAQVQVGDGEIDCASVQVTREQMSVTISGLRRDYRWAEADRHLWIADERGT
WHLREAEEHKIHRAVGARPAEVVSPMPGSVIAVQVESGSQISAGDVVVVVEAMKMEHS
LEAPVSGRVQVLVSVGDQVKVEQVLARIKD"
CDS complement(2788141..2789730)
/codon_start=1
/note="gene_2557"
/transl_table=11
/translation="VTTPSIAIAPSFADEHRRLVAELNNKLAAAALGGNERARKRHVS
RGKLLPRERVDRLLDPGSPFLELAPLAAGGMYSDESPGAGIITGIGRVSGRQCVIVAN
DATVKGGTYYPMTVKKHLRAQEVALQNMLPCIYLVDSGGAFLPRQDEVFPDREHFGRI
FYNQATMSAKGIPQVAAVLGSCTAGGAYVPAMSDEAVIVREQGTIFLGGPPLVKAATG
EIVSAEELGGGDLHSRTSGVTDHLADDDEDALRIVRAIADTFGPCEPAQWDVRRSVEP
KYPQAELYDVVPPDPRVPYDVHEVVVRIVDGSEFSEFKAKYGKTLVTAFARVHGHPVG
IVANNGVLLSESALKGAHFIELCDKRKIPLLFLQNIAGFMVGRDYEAGGIAKHGAKMV
TAVACARVPKLTVVIGGSYGAGNYSMCGRAYSPRFLWMWPNARISVMGGEQAASVLAT
VRGEQLSAAGTPWSPDEEEAFKAPIRAQYEDQGNPYYSTARLWDDGIIDPADTRTVVG
LALSLCAHAPLDQVGYGVFRM"
CDS complement(2789727..2790383)
/codon_start=1
/note="gene_2558"
/transl_table=11
/translation="MSAPGWSRDEMAARVAAEFEDGQYVNLGIGMPTLIPNHIPDGVH
VVLHSENGILGVGPYPRREDVDADLINAGKETVTTLPGAAFFSSSTSFGIIRGGHLDV
AVLGAMQVSVTGDLANWMIPGKMVKGMGGAMDLVHGARKVIVMMEHTAKDGSPKILER
CTLPLTGVGCVDRIVTELAVIDVCADGLHLVQTAPGVSVDEVVAKTQPPLVLRDLATQ
"
CDS complement(2790380..2791126)
/codon_start=1

```
                     /gene="pcaI"
                     /note="gene_2559"
                     /transl_table=11
                     /translation="MDKVVATAAEAVADIANGSSLAVGGFGLCGIPEALIAALVDSGV
                     TDLETVSNNCGIDGVGLGLLLQHKRIRRTVSSYVGENKEFARQFLAGELEVELTPQGT
                     LAERLRAGGMGIPAFYTPAGVGTQVADGGLPWRYDASGGVAVVSPAKETREFDGVTYV
                     LERGIRTDFALVHAWQGDRHGNLMYRHAAANFNPECASAGRITIAEVEHLVEPGEIDP
                     ATVHTPGVFVHRVVHVPNPAKKIERETVRQ"
CDS                  complement(2791209..2792852)
                     /codon_start=1
                     /note="gene_2560"
                     /transl_table=11
                     /translation="VAAAEVVDPNRLSYDRGPSAPSLLESTIGANLAATAARYGHREA
                     LVDMVARRRFNYSELLTDVHRLATGLVRAGIGPGDRVGIWAPNRWEWVLVQYATAEIG
                     AILVTINPAYRVREVEYALRQSGVAMVIAVASFKDADYAAMLAEVGPRCPDLADVILL
                     ESDRWDALAGAEPDLPALQQTAARLDGSDPVNIQYTSGTTAYPKGVTLSHRNILNNGY
                     LVGELLGYTAQDRICIPVPFYHCFGMVMGNLAATSHGAAMVIPAPGFDPAATLRAVQD
                     ERCTSLYGVPTMFIAELGLPDFTDYELGSLRTGIMAGAACPVEVMRKVISRMHMPGVS
                     ICYGMTETSPVSTQTRADDSVDRRVGTVGRVGPHLEIKVVDPATGEAVPRGVVGEFCT
                     RGYSVMAGYWNDPQKTAEVIDADGWMHTGDLAEMDPSGYVRIAGRIKDLVVRGGENIS
                     PREIEELLHTHPDIVDGHVIGVPDAKYGEELMAVVKLRNDAPELTIERLREYCMGRIA
                     RFKIPRYLWIVDEFPMTVTGKVRKVEMRQQALEYLRGQQ"
CDS                  2793103..2793615
                     /codon_start=1
                     /note="gene_2561"
                     /transl_table=11
                     /translation="VRLEDIGAAAGVSGPAIYRHFPNKESLLVELLVGVSARLLAGAR
                     DVTTRSANLAAALDGLIEFHLDFALGEADLIRIQDRDLAHLPAVAERQVRKAQRQYVE
                     VWVGVLRELNPGLAEADARLMAHAVFGLLNSTPHSMKAADSKPARTVRARAVLRAMTV
                     AALSAADRCL"
CDS                  2793694..2794515
                     /codon_start=1
                     /note="gene_2562"
                     /transl_table=11
                     /translation="MNDPRRPQRFGPPLSGYGPTGPQVPPNPPTADPAYADQSPYAST
                     YGGYVSPPWSPGGPPPRPPQWPPGPHEASPTQQLPQYWQYDQPPPGGFPPDGLTPPPP
                     QGPRTPRWLWFAAGSAVLLVVALVIALVIANGSVKKQTAIEPLPPMPGPSPTRPTTTT
                     PTPPSPSAAPAPTTTTGTPSETVAGAMQTVVYDVTGEGRAISITYMDSGNVIQTEFNV
                     ALPWRKEVSLSKSSLHPASVTIVNIGHNVTCSVTVAGVQVRQRTGAGLTICDAPS"
CDS                  complement(2794512..2795858)
                     /codon_start=1
                     /note="gene_2563"
                     /transl_table=11
                     /translation="VPDMNNPGSRAGTLLHFRVVAWAMWDCGSTGLNAIVTTFVFSVY
                     LTSAVGQGLPGGTSPASWLGRAGAVAGLTIGVLAPVVGVWVESPHRRRVALSVLTGTA
                     VALTCAMFLIRDDPRYLWAGLVLLAATAASSDLSSVPYNAMLRQLSTPSTAGRISGFG
                     WASGYVGSVALLLVIYLGFMSGSGSQRGLLQLPVANGLNVRMAMLVAAAWLALLGLPL
                     LLVAHRLPDSGAASHPSTGLLGGYRKLWTEISAEWRRDRNLVYFLVASAIFRDGLAAI
                     FAFGAVLGVNAYGLTQADVLIFGAAASVVAAVGAVLGGFVDHRIGSKPVIVGSLAAII
                     AAALTLLTLSGPTAFWACGLLLCVFIGPAQSSARALLLHMAQHGKEGVAFGLYTMTGR
                     AVSFLGPWLFSVFVDVFHTVRAGLGGVCLVLTTGLLLMLRVQVSRHGGALTTAQSS"
```

```
CDS             2795934..2796740
                /codon_start=1
                /note="gene_2564"
                /transl_table=11
                /translation="MPIPAPSPDARAVVTGASQNIGAALATELAARGHHLIVTARRED
                VLTELAARLADKYRVTVDVRPADLADPQERSKLADELAARPISILCANAGTATFGPIA
                SLDLAGEKTQVQLNAVAVHDLTLAVLPGMIERKAGGILISGSAAGNSPIPYNATYAAT
                KAFVNTFSESLRGELRGSGVHVTVLAPGPVRTELPDASEASLVEKLVPDFLWISTEHT
                ARVSLNALERNKMRVVPGLTSKAMSVASQYAPRAIVAPIVGAFYKRLGGS"
CDS             complement(2796744..2798345)
                /codon_start=1
                /note="gene_2565"
                /transl_table=11
                /translation="MGTESAAGGPGGPAQRIAAGYTVEGQALQLGTVVVDGEPDPSAQ
                IRIPLATVNRHGLVAGATGTGKTKTLQLIAEQLSAAGVAVLMADVKGDLSGLARPGEA
                ADKTAARAKDTGDDWVPTAFPVEFLSLGASGVGVPVRATISSFGPILLAKVLGLNATQ
                ESTLGLIFHWADQRGLPLLDLKDLRAVITHLTSDEGKVELKSLGAVSPTTAGVILRAL
                VNLEAEGADTFFGEPELRPEDLLRVDSQGRGIISLLEFGSQALRPAMFSTFLMWVLAD
                LFTFLPEVGDLDKPKLVFFFDEAHLLFTDASKAFLEQVEQTVKLIRSKGVGVFFCTQL
                PTDLPNDVLSQLGARIQHALRAFTPDDHKALRKTVRTYPKTDVYDLESALTSLGTGEA
                VVTVLSEKGAPTPVAWTRMRAPRSLMAAIGAEAIGAAAQASSLQAVYGQTIDRPSAHE
                ILSAKLAPAQEAPAQEAPAPRGQYDPLPWPDDFEVPPMPAPVEPQGPAVWEEILKNPT
                VKSVLNTTAREITRSIFGTGRRRRK"
CDS             2798362..2799060
                /codon_start=1
                /note="gene_2566"
                /transl_table=11
                /translation="LPGGDSRDGSHAPTLSAVQDELVWIDCEMTGLDLGSDKLIEIAA
                LVTDADLNILGDGVDVVMHADDAALSGMIDVVAEMHSRSGLIDEVKASTVDLATAEAM
                VLDYINEHVKQPKTAPLAGNSIATDRAFIARDMPTLDSFLHYRMIDVSSIKELCRRWY
                PRIYFGQPPKGLTHRALADIHESIRELRFYRRTAFVPQPGPSTSEIAAVVAELSDGAG
                AQEETDSAEAPQSG"
tRNA            2799110..2799185
                /note="identified by tRNAscan-SE; putative"
CDS             complement(2799812..2801059)
                /codon_start=1
                /note="gene_2567"
                /transl_table=11
                /translation="MTSSHLIDAEQLLADQLAQASPDLLRGLLSTFIAALMGAEADAL
                CGAGYRERSDERSNQRNGYRHRDFDTRAATIDVAIPKLRQGSYFPDWLLQRRKRAERA
                LTSVVATCYLLGVSTRRMERLVETLGVTKLSKSQVSIMAKELDEAVEAFRTRPLDAGP
                YTFLAADALVLKVREAGRVVGVHTLIATGVNAEGYREILGIQVTSAEDGAGWLAFFRD
                LVARGLSGVALVTSDAHAGLVAAIGATLPAAAWQRCRTHYAANLMAATPKPSWPWVRT
                LLHSIYDQPDAESVVAQYDRVLDALTDKLPAVAEHLDTARTDLLAFTAFPKQIWRQIW
                SNNPQERLNREVRRRTDVVGIFPDRASIIRLVGAVLAEQHDEWIEGRRYLGLEVLTRA
                RAALTSTEEPAKQQTTNTPALTT"
CDS             2801417..2801839
                /codon_start=1
                /note="gene_2568"
                /transl_table=11
                /translation="VDDIAAFKLDSLPDITFTVTRAISSGGENPAGFLNFAARREQPE
```

ILGGGGRPGPVGPEAVDTPRIRGGKVPFVFRTLPGYTFYASQIEPRVGDPEGPTLLAG
FGNIPETSQRSPGWIRITCKGPDDDEELEFFGFAGPES"
CDS complement(2802133..2802351)
/codon_start=1
/note="gene_2569"
/transl_table=11
/translation="LRLHPNMVRQGGRRSAADPFVIALAMVNNATVVTQETASGNIEK
PRIPDVCDALGVPWLTLMGYIEAQGWTF"
CDS 2802340..2802603
/codon_start=1
/gene="HIMB100_00000290"
/note="gene_2570"
/transl_table=11
/translation="VQPQDVPRRLLDLFIQRTEQASLPVRPPLRVIITPGEFALNLID
RPDLTGADRILNPAPQTAKHRWPKQITPSVQDGAGIEGIEHSG"
CDS complement(2802600..2803829)
/codon_start=1
/note="gene_2571"
/transl_table=11
/translation="MWVGWCIIIAMRSIPASVESSVLRWARESCGLTEVAAARKLGLP
DDRVAAWEVGEVVPTIAQLRKAAEVYKRSLAVFFLSEPPEGFDTLRDFRRLDGAASGQ
WTPGLHEEFRRAHTQRDFALELADAEDREIPGAWRLPLSGDEADADIAARIRKALIEV
SPLPIPVASVDPYEHLNAWVSAIETSGVLVLATRGGKVAIDEMRGMCLYFDELPVIVL
NGSDHPRPRLFSLLHEFVHVVLHTEGLCDVIADAHPSTQDRSLEARCNAIAAAVLMPA
DVVRARPEVIVRSETPSSWDYESLRPVAAHFGVSAEAFLRRLSTLGIVPVEVYRQRRA
EFIAAHEDEAERARSAGGGNWYRNTVRDLGKGYVRAVTDAHRRRVIDSNTAAIYLDAK
VSQIPKLAESAELRSVV"
CDS complement(2803966..2804769)
/codon_start=1
/note="gene_2572"
/transl_table=11
/translation="VTADWVVTFTFDADPSMETMDAWETQLEGFDALVSRVPGHGIDV
TVYAPGDWSVFDALAKMAGEVMPVVQAKSPIAVQIISEPEHRLRAEAFTTPELMSAAE
IADELGVSRQRVHQLRSTAGFPAPLADLRGGAVWDAAAVRRFAETWERKPGRPHTGTA
KFAYSWAVGPAVGRSGKAPNVRWRVENPDKIRFVLRNIGDDIAEDVEIDLSRIDAITR
NVPKKTVIRPGEGLNMVLIAAWGHPLPNQLYVRWAGQDEWAAVPLHPAH"
CDS complement(2804766..2805017)
/codon_start=1
/note="gene_2573"
/transl_table=11
/translation="VNSAIIKIAKWAQSQQWTVEDDASGYTRFYNPQGVYIARFPATP
SNEYRRMRDLLGALKKAGLTWPPPSKKERRAQHRKEGAQ"
CDS complement(2805365..2806507)
/codon_start=1
/note="gene_2574"
/transl_table=11
/translation="MIGAVACGSGRGPAPIKVIADKGTPFADLLVPKLTASVTDGAVG
VTVDAPVSVTAADGVLAAVTMVNDNGRPVAGRLSPDGLRWSTTEQLGYNRRYTLNATA
LGLGGAATRQLTFQTSSPAHLTMPYVMPGDGEVVGVGEPVAIRFDENIADRGAAEKAI
KITTNPPVEGAFYWLNNREVRWRPEHFWKPGTAVDVAVNTYGVDLGEGMFGEDNVQTH
FTIGDEVIATADDNTKILTVRVNGEVVKSMPTSMGKDSTPTANGIYIVGSRYKHIIMD

SSTYGVPVNSPNGYRTDVDWATQISYSGVFVHSAPWSVGAQGHTNTSHGCLNVSPSNA
QWFYDHVKRGDIVEVVNTVGGTLPGIDGLGDWNIPWDQWRAGNAKA"
tRNA 2806750..2806822
/note="identified by tRNAscan-SE; putative"
CDS 2807041..2808519
/codon_start=1
/note="gene_2575"
/transl_table=11
/translation="VSRLIVAPDWLASAAAEVQSIGSALSAANAAAAAPTTLLVAAAE
DEVSAAAAALFANYGREYQTLSVRFASLDQQFAQALNSAAASYQTAEATGASLVQTAT
QGVLGVINAPTEFMFGRSLIGDGADGTAASPIGEPGGILYGDGGNGYSQTTPGAVGGA
GGSAGFIGNGGAGGAGGPGAGGGTGGLGGWLWGNNGAAGTGDPVNVAVPLRVENNFPL
VNLLVNRGPTVPILLDTGSSSLVIPFWKIGWQNLGLPTGFDVVHYGNGVSIVYADVPT
TVDFGGGAATTPTSVHVGILPYPRNLDSLVLIASGGAFGPNGNGILGIGPNVGSYAVS
GPGNVVTTDLPGQLNEGTLIDIPGGYMQFGPNTGTPITSVTGAPITVLNVQIGGYDPN
GGYWSLPSIFDSGGNHGTLPAVILGTGQTTGYAPPGTVISISIHDNQTLLYQYTTTAS
NSPVVTADPRLNTGLTPFLLGPVYISNNPSGVGTVVFNYPPP"
CDS complement(2808644..2808871)
/codon_start=1
/note="gene_2576"
/transl_table=11
/translation="VVDRDPNTIKQEIDQTRDQLAATIDSLAERANPRRLADDAKTRV
IAFLRKPIVTVSLVGIGSVVVVVVIHKIRNR"
CDS 2808940..2809413
/codon_start=1
/note="gene_2577"
/transl_table=11
/translation="VTKTTRLTPGDKAPAFTLPDADGNNVSLADYRGRRVIVYFYPAA
STPGCTKQACDFRDNLGDFTTAGLNVVGISPDKPEKLATFRDAQGLTFPLLSDPDREV
LTAWGAYGEKQMYGKTVQGVIRSTFVVDEDGKIVVAQYNVKATGHVAKLRRDLSV"
CDS complement(2809385..2810695)
/codon_start=1
/note="gene_2578"
/transl_table=11
/translation="VRCDLEELVRIESVWADPDRRDEVHRSARAVADLLSQAGFDDVR
IVSERGAPAVIARYPAPPGAPTVLLYAHHDVQPEGDRGQWVSPPFEPTERGGRLYGRG
TADDKAGIATHVAAFWAHGGRPPVGVTVFVEGEEESGSPSLGRLLAAHRDALAADVIV
IADSDNWSTDIPALTVSLRGMADCVVEVATLDHGLHSGLWGGVVPDALTVLVRLLASL
HDDDGNVAVAGMHESTAARVDYPAGRVRAESGLLDGVSEIGTGSVPQRLWAKPAITVI
GIDTTSVAAASNTLIPRARAKISIRVAPGGDATAHLDAVEAHLRRHAPWGAQVTVTRG
EVGQPYAIEASGPVYDAARSAFRQAWGADPIDMGMGGSIPFIAEFAAAFPQATILVTG
VEDPGTQAHSVNESLHLGVLERAATAEALLLAKLAAIPTGRAEA"
CDS complement(2810794..2811186)
/codon_start=1
/gene="acpS"
/note="gene_2579"
/transl_table=11
/translation="MGIVGVGIDLVSIPDFAEQVDQPGTVFAETFTPGERRDASDKSS
SAARHLAARWAAKEAVIKAWSGSRFAQRPVLPEDIHRDIEVVTDMWGRPRVRLTGAIA
EYLADVTIHVSLTHEGDTAAAVAILEAP"
CDS complement(2811379..2820588)

/codon_start=1
/note="gene_2580"
/transl_table=11
/translation="VTIHEHDRVSADRGGDSPHTTHALVDRLMAGEPYAVAFGGQGSA
WLETLEELVSATGIETELATLVGEAELLLDPVTDELIVVRPIGFEPLQWVRALAAEDP
VPSDKHLTSAAVSVPGVLLTQIAATRALARQGMDLVATPPVAMAGHSQGVLAVEALKA
GGARDVELFALAQLIGAAGTLVARRRGISVLGDRPPMVSVTNADPERIGRLLDEFAQD
VRTVLPPVLSIRNGRRAVVITGTPEQLSRFELYCRQISEKEEADRKNKVRGGDVFSPV
FEPVQVEVGFHTPRLSDGIDIVAGWAEKAGLDVALARELADAILIRKVDWVDEITRVH
AAGARWILDLGPGDILTRLTAPVIRGLGIGIVPAATRGGQRNLFTVGATPEVARAWSS
YAPTVVRLPDGRVKLSTKFTRLTGRSPILLAGMTPTTVDAKIVAAAANAGHWAELAGG
GQVTEEIFGNRIEQMAGLLEPGRTYQFNALFLDPYLWKLQVGGKRLVQKARQSGAAID
GVVISAGIPDLDEAVELIDELGDIGISHVVFKPGTIEQIRSVIRIATEVPTKPVIMHV
EGGRAGGHHSWEDLDDLLLATYSELRSRANITVCVGGGIGTPRRAAEYLSGRWAQAYG
FPLMPIDGILVGTAAMATKESTTSPSVKRMLVDTQGTDQWISAGKAQGGMASSRSQLG
ADIHEIDNSASRCGRLLDEVAGDAEAVAERRDEIIAAMAKTAKPYFGDVADMTYLQWL
RRYVELAIGEGNSTADTASVGSPWLADTWRDRFEQMLQRAEARLHPQDFGPIQTLFTD
AGLLDNPQQAIAALLARYPDAETVQLHPADVPFFVTLCKTLGKPVNFVPVIDQDVRRW
WRSDSLWQAHDARYDADAVCIIPGTASVAGITRMDEPVGELLDRFEQAAIDEVLGAGV
EPKDVASRRLGRADVAGPLAVVLDAPDVRWAGRTVTNPVHRIADPAEWQVHDGPENPR
ATHSSTGARLQTHGDDVALSVPVSGTWVDIRFTLPANTVDGGTPVIATEDATSAMRTV
LAIAAGVDSPEFLPAVANGTATLTVDWHPERVADHTGVTATFGEPLAPSLTNVPDALV
GPCWPAVFAAIGSAVTDTGEPVVEGLLSLVHLDHAARVVGQLPTVPAQLTVTATAANA
TDTDMGRVVPVSVVVTGADGAVIATLEERFAILGRTGSAELADPARAGGAVSANATDT
PRRRRRDVTITAPVDMRPFAVVSGDHNPIHTDRAAALLAGLESPIVHGMWLSAAAQHA
VTATDGQARPPARLVGWTARFLGMVRPGDEVDFRVERVGIDQGAEIVDVAARVGSDLV
MSASARLAAPKTVYAFPGQGIQHKGMGMEVRARSKAARKVWDTADKFTRDTLGFSVLH
VVRDNPTSIIASGVHYHHPDGVLYLTQFTQVAMATVAAAQVAEMREQGAFVEGAIACG
HSVGEYTALACVTGIYQLEALLEMVFHRGSKMHDIVPRDELGRSNYRLAAIRPSQIDL
DDADVPAFVAGIAESTGEFLEIVNFNLRGSQYAIAGTVRGLEALEAEVERRRELTGGR
RSFILVPGIDVPFHSRVLRVGVAEFRRSLDRVMPRDADPDLIIGRYIPNLVPRLFTLD
RDFIQEIRDLVPAEPLDEILADYDTWLRERPREMARTVFIELLAWQFASPVRWIETQD
LLFIEEAAGGLGVERFVEIGVKSSPTVAGLATNTLKLPEYAHSTVEVLNAERDAAVLF
ATDTDPEPEPEEDEPVAESPAPDVVSEAAPVAPAASSAGPRPDDLVFDAADATLALIA
LSAKMRIDQIEELDSIESITDGASSRRNQLLVDLGSELNLGAIDGAAESDLAGLRSQV
TKLARTYKPYGPVLSDAINDQLRTVLGPSGKRPGAIAERVKKTWELGEGWAKHVTVEV
ALGTREGSSVRGGAMGHLHEGALADAASVDKVIDAAVASVAARQGVSVALPSAGSGGG
ATIDAAALSEFTDQITGREGVLASAARLVLGQLGLDDPVNALPAAPDSELIDLVTAEL
GADWPRLVAPVFDPKKAVVFDDRWASAREDLVKLWLTDEGDIDADWPRLAERFEGAGH
VVATQATWWQGKSLAAGRQIHASLYGRIAAGAENPEPGRYGGEVAVVTGASKGSIAAS
VVARLLDGGATVIATTSKLDEERLAFYRTLYRDHARYGAALWLVAANMASYSDVDALV
EWIGTEQTESLGPQSIHIKDAQTPTLLFPFAAPRVVGDLSEAGSRAEMEMKVLLWAVQ
RLIGGLSTIGAERDIASRLHVVLPGSPNRGMFGGDGAYGEAKSALDAVVSRWHAESSW
AARVSLAHALIGWTRGTGLMGHNDAIVAAVEEAGVTTYSTDEMAALLLDLCDAESKVA
AARSPIKADLTGGLAEANLDMAELAAKAREQMSAAAAVDEDAEAPGAIAALPSPPRGF
TPAPPPQWDDLDVDPADLVVIVGGAEIGPYGSSRTRFEMEVENELSAAGVLELAWTTG
LIRWEDDPQPGWYDTESGEMVDESELVQRYHDAVVQRVGIREFVDDGAIDPDHASPLL
VSVFLEKDFAFVVSSEADARAFVEFDPEHTVIRPVPDSTDWQVIRKAGTEIRVPRKTK
LSRVVGGQIPTGFDPTVWGISADMAGSIDRLAVWNMVATVDAFLSSGFSPAEVMRYVH
PSLVANTQGTGMGGGTSMQTMYHGNLLGRNKPNDIFQEVLPNIIAAHVVQSYVGSYGA
MIHPVAACATAAVSVEEGVDKIRLGKAQLVVAGGLDDLTLEGIIGFGDMAATADTSMM

RGRGIHDSKFSRPNDRRRLGFVEAQGGGTILLARGDLALRMGLPVLAVVAFAQSFGDG
VHTSIPAPGLGALGAGRGGKDSPLARALAKLGVAADDVAVISKHDTSTLANDPNETEL
HERLADALGRSEGAPLFVVSQKSLTGHAKGGAAVFQMMGLCQILRDGVIPPNRSLDCV
DDELAGSAHFVWVRDTLRLGGKFPLKAGMLTSLGFGHVSGLVALVHPQAFIASLDPAQ
RADYQRRADARLLAGQRRLASAIAGGAPMYQRPGDRRFDHHAPERPQEASMLLNPAAR
LGDGEAYIG"

CDS complement(2821108..2821830)
/codon_start=1
/note="gene_2581"
/transl_table=11
/translation="MSVSRRDVLKFAAATPGVLGLGVVASSLRAAPASAGSLGTLLDY
AAGVIPASQIRAAGAVGAIRYVSDRRPGGAWMLGKPIQLSEARDLSGNGLKIVSCYQY
GKGSTADWLGGASAGVQHARRGSELHAAAGGPTSAPIYASIDDNPSYEQYKNQIVPYL
RSWESVIGHQRTGVYANSKTIDWAVNDGLGSYFWQHNWGSPKGYTHPAAHLHQVEIDK
RKVGGVGVDVNQILKPQFGQWA"

CDS 2822347..2822574
/codon_start=1
/note="gene_2582"
/transl_table=11
/translation="MTVKRTTIELDEDLVRAAQAVTGETLRATVERALQQLVAAAAEQ
AAARRRRIVDHLAHAGTHVDADVLLSEQAWR"

CDS 2822571..2822972
/codon_start=1
/note="gene_2583"
/transl_table=11
/translation="MTTWILDKSAHVRLVAGATPPAGIDLTDLAICDIGELEWLYSAR
SATDYDSQQTSLRAYQILRAPSDIFDRVRHLQRDLAHHRGMWHRTPLPDLFIAETALH
HRAGVLHHDRDYKRIAVVRPGFQACELSRGR"

CDS complement(2823007..2823927)
/codon_start=1
/note="gene_2584"
/transl_table=11
/translation="MTIPDAQTLMRPILAYLADGQAKSAKDVIAAMSDEFGLSDDERA
QMLPSGRQRTMYDRVHWSLTHMSQAGLLDRPTRGHVQVTDTGRQVLKAHLERVDMAVL
REFPSYIAFRERTKAKQPVDATAKRPSGDDVQVSPEDLIDAALAENRAAVEGEILKKA
LTLSPTGFEDLVIRLLEAMGYGRAGAVERTSASGDAGIDGIISQDPLGLDRIYVQAKR
YAVDQTIGRPKIHEFAGALLGKQGDRGVYITTSSFSRGAREEAERINARIELIDGARL
AELLVRYRVGVQAVQTVELLRLDEDFFDGL"

CDS complement(2824268..2824492)
/codon_start=1
/note="gene_2585"
/transl_table=11
/translation="VTVPKAVRDALGLQSGDRVVFRVEGNRPVLARTPDFLAPAGTIR
WPSFDRSVDRLKTIEGVEPPPVGSRRSRGP"

CDS 2824572..2825522
/codon_start=1
/note="gene_2586"
/transl_table=11
/translation="VELLVAANPAEDSRLPYLIRLPVGAGLVFATSDVWPRTKALYCH
RLDIADWPADPVVVDRVELRSCSRRGAAIDVVAARARENRSQLVHTMARGRQVVFWQS
PKTRKQSRPGVRTPTARAAGIPELHIVVDAHERYPYTFADKPAKTTREALPCGDYGLK

VAGQLVAAVERKALADLTSGVLNGNLKYQLTELAALPRAAVVVEDRYSEIFAHSFARP
TAIADGLAELQIGFPNVPIVFCQTRKLAQEYTYRYLAAALTWFVDDADATTVFEPAAA
EPEPSSAELRAWAKSVGLPVSDRGRLRPQILQAWRAAHPR"

```
CDS             complement(2825523..2825942)
                /codon_start=1
                /note="gene_2587"
                /transl_table=11
                /translation="VTALLDVNVLIALGWPNHVHHAAAQRWFTQFSSNGWATTPITEA
                GYVRISSNRSVMQVSTTPAIAIAQLAAMTSLAGHTFWPDDVPLIVGSAGDRDAVSNHR
                RVTDCHLIALAARYGGRLVTFDAALADSASAGLVEVL"
CDS             complement(2825939..2826163)
                /codon_start=1
                /note="gene_2588"
                /transl_table=11
                /translation="MRTTLQIDDDVLEDARSIARSEGKSVGAVISELARRSLRPVGIV
                EVDGFPVFDVPPDAPTVTSEDVVRALEDDV"
CDS             complement(2826194..2829037)
                /codon_start=1
                /note="gene_2589"
                /transl_table=11
                /translation="MNPNSVRPRRLHVSALAAVANPSYTRLDTWNLLDDACRHLAEVD
                LAGLDTTHDVARAKRLMDRIGAYERYWLYPGAQNLATFRAHLDSHSTVRLTEEVSLAV
                RLLSEYGDRTALFDTSASLAEQELVAQAKQQQFYTVLLADDSPATAPDSLAECLRQLR
                NPADEVQFELLVVASIEDAITAVALNGEIQAAIIRHDLPLRSRDRVPLMTTLLGTDGD
                EAVANETHDWVECAEWIRELRPHIDLYLLTDESIAAETQDEPDVYDRTFYRLNDVTDL
                HSTVLAGLRNRYATPFFDALRAYAAAPVGQFHALPVARGASIFNSKSLHDMGEFYGRN
                IFMAETSTTSGGLDSLLDPHGNIKTAMDKAAVTWNANQTYFVTNGTSTANKIVVQALT
                RPGDIVLIDRNCHKSHHYGLVLAGAYPMYLDAYPLPQYAIYGAVPLRTIKQALLDLEA
                AGQLHRVRMLLLTNCTFDGVVYNPRRVMEEVLAIKPDICFLWDEAWYAFATAVPWARQ
                RTAMIAAERLEQMLSTAEYAEEYRNWCASMDGVDRSEWVDHRLLPDPNRARVRVYATH
                STHKSLSALRQASMIHVRDQDFKALTRDAFGEAFLTHTSTSPNQQLLASLDLARRQVD
                IEGFELVRHVYNMALVFRHRVRKDRLISKWFRILDESDLVPDAFRSSTVSSYRQVRQG
                ALADWNEAWRSDQFVLDPTRLTLFIGATGMNGYDFREKILMERFGIQINKTSINSVLL
                IFTIGVTWSSVHYLLDVLRRVAIDLDRSQKAASGADLALHRRHVEEITQDLPHLPDFS
                EFDLAFRPDDASSFGDMRSAFYAGYEEADREYVQIGLAGRRLAEGKTLVSTTFVVPYP
                PGFPVLVPGQLVSKEIIYFLAQLDVKEIHGYNPDLGLSVFTQAALARMEAARNAVATV
                GAALPAFEVPRDASALNGTVNGDSVLQGVAEDA"
CDS             complement(2829109..2829510)
                /codon_start=1
                /note="gene_2590"
                /transl_table=11
                /translation="MTRLELRVVVAAVLAATVVLGAVVCAAYGLTIVASAMSIYALGV
                GAWLYHAIERLILARRISTVRTAAKPLQPLLPVMAAIMGLTQAVVRSLGDVTDLPARR
                RELSQLPVLRWVDNSGNRANRRIADSDDLAD"
CDS             complement(2829510..2829929)
                /codon_start=1
                /gene="nusB"
                /note="gene_2591"
                /transl_table=11
                /translation="VDLLFEAEVRGISAAEVVDTRAALAEAKPDIARLHPYTAAVARG
                VSEHAAHIDDLITAHLRGWTLDRLPAVDRAILRVSVWELLHAADVPEPVVVDEAVQLA
```

```
                     KELSTDDSPGFVNGVLGQVMLVTPQLRAAAQAVRGGA"
     CDS             complement(2829983..2830546)
                     /codon_start=1
                     /note="gene_2592"
                     /transl_table=11
                     /translation="VATTADFKNGLVLVIDGQLWTITEFQHVKPGKGPAFVRTKLKNV
                     LSGKVVDKTFNAGVKVDTATVDRRDTTYLYRDGSDFVFMDSQDYEQHPLPEALVGDAA
                     RFLLEGMPVQVAFHNGVPLYIELPVTVELEVTHTEPGLQGDRSSAGTKPATLQTGAQI
                     NVPLFINTGDKLKVDSRDGSYLGRVNA"
     CDS             complement(2830556..2831611)
                     /codon_start=1
                     /note="gene_2593"
                     /transl_table=11
                     /translation="MLISDLINVRYLSGFSGSNGALLVFADERDAVLATDGRYRTQAA
                     SQAPDLEVAIERAVGRYLAGRAGEAGVGKLGFESHVVTVDGLDALAGALEGKNTELVR
                     ASGTVESLREVKDAGELALLRLACEAADAALTDLVARGGLRPGRTERQVSRELEALML
                     DHGADAVSFETIVAAGANSAIPHHRPTDAVLQVGDFVKIDFGALVAGYHSDMTRTFVL
                     GKAADWQLEIYQLVAEAQQAGRQALLPGAELRGVDAAARQLIADAGYGEHFGHGLGHG
                     VGLQIHEAPGIGVTSAGTLLAGSVVTVEPGVYLPGRGGVRIEDTLVVAGGTPKMPETA
                     GQTPELLTRFPKELAIL"
     CDS             2831702..2832400
                     /codon_start=1
                     /note="gene_2594"
                     /transl_table=11
                     /translation="MPMTNWMLRGLAFAAAMVVLRLFQGALINAWQMLSGLISLVLLL
                     LFAIGGVVWGVMDGRADAKASPDPDRRQDLAMTWLLAGLVAGALSGAVAWLISLFYKA
                     IYTGGPINELTTFAAFTALIVFLVGIVGVAVGRWLVDRQLAKAPVRHHGLAAEHERAA
                     DTDVFSAVRADDSPTGEMQVAQPEAQTAAVATVEREAPTEVIRTTESDTPTEVIRTDT
                     EADQTKPGDEPKKD"
     CDS             complement(2832404..2832847)
                     /codon_start=1
                     /note="gene_2595"
                     /transl_table=11
                     /translation="MSELIVNVINGPNLGRLGRREPAVYGGTTHDELVALIEREAAEL
                     GLKAVVRQSDSEAQLLDWIHQAADAAEPVILNAGGLTHTSVALRDACAELSAPLIEVH
                     ISNVHAREEFRRHSYLSPIATGVIVGLGIQGYLLALRYLAEHVGT"
     CDS             complement(2832844..2833932)
                     /codon_start=1
                     /gene="aroB"
                     /note="gene_2596"
                     /transl_table=11
                     /translation="MTDIGAPVTVQVAVDPPYPVVIGTGLLDELEDLLADRHKVAVVH
                     QPGLAETAEEIRKRLAGKGVDAHRIEIPDAEAGKDLPVVGFIWEVLGRIGIGRKDALV
                     SLGGGAATDVAGFAAATWLRGVSIVHLPTTLLGMVDAAVGGKTGINTDAGKNLVGAFH
                     QPLAVLVDLATLQTLPRDEMICGMAEVVKAGFIADPVILDLIEADPQAALDPAGDVLP
                     ELIRRAITVKAEVVAADEKESELREILNYGHTLGHAIERRERYRWRHGAAVSVGLVFA
                     AELARLAGRLDDATAQRHRTILSSLGLPVSYDPDALPQLLEIMAGDKKTRAGVLRFVV
                     LDGLAKPGRMVGPDPGLLVTAYAGVCAP"
     CDS             complement(2833929..2834459)
                     /codon_start=1
                     /gene="aroK"
```

/note="gene_2597"
/transl_table=11
/translation="MAPKAVLVGLPGSGKSTIGRRLAKALGVGLLDTDVAIEQRTGRS
IADIFATDGEQEFRRIEEDVVRAALADHDGVLSLGGGAVTSPGVRAALAGHTVVYLEI
SAAEGVRRTGGNTVRPLLAGPDRAEKYRALMAKRAPLYRRVATMRVDTNRRNPGAVVR
HILSRLQVPSPSEAAT"
CDS complement(2834463..2835668)
/codon_start=1
/note="gene_2598"
/transl_table=11
/translation="VLRWITAGESHGRALVAVVEGMVAGVHVTSADIADQLARRRLGY
GRGARMTFERDAVTVLSGIRHGSTLGGPIAIEIGNTEWPKWETVMAADPVDPAELADV
ARNAPLTRPRPGHADYAGMLKYGFDDARPVLERASARETAARVAAGTVARAFLRQALG
VEVLSHVISIGASAPYEGPPPRAEDLPAIDASPVRAYDKAAEADMIAQIEAAKKDGDT
LGGVVEAVALGLPVGLGSFTSGDHRLDSQLAAAVMGIQAIKGVEIGDGFQTARRRGSR
AHDEMYPGPDGVVRSTNRAGGLEGGMTNGQPLRVRAAMKPISTVPRALATVDLATGDE
AVAIHQRSDVCAVPAAGVVVETMVALVLARAALEKFGGDSLAETQRNIAAYQRSVADR
EAPAARVSG"
CDS 2835776..2836090
/codon_start=1
/note="gene_2599"
/transl_table=11
/translation="VEVAGGTRARLRVTADGLQALAGRCATLAGELSAAVAPSGAVLS
WQANAVAVNAAHARAGAAAAAVSARMRATAAALGQAARRYAGQDTAAAAALGAVRPWG
TH"
CDS 2836386..2837597
/codon_start=1
/note="gene_2600"
/transl_table=11
/translation="VLDAVSDARRDGFAVGEDYTVTDRSTGGSRQQRAARLGQAQGHA
DFIRHRVGALLATDRDIATRVSAATQGLDELAFEDVPGVDTPAEDGVQAVDFRQAPPP
GAPGGMSSGDIDAIDAANRALLQDMLAEYSRLPDGQVKTDRLADIAAIQEALRVPDSH
LIYVARPDDPADMIPAVTAVGDPFTADHVSVTVPGVSGTTRQTIATMTQEARGLREEA
RVIAHSVGESENVATIAWVGYQPPPVLASWNTVDDDLAQAGAPKLEAFLRDLQAGSHN
PGHTTALFGHSYGSLLSGIALKDGASSLVDNAVLYGSPGFDATSPAKLGMNDHNFFVM
TTPDDPIRYPARLAPLHGWGSDGADTIGTVGRQGTPARVGIRPQRDHRRIPGPLPLHP
SADRRGIHSAG"
CDS 2837826..2838383
/codon_start=1
/note="gene_2601"
/transl_table=11
/translation="MDHNPDTSRRLTGEQKIQLIDSMRNKGSYEAARERLTATARIIA
DRVSAAIPGQTWKFDDDPNIQQSDRNGALCDKLTADIARRPIANSVMFGATFSAEDFK
IAANIVREEAAKYGATTESSLFNESAKRDYDVQGNGYEFRLLQIKFATLNITGDCFLL
QKVLDLPAGQLPPEPPIWPTTSTPH"
CDS 2838482..2839039
/codon_start=1
/note="gene_2602"
/transl_table=11
/translation="MDQSPDTSRRLTDEQKIQLIDSMRNKGSYEAARERLTATARIIA
DRVSAAIPGQTWKFTEDPAGRKADREGLSCKELTGDIARRPIADAVIFGTAFSAEDFK

VVTNIVREEAAKYGATTESSLFNESAKRDYDVQGNGYEFRLLQIKFATLNITGDCFLL
QKVLDLPAGQLPPEPPIWPTTSTPH"
CDS 2839138..2839698
/codon_start=1
/note="gene_2603"
/transl_table=11
/translation="MGQNPDKSPHLTGEQKIQLIDSMRHKGSYEAARERLTATAQIIA
DRVSAAIPGQTWKFNDDSYGQDFYRNGSLCKELSADIARRPMAKPVDFGSTFSAEDFK
IAANIVREEAAKYGVTTESSLFNESAKRDYDVQGNGYEFNLGQIKFATLNITGDCFLL
QKVLDLPAGQLPPEPPIWPTTSTPTP"
CDS 2839668..2839973
/codon_start=1
/note="gene_2604"
/transl_table=11
/translation="LADDLDANPVSTTIVAGVIQGHLPVILPTRRRARDLGHTTALFR
AQTLQCIYLSIEYLYVCSMSRRTTIDIDDILLARAQAALGTTGLKDRVDAALRAAVR"
CDS 2840063..2840479
/codon_start=1
/note="gene_2605"
/transl_table=11
/translation="VMVFCVDTSAWHHAARPEVARRWLAALSADQIGICDHVRLEILY
SANSATDYDALADELDGLARIPVGAETFTRACQVQRELAHVAGLHHRSVKIADLVIAA
AAELSGTIVWHYDENYDRVAAITGQPTEWIVPRGTL"
CDS 2840503..2840775
/codon_start=1
/note="gene_2606"
/transl_table=11
/translation="MYGDVMRTQVTLGKEELELLDRAAKASGASRSELIRRAIHRAYG
TGSKQERLAALDHSRGSWRGRDFTGTEYVDAIRGDLNERLARLGLA"
CDS 2840772..2841149
/codon_start=1
/note="gene_2607"
/transl_table=11
/translation="VKLIDTTIAVDHLRGEPAAAVLLAELINNGEEIAASELVRFELL
AGVRESELAALEAFFSAVVWTLVTEDIARIGGRLARRYRSSHRGIDDVDYLIAATAIV
VDADLLTTNVRHFPMFPDLQPPY"
CDS complement(2841165..2841539)
/codon_start=1
/note="gene_2608"
/transl_table=11
/translation="VLPENLEQRVTALESQVRELADRVRASEQDAAAARVLAGAADRD
VTEFVGEFRDFRRATIGSFNALREDFTALREEMTERFSHVEERFSRVDDGFTEMRGKL
DGAAAGQQRIVELIEQLIADQG"
CDS complement(2841639..2842034)
/codon_start=1
/note="gene_2609"
/transl_table=11
/translation="MIFVDTSFWAALGNAGDARHGTAKRLWASKPPVVMTSNHVLGET
WTLLNRRCGHRAAVAAAAIRLSTVVRVEHVTADLEEQAWEWLVRHDEREYSFVDATSF
AVMRKKGIQNAYAFDGDFSAAGFVEVRPE"
CDS complement(2842031..2842276)

/codon_start=1
/note="gene_2610"
/transl_table=11
/translation="MLVAYICHVKRLQIYIDEDVDRALAVEARRRRTSKAALIREYVA
EHLRQPGPDPVDAFVGSFVGEADLSASVDDVVYGKHE"
CDS complement(2842687..2843106)
/codon_start=1
/note="gene_2611"
/transl_table=11
/translation="MLAAAVLAWMGVLCVCDVRQRRLPNWLTLPGAGVILLFAGLAGR
GVPALAGAAALAGVYLLVHLALPAAMGAGDVKLAIGLGGLTGCFGVEVWFLAALAAPL
LTAVCGVMVTPWGVRTLPHGPSMCVASLGAVGLALLG"
CDS complement(2843118..2843900)
/codon_start=1
/gene="aroE"
/note="gene_2612"
/transl_table=11
/translation="VLGSPIAHSRSPQLHLAAYRALGLHDWTYERIECGAAELPVVVG
GFGPEWVGVSVTMPGKFAALRFADERTARADLVGSANTLVRTPHGWRADNTDIDGVAG
ALGAAAGHALVLGSGGTAPAAVVGLAELGVTDITVVARNSDKAARLVDLGTRVGVATR
FCAFDSGGLADAVAAAEVLVSTIPAEVAAGYAGTLAAIPVLLDAIYDPWPTPLAAAVG
SAGGRVISGLQMLLHQAFAQVEQFTGLPAPREAMTCALAALD"
CDS complement(2843923..2845176)
/codon_start=1
/note="gene_2613"
/transl_table=11
/translation="MPDGGHRHRAQPVSVRPNRHRRTRVSRAQRRHAQQIRRRRRVAG
GFALSLLVVVVVVAVVVGAKLWQTMLGFGNDYTGPGKRDIVIQIRAGDSTTAVGETLL
KHGVVATVRAFVDAAHGNTAISSIQPGFYRMRTEISAASAVARLTDPHNRVGKLVIPE
GRQLDDTTDMKTNVVNPGIFALISRATCVDLDGTQRCVSVADLRAAASRSTPTMLSVP
RWAVGPVMELGTDHRRIEGLIAPGTFNIDPSASAETILATLISAGAVEYMKSGLVDTA
KSLGLSPYDILVVASLVQQEANTQDFPKVARVIYNRLHEHRTLEFDSTVNYPLDRREV
ATSDTDRAQRTPWNTYMAQGLPATAICSPGVDALRAAEHPVPGDWLYFVTIDSQGTTL
FTRDYQQHLANIELAKHNGVLDSAR"
CDS complement(2845169..2845597)
/codon_start=1
/note="gene_2614"
/transl_table=11
/translation="VGAARIGVACSDPDAILATPVETVRRDRSGKHLRRLAALAAELE
AVEVIVGLPRTLADRIGRSAQDAIELAEALARRVSPTPVRLADERLTTVSAQRSLRQA
GVRASEQRAVIDQAAAVAILQSWLDERLAAMAGTQEGSDA"
CDS complement(2845682..2848396)
/codon_start=1
/gene="alaS"
/note="gene_2615"
/transl_table=11
/translation="VQTHEIRKRFLDHFVKAGHTEVPSASVILDDPNLLFVNAGMVQF
VPFFLGQRTPPYPTATSIQKCIRTPDIDEVGITTRHNTFFQMAGNFSFGDYFKRGAIE
LAWALLTNSLAAGGYGLDPERIWTTVYFDDDEAVRLWQEVAGLPAERIQRRGMADNYW
SMGIPGPCGPSSEIYYDRGPEFGPAGGPIVSEDRYLEVWNLVFMQNERGEGTTKEDYQ
ILGPLPRNNIDTGMGVERIALVLQDVHNVYETDLLRPVIDTVARVAARAYDVGNHEDD

VRYRIIADHSRTAAILIGDGVSPGNDGRGYVLRRLLRRVIRSAKLLGIDAAIVGDLMA
TVRNAMGPSYPELVADFERISRIAVAEETAFNRTLASGSRLFEEVASSTKKSGATVLS
GSDAFTLHDTYGFPIELTLEMAAETGLQVDEIGFRELMAEQRRRAKADAAARKHAHAD
LSAYRELVDAGATEFTGFDELRSQARILGIFVDGKRVPVVAHGVAGGAGEGQRVELVL
DRTPLYAESGGQIADEGTISGTGSSEAARAAVTDVQKIAKTLWVHRVNVESGEFVEGD
TVIAAVDPGWRRGATQGHSGTHMVHAALRQVLGPNAVQAGSLNRPGYLRFDFNWQGPL
TDDQRTQVEEVTNEAVQADFEVRTFTEQLDKAKAMGAIALFGESYPDEVRVVEMGGPF
SLELCGGTHVSNTAQIGPVTILGESSIGSGVRRVEAYVGLDSFRHLAKERALMAGLAS
SLKVPSEEVPARVANLVERLRAAEKELERVRMASARAAATNAAAGAQRIGNVRLVAQR
MSGGMTAADLRSLIGDIRGKLGSEPAVVALIAEGESQTVPYAVAANPAAQDLGIRAND
LVKQLAVAVEGRGGGKADLAQGSGKNPTGIDAALDAVRSEIAVIARVG"

CDS complement(2848487..2848888)
/codon_start=1
/note="gene_2616"
/transl_table=11
/translation="VDTDVLDVDTARRRIVDLTDAVRAFCTAHDDGLCNVFVPHATAG
VAIIETGAGSDEDLVDTLVRLLPRDDRYRHAHGSYGHGADHLLPAFVAPSVTVPVSGG
QPLLGTWQSIVLVDLNQDNPRRSVRLSFVEG"

CDS 2848983..2849657
/codon_start=1
/note="gene_2617"
/transl_table=11
/translation="LTGGATGALPRTMKEGWIVYARSTTIQAQSECIDTGIAHVRDVV
MPALQGMDGCIGVSLLVDRQSGRCIATSAWETAEAMHASREQVTPIRDRCAEMFGGTP
AVEEWEIAAMHRDHRSAEGACVRATWVKVPADQVDQGIEYYKSSVLPQIEGLDGFCSA
SLLVDRTSGRAVSSATFDSFDAMERNRDQSNALKATSLREAGGEELDECEFELALAHL
RVPELV"

CDS 2849829..2850452
/codon_start=1
/note="gene_2618"
/transl_table=11
/translation="VVYARSTTIEAQPLSVDIGIAHVRDVVMPALQEIDGCVGVSLLVD
RQSGRCIATSAWETLEAMRASVERVAPIRDRAALMFAGSARVEEWDIALLHRDHPSHE
GACVRATWLKVVPDQLGRSLEFYRTSVLPELESLDGFCSASLMVDHPACRRAVSCSTF
DSMDAMARNRDRASELRSRRVRELGAEVLDVAEFELAIAHLRVPELV"

CDS complement(2850482..2851840)
/codon_start=1
/note="gene_2619"
/transl_table=11
/translation="MPEAVSDGLFDVPGVPMTSGHDLGASAGAPLAVRMRPASLDEVV
GQDHLLAPGSPLRRLVEGSGVASVILYGPPGSGKTTLAALISQATGRRFEALSALSAG
VKEVRAVIENSRKALLHGEQTVLFIDEVHRFSKTQQDALLSAVEHRVVLLVAATTENP
SFSVVAPLLSRSLILQLRPLTAEDTRAVVQRAIDDPRGLGRAVAVAPEAVDLLVQLAA
GDARRALTALEVAAEAAQAAGELVSVQTIERSVDKAAVRYDRDGDQHYDVVSAFIKSV
RGSDVDAALHYLARMLVAGEDPRFIARRLMILASEDIGMADPSALQVAVAAAQTVALI
GMPEAQLTLAHATIHLATAPKSNAVTTALAAAMNDIKAGKAGLVPAHLRDGHYSGAAA
LGNAQGYKYSHDDPDGVVAQQYPPDELVDVDYYRPTGRGGEREIAGRLDRLRAIIRKK
RG"

CDS 2851986..2852963
/codon_start=1
/note="gene_2620"

```
                     /transl_table=11
                     /translation="MSQPPEHPGNPADPQGGNQGAGSYPPPGYGAPPPPPGYGPPPGT
                     YLPPGYNAPPPPPGYGPPPGPPPPGYPTHLQSSGFSVGDAISWSWNRFTQNAVTLVVP
                     VLAYAVALAAVIGATAGLVVALSDRATTAYTNTSGVSSESVDITMTPAAGIVMFLGYI
                     ALFALVLYMHAGILTGCLDIADGKPVTIATFFRPRNLGLVLVTGLLIVALTFIGGLLC
                     VIPGLIFGFVAQFAVAFAVDRSTSPIDSVKASIETVGSNIGGSVLSWLAQLTAVLVGE
                     LLCFVGMLIGIPVAALIHVYTYRKLSGGQVVEAVRPAPPVGWPPGPQLA"
     CDS             complement(2852876..2853199)
                     /codon_start=1
                     /gene="TMLG_02289"
                     /note="gene_2621"
                     /transl_table=11
                     /translation="VAAAGSVDCIDHASDQISELSNYVHIGRNLGSIPILEVIYVNLW
                     ESHCPNAFQAPDANSGERVTNPAGQDGQPASAGADYASWGPGGQPTGGAGRTASTTWP
                     PDSFR"
     CDS             2853320..2854057
                     /codon_start=1
                     /note="gene_2622"
                     /transl_table=11
                     /translation="MGIQRAVLLIADIGGYTNYMHWNRKHLAHAQWTVAQLLESVIDA
                     AKGMKLAKLEGDAAFFWAPGGNTSVLVCDRPPQMRQRFRTRREQIKKDHPCDCKSCEQ
                     RDNLSIKFVAHEGEVAEQKVKRNVELAGVDVILVHRMLKNEVPVSEYLFMTDVVAQCL
                     DESVRKLATPLTHDFEGIGETSTHYTDLATSDMPPAVPDHSFFGLLWADVKFEWHALP
                     YLLGFKKACAGFRSLGRGATEEPAEMG"
     CDS             2854200..2855249
                     /codon_start=1
                     /note="gene_2623"
                     /transl_table=11
                     /translation="MLFAALRDVQWRKRRLVIAIVSTGLVFAMTLVLTGLVNGFRVEA
                     ERTVDSMGVDAFVVKAGAAGPFLGSTPFAQIDLPQVARAPGVLAAAPLATAPSTIRQG
                     TSARNVTAFGAPEHGPGMPRVSDGRAPSTPDEVAVSSTLGRNLGDDLQVGARTLRIVG
                     IVPESTALAKIPNIFLTTEGLQQLAYNGQPTISSIGIDGMPRQLPDGYQTVNRADAVS
                     DLMRPLKVAVDAITVVAVLLWIVAALIVGSVVYLSALERLRDFAVFKAIGVPTRSILA
                     GLALQAVVVALLAAVVGGILSLLLAPLFPMTVVVPLSAFVALPAIATVIGLLASVAGL
                     RRVVAIDPALAFGGP"
     CDS             2855252..2856244
                     /codon_start=1
                     /note="gene_2624"
                     /transl_table=11
                     /translation="MGGLTISDLVVEYSSGGYAVRPIDGLSLDVAPGSLVILLGPSGC
                     GKTTLLSCLGGILRPKSGSIKFDDVDITTLEGAALAKYRRDKVGIVFQAFNLVSSLTA
                     LENVMVPLRAAGVSRAAARKRAEDLLIRVNLGERMKHRPGDMSGGQQQRVAVARAIAL
                     DPQLILADEPTAHLDFIQVEEVLRLIRSLAQGDRVVVVATHDSRMLPLADRVLELMPA
                     QVSPNQPPETVHVKAGEVLFEQSTMGDLIYVVSEGEFEIVRELADGGEELVKTAAPGD
                     YFGEIGVLFHLPRSATVRARSDATAVGYTAQAFRERLGVTRVADLIEHRELASE"
     CDS             2856521..2858272
                     /codon_start=1
                     /note="gene_2625"
                     /transl_table=11
                     /translation="MTTARRRPKRRGTDARTALRNVPILADIDDEQLERLATTVERRH
                     VPANQWLFHAGEPADSIYIVDSGRFVAVAPEGHVFAEMASGDSIGDLGVIAGAARSAG
```

VRALRDGVVWRIAAETFTDMLEATPLLQSAMLRAMARMLRQSRPAKTARRPRVIGVVS
NGDTAAAPMVDAIATSLDSHGRTAVIAPPVETTSAVQEYDELVEAFSETLDRAERSND
WVLVVADRGAGDLWRHYVSAQSDRLVVLVDQRYPPDAVDSLATQRPVHLITCLAEPDP
SWWDRLAPVSHHPANSDGFGALARRIAGRSLGLVMAGGGARGLAHFGVYQELTEAGVV
IDRFGGTSSGAIASAAFALGMDAGDAIAAAREFIAGSDPLGDYTIPISALTRGGRVDR
LVQGFFGNTLIEHLPRGFFSVSADMITGDQIIHRRGSVSGAVRASISIPGLIPPVHNG
EQLLVDGGLLNNLPANVMCADTDGEVICVDLRRTFVPSKGFGLLPPIVTPPGLLRRLL
TGTDNALPPLQETLLRAFDLAASTANLRELPRVAAIIEPDVSKIGVLNFKQIDAALEA
GRMAARAALQAQPDLVR"

CDS 2858364..2859884
/codon_start=1
/note="gene_2626"
/transl_table=11
/translation="MSIKVALEHRTSYTFDRLVRVYPHIVRLRPAPHSRTSIEAYSLR
IEPADHFINWQQDALGNFLARLVFPNPMRQLRITVGLIADLKVINPFDFFIEDWAEIW
PCAGMAYPKALADDLRPYLRPVDEDGDGSGPGELTQAWVRNFTVPDGTRTIDFLVALN
RAINADVGYCVRMEPGVQTPDFTLRTGVGSCRDSAWLLVSILRQFGLAARFVSGYLVQ
LASDIEALDGPSGPAADFTDLHAWSEAYIPGAGWIGLDPTSGLLAGEGHIPLAATPHP
ASAAPISGGTDLCDTVLEFSNTVTRVHEDPRVTLPYTDESWKTICEVGQRVDERLAAA
DVRLTVGGEPTFVSVDNQVAEEWRTAADGPHKRERASDLAARLKAVWAPQGLIHRGQG
RWYPGEPLPRWQIALYWRTDGRPLWTNDALLADPWGAPPADPVDDDAAYRVLAGIADG
LGLPISQVRPAYEDPLSRLAAAVRMPAGDPVESGDDLGCDTNPDTPTGRAALLAPR"

CDS 2859910..2861703
/codon_start=1
/note="gene_2627"
/transl_table=11
/translation="VLPLHRRDDGQGWASANWRLRRGRIVLLEGDSPAGLRLPLDSIS
WRPPRASFDADPVAVRSTLPAEPHTDRAVVEDPETAPTTALVAEVRGGLVHIFLPPTD
ALEHFIDLVARVEAAATTANCPVVIEGYGPPPDPRLTSTTITPDPGVIEVNIAPTASF
AEQRQQLETLYQQARLARLTTEAFDVDGTHGGTGGGNHITLGGVTPADSPLLRRPDLL
VSLLTYWQRHPSLSYLFAGRFVGTTSQAPRVDEGRAEALYELEIAFAEILRLSPSSGG
GRPQPWVTDRALRHLLTDITGNTHRAEFCIDKLYSPDSARGRLGLLELREFEMPPHLH
MAMVQSLLVRSLVAWFWDQPLRAPLIRHGANLHGRYLLPHFLIHDIADVAADLRAHGI
AFETSWLDPFTEFRFPRIGTAVFDGIEIELRGAIEPWHTLGEEATAAGTARYVDSSVE
RIQVRIIGADRHRYVVTCNGYPMPLLATDNPDIHVGGVRFKAWQPPSALHPTITVDGP
LRFELIDIATATSCGGCTYHVAHPGGRAYDEPPVNAVEAEARRARRFEATGFTPGKLD
LSDIREKQARISTDIGAPGILDLRRVRTVQQ"

CDS 2861703..2864357
/codon_start=1
/note="gene_2628"
/transl_table=11
/translation="MAPSASAATNGYDVDRLLAGYRTARAQETLFDLRDGPGAGYDEF
VDDDGNVRPTWTELADAVAERGKAGLDRLRSVVHSLIDHDGITYTAIDAHRDALTGDH
DLEPGPWRLDPLPLVISAADWEVLEAGLVQRSRLLDAILADLYGPRSMLTEGVLPPEM
LFAHPGYVRAANGIQMPGRHQLFMHACDLSRLPDGTFQVNADWTQAPSGSGYAMADRR
VVAHAVPDLYEELAPRPTTPFAQALRLALIDAAPDVAQDPVVVVLSPGIYSETAFDQA
YLATLLGFPLVESADLVVRDGKLWMRSLGTLKRVDVVLRRVDAHYADPLDLRADSRLG
VVGLVEAQHRGTVTVVNTLGSGILENPGLLRFLPQLSERLLDESPLLHTAPVYWGGIA
SERSHLLANVSSLLIKSTVSGETLVGPTLSSAQLADLAVRIEAMPWQWVGQELPQFSS
APTNHAGVLSSAGVGMRLFTVAQRSGYAPMIGGLGYVLAPGPAAYTLKTVAAKDIWVR
PTERAHAEVITVPVLAPPAKTGAGTWAVSSPRVLSDLFWMGRYGERAENMARLLIVTR

ERYHVFRHQQDTDESECVPVLMAALGKITGYDTATGAGSAYDRADMIAVAPSTLWSLT
VDPDRPGSLVQSVEGLALAARAVRDQLSNDTWMVLANVERAVEHKSDPPQSLAEADAV
LASAQAETLAGMLTLSGVAGESMVHDVGWTMMDIGKRIERGLWLTALLQATLSTVRHP
AAEQAIIEATLVACESSVIYRRRTVGKFSVAAVTELMLFDAQNPRSLVYQLERLRADL
KDLPGSSGSSRPERMVDEMNTRLRRSHPEELEEVSADGLRAELAELLAGIHASLRDVA
DVLTATQLALPGGMQPLWGPDQRRVMPA"

CDS complement(2864354..2865379)
/codon_start=1
/note="gene_2629"
/transl_table=11
/translation="MRDFHCPNCGQRLAFENSACLSCGSALGFSLGRMALLVIADDAD
VQLCANLHLAQCNWLVPSDQLGGLCSSCVLTIERPSDTNTAGLAEFARAEGAKRRLIA
ELHELKLPIVGRDQDPDHGLAFRLLSSAHENVTTGHQNGVITLDLAEGDDVHREQLRV
EMDEPYRTLLGHFRHEIGHYYFYRLIASSSDYLSRFNELFGDPDADYSQALDRHYRGG
PPEGWQDSFVSSYATMHASEDWAETFAHYLHIRDALDTAAWCGLAPASATFDRPALGP
SAFNTIIDKWLPLSWSLNMVNRSMGHDDLYPFVLPAAVLEKMRFIHTVVDEVAPDFEP
AHSRRTV"

CDS complement(2865372..2866292)
/codon_start=1
/note="gene_2630"
/transl_table=11
/translation="LPLSGTHRYRVTHRTEYRYSDVVTSSYGRGFLTPRNSLRQRCVA
HRLTIDPAPADRSTSRDGYGNISSYFHVTEPHRTLTITSDSIVDVSPPPPGLYTSGPA
LQPWEAARPAGLPGSLATEFTLDLNPPEITDAVREYAAPSFLPKRPLVEVLRDLASRI
YTDFTYRSGSTTISTGVNEVLLAREGVCQDFARLAIACLRANGLAACYVSGYLATDPP
PGKDRMIGIDATHAWASVWTPQQPGRFEWLGLDPTNDQLVDQRYIVVGRGRDYADVPP
LRGIIYTNSENSVIDVSVDVVPFEGDALHA"

CDS 2866420..2866809
/codon_start=1
/note="gene_2631"
/transl_table=11
/translation="VATWDDVARIVGGLPLTAEQAPHDWRVGRKLLAWERPLRKSDRE
ALTRAGSEPPSGDIVGVRVSDEGVKFALIADEPGVYFTTPHFDGYPAVLVRLAEIEVR
DLEELITEAWLMQAPKQLVQAFLANSG"

CDS complement(2866801..2867868)
/codon_start=1
/note="gene_2632"
/transl_table=11
/translation="MSASLLVRTACGGRAVAQRLRTVLWPITQTSVVAGLAWYLTHDV
FNHPQAFFAPISAVVCMSATNVLRARRAQQMIVGVALGIVLGAGVHALLGSGPIAMGV
VVFIALSVAVLCARGLVAQGLMFINQAAVSAVLVLVFASNGSVVFERLFDALVGGGLA
IVFSILLFPPDPVVMLCSARADVLAAVRDILAELVNTVSDPTSAPPDWPMAAADRLHQ
QLNGLIEVRANAAMVARRAPRRWGVRSTVRDLDQQAVYLALLVSSVLHLARTIAGPGG
DKLPTPVHAVLTDLAAGTGLADADPTAANEHAAAARATASTLQSAACGSNEVVRADIV
QACVTDLQRVIERPGPSGMSA"

CDS complement(2867875..2869710)
/codon_start=1
/gene="aspS"
/note="gene_2633"
/transl_table=11
/translation="VFVLRSHAAGLLREGDAGQQVTLAGWVARRRDHGGVIFIDLRDA

SGIAQVVFRDPQDTEVLAQAHRLRAEFCVSVAGVVEIRPEGNANPEIATGEIEVNATS
LTVLGECAPLPFQLDEPAGEELRLKYRYLDLRRDDPAAAIRLRSRVNAAARAVLARHD
FVEIETPTITRSTPEGARDFLVPARLHPGSFYALPQSPQLFKQLLMVAGMERYYQIAR
CYRDEDFRADRQPEFTQLDMEMSFVDAEDIIAISEEVLTELWALIGYRIPTPIPRIGY
AEAMRRFGTDKPDLRFGLELVECTDFFSDTTFRVFQAPYVGAVVMPGGASQPRRTLDG
WQDWAKQRGHRGLAYVLVAEDGTLGGPVAKNLTEAERTGLADHVGAKPGDCIFFSAGP
VKSSRALLGAARVEIANRLGLIDPDAWAFVWVVDPPLFEPADEATAAGEVAVGSGAWT
AVHHAFTAPKPEWEDRIESDTGSVLADAYDIVCNGHEIGGGSVRIHRRDIQERVFAVM
GLDKAEVEEKFGFLLEAFMFGAPPHGGIAFGWDRTTALLAGMDSIREVIAFPKTGGGV
DPLTDAPAPITAQQRKESGIDAQPKRVQRHDQIFAVTTSHPSVNK"

CDS 2869863..2870690
/codon_start=1
/note="gene_2634"
/transl_table=11
/translation="LHKAGYSPLLCGHTPRAGIELRRDGADPIVVPGPVHTSPREVAG
PVDVLILAVKATQNDAARPWLTRLCDERTVVAVLQNGVEQVEQVQPHCPSSAVVPAIV
WCSAETQPQGWVRLRGEAALVVPTGPAAEQFAGLLRGAGATVDCDPDFTTAAWRKLLV
NALAGFMVLSGRRSAMFRRDDVAALSRRYVAECLAVARAEGARLDDDVVDEVVRLVRS
APQDMGTSMLADRAAHRPLEWDLRNGVIVRKARAHGLATPISDVLVPLLAAASDGPG"

CDS 2870713..2871216
/codon_start=1
/note="gene_2635"
/transl_table=11
/translation="MYPCERVGLSFTETAPYLFRNTVDLAITPEQLFEVLADPQAWPR
WATVITKVTWTSPEPFGAGTTRIVEMRGGIVGDEEFISWEPFTRMAFRFNECSTRAVG
AFAEDYRVQAIPGGCRLTWTMAQKLAGPARPALFVFRPLLNLALRRFLRNLRRYTDAR
FAAAQQS"

CDS 2871246..2872127
/codon_start=1
/note="gene_2636"
/transl_table=11
/translation="MTFNEGVQIDTSTTSTSGSGGGRRLAIGGGLGGLLVVVVAMLLG
VDPGGVLSQQPLDTRDHVAPGFDLSQCRTGADANRFVQCRVVATGNSVDAVWKPLLPG
YTRPHMRLFSGQVGTGCGPASSEVGPFYCPVDKTAYFDTDFFQVLVTQFGSSGGPFAE
EYVVAHEYGHHVQNLLGVLGRAQQGAQGAAGSGVRTELQADCYAGVWAYYASTVKQES
TGVPYLEPLSDKDIQDALAAAAAVGDDRIQQQTTGRTNPETWTHGSAAQRQKWFTVGY
QTGDPNICDTFSAADLG"

CDS complement(2872133..2872597)
/codon_start=1
/note="gene_2637"
/transl_table=11
/translation="VPAGVGNASGSVLDMTSVRTVPSAVALVTFAGAALSGVIPAIAR
ADPVGHQVTYTVTTTSDLMANIRYMSADPPSMAAFNADSSKYMITLHTPIAGGQPLVY
TATLANPSQWAIVTASGGLRVNPEFHCEIVVDGQVVVSQDGGSGVQCSTRPW"

CDS 2873017..2874414
/codon_start=1
/note="gene_2638"
/transl_table=11
/translation="VGGLHLQFGRNASTEMVVSWHTTDTVGNPRVMLGTPTSGFGSVV
VAETRSYRDAKSNTEVRVNHAHLTNLTPDTDYVYAAVHDGTTPELGTARTAPSGRKPL
RFTSFGDQSTPALGRLADGRYVSDNIGSPFAGDITIAIERIAPLFNLINGDLCYANLA

QDRIRTWSDWFDNNTRSARYRPWMPAAGNHENEVGNGPIGYDAYQTYFAVPDSGSSPQ
LRGLWYSFTAGSVRVISLHNDDVCYQDGGNSYVRGYSGGEQRRWLQAELANARRDSEI
DWVVVCMHQTAISTADDNNGADLGIRQEWLPLFDQYQVDLVVCGHEHHYERSHPLRGA
LGTDTRTPIPVDTRSDLIDSTRGTVHLVIGGGGTSKPTNALLFPQPRCQVITGVGDFD
PAIRRKPSIFVLEDAPWSAFRDRDNPYGFVAFDVDPGQPGGTTSIKATYYAVTGPFGG
LTVIDQFTLTKPRGG"

CDS complement(2874416..2875438)
/codon_start=1
/note="gene_2639"
/transl_table=11
/translation="MRWARQAVAVNGMPVDDGALPGLQRIGLVRSVRAPQFDGITFHE
VLCKSALNKVPNAAALPFRYTVNGYRGCSHACRYCFARPTHEYLDFNPGTDFDTQVVV
KTNVAAVLRHELRRPSWRRETVALGTNTDPYQRAEGRYALMPGIIGALAASGTPLSIL
TKGTLLRRDLPLIAEAAQQVPVSVAVSLAVGDPELHRDVESGTPTPQARLALITAIRA
AGLDCHVMVAPVLPQLTDSGEHLDQLLGQIAAAGATGVTVFGLHLRGSTRGWFMCWLA
RAHPELVSRYRELYRRGPYLPPSYREMLRERVAPLIAKYRLAGDHRPAPPETEAALVP
VQATLF"

CDS 2875546..2876448
/codon_start=1
/note="gene_2640"
/transl_table=11
/translation="MTAFGVEPYGQPKYLEIAGKRMAYIDEGKGDAIVFQHGNPTSSY
LWRNIMPHLEGLGRLVACDLIGMGASDKLSPSGPDRYSYGEQRDFLFALWDALDLGDH
VVLVLHDWGSALGFDWANQHRDRVQGIAFMEAIVTPMTWADWPPAVRGVFQGFRSPQG
EPMALEHNIFVERVLPGAILRQLSDEEMNHYRRPFVNGGEDRRPTLSWPRNLPIDGEP
AEVVALVNEYRSWLEETDMPKLFINAEPGAIITGRIRDYVRSWPNQTEITVPGVHFVQ
EDSPEEIGAAIAQFVRQLRSAAGV"

CDS complement(2876728..2877999)
/codon_start=1
/gene="hisS"
/note="gene_2641"
/transl_table=11
/translation="VTEFSSFSAPKGVPDYVPPDSAQFVAVRDGLLAAARQAGYSHIE
LPIFEDTALFARGVGESTDVVSKEMYTFADRGDRSVTLRPEGTAGVVRAVIEHGLDRG
ALPVKLCYAGPFFRYERPQAGRYRQLQQVGVEAIGVDDPALDAEVIAIADAGFRSLGL
DGFRLEITSLGDESCRPQYRELLQEFLFGLDLDEDTRRRAGINPLRVLDDKRPELRAM
TASAPVLLDHLSDVAKQHFDTVLAHLDALGVPYVINPRMVRGLDYYTKTAFEFVHDGL
GAQSGIGGGGRYDGLMHQLGGQDLSGIGFGLGVDRTVLALRAEGKTAGDSARCDVFGV
PLGEAAKLRLAVLAGRLRAAGVRVDLAYGDRGLKGAMRAAARSGARVALVAGDRDIEA
GTVAVKDLTTGEQVSVSMDSVVAEVISRLAG"

CDS complement(2877996..2878670)
/codon_start=1
/note="gene_2642"
/transl_table=11
/translation="VLITGFPAGLLACNCYVLAERPGTDAVIVDPGQGAMGTLRRILD
KNRLTPAAVLLTHGHIDHIWSAQKVSDTFGCPTYVHPADRFMLTDPIYGLGPRIAQLV
AGAFFREPKQVVELDRDGDKIDLGGISVNIDHTPGHTRGSVVFRVLQATNNDKDIVFT
GDTLFERAIGRTDLAGGSGRDLLRSIVDKLLVLDDSTVVLPGHGNSTTIGAERRFNPF
LEGLSR"

CDS 2878721..2879647
/codon_start=1

/note="gene_2643"
/transl_table=11
/translation="MGHLTPVAAPRLACAFVPTNAQRRATAKRKLERQLERRAKQAKR
RRILTIVGGSLAAVAVIVAVVFTVVVNKDDHQSTTSATPTDSASTSPPQAATAPPLPP
FKPSANLGANCQYPPSPDKAVKPVKLPRTGKVPTDPAQVSVSMVTNQGNIGLMLANNE
SPCTVNSFVSLAQQGFFKGTTCHRLTTSPMLAVLQCGDPKGDGTGGPGYQFANEYPTD
QYSANDPKLNEPVIYPRGTLAMANAGPNTNSSQFFMVYRDSKLPPQYTVFGTIQADGL
TTLDKIAKAGVAGGGEDGKPATEVTITSVLLD"

CDS complement(2879733..2882105)
/codon_start=1
/note="gene_2644"
/transl_table=11
/translation="VAEDQLTAQAVAPPTEASAALEPALETPESPVETLKTSISASRR
VRARLARRMTAQRSTTNPVLEPLVAVHREIYPKADLSILQRAYEVADQRHASQLRQSG
DPYITHPLAVANILAELGMDTTTLVAALLHDTVEDTGYTLEALTEEFGEEVGHLVDGV
TKLDRVVLGSAAEGETIRKMITAMARDPRVLVIKVADRLHNMRTMRFLPPEKQARKAR
ETLEVIAPLAHRLGMASVKWELEDLSFAILHPKKYEEIVRLVAGRAPSRDTYLAKVRA
EIVNTLTASKIKATVEGRPKHYWSIYQKMIVKGRDFDDIHDLVGVRILCDEIRDCYAA
VGVVHSLWQPMAGRFKDYIAQPRYGVYQSLHTTVVGPEGKPLEVQIRTRDMHRTAEYG
IAAHWRYKEAKGRNGVLHPHAAAEIDDMAWMRQLLDWQREAADPGEFLESLRYDLAVQ
EIFVFTPKGDVITLPTGSTPVDFAYAVHTEVGHRCIGARVNGRLVALERKLENGEVVE
VFTSKAPNAGPSRDWQQFVVSPRAKTKIRQWFAKERREEALETGKDAMAREVRRGGLP
LQRLVNGESMAAVARELHYADVSALYTAIGEGHVSAKHVVQRLLAELGGIDQAEEELA
ERSTPATMPRRPRSTDDVGVSVPGAPGVLTKLAKCCTPVPGDVIMGFVTRGGGVSVHR
TDCTNAASLQQQAERIIEVLWAPSPSSVFLVAIQVEALDRHRLLSDVTRALADEKVNI
LSASVTTSGDRVAISRFTFEMGDPKHLGHLLNAVRNVEGVYDVYRVTSAA"

CDS complement(2882136..2882807)
/codon_start=1
/note="gene_2645"
/transl_table=11
/translation="VCHGGTWAGDYVLNVIATGLSLKARGKRRRQRWVDDGRVLALGE
SRRSSAISVADVVASLTRDVADFPVPGVEFKDLTPLFADRRGLAAVTEALADRASGAD
LVAGVDARGFLVAAAVATRLEVGVLAVRKGGKLPRPVLSEEYYREYGAATLEILAEGI
EVAGRRVVIIDDVLATGGTIGATRRLLERGGANVAGAAVVVELAGLSGRAALAPLPVH
SLSRL"

CDS complement(2882911..2884584)
/codon_start=1
/note="gene_2646"
/transl_table=11
/translation="VAPRRRRHTRIAGLRVVGTATLVAATTLTACSGSAAAQIDYVVD
GALVTYNTNTVIGAASAGAQAFARTLTGFGYHGPDGQVVADRDFGTVSVVEGSPLILD
YQISDDAVYSDGRPVTCDDLVLAWAAQSGRFPGFDAATQAGYVDIANIECTAGQKKAR
VSFIPDRSVVDHSQLFTATSLMPSHVIADQLHIDVTAALLSNNVSAVEQIARLWNSTW
DLKPGRSHDEVRSRFPSSGPYKIESVLDDGAVVLVANDRWWGTKAITKRITVWPQGAD
IQDRVNNRSVDVVDVAAGSSGSLVTPDSYQRTDYPSAGIEQLIFAPQGSLAQSRTRRA
LALCVPRDAIARDAGVPIANSRLSPATDDALTDADGAAEARQFGRVDPAAARDALGGT
PLTVRIGYGRPNARLAATIGTIADACAPAGITVSDVTVDTPGPQALRDGKIDVLLAST
GGATGSGSSGSSAMDAYDLHSGNGNNLSGYANAQIDGIISALAVSADPAERARLLAEA
APVLWDEMPTLPLYRQQRTLLMSTKMYAVSRNPTRWGAGWNMDRWALAR"

CDS complement(2884590..2885918)
/codon_start=1

```
                     /gene="secF"
                     /note="gene_2647"
                     /transl_table=11
                     /translation="MASKAKTGRDDEATSAVELTEATESAVARTDGDSTTDTASKLGH
                     HSFLSRLYTGTGAFEVVGRRRLWFGVSGAIVAVAIASIVFRGFTFGIDFKGGTTVSFP
                     RGSTQVAQVEDVYYRALGSEPQSVVIVGAGASATVQIRSETLTSDQTAKLRDALFEAF
                     GPKGTDGQPSKQAISDSAVSETWGGQITKKAVIALVVFLVLVALYITVRYERYMTISA
                     ITAMLFDLTVTAGVYSLVGFEVTPATVIGLLTILGFSLYDTVIVFDKVEENTHGFQHT
                     TRRTFAEQANLAINQTFMRSINTSLIGVLPVLTLMVVAVWLLGVGTLKDLALVQLIGI
                     IIGTYSSIFFATPLLVTLRERTELVRNHTRRVLKRRNSGSPAGSEDASTDGGEQPAAA
                     DEQSLVGITQASSQSAPRAAQGSSKPAPGARPVRPVGTRRPPGKRNAGRR"
     CDS             complement(2885922..2887643)
                     /codon_start=1
                     /gene="secD"
                     /note="gene_2648"
                     /transl_table=11
                     /translation="VASSSAPVHPARYLSVFLVMLIGIYLLVFFTGDKHTAPKLGIDL
                     QGGTRVTLTARTPDGSAPSREALAQAQQIISARVNGLGVSGSEVVVDGDNLVITVPGN
                     DGSEARNLGQTARLYIRPVLNSMPAQPAAEEPQPAPSAEPQPPGQPAAPPPAQSGAPA
                     SPQPGAQPRPYPQDPAPSPNPTSPASPPPAPPAEAPATDPRKDLAERIAQEKKLRQST
                     NQYMQMVALQFQATRCESDDILAGNDDPKLPLVTCSTDHKTAYLLAPSIISGDQIQNA
                     TSGMDQRGIGYVVDLQFKGPAANIWADYTAAHIGTQTAFTLDSQVVSAPQIQEAIPGG
                     RTQISGGDPPFTAATARQLANVLKYGSLPLSFEPSEAQTVSATLGLSSLRAGMIAGAI
                     GLLLVLVYSLLYYRVLGLLTALSLVASGSMVFAILVLLGRYINYTLDLAGIAGLIIGI
                     GTTADSFVVFFERIKDEIREGRSFRSAVPRGWARARKTIVSGNAVTFLAAAVLYFLAI
                     GQVKGFAFTLGLTTILDLVVVFLVTWPLVYLASKSSLLAKPAYNGLGAVQQVARERRA
                     MARTGRG"
     CDS             complement(2887753..2888100)
                     /codon_start=1
                     /gene="yajC"
                     /note="gene_2649"
                     /transl_table=11
                     /translation="MESFVLFLPFLLIMGGFMYFASRRQRRAMQATIDLHDSLQPGER
                     VHTTSGLEATIVAIADDTIDLEIAPGVVTTWMKLAIRDRILPDDDIDEELNEDLDKDV
                     DDVAGERRVTNDS"
     CDS             2888267..2889616
                     /codon_start=1
                     /gene="gabT"
                     /note="gene_2650"
                     /transl_table=11
                     /translation="VASLQQSRRLVTEIPGPASQALTHRRAAAVSSGVGVTLPVFVAR
                     AGGGIVEDVDGNRLIDLGSGIAVTTIGNSSPRVVDAVRTQVAEFTHTCFMVTPYEGYV
                     AVAEQLNRITPGSGPKRSVLFNSGAEAVENAVKIARSYTGKPAVVAFDHAYHGRTNLT
                     MALTAKSMPYKSGFGPFAPEIYRAPLSYPYRDGLLDKQLATNGELAAARAIGVIDKQV
                     GANNLAALVIEPIQGEGGFIVPAEGFLPALLDWCRKNHVVFIADEVQTGFARTGAMFA
                     CEHEGPDGLEPDLICTAKGIADGLPLSAVTGRAEIMNAPHVGGLGGTFGGNPVACAAA
                     LATIATIESDGLIERARQIERLVTDRLTTLQAVDDRIGDVRGRGAMIAVELVKSGTTE
                     PDAGLTERLATAAHAAGVIILTCGMFGNIIRLLPPLTIGDELLSEGLDIVCAILADL"
     CDS             2889778..2893284
                     /codon_start=1
                     /note="gene_2651"
```

```
                     /transl_table=11
                     /translation="MSINDQRLTRRVEDLYASDAQFAAASPNEAITQAIDQPGVALPQ
                     LIRMVMEGYADRPALGQRALRFVTDPDSGRTMVELLPRFETITYRELWARAGTLATAL
                     SAEPAIRPGDRVCVLGFNSVDYTTIDIALIRLGAVSVPLQTSAPVTGLRPIVTETEPT
                     MIATSIDNLGDAVEVLAGHAPARLVVFDYHGKVDTHREAVEAARARLAGSVTIDTLAE
                     LIERGRALPATPIADSADDALALLIYTSGSTGAPKGAMYRESQVMSFWRKSSGWFEPS
                     GYPSITLNFMPMSHVGGRQVLYGTLSNGGTAYYVAKSDLSTLFEDLALVRPTELCFVP
                     RIWDMVFAEFHSEVDRRLVDGADRAALEAQVKAELRENVLGGRFVMALTGSAPISAEM
                     TAWVESLLADVHLVEGYGSTEAGMVLNDGMVRRPAVIDYKLVDVPELGYFGTDQPYPR
                     GELLVKTQTMFPGYYQRPDVTAEVFDPDGFYRTGDIMAKVGPDQFVYLDRRNNVLKLS
                     QGEFIAVSKLEAVFGDSPLVRQIFIYGNSARAYPLAVVVPSGDALSRHGIENLKPVIS
                     ESLQEVARAAGLQSYEIPRDFIIETTPFTLENGLLTGIRKLARPQLKKFYGERLERLY
                     TELADSQSNELRELRQSGPDAPVLPTLCRAAAALLGSTAADVRPDAHFADLGGDSLSA
                     LSLANLLHEIFGVDVPVGVIVSPASDLRALADHIEAARTGVRRPSFASIHGRSATEVH
                     ASDLTLDKFIDAATLAAAPNLPAPSAQVRTVLLTGATGFLGRYLALEWLDRMDLVNGK
                     LICLVRARSDEEAQARLDATFDSGDPYLVRHYRELGAGRLEVLAGDKGEADLGLDRVT
                     WQRLADTVDLIVDPAALVNHVLPYSQLFGPNAAGTAELLRLALTGKRKPYIYTSTIAV
                     GEQIPPEAFTEDADIRAISPTRRIDDSYANGYANSKWAGEVLLREAHEQCGLPVTVFR
                     CDMILADTSYTGQLNLPDMFTRLMLSLAATGIAPGSFYELDAHGNRQRAHYDGLPVEF
                     VAEAICTLGTHSPDRFVTYHVMNPYDDGIGLDEFVDWLNSPTSGSGCTIQRIADYGEW
                     LQRFETSLRALPDRQRHASLLPLLHNYREPAKPICGSIAPTDQFRAAVQEAKIGPDKD
                     IPHLTAAIIAKYISNLRLLGLL"
     CDS             2893458..2895098
                     /codon_start=1
                     /note="gene_2652"
                     /transl_table=11
                     /translation="MSFVTAAPEMLATAAQNVANIGTSLSAANATAAASTTSVLAAGA
                     DEVSQAIARLFSDYATHYQSLNAQAAAFHHSFVQTLNAAGGAYSSAEAANASAQALEQ
                     NLLAVINAPAQALFGRPLIGNGANGTAASPNGGDGGILYGNGGNGFSQTTAGVAGGAG
                     GSAGLIGNGGNGGAGGAGAAGGAGGAGGWLLGNGGAGGPGGPTDVPAGTGGAGGAGGD
                     APLIGWGGNGGPGGFAAFGNGGAGGNGGASGSLFGVGGAGGVGGSSEDVGGTGGAGGA
                     GRGLFLGLGGDGGAGGTSNNNGGDGGAGGTAGGRLFSLGGDGGNGGAGTAIGSNAGDG
                     GAGGDSSALIGYAQGGSGGLGGFGESTGGDGGLGGAGAVLIGTGVGGFGGLGGGSNGT
                     GGAGGAGGTGATLIGLGAGGGGSIGGFAVNVGNGVGGLGGQGGQGAALIGLGAGGAGG
                     AGGATVVGLGGNDGDGGDGGGLFSIGVGGDGGNAGNGAMPANGGNGGNAGVIANGSFA
                     PSFVGFGGNGGNGVNGGTGGTGGSGGILFGANGANGPS"
     CDS             complement(2895115..2896149)
                     /codon_start=1
                     /gene="ruvB"
                     /note="gene_2653"
                     /transl_table=11
                     /translation="MTERSDRDVSPALTVGEGDIDVSLRPRSLREFIGQPRVREQLQL
                     VIEGAKNRGGTPDHILLSGPPGLGKTSLAMIIAAELGSSLRVTSGPALERAGDLAAML
                     SNLVEHDVLFIDEIHRIARPAEEMLYLAMEDFRVDVVVGKGPGATSIPLEVAPFTLVG
                     ATTRSGALTGPLRDRFGFTAHMDFYEPAELERVLARSAGILGIELGADAGAEIARRSR
                     GTPRIANRLLRRVRDFAEVRADGVITRDVAKAALEVYDVDELGLDRLDRAVLSALTRS
                     FGGGPVGVSTLAVAVGEEAATVEEVCEPFLVRAGMVARTPRGRVATALAWTHLGMTPP
                     VGASQPGLFE"
     CDS             complement(2896146..2896736)
                     /codon_start=1
                     /gene="ruvA"
```

```
                     /note="gene_2654"
                     /transl_table=11
                     /translation="MIASVRGEVLEVALDHVVIEAAGVGYRVNATPATLATLRQGTEA
                     RLITAMIVREDSMTLYGFPDGETRDLFLTLLSVSGVGPRLAMAALAVHDAPALRQVLA
                     DGNVAALTRVPGIGKRGAERMVLELRDKVGVAATGGALSTNGHAVRSPVVEALVGLGF
                     AAKQAEEATDTVLAANHDATTSSALRSALSLLGKAR"
     CDS             complement(2896733..2897290)
                     /codon_start=1
                     /gene="ruvC"
                     /note="gene_2655"
                     /transl_table=11
                     /translation="MGVDPGLTRCGLSLIESGRGRQLTALDVDVVRTPSDAALAQRLL
                     AISDAVEHWLDTHHPEVVAIERVFSQLNVTTVMGTAQAGGVIALAAAKRGVDVHFHTP
                     SEVKAAVTGNGSADKAQVTAMVTKILALQAKPTPADAADALALAICHCWRAPTIARMA
                     EATSRAEARAAQQRHAYLAKLKAAR"
     CDS             2897441..2897653
                     /codon_start=1
                     /note="gene_2656"
                     /transl_table=11
                     /translation="VIPKRIRERLGLRGNDQVEITERDGRIEIEPAPTGVELVREGSV
                     LVARPERPLPPLTDEIVRETLDRTRR"
     CDS             2897650..2898054
                     /codon_start=1
                     /note="gene_2657"
                     /transl_table=11
                     /translation="VIAPDTSVLVAGFATWHEGHEAAVRALNRGVHLIAHAAVETYSV
                     LTRLPPPHRIAPVAVHAYLADITSSNYLALDARSYRGLTDHLAEHDVTGGATYDALVG
                     FTAKAAGAKLLTRDLRAVETYERLRVEVELVT"
     CDS             2898271..2898891
                     /codon_start=1
                     /note="gene_2658"
                     /transl_table=11
                     /translation="VGNLLVVIAVALFIAAIVVLVVAIRRPKTPATPGGRRDPLAFDA
                     MPQFGPRQLGPGAIVSHGGIDYVVRGSVTFREGPFVWWEHLLEGGDTPTWLSVQEDDG
                     RLELAMWVKRTDLGLQPGGQHVIDGVTFQETERGHAGYTTEGTTGLPAGGEMDYVDCA
                     SAGQGADESMLLSFERWAPDMGWEIATGKSVLAGELTVYPAPPVSA"
     CDS             complement(2898941..2899141)
                     /codon_start=1
                     /gene="PHACADRAFT_196521"
                     /note="gene_2659"
                     /transl_table=11
                     /translation="VGGLGQIAAPAAGHAGDFLAEASLDGDDMRRAEDAELQCAAVGV
                     GQSVGGQWPRRGCVQHESKQCP"
     CDS             2899100..2899396
                     /codon_start=1
                     /note="gene_2660"
                     /transl_table=11
                     /translation="VARSRGGDLPESTHAPGYHLQSHTETHDEAAFRRLARHLRERCT
                     RATGWLGGVFPGDDAALTALAAEPDGTGWRWRTWHLYPSASGGTVVHTTSRWRP"
     CDS             2899393..2899824
                     /codon_start=1
```

/note="gene_2661"
/transl_table=11
/translation="MSRNRLFLVAGILAVAAAVSLISGITLLNRDVGSYIASHYRQES
RDVNGTRYLCTGSPKQVATTLVKYQTPAARASHTDTEYLRYRNNIVTVGPDGTYPCII
RVENLSAGYNHGAYVFLGPGFTPGSPSGGSGGSPGGPGGSK"
CDS 2899849..2900112
/codon_start=1
/note="gene_2662"
/transl_table=11
/translation="MYQAGVDFGTISLTPILHGVVATVLYFLVGAAVLVAGFLMVNLL
TPGDLRRLVFIDRRPNAVVLAATMYVALAIVTIAAIYASWPRA"
CDS 2900118..2900297
/codon_start=1
/note="gene_2663"
/transl_table=11
/translation="VAVYGIVGVALQGVALVILEIAVPGRFREHIDAPALHPAVFATA
VMLLAVAGVIAAALS"
CDS 2900294..2901865
/codon_start=1
/note="gene_2664"
/transl_table=11
/translation="MTSTRQAGEATEASVRWRAVLLAAVAACAACGLVYELALLTLAA
SLNGGGIVATSLIVAGYIAALGAGALLIKPLLAHAAIAFIAVEAVLGIIGGLSAAALY
AAFAFLDELDGSTLVLAVGTALIGGLVGAEVPLLMTLLQRGRVAGAADAGRTLANLNA
ADYLGALVGGLAWPFLLLPQLGMIRGAAVTGIVNLAAAGVVSIFLLRHVVSGRQLVTA
LCALAAALGLIATLLVHSHDIETTGRQQLYADPIIAYRHSAYQEIVVTRRGDDLRLYL
DGGLQFCTRDEYRYTESLVYPAVSDGARSVLVLGGGDGLAARELLRQPGIEQIVQVEL
DPAVIELARTTLRDVNAGSLDNPRVHVVIDDAMSWLRGAAVPPAGFDAVIVDLRDPDT
PVLGRLYSTEFYALAARALAPGGLMVVQAGSPYSTPTAFWRIISTIRSAGYAVTPYHV
HVPTFGDWGFALARLTDIAPTPAVPSTAPALRFLDQQVLEAATVFSGDIRPRTLDPST
LDNPHIVEDMRHGWD"
CDS 2901976..2902263
/codon_start=1
/note="gene_2665"
/transl_table=11
/translation="MKTTLDLPDELMRAIKVRAAQQGRKMKDVVTELLRSGLSQTHSG
APIPTPRRVQLPLVHCGGAATREQEMTPERVAAALLDQEAQWWSGHDDAAL"
CDS 2902250..2902690
/codon_start=1
/note="gene_2666"
/transl_table=11
/translation="MLLCDTNIWLALALSGHVHHRASRAWLDTINAPGVIHFCRATQQ
SLLRLLTNRTVLGAYGSPPLTNREAWAAYAAFLDDDRIVLAGAEPDGLEAQWRAFAVR
QSPAPKVWMDAYLAAFALTGGFELVTTDTAFTQYGGIELRLLAK"
CDS complement(2902711..2903466)
/codon_start=1
/note="gene_2667"
/transl_table=11
/translation="MSGHSKWATTKHKKAVVDARRGKMFARLIKNIEVAARVGGGDPA
GNPTLYDAIQKAKKSSVPNENIERARKRGAGEEAGGADWQTIMYEGYAPNGVAVLIEC
LTDNRNRAASEVRVAMTRNGGTMADPGSVSYLFSRKGVVTLEKNGLTEDDVLAAVLEA

GAEDVNDLGDSFEVISEPAELVAVRSALQDAGIDYESAEASFQPSVSVPVDLDGARKV
FKLVDALEDSDDVQNVWTNVDVSDEVLAALDDE"
CDS complement(2903599..2904195)
/codon_start=1
/note="gene_2668"
/transl_table=11
/translation="VSVPRVGVLALQGDTREHLAALRECGAEPMTVRRRDELDAVDAL
VIPGGESTTMSHLLLDLDLLGPLRARLADGLPAYGSCAGMILLASEILDAGAAGRQAL
PLRAMNMTVRRNAFGSQVDSFEGDIEFAGLDDPVRAVFIRAPWVERVGDGVQVLARAA
GHIVAVRQGAVLATAFHPEMTGDRRIHQLFVDIVTSAA"
CDS complement(2904203..2905048)
/codon_start=1
/note="gene_2669"
/transl_table=11
/translation="VSIEEILDLEQLEVNIYRGSVFSPESGFLQRTFGGHVAGQSLVS
AVRTVDPRYMVHSLHGYFLRPGDAKERTVFLVERIRDGGSLCTRRVNAVQHGETIFSM
AASFQTEQEGITHQDVMPAAPPPDGLPGLNSIKVFDDAGFRQFDEWDVCIVPRERLRL
LPGKASQQQVWLRHRDPLPDDPVLHICALAYMSDLTLLGSAQVNHLDVRDQLQVASLD
HAMWFMRPFRADEWLLYDQSSPSASGGRALTRGEIFTRSGEMVAAVMQEGLTRHRRGH
RSVGQ"
CDS complement(2905077..2905976)
/codon_start=1
/note="gene_2670"
/transl_table=11
/translation="MDPAGNPATGTARVKRGMAEMLKGGVIMDVVTPEQARIAEGAGA
VAVMALERVPADIRAQGGVSRMSDPDMIEGIIAAVTIPVMAKVRIGHFVEAQILQTLG
VDYIDESEVLTPADYAHHIDKWNFTVPFVCGATNLGEALRRISEGAAMIRSKGEAGTG
DVSNATTHMRAIGGEIRRLTSMSEDELFVAAKELQAPYELVAEVARAGKLPVTLFTAG
GIATPADAAMMMQLGAEGVFVGSGIFKSGAPEHRAAAIVKATTFFDDPDVLAKVSRGL
GEAMVGINVDEIAVGHRLAQRGW"
CDS 2906104..2906778
/codon_start=1
/note="gene_2671"
/transl_table=11
/translation="MDDDAQMVAIDKDQLARMRGEYGPEKDGCGDLDFDWLDDGWLTL
LRRWLNDAQRAGVSEPNAMVLATVADGKPVTRSVLCKILDESGVAFFTSYTSAKGEQL
AVTPYASATFPWYQLGRQAHVQGPVSKVSTEEIFTYWSMRPRGAQLGAWASQQSRPVG
SRAQLDNQLAEVTRRFADQDQIPVPPGWGGYRIAPEIVEFWQGRENRMHNRIRVANGR
LERLQP"
CDS 2906952..2908694
/codon_start=1
/note="gene_2672"
/transl_table=11
/translation="MNFAVLPPEVNSARIFAGAGLGPMLAAASAWDGLAEELHAAAGS
FASVTTGLAGDAWHGPASLAMTRAASPYVGWLNTAAGQAAQAAGQARLAASAFEATLA
ATVSPAMVAANRTRLASLVAANLLGQNAPAIAAAEAEYEQIWAQDVAAMFGYHSAASA
VATQLAPIQEGLQQQLQNVLAQLASGNLGSGNVGVGNIGNDNIGNANIGFGNRGDANI
GIGNIGDRNLGIGNTGNWNIGIGITGNGQIGFGKPANPDVLVVGNGGPGVTALVMGGT
DSLLSLPNIPLLEYAARFITPVHPGYTATFLETPSQFFPFTGLNSLTYDVSVAQGVTN
LHTAIMAQLAAGNEVVVFGTSQSATIATFEMRYLQSLPAHLRPGLDELSFTLTGNPNR
PDGGILTRFGFSIPQLGFTLSGATPADAYPTVDYAFQYDGVNDFPKYPLNVFATANAI

AGILFLHSGLIALPPDLASGVVQPVSSPDVLTTYILLPSQDLPLLVPLRAIPLLGNPL
ADLIQPDLRVLVELGYDRTAHQDVPSPFGLFPDVDWAEVAADLQQGAVQGVNDALSGL
GLPPPWQPALPRLF"

```
CDS             complement(2908716..2909771)
                /codon_start=1
                /note="gene_2673"
                /transl_table=11
                /translation="MTWLVLAGAVLLVVLVAFGAWGYQTANRLNRLNVRYDLSWQSLD
                SALARRAVVARAVAIDAYGGAPQGSRLAALADAAEGAPRHARENAENELSAALAMVNP
                ASLPAALIAELADAEARVLLARRFHNDAVRDTLALGERRLVRLLRLGGTAVLPTYFEI
                VERPHALVHGDQGASGRRTSARVVLLDDSGAVLLLCGSDPANPAFRDGAAPKWWFTVG
                GQVRPGERLAQAAARELAEETGLRVAPADMIGPIWRRDEVFEFNGSLIDSEEFYLVHR
                TRRFEPAVQGRTELERRYIRDARWCDANDIAQLVAAGERVYPLQLGELLPAANRLVDV
                ALDNGAARDAGVPQPIR"
CDS             complement(2909771..2910907)
                /codon_start=1
                /note="gene_2674"
                /transl_table=11
                /translation="MRIGMICPYSFDVPGGVQSHVLQLAEVMRTRGHLVSVLAPASPH
                AALPDYFVSGGRAVPIPYNGSVARLRFGPATHRKVKKWLAHGDFDVLHLHEPNAPSLS
                MLALNIAEGPIVATFHTSTTKSLTLTVFQGILRPMHEKIVGRIAVSDLARRWQMEALG
                SDAVEIPNGVDVDSFASAARLDGYPRQGKTVLFLGRYDEPRKGMAVLLDALPKVVQRF
                PDVQLLIVGHGDADQLRGQAGRLAAHLRFLGQVDDAGKASAMRSADVYCAPNTGGESF
                GIVLVEAMAAGTAVVASDLDAFRRVLRDGEVGHLVPVDPPDLQAAALADGLIAVLEND
                VLRERYVAAGNAAVRRYDWSVVASQIMRVYETVAGSGAKVQVAS"
CDS             complement(2910918..2911817)
                /codon_start=1
                /note="gene_2675"
                /transl_table=11
                /translation="VTRTATDWAYAAGWMAVRALPEFAVRNAFDTGARYFARHGGPEQ
                LRKNLARVLGVPPAAVPDPLMCASLESYGRYWREVFRLPTMNHRKLARQLDRVIGGLD
                HLDAALAAGLGAVLALPHSGNWDMAGMWLVQRHGTFTTVAERLKPESLYQRFIDYRES
                LGFEVLPLSGGERPPFEVLCERLRNNRVVCLMAERDLTRTGVEVDFFGEPTRMPVGPA
                KLAVETGAALLPTHCWFEGRGWGFQVYPALDCTSGDVAAITQALADRFAQNIAAHPAD
                WHMLQPQWLADLSESRRAQLRSR"
CDS             complement(2911865..2912518)
                /codon_start=1
                /note="gene_2676"
                /transl_table=11
                /translation="MSKLPFLSRAAFARITTPIARGLLRVGLTPDVVTILGTTASVAG
                ALTLFPMGKLFAGACVVWFFVLFDMLDGAMARERGGGTRFGAVLDATCDRISDGAVFC
                GLLWWIAFHMRDRPLVIATLICLVTSQVISYIKARAEASGLRGDGGFIERPERLIIVL
                TGAGVSDFPFVPWPPALSVGMWLLAVASVITCVQRLHTVWTSPGAIDRMAIPGKGDR"
CDS             complement(2912515..2913102)
                /codon_start=1
                /note="gene_2677"
                /transl_table=11
                /translation="VSDEDRTDRATEDHTIFDRGVGQRDQLQRLWTPYRMNYLAEAPV
                KRDPNSSASPAQPFTEIPQLSDEEGLVVARGKLVYAVLNLYPYNPGHLMVVPYRRVSE
                LEDLTDLESAELMAFTQKAIRVIKNVSRPHGFNVGLNLGTSAGGSLAEHLHVHVVPRW
                GGDANFITIIGGSKVIPQLLRDTRRLLATEWARQP"
```

```
CDS             complement(2913095..2915173)
                /codon_start=1
                /gene="thrS"
                /note="gene_2678"
                /transl_table=11
                /translation="MSAPAQPAPGVDGGDPSQARIRVPAGTTAATAVGEAGLPRRGTP
                DAIVVVRDADGNLRDLSWVPDVDTDITPVAANTDDGRSVIRHSTAHVLAQAVQELFPQ
                AKLGIGPPITDGFYYDFDVPEPFTPEDLAALEKRMRQIVKEGQLFDRRVYESTEQARA
                ELANEPYKLELVDDKSGDAEIMEVGGDELTAYDNLNPRTRERVWGDLCRGPHIPTTKH
                IPAFKLTRSSAAYWRGDQKNASLQRIYGTAWESQEALDRHLEFIEEAQRRDHRKLGVE
                LDLFSFPDEIGSGLAVFHPKGGIVRRELEDYSRRKHTEAGYQFVNSPHITKAQLFHTS
                GHLDWYADGMFPPMHIDAEYNADGSLRKPGQDYYLKPMNCPMHCLIFRARGRSYRELP
                LRLFEFGTVYRYEKSGVVHGLTRVRGLTMDDAHIFCTRDQMRDELRSLLRFVLDLLAD
                YGLTDFYLELSTKDPEKFVGAEEVWEEATTVLAEVGAESGLELVPDPGGAAFYGPKIS
                VQVKDALGRTWQMSTIQLDFNFPERFGLEYTAADGTRHRPVMIHRALFGSIERFFGIL
                TEHYAGAFPAWLAPVQVVGIPVADEHVAYLEEVATQLKSHGVRAEVDASDDRMAKKIV
                HHTNHKVPFMVLAGDRDVAAGAVSFRFGDRTQINGVARDDAVAAIVAWIADRENAVPT
                AELVKVAGRE"
CDS             complement(2915506..2916675)
                /codon_start=1
                /note="gene_2679"
                /transl_table=11
                /translation="VLALSQAGSTYAVAEAASATPLQNVLDAINAPVQSLTGRPLIGD
                GANGIDGTGQAGGNGGWLWGNGGNSGSGAPGQAGGAGGAAGLIGNGGAGGAGGQGLPF
                EAGANGGAGGAGGWLFGNGGAGGNGGIGGAGTNLAIGGHGGNGGNAGLIGAGGTGGAG
                GTGGGEPSAGASGGNGGNGGNGGLLIGNSGDGGAAGNGAGISQNGPASGFGGNGGHAG
                TTGLIGNGGNGGAGGAGGDVSADFGGVGFGGQGGNGGAGGLLYGNGGAGGNGGAAGSP
                GSVTAFGGNGGSGGSGGNGGNALIGNAGAGGSAGAGGNGASAGTAGGSGGDGGKGGNG
                GSVGLIGNGGNGGNGGNGGAGSLFNGAPGFGGPGGSGGASLLGPPGLAGTNGADG"
CDS             2916730..2916963
                /codon_start=1
                /gene="MULP_01060"
                /note="gene_2680"
                /transl_table=11
                /translation="VAAEQRRVDTSSWAATNTWVVAAAAVLAVLIAEPIRAASAAAVD
                TNCGATLTNDIEILLTATMLRCGRPTACYPCGG"
CDS             2917238..2917738
                /codon_start=1
                /note="gene_2681"
                /transl_table=11
                /translation="VDLNALADLPLTYPEVGATATGRLPAGYNHLDVSTQIGTGRQRF
                EQAADAVMHWGMQRNAGLRVRASSETAIVSAVVLVGIAFLRAPCRVVYVIDEPDVRGF
                GYGTLPGHPVSGEERFAVRCDPMTSVVFAEVLSFSRPATWASKAAGPLGAVTQRFIAQ
                RYLRAV"
CDS             complement(2917755..2918195)
                /codon_start=1
                /note="gene_2682"
                /transl_table=11
                /translation="MSIRPTTSPALADQLKDPAYSAYVLLRTLFTVAPILFGLDKFFN
                LLTHPQHWNMYLAGWINDLVPGTADQCMYLVGAIEIVAGVLVAVAPRIGAWVVAAWLA
                GIILNLVTGPGFYDIALRDFGLLVGAIALARLAQGVHSGGIGRP"
```

```
CDS             2918291..2919019
                /codon_start=1
                /note="gene_2683"
                /transl_table=11
                /translation="VDGRASFERDVAGIGALVDPVRRQLYQFVCSQSMPVSRDQAADA
                VGIPRHQAKFHLDRLTAEGLLDTEYARLTGRSGPGAGRTAKLYRRAGRDIALSLPQRE
                YELAGRLMAAAIVLSATTGEPTVEVLNRIAHDYGQAMGAAATTRPPADPAAALELTLD
                VLRKYGYEPRRPAGPGDDEVELVNCPFHALAREQTELACNMNHALITGVADALAPHSP
                AVRLAPGPARCCVVLKRCSAHDPE"
CDS             complement(2919004..2919357)
                /codon_start=1
                /note="gene_2684"
                /transl_table=11
                /translation="MESISLTSLAAEKLAEAQQTHSGRAAHTIHGGHTHELRQTVLAL
                LAGHDLSEHDSPGEATLQVLQGHVCLTAGEDAWNGRAGDYVAIPPTRHALHAVEDSVI
                MLTVLKSLPDAHSGS"
CDS             complement(2919370..2919795)
                /codon_start=1
                /note="gene_2685"
                /transl_table=11
                /translation="VSAGPAIEVAVAFVWLGMVVAISFLEAPLKFRAAGVTLQIGLGI
                GRLVFRALNTVEVGFALVILAIVVVGSTPARIAAAFSVALAALAVQLIAVRPRLTRRS
                NQVLAGLQAPRSRGHHIYVGLEIVKVVALLVAGILLLNG"
CDS             complement(2919792..2920463)
                /codon_start=1
                /note="gene_2686"
                /transl_table=11
                /translation="MGVSVIIRSLQEPVGRRRAVLRALCASRVPMSIAAIAGKLGVHP
                NTVRFHLDNLVADGQVERVEPGRGRPGRPPLMFRAVRRTDSTGTRRYRLLAEILASGL
                AAERDSRAMALSAGRAWGRQLEAPPAGADTEETIDHLVAVLDDLGFAPERRASNGRQQ
                VGLRHCPFLELAETQAGVVCPVHLGIMRGALQTWAPVTVDRLDAFVEPDLCLAHFTPL
                EGAIR"
CDS             2920541..2921362
                /codon_start=1
                /note="gene_2687"
                /transl_table=11
                /translation="MANKRGNAGQPLPLSDRDDDHMQGHWLLARLGKRVLRPGGVELT
                RTLLARAEVTDADVLELAPGLGRTAAEILARNPRSYVGAESDPNAANLVRHVLAGRGD
                VRVTDAADTGLSDASADVVIGEAMLTMQGNAAKHTIVAEAARVLRPGGRYAIHELALV
                PDDVAEQVRTDLRQSLARALKVNARPLTVAEWSHLLAGHGLVVEHVVTASMALLQPRR
                VIADEGLLGALRFAGNLLIHRAARRRVLLMRHTFRRHRERLTAVAIVAHKPHVDS"
CDS             2921498..2922391
                /codon_start=1
                /note="gene_2688"
                /transl_table=11
                /translation="MSSGNSSLGIIVGIDDSPAAQVAVRWAARDAELRKIPLTLVHAV
                SPEVATWLEVPLPPGVLRWQQDHGRHLIDDALKVVEQASLRAGPPTVHSEIVPAAAVP
                TLVDMSKDAVLMVVGCLGSGRWPGRLLGSVSSGLLRHAHCPVVIIHDEDSVMPHPQQA
                PVLVGVDGSSASELATAIAFDEASRRNVDLVALHAWSDVDVSEWPGIDWPATQSMAEQ
                VLAERLAGWQERYPNVAITRVVVRDQPARQLVQRSEEAQLVVVGSRGRGGYAGMLVGS
                VGETVAQLARTPVIVARESLT"
```

CDS complement(2922394..2923188)
/codon_start=1
/note="gene_2689"
/transl_table=11
/translation="MKTIIVGIDGSHAAITAALWGVDEAISRAVPLRLVSVIKPTHPS
PDDYDRDLAHAERSLREAQSAVEAAGKLVKIETDIPRGPAGPVLVEASRDAEMICVGS
VGIGRYASSILGSTATELAEKAHCPVAVMRSKVDQPASDINWIVVRMTDAPDNEAVLE
YAAREAKLRQAPILALGGRPEELREIPDGEFERRVQDWHHRHPDVRVYPITTHTGIAR
FLADHDERVQLAVIGGGEAGQLARLVGPSGHPVFRHAECSVLVVRR"
CDS complement(2923227..2924408)
/codon_start=1
/note="gene_2690"
/transl_table=11
/translation="MRDAIPLGRIAGFVVNVHWSVLVILWLFTWSLATMLPGTVGGYP
AVVYWLLGAGGAVMLLASLLAHELAHAVVARRAGVSVESVTLWLFGGVTALGGEAKTP
KAAFRIAFAGPATSLALSATFGALAITLAGVRTPAIVISVAWWLATVNLLLGLFNLLP
GAPLDGGRLVRAYLWRRHGDSVRAGIGAARAGRVVALVLIALGLAEFVAGGLVGGVWL
AFIGWFIFAAAREEETRISTQQLFAGVRVADAMTAQPHTAPGWINVEDFIQRYVLGER
HSAYPVADRDGSITGLVALRQLRDVAPSRRSTTSVGDIALPLHSVPTARPQEPLTALL
ERMAPLGPRSRALVTEGSAVVGIVTPSDVARLIDVYRLAQPEPTFTTSPQDADRFSDA
G"
CDS complement(2924467..2924898)
/codon_start=1
/note="gene_2691"
/transl_table=11
/translation="MTTARDIMNAGVTCVGEHETLTAAAQYMREHDIGALPICGDDDR
LHGMLTDRDIVIKGLAAGLDPNTATAGELARDSIYYVDANASIQEMLNVMEEHQVRRV
PVISEHRLVGIVTEADIARHLPEHAIVQFVKAICSPMALAS"
CDS complement(2925412..2926653)
/codon_start=1
/note="gene_2692"
/transl_table=11
/translation="MASSASDGTHERSAFRLSPPVLSGAMGPFMHTGLYVAQSWRDYL
GQQPDKLPIARPTIALAAQAFRDEIVLLGLKARRPVSNHRVFERISQEVAAGLEFYGN
RGWLEKPSGFFAQPPPLTEVAVRKVKDRRRSFYRIFFDSGFTPHPGEPGSQRWLSYTA
NNREYALLLRHPEPRPWLVCVHGTEMGRAPLDLAVFRAWKLHDELGLNIVMPVLPMHG
PRGQGLPKGAVFPGEDVLDDVHGTAQAVWDIRRLLSWIRSQEEESLIGLNGLSLGGYI
ASLVASLEEGLACAILGVPVADLIELLGRHCGLRHKDPRRHTVKMAEPIGRMISPLSL
TPLVPMPGRFIYAGIADRLVHPREQVTRLWEHWGKPEIVWYPGGHTGFFQSRPVRRFV
QAALEQSGLLDAPRTQRDRSA"
CDS 2927071..2927325
/codon_start=1
/note="gene_2693"
/transl_table=11
/translation="MNLAIWHPRKVQSATIYQVTDRLHDGRTARVPGDEITSTVSGWL
SELGTQSPLADELARAVRIGDWPAAYAIGEHLSVEIAVAV"
CDS 2927726..2928796
/codon_start=1
/note="gene_2694"
/transl_table=11
/translation="VYFDDSHDTLDAVERREATWRDVRKHLESRDAKQELIDSLEEAV

RDSRPAVGQRGRALIATGEQVLVNEHLIGPPPATVIRLSDYPYVVPLIDLEMRRPTYV
FAAVDHTGADVKLYQGATISSTKIDGVGYPVHKPVTAGWNGYGDFQHTTEEAIRMNCR
AVADHLTRLVDAADPEVVFVSGEVRSRTDLLSTLPQRVAVRVSQLHAGPRKSALDEEE
IWDLTSAEFTRRRYAEITNVAQQFEAEIGRGSGLAAQGLAEVCAALRDGDVDTLIVGE
LGEATVVTGKARTTVARDADMLSELGEPVDRVARADEALPFAAIAVGAALVRDDNRIA
PLDGVGALLRYAATNRLGSHRS"

CDS 2928945..2929337
/codon_start=1
/note="gene_2695"
/transl_table=11
/translation="VPHTADLRIEAWAPTRDGCIRQAVLGTVESFLDLESAHAVHTRL
RRLTADRDDDLLVAVLEEVIYLLDTVGETPVDLRLRDVDGGVDVTFATTDASTLVQVG
AVPKAVSLNELRFSQGRHGWRCAVTLDV"

CDS 2929353..2930774
/codon_start=1
/note="gene_2696"
/transl_table=11
/translation="MKIVEETPYRFRIEQEGAMRVPGIVFASRSLLPRDEGDMALMQV
VNVATLPGIVRASYAMPDVHWGYGFPIGGVAATDVDNDGVVSPGGVGFDISCGVRLLV
GEGLDREELQPRLPAVMDRLDRAIPRGVGTAGVWRLPDRNTLQEVLTGGARFAVEQGH
GVALDLERCEDGGVMTGADAAKISDRALQRGLGQIGSLGSGNHFLEVQAVDRVYDPVA
AAPMGLAEGTVCVMIHTGSRGLGHQICTDHVRQMEQAMGRYGIAVPDRQLACVPVHSP
DGQAYLAAMAAAANYGRANRQLLTEATRRVFADATGTPLDLLYDVSHNLAKIETHPID
GQLRSVCVHRKGATRSLPPHHHELPAELAAVGQPVLIPGTMGTASYVLAGVTGNPAFF
STAHGAGRVLSRHQAARHTSGEAIRASLAKRGIIVRGTSRRGIAEEKPEAYKDVDEVI
EASHQSGLARKVARLVPLGCVKG"

CDS complement(2930813..2931166)
/codon_start=1
/note="gene_2697"
/transl_table=11
/translation="LDRYRVQLAGQIGGRKRKSDREAAMTDSEHVGKTCQIDVLIEEH
DERTRAKARLSWAGRQMVGVGLARLDPADEPVAQIGDELAIARALSDLANQLFALTSS
DIEASTHQPVTGLHH"

CDS complement(2931239..2931724)
/codon_start=1
/note="gene_2698"
/transl_table=11
/translation="VNAYDVLKRHHTVLKGLGRKVGEAPVNSEERHVLFDEMLIELDI
HFRIEDDLYYPALSAAGKPITGTHAEHRQVVDQLATLLRTPQRAPGYEEEWNVFRTVL
EAHADVEERDMIPAPTPVHITDAELEELGDKMAARIEQLRGSPLYTLRTKGKADLLKA
I"

CDS complement(2931751..2932008)
/codon_start=1
/note="gene_2699"
/transl_table=11
/translation="LLPLGANITLAELPEPELRQLFPHEELQIPVSCGGLGAGAGGRM
DMRAVGLVPVRRASRYCTGFFVLIHSYLTLMGRGARLRLSR"

CDS complement(2932009..2934411)
/codon_start=1
/note="gene_2700"
/transl_table=11

/translation="MSFVIAVPEALTMAASDLANIGSTINAANAAAALPTTGVVAAAA
DEVSAAVAALFGSYAQSYQAFGAQLSAFHAQFVQSLTNGARSYVVAEATSAAPLQDLL
GVVNAPAQALLGRPLIGNGANGADGTGAPGGPGGLLLGNGGNGGSGAPGQPGGAGGDA
GLIGNGGTGGKGGDGLVGSGAAGGVGGRGGWLLGNGGTGGAGGAAGATLVGGTGGVGG
ATGLIGSGGFGGAGGAAAGVGTTGGVGGSGGVGGVFGNGGFGGAGGLGAAGGVGGAAS
YFGTGGGGGVGGDGAPGGDGGAGPLLIGNGGVGGLGGAGAAGGNGGAGGMLLGDGGAG
GQGGPAVAGVLGGMPGAGGNGGNANWFGSGGAGGQGGTGLAGTNGVNPGSIANPNTGA
NGTDNSGNGNQTGGNGGPGPAGGVGEAGGVGGQGGLGESLDGNDGTGGKGGAGGTAGT
DGGAGGAGGAGGIGETDGSAGGVATGGEGGDGATGGVDGGVGGAGGKGGQGHNTGVGD
AFGGDGGIGGDGNGALGAAGGNGGTGGAGGNGGRGGMLIGNGGAGGAGGTGGTGGGGA
AGFAGGVGGAGGEGLTDGAGTAEGGTGGLGGLGGVGGTGGMGGSGGVGGNGGAAGSLI
GLGGGGGAGGVGGNGGAAGSLIGLGGGGGAGGVGGTGGIGGIGGAGGNGGAGGAGTTT
GGGATIGGGGGTGGVGGAGGTGGTGGAGGTTGGSGGAGGLIGWAGAAGGTGAGGTGGQ
GGLGGQGGNGGNGGTGATGGQGGDFALGGNGGAGGAGGSPGGSSGIQGNMGPPGTQGA
DG"

CDS 2934302..2934682
/codon_start=1
/note="gene_2701"
/transl_table=11
/translation="VVGNAAAAFAALIVDPMLARSEAAIVNASGTAITNDICDLLDRT
TRMVAADHRALGSNKSYPASQTAEAIWPPARTLRYDRQSPWLATGFDRRMSQTVTGVG
VQNCAVSKRRCSAVDHSSRTPYRR"

CDS 2934731..2935360
/codon_start=1
/note="gene_2702"
/transl_table=11
/translation="MAGMPEGKLILLNGGSSAGKTSLALAFQDLAAECWMHIGIDLFW
FALPPEQLDLARVRPEYYTWDSAVEADGLEWFTVHPGPILDLAMHSRYRAIRAYLDNG
MNVIADDVIWTREWLVDALRVFEGCRVWMVGVHVSDEEGARRELERGDRHPGWNRGSA
RAAHADAEYDFELDTTATPVHELARELHESYQACPYPMAFNRLRKRFLS"

CDS 2935556..2936212
/codon_start=1
/note="gene_2703"
/transl_table=11
/translation="MDVEALLQSIPPLMVYLVVGAVVGIESLGIPLPGEIVLVSAAVL
SSHPELAVNPIGVGGAAVIGAVVGDSIGYSIGRRFGLPLFDRLGRRFPKHFGPGHVAL
AERLFNRWGVRAVFLGRFIALLRIFAGPLAGALKMPYPRFLAANVTGGICWAGGTTAL
VYFAGMAAQHWLERFSWIALVIAVIAGITAAILLRERTSRAIAELEAEHCRKAGTTAA
"

CDS 2936375..2936821
/codon_start=1
/note="gene_2704"
/transl_table=11
/translation="MGLITTEPRSSPHPLSPRLVHELGDPHSTLRATTDGSGAALLIH
AGGEIDGRNEHLWRQLVTEAAAGVTAPGPLIVDVTGLDFMGCCAFAALADEAQRCRCR
GIDLRLVSHQPIVARIAEAGGLSRVLPIYPTVDTALGKGTAGPARC"

CDS complement(2936996..2937328)
/codon_start=1
/note="gene_2705"
/transl_table=11
/translation="MVVRSILLFVLAAVAEIGGAWLVWQGVREQRGWLWAGLGVIALG

VYGFFATLQPDAHFGRVLAAYGGVFVAGSLAWGMALDGFRPDRWDVIGALGCMAGVAV
IMYAPRGH"
CDS complement(2937448..2937807)
/codon_start=1
/note="gene_2706"
/transl_table=11
/translation="MPKSLPVIDISAPVCCAPVAAGPMSDGDALAVALRLKALADPAR
VKIMSYLFSSPAGEQVSGQLAAALSLSDGTVSHHLAQLRKAGLVISDRRGMHVFHRVH
PEALQALCTVLNPNCCA"
CDS 2937909..2938367
/codon_start=1
/note="gene_2707"
/transl_table=11
/translation="MSRVQLALNVDDLEAAITFYSRLFNAEPAKRKPGYANFAIADPP
LKLVLLENPGTGGTLNHLGVEVGSSNTVHAEIARLTEAGLVTEKEIGTTCCFATQDKV
WVTGPGGERWEVYTVLADSETFGSGPRHNDTSDGEASMCCDGQVAVGASG"
CDS 2938503..2938883
/codon_start=1
/note="gene_2708"
/transl_table=11
/translation="MSNLHPLPEVASCVVAPLVREPLNPPAAAEMAARFKALADPVRL
QLLSSVASRAGGEACVCDISAGVEVSQPTISHHLKVLRDAGLLTSRRRASWVYYAVVP
EALTVLSNLLSVHADAAPALGAPA"
CDS 2938880..2940376
/codon_start=1
/note="gene_2709"
/transl_table=11
/translation="MTETVTRTAAPAVVGKLSTLDRFLPVWIGSAMAAGLLLGRWIPG
LHTALEGVQLDGISLPIALGLLIMMYPVLAKVRYDRLDTVTGDRKLLLSSLLLNWVLG
PALMFALAWLLLADLPEYRTGLIIVGLARCIAMVIIWNDLACGDREAAAVLVALNSIF
QVAMFAALGWFYLSVLPGWLGLEQTTIATSPWQIAKSVLIFLGIPLLAGYLSRRIGEK
TKGRNWYESRFLPKVGPWALYGLLFTIVILFALQGDQITGRPLDVARIALPLLAYFAI
MWVGGYLLGAALRLGYRRTTTLAFTAASNNFELAIAVAIATYGATSGQALAGVVGPLI
EVPVLVGLVYVSLALRNRLAGPNATHDADKPSVLFVCVHNAGRSQMAAGLLTHLAGDR
IEVRSAGTEPAGQVNPTAVAAMAEMGIDITANAPTLLTGGQVQSSDVVITMGCGDACP
YFPGASYRNWKLPDPAGQPLDVVRMIRDDIADRVQALIAELLATAKTR"
CDS 2940494..2940802
/codon_start=1
/note="gene_2710"
/transl_table=11
/translation="VAALACVERLVKKLGPVRPYGFLLICPVRSLDKAAARDRVRRYR
ERLRQRGLRPIQIWVPDVNAPEFVGEAHRPSALVAAREYEDDDQAFVDAVSVDWDDAT
"
tRNA complement(2941464..2941538)
/note="identified by tRNAscan-SE; putative"
tRNA 2941723..2941798
/note="identified by tRNAscan-SE; putative"
tRNA 2941825..2941898
/note="identified by tRNAscan-SE; putative"
tRNA 2941912..2941986
/note="identified by tRNAscan-SE; putative"

CDS 2942319..2942477
/codon_start=1
/gene="TBEG_01450"
/note="gene_2711"
/transl_table=11
/translation="MSGGWLAEHLGLSTNRLRHELADRLDAHYGPPAQNRELARPSLR
IINEGTDG"
CDS 2942841..2943074
/codon_start=1
/note="gene_2712"
/transl_table=11
/translation="VEVRASARKHGINDDAMLHAYRNALRYVELEYHGEVQLLVIGPD
QTGRLLELVIPADEPPRIIHANVLRPKFYDYLR"
CDS 2943085..2943339
/codon_start=1
/note="gene_2713"
/transl_table=11
/translation="VKHKTDIDEWLDTIEPNPADAHDASHLRRIIAAKEAVQTAESEL
RAAVNAARAAGDTWAAIGVALGITRQAAFQRFGPHSTASP"
CDS 2944059..2944862
/codon_start=1
/note="gene_2714"
/transl_table=11
/translation="MTSSHLIDTEQLLADQLAQASPDLLRGLLSTFIAALMGAEADAL
CGAGYRERSDERSNQRNGYRHRDFDTRAATIDVAIPKLRQGSYFPDWLLQRRKRAERA
LTSVVATCYLLGVSTRRMERLVETLGVTKLSKSQVSIMAKELDEAVEAFRTRPLDAGP
YTFLAADALVLKVREAGRVVGVHTLIATGVNAEGYREILGIQVTSAEDGAGWLAFFRD
LVARGLSGVALVTSDAHAGLVAAIGATLPAAAWQRCRTHYAANHGRHNA"
CDS 2944884..2945642
/codon_start=1
/note="gene_2715"
/transl_table=11
/translation="MPEPTPTAYPVRLDELINAIKRVHSDVLDQLSDAVLAAEHLGEI
ADHLIGHFVDQARRSGASWSDIGKSMGVTKQAAQKRFVPRAEATTLDSNQGFRRFTPR
ARNAVVAAQNAAHGATSSEITPDHLLLGVLTDPAALATALLQQQEIDIATLRTAVTLP
PAVTEPPQPIPFSGPARKVLELTFREALRLGHNYIGTEHLLLALLELEDGDGPLHRSG
VDKSRAEADLITTLASLTGANAAGATDAGATDAG"
CDS 2945721..2946242
/codon_start=1
/note="gene_2716"
/transl_table=11
/translation="MRRWLIVLATLLVAAAGVAAANDVPRAWAGDAPIGHIGDTLRVD
TGTYVADVTVSSVVPVDPPPGFAYTRSGVPVKSFPDSSVTRADVTVRAVRVPNSFILA
TNFSFTGVTPFADAYKPRPCDASDWLDAALGNAPQGSIVRGGVYWDAYRDPVSVVVLL
DKKTGQHLAQWNL"
CDS 2946271..2946741
/codon_start=1
/note="gene_2717"
/transl_table=11
/translation="VTDADELAAVAARTFPLACPPAVAPEHIASFVDANLSSARFAEY
LTDPRRAILTARHDGRIVGYAMLIRGDDRDVELSKLYLLPGYHGTGAAAALMHKVLAT

AADWGALRVWLGVNQKNQRAQRFYAKTGFKINGTRTFRLGAHHENDYVMVRELV"
CDS complement(2946719..2947828)
/codon_start=1
/note="gene_2718"
/transl_table=11
/translation="MTLIAARRYSATMHGSASEACGSVDHLVDRHPTVSPVRLIAQLR
PPPTFAEVSFATYRPDPVEPTQAAAVVACQDFCRQAVERRAGRKKWFGKRDVLPGVGL
YLDGGFGVGKTHLLASAYYQLPGTGPDAPTCPKAFATFGELTQLAGVFGFADCIDLLA
NYTALCIDEFELDDPGNTTLISRLLSALVERGVSVAATSNTLPEQLGEGRFAAQDFLR
EINTLASIFTTVRIEGPDYRHRDLPPAPAPLSDEEVAARAARVEGATLDDFDALCAHL
ATMHPSRYLTLIEGVTAVFLTGVHGIDDQNVALRLVALVDRLYDAGIPVVASGAKLDT
IFSEEMLAGGYRKKYLRATSRLLALTAGVIQAREP"
CDS 2947827..2948603
/codon_start=1
/note="gene_2719"
/transl_table=11
/translation="MPDSGQLGAADTPLRLLSSVHYLTDGELPQLYDYPDDGTWLRAN
FISSLDGGATVDGTSGAMAGPGDRFVFNLLRELADVIVVGVGTVRIEGYSGVRMGVVQ
RQHRQARGQSEVPQLAIVTRSGRLDRDMAVFTRTEMAPLVLTTTAVADDTRQRLAGLA
EVIACSGDDPGTVDEAVLVSQLAARGLRRILTEGGPTLLGTFVERDVLDELCLTIAPY
VVGGLARRIVTGPGQVLTRMRCAHVLTDDSGYLYTRYVKT"
CDS 2948700..2950256
/codon_start=1
/note="gene_2720"
/transl_table=11
/translation="MTSTAMLVALTCSATVLAACVPAFGADPRFATYSGAGPQGAATT
TPPPAGPPPLAAPKNDLSWHDCTSRVYSNAGIPAAPGVKLECASYDTDLDPLVGGSTA
VSIGVVRARSNQTPSDAGPLVFTTGSDLPSSTQLPVWLAHAGIDVLRSHPIVAVDRRG
MGMSSPIDCRDHFDRDEMRDQAQFQAGDDPVANLSDISNTATTDCTDAIAPGESAYDN
THAASDIERLRKLWDVPALAFVGIGNGTQVALAYAASRPDNVARLILDSPIALGVSAE
AAAEQQVQGQQAALDAFAAQCVAVNCALGSDPKGAVSALLSAARSGDGPGGASVAAVA
NAVATALGFPDSGRVDSTTKLADALAAARSGDMNLLSALINRADTTRDTDGQFISSCS
DAVNRPTPDRVRELVVAWGKLYPQFGAVAALNLVKCVHWPSSSPPQPPKDLKVDVLLL
GVQNDPIVGNEGVAATAATAINANAASKRVMWQGIGHGASIYSSCAVPPLVAYLDTGK
LPDTDTYCPA"
CDS 2950279..2951580
/codon_start=1
/note="gene_2721"
/transl_table=11
/translation="VYGALVTAADSIRTGLGASLLAGFRPRTGAPSTATILRSALWPA
AVLSVLHRSIVLTTNGNITDDFKPVYRAVLNFRRGWDIYNEHFDYVDPHYLYPPGGTL
LMAPFGYLPFAPSRYLFISINTAAILVAAYLLLRMFNFTLTSVAAPALILAMFATETV
TNTLVFTNINGCILLLEVLFLRWLLDGRASRQWCGGLAIGLTLVLKPLLGPLLLLPLL
NRQWRALVAAVVVPVVVNVAALPLVSDPMSFFTRTLPYILGTRDYFNSSILGNGVYFG
LPTWLILFLRILFTAITFGALWLLYRYYRTGDPLFWFTTSSGVLLLWSWLVMSLAQGY
YSMMLFPFLMTVVLPNSVIRNWPAWLGVYGFMTLDRWLLFNWMRWGRALEYLKITYGW
SLLLIVTFTVLYFRYLDAKADNRLDGGIDPAWLTPEREGQR"
CDS 2951749..2952159
/codon_start=1
/note="gene_2722"
/transl_table=11

/translation="MTRPKLELSDDEWRQKLTPQEFHVLRRAGTERPFTGEYTDTTTA
GIYQCRACGAELFRSTEKFESHCGWPSFFDPKSSDAVTLRPDHSLGMTRTEVLCANCD
SHLGHVFAGEGYPTPTDKRYCINSISLRLVPGSV"
CDS complement(2952227..2952979)
/codon_start=1
/note="gene_2723"
/transl_table=11
/translation="MTAQFDPADPTRFEEMYRDDRVAHGLPAATPWDIGGPQPVVQQL
VALGAIRGEVLDPGTGPGHHAIYYAAKGYAATGIDGSVAAIERARDNARKAGVSVNFQ
VGDATTLDGLDGRFDTVVDCAFYHTFSTAPELQRCYVRALRRASKPGARLYMFEFGEH
NVNGFSMPRSLSEDDFRQVLPVGGWEITYLGTTTYQVNLSVEALELMAARNPDMADQV
RCVLERFRAIKPWLVGGRVHAPFWEVHATRVD"
CDS complement(2952976..2953671)
/codon_start=1
/note="gene_2724"
/transl_table=11
/translation="MARLDYDALNATLRYLMFSVFSVSPGALGDQRDAIIDDASTFFK
QQEERGVVVRGLYDVAGLRADADFMVWTHAERVEALQATYADFRRTTTLGRACTPVWS
GVGLHRPAEFNKSHIPAFLAGEEPGAYICVYPFVRSYEWYLLPDEERRRMLAEHGMAA
RGYKDVRANTVPAFALGDYEWILAFEAPELDRIVDLMRELRATDARRHTRAETPFFTG
PRVPVEQLVHSLP"
CDS complement(2953677..2954990)
/codon_start=1
/note="gene_2725"
/transl_table=11
/translation="LTSAYRLRQAVGDDATITLFEPADRLGGVLRTEHIGGQPMDLGA
EAFVLRRPEMPALLAELGLSDRQLASTGARPLIYSQQRLHPLPPQTVVGIPSSAGSMA
GLVDDATLARIDAEAARPFTWQVGSDPAVADLVADRFGDQVVARSVDPLLSGVYAGSA
ATIGLRAAAPSVAAALDRGATSVTDAVRQALPPGSGGPVFGALDGGYQVLLDGLVRRS
RVHWVRARVVQLERGWVLRDETGGRWQADAVILAVPAPRLARLVDGIAPRTHAAARQI
VSASSAVVALAVPGGTAFPHCSGVLVAGDESPHAKAITLSSRKWGQRGDVALLRLSFG
RFGDEPALTASDDQLLAWAADDLVTVFGVAVDPVDVRVRRWIEAMPQYGPGHADVVAE
LRAGLPPTLAVAGSYLDGIGVPACVGAAGRAVTSVIEALDAQVAR"
CDS complement(2955032..2956105)
/codon_start=1
/note="gene_2726"
/transl_table=11
/translation="MSTRRDLPQSPYLAAVTGRKPSRVPVWFMRQAGRSLPEYRALRE
RYSMLAACFEPDVACEITLQPIRRYDVDAAILFSDIVVPLRAAGVDLDIVADVGPVIA
DPVRTAADVAAMKPLDPQAIQPVLVAASLLVAELGDVPLIGFAGAPFTLASYLVEGGP
SRHHAHVKAMMLAEPASWHALMAKLTDLTIAFLVGQIDAGVDAIQVFDSWAGALSPID
YRQYVLPHSARVFAALGEHGVPMTHFGVGTAELLGAMSEAVTAGERPGRGAVVGVDWR
TPLTDAAARVVPGTALQGNLDPAVVLAGWPAVERAARAVVDDGRRAVDAGAAGHIFNL
GHGVLPESDPAVLADLVSLVHSL"
CDS 2956020..2956988
/codon_start=1
/note="gene_2727"
/transl_table=11
/translation="MNHTGTRLGLRPVTAARYGDCGRSRRVLIELNAATTATPHLRNI
DPMPVTYDDFPSLRCEIHDQPGHEGVLELVLDSPGLNSVGPHMHRDLADIWPVIDRDP
AVRVVLVRGEGKAFSSGGSFDLIAETIGDYQGRLRIMREARDLVLNLVNFDKPVVSAI

RGPAVGAGLVVALLADISVAGRAAKIIDGHTKLGVAAGDHAAICWPLLVGMAKAKYYL
LTCEPLSGEEAERIGLVSICVDDDDVLPTATRLAERLAAGAQNAIRWTKRSLNHWYRM
FGPAFETSLGLEFIGFGGPDVREGLAAHREKRPARFGADPDPGAGS"

```
CDS             2957097..2957933
                /codon_start=1
                /note="gene_2728"
                /transl_table=11
                /translation="LTSAGDDAERSDEEERRLTSAGDDAERSDEEERRLTSAGDDAER
                SDEEERRLTSAGDDAERSDEEERRLTSAGDDAERSDEEERRLTSAEPALFREAVAAMN
                AVTVRPEIELGPIRPPQRLAPYSYALGAEIKHPELDVIPERSEGDAFGRLIMLYDPDG
                SDAWDGTIRLVAYVQADLDSSEAVDPLLPEVAWSWLVDALTARTDQVRALGGTVTATT
                SVRYGDISGPPRAHQLELRASWTATTPDLGAHVQAFCDVLEHAAGLPPAGVTDLGSRS
                RA"
variation       2957394..2957444
                /note="supplemented from BCG moreau"
CDS             2957935..2959251
                /codon_start=1
                /note="gene_2729"
                /transl_table=11
                /translation="MCPEPSHAGAAESEGTESEPTPLLRPAGGIPDLCVTVGEIAAAA
                ELLDRGRGPFAVDAERASGFRYSGRAYLIQIRRAEAGTVLIDPVSHGGDPLTVLAPVA
                EVLSTNEWILHSADQDLPCLAEVGMRPPALYDTELAGRLAGFDRVNLAAMVERLLGLG
                LTKGHGAADWSKRPLPSAWLNYAALDVELLIELRAVISRVLAEQGKTDWAAQEFEHLR
                SFESRPPPAAARQDRWRRTSGIHKVHDRRGLAAVRELWTARDRIAQRRDIAPRRILPD
                SAIIDAAIADPKSVDDLVALPVFGGRNQRRSAAVWWAALAAARESPDPPEIAEPANGP
                PPPGRWVRRKPAAAARLDAARAALTEVSQRVRVPTENLVSPDLVRRLCWEWEDISQSS
                PDPIAAVEAYLRTGQARAWQLELVVPILTAALTGAPDAGAQGDDGS"
CDS             complement(2959248..2961164)
                /codon_start=1
                /note="gene_2730"
                /transl_table=11
                /translation="MLQQIRGPADLQHLSQAQLRELAAEIREFLIHKVAATGGHLGPN
                LGVVELTLALHRVFDSPHDPIIFDTGHQAYVHKMLTGRSQDFATLRKKGGLSGYPSRA
                ESEHDWVESSHASAALSYADGLAKAFELTGHRNRHVVAVVGDGALTGGMCWEALNNIA
                ASRRPVIIVVNDNGRSYAPTIGGVADHLATLRLQPAYEQALETGRDLVRAVPLVGGLW
                FRFLHSVKAGIKDSLSPQLLFTDLGLKYVGPVDGHDERAVEVALRSARRFGAPVIVHV
                VTRKGMGYPPAEADQAEQMHSTVPIDPATGQATKVAGPGWTATFSDALIGYAQKRRDI
                VAITAAMPGPTGLTAFGQRFPDRLFDVGIAEQHAMTSAAGLAMGGLHPVVAIYSTFLN
                RAFDQIMMDVALHKLPVTMVLDRAGITGSDGASHNGMWDLSMLGIVPGIRVAAPRDAT
                RLREELGEALDVDDGPTALRFPKGDVGEDISALERRGGVDVLAAPADGLNHDVLLVAI
                GAFAPMALAVAKRLHNQGIGVTVIDPRWVLPVSDGVRELAVQHKLLVTLEDNGVNGGA
                GSAVSAALRRAEIDVPCRDVGLPQEFYEHASRSEVLADLGLTDQDVARRITGWVAALG
                TGVCASDAIPEHLD"
CDS             2961308..2961805
                /codon_start=1
                /note="gene_2731"
                /transl_table=11
                /translation="MKVNIDPTAPTFATYRRDMRAEQMAEDYPVVSIDSDALDAARML
                AEHRLPGLLVTAGAGKQYAVLPASQVVRFIVPRYVQDDPSLAGVLNESTADRCAERLS
                GKKVRDVLPDHLVEVPPANADDTIIEVAAVMARLRSPLLAVVKDGSLLGVVTASRLLA
                AALKT"
```

CDS   2961810..2963099
                /codon_start=1
                /gene="arsA"
                /note="gene_2732"
                /transl_table=11
                /translation="VSVVAVTIFVAAYVLIASDRVNKTMVALTGAAAVVVLPVITSHD
                IFYSHDTGIDWDVIFLLVGMMIIVGVLRQTGVFEYTAIWAAKRARGSPLRIMILLVLV
                SALASALLDNVTTVLLIAPVTLLVCDRLNINTTSFLMAEVFASNIGGAATLVGDPPNI
                IVASRAGLTFNDFMLHLTPLVVIVLIALIAVLPRLFGSITVEADRIADVMALDEGEAI
                RDRGLLVKCGAVLVLVFAAFVAHPVLHIQPSLVALLGAGMLIVVSGLTRSEYLSSVEW
                DTLLFFAGLFIMVGALVKTGVVNDLARAATQLTGGNIVATAFLILGVSAPISGIIDNI
                PYVATMTPLVAELVAVMGGQPSTDTPWWALALGADFGGNLTAIGASANVVMLGIARRA
                GAPISFWEFTRKGAVVTAVSIALAAIYLWLRYFVLLH"
CDS   2963179..2964465
                /codon_start=1
                /gene="arsB1"
                /note="gene_2733"
                /transl_table=11
                /translation="MSIIAITVFVAGYALIASDRVSKTRVALTCAAIMVGAGIVGSDD
                VFYSHEAGIDWDVIFLLLGMMIIVSVLRHTGVFEYVAIWAVKRANAAPLRIMILLVLV
                TALGSALLDNVTTVLLIAPVTLLVCDRLGVNSTPFLVAEVFASNVGGAATLVGDPPNI
                IIASRAGLTFNDFLIHMAPAVLVVMIALIGLLPWLLGSVTAEPDRVADVLSLNEREAI
                HDRGLLIKCGVVLVLVFAAFIAHPVLHIQPSLVALLGAGVLVRFSGLERSDYLSSVEW
                DTLLFFAGLFVMVGALVKTGVVEQLARAATELTGGNELLTVGLILGISAPVSGIIDNI
                PYVATMTPIVTELVAAMPGHVHPDTFWWALALSADFGGNLTAVGASANVVMLGIARRS
                GTPISFWKFTRKGAVVTAVSLVLSAVYLWLRYFVFG"
CDS   complement(2964476..2965198)
                /codon_start=1
                /note="gene_2734"
                /transl_table=11
                /translation="LAAFGRNDIRGTYRDPLLVMLVIAPVIWTTGVALLTPLFTEMLA
                RRYGFDLVGYYPLILTAFLLLTSIIVAGALAAFLVLDDVDAGTMTALRVTPVPLSVFF
                GYRAATVMVVTTIYVVATMSCSGILEPGLVSSLIPIGLVAGLSAVVTLLLILAVANNK
                IQGLAMVRALGMLIAGLPCLPWFISSNWNLAFGVLPPYWAAKAFWVASDHGTWWPYLV
                GGAVYNLAIVWVLFRRFRAKHA"
CDS   complement(2965231..2965944)
                /codon_start=1
                /note="gene_2735"
                /transl_table=11
                /translation="MTRLVPALRLELTLQVRQKFLHAAVFSGLIWLAVLLPMPVSLRP
                VAEPYVLVGDIAIIGFFFVGGTVFFEKQERTIGAIVSTPLRFWEYLAAKLTVLLAISL
                FVAVVVATIVHGLGYHLLPLVAGIVLGTLLMLLVGFSSSLPFASVTDWFLAAVIPLAI
                MLAPPVVHYSGLWPNPVLYLIPTQGPLLLLGAAFDQVSLAPWQVGYAVVYPIVCAAGL
                CRAAKALFGRYVVQRSGVL"
CDS   complement(2965941..2966846)
                /codon_start=1
                /note="gene_2736"
                /transl_table=11
                /translation="MTALNRAVASARVGTEVIRVRGLTFRYPKAAEPAVRGMEFTVGR
                GEIFGLLGPSGAGKSTTQKLLIGLLRDHGGQATVWDKEPAEWGPDYYERIGVSFELPN
                HYQKLTGYENLRFFASLYAGATADPMQLLAAVGLADDAHTLVGKYSKGMQMRLTFARS

LINDPELLFLDEPTSGLDPVNARKIKDIIVDLKARGRTIFLTTHDMATADELCDRVAF
VVDGRIVALDSPTELKIARSRRRVRVEYRGDGGGLETAEFGMDGLADDPAFHSVLRNH
HVETIHSREASLDDVFVEVTGRQLT"
CDS complement(2967041..2968258)
/codon_start=1
/note="gene_2737"
/transl_table=11
/translation="VTRAGDDAVNLTLVTGAPANGGSCVAHHEGRVVFVRYALPGERV
RARVTAQRGSYWHAEAFEVIDPSPDRIGSLCSIAGADGAGCCDLAFAAPEAARTLKAQ
VVANQLERLGRHSWQGEAQPLSDAGPTGWRIRVRLDVGADRRPGFHRYHSGELVTDLD
CGQLPVGMLDGLVAADWPPEAQLYVALDDDGERHVVCSVRQGPRNRTRTVTNVVEGAY
HAHQRVHRRSWRVPVTAFWQAHRDAAAVYSDLIADWAQPAPGMTAWDLYGGAGVFAAV
LGEAVGESGRVLTVDTSRLASGAARAALVDLPQVEVVTGSVRRVLAVQPAGADLAVLD
PPRSGAGREVVDLLAGAGVPRLIHIGCEAASFARDIGLYRGHGYAVEKIKVFDAFPLT
HYVECVALLTRKV"
CDS complement(2968414..2970414)
/codon_start=1
/note="gene_2738"
/transl_table=11
/translation="LALVSKLSTAARRLLIGRPFRSDRLSHTLLPKRIALPVFASDAM
SSIAYAPEEIFLVLSVAGLAAYSMAPLIGLAVAAVLLVVVSSYRQNVHAYPSGGGDYE
VVTTNLGATGGLVVASALMVDYVLTVAVSISSAASNIGSVSPFVYEHKVLFAVGAIVL
IMAMNLRGVRESGLAFAIPTYAFIAGIGTMLVWGLFRIFVLGNPVRAESAAFEMHAEH
GQIVGFALVFLVARSFSSGCAALTGVEAISNGVPAFQKPKSRNAATTLLMLGIIAVSM
FMGMIVLAVETGVQVVDDPDTQLTGAPPGYQQKTLVAQLAQAVFGGFYLGFLLIAAVT
ALILVLAANTAFNGFPVLGSVLAQHSYLPRQLHTRGDRLAFSNGILFLAAAAIGAVVA
FRAELTALIQLYIVGVFISFTMSQVGMVRHWTRLLSAETDPRARRAMLRSRAVNTVGF
VSTGTVLLIVLVTKFLAGAWIAIVAMGGFFMMMKLIHRHYDAVNRELAEQAEEAEITL
PSRNHAVVLVSKLHLPTLRALTYARATRPDVLEAVTVNVDDAETRELVRQWQDSDVSV
PLKVIASPYREITRPVLDYVKRVSKESPRTVVTVFIPEYVVGRWWEQLLHNQSALRLK
GRLLFMPGVMVTSVPWQLTSSERIKTLQPHAAPGDTRRGIFD"
CDS 2970540..2971223
/codon_start=1
/note="gene_2739"
/transl_table=11
/translation="MRVVVMGCGRVGASVADGLSRIGHEVAIIDRDSAAFNRLSPQFA
GERVLGQGFDRDVLLRAGIQGADAFAAVSSGDNSNIISARLARETFGVPRVVARIYDA
KRAEVYERLGIPTIATVPWTTDRLLNALMQDTETAKWRDPTGTVAVAEVVLHEDWVGH
RATDLEQATGARIAFLIRFGTGVLPEPKTVLQAGDKVYIAAISGRAAEAAAIAALPPS
EDFESGARR"
CDS 2971220..2971882
/codon_start=1
/note="gene_2740"
/transl_table=11
/translation="MKVAVAGAGAVGRSVTRELVENGHDITLIERNPDHLDAAAIPEA
HWRLGDACELSLLESIHLEEFDVVVAATGDDKVNVVLSLLAKTEFAVPRVVARVNDPR
NEWLFNDAWGVDVAVSTPRMLASLIEEAVTVGDLVRLMEFRTGQANLVEITLPDNTPW
GGKPVRKLQLPRDAALVTILRGPRVIVPEADEPLEGGDELLFVAVTEAEEELSRLLLP
SM"
CDS complement(2971893..2972564)
/codon_start=1

/note="gene_2741"
/transl_table=11
/translation="VNANRTSAQRLLAQAGGVSGLVYSSLPVVTFVVASSAAGLLPAI
GFALSMAGLILLWRLLRRESARPVVAGFCGVAVCALIAYLVGQSKGYFLLGIWMSLLW
AVVFTLSILIRRPIVGYLWSWLSGRDRAWRDVSRAVFAFDVATLGWTLVFAARFIVQR
HLYDADKTGWLGVARIGMGWPLTALAALATYAAIKAAQRAILASHDAAAVGGAAEFDA
DAGRE"
CDS complement(2972595..2972963)
/codon_start=1
/note="gene_2742"
/transl_table=11
/translation="MGAQGYLRRLTRRLTEDLEQRDVEELSDEVLNAGAQRAIDCQRG
QEVTVVGTLRSVETNGKGCSGGVSAELFDGSDTVTLVWLGQRRIPGIDTGRTLRVRGR
LGKLENGTKAIYNPHYEIQR"
CDS 2973112..2973819
/codon_start=1
/note="gene_2743"
/transl_table=11
/translation="MAVDLDGVTTVLLPGTGSDNDYVRRAFSAPLRRAGAVLVTPVPH
PGRLIDGYRAALDDAARDGPVVVGGVSLGAAVAAAWALEHPDRAVAVLAALPAWTGEP
ELAPAAQAARYTAARLRCDGLAATTTRMRASSPVWLAEELTRSWRVQWPELPDAMEEA
AAYVAPSRAELARLVAPLAVAAAVDDPIHPLQVAADWVSVAPHAALRTVTLDEIGADA
AALGSACLAALAEVSGA"
CDS complement(2974025..2974804)
/codon_start=1
/note="gene_2744"
/transl_table=11
/translation="MAFGRRTGKDGGKRKAGHAPVQPADEHVRPEDTVVASAAAASGV
EDQEELQGPFDIDDFDDPSVAVLARLDLGSVLIPMPAAGQVQVELTESGVPSAVWVIT
PNGRYSIAAYAAPKTGGLWREVAGELADSLRKDSAKVSIKDGPWGREVIGIAAGVVRF
IGVNGYRWMIRCVVNGPQETVDALTEEAREALADTVVRRGDTPLPVRTPLPVHLPEPM
AAQLREAAAAQADTQRQAAAGVARRGAQGSAMQQLRSTTGG"
CDS complement(2974879..2975175)
/codon_start=1
/gene="dut"
/note="gene_2745"
/transl_table=11
/translation="MVGLVHPRSGLATRVGLSIVNSPGTIDAGYRGEIKVALINLDPA
APIVVHRGDRIAQLLVQRVELVELVEVSSFDEAGLASTSRGDGGHGSSGGHASL"
CDS 2975158..2975364
/codon_start=1
/gene="Caci_3048"
/note="gene_2746"
/transl_table=11
/translation="MDQPDHAERDGDRNTRPYQGAAPRCQLDVFGAVEINAGVAVVSA
AGQREPGVEADDRQSGRHGATDYP"
CDS 2975333..2975854
/codon_start=1
/note="gene_2747"
/transl_table=11
/translation="VVDTGPQTTLDRVSGTRLAPHSVRYRERLWVPWWWWPLAFALAA

LIAFEVNLGVAALPDWVPFATLFTVAAGTLLWLGRVEIRVTAGSADGAGVKLWAGPAH
LPVAVIARSAEIPATAKSAALGRQLDPAAYVLHRAWVGPMVLVVLDDPNDPTPYWLVS
CRHPERVLSALRS"

CDS complement(2975859..2976161)
/codon_start=1
/note="gene_2748"
/transl_table=11
/translation="MPTDYDAPRRTETDDVSEDSLEELKARRNEAASAVVDVDESESA
ESFELPGADLSGEELSVRVVPKQADEFTCSSCFLVQHRSRLASEKNGVMICTDCAA"

CDS 2976399..2977049
/codon_start=1
/note="gene_2749"
/transl_table=11
/translation="VVAQITEGTAFDKHGRPFRRRNPRPAIVVVAFLVVVTCVMWTLA
LTRPPDVREAAVCNPPPQPAGSAPTNLGEQVSRTDMTDVAPAKLSDTKVHVLNASGRG
GQAADIAGALQDLGFAQPTAANDPIYAGTRLDCQGQIRFGTAGQATAAALWLVAPCTE
LYHDSRADDSVDLALGTDFTTLAHNDDIDAVLANLRPGATEPSDPALLAKIHANSC"

CDS complement(2977059..2977931)
/codon_start=1
/note="gene_2750"
/transl_table=11
/translation="VTRPDNEPARLRSVAENLAAEAAAFVRGRRAEVFGISRAGDGDG
AVRAKSSPTDPVTVVDTDTERLLRDRLAQLRPGDPILGEEGGGPADVTATPSDRVTWV
LDPIDGTVNFVYGIPAYAVSIGAQVGGITVAGAVADVAARTVYSAATGLGAHLTDERG
RHVLRCTGVDELSMALLGTGFGYSVRCREKQAELLAHVVPLVRDVRRIGSAALDLCMV
AAGRLDAYYEHGVQVWDCAAGALIAAEAGARVLLSTPRAGGAGLVVVAAAPGIADELL
AALQRFNGLEPIPD"

CDS 2978054..2978851
/codon_start=1
/note="gene_2751"
/transl_table=11
/translation="MTSTGPETSETPGATTQRHGFGIDVGGSGIKGGIVDLDTGQLIG
DRIKLLTPQPATPLAVAKTIAEVVNGFGWRGPLGVTYPGVVTHGVVRTAANVDKSWIG
TNARDTIGAELGGQQVTILNDADAAGLAETRYGAGKNNPGLVVLLTFGTGIGSAVIHN
GTLIPNTEFGHLEVGGKEAEERAASSVKEKNDWTYPKWAKQVTRVLIAIENAIWPDLF
IAGGGISRKADKWVPLLENRTPVVPAALQNTAGIVGAAMASVADTTH"

CDS 2979025..2980617
/codon_start=1
/note="gene_2752"
/transl_table=11
/translation="VYVAATKASTATDEPVKRTATKSPAASASGAKTGAKRTAAKSAS
GSPPAKRATKPAARSVKPASAPQDTTTSTIPKRKTRAAAKSAAAKAPSARGHATKPRA
PKDAQHEAATDPEDALDSVEELDAEPDLDVEPGEDLDLDAADLNLDDLEDDVAPDADD
DLDSGDDEDHEDLEAEAAVAPGQTADDDEEIAEPTEKDKASGDFVWDEDESEALRQAR
KDAELTASADSVRAYLKQIGKVALLNAEEEVELAKRIEAGLYATQLMTELSERGEKLP
AAQRRDMMWICRDGDRAKNHLLEANLRLVVSLAKRYTGRGMAFLDLIQEGNLGLIRAV
EKFDYTKGYKFSTYATWWIRQAITRAMADQARTIRIPVHMVEVINKLGRIQRELLQDL
GREPTPEELAKEMDITPEKVLEIQQYAREPISLDQTIGDEGDSQLGDFIEDSEAVVAV
DAVSFTLLQDQLQSVLDTLSEREAGVVRLRFGLTDGQPRTLDEIGQVYGVTRERIRQI
ESKTMSKLRHPSRSQVLRDYLD"

CDS 2980654..2981082

```
                     /codon_start=1
                     /note="gene_2753"
                     /transl_table=11
                     /translation="MSASRTMVSSGSEFESAVGYSRAVRIGPLVVVAGTTGSGDDIAA
                     QTRDALRRIEIALGQAGATLADVVRTRIYVTDISRWREVGEVHAQAFGKIRPVTSMVE
                     VTALIAPGLLVEIEADAYVGSAVADRNSGAGPKDPSPAGG"
     CDS             complement(2981010..2981393)
                     /codon_start=1
                     /note="gene_2754"
                     /transl_table=11
                     /translation="MTPDPAMLVHLCGVQEWSHARERGGIYPESDKTGYIHLSTLEQV
                     HLPANRLYRGRADLVLLYIDPAALDSPVRWEPGVPTDPRSMLFPHLYGPLPVRAVIGA
                     AAYPPAGDGSFGPAPEFRSATADPT"
     CDS             2981519..2981707
                     /codon_start=1
                     /gene="TMKG_03137"
                     /note="gene_2755"
                     /transl_table=11
                     /translation="MNATLTSPELTRADRCDRCGAAARVRAKLPSGAELLFCQHHANE
                     HEAKLTEMSAVLEVSGSE"
     CDS             2981769..2982743
                     /codon_start=1
                     /note="gene_2756"
                     /transl_table=11
                     /translation="MSDQVPKPHRHHIWRITRRTLSKSWDDSIFSESAQAAFWSALSL
                     PPLLLGMLGSLAYVAPLFGPDTLPAIEKSALSTAHSFFSPSVVNEIIEPTIGDITNNA
                     RGEVASLGFLISLWAGSSAISAFVDAVVEAHDQTPLRHPVRQRFFALFLYVVMLVFLV
                     ATAPVMVVGPRKVSEHIPESLANLLRYGYYPALILGLTVGVILLYRVALPVPLPTHRL
                     VLGAVLAIAVFLIATLGLRVYLAWITRTGYTYGALATPIAFLLFAFFGGFAIMLGAEL
                     NAAVQEEWPAPATHAHRLGNWLKARIGVGTTTYSSTAQHSAVAAEPPS"
     CDS             complement(2982744..2982983)
                     /codon_start=1
                     /note="gene_2757"
                     /transl_table=11
                     /translation="MQTQTIERTDADERVDDGTGSDTPKYFHYVKKDKIAESAVMGSH
                     VVALCGEVFPVTRAPKPGSPVCPDCKRIYDTLKKG"
     CDS             2983053..2983481
                     /codon_start=1
                     /note="gene_2758"
                     /transl_table=11
                     /translation="MKHGLRLGFNGQFDDFDDFDDKGRPVLISAAAPSYEVEHRTRVR
                     KYLTLMAFRVPALILAAIAYGAWHNGLISLLIVAASVPLPWMAVLIANDRPPRRADEP
                     RRFDVARRRIPLFPTAERPALEPRRQPAERSAPRGFADHG"
     CDS             2983657..2984628
                     /codon_start=1
                     /note="gene_2759"
                     /transl_table=11
                     /translation="MADAPTRATTSRVDSDLDAQSPAADLVRVYLNGIGKTALLNAAG
                     EVELAKRIEAGLYAEHLLETRKRLGENRKRDLAAVVRDGEAARRHLLEANLRLVVSLA
                     KRYTGRGMPLLDLIQEGNLGLIRAMEKFDYTKGFKFSTYATWWIRQAITRGMADQSRT
                     IRLPVHLVEQVNKLARIKREMHQHLGREATDEELAAESGIPIDKINDLLEHSRDPVSL
```

DMPVGSEEEAPLGDFIEDAEAMSAENAVIAELLHTDIRSVLATLDEREHQVIRLRFGL
DDGQPRTLDQIGKLFGLSRERVRQIERDVMSKLRHGERADRLRSYAS"
CDS 2984761..2985453
/codon_start=1
/note="gene_2760"
/transl_table=11
/translation="MNELVDTTEMYLRTIYDLEEEGVTPLRARIAERLDQSGPTVSQT
VSRMERDGLLRVAGDRHLELTEKGRALAIAVMRKHRLAERLLVDVIGLPWEEVHAEAC
RWEHVMSEDVERRLVKVLNNPTTSPFGNPIPGLVELGVGPEPGADDANLVRLTELPAG
SPVAVVVRQLTEHVQGDIDLITRLKDAGVVPNARVTVETTPGGGVTIVIPGHENVTLP
HEMAHAVKVEKV"
CDS complement(2985466..2986548)
/codon_start=1
/note="gene_2761"
/transl_table=11
/translation="VPVVSVAFMTKYRGQFELNRPATLIAALPAILGFVPEKSLVLVS
LAAGELGSVMRADLCDELADRVGHLAELVAAANPAAAIAVIVDANGAQCPRCNEEYRQ
LCAALAAALSQRDIVLWAAHVVDRVAAGGRWHCVDGCGCSGVIDDPSASPLAMAAVLD
GRQLYPRRSDLQAVIAVDDPVRSAELAVALGHQAADREIAHRADSVGCSRQDVENALA
AAARVADGQSLSDTELARLGCALGDARVRDMLYALAVGENAGAAESLWALLARVLPEP
WRVEALVLLAFSAYARGDGPLAGVSLQAALCCEPGHRMAGMLDTALQSGLRPEHIRDI
AVTGYQRAEQLGIRLPPRRAFGQRAG"
CDS 2986637..2988043
/codon_start=1
/note="gene_2762"
/transl_table=11
/translation="MREYDIVVIGSGPGGQKAAIASAKLGKSVAIVERGRMLGGVCVN
TGTIPSKTLREAVLYLTGMNQRELYGASYRVKDRITPADLLARTQHVIGKEVDVVRNQ
LMRNRVDLIVGHGRFIDPHTILVEDQARREKTTVTGDYIIIATGTRPARPSGVEFDEE
RVLDSDGILDLKSLPSSMVVVGAGVIGIEYASMFAALGTKVTVVEKRDNMLDFCDPEV
VEALKFHLRDLAVTFRFGEEVTAVDVGSAGTVTTLASGKQIPAETVMYSAGRQGQTDH
LDLHNAGLEVQGRGRIFVDDRFQTKVDHIYAVGDVIGFPALAATSMEQGRLAAYHAFG
EPTDGITELQPIGIYSIPEVSYVGATEVELTKSSIPYEVGVARYRELARGQIAGDSYG
MLKLLVSTEDLKLLGVHIFGTSATEMVHIGQAVMGCGGSVEYLVDAVFNYPTFSEAYK
NAALDVMNKMRALNQFRR"
CDS 2988261..2989235
/codon_start=1
/note="gene_2763"
/transl_table=11
/translation="MARDQGADEAREYEPGQPGMYELEFPAPQLSSSDGRGPVLVHAL
EGFSDAGHAIRLAAAHLKAALDTELVASFAIDELLDYRSRRPLMTFKTDHFTHSDDPE
LSLYALRDSIGTPFLLLAGLEPDLKWERFITAVRLLAERLGVRQTIGLGTVPMAVPHT
RPITMTAHSNNRELISDFQPWISEIQVPGSASNLLEYRMAQHGHEVVGFTVHVPHYLT
QTDYPAAAQALLEQVAKTGSLQLPLAALAEAAAEVQAKIDEQVQASAEVAQVVAALER
QYDAFIDAQENRSLLTRDEDLPSGDELGAEFERFLAQQAEKKSDDDPT"
CDS 2989324..2990319
/codon_start=1
/note="gene_2764"
/transl_table=11
/translation="VRDVAPPTLQFRTVHGYRRAFRIAGSGPAILLIHGIGDNSTTWN
GVHAKLAQRFTVIAPDLLGHGQSDKPRADYSVAAYANGMRDLLSVLDIERVTIVGHSL

GGGVAMQFAYQFPQLVDRLILVSAGGVTKDVNIVFRLASLPMGSEAMALLRLPLVLPA
VQIAGRIVGKAIGTTSLGHDLPNVLRILDDLPEPTASAAFGRTLRAVVDWRGQMVTML
DRCYLTEAIPVQIIWGTKDVVLPVRHAHMAHAAMPGSQLEIFEGSGHFPFHDDPARFI
DIVERFMDTTEPAEYDQAALRALLRRGGGEATVTGSADTRVAVLNAIGSNERSAT"

CDS 2990368..2991054
/codon_start=1
/note="gene_2765"
/transl_table=11
/translation="MAIEVSVLRVFTDSDGNFGNPLGVINASKVEHRDRQQLAAQSGY
SETIFVDLPSPGSTTAHATIHTPRTEIPFAGHPTVGASWWLRERGTPINTLQVPAGIV
QVSYHGDLTAISARSEWAPEFAIHDLDSLDALAAADPADFPDDIAHYLWTWTDRSAGS
LRARMFAANLGVTEDEATGAAAIRITDYLSRDLTITQGKGSLIHTTWSPEGWVRVAGR
VVSDGVAQLD"

CDS complement(2991063..2991581)
/codon_start=1
/note="gene_2766"
/transl_table=11
/translation="MPVPRKRAMTRDLAPALQALSPLLGSWAGRGAGKYPTIRPFEYL
EEVVFAHVGKPFLTYTQQTRAVADGKPLHSETGYLRVCRPGCVELVLAHPSGITEIEV
GTYSVTGDVIELELSTRADGSIGLAPTAKEVTALDRSYRIDGDELSYSLQMRAVGQPL
QDHLAAVLHRQR"

CDS complement(2991609..2992073)
/codon_start=1
/gene="nrdR"
/note="gene_2767"
/transl_table=11
/translation="MHCPFCRHPDSRVIDSRETDEGQAIRRRRSCPECGRRFTTVETA
VLAVVKRSGVTEPFSREKVISGVRRACQGRQVDDDALNLLAQQVEDSVRAAGSPEIPS
HDVGLAILGPLRELDEVAYLRFASVYRSFSSADDFAREIEALRAHRNLSAHS"

CDS complement(2992236..2992526)
/codon_start=1
/note="gene_2768"
/transl_table=11
/translation="VGLALLAAAITLWLGLVAQFGQMITGGSADGSADSTGRVPDRLA
VVRVETGESLHDVAVRVAPNAPTRQVADRIRELNGLQTPALAVGQTLIAPVG"

CDS 2992984..2993694
/codon_start=1
/note="gene_2769"
/transl_table=11
/translation="MNDSNDTSVAGGAAGADSRVLSADSALTERQRTILDVIRASVTS
RGYPPSIREIGDAVGLTSTSSVAHQLRTLERKGYLRRDPNRPRAVNVRGADDAALPPV
TEVAGSDALPEPTFAPVLGRIAAGGPILAEEAVEDVFPLPRELVGEGTLFLLKVIGDS
MVEAAICDGDWVVVRQQNVADNGDIVAAMIDGEATVKTFKRAGGQVWLMPHNPAFDPI
PGNDATVLGKVVTVIRKV"

CDS complement(2993716..2995815)
/codon_start=1
/note="gene_2770"
/transl_table=11
/translation="VNGQRGQLSTLIGRTLLGLAATAVTAVLLAPTVAASPMGDAEDA
MMAAWEKAGGDTSTLGVRKGDVYPIGDGFALDFAGGKMFFTPATGAKYLYGPLLDKYE
SLGGAADSDLGFPTINEVPGLAGPDSRVSTFSAADNPVIFWTPEHGAFVVRGALNAAW

DKLGSSGGVLGAPVGDETYDGEVTAQKFSGGEVSWNRATKEFTTVPAVLAEQLKGLQV
AIDPSAAINMAWRAAGGAAGPLGAKKGGQYPISGDGIAQDFVGGKVFFSPATGANAVE
GEILAKYESLGGPVSSDLGFPIANETDGGFGPSSRIVRFSAADKPVIFWAPDHGAFVV
RGAMVAAWDKLRGPNGKLGAPVGDQTVDGDVVSQKFTGGMISWNRAKNTFTTDPANLA
PLLSGLQVSGQNQPSTSAMPPPGKKFTWHWWWLGAAALGVLLVVMVALVVFGLRRRRR
GYDAAAYDDDRAGDVEYGTAADGDWPPDEDFGSEHFGFGDQFPPEPVAPDAGSTPRVS
WPRGAGAAVGDAEHLPGEEGYGSDLLSGPSNVGVEEEDTDAVDTTPTPVVSQADLSEV
GPDLIVPERVVPETFVPQAFVPEAVAPEAVPPDVHAADLADTGLPAAAVSAAEDRGGR
HAAAEPPEPPSAGVRPAIHLPLEDPYQMPNGYPVKASVSFGLYYPPGSALYHDTLAEL
WFASEEVAQVNGFIRAD"

CDS 2996105..2997298
/codon_start=1
/note="gene_2771"
/transl_table=11
/translation="MGASGLVWTLTIVLIAGLMLVDYVLHVRKTHVPTLRQAVIQSAT
FVGIAILFGIAVVVFGGSELAVEYFACYLTDEALSVDNLFVFLVIISSFGVPRLAQQK
VLLFGIAFALVTRTGFIFVGAALIENFNSAFYLFGLVLLVMAGNLARPTGLESRDAET
LKRSVIIRLADRFLRTSQDYNGDRLFTVSNNKRMMTPLLLVMIAVGGTDILFAFDSIP
ALFGLTQNVYLVFAATAFSLLGLRQLYFLIDGLLDRLVYLSYGLAVILGFIGVKLMLE
ALHDNKIPFINGGKPVPTVEVSTTQSLTVIIIVLLITTAASFWSARGRAQNAMARARR
YATAYLDLHYETESAERDKIFTALLAAERQINTLPTKYRMQPGQDDDLMTLLCRAHAA
RDAHM"

CDS complement(2997327..2998487)
/codon_start=1
/note="gene_2772"
/transl_table=11
/translation="MGSATKYQRTLFEPEHELFRESYRAFLDRHVAPYHDEWEKTKIV
DRGVWLEAGKQGFLGMAVPEEYGGGGNADFRYNTVITEETCAGRYSGIGFGLHNDIVA
PYLLALATEEQKRRWFPNFCTGELTTAIAMTEPGTGSDLQGITTRAVKHGDHYVLNGS
KTFITNGINSDLVIVVAQTDPEKGAQGFSLLVVERGMAGFERGRQLDKIGLDAQDTAE
LSFTDVAVPAENLLGQEGMGFIYLMQNLPQERISIAIMAAAGMESVLEQTLQYAKERK
AFGRSIGSFQNSRFLLAELATEATVVRIMVDEFIKLHLAGKLTAEQAAMAKWYATEKQ
VYLNDRCLQLHGGYGYMREYPVARAYLDSRVQTIYGGTTEIMKEIIGRGLGV"

CDS complement(2998623..3000053)
/codon_start=1
/note="gene_2773"
/transl_table=11
/translation="MTYPDPPQTGLSDFTPSLGELALEDRSALRRVAGLSTELADVSE
VEYRQLRLERVVLVGVWTEGSAADNRASLAELAALAETAGSQVLEGLIQRRDKPDPST
YIGSGKAAELREVIVATGADTVICDGELSPAQLTALEKAVQVKVIDRTALILDIFAQH
ATSREGKAQVSLAQMEYMLPRLRGWGESMSRQAGGRAGDSGGGVGLRGPGETKIETDR
RRIRERMAKLRRDIRAMKQVRDTQRSRRRHSDVPSIAIVGYTNAGKSSLLNALTGAGV
LVQDALFATLEPTTRRAEFGDGRPVVLTDTVGFVRHLPTQLVEAFRSTLEEVVHADLL
VHVVDGSDGHPLAQIDAVRQVISEVIADHDGDPPPELLVVNKVDVASDLMLAKLRHGL
PGAVFVSARTGDGIDALRRRMAELVVPADTAVDVVIPYDRGDLVARVHADGRIQQAEH
KPEGTRIKARVPEALAATLREFAPRA"

CDS complement(3000127..3000996)
/codon_start=1
/gene="dapF"
/note="gene_2774"
/transl_table=11

```
                     /translation="MIFAKGHGTQNDFVLLPDVDAELVLTAARVAALCDRRKGLGADG
                     VLRVTTAGAAQAVGVLDSLPEGVRVTDWYMDYRNADGSAAQMCGNGVRVFAHYLRASG
                     LEVRDEFVVGSLAGPRPVTCHHVEAAYADVSVDMGKANRLGAGEAVVGGRRFHGLAVD
                     VGNPHLACVDSQLTVDGLAALDVGAPVSFDGAQFPDGVNVEVLTAPVDGAVWMRVHER
                     GVGETRSCGTGTVAAAVAALAAVGSPTGTLTVHVPGGEVVVTVTDATSFLRGPSVLVA
                     RGDLADDWWNAMG"
CDS                  complement(3001021..3001965)
                     /codon_start=1
                     /gene="miaA"
                     /note="gene_2775"
                     /transl_table=11
                     /translation="VRPLAIIGPTGAGKSQLALDVAARLGARVSVEIVNADAMQLYRG
                     MDIGTAKLPVSERRGIPHHQLDVLDVTETATVARYQRAAAADIEAIAARGAVPVVVGG
                     SMLYVQSLLDDWSFPATDPSVRARWERRLAEVGVDRLHAELARRDPAAAAAILPTDAR
                     RTVRALEVVELTGQPFAASAPRIGAPRWDTVIVGLDCQTTILDERLARRTDLMFDQGL
                     VEEVRTLLRNGLREGVTASRALGYAQVIAALDAGAGADMMRAAREQTYLGTRRYVRRQ
                     RSWFRRDHRVHWLDAGVASSPDRARLVDDAVRLWRHVT"
CDS                  complement(3001962..3002657)
                     /codon_start=1
                     /note="gene_2776"
                     /transl_table=11
                     /translation="VLSAIGIVPSAPVLVPELAGAAAAELADLGAAVIAAASLLPKSW
                     IAVGTGRADDVVRPTDVGTFAGFGADVRVGLAPQDGDGVAVPVELPLCALLTAWVRGQ
                     ARPEARAQVHVYASDHGSDAAVARGRQLRADIDREPDPIGVLVVADGLNTLTPRAPGG
                     YDPDGAGMQRALDDALASGDLAVLTRLPAQVLGRVAFQVLAGLAEPGPRSAKEFYRGA
                     PHGVGYFAGVWQP"
CDS                  complement(3002766..3003671)
                     /codon_start=1
                     /note="gene_2777"
                     /transl_table=11
                     /translation="MASVEFATILALGAALLAGIGYVTLQRSARQVTAEEYVGHLTLF
                     HLSLRHALWWLGSLAAVASFTLQAIALTMGSVVLVQSLQATALLFALLIDARLTHHRC
                     TPREWMWAVLLAGAVAVIVMSGNPAAGTTRAPFSTWAVVAVVVVPAVVLCVVGARIAS
                     GSLSAVLLAVASSATLAVFTVLTKGVVTELGEGFATLIRTPELYAWILVLPIGLMLQQ
                     SSLRVGALTASLPTITVARPVIASVLGITVLDEVLHTGRVALVALVAVVVVVVVATVA
                     LARDEVAMMTVSAGELGAAGQLAVR"
CDS                  3004177..3005574
                     /codon_start=1
                     /note="gene_2778"
                     /transl_table=11
                     /translation="MRGIRAKGCKVGDKRMTADEPRSDDSSGSAPQPAATPVPRPGPR
                     PGPRPVPRPTSYPVGAHPPSDPHRFGRIDDDGTVWLVSASGERIVGSWQAGDPEAAFA
                     HFGRRFDDLSTEIMLMDERLASGTGDARKIKAHAIALAETLPTACVLGDVDALADRLT
                     SIRDRAEVIAAADRSRREEHRAAQTARKEALAAEAEELAANATQWKVAGDRLRAILDE
                     WKTISGVDRKVDDALWKRYSTARDTFNRRRGSHFAELDRERSGVRQSKERLCERAEEL
                     SESTDWTATSAEFRKLLADWKAAGRASKDVDDALWRRFKAAQDSFFTARNAATAEKEA
                     ELRANADAKEALLAEAERLDTTNHEAARAALRSIAEKWDAIGKVSRERAAELERRLRA
                     VEKKVREAGEADWSDPQARARAEQFRARAEQFEHQAEKAAAAGRTKEADEAKANAEQW
                     RQWAEAAADALTRRP"
CDS                  complement(3005571..3006182)
                     /codon_start=1
```

/note="gene_2779"
/transl_table=11
/translation="MSHEHDAGDLDALRAEIEAAERRVAREIEPGARALVVAILVFVL
LGSFILPHTGSVRGWDVLFSSHGAGRAAVALPSRVFAWLALVFGVGFSMLALLTRRWA
LAWVALAGSAMASGTGLLAVWSRQTVAAGHPGPGIGLIVAWITAIVLTFHWAQVVWSR
TIVQLAAEERRRRVVAQQQCKTLLDHVQTDSEAGTTPDRGTDR"
CDS complement(3006182..3007720)
/codon_start=1
/note="gene_2780"
/transl_table=11
/translation="MVAHDAAAGVTGEGAGPPVRRAPARTYQVRTYGCQMNVHDSERL
AGLLEAAGYRRATDGSEADVVVFNTCAVRENADNRLYGNLSHLAPRKRANPDMQIAVG
GCLAQKDRDAVLRRAPWVDVVFGTHNIGSLPTLLERARHNKVAQVEIAEALQQFPSSL
PSSRESAYAAWVSISVGCNNSCTFCIVPSLRGREVDRSPADILAEVRSLVNDGVLEVT
LLGQNVNAYGVSFADPALPRNRGAFAELLRACGDIDGLERVRFTSPHPAEFTDDVIEA
MAQTRNVCPALHMPLQSGSDRILRAMRRSYRAERYLGIIERVRAAIPHAAITTDLIVG
FPGETEEDFAATLDVVRRARFAAAFTFQYSKRPGTPAAQLDGQLPKAVVQERYERLIA
LQEQISLEANRALVGQAVEVLVATGEGRKDTVTARMSGRARDGRLVHFTAGQPRVRPG
DVITTKVTEAAPHHLIADAGVLTHRRTRAGDAHTAGQPGRAVGLGMPGVGLPVSAAKP
GGCR"
CDS 3008017..3008871
/codon_start=1
/note="gene_2781"
/transl_table=11
/translation="MSDRSAIEWTGATWNPVTGCDRVSPGCDHCYAMTLAKRLKAMGS
DKYQTDGDPRTSGPGFGVTIHPRSLDEPFRWRSPRTVFVNSMADLFHARVALWFIREV
FEVMRATPQHTYQILTKRSLRLRRLAHKLEWPSNVWMGVSVENVDAFRRIEDLRQVPA
AVRFLSCEPLLGPLDGINLGSIDWVIAGGESGPNFRPIDPQWVRHIRDTCTAADVPFF
FKQWGGRTPKAFGRELDGRCWDEMPLIEIRNPDPRTTSRVHADPMLATAPTESAQRSN
PGQLVRQR"
CDS complement(3008756..3009748)
/codon_start=1
/note="gene_2782"
/transl_table=11
/translation="MAREWSYWTRNKLEILAGYLPAFNRASQTSRERIYLDLMAGQPE
NIDRDMGEKFDGSSLIAMKADPPFTRLRFCELNPLASELDVALRTRFPGDGRYRVVAG
DSNVTIDETLAELGPWRWAPTFAFIDQQAAEVHWETINKVAAFRQNPRNLKTELWMLM
SPTMIARGVKGTNAELFIEQVTRMYGDADWKRIQAARWRHHLTAPAYRAEMVNLMRVK
LEYELGYKYSHRIPMQMHNKVTIFDMVFATDHWAGDAIMCHLYNRAAQKEPEMMRQAK
SAKQQKESEDRGEMGLFSVGELAVQDSNAGQILWAPSPTWDPRARGWWSEDPGF"
CDS complement(3009758..3010282)
/codon_start=1
/note="gene_2783"
/transl_table=11
/translation="MTVSCPPPSTSEREEQARALCLRLLTARSRTRAELAGQLAKRGY
PEDIGNRVLDRLAAVGLVDDTDFAEQWVQSRRANAAKSKRALAAELHAKGVDDDVITT
VLGGIDAGAERGRAEKLVRARLRREVLIDDGTDEARVSRRLVAMLARRGYGQTLACEV
VIAELAAERERRRV"
CDS complement(3010248..3012620)
/codon_start=1
/note="gene_2784"

/transl_table=11
/translation="MTQTPDREKALELAVAQIEKSYGKGSVMRLGDEARQPISVIPTG
SIALDVALGIGGLPRGRVIEIYGPESSGKTTVALHAVANAQAAGGVAAFIDAEHALDP
DYAKKLGVDTDSLLVSQPDTGEQALEIADMLIRSGALDIVVIDSVAALVPRAELEGEM
GDSHVGLQARLMSQALRKMTGALNNSGTTAIFINQLRDKIGVMFGSPETTTGGKALKF
YASVRMDVRRVETLKDGTNAVGNRTRVKVVKNKCLAEGTRIFDPVTGTTHRIEDVVDG
RKPIHVVAAAKDGTLHARPVVSWFDQGTRDVIGLRIAGGAIVWATPDHKVLTEYGWRA
AGELRKGDRVAQPRRFDGFGDSAPIPADHARLLGYLIGDGRDGWVGGKTPINFINVQR
ALIDDVTRIAATLGCAAHPQGRISLAIAHRPGERNGVADLCQQAGIYGKLAWEKTIPN
WFFEPDIAADIVGNLLFGLFESDGWVSREQTGALRVGYTTTSEQLAHQIHWLLLRFGV
GSTVRDYDPTQKRPSIVNGRRIQSKRQVFEVRISGMDNVTAFAESVPMWGPRGAALIQ
AIPEATQGRRRGSQATYLAAEMTDAVLNYLDERGVTAQEAAAMIGVASGDPRGGMKQV
LGASRLRRDRVQALADALDDKFLHDMLAEELRYSVIREVLPTRRARTFDLEVEELHTL
VAEGVVVHNCSPPFKQAEFDILYGKGISREGSLIDMGVDQGLIRKSGAWFTYEGEQLG
QGKENARNFLVENADVADEIEKKIKEKLGIGAVVTDDPSNDGVLPAPVDF"
CDS 3012815..3012988
/codon_start=1
/note="gene_2785"
/transl_table=11
/translation="VRPDLRARLVRITDDLLNTASLAGSGVLTGPDLTFRRRSCCLFY
RVPAGGKCGDCPL"
CDS complement(3013002..3013208)
/codon_start=1
/note="gene_2786"
/transl_table=11
/translation="MLAGVRLTEFHERVALHFGAAYGSSVLLDHVLTGFDGRSAAQAI
EDGVEPRDVWRALCADFDVPHDRW"
CDS complement(3013219..3014385)
/codon_start=1
/note="gene_2787"
/transl_table=11
/translation="MRVAVVAGPDPGHSFPAIALCQRFRAAADTPTLFTGVEWLEAAR
AAGIDAVELDGLAATDRDLDAGARIHRRAAQMAVLNVPRLRALEPELVVSDVITACGG
MAAELLGIPWVELNPHPLYLPSKGLPPIGSGLAAGTGIRGRLRDATMRALTGRSWRAG
LRQRAAVRVEIGLPARDPGPLRRLIATLPALEVPRPDWPAEAVVVGPLHFEPTDRVLA
IPAGTGPVVVVAPSTALTGTAGLTEVALQSLTPGETVPSGSRLVVSRLSGADLTVPPW
AVAGLGSQAELLTRADLVICGGGHGMVAKTLLAGVPMVVVPGGGDQWEIANRVVRQGS
AVLIRPLTADALVAAVNEVLSSPRFREAARRAAASVAGAADPVRVCHDALALAG"
CDS 3014429..3014878
/codon_start=1
/gene="ephG"
/note="gene_2788"
/transl_table=11
/translation="MAELTETSPETPETTEAIRAVEAFLNALQNEDFDTVDAALGDDL
VYENVGFSRIRGGRRTATLLRRMQGRVGFEVKIHRIGADGAAVLTERTDALIIGPLRV
QFWVCGVFEVDDGRITLWRDYFDVYDMFKGLLRGLVALVVPSLKATL"
CDS 3015154..3016602
/codon_start=1
/note="gene_2789"
/transl_table=11
/translation="MDLASIGSTVSAASAAASAPTVAILAAGADEVSIAVAALFGMHG

```
                     QAYQALSVQASAFHQQFVQALTAGAYSYASAEAAAVTPLQQLVDVINAPFRSALGRPL
                     IGNGANGKPGTGQDGGAGGLLYGSGGNGGSGLAGSGQKGGNGGAAGLFGNGGAGGAGA
                     SNQAGNGGAGGNGGAGGLIWGTAGTGGNGGFTTFLDAAGGAGGAGGAGGLFGAGGAGG
                     VGGAALGGGAQAAGGNGGAGGVGGLFGAGGAGGAGGFGDTGGTGGDGGSGGLFGVGGA
                     GGHGGFGSAAGGDGGAGGAGGTVFGSGGAGGAGGVATVAGHGGHGGNAGLLYGTGGAG
                     GAGGFGGFGGDGGDDGIGGLVGSGGAGGSGGTGTLSGGRGGAGGNAGTFYGSGGAGGA
                     GGESDNGDGGNGGVGGKAGLVGEGGNGGDGGATIAGKGGSGGNGGNAWLTGQGGNGGN
                     AAFGKAGTGSVGVGGAGGLLEGQNGENGLLPS"
     variation       3016136..3016309
                     /note="supplemented from AL2122/97"
     CDS             3016940..3017149
                     /codon_start=1
                     /note="gene_2790"
                     /transl_table=11
                     /translation="LARHPVGHPPQIPATERTETPTFGPLRIRFWACGIFEVQRARYG
                     ITTSASKALVRGLTASAFSSPRATL"
     CDS             3017153..3017557
                     /codon_start=1
                     /note="gene_2791"
                     /transl_table=11
                     /translation="MSDNAIRPRPNPWQYIRYCYGARLPDSMRDWVRNDLAGKGAAIR
                     MMIRVAVPAVLVLAPFWLIPTSLDVHLSMTLPILIPFVYFSHALNKVWRRHMLRVHNL
                     DPELVDEHARQRDAHIHRAYIERYGPRPDPND"
     CDS             complement(3017554..3018366)
                     /codon_start=1
                     /note="gene_2792"
                     /transl_table=11
                     /translation="MAVKAGQRRPWRSLLQRGVDTAGDLADLVAQKISVAIDPRARLL
                     RRRRRALRWGLVFTAGCLLWGLVTALLAAWGWFTSLLVITGTIAVTQAIPATLLLLRY
                     RWLRSEPLPVRRPASVRRLPPPGSAARPAMSALGASERGFFSLLGVMERGAMLPADEI
                     RDLTAAANQTSAAMVATAAEVVSMERAVQCSAASRSYLVPTINAFTAQLSTGVRQYNE
                     MVTAAAQLVSSANGAGGAGPGQQRYREELAGATDRLVAWAQAFDELGGLPRR"
     CDS             complement(3018385..3019197)
                     /codon_start=1
                     /note="gene_2793"
                     /transl_table=11
                     /translation="MANPFVKAWKYLMALFSSKIDEHADPKVQIQQAIEEAQRTHQAL
                     TQQAAQVIGNQRQLEMRLNRQLADIEKLQVNVRQALTLADQATAAGDAAKATEYNNAA
                     EAFAAQLVTAEQSVEDLKTLHDQALSAAAQAKKAVERNAMVLQQKIAERTKLLSQLEQ
                     AKMQEQVSASLRSMSELAAPGNTPSLDEVRDKIERRYANAIGSAELAESSVQGRMLEV
                     EQAGIQMAGHSRLEQIRASMRGEALPAGGTTATPRPATETSGGAIAEQPYGQ"
     CDS             complement(3019327..3019665)
                     /codon_start=1
                     /note="gene_2794"
                     /transl_table=11
                     /translation="MAALVREVVGDVLRGARMSQGRTLREVSDSARVSLGYLSEIERG
                     RKEPSSELLSAICTALQLPLSVVLIDAGERMARQERLARATPAGRATGATIDASTKVV
                     IAPVVSLAVA"
     CDS             complement(3019736..3020335)
                     /codon_start=1
                     /note="gene_2795"
```

/transl_table=11
/translation="VSAQPETGQIAGRARIANLANILTLLRLVMVPVFLLALFYGGGH
HSAARVVAWAIFATACITDRFDGLLARNYGMATEFGAFVDPIADKTLIGSALIGLSML
GDLPWWVTVLILTRELGVTVLRLAVIRRGVIPASWGGKLKTFVQAVAIGLFVLPLSGP
LHVAAVVVMAAAILLTVITGVDYVARALRDIGGIRQTAS"
CDS 3020426..3020920
/codon_start=1
/note="gene_2796"
/transl_table=11
/translation="VVRRARTSDVPAIKQLVDTYAGKILLEKNLVTLYEAVQEFWVAE
HPDLYGKVVGCGALHVLWSDLGEIRTVAVDPAMTGHGIGHAIVDRLLQVARDLQLQRV
FVLTFETEFFARHGFTEIEGTPVTAEVFDEMCRSYDIGVAEFLDLSYVKPNILGNSRM
LLVL"
CDS complement(3020989..3023292)
/codon_start=1
/note="gene_2797"
/transl_table=11
/translation="MRAVWMMAAKGTGGAARSIGRARDIEPGHRRDGIALVLLGLAVV
VAASSWFDAARPLGAWVDALLRTFIGSAVVMLPLVAAAVAVVLMRTSPNPDSRPRLIL
GASLIGLSFLGLCHLWAGSPEAPESRLRAAGFIGFAIGGPLSDGLTAWIAAPLLFIGA
LFGLLLLAGITIREVPDAMRAMFGTRLLPREYADDFEDFADFDGDDADTVEVARQDFS
DGYYDEVPLCSDDGPPAWPSAEVPQDDTATIPEASAGRGSGRRGRRKDTQVLDRIVEG
PYTLPSLDLLISGDPPKKRSAANTHMAGAIGEVLTQFKVDAAVTGCTRGPTVTRYEVE
LGPGVKVEKITALQRNIAYAVATESVRMLAPIPGKSAVGIEVPNTDREMVRLADVLTA
RETRRDHHPLVIGLGKDIEGDFISANLAKMPHLLVAGSTGSGKSSFVNSMLVSLLTRA
TPEEVRMILIDPKMVELTPYEGIPHLITPIITQPKKAAAALAWLVDEMEQRYQDMQAS
RVRHIDDFNDKVRSGAITAPLGSQREYRPYPYVVAIVDELADLMMTAPRDVEDAIVRI
TQKARAAGIHLVLATQRPSVDVVTGLIKTNVPSRLAFATSSLTDSRVILDQAGAEKLI
GMGDGLFLPMGASKPLRLQGAYVSDEEIHAVVTACKEQAEPEYTEGVTTAKPTAERTD
VDPDIGDDMDVFLQAVELVVSSQFGSTSMLQRKLRVGFAKAGRLMDLMETRGIVGPSE
GSKAREVLVKPDELAGTLAAIRGDGGE"
CDS 3023639..3023953
/codon_start=1
/note="gene_2798"
/transl_table=11
/translation="MPVVVVATLTAKPESVDTVRDILTRAVDDVHREPGCQLYALHET
GETFIFVEQWADAEALKAHSGAPAVATMFTAAGEHLVGAPDIKLLQPVPAGDPSKGQL
RR"
CDS 3023950..3024768
/codon_start=1
/note="gene_2799"
/transl_table=11
/translation="VIDRPLEGKVAFITGAARGLGRAHAVRLAADGANIIAVDICEQI
ASVPYPLSTADDLAATVELVEDAGGGIVARQGDVRDRASLSVALQAGLDEFGRLDIVV
ANAGIAMMQAGDDGWRDVIDVNLTGVFHTVQVAIPTLIEQGTGGSIVLISSAAGLVGI
GSSDPGSLGYAAAKHGVVGLMRAYANHLAPQNIRVNSVHPCGVDTPMINNEFFQQWLT
TADMDAPHNLGNALPVELVQPTDIANAVAWLASEEARYVTGVTLPVDAGFVNKR"
CDS 3024772..3025662
/codon_start=1
/note="gene_2800"
/transl_table=11

/translation="MARNPAAQTAFGPMVLAAVEQNEPPGRRLVDDDLADLFLPRPLR
WLAGATRSAVLRRLLISASEWSGRGLWANLACRKRFIGDKLDEALGDIDAVVILGAGL
DTRAYRLTRRVRMPVFEVDLPVNIARKAKTVRRVLGELPLSVRLVALDFEHDDLLTAL
AEHGYRTEYRVFFVCEGVTQYLTERAVRRTLEGLRAAAPGSRMVFTYVRRDFIDGTNR
YGTRTLYHTVRQRRQLWHFGLDPEEVAGFLADYGWRLTEQAGPEELVQRYVEPTGRNL
NASQIEWSAYAEKSEPVTPR"

CDS complement(3025649..3027325)
/codon_start=1
/note="gene_2801"
/transl_table=11
/translation="VDVDLPPPGPLTSGGLRVTALGGINEIGRNMTVFEHLGRLLIID
CGVLFPGHDEPGVDLILPDMRHVEDRLDDIEALVLTHGHEDHIGAIPFLLKLRPDIPV
VGSKFTLALVAEKCREYRITPVFVEVREGQSTRHGVFECEYFAVNHSTPDALAIAVYT
GAGTILHTGDIKFDQLPPDGRPTDLPGMSRLGDTGVDLLLCDSTNAEIPGVGPSESEV
GPTLHRLIRGADGRVIVACFASNVDRVQQIIDAAVALGRRVSFVGRSMVRNMRVARQL
GFLRVADSDLIDIAAAETMAPDQVVLITTGTQGEPMSALSRMSRGEHRSITLTAGDLI
VLSSSLIPGNEEAVFGVIDALSKIGARVVTNAQARVHVSGHAYAGELLFLYNGVRPRN
VMPVHGTWRMLRANAKLAASTGVPQESILLAENGVSVDLVAGKASISGAVPVGKMFVD
GLIAGDVGDITLGERLILSSGFVAVTVVVRRGTGQPLAAPHLHSRGFSEDPKALEPAV
RKVEAELESLVAANVTDPIRIAQGVRRTVGKWVGETYRRQPMIVPTVIEV"

CDS complement(3027356..3028258)
/codon_start=1
/note="gene_2802"
/transl_table=11
/translation="VTTVGFDVAARLGTLLTAMVTPFSGDGSLDTATAARLANHLVDQ
GCDGLVVSGTTGESPTTTDGEKIELLRAVLEAVGDRARVIAGAGTYDTAHSIRLAKAC
AAEGAHGLLVVTPYYSKPPQRGLQAHFTAVADATELPMLLYDIPGRSAVPIEPDTIRA
LASHPNIVGVKDAKADLHSGAQIMADTGLAYYSGDDALNLPWLAMGATGFISVIAHLA
AGQLRELLSAFGSGDIATARKINIAVAPLCNAMSRLGGVTLSKAGLRLQGIDVGDPRL
PQVAATPEQIDALAADMRAASVLR"

CDS complement(3028327..3029079)
/codon_start=1
/gene="thyX"
/note="gene_2803"
/transl_table=11
/translation="VAETAPLRVQLIAKTDFLAPPDVPWTTDADGGPALVEFAGRACY
QSWSKPNPKTATNAGYLRHIIDVGHFSVLEHASVSFYITGISRSCTHELIRHRHFSYS
QLSQRYVPEKDSRVVVPPGMEDDADLRHILTEAADAARATYSELLAKLEAKFADQPNA
ILRRKQARQAARAVLPNATETRIVVTGNYRAWRHFIAMRASEHADVEIRRLAIECLRQ
LAAVAPAVFADFEVTTLADGTEVATSPLATEA"

CDS complement(3029595..3031217)
/codon_start=1
/note="gene_2804"
/transl_table=11
/translation="MPPRKKQAPQAPSTMKELKDTLWKAADKLRGSLSASQYKDVILG
LVFLKYVSDAYDERREAIRAELAAEGMEESQIEDLIDDPEQYQGYGVFVVPVSARWKF
LAENTKGKPAVGGEPAKNIGQLIDEAMDAVMKANPTLGGTLPRLYNKDNIDQRRLGEL
IDLFNSARFSRQGEHRARDLMGEVYEYFLGNFARAEGKRGGEFFTPPSVVKVIVEVLE
PSSGRVYDPCCGSGGMFVQTEKFIYEHDGDPKDVSIYGQESIEETWRMAKMNLAIHGI
DNKGLGARWSDTFARDQHPDVQMDYVMANPPFNIKDWARNEEDPRWRFGVPPANNANY
AWIQHILYKLAPGGRAGVVMANGSMSSNSNGEGDIRAQIVEADLVSCMVALPTQLFRS

TGIPVCLWFFAKDKAAGKQGSIDRCGQVLFIDARELGDLVDRAERALTNEEIVRIGDT
FHAWRGSKSAAVKGIMYEDVPGFCKSATLAEIKATDYALTPGRYVGTPAVEDDGEPID
EKMARLSKALLEAFDESARLERVVREQLGRLR"
CDS complement(3031304..3031720)
/codon_start=1
/note="gene_2805"
/transl_table=11
/translation="MTTRYLLDKSAAYRAHLPAVRHRLEPLMERGLLARCGITDLEFG
VSARSREDHRTLGTYRRDALEYVNTPDTVWVRAWEIQEALTDKGFHRSVKIPDLIIAA
VAEHHGIPVMHYDQDFERIAAITRQPVEWVVAPGTA"
CDS complement(3031717..3031944)
/codon_start=1
/note="gene_2806"
/transl_table=11
/translation="VTRTMIDIDDDLLARAAKELGTTTKKDTVHAALRAALRASAARS
LMNRMAENATGTQDEALVNAMWRDGHPENTA"
CDS complement(3032009..3032404)
/codon_start=1
/note="gene_2807"
/transl_table=11
/translation="VIVDTSAIVAIVSGESGAQVLKEALERSPNSRMSAPNYVELCAI
MQRRDRPEISRLVDRLLDDYGIQVEAVDADQARVAAQAYRDYGRGSGHPARLNLGDTY
SYALAQVTGEPLLFRGDDFTHTDIRPACT"
CDS complement(3032401..3032670)
/codon_start=1
/note="gene_2808"
/transl_table=11
/translation="MSLNIKSQRTVALVRELAARTGTNQTAAVEDAVARRLSELDRED
RARAEARRAAAEQTLRDLDKLLSDDDKRLIRRHEVDLYDDSGLPR"
CDS complement(3032680..3033774)
/codon_start=1
/note="gene_2809"
/transl_table=11
/translation="MSRVEKVEKVRLGDHLDFSNGHTSGHTSPASEPGGRYPVYGANG
VIGYSAQHNARGPLIVVGRVGSYCGSLRYCDSDVWVTDNALACRAKKPEETRYWYYAL
LGFGLNRYRAGSGQPLLSQGVLRNVSVSAVAAPDRPRIGEILGAFDDKIAANDRVIEA
AEALMLAIVGRLSAYVPLSSLASRSTACLDAQHFDSTVAHYSFAAFDGGAQPSRVGGR
TIRSAKLVVSQPCVLFPKLNPRIPRIWNITSLPSEMALASTEFVVLRPVGVDTSALWA
ALRQPDVLAELRQLVGGMTGSRQRIQPTQLLRVWVRDVRRLTPGHAAAIANLGALCNE
RRIESARLASCRDALLPLLMSGIDGLPAGR"
CDS complement(3033771..3034043)
/codon_start=1
/note="gene_2810"
/transl_table=11
/translation="VAAKRRHLYYVRPLDGHPVARVDRKTDRAADSLPVAGVLGELDI
PPVTVAEGLAGELASMASWLGLGGIAVSTRGDLAGELCAATKRTNG"
CDS complement(3034264..3034743)
/codon_start=1
/note="gene_2811"
/transl_table=11
/translation="MVGLIWAQATSGVIGRGGDIPWRLPEDQAHFREITMGHTIVMGR

RTWDSLPAKVRPLPGRRNVVLSRQADFMASGAEVVGSLEEALTSPETWVIGGGQVYAL
ALPYATRCEVTEVDIGLPREAGDALAPVLDETWRGETGEWRFSRSGLRYRLYSYHRS"
CDS complement(3034814..3035614)
/codon_start=1
/gene="thyA"
/note="gene_2812"
/transl_table=11
/translation="VSIVTPYEDLLRFVLETGTPKSDRTGTGTRSLFGQQMRYDLSAG
FPLLTTKKVHFKSVAYELLWFLRGDSNIGWLHEHGVTIWDEWASDTGELGPIYGVQWR
SWPAPSGEHIDQISAALDLLRTDPDSRRIIVSAWNVGEIERMALPPCHAFFQFYVADG
RLSCQLYQRSADLFLGVPFNIASYALLTHMMAAQAGLSVGEFIWTGGDCHIYDNHVEQ
VRLQLSREPRPYPKLLLADRDSIFEYTYEDIVVKNYDPHPAIKAPVAV"
CDS 3035770..3036507
/codon_start=1
/note="gene_2813"
/transl_table=11
/translation="MPKTTDTAATPDGTCAVRLFTPDGPGRWPGVVMFPDAGGVRDTF
DRMAAKLAGFGYVVLLPDVYYREGDWAPFDMKTAFGDPQERARIMFMIGTLTPDRVTR
DADALLNYLASRPEVIGDRFGVCGYCIGGRMSVVVAGRLPDRVAAAAAFHPGGLVANS
PDSPHLLADRISATVYIGGAENDPSFTADHAEKLDKAFSAAGVPHRIECYPAAHGFAV
PDNPSYDAAADERHWAAMTETFGAALN"
CDS complement(3036722..3037504)
/codon_start=1
/gene="fabG"
/note="gene_2814"
/transl_table=11
/translation="MTSLDLTGRTAIITGASRGIGLAIAQQLAAAGAHVVLTARRQEA
ADEAAAQVGDRALGVGAHAVDEDAARRCVDLTLERFGSVDILINNAGTNPAYGPLLEQ
DHARFAKIFDVNLWAPLMWTSLVVTAWMGEHGGAVVNTASIGGMHQSPAMGMYNATKA
ALIHVTKQLALELSPRIRVNAICPGVVRTRLAEALWKDHEDPLAATIALGRIGEPADI
ASAVAFLVSDAASWITGETMIIDGGLLLGNALGFRAAPSTEH"
CDS complement(3038028..3039170)
/codon_start=1
/note="gene_2815"
/transl_table=11
/translation="MYAGAGAAPLMAAGATWNGLAVELSTTASSVESVIMQLTTEQWL
GPASMSMVVAAQPYLAWLTYTAESAAHAAAQAMASAAAFEAAFAMTVPPAEVAANRAL
LAALVATNVLGQNTPAIMATEAHYGEMWAQDALAMYGYAASSAAAGRLNPLITPSQTA
NMAGLAGQAAAVSHAAAASTVQQVGLGSLISNLPNAVMGFASPLTSAADAAGLGGIIQ
DIEELLGITFVQNAINGAVNTTAWFVMATIRNAVFLGHAFAALNPATVTAAADAVPAA
AAAAGLAHTVTPVGVGGASLTASLGEASSVGGLSVPAGWSTAAPAMTSGTTALEGSGW
AVPEEAGPVAAMPGMAGISGAAKGAGAYAGPRYGFKPIVMPKQVVV"
CDS complement(3039292..3040119)
/codon_start=1
/note="gene_2816"
/transl_table=11
/translation="MSFLTTQPEELAAAAGKLETIGSAMVAQNAAAAAPTTTGVIPAA
ADEISVLQASLFTAYGTLYQQVSAEAAAVYDLFVKTLGVSAGTYAATEAANSSAAASP
LSGIASILGSTPGKVPSWISDIANIFNIGAGNWASAASDLLGLASGGLLPAAEEAALE
EGLEGAGLSELGAAEAAVGEAPIAAGLGAAPLAAGLSRASSIGALSVPPSWAGQANLV
SSTSTLQGAGWTTAAPHGAAGTVIPGMPGLASATRSSAGFGAPRYGAKPIVMPKPAV"

```
CDS             complement(3040443..3041651)
                /codon_start=1
                /note="gene_2817"
                /transl_table=11
                /translation="MAASDGAPATTALDQKRNASVDFGALPPEVNSARMYGGAGAADL
                LAAAAAWNGIAVEVSTAASSVGSVITRLSTEHWMGPASLSMAAAVQPYLVWLTCTAES
                SALAAAQAMASAAAFETAFALTVPPAEVVANRALLAELTATNILGQNVSAIAATEARY
                GEMWAQDASAMYGYAAASAVAARLNPLTRPSHITNPAGLAHQAAAVGQAGASASARQV
                GLSHLISDVADAVLSFASPVMSAADTGLEAVRQFLNLDVPLFVESAFHGLGGVADFAT
                AAIGNMTLLADAMGTVGGAAPGGGAAAAVAHAVAPAGVGGTALTADLGNASVVGRLSV
                PASWSTAAPATAAGAALDGTGWAVPEEDGPIAVMPPAPGMVVAANSVGADSGPRYGVK
                PIVMPKHGLF"
CDS             complement(3041715..3042167)
                /codon_start=1
                /note="gene_2818"
                /transl_table=11
                /translation="VRRLLIVHHTPSPHMQEMFEAVVSGATDPEIEGVEVVRRPALTV
                SPIEMLEADGYLLGTPANLGYISGALKHAFDVCYYPCLDTTRGRSFGAYIHGNEGTEG
                AERAVDAITTGLGWVQAAETVVVMGKPSKADIEACWNLGATVAAQLMG"
CDS             complement(3042253..3042726)
                /codon_start=1
                /note="gene_2819"
                /transl_table=11
                /translation="MTRRTLYVQLIIAFMCVAMVAYLVMLGRVAVAMIGSGRAAAAGL
                GLALLILPVIGLWAMIATLRAGFAYQRLARLIAEDGLDIDASALPRRASGRIQRDAAD
                ALFAAVRTELEDDADDWRRWYRLARAYDYAGDRRRAREAMKTALQLEGRARPGAR"
CDS             complement(3042738..3043484)
                /codon_start=1
                /note="gene_2820"
                /transl_table=11
                /translation="VKAMRVGVLGAKGKVGATMVRAVAAADDLTLSAELDAGDPLSLL
                TDGNTEVVIDFTHPDVVMGNLEFLIDNGIHAVVGTTGFTAERFQQVESWLVAKPNTSV
                LIAPNFAIGAVLSMHFAKQAARFFDSAEVIELHHPHKADAPSGTAARTAKLIAEARKG
                LPPNPDATSTSLPGARGADVDGIPVHAVRLAGLVAHQEVLFGTEGEILTIRHDSLDRT
                SFVPGVLLAVRRIAERPGLTVGLEPLLDLH"
CDS             complement(3043486..3043767)
                /codon_start=1
                /note="gene_2821"
                /transl_table=11
                /translation="MVDFAPVTGCVAVGSTMGAVPALLRVRFPWPPFEPDVRLSPYLA
                LHGICRWGGSDSCDRTTVQVFHLHSINKRLTAHAGFGAAAVVGLEDGPV"
CDS             3044043..3044504
                /codon_start=1
                /note="gene_2822"
                /transl_table=11
                /translation="MHYPVWRQSWTGILDPYLLDMIGSPKLWVEESYPQSLKRGGWSM
                WIAESGGQPIGMTMFGPDIAHPDRIQIDALYVAENSQRHGIGGRLLNRALHSHPSADM
                ILWCAEKNSKARGFYEKKDFHIDGRTFTWKPLSGVNVPHVGYRLYRSAPPG"
CDS             complement(3044508..3045599)
                /codon_start=1
                /note="gene_2823"
```

```
                     /transl_table=11
                     /translation="MAERTVPETSWASRPADLYGRRSRDRFFTALWGVRALLGGLGAA
                     SRWEPSRVQPVRRTNPAVVTKRELVAPDVVALTLADPGGGLLPAWSPGGHIDVQLPSG
                     RRRQYSLCGVPGRRTDYRIAIRRIADGGGGSIEMHEAFDVGDTCEFEGPRNAFHLGLA
                     ERDVLFVIGGIGVTPILPMIRAAEQRGIDWRAIYAGRGREYMPFLDEVVAVAPGRVTV
                     WADDEHGRFASVDELLAGAGPTTAVYVCGPPGMLEAVRVARNQHADAPLHYERFSPPP
                     VVDGVPFELELARSRRVLRVPANRSALDVMLDWDPTTAYSCQQGFCGTCKVRVLAGQV
                     DRRGRIIEGDNEMLVCVSRAVSGRVVIDA"
     CDS             complement(3045619..3046536)
                     /codon_start=1
                     /note="gene_2824"
                     /transl_table=11
                     /translation="MVSVDDRATGPHSSESRAADHERLVLEPRDVEFDWTNLPFHYVP
                     NEPMATHVLNVLHMLLPAGEEFFVRVFKKTLPLIKDDQLRLDVQGFIGQEAMHSQAHS
                     GVVDHFDAQGVDVTAFTNQIRWLFEKLLGESPRRSPRRQYSWLLEQVSFIAAIEHYTA
                     VMGEWILNSPQLDAVGADPVMLDMLRWHGAEEVEHKAVAFDTMKHLRAGYWRQVRAQL
                     TVTPVMLLLWIRGVRFMYSVDPYLPPGTKPRWRDYFKAARRGLVPGLPRLLRVVGHYY
                     KPGFHPSQLGGLGAAVDYLAVSPAARASH"
     CDS             complement(3046847..3047317)
                     /codon_start=1
                     /note="gene_2825"
                     /transl_table=11
                     /translation="MPDPDGPSVTVTVEIDANPDLVYGLITDLPTLASLAEEVVAMQL
                     RKGDDVRKGAVFVGRNENGGRRWTTTCTVTDADPGRVFAFDVRSGIIPISRWQYGIVA
                     TEHGCRVTESTWDRRPSWFRAVARMATGVKDRASVNTEHIRRTLQRLKDRAEAG"
     CDS             complement(3047349..3047744)
                     /codon_start=1
                     /note="gene_2826"
                     /transl_table=11
                     /translation="MPNNALADTVGIAPSTCHGRVRRLVDLGVIRGFYTDIDPVAVGL
                     PLQAMISVNLQSSARGKIRSFIQQIRRKRQVMDVYFLAGADDFILHVAARDTEDLRSF
                     VVENLNADADVAGTQTSLIFEHLRGAAPI"
     CDS             3048223..3049068
                     /codon_start=1
                     /note="gene_2827"
                     /transl_table=11
                     /translation="LFTFLHLAASRACTDALLDSGTTSIAYETVQTADGALPLLAPMS
                     EVAGRLAAQVGAYHLMRTQGGRGVLMGGVPGVEPADVVVIGAGTAGYNAARIANGMGA
                     TVTVLDINIDKLRQLDAEFCGRIHTRYSSAYELEGAVKRADLVIGAVLVPGAKAPKLV
                     SNSLVAHMKPGAVLVDIAIDQGGCFEGSRPTTYDHPTFAVHDTLFYCVANMPASVPKT
                     STYALTNATMPYVLELADHGWRAACRSNPALAKGLSTHEGALLSERVATDLGVPFTEP
                     ASVLA"
     CDS             complement(3049083..3050117)
                     /codon_start=1
                     /note="gene_2828"
                     /transl_table=11
                     /translation="MVLGFWDIAVPIVGAPMAGGPSTPALAAAVSNAGGLGFVAGGYL
                     SADRLADDIAAARAATTGPIGANLFVPQPSVADWAQLEYYADELEEVAEYYHTEVGQP
                     VYGDDDDWVRKLEVVADVRPEVVSFTFGAPPPDVVQRLSALGLLVSITVTSVYEAGVA
                     IAAGADSLVVQGPAAGGHRGTFAPDMEPGTESLHQLLDRIGSAHDVPLVAAGGLGTAE
                     DVAAVLRRGAIAAQVGTALLLADEAGTNAAHRAALKNPEFDATLVTRAFSGRYARGLA
```

NNFTRLLDHVAPLGYPEVHQMTKPIRAAAVQADDPHGTNLWAGSAHRKTRPGPAADII
ASLTPDVCSA"
CDS complement(3050178..3051419)
/codon_start=1
/note="gene_2829"
/transl_table=11
/translation="VVTEFLPAVHSASVGVWVGVGSRDEGATVAGAAHFLEHLLFKST
PTRSAVDIAQAMDAVGGELNAFTAKEHTCYYAHVLGSDLPLAVDLVADVVLNGRCAAD
DVEVERDVVLEEIAMRDDDPEDALADMFLAALFGDHPVGRPVIGSAQSVSVMTRAQLQ
SFHLRRYTPERMVVAAAGNVDHDGLVALVREHFGSRLVRGRRPVAPRKGTGRVNGSPR
LTLVSRDAEQTHVSLGIRTPGRGWEHRWALSVLHTALGGGLSSRLFQEVRETRGLAYS
VYSALDLFADSGALSVYAACLPERFADVMRVTADVLESVARDGITEAECGIAKGSLRG
GLVLGLEDSSSRMSRLGRSELNYGKHRSIEHTLRQIEQVTVEEVNAVARHLLSRRYGA
AVLGPHGSKRSLPQQLRAMVG"
CDS complement(3051472..3053703)
/codon_start=1
/note="gene_2830"
/transl_table=11
/translation="VFETTATIDNGSFGTRTIRFETGRLALQAAGAVVAYLDDDNMLL
SATTASKNPKEHFDFFPLTVDVEERMYAAGRIPGSFFRREGRPSTDAILTCRLIDRPL
RPSFVDGLRNEIQIVVTILSLDPGDLYDVLAINAASASTQLGGLPFSGPIGGVRVALI
DGTWVGFPTVDQIERAVFDMVVAGRIVEGDVAIMMVEAEATENVVELVEGGAQAPTES
VVAAGLEAAKPFIAALCTAQQELADAAGKSGKPTVDFPVFPDYGEDVYYSVSSVATDE
LAAALTIGGKAERDQRIDEIKTQVVQRLADTYEGREKEVGAALRALTKKLVRQRILTD
HFRIDGRGITDIRALSAEVAVVPRAHGSALFERGETQILGVTTLDMIKMAQQIDSLGP
ETSKRYMHHYNFPPFSTGETGRVGSPKRREIGHGALAERALVPVLPSVEEFPYAIRQV
SEALGSNGSTSMGSVCASTLALLNAGVPLKAPVAGIAMGLVSDDIQVEGAVDGVVERR
FVTLTDILGAEDAFGDMDFKVAGTKDFVTALQLDTKLDGIPSQVLAGALEQAKDARLT
ILEVMAEAIDRPDEMSPYAPRVTTIKVPVDKIGEVIGPKGKVINAITEETGAQISIED
DGTVFVGATDGPSAQAAIDKINAIANPQLPTVGERFLGTVVKTTDFGAFVSLLPGRDG
LVHISKLGKGKRIAKVEDVVNVGDKLRVEIADIDKRGKISLILVADEDSTAAATDAAT
VTS"
CDS complement(3054084..3054599)
/codon_start=1
/note="gene_2831"
/transl_table=11
/translation="MRAWLAAATTALFVVATGCSSATNVAELKVGDCVKLAGTPDRPQ
ATKAECGSPASNFKVVAVVQEDHAECPADVDSTYSMRNAFNGSTNTICLDIDWVIGGC
MSVDPTHNTDPFRVDCDDASVPHRQRATQILKDLDSPVSVDQCASGVGYVYTQRRFAV
CVEDVTGGPRS"
CDS complement(3054612..3054881)
/codon_start=1
/gene="rpsO"
/note="gene_2832"
/transl_table=11
/translation="VALTAEQKKEILRSYGLHETDTGSPEAQIALLTKRIADLTEHLK
VHKHDHHSRRGLLLLVGRRRRLIKYISQIDVERYRSLIERLGLRR"
CDS complement(3055038..3055961)
/codon_start=1
/gene="ribF"
/note="gene_2833"

/transl_table=11
/translation="VLTIGVFDGVHRGHAELIAHAVKAGRARGVPAVLMTFDPHPMEV
VYPGSHPAQLTTLTRRAELVQDLGIEVFLVMPFTTDFMKLTPDRFIHELLVEHLHVVE
VVVGENFTFGKKAAGNVDTLRRAGERFGFAVESMSLVSEHHSNETVTFSSTYIRSCVD
AGDMVAAMEALGRPHRVEGVVVRGEGRGAELGFPTANVAPPMYSAIPADGVYAAWFTV
LGHGPVTGTVVPGERYQAAVSVGTNPTFSGRTRTVEAFVLDTTADLYGQHVALDFVGR
IRGQKKFESVRQLVAAMGADTERARDLLSTG"

CDS 3056271..3058007
/codon_start=1
/note="gene_2834"
/transl_table=11
/translation="MFDAAVKSYQSGDLANARAAFGRLTVENPDMSDGWLGLLACGDH
HLDTLAGAHQHSEALYSETRRVGLPDGELSAVVMAPMYLGLRVWSRATIGLAYASALI
IADRHDEAAATLDDPVITEDTGAAQYRQFVMATLFHKTRSWSNLLKVTEISPPSGATD
VRDEVADAVAALASTAAASLGQFQFALELAEQVSTTNPRVTADVTLTRAWCLRELGDD
DAARVALSATTTGDAPRTNTTAEQAGSPQPKFRHPYDDGRDLLVARRRPPAGDGWRKA
VTKMTFGRVNPEPSAKREQTDELIQRICAPLADVHKLAFVSAKGGVGKTTMTVLVGNA
VARLRGDRVMAVDVDADLGDLSARFSERGGPQTNIEHFVSSQHTKRYADVRVHTVMNK
DRLEMLGAQNDPRSTYKFGPEDYGAAMQILETHCNVILLDCGTPVNGPLFSNILNDVT
GLVVVASEDVRGVEGALVTLDWLGAHGFGRLLQHTVVVLNAIQKTRSLVDCGAAENQF
RKRVPDFFRIPYDPHLATGLAVDFSSLKRRTRNAVLDLAGGLAQHYPASRVRPRGEDS
WKTWIETMRQVG"

CDS 3058092..3058778
/codon_start=1
/note="gene_2835"
/transl_table=11
/translation="VRADEEPGDLSAVAQDYLKVIWTAQEWSQDKVSTKMLAERIGVS
ASTASESIRKLAEQGLVDHEKYGAVTLTDSGRRAALAMVRRHRLLETFLVNELGYRWD
EVHDEAEVLEHAVSDRLMARIDAKLGFPQRDPHGAPIPGADGQVPTPPARQLWACRDG
DTGTVARISDADPQMLRYFASIGISLDSRLRVLARREFAGMISVAIDSADGATVDLGS
PAAQAIWVVS"

CDS complement(3058839..3060071)
/codon_start=1
/note="gene_2836"
/transl_table=11
/translation="MFEWSDTDLMVRDAVRQFIDKEIRPHQDALETGELSPYPIARKL
FSQFGLDVLLAESVNQMLDGERAKREKRDSSGSFGLADQASMVAVLVSELAGVSIGLL
STVAVSLGLGAATIMSRGTLAQQERWVPTLVTLEKIAAWAITEPDSGSDAFGGMKTHV
TRDGEDYILNGHKTFITNGPYADVLVVYAKLADGEPASDWRNRPVLVFVLDAGMPGLT
QGKPFKKMGMMSSPTGELFFDNVRLTPDRLLCAEGDGRDSARANFAVERLGVALMSLG
IINECHRLCVDYAKTRTLWGRNIGQFQLIQLKLAKMEVARINVQNMVFQAIERLKAGK
QLTLAEASAIKLYSSEAATDVAMEAVQLFGGNGYMAEYRVEQLARDAKSLMIYAGSNE
VQVTHIAKGLLGEPASRA"

CDS complement(3060097..3061260)
/codon_start=1
/note="gene_2837"
/transl_table=11
/translation="VGMTKFEKPGRREGWDYPDMARESGTKALRDAGIDYREVEQGYV
GYVYGESTSGQRALYELGMTGIPIVNVNNNCSTGSTALYLGAQAIRGGLADCVLALGF
EKMQPGALGGGADDRESPLGRHVKALAEIDEFGFPVAPWMFGAAGREHMKKYGTTAEH
FAKIGYKNHKHSVNNPYAQFQDEYTLDDILASKMISDPLTKLQCSPTSDGSAAVVLAS

EDYLANHNLAGRAVEIVGQAMTTDFASTFDGSARNIIGYDMTVQAAQRVYQQSGLGPK
DFGVIELHDCFSANELLLYEALGLCGPGEAPELIDDNQTTYGGRWVVNPSGGLISKGH
PLGATGLAQCAELTWQLRGTAEARQVDNVTAALQHNIGLGGAAVVTAYQRAER"
CDS complement(3061335..3062714)
/codon_start=1
/note="gene_2838"
/transl_table=11
/translation="MAKFEIPEGWMVQAFRFTLDPTAEQARALARHFGARRKAYNWTV
ATLKADIDAWQATGIQTAKPSLRVLRKRWNTVKNDVCVNIETGVVWWPECSKEAYADG
IDGAVDAYWNWQNSRSGKRDGKRMGFPRFKKKGRDPDRVTFTTGAMRVEPDRRHLTLP
VIGTVRTHENTRRVERLIAKGRSRVLAITVRRNGTRIDASVRVLVQRPQQPKVTDPGS
RVGVDVGVRRLATVATADGAVLERVPNPRPLDAALNELRHVCRARSRCTKGSRRYRER
TTEISRLHRRVNDVRTHHLHCLTTHLAKTHGRIVVEGLDAAGMLRQQGLSGARARRRG
LSDAALGTPRRHLSYKTGWYGSQLVVADRWFPSSKTCHVCGHVQEIGWAEHWQCDSCS
ASHQRDDCAAINLARYEDTSSVVGPVGAAVKRGADRKTRPGRAGGREARKGSSRKAAE
QPRDGVQVA"
CDS complement(3062714..3063295)
/codon_start=1
/note="gene_2839"
/transl_table=11
/translation="MNLAVWAERNGVARVTAYRWFHAGLLPVPARKAGRLILVDDQPA
DRSRRARTAVYARVSSADQKPDLDRQVARVTAWATTEQIAVDKVVTEVGSALNGHRRK
FLALLRDPSVKRIVVEHRDRFCRFGSEYVEAALAAQGRELVVVDSAEVDDDLVRDMTE
ILTSMCARLYGKRAAQNRAKRALAAAAEESEAA"
CDS complement(3063497..3064393)
/codon_start=1
/gene="truB"
/note="gene_2840"
/transl_table=11
/translation="MSATGPGIVVIDKPAGMTSHDVVGRCRRIFATRRVGHAGTLDPM
ATGVLVIGIERATKILGLLTAAPKSYAATIRLGQTTSTEDAEGQVLQSVPAKHLTIEA
IDAAMERLRGEIRQVPSSVSAIKVGGRRAYRLARQGRSVQLEARPIRIDRFELLAARR
RDQLIDIDVEIDCSSGTYIRALARDLGDALGVGGHVTALRRTRVGRFELDQARSLDDL
AERPALSLSLDEACLLMFARRDLTAAEASAAANGRSLPAVGIDGVYAACDADGRVIAL
LRDEGSRTRSVAVLRPATMHPG"
CDS complement(3064390..3065073)
/codon_start=1
/gene="pptT"
/note="gene_2841"
/transl_table=11
/translation="MTVGTLVASVLPATVFEDLAYAELYSDPPGLTPLPEEAPLIARS
VAKRRNEFITVRHCARIALDQLGVPPAPILKGDKGEPCWPDGVVGSLTHCAGYRGAVV
GRRDAVRSVGIDAEPHDVLPNGVLDAISLPAERADMPRTMPAALHWDRILFCAKEATY
KAWFPLTKRWLGFEDAHITFETDSTGWTGRFVSRILIDGSTLSGPPLTTLRGRWSVER
GLVLTAIVL"
CDS complement(3065070..3066044)
/codon_start=1
/note="gene_2842"
/transl_table=11
/translation="VTWKGSGQETVGAEPTLWAISDLHTGHLGNKPVAESLYPSSPDD
WLIVAGDVAERTDEIRWSLDLLRRRFAKVIWVPGNHELWTTNRDPMQIFGRARYDYLV

NMCDEMGVVTPEHPFPVWTERGGPATIVPMFLLYDYSFLPEGANSKAEGVAIAKERNV
VATDEFLLSPEPYPTRDAWCHERVAATRARLEQLDWMQPTVLVNHFPLLRQPCDALFY
PEFSLWCGTTKTADWHTRYNAVCSVYGHLHIPRTTWYDGVRFEEVSVGYPREWRRRKP
YSWLRQVLPDPQYAPGYLNDFGGHFVITPEMRTQAAQFRERLRQRQSR"
CDS complement(3066189..3066752)
/codon_start=1
/gene="lppV"
/note="gene_2843"
/transl_table=11
/translation="MRWPTAWLLALVCVMATGCGPSGHGTRAGEEGPLSPEKVAELEN
PLRAKPPLEDAKDQYRAAVTQLANAITALVPGLTWRTDMDTWTGCGGEYEWTRAKAAY
FMIVFSGPIPDDKWLQAVQIVKDGVEQFGATGFGVMKNKPADHDVYFAGHGGVEFKFS
TQKAAVLTAQSDCRISRTDTPKPSPTP"
CDS complement(3066752..3068440)
/codon_start=1
/note="gene_2844"
/transl_table=11
/translation="VPLTVADIDRWNAQAVREVFHAASARAEVTFEASRQLAALSIFA
NSGGKTAEAAAHHNAGIRRDLDAHGNEALAVARAADRAADGIVKVQSELAALRHAAAA
AELTIDALINRVVPIPGLRSTEAQWARTLAKQTELQAELDAIMAEANAVDEELASAVN
MADGDAPIPADSGPPVGPEGLTPTQLASDANEERLREERARLQAHLERLQAEYDQLSV
RAARDYHNGILDGDAVGRLAALTDELSAARGRLGELDAVDEALSRAPETYLTQLQIPE
DPNQQVLAAVAVGNPDTAANVSVTVPGVGSTTRGALPGMVTEARDLRSEVIRQLNAAG
KPASVATIAWMGYHPPPNPLDTGSAGDLWQTMTDGQAHAGAADLSRYLQQVRANNPSG
HLTVLGHSYGSLTASLALQDLDAQSAHPVNDVVFYGSPGLELYSPAQLGLDHGHAYVM
QAPHDLITNLVAPLAPLHGWGLDPYLTPGFTELSSQAGFDPGGIWRDGVYAHGDYPRS
FLDAAGQPQLRMSGYNLAAIAAGLPDNTVGPPLLPPILGGGMPAAPGPALRGGR"
CDS complement(3068444..3068770)
/codon_start=1
/note="gene_2845"
/transl_table=11
/translation="VFQISPEQWMHSAAQVTTQGEGLAVGHLSSDYRMQAAQFGWQGA
SAMALNAKMDDWLDASRALLTRIGDHAFGLQEAAIQHAAAEAERAQALAQVGVSADVV
AGPRGV"
CDS 3068943..3069530
/codon_start=1
/note="gene_2846"
/transl_table=11
/translation="VFTRVRLIGGLGALTAAVVVVGTVGWQGIPPAPTGGDAVQLRST
AAPMSTTMKSPIVATTDPSPFDPCRDIPFDVIQRLGLAYTPPEAEEGLRCHFDAGNYQ
MAVEPIIWRTYAQTLPPDAIETTIAGHRAAQYWVRKPTYHNSFWYSSCMVTFKTSYGV
IQQSLFYSTVYSEPDVDCPSTNLQRANDLVPYYRF"
CDS 3069549..3071198
/codon_start=1
/note="gene_2847"
/transl_table=11
/translation="VSTTSARPERPKLRALTGRVGGQALGGLLGLPRATTRYTVGHVR
VPMRDGVQLVADHYAPATSQPVGTLLVRGPYGRRFPFSLVFARIYAARGYHVVLQSVR
GTFGSGGVFEPMVNEAADGADTVAWLREQPWFTGRFGTIGLPYLGFTQWALLHDPPPE
LAAAVITVGPHDFRASVWGTGSFTVNDFLGWSDLVSHQEDPGRIRAGIRQLTAPRRVA
RTAATLPLGESARTLLGTGAPWFESWVEHTDRDDPFWDRLRFPAALDRVQVPVLLVGG

WQDIFLRQTLQQYRHLRDRGVHVALTVGPWTHTQMLTKGLATGARESLDWLDAHLGRA
PALRPSPVRVFVTGQGWRHLPDWPPATTERAWYLQPGGRLGESAPASGTPPATFRYHP
ADPTPTTGGPLLSSNGGYRDDSRLATRADVLCFTGAPLTHDLCVHGNPVVELVHSSDN
PYVDVFVRVSEVDAKGRSRNVSDGYRRLGDAPELVRVELDAIAHRFRADSRIRVLIAG
SWFPRYARNLGTPEPILTGRQLKPATHAVHFGRSRLLLPVG"

CDS complement(3071300..3071653)
/codon_start=1
/note="gene_2848"
/transl_table=11
/translation="MRRGEIWQVDLDPARGSEANNQRPAVVVSNDRANATATRLGRGV
ITVVPVTSNIAKVYPFQVLLSATTTGLQVDCKAQAEQIRSIATERLLRPIGRVSAAEL
AQLDEALKLHLDLWS"

CDS complement(3071640..3071870)
/codon_start=1
/note="gene_2849"
/transl_table=11
/translation="VKLSVSLSDDDVAILDAYVKRAGLPSRSAGLQHAIRVLRYPTLE
DDYANAWQEWSAAGDTDAWEQTVGDGVGDAPR"

CDS complement(3071913..3072929)
/codon_start=1
/note="gene_2850"
/transl_table=11
/translation="VARAAQAALARQRFVSAIDVLLGLGWLAPSHVDQWRQGRVDSLE
QVVQANLSKITAVMAALRRWARDRGLNPSETDYVARTRDRRRLRFSVTGEDAIERAYR
THWVSPELSERAVARQSRRPDLVVIMPVNDWSCASCGGSGDLMFLEDAGPLCLDCADL
GHLVFLPSGDAALTRRAKRASRLSAVVVRWSRARKRYERQGILVEAEALERAENECLA
DAEVRARRRERDEARRANEDLRLQAEFGAAIRTLFPNCPAGRAEAIARHAATRGSGRI
GRSAAGRALDPEAVRLAVAASVRHIDTSFDELLMSGVDRETARHRVGEHVEEVLRDWR
ATSR"

CDS 3072955..3073422
/codon_start=1
/note="gene_2851"
/transl_table=11
/translation="MTCPSLVGLRTEAAELSYSDQPDALGVAMRERREQQNLVRPPRR
NASRRINTDQTSTKYVYITYMPETLTGRLNFRLSPEQEQALRHAAALTGQSLSGFVLS
AAVDHAHDLLARANRIELSEAAFRRFVAALDEPDEAAPELVRLARRKSRIPPH"

CDS complement(3073598..3073789)
/codon_start=1
/note="gene_2852"
/transl_table=11
/translation="VPGTGHGSDEGIEEKIATETGALLLPVERQASEDEHWDRVGSGW
PRPGRDGTRIRSMLPMASA"

CDS 3073772..3073927
/codon_start=1
/note="gene_2853"
/transl_table=11
/translation="VAGARHYGARALVVDAIDDRAAEFYGHHGFLPLEGRRLYRRISD
IARALGV"

CDS 3074000..3074404
/codon_start=1
/note="gene_2854"

/transl_table=11
/translation="MGRDNGKILDPVVATTGMGRSTARQMLTGPRLPGPAEQVDGRSL
RPRGFSDEARALLEHVWALMGMPCGKYLVVMHDLWLPLLTAAGDLDKPLVTEASVAEL
KATALPGANRMPHWAAGTLPDGFPARAVRTRT"
CDS 3074401..3074592
/codon_start=1
/note="gene_2855"
/transl_table=11
/translation="VKTNPRYGPAFYSVMTVLFLALFVLNVCTHGSTLGLISTGGLAV
LMGYIGYRGWSGKRHINRQ"
CDS 3074833..3075945
/codon_start=1
/note="gene_2856"
/transl_table=11
/translation="MLTGPGLPEPAEQVDGRRLRARGFSDDARALLEHVWALMGMPCG
KYLVVMLELWLPLVAAAGDLDKPFATEAAVAELKAMSAATVDRYLKPARERMRIKGIS
TTKPSPLLRNSITIHTCSDEAPKVPGVIEADTVAHCGPSLIGEFARTLTMTDLVTGWT
ENASIRNNAAKWILEGIKECQQRFPFPMTVFDSDCGGEFINHDVAGWLQARDIAQTRS
RPYQKNDQAHVESKNNHVVRKHAFYWRYDTGEELELLNRLWPLVSLRCNFFTPTKKPV
GYTSTVNGRRKRIYDKPATPWQRLQASGVLDAQQLSTVAARIEGFNPADLTRQINAIQ
TQLLDLAKTKTEALATARHIDLQSLQPSINRLAKAK"
CDS 3076541..3076852
/codon_start=1
/note="gene_2857"
/transl_table=11
/translation="MTYAARDDTTLPKLLAQMRWVVLVDKRQLAVLLLENEGPVASAT
DPLDTRGDSDYENQPVDAVERLCRRLADQAVRQWGFMQGLKQKLGPGVDVRMKLVEWN
R"
CDS complement(3076874..3077041)
/codon_start=1
/note="gene_2858"
/transl_table=11
/translation="VLAYFDHHASNGPTEAINGRLEALCRNALGFRNLTHYRIRSLLH
CGNLAQLIHAL"
CDS 3077452..3077880
/codon_start=1
/note="gene_2859"
/transl_table=11
/translation="VTHVLLPVSALLRRADTAAVIVSALAAKATSRVGFRRIATDVAR
PAETVRGWLRRFAERVEAVRSVFTVWLCAVDADPVMPDAGGGGFVDAVVAIGALAAAI
GRRFSLPTVSLAETAVAVSGGRLLAPGWPGEWVQHESTLP"
CDS 3077951..3079360
/codon_start=1
/note="gene_2860"
/transl_table=11
/translation="VAVGDDEEKVRAERARAIGLFRYQLIWEAADAAHSTKQRGKMVR
ELASREHTDPFGRRVRISRQTIDRWIRGWRAGGFDALVPNPRQCTPRTPAEVLELAVA
LRRENPQRTAAAIRRILRTQLGWAPDERTLQRNFHRLGLTGATTGSAPAVFGRFEAEH
PNALWTGDVLHGIRIDLRKTYLFAFLDDHSRLVPGYRWGHAEDTVRLAAALRPALASR
GVPNAVYVDNGSPYVDAWLLRACAKLGVRLVHSTPGRPQGRGKIERFFRTVREQFLVE
ITGEPDVVGRHYVADLAELNRLFTAWVETVYHRSVHSETGQTPLARWSAGGPIPLPAP

ETLTEAFLWEEHRRVTKTATVSLHGNRYEIDPALVGRKVELVFDPFDLTRIEVRLAGA
PMGRAIPYHIGRHSHPKAKPETPTAPPKPSGIDYAQLIETAHAAELARGVNYTALTGA
ADQIPGQLDLLTGQEAQPK"

```
CDS             3079357..3080169
                /codon_start=1
                /note="gene_2861"
                /transl_table=11
                /translation="MMHKLISYYGFSRMPFGRDLAPGMLHRHSAHNEAVARIGWCIAD
                RRIGVITGEVGAGKTVAVRAALASLDRSRHTVIYLPDPTVGVQGIHHRIVASLGGQPL
                THHATLAPQAADALAAEQAERGRTPVVVVEEAHLLGYDQLEALRLLTNHDLDSSSPFA
                CLLIGQPTLRRRMKLGVLAALDQRIGLRYAMPPMTDTNTGSYLRHHLKLAGRDDALFS
                DDAIGLIHQTSRGYPRAVNNLALQALVAAFAADKAIVDESTTRTAIAEVTAD"
CDS             complement(3081772..3082656)
                /codon_start=1
                /note="gene_2862"
                /transl_table=11
                /translation="LRWGVESICTQLTELGVPIAPSTYYDHINREPSRRELRDGELKE
                HISRVHAANYGVYGARKVWLTLNREGIEVARCTVERLMTKLGLSGTTRGKARRTTIAD
                PATARPADLVQRRFGPPAPNRLWVADLTYVSTWAGFAYVAFVTDAYARRILGWRVAST
                MATSMVLDAIEQAIWTRQQEGVLDLKDVIHHTDRGSQYTSIRFSERLAEAGIQPSVGA
                VGSSYDNALAETINGLYKTELIKPGKPWRSIEDVELATARWVDWFNHRRLYQYCGDVP
                PVELEAAYYAQRQRPAAG"
CDS             complement(3082707..3083033)
                /codon_start=1
                /note="gene_2863"
                /transl_table=11
                /translation="MSGGSSRRYPPELRERAVRMVAEIRGQHDSEWAAISEVARLLGV
                GCAETVRKWVRQAQVDAGARPGTTTEESAELKRLRRDNAELRRANAILKTASAFFAAE
                LDRPAR"
CDS             complement(3085287..3085628)
                /codon_start=1
                /note="gene_2864"
                /transl_table=11
                /translation="MPTRSREEYFNLPLKVDESSGTIGKMFVLVIYDISDNRRRASLA
                KILAGFGYRVQESAFEAMLTKGQLAKLVARIDRFAIDCDNIRIYKIRGVAAVTFYGRG
                RLVSAEEFVFF"
CDS             complement(3085629..3086645)
                /codon_start=1
                /note="gene_2865"
                /transl_table=11
                /translation="MVQLYVSDSVSRISFADGRVIVWSEELGESQYPIETLDGITLFG
                RPTMTTPFIVEMLKRERDIQLFTTDGHYQGRISTPDVSYAPRLRQQVHRTDDPAFCLS
                LSKRIVSRKILNQQALIRAHTSGQDVAESIRTMKHSLAWVDRSGSLAELNGFEGNAAK
                AYFTALGHLVPQEFAFQGRSTRPPLDAFNSMVSLGYSLLYKNIIGAIERHSLNAYIGF
                LHQDSRGHATLASDLMEVWRAPIIDDTVLRLIADGVVDTRAFSKNSDTGAVFATREAT
                RSIARAFGNRIARTATYIKGDPHRYTFQYALDLQLQSLVRVIEAGHPSRLVDIDITSE
                PSGA"
CDS             complement(3086895..3087905)
                /codon_start=1
                /note="gene_2866"
                /transl_table=11
```

/translation="MILFSPIGTADPITALGDGPMLHIVRHYRPIVVVLFLSAEIAAF
ENADRRYSAAITRLAPETDVRIVTYTNPSVHRFDLFVPVFRNHLVELSAEFPDRTILL
NTSSGTPAMQAALVAINVFGIPRTTAVQVSTPARALSKPGDRESPDAYDLELMWDAND
DNQPGAPNRCFEATSAALGALLERANLKQLIVSYDYSAAVTIAADSRLPDQVSNLIRG
AMHRSRLEHLVAPKFFKDTAFTYDPANKVAEYISALALLAKREQWAEFARSATPAITI
VLRAAVAKHLPEDRYLDDMGRVDRRKLEREPEIRCALKHPPKSPNAEWYLYTKDWLAL
LR"
CDS complement(3087902..3089029)
/codon_start=1
/gene="csm5"
/note="gene_2867"
/transl_table=11
/translation="MNTYLKPFELTLRCLGPVFIGSGEKRTSKEYHVEGDRVYFPDME
LLYADIPAHKRKSFEAFVMNTDGAQATAPLKEWVEPNAVKLDPAKHRGYEVKIGSIEP
RRASRGRGGRMTRKKLTLNEIHAFIKDPLGRPYVPGSTVKGMLRSIYLQSLVHKRTAQ
PVRVPGHQTREHRQYGERFERKELRKSGRPNTRPQDAVNDLFQAIRVTDSPALRTSDL
LICQKMDMNVHGKPDGLPLFRECLAPGTSISHRVVVDTSPTARGGWREGERFLETLAE
TAASVNQARYAEYRAMYPGVNAIVGPIVYLGGGAGYRSKTFVTDQDDMAKVLDAQFGK
VVKHVDKTRELRVSPLVLKRTKIDNICYEMGQCELSIRRAE"
CDS complement(3089026..3089934)
/codon_start=1
/note="gene_2868"
/transl_table=11
/translation="MNSRLFRFDFDRTHFGDHGLESSTISCPADTLYSALCVEALRMG
GQQLLGELVACSTLRLTDLLPYVGPDYLVPKPLHSVRSDGSSMQKKLAKKIGFLPAAQ
LGSFLDGTADLKELAARQTKIGVHAVSAKAAIHNGKKDADPYRVGYFRFELDAGLWLL
ATGSESELGLLTRLLKGISALGGERTSGFGAFNLTESEAPAALTPTVDAASLMTLTTS
LPTDDELEAALAGATYRLVKRSGFVASSTYADMPLRKRDIYKFAAGSVFSRPFQGGIL
DVSLGGNHPVYSYARPLFLALPESAA"
CDS complement(3089915..3090625)
/codon_start=1
/note="gene_2869"
/transl_table=11
/translation="MTTSYAKIEITGTLTVLTGLQIGAGDGFSAIGAVDKPVVRDPLS
RLPMIPGTSLKGKVRTLLSRQYGADTETFYRKPNEDHAHIRRLFGDTEEYMTGRLVFR
DTKLTNKDDLEARGAKTLTEVKFENAINRVTAKANLRQMERVIPGSEFAFSLVYEVSF
GTPGEEQKASLPSSDEIIEDFNAIARGLKLLELDYLGGSGTRGYGQVKFSNLKARAAV
GALDGSLLEKLNHELAAV"
CDS complement(3090635..3091009)
/codon_start=1
/note="gene_2870"
/transl_table=11
/translation="MSVIQDDYVKQAEVIRGLPKKKNGFELTTTQLRVLLSLTAQLFD
EAQQSANPTLPRQLKEKVQYLRVRFVYQSGREDAVKTFVRNAKLLEALEGIGDSRDGL
LRFCRYMEALAAYKKYLDPKDK"
CDS complement(3091006..3093444)
/codon_start=1
/note="gene_2871"
/transl_table=11
/translation="MNPQLIEAIIGCLLHDIGKPVQRAALGYPGRHSAIGRAFMKKVW
LRDSRNPSQFTDEVDEADIGVSDRRILDAISYHHSSALRTAAENGRLAADAPAYIAYI

ADNIAAGTDRRKADSDDGHGASTWDPDTPLYSMFNRFGSGTANLAFAPEMLDDRKPIN
IPSPRRIEFDKDRYAAIVNKLKAILVDLERSDTYLASLLNVLEATLSFVPSSTDASEV
VDVSLFDHLKLTGALGACIWHYLQATGQSDFKSALFDKQDTFYNEKAFLLTTFDVSGI
QDFIYTIHSSGAAKMLRARSFYLEMLTEHLIDELLARVGLSRANLNYSGGGHAYLLLP
NTESARKSVEQFEREANDWLLENFATRLFIATGSVPLAANDLMRRPNESASQASNRAL
RYSGLYRELSEQLSAKKLARYSADQLRELNSRDHDGQKGDRECSVCHTVNRTVSADDE
PKCSLCQALTAASSQIQSESRRFLLISDGATKGLPLPFGATLTFCSRADADKALQQPQ
TRRRYAKNKFFAGECLGTGLWVGDYVAQMEFGDYVKRASGIARLGVLRLDVDNLGQAF
THGFMEQGNGKFNTISRTAAFSRMLSLFFRQHINYVLARPKLRPITGDDPARPREATI
IYSGGDDVFVVGAWDDVIEFGIELRERFHEFTQGKLTVSAGIGMFPDKYPISVMAREV
GDLEDAAKSLPGKNGVALFDREFTFGWDELLSKVIEEKYRHIADYFSGNEERGMAFIY
KLLELLAERDDRITKARWVYFLTRMRNPTGDTAPFQQFANRLHQWFQDPTDAKQLKTA
LHLYIYRTRKEESE"

CDS complement(3093441..3094229)
/codon_start=1
/note="gene_2872"
/transl_table=11
/translation="MTEHLSRLTLTLEVDAPLERARVATLGPHLHGVLMESIPADYVQ
TLHTVPVNPYSQYALARSTTSLEWKISTLTNEARQQIVGPINDAAFAGFRLRASGIAT
QVTSRSLEQNPLSQFARIFYARPETRKFRVEFLTPTAFKQSGEYVFWPDPRLVFQSLA
QKYGAIVDGEEPDPGLIAEFGQSVRLSAFRVASAPFAVGAARVPGFTGSATFTVRGVD
TFASYIAALLWFGEFSGCGIKASMGMGAIRVQPLAPREKCVPKP"

CDS complement(3094563..3095210)
/codon_start=1
/note="gene_2873"
/transl_table=11
/translation="MELPGAKRLGDDRRPLGTLRCWRHSDIGPARGIVVTPALKEWSA
AVHALLDGRQTVLLRKGGIGEKRFEVAAHEFLLFPTVAHSHAERVRPEHRDLLGPAAA
DSTDECVLLRAAAKVVAALPVNRPEGLDAIEDLHIWTAESVRADRLDFRPKHKLAVLV
VCAIPLAEPVRLARRPEYGGCTSWVQLPLTPQLAEPVHDEAALAEVAARVREAVG"

CDS complement(3095380..3096264)
/codon_start=1
/note="gene_2874"
/transl_table=11
/translation="VAGLTRALVARHALGRAEAYDAALLDVAQDHLLYLLSQTVQFGD
NRLVFKGGTSLRKCRLGNVGRFSTDLDFSAPDDEVVLEVCELIDGARVGGFEFGVQST
RGDGRHWQLRVRHTELGEPRIVASVEFARRPLALPSELLAFIQLPIHKAYGFGLPTLP
VVAEAEACAEKLARYRRVALARDLYDLNHFASRTIDEPLVRRLWVLKVWGDVVDDRRG
TRPLRVEDVLAARSEHDFQPDSIGVLTRPVAMAAWEARVRKRFAFLTDLDADEQRWAA
CDERHRREVENALAVLRS"

CDS complement(3096267..3097103)
/codon_start=1
/note="gene_2875"
/transl_table=11
/translation="VVSGLARDRPVVVTKEDLTQRLTEAGCGRDPDSAIRELRRIGWL
VQLPVKGTWAFIPPGEAAISDPYLPLRSWLARDQNAGFMLAGASAAWHLGYLDRQPDG
RIPIWLPPAKRLPDGLASYVSVVRIPWNAADTALLAPRPALLVRRRLDLVAWATGLPA
LGPEALLVQIATRPASFGPWADLVPHLDDLVADCSDERLERLLSGRPTSAWQRASYLL
DSGGEPARGQALLAKRHTEVMPVTRFTTAHSRDRGESVWAPEYQLVDELVVPLLRVIG
KA"

CDS complement(3097459..3098004)

/codon_start=1
/note="gene_2876"
/transl_table=11
/translation="VTPALKEWSAAVHALLDGRQTVLLRKGGIGEKRFEVAAHEFLLF
PTVAHSHAERVRPEHRDLLGPAAADSTDECVLLRAAAKVVAALPVNRPEGLDAIEDLH
IWTAESVRADRLDFRPKHRLAVLVVSAIPLAEPVRLARTPEYGGCTSWVQLPVTPTLA
APVHDEAALAEVAARVREAVG"
CDS complement(3098001..3098270)
/codon_start=1
/note="gene_2877"
/transl_table=11
/translation="MCRNITELRGLQPPATPVEIAAAARQYVRKVSGITHPSAATAEA
FEAAVAEVTATTTRLLDALPPRRQPPKTVPPLRRPDVAARLAGSR"
CDS complement(3098291..3098683)
/codon_start=1
/note="gene_2878"
/transl_table=11
/translation="MTTVLLDSHVAYWWSAEPQRLSMAASQAIEHADELAVAAISWFE
LAWLAEQERIQLAIPVLSWLQQLAEHVRTVGITPSVAATAVALPSSFPGDPADRLIYA
TAIEHGWRLVTKDRRLRSHRHPRPVTVW"
CDS complement(3098680..3098907)
/codon_start=1
/note="gene_2879"
/transl_table=11
/translation="MTRKMTATEVKAKILSLLDEVAQGEEIEITKHGRTVARLVAATG
PHALKGRFSGVAMAAVDDDELFTTGVSWNVS"
CDS 3098942..3099691
/codon_start=1
/note="gene_2880"
/transl_table=11
/translation="MTDDILLIDTDERVRTLTLNRPQSRNALSAALRDRFFAALADAE
ADDDIDVVILTGADPVFCAGLDLKELAGQTALPDISPRWPAMTKPVIGAINGAAVTGG
LELALYCDILIASEHARFADTHARVGLLPTWGLSVRLPQKVGIGLARRMSLTGDYLSA
TDALRAGLVTEVVAHDQLLPTARRVAASIVGNNQNAVRALLASYHRIDESQTAAGLWL
EACAAKQFRTSGDTIAANREAVLQRGRAQVR"
CDS complement(3099770..3100852)
/codon_start=1
/note="gene_2881"
/transl_table=11
/translation="MANVQYSAVTQRYPGADAPTVDNLDLDIADGEFLVLVGPSGCGK
STTLRVLAGLEPIESGRISIGDVDVTHLPPRARDVAMVFQNYALYPNMTVAANMGFAL
RNAGMSRADTRRRVLEVADMLELTDLLDRKPAKLSGGQRQRVAMGRAIVRRPRVFCMD
EPLSNLDAKLRVSTRSQISGLQRRLGTTTVYVTHDQVEAMTMGDRVAVLKDGVLQQVD
TPRALYDDPVNTFVATLIGAPAMNLIDAAVAHGVVRAPDLAIPVPDPAAERVLVGVRP
ESWDVASIGTPGSLTVHVELVEELGFESFVYATPVDQRGWSSRAPRIVFRTDRRTAVR
VGESLAIVPHSQEVRLFNSRTETRLR"
CDS complement(3100845..3101477)
/codon_start=1
/note="gene_2882"
/transl_table=11
/translation="LTLTEPATIAAGNFYRNSIHGKGYAAVANDIANEFATGILASAV

```
                     ASTGSLAGITASARFDFGAAPLPTGPDAAPACPTGGAGLAIPAKLSEERKVNALKFIA
                     FVTNPTNTAYFSQQTGYLPVRKSAVDDASERHYLADNPRARVALDQLPHTRTQDYARV
                     FLPGGDRIISAGLESIGLRGADVTKTFTNIQKRLQVILDRQIMRKLAGHG"
     CDS             complement(3101523..3102155)
                     /codon_start=1
                     /note="gene_2883"
                     /transl_table=11
                     /translation="MDPLNRRQFLALAAAAAGVTAGCAGMGGGGSVKSGSGPIDFWSS
                     HPGQSSAAERELIGRFQDRFPTLSVKLIDAGKDYDEVAQKFNAALIGTDVPDVVLLDD
                     RWWFHFALIGVLTALDDLFGQVGVDTTDYVDSLLADYEFNGRHYAVPYARSTPLFYYN
                     KAAWQQAGLPDRGLQSWSEFDEWGPELQRVVGAGRSAHGWANADLISWTF"
     CDS             complement(3102158..3102943)
                     /codon_start=1
                     /note="gene_2884"
                     /transl_table=11
                     /translation="MLLVVTLIAGPLLFVFFTSFKDQPDIYAQPTSWWPLRWYPQNYR
                     TATEQIPFWTFLRNSLIITSVLAVVKFTLGVLSAFGLVFVRFPGRTAVFLVIIAALMV
                     PNQITVISNYALISHLGLRNTFAGIILPLAGVAFGTFLMRNHFLSLPAEIIEAARMDG
                     ARWWQLLLRVVLPMSRPTMVAVGVITVVNEWNEYLWPFLMSDDESVAPLPIGLTFLQQ
                     AEGVTNWGPVMAVTLLAMLPILLVFIALQRQMIKGLTSGAVKG"
     CDS             complement(3102982..3103848)
                     /codon_start=1
                     /note="gene_2885"
                     /transl_table=11
                     /translation="VRDYALFVVLVGPNVALLLLFVYRPLADNIRLSFFDWNVSDPSA
                     RFVGLSNYTEWFTRSDTRQIVFNTAVFTGAAVVGSMVLGLALAMLLDRPLRGRNLVRS
                     TVFAPFVISGAAVGLAAQFVFDPHFGLIQDLLRRIGVGVPDFYQDARWALFMVTITYV
                     WKNLGYTFVIYLAALQGVRRDLLEAAEIDGASRWAVFRRVLLPQLRPTTFFLSITVLI
                     NSLQVFDVINVMTRGGPEGTGTTTMVYQVYVETFRNFRAGYGATVATIMFLVLLAVTY
                     YQVRVMDRGQRQ"
     CDS             complement(3103980..3105299)
                     /codon_start=1
                     /note="gene_2886"
                     /transl_table=11
                     /translation="LSQVGHRAGGRQIAQLALPALGVLAAEPLYLLFDIAVVGRLGAI
                     SLAGLAIGSLVLGLVGSQATFLSYGTTARAARRYGAGNRVAAVTEGVQATWLALGLGA
                     LVVVVVEATATPLVSAIASGDGITAAALPWLRIAILGTPAILVSLAGNGWLRGVHDTV
                     RPLRYVVAGFGSSALLCPLLVYGWLGLPRWGLTGSAVANLVGQWLAALLFAGALLAER
                     VSLRPDRAVLGAQLMMARDLIVRTLAFQVCYVSAAAVAARFGAAALAAHQVVLQLWGL
                     LALVLDSLAIAAQSLVGAALGAGDAGHAKAVAWRVTAFSLLAAGILAAALGLGSSVLP
                     GLFTDDRSVLAAIGVLWWFMVVQLPFAGIVFAVDGVLLGAGDAAFMRTATVASALVGF
                     LPLVWLSLAYGWGLAGIWSGLGTFIVLRLIFVGWRAYSGRWAVTGAA"
     CDS             complement(3105306..3106316)
                     /codon_start=1
                     /note="gene_2887"
                     /transl_table=11
                     /translation="VTTIDPRSELVDGRRRAGARVDAVGAAALLSAAARVGVVCHVHP
                     DADTIGAGLALALVLDGCGKRVEVSFAAPATLPESLRSLPGCHLLVRPEVMRRDVDLV
                     VTVDIPSVDRLGALGDLTDSGRELLVIDHHASNDLFGTANFIDPSADSTTTMVAEILD
                     AWGKPIDPRVAHCIYAGLATDTGSFRWASVRGYRLAARLVEIGVDNATVSRTLMDSHP
                     FTWLPLLSRVLGSAQLVSEAVGGRGLVYVVVDNREWVAARSEEVESIVDIVRTTQQAE
```

VAAVFKEVEPHRWSVSMRAKTVNLAAVASGFGGGGHRLAAGYTTTGSIDDAVASLRAA
LG"
CDS complement(3106291..3106842)
/codon_start=1
/gene="rbfA"
/note="gene_2888"
/transl_table=11
/translation="MADAARARRLAKRIAAIVASAIEYEIKDPGLAGVTITDAKVTAD
LHDATVYYTVMGRTLHDEPNCAGAAAALERAKGVLRTKVGAGTGVRFTPTLTFTLDTI
SDSVHRMDELLARARAADADLARVRVGAKPAGEADPYRDNGSVAQSPAPGGLGIRTSD
GPEAVEAPLTCGGDTGDDDRPKE"
CDS complement(3106842..3109544)
/codon_start=1
/gene="infB"
/note="gene_2889"
/transl_table=11
/translation="VAAGKARVHELAKELGVTSKEVLARLSEQGEFVKSASSTVEAPV
ARRLRESFGGSKPAPAKGTAKSPGKGPDKSLDKALDAAIDMAAGNGKATAAPAKAADS
GGAAIVSPTTPAAPEPPTAVPPSPQAPHPGMAPGARPGPVPKPGIRTPRVGNNPFSSA
QPADRPIPRPPAPRPGTARPGVPRPGASPGSMPPRPGGAVGGARPPRPGAPRPGGRPG
APGAGRSDAGGGNYRGGGVGAAPGTGFRGRPGGGGGGRPGQRGGAAGAFGRPGGAPRR
GRKSKRQKRQEYDSMQAPVVGGVRLPHGNGETIRLARGASLSDFADKIDANPAALVQA
LFNLGEMVTATQSVGDETLELLGSEMNYNVQVVSPEDEDRELLESFDLSYGEDEGGEE
DLQVRPPVVTVMGHVDHGKTRLLDTIRKANVREAEAGGITQHIGAYQVAVDLDGSQRL
ITFIDTPGHEAFTAMRARGAKATDIAILVVAADDGVMPQTVEAINHAQAADVPIVVAV
NKIDKEGADPAKIRGQLTEYGLVPEEFGGDTMFVDISAKQGTNIEALEEAVLLTADAA
LDLRANPDMEAQGVAIEAHLDRGRGPVATVLVQRGTLRVGDSVVAGDAYGRVRRMVDE
HGEDVEVALPSRPVQVIGFTSVPGAGDNFLVVDEDRIARQIADRRSARKRNALAARSR
KRISLEDLDSALKETSQLNLILKGDNAGTVEALEEALMGIQVDDEVVLRVIDRGVGGI
TETNVNLASASDAVIIGFNVRAEGKATELASREGVEIRYYSVIYQAIDEIEQALRGLL
KPIYEENQLGRAEIRALFRSSKVGLIAGCLVTSGVMRRNAKARLLRDNIVVAENLSIA
SLRREKDDVTEVRDGFECGLTLGYADIKEGDVIESYELVQKERA"
CDS complement(3110056..3111099)
/codon_start=1
/gene="nusA"
/note="gene_2890"
/transl_table=11
/translation="MNIDMAALHAIEVDRGISVNELLETIKSALLTAYRHTQGHQTDA
RIEIDRKTGVVRVIARETDEAGNLISEWDDTPEGFGRIAATTARQVMLQRFRDAENER
TYGEFSTREGEIVAGVIQRDSRANARGLVVVRIGTETKASEGVIPAAEQVPGESYEHG
NRLRCYVVGVTRGAREPLITLSRTHPNLVRKLFSLEVPEIADGSVEIVAVAREAGHRS
KIAVRSNVAGLNAKGACIGPMGQRVRNVMSELSGEKIDIIDYDDDPARFVANALSPAK
VVSVSVIDQTARAARVVVPDFQLSLAIGKEGQNARLAARLTGWRIDIRGDAPPPPPGQ
PEPGVSRGMAHDR"
CDS complement(3111096..3111647)
/codon_start=1
/note="gene_2891"
/transl_table=11
/translation="VTTGLPSQRQVIELLGADFACAGYEIEDVVIDARARPPRIAVIA
DGDAPLDLDTIAALSRRASALLDGLDGANKIRGRYLLEVSSPGVERPLTSEKHFRRAR
GRKVELVLSDGSRLTGRVGEMRAGTVALVIREDRGWAVREIPLAEIVKAVVQVEFSPP

APAELELAQSSEMGLARGTEAGA"
CDS 3111902..3112387
/codon_start=1
/note="gene_2892"
/transl_table=11
/translation="VLAGGAALAALGVVSACGESAPKAPAVEELRSPLDQARHDGALA
AAAATAIGIPPQVAAALTVVATQRTSHARALATEIARAAGKLVSATSETSSSSPSPTD
PAAPPPAVSDVIDSLRTSAGEASRLVATTSGYRAGLLASIAASCTASYTVALVPSGPS
I"
CDS 3112384..3112872
/codon_start=1
/note="gene_2893"
/transl_table=11
/translation="MTSSEPAHGATPKRSPSEGSADNAALCDALAVEHATIYGYGIVS
ALSPPGVNFLVADALKQHRHRRDDVIVMLSARGVTAPIAAAGYQLPMQVSSAADAARL
AVRMENDGATAWRAVVEHAETADDRVFASTALTESAVMATRWNRVLGAWPITAAFPGG
DE"
CDS complement(3112873..3114621)
/codon_start=1
/note="gene_2894"
/transl_table=11
/translation="VITRMSELFLRTLRDDPADAEVASHKLLIRAGYIRPVAPGLYSW
LPLGLRVLRNIERVIRDEMNAIGGQEILFPALLPRAPYETTNRWTQYGDSVFRLKDRR
GNDYLLGPTHEELFTLTVKGEYSSYKDFPLTLYQIQTKYRDEARPRAGILRAREFVMK
DSYSFDIDAAGLKAAYRAHREAYQRIFDRLQVRYVIVSAVSGAMGGSASEEFLAESPS
GEDAFVRCLESGYTANVEAVVTARPDTLPIDGLPEAVVHDTGDTPTIASLVAWANEAD
LGRTVTAADTLKNVLIKVRQPGGDTELLAIGVPGDREVDDKRLGAALEPADYALLDDD
DFAKHPFLVKGYIGPKALRENNVRYLVDPRIVDGTSWITGADQPGRHVVGLVAGRDFT
ADGTIEAAEVREGDPSPDGAGPLVMARGIEIGHIFQLGSKYTDAFTADVLGEDGKPVR
LTMGSYGIGVSRLVAVVAEQHHDELGLRWPSTVAPFDVHLVIANKDAQARAGATALAA
DLDRLGVEVLLDDRQASPGVKFKDAELLGMPWIVVVGRGWADGVVELRDRFSGQTREL
VAGASLATDIAAAVTG"
CDS complement(3114710..3116302)
/codon_start=1
/note="gene_2895"
/transl_table=11
/translation="MTALNDTERAVRNWRAGRPHRPAPMRPPRSEETASERPSRYYPT
WLPSRSFIAAVIAIGGMQLLATMDSTVAIVALPKIQNELSLSDAGRSWVITAYVLTFG
GLMLLGGRLGDTIGRKRTFIVGVALFTISSVLCAVAWDEATLVIARLSQGVGSAIASP
TGLALVATTFPKGPARNAATAVFAAMTAIGSVMGLVVGGALTEVSWRWAFLVNVPIGL
VMIYLARTALRETNKERMKLDATGAILATLACTAAVFAFSIGPEKGWMSGITIGSGLV
ALAAAVAFVIVERTAENPVVPFHLFRDRNRLVTFSAILLAGGVMFSLTVCIGLYVQDI
LGYSALRAGVGFIPFVIAMGIGLGVSSQLVSRFSPRVLTIGGGYLLFGAMLYGSFFMH
RGVPYFPNLVMPIVVGGIGIGMAVVPLTLSAIAGVGFDQIGPVSAIALMLQSLGGPLV
LAVIQAVITSRTLYLGGTTGPVKFMNDVQLAALDHAYTYGLLWVAGAAIIVGGMALFI
GYTPQQVAHAQEVKEAIDAGEL"
CDS complement(3116325..3117542)
/codon_start=1
/note="gene_2896"
/transl_table=11
/translation="VTENPYLVGLRLAGKKVVVVGGGTVAQRRLPLLIASGADVHVIA

PSVTPAVEAMDQITLSVRDYRDGDLDGAWYAIAATDDARVNVAVVAEAERRRIFCVRA
DIAVEGTAVTPASFSYAGLSVGVLAGGEHRRSAAIRSAIREALQQGVITAQSSDVLSG
GVALVGGGPGDPELITVRGRRLLAQADVVVADRLAPPELLAELPPHVEVIDAAKIPYG
RAMAQDAINAVLIERARSGNFVVRLKGGDPFVFARGYEEVLACAHAGIPVTVVPGVTS
AIAVPAMAGVPVTHRAMTHEFVVVSGHLAPGHPESLVNWDALAALTGTIVLLMAVERI
ELFVDVLLKGGRTADTPVLVVQHGTTAAQQTLRATLADTPEKVRAAGIRPPAIIVIGA
VVGLSGVRGLNNS"

CDS complement(3117539..3117901)
/codon_start=1
/gene="HMPREF0591_3779"
/note="gene_2897"
/transl_table=11
/translation="MRPAILHERNGTAKSRAGNSQWRYIRGQRGGDPRRPVPALAIAT
GPDGGDPRRPVPALAIATGPDGGDPRRPVPALAIATGPDGGDPRRPVPALAIATGPDG
GDPRRPVPALAIATRLGG"

CDS complement(3117873..3119231)
/codon_start=1
/note="gene_2898"
/transl_table=11
/translation="VAVAAPASGSGKTTIATGLIGALRQAGHTVAPFKVGPDFIDPGY
HALAAGRPGRNLDPVLVGERLIGPLYAHGVAGADIAVIEGVLGLFDGRIGPAGGAPAA
GSTAHVAALLGAPVILVVDARGQSHSVAALLHGFSTFDTATRIAGVILNRVGSARHEQ
VLRQACDQAGVAVLGAIPRTAELELPTRYLGLVTAVEYGRRARLAVQAMTAVVARHVD
LAAVIACAGSQAAHPPWDPVIAVGNTARQPATVAIAAGRAFTFGYAEHAEMLRAAGAE
VVEFDPLSETLPEGTDAVVLPGGFPEQFTAELSANDTVRRQINELAAAGAPVHAECAG
LLYLVSELDGHPMCGVVAGSARFTQHLKLGYRDAVAVVDSALYSVGERVVGHEFHRTA
VTFADSYQPAWVYQGQDVDDVRDGAVHSGVHASYLHTHPAATPGAVARFVAHAACNTP
RA"

CDS complement(3119246..3119869)
/codon_start=1
/note="gene_2899"
/transl_table=11
/translation="MPQGNPLAVPNDGLTTRARRNMPILAVHTGEGKGKSTAAFGMAL
RAWNAGLDIAVFQFVKSAKWKVGEEAAFRQLGRLHDQHGIGGAVEWHKMGAGWSWTRT
SRKAGTDVDRAAAAADGCAEIALRLATQRHDFYLLDEFTYPLKWGWLDVDEVVDVLRA
RPGHQHVVITGRDAPQRLVAAADLVTEMTKVKHPMDAGRKGQKGIEW"

CDS complement(3119890..3121779)
/codon_start=1
/note="gene_2900"
/transl_table=11
/translation="VKPYPFSAIVGHDRLRLALLLCAVRPEIGGALIRGEKGTAKSTA
VRGLAALLSVATGSTETGLVELPLGATEDRVVGSLDLQRVMRDGEHAFSPGLLARAHG
GVLYVDEVNLLHDHLVDILLDAAAMGRVHVERDGISHSHEARFVLIGTMNPEEGELRP
QLLDRFGLTVDVQASRDIDVRVQVIRRRMAYEADPDAFVARYADADAELAHRIAAARA
TVDDVVLGDNELRRIAALCAAFDVDGMRADLVVARTAAAHAAWRGVRTVEEQDIQAAA
ELALPHRRRRDPFDDHGIDRDQLDEALALASVDPEPEPDPPGGGQSANEPASQPNSRS
KSTEPGAPSSMGDDPPRPASPRLRSSPRPSAPPSKIFRTRALRVPGVGTGAPGRRSRA
RNASGSVVAAAEVSDPDAHGLHLFATLLAAGERAFGAGPLRPWPDDVRRAIRESREGN
LVIFVVDASGSMAARDRMAAVSGATLSLLRDAYQRRDKVAVITFRQHEATLLLSPTSS
AHIAGRRLARFSTGGKTPLAEGLLAARALIIREKVRDRARRPLVVVLTDGRATAGPDP
LGRSRTAAAGLVAEGAAAVVVDCETSYVRLGLAAQLARQLGAPVVRLEQLHADYLVHA

VRGVA"
CDS complement(3121776..3122246)
/codon_start=1
/note="gene_2901"
/transl_table=11
/translation="MTEALRRVWAKDLDARALYELLKLRVEVFVVEQACPYPELDGRD
LLAETRHFWLETPDGEVTCTLRLMEEHAGGEKVFRIGRLCTKRDARGQGHSNRLLCAA
LAEVGDYPCRIDAQAYLTAMYAQHGFVRDGDEFLDDGIPHVPMLRPGSGQVERP"
CDS complement(3122305..3123786)
/codon_start=1
/note="gene_2902"
/transl_table=11
/translation="VSDLARTDVVLIGAGIMSATLGVLLRRLEPNWSITLIERLDAVA
AESSGPWNNAGTGHSALCEMNYTPEMPDGSIDITKAVRVNEQFQVTRQFWAYAAENGI
LTDVRSFLNPVPHVSFVHGSRGVEYLRRRQKALAGNPLFAGTEFIESPDEFARRLPFM
AAKRAFSEPVALNWAADGTDVDFGALAKQLIGYCVQNGTTALFGHEVRNLSRQSDGSW
TVTMCNRRTGEKRKLNTKFVFVGAGGDTLPVLQKSGIKEVKGFAGFPIGGRFLRAGNP
ALTASHRAKVYGFPAPGAPPLGALHLDLRFVNGKSWLVFGPYAGWSPKFLKHGQISDL
PRSIRPDNLLSVLGVGLTERRLLNYLISQLRLSEPERVSALREFAPSAIDSDWELTIA
GQRVQVIRRDERNGGVLEFGTTVIGDADGSIAGLLGGSPGASTAVAIMLDVLQKCFAN
RYQSWLPTLKEMVPSLGVQLSNEPALFDEVWSWSTKALKLGAA"
CDS 3123993..3125840
/codon_start=1
/note="gene_2903"
/transl_table=11
/translation="MLYVVASPDLMTAAATNLAEIGSAISTANGAAALPTVEVVAAAA
DEVSTQIAALFGAHARSYQTLSTQAAAFHSRFVQALTTAAASYASVEAANASPLQVAL
DVINAPAQTLLGRPLIGNGADGSTPGQAGGPGGLLYGNGGNGAAGGPNQAGGAGGNAG
LIGNGGAGGAGGVGAVGGNGGTGDLLFGNGGAGGQGGLGLAGINGGSGGQGGHGGNAI
LFGQGGAGGPGGTGAMGVAGTNPTPIGTAAPGSDGVNQIGNGGNTDLTGGAGGDGNAG
STTVNGGNGGTGGAARNSSGGTGNSFGGAGGAGGDGANGGDGGAGGEALTEGGATAVS
GAGGKGGNAEASGGAGGNGGKGGFAQATTSVTGGNGGNGGNGHDSTAPGGAGGSGGVG
GDGGRGGLLAGNGGTGGAGGNGGTGGAGAPGGAGGAGGKADIANSLGDNATVTGGNGG
TGGDGGSALGTGGAGGAGGLGGHGGAGGLLIGNGGAGGAGGLGGAGGAGGAGGEGGAG
GAGGEAIPGGASTNSAGGDGGAGGTGGNGGDGGAGGAPGLGGAGGAGGWLIGQSGSTG
GGGAGGAGGAGGAGGAGGSGGAGGHGDTTSGKNGSSGTAGFDGNPGQPG"
variation 3125211..3125264
/note="supplemented from AL2122/97"
CDS 3125877..3126917
/codon_start=1
/note="gene_2904"
/transl_table=11
/translation="VTGWVPDVLPGYWQCTIPLGPDPDDEGDIVATLVGRGPQTGKAR
GDTTGAHHTVLAVHGYTDYFFHTELADHFANRGFAFYALDLRKCGRSRAPGQTPHFIT
DLARYDTELEHSLSIINEQNRSAKVLVYGHSAGGLIVSLWLDRLRQRGEITRAGVTGL
VLNSPFLDLQGPAILRLPLTSAFFAAMARMRPKWVARPPKEGGYGCTLHRDYDGEFDY
NLQWKPVGGFPVTFGWIHASRRGHARLHRGIDVGVPNLILCSDHTVREKADPATLHRG
DAVLDVTHITRWAGCIGNRSTVIAVADAKHDVFLSLPQPRQMAYRRLDLWLDDYLGTH
NDTDASASSGKG"
CDS 3126930..3128309
/codon_start=1

/note="gene_2905"
/transl_table=11
/translation="METYDIAIIGTGSGNSILDERYASKRAAICEQGTFGGTCLNVGC
IPTKMFVYAAEVAKTIRGASRYGIDAHIDRVRWDDVVSRVFGRIDPIALSGEDYRRCA
PNIDVYRTHTRFGPVQADGRYLLRTDAGEEFTAEQVVIAAGSRPVIPPAILASGVDYH
TSDTVMRIAELPEHIVIVGSGFIAAEFAHVFSALGVRVTLVIRGSCLLRHCDDTICER
FTRIASTKWELRTHRNVVDGQQRGSGVALRLDDGCTINADLLLVATGRVSNADLLDAE
QAGVDVEDGRVIVDEYQRTSARGVFALGDVSSPYLLKHVANHEARVVQHNLLCDWEDT
QSMIVTDHRYVPAAVFTDPQIAAVGLTENQAVAKGLDISVKIQDYGDVAYGWAMEDTS
GIVKLITERGSGRLLGAHIMGYQASSLIQPLIQAMSFGLTAAEMARGQYWIHPALPEV
VENALLGLR"
CDS 3128409..3129527
/codon_start=1
/note="gene_2906"
/transl_table=11
/translation="MASSQLDRQRSRSAKMNRALTAAEWWRLGLMFAVIVALHLVGWL
TVTLLVEPARLSLGGKAFGIGVGLTAYTLGLRHAFDADHIAAIDNTTRKLMSDGHRPL
AVGFFFSLGHSTVVFGLAVMLVTGLKAIVGPVENDSSTLHHYTGLIGTSISGAFLYLI
GILNVIVLVGIVRVFAHLRRGDYDEAELEQQLDNRGLLIRFLGRFTKSLTKSWHMYPV
GFLFGLGFDTATEIALLVLAGTSAAAGLPWYAILCLPVLFAAGMCLLDTIDGSFMNFA
YGWAFSSPVRKIYYNITVTGLSVAVALLIGSVELLGLIANQLGWQGPFWDWLGGLDLN
TVGFVVVAMFALTWAIALLVWHYGRVEERWTPAPDRTT"
CDS 3129593..3129925
/codon_start=1
/note="gene_2907"
/transl_table=11
/translation="VLEKIFAENDVRANVNRAAFENNGIRALDLMSSPGSGKTTVLGA
ALDEHADQFAIGVIEGDITTDLDAANGRGTQVSLLNNQHGFCAECHLDAPMVNRALAG
APDGVRRR"
CDS 3129937..3130152
/codon_start=1
/note="gene_2908"
/transl_table=11
/translation="MVSSVTEGKDKPLMYPATFRSRDVVLLDKIDLVPFLDADVDAYI
AHVREVNAAATILPTSTRTGAGMGSWS"
CDS complement(3130308..3131081)
/codon_start=1
/note="gene_2909"
/transl_table=11
/translation="MDLSQRLAGRVAVITGGGSGIGLAAGRRMRAEGATIVVGDVDVE
AGGAAADELSGLFVPTDVCDEDAVNGLFDGAAETYGRIDIAFNNAGISPPEDNLIENT
ELAAWQRVQDVNLKSVYLCCRAALRHMVLAGKGSIVNTASFVAVMGSATSQISYTASK
GGVLAMSRELGVQFARQGIRVNALCPGPVNTPLLQELFAKNPERAARRMVHVPLGRFA
EPDEIAAAVAFLASDDASFITASTFLVDGGISSAYVTPL"
CDS complement(3131081..3132448)
/codon_start=1
/note="gene_2910"
/transl_table=11
/translation="MSTTQLINPATEEVLASVDHTDANAVDDAVQRARAAQRRWARLA
PAQRAAGLRAFAAAVQAHLDELAALEVANSGHPIVSAEWEAGHVRDVLAFYAASPERL
SGRQIPVAGGVDVTFNEPMGVVGVITPWNFPMVIASWAIAPALAAGNAVLVKPAELTP

LTTMRLGELAVEAGLDEDLLQVLPGKGTVVGERFVTHPDIRKIVFTGSTEVGKRVMAG
AAAQVKRVTLELGGKSANIVFHDCDLERAATTAPAGVFDNAGQDCCARSRILVQRSVY
DRFMELLEPAVHSIVVGDPGSRATEMGPLVSRAHRDKVAGYVPDDAPVAFRGTAPAGR
GFWFPPTVLTPKRGDRTVTDEIFGPVVVVLTFDDEADAISLANDTAYGLSGSIWTDDL
SRALRVARAVESGNLSVNSHSSVRFNTPFGGFKQSGVGRELGPDAPLQFTETKNVFIA
VGEEM"

CDS complement(3132445..3133371)
/codon_start=1
/note="gene_2911"
/transl_table=11
/translation="VDLSASRSDGGDPLRPASPRLRSPVSDGGDPLRPASPRLRSPVS
DGGDPLRPASPRLRSPLGASRPVVGLTAYLEQVRTGVWDIPAGYLPADYFEGITMAGG
VAVLLPPQPVDPESVGCVLDSLHALVITGGYDLDPAAYGQEPHPATDHPRPGRDAWEF
ALLRGALQRGMPVLGICRGTQVLNVALGGTLHQHLPDILGHSGHRAGNGVFTRLPVHT
ASGTRLAELIGESADVPCYHHQAIDQVGEGLVVSAVDVDGVIEALELPGDTFVLAVQW
HPEKSLDDLRLFKALVDAASGYAGRQSQAEPR"

CDS complement(3133352..3134725)
/codon_start=1
/note="gene_2912"
/transl_table=11
/translation="VTGPGSPPLAWTELERLVAAGDVDTVIVAFTDMQGRLAGKRISG
RHFVDDIATRGVECCSYLLAVDVDLNTVPGYAMASWDTGYGDMVMTPDLSTLRLIPWL
PGTALVIADLVWADGSEVAVSPRSILRRQLDRLKARGLVADVATELEFIVFDQPYRQA
WASGYRGLTPASDYNIDYAILASSRMEPLLRDIRLGMAGAGLRFEAVKGECNMGQQEI
GFRYDEALVTCDNHAIYKNGAKEIADQHGKSLTFMAKYDEREGNSCHIHVSLRGTDGS
AVFADSNGPHGMSSMFRSFVAGQLATLREFTLCYAPTINSYKRFADSSFAPTALAWGL
DNRTCALRVVGHGQNIRVECRVPGGDVNQYLAVAALIAGGLYGIERGLQLPEPCVGNA
YQGADVERLPVTLADAAVLFEDSALVREAFGEDVVAHYLNNARVELAAFNAAVTDWER
IRGFERL"

CDS complement(3134885..3135742)
/codon_start=1
/note="gene_2913"
/transl_table=11
/translation="MPSRTALSPGVLSPTRPVPNWIARPEYVGKPAAQEGSEPWVQTP
EVIEKMRVAGRIAAGALAEAGKAVAPGVTTDELDRIAHEYLVDNGAYPSTLGYKGFPK
SCCTSLNEVICHGIPDSTVITDGDIVNIDVTAYIGGVHGDTNATFPAGDVADEHRLLV
DRTREATMRAINTVKPGRALSVIGRVIESYANRFGYNVVRDFTGHGIGTTFHNGLVVL
HYDQPAVETIMQPGMTFTIEPMINLGALDYEIWDDGWTVVTKDRKWTAQFEHTLLVTD
TGVEILTCL"

CDS complement(3135784..3136368)
/codon_start=1
/note="gene_2914"
/transl_table=11
/translation="MTETGGDMVALRVSDADPNGTMRRLHNAVALGLINIDEFEQRSS
RVSFARTRSELDGLVGDLPRPGAIVTSAADRVELRGWAGSLKRHGEWIVPTRLALVRR
LGSIELDLVKARFAGPVVVIELDMMFGSLEVRLPNGASASIDDVEVYVGSASDRRKDA
PAEGTPHVVLTGRMVCGSVVIKGPRRALLRRHRG"

CDS 3136717..3137097
/codon_start=1
/note="gene_2915"
/transl_table=11

```
                     /translation="MIFVDTNVFMYAVGRDHPLRMPAREFLEHSLEHQDRLVTSAEAM
                     QELLNAYVPVGRNSTLDSALTLVRALTEIWPVEAADVAHARTLHHRHPGLGARDLLHL
                     ACCQRRGVTRIKTFDHTLASAFRS"
     CDS             complement(3137179..3138951)
                     /codon_start=1
                     /note="gene_2916"
                     /transl_table=11
                     /translation="LLLLAVVAMSGCTPRPQGPGPAAEKFFAALAIGDTASAAQLSDN
                     PNEAREALNAAWAGLQAAHLDAQVLSAKYAEDTGTVAYRFSWHLPKDRIWTYDGQLKM
                     ARDEGRWHVRWTTSGLHPKLGEHQTFALRADPPRRASVNEVGGTDVLVPGYLYHYSLD
                     AGQAGRELFGTAHAVVGALHPFDDTLNDPQLLAEQASSSTQPLDLVTLHADDSNRVAA
                     AIGQLPGVVITPQAELLPTDKHFAPAVLNDVKKAVVDELDGKAGWRVVSVNQNGVDVS
                     VLHEVAPSPASSVSITLDRVVQNAAQHAVNTRGGKAMIVVIKPSTGEILAIAQNAGAD
                     ADGPVATTGLYPPGSTFKMITAGAAVERDLATPETLLGCPGEIDIGHRTIPNYGGFDL
                     GVVPMSRAFASSCNTTFAELSSRLPPRGLTQAARRYGIGLDYQVDGITTVTGSVPPTV
                     DLAERTEDGFGQGKVLASPFGMALVAATVAAGKTPVPQLIAGRPTAVEGDATPISQKM
                     IDALRPMMRLVVTNGTAKEIAGCGEVFGKTGEAEFPGGSHSWFAGYRGDLAFASLIVG
                     GGSSEYAVRMTKVMFESLPPGYLA"
     CDS             3138857..3139174
                     /codon_start=1
                     /note="gene_2917"
                     /transl_table=11
                     /translation="MASAAKNFSAAGPGPWGRGVQPDMATTASSSKPEVAEANVVLVT
                     IVADVKNCDGDTGRDTPRPNRYAETRSRMEHHRENRGQKSQPRYARDRSIEQGADRKH
                     QQT"
     CDS             3139262..3139543
                     /codon_start=1
                     /note="gene_2918"
                     /transl_table=11
                     /translation="MRILPISTIKGKLNEFVDAVSSTQDQITITKNGAPAAVLVGADE
                     WESLQETLYWLAQPGIRESIAEADADIASGRTYGEDEIRAEFGVPRRPH"
     CDS             3139547..3139810
                     /codon_start=1
                     /note="gene_2919"
                     /transl_table=11
                     /translation="VPYTVRFTTTARRDLHKLPPRILAAVVEFAFGDLSREPLRVGKP
                     LRRELAGTFSARRGTYRLLYRIDDEHTTVVILRVDHRADIYRR"
     CDS             complement(3140183..3141037)
                     /codon_start=1
                     /note="gene_2920"
                     /transl_table=11
                     /translation="MSAPPISRLVGERQVSVVRDAAAVWRVLDDDPIESCMVAARVAD
                     HGIDPNAIGGELWTRRGAHESLCFAGANLIPLRGGPIDLNAFADVAMSTPRRCSSLVG
                     RADLVLPMWQRLEPVWGPARDVRDNQPLMALATHPSCAIDTGVRQVRPEELDSYLVAA
                     VDMFIGEVGVDPRLGDGGRGYRRRVAGLIAAGRAWARFEHGQVIFKAEVGSQSPAVGQ
                     IQGVWVHPEWRGIGLGTAGTATLAAVIVGSGRIASLYVNSFNTVARAAYARVGFKEIG
                     TFATVLLD"
     CDS             complement(3141093..3142256)
                     /codon_start=1
                     /gene="ispG"
                     /note="gene_2921"
```

/transl_table=11
/translation="VTVGLGMPQPPAPTLAPRRATRQLMVGNVGVGSDHPVSVQSMCT
TKTHDVNSTLQQIAELTAAGCDIVRVACPRQEDADALAEIARHSQIPVVADIHFQPRY
IFAAIDAGCAAVRVNPGNIKEFDGRVGEVAKAAGAAGIPIRIGVNAGSLDKRFMEKYG
KATPEALVESALWEASLFEEHGFGDIKISVKHNDPVVMVAAYELLAARCDYPLHLGVT
EAGPAFQGTIKSAVAFGALLSRGIGDTIRVSLSAPPVEEVKVGNQVLESLNLRPRSLE
IVSCPSCGRAQVDVYTLANEVTAGLDGLDVPLRVAVMGCVVNGPGEAREADLGVASGN
GKGQIFVRGEVIKTVPEAQIVETLIEEAMRLAAEMGEQDPGATPSGSPIVTVS"
CDS complement(3142273..3143487)
/codon_start=1
/note="gene_2922"
/transl_table=11
/translation="MMFVTGIVLFALAILISVALHECGHMWVARRTGMKVRRYFVGFG
PTLWSTRRGETEYGVKAVPLGGFCDIAGMTPVEELDPDERDRAMYKQATWKRVAVLFA
GPGMNLAICLVLIYAIALVWGLPNLHPPTRAVIGETGCVAQEVSQGKLEQCTGPGPAA
LAGIRSGDVVVKVGDTPVSSFDEMAAAVRKSHGSVPIVVERDGTAIVTYVDIESTQRW
IPNGQGGELQPATVGAIGVGAARVGPVRYGVFSAMPATFAFTGDLTVEVGKALAALPT
KVGALVRAIGGGQRDPQTPISVVGASIIGGDTVDHGLWVAFWFFLAQLNLILATINLL
PLLPFDGGHIAVAVFERIRNMVRSARGKVAAAPVNYLKLLPATYVVLVLVVGYMLLTV
TADLVNPIRLFQ"
CDS complement(3143495..3144784)
/codon_start=1
/note="gene_2923"
/transl_table=11
/translation="VIRRRGDNEVVAHNDEVTNSTDGRADGRLRVVVLGSTGSIGTQA
LQVIADNPDRFEVVGLAAGGAHLDTLLRQRAQTGVTNIAVADEHAAQRVGDIPYHGSD
AATRLVEQTEADVVLNALVGALGLRPTLAALKTGARLALANKESLVAGGSLVLRAARP
GQIVPVDSEHSALAQCLRGGTPDEVAKLVLTASGGPFRGWSAADLEHVTPEQAGAHPT
WSMGPMNTLNSASLVNKGLEVIETHLLFGIPYDRIDVVVHPQSIIHSMVTFIDGSTIA
QASPPDMKLPISLALGWPRRVSGAAAACDFHTASSWEFEPLDTDVFPAVELARQAGVA
GGCMTAVYNAANEEAAAAFLAGRIGFPAIVGIIADVLHAADQWAVEPATVDDVLDAQR
WARERAQRAVSGMASVAIASTAKPGAAGRHASTLERS"
CDS 3144863..3145120
/codon_start=1
/note="gene_2924"
/transl_table=11
/translation="VRTTIRIDDELYREVKAKAARSGRTVAAVLEDAVRRGLNPPKPQ
AAGRYRVQPSGKGGLRPGVDLSSNAALAEAMNDGVSVDAVR"
CDS 3145107..3145550
/codon_start=1
/note="gene_2925"
/transl_table=11
/translation="MLCVDVNVLVYAHRADLREHADYRGLLERLANDDEPLGLPDSVL
AGFIRVVTNRRVFTEPTSPQDAWQAVDTLLAAPAAMRLRPGERHWMAFRQLASDVDAN
GNDIADAHLAAYALENNATWLSADRGFARFRRLRWRHPLDGQTHL"
CDS 3145630..3146292
/codon_start=1
/note="gene_2926"
/transl_table=11
/translation="MINVQAKPAAAASLAAIAIAFLAGCSSTKPVSQDTSPKPATSPA
APVTTAAMADPAADLIGRGCAQYAAQNPTGPGSVAGMAQDPVATAASNNPMLSTLTSA

LSGKLNPDVNLVDTLNGGEYTVFAPTNAAFDKLPAATIDQLKTDAKLLSSILTYHVIA
GQASPSRIDGTHQTLQGADLTVIGARDDLMVNNAGLVCGGVHTANATVYMIDTVLMPP
AQ"
CDS 3146388..3146579
/codon_start=1
/note="gene_2927"
/transl_table=11
/translation="MTVTPRPAQADPRSMPAEVASRELRNNTAGLLRRVQAGEDITIT
ANGKPVALLTAGSPHGADG"
CDS 3146911..3148659
/codon_start=1
/note="gene_2928"
/transl_table=11
/translation="VLTLALVGFLGGLITGISPCILPVLPVIFFSGAQSVDAAQVAKP
EGAVAVRRKRALSATLRPYRVIGGLVLSFGMVTLLGSALLSVLHLPQDAIRWAALVAL
VAIGAGLIFPRFEQLLEKPFSRIPQKQIVTRSNGFGLGLALGVLYVPCAGPILAAIVV
AGATATIGLGTVVLTATFALGAALPLLFFALAGQRIAERVGAFRRRQREIRIATGSVT
ILLAVALVFDLPAALQRAIPDYTASLQQQISTGTEIREQLNLGGIVNAQNAQLSNCSD
GAAQLESCGTAPDLKGITGWLNTPGNKPIDLKSLRGKVVLIDFWAYSCINCQRAIPHV
VGWYQAYKDSGLAVIGVHTPEYAFEKVPGNVAKGAANLGISYPIALDNNYATWTNYRN
RYWPAEYLIDATGTVRHIKFGEGDYNVTETLVRQLLNDAKPGVKLPQPSSTTTPDLTP
RAALTPETYFGVGKVVNYGGGGAYDEGSAVFDYPPSLAANSFALRGRWALDYQGATSD
GNDAAIKLNYHAKDVYIVVGGTGTLTVVRDGKPATLPISGPPTTHQVVAGDRLASETL
EVRPSKGLQVFSFTYG"
CDS 3148755..3149336
/codon_start=1
/note="gene_2929"
/transl_table=11
/translation="MKVKNTIAATSFAAAGLAALAVAVSPPAAAGDLVGPGCAEYAAA
NPTGPASVQGMSQDPVAVAASNNPELTTLTAALSGQLNPQVNLVDTLNSGQYTVFAPT
NAAFSKLPASTIDELKTNSSLLTSILTYHVVAGQTSPANVVGTRQTLQGASVTVTGQG
NSLKVGNADVVCGGVSTANATVYMIDSVLMPPA"
CDS 3149388..3149702
/codon_start=1
/note="gene_2930"
/transl_table=11
/translation="MFGQWEFDVSPTGGIAVASTEVEHFAGSQHEVDTAEVPSAAWGR
SRIDHRTWHIVGLCIFGFLLAMLRGNHVGHVEDWFLITFAAVVLFVLARDLWGRRRDW
IR"
CDS complement(3149733..3150596)
/codon_start=1
/note="gene_2931"
/transl_table=11
/translation="VNEALIGLAFAAGLVAALNPCGFAMLPAYLLLVVHGQDSAGRTG
PLSAVGRAAAATVGMALGFLTVFGIFGALTISAATAVQRYLPYATVLIGLALIALGGW
LLLGRGLTALTPRSLGVRWAPTVRLGSMYGYGISYAVASLSCTIGPFLAVTGAGLRGG
SVVGSVAIYLAYVAGLTLVVGVLAVAAATASSALADRLRRILPFVNRISGALLVVVGL
YVGYYGLYELRLIAGVGANPQDAVIAAAGRLQGALAGWVNQHGAWPWAVLLVVLVVGA
FAGTWFRRVRR"
CDS complement(3150601..3151122)
/codon_start=1

/note="gene_2932"
/transl_table=11
/translation="MSLRLVSPIKAFADGIVAVAIAVVLMFGLANTPRAVAADERLQF
TATTLSGAPFDGASLQGKPAVLWFWTPWCPFCNAEAPSLSQVAAANPAVTFVGIATRA
DVGAMQSFVSKYNLNFTNLNDADGVIWARYNVPWQPAFVFYRADGTSTFVNNPTAAMS
QDELSGRVAALTS"
CDS complement(3151308..3152402)
/codon_start=1
/note="gene_2933"
/transl_table=11
/translation="MVPELMFDEPRPGRPPRHLADLDAAGRASAVAELGLPAFRAKQL
AHQYYGRLIADPRQMTDLPAAVRDRIAGAMFPNLLTASADITCDAGQTRKTLWRAVDG
TMFESVLMRYPRRNTVCISSQAGCGMACPFCATGQGGLTRNLSTAEILEQVRAGAAAL
RDDFGDRLSNVVFMGMGEPLANYARVLAAVQRITARPPSGFGISARAVTVSTVGLAPA
IRNLADARLGVTLALSLHAPDDGLRDTLVPVNNRWRISEALDAARYYANVTGRRVSIE
YALIRDVNDQPWRADLLGKRLHRVLGPLAHVNLIPLNPTPGSDWDASPKPVEREFVKR
VRAKGVSCTVRDTRGREISAACGQLAAVGG"
CDS complement(3152425..3153345)
/codon_start=1
/note="gene_2934"
/transl_table=11
/translation="VTTNDAGTGNPAEQPARGAKQQPATETSRAGRDLRAAIVVGLSI
GLVLIAVLVFVPRVWVAIVAVATLVATHEVVRRLREAGYLIPVIPLLIGGQAAVWLTW
PFGAVGALAGFGGMVVVCMIWRLFMQDSVTRPTTGGAPSPGNYLSDVSATVFLAVWVP
LFCSFGAMLVYPENGSGWVFCMMIAVIASDVGGYAVGVLFGKHPMVPTISPKKSWEGF
AGSLVCGITATIITATFLVGKTPWIGALLGVLFVLTTALGDLVESQVKRDLGIKDMGR
LLPGHGGLMDRLDGILPSAVAAWIVLTLLP"
CDS complement(3153368..3153925)
/codon_start=1
/gene="frr"
/note="gene_2935"
/transl_table=11
/translation="MIDEALFDAEEKMEKAVAVARDDLSTIRTGRANPGMFSRITIDY
YGAATLITQLASINVPEARLVVIKPYEANQLRAIETAIRNSDLGVNPTNDGALIRVAV
PQLTEERRRELVKQAKHKGEEAKVSVRNIRRKAMEELHRIRKEGEAGEDEVGRAEKDL
DKTTHQYVTQIDELVKHKEGELLEV"
CDS complement(3154097..3154882)
/codon_start=1
/gene="pyrH"
/note="gene_2936"
/transl_table=11
/translation="MTEPDVAGAPASKPEPASTGAASAAQLSGYSRVLLKLGGEMFGG
GQVGLDPDVVAQVARQIADVVRGGVQIAVVIGGGNFFRGAQLQQLGMERTRSDYMGML
GTVMNSLALQDFLEKEGIVTRVQTAITMGQVAEPYLPLRAVRHLEKGRVVIFGAGMGL
PYFSTDTTAAQRALEIGADVVLMAKAVDGVFAEDPRVNPEAELLTAVSHREVLDRGLR
VADATAFSLCMDNGMPILVFNLLTDGNIARAVRGEKIGTLVTT"
CDS 3155174..3155875
/codon_start=1
/note="gene_2937"
/transl_table=11
/translation="LEVLLLTDEADLESALPELESFAQSVQRAPLDDPGAAKGADADV

AIIDARADLAAARRVCRRLTTSAPALAVVAVVAPANFVAVDGDWIFDDVLLNAAGGAE
LQARLRLAITRRRSTLAGTLQFGDLVLHPASYTASLGDRDLGLTLTEFKLMNFLVQHA
GRAFTRTRLMREVWGYECHGRIRTVDVHVRRLRAKLGAEHESMIDTVRGVGYMAVTPP
QPRWIISESILNRCK"

CDS complement(3155954..3157270)
/codon_start=1
/note="gene_2938"
/transl_table=11
/translation="MARLKVPEGWCVQAFRFTLNPTQTQAASLARHFGARRKAFNWTV
TALKADIKAWRADGTESAKPSLRVLRKRWNTVKDQVCVNAQTGQVWWPECSKEAYADG
IAGAVDAYWNWQSCRAGKRAGKTVGVPRFKKKGRDADRVCFTTGAMRVEPDRRHLTLP
VIGTIRTYENTRRVERLIAKGRARVLAITVRRNGTRLDASVRVLVQRPQQRRVALPDS
RVGVDVGVRRLATVADAEGTVLEQVPNPRPLDAALRGLRRVSRARSRCTKGSRRYCER
TTELSRLHRRVNDVRTHHLHVLTTRLAKTHGRIVVEGLDAAGMLRQKGLPGARARRRA
LSDAALATPRRHLSYKTGWYGSSLVVADRWFPSSKTCHACRHVQDIGWDEIWQCDGCS
ITHQRDDNAAINLARYEEPPSVVGPVGAAVKRGADRKTGPGPAGWP"

CDS complement(3157270..3157857)
/codon_start=1
/note="gene_2939"
/transl_table=11
/translation="MNLVVWAEGNGVARVIAYRWLRVGRLPVPARRVGRVILVDEPAG
QPGRWGRTAVCARLSSADQKVDLDRQVVGVTAWATAEQIPVGKVVTEVGSALYGRRRT
FLTLLGDPTVRRIVMKRRDRLGRFGFECVQAVLAADGRELVVVDSADVDDDVVGDITE
ILTSICARLYGKRAAGNRAARAVAAAARAGGHEAR"

CDS 3158156..3158575
/codon_start=1
/note="gene_2940"
/transl_table=11
/translation="MGLADDAPLGYLLYRVGAVLRPEVSAALSPLGLTLPEFVCLRML
SQSPGLSSAELARHASVTPQAMNTVLRKLEDAGAVARPASVSSGRSLPATLTARGRAL
AKRAEAVVRAADARVLARLTAPQQREFKRMLEKLGSD"

CDS complement(3158589..3159926)
/codon_start=1
/note="gene_2941"
/transl_table=11
/translation="VGRADVVEAAIARAEAVNPALNALAYAAFDVARDAAAMGTGQEA
FFSGVPTFIKDNVDVAGQPSMHGTDAWEPYAAVADSEITRVVLGTGLVSLGKTQLSEF
GFSAVAEHPRLGPVRNPWNTDYTAGASSSGSGALVAAGVVPIAHANDGGGSIRIPAAC
NGLVGLKPSRGRLPLEPEYRRLPVGIVANGVLTRTVRDTAAFYREAERLWRNHQLPPV
GDVTSPVKQRLRIAVVTRSVLREASPEVRQLTLKLAGLLEELGHRVEHVDHPPAPASF
VDDFVLYWGFLALAQVRSGRRTFGRTFDPTRLDELTLGLARHTGRNLHRLPLAIMRLR
MLRRRSVRFFGTYDVLLTPTVAEATPQVGYLAPTDYQTVLDRLSSWVVFTPVQNVTGV
PAISLPLAQSADGMPVGMMLSADTGREALLLLELAYELEEARPWARIHAPNIAE"

CDS complement(3160017..3160832)
/codon_start=1
/gene="tsf"
/note="gene_2942"
/transl_table=11
/translation="MANFTAADVKRLRELTGAGMLACKNALAETDGDFDKAVEALRIK
GAKDVGKRAERATAEGLVAAKDGALIELNCETDFVAKNAEFQTLADQVVAAAAAAKPA
DVDALKGASIGDKTVEQAIAELSAKIGEKLELRRVAIFDGTVEAYLHRRSADLPPAVG

VLVEYRGDDAAAAHAVALQIAALRARYLSRDDVPEDIVASERRIAEETARAEGKPEQA
LPKIVEGRLNGFFKDAVLLEQASVSDNKKTVKALLDVAGVMVTRFVRFEVGQA"
CDS complement(3160844..3161707)
/codon_start=1
/gene="rpsB"
/note="gene_2943"
/transl_table=11
/translation="MAVVTMKQLLDSGTHFGHQTRRWNPKMKRFIFTDRNGIYIIDLQ
QTLTFIDKAYEFVKETVAHGGSVLFVGTKKQAQESVAAEATRVGMPYVNQRWLGGMLT
NFSTVHKRLQRLKELEAMEQTGGFEGRTKKEILGLTREKNKLERSLGGIRDMAKVPSA
IWVVDTNKEHIAVGEARKLGIPVIAILDTNCDPDEVDYPIPGNDDAIRSAALLTRVIA
SAVAEGLQARAGLGRADGKPEAEAAEPLAEWEQELLASATASATPSATASTTALTDAP
AGATEPTTDAS"
CDS complement(3162519..3163745)
/codon_start=1
/note="gene_2944"
/transl_table=11
/translation="LDFGVLPPEINSGRMYAGPGSGPMMAAAAAWDSLAAELGLAAGG
YRLAISELTGAYWAGPAAASMVAAVTPYVAWLSATAGQAEQAGMQARAAAAAYELAFA
MTVPPPVVVANRALLVALVATNFFGQNTPAIAATEAQYAEMWAQDAAAMYAYAGSAAI
ATELTPFTAAPVTTSPAALAGQAAATVSSTVPPLATTAAVPQLLQQLSSTSLIPWYSA
LQQWLAENLLGLTPDNRMTIVRLLGISYFDEGLLQFEASLAQQAIPGTPGGAGDSGSS
VLDSWGPTIFAGPRASPSVAGGGAVGGVQTPQPYWYWALDRESIGGSVSAALGKGSSA
GSLSVPPDWAARARWANPAAWRLPGDDVTALRGTAENALLRGFPMASAGQSTGGGFVH
KYGFRLAVMQRPPFAG"
CDS 3164205..3165122
/codon_start=1
/note="gene_2945"
/transl_table=11
/translation="LPRAGTQMRAFAQAVEAAGFDVLAFPDHLVPSVSPFAGATAAAM
ATQRLHTGTLVLNNDFRHPVDTAREAAGVATLAEGRFELGLGAGHRRSEYDAAGITFD
SGATRVARLIESAHLIRALLDAEPVDFDGQHYRVHAEAGSLVAPPKVRVPLLVGGNGT
EVLRLGGRIADIVGLAGISHNRDATQVRFTHFDADGLADRIAVVRHAAGDRFEAIELN
ALIQAVVCTNDRNAAAAELAATLGGITPEQVLESPFLLLGTHEQMAEALAARQRRFGV
SYWTVFDEWAGRASAMRDIAEVIALLRYG"
CDS complement(3165119..3166015)
/codon_start=1
/gene="xerC"
/note="gene_2946"
/transl_table=11
/translation="VQAILDEFDEYLALQCGRSVHTRRAYLGDLRSLFAFLADRGSSL
DALTLSVLRSWLAATAGAGAARTTLARRTSAVKAFTAWAVRRGLLAGDPAARLQVPKA
RRTLPAVLRQDQALRAMAAAESGAEQGDPLALRDRLIVELLYATGIRVSELCGLDVDD
IDTGHRLVRVLGKGNKQRTVPFGQPAADALHAWLVDGRRALVTAESGHALLLGARGRR
LDVRQARTAVHQTVAAVDGAPDMGPHGLRHSAATHLLEGGADLRVVQELLGHSSLATT
QLYTHVAVARLRAVHERAHPRA"
CDS complement(3166106..3166957)
/codon_start=1
/note="gene_2947"
/transl_table=11
/translation="VAGRPLHAFEVVATRHLAPHMVRVVLGGSGFDTFVPSDFTDSYI

KLVFVDDDVDVGRLPRPLTLDSFADLPTAKRPPVRTMTVRHVDAAAREIAVDIVLHGE
HGVAGPWAAGAQRGQPIYLMGPGGAYAPDPAADWHLLAGDESAIPAIAAALEALPPDA
IGRAFIEVAGPDDEIGLTAPDAVEVNWVYRGGRADLVPEDRAGDHAPLIEAVTTTAWL
PGQVHVFIHGEAQAVMHNLRPYVRNERGVDAKWASSISGYWRRGRTEEMFRKWKKELA
EAEAGTH"

CDS complement(3166990..3168159)
/codon_start=1
/note="gene_2948"
/transl_table=11
/translation="MIDPTARAWAYLSRVAEPPCAQLAALVRCVGPVEAADRVRRGQV
GNELAQHTGARRGIDRAADDLELLMRRGGRLITPDDDEWPVLAFAAFSGAGARARPCG
HSPLVLWALGPARLDEVAPRAAAVVGTRAATAYGEHVAADLAAGLAERDVAVVSGGAY
GIDGAAHRAALDSEGITVAVLAGGFDIPYPAGHSALLHRIAQHGVLFTEYPPGVRPAR
HRFLTRNRLVAAVARAAVVVEAGLRSGAANTAAWARALGRVVAAVPGPVTSSASAGCH
TLLRHGAELVTRADDIVEFVGHIGELAGDEPRPGAALDVLSEAERQVYEALPGRGAAT
IDEIAVGSGLLPAQVLGPLAILEVAGLAECRDGRWRILRAGAGQAAAKGAAARLV"

CDS complement(3168156..3169667)
/codon_start=1
/note="gene_2949"
/transl_table=11
/translation="MALGRAFSVAVRGLDGEIVEIEADITSGLPGVHLVGLPDAALQE
SRDRVRAAVTNCGNSWPMARLTLALSPATLPKMGSVYDIALAAAVLSAQQKKPWERLE
NTLLLGELSLDGRVRPVRGVLPAVLAAKRDGWPAVVVPADNLPEASLVDGIDVRGVRT
LGQLQSWLRGSTGLAGRITTADTTPESAADLADVVGQSQARFAVEVAAAGAHHLMLTG
PPGVGKTMLAQRLPGLLPSLSGSESLEVTAIHSVAGLLSGDTPLITRPPFVAPHHSSS
VAALVGGGSGMARPGAVSRAHRGVLFLDECAEISLSALEALRTPLEDGEIRLARRDGV
ACYPARFQLVLAANPCPCAPADPQDCICAAATKRRYLGKLSGPLLDRVDLRVQMHRLR
AGAFSAADGESTSQVRQRVALAREAAAQRWRPHGFRTNAEVSGPLLRRKFRPSSAAML
PLRTALDRGLLSIRGVDRTLRVAWSLADLAGRTSPGIDEVAAALSFRQTGARR"

CDS complement(3169667..3170011)
/codon_start=1
/note="gene_2950"
/transl_table=11
/translation="MGEALAVDYLTSMGLRILNRNWRCRYGELDVIACDAATRTVVFV
EVKTRTGDGYGGLAHAVTERKVRRLRRLAGLWLADQEERWAAVRIDVIGVRVGPKNSG
RTPELTHLQGIG"

CDS complement(3170301..3171131)
/codon_start=1
/gene="fdhD"
/note="gene_2951"
/transl_table=11
/translation="VGYATAHRRVRHLSADQVITRPETLAVEEPLEIRVNGTPVTVTM
RTPGSDFELVQGFLLAEGVVAHREDVLTVSYCGRRVEGNATGASTYNVLDVALAPGVK
PPDVDVTRTFYTTSSCGVCGKASLQAVSQVSRFAPGGDPATVAADTLKAMPDQLRRAQ
KVFARTGGLHAAALFGVDGAMLAVREDIGRHNAVDKVIGWAFERDRIPLGASVLLVSG
RASFELTQKALMAGIPVLAAVSAPSSLAVSLADASGITLVAFLRGDSMNVYTRADRIT
"

CDS complement(3171131..3173455)
/codon_start=1
/note="gene_2952"
/transl_table=11

/translation="VRWQRSAASRDVLADYDEQAVTVAPRKREAAGVRAVMVSLQRGM
QQMGALRTAAALARLNQRNGFDCPGCAWPEEPGGRKLAEFCENGAKAVAEEATKRTVT
AEFFARHSVAELSAKPEYWLSQQGRLAHPMVLRPGDDHYRPISWDAAYQLIAEQLNGL
DSPDRAVFYTSGRTSNEAAFCYQLLVRSFGTNNLPDCSNMCHESSGAALTDSIGIGKG
SVTIGDVEHADLIVIAGQNPGTNHPRMLSVLGKAKANGAKIIAVNPLPEAGLIRFKDP
QKVNGVVGHGIPIADEFVQIRLGGDMALFAGLGRLLLEAEERVPGSVVDRSFVDNHCA
GFDGYRRRTLQVGLDTVMDATGIELAQLQRVAAMLMASQRTVICWAMGLTQHAHAVAT
IGEVTNVLLLRGMIGKPGAGVCPVRGHSNVQGDRTMGIWEKMPEQFLAALDREFGITS
PRAHGFDTVAAIRAMRDGRVSVFMGMGGNFASATPDTAVTEAALRRCALTVQVSTKLN
RSHLVHGATALILPTLGRTDRDTRNGRKQLVSVEDSMSMVHLSRGSLHPPSDQVRSEV
QIICQLARALFGPGHPVPWERFADDYDTIRDAIAAVVPGCDDYNHKVRVPDGFQLPHP
PRDAREFRTSTGKANFAVNPLQWVPVPPGRLVLQTLRSHDQYNTTIYGLDDRYRGVKG
GRRVVFINPADIETFGLTAGDRVDLVSEWTDGQGGLQERRAKDFLVVAYSTPVGNAAA
YYPETNPLVPLDHTAAQSNTPVSKAIIVRLEPTA"
CDS complement(3173528..3173833)
/codon_start=1
/note="gene_2953"
/transl_table=11
/translation="MSAEDLEKYETEMELSLYREYKDIVGQFSYVVETERRFYLANSV
EMVPRNTDGEVYFELRLADAWVWDMYRPARFVKQVRVVTFKDVNIEEVEKPELRLPE"
CDS complement(3173887..3174624)
/codon_start=1
/gene="rnhB"
/note="gene_2954"
/transl_table=11
/translation="MRTLESALHRGGLGPVAGVDEVGRGACAGPLVVAACVLGPGRIA
SLAALDDSKKLSEQAREKLFPLICRYAVAYHVVFIPSAEVDRRGVHVANIEGMRRAVA
GLAVRPGYVLSDGFRVPGLPMPSLPVIGGDAAAACIAAASVLAKVSRDRVMVALDADH
PGYGFAEHKGYSTPAHSRALARLGPCPQHRYSFINVRRVASGSNTAEVADGQPDPRDG
TAQTGEGRWSKSSHPATMRATGRAQGT"
CDS complement(3174695..3175579)
/codon_start=1
/note="gene_2955"
/transl_table=11
/translation="VTETTDSPSERQPGPAEPELSSRDPDIAGQVFDAAPFDAAPDAD
SEGDSKAAKTDEPRPAKRSTLREFAVLAVIAVVLYYVMLTFVARPYLIPSESMEPTLH
GCSTCVGDRIMVDKLSYRFGSPQPGDVIVFRGPPSWNVGYKSIRSHNVAVRWVQNALS
FIGFVPPDENDLVKRVIAVGGQTVQCRSDTGLTVNGRPLKEPYLDPATMMADPSIYPC
LGSEFGPVTVPPGRVWVMGDNRTHSADSRAHCPLLCTNDPLPGTVPVANVIGKARLIV
WPPSRWGVVRSVNPQQGR"
CDS complement(3175637..3175978)
/codon_start=1
/note="gene_2956"
/transl_table=11
/translation="MNRLDFVDKPSLRDDIPAFNPGDTINVHVKVIEGAKERLQVFKG
VVIRRQGGGIRETFTVRKESYGVGVERTFPVHSPNIDHIEVVTRGDVRRAKLYYLREL
RGKKAKIKEKR"
CDS 3176353..3177297
/codon_start=1
/note="gene_2957"
/transl_table=11

/translation="MRARPLTLLTALAAVTLVVVAGCEARVEAEAYSAADRISSRPQA
RPQPQPVELLLRAITPPRAPAASPNVGFGELPTRVRQATDEAAAMGATLSVAVLDRAT
GQLVSNGNTQIIATASVAKLFIADDLLLAEAEGKVTLSPEDHHALDVMLQSSDDGAAE
RFWSQDGGNAVVTQVARRYGLRSTAPPSDGRWWNTISSAPDLIRYYDMLLDGSGGLPL
DRAAVIIADLAQSTPTGIDGYPQRFGIPDGLYAEPVAVKQGWMCCIGSSWMHLSTGVI
GPERRYIMVIESLQPADDATARATITQAVRTMFPNGRI"
CDS complement(3177390..3178082)
/codon_start=1
/gene="trmD"
/note="gene_2958"
/transl_table=11
/translation="VRIDIVTIFPACLDPLRQSLPGKAIESGLVDLNVHDLRRWTHDV
HHSVDDAPYGGGPGMVMKAPVWGEALDEICSSETLLIVPTPAGVLFTQATAQRWTTES
HLVFACGRYEGIDQRVVQDAARRMRVEEVSIGDYVLPGGESAAVVMVEAVLRLLAGVL
GNPASHQDDSHSTGLDGLLEGPSYTRPASWRGLDVPEVLLSGDHARIAAWRREVSLQR
TRERRPDLSHPD"
CDS complement(3178086..3178616)
/codon_start=1
/gene="rimM"
/note="gene_2959"
/transl_table=11
/translation="MELVVGRVVKSHGVTGEVVVEIRTDDPADRFAPGTRLRAKGPFD
GGAEGSAVSYVIESVRQHGGRLLVRLAGVADRDAADALRGSLFVIDADDLPPIDEPDT
YYDHQLVGLMVQTATGEGVGVVTEVVHTAAGELLAVKRDSDEVLVPFVRAIVTSVSLD
DGIVEIDPPHGLLNLE"
CDS complement(3178630..3178872)
/codon_start=1
/note="gene_2960"
/transl_table=11
/translation="MSAVVVDAVEHLVRGIVDNPDDVRVDLITSRRGRTVEVHVHPDD
LGKVIGRGGRTATALRTLVAGIGGRGIRVDVVDTDQ"
CDS complement(3178880..3179368)
/codon_start=1
/gene="rpsP"
/note="gene_2961"
/transl_table=11
/translation="MAVKIKLTRLGKIRNPQYRVAVADARTRRDGRAIEVIGRYHPKE
EPSLIEINSERAQYWLSVGAQPTEPVLKLLKITGDWQKFKGLPGAQGRLKVAAPKPSK
LEVFNAALAAADGGPTTEATKPKKKSPAKKAAKAAEPAPQPEQPDTPALGGEQAELTA
ES"
CDS complement(3179552..3179995)
/codon_start=1
/note="gene_2962"
/transl_table=11
/translation="MCAVLDRSMLSVAEISDRLEIQQLLVDYSSAIDQRRFDDLDRVF
TPDAYIDYRALGGIDGRYPKIKQWLSQVLGNFPVYAHMLGNFSVRVDGDTASSRVICF
NPMVFAGDRQQVLFCGLWYDDDFVRTPDGWRIIRRVETKCFQKMM"
CDS 3180064..3180939
/codon_start=1
/note="gene_2963"
/transl_table=11

/translation="MRKLMTATAALCACAVTVSAGAAWADADVQPAGSVPIPDGPAQT
WIVADLDSGQVLAGRDQNVAHPPASTIKVLLALVALDELDLNSTVVADVADTQAECNC
VGVKPGRSYTARQLLDGLLLVSGNDAANTLAHMLGGQDVTVAKMNAKAATLGATSTHA
TTPSGLDGPGGSGASTAHDLVVIFRAAMANPVFAQITAEPSAMFPSDNGEQLIVNQDE
LLQRYPGAIGGKTGYTNAARKTFVGAAARGGRRLVIAMMYGLVKEGGPTYWDQAATLF
DWGFALNPQASVGSL"

CDS complement(3180999..3181586)
/codon_start=1
/note="gene_2964"
/transl_table=11
/translation="MARTQQQRREETVARLLQASIDTIIEVGYARASAAVITKRAGVS
VGALFRHFETMGDFMAATAYEVLRRQLETFTKQVAEIPADRPALPAALTILRDITAGS
TNAVLYELMVAARTDEKLKETLQNVLGQYSAKIHDAARALPGAESFPEETFPVIVALM
TNVFDGAAIVRGVLPQPELEEQRIPMLTALLTAGL"

CDS complement(3181588..3183423)
/codon_start=1
/note="gene_2965"
/transl_table=11
/translation="MLAWRQLNDLEETVTYDVIIRDGLWFDGTGNAPLTRTLGIRDGV
VATVAAGALDETGCPEVVDAAGKWVVPGFIDVHTHYDAEVLLDPGLRESVRHGVTTVL
LGNCSLSTVYANSEDAADLFSRVEAVPREFVLGALRDNQTWSTPAEYIEAIDALPLGP
NVSSLLGHSDLRTAVLGLDRATDDTVRPTEAELAKMAKLLDEALEAGMLGMSGMDAAI
DKLDGDRFRSRALPSTFATWRERRKLISVLRHRGRILQSAPDVDNPVSALLFFLASSR
IFNRRKGVRMSMLVSADAKSMPLAVHVFGLGTRVLNKLLGSQVRFQHLPVPFELYSDG
IDLPVFEEFGAGTAALHLRDQLQRNELLADRSYRRSFRREFDRIKLGPSLWHRDFHDA
VIVECPDKSLIGKSFGAIADERGLHPLDAFLDVLVDNGERNVRWTTIVANHRPNQLNK
LAAEPSVHMGFSDAGAHLRNMAFYNFGLRLLKRARDADRAGQPFLSIERAVYRLTGEL
AEWFGIGAGTLRQGDRADFAVIDPTHLDESVDGYHEEAVPYYGGLRRMVNRNDATVVA
TGVGGTVVFRGGQFGGQFRDGYGQNVKSGRYLRAGELGAALSRSA"

CDS complement(3183492..3185249)
/codon_start=1
/note="gene_2966"
/transl_table=11
/translation="MALASGVTFAGYTVVRMLGCSAMGEVYLVQHPGFPGWQALKVLS
PAMAADDEFRRRFQRETEVAARLFHPHILEVHDRGEFDGQLWIAMDYVDGIDATQHMA
DRFPAVLPVGEVLAIVTAVAGALDYAHQRGLLHRDVNPANVVLTSQSAGDQRILLADF
GIASQPSYPAPELSAGADVDGRADQYALALTAIHLFAGAPPVDRSHTGPLQPPKLSAF
RPDLARLDGVLSRALATAPADRFGSCREFADAMNEQAGVAIADQSSGGVDASEVTAAA
GEEAYVVDYPAYGWPEAVDCKEPSARAPAPAAPTPQRRGSMLQSAAGVLARRLDNFST
ATKAPASPTRRRPRRILVGAVAVLLLAGLFAVGIVIGRKTNTTATEVARPPTSGSAVP
SAPTTTVAVTAPVPLDGTYRIEIQRSKQTYDYTPTPQPPDVNTWWAFRTSCTPTECLA
AATMLDDNDHTQAKTPPVRPFLMQFGEGQWKSRPETVQFPCVGPNGSPSTQATTQLLA
LRPQPQGDLVGEMVVTVHSNECGQQGAVIRIPAVASRSGDLPPAVTVPDPATIPDTPD
TTSTATLTPPTTTAPGPGR"

CDS complement(3185293..3186420)
/codon_start=1
/note="gene_2967"
/transl_table=11
/translation="VRRSRVKRVDTIRPRSRAVRLHVRGLGLPDETAIQLWIVDGRIS
TEPVAGADTVFDGGWILPGLVDAHCHVGLGKHGNVELDEAIAQAETERDVGALLLRDC
GSPTDTRGLDDHEDLPRIIRAGRHLARPKRYIAGFAVELEDESQLPAAVAEQARRGDG

WVKLVGDWIDRQIGDLAPLWSDDVLKAAIDTAHAQGARVTAHVFSEDALPGLINAGID
CIEHGTGLTDDTIALMLEHGTALVPTLINLENFPGIADAAGRYPTYAAHMRDLYARGY
GRVAAAREAGVPVYAGTDAGSTIEHGRIADEVAALQRIGMTAHEALGAACWDARRWLG
RPGLDDRASADLLCYAQDPRQGPGVLQHPDLVILRGRTFGP"
CDS complement(3186433..3188010)
/codon_start=1
/note="gene_2968"
/transl_table=11
/translation="VFESLSDRLTAALQGLRGKGRLTDADIDATTREIRLALLEADVS
LPVVRAFIHRIKERARGAEVSSALNPAQQVVKIVNEELISILGGETRELAFAKTPPTV
VMLAGLQGSGKTTLAGKLAARLRGQGHTPLLVACDLQRPAAVNQLQVVGERAGVPVFA
PHPGASPESGPGDPVAVAAAGLAEARAKHFDVVIVDTAGRLGIDEELMAQAAAIRDAI
NPDEVLFVLDAMIGQDAVTTAAAFGEGVGFTGVALTKLDGDARGGAALSVREVTGVPI
LFASTGEKLEDFDVFHPDRMASRILGMGDVLSLIEQAEQVFDAQQAEEAAAKIGAGEL
TLEDFLEQMLAVRKMGPIGNLLGMLPGAAQMKDALAEVDDKQLDRVQAIIRGMTPQER
ADPKIINASRRLRIANGSGVTVSEVNQLVERFFEARKMMSSMLGGMGIPGIGRKSATR
KSKGAKGKSGKKSKKGTRGPTPPKVKSPFGVPGMPGLAGLPGGLPDLSQMPKGLDELP
PGLADFDLSKLKFPGKK"
CDS 3188160..3189968
/codon_start=1
/note="gene_2969"
/transl_table=11
/translation="MLHFTAATSRFRLGRERANSVRSDGGWGVLQPVSATFNPPLRGW
QRRALVQYLGTQPRDFLAVATPGSGKTSFALRIAAELLRYHTVEQVTVVVPTEHLKVQ
WAHAAAAHGLSLDPKFANSNPQTSPEYHGVMVTYAQVASHPTLHRVRTEARKTLVVFD
EIHHGGDAKTWGDAIREAFGDATRRLALTGTPFRSDDSPIPFVSYQPDADGVLRSQAD
HTYGYAEALADGVVRPVVFLAYSGQARWRDSAGEEYEARLGEPLSAEQTARAWRTALD
PEGEWMPAVITAADRRLRQLRAHVPDAGGMIIASDRTTARAYARLLTTMTAEEPTVVL
SDDPGSSARITEFAQGTSRWLVAVRMVSEGVDVPRLSVGVYATNASTPLFFAQAIGRF
VRSRRPGETASIFVPSVPNLLQLASALEVQRNHVLGRPHRESAHDPLDGDPATRTQTE
RGGAERGFTALGADAELDQVIFDGSSFGTATPTGSDEEADYLGIPGLLDAEQMRALLH
RRQDEQLRKRAQLQKGATQPATSGASASVHGQLRDLRRELHTLVSIAHHRTGKPHGWI
HDELRRRCGGPPIAAATRAQIKARIDALRQLNSERS"
CDS complement(3189979..3192405)
/codon_start=1
/note="gene_2970"
/transl_table=11
/translation="MEAESPCAASDLAVARRELLSGNHRELDPVGLRQTWLDLHESWL
IDKADEIGIADASGFAIVGVGGLGRRELLPYSDLDVLLLHDGKPADILRPVADRLWYP
LWDANIRLDHSVRTVSEALTIANSDLMAALGMLEARHIAGDQQLSFALIDGVRRQWRN
GIRSRMGELVEMTYARWRRCGRIAQRAEPDLKLGRGGLRDVQLLDALALAQLIDRHGI
GHTDLPAGSLDGAYRTLLDVRTELHRVSGRGRDHLLAQFADEISAALGFGDRFDLART
LSSAGRTIGYHAEAGLRTAANALPRRGISALVRRPKRRPLDEGVVEYAGEIVLARDAE
PEHDPGLVLRVAAASADTGLPIGAATLSRLAASVPDLPTPWPQEALDDLLVVLSAGPT
TVATIEALDRTGLWGRLLPEWEPIRDLPPRDVAHKWTVDRHVVETAVHAAPLATRVAR
PDLLALGALLHDIGKGRGTDHSVLGAELVIPVCTRLGLSPPDVWTLSKLVRHHLLLPI
TATRRDLNDPKTIEAVSEALGGDPQLLEVLHALSEADSKATGPGVWSDWKASLVDDLV
RRCRMVMAGESLPQAEPTAPHYLSLAADHGVHVEISPRDGERIDAVIVAPDERGLVSK
AAAVLALNSLRVHSASVNVHQGVAITEFVVSPLFGSPPAAELVRQQFVGALNGDVDVL
GMLQKRDSDAASLVSARAGDVQAGVPVTRTAAPPRILWLDTAAPAKLILEVRAMDRAG
LLALLAGALEGAGAGIVWAKVNTFGSTAADVFCVTVPAELDARAAVEQHLLEVLGASV

DVVVDEPVGD"
CDS complement(3192463..3192801)
/codon_start=1
/note="gene_2971"
/transl_table=11
/translation="MKLITAIVKPFTLDDVKTSLEDAGVLGMTVSEIQGYGRQKGHTE
VYRGAEYSVDFVPKVRIEVVVDDSIVDKVVDSIVRAARTGKIGDGKVWVSPVDTIVRV
RTGERGHDAL"
CDS complement(3192798..3194231)
/codon_start=1
/note="gene_2972"
/transl_table=11
/translation="VDQFPIMGVPDGGDTAWMLVSSALVLLMTPGLAFFYGGMVRSKS
VLNMIMMSISAMGVVTVLWALYGYSIAFGDDVGNIAGNPSQYWGLKGLIGVNAVAADP
STQTAAVNIPLAGTLPATVFVAFQLMFAIITVALISGAVADRLKFGAWLLFAGLWATF
VYFPVAHWVFAFDGFAAEHGGWIANKLHAIDFAGGTAVHINAGVAALMLAIVLGKRRG
WPATLFRPHNLPFVMLGAALLWFGWYGFNAGSATTANGVAGATFVTTTIATAAAMLGW
LLTERVRDGKATTLGAASGIVAGLVAITPSCSSVNVLGALAVGVSAGVLCALAVGLKF
KLGFDDSLDVVGVHLVGGLVGTLLVGLLAAPEAPAINGVAGVSKGLFYGGGFAQLERQ
ALGACSVLVYSGIITLILALILKFTIGLRLDAEQESTGIDEAEHAESGYDFAVASGSV
LPPRVTVEDSRNGIQERIGQKVEAEPK"
CDS complement(3194708..3195976)
/codon_start=1
/note="gene_2973"
/transl_table=11
/translation="VWEGLWIATAVIAALVVIAALTLGLVLYRRRRISLSPRPERGVV
DRSGGYTASSGITFSQTPTTQPAERIDTSGLPAVGDDATVPRDAPKRTIADVHLPEFE
PEPQAPEVPEADAIAPPEGRLERLRGRLARSQNALGRGLLGLIGGGDLDEDSWQDVED
TLLVADLGPAATASVVSQLRSRLASGNVRTEADARAVLRDVLINELQPGMDRSIRALP
HAGHPSVLLVVGVNGTGKTTTVGKLARVLVADGRRVVLGAADTFRAAAADQLQTWAAR
VGAAVVRGPEGADPASVAFDAVDKGIAAGADVVLIDTAGRLHTKVGLMDELDKVKRVV
TRRASVDEVLLVLDATIGQNGLAQARVFAEVVDISGAVLTKLDGTAKGGIVFRVQQEL
GVPVKLVGLGEGPDDLAPFEPAAFVDALLG"
CDS complement(3196026..3199643)
/codon_start=1
/note="gene_2974"
/transl_table=11
/translation="VYLKSLTLKGFKSFAAPTTLRFEPGITAVVGPNGSGKSNVVDAL
AWVMGEQGAKTLRGGKMEDVIFAGTSSRAPLGRAEVTVSIDNSDNALPIEYTEVSITR
RMFRDGASEYEINGSSCRLMDVQELLSDSGIGREMHVIVGQGKLEEILQSRPEDRRAF
IEEAAGVLKHRKRKEKALRKLDTMAANLARLTDLTTELRRQLKPLGRQAEAAQRAAAI
QADLRDARLRLAADDLVSRRAEREAVFQAEAAMRREHDEAAARLAVASEELAAHESAV
AELSTRAESIQHTWFGLSALAERVDATVRIASERAHHLDIEPVAVSDTDPRKPEELEA
EAQQVAVAEQQLLAELDAARARLDAARAERADRERRAAEADRAHLAAVREEADRREGL
ARLAGQVETMRARVESIDESVARLSERIEDAAMRAQQTRAEFETVQGRIGELDQGEVG
LDEHHERTVAALRLADERVAELQSAERAAERQVASLRARIDALAVGLQRKDGAAWLAH
NRSGAGLFGSIAQLVKVRSGYEAALAAALGPAADALAVDGLTAAGSAVSALKQADGGR
AVLVLSDWPAPQAPQSASGEMLPSGAQWALDLVESPPQLVGAMIAMLSGVAVVNDLTE
AMGLVEIRPELRAVTVDGDLVGAGWVSGGSDRKLSTLEVTSEIDKARSELAAAEALAA
QLNAALAGALTEQSAGQDAAEQALAALNESDTAISAMYEQLGRLGQEARAAEEEWNRL
LQQRTEQEAVRTQTLDDVIQLETQLRKAQETQRVQVAQPIDRQAISAAADRARGVEVE

ARLAVRTAEERANAVRGRADSLRRAAAAEREARVRAQQARAARLHAAAVAAAVADCGR
LLAGRLHRAVDGASQLRDASAAQRQQRLAAMAAVRDEVNTLSARVGELTDSLHRDELA
NAQAALRIEQLEQMVLEQFGMAPADLITEYGPHVALPPTELEMAEFEQARERGEQVIA
PAPMPFDRVTQERRAKRAERALAELGRVNPLALEEFAALEERYNFLSTQLEDVKAARK
DLLGVVADVDARILQVFNDAFVDVEREFRGVFTALFPGGEGRLRLTEPDDMLTTGIEV
EARPPGKKITRLSLLSGGEKALTAVAMLVAIFRARPSPFYIMDEVEAALDDVNLRRLL
SLFEQLREQSQIIIITHQKPTMEVADALYGVTMQNDGITAVISQRMRGQQVDQLVTNS
S"
CDS complement(3199655..3199936)
/codon_start=1
/note="gene_2975"
/transl_table=11
/translation="MSAPDVRLTAWVHGWVQGVGFRWWTRCRALELGLTGYAANHADG
RVLVVAQGPRAACQKLLQLLQGDTTPGRVAKVVADWSQSTEQITGFSER"
CDS complement(3199923..3200336)
/codon_start=1
/note="gene_2976"
/transl_table=11
/translation="MTQLWVERTGTRRYIGRSTRGAQVLVGSEDVDGVFTPGELLKIA
LAACSGMASDQPLARRLGDDYQAVVKVSGAADRDQERYPLIEETMELDLSGLTEDEKE
RLLVVINRAVELACTVGRTLKSGTTVNLEVVDVGA"
CDS complement(3200438..3201307)
/codon_start=1
/note="gene_2977"
/transl_table=11
/translation="MPELPEVEVVRRGLQAHVTGRTITEVRVHHPRAVRRHDAGPADL
TARLRGARINGTDRRGKYLWLTLNTAGVHRPTDTALVVHLGMSGQMLLGAVPCAAHVR
ISALLDDGTVLSFADQRTFGGWLLADLVTVDGSVVPVPVAHLARDPLDPRFDCDAVVK
VLRRKHSELKRQLLDQRVVSGIGNIYADEALWRAKVNGAHVAATLRCRRLGAVLHAAA
DVMREALAKGGTSFDSLYVNVNGESGYFERSLDAYGREGENCRRCGAVIRRERFMNRS
SFYCPRCQPRPRK"
CDS complement(3201611..3202333)
/codon_start=1
/gene="rnc"
/note="gene_2978"
/transl_table=11
/translation="MIRSRQPLLDALGVDLPDELLSLALTHRSYAYENGGLPTNERLE
FLGDAVLGLTITDALFHRHPDRSEGDLAKLRASVVNTQALADVARRLCAEGLGVHVLL
GRGEANTGGADKSSILADGMESLLGAIYLQHGMEKAREVILRLFGPLLDAAPTLGAGL
DWKTSLQELTAARGLGAPSYLVTSTGPDHDKEFTAVVVVMDSEYGSGVGRSKKEAEQK
AAAAAWKALEVLDNAMPGKTSA"
CDS complement(3202330..3202869)
/codon_start=1
/note="gene_2979"
/transl_table=11
/translation="MTVDIARLGRRPGAMFELHDTVHSPARIGLELIAIDQGALLDLD
LRVESVSEGVLVTGTVAAPTVGECARCLSPVRGRVQVALTELFAYPDSATDETTEEDE
VGRVVDETIDLEQPIIDAVGLELPFSPVCRPDCPGLCPQCGVPLASEPGHRHEQIDPR
WAKLVEMLGPESDTLRGER"
CDS complement(3203004..3203747)
/codon_start=1

/note="gene_2980"
/transl_table=11
/translation="VAVYRVFEALDELSAIVEEARGVPMTAGCVVPRGDVLELIDDIK
DAIPGELDDAQDVLDARDSMLQDAKTHADSMVSSATTEAESILNHARTEADRILSDAK
AQADRMVSEARQHSERMVADAREEAIRIATAAKREYEASVSRAQAECDRLIENGNISY
EKAVQEGIKEQQRLVSQNEVVAAANAESTRLVDTAHAEADRLRGECDIYVDNKLAEFE
EFLNGTLRSVGRGRHQLRTAAGTHDYAVR"
CDS 3203980..3204765
/codon_start=1
/note="gene_2981"
/transl_table=11
/translation="MLARHGPRYGGSVNGHSDDSSGDAKQAAPTLYIFPHAGGTAKDY
VAFSREFSADVKRIAVQYPGQHDRSGLPPLESIPTLADEIFAMMKPSARIDDPVAFFG
HSMGGMLAFEVALRYQSAGHRVLAFFVSACSAPGHIRYKQLQDLSDREMLDLFTRMTG
MNPDFFTDDEFFVGALPTLRAVRAIAGYSCPPETKLSCPIYAFIGDKDWIATQDDMDP
WRDRTTEEFSIRVFPGDHFYLNDNLPELVSDIEDKTLQWHDRA"
CDS complement(3205761..3205970)
/codon_start=1
/note="gene_2982"
/transl_table=11
/translation="VLTRESRHLSRRVQIKQINLNYDRGRLPVVAGCVFRYIAYHGRS
RENGYRLGILQHRRNAVIVNAILWS"
CDS 3206031..3207230
/codon_start=1
/note="gene_2983"
/transl_table=11
/translation="VSHTNVIANVTQSMYGYFGDPAKIPTGTVVSWLPLYHDMGLILG
ICAPLVARRRAVLMSPMSFLRRPARWMQLLATSGRCFSAAPNFAFELAVRRTSDQDMA
GLDLRDVVGIVSGSERIHVATVRRFIERFAPYNLSPTAIRPSYGLAEATLYVAAPEAG
AAPKTVRFDYEQLTAGQARPCGTDGSVGTELISYGSPDPSSVRIVNPETMVENPPGVV
GEIWVHGDHVTMGYWQKPKQTAQVFDAKLVDPAPAAPEGPWLRTGDLGVISDGELFIM
GRIKDLLIVDGRNHYPDDIEATIQEITGGRAAAIAVPDDITEQLVAIIEFKRRGSTAE
EVMLKLRSVKREVTSAISKSHSLRVADLVLVSPGSIPITTSGKIRRSACVERYRSDGF
KRLDVAV"
CDS 3207227..3212857
/codon_start=1
/note="gene_2984"
/transl_table=11
/translation="MTGSISGEADLRHWLIDYLVTNIGCTPDEVDPDLSLADLGVSSR
DAVVLSGELSELLGRTVSPIDFWEHPTINALAAYLAAPEPSPDSDAAVKRGARNSLDE
PIAVVGMGCRFPGGISCPEALWDFLCERRSSISQVPPQRWQPFEGGPPEVAAALARTT
RWGSFLPDIDAFDAEFFEISPSEADKMDPQQRLLLEVAWEALEHAGIPPGTLRRSATG
VFAGACLSEYGAMASADLSQVDGWSNSGGAMSIIANRLSYFLDLRGPSVAVDTACSSS
LVAIHLACQSLRTQDCHLAIAAGVNLLLSPAVFRGFDQVGALSPTGQCRAFDATADGF
VRGEGAGVVVLKRLTDAQRDGDRVLAVICGSAVTQDGRSNGLMAPNPAAQMAVLRAAY
TNAGMQPSEVDYVEAHGTGTLLGDPIEARALGTVLGRGRPEDSPLLIGSVKTNLGHTE
AAAGIAGFIKTVLAVQHGQIPPNQHFETANPHIPFTDLRMKVVDTQTEWPATGHPRRA
GVSSFGFGGTNAHVVIEQGQEVRPAPGQGLSPAVSTLVVAGKTMQRVSATAGMLADWM
EGPGADVALADVAHTLNHHRSRQPKFGTVVARDRTQAIAGLRALAAGQHAPGVVNPAE
GSPGPGTVFVYSGRGSQWAGMGRQLLADEPAFAAAVAELEPVFVEQAGFSLHDVLANG
EELVGIEQIQLGLIGMQLALTELWCSYGVQPDLVIGHSMGEVAAAVVAGALTPAEGLR

VTATRSRLMAPLSGQGGMALLELDAPTTEALIADFPQVTLGIYNSPRQTVIAGPTEQI
DELITRVRARDRFASRVNIEVAPHNPAMDALQPAMRSELADLTPRTPTIGIISTTYAD
LHTQPVFDAEHWATNMRNPVHFQQAIASAGSGADGAYHTFIEISAHPLLTQAIIDTLH
SAQPGARYTSLGTLQRDTDDVVTFRTNLNKAHTIHPPHTPHPPEPHPPIPTTPWQHTR
HWITTKYPAGSVGSAPRAGTLLGQHTTVATVSASPPSHLWQARLAPDAKPYQGGHRFH
QVEVVPASVVLHTILSAATELGYSALSEVRFEQPIFADRPRLIQVVADNRAISLASSP
AAGTPSDRWTRHVTAQLSSSPSDSASSLNEHHRANGQPPERAHRDLIPDLAELLAMRG
IDGLPFSWTVASWTQHSSNLTVAIDLPEALPEGSTGPLLDAAVHLAALSDVADSRLYV
PASIEQISLGDVVTGPRSSVTLNRTAHDDDGITVDVTVAAHGEVPSLSMRSLRYRALD
FGLDVGRAQPPASTGPVEAYCDATNFVHTIDWQPQTVPDATHPGAEQVTHPGPVAIIG
DDGAALCETLEGAGYQPAVMSDGVSQARYVVYVADSDPAGADETDVDFAVRICTEITG
LVRTLAERDADKPAALWILTRGVHESVAPSALRQSFLWGLAGVIAAEHPELWGGLVDL
AINDDLGEFGPALAELLAKPSKSILVRRDGVVLAPALAPVRGEPARKSLQCRPDAAYL
ITGGLGALGLLMADWLADRGAHRLVLTGRTPLPPRRDWQLDTLDTELRRRIDAIRALE
MRGVTVEAVAADVGCREDVQALLAARDRDGAAPIRGIIHAAGITNDQLVTSMTGDAVR
QVMWPKIGGSQVLHDAFPPGSVDFFYLTASAAGIFGIPGQGSYAAANSYLDALARARR
QQGCHTMSLDWVAWRGLGLAADAQLVSEELARMGSRDITPSEAFTAWEFVDGYDVAQA
VVVPMPAPAGADGSGANAYLLPARNWSVMAATEVRSELEQGLRRIIAAELRVPEKELD
TDRPFAELGLNSLMAMAIRREAEQFVGIELSATMLFNHPTVKSLASYLAKRVAPHDVS
QDNQISALSSSAGSVLDSLFDRIESAPPEAERSV"

CDS 3212857..3217470
/codon_start=1
/note="gene_2985"
/transl_table=11
/translation="MRTAFSRISGMTAQQRTSLADEFDRVSRIAVAEPVAVVGIGCRF
PGDVDGPESFWDFLVAGRNAISTVPADRWDAEAFYHPDPLTPGRMTTKWGGFVPDVAG
FDAEFFGITPREAAAMDPQQRMLLEVAWEALEHAGIPPDSLGGTRTAVMMGVYFNEYQ
SMLAASPQNVDAYSGTGNAHSITVGRISYLLGLRGPAVAVDTACSSSLVAVHLACQSL
RLRETDLALAGGVSITLRPETQIAISAWGLLSPQGRCAAFDAAADGFVRGEGAGVVVL
KRLTDAVRDGDQVLAVVRGSAVNQDGRSNGVTAPNTAAQCDVIADALRSGDVAPDSVN
YVEAHGTGTVLGDPIEFEALAATYGHGGDACALGAVKTNIGHLEAAAGIAGFIKATLA
VQRATIPPNLHFSQWNPAIDAASTRFFVPTQNSPWPTAEGPRRAAVSSFGLGGTNAHV
IIEQGSELAPVSEGGEDTGVSTLVVTGKTAQRMAATAQVLADWMEGPGAEVAVADVAH
TVNHHRARQATFGTVVARDRAQAIAGLRALAAGQHAPGVVSHQDGSPGPGTVFVYSGR
GSQWAGMGRQLLADEPAFAAAVAELEPVFVEQAGFSLRDVIATGKELVGIEQIQLGLI
GMQLTLTELWRSYGVQPDLVIGHSMGEVAAAVVAGALTPAEGLRVTATRARLMAPLSG
QGGMALLGLDAAATEALIADYPQVTVGIYNSPRQTVIAGPTEQIDELIARVRAQNRFA
SRVNIEVAPHNPAMDALQPAMRSELADLTPRTPTIGIISTTYADLHTQPIFDAEHWAT
NMRNPVRFQQAIASAGSGADGTYHTFIEISAHPLLTQAIADTLEDAHRPTKSAAKYLS
IGTLQRDADDTVTFRTNLYTADIAHPPHTCHPPEPHPTIPTTPWQHTHHWIATTHPST
AAPEDPGSNKVVVNGQSTSESRALEDWCHQLAWPIRPAVSADPPSTAAWLVVADNELC
HELARAADSRVDSLSPPALAAGSDPAALLDDALRGVDNVLYAPPVPGELLDIESAYQVF
HATRRLAAAMVASSATAISPPKLFIMTRNAQPISEGDRANPGHAVLWGLGRSLALEHP
EIWGGIIDLDDSMPAELAVRHVLTAAHGTDGEDQVVYRSGARHVPRLQRRTLPGKPVT
LNADASQLVIGATGNIGPHLIRQLARMGAKTIVAMARKPGALDELTQCLAATGTDLIA
VAADATDPAAMQTLFDRFGTELPPLEGIYLAAFAGRPALLSEMTDDDVTTMFRPKLDA
LALLHRLSLKSPVRHFVLFSSVSGLLGSRWLAHYTATSAFLDSFAGARRTMGLPATVV
DWGLWKSLADVQKDATQISAESGLQPMADEVAIGALPLVMNPDAAVATVVVAADWPLL
AAAYRTRGALRIVDDLLPAPEDVGKGESEFRTSLRSCPAEKRRDMLFDHVGALAATVM
GMPPTEPLDPSAGFFQLGMDSLMSVTLQRALSESLGEFLPASVVFDYPTVYSLTDYLA
TVLPELLEIGATAVATQQATDSYHELTEAELLEQLSERLRGTQ"

CDS 3217467..3224033
/codon_start=1
/gene="ppsC"
/note="gene_2986"
/transl_table=11
/translation="MTAATPDRRAIITEALHKIDDLTARLEIAEKSSSEPIAVIGMGC
RFPGGVNNPEQFWDLLCAGRSGIVRVPAQRWDADAYYCDDHTVPGTICSTEGGFLTSW
QPDEFDAEFFSISPREAAAMDPQQRLLIEVAWEALEDAGVPQHTIRGTQTSVFVGVTA
YDYMLTLAGRLRPVDLDAYIPTGNSANFAAGRLAYILGARGPAVVIDTACSSSLVAVH
LACQSLRGRESDMALVGGTNLLLSPGPSIACSRWGMLSPEGRCKTFDASADGYVRGEG
AAVVVLKRLDDAVRDGNRILAVVRGSAVNQDGASSGVTVPNGPAQQALLAKALTSSKL
TAADIDYVEAHGTGTPLGDPIELDSLSKVFSDRAGSDQLVIGSVKTNLGHLEAAAGVA
GLMKAVLAVHNGYIPRHLNFHQLTPHASEAASRLRIAADGIDWPTTGRPRRAGVSSFG
VSGTNAHVVIEQAPDPMAAAGTEPQRGPVPAVSTLVVFGKTAPRVAATASVLADWLDG
PGAAVPLADVAHTLNHHRARQTRFGTVAAVDRRQAVIGLRALAAGQSAPGVVAPREGS
IGGGTVFVYSGRGSQWAGMGRQLLADEPAFAAAIAELEPEFVAQGGFSLRDVIAGGKE
LVGIEQIQLGLIGMQLALTALWRSYGVTPDAVIGHSMGEVAAAVVAGALTPAQGLRVT
AVRSRLMAPLSGQGTMALLELDAEATEALIADYPEVSLGIYASPRQTVISGPPLLIDE
LIDKVRQQNGFATRVNIEVAPHNPAMDALQPAMRSELADLTPQPPTIPIISTTYADLG
ISLGSGPRFDAEHWATNMRNPVRFHQAIAHAGADHHTFIEISAHPLLTHSISDTLRAS
YDVDNYLSIGTLQRDAHDTLEFHTNLNTTHTTHPPQTPHPPEPHPVLPTTPWQHTQHW
ITATSAAYHRPDTHPLLGVGVTDPTNGTRVWESELDPDLLWLADHVIDDLVVLPGAAY
AEIALAAATDTFAVEQDQPWMISELDLRQMLHVTPGTVLVTTLTGDEQRCQVEIRTRS
GSSGWTTHATATVARAEPLAPLDHEGQRREVTTADLEDQLDPDDLYQRLRGAGQQHGP
AFQGIVGLAVTQAGVARAQVRLPASARTGSREFMLHPVMMDIALQTLGATRTATDLAG
GQDARQGPSSNSALVVPVRFAGVHVYGDITRGVRAVGSLAAAGDRLVGEVVLTDANGQ
PLLVVDEVEMAVLGSGSGATELTNRLFMLEWEPAPLEKTAEATGALLLIGDPAAGDPL
LPALQSSLRDRITDLELASAADEATLRAAISRTSWDGIVVVCPPRANDESMPDEAQLE
LARTRTLLVASVVETVTRMGARKSPRLWIVTRGAAQFDAGESVTLAQTGLRGIARVLT
FEHSELNTTLVDIEPDGTGSLAALAEELLAGSEADEVALRDGQRYVNRLVPAPTTTSG
DLAAEARHQVVNLDSSGASRAAVRLQIDQPGRLDALNVHEVKRGRPQGDQVEVRVVAA
GLNFSDVLKAMGVYPGLDGAAPVIGGECVGYVTAIGDEVDGVEVGQRVIAFGPGTFGT
HLGTIADLVVPIPDTLADNEAATFGVAYLTAWHSLCEVGRLSPGERVLIHSATGGVGM
AAVSIAKMIGARIYTTAGSDAKREMLSRLGVEYVGDSRSVDFADEILELTDGYGVDVV
LNSLAGEAIQRGVQILAPGGRFIELGKKDVYADASLGLAALAKSASFSVVDLDLNLKL
QPARYRQLLQHILQHVADGKLEVLPVTAFSLHDAADAFRLMASGKHTGKIVISIPQHG
SIEAIAAPPPLPLVSRDGGYLIVGGMGGLGFVVARWLAEQGAGLIVLNGRSAPSDEVA
AAIAELNASGSRIEVITGDITEPDTAERLVRAVEDAGFRLAGVVHSAMVLADEIVLNM
TDSAARRVFAPKVTGSWRLHVATAARDVDWWLTFSSAAALLGTPGQGAYAAANSWVDG
LVAHRRSAGLPAVGINWGPWADVGRAQFFKDLGVEMINAEQGLAAMQAVLTADRGRTG
VFSLDARQWFQSFPAVAGSSLFAKLHDSAARKSGQRRGGGAIRAQLDALDAAERPGHL
ASAIADEIRAVLRSGDPIDHHRPLETLGLDSLMGLELRNRLEASLGITLPVALVWAYP
TISDLATALCERMDYATPAAAQEISDTEPELSDEEMDLLADLVDASELEAATRGES"
CDS 3224030..3229513
/codon_start=1
/note="gene_2987"
/transl_table=11
/translation="MTSLAERAAQLSPNARAALARELVRAGTTFPTDICEPVAVVGIG
CRFPGNVTGPESFWQLLADGVDTIEQVPPDRWDADAFYDPDPSASGRMTTKWGGFVSD
VDAFDADFFGITPREAVAMDPQHRILLEVAWEALEHAGIPPDSLSGTRTGVMMGLSSW
DYTIVNIERRADIDAYLSTGTPHCAAVGRIAYLLGLRGPAVAVDTACSSSLVAIHLAC

QSLRLRETDVALAGGVQLTLSPFTAIALSKWSALSPTGRCNSFDANADGFVRGEGCGV
VVLKRLADAVRDQDRVLAVVRGSATNSDGRSNGMTAPNALAQRDVITSALKLADVTPD
SVNYVETHGTGTVLGDPIEFESLAATYGLGKGQGESPCALGSVKTNIGHLEAAAGVAG
FIKAVLAVQRGHIPRNLHFTRWNPAIDASATRLFVPTESAPWPAAAGPRRAAVSSFGL
SGTNAHVVVEQAPDTAVAAAGGMPYVSALNVSGKTAARVASAAAVLADWMSGPGAAAP
LADVAHTLNRHRARHAKFATVIARDRAEAIAGLRALAAGQPRVGVVDCDQHAGGPGRV
FVYSGQGSQWASMGQQLLANEPAFAKAVAELDPIFVDQVGFSLQQTLIDGDEVVGIDR
IQPVLVGMQLALTELWRSYGVIPDAVIGHSMGEVSAAVVAGALTPEQGLRVITTRSRL
MARLSGQGAMALLELDADAAEALIAGYPQVTLAVHASPRQTVIAGPPEQVDTVIAAVA
TQNRLARRVEVDVASHHPIIDPILPELRSALADLTPQPPSIPIISTTYESAQPVADAD
YWSANLRNPVRFHQAVTAAGVDHNTFIEISPHPVLTHALTDTLDPDGSHTVMSTMNRE
LDQTLYFHAQLAAVGVAASEHTTGRLVDLPPTPWHHQRFWVTDRSAMSELAATHPLLG
AHIEMPRNGDHVWQTDVGTEVCPWLADHKVFGQPIMPAAGFAEIALAAASEALGTAAD
AVAPNIVINQFEVEQMLPLDGHTPLTTQLIRGGDSQIRVEIYSRTRGGEFCRHATAKV
EQSPRECAHAHPEAQGPATGTTVSPADFYALLRQTGQHHGPAFAALSRIVRLADGSAE
TEISIPDEAPRHPGYRLHPVVLDAALQSVGAAIPDGEIAGSAEASYLPVSFETIRVYR
DIGRHVRCRAHLTNLDGGTGKMGRIVLINDAGHIAAEVDGIYLRRVERRAVPLPLEQK
IFDAEWTESPIAAVPAPEPAAETTRGSWLVLADATVDAPGKAQAKSMADDFVQQWRSP
MRRVHTADIHDESAVLAAFAETAGDPEHPPVGVVVFVGGASSRLDDELAAARDTVWSI
TTVVRAVVGTWHGRSPRLWLVTGGGLSVADDEPGTPAAASLKGLVRVLAFEHPDMRTT
LVDLDITQDPLTALSAELRNAGSGSRHDDVIAWRGERRFVERLSRATIDVSKGHPVVR
QGASYVVTGGLGGLGLVVARWLVDRGAGRVVLGGRSDPTDEQCNVLAELQTRAEIVVV
RGDVASPGVAEKLIETARQSGGQLRGVVHAAAVIEDSLVFSMSRDNLERVWAPKATGA
LRMHEATADCELDWWLGFSSAASLLGSPGQAAYACASAWLDALVGWRRASGLPAAVIN
WGPWSEVGVAQALVGSVLDTISVAEGIEALDSLLAADRIRTGVARLRADRALVAFPEI
RSISYFTQVVEELDSAGDLGDWGGPDALADLDPGEARRAVTERMCARIAAVMGYTDQS
TVEPAVPLDKPLTELGLDSLMAVRIRNGARADFGVEPPVALILQGASLHDLTADLMRQ
LGLNDPDPALNNADTIRDRARQRAAARHGAAMRRRPKPAVQGG"

CDS 3229519..3233985
/codon_start=1
/note="gene_2988"
/transl_table=11
/translation="VSIPENAIAVVGMAGRFPGAKDVSAFWSNLRRGKESIVTLSEQE
LRDAGVSDKTLADPAYVRRAPLLDGIDEFDAGFFGFPPLAAQVLDPQHRLFLQCAWHA
LEDAGADPARFDGSIGVYGTSSPSGYLLHNLLSHRDPNAVLAEGLNFDQFSLFLQNDK
DFLATRISHAFNLRGPSIAVQTACSSSLVAVHLACLSLLSGECDMALAGGSSLCIPHR
VGYFTSPGSMVSAVGHCRPFDVRADGTVFGSGVGLVVLKPLAAAIDAGDRIHAVIRGS
AINNDGSAKMGYAAPNPAAQADVIAEAHAVSGIDSSTVSYVECHGTGTPLGDPIEIQG
LRAAFEVSQTSRSAPCVLGSVKSNIGHLEVAAGIAGLIKTILCLKNKALPATLHYTSP
NPELRLDQSPFVVQSKYGPWECDGVRRAGVSSFGVGGTNAHVVLEEAPAEASEVSAHA
EPAGPQVILLSAQTAAALGESRTALAAALETQDGPRLSDVAYTLARRRKHNVTMAAVV
HDREHAATVLRAAEHDNVFVGEAAHDGEHGDRADAAPTSDRVVFLFPGQGAQHVGMAK
GLYDTEPVFAQHFDTCAAGFRDETGIDLHAEVFDGTATDLERIDRSQPALFTVEYALA
KLVDTFGVRAGAYIGYSTGEYIAATLAGVFDLQTAIKTVSLRARLMHESPPGAMVAVA
LGPDDVTQYLPPEVELSAVNDPGNCVVAGPKDQIRALRQRLTEAGIPVRRVRATHAFH
TSAMDPMLGQFQEFLSRQQLRPPRTPLLSNLTGSWMSDQQVVDPASWTRQISSPIRFA
DELDVVLAAPSRILVEVGPGGSLTGSAMRHPKWSTTHRTVRLMRHPLQDVDDRDTFLR
ALGELWSAGVEVDWTPRRPAVPHLVSLPGYPFARQRHWVEPNHTVWAQAPGANNGSPA
GTADGSTAATVDAARNGESQTEVTLQRIWSQCLGVSSVDRNANFFDLGGDSLMAISIA
MAAANEGLTITPQDLYEYPTLASLTAAVDASFASSGLAKPPEAQANPAVPPNVTYFLD
RGLRDTGRCRVPLILRLDPKIGLPDIRAVLTAVVNHHDALRLHLVGNDGIWEQHIAAP

AEFTGLSNRSVPDGVAAGSPEERAAVLGILAELLEDQTDPNAPLAAVHIAAAHGGPHY
LCLAIHAMVTDDSSRQILATDIVTAFGQRLAGEEITLEPVSTGWREWSLRCAALATHP
AALDTRSYWIENSTKATLWLADALPNAHTAHPPRADELTKLSSTLSVEQTSELDDGRR
RFRRSIQTILLAALGRTIAQTVGEGVVAVELEGEGRSVLRPDVDLRRTVGWFTTYYPV
PLACATGLGALAQLDAVHNTLKSVPHYGIGYGLLRYVYAPTGRVLGAQRTPDIHFRYA
GVIPELPSGDAPVQFDSDMTLPVREPIPGMGHAIELRVYRFGGSLHLDWWYDTRRIPA
ATAEALERTFPLALSALIQEAIAAEHTEHDDSEIVGEPEAGALVDLSSMDAG"

CDS 3233996..3234991
/codon_start=1
/note="gene_2989"
/transl_table=11
/translation="MRNDDMAVVVNGVRKTYGKGKIVALDDVSFKVRRGEVIGLLGPN
GAGKTTMVDILSTLTRPDAGSAIIAGYDVVSEPAGVRRSIMVTGQQVAVDDALSGEQN
LVLFGRLWGLSKSAARKRAAELLEQFSLVHAGKRRVGTYSGGMRRRIDIACGLVVQPQ
VAFLDEPTTGLDPRSRQAIWDLVASFKKLGIATLLTTQYLEEADALSDRIILIDHGII
IAEGTANELKHRAGDTFCEIVPRDLKDLDAIVAALGSLLPEHHRAMLTPDSDRITMPA
PDGIRMLVEAARRIDEARIELADIALRRPSLDDVFLAMTTDPTESLTHLVSGSAR"

CDS 3234988..3235857
/codon_start=1
/note="gene_2990"
/transl_table=11
/translation="MSGPAIDASPALTFNQSSASIQQRRLSTGRQMWVLYRRFAAPSL
LNGEVLTTVGAPIIFMVGFYIPFAIPWNQFVGGASSGVASNLGQYITPLVTLQAVSFA
AIGSGFRAATDSLLGVNRRFQSMPMAPLTPLLARVWVAVDRCFTGLVISLVCGYVIGF
RFHRGALYIVGFCLLVIAIGAVLSFAADLVGTVTRNPDAMLPLLSLPILIFGLLSIGL
MPLKLFPHWIHPFVRNQPISQFVAALRALAGDTTKTASQVSWPVMAPTLTWLFAFVVI
LALSSTIVLARRP"

CDS 3235854..3236684
/codon_start=1
/note="gene_2991"
/transl_table=11
/translation="MITTTSQEIELAPTRLPGSQNAARLFVAQTLLQTNRLLTRWARD
YITVIGAIVLPILFMVVLNIVLGNLAYVVTHDSGLYSIVPLIALGAAITGSTFVAIDL
MRERSFGLLARLWVLPVHRASGLISRILANAIRTLVTTLVMLGTGVVLGFRFRQGLIP
SLMWISVPVILGIAIAAMVTTVALYTAQTVVVEGVELVQAIAIFFSTGLVPLNSYPGW
IQPFVAHQPVSYAIAAMRGFAMGGPVLSPMIGMLVWTAGICVVCAVPLAIGYRRASTH
"

CDS 3236731..3237999
/codon_start=1
/note="gene_2992"
/transl_table=11
/translation="MFPGSVIRKLSHSEEVFAQYEVFTSMTIQLRGVIDVDALSDAFD
ALLETHPVLASHLEQSSDGGWNLVADDLLHSGICVIDGTAATNGSPSGNAELRLDQSV
SLLHLQLILREGGAELTLYLHHCMADGHHGAVLVDELFSRYTDAVTTGDPGPITPQPT
PLSMEAVLAQRGIRKQGLSGAERFMSVMYAYEIPATETPAVLAHPGLPQAVPVTRLWL
SKQQTSDLMAFGREHRLSLNAVVAAAILLTEWQLRNTPHVPIPYVYPVDLRFVLAPPV
APTEATNLLGAASYLAEIGPNTDIVDLASDIVATLRADLANGVIQQSGLHFGTAFEGT
PPGLPPLVFCTDATSFPTMRTPPGLEIEDIKGQFYCSISVPLDLYSCAVYAGQLIIEH
HGHIAEPGKSLEAIRSLLCTVPSEYGWIME"

CDS complement(3238162..3244497)
/codon_start=1

/gene="mas-2"
/note="gene_2993"
/transl_table=11
/translation="MESRVTPVAVIGMGCRLPGGINSPDKLWESLLRGDDLVTEIPPD
RWDADDYYDPEPGVPGRSVSRWGGFLDDVAGFDAEFFGISEREATSIDPQQRLLLETS
WEAIEHAGLDPASLAGSSTAVFTGLTHEDYLVLTTTAGGLASPYVVTGLNNSVASGRI
AHTLGLHGPAMTFDTACSSGLMAVHLACRSLHDGEADLALAGGCAVLLEPHACVAASA
QGMLSSTGRCHSFDADADGFVRSEGCAMVLLKRLPDALRDGNRIFAVVRGTATNQDGR
TETLTMPSEDAQVAVYRAALAAAGVQPETVGVVEAHGTGTPIGDPIEYRSLARVYGAG
TPCALGSAKSNMGHSTASAGTVGLIKAILSLRHGVVPPLLHFNRLPDELSDVETGLFV
PQAVTPWPNGNDHTPKRVAVSSFGMSGTNVHAIVEEAPAEASAPESSPGDAEVGPRLF
MLSSTSSDALRQTARQLATWVEEHQDCVAASDLAYTLARGRAHRPVRTAVVAANLPEL
VEGLREVADGDALYDAAVGHGDRGPVWVFSGQGSQWAAMGTQLLASEPVFAATIAKLE
PVIAAESGFSVTEAITAQQTVTGIDKVQPAVFAVQVALAATMEQTYGVRPGAVVGHSM
GESAAAVVAGALSLEDAARVICRRSKLMTRIAGAGAMGSVELPAKQVNSELMARGIDD
VVVSVVASPQSTVIGGTSDTVRDLIARWEQRDVMAREVAVDVASHSPQVDPILDDLAA
ALADIAPMTPKVPYYSATLFDPREQPVCDGAYWVDNLRNTVQFAAAVQAAMEDGYRVF
AELSPHPLLTHAVEQTGRSLDMSVAALAGMRREQPLPHGLRGLLTELHRAGAALDYSA
LYPAGRLVDAPLPAWTHARLFIDDDGQEQRAQGACTITVHPLLGSHVRLTEEPERHVW
QGDVGTSVLSWLSDHQVHNVAALPGAAYCEMALAAAAEVFGEAAEVRDITFEQMLLLD
EQTPIDAVASIDAPGVVNFTVETNRDGETTRHATAALRAAEDDCPPPGYDITALLQAH
PHAVNGTAMRESFAERGVTLGAAFGGLTTAHTAEAGAATVLAEVALPASIRFQQGAYR
IHPALLDACFQSVGAGVQAGTATGGLLLPLGVRSLRAYGPTRNARYCYTRLTKAFNDG
TRGGEADLDVLDEHGTVLLAVRGLRMGTGTSERDERDRLVSERLLTLGWQQRALPEVG
DGEAGSWLLIDTSNAVDTPDMLASTLTDALKSHGPQGTECASLSWSVQDTPPNDQAGL
EKLGSQLRGRDGVVIVYGPRVGDPDEHSLLAGREQVRHLVRITRELAEFEGELPRLFV
VTRQAQIVKPHDSGERANLEQAGLRGLLRVISSEHPMLRTTLIDVDEHTDVERVAQQL
LSGSEEDETAWRNGDWYVARLTPSPLGHEERRTAVLDPDHDGMRVQVRRPGDLQTLEF
VASDRVPPGPGQIEVAVSMSSINFADVLIAFGRFPIIDDREPQLGMDFVGVVTAVGEG
VTGHQVGDRVGGFSEGGCWRTFLTCDANLAVTLPPGLTDEQAITAATAHATAWYGLND
LAQIKAGDKVLIHSATGGVGQAAISIARAKGAEIFATAGNPAKRAMLRDMGVEHVYDS
RSVEFAEQIRRDTDGYGVDIVLNSLTGAAQRAGLELLAFGGRFVEIGKADVYGNTRLG
LFPFRRGLTFYYLDLALMSVTQPDRVRELLATVFKLTADGVLTAPQCTHYPLAEAADA
IRAMSNAEHTGKLVLDVPRSGRRSVAVTPEQAPLYRRDGSYIITGGLGGLGLFFASKL
AAAGCGRIVLTARSQPNPKARQTIEGLRAAGADIVVECGNIAEPDTADRLVSAATATG
LPLRGVLHSAAVVEDATLTNITDELIDRDWSPKVFGSWNLHRATLGQPLDWFCLFSSG
AALLGSPGQGAYAAANSWVDVFAHWRRAQGLPVSAIAWGAWGEVGRATFLAEGGEIMI
TPEEGAYAFETLVRHDRAYSGYIPILGAPWLADLVRRSPWGEMFASTGQRSRGPSKFR
MELLSLPQDEWAGRLRRLLVEQASVILRRTIDADRSFIEYGLDSLGMLEMRTHVETET
GIRLTPKVIATNNTARALAQYLADTLAEEQAAAPAAS"

CDS 3245117..3246859
/codon_start=1
/note="gene_2994"
/transl_table=11
/translation="MSVRSLPAALRACARLQPHDPAFTFMDYEQDWDGVAITLTWSQL
YRRTLNVARELSRCGSTGDRVVISAPQGLEYVVAFLGALQAGRIAVPLSVPQGGVTDE
RSDSVLSDSSPVAILTTSSAVDDVVQHVARRPGESPPSIIEVDLLDLDAPNGYTFKED
EYPSTAYLQYTSGSTRTPAGVVMSHQNVRVNFEQLMSGYFADTDGIPPPNSALVSWLP
FYHDMGLVIGICAPILGGYPAVLTSPVSFLQRPARWMHLMASDFHAFSAAPNFAFELA
ARRTTDDDMAGRDLGNILTILSGSERVQAATIKRFADRFARFNLQERVIRPSYGLAEA
TVYVATSKPGQPPETVDFDTESLSAGHAKPCAGGGATSLISYMLPRSPIVRIVDSDTC

```
                     IECPDGTVGEIWVHGDNVANGYWQKPDESERTFGGKIVTPSPGTPEGPWLRTGDSGFV
                     TDGKMFIIGRIKDLLIVYGRNHSPDDIEATIQEITRGRCAAISVPGDRSTEKLVAIIE
                     LKKRGDSDQDAMARLGAIKREVTSALSSSHGLSVADLVLVAPGSIPITTSGKVRRGAC
                     VEQYRQDQFARLDA"
     CDS             3247014..3249614
                     /codon_start=1
                     /gene="mmpL7"
                     /note="gene_2995"
                     /transl_table=11
                     /translation="VIANLLLTFTQAEPHDTSPALLPQDAKTAAATSRIAQAFPGTGS
                     NAIAYLVVEGGSTLEPQDQPYYDAAVGALRADTRHVGSVLDWWSDPVTAPLGTSPDGR
                     SATAMVWLRGEAGTTQAAESLDAVRSVLRQLPPSEGLRASIVVPAITNDMPMQITAWQ
                     SATIVTVAAVIAVLLLLRARLSVRAAAIVLLTADLSLAVAWPLAAVVRGHDWGTDSVF
                     SWTLAAVLTIGTITAATMLAARLGSDAGHSAAPTYRDSLPAFALPGACVAIFTGPLLL
                     ARTPALHGVGTAGLGVFVALAASLTVLPALIALAGASRQLPAPTTGAGWTGRLSLPVS
                     SASALGTAAVLAICMLPIIGMRWGVAENPTRQGGAQVLPGNALPDVVVIKSARDLRDP
                     AALIAINQVSHRLVEVPGVRKVESAAWPAGVPWTDASLSSAAGRLADQLGQQAGSFVP
                     AVTAIKSMKSIIEQMSGAVDQLDSTVNVTLAGARQAQQYLDPMLAAARNLKNKTTELS
                     EYLETIHTWIVGFTNCPDDVLCTAMRKVIEPYDIVVTGMNELSTGADRISAISTQTMS
                     ALSSAPRMVAQMRSALAQVRSFVPKLETTIQDAMPQIAQASAMLKNLSADFADTGEGG
                     FHLSRKDLADPSYRHVRESMFSSDGTATRLFLYSDGQLDLAAAARAQQLEIAAGKAMK
                     YGSLVDSQVTVGGAAQIAAAVRDALIHDAVLLAVILLTVVALASMWRGAVHGAAVGVG
                     VLASYLAALGVSIALWQHLLDRELNALVPLVSFAVLASCGVPYLVAGIKAGRIADEAT
                     GARSKGAVSGRGAVAPLAALGGVFGAGLVLVSGGSFSVLSQIGTVVVLGLGVLITVQR
                     AWLPTTPGRR"
     CDS             3250246..3251487
                     /codon_start=1
                     /note="gene_2996"
                     /transl_table=11
                     /translation="MLTVEDWAEIRRLHRAEGLPIKMIARVLGISKNTVKSALESNQQ
                     PKYERAPQGSIVDAVEPRIRELLQAYPTMPATVIAERIGWERSIRVLSARVAELRPVY
                     LPPDPASRTTYVAGEIAQCDFWFPPIELPVGFGQTRTAKQLPVLTMVCAYSRWLLAML
                     LPSRCAEDLFAGWWRLIEALGAVPRVLVWDGEGAIGRWRGGRSELTTECQAFRGTLAA
                     KVLICRPADPEAKGLIERAHDYLERSFLPGRVFASPADFNAQLGAWLALVNTRTRRAL
                     GCAPTDRIGADRAAMLSLPPVAPATGWCTSLRLPRDHYVRCDSNDYSVHPGVIGHRVL
                     VRADLERVHVFCDGELVADHERIWAVHQTVSDPAHVEAAKVLRRRHFSAASPVVEPQV
                     QVRSLSDYDDALGVDIDGGVA"
     CDS             complement(3251661..3251846)
                     /codon_start=1
                     /gene="MGAST_01285"
                     /note="gene_2997"
                     /transl_table=11
                     /translation="VPGGAQKHDVIAGGDEIQGAQMCDGVAFEATSMLKVELFQRLPS
                     REAGGADAALTTMGLPG"
     CDS             3251905..3252288
                     /codon_start=1
                     /note="gene_2998"
                     /transl_table=11
                     /translation="VLFATAAEWVARLAEAHHAGRIYAELTRLCRYPLLVVDEVGYIP
                     FEPEAANLFFQLVSSRYERASLIVTSNKAFGRWGEVFGGDDVVAAAMIDRLVHHAEVV
                     ALKGDSYRLKDRDLGRVPPAGTTEE"
```

```
CDS             complement(3252406..3253107)
                /codon_start=1
                /note="gene_2999"
                /transl_table=11
                /translation="MNDGKRAVTSAVLVVLGACLALWLSGCSSPKPDAEEQGVPVSPT
                ASDPALLAEIRQSLDATKGLTSVHVAVRTTGKVDSLLGITSADVDVRANPLAAKGVCT
                YNDEQGVPFRVQGDNISVKLFDDWSNLGSISELSTSRVLDPAAGVTQLLSGVTNLQAQ
                GTEVIDGISTTKITGTIPASSVKMLDPGAKSARPATVWIAQDGSHHLVRASIDLGSGS
                IQLTQSKWNEPVNVD"
CDS             complement(3253129..3253407)
                /codon_start=1
                /gene="PAL_GLEAN10019973"
                /note="gene_3000"
                /transl_table=11
                /translation="LAGPGDGERRNGASEEAGNLAGPGDGERRNGASEEAGNLAGPGD
                GERRNGASEEAGNLAGPGDGERRNGASEEAGSHAQRDPINLHSACGPI"
CDS             complement(3253456..3259563)
                /codon_start=1
                /note="gene_3001"
                /transl_table=11
                /translation="VVSEFPTDRGWDVEGLYDPDPDAEGKTYTRWGAFLDDATGFDAG
                FFGIAPSEVLAMDPQQRLMLEVSWEALEHAGIDPLSLRGSATGVYTGIFAASYGNRDT
                GGLQGYGLTGTSISVASGRVSYVLGLQGPAVSVDTACSSSLVAIHWAMSSLRSGECDL
                ALAGGVTVMGLPSIFVGFSRQRGLAADGRCKAFAAAADGTGWGEGAGVVVLERLSDAR
                RLGHSVLAVVRGSAVNQDGASNGLTAPNGLAQQRVIQAALANAGLSAADVDVVEAHGT
                ATTLGDPIEAQALLSTYGQGRPAEQPLWVGSIKSNMGHTQAAAGVAGVIKMVQAMRHG
                VMPATLHVDEPSPRVDWTSGAVSVLTEAREWSVDGRPRRAAVSSFGISGTNAHLILEE
                APVPAPAEAPVEASESTGGPRPSMVPWVISARSAEALTAQAGRLMAHVQANPGLDPID
                VGCSLASRSVFEHRAVVVGASREQLIAGLAGLAAGEPGAGVAVGQPGSVGKTVVVFPG
                QGAQRIGMGRELYGELPVFAQAFDAVADELDRHLRLPLRDVIWGADADLLDSTEFAQP
                ALFAVEVASFAVLRDWGVLPDFVMGHSVGELAAAHAAGVLTLADAAMLVVARGRLMQA
                LPAGGAMVAVAASEDEVEPLLGEGVGIAAINAPESVVISGAQAAANAIADRFAAQGRR
                VHQLAVSHAFHSPLMEPMLEEFARVAARVQAREPQLGLVSNVTGELAGPDFGSAQYWV
                DHVRRPVRFADSARHLQTLGATHFIEAGPGSGLTGSIEQSLAPAEAMVVSMLGKDRPE
                LASALGAAGQVFTTGVPVQWSAVFAGSGGRRVQLPTYAFQRRRFWETPGADGPADAAG
                LGLGATEHALLGAVVERPDSDEVVLTGRLSLADQPWLADHVVNGVVLFPGAGFVELVI
                RAGDEVGCALIEELVLAAPLVMHPGVGVQVQVVVGAADESGHRAVSVYSRGDQSQGWL
                LNAEGMLGVAAAETPMDLSVWPPEGAESVDISDGYAQLAERGYAYGPAFQGLVAIWRR
                GSELFAEVVAPGEAGVAVDRMGMHPAVLDAVLHALGLAVEKTQASTETRLPFCWRGVS
                LHAGGAGRVRARFASAGADAISVDVCDATGLPVLTVRSLVTRPITAEQLRAAVTAAGG
                ASDQGPLEVVWSPISVVSGGANGSAPPAPVSWADFCAGSDGDASVVVWELESAGGQAS
                SVVGSVYAATHTALEVLQSWLGADRAATLVVLTHGGVGLAGEDISDLAAAAVWGMARS
                AQAENPGRIVLIDTDAAVDASVLAGVGEPQLLVRGGTVHAPRLSPAPALLALPAAESA
                WRLAAGGGGTLEDLVIQPCPEVQAPLQAGQVRVAVAAVGVNFRDVVAALGMYPGQAPP
                LGAEGAGVVLETGPEVTDLAVGDAVMGFLGGAGPLAVVDQQLVTRVPQGWSFAQAAAV
                PVVFLTAWYGLADLAEIKAGESVLIHAGTGGVGMAAVQLARQWGVEVFVTASRGKWDT
                LRAMGFDDDHIGDSRTCEFEEKFLAVTEGRGVDVVLDSLAGEFVDASLRLLVRGGRFL
                EMGKTDIRDAQEIAANYPGVQYRAFDLSEAGPARMQEMLAEVRELFDTRELHRLPVTT
                WDVRCAPAAFRFMSQARHIGKVVLTMPSALADRLADGTVVITGATGAVGGVLARHLVG
                AYGVRHLVLASRRGDRAEGAAELAADLTEAGAKGQVVACDVADRAAVAGLFAQLSREY
                PPVRGVIHAAGVLDDAVITSLTPDRIDTVLRAKVDAAWNLHQATSDLDLSMFVLCSSI
```

AATVGSPGQGNYSAANAFLDGLAAHRQAAGLAGISLAWGLWEQPGGMTAHLSSRDLAR
MSRSGLAPMSPAEAVELFDAALAIDHPLAVATLLDRAALDARAQAGALPALFSGLARR
PRRRQIDDTGDATSSKSALAQRLHGLAADEQLELLVGLVCLQAAAVLGRPSAEDVDPD
TEFGDLGFDSLTAVELRNRLKTATGLTLPPTVIFDHPTPTAVAEYVAQQMSGSRPTES
GDPTSQVVEPAAAEVSVHA"

CDS complement(3259791..3261908)
/codon_start=1
/note="gene_3002"
/transl_table=11
/translation="MRNGNLAGLLAEQASEAGWYDRPAFYAADVVTHGQIHDGAARLG
EVLRNRGLSSGDRVLLCLPDSPDLVQLLLACLARGVMAFLANPELHRDDHALAARNTE
PALVVTSDALRDRFQPSRVAEAAELMSEAARVAPGGYEPMGGDALAYATYTSGTTGPP
KAAIHRHADPLTFVDAMCRKALRLTPEDTGLCSARMYFAYGLGNSVWFPLATGGSAVI
NSAPVTPEAAAILSARFGPSVLYGVPNFFARVIDSCSPDSFRSLRCVVSAGEALELGL
AERLMEFFGGIPILDGIGSTEVGQTFVSNRVDEWRLGTLGRVLPPYEIRVVAPDGTTA
GPGVEGDLWVRGPAIAKGYWNRPDSPVANEGWLDTRDRVCIDSDGWVTYRCRADDTEV
IGGVNVDPREVERLIIEDEAVAEAAVVAVRESTGASTLQAFLVATSGATIDGSVMRDL
HRGLLNRLSAFKVPHRFAVVDRLPRTPNGKLVRGALRKQSPTKPIWELSLTEPGSGVR
AQRDDLSASNMTIAGGNDGGATLRERLVALRQERQRLVVDAVCAEAAKMLGEPDPWSV
DQDLAFSELGFDSQMTVTLCKRLAAVTGLRLPETVGWDYGSISGLAQYLEAELAGGHG
RLKSAGPVNSGATGLWAIEEQLNKVEELVAVIADGEKQRVADRLRALLGTIAGSEAGL
GKLIQAASTPDEIFQLIDSELGK"

CDS complement(3261925..3262539)
/codon_start=1
/note="gene_3003"
/transl_table=11
/translation="MWGKNMTECFLSDQEIRKLNRDLRILIAANGTLTRVLNIVADDE
VIVQIVKQRIHDVSPKLSEFEQLGQVGVGRVLQRYIILKGRNSEHLFVAAESLIAIDR
LPAAIITRLTQTNDPLGEVMAASHIETFKEEAKVWVGDLPGWLALHGYQNSRKRAVAR
RYRVISGGQPIMVVTEHFLRSVFRDAPHEEPDRWQFSNAITLAR"

CDS complement(3262550..3264409)
/codon_start=1
/note="gene_3004"
/transl_table=11
/translation="MKTNSSFHAAGEVATQPAWGTGEQAAQPLNGSTSRFAMSESSLA
DLLQKAASQYPNRAAYKFIDYDTDPAGFTETVTWWQVHRRAMIVAEELWIYASSGDRV
AILAPQGLEYIIAFMGVLQAGLIAVPLPVPQFGIHDERISSALRDSAPSIILTTSSVI
DEVTTYAPHACAAQGQSAPIVVAVDALDLSSSRALDPTRFERPSTAYLQYTSGSTRAP
AGVVLSHKNVITNCVQLMSDYIGDSEKVPSTPVSWLPFYHDMGLMLGIILPMINQDTA
VLMSPMAFLQRPARWMQLLAKHRAQISSAPNFGFELAVRRTSDDDMAGLDLGHVRTIV
TGAERVNVATLRRFTERFAPFNLSETAIRPSYGLAEATVYVVATAGPGRAPKSVCFDYQ
QLSVGQAKRTENGSEGANLVSYGAPRASTVRIVDPETRMENPAGTVGEIWVQGDNVGL
GYWRNPQQTEATFRARLVTPSPGTSEGPWLRTGDLGVIFEGELFITGRIKELLVVDGA
NHYPEDIEATIQEITGGRVVAIAVPDDRTEKLVTIIELMKRGRTDEEEKNRLRTVKRE
VASAISRSHRLRVADVVMVAPGSIPVTTSGKVRRSASVERYLHHEFSRLDAMA"

CDS complement(3265057..3266202)
/codon_start=1
/note="gene_3005"
/transl_table=11
/translation="VGGLRFGFVDALVHSRLPPTLPARSSMAAATVMGADSYWVGDHL
NALVPRSIATSEYLGIAAKFVPKIDANYEPWTMLGNLAFGLPSRLRLGVCVTDAGRRN

PAVTAQAAATLHLLTRGRAILGIGVGEREGNEPYGVEWTKPVARFEEALATIRALWNS
NGELISRESPYFPLHNALFDLPPYRGKWPEIWVAAHGPRMLRATGRYADAWIPIVVVR
PSDYSRALEAVRSAASDAGRDPMSITPAAVRGIITGRNRDDVEEALESVVVKMTALGV
PGEAWARHGVEHPMGADFSGVQDIIPQTMDKQTVLSYAAKVPAALMKEVVFSGTPDEV
IDQVAEWRDHGLRYVVLINGSLVNPSLRKTVTAVLPHAKVLRGLKKL"

CDS 3266395..3267207
/codon_start=1
/note="gene_3006"
/transl_table=11
/translation="MAFSRTHSLLARAGSTSTYKRVWRYWYPLMTRGLGNDEIVFINW
AYEEDPPMDLPLEASDEPNRAHINLYHRTATQVDLGGKQVLEVSCGHGGGASYLTRTL
HPASYTGLDLNQAGIKLCKKRHRLPGLDFVRGDAENLPFDDESFDVVLNVEASHCYPH
FRRFLAEVVRVLRPGGYFPYADLRPNNEIAAWEADLAATPLRQLSQRQINAEVLRGIG
NNSQKSRDLVDRHLPAFLRFAGREFIGVQGTQLSRYLEGGELSYRMYCFTKD"

CDS 3267233..3268489
/codon_start=1
/note="gene_3007"
/transl_table=11
/translation="MSPAEREFDIVLYGATGFSGKLTAEHLAHSGSTARIALAGRSSE
RLRGVRMMLGPNAADWPLILADASQPLTLEAMAARAQVVLTTVGPYTRYGLPLVAACA
KAGTDYADLTGELMFCRNSIDLYHKQAADTGARIILACGFDSIPSDLNVYQLYRRSVE
DGTGELCDTDLVLRSFSQRWVSGGSVATYSEAMRTASSDPEARRLVTDPYTLTTDRGA
EPELGAQPDFLRRPGRDLAPELAGFWTGGFVQAPFNTRIVRRSNALQEWAYGRRFRYS
ETMSLGKSMAAPILAAAVTGTVAGTIGLGNKYFDRLPRRLVERVTPKPGTGPSRKTQE
RGHYTFETYTTTTTGARYRATFAHNVDAYKSTAVLLAQSGLALALDRDRLAELRGVLT
PAAAMGDALLARLPGAGVVMGTTRLS"

CDS complement(3268620..3269345)
/codon_start=1
/note="gene_3008"
/transl_table=11
/translation="MRLPGMLRPTAERHFHSIFYLRHNARRQEHLATLGLDLGNKSVL
EVGAGIGDHTQFFLDRGCKVLCTEPRGENLDVIRQRFGSNPNVTVDHLDLDGDLPAEA
HQYDVVYCYGVLYHLSRPAEALAWMCDRAVDLLLLETCVSYSGEDEPFLVSERASSPS
QAITGTGCRPSRVWVMNRLREKMPHVYVTATQPQHRQFPLDWRANGPIASTGLARAVF
VASRAPLNLPTLVEELPMVQRRC"

CDS complement(3269534..3270277)
/codon_start=1
/note="gene_3009"
/transl_table=11
/translation="LPPHRWRLAPWYWRGLQVTLEPGSAIAWIVRLTGGFEETEIDIA
AALYSALYPDRCILDVGANVGIHSLAWARLAPVVALEPAPGTHSRLEANVAANGLQDR
IRTLRTAAGDAVGEVDFFVAADSAFSSLNDTGRIRIRERTRVPCTTLDALAAELPLPV
GLLKIDVEGLERAVIAGAAELLRRDRPVLLVEIYGGAASNPDPERTIADIRAYGYEPF
VYADDAGLQPYQRHRDDRYCYFFIPSRKG"

CDS 3270622..3271353
/codon_start=1
/note="gene_3010"
/transl_table=11
/translation="VKSLKLARFIARSAAFEVSRRYSERDLKHQFVKQLKSRRVDVVF
DVGANSGQYAAGLRRAAYKGRIVSFEPLSGPFTILESKASTDPLWDCRQHALGDSDGT
VTINIAGNAGQSSSVLPMLKSHQNAFPPANYVGTQEASIHRLDSVAPEFLGMNGVAFL

```
                     KVDVQGFEKQVLAGGKSTIDDHCVGMQLELSFLPLYEGGMLIPEALDLVYSLGFTLTG
                     LLPCFIDANNGRMLQADGTFFREDD"
     CDS             3271481..3272251
                     /codon_start=1
                     /note="gene_3011"
                     /transl_table=11
                     /translation="MAAPMFSIIIPTLNVAAVLPACLDSIARQTCGDFELVLVDGGST
                     DETLDIANIFAPNLGERLIIHRDTDQGVYDAMNRGVDLATGTWLLFLGADDSLYEADT
                     LARVAAFIGEHEPSDLVYGDVIMRSTNFRWGGAFDLDRLLFKRNICHQAIFYRRGLFG
                     TIGPYNLRYRVLADWDFNIRCFSNPALVTRYMHVVVASYNEFGGLSNTIVDKEFLKRL
                     PMSTRLGIRLVIVLVRRWPKVISRAMVMRTVISWRRRR"
     CDS             complement(3272399..3272605)
                     /codon_start=1
                     /note="gene_3012"
                     /transl_table=11
                     /translation="VSQFKTLKYRNDSPERFDSIEHARTWPGHMGAAMANAYSANPNP
                     FGVSPQPPKPATAAWINPPTPDPK"
     CDS             complement(3272855..3273955)
                     /codon_start=1
                     /note="gene_3013"
                     /transl_table=11
                     /translation="MEETSVAGDPGPDAGTSTAPNAAPEPVARRQRILFVGEAATLAH
                     VVRPFVLARSLDPSRYEVHFACDPRFNKLLGPLPFPHHPIHTVPSEEVLLKIAQGRLF
                     YNTRTLRKYIAADRKILNEIAPDVVVGDNRLSLSVSARLAGIPYIAIANAYWSPQARR
                     RFPLPDVPWTRFFGVRPVSILYRLYRPLIFALYCLPLNWLRRKHGLSSLGWDLCRIFT
                     DGDYTLYADVPELVPTYNLPANHRYLGPVLWSPDVKPPTWWHSLPTDRPIIYATLGSS
                     GGKNLLQVVLNALGRFTRDGDRGHRWPEPPEERAGQRLRRGLPAGRSGCSALRRGALQ
                     RRQPDDAAGVGGRGAGDRAPQQHGPALEHGGP"
     CDS             complement(3274056..3274793)
                     /codon_start=1
                     /note="gene_3014"
                     /transl_table=11
                     /translation="MGLVWRSRTSLVGQLIGLVRLVASFAAQLFYRPSDAVAEEYHKW
                     YYGNLVWTKTTYMGINCWKSVSDMWNYQEILSELQPSLVIEFGTRYGGSAVYFANIMR
                     QIGQPFKVLTVDNSHKALDPRARREPDVLFVESSSTDPAIAEQIQRLKNEYPGKIFAI
                     LDSDHSMNHVLAEMKLLRPLLSAGDYLVVEDSNINGHPVLPGFGPGPYEAIEAYEDEF
                     PNDYKHDAERENKFGWTSAPNGFLIRN"
     CDS             complement(3274908..3275258)
                     /codon_start=1
                     /note="gene_3015"
                     /transl_table=11
                     /translation="MRIAVLHGVIQDDAIVVVDDLGLVPELDRPRPSAVGRNATAVVS
                     LPVVALSPRAGQAGYLWQSITRGLRVTPICCYHPPCGGGVQKMLSRKLGRVCPAPSPK
                     DAARGAHNVGANAV"
     CDS             3275238..3275627
                     /codon_start=1
                     /note="gene_3016"
                     /transl_table=11
                     /translation="VEHGNPHDAPQLAPAVERITTRAGRPPGTVTADRGYGEKRVEDD
                     LHDLGVRTVAIPRKGRPSQARRAEEQRPSFRRTVKWRTGSEGRISTLKRNYGWNRSCI
                     DGTEGTRIWTRHGILTHNLIKISSLAA"
```

```
CDS             complement(3275728..3276924)
                /codon_start=1
                /note="gene_3017"
                /transl_table=11
                /translation="VTLAHVVRPFALAQSLDPSRYEVHFACDPRYNQLLGPLPFRHHA
                IHTIPSERFFGNLTQGRFYAMRTLRKYVEADLRVLDEIAPDLVVGDLRISLSVSARLA
                GIPYIAIANAYWSPYAQRRFPLPDVIWTRLFGVRLVKLLYRLERPLLFALQCMPLNWV
                RRRHGLSSLGWNLCRIFTDGDHTLYADVPELMPTYDLPANHEYLGPVLWSPAGKPPTW
                WDSLPTDRPIVYATLGTSGGRNLLQLVLNALAELPVTVIAATAGRSDLKTVPANAFVA
                DYLPGEAAAARSAVVVCNGGSLTTQQALVAGVPVIGVAGNLDQHLNMEAVERAGAGVL
                LRTERLKSQRVAGAVMQVISRSEYRQAAARLADAFGRDRVGFPQHVENALRLMPENRP
                RTWLAS"
CDS             3277191..3278411
                /codon_start=1
                /note="gene_3018"
                /transl_table=11
                /translation="VTSTKVEDRVTAAVLGAIGHALALTASMTWEILWALILGFALSA
                VVQAVVRRSTIVTLLGDDRPRTLVIATGLGAASSSCSYAAVALARSLFRKGANFTAAM
                AFEIGSTNLVVELGIILALLMGWQFTAAEFVGGPIMILVLAVLFRLFVGARLIDAARE
                QAERGLAGSMEGHAAMDMSIKREGSFWRRLLSPPGFTSIAHVFVMEWLAILRDLILGL
                LIAGAIAAWVPESFWQSFFLANHPAWSAVWGPIIGPIVAIVSFVCSIGNVPLAAVLWN
                GGISFGGVIAFIFADLLILPILNIYRKYYGARMMLVLLGTFYASMVVAGYLIELLFGT
                TNLIPSQRSATVMTAEISWNYTTWLNVIFLVIAAALVVRFITSGGLPMLRMMGGSPDA
                PHDHHDRHDDHLGH"
CDS             3278622..3279416
                /codon_start=1
                /gene="purU"
                /note="gene_3019"
                /transl_table=11
                /translation="VSTFLARAGANIISLDQHSTAPEGGTFLQRAIFHLPGLTAAVDE
                LQRDFGSTVADKFGIDYRFAEAAKPKRVAIMASTEDHCLLDLLWRNRRGELEMSVVMV
                IANHPDLAAHVRPFGVPFIHIPATRDTRTEAEQRQLQLLSGNVDLVVLARYMQILSPG
                FLEAIGCPLINIHHSFLPAFTGAAPYQRARERGVKLIGATAHYVTEVLDEGPIIEQDV
                VRVDHTHTVDDLVRVGADVERAVLSRAVLWHCQDRVIVHHNQTIVF"
CDS             3280056..3280241
                /codon_start=1
                /note="gene_3020"
                /transl_table=11
                /translation="MFTARIRALAGMSLLASAIGLAAFGAATGTANAAPTHQPEWGTY
                TCYDYATQTFYECFDPS"
CDS             complement(3280285..3280770)
                /codon_start=1
                /gene="coaD"
                /note="gene_3021"
                /transl_table=11
                /translation="MTGAVCPGSFDPVTLGHVDIFERAAAQFDEVVVAILVNPAKTGM
                FDLDERIAMVKESTTHLPNLRVQVGHGLVVDFVRSCGMTAIVKGLRTGTDFEYELQMA
                QMNKHIAGVDTFFVATAPRYSFVSSSLAKEVAMLGGDVSELLPEPVNRRLRDRLNTER
                T"
CDS             complement(3280856..3281341)
                /codon_start=1
```

/note="gene_3022"
/transl_table=11
/translation="VRESLFNIVTARRDLTGLAVLDLYAGSGALGLEALSRGAASVLF
VESDQRSAAVIARNIEALGLSGATLRRGAVAAVVAAGTTSPVDLVLADPPYNVDSADV
DAILAALGTNGWTREGTVAVVERATTCAPLTWPEGWRRWPQRVYGDTRLELAERLFAN
V"
CDS complement(3281618..3285001)
/codon_start=1
/gene="pca"
/note="gene_3023"
/transl_table=11
/translation="VFSKVLVANRGEIAIRAFRAAYELGVGTVAVYPYEDRNSQHRLK
ADESYQIGDIGHPVHAYLSVDEIVATARRAGADAIYPGYGFLSENPDLAAACAAAGIS
FVGPSAEVLELAGNKSRAIAAAREAGLPVLMSSAPSASVDELLSVAAGMPFPLFVKAV
AGGGGRGMRRVGDIAALPEAIEAASREAESAFGDPTVYLEQAVINPRHIEVQILADNL
GDVIHLYERDCSVQRRHQKVIELAPAPHLDAELRYKMCVDAVAFARHIGYSCAGTVEF
LLDERGEYVFIEMNPRVQVEHTVTEEITDVDLVASQLRIAAGETLEQLGLRQEDIAPH
GAALQCRITTEDPANGFRPDTGRISALRTAGGAGVRLDGSTNLGAEISPYFDSMLVKL
TCRGRDLPTAVSRARRAIAEFRIRGVSTNIPFLQAVLDDPDFRAGRVTTSFIDERPQL
LTARASADRGTKILNFLADVTVNNPYGSRPSTIYPDDKLPDLDLRAAPPAGSKQRLVK
LGPEGFARWLRESAAVGVTDTTFRDAHQSLLATRVRTSGLSRVAPYLARTMPQLLSVE
CWGGATYDVALRFLKEDPWERLATLRAAMPNICLQMLLRGRNTVGYTPYPEIVTSAFV
QEATATGIDIFRIFDALNNIESMRPAIDAVRETGSAIAEVAMCYTGDLTDPGEQLYTL
DYYLKLAEQIVDAGAHVLAIKDMAGLLRPPAAQRLVSALRSRFDLPVHLHTHDTPGGQ
LASYVAAWHAGADAVDGAAAPLAGTTSQPALSSIVAAAAHTEYDTGLSLSAVCALEPY
WEALRKVYAPFESGLPGPTGRVYHHEIPGGQLSNLRQQAIALGLGDRFEEIEEAYAGA
DRVLGRLVKVTPTSKVVGDLALALVGAGVSADEFASDPARFGIPESVLGFLRGELGDP
PGGWPEPLRTAALAGRGAARPTAQLAADDEIALSSVGAKRQATLNRLLFPSPTKEFNE
HREAYGDTSQLSANQFFYGLRQGEEHRVKLERGVELLIGLEAISEPDERGMRTVMCIL
NGQLRPVLVRDRSIASAVPAAEKADRGNPGHIAAPFAGVVTVGVCVGERVGAGQTIAT
IEAMKMEAPITAPVAGTVERVAVSDTAQVEGGDLLVVVS"
CDS complement(3285026..3285658)
/codon_start=1
/note="gene_3024"
/transl_table=11
/translation="VVAARPAERSGDPAAVRVPVPSAWWVLIGGVIGLFASMTLTVEK
VRILLDPIYVPSCNVNPIVSCGSVMTTPQASLLGFPNPLLGIAGFTVVVVTGVLAVAK
VPLPRWYWIGLAVGILVGVAFVHWLIFQSLYRIGALCPYCMVVWAVIATLLVVVASIV
FGPMRENRGSQERVGARLLYQWRWSLATLWFTTVFLLIMVRFWDYWSTLI"
CDS complement(3285664..3286431)
/codon_start=1
/note="gene_3025"
/transl_table=11
/translation="VADKSKRPPRFDLKSADGSFGRLVQIGGTTIVVVFAVVLVFYIV
TSRDDKKDGVAGPGDAVRVTSSKLVTQPGTSNPKAVVSFYEDFLCPACGIFERGFGPT
VSKLVDIGAVAADYTMVAILDSASNQHYSSRAAAAAYCVADESIEAFRRFHAALFSKD
IQPAELGKDFPDNARLIELAREAGVVGKVPDCINSGKYIEKVDGLAAAVNVHATPTVR
VNGTEYEWSTPAALVAKIKEIVGDVPGIDSAAATATS"
CDS complement(3286528..3287586)
/codon_start=1
/note="gene_3026"

/transl_table=11
/translation="VQGTVVNVGVKVLPWIPTPAKRILSAGRSVIIDGNTLDPTLQLM
LSTSRIFGVDGLAVDDDIVASRAHMRAICEAMPGPQIHVDVTDLSIPGPAGEIPARHY
RPSGGGATPLLVFYHGGGWTLGDLDTHDALCRLTCRDADIQVLSIDYRLAPEHPAPAA
VEDAYAAFVWAHEHASDEFGALPGRVAVGGDSAGGNLSAVVCQLARDKARYEGGPTPV
LQWLLYPRTDFTAQTRSMGLFGNGFLLTKRDIDWFHTQYLRDSDVDPADPRLSPLLAE
SLSGLAPALIAVAGFDPLRDEGESYAKALRAAGTAVDLRYLGSLTHGFLNLFQLGGGS
AAGTNELISALRAHLSRV"

CDS 3288056..3288904
/codon_start=1
/note="gene_3027"
/transl_table=11
/translation="VTGESGAAAAPSITLNDEHTMPVLGLGVAELSDDETERAVSAAL
EIGCRLIDTAYAYGNEAAVGRAIAASGVAREELFVTTKLATPDQGFTRSQEACRASLD
RLGLDYVDLYLIHWPAPPVGKYVDAWGGMIQSRGEGHARSIGVSNFTAEHIENLIDLT
FVTPAVNQIELHPLLNQDELRKANAQHTVVTQSYCPLALGRLLDNPTVTSIASEYVKT
PAQVLLRWNLQLGNAVVVRSARPERIASNFDVFDFELAAEHMDALGGLNDGTRVREDP
LTYAGT"

CDS complement(3288978..3289691)
/codon_start=1
/note="gene_3028"
/transl_table=11
/translation="MNRRTLLWLSAIAALALVVAYQTLGSSAGRHADEFAARAGVPTV
QPGADVLAGIAVLPKRIHRYDYRRSAFGHPWDDRNDAPGGHNGCDTRDDILDRDLVDK
TYVSIKRCPNAVATGTLRDPYTNTTVAFQRGASVGQSVQIDHIVPLSYAWDMGAYRWP
NSERMRFANDPANLLAVQGQANQDKGDSPPAQWMPPNKAFACQYAMQFIAVLRGYSLP
VDQPSSDVLRQAAATCPTG"

CDS complement(3289688..3291901)
/codon_start=1
/note="gene_3029"
/transl_table=11
/translation="VASLSARLDRVLGATAADALDEQFGMRTVDDLLRHYPRSYVEGA
ARVGIGDARPEAGEHITIVDVITDTYSFPMKKKPNRKCLRITVGGGRNKVTATFFNAD
YIMRDLTKHTKVMLSGEVGYYKGAMQLTHPAFLILDSPDGKNHGTRSLKSIADASKAI
SGELVVEEFERRFFPIYPASTKVQSWDIFKCVRQVLDVLDRVDDPLPAELRAKHGLIP
EDEALRAIHLAESQSLRERARERLTFDEAVGLQWALVARRHGELSESGPSAAWKSNGL
AAELLRRLPFELTAGQREVLDVLSDGLAANRPLNRLLQGEVGSGKTIVAVLAMLQMVD
AGYQCALLAPTEVLAAQHLRSIRDVLGPLAMGGQLGGAENATRVALLTGSMTAGQKKQ
VRAEIASGQVGIVIGTHALLQEAVDFHNLGMVVVDEQHRFGVEQRDQLRAKAPAGITP
HLLVMTATPIPRTVALTVYGDLETSTLRELPLGRQPIATNVIFVKDKPAWLDRAWRRI
IEEAAAGRQAYVVAPRIDESDDTDVQGGVRPSATAEGLFSRLRSAELAELRLALMHGR
LSADDKDAAMAAFRAGEVDVLVCTTVIEVGVDVPNATVMLVMDADRFGISQLHQLRGR
IGRGEHPSVCLLASWVPPDTPAGQRLRAVAGTMDGFALADLDLKERKEGDVLGRNQSG
KAITLRLLSLAEHEEYIVAARDFCIEAYKNPTDPALALMAARFTSTDRIEYLDKS"

CDS complement(3291904..3293565)
/codon_start=1
/note="gene_3030"
/transl_table=11
/translation="VGTADRPLDASALRDWAHAVVSDLILHIDEINRLNVFPVADSDT
GVNMLFTMRAAVVEADLHANSQADAEDVARVAAALAAGALNGARGNSGVILSQILRGI
AEVTATAAAASGAVLRAVDANALGAALWRGVELVVASMGGVEVPGTIVSVLRAAAGAV

DQCAHEGLAGAVTAAGDAAVIALEKTPEQLDVLADAGAVDAGGRGLLVLLDALRSTIC
GQAPARAVYEPSPRALPTDTATQRPAPQFEVMYLLAVCDAAAADQLRDRLKELGESVA
IAAAPPDSYSVHVHTDDAGAAVEAGLAVGRVSRIVISALGSGTSGLPAGGWTRGRAVL
AVVDGDGAAELFAGEGACVLRPGPDAVTPAADISAHQLVRAVVDTGAAHVMVLPNGYV
AAEELVAGCTAAIGWGVDVVPVPTGSMVQGLAALAVHDAARQAVDDGYSMARAAGASR
HGSVRIATQKALTWAGTCKPGDGLGIAGDEVLIVADDVAAAAIGLVDLLLASGGDLVT
VLIGAGVTEDVAVVLERHVHDHHPGTELVSYRTGHRGDALLIGVE"
CDS 3293807..3294001
/codon_start=1
/gene="rpmB"
/note="gene_3031"
/transl_table=11
/translation="MAAVCDICGKGPGFGKSVSHSHRRTSRRWDPNIQTVHAVTRPGG
NKKRLNVCTSCIKAGKITRG"
CDS complement(3294024..3294707)
/codon_start=1
/note="gene_3032"
/transl_table=11
/translation="VTARPLSELVERGWAAALEPVADQVAHMGQFLRAEIAAGRRYLP
AGSNVLRAFTFPFDNVRVLIVGQDPYPTPGHAVGLSFSVAPDVRPWPRSLANIFDEYT
ADLGYPLPSNGDLTPWAQRGVLLLNRVLTVRPSNPASHRGKGWEAVTECAIRALAARA
APLVAILWGRDASTLKPMLAAGNCVAIESPHPSPLSASRGFFGSRPFSRANELLVGMG
AEPIDWRLP"
CDS complement(3294740..3295741)
/codon_start=1
/note="gene_3033"
/transl_table=11
/translation="VTTKDHSLATESPTLQQLGEFAVIDRLVRGRRQPATVLLGPGDD
AALVSAGDGRTVVSTDMLVQDSHFRLDWSTPQDVGRKAIAQNAADIEAMGARATAFVV
GFGAPAETPAAQASALVDGMWEEAGRIGAGIVGGDLVSCRQWVVSVTAIGDLDGRAPV
LRSGAKAGSVLAVVGELGRSAAGYALWCNGIEDFAELRRRHLVPQPPYGHGAAAAAVG
AQAMIDVSDGLLADLRHIAEASGVRIDLSAAALAADRDALTAAATALGTDPWPWVLSG
GEDHALVACFVGPVPAGWRTIGRVLDGPARVLVDGEEWTGYAGWQSFGEPDNQGSLG"
CDS complement(3295738..3297117)
/codon_start=1
/note="gene_3034"
/transl_table=11
/translation="MPKFEVPDGWTVQAFRFTLDPTEDQAKALARHFGARRKAYNWTV
ATLKADIQAWHASGTVTAKPSLRVLRKRWNTVKDDVCVNTETGVAWWPECSKEAYADG
IAGAVEAYWNWQTSRAGKRAGKRVGFPRFKRKGRDQDRVSFTTGAMRVEPDRRHLTLP
VIGTVRTHENTRRIERLIKAGRARVLAISVRRNGTRLDASVRVLVQRPQQPKVVHPGS
RVGVDVGVRRLATVATADGTAIEQVENPRPLGAALRELRHVCRARSRCTKGSRRYRER
TTQISRLHRRVNDVRTHHLHVLTTRLAQTHGRIVVEGLDATEMLRQKGLPGARARRRG
LSDAALGTPRRHLSYKTVWYGSALVVADRWFPSSKTCHACRHVQDIGWDEQWQCDRCS
VVHQRDDCAAINLARYEETSSIVGPVGAAVKRGADRKTGPRPAGGCEARKGSSPKAAE
QPRDGVQVA"
CDS complement(3297117..3297701)
/codon_start=1
/note="gene_3035"
/transl_table=11
/translation="MNLATWAERNGVARGTAYRWFRAGLLSVMARRVGRLILVDEPAG

DAGMRSPTAVYARVSSADQKADLDRQVARVTAWAAAQQMPVDKVVTEVGSAFNEHRRK
FLSLLRDPSVHRIVVEHRDRFCRLGSKYVQAAFAAQGRELVVVDSAEVGDDLVRDMTE
ILTSMCARLYGKRAAENRTKRALAAAAGEDHEAA"
CDS 3297901..3298458
/codon_start=1
/note="gene_3036"
/transl_table=11
/translation="VAAQVTGESDGPPRAVLIAAAALAAAVIGVILVVAANRQPPERP
VVIPAVPAPQATGPGCKALLAALPQRLGEYRRAPVAEPTTAGATAWRTGPNSTPVILR
CGLDRPAEFVVGSAIQVVDRVQWFQVAAQNPDEPGRSTWYTVDRPVYVALTLPSGSGP
TAIQELSDVIDHTIPAVPIDPAPAR"
variation 3298798..3298915
/note="supplemented from BCG moreau"
CDS complement(3299044..3300165)
/codon_start=1
/gene="ddl"
/note="gene_3037"
/transl_table=11
/translation="VSANDRRDRRVRVAVVFGGRSNEHAISCVSAGSILRNLDSRRFD
VIAVGITPAGSWVLTDANPDALTITNRELPQVKSGSGTELALPADPRRGGQLVSLPPG
AGEVLESVDVVFPVLHGPYGEDGTIQGLLELAGVPYVGAGVLASAVGMDKEFTKKLLA
ADGLPVGAYAVLRPPRSTLHRQECERLGLPVFVKPARGGSSIGVSRVSSWDQLPAAVA
RARRHDPKVIVEAAISGRELECGVLEMPDGTLEASTLGEIRVAGVRGREDSFYDFATK
YLDDAAELDVPAKVDDQVAEAIRQLAIRAFAAIDCRGLARVDFFLTDDGPVINEINTM
PGFTTISMYPRMWAASGVDYPTLLATMIETALARGVGLH"
CDS complement(3300243..3301247)
/codon_start=1
/gene="gpsA"
/note="gene_3038"
/transl_table=11
/translation="MAGIASTVAVMGAGAWGTALAKVLADAGGEVTLWARRAEVADQI
NTTRYNPDYLPGALLPPSIHATADAEEALGGASTVLLGVPAQTMRANLERWAPLLPEG
ATLVSLAKGIELGTLMRMSQVIISVTGAEPAQVAVISGPNLASEIAECQPAATVVACS
DSGRAVALQRALNSGYFRPYTNADVVGTEIGGACKNIIALACGMAVGIGLGENTAAAI
ITRGLAEIIRLGTALGANGATLAGLAGVGDLVATCTSPRSRNRSFGERLGRGETLQSA
GKACHVVEGVTSCESVLALASSYDVEMPLTDAVHRVCHKGLSVDEAITLLLGRRTKPE
"
CDS 3301366..3302010
/codon_start=1
/note="gene_3039"
/transl_table=11
/translation="MSGTPDDGDIGLIIAVKRLAAAKTRLAPVFSAQTRENVVLAMLV
DTLTAAAGVGSLRSITVITPDEAAAAAAAGLGADVLADPTPEDDPDPLNTAITAAERV
VAEGASNIVVLQGDLPALQTQELAEAISAARHHRRSFVADRLGTGTAVLCAFGTALHP
RFGPDSSARHRRSGAVELTGAWPGLRCDVDTPADLTAARQLGVGPATARAVAHR"
CDS 3302102..3304330
/codon_start=1
/gene="ppk"
/note="gene_3040"
/transl_table=11
/translation="MMSNDRKVTEIENSPVTEVRPEEHAWYPDDSALAAPPAATPAAI

SDQLPSDRYLNRELSWLDFNARVLALAADKSMPLLERAKFLAIFASNLDEFYMVRVAG
LKRRDEMGLSVRSADGLTPREQLGRIGEQTQQLASRHARVFLDSVLPALGEEGIYIVT
WADLDQAERDRLSTYFNEQVFPVLTPLAVDPAHPFPFVSGLSLNLAVTVRQPEDGTQH
FARVKVPDNVDRFVELAAREASEEAAGTEGRTALRFLPMEELIAAFLPVLFPGMEIVE
HHAFRITRNADFEVEEDRDEDLLQALERELARRRFGSPVRLEIADDMTESMLELLLRE
LDVHPGDVIEVPGLLDLSSLWQIYAVDRPTLKDRTFVPATHPAFAERETPKSIFATLR
EGDVLVHHPYDSFSTSVQRFIEQAAADPNVLAIKQTLYRTSGDSPIVRALIDAAEAGK
QVVALVEIKARFDEQANIAWARALEQAGVHVAYGLVGLKTHCKTALVVRREGPTIRRY
CHVGTGNYNSKTARLYEDVGLLTAAPDIGADLTDLFNSLTGYSRKLSYRNLLVAPHGI
RAGIIDRVEREVAAHRAEGAHNGKGRIRLKMNALVDEQVIDALYRASRAGVRIEVVVR
GICALRPGAQGISENIIVRSILGRFLEHSRILHFRAIDEFWIGSADMMHRNLDRRVEV
MAQVKNPRLTAQLDELFESALDPCTRCWELGPDGQWTASPQEGHSVRDHQESLMERHR
SP"

CDS 3304413..3305366
/codon_start=1
/note="gene_3041"
/transl_table=11
/translation="VSIQNSSARRRSAGRIVYAAGAVLWRPGSADSEGPVEIAVIHRP
RYDDWSLPKGKVDPGETAPVGAVREILEETGHRANLGRRLLTVTYPTDSPFRGVKKVH
YWAARSTGGEFTPGSEVDELIWLPVPDAMNKLDYAQDRKVLCRFAKHPADTQTVLVVR
HGTAGSKAHFSGDDSKRPLDKRGRAQAEALVPQLLAFGATDVYAADRVRCHQTMEPLA
AELNVTIHNEPTLTEESYANNPKRGRHRVLQIVEQVGTPVICTQGKVIPDLITWWCER
DGVHPDKSRNRKGSTWVLSLSAGRLVTADHIGGALAANVRA"

CDS complement(3305424..3306068)
/codon_start=1
/note="gene_3042"
/transl_table=11
/translation="MNKAELIDVLTQKLGSDRRQATAAVENVVDTIVRAVHKGDSVTI
TGFGVFEQRRRAARVARNPRTGETVKVKPTSVPAFRPGAQFKAVVSGAQRLPAEGPAV
KRGVGASAAKKVAKKAPAKKATKAAKKAATKAPARKAATKAPAKKAATKAPAKKAVKA
TKSPAKKVTKAVKKTAVKASVRKAATKAPAKKAAAKRPATKAPAKKATARRGRK"

variation 3305569..3305595
/note="supplemented from AL2122/97"

CDS complement(3306281..3306877)
/codon_start=1
/gene="leuD"
/note="gene_3043"
/transl_table=11
/translation="MEAFHTHSGIGVPLRRSNVDTDQIIPAVFLKRVTRTGFEDGLFA
GWRSDPAFVLNLSPFDRGSVLVAGPDFGTGSSREHAVWALMDYGFRVVISSRFGDIFR
GNAGKAGLLAAEVAQDDVELLWKLIEQSPGLEITANLQDRIITAATVVLPFKIDDHSA
WRLLEGLDDIALTLRKLDEIEAFEGACAYWKPRTLPAP"

CDS complement(3306902..3308323)
/codon_start=1
/note="gene_3044"
/transl_table=11
/translation="MALQTGEPRTLAEKIWDDHIVVSGGGCAPDLIYIDLHLVHEVTS
PQAFDGLRLAGRRVRRPELTLATEDHNVPTVDIDQPIADPVSRTQVETLRRNCAEFGI
RLHSMGDIEQGIVHVVGPQLGLTQPGMTIVCGDSHTSTHGAFGALAMGIGTSEVEHVL
ATQTLPLRPFKTMAVNVDGRLPDGVSAKDIILALIAKIGTGGGQGHVIEYRGSAIESL
SMEGRMTICNMSIEAGARAGMVAPDETTYAFLRGRPHAPTGAQWDTALVYWQRLRTDV

GAVFDTEVYLDAASLSPFVTWGTNPGQGVPLAAAVPDPQLMTDDAERQAAEKALAYMD
LRPGTAMREIAVDAVFVGSCTNGRIEDLRVVAEVLRGRKVADGVRMLIVPGSMRVRAQ
AEAEGLGEIFTDAGAQWRQAGCSMCLGMNPDQLASGERCAATSNRNFEGRQGAGGRTH
LVSPAVAAATAVRGTLSSPADLN"

```
CDS             3308440..3309096
                /codon_start=1
                /note="gene_3045"
                /transl_table=11
                /translation="VLHAVAESPCGLAELCDRTDLPRATAYRLAAALEVHRLLGRGQD
                GHWRLGPAITELATHVDDPLLVACAAVLPQLRDATGESVQVYRREGTSRVCVAALEPA
                AGLRDTVPVGARLPMTAGSGAKVLLAHTDAATQAAVLPKAVFSARALAEVCRRGWAQS
                VAEREPGVASVSAPVRDGRGVVIAAISVSGPIDRMGRRPGVRWAADLLSAADALTRRL
                "
CDS             complement(3309107..3309967)
                /codon_start=1
                /note="gene_3046"
                /transl_table=11
                /translation="MCVTWAEMPKIAALIRHIEDLHARHGRSYILRAGISSLFRYIEG
                VHGERPWGTVLDAGTGVKSLQWIQTLPTERWTAVTAARSLADKTRAALGSAMRPQDRL
                LVGNWVDDSLLAGETFDTILVDYLVGAIEGFAPYWQDRVFERLRPHLADHGRLYLVGL
                EPYVQFEPETESGKIIWEIGRVRDACLLLAGERPYREFPLDWMLGRLGLAGFRILEAR
                RFPIRYRARYVNGQLNMCLARIERFSSNGLGMAMRAYVEELRARALQLNERQDGLWHG
                NDYVIAVEPM"
CDS             3310230..3310721
                /codon_start=1
                /note="gene_3047"
                /transl_table=11
                /translation="MGTKQRADIVMSEAEIADFVNSSRTGTLATIGPDGQPHLTAMWY
                AVIDGEIWLETKAKSQKAVNLRRDPRVSFLLEDGDTYDTLRGVSFEGVTEIVEEPEAL
                HRVGVSVWERYTGPYTDECKPMVDQMMNKRVGVRIVARRTRSWDHRKLGLPHMSVGGS
                TAP"
tRNA            complement(3310792..3310867)
                /note="identified by tRNAscan-SE; putative"
tRNA            complement(3310907..3310978)
                /note="identified by tRNAscan-SE; putative"
CDS             complement(3311053..3312525)
                /codon_start=1
                /gene="gltX"
                /note="gene_3048"
                /transl_table=11
                /translation="VTATETVRVRFCPSPTGTPHVGLVRTALFNWAYARHTGGTFVFR
                IEDTDAQRDSEESYLALLDALRWLGLDWDEGPEVGGPYGPYRQSQRAEIYRDVLARLL
                AAGEAYHAFSTPEEVEARHVAAGRNPKLGYDNFDRHLTDAQRAAYLAEGRQPVVRLRM
                PDDDLAWNDLVRGPVTFAAGSVPDFALTRASGDPLYTLVNPCDDALMKITHVLRGEDL
                LPSTPRQLALHQALIRIGVAERIPKFAHLPTVLGEGTKKLSKRDPQSNLFAHRDRGFI
                PEGLLNYLALLGWSIADDHDLFGLDEMVAAFDVADVNSSPARFDQKKADALNAEHIRM
                LDVGDFTVRLRDHLDTHGHHIALDEAAFAAAAELVQTRIVVLGDAWELLKFFNDDQYV
                IDPKAAAKELGPDGAAVLDAALAALTSATDWTAPLIEAALKDALIEGLALKPRKAFSP
                IRVAATGTTVSPPLFESLELLGRDRSMQRLRAARQLVGHA"
CDS             complement(3312522..3313367)
                /codon_start=1
```

/note="gene_3049"
/transl_table=11
/translation="VPARIVARQGPAKLIQMRIGRIASPDGVAFASIDGELGEPSEMT
AREIAEHPFGTPTFTGRSWPLADVRLLAPILASKVVCVGKNYADHIAEMGGRPPADPV
IFLKPNTAIIGPNTPIRLPANASPVHFEGELAIVIGRACKDVPAAQAVDNILGYTIGN
DVSARDQQQSDGQWTRAKGHDTFCPVGPWIVTDLAPFDPADLELRTVVNGDVKQHART
SLMIHDIGAIVEWISAIMTLLPGDLILTGTPAGVGPIEDGDTVSITIEGIGTLTNPVV
RKGKP"

variation 3313382..3313437
/note="supplemented from AL2122/97"

CDS complement(3314190..3314405)
/codon_start=1
/gene="CH063_05233"
/note="gene_3050"
/transl_table=11
/translation="MRDPTWSDQRPTATRPSAPSSCDMVTKTPACATDQPWWLISQTN
MNVTVTVCGIISNADAACIRHRIDDPR"

CDS complement(3314762..3315772)
/codon_start=1
/gene="leuB"
/note="gene_3051"
/transl_table=11
/translation="MKLAIIAGDGIGPEVTAEAVKVLDAVVPGVQKTSYDLGARRFHA
TGEVLPDSVVAELRNHDAILLGAIGDPSVPSGVLERGLLLRLRFELDHHINLRPARLY
PGVASPLSGNPGIDFVVVREGTEGPYTGNGGAIRVGTPNEVATEVSVNTAFGVRRVVA
DAFERARRRRKHLTLVHKTNVLTLAGGLWLRTVDEVGECYPDVEVAYQHVDAATIHMI
TDPGRFDVIVTDNLFGDIITDLAAAVCGGIGLAASGNIDATRANPSMFEPVHGSAPDI
AGQGIADPTAAIMSVALLLSHLGEHDAAARVDRAVEAHLATRGSERLATSDVGERIAA
AL"

CDS complement(3315787..3317373)
/codon_start=1
/note="gene_3052"
/transl_table=11
/translation="VSLPVVLIADKLAPSTVAALGDQVEVRWVDGPDRDKLLAAVPEA
DALLVRSATTVDAEVLAAAPKLKIVARAGVGLDNVDVDAATARGVLVVNAPTSNIHSA
AEHALALLLAASRQIPAADASLREHTWKRSSFSGTEIFGKTVGVVGLGRIGQLVAQRI
AAFGAYVVAYDPYVSPARAAQLGIELLSLDDLLARADFISVHLPKTPETAGLIDKEAL
AKTKPGVIIVNAARGGLVDEAALADAITGGHVRAAGLDVFATEPCTDSPLFELAQVVV
TPHLGASTAEAQDRAGTDVAESVRLALAGEFVPDAVNVGGGVVNEEVAPWLDLVRKLG
VLAGVLSDELPVSLSVQVRGELAAEEVEVLRLSALRGLFSAVIEDAVTFVNAPALAAE
RGVTAEICKASESPNHRSVVDVRAVGADGSVVTVSGTLYGPQLSQKIVQINGRHFDLR
AQGINLIIHYVDRPGALGKIGTLLGTAGVNIQAAQLSEDAEGPGATILLRLDQDVPDD
VRTAIAAAVDAYKLEVVDLS"

CDS 3317403..3318845
/codon_start=1
/note="gene_3053"
/transl_table=11
/translation="VDVTVVGSGPNGLATAVICARAGLNVQVVEAQATFGGGARSAAD
FEFPEVLHDVCSAVHPLALASPFFAEFDLPARGVTLTVPDIAYANPLPGRPAAIAYHD
LAHTSAKLDDGASWRRLLGPLVAHSETVVEFMLSDKRSLPTALGSVLRLGLRMLAQGT
PAWRSLAGEDARALFTGVAAHAISPLPSLVSAGAGLMLATLAHSVGWPIPVGGTQAIA

DALIADLRAHGGRLAAGVEITEPQRSVVVFDTAPTALLRVYRDKLPHRYAKALRRYRF
RAGIAKVDFVLSDEIPWSDPRLRRAATLHLGGTRDQMARAEADVAAGRHADWPMVLAA
CPHVADPGRIDETGRRPFWTYAHVPSGSTLDATETVTSVLERFAPGFRDIVVAARAVP
AARMADHNANYVGGDITVGANSTWRAIAGPTPRLNPWRTPIPKVYLCSAATPPGAGVH
GMCGWYAARTLLRTEFGITRMPPLGHELRP"
CDS complement(3319037..3319282)
/codon_start=1
/note="gene_3054"
/transl_table=11
/translation="VENRPSVLSPASLIDMRVTRVTIDPDVMGGMPCIRGLRIPVATV
VAMVADQMTSTEILAELPELEEADVTEALRYAAVGSQ"
CDS 3319167..3319580
/codon_start=1
/note="gene_3055"
/transl_table=11
/translation="MRKPRMHGMPPITSGSMVTRVTRMSIRLAGDSTLGRFSTSRLGL
SSAKSKPEGDFGTACGAVSGGDAGVVALAEGVDDGQSKPGAAGGARGVGGFRESRADC
GEQFGVASWTPQGEFEFGGQEAKGVRSSWPASLTN"
CDS 3319891..3320871
/codon_start=1
/note="gene_3056"
/transl_table=11
/translation="MEGTRMAGAKHAGRIVAITTAAAVILAACSSGSKGGAGSGHAGK
ARSAVTTTDADWKPVADALGRSGKLGDNNTAYRINLPRNDLHITSYGVDIKPGLSLGG
YAAFARYDNNETLLMGDLVITEEELPKVTDALQAHGIAQTALHKHLLQQDPPVWWTHI
HGMGDAARLAQGLKAALDATTIGPPTPPPARQPPVDIDVAGVDQALGRKGTQDGGLLK
YSIPRKDTIIEDGHVLPAVSLNLTTVINFQPVGRGRAAINGDFILIAPEVQEVIRAMR
AGNITIVELHNHGLTEEPRLFYMHYWAVDDAVTLARALRPAMDATNLQSS"
CDS 3320916..3321575
/codon_start=1
/note="gene_3057"
/transl_table=11
/translation="MAVHGFLLERVSVVRDEATVLRQVSAHFPAGRCSAVRGASGSGK
TTLLRLLNRLIDPTSGKVWLDGVPLTDLDVLVLRRRVGLVAQAPVVLTDAVLNEVRVG
RPDLPEGRVTELLARLCLGQSAREAFLPHQRSALRTALIPAIDSTKVVGLISLPGAMS
GLILAGVDPLTAIRYQIVVMYLLLAATAVAALTCARLAERALFDRAHRLVSLPAATRR
A"
CDS complement(3321889..3322902)
/codon_start=1
/gene="ilvC"
/note="gene_3058"
/transl_table=11
/translation="VALEMFYDDDADLSIIQGRKVGVIGYGSQGHAHSLSLRDSGVQV
RVGLKQGSRSRPKVEEQGLDVDTPAEVAKWADVVMVLAPDTAQAEIFAGDIEPNLKPG
DALFFGHGLNVHFGLIKPPADVAVAMVAPKGPGHLVRRQFVDGKGVPCLVAVEQDPRG
DGLALALSYAKAIGGTRAGVIKTTFKDETETDLFGEQTVLCGGTEELVKAGFEVMVEA
GYPAELAYFEVLHELKLIVDLMYEGGLARMYYSVSDTAEFGGYLSGPRVIDAGTKERM
RDILREIQDGSFVHKLVADVEGGNKQLEELRRQNAEHPIEVVGKKLRDLMSWVDRPIT
ETA"
CDS complement(3322928..3323434)
/codon_start=1

/gene="ilvH"
/note="gene_3059"
/transl_table=11
/translation="MSPKTHTLSVLVEDKPGVLARVAALFSRRGFNIESLAVGATECK
DRSRMTIVVSAEDTPLEQITKQLNKLINVIKIVEQDDEHSVSRELALIKVQADAGSRS
QVIEAVNLFRANVIDVSPESLTVEATGNRGKLEALLRVLEPFGIREIAQSGMVSLSRG
PRGIGTAK"
CDS complement(3323434..3325290)
/codon_start=1
/note="gene_3060"
/transl_table=11
/translation="VSAPTKPHSPTFKPEPHSAANEPKHPAARPKHVALQQLTGAQAV
IRSLEELGVDVIFGIPGGAVLPVYDPLFDSKKLRHVLVRHEQGAGHAASGYAHVTGRV
GVCMATSGPGATNLVTPLADAQMDSIPVVAITGQVGRGLIGTDAFQEADISGITMPIT
KHNFLVRSGDDIPRVLAEAFHIAASGRPGAVLVDIPKDVLQGQCTFSWPPRMELPGYK
PNTKPHSRQVREAAKLIAAARKPVLYVGGGVIRGEATEQLRELAELTGIPVVTTLMAR
GAFPDSHRQNLGMPGMHGTVAAVAALQRSDLLIALGTRFDDRVTGKLDSFAPEAKVIH
ADIDPAEIGKNRHADVPIVGDVKAVITELIAMLRHHHIPGTIEMADWWAYLNGVRKTY
PLSYGPQSDGSLSPEYVIEKLGEIAGPDAVFVAGVGQHQMWAAQFIRYEKPRSWLNSG
GLGTMGFAIPAAMGAKIALPGTEVWAIDGDGCFQMTNQELATCAVEGIPVKVALINNG
NLGMVRQWQSLFYAERYSQTDLATHSHRIPDFVKLAEALGCVGLRCEREEDVVDVINQ
ARAINDCPVVIDFIVGADAQVWPMVAAGTSNDEIQAARGIRPLFDDITEGHA"
CDS 3325613..3325990
/codon_start=1
/note="gene_3061"
/transl_table=11
/translation="VATTSPVVIKVSPMAHFAVGFLTLGLLVPVLTWPVSAPLLVIPV
ALSASIIRLRTLADERGVTVRTLVGSRAVRWDDIDGLRFHRGSWARATLKDGTELRLP
AVTFATLPHLTEASSGRVPNPYR"
CDS complement(3325997..3326782)
/codon_start=1
/note="gene_3062"
/transl_table=11
/translation="MPGRPVSASLVDPEDDLTPARYAGDFGSGTTTVIPPYDAASSGV
GNSGYSLIEAAEPLPYVQPQPGRQVPAGSAGIDMDDDERVRAAGRRGTQNLGLLILRV
GLGAVLIAHGLQKLFGWWDGQGLAGFQNSLSDIGYQHAEILAYVSAGGEIVAGVLLVL
GLFTPLAAAGALAFLINGLLAGISAQHSRPVAYFLQDGHEYQITLVVMAVAVILSGPG
RYGLDAARGWAHRPFIGSFVALLGGIAAGIAVWVLLNGANPLA"
CDS complement(3327108..3327353)
/codon_start=1
/gene="PMI09_03997"
/note="gene_3063"
/transl_table=11
/translation="MTVFTFGSALIEIESSTAPVMRSATSAVLLSMPMIKPLVLSRQPC
ITPGAGHSLGKGTGGSGGGGGGVELGWGRSSGSVVNG"
CDS 3327256..3328134
/codon_start=1
/note="gene_3064"
/transl_table=11
/translation="MGIDSKTALVAERITGAVEEISISAEPKVKTVIPVDPAGDGGLM
DIVLSPTYSQDRLMYAYISTPTDNRVVRVADGDIPKDILTGIPKGAAGNTGALIFTSP

TTLVVMTGDAGDPALAADPQSLAGKVLRIEQPTTIDQTPPTTALSGIGSGGGLCIDPV
DGSLYVADRTPTADRLQRITKNSEVSTVWTWPDKPGVAGCAAMDGTVLVNLINTKLTV
AVRLAPSTGAVTGEPDVVRKDTHAHAWALRMSPDGNVWGATVNKTAGDAEKLDDVVFP
LFPQGGGFPRNNDDKT"
CDS complement(3328140..3328718)
/codon_start=1
/note="gene_3065"
/transl_table=11
/translation="VIDESFDELMGMLDHPVFVVTTQADGHPAGCLVSFATQTSVQPP
SFMVGLPRSTGTSEVASRSEHLAVHVLSQRQHVLAELFGSQTEEEVNKFARCSWRAGP
CGMPILDDAAAWFIGRTASRSDVGDYVAYLLEPVSVWAPECSEDLLYLSDLDFDVDDI
DPGKEASPRFYERERGDETRRYGVVRFTLDVP"
CDS 3328948..3329571
/codon_start=1
/note="gene_3066"
/transl_table=11
/translation="MLTVVAVIGILECGLVLHMPDNDLWYCGPWTLWVMAGRGVASGA
GVWRGDRVATPLAVAITAAGLVSGARIGPGAAAKRDPQLAQWNEIRSHYQEIAEWIDH
DTATAHPAVAATQISAAGSFGRANMVDYLGLLDSRADETVRRDEFSRWLSAKPDYLVT
TEQSVDAATIALPEFRHAYDRAATIGTLNVYRRNSPDGDEPLPADGN"
CDS complement(3329568..3331097)
/codon_start=1
/gene="gatB"
/note="gene_3067"
/transl_table=11
/translation="MTVAAGAAKAAGAELLDYDEVVARFQPVLGLEVHVELSTATKMF
CGCTTTFGGEPNTQVCPVCLGLPGSLPVLNRAAVESAIRIGLALNCEIVPWCRFARKN
YFYPDMPKNYQISQYDEPIAINGYLDAPLEDGTTWRVEIERAHMEEDTGKLTHIGSET
GRIHGATGSLIDYNRAGVPLIEIVTKPIVGAGARAPQIARSYVTALRDLLRALDVSDV
RMDQGSMRCDANVSLKPAGTTEFGTRTETKNVNSLKSVEVAVRYEMQRQGAILASGGR
ITQETRHFHEAGYTSAGRTKETAEDYRYFPEPDLEPVAPSRELVERLRQTIPELPWLS
RRRIQQEWGVSDEVMRDLVNAGAVELVAATVEHGASSEAARAWWGNFLAQKANEAGIG
LDELAITPAQVAAVVALVDEGKLSNSLARQVVEGVLAGEGEPEQVMTARGLALVRDDS
LTQAAVDEALAANPDVADKIRGGKVAAAGAIVGAVMKATRGQADAARVRELVLEACGQ
G"
CDS complement(3331127..3332158)
/codon_start=1
/note="gene_3068"
/transl_table=11
/translation="MRIGVLTGGGDCPGLNAVIRAVVRTCHARYGSSVVGFQNGFRGL
LENRRVQLHNDDRNDRLLAKGGTMLGTARVHPDKLRAGLPQIMQTLDDNGIDVLIPIG
GEGTLTAASWLSEENVPVVGVPKTIDNDIDCTDVTFGHDTALTVATEAIDRLHSTAES
HERVMLVEVMGRHAGWIALNAGLASGAHMTLIPEQPFDIEEVCRLVKGRFQRGDSHFI
CVVAEGAKPAPGTIMLREGGLDEFGHERFTGVAAQLAVEVEKRINKDVRVTVLGHIQR
GGTPTAYDRVLATRFGVNAADAAHAGEYGQMVTLRGQDIGRVPLADAVRKLKLVPQSR
YDDAAAFFG"
CDS complement(3332254..3333738)
/codon_start=1
/gene="gatA"
/note="gene_3069"
/transl_table=11

```
                     /translation="VTDIIRSDAATLAAKIAIKEVSSTEITRACLDQIEATDETYHAF
                     LHVAADEALAAAAAVDKQVAAGEPLPSALAGVPLALKDVFTTSDMPTTCGSKILEGWR
                     SPYDATLTARLRAAGIPILGKTNMDEFAMGSSTENSAYGPTRNPWNLDRVPGGSGGGS
                     AAALAAFQAPLAIGSDTGGSIRQPAALTATVGVKPTYGTVSRYGLVACASSLDQGGPC
                     ARTVLDTALLHQVIAGHDPRDSTSVDAEVPDVVGAARAGAVGDLRGVRVGVVRQLHGG
                     EGYQPGVLASFEAAVEQLTALGAEVSEVDCPHFDHALAAYYLILPSEVSSNLARFDAM
                     RYGLRVGDDGTRSAEEVMAMTRAAGFGPEVKRRIMIGTYALSAGYYDAYYNQAQKVRT
                     LIARDLDAAYRSVDVLVSPTTPTTAFRLGEKVDDPLAMYLFDLCTLPLNLAGHCGMSV
                     PSGLSPDDGLPVGLQIMAPALADDRLYRVGAAYEAARGPLLSAI"
CDS                  complement(3333735..3334034)
                     /codon_start=1
                     /gene="gatC"
                     /note="gene_3070"
                     /transl_table=11
                     /translation="VSQISRDEVAHLARLARLALTETELDSFAGQLDAILTHVSQIQA
                     VDVTGVQATDNPLKDVNVTRPDETVPCLTQRQVLDQAPDAVDGRFAVPQILGDEQ"
CDS                  3334110..3334775
                     /codon_start=1
                     /note="gene_3071"
                     /transl_table=11
                     /translation="VLAVRSYLLRIELADRPGSLGSLAVALGSVGADILSLDVVERGN
                     GYAIDDLVVELPPGAMPDTLITAAEALNGVRVDSVRPHTGLLEAHRELELLDHVAAAE
                     GATARLQVLVNEAPRVLRVSWCTVLRSSGGELHRLAGSPGAPETRANSAPWLPIERAA
                     ALDGGADWVPQAWRDMDTTMVAAPLGDTHTAVVLGRPGPEFRPSEVARLGYLAGIVAT
                     MLR"
CDS                  complement(3334849..3336924)
                     /codon_start=1
                     /gene="ligA"
                     /note="gene_3072"
                     /transl_table=11
                     /translation="VSSPDADQTAPEVLRQWQALAEEVREHQFRYYVRDAPIISDAEF
                     DELLRRLEALEEQHPELRTPDSPTQLVGGAGFATDFEPVDHLERMLSLDNAFTADELA
                     AWAGRIHAEVGDAAHYLCELKIDGVALSLVYREGRLTRASTRGDGRTGEDVTLNARTI
                     ADVPERLTPGDDYPVPEVLEVRGEVFFRLDDFQALNASLVEEGKAPFANPRNSAAGSL
                     RQKDPAVTARRRLRMICHGLGHVEGFRPATLHQAYLALRAWGLPVSEHTTLATDLAGV
                     RERIDYWGEHRHEVDHEIDGVVVKVDEVALQRRLGSTSRAPRWAIAYKYPPEEAQTKL
                     LDIRVNVGRTGRITPFAFMTPVKVAGSTVGQATLHNASEIKRKGVLIGDTVVIRKAGD
                     VIPEVLGPVVELRDGSEREFIMPTTCPECGSPLAPEKEGDADIRCPNARGCPGQLRER
                     VFHVASRNGLDIEVLGYEAGVALLQAKVIADEGELFALTERDLLRTDLFRTKAGELSA
                     NGKRLLVNLDKAKAAPLWRVLVALSIRHVGPTAARALATEFGSLDAIAAASTDQLAAV
                     EGVGPTIAAAVTEWFAVDWHREIVDKWRAAGVRMVDERDESVPRTLAGLTIVVTGSLT
                     GFSRDDAKEAIVARGGKAAGSVSKKTNYVVAGDSPGSKYDKAVELGVPILDEDGFRRL
                     LADGPASRT"
CDS                  complement(3336955..3337968)
                     /codon_start=1
                     /note="gene_3073"
                     /transl_table=11
                     /translation="VSVFATATGIGSWPGTAAREAAQVVVGELAGALAYLTELPARGV
                     GADMLGRAGGLLVDVAIDTVPRGYRIAARPGAVTRRAASLLDEDMDALEEAWETAGLR
                     GCGRAVKVQAPGPVTLVAGLELANGHRAITDPGAVRDLAASLAEGVAAHRAALARRLD
                     TPVVVQFDEPSLPAALGGRLTGVTALSPVAPLDETVAEALLDTCIAAVDADVALHSCS
```

PDLPWDLLQRSRISAVSVDASTLQAADLDAVAAFVESGRTVVLGLVPVTAPERAPSME
EVAAAAVAVTDRLGVPRSALRDRLGVSPACGLANATGQWARTAVGLARDVAEAFARDP
EAI"
CDS 3338062..3338691
/codon_start=1
/note="gene_3074"
/transl_table=11
/translation="MVGLTRPLLLCGATLLIAACTRVVGGTASATFGGDRQGMLDVAT
ILLDQSRMQAITGSGDDLTIIPTMDTTYPVDVDDFAQPIPRECRFIYAETAVFGSEIE
AFHKTTFQDRPDGSLISEAAAAYRDAGTARRAFDTLAVTVHDCAASPAGWLFVSRWTA
GGNSLHIRAGDCGRDYRVLSAALLEVTFCGFPESVSDIVMTNIAANVPG"
CDS complement(3338794..3339159)
/codon_start=1
/note="gene_3075"
/transl_table=11
/translation="VVSQSMYSYPAMTANVGDMAGYTGTTQSLGADIASERTAPSRAC
QGDLGMSHQDWQAQWNQAMEALARAYRRCRRALRQIGVLERPVGDSSDCGTIRVGSFR
GRWLDPRHAGPATAADAGD"
CDS complement(3339243..3340580)
/codon_start=1
/note="gene_3076"
/transl_table=11
/translation="LIPLAGDPVSSHRTPVWLASPPEVHSALLSAGPGPGSLQAAAAG
WSALSAEYAAVAQELSAVVAAVGAGVWQGPSAELFVAAYVPYVAWLVQASADSAAAAG
EHEAAAAGYVCALAEMPTLPELAANHLTHAVLVATNFFGINTIPIALNEADYVRMWVQ
AATVMSAYEAVVGAALVATPHTGPAPVIVKPGANEASNAVAAATITPFPWHEIVQFLE
ETFAAYDQYLSALLSELPAVAWVWFQLFVDILGFNIIGFIITLASNAQLLTEFAINAS
YVAVGLLYAIAGVIDIVVEWVIGNLFGVVPLLGGPLLGALAAAVVPGVAGLAGVAGLA
ALPAVGAAAGAPAALVGSVAPVSGGVVSPQARLVSAVEPAPASTSVSVLASDRGAGAL
GFVGTAGKESVGQPAGLTVLADEFGDGAPVPMLPGSWGPDLVGVAGDGGLVSV"
CDS complement(3340959..3341249)
/codon_start=1
/note="gene_3077"
/transl_table=11
/translation="MSQIMYNYPAMMAHAGDMAGYAGTLQSLGADIASEQAVLSSAWQ
GDTGITYQGWQTQWNQALEDLVRAYQSMSGTHESNTMAMLARDGAEAAKWGG"
CDS complement(3341284..3341577)
/codon_start=1
/note="gene_3078"
/transl_table=11
/translation="MSLLDAHIPQLIASHTAFAAKAGLMRHTIGQAEQQAMSAQAFHQ
GESAAAFQGAHARFVAAAAKVNTLLDIAQANLGEAAGTYVAADAAAASSYTGF"
CDS complement(3341624..3342931)
/codon_start=1
/note="gene_3079"
/transl_table=11
/translation="VTAPVWLASPPEVHSALLSAGPGPGSLQAAAAGWSALSAEYAAV
AQELSAVVAAVGAGVWQGPSAELFVAAYVPYVAWLVQASADSAAAAGEHEAAAAGYVC
ALAEMPTLPELAANHLTHAVLVATNFFGINTIPIALNEADYVRMWVQAATVMSAYEAV
VGAALVATPHTGPAPVIVKPGANEASNAVAAATITPFPFGELAKFLEMAAQAFTEVGE
LIMKSAEAWAVGFVELITGLVNFEPWLVLTGMIDMFFATVGFALGVFVLVPLLEFAVV

```
                     LELAILSIGWIISNIFGAIPVLAGPLLGALAAAVVPGVAGVTGLAGLAAVPAVGAAAG
                     APAALVGSVAPVSGGVVSPQARLVSAVEPAPASTSVSVLASDRGAGALGFVGTAGKES
                     VGQPAGLTVLADEFGDGAPVPMLPGSWGPDLVGVAGDGGLVSV"
CDS                  complement(3342928..3343245)
                     /codon_start=1
                     /note="gene_3080"
                     /transl_table=11
                     /translation="VVTLRVVPEGLAAASAAVEALTARLAAAHAGAAPAITAVVAPAA
                     DPVSLQSAVGFSALGSEHAAIAGEGVEELGRSGVAVGESGIGYAAGDAVAAATYLVSG
                     GSL"
CDS                  complement(3343624..3344871)
                     /codon_start=1
                     /note="gene_3081"
                     /transl_table=11
                     /translation="MTSSHLIDTEQLLADQLAQASPDLLRGLLSTFIAALMGAEADAL
                     CGAGYRERSDERSNQRNGYRHRDFDTRAATIDVAIPKLRQGSYFPDWLLQRRKRAERA
                     LTSVVATCYLLGVSTRRMERLVETLGVTKLSKSQVSIMAKELDEAVEAFRTRPLDAGP
                     YTFLAADALVLKVREAGRVVGVHTLIATGVNAEGYREILGIQVTSAEDGAGWLAFFRD
                     LVARGLSGVALVTSDAHAGLVAAIGATLPAAAWQRCRTHYAANLMAATPKPSWPWVRT
                     LLHSIYDQPDAESVVAQYDRVLDALTDKLPAVAEHLDTARTDLLAFTAFPKQIWRQIW
                     SNNPQERLNREVRRRTDVVGIFPDRASIIRLVGAVLAEQHDEWIEGRRYLGLEVLTRA
                     RAALTSTEEPAKQQTTNTPALTT"
CDS                  complement(3345034..3346137)
                     /codon_start=1
                     /note="gene_3082"
                     /transl_table=11
                     /translation="MKVLAAMSGGVDSSVAAARMVDAGHEVVGVHMALSTAPGTLRTG
                     SRGCCSKEDAADARRVADVLGIPFYVWDFAEKFKEDVINDFVSSYARGETPNPCVRCN
                     QQIKFAALSARAVALGFDTVATGHYARLSGGRLRRAVDRDKDQSYVLAVLTAQQLRHA
                     AFPIGDTPKRQIRAEAARRGLAVANKPDSHDICFIPSGNTKAFLGERIGVRRGVVVDA
                     DGVVLASHDGVHGFTIGQRRGLGIAGPGPNGRPRYVTAIDADTATVHVGDVTDLDVQT
                     LTGRAPVFTAGAAPSGPVDCVVQVRAHGETVSAVAELIGDALFVQLHAPLRGVARGQT
                     LVLYRPDPAGDEVLGSATIAGASGLSTGGNPGA"
CDS                  complement(3346134..3347315)
                     /codon_start=1
                     /note="gene_3083"
                     /transl_table=11
                     /translation="MAYLDHAATTPMHPAAIEAMAAVQRTIGNASSLHTSGRSARRRI
                     EEARELIADKLGARPSEVIFTAGGTESDNLAVKGIYWARRDAEPHRRRIVTTEVEHHA
                     VLDSVNWLVEHEGAHVTWLPTAADGSVSATALREALQSHDDVALVSVMWANNEVGTIL
                     PIAEMSVVAMEFGVPMHSDAIQAVGQLPLDFGASGLSAMSVAGHKFGGPPGVGALLLR
                     RDVTCVPLMHGGGQERDIRSGTPDVASAVGMATAAQIAVDGLEENSARLRLLRDRLVE
                     GVLAEIDDVCLNGADDPMRLAGNAHFTFRGCEGDALLMLLDANGIECSTGSACTAGVA
                     QPSHVLIAMGVDAASARGSLRLSLGHTSVEADVDAALEVLPGAVARARRAALAAAGAS
                     R"
CDS                  complement(3347412..3348326)
                     /codon_start=1
                     /note="gene_3084"
                     /transl_table=11
                     /translation="MSAPAVTEHSWLPRATCGVSCVSVGDAAQVRRPLVVLRVALRVM
                     LALLLVPGVPLVVMPLPGRTRVQRIYCRLVLRLFGVRITVSGSPVRNLRGVLVVSGHV
```

SWLDVFCIGSVLPGSFVARADMFTGRTIGIVARILKIIPIERASLRRLPGVVDTIARR
LRAGQTVVAFPEGTTWCGRPGDDAGRPAARAGAGCSHRGCGAFYPAMFQAAIDAGRPV
QPLRLTYHHVDGTVSTAPAFVGDDTLVRSVCRLLTVRRTLAWVRVESLQLPGTDRRNL
ARRCQSAVLAGALGQSGQRPGRRHVPAT"
CDS complement(3348323..3349168)
/codon_start=1
/note="gene_3085"
/transl_table=11
/translation="MSIASVLIPSDKPHGVATGSSTGPRYSLLLSTDPSMVEAAQRLR
YDVFSTTPGFALPAAADTRRDGDRFDEYCDHLLVRDDDTGELVGCYRMLAPAGAIAAG
GLYTATEFDVCAFDPLRPSLVEMGRAVVREGHRNGGVVLLMWAGILAYLDRYGYDYVT
GCVSVPIGGDGETPGSRLRGVRDFILNRHAAPPQCQVYPYRPVRVDGRSLDDILPPPR
PAVPPLMRGYLRLGARACGEPAHDPDFGVGDFCLLLDKDHADTRYLRRLRSVAAASEM
VNDAR"
CDS complement(3349324..3350280)
/codon_start=1
/note="gene_3086"
/transl_table=11
/translation="MAEVLVLVEHAEGALKKVSAELITAARALGEPAAVVVGVPGTAA
PLVDGLKAAGAAKIYVAESDLVDKYLITPAVDVLAGLAESSAPAGVLIAATADGKEIA
GRLAARIGSGLLVDVVDVREGGVGVHSIFGGAFTVEAQANGDTPVITVRAGAVEAEPA
AGAGEQVSVEVPAAAENAARITAREPAVAGDRPELTEATIVVAGGRGVGSAENFSVVE
ALADSLGAAVGASRAAVDSGYYPGQFQVGQTGKTVSPQLYIALGISGAIQHRAGMQTS
KTIVAVNKDEEAPIFEIADYGVVGDLFKVAPQLTEVIKARKG"
CDS complement(3350319..3351119)
/codon_start=1
/note="gene_3087"
/transl_table=11
/translation="MTNIVVLIKQVPDTWSERKLTDGDFTLDREAADAVLDEINERAV
EEALQIREKEAADGIEGSVTVLTAGPERATEAIRKALSMGADKAVHLKDDGMHGSDVI
QTGWALARALGTIEGTELVIAGNESTDGVGGAVPAIIAEYLGLPQLTHLRKVSIEGGK
ITGERETDEGVFTLEATLPAVISVNEKINEPRFPSFKGIMAAKKKEVTVLTLAEIGVE
SDEVGLANAGSTVLASTPKPAKTAGEKVTDEGEGGNQIVQYLVAQKII"
CDS 3351350..3352174
/codon_start=1
/note="gene_3088"
/transl_table=11
/translation="MCAFVPHVPRHSRGDNPPSASTASPAVLTLTGERTIPDLDIENY
WFRRHQVVYQRLAPRCTARDVLEAGCGEGYGADLIACVARQVIAVDYDETAVAHVRSR
YPRVEVMQANLAELPLPDASVDVVVNFQVIEHLWDQARFVRECARVLRGSGLLMVSTP
NRITFSPGRDTPINPFHTRELNADELTSLLIDAGFVDVAMCGLFHGPRLRDMDARHGG
SIIDAQIMRAVAGAPWPPELAADVAAVTTADFEMVAAGHDRDIDDSLDLIAIAVRP"
CDS 3352171..3353751
/codon_start=1
/note="gene_3089"
/transl_table=11
/translation="LNTSASPVPGLFTLVLHTHLPWLAHHGRWPVGEEWLYQSWAAAY
LPLLQVLAALADENRHRLITLGMTPVVNAQLDDPYCLNGVHHWLANWQLRAEEAASVR
YARQSKSADYPSCTPEALRAFGIRECADAARALDNFATRWRHGGSPLLRGLIDAGTVE
LLGGPLAHPFQPLLAPRLREFALREGLADAQLRLAHRPKGIWAPECAYAPGMEVDYAT
AGVSHFMVDGPSLHGDTALGRPVGKTDVVAFGRDLQVSYRVWSPKSGYPGHAAYRDFH

TYDHLTGLKPARVTGRNVPSEQKAPYDPERADRAVDVHVADFVDVVRNRLLSESERIG
RPAHVIAAFDTELFGHWWYEGPTWLQRVLRALPAAGVRVGTLSDAIADGFVGDPVELP
PSSWGSGKDWQVWSGAKVADLVQLNSEVVDTALTTIDKALAQTASLDGPLPRDHVADQ
ILRETLLTVSSDWPFMVSKDSAADYARYRAHLHAHATREIAGALAAGRRDTARRLAEG
WNRADGLFGALDARRLPK"

CDS 3353795..3355027
/codon_start=1
/note="gene_3090"
/transl_table=11
/translation="MVSWEYPPVVIGGLGRHVHHLSTALAAAGHDVVVLSRCPSGTDP
STHPSSDEVTEGVRVIAAAQDPHEFTFGNDMMAWTLAMGHAMIRAGLRLKKLGTDRSW
RPDVVHAHDWLVAHPAIALAQFYDVPMVSTIHATEAGRHSGWVSGALSRQVHAVESWL
VRESDSLITCSASMNDEITELFGPGLAEITVIRNGIDAARWPFAARRPRTGPAELLYV
GRLEYEKGVHDAIAALPRLRRTHPGTTLTIAGEGTQQDWLIDQARKHRVLRATRFVGH
LDHTELLALLHRADAAVLPSHYEPFGLVALEAAAAGTPLVTSNIGGLGEAVINGQTGV
SCAPRDVAGLAAAVRSVLDDPAAAQRRARAARQRLTSDFDWPTVATATAQVYLAAKRG
ERQPQPRLPIVEHALPDR"

CDS 3355061..3355450
/codon_start=1
/note="gene_3091"
/transl_table=11
/translation="VKPQDQGLHFPYRYDLRLAPMWLPFRWPGSQGVTVTEDGRFVAR
YGPFRVEAPLSSVRDAHITGPYRWWTAVGPRLSMVDDGLTFGTNAAAGVCIHFEPRIH
RVIGLRDHSALTVTVADPEGLVAALSS"

CDS 3355629..3356177
/codon_start=1
/note="gene_3092"
/transl_table=11
/translation="MAHSIVRTLLASGAATALIAIPTACSFSIGTSHSHSVSKAEVAR
QITAKMTDAAGNKPESVTCPSDLPAEVGAELNCEMKIKDRTFNVNVTVTSVDGSDVKF
DMVETVDKNQVANIISDKLFQRVGARPDSVTCPDNLKGVEGAKLRCRLTDGSKTYGIS
VIVTSVDAGDVNFDFKVDDHPE"

CDS complement(3356268..3357137)
/codon_start=1
/note="gene_3093"
/transl_table=11
/translation="VVWGRVPITAPAGAATGVTSRADAHSQMRRYAQTGPTAKLSSAP
MTTMWGAPLHRRWRGSRLRDPRQAKFLTLASLKWVLANRAYTPWYLVRYWRLLRFKLA
NPHIITRGMVFLGKGVEIHATPELAQLEIGRWVHIGDKNTIRAHEGSLRFGDKVVLGR
DNVINTYLDIEIGDSVLMADWCYICDFDHRMDDITLPIKDQGIIKSPVRIGPDTWIGV
KVSVLRGTTIGRGCVLGSHAVVRGAIPDYSIAVGAPAKVVKNRQLSWEASAAQRAELA
AALADIERKKAAR"

CDS 3357703..3358710
/codon_start=1
/note="gene_3094"
/transl_table=11
/translation="MEWQNDNNGRQRWCVRLVQGGGFAGPLFDGFDNLYVGQPGAIIS
FPPTQWTRWRQPVIGMPSTPRFLGHGRLLVSTHLGQLLVFDTRRGMVVGSPVDLVDGI
DPTDATRGLADCAPARPGCPVAAAPAFSSVNGTVVVSVWQPGEPAAKLVGLKYHAEQL
VREWTSDAVSAGVLASPVLSADGSTVYVNGRDHRLWALNAADGKAKWSAPLGFLAQTP
PALTPHGLIVSGGGPDTALAAFRDAGDHAEGAWRRDDVTALSTASLAGTGVGYTVISG

PNHDGTPGLSLLVFDPANGHTVNSYPLPGATGYPVGVSVGNDRRVVTATSDGQVYSFA
P"
CDS complement(3358707..3359390)
/codon_start=1
/note="gene_3095"
/transl_table=11
/translation="MRYLIATAVLVAVVLVGWPAAGAPPSCAGLGGTVQAGQICHVHA
SGPKYMLDMTFPVDYPDQQALTDYITQNRDGFVNVAQGSPLRDQPYQMDATSEQHSSG
QPPQATRSVVLKFFQDLGGAHPSTWYKAFNYNLATSQPITFDTLFVPGTTPLDSIYPI
VQRELARQTGFGAAILPSTGLDPAHYQNFAITDDSLIFYFAQGELLPSFVGACQAQVP
RSAIPPLAI"
CDS complement(3359463..3360539)
/codon_start=1
/note="gene_3096"
/transl_table=11
/translation="VRARFGARAPWLVETTLLRRRAAGKLGELCPNVGVSQWLFTDEA
LQQATAAPVARHRARRLAGRVVHDATCSIGTELAALRELAVRAVGSDIDPVRLAMARH
NLAALGMEADLCRADVLHPVTRDAVVVIDPARRSNGRRRFHLADYQPGLGPLLDRYRG
RDVVVKCAPGIDFEEVGRLGFEGEIEVISYRGGVREACLWSAGLAGSGIRRRASILDS
GEQIGDDEPDDCGVRPAGKWIVDPDGAVVRAGLVRNYGARHGLWQLDPQIAYLSGDRL
PPALRGFEVLEQLAFDERRLRQVLSALDCGAAEILVRGVAIDPDALRRRLRLRGSRPL
AVVITRIGAGSLSHVTAYVCRPSR"
CDS complement(3360674..3361687)
/codon_start=1
/note="gene_3097"
/transl_table=11
/translation="VTGAAVRLAYMTRSSNIPADATPNPHATAEQVAAARHDSKLAQV
LYHDWEAENYDEKWSISYDQRCVDYARGRFDAIVPDEVIAQLPYDRALELGCGTGFFL
LNLIQAGVARRGSVTDLSPGMVKVATRNGQALGLDIDGRVADAEGIPYDDDAFDLVVG
HAVLHHIPDVELSLREVVRVLKPGGRFVFAGEPTTVGDGYARTLSTLTWRVVTNATKL
PGLRGWRRPQGELDESSRAAALEALVDLHTFTPQDLQRIAHNAGAVEVQTATEEFTAA
MLGWPLRTFECTVPPGRLGWGWARFAFTSWKTLGWVDANVWRHVVPKGWFYNVMITGV
KPS"
CDS complement(3361668..3362453)
/codon_start=1
/gene="echA17"
/note="gene_3098"
/transl_table=11
/translation="VAAVTPTVPEFVNVVVSDGSQDAGLAMLLLSRPPTNAMTRQVYR
EVVAAANELGRRDDVAAVILYGGHEIFSAGDDMPELRTLSAQEADTAARIRQQAVDAV
AAIPKPTVAAITGYALGAGLTLALAADWRVSGDNVKFGATEILAGLIPSGDGMARLTR
AAGPSRAKELVFSGRFFDAEEALALGLIDDMVAPDDVYDAAAAWARRFLDGPPHALAA
AKAGISDVYELAPAERIAAERRRYVEVFAAGQGGGSKGDRGGR"
CDS complement(3362441..3363175)
/codon_start=1
/note="gene_3099"
/transl_table=11
/translation="MDFAAGVMVFPGGGVDDRDRDADLGRLGAWAGPPPQWWAQRFGI
EPDLAEALVCAAARETFEESGVLFAGPVDQDHSAPNSIVSDASVYGDARRALADRTLS
FADFLQREKLVLRSDLLRPWANWVTPEAELTRRYDTYFFVGALPEGQRADGENTESDR
AGWVLPADAIADFAAGRNFLLPPTWTQLDSLAGHTVADVLAVERQIVPVQPQLARNGD

NWEIEFFDSDRYNQARRSGGSTGWPL"
CDS complement(3363304..3364167)
/codon_start=1
/note="gene_3100"
/transl_table=11
/translation="MRHDSRVLDNGGPDAADPDLLIDFRNVSLRRNGRTLVGPLDWAV
ELDERWVIVGPNGAGKTSLLRIAAAAEHPSSGVAFVLGERLGRVDVSELRARVGLSSS
ALAERVPGDERVRDLVVSAGYAVLGRWRERYEAVDYHRAIDMLESLGAEHLANRTYGT
LSEGERKRVLIARALMTDPELLLLDEPAAGLDLGGREELVARLADLAADPDAPALVLV
THHVEEIPPGFSHCLLLSEARVVAAGLLPDALTAENLSTAFGQEITLEVADGRYFARR
RRSRAAHRRQS"
CDS complement(3364182..3365411)
/codon_start=1
/note="gene_3101"
/transl_table=11
/translation="MPAKVSVLITVTGMDQPGVTSALFEVLAQHGVELLNVEQVVIRG
RLTLGVLVSCPLDVADGTALRDDVASAIHGVGLDVAIERSDDLPIIRQPSTHTIFVLG
RPITAGAFSAVAREVAALGVNIDFIRGISDYPVTGLELRVSVPPGCVGPLQIALTKVA
AEEHVDVAVEDYGLAWRTKRLIVFDVDSTLVQGEVIEMLAARAGAQGQVAAITEAAMR
GELDFAESLQRRVATLAGLPATVIDDVAEQLELMPGARTTIRTLRRLGFRCGVVSGGF
RRIIEPLARELMLDFVASNELEIVDGILTGRVVGPIVDRPGKAKALRDFASQYGVPME
QTVAVGDGANDIDMLGAAGLGIAFNAKPALREVADASLSHPYLDTVLFLLGVTRGEIE
AADAGDCGVRRVEIPAD"
CDS complement(3365449..3367170)
/codon_start=1
/note="gene_3102"
/transl_table=11
/translation="LTAEAPPLGELEAIRPYPARTGPKGSLVYKLITTTDHKMIGIMY
CVACISFFFIGGLLALLMRTELAAPGLQFLSNEQFNQLFTMHGTIMLLFYATPIVFGF
ANLVLPLQIGAPDVAFPRLNAFSFWLFVFGATIGAAGFITPGGAADFGWTAYTPLTDA
IHSPGAGGDLWIMGLIVAGLGTILGAVNMITTVVCMRAPGMTMFRMPIFTWNIMVTSI
LILIAFPLLTAALFGLAADRHLGAHIYDAANGGVLLWQHLFWFFGHPEVYIIALPFFG
IVSEIFPVFSRKPIFGYTTLVYATLSIAALSVAVWAHHMFATGAVLLPFFSFMTYLIA
VPTGIKFFNWIGTMWKGQLTFETPMLFSVGFMVTFLLGGLTGVLLASPPLDFHVTDSY
FVVAHFHYVLFGTIVFATFAGIYFWFPKMTGRLLDERLGKLHFWLTFIGFHTTFLVQH
WLGDEGMPRRYADYLPTDGFQGLNVVSTIGAFILGASMFPFVWNVFKSWRYGEVVTVD
DPWGYGNSLEWATSCPPPRHNFTELPRIRSERPAFELHYPHMVERLRAEAHVGRHHDE
PAMVTSS"
CDS 3367481..3368464
/codon_start=1
/note="gene_3103"
/transl_table=11
/translation="MITPTTQIAGAGVLGNDRKPDESCARAAAAADPGPPTRPAHNAA
GVSPEMVQVPAEAQRIVVLSGDQLDALCALGLQSRIVAAALPNSSSSQPSYLGTTVHD
LPGVGTRSAPDLRAIAAAHPDLILGSQGLTPQLYPQLAAIAPTVFTAAPGADWENNLR
GVGAATARIAAVDALITGFAEHATQVGTKHDATHFQASIVQLTANTMRVYGANNFPAS
VLSAVGVDRPPSQRFTDKAYIEIGTTAADLAKSPDFSAADADIVYLSCASEAAAERAA
VILDSDPWRKLSANRDNRVFVVNDQVWQTGEGMVAARGIVDDLRWVDAPIN"
CDS 3368534..3369574
/codon_start=1
/note="gene_3104"

```
                     /transl_table=11
                     /translation="MSTVAAYAAMSATEPLTKTTITRRDPGPHDVAIDIKFAGICHSD
                     IHTVKAEWGQPNYPVVPGHEIAGVVTAVGSEVTKYRQGDRVGVGCFVDSCRECNSCTR
                     GIEQYCKPGANFTYNSIGKDGQPTQGGYSEAIVVDENYVLRIPDVLPLDVAAPLLCAG
                     ITLYSPLRHWNAGANTRVAIIGLGGLGHMGVKLGAAMGADVTVLSQSLKKMEDGLRLG
                     AKSYYATADPDTFRKLRGGFDLILNTVSANLDLGQYLNLLDVDGTLVELGIPEHPMAV
                     PAFALALMRRSLAGSNIGGIAETQEMLNFCAEHGVTPEIELIEPDYINDAYERVLASD
                     VRYRFVIDISAL"
     CDS             complement(3369563..3369937)
                     /codon_start=1
                     /note="gene_3105"
                     /transl_table=11
                     /translation="VTKTFSHPHFFRSVLRWLQVGYPEGVPGPDRVALLSLLRSTPLT
                     EEQIGEVVRHFTENGSPAVADRVIDRDEIAEFISEVTHHDAGPENIQRVAGILAAAGW
                     PLAGVDVGESESGSDRAPASQG"
     CDS             complement(3370271..3370567)
                     /codon_start=1
                     /note="gene_3106"
                     /transl_table=11
                     /translation="LIFDMGGPFDADAEAHFDEVAEAFAKLTNVDRDVGVDLEKELCM
                     TVEADDRSDALVTRRLLPRVPRCIPLAARLAPGTIGCPSFWNPIATGGASRQAL"
     CDS             complement(3370653..3371627)
                     /codon_start=1
                     /gene="nrdF2"
                     /note="gene_3107"
                     /transl_table=11
                     /translation="VTGNAKLIDRVSAINWNRLQDEKDAEVWDRLTGNFWLPEKVPVS
                     NDIPSWGTLTAGEKQLTMRVFTGLTMLDTIQGTVGAVSLIPDALTPHEEAVLTNIAFM
                     ESVHAKSYSQIFSTLCSTAEIDDAFRWSEENRNLQRKAEIVLQYYRGDEPLKRKVAST
                     LLESFLFYSGFYLPMYWSSRAKLTNTADMIRLIIRDEAVHGYYIGYKFQRGLALVDDV
                     TRAELKDYTYELLFELYDNEVEYTQDLYDEVGLTEDVKKFLRYNANKALMNLGYEALF
                     PRDETDVNPAILSALSPNADENHDFFSGSGSSYVIGKAVVTEDDDWDF"
     CDS             complement(3371758..3373332)
                     /codon_start=1
                     /note="gene_3108"
                     /transl_table=11
                     /translation="VSIADTAAKPSTPSPANQPPVRTRAVIIGTGFSGLGMAIALQKQ
                     GVDFVILEKADDVGGTWRDNTYPGCACDIPSHLYSFSFEPKADWKHLFSYWDEILGYL
                     KGVTDKYGLRRYIEFNSLVDRGYWDDDECRWHVFTADGREYVAQFLISGAGALHIPSF
                     PEIAGRDEFAGPAFHSAQWDHSIDLTGKRVAIVGTGASAIQIVPEIVGQVAELQLYQR
                     TPPWVVPRTNEELPVSLRRALRTVPGLRALLRLGIYWAQEALAYGMTKRPNTLKIIEA
                     YAKYNIRRSVKDRELRRKLTPRYRIGCKRILNSSTYYPAVADPKTELITDRIDRITHD
                     GIVTADGTGREVFREADVIVYATGFHVTDSYTYVQIKGRHGEDLVDRWNREGIGAHRG
                     ITVANMPNLFFLLGPNTGLGHNSVVFMIESQIHYVADAIAKCDRMGVQALAPTREAQD
                     RFNQELQRRLAGSVWNSGGCRSWYLDEHGKNTVLWCGYTWQYWLTTRSVNPAEYRFFG
                     IGNGLSSDRATVAAAN"
     CDS             complement(3373466..3374206)
                     /codon_start=1
                     /note="gene_3109"
                     /transl_table=11
                     /translation="VVRIPRPHPSAKPGVKVDARSERWREHRKKVRNEIVDAAFRAID
```

RLGPELSVRQIAEEAGTAKPKIYRHFTDKSDLLEAIGMRLRDMLWAAIFPSLDLATDS
AREVIRRSVEEYVNLVDQHPNVLRVFIQGRSAKQSEATVRTLNEGREITLAMAEMFNN
ELREMELNRAALELAAFAAFGSAASATEWWLGPEPDSPRRMPREQFVAHLTTIMMGVI
VGTAEALGIAVDPDQPIHDAVPNNPAVR"

CDS complement(3374334..3376511)
/codon_start=1
/note="gene_3110"
/transl_table=11
/translation="VPPTVIAEPVASGAHASYSGGPGETDYHALNAMLNLYDADGKIQ
FDKDREAAHQYFLQHVNQNTVFFHNQDEKLDYLIRENYYEREVLDQYSRNFVKTLLDR
AYAKKFRFPTFLGAFKYYTSYTLKTFDGKRYLERFEDRVVMVALTLAAGDTALAELLV
DEIIDGRFQPATPTFLNSGKKQRGEPVSCFLLRVEDNMESIGRSINSALQLSKRGGGV
ALLLTNIREHGAPIKNIENQSSGVIPIMKLLEDAFSYANQLGARQGAGAVYLHAHHPD
IYRFLDTKRENADEKIRIKTLSLGVVIPDITFELAKRNDDMYLFSPYDVERVYGVPFA
DISVTEKYYEMVDDARIRKTKIKAREFFQTLAELQFESGYPYIMFEDTVNRANPIDGK
ITHSNLCSEILQVSTPSLFNEDLSYAKVGKDISCNLGSLNIAKTMDSPDFAQTIEVAI
RALTAVSDQTHIKSVPSIEQGNNDSHAIGLGQMNLHGYLARERIFYGSDEGIDFTNIY
FYTVLYHALRASNRIAIERGTHFKGFERSKYASGEFFDKYTDQIWEPKTQKVRQLFAD
AGIRIPTQDDWRRLKESVQAHGIYNQNLQAVPPTGSISYINHSTSSIHPIVSKVEIRK
EGKIGRVYYPAPYMTNDNLEYYEDAYEIGYEKIIDTYAAATQHVDQGLSLTLFFKDTA
TTRDVNKAQIYAWRKGIKTLYYIRLRQMALEGTEVEGCVSCML"

CDS complement(3376481..3376933)
/codon_start=1
/gene="nrdI"
/note="gene_3111"
/transl_table=11
/translation="MDIAGRSLVYFSSVSENTHRFVQKLGIPATRIPLHGRIEVDEPY
VLILPTYGGGRANPGLDAGGYVPKQVIAFLNNDHNRAQLRGVIAAGNTNFGAEFCYAG
DVVSRKCSVPYLYRFELMGTEDDVAAVRTGLAEFWKEQTCHQPSLQSL"

CDS complement(3376968..3377207)
/codon_start=1
/note="gene_3112"
/transl_table=11
/translation="MTVTVYTKPACVQCSATSKALDKQGIAYQKVDISLDSEARDYVM
ALGYLQAPVVVAGNDHWSGFRPDRIKALAGAALTA"

CDS complement(3377670..3378224)
/codon_start=1
/note="gene_3113"
/transl_table=11
/translation="VSDTKSDIKILALVGSLRAASFNRQIAELAAKVAPDGVTVTMFE
GLGDLPFYNEDIDTATEVPAPVSALREAASDAHAALVVTPEYNGSIPAVIKNAIDWLS
RPFGDGALKDKPLAVIGGSMGRYGGVWAHDETRKSFSIAGTRVVDAIKLSVPFQTLGK
SVADDAGLAANVRDAVGNLAAEVG"

CDS 3378316..3378930
/codon_start=1
/note="gene_3114"
/transl_table=11
/translation="VSGAERLGDLPVFARQEPVPERGDAARNRALLLEAARRLIARSG
ADAITMDDVAAAAGVGKGTLFRRFGSRAGLMMVLLDEDERASQQAFLFGPPPLGPDAP
PLDRLIAFGRERMRFVHAHHQLLSEANRDPQTRHSAALSVLRTHLRVLLASAPTTGDL
DAQTDALLALLDVDYVEHQLNAGGHTLQTLGDAWESLARKLCGR"

```
CDS             3378940..3379980
                /codon_start=1
                /note="gene_3115"
                /transl_table=11
                /translation="MPTAAPRWILHVDLDQFLASVELLRHPELAGLPVIVGGNGDPTE
                PRKVVTCASYEARAYGVRAGMPLRTAARRCPEATFLPSNPAAYNAASEEVVALLRDLG
                YPVEVWGWDEAYLAVAPGTPDDPIEVAEEIRKVILSQTGLSCSIGISDNKQRAKIATG
                LAKPAGIYQLTDANWMAIMGDRTVEALWGVGPKTTKRLAKLGINTVYQLAHTDSGLLM
                STFGPRTALWLLLAKGGGDTEVSAQAWVPRSRSHAVTFPRDLTCRSEMESAVTELAQR
                TLNEVVASSRTVTRVAVTVRTATFYTRTKIRKLQAPSTDPDVITAAARHVLDLFELDR
                PVRLLGVRLELA"
CDS             complement(3380034..3380897)
                /codon_start=1
                /note="gene_3116"
                /transl_table=11
                /translation="MLQRGAGQYFAGKRCFVTGAASGIGRATALRLAAQGAELYLTDR
                DRDGLAQTVCDARALGAQVPEHRVLDVSDYQDVAAFAADIHARHPSMDVVLNIAGVSA
                WGTVDQLTHDQWSRMVAINLMGPIHVIETLVPPMVAAGRGGHLVNVSSAAGLVGLPWH
                AAYSASKYGLRGLSEVLRFDLARHGIGVSVVVPGAVKTPLVNTVEIAGVDRDDPRVNR
                WVERFSGHAVTPEKAADKILAGVTRNRYLVYTSADIRALYAFKRYAWWPYTLVMRRVN
                VFFTRALRPGP"
CDS             complement(3380961..3381611)
                /codon_start=1
                /note="gene_3117"
                /transl_table=11
                /translation="VTSHAADEKQAAPPMRRRGDRHRQAILRAARELLEETPFAELSV
                RAISLRAGVARSGFYFYFDSKYSVLAQILAEATEELEEASQHFSARQPGESPEQFVNR
                MIGSVAAVYANNDPVLRACNAARQSDMEIRDILERQFQVLLRETIGVFEAEVKAGTAH
                PISEDLPTLVRTLAATTALMLTGDALLVGPDSDAARRVRVLEQMWLNALWGGGKAP"
CDS             3381727..3383205
                /codon_start=1
                /note="gene_3118"
                /transl_table=11
                /translation="MATIHPPAYLLDQAKRRFTPSFNNFPGMSLVEHMLLNTKFPEKE
                LAEPPPGSGLKPVVGDAGLPILGHMIEMLRGGPDYLMFLYKTKGPVVFGDSAVLPGVA
                ALGPDAAQVIYSNRNKDYSQQGWVPVIGPFFHRGLMLLDFEEHMFHRRIMQEAFVRSR
                LAGYLEQMDRVVSRVVADDWVVNDARFLVYPAMKALTLDIASMVFMGHEPGTDHELVT
                KVNKAFTITTRAGNAVIRTSVPPFTWWRGLRARELLENYFTARVKERREASGNDLLTV
                LCQTEDDDGNRFSDADIVNHMIFLMMAAHDTSTSTATTMAYQLAAHPEWQQRCRDESD
                RHGDGPLDIESLEQLESLDLVMNESIRLVTPVQWAMRQTVRDTELLGYYLPKGTNVIA
                YPGMNHRLPEIWTDPLTFDPERFTEPRNEHKRHRYAFTPFGGGVHKCIGMVFGQLEIK
                TILHRLLRRYRLELSRPDYQPRWDYSAMPIPMDGMPIVLRPR"
CDS             complement(3383976..3385448)
                /codon_start=1
                /note="gene_3119"
                /transl_table=11
                /translation="MSTEPDAVWTDKRASKIARRIEADIVRRGWPIGASLGSESALQQ
                RFCVSRSVLREAVRLVEHHQVARMRRGPNGGLFICEPNAGPATRAVVIYLEYLGTTIG
                DLLGARLVLEPLAASLAAEHIDEPGIERLRAVLRAEERWRPGLPPPPEQFYRVLAEQS
                KNPVLQLFIDILMRLTKRYVQKSGTQSAGEAVEAAGQVHNEHSDIVAAVTAGDSAWAK
                TLSERHVEAVAGWLQQHQRGNDAAVRNGGRAREPRRAQQLILGAPRGKLAEVLAATIG
```

DDIAASGWQVGSVFGTETALLERYQVSRAVLREAVRLLEYHAIAHMRRGPGGGLVVTT
PQPQASIDTIALYLQYRKPSREDLRCVRDAIEIDNVAKVVKRRSEPEVASFLDTLGRP
RLDNPTDDVRAAAVEEFRFHVGLARAAGNTMLDLFLLILVELFRRHLSSTEQALPTWS
DVVAVGHAHVRILEAIGSGDDSLARCRTRRHLDAAASWWL"

CDS complement(3385565..3387730)
/codon_start=1
/note="gene_3120"
/transl_table=11
/translation="MGIALTDDHRELSGVARAFLTSQKVRWAARASLDAAGDARPPFW
QNLAELGWLGLHIDERHGGSGYGLSELVVVIEELGRAVAPGLFVPTVIASAVVAKEGT
DDQRARLLPALIDGTLTAGVGLDSQVQVTDGVADGEAGIVLGAGLAELLLVAAGDDVL
VLERGRKGVSVDVPENFDPTRRSGRVRLDNVRVTTDDILLGAYESALARARTLLAAEA
VGGAADCVDSAVAYAKVRQQFGRTIATFQAVKHHCANMLVAAESAIAAVWDAARAAAE
DEEQFRLAAAVAAALAFPAYARNAELNIQVHGGIGFTWEHDAHLHLRRALVTVGLFGG
DAPVRDVFERTAAGVTRAISLDLPAQAEELRARIRSDAAEIAALEKDAQRDKLIETGY
VMPHWPRPWGRAAGAVEQLVIEEEFSAAGIERPDYSITGWVILTLIQHGTPWQIERFV
EKALRQQEIWCQLFSEPDAGSDAASVKTRATRVEGGWEINGQKVWTCGAQYCARGLAT
VRTDPDAPKHAGITTVIIDMLAPGVEVRPLRQITGDSEFNEVFFNDVFVPDEDVVGAP
NSGWTVARATLGNERVSIGGSGSYYEAMAAKLVQLVQRRSDAFAGAPIRVGAFLAEDH
ALRLLNLRRAARSVEGAGPGPEGNITKLKVAEHMIEGAAIAAALWGPEIALLDGPGRV
IGRTVMGARGMAIAGGTSEVTRNQIAERILGMPRDPLIS"

CDS 3387887..3389410
/codon_start=1
/gene="ligB"
/note="gene_3121"
/transl_table=11
/translation="VLLHDVAITSMDVAATSSRLTKVARIAALLHRAAPDTQLVTIIV
SWLSGELPQRHIGVGWAALRSLPPPAPQPALTVTGVDATLSKIGTLSGKGSQAQRAAL
VAELFSAATEAEQTFLLRLLGGELRQGAKGGIMADAVAQAAGLPAATVQRAAMLGGDL
AAAAAAGLSGAALDTFTLRVGRPIGPMLAQTATSVHDALERHGGTTIFEAKLDGARVQ
IHRANDQVRIYTRSLDDVTARLPEVVEATLALPVRDLVADGEAIALCPDNRPQRFQVT
ASRFGRSVDVAAARATQPLSVFFFDILHRDGTDLLEAPTTERLAALDALVPARHRVDR
LITSDPTDAANFLDATLAAGHEGVMAKAPAARYLAGRRGAGWLKVKPVHTLDLVVLAV
EWGSGRRRGKLSNIHLGARDPATGGFVMVGKTFKGMTDAMLDWQTTRFHEIAVGPTDG
YVVQLRPEQVVEVALDGVQRSSRYPGGLALRFARVVRYRADKDPAEADTIDAVRALY"

CDS 3389546..3391822
/codon_start=1
/note="gene_3122"
/transl_table=11
/translation="VAAPTPSNRIEERSGHASCVRADADLPPVAILGRSPITLRHKIF
FVAVAVIGALAWTVVAFFRNEPVNAVWIVVAAGCTYIIGFRFYARLIEMKVVRPRDDH
ATPAEILDDGTDYVPTDRRVVFGHHFAAIAGAGPLVGPVLATQMGYLPSSIWIVVGAV
LAGCVQDYLVLWISVRRRGRSLGQMVRDELGATAGVAALVGIPVIITIVIAVLALVVV
RALAKSPWGVFSIAMTIPIAIFMGCYLRFLRPGRVSEVSLIGIGLLLLAVVSGDWVAH
TSWGAAWFSLSPVTLCWLLISYGFAASVLPVWLLLAPRDYLSTFMKVGTIALLAIGVC
AAHPIIEAPAVSKFAGSGNGPVFAGSLFPFLFITIACGALSGFHALICSGTTPKMLEK
EGQMRVIGYCGMMTESFVAVIALLTAAILDQHLYFTLNAPSLHTHDSAATAAKYVNGL
GLTGSPVTPDHISQAAASVGEQTIVSRTGGAPTLAFGMAEMLHRVVGGVGLKAFWYHF
AIMFEALFILTTVDAGTRAARFMISDALGNFGGVLRKLQNPSWRPGAWACSLVVVAAW
GSILLLGVTDPLGGINTLFPLFGIANQLLAGIAPTVITVVVIKKGRLKWAWIPGIPLL
WDLAVTLTASWQKIFSADPSVGYWTQHAHYAAAQHAGETAFGSATNADEINDVVRNTF

VQGTLSIVFVVVVVLVVVAGVIVALKTIRGRGIPLAEDDPAPSTLFAPAGLIPTAAER
KLQRRLGAPASASVAAPD"
CDS complement(3392128..3392553)
/codon_start=1
/note="gene_3123"
/transl_table=11
/translation="MVKDLDRRLAGCLPAVLSLFRLVYGLLFAGYGSMILFGWPVTSA
QPVEFGSWPGWYAGVIELVAGLLIATGLFTRAVAFVASGEMAVAYFWMHQPYALWPIG
GPPDGNGGTPAILFCFGFFLLVFTGGGIYSIDARRTVTA"
CDS 3392690..3393013
/codon_start=1
/note="gene_3124"
/transl_table=11
/translation="LIYLYLLCAIFAEVVATSLLKSTEGFTRLWPTVGCLVGYGIAFA
LLALSISHGMQTDVAYALWSAIGTAAIVLVAVLFLGSPISVMKVVGVGLIVVGVVTLN
LAGAH"
CDS 3393010..3393618
/codon_start=1
/note="gene_3125"
/transl_table=11
/translation="LTAGSDRRPRDPAGRRQAIVEAAERVIARQGLGGLSHRRVAAEA
NVPVGSTTYYFNDLDALREAALAHAANASADLLAQWRSDLDKDRDLAATLARLTTVYL
ADQDRYRTLNELYMAAAHRPELQRLARLWPDGLLALLEPRIGRRAANAVTVFFDGATL
HALITGTPLSTDELTDAIARLVADGPEQREVGQSAHAGRTPD"
CDS 3393596..3394141
/codon_start=1
/note="gene_3126"
/transl_table=11
/translation="MRDEPPTDTAAAPTTGAAPEIDTAREYEVTAEYQSWRVVWGSAA
ALLAVGVGIGAAILLGWFTLAHRHPDQPGAAATPPPAGLTTRSAPTAAPPSTLQSPDL
DSVFLGNLHDRGISFTNPDAAVYNGKMVCTNLGGGMTVQQVVEALQSSSPALGDRTTA
YVAVSIRTYCPKYDAVLPPGS"
tRNA complement(3394143..3394215)
/note="identified by tRNAscan-SE; putative"
CDS complement(3394282..3395925)
/codon_start=1
/note="gene_3127"
/transl_table=11
/translation="MVANPRAGQPAQPEDLVDLPHLVTAYYSIEPDPDDLAQQVAFGT
SGHRGSALTGTFNELHILAITQAIVEYRAAQGTTGPLFIGRDTHGLSEPAWVSALEVL
AANQVVAVVDSRDRYTPTPAISHAILTYNRGRTEALADGIVVTPSHNPPSDGGIKYNP
PNGGPADTAATTAIAKRANEILLARSMVKRLPLARALRTAQRHDYLGHYVDDLPNVVD
IAAIREAGVRIGADPLGGASVDYWGEIAHRHGLDLTVVNPLVDATWRFMTLDTDGKIR
MDCSSPDAMAGLIRTMFGNRERYQIATGNDADADRHGIVTPDEGLLNPNHYLAVAIEY
LYTHRPSWPAGIAVGKTVVSSSIIDRVVAGIGRQLVEVPVGFKWFVDGLIGATLGFGG
EESAGASFLRRDGSVWTTDKDGIIMALLAAEILAVTGATPSQRYHALAGEYGGPCYAR
IDAPADREQKARLARLSADQVSATELAGEPITAKLTTAPGNGAALGGLKVTTANAWFA
ARPSGTEDVYKIYAESFRGPQHLVEVQQTAREVVDRVIG"
CDS 3395995..3396393
/codon_start=1
/gene="ccrB"

/note="gene_3128"
/transl_table=11
/translation="VPNHDYRELAAVFAGGALGALARAALSALAIPDPARWPWPTFTV
NVVGAFLVGYFTTRLLERLPLSSYRRPLLGTGLCGGLTTFSTMQVETISMIEHGHWGL
AAAYSVVSITLGLLAVHLATVLVRRVRIRR"
CDS 3396390..3396770
/codon_start=1
/gene="ccrB"
/note="gene_3129"
/transl_table=11
/translation="MTASTALTVAIWIGVMLIGGIGSVLRFLVDRSVARRLARTFPYG
TLTVNITGAALLGFLAGLALPKDAALLAGTGFVGAYTTFSTWMLETQRLGEDRQMVSA
LANIVVSVVLGLAAALLGQWIAQI"
CDS 3396767..3397876
/codon_start=1
/note="gene_3130"
/transl_table=11
/translation="MNEQCLKLTAYFGERQRAVGGAGRFLADAMLDLFGSHNVATSVM
LRGTTSFGPKHEFRCDQSLSLSEDPPVTVAAVDIESKIRSLVDDVTAMTDRGLVTLER
ARLVTRHSGAEEFGDIDSRNGDAAKLTIYAGRQVRVAGAPAYYTICELLHRHGFAGAT
VLLGVDGTAHGRRRRARFFGRNVNVPLMIIAVGTPAQVAVAAMELTAALPNPLLTIER
VRLCKRDGELFARPQQLPQTDDQGRTLWQKLMVHTAEATHHEGLPIHRALVHRLMQSE
TARGATALRGIWGFYGDHKPHGDKLFQLVRRVPVTTIIVDTPQAIARSFDIVDELTNW
HGLVTSEMVPAAVSLTGSRDGTQKTGETPLARYDY"
CDS complement(3398101..3398598)
/codon_start=1
/note="gene_3131"
/transl_table=11
/translation="VTGTARAGIGAGADPAVVDAVAVAADDCGFATLWVGEHVVMVDR
PASRYPYSRDGVIAVPAQADWLDPMIALSFAAAASSRVDVATGVLLLPEHNPVIVAKE
AASLDRLSGRRLTLGVASDGPRRSSTRSECHSSGAQSAPPNTSLQCAHYGATTSHRST
ATVGS"
CDS complement(3398632..3398988)
/codon_start=1
/note="gene_3132"
/transl_table=11
/translation="MVRETRVRVARVYEDIDPDDGQRVLVDRIWPHGIRKDDQRVGIW
CKDVAPSKELREWYHHQPERFDEFASRYQEELHDSAALAELRKLTGRSVVTPVTATRH
VARSHAAVLAQLLNGR"
CDS 3399082..3400356
/codon_start=1
/note="gene_3133"
/transl_table=11
/translation="MFETLTAIDPDAEEAALIERIAELERLKSAAAAGQARAAAAVDA
ARRAAEGAAGVPAARRGRGLASEIALARRDSPARGSRHLGFAKALVYEMPHTLAALDC
GALSEWRATLIVRESACLDVADRRALDAELCGDPGDLEGMGDARVVAAARAIAYRLDP
QAVVDRAANAENDRTVTIRPAPDTMTYLTALLPVAQGVSVYAALTRAADTRCDGRSRG
QVMADTLVERVTGRDAAVPTPIAVNLVMSDETLLGAANTPAQLCGYGPIPAAVARTMV
ASAVTDQRSRATLRRLYAHPQAGALVSMESRARLFPRGLAAFIELRDQRCRTPYCDAP
IRHRDHAHPWADGGPTSAHNGLGTCERCNYAKQAPGWRVSTSVDENHTHTAEFITPTG
SRHRSGAPPHLPAVTVSELEVRIGIALARYAA"

```
CDS             complement(3400353..3401276)
                /codon_start=1
                /note="gene_3134"
                /transl_table=11
                /translation="MTSMYEQVDTNTADPVAGSRIDPVLARSWLLVNGAHGDRFESAA
                HSRADIVVLDIEDAVAPKDKHAARDNAVRWFGDGNADWVRINGFGTPWWADDLAMLAD
                SPVGGVMLAMVESVDHVTETAKRLPNVPIVALVETARGLERINEIAAAKGTFRLAFGI
                GDFRRDTGFGEDPATLAYARSRFTIAARAAGLPSAIDGPTIGSNALKLIEATAVSAEF
                GMTGKICLSPDQCPVVNEGLSPSQDEIVWAKEFFAEFARDGGEIRNGSDLPRIARATK
                ILDLARAYGIEVSDFEDEPVHMPAPTDTYHY"
CDS             3401375..3401851
                /codon_start=1
                /note="gene_3135"
                /transl_table=11
                /translation="MVLDGVVSDTRRSRTIAARQQTIWDVLADFGSLSSWVEGVDHSC
                VLNHGPDGGALGSTRRVQVGRNTLVERVIEFDPPTTLAYRIEGLPARLRKVTNRWTLR
                PADPVGAVTVVTLTSTIEIGGNPLARLAELVVGRAMAKRSNTMLAGLAQRLEDKHG"
CDS             3401844..3403655
                /codon_start=1
                /note="gene_3136"
                /transl_table=11
                /translation="MANRPDIIIVMTDEERAVPPYESAEVLAWRQRSLTGRRWFDEHG
                ISFTRHYTGSLACVPSRPTIFTGQYPDLHGVTQTDGIGKRFDDSRLRWLRAGEVPTLG
                NWFRAAGYDTHYDGKWHISHADLEDPATGAPLATNDNEGVVDSAAVRRYLDADPLGPY
                GFSGWVGPEPHGAGLANSGFRRDPLVADRVVAWLTERYARRRAGDTAAMRPFLLVASF
                VNPHDIVLFPAWVWRSPLKPSPLDPPHVPAAPTADEDLSTKPAAQVAYREAYYSGYGL
                TRMVSRNYARNAQRYRDLYYRLHAEVDGPIDRVRRAVTEGGSEDAMLVRTSDHGDLLG
                AHGGLHQKWFNLYDEATRVPFVIARIGEKATQPRTVSAPTSHVDLVPTLLSAAGVDVD
                VVAAALAESFSEVHPLPGRDLMPVVDGASADEGRAIYLMTRDNVLEGDTGASLLSRQL
                GRIVNPPAPLRIKVPAHVAANFEGLVVRVDDTDAAGGAGHLWKLVRTFDDPATWTEPG
                VRHLATNGMGGDAYRTDPLDDQWELYDLTADPIEAYNRWTDPQLHELRQHLRMLLKQQ
                RAVSVPERNQPWPYAHRLPPSGASNGLVRRVLGRFVR"
CDS             3403656..3404057
                /codon_start=1
                /note="gene_3137"
                /transl_table=11
                /translation="LQKLLFTIGLALFLIGLLTGLVIPALKNPRMALSSHLEGVLNGM
                FLVVLGLLWPHIDLPEAWQVIAVALIVYSAYANWLATLLAAAWGAGRKFAPIATGDHK
                APAAKEGFVSFLLLSLSVAIVIGVVIVIIGL"
CDS             complement(3404073..3404900)
                /codon_start=1
                /note="gene_3138"
                /transl_table=11
                /translation="MQFGVLTFVTDEGIGPAELGAALEHRGFESLFLAEHTHIPVNTQ
                SPYPGGGPIPEKYYRTLDPFVALAAAAATTQSLVLGTGIALIPERDPIVTAKEVASLD
                LVSQGRFRFGVGVGWLREEVANHGVDPAVRGRVIDERLRAIIEIWTQEQAEFHGTYVD
                FDPIYCWPKPVTKPYPPLYVGGGPANFPRIARLNAGWIAISPSPQRLSGPLQRLRAMA
                GGDVPVTVCQWGEAAAKDLEGYRHLGVERVLLELPTEPRDPTLRYLDKLQAELARLA"
CDS             complement(3404959..3408291)
                /codon_start=1
                /note="gene_3139"
```

/transl_table=11
/translation="MTDVDPHATRRDLVPNIPAELLEAGFDNVEEIGRGGFGVVYRCV
QPSLDRAVAVKVLSTDLDRDNLERFLREQRAMGRLSGHPHIVTVLQVGVLAGGRPFIV
MPYHAKNSLETLIRRHGPLDWRETLSIGVKLAGALEAAHRVGTLHRDVKPGNILLTDY
GEPQLTDFGIARIAGGFETATGVIAGSPAFTAPEVLEGASPTPASDVYSLGATLFCVL
TGHAAYERRSGERVIAQFLRITSQPIPDLRKQGLPADVAAAIERAMARHPADRPATAA
DVGEELRDVQRRNGVSVDEMPLPVELGVERRRSPEAHAAHRHTGGGTPTVPTPPTPAT
KYRPSVPTGSLVTRSRLTDILRAGGRRRLILIHAPSGFGKSTLAAQWREELSRDGAAV
AWLTIDNDDNNEVWFLSHLLESIRRVRPTLAESLGHVLEEHGDDAGRYVLTSLIDEIH
ENDDRIAVVIDDWHRVSDSRTQAALGFLLDNGCHHLQLIVTSWSRAGLPVGRLRIGDE
LAEIDSAALRFDTDEAAALLNDAGGLRLPRADVQALTTSTDGWAAALRLAALSLRGGG
DATQLLRGLSGASDVIHEFLSENVLDTLEPELREFLLVASVTERTCGGLASALAGITN
GRAMLEEAEHRGLFLQRTEDDPNWFRFHQMFADFLHRRLERGGSHRVAELHRRASAWF
AENGYLHEAVDHALAAGDPARAVDLVEQDETNLPEQSKMTTLLAIVQKLPTSMVVSRA
RLQLAIAWANILLQRPAPATGALNRFETALGRAELPEATQADLRAEADVLRAVAEVFA
DRVERVDDLLAEAMSRPDTLPPRVPGTAGNTAALAAICRFEFAEVYPLLDWAAPYQEM
MGPFGTVYAQCLRGMAARNRLDIVAALQNFRTAFEVGTAVGAHSHAARLAGSLLAELL
YETGDLAGAGRLMDESYLLGSEGGAVDYLAARYVIGARVKAAQGDHEGAADRLSTGGD
TAVQLGLPRLAARINNERIRLGIALPAAVAADLLAPRTIPRDNGIATMTAELDEDSAV
RLLSAGDSADRDQACQRAGALAAAIDGTRRPLAALQAQILHIETLAATGRESDARNEL
APVATKCAELGLSRLLVDAGLA"
CDS 3408343..3409581
/codon_start=1
/note="gene_3140"
/transl_table=11
/translation="VTPHYRQAAASRLDTHRTQKLRSQTNGGKDRHQLTYEQFARMLT
LMGPSDLWTVERAARHWGVSASRARAILSSRHIHRVSGYPAQAIKAVTLRQGARTDLK
TANHLAPAAQAFTMAETGAAIGETEDERARLRIFFEFLRGADETGTSALDLIVDEPAL
IGEHRFDALLAAAAEYISARWGRPGPLWSVSIERFLDTAWWVSDLPSARAFAAVWTPA
PFRRRGIYLDRHDLTSDGVCVMPEPVFNRTELQRAFTALAAKLERRGVVGQVHVVGGA
AMLLAYNSRVTTRDIDALFSTDGPMLEAIREVADEMGWPRTWLNNQASGYVSRTPGEG
APVFDHPFLHVVATPAQHLLAMKVVAARGVRDGEDIRLLLDRLRITSAAGVWEIVARY
FPAETITDRSRLLVEDLLNQ"
CDS complement(3409707..3410729)
/codon_start=1
/note="gene_3141"
/transl_table=11
/translation="MELGSLIRATNLWGYTDLMRELGADPLPFLRRFDIPPGIEHQED
AFMSLAGFVRMLEASAAELDCPDFGLRLARWQGLGILGPVAVIARNAATLFGGLEAIG
RYLYVHSPALTLTVSSTTARSNVRFGYEVTEPGIPYPLQGYELSMANAARMIRLLGGP
QARARVFSFRHAQLGTDAAYREALGCTVRFGRTWCGFEVDHRLAGRPIDHADPETKRI
ATKYLESQYLPSDATLSERVVGLARRLLPTGQCSAEAIADQLDMHPRTLQRRLAAEGL
RCHDLIERERRAQAARYLAQPGLYLSQIAVLLGYSEQSALNRSCRCWFGMTPRQYRAY
GGVSGR"
CDS 3410807..3412294
/codon_start=1
/note="gene_3142"
/transl_table=11
/translation="MNQHFDVLIIGAGLSGIGTACHVTAEFPDKTIALLERRERLGGT
WDLFRYPGVRSDSDMFTFGYKFRPWRDVKVLADGASSRQYIADTATEFGIDEKIHYGL
KVNTAEWSSRQCRWTVAGVHEATGETRTYTCDYLISCTGYYNYDAGYLPDFPGVHRFG

GRCVHPQHWPEDLDYSGKKVVVIGSGATAVTLVPAMAGSNPGSAAHVTMLQRSPSYIF
SLPAVDKISEVLGRFLPDRWVYEFGRRRNIAIQRKLYQACRRWPKLMRRLLLWEVRRR
LGRSVDMSNFTPNYLPWDERLCAVPNGDLFKTLASGAASVVTDQIETFTEKGILCKSG
REIEADIIVTATGLNIQMLGGMRLIVDGAEYQLPEKMTYKGVLLENAPNLAWIIGYTN
ASWTLKSDIAGAYLCRLLRHMADNGYTVATPRDAQDCALDVGMFDQLNSGYVKRGQDI
MPRQGSKHPWRVLMHYEKDAKILLEDPIDDGVLHFAAAAQDHAAA"

CDS 3412300..3413226
/codon_start=1
/note="gene_3143"
/transl_table=11
/translation="MNLRKNVIRSVLRGARPLFASRRLGIAGRRVLLATLTAGARAPK
GTRFQRVSIAGVPVQRVQPPHAATSGTLIYLHGGAYALGSARGYRGLAAQLAAAAGMT
ALVPDYTRAPHAHYPVALEEMAAVYTRLLDDGLDPKTTVIAGDSAGGGLTLALAMALR
DRGIQAPAALGLICPWADLAVDIEATRPALRDPLILPSMCTEWAPRYVGSSDPRLPGI
SPVYGDMSGLPPIVMQTAGDDPICVDADKIETACAASKTSIEHRRFAGMWHDFHLQVG
LLPEARDAIADLGARLRGHLHQSQGQPRGVVK"

CDS 3413223..3414053
/codon_start=1
/note="gene_3144"
/transl_table=11
/translation="MSSFEGKVAVITGAGSGIGRALALNLSEKRAKLALSDVDTDGLA
KTVRLAQALGAQVKSDRLDVAEREAVLAHADAVVAHFGTVHQVYNNAGIAYNGNVDKS
EFKDIERIIDVDFWGVVNGTKAFLPHVIASGDGHIVNISSLFGLIAVPGQSAYNAAKF
AVRGFTEALRQEMLVARHPVKVTCVHPGGIKTAVARNATVADGEDQQTFAEFFDRRLA
LHSPEMAAKTIVNGVAKGQARVVVGLEAKAVDVLARIMGSSYQRLVAAGVAKFFPWAK
"

CDS 3414084..3415190
/codon_start=1
/note="gene_3145"
/transl_table=11
/translation="MKTTAAVLFEAGKPFELMELDLDGPGPGEVLVKYTAAGLCHSDL
HLTDGDLPPRFPIVGGHEGSGVIEEVGAGVTRVKPGDHVVCSFIPNCGTCRYCCTGRQ
NLCDMGATILEGCMPDGSFRFHSQGTDFGAMCMLGTFAERATVSQHSVVKVDDWLPLE
TAVLVGCGVPSGWGTAVNAGNLRAGDTAVIYGVGGLGINAVQGATAAGCKYVVVVDPV
AFKRETALKFGATHAFADAASAAAKVDELTWGQGADAALILVGTVDDEVVSAATAVIG
KGGTVVITGLADPAKLTVHVSGTDLTLHEKTIKGSLFGSCNPQYDIVRLLRLYDAGQL
MLDELVTTTYNLEQVNQGYQDLRDGKNIRGVIVH"

CDS 3415228..3416646
/codon_start=1
/note="gene_3146"
/transl_table=11
/translation="MRRLNGVDALMLYLDGGSAYNHTLKISVLDPSTDPDGWSWPKAR
QMFEERAHLLPVFRLRYLPTPLGLHHPIWVEDPEFDLDAHVRRVVCPAPGGMAEFCAL
VEQIYAHPLDRDRPLWQTWVVEGLDGGRVALVTLLHHAYSDGVGVLDMLAAFYNDTPD
EAPVVAPPWEPPPLPSTRQRLGWALRDLPSRLGKIAPTVRAVRDRVRIEREFAKDGDR
RVPPTFDRSAPPGPFQRGLSRSRRFSCESFPLAEVREVSKTLGVTINDVFLACVAGAV
RRYLERCGSPPTDAMVATMPLAVTPAAERAHPGNYSSVDYVWLRADIADPLERLHATH
LAAEATKQHFAQTKDADVGAVVELLPERLISGLARANARTKGRFDTFKNVVVSNVPGP
REPRYLGRWRVDQWFSTGQISHGATLNMTVWSYCDQFNLCVMADAVAVRNTWELVGGF
RASHEELLAAARAQATPKEMAT"

CDS 3416643..3418067

/codon_start=1
/note="gene_3147"
/transl_table=11
/translation="MTRINPIDLSFLLLERANRPNHMAAYTIFEKPKGQKSSFGPRLF
DAYRHSQAAKPFNHKLKWLGTDVAAWETVEPDMGYHIRHLALPAPGSMQQFHETVSFL
NTGLLDRGHPMWECYIIDGIERGRIAILLKVHHALIDGEGGLRAMRNFLSDSPDDTTL
AGPWMSAQGADRPRRTPATVSRRAQLQGQLQGMIKGLTKLPSGLFGVSADAADLGAQA
LSLKARKASLPFTARRTLFNNTAKSAARAYGNVELPLADVKALAKATGTSVNDVVMTV
IDDALHHYLAEHQASTDRPLVAFMPMSLREKSGEGGGNRVSAELVPMGAPKASPVERL
KEINAATTRAKDKGRGMQTTSRQAYALLLLGSLTVADALPLLGKLPSANVVISNMKGP
TEQLYLAGAPLVAFSGLPIVPPGAGLNVTFASINTALCIAIGAAPEAVHEPSRLAELM
QRAFTELQTEAGTTSPTTSKSRTP"
CDS 3418064..3419575
/codon_start=1
/note="gene_3148"
/transl_table=11
/translation="MKNIGWMLRQRATVSPRLQAYVEPSTDVRMTYAQMNALANRCAD
VLTALGIAKGDRVALLMPNSVEFCCLFYGAAKLGAVAVPINTRLAAPEVSFILSDSGS
KVVIYGAPSAPVIDAIRAQADPPGTVTDWIGADSLAERLRSAAADEPAVECGGDDNLF
IMYTSGTTGHPKGVVHTHESVHSAASSWASTIDVRYRDRLLLPLPMFHVAALTTVIFS
AMRGVTLISMPQFDATKVWSLIVEERVCIGGAVPAILNFMRQVPEFAELDAPDFRYFI
TGGAPMPEALIKIYAAKNIEVVQGYALTESCGGGTLLLSEDALRKAGSAGRATMFTDV
AVRGDDGVIREHGEGEVVIKSDILLKEYWNRPEATRDAFDNGWFRTGDIGEIDDEGYL
YIKDRLKDMIISGGENVYPAEIESVIIGVPGVSEVAVIGLPDEKWGEIAAAIVVADQN
EVSEQQIVEYCGTRLARYKLPKKVIFAEAIPRNPTGKILKTVLREQYSATVPK"
CDS complement(3420072..3420374)
/codon_start=1
/gene="TBCG_03025"
/note="gene_3149"
/transl_table=11
/translation="VRLSSIAFGAEYWLAPSGFTRAPQNSVDRFLFNVPGAVDALCVT
QSRRREEGLHGEQLGPSADPGDGPAHHAIFGEHTLVSMEGTAPTGATPARPSAERR"
CDS 3420514..3421401
/codon_start=1
/note="gene_3150"
/transl_table=11
/translation="MTWQIVFVVICVIVAGVAALFWRLPSDDTTRSRAKTVTIAAVAA
AAVFFFLGCFTIVGTRQFAIMTTFGRPTGVSLNNGFHGKWPWQMTHPMDGAVQIDKYV
KEGNTDQRITVRLGNQSTALADVSIRWQLKQAAAPELFQQYKTFDNVRVNLIERNLSV
ALNEVFAGFNPLDPRNLDVSPLPSLAKRAADILRQDVGGQVDIFDVNVPTIQYDQSTE
DKINQLNQQRAQTSIALEAQRTAEAQAKANEILSRSISDDPNVVVQNCITAAINKGIS
PLGCWPGSSALPTIAVPGR"
CDS 3421419..3423110
/codon_start=1
/note="gene_3151"
/transl_table=11
/translation="MPIPFADGMLSRLGRRGAALDLIEEFEDESGEPPASLSPADLLA
AEPALLLQKMENRLVRHHLANPDVLSGEQLRKLRYILNFARLADFEPGAAGPGGSRGR
GDISVGGQVAPWRSRVVDALYAPLREEPDPVTALEGAKDVLATLVDDQDDQRRVLIER
HGSDFSATELDAEVGYKKLVTVLGGGGGAGFVYIGGMQRLLAAGQVPDYMIGSSFGSI
IGSLVARELPVPIDEYAEWAKTVSYRAILGPERRRSRHGLAGMFTLRFDQFAHTLLSR

ADGERMRMSDLAIPFDVVVAGVRRQPYAALPSRFRHRERSTLTLRSLPFLPIGIGPWV
AARMWQVAAFIDLRVVKPIVISADGATRDVNVVDAASFSSAIPGVLHHETSDPRMLPI
LDELCADQDVAAMVDGGAASNVPVELAWERVRDGRLGTRNACYLAFDCFHPHWDPRHL
WLVPITQAVQLQMVRNLPYADHLVRFEPTLSPVNLAPSAAAIDRACRWGRDSVEPAIA
VTSALLEPTWWEGDRPPAAEPKERTKSAASSMSAVMAAIQAPTGRFRRWRSRHLT"

CDS complement(3423117..3424037)
/codon_start=1
/note="gene_3152"
/transl_table=11
/translation="MSGGLFGLLDHVAVLARLAAASIDDIGAAAGRATAKAAGVVIDD
TAVTPQYVHRITAERELPIIKRIAIGSVRNKLLLILPGALLLSQLVPWLLTPLLMLGA
TYLCYEGAEKVCGVIGGRGHDAAPQVAERELVAGAIRTDFILSAEIMVIALNEVADQP
FVPRLIVLVIVALVITAAVYGVVAVIVQMDDVGLRLTQTASRFGQRIGGGLVAGMPKL
LSALSAVGMGAMLWVGGHIVLVGSDHLGWHAPYRLVHHLDDHLVGSAGGALTWLVSTA
ACAATGLVIGIVVVALVHLVCFRPPRSRSL"

CDS complement(3424063..3425067)
/codon_start=1
/note="gene_3153"
/transl_table=11
/translation="MTDIEVALPFWLDRPDHEATDVALAAADTGFAALWIGEMATYDA
FALATSIGLRTPNMTLKVGPLAVGVRGPVGLALGVSSVASLTGCRVDLALGASSPAIV
AGWHGRPWAHHVPVMRETIECLRSIFTGARVEYSGRHVNSRGFRLRGAAPDTRIALGA
FGPGMIRLAAQHADEVVLNLASPFRVGRVRAAIDSAAAAAGRAAPRLTVWVPVAVNPG
AAAHSQLAAQLAVYLAPPGYGEMFSALGFDGLVRSARSRATRRELAVAVPSELLDRVC
ALGSPDRVAARLRAYADAGADCVAVVPATAEDPGGRVALRALRPGGLYGTAGDNDGRR
"

CDS complement(3425064..3426194)
/codon_start=1
/note="gene_3154"
/transl_table=11
/translation="VNQSETEIEILAEKIARWARARSAEIERDRRLPDELVTRLREAG
LLRATMPREVAAPELAPGRALRCAEAVARGDASAGWCVSIAITSALLVAYLPARSREE
MFGGGRGVAAGVWAPRGTARSVDGGVVVSGRWPFCSGINHADIMFAGCFVDDRQVPSV
VALNKDELQVLDTWHTLGLRGTGSHDCVADDVFVPADRVFSVFDGPIVDRPLYRFPVF
GFFALSIGAAALGNARAAIDDLVELAGGKKGLGSTRTLAERSATQAAAATAESALGAA
RALFYEVIEAAWQVSHDAEAVPVTMRNRLRLAATHAVRTSADVVRSMYDLAGGTAIYD
NAPLQRRFRDAFTATAHFQVNEASRELPGRVLLDQPADVSML"

CDS 3426276..3426752
/codon_start=1
/note="gene_3155"
/transl_table=11
/translation="MAVSDLSHRFEGESVGRALELVGERWTLLILREAFFGVRRFGQL
ARNLGIPRPTLSSRLRMLVEVGLFDRVPYSSDPERHEYRLTEAGRDLFAAIVVLMQWG
DEYLPRPEGPPIKLRHHTCGEHADPRLICTHCGEEITARNVTPEPGPGFKAKLASS"

CDS 3426850..3427989
/codon_start=1
/note="gene_3156"
/transl_table=11
/translation="VHRRTALKLPLLLAAGTVLGQAPRAAAGEPGRWSADRAHRWYQA
HGWLVGANYITSNAINQLEMFQPGTYDPRRIDNELGLARFHGFNTVRVFLHDLLWAQD
APGFQTRLAQFVAIAARYHIKPLFVLFDSCWDPLPRPGRQRAPRAGVHNSGWVQSPGA

ERLDDRRYASTLYNYVTGVLGQFRNDDRVLGWDLWNEPDNPARVYRKVERKDKLERVA
ELLPQVFRWARTVDPVQPLTSGVWQGNWGDPGRRSTISAIQLDNADVITFHSYAAPAE
FEGRIAELAPLQRPILCTEYLARSQGSTVEGILPIAKRHNVGAFNWGLVAGKTQTYLP
WDSWDHPYRAPPKVWFHDLLHPNGRPYRDGEVQTIRKLNGMPSQD"

CDS complement(3428081..3429394)
/codon_start=1
/note="gene_3157"
/transl_table=11
/translation="VVSYVVALPEVMSAAATDVASIGSVVATASQGVAGATTTVLAAA
EDEVSAAIAALFSAHGQDYQALSAQLAVFHERFVQALTGAAKGYAAAELANASLLQSE
FASGIGNGFATIHQEIQRAPTALAAGFTQVPPFAAAQAGIFTGTPSGAAGFDIASLWP
VKPLLSLSALETHFAIPNNPLLALIASDIPPLSWFLGNSPPPLLNSLLGQTVQYTTYD
GMSVVQITPAHPTGEYVVAIHGGAFILPPSIFHWLNYSVTAYQTGATVQVPIYPLVQE
GGTAGTVVPAMAGLISTQIAQHGVSNVSVVGDSAGGNLALAAAQYMVSQGNPVPSSMV
LLSPWLDVGTWQISQAWAGNLAVNDPLVSPLYGSLNGLPPTYVYSGSLDPLAQQAVVL
EHTAVVQGAPFSFVLAPWQIHDWILLTPWGLLSWPQINQQLGIAA"

CDS 3429715..3429915
/codon_start=1
/note="gene_3158"
/transl_table=11
/translation="MTATIGFRPTEKDEQIIKAAMRSGERKSDVIRRALQLLEREVWI
KQARTDAERLRDEDVSTEPDAW"

CDS 3429909..3430229
/codon_start=1
/note="gene_3159"
/transl_table=11
/translation="VVIRGAVYRVDFGDAKRGHEQRGRRYAVVISPGSMPWSVVTVVP
TSTSAQPAVFRPELEVMGTKTRFLVDQIRTIGIVYVHGDPVDYLDRDQMAKVEHAVAR
YLGL"

CDS complement(3430716..3431567)
/codon_start=1
/note="gene_3160"
/transl_table=11
/translation="VTTPGRPLTTLDKSDVLAGLFAVWHSLDALLDGLLETDWQATSP
LPGWDVKAVVSHIIGTESFLLGIAAPEPDTDVSALAHVRNPIGVMNECWVRHLGTESG
VGLLERFRAVTSQRRKVLASLSDDEWNAPTTTPSGPDSYGRFMRIRIFDCWMHEQDIR
AAVQRPSSDDELGGPASPLVLDEIAATMGFVVGKLAKAPDGSRVLLELTGPLSRSIRV
SVDGRARVVDDFGGPAPTATIRLDGLQFTRLAGGRPMSPARSQDVELGGDKELAGHIL
ERLNFVI"

CDS complement(3431604..3432086)
/codon_start=1
/gene="smpB"
/note="gene_3161"
/transl_table=11
/translation="VSKSSRGGRQIVASNRKARHNYSIIEVFEAGVALQGTEVKSLRE
GQASLADSFATIDDGEVWLRNAHIPEYRHGSWTNHEPRRNRKLLLHRRQIDTLVGKIR
EGNFALVPLSLYFAEGKVKVELALARGKQARDKRQDMARRDAQREVLRELGRRAKGMT
"

CDS complement(3432089..3432982)
/codon_start=1
/note="gene_3162"

/transl_table=11
/translation="VRFGFLLNEVLTGFRRNVTMTIAMILTTAISVGLFGGGMLVVRL
ADSSRAIYLDRVESQVFLTEDVSANDSSCDTTACKALREKIETRSDVKAVRFLNRQQA
YDDAIRKFPQFKDVAGKDSFPASFIVKLENPEQHKDFDTAMKGQPGVLDVLNQKELID
RLFAVLDGLSSAAFAVALVQAIGAILLIANMVQVAAYTRRTEIGIMRLVGASRWYTQL
PFLVEAMLAATMGVGIAVAGLMVVRALFLENALNQFYQANLIAKVDYADILFITPWLL
LLGVAMSGLTAYLTLRLYVRR"
CDS complement(3432983..3433672)
/codon_start=1
/note="gene_3163"
/transl_table=11
/translation="MITLDHVTKQYKSSARPALDDINVKIDKGEFVFLIGPSGSGKST
FMRLLLAAETPTSGDVRVSKFHVNKLRGRHVPKLRQVIGCVFQDFRLLQQKTVYDNVA
FALEVIGKRTDAINRVVPEVLETVGLSGKANRLPDELSGGEQQRVAIARAFVNRPLVL
LADEPTGNLDPETSRDIMDLLERINRTGTTVLMATHDHHIVDSMRQRVVELSLGRLVR
DEQRGVYGMDR"
CDS complement(3433716..3434153)
/codon_start=1
/note="gene_3164"
/transl_table=11
/translation="VKLSNQKRHWPGYLFGRIRTSTLVLIAAFLAVWWIYETYRPQAP
GPGDSPPTQVVPPGFVPDPDYTWVPRTRVQPPTVKATPTTTSSTPPVSPPETTTDSAV
PPPFELPPPFGPGTTTPTPPAPLPQPGPGPTAGTYPKSEPPTR"
CDS complement(3434155..3435081)
/codon_start=1
/note="gene_3165"
/transl_table=11
/translation="MTTSGTVLATSIAQHWHNFWRGEIGDWILNRGLRIVMLLIAAVL
AARFVTWLANRVTRRLDLGFTESDALVRSEATKHRQAVASVISWVSIVLIYVVVVYEV
IDVLPVPVGALVGPAAVLGAALGFGAQRLVQDLLAGFFIIVEKQYGFGDLVELSMVGS
PENAAGTVEDVTLRVTKLRSSEGEVFTVPNGNIVKSVNLSKDWARAVVDIPVPTSADL
GRVNEVLHQECEHARHDSLLGELLLDEPTVMGVERIEVDTVTLRLVARTLPGKQFEAG
RQLRVLVIRALTRAGIVTAADARAAVAESPEQ"
CDS complement(3435071..3436207)
/codon_start=1
/gene="prfB"
/note="gene_3166"
/transl_table=11
/translation="MPVTLAAVDPDRQADIAALDCTLTTVERVLDVEGLRSRIEKLEH
EASDPHLWDDQTRAQRVTSELSHTQGELRRVEELRRRLDDLPVLYELAAEEAGAAAAD
AVAEADAELKSLRADIEATEVRTLLSGEYDEREALVTIRSGAGGVDAADWAEMLMRMY
IRWAEQHKYPVEVFDTSYAEEAGIKSATFAVHAPFAYGTLSVEQGTHRLVRISPFDNQ
SRRQTSFAEVEVLPVVETTDHIDIPEGDVRVDVYRSSGPGGQSVNTTDSAVRLTHIPS
GIVVTCQNEKSQLQNKIAAMRVLQAKLLERKRLEERAELDALKADGGSSWGNQMRSYV
LHPYQMVKDLRTEYEVGNPAAVLDGDLDGFLEAGIRWRNRRNDD"
CDS 3436311..3437681
/codon_start=1
/note="gene_3167"
/transl_table=11
/translation="MRPYYIAIVGSGPSAFFAAASLLKAADTTEDLDMAVDMLEMLPT
PWGLVRSGVAPDHPKIKSISKQFEKTAEDPRFRFFGNVVVGEHVQPGELSERYDAVIY

AVGAQSDRMLNIPGEDLPGSIAAVDFVGWYNAHPHFEQISPDLSGARAVVIGNGNVAL
DVARILLTDPDVLARTDIADHALESLRPRGIQEVVIVGRRGPLQAAFTTLELRELADL
DGVDVVIDPAELDGITDEDAAAVGKVCKQNIKVLRGYADREPRPGHRRMVFRFLTSPI
EIKGKRKVERIVLGRNELVSDGSGRVAAKDTGEREELPAQLVVRSVGYRGVPTPGLPF
DDQSGTIPNVGGRINGSPNEYVVGWIKRGPTGVIGTNKKDAQDTVDTLIKDLGNAKEG
AECKSFPEDHADQVADWLAARQPKLVTSAHWQVIDAFERAAGEPHGRPRVKLASLAEL
LRIGLG"

CDS complement(3437682..3439265)
/codon_start=1
/note="gene_3168"
/transl_table=11
/translation="MRSWWGWGTVEDALSDQETQALQSRVAALVSGHDLSDHPPPDLT
ALGLAAPRVSPPASLAALCSSDLVDRAGHARGKAYRDIARNLQGQLDHLPDLIARPRS
EQDVIDVLDWCAREGIAVIPYGGGSSVVGGVEPRFDEPVVTVDVTAMSAVLEIDRVSR
AARIQAGAFGPSIEHQLRPHDLTLRHFPQSFGFSTLGGWLATRSGGHFATLYTHIDDL
TESLRIVTPVGISESRRLPGSGAGPSPDRLFLGSEGTLGIITEAWMRLQHRPRWQVTV
SVVFDDWAAAVAATRTIAQAGLYPANCRLLDPAEALLNAGTSVGGGLLVLAFESADHP
IDPWLHRAVAITAEHGGTVTAQRSRGTTSDATEHNAAANWRSAFLRMPYQRDALVRRG
VIAETFETACTWDGFDTLHAAVTDAARTAIWKVCGTGVVTCRFTHVYPDGPAPYYGIY
AGGRWGSLDAQWDEIKAAVSEAISASGGTITHHHAVGRDHRAWYDRQRPDPFAAALRA
AKSALDPAGILNPGVLLGR"

CDS complement(3439276..3439479)
/codon_start=1
/note="gene_3169"
/transl_table=11
/translation="MPGKEIDRVRATSALAVIRQHPVMVFFALSPVLAALGVMWWLAG
AGWAIVAALVLVVVGGAMIVLKR"

CDS 3440085..3441032
/codon_start=1
/gene="moaA1"
/note="gene_3170"
/transl_table=11
/translation="MPEEHYTWLPRQDLLSVKEISAIVDVFLSVGVSKVRITGGEPLI
RPDLPEIVRTLSAKVGEDSGLRDLAITTNGVLLADRVDGLKAAGMKRITVSLDTLQPE
RFKAISQRNSHDKVIAGIKAVAAAGFTDTKIDTTVMRGANHDELADLIEFARTVNAEV
RFIEYMDVGGATHWAWEKVFTKANMLESLEKRYGRIEPLPKHDTAPANRYALPDGTTF
GIIASTTEPFCATCDRSRLTADGLWLHCLYAISGINLREPLRAGATHDDLVETVTTGW
RRRTDRGAEQRLAQRERGVFLPLSTLKADPHLEMHTRGG"

CDS 3441083..3441478
/codon_start=1
/note="gene_3171"
/transl_table=11
/translation="MTVSTPEQHEQRASHDASEGKHNVCQGRLAALADAAVSEKLGAL
PGWQLLDMRLSRAFQCTNFDQSIDFMNRVASIANDINHHPDIAVLDKRSVRVTAWTRK
LGYLTDIDFDLAASVEAMYATEFADRPAR"

CDS 3441475..3441987
/codon_start=1
/gene="moaC"
/note="gene_3172"
/transl_table=11
/translation="MIDHALALTHIDERGAARMVDVSEKPVTLRVAKASGLVIMKPST

LRMISDGAAAKGDVMAAARIAGIAAAKRTGDLIPLCHPLGLDAVSVTITPCEPDRVKI
LATTTTLGRTGVEMEALTAVSVAALTIYDMCKAVDRAMEISQIVLQEKSGGRSGVYRR
SASDLACQSR"
CDS 3442004..3442255
/codon_start=1
/note="gene_3173"
/transl_table=11
/translation="MIKVNVLYFGAVREACDETPREEVEVQNGTDVGNLVDQLQQKYP
RLRDHCQRVQMAVNQFIAPLSTVLGDGDEVAFIPQVAGG"
CDS 3442377..3443045
/codon_start=1
/note="gene_3174"
/transl_table=11
/translation="MTSRDGFTIVCDWNGTLCDDRTILLDAVGQTLVNEGFEPLSQQQ
LIQRFARPLRTFFENACGRDLLTSEWERVQSTFRRIYRSREAEVTLVEDAYDVLAQGN
RSAAGQFLLSLAPHDELMHFVQKYGIAKWFNEIRGRTRPDQEKPMMLAELIMQRSLNP
TRVVHIGDSLEDAAAASAVGAISVLVTGASRQPPDRVMLKQLQPFVASSLKQALQYAG
GDGD"
CDS 3443029..3443592
/codon_start=1
/note="gene_3175"
/transl_table=11
/translation="VATVIDDEGTQVVAARLPFGWPADSGVTADIIEAAMELAIDTAR
HATAPFGAALLDVTTLRAFSGGNTYFESGDRFAHAETNVLRAAMSTLPELSNHVLIST
AEPCPMCAAASVLSGVRAIIFGTSIETLIQCGWFQIRISASDVVAASTRPTRPSVYSG
FLSHKTDLLYRNSENRRAMNPWTDPSH"
CDS 3443754..3445001
/codon_start=1
/note="gene_3176"
/transl_table=11
/translation="MTSSHLIDTEQLLADQLAQASPDLLRGLLSTFIAALMGAEADAL
CGAGYRERSDERSNQRNGYRHRDFDTRAATIDVAIPKLRQGSYFPDWLLQRRKRAERA
LTSVVATCYLLGVSTRRMERLVETLGVTKLSKSQVSIMAKELDEAVEAFRTRPLDAGP
YTFLAADALVLKVREAGRVVGVHTLIATGVNAEGYREILGIQVTSAEDGAGWLAFFRD
LVARGLSGVALVTSDAHAGLVAAIGATLPAAAWQRCRTHYAANLMAATPKPSWPWVRT
LLHSIYDQPDAESVVAQYDRVLDALTDKLPAVAEHLDTARTDLLAFTAFPKQIWRQIW
SNNPQERLNREVRRRTDVVGIFPDRASIIRLVGAVLAEQHDEWIEGRRYLGLEVLTRA
RAALTSTEEPAKQQTTNTPALTT"
CDS 3445079..3446248
/codon_start=1
/note="gene_3177"
/transl_table=11
/translation="MTEALIPAPSQISLTRDEVRRYSRHLIIPDIGVNGQQRLKDARV
LCIGAGGLGSPALLYLAAAGVGTIGIIDGDHVDESNLQRQIIHGTSDVGRPKVESAAE
AVAEINPHVRVTQYREMLTHDNALEIFGDHDLIVDGTDNFTTRYLINDAAVLAGKPYV
WGSIYRFNGQTSVFWPGRGPCYRCLHPAPPPPGLVPSCAEGGVLGAICATIASIQVTE
VLKLLTGVGTPLVGRLLMYEALDATYHQIRIAKNPDCAICGDAPTITELVDDSVSCAS
TQSVDPELVISCDELRTKQQSDQNFLLVDVREPAEFDIAHIPGSILIPKGEIGSAAGL
AQLPLDKEIVLYCKSGIRSAQALTTLKAAGLHNVKHLDGGIAEWTRTIDSSLLVY"
CDS 3446277..3447239
/codon_start=1

/gene="cysA3"
/note="gene_3178"
/transl_table=11
/translation="MARCDVLVSADWAESNLHAPKVVFVEVDEDTSAYDRDHIAGAIK
LDWRTDLQDPVKRDFVDAQQFSKLLSERGIANEDTVILYGGNNNWFAAYAYWYFKLYG
HEKVKLLDGGRKKWELDGRPLSSDPVSRPVTSYTASPPDNTIRAFRDEVLAAINVKNL
IDVRSPDEFSGKILAPAHLPQEQSQRPGHIPGAINVPWSRAANEDGTFKSDEELAKLY
ADAGLDNSKETIAYCRIGERSSHTWFVLRELLGHQNVNIAFGYGPHACPASAYSRMCL
TTFFTSLTQRFPQLQLARPFEDLERRGKGLHSVGIKELLVTWPT"

CDS 3447662..3448087
/codon_start=1
/note="gene_3179"
/transl_table=11
/translation="VGEDSLEDLEQRRARLYDQLAATGDFRRGSISENYRRCGKPNCV
CAQEGHPGHGPRYLWTRTVAGRGTKGRQLSVEEVDKVRAELANYHRFAQVSEQIVAVN
EAICEARPPNPAATAPPAGTTGHKKGGSATRSRRSSPPS"

CDS 3448265..3448591
/codon_start=1
/note="gene_3180"
/transl_table=11
/translation="VVRRLPRQEPRYRAGPGPVAPRLLPLPHLRAWDGAPWIWNLATA
ILPEATPIVDLYHARQHVHDLAGQLAPALGEHHSDWLTARLVDLDSGDIETLVQQPIG
QHTGHT"

CDS 3449034..3449903
/codon_start=1
/note="gene_3181"
/transl_table=11
/translation="VQFNVLGPLELNLRGTKLPLGTPKQRAVLAMLLLSRNQVVAADA
LVQAIWEKSPPARARRTVHTYICNLRRTLSDAGVDSRNILVSEPPGYRLLIGDRQQCD
LDRFVAAKESGLRASAKGYFSEAIRYLDSALQNWRGPVLGDLRSFMFVQMFSRALTGD
ELLVHTKLAEAAIACGRADVVIPKLERLVAMHPYRESLWKQLMLGYYVNEYQSAAIDA
YHRLKSTLAEELGVEPAPTIRALYHKILRQLPMDDLVGRVTRGRVDLRGGNGAKVEEL
TESDKDLLPIGLA"

CDS 3450047..3450283
/codon_start=1
/gene="CHLNCDRAFT_141452"
/note="gene_3182"
/transl_table=11
/translation="VTCQPAPSRSISPPGIPAPAPLILIGISIIGIPPPAAPAACVSA
TGLGGTPNPDIAEEAIEMGIDPCHVGGTDIDPTN"

CDS complement(3450330..3451178)
/codon_start=1
/note="gene_3183"
/transl_table=11
/translation="MVLGFSWLPPEINSARMFAGAGSGPLFAAASAWEGLAVDLWASA
SSFESVLAALTTGPWTGPASMSMAAAASPYVGWLSTVASQAQLAAIQARAAATAFEAA
LAATVHPTAVTANRVSLASLIAANVLGQNTPAIAATEFDYLEMWAQDVAAMVGYHAGA
KSVAATLAPFSLPPVSLAGLAAQVGTQVAGMATTASAAVTPVVEGAMASVPTVMSGMQ
SLVSQLPLQHASMLFLPVRILTSPITTLASMARESATRLGPPAGGLAAANTPNPVVRL
FRRSSR"

CDS 3451629..3452708

/codon_start=1
/note="gene_3184"
/transl_table=11
/translation="LIKPDDHTVNVVDIQVLKNAVLLACRAPSVHNSQPWRWVAESGS
EHTTVHLFVNRHRTVPATDHSGRQAIISCGAVLDHLRIAMTAAHWQANITRFPQPNQP
DQLATVECSPIDHVTAGQRNRAQAILQRRTDRLPFDSPMYWHLFEPALRDAVDKDVAM
LDVVSDDQRTRLVVASQLSEVLRRDDPYYHAELEWWTSPFVLAHGVPPDTLASDAERL
RVDLGRDFPVRSYQNRRAELADDRSKVLVLSTPSDTRADALRCGEVLSTILLECTMAG
MATCTLTHLIESSDSRDIVRGLTRQRGEPQALIRVGIAPPLAAVPAPTPRRPLDSVLQ
IRQTPEKGRNASDRNARETGWFSPP"
CDS complement(3452695..3453045)
/codon_start=1
/note="gene_3185"
/transl_table=11
/translation="VLNRMWKLVNDRLNYLTPTIKPIGYASSADGRRRRLYDAPQTPL
DRPLAARVLSAAQQADLITYRDSLNPAQIGRKIADLQNRLLILAKEKTEQLYLANIPT
ALPDIHKGILIKAG"
CDS complement(3453127..3453720)
/codon_start=1
/note="gene_3186"
/transl_table=11
/translation="VLQRVWSASGGQCGKYLAASMVLQLDGLERHGVLEFGRDRYGPE
VREELLAMSAASIDRYLKTAKAKDQISGVSTTKPSPLLRNSIKVRRAGDEVEAEPGFF
EGDTVAHCGPTLKGEFAHTLNLTDVHIGWVFTRTVRNNARTHILAGLKASVTEIPHGI
TGLDFDNGTVFLNKPVISWAGDNGIYFTRFRPYKKNH"
CDS 3454187..3454519
/codon_start=1
/note="gene_3187"
/transl_table=11
/translation="VVQGRTVLFRTAEGAKLFSAVAKCAVAFEADDHNVAEGWSVIVK
VRAQVLTTDAGVREAERAQLLPWTATLKRHCVRVIPWEITGRHFRFGPEPDRSQTFAC
EASSHNQR"
CDS complement(3454502..3455893)
/codon_start=1
/note="gene_3188"
/transl_table=11
/translation="MNHLTTLDAGFLKAEDVDRHVSLAIGALAVIEGPAPDQEAFLSS
LAQRLRPCTRFGQRLRLRPFDLGAPKWVDDPDFDLGRHVWRIALPRPGNEDQLFELIA
DLMARRLDRGRPLWEVWVIEGLADSKWAILTKLHHCMADGIAATHLLAGLSDESMSDS
FASNIHTTMQSQSASVRRGGFRVNPSEALTASTAVMAGIVRAAKGASEIAAGVLSPAA
SSLNGPISDLRRYSAAKVPLADVEQVCRKFDVTINDVALAAITESYRNVLIQRGERPR
FDSLRTLVPVSTRSNSALSKTDNRVSLMLPNLPVDQENPLQRLRIVHSRLTRAKAGGQ
RQFGNTLMAIANRLPFPMTAWAVGLLMRLPQRGVVTVATNVPGPRRPLQIMGRRVLDL
YPVSPIAMQLRTSVAMLSYADDLYFGILADYDVVADAGQLARGIEDAVARLVAISKRR
KVTRRRGALSLVV"
CDS 3456078..3457076
/codon_start=1
/note="gene_3189"
/transl_table=11
/translation="MNTHFPDAETVRTVLTLAVRAPSIHNTQPWRWRVCPTSLELFSR
PDMQLRSTDPDGRELILSCGVALHHCVVALASLGWQAKVNRFPDPKDRCHLATIGVQP

LVPDQADVALAAAIPRRRTDRRAYSCWPVPGGDIALMAARAARGGVMLRQVSALDRMK
AIVAQAVLDHVTDEEYLRELTIWSGRYGSVAGVPARNEPPSDPSAPIPGRLFAGPGLS
QPSDVLPADDGAAILALGTETDDRLARLRAGEAASIVLLTATAMGLACCPITEPLEIA
KTRDAVRAEVFGAGGYPQMLLRVGWAPINADPLPPTPRRELSQVVEWPEELLRQRC"
CDS complement(3457056..3458792)
/codon_start=1
/note="gene_3190"
/transl_table=11
/translation="MTTGGLVDENDGAAMRPLRHTLSQLRLHELLVEVQDRVEQIVEG
RDRLDGLVEAMLVVTAGLDLEATLRAIVHSATSLVDARYGAMEVHDRQHRVLHFVYEG
IDEETVRRIGHLPKGLGVIGLLIEDPKPLRLDDVSAHPASIGFPPYHPPMRTFLGVPV
RVRDESFGTLYLTDKTNGQPFSDDDEVLVQALAAAAGIAVANARLYQQAKARQSWIEA
TRDIATELLSGTEPATVFRLVAAEALKLTAADAALVAVPVDEDMPAADVGELLVIETV
GSAVASTVGRTIPVAGAVLREVFVNGIPRRVDRVDLEGLDELADAGPALLLPLRARGT
VAGVVVVLSQGGPGAFTDEQLEMMAAFADQAALAWQLATSQRRMRELDVLTDRDRIAR
DLHDHVIQRLFAIGLALQGAVPHERNPEVQQRLSDVVDDLQDVIQEIRTTIYDLHGAS
QGITRLRQRIDAAVAQFADSGLRTSVQFVGPLSVVDSALADQAEAVVREAVSNAVRHA
KASTLTVRVKVDDDLCIEVTDNGRGLPDEFTGSGLTNLRQRAEQAGGEFTLASVPGAS
GTVLRWSAPLSQ"
CDS complement(3458789..3459442)
/codon_start=1
/note="gene_3191"
/transl_table=11
/translation="VVKVFLVDDHEVVRRGLVDLLGADPELDVVGEAGSVAEAMARVP
AARPDVAVLDVRLPDGNGIELCRDLLSRMPDLRCLILTSYTSDEAMLDAILAGASGYV
VKDIKGMELARAVKDVGAGRSLLDNRAAAALMAKLRGAAEKQDPLSGLTDQERTLLGL
LSEGLTNKQIADRMFLAEKTVKNYVSRLLAKLGMERRTQAAVFATELKRSRPPGDGP"
CDS complement(3459470..3460276)
/codon_start=1
/note="gene_3192"
/transl_table=11
/translation="MSDPRPARAVVVGIDGSRAATHAALWAVDEAVNRDIPLRLVYVI
DPSQLSAAGEGGGQSAARAALHDASRKVEATGQPVKIETEVLCGRPLTKLMQESRSAA
MLCVGSVGLDHVRGRRGSVAATLAGSALCPVAVIHPSPAEPATTSQVSAVVAEVDNGV
VLRHAFEEARLRGVPLRAVAVHAAETPDDVEQGSRLAHVHLSRRLAHWTRLYPEVRVD
RAIAGGSACRHLAANAKPGQLFVADSHSAHELCGAYQPGCAVLTVRSANL"
CDS 3460861..3462006
/codon_start=1
/note="gene_3193"
/transl_table=11
/translation="MDYAFLPPEINSARMYSGPGPNSMLVAAASWDALAAELASAAEN
YGSVIARLTGMHWWGPASTSMLAMSAPYVEWLERTAAQTKQTATQARAAAAAFEQAHA
MTVPPALVTANRAELKALIASNLLGQNTAAIAAIEAQYAEMWAQDAAAMYGYATTSAA
ARQLTPFSSPQQTTNPAGLAAQNAAVTQAATNSAGNTPTALSQLSSFLSQAVEAPTGW
PNILPDDFTILDGILAAYATVGVTQDIESICAGIIGAGNNLGLLGAASENPAELAPGA
FGIDAALSSAEKGAAASMHDAVLASAGRAGSIGPMSVPPSWATPSSTPVSALSGAGLT
TLDGTDVAEHGTPGLPGVPAGTDKRASGVIPRYGVRLTVMSRPPAAG"
CDS 3462658..3463800
/codon_start=1
/note="gene_3194"
/transl_table=11

/translation="MDFALLPPEVNSARMYTGPGAGSLLAAAGGWDSLAAELATTAEA
YGSVLSGLAALHWRGPAAESMAVTAAPYIGWLYTTAEKTQQTAIQARAAALAFEQAYA
MTLPPPVVAANRIQLLALIATNFFGQNTAAIAATEAQYAEMWAQDAAAMYGYATASAA
AALLTPFSPPRQTTNPAGLTAQAAAVSQATDPLSLLIETVTQALQALTIPSFIPEDFT
FLDAIFAGYATVGVTQDVESFVAGTIGAESNLGLLNVGDENPAEVTPGDFGIGELVSA
TSPGGGVSASGAGGAASVGNTVLASVGRANSIGQLSVPPSWAAPSTRPVSALSPAGLT
TLPGTDVAEHGMPGVPGVPVAAGRASGVLPRYGVRLTVMAHPPAAG"
CDS complement(3463809..3464141)
/codon_start=1
/gene="PPE"
/note="gene_3195"
/transl_table=11
/translation="VGWEFGVLLILIAVLAVFLAPRLIPRGPRGDLASGTLLVTGVSP
RPDAGGQQYVTIAGIITGPTVNEYAVYQRMAVDVDQWPTVGQILPVVYSPKNPDNWTF
TPNGPPVG"
CDS 3464257..3465039
/codon_start=1
/note="gene_3196"
/transl_table=11
/translation="VSHDDLMLALALADRADELTRVRFGALDLRIDTKPDLTPVTDAD
RAVESDVRQTLGRDRPGDGVLGEEFGGSTTFTGRQWIVDPIDGTKNFVRGVPVWASLI
ALLEDGVPSVGVVSAPALQRRWWAARGRGAFASVDGARPHRLSVSSVAELHSASLSFS
SLSGWARLGLRERFIGLTDTVWRVRAYGDFLSYCLVAEGAVDIAAEPQVSVWDLAALD
IVVREAGGRLTSLDGVAGPHGGSAVATNGLLHDEVLTRLNAG"
CDS 3465059..3466147
/codon_start=1
/note="gene_3197"
/transl_table=11
/translation="MSDPFTIATKHWHRLHDSRIQCDVCPRACKLHEGQRGLCFVRGR
FDDQVKLTSYGRSSGFCVDPIEKKPLNHFLPGSATLSFGTAGCNLACKFCQNWDISKS
REIDVLANRAAPADIARTAHELGCRSVAFTYNDPTIFWEYAADVADACHDQGIKAVAV
TAGYMCPEPRAEFYRRVDAANVDLKAFTEDFYRKVCVSHLRNVLDTLAYLRHQTNVWL
EITTLLIPGRNDSDAEVAAECRWIRENLGVDVPVHFTAFHPDYKMMDTPATPPATLTR
AREIGIGEGLRFVYTGNVHDAVGGSTSCPGCRATVIVRDWYSIRHYALTEDGRCQACG
YQMPGVYDGPAGHWGQRRLPLLTSLSRM"
CDS 3466227..3467633
/codon_start=1
/note="gene_3198"
/transl_table=11
/translation="MTNTTSAANAAKPSGARTDRRGRTTGVGLAPHKRTGIDVALALL
TPIVGQEFLDKYRLRDPLNRSLRYGVKTMFATAGAATRQFQRVQGLRGGPTRLKSSGR
DYFDLTPDDDQKLIIETVDEFAEEVLRPAAHDADDAATYPSDLTAKAAELGITAINIP
EDFDGIAEHRSSVTNVLVAEALAYGDMGLALPILAPGGVASALTHWGSADQQATYLKE
FAGENVPQACVAITEPQPLFDPTRLKTTAVRTPSGYRLDGVKSLIPAAADAELFIVGA
QLGGKPALFIVESAASGLTVKADPSMGIRGAALGQVELCGVSVPLNARLGEDEASDND
YSEALALARLGWAALAVGTSHAVLDYVVPYVKQRQAFGEPIAHRQAVAFMCANIAIEL
DGLRLITWRGASRAEQGLPFAREAALAKRLGSDKGMQIGLDGVQLLGGHGYTKEHPVE
RWYRDLRAIGVAEGVVVI"
CDS 3467654..3468859
/codon_start=1
/note="gene_3199"

/transl_table=11
/translation="MAINLELPRKLQAIIVKTHQGAAEMMRPIARKYDLKEHAYPVEL
DTLINLFEGAAESFNFAGAHSLRDEDEGKDENHNGANMAAVVQTMEASWGDVAMMLSL
PYQGLGNAAISAVATDEQLERLGKVWAAMAITEPEFGSDSAAVSTTATLDGDEYVING
EKIFVTAGSRATHIVVWATLDKSLGRPAIKSFIVPREHPGVTVERLEHKLGIKGSDTA
VIRFDNARIPKGNLLGNPEIEVGKGFAGVMETFDNTRPIVAAMAVGIGRAALEEIRSV
LTGAGVEISYDKPSHTQSAAAAEFLRMEADWEASYLLSLRAAWQADNNIPNSKEASMS
KAKAGRMASDVTCKTVELAGTTGYSEQSLLEKWARDSKILDIFEGTQQIQQLVVARRL
LGLSSSELK"
CDS 3468911..3469930
/codon_start=1
/note="gene_3200"
/transl_table=11
/translation="LRLLAPVCTPASRLVGMRAVRVTRLEGPDAVEVAEVEEPTSAGV
VIEVHAAGVAFPDALLTRGRYQYRPEPPFVLGAEIAGVVRSAPDNSQVRSGDRVVGLT
MLTGGMAEVAVLSPERVFKLPDNMTFEAGAGVLFNDLTVYFALAVRGRLQAGETVLVH
GAAGGIGTSTLRLAPALGASRTVAVVSTQEKAELATVAGATDVVLAEGFKDAVQELTN
GRGVDIVVDPVGGDRFTDSLRSLAAGGRLLVIGFTGGEIPTVKVNRLLLNNIDVVGVG
WGAWSLTHPDALAQQWSQLERLLRSGKLPPPEPVVYPLDQAAAAIASLENRTAKGKVV
LRVRD"
CDS complement(3469982..3470410)
/codon_start=1
/note="gene_3201"
/transl_table=11
/translation="MTEQEMTEQWLEGCAVQRIMFRDGLVLNFDDYNELVISVPLQLT
LPAIETSPAEVVAIDPNDPADHERPLFDFAGATCTAFVWYDTGDLHLEFSDGHQIDVH
PDDRVTAWELYGKYHGYAACLAPGKLRVVRHDVADANGDQ"
CDS 3470518..3470919
/codon_start=1
/note="gene_3202"
/transl_table=11
/translation="VPDSSTALRILVYSDNVQTRERVMRALGKRLHPDLPDLTYVEVA
TGPMVIRQMDRGGIDLAILDGEATPTGGMGIAKQLKDELASCPPILVLTGRPDDTWLA
SWSRAEAAVPHPVDPIVLGRTVLSLLRAPAH"
CDS complement(3470952..3472181)
/codon_start=1
/note="gene_3203"
/transl_table=11
/translation="VSFVVLPPEINSLRMFIGAGTAPMLAAAAAWDGLAEELGTAAQS
FASVTAGLAGQAWQGPAALAMAAAAAPYAGWLTAAAAQSAGAAGQARAVASIFEAAQA
ATVLPAAVAANRDAFVQLVMTNLFGQNAPLIAAAEGVYEEMWAADVAAMSGYYSGASA
IAAQVVPWASLLQRFPGLGAGATGATGGESVGTGATGGESVGTGGGESVGTGGATASG
GGVGYVGGGVASAGLAAGDPAHGSVGQGNFGGGDVGAGDVVASSATSAHAGVVSPGFI
GAPLALAALGQMARGGTNSAPGTATESARAPEPAASAPPEAVVEVPELEVPAMGVLPT
VDPKVAAKAAPLSTTRVGQSAGSGIPESTLRTAQGQQASETSAAEETAPSLRPEAAAG
QLRPRVRQDPKIQMRGG"
CDS 3472441..3472932
/codon_start=1
/note="gene_3204"
/transl_table=11
/translation="MLKGTVTARSPGSSRQPGHRPAAALAAPYDGSWSELNVYIPILV

LAALAAAFAVVSVVIASLVGPSRFNRSKQAAYECGIEPASTGARTSIGPGAASGQRFP
IKYYLTAMLFIVFDIEIVFLYPWAVSYDSLGTFALVEMAIFMLTVFVAYAYVWRRGGL
TWD"
CDS 3472941..3473495
/codon_start=1
/note="gene_3205"
/transl_table=11
/translation="VGLEEQLPGGILLSTVEKVAGYVRKNSLWPATFGLACCAIEMMA
TAGPRFDIARFGMERFSATPRQADLMIVAGRVSQKMAPVLRQIYDQMAEPKWVLAMGV
CASSGGMFNNYAIVQGVDHVVPVDIYLPGCPPRPEMLLHAILKLHEKIQQMPLGINRE
RAIAEAEEAALLARPTIEMRGLLR"
CDS 3473492..3474202
/codon_start=1
/note="gene_3206"
/transl_table=11
/translation="MSPPNQDAQEGRPDSPTAEVVDVRRGMFGVSGTGDTSGYGRLVR
QVVLPGSSPRPYGGYFDDIVDRLAEALRHERVEFEDAVEKVVVYRDELTLHVRRDLLP
RVAQRLRDEPELRFELCLGVSGVHYPHETGRELHAVYPLQSITHNRRLRLEVSAPDSD
PHIPSLFAIYPTNDWHERETYDFFGIIFDGHPALTRIEMPDDWQGHPQRKDYPLGGIP
VEYKGAQIPPPDERRGYN"
CDS 3474202..3475524
/codon_start=1
/note="gene_3207"
/transl_table=11
/translation="MTAIADSAGGAGETVLVAGGQDWQQVVDAARSADPGERIVVNMG
PQHPSTHGVLRLILEIEGETVVEARCGIGYLHTGIEKNLEYRYWTQGVTFVTRMDYLS
PFFNETAYCLGVEKLLGITDEIPERVNVIRVLMMELNRISSHLVALATGGMELGAMTP
MFVGFRAREIVLTLFEKITGLRMNSAYIRPGGVAQDLPPNAATEIAEALKQLRQPLRE
MGELLNENAIWKARTQGVGYLDLTGCMALGITGPILRSTGLPHDLRKSEPYCGYQHYE
FDVITDDSCDAYGRYMIRVKEMWESMKIVEQCLDKLRPGPTMISDRKLAWPADLQVGP
DGLGNSPKHIAKIMGSSMEALIHHFKLVTEGIRVPAGQVYVAVESPRGELGVHMVSDG
GTRPYRVHYRDPSFTNLQSVAAMCEGGMVADLIAAVASIDPVMGGVDR"
CDS 3475521..3476279
/codon_start=1
/note="gene_3208"
/transl_table=11
/translation="VTQPPGQPVFIRLGPPPDEPNQFVVEGAPRSYPPDVLARLEVDA
KEIIGRYPDRRSALLPLLHLVQGEDSYLTPAGLRFCADQLGLTGAEVSAVASFYTMYR
RRPTGEYLVGVCTNTLCAVMGGDAIFDRLKEHLGVGHDETTSDGVVTLQHIECNAACD
YAPVVMVNWEFFDNQTPESARELVDSLRSDTPKAPTRGAPLCGFRQTSRILAGLPDQR
PDEGQGGPGAPTLAGLQVARKNDMQAPPTPGADE"
CDS 3476276..3477613
/codon_start=1
/note="gene_3209"
/transl_table=11
/translation="MTTQATPLTPVISRHWDDPESWTLATYQRHDRYRGYQALQKALT
MPPDDVISIVKDSGLRGRGGAGFATGTKWSFIPQGDTGAAAKPHYLVVNADESEPGTC
KDIPLMLATPHVLIEGVIIAAYAIRAHHAFVYVRGEVVPVLRRLHNAVAEAYAAGFLG
RNIGGSGFDLELVVHAGAGAYICGEETALLDSLEGRRGQPRLRPPFPAVAGLYGCPTV
INNVETIASVPSIILGGIDWFRSMGSEKSPGFTLYSLSGHVTRPGQYEAPLGITLREL
LDYAGGVRAGHRLKFWTPGGSSTPLLTDEHLDVPLDYEGVGAAGSMLGTKALEIFDET

TCVVRAVRRWTEFYKHESCGKCTPCREGTFWLDKIYERLETGRGSHEDIDKLLDISDS
ILGKSFCALGDGAASPVMSSIKHFRDEYLAHVEGGGCPFDPRDSMLVANGVDA"
CDS 3477610..3480030
/codon_start=1
/note="gene_3210"
/transl_table=11
/translation="VTQAADTDIRVGQPEMVTLTIDGVEISVPKGTLVIRAAELMGIQ
IPRFCDHPLLEPVGACRQCLVEVEGQRKPLASCTTVATDDMVVRTQLTSEIADKAQHG
VMELLLINHPLDCPMCDKGGECPLQNQAMSNGRTDSRFTEAKRTFAKPINISAQVLLD
RERCILCARCTRFSDQIAGDPFIDMQERGALQQVGIYADEPFESYFSGNTVQICPVGA
LTGTAYRFRARPFDLVSSPSVCEHCASGCAQRTDHRRGKVLRRLAGDDPEVNEEWNCD
KGRWAFTYATQPDVITTPLIRDGGDPKGALVPTSWSHAMAVAAQGLAAARGRTGVLVG
GRVTWEDAYAYAKFARITLGTNDIDFRARPHSAEEADFLAARIAGRHMAVSYADLESA
PVVLLVGFEPEDESPIVFLRLRKAARRHRVPVYTIAPFATGGLHKMSGRLIKTVPGGE
PAALDDLATGAVGDLLATPGAVIMVGERLATVPGGLSAAARLADTTGARLAWVPRRAG
ERGALEAGALPTLLPGGRPLADEVARAQVCAAWHIAELPAAAGRDADGILAAAADETL
AALLVGGIEPADFADPDAVLAALDATGFVVSLELRHSAVTERADVVFPVAPTTQKAGA
FVNWEGRYRTFEPALRGSTLQAGQSDHRVLDALADDMGVHLGVPTVEAAREELAALGI
WDGKHAAGPHIAATGPTQPEAGEAILTGWRMLLDEGRLQDGEPYLAGTARTPVVRLSP
DTAAEIGAADGEAVTVSTSRGSITLPCSVTDMPDRVVWLPLNSAGSTVHRQLRVTIGS
IVKIGAGS"
CDS 3480146..3481378
/codon_start=1
/note="gene_3211"
/transl_table=11
/translation="MTTFGHDTWWLVAAKAIAVFVFLMLTVLVAILAERKLLGRMQLR
PGPNRVGPKGALQSLADGIKLALKESITPGGIDRFVYFVAPIISVIPAFTAFAFIPFG
PEVSVFGHRTPLQITDLPVAVLFILGLSAIGVYGIVLGGWASGSTYPLLGGVRSTAQV
ISYEVAMGLSFATVFLMAGTMSTSQIVAAQDGVWYAFLLLPSFVIYLISMVGETNRAP
FDLPEAEGELVAGFHTEYSSLKFAMFMLAEYVNMTTVSALAATLFFGGWHAPWPLNMW
ASANTGWWPLIWFTAKVWGFLFIYFWLRATLPRLRYDQFMALGWKLLIPVSLVWVMVA
AIIRSLRNQGYQYWTPTLVFSSIVVAAAMVLLLRKPLSAPGARASARQRGDEGTSPEP
AFPTPPLLAGATKENAGG"
CDS 3481371..3482006
/codon_start=1
/note="gene_3212"
/transl_table=11
/translation="VANTDRPALPHKRAVPPSRADSGPRRRRTKLLDAVAGFGVTLGS
MFKKTVTEEYPERPGPVAARYHGRHQLNRYPDGLEKCIGCELCAWACPADAIYVEGAD
NTEEERFSPGERYGRVYQINYLRCIGCGLCIEACPTRALTMTYDYELADDNRADLIYE
KDRLLAPLLPEMAAPPHPRAPGATDKDYYLGNVTAEGLRGVRESQTTGDSR"
CDS 3482003..3482791
/codon_start=1
/note="gene_3213"
/transl_table=11
/translation="MTAVLASDVIVRTSTGEAVMFWVLSALALLGAVGVVLAVNAVYS
AMFLAMTMIILAVFYMAQDALFLGVVQVVVYTGAVMMLFLFVLMLIGVDSAESLKETL
RGQRVAAVLTGVGFGVLLISTIGQVATRGFAGLTVANANGNVEGLAALIFSRYLWAFE
LTSALLITAAVGAMVLAHRERFERRKTQRELSQERFRPGGHPTPLPNPGVYARHNAVD
VAALLPDGSYSELSVPRMLRTRGADGLQTPSPGAVSGSLEGGAS"
CDS 3482788..3483087

/codon_start=1
/note="gene_3214"
/transl_table=11
/translation="MNPANYLYLSVLLFTIGASGVLLRRNAIVMFMCVELMLNAVNLA
FVTFARMHGHLDAQMIAFFTMVVAACEVVVGLAIIMTIFRTRKSASVDDANLLKG"

CDS 3483098..3484999
/codon_start=1
/note="gene_3215"
/transl_table=11
/translation="VTTSLGTHYTWLLVALPLAGAAILLFGGRRTDAWGHLLGCAAAL
AAFGVGAMLLADMLGRDGLERAIHQQVFTWIPAGGLQVDFGLQIDQLSMCFVLLISGV
GSLIHIYSVGYMAEDPDRRRFFGYLNLFLASMLLLVVADNYVLLYVGWEGVGLASYLL
IGFWYHKPSAATAAKKAFVMNRVGDAGLAVGMFLTFSTFGTLSYAGVFAGVPAASRAV
LTAIGLLMLLGACAKSAQVPLQAWLGDAMEGPTPVSALIHAATMVTAGVYLIVRSGPL
YNLAPTAQLAVVIVGAVTLLFGAIIGCAKDDIKRALAASTISQIGYMVLAAGLGPAGY
AFAIMHLLTHGFFKAGLFLGSGAVIHAMHEEQDMRRYGGLRAALPVTFATFGLAYLAI
IGVPPFAGFFSKDAIIEAALGAGGIRGSLLGGAALLGAGVTAFYMTRVMLMTFFGEKR
WTPGAHPHEAPAVMTWPMILLAVGSVFSGGLLAVGGTLRHWLQPVVGSHEEATHALPT
WVATTLALGVVAVGIAVAYRMYGTAPIPRVAPVRVSALTAAARADLYGDAFNEEVFMR
PGAQLTNAVVAVDDAGVDGSVNALATLVSQTSNRLRQMQTGFARNYALSMLVGAVLVA
AALLVVQLW"

CDS 3484993..3486657
/codon_start=1
/gene="nuoM"
/note="gene_3216"
/transl_table=11
/translation="VVNNVPWLSVLWLVPLAGAVLIILLPPGRRRLAKWAGMVVSVLT
LAVSIVVAAEFKPSAEPYQFVEKHSWIPAFGAGYTLGVDGIAVVLVLLTTVLIPLLLV
AGWNDATDADDLSPASGRYPQRPAPPRLRSSGGERTRGVHAYVALTLAIESMVLMSVI
ALDVLLFYVFFEAMLIPMYFLIGGFGQGAGRSRAAVKFLLYNLFGGLIMLAAVIGLYV
VTAQYDSGTFDFREIVAGVAAGRYGADPAVFKALFLGFMFAFAIKAPLWPFHRWLPDA
AVESTPATAVLMMAVMDKVGTFGMLRYCLQLFPDPSTYFRPLIVTLAIIGVIYGAIVA
IGQTDMMRLIAYTSISHFGFIIAGIFVMTTQGQSGSTLYMLNHGLSTAAVFLIAGFLI
ARRGSRSIADYGGVQKVAPILAGTFMVSAMATVSLPGLAPFISEFLVLLGTFSRYWLA
AAFGVTALVLSAVYMLWLYQRVMTGPIAEGNERIGDLVGREMIVVAPLIALLLVLGVY
PKPVLDIINPAVENTMTTIGQHDPAPSVAHPVPAVGASRTAEGPHP"

CDS 3486654..3488249
/codon_start=1
/note="gene_3217"
/transl_table=11
/translation="MILPAPHVEYFLLAPMLIVFSVAVAGVLAEAFLPRRWRYGAQVT
LALGGSAVALIAVIVVARSIHGSGHAAVLGAIAVDRATLFLQGTVLLVTIMAVVFMAE
RSARVSPQRQNTLAVARLPGLDSFTPQASAVPGSDAERQAERAGATQTELFPLAMLSV
GGMMVFPASNDLLTMFVALEVLSLPLYLMCGLARNRRLLSQEAAMKYFLLGAFSSAFF
LYGVALLYGATGTLTLPGIRDALAARTDDSMALAGVALLAVGLLFKVGAVPFHSWIPD
VYQGAPTPITGFMAAATKVAAFGALLRVVYVALPPLHDQWRPVLWAIAILTMTVGTVT
AVNQTNVKRMLAYSSVAHVGFILTGVIADNPAGLSATLFYLVAYSFSTMGAFAIVGLV
RGADGSAGSEDADLSHWAGLGQRSPIVGVMLSMFLLAFAGIPLTSGFVSKFAVFRAAA
SAGAVPLVIVGVISSGVAAYFYVRVIVSMFFTEESGDTPHVAAPGVLSKAAIAVCTVV
TVVLGIAPQPVLDLADQAAQLLR"

CDS complement(3488255..3490024)

/codon_start=1
/note="gene_3218"
/transl_table=11
/translation="MNYSVLPPEINSLRMFTGAGSAPMLAASVAWDRLAAELAVAASS
FGSVTSGLAGQSWQGAAAAMAAAAAPYAGWLAAAAARAAGASVQAKAVASAFEAARAA
TVHPMLVAANRNAFVQLVLSNLFGQNAPAIAAAEAMYEQMWAADVAAMVGYHGGASAA
AAQLSSWSIGLQQALPAAPSALAAAIGLGNIGVGNLGGGNTGEYNLGSGNSGNANVGS
GNSGNANVGSGNDGATNLGSGNIGNTNLGSGNVGNVNLGSGNRGFGNLGNGNFGSGNL
GSGNTGSTNFGGGNLGSFNLGSGNIGSSNIGFGNNGDNNLGLGNNGNNNIGFGLTGDN
LVGIGALNSGIGNLGFGNSGNNNIGFFNSGNNNVGFFNSGNNNFGFGNAGDINTGFGN
AGDTNTGFGNAGFFNMGIGNAGNEDMGVGNGGSFNVGVGNAGNQSVGFGNAGTLNVGF
ANAGSINTGFANSGSINTGGFDSGDRNTGFGSSVDQSVSSSGFGNTGMNSSGFFNTGN
VSAGYGNNGDVQSGINNTNSGGFNVGFYNSGAGTVGIANSGLQTTGIANSGTLNTGVA
NTGDHSSGGFNQGSDQSGFFGQP"
CDS complement(3490272..3492332)
/codon_start=1
/note="gene_3219"
/transl_table=11
/translation="VAEGGVLLTGTRIFTKSPLFVAPFSYSLFYEYRDVHRCLSHWPG
RPVGEGAGLMNYSVLPPEINSLRMFTGAGSAPMLAASVAWDGLAAELAVAASSFGSVT
SGLAGQSWQGAAAAAMAAAAAPYAGWLAAAAARAAGASAQAKAVASAFEAARAATVHP
MLVAANRNAFVQLVLSNLFGQNAPAIAAAEAMYEQMWAADVAAMVGYHGGASAAAAAL
PSWQQALRGLPGLGQVASAISGGAASMFAAPAAATAAVTPPALNTGLGNIGSWNLGGG
NVGLLNLGSGNFGSLNLGGGNTGNANLGGGNWGFANLGSGNIGNTNFGNGNQGNLNFG
SGNLLGNGNFGFGNAFGDGNLGSGNVGSTNLGSGNFGSFNVGSGNMGMSNIGFGNLGN
NNLGFGNNGNNNIGFGLTGDNLVGIGALNSGIGNMGFGNSGNNNIGFFNSGNGNVGFF
NSGDGNTGFGNAGDVNTGFWNGGPFNTGFGNGGNTNFGFGNAGFQNMGHGNAGGVNVG
SGNAGLANTGDFNSGGVVSGIGGNTGSFNSGNLNTGFGNAGDLNTGLFNSGDVNTGIG
STVDQPGSVSGFGNTGTSVSGFNNSGNLTSGFGNMNSNVFDSTSGFQNIGDANVGFFN
SGNSNEGFFNTGMFNNGIYNSGVASTGIANSGNASSGVANSGDNSSGAFNQGDNQAGF
FGQP"
CDS complement(3492348..3492989)
/codon_start=1
/note="gene_3220"
/transl_table=11
/translation="MPRQAGRWSPTALRILGAAAELIALRGYSSTSTRDIAAAVGVEQ
PAIYKHFSAKRDILAALVRLAVEWPLELFGHITAMPVPAVVKLHRWLTESLDHLHASP
YVLVSILITPDLHQESFVAERELVAEMERALVGLIETGQGEGDVRAMHPLSAARLVQA
LFDALALPEFAVSPDEIVEFAMTALLSDPDRLAEIRAAADALEIQTAPPDRGL"
CDS complement(3493000..3494148)
/codon_start=1
/note="gene_3221"
/transl_table=11
/translation="MLSTDNRAELGDILTDIGDYLDDNPPALSLPPAAYTSSELWQLE
RERIFNRSWMLVAHVDQLAKTGDYVTVSVAGEPVMVVRDVDGQLHALSPICRHRLMLM
VEPGAGRIDTLTCQYHLWRYGLDGRLRGAPHMAANLDFNRRECRLPQFAVATWNGLVW
INLDADAEPIAAHLDLTDDEFAGYRLGEMVQVESWSHEWRANWKVAAENGHENYHVLG
LHRQTLEPFVPGGGDLDVRQYSRWALRLRVPFTVPVEAKSLQLNEVQKSNLVVLWTFP
NSALAIAGERVVWFGFIPQSIDRVQVLGGVLTTPELAADAAATAQTSQFVMAMINDED
RLGLEAVQVGAGSRFAERGHLSSKEWPGMLAFYRNLAMALVGDHPGAS"
CDS 3494296..3494673

```
                     /codon_start=1
                     /gene="HSISM1_445"
                     /note="gene_3222"
                     /transl_table=11
                     /translation="LQRERRRSQPSERETNGGNGRRPAPGDNTGPVSAHQVDGGANPD
                     NAASTCVGPDTTIITTDNTTASVAARRNTAATPTAITAASPTTTASPCDPTAADPTII
                     SPNTVESPRVPGCANEVMAASPG"
     CDS             complement(3494651..3495922)
                     /codon_start=1
                     /note="gene_3223"
                     /transl_table=11
                     /translation="VIQTCEVELRWRASQLTLAIATCAGVALAAAVVAGRWQLIAFAA
                     PLLGVLCSISWQRPVPVIQVHGDPDSQRCFENEHVRVTVWVTTESVDAAVELTVSALA
                     GMQFEALESVSRRTTTVSAVAQRWGRYPIRARVAVVARGGLLMGAGTVDAAEIVVFPL
                     TPPQSTPLPQTELLDRLGAHLTRHVGPGVEYADIRPYVPGDQLRAVNWVVSARRGRLH
                     VTRRLTDRAADVVVLIDMYRQPAGPATEATERVVRGAAQVVQTALRNGDRAGIVALGG
                     NRPRWLGADIGQRQFYRVLDTVLGAGEGFENTTGTLAPRAAVPAGAVVIAFSTLLDTE
                     FALALIDLRKRGHVVVAVDVLDSCPLQDQLDPLVVRMWALQRSAMYRDMATIGVDVLS
                     WPADHSLQQSMGALPNRRRRGRGRASRARLP"
     CDS             complement(3495952..3496914)
                     /codon_start=1
                     /note="gene_3224"
                     /transl_table=11
                     /translation="MIMPAATTTAHCEAVLDEIERVVVGKRSALTLILTAVLARGHVL
                     IEDLPGLGKTLIARSFAAALGLDFTRVQFTPDLLPADLLGSTIYDMQSGRFEFRAGPI
                     FTNLLLADEINRTPPKTQAALLEAMAEGQVSIDGQTHKLAMPFIVLATDNPIEYEGTY
                     PLPEAQLDRFAIRLELRYLSERDETSMLRRRLERGSADPTVNQVVDCHDLLAMRESVE
                     QVTVHEDVLHYVVSLANATRHHPQVAVGASPRAELDLVQLSRARALLLGRDYVIPEDV
                     KELATAAVAHRITLRPEMWVRKIAGADVVSELLRRLPVPRISGT"
     CDS             complement(3496922..3497404)
                     /codon_start=1
                     /note="gene_3225"
                     /transl_table=11
                     /translation="MKRLIALGIFLIVGIELLALILHDRRLVLAGSGLALALVLLNVR
                     RMLGNRDELTAAPDSDDLGEGLRRWLSNTETTIRWSESTRADWDRHLRPMLARRFEIA
                     TGHRQAKDPVAFAATGRMLFGDELWEWVNPNNVTHTGDRQPGPGRAALEEILQKLEQV
                     "
     CDS             complement(3497401..3498309)
                     /codon_start=1
                     /note="gene_3226"
                     /transl_table=11
                     /translation="VIVLLMLAGAALRGHLPADDGAPLAAAGGSRAALMFIVAALAAT
                     LALIALAIITRLRHPLPVAPSAGELSAMLGGAAGRPNWRVLLLGLGTILAWLLIAILL
                     ARLFVPDDVGPAAPIPDSTATPDASSTTPSRPQPPQDNNDDVLGILFASTIGLFLMVV
                     AGSLITSRRQRKSAPARISGDRIEAPAPSARSESLARAAEIGLAEMADLRREPREAII
                     ACYVAMERELSHVPGVAPQDFDTPTEVLARAVEHRALHGASAAALVSLFAEARFSPHV
                     MNEEHREVAMRLLRLVLDELSTRTAI"
     CDS             complement(3498440..3499066)
                     /codon_start=1
                     /note="gene_3227"
                     /transl_table=11
```

/translation="MKADLPSLDKAPGAGRPRDPRIDSAILSATAELLVQIGYSNLSL
AAVAERAGTTKSALYRRWSSKAELVHEAAFPAAPTALQAAAGDIAADIRMMIAATRDV
FTTPVVRAALPGLVADMTADAELNARVLARFADLFAAVRMRLREAVDRGEAHPDVDPD
RLIELIGGATMLRMLLYPDDMLDDAWVDQTTAIVVRGVHRAAPGGSVV"
CDS 3499111..3500247
/codon_start=1
/note="gene_3228"
/transl_table=11
/translation="VANEPAIGAIDRLQRSSRDVTTLPAVISRWLSSVLPGGAAPEVT
VESGVDSTGMSSETIILTARWQQDGRSIQQKLVARVAPAAEDVPVFPTYRLDHQFEVI
RLVGELTDVPVPRVRWIETTGDVLGTPFFLMDYVEGVVPPDVMPYTFGDNWFADAPAE
RQRQLQDATVAALATLHSIPNAQNTFSFLTQGRTSDTTLHRHFNWVRSWYDFAVEGIG
RSPLLERTFEWLQSHWPDDAAAREPVLLWGDARVGNVLYRDFQPVAVLDWEMVALGPR
ELDVAWMIFAHRVFQELAGLATLPGLPEVMREDDVRATYQALTGVELGDLHWFYVYSG
VMWACVFMRTGARRVHFGEIEKPDDVESLFYHAGLMKHLLGEEH"
CDS 3500247..3501371
/codon_start=1
/note="gene_3229"
/transl_table=11
/translation="MPQMLGPLDEYPLHQLPQPIAWPGSSDRNFYDRSYFNAHDRTGN
IFLITGIGYYPNLGVKDAFVLIRRADIQTAVHLSDAIDSDRLHQHVNGYRVEVVEPLR
KLRIVLDETEGVAADLTWEGLFDVVQEQPHVLRSGNRVTLDAQRFAQLGTWSGRIVVD
GERIAVDPATWLGSRDRSWGIRPVGEPEPAGRPADPPFEGMWWLYVPLAFDDFAVVLI
IQEEPDGFRSLNDCTRIWRDGHVEQLGWPRVRIHYRSGTRIPTGATIEASTPDGAPVH
FDVESKLAVPTHVGGGYGGDSDWSHGMWKGEKFVERRTYDMTDPTIIARAGFGVIDHV
GRALCRDGDGNPVQGWGLFEHGALGRHDPSGFADWSTLAP"
CDS 3501547..3502860
/codon_start=1
/note="gene_3230"
/transl_table=11
/translation="VVGAGFAGLAAARELTRQGHEVLVFEGRDRVGGRSLTGRVAGVP
ADMGGSFIGPTQDAVLALATELGIPTTPTHRDGRNVIQWRGSARSYRGTIPKLSLTGL
IDIGRLRWQFERIARGVPVAAPWDARRARELDDVSLGEWLRLVRATSSSRNLMAIMTR
VTWGCEPDDVSMLHAARYVRAAGGLDRLLDVKNGAQQDRVPGGTQQIAQAAAAQLGAR
VLLNAAVRRIDRHGAGVTVTSDQGQAEAGFVIVAIPPAHRVAIEFDPPLPPEYQQLAH
HWPQGRLSKAYAAYSTPFWRASGYSGQALSDEAPVFITFDVSPHADGPGILMGFVDAR
GFDSLPIEERRRDALRCFASLFGDEALDPLDYVDYRWGTEEFAPGGPTAAVPPGSWTK
YGHWLREPVGPIHWASTETADEWTGYFDGAVRSGQRAAAEVAALL"
CDS complement(3502855..3503754)
/codon_start=1
/note="gene_3231"
/transl_table=11
/translation="LTVRAADGTPLHTQVFGPPHGYPIVLTHGFVCAIRAWAYQIADL
AGDYRVIAFDHRGHGRSGVPRRGAYSLNHLAADLDSVLDATLAPRERAVVAGHSMGGI
TIAAWSDRYRHKVRRRTDAVALINTTTGDLVRKVKLLSVPRELSPVRVLAGRSLVNTF
GGFPLPGAARALSRHVISTLAVAADADPSATRLVYELFTQMSAAGRGGCAKMLVEEVG
SAHLNLDGLTVPTLVIGGVRDRLTPISQSRRIARTAPNVVGLVELPGGHCSMLERHQE
VNSHLRALAESVTRHVRDRRISS"
CDS complement(3503997..3504176)
/codon_start=1
/note="gene_3232"

```
                     /transl_table=11
                     /translation="MGRGPDKVAGRVWITTSRPGEEPTRIEVVLIAAYRNGRIHRIWE
                     TTWPSWRNVAALDDY"
     CDS             complement(3504452..3504988)
                     /codon_start=1
                     /note="gene_3233"
                     /transl_table=11
                     /translation="LKAAAELFYCEGINATGVELIANKASVSKRTLYQHFPSKSALVE
                     EYLRGLRQAAGEADKMPKASNATPRERLLALFDRPNRGDGRMRGCPFHNAAVEAAGEM
                     PGVERIVHSHKRDYIKGLARLAREAGAAHPRSLGNQLAVLFEGAAALSTSLDDAGPWA
                     HARAAAEVLIDQATARPV"
     CDS             3505147..3505854
                     /codon_start=1
                     /note="gene_3234"
                     /transl_table=11
                     /translation="MTSLAERTALVTGANRGMGREYVAQLLGRKVAKVYAATRNPLAI
                     DVSDPRVIPLQLDVTDAVSVAEAADLATDVGILINNAGISRASSVLDKDTSALRGELE
                     TNLFGPLALASAFADRIAERSGAIVNVSSVLAWLPLGMSYGVSKAAMWSATESMRIEL
                     APRGVQVVGVYVGLVDTDMGRFADAPKSDPADVVRQVLDGIEAGKEDVLADEMSRQVR
                     ASLNVPARERIARLMGN"
     CDS             3505869..3507356
                     /codon_start=1
                     /note="gene_3235"
                     /transl_table=11
                     /translation="MAMSAKASDDIAWLPATAQLAVLAAKKVSSAELVELYLSRIDTY
                     NASLNAIVTVDPDAARRVAKRSDAARARGDELGPLHGLPITVKDSYETAGMRTTCGRR
                     DLADYVPTQDAEAVARLRRAGAIIMGKTNMPTGNQDVQASNPVFGRTNNPWDAARTSG
                     GSAGGGAAATAAGLTSFDYGSEIGGSTRIPAHYCGLYGHKSTWRSVPLVGHIPSAPGN
                     PGRWGQADMACAGVQVRGARDIIPALEATVGPMRADGGFSYALAPPRAGALKDFRVAV
                     WAEDPHCPIDADVRRAMDDAVAALRAAGAHVVEQPATIPVDMAVSHNIFQSLVFGAFA
                     VDRSTLSPASAAALGLRAVRHPRGEAANALGATLQSHRAWLFADAARHEMRDRWAGFF
                     NEFDVLLLPVTPTPAPLHHNKDHDRLGRTIDVDGVSRSYWDQLKWNALANIAGTPATT
                     MPITTTATGLPIGIQAMGPAGGDRTTVEFAALLTEVLGGFRVPPL"
     CDS             complement(3507353..3507670)
                     /codon_start=1
                     /note="gene_3236"
                     /transl_table=11
                     /translation="MKELHDAISRRDGVRVLPATAGFVDEHREHAARWDLARIISALG
                     DEVAFGVVGSAEDPFEGEQLRLARERLADSVEITELAGGHLTTAEQPDRLAEVIAALP
                     ERS"
     CDS             complement(3507682..3508308)
                     /codon_start=1
                     /note="gene_3237"
                     /transl_table=11
                     /translation="MTHRASALISAQEWFSAGERVGYDAERPGINPRSPLRAFIRRAA
                     GTGVTRTFLPGWPDGSYGWAKVEAFLSSRFHFPRIYLDYIGHGDSDKPRDYPYSTFER
                     ADLVEALWHAEGIAQTVVVAFDYSCIVSLELLARRIDRERAGNDQRTRITACLLANGG
                     IFADGHTHAWYTTPLLTSPLGAAITPIGQRSWRMFAPFLRPSSRADTH"
     CDS             3508455..3509315
                     /codon_start=1
                     /note="gene_3238"
```

/transl_table=11
/translation="VPQRQAGDIGATYQDAPTKSINVGGTRFVYRRLGADAGVPVIFL
HHLGAVLDNWDPRVVDGIAAKHPVVTFDNRGVGASEGQTPDTVTTMADDAIAFVRALG
FDQVDLLGFSLGGFVAQVIAQQEPQLVRKIILAGTGPAGGVGIGKVTFGTIRESIKAT
LTFRDPKELRFFTRTDSGKSAARQFVKRLKERKDNRDKSITVRAFRSQLKAIHAWGTQ
KPSDLTSIGHPVLIANGDDDTMVPTSNSLDLADRLPDATLRIYPDAGHGGIFQHHAQF
VDDALQFLES"
CDS 3509446..3509805
/codon_start=1
/note="gene_3239"
/transl_table=11
/translation="LRLGAGFRKPVPTLLLEHRSRKSGKNFVAPLLYITDRNNVIVVA
SALGQAENPQWYRNLPPNPDTHIQIGSDRRPVRAVVASSDERARLWPRPVDAYADFDS
CQSWTERGIPVIILRPR"
CDS 3510045..3510338
/codon_start=1
/note="gene_3240"
/transl_table=11
/translation="VTAAEDKEERLRLSGTRIELEELLQLPVDVAYEGLLTDDVSESV
RKKLITLRAGPSRTACSNLRNPAGVGADDGARRDVVHQRDIADGTGVEPATHR"
CDS 3510626..3511915
/codon_start=1
/note="gene_3241"
/transl_table=11
/translation="VVHDEAGHELIERHMLEQLREVAEYTRVVLINGPRQAGKTTLLQ
QLHAELGGWLRSLDVDVERASARADPEGYIMSAPRPTFLDEVQCAGDPLILAIKTATD
RDRRPRQFFLSGSTRFLTVPTLSESLAGRVAILDLWPLSVAERSGVRPEIIAQLFTEP
QVVLGTEPAPVTRHEYLQLACAGGFPEVVQRPAGRARSRWFSDYLRTVTQRDVRELKR
IEQTDRLPRFMRYLAAITAQELNVAEAARVIGVDAGTIRSDLALFETVYLVHRLPAWS
RNLTAKIKKRSKIHVVDSGFAAWLRGQSADSLARPTAEGAGPIMETFVINELMKLRAA
TELEVDLYHFRDRDGREIDCILQTPDSRVVGVEVKASATVNVHDFRHLSFARDRLGDE
FITGVLFYTGARALPFGDRLMALPINLLWNGQSVSSL"
CDS complement(3512262..3512696)
/codon_start=1
/note="gene_3242"
/transl_table=11
/translation="VPLVYFDASAFVKLLTTETGSSLASALWDGCDAALSSRLAYPEV
RAALAAAARNHDLTESELADAERDWEDFWAATRPVELTATVEQHAGHLARAHALRGAD
AVHLASALAVGDPGLVVAVWDRRLHTGAHAAGCRVAPAQLDP"
CDS complement(3512699..3512947)
/codon_start=1
/note="gene_3243"
/transl_table=11
/translation="VTELRAHLSDWLDRARAGGEVVITERGIPIARLAALDSTDTLER
LTAEGVIGKATAQRPVAAGRPRPRPQRPVSDRVSDQRR"
CDS 3513388..3513726
/codon_start=1
/note="gene_3244"
/transl_table=11
/translation="VILLPQVERWFFALNRDAMASVTGAIDLLEMEGPTLGRPVVDKV
NDSTFHNMKELRPAGTSIRILFAFDPARQAILLLGGDKAGNWKRWYDNNIPIADQRSE

```
                     NWLASEHGGG"
     CDS             3513723..3514052
                     /codon_start=1
                     /note="gene_3245"
                     /transl_table=11
                     /translation="MTMARNWRDIRADAVAQGRVDLQRAAVAREEMRDAVLAHRLAEI
                     RKALGHARQADVAALMGVSQARVSKLESGDLSHTELGTLQAYVAALGGHLRIVAEFGE
                     NTVELTA"
     CDS             3514590..3514937
                     /codon_start=1
                     /note="gene_3246"
                     /transl_table=11
                     /translation="MAVTLDRAVEASEIVDALKPFGVTQVDVAAVIQVSDRAVRGWRT
                     GDIRPERYDRLAQLRDLVLLLSDSLTPRGVGQWLHAKNRLLDGQRPVDLLAKDRYEDV
                     RSAAESFIDGAYV"
     CDS             3514934..3515554
                     /codon_start=1
                     /note="gene_3247"
                     /transl_table=11
                     /translation="VKLADAIATAPRRTLKGTYWHQGPTRHPVTSCADPARGPGRYHR
                     TGEPGVWYASNKEQGAWAELFRHFVDDGVDPFEVRRRVGRVAVTLQVLDLTDERTRSH
                     LGVDETDLLSDDYTTTQAIAAARDANFDAVLAPAAALPGCQTLAVFVHALPNIEPERS
                     EVRQPPPRLANLLPLIRPHEHMPDSVRRLLATLTRAGAEAIRRRRR"
     CDS             complement(3515714..3516766)
                     /codon_start=1
                     /note="gene_3248"
                     /transl_table=11
                     /translation="MLPATEVDQQYRDWGEQRTRRVTRLVLVVPFEGHKDIFNLRPDQ
                     FTTMPPQVLRLQGHEIHLAIDNPSNDAAAINAAFHKQIANIEKYLGWSRRQIDLHNQG
                     LRNELPGMVARRREQLLATRNLQAEIGFPVRRRKDADTYAAPISRKSVRPRPHRPAGA
                     RAAFKPEPAMQDEDYQSALRVLRNQRNALERTPSVAAKLDGEEIRDMLLVGLNAQFEG
                     DAGGELFNGAGKTDILIRVDDRNIFIGECKVWSGPRTMDDALKQLFGYLVWRDTKAAI
                     LLFIRNKDVTAVIDNAIAKIKEHPNHKRCPAHRAGADQYEFTMHADGDPEREIHLTLI
                     PFALRPTAEVPTTTIP"
     CDS             3516665..3516925
                     /codon_start=1
                     /gene="J112_17100"
                     /note="gene_3249"
                     /transl_table=11
                     /translation="MPLEGHHENEPRHASGALFAPIPVLLVDLGGWQHESVVDAQIGA
                     FDRTQFDELFDDVINGRVQQVVGVPALKTLRRGLARLSEKSG"
     CDS             3517147..3517356
                     /codon_start=1
                     /note="gene_3250"
                     /transl_table=11
                     /translation="MITVLDMNGFKDARPDRLPLSASVWDIAQRYNKGGPTVTEALYE
                     ALKELEAQVIALQRSEGKGLLSRLS"
     CDS             3517391..3517576
                     /codon_start=1
                     /gene="TBJG_01937"
                     /note="gene_3251"
```

/transl_table=11
/translation="MDHPTEREWASIAEHTRASNFTGDLLRMPPYPLILTLRTLVGSA
EVVTASHTLFLSAATEY"
CDS complement(3517603..3518859)
/codon_start=1
/note="gene_3252"
/transl_table=11
/translation="VLSSSRRVKKGPGRRPQSAKRQRFMELRARGWSISAAGREVGVS
RTAANNWARGYKTYRRGEAVGFVPALDRLAVRQISSRYLSEEERINIADLRRSGLSIR
KIADQLGRAPSTVSRELRRNSRRDGQYRPFEAHRWAVQRRVRRHRRRIDKNPDLCELI
AELLAQRWSPQQIARHLRRKYPDDRSMWLCHESIYQAVYQPQSRLIRPPQVKSPHRGP
LRTGRTHRRAHLRPGRRRPRFAQPMLSIHQRPFDPADRSEPGHWEGDLIVGKNQGSAI
GTLVERQTRLIRLLHLPTHDAYCLRIAITETMSDLPVTLVRSITWDQGIEMARHIDIT
ADLGAPVYFCDSRSPWQRASNENSNGLLRQYFPKGTSLSTYTPDHLRAVEYEINNRPR
QVLGHRSPAELFTALLTSPDHQLLRR"
CDS complement(3518921..3519142)
/codon_start=1
/note="gene_3253"
/transl_table=11
/translation="VLRNGSSTVVRRAPTANRDAAGGGIKMPPPAAPDAPVQRPDSET
DWDALRAAYPRDAERVKRAMFIVSAATEN"
CDS complement(3519087..3519269)
/codon_start=1
/note="gene_3254"
/transl_table=11
/translation="VVAPIRFAAPRAAPGMSECAAIVTRREDREMGKQGDRKARKQRA
QEREQHRRQASANREP"
tRNA complement(3519656..3519732)
/note="identified by tRNAscan-SE; putative"
CDS 3519852..3520313
/codon_start=1
/note="gene_3255"
/transl_table=11
/translation="MIPQPLSQLGDLARRPGRRVLCSPKTAAPSISNATVASPAAPGL
ELSTGIALAFPRGPFVPAAAAWELQEATSGKFQLGLGTQVRKNVVHRYGMAFHRPGPR
LRYLLAVKACFAVFQTGTPDHHGEFDNPDFITAQWSPARIDPPGPSPAGPR"
CDS complement(3520483..3523437)
/codon_start=1
/note="gene_3256"
/transl_table=11
/translation="MPKLTRRSRILIMIALGVIVLLLAGPRLIDAYVDWLWFGELGYR
SVFTTMLATRIVVCLVAGVVVGGIVFGGLALAYRTRPVFVPDADNDPVARYRAVVLAR
LRLVGIGIPAAIGLLAGIVAQSYWARIQLFLHGGDFGVRDPQFGRDLGFYAFELPFYR
LMLSYMLVSVFLAFVANLVAHYIFGGIRLSGRTGALSRSARVQLVSLVGVLVLLKAVA
YWLDRYELLSHTRGGKPFTGAGYTDINAVLPAKLILMAIALICAAAVFSAIALRDLRI
PAIGLVLLLLSSLIVGAGWPLIVEQISVKPNAAQKESEYISRSITATRQAYGLTSDVV
TYRNYSGDSPATAEQVAADRATTSNIRLLDPTIVSPAFTQFQQGKNFYYFPDQLSIDR
YLDRNGNLRDYVVAARELNPDRLIDNQRDWINRHTVYTHGNGFIASPANTVRGIANDP
NQNGGYPEFLVNVVGANGTVVSDGPAPLDQPRIYFGPVISNTSADYAIVGRNGDDREY
DYETNIDTKRYTYTGSGGVPLGGWLARSVFAAKFAERNFLFSNVIGSNSKILFNRDPA
QRVEAVAPWLTTDSAVYPAIVNKRLVWIVDGYTTLDNYPYSELTSLSSATADSNEVAF

NRLVPDKKVSYIRNSVKATVDAYDGTVTLYQQDEKDPVLKAWMQVFPGTVKPKSDIAP
ELAEHLRYPEDLFKVQRMLLAKYHVNDPVTFFSTSDFWDVPLDPNPTASSYQPPYYIV
AKNIAKDDNSASYQLISAMNRFKRDYLAAYISASSDPATYGNLTVLTIPGQVNGPKLA
NNAITTDPAVSQDLGVIGRDNQNRIRWGNLLTLPVAQGGLLYVEPVYASPGASDAASS
YPRLIRVAMMYNDKVGYGPTVRDALTGLFGPGAGATATGIAPTEAAVPPSPAANPPPP
ASGPQPPPVTAAPPVPVGAVTLSPAKVAALQEIQAAIGAARDAQKKGDFAAYGSALQR
LDEAITKFNDAG"

CDS complement(3523553..3524551)
/codon_start=1
/note="gene_3257"
/transl_table=11
/translation="MVALVPIVVFGVLLAVVTVPFVALGPGPTFDTLGEIDGKQVVQI
VGTQTYPTSGHLNMTTVSQRDGLTLGEALALWLSGQEQLMPRDLVYPPGKSREEIEND
NAADFKRSEAAAEYAALGYLKYPKAVTVASVMDPGPSVDKLQAGDAIDAVDGTPVGNL
DQFTALLKNTKPGQEVTIDFRRKNEPPGIAQITLGKNKDRDQGVLGIEVVDAPWAPFA
VDFHLANVGGPSAGLMFSLAVVDKLTSGHLVGSTFVAGTGTIAVDGKVGQIGGITHKM
AAARAAGATVFLVPAKNCYEASSDSPPGLKLVKVETLSQAVDALHAMTSGSPTPSC"

CDS 3524671..3526071
/codon_start=1
/note="gene_3258"
/transl_table=11
/translation="MGDLPFGFSSGDDPPEDPSGRDKRGKDGADSGSGANPLGAFGIG
GEFNMADLGQIFTRLGEMFGGVGTAMAAGKTSGPVNYDLARQVASSSIGFIAPIPAAT
NSAIADAVHLADTWLDGATSLPAGATKAVGWSPTDWVDNTLATWKRLCDPMAQQISTV
WASSLPEEAKSMAGPLLSIMSQMGGIAFGSQLGQALGRLSREVLTSTDIGLPLGPKGV
AAILPGAVESFAAGLEQPRSEILTFLATREAAHHRLFSHVPWLASQLLGAVEAYAMGM
KIDMTGIEELARDINPTSLADPAAMEQLLSQGVFEPKATPAQTQALERLETLLALIEG
WVQTVVTAALGERIPGEAALSETLRRRRASGGPAEQTFATLVGLELRPRKLREAGALW
ERLTRAVGMDARDAVWQHPDLLPATDDLDDPAAFIDRVIGGDTSGIDEAIAELERDQQ
ARGADDSGHDGGPVDN"

CDS 3526344..3526976
/codon_start=1
/note="gene_3259"
/transl_table=11
/translation="MANLVAQLVGAGVATPLANPGNLDSRRRAASIRVHGRGPLSDLL
VQALRCSGARIRHSSQPHAAVTPAGVDLVVLSDYLVADPHMVRDLHTERVPHLPVRVR
DGTGMVGPLVVPGVTSCLGCADLHRSDRDAAWPAIAAQLRDTVGVADRATLLATAALA
LSQVNRVIAAVRGQEATPEPPSALNTTLEFDLNAGSIVARQWTRHPRCFC"

CDS complement(3526985..3527185)
/codon_start=1
/note="gene_3260"
/transl_table=11
/translation="VQEGGPQETMSARSTQHDAADALFRAIIETLDKHRNERTLTEDV
LDTLARAYASISTNVPEQGRLG"

CDS 3527323..3528666
/codon_start=1
/note="gene_3261"
/transl_table=11
/translation="MDDGSVSDIKRGRAARNAKLASIPVGFAGRAALGLGKRLTGKSK
DEVTAELMEKAANQLFTVLGELKGGAMKVGQALSVMEAAIPDEFGEPYREALTKLQKD
APPLPASKVHRVLDGQLGTKWRERFSSFNDTPVASASIGQVHKAIWSDGREVAVKIQY

PGADEALRADLKTMQRMVGVLKQLSPGADVQGVVDELVERTEMELDYRLEAANQRAFA
KAYHDHPRFQVPHVVASAPKVVIQEWIEGVPMAEIIRHGTTEQRDLIGTLLAELTFDA
PRRLGLMHGDAHPGNFMLLPDGRMGIIDFGAVAPMPGGFPIELGMTIRLAREKNYDLL
LPTMEKAGLIQRGRQVSVREIDEMLRQYVEPIQVEVFHYTRKWLQKMTVSQIDRSVAQ
IRTARQMDLPAKLAIPMRVIASVGAILCQLDAHVPIKALSEELIPGFAEPDAIVV"

CDS complement(3529408..3531510)
/codon_start=1
/note="gene_3262"
/transl_table=11
/translation="MSIASDPLIAGLDDQQREAVLAPRGPVCVLAGAGTGKTRTITHR
IASLVASGHVAAGQVLAVTFTQRAAGEMRSRLRALDAAARTGSGVGAVQALTFHAAAY
RQLRYFWSRVIADTGWQLLDSKFAVVARAASRTRLHASTDDVRDLAGEIEWAKASLIG
PEEYVTAVAAARRDPPLDAAQIAAVYSEYEALKARGDGVTLLDFDDLLLHTAAAIEND
AAVAEEFQDRYRCFVVDEYQDVTPLQQRVLSAWLGDRDDLTVVGDANQTIYSFTGASP
RFLLDFSRRFPDAAVVRLERDYRSTPQVVSLANRVIAAARGRVAGSKLRLSGQREPGP
VPSFHEHSDEPAEAATVAASIARLIASGTPPSEVAILYRVNAQSEVYEEALTQAGIAY
QVRGGEGFFNRQEIKQALLALQRVSERDTDAALSDVVRAVLAPLGLTAQPPVGTRARE
RWEALTALAELVDDELAQRPALQLPGLLAELRRRAEARHPPVVQGVTLASLHAAKGLE
WDAVFLVGLADGTLPISHALAHGPNSEPVEEERRLLYVGITRARVHLALSWALSRSPG
GRQSRKPSRFLNGIAPQTRADPVPGTSRRNRGAAARCRICNNELNTSAAVMLRRCETC
AADVDEELLLQLKSWRLSTAKEQNVPAYVVFTDNTLIAIAELLPTDDAALIAIPGIGA
RKLEQYGSDVLQLVRGRT"

CDS 3531634..3531888
/codon_start=1
/note="gene_3263"
/transl_table=11
/translation="MITAALTIYTTSWCGYCLRLKTALTANRIAYDEVDIEHNRAAAE
FVGSVNGGNRTVPTVKFADGSTLTNPSADEVKAKLVKIAG"

CDS complement(3531901..3532842)
/codon_start=1
/gene="nudC"
/note="gene_3264"
/transl_table=11
/translation="VTNVSGVDFQLRSVPLLSRVGADRADRLRTDMEAAAAGWPGAAL
LRVDSRNRVLVANGRVLLGAAIELADKPPPEAVFLGRVEGGRHVWAVRAALQPIADPD
IPAEAVDLRGLGRIMDDTSSQLVSSASALLNWHDNARFSALDGAPTKPARAGWSRVNP
ITGHEEFPRIDPAVICLVHDGADRAVLARQAAWPERMFSLLAGFVEAGESFEVCVARE
IREEIGLTVRDVRYLGSQPWPFPRSLMVGFHALGDPDEEFSFSDGEIAEAAWFTRDEV
RAALAAGDWSSASESKLLLPGSISIARVIIESWAACE"

CDS complement(3532901..3533947)
/codon_start=1
/note="gene_3265"
/transl_table=11
/translation="LRGLDEKLTAQPGYALVGVLRIPQRRASPARVISRRVVVAVVAL
LLTAGIVYVDRDGYLDAQGDRLTFLDCLYYAAVTLSTTGYGDITPISEFARAINIFVI
TPLRIAFLILLVGTTLEVLTETSRQAYKIQRWRSRVRNHTVVIGYGTKGKTAVAAMVS
DELVPGEIVVVDTDSGVLERAAAAGLVTVHGDATKSDVLRLAGTQHASSIIVATSRDD
TAVLVTLTAREIAPKAKIVASIREAENQHLLRQSGADTVVVSSETAGRLLGIATTTPS
VVEMIEDLLTPEAGLAVAEREVEQAEVGGSPRHLRDIVLGVVRDGQLLRIGAPEVDAI
EASDRLLYIRQVGR"

CDS complement(3534030..3537335)

/codon_start=1
/note="gene_3266"
/transl_table=11
/translation="MTQTAAPARYSPAELACALGLFPPTAEQAAVIAAPPGPLVVIAG
AGAGKTETMAARVVWLVANGYAEPGQVLGLTFTRKAAGQLLRRVRSRLARLAGIGLGC
GDPAACAPVVSTYHAFAGSLLRDYGLLLPLEPDTRLLSETELWQLAFDVVSGYDGVLC
TDKSPAAVTSIVVRLWGQLGEHLVDTRALRDTHVELERLVHALPAGRYQRDRGPSQWL
LRMLATQTQRAELVPLLDALGERMHAGKVMDFAMQMASAARLAATSPQVGQDLRRRYR
VVLLDEYQDTGHAQRVVLSSLFGGGVDDGLALTAVGDPIQSIYGWRGASATNLPRFTT
DFPLSDGTPAPVLELLTSWRNPPQALRVANGISAEARRRSVAVRALRPRPDAPPGAVR
CALLPDVQAEREWIADHLRMRYQRAEADGVKPPTAAVLVRRNADAAAIADTLRARGIP
AEVVGLAGLLSIPEVAEVVAMLRLVADPTAGAAAMRVLTGPRWRLGARDLAALWRRAL
TLSGESPSTASPESIAMAASADADNPCLADAISDPGSAEGYSVAGYGRIGALAGELSA
LRGRLGHSLPDLVAEVRRVLGVDCEVRASAPVSGGWAGPEHLDAFADVVAGYAERASA
RSSEASVAGLLAYLDVAEVVENGLPPAELTVACDRVQVLTVHAAKGLEWQVVAVAHLS
RGVFPSTVSRSSWLTDPAELPPLLRGDRASAGAHGIPVLDTSAVADRKQLSDKISEHR
RLLDRRRVDEERRLLYVAVTRAEDTLLVSGHHWGPTGTKPRGPSEFLCELKDIIDRSA
AAGDPCGVVEQWASAPAGDERNPLCDNAIEAVWPADPLAARRGDVERGAALVAAAMSA
DLPGSTTDIDHPPRPGDAPWSTDVDALLAERAHAARGAPARGLPNHLSVSSLVELVGD
PVGARQRLMCRLPKRPDPHAWLGDAFHAWVQQFYGAELLFDLGDLPGAADREVGDPEE
LAALQRAFTASSWAARTPAAVEVPFEMPIGDTVVRGRIDAVFVDPDGGATVVDWKTGK
PPHGPAAMRQAAVQLAVYRLAWAALRGCPTSSVRTAFYYVRSGITVVPDELPAPGELA
MLLTDCAGRRSDT"

CDS complement(3537332..3540499)
/codon_start=1
/note="gene_3267"
/transl_table=11
/translation="MSHIWGVEAGAALAPGLRGPVLVLGGPGTGKSTLLVEAAVAHIG
AGTDPESVLLLTGSGRMGMRARSALTTALLRSRTNGPCRAAIREPVVRTVHSYAYAVL
RKAAQRAGDALPRLLTSAEQDAIIRELLAGDAEDGPAATTTWPAHLRPALTTAGFATE
LRNLLARCAERGLDPLELQQLGRRRGRPEWIAAGQFAQRYEQVMLLRGAVGLAAPQAT
APALSAAELVGAALEAFAVDPELLAAERARVRTLLVDDAQQLDPQAARLVRMLAAGTE
LALIAGDPNQAVFGFRGGEPTGLLADDPPPAGGAPIPSVTLTVSHRCAPAVARAVTGI
ARRLPGRSVGRRIEGTGTEVGSVTVRLAGSAHAEAAMIADALRRAHLIDGVPWSQMAV
IVRSVPRAVRLPRALAAAGVPVAPPAVGGPLSAEPAVRALLTVLEATADGLDGDQALL
LLTGPIGGVDPVSLRQLRRTLQRARPGQTSRKFGDLLVEVLGGDAPPSGPGSRALRRV
RAVLTAAARCHRSGSLGGQDPRHTLWAAWQRSGLQRRWLAASEHGGAAAVQATRDLET
VTALFDITDHYVSRTSGASLRGLVEHVTALQLPVVRPEPAAPTEQVMVLSAHAALGHE
WDLVVIAGLQDGLWPNTVPRGGVLGTQRLLDELDGVTKDASMRAPLLAEERRLLVTAM
GRARRRLLVTAVDSDAGGGGHEAVLPSAFFFEIAQWADGDGEPVAMQPVSAPRVLSAA
AVVGRLRAVVCAPACAVDDADRDCAATQLARLAKAGVPGADPSEWHGLAPVSTSDPLC
DSDDLVTLTPSTLQALNDCPLRWLAERHGGTNTRELPSAVGSVLHALFAEPGRSESQL
LAELDRVWGHLPFGAQWYSANELARHRAMIQAFVQWRAQSRSELTEVGVEVDIDGALE
DGSGQARKIRLRGRADRLERDPAGRLVIVDIKTGKTPVSKDDAQQHAQLAMYQLAVAE
GLVRAGDEPGGARLVYVGKSGAAGVAERKQDPLTPAARDEWRNLVRQLAAATAGPQFI
ARRNDGCTHCPLRPGCPAHVRGSAP"

CDS 3540585..3540794
/codon_start=1
/gene="TBCG_03138"
/note="gene_3268"
/transl_table=11

```
                     /translation="MAAVVGGGPQDEIPEADAVEQGRAVDFDDEAGLDTAYLSGGAGD
                     RDASEADVVDQAFVVPVADDEEIDR"
     CDS             3540825..3541610
                     /codon_start=1
                     /note="gene_3269"
                     /transl_table=11
                     /translation="VIIDLHVQRYGPSGPARVLTIHGVTEHGRIWHRLAHHLPEIPIA
                     APDLLGHGRSPWAAPWTIDANVSALAALLDNQGDGPVVVVGHSFGGAVAMHLAAARPD
                     QVAALVLLDPAVALDGSRVREVVDAMLASPDYLDPAEARAEKATGAWADVDPPVLDAE
                     LDEHLVALPNGRYGWRISLPAMVCYWSELARDIVLPPVGTATTLVRAVRASPAYVSDQ
                     LLAALDKRLGADFELLDFDCGHMVPQAKPTEVAAVIRSRLGPR"
     CDS             3541613..3541918
                     /codon_start=1
                     /note="gene_3270"
                     /transl_table=11
                     /translation="MAPVTDEQVELVRSLVAAIPLGRVSTYGDIAALAGLSSPRIVGW
                     IMRTDSSDLPWHRVIRASGRPAQHLATRQLELLRAEGVLSVDGRVALSEIRYEFPPG"
     CDS             complement(3541925..3542803)
                     /codon_start=1
                     /note="gene_3271"
                     /transl_table=11
                     /translation="MGSTRLTGVNVEPPPEHVLVAFGLAGAQPILLGAGWEGGWRCGE
                     VVLSMVADNARAAWSARVRETLFVDGVRLARPVRSTDGRYVVSGWRADTFVAGAPEPR
                     HDEVVSAAVRLHEATGKLERPRFLTQGPAAPWAEIDVFVAADRAGWEERPLQSVPPGV
                     PTAPPAADPQRSIDLINQLAGLRKPTKSPNQLVHGDLYGTVLFAGTAPPGITDITPYW
                     RPASWAAGVAVVDALSWGAADDGLIERWNALPEWPQMLLRALMFRLAVYALHPRSTAE
                     AFPGLAHTAALVRLVL"
     CDS             complement(3542830..3544008)
                     /codon_start=1
                     /note="gene_3272"
                     /transl_table=11
                     /translation="VSTSLPPLVEPASALSREEVARYSRHLIIPDLGVDGQKRLKNAR
                     VLVIGAGGLGAPTLLYLAAAGVGTIGIVDFDVVDESNLQRQVIHGVADVGRSKAQSAR
                     DSIVAINPLIRVRLHELRLAPSNAVDLFKQYDLILDGTDNFATRYLVNDAAVLAGKPY
                     VWGSIYRFEGQASVFWEDAPDGLGVNYRDLYPEPPPPGMVPSCAEGGVLGIICASVAS
                     VMGTEAIKLITGIGETLLGRLLVYDALEMSYRTITIRKDPSTPKITELVDYEQFCGVV
                     ADDAAQAAKGSTITPRELRDWLDSGRKLALIDVRDPVEWDIVHIDGAQLIPKSLINSG
                     EGLAKLPQDRTAVLYCKTGVRSAEALAAVKKAGFSDAVHLQGGIVAWAKQMQPDMVMY
                     "
     CDS             complement(3544099..3545085)
                     /codon_start=1
                     /note="gene_3273"
                     /transl_table=11
                     /translation="VLRDEWREPLRALRDPLAATDRRVRARRDRKRQWRKQTWLGRFV
                     STYGWRAYALPVLMVLTTVVVYQTVTGTSTPRPAAAQTVRDSPAIGVVGTAILDAPPR
                     GLAVFDANLPAGTLPDGGPFTEAGDKTWRVVPGTTPQVGQGTVKVFRYTVEIENGLDP
                     TMYGGDNAFAQMVDQTLTNPKGWTHNPQFAFVRIDSGKPNFRISLVSPTTVRGGCGYE
                     FRLETSCYNPSFGGMDRQSRVFINEARWVRGAVPFEGDVGSYRQYVINHEVGHAIGYL
                     RHEPCDQQGGLAPVMMQQTFSTSNDDAAKFDPDFVKADGKTCRFNPWPYPIP"
     CDS             3545302..3545988
                     /codon_start=1
```

/note="gene_3274"
/transl_table=11
/translation="MSDLAKTAQRRALRSSGSARPDEDVPAPNRRGNRLPRDERRGQL
LVVASDVFVDRGYHAAGMDEIADRAGVSKPVLYQHFSSKLELYLAVLHRHVENLVSGV
HQALSTTTDNRQRLHVAVQAFFDFIEHDSQGYRLIFENDFVTEPEVAAQVRVATESCI
DAVFALISADSGLDPHRARMIAVGLVGMSVDCARYWLDADKPISKSDAVEGTVQFAWG
GLSHVPLTRS"

CDS complement(3545975..3546268)
/codon_start=1
/gene="TB9"
/note="gene_3275"
/transl_table=11
/translation="MPMEGATVEVKIGITDSPRELVFSSAQTPSEVEELVSNALRDDS
GLLTLTDERGRRFLIHTARIAYVEIGVADARRVGFGVGVDAAAGSAGKVATSG"

CDS 3546731..3547132
/codon_start=1
/note="gene_3276"
/transl_table=11
/translation="LRPQLPPETVTTVAPPGTGPTTVPTRTPTAAPPQTAVPPPAPLN
PRTVVYRVTGTKQLFDLVNVVYTDARGFPVTDFNVSLPWTKMVVLNPGVQTESVVATS
LYSRLNCSIVNTGAQTVVASTNNAIIATCTR"

CDS complement(3547142..3547837)
/codon_start=1
/note="gene_3277"
/transl_table=11
/translation="MPSPSSADQVADSPRPRLPADHPGVNELFALLAYGEVAAFYRLT
DEARMAPDLRGRISMASMAAAEMGHYELLRNALERRGVDVVSAMSKYTSALENYHRLT
TPSTWLEALVKTYVADALAADLYLEIADGLPDEVADVVRAALSETGHSQFVVAEVRAA
VTASGKQRSRLALWSRRLLGEAITQAQLVLADHDELVDLVVSGSGGLSQLGAFFDRLQ
QTHDQRMRELGLS"

CDS 3548105..3549679
/codon_start=1
/note="gene_3278"
/transl_table=11
/translation="VKHTTESTFAKLGVRDEIVRALGEEGIKRPFAIQELTLPLALDG
EDVIGQARTGMGKTFAFGVPLLQRITSGDGTRPLTGAPRALVVVPTRELCLQVTDDLA
TAGKYLTAGPDTDDAAAVRRRLSVVSIYGGRPYEPQIEALRAGADVVVGTPGRLLDLC
QQGHLQLGGLSVLVLDEADEMLDLGFLPDIERILRQIPADRQSMLFSATMPDPIITLA
RTFMVRPTHIRAEAPHSSAVHDATEQFVYRAHALDKVELVSRVLQARDRGATMIFTRT
KRTAQKVADELTERGFAVGAVHGDLGQLAREKALKAFRTGGIDVLVATDVAARGIDID
DVTHVINYQCPEDEKMYVHRIGRTGRAGRTGVAVTLVDWDELPRWSMIDQALGLGSPD
PAETYSNSPHLYAELAIPATAGGTVGPARKSQGRRRDTDCDGQKTAQHARNTPRRRRT
RGGKPVTGHPGTNPISSPIVGGDATSEPGSGTASDSGSDVVSGSRSGNGEAARRRRRR
RRRPTHAQDGFAARAN"

CDS 3549692..3550915
/codon_start=1
/note="gene_3279"
/transl_table=11
/translation="MVKPERRTKTDIAAAATIAVVVAVAASLIWWTSDARATISRPAA
VAVPTPAPAREVPTSLKQLWTAASPATRVPVVVGGTVATGDGRQVDGRDPATGESLWS
YARDTDLCGVTWVYHYAVAVYRYDRGCGQVSTIDGSTGRRGAARSGYADPRVRLFSDG

TTVLSAGDTRLELWRSDMVRMLAYGEIDARVKPSNRGLQSGCTLESAAASSAAVSVLE
ACTNQAGLRLVLLRPGKEDDEPIQRIVPEPGARPGSGARVLVVSQNNTAVYLPARSGA
QPRVDVIDETGATVSSTLLAKPPSTSAVASRTGNLVTWWTGDALLVFDAGNLTQRYTI
AAGETTAPVGPGVMMAGQLLVPVTGGIGVYDPVSGANNRYIPVTRPPSTSAVIPAVSG
SRVIEQRGDTLVALG"

```
CDS             complement(3550991..3551791)
                /codon_start=1
                /note="gene_3280"
                /transl_table=11
                /translation="MTDTRVLAVANQKGGVAKTTTVASLGAAMVEKGRRVLLVDLDPQ
                GCLTFSLGQDPDKLPVSVHEVLLGEVEPNAVLVTTMEGMTLLPANIDLAGAEAMLLMR
                AGREYALKRALAKFSDRFDVVIIDCPPSLGVLTLNGLTAADEAIVPLQCEMLAHRGVG
                QFLRTVADVQQITNPNLRLLGALPTLYDSRTTHTRDVLLDVADRYDLQVLAPPIPRTV
                RFAEASASGSSVMAGRKNKGAVAYRELAQALLKHWKTGRPLPTFTVDL"
CDS             3551945..3552556
                /codon_start=1
                /note="gene_3281"
                /transl_table=11
                /translation="MGVRNHRLLLLRHGETAWSTLGRHTGGTEVELTDTGRTQAELAG
                QLLGELELDDPIVICSPRRRTLDTAKLAGLTVNEVTGLLAEWDYGSYEGLTTPQIRES
                EPDWLVWTHGCPAGESVAQVNDRADSAVALALEHMSSRDVLFVSHGHFSRAVITRWVQ
                LPLAEGSRFAMPTASIGICGFEHGVRQLAVLGLTGHPQPIAAG"
CDS             3552745..3553671
                /codon_start=1
                /note="gene_3282"
                /transl_table=11
                /translation="MVPDGVLRARKLPDWPTGPLPKVRVAAALPPPADYLTRIGRARD
                LLAAFDGPLHKVVLARAVQLTADAPLDARVLLRRLVVADPTAYGYLVDLTSAGNDDTG
                AALVGASPELLVARSGNRVMCKPFAGSAPRAADPKLDAANAAALASSAKNRHEHQLVV
                DTMRVALEPLCEDLTIPAQPQLNRTAAVWHLCTAITGRLRNISTTAIDLALALHPTPA
                VGGVPTKAATELIAELEGDRGFYAGAVGWCDGRGDGHWVVSIRCAQLSADRRAALAHA
                GGGIVAESDPDDELEETTTKFATILTALGVEQ"
CDS             3553819..3554151
                /codon_start=1
                /note="gene_3283"
                /transl_table=11
                /translation="MRGHVAEVNGGVAAMALWFLNFSTWDGVAGIYVEDLFVWPRFRR
                RGLARGLLSTLARECVDNRYTRLAWSVLNWNSDAIALYDRIGGQPQHEWTIYRLSGPR
                LAALAAPR"
CDS             complement(3554103..3554510)
                /codon_start=1
                /note="gene_3284"
                /transl_table=11
                /translation="VRGAGLIVAVQGGAALVVAAALLVRGLAGADQHIVNGLGTAGWF
                VLVGGAVLAAGCRLAVGKLWGRGLAVFAQLLLLPVAWYLIVGSHQPAIGIPVGIIALG
                VLVLLFSPPSIRWAAGRDQRGAASAANRGPDSR"
variation       3554661..3554716
                /note="supplemented from AL2122/97"
CDS             3554823..3555788
                /codon_start=1
                /note="gene_3285"
```

```
                     /transl_table=11
                     /translation="MRAVLIVNPTATATTPAGRDLLAHALESRLQLTVEHTNHRGHGT
                     ELGQAAVADGVDLVVVHGGDGTVSAVVNGMLGRPGTTPVRPVPAVAVVPGGSANVLAR
                     ALGISADPIAATNQLIQLLDDYGRHQQWRRIGLIDCGERWAVFNAGMGVDAEVVAAVE
                     AERDKGGKVTAWRYIRAAVRAVLACTRREPALTLQLPNRDPITGVHFVFVSNSSPWTY
                     ANNRPVWTNPDCRFESGLGVFATTSMKVVPTLRVVRQMFAKQPKFEFNHVINNDDVAC
                     LRVTSMGPPIASQFDGDYLGVRETMTFRAVPDALAVVAPPARKRI"
     CDS             3556068..3556322
                     /codon_start=1
                     /note="gene_3286"
                     /transl_table=11
                     /translation="MDWRHKAVCRDEDPELFFPVGNSGPALAQIADAKLVCNRCPVTT
                     ECLSWALNTGQDSGVWGGMSEDERRALKRRNARTKARTGV"
     CDS             complement(3556384..3557889)
                     /codon_start=1
                     /note="gene_3287"
                     /transl_table=11
                     /translation="MSTLGDLLAEHTVLPGSAVDHLHAVVGEWQLLADLSFADYLMWV
                     RRNDGVLVCVAQCRPNTGPTVVHTDAVGTVVAANSMPLVAATFSGGVPGREGAVGQQN
                     SCQHDGHSVEVSPVRFGDQVVAVLTRHQPELAARRRSGHLETAYRLCATDLLRMLAEG
                     TFPDAGDVAMSRSSPRAGDGFIRLDVDGVVSYASPNALSAYHRMGLTTELEGVNLIDA
                     TRPLISDPFEAHEVDEHVQDLLAGDGKGMRMEVDAGGATVLLRTLPLVVAGRNVGAAI
                     LIRDVTEVKRRDRALISKDATIREIHHRVKNNLQTVAALLRLQARRTSNAEGREALIE
                     SVRRVSSIALVHDALSMSVDEQVNLDEVIDRILPIMNDVASVDRPIRINRVGDLGVLD
                     SDRATALIMVITELVQNAIEHAFDPAAAEGSVTIRAERSARWLDVVVHDDGLGLPQGF
                     SLEKSDSLGLQIVRTLVSAELDGSLGMRDARERGTDVVLRVPVGRRGRLML"
     CDS             complement(3557906..3558145)
                     /codon_start=1
                     /note="gene_3288"
                     /transl_table=11
                     /translation="MAEKSGVMMAEDVRAEIVASVLEVVVNEGDQIDKGDVVVLLESM
                     KMEIPVLAEAAGTVSKVAVSVGDVIQAGDLIAVIS"
     CDS             complement(3558406..3558711)
                     /codon_start=1
                     /note="gene_3289"
                     /transl_table=11
                     /translation="VSENCGPTDAHADHDDSHGGMGCAEVIAEVWTLLDGECTPETRE
                     RLRRHLEACPGCLRHYGLEERIKALIGTKCRGDRAPEGLRERLRLEIRRTTIIRGGP"
     CDS             complement(3558708..3559259)
                     /codon_start=1
                     /note="gene_3290"
                     /transl_table=11
                     /translation="MSSPVSSRRLANLVKESLQGSVLGGVVSDAVLPAVSDDVKPGAG
                     EDAYRVPVVVAAGSGAVVQVGGLEVGSAAVAGEVADTVAELFVCRPTEPDVGDFVGLA
                     GGAGDAGQAGQQFGLGVGVRGESFGARRRLALSTVGASGATAGLRKTHDGHHGCQARG
                     ALTQRRLYIGNPSEITDTRMVHQ"
     CDS             complement(3559256..3560023)
                     /codon_start=1
                     /note="gene_3291"
                     /transl_table=11
                     /translation="VSTATSLLGEKRLARMLARPVSAPVLSGDTANEGTQLIKMADID
```

GVTGSAGLQPGPSEETDEELTARFERDAIPLLDQLYGGALRMTRNPADAEDLLQETMV
KAYAGFRSFRHGTNLKAWLYRILTNTYINSYRKKQRQPAEYPTEQITDWQLASNAEHS
STGLRSAEVEALEALPDTEIKEALQALPEEFRMAVYYADVEGFPYKEIAEIMDTPIGT
VMSRLHRGRRQLRGLLADVARDRGFARGEQAHEGVSS"

CDS 3560206..3561054
/codon_start=1
/note="gene_3292"
/transl_table=11
/translation="MSLNGKTMFISGASRGIGLAIAKRAARDGANIALIAKTAEPHPK
LPGTVFTAAKELEEAGGQALPIVGDIRDPDAVASAVATTVEQFGGIDICVNNASAINL
GSITEVPMKRFDLMNGIQVRGTYAVSQACIPHMKGRENPHILTLSPPILLEKKWLRPT
AYMMAKYGMTLCALGIAEEMRADGIASNTLWPRTMVATAAVQNLLGGDEAMARSRKPE
VYADAAYVIVNKPATEYTGKTLLCEDVLVESGVTDLSVYDCVPGATLGVDLWVEDANP
PGYLPA"

CDS complement(3561371..3562795)
/codon_start=1
/note="gene_3293"
/transl_table=11
/translation="VRFAKLSDGLSDGIVTLSPLCLDDVDAHLAGGDERLVRWLSGMP
STRASVEAYIRHCREQWVTGGPLRSFGIRTVAETIVGTIDLRFDGEGLASGQVNVAYG
LYPSWRGRGLATRAVDLVCQYAAEHGATEAVIKVEPENSASARVALRAGFAFVRRICE
QDGTVFDRYERVLRAKMHADEVDIDEDLVRRLLRAQFPQWADLPIAPVRSAGTDNAMY
RLGEDLAVRIPRIGWAIESLRTEQQWLPRIAAHLGVASPVPVGLGSPAEGFGWPWSVC
RWVAGENPSAAEFVEPNRAVEDLADFITTLRATDPMGGPPAKRGAPLGEQDAEVRAAL
AALDGIIDVHAATAAWESALRVPPYAGPPMWFHGDLSRFNILTAQGRLTGVIDFGLMG
VGDPSVDLIIAWNLLSAPARAQFRVAVGAADDDWMRGRGRALAIALIALPYYQDTNPP
LAASARYAIGEVLADFRYGARPGC"

CDS complement(3562919..3563677)
/codon_start=1
/note="gene_3294"
/transl_table=11
/translation="MCGRFAVTTDPAQLAEKITAIDEATGCGGGKTSYNVAPTDTIAT
VVSRHSEPDDEPTRRVRLMRWGLIPSWIKAGPGGAPDAKGPPLINARADKVATSPAFR
SAVRSKRCLVPMDGWYEWRVDPDATPGRPNAKTPFFLHRHDGALLFTAGLWSVWKSYR
SAPPLLSCTVITTDAVGELAEIHDRMPLLLAEEDWDDWLNPDAPPDPELLARPPDVRD
IALRQVSTLVNNVRNNGPELLEPARSQPEQIQLL"

CDS 3563780..3565084
/codon_start=1
/note="gene_3295"
/transl_table=11
/translation="VTVPGSKSQTNRALVLAALAAAQGRGASTISGALRSRDTELMLD
ALQTLGLRVDGVGSELTVSGRIEPGPGARVDCGLAGTVLRFVPPLAALGSVPVTFDGD
QQARGRPIAPLLDALRELGVAVDGTGLPFRVRGNGSLAGGTVAIDASASSQFVSGLLL
SAASFTDGLTVQHTGSSLPSAPHIAMTAAMLRQAGVDIDDSTPNRWQVRPGPVAARRW
DIEPDLTNAVAFLSAAVVSGGTVRITGWPRVSVQPADHILAILRQLNAVVIHADSSLE
VRGPTGYDGFDVDLRAVGELTPSVAALAALASPGSVSRLSGIAHLRGHETDRLAALST
EINRLGGTCRETPDGLVITATPLRPGIWRAYADHRMAMAGAIIGLRVAGVEVDDIAAT
TKTLPEFPRLWAEMVGPGQGWGYPQPRSGQRARRATGQGSGG"

CDS 3565081..3566073
/codon_start=1
/note="gene_3296"

/transl_table=11
/translation="LRPGDYDESDVKVRSGRSSRPRTKTRPEHADAEAAMVVSVDRGR
WGCVLGGRPDRRITAMRARELGRTPIVVGDDVDVVGDLSGRPDTLARIVRRAPRRTVL
RRTADDTDPTERVVVANADQLLIVVALADPPPRTGLVDRALIAAYAGGLTPILCLTKT
DLAPAEPFGKQFADLELTVTAAGVDDPLLAVADLLAGKITVLLGHSGVGKSTLVNRLV
PEADRAVGEVTEIGRGRHTSTRSVALPLGDTLSGSGWVIDTPGIRSFGLAHIQPDNVL
LAFSDLAEATRECPRGCGHMGPPADPECALDTLSGPAARRAAAARRLLAVLSQT"
CDS complement(3566106..3567389)
/codon_start=1
/note="gene_3297"
/transl_table=11
/translation="MAITDVDVFAHLTDADIENLAAELDAIRRDVEESRGERDARYIR
RTIAAQRALEVSGRLLLAGSSRRLAWWTGALTLGVAKIIENMEIGHNVMHGQWDWMND
PEIHSSTWEWDMSGSSKHWRYTHNFVHHKYTNILGMDDDVGYGMLRVTRDQRWKRYNI
FNVVWNTILAIGFEWGVALQHLEIGKIFKGRADREAAKTRLREFSAKAGRQVFKDYVA
FPALTSLSPGATYRSTLTANVVANVIRNVWSNAVIFCGHFPDGAEKFTKTDMIGEPKG
QWYLRQMLGSANFNAGPALRFMSGNLCHQIEHHLYPDLPSNRLHEISVRVREVCDRYD
LPYTTGSFLVQYGKTWRTLAKLSLPDKYLRDNADDAPETRSERMFAGLGPGFAGADPV
TGRRRGLKTAIAAVRGRRRSKRMAKSVTEPDDLAA"
CDS complement(3567467..3568609)
/codon_start=1
/note="gene_3298"
/transl_table=11
/translation="MSKKHTTLNASIIDTRRPTVAGADRHPGWHALRKIAARITTPLL
PDDYLHLANPLWSARELRGRILGVRRETEDSATLFIKPGWGFSFDYQPGQYIGIGLLV
DGCWRWRSYSLTSSPAASGSARMVTVTVKAMPEGFLSTHLVAGVKPGTIVRLAAPQGN
FVLPDPAPPLILFLTAGSGITPVMSMLRTLVRRNQITDVVHLHSAPTAADVMFGAELA
ALAADHPGYRLSVRETRAQGRLDLTRIGQQVPDWRERQTWACGPEGVLNQADKVWSSA
GASDRLHLERFAVSKTAPAGAGGTVTFARSGKSVAADAATSLMDAGEGAGVQLPFGCR
MGICQSCVVDLVEGHVRDLRTGQRHEPGTRVQTCVSAASGDCVLDI"
CDS complement(3568719..3569228)
/codon_start=1
/note="gene_3299"
/transl_table=11
/translation="VTQVYIPATLAMLQRLVADGALWPVNGTAFAVTPTLRESYAEGD
DEELAEVALREAALASLRLLAADIGATADALPPRRAVLAAEVDDATYRPDLDDAVVRL
AGPITIDQVVAAYVDNAGAEPAVMAAIAVIDAADLGDEDAELVVGDAQDHDLAWYANQ
ELPFLLDLL"
CDS complement(3569225..3570112)
/codon_start=1
/note="gene_3300"
/transl_table=11
/translation="VDIPSVDVSTATNDGASSRAKGHRSAAPGRRKISDAVYQAELFR
LQTEFVKLQEWARHSGARLVVIFEGRDGAGKGGAIKRITEYLNPRVARIAALPAPTDR
ERGQWYYQRYIAHLPAKGEIVLFDRSWYNRAGVEKVMGFCTPQEYVLFLRQTPIFEQM
LIDDGILLRKYWFSVSDAEQLRRFKARRNDPVRQWKLSPMDLESVYRWEDYSRAKDEM
MVHTDTPVSPWYVVESDIKKHARLNMMAHLLSTIDYADVEKPKVKLPPRPLVSGNYRR
PPRELSTYVDDYVATLIAR"
CDS complement(3570136..3570570)
/codon_start=1
/note="gene_3301"

/transl_table=11
/translation="LVDLPVGEGNAVVRLSQIAHATESNPTAASLVDARTIVTLSGLA
PATLHAMGVRVATSFSARLFNLLITNAPGTQSQMYIAGTKLLETYSVPPLLHNQALAI
SVTSYNGMLYFGINADRDAMSDVDLLPGLLSQALDELLEASR"
CDS complement(3570563..3571546)
/codon_start=1
/note="gene_3302"
/transl_table=11
/translation="VVTRLSASDASFYQLENTATPMYVGLLLILRRPRAGLSYEALLE
TVEQRLPQIPRYRQKVQEVKLGLARPVWIDDRDFDITYHVRRSALPSPGSDEQLHELI
ARLAARPLDKSRPLWEMYLVEGLEKNRIALYTKSHQALINGVTALAIGHVIADRTRRP
PAFPEDIWVPERDPGTTRLLLRAVGDWLVRPGAQLQAVGSAVAGLVTNSGQLVETGRK
VLDIARTVARGTAPSSPLNATVSRNRRFTVARASLDDYRTVRARYDCDVHDVVLTVIA
GALGNWLMSRGEAVAPTATVRAMAPLSVYADDQLDSTGPGQAYQPGDTVLG"
CDS 3571747..3572298
/codon_start=1
/note="gene_3303"
/transl_table=11
/translation="LTISPIANSPGDTFAVTPVVEYEPPPRNIPPCGQSSHAARRPHT
PQLARRQPIRPSGRAPAAVTSTAKSPRLRQAGTFADAALRRVLEVIDRRRPVGQLRPL
LAPGLVDSVLAVSRTAAGHQQGAAMLRRIRLTPAGPDTADTAAEVFGTYSRGDRIHAI
ACRVEQRPAGNETRWLMVALHIG"
CDS complement(3572316..3573473)
/codon_start=1
/note="gene_3304"
/transl_table=11
/translation="VEVSRALLFELGVLLAVLAVLGAVARRFALSPIPVYLLAGLSLG
NGGILGVAAAGEFIATGAPIGVVLLLLALGLEFSATEFASSLRHHLPSAGVDIVLNAT
PGAVAGWLLGLDGVAILGLAGVTYISSSGVIARLLEDLRRLGNRETPAVLSVLVLEDF
AMAAYLPLFAVLATDGSWLEAVVGMTVAIAALLGAFAASYRWGHHVGRLVTHPDSEQL
LLRVLGITLIVAAVAESLHASAAVGAFLVGLTLTGETADRARMVLTPLRDLFATIFFL
GIGLSVDPGKLVSMLPVALALAAVTAATKVATGMFAARREGVARRGQLRAGTALVARG
EFSLIIIGLAGASIPGVAALATAYVFVMAIVGPILARYTGGGLPAAAVASN"
CDS complement(3573478..3573960)
/codon_start=1
/note="gene_3305"
/transl_table=11
/translation="MDVKEVLLPGVGLRYEFTSYRGDRIGIVARRSGGFDVVLYGRDD
PDEARPVLRLTDEEAEAVAQILGAPRIAERFTELTREVPGLKAGQIHIRAGSLFVDRP
LGDTRARTRTGASIVAIVRDEDVLASPGPTDVLRAGDVLIVIGTEDGIAGVEQIVEKG
"
CDS complement(3574021..3574755)
/codon_start=1
/note="gene_3306"
/transl_table=11
/translation="MKRYLTIIYGAASYLVFLVAFGYAIGFVGDVVVPRTVDHAIAAP
IGQAVVVNLVLLGVFAVQHSVMARQGFKRWWTRFVPPSIERSTYVLLASVALLLLYWQ
WRTMPAVIWDVRQPAGRVALWALFWLGWATVLTSTFMINHFELFGLRQVYLAWRGKPY
TEIGFQAHLLYRWVRHPIMLGFVVAFWATPMMTAGHLLFAIGATGYILVALQFEERDL
LAALGDQYRDYRREVSMLLPWPHRHT"
CDS complement(3574814..3578020)

/codon_start=1
/note="gene_3307"
/transl_table=11
/translation="VYRFLSSQKSLPGTPRAPARMHISLHGGKGFANLTRRRRPSSAS
VLLVAGFGAFLAFLDSTIVNIAFPDIQRSFPSYDIGSLSWILNGYNIVFAAFMVAAGR
LADLLGRRRTFLSGVLVFTIASGLCAVAGSVEQLVAFRVLQGIGAAILVPASLALVVE
GFDAARRAHAIGLWGAAAAIAAGLGPPIGGLLVEWAGWRWVLLVNVPLGIVAAIATKR
MLVESRASGRRRMPDLRGALLLAVTLGLVTLGLVKGPDWGWLSVATVGSFLASVLTSV
GFVHSSRSHPAPLVEPALLRSRSFVAGNLLTLVAAAGFYCYGLTHVLYLNYVWHYSLL
KAGFAIAPAAVVAAVVAAALGRVAGRHGHRVIVLVGALVWAGSLVWYLQRVGSEPDFL
RVWLPGQLLQGIGVGATLPVLSSAALAEVAKGGSYATSSAVVSTTRQLGAVLGVAVMV
ILIGKPEHGTAEEALRRGWAMAAICFIAVAVAAAVLGRTNRNPVQMPAPEPAIAPRLE
PPIPQPAAAPIEHWAAGDADPLGNLPLFAGLDAATLAQLGEHVEDVELEAGCYLFHEG
DPSDSLYVIRTGRVQVLQDSIVLKELGRGEVLGELGLLIDAPRSATVRALRDTKLVRL
TKAQFDEIADHGALAALVKVLATRLREAPPPATDSTSPEVVVSVIGVSGDAPVPAVAA
GLLTALSARLRAVDPGRVDRDGLDRAERVADKVVLHAAVEDAGWRDFCLRVADRIVLV
AGDPNPQAARLPARARGADLVLAGPAASREHRRQWEELITPRSVHVVHYRRILENVRP
LAARIAGRSIGLVLGGGGARGFAHLGVLDELERVGVTIDRFAGTSMGAVIAVFGACGM
DAATADAYAYEYFIRHNPLSDYAFPVRGLVRGRRTLTLLEAAFGDRLVEELPKEFRCV
SVDLLARRPVVHRRGRLVDVIGCSLRLPGIYPPQVYNGRLHVDGGVLDNLPVSTRASP
DGPLIAVSIGLGGGGPGSARQDGSPKVPGIGDTLMRTMTIGSQRGADAALSLAQVVIR
PDTGAVGLLEFHQIDAAREAGRVAAREAMPHIMALLNR"
CDS complement(3578039..3580831)
/codon_start=1
/note="gene_3308"
/transl_table=11
/translation="VADYVGTLSDDVEKLTDAELRAKTDEFKRRLADQKNPETLDDLL
PEAFAVAREAAWRVLDQRPFDVQVMGAAALHLGNVAEMKTGEGKTLTCVLPAYLNALA
GNGVHIVTVNDYLAKRDSEWMGRVHRFLGLQVGVILATMTPDERRVAYNADITYGTNN
EFGFDYLRDNMAHSLDDLVQRGHHYAIVDEVDSILIDEARTPLIISGPADGASNWYTE
FARLAPLMEKDVHYEVDLRKRTVGVHEKGVEFVEDQLGIDNLYEAANSPLVSYLNNAL
KAKELFSRDKDYIVRDGEVLIVDEFTGRVLIGRRYNEGMHQAIEAKEHVEIKAENQTL
ATITLQNYFRLYDKLAGMTGTAQTEAAELHEIYKLGVVSIPTNMPMIREDQSDLIYKT
EEAKYIAVVDDVAERYAKGQPVLIGTTSVERSEYLSRQFTKRRIPHNVLNAKYHEQEA
TIIAVAGRRGGVTVATNMAGRGTDIVLGGNVDFLTDQRLRERGLDPVETPEEYEAAWH
SELPIVKEEASKEAKEVIEAGGLYVLGTERHESRRIDNQLRGRSGRQGDPGESRFYLS
LGDELMRRFNGAALETLLTRLNLPDDVPIEAKMVTRAIKSAQTQVEQQNFEVRKNVLK
YDEVMNQQRKVIYAERRRILEGENLKDQALDMVRDVITAYVDGATGEGYAEDWDLDAL
WTALKTLYPVGITADSLTRKDHEFERDDLTREELLEALLKDAERAYAAREAELEEIAG
EGAMRQLERNVLLNVIDRKWREHLYEMDYLKEGIGLRAMAQRDPLVEYQREGYDMFMA
MLDGMKEESVGFLFNVTVEAVPAPPVAPAAEPAELAEFAAAAAAAAQQRSAVDGGARE
RAPSALRAKGVASESPALTYSGPAEDGSAQVQRNGGGAHKTPAGVPAGASRRERREAA
RRQGRGAKPPKSVKKR"
CDS complement(3580967..3581626)
/codon_start=1
/note="gene_3309"
/transl_table=11
/translation="MSRLAVDSGQVLAEPKSNAEIVFKGRNVEIPDHFRIYVSQKLAR
LERFDRTIYLFDVELDHERNRRQRKSCQRVEITARGRGPVVRGEACADSFYAALESAV
VKLESRLRRGKDRRKVHYGDKTPVSLAEATAVVPAPENGFNTRPAEAHDHDGAVVERE
PGRIVRTKEHPAKPMSVDDALYQMELVGHDFFLFYDKDTERPSVVYRRHAYDYGLIRL

A"
CDS complement(3581927..3582427)
/codon_start=1
/note="gene_3310"
/transl_table=11
/translation="VFALGRYAGVRRQAILAMKEHGRRDLVAPLACALIVGVDHLSSW
GMLENPLTMVPAPTRRWAARRRGGDPVSRMARIAGATLGRHHDVTVVPALRMRALARD
SVGLGASARERNITGRVLLRGQRPRNEVVLVDDIITTGATARESVRVLQAAGVRVGAV
LAVAAA"
CDS 3582308..3582646
/codon_start=1
/gene="BURMUCF2_B0608"
/note="gene_3311"
/transl_table=11
/translation="VVDTDNQRARQWRDEVATAVLFHSEDRLTTHARVAAEREHRHLW
VNTRTHHVRFTGSHRQLGGTGGTPAGRRRTAATAFQRQDEVKHTPSVAVTGDSSAVNR
RRCAAAARQS"
CDS complement(3582606..3583448)
/codon_start=1
/note="gene_3312"
/transl_table=11
/translation="MSPRVPRLRWDDPFRALDMLASLWSSTGMSLVSAGAAQAVAAPY
RTLFTTLQQLLIGKEVTVRIGDHDVVLTVTELDSALEPQGLAVGQLGEVRVAARGISW
DQHHLHSAVAVLRNVHIRPGVPPLVIAAPVELSSALPTEIFDDVLRQATPQLRGELSE
SGAARLRWARRPDWGGLEVDVDVAGTTSQTTLWLRPRTVITGQRRWTLPARTPAYRVP
LPELPHGLRITDVSLAADCLQLSALLPEWRTELPLRYLESVITQLSQGALSFVWPPLR
SGAD"
CDS complement(3583516..3585267)
/codon_start=1
/note="gene_3313"
/transl_table=11
/translation="MRLTILLFLGAVLAGCASVPSTSAPQAIGTVERPVPSNLPKPSP
GMDPDVLLREFLKATADPANRHLAARQFLTESASNAWDDAGSALLIDHVVFVETRSAE
KVSVTMRADILGSLSDVGVFETAEGQLPDPGPIELVKTSGGWRIDRLPNGVFLDWQQF
QETYKRNTLYFADPTGKTVVPDPRYVAVSDRDQLATELVSKLLAGPRPEMARTVRNLL
APPLRLRGPVTRADGGKSGIGRGYGGARVDMEKLSTTDPHSRQLLAAQIIWTLARADI
RGPYVINADGAPLEDRFAEGWTTSDVAATDPGVADGAAAGLHALVNGSLVAMDAQRVT
PVPGAFGRMPEQTAAAVSRSGRQVASVVTLGRGAPDEAASLWVGDLGGEAVQSADGHS
LLRPSWSLDDAVWVVVDTNVVLRAIQDPASGQPARIPVDSTAVASRFPGAINDLQLSR
DGTRAAMVIGGQVILAGVEQTQAGQFALTYPRRLGFGLGSSVVSLSWRTGDDIVVTRT
DAAHPVSYVNLDGVNSDAPSRGLQTPLTAIAANPSTVYVAGPQGVLMYSASVESRPGW
ADVPGLMVPGAAPVLPG"
CDS complement(3585267..3586970)
/codon_start=1
/note="gene_3314"
/transl_table=11
/translation="MIFGSRRRIRGRRGRSGPMTRGLSALSRAVAVAWRRSLQLRVVA
LTLGLSLAVILALGFVLTSQVTNRVLDIKVRAAIDQIERARTTVSGIVNGEETRSLDS
SLQLARNTLTSKTDPASGAGLAGAFDAVLMVPGDGPRAASTAGPVDQVPNALRGFVKA
GQAAYQYATVQTEGFSGPALIIGTPTLSRVANLELYLIFPLASEQATITLVRGTMATG
GLVLLVLLAGIALLVSRQVVVPVRSASRIAERFAEGHLSERMPVRGEDDMARLAVSFN

```
                     DMAESLSRQIAQLEEFGNLQRRFTSDVSHELRTPLTTVRMAADLIYDHSADLDPTLRR
                     STELMVSELDRFETLLNDLLEISRHDAGVAELSVEAVDLRTTVNNALGNVGHLAEEAG
                     IELLVDLPAEQVIAEVDARRVERILRNLIANAIDHAEHKPVRIRMAADEDTVAVTVRD
                     YGVGLRPGEEKLVFSRFWRSDPSRVRRSGGTGLGLAISVEDARLHQGRLEAWGEPGEG
                     ACFRLTLPLVRGHKVTTSPLPMKPIPQPVLQPVAQPNPQPMPPEYKERQRPREHAEWS
                     G"
     CDS             complement(3587020..3587706)
                     /codon_start=1
                     /note="gene_3315"
                     /transl_table=11
                     /translation="MDTMRQRILVVDDDASLAEMLTIVLRGEGFDTAVIGDGTQALTA
                     VRELRPDLVLLDLMLPGMNGIDVCRVLRADSGVPIVMLTAKTDTVDVVLGLESGADDY
                     IMKPFKPKELVARVRARLRRNDDEPAEMLSIADVEIDVPAHKVTRNGEQISLTPLEFD
                     LLVALARKPRQVFTRDVLLEQVWGYRHPADTRLVNVHVQRLRAKVEKDPENPTVVLTV
                     RGVGYKAGPP"
     CDS             complement(3587776..3588420)
                     /codon_start=1
                     /note="gene_3316"
                     /transl_table=11
                     /translation="VLIAIEGVDGAGKRTLVEKLSGAFRAAGRSVATLAFPRYGQSVA
                     ADIAAEALHGEHGDLASSVYAMATLFALDRAGAVHTIQGLCRGYDVVILDRYVASNAA
                     YSAARLHENAAGKAAAWVQRIEFARLGLPKPDWQVLLAVSAELAGERSRGRAQRDPGR
                     ARDNYERDAELQQRTGAVYAELAAQGWGGRWLVVGADVDPGRLAATLAPPDVPS"
     CDS             complement(3588517..3590004)
                     /codon_start=1
                     /note="gene_3317"
                     /transl_table=11
                     /translation="MTGNLVTKNSLTPDVRNGIDFKIADLSLADFGRKELRIAEHEMP
                     GLMSLRREYAEVQPLKGARISGSLHMTVQTAVLIETLTALGAEVRWASCNIFSTQDHA
                     AAAVVVGPHGTPDEPKGVPVFAWKGETLEEYWWAAEQMLTWPDPDKPANMILDDGGDA
                     TMLVLRGMQYEKAGVVPPAEEDDPAEWKVFLNLLRTRFETDKDKWTKIAESVKGVTEE
                     TTTGVLRLYQFAAAGDLAFPAINVNDSVTKSKFDNKYGTRHSLIDGINRGTDALIGGK
                     KVLICGYGDVGKGCAEAMKGQGARVSVTEIDPINALQAMMEGFDVVTVEEAIGDADIV
                     VTATGNKDIIMLEHIKAMKDHAILGNIGHFDNEIDMAGLERSGATRVNVKPQVDLWTF
                     GDTGRSIIVLSEGRLLNLGNATGHPSFVMSNSFANQTIAQIELWTKNDEYDNEVYRLP
                     KHLDEKVARIHVEALGGHLTKLTKEQAEYLGVDVEGPYKPDHYRY"
     CDS             complement(3590109..3590744)
                     /codon_start=1
                     /note="gene_3318"
                     /transl_table=11
                     /translation="VSTPSATVAPVKRIPYAEASRALLRDSVLDAMRDLLLTRDWSAI
                     TLSDVARAAGISRQTIYNEFGSRQGLAQGYALRLADRLVDNVHASLDANVGNFYEAFL
                     QGFRSFFAESAADPLVISLLTGVAKPDLLQLITTDSAPIITRASARLAPAFTDTWVAT
                     TDNDANVLSRAIVRLCLSYVSMPPEADHDVAADLARLITPFAERHGVINVP"
     CDS             complement(3590741..3590923)
                     /codon_start=1
                     /note="gene_3319"
                     /transl_table=11
                     /translation="VNDYKLFRCIQCGFEYDEALGWPEDGIAAGTRWDDIPDDWSCPD
                     CGAAKSDFEMVEVARS"
     CDS             complement(3590928..3591098)
```

/codon_start=1
/note="gene_3320"
/transl_table=11
/translation="MMAAYRCPVCDYVYDEANGDAREGFPAGTGWDQIPDDWCCPDCA
VREKVDFEKIGG"
CDS complement(3591095..3592345)
/codon_start=1
/note="gene_3321"
/transl_table=11
/translation="MTTQIGSGGPEAPRPPEVEEWRDKKRYLWLMGLIAPTALVVMLP
LIWGMNQLGWHAAAQVPLWIGPILLYVLLPLLDLRFGPDGQNPPDEVTDRLENDKYYR
YCTYIYIPFQYLSVVLGAYLFTAANLSWLGFDGALSWAGKLGVALSVGVLGGVGINTA
HEMGHKKDSLERWLSKITLAQTCYGHFYIEHNRGHHVRVSTPEDPASARFGETLWEFL
PRSVIGGLRSAVHLEAQRLRRLGVSPWNPMTYLRNDVLNAWLMSVVLWGGLIAVFGPA
LIPFVIIQAVFGFSLLEAVNYLEHYGLLRQKSANGRYERCAPVHSWNSDHIVTNLFLY
HLQRHSDHHANPTRRYQTLRSMAGAPNLPSGYASMISLTYFPPLWRKVMDHRVLEHYG
GDITRVNLHPRVREKALARYGASA"
CDS complement(3592454..3593941)
/codon_start=1
/note="gene_3322"
/transl_table=11
/translation="MAGRRRMKSVEQSIADTDEPTTRLRKDLTWWDLVVFGVSVVIGA
GIFTVTASTAGDITGPAIWISFLIAAATCALAALCYAEFASTLPVAGSAYTFSYATFG
EFLAWVIGWNLVLELAMGAAVVAKGWSSYLGTVFGFGNGTGHLGSLQLDWGALVIVTL
VATLIALGTKLSSRFSAVVTAIKVSVVVLVVVVGAFYIRAANYSPFIPEPEVQHHGGG
LDQSVFSLLTGAQGSHYGWYGVLAGASIVFFAFIGFDIVATMAEETKRPQRDVPRGIL
ASLGVVTLLYVAVSVVLSGMVPYTQLRTVPGRGPANLATAFQANGVYWASGIISVGAL
AGLTTVVMVLMLGQCRVLFAMARDGLVPRQLAKTGSRGTPVRVTVLVAVLVATTASVF
PITKLEEMVNVGTLFAFILVSAGVVVLRRTRPDLQRGFTAPWVPLLPIAAVCACLWLM
LNLTALTWIRFGIWLVAGTAIYVGYGRRHSAQGLRQARESATRRC"
CDS 3594032..3595420
/codon_start=1
/note="gene_3323"
/transl_table=11
/translation="VVIGASIAGLCAARVLSDFYSTVTVFERDELPEAPANRATVPQD
RHLHMLMARGAQEFDSLFPGLLHDMVAAGVPMLENRPDCIYLGAAGHVLGTGHTLRKE
FTAYVPSRPHLEWQLRRRVLQLSNVQIVRRLVTEPQFERRQQRVVGVLLDSPGSGQDR
EREEFIAADLVVDAAGRGTRLPVWLTQWGYRRPAEDTVDIGISYASHQFRIPDGLIAE
KVVVAGASHDQSLGLGMLCYEDGTWVLTTFGVADAKPPPTFDEMRALADKLLPARFTA
ALAQAQPIGCPAFHAFPASRWRRYDKLERFPRGIVPFGDAVASFNPTFGQGMTMTSLQ
AGHLRRALKARNSAMKGDLAAELNRATAKTTYPVWMMNAIGDISFHHATAEPLPRWWR
PAGSLFDQFLGAAETDPVLAEWFLRRFSLLDSLYMVPSVPIIGRAIAHNLRLWLKEQR
ERRQPVTTRRSP"
CDS complement(3595398..3596624)
/codon_start=1
/note="gene_3324"
/transl_table=11
/translation="VELLRGALRTYAWGSRTAIAEFTGRPVPAAHPEAELWFGAHPGD
PAWLQTPHGQTSLLEALVADPEGQLGSASRARFGDVLPFLVKVLAADEPLSLQAHPSA
EQAVEGYLREERMGIPVSSPVRNYRDTSHKPELLVALQPFEALAGFREAARTTELLRA
LAVSDLDPFIDLLSEGSDADGLRALFTTWITAPQPDIDVLVPAVLDGAIQYVSSGATE

FGAEAKTVLELGERYPGDAGVLAALLLNRISLAPGEAIFLPAGNLHAYVRGFGVEVMA
NSDNVLRGGLTPKHVDVPELLRVLDFAPTPKARLRPPIRREGLGLVFETPTDEFAATL
LVLDGDHLGHEVDASSGHDGPQILLCTEGSATVHGKCGSLTLQRGTAAWVAADDGPIR
LTAGQPAKLFRATVGL"

CDS complement(3596632..3597672)
/codon_start=1
/note="gene_3325"
/transl_table=11
/translation="VNVARAIDLEDTEGLIAADRGALLRAASMAGAQVRAIAAAADEG
ELDLLRGSDRPRSVIWVTGRGTAETAGTILASTLGAGAAEPIVLASAAPPWVGPLDVL
IVAGDDPGDPALVGAAAIGVRRGARVVVVAPYEGPLRDSTAGRVAVLEPRLRVPDEFG
LSRYLAAGLAALQTVDPKLRIDLASLADELDAEALRNSAGREVFTNPAKALAARVSGC
QLALAGDNAATLALARHGSSVMLRIANQVVAATRLSDAVVALRAGTPPDALFHDEEID
GPAPQRLRVLALALAGERTVVAARVAGLDDAYLVAAEDVPELLDAPVGSGGAVLAVRL
EMAAVYLRLVRG"

CDS complement(3597669..3599066)
/codon_start=1
/gene="manB"
/note="gene_3326"
/transl_table=11
/translation="MSWPAAAVDRVIKAYDVRGLVGEEIDESLVTDLGAAFARLMRTE
DARPVVIGHDMRDSSPSLADAFAAGVTGQGLDVVRVGLASTDQLYFASGLLDCPGAMF
TASHNPAAYNGIKMCRAAAKPVGADTGLTAIRDDLIAGVARYDGTPGTIADQDVLVDY
GAFLRSLVDTSGLRPLRVAVDAGNGMAGHTAPAVLGVIDSITLLPLYFELDGSFPNHE
ANPLDPANLVDLQAYVRDTGADIGLAFDGDADRCFVVDERGQPVSPSTVTALVAAREL
NREIGATIIHNVITSRAVPELVAERGGTPLRSRVGHSYIKALMAETGAIFGGEHSAHY
YFRDFWGADSGMLAALHVLAALGEQSRPLSELTADYQRYESSGEINFTVVDSSACVEA
VLKSFGNRIVSIDHLDGVTVDLGDDSWFNLRSSNTEPLLRLNVEGRSVGDVDAVVRQV
SAEIAAQSAHAKAGP"

CDS complement(3599168..3599560)
/codon_start=1
/note="gene_3327"
/transl_table=11
/translation="VATLTFVYSDSTAVIGPLATAREPHSWDLCVGHAGRITAPRGWE
LVRHAGPLPSHPDEDDLVALADAVREGGPSAGRRHHPGGNGAPLHGFDDFPAAATGAP
TGGGVLAPPEPGAGRRRGHLRVLPDPAD"

CDS 3599782..3600201
/codon_start=1
/note="gene_3328"
/transl_table=11
/translation="MRGPLLPPTVPGWRSRAERFDMAVLEAYEPIERRWQERVSQLDI
AVDEIPRIAAKDPESVQWPPEVIADGPIALARLIPAGVDVRGNATRARIVLFRKPIER
RAKDTEELGELLHEILVAQVAIYLDVDPSVIDPTIDD"

CDS complement(3600229..3600600)
/codon_start=1
/note="gene_3329"
/transl_table=11
/translation="MSYEHLRGVMGGTPHTTTGSATASATAVLRPHLSLVPEAPAPFE
EPLPPEATDQWQDRALCAQTDPEAFFPEKGGSTREAKKICMGCEVRHECLEYALAHDE
RFGIWGGLSERERRRLKRGII"

CDS complement(3600765..3601016)

/codon_start=1
/gene="MRGA327_20070"
/note="gene_3330"
/transl_table=11
/translation="VVGVRVGRIGSKLAQAEQLLDPEQEAGAADSTGQNGDLHIAGQY
LPAPITKRWLRVTLDTPGPNHNRTAEILLRDDMEMRRHT"
CDS 3600900..3601895
/codon_start=1
/note="gene_3331"
/transl_table=11
/translation="VKVTVLAGGVGGARFLLGVQQLLGLGQFAANSAHSDADHQLSAV
VNVGDDAWIHGLRVCPDLDTCMYTLGGGVDPQRGWGQRDETWHAMQELVRYGVQPDWF
ELGDRDLATHLVRTQMLQAGYPLSQITEALCDRWQPGARLLPATDDRCETHVVITDPV
DESRKAIHFQEWWVRYRAQVPTHSFAFVGAEKSSAATEAIAALADADIIMLAPSNPVV
SIGAILAVPGIRAALREATAPIVGYSPIIGEKPLRGMADTCLSVIGVDSTAAAVGRHY
GARCATGILDCWLVHDGDHAEIDGVTVRSVPLLMTDPNATAEMVRAGCDLAGVVA"
CDS 3601892..3603238
/codon_start=1
/note="gene_3332"
/transl_table=11
/translation="LTGPEHGSASTIEILPVIGLPEFRPGDDLSAAVAAAAPWLRDGD
VVVVTSKVVSKCEGRLVPAPEDPEQRDRLRRKLIEDEAVRVLARKDRTLITENRLGLV
QAAAGVDGSNVGRSELALLPVDPDASAATLRAGLRERLGVTVAVVITDTMGRAWRNGQ
TDAAVGAAGLAVLRNYAGVRDPYGNELVVTEVAVADEIAAAADLVKGKLTATPVAVVR
GFGVSDDGSTARQLLRPGANDLFWLGTAEALELGRQQAQLLRRSVRRFSTDPVPGDLV
EAAVAEALTAPAPHHTRPTRFVWLQTPAIRARLLDRMKDKWRSDLTSDGLPADAIERR
VARGQILYDAPEVVIPMLVPDGAHSYPDAARTDAEHTMFTVAVGAAVQALLVALAVRG
LGSCWIGSTIFAADLVRDELDLPVDWEPLGAIAIGYADEPSGLRDPVPAADLLILK"
CDS 3603606..3605195
/codon_start=1
/note="gene_3333"
/transl_table=11
/translation="MTTFAGKTAASADKVRGGYYTPPAVARFLAHWVHQAGPKILEPS
CGDGRILRELSAITDHAHGVELVAREAKKSRDFASVDTENLFTWLHKTQLGSWDGVAG
NPPYIRFGNWASEQRDPALELMRRVGLRPTKLTNAWVPFVVASTTLARDGGRVGLVVP
AELLQVTYAAQLREFLLSRYREITLVTFERLVFDGILQEVVLFCGVVGPGPAHIRTVR
LGDANDLNALGDKDFTNESAPALLHEKEKWTKYFLDPAQIRLLRGLKQSATMIRLGEL
ADVDVGIVTGRNSFFTFTDAKAQALGLRAHCVPLVSRSAQLSGLIYDEDCRACDVAGN
HRTWLLDAADYPTDPALVAHITAGEAAGVHLGYKCSIRKPWWSTPSLWMPDLFMLRQI
HFAPRLTVNAAAATSTDTVHRVRLDPNVDPATLAAVFHNSATFAFAEIMGRSYGGGIL
ELEPREAEQLPMPPPAYGSAELAQDVDLLLKANEIDKALDVVDRHVLIDGLGLSPRLV
AGCRAAWLTLRDRRTKRGSRR"
CDS complement(3605255..3606334)
/codon_start=1
/note="gene_3334"
/transl_table=11
/translation="LATHQVDAVVLVGGKGTRLRPLTLSAPKPMLPTAGLPFLTHLLS
RIAAAGIEHVILGTSYKPAVFEAEFGDGSALGLQIEYVTEEHPLGTGGGIANVAGKLR
NDTAMVFNGDVLSGADLAQLLDFHRSNRADVTLQLVRVGDPRAFGCVPTDEEDRVVAF
LEKTEDPPTDQINAGCYVFERNVIDRIPQGREVSVEREVFPALLADGDCKIYGYVDAS
YWRDMGTPEDFVRGSADLVRGIAPSPALRGHRGEQLVHDGAAVSPGALLIGGTVVGRG

AEIGPGTRLDGAVIFDGVRVEAGCVIERSIIGFGARIGPRALIRDGVIGDGADIGARC
ELLSGARVWPGVFLPDGGIRYSSDV"
CDS complement(3606336..3607247)
/codon_start=1
/note="gene_3335"
/transl_table=11
/translation="VTDVLPVVAVTYSPGPHLERFLASLSLATERPVSVLLADNGSTD
GTPQAAVQRYPNVRLLPTGANLGYGTAVNRTIAQLGEMAGDAGEPWVDDWVIVANPDV
QWGPGSIDALLDAASRWPRAGALGPLIRDPDGSVYPSARQMPSLIRGGMHAVLGPFWP
RNPWTTAYRQERLEPSERPVGWLSGSCLLVRRSAFGQVGGFDERYFMYMEDVDLGDRL
GKAGWLSVYVPSAEVLHHKAHSTGRDPASHLAAHHKSTYIFLADRHSGWWRAPLRWTL
RGSLALRSHLMVRRSRRRKLKLVEGRH"
CDS complement(3607240..3608154)
/codon_start=1
/note="gene_3336"
/transl_table=11
/translation="MAGRSERLVITGAGGQLGSHLTAQAAREGRDMLALTSSQWDITD
PAAAERIIRHGDVVINCAAYTDVDGAESNEAVAYAVNATGPQHLARACARVGARLIHV
STDYVFDGDFGGAEPRPYEPTDETAPQGVYARSKLAGEQAVLAAFPEAAVVRTAWVYT
GGTGKDFVAVMRRLAAGHGRVDVVDDQTGSPTYVADLAEALLALADAGVRGRVLHAAN
EGVVSRFGQARAVFEECGADPQRVRPVSSAQFPRPAPRPSYSALSSRQWALAGLTPLR
HWRSALATALAAPANSTSIDRRLPSTRD"
CDS 3608230..3609726
/codon_start=1
/note="gene_3337"
/transl_table=11
/translation="VMSAQRVVRTVRTARAISTALAVAIVLGTGVAWSSVRSFEDGIF
HMSAPSLGHGGDDGAIDILLVGLDSRTDAHGNPLSAEELATLHAGDEEATNTDTIILI
RVPNNGKSATAISIPRDSYVAAPGLGKTKINGVYGQTRETKRAGLVQAGASPTEAAAA
GTEAGREALIKTVADLTGVTVDHYAEIGLLGFALIADALGGVDVCLKEPVYEPLSGAD
FPAGRQKLNGPQALSFVRQRHDLPRGDLDRVVRQQAVMAALAHRVISGQTLSSPATLK
RLEQAVQRSVVLSSGWDIMDFVRQLQKLAGGNVAFATIPVLDGAGWSDDGMQSVVRVD
PRQVQDWVVGLLHEQDQGKTDELAYTPAKTTANVVNDTDINGLAAAVSKVLSSKGFTT
GSVGNNDGDHVPGSQVRAAKADDLGAQQVAKELGGLPVVADASIAPGSVRVVLANDYS
GPGSGLGGSDPNGVVSPARAFNLGSADDTTPPPSPILTAGSDAPECIN"
CDS 3609714..3610454
/codon_start=1
/note="gene_3338"
/transl_table=11
/translation="VHQLTTPTTLSGAILDPMLRADPVGPRITYYDDATGERIELSAV
TLANWAAKTGNLLRDELAAGPASRVAILLPAHWQTAAVLFGVWWIGAQAILDDSPADV
ALCTADRLAEADAVVNSAAVAGEVAVLSLDPFGRPATGLPVGVTDYATAVRVHGDQIV
PEHNPGPVLAGRSVEQILRDCAASAAARGLTAADRVLSTASWAGPDELVDGLLAILAA
GASLVQVANPDPAMLQRRIATEKVTRVL"
CDS 3610579..3610860
/codon_start=1
/note="gene_3339"
/transl_table=11
/translation="MAIQVFLAKATTTVITGLAGVTAYEILKKAAAKAPLRQTAVSAA
ALGLRGTRKAEEAAESARLKVADVMAEARERIGEESPTPAISDLHDHDH"
CDS 3610871..3613027

```
                     /codon_start=1
                     /note="gene_3340"
                     /transl_table=11
                     /translation="MTLEVVSDAAGRMRVKVDWVRCDSRRAVAVEEAVAKQNGVRVVH
                     AYPRTGSVVVWYSPRRADRAAVLAAIKGAAHVAAELIPARAPHSAEIRNTDVLRMVIG
                     GVALALLGVRRYVFARPPLLGTTGRTVATGVTIFTGYPFLRGALRSLRSGKAGTDALV
                     SAATVASLILRENVVALTVLWLLNIGEYLQDLTLRRTRRAISELLRGNQDTAWVRLTD
                     PSAGSDAATEIQVPIDTVQIGDEVVVHEHVAIPVDGEVVDGEAIVNQSAITGENLPVS
                     VVVGTRVHAGSVVVRGRVVVRAHAVGNQTTIGRIISRVEEAQLDRAPIQTVGENFSRR
                     FVPTSFIVSAIALLITGDVRRAMTMLLIACPCAVGLSTPTAISAAIGNGARRGILIKG
                     GSHLEQAGRVDAIVFDKTGTLTVGRPVVTNIVAMHKDWEPEQVLAYAASSEIHSRHPL
                     AEAVIRSTEERRISIPPHEECEVLVGLGMRTWADGRTLLLGSPSLLRAEKVRVSKKAS
                     EWVDKLRRQAETPLLLAVDGTLVGLISLRDEVRPEAAQVLTKLRANGIRRIVMLTGDH
                     PEIAQVVADELGIDEWRAEVMPEDKLAAVRELQDDGYVVGMVGDGINDAPALAAADIG
                     IAMGLAGTDVAVETADVALANDDLHRLLDVGDLGERAVDVIRQNYGMSIAVNAAGLLI
                     GAGGALSPVLAAILHNASSVAVVANSSRLIRYRLDR"
     CDS             complement(3613024..3613638)
                     /codon_start=1
                     /note="gene_3341"
                     /transl_table=11
                     /translation="VAREAEWQRNVRWARWLAWVSLAVLLTEGAVGLWQGIAVGSVAL
                     TGWALGGGSEGLASAMVLWRFTGDRTWSATAEHRAQRGVAVSFWLTAPYLVAESIRHL
                     AGEHRAETSVIGIGLTAIALLLMPVLGWANHRVGERLGSGATAGEGTQNYLCAAQAAA
                     VLLGLAITAVWSNGWWIDPAIGLAIAGIAVWQGIRTWRGHGCGC"
     CDS             3613793..3614977
                     /codon_start=1
                     /note="gene_3342"
                     /transl_table=11
                     /translation="MPTSNPAKPLDGFRVLDFTQNVAGPLAGQVLVDLGAEVIKVEAP
                     GGEAARQITSVLPGRPPLATYFLPNNRGKKSVTVDLTTEQAKQQMLRLADTADVVLEA
                     FRPGTMEKLGLGPDDLRSRNPNLIYARLTAYGGNGPHGSRPGIDLVVAAEAGMTTGMP
                     TPEGKPQIIPFQLVDNASGHVLAQAVLAALLHRERNGVADVVQVAMYDVAVGLQANQL
                     MMHLNRAASDQPKPEPAPKAKRRKGVGFATQPSDAFRTADRYIVISAYVPKHWQKLCY
                     LIGRPDLVEDQRFAEQRSRSINYAELTAELELALASKTATEWVQLLQANGLMACLAHT
                     WKQVVDTPLFAESDLTLEVGRGADTITVIRTPARYASFRAVVTDPPPTAGEHNAVFLA
                     RP"
     CDS             3615006..3617276
                     /codon_start=1
                     /note="gene_3343"
                     /transl_table=11
                     /translation="MSTAVNSCTEAPASRSQWMLANLRHDVPASLVVFLVALPLSLGI
                     AIASGAPIIAGVIAAVVGGIVAGAVGGSPVQVSGPAAGLTVVVAELIDELGWPMLCLM
                     TIAAGALQIVFGLSRMARAALAIAPVVVHAMLAGIGITIALQQIHVLLGGTSHSSAWR
                     NIVALPDGILHHELHEVIVGGTVIAILLMWSKLPAKVRIIPGPLVAIAGATVLALLPV
                     LQTERIDLQGNFFDAIGLPKLAEMSPGGQPWSHEISAIALGVLTIALIASVESLLSAV
                     GVDKLHHGPRTDFNREMVGQGSANVVSGLLGGLPITGVIVRSSANVAAGARTRMSTIL
                     HGVWILLFASLFTNLVELIPKAALAGLLIVIGAQLVKLAHIKLAWRTGNFVIYAITIV
                     CVVFLNLLEGVAIGLVVAIVFLLVRVVRAPVEVKPVGGEQSKRWRVDIDGTLSFLLLP
                     RLTTVLSKLPEGSEVTLNLNADYIDDSVSEAISDWRRAHETRGGVVAIVETSPAKLHH
                     AHARPPKRHFASDPIGLVPWRSARGKDRGSASVLDRIDEYHRNGAAVLHPHIAGLTDS
                     QDPYELFLTCADSRILPNVITASGPGDLYTVRNLGNLVPTDPDDRSVDAALDFAVNQL
```

GVSSVVVCGHSSCAAMTALLEDDPANTTTPMMRWLENAHDSLVVFRNHHPARRSAESA
GYPEADQLSIVNVAVQVERLTRHPILATAVAAADLQVIGIFFDISTARVYEVGPNGII
CPDEPADRPVDHESAQ"
CDS complement(3617265..3618434)
/codon_start=1
/note="gene_3344"
/transl_table=11
/translation="MVGWAGNPSFDLFKLPEEHDEMRSAIRALAEKEIAPHAAEVDEK
ARFPEEALVALNSSGFNAVHIPEEYGGQGADSVATCIVIEEVARVDASASLIPAVNKL
GTMGLILRGSEELKKQVLPALAAEGAMASYALSEREAGSDAASMRTRAKADGDHWILN
GAKCWITNGGKSTWYTVMAVTDPDRGANGISAFMVHKDDEGFTVGPKERKLGIKGSPT
TELYFENCRIPGDRIIGEPGTGFKTALATLDHTRPTIGAQAVGIAQGALDAAIAYTKD
RKQFGESISTFQAVQFMLADMAMKVEAARLMVYSAAARAERGEPDLGFISAASKCFAS
DVAMEVTTDAVQLFGGAGYTTDFPVERFMRDAKITQIYEGTNQIQRVVMSRALLR"
CDS complement(3618459..3618983)
/codon_start=1
/note="gene_3345"
/transl_table=11
/translation="MTPAGERPRVGVIMGSDSDWPVMADAAAALAEFDIPAEVRVVSA
HRTPEAMFSYARGAAERGLEVIIAGAGGAAHLPGMVAAATPLPVIGVPVPLGRLDGLD
SLLSIVQMPAGVPVATVSIGGARNAGLLAVRMLGAANPQLRARIVAFQDRLADVVAAK
DAELQRLAGKLTRD"
CDS complement(3618980..3620269)
/codon_start=1
/note="gene_3346"
/transl_table=11
/translation="MMAVASSRTPAVTSFIAPLVAMVGGGQLARMTHQAAIALGQNLR
VLVTSADDPAAQVTPNVVIGSHTDLAALRRVAAGADVLTFDHEHVPNELLEKLVADGV
NVAPSPQALVHAQDKLVMRQRLAAAGVAVPRYAGIKDPDEIDVFAARVDAPIVVKAVR
GGYDGRGVRMARDVADARDFARECLADGVAVLVEERVDLRRELSALVARSPFGQGAAW
PVVQTVQRDGTCVLVIAPAPALPDDLATAAQRLALQLADELGVVGVLAVELFETTDGA
LLVNELAMRPHNSGHWTIDGARTSQFEQHLRAVLDYPLGDSDAVVPVTVMANVLGAAQ
PPAMSVDERLHHLFARMPDARVHLYGKAERPGRKVGHINFLGSDVAQLCERAELAAHW
LSHGRWTDGWDPHRASDDAVGVPPACGGRSDEEERRL"
CDS 3620223..3621041
/codon_start=1
/note="gene_3347"
/transl_table=11
/translation="MNEVTAGVRELATAIMVSRHLTGVLAGHGSQTVTYHFASILCSS
VHSLVVSFADATIARLPGVVQPYAQRHHELIKFAIVGGTTFIIDTAIFYTLKLTVLEP
KPVTAKVIAGIVAVIASYVLNREWSFRDRGGRERHHEALLFFAFSGVGVLLSMAPLWF
SSYILQLRVPTVSLTMENIADFISAYIIGNLLQMAFRFWAFRRWVFPDEFARNPDKAL
ESALTAGGIAEVFEDVLEGGFEDGNVTLLRAWRNRANRFAQLGDSSEPRVSKTL"
CDS complement(3620996..3621514)
/codon_start=1
/note="gene_3348"
/transl_table=11
/translation="MSYPENVLAAGEQVVLHRHPHWNRLIWPVVVLVLLTGLAAFGSG
FVNSTPWQQTAKNVIHAVIWGIWLVIVGWLTLWPFLSWLTTHFVVTNRRVMFRHGVLT
RSGIDIPLARINSVEFRDRIFERIFRTGTLIIESASQDPLEFYNIPRLREVHALLYHK
VFDTLGSDESPS"

```
CDS             complement(3621557..3622357)
                /codon_start=1
                /note="gene_3349"
                /transl_table=11
                /translation="VTDRDRLRPPLDERSLRDQLIGAGSGWRQLDVVAQTGSTNADLL
                ARAASGADIDGVVLIAEHQTAGRGRHGRGWAATARAQIILSVGVRVVDVPVQAWGWLS
                LAAGLAVLDSVAPLIAVPPAETGLKWPNDVLARGGKLAGILAEVAQPFVVLGVGLNVT
                QAPEEVDPDATSLLDLGVAAPDRNRIASRLLRELEARIIQWRNANPQLAANYRARSLT
                IGSRVRVELPGGQDVVGIARDIDDQGRLCLDVGGRTVVVSAGDVVHLR"
CDS             3622407..3624053
                /codon_start=1
                /note="gene_3350"
                /transl_table=11
                /translation="MTSVTDRSAHSAERSTEHTIDIHTTAGKLAELHKRREESLHPVG
                EDAVEKVHAKGKLTARERIYALLDEDSFVELDALAKHRSTNFNLGEKRPLGDGVVTGY
                GTIDGRDVCIFSQDATVFGGSLGEVYGEKIVKVQELAIKTGRPLIGINDGAGARIQEG
                VVSLGLYSRIFRNNILASGVIPQISLIMGAAAGGHVYSPALTDFVIMVDQTSQMFITG
                PDVIKTVTGEEVTMEELGGAHTHMAKSGTAHYAASGEQDAFDYVRELLSYLPPNNSTD
                APRYQAAAPTGPIEENLTDEDLELDTLIPDSPNQPYDMHEVITRLLDDEFLEIQAGYA
                QNIVVGFGRIDGRPVGIVANQPTHFAGCLDINASEKAARFVRTCDCFNIPIVMLVDVP
                GFLPGTDQEYNGIIRRGAKLLYAYGEATVPKITVITRKAYGGAYCVMGSKDMGCDANL
                AWPTAQIAVMGASGAVGFVYRQQLAEAAANGEDIDKLRLRLQQEYEDTLVNPYVAAER
                GYVDAVIPPSHTRGYIGTALRLLERKIAQLPPKKHGNVPL"
CDS             3624034..3624504
                /codon_start=1
                /note="gene_3351"
                /transl_table=11
                /translation="MGTCPCESSERNEPVSRVSGTNEVSDGNETNNPAEVSDGNETNN
                PAEVSDGNETNNPAPVSRVSGTNEVSDGNETNNPAPVTEKPLHPHEPHIEILRGQPTD
                QELAALIAVLGSISGSTPPAQPEPTRWGLPVDQLRYPVFSWQRITLQEMTHMRR"
CDS             3624501..3625169
                /codon_start=1
                /note="gene_3352"
                /transl_table=11
                /translation="MTRLVLGSASPGRLKVLRDAGIEPLVIASHVDEDVVIAALGPDA
                VPSDVVCVLAAAKAAQVATTLTGTQRIVAADCVVVACDSMLYIEGRLLGKPASIDEAR
                EQWRSMAGRAGQLYTGHGVIRLQDNKTVYRSAETAITTVYFGTPSASDLEAYLASGES
                LRVAGGFTLDGLGGWFIDGVQGNPSNVIGLSLPLLRSLVQRCGLSVAALWAGNAGGPA
                HKQQ"
CDS             3625210..3626103
                /codon_start=1
                /note="gene_3353"
                /transl_table=11
                /translation="VPLPADPSPTLSAYAHPERLVTADWLSAHMGAPGLAIVESDEDV
                LLYDVGHIPGAVKIDWHTDLNDPRVRDYINGEQFAELMDRKGIARDDTVVIYGDKSNW
                WAAYALWVFTLFGHADVRLLNGGRDLWLAERRETTLDVPTKTCTGYPVVQRNDAPIRA
                FRDDVLAILGAQPLIDVRSPEEYTGKRTHMPDYPEEGALRAGHIPTAVHIPWGKAADE
                SGRFRSREELERLYDFINPDDQTVVYCRIGERSSHTWFVLTHLLGKADVRNYDGSWTE
                WGNAVRVPIVAGEEPGVVPVV"
CDS             3626100..3626531
                /codon_start=1
```

/note="gene_3354"
/transl_table=11
/translation="MTAPASLPAPLAEVVSDFAEVQGQDKLRLLLEFANELPALPSHL
AESAMEPVPECQSPLFLHVDASDPNRVRLHFSAPAEAPTTRGFASILAAGLDEQPAAD
ILAVPEDFYTELGLAALISPLRLRGMSAMLARIKRRLREAD"
CDS 3626639..3628441
/codon_start=1
/note="gene_3355"
/transl_table=11
/translation="VASHAGSRIARISKVLVANRGEIAVRVIRAARDAGLPSVAVYAE
PDAESPHVRLADEAFALGGQTSAESYLDFAKILDAAAKSGANAIHPGYGFLAENADFA
QAVIDAGLIWIGPSPQSIRDLGDKVTARHIAARAQAPLVPGTPDPVKGADEVVAFAEE
YGLPIAIKAAHGGGGKGMKVARTIDEIPELYESAVREATAAFGRGECYVERYLDKPRH
VEAQVIADQHGNVVVAGTRDCSLQRRYQKLVEEAPAPFLTDFQRKEIHDSAKRICKEA
HYHGAGTVEYLVGQDGLISFLEVNTRLQVEHPVTEETAGIDLVLQQFRIANGEKLDIT
EDPTPRGHAIEFRINGEDAGRNFLPAPGPVTKFHPPSGPGVRVDSGVETGSVIGGQFD
SMLAKLIVHGADRAEALARARRALNEFGVEGLATVIPFHRAVVSDPAFIGDANGFSVH
TRWIETEWNNTIEPFTDGEPLDEDARPRQKVVVEIDGRRVEVSLPADLALSNGGGCDP
VGVIRRKPKPRKRGAHTGAAASGDAVTAPMQGTVVKFAVEEGQEVVAGDLVVVLEAMK
MENPVTAHKDGTITGLAVEAGAAITQGTVLAEIK"
CDS complement(3628451..3629236)
/codon_start=1
/note="gene_3356"
/transl_table=11
/translation="VTARAAGGSASRANEYADVPEMFRELVGLPAGSPEFQRHRDKIV
QRCLPLADHIARRFEGRGEPRDDLIQVARVGLVNAAVRFDVKTGSDFVSFAVPTIMGE
VRRHFRDNSWSVKVPRRLKELHLRLGTATADLSQRLGRAPSASELAAELGMDRAEVIE
GLLAGSSYHTLSIDSGGGSDDDARAITDTLGDVDAGLDQIENREVLRPLLEALPERER
TVLVLRFFDSMTQTQIAERVGISQMHVSRLLAKSLARLRDQLE"
CDS complement(3629233..3629670)
/codon_start=1
/note="gene_3357"
/transl_table=11
/translation="MADSDLPTKGRQRGVRAVELNVAARLENLALLRTLVGAIGTFED
LDFDAVADLRLAVDEVCTRLIRSALPDATLRLVVDPRKDEVVVEASAACDTHDVVAPG
SFSWHVLTALADDVQTFHDGRQPDVAGSVFGITLTARRAASSR"
CDS complement(3629868..3630179)
/codon_start=1
/note="gene_3358"
/transl_table=11
/translation="MGDTYRDPVDHLRTTRPLAGESLIDVVHWPGYLLIVAGVVGGVG
ALAAFGTGHHAEGMTFGVVAIVVTVVGLAWLAFEHRRIRKIADRWYTEHPEVRRQRLA
G"
CDS complement(3630316..3630693)
/codon_start=1
/note="gene_3359"
/transl_table=11
/translation="MHEVGGPSRGDRLGRDDSEVHSAIRFAVVAAVVGVGFLIMGALL
VSTCSGVDTAACGPPQRILLALGGPLILCAAGLWAFLRTYRVWRAEGTWWGWHGAGWF
LLTLMVLTLCIGVPPIAGPVMAP"
CDS complement(3630727..3631995)

```
                     /codon_start=1
                     /note="gene_3360"
                     /transl_table=11
                     /translation="MLVDGLDIVLDLTRSGGSYLVDAITGRRYLDMFTFVASSALGMN
                     PPALVDDREFHAELMQAALNKPSNSDVYSVAMARFVETFARVLGDPALPHLFFVEGGA
                     LAVENALKAAFDWKSRHNQAHGIDPALGTQVLHLRGAFHGRSGYTLSLTNTKPTITAR
                     FPKFDWPRIDAPYMRPGLDEPAMAALEAEALRQARAAFETRPHDIACFVAEPIQGEGG
                     DRHFRPEFFAAMRELCDEFDALLIFDEVQTGCGLTGTAWAYQQLDVAPDIVAFGKKTQ
                     VCGVMAGRRVDEVADNVFAVPSRLNSTWGGNLTDMVRARRILEVIEAEGLFERAVQHG
                     KYLRARLDELAADFPAVVLDPRGRGLMCAFSLPTTADRDELIRQLWQRAVIVLPAGAD
                     TVRFRPPLTVSTAEIDAAIAAVRSALPVVT"
     CDS             complement(3632127..3632579)
                     /codon_start=1
                     /note="gene_3361"
                     /transl_table=11
                     /translation="MNEALDDIDRILVRELAADGRVTLSELATRAGLSVSAVQSRVRR
                     LESRGVVQGYSARINPEAVGHLLSAFVAITPLDPSQPDDAPARLEHIEEVESCYSVAG
                     EESYVLLVRVASARALEDLLQRIRTTANVRTRSTIILNTFYSDRQHIP"
     CDS             3632610..3633857
                     /codon_start=1
                     /note="gene_3362"
                     /transl_table=11
                     /translation="MSRSKRLQTGQLRARFAAGLSAMYAAEVPAYGTLVEVCAQVNSD
                     YLTRHRRAERLGSLQRVTAERHGAIRVGNPAELAAVADLFAAFGMLPVGYYDLRTAES
                     PIPVVSTAFRPIDANELAHNPFRVFTSMLAIEDRRYFDADLRTRVQTFLARRQLFDPA
                     LLAQARAIAADGGCDADDAPAFVAAAVAAFALSREPVEKSWYDELSRVSAVAADIAGV
                     GSTHINHLTPRVLDIDDLYRRMTERGITMIDTIQGPPRTDGPDVLLRQTSFRALAEPR
                     MFRDEDGTVTPGILRVRFGEVEARGVALTPRGRERYEAAMAAADPAAVWATHFPSTDA
                     EMAAQGLAYYRGGDPSAPIVYEDFLPASAAGIFRSNLDRDSQTGDGPDDAGYNVDWLA
                     GAIGRHIHDPYALYDALAQEERR"
     CDS             3633884..3635368
                     /codon_start=1
                     /note="gene_3363"
                     /transl_table=11
                     /translation="VLEACQAIGVTAALGEPGEHSLPASTPITGDVLFSIAPTTPEQA
                     DHAIAAAAATFTAWRSTPAPVRGALVARLGELLTAHQQDLATLVTVEVGKITAEARGE
                     VQEMIDVCQFSVGLSRQLYGRTIASERAGHRLLETWHPLGVVGVITAFNFPVAVWAWN
                     TAVALVCGDTVVWKPSELTPLTALACQALLSRAAADVGAPAAVGGLLLGGAERGAQLV
                     DDPRVALLSATGSVRMGQQVGPRVARRFGRVLLELGGNNAAIVAPSADLELAVRCIVF
                     AAAGTAGQRCTSLRRLIVHRSVADDVVARVVGAYRQLAIGDPSAPDTLVGPLIHEAAY
                     RDMVAALERARTDGGEVIGGDRREVGSPGAYYVAPAVVRMPSQTAIVATETFAPILYV
                     LTYDDLDEAIALNNAVPQGLSSSIFTTDLREAEHFLDQSDCGIANVNIGTSGAEIGGA
                     FGGEKQTGGGRESGSDAWKAYMRRATNTVNYSSELPLAQGVKFG"
     CDS             3635381..3636016
                     /codon_start=1
                     /gene="MRGA327_20270"
                     /note="gene_3364"
                     /transl_table=11
                     /translation="VRLGIIDARLGLGGVEFIHFIQCPLRIPKLPRRRPEDVDGDGGE
                     AVAEWLGLWAGVQDHAAGEACADFVAESADRAGVSTTEGCRRLDFDRGNEAVCGFQYE
                     VYFGAVSIAVVEQSRCLLRPSELSPEFCDHESFDDGSGRVGVGMWTGSGRRDIDEASG
```

QSGVEKEDFRSIDDPLGKVGRPRGYAVDEIQGLEEVEVRRQGTYGDFGVGS"
CDS 3636321..3637013
/codon_start=1
/note="gene_3365"
/transl_table=11
/translation="VVYVSNICHMATARRRLSPQDRRAELLALGAEVFGKRPYDEVRI
DEIAERAGVSRALMYHYFPDKRAFFAAVVKDEADRLYAATNKAPAPGMTMFEEIRTGV
LAYMAYHQQNPEAAWAAYVGLGRSDPVLLGIDDEAKNRQMEHIMSRIAEVVSGIDRDN
TLDPEVERDLRVIIHGWLAFTFELCRQRIMDPSTDAERLADACAHALLDAISRLPQIP
AELADAMATARM"
CDS 3637057..3641598
/codon_start=1
/note="gene_3366"
/transl_table=11
/translation="VRFAQPSALSRFSALTRDWFTSTFAAPTAAQASAWAAIADGDNT
LVIAPTGSGKTLAAFLWALDSLAGSEPMSERPAATRVLYVSPLKALAVDVERNLRTPL
AGLTRLAERQGLPAPQIRVGVRSGDTPPALRRQLVSQPPDVLITTPESLFLMLTSAAR
QTLTGVQTVIIDEIHAIAATKRGAHLALSLERLDDLSSRRRAQRIGLSATVRPPEELA
RFLSGQSPTTIVAPPAAKTVELSVQVPVPDMANLTDNTIWPDVEARLVDLIESHNSTI
VFANSRRLAERLTARLNEIHAARCGIELAPDTNQQVAGGAPAHIMGSGQTFGAPPVLA
RAHHGSISKEQRAVVEEDLKRGQLKAVVATSSLELGIDMGAVDLVIQVQAPPSVASGL
QRIGRAGHQVGEISRGVLFPKHRTDLLGCAVSVQRMLAGEIETMRVPANPLDILAQHT
VAAAALEPLDADAWFDTVRRAAPFATLPRSLFEATLDLLSGKYPSTEFAELRPRLVYD
RDTGTLTARPGAQRLAVTSGGAIPDRGLFAVYLATERPSRVGELDEEMVYESRPGDVI
SLGATSWRITEITHDRVLVIPAPGQPARLPFWRGDDAGRPAELGAALGALTGELAALD
RTAFGTRCAGLGFDDYATDNLWRLLDDQRTATAVVPTDSTLLVERFRDELGDWRVILH
SPYGLRVHGPLALAVGRRLRDRYGIDEKPTASDNGIMVRLPDTVSAGEDSPPGAELFV
FDADEIDPIVTTEVAGSALFASRFRESAARALLLPRRHPGRRSPLWQQRQRAARLMEV
ARKYPDFPIVLETVRECLQNVYDVPILVELMARIAQRRVRVAEAETAKPSPFAASLLF
GYVGAFMYEGDTPLAERRAAALALDGTLLAELLGRVELRELLDPDVIAATSRQLQHLA
ADRVARDAEGVADLLRLLGPLTEDEIAARAGAPEVSGWLDGLRAAKRALVVSFAGRSW
WVAVEDMGRLRDGVGAAVPVGLPASFTEAVADPLGELLGRYARTHTPFTTAAAAARFG
LGLRVTADVLGRLASDGRLVRGEFVAAAEGSAGGEQWCDAEVLRILRRRSLAALRAQA
EPVSTAAYGRFLPAWQHVSAGNSGIDGLAAVIDQLAGVRIPASAIEPLVLAPRIRDYS
PAMLDELLASGDVTWSGAGSISGSDGWIALHPADSAPMTLAEPAEIDFTDAHRAILAS
LGTGGAYFFRQLTHDGLTEAELKAALWELIWAGRVTGDTFAPVRAVLGGAGTRKRAAP
AHGGHRPPRLSRYRLTHAQARNADPTVAGRWSALPLPEPDSTLRAHYQAELLLNRHGV
LTKDAVAAEGVAGGFATLYKVLSAFEDAGRCQRGYFIESLGGAQFAVASTVDRLRSYL
DGVDPEQPDYHAVVLAAADPANPYGAALPWPASSADGTARPGRKAGALVVLVDGELAW
FLERGGRSLLTFTDDPEANHAAAIGLADLVTAGRVASILVERADGMPVLQPGGRASAA
LTALLAAGFVRTPRGLRRR"
CDS 3641602..3642369
/codon_start=1
/note="gene_3367"
/transl_table=11
/translation="MPEGDTVWHTAATLRRHLAGRTLTRCDIRVPRFAAVDLTGEVVD
EVISRGKHLFIRTGTASIHSHLQMDGSWRVGNRPVRVDHRARIILEANQQEQAIRVVG
VDLGLLEVIDRHNDGAVVAHLGPDLLADDWDPQRAAANLIVAPDRPIAEALLDQRVLA
GIGNVYCNELCFVSGVLPTAPVSAVADPRRLVTRARDMLWVNRFRWNRCTTGDTRAGR
RLWVYGRAGQGCRRCGTLIAYDTTDERVRYWCPACQR"
CDS complement(3642392..3643306)

```
                     /codon_start=1
                     /note="gene_3368"
                     /transl_table=11
                     /translation="MPWARMLSLIVLMVCLAGCGGDQLLARHASSVATFQFGGLTRSY
                     RLHVPPAEPSGLVISLHGGGGTGAGQEALTDFDAVADAADLLVVYPDGYDKSWADGRG
                     ASPADRRHLDDVGFLVALAAKLVHDFDIAPGHVFATGMSNGGFMSNRLACDRADIFAA
                     VAPVAGTLGVGVTCNPSRPVSVLEAHGTADPLVPFNGGAVRGRGGLSHSISVASLVDR
                     WRAVDGCQGDPSAAELPDVGDGTMVHLFDSSSCAAGTEVISYQIDNGGHTWPGGRQYL
                     PKAVIGATTRAFDGSQVIAQFFATHGRD"
     CDS             complement(3643333..3646182)
                     /codon_start=1
                     /note="gene_3369"
                     /transl_table=11
                     /translation="MSEDNALVLVAGYQDLDSARHDFQTLVDAAKDKSIPLQGAVLIG
                     KDAEGSPVLVDTGNRLGRRGAAWGAGVGLAIGLFSPALLASAALGAATGALAGTFAHH
                     RIKTGLADKIGQALAAGRAVVIAVTEAQGRLEAGQALASSPMKSVAELSRSTLRSLGA
                     ALREAMGKFNPDRTRLPLPQRRFGGVVGRTMAESVGDWSIVPSPFPPDDAPNVLIVLI
                     DDAGFGGPDTFGGAIRTPTLSRLAQNGLIYNRFHVTAVCSPTRAALLTGRNHHRVGFG
                     SVCEFPGPYPGYSAVRPRSCAALPRILRDNGYVTGAFGKWHLTPDFWGFPSGAAGQYD
                     PIISQDNSVIGIPEGSGEDGRPYYFPDDLTDKAIEWLHTVRAQNATKPWMLYYATGAT
                     HAPHHVFKEWADKYRGEFDDGWDVYRQKTFERQKRLGIIPPDAELTERPDLFPAWDSM
                     SEAQKRLFARQMEVFAGFSENADWNVGRLLDAIEDLGESDNTLVFYIWGDNGASMEGT
                     NTGSFNEMTFLNGLDLDAERQLELIEQYGGIAALGDEFTAPHFASAWAHASNTPLQWG
                     KQMASHLGGTRDPLVVAWPARIRPDGRVRSQFTHCIDIAPTVLAAIGLPEPTHVDGFE
                     QEPMDGTSFVRTFDDAEAEDRHTVQYFENFGSRAIYKDGWWACARLDKAPWDLSPETM
                     RRFAPGTYDPDQDVWELYYLPDDFSQAKNLAAEHPDKVAELTQLWWQEAERNRVLPLL
                     GGLAVMFGDLPPLPTTARFSFKGDVQNIQRGMVPRICGRSYAIEARLHIPDGGAQGVI
                     VANADFMGGFALWVDEQRHLHHTYSFLGVETYRQVSSEPLPTGDVTVRMLFDSHQPVA
                     ASGGRVTLWADDRLIGEGELPQTVPLAFTSYAGMDIGRDNGLVVDRGYEDKAPYAFTG
                     TVTEVIFDLKPVHPEAARALHEHASVQAVGQGAAG"
     variation        3644692..3665335
                     /note="supplemented from BCG moreau"
     CDS             complement(3646205..3647122)
                     /codon_start=1
                     /note="gene_3370"
                     /transl_table=11
                     /translation="VALRPEDRLLSVHDVLGPVRVRLLGGSVLAELTARFGVAARAKV
                     LAGEVVDDDGAVVDSGTVLPPGSVVHLYRDLPDEVPVPFDVPVLHQDADIVVVDKPHF
                     LATMPRGRHVAQTALVRLRRELGLPELSPAHRLDRLTAGVLLFTTRREVRGSYQTMFA
                     RGLVRKTYLARAPVAPGLALPRLVRSRIVKRRGHLQAVCEPGVPNAETLVERIARDGL
                     YRLTPTTGRTHQLRVHMAALGIPIMGDPLYPNVISVAAHDFSTPLQLLAQRIEFDDPL
                     TGSHREFASTRTLTGATLPTWSAAADCRP"
     CDS             complement(3647134..3647799)
                     /codon_start=1
                     /gene="phoY1_3"
                     /note="gene_3371"
                     /transl_table=11
                     /translation="MRTVYHQRLTELAGRLGEMCSLAGIAMKRATQALLEADIGAAEQ
                     VIRDHERIVAMRAQVEKEAFALLALQHPVSGELREIFSAVQIIADTERMGALAVHIAK
                     ITRREYPNQVLPEEVRNCFADMAKVAIALGDSARQVLVNRDPQEAAQLHDRDDAMDDL
                     HRHLLSVLIDREWRHGVRVGVETALLGRFFERFADHAVEVGRRVIFMVTGVLPTEDEI
```

```
                     STY"
CDS                  complement(3647907..3649664)
                     /codon_start=1
                     /gene="glpD2_3"
                     /note="gene_3372"
                     /transl_table=11
                     /translation="VSNPIQAPDGGQGWPAAALGPAQRAVAWKRLGTEQFDVVVIGGG
                     VVGSGCALDAATRGLKVALVEARDLASGTSSRSSKMFHGGLRYLEQLEFGLVREALYE
                     RELSLTTLAPHLVKPLPFLFPLTKRWWERPYIAAGIFLYDRLGGAKSVPAQRHFTRAG
                     ALRLSPGLKRSSLIGGIRYYDTVVDDARHTMTVARTAAHYGAVVRCSTQVVALLREGD
                     RVIGVGVRDSENGAVAEVRGHVVVNATGVWTDEIQALSKQRGRFQVRASKGVHVVVPR
                     DRIVSDVAMILRTEKSVMFVIPWGSHWIIGTTDTDWNLDLAHPAATKADIDYILGTVN
                     AVLATPLTHADIDGVYAGLRPLLAGESDDTSKLSREHAVAVPAAGLVAIAGGKYTTYR
                     VMAADAIDAAVQFIPARVAPSITEKVSLLGADGYFALVNQAEHVGALQGLHPYRVRHL
                     LDRYGSLISDVLAMAASDPSLLSPITEAPGYLKVEAAYAAAAEGALHLEDILARRMRI
                     SIEYPHRGVDCAREVAEVVAPVLGWTAADIDREVANYMARVEAEVLSQAQPDDVSADM
                     LRASAPEARAEILEPVPLD"
CDS                  complement(3649745..3651160)
                     /codon_start=1
                     /gene="lpdA_3"
                     /note="gene_3373"
                     /transl_table=11
                     /translation="VVTRIVILGGGPAGYEAALVAATSHPETAQVTVIDCDGIGGAAV
                     LDDCVPSKTFIASTGLRTELRRAPHLGFHIDFDDAKISLPQIHARVKTLAAAQSADIT
                     AQLLSMGVQVIAGRGELIDSTPGLARHRIKATAADGSTSEHEADVVLVATGASPRILP
                     SAQPDGERILTWRQLYDLDALPDHLIVVGSGVTGAEFVDAYTELGVPVTVVASQDHVL
                     PYEDADAALVLEESFAERGVRLFKNARAASVTRTGAGVLVTMTDGRTVEGSHALMTIG
                     SVPNTSGLGLERVGIQLGRGNYLTVDRVSRTSATGIYAAGDCTGLLPLASVAAMQGRI
                     AMYHALGEGVSPIRLRTVAATVFTRPEIAAVGVPQSVIDAGSVAARTIMLPLRTNARA
                     KMSEMRHGFVKIFCRRSTGVVIGGVVVAPIASELILPIAVAVQNRITVNELAQTLAVY
                     PSLSGSITEAARRLMAHDDLD"
CDS                  3651243..3651842
                     /codon_start=1
                     /note="gene_3374"
                     /transl_table=11
                     /translation="MHAAQRREEEPSSQAQLDDDPHRSAARWVPPEPPPSKLSPVPLY
                     AAYGSNMHPEQMLERAPHSPMAGTGWLPGWRLTFGGEDIGWEGALATVVEDPDSKVFV
                     VLYDMTPADEKNLDRWEGSEFGIHQKIRCRVERISSDTTTDPVLAWLYVLDAWEGGLP
                     SARYLGVMADAAEIAGAPSDYVHDLRTRPARNIGPGTIA"
CDS                  complement(3651861..3653030)
                     /codon_start=1
                     /gene="amiA1_3"
                     /note="gene_3375"
                     /transl_table=11
                     /translation="MSLADAAESWLAAHHDDLVGWRRHIHRYPELGRQEYATTQFVAE
                     RLADAGLNPKVLPGGTGLTCDFGPQHQPRIALRADMDALPMAERTGAPYASTMPNVAH
                     ACGHDAHTAILLGAALALASVPELPVGVRLIFQAAEELMPGGAIDAIAAGALAGVSRI
                     FALHCDPRLEVGKVAVRQGPITSAADSIEITLYSPGGHTSRPHLTADLVYGLGTLVTG
                     LPGVLSRRIDPRNSTVLVWGAVNAGMAANAIPQTGVLSGTVRTASRQTWVDLEELVRQ
                     AISALLLPLAIEHTLQYRRGVPPVVNEEISTRILAHAIEAIGPGVLADTRQSGGGEDF
                     SWYLEEVPGAMARLGVWSGDGLQLDLHQPTFDIDERALAIGLRVMVNIIEQAAAH"
```

CDS complement(3653027..3654211)
/codon_start=1
/gene="amiB1_3"
/note="gene_3376"
/transl_table=11
/translation="MPAASASDRVEELVRRRGGELVELSHAIHAEPELAFAEHRSCAK
AQALVAERGFEITTAAGGLDTAFRADYGSGPLVVGVCAEYDALPGIGHACGHNIIAAS
AVGTALALAEVADDLGLTVALLGTPAEESGGGKALMLQAGTFDDVAVAVMVHPGPTDI
AGARSLALSEVTVRYRGKESHAAVAPHLGVNAADAVTVAQVAIGVLRQQLAPGQMVHG
IVTDGGQAVNVIPGQARLQYAMRAVESDSLRELQTRMFACFAAGALAAGCEYEIDEAA
PAYAELKPDPWLADVCREEMQRLGREPLLPALEAELPLGSTDMGNVTQVLPGIHPVIG
LDAGAATVHQRAFTVASAGASADRAVVDGAIMLARTVVRLAQTPDERDRVLAAQQRRA
AR"
CDS 3654276..3655082
/codon_start=1
/gene="deoD_3"
/note="gene_3377"
/transl_table=11
/translation="VADPRPDPDELARRAAQVIADRTGIGEHDVAVVLGSGWLPAVAA
LGSPTTVLPQAELPGFVPPTAAGHAGELLSVPIGAHRVLVLAGRIHAYEGHDLRYVVH
PVRAARAAGAQIMVLTNAAGGLRADLQVGQPVLISDHLNLTARSPLVGGEFVDLTDAY
SPRLRELARQSDPQLAEGVYAGLPGPHYETPAEIRMLQTLGADLVGMSTVHETIAARA
AGAEVLGVSLVTNLAAGITGEPLSHAEVLAAGAASATRMGALLADVIARF"
CDS 3655086..3656690
/codon_start=1
/gene="pmmB_3"
/note="gene_3378"
/transl_table=11
/translation="VTPENWIAHDPDPQTAAELAACGPDELKARFSRPLAFGTAGLRG
HLRGGPDAMNLAVVLRATWAVARVLTDRGLAGSPVIVGRDARHGSPAFAAAAAEVLAA
AGFSVLLLPDPAPTPVVAFAVRHTGAAAGIQITASHNPATDNGYKVYVDGGLQLLAPT
DRQIEAAMATAPPADQIARKTVNPSENRASDLIDRYIQRAAGVRRCAGSVRVALTPLH
GVGGAMAVETLRRAGFTEVHTVATQFAPNPDFPTVTLPNPEEPGATDALLTLATDVDA
DVAIALDPDADRCAVGIPTVSGWRMLSGDETGWLLGDYILSQTDDRASPPETRVVAST
VVSSRMLAAIAAHHAAVHVETLTGFKWLARADANLPGTLVYAYEEAIGHCVDPTAVRD
KDGISAAVLVCDLVAALKGQGRSVTDALDELARCYGVHEVAALSRPVGGAVETTDLMR
RLREDPPRRLAGFPATVTDIGDTLILTGGDDNMLVRVAVRPSGTEPKLKCYLEIRCAV
TGDLPAARQLVRARIDELSASVRRWW"
CDS complement(3656692..3657315)
/codon_start=1
/gene="upp"
/note="gene_3379"
/transl_table=11
/translation="VQVHVVDHPLAAARLTTLRDERTDNAGFRAALRELTLLLIYEAT
RDAPCEPVPIRTPLAETVGSRLTKPPLLVPVLRAGLGMVDEAHAALPEAHVGFVGVAR
DEQTHQPVPYLDSLPDDLTDVPVMVLDPMVATGGSMTHTLGLLISRGAADITVLCVVA
APEGIAALQKAAPNVRLFTAAIDEGLNEVAYIVPGLGDAGDRQFGPR"
CDS 3657420..3658319
/codon_start=1
/gene="sapM_3"
/note="gene_3380"

```
                     /transl_table=11
                     /translation="MLRGIQALSRPLTRVYRALAVIGVLAASLLASWVGAVPQVGLAA
                     SALPTFAHVVIVVEENRSQAAIIGNKSAPFINSLAANGAMMAQAFAETHPSEPNYLAL
                     FAGNTFGLTKNTCPVNGGALPNLGSELLSAGYTFMGFAEDLPAVGSTVCSAGKYARKH
                     VPWVNFSNVPATLSVPFSAFPKPQNYPGLPTVSFVIPNADNDMHDGSIAQGDAWLNRH
                     LSAYANWAKTNNSLLVVTWDEDDGSSRNQIPTVFYGAHVRPGTYNETISHYNVLSTLE
                     QIYGLPKTGYATNAPPITDIWGD"
     CDS             3658307..3659605
                     /codon_start=1
                     /note="gene_3381"
                     /transl_table=11
                     /translation="LGRLAAVAILCRMVADLVPIRLSLSAGDRYTLWAPRWRDAGDEW
                     EAFLGKDDDLYGFESVSDLVAFVRTDTENDLVDHPAWQDLTGAHAHNLNPAEDNQFDL
                     VVVEELLAEKPTAESVAALAASLAIVSAIGSVCELAAVSKFFNGNPILGTVSGGLEHF
                     TGKAGNKRWNSIAEVIGRSWDDVLAAIDEIISTPEVDAELSEKVAEELAEEPEGAEEV
                     AAEVEATQDTQEAAESDDEEADAPGDSVVLGGDRDFWLQVGIDPIQIMTGTATFYTLR
                     CYLDDRPIFLGRNGRISVFGSERALARYLADEHDHDLSDLSTYDDIRTAATDGSLAVA
                     VTDDNVYVLSGLVDDFADGPDAVDREQLDLAVELLRDIGDYSEDSAVDKALETTRPLG
                     QLVAYVLDPHSVGKPTAPYAAAVREWEKLERFVESRLRRE"
     CDS             complement(3659626..3660558)
                     /codon_start=1
                     /note="gene_3382"
                     /transl_table=11
                     /translation="VLLTGPPPSLPERIRTDEADVLMLPDGRALAYLEWGDSTGYPAF
                     YFHGTPSSRLEGAFADGAARRTGFRLIAIDRPGYGRSTFQAGRNFRDWPADVCALADA
                     FELEEFGVVGHSGAGPHLFACGAVIPRTRLAFVGALGPWGPLATPDIMRSLNAADRCY
                     ARLARSGPRLFGALFAPLGWCAKYTPGLFSTLLAAAVPAADKHLLSDERFGRHLRAIQ
                     LEAFRQGSRGAAYESFLQFRPWGFDLAEVAVPTHIWLGDRDSFVPRAMGEYLQRAIPH
                     VDLHWAHGKGHFNIEDWDAILAACALDIGKRRGG"
     CDS             complement(3660578..3660748)
                     /codon_start=1
                     /note="gene_3383"
                     /transl_table=11
                     /translation="MNGDSASTIDIDKAVTRTPVRRIVRSALDRLWLRQSQPRRYRFL
                     EDSCMARALNRL"
     CDS             complement(3660927..3661238)
                     /codon_start=1
                     /note="gene_3384"
                     /transl_table=11
                     /translation="MYRFACRTLMLAACILATGVAGLGVGAQSAAQTAPVPDYYWCPG
                     QPFDPAWGPNWDPYTCHDDFHRDSDGPDHSRDYPGPILEGPVLDDPGAAPPPPAAGGG
                     A"
     CDS             complement(3661309..3662406)
                     /codon_start=1
                     /gene="add_3"
                     /note="gene_3385"
                     /transl_table=11
                     /translation="MTAAPTLQTIRLAPKALLHDHLDGGLRPATVLDIAGQVGYDDLP
                     ATDVDALASWFRTQSHSGSLERYLEPFSHTVAVMQTPEALYRVAFECAQDLAADSVVY
                     AEVRFAPELHISCGLSFDDVVDTVLTGFAAGEKACAADGQPITVRCLVTAMRHAAMSR
                     EIAELAIRFRDKGVVGFDIAGAEAGHPPTRHLDAFEYMRDHNARFTIHAGEAFGLPSI
```

HEAIAFCGADRLGHGVRIVDDIDVDADGGFQLGRLAAILRDKRIPLELCPSSNVQTGA
VASIAEHPFDLLARARFRVTVNTDNRLMSDTSMSLEMHRLVEAFGYGWSDLARFTVNA
MKSAFIPFDQRLAIIDEVIKPRFAALMGHSE"

CDS complement(3662406..3663689)
/codon_start=1
/gene="deoA"
/note="gene_3386"
/transl_table=11
/translation="VTDFAFDAPTVIRTKRDGGRLSDAAIDWVVKAYTDGRVADEQMS
ALLMAIVWRGMDRGEIARWTAAMLASGARLDFTDLPLATVDKHSTGGVGDKITLPLVP
VVAACGGAVPQASGRGLGHTGGTLDKLESITGFTANLSNQRVREQLCDVGAAIFAAGQ
LAPADAKLYALRDITGTVESLPLIASSIMSKKLAEGAGALVLDVKVGSGAFMRSPVQA
RELAHTMVELGAAHGVPTRALLTEMNCPLGRTVGNALEVAEALEVLAGGGPPDVVELT
LRLAGEMLELAGIHGRDPAQTLRDGTAMDRFRWLVAAQGGDLSKPLPIGSHSETVTAG
ASGTMGDIDAMAVGLAAWRLGAGRSRPGARVQHGAGVRIHRRPGEPVVVGEPLFTLYT
NAPERFGAARAELAGGWSIRDSPPQVRPLIVDRIV"

CDS complement(3663686..3664087)
/codon_start=1
/gene="cdd_3"
/note="gene_3387"
/transl_table=11
/translation="MPDVDWNMLRGNATQAAAGAYVPYSRFAVGAAALVDDGRVVTGC
NVENVSYGLTLCAECAVVCALHSTGGGRLLALACVDGHGSVLMPCGRCRQVLLEHGGS
ELLIDHPVRPRRLGDLLPDAFGLDDLPRERR"

CDS 3664324..3664662
/codon_start=1
/gene="sdhC_3"
/note="gene_3388"
/transl_table=11
/translation="MWSWVCHRISGATIFFFLFVHVLDAAMLRVSPQTYNAVLATYKT
PIVGLMEYGLVAAVLFHALNGIRVILIDFWSEGPRYQRLMLWIIGSVFLLLMVPAGVV
VGIHMWEHFR"

CDS 3664740..3665093
/codon_start=1
/gene="sdhD_3"
/note="gene_3389"
/transl_table=11
/translation="MPNFEKFAWLFMRFSGVVLVFLAIGHLFIMLMWDNGVYRLDFNF
VAQRWASPFWQTWDLLLLWLAQLHGGNGLRTIIDDYSRKDTTRFWLNSLLVLSMLFTL
MLGTYVIVTFDPNIS"

CDS complement(3665057..3666886)
/codon_start=1
/note="gene_3390"
/transl_table=11
/translation="MSEDNALVLVAGYQDLDSARHDFQTLVDAAKDKSIPLQGAVLIG
KDAEGSPVLVDTGNRLGRRGAAWGAGVGLAIGLFSPALLASAALGAATGALAGTFAHH
RIKTGLADKIGQALAAGRAVVIAVTEAQGRLEAGQALASSPMKSVAELSRSTLRSLGA
ALREAMGKFNPDRTRLPLPQRRFGGVVGRTMAESVGDWSIVPSPFPPDDAPNVLIVLI
DDAGFGGPDTFGGAIRTPTLSRLAQNGLIYNRFHVTAVCSPTRAALLTGRNHHRVGFG
SVCEFPGPYPGYSAVRPRSCAALPRILRDNGYVTGAFGKWHLTPDNVQGAAGPFDNWP
LGWGFDHFWGFPSGAAGQYDPIISQDNSVIGIPEGSGEDGRPYYFPDDLTDKAIEWLH

TVRAQNATKPWMLYYATGATHAPHHVFKEWADKYRGEFDDGWDVYRQKTFERQKRLGI
IPPDAELTERPDLFPAWDSMSEAQKRLFARQMEVFAGFSENADWNVGRLLDAIEDLGE
SDNTLVFYIWGDNGASMEGTNTGSFNEMTFLNGLDLDAERQLELIEQYGGIAALGDEF
TAPHFASAWAHGGGTARLRGRGGVIMCSFRAFQEMFGSNVTIT"

CDS complement(3666909..3667826)
/codon_start=1
/note="gene_3391"
/transl_table=11
/translation="VALRPEDRLLSVHDVLGPVRVRLLGGSVLAELTARFGVAARAKV
LAGEVVDDDGAVVDSGTVLPPGSVVHLYRDLPDEVPVPFDVPVLHQDADIVVVDKPHF
LATMPRGRHVAQTALVRLRRELGLPELSPAHRLDRLTAGVLLFTTRREVRGSYQTMFA
RGLVRKTYLARAPVAPGLALPRLVRSRIVKRRGHLQAVCEPGVPNAETLVERIARDGL
YRLTPTTGRTHQLRVHMAALGIPIMGDPLYPNVISVAAHDFSTPLQLLAQRIEFDDPL
TGSHREFASTRTLTGATLPTWSAAADCRP"

CDS complement(3667838..3668503)
/codon_start=1
/gene="phoY1_3"
/note="gene_3392"
/transl_table=11
/translation="MRTVYHQRLTELAGRLGEMCSLAGIAMKRATQALLEADIGAAEQ
VIRDHERIVAMRAQVEKEAFALLALQHPVSGELREIFSAVQIIADTERMGALAVHIAK
ITRREYPNQVLPEEVRNCFADMAKVAIALGDSARQVLVNRDPQEAAQLHDRDDAMDDL
HRHLLSVLIDREWRHGVRVGVETALLGRFFERFADHAVEVGRRVIFMVTGVLPTEDEI
STY"

CDS complement(3668611..3670368)
/codon_start=1
/gene="glpD2_3"
/note="gene_3393"
/transl_table=11
/translation="VSNPIQAPDGGQGWPAAALGPAQRAVAWKRLGTEQFDVVVIGGG
VVGSGCALDAATRGLKVALVEARDLASGTSSRSSKMFHGGLRYLEQLEFGLVREALYE
RELSLTTLAPHLVKPLPFLFPLTKRWWERPYIAAGIFLYDRLGGAKSVPAQRHFTRAG
ALRLSPGLKRSSLIGGIRYYDTVVDDARHTMTVARTAAHYGAVVRCSTQVVALLREGD
RVIGVGVRDSENGAVAEVRGHVVVNATGVWTDEIQALSKQRGRFQVRASKGVHVVVPR
DRIVSDVAMILRTEKSVMFVIPWGSHWIIGTTDTDWNLDLAHPAATKADIDYILGTVN
AVLATPLTHADIDGVYAGLRPLLAGESDDTSKLSREHAVAVPAAGLVAIAGGKYTTYR
VMAADAIDAAVQFIPARVAPSITEKVSLLGADGYFALVNQAEHVGALQGLHPYRVRHL
LDRYGSLISDVLAMAASDPSLLSPITEAPGYLKVEAAYAAAAEGALHLEDILARRMRI
SIEYPHRGVDCAREVAEVVAPVLGWTAADIDREVANYMARVEAEVLSQAQPDDVSADM
LRASAPEARAEILEPVPLD"

CDS complement(3670449..3671864)
/codon_start=1
/gene="lpdA_3"
/note="gene_3394"
/transl_table=11
/translation="VVTRIVILGGGPAGYEAALVAATSHPETAQVTVIDCDGIGGAAV
LDDCVPSKTFIASTGLRTELRRAPHLGFHIDFDDAKISLPQIHARVKTLAAAQSADIT
AQLLSMGVQVIAGRGELIDSTPGLARHRIKATAADGSTSEHEADVVLVATGASPRILP
SAQPDGERILTWRQLYDLDALPDHLIVVGSGVTGAEFVDAYTELGVPVTVVASQDHVL
PYEDADAALVLEESFAERGVRLFKNARAASVTRTGAGVLVTMTDGRTVEGSHALMTIG
SVPNTSGLGLERVGIQLGRGNYLTVDRVSRTSATGIYAAGDCTGLLPLASVAAMQGRI

AMYHALGEGVSPIRLRTVAATVFTRPEIAAVGVPQSVIDAGSVAARTIMLPLRTNARA
KMSEMRHGFVKIFCRRSTGVVIGGVVVAPIASELILPIAVAVQNRITVNELAQTLAVY
PSLSGSITEAARRLMAHDDLD"
CDS 3671947..3672546
/codon_start=1
/note="gene_3395"
/transl_table=11
/translation="MHAAQRREEEPSSQAQLDDDPHRSAARWVPPEPPPSKLSPVPLY
AAYGSNMHPEQMLERAPHSPMAGTGWLPGWRLTFGGEDIGWEGALATVVEDPDSKVFV
VLYDMTPADEKNLDRWEGSEFGIHQKIRCRVERISSDTTTDPVLAWLYVLDAWEGGLP
SARYLGVMADAAEIAGAPSDYVHDLRTRPARNIGPGTIA"
CDS complement(3672565..3673734)
/codon_start=1
/gene="amiA1_3"
/note="gene_3396"
/transl_table=11
/translation="MSLADAAESWLAAHHDDLVGWRRHIHRYPELGRQEYATTQFVAE
RLADAGLNPKVLPGGTGLTCDFGPQHQPRIALRADMDALPMAERTGAPYASTMPNVAH
ACGHDAHTAILLGAALALASVPELPVGVRLIFQAAEELMPGGAIDAIAAGALAGVSRI
FALHCDPRLEVGKVAVRQGPITSAADSIEITLYSPGGHTSRPHLTADLVYGLGTLVTG
LPGVLSRRIDPRNSTVLVWGAVNAGMAANAIPQTGVLSGTVRTASRQTWVDLEELVRQ
AISALLLPLAIEHTLQYRRGVPPVVNEEISTRILAHAIEAIGPGVLADTRQSGGGEDF
SWYLEEVPGAMARLGVWSGDGLQLDLHQPTFDIDERALAIGLRVMVNIIEQAAAH"
CDS complement(3673731..3674915)
/codon_start=1
/gene="amiB1_3"
/note="gene_3397"
/transl_table=11
/translation="MPAASASDRVEELVRRRGGELVELSHAIHAEPELAFAEHRSCAK
AQALVAERGFEITTAAGGLDTAFRADYGSGPLVVGVCAEYDALPGIGHACGHNIIAAS
AVGTALALAEVADDLGLTVALLGTPAEESGGGKALMLQAGTFDDVAVAVMVHPGPTDI
AGARSLALSEVTVRYRGKESHAAVAPHLGVNAADAVTVAQVAIGVLRQQLAPGQMVHG
IVTDGGQAVNVIPGQARLQYAMRAVESDSLRELQTRMFACFAAGALAAGCEYEIDEAA
PAYAELKPDPWLADVCREEMQRLGREPLLPALEAELPLGSTDMGNVTQVLPGIHPVIG
LDAGAATVHQRAFTVASAGASADRAVVDGAIMLARTVVRLAQTPDERDRVLAAQQRRA
AR"
CDS 3674980..3675786
/codon_start=1
/gene="deoD_3"
/note="gene_3398"
/transl_table=11
/translation="VADPRPDPDELARRAAQVIADRTGIGEHDVAVVLGSGWLPAVAA
LGSPTTVLPQAELPGFVPPTAAGHAGELLSVPIGAHRVLVLAGRIHAYEGHDLRYVVH
PVRAARAAGAQIMVLTNAAGGLRADLQVGQPVLISDHLNLTARSPLVGGEFVDLTDAY
SPRLRELARQSDPQLAEGVYAGLPGPHYETPAEIRMLQTLGADLVGMSTVHETIAARA
AGAEVLGVSLVTNLAAGITGEPLSHAEVLAAGAASATRMGALLADVIARF"
CDS 3675790..3677394
/codon_start=1
/gene="pmmB_3"
/note="gene_3399"
/transl_table=11

/translation="VTPENWIAHDPDPQTAAELAACGPDELKARFSRPLAFGTAGLRG
HLRGGPDAMNLAVVLRATWAVARVLTDRGLAGSPVIVGRDARHGSPAFAAAAAEVLAA
AGFSVLLLPDPAPTPVVAFAVRHTGAAAGIQITASHNPATDNGYKVYVDGGLQLLAPT
DRQIEAAMATAPPADQIARKTVNPSENRASDLIDRYIQRAAGVRRCAGSVRVALTPLH
GVGGAMAVETLRRAGFTEVHTVATQFAPNPDFPTVTLPNPEEPGATDALLTLATDVDA
DVAIALDPDADRCAVGIPTVSGWRMLSGDETGWLLGDYILSQTDDRASPPETRVVAST
VVSSRMLAAIAAHHAAVHVETLTGFKWLARADANLPGTLVYAYEEAIGHCVDPTAVRD
KDGISAAVLVCDLVAALKGQGRSVTDALDELARCYGVHEVAALSRPVGGAVETTDLMR
RLREDPPRRLAGFPATVTDIGDTLILTGGDDNMLVRVAVRPSGTEPKLKCYLEIRCAV
TGDLPAARQLVRARIDELSASVRRWW"

CDS complement(3677396..3678019)
/codon_start=1
/gene="upp"
/note="gene_3400"
/transl_table=11
/translation="VQVHVVDHPLAAARLTTLRDERTDNAGFRAALRELTLLLIYEAT
RDAPCEPVPIRTPLAETVGSRLTKPPLLVPVLRAGLGMVDEAHAALPEAHVGFVGVAR
DEQTHQPVPYLDSLPDDLTDVPVMVLDPMVATGGSMTHTLGLLISRGAADITVLCVVA
APEGIAALQKAAPNVRLFTAAIDEGLNEVAYIVPGLGDAGDRQFGPR"

CDS 3678124..3679023
/codon_start=1
/gene="sapM_3"
/note="gene_3401"
/transl_table=11
/translation="MLRGIQALSRPLTRVYRALAVIGVLAASLLASWVGAVPQVGLAA
SALPTFAHVVIVVEENRSQAAIIGNKSAPFINSLAANGAMMAQAFAETHPSEPNYLAL
FAGNTFGLTKNTCPVNGGALPNLGSELLSAGYTFMGFAEDLPAVGSTVCSAGKYARKH
VPWVNFSNVPATLSVPFSAFPKPQNYPGLPTVSFVIPNADNDMHDGSIAQGDAWLNRH
LSAYANWAKTNNSLLVVTWDEDDGSSRNQIPTVFYGAHVRPGTYNETISHYNVLSTLE
QIYGLPKTGYATNAPPITDIWGD"

CDS 3679011..3680309
/codon_start=1
/note="gene_3402"
/transl_table=11
/translation="LGRLAAVAILCRMVADLVPIRLSLSAGDRYTLWAPRWRDAGDEW
EAFLGKDDDLYGFESVSDLVAFVRTDTENDLVDHPAWQDLTGAHAHNLNPAEDNQFDL
VVVEELLAEKPTAESVAALAASLAIVSAIGSVCELAAVSKFFNGNPILGTVSGGLEHF
TGKAGNKRWNSIAEVIGRSWDDVLAAIDEIISTPEVDAELSEKVAEELAEEPEGAEEV
AAEVEATQDTQEAAESDDEEADAPGDSVVLGGDRDFWLQVGIDPIQIMTGTATFYTLR
CYLDDRPIFLGRNGRISVFGSERALARYLADEHDHDLSDLSTYDDIRTAATDGSLAVA
VTDDNVYVLSGLVDDFADGPDAVDREQLDLAVELLRDIGDYSEDSAVDKALETTRPLG
QLVAYVLDPHSVGKPTAPYAAAVREWEKLERFVESRLRRE"

CDS complement(3680330..3681262)
/codon_start=1
/note="gene_3403"
/transl_table=11
/translation="VLLTGPPPSLPERIRTDEADVLMLPDGRALAYLEWGDSTGYPAF
YFHGTPSSRLEGAFADGAARRTGFRLIAIDRPGYGRSTFQAGRNFRDWPADVCALADA
FELEEFGVVGHSGAGPHLFACGAVIPRTRLAFVGALGPWGPLATPDIMRSLNAADRCY
ARLARSGPRLFGALFAPLGWCAKYTPGLFSTLLAAAVPAADKHLLSDERFGRHLRAIQ
LEAFRQGSRGAAYESFLQFRPWGFDLAEVAVPTHIWLGDRDSFVPRAMGEYLQRAIPH

```
                     VDLHWAHGKGHFNIEDWDAILAACALDIGKRRGG"
     CDS             complement(3681282..3681452)
                     /codon_start=1
                     /note="gene_3404"
                     /transl_table=11
                     /translation="MNGDSASTIDIDKAVTRTPVRRIVRSALDRLWLRQSQPRRYRFL
                     EDSCMARALNRL"
     CDS             complement(3681631..3681942)
                     /codon_start=1
                     /note="gene_3405"
                     /transl_table=11
                     /translation="MYRFACRTLMLAACILATGVAGLGVGAQSAAQTAPVPDYYWCPG
                     QPFDPAWGPNWDPYTCHDDFHRDSDGPDHSRDYPGPILEGPVLDDPGAAPPPPAAGGG
                     A"
     CDS             complement(3682013..3683110)
                     /codon_start=1
                     /gene="add_3"
                     /note="gene_3406"
                     /transl_table=11
                     /translation="MTAAPTLQTIRLAPKALLHDHLDGGLRPATVLDIAGQVGYDDLP
                     ATDVDALASWFRTQSHSGSLERYLEPFSHTVAVMQTPEALYRVAFECAQDLAADSVVY
                     AEVRFAPELHISCGLSFDDVVDTVLTGFAAGEKACAADGQPITVRCLVTAMRHAAMSR
                     EIAELAIRFRDKGVVGFDIAGAEAGHPPTRHLDAFEYMRDHNARFTIHAGEAFGLPSI
                     HEAIAFCGADRLGHGVRIVDDIDVDADGGFQLGRLAAILRDKRIPLELCPSSNVQTGA
                     VASIAEHPFDLLARARFRVTVNTDNRLMSDTSMSLEMHRLVEAFGYGWSDLARFTVNA
                     MKSAFIPFDQRLAIIDEVIKPRFAALMGHSE"
     CDS             complement(3683110..3684393)
                     /codon_start=1
                     /gene="deoA"
                     /note="gene_3407"
                     /transl_table=11
                     /translation="VTDFAFDAPTVIRTKRDGGRLSDAAIDWVVKAYTDGRVADEQMS
                     ALLMAIVWRGMDRGEIARWTAAMLASGARLDFTDLPLATVDKHSTGGVGDKITLPLVP
                     VVAACGGAVPQASGRGLGHTGGTLDKLESITGFTANLSNQRVREQLCDVGAAIFAAGQ
                     LAPADAKLYALRDITGTVESLPLIASSIMSKKLAEGAGALVLDVKVGSGAFMRSPVQA
                     RELAHTMVELGAAHGVPTRALLTEMNCPLGRTVGNALEVAEALEVLAGGGPPDVVELT
                     LRLAGEMLELAGIHGRDPAQTLRDGTAMDRFRWLVAAQGGDLSKPLPIGSHSETVTAG
                     ASGTMGDIDAMAVGLAAWRLGAGRSRPGARVQHGAGVRIHRRPGEPVVVGEPLFTLYT
                     NAPERFGAARAELAGGWSIRDSPPQVRPLIVDRIV"
     CDS             complement(3684390..3684791)
                     /codon_start=1
                     /gene="cdd_3"
                     /note="gene_3408"
                     /transl_table=11
                     /translation="MPDVDWNMLRGNATQAAAGAYVPYSRFAVGAAALVDDGRVVTGC
                     NVENVSYGLTLCAECAVVCALHSTGGGRLLALACVDGHGSVLMPCGRCRQVLLEHGGS
                     ELLIDHPVRPRRLGDLLPDAFGLDDLPRERR"
     CDS             3685028..3685366
                     /codon_start=1
                     /gene="sdhC_3"
                     /note="gene_3409"
```

/transl_table=11
/translation="MWSWVCHRISGATIFFFLFVHVLDAAMLRVSPQTYNAVLATYKT
PIVGLMEYGLVAAVLFHALNGIRVILIDFWSEGPRYQRLMLWIIGSVFLLLMVPAGVV
VGIHMWEHFR"
CDS 3685444..3685797
/codon_start=1
/gene="sdhD_3"
/note="gene_3410"
/transl_table=11
/translation="MPNFEKFAWLFMRFSGVVLVFLAIGHLFIMLMWDNGVYRLDFNF
VAQRWASPFWQTWDLLLLWLAQLHGGNGLRTIIDDYSRKDTTRFWLNSLLVLSMLFTL
MLGTYVIVTFDPNIS"
CDS complement(3685761..3687590)
/codon_start=1
/note="gene_3411"
/transl_table=11
/translation="MSEDNALVLVAGYQDLDSARHDFQTLVDAAKDKSIPLQGAVLIG
KDAEGSPVLVDTGNRLGRRGAAWGAGVGLAIGLFSPALLASAALGAATGALAGTFAHH
RIKTGLADKIGQALAAGRAVVIAVTEAQGRLEAGQALASSPMKSVAELSRSTLRSLGA
ALREAMGKFNPDRTRLPLPQRRFGGVVGRTMAESVGDWSIVPSPFPPDDAPNVLIVLI
DDAGFGGPDTFGGAIRTPTLSRLAQNGLIYNRFHVTAVCSPTRAALLTGRNHHRVGFG
SVCEFPGPYPGYSAVRPRSCAALPRILRDNGYVTGAFGKWHLTPDNVQGAAGPFDNWP
LGWGFDHFWGFPSGAAGQYDPIISQDNSVIGIPEGSGEDGRPYYFPDDLTDKAIEWLH
TVRAQNATKPWMLYYATGATHAPHHVFKEWADKYRGEFDDGWDVYRQKTFERQKRLGI
IPPDAELTERPDLFPAWDSMSEAQKRLFARQMEVFAGFSENADWNVGRLLDAIEDLGE
SDNTLVFYIWGDNGASMEGTNTGSFNEMTFLNGLDLDAERQLELIEQYGGIAALGDEF
TAPHFASAWAHGGGTARLRGRGGVIMCSFRAFQEMFGSNVTIT"
CDS complement(3687613..3688530)
/codon_start=1
/note="gene_3412"
/transl_table=11
/translation="VALRPEDRLLSVHDVLGPVRVRLLGGSVLAELTARFGVAARAKV
LAGEVVDDDGAVVDSGTVLPPGSVVHLYRDLPDEVPVPFDVPVLHQDADIVVVDKPHF
LATMPRGRHVAQTALVRLRRELGLPELSPAHRLDRLTAGVLLFTTRREVRGSYQTMFA
RGLVRKTYLARAPVAPGLALPRLVRSRIVKRRGHLQAVCEPGVPNAETLVERIARDGL
YRLTPTTGRTHQLRVHMAALGIPIMGDPLYPNVISVAAHDFSTPLQLLAQRIEFDDPL
TGSHREFASTRTLTGATLPTWSAAADCRP"
CDS complement(3688542..3689207)
/codon_start=1
/gene="phoY1_3"
/note="gene_3413"
/transl_table=11
/translation="MRTVYHQRLTELAGRLGEMCSLAGIAMKRATQALLEADIGAAEQ
VIRDHERIVAMRAQVEKEAFALLALQHPVSGELREIFSAVQIIADTERMGALAVHIAK
ITRREYPNQVLPEEVRNCFADMAKVAIALGDSARQVLVNRDPQEAAQLHDRDDAMDDL
HRHLLSVLIDREWRHGVRVGVETALLGRFFERFADHAVEVGRRVIFMVTGVLPTEDEI
STY"
CDS complement(3689315..3691072)
/codon_start=1
/gene="glpD2_3"
/note="gene_3414"

```
                     /transl_table=11
                     /translation="VSNPIQAPDGGQGWPAAALGPAQRAVAWKRLGTEQFDVVVIGGG
                     VVGSGCALDAATRGLKVALVEARDLASGTSSRSSKMFHGGLRYLEQLEFGLVREALYE
                     RELSLTTLAPHLVKPLPFLFPLTKRWWERPYIAAGIFLYDRLGGAKSVPAQRHFTRAG
                     ALRLSPGLKRSSLIGGIRYYDTVVDDARHTMTVARTAAHYGAVVRCSTQVVALLREGD
                     RVIGVGVRDSENGAVAEVRGHVVVNATGVWTDEIQALSKQRGRFQVRASKGVHVVVPR
                     DRIVSDVAMILRTEKSVMFVIPWGSHWIIGTTDTDWNLDLAHPAATKADIDYILGTVN
                     AVLATPLTHADIDGVYAGLRPLLAGESDDTSKLSREHAVAVPAAGLVAIAGGKYTTYR
                     VMAADAIDAAVQFIPARVAPSITEKVSLLGADGYFALVNQAEHVGALQGLHPYRVRHL
                     LDRYGSLISDVLAMAASDPSLLSPITEAPGYLKVEAAYAAAAEGALHLEDILARRMRI
                     SIEYPHRGVDCAREVAEVVAPVLGWTAADIDREVANYMARVEAEVLSQAQPDDVSADM
                     LRASAPEARAEILEPVPLD"
     CDS             complement(3691153..3692568)
                     /codon_start=1
                     /gene="lpdA_3"
                     /note="gene_3415"
                     /transl_table=11
                     /translation="VVTRIVILGGGPAGYEAALVAATSHPETAQVTVIDCDGIGGAAV
                     LDDCVPSKTFIASTGLRTELRRAPHLGFHIDFDDAKISLPQIHARVKTLAAAQSADIT
                     AQLLSMGVQVIAGRGELIDSTPGLARHRIKATAADGSTSEHEADVVLVATGASPRILP
                     SAQPDGERILTWRQLYDLDALPDHLIVVGSGVTGAEFVDAYTELGVPVTVVASQDHVL
                     PYEDADAALVLEESFAERGVRLFKNARAASVTRTGAGVLVTMTDGRTVEGSHALMTIG
                     SVPNTSGLGLERVGIQLGRGNYLTVDRVSRTSATGIYAAGDCTGLLPLASVAAMQGRI
                     AMYHALGEGVSPIRLRTVAATVFTRPEIAAVGVPQSVIDAGSVAARTIMLPLRTNARA
                     KMSEMRHGFVKIFCRRSTGVVIGGVVVAPIASELILPIAVAVQNRITVNELAQTLAVY
                     PSLSGSITEAARRLMAHDDLD"
     CDS             3692651..3693250
                     /codon_start=1
                     /note="gene_3416"
                     /transl_table=11
                     /translation="MHAAQRREEEPSSQAQLDDDPHRSAARWVPPEPPPSKLSPVPLY
                     AAYGSNMHPEQMLERAPHSPMAGTGWLPGWRLTFGGEDIGWEGALATVVEDPDSKVFV
                     VLYDMTPADEKNLDRWEGSEFGIHQKIRCRVERISSDTTTDPVLAWLYVLDAWEGGLP
                     SARYLGVMADAAEIAGAPSDYVHDLRTRPARNIGPGTIA"
     CDS             complement(3693269..3694438)
                     /codon_start=1
                     /gene="amiA1_3"
                     /note="gene_3417"
                     /transl_table=11
                     /translation="MSLADAAESWLAAHHDDLVGWRRHIHRYPELGRQEYATTQFVAE
                     RLADAGLNPKVLPGGTGLTCDFGPQHQPRIALRADMDALPMAERTGAPYASTMPNVAH
                     ACGHDAHTAILLGAALALASVPELPVGVRLIFQAAEELMPGGAIDAIAAGALAGVSRI
                     FALHCDPRLEVGKVAVRQGPITSAADSIEITLYSPGGHTSRPHLTADLVYGLGTLVTG
                     LPGVLSRRIDPRNSTVLVWGAVNAGMAANAIPQTGVLSGTVRTASRQTWVDLEELVRQ
                     AISALLLPLAIEHTLQYRRGVPPVVNEEISTRILAHAIEAIGPGVLADTRQSGGGEDF
                     SWYLEEVPGAMARLGVWSGDGLQLDLHQPTFDIDERALAIGLRVMVNIIEQAAAH"
     CDS             complement(3694435..3695619)
                     /codon_start=1
                     /gene="amiB1_3"
                     /note="gene_3418"
                     /transl_table=11
```

/translation="MPAASASDRVEELVRRRGGELVELSHAIHAEPELAFAEHRSCAK
AQALVAERGFEITTAAGGLDTAFRADYGSGPLVVGVCAEYDALPGIGHACGHNIIAAS
AVGTALALAEVADDLGLTVALLGTPAEESGGGKALMLQAGTFDDVAVAVMVHPGPTDI
AGARSLALSEVTVRYRGKESHAAVAPHLGVNAADAVTVAQVAIGVLRQQLAPGQMVHG
IVTDGGQAVNVIPGQARLQYAMRAVESDSLRELQTRMFACFAAGALAAGCEYEIDEAA
PAYAELKPDPWLADVCREEMQRLGREPLLPALEAELPLGSTDMGNVTQVLPGIHPVIG
LDAGAATVHQRAFTVASAGASADRAVVDGAIMLARTVVRLAQTPDERDRVLAAQQRRA
AR"

CDS 3695684..3696490
/codon_start=1
/gene="deoD_3"
/note="gene_3419"
/transl_table=11
/translation="VADPRPDPDELARRAAQVIADRTGIGEHDVAVVLGSGWLPAVAA
LGSPTTVLPQAELPGFVPPTAAGHAGELLSVPIGAHRVLVLAGRIHAYEGHDLRYVVH
PVRAARAAGAQIMVLTNAAGGLRADLQVGQPVLISDHLNLTARSPLVGGEFVDLTDAY
SPRLRELARQSDPQLAEGVYAGLPGPHYETPAEIRMLQTLGADLVGMSTVHETIAARA
AGAEVLGVSLVTNLAAGITGEPLSHAEVLAAGAASATRMGALLADVIARF"

CDS 3696494..3698098
/codon_start=1
/gene="pmmB_3"
/note="gene_3420"
/transl_table=11
/translation="VTPENWIAHDPDPQTAAELAACGPDELKARFSRPLAFGTAGLRG
HLRGGPDAMNLAVVLRATWAVARVLTDRGLAGSPVIVGRDARHGSPAFAAAAAEVLAA
AGFSVLLLPDPAPTPVVAFAVRHTGAAAGIQITASHNPATDNGYKVYVDGGLQLLAPT
DRQIEAAMATAPPADQIARKTVNPSENRASDLIDRYIQRAAGVRRCAGSVRVALTPLH
GVGGAMAVETLRRAGFTEVHTVATQFAPNPDFPTVTLPNPEEPGATDALLTLATDVDA
DVAIALDPDADRCAVGIPTVSGWRMLSGDETGWLLGDYILSQTDDRASPPETRVVAST
VVSSRMLAAIAAHHAAVHVETLTGFKWLARADANLPGTLVYAYEEAIGHCVDPTAVRD
KDGISAAVLVCDLVAALKGQGRSVTDALDELARCYGVHEVAALSRPVGGAVETTDLMR
RLREDPPRRLAGFPATVTDIGDTLILTGGDDNMLVRVAVRPSGTEPKLKCYLEIRCAV
TGDLPAARQLVRARIDELSASVRRWW"

CDS complement(3698100..3698723)
/codon_start=1
/gene="upp"
/note="gene_3421"
/transl_table=11
/translation="VQVHVVDHPLAAARLTTLRDERTDNAGFRAALRELTLLLIYEAT
RDAPCEPVPIRTPLAETVGSRLTKPPLLVPVLRAGLGMVDEAHAALPEAHVGFVGVAR
DEQTHQPVPYLDSLPDDLTDVPVMVLDPMVATGGSMTHTLGLLISRGAADITVLCVVA
APEGIAALQKAAPNVRLFTAAIDEGLNEVAYIVPGLGDAGDRQFGPR"

CDS 3698828..3699727
/codon_start=1
/gene="sapM_3"
/note="gene_3422"
/transl_table=11
/translation="MLRGIQALSRPLTRVYRALAVIGVLAASLLASWVGAVPQVGLAA
SALPTFAHVVIVVEENRSQAAIIGNKSAPFINSLAANGAMMAQAFAETHPSEPNYLAL
FAGNTFGLTKNTCPVNGGALPNLGSELLSAGYTFMGFAEDLPAVGSTVCSAGKYARKH
VPWVNFSNVPATLSVPFSAFPKPQNYPGLPTVSFVIPNADNDMHDGSIAQGDAWLNRH

LSAYANWAKTNNSLLVVTWDEDDGSSRNQIPTVFYGAHVRPGTYNETISHYNVLSTLE
QIYGLPKTGYATNAPPITDIWGD"
CDS 3699715..3701013
/codon_start=1
/note="gene_3423"
/transl_table=11
/translation="LGRLAAVAILCRMVADLVPIRLSLSAGDRYTLWAPRWRDAGDEW
EAFLGKDDDLYGFESVSDLVAFVRTDTENDLVDHPAWQDLTGAHAHNLNPAEDNQFDL
VVVEELLAEKPTAESVAALAASLAIVSAIGSVCELAAVSKFFNGNPILGTVSGGLEHF
TGKAGNKRWNSIAEVIGRSWDDVLAAIDEIISTPEVDAELSEKVAEELAEEPEGAEEV
AAEVEATQDTQEAAESDDEEADAPGDSVVLGGDRDFWLQVGIDPIQIMTGTATFYTLR
CYLDDRPIFLGRNGRISVFGSERALARYLADEHDHDLSDLSTYDDIRTAATDGSLAVA
VTDDNVYVLSGLVDDFADGPDAVDREQLDLAVELLRDIGDYSEDSAVDKALETTRPLG
QLVAYVLDPHSVGKPTAPYAAAVREWEKLERFVESRLRRE"
CDS complement(3701034..3701966)
/codon_start=1
/note="gene_3424"
/transl_table=11
/translation="VLLTGPPPSLPERIRTDEADVLMLPDGRALAYLEWGDSTGYPAF
YFHGTPSSRLEGAFADGAARRTGFRLIAIDRPGYGRSTFQAGRNFRDWPADVCALADA
FELEEFGVVGHSGAGPHLFACGAVIPRTRLAFVGALGPWGPLATPDIMRSLNAADRCY
ARLARSGPRLFGALFAPLGWCAKYTPGLFSTLLAAAVPAADKHLLSDERFGRHLRAIQ
LEAFRQGSRGAAYESFLQFRPWGFDLAEVAVPTHIWLGDRDSFVPRAMGEYLQRAIPH
VDLHWAHGKGHFNIEDWDAILAACALDIGKRRGG"
CDS complement(3701986..3702156)
/codon_start=1
/note="gene_3425"
/transl_table=11
/translation="MNGDSASTIDIDKAVTRTPVRRIVRSALDRLWLRQSQPRRYRFL
EDSCMARALNRL"
CDS complement(3702335..3702646)
/codon_start=1
/note="gene_3426"
/transl_table=11
/translation="MYRFACRTLMLAACILATGVAGLGVGAQSAAQTAPVPDYYWCPG
QPFDPAWGPNWDPYTCHDDFHRDSDGPDHSRDYPGPILEGPVLDDPGAAPPPPAAGGG
A"
CDS complement(3702717..3703814)
/codon_start=1
/gene="add_3"
/note="gene_3427"
/transl_table=11
/translation="MTAAPTLQTIRLAPKALLHDHLDGGLRPATVLDIAGQVGYDDLP
ATDVDALASWFRTQSHSGSLERYLEPFSHTVAVMQTPEALYRVAFECAQDLAADSVVY
AEVRFAPELHISCGLSFDDVVDTVLTGFAAGEKACAADGQPITVRCLVTAMRHAAMSR
EIAELAIRFRDKGVVGFDIAGAEAGHPPTRHLDAFEYMRDHNARFTIHAGEAFGLPSI
HEAIAFCGADRLGHGVRIVDDIDVDADGGFQLGRLAAILRDKRIPLELCPSSNVQTGA
VASIAEHPFDLLARARFRVTVNTDNRLMSDTSMSLEMHRLVEAFGYGWSDLARFTVNA
MKSAFIPFDQRLAIIDEVIKPRFAALMGHSE"
CDS complement(3703814..3705097)
/codon_start=1

```
                     /gene="deoA"
                     /note="gene_3428"
                     /transl_table=11
                     /translation="VTDFAFDAPTVIRTKRDGGRLSDAAIDWVVKAYTDGRVADEQMS
                     ALLMAIVWRGMDRGEIARWTAAMLASGARLDFTDLPLATVDKHSTGGVGDKITLPLVP
                     VVAACGGAVPQASGRGLGHTGGTLDKLESITGFTANLSNQRVREQLCDVGAAIFAAGQ
                     LAPADAKLYALRDITGTVESLPLIASSIMSKKLAEGAGALVLDVKVGSGAFMRSPVQA
                     RELAHTMVELGAAHGVPTRALLTEMNCPLGRTVGNALEVAEALEVLAGGGPPDVVELT
                     LRLAGEMLELAGIHGRDPAQTLRDGTAMDRFRWLVAAQGGDLSKPLPIGSHSETVTAG
                     ASGTMGDIDAMAVGLAAWRLGAGRSRPGARVQHGAGVRIHRRPGEPVVVGEPLFTLYT
                     NAPERFGAARAELAGGWSIRDSPPQVRPLIVDRIV"
     CDS             complement(3705094..3705495)
                     /codon_start=1
                     /gene="cdd_3"
                     /note="gene_3429"
                     /transl_table=11
                     /translation="MPDVDWNMLRGNATQAAAGAYVPYSRFAVGAAALVDDGRVVTGC
                     NVENVSYGLTLCAECAVVCALHSTGGGRLLALACVDGHGSVLMPCGRCRQVLLEHGGS
                     ELLIDHPVRPRRLGDLLPDAFGLDDLPRERR"
     CDS             3705732..3706070
                     /codon_start=1
                     /gene="sdhC_3"
                     /note="gene_3430"
                     /transl_table=11
                     /translation="MWSWVCHRISGATIFFFLFVHVLDAAMLRVSPQTYNAVLATYKT
                     PIVGLMEYGLVAAVLFHALNGIRVILIDFWSEGPRYQRLMLWIIGSVFLLLMVPAGVV
                     VGIHMWEHFR"
     CDS             3706148..3706501
                     /codon_start=1
                     /gene="sdhD_3"
                     /note="gene_3431"
                     /transl_table=11
                     /translation="MPNFEKFAWLFMRFSGVVLVFLAIGHLFIMLMWDNGVYRLDFNF
                     VAQRWASPFWQTWDLLLLWLAQLHGGNGLRTIIDDYSRKDTTRFWLNSLLVLSMLFTL
                     MLGTYVIVTFDPNIS"
     CDS             complement(3706465..3708294)
                     /codon_start=1
                     /note="gene_3432"
                     /transl_table=11
                     /translation="MSEDNALVLVAGYQDLDSARHDFQTLVDAAKDKSIPLQGAVLIG
                     KDAEGSPVLVDTGNRLGRRGAAWGAGVGLAIGLFSPALLASAALGAATGALAGTFAHH
                     RIKTGLADKIGQALAAGRAVVIAVTEAQGRLEAGQALASSPMKSVAELSRSTLRSLGA
                     ALREAMGKFNPDRTRLPLPQRRFGGVVGRTMAESVGDWSIVPSPFPPDDAPNVLIVLI
                     DDAGFGGPDTFGGAIRTPTLSRLAQNGLIYNRFHVTAVCSPTRAALLTGRNHHRVGFG
                     SVCEFPGPYPGYSAVRPRSCAALPRILRDNGYVTGAFGKWHLTPDNVQGAAGPFDNWP
                     LGWGFDHFWGFPSGAAGQYDPIISQDNSVIGIPEGSGEDGRPYYFPDDLTDKAIEWLH
                     TVRAQNATKPWMLYYATGATHAPHHVFKEWADKYRGEFDDGWDVYRQKTFERQKRLGI
                     IPPDAELTERPDLFPAWDSMSEAQKRLFARQMEVFAGFSENADWNVGRLLDAIEDLGE
                     SDNTLVFYIWGDNGASMEGTNTGSFNEMTFLNGLDLDAERQLELIEQYGGIAALGDEF
                     TAPHFASAWAHGGGTARLRGRGGVIMCSFRAFQEMFGSNVTIT"
     CDS             complement(3708317..3709234)
```

/codon_start=1
/note="gene_3433"
/transl_table=11
/translation="VALRPEDRLLSVHDVLGPVRVRLLGGSVLAELTARFGVAARAKV
LAGEVVDDDGAVVDSGTVLPPGSVVHLYRDLPDEVPVPFDVPVLHQDADIVVVDKPHF
LATMPRGRHVAQTALVRLRRELGLPELSPAHRLDRLTAGVLLFTTRREVRGSYQTMFA
RGLVRKTYLARAPVAPGLALPRLVRSRIVKRRGHLQAVCEPGVPNAETLVERIARDGL
YRLTPTTGRTHQLRVHMAALGIPIMGDPLYPNVISVAAHDFSTPLQLLAQRIEFDDPL
TGSHREFASTRTLTGATLPTWSAAADCRP"

CDS complement(3709246..3709911)
/codon_start=1
/gene="phoY1_3"
/note="gene_3434"
/transl_table=11
/translation="MRTVYHQRLTELAGRLGEMCSLAGIAMKRATQALLEADIGAAEQ
VIRDHERIVAMRAQVEKEAFALLALQHPVSGELREIFSAVQIIADTERMGALAVHIAK
ITRREYPNQVLPEEVRNCFADMAKVAIALGDSARQVLVNRDPQEAAQLHDRDDAMDDL
HRHLLSVLIDREWRHGVRVGVETALLGRFFERFADHAVEVGRRVIFMVTGVLPTEDEI
STY"

CDS complement(3710019..3711776)
/codon_start=1
/gene="glpD2_3"
/note="gene_3435"
/transl_table=11
/translation="VSNPIQAPDGGQGWPAAALGPAQRAVAWKRLGTEQFDVVVIGGG
VVGSGCALDAATRGLKVALVEARDLASGTSSRSSKMFHGGLRYLEQLEFGLVREALYE
RELSLTTLAPHLVKPLPFLFPLTKRWWERPYIAAGIFLYDRLGGAKSVPAQRHFTRAG
ALRLSPGLKRSSLIGGIRYYDTVVDDARHTMTVARTAAHYGAVVRCSTQVVALLREGD
RVIGVGVRDSENGAVAEVRGHVVVNATGVWTDEIQALSKQRGRFQVRASKGVHVVVPR
DRIVSDVAMILRTEKSVMFVIPWGSHWIIGTTDTDWNLDLAHPAATKADIDYILGTVN
AVLATPLTHADIDGVYAGLRPLLAGESDDTSKLSREHAVAVPAAGLVAIAGGKYTTYR
VMAADAIDAAVQFIPARVAPSITEKVSLLGADGYFALVNQAEHVGALQGLHPYRVRHL
LDRYGSLISDVLAMAASDPSLLSPITEAPGYLKVEAAYAAAAEGALHLEDILARRMRI
SIEYPHRGVDCAREVAEVVAPVLGWTAADIDREVANYMARVEAEVLSQAQPDDVSADM
LRASAPEARAEILEPVPLD"

CDS complement(3711857..3713272)
/codon_start=1
/gene="lpdA_3"
/note="gene_3436"
/transl_table=11
/translation="VVTRIVILGGGPAGYEAALVAATSHPETAQVTVIDCDGIGGAAV
LDDCVPSKTFIASTGLRTELRRAPHLGFHIDFDDAKISLPQIHARVKTLAAAQSADIT
AQLLSMGVQVIAGRGELIDSTPGLARHRIKATAADGSTSEHEADVVLVATGASPRILP
SAQPDGERILTWRQLYDLDALPDHLIVVGSGVTGAEFVDAYTELGVPVTVVASQDHVL
PYEDADAALVLEESFAERGVRLFKNARAASVTRTGAGVLVTMTDGRTVEGSHALMTIG
SVPNTSGLGLERVGIQLGRGNYLTVDRVSRTSATGIYAAGDCTGLLPLASVAAMQGRI
AMYHALGEGVSPIRLRTVAATVFTRPEIAAVGVPQSVIDAGSVAARTIMLPLRTNARA
KMSEMRHGFVKIFCRRSTGVVIGGVVVAPIASELILPIAVAVQNRITVNELAQTLAVY
PSLSGSITEAARRLMAHDDLD"

CDS 3713355..3713954
/codon_start=1

/note="gene_3437"
/transl_table=11
/translation="MHAAQRREEEPSSQAQLDDDPHRSAARWVPPEPPPSKLSPVPLY
AAYGSNMHPEQMLERAPHSPMAGTGWLPGWRLTFGGEDIGWEGALATVVEDPDSKVFV
VLYDMTPADEKNLDRWEGSEFGIHQKIRCRVERISSDTTTDPVLAWLYVLDAWEGGLP
SARYLGVMADAAEIAGAPSDYVHDLRTRPARNIGPGTIA"
CDS complement(3713973..3715142)
/codon_start=1
/gene="amiA1_3"
/note="gene_3438"
/transl_table=11
/translation="MSLADAAESWLAAHHDDLVGWRRHIHRYPELGRQEYATTQFVAE
RLADAGLNPKVLPGGTGLTCDFGPQHQPRIALRADMDALPMAERTGAPYASTMPNVAH
ACGHDAHTAILLGAALALASVPELPVGVRLIFQAAEELMPGGAIDAIAAGALAGVSRI
FALHCDPRLEVGKVAVRQGPITSAADSIEITLYSPGGHTSRPHLTADLVYGLGTLVTG
LPGVLSRRIDPRNSTVLVWGAVNAGMAANAIPQTGVLSGTVRTASRQTWVDLEELVRQ
AISALLLPLAIEHTLQYRRGVPPVVNEEISTRILAHAIEAIGPGVLADTRQSGGGEDF
SWYLEEVPGAMARLGVWSGDGLQLDLHQPTFDIDERALAIGLRVMVNIIEQAAAH"
CDS complement(3715139..3716323)
/codon_start=1
/gene="amiB1_3"
/note="gene_3439"
/transl_table=11
/translation="MPAASASDRVEELVRRRGGELVELSHAIHAEPELAFAEHRSCAK
AQALVAERGFEITTAAGGLDTAFRADYGSGPLVVGVCAEYDALPGIGHACGHNIIAAS
AVGTALALAEVADDLGLTVALLGTPAEESGGGKALMLQAGTFDDVAVAVMVHPGPTDI
AGARSLALSEVTVRYRGKESHAAVAPHLGVNAADAVTVAQVAIGVLRQQLAPGQMVHG
IVTDGGQAVNVIPGQARLQYAMRAVESDSLRELQTRMFACFAAGALAAGCEYEIDEAA
PAYAELKPDPWLADVCREEMQRLGREPLLPALEAELPLGSTDMGNVTQVLPGIHPVIG
LDAGAATVHQRAFTVASAGASADRAVVDGAIMLARTVVRLAQTPDERDRVLAAQQRRA
AR"
CDS 3716388..3717194
/codon_start=1
/gene="deoD_3"
/note="gene_3440"
/transl_table=11
/translation="VADPRPDPDELARRAAQVIADRTGIGEHDVAVVLGSGWLPAVAA
LGSPTTVLPQAELPGFVPPTAAGHAGELLSVPIGAHRVLVLAGRIHAYEGHDLRYVVH
PVRAARAAGAQIMVLTNAAGGLRADLQVGQPVLISDHLNLTARSPLVGGEFVDLTDAY
SPRLRELARQSDPQLAEGVYAGLPGPHYETPAEIRMLQTLGADLVGMSTVHETIAARA
AGAEVLGVSLVTNLAAGITGEPLSHAEVLAAGAASATRMGALLADVIARF"
CDS 3717198..3718802
/codon_start=1
/gene="pmmB_3"
/note="gene_3441"
/transl_table=11
/translation="VTPENWIAHDPDPQTAAELAACGPDELKARFSRPLAFGTAGLRG
HLRGGPDAMNLAVVLRATWAVARVLTDRGLAGSPVIVGRDARHGSPAFAAAAAEVLAA
AGFSVLLLPDPAPTPVVAFAVRHTGAAAGIQITASHNPATDNGYKVYVDGGLQLLAPT
DRQIEAAMATAPPADQIARKTVNPSENRASDLIDRYIQRAAGVRRCAGSVRVALTPLH
GVGGAMAVETLRRAGFTEVHTVATQFAPNPDFPTVTLPNPEEPGATDALLTLATDVDA

DVAIALDPDADRCAVGIPTVSGWRMLSGDETGWLLGDYILSQTDDRASPPETRVVAST
VVSSRMLAAIAAHHAAVHVETLTGFKWLARADANLPGTLVYAYEEAIGHCVDPTAVRD
KDGISAAVLVCDLVAALKGQGRSVTDALDELARCYGVHEVAALSRPVGGAVETTDLMR
RLREDPPRRLAGFPATVTDIGDTLILTGGDDNMLVRVAVRPSGTEPKLKCYLEIRCAV
TGDLPAARQLVRARIDELSASVRRWW"

CDS complement(3718804..3719427)
/codon_start=1
/gene="upp"
/note="gene_3442"
/transl_table=11
/translation="VQVHVVDHPLAAARLTTLRDERTDNAGFRAALRELTLLLIYEAT
RDAPCEPVPIRTPLAETVGSRLTKPPLLVPVLRAGLGMVDEAHAALPEAHVGFVGVAR
DEQTHQPVPYLDSLPDDLTDVPVMVLDPMVATGGSMTHTLGLLISRGAADITVLCVVA
APEGIAALQKAAPNVRLFTAAIDEGLNEVAYIVPGLGDAGDRQFGPR"

CDS 3719532..3720431
/codon_start=1
/gene="sapM_3"
/note="gene_3443"
/transl_table=11
/translation="MLRGIQALSRPLTRVYRALAVIGVLAASLLASWVGAVPQVGLAA
SALPTFAHVVIVVEENRSQAAIIGNKSAPFINSLAANGAMMAQAFAETHPSEPNYLAL
FAGNTFGLTKNTCPVNGGALPNLGSELLSAGYTFMGFAEDLPAVGSTVCSAGKYARKH
VPWVNFSNVPATLSVPFSAFPKPQNYPGLPTVSFVIPNADNDMHDGSIAQGDAWLNRH
LSAYANWAKTNNSLLVVTWDEDDGSSRNQIPTVFYGAHVRPGTYNETISHYNVLSTLE
QIYGLPKTGYATNAPPITDIWGD"

CDS 3720419..3721717
/codon_start=1
/note="gene_3444"
/transl_table=11
/translation="LGRLAAVAILCRMVADLVPIRLSLSAGDRYTLWAPRWRDAGDEW
EAFLGKDDDLYGFESVSDLVAFVRTDTENDLVDHPAWQDLTGAHAHNLNPAEDNQFDL
VVVEELLAEKPTAESVAALAASLAIVSAIGSVCELAAVSKFFNGNPILGTVSGGLEHF
TGKAGNKRWNSIAEVIGRSWDDVLAAIDEIISTPEVDAELSEKVAEELAEEPEGAEEV
AAEVEATQDTQEAAESDDEEADAPGDSVVLGGDRDFWLQVGIDPIQIMTGTATFYTLR
CYLDDRPIFLGRNGRISVFGSERALARYLADEHDHDLSDLSTYDDIRTAATDGSLAVA
VTDDNVYVLSGLVDDFADGPDAVDREQLDLAVELLRDIGDYSEDSAVDKALETTRPLG
QLVAYVLDPHSVGKPTAPYAAAVREWEKLERFVESRLRRE"

CDS complement(3721738..3722670)
/codon_start=1
/note="gene_3445"
/transl_table=11
/translation="VLLTGPPPSLPERIRTDEADVLMLPDGRALAYLEWGDSTGYPAF
YFHGTPSSRLEGAFADGAARRTGFRLIAIDRPGYGRSTFQAGRNFRDWPADVCALADA
FELEEFGVVGHSGAGPHLFACGAVIPRTRLAFVGALGPWGPLATPDIMRSLNAADRCY
ARLARSGPRLFGALFAPLGWCAKYTPGLFSTLLAAAVPAADKHLLSDERFGRHLRAIQ
LEAFRQGSRGAAYESFLQFRPWGFDLAEVAVPTHIWLGDRDSFVPRAMGEYLQRAIPH
VDLHWAHGKGHFNIEDWDAILAACALDIGKRRGG"

CDS complement(3722690..3722860)
/codon_start=1
/note="gene_3446"
/transl_table=11

/translation="MNGDSASTIDIDKAVTRTPVRRIVRSALDRLWLRQSQPRRYRFL
EDSCMARALNRL"
CDS complement(3723039..3723350)
/codon_start=1
/note="gene_3447"
/transl_table=11
/translation="MYRFACRTLMLAACILATGVAGLGVGAQSAAQTAPVPDYYWCPG
QPFDPAWGPNWDPYTCHDDFHRDSDGPDHSRDYPGPILEGPVLDDPGAAPPPPAAGGG
A"
CDS complement(3723421..3724518)
/codon_start=1
/gene="add_3"
/note="gene_3448"
/transl_table=11
/translation="MTAAPTLQTIRLAPKALLHDHLDGGLRPATVLDIAGQVGYDDLP
ATDVDALASWFRTQSHSGSLERYLEPFSHTVAVMQTPEALYRVAFECAQDLAADSVVY
AEVRFAPELHISCGLSFDDVVDTVLTGFAAGEKACAADGQPITVRCLVTAMRHAAMSR
EIAELAIRFRDKGVVGFDIAGAEAGHPPTRHLDAFEYMRDHNARFTIHAGEAFGLPSI
HEAIAFCGADRLGHGVRIVDDIDVDADGGFQLGRLAAILRDKRIPLELCPSSNVQTGA
VASIAEHPFDLLARARFRVTVNTDNRLMSDTSMSLEMHRLVEAFGYGWSDLARFTVNA
MKSAFIPFDQRLAIIDEVIKPRFAALMGHSE"
CDS complement(3724518..3725801)
/codon_start=1
/gene="deoA"
/note="gene_3449"
/transl_table=11
/translation="VTDFAFDAPTVIRTKRDGGRLSDAAIDWVVKAYTDGRVADEQMS
ALLMAIVWRGMDRGEIARWTAAMLASGARLDFTDLPLATVDKHSTGGVGDKITLPLVP
VVAACGGAVPQASGRGLGHTGGTLDKLESITGFTANLSNQRVREQLCDVGAAIFAAGQ
LAPADAKLYALRDITGTVESLPLIASSIMSKKLAEGAGALVLDVKVGSGAFMRSPVQA
RELAHTMVELGAAHGVPTRALLTEMNCPLGRTVGNALEVAEALEVLAGGGPPDVVELT
LRLAGEMLELAGIHGRDPAQTLRDGTAMDRFRWLVAAQGGDLSKPLPIGSHSETVTAG
ASGTMGDIDAMAVGLAAWRLGAGRSRPGARVQHGAGVRIHRRPGEPVVVGEPLFTLYT
NAPERFGAARAELAGGWSIRDSPPQVRPLIVDRIV"
CDS complement(3725798..3726199)
/codon_start=1
/gene="cdd_3"
/note="gene_3450"
/transl_table=11
/translation="MPDVDWNMLRGNATQAAAGAYVPYSRFAVGAAALVDDGRVVTGC
NVENVSYGLTLCAECAVVCALHSTGGGRLLALACVDGHGSVLMPCGRCRQVLLEHGGS
ELLIDHPVRPRRLGDLLPDAFGLDDLPRERR"
CDS 3726436..3726774
/codon_start=1
/gene="sdhC_3"
/note="gene_3451"
/transl_table=11
/translation="MWSWVCHRISGATIFFFLFVHVLDAAMLRVSPQTYNAVLATYKT
PIVGLMEYGLVAAVLFHALNGIRVILIDFWSEGPRYQRLMLWIIGSVFLLLMVPAGVV
VGIHMWEHFR"
CDS 3726852..3727205

```
                     /codon_start=1
                     /gene="sdhD_3"
                     /note="gene_3452"
                     /transl_table=11
                     /translation="MPNFEKFAWLFMRFSGVVLVFLAIGHLFIMLMWDNGVYRLDFNF
                     VAQRWASPFWQTWDLLLLWLAQLHGGNGLRTIIDDYSRKDTTRFWLNSLLVLSMLFTL
                     MLGTYVIVTFDPNIS"
     CDS             3727485..3729107
                     /codon_start=1
                     /gene="sdhA"
                     /note="gene_3453"
                     /transl_table=11
                     /translation="MCAALANVEDDNWEWHTFDTVKGGDYLADQDAVEIMCKEAIDAV
                     LDLEKMGMPFNRTPEGRIDQRRFGGHTRDHGKAPVRRACYAADRTGHMILQTLYQNCV
                     KHDVEFFNEFYALDLALTQTPSGPVATGVIAYELATGDIHVFHAKAVVIATGGSGRMY
                     KTTSNAHTLTGDGIGIVFRKGLPLEDMEFHQFHPTGLAGLGILISEAVRGEGGRLLNG
                     EGERFMERYAPTIVDLAPRDIVARSMVLEVLEGRGAGPLKDYVYIDVRHLGEEVLEAK
                     LPDITEFARTYLGVDPVTELVPVYPTCHYLMGGIPTTVTGQVLRDNTSVVPGLYAAGE
                     CACVSVHGANRLGTNSLLDINVFGRRAGIAAASYAQGHDFVDMPPNPEAMVVGWVSDI
                     LSEHGNERVADIRGALQQSMDNNAAVFRTEETLKQALTDIHALKERYSRITVHDKGKR
                     FNTDLLEAIELGFLLELAEVTVVGALNRKESRGGHAREDYPNRDDVNYMRHTMAYKEI
                     GADKEGPELRSDVRLDFKPVVQTRYEPKERKY"
     CDS             3729107..3729898
                     /codon_start=1
                     /gene="sdhB"
                     /note="gene_3454"
                     /transl_table=11
                     /translation="MSVEPDVETLDPPLPPVPDGAVMVTVKIARFNPDEPDAFAATGG
                     WQSFRVPCLPSDRLLNLLIYIKGYLDGTLTFRRSCAHGVCGSDTMRINGVNRLACKVL
                     MRDLLPKKKGKSLTVTVEPIRGLPVEKDLVVDMEPFFDAYRAIKPYLITSGNPPTRER
                     IQSPTDRARYDDTTKCILCACCTTSCPVFWHEGSYFGPAAIVNAHRFIFDSRDEAAAE
                     RLDILNEVDGVWRCRTTFNCTESCPRGIEVTKAIQEVKRALMFTR"
     CDS             complement(3729977..3730405)
                     /codon_start=1
                     /note="gene_3455"
                     /transl_table=11
                     /translation="MRALLDVNVLLALLDRDHVDHERARAWITGQIERGWASCAITQN
                     GFVRVISQPRYPSPISVAHAIDLLARATHTRYHEFWSCTVSILDSKVIDRSRLHSPKQ
                     VTDAYLLALAVAHDGRFVTFDQSIALTAVPGATKQHLATL"
     CDS             complement(3730409..3730651)
                     /codon_start=1
                     /note="gene_3456"
                     /transl_table=11
                     /translation="MRTTLSIDDDVLLAVKERARREKRTAGEILSDLARQALTNQNPQ
                     PAASQEDAFHGFEPLPHRGGAVSNALIDRLRDEEAV"
     CDS             complement(3730773..3731387)
                     /codon_start=1
                     /note="gene_3457"
                     /transl_table=11
                     /translation="MSVQTDPALREHPNRVDWNARYERAGSAHAPFAPVPWLADVLRA
                     GVPDGPVLELASGRSGTALALAAHGRQVTAIDVSDVALLQLDSEAVRRGVADRLNLVQ
```

ADLGCWEPGETRFALVLSRLFWDAAIFHRACEAVMPGGVLAWESLALSGAEAGTASAK
RRVKPGEPACLLPADFTVVHEGQGNCDSAPSRIMIARRSPLPGA"

CDS complement(3731384..3732049)
/codon_start=1
/note="gene_3458"
/transl_table=11
/translation="MITVNVLYFGAVREACKVAHEKISLESGTTVDGLVDQLQIDYPP
LADFRKRVRMAVNESIAPASTILDDGDTVAFIPQVAGGSDVYCRLTDEPLSVDEVLNA
ISGPSQGGAVIFVGTVRNNNNGHEVTKLYYEAYPAMVHRTLMDIIEECERQADGVRVA
VAHRTGELRIGDAAVVIGASAPHRAAAFDAARMCIERLKQDVPIWKKEFALDGVEWVA
NRP"

CDS complement(3732050..3732583)
/codon_start=1
/gene="moaC"
/note="gene_3459"
/transl_table=11
/translation="VNDHDGVLTHLDEQGAARMVDVSAKAVTLRRARASGAVLMKPST
LDMICHGTAAKGDVIATARIAGIMAAKRTGELIPLCHPLGIEAVTVTLEPQGADRLSI
AATVTTVARTGVEMEALTAVTVTALTVYDMCKAVDRAMTITDIRLDEKSGGRSGHYRR
HDADVKPSDGGSTEDGC"

CDS complement(3732580..3732954)
/codon_start=1
/note="gene_3460"
/transl_table=11
/translation="MAHGNVSRCEESSLHDVCCGRLSALTDRELSERLTALPGWELVD
GKLRHTFGFGSFDQSMKFVAKIAAIADKFNHHPDICVHNKRSVRLTCWTRQMHCLTRV
DFDLAEAFSAVHDEQCSQQVAR"

CDS complement(3733051..3734055)
/codon_start=1
/gene="moaA3"
/note="gene_3461"
/transl_table=11
/translation="MGDLRLSVIDQCNLRCRYCMPEAEYAWLPRADLLSVDEISLIVD
AFIAVGVDKIRLTGGEPLIRSDLAAIIEVISAKVGDGSGLQDLAITTNGVLLADQARK
LKSAGMRRITISLDTLRPDRFKAISQRGTHYKVIEGIEAVAAAGFTDTKLDSVVIRGF
NDDELSDLIEFARNVNAEVRFIEYMDVGGATQWSMDKVFTKAQMLSTLGKKYGPIAAL
PKYDSAPANRYRLPDGTTFGIIASTTEPFCATCDRSRLTADGIWLHCLYALSGINLRE
SVRAGASANDVVQILQRGWRDRANRGAEQRLAQRTRQVFLPVSRLKRDPHLEMHTRGG
"

CDS 3734760..3735905
/codon_start=1
/note="gene_3462"
/transl_table=11
/translation="MARNELRFGILGPLEISAGFRSLPLGTPKQRAVLATLIIHRNRP
VGIDSLIDAAWEQDRPEGSRATVYTYVSNLRRLVSTTGADSHSILASAPPGYRLAVAD
NQYDVARFISQRSAGLRAAAAGSFEQASDHLSAALAEWRGPVLDDLREFSFVTRLANS
LVEDKIIAHTALAEAEIACGRADSVISELEELILEHPYHEALWRQLIAAYYVSERQSD
ALDAYRRLKTSLAEDLGVDPGPKVRTLYEQVLRQQALDTRVVVQAAAGDIIRALEHSP
GMTDRSPRAAIRDAAGHRSPLGRLPLRIGRSKSNDMVLPDGKVSPYHAVIVNTGESFM
ITDLRSVNGVYVRGRRIATTATLNDGDHIRIGDHELTFEVIPHESGR"

CDS complement(3736000..3736221)

/codon_start=1
/note="gene_3463"
/transl_table=11
/translation="VEPPGAAVAEVIARHSASFDMYTGRLFAVSLLPGSPDRLVLTAS
RLCVDDASWQTVVEDLVRQYDESVLVPAR"
CDS 3736812..3738524
/codon_start=1
/note="gene_3464"
/transl_table=11
/translation="MVVVGTDAHKYSHTFVATDEVGRQLGEKTVKATTAGHATAIMWA
REQFGLELIWGIEDCRNMSARLERDLLAAGQQVVRVPTKLMAQTRKSARSRGKSDPID
ALAVARAVLRETDLPLATHDETSRELKLLTDRRDVLVAQRTSAINRLRWLVHELDPER
APAARSLDAAKHQQALRTWLDTQPGLVAELARAELTDIIRLTGEINTLAQRISARVHQ
VAPALLEIPGCAELTAAKIVGEAAGVTRFKSEAAFACHAAVAPIPVWSGNTAGQMRLS
RSGNRQLNAALHRIALTQIRMTDSRGQAYYQRLQDAGKTKRAALRCLKRRLARTVFQA
LRTVHQPSSEHTQPAAACHRSYCSSHLGEPPRLTDMTQKTRIQPLPPKRAGLLIRALY
RIAKRRFGEVPEPFTVTAHHRRLLIANVVHEALLQRASRKLPPSVRELAVFWTARSIG
CSWCVDFGAMLQRLDGLDVDRLTDIDNYATSSKFSDDERAAIAYAEAMTADPHSVTDE
QVADLRARFGEAGVIELTYQIGVENMRARMNSALGITEQGFNSGDACRVPWAAPDVPS
AESR"
CDS complement(3738457..3739404)
/codon_start=1
/note="gene_3465"
/transl_table=11
/translation="MTSMEVSEFEALRQHLMSVAYRLTGTVADAEDIVQEAWLRWDSQ
DTVIADPRAWLTTVVSRLGLDKLRSAAHRRETYTGTWLPEPVVTGLDATDPLAAVVAA
EDARFAAMVVLERLRPDQRVAFVLHDGFAVPFAEVAEVLGTSEAAARQLASRARKAVT
AQPALISGDPDPAHNEVVGRLMAAMAAGDLDTVVSLLHPDVTFTGDSNGKAPTAVRAV
RGSDKVVRFILGLVQRYGPGLFGANQLALVNGELGAYTAGLPGVDGYRAMAPRITAIT
VRDGKVCALWDIANPDKFTGSPLKERRAQPTGRGRHHRN"
CDS 3739455..3740771
/codon_start=1
/note="gene_3466"
/transl_table=11
/translation="LHFARHGAGIQHPVIVRGDGVTIFDDRGKSYLDALSGLFVVQVG
YGRAELAEAAARQAGTLGYFPLWGYATPPAIELAERLARYAPGDLNRVFFTSGGTEAV
ETAWKVAKQYFKLTGKPGKHKVISRSIAYHGTTQGALAITGLPLFKAPFEPLTPGGFR
VPNTNFYRAPLHTDLKEFGRWAADRIAEAIEFEGPDTVAAVFLEPVQNAGGCIPAPPG
YFERVREICDRYDVLLVSDEVICAFGRIGSMFACEDLGYVPDMITCAKGLTSGYSPLG
AMIASDRLFEPFNDGETMFAHGYTFGGHPVSAAVGLANLDIFEREGLSDHVKRNSPAL
RATLEKLYDLPIVGDIRGEGYFFGIELVKDQATKQTFTDDERARLLGQVSAALFEAGL
YCRTDDRGDPVVQVAPPLISGQPEFDTIETILRSVLTDTGRKYLHL"
CDS 3740879..3742057
/codon_start=1
/note="gene_3467"
/transl_table=11
/translation="VFAVGTGIGLPTAAGEPNAAPAACPYKVSTPPAVDSSEVPAAGE
PPLPLVVPPTPVGGNALGGCGIITAPGSAPAPGDVSAEAWLVADLDSGAVIAARDPHG
RHRPASVIKVLVAMASINTLTLNKSVAGTADDAAVEGTKVGVNTGGTYTVNQLLHGLL
MHSGNDAAYALARQLGGMPAALEKINLLAAKLGGRDTRVATPSGLDGPGMSTSAYDIG
LFYRYAWQNPVFADIVATRTFDFPGHGDHPGYELENDNQLLYNYPGALGGKTGYTDDA

GQTFVGAANRDGRRLMTVLLHGTRQPIPPWEQAAHLLDYGFNTPAGTQIGTLIEPDPS
LMSTDRNPADRQRVDPQAAARISAADALPVRVGVAVIGALIVFGLIMVARAMNRRPQH
"
CDS 3742426..3743661
/codon_start=1
/note="gene_3468"
/transl_table=11
/translation="LLTTTAVLGQIAGALGGGILANAIGRKKSVVLIVAGYAVFALLG
ATSVSVPMLVVARLLLGVTIGLSVVVVPVYVAESAPAAVRGSLVTAYQLATLSGIVVG
YLVGYLLAGSHGWRAMFGLAAAPATLLLPLLWRMPDTARWYLLKGRIADARSALRRIQ
PEADIDAELADMAAAVDERGGGIGEMVRRPYLRATLFVIALGFLVQITGINAIIYYSP
RLFAAMGFAGYFAMLALPAMVQVAGLAAVCASLFLVDRLGRRPILLSGIATMITADAV
LITVFANDSDGGTGLVLGFAGVLLFIIGFNFGFGSLVWVYAAESFPSRLRSMGSSLML
TSTLTANAIVAAFSLTMLRVLGGAGVFAVFGTFAVVAFVVVYRFAPETKGRKLEEIRH
FWENGGRWPAERSPAADEP"
CDS 3743658..3744809
/codon_start=1
/note="gene_3469"
/transl_table=11
/translation="VTVLGADAVVIDGRICRPGWVHTADGRILSGGAGAPPMPADAEF
PDAIVVPGFVDMHVHGGGGASFADGNAADIARAAEFHLRHGTTTTLASLVTAGPAELL
SAVGALAEATRDGVVAGIHLEGPWLSPARCGAHDHTRMRAPDPAEIESVLAAADGAVR
MVTLAPELPGSDAAIRRFRDAEVVVAVGHTDATYTQTRHAIDLGATVGTHLFNAMPPL
DHRAPGPVLALLCDPRVTVEIIADGVHVHPAVVHAVIEAVGPDRVAVVTDAIAAAGCG
DGAFRLGTMPIEVESSVARVAGASTLAGSTTTMDQLFRTVAGLGSKSDSAGDVALAAA
VQVTSATPARALGLTGVGRLAAGYAANLVVLDRDLRVTAVMVNDDWRVG"
CDS complement(3745000..3745845)
/codon_start=1
/note="gene_3470"
/transl_table=11
/translation="MFTGIASHAGALGAALVVLIGAAILHDGPAAADPNQDDRFLALL
EKKEIPAVANVPRVIDAAHKVCRKLDGGMPVNDIVDGLRNDAYNIDPVMRLYPVRLTT
TMTRFISAAVEIYCPNHHSKMAFAMANFEPGSNEPTHRVAASTRSAVNSGSDLRASVS
DMTIMSPGWREPTGAMLASVLGAVRAGDPLIPNPPPIPVPPPAAQTLIPPPPIVAPPP
PRPAPPQQPPPPPPEVEPPAGVPQSGGAAGSGGAGSGGGGGGDGPVEPSPARPMPPGF
IRLAP"
CDS 3745990..3746301
/codon_start=1
/gene="TBLG_04069"
/note="gene_3471"
/transl_table=11
/translation="MGALRSQIQRTTPEPLAEAHPQAFDPAPVVGMGACRRNQRMVIV
ASGARPARAHAVRCRWLFSSGNSFPQAAIQRYGLTEIRLVTIARTAGGSHVPSVNLLV
H"
CDS 3746320..3746760
/codon_start=1
/note="gene_3472"
/transl_table=11
/translation="MKISEVAALTNTSTKTLRFYENSGLLPPPARTASGYRNYGPEIV
DRLRFIHRGQAAGLALQEVRQILAIHDRGEAPCAHVRQLLSTRIDEVRAQIAELIALE
GHLQTLLDHASYGPPTEHDHSTVCWILESDLDEPTAIEVSDIHA"

CDS complement(3746794..3747663)
/codon_start=1
/note="gene_3473"
/transl_table=11
/translation="MGELAEPGVLDRLRARFGWLDHVVRAFTRFNDRNGSLFAAGLTY
YTIFAIFPLLMVGFGVGGFALSRRPELLTTLEERIRTSVSGVVGQQLVDLMNSAIDAR
ASVGVIGLATAAWVGLGWMWHLREALSQMWAHPVAPAGYLRTKLSDLAAMVGTFVVIV
ATIALTVLGHARPMAAVLRWLEIPQFSVFDEIFRGISVLVSVLVSWVLFTWMIGRLPR
EPVGLVTAARAGLMAAVGFELFKQVGAIYLQIVLRSPAGAVFGPVLGLMVFAFVTAWL
ILFATAWAATASA"
CDS complement(3747684..3748694)
/codon_start=1
/note="gene_3474"
/transl_table=11
/translation="MSTPTGSRRIFSGVQPTSDSLHLGNALGAVAQWVGLQDDHDAFF
CVVDLHAITIPQDPEALRRRTLITAAQYLALGIDPGRATIFVQSQVPAHTQLAWVLGC
FTGFGQASRMTQFKDKSARQGSEATTVGLFTYPVLQAADVLAYDTELVPVGEDQRQHL
ELARDVAQRFNSRFPGTLVVPDVLIPKMTAKIYDLQDPTSKMSKSAGTDAGLINLLDD
PALSAKKIRSAVTDSERDIRYDPDVKPGVSNLLNIQSAVTGTDIDVLVDGYAGHGYGD
LKKDTAEAVVEFVNPIQARVDELTADPAELEAVLAAGAQRAHDVASKTVQRVYDRLGF
LL"
CDS 3748863..3749612
/codon_start=1
/note="gene_3475"
/transl_table=11
/translation="VWEKVAQQLADTGRFEVFAPTMAGHNGGPASGTRFLSSAVLADH
VERQLDELGWETSHIVGNSLGGWVAFELERRGRARSVTGIAPAGGWTRWSPVKFEVIA
KFIAGAPILAVAHILGQRALRLPFSRLLATLPISATPDGVSERELSGIIDDAAHCPAY
FQLLVKALVLPGLQELEHTAVPSHVVLCEQDRVVPPSRFSRHFTDSLPAGHRLTVLDG
VGHVPMFEAPGRITELITSFIEECCPHVRAS"
CDS complement(3749679..3750908)
/codon_start=1
/note="gene_3476"
/transl_table=11
/translation="MSNAPKIKVSGPVVELDGDEMTRVIWKLIKDMLILPYLDIRLDY
YDLGIEHRDATDDQVTIDAAYAIKKHGVGVKCATITPDEARVEEFNLKKMWLSPNGTI
RNILGGTIFREPIVISNVPRLVPGWTKPIVIGRHAFGDQYRATNFKVDQPGTVTLTFT
PADGSAPIVHEMVSIPEDGGVVLGMYNFKESIRDFARASFSYGLNAKWPVYLSTKNTI
LKAYDGMFKDEFERVYEEEFKAQFEAAGLTYEHRLIDDMVAACLKWEGGYVWACKNYD
GDVQSDTVAQGYGSLGLMTSVLMTADGKTVEAEAAHGTVTRHYRQYQAGKPTSTNPIA
SIFAWTRGLQHRGKLDGTPEVIDFAHKLESVVIATVESGKMTKDLAILIGPEQDWLNS
EEFLDAIADNLEKELAN"
CDS complement(3750948..3751274)
/codon_start=1
/note="gene_3477"
/transl_table=11
/translation="VLTSVYLFGFERPMRGRIGVGAIAVGAHELSPSGQGPVMADPRL
PRSRRWLLNLVITRGTPPRLEGCRPASRGLTLALMTDSKPSAWQLLSANSKHNSGGLN
WPFTGR"
CDS 3751191..3752540
/codon_start=1

```
                     /note="gene_3478"
                     /transl_table=11
                     /translation="MSADSNSTDADPTAHWSFETKQIHAGQHPDPTTNARALPIYATT
                     SYTFDDTAHAAALFGLEIPGNIYTRIGNPTTDVVEQRIGALEGGVAALFLSSGQAAET
                     FAILNLAGAGDHIVSSPRLYGGTYNLFHYSLAKLGIEVSFVDDPDDLDTWQAAVRPNT
                     KAFFAETISNPQIDLLDTPAVSEVAHRNGVPLIVDNTIATPYLIQPLAQGADIVVHSA
                     TKYLGGHGAAIAGVIVDGGNFDWTQGRFPGFTTPDPSYHGVVFAELGPPAFALKARVQ
                     LLRDYGSAASPFNAFLVAQGLETLSLRIERHVANAQRVAEFLAARDDVLSVNYAGLPS
                     SPWHERAKRLAPKGTGAVLSFELAGGIEAGKAFVNALKLHSHVANIGDVRSLVIHPAS
                     TTHAQLSPAEQLATGVSPGLVRLAVGIEGIDDILADLELGFAAARRFSADPQSVAAF"
     CDS             3752552..3753691
                     /codon_start=1
                     /gene="metX"
                     /note="gene_3479"
                     /transl_table=11
                     /translation="MTISDVPTQTLPAEGEIGLIDVGSLQLESGAVIDDVCIAVQRWG
                     KLSPARDNVVVVLHALTGDSHITGPAGPGHPTPGWWDGVAGPGAPIDTTRWCAVATNV
                     LGGCRGSTGPSSLARDGKPWGSRFPLISIRDQVQADVAALAALGITEVAAVVGGSMGG
                     ARALEWVVGYPDRVRAGLLLAVGARATADQIGTQTTQIAAIKADPDWQSGDYHETGRA
                     PDAGLRLARRFAHLTYRGEIELDTRFANHNQGNEDPTAGGRYAVQSYLEHQGDKLLSR
                     FDAGSYVILTEALNSHDVGRGRGGVSAALRACPVPVVVGGITSDRLYPLRLQQELADL
                     LPGCAGLRVVESVYGHDGFLVETEAVGELIRQTLGLADREGACRR"
     CDS             3753709..3754419
                     /codon_start=1
                     /note="gene_3480"
                     /transl_table=11
                     /translation="MSLSFGSAVGAYERGRPSYPPEAIDWLLPAAARRVLDLGAGTGK
                     LTTRLVERGLDVVAVDPIPEMLDVLRAALPQTVALLGTAEEIPLDDNSVDAVLVAQAW
                     HWVDPARAIPEVARVLRPGGRLGLVWNTRDERLGWVRELGEIIGRDGDPVRDRVTLPE
                     PFTTVQRHQVEWTNYLTPQALIDLVASRSYCITSPAQVRTKTLDRVRQLLATHPALAN
                     SNGLALPYVTVCVRATLA"
     CDS             complement(3754428..3758444)
                     /codon_start=1
                     /note="gene_3481"
                     /transl_table=11
                     /translation="MSFVVMPPEINSLLIYTGAGPGPLLAAAAAWDELAAELGSAAAA
                     FGSVTSGLVGGIWQGPSSVAMAAAAAPYAGWLSAAAASAESAAGQARAVVGVFEAALA
                     ATVDPFVIAANRSRLVSLALSNLFGQNTPAIAAAEFDYELMWAQDVAAMLGYHTGASA
                     AAEALAPFGSPLASLAAAAEPAKSLAVNLGLANVGLFNAGSGNVGSYNVGAGNVGSYN
                     VGGGNIGGNNVGLGNVGFGNVGLANSGLTPGLMGLGNIGFGNAGSYNFGLANMGVGNI
                     GFANTGSGNFGIGLTGDNLTGFGGFNTGSGNVGLFNSGTGNVGFFNSGTGNWGVFNSG
                     SYNTGIGNSGIASTGLFNAGGFNTGVVNAGSYNTGSFNAGQANTGGFNPGSVNTGWLN
                     TGDTNTGVANSGDVNTGAFISGNYSNGVLWRGDYQGLLGFSYRPAVLPQTPFLDLTLT
                     GGLGSVVIPAIDIPAIRPEFSANVAIDSFTVPSIPIPQIDLAATTVSVGLGPITVPHL
                     DIPRAPVTLNYLFGSQPGGPLKIGPITGLFNTPIGLTPLALSQIVIGASSSQGTITAF
                     LANLPFSTPVVTIDEIPLLASITGHSEPVDIFPGGLTIPAMNPLSINLSGGTGAVTIP
                     AITIGEIPFDLVAHSTLGPVHILIDLPAVPGFGNTTGAPSSGFFNSGAGGVSGFGNVG
                     AMVSGGWNQAPSALLGGGSGVFNAGTLHSGVLNFGSGMSGLFNTSVLGLGAPALVSGL
                     GSVGQQLSGLLASGTALHQGLVLNFGLADVGLGNVGLGNVGDFNLGAGNVGGFNVGGG
                     NIGGNNVGLGNVGWGNFGLGNSGLTPGLMGLGNIGFGNAGSYNFGLANMGVGNIGFAN
                     TGSGNFGIGLTGDNLTGFGGFNTGSGNVGLFNSGTGNVGFFNSGTGNWGVFNSGSYNT
```

GIGNSGIASTGLFNAGGFNTGVVNAGSYNTGSFNAGQANTGGFNPGSVNTGWLNTGDT
NTGVANSGDVNTGAFISGNYSNGAFWRGDYQGLLGFSYTSTIIPEFTVANIHASGGAG
PIIVPSIQFPAIPLDLSATGHIGGFTIPPVSISPITVRIDPVFDLGPITVQDITIPAL
GLDPATGVTVGPIFSSGSIIDPFSLTLLGFINVNVPAIQTAPSEILPFTVLLSSLGVT
HLTPEITIPGFHIPVDPIHVELPLSVTIGPFVSPEITIPQLPLGLALSGATPAFAFPL
EITIDRIPVVLDVNALLGPINAGLVIPPVPGFGNTTAVPSSGFFNIGGGGGLSGFHNL
GAGMSGVLNAISDPLLGSASGFANFGTQLSGILNRGADISGVYNTGALGLITSALVSG
FGNVGQQLAGLIYTGTGP"

CDS complement(3758493..3764117)
/codon_start=1
/note="gene_3482"
/transl_table=11
/translation="MSLMVAPELVAAAAADLTGIGQAISAANAAAAGPTTQVLAAAGD
EVSAAIAALFGTHAQEYQALSARVATFHEQFVRSLTAAGSAYATAEAANASPLQALEQ
QVLGAINAPTQLWLGRPLIGDGVHGAPGTGQPGGAGGLLWGNGGNGGSGAAGQVGGPG
GAAGLFGNGGSGGSGGAGAAGGVGGSGGWLNGNGGAGGAGGTGANGGAGGNAWLFGAG
GSGGAGTNGGVGGSGGFVYGNGGAGGIGGIGGIGGNGGDAGLFGNGGAGGAGAAGLPG
AAGLNGGDGSDGGNGGTGGNGGRGGLLVGNGGAGGAGGVGGDGGKGGAGDPSFAVNNG
AGGNGGHGGNPGVGGAGGAGGLLAGAHGAAGATPTSGGNGGDGGIGATANSPLQAGGA
GGNGGHGGLVGNGGTGGAGGAGHAGSTGATGTALQPTGGNGTNGGAGGHGGNGGNGGA
QHGDGGVGGKGGAGGSGGAGGNGFDAATLGSPGADGGMGGNGGKGGDGGGGGAGGTAV
AGTAGKAGDGGAGGDGGKAGDGGAGAAGDVTLAVNQGAGGDGGNGGEVGVGGKGGAGG
VSANPALNGSAGANGTAPTSGGNGGNGGAGATPTVAGENGGAGGNGGHGGSVGNGGAG
GAGGNGVAGTGLALNGGNGGIGGNGGSAAGTGGDGGKGGNGGAGANGQDFSASANGAN
GGQGGNGGNGGIGGKGGDAFATFAKAGNGGAGGNGGAAGNGGGGAAGDVTLAINQGAG
GAGGNGGNVGVAGQGGAGGKGAIPAMKGATGADGTAPTSGGDGGNGGNGASPTVAGGN
GGDGGKGGSGGNVGNGGNGGAGGNGAAGQAGTPGPTSGDSGTSGTDGGAGGNGGAGGA
GGTLAGHGGNGGKGVNGGQGGIGGAGERGADGAGPNANGANGANGENGGSGGNGGDGGAGG
NGGAGGKAQAAGYTDGATGTGGDGGNGGDGGKAGDGGAGANGLNSGAMLPGGGTVGNP
GTGGNGGNGGNAGVGGTGGKAGTGSLTGLDGTDGITPNGGNGGNGGNGGKGGTAGNGS
GAAGGNGGNGGSGLNGGDAGNGGNGGGALNQAGFFGTGGKGGNGGNGGAGMINGGLGG
FGGAGGGGAVDVAATTGGAGGNGGAGGFASTGLGGPGGAGGPGGAGDFASGVGGVGGA
GGDGGAGGVGGFGGQGGIGGEGRTGGNGGSGGDGGGGISLGGQGGNGGFGGAGGNGGI
GTDAGGAGGAGGAGGNGGSSKSTTTGNAGSGGAGGNGGTGLNGAGGAGGAGGNAGVAG
VSFGNAVGGDGGNGGNGGHGGDGTTGGAGGKGGNGSSGAASGSGVVNVTAGHGGNGGN
GGNSGNSTGVAGLAGGAAGAGGNGGGTSSAAGHGGSGGNGGSGGSGGSGTTGGAGAAG
GNGGAGAGGGSLSTGQSGGHGGSGGAGGNGGAGSAGNGGAGGAGGNGGAGGNGGGGDA
GNAGSGGNGGKGGDGVGPGSTGGAGGKGGAGANGGSSNGNARGGNAGNGGHGGAGGSG
DTGGAGGAGGQGGFGGTGGSGSGIGGGAGGNGGNGGAGGTGVVLGGKGGDGGNGDHGG
PATNPGSGSRGGAGGSGGNGGAGGNATGSGGKGGAGGNGGDGSFGATSGPASIGVTGA
PGGNGGKGGAGGSNPNGSGGDGGKGGNGGAGGNGGSIGANSGIVGGSGGAGGAGGAGG
NGSLSSGEGGKGGDGGHGGDGVGGNSSVTQGGSGGGGGAGGAGGSGFFGGKGGFGGDG
GQGGPNGGGTVGTVAGGGGNGGVGGRGGDGVFAGAGGQGGLGGQGGNGGGSTGGNGGL
GGAGGGGGNAPDGGFGGNGGKGGQGGIGGGTQSATGLGGDGGDGGDGGNGGNSGAKAG
GAGGKGQAGQPNSGTEPGFGGDGGLGGAGATP"

CDS complement(3764547..3764804)
/codon_start=1
/note="gene_3483"
/transl_table=11
/translation="MTVRAVLRRTVGAQWPILAGVNFWRRGALLIGIGVGVAAVLRLV
LSEERAGLLVVRSKGIDFVTTVTVAAAMVYIASTIDPLGTG"

CDS complement(3765060..3768335)
/codon_start=1
/note="gene_3484"
/transl_table=11
/translation="MGVGNIGFANTGTNNLGIGLAGDNQTGIGGLNSGAGNIGLFNSG
TGNIGFFNSGTGNWGLFNSGSFNTGIGNSGTGSTGLFNAGGFTTGLANAGSYNTGSFN
VGDTNTGGFNPGSINTGWFNTGNANTGIANSGNVDTGALMSGNFSNGILWRGNYEGLF
SYSYSLDVPRITILDAHFTGAFGPVVVPPIPVPAINAHLTGNAAMGAFTIPQIDIPAL
NPNVTGSVGFGPIAVPSVTIPALTAARAVLDMAASVGATSEIEPFIVWTSSGAIGPTW
YSVGRIYNAGDLFVGGNIISGIPTLSTTGPVHAVFNAASQAFNTPALNIHQIPLGFQV
PGSIDAITLFPGGLTFPANSLLNLDVFVGTPGATIPAITFPEIPANADGELYVIAGNI
PLINIPPTPGIGNTTTVPSSGFFNTGAGGGSGFGNFGANMSGWWNQAHTALAGAGSGI
ANVGTLHSGVLNLGSGLSGIYNTSTLPLGTPALVSGLGNVGDHLSGLLASNVGQNPIT
IVNIGLANVGNGNVGLGNIGNLNLGAANIGDVNLGFGNIGDVNLGFGNIGGGNVGFGN
IGDANFGFGNSGLAAGLAGMGNIGLGNAGSGNVGWANMGLGNIGFGNTGTNNLGIGLT
GDNQSGIGGLNSGTGNIGLFNSGTGNIGFFNSGTANFGLFNSGSYNTGIGNSGVASTG
LVNAGGFNTGVANAGSYNTGSFNAGDTNTGGFNPGSTNTGWFNTGNANTGVANAGNVN
TGALITGNFSNGILWRGNYEGLAGFSFGYPIPLFPAVGADVTGDIGPATIIPPIHIPS
IPLGFAAIGHIGPISIPNIAIPSIHLGIDPTFDVGPITVDPITLTIPGLSLDAAVSEI
RMTSGSSSGFKVRPSFSFFAVGPDGMPGGEVSILQPFTVAPINLNPTTLHFPGFTIPT
GPIHIGLPLSLTIPGFTIPGGTLIPQLPLGLGLSGGTPPFDLPTVVIDRIPVELHAST
TIGPVSLPIFGFGGAPGFGNDTTAPSSGFFNTGGGGGSGFSNSGSGMSGVLNAISDPL
LGSASGFANFGTQLSGILNRGAGISGVYNTGTLGLVTSAFVSGFMNVGQQLSGLLFAG
TGP"
CDS complement(3768242..3774532)
/codon_start=1
/note="gene_3485"
/transl_table=11
/translation="LNFPVLPPEINSVLMYSGAGSSPLLAAAAAWDGLAEELGSAAVS
FGQVTSGLTAGVWQGAAAAAMAAAAAPYAGWLGSVAAQAVAVAGQARAAVAAFEAALA
ATVDPAAVAVNRMAMRALAMSNLLGQNAAAIAAVEAEYELMWAADVAAMAGYHSGASA
AAAALPAFSPPAQALGGGVGAFLNAIFAGPAKMLRLNAGLGNVGNYNVGLGNVGIFNL
GAANVGAQNLGAANAGSGNFGFGNIGNANFGFGNSGLGLPPGMGNIGLGNAGSSNYGL
ANLGVGNIGFANTGSNNIGIGLTGDNLTGIGGLNSGTGNLGLFNSGTGNIGFFNSGTG
NFGVFNSGSYNTGVGNAGTASTGLFNVGGFNTGVANVGSYNTGSFNAGNTNTGGFNPG
NVNTGWLNTGNTNTGIANSGNVNTGAFISGNFSNGVLWRGDYEGLWGLSGGSTIPAIP
IGLELNGGVGPITVLPIQILPTIPLNIHQTFSLGPLVVPDIVIPAFGGGTAIPISVGP
ITISPITLFPAQNFNTTFPVGPFFGLGVVNISGIEIKDLAGNVTLQLGNLNIDTRINQ
SFPVTVNWSTPAVTIFPNGISIPNNPLALLASASIGTLGFTIPGFTIPAAPLPLTIDI
DGQIDGFSTPPITIDRIPLNLGASVTVGPILINGVNIPATPGFGNTTTAPSSGFFNSG
DGGVSGFGNFGAGSSGWWNQAQTEVAGAGSGFANFGSLGSGVLNFGSGVSGLYNTGGL
PPGTPAVVSGIGNVGEQLSGLSSAGTALNQSLIINLGLADVGSVNVGFGNVGDFNLGA
ANIGDLNVGLGNVGGGNVGFGNIGDANFGLGNAGLAAGLAGVGNIGLGNAGSGNVGFG
NMGVGNIGFGNTGTNNLGIGLTGDNQTGIGGLNSGAGNIGLFNSGTGNVGLFNSGTGN
FGLFNSGGYNTGIGNGGTGSTGLFNAGNFNTGVANPGSYNTGSFNVGDTNTGGFNPGS
INTGWFNTGNANTGVANSGTVNTGAFITGNDSNGILWRGNFEGLFGLNVGITIPEFPI
HWTSTGGIGPIIIPDTTILPPIHLGLTGQANYGFAVPDIPIPAIHIDFDGAADAGFTA
PATTLLSALGITGQFRFGPITVSNVQLNPFNVNLKLQFLHDAFPNEFPDPTISVQIQV
AIPLTSATLGGLALPLQQTIDAIELPAISFSQSIPIDIPPIDIPASTINGISMSEVVP
IDVSVDIPAVTITGTRIDPIPLNFDVLSSAGPINISIIDIPALPGFGNSTELPSSGFF
NTGGGGGSGIANFGAGVSGLLNQASSPMVGTLSGLGNAGSLASGVLNSGVDISGMFNV

STLGSAPAVISGFGNLGNHVSGVSIDGLLAMLTSGGSGGSGQPSIIDAAIAELRHLNP
LNIVNLGNVGSYNLGFANVGDVNLGAGNLGNLNLGGGNLGGQNLGLGNLGDGNVGFGN
LGHGNVGFGNSGLGALPGIGNIGLGNAGSNNVGFGNMGLGNIGFGNTGTNNLGIGLTG
DNQTGFGGLNSGAGNLGLFNSGTGNIGFFNTGTGNWGLFNSGSYNTGIGNSGTGSTGL
FNAGSFNTGLANAGSYNTGSLNAGNTNTGGFNPGNVNTGWFNAGHTNTGGFNTGNVNT
AAFNSGSFNNGALWTGDHHGLVGFSYSIEITGSTLVDINETLNLGPVHIDQIDIPGMS
LFDIHELVNIGPFRIEPIDVPAVVLDIHETMVIPPIVFLPSMTIGGQTYTIPLDTPPA
PAPPPFRLPLLFVNALGDNWIVGASNSTGMSGGFVTAPTQGILIHTGPSSATTGSLAL
TLPTVTIPTITTSPIPLKIDVSGGLPAFTLFPGGLNIPQNAIPLTIDASGVLDPITIF
PGGFTIDPLPLSLALNISVPDSSVPIIIVPPTPGFGNATATPSSGFFNSGAGGVSGFG
NFGAGSSGWWNQAHAALAGAGSGVLNVGTLNSGVLNVGSGISGLYNTAIVGLGTPALV
SGAGNVGQQLSGVLAAGTALTQSPIINLGLADVGNYNLGLGNVGDFNLGAANLGDLNL
GLGNIGNANVGFGNIGHGNVGFGNSGLGRRSASAISGWAMRAAPTLAWPTWVWATSGS
PTPAPTTSGLGWPATTRPASAA"

CDS 3775112..3775603
/codon_start=1
/note="gene_3486"
/transl_table=11
/translation="VTAENPGRSRRTLVGIDAAITACHHIAIRDDVGARSIRFSVEPT
LAGLRTLTDKLSGYDDIDATVEPTSMTWLPLTIAVENAGDTMHMAGARHCARLRGAIV
GKSKSDVIDAEVLTRASEVFDLTPLTLPTPAQLALRRSVIRRAGAVIDANRSWRRLMS
LAR"

CDS complement(3775640..3776380)
/codon_start=1
/note="gene_3487"
/transl_table=11
/translation="VAIDPAAAYASAIRTPGLLPNAKLVVDHFHVTTLANDALTAVRR
RVTWAFHDRRGRKIDPQWANRRRLLTARERLSDKSFAKMRNRINAVDPRAQILSAWIA
KEELRTLLSTVRTGGDPHLARHHLHRFLPGASTRRSPNCSPWPPPLTSHPRSTPSWSP
ASPTRASVVGEVAEMLGDIDGQCVQVEVPVPERGPAGCGGLDGLGRAGVSATPRVCAA
MTAVNVAGRCAGQQADVGPTPQHRCRGR"

CDS complement(3776670..3776906)
/codon_start=1
/note="gene_3488"
/transl_table=11
/translation="VKEYAVTSPKDLPYGEDRIMVRWNKIRWRCREDYCKLGPFIEAI
TQVPARVRSTLRLRRQMAKAIGDAARSVGRGRPG"

CDS complement(3777294..3780953)
/codon_start=1
/note="gene_3489"
/transl_table=11
/translation="VLNVGTLGSGVLNVGSGVSGIYNTSVLPLGTPAVLSGLGNVGHQ
LSGVSAAGTALNQIPILNIGLADVGNFNVGFGNVGDVNLGAANLGAQNLGLGNVGTGN
LGFANVGHGNIGFGNSGLTAGAAGLGNTGFGNAGSANYGFANQGVRNIGLANTGTGNI
GIGLVGDNLTGIGGLNSGAGNIGLFNSGTGNIGFFNSGTGNFGIGNSGSFNTGIGNSG
TGSTGLFNAGSFNTGVANAGSYNTGSFNAGDTNTGGFNPGTINTGWFNTGHTNTGIAN
SGNVGTGAFMSGNFSNGLLWRGDHEGLFSLFYSLDVPRITIVDAHLDGGFGPVVLPPI
PVPAVNAHLTGNVAMGAFTIPQIDIPALTPNITGSAAFRIVVGSVRIPPVSVIVEQII
NASVGAEMRIDPFEMWTQGTNGLGITFYSFGSADGSPYATGPLVFGAGTSDGSHLTIS
ASSGAFTTPQLETGPITLGFQVPGSVNAITLFPGGLTFPATSLLNLDVTAGAGGVDIP
AITWPEIAASADGSVYVLASSIPLINIPPTPGIGNSTITPSSGFFNAGAGGGSGFGNF

GAGTSGWWNQAHTALAGAGSGFANVGTLHSGVLNLGSGVSGIYNTSTLGVGTPALVSG
LGNVGHQLSGLLSGGSAVNPVTVLNIGLANVGSHNAGFGNVGEVNLGAANLGAHNLGF
GNIGAGNLGFGNIGHGNVGVGNSGLTAGVPGLGNVGLGNAGGNNWGLANVGVGNIGLA
NTGTGNIGIGLTGDYQTGIGGLNSGAGNLGLFNSGAGNVGFFNTGTGNFGLFNSGSFN
TGVGNSGTGSTGLFNAGSFNTGVANAGSYNTGSFNVGDTNTGGFNPGSINTGWLNAGN
ANTGVANAGNVNTGAFVTGNFSNGILWRGDYQGLAGFAVGYTLPLFPAVGADVSGGIG
PITVLPPIHIPPIPVGFAAVGGIGPIAIPDISVPSIHLGLDPAVHVGSITVNPITVRT
PPVLVSYSQGAVTSTSGPTSEIWVKPSFFPGIRIAPSSGGGATSTQGAYFVGPISIPS
GTVTFPGFTIPLDPIDIGLPVSLTIPGFTIPGGTLIPTLPLGLALSNGIPPVDIPAIV
LDRILLDLHADTTIGPINVPIAGFGGAPGFGNSTTLPSSGFFNTGAGGGSGFSNTGAG
MSGLLNAMSDPLLGSASGFANFGTQLSGILNRGAGISGVYNTGALGVVTAAVVSGFGN
VGQQLSGLLFTGVGP"

CDS complement(3780947..3787081)
/codon_start=1
/note="gene_3490"
/transl_table=11
/translation="VLNGDIGPITIQPIPILPTIPLSIHQTVNLGPLVVPDIVIPAFG
GGIGIPINIGPLTITPITLFAQQTFVNQLPFPTFSLGKITIPQIQTFDSNGQLVSFIG
PIVIDTTIPGPTNPQIDLTIRWDTPPITLFPNGISAPDNPLGLLVSVSISNPGFTIPG
FSVPAQPLPLSIDIEGQIDGFSTPPITIDRIPLTVGGGVTIGPITIQGLHIPAAPGVG
NTTTAPSSGFFNSGAGGVSGFGNVGAGSSGWWNQAPSALLGAGSGVGNVGTLGSGVLN
LGSGISGFYNTSVLPFGTPAAVSGIGNLGQQLSGVSAAGTTLRSMLAGNLGLANVGNF
NTGFGNVGDVNLGAANIGGHNLGLGNVGDGNLGLGNIGHGNLGFANLGLTAGAAGVGN
VGFGNAGINNYGLANMGVGNIGFANTGTGNIGIGLVGDHRTGIGGLNSGIGNIGLFNS
GTGDVGFFNSGTGNFGIGNSGRFNTGIGNSGTASTGLFNAGSFSTGIANTGDYNTGSF
NAGDTNTGGFNPGGINTGWFNTGHANTGLANAGTFGTGAFMTGDYSNGLLWRGGYEGL
VGVRVGPTISQFPVTVHAIGGVGPLHVAPVPVPAVHVEITDATVGLGPFTVPPISIPS
LPIASITGSVDLAANTISPIRALDPLAGSIGLFLEPFRLSDPFITIDAFQVVAGVLFL
ENIIVPGLTVSGQILVTPTPIPLTLNLDTTPWTLFPNGFTIPAQTPVTVGMEVANDGF
TFFPGGLTFPRASAGVTGLSVGLDAFTLLPDGFTLDTVPATFDGTILIGDIPIPIIDV
PAVPGFGNTTTAPSSGFFNTGGGGGSGFANVGAGTSGWWNQGHDVLAGAGSGVANAGT
LSSGVLNVGSGISGWYNTSTLGAGTPAVVSGIGNLGQQLSGFLANGTVLNRSPIVNIG
WADVGAFNTGLGNVGDLNWGAANIGAQNLGLGNLGSGNVGFGNIGAGNVGFANSGPAV
GLAGLGNVGLSNAGSNNWGLANLGVGNIGLANTGTGNIGIGLVGDYQTGIGGLNSGSG
NIGLFNSGTGNVGFFNTGTGNFGLFNSGSFNTGIGNSGTGSTGLFNAGNFNTGIANPG
SYNTGSFNVGDTNTGGFNPGDINTGWFNTGIMNTGTRNTGALMSGTDSNGMLWRGDHE
GLFGLSYGITIPQFPIRITTTGGIGPIVIPDTTILPPLHLQITGDADYSFTVPDIPIP
AIHIGINGVVTVGFTAPEATLLSALKNNGSFISFGPITLSNIDIPPMDFTLGLPVLGP
ITGQLGPIHLEPIVVAGIGVPLEIEPIPLDAISLSESIPIRIPVDIPASVIDGISMSE
VVPIDASVDIPAVTITGTTISAIPLGFDIRTSAGPLNIPIIDIPAAPGFGNSTQMPSS
GFFNTGAGGGSGIGNLGAGVSGLLNQAGAGSLVGTLSGLGNAGTLASGVLNSGTAISG
LFNVSTLDATTPAVISGFSNLGDHMSGVSIDGLIAILTFPPAESVFDQIIDAAIAELQ
HLDIGNALALGNVGGVNLGLANVGEFNLGAGNVGNINVGAGNLGGSNLGLGNVGTGNL
GFGNIGAGNFGFGNAGLTAGAGGLGNVGLGNAGSGSWGLANVGVGNIGLANTGTGNIG
IGLTGDYRTGIGGLNSGTGNLGLFNSGTGNIGFFNTGTGNFGLFNSGSYSTGVGNAGT
ASTGLFNAGNFNTGLANAGSYNTGSLNVGSFNTGGVNPGTVNTGWFNTGHTNTGLFNT
GNVNTGAFNSGSFNNGALWTGDYHGLVGFSFSIDIAGSTLLDLNETLNLGPIHIEQID
IPGMSLFDVHEIVEIGPFTIPQVDVPAIPLEIHESIHMDPIVLVPATTIPAQTRTIPL
DIPASPGSTMTLPLISMRFEGEDWILGSTAAIPNFGDPFPAPTQGITIHTGPGPGTTG
ELKISIPGFEIPQIATTRFLLDVNISGGLPAFTLFAGGLTIPTNAIPLTIDASGALDP
ITIFPGGYTIDPLPLHLALNLTVPDSSIPIIDVPPTPGFGNTTATPSSGFFNSGAVGC

RGSETSGRTCRAGGTRRRARWRGRDRGC"
CDS complement(3787139..3788464)
/codon_start=1
/note="gene_3491"
/transl_table=11
/translation="VSGWEGEWVMEFPVLPPEINSVLMYSGAGSSPLLAAAAAWDGLA
EELGSAAVSFGQVTSGLTAGVWQGAAAAAMAAAAAPYAGWLGSVAAAAEAVAGQARVV
VGVFEAALAATVDPALVAANRARLVALAVSNLLGQNTPAIAAAEAEYELMWAADVAAM
AGYHSGASAAAAALPAFSPPAQALGGGVGAFLTALFASPAKALSLNAGLGNVGNYNVG
LGNVGVFNLGAGNVGGQNLGFGNAGGTNVGFGNLGNGNVGFGNSGLGAGLAGLGNIGL
GNAGSSNYGFANLGVGNIGFGNTNNVGVGLTGNHLTGIGGLNSGTGNIGLFNSGTGNV
GFFNSGTGNFGVFNSGNYNTGVGNAGTASTGLFNAGNFNTGVVNVGSYNTGSFNAGDT
NTGGFNPGGVNTGWLNTGNTNTGIANSGNVNTGAFISGNFNNGVLWVGD"
CDS complement(3788681..3789475)
/codon_start=1
/note="gene_3492"
/transl_table=11
/translation="VLASCPARSGAAVADAIKSAVGVQPSGVEHKTLRRMDLVRYLAG
GHTTYPPEGFVAGSDVIGTTNPAAAQAIVAAIGTWPPAAGRASALIDSLGGAVGDMDP
EGSAFPWCRQSAVVQWYVNTPSDGQVATANKWLSDAHHAVQHFSVGGYVNYLEANAAA
SQYFGANLSRLTTVRRKYDPDRIMYSGLDFSTRQVAERLLPALGFRVRFGVLVIRCAL
CTDTVKRLGTLPNLTWSRLKVNVAVTQEQAGVMDLPALPVRRTPRR"
CDS complement(3789557..3789928)
/codon_start=1
/note="gene_3493"
/transl_table=11
/translation="VSAATDLYAVHQALAGESRAIPTGSCPTVGVAGLTLGGGLGADS
RHAGLTCDALKSATVVLPGGDAVSASADDHAELFWALRGGGGGNFGVTTSMTFARFPT
ADCDVVRVDFAPSAAAQVLVG"
CDS complement(3790071..3790331)
/codon_start=1
/note="gene_3494"
/transl_table=11
/translation="MSRQTFLRGAVGAPATSAVFPTILARATPGDGWASLASSIGGQV
LLPANGRAFTSGKQIFNSNYSGLNPAAVVTVASQADVRKAVS"
CDS 3790446..3790835
/codon_start=1
/note="gene_3495"
/transl_table=11
/translation="MNLRRHQTLTLRLLAASAGILSAAAFAAPAQANPVDDAFIAALN
NAGVNYGDPVDAKALGQSVCPILAEPGGSFNTAVASVVARAQGMSQDMAQTFTSIAIS
MYCPSVMADAASGNLPALPDMPGLPGS"
CDS complement(3790849..3791142)
/codon_start=1
/note="gene_3496"
/transl_table=11
/translation="MTVRAVFRRTVGAQWPILLVGSIFAVGFVLAGANFWRRGALLIG
IGVGVAAVLRLVLSEERAGLLVVRSKGIDFVTTVTVAAAMVYIASTIDPLGTG"
CDS complement(3791139..3792056)
/codon_start=1
/note="gene_3497"

/transl_table=11
/translation="LSVRWARERVVTIGTADTHARLAGVGAIMLDGKATRDEIFGDLK
PRVAALDAAGRTPGLGTILVGDDPGSQAYVRGKHADCAKVGITSIRRDLPADISTATL
NETIDELNANPDCTGYIVQLPLPKHLDENAALERVDPAKDADGLHPTNLGRLVLGTPA
PLPCTPRGIVHLLRRYDISIAGAHVVVIGRGVTVGRPLGLLLTRRSENATVTLCHTGT
RDLPALTRQADIVVAAVGVAHLLTADMVRPGAAVIDVGVSRTDDGLVGDVHPDVWELA
GHVSPNPGGVGPLTRAFLLTNVVELAERR"
CDS 3792108..3792383
/codon_start=1
/note="gene_3498"
/transl_table=11
/translation="MSISASEARQRLFPLIEQVNTDHQPVRITSRAGDAVLMSADDYD
AWQETVYLLRSPENARRLMEAVARDKAGHSAFTKSVDELREMAGGEE"
CDS 3792380..3792637
/codon_start=1
/note="gene_3499"
/transl_table=11
/translation="VRSVNFDPDAWEDFLFWLAADRKTARRITRLIGEIQRDPFSGIG
KPEPLQGELSGYWSRRIDDEHRLVYRAGDDEVTMLKARYHY"
CDS 3792679..3793869
/codon_start=1
/note="gene_3500"
/transl_table=11
/translation="VAPGSCEAPDVFNPAKLGPLTLRNRVIKAATFEARTPDALVTDD
LIEYHRLPAAGGVAMTTVAYCAVSPGGRTGGNQIWMRPHAVPGLRRLTEAIHAEGAAI
SAQIGHAGPVADARSNQATALAPVRFFNPIAMRFAQKATREDIDDVLAAHAHAARLAV
DAGFDAVEIHLGHNYLASAFLSPLLNRRDDEFGGSLQNRAKVARGLVMAVRRAVRQQV
AVTAKLNMTDGIRGGITVDEALTTARWLQDDGGLDAIELTAGSSLVNPMYLFRGDAPV
KEFAAAFKPPLRWGIRMTGHRFFREYPYRDAYLLREARLFRAELTIPLILLGGITNRT
TMDLAMAEGFEFVAMARALLAEPDLVNRIAAEGSQVRSACTHCNQCMATIYRRTHCVV
TGAP"
CDS 3794007..3794354
/codon_start=1
/note="gene_3501"
/transl_table=11
/translation="VLTVRSDRSQQCFAAGRDVVVGSDLRADMRVAHPLIARAHLLLR
FDRGNWIAIDNDSQSGMFVDGQRVSEVDIYDGLTINIGKPTGPWITFEVGHHQGIIGR
LSRTPSSRPGSPI"
CDS complement(3794351..3794902)
/codon_start=1
/note="gene_3502"
/transl_table=11
/translation="LQQWVDCEFTGRDFRDEDLSRLHTERAMFSECDFSGVNLAESQH
RGSAFRNCTFERTTLWHSTFAQCSMLGSVFVACRLRPLTLDDVDFTLAVLGGNDLRGL
NLTGCRLRETSLVDTDLRKCVLRGADLSGARTTGARLDDADLRGATVDPVLWRTASLV
GARVDVDQAVAFAAAHGLCLAGG"
CDS complement(3794909..3795490)
/codon_start=1
/gene="cvnD6"
/note="gene_3503"
/transl_table=11

/translation="VALKHSEASGTASTKIVIAGGFGSGKTTFVGAVSEIMPLRTEAM
VTDASAGVDMLEATPDKRSTTVAMDFGRITLGEDLVLYLFGTPGQRRFWFMWDDLVRG
AIGAIVLVDCRRLQDSFAAVDFFEHRNLPFLIAINEFDSAPRYPVSAVRDALTLPAHI
PVINVDARNRRSATDALIAVSEYALATLSPAGG"
CDS complement(3795471..3795839)
/codon_start=1
/note="gene_3504"
/transl_table=11
/translation="LFNPAGDRPKAGLVRPYTLTAGRTGTDVDLPLQAPVQTLPAGPA
GRWPAYDMRRRILQLCIGSPSVAEISARLDLPVGVARVLVGDLVTSGYLRVHATLTDR
STRDERHELIGRTLRGLKAL"
CDS complement(3795817..3796209)
/codon_start=1
/note="gene_3505"
/transl_table=11
/translation="MKARLPDSPLDWLVSKFAREVLGVAHALLVSVDGLPVAASEHLP
RERADQLAAVTSGLASLAGGAAQLFDGGQVLQSVVEMQNGYLLLMQVGDGSALAALAA
TGCDIGQIGYEMAILVERVGGVVQSCRR"
CDS complement(3796206..3798698)
/codon_start=1
/note="gene_3506"
/transl_table=11
/translation="VFTIALLPLVVAMVLAGLRVEAAMASTSGLRLVAARAEMIPAIT
KYMSALDVAVLASSTGHDVEGAQKNFTARKYELQTRLADTDVIADVRSGVNTLLNGGQ
ALLDKVLADSIGLRDRVTAYAPLLLTAQNVIDASVRVDSEQIRTQVQGLSRAVGARGQ
MTMQEILVTRGADLAEPQLRSAMVTLAGTEPSTLFGMSAALGAGSPDTKNLQQQMVTR
MAIMSDPAVALVNNPELLHSIQITRDIAEQVITDTTEAVTKSVQSQATDRRDAAIRDA
VLVLAAIATAIVVVLVVARTLVGPMRVLRDGALKVAHTDLDGEIAAVRAGDEPIPEPL
AVYTTEEIGQVAHAVDELHTRALLLAGEETRLRLLVNEMFETMSRRSRSLVDQQLSVI
DQLERNEEDPARLDSLFRLDHLAARLRRNSANLLVLAGAQITRDHREPVPLSTVISAA
VSEVEDYRRVDIARVPDCAVVGAAAGGVIHLLAELIDNALRYSSPTTPVRVAAAIGSE
GSVLLRISDSGLGMTDADRRMANMRLRAGGEVTPDSARHMGLFVVGRLAGRHGIRVGL
RGPVTGEQGTGTTAEVYLPLAVLEGTAPAQPPKPRVFAIKPPCPEPAAADPTDVPAAI
GPLPPVTLLPRRTPGSSGIADVPAQPMQQRRRELKTPWWEDRFQQEPKQPPAPEPRPA
PPPAKPAPPAGPVDDDVIYRRMLSEMVGDPHELAHSPDLDWKSVWDHGWSAAAEAADK
PVQSRTDYGLPVREPGARLVPGAAVPEGPDREHPGAALASNGGLHPGRAPRHAAAVRD
PDAVRASISSHFGGVRTGRSHARESSQGPNQQ"
CDS 3799072..3799536
/codon_start=1
/note="gene_3507"
/transl_table=11
/translation="MFRLLFVSPRIAPNTGNAIRTCAATGCELHLVEPLGFDLSEPKL
RRAGLDYHDLASVTVHASLAHAWEALSPARVFAFTAQATTLFTNVGYRAGDVLMFGPE
PTGLDEATLADTHITGQVRIPMLAGRRSLNLSNAAAVAVYEAWRQHGFAGAV"
CDS 3799876..3801783
/codon_start=1
/note="gene_3508"
/transl_table=11
/translation="VLAAWGWVVMSFVVAVPEALAAAASDVANIGSALSAANAAAAAG
TTGLLAAGADEVSAALASLFSGHAVSYQQVAAQATALHDQFVQALTGAGGSYALTEAA
NVQQNLLNAINAPTQALLGRPLIGDGAVGTASSPDGQDGGLLFGNGGAGYNSAATPGM

```
                     AGGNGGNAGLIGNGGTGGSGGAGAAGGAGGSGGWLYGNGGNGGIGGNAIVAGGAGGNG
                     GAGGAAGLWGSGGSGGQGGNGLTGNDGVNPAPVTNPALNGAAGDSNIEPQTSVLIGTQ
                     GGDGTPGGAGVNGGNGGAGGDANGNPANTSIANAGAGGNGAAGGDGGANGGAGGAGGQ
                     AASAGSSVGGDGGNGGAGGTGTNGHAGGAGGAGGQAASAGSSVGGDGGNGGAGGTGTN
                     GHAGGAGGAGGAGGRGGWLVGSGGNGGNGGNGAAGGNGAIGGTGGAGGVPANQGGNSA
                     LGTQPVSGDGGDGGNGGTGGTGGRGGDGGSGGAGGASGWLMGNGGNGGNGGTGGSGGV
                     GGNGGIGGDGAGGGNATSTSSIPFDAHGGNGGAGGDAGHGGTGGDGGDGGHAGTGGRG
                     GLLAGQHANSGNGGGGGTGGAGGTHGTPGSGNAGGTGTGNADSTNGGPGSDGLGGDAF
                     NGSRGTDGNPG"
variation            3800876..3800980
                     /note="supplemented from AL2122/97"
CDS                  complement(3801784..3802428)
                     /codon_start=1
                     /note="gene_3509"
                     /transl_table=11
                     /translation="MTLNLSVDEVLTTTRSVRKRLDFDKPVPRDVLMECLELALQAPT
                     GSNSQGWQWVFVEDAAKKKAIADVYLANARGYLSGPAPEYPDGDTRGERMGRVRDSAT
                     YLAEHMHRAPVLLIPCLKGREDESAVGGVSFWASLFPAVWSFCLALRSRGLGSCWTTL
                     HLLDNGEHKVADVLGIPYDEYSQGGLLPIAYTQGIDFRPAKRLPAESVTHWNGW"
CDS                  3802427..3802861
                     /codon_start=1
                     /note="gene_3510"
                     /transl_table=11
                     /translation="MWAGYRWAMSVELTQEVSARLTSDLYGWLTTVARSGQPVPRLVW
                     FYFDGTDLTVYSMPQAAKVAHITAHPQVSLNLDSDGNGAGIIVVGGTAAVVATDVDCR
                     DDAPYWAKYREDAAKFGLTEAIAAYSTRLKITPTRVWTTPTG"
CDS                  complement(3802950..3806189)
                     /codon_start=1
                     /gene="dnaE"
                     /note="gene_3511"
                     /transl_table=11
                     /translation="MERVLNGKPRHAGVPAFDADGDVPRSRKRGAYQPPGRERVGSSV
                     AYAELHAHSAYSFLDGASTPEELVEEAARLGLCALALTDHDGLYGAVRFAEAAAELDV
                     RTVFGAELSLGATARTERPDPPGPHLLVLARGPEGYRRLSRQLAAAHLAGGEKGKPRY
                     DFDALTEAAGGHWHILTGCRKGHVRQALSQGGPAAAQRALADLVDRFTPSRVSIELTH
                     HGHPLDDERNAALAGLAPRFGVGIVATTGAHFADPSRGRLAMAMAAIRARRSLDSAAG
                     WLAPLGGAHLRSGEEMARLFAWCPEAVTAAAELGERCAFGLQLIAPRLPPFDVPDGHT
                     EDSWLRSLVMAGARERYGPPKSAPRAYSQIEHELKVIAQLRFPGYFLVVHDITRFCRD
                     NDILCQGRGSAANSAVCYALGVTAVDPVANELLFERFLSPARDGPPDIDIDIESDQRE
                     KVIQYVYHKYGRDYAAQVANVITYRGRSAVRDMARALGFSPGQQDAWSKQVSHWTGQA
                     DDVDGIPEQVIDLATQIRSLPRHLGIHSGGMVICDRPIADVCPVEWARMANRSVLQWD
                     KDDCAAIGLVKFDLLGLGMLSALHYAKDLVAEHKGIEVDLARLDLSEPAVYEMLARAD
                     SVGVFQVESRAQMATLPRLKPRVFYDLVVEVALIRPGPIQGGSVHPYIRRRNGVDPVI
                     YEHPSMAPALRKTLGVPLFQEQLMQLAVDCAGFSAAEADQLRRAMGSKRSTERMRRLR
                     GRFYDGMRALHGAPDEVIDRIYEKLEAFANFGFPESHALSFASLVFYSAWFKLHHPAA
                     FCAALLRAQPMGFYSPQSLVADARRHGVAVHGPCVNASLAHATCENAGTEVRLGLGAV
                     RYLGAELAEKLVAERTANGPFTSLPDLTSRVQLSVPQVEALATAGALGCFGMSRREAL
                     WAAGAAATGRPDRLPGVGSSSHIPALPGMSELELAAADVWATGVSPDSYPTQFLRADL
                     DAMGVLPAERLGSVSDGDRVLIAGAVTHRQRPATAQGVTFINLEDETGMVNVLCTPGV
                     WARHRKLAHTAPALLIRGQVQNASGAITVVAERMGRLTLAVGARSRDFR"
CDS                  3806381..3807721
```

/codon_start=1
/note="gene_3512"
/transl_table=11
/translation="MAQLTALDAGFLKSRDPERHPGLAIGAVAVVNGAAPSYDQLKTV
LTERIKSIPRCTQVLATEWIDYPGFDLTQHVRRVALPRPGDEAELFRAIALALERPLD
PDRPLWECWIIEGLNGNRWAILIKIHHCMAGAMSAAHLLARLCDDADGSAFANNVDIK
QIPPYGDARSWAETLWRMSVSIAGAVCTAAARAVSWPAVTSPAGPVTTRRRYQAVRVP
RDAVDAVCHKFGVTANDVALAAITEGFRTVLLHRGQQPRADSLRTLEKTDGSSAMLPY
LPVEYDDPVRRLRTVHNRSQQSGRRQPDSLSDYTPLMLCAKMIHALARLPQQGIVTLA
TSAPGPRHQLRLMGQKMDQVLPIPPTALQLSTGVAVLSYGDELVFGITADYDAASEMQ
QLVNGIELGVARLVALSDDSVLLFTKDRRKRSSRALPSAARRGRPSVPTARARH"
CDS 3807763..3808938
/codon_start=1
/note="gene_3513"
/transl_table=11
/translation="VRKLGPVTIDPRRHDAVLFDTTLDATQELVRQLQEVGVGTGVFG
SGLDVPIVAAGRLAVRPGRCVVVSAHSAGVTAARESGFALIIGVDRTGCRDALRRDGA
DTVVTDLSEVSVRTGDRRMSQLPDALQALGLADGLVARQPAVFFDFDGTLSDIVEDPD
AAWLAPGALEALQKLAARCPIAVLSGRDLADVTQRVGLPGIWYAGSHGFELTAPDGTH
HQNDAAAAAIPVLKQAAAELRQQLGPFPGVVVEHKRFGVAVHYRNAARDRVGEVAAAV
RTAEQRHALRVTTGREVIELRPDVDWDKGKTLLWVLDHLPHSGSAPLVPIYLGDDITD
EDAFDVVGPHGVPIVVRHTDDGDRATAALFALDSPARVAEFTDRLARQLREAPLRAT"
CDS 3809190..3810065
/codon_start=1
/note="gene_3514"
/transl_table=11
/translation="MTKMDEASNPCGGDIEAEMCQLMREQPPAEGVVDRVALQRHRNV
ALITLSHPQAQNALNLASWRRLKRLLDDLAGESGLRAVVLRGAGDKAFAAGADIKEFP
NTRMSAADAAEYNESLAVCLRALTTMPIPVIAAVRGLAVGGGCELATACDVCIATDDA
RFGIPLGKLGVTTGFTEADTVARLIGPAALKYLLFSGELIGIEEAARWGLVQKVVAPQ
DLAAATAKLVGQVCRQSAVTMRAAKVVANMHGRALTGADTDALIRFGVEAYEGADLRE
GVAAFSQGRPPKFDD"
CDS 3810070..3811497
/codon_start=1
/note="gene_3515"
/transl_table=11
/translation="MTDADSAVPPRLDEDAISKLELTEVADLIRTRQLTSAEVTESTL
RRIERLDPQLKSYAFVMPETALAAARAADADIARGHYEGVLHGVPIGVKDLCYTVDAP
TAAGTTIFRDFRPAYDATVVARLRAAGAVIIGKLAMTEGAYLGYHPSLPTPVNPWDPT
AWAGVSSSGCGVATAAGLCFGSIGSDTGGSIRFPTSMCGVTGIKPTWGRVSRHGVVEL
AASYDHVGPITRSAHDAAVLLSVIAGSDIHDPSCSAEPVPDYAADLALTRIPRVGVDW
SQTTSFDEDTTAMLADVVKTLDDIGWPVIDVKLPALAPMVAAFGKMRAVETAIAHADT
YPARADEYGPIMRAMIDAGHRLAAVEYQTLTERRLEFTRSLRRVFHDVDILLMPSAGI
ASPTLETMRGLGQDPELTARLAMPTAPFNVSGNPAICLPAGTTARGTPLGVQFIGREF
DEHLLVRAGHAFQQVTGYHRRRPPV"
CDS 3811605..3812258
/codon_start=1
/note="gene_3516"
/transl_table=11
/translation="LSISAVVFDRDGVLTSFDWTRAEEDVRRITGLPLEEIERRWGGW
LNGLTIDDAFVETQPISEFLSSLARELELGSKARDELVRLDYMAFAQGYPDARPALEE

ARRRGLKVGVLTNNSLLVSARSLLQCAALHDLVDVVLSSQMIGAAKPDPRAYQAIAEA
LGVSTTSCLFFDDIADWVEGARCAGMRAYLVDRSGQTRDGVVRDLSSLGAILDGAGP"
CDS complement(3812350..3813801)
/codon_start=1
/note="gene_3517"
/transl_table=11
/translation="METFRTLLAKAALGNGISSTAYDTAWVAKLGQLDDELSDLALNW
LCERQLPDGSWGAEFPFCYEDRLLSTLAAMISLTSNKHRRRRAAQVEKGLLALKNLTS
GAFEGPQLDIKDATVGFELIAPTLMAEAARLGLAICHEESILGELVGVREQKLRKLGG
SKINKHITAAFSVELAGQDGVGMLDVDNLQETNGSVKYSPSASAYFALHVKPGDKRAL
AYISSIIQAGDGGAPAFYQAEIFEIVWSLWNLSRTDIDLSDPEIVRTYLPYLDHVEQH
WVRGRGVGWTGNSTLEDCDTTSVAYDVLSKFGRSPDIGAVLQFEDADWFRTYFHEVGP
SISTNVHVLGALKQAGYDKCHPRVRKVLEFIRSSKEPGRFCWRDKWHRSAYYTTAHLI
CAASNYDDALCSDAVGGFLIRRGPMARGDFSTAKRLRKRQHIAFKLWRIGRGTAAHPC
RRRSVARVGGYRNTANRHTRRCGLPRHFTARRR"
CDS complement(3813806..3814696)
/codon_start=1
/note="gene_3518"
/transl_table=11
/translation="MNLVSEKEFLDLPLVSVAEIVRCRGPKVSVFPFDGTRRWFHLEC
NPQYDDYQQAALRQSIRILKMLFEHGIETVISPIFSDDLLDRGDRYIVQALEGMALLA
NDEEILSFYKEHEVHVLFYGDYKKRLPSTAQGAAAVKSFDDLTISTSSNTEHRLCFGV
FGNDAAESVAQFSISWNETHGKPPTRREIIEGYYGEYVDKADMFIGFGRFSTFDFPLL
SSGKTSLYFTVAPSYYMTETTLRRILYDHIYLRHFRPKPDYSAMSADQLNVLRNRYRA
QPDRVFGVGCVHDGIWFAEG"
CDS complement(3814705..3816543)
/codon_start=1
/note="gene_3519"
/transl_table=11
/translation="MTTTIRNGRGDLITAIGGPCDVQALPESQLPELAVQMRRRLIET
VTATGGHLGAGLGMVELTIALHRVFTSPHDIVVFDTGHQTYPHKLLTGRGKDFATLRQ
ADGLSGYPNRHESPHDWVENSHASVSLAWVDGIAKALALQGQCDRRVIAVIGDGALTG
GVAWEGLNNLGAATRPVIVVLNDNGRSYDPTAGALAAHLEELRVGTPRGPNLFENMGF
TYIGPVDGHNIPDTCAVLRKAAAAARPVVVHAVTSKGRGYPPAEADERDHMHAYGVVD
IATGLASTPSQRSWTDVFEDEIARIADDRSDVVGLTAAMRLPTGLGALSRRYPHRVFD
SGIAEQHLLASAAGLAAAGTHPVVAVYSTFLHRAFDQLLFDIGLHRLPVTLVLDRAGV
TGPDGPSHHGLWDLALLACVPGFQIACPRDAPRLRQQLRTAIATAAPTAVRFPKGAPG
EPITAEHTIGGLDVLHTPPPHWRPDVLLVAVGAMSRPCMDAARCLSEEQIGVTVVDPQ
WVWPISPALTELAGRHRITVCVEDAIADVGIGAHLSHHIGRTHPRTRTYTLGLPPAYI
PHASRDHILSSHGLTGPAIRIRCKSLLNTLHEVPGPEDHPDSGDSY"
CDS complement(3816540..3817535)
/codon_start=1
/note="gene_3520"
/transl_table=11
/translation="VLMAEVFVGPVAQGYASGEVTVLLASPRSFCAGVERAIETVKRV
LDVAEGPVYYVRKQIVHNTVVVAELRDRGAVFVEDLDEIPDPPPPGAVVVFSAHGVSPA
VRAGADERGLQVVDATCPLVAKVHAEAARFAARGDTVVFIGHAGHEETEGTLGVAPRS
TLLVQTPADVAALNLPEGTQLSYLTQTTLALDETADVIDALRARFPTLGQPPSEDICY
ATTNRQRALQSMVGECDVVLVIGSCNSSNSRRLVELAQRSGTPAYLIDGPDDIEPEWL
SSVSTIGVTAGASAPPRLVGQVIDALRGYASITVVERSIATETVRFGLPKQVRAQ"
CDS complement(3817529..3818302)

```
                     /codon_start=1
                     /note="gene_3521"
                     /transl_table=11
                     /translation="VSAAVELVHNFTLLHDDVMDGDATRRGRPTVWSVWGVGGAILLG
                     DALHATAVRILTGLTDECVAVRAIRRLQMSCLDLCIGQFEDCLLEGQPEVTVDDYLRM
                     AAGKTAALTGCCCALGALVANADDATIAALERFGHELGLAFQCVDDLIGIWGDPGVTG
                     KPVGNDLARRKATLPVVAALNSRSEAATELAALYQAPAAMTASDVERATALVKVAGGG
                     HVAQRCADERIQAAIAALPDAVRSPDLIALSQLICRREC"
     CDS             complement(3819335..3819727)
                     /codon_start=1
                     /note="gene_3522"
                     /transl_table=11
                     /translation="MAAIYLDSSAIVKLAVREPESDALRRYLRTRHPRVSSALARAEV
                     MRALLDKGESARKAGRRALAHLDLLRVDKRVLDLAGGLLPFELRTLDAIHLATAQRLG
                     VDLGRLCTYDDRMRDAAKTLGMAVIAPS"
     CDS             complement(3819727..3820002)
                     /codon_start=1
                     /note="gene_3523"
                     /transl_table=11
                     /translation="MTSVGVRALRQRASELLRRVEAGETIEITDRGRPVALLSPLPQG
                     GPYEQLLASGEIERATLDVVDLPEPLDLDAGVELPSVTLARLREHER"
     CDS             3820109..3820888
                     /codon_start=1
                     /note="gene_3524"
                     /transl_table=11
                     /translation="LFLGWLALSWEDGLNALVAEGIEAIVFRTVGDQASLWESVLPEE
                     LRRLPEELARVDALLDDSAFFCPFVPFFDPRMGRPSIPMETYLRLMFLKFRYRLGYES
                     LCREVTDSITWRRFCRIPLEGSVPHPTTLMKLTTRCGEDAVAGLNEALLAKAASEKLL
                     RTNKVRADTTVVEGDVGYPTDTGLLAKAVGSMARTVARIKAADAGSAPLGGSSGPRDR
                     LQAAVTRRAATRSGAGLRAPDHRGASRDRRAGADRGCRGGT"
     CDS             3820815..3821555
                     /codon_start=1
                     /note="gene_3525"
                     /transl_table=11
                     /translation="VRRITGELAGIAEQALTEAAAVVRNAQRAVRRASGRRKAWLRQA
                     INHLEKLIGRTERVVDQARSRLAGVMPDSSSRLVSLHDADARPIRKGRLGKPVEFGYK
                     AQVVDNADGVILDHSVELGNPADAPQLAPAIERISRRTGRPPRAVTADRGCGDASVED
                     DLHQLGVRNVAIPRKSKPSATRRAFEHRRAFRDKIKWRTGSEGRINHLKRSYGWNRTE
                     LTGITGARTWCGHGVFAHNLVKISTLAA"
     CDS             3821745..3823997
                     /codon_start=1
                     /note="gene_3526"
                     /transl_table=11
                     /translation="MSFVIANPEMLAAAATDLAGIRSAISAATAAAAAPTIQVAAAGA
                     DEVSLAISALFGQHAQAYQALSAQATIFHDQFVQALTSGGNLYAAAESHTVEQMVLNA
                     INAPTQTLFGRPLIGDGANGTAENPDGQNGGLLFGNGGNGFTQTTAGVAGGNGGSAGL
                     IGNGGAGGIGGAGTGTGGHGGAGGAGGRAWLWGTGGAGGAGAAAAIGNAVTPGGAGGA
                     GGAGGDGGWLFGDGGAGGTGGNGGSGFNSLTSSVGGAGGAGGHAGLFGAGGTGGTGGI
                     GGQNTETGPAASNGGAGGAGGGGGYLVGDGGAGGTGGAGGKNSSGGATLTGGTGGTGG
                     AGGAAGWLYGSGGAGGAGGLNNAGGATGGTGGTGGAGGSGAWLYGNGGAAGAGGNGGN
                     NTSAGTGGVGASGGTGGNAGLIGAGGHGGAGGAGGNQTGGVGNGGAGGNGGAGGAGGQ
```

LYGNGGDGGNGGAGGANIAGGNGSDGGAAGHGGAGGSARLIGAGGHGGDGGAGGNTAG
RRADAIAGTGGDGGNGGNGGLLSGNAGAGGHGGAGGSSTATTTTGTPPTGATGGNGGN
GGAGGTAGFTGSGGIGGNGGAGGTGGNAGVALSVGSTGGLGGNGGSGGLGGGGGSLFG
NGGAGGVGATGGNAGSGIGPASVGGNGGKGGVGAAGGLAGQIGNGGSGGSGGAGGNGG
TGDTAGNGGNGGAGAVGGNAQLIGNGGNGGGGGNGGTGATPGTGGAGAAGGTGGTLFG
APGTTGADGT"

```
CDS             complement(3824068..3824940)
                /codon_start=1
                /note="gene_3527"
                /transl_table=11
                /translation="MAIDPNSIGAVTEPMLFEWTDRDTLLYAIGVGAGTGDLAFTTEN
                SHGIDQQVLPTYAVICCPAFGAAAKVGTFNPAALLHGSQGIRLHAPLPAAGKLSVVTE
                VADIQDKGEGKNAIVVLRGRGCDPESGSLVAETLTTLVLRGQGGFGGARGERPAAPEF
                PDRHPDARIDMPTREDQALIYRLSGDRNPLHSDPWFATQLAGFPKPILHGLCTYGVAG
                RALVAELGGGVAANITSIAARFTKPVFPGETLSTVIWRTEPGRAVFRTEVAGSDGAEA
                RVVLDDGAVEYVAG"
CDS             3824939..3825724
                /codon_start=1
                /note="gene_3528"
                /transl_table=11
                /translation="MQLSSRLENHRKPFPRTVSTGQAIAMAKRTPVRKACTVLAVLAA
                TLLLGACGGPTQPRSITLTFIRNAQSQANADGIIDTDMPGSGLSADGKAEAQQVAHQV
                SRRDVDSIYSSPMAADQQTAGPLAGELGKQVEILPGLQAINAGWFNGKPESMANSTYM
                LAPADWLAGDVHNTIPGSISGTEFNSQFSAAVRKIYDSGHNTPVVFSQGVAIMIWTLM
                NARNSRDSLLTTHPLPNIGRVVITGNPVTGWRLVEWDGIRNFT"
CDS             3825770..3827722
                /codon_start=1
                /note="gene_3529"
                /transl_table=11
                /translation="MRYVVTGGTGFIGRHVVSRLLDGRPEARLWALVRRQSLSRFERL
                AGQWGDRVRPLVGDLTELELSERTIAELGDIDHVLHCAAVHDTTWADATRAVIELAAR
                LDATFHHVSSIAVAGDFAGHYTEADFDVGQRLPTPYHRMTFEAERLVRSTPGLRYRIY
                RPAVVVGDSRTGEMDTIDGPYYLFGVLAKLAVLPSFTPMLLPDIGRTNIVPVDYVADA
                LVALMHADGRDGQTFHLTAPTAIGLRGIYRGIAGAAGLPPLLGTLPGFVAAPVLNARG
                RAKVLRNMAATQLGIPAEIFDVVGCAPTFTSDTTREALRGTGIHVPEFATYAPGLWRY
                WAEHLDPDRARRNDPLLGRHVIITGASSGIGRASAIAVAKRGATVFALARNGNALDEL
                VTEIRAHGGQAHAFTCDVTDSASVEHTVKDILGRFDHVDYLVNNAGRSIRRSVVNSTD
                RLHDYERVMAVNYFGAVRMVLALLPHWRERRFGHVVNVSSAGVQARNPKYSSYLPTKA
                ALDAFADVVASETLSDHITFTNIHMPLVATPMIVPSRRLNPVRAISAERAAAMVIRGL
                VEKPARIDTPLGTLAEAGNYVAPRLSRRILHQLYLGYPDSAAAQGISRPDADRPPAPR
                RPRRSARAGVPRPLRRLGRLVPGVHW"
CDS             complement(3827723..3828586)
                /codon_start=1
                /note="gene_3530"
                /transl_table=11
                /translation="MPDELKPHFANVQAHYDLSDDFFRLFLDPTQTYSCAYFERDDMT
                LQEAQIAKIDLALGKLGLQPGMTLLDVGCGWGATMMRAVEKYDVNVVGLTLSKNQANH
                VQQLVANSESLRSKRVLLAGWEQFDEPVDRIVSIGAFEHFGHERYDAFFSLAHRLLPA
                DGVMLLHTITGLHPKEIHERGLPMSFTFARFLKFIVTEIFPGGRLPSIPMVQECASAN
                GFTVTRVQSLQPHYAKTLDLWSAALQANKGQAIALQSEEVYERYMKYLTGCAEMFRIG
                YIDVNQFTCQK"
```

```
CDS             3828610..3829536
                /codon_start=1
                /note="gene_3531"
                /transl_table=11
                /translation="VSVVFADVDTGIDDALAVIYLLASPDADLVGIASTGGNIAVGQV
                CANNLSLLELCGAADIPVSKGADEPLGGRWPDHPKFHGPKGIGYAELPASNRRLTDYD
                ATTAWIAAAHSHAGDLIGLVTGPLTNLALALRAEPALPRLLRRLVIMGGMFDGQPITE
                WNIRVDPEAASEVFTAWAGQRQLPIVCGLDLTRRVAMTPDILARLASVCGSSPVMRVI
                EDALRFYFESHEARGHGYLAYMHDPLAAAVAMDPELLTTRTATVDVDPTGATVTDWSG
                KRNPNARIGMSVDPAVFFDRFVERIGRFARRT"
CDS             complement(3829591..3831171)
                /codon_start=1
                /note="gene_3532"
                /transl_table=11
                /translation="MASARVLAIWCMDWPAVAAAAAAGLSATAPVAVTLANRVIACSA
                TARAAGVRRGLRRREAAARCPQLFIATADADRDARLFEGVIAAVDDLVPRAELLRPGL
                LVLPVRGPARFFGSEQMAAERLIDAVAAAGAECQVGIADRLSTAVFAARAGRIVEPGG
                DARFLSLLSIRQLATEPSLSGPGRDDLTDLLWRMGIRTIGQFAALSRTDVASRFGADA
                VAAHRFARGEPERAPCGREPPPDLAAELACDPPIDRVDAAAFAGRSLAAELHRALMAA
                GVGCTRLAIHAVTANGEERSRVWRCAEPLTEDATADRVRWQLDGWLNNRNARDRPTAA
                VTLLRLQAVETVSASEGLQLPLWGGLGEQDRLRARRALVRVQGLLGPEAVRVPVLSGG
                HGPAERITLTVLGLVAPEPVPQADPGQPWPGRLPDPSPAVLFDDPVDLLDAQGNPIRV
                TSRGMFSADPARLRVRGRDDRLRWWAGPWPDDERWWDPDRASGRTARAQVLLDGDPGT
                ALLLCYRQRRWYLEGSYE"
CDS             complement(3831171..3831785)
                /codon_start=1
                /note="gene_3533"
                /transl_table=11
                /translation="MTVAFASDQRLENGAEQLESLRRQMALLSEKVSGGPSRSGDLVP
                AGPVSLPPGTVGVLSGARSLLLSMVASVTAAGGNAAIVGQPDIGLLAAVEMGADLSRL
                AMIPDPGTDPVEVAAVLIDGMDLVVLGLGGRRVTRARARAVVARARQKGCTLLVTDGD
                WQGVSTRLAARVCGYEITPALRGVPTPGLGRISGVRLQINGRGR"
CDS             3831784..3832494
                /codon_start=1
                /note="gene_3534"
                /transl_table=11
                /translation="MTSLLFENMFEVSKHPSLESVKVMRGCLVQSRKTTSVLAAALLF
                CGLLGPGTAPPATGGGPACRPAELFATDNTTDGFELPAVATIALTGTVVTGSTLVDGV
                FWSNERQQIGYERSREFHLCVVDAPTLHNAAEALHRQFNQEAVLTFDYLPQNAPEADA
                ILITVPDIGIARFRDAFASDLAAHHRLRGGSVTTADHTLILVAGNGDLDVARRLVEEA
                GGDWNATTIAHGRREFVN"
CDS             complement(3832650..3834227)
                /codon_start=1
                /note="gene_3535"
                /transl_table=11
                /translation="VVQPADIDVPETPARPVLVVDFGAQYAQLIARRVREARVFSEVI
                PHTASIEEIRARQPVALVLSGGPASVYADGAPKLDPALLDLGVPVLGICYGFQAMAQA
                LGGIVAHTGTREYGRTELKVLGGKLHSDLPEVQPVWMSHGDAVTAAPDGFDVVASSAG
                APVAAFEAFDRRLAGVQYHPEVMHTPHGQQVLSRFLHDFAGLGAQWTPANIANALIEQ
                VRTQIGDGHAICGLSGGVDSAVAAALVQRAIGDRLTCVFVDHGLLRAGERAQVQRDFV
                AATGANLVTVDAAETFLEALSGVSAPEGKRKIIGRQFIRAFEGAVRDVLDGKTAEFLV
```

QGTLYPDVVESGGGSGTANIKSHHNVGGLPDDLKFTLVEPLRLLFKDEVRAVGRELGL
PEEIVARQPFPGPGLGIRIVGEVTAKRLDTLRHADSIVREELTAAGLDNQIWQCPVVL
LADVRSVGVQGDGRTYGHPIVLRPVSSEDAMTADWTRVPYEVLERISTRITNEVAEVN
RVVLDITSKPPATIEWE"

CDS complement(3834239..3835147)
/codon_start=1
/note="gene_3536"
/transl_table=11
/translation="MTEIEQAYRITESITRTAARNFYYGIRLLPREKRAALSAVYALG
RRIDDVADGELAPETKITELDAIRKSLDNIDDSSDPVLVALADAARRFPVPIAMFAEL
IDGARMEIDWTGCRDFDELIVYCRRGAGTIGKLCLSIFGPVSTATSRYAEQLGIALQQ
TNILRDVREDFLNGRIYLPRDELDRLGVRLRLDDTGALDDPDGRLAALLRFSADRAAD
WYSLGLRLIPHLDRRSAACCAAMSGIYRRQLALIRASPAVVYDRRISLSGLKKAQVAA
AALASSVTCGPAHGPLPADLGSHPSH"

CDS complement(3835176..3836255)
/codon_start=1
/note="gene_3537"
/transl_table=11
/translation="MRGTDEKYGLPPQPDSDRMTRRTLPVLGLAHELITPTLRQMADR
LDPHMRPVVSYHLGWSDERGRPVNNNCGKAIRPALVFVAAEAAGADPHSAIPGAVSVE
LVHNFSLVHDDLMDRDEHRRHRPTVWALWGDAMALLAGDAMLSLAHEVLLDCDSPHVG
AALRAISEATRELIRGQAADTAFESRTDVALDECLKMAEGKTAALMAASAEVGALLAG
APRSVREALVAYGRHIGLAFQLVDDLLGIWGRPEITGKPVYSDLRSRKKTLPVTWTVA
HGGSAGRRLAAWLVDETGSQTASDDELAAVAELIECGGGRRWASAEARRHVTQGIDMV
ARIGIPDRPAAELQDLAHYIVDRQA"

CDS 3836335..3837324
/codon_start=1
/note="gene_3538"
/transl_table=11
/translation="MAEALLEIAGQTINQKDLGRSGRMTRTDNDTWDLASSVGATATM
IATARALASRAENPLINDPFAEPLVRAVGIDLFTRLASGELRLEDIGDHATGGRWMID
NIAIRTKFYDDFFGDATTAGIRQVVILAAGLDTRAYRLPWPPGTVVYEIDQPAVIKFK
TRALANLNAEPNAERHAVAVDLRNDWPTALKNAGFDPARPTAFSAEGLLSYLPPQGQD
RLLDAITALSAPDSRLATQSPLVLDLAEEDEKKMRMKSAAEAWRERGFDLDLTELIYF
DQRNDVADYLAGSGWQVTTSTGKELFAAQGLPPFEDDHITRFADRRYISAVLK"

CDS 3837388..3838176
/codon_start=1
/note="gene_3539"
/transl_table=11
/translation="MANWYRPNYPEVRSRVLGLPEKVRACLFDLDGVLTDTASLHTKA
WKAMFDAYLAERAERTGEKFVPFDPAADYHTYVDGKKREDGVRSFLSSRAIEIPDGSP
DDPGAAETVYGLGNRKNDMLHKLLRDDGAQVFDGSRRYLEAVTAAGLGVAVVSSSANT
RDVLATTGLDRFVQQRVDGVTLREEHIAGKPAPDSFLRAAELLGVTPDAAAVFEDALS
GVAAGRAGNFAVVVGINRTGRAAQAAQLRRHGADVVVTDLAELL"

CDS 3838191..3840551
/codon_start=1
/note="gene_3540"
/transl_table=11
/translation="MITEDAFPVEPWQVRETKLNLNLLAQSESLFALSNGHIGLRGNL
DEGEPFGLPGTYLNSFYEIRPLPYAEAGYGYPEAGQTVVDVTNGKIFRLLVGDEPFDV
RYGELISHERILDLRAGTLTRRAHWRSPAGKQVKVTSTRLVSLAHRSVAAIEYVVEAI

```
                     EEFVRVTVQSELVTNEDVPETSADPRVSAILDRPLQAVEHERTERGALLMHRTRASAL
                     MMAAGMEHEVEVPGRVEITTDARPDLARTTVICGLRPGQKLRIVKYLAYGWSSLRSRP
                     ALRDQAAGALHGARYSGWQGLLDAQRAYLDDFWDSADVEVEGDPECQQAVRFGLFHLL
                     QASARAERRAIPSKGLTGTGYDGHAFWDTEGFVLPVLTYTAPHAVADALRWRASTLDL
                     AKERAAELGLEGAAFPWRTIRGQESSAYWPAGTAAWHINADIAMAFERYRIVTGDGSL
                     EEECGLAVLIETARLWLSLGHHDRHGVWHLDGVTGPDEYTAVVRDNVFTNLMAAHNLH
                     TAADACLRHPEAAEAMGVTTEEMAAWRDAADAANIPYDEELGVHQQCEGFTTLAEWDF
                     EANTTYPLLLHEAYVRLYPAQVIKQADLVLAMQWQSHAFTPEQKARNVDYYERRMVRD
                     SSLSACTQAVMCAEVGHLELAHDYAYEAALIDLRDLHRNTRDGLHMASLAGAWTALVV
                     GFGGLRDDEGILSIDPQLPDGISRLRFRLRWRGFRLIVDANHTDVTFILGDGPGTQLT
                     MRHAGQDLTRHTDTPSTIAVRTRKPLLPPPPQPPGREPVHRRALAR"
     variation        3840593..3840704
                     /note="supplemented from AL2122/97"
     CDS             complement(3841279..3842259)
                     /codon_start=1
                     /note="gene_3541"
                     /transl_table=11
                     /translation="MLKLEPGGSTIPKIPFIRPSFPGPAELAEDFVQIAQANWYTNFG
                     PNERRFARALRDYLGPHLHVATLANGTLALLAALHVSFGAGTRDRYLLMPSFTFVGVA
                     QAALWTGYRPWFIDIDANTWQPCVHSARAVIERFRDRIAGILLANVFGVGNPQISVWE
                     ELAAEWELPIVLDSAAGFGSTYADGERLGGRGACEIFSFHATKPFAVGEGGALVSRDP
                     RLVEHAYKFQNFGLVQTRESIQLGMNGKLSEISAAIGLRQLVGLDRRLASRRKVLECY
                     RTGMADADGVRFQDNANVASLCFASACCTSADHKAAVLGSLRRHAIEARDY"
     CDS             complement(3842702..3844276)
                     /codon_start=1
                     /note="gene_3542"
                     /transl_table=11
                     /translation="MITPPTFDVAFIGSGAACSMTLLEMADALLSSPSASPKLRIAVV
                     ERDEQFWCGIPYGQRSSIGSLAIQKLDDFADEPEKAAYRIWLEQNKQRWLAFFQAEGG
                     AAAARWICDNRDALDGNQWGELYLPRFLFGVFLSEQMIAAIAALGERDLAEIVTIRAE
                     AMSAHSADGHYRIGLRPSGNGPTAIAAGKVVVAIGSPPTKAILASDSEPAFTYINDFY
                     SPGGESNVARLRDSLDRVESWEKRNVLVVGSNATSLEALYLMRHDARIRARVRSITVI
                     SRSGVLPYMICNQPPEFDFPRLRTLLCTEAIAAADLMSAIRDDLATAEERSLNLADLY
                     DAVAALFGQALHKMDLVQQEEFFCVHGMNFTKLVRRAGRDCRQASEELAADGTLSLLA
                     GEVLRVDACASGQPFATMTYRAAGAEHTHPVPFAAVVNCGGFEELDTCSSPFLVSAMQ
                     NGLCRPNRTNRGLLVNDDFEASPGFCVIGPLVGGNFTPKIRFWHVESAPRVRSLAKSL
                     AASLLASLQPVALAPC"
     CDS             complement(3844320..3845024)
                     /codon_start=1
                     /note="gene_3543"
                     /transl_table=11
                     /translation="VTILILTDNVHAHALAVDLQARHGDMDVYQSPIGQLPGVPRCDV
                     AERVAEIVERYDLVLSFHCKQRFPAALIDGVRCVNVHPGFNPYNRGWFPQVFSIIDGQ
                     KVGVTIHEIDDQLDHGPIIAQRECAIESWDSSGSVYARLMDIERELVLEHFDAIRDGS
                     YTAKSPATEGNLNLKKDFEQLRRLDLNERGTFGHFLNRLRALTHDDFRNAWFVDASGR
                     KVFVRVVLEPEKPAEA"
     CDS             complement(3845142..3845708)
                     /codon_start=1
                     /note="gene_3544"
                     /transl_table=11
                     /translation="MTTRPATDRRKMPTGREEVAAAILQAATDLFAERGPAATSIRDI
```

```
                     AARSKVNHGLVFRHFGTKDQLVGAVLDHLGTKLTRLLHSEAPADIIERALDRHGRVLA
                     RALLDGYPVGQLQQRFPNVAELLDAVRPRYDSDLGARLAVAHALALQFGWRLFAPMLR
                     SATGIDELTGDELRLSVNDAVARILEPH"
variation             3845462..3845483
                     /note="supplemented from BCG moreau"
CDS                   3845770..3846657
                     /codon_start=1
                     /note="gene_3545"
                     /transl_table=11
                     /translation="MTDLITVKKLGSRIGAQIDGVRLGGDLDPAAVNEIRAALLAHKV
                     VFFRGQHQLDDAEQLAFAGLLGTPIGHPAAIALADDAPIITPINSEFGKANRWHTDVT
                     FAANYPAASVLRAVSLPSYGGSTLWANTAAAYAELPEPLKCLTENLWALHTNRYDYVT
                     TKPLTAAQRAFRQVFEKPDFRTEHPVVRVHPETGERTLLAGDFVRSFVGLDSHESRVL
                     FEVLQRRITMPENTIRWNWAPGDVAIWDNRATQHRAIDDYDDQHRLMHRVTLMGDVPV
                     DVYGQASRVISGAPMEIAG"
CDS                   3846692..3846991
                     /codon_start=1
                     /note="gene_3546"
                     /transl_table=11
                     /translation="MRATVGLVEAIGIRELRQHASRYLARVEAGEELGVTNKGRLVAR
                     LIPVQAAERSREALIESGVLIPARRPQNLLDVTAEPARGRKRTLSDVLNEMRDEQ"
CDS                   3846988..3847398
                     /codon_start=1
                     /note="gene_3547"
                     /transl_table=11
                     /translation="VIYMDTSALTKLLISEPETTELRTWLTAQSGQGEDAATSTLGRV
                     ELMRVVARYGQPGQTERARYLLDGLDILPLTEPVIGLAETIGPATLRSLDAIHLAAAA
                     QIKRELTAFVTYDHRLLSGCREVGFVTASPGAVR"
CDS                   complement(3847431..3849167)
                     /codon_start=1
                     /note="gene_3548"
                     /transl_table=11
                     /translation="MKPDYDVLIIGSGFGGSVTALRLTEKGYRVGVLEAGRRFSDEEF
                     AKTSWDLRKFLWAPRLGCYGIQRIHPLRNVMILAGAGVGGGSLNYANTLYVPPEPFFA
                     DQQWSHITDWRGELMPHYQQAQRMLGVVQNPTFTDADRIVKEVADEMGFGDTWVPTPV
                     GVFFGPDGTKTPGKTVPDPYFGGAGPARTGCLECGCCMTGCRHGAKNTLVKNYLGLAE
                     SAGAQVIPMTTVKGFERRSDGLWEVRTVRTGSWLRRDRRTFTATQLVLAAGTWGTQHL
                     LFKMRDRGRLPGLSKRLGVLTRTNSESIVGAATLKVNPDLDLTHGVAITSSIHPTADT
                     HIEPVRYGKGSNAMGLLQTLMTDGSGPQGTDVPRWRQLLQTASQDPRGTIRMLNPRQW
                     SERTVIALVMQHLDNSITTFTKRGKLGIRWYSSKQGHGEPNPTWIPIGNQVTRRIAAK
                     IDGVAGGTWGELFNIPLTAHFLGGAVIGDDPEHGVIDPYHRVYGYPTLYVVDGAAISA
                     NLGVNPSLSIAAQAERAASLWPNKGETDRRPPQGEPYRRLAPIQPAHPVVPADAPGAL
                     RWLPIDPVSNAG"
CDS                   complement(3849223..3850350)
                     /codon_start=1
                     /note="gene_3549"
                     /transl_table=11
                     /translation="MVEIGMGRTARRTYELSEISIVPSRRTRSSKDVSTAWQLDAYRF
                     EIPVVAHPTDALVSPEFAIELGRLGGLGVLNGEGLIGRHLDVEAKIAQLLEAAAADPE
                     PSTAIRLLQELHAAPLNPDLLGAAVARIREAGVTTAVRVSPQNAQWLTPVLVAAGIDL
                     LVIQGTIVSAERVASDGEPLNLKTFISELDIPVVAGGVLDHRTALHLMRTGAAGVIVG
```

YGSTQGVTTTDEVLGISVPMATAIADAAAARRDYLDETGGRYVHVLADGDIHTSGELA
KAIACGADAVVLGTPLAESAEALGEGWFWPAAAAHPSLPRGALLQIAVGERPPLARVL
GGPSDDPFGGLNLVGGLRRSMAKAGYCDLKEFQKVGLTVGG"
CDS complement(3850370..3851959)
/codon_start=1
/note="gene_3550"
/transl_table=11
/translation="MSRGMSGLEDSSDLVVSPYVRMGGLTTDPVPTGGDDPHKVAMLG
LTFDDVLLLPAASDVVPATADTSSQLTKKIRLKVPLVSSAMDTVTESRMAIAMARAGG
MGVLHRNLPVAEQAGQVEMVKRSEAGMVTDPVTCRPDNTLAQVDALCARFRISGLPVV
DDDGALVGIITNRDMRFEVDQSKQVAEVMTKAPLITAQEGVSASAALGLLRRNKIEKL
PVVDGRGRLTGLITVKDFVKTEQHPLATKDSDGRLLVGAAVGVGGDAWVRAMMLVDAG
VDVLVVDTAHAHNRLVLDMVGKLKSEVGDRVEVVGGNVATRSAAAALVDAGADAVKVG
VGPGSICTTRVVAGVGAPQITAILEAVAACRPAGVPVIADGGLQYSGDIAKALAAGAS
TAMLGSLLAGTAEAPGELIFVNGKQYKSYRGMGSLGAMRGRGGATSYSKDRYFADDAL
SEDKLVPEGIEGRVPFRGPLSSVIHQLTGGLRAAMGYTGSPTIEVLQQAQFVRITPAG
LKESHPHDVAMTVEAPNYYAR"
CDS 3852166..3852576
/codon_start=1
/note="gene_3551"
/transl_table=11
/translation="VRDHLPPGLPPDPFADDPCDPSAALEAVEPGQPLDQQERMAVEA
DLADLAVYEALLAHKGIRGLVVCCDECQQDHYHDWDMLRSNLLQLLIDGTVRPHEPAY
DPEPDSYVTWDYCRGYADASLNEAAPDADRFRRR"
CDS complement(3852586..3853485)
/codon_start=1
/note="gene_3552"
/transl_table=11
/translation="MREFGNPLGDRPPLDELARTDLLLDALAEREEVDFADPRDDALA
ALLGQWRDDLRWPPASALVSQDEAVAALRAGVAQRRRARRSLAAVGSVAAALLVLSGF
GAVVADARPGDLLYGLHAMMFNRSRVSDDQIVLSAKANLAKVEQMIAQGQWAEAQDEL
AEVSSTVQAVTDGSRRQDLINEVNLLNTKVETRDPNATLRPGSPSNPAAPGSVGNSWT
PLAPVVEPPTPPTPASAAEPSMSAGVSESPMPNSTSTVAASPSTPSSKPEPGSIDPSL
EPADEATNPAGQPAPETPVSPTH"
CDS complement(3853478..3854053)
/codon_start=1
/note="gene_3553"
/transl_table=11
/translation="MTMQGERLDAVVAEAVAGDRNALREVLETIRPIVVRYCRARVGT
VERSGLSADDVAQEVCLATITALPRYRDRGRPFLAFLYGIAAHKVADAHRAAGRDRAY
PAETLPERWSADAGPEQMAIEADSVTRMNELLEILPAKQREILILRVVVGLSAEETAA
AVGSTTGAVRVAQHRALQRLKDEIVAAGDYA"
CDS complement(3854134..3854925)
/codon_start=1
/note="gene_3554"
/transl_table=11
/translation="VLVAAAFGNQPGSWPLPTAITPHHLWLRAVAAGGQGRYAHAYGD
LSVLRRLVPAGPLASLAHSTQGSLLRQLGWHTLARGWDGRALALAGADREAGADALIG
LAADALGVGRFAAAGALLDRADPLVVSPLVADRLAVRRRWVAAELAMATGDGATAVRH
AEEAVELTQAMAVASARHRVKSDVVLAAALCSAGAVARARAVGEEALDATARFGLLPL
RWALACLLIDIGTVTFSAQQLRELTKIRNICAGQVRRAGGCWRTA"

```
CDS             3855332..3855640
                /codon_start=1
                /note="gene_3555"
                /transl_table=11
                /translation="MPQPEQLPGPNADIWNWQLQGLCRGMDSSMFFHPDGERGRARTQ
                REQRAKEMCRRCPVIEACRSHALEVGEPYGVWGGLSESERDLLLKGTMGRTRGIRRTA
                "
CDS             complement(3855712..3857331)
                /codon_start=1
                /gene="groEL"
                /note="gene_3556"
                /transl_table=11
                /translation="MSKLIEYDETARRAMEVGMDKLADTVRVTLGPRGRHVVLAKAFG
                GPTVTNDGVTVAREIELEDPFEDLGAQLVKSVATKTNDVAGDGTTTATILAQALIKGG
                LRLVAAGVNPIALGVGIGKAADAVSEALLASATPVSGKTGIAQVATVSSRDEQIGDLV
                GEAMSKVGHDGVVSVEESSTLGTELEFTEGIGFDKGFLSAYFVTDFDNQQAVLEDALI
                LLHQDKISSLPDLLPLLEKVAGTGKPLLIVAEDVEGEALATLVVNAIRKTLKAVAVKG
                PYFGDRRKAFLEDLAVVTGGQVVNPDAGMVLREVGLEVLGSARRVVVSKDDTVIVDGG
                GTAEAVANRAKHLRAEIDKSDSDWDREKLGERLAKLAGGVAVIKVGAATETALKERKE
                SVEDAVAAAKAAVEEGIVPGGGASLIHQARKALTELRASLTGDEVLGVDVFSEALAAP
                LFWIAANAGLDGSVVVNKVSELPAGHGLNVNTLSYGDLAADGVIDPVKVTRSAVLNAS
                SVARMVLTTETVVVDKPAKAEDHDHHHGHAH"
CDS             complement(3857426..3857728)
                /codon_start=1
                /gene="groES"
                /note="gene_3557"
                /transl_table=11
                /translation="VAKVNIKPLEDKILVQANEAETTTASGLVIPDTAKEKPQEGTVV
                AVGPGRWDEDGEKRIPLDVAEGDTVIYSKYGGTEIKYNGEEYLILSARDVLAVVSK"
CDS             complement(3857995..3859029)
                /codon_start=1
                /note="gene_3558"
                /transl_table=11
                /translation="MTTVLGIETSCDETGVGIARLDPDGTVTLLADEVASSVDEHVRF
                GGVVPEIASRAHLEALGPAMRRALAAAGLKQPDIVAATIGPGLAGALLVGVAAAKAYS
                AAWGVPFYAVNHLGGHLAADVYEHGPLPECVALLVSGGHTHLLHVRSLGEPIIELGST
                VDDAAGEAYDKVARLLGLGYPGGKALDDLARTGDRDAIVFPRGMSGPADDRYAFSFSG
                LKTAVARYVESHAADPGFRTADIAAGFQEAVADVLTMKAVRAATALGVSTLLIAGGVA
                ANSRLRELATQRCGEAGRTLRIPSPRLCTDNGAMIAAFAAQLVAAGAPPSPLDVPSDP
                GLPVMQGQVR"
CDS             complement(3859026..3859502)
                /codon_start=1
                /note="gene_3559"
                /transl_table=11
                /translation="VTADTEPVTIGALTRADAQRCAELEAQLFVGDDPWPPAAFNREL
                ASPHNHYVGARSGGTLVGYAGISRLGRTPPFEYEVHTIGVDPAYQGRGIGRRLLRELL
                DFARGGVVYLEVRTDNDAALALYRSVGFQRVGLRRRYYRVSGADAYTMRRDSGDPS"
CDS             complement(3859499..3860152)
                /codon_start=1
                /note="gene_3560"
                /transl_table=11
```

/translation="LVVGPVMSRVQISTVLAIDTATPAVTAGIVRRHDLVVLGERVTV
DARAHAERLTPNVLAALADAALTMADLDAVVVGCGPGPFTGLRAGMASAAAYGHALGI
PVYGVCSLDAIGGQTIGDTLVVTDARRREVYWARYCDGIRTVGPAVNAAADVDPGPAL
AVAGAPEHAALFALPCVEPSRPSPAGLVAAVNWADKPAPLVPLYLRRPDAKPLAVCT"
CDS complement(3860131..3860637)
/codon_start=1
/note="gene_3561"
/transl_table=11
/translation="LSREGIRRRPKARAGLTGGGTATLPRVEDTLTLGSRLGEQLCAG
DVVVLSGPLGAGKTVLAKGIAMAMDVEGPITSPTFVLARMHRPRRPGTPAMVHVDVYR
LLDHNSADLLSELDSLDLDTDLEDAVVVVEWGEGLAERLSQRHLDVRLERVSHSDTRI
ATWSWGRS"
CDS complement(3860634..3861746)
/codon_start=1
/gene="alr"
/note="gene_3562"
/transl_table=11
/translation="MVDLGAIEHNVRVLREHAGHAQLMAVVKADGYGHGATRVAQTAL
GAGAAELGVATVDEALALRADGITAPVLAWLHPPGIDFGPALLADVQVAVSSLRQLDE
LLHAVRRTGRTATVTVKVDTGLNRNGVGPAQFPAMLTALRQAMAEDAVRLRGLMSHMV
YADKPDDSINDVQAQRFTAFLAQAREQGVRFEVAHLSNSSATMARPDLTFDLVRPGIA
VYGLSPVPALGDMGLVPAMTVKCAVALVKSIRAGEGVSYGHTWIAPRDTNLALLPIGY
ADGVFRSLGGRLEVLINGRRCPGVGRICMDQFMVDLGPGPLDVAEGDEAILFGPGIRG
EPTAQDWADLVGTIHYEVVTSPRGRITRTYREAENR"
CDS 3862679..3863215
/codon_start=1
/gene="PPE59"
/note="gene_3563"
/transl_table=11
/translation="MHPMIPAEYISNIIYEGPGADSLFFASGQLRELAYSVETTAESL
EDELDELDENWKGSSSDLLADAVERYLQWLSKHSSQLKHAAWVINGLANAYNDTRRKV
VPPEEIAANREEVHRLIASNVAGVNTPAIAGLDAQYQQYRAQNIAVMNDYQSTARFIL
AYLPRWQEPPQIYGGGGG"
CDS complement(3863156..3864319)
/codon_start=1
/note="gene_3564"
/transl_table=11
/translation="VIDTAIEEMIPLIGVRAACAATGRAPASYYRAHSKRLSAQSDTF
TSTAVTDPSGPRESAQPRALSAAEREHVLAVLNSQRFADMAPAVVYATLLDEGIYLCS
ESTMYRLLRERGQTGDRRRQATHPAAVKPELVAHQPNSVWSWDITKLRGPAKWSYYYL
YVILDIFSRYVVGWMVASRESKVLAERLIAQTLAAQHISADQLTLHADRGSSMSSKPV
ALLLADLGVTKSHSRPHTSNDNPLSEAQFKTLKYRPDFPKRFESIEAARVHCDRFFGW
YNHEHKHSGIGLHTPADVHYGRADQIRRHRATVLDTAYRDHLERIRSQTTRATRATGL
QRDQPTTEGGPADSINPRKSCLRNVDRFRPGLLDLPAPAPVDLRRLLPSGQIR"
CDS complement(3864358..3864513)
/codon_start=1
/note="gene_3565"
/transl_table=11
/translation="LAALSARRGPKPGKAGANAADAEIAWLRAELDTAREVIRVQGEL
SALLERL"
CDS complement(3864538..3864747)

/codon_start=1
/note="gene_3566"
/transl_table=11
/translation="LTNRAGTGRRGSSVTVDEVPDPQVPERAQRRTFTVKYKLAILDE
YDRADRTERGAILRRENLYSSLLTE"
CDS complement(3864808..3865653)
/codon_start=1
/note="gene_3567"
/transl_table=11
/translation="VFAELIRAGLQALIEAEATEAIGAGRYERSDGRIVHRNGHRPKT
VSTTAGDIEVQIPKLRAGSFFPSLLERRRRIDKALHAVIMEAYVHGVSTRSVDDLVAA
MGVQAGVSKSEVSRICAGLDTEIEAFRTRSLTHTEFPYVFCDATFCKVRVGAHVVSQA
LVVATGVSIDGTREVLGTAVGDSESYEFWREFLASLKARGLTGVHLVISDAHAGLKAA
VAQQFSGASWQRCRVHFMRNLYTAVAAKHAPAVTVAVKTIFAHTDPEEVGAQWDRVAD
PLCQP"
CDS complement(3865886..3867268)
/codon_start=1
/note="gene_3568"
/transl_table=11
/translation="VSRSHPSVPAHSIAPAYTGRMFTAPVPALRMPDESMDPEAAYRF
IHDELMLDGSSRLNLATFVTTWMDPEAEKLMAETFDKNMIDKDEYPATAAIEARCVSM
VADLFHAEGLRDHDPTSATGVSTIGSSEAVMLGGLALKWRWRQRVGSWKGRMPNLVMG
SNVQVVWEKFCRYFDVEPRYLPMERGRYVITPEQVLAAVDENTIGVVAILGTTYTGEL
EPIAEICAALDKLAAGGGVDVPVHVDAASGGFVVPFLHPDLVWDFRLPRVVSINVSGH
KYGLTYPGVGFVVWRGPEHLPEDLVFRVNYLGGDMPTFTLNFSRPGNQVVGQYYNFLR
LGRDGYTKVMQALSHTARWLGDQLREVDHCEVISDGSAIPVVSFRLAGDRGYTEFDVS
HELRTFGWQVPAYTMPDNATDVAVLRIVVREGLSADLARALHDDAVTALAALDKVKPG
GHFDAQHFAH"
CDS complement(3867306..3868727)
/codon_start=1
/note="gene_3569"
/transl_table=11
/translation="MRHYYSVDTIRAAEAPLLASLPDGALMRRAAFGLATEIGRELTA
RTGGVVGRRVCAVVGSGDNGGDALWAATFLRRRGAAADAVLLNPDRTHRKALAAFTKS
GGRLVESVSAATDLVIDGVVGISGSGPLRPAAAQVFAAVQAAAIPVVAVDIPSGIDVA
TGAITGPAVHAALTVTFGGLKPVHALADCGRVVLVDIGLDLAHTDVLGFEATDVAARW
PVPGPRDDKYTQGVTGVLAGSSTYPGAAVLCTGAAVAATSGMVRYAGTAHAEVLAHWP
EVIASPTPAAAGRVQAWVVGPGLGTDEAGAAALWFALDTDLPVLVDADGLTMLADHPD
LVAGRNAPTVLTPHAGEFARLAGAPPGDDRVGACRQLADALGATVLLKGNVTVIADPG
GPVYLNPAGQSWAATAGSGDVLSGMIGALLASGLPSGEAAAAAAFVHARAAAAAAADP
GPGDAPTSASRISGHIRAALAAL"
CDS complement(3868729..3869442)
/codon_start=1
/note="gene_3570"
/transl_table=11
/translation="VADASVVARLRSWALAVWHFVSNAPLTYAWLVVLVITTIIQNNL
TGSQLHFVLLHRSTNIAELGRDPLEVLFSSLLWIDGRNLEPYLLLFTLFLAPAEHWLG
HLRWLTVGLTAHIGATYLSEGLLYLAIQHRDASERMVHARDIGVSYFLVGVMAVLTYH
IAKPWRWGYLGVLLVIFGFPLIAMDKAELDFTAVGHFASILIGLLFYPMARERDGRLW
NPARIKSLLHRRGTRGRRA"
CDS complement(3869453..3870391)

/codon_start=1
/note="gene_3571"
/transl_table=11
/translation="VAWRWHPLTEGSRGYNFRAGTHKWAGSELGRILRVVVGLVLVIA
AYVTVIALYHSTGLGRPHEVAHGRPTADGTTVTLHVEQLQTIKGVLVANLAVSPGTEL
LDSQTQGLKDDLTVTVTSVVTPTKRTWSSGSLPGVFPVPLTISGDPANWPFDHYRSGP
ITVQLYRGAAHAPERVSVTFVDRLPGWNVDISGVGDANVPAPYRVGLHRSPSSVAFGT
VIVGVLIALAGVGLFVAVQTARGRRQFQPPMTTWYAAMLFAVIPLRNALPDAPPIGFW
IDVTVVLWVVVALVTSMVLYILCWWWHLKPDVDETM"

CDS complement(3870529..3872403)
/codon_start=1
/note="gene_3572"
/transl_table=11
/translation="VCGIVGYVGRRPAYVVVMDALRRMEYRGYDSSGIALVDGGTLTV
RRRAGRLANLEEAVAEMPSTALSGTTGLGHTRWATHGRPTDRNAHPHRDAAGKIAVVH
NGIIENFAVLRRELETAGVEFASDTDTEVAAHLVARAYRHGETADDFVGSVLAVLRRL
EGHFTLVFANADDPGTLVAARRSTPLVLGIGDNEMFVGSDVAAFIEHTREAVELGQDQ
AVVITADGYRISDFDGNDGLQAGRDFRPFHIDWDLAAAEKGGYEYFMLKEIAEQPAAV
ADTLLGHFVGGRIVLDEQRLSDQELREIDKVFVVACGTAYHSGLLAKYAIEHWTRLPV
EVELASEFRYRDPVLDRSTLVVAISQSGETADTLEAVRHAKEQKAKVLAICNTNGSQI
PRECDAVLYTRAGPEIGVASTKTFLAQIAANYLLGLALAQARGTKYPDEVEREYHELE
AMPDLVARVIAATGPVAELAHRFAQSSTVLFLGRHVGYPVALEGALKLKELAYMHAEG
FAAGELKHGPIALIEDGLPVIVVMPSPKGSATLHAKLLSNIREIQTRGAVTIVIAEEG
DETVRPYADHLIEIPAVSTLLQPLLSTIPLQVFAASVARARGYDVDKPRNLAKSVTVE
"

CDS 3872500..3872901
/codon_start=1
/note="gene_3573"
/transl_table=11
/translation="LSNPYAQHQLKLIRHTGALILWQQRTYVVSGTREQCEAAYKSAQ
TYNLLVGWWSLVSLPAMNWIALISNFNAIRRVRAAADGASVPHGPHAIAHPAVPRGPI
PAGWYPDPSGAGLRYWDGATWTHWTHPPRHR"

CDS 3872911..3873753
/codon_start=1
/note="gene_3574"
/transl_table=11
/translation="VPRIRKLVAALHRRGPHRVLRGDLAFAGLPGVVYTPEAGLHLPG
VAFGHDWLTGTSRYSGLLEHLASWGIVAAAPDSERGLAPSVLNLAFDLGVALDIVAGV
RLGPGKISVHPAKLGLVGHGFGGSAAVFAAAGLTGTHVKSVAAIFPTVTNPAAEQPAA
TLDVPGLILTAPGDPKTLTSNALGLSRAWDKATLRIVSKARAGGLVEGRRLTKVLGLP
GPHRRTQRSVRALLTGYLLYTLGGDKTYRRFADPDLQLPKTDPIDPEAPPITPGEKIV
TLLK"

CDS complement(3873773..3875176)
/codon_start=1
/note="gene_3575"
/transl_table=11
/translation="VADRLNVAERLAEGRPAAEHTQSYVRACHLVGYQHPDLTAYPAQ
IHDWYGSEDGLDLHALDADCAQLRAAASVLMEALRMERSQVAVLAAAWTGSGADAAVH
FVQRHCETGNSVVTEVRAAAQRCESLRDNLWQLVDSKVATAIAIDERALAQRPAWLAA
AEALTTEGADRPTAVEVVRQQIQPYVDDDVRNDWLTTMRSTTAGVAASYDAVTDQLAS
APRAHFEIPDDLGPGRQPSPASVPAQPSATAAITPAAALPPPDPVPAVTSRPVTPSDF

```
                     GSAPGDGSATPAGVGSAGGFGDAGGTGGLGGFAGLAGLANRIVDAVDSLLGSVAEQLG
                     DPLAADNPPGAVDPFAEDAADNADDGDDAHPEEADEAAEPKEATEPDEADEVDDADES
                     VPAERAQDVAEEATLPPVAEPPPPAAPPVAEPPPPVAAPAPPGAPEPANGPSPEALSE
                     GATPCEIAADELPQAGP"
     CDS             complement(3875179..3875490)
                     /codon_start=1
                     /note="gene_3576"
                     /transl_table=11
                     /translation="MRPDSVNSAGIDIAAVYAVADRFSAAAELIDDAIGNHLTRLAFG
                     GACAGRGHASRGDALRCRLDRLAGELSVWSRAAVQIAFALRAGANRYAEADLCAAARI
                     G"
     CDS             complement(3875538..3876884)
                     /codon_start=1
                     /note="gene_3577"
                     /transl_table=11
                     /translation="MGRLFGTDGVRGVANRELTAELALALGAAAARRLSRSGAPGRRV
                     AVLGRDPRASGEMLEAAVIAGLTSEGVDALRVGVLPTPAVAYLTGAYDADFGVMISAS
                     HNPMPDNGIKIFGPGGHKLDDDTEDQIEDLVLGVSRGPGLRPAGAGIGRVIDAEDATE
                     RYLRHVAKAATARLDDLAVVVDCAHGAASSAAPRAYRAAGARVIAINAEPNGRNINDG
                     CGSTHLDPLRAAVLAHRADLGLAHDGDADRCLAVDANGDLVDGDAIMVVLALAMKEAG
                     ELACNTLVATVMSNLGLHLAMRSAGVTVRTTAVGDRYVLEELRAGDYSLGGEQSGHIV
                     MPALGSTGDGIVTGLRLMTRMVQTGSSLSDLASAMRTLPQVLINVEVVDKATAAAAPS
                     VRTAVEQAAAELGDTGRILLRPSGTEPMIRVMVEAADEGVAQRLAATVADAVSTAR"
     CDS             complement(3877009..3877464)
                     /codon_start=1
                     /gene="rpsI"
                     /note="gene_3578"
                     /transl_table=11
                     /translation="MTETTPAPQTPAAPAGPAQSFVLERPIQTVGRRKEAVVRVRLVP
                     GTGKFDLNGRSLEDYFPNKVHQQLIKAPLVTVDRVESFDIFAHLGGGGPSGQAGALRL
                     GIARALILVSPEDRPALKKAGFLTRDPRATERKKYGLKKARKAPQYSKR"
     CDS             complement(3877461..3878048)
                     /codon_start=1
                     /gene="rplM"
                     /note="gene_3579"
                     /transl_table=11
                     /translation="MLVGKLLGWRAVRRDLGSMSVAETNPPFTPDRRPGHPGIHPREK
                     KVSAVPTYAPKAGDTTRSWYVIDATDVVLGRLAVAAANLLRGKHKPTFAPNVDGGDFV
                     IVINADKVAISGDKLQHKMVYRHSGYPGGLHKRTIGELMQRHPDRVVEKAILGMLPKN
                     RLSRQIQRKLRVYAGPEHPHSAQQPVPYELKQVAQ"
     CDS             complement(3878137..3878439)
                     /codon_start=1
                     /note="gene_3580"
                     /transl_table=11
                     /translation="MNADPVLSYNFDAIEYSVRQEIHTTAARFNAALQELRSQIAPLQ
                     QLWTREAAAAYHAEQLKWHQAASALNEILIDLGNAVRHGADDVAHADRRAAGAWAR"
     CDS             complement(3878460..3878777)
                     /codon_start=1
                     /note="gene_3581"
                     /transl_table=11
                     /translation="VSTPNTLNADFDLMRSVAGITDARNEEIRAMLQAFIGRMSGVPP
```

SVWGGLAAARFQDVVDRWNAESTRLYHVLHAIADTIRHNEAALREAGQIHARHIAAAG
GDL"
CDS complement(3878830..3880044)
/codon_start=1
/note="gene_3582"
/transl_table=11
/translation="VSPHRAVIEAGPGAIRRLCCGADVVADTAVSAAALAAIDDQVAL
LDERPVAVDSLWFDALRSVAVDHRDGPVVVHPSWWSAARVEVVTAAARTLTRDVVVHP
RSWLLRQASSGVSAATVVVEIAERLVLVAGAEVAAVARRTDAESVAGQVGSVIARMTR
GITAVVLIDVPSTVAGAAALAAAIAGAVRGTGSSVVEIDGVRLARLARAALPPSDEPA
DPAARPATRSRVPTLARVAAAGVALALLAPAAVVRHGATTLQRPPTTLLVEGRVALTI
PADWSTQRVVSGPGSARVQVTSPADPEVALHVTQSPVPGETLPGTAQRLKRAIDASPA
GVFVDFNPSDIRAGRPAVTYREVRAGHQVRWTILLDGAVRISVGCQSGPGHEDLLREV
CAQAVRSVHAVG"
CDS complement(3880041..3883550)
/codon_start=1
/note="gene_3583"
/transl_table=11
/translation="MLVSLVVTAVTGRGRRHVSGIHNDRVDYLGYLSVLRTSVTQTAA
AQHVSLNWTHPDPATLWTLIGGPRMWERRPGAADFCRIRVGVGSAPLATRLVVGQLPP
AQRADPVTRAALRCFLAAHATIADAPIAIPLRVGGPIAIDGDPTKVRGLLRAMICQLA
VWHSPEELLIAGVVSDRNRAHWDWLKWLPHNQHPNACDALGPAPMVYSTLAEMQNALA
ATVLAHVVAIVDTAERGNGAITGVITIEVGARRDGAPPVVRCAGEVTALACPDQLEPQ
DALVCARRLAAHRVGHSGRTFIRGSGWAELVGIGDVAAFDPSTLWRNVNQHDRLRVPI
GVTPDGTAVQLDIKEAAEQGMGPHGLCVGATGSGKSELLRTIALGMMARNSPEVLNLL
LVDFKGGATFLDLAGAPHVAAVITNLAEEAPLVARMQDALAGEMSRRQQLLRMAGHLV
SVTAYQRARQTGAQLPCLPILFIVVDEFSELLSQHPEFVDVFLAIGRVGRSLGMHLLL
ASQRLDEGRLRGLETHLSYRMCLKTWSASESRNVLGTQDAYQLPNTPGAGLLQTGTGE
LIRFQTAFVSGPLRRASPSAVHPVAPPSVRPFTTHAAAPVTAGPVGGTAEVPTPTVLH
AVLDRLVGHGPAAHQVWLPPLDEPPMLGALLRDAEPAQAELAVPIGIVDRPFEQSRVP
LTIDLSGAAGNVAVVGAPQTGKSTALRTLIMALAATHDAGRVQFYCLDFGGGALAQVD
ELPHVGAVAGRAQPQLASRMLAELESAVRFREAFFRDHGIDSVARYRQLRAKSAAESF
ADIFLVIDGWASLRQEFAALEESIVALAAQGLSFGVHVALSAARWAEIRPSLRDQIGS
RIELRLADPADSELDRRQAQRVPVDRPGRGLSRDGMHMVIALPDLDGVALRRRSGDPV
APPIPLLPARVDYDSVVARAGDELGAHILLGLEERRGQPVAVDFGRHPHLLVLGDNEC
GKTAALRTLCREIVRTHTAARAQLLIVDFRHTLLDVIESEHMGGYVSSPAALGAKLSS
LVDLLQARMPAPDVSQAQLRARSWWSGPDIYVVVDDYDLVAVSSGNPLMVLLEYLPHA
RDLGLHLVVARRSGGAARALFEPVLASLRDLGCRALLMSGRPDEGALFGSSRPMPLPP
GRGILVTGAGDEQLVQVAWSPPP"
CDS 3883483..3883770
/codon_start=1
/gene="MRGA423_21730"
/note="gene_3584"
/transl_table=11
/translation="LWIPETCRRPRPVTAVTTNETSIIIGKARNVGWRVAGEPGKNTV
TMTPTVATDMTTGSRRISRLDGSDRRNSGGGATRMSAVAHAGPEFMLGDGM"
CDS 3883865..3885268
/codon_start=1
/note="gene_3585"
/transl_table=11
/translation="MPTSDPGLRRVTVHAGAQAVDLTLPAAVPVATLIPSIVDILGDR

GASPATAARYQLSALGAPALPNATTLAQCGIRDGAVLVLHKSSAQPPTPRCDDVAEAV
AAALDTTARPQCQRTTRLSGALAASCITAGGGLMLVRNALGTNVTRYSDATAGVVAAA
GLAALLFAVIACRTYRDPIAGLTLSVIATIFGAVAGLLAVPGVPGVHSVLVAAMAAAA
TSVLAMRITGCGGITLTAVACCAVVVAAATLVGAITAAPVPAIGSLATLASFGLLEVS
ARMAVLLAGLSPRLPPALNPDDADALPTTDRLTTRANRADAWLTSLLAAFAASATIGA
IGTAVATHGIHRSSMGGIALAAVTGALLLLRARSADTRRSLVFAICGITTVATAFTVA
ADRALEHGPWIAALTAMLAAVAMFLGFVAPALSLSPVTYRTIELLECLALIAMVPLTA
WLCGAYSAVRHLDLTWT"

CDS 3885259..3886632
/codon_start=1
/note="gene_3586"
/transl_table=11
/translation="MDMTTSRTLRLLVVSALATLSGLGTPVAHAVSPPPIDERWLPES
ALPAPPRPTVQREVCTEVTAESGRAFGRAERSAQLADLDQVWRLTRGAGQRVAVIDTG
VARHRRLPKVVAGGDYVFTGDGTADCDAHGTLVAGIIAAAPDAQSDNFSGVAPDVTLI
SIRQSSSKFAPVGDPSSTGVGDVDTMAKAVRTAADLGASVINISSIACVPAAAAPDDR
ALGAALAYAVDVKNAVIVAAAGNTGGAAQCPPQAPGVTRDSVTVAVSPAWYDDYVLTV
GSVNAQGEPSAFTLAGPWVDVAATGEAVTSLSPFGDGTVNRLGGQHGSIPISGTSYAA
PVVSGLAALIRARFPTLTARQVMQRIESTAHHPPAGWDPLVGNGTVDALAAVSSDSIP
QAGTATSDPAPVAVPVPRRSTPGPSDRRALHTAFAGAAICLLALMATLATASRRLRPG
RNGIAGD"

CDS complement(3886597..3888009)
/codon_start=1
/note="gene_3587"
/transl_table=11
/translation="VPSPATTWLHVSGYRFLLRRIECALLFGDVCAATGALRARTTSL
ALGCVLAIVAAMGCAFVALLRPQSALGQAPIVMGRESGALYVRVDDVWHPVLNLASAR
LIAATNANPQPVSESELGHTKRGPLLGIPGAPQLLDQPLAGAESAWAICDSDNGGSTT
VVVGPAEDSSAQVLTAEQMILVATESGSPTYLLYGGRRAVVDLADPAVVWALRLQGRV
PHVVAQSLLNAVPEAPRITAPRIRGGGRASVGLPGFLVGGVVRITRASGDEYYVVLED
GVQRIGQVAADLLRFGDSQGSVNVPTVAPDVIRVAPIVNTLPVSAFPDRPPTPVDGSP
GRAVTTLCVTWTPAQPGAARVAFLAGSGPPVPLGGVPVTLAQADGRGPALDAVYLPPG
RSAYVAARSLSGGGTGTRYLVTDTGVRFAIHDDDVAHDLGLPTAAIPAPWPVLATLPS
GPELSRANASVARDTVAPGP"

CDS 3888175..3888918
/codon_start=1
/gene="cut3"
/note="gene_3588"
/transl_table=11
/translation="LGAGALITAVVLLIALGAVWTPVAFADGCPDAEVTFARGTGEPP
GIGRVGQAFVDSLRQQTGMEIGVYPVNYAASRLQLHGGDGANDAISHIKSMASSCPNT
KLVLGGYSQGATVIDIVAGVPLGSISFGSPLPAAYADNVAAVAVFGNPSNRAGGSLSS
LSPLFGSKAIDLCNPTDPICHVGPGNEFSGHIDGYIPTYTTQAASFVVQRLRAGSVPH
LPGSVPQLPGSVLQMPGTAAPAPESLHGR"

CDS 3888965..3889645
/codon_start=1
/note="gene_3589"
/transl_table=11
/translation="VIPRPQPHSGRWRAGAARRLTSLVAAAFAAATLLLTPALAPPAS
AGCPDAEVVFARGTGEPPGLGRVGQAFVSSLRQQTNKSIGTYGVNYPANGDFLAAADG
ANDASDHIQQMASACRATRLVLGGYSQGAAVIDIVTAAPLPGLGFTQPLPPAADDHIA

AIALFGNPSGRAGGLMSALTPQFGSKTINLCNNGDPICSDGNRWRAHLGYVPGMTNQA
ARFVASRI"
CDS 3889703..3889942
/codon_start=1
/gene="TBJG_02218"
/note="gene_3590"
/transl_table=11
/translation="VTAADWHTAYHGLMLAPDPARQARRPSTQPNAHLSAECAAMNRN
HFTGREVCGHANRTLGLTSTAIALRRVDAGRHNKQ"
CDS 3889917..3891602
/codon_start=1
/note="gene_3591"
/transl_table=11
/translation="MPGVITNSESPTAADHDRITATRETLEDYTLRLAPRSYRRWPPA
VVGISALGGIAYLADFAIGANVGITWGTANALCGIAIFALVVFVTGLPLAYYAARYNI
DLDLITRGSGFGYYGSVVTNVIFATFTFIFFALEGSIMAQGLKLGLHIPLWAGYACST
LIIFPLVVYGMKVLSQLQLWTTPLWLILMAAPFGYLVVSHPDSIGQFFSYAGKDGHGG
LSFGSVLLAAGVCLSLIAQIAEQIDYLRFMPPRTPENANRWWTWTLLAGPGWVAFGAT
KQIIGLFLAVYLMANIPGSSTIANQPVHQFMQIYRTFVPGWLALTLAVILVVLSQIKI
NVTNAYSGSLAWTNSFTRLTKHYPGRVVFLGVNLAIALILMEANMFDFLNTILGCYAN
CGMAWVVAVASDIGFNKYLLGLSPKTPEFRRGMLYAINPVGFGSLLLAAGLSIVTFFG
GLGAALQPYSPLVAIVTALVMPPILAAATKGKYYLRRTHDGIDLPMYDEHGNPSAAVL
TCHVCHQDFERPDMLACQTHGAHVCSLCLSTDKQAEHVLPGLARAHIPGDQVP"
CDS complement(3891564..3892334)
/codon_start=1
/gene="truA"
/note="gene_3592"
/transl_table=11
/translation="VGQRTVAGDLDAALTTIFRTPVRLRAAGRTDAGVHASGQVAHVD
VPADALPNAYPRAGHVGDPEFLPLLRRLGRFLPADVRILDITRAPAGFDARFSALRRH
YVYRLSTAPYGVEPQQARYITAWPRELDLDAMTAASRDLMGLHDFAAFCRHREGATTI
RDLQRLDWSRAGTLVTAHVTADAFCWSMVRSLVGALLAVGEHRRATTWCRELLTATGR
SSDFAVAPAHGLTLIQVDYPPDDQLASRNLVTRDVRSG"
CDS complement(3892402..3892944)
/codon_start=1
/gene="rplQ"
/note="gene_3593"
/transl_table=11
/translation="MPKPTKGPRLGGSSSHQKAILANLATSLFEHGRITTTEPKARAL
RPYAEKLITHAKKGALHNRREVLKKLRDKDVVHTLFAEIGPFFADRDGGYTRIIKIEA
RKGDNAPMAVIELVREKTVTSEANRARRVAAAQAKAKKAAAMPTEESEAKPAEEGDVV
GASEPDAKAPEEPPAEAPEN"
CDS complement(3892976..3894019)
/codon_start=1
/note="gene_3594"
/transl_table=11
/translation="MLISQRPTLSEDVLTDNRSQFVIEPLEPGFGYTLGNSLRRTLLS
SIPGAAVTSIRIDGVLHEFTTVPGVKEDVTEIILNLKSLVVSSEEDEPVTMYLRKQGP
GEVTAGDIVPPAGVTVHNPGMHIATLNDKGKLEVELVVERGRGYVPAVQNRASGAEIG
RIPVDSIYSPVLKVTYKVDATRVEQRTDFDKLILDVETKNSISPRDALASAGKTLVEL
FGLARELNVEAEGIEIGPSPAEADHIASFALPIDDLDLTVRSYNCLKREGVHTVGELV

ARTESDLLDIRNFGQKSIDEVKIKLHQLGLSLKDSPPSFDPSEVAGYDVATGTWSTEG
AYDEQDYAETEQL"
CDS complement(3894170..3894775)
/codon_start=1
/gene="rpsD"
/note="gene_3595"
/transl_table=11
/translation="MARYTGPVTRKSRRLRTDLVGGDQAFEKRPYPPGQHGRARIKES
EYLLQLQEKQKARFTYGVMEKQFRRYYEEAVRQPGKTGEELLKILESRLDNVIYRAGL
ARTRRMARQLVSHGHFNVNGVHVNVPSYRVSQYDIVDVRDKSLNTVPFQIARETAGER
PIPSWLQVVGERQRVLIHQLPERAQIDVPLTEQLIVEYYSK"
CDS complement(3894784..3895203)
/codon_start=1
/note="gene_3596"
/transl_table=11
/translation="MPPAKKGPATSARKGQKTRRREKKNVPHGAAHIKSTFNNTIVTI
TDPQGNVIAWASSGHVGFKGSRKSTPFAAQLAAENAARKAQDHGVRKVDVFVKGPGSG
RETAIRSLQAAGLEVGAISDVTPQPHNGVRPPNRRRV"
CDS complement(3895207..3895581)
/codon_start=1
/gene="rpsM"
/note="gene_3597"
/transl_table=11
/translation="MARLVGVDLPRDKRMEVALTYIFGIGRTRSNEILAATGIDRDLR
TRDLTEEQLIHLRDYIEANLKVEGDLRREVQADIRRKIEIGCYQGLRHRRGMPVRGQR
TKTNARTRKGPKRTIAGKKKAR"
CDS complement(3895943..3896164)
/codon_start=1
/note="gene_3598"
/transl_table=11
/translation="MAKKDGAIEVEGRVVEPLPNAMFRIELENGHKVLAHISGKMRQH
YIRILPEDRVVVELSPYDLSRGRIVYRYK"
CDS 3896418..3897275
/codon_start=1
/note="gene_3599"
/transl_table=11
/translation="MTNCAAGKPSSGPNLGRFGSFGRGVTPQQATEIEALGYGAVWVG
GSPPAALSWVEPILQATTTLCVATGIVNIWSAPAQRVAESFHRIEAAYPGRFLLGIGV
GHAEMISEYRKPYNALVEYLDRLDDYGVPANRRVVAALGPRVLGLSARRSAGAHPYLT
TPEHTARARELIGPSAFLAPEHKVVLTTDSARARTVGRQALDMYFNLANYRNNWKRLG
FTDDEVSRPGSDRLVDAVVAYGTPDAIAARLNEHLLAGADHVPIQVLTEDDNLVSALT
ELAKPLRLT"
CDS 3897348..3898343
/codon_start=1
/note="gene_3600"
/transl_table=11
/translation="MRLLVTGGAGFIGTNFVHSAVREHPDDAVTVLDALTYAGRRESL
ADVEDAIRLVQGDITDAELVSQLVAESDAVVHFAAESHVDNALDNPEPFLHTNVIGTF
TILEAVRRHGVRLHHISTDEVYGDLELDDRARFTESTPYNPSSPYSATKAGADMLVRA
WVRSYGVRATISNCSNNYGPYQHVEKFIPRQITNVLTGRRPKLYGAGANVRDWIHVDD
HNSAVRRILDRGRIGRTYLISSEGERDNLTVLRTLLRLMDRDPDDFDHVTDRVGHDLR

YAIDPSTLYDELCWAPKHTDFEEGLRTTIDWYRDNESWWRPLKDATEARYQERGQ"
CDS 3898345..3898953
/codon_start=1
/note="gene_3601"
/transl_table=11
/translation="MKARELDVPGAWEITPTIHVDSRGLFFEWLTDHGFRAFAGHSLD
VRQVNCSVSSAGVLRGLHFAQLPPSQAKYVTCVSGSVFDVVVDIREGSPTFGRWDSVL
LDDQDRRTIYVSEGLAHGFLALQDNSTVMYLCSAEYNPQREHTICATDPTLAVDWPLV
DGAAPSLSDRDAAAPSFEDVRASGLLPRWEQTQRFIGEMRGT"
CDS 3899469..3900428
/codon_start=1
/note="gene_3602"
/transl_table=11
/translation="VDVSTRQAAEADLAGKAAQYRPDELARYAQRVMDWLHPDGDLTD
TERARKRGITLSNQQYDGMSRLSGYLTPQARATFEAVLAKLAAPGATNPDDHTPVIDT
TPDAAAIDRDTRSQAQRNHDGLLAGLRALIASGELGQHNGLPVSIVVTTTLTDLQTGA
GKGFTGGGTLLPMADVIRMTSHAHHYSPASGRYPQAIFDHGTPLALYHTKRLASPAQR
IMLFANDRGCTKPGCDAPAYHSQAHHVTGWTSTGRTDITELTLACDPDNRLAEKGWTT
RKNTHGHTEWLPPPHLDHGQPRTNTFHHPERFLHNQDDDDEPD"
CDS complement(3900486..3901580)
/codon_start=1
/note="gene_3603"
/transl_table=11
/translation="VGTHAATMRVRAGVRSSPLLLHAGTPPTAAAAESGMRTLVTGSS
GHLGEALVRTLRARGADVVSLDSRPSRYTNIVGCVSDRALLRDVMAGVEVVFHAAAHH
KPQLAFLPRQAFLDTNIIGTQTVLDAAVAANVRAFVMTSSTTVFGDALTPPADQPAAW
IDESVTPIPKNIYGVTKASSEDLCQLAHRNDGLACVVLRVARFFVEGDDMPDLYDGRS
QDNIKANEYACRRVALEDAVDAHLNAAQRAPQLGFGRYLVSATTPFTRDDLTQLRTDA
ASVFARRVPLAAAVWTQRGWRFPDRLDRVYVNSRARRDLNWRPRFDLNAVAARLARGQ
SVHTPLSQLVGSKAYAHSSYHRGVFAPARP"
CDS complement(3901584..3902594)
/codon_start=1
/note="gene_3604"
/transl_table=11
/translation="MLMTATHREPIVLDTTVRDGSYAVNFQYTDDDVRRIVGDLDAAG
IPYIEIGHGVTIGAAAAQGPAAHTDEEYFRAARSVVRNARLGAVIVPALARIETVDLA
GDYLDFLRICVIATEFELVMPFVERAQSKGLEVSIQLVKSHLFEPDVLAAAGKRARDV
GVRIVYVVDTTGTFLPEDARRYVEALRGASDVSVGFHGHNNLAMAVANTLEAFDAGAD
FLDGTLMGFGRGAGNCQIECLVAALQRRGHLAAVGLDRIFDAARSDMLGRSPQSYGID
PWEISFGFHGLDSLQVEHLRAAAQQAGLSVSHVIRQTAKSHAGQWLSPQDIDRVVVGM
RA"
CDS complement(3902655..3904328)
/codon_start=1
/note="gene_3605"
/transl_table=11
/translation="MAVTPVTVGDHLVARMRAAGISVVCGLPTSRLDSLLVRLSRDAG
FQIVLARHEGGAGYLADGFARASGKSAAVFVAGPGATNVISAVANASVNQVPMLILTG
EVAVGEFGLHSQQDTSDDGLGLGATFRRFCRCSVSIESIANARSKIDSAFRALASIPR
GPVHIALPRDLVDERLPAHQLGTAAAGLGGLRTLAPCGPDVADEVIGRLDRSRAPMLV
LGNGCRLDGIGEQIVAFCEKAGLPFATTPNGRGIVAETHPLSLGVLGIFGDGRADEYL
FDTPCDLLIAVGVSFGGLVTRSFSPRWRGLKADVVHVDPDPSAVGRFVATSLGITTSG

RAFVNALNCGRPPRFCRRVGVRPPAPAALPGTPQARGESIHPLELMHELDRELAPNAT
ICADVGTCISWTFRGIPVRRPGRFFATVDFSPMGCGIAGAIGVALARPEEHVICIAGD
GAFLMHGTEISTAVAHGIRVTWAVLNDGQMSASAGPVSGRMDPSPVARIGANDLAAMA
RALGAEGIRVDTRCELRAGVQKALAATGPCVLDIAIDPEINKPDIGLGR"
CDS complement(3904319..3904804)
/codon_start=1
/note="gene_3606"
/transl_table=11
/translation="VLQATVALSAGHKPAFRGFVKDPPRARAHAAAMFVSNAREAEPF
VAPDLSEIRVLVDRATVGVASVSLAHATVAAGAETVWHRLQATDEIYFVLSGRGLVSV
GDESGEVGPGDAVWIPAGVPQKIRALGSVPLTFLCACGPAYLPERDQRMGEAAVIGAW
P"
CDS 3904873..3905379
/codon_start=1
/note="gene_3607"
/transl_table=11
/translation="MRPVDEQWIEILRIQALCARYCLTIDTQDGEGWAGCFTEDGAFE
FDGWVIRGRPALREYADAHARVVRGRHLTTDLLYEVDGDVATGRSASVVTLATAAGYK
ILGSGEYQDRLIKQDGQWRIAYRRLRNDRLVSDPSVAVNVADADVAAVVGHLLAAARR
LGTQMSDT"
CDS complement(3905459..3906361)
/codon_start=1
/note="gene_3608"
/transl_table=11
/translation="MVLDPLMDPISMAAESFSVHGPGGARIVADRLGDPRARAVVFLH
GGGQTRRSWGRAAAAVAERGWQAVTIDLRGHGESDWSSEGDYRLVSFAGDIQEVLRNL
PGQPALVGASLGGFAAMLLAGELSPGIASAVVLVDIVPNMDLAGASRIHAFMAERVES
GFGSLDEVADVIANYNPHRPRPSDPDGLVANLRRRGDRWYWHWDPQFIGGIAAFPPVE
VTDVDRMNAAVATILRDEVPVLLVRGQVSDIVRQESADQFLSRFPQVEFTDVRGAGHM
VAGDRNDAFAGAVLDFLARHVGVR"
CDS complement(3906524..3907873)
/codon_start=1
/note="gene_3609"
/transl_table=11
/translation="MTVSIAPPSRPSQAETRRAIWNTIRGSSGNLVEWYDVYVYTVFA
TYFEDQFFDRADRNSTVYVYAIFAVTFVTRPVGSWFFGRFADRRGRRAALTFSVSLMA
ACSLIVALVPSRSSIGVAAPILLILCRLVQGFATGGEYGTSVTYMSEAATRERRGYFS
SFQYVTLVGGHVLAQFTLLVILAVFTREQVHEFGWRIGFAVGGGAAIVVFWLRRTMDE
SLSQERLTAIKAGRDHDSGSLRELATHYWKPLLLCFLVTLGGTVAFYTYSVNAPAIVK
SVYGSQAMTATWINLVGLILLMMLQPIGGMISDKIGRKPLLLWFGVGGLIYTYVLVTY
LPETRSPTMSFLLVAVGYVILTGYCSINALVKSELFPAHVRALGVGVGYALANSVFGG
TAPLIYQALKERDQVPMFIAYVTACIAVSLIVYVFFIKNKADTYLDREQGFAFYGHA"
CDS 3908246..3908542
/codon_start=1
/note="gene_3610"
/transl_table=11
/translation="VSFTAQPEMLAAAAGELRSLGATLKASNAAAAVPTTGVVPPAAD
EVSLLLATQFRTHAATYQTASAKAAVIHEQFVTTLATSASSYADTEAANAVVTG"
CDS 3908579..3909760
/codon_start=1
/note="gene_3611"

/transl_table=11
/translation="VVDFGALPPEINSARMYAGPGSASLVAAAKMWDSVASDLFSAAS
AFQSVVWGLTVGSWIGSSAGLMAAAASPYVAWMSVTAGQAQLTAAQVRVAAAAYETAY
RLTVPPPVIAENRTELMTLTATNLLGQNTPAIEANQAAYSQMWGQDAEAMYGYAATAA
TATEALLPFEDAPLITNPGGLLEQAVAVEEAIDTAAANQLMNNVPQALQQLAQPAQGV
VPSSKLGGLWTAVSPHLSPLSNVSSIANNHMSMMGTGVSMTNTLHSMLKGLAPAAAQA
VETAAENGVWAMSSLGSQLGSSLGSSGLGAGVAANLGRAASVGSLSVPPAWAAANQAV
TPAARALPLTSLTSAAQTAPGHMLGGLPLGHSVNAGSGINNALRVPARAYAIPRTPAA
G"
CDS 3909790..3911274
/codon_start=1
/note="gene_3612"
/transl_table=11
/translation="LFPAAVGVLWQSGLRDPTPPGGPHGIEGLSMAFEKPSPVTALTQ
ELRFATTMTGGVSLAIWMAGVTREINLLAQASQWRRLGGTFPTNSQLTNESAASLRLY
AQLIDLLDMVVDVDILSGTSAGGINAALLASSRVTGSDLGGIRDLWLDLGALTELLRD
PRDKKTPSLLYGDERIFAALAKRLPKLATGPFPPTTFPEAARTPSTTLYITTTLLAGE
TSRFTDSFGTLVQDVDRRGLFTFTETDLARPDTAPALALAARSSASFPLAFEPSFLPF
TKGTAKKGEVPARPAMAPFTSLTRPHWVSDGGLLDNRPIGVLFKRIFDRPARRPVRRV
LLFVVPSSGPAPDPMHEPPPDNVDEPLGLIDGLLKGLAAVTTQSIAADLRAIRAHQDC
MEARTDAKLRLAELAATLRNGTRLLTPSLLTDYRTREATKQAQTLTSALLRRLSTCPP
ESGPATESLPKSWSAELTVGGDADKVCRQLASFRCVLQEVMASQ"
CDS 3911279..3912325
/codon_start=1
/note="gene_3613"
/transl_table=11
/translation="MWGRLDGAGWLVHVLLDPRRVRWIVGERADTNGPQSGAQWFLGK
LKELGAPDFPSPGYPLPAVGGGPAQHLTEDMLLDELGFLDDPAKPLPASIPWTALWLS
QAWQQRVLEEELDGLANTVLDPQPGKLPDWSPTSSRTWATKVLAAHPGDAKYALLNEN
PIAGETFASDKGSPLMAHTVAKAAATAAGAAGSVRQLPSVLKPPLITLRTLTLSGYRV
VSLTKGIARSTIIAGALLLVLGVAAAIQSVTVFGVTGLIAAGTGGLLVVLGTWQVSGR
LLFALLSFSVVGAVLALATPVVREWLFGTQQQPGWVGTHAYWLGAQWWHPLVVVGLIA
LVAIMIAAANPGRR"
CDS complement(3912349..3913842)
/codon_start=1
/note="gene_3614"
/transl_table=11
/translation="VSQTARRLGPQDMFFLYSESSTTMMHVGALMPFTPPSGAPPDLL
RQLVDESKASEVVEPWSLRLSHPELLYHPTQSWVVDDNFDLDYHVRRSALASPGDERE
LGIPVSRLHSHALDLRRPPWEVHFIEGLEGGRFAIYIKMHHSLIDGYTGQKMLARSLS
TDPHDTTHPLFFNIPTPGRSPADTQDSVGGGLIAGAGNVLDGLGNVVRGLGGLVSGVG
SVLGSVAGAGRSTFELTKALVNAQLRSDHEYRNLVGSVQAPHCILNTRISRNRRFATQ
QYPLDRLKAIGAQYDATINDVALAIIGGGLRRFLDELGELPNKSLIVVLPVNVRPKDD
EGGGNAVATILATLGTDVADPVQRLAAVTASTRAAKAQLRSMDKDAILAYSAALMAPY
GVQLASTLSGVKPPWPYTFNLCVSNVPGPEDVLYLRGSRMEASYPVSLVAHSQALNVT
LQSYAGTLNFGFIGCRDTLPHLQRLAVYTGEALDQLAAADGAAGLGS"
CDS complement(3913933..3914679)
/codon_start=1
/note="gene_3615"
/transl_table=11
/translation="LGARNFVELGRSVVTEAVSMRGLLPVAGHWVSVLTGLVPLALVI

ALSPLSVIPAVLVVHSPQPRPSSLAFLGGWLLGLAVVTAVFVAASGALGGLSTTSPAW
ASWLRVVLGSALIVFGVLRWLTRHRHTEMPGWMRAFASFTPARAGLVGAVLVVVRPEV
LIICAAAGLAIGSGGHGAAGSWIYTAFFAMLAASTVAIPILAYVAAGDRLDDSLERLK
DWMEKNHAGMVAAILVVIGLLLLYNGVHAM"

```
CDS             complement(3915591..3916253)
                /codon_start=1
                /note="gene_3616"
                /transl_table=11
                /translation="VSDEIDPDWPAPAYQPSDDVDTTPPAPGGSWPTAWLVALVVLAC
                VAAAVVAYAGMHRVRPGANQAAPATTSAPARPTSPASQVGPCGPDEATAVRAALAQLA
                PDSKTGRPWNSTPEDSNYDPCADLSAVLVTVQDATNSSPDQALMFHRGTFVGTATPRA
                YPFTNLIGPASTNDIVVLSYRTRQSCDGCQDGILTIVGFAWRGDHVQILDSLPELFDA
                PP"
CDS             3916558..3918057
                /codon_start=1
                /gene="cpsA"
                /note="gene_3617"
                /transl_table=11
                /translation="VPQPSRIRKRLSRGVMTLVSVVALLMTGAGYWVAHGALGGITIS
                QALTPEDPRSSGNNMNILLIGLDSRKDQEGNDLPWSVLKQLHAGDSDDGGYNTNTLIL
                VHVGADGKVVAFSIPRDDWVPFTGVPGYNHIKIKEAYGLTKQYVAEQLANQGVSDRKE
                LETRGREAARAATLRAVRSLTGVPIDYFAEINLAGFYDLAQTLGGVDVCLNHAVYDSY
                SGADFPAGRQRLNAAQALAFVRQRHGLDNGDLDRTHRQQAFLSSVMRELQDSGTFTNL
                DRLDNLMAVARKDVVLSAGWDEDLFRRMGDLAGGNVEFRTLPVVRYDNIDGQDVNIID
                PTAIRAEVAAAFGSAPPTSQTAAAAKPNPSTVVDVVNAGSISGLASQVSGALLKRGYT
                AGQVRDRESGDPFTTAIEYGAGAETDAQNVADLLGIDAPNHPDPAVAPGHIRVTVDTN
                FSLPAPDEATAAATSTETSTYPLYGGGTTTDPTPDQGAPIDGGGVPCVN"
CDS             complement(3918063..3918944)
                /codon_start=1
                /note="gene_3618"
                /transl_table=11
                /translation="MVQNGENLFQFRREGPQVQLSFQDRTYLVTGGGSGIGKGVAAGL
                VAAGAAVMIVGRNPDKLAAAVKDIEALKTGAIGYEPADITDEEQTLRVVDAATAWHGR
                LHGVVHCAGGSQTIGPITQIDSQAWRRTVDLNVNGTMYVLKHAARELVRGGGGSFVGI
                SSIAASNTHRWFGAYGVTKSAVDHMMKLAADELGPSWVRVNSIRPGLIRTDLVVPVTE
                SPELSADYRVCTPLPRVGEVEDVANLAMFLLSDAASWITGQVINVDGGHMLRRGPDFS
                PMLEPVFGADGLRGVVG"
CDS             3919258..3919662
                /codon_start=1
                /note="gene_3619"
                /transl_table=11
                /translation="VGLVFLSEGIQKFMYPDQLGPGRFERIGIPAATFFADLDGVVEI
                VCGTLVLLGLLTRVAAVPLLIDMVGAIVLTKLRALQPGGFLGVEGFWGMAHAARTDLS
                MLLGLIFLLWSGPGRWSLDRRLSKRATACGAR"
CDS             complement(3919615..3920736)
                /codon_start=1
                /note="gene_3620"
                /transl_table=11
                /translation="MSSYYARRPLQSSGCSNSDSCWDGAPIEITESGPSVAGRLAALA
                SRMTIKPLMTVGSYLSPLPLPLGFVDFACRVWRPGQGTVRTTINLPNATAQLVRAPGV
                RAADGAGRVVLYLHGGAFVMCGPNSHSRIVNALSGFAESPVLIVDYRLIPKHSLGMAL
```

DDCHDAYQWLRARGYRPEQIVLAGDSAGGYLALALAQRLQCDDEKPAAIVAISPLLQL
AKGPKQDHPNIGTDAMFPARAFDALAAWVRAAAAKNMVDGRPEDLYEPLDHIESSLPP
TLIHVSGSEVLLHDAQLGAGKLAAAGVCAEVRVWPGQAHLFQLATPLVPEATRSLRQI
GQFIRDATADSSLSPVHRSRYVAGSPRAASRGAFGQSPI"

CDS 3921108..3921431
/codon_start=1
/note="gene_3621"
/transl_table=11
/translation="MREFQRAAVRLHILHHAADNEVHGAWLTQELSRHGYRVSPGTLY
PTLHRLEADGLLVSEQRVVDGRARRVYRATPAGRAALTEDRRALEELAREVLGRQSHT
AGNGT"

CDS 3921495..3921677
/codon_start=1
/note="gene_3622"
/transl_table=11
/translation="LAYSYVMSTKSDHGEIGDVEPLADSTASQARRVVAAYANDADEC
RIFLSMLGIGPAKLES"

CDS 3921677..3923179
/codon_start=1
/note="gene_3623"
/transl_table=11
/translation="MAPSGGQEAQICDSETFGDSDFVVVANRLPVDLERLPDGSTTWK
RSPGGLVTALEPVLRRRRGAWVGWPGVNDDGAEPDLHVLDGPIIQDELELHPVRLSTT
DIAQYYEGFSNATLWPLYHDVIVKPLYHREWWDRYVDVNQRFAEAASRAAAHGATVWV
QDYQLQLVPKMLRMLRPDLTIGFFLHIPFPPVELFMQMPWRTEIIQGLLGADLVGFHL
PGGAQNFLILSRRLVGTDTSRGTVGVRSRFGAAVLGSRTIRVGAFPISVDSGALDHAA
RDRNIRRRAREIRTELGNPRKILLGVDRLDYTKGIDVRLKAFSELLAEGRVKRDDTVL
VQLATPSRERVESYQTLRNDIERQVGHINGEYGEVGHPVVHYLHRPAPRDELIAFFVA
SDVMLVTPLRDGMNLVAKEYVACRSDLGGALVLSEFTGAAAELRHAYLVNPHDLEGVK
DGIEEALNQTEEAGRRRMRSLRRQVLAHDVDRWAQSFLDALAGAHPRGQG"

CDS 3923331..3923909
/codon_start=1
/note="gene_3624"
/transl_table=11
/translation="VNIRCGLAAGAVICSAVALGIALHSGDPARALGPPPDGSYSFNQ
AGVSGVTWTITALCDQPSGTRNMNDYSDPIVWAFNCALNVVSTTPQQITRTDRLQNFS
GRARMSSMLWTFQVNQADGVACPDGSTAPSSETYAFSDETLTGTHTTVHGAVCGLQPK
LSKQPFSLQLIGPPPSPVQRYPLYCNNIAMCY"

CDS complement(3923906..3924388)
/codon_start=1
/note="gene_3625"
/transl_table=11
/translation="MRRLISVAYALMVATIVGLSAAGGWFYWDRVQTGGEASARALLP
KLAMQEIPQVFGYDYQTVERSLTAVYPLLTPDYRQEFQKSANAQIIPEAKKREVVVQA
NVVGVGVMDAKRDCASVMVYLNRTVTDKTRQPLYDGSRLRVDFQRIDGKWLIAYITPI
"

CDS complement(3924388..3925116)
/codon_start=1
/note="gene_3626"
/transl_table=11
/translation="VAADTGVAGGQQSTTRRARRKASRPAGPAEGESSRPAQGAATVR

AAARTESKPAKAAKPALRPVKPPPRRPAHRVLVGWLSLAAGLLAIAALAWGVTALVMQ
NRDADARQARNQRFVDAATQTVVNMFSYTPDTIDESVNRFVNGTSGPLRGMLNANNNV
DNLKGLFRATNATSEAVVNGAALEGIDEISDNASVLVSVRVTVADIDGVNKPSMPYRL
RVIVHEDENGRMTGYDLKYPDGGN"

CDS complement(3925116..3926810)
/codon_start=1
/note="gene_3627"
/transl_table=11
/translation="MIDRLAKIQLSIFAVITVITLSVMAIFYLRLPATFGIGTYGVSA
DFVAGGGLYKNANVTYRGVAVGRVESVGLNPNGVTAHMRLNSGTAIPSNVTATVRSVS
AIGEQYIDLVPPENPSSTKLRNGFRIQRQNTRIGQDVADLLRQAETLLGSLGDTRLRE
LLHEAFIATNGAGPELARLIESARLLVDEANANYPQVSQLIDQAGPFLQAQIRAGGDI
KSLADGLARFTWQLRAADPRLRDTLAGAPDAIDEANTAFSGIRPSFPALAASLANLGR
VGVIYHKSIEQLLVVFPALFAAIITSAGGVPQDEGAKLDFKIDLHDPPPCMTGFLPPP
LVRSPADESVREIPRDMYCKTAQNDPSTVRGARNYPCQEFPGKRAPTVQLCRDPRGYV
PVGTNPWRGPPIPYGTEVTDGRNILPPNKFPYIPPGADPDPGVPIVGPPPPGQVAGPG
PAPHQPAQPAPPPNDNGPPPPFTSWMPPGYPPEPPQVPYPATIPPPPPPEGTGPPPGP
APGPQPQASGPAYTIYDQLSGAFADPAGGTGIFAPGMTGASSAENWVDLMRDPRQL"

CDS complement(3926821..3927975)
/codon_start=1
/note="gene_3628"
/transl_table=11
/translation="MNRIWLRAIILTASSALLAGCQFGGLNSLPLPGTAGHGEGAYSV
TVEMADVATLPQNSPVMVDDVTVGSVAGIVAVQRPDGSFYAAVKLDLDKNVLLPANAV
AKVSQTSLLGSLHVELAPPTDRPPTGRLVDGSRITEANTDRFPTTEEVFSALGVVVNK
GNVGALEEIIDETHQAVAGRQAQFVNLVPRLAELTAGLNRQVHDIIDALDGLNRVSAI
LARDKDNLGRALDTLPDAVRVLNQNRDHIVDAFAALKRLTMVTSHVLAETKVDFGEDL
KDLYSIVKALNDDRKDFVTSLQLLLTFPFPNFGIKQAVRGDYLNVFTTFDLTLRRIGE
TFFTTAYFDPNMAHMDEILNPPDFLIGELANLSGQAADPFKIPPGTASGQ"

CDS complement(3927972..3929324)
/codon_start=1
/note="gene_3629"
/transl_table=11
/translation="MGRVAMLTGSRGLRYATVIALVAALVGGVYVLSSTGNKRTIVGY
FTSAVGLYPGDQVRVLGVPVGEIDMIEPRSSDVKITMSVSKDVKVPVDVQAVIMSPNL
VAARFIQLTPVYTGGAVLPDNGRIDLDRTAVPVEWDEVKEGLTRLAADLSPAAGELQG
PLGAAINQAADTLDGNGDSLHNALRELAQVAGRLGDSRGDIFGTVKNLQVLVDALSES
DEQIVQFAGHVASVSQVLADSSANLDQTLGTLNQALSDIRGFLRENNSTLIETVNQLN
DFAQTLSDQSENIEQVLHVAGPGITNFYNIYDPAQGTLNGLLSIPNFANPVQFICGGS
FDTAAGPSAPDYYRRAEICRERLGPVLRRLTVNYPPIMFHPLNTITAYKGQIIYDTPA
TEAKSETPVPELTWVPAGGGAPVGNPADLQSLLVPPAPGPAPAPPAPGAGPGEHGGGG
"

CDS complement(3929324..3930397)
/codon_start=1
/note="gene_3630"
/transl_table=11
/translation="LLNRKPSSKHERDPLRTGIFGLVLVICVVLIAFGYSGLPFWPQG
KPYDAYFTDAGGITPGNSVYVSGLKVGAVSAVSLAGNSAKVTFSVDRSIVVGDQSLAA
IRTDTILGERSIAVSPAGSGKSTTIPLSRTTTPYTLNGVLQDLGRNANDLNRPQFEQA
LNVFTQALHDATPQVRGAVDGLTSLSRALNRRDEALQGLLAHAKSVTSVLSERAEQVN
KLVEDGNQLFAALDARRAALSALISGIDDVAAQISGFVADNRKEFGPALSKLNLVLAN

LNERRDYITEALKRLPTYATTLGEVVGSGPGFNVNVYSVLPGPLVATVFDLVFQPGKL
PDSLADYLRGFIQERWIIRPKSP"
CDS complement(3930387..3931439)
/codon_start=1
/note="gene_3631"
/transl_table=11
/translation="MAGSGVPSHRSMVIKVSVFAVVMLLVAAGLVVVFGDFRFGPTTV
YHATFTDASRLKAGQKVRIAGVPVGSVKAVKLNPDHSIDVAFAIDRSYTLYSSTRAVI
RYENLVGDRFLEITSGPGELRKLPPGGTINVAHTQPALDLDALLGGLRPVLKGFDADK
INTITSAVIELLQGQGGPLANVLADTGAFSAALGARDQLIGEVITNLNAVLATVDAKS
AQFSASVDQLQQLVSGLAKNRDPIAGAISPLASTTTDLTELLRNSRRPLQGILENARP
LATELDNRKAEVNNDIEQLGEDYLRLSALGSYGAFFNIYFCSVTIKINGPAGSDILLP
IGGQPDPSKGRCAFAK"
CDS complement(3931439..3932641)
/codon_start=1
/note="gene_3632"
/transl_table=11
/translation="MSGGGSRRTSVRVAAALLAGLMVGSAVLTYLSYTAAFTSTDTVT
VSSPRAGLVMEKGAKVKYRGIQVGKVTDISYSGNQARLKLAIDSGEMGFIPSNATVRI
AGNTIFGAKSVEFIPPKTPSPKPLSPNAHVAASQVQLEVNTLFQSLIDLLHKIDPLET
NATLSALSEGLRGHGDDLGALLSGLNTLTRQANPKLPALQEDFRKAAVVANVYADAAG
DLNTVFDNLPTINKTIVDQKDNLNDTLLATIGLSNNAYETLAPAEQNFIDAINRLRAP
LKVTSDYSPVFGCLFKGIARGVKEFAPLIGVRKAGLFTSSSFVLGAPSYTYPESLPIV
NASGGPNCRGLPDIPTKQTGGSFYRAPFLVTDNALIPYQPFTELQVDAPSTLQFLFNG
AFAERDDF"
CDS complement(3932661..3933503)
/codon_start=1
/note="gene_3633"
/transl_table=11
/translation="MSYDVTIRFRRFFSRLQRPVDNFGEQALFYGETMRYVPNAITRY
RKETVRLVAEMTLGAGALVMIGGTVGVAAFLTLASGGVIAVQGYSSLGDIGIEALTGF
LSAFLNVRVVAPVIAGIALAATIGAGATAQLGAMRVSEEIDAVECMAVHSVSYLVSTR
LIAGLVAIIPLYSLSVLAAFFAARFTTVFVNGQSAGLYDHYFNTFLIPSDLLWSFMQA
IAMSIAVMLVHTYYGYNASGGSVGVGVAVGQAVRTSLIVVVVITLFISLAVYGASGNF
NLSG"
CDS complement(3933538..3934302)
/codon_start=1
/note="gene_3634"
/transl_table=11
/translation="LIQQLAVPARAVGGFFEMSMDTARAAFRRPFQFREFLDQTWMVA
RVSLVPTLLVSIPFTVLVAFTLNILLREIGAADLSGAGTAFGTITQLGPVVTVLVVAG
AGATAICADLGARTIREEIDAMRVLGIDPIQRLVVPRVLASTLVALLLNGLVCAIGLS
GGYAFSVFLQGVNPGAFINGLTVLTGLRELILAEIKALLFGVMAGLVGCYRGLTVKGG
PKGVGNAVNETVVYAFICLFVINVVMTAIGVRISAQ"
CDS complement(3934528..3935412)
/codon_start=1
/gene="fabG"
/note="gene_3635"
/transl_table=11
/translation="VVTGAAAGLGRAEALGLARLGATVVVNDVASALDASDVVDEIGA
AAADAGAKAVAVAGDISQRATADELLASAVGLGGLDIVVNNAGITRDRMLFNMSDEEW

DAVIAVHLRGHFLLTRNAAAYWRDKAKDAEGGSVFGRLVNTSSEAGLVGPVGQANYAA
AKAGITALTLSAARALGRYGVCANVICPRARTAMTADVFGAAPDVEAGQIDPLSPQHV
VSLVQFLASPAAAEVNGQVFIVYGPQVTLVSPPHMERRFSADGTSWDPTELTATLRDY
FAGRDPEQSFSATDLMRQ"
CDS complement(3935506..3935697)
/codon_start=1
/note="gene_3636"
/transl_table=11
/translation="VRVIVDRDRCEGNAVCLGIAPDIFDLDDEDYAVVKTDPIPVDQE
DLAEQAIAECPRAALSRGE"
CDS 3935912..3937114
/codon_start=1
/note="gene_3637"
/transl_table=11
/translation="MRISYTPQQEELRRELRSYFATLMTPERREALSSVQGEYGVGNV
YRETIAQMGRDGWLALGWPKEYGGQGRSAMDQLIFTDEAAIAGAPVPFLTINSVAPTI
MAYGTDEQKRFFLPRIAAGDLHFSIGYSEPGAGTDLANLRTTAVRDGDDYVVNGQKMW
TSLIQYADYVWLAVRTNPESSGAKKHRGISVLIVPTTAEGFSWTPVHTMAGPDTSATY
YSDVRVPVANRVGEENAGWKLVTNQLNHERVALVSPAPIFGCLREVREWAQNTKDAGG
TRLIDSEWVQLNLARVHAKAEVLKLINWELASSQSGPKDAGPSPADASAAKVFGTELA
TEAYRLLMEVLGTAATLRQNSPGALLRGRVERMHRACLILTFGGGTNEVQRDIIGMVA
LGLPRANR"
CDS 3937139..3938260
/codon_start=1
/note="gene_3638"
/transl_table=11
/translation="MDFTTTEAAQDLGGLVDTIVDAVCTPEHQRELDKLEQRFDRELW
RKLIDAGILSSAAPESLGGDGFGVLEQVAVLVALGHQLAAVPYLESVVLAAGALARFG
SPELQQGWGVSAVSGDRILTVALDGEMGEGPVQAAGTGHGYRLTGTRTQVGYGPVADA
FLVPAETDSGAAVFLVAAGDPGVAVTALATTGLGSVGHLELNGAKVDAARRVGGTDVV
VWLGTLSTLSRTAFQLGVLERGLQMTAEYARTREQFDRPIGSFQAVGQRLADGYIDVK
GLRLTLTQAAWRVAEDSLASRECPQPADIDVATAGFWAAEAGHRVAHTIVHVHGGVGV
DTDHPVHRYFLAAKQTEFALGGATGQLRRIGRELAETPA"
CDS 3938331..3939839
/codon_start=1
/gene="fadD17"
/note="gene_3639"
/transl_table=11
/translation="MTPTHPTVTELLLPLSEIDDRGVYFEDSFTSWRDHIRHGAAIAA
ALRERLDPARPPHVGVLLQNTPFFSATLVAGALSGIVPVGLNPVRRGAALAGDIAKAD
CQLVLTGSGSAEVPADVEHINVDSPEWTDEVAAHRDTEVRFRSADLADLFMLIFTSGT
SGDPKAVKCSHRKVAIAGVTITQRFSLGRDDVCYVSMPLFHSNAVLVGWAVAAACQGS
MALRRKFSASQFLADVRRYGATYANYVGKPLSYVLATPELPDDADNPLRAVYGNEGVP
GDIDRFGRRFGCVVMDGFGSTEGGVAITRTLDTPAGALGPLPGGIQIVDPDTGEPCPT
GVVGELVNTAGPGGFEGYYNDEAAEAERMAGGVYHSGDLAYRDDAGYAYFAGRLGDWM
RVDGENLGTAPIERVLMRYPDATEVAVYPVPDPVVGDQVMAALVLAPGTKFDADKFRA
FLTEQPDLGHKQWPSYVRVSAGLPRTMTFKVIKRQLSAEGVACADPVWPIRR"
CDS 3940010..3944128
/codon_start=1
/gene="PE_PGRS53"
/note="gene_3640"

/transl_table=11
/translation="MSFVLVSPETVAAVATDLKRIGASLAHENASAAASTTAVVSAAA
DEVSTAVAALFSQHAQGYQAAAAQVAAFHSRFVQALTAGAGAYAFAEAANASPLQSAM
GAVSASAQTLLSRPLIGNGANATTPGGNGGDGGWLFGSGGNGAPGAAGQSGGNGGSAG
LWGNGGAGGAGGSGGAAGGNGGNGGWLFGAGGTGGIGGTGAPGAMGGTGGNGGNGALL
IGGGGLGGAGGMGGTGGGTGGTGGNGGNGALLIGAGGVGGAGGIGGQGTGAGGAAGAG
GTGGNGGAGGLFMNGGDGGAGGQGGDGAAGDAAASAGGTGGKGGQGGDGGTGGAGGAG
PVLFGHGGAGGMGGQGGTGGMGGAGGDGTTVIAAGTGGEGGTGGTGGTGGNGADAAAV
VGFGANGDPGFAGGKGGNGGIGGAAVTGGVAGDGGTGGKGGTGGAGGAGNDAGSTGNP
GGKGGDGGIGGAGGAGGAAGTGNGGHAGNTGDGGDGGTGGNGGNGTGGVNGADNTLNP
DTPGGAGEPGGAGGAGGAGGAAGGPGGTGGTGGNGGNGGNGGNGGNAGNNSTNAPVGG
EGGAGGDGGAGGAGGAANGGTAGSQGTGGVGGDGGAGGNGGGGKAGTGNSGNFGVDGE
AGFSGGAGGNGGVGGAAGANGGTGGSGGNGGDGGAGGIGGAGGNGIPGTGTEPAGGTG
AKGGDGGDGGAGGAGGNAGGAGGNGGAGGQGGNAGQGGAGGAGGNAVIPGDGVGKAPH
GGAGGSGGDGGKGGQGGSGGTGGSGAPIGGGAGGTGGSGGHAGKGGAGGIGAQGTTIT
VPGNGGNAGDGGNGGGGGAGGTGGDGATGTPAGNGGDGGNGGNAGDGGNGGSGDFGGN
TTSGASGSGGNGGNAGTAGSGGAGGTGGTGLSGGNGGNGGDGGNGAHGTVGAQFVPAT
SLPTPNGGAGGNGGTGSNGGAPGPAGAPGPTTGGNAGSQGIGGDGGNGGDGGKGGDGA
DAVNVVFMPTEPQAATGTAGSAGDPTGGNGGPGTPGSPMVAPPPPTPITQVQQGGDGG
AGGTGSTNANDGTATGGKGGEGGVGSILGGPGGNGGTGGNASATGTNGVANAGNGGKG
GDGGQFGAGGNGGAGGSVTDGSAGSTAGNGGNGGNATNGTIAGQPAGGNGSAGGKGGD
GGNIAAGATGTAGNGGNGGNGNDGAVNAGTGGSGGNGGNAGGGGANGGDGGAGGAGGA
GGRGGKGIDGGFGGDGGNGGSNNGTGAGGNGGNGGTGGVGSVGAAGGDGGNGGTGGFA
GFGGTAGNGGSGGTGGAGGDGGTGGGGGNGGTGVIAGGGGTGGNGGASGAGGAGGTGG
FAGNGNAGGNGGTGGASEDGDNGNAGSGATGGTGGNGGTGGDGGAAGLGGVA"

CDS 3944419..3950694
/codon_start=1
/note="gene_3641"
/transl_table=11
/translation="MSFVLIAPEFVTAAAGDLTNLGSSISAANASAASATTQVLAAGA
DEVSARIAALFGGFGLEYQAISAQVAAYHQRFVQALSTGAGAYASAEATAAEQIVLGV
INAPTQALLGRPLIGDGANATTPGGAGGAGGLLFGNGGAGAAGAPGQAGGPGGPAGLW
GNGGPGGAGGSGGGTGGAGGAGGWLFGVGGAGGVGGAGGGTGGAGGPGGLIWGGGGAG
GVGGAGGGTGGAGGRAELLFGAGGAGGAGGAGTDGGPGATGGTGGHGGVGGDGGWLAP
GGAGGAGGQGGAGGAGSDGGALGGTGGTGGTGGAGGAGGRGALLLGAGGQGGLGGAGG
QGGTGGAGGDGVLGGVGGTGGKGGVGGVAGLGGAGGAAGQLFSAGGAAGAVGVGGTGG
QGGAGGAGAAGADAPASTGLTGGTGFAGGAGGVGGQGGNAIAGGINGSGGAGGTGGQG
GAGGMGGSGADNASGIGADGGAGGTGGNAGAGGAGGAAGTGGTGGVVGAAGKAGIGGT
GGQGGACGAGSSGTDATATGATGGTGFSLGACGAGGAGGNTGVGGTNGSGGQDGTTGA
GGACGAGGVGADNPTGIGGAGGTGGAGGTGGTGGAAGAGGAGGAVGTGGTGGVVGDVG
NAGIGGTGGKGGAGGAGGAGADATATGATGGTGFAGGAGGAGGQGGSSGAGGTNGSGG
AGGTGGQGGAGGAGGAGADNPTGIGGAGGTGGTGGAAGAGGAGGAIGTGGTGGAVGSV
GNAGIGGTGGTGGVGGAGGAGAAAAAGSSATGGAGFAGGAGGEGGAGGNSGVGGTNGS
GGAGGAGGKGGTGGAGGSGADNPTGAGFAGGAGGTGGAAGAGGAGGATGTGGTGGVVG
ATGSAGIGGAGGRGGDGGDGASGLGLGLSGFDGGQGGQGGDGGSAGAGGINGAGGAGG
DGGDGGDGATGAAGLGDNGGVGGDGGAGGAAGNGGNAGVGLTAKAGDGGAAGNGGNGG
AGGAGGAGDNNFNGGQGGAGGQGGQGGLGGASTTSINANGGAGGNGGTGGKGGAGGAG
TLGVGGSGGTGGDGGDAGAGGGGGFGGAAGKAGGGGNGGVGGDGGDGATGAAGLGDNG
GVGGDGGAGGAAGNGGNAGVGLTAKAGDGGAAGNGGNGGAGGAGGAGDNNFNGGQGGA
GGQGGQGGLGGASTTSINANGGAGGNGGTGGKGGAGGAGTLGVGGSGGTGGDGGDAGA
GGGGGFGGAAGKAGGGGNGGVGGDGGEGASGLGLDLSGFDGGQGGQGGAGGNAGAGGI

NGAGGAGGTGGAGGDGAPATLIGGPDGGDGGQGGIGGDGGNAGFGAGVPGDGGIGGTG
GAGGAGGAGADGDPSIDGGQGGAGGHGGQGGKGGLNSTGLASAASGDGGNGGAGGAGG
NGGDGDGFIGGSGGTGGTGGDAGVGGLANTGGTAGNAGIGGAGGRGGDGGAGDSGALS
QDGNGFAGGQGGQGGAGGNAGAGGINGAGGTGGTGGAGGDGAPATLIGGPDGGDGGQG
GIGGDGGNAGFGAGVPGDGGIGGTGGAGGAGGAGADGDPSIDGGQGGAGGHGGQGGKG
GLNSTGLASAASGDGGNGGAGGAGGNGGDGDGFIGGSGGTGGTGGDAGVGGLANTGGT
AGNAGIGGAGGRGGDGGAGDSGALSQDGNGFAGGQGGQGGAGGNAGAGGINGAGGTGG
TGGAGGDGQNGTTGVASEGGAGGQGGDGGDGGQGGIGGDGGNAGFGAGVPGDGGIGGT
GGAGGAGGAGADGDPSIDGGQGGAGGHGGQGGKGGLNSTGLASAASGDGGNGGAGGAG
GNGGDGDGFIGGSGGTGGTGGDAGVGGLANTGGTAGNAGIGGAGGRGGDGGAGDSGAL
SQDGNGFAGGQGGQGGAGGNAGAGGINGAGGTGGTGGAGGDGAPATLIGGPDGGDGGD
GGQGGGAGFGSGVAGAAGAGGNGGKGGDGGTGGTGGTNFAGGQGGAGGRGGAGGNGAN
GVGDNAAGGDGGNGGAGGLGGGGGTGGTNGNGGLGGGGGNGGAGGAGGTPTGSGTEGT
GGDGGDAGAGGNGGSATGVGNGGNGGDGGNGGDGGNGAPGGFGGGAGAGGLGGSGAGG
GTDGDDGNGGSPGTDGS"

variation 3946503..3946580
/note="supplemented from BCG moreau"

variation 3946793..3946837
/note="supplemented from BCG moreau"

CDS complement(3950861..3952408)
/codon_start=1
/note="gene_3642"
/transl_table=11
/translation="VNGAQALINTLVDGGVDVCFANPGTSEMHFVAALDAVPRMRGML
TLFEGVATGAADGYARIAGRPAAVLLHLGPGLGNGLANLHNARRARVPMVVVVGDHAT
YHKKYDAPLESDIDAVAGTVSGWVRRTEAAADVGADAEAAIAASRSGSQIATLILPAD
VCWSDGAHAAAGVPAQAAAAPVDVGPVAGVLRSGEPAMMLIGGDATRGPGLTAAARIV
QATGARWLCETFPTCLERGAGIPAVERLAYFAEGAAAQLDGVKHLVLAGARSPVSFFA
YPGMPSDLVPAGCEVHVLAEPGGAADALAALADEVAPGTVAPVAGASRPQLPTGDLTS
VSAADVVGALLPERAIVVDESNTCGVLLPQATAGAPAHDWLTLTGGAIGYGIPAAVGA
AVAAPDRPVLCLESDGSAMYTISGLWSQARENLDVTTVIYNNGAYDILRIELQRVGAG
SDPGPKALDLLDISRPTMDFVKIAEGMGVPARRVTTCEEFADALRAAFAEPGPHLIDV
VVPSLVG"

CDS complement(3952405..3953241)
/codon_start=1
/note="gene_3643"
/transl_table=11
/translation="MTIDVWMQHPTQRFLHGDMFASLRRWTGGSIPETDIPIEATVSS
MDAGGVTLGLLSAWRGPNGQDLISNDAVAEWVRLYPNRFAGLAAVDLDRPMAAVRELR
RRVGEGFVGLRVVPWLWGAPPTDRRYYPLFAECVQSAVPFCTQVGHTGPLRPSETGRP
IPYIDQVALDFPELVIVCGHVGYPWTEEMVAVARKHENVYIDTSAYTIKRLPGKLVRF
MKTDTGQRKVLFGTNYPMIAHTHALTGLDELGLSDEARRDFLHGNAVRVFKLDPRGKV
QT"

CDS 3953601..3959417
/codon_start=1
/note="gene_3644"
/transl_table=11
/translation="VSFVLISPEVVSAAAGDLANVGSTISAANKAAAAATTQVLAAGA
DEVSARIAALFGMYGLEYQAISAQVAAYHQQFVQTLRTGAASYMLAEATNVEQNLLNL
INAPTQTLLGRPLIGDGANATTPGGAGGDGGLLFGSGGNGAPGAPGQAGGAGGSAGLL
GNGGSGGAGGTGAPGGNGGNAGWLYGRGGVGGAGGIGGGTGGAGGHAWLFGHGGTGGI

GGGPGGNGGWLLGNGGHGGAGGIGGGSGGAGGNGGWLLGNGGIGGAGGTGGGAGGTGG
NAAWLLGGGGTGGAGGIGGGNGGHGGNGGWLLGNGGNGGLGGDGDGGTGGGHGGNGGN
PGWLLGTAGGGGNGGAGSTGTAGGGSGGTGGDGGTGGRGGLLMGAGAGGHGGTGGAGG
AGVDGGGAGGAGGAGGNGGAGGQAALLFGRGGTGGAGGYGGDGGGGGDGFDGTMAGLG
GTGGSGGTGGDGGAPGNGGAGGAGQLLSHSGVAGASGKGGAGGTGGNGGAGSAGADAP
AGSGAMGSTGFAGGAGGDGGNGGGSGASQGNGGNGGNGGTGGKGGTGGAGMNSLDPLL
AAQDGGQGGTGGTGGNAGAGGTGFTQGADGNAGNGGDGGVGGNGGNGADNTTTAAAGT
TGGAGGAGGAGGTGGTGGAAGTGTGGQQGNGGNGGNGGTGGKGGTGGAGMNSLDPLLA
AQDGGQGGTGGTGGNAGAGGTGFTQGADGNAGNGGDGGVGGNGGNGADNTTTAAAGTT
GGDGGAGGAGGTGGTGGAAGTGTGGQQGNGGNGGNGGTGGKGGTGGAGMNSLDPLLAA
QDGGQGGTGGTGGNAGAGGTGFTQGADGNAGNGGDGGVGGNGGNGADNTTTAAAGTTG
GDGGAGGAGGTGGTGGAAGTGTGGQQGNGGNGGNGGTGGKGGTGGDGALAGSSGGAGG
KGGNGGDAGKAGTGSAPGTAGTGGDGGKGGNGGIGAAGTTGPVGTGASGGTGGSGGAG
GTGGDGGAANGGTAGAGGAGGNGGKGGDGGAGVTSSTAGNSGGAGGSGGKGGDAGAGG
AGATPGANGIAGNGGDGGDGAAGAVGISGATGAGDGGHGGTGGAGGNGGTGGAGGSGI
DGVGGGTGGTGGNGGNGAIGGAGGDAGGSGNSGGNGGTGGKGGNAGAGGAAGSNGGTV
GANGTGGDGGNGGAAGAATAGSNGGAGTGSAGGNGGTGGRGGSGGAGGDGIGGVGGGK
GGNGADGEVGGAGGAGGSGPNTSPGGNGGQGGQGGSGGAGGAAGAGGAGGGANGTAGN
GGQGGAGGTGGAGAASSATNGGSGGAGGTGGAGGTGGAGGDGVGGAGGGNGGHGGDAG
DGGNGANGNNRSSGSFLAAGGTGGAAGDGGQGGQGGAGGGAGGQGGAGGAGGTGGNGG
NITGGTAGTAGAAGNGGAAGKGGAGGQGGTGGGTGGQGGAGGDGGAGGTGGDRTVGGG
TVPAGSGGQGGNAGGGGAGGQGGADGGSGGDGGDAGTGGNGGNGGNRNSGNGTGGAGG
NGGGGANGGAGGAGGSGGGTGGNGGAGGDAGDAGNGGNGNGTGNGGNGGNGGIAGMGG
NGGAGTGSGNGGNGGSGGNGGNAGMGGNSGTGSGDGGAGGNGGAAGTGGTGGDGGLTG
TGGTGGSGGTGGDGGNGGNGGNGADNTANMTAQAGGDGGNGGDGGFGGGAGAGGGGLT
AGANGTGGQGGAGGDGGNGAIGGHGPLTDDPGGNGGTGGNGGTGGTGGAGIGSLGGGT
GGDGGNGGTGGNGGTGGEGGEVGGAGGTGGAAGNGGDGGTGGTGGGDGGAGGTGGTGG
TGGLGDPRVGGSGGDGGTGGSGGAAGNGGNGGNAGAGGNGNGGTGGAGGIGGTGGNGG
DAEPGVPPGAGGAGGAGTTGGKGGTGGNGSGTGSGGTGGDGGTGGGGGNGGTGWNGGK
GDTGSGGGAGDGGKAPAGGTGGAGGDGGAGGKGGSGGV"

variation 3955397..3955741
/note="supplemented from AL2122/97"

variation 3958640..3958777
/note="supplemented from AL2122/97"

variation 3958922..3959167
/note="supplemented from AL2122/97"

CDS complement(3959546..3960070)
/codon_start=1
/note="gene_3645"
/transl_table=11
/translation="VRPARTLAKKGNIPVGYYKDEKKTAETFRTINGVRYAIPGDYAQ
VEEDGTVTMLGRGSVSINSGGEKVYPEEVEAALKGHPDVFDALVVGVPDPRYGQQVAA
VVQARPGCRPSLAELDSFVRSEIAGYKVPRSLWFVDEVKRSPAGKPDYRWAKEQTEAR
PADDVHAGHVTSGS"

CDS 3960248..3962953
/codon_start=1
/note="gene_3646"
/transl_table=11
/translation="MSFVLIAPEFVTAAAGDLTNLGSSISAANASAASATTQVLAAGA
DEVSARIAALFGGFGLEYQAISAQVAAYHQRFVQASSTGAGAYASAEATAAEQIVLGV
INAPTQALLGRPLIGDGANATTPGGAGGAGGSGGGTGGAGGAGGWLFGVGGAGGVGGA

GGGTGGAGGPGGLIWGGGGAGGVGGAGGGTGGAGGRAELLFGAGGAGGAGGAGTDGGP
GATGGTGGHGGVGGDGGWLAPGGAGGAGGQGGAGGAGSDGGALGGTGGTGGTGGAGGA
GGRGALLLGAGGQGGLGGAGGQGGMGGAGGAGADNPTGIGGTGGDGGTGGSAGEGGAG
GAAGQLFSASGAAGNAGVGGAGGQGGDGGAGGAGADADQPGATGGTGFAGGAGGAGGA
GGSSGAGGTNGSGGAGGTGGQGGMGGAGGAGADNPTGIGGTGGDGGTGGAAGAGGAGG
AAGTGGTGGMIGTTGNAGVGGAGGQGGDGGAGGAGADADQPGATGGTGFAGGAGGAGG
AGGSSGAGGTNGSGGAGGTGGQGGAGGAGGAGADNPTGIGGTGGDGGTGGAAGAGGAG
GAAGTGGTGGMIGTTGNAGVGGAGGQGGDGGAGGAGGAGGSSGAGGTNGSGGAGGTGG
QGGMGGAGGAGADNPTGIGGTGGDGGTGGAAGAGGAGGAAGTGGTGGMIGTTGNAGVG
GAGGQGGDGGAGGAGADADQPGATGGTGFAGGAGGAGGAGGSSGAGGTNGSGGAGGTG
GQGGAGGAGGAGADNPTGIGGTGGDGGTGGAAGAGGAGGAAGTGGTGGMIGTTGNAGV
GGAGGQGGDGGAGGAGGAGGSSGAGGTNGSGGAGGTGGQGGAGGAGGAGADNPTGIGG
TGGDGGTGGSAGARRGRRSGRHRRHRRHDRHHRQRRCRRGRRPRR"

variation 3961492..3962127
/note="supplemented from AL2122/97"

CDS 3962898..3964112
/codon_start=1
/gene="emb-9"
/note="gene_3647"
/transl_table=11
/translation="MIGTTGNAGVGGAGGQGGDGGAGGAGADADQPGATGGTGFAGGA
GGAGGAGGAGGSSGAGGTNGSGGAGGTGGQGGAGGAGGAGADNPTGIGGTGGDGGTGG
AAGAGGAGGAAGTGGTGGMIGTTGNAGVGGAGGQGGDGGAGGAGADADQPGATGGTGF
AGGAGGAGGAGGSSGAGGTNGSGGAGGTGGQGGAGGAGISFSNGSNGGTGGTGGVGGT
GGDGGNAGTGAGDPGKGGTGGTGGSGGAGGSGGANFNGGTGGTGGTGGTGGKGGMGGI
AGDGGPGGDGGNAGVGGKGGTNGNGGSGGTGGTGGPGGSGGAPTGSGTGGKGGAGGDG
GDGADGGAATGVGDGGDGGNGGNGGNGGTGVGSPGGLGGAGGTGGLGGAGAGGGADGD
DGDDGQPGNNGS"

CDS complement(3964673..3966319)
/codon_start=1
/gene="fadD19"
/note="gene_3648"
/transl_table=11
/translation="VAVALNIADLAEHAIDAVPDRVAVICGDEQLTYAQLEDKANRLA
HHLIDQGVQKDDKVGLYCRNRIEIVIAMLGIVKAGAILVNVNFRYVEGELRYLFDNSD
MVALVHERRYADRVANVLPDTPHVRTILVVEDGSDQDYRRYGGVEFYSAIAAGSPERD
FGERSADAIYLLYTGGTTGFPKGVMWRHEDIYRVLFGGTDFATGEFVKDEYDLAKAAA
ANPPMIRYPIPPMIHGATQSATWMALFSGQTTVLAPEFNADEVWRTIHKHKVNLLFFT
GDAMARPLVDALVKGNDYDLSSLFLLASTAALFSPSIKEKLLELLPNRVITDSIGSSE
TGFGGTSVVAAGQAHGGGPRVRIDHRTVVLDDDGNEVKPGSGMRGVIAKKGNIPVGYY
KDEKKTAETFRTINGVRYAIPGDYAQVEEDGTVTMLGRGSVSINSGGEKVYPEEVEAA
LKGHPDVFDALVVGVPDPRYGQQVAAVVQARPGCRPSLAELDSFVRSEIAGYKVPRSL
WFVDEVKRSPAGKPDYRWAKEQTEARPADDVHAGHVTSGG"

CDS 3966384..3967184
/codon_start=1
/note="gene_3649"
/transl_table=11
/translation="VATVESGPDALVERRGHTLIVTMNRPAARNALSTEMMRIMVQAW
DRVDNDPDIRCCILTGAGGYFCAGMDLKAATQKPPGDSFKDGSYDPSRIDALLKGRRL
TKPLIAAVEGPAIAGGTEILQGTDIRVAGESAKFGISEAKWSLYPMGGSAVRLVRQIP
YTLACDLLLTGRHITAAEAKEMGLIGHVVPDGQALTKALELADAISANGPLAVQAILR

SIRETECMPENEAFKIDTQIGIKVFLSDDAKEGPRAFAEKRAPNFQNR"
CDS 3967280..3968119
/codon_start=1
/note="gene_3650"
/transl_table=11
/translation="VIEPFLGSEAIASGALTRHRLRSAYATIHPDVYVSPGADLTAWS
RAQAAWLWSRRRGVIAGQSAAAMHGAKWVDARQAAELLYDHRRPPAGIHTWSDRVADD
EIQPISGMNTTTPARTALDLARRYPVGKAVAAIDALARATDLKLADVEMLAERYRGSR
GIRNARIALDLVDPGAESPRETWLRLLLIRAGFPRPQTQIPVYDEYGQLVAVIDMGWA
GIKVGVDYEGDHHRTDRRTFNKDIKRAEALTELGWTDVRVTVEDTEGGIIWRVSAAWQ
RRT"
CDS complement(3968174..3968761)
/codon_start=1
/note="gene_3651"
/transl_table=11
/translation="VLVSSEVDGERLSDDELVMETLLILIGGDETTRHTLSGGTEQLL
RNRDQWDLLQRDPSLLPGAIEEMLRWTAPVKNMCRVLTADTEFHGTALCAGEKMMLLF
ESANFDEAVFCEPEKFDVQRNPNSHLAFGFGTHFCLGNQLARLELSLMTERVLRRLPD
LRLVADDSVLPLRPANFVSGLESMPVVFTPSPPLG"
CDS complement(3968752..3969369)
/codon_start=1
/note="gene_3652"
/transl_table=11
/translation="MTEAPDVDLADGNFYASREARAAYRWMRANQPVFRDRNGLAAAS
TYQAVIDAERQPELFSNAGGIRPDQPALPMMIDMDDPAHLLRRKLVNAGFTRKRVKDK
EASIAALCDTLIDAVCERGECDFVRDLAAPLPMAVIGDMLGVRPEQRDMFLRWSDDLV
TFLSSHVSQEDFQITMDAFAAYNDFTRATIAARRADPPTTWSACW"
CDS 3969398..3970108
/codon_start=1
/note="gene_3653"
/transl_table=11
/translation="MPVSQHTIAGTVLTMPVRIRTANLHSAMFSVPADPAQRLIDYSG
LRVCEYLPGKAIVMQMLVRYVDGDLGRYHEYGTAIMVNPPGTQRRGPRALTRAAAFIH
HLPVDQVFTLEAGRTIWGFPKIMADFNVTDGRRFGFDVSADGRLIAGIEFSTGLPVPT
LGWQMLKTYSHHDGVTREIPWEMKVSGLRARLGGARLRLGDHPYAKELASLGLPKRAL
LSQSAANVEMTFGDGHPI"
CDS complement(3970173..3971204)
/codon_start=1
/note="gene_3654"
/transl_table=11
/translation="MKLGLQLGYWGAQPPQNHAELVAAAEDAGFDTVFTAEAWGSDAY
TPLAWWGSSTQRVRLGTSVIQLSARTPTACAMAALTLDHLSGGRHILGLGVSGPQVVE
GWYGQRFPKPLARTREYIDIVRQVWARESPVTSAGPHYRLPLTGEGTTGLGKALKPIT
HPLRADIPIMLGAEGPKNVALAAEICDGWLPIFYSPRMAGMYNEWLDEGFARPGARRS
REDFEICATAQVVITDDRAAAFAGIKPFLALYMGGMGAEETNFHADVYRRMGYTQVVD
EVTKLFRSGRKDEAAEIIPDELVDDAVIVGDIDHVRKQMAVWEAAGVTMMVVTAGSAE
QVRDLAALV"
CDS 3971276..3972280
/codon_start=1
/note="gene_3655"
/transl_table=11

```
                     /translation="VTASSSGPALIDHSEPPLSAPLTLSFDYTRSVGPTLSRFFTALR
                     ARRIVGVRGSDGRVHVPPVEYDPVTYEPLSEMVPVSSVGTVASWTWQPEPLAGQPLDR
                     PFAWALIKLDGADTLLMHAVDVGTAGPSAIHTGARVHAHWADQPVGAITDIACFALGE
                     TAEPVAAHKTEDARDPVTMIVTPIQLEIQHTASHEESAYLRAIAQGKLVGARTGKTGK
                     VYFPPHGADPATGKPTSEFVELPDKGTVTTFAIVNIPFLGQRIKPPYVAAYVLLDGAD
                     IPFLHLVSDVDAHQVRMGMRVEAVWKPRERWGLGIDNIEYFRPTGEPDADYDTYKHHL
                     "
CDS                  3972296..3973360
                     /codon_start=1
                     /note="gene_3656"
                     /transl_table=11
                     /translation="MSVRDIAVVGFAHAPHVRRTDGTTNGVEMLMPCFAQLYDELGIT
                     KADIGFWCSGSSDYLAGRAFSFISAIDSIGAVPPINESHVEMDAAWALYEAYIKLLTG
                     EVDTALVYGFGKSSAGTLRRVLSRQTDPYTVAPLWPDSVSMAGLQARLGLDSGKWTHE
                     QMARVAFDSFTNARRVDSVEPPITVGELLARPFFADPLRRHDIAPITDGAAAVVLAAD
                     NRARELRENPAWITGIEHRIESPALGARDITESPSTKLAAKIATGGHTGDIDVAEIHG
                     PFTHQHLIVAEAIRIPGKTKVNPSGGPLAANPMFAAGLERIGFAAQHIWDGSARRVLA
                     HATSGPALQQNLVAVMEGRG"
CDS                  3973377..3974561
                     /codon_start=1
                     /note="gene_3657"
                     /transl_table=11
                     /translation="MAGKLAAVLGTGQTKYVAKRQDVSMNGLVREAIDRALADSGSTF
                     DDIDAVVVGKAPDFFEGVMMPELFMADAMGATGKPLIRVHTAGSVGGSTGVVAASLVQ
                     SGKYRRVLALAWEKQSESNAMWALSIPVPFTKPVGAGAGGYFAPHVRAYIRRSGAPAH
                     IGAMVAVKDRLNGSRNPLAHLQQPDITLEKVMASQMLWDPIRFDETCPSSDGACAVVV
                     GDEEIADARLAQGHPVAWIHGTALRTEPLAFAGRDQVNPQAGRDAAAALWKAAGITSP
                     IDEIDAAEIYVPFSWFEPMWLENLGFAREGEGWKLTEAGETAIGGRLPVNPSGGVLSA
                     NPIGASGLIRFAEAAIQVMGKAEARQVPGARKALGHAYGGGSQYFSMWVVGCEKPKQA
                     AA"
CDS                  3974900..3975634
                     /codon_start=1
                     /note="gene_3658"
                     /transl_table=11
                     /translation="VPIDLWNPDGVTVDLADAVYVADSGHKRLLKLPAGSNTPTTLPF
                     TDTIGPGGVAVNSNRDVYVIDEDSHHVLKLAAGIEPPVELPFGSLGDAHGLAVDRSDS
                     VYVVDYDNAKVLKLPPGADTPTELPFVGLDHPYDVAVDGAGTVYVTDSGHNRVVALTA
                     GSATPVHLPFADLSFPAGVTVDRDDSVYVADLNNNRVLKLAAGSNAQSQLPFTGLFSP
                     TDVAVDNDGAVYVIDFYNRMLKLPTA"
CDS                  complement(3975648..3976172)
                     /codon_start=1
                     /note="gene_3659"
                     /transl_table=11
                     /translation="MPLFSFEGRSPRIDPTAFVAPTATLIGDVTIEAGASVWFNAVLR
                     GDYAPVVVREGANVQDGAVLHAPPGIPVDIGPGATVAHLCVIHGVHVGSEALIANHAT
                     VLDGAVIGARCMIAAGALVVAGTQIPAGMLVTGAPAKVKGPIEGTGAEMWVNVNPQAY
                     RDLAARHLAGLEPM"
CDS                  3976287..3977447
                     /codon_start=1
                     /gene="kshA"
                     /note="gene_3660"
```

/transl_table=11
/translation="VSTDTSGVGVREIDAGALPTRYARGWHCLGVAKDYLEGKPHGVE
AFGTKLVVFADSHGDLKVLDGYCRHMGGDLSEGTVKGDEVACPFHDWRWGGDGRCKLV
PYARRTPRMARTRSWTTDVRSGLLFVWHDHEGNPPDPAVRIPEIPEAASDEWTDWRWN
RILIEGSNCRDIIDNVTDMAHFFYIHFGLPTYFKNVFEGHIASQYLHNVGRPDVDDLG
TSYGEAHLDSEASYFGPSFMINWLHNRYGNYKSESILINCHYPVTQNSFVLQWGVIVE
KPKGMSEEMTDKLSRVFTEGVSKGFLQDVEIWKHKTRIDNPLLVEEDGAVYQLRRWYE
QFYVDVADIKPEMVERFEIEVDTKRANEFWNAEVEKNLKSREVSDDVPAEQH"
CDS 3977453..3977902
/codon_start=1
/note="gene_3661"
/transl_table=11
/translation="MPDDQPAVPDVDRLARSMLLLHGDHHDHNDSPEQHRTCGSWSKS
RDFADDPQRAAAVREASRAERDRYLTSGLQPVDCRFCHVTVTVKRLGPGHTAVQWNTE
ASRRCAYFTELRARGGDSARTRSCPRLTDSIEHAVAEGYLEHHDPNR"
CDS complement(3978327..3979040)
/codon_start=1
/note="gene_3662"
/transl_table=11
/translation="MMLDRLRQGGYWLVRGKINLIDRAFTSCRIESFADLGAVWGVEG
AYTFRALDKYPVKEAVLVDGRITPTVAARANSYPQLRVIEGNFGDQEIADKVGNVDAL
FLFDVLLHQVSPDWDTILDMYAKNVRCLLIYNQQWIGSTTTVRLLDLGEKHYFRNVPH
SKLNKAYRDLFQKLDKKHPDHDKPWRDIPDIWQWGITDADLESKASELGFKLLYKEDC
RGFGWLPNIQNRAFLFARQ"
CDS 3979271..3979468
/codon_start=1
/note="gene_3663"
/transl_table=11
/translation="MAFREVSMNKIREVLRVWLGVAGLPAPGCRTIAAHCGMDRKTVR
RYVEARAGTRSAPRRRCQRYR"
CDS complement(3979732..3980886)
/codon_start=1
/note="gene_3664"
/transl_table=11
/translation="MTRRPDRKDVATVDELHASATKLVGLDDFGTDDDNYREALGVLL
DAYQGEAGLTVLGSKMNRFFLRGALVARLLSQSAWKQYPEHVDVAIKRPIFVTGLVRT
GTTALHRLLGADPAHQGLHMWLAEYPQPRPPRETWESNPLYRQLDAQFTQHHAENPGY
TGLHFMAAYELEECWQLLRQSLHSVSYEALAHVPSYADWLSRQDWTPSYCRHRRNLQL
IGLNDAEKRWVLKNPSHLFALDALMATYPDALVVQTHRPVETIMASMCSLAQHTTEGW
STKFVGAQIGADAMDTWSRGLERFNAARAKYDSAQFYDVDYHDLIADPLGTVADIYRH
FGLTLSDEARQAMTTVHAESQSGARAPKHSYSLADYGLTVEMVKERFAGL"
CDS complement(3980886..3981668)
/codon_start=1
/note="gene_3665"
/transl_table=11
/translation="MTGMLKRKVIVVSGVGPGLGTTLAHRCARDGADLVLAARSAERL
DDVAKQIIDTGRRAVAVRTDITDDDDVSNLVQATLAAYGKADVLINNAFRVPSMKPLA
GTTFEHIRDAIELSALGTLRLIQAFTPALAQSHGAIVNVNSMVIRHSQPKYGTYKMAK
SVLLAMSHSLATELGEQGIRVNSVAPGYIWGDTLKSYFDHQAGKYGTTVDQIYQATAA
NSDLKRLPTEDEVASAILFLASDLASGITGQTLDVNCGEYHT"
CDS complement(3981665..3982792)

/codon_start=1
/note="gene_3666"
/transl_table=11
/translation="VYSDPLREAIAEAEQLVAAAPHIETEADLLEGLQYLAGCIAGCM
HLAFDYERDHPFLQSGTGPFTKMGLDNPDTLYFGTRLQANRDYVVSGRRGTTTDLSFQ
LLGGEYTDYNVPASQAAFDDRELDIAADGSFEWRLRPSAPGQLVIREVYGDWSQQRGT
LAIARLDTVGTAPPPLTRELMEKRYATAGSQLVNRVKTWLQFPQWFYLNIPVNTMVAP
RLTPGGLATQYSSAGHFELRPGQALVITVPVSDAPYLGFQLGSMWYISLDYINHQTSL
NASQAQADPDGKVRIVVAEQNPGVTNWVETLGHRRGFLQFRWQRVSRELTEADGPTVE
LVDFDAIPAALPHYQHNKISEDDWRARIALRQRQIATRMLG"
CDS 3983191..3984411
/codon_start=1
/note="gene_3667"
/transl_table=11
/translation="MFMDFAMLPPEVNSTRMYSGPGAGSLWAAAAAWDQVSAELQSAA
ETYRSVIASLTGWQWLGPSSVRMGAAVTPYVEWLTTTAAQARQTATQITAAATGFEQA
FAMTVPPPAIMANRAQVLSLIATNFFGQNTAAIAALETQYAEMWEQDATAMYDYAATS
AAARTLTPFTSPQQDTNSAGLPAQSAEVSRATANAGAADGNWLGNLLEEIGILLLPIA
PELTPFFLEAGEIVNAIPFPSIVGDEFCLLDGLLAWYATIGSINNINSMGTGIIGAEK
NLGILPELGSAAAAAAPPPADIAPAFLAPLTSMAKSLSDGALRGPGEVSAAMRGAGTI
GQMSVPPAWKAPAVTTVRAFDATPMTTLPGGDAPAAGVPGLPGMPASGAGRAGVVPRY
GVRLTVMTRPLSGG"
CDS complement(3984553..3986301)
/codon_start=1
/note="gene_3668"
/transl_table=11
/translation="MNYAVLPPELNSLRMFTGAGSAPMLAAAVAWDGLAAELGSAASS
FGSVTSDLASQAWQGPAAAAMAAAAAPYAGWLSAAAARAAGAAAQAKAVASAFEAARA
ATVHPLLVAANRNAFAQLVMSNWFGLNAPLIAAVEGAYEQMWAADVAAMVGYHSGASA
AAEQLVPFQQALQQLPNLGIGNIGNANLGGGNTGDLNTGNGNIGNTNLGSGNRGDANL
GSGNIGNSNVGGGNVGNGNFGSGNGRAGLPGSGNVGNGNLGNSNLGSGNTGNSNVGFG
NTGNNNVGTGNAGSGNIGAGNTGSSNWGFGNNGIGNIGFGNTGNGNIGFGLTGNNQVG
IGGLNSGSGNIGLFNSGTNNVGFFNSGNGNLGIGNSSDANVGIGNSGATVGPFVAGHN
TGFGNSGSLNTGMGNAGGVNTGFGNGGAINLGFGNSGQLNAGSFNAGSINTGNFNSGQ
GNTGDFNAGVRNTGWSNSGLTNTGAFNAGSLNTGFGAVGTGSGPNSGFGNAGTNNSGF
FNTGVGSSGFQNGGSNNSGLQNAVGTVIAAGFGNTGAQTVGIANSGVLNSGFFNSGVH
NSGGFNSENQRSGFGN"
CDS complement(3986400..3987440)
/codon_start=1
/note="gene_3669"
/transl_table=11
/translation="MTDMWDVRITDTSLRDGSHHKRHQFTKDEVGAIVAALDAAGVPV
IEVTHGDGLGGSSFNYGFSKTPEQELIKLAAATAKEARIAFLMLPGVGTKDDIKEARD
NGGSICRIATHCTEADVSIQHFGLARELGLETVGFLMMAHTIAPEKLAAQARIMADAG
CQCVYVVDSAGALVLDGVADRVSALVAELGEDAQVGFHGHENLGLGVANSVAAVRAGA
KQIDGSCRRFGAGAGNAPVEALIGVFDKIGVKTGIDFFDIADAAEDVVRPAMPAECLL
DRNALIMGYSGVYSSFLKHAVRQAERYGVPASALLHRAGQRKLIGGQEDQLIDIALEI
KRELDSGAAVTH"
CDS complement(3987437..3988348)
/codon_start=1
/note="gene_3670"

/transl_table=11
/translation="MPSKAKVAIVGSGNISTDLLYKLLRSEWLEPRWMVGIDPESDGL
ARAAKLGLETTHEGVDWLLAQPDKPDLVFEATSAYVHRDAAPKYAEAGIRAIDLTPAA
VGPAVIPPANLREHLDAPNVNMITCGGQATIPIVYAVSRIVEVPYAEIVASVASVSAG
PGTRANIDEFTKTTARGVQTIGGAARGKAIIILNPADPPMIMRDTIFCAIPTDADREA
IAASIHDVVKEVQTYVPGYRLLNEPQFDEPSINSGGQALVTTFVEVEGAGDYLPPYAG
NLDIMTAAATKVGEEIAKETLVVGGAR"
CDS complement(3988359..3989144)
/codon_start=1
/note="gene_3671"
/transl_table=11
/translation="MLRDATRDELAADLAQAERSRDPIGQLTAAHPEIDVVDAYEIQL
INIRQRVAEGARVVGHKVGLSSPIMQQMMGVDEPDYGHLLDDMQVFEDTPVQASRYLS
PRVEVEVGFILAADLPGAGCTEDDVLAATEALVPAIELIDTRIKDWQIKICDTIADNA
SAAGFVLGAARVPPADLDVRAIDAKLTRNGEVVAEGRSDAVLGNPATAVAWLAGKVES
FGVRLRKGDIVLPGSCTFAVEARAGDEFVADFTGLGLVRLSFE"
CDS 3989208..3990908
/codon_start=1
/note="gene_3672"
/transl_table=11
/translation="VFYMTVQEFDVVVVGSGAAGMVAALVAAHRGLSTVVVEKAPHYG
GSTARSGGGVWIPNNEVLKRRGVRDTPEAARTYLHGIVGEIVEPERIDAYLDRGPEML
SFVLKHTPLKMCWVPGYSDYYPEAPGGRPGGRSIEPKPFNARKLGADMAGLEPAYGKV
PLNVVVMQQDYVRLNQLKRHPRGVLRSMKVGARTMWAKATGKNLVGMGRALIGPLRIG
LQRAGVPVELNTAFTDLFVENGVVSGVYVRDSHEAESAEPQLIRARRGVILACGGFEH
NEQMRIKYQRAPITTEWTVGASANTGDGILAAEKLGAALDLMDDAWWGPTVPLVGKPW
FALSERNSPGSIIVNMSGKRFMNESMPYVEACHHMYGGEHGQGPGPGENIPAWLVFDQ
RYRDRYIFAGLQPGQRIPSRWLDSGVIVQADTLAELAGKAGLPADELTATVQRFNAFA
RSGVDEDYHRGESAYDRYYGDPSNKPNPNLGEVGHPPYYGAKMVPGDLGTKGGIRTDV
NGRALRDDGSIIDGLYAAGNVSAPVMGHTYPGPGGTIGPAMTFGYLAALHIADQAGKR
"
CDS 3990910..3991770
/codon_start=1
/note="gene_3673"
/transl_table=11
/translation="MPIDLDVALGAQLPPVEFSWTSTDVQLYQLGLGAGSDPMNPREL
SYLADDTPQVLPTFGNVAATFHLTTPPTVQFPGIDIELSKVLHASERVEVPAPLPPSG
SARAVTRFTDIWDKGKAAVICSETTATTPDGLLLWTQKRSIYARGEGGFGGKRGPSGS
DVAPERAPDLQVAMPILPQQALLYRLCGDRNPLHSDPEFAAAAGFPRPILHGLCTYGM
TCKAIVDALLDSDATAVAGYGARFAGVAYPGETLTVNVWKDGRRLVASVVAPTRDNAV
VLSGVELVPA"
CDS 3991952..3993346
/codon_start=1
/note="gene_3674"
/transl_table=11
/translation="MYAGGGPGSLSAAAAAWDELAAELWLAAASFESVCSGLADRWWQ
GPSSRMMAAQAARHTGWLAAAATQAEGAASQAQTMALAYEAAFAATVHPALVAANRAL
VAWLAGSNVFGQNTPAIAAAEAIYEQMWAQDVVAMLNYHAVASAVGARLRPWQQLLHE
LPRRLGGEHSDSTNTELANPSSTTTRITVPGASPVHAATLLPFIGRLLAARYAELNTA
IGTNWFPGTTPEVVSYPATIGVLSGSLGAVDANQSIAIGQQMLHNEILAATASGQPVT
VAGLSMGSMVIDRELAYLAIDPNAPPSSALTFVELAGPERGLAQTYLPVGTTIPIAGY

TVGNAPESQYNTSVVYSQYDIWADPPDRPWNLLAGANALMGAAYFHDLTAYAAPQQGI
EIAAVTSSLGGTTTTYMIPSPTLPLLLPLKQIGVPDWIVGGLNNVLKPLVDAGYSQYA
PTAGPYFSHGNLVW"

CDS complement(3993347..3994507)
/codon_start=1
/note="gene_3675"
/transl_table=11
/translation="VLSGQAAIVGIGATDFSKNSGRSELRLAAEAVLDALADAGLSPT
DVDGLTTFTMDTNTEIAVARAAGIGELTFFSKIHYGGGAACATVQHAAMAVATGVADV
VVAYRAFNERSGMRFGQVQTRLTENADSTGVDNSFSYPHGLSTPAAQVAMIARRYMHL
SGATSRDFGAVSVADRKHAANNPKAYFYGKPITIEDHQNSRWIAEPLRLLDCCQETDG
AVAIVVTSAARARDLKQRPVVIEAAAQGCSPDQYTMVSYYRPELDGLPEMGLVGRQLW
AQSGLTPADVQTAVLYDHFTPFTLIQLEELGFCGKGEAKDFIADGAIEVGGRLPINTH
GGQLGEAYIHGMNGIAEGVRQLRGTSVNPVAGVEHVLVTAGTGVPTSGLILG"

CDS complement(3994507..3994896)
/codon_start=1
/note="gene_3676"
/transl_table=11
/translation="MTVVGAVLPELKLYGDPTFIVSTALATRDFQDVHHDRDKAVAQG
SKDIFVNILTDTGLVQRYVTDWAGPSALIKSIGLRLGVPWYAYDTVTFSGEVTAVNDG
LITVKVVGRNTLGDHVTATVELSMRDS"

CDS complement(3994893..3995828)
/codon_start=1
/note="gene_3677"
/transl_table=11
/translation="VTGVSDIQEAVAQIKAAGPSKPRLARDPVNQPMINNWVEAIGDR
NPIYVDDAAARAAGHPGIVAPPAMIQVWTMMGLGGVRPKDDPLGPIIKLFDDAGYIGV
VATNCEQTYHRYLLPGEQVSISAELGDVVGPKQTALGEGWFINQHIVWQVGDEDVAEM
NWRILKFKPAGSPSSVPDDLDPDAMMRPSSSRDTAFFWDGVKAHELRIQRLADGSLRH
PPVPAVWQDKSVPINYVVSSGRGTVFSFVVHHAPKVPGRTVPFVIALVELEEGVRMLG
ELRGADPARVAIGMPVRATYIDFPDWSLYAWEPDE"

CDS complement(3995825..3996988)
/codon_start=1
/note="gene_3678"
/transl_table=11
/translation="MFIDLTPEQRQLQAEIRQYFSNLISPDERTEMEKDRHGPAYRAV
IRRMGRDGRLGVGWPKEFGGLGFGPIEQQIFVNEAHRADVPLPAVTLQTVGPTLQAHG
SELQKKKFLPAILAGEAHFAIGYTEPEAGTDLASLRTTAVRDGDHYIVNGQKVFTTGA
HDADYIWLACRTDPNAAKHKGISILIVDTKDPGYSWTPIILADGAHHTNATYYNDVRV
PVDMLVGKENDGWRLITTQLNNERVMLGPAGRFASIYDRVHAWASVPGGNGVTPIDHD
DVKRALGEIRAIWRINELLNWQVASAGEDINMADAAATKVFGTERVQRAGRLAEEIVG
KYGNPAEPDTAELLRWLDAQTKRNLVITFGGGVNEVMREMIAASGLKVPRVPR"

CDS complement(3996973..3997992)
/codon_start=1
/note="gene_3679"
/transl_table=11
/translation="MDFDPTAEQQAVADVVTSVLERDISWEALVCGGVTALPVPERLG
GDGVGLFEVGALLTEVGRHGAVTPALATLGLGVVPLLELASAEQQDRFLAGVAKGGVL
TAALNEPGAALPDRPATSFVGGRLSGTKVGVGYAEQADWMLVTADNAVVVVSPTADGV
RMVRTPTSNGSDEYVMTMDGVAVADCDILADVAAHRVNQLALAVMGAYADGLVAGALR
LTADYVANRKQFGKPLSTFQTVAAQLAEVYIASRTIDLVAKSVIWRLAEDLDAGDDLG

VLGYWVTSQAPPAMQICHHLHGGMGMDVTYPMHRYYSTIKDLTRLLGGPSHRLELLGA
RCSLT"
CDS complement(3997992..3999245)
/codon_start=1
/gene="cyp125A7"
/note="gene_3680"
/transl_table=11
/translation="VPSPNLPPGFDFTDPAIYAERLPVAEFAELRSAAPIWWNGQDPG
KGGGFHDGGFWAITKLNDVKEISRHSDVFSSYENGVIPRFKNDIAREDIEVQRFVMLN
MDAPHHTRLRKIISRGFTPRAVGRLHDELQERAQKIAAEAAAAGSGDFVEQVSCELPL
QAIAGLLGVPQEDRGKLFHWSNEMTGNEDPEYAHIDPKASSAELIGYAMKMAEEKAKN
PADDIVTQLIQADIDGEKLSDDEFGFFVVMLAVAGNETTRNSITQGMMAFAEHPDQWE
LYKKVRPETAADEIVRWATPVTAFQRTALRDYELSGVQIKKGQRVVMFYRSANFDEEV
FQDPFTFNILRNPNPHVGFGGTGAHYCIGANLARMTINLIFNAVADHMPDLKPISAPE
RLRSGWLNGIKHWQVDYTGRCPVAH"
CDS 3999405..4000580
/codon_start=1
/note="gene_3681"
/transl_table=11
/translation="MGYPVIVEATRSPIGKRNGWLSGLHATELLGAVQKAVVDKAGIQ
SGLHAGDVEQVIGGCVTQFGEQSNNISRVAWLTAGLPEHVGATTVDCQCGSGQQANHL
IAGLIAAGAIDVGIACGIEAMSRVGLGANAGPDRSLIRAQSWDIDLPNQFEAAERIAK
RRGITREDVDVFGLESQRRAQRAWAEGRFDREISPIQAPVLDEQNQPTGERRLVFRDQ
GLRETTMAGLGELKPVLEGGIHTAGTSSQISDGAAAVLWMDEAVARAHGLTPRARIVA
QALVGAEPYYHLDGPVQSTAKVLEKAGMKIGDIDIVEINEAFASVVLSWARVHEPDMD
RVNVNGGAIALGHPVGCTGSRLITTALHELERTDQSLALITMCAGGALSTGTIIERI"
CDS 4000692..4001147
/codon_start=1
/gene="ddn"
/note="gene_3682"
/transl_table=11
/translation="MPKSPPRFLNSPLSDFFIKWMSRINTWMYRRNDGEGLGGTFQKI
PVALLTTTGRKTGQPRVNPLYFLRDGGRVIVAASKGGAEKNPMWYLNLKANPKVQVQI
KKEVLDLTARDATDEERAEYWPQLVTMYPSYQDYQSWTDRTIPIVVCEP"
CDS complement(4001230..4002144)
/codon_start=1
/note="gene_3683"
/transl_table=11
/translation="MGLVDGRVVIVTGAGGGIGRAHALAFAAEGARVVVNDIGVGLDG
SPASGGSAAQDVVDEILAAGGQAVADGSDISDWDQAANLIQAAVETYGGVDVLVNNAG
IVRDRMIANTSEEEFDAVIAVHLKGHFATMRHAASHWRGLSKAGKAPKDIDARIINTS
SGAGLQGSVGQGNYSAAKAGIAALTLVGAAEMRRYGVTVNAIAPAARTRMTETVFAEV
MAKPQEGFDAMAPENVSPLVVWLGSAESRDVTGKVFEVEGGIIRVAEGWAHGPQVDKG
VKWDPAELGPVVSDLLAKSRPPVPVYGA"
CDS complement(4002167..4002946)
/codon_start=1
/note="gene_3684"
/transl_table=11
/translation="VTLAEAADAINFGLAGRVVLVTGGVRGVGAGISSVFAEQGATVI
TCARRAVDGQPYEFHRCDIRDEDSVKRLVGEIGERHGRLDMLVNNAGGSPYALAAEAT
HNFHRKIVELNVLAPLLVSQHANVLMQAQPNGGSIVNICSVSGRRPTPGTAAYGAAKA

GLENLTTTLAVEWAPKVRVNAVVVGMVETERSELFYGDAESIARVAATVPLGRLARPA
DIGWAAAFLASDAASYISGATLEVHGGGEPPPYLGASSANK"
CDS 4003001..4003744
/codon_start=1
/note="gene_3685"
/transl_table=11
/translation="MPITSTTPEPGIVAVTVDYPPVNAIPSKAWFDLADAVTAAGANS
DTRAVILRAEGRGFNAGVDIKEMQRTEGFTALIDANRGCFAAFRAVYECAVPVIAAVN
GFCVGGGIGLVGNSDVIVASEDATFGLPEVERGALGAATHLSRLVPQHLMRRLFFTAA
TVDAATLQHFGSVHEVVSRDQLDEAALRVARDIAAKDTRVIRAAKEALNFIDVQRVNA
SYRMEQGFTFELNLAGVADEHRDAFVKKS"
CDS 4003744..4004622
/codon_start=1
/note="gene_3686"
/transl_table=11
/translation="VPDKRTSLDDAVAQLRSGMTIGIAGWGSRRKPMAFVRAILRSDV
TDLTVVTYGGPDLGLLCSAGKVKRVYYGFVSLDSPPFYDPWFAHARTSGAIEAREMDE
GMLRCGLQAAAQRLPFLPIRAGLGSSVPQFWAGELQTVTSPYPAPGGGYETLIAMPAL
RLDAAFAHLNLGDSHGNAAYTGIDPYFDDLFLMAAERRFLSVERIVATEELVKSVPPQ
ALLVNRMMVDAIVEAPGGAHFTTAAPDYGRDEQFQRHYAEAASTQVGWQQFVHTYLSG
TEADYQAAVHNFGASR"
CDS 4004619..4005371
/codon_start=1
/note="gene_3687"
/transl_table=11
/translation="VSTRAEVCAVACAELFRDAGEIMISPMTNMASVGARLARLTFAP
DILLTDGEAQLLADTPALGKTGAPNRIEGWMPFGRVFETLAWGRRHVVMGANQVDRYG
NQNISAFGPLQRPTRQMFGVRGSPGNTINHATSYWVGNHCKRVFVEAVDVVSGIGYDK
VDPDNPAFRFVNVYRVVSNLGVFDFGGPDHSMRAVSLHPGVTPGDVRDATSFEVHDLD
AAEQTRLPTDDELHLIRAVIDPKSLRDREIRS"
CDS 4005469..4006536
/codon_start=1
/note="gene_3688"
/transl_table=11
/translation="MRLRTPLTELIGIEHPVVQTGMGWVAGARLVSATANAGGLGILA
SATMTLDELAAAITKVKAVTDKPFGVNIRADAADAGDRVELMIREGVRVASFALAPKQ
QLIARLKEAGAVVIPSIGAAKHARKVAAWGADAMIVQGGEGGGHTGPVATTLLLPSVL
DAVAGTGIPVIAAGGFFDGRGLAAALCYGAAGVAMGTRFLLTSDSTVPDAVKRRYLQA
GLDGTVVTTRVDGMPHRVLRTELVEKLESGSRARGFAAALRNAGKFRRMSQMTWRSMI
RDGLTMRHGKELTWSQVLMAANTPMLLKAGLVDGNTEAGVLASGQVAGILDDLPSCKE
LIESIVLDAITHLQTASALVE"
CDS 4006587..4008590
/codon_start=1
/note="gene_3689"
/transl_table=11
/translation="MDQAAPESAAHHPLPDPGESVPRLALPTIGIFLATLTAFVGSTT
AYISGWIPFWVTIPVNAAVTFVMFTVVHDASHYAISSIRWVNGLFGRLAWLFVGPVVA
FPAFGYIHIQHHRHSNDDEQDPDTFASHGSLWVLPLRWSMVEYFYIKYYLPRGRSRPV
IEVAETLVMMTLFLTGLIVAIVTGNFWTLAIVFLIPQRIGLTVLAWWFDWLPHHGLED
TQRSNRYRATRNRVGAEWLFTPVLLSQNYHLVHHLHPSVPFYRYLRTWRRNEEAYLER
NAAISTVFGQQLNPDEYRQWKELNGRLARLLPVRMPARSSSPHAVLHRIPVASVDPIT

ADATLVTFAVPEALRDAFRFEPGQHVTVRTDLGGQGIRRNYSICAPATRAQLRIAVKH
IPGGAFSTFVANELKAGDVLELMTPTGRFGTPLDPLHRKHYVGLVAGSGITPVLSILA
TTLEIETESRFTLIYGNRTKESTMFRAELDRLESRYADRLEILHVLSSEPLHTPELRG
RIDRDKLTRWLTSTLRPAGVDEWFICGPLAMATAVRETLIEHGVDSERIHLELFYGFD
TPPATRPSYAGATVTFTLSGQRAIFDLVPGDSILEGALGLRSDAPYACMGGACGTCRA
KLIEGNVEMDHNFALRKAELDAGYILTCQSHPTTPFVAVDYDA"

CDS complement(4008678..4009547)
/codon_start=1
/note="gene_3690"
/transl_table=11
/translation="MDELPWPVLGSEVLAAKAIPERAMRQLYEPVYPGVYAPAGVELT
ARQRAHAAWLWSRRRAVVAGNSAAALLGAKWVNPALDAELVHANRKPPPRIVVHTDRL
APHETVAVDGVAVTTPARTAFDIGRRTPSRLQAVQRLDALANSTDVKVADVQAVIAEH
TGARGLVRLRAVLPLIDGGAESPQETWTRLVLIDAGLPKPQTQIRVFDDYGDFVARID
LGYEQLRVGVEYDGPQHWTDPAQRARDIERSTALLDLGWTIIRVTSELLRYRRGTFVG
RVDAAMRAAGWRP"

CDS complement(4009652..4010875)
/codon_start=1
/note="gene_3691"
/transl_table=11
/translation="MPRVDDDAVGVPLTGNGRGAVMTEAYVIDAVRTAVGKRGGALAG
IHPVDLGALAWRGLLDRTDIDPAAVDDVIAGCVDAIGGQAGNIARLSWLAAGYPEEVP
GVTVDRQCGSSQQAISFGAQAIMSGTADVIVAGGVQNMSQIPISSAMTVGEQFGFTSP
TNESKQWLHRYGDQEISQFRGSELIAEKWNLSREEMERYSLTSHERAFAAIRAGHFEN
EIITVETESGPFRVDEGPRESSLEKMAGLQPLVEGGRLTAAMASQISDGASAVLLASE
RAVKDHGLRPRARIHHISARAADPVFMLTGPIPATRYALDKTGLAIDDIDTVEINEAF
APVVMAWLKEIKADPAKVNPNGGAIALGHPLGATGAKLFTTMLGELERIGGRYGLQTM
CEGGGTANVTIIERL"

CDS complement(4010877..4011479)
/codon_start=1
/note="gene_3692"
/transl_table=11
/translation="MDRVAGQVNSRRGELLELAAAMFAERGLRATTVRDIADGAGILS
GSLYHHFASKEEMVDELLRGFLDWLFARYRDIVDSTANPLERLQGLFMASFEAIEHHH
AQVVIYQDEAQRLASQPRFSYIEDRNKQQRKMWVDVLNQGIEEGYFRPDLDVDLVYRF
IRDTTWVSVRWYRPGGPLTAQQVGQQYLAIVLGGITKEGV"

CDS 4011828..4013486
/codon_start=1
/note="gene_3693"
/transl_table=11
/translation="MAHFSVLPPEINSLRMYLGAGSAPMLQAAAAWDGLAAELGTAAS
SFSSVTTGLTGQAWQGPASAAMAAAAAPYAGFLTTASAQAQLAAGQAKAVASVFEAAK
AAIVPPAAVAANREAFLALIRSNWLGLNAPWIAAVESLYEEYWAADVAAMTGYHAGAS
QAAAQLPLPAGLQQFLNTLPNLGIGNQGNANLGGGNTGSGNIGNGNKGSSNLGGGNIG
NNNIGSGNRGSDNFGAGNVGTGNIGFGNQGPIDVNLLATPGQNNVGLGNIGNNNMGFG
NTGDANTGGGNTGNGNIGGGNTGNNNFGFGNTGNNNIGIGLTGNNQMGINLAGLLNSG
SGNIGIGNSGTNNIGLFNSGSGNIGVFNTGANTLVPGDLNNLGVGNSGNANIGFGNAG
VLNTGFGNASILNTGLGNAGELNTGFGNAGFVNTGFDNSGNVNTGNGNSGNINTGSWN
AGNVNTGFGIITDSGLTNSGFGNTGTDVSGFFNTPTGPLAVDVSGFFNTASGGTVING
QTSGIGNIGVPGTLFGSVRSGLNTGLFNMGTAISGLFNLRQLLG"

CDS complement(4013495..4014283)

/codon_start=1
/note="gene_3694"
/transl_table=11
/translation="MNLSVAPKEIAGHGLLDGKVVVVTAAAGTGIGSATARRALAEGA
DVVISDHHERRLGETAAELSALGLGRVEHVVCDVTSTAQVDALIDSTTARMGRLDVLV
NNAGLGGQTPVADMTDDEWDRVLDVSLTSVFRATRAALRYFRDAPHGGVIVNNASVLG
WRAQHSQSHYAAAKAGVMALTRCSAIEAAEYGVRINAVSPSIARHKFLDKTASAELLD
RLVAGEAFGRAAEPWEVAATIAFLASDYSSYLTGEVISVSCQHP"
CDS complement(4014280..4015437)
/codon_start=1
/note="gene_3695"
/transl_table=11
/translation="MQDVEEFRAQVRGWLADNLAGEFAALKGLGGPGREHEAFEERRA
WNQRLAAAGLTCLGWPEEHGGRGLSTAHRVAFYEEYARADAPDKVNHFGEELLGPTLI
AFGTPQQQRRFLPRIRDVTELWCQGYSEPGAGSDLASVATTAELDGDQWVINGQKVWT
SLAHLSQWCFVLARTEKGSQRHAGLSYLLVPLDQPGVQIRPIVQITGTAEFNEVFFDD
ARTDADLVVGAPGDGWRVAMATLTFERGVSTLGQQIVYARELSNLVELARRTAAADDP
LIRERLTRAWTGLRAMRSYALATMEGPAVEQPGQDNVSKLLWANWHRNLGELAMDVIG
KPGMTMPDGEFDEWQRLYLFTRADTIYGGSNEIQRNIIAERVLGLPREAKG"
CDS 4015485..4017008
/codon_start=1
/note="gene_3696"
/transl_table=11
/translation="LINDLRTVPAALDRLVRQLPDHTALIAEDRRFTSTELRDAVYGA
AAALIALGVEPADRVAIWSPNTWHWVVACLAIHHAGAAVVPLNTRYTATEATDILDRA
GAPVLFAAGLFLGADRAAGLDRAALPALRHVVRVPVEADDGTWDEFIATGAGALDAVA
ARAAAVAPQDVSDILFTSGTTGRSKGVLCAHRQSLSASASWAANGKITSDDRYLCINP
FFHNFGYKAGILACLQTGATLIPHVTFDPLHALRAIERHRITVLPGPPTIYQSLLDHP
ARKDFDLSSLRFAVTGAATVPVVLVERMQSELDIDIVLTAYGLTEANGMGTMCRPEDD
AVTVATTCGRPFADFELRIADDGEVLLRGPNVMVGYLDDTEATAAAIDADGWLHTGDI
GAVDQAGNLRINDRLKDMYICGGFNVYPAEVEQVLARMDGVADAAVIGVPDQRLGEVG
RAFVVARPGTGLDEASVIAYTREHLANFKTPRSVRFVDVLPRNAAGKVSKPQLRELG"
CDS 4017009..4018142
/codon_start=1
/note="gene_3697"
/transl_table=11
/translation="MDLNFDDETLAFQAEVREFLAANAASIPTKSYDNAEGFAQHRYW
DRVLFDAGLSVITWPAKYGGRDAPLLHWIVFEEEYFRAGAPGRASANGTSMLAPTLFA
HGTAEQLDRILPKMASGEQIWAQAWSEPESGSDLASLRSTASKVDGGWLLNGQKIWSS
RAPFADMGFGLFRSDPAVERHRGLTYFMFDLKAKGVTVRPIAQLGGDTGFGEIFLDDV
FVPDRDVIGAPNDGWRAAMSTSSNERGMSLRSPARFLASAERLVQLWKDRGSPPEFAD
RVADAWIKAQAYRLQTFGTVTRLAAGGELGAESSVTKVFWSELDVHLHQTALDLRGAD
GELAGPWTEGLLFALGGPIYAGTNEIQRNIIAERLLGLPREKT"
CDS 4018145..4019098
/codon_start=1
/note="gene_3698"
/transl_table=11
/translation="MEFALNEQQRDFAASIDAALGAADLPGVVRAWAAGDVAPGRKVW
QQLANLGVTALGVAEKFDGLGASPVDLVVALERLGRWCVPGPVTESIAVAPILLAHDD
RAERSHGLASGELIATVAMPPRVPRAVDADTAGLVLLAGDGSVTEGTPGDCHRSVDPS
RRLYEVAASGQAWRAPKDVVARAYEFGALATAAQLVGAGQALLEAAVNYAKQRTQFGR

AIGSYQAIKHKLADVHIAIELACPLVYGAAVSLEPRDVSAAKAAASEAALLAARSALQ
THGAIGFTCEHDLSLWLLRVQALHSAWGTPQEHRRRVLEAL"
CDS 4019095..4020051
/codon_start=1
/note="gene_3699"
/transl_table=11
/translation="MTPPEERQMLRETVASLVAKHAGPAAVRAAMASDRGYDESLWRL
LCEQVGAAALVIPEELGGAGGELADAAIVVQELGRALVPSPLLGTTLAELALLAAAKP
DAQALTELAQGSAIGALVLDPDYVVNGDIADIVVAATSGQLTRWTRFSAQPVATMDPT
RRLARLQSEETEPLCPDPGIADTAAILLAAEQIGAAERCLQLTVEYAKSRVQFGRPIG
SFQALKHRMADLYVTIAAARAVVADACHAPTPTNAATARLAASEALSTAAAEGIQLHG
GIAITWEHDMHLYFKRAHGSAQLLESPREVLRRLESEVWESP"
CDS 4020036..4021214
/codon_start=1
/note="gene_3700"
/transl_table=11
/translation="VGVAVTDRVALRAGVPPFYVMDVWLAAAERQRTHGDLVNLSAGQ
PSAGAPEPVRAAAAAALHLNQLGYSVALGIPELRDAIAADYQRRHGITVEPDAVVITT
GSSGGFLLAFLACFDAGDRVAMASPGYPCYRNILSALGCEVVEIPCGPQTRFQPTAQM
LAEIDPPLRGVVVASPANPTGTVIPPEELAAIASWCDASDVRLISDEVYHGLVYQGAP
QTSCAWQTSRNAVVVNSFSKYYAMTGWRLGWLLVPTVLRRAVDCLTGNFTICPPVLSQ
IAAVSAFTPEATAEADGNLASYAINRSLLLDGLRRIGIDRLAPTDGAFYVYADVSDFT
SDSLAFCSKLLADTGVAIAPGIDFDTARGGSFVRISFAGPSGDIEEALRRIGSWLPSQ
"
CDS complement(4021179..4022030)
/codon_start=1
/note="gene_3701"
/transl_table=11
/translation="MALDLTAYFDRINYRGATDPTLDVLQDLVTVHSRTIPFENLDPL
LGVPVDDLSPQALADKLVLRRRGGYCFEHNGLMGYVLAELGYRVRRFAARVVWKLAPD
APLPPQTHTLLGVTFPGSGGCYLVDVGFGGQTPTSPLRLETGAVQPTTHEPYRLEDRV
DGFVLQAMVRDTWQTLYEFTTQTRPQIDLKVASWYASTHPASKFVTGLTAAVITDDAR
WNLSGRDLAVHRAGGTEKIRLADAAAVVDTLSERFGINVADIGERGALETRIDELLAR
QPGADAP"
CDS complement(4022567..4023130)
/codon_start=1
/note="gene_3702"
/transl_table=11
/translation="MSAQIDPRTFRSVLGQFCTGITVITTVHDDVPVGFACQSFAALS
LEPPLVLFCPTKVSRSWQAIEASGRFCVNVLTEKQKDVSARFGSKEPDKFAGIDWRPS
ELGSPIIEGSLAYIDCTVASVHDGGDHFVVFGAVESLSEVPAVKPRPLLFYRGDYTGI
EPEKTTPAHWRDDLEAFLTTTTQDTWL"
CDS complement(4023145..4024047)
/codon_start=1
/note="gene_3703"
/transl_table=11
/translation="MSIRSLGYLRIEATDMAAWREYGLKVLGMVEGKGAPEGALYLRM
DDFPARLVVVPGEHDRLLEAGWECANAEGLQEIRNRLDLEGTPYKEATAAELADRRVD
EMIRFADPSGNCLEVFHGTALEHRRVVSPYGHRFVTGEQGMGHVVLSTRDDAEALHFY
RDVLGFRLRDSMRLPPQMVGRPADGPPAWLRFFGCNPRHHSLAFLPMPTSSGIVHLMV
EVEQADDVGLCLDRALRRKVPMSATLGRHVNDLMLSFYMKTPGGFDIEFGCEGRQVDD

RDWIARESTAVSLWGHDFTVGARG"
CDS complement(4024044..4024919)
/codon_start=1
/note="gene_3704"
/transl_table=11
/translation="MTATEELTFESTSRFAEVDVDGPLKLHYHEAGVGNDQTVVLLHG
GGPGAASWTNFSRNIAVLARHFHVLAVDQPGYGHSDKRAEHGQFNRYAAMALKGLFDQ
LGLGRVPLVGNSLGGGTAVRFALDYPARAGRLVLMGPGGLSINLFAPDPTEGVKRLSK
FSVAPTRENLEAFLRVMVYDKNLITPELVDQRFALASTPESLTATRAMGKSFAGADFE
AGMMWREVYRLRQPVLLIWGREDRVNPLDGALVALKTIPRAQLHVFGQCGHWVQVEKF
DEFNKLTIEFLGGGR"
CDS complement(4024934..4026118)
/codon_start=1
/note="gene_3705"
/transl_table=11
/translation="VTSIQQRDAQSVLAAIDDLLPEIRDRAQATEDLRRLPDETVKAL
DDVGFFTLLQPQQWGGLQCDPALFFEATRRLASVCGSTGWVSSIVGVHNWHLALFDQR
AQEEVWGEDPSTRISSSYAPMGAGVVVDGGYLVNGSWNWSSGCDHASWTFVGGPVIKD
GRPVDFGSFLIPRSEYEIKDVWYVVGLRGTGSNTLVVKDVFVPRHRFLSYKAMNDHTA
GGLATNSAPVYKMPWGTMHPTTISAPIVGMAYGAYAAHVEHQGKRVRAAFAGEKAKDD
PFAKVRIAEAASDIDAAWRQLIGNVSDEYALLAAGKEIPFELRARARRDQVRATGRSI
ASIDRLFEASGATALSNEAPIQRFWRDAHAGRVHAANDPERAYVIFGNHEFGLPPGDT
MV"
CDS 4026265..4027341
/codon_start=1
/note="gene_3706"
/transl_table=11
/translation="LTEAIGDEPLGDHVLELQIAEVVDETDEARSLVFAVPDGSDDPE
IPPRRLRYAPGQFLTLRVPSERTGSVARCYSLCSSPYTDDALAVTVKRTADGYASNWL
CDHAQVGMRIHVLAPSGNFVPTTLDADFLLLAAGSGITPIMSICKSALAEGGGQVTLL
YANRDDRSVIFGDALRELAAKYPDRLTVLHWLESLQGLPSASALAKLVAPYTDRPVFI
CGPGPFMQAARDALAALKVPAQQVHIEVFKSLESDPFAAVKVDDSGDEAPATAVVELD
GQTHTVSWPRTAKLLDVLLAAGLDAPFSCREGHCGACACTLRAGKVNMGVNDVLEQQD
LDEGLILACQSRPESDSVEVTYDE"
CDS 4027359..4027889
/codon_start=1
/note="gene_3707"
/transl_table=11
/translation="MTRLIPGCTLVGLMLTLLPAPTSAAGSNTATTLFPVDEVTQLET
HTFLDCHPNGSCDFVAGANLRTPDGPTGFPPGLWARQTTEIRSTNRLAYLDAHATSQF
ERVMKAGGSDVITTVVYFGEGPPDKYQTTGVIDSTNWSTGQPMTDVNVIVCTHMQVVYP
GVNLTSPSTCAQANFS"
CDS complement(4027925..4030060)
/codon_start=1
/note="gene_3708"
/transl_table=11
/translation="VVATVTDEQSAARELVRGWARTAASGAAATAAVRDMEYGFEEGN
ADAWRPVFAGLAGLGLFGVAVPEDCGGAGGSIEDLCAMVDEAARALVPGPVATTAVAT
LVVSDPKLRSALASGERFAGVAIDGGVQVDPKTSTASGTVGRVLGGAPGGVVLLPADG
NWLLVDTACDEVVVEPLRATDFSLPLARMVLTSAPVTVLEVSGERVEDLAATVLAAEA
AGVARWTLDTAVAYAKVREQFGKPIGSFQAVKHLCAQMLCRAEQADVAAADAARAAAD

SDGTQLSIAAAVAASIGIDAAKANAKDCIQVLGGIGCTWEHDAHLYLRRAHGIGGFLG
GSGRWLRRVTALTQAGVRRRLGVDLAEVAGLRPEIAAAVAEVAALPEEKRQVALADTG
LLAPHWPAPYGRGASPAEQLLIDQELAAAKVERPDLVIGWWAAPTILEHGTPEQIERF
VPATMRGEFLWCQLFSEPGAGSDLASLRTKAVRADGGWLLTGQKVWTSAAHKARWGVC
LARTDPDAPKHKGITYFLVDMTTPGIEIRPLREITGDSLFNEVFLDNVFVPDEMVVGA
VNDGWRLARTTLANERVAMATGTALGNPMEELLKVLGDMELDVAQQDRLGRLILLAQA
GALLDRRIAELAVGGQDPGAQSSVRKLIGVRYRQALAEYLMEVSDGGGLVENRAVYDF
LNTRCLTIAGGTEQILLTVAAERLLGLPR"

CDS 4030326..4030931
/codon_start=1
/note="gene_3709"
/transl_table=11
/translation="MKVAVLAESELGSEAQRERRKRILDATMAIASKGGYEAVQMRAV
ADRADVAVGTLYRYFPSKVHLLVSALGREFSRIDAKTDRSAVAGATPFQRLNFMVGKL
NRAMQRNPLLTEAMTRAYVFADASAASEVDQVEKLIDSMFARAMANGEPTEDQYHIAR
VISDVWLSNLLAWLTRRASATDVSKRLDLAVRLLIGDQDSA"

CDS complement(4030937..4031959)
/codon_start=1
/note="gene_3710"
/transl_table=11
/translation="VSRTTVSNAFNRPDQLSADLRERVLATAKRLGYAGPDPVARSLR
TRKAGAVGLVMAEPLTYFFSDPAARDFVAGVAQSCEELGQGLQLVSVGSSRSLADGTA
AVLGAGVDGFVVYSVGDDDPYLQVVLQRRLPVVVVDQPKDLSGVSRVGIDDRAAMREL
AGYVLGLGHRELGLLTMRLGRDRRQDLVDAERLRSPTFDVQRERIVGVWEAMTAAGVD
PDSLTVVESYEHLPTSGGTAAKVALQANPRLTALMCTADILALSAMDYLRAHGIYVPG
QMTVTGFDGVPEALSRGLTTVAQPSLHKGHRAGELLLKPPRSGLPVIEVLDTELVRGR
TAGPPA"

CDS 4032206..4032919
/codon_start=1
/note="gene_3711"
/transl_table=11
/translation="VGKQLAALAALVGACMLAAGCTNVVDGTAVAADKSGPLHQDPIP
VSALEGLLLDLSQINAALGATSMKVWFNAKAMWDWSKSVADKNCLAIDGPAQEKVYAG
TGWTAMRGQRLDDSINDSKKRDHYAIQAVVGFPTAHDAEEFYSSSVQSWSSCSNRRFV
EVTPGQDDAAWTVADVVNDNGMLSSSQVQEGGDGWTCQRALTARNNVTIDIVTCAYSQ
PDLVAIGIANQIAAKVAKQ"

CDS 4033152..4033976
/codon_start=1
/note="gene_3712"
/transl_table=11
/translation="MGVATLLVDDGSSALMTDGYFSRPGLARVAAGKVSPSAERVDGC
LARANVSRLTAVIPVHTHIDHAMDSALVADRTGAQLVGGESAANVGRGYGLPEESLVV
AVPGEPIQLGAFDVTLVESHHCPPDRFPGVISAPLTPPVKASAYRCGEAWSTLVHHRP
SGRRLLIQDSAGFVSGALAGYRADAAYLSVGQLGLQPPSYLLEYWTETVRTVGVRRVI
LIHWDDFFRPLSKPLRALPYAADDLDLSIRILDELAAQDGVALQMPTVWRREDPWM"

CDS 4033990..4035231
/codon_start=1
/note="gene_3713"
/transl_table=11
/translation="LTLAVALILLAVVLGFAVARPRGWPEAAAAVPAAVILLAIGAIS
PQQAMAQVSGLARVVAFLGAVLVLAKLCDDEGLFEAAGAAMARASAESHRLLRQVFAV

SAAITAALCLDATVVLLTPVVLATVRRLRTPVRPYAYATAHLANAASLLLPVSNLTNL
LAYHGAGISFTKFTLLMALPWLSAVAAVYVVFRWFFARDLRVVPDRQQLKPAPRLPMF
VLVVVALTLGGFAVAESVGLAPTWAALAGAAVLALRSLRRGHTSVLRIARAVNVSFLV
FVLALGVVVHAVMLNGMAARMSAVLPTGSGLPALLGIAALASVLANVVNNLPATLVLV
PLVAAGGPAAVLAVLLGVNIGPNLTYAGSLSNLLWRGVLRRHNVDASVGEYTRLGLCT
VPAALAMAVLALWASAQVLGI"
CDS complement(4035273..4036241)
/codon_start=1
/note="gene_3714"
/transl_table=11
/translation="MPGNSRRRGAVRKSGTKKGAGVGSGGQRRRGLEGRGPTPPAHLR
PHHPAAKRARAQPRRPVKRADETETVLGRNPVLECLRAGVPATALYVALGTEADERLT
ECVARAADSGIAIVELLRADLDRMTANHLHQGIALQVPPYNYAHPDDLLAAALDQPPA
LLVALDNLSDPRNLGAIVRSVAAFGGHGVLIPQRRSASVTAVAWRTSAGAAARIPVAR
ATNLTRTLKGWADRGVRVIGLDAGGGTALDDVDGTDSLVVVVGSEGKGLSRLVRQNCD
EVVSIPMAAQAESLNASVAAGVVLAAIARQRRRPREPREQTQNRMI"
CDS complement(4036242..4037651)
/codon_start=1
/gene="cysS"
/note="gene_3715"
/transl_table=11
/translation="VTDRARLRLHDTAAGVVRDFVPLRPGHVSIYLCGATVQGLPHIG
HVRSGVAFDILRRWLLARGYDVAFIRNVTDIEDKILAKAAAAGRPWWEWAATHERAFT
AAYDALDVLPPSAEPRATGHITQMIEMIERLIQAGHAYTGGGDVYFDVLSYPEYGQLS
GHKIDDVHQGEGVAAGKRDQRDFTLWKGEKPGEPSWPTPWGRGRPGWHLECSAMARSY
LGPEFDIHCGGMDLVFPHHENEIAQSRAAGDGFARYWLHNGWVTMGGEKMSKSLGNVL
SMPAMLQRVRPAELRYYLGSAHYRSMLEFSETAMQDAVKAYVGLEDFLHRVRTRVGAV
CPGDPTPRFAEALDDDLSVPIALAEIHHVRAEGNRALDAGDHDGALRSASAIRAMMGI
LGCDPLDQRWESRDETSAALAAVDVLVQAELQNREKAREQRNWALADEIRGRLKRAGI
EVTDTADGPQWSLLGGDTK"
CDS complement(4037716..4038195)
/codon_start=1
/gene="ispF"
/note="gene_3716"
/transl_table=11
/translation="VNQLPRVGLGTDVHPIEPGRPCWLVGLLFPSADGCAGHSDGDVA
VHALCDAVLSAAGLGDIGEVFGVDDPRWQGVSGADMLRHVVVLITQHGYRVGNAVVQV
IGNRPKIGWRRLEAQAVLSRLLNAPVSVSATTTDGLGLTGRGEGLAAIATALVVSLR"
CDS complement(4038192..4038887)
/codon_start=1
/gene="ispD"
/note="gene_3717"
/transl_table=11
/translation="VVREAGEVVAIVPAAGSGERLAVGVPKAFYQLDGQTLIERAVDG
LLDSGVVDTVVVAVPADRTDEARQILGHRAMIVAGGSNRTDTVNLALTVLSGTAEPEF
VLVHDAARALTPPALVARVVEALRDGYAAVVPVLPLSDTIKAVDANGVVLGTPERAGL
RAVQTPQGFTTDLLLRSYQRGSLDLPAAEYTDDASLVEHIGGQVQVVDGDPLAFKITT
KLDLLLAQAIVRG"
CDS complement(4038904..4039392)
/codon_start=1
/note="gene_3718"

/transl_table=11
/translation="MIFKVGDTVVYPRHGAALVEAIETRTIKGEQKEYLVLKVAQGDL
TVRVPAENAEYVGVRDVVGQEGLDKVFQVLRAPHTEEPTNWSRRYKANLEKLASGDVN
KVAEVVRDLWRRDQERGLSAGEKRMLAKARQILVGELALAESTDDAKAETILDEVLAA
AS"
CDS 4039677..4040225
/codon_start=1
/note="gene_3719"
/transl_table=11
/translation="VNRCNIRLRLAGMTTWVASIALLAAALSGCGAGQISQTANQKPA
VNGNRLTINNVLLRDIRIQAVQTSDFIQPGKAVDLVLVAVNQSPDVSDRLVGITSDIG
SVTVAGDARLPASGMLFVGTPDGQIVAPGPLPSNQAAKATVNLTKPIANGLTYNFTFK
FEKAGQGSVMVPISAGLATPHE"
CDS 4040291..4041733
/codon_start=1
/note="gene_3720"
/transl_table=11
/translation="VANARSQYRCSECRHVSAKWVGRCLECGRWGTVDEVAVLSAVGG
TRRRSVAPASGAVPISAVDAHRTRPCPTGIDELDRVLGGGIVPGSVTLLAGDPGVGKS
TLLLEVAHRWAQSGRRALYVSGEESAGQIRLRADRIGCGTEVEEIYLAAQSDVHTVLD
QIETVQPALVIVDSVQTMSTSEADGVTGGVTQVRAVTAALTAAAKANEVALILVGHVT
KDGAIAGPRSLEHLVDVVLHFEGDRNGALRMVRGVKNRFGAADEVGCFLLHDNGIDGI
VDPSNLFLDQRPTPVAGTAITVTLDGKRPLVGEVQALLATPCGGSPRRAVSGIHQARA
AMIAAVLEKHARLAIAVNDIYLSTVGGMRLTEPSADLAVAIALASAYANLPLPTTAVM
IGEVGLAGDIRRVNGMARRLSEAARQGFTIALVPPSDDPVPPGMHALRASTIVAALQY
MVDIADHRGTTLATPPSHSGTGHVPLGRGT"
CDS 4041738..4042814
/codon_start=1
/note="gene_3721"
/transl_table=11
/translation="MHAVTRPTLREAVARLAPGTGLRDGLERILRGRTGALIVLGHDE
NVEAICDGGFSLDVRYAATRLRELCKMDGAVVLSTDGSRIVRANVQLVPDPSIPTDES
GTRHRSAERAAIQTGYPVISVSHSMNIVTVYVRGERHVLTDSATILSRANQAIATLER
YKTRLDEVSRQLSRAEIEDFVTLRDVMTVVQRLELVRRIGLVIDYDVVELGTDGRQLR
LQLDELLGGNDTARELIVRDYHANPEPPSTGQINATLDELDALSDGDLLDFTALAKVF
GYPTTTEAQDSALSPRGYRAMAGIPRLQFAHADLLVRAFGTLQGLLAASAGDLQSVDG
IGAMWARHVRDGLSQLAESTISDQ"
CDS complement(4042815..4043630)
/codon_start=1
/note="gene_3722"
/transl_table=11
/translation="VTWPYGVIVLDLEPRGPLPTEIYWRRRGLALGIAVVVVGIAVAI
VIAFVDSSAGAKPVSADKPASAQSHPGSPAPQAPQPAGQTEGNAAAAPPQGQNPETPT
PTAAVQPPPVLKEGDDCPDSTLAVKGLTNAPQYYVGDQPKFTMVVTNIGLVSCKRDVG
AAVLAAYVYSLDNKRLWSNLDCAPSNETLVKTFSPGEQVTTAVTWTGMGSAPRCPLPR
PAIGPGTYNLVVQLGNLRSLPVPFILNQPPPPGPVPAPGPAQAPPPESPAQGG"
CDS complement(4043715..4044338)
/codon_start=1
/note="gene_3723"
/transl_table=11
/translation="MPNTNPVAAWKALKEGNERFVAGRPQHPSQSVDHRAGLAAGQKP

TAVIFGCADSRVAAEIIFDQGLGDMFVVRTAGHVIDSAVLGSIEYAVTVLNVPLIVVL
GHDSCGAVNAALAAINDGTLPGGYVRDVVERVAPSVLLGRRDGLSRVDEFEQRHVHET
VAILMARSSAISERIAGGSLAIVGVTYQLDDGRAVLRDHIGNIGEEV"
CDS 4044484..4045251
/codon_start=1
/note="gene_3724"
/transl_table=11
/translation="MLQQTPAARVLAIWPDWVRRWPTPSATATASTADVLRAWGKLGY
PRRAKRLHECATVIARDHNDVVPDDIEILVTLPGVGSYTARAVACFAYRQRVPVVDTN
VRRVVARAVHGRADAGAPSVPRDHADVLALLPHRETAPEFSVALMELGATVCTARTPR
CGLCPLDWCAWRHAGYPPSDGPPRRGQAYTGTDRQVRGRLLDVLRAAEFPVTRAELDV
AWLTDTAQRDRALESLLADALVTRTVDGRFALPGEGF"
CDS complement(4045248..4047002)
/codon_start=1
/note="gene_3725"
/transl_table=11
/translation="MSFVIVAPEALMSVASEVAGIGSALNAANAAAAAPTTGVLAAAA
DEVSAAMAALFGAHAQEYQRLSAQAAGFHAQFVQALNAGVNSYASAEAANASPLQAVE
QQVLGLINGPAQTLLGRPLIGNGADGAPGTGQPGGPGGLLWGNGGNGGSGVAGVGGPG
GSGGAAGLFGHGGNGGAGGSNAAGAGGVGGAGGAGWLVGNGGAGGFGGVGTTVSGNGG
AGGAAGAFGNGGVGGAGGAAVIGGLPGNGGAGGNAGLIGAGGDGGVGGVGAPGTNGMN
PPPNQTSQAANGSPGANNGAGSGGAGLPGNPGAVPGRAGGAGGLGGSGSDTSEGPVTG
GNGGNGGDGGPGAPGGNGAPGGIGVNTGTGWAYGGNGGNGGDGGAGARGGDGGNGGNG
LALNGGNGIGGNGGAGGRGGTGAAGGNGGIGGGATGTLTFFGSGGDGGPGGAGANTAG
TGGVGGVGGAGGQGGLLFGDGGNGGAGGAGGIGGTGASGGAGGKGGSGLVGGDGGNGG
AGGAGGNGGKGGAGGAGGGAGMFSQPGVHGAGGTGGQGGAGGAGGAGGAAGAGTVVAG
NPGDPGGFGAAGADGLPG"
CDS complement(4047113..4047886)
/codon_start=1
/note="gene_3726"
/transl_table=11
/translation="VPRMPANLLTHRGGRGEPLVLVHGLMGRGSTWARQLPWLTLLGA
VYTYDAPWHRGRDVADPHPISTERFVADLGDAVSALGAPTRMVGHSMGALHSWCLAAE
RPELVSALVVEDMAPDFRGRTTGPWEPWLRALPVEFDSAEQVFAEFGPVAGRYFLDAF
DRTATGWRLHGRTARWIEIAAEWGTRDYWAQWRAVRSPALLIEAGDGVTPPGQMRAMA
ERDYPTAYLRVPDAGHLVHDEAPQVYRRAVESFLAGLTP"
CDS 4047901..4048218
/codon_start=1
/note="gene_3727"
/transl_table=11
/translation="MPVVKINAIEVPAGAGPELEKRFAHRAHAVENSPGFLGFQLLRP
VKGEERYFVVTHWESDEAFQAWANGPAIAAHAGHRANPVATGASLLEFEVVLDVGGTG
KTA"
CDS 4048238..4049554
/codon_start=1
/note="gene_3728"
/transl_table=11
/translation="MLALSAAAALIVALASGCSSAPTPSANAANHGHRIDTRTPPGLR
AQQTMDMLNSDWPIGEIGVGTLAAPGQVDTVKTTMEALWWDRPFALAGVDIGASVAAL
HLISSYGAQQDIRIHTDDDGWVDRFDVETQAPSIASWRDVDAVLSKTGARYSFQVAKV
DNGRCDPVAGTSTGESLPLASIFKLYVLHALAGAVQHNTVSWDDLLTVTAKSKAVGSS

GLELPVGARVSVRTAAEKMIATSDNMATDLLIERLGTRAIEEALASAGHHDPASMTPF
PTMYELFSVGWGKPDLRDQWKHATQQVRAQILRQTNSTPYQPDPTRAHTPASNYGAEW
YGSAEDICRVHAALRADAVGPASPVRQIMSAVPGIQLDRSVWPYIGAKAGGLPGDLTF
SWYAVDKTGQPWVVSFQLNWPRDHGPTVTGWMLQVARQVFALIAPQ"
CDS 4049701..4050528
/codon_start=1
/note="gene_3729"
/transl_table=11
/translation="VGWIGDPIWLEEVLRPALGERLRVLDGWRERGHGDFRDIRGVMW
HHTGNSRETAKSIARGRPDLPGPLANLHIAHSGVVTIVAVGVCWHAGRGSYPWLPTDN
ANWHMIGVECAWPTIRRDGSYDAGERWPDAQIVSMRDVAAALTLKLGYGPERNIGHKE
YAGAAQGKWDPGNLSMDWFRAEVAKDTRGEFDHPLTPPPAVIARPPILPKPRNPRDDR
ILLEEVWDQLRGIEGRGWPVLGDKTIVDYLAELGNKVDALAAKLDAREGLDRPSDTR"
CDS complement(4050442..4051920)
/codon_start=1
/note="gene_3730"
/transl_table=11
/translation="MSFVIAVPEFLSAAATDLANLGSTISAANAAASIPTTGVLAAGA
DDVSAAIAALFGAHAQAYQTISAQAATFHAQFVQTLSAGAGAYANAEAANVQQSLLNA
INAPTQALLGRPLIGDGADGTAPGQNGGAGGLLYGNGGNGAAGVNAGIAGGSGGAAGL
IGNGGSGGAGGAGAAGGSGGQGGLLYGNGGAGGNGGAATIPGGNGGAGGAGGNAWLFG
NGGAGGLGAAGAAGAAGVNPLTVPAGQGSMGNNGEPGGPASQAPSSGKPVAPVALAVL
ACPLAGPAGPAETVAPARPAAPGGPAGPAALAVGVGSWSATAAQVASGAQGAKAASAP
GAAPAVRAEWAAPGNQALGVTLVTGVTAGSAVTAAPAETAARAARAARAACSASPVAP
GWAVPPVAGVMAGAVVSPVWPAPRALGLRAVAATATSASSALKVHPASPASRASPVDI
VAAGPSAPRRLLEQLPSVTGPVEALARVEFCGQGVDLIAELG"
CDS complement(4052028..4054574)
/codon_start=1
/note="gene_3731"
/transl_table=11
/translation="MFERFTDRARRVVVLAQEEARMLNHNYIGTEHILLGLIHEGEGV
AAKSLESLGISLEGVRSQVEEIIGQGQQAPSGHIPFTPRAKKVLELSLREALQLGHNY
IGTEHILLGLIREGEGVAAQVLVKLGAELTRVRQQVIQLLSGYQGKEAAEAGTGGRGG
ESGSPSTSLVLDQFGRNLTAAAMEGKLDPVIGREKEIERVMQVLSRRTKNNPVLIGEP
GVGKTAVVEGLAQAIVHGEVPETLKDKQLYTLDLGSLVAGSRYRGDFEERLKKVLKEI
NTRGDIILFIDELHTLVGAGAAEGAIDAASILKPKLARGELQTIGATTLDEYRKYIEK
DAALERRFQPVQVGEPTVEHTIEILKGLRDRYEAHHRVSITDAAMVAAATLADRYIND
RFLPDKAIDLIDEAGARMRIRRMTAPPDLREFDEKIAEARREKESAIDAQDFEKAASL
RDREKTLVAQRAEREKQWRSGDLDVVAEVDDEQIAEVLGNWTGIPVFKLTEAETTRLL
RMEEELHKRIIGQEDAVKAVSKAIRRTRAGLKDPKRPSGSFIFAGPSGVGKTELSKAL
ANFLFGDDDALIQIDMGEFHDRFTASRLFGAPPGYVGYEEGGQLTEKVRRKPFSVVLF
DEIEKAHQEIYNSLLQVLEDGRLTDGQGRTVDFKNTVLIFTSNLGTSDISKPVGLGFS
KGGGENDYERMKQKVNDELKKHFRPEFLNRIDDIIVFHQLTREEIIRMVDLMISRVAG
QLKSKDMALVLTDAAKALLAKRGFDPVLGARPLRRTIQREIEDQLSEKILFEEVGPGQ
VVTVDVDNWDGEGPGEDAVFTFTGTRKPPAEPDLAKAGAHSAGGPEPAAR"
CDS complement(4054851..4055189)
/codon_start=1
/note="gene_3732"
/transl_table=11
/translation="MAKKVTVTLVDDFDGSGAADETVEFGLDGVTYEIDLSTKNATKL
RGDLKQWVAAGRRVGGRRRGRSGSGRGRGAIDREQSAAIREWARRNGHNVSTRGRIPA

DVIDAYHAAT"
CDS complement(4055293..4056810)
/codon_start=1
/gene="lysS"
/note="gene_3733"
/transl_table=11
/translation="VSAADTAEDLPEQFRIRRDKRARLLAQGRDPYPVAVPRTHTLAE
VRAAHPDLPIDTATEDIVGVAGRVIFARNSGKLCFATLQDGDGTQLQVMISLDKVGQA
ALDAWKADVDLGDIVYVHGAVISSRRGELSVLADCWRIAAKSLRPLPVAHKEMSEESR
VRQRYVDLIVRPEARAVARLRIAVVRAIRTALQRRGFLEVETPVLQTLAGGAAARPFA
THSNALDIDLYLRIAPELFLKRCIVGGFDKVFELNRVFRNEGADSTHSPEFSMLETYQ
TYGTYDDSAVVTRELIQEVADEAIGTRQLPLPDGSVYDIDGEWATIQMYPSLSVALGE
EITPQTTVDRLRGIADSLGLEKDPAIHDNRGFGHGKLIEELWERTVGKSLSAPTFVKD
FPVQTTPLTRQHRSIPGVTEKWDLYLRGIELATGYSELSDPVVQRERFADQARAAAAG
DDEAMVLDEDFLAALEYGMPPCTGTGMGIDRLLMSLTGLSIRETVLFPIVRPHSN"
CDS complement(4056911..4057729)
/codon_start=1
/note="gene_3734"
/transl_table=11
/translation="VLLAIDVRNTHTVVGLLSGMKEHAKVVQQWRIRTESEVTADELA
LTIDGLIGEDSERLTGTAALSTVPSVLHEVRIMLDQYWPSVPHVLIEPGVRTGIPLLV
DNPKEVGADRIVNCLAAYDRFRKAAIVVDFGSSICVDVVSAKGEFLGGAIAPGVQVSS
DAAAARSAALRRVELARPRSVVGKNTVECMQAGAVFGFAGLVDGLVGRIREDVSGFSV
DHDVAIVATGHTAPLLLPELHTVDHYDQHLTLQGLRLVFERNLEVQRGRLKTAR"
CDS complement(4057732..4058151)
/codon_start=1
/note="gene_3735"
/transl_table=11
/translation="MLRTMLKSKIHRATVTCADLHYVGSVTIDADLMDAADLLEGEQV
TIVDIDNGARLVTYAITGERGSGVIGINGAAAHLVHPGDLVILIAYATMDDARARTYQ
PRIVFVDAYNKPIDMGHDPAFVPENAGELLDPRLGVG"
CDS complement(4058151..4059080)
/codon_start=1
/gene="panC"
/note="gene_3736"
/transl_table=11
/translation="MTIPAFHPGELNVYSAPGDVADVSRALRLTGRRVMLVPTMGALH
EGHLALVRAAKRVPGSVVVVSIFVNPMQFGAGEDLDAYPRTPDDDLAQLRAEGVEIAF
TPTTAAMYPDGLRTTVQPGPLAAELEGGPRPTHFAGVLTVVLKLLQIVRPDRVFFGEK
DYQQLVLIRQLVADFNLDVAVVGVPTVREADGLAMSSRNRYLDPAQRAAAVALSAALT
AAAHAATAGAQAALDAARAVLDAAPGVAVDYLELRDIGLGPMPLNGSGRLLVAARLGT
TRLLDNIAIEIGTFAGTDRPDGYRAILESHWRN"
CDS complement(4059077..4059988)
/codon_start=1
/note="gene_3737"
/transl_table=11
/translation="MERFDGLRPARLKVGIISAGRVGTALGVALQRADHVVVACSAIS
HASRRRAQRRLPDTPVLPPLDVAASAELLLLAVTDSELAGLVSGLAATSAVRPQTIVA
HTSGANGIGILAPLAQQGCIPLAIHPAMTFTGSDEDISRLPDTCFGITAADDVGYAIG
QSLVLEMGGEPFCVREDARILYHAALAHASNHIVTVLADALEALRAALSGGELLGQQT
VDDQPGGIVERIVGPLARAALENTLQRGQAALTGPVARGDAAAVADHLAALADVDAAL

AQAYRINALRTAQRAHAPADVVEVLTA"
CDS complement(4060173..4061366)
/codon_start=1
/note="gene_3738"
/transl_table=11
/translation="MTVLSRGARVRRGGRRPGWVLLTALLVLAIGASSALVFTDRVEL
LKLAVLLALWAAVAGAFVSVLYRRQSDVDQARVRDLKLVYDLQLDREISARREYELTL
ESQLRRELASELRAPAADEVAALRAELAALRTSLEILFDADLEHRPALGTGEKEARAA
RALDGESPPADWVSSDRVMAVRGGDGASRTDEASIIDVPEVGVPPVSGGPRHYEAPPP
PQPEPLFEPRHRPPPLPPQQERPVWQPVTSHGQWLPAETPGSQWASVEPETTPAAPPP
GRRRRARHASPADQAYNPPAYVELAAQYGESGRRSRHSAEHRDHDIGGSGAGTGERPP
SPPMAPPPPAEPTRRHRTADTPPDDSGGLHARDPLTGGQSVADLMARLQVESTGGGRR
RRRGE"
CDS complement(4061575..4062051)
/codon_start=1
/note="gene_3739"
/transl_table=11
/translation="MGPTRKRDLTAAVVGAAAVGYLLVAVLYRWFPPITVWTGLSLLA
VAVAEALWARYVRVKISDGEIGDGPGWLHPLVVARSLMVAKASAWVGALVTGWWIGVL
AYFLPRRSWLRAAAEDTTGTVVAAGSALALVVAALWLQHCCKSPQDPTEHADGAES"
CDS complement(4062051..4062617)
/codon_start=1
/note="gene_3740"
/transl_table=11
/translation="MTRVVLSVGSNLGDRLARLRSVADGLGDALIAASPIYEADPWGG
VEQGQFLNAVLIADDPTCEPREWLRRAEEFERAAGRVRGQRWGPRNLDVDLIACYQTS
ATEALVEVTARENHLTLPHPLAHLRAFVLIPWIAVDPTAQLTVAGCPRPVTRLLAELE
PADRDSVRLFRPSFDLNSRHPVSRAPES"
CDS complement(4062614..4063015)
/codon_start=1
/note="gene_3741"
/transl_table=11
/translation="MADRIELRGLTVHGRHGVYDHERVAGQRFVIDVTVWIDLAEAAN
SDDLADTYDYVRLASRAAEIVAGPPRKLIETVGAEIADHVMDDQRVHAVEVAVHKPQA
PIPQTFDDVAVVIRRSRRGGRGWVVPAGGAV"
CDS complement(4063008..4063835)
/codon_start=1
/note="gene_3742"
/transl_table=11
/translation="VQVMGVLNVTDDSFSDGGCYLDLDDAVKHGLAMAAAGAGIVDVG
GESSRPGATRVDPAVETSRVIPVVKELAAQGITVSIDTMRADVARAALQNGAQMVNDV
SGGRADPAMGPLLAEADVPWVLMHWRAVSADTPHVPVRYGNVVAEVRADLLASVADAV
AAGVDPARLVLDPGLGFAKTAQHNWAILHALPELVATGIPVLVGASRKRFLGALLAGP
DGVMRPTDGRDTATAVISALAALHGAWGVRVHDVRASVDAIKVVEAWMGAERIERDG"
CDS complement(4063847..4064455)
/codon_start=1
/gene="folE"
/note="gene_3743"
/transl_table=11
/translation="MSQLDSRSASARIRVFDQQRAEAAVRELLYAIGEDPDRDGLVAT
PSRVARSYREMFAGLYTDPDSVLNTMFDEDHDELVLVKEIPMYSTCEHHLVAFHGVAH

```
                     VGYIPGDDGRVTGLSKIARLVDLYAKRPQVQERLTSQIADALMKKLDPRGVIVVIEAE
                     HLCMAMRGVRKPGSVTTTSAVRGLFKTNAASRAEALDLILRK"
     CDS             complement(4064471..4066753)
                     /codon_start=1
                     /note="gene_3744"
                     /transl_table=11
                     /translation="MNRKNVTRTITAIAVVVLLGWSFFYFSDDTRGYKPVDTSVAITQ
                     INGDNVKSAQIDDREQQLRLILKKGNNETDGSEKVITKYPTGYAVDLFNALSAKNAKV
                     STVVNQGSILGELLVYVLPLLLLVGLFVMFSRMQGGARMGFGFGKSRAKQLSKDMPKT
                     TFADVAGVDEAVEELYEIKDFLQNPSRYQALGAKIPKGVLLYGPPGTGKTLLARAVAG
                     EAGVPFFTISGSDFVEMFVGVGASRVRDLFEQAKQNSPCIIFVDEIDAVGRQRGAGLG
                     GGHDEREQTLNQLLVEMDGFGDRAGVILIAATNRPDILDPALLRPGRFDRQIPVSNPD
                     LAGRRAVLRVHSKGKPMAADADLDGLAKRTVGMTGADLANVINEAALLTARENGTVIT
                     GPALEEAVDRVIGGPRRKGRIISEQEKKITAYHEGGHTLAAWAMPDIEPIYKVTILAR
                     GRTGGHAVAVPEEDKGLRTRSEMIAQLVFAMGGRAAEELVFREPTTGAVSDIEQATKI
                     ARSMVTEFGMSSKLGAVKYGSEHGDPFLGRTMGTQPDYSHEVAREIDEEVRKLIEAAH
                     TEAWEILTEYRDVLDTLAGELLEKETLHRPELESIFADVEKRPRLTMFDDFGGRIPSD
                     KPPIKTPGELAIERGEPWPQPVPEPAFKAAIAQATQAAEAARSDAGQTGHGANGSPAG
                     THRSGDRQYGSTQPDYGAPAGWHAPGWPPRSSHRPSYSGEPAPTYPGQPYPTGQADPG
                     SDESSAEQDDEVSRTKPAHG"
     CDS             4066848..4067648
                     /codon_start=1
                     /gene="TMJG_00858"
                     /note="gene_3745"
                     /transl_table=11
                     /translation="VQRVATISPVAIANPAPGDNAARKTGRGGARQSPQSRVQRVATI
                     SPVAIANPAPGDNAARKTGRGGARQSPQSRVQRVATISPVAIANPAPGDNAARKTGRG
                     GARQSPQSRVQRVATISPVAIANPAPGDNAARKTGRGGARQSPQSRVQRVATISPVAI
                     ANPAPGDNAARKTGRGGARQSPQSRVQRVATISPVAIANPAPGDNAARKTGRGGARQS
                     PQSRVQRVATISPVAIANPAPGDNAARKTGRGGARQSPQSRVQRVATG"
     variation       4066848..4067069
                     /note="supplemented from AL2122/97"
     variation       4067267..4067377
                     /note="supplemented from BCG moreau"
     CDS             complement(4068234..4068806)
                     /codon_start=1
                     /note="gene_3746"
                     /transl_table=11
                     /translation="MNSEGTVDLPGNDFDSNDFDAVDLWGADGAEGWTADPIIGVGSA
                     ATPDTGPDLDNAHGQAETDTEQEIALFTVTNPPRTVSVSTLMDGRIDHVELSARVAWM
                     SESQLASEILVIADLARQKAQSAQYAFILDRMSQQVDADEHRVALLRKTVGETWGLPS
                     PEEAAAAEAEVFATRYSDDCPAPDDESDPW"
     CDS             complement(4068904..4069215)
                     /codon_start=1
                     /note="gene_3747"
                     /transl_table=11
                     /translation="MTENLTVQPERLGVLASHHDNAAVDASSGVEAAAGLGESVAITH
                     GPYCSQFNDTLNVYLTAHNALGSSLHTAGVDLAKSLRIAAKIYSEADEAWRKAIDGLF
                     T"
     CDS             complement(4069289..4070467)
                     /codon_start=1
```

/note="gene_3748"
/transl_table=11
/translation="MSRVFIIDPTISAIDGLYDLLGIGIPNQGGILYSSLEYFEKALE
ELAAAFPGDGWLGSAADKYAGKNRNHVNFFQELADLDRQLISLIHDQANAVQTTRDIL
EGAKKGLEFVRPVAVDLTYIPVVGHALSAAFQAPFCAGAMAVVGGALAYLVVKTLINA
TQLLKLLAKLAELVAAAIADIISDVADIIKGILGEVWEFITNALNGLKELWDKLTGWV
TGLFSRGWSNLESFFAGVPGLTGATSGLSQVTGLFGAAGLSASSGLAHADSLASSASL
PALAGIGGGSGFGGLPSLAQVHAASTRQALRPRADGPVGAAAEQVGGQSQLVSAQGSQ
GMGGPVGMGGMHPSSGASKGTTTKKYSEGAAAGTEDAERAPVEADAGGGQKVLVRNVV
"

CDS 4070933..4071448
/codon_start=1
/note="gene_3749"
/transl_table=11
/translation="MNQTNDRQQAVIDALVGAGLDRKDIRTTRVTVAPQYSNPEPAGT
ATITGYRADNDIEVKIHPTDAASRLLALVVSTGGDATRISSVSYSIGDDSQLVKDARA
RAFQDAKNRADQYAQLSGLRLGKVISISEASGAAPTHEAPAPPRGLSAVPLEPGQQTV
GFSVTVVWELT"

CDS complement(4071453..4072091)
/codon_start=1
/note="gene_3750"
/transl_table=11
/translation="LVVGPAAWHAVHVTQSSSAITPGQTAELYPGDIKSVLLTAEQIQ
ARIAELGEQIGNDYRELSATTGQDLLLITVLKGAVLFVTDLARAIPVPTQFEFMAVSS
YGSSASSSGVVRILKDLDRDIHGRDVLIVEDVVDSGLTLSWLSRNLTSRNPRSLRVCT
LLRKPDAVHANVEIAYVGFDIPNDFVVGYGLDYDERYRDLSYIGTLDPRVYQ"

CDS complement(4072100..4073071)
/codon_start=1
/note="gene_3751"
/transl_table=11
/translation="MDRQSAVAQLRAAAEQFARVHLDACDRWSVGLSGGPDSLALTAV
AARLWPTTALIVDHGLQPGSATVAETARIQAISLGCVDARVLCVQVGAAGGREAAARS
ARYSALEEHRDGPVLLAHTLDDQAETVLLGLGRGSGARSIAGMRPYDPPWCRPLLGVR
RSVTHAACRELGLTAWQDPHNTDRRFTRTRLRTEVLPLLEDVLGGGVAEALARTATAL
REDTDLIDTIAAQALPGAAVAGSRGQELSTSALTALPDAVRRRVIRGWLLAGGATGLT
DRQIRGVDRLVTAWRGQGGVAVGSTLRGQRLVAGRRDGVLVLRREPV"

CDS complement(4073050..4074123)
/codon_start=1
/note="gene_3752"
/transl_table=11
/translation="VVLRVHDVTGASELTLGNTVDWEFAASVGERLARPAPPSTEYTR
RQVIDELTVAAEKAEPPVRDVTGLIADGVVPPARVVDRPAWIRSAAESMRAMTHGSAK
PRGFLTGRITGAQTGAVLAFVASGILGQYDPFGAAGEGCLLLVYPNVIAVERQLRVEP
SDFRLWVCLHEVTHRVQFTANPWLSGYMSQALNLLTFEPVDDIGRVVSRLADFIRSRG
HGTDDSEVNPSGILGLVRAVQSEPQRKALDQLLVLGTLLEGHAEHVMDAVGPMVVPSV
ATIRRRFDDRRHHKQPPLQRLVRALLGFDAKLSQYTRGKAFVDHVVDRAGMKLFNTIW
SGPETLPLPAEIENPQRWIDRVL"

CDS complement(4074099..4075484)
/codon_start=1
/note="gene_3753"
/transl_table=11

/translation="MGPTRWRKSTHVVVGAAVLAFVAVVVAAAALVTTGGHRAGVRAP
VPPPRPPTVKAGVVPVADTAATPSAAGVTAALAVVAVDPDLGKLAGRITDALTGQELW
QRLDDVPLVPASTNKILTAAAALLTLDRQARISTRVVAGGQNPQGPVVLVGAGDPTLS
AAPPGQDTWYHGAARIGDLVEQIRRSGVTPTAVQVDASAFSGPTMAPGWDPADIDNGD
IAPIEAAMIDAGRIQPTTVNSRRSRTPALDAGRELAKALGLDPAAVTIASAPAGARQL
AVVQSAPLIQRLSQMMNASDNVMAECIGREVAVAINRPQSFSGAVDAVTSRLNTAHID
TAGAALVDSSGLSLDNRLTARTLDATMQAAAGPDQPALRPLLDLLPIAGGSGTLGERF
LDAATDQGPAGWLRAKTGSLTAINSLVGVLTDRSGRVLTFAFISNEAGPNGRNAMDAL
ATKLWFCGCTT"

CDS 4075622..4076110
/codon_start=1
/note="gene_3754"
/transl_table=11
/translation="VQFDVTIEIPKGQRNKYEVDHETGRVRLDRYLYTPMAYPTDYGF
IEDTLGDDGDPLDALVLLPQPVFPGVLVAARPVGMFRMVDEHGGDDKVLCVPAGDPRW
DHVQDIGDVPAFELDAIKHFFVHYKDLEPGKFVKAADWVDRAEAEAEVQRSVERFKAG
TH"

CDS complement(4076156..4077253)
/codon_start=1
/note="gene_3755"
/transl_table=11
/translation="MSTFRIFGFSLLMTVVALVTGYLHGGPTALFLLAVLALLEVSLS
FDNAIINAAILQRMSPFWQRMFLTIGILIAVFGMRLVFPLAIIWTTAGLDPVRAMELA
LRPPAHGALEFADGSPSYEKLITAAHPQIAAFGGMFLLMLFLDFVVHDRDIKWLKWIE
VPFARIGRLGQVPVIVASVGLVLAGALLTHSSDQRGTVLIAGLLGMVTYLVVNGISRA
FRPAGLGEATPGVQARQAAGKAGCALFLYLEVLDAAFSFDGVTGAFAITTDPIIIALG
LGVVGAMFVRSITIYLVRQDTLDRYVYLEHGAHWAIGALAIILLLSIDHRFAVPEWVT
ASVGVVFIGAAFTESVRRNRLTVRSPTKFGS"

CDS 4077374..4078669
/codon_start=1
/note="gene_3756"
/transl_table=11
/translation="LAVGAAAVTEVGDTASPVGSSGASGGAIASGSVARVGTATAVTA
LCGYAVIYLAARNLAPNGFSVFGVFWGAFGLVTGAANGLLQETTREVRSLGYLDVSAD
GRRTHPLRVSGMVGLGSLVVIAGSSPLWSGRVFAEARWLSVALLSIGLAGFCLHATLL
GMLAGTNRWTQYGALMVADAVIRVVVAAATFVIGWQLVGFIWATVAGSVAWLIMLMTS
PPTRAAARLMTPGATATFLRGAAHSIIAAGASAILVMGFPVLLKLTSNELGAQGGVVI
LAVTLTRAPLLVPLTAMQGNLIAHFVDERTERIRALIAPAALIGGVGAVGMLAAGVVG
PWIMRVAFGSEYQSSSALLAWLTAAAVAIAMLTLTGAAAVAAALHRAYSLGWVGATVG
SGLLLLLPLSLETRTVVALLCGPLVGIGVHLVALARTDE"

CDS 4078713..4079438
/codon_start=1
/note="gene_3757"
/transl_table=11
/translation="MASKMDTETHYSDVWVVIPAFNEAAVIGKVVTDVRSVFDHVVCV
DDGSTDGTGDIARRSGAHLVRHPINLGQGAAIQTGIEYARKQPGAQVFATFDGDGQHR
VKDVAAMVDRLGAGDVDVVIGTRFGRPVGKASASRPPLMKRIVLQTGARLSRRGRRLG
LTDTNNGLRVFNKTVADGLNITMSGMSHATEFIMLIAENHWRVAEEPVEVLYTEYSKS
KGQPLLNGVNIIFDGFLRGRMPR"

CDS 4079435..4079779
/codon_start=1

/note="gene_3758"
/transl_table=11
/translation="MNWIQVLLIASIIGLLFYLLRSRRSARSRAWVKVGYVLFVLAGI
YAVLRPDDTTVVANWFGVRRGTDLMLYALVMAFSFTTLSTYMRFKDLELRYARIARAL
ALEGAQAPEQCR"
CDS 4079990..4080865
/codon_start=1
/note="gene_3759"
/transl_table=11
/translation="MTQSSSVERLVGEIDEFGYTVVEDVLDADSVAAYLADTRRLERE
LPTVIANSTTVVKGLARPGHVPVDRVDHDWVRIDNLLLHGTRYEALPVHPKLLPVIEG
VLGRDCLLSWCMTSNQLPGAVAQRLHCDDEMYPLPRPHQPLLCNALIALCDFTADNGA
TQVVPGSHRWPERPSPPYPEGKPVEINAGDALIWNGSLWHTAAANRTDAPRPALTINF
CVGFVRQQVNQQLSIPRELVRCFEPRLQELIGYGLYAGKMGRIDWRPPADYLDADRHP
FLDAVADRLQTSVRL"
CDS complement(4080866..4081810)
/codon_start=1
/note="gene_3760"
/transl_table=11
/translation="VRALVTGAAGFIGSTLVDRLLADGHSVVGLDNFATGRATNLEHL
ADNSAHVFVEADIVTADLHAILEQHRPEVVFHLAAQIDVRRSVADPQFDAAVNVIGTV
RLAEAARQTGVRKIVHTSSGGSIYGTPPEYPTPETAPTDPASPYAAGKVAGEIYLNTF
RHLYGLDCSHIAPANVYGPRQDPHGEAGVVAIFAQALLSGKPTRVFGDGTNTRDYVFV
DDVVDAFVRVSADVGGGLRFNIGTGKETSDRQLHSAVAAAVGGPDDPEFHPPRLGDLK
RSCLDIGLAERVLGWRPQIELADGVRRTVEYFRHKHTD"
CDS 4081848..4083608
/codon_start=1
/note="gene_3761"
/transl_table=11
/translation="MPRVALVAVLLITVQLVVRVVLAFGGYFYWDDLILVGRAGTGGL
LSPSYLFDDHDGHVMPGAFLVAGAIIRVAPLVWTGPAISLVVLQLLESLALLRALYVI
SSWRPVLLIPLTFALFTPLAVPGFAWWAAALNSLPMLAALAWVCADAILLVRTGNHRY
AVTGVLVYLGGLLFFEKAAVIPFVSFAVAALQCHVRGDRSALATVWRAGVRLWTPSLA
LTVGWVALYLAVVDQRRWSSDLSMTWDLLCRSVTHGIVPALAGGPWDWARWAPASPWA
TPPAVVMVLGWLVLIAVLALSLVRKRRIGPVWLTAAGYAVACQVPIFLMRSSPFTALE
LAQTLRYFPDLVVVLALLAAVALQAPNRAGTRWLDASPARAVATVASAVLFLTSSLYS
TATFLASWRDNPTEGYLKNAQASLAAAASGAPLLDQEVDPLVLQRVAWPENLASHMFA
LLRVRPEFATTTTQLRMFTSTGRLVDAKVTWVRTIIAGPVPQCGYFVQPDRPERLILD
GPLLPGDWTVELNYLANSDGSMALALSDGPERKVPVHPGLNRVYARLPGAGDAITVRA
NTTALSLCIGAAPVGFLAPA"
CDS 4083951..4084298
/codon_start=1
/note="gene_3762"
/transl_table=11
/translation="VLSVEDWAEIRRLRRSERLPISEIARVLKISRNTVKSALASDGP
PKYQRAAKGSVADEAEPRIRELLAAYPRMPATVIAERIGWWYSIRTLSGRVRELRPLY
LPPDPASRDICGR"
CDS 4084683..4085183
/codon_start=1
/note="gene_3763"
/transl_table=11

```
                     /translation="LPGRVFASPADFNTQLQAWLVRANHRQHRVLGCRPADRIEADTA
                     AMLTLPPVGPSIGWRTSTRLPRDHYVRLDGNDYSVHPVAIGRRIEITADLSRVRVWCG
                     GTLVADHDRIWAKHQTISDPEHVVAAKLLRRKRFDIVGPPHHVEVEQRLLTTYDTVLG
                     LDGPVA"
     CDS             4085183..4085929
                     /codon_start=1
                     /note="gene_3764"
                     /transl_table=11
                     /translation="MAAKTATNSRDVAAELAYLTRALKAPTLRGAIEQLADRARTKTW
                     SYEEFLAACLQREVSARESHGGEGRIRAARFPSRKSLEEFDFDHARGLKRDTIAHLGT
                     LDFVTLAIGIAIRACQAGHRVLFATASQWVDRLAAAHHSGTLQSELIRLARYPLLVVD
                     EVGYIPFEPEAANLFFQLVSSRYERASLIVTSNKPFGRWGEVFGDDVVAAAMIDRLVH
                     HAEVIALKGDSYRIKDRDLGRVPTVTADDQ"
     CDS             complement(4086083..4086649)
                     /codon_start=1
                     /note="gene_3765"
                     /transl_table=11
                     /translation="MAGLFTPPASGAATLQRAARDAAPDARWLLAVSDRNGIVSTSAT
                     TCNYPPAANDSAQDGFRHALAAAIAADIDEALRHGYGDLLELAYPLMSWPRRGVFGGP
                     TPAPRGLATRQCPPRTVHVDRVRPNGAERALRARFRPILRPQFTLGDGANGLPLAACT
                     KTGAYVPHLPYSPIAVDPQPSAGQQGPS"
     CDS             complement(4086719..4087948)
                     /codon_start=1
                     /note="gene_3766"
                     /transl_table=11
                     /translation="MALPQSALSELLDAFRTGDGVDLIRDAVRLVLQELSELEATERI
                     GAARYERSDTRVTDRNGARSRVLSTQAGDVELRIPKLRKGSFFPAILEPRRRIDQALY
                     AVVMEAYVHGISTRAVDDLVEAMGVETGISKSEVSRICAGLDEIVGAFRTRTLGHIEF
                     PYVYLDATYLNVRNGTGQVVSMAVIVASGIAADGSREILGLDVGDSEDETFWRGFLTS
                     LKGRGLGGVRLVISDQHAGLVKALKRCFQGAGHQRCRVHFARNLLAHVPKDKADMVAS
                     MFRMIFSAPDAEAVHATWEEVRDRLAASFPKIGPLMDDARAEVLAFTAFPKAHWQKIW
                     STNPLERINKEIKRRSRVVGIFPNPAAVIRLVGAVLADMHDEWQASERRYLSEASMAL
                     LYPDSDNAVVAAISGGQ"
     CDS             complement(4088124..4088759)
                     /codon_start=1
                     /note="gene_3767"
                     /transl_table=11
                     /translation="VPHPWDTGDHERNWQGYFIPAMSVLRNRVGARTHAELRDAENDL
                     VEARVIELREDPNLLGDRTDLAYLRAIHRQLFQDIYVWAGDLRTVGIEKEDESFCAPG
                     GISRPMEHVAAEIYQLDRLRAVGEGDLAGQVAYRYDYVNYAHPFREGNGRSTREFFDL
                     LLSERGSGLDWGKTDLEELHGACHVARANSDLTGLVAMFKGILDAEPTYDF"
     CDS             complement(4088770..4088964)
                     /codon_start=1
                     /note="gene_3768"
                     /transl_table=11
                     /translation="VFVQATELQKVKRRFRNVRATRRNTELEGTRSTAATRADQNDYA
                     RGKITAAELGERVRRRYNIQ"
     CDS             4089129..4089263
                     /codon_start=1
                     /gene="MORY_19295"
                     /note="gene_3769"
```

```
                     /transl_table=11
                     /translation="VTGRKAGGAPYTVTIPEFGAAALREQRALVIPFDPVFPARRGTR
                     "
     CDS             4089359..4089550
                     /codon_start=1
                     /note="gene_3770"
                     /transl_table=11
                     /translation="VERSIGLEAAAQQAGHSGSEITRRHYVERSVTVPDYTAALDEYS
                     RPIRAFRPLKSNRPGDIPT"
     tRNA            complement(4089561..4089636)
                     /note="identified by tRNAscan-SE; putative"
     CDS             complement(4089715..4090923)
                     /codon_start=1
                     /note="gene_3771"
                     /transl_table=11
                     /translation="MMSGVFTRLVGQQAVEAELLATAKAARRDSAHSAGGGGTMTHAW
                     LLTGPPGSGRSVAALCFAAALQCTSGGEPGCGRCRACTTTLAGTHADVRRVIPEGLSI
                     GVDEMRAIVQIAARRPTTGHWQIVVIEDADRLTEGAANALLKVVEEPPPSTVFLLCAP
                     SVDPEDIAVTLRSRCRHVALVTPSTHAIAQVLSDGDGLDPDTANWAASVSGGHVGRAR
                     RLATDPQARQRRERALGLARDAATPSRAYAAAEELVAGAEAEALALTAQRIEAETEEL
                     RTALGAGGTGKGTGAALRGATGAMKDLERRQKSRQTRASRDALDRALIDLATYFRDAL
                     LVAAHAGGVRANHPDMADRVAALAAHAPPERLLRCIEAVLACREALAVNVKPKFAVDA
                     MVATIGQELR"
     CDS             4091066..4092655
                     /codon_start=1
                     /note="gene_3772"
                     /transl_table=11
                     /translation="MATTAALPRRIHAFVRWVVRTPWPLFSLSMLQSDIIGALFVLGF
                     LRYGLPPQDNIQLQDLPPVNLLIFVSTVIILFLAGAVVNLKLLMPVFRWQRRDNLLTE
                     PDPAATELARSRALRMPLYRTLISLAVWATGGGVFILASWSVAKHAAPVVAVATALGA
                     TATAIIGYLQSERVLRPVAVAALRSGVPENVNAPGVILRLMLAWIPSTGVPLLAIVLA
                     VAADKIALLHATPEALFNPILMMALAALGIGSVSTLLVAMSIADPLRQLRWALSEVQR
                     GNYNAHMQIYDASELGLLQAGFNDMVRELSERQRLRDLFGRYVGEDVARRALERGTEL
                     GGQERDVAVLFVDLVGSTQLAATRPPAEVVQLLNEFFRVVVETVARHGGFVNKFQGDA
                     ALAIFGAPIEHPDGAGAALSAARELHDELIPVLGSAEFGIGVSAGRAIAGHIGAQARF
                     EYTVIGDPVNEAARLTELAKLEDGHVLASAIAVSGALDAEALCWDVGEVVELRGRAAP
                     TQLARPMNLAAPEEVSSEVRG"
     CDS             complement(4092652..4095456)
                     /codon_start=1
                     /note="gene_3773"
                     /transl_table=11
                     /translation="LADPKTKGRGSGGNGSGRRLVIVESPTKARKLASYLGSGYIVES
                     SRGHIRDLPRAASDVPAKYKSQPWARLGVNVDADFEPLYIISPEKRSTVSELRGLLKD
                     VDELYLATDGDREGEAIAWHLLETLKPRIPVKRMVFHEITEPAIRAAAEHPRDLDIDL
                     VDAQETRRILDRLYGYEVSPVLWKKVAPKLSAGRVQSVATRIIVARERDRMAFRSAAY
                     WDILAKLDASVSDPDAAPPTFSARLTAVAGRRVATGRDFDSLGTLRKGDEVIVLDEGS
                     ATALAAGLDGTQLTVASAEEKPYARRPYPPFMTSTLQQEASRKLRFSAERTMSIAQRL
                     YENGYITYMRTDSTTLSESAINAARTQARQLYGDEYVAPAPRQYTRKVKNAQEAHEAI
                     RPAGETFATPDAVRRELDGPNIDDFRLYELIWQRTVASQMADARGMTLSLRITGMSGH
                     QEVVFSATGRTLTFPGFLKAYVETVDELVGGEADDAERRLPHLTPGQRLDIVELTPDG
                     HATNPPARYTEASLVKALEELGIGRPSTYSSIIKTIQDRGYVHKKGSALVPSWVAFAV
```

TGLLEQHFGRLVDYDFTAAMEDELDEIAAGNERRTNWLNNFYFGGDHGVPDSVARSGG
LKKFVGINLEGIDAREVNSIKLFDDTHGRPIYVRVGKNGPYLERLVAGDTGEPTPQRA
NLSDSITPDELTLQVAEELFATPQQGRTLGLDPETGHEIVAREGRFGPYVTEILPEPA
ADAAAAAQGVKKRQKAAGPKPRTGSLLRSMDLQTVTLEDALRLLSLPRVVGVDPASGE
EITAQNGRYGPYLKRGNDSRSLVTEDQIFTITLDEALKIYAEPKRRGRQSASAPPLRE
LGTDPASGKPMVIKDGRFGPYVTDGETNASLRKGDDVASITDERAAELLADRRARGPA
KRPARKAARKVPAKKAAKRD"

CDS complement(4095809..4096387)
/codon_start=1
/note="gene_3774"
/transl_table=11
/translation="VSQLSFFAAESVPPAVADLSGVLAGPGQIVLVGCGARLSVVVAE
SWRASALAEMIQEAGLVPEVARTDENTPLVRTAVDPLLCGIAAEWTRGAVKTVPPRWL
PGPRELRAWTLAAGSPEADRYLLGLDPHAPDTHSPLASALMRVGIAPTLIGTRGTRPA
LRISGRRRLSRLVENVGEPPDGAEAWVQWPRT"

CDS complement(4096527..4096730)
/codon_start=1
/note="gene_3775"
/transl_table=11
/translation="MPQGTVKWFNAEKGFGFIAPEDGSADVFVHYTEIQGTGFRTLEE
NQKVEFEIGHSPKGPQATGVRSL"

CDS 4096980..4099295
/codon_start=1
/note="gene_3776"
/transl_table=11
/translation="MASFGSHLLAAAVAGTPPGERPLRHVAELPPQAGRPRGWPEWAE
PDVVDAFADRGISSPWSHQAEAAELAYAGRHVVIGTGPASGKSLAYQLPVLNALATDS
RARALYLSPTKALGHDQLRAAHALAAAVPRLADVAPTAYDGDSPDEVRRFARERSRWL
FSNPEMTHLSVLRNHARWAVLLRNLRFVIVDECHYYRGVFGSNVAMVLRRLLRLCARY
SAHPTVIFASATTASPGATAADLIGQPVVEVTEDGSPRGARTVALWEPALRSDVIGEH
GAPVRRSAGAEAARVMADLIVEGAQTLTFVRSRRAAELTALGARARLVDIAPELSDTV
ASYRAGYLAEDRSALHQALAEGQLRGLATTNALELGVDIAGLDAVVLAGFPGTVASFW
QQAGRSGRRGQGALVVLIARDDPLDTYLVHHPAALLDKPVERVVIDPVNPHLLGPQLL
CAATELPLDDAEVRSWGAVEVAESLVDDGLLRRRNGRYFPAPGVKPHAAVDVRGAIGG
QIVIVEAGTGRLLGSVGVGQAPAAAHPGAVYLHQGETYVVDSLDFQDGIAFVHAEDPG
YATFAREVTDIAVTGTGERLVFGPVALGLVPVTVTNHVVGYLRRQLSGEVLDFVELDM
PEHTLPTTAVMYTITSDALVRSGIEATRIPGSLHAAEHAAIGLLPLVASCDRDDIGGM
STATGPEGLPSVFVYDGYPGGAGFAERGFRRARTWLGATAEAIEACECPSGCPSCVQS
PKCGNGNDPLDKAGAVRVLRLVLAELSEESP"

CDS 4099432..4099716
/codon_start=1
/note="gene_3777"
/transl_table=11
/translation="VSFVIAAPEALDSAATDLVVLGSTLGAATAAAAAQTTGIVAAAH
DEVSAAIAALFSAHGQAYQAASAQAAAFHTRFIRARSRHPQQETTCRRVR"

CDS 4100040..4101077
/codon_start=1
/note="gene_3778"
/transl_table=11
/translation="MTHDWLLVETLGDEPAVVARGRELKKLVPITTFLRRSPYLAAVR
TAIAETLQTGQSLTSITPKHDRVIRTEPVIMTDGRMHGVQVWSGPTDAEPPDRPIPGP

LKWDLTRGVATDTPESLTNSGKNPEVEITYGRAFAEDLPARELNPNETQVLAMAVKAK
PGKTLCSIWDLTDWQGTPIRIGFVARSALEPGPNGRDHLVARAMNWRAETKAPAVPVD
DLAQRILIGLAQAGVHRALVDLKTWTLLKWLDQPCSFYDWRRSAADGPRLHPDDQHVI
DAMTRDLANGSASHVLRLPGHDVDWVPVHVTVNRIELEPDTFAGLVALRLPTDEELAD
AGLPKATDVTT"
CDS 4101998..4102834
/codon_start=1
/note="gene_3779"
/transl_table=11
/translation="MHGQTYQALSARAAAFHERFVQALATGGGAYAAAEAASVSPLQS
ALDLLNAPTQALLGRPLVGNGANGAPGTGANGGDGGILFGSGGAGGSGAAGMAGGNGG
AAGLFGNGGAGGAGGSATAGAAGAGGNGGAGGLLFGTAGAGGNGGLSLGLGVAGGAGG
AGGSGGSDTAGHGGTGGAGGLLFGAGGAGGAGGLGGFRGAGGTGGAGGDGGNAGLFGD
GGAGEDGTTPGGNGGAGGVAGLFGDGGNGGNAGVGTPAGNVGAGGTGGLLLGQDGMTG
LT"
CDS complement(4102951..4103328)
/codon_start=1
/note="gene_3780"
/transl_table=11
/translation="LLLGQFRWPSRGDRGSATVLAVAMVAVLLCVTGAGAYLGSAVVA
RHRAQAAADLASLAAAARLPSGLAAACARATLVARAMRVEHAQCRVVDLDVVVTVEVA
VAFAGVATATARAGPAKVPTTPG"
CDS complement(4103291..4103578)
/codon_start=1
/note="gene_3781"
/transl_table=11
/translation="LAIATLVLVLVLCLAGVTAVSMQVRCIDAAREAARLAARGDVRS
ATDVARSIAPRAALVQVHRDGEFVVATVTAHSNLLPTLDIAARAISVAEPG"
CDS complement(4103614..4103820)
/codon_start=1
/note="gene_3782"
/transl_table=11
/translation="MLVITMFRVLVARMTALAVDESGMSTVEYAIGTIAAAAFGAILY
TVVTGDSIVSALNRIIGRALSTKV"
CDS complement(4103830..4104432)
/codon_start=1
/note="gene_3783"
/transl_table=11
/translation="VSAAAVLLAMALWLGAGPSVVRARAGRPPRAHRPHQGLLLGRTD
VADPLAVAASLDVLAVCLAAGMAVSTAAAATAAVAPPRLARVLRRAADLLALGADPNI
AWSRPPDLPPGTHDAQTDAVLRLARRSAASGAALADGIVELAVQVRHDAAQAAAAAAE
RAGVLIAGPLGLCFLPAFLCVGIVPLVVGLAGDVLQFGLV"
CDS complement(4104429..4105229)
/codon_start=1
/note="gene_3784"
/transl_table=11
/translation="MSGIASAALILSLALVVLPGSPRCRLTPDDTGRRVLLVGARRVA
WGVGCVAVGVAALLPLPTVVAVAVLGATLGLRYRRRRRYLRRSREGQALEAALELVVG
ELRAGAHPVRAFSIAADETGGPVAVALRAVAARARLGADVTAGLLAAARSSALPAYWE
RLAVCWQLGSDHGLAIASLMRAAQRDVAERQRFSARVSAGMAGARASAAILAILPLLG
VLLGQLIGARPLSFLLTGRVGGWLLVVGLTLACAGLLWSDRITDRPVL"

```
CDS             complement(4105226..4106389)
                /codon_start=1
                /gene="cpaF"
                /note="gene_3785"
                /transl_table=11
                /translation="MTGSLIERVRERLASESGPLRPSVVAAAIRAESGGMLGDTEVLA
                NLRVLQTELTGAGILEPLLSADGTTDVLVTAPDSVWVDDGNGLRRSQIRFADESAVRR
                LAQRLALAAGRRLDDAQPWVDGQLTGIGVGGFAVRLHAVLPPVATQGTCLSLRVLRPA
                TQDLAALAAAGAIDPAAAALVADIVTARLAFLVCGGTGAGKTTLLAAMLGAVSPDERI
                VCVEDAAELAPRHPHLVKLVARRANVEGIGEVTVRQLVRQALRMRPDRIVVGEVRGAE
                VVDLLAALNTGHEGGAGTVHANNPGEVPARMEALGALGGLDRAALHSQLAAAVQVLLH
                VARDRAGRRRLAEIAVLRQAEGRVQAVTVWHADRGMSDDAAALHDLLRSRASA"
CDS             complement(4106386..4107459)
                /codon_start=1
                /note="gene_3786"
                /transl_table=11
                /translation="VGSGVLAMLTDPGLRDELDRVAAAVGVRVVHLGGRHLVSRKTWS
                AAAAVVLDHAAADRCGRLALPRRTHVSVLTGTEAATATWAAAITVGAQHVLRMPEQEG
                ELVRELAEAAESARDDGICGAVVAVIGGRGGAGASLFAVALAQAAADALLVDLDPWAG
                GIDLLVGGETAPGLRWPDLALQGGRLNWSAVRAALPRPRGISVLSGTRRGYELDAGPV
                DAVIDAGRRGGVTVVCDLPRRLTDATQAALDAADLVVLVSPCDVRACAAAATMAPVLT
                AINPNLGLVVRGPSPGGLRAAEVADVAGVPLLASMRAQPRLAEQLEHGGLRLRRRSVL
                ASAARRVLGVLPRAGSGRHGRAA"
CDS             4107938..4108801
                /codon_start=1
                /note="gene_3787"
                /transl_table=11
                /translation="VTVSDSPAQRQTPPQTPGGTAPRARTAAFFDLDKTIIAKPSTLA
                FSKPFFAQGLLNRRAVLKSSYAQFIFLLSGADHDQMDRMRTHLTNMCAGWDVAQVRSI
                VNETLHDIVTPLVFAEAADLIAAHKLCGRDVVVVSASGEEIVGPIARALGATHAMATR
                MIVEDGKYTGEVAFYCYGEGKAQAIRELAASEGYPLEHCYAYSDSITDLPMLEAVGHA
                SVVNPDRGLRKEASVRGWPVLSFSRPVSLRDRIPAPSAAAIATTAAVGISALAAGAVT
                YALLRRFAFQP"
CDS             complement(4109556..4110326)
                /codon_start=1
                /note="gene_3788"
                /transl_table=11
                /translation="VTVDPLAPLMELPGVAAASDRVRDALSRVHRHRTNLRGWPVAAA
                EASLRAARASSVLDGGPARLHDAGAPTSGKPALSDPVFAGALRVGQALEGGAGPVVGV
                WRRAPLQALARLHMLAAADQVDDDRLGRPRSDADVGPRLELLADVVTHPTLASAPVVA
                AVAHGELLTLRPFGCADGVVARAVSRLVTIATGLDPHGLGVPEVIWMRQPAEYHDAAR
                RFAGGTPDGVAGWLLLCCGAMLDGAREALSIAESLSPG"
CDS             complement(4110323..4111978)
                /codon_start=1
                /note="gene_3789"
                /transl_table=11
                /translation="MAGMSVPAAPLLSVEGLEVTFGTDAPAVCGVDLAVRSGQTVAVV
                GESGSGKSTTAAAILGLLPAGGRITAGRVVFDGRDITGADAKRLRSIRGREIGYVPQD
                PMTNLNPVWKVGFQVTEALRANTDGRAARRRAVELLAEAGLPDPAKQAGRYPHQLSGG
                MCQRALIAIGLAGRPRLLIADEPTSALDVTVQRQVLDHLQGLTDELGTALLLITHDLA
                LAAQRAEAVVVVRRGVVVESGAAQSILQSPQHEYTRRLVAAAPSLTARSRRPPESRSR
```

ATTQAGDILVVSELTKIYRESRGAPWRRVESRAVDGVSFRLPRASTLAIVGESGSGKS
TLARMVLGLLQPTSGTVVFDGTYDVGALARDQVLAFRRRVQPVFQNPYSSLDPMYSVF
RAIEEPLRVHHVGDRRQRQRAVRELVDQVALPSSILGRRPRELSGGQRQRVAIARALA
LRPEVLVCDEAVSALDVLVQAQILDLLADLQADLGLTYLFISHDLAVIRQIADDVLVM
RAGRVVEHASTEEVFSRPRHEYTRQLLQAIPGAPSAPRKVGNL"
CDS complement(4111966..4112829)
/codon_start=1
/note="gene_3790"
/transl_table=11
/translation="MAEHTGFWLDAWRGLRRRPKFVIAAALILLILVVAAFPSLFTAA
DPTYADPSQSMLAPSAAHWFGTDLQGHDIYSRTVYGARASVTVGLGATLAVFVVGGAL
GALAGFYGSWIDAVVSRVTDVFLGLPLLLAAIVLMQVMHHRTVWTVIAILALFGWPQV
ARIARGAVLEVRASDYVLAAKALGLNRFQILLRHALPNAVGPVIAVATVALGIFIVTE
ATLSYLGVGLPTSVVSWGGDINVAQTRLRSGSPILFYPAGALAITVLAFMMMGDALRD
ALDPASRAWRA"
CDS complement(4112822..4113748)
/codon_start=1
/note="gene_3791"
/transl_table=11
/translation="MGWYVARRVAVMVPVFLGATLLIYGMVFLLPGDPVAALAGDRPL
TPAVAAQLRSHYHLDDPFLVQYLRYLGGILHGDLGRAYSGLPVSAVLAHAFPVTIRLA
LIALAVEAVLGIGFGVIAGLRQGGIFDSAVLVTGLVIIAIPIFVLGFLAQFLFGVQLE
IAPVTVGERASVGRLLLPGIVLGAMSFAYVVRLTRSAVAANAHADYVRTATAKGLSRP
RVVTVHILRNSLIPVVTFLGADLGALMGGAIVTEGIFNIHGVGGVLYQAVTRQETPTV
VSIVTVLVLIYLITNLLVDLLYAALDPRIRYG"
CDS complement(4113750..4115375)
/codon_start=1
/note="gene_3792"
/transl_table=11
/translation="MVRRMRAALAALATGLLVLAPVAGCGGGVLSPDVVLVNGGEPPN
PLIPTGTNDSNGGRIIDRLFAGLMSYDAVGKPSLEVAQSIESADNVNYRITVKPGWKF
TDGSPVTAHSFVDAWNYGALSTNAQLQQHFFSPIEGFDDVAGAPGDKSRTTMSGLRVV
NDLEFTVRLKAPTIDFTLRLGHSSFYPLPDSAFRDMAAFGRNPIGNGPYKLADGPAGP
AWEHNVRIDLVPNPDYHGNRKPRNKGLRFEFYANLDTAYADLLSGNLDVLDTIPPSAL
TVYQRDLGDHATSGPAAINQTLDTPLRLPHFGGEEGRLRRLALSAAINRPQICQQIFA
GTRSPARDFTARSLPGFDPNLPGNEVLDYDPQRARRLWAQADAISPWSGRYAIAYNAD
AGHRDWVDAVANSIKNVLGIDAVAAPQPTFAGFRTQITNRAIDSAFRAGWQGDYPSMI
EFLAPLFTAGAGSNDVGYINPEFDAALAAAEAAPTLTESHELVNDAQRILFHDMPVVP
LWDYISVVGWSSQVSNVTVTWNGLPDYENIVKA"
CDS 4116733..4118037
/codon_start=1
/gene="acsA2"
/note="gene_3793"
/transl_table=11
/translation="VVRRTGIEMAWSEGRDLWWHHVVGSASPAHTPEPFDSEHPLFLL
YTSGTTGKPKGIMHTSGGYLTQCCYTMRTIFDVKPDSDVFWCTADIGWVTGHTYGVYG
PLCNGVTEVLYEGTPDTPDRHRHFQIIEKYGVTIYYTAPTLIRMFMKWGREIPDSHDL
SSLRLLGSVGEPINPEAWRWYRDVIGGGRTPLVDTWWQTETGSAMISPLPGIAAAKPG
SAMTPLPGISAKIVDDHGDPLPPHTEGAQHVTGYLVLDQPWPSMLRGIWGDPARYWHS
YWSKFSDKGYYFAGDGARIDPDGAIWVLGRIDDVMNVSGHRISTAEVESALVAHSGVA
EAAVVGVTDETTTQAICAFVVLRANYAPHDRTAEELRTEVARVISPIARPRDVHVVPE

LPKTRSGKIMRRLLRDVAENRELGDTSTLLDPTVFDAIRAAK"
CDS complement(4118073..4118771)
/codon_start=1
/note="gene_3794"
/transl_table=11
/translation="LQTAHRRFAAAFAAVLLAVVCLPANTAAADDKLPLGGGAGIVVN
GDTMCTLTTIGHDKNGDLIGFTSAHCGGPGAQIAAEGAENAGPVGIMVAGNDGLDYAV
IKFDPAKVTPVAVFNGFAINGIGPDPSFGQIACKQGRTTGNSCGVTWGPGESPGTLVM
QVCGGPGDSGAPVTVDNLLVGMIHGAFSDNLPSCITKYIPLHTPAVVMSINADLADIN
AKNRPGAGFVPVPA"
CDS 4119117..4119635
/codon_start=1
/note="gene_3795"
/transl_table=11
/translation="VSKIDRKNGVPSTLTTIPLADPHAGPAEPSIGDLIKDATTQMST
LVRAEVELARAEITRDVKKGLTGSVFFISSLVVGFYSTFFFFFFVAELLDTWIWRWVA
FLLVFAIMVVVTAVLALLGFLKVRRIRGPRQTIASVKETRTALTPGHDKTPVTPKPVT
SDRATPVDPSGW"
CDS 4119657..4120619
/codon_start=1
/note="gene_3796"
/transl_table=11
/translation="MTRIAGPWRHLDVHANGIRFHVVEAVPSGQPEGPDAATPPMQPA
LARPLVILLHGFGSFWWSWRHQLCGLTGARVVAVDLRGYGGSDKPPRGYDGWTLAGDT
AGLIRALGHPSATLVGHADGGLACWTTALLHSRLVRAIALISSPHPAALRRSTLTRRD
QRHALLPTLLRYQLPIWPERLLTRNNAAEIERLVRARGCAKWLASEDFSQAIDHLRQA
IQIPAAAHCALEYQRWAVRSQLRSEGRRFIRAMTQQLGMPLLHLRGDADPYVLADPVE
RTQRYAPHGRYISIAGAGHFSHEEAPEEVNRHLMRFLEQVHQLS"
CDS complement(4120612..4121814)
/codon_start=1
/note="gene_3797"
/transl_table=11
/translation="MNSMTPSQWLDIAVLAVAFIAAISGWRAGALGSMLSFGGVLLGA
TAGVLLAPHIVSQISAPRAKLFAALFLILALVVVGEVAGVVLGRAVRGAIRNRPIRLI
DSVIGVGVQLVVVLTAAWLLAMPLTQSKEQPELAAAVKGSRVLARVNEAAPTWLKTVP
KRLSALLTTSGLPAVLEPFSRTPVIPVASPDPALVNNPVVAATEPSVVKIRSLAPRCQ
KVLEGTGFVISPDRVMTNAHVVAGSNNVTVYAGDKPFEATVVSYDPSVDVAILAVPHL
PPPPLVFAAEPAKTGADVVVLGYPGGGNFTATPARIREAIRLSGPDIYGDPEPVTRDV
YTIRADVEQGDSGGPLIDLNGQVLGVVLGAAVDDAETGFVLTAGEVAGQLAKIGATQP
VGTGACVS"
CDS complement(4121811..4122632)
/codon_start=1
/note="gene_3798"
/transl_table=11
/translation="MSAGGTPLQAGATPTGSRGTVALRPDAGPSWLRPLVDNVGQIPD
AYRRRLPADVLAMVTAAGAVSAMTSSRRDHREAAVLVLFSGPEAGPGDGGVPDDADLL
LTVRASTLRHHAGQAAFPGGVVDPADDGPVATALREANEETGIDPSRLHPLATMERTF
IAPSRFHVVPVLAYSPDPGPVAVVNEAETAIVARVPVRAFINPANRLMVYRRPHTRRW
AGPAFLLNQMLVWGFTGQVISAVLDVAGWAQPWDTGDIRELDAAMVLIDDESDPR"
CDS complement(4122764..4123411)
/codon_start=1

/note="gene_3799"
/transl_table=11
/translation="MTTLTGKTRWTIAILAVVAALMAALVAQLHDYSASSTISQRPAP
REHRDGDTPEALAWSRQRANLPPCPAAGNGPGAAALRGVVVVCAGDGSAVDVARALAG
RRVVINLWAHWCAPCMTELPVMAEYQRRVGPAVLVVTVHQGQNEAAALSRLADLGVRL
PTLQDDRRRVAAALRVANVMPATVVLRPDGSVAQTLPRAFGSADEIVAAVGNDAG"
CDS complement(4123447..4124148)
/codon_start=1
/note="gene_3800"
/transl_table=11
/translation="LVRRARRMNRALAQAFPHVYCELDFTTPLELAVATILSAQSTDK
RVNLTTPALFARYRTARDYAQADRTELESLIRPTGFYRNKAASLIGLGQALVERFGGE
VPATMDKLVTLPGVGRKTANVILGNAFGIPGITVDTHFGRLVRRWRWTTAEDPVKVEQ
AVGELIERKEWTLLSHRVIFHGRRVCHARRPACGVCVLAKDCPSFGLGPTEPLLAAPL
VQGPETDHLLALAGL"
CDS 4124042..4124260
/codon_start=1
/note="gene_3801"
/transl_table=11
/translation="MVATASSSGVVKSSSQYTCGNACAKARFIRRARRTKASRVSADQ
RPGTSAARAAGFDLDDFAAVTYDRVLIS"
CDS 4124292..4124669
/codon_start=1
/note="gene_3802"
/transl_table=11
/translation="MFTLLVSWLLVACVPGLLMLATLGLGRLERFLARDTVTATDVAE
FLEQAEAVDVHTLARNGMPEALDYLHRRQARRITDSPPLGSGAGPRYAGPLFVTDLDS
PVEPPRHGQPNPQFRTARHANHV"
CDS 4124768..4125442
/codon_start=1
/note="gene_3803"
/transl_table=11
/translation="VDEILARAGIFQGVEPSAIAALTKQLQPVDFPRGHTVFAEGEPG
DRLYIIISGKVKIGRRAPDGRENLLTIMGPSDMFGELSIFDPGPRTSSATTITEVRAV
SMDRDALRSWIADRPEISEQLLRVLARRLRRTNNNLADLIFTDVPGRVAKQLLQLAQR
FGTQEGGALRVTHDLTQKEIAQLAGASRETVNKALADFAHRGWIRLEGKSVLISDSER
LARRAR"
CDS complement(4125548..4126342)
/codon_start=1
/note="gene_3804"
/transl_table=11
/translation="MSKTAESLTHPAYGQLRAVTDTASVLLADNPGLLTLDGTNTWVL
RGPLSDELVVVDPGPDDDEHLARVAALGRIALVLISHRHGDHTSGIDKLVALTGAPVR
AADPQFLRRDGETLTDGEVIDVAGLTITVLATPGHTADSLSFVLDDAVLTADTVLGCG
TTVIDKEDGSLADYLESLHRLRGLGRRTVLPGHGPDLLDLEAIASGYLLHRHERLEQI
RAALRDLGDDATVREVVEHVYLDVDEKLWNAAEWSVQAQLDYLRTR"
CDS complement(4126349..4126804)
/codon_start=1
/note="gene_3805"
/transl_table=11
/translation="MSAKARLGQLGVTLPQVAAPLAAYVPAVRTGNLVYTAGQLPLEA

GKLVRTGKLGADVNPEEGKTLARICALNALAAVDSLVDLDAVTRVVKVVGFVASAPGF
HGQPSVINGASDLLAEVFGDSGAHARSAVGVSELPLDAPVEVELIVEVG"
CDS complement(4126820..4126981)
/codon_start=1
/note="gene_3806"
/transl_table=11
/translation="MTQPTAWEYATVPLLTHATKQILDQWGADGWELVAVLPGPTGEQ
HVAYLKRPK"
CDS 4127117..4128088
/codon_start=1
/note="gene_3807"
/transl_table=11
/translation="LSGVRLHLVTGKGGTGKSTIAAALALTLAAGGRKVLLVEVEGRQ
GIAQLFDVPPLPYQELKIATAERGGQVNALAIDIEAAFLEYLDMFYNLGIAGRAMRRI
GAVEFATTIAPGLRDVLLTGKIKETVVRLDKNKLPVYDAIVVDAPPTGRIARFLDVTK
AVSDLAKGGPVHAQSEGVVKLLHSNQTAIHLVTLLEALPVQETLEAIEELAQMELPIG
SVIVNRNIPAHLEPQDLAKAAEGEVDADSVRAGLLTAGVKLPDADFAGLLTETIQHAT
RITARAEIAQQLDALQVPRLELPTVSDGVDLGSLYELSESLAQQGVR"
CDS 4128085..4129245
/codon_start=1
/note="gene_3808"
/transl_table=11
/translation="MSVTPKTLDMGAILADTSNRVVVCCGAGGVGKTTTAAALALRAA
EYGRTVVVLTIDPAKRLAQALGINDLGNTPQRVPLAPEVPGELHAMMLDMRRTFDEMV
MQYSGPERAQSILDNQFYQTVATSLAGTQEYMAMEKLGQLLSQDRWDLIVVDTPPSRN
ALDFLDAPKRLGSFMDSRLWRLLLAPGRGIGRLITGVMGLAMKALSTVLGSQMLADAA
AFVQSLDATFGGFREKADRTYALLKRRGTQFVVVSAAEPDALREASFFVDRLSQESMP
LAGLVFNRTHPMLCALPIERAIDAAETLDAETTDSDATSLAAAVLRIHAERGQTAKRE
IRLLSRFTGANPTVPVVGVPSLPFDVSDLEALRALADQLTTVGNDAGRAAGR"
CDS complement(4129488..4129844)
/codon_start=1
/note="gene_3809"
/transl_table=11
/translation="VSGTRPAARRTNLTAAQNVVRSVDAEERIAWVSKALCRTTDPDE
LFVRGAAQRKAAVICRHCPVMQECAADALDNKVEFGVWGGMTERQRRALLKQHPEVVS
WSDYLEKRKRRTGTAG"
CDS 4130206..4132638
/codon_start=1
/note="gene_3810"
/transl_table=11
/translation="MPERLPAAITVLKLAGCCLLASVVATALTFPFAGGLGLMSNRAS
EVVANGSAQLLEGQVPAVSTMVDAKGNTIAWLYSQRRFEVPSDKIANTMKLAIVSIED
KRFADHSGVDWKGTLTGLAGYASGDLDTRGGSTLEQQYVKNYQLLVTAQTDAEKRAAV
ETTPARKLREIRMALTLDKTFTKSEILTRYLNLVSFGNNSFGVQDAAQTYFGINASDL
NWQQAALLAGMVQSTSTLNPYTNPDGALARRNVVLDTMIENLPGEAEALRAAKAEPLG
VLPQPNELPRGCIAAGDRAFFCDYVQEYLSRAGISKEQVATGGYLIRTTLDPEVQAPV
KAAIDKYASPNLAGISSVMSVIKPGKDAHKVLAMASNRKYGLDLEAGETMRPQPFSLV
GDGAGSIFKIFTTAAALDMGMGINAQLDVPPRFQAKGLGSGGAKGCPKETWCVVNAGN
YRGSMNVTDALATSPNTAFAKLISQVGVGRAVDMAIKLGLRSYANPGTARDYNPDSNE
SLADFVKRQNLGSFTLGPIELNALELSNVAATLASGGVWCPPNPIDQLIDRNGNEVAV
TTETCDQVVPAGLANTLANAMSKDAVGSGTAAGSAGAAGWDLPMSGKTGTTEAHRSAG

FVGFTNRYAAANYIYDDSSSPTDLCSGPLRHCGSGDLYGGNEPSRTWFAAMKPIANNF
GEVQLPPTDPRYVDGAPGSRVPSVAGLDVDAARQRLKDAGFQVADQTNSVNSSAKYGE
VVGTSPSGQTIPGSIVTIQISNGIPPAPPPPPLPEDGGPPPPVGSQVVEIPGLPPITI
PLLAPPPPAPPP"

CDS 4132707..4133666
/codon_start=1
/note="gene_3811"
/transl_table=11
/translation="MAAVLPTLIRTGAVALGSAIAGIGYAALVERNAFVLREVTMPVL
TPGSTPLRVLHISDLHMLPNQHRKQAWLRELASWEPDLVVNTGDNLAHPKAVPAVVQT
LSDLLSRPGVFVFGSNDYFGPRLKNPMNYLTSPDHRVRGAALPWQDLRAAFTERGWLD
LTHTRREFEVAGLHIAAAGVDDPHIDRDRYDTIAGPASPAANLRLGLTHSPEPRVLDR
FAADGYQLVLAGHTHGGQLCLPLYGALVTNCGLDRSRAKGASHWGANMRLHVSAGIGT
SPFAPVRFCCRPEATLLTLIATPMGGRDSSSNLGRSQPTVSVR"

CDS 4133663..4134769
/codon_start=1
/note="gene_3812"
/transl_table=11
/translation="LSGGACIAVRSLSRSWTDNAIRLIEADARRSADTHLLRYPLPAA
WCTDVDVELYLKDETTHITGSLKHRLARSLFLYALCNGWINENTTVVEASSGSTAVSE
AYFAALLGLPFIAVMPAATSASKIALIESQGGRCHFVQNSSQVYAEAERVAKETGGHY
LDQFTNAERATDWRGNNNIAESIYVQMREEKHPTPEWIVVGAGTGGTSATIGRYIRYR
RHATRLCVVDPENSAFFPAYSEGRYDIVMPTSSRIEGIGRPRVEPSFLPGVVDRMVAV
PDAASIAAARHVSAVLGRRVGPSTGTNLWGAFGLLAEMVKQGRSGSVVTLLADSGDRY
ADTYFSDEWVSAQGLDPAGPAAALVEFERSCRWT"

tRNA 4134831..4134907
/note="identified by tRNAscan-SE; putative"

CDS complement(4135585..4136946)
/codon_start=1
/note="gene_3813"
/transl_table=11
/translation="VAAFAVRREHAPDALGGPPGLPAPRGFRAAFAAAYAVAYLAGGE
RRMLRLIRRYGPIMTMPILSLGDVAIVSDSALAKEVFTAPTDVLLGGEGVGPAAAIYG
SGSMFVQEEPQHLRRRKLLTPPLHGAALDRYVPIIENSTRAAMHTWPVDRPFAMLTVA
RSLMLDVIVKVIFGVDDPEEVRRLGRPFERLLNLGVSEQLTVRYALRRLGALRVWPAR
ARANTEIDDVVMALIAQRRADPRLGERHDVLSLLVSARGESGEQLSDSEIRDDLITLV
LAGHETTATTLAWAFDLLLHHPDALRRVRAEAVGGGEAFTTAVINETLRVRPPAPLTA
RVAAQPLTIGGYRVEAGTRIVVHIIAINRSAEVYEHPHEFRPERFLGTRPQTYAWVPF
GGGVKRCLGANFSMRELITVLHVLLREGEFTAVDDEPERIVRRSIMLVPRRGTRVRFR
PAR"

CDS complement(4137041..4137406)
/codon_start=1
/note="gene_3814"
/transl_table=11
/translation="LIGNLLSLGVVMVYTGSDAGDHASAPQPSGSGSVPASVNVPGLV
VAAVWAVGLVAGLVALTIGHLAVAAAALVVAVMAPWCRVAYIAHGQHRVCGETLRGTP
AGETASFPTGWRGLRFSTR"

CDS complement(4137613..4137858)
/codon_start=1
/note="gene_3815"
/transl_table=11

/translation="VADNPTALVIDLSAVEFLGSVGLKILAATSEKIGQSVKFGVVAR
GSVTRRPIHLMGLDKTFRLFSTLHDALTGVRGGRIDR"
CDS complement(4138183..4138647)
/codon_start=1
/note="gene_3816"
/transl_table=11
/translation="MAELKSQLRSDLTQAMKTQDKLRTATIRMLLAAIQTEEVSGKQA
RELSDDEVIKVLARESRKRGEAAEIYTQNGRGELAATEHAEARIIDEYLPTPLTEGEL
ADVADTAIAEVAEELGHRPSMKQMGLVMKAATVIAAGKADGARLSAAVKERL"
CDS 4138647..4140002
/codon_start=1
/note="gene_3817"
/transl_table=11
/translation="LHKRYAPQRPKPDTETYIEKCTDRRQDGGHDERRQLLRPVSMLP
PGYPVEPPPVAPGYAPAGYPPYPATPPGYGPPGYGAPPSYGPPPGYGPPLGYPAAPPG
CGPPPGYGPPLGYGPPLAPGAVKPGIIPLRPLTLSDIFNGAVGYIRANPKATLGLTAM
VVVTLQIISLVALFGPMTAFGDIVTGEPDELTGAVVGGWSASFGASLLVSWLAGVLLS
GMLTVIVGRAVFGSPITVGEAWAKVRGRLLALFGLALLEAAGVVAVLGLAVVILSGVA
AAANEAAAALLGFPLLLVVGVSLAYLYVVLLFAPVLIVLERLPIVEAITRSFALVRHG
FWRVLGIRLLTVLVVGVVGNAIAAPFMIVGEIVTAVTASDGSVTMRLVGATLSAIGVT
IGQIVTAPFSAGVVVLLYTDRRIRAEAFDLVLQTGLEAGPAGGPAPVESTDNLWLTRP
F"
CDS 4139981..4140682
/codon_start=1
/note="gene_3818"
/transl_table=11
/translation="MAHAAFLKGVSEDRLTVPSIDIDREAAHQAAQRELDKPIYPKDS
LTKELTDWIDEQLYRILEKGSSIPGGWFTITVLLILLMIAVTAAVQIARRTMRTNRGG
DYQLFDAGQLTAAQHRSTAESYAAEGNWAAAIRHRLQAVARELEETGMLNPAAGRTAN
ELASDAGEVLPHLAGELTQAATAFNDVTYGERPGTQGAYQMIADLDDHLRSRSPAVVS
AVQHPAVFDSWAQVR"
CDS 4140679..4141809
/codon_start=1
/note="gene_3819"
/transl_table=11
/translation="VIPTRLATVRRRRPWRGVLLTLAAVAVVASIGTYLTAPRPGGAM
APASTSSTGGHALATLLGNHGVEVVVADSIADVEAAARPDSLLLVAQTQYLVDNALLD
RLAKAPGDLLLVAPTSRTRTALTPQLRIAAASPFNSQPNCTLREANRAGSVQWGPSDT
YQATGDLVLTSCYGGALVRFRAEGRTITVVGSSNFMTNGGLLPAGNAALAMNLAGNRP
RLVWYAPDHIEGEMSSPSSLSDLIPENVHWTIWQLWLVVLLVALWKGRRIGPLVAEEL
PVVIRASETVEGRGRLYRSRRARDRAADALRTATLQRLRPRLGVGAGAPAPAVVTTIA
QRSKADPPFVAYHLFGPAPATDNDLLQLARALDDIERQVTHS"
CDS 4141959..4142882
/codon_start=1
/note="gene_3820"
/transl_table=11
/translation="LLALRAEVAKAVVGQDDGVISGLVIALLCRGHVLLEGVPGVAKTL
IVRAMSAALQLEFKRVQFTPDLMPGDVTGSLVYDARTAEFVFRPGPVFTNLLLADEIN
RTPPKTQAALLEAMEERQVSVEGEPKPLPNPFIVAATQNPIEYEGTYQLPEAQLDRFL
LKLNVTLPARDSEIAILDRHAHGFDPRDLSAINPVAGPAELAAGREAVRHVLVANEVL
GYIVDIVGATRSSPALQLGVSPRGATALLGTARSWAWLSGRDYVTPDDVKAMARPTLR

HRVMLRPEAELEGATPDGVLDGILASVPVPR"
CDS 4143016..4144338
/codon_start=1
/note="gene_3821"
/transl_table=11
/translation="MILTGRTGLLALICVLPIALSPWPARAFVMLLVALAVAVTVDTL
LAASTRKLRFTRSPYTSARLGQPVDASLLLCNGGRRRFRGQVRDAWPPSARAQPHTHD
VDVAAGQRQQVHTALRPVRRGDQRAAMVTARSIGPLGLAGRQSSQSVPGLVRVLPPFL
SRKHLPSRLAKLREIDGVLPTLIRGQGTEFDSLREYVVGDDVRSIDWRASARRPDVMV
RTWRPERDRRVVIVLDTGRMAAGRVGVDPTAADPAGWPRLDWSMDAALLLAALASRAG
DHVDFLAHDRISRAGVFGASRSELLAQLVDAMAPLRPALIESDWHAMIATILRRTRRR
SLVVLLTDLNATALDEGLLPVLPQLSARHHVLVAAVADPRVDQLAAGRSDAAAVYDAA
AAERARNDRRAIASQLRRGGVDVIDAPPAEIAPGLADRYLAMKATGRL"
CDS complement(4144412..4145404)
/codon_start=1
/note="gene_3822"
/transl_table=11
/translation="VDVDAFLLTNRGTWDRLDHLIKKRHSLSGAEIDELVELYQRVST
HLSMLRSASSDQLMTGRLSSLVARARSAVTGAHAPLTRTFIRFWTVSFPVVAYRTWRW
WLATAVAFFAVVVLIGFWVAGSHEVQSAIGTPTEIDELVSHDVQSYYSEHPAASFALQ
VWVNNSWVATTCIAMSVVLGLPIPLVLFDNAANVGLIAGLMFQAGKGDFLLGLLLPHG
LLELTAVFLAAAIGMRLGWSVISAGNRPRGQVLAEQGRGVVSVAVGLVGVFLVAGLIE
AVVTPSPLPTFVRIAVGIIAEAVFLSYIGYFGRRAAQAGETGDMEDAPDVVPTG"
CDS 4145496..4146428
/codon_start=1
/note="gene_3823"
/transl_table=11
/translation="MSEVVTGDAVVLDVQIAQLPVRAVSAVIDITIIFIGYILGLMLW
ATALTQFDEALTTAFLIIFTVLALVGYPLVWETATRGRSVGKIVMGLRVVSDDGGPER
FRQALFRALASVVEIWMLLGSPAVICSMLSPKAKRVGDVFAGTVVVSERGPRLGPPPV
MPPSLAWWASSLQLSGLTAGQAEVARQFLVRAPQLDPALREQMAYRIAGDVVARIAPP
PPPGVPPQLVLAAVLAERHRRELLRLRPTLPPAGQAPWAQMAPHRGWPPGLSGATPWS
PQQPVIPWPEPDPPPQAAPWPQQAPDGPGFSPPG"
CDS complement(4146492..4148048)
/codon_start=1
/gene="glpK"
/note="gene_3824"
/transl_table=11
/translation="VSDAILGEQLAESSDFIAAIDQGTTSTRCMIFDHHGAEVARHQL
EHEQILPRAGWVEHNPVEIWERTASVLISVLNATNLSPKDIAALGITNQRETTLVWNR
HTGRPYYNAIVWQDTRTDRIASALDRDGRGNLIRRKAGLPPATYFSGGKLQWILENVD
GVRAAAENGDALFGTPDTWVLWNLTGGPRGGGVHVTDVTNASRTMLMDLETLDWDDEL
LSLFSIPRAMLPEIASSAPSEPYGVTLATGPVGGEVPITGVLGDQHAAMVGQVCLAPG
EAKNTYGTGNFLLLNTGETIVRSNNGLLTTVCYQFGNAKPVYALEGSIAVTGSAVQWL
RDQLGIISGAAQSEALARQVPDNGGMYFVPAFSGLFAPYWRSDARGAIVGLSRFNTNA
HLARATLEAICYQSRDVVDAMEADSGVRLQVLKVDGGITGNDLCMQIQADVLGVDVVR
PVVAETTALGAAYAAGLAVGFWAAPSDLRANWREDKRWTPTWDDDERAAGYAGWRKAV
QRTLDWVDVS"
CDS complement(4148098..4148556)
/codon_start=1
/note="gene_3825"

```
                     /transl_table=11
                     /translation="LGDFGPWVSETFDVDVLVHATHRASPFHDKAKTLVERFLARPGL
                     VYLLWPVALGYLRVVTHPTLLGAPLAPEVAVENIEQFTSRPHVRQVGEANGFWPVYRR
                     VADPVKPRGNLVPDAHLVALMRHHGIATIWSHDRDFRKFEGIRIRDPFSG"
     CDS             complement(4148532..4148756)
                     /codon_start=1
                     /note="gene_3826"
                     /transl_table=11
                     /translation="MRTTIDLDDDILRALKRRQREERKTLGQLASELLAQALAAEPPP
                     NVDIRWSTADLRPRVDLDDKDAVWAILDRG"
     CDS             4148843..4150315
                     /codon_start=1
                     /note="gene_3827"
                     /transl_table=11
                     /translation="VGEEVTRTTYSREHQREYRRKVRLCLDVFETMLAQTRFEADRPL
                     TGIEIECNLVDADYQPAMSNRYVLDAIADPAYQTELGAYNIEFNVPPRPLPGRTCLEL
                     EDEVRASLNDAETKASCSGAHIVMIGILPTLMPEHLTDGWMSASARYAALNESIFKAR
                     GEDIPINIAGPEPLSCHAGSIAPESACTSVQLHLQLAPADFPANWNAAQVLAGRQLAL
                     GANSPYFFGHQLWSETRIELFTQSTDARPEELKSRGVRPRVWFGERWITSALDLFQEN
                     IRYFPTLLPEVSDEDPLAELSAGRIPHLSELRLHNGTVYRWNRPVYDVVDGRPHLRLE
                     NRVLPAGPTVVDMLANHAFYYGALRGLSEADPPLWTQMNFAAAQANFLAAARYGMDAQ
                     LDWPGLGEVTTRELVLGTLLPMAHEGLRRWGVDAEVRDRFLGVIGGRAQTGRNGARWQ
                     VATVAALQDGGLTRPAALAEMLRRYCEHMHSNEPVHTWDT"
     CDS             4150337..4151038
                     /codon_start=1
                     /note="gene_3828"
                     /transl_table=11
                     /translation="MTDEVMDWDSAYREQGAFEGPPPWNIGEPQPELATLIAAGKVRS
                     DVLDAGCGYAELSLALAADGYTVVGIDLTPTAVAAATKAAEERGLTTASFVQADITEF
                     AAYPAGSAGRFSTVIDSTLFHSLPVDSRDRYLSSVHRAAAPGASYYVLVFAKGAFPAE
                     LEVKPNEVDEDELRAAVSKYWKIDEIRPAFIHVNPVTIPPQLAGAPVEFPPYDHDEKG
                     RVKFPAYLLTAHKAG"
     CDS             complement(4151041..4152228)
                     /codon_start=1
                     /note="gene_3829"
                     /transl_table=11
                     /translation="MQDEAMRRSGANSPAGDSLADRWRAARPPVAGLHLDSAACSRQS
                     FAALDAAAQHARHEAEVGGYVAAEAAAAVLDAGRAAVAALSGLPDAEVVFTTGSLHAL
                     DLLLGSWPGENRTLACLPGEYGPNLAVMAAHGFDVRPLPTLQDGRVALDDAAFMLADD
                     PPDLVHLTVVASHRGVAQPLAMVAQLCTELKLPLVVDAAQGLGHVDCAVGADVTYASS
                     RKWIAGPRGVGVLAVRPELMERLRARLPAPDWMPPLTVAQQLGFGEANVAARVGFSVA
                     LGEHLACGPQAIRARLAELGDIARTVLADVSGWRVVEAVDEPSAITTLAPIDGADPAA
                     VRAWLLSQRRIVTTYAGVERAPLELPAPVLRISPHVDNTADDLDAFAEALVAATAATS
                     GER"
     CDS             complement(4152244..4153209)
                     /codon_start=1
                     /note="gene_3830"
                     /transl_table=11
                     /translation="MRVSVANHLGEDAGHLALRRDVYSGLQKTPKSLPPKWFYDTVGS
                     ELFDQITRLPEYYPTRAEAEILRARSAEVASACRADTLVELGSGTSEKTRMLLDALRH
                     RGSLRRFVPFDVDASVLSATATAIQREYSGVEINAVCGDFEEHLTEIPRGGRRLFVFL
```

GSTIGNLTPGPRAQFLTALAGVMRPGDSLLLGTDLVKDAARLVRAYDDPGGVTAQFNR
NVLAVINRELEADFDVDAFQHVARWNSAEERIEMWLRADGRQRVRVGALDLTVDFDAG
EEMLTEVSCKFRPQAVGAELAAAGLHRIRWWTDEAGDFGLSLAAK"
CDS complement(4153206..4153907)
/codon_start=1
/note="gene_3831"
/transl_table=11
/translation="MCRHLGWLGAQVAVSSLVLDPPQGLRVQSYAPRRQKHGLMNADG
WGVGFFDGAIPRRWRSPAPLWGDTSFHSVAPALRSHCILAAVRSATVGMPIEVSATPP
FTDGHWLLAHNGVVDRAVLPAGPAAESVCDSAILAATIFAHGLDALGDTIVKVGAADP
NARLNILAANGSRLIATTWGDTLSILRRADGVVLASEPYDDDSGWGDVPDRHLVEVTQ
KGVTLTALDRAKGPR"
CDS complement(4153907..4155184)
/codon_start=1
/note="gene_3832"
/transl_table=11
/translation="VTSPEQLACHLARARARTLRLVDFDDAELCCQYDPLMSPLVWDL
AHIGQQEELWLLRGGDPGQPGLLPPAVEGLYDAFEHSRASRVELPLLSPARARSYCAT
VRSAALDALAALPEDGDSFVFAMVISHENQHDETMLQALNLRTGSPLLAATSALPAGR
PRMAGTSVLVAGGPFVLGVDAADEPCSLDNERPAHVVDVPAFRIGRVPVTNGEWQDFI
DDGGYTQSRWWSERGWQHRQRAGLTAPQFWRSGGRTRTRFGHVEDIPADEPVQHVSYF
EAEAYAAWAGARLPTEVEWEKACAWDPATGSRRRYPWGTEEPTDTYANLGGQTLRPAP
VGAYPAGASACGAEQMLGDVWEWTTSPLRPWPGFVPMVYERYSQPFFGGDYRVLRGGS
WAVEPAILRPSFRNWDHPYRRQIFAGVRLAWDI"
CDS complement(4155181..4156479)
/codon_start=1
/note="gene_3833"
/transl_table=11
/translation="MTLAAMTAAASQLDNAAPDDVEITDSSAAAEYIADGCLVDGPLG
RVGLEMEAHCFDPADPFRRPSWEEITEVLEWLSPLPGGSVVSVEPGGAVELSGPPADG
VLAAIGAMTRDQAVLRSALANAGLGLVFLGADPLRSPVRVNPGARYRAMEQFFAASHS
GVPGAAMMTSTAAIQVNLDAGPQEGWAERVRLAHALGPTMIAIAANSPMLGGRFSGWQ
STRQRVWGQMDSARCGPILGASGDHPGIDWAKYALKAPVMMVRSPDTQDTRAVTDYVP
FTDWVDGRVLLDGRRATVADLVYHLTTLFPPVRPRQWLEIRYLDSVPDEVWPAVVFTL
VTLLDDPVAADLAVDAVEPVATAWDTAARIGLADRRLYLAANRCLAIAARRVPTELIG
AMQRLVDHVDRGVCPADDFSDRVIAGGIASAVTGMMHGAS"
CDS complement(4156611..4157255)
/codon_start=1
/note="gene_3834"
/transl_table=11
/translation="MRIAAAVVSIGLAVIAGFAVPVADAHPSEPGVVSYAVLGKGSVG
NIVGAPMGWEAVFTRPFQAFWVELPACNNWVDIGLPEVYDDPDLASFNGATTQTSATD
QTHLVKQAVGVFASNDAADRAFHRVVDRTVGCSGQTTAIHLDDGTTQVWSFAGGPSTG
TDEAWTKQEAGTDRRCFVQTRLRENVLLQAKVCQSGNAGPAVNVLAGAMQNTLG"
CDS complement(4157884..4158204)
/codon_start=1
/note="gene_3835"
/transl_table=11
/translation="MRHMSETSETPTPPPHQTPKVFKAAAWVAIAAGTVFIVAVIFFT
GYILGKHAGHGGFHHRQHHQHPAMMLRPGSPHGGPAAVRPGPGPGGPGQVPSSVSPPA
TPAP"

```
CDS             complement(4158323..4159471)
                /codon_start=1
                /note="gene_3836"
                /transl_table=11
                /translation="MRVCSAWVVRCSLVVSFLVPQCVWYARADPPAPAPRPILPPLAP
                GQVLRIGPTAGTGTPTGDYGIGATDLCEFVEFPSQLLQVCGDSFAGQGVGFGGWYAPV
                ALHVDTESIDDPAGVRYTGVTGVGTPLLADPTPPGDSQLPAGVVQINRRNYLMVTTTK
                DLQPQNSRLVRAEAARGGWQTVSGSRRNAAYQDGRQTQISGYYDPVPTPDSPTGWVYI
                VADSFTRGEPAVLYRATPESFTDRSRWQGWAGGPDGGWNKPPTPLWPDQLGEMSIRQI
                DGQTVLSYFNASTGNMEVRVAHHPTSLGAAPVTTVVRHDEWPEPAESLPPPYDNRLAQ
                PYGGYISPGSTIDELRIFVSQWDTRARQNGPYRVIQFAVNPFKPWSDP"
CDS             complement(4159473..4160510)
                /codon_start=1
                /note="gene_3837"
                /transl_table=11
                /translation="MGLSIGIVGATGQVGQVMRTLLDERDFPASAVRFFASARSQGRK
                LAFRGQEIEVEDAETADPSGLDIALFSAGSAMSKVQAPRFAAAGVTVIDNSSAWRKDP
                DVPLVVSEVNFERDAHRRPKGIIANPNCTTMAAMPVLKVLHDEARLVRLVVSSYQAVS
                GSGLAGVAELAEQARAVIGGAEQLVYDGGALEFPPPNTYVAPIAFNVVPLAGSLVDDG
                SGETDEDQKLRFESRKILGIPDLLVSGTCVRVPVFTGHSLSINAEFAQPLSPERAREL
                LDGATGVQLVDVPTPLAAAGVDESLVGRIRRDPGVPDGRGLALFVSGDNLRKGAALNT
                IQIAELLTADL"
CDS             complement(4160511..4161776)
                /codon_start=1
                /note="gene_3838"
                /transl_table=11
                /translation="VALVVQKYGGSSVADAERIRRVAERIVATKKQGNDVVVVVSAMG
                DTTDDLLDLAQQVCPAPPPRELDMLLTAGERISNALVAMAIESLGAHARSFTGSQAGV
                ITTGTHGNAKIIDVTPGRLQTALEEGRVVLVAGFQGVSQDTKDVTTLGRGGSDTTAVA
                MAAALGADVCEIYTDVDGIFSADPRIVRNARKLDTVTFEEMLEMAACGAKVLMLRCVE
                YARRHNIPVHVRSSYSDRPGTVVVGSIKDVPMEDPILTGVAHDRSEAKVTIVGLPDIP
                GYAAKVFRAVADADVNIDMVLQNVSKVEDGKTDITFTCSRDVGPAAVEKLDSLRNEIG
                FSQLLYDDHIGKVSLIGAGMRSHPGVTATFCEALAAVGVNIELISTSEIRISVLCRDT
                ELDKAVVALHEAFGLGGDEEATVYAGTGR"
CDS             4162153..4164480
                /codon_start=1
                /note="gene_3839"
                /transl_table=11
                /translation="MPVNRYRPFAEEVEPIRLRNRTWPDRVIDRAPLWCAVDLRDGNQ
                ALIDPMSPARKRRMFDLLVRMGYKEIEVGFPSASQTDFDFVREIIEQGAIPDDVTIQV
                LTQCRPELIERTFQACSGAHRAIVHFYNSTSILQRRVVFRANRAEVQAIATDGARKCV
                EQAAKYPGTQWRFEYSPESYTGTELEYAKQVCDAVGEVIAPTPERPIIFNLPATVEMT
                TPNVYADSIEWMSRNLANRESVILSLHPHNDRGTAVAAAELGFAAGADRIEGCLFGNG
                ERTGNVCLVTLGLNLFSRGVDPQIDFSNIDEIRRTVEYCNQLPVHERHPYGGDLVYTA
                FSGSHQDAINKGLDAMKLDADAADCDVDDMLWQVPYLPIDPRDVGRTYEAVIRVNSQS
                GKGGVAYIMKTDHGLSLPRRLQIEFSQVIQKIAEGTAGEGGEVSPKEMWDAFAEEYLA
                PVRPLERIRQHVDAADDDGGTTSITATVKINGVETEISGSGNGPLAAFVHALADVGFD
                VAVLDYYEHAMSAGDDAQAAAYVEASVTIASPAQPGEAGRHASDPVTIASPAQPGEAG
                RHASDPVTIASPAQPGEAGRHASDPVTIASPAQPGEAGRHASDPVTIASPAQPGEAGR
                HASDPVTIASPAQPGEAGRHASDPVTIASPAQPGEAGRHASDPVTIASPAQPGEAGRH
                ASDPVTIASPAQPGEAGRHASDPVTIASPAQPGEAGRHASDPVTIASPAQPGEAGRHA
```

```
                     SDPVTSKTVWGVGIAPSITTASLRAVVSAVNRAAR"
     CDS             complement(4164546..4165535)
                     /codon_start=1
                     /gene="dnaQ"
                     /note="gene_3840"
                     /transl_table=11
                     /translation="VSHTWGRPASHQDRGWAVIDVETSGFRPGQARIISLAVLGLDAA
                     GRLEQSVVSLLNPKVDPGPTHVHGLTAAMLDDQPQFADIAGEVVDVLRGRTLVAHNVA
                     FDYAFLAAEAEIAEAELPVDFVMCTVELARRLQLGVDNLRLETLAAHWGVPQQRPHDA
                     FDDARVLTGILAAALESARELDVWLPVHPVTRRRWPNGRVTHDELRPLKALAARMACP
                     YLNPGRYVQGRPLVQGMRVGLAAEVKRTHEELVERILHAGLAYSDVVDRDTSLVVCNA
                     TAPEHGKGYHALQLGVPVMPEARFMECIGAVVGGASVEDFTDVAPVEKQLALF"
     CDS             4165651..4165709
                     /note="supplemented from AL2122/97"
     variation       4165651..4165709
                     /note="supplemented from AL2122/97"
     CDS             4165728..4166969
                     /codon_start=1
                     /note="gene_3841"
                     /transl_table=11
                     /translation="VVTTRARLALAAGAGARWASRVTGRGAGAMIGGLVAMTLDRSIL
                     RQLGMGRRTVVVTGTNGKSTTTRMTAAALGTLGAVATNAEGANMDAGLVAALAAHRDA
                     ELAVLEVDEMHVPHISDAVDPAVVVLLNLSRDQLDRVGEINVIERTLRAGLARHPDAV
                     VVANCDDVLMTSAAYDSPNVVWVAAGGAWSNDSVSCPRSSEVIVRKAPSQEDHWYSTG
                     ADFKRPAPHWWFDDATLYGPDGLALPMRLALPGSVNRGNAAQAVAAAVALGADPAVAV
                     AAVCQVDEVAGRYRTVRIGAHQARILLAKNPAGWQEALAMVDKHADGVVIAVNGRVPD
                     GEDLSWLWDVRFEHFETTRVVAAGERGTDLAVRLGYAGVEHTLVHDTVAAIASCPPGR
                     VEVVANYTAFLQLQRALARRG"
     CDS             4166974..4167669
                     /codon_start=1
                     /note="gene_3842"
                     /transl_table=11
                     /translation="VVRIGLVLPDVMGTYGDGGNAVVLRQRLLLRGIAAEIVEITLAD
                     PVPDSLDLYTLGGAEDYAQRLATRHLRRYPGLQRAAGRGAPVLAICAAIQVLGHWYET
                     SSGDRVDGVGLLDVTTSPQDARTIGELVSKPLLAGLTQPLTGFENHRGGTVLGPGTSP
                     LGAVVKGAGNRAGDGFDGAVAGSVVATYMHGPCLARNPELADLLLSKVVGELAPLDLP
                     EVDLLRRERLSAR"
     CDS             complement(4167678..4168532)
                     /codon_start=1
                     /note="gene_3843"
                     /transl_table=11
                     /translation="MSVMGDVFIGSEAITAGRLTRHELQRWYQPMFRGVYVSRRSVPT
                     LWDRTVGAWLATRRHGVIAGNAASALHGAQWVDVDVAIELISPTTRPQHGLVIRRETL
                     CDDEITRVVGLPVTTLARTAYDLGRHLSRGEAVARLDALMRATPFSRDDVLLLAKRHA
                     GARGVRRLRDVLPLVDGGAASPKETWLRLLLIDAGLPVPTTQIPVVHRWRNVGVLDMG
                     WEKYMVAAEYDGDQHRSDRGRYVKDQRRLRKLAELGWIVIRVIAEDNPDDVVNRVRAA
                     LLARGWRP"
     CDS             complement(4168636..4169247)
                     /codon_start=1
                     /gene="recR"
                     /note="gene_3844"
```

/transl_table=11
/translation="MFEGPVQDLIDELGKLPGIGPKSAQRIAFHLLSVEPSDIDRLTG
VLAKVRDGVRFCAVCGNVSDNERCRICSDIRRDASVVCIVEEPKDIQAVERTREFRGR
YHVLGGALDPLSGIGPDQLRIRELLSRIGERVDDVDVTEVIIATDPNTEGEATATYLV
RMLRDIPGLTVTRIASGLPMGGDLEFADELTLGRALAGRRVLA"
CDS complement(4169259..4169660)
/codon_start=1
/note="gene_3845"
/transl_table=11
/translation="MQPGGDMSALLAQAQQMQQKLLEAQQQLANSEVHGQAGGGLVKV
VVKGSGEVIGVTIDPKVVDPDDIETLQDLIVGAMRDASQQVTKMAQERLGALAGAMRP
PAPPAAPPGAPGMPGMPGMPGAPGAPPVPGI"
CDS 4169759..4170520
/codon_start=1
/note="gene_3846"
/transl_table=11
/translation="VDLRVGPRVGFAMIVGVLVAAATPIISSASATPANIAGMVVFID
PGHNGANDASIGRQVPTGRGGTKNCQASGTSTNSGYPEHTFTWETGLRLRAALNALGV
RTALSRGNDNALGPCVDERANMANALRPNAIVSLHADGGPASGRGFHVNYSAPPLNAI
QAGPSVQFARIMRDQLQASGIPKANYIGQDGLYGRSDLAGLNLAQYPSILVELGNMKN
PADSALMESAEGRQKYANALVRGVAGFLATQGQAR"
CDS complement(4170562..4171005)
/codon_start=1
/note="gene_3847"
/transl_table=11
/translation="MGQVSAASTILINAEPTATLDALADYETVRPKILSPHYSEYQVL
EGGKGRGTVAKWRLQATQSRVRDVQVNVDVAGHTVIEKDMNSSMVTNWTVAPAGPGSS
VTVKTTWTGAGGVKGFFEKTFAPLGLKKIQAEVLSNLKTELEGDA"
CDS 4171053..4172465
/codon_start=1
/note="gene_3848"
/transl_table=11
/translation="MQGQLSRTRVYAVPVPGSAQSAYACGVERLLASYRSIPATASIR
LAKPTSNLFRARVKHDARGLDASGLTGVIGIDPEARTADVAGMCTYEDLIAATLHYGL
SPLVVPQLRTITLGGAVTGLGIESASFRNGLPHESVLEMDILTGAGELLTVSPGQHSD
LYRAFPNSYGTLGYSTRLRIQLEPVRPFVALRHIRFSSLTAMVAAMERIIDTGGLDGE
SVDYLDGVVFSADESYLCIGMQTSVPGPVSDYTGQDIYYRSIQHEAGIKEDRLTIHDY
FWRWDTDWFWCSRSFGAQNPRLRRWWPRRYRRSSVYWRLMALDQRFGIADRFENSRGR
PARERVVQDIEVPIERTCEFLEWFGENVPISPIWLCPLRLRDHAGWPLYPIRPDRSYV
NIGFWSSVPVGATEGATNRKIENKVSALDGHKSLYSDSFYTREEFDELYGGETYNTVK
KAYDPDSRLLDLYAKAVQRR"
CDS 4172462..4173745
/codon_start=1
/note="gene_3849"
/transl_table=11
/translation="MTTGRLSMAEILEIFTATGQHPLKFTAYDGSTAGQDDATLGLDL
RTPRGATYLATAPGELGLARAYVSGDLQAHGVHPGDPYELLKTLTERVDFKRPSARVL
ANVVRSIGVEHILPIAPPPQEARPRWRRMANGLLHSKTRDAEAIHHHYDVSNNFYEWV
LGPSMTYTCAVFPNAEASLEQAQENKYRLIFEKLRLEPGDRLLDVGCGWGGMVRYAAR
RGVRVIGATLSAEQAKWGQKAVEDEGLSDLAQVRHSDYRDVAETGFDAVSSIGLTEHI
GVKNYPFYFGFLKSKLRTGGLLLNHCITRHDNRSTSFAGGFTDRYVFPDGELTGSGRI

TTEIQQVGLEVLHEENFRHHYAMTLRDWCGNLVEHWDDAVAEVGLPTAKVWGLYMAAS
RVAFERNNLQLHHVLATKVDPRGDDSLPLRPWWQP"
CDS complement(4173742..4175478)
/codon_start=1
/note="gene_3850"
/transl_table=11
/translation="VALYRKYRPASFAEVVGQEHVTAPLSVALDAGRINHAYLFSGPR
GCGKTSSARILARSLNCAQGPTANPCGVCESCVSLAPNAPGSIDVVELDAASHGGVDD
TRELRDRAFYAPVQSRYRVFIVDEAHMVTTAGFNALLKIVEEPPEHLIFIFATTEPEK
VLPTIRSRTHHYPFRLLPPRTMRALLARICEQEGVVVDDAVYPLVIRAGGGSPRDTLS
VLDQLLAGAADTHVTYTRALGLLGVTDVALIDDAVDALAACDAAALFGAIESVIDGGH
DPRRFATDLLERFRDLIVLQSVPDAASRGVVDAPEDALDRMREQAARIGRATLTRYAE
VVQAGLGEMRGATAPRLLLEVVCARLLLPSASDAESALLQRVERIETRLDMSIPAPQA
VPRPSAAAAEPKHQPAREPRPVLAPTPASSEPTVAAVRSMWPTVRDKVRLRSRTTEVM
LAGATVRALEDNTLVLTHESAPLARRLSEQRNADVLAEALKDALGVNWRVRCETGEPA
AAASPVGGGANVATAKAVNPAPTANSTQRDEEEHMLAEAGRGDPSPRRDPEEVALELL
QNELGARRIDNA"
CDS complement(4175568..4176875)
/codon_start=1
/note="gene_3851"
/transl_table=11
/translation="VSFDSLSPQELAALHARHQQDYAALQGMKLALDLTRGKPSAEQL
DLSNQLLSLPGDDYRDPEGTDTRNYGGQHGLPGLRAIFAELLGIAVPNLIAGNNSSLE
LMHDIVAFSMLYGGVDSPRPWIQEQDGIKFLCPVPGYDRHFAITETMGIEMIPIPMLQ
DGPDVDLIEELVAVDPAIKGMWTVPVFGNPSGVTYSWETVRRLVQMRTAAPDFRLFWD
NAYAVHTLTLDFPRQVDVLGLAAKAGNPNRPYVFASTSKITFAGGGVSFFGGSLGNIA
WYLQYAGKKSIGPDKVNQLRHLRFFGDADGVRLHMLRYQQILAPKFALVAEVLDQRLS
ESKIASWTEPKGGYFISLDVLPGTARRTVALAKDVGIAVTEAGASFPYRKDPDDKNIR
IAPSFPSVPDLRNAVDGLATCALLAATETLLNQGLASSAPNVR"
tRNA 4177092..4177180
/note="identified by tRNAscan-SE; putative"
CDS 4177283..4178047
/codon_start=1
/note="gene_3852"
/transl_table=11
/translation="MGRKVAVLWHASFSIGAGVLYFYFVLPRWPELMGDTGHSLGTGL
RIATGALVGLAALPVVFTLLRTRKPELGTPQLALSMRIWSIMAHVLAGALIVGTAISE
VWLSLDAAGQWLFGIYGAAAAIAVLGFFGFYLSFVAELPPPPPKPLKPKKPKQRRLRR
KKTAKGDEAEPEAAEEAENTELAAQEDEEAVEAPPESIESPGGEPESATREAPAAETA
TAEEPRGGLRNRRPTGKTSHRRRRTRSGVQVAKVDE"
CDS 4178214..4178915
/codon_start=1
/gene="cut5b"
/note="gene_3853"
/transl_table=11
/translation="MDVIRWARRLAVVAGTAAAVTTPGLLSAHVPMVSAEPCPDVEVV
FARGTGEPPGIGSVGGLFVDALRSQVGAKSLGVYAVNYPASNDFASSDFPKTVIDGIR
DAGSHIQSMAMSCPQTRQVLGGYSQGAAVAGYVTSAVVPPAVPVQAVPAPMAPEVANH
VAAVTLFGAPSAQFLGQYGAPPIAIGPLYQPKTLQLCADGDSICGDGNSPVAHGLYAV
NGMVGQGANFAASRL"
CDS 4178960..4179961

/codon_start=1
/note="gene_3854"
/transl_table=11
/translation="MQNATMRVLVTGGTGFVGGWTAKAIADAGHSVRFLVRNPARLKT
SVAKLGVDVSDFAVADISDRDSVREALNGCDAVVHSAALVATDPRETSRMLSTNMAGA
QNVLGQAVELGMDPIVHVSSFTALFRPNLATLSADLPVAGGTDGYGQSKAQIEIYARG
LQDAGAPVNITYPGMVLGPPVGDQFGEAGEGVRSALWMHVIPGRGAAWLIVDVRDVAA
LHAALLESGRGPRRYTAGGHRIPVPELAKILGEVAGTTMLAVPVPDSALRVAGSVLDQ
AGPYLPFNTPFTAAGMQYYTQMPEPDDSPSEKELGITYRDPRDTVADTVTALRGLGS"
CDS complement(4179958..4180128)
/codon_start=1
/note="gene_3855"
/transl_table=11
/translation="VRRGGAVTMDYVATAIVVTLVILLGVGIVVNELLRLKQWLKNSR
PDAEPAEPPDGS"
CDS 4180168..4181361
/codon_start=1
/note="gene_3856"
/transl_table=11
/translation="MKAVTCTNAKLEVVDRPSPAPAKGQLLLDVLRCGICGSDLHARL
HCDELADVMAESGYHAFMRSNQQVVFGHEFCGEVVDYGPGTRRTPRRGTPVVAMPLLR
RGNKEVHGIGLSTMAPGAYAERLVVEQSLTFPVPNGLAPEIAALTEPMAVGWHAVRRG
EVGKGDVAIVIGCGPIGLAVICMLKSRGVHTVIASDFSPGRRALATACGADSVVDPVQ
DSPYAVAAGLGQGNRHLQSILDAFDLAVGTVESLQRLRLPWWHLWRAAEAAGAATPKR
PVIFECVGVPGIIDGIIASAPLFSRVVVVGVCMGSDHIRPAMAINKEINLRFVLGYTP
LEFRDTLHMLADGKVNAAPLITGTVGLPGVAAAFDALGDPEAHAKIMIDPKSNAASPQ
PFRVE"
CDS 4181696..4183510
/codon_start=1
/note="gene_3857"
/transl_table=11
/translation="LQMKPSPADTHVVIAGAGIAGLAAAMILAEAGVRVTLCEAASEA
GGKAKSLRLADGHPTEHSLRVYTDTYQTLLTLFSRIPTEHDRTMLDNLVGVSMVSATA
QGVIGRIAAPVALQRRRPTFARIIGKVVEPPRQLVRILLRGPMVIVGLAQRGVPATDV
LHYLYAHLRLLWMCRERLLAELGDISYADYLQLGCKSAQAQEFFSAVPRIYVAARTSA
EAAAIAPIVLKGLFRLKSNCPSALNDAKLPAIMMMDGPTSERMVDPWIRHLTRLGVDI
HFNTRVGDLEFDDGRVTALISSDGRRFACDYALLAVPYLTLRELAKSAHVKRYLPQLT
QQHALALEASNGIQCFLRDLPATWPPFIRPGVVTTHLQSQWSLVCVLQGEGFWKNVRL
PEGTRYVLSITWSDVETPGPVFDRPLSECTPDEILTECLTQCGLDKSNVLGWRIDHEL
KHLDEAEYEKVASELPPHLVSAPARGQRMVNFSPLTVLMPGARHRSPGICTSVPNLLL
AGEVIYSPDLTLFVPTMEKAACSGYLAARQIMNMVASHAAPLRIDFRDPAPFAVLRRV
DRWFWSRRRRPPDRSTFATPPTAMPAPSHLTDVDRSAS"
CDS 4183743..4186817
/codon_start=1
/note="gene_3858"
/transl_table=11
/translation="VLLVAAFGAFLAFLDSTIVNVAFPDIQRHFHSDISDLSWMLNAY
NIVFAAFLVAAGRLADLMGRKRVFILGVALFTVASGLCAIAESVGELVAFRVLQGIGA
AVLVPASLGLVVEAFPAERRAHGVNLWGAAGAIAAGLGPPIGGALIEADGWRWVFLVN
LPLGVFAVLAARRALVENRAAGRRRVPDVRGAVLLAFALGLLTLGLIKGPDWGWASLP
TSGSLLAAAVAMVGFVMSSRHHPAPMVEPTLLRIQSFVAGTGLTAVASAGFYAYLLTH

VLFLNYVWGYTLLEAGMAVAPAALVAAVVAAVLGRVADRHGYRFIVGIGALIWAASLL
WYLKVVGSQPDFLGEWLPGQILQGIGVGATFPLLGSAALARLAKGGSYATASAVTGTI
RQVGAVIGVAVLVILVGTPAPGAAEEALRHRWALAAICFVAVGIGALSLGRIRPVPAA
VEPPPGPPVAPLGARRPPRPAPVASPAAAVAPTPKTSREVNLLEALRFARPDTQQIEL
QAGSYLFHAGDVSDALYVVRSGRLQVLAGDGAKDEVVAELGRGQVVGELGVLLDAPRS
ASVRAVRDSSLMRVTKAEFAKIADAGVLGALAGVLAKRQHQTRVASQRTTPEVVVAVV
GVDANAPVAMVATELCRALSTRLRAVAPGRVDCDGLERAEQTADRVVLHAAVGDARWR
EFCLRVADRVVLVASNPAVPVAPLPTRATGADLVLAGRPAGREHRRAWEQLITPRSMH
VVRREFVADDLRVLATRIAGRSVGLVLSGGAARACAHLGVLEELEAAGVTVDRFAGTS
MGAIIAALAASGLDAAGVDAQIYEHFVRKSHGDYTLPSKGLIRGKRTQSTLRTIFGDH
LVEELPKHFRCVSVDLLARRPVVHRQGPLADVVGCSMRLPFLYAPLPYGGTLHVDGGV
LDNVPVTTLVGKDGPLIAVNVASGGNPSPASGGHRRGKPRVPGLTDTLLRTMTISSAM
ASEKVLAQADLVIKPNPIGVGLMEYHQIDRAREAGRIAAREALPQIMELVHG"

CDS 4186996..4189362
/codon_start=1
/note="gene_3859"
/transl_table=11
/translation="MTRADGKRDRDEMFVEYTKSICPVCKVVVDAQVNIRHDKVYLRK
RCREHGSFEALVYGDAQMYLESARFNKPGTFPLRFQTEVRDGCPSDCGLCPDHKQHAC
LGLIEVNTHCNLDCPICFADSGHQPDGYAITAAQCERMLDTLVAAEGEPEVVMFSGGE
PTIHKQLLEFVDAAQARPVKTIIINTNGIRLASDRRFVDQLATRNRPGHPVHIYLQFD
GLDEATHRRIRGHDLRDVKQRALDNCAAAGLTVSLVAAVERGLNEHELGAVIRHGMAQ
PGVQSVVFQPVTHAGRHVQFDPLTRLTNSDIIACITAQLPEWFRPGDFFPVPCCFPSC
RSITYLLTDGEHVVPIPRLLNVEDYLDYVSNRVIPDLAIREALENLWSASAVPGTDTM
TAQLQRATAALNCAEGCGINLPEALTHLTDRVFAIVIQDFQDPYTLNVKQLMKCCVQQ
ITPDGRLIPFCAYNSVGYREQVREQLTGVPVPDIVPNAIPLAGLLADAPHGSKQANTG
GSIARLAGPTRGAPMALPPQQIKACCADAYSRDIVALLLGDSFHPGGATLTRRLADQL
GLRSTGDPRRVADIAAGPGASARLLASDYGVAVDGVDISEINVKRAQAAVAQTGLTER
VRFHLGDAESVPLPDDTFDALVCECAFCTFPDKNAAAQQFARILRPGGLAGITDVTVG
DGGLPAELTPLAAWVACIADARTVTDYTDILEGAGLRTRHIESHDESLLDMIDRIDAR
ITALHVAAPEILADNGIRHDSVRDFTALARAAVQTGRIGYTLMIAEKP"

CDS complement(4189427..4190611)
/codon_start=1
/note="gene_3860"
/transl_table=11
/translation="MERNPSHLGSYDASGGIAVTDLASIGDTGGMTGNCMCSFLRAAC
WNSDMAAAAEELDVDGIAVRLTSPDRMYFPKLGSHGTKRRLVEYYFAVAGGPMLTALR
DRPTHLQRFPDGVDGEQIYQKRIPRHRPDYLQTCRVTFPSGRMADALKVTHPAAIVWA
AQMGTITLHPWQVRCPDTEHPDELRIDLDPQPGTGFVEARTVAVDVLRSVLDDLGLVG
YPKTSGGRGIHVFLRIATDWDFVEVRRAGIALAREVERRAPDAVTTSWWKEERGARIF
IDFNQNARDRTMASAYSVRPTPIATVSMPLTWEELAGADPDDYTMTTVPELVKIRDDP
WAGMDDVAQSIAPLLDLAAADEERGLGDMPYPPNYPKMPGEPKRVQPSRDTDLKGGNT
SK"

CDS 4190505..4191581
/codon_start=1
/note="gene_3861"
/transl_table=11
/translation="MQLPVMPPVSPMLAKSVTAIPPDASYEPKWDGFRSICFRDGDQV
ELGSRNERPMTRYFPELVAAIRAELPHRCVIDGEIIIATDHGLDFEALQQRIHPAESR
VRMLADRTPASFIAFDLLALGDDDYTGRPFSERRAALVDAVTGSGADADLSIHVTPAT
TDMATAQRWFSEFEGAGLDGVIAKPPHITYQPDKRVMFKIKHLRTADCVVAGYRVHKS

GSDAIGSLLLGLYQEDGQLASVGVIGAFPMAERRRLLTELQPLVTSFDDHPWNWAAHV
AGQRTPRKNEFSRWNVGKDLSFVPLRPERVVEVRYDHMEGARFRHTAQFNRWRPDRDP
RSCSYAQLERPLTVSLSDIVPGLR"
CDS 4191681..4192739
/codon_start=1
/note="gene_3862"
/transl_table=11
/translation="MAVLPACRLGLVVCVATAVITATMVLATPSYACACGAAVTAHGS
QATLNHEVALLHWDGTTETIVMQLAMNADTDNVALVVPTPTPAIVTTADQSTFGELDT
LSAPLIEHQRHWSLRRGVGASGPQEAAARAPHVLNQVRLGPLEATTLTGGDLSGLQTW
LSDNGYAIRPAVSAALDPYVRDGWAFVAIRLTSTDLIVGGLDPVRMTFRSSRLVYPMR
LSVAAQEPQHVTIFTLSDHRQQRTDADAATQTTHVRFAGDMSTAVRDPLLRELIGNHG
SYLTKVEVDIYQTSRISSDFTFGNAPNDDPYRQVVTVYDDVALPPLLLVVVSAIAVGA
AGGAVVVVLRRRRRAHTG"
CDS complement(4192759..4193259)
/codon_start=1
/note="gene_3863"
/transl_table=11
/translation="MPKLSAGVLLYRARAGVVDVLLAHPGGPFWAGKDDGAWSIPKGE
YTGGEDPWLAARREFSEEIGLCVPDGPRIDFGSLKQSGGKVVTVFGVRADLDITDARS
STFELDWPKGSGKMRKFPEVDRVSWFPVARARTKLLKGQRGFLDRLMAHPAVAGLSEG
PESLPR"
CDS complement(4193273..4194637)
/codon_start=1
/note="gene_3864"
/transl_table=11
/translation="MDLMMPNDSMFLFIESREHPMHVGGLSLFEPPQGAGPEFVREFT
ERLVANDEFQPMFRKHPATIGGGIARVAWAYDDDIDIDYHVRRSALPSPGRVRDLLEL
TSRLHTSLLDRHRPLWELHVVEGLNDGRFAMYTKMHHALIDGVSAMKLAQRTLSADPD
DAEVRAIWNLPPRPRTRPPSDGSSLLDALFKMAGSVVGLAPSTLKLARAALLEQQLTL
PFAAPHSMFNVKVGGARRCAAQSWSLDRIKSVKQAAGVTVNDAVLAMCAGALRYYLIE
RNALPDRPLIAMVPVSLRSKEDADAGGNLVGSVLCNLATHVDDPAQRIQTISASMDGN
KKVLSELPQLQVLALSALNMAPLTLAGVPGFLSAVPPPFNIVISNVPGPVDPLYYGTA
RLDGSYPLSNIPDGQALNITLVNNAGNLDFGLVGCRRSVPHLQRLLAHLESSLKDLEQ
AVGI"
CDS 4194836..4195324
/codon_start=1
/note="gene_3865"
/transl_table=11
/translation="MSLAWDVVSVDKPDDVNVVIGQAHFIKAVEDLHEAMVGVSPSLR
FGLAFCEASGPRLVRHTGNDGDLVELATRTALAIAAGHSFVIFLREGFPINILNPVQA
VPEVCTIYCATANPVDVVVAVTPHGRGIVGVVDGQTPLGVETDRDIAQRRDLLRAIGY
KL"
CDS 4195381..4196442
/codon_start=1
/note="gene_3866"
/transl_table=11
/translation="MSVVRGTALANYPSLVAGLGGDPATLLRAAGVRDQDVGNYDAFI
SIRAAIRAIESAAAVTATMDFGRRLAQRQGIKILGPVGVAARTAATVGDALAIFNTFM
AAYSPVIAIRITPLAGQRSFIALEFLLDEPASYPQTMELALGVALGVIRLLLGADYAP
LAVHLPHDPLTPEAFYLQYFGCRPYFAERVGGFTMRTADLSRPLNRDDVAHRVVVDYL

SSITPLGEGIVESVRTIVRQLLPTGAATLNVVAEQFHLHPKTLQRRLAEENTTFVILV
DRVRKDVADRYLRTTGIGLTHLARELGYAEQSVLTRSCKRWFGTGPAAYRNQARLQTT
VSAPGSGRGPNPGNVSVSC"

CDS 4196446..4198035
/codon_start=1
/note="gene_3867"
/transl_table=11
/translation="MDQDRSDNTALRRGLRIALRGRRDPLPVAGRRSRTSGGIGDLHT
RKVLDLTIRLAEVMLSSGSGTADVVATAQDVAQAYQLTDCVVDITVTTIIVSALATTD
TPPVTIMRSVRTRSTDYSRLAELDRLVQRITSGGVAVDQAHEAMDELTERPHPYPRWL
ATAGAAGFALGVAMLLGGTWLTCVLAAVTSGVIDRLGRLLNRIGTPLFFQRVFGAGIA
TLVAVAAYLIAGQDPTALVATGIVVLLSGMTLVGSMQDAVTGYMLTALARLGDALFLT
AGIVVGILISLRGVTNAGIQIELHVDATTTLATPGMPLPILVAVSGAALSGVCLTIAS
YAPLRSVATAGLSAGLAELVLIGLGAAGFGRVVATWTAAIGVGFLATLISIRRQAPAL
VTATAGIMPMLPGLAVFRAVFAFAVNDTPDGGLTQLLEAAATALALGSGVVLGEFLAS
PLRYGAGRIGDLFRIEGPPGLRRAVGRVVRLQPAKSQQPTGTGGQRWRSVALEPTTAD
DVDAGYRGDWPATCTSATEVR"

CDS complement(4198032..4198409)
/codon_start=1
/note="gene_3868"
/transl_table=11
/translation="VLRVALGLTKASPVYLPGVKGRITADVAPVAVRPAAAPPLRESA
AVRPEARLVSAVAPAPAGTSASVLASDRGAGVLGFAGTAGKESVGRPAGLTTLAGGEF
GGSPSVPMVPASWEQLVGAGEAG"

CDS complement(4198414..4199769)
/codon_start=1
/note="gene_3869"
/transl_table=11
/translation="MELMSPIDALFLSAESREHPLHVGALQLFEPPAGAGRGFVRETY
QAMLQCREIAPLFRKRPTSLHGALINLGWSTDADVDLGYHARRSALPAPGRVRELLEL
TSRLHSNLLDRHRPLWETHVIEGLRDGRFAIYSKMHHALVDGVSGLTLMRQPMTTDPI
EGKLRTAWSPATQHTAIKRRRGRLQQLGGMLGSVAGLAPSTLRLARSALIEQQLTLPF
GAPHTMLNVAVGGARRCAAQSWPLDRVKAVKDAAGVSLNDVVLAMCAGALREYLDDND
ALPDTPLVAMVPVSLRTDRDSVGGNMVGAVLCNLATHLDDPADRLNAIHASMRGNKNV
LSQLPRAQALAVSLLLLSPAALNTLPGLAKATPPPFNVCISNVPGAREPLYFNGARMV
GNYPMSLVLDGQALNITLTSTADSLDFGVVGCCRSVPHVQRVLSHLETSLKELERAVG
L"

CDS complement(4199760..4200434)
/codon_start=1
/note="gene_3870"
/transl_table=11
/translation="MIGRDRAYAVTRRKDIAKQRLVWRLCQRYPRAARRLIRHLNAKQ
LAAGYPADEHFKPVYNPWDQRLCAVPDADMFKAIRDGRASVVTEAIDTFTENGIRLQS
GRELAADISITATGLNLLAFGGINLSVDGVAVDVAEKVAFKGFLLSDVSNFAGPHGRT
RAHHLLSAAARSHADPAAAGRRSPLADLKVLREGPVDDDHLRFTTSASASRLTVKRIT
RSTPWN"

CDS complement(4200431..4200916)
/codon_start=1
/note="gene_3871"
/transl_table=11
/translation="VLVHSEFGDAHHPRLTTKSRVSLRPNRKYAVHSEQSASIEHVDV

LIVGAGISGTGAAYYLKTMQPAKTFAIVEARYPAIRSDSDLHTFSYEFKPWQHEKATA
SADAIMVHRGRSLAGGDRTLRHRRTRHHELRMVIIGSGATAVTLVPAMAQTAGAVTMP
K"
CDS complement(4200972..4202906)
/codon_start=1
/note="gene_3872"
/transl_table=11
/translation="LVLARRTKLFALSEMRWAALALGLFSAGLLTQLCGAPQWVRWAL
FLACYATGGWEPGLAGLQALQRRTLDVDLLMVVAAIGAAAIGQIAEGALLIVIFATSG
ALEALVTARTADSVRGLMGLAPGTATRVGAGGGEETVNAADLRIGDIVLVRPGERISA
DATVLAGGSEVDQATVTGEPLPVDKSIGDQVFAGTVNGTGALRIRVDRLARDSVVARI
ATLVEQASQTKARTQLFIEKVEQRYSIGMVAVTLAVFAVPPLWGETLQRALLRAMTFM
IVASPCAVVLATMPPLLAAIANAGRHGVLAKSAIVMEQLGTTTRIAFDKTGTLTRGTP
ELAGIWVYERRFTDDELLRLAAAAEYPSEHPLGAAIVKAAQSRRIRLPTVGEFTAHPG
CRVTARVDGHVIAVGSATALLGTAGAAALEASMITAVDFLQGEGYTVVVVVCDSHPVG
LLAITDQLRPEAAAAISAATKLTGAKPVLLTGDNRATADRLGVQVGIDDVRAGLLPDD
KVAAVRQLQAGGARLTVVGDGINDAPALAAAHVGIAMGSARSELTLQTADAVVVRDDL
TTIPTVIAMSRRARRIVVANLIVAVTFIAGLVVWDLAFTLPLPLGVARHEGSTIIVGL
NGLRLLRHTAWRRAAGTAHR"
CDS 4203021..4203383
/codon_start=1
/note="gene_3873"
/transl_table=11
/translation="MGHGVEGRNRPSAPLDSQAAAQVASTLQALATPSRLMILTQLRN
GPLPVTDLAEAIGMEQSAVSHQLRVLRNLGLVVGDRAGRSIVYSLYDTHVAQLLDEAI
YHSEHLHLGLSDRHPSAG"
CDS complement(4203467..4203679)
/codon_start=1
/note="gene_3874"
/transl_table=11
/translation="MSDCNVLGGALEQGGTDPLTGFYRDGCCATGPEDLGWHTICAVM
TTEFLAHQRSVGNDLSIARPPRWLRP"
CDS complement(4203752..4204087)
/codon_start=1
/note="gene_3875"
/transl_table=11
/translation="MQSMSFDPAVADIGSQVVNNAFQGLQAGAVAWVSLSSLLPAGAE
EVSAWAVTAFTTAATGLLALNQAAQEELRKAGEVFTAIARMYSDADVRAAACLLEAIP
RPGQTLARE"
CDS 4204305..4204688
/codon_start=1
/note="gene_3876"
/transl_table=11
/translation="VILTGAFLADAAAAVDNKLNVQGGVLSRFAVGPDRLARFVLVVL
TQAEPDSSDRDITVEMRPPTDDEPIRLNFEAPEAAVAEFPGFAFFEIQLRLPVNGRWV
LVVTGGTGAISLPVLVSDMPATIGF"
CDS 4204818..4205177
/codon_start=1
/note="gene_3877"
/transl_table=11
/translation="VIVGAFLAEAASVVDNKLNVSGGVLYRFAVDPDRSAQFLLVVLT

QAETDDPDRRVDVEVWPPTGDDAHHIEFELPEAAVAAEVGFAIFRIEVNLPVDGRWVL
VVTGDAGTISLPLIVTG"
CDS complement(4205210..4205560)
/codon_start=1
/note="gene_3878"
/transl_table=11
/translation="MNNHYKDRHVLAAAVRERAEVIVTTNLKHFPDDALKPYQIKALH
PDDFLLDQLDLYEEATKAVILGMVDAYIDPPFTPHSLLDALGEQVPQFAAKARRLFPS
GSPFGLGVLLPFDQ"
CDS complement(4205787..4206233)
/codon_start=1
/note="gene_3879"
/transl_table=11
/translation="MTAAVQERTVLPPRDPQDMTSLLEVLGAPEVSVCGNAGQPMTLP
EPVRDALYNVVLALSQGKGISLVPRHLKLTTQEAADLLNISRPTLVRLLEDGRIPFEK
PGRHRRVSLDALLEYQQETRSNRRAALGELSRDALGELQAALAEKK"
CDS complement(4206337..4206537)
/codon_start=1
/gene="MT3858"
/note="gene_3880"
/transl_table=11
/translation="LTPADTAKLAAVCRKSCGVICWTFARFTAPQIRPRVGFGRGRRA
PVSSSDQLVRSAGLDVTVLGGV"
CDS 4206455..4206670
/codon_start=1
/note="gene_3881"
/transl_table=11
/translation="VKRAKVQQITPHDLRHTAASLAVSAGVNVLALQRILGHKSAKVT
LDTYADLFDADLDAVAVTLGKDADQQT"
tRNA complement(4206709..4206798)
/note="identified by tRNAscan-SE; putative"
CDS complement(4206828..4207286)
/codon_start=1
/note="gene_3882"
/transl_table=11
/translation="VTTDEDLIRAALAVAATAGPRDVPVGAVVVGADGTELARAVNAR
EALGDPTAHAEILAMRLAAGVLGDGWRLEGTTLAVTVEPCTMCAGALVLARVARLVFG
AWEPKTGAVGSLWDVVRDRRLNHRPEVRGGVLARECAAPLEAFFARQRLG"
CDS complement(4207302..4207823)
/codon_start=1
/note="gene_3883"
/transl_table=11
/translation="MGAQRASTQRPAADTPDGFGVAVVREEGRWRCSPMGPKALTSLR
AAETELRELRSAGAVFGLLDVDDEFFVIVRPAPSGTRLLLSDATAALDYDIAAEVLDN
LDAEIDPEDLEDADPFEEGDLGLLSDIGLPEAVLGVILDETDLYADEQLGRIAREMGF
ADQLSAVIDRLGR"
CDS 4207969..4208907
/codon_start=1
/note="gene_3884"
/transl_table=11
/translation="VLGLGLIGGSIMRAAAAAGREVFGYNRSVEGAHGARSDGFDAIT

DLNQTLTRAAATEALIVLAVPMPALPGMLAHIRKSAPGCPLTDVTSVKCAVLDEVTAA
GLQARYVGGHPMTGTAHSGWTAGHGGLFNRAPWVVSVDDHVDPTVWSMVMTLALDCGA
MVVPAKSDEHDAAAAAVSHLPHLLAEALAVTAAEVPLAFALAAGSFRDATRVAATAPD
LVRAMCEANTGQLAPAADRIIDLLSRARDSLQSHGSIADLADAGHAARTRYDSFPRSD
IVTVVIGADKWREQLAAAGRAGGVITSALPSLDSPQ"
CDS complement(4208870..4209391)
/codon_start=1
/note="gene_3885"
/transl_table=11
/translation="MESVRVQLSGKRIRANGRIVAAATANNPAFGAHYDLQTDETGAT
KRFGLTVTLAERERQLAIARDEENMWLVTDHQGERRAAYNGALDIDLVFSPFFNALPI
RRLGLHERAESIALPVVYVNVPEMSVDAATVSYTSEGRLDGIKLRSPVADTTVTVDSD
GFIVDYPGLAERM"
CDS complement(4209475..4210194)
/codon_start=1
/note="gene_3886"
/transl_table=11
/translation="MNFLQQALSYLLTASNWTGPVGLAVRTCEHLEYTAVAVAASALI
AVPVGLLIGHTGRGTLLVVGAVNGLRALPTLGVLLLGVLLFGLGLGPPLVALMLLGIP
SLLASTYAGIASVDPLVVDAARAMGMTESQVLLRVEVPNALPLMLGGLRSATLQVVAT
ATVAAYASLGGLGGYLIDGIKERRFHIALVGAMMVAALALTLDGLLALAGWVSVPGTG
RMRKLAAVVDKPAAGGGHALR"
CDS complement(4210191..4210880)
/codon_start=1
/note="gene_3887"
/transl_table=11
/translation="MHYLMTHPGAAWALTVVHLRLSLLPVLIGLMSAVPLGLLVQRAP
LLRRLTTATASVIFTIPSLALFVVLPLIIGTRILDEANVIVALAAYTTALLVRAVLEA
LDAVPAQVHDAATAIGYSRIAQMLKVELPLSIPVLVAGLRVVAVTNIAMVSVGSVIGI
GGLGTWFTAGYQTNKSDQIVAGVVAMFLLAIVVDVVINLAGRLATPWERAPRAARRRR
QVAAPITGGAR"
CDS complement(4210868..4211998)
/codon_start=1
/note="gene_3888"
/transl_table=11
/translation="LICFDDVSKVYAHGATAVDRLTLEVPNGMLTVFVGPSGCGKTTA
LRMINRMVDPTSGTITVDGTDVSTVNAVKLRLGIGYVIQNAGLMPHQRVIDNVATVPV
LKGQPRRAARKAGYEVLERVGLDPKVATRYPAQLSGGEQQRVGVARALAADPPILLMD
EPFSAVDPVVRHELQNEILRLQAELHKTIVFVTHDIDEALKLADLVAVFAPGGALAQY
DETARLLSSPANDFVSKFIGLGRGYRWLQLFDAAGLPVRDIEQVSVNGLSDARDRQVR
DGWVLVVDGAGAPLGWIDADGRRRHRGGAALSDAMTVGGSVFRPNGNLSQALDAALSS
PSGVGVAVDGGGKVIGGILAADVLAEFQKGKKAGGGAKPCTT"
CDS complement(4212007..4212909)
/codon_start=1
/note="gene_3889"
/transl_table=11
/translation="VWLATVCLVASCANADPLGSATGSVKSIVVGSGDFPESQVIAEI
YAQVLQANGFDVGRRLGIGSRETYIPALKDHSIDLVPEYIGNLLLYFQPDATVTMLDA
VELELYKRLPGDLSILTPSPASDTDTVTVTAATAARWNLKTIADLAPHSADVKFAAPS
VFQTRPSGLPGLRHKYSLDIAPGNFVTINDGGGAVTVRALVEGTATAANLFSTSAAIP
QNHLVVLEDPEHNFLAGNIVPLVNSRKKSDHLKDVLDAVSAKLTTAGLAELNAAVSGN

SGVDPDQAARKWVRDNGFDHPVRQ"
CDS 4213119..4213421
/codon_start=1
/note="gene_3890"
/transl_table=11
/translation="VPGSVPGKAPEEPPVKFTRAAAVWSALIVGFLILILLLIFIAQN
TASAQFAFFGWRWSLPLGVAILLAAVGGGLITVFAGTARILQLRRAAKKTHAAALR"
CDS complement(4213443..4214498)
/codon_start=1
/note="gene_3891"
/transl_table=11
/translation="VTSVDRLDGLDLGALDRYLRSLGIGRDGELRGELISGGRSNLTF
RVYDDASSWLVRRPPLHGLTPSAHDMAREYRVVAALGDTPVPVARTISLCQDDSVLGA
PFQVVEFVAGQVVRRRAELEALGSRSVIEGCVDALIRVLVDLHSIDPKAVGLSDFGKP
DGYLERQVRRWGSQWELVRLPDDHRDADISRLHLALQQAIPQQSRTSIVHGDYRIDNT
ILDTDDPCHVRAVVDWELSTLGDPLSDAALMCVYRDPALDLIVHAQAAWTSPLLPAAD
ELADRYSLVSGQPLGHWEFYMALAYFKLAIIAAGIDYRRRMSEQAEGKDTAAESVPDV
VAPLIARGLAEIAKKSG"
CDS complement(4214577..4216451)
/codon_start=1
/note="gene_3892"
/transl_table=11
/translation="MEHKPPTAVIQAAHGEHSLPLHDTTDFDDADRGFIAALSPCVIK
AADGRVVWDNDAYSFLDGAAPTSVHPSLWRQSQLTAKQGLYQVVPGIYQVRGFDISNI
SFVEGDTGLIVIDPLVSTEVAAAALDLYRAHRGADRPVVAVIYTHSHVDHFGGVLGVT
TQADVDAGKVAVLAPEGFTAHAVQENIYAGSAMMRRAGYMYGTVLARGLRGHVGCGLG
QTLSTGEVSLVVPTVDITETGETHTIDGVEIEFQMAPGTEAPAEMHFYFPRFRALCMA
ENATHNLHNLLTLRGALVRDPRAWSGYLTEAIDTFADRTDVVFASHHWPTWGREKIVE
FLSQQRDMHSYLHDQTLRLLNQGYTGVEIAEMFQLPPALQRAWHTHGYYGSVSHNVKA
IYQRYMGWFDGNPGWLWPHPPEALAPRYVDALGGIDRVLELAREAFDAGDFRWAATLL
DHAVFADSEHAAARGLYADTLEQLAYGAECATWRNFFLTGAAELRDGNPGSSGQVPAP
TFFAQLTPDQIFDVLAISINGPRAWDLDLAIDFTFTEPDVNYRLTLRNGVLIHRKLPA
DPATANATVTVGDKVRLVAAALGDISSPGFEVFGDRTVLQTFLSVLDRPDSAFNIVTP
"
CDS 4216628..4217107
/codon_start=1
/note="gene_3893"
/transl_table=11
/translation="VKRGLTVAVAGAAILVAGLSGCSSNKSTTGSGETTTAAGTTASP
GAASGPKVVIDGKDQNVTGSVVCTTAAGNVNIAIGGAATGIAAVLTDGNPPEVKSVGL
GNVNGVTLGYTSGTGQGNASATKDGSHYKITGTATGVDMANPMSPVNKSFEIEVTCS"
CDS complement(4217163..4218599)
/codon_start=1
/note="gene_3894"
/transl_table=11
/translation="VVCVGITAATEMALRRHLVAQLDNQLGGTSYRSVLMYPEKMPRP
PWRRETHNYIRSGPGPRFLDAPGQPAGMVAAVVSDGTTVAAGYLTGSGSRAALTSTGR
SQLERIAGSRTPLTLDLDGLGRYRVLAAPSRNGHDVIVTGLSMGNVDATMLQMLIIFG
IVTVIALVAATTAGIVIIKRALAPLRRVAQTASEVVDLPLDRGEVKLPVRVPEPDANP
STEVGQLGSALNRMLDHIAAALSARQASETRVRQFVADASHELRTPLAAIRGYTELTQ
RIGDDPEAVAHAMSRVASETERITRLVEDLLLLARLDSGRPLERGPVDMSRLAVDAVS

DAHVAGPDHQWALDLPPEPVVIPGDAARLHQVVTNLLANARVHTGPGTIVTTRLSTGP
THVVLQVIDNGPGIPAALQSEVFERFARGDTSRSRQAGSTGLGLAIVSAVVKAHNGTI
TVSSSPGYTEFAVRLPLDGWQPLESSPR"

```
CDS             complement(4218661..4219365)
                /codon_start=1
                /note="gene_3895"
                /transl_table=11
                /translation="MRRADGQPVTVLVVDDEPVLAEMVSMALRYEGWNITTAGDGSSA
                IAAARRQRPDVVVLDVMLPDMSGLDVLHKLRSENPGLPVLLLTAKDAVEDRIAGLTAG
                GDDYVTKPFSIEEVVLRLRALLRRTGVTTVDSGAQLVVGDLVLDEDSHEVMRAGEPVS
                LTSTEFELLRFMMHNSKRVLSKAQILDRVWSYDFGGRSNIVELYISYLRKKIDNGREP
                MIHTLRGAGYVLKPAR"
CDS             4219739..4220563
                /codon_start=1
                /note="gene_3896"
                /transl_table=11
                /translation="VTDTLSADVSEYQVPVNNSYPYRVLSIRVCDGTYRDRNFAHNYR
                WMRSAFDSGRLTFGIVYTYARPNWWANANTVRSMIDAAGGLHPRVALMLDVESGGNPP
                GDGSSWINRLYWNLADYAGSPVRIIGYANAYDFFNMWRVRPAGLRVIGAGYGSNPNLP
                GQVAHQYTDGSGYSPNLPQGAPPFGRCDMNSANGLTPQQFAAACGVTTTGGPLMALTD
                EEQTELLTKVREIWDQLRGPNGAGWPQLGQNEQGQDLTPVDAIAVIKNDVAAMLAE"
CDS             complement(4220577..4221521)
                /codon_start=1
                /note="gene_3897"
                /transl_table=11
                /translation="VPRTDNDSWAITESVGATALGVAAARAAETESDNPLINDPFARI
                FVDAAGDGIWSMYTNRTLLAGATDLDPDLRAPIQQMIDFMAARTAFFDEYFLATADAG
                VRQVVILASGLDSRAWRLPWPDGTVVYELDQPKVLEFKSATLRQHGAQPASQLVNVPI
                DLRQDWPKALQKAGFDPSKPCAWLAEGLVRYLPARAQDLLFERIDALSRPGSWLASNV
                PGAGFLDPERMRRQRADMRRMRAAAAKLVETEISDVDDLWYAEQRTAVAEWLRERGWD
                VSTATLPELLARYGRSIPHSGEDSIPPNLFVSAQRATS"
CDS             4221651..4222010
                /codon_start=1
                /note="gene_3898"
                /transl_table=11
                /translation="MGSTPPRTPQEVFAHHGQALAAGDLDEIVADYADDSFVITPAGI
                ARGKEGIRQLFVKLLDDIPNALWDLKTQIFEGDILFLEWTANSAVSRVDDGVDTFVFR
                DGTIWAHTVRYTPHPKT"
CDS             4222196..4222468
                /codon_start=1
                /note="gene_3899"
                /transl_table=11
                /translation="VTTLKELGARVAALEANQADYRAVLAAVNPPGANQREIATTVRE
                HTGRLDRVTTKVGQLAAKSDDTNARVRSLEEGQAEIKDLLLRALDK"
CDS             complement(4222781..4223356)
                /codon_start=1
                /note="gene_3900"
                /transl_table=11
                /translation="MLSGIQQNTLMDNDPLAHGYYVADLLVALAVVVLMLRARRTRPE
                LARMLLLGTLIGLVWELPVFGLSAWTNTPIIEWATPLPLPTVVFLLAHSVWDGALLTM
                GWLLARALTGEPAGALGLTVQVLWGQLTALAVELSAILAGTWSYVDDLWFNPVMFWFR
```

```
                     GHPVTAAMQLTWLLAPLCFAALVRRLALTAR"
     CDS             complement(4223462..4223644)
                     /codon_start=1
                     /note="gene_3901"
                     /transl_table=11
                     /translation="VGSTPWCPNPCQCTLRTPVEVLELAVALRPENPDRTAGAIQRIL
                     RAQLAGDRIALRGRGS"
     CDS             complement(4223659..4223850)
                     /codon_start=1
                     /note="gene_3902"
                     /transl_table=11
                     /translation="VRAERARAIGLFRYQLIREAADAAHSTKERGKMVRELASREHTD
                     PFGRKVRISRHTIDRWIRN"
     CDS             complement(4223985..4224311)
                     /codon_start=1
                     /note="gene_3903"
                     /transl_table=11
                     /translation="VPAPAEKALSQVGFRRIAADLARPAETVRGWLRRFAERAEAVRS
                     VFTVMLRAVDPDPVMPDAAVGVFAYAVTVIAAVVTVIECQFALSTVSLAETAVAVSGG
                     RLVAPG"
     tRNA            complement(4224446..4224518)
                     /note="identified by tRNAscan-SE; putative"
     tRNA            complement(4224549..4224637)
                     /note="identified by tRNAscan-SE; putative"
     CDS             4224715..4225776
                     /codon_start=1
                     /note="gene_3904"
                     /transl_table=11
                     /translation="VTARLRPELAGLPVYVPGKTVPGAIKLASNETVFGPLPSVRAAI
                     DRATDTVNRYPDNGCVQLKAALARHLGPDFAPEHVAVGCGSVSLCQQLVQVTASVGDE
                     VVFGWRSFELYPPQVRVAGAIPIQVPLTDHTFDLYAMLAAVTDRTRLIFVCNPNNPTS
                     TVVGPDALARFVEAVPAHILIAIDEAYVEYIRDGMRPDSLGLVRAHNNVVVLRTFSKA
                     YGLAGLRIGYAIGHPDVITALDKVYVPFTVSSIGQAAAIASLDAADELLARTDTVVAE
                     RARVSAELRAAGFTLPPSQANFVWLPLGSRTQDFVEQAADARIVVRPYGTDGVRVTVA
                     APEENDAFLRFARRWRSDQ"
     CDS             complement(4225822..4226223)
                     /codon_start=1
                     /note="gene_3905"
                     /transl_table=11
                     /translation="MVLGGMVGAEFSLRADIDSVERLVSGAARSALDAWHRHGLEGDV
                     SLGPGSMSAKVAVSVFSVEFLVHAWDYAVAVGSELKAADSLAEYVLELARKLIKPEER
                     SVAGFNEPVDVPEDGGALERLIAFTGRNPAR"
     CDS             4226371..4227255
                     /codon_start=1
                     /note="gene_3906"
                     /transl_table=11
                     /translation="VGNLVRVAIQAAYWPRYGLGMGETYESVTVETKDQVAQVTLIGP
                     GKGNAMGPAFWSEMPEVFHALDADREVRAIVITGSGKNFSYGLDVPAMGGMFAPLIAD
                     GALARPRTDFHTEILRMQKAINAVADCRTPTIAAVQGWCIGGAVDLISAVDIRYASAD
                     AKFSVREVKLAIVADMGSLARLPLILSDGHLRELALTGKNIDAARAEKIGLVNDVYDD
                     ADQTLAAAHATAAEIAANPPLAVYGIKDVLDQQRTSAVSENLRYVAAWNAAFLPSKDL
```

TEGISATFAKRPPQFTGE"
CDS 4227267..4228514
/codon_start=1
/note="gene_3907"
/transl_table=11
/translation="MRAGDGKIRVPADLDAVTATGEEDHSEIDGAAVDRIWRAARHWY
RAGMHPAIQLCIRHHGRVVLNRAIGHGWGNAPTDEADAEKIPVTTDTPFCVYSAAKAI
TATVVHMLVERGHFALDDRVCEYLPSYTSHGKHRTTIRHVLTHSAGVPFPTGPRPDVR
RADDHEYAVERLGELRPLYRPGLVHIYHALTWGPLMREIVYAATGKEIREILATEILD
PLGFRWTNFGVAERDVPLVAPSHATGRQLPPVIAAVFRKAIGGTVHEIIPYTNTPFFL
STILPSSNTVSTANELSRFMEILRRGGELDGVRVLSPETLRGAVTECRRLRPDFATGL
MPLRWGTGFMLGSAKYGPFGRNAPAAFGHLGLVNIAVWADPERALSGGLISSGKPGRD
PEAGRYGALLNAITAEIPRASSG"
CDS 4228686..4230230
/codon_start=1
/note="gene_3908"
/transl_table=11
/translation="LSDPVELRDADDAALLAAIEDCARAEVAAGARRLSAIAELTSRR
TGNDQRADWACDGWDCAAAEVAAALTVSHRKASGQMHLSLTLNRLPQVAALFLAGQLS
ARLVSIIAWRTYLVRDPEALSLLDAALAKHATAWGPLSAPKLEKAIDSWIDRYDPAAL
RRTRISARSRDLCIGDPDEDAGTAALWGRLFATDAAMLDKRLTQLAHGVCDDDPRTIA
QRRADALGALAAGADRLTCGCGNSDCPSSAGNHRQATGVVIHVVADAAALGAAPDPRL
SGPEPALAPEAPATPAVKPPAALISGGGVVPAPLLAELIRGGAALSRVRHPGDLRSEP
HYRPSAKLAEFVRIRDMTCRFPGCDQPTEFCDIDHTLPYPLGPTHPSNLKCLCRKHHL
LKTFWTGWRDVQLPDGTIIWTAPNGHTYTTHPDSRIFLPSWHTTTAALPPAPSPPAIG
PTHTLLMPRRRRTRAAELAHRIKRERAHVTQRNKPPPSGGDTAVAEGFEPPDGVSRLS
LSRRVH"
tRNA complement(4230163..4230249)
/note="identified by tRNAscan-SE; putative"
CDS 4230276..4231262
/codon_start=1
/note="gene_3909"
/transl_table=11
/translation="VTIMRAVVAESSDRLVWQEVPDVSAGPGEVLIKVAASGVNRADV
LQAAGKYPPPPGVSDIIGLEVSGIVAAVGPGVTEWSAGQEVCALLAGGGYAEYVAVPA
DQVLPIPPSVNLVDSAALPEVACTVWSNLVMTAHLRPGQLVLIHGGASGIGSHAIQVA
RALAARVAITAGSPEKLELCRDLGAQITINYRDEDFVARLKQETDGSGADIILDIMGA
SYLDRNIDALATDGQLIVIGMQGGVKAELNLGKLLTKRARVIGTTLRARPVSGPHGKA
AIAQAVAASVWPMIAANRVRPVIGTRLPIQQAAQAHELMLSGKTFGKILLTV"
CDS complement(4231281..4232477)
/codon_start=1
/note="gene_3910"
/transl_table=11
/translation="MAYDVARVRGLHPSLGDGWVHFDAPAGMLIPDSVATTVSTAFRR
SGASTVGAHPSARRSAAVLDAAREAVADLVNADPGGVVLGADRAVLLSLLAEASSSRA
GLGYEVIVSRLDDEANIAPWLRAAHRYGAKVKWAEVDIETGELPTWQWESLISKSTRL
VAVNSASGTLGGVTDLRAMTKLVHDVGALVVVDHSAAAPYRLLDIRETDADVVTVNAH
AWGGPPIGAMVFRDPSVMNSFGSVSTNPYATGPARLEIGVHQFGLLAGVVASIEYLAA
LDESARGSRRERLAVSMQSADAYLNRVFDYLMVSLRSLPLVMLIGRPEAQIPVVSFAV
HKVPADRVVQRLADNGILAIANTGSRVLDVLGVNDVGGAVTVGLAHYSTMAEVDQLVR
ALASLG"

CDS 4232567..4234567
/codon_start=1
/note="gene_3911"
/transl_table=11
/translation="VGLWFGTLIALILLIAPGAMVARIAQLRWPVAIAVGPALTYGVV
ALAIIPYGALGIPWNGWTALAALAVTCAVATGLQLLLARFRDLDAEALAVSRWPAVTV
AAGVLLGALLIGWAAYRGIPHWQSIPSTWDAVWHANTVRFILDTGQASSTHMGELRNV
ETHAPLYYPSVFHGLVAVFCQLTGAAPTTGYTLSSLAASVWLFPVSAAVLTWRAVRSH
PGALWSASCASAEWRAAGAAGTAAALSASFTAVPYVEFDTAAMPNLAAYGIAVPTMVL
ITSTLRHRDRIPVAVLALVGVFSLHITGGIVVALLVSAWWLFEALRHPVRSRLADLLT
LAGVAAMAGLVMLPQFLSVRQQEDIIAGHAFPTYLSKKRGLFDAVFQHSRHLNDFPVQ
YALIVLAAIGGLILLVKKIWWPLAVWLLLIVMNVDAGTPLGGPIGGVAGALGEFFYHD
PRRIAAATTLLLMLMAGVALFATVMLLVAAAKRLTDRFRPQPVSVWASATATLLIGAT
LVSAWHYFPRHRFLFGDKYDSVMIDQKDLDAMAYLASLPGARDTLIGNANTDGTAWMY
AVAGLHPLWTHYDYPLQQGPGYHRFIFWAYGRNGESDPRVLEAIQVLRIRYILTSTPT
VRGFAVPDGLVSLETSRSWAKIYDNGEARIYEWRGTAAATHS"
CDS 4234583..4235107
/codon_start=1
/note="gene_3912"
/transl_table=11
/translation="MVIGLSTGSDDDDVEVIGGVDPRLIAVQENDSDESSLTDLVEQP
AKVMRIGTMIKQLLEEVRAAPLDEASRNRLRDIHATSIRELEDGLAPELREELDRLTL
PFNEDAVPSDAELRIAQAQLVGWLEGLFHGIQTALFAQQMAARAQLQQMRQGALPPGV
GKSGQHGHGTGQYL"
CDS 4235111..4235932
/codon_start=1
/note="gene_3913"
/transl_table=11
/translation="VSDPHHPHIQTHNAWVEFPIFDAKSRSLKKAVLGKAGGTIGRNN
SNVVVIEALRDITMELNLGDRVGLVGHNGAGKSTLLRLLSGIYEPTRGWAKVTGRVAP
VFDLGIGMDPEISGYENIIIRGLFLGQTRKQMQAKVDEIAEFTELGEYLSMPLRTYST
GMRVRLAMGVVTSIDPEILLLDEGIGAVDADFLRKAQSRLQNLVERSGILVFASHSNE
FLARLCKTAIWIDHGVIRLAGGIEEVVRAYEGEDAARHVREVLAETQADRQNVQG"
CDS 4235929..4236843
/codon_start=1
/note="gene_3914"
/transl_table=11
/translation="MTESVFAVVVTHRRPDELAKSLDVLTAQTRLPDHLIVVDNDGCG
DSPVRELVAGQPIATTYLGSRRNLGGAGGFALGMLHALAQGADWVWLADDDGHAQDAR
VLATLLACAEKYSLAEVSPMVCNIDDPTRLAFPLRRGLVWRRRASELRTEAGQELLPG
IASLFNGALFRASTLAAIGVPDLRLFIRGDEVEMHRRLIRSGLPFGTCLDAAYLHPCG
SDEFKPILCGRMHAQYPDDPGKRFFTYRNRGYVLSQPGLRKLLAQEWLRFGWFFLVTR
RDPKGLWEWIRLRRLGRREKFGKPGGSA"
CDS 4236840..4237682
/codon_start=1
/note="gene_3915"
/transl_table=11
/translation="MTFMDAQASFQTQSRTLARVRGDLVDGFRRHELWLHLGWQDIKQ
RYRRSVLGPFWITIATGTTAVAMGGLYSKLFRLELSEHLPYVTLGLIVWNLINAAILD
GAEVFVANEGLIKQLPAPLSVHVYRLVWRQMIFFAHNIVIYFVIAIIFPKPWSWADLS
FLPALALIFLNCVWVSLCFGILATRYRDIGPLLFSVVQLLFFMTPIIWNDETLRRQGA

GRWSSIVELNPLLHYLDIVRAPLLGAHQELRHWLVVLVLTVVGWMLAAFAMRQYRARV
PYWV"
CDS 4237838..4238818
/codon_start=1
/note="gene_3916"
/transl_table=11
/translation="MEILVTGGAGFQGSHLTESLLANGHWVTVLDKSSRNAVRNMQGF
RSHDRAAFISGSVTDGQTIDRAVRDHHVVFHLAAHVNVDQSLGDPESFLETNVMGTYR
VLEAVRRYRNRLIYVSTCEVYGDGHNLKEGERLDEHAELKPNSPYGASKAAADRLCYS
YFRSYGLDVTIVRPFNIFGVRQKAGRFGALIPRLVRQGINGEGLTIFGAGSATRDYLY
VSDIVGAYNLVLRTPTLRGQAINFASGKDTRVRDIVEYVADKFGARIEHRDARPGEVQ
RFPADISLAKSIGFQPQVEIWDGIDRYINWAKDQPQYPYEQDGFSGSSVL"
CDS 4238902..4239975
/codon_start=1
/note="gene_3917"
/transl_table=11
/translation="MVTVARRPVCPVTLTPGDPALASVRDLVDAWSAHDALAELVTMF
GGAFPQTDHLEARLASLDKFSTAWDYRARARAARALHGEPVRCQDSGGGARWLIPRLD
LPAKKRDAIVGLAQQLGLTLESTPQGTTFDHVLVIGTGRHSNLIRARWARELAKGRQV
GHIVLAAASRRLLPSEDDAVAVCAPGARTEFELLAAAARDAFGLDVHPAVRYVRQRDD
NPHRDSMVWRFAADTNDLGVPITLLEAPSPEPDSSRATSADTFTFTAHTLGMQDSTCL
LVTGQPFVPYQNFDALRTLALPFGIQVETVGFGIDRYDGLGELDQQHPAKLLQEVRST
IRAARALLERIEAGERMATDPRR"
CDS complement(4239956..4241179)
/codon_start=1
/note="gene_3918"
/transl_table=11
/translation="MRILAMTRAHNAGRTLAATLDSLAVFSDDIYVIDDRSTDDTAEI
LANHPAVTNVVRARPDLPPTPWLIPESAGLELLYRMADFCRPDWVMMVDADWLVETDI
DLRAVLARTPDDIVALMCPMVSRWDDPEYPDLIPVMGTAEALRGPLWRWYPGLRAGGK
LMHNPHWPANITDHGRIGQLPGVRLVHSGWSTLAERILRVEHYLRLDPDYRFNFGVAY
DRSLLFGYALDEVDLLKADYRRRIRGDFDPLEPGGRLPIDREPRAIGRGYGPHAGGFH
PGVDFATDPGTPVYAVASGAVSAIDEVDGLVSLTIARCELDVVYVFRPGDEGRLVLGD
RIAAGAQLGTIGAQGESADGYLHFEVRTQDGHVNPVRYLANMGLRPWPPPGRLRAVSG
SYPPATPCTITAEDR"
CDS complement(4241192..4242118)
/codon_start=1
/note="gene_3919"
/transl_table=11
/translation="VARTDDDSWDLATGVGATATLVAAGRARAARAAQPLIDDPFAEP
LVRAVGVEFLTRWATGELDAADVDDPDAAWGLQRMTTELVVRTRYFDQFFLDAAAAGV
RQAVILASGLDARGYRLPWPADTTVFEVDQPRVLEFKAQTLAGLGAQPTADLRMVPAD
LRHDWPDALRRGGFDAAEPAAWIAEGLFGYLPPDAQNRLLDHVTDLSAPGSRLALEAF
LGSADRDSARVEEMIRTATRGWREHGFHLDIWALNYAGPRHEVSGYLDNHGWRSVGTT
TAQLLAAHDLPAAPALPAGLADRPNYWTCVLG"
CDS 4242362..4242847
/codon_start=1
/note="gene_3920"
/transl_table=11
/translation="VSEKVESKGLADAARDHLAAELARLRQRRDRLEVEVKNDRGMIG
DHGDAAEAIQRADELAILGDRINELDRRLRTGPTPWSGSETLPGGTEVTLRFPDGEVV

TMHVISVVEETPVGREAETLTARSPLGQALAGHQPGDTVTYSTPQGPNQVQLLAVKLP
S"
CDS 4242893..4243321
/codon_start=1
/note="gene_3921"
/transl_table=11
/translation="MIRSESGAAPPRQHLHLSAQVMRFVVTGGLAGIVDFGLYVVLYK
VAGLQVDLSKAISFIVGTITAYLINRRWTFQAEPSTARFVAVMLLYGITFAVQVGLNH
LCLALLHYRAWAIPVAFVIAQGTATVINFIVQRAVIFRIR"
CDS 4243460..4244746
/codon_start=1
/note="gene_3922"
/transl_table=11
/translation="MIVKAVARVAESGGGRGAIARGLGRSYGDNAQNGGGLVIDMTPL
NTIHSIDADTKLVDIDAGVNLDQLMKAALPFGLWVPVLPGTRQVTVGGAIACDIHGKN
HHSAGSFGNHVRSMDLLTADGEIRHLTPTGEDAELFWATVGGNGLTGIIMRATIEMTP
TSTAYFIADGDVTASLDETIALHSDGSEARYTYSSAWFDAISAPPKLGRAAVSRGRLA
TVEQLPAKLRSEPLKFDAPQLLTLPDVFPNGLANKYTFGPIGELWYRKSGTYRGKVQN
LTQFYHPLDMFGEWNRAYGPAGFLQYQFVIPTEAVDEFKKIIGVIQASGHYSFLNVFK
LFGPRNQAPLSFPIPGWNICVDFPIKDGLGKFVSELDRRVLEFGGRLYTAKDSRTTAE
TFHAMYPRVDEWISVRRKVDPLRVFASDMARRLELL"
CDS 4244747..4245511
/codon_start=1
/note="gene_3923"
/transl_table=11
/translation="MVLDAVGNPQTVLLLGGTSEIGLAICERYLHNSAARIVLACLPD
DPRREDAAAAMKQAGARSVELIDFDALDTDSHPKMIEAAFSGGDVDVAIVAFGLLGDA
EELWQNQRKAVQIAEINYTAAVSVGVLLAEKMRAQGFGQIIAMSSAAGERVRRANFVY
GSTKAGLDGFYLGLSEALREYGVRVLVIRPGQVRTRMSAHLKEAPLTVDKEYVANLAV
TASAKGKELVWAPAAFRYVMMVLRHIPRSIFRKLPI"
CDS 4245553..4247445
/codon_start=1
/note="gene_3924"
/transl_table=11
/translation="MGAFTSARAREVLVALGQLAAAVVVAVGVAVVSLLAIARVEWPA
FPSSNQLHALTTVGQVGCLAGLVGIGWLWRHGRFRRLARLGGLVLVSAFTVVTLGMPL
GATKLYLFGISVDQQFRTEYLTRLTDTAALRDMTYIGLPPFYPPGWFWIGGRAAALTG
TPAWEMFKPWAITSMAIAVAVALVLWWRMIRFEYALLVTVATAAVMLAYSSPEPYAAM
ITVLLPPMLVLTWSGLGARDRQGWAAVVGAGVFLGFAATWYTLLVAYGAFTVVLMALL
LAGSRLQSGIKAAVDPLCRLAVVGAIAAAIGSTTWLPYLLRAARDPVSDTGSAQHYLP
ADGAALTFPMLQFSLLGAICLLGTLWLVMRARSSAPAGALAIGVLAVYLWSLLSMLAT
LARTTLLSFRLQPTLSVLLVAAGAFGFVEAVQALGKRGRGVIPMAAAIGLAGAIAFSQ
DIPDVLRPDLTIAYTDTDGYGQRGDRRPPGSEKYYPAIDAAIRRVTGKRRDRTVVLTA
DYSFLSYYPYWGFQGLTPHYANPLAQFDKRATQIDSWSGLSTADEFIAALDKLPWQPP
TVFLMRHGAHNSYTLRLAQDVYPNQPNVRRYTVDLRTALFADPRFVVEDIGPFVLAIR
KPQESA"
CDS 4247445..4250729
/codon_start=1
/note="gene_3925"
/transl_table=11
/translation="MATEAAPPRIAVRLPSTSVRDAGANYRIARYVAVVAGLLGAVLA

IATPLLPVNQTTAQLNWPQNGTFASVEAPLIGYVATDLNITVPCQAAAGLAGSQNTGK
TVLLSTVPKQAPKAVDRGLLLQRANDDLVLVVRNVPLVTAPLSQVLGPTCQRLTFTAH
ADRVAAEFVGLVQGPNAEHPGAPLRGERSGYDFRPQIVGVFTDLAGPAPPGLSFSASV
DTRYSSSPTPLKMAAMILGVALTGAALVALHILDTADGMRHRRFLPARWWSIGGLDTL
VIAVLVWWHFVGANTSDDGYILTMARVSEHAGYMANYYRWFGTPEAPFGWYYDLLALW
AHVSTASIWMRLPTLAMALTCWWVISREVIPRLGHAVKTSRAAAWTAAGMFLAVWLPL
DNGLRPEPIIALGILLTWCSVERAVATSRLLPVAIACIIGALTLFSGPTGIASIGALL
VAIGPLRTILHRRSRRFGVLPLVAPILAAATVTAIPIFRDQTFAGEIQANLLKRAVGP
SLKWFDEHIRYERLFMASPDGSIARRFAVLALVLALAVSVAMSLRKGRIPGTAAGPSR
RIIGITIISFLAMMFTPTKWTHHFGVFAGLAGSLGALAAVAVTGAAMRSRRNRTVFAA
VVVFVLALSFASVNGWWYVSNFGVPWSNSFPKWRWSLTTALLELTVLVLLLAAWFHFV
ANGDGRRTARPTRFRARLAGIVQSPLAIATWLLVLFEVVSLTQAMISQYPAWSVGRSN
LQALAGKTCGLAEDVLVELDPNAGMLAPVTAPLADALGAGLSEAFTPNGIPADVTADP
VMERPGDRSFLNDDGLITGSEPGTEGGTTAAPGINGSRARLPYNLDPARTPVLGSWRA
GVQVPAMLRSGWYRLPTNEQRDRAPLLVVTAAGRFDSREVRLQWATDEQAAAGHHGGS
MEFADVGAAPAWRNLRAPLSAIPSTATQVRLVADDQDLAPQHWIALTPPRIPRVRTLQ
NVVGAADPVFLDWLVGLAFPCQRPFGHQYGVDETPKWRILPDRFGAEANSPVMDHNGG
GPLGITELLMRATTVASYLKDDWFRDWGALQRLTPYYPDAQPADLNLGTVTRSGLWSP
APLRRG"

CDS 4250815..4254099
/codon_start=1
/note="gene_3926"
/transl_table=11
/translation="VPHDGNERSHRIARLAAVVSGIAGLLLCGIVPLLPVNQTTATIF
WPQGSTADGNITQITAPLVSGAPRALDISIPCSAIATLPANGGLVLSTLPAGGVDTGK
AGLFVRANQDTVVVAFRDSVAAVAARSTIAAGGCSALHIWADTGGAGADFMGIPGGAG
TLPPEKKPQVGGIFTDLKVGAQPGLSARVDIDTRFITTPGALKKAVMLLGVLAVLVAM
VGLAALDRLSRGRTLRDWLTRYRPRVRVGFASRLADAAVIATLLLWHVIGATSSDDGY
LLTVARVAPKAGYVANYYRYFGTTEAPFDWYTSVLAQLAAVSTAGVWMRLPATLAGIA
CWLIVSRFVLRRLGPGPGGLASNRVAVFTAGAVFLSAWLPFNNGLRPEPLIALGVLVT
WVLVERSIALGRLAPAAVAIIVATLTATLAPQGLIALAPLLTGARAIAQRIRRRRATD
GLLAPLAVLAAALSLITVVVFRDQTLATVAESARIKYKVGPTIAWYQDFLRYYFLTVE
SNVEGSMSRRFAVLVLLFCLFGVLFVLLRRGRVAGLASGPAWRLIGTTAVGLLLLTFT
PTKWAVQFGAFAGLAGVLGAVTAFTFARIGLHSRRNLTLYVTALLFVLAWATSGINGW
FYVGNYGVPWYDIQPVIASHPVTSMFLTLSILTGLLAAWYHFRMDYAGHTEVKDNRRN
RILASTPLLVVAVIMVAGEVGSMAKAAVFRYPLYTTAKANLTALSTGLSSCAMADDVL
AEPDPNAGMLQPVPGQAFGPDGPLGGISPVGFKPEGVGEDLKSDPVVSKPGLVNSDAS
PNKPNAAITDSAGTAGGKGPVGINGSHAALPFGLDPARTPVMGSYGENNLAATATSAW
YQLPPRSPDRPLVVVSAAGAIWSYKEDGDFIYGQSLKLQWGVTGPDGRIQPLGQVFPI
DIGPQPAWRNLRFPLAWAPPEADVARIVAYDPNLSPEQWFAFTPPRVPVLESLQRLIG
SATPVLMDIATAANFPCQRPFSEHLGIAELPQYRILPDHKQTAASSNLWQSSSTGGPF
LFTQALLRTSTIATYLRGDWYRDWGSVEQYHRLVPADQAPDAVVEEGVITVPGWGRPG
PIRALP"

CDS 4254180..4257392
/codon_start=1
/note="gene_3927"
/transl_table=11
/translation="VATIAGLIGFVLSVATPLLPVVQTTAMLDWPQRGQLGSVTAPLI
SLTPVDFTATVPCDVVRAMPPAGGVVLGTAPKQGKDANLQALFVVVSAQRVDVTDRNV
VILSVPREQVTSPQCQRIEVTSTHAGTFANFVGLKDPSGAPLRSGFPDPNLRPQIVGV
FTDLTGPAPPGLAVSATIDTRFSTRPTTLKLLAIIGAIVATVVALIALWRLDQLDGRG

SIAQLLLRPFRPASSPGGMRRLIPASWRTFTLTDAVVIFGFLLWHVIGANSSDDGYIL
GMARVADHAGYMSNYFRWFGSPEDPFGWYYNLLALMTHVSDASLWMRLPDLAAGLVCW
LLLSREVLPRLGPAVAASKPAYWAAAMVLLTAWMPFNNGLRPEGIIALGSLVTYVLIE
RSMRYSRLTPAALAVVTAAFTLGVQPTGLIAVAALVAGGRPMLRILVRRHRLVGTLPL
VSPMLAAGTVILTVVFADQTLSTVLEATRVRAKIGPSQAWYTENLRYYYLILPTVDGS
LSRRFGFLITALCLFTAVFIMLRRKRIPSVARGPAWRLMGVIFGTMFFLMFTPTKWVH
HFGLFAAVGAAMAALTTVLVSPSVLRWSRNRMAFLAALFFLLALCWATTNGWWYVSSY
GVPFNSAMPKIDGITVSTIFFALFAIAAGYAAWLHFAPRGAGEGRLIRALTTAPVPIV
AGFMAAVFVASMVAGIVRQYPTYSNGWSNVRAFVGGCGLADDVLVEPDTNAGFMKPLD
GDSGSWGPLGPLGGVNPVGFTPNGVPEHTVAEAIVMKPNQPGTDYDWDAPTKLTSPGI
NGSTVPLPYGLDPARVPLAGTYTTGAQQQSTLVSAWYLLPKPDDGHPLVVVTAAGKIA
GNSVLHGYTPGQTVVLEYAMPGPGALVPAGRMVPDDLYGEQPKAWRNLRFARAKMPAD
AVAVRVVAEDLSLTPEDWIAVTPPRVPDLRSLQEYVGSTQPVLLDWAVGLAFPCQQPM
LHANGIAEIPKFRITPDYSAKKLDTDTWEDGTNGGLLGITDLLLRAHVMATYLSRDWA
RDWGSLRKFDTLVDAPPAQLELGTATRSGLWSPGKIRIGP"

CDS 4257460..4258587
/codon_start=1
/note="gene_3928"
/transl_table=11
/translation="MLLGMHQAGHVGTHERRAAATRRSALTAAGLAVVGAGVLGASAC
SPQKSPQPSSPRLPDNALITLGVAAGPPPTPSRVGISSVLKIGRDLYVIDCGLGSLNA
FTNAGLQFDDLKAMFITHLHTDHIVDYYNFFLSGGFLAPPGRAPVLVYGPGPAGGLPP
SEVGNPNPATVNPANPTPGLAAATEALHRAFAYTSNIFIRDYGIDNVADLVKVTEIGL
PPGSDYRNRAPKMSPFSVASDDNVSVTATLVSHYDVYPAFGFRFDLKKSGVSVTFSGD
TTKSDNLITLAQGTDILVHEAVFSLDTAYFGNAFPPNYLVNSHISAEQVGEVAAAAKP
KQLILSHYAPDDLPDSQWLDKIKKNYSGMTTIARDGQVFAL"

CDS 4258667..4260448
/codon_start=1
/note="gene_3929"
/transl_table=11
/translation="MPEYDLEAVDKLPFSTPEKAQRYQTENYRGAMGLNWYLTDPTLQ
FIMAYYLRPDELAFAEPHLTRIGELTGGPVTRWAEETDRNPPRLERYDRWGHDISRVV
LPESFIQSKRAVIEARQAVRDDAARAGVKPSLALFAADYLLNQADIGMACALATGGNM
VRSLVTAYAPPDVREFVLGKLNSGEWDGEAAQLLTERAGGSDLGALETTATRSGDVWL
LNGFKWFASNCAGEAFVVLAKPEGAPDSTRGVATFLVLRTRRDGSRNGVRIRRLKDKL
GTRSVASGEIEFVDAEAFLLSGEPSADAGPSDGKGLTRMMELTNRLRLGTASFALGNA
RRALVESLCYAGQRRAFGGALIDKPLMRRKLAEMVVDVEAALAMVFDGFGAANHRQPR
CLPQRIAVPVTKLKTCRLGITVASDAIEIHGGNGYIETWPVARLLRDAQVNTIWEGPD
NILCLDVRRGIEQTRAHETLLARLRDAVSVSDDDDTTRLVSRRIEDLDAAITAWTKLD
RQLAEARLFPLAQFMGDVYAGALLTEQAAWERATRGTDRKALVARLYARRYLADQGPL
RGIDADCDEALQRFDELVAGAFTAEQT"

CDS 4260575..4261909
/codon_start=1
/note="gene_3930"
/transl_table=11
/translation="VRNVRLFRALLGVDKRTVIEDIEFEEDDAGDGARVIARVRPRSA
VLRRCGRCGRKASWYDRGAGLRQWRSLDWGTVEVFLEAEAPRVNCPTHGPTVVAVPWA
RHHAGHTYAFDDTVAWLAVACSKTAVCELMRIAWRTVGAIVARVWADTEKRIDRFANL
RRIGIDEISYKRHHRYLTVVVDHDSGRLVWAAPGHDKATLGLFFDALGAERAAQITHV
SADAADWIADVVTERCPDAIQCADPFHVVAWATEALDVERRRAWNDARAIARTEPKWG
RGRPGKNAAPRPGRERARRLKGARYALWKNPEDLTERQSAKLAWIAKTDPRLYRAYLL

KESLRHVFSVKGEEGKQALDRWISWAQRCRIPVFVELAARIKRHRVAIDAALDHGLSQ
GLIESTNTKIRLLTRIAFGFRSPQALIALAMLTLAGHRPTLPGRHNHPQISQ"
CDS complement(4261962..4263530)
/codon_start=1
/note="gene_3931"
/transl_table=11
/translation="VTVTEPVLHTTAEKLAELRERLELAKEPGGEKAAAKRDKKGIPS
ARARIYELVDPGSFMEIGALCRTPGDPNALYGDGVVTGHGLINGRPVGVFSHDQTVFG
GTVGEMFGRKVARLMEWCAMVGCPIVGINDSGGARIQDAVTSLAWYAELGRRHELLSG
LVPQISIILGKCAGGAVYSPIQTDLVVAVRDQGYMFVTGPDVIKDVTGEDVSLDELGG
ADHQASYGNIHQVVESEAAAYQYVRDFLSFLPSNCFDKPPVVNPGLEPEITGHDLELD
SIVPDSDNMAYDMHEVLLRIFDDGDFLDVAAQAGQAIITGYARVDGRTVGVVANQPMH
MSGAIDNEASDKAARFIRFSDAFDIPLVFVVDTPGFLPGVEQEKNGIIKRGGRFLYAV
VEADVPKVTITIRKSYGGAYAVMGSKQLTADLNFAWPTARIAVIGADGAAQLLMKRFP
DPNAPEAQAIRKSFVENYNLNMAIPWIAAERGFIDAVIDPHETRLLLRKSMHLLRDKQ
LWWRVGRKHGLIPV"
CDS complement(4263527..4268587)
/codon_start=1
/note="gene_3932"
/transl_table=11
/translation="MVELGLSSRDAVAMAADIEDLTGVTLSVAVAFAHPTIESLATRI
IEGEPETDLAGDDAEDWSRTGPAERVDIAIVGLSTRFPGEMNTPEQTWQALLEGRDGI
TDLPDGRWSEFLEEPRLAARVAGARTRGGYLKDIKGFDSEFFAVAKTEADNIDPQQRM
ALELTWEALEHARIPASSLRGQAVGVYIGSSTNDYSFLAVSDPTVAHPYAITGTSSSI
IANRVSYFYDFHGPSVTIDTACSSSLVAIHQGVQALRNGEADVVVAGGVNALITPMVT
LGFDEIGAVLAPDGRIKSFSADADGYTRSEGGGMLVLKRVDDARRDGDAILAVIAGSA
VNHDGRSNGLIAPNQDAQADVLRRAYKDAGIDPRTVDYIEAHGTGTILGDPIEAEALG
RVVGRGRPADRPALLGAVKTNVGHLESAAGAASMAKVVLALQHDKLPPSINFAGPSPY
IDFDAMRLKMITTPTDWPRYGGYALGGVSSFGFGGANAHVVVREVLPRDVVEKEPEPE
PEPKAAAEPAEAPTLAGHALRFDEFGNIITDSAVAEEPEPELPGVTEEALRLKEAALE
ELAAQEVTAPLVPLAVSAFLTSRKKAAAAELADWMQNPEGQASSLESIGRSLSRRNHG
RSRAVVLAHDHDEAIKGLRAVAAGKQAPNVFSVDGPVTTGPVWVLAGFGAQHRKMGKS
LYLRNEVFAAWIEKVDALVQDELGYSVLELILDDAQDYGIETTQVTIFAIQIALGELL
RHHGAKPAAVIGQSLGEAASAYFAGGLSLRDATRAICSRSHLMGEGEAMLFGEYIRLM
ALVEYSADEIREVFSDFPDLEVCVYAAPTQTVIGGPPEQVDAILARAEAEGKFARKFA
TKGASHTSQMDPLLGELTAELQGIKPTSPTCGIFSTVHEGRYIKPGGEPIHDVEYWKK
GLRHSVYFTHGIRNAVDSGHTTFLELAPNPVALMQVALTTADAGLHDAQLIPTLARKQ
DEVSSMVSTMAQLYVYGHDLDIRTLFSRASGPQDYANIPPTRFKRKEHWLPAHFSGDG
STYMPGTHVALPDGRHVWEYAPRDGNVDLAALVRAAAAHVLPDAQLTAAEQRAVPGDG
ARLVTTMTRHPGGASVQVHARIDESFTLVYDALVSRAGSESVLPTAVGAATAIAVADG
APVAPETPAEDADAETLSDSLTTRYMPSGMTRWSPDSGETIAERLGLIVGSAMGYEPE
DLPWEVPLIELGLDSLMAVRIKNRVEYDFDLPPIQLTAVRDANLYNVEKLIEYAVEHR
DEVQQLHEHQKTQTAEEIARAQAELLHGKVGKTEPVDSEAGVALPSPQNGEQPNPTGP
ALNVDVPPRDAAERVTFATWAIVTGKSPGGIFNELPRLDDEAAAKIAQRLSERAEGPI
TAEDVLTSSNIEALADKVRTYLEAGQIDGFVRTLRARPEAGGKVPVFVFHPAGGSTVV
YEPLLGRLPADTPMYGFERVEGSIEERAQQYVPKLIEMQGDGPYVLVGWSLGGVLAYA
CAIGLRRLGKDVRFVGLIDAVRAGEEIPQTKEEIRKRWDRYAAFAEKTFNVTIPAIPY
EQLEELDDEGQVRFVLDAVSQSGVQIPAGIIEHQRTSYLDNRAIDTAQIQPYDGHVTL
YMADRYHDDAIMFEPRYAVRQPDGGWGEYVSDLEVVPIGGEHIQAIDEPIIAKVGEHM
SRALGQIEADRTSEVGKQ"
CDS complement(4268735..4270624)

/codon_start=1
/note="gene_3933"
/transl_table=11
/translation="MAYHNPFIVNGKIRFPANTNLVRHVEKWAKVRGDKLAYRFLDFS
TERDGVARDILWSDFSARNRAVGARLQQVTQPGDRVAILCPQNLDYLISFFGALYSGR
IAVPLFDPAEPGHVGRLHAVLDDCAPSTILTTTDSAEGVRKFIRARSAKERPRVIAVD
AVPTEVAATWQQPEANEETVAYLQYTSGSTRIPSGVQITHLNLPTNVVQVLNALEGQE
GDRGVSWLPFFHDMGLITVLLASVLGHSFTFMTPAAFVRRPGRWIRELARKPGETGGT
FSAAPNFAFEHAAVRGVPRDDEPPLDLSNVKGILNGSEPVSPASMRKFFEAFAPYGLK
QTAVKPSYGLAEATLFVSTTPMDEVPTVIHVDRDELNNQRFVEVAADAPNAVAQVSAG
KVGVSEWAVIVDADTASELPDGQIGEIWLHGNNLGTGYWGKEEESAQTFKNILKSRIS
ESRAEGAPDDALWVRTGDYGTYFKDHLYIAGRIKDLVIIDGRNHYPQDLECTAQESTK
ALRVGYVAAFSVPANQLPQTVFDDSHAGLKFDPEDTSEQLVIVGERAAGTHKLDHQPI
VDDIRAAIAVGHGVTVRDVLLVSAGTIPRTSSGKIGRRACRAAYLDGSLRSGVGSPTV
FATSD"
CDS complement(4270937..4271947)
/codon_start=1
/note="gene_3934"
/transl_table=11
/translation="MAKNSRRKRHRILAWIAAGAMASVVALVIVAVVIMLRGAESPPS
AVPPGVLPPGPTPAHPHKPRPAFQDASCPDVQMISVPGTWESSPQQNPLNPVQFPKAL
LLKVTGPIAQQFAPARVQTYTVAYTAQFHNPLTTDNQMSYNDSRAEGTRAMVAAMTDM
NNRCPLTSYVLIGFSQGAVIAGDVASDIGNGRGPVDEDLVLGVTLIADGRRQQGVGNQ
VPPSPRGEGAEITLHEVPVLSGLGLTMTGPRPGGFGALDGRTNEICAQGDLICAAPAQ
AFSPANLPTTLNTLAGGAGQPVHAMYATPEFWNSDGEPATEWTLNWAHQLIENAPHPK
HR"
CDS complement(4272145..4273044)
/codon_start=1
/note="gene_3935"
/transl_table=11
/translation="MKGRSALLRALWIAALSFGLGGVAVAAEPTAKAAPYENLMVPSP
SMGRDIPVAFLAGGPHAVYLLDAFNAGPDVSNWVTAGNAMNTLAGKGISVVAPAGGAY
SMYTNWEQDGSKQWDTFLSAELPDWLAANRGLAPGGHAAVGAAQGGYGAMALAAFHPD
RFGFAGSMSGFLYPSNTTTNGAIAAGMQQFGGVDTNGMWGAPQLGRWKWHDPWVHASL
LAQNNTRVWVWSPTNPGASDPAAMIGQAAEAMGNSRMFYNQYRSVGGHNGHFDFPASG
DNGWGSWAPQLGAMSGDIVGAIR"
CDS complement(4273224..4274240)
/codon_start=1
/note="gene_3936"
/transl_table=11
/translation="MQLVDRVRGAVTGMSRRLVVGAVGAALVSGLVGAVGGTATAGAF
SRPGLPVEYLQVPSPSMGRDIKVQFQSGGANSPALYLLDGLRAQDDFSGWDINTPAFE
WYDQSGLSVVMPVGGQSSFYSDWYQPACGKAGCQTYKWETFLTSELPGWLQANRHVKP
TGSAVVGLSMAASSALTLAIYHPQQFVYAGAMSGLLDPSQAMGPTLIGLAMGDAGGYK
ASDMWGPKEDPAWQRNDPLLNVGKLIANNTRVWVYCGNGKPSDLGGNNLPAKFLEGFV
RTSNIKFQDAYNAGGGHNGVFDFPDSGTHSWEYWGAQLNAMKPDLQRALGATPNTGPA
PQGA"
CDS complement(4274535..4276532)
/codon_start=1
/note="gene_3937"
/transl_table=11

/translation="LGPLLPSASLKLNVLPVGVLPRRPVVGRVGRPVFPYEPMVRVSL
WLSVTAVAVLFGWGSWQRRWIADDGLIVLRTVRNLLAGNGPVFNQGERVEANTSTAWT
YLLYVGGWVGGPMRLEYVALALAMVLSLLGMVLLMLGTGRLYAPSLRGRRAIMLPAGA
LVYIAVPPARDFATSGLESGLVLAYLGLLWWMMVCWSQPLRARPDSQMFLGALAFVAG
CSVLVRPEFALIGGLALIMMLIAARTWRRRVLIVLAGGFLPVAYQIFRMGYYGLLVPS
TALAKDAAGDKWSQGMIYVSNFNRPYALWVPLVLSVPLGLLLMTARRRPSFLRPVLAP
DYGRVARAVQSPPAVVAFIVGSGVLQALYWVRQGGDFMHGRVLLAPLFCLLAPVGVIP
ILLPDGKDFSRETGRWLVGALSGLWLGIAGWSLWAANSPGMGDDATRVTYSGIVDERR
FYAQATGHAHPLTAADYLDYPRMAAVLTALNNTPEGALLLPSGNYNQWDLVPMIRPSS
GTAPGGKPAPKPQHAVFFTNMGMLGMNVGLDVRVIDQIGLVNPLAAHTERLKHARIGH
DKNLFPDWVIADGPWVKWYPGIPGYIDQQWVTQAEAALQCPATRAVLNSVRAPITLHR
FLSNVLHSYEFTRYRIDRVPRYELVRCGLDVPDGPGPPPRE"

CDS complement(4276507..4277415)
/codon_start=1
/note="gene_3938"
/transl_table=11
/translation="MSEDVVTQPPANLVAGVVKAIRPRQWVKNVLVLAAPLAALGGGV
RYDYIEVLSKVSMAFVVFSLAASAVYLVNDVRDVEADREHPTKRFRPIAAGVVPEWLA
YTVAVVLGVTSLAGAWMLTPNLALVMVVYLAMQLAYCFGLKHQAVVDICVVSSAYLIR
AIAGGVATKIPLSKWFLLIMAFGSLFMVAGKRYAELHLAERTGAAIRKSLESYTSTYL
RFVWTLSATAVVLCYGLWAFERDGYSGSWFAVSMIPFTIAILRYAVDVDGGLAGEPED
IALRDRVLQLLALAWIATVGAAVAFG"

CDS complement(4277422..4277955)
/codon_start=1
/note="gene_3939"
/transl_table=11
/translation="MAERAPRGEVAVMVAVQSALVDRPGMLATARGLSHFGEHCIGWL
ILALLGAIALPRRRREWLVAGAGAFVAHAIAVLIKRLVRRQRPDHPAIAVNVDTPSQL
SFPSAHATSTTAAALLMGRATGLPLPVVLVPPMALSRILLGVHYPSDVAVGVALGATV
GAIVDSVGGGRQRARKR"

CDS complement(4277948..4279861)
/codon_start=1
/note="gene_3940"
/transl_table=11
/translation="MSELAASLLSRVILPRPGEPLDVRKLYLEESTTNARRAHAPTRT
SLQIGAESEVSFATYFNAFPASYWRRWTTCKSVVLRVQVTGAGRVDVYRTKATGARIF
VEGHDFTGTEDQPAAVETEVVLQPFEDGGWVWFDITTDTAVTLHSGGWYATSPAPGTA
NIAVGIPTFNRPADCVNALRELTADPLVDQVIGAVIVPDQGERKVRDHPDFPAAAARL
GSRLSIHDQPNLGGSGGYSRVMYEALKNTDCQQILFMDDDIRLEPDSILRVLAMHRFA
KAPMLVGGQMLNLQEPSHLHIMGEVVDRSIFMWTAAPHAEYDHDFAEYPLNDNNSRSK
LLHRRIDVDYNGWWTCMIPRQVAEELGQPLPLFIKWDDADYGLRAAEHGYPTVTLPGA
AIWHMAWSDKDDAIDWQAYFHLRNRLVVAAMHWDGPKAQVIGLVRSHLKATLKHLACL
EYSTVAIQNKAIDDFLAGPEHIFSILESALPQVHRIRKSYPDAVVLPAASELPPPLHK
NKAMKPPVNPLVIGYRLARGIMHNLTAANPQHHRRPEFNVPTQDARWFLLCTVDGATV
TTADGCGVVYRQRDRAKMFALLWQSLRRQRQLLKRFEEMRRIYRDALPTLSSKQKWET
ALLPAANQEPEHG"

CDS complement(4279858..4281057)
/codon_start=1
/note="gene_3941"
/transl_table=11
/translation="MQPMTARFDLFVVGSGFFGLTIAERVATQLDKRVLVLERRPHIG

GNAYSEAEPQTGIEVHKYGAHLFHTSNKRVWDYVRQFTDFTDYRHRVFAMHNGQAYQF
PMGLGLVSQFFGKYFTPEQARQLIAEQAAEIDTADAQNLEEKAISLIGRPLYEAFVKG
YTAKQWQTDPKELPAANITRLPVRYTFDNRYFSDTYEGLPTDGYTAWLQNMAADHRIE
VRLNTDWFDVRGQLRPGSPAAPVVYTGPLDRYFDYAEGRLGWRTLDFEVEVLPIGDFQ
GTAVMNYNDLDVPYTRIHEFRHFHPERDYPTDKTVIMREYSRFAEDDDEPYYPINTEA
DRALLATYRARAKSETASSKVLFGGRLGTYQYLDMHMAIASALNMYDNVLAPHLRDGV
PLLQDGA"
CDS 4281321..4282175
/codon_start=1
/note="gene_3942"
/transl_table=11
/translation="VPNRRRRKLSTAMSAVAALAVASPCAYFLVYESTETTERPEHHE
FKQAAVLTDLPGELMSALSQGLSQFGINIPPVPSLTGSGDASTGLTGPGLTSPGLTSP
GLTSPGLTDPALTSPGLTPTLPGSLAAPGTTLAPTPGVGANPALTNPALTSPTGATPG
LTSPTGLDPALGGANEIPITTPVGLDPGADGTYPILGDPTLGTIPSSPATTSTGGGGL
VNDVMQVANELGASQAIDLLKGVLMPSIMQAVQNGGAAAPAASPPVPPIPAAAAVPPT
DPITVPVA"
CDS 4282338..4283999
/codon_start=1
/note="gene_3943"
/transl_table=11
/translation="VPSRTRAPTTLLTAMAATVVIVAWIANRPPASSHEPSPTPNTQL
AEQPLIGLGGGVTVRELTQDTPFSLVALTGDLAGTSARVRAKRPDGDWGPWYQTEYET
EPRDPAGTDGSVELGGLNPGPRSTDPVFVGTTTTVQVAVTRPIDAPITQPPAGRPPND
LLDSGLGYRPATKEQPFGQNISAILISPPQAPPGTQWTPPTAVTMAGQPPAIISRAEW
GADESLRCETPEYDRGVRAAVVHHTAGSNDYSPLESAGIVKAIYTYHSKTLGWCDIAY
NALVDKYGQVFEGSAGGLTKPVEGFHTGGFNRNTWGVAMIGNFDDVAPTPIQIRTVGR
LLGWRLGMDDVDPRSMVDLQSAGSSYTTFPGGAIARLPAIFTHRDVGNTDCPGNAAYA
VMDEIRDIAAHFNDPPEELIKALEGGAIYQRWQALGGMNSALGAPTSPEADAADGARY
ATFAKGAMYWSPVTDAQPITGAIYEAWASQSYERGPLGLPTSAEIQEPLQITQNFQHG
TLNFERLTGNVTEVVDGITTPLATRPPSGPTVPPEHFTLPTHPIT"
CDS 4284153..4285667
/codon_start=1
/note="gene_3944"
/transl_table=11
/translation="VSFVVTVPEAVAAAAGDLAAIGSTLREATAAAAGPTTGLAAAAA
DDVSIAVSQLFGRYGQEFQTVSNQLAAFHTEFVRTLNRGAAAYLNTESANGGQLFGQI
EAGQRAVSAAAAAAPGGAYGQLVANTATNLESLYGAWSANPFPFLRQIIANQQVYWQQ
IAAALANAVQNFPALVANLPAAIDAAVQQFLAFNAAYYIQQIISSQIGFAQLFATTVG
QGVTSVIAGWPNLAAELQLAFQQLLVGDYNAAVANLGKAMTNLLVTGFDTSDVTIGTM
GTTISVTAKPKLLGPLGDLFTIMTIPAQEAQYFTNLMPPSILRDMSQNFTNVLTTLSN
PNIQAVASFDIATTAGTLSTFFGVPLVLTYATLGAPFASLNAIATSAETIEQALLAGN
YLGAVGALIDAPAHALDGFLNSATVLDTPILVPTGLPSPLPPTVGITLHLPFDGILVP
PHPVTATISFPGAPVPIPGFPTTVTVFGTPFMGMAPLLINYIPQQLALAIKPAA"
CDS complement(4285664..4285816)
/codon_start=1
/note="gene_3945"
/transl_table=11
/translation="MRGYDAVHCASAEQLDDDEVVAAAADQRLLTAWLELGMATYDTN
QRATPR"
CDS complement(4285976..4286785)

/codon_start=1
/note="gene_3946"
/transl_table=11
/translation="VPALVACDVDGTLLDDGETVTKRTRDAVHAAVDAGTHFILATGR
PPRWVRPIVDALGFAPMAVCANGAVIYDPGTDRVTSVRTLPVDALATLAEVATRVIPG
AGLAVERIGERAHDTATPQFVSSPGYEHAWLNPDNTEVSIDHLLSAPAIKLLIRKAGA
ASADMAAELAKHVGFEGDITYSTNNGLVEIVPLGISKATGVDEIARPLGISDAEVVAF
GDMPNDVPMLLRAGLGVAMGNAHPDALAVADEVTAPNSEDGVARVLERWWS"
CDS complement(4286812..4287594)
/codon_start=1
/note="gene_3947"
/transl_table=11
/translation="MAEPFFRMMEILVPSIVAANGNKITFEGLENIPERGGALIALNH
TSYVDWVPASIAAHHRRRLRFMIKAEMQDVRAVNYVIKHAQLIPVDRSVGADAYAVAV
QRLRAGELVGLHPEATISRSLELREFKTGAARMALEAQVPIIPMIVWGAHRIWPKDHP
KNLFRNKIPIVAAIGSPVRPEGNAEQLNAVLRQAMNAILYRVQEEYPHPKGEHWVPRR
LGGGAPTVEESRQLRIAELAKRRQKRGYDGVTSSRRSQVGPH"
CDS complement(4287612..4288367)
/codon_start=1
/note="gene_3948"
/transl_table=11
/translation="MAEPTYRVLEILAQLLVLATGTRITYVGEENVPDQGGAVVAINH
TSYVDWLPAALAMHRRRRRMRFMIKAEMQRVRLVNFLIRHTRTIPVDRGAGGSAYAVA
VQRLREGELVGVYPEATISRSFELKGFKTGAARMAAEADVPIVPVVVWGAQRIWTKDH
PRQIGRAKVPVTVQVGRPLRAAAGIEQTNAALRESMTALLWQAQERYPHPAGAYWVPR
RLGGGAPTLAEAARMEADEAAARAASRTPHESR"
CDS complement(4288371..4289150)
/codon_start=1
/note="gene_3949"
/transl_table=11
/translation="VEPVYGTVIRLARLSWRIQGLKITVTGVDNLPTSGGAVVAINHT
SYLDFTFAGLPAYQQGLGRKVRFMAKQEVFDHKITGPIMRSLRHIPVDRQDGSASYDA
AVRMLKAGELVGVYPEATISRSFEIKEFKTGAARMAIEAGVPIVPHIVWGAQRIWTKD
RPKKLFRPKVPVTIVVGERIEPTLPTAELNGLLHSRMQHLLERAQELYGPHPAGEFWV
PHRLGGGAPSLAEAARLDAQEAAVRAARRAQRAHPAGAPEQ"
CDS 4289226..4289981
/codon_start=1
/note="gene_3950"
/transl_table=11
/translation="VSFPSSPPALPAIVARFAVGRPVRAVWVNELGGVTFRVDSGMGA
GCEFIKVARRGTADFANEARRLRWAAPYLAVPRVLGVGVDGDWAWLHTDALPGLSAVH
PRWRASPQVAVPALGAGLRTLHDSLPVHSCPFDWSTASRLAKLAPARRAELGDSPPVD
RLVVCHGDACSPNTILDDTGRCCGHVDFGNLGVADRWADLAVATLSLQWNFPDYPGQV
RDDEFFAAYGVAPDPARIDYYRRLWQAEDDSSR"
CDS 4290028..4291578
/codon_start=1
/note="gene_3951"
/transl_table=11
/translation="VQVTSVGHAGFLIQTQAGSILCDPWVNPAYFASWFPFPDNSGLD
WGALGECDYLYVSHLHKDHFDAENLRAHVNKDAVVLLPDFPVPDLRNELQKLGFHRFF
ETTDSVKHRLRGPNGDLDVMIIALRAPADGPIGDSALVVADGETTAFNMNDARPVDLD

VLASEFGHIDVHMLQYSGAIWYPMVYDMPARAKDAFGAQKRQRQMDRARQYIAQVGAT
WVVPSAGPPCFLAPELRHLNDDGSDPANIFPDQMVFLDQMRAHGQDGGLLMIPGSTAD
FTGTTLNSLRHPLPAEQVEAIFTTDKAAYIADYADRMAPVLAAQKAGWAAAAGEPLLQ
PLRTLFEPIMLQSNEICDGIGYPVELAIGPETIVLDFPKRAVREPIPDERFRYGFAIA
PELVRTVLRDNEPDWVNTIFLSTRFRAWRVGGYNEYLYTFFKCLTDERIAYADGWFAE
AHDDSSSITLNGWEIQRRCPHLKADLSKFGVVEGNTLTCNLHGWQWRLDDGRCLTARG
HQLRSSRP"

CDS 4291578..4291910
/codon_start=1
/note="gene_3952"
/transl_table=11
/translation="MQFYDDGVVQLDRAALTLRRYHFPSGTAKVIPLDQIRGYQAESL
GFLMARFNIWGRPDLRRWLPLDVYRPLKSTLVTLDVPGMRPKPACTPTRPKEFIALLD
ELLALHRT"

CDS complement(4291998..4293224)
/codon_start=1
/note="gene_3953"
/transl_table=11
/translation="MSRLMIFTWDLPGRCNIRAMNYAINAHLRRHDTYHSWFEFDNAE
HIVRHTIADPADIEVVQAEHQNMTSAELRHHIATPQPLQWDCFLFGIIQSDDHFTFYA
SIAHLCVDPMIVGVLFIEIHMMYSALVGGDPPIELPPAGRYDDHCVRQYADTAALTLD
SARVRRWVEFAANNDGTLPHFPLPLGDLSVPHTGKLLTETLMDEQQGERFEAACVAAG
ARFSGGVFACAALAERELTNCETFDVVTTTDTRRTPTELRTTGWFTGLVPITVPVASG
LFDSAARVAQISFDSGKDLATVPFDRVLELARPETGLRPPRPGNFVMSFLDASIAPLS
TVANSDLNFRIYDEGRVSHQVSMWVNRYQHQTTVTVLFPDNPIASESVANYIAAMKSI
YIRTADGTLAILKPGT"

CDS 4293468..4294265
/codon_start=1
/note="gene_3954"
/transl_table=11
/translation="VTLKPAGVISSDDAPQTTIVSGGVRLLTMWSTVLVLALSVICEP
VRIGLVVLMLNRRRPLLHLLTFLCGGYTMAGGVAMVTLVVLGATPLAGHFSVAEVQIG
TGLIALLIAFALTTNVIGKHVRRATHARVGDNGGRVLRESVPPSGTHKLAVRARCFLQ
GDSLYVAGVSGLGAALPSANYMGAMAAILASGATPATQALAVVTFNVVAFTVAEVPLV
SYLAAPRKTRAFMAALQSWLRSRSRRDAALLVAAGGCLMLTLGLSNL"

CDS 4294351..4294668
/codon_start=1
/note="gene_3955"
/transl_table=11
/translation="VFAIAAGLRWSAAVPPLHKGDAVTKLLVGAIAGGMLAYAAILGD
GIASADTALIVPGTAPSPYGPLRSLYHFNPAMQPQIGANYYTPTATRHVVSYPGSFWP
VTG"

CDS 4294752..4295513
/codon_start=1
/note="gene_3956"
/transl_table=11
/translation="VAGLSQGTLVLDREQARLANDPTAPPPGQLTFIKAGDPNNLLWR
AFRPGTHVPIIDYTVPAPVESQYDTINIVGQYDIFSDPPNRPGNLLADLNAIAAGGYY
GHSATAFSDPARVAPRDITTTTNSLGATTTTYFIRTDQLPLVRALVDMAGLPPQAAGT
VDAALRPIIDRAYQPGPAPAVNPRDLVQGIRGIPAIAPAIAIPIGSTTGASAATSTAA
ATAAATNALRGANVGPGANKALSMVRGLLPKGKKH"

```
CDS             complement(4295838..4298975)
                /codon_start=1
                /note="gene_3957"
                /transl_table=11
                /translation="VVIAFWVALAGLLAPTVPSLDAISQRHPVAILPSDAPVLVSTRQ
                MTAAFREAGLQSVAVVVLSDAKGLGAADERSYKELVDALRRDTRDVVMLQDFVTTPPL
                RELMTSKDNQAWILPVGLPGDLGSTQSKQAYARVADIVEHQVAGSTLTANLTGPAATV
                ADLNLTGQRDRSRIEFAITILLLVILLIIYRNPITMVLPLITIGMSVVVAQRLVAIAG
                LAGLGIANQSIIFMSGMMVGAGTDYAVFLISRYHDYLRQGADSDQAVKKALTSIGKVI
                AASAATVAITFLGMVFTQLGILKTVGPMLGISVAVVFFAAVTLLPALMVLTGRRGWIA
                PRRDLTRRFWRSSGVHIVRRPKTHLLASALVLVILAGCAGLARYNYDDRKTLPASVES
                SIGYAALDKHFPSNLIIPEYLFIQSSTDLRTPKALADLEQMVQRVSQVPGVAMVRGIT
                RPAGRSLEQARTSWQAGEVGSKLDEGSKQIAAHTGDIDKLAGGANLMASKLGDVRAQV
                NRAISTVGGLIDALAYLQDLLGGNRVLGELEGAEKLIGSMRALGDTIDADASFVANNT
                EWASPVLGALDSSPMCTADPACASARTELQRLVTARDDGTLAKISELARQLQATRAVQ
                TLAATVSGLRGALATVIRAMGSLGMSSPGGVRSKINLVNKGVNDLADGSRQLAEGVQL
                LVDQVKKMGFGLGEASAFLLAMKDTATTPAMAGFYIPPELLSYATGESVKAETMPSEY
                RDLLGGLNVDQLKKVAAAFISPDGHSIRYLIQTDLNPFSTAAMDQIDAITAAARGAQP
                NTALADAKVSVVGLPVVLKDTRDYSDHDLRLIIAMTVCIVLLILIVLLRAIVAPLYLI
                GSVIVSYLAALGIGVIVFQFLLGQEMHWSIPGLTFVILVAVGADYNMLLISRLREEAV
                LGVRSGVIRTVASTGGVITAAGLIMAASMYGLVFASLGSVVQGAFVLGTGLLLDTFLV
                RTVTVPAIAVLVGQANWWLPSSWRPATWWPLGRRRGRAQRTKRKPLLPKEEEEQSPPD
                DDDLIGLWLHDGLRL"
CDS             complement(4299217..4300638)
                /codon_start=1
                /note="gene_3958"
                /transl_table=11
                /translation="VSAVPPSYVQARQIRSFSEQAARGLDHSRLLIASVEVFGHCDLR
                AMTYVINAHLRRHDTYRSWFELRDTDHIVRHSIADPADIEFVPTTHGEMTSADLRQHI
                VATPDSLHWDCFSFGVIQRADSFTFYASIDHLHADGQFVGVGLMEFQSMYTALIMGEP
                PIGLSEAGSYVDFCVRQHEYTSALTVDSPEVRAWIDFAEINNGTFPEFPLPLGDPSVR
                CGGDLLSMMLMDEQQTQRFESACMAANARFIGGMLACIAIAIHELTGADTYFGITPKD
                IRTPADLMTQGWFTGQIPVTVPVAGLSFNEIARIAQTSFDTGADLAKVPFERVVELSP
                SLRRPQPLFSLVNFFDAQVGPLSAVTKLFEGLNVGTYSDGRVTYPLSTMVGRFDETAA
                SVLFPDNPVARESVTAYLRAIRSVCMRIANGGTAERVGNVVALSPGRRNNIERMTWRS
                CRAGDFIDICNLKVANVTVDREA"
CDS             complement(4300803..4307090)
                /codon_start=1
                /gene="pks2"
                /note="gene_3959"
                /transl_table=11
                /translation="MACRLPGGIDSPELLWKALLRGDDLITEVPPDRWDCDEFYDPQP
                GVPGRTVCKWGGFLDNPADFDCEFFGIGEREAIAIDPQQRLLLETSWEAMEHAGLTQQ
                TLAGSATGVFAGVTHGDYTMVAADAKQLEEPYGYLGNSFSMASGRVAYAMRLHGPAIT
                VDTACSSGLTAVHMACRSLHEGESDVALAGGVALMLEPRKAAAGSALGMLSPTGRCRA
                FDVAADGFVSGEGCAVVVLKRLPDALADGDRILAVIRGTSANQDGHTVNIATPSQPAQ
                VAAYRAALAAGGVDAATVGMVEAHGPGTPIGDPIEYASVSEVYGVDGPCALASVKTNF
                GHTQSTAGVLGLIKVVLALKHGVVPRNLHFTRLPDEIAGITTNLFVPEVTTPWPTNGR
                QVPRRAAVSSYGFSGTNVHAVVEQAPQTEAQPHAASTPPTGTPALFTLSASSADALRQ
                TAQRLTDWIQQHADSLVLSDLAYTLARRRTHRSVRTAVIASSVDELIAGLGEVADGDT
                VYQPAVGQDDRGPVWLFSGQGSQWAAMGADLLTNESVFAATVAELEPLIAAESGFSVT
```

EAMTAPETVTGIDRVQPTIFAMQVALAATMAAYGVRPGAVIGHSMGESAAAVVAGVLS
AEDGVRVICRRSKLMATIAGSAAMASVELPALAVQSELTALGIDDVVVAVVTAPQSTV
IAGGTESVRKLVDIWERRDVLARAVAVDVASHSPQVDPILDELIAALADLNPKAPEIP
YYSATLFDPREAPACDARYWADNLRHTVRFSAAVRSALDDGYRVFAELSPHPLLTHAG
DQIAGSVGMPVAALAGMRREQPLPLGLRRLLTDLHNAGAAVDFSVLCPQGRLVDAPLP
AWSHRFLFYDREGVDNRSPGGSTVAVHPLLGAHVRLPEEPERHAWQADVGTATLPWLG
DHRIHNVAALPGAAYCEMALSAARAVLGEQSEVRDMRFEAMLLLDDQTPVSTVATVTS
PGVVDFAVEALQEGVGHHLRRASAVLQQVSGECEPPAYDMASLLEAHPCRVDGEDLRR
QFDKHGVQYGPAFTGLAVAYVAEDATATMLAEVALPGSIRSQQGLYAIHPALLDACFQ
SVGAHPDSQSVGSGLLVPLGVRRVRAYAPVRTARYCYTRVTKVELVGVEADIDVLDAH
GTVLLAVCGLRIGTGVSERDKHNRVLNERLLTIEWHQRELPEMDPSGAGKWLLISDCA
ASDVTATRLADAFREHSAACTTMRWPLHDDQLAAADQLRDQVGSDEFSGVVVLTGSNT
GTPHQGSADRGAEYVRRLVGIARELSDLPGAVPRMYVVTRGAQRVLADDCVNLEQGGL
RGLLRTIGAEHPHLRATQIDVDEQTGVEQLARQLLATSEEDETAWRDNEWYVARLCPT
PLRPQERRTIVADHQQSGMRLQIRTPGDMQTIELAAFHRVPPGPGQIEVAVRASSVNF
ADVLIAFGRYPSFEGHLPQLGTDFAGVVTAVGPGVTDHKVGDHVGGMSPNGCWGTFVT
CDARLAATLPPGLGDAQAAAVTTAHATAWYGLHELARIRAGDTVLIHSGTGGVGQAAI
AIARAAGAEIFATAGTPQRRELLRNMGIEHVYDSRSIEFAEQIRRDTNGRGVDVVLNS
VTGAAQLAGLKLLAFRGRFVEIGKRDIYGDTKLGLFPFRRNLSFYAVDLGLLSATHPE
ELRDLLGTVYRLTAAGELPMPQSTHYPLVEAATAIRVMGNAEHTGKLVLHIPQTGKSL
VTLPPEQAQVFRPDGSYIITGGLGGLGLFLAEKMAAAGCGRIVLNSRTQPTQKMRETI
EAIAAMGSEVVVECGDIAQPGTAERLVATAVATGLPVRGVLHAAAVVEDATLANITDE
LLARDWAPKVHGAWELHEATSGQPLDWFCLFSSAAALTGSPGQSAYSAANSWLDAFAH
WRQAQGLPATAIAWGAWSDIGQLGWWSASPARASALEESNYTAITPDEGAYAFEALLR
HNRVYTGYAPVIGAPWLVAFAERSRFFEVFSSSNGSGTSKFRVELNELPRDEWPARLR
QLVAEQVSLILRRTVDPDRPLPEYGLDSLGALELRTRIETETGIRLAPKNVSATVRGL
ADHLYEQLAPDDAPAAALSSQ"

CDS complement(4307636..4307827)
/codon_start=1
/note="gene_3960"
/transl_table=11
/translation="LIDRNRWRAGNTGRWRNARDDIAHYRGCCQNDIGFRVTHYRVNT
FIAGAAERSRKWNRNKTGL"

CDS 4307933..4309144
/codon_start=1
/note="gene_3961"
/transl_table=11
/translation="MITYKNILANFQQMISAYFADTGAVPPLDLFIMSWLPFYHDMGL
VLGVCAPIIVGCGAVLTSPVAFLQRPARWLQLMAREGQAFSAAPNFAFELTAAKAIDD
DLAGLDLGRIKTILCGSERVHPATLKRFVDRFSRFNLREFAIRPAYGLAEATVYVATS
QAGQPPEIRYFEPHELSAGQAKPCATGAGTALVSYPLPQSPIVRIVDPNTNTECPPGT
IGEIWVHGDNVAGGYWEKPDETERTFGGALVAPSAGTPVGPWLRTGDSGFVSEDKFFI
IGRIKDLLIVYGRNHSPDDIEATIQEITRGRCAAIAVPSNGVEKLVAIVELNNRGNLD
TERLSFVTREVTSAISTSHGLSVSDLVLVAPGSIPITTSGKVRRAECVKLYRHNEFTR
LDAKPLQASDL"

CDS complement(4309141..4310364)
/codon_start=1
/note="gene_3962"
/transl_table=11
/translation="MARFEVPEGWCVQAFRFTLDPTEDQARALARHFGARRKAYNWAV
ATLKADIEAWRVTGIGTVKPSLRVLRKRWNTVKDEVCVNAETGAVWWPECSKEAYADG

IGGAVDAYWNWQNSRSGKREGKTMGFPRFKKKGRDQDRVTFTTGAMRVEPDRRHLTLP
VVGTVRTHENTRRIERLIATGRARVLAISVRRNGTRLDASVRVLVQRPQQPNVAQPGS
RVGVDVGVRRLATVANEAGAVLEEVPNPRPLDAALKELRYASRARSRCTKGSRRYRER
TTEISRLHRRVNDVRTHHLHVLTTRLAQTHGHIVVEGLDAAGMLRQKGLPGARARRRG
LSDSALGTPRRHLSYKTGWYGSALVVADRWFPSLSVEPTVRPGLARLVAVKRGREAAA
WLPNNPETGCKSRDH"

CDS complement(4310364..4310945)
/codon_start=1
/note="gene_3963"
/transl_table=11
/translation="MNLAVWAERNGVAWVIAYRWFRAGLLPVPAQRVGRLILVNDPAV
EESGRGRTLVYARVSSADQRSDLDRRVARVTAWATSQHLSVDKVVAEGGWALNGHRRK
FFALLGDPVVTRIVVEHRDRFCWFGSEYVEAALVAQGRELVVVDLAEVDDDLVGDMTE
ILTSMCARLYGERAAQNGAKRALAAAVGDAEAA"

CDS complement(4310976..4312586)
/codon_start=1
/note="gene_3964"
/transl_table=11
/translation="MTGYDAIVIGAGHNGLTAAVLLQRAGLRTACLDAKRYAGGMAST
VELFDGYRFDIAGSVQFPTSSAVSSELGLDSLPTVDLEVMSVALRGVGDDPVVQFTDP
TKMLTHLHRVHGADAVTGMAGLLAWSQAPTRALGRFEAGTLPKSFDEMYACATNEFER
SAIDDMLFGSVTDVLDRHFPDREKHGALRGSMTVLAVNTLYRGPATPGSAAALAFGLG
VPEGDFVRWKKLRGGIGALTTHLSQLLERTGGEVRLRSKVTEIVVDNSRSSARVRGVR
TAAGDTLTSPIVVSAIAPDVTINELIDPAVLPSEIRDRYLRIDHRGSYLQMHFALAQP
PAFAAPYQALNDPSMQASMGIFCTPEQVQQQWEDCRRGIVPADPTVVLQIPSLHDPSL
APAGKQAASAFAMWFPIEGGSKYGGYGRAKVEMGQNVIDKITRLAPNFKGSILRYTTF
TPKHMGVMFGAPGGDYCHALLHSDQIGPNRPGPKGFIGQPIPIAGLYLGSAGCHGGPG
ITFIPGYNAARQALADRRAANCCVLSGR"

CDS complement(4312634..4313263)
/codon_start=1
/note="gene_3965"
/transl_table=11
/translation="MVRPPQTARSERTREALRQAALVRFLAQGVEATSAEQIAEDAGV
SLRTFYRHFRSKHDLLFADYDAGLHWFRAALDARPADESIIDSVQAAIFSFPYDVDAV
TKIASLRRGELEPSRIVRHMREVEADFADAIQAQLRRRNCDIAGAPDARLHIAVTARC
VAAAVFGAMEAWMLGSDRSLGELARVCHVALESLRVGISDTWTTLTVSS"

CDS 4313335..4313817
/codon_start=1
/note="gene_3966"
/transl_table=11
/translation="MVSLLVHAALGVVVIGWIVSSNPKVFTRPAGGSWFSLPECVYYV
VGIASIALGWYFNIRFVQQYAHGAANPLWGPGSWAEYVRLMFTNPAASSAGQDYTIAN
VILLPLFSTTDGYRRGLRRPWLYFVSSLFTSFAFAFAFYFATIERQHRHERSRATVGA
"

CDS complement(4313814..4314332)
/codon_start=1
/note="gene_3967"
/transl_table=11
/translation="VANQLLPWALQHVELGPRTLEIGPGYGATLQALLGLTASLTAVE
VDNSMVERLNRRYGQRARIIRGDGTQTGLPDDHFTSVVCFTMLHHVASAQLQDQLFAE
AYRVLQPGGVFAGSDGVPSLPFRLIHIADTYTPIAPADLPGRLRAVGFTDIHVDVAGA

RLRWRATKPVAA"
CDS 4314445..4315236
/codon_start=1
/note="gene_3968"
/transl_table=11
/translation="MSENSHHRLATTSLTLPPGARIERHRHPSHQIVYPSAGAVSVTT
HAGTWITPVNRAIWIPAGCWHQHKFHGHTQFHGVALDPQRYRGGPATPTVLAVNPLMR
ELIIACSQADRTDTDEHHRMLAVLQDQLPTTSIREPLWVPSPTDRRLRHACALIADNL
TQPLTLQQIGGRIGVSQRTLSRLFSDELGMTFPQWRTQLRLQHALVLLAERHDVTSVA
SECGWATPSAFIDTYRQAFGHTPGQAAKPMAATRLTRLRRARDRR"
CDS complement(4315233..4316492)
/codon_start=1
/note="gene_3969"
/transl_table=11
/translation="VIDLKLLRENPDAVRRSQLSRGEDPALVDALLTADAARRAVIST
ADSLRAEQKAASKSVGGASPEERPPLLRRAKELAEQVKAAEADEVEAEAAFTAAHLAI
SNVIVDGVPAGGEDDYAVLDVVGEPSYLENPKDHLELGESLGLIDMQRGAKVSGSRFY
FLTGRGALLQLGLLQLALKLAVDNGFVPTIPPVLVRPEVMVGTGFLGAHAEEVYRVEG
DGLYLVGTSEVPLAGYHSGEILDLSRGPLRYAGWSSCFRREAGSHGKDTRGIIRVHQF
DKVEGFVYCTPADAEHEHERLLGWQRQMLARIEVPYRVIDVAAGDLGSSAARKFDCEA
WIPTQGAYRELTSTSNCTTFQARRLATRYRDASGKPQIAATLNGTLATTRWLVAILEN
HQRPDGSVRVPDALVPFVGVEVLEPVA"
CDS 4316625..4317974
/codon_start=1
/note="gene_3970"
/transl_table=11
/translation="MLDAPEQDPVDPGDPASPPHGEAEQPLPGPRWPRALRASATRRA
LLLTALGGLLIAGLVTAIPAVGRAPERLAGYIASYPVPSTGAKINASFNRVASGDCLM
WPDGTPESAAIVSCADEHRFEVAESIDMRTFPGMEYGQNAAPPSPARIQQISEEQCEA
AVRRYLGTKFDPNSKFTISMLWPGDRAWRQAGERRMLCGLQSPGPNNQQLAFKGKVAD
IDQSKVWPAGTCLGIDATTNQPIDVPVDCAAPHAMEVSGTVNLAERFPDALPSEPEQD
GFIKDACTRMTDAYLAPLKLRTTTLTLIYPTLTLPSWSAGSRVVACSIGATLGNGGWA
TLVNSAKGALLINGQPPVPPPDIPEERLNLPPIPLQLPTPRPAPPAQQLPSTPPGTQH
LPAQQPVVTPTRPPESHAPASAAPAETQPPPPDAGAPPATQSPEATPPGPAEPAPAG"
CDS 4317991..4318329
/codon_start=1
/note="gene_3971"
/transl_table=11
/translation="MDPQRFDELVSDALDLIPPELADAMDNVVVLVANRHPQHENLLG
QYEGVALTERGSDYAGSLPDAITIYREALLDACDSEDEVVDQVAITVIHEVAHHFGID
DERLDQLGWA"
CDS complement(4318588..4319286)
/codon_start=1
/note="gene_3972"
/transl_table=11
/translation="MSGRLVLLRHGQSYGNVERRLDTLPPGTALTPLGRDQARAFARS
GCRRPALLAHSVAIRAYQTAAVVAAELDMVAHEVAGIHEVQVGELENRNDDEAVAEFN
ATYSRWHRGELDVPLPGGETANDVLDRYLPVLADLRMRYLDDGDWDGDIVVVSHSAAI
RLAAAVLAGVDGNFVLDNHLENVESVVLAPITDGRWSCVQWGLRKPPFCPDPAEAAAS
PVTHAVTSSTDPMG"
CDS complement(4319283..4320248)

```
                     /codon_start=1
                     /note="gene_3973"
                     /transl_table=11
                     /translation="VVRIAYLGPEGTFTEAALVRMVAAGLVPETGPDALQRMPVESAP
                     AALAAVRDGGADYACVPIENSIDGSVLPTLDSLAIGVRLQVFAETTLDVTFSIVVKPG
                     RNAADVRTLAAFPVAAAQVRQWLAAHLPAADLRPAYSNADAARQVADGLVDAAVTSPL
                     AAARWGLAALADGVVDESNARTRFVLVGRPGPPPARTGADRTSAVLRIDNQPGALVAA
                     LAEFGIRGIDLTRIESRPTRTELGTYLFFVDCVGHIDDEAVAEALKAVHRRCADVRYL
                     GSWPTGPAAGAQPPLVDEASRWLARLRAGKPEQTLVRPDDQGAQA"
     CDS             4320344..4321120
                     /codon_start=1
                     /note="gene_3974"
                     /transl_table=11
                     /translation="MPPLTSLAPTTAERIRSACARAGGALLVVEREDPVPVPIHHLLY
                     DGSFAVAVPVDRGEVSGSQALLELTDYAPLPVREPVRSLVWIRGCLHQIPPAELVETL
                     DLIATDNPNPALLQVETPRSGPADAAETRYTMQRLEIESVVVTDATGAEPVTVADLLA
                     ARPDPFCEIESTLLWHLATAHDDVVARLVSRLPAPLRRGQIRPLGLDRYGVRFRIEAR
                     DGDRDIRLPFHKPVDDMTGLSQAIRVLMGCPFRNGLRARR"
     CDS             4321197..4321559
                     /codon_start=1
                     /note="gene_3975"
                     /transl_table=11
                     /translation="LASDNPTTLISIPRDSYVPIPGHGRDKINAAFALGGGRLLTQTV
                     ELATGLHLDHYAEVGFSEFADLVDAFDPLAGVDLPAGCQTLDGRAALGYVRTRATPRA
                     DLEGSDVPVPAAAFETQP"
     CDS             4321757..4322302
                     /codon_start=1
                     /note="gene_3976"
                     /transl_table=11
                     /translation="MTEYEGPKTKFHALMQEQIHNEFTAAQQYVAIAVYFDSEDLPQL
                     AKHFYSQAVEERNHAMMLVQHLLDRDLRVEIPGVDTVRNQFDRPREALALALDQERTV
                     TDQVGRLTAVARDEGDFLGEQFMQWFLQEQIEEVALMATLVRVADRAGANLFELENFV
                     AREVDVAPAASGAPHAAGGRL"
     CDS             complement(4322317..4323147)
                     /codon_start=1
                     /note="gene_3977"
                     /transl_table=11
                     /translation="VDMTWADEVLAGHPFVVAHRGASAARPEHTLAAYDLALKEGADG
                     VECDVRLTRDGHLVCVHDRRLDRTSTGAGLVSTMTLAQLRELEYGAWHDSWRPDGSHG
                     DTSLLTLDALVSLVLDWHRPVKIFVETKHPVRYGSLVENKLLALLHRFGIAAPASADR
                     SRAVVMSFSAAAVWRIRRAAPLLPTVLLGKTPRYLTSSAATAVGATAVGPSLPALKEY
                     PQLVDRSAAQGRAVYCWNVDEYEDIDFCREVGVAWIGTHHPGRTKAWLEDGRANGTTR
                     "
     CDS             complement(4323147..4323701)
                     /codon_start=1
                     /note="gene_3978"
                     /transl_table=11
                     /translation="VVASASVWLGVLVSLAAIAAAGTTIVLLVRWLVARRAAAFMHQG
                     LPERRSARELWAGCLLPMVNLLWAPLYVIELALVEDRYTRLRRPIVVWWIVWIVSNAI
                     SMFAFATSWVTDAQGIANNTTMMVLAYLCAAAAVAAAARVFEGFEQKPVERPAHRWVV
                     VNTDGRSAPASSVAVELDGQEPAA"
```

CDS 4323634..4323900
/codon_start=1
/gene="DS2_01918"
/note="gene_3979"
/transl_table=11
/translation="VPAAAIAAKLTKTPSQTEALATTEFNNVFRLMTSNTYRTTNTTT
AMLNTSRLTNSVVRTAAGDGPAFAEAGTGAWSTWSVSPHRGIPA"
CDS 4326353..4326844
/codon_start=1
/note="gene_3980"
/transl_table=11
/translation="VTAENPGRSRRTLVGIDAAITACHHIAIRDDVGARSIRFSVEPT
LAGLRTLTDKLSGYDDIDATVEPTSMTWLPLTIAVENAGDTMHMAGARHCARLRGAIV
GKSKSDVIDAEVLTRASEVFDLTPLTLPTPAQLALRRSVIRRAGAVIDANRSWRRLMS
LAR"
CDS 4326859..4327218
/codon_start=1
/note="gene_3981"
/transl_table=11
/translation="VDRVRRVVTDRDSGAGALARHPLAGRRTDPQLAAFYHRLMTTQR
HCHTQATIAVARKLAERTRVTITTGRPYQLRDTNGDPVTARGAKELIDAHYHVDTRTH
PHNRAHTDTMQNSKPAR"
CDS complement(4327832..4327996)
/codon_start=1
/note="gene_3982"
/transl_table=11
/translation="MGWVGKKKSTAGQLAGTANELTKEVLERAVHRESPVIRPDVVVG
IPAVDRRPKQ"
CDS 4328282..4328905
/codon_start=1
/note="gene_3983"
/transl_table=11
/translation="VAEYTLPDLDWDYGALEPHISGQINELHHSKHHATYVKGANDAV
AKLEEARAKEDHSAILLNEKNLAFNLAGHVNHTIWWKNLSPNGGDKPTGELAAAIADA
FGSFDKFRAQFHAAATTVQGSGWAALGWDTLGNKLLIFQVVYDHQTNFPLGIVPLLLLD
MWEHAFYLQYKNVKVDFAKAFWNVVNWADVQSRYAAATSQTKGLTFG"
CDS 4329116..4329649
/codon_start=1
/note="gene_3984"
/transl_table=11
/translation="MGTGSGGPIGVSPFHSRGALKGFVISGRWPDSTKEWAQLLMVAV
RVASLPGLLSTTTVFGAREELPDEPEPGTVGLVLAEGTVFGESAIQPGYFADHQPPAL
LMLHPPSETTPSLPECTGAASGCVLLPGLPYLGLEHRAAWVEAEADGTITSMVSRVGV
DPISHPDTAILAMLLAA"
CDS 4329904..4330812
/codon_start=1
/note="gene_3985"
/transl_table=11
/translation="MLAATLLSLGAVFLAELGDRSQLITMTYTLRYRWWVVLTGVAIA
AFTVHGVAVAIGHFLGSTVPARPAACVSAIAFLIFAVWVWREDTASDSETSPTAAEPR
LALFTVVSSFALAELGDKTTLATVTLASDHHWAGVWIGTTLGMILADGLAIGAGLLLH

RRLPERLLQVLTGLLFLLFGLWLLFDDALGFRSIAIAVTAAVVLAAATTAVSVRVAQT
RRRRPTAAATPEDDSTRPERSSVAPGHPGSILLPLPEVSLRGRRPPSGSPDERCADPG
SKGGSRRISVGCWLPGVGRIRPTRSS"
CDS 4331077..4331475
/codon_start=1
/note="gene_3986"
/transl_table=11
/translation="MSTTFAARLNRLFDTVYPPGRGPHTSAEVIAALKAEGITMSAPY
LSQLRSGNRTNPSGATMAALANFFRIKAAYFTDDEYYEKLDKELQWLCTMRDDGVRRI
AQRAHGLPSAAQQKVLDRIDELRRAEGIDA"
CDS 4331593..4332249
/codon_start=1
/note="gene_3987"
/transl_table=11
/translation="MGLFGKRKSRATRRAEARAIKARAKLEAKLSAKNEARRIKAAQR
AESKALKAQLKARRDSDRAALKVAEAELKVAREGKLLSPTRIRRLLTVSRLLAPILTP
VIYRAAMAARGLIDQRRADQLGVPLAQIGRFSGHGARLSARVGGAERSLRMVQEKKPK
DVETKQFVSAVTNRLTDLSAAVAAAEHMPAKRRRTAHSAISSQLDGIEADLMARLGLT
"
CDS 4332261..4332545
/codon_start=1
/note="gene_3988"
/transl_table=11
/translation="MTAIGMSHPPRVHRRVGGQRTALTAGIGLLLAALVLTTIANPPA
AFAHTAQLSTATPAPAVAATDANDVPTWPFVVGTVAAVAVAALWAVRRGR"
CDS 4332652..4333056
/codon_start=1
/note="gene_3989"
/transl_table=11
/translation="MPDPQDRPDSEPSDASTPPAKKLPAKKAAKKAPARKTPAKKAPA
KKTPAKGAKSAPPKPAEAPVSLQQRIETNGQLAAAAKDAAAQAKSTVEGANDALARNA
SVPAPSHSPVPLIVAVTLSLLALLLIRQLRRR"
CDS 4333073..4333546
/codon_start=1
/note="gene_3990"
/transl_table=11
/translation="VAISFRPTADLVDDIGPDVRSCDLQFRQFGGRSQFAGPISTVRC
FQDNALLKSVLSQPSAGGVLVIDGAGSLHTALVGDVIAELARSTGWTGLIVHGAVRDA
AALRGIDIGIKALGTNPRKSTKTGAGERDVEITLGGVTFVPGDIAYSDDDGIIVV"
CDS complement(4333582..4335051)
/codon_start=1
/note="gene_3991"
/transl_table=11
/translation="MTEHLDVVIVGAGISGVSAAWHLQDRCPTKSYAILEKRESMGGT
WDLFRYPGIRSDSDMYTLGFRFRPWTGRQAIADGKPILEYVKSTAAMYGIDRHIRFHH
KVISADWSTAENRWTVHIQSHGTLSALTCEFLFLCSGYYNYDEGYSPRFAGSEDFVGP
IIHPQHWPEDLDYDAKNIVVIGSGATAVTLVPALADSGAKHVTMLQRSPTYIVSQPDR
DGIAEKLNRWLPETMAYTAVRWKNVLRQAAVYSACQKWPRRMRKMFLSLIQRQLPEGY
DVRKHFGPHYNPWDQRLCLVPNGDLFRAIRHGKVEVVTDTIERFTATGIRLNSGRELP
ADIIITATGLNLQLFGGATATIDGQQVDITTTMAYKGMMLSGIPNMAYTVGYTNASWT
LKADLVSEFVCRLLNYMDDNGFDTVVVERPGSDVEERPFMEFTPGYVLRSLDELPKQG

SRTPWRLNQNYLRDIRLIRRGKIDDEGLRFAKRPAPVGV"
CDS 4335127..4335777
/codon_start=1
/note="gene_3992"
/transl_table=11
/translation="VTTSAASQASLPRGRRTARPSGDDRELAILATAENLLEDRPLAD
ISVDDLAKGAGISRPTFYFYFPSKEAVLLTLLDRVVNQADMALQTLAENPADTDRENM
WRTGINVFFETFGSHKAVTRAGQAARATSVEVAELWSTFMQKWIAYTAAVIDAERDRG
AAPRTLPAHELATALNLMNERTLFASFAGEQPSVPEARVLDTLVHIWVTSIYGENR"
CDS complement(4335979..4336986)
/codon_start=1
/note="gene_3993"
/transl_table=11
/translation="MDPVTALRQIAYYKDRNRHDPRRVMAYRNAADIIEGLDDAARQR
HGQANSWQSLAGIGPKTAKVIAQAWSGREPDLLAELRADAEDLGGGAIRAALRGDLHL
HSNWSDGSAPIEEMMATAAALGHQYCALTDHSPRLTIANGLSPDRLRKQLDVIDELRE
KFAPLRILTGIEVDILEDGSLDQEPEMLDRLDIVVASVHSKLSMDSAAMTRRMVRAVA
NGHTDVLGHCTGRLIAGNRGIRPESKFDAEAVFTACREHGTAVEINSRPERRDPPTRL
LHLARDIGCVFSIDTDAHAPGQLDFLGYGAQRALDAEVPADRIVNTWPADTLLAWTGS
H"
CDS complement(4336995..4337192)
/codon_start=1
/note="gene_3994"
/transl_table=11
/translation="MNCALGFDTKPILLASYVTHGARRATANQFERPAKGAGVLMALL
ILGEMAGFAVVVTGVVFGQLV"
CDS complement(4337617..4339083)
/codon_start=1
/gene="gltD"
/note="gene_3995"
/transl_table=11
/translation="MADPGGFLKYTHRKLPKRRPVPLRLRDWREVYEEFDNESLRQQA
TRCMDCGIPFCHNGCPLGNLIPEWNDLVRRGRWRDAIERLHATNNFPDFTGRLCPAPC
EPACVLGINQDPVTIKQIELEIIDKAFDEGWVQPRPPRKLTGQTVAVVGSGPAGLAAA
QQLTRAGHTVTVFEREDRIGGLLRYGIPEFKMEKRHLDRRLDQMRSEGTEFRPGVNVG
VDISAEKLRADFDAVVLAGGATAWRELPIPGRELEGVHQAMEFLPWANRVQEGDDVLD
EDGQPPITAKGKKVVIIGGGDTGADCLGTVHRQGAIAVHQFEIMPRPPDARAESTPWP
TYPLMYRVSAAHEEGGERVFSVNTEAFVGTDGRVSALRAHEVNMLDGKFVKVEGSDFE
LEADLVLLAMGFVGPERAGLLTALGVKFTEHGNVARGDDFDTSVPGVFVAGDMGRGQS
LIVWAIAEGRAAAAAVDRYLMGSSALPAPVKPTAAPLQ"
CDS complement(4339076..4343659)
/codon_start=1
/note="gene_3996"
/transl_table=11
/translation="MTPKRVGLYNPAFEHDSCGVAMVVDMHGRRSRDIVDKAITALLN
LEHRGAQGAEPRSGDGAGILIQVPDEFLREAVDFELPAPGSYATGIAFLPQSSKDAAA
ACAAVQKIAEAEGLQVLGWRSVPTDDSSLGALSRDAMPTFRQVFLAGASGMALERRCY
VVRKRAEHELGTKGPGQDGPGRETVYFPSLSGQTLVYKGMLTTPQLKAFYLDLQDERL
TSALGIVHSRFSTNTFPSWPLAHPFRRIAHNGEINTVTGNENWMRAREALIKTDIFGS
AADVEKLFPICTPGASDTARFDEVLELLHLGGRSLAHAVLMMIPEAWERHESMDPARR
AFYQYHASLMEPWDGPASMTFTDGTVVGAVLDRNGLRPSRIWVTDDGLVVMASEAGVL

DLHPSTVVRRMRLQPGRMFLVDTAQGRIVSDEEIKADLAAEHPYQEWLDNGLVPLDEL
PEGKDVRMPHHRIVMRQLAFGYTYEELNLLVAPMARLGAEPIGSMGTDTPVAVLSQRP
RMLYDYFHQLFAQVTNPPLDAIREEVVTSLQGTTGGERDLLNPDENSCHQIVLPQPIL
RNHELAKLVSLDPNDKVNGRPHGLRSKVIRCLYRVSEGGAGLAAALEEVRGAAAAAIA
DGARIIILSDRESDEEMAPIPSLLAVAGVHHHLVRERTRTQVGLVVESGDAREVHHMA
ALVGFGAAAINPYLVFESIEDMLDRGVIEGIDRTAALNNYIKAAGKGVLKVMSKMGIS
TLASYTGAQLFQAVGISEQVLDEYFTGLTCPTGGITLDDIAADVAARHRLAYLDRPDE
RAHRELEVGGEYQWRREGEYHLFNPETVFKLQHSTRTGQYKIFKEYTRLVDDQSERMA
SLRGLLKFRTGVRPPVPLDEVEPASEIVKRFSTGAMSYGSISAEAHETLAIAMNRLGA
RSNCGEGGEDVKRFDRDPNGDWRRSAIKQVASARFGVTSHYLTNCTDLQIKMAQGAKP
GEGGQLPGHKVYPWVAEVRHSTPGVGLISPPPHHDIYSIEDLAQLIHDLKNANPSARV
HVKLVSENGVGTVAAGVSKAHADVVLISGHDGGTGATPLTSMKHAGAPWELGLAETQQ
TLLLNGLRDRIVVQVDGQLKTGRDVMIATLLGAEEFGFATAPLVVAGCIMMRVCHLDT
CPVGVATQNPLLRERFTGKPEFVENFFMFIAEEVREYLAQLGFRTVNEAVGQAGALDT
TLARAHWKAHKLDLAPVLHEPESAFMNQDLYCSSRQDHGLDKALDQQLIVMSREALDS
GKPVRFSTTIGNVNRTVGTMLGHELTKAYGGQGLPDGTIDITFDGSAGNSFGAFVPKG
ITLRVYGDANDYVGKGLSGGRIVVRPSDDAPQDYVAEDNIIGGNVILFGATSGEVYLR
GVVGERFAVRNSGAHAVVEGVGDHGCEYMTGGRVVILGRTGRNFAAGMSGGVAYVYDP
DGELPANLNSEMVELETLDEDDADWLHGTIQVHVDATDSAVGQRILSDWSGQQRHFVK
VMPRDYKRVLQAIALAERDGVDVDKAIMAAAHG"

CDS 4344652..4345416
/codon_start=1
/note="gene_3997"
/transl_table=11
/translation="MVRLVTFGLVGLGRSGMQRQEAQFEATIRTVLHGNHKVAVLGKG
GVGKTSVAACVGSILAELRQQDRIVGIDADTAFGRLSSRIDPRAAGSFWELTTDTNLR
SFTDITARLGRNSAGLYVLAGQPASGPRRALDPAIYREAALRLDHHFAISVIDCGSSM
EAAVTQEVLRDVDALIVVSSPWADGASAAANTIEWLSDYGLTGLLRRSIVVLNDSDGH
ADKRTKSLLAQEFIDHGQPVVEVPFDPHLRPGGSSI"

CDS complement(4345748..4346038)
/codon_start=1
/note="gene_3998"
/transl_table=11
/translation="MTVTALYEVPLGVCTQDPDRWTTTPDDEAKTLCRACPRRWLCAR
DAVESAGAEGLWAGVVIPESGRARAFALGQLRSLAERNGYPVRDHRVSAQSA"

CDS 4346734..4347603
/codon_start=1
/note="gene_3999"
/transl_table=11
/translation="VTVSDKTLIRFGDPPGGKALTFEVVRPSDSAAQHGRVQPSADLS
DDPAHNAAPVAPDPGVVRAGAAAAARRRELDISQRSLAADGIINAGALIAFEKGRSWP
RERTRAKLEEVLQWPAGTIARIRRGEPTEPATNPDASPGLRPADGPASLIAQAVTAAV
DGCSLAIAALPATEDPEFTERAAPILADLRQLEAIAVQATRISRITPELIKALGAVRR
HHDELMRLGATAPGATLAQRLYAARRRANLSTLETAQAAGVAEEMIVGAEAEEELPAE
ATEAIEALIRQIN"

CDS 4347846..4349102
/codon_start=1
/gene="espE"
/note="gene_4000"
/transl_table=11
/translation="MASGSGLCKTTSNFIWGQLLLLGEGIPDPGDIFNTGSSLFKQIS

DKMGLAIPGTNWIGQAAEAYLNQNIAQQLRAQVMGDLDKLTGNMISNQAKYVSDTRDV
LRAMKKMIDGVYKVCKGLEKIPLLGHLWSWELAIPMSGIAMAVVGGALLYLTIMTLMN
ATNLRGILGRLIEMLTTLPKFPGLPGLPSLPDIIDGLWPPKLPDIPIPGLPDIPGLPD
FKWPPTPGSPLFPDLPSFPGFPGFPSLPGFPGLPGFPEFPAIPGFPALPGLPSIPNLF
PGLPGLGDLLPGVGDLGKLPTWTELAALPDFLGGFAGLPSLGFGNLLSFASLPTVGQV
TATMGQLQQLVAAGGGPSQLASMGSQQAQLISSQAQQGGQQHATLVSDKKEDEEGAAA
GVAEAERAPIDAGTAASQRGQEGTVL"

CDS 4349190..4349501
/codon_start=1
/note="gene_4001"
/transl_table=11
/translation="MTGFLGVVPSFLKVLAGMHNEIVGDIKRATDTVAGISGRVQLTH
GSFTSKFNDTLQEFETTRSSTGTGLQGVTSGLANNLLAAAGAYLKADDGLAGVIDKIF
G"

CDS 4349504..4350355
/codon_start=1
/note="gene_4002"
/transl_table=11
/translation="MTGPSAAGRAGTADNVVGVEVTIDGMLVIADRLHLVDFPVTLGI
RPNIPQEDLRDIVWEQVQRDLTAQGVLDLHGEPQPTVAEMVETLGRPDRTLEGRWWRR
DIGGVMVRFVVCRRGDRHVIAARDGDMLVLQLVAPQVGLAGMVTAVLGPAEPANVEPL
TGVATELAECTTASQLTQYGIAPASARVYAEIVGNPTGWVEIVASQRHPGGTTTQTDA
AAGVLDSKLGRLVSLPRRVGGDLYGSFLPGTQQNLERALDGLLELLPAGAWLDHTSDH
AQASSRG"

CDS 4350394..4350945
/codon_start=1
/gene="espH"
/note="gene_4003"
/transl_table=11
/translation="MVDPPGNDDDHGDLDALDFSAAHTNEASPLDALDDYAPVQTDDA
EGDLDALHALTERDEEPELELFTVTNPQGSVSVSTLMDGRIQHVELTDKATSMSEAQL
ADEIFVIADLARQKARASQYTFMVENIGELTDEDAEGSALLREFVGMTLNLPTPEEAA
AAEAEVFATRYDVDYTSRYKADD"

CDS 4350989..4352659
/codon_start=1
/gene="eccA1"
/note="gene_4004"
/transl_table=11
/translation="MSEARSLDLFTEITNYDESACDAWIGRIRCGDTDRVTLFRAWYS
RRNFGQLSGSVQISMSTLNARIAIGGLYGDITYPVTSPLAITMGFAACEAAQGNYADA
MEALEAAPVAGSEHLVAWMKAVVYGAAERWTDVIDQVKSAGKWPDKFLAGAAGVAHGV
AAANLALFTEAERRLTEANDSPAGEACARAIAWYLAMARRSQGNESAAVALLEWLQTT
HPEPKVAVALKDPSYRLKTTTAEQIASRADPWDPGSVVTDNSGRERLLAEAQAELDRQ
IGLTRVKNQIERYRAATLMARVRAAKGMKVAQPSKHMIFTGPPGTGKTTIARVVANIL
AGLGVIAEPKLVETSRKDFVAEYEGQSAVKTAKTIDQALGGVLFIDEAYALVQERDGR
TDPFGQEALDTLLARMENDRDRLVVIIAGYSSDIDRLLETNEGLRSRFATRIEFDTYS
PEELLEIANVIAAADDSALTAEAAENFLQAAKQLEQRMLRGRRALDVAGNGRYARQLV
EASEQCRDMRLAQVLDIDTLDEDRLREINGSDMAEAIAAVHAHLNMRE"

CDS 4352663..4354105
/codon_start=1
/note="gene_4005"

```
                     /transl_table=11
                     /translation="MGLRLTTKVQVSGWRFLLRRLEHAIVRRDTRMFDDPLQFYSRSI
                     ALGIVVAVLILAGAALLAYFKPQGKLGGTSLFTDRATNQLYVLLSGQLHPVYNLTSAR
                     LVLGNPANPATVKSSELSKLPMGQTVGIPGAPYATPVSAGSTSIWTLCDTVARADSTS
                     PVVQTAVIAMPLEIDASIDPLQSHEAVLVSYQGETWIVTTKGRHAIDLTDRALTSSMG
                     IPVTARPTPISEGMFNALPDMGPWQLPPIPAAGAPNSLGLPDDLVIGSVFQIHTDKGP
                     QYYVVLPDGIAQVNATTAAALRATQAHGLVAPPAMVPSLVVRIAERVYPSPLPDEPLK
                     IVSRPQDPALCWSWQRSAGDQSPQSTVLSGRHLPISPSAMNMGIKQIHGTATVYLDGG
                     KFVALQSPDPRYTESMYYIDPQGVRYGVPNAETAKSLGLSSPQNAPWEIVRLLVDGPV
                     LSKDAALLEHDTLPADPSPRKVPAGASGAP"
     CDS             4354105..4356348
                     /codon_start=1
                     /note="gene_4006"
                     /transl_table=11
                     /translation="MTTKKFTPTITRGPRLTPGEISLTPPDDLGIDIPPSGVQKILPY
                     VMGGAMLGMIAIMVAGGTRQLSPYMLMMPLMMIVMMVGGLAGSTGGGGKKVPEINADR
                     KEYLRYLAGLRTRVTSSATSQVAFFSYHAPHPEDLLSIVGTQRQWSRPANADFYAATR
                     IGIGDQPAVDRLLKPAVGGELAAASAAPQPFLEPVSHMWVVKFLRTHGLIHDCPKLLQ
                     LRTFPTIAIGGDLAGAAGLMTAMICHLAVFHPPDLLQIRVLTEEPDDPDWSWLKWLPH
                     VQHQTETDAAGSTRLIFTRQEGLSDLAARGPHAPDSLPGGPYVVVVDLTGGKAGFPPD
                     GRAGVTVITLGNHRGSAYRIRVHEDGTADDRLPNQSFRQVTSVTDRMSPQQASRIARK
                     LAGWSITGTILDKTSRVQKKVATDWHQLVGAQSVEEITPSRWRMYTDTDRDRLKIPFG
                     HELKTGNVMYLDIKEGAEFGAGPHGMLIGTTGSGKSEFLRTLILSLVAMTHPDQVNLL
                     LTDFKGGSTFLGMEKLPHTAAVVTNMAEEAELVSRMGEVLTGELDRRQSILRQAGMKV
                     GAAGALSGVAEYEKYRERGADLPPLPTLFVVVDEFAELLQSHPDFIGLFDRICRVGRS
                     LRVHLLLATQSLQTGGVRIDKLEPNLTYRIALRTTSSHESKAVIGTPEAQYITNKESG
                     VGFLRVGMEDPVKFSTFYISGPYMPPAAGVETNGEAGGPGQQTTRQAARIHRFTAAPV
                     LEEAPTP"
     CDS             4356451..4358226
                     /codon_start=1
                     /note="gene_4007"
                     /transl_table=11
                     /translation="MTAEPEVRTLREVVLDQLGTAESRAYKMWLPPLTNPVPLNELIA
                     RDRRQPLRFALGIMDEPRRHLQDVWGVDVSGAGGNIGIGGAPQTGKSTLLQTMVMSAA
                     ATHSPRNVQFYCIDLGGGGLIYLENLPHVGGVANRSEPDKVNRVVAEMQAVMRQRETT
                     FKEHRVGSIGMYRQLRDDPSQPVASDPYGDVFLIIDGWPGFVGEFPDLEGQVQDLAAQ
                     GLAFGVHVIISTPRWTELKSRVRDYLGTKIEFRLGDVNETQIDRITREIPANRPGRAV
                     SMEKHHLMIGVPRFDGVHSADNLVEAITAGVTQIASQHTEQAPPVRVLPERIHLHELD
                     PNPPGPESDYRTRWEIPIGLRETDLTPAHCHMHTNPHLLIFGAAKSGKTTIAHAIARA
                     ICARNSPQQVRFMLADYRSGLLDAVPDTHLLGAGAINRNSASLDEAVQALAVNLKKRL
                     PPTDLTTAQLRSRSWWSGFDVVLLVDDWHMIVGAAGGMPPMAPLAPLLPAAADIGLHI
                     IVTCQMSQAYKATMDKFVGAAFGSGAPTMFLSGEKQEFPSSEFKVKRRPPGQAFLVSP
                     DGKEVIQAPYIEPPEEVFAAPPSAG"
     variation        4357889..4367390
                     /note="supplemented from AL2122/97"
     CDS             4358369..4358665
                     /codon_start=1
                     /note="gene_4008"
                     /transl_table=11
                     /translation="MEKMSHDPIAADIGTQVSDNALHGVTAGSTALTSVTGLVPAGAD
                     EVSAQAATAFTSEGIQLLASNASAQDQLHRAGEAVQDVARTYSQIDDGAAGVFA"
```

CDS 4358690..4359805
/codon_start=1
/note="gene_4009"
/transl_table=11
/translation="VITMLWHAMPPELNTARLMAGAGPAPMLAAAAGWQTLSAALDAQ
AVELTARLNSLGEAWTGGGSDKALAAATPMVVWLQTASTQAKTRAMQATAQAAAYTQA
MATTPSLPEIAANHITQAVLTATNFFGINTIPIALTEMDYFIRMWNQAALAMEVYQAE
TAVNTLFEKLEPMASILDPGASQSTTNPIFGMPSPGSSTPVGQLPPAATQTLGQLGEM
SGPMQQLTQPLQQVTSLFSQVGGTGGGNPADEEAAQMGLLGTSPLSNHPLAGGSGPSA
GAGLLRAESLPGAGGSLTRTPLMSQLIEKPVAPSVMPAAAAGSSATGGAAPVGAGAMG
QGAQSGGSTRPGLVAPAPLAQEREEDDEDDWDEEDDW"
CDS 4359898..4360200
/codon_start=1
/note="gene_4010"
/transl_table=11
/translation="MAEMKTDAATLAQEAGNFERISGDLKTQIDQVESTAGSLQGQWR
GAAGTAAQAAVVRFQEAANKQKQELDEISTNIRQAGVQYSRADEEQQQALSSQMGF"
CDS 4360233..4360520
/codon_start=1
/note="gene_4011"
/transl_table=11
/translation="MTEQQWNFAGIEAAASAIQGNVTSIHSLLDEGKQSLTKLAAAWG
GSGSEAYQGVQQKWDATATELNNALQNLARTISEAGQAMASTEGNVTGMFA"
CDS complement(4360645..4361046)
/codon_start=1
/note="gene_4012"
/transl_table=11
/translation="MGIGGVGGLGGAGSGPAMGMGGVGGLGGAGSGPAMGMGGVGGLD
AAGSGEGGSPAAIGIGVGGGGGGGGGGGGGADTNRSDRSSDVGGGVWPLGFGRFADAG
AGGNEALGSKNGCAAISSGASIPSCGRKSLS"
CDS 4361708..4362634
/codon_start=1
/note="gene_4013"
/transl_table=11
/translation="VKKVKPQKPKATKPPKVVSQRGWRHWVHALTRINLGLSPDEKYE
LDLHARVRRNPRGSYQIAVVGLKGGAGKTTLTAALGSTLAQVRADRILALDADPGAGN
LADRVGRQSGATIADVLAEKELSHYNDIRAHTSVNAVNLEVLPAPEYSSAQRALSDAD
WHFIADPASRFYNLVLADCGAGFFDPLTRGVLSTVSGVVVVASVSIDGAQQASVALDW
LRNNGYQDLASRACVVINHIMPGEPNVAVKDLVRHFEQQVQPGRVVVMPWDRHIAAGT
EISLDLLDPIYKRKVLELAAALSDDFERAGRR"
CDS 4362703..4364166
/codon_start=1
/note="gene_4014"
/transl_table=11
/translation="VTILTGRRMTDLVLPAAVPMETYIDDTVAVLSEVLEDTPADVLG
GFDFTAQGVWAFARPGSPPLKLDQSLDDAGVVDGSLLTLVSVSRTERYRPLVEDVIDA
IAVLDESPEFDRTALNRFVGAAIPLLTAPVIGMAMRAWWETGRSLWWPLAIGILGIAV
LVGSFVANRFYQSGHLAECLLVTTYLLIATAAALAVPLPRGVNSLGAPQVAGAATAVL
FLTLMTRGGPRKRHELASFAVITAIAVIAAAAAFGYGYQDWVPAGGIAFGLFIVTNAA
KLTVAVARIALPPIPVPGETVDNEELLDPVATPEATSEETPTWQAIIASVPASAVRLT
ERSKLAKQLLIGYVTSGTLILAAGAIAVVVRGHFFVHSLVVAGLITTVCGFRSRLYAE

RWCAWALLAATVAIPTGLTAKLIIWYPHYAWLLLSVYLTVALVALVVVGSMAHVRRVS
PVVKRTLELIDGAMIAAIIPMLLWITGVYDTVRNIRF"

CDS 4364317..4365159
/codon_start=1
/note="gene_4015"
/transl_table=11
/translation="MAEPLAVDPTGLSAAAAKLAGLVFPQPPAPIAVSGTDSVVAAIN
KTMPSIESLVSDGLPGVKAALTRTASNMNAAADVYAKTDQSLGTSLSQYAFGSSGEGL
AGVASVGGQPSQATQLLSTPVSQVTTQLGETAAELAPRVVATVPQLVQLAPHAVQMSQ
NASPIAQTISQTAQQAAQSAQGGSGPMPAQLASAEKPATEQAEPVHEVTNDDQGDQGD
VQPAEVVAAARDEGAGASPGQQPGGGVPAQAMDTGAGARPAASPLAAPVDPSTPAPST
TTTL"

CDS complement(4365217..4367451)
/codon_start=1
/note="gene_4016"
/transl_table=11
/translation="MSITRPTGSYARQMLDPGGWVEADEDTFYDRAQEYSQVLQRVTD
VLDTCRQQKGHVFEGGLWSGGAANAANGALGANINQLMTLQDYLATVITWHRHIAGLI
EQAKSDIGNNVDGAQREIDILENDPSLDADERHTAINSLVTATHGANVSLVAETAERV
LESKNWKPPKNALEDLLQQKSPPPPDVPTLVVPSPGTPGTPGTPGTPITPGTPITPGT
PITPGTPITPGTPITPIPGAPVTPITPTPGTPVTPVTPGKPVTPVTPVKPGTPGEPTP
ITPVTPPVAPATPATPATPVTPAPAPHPQPAPAPAPSPGPQPVTPATPGPSGPATPGT
PGGEPAPHVKPAALAEQPGVPGQHAGGGTQSGPAHADESAASVTPAAASGVPGARAAA
AAPSGTAVGAGARSSVGTAAASGAGSHAATGRAPVATSDKAAAPSTRAASARTAPPAR
PPSTDHIDKPDRSESADDGTPVSMIPVSAARAARDAATAAASARQRGRGDALRLARRI
AAALNASDNNAGDYGFFWITAVTTDGSIVVANSYGLAYIPDGMELPNKVYLASADHAI
PVDEIARCATYPVLAVQAWAAFHDMTLRAVIGTAEQLASSDPGVAKIVLEPDDIPESG
KMTGRSRLEVVDPSAAAQLADTTDQRLLDLLPPAPVDVNPPGDERHMLWFELMKPMTS
TATGREAAHLRAFRAYAAHSQEIALHQAHTATDAAVQRVAVADWLYWQYVTGLLDRAL
AAAS"

CDS complement(4367868..4368215)
/codon_start=1
/gene="espL"
/note="gene_4017"
/transl_table=11
/translation="VSMDELDPHVARALTLAARFQSALDGTLNQMNNGSFRATDEAET
VEVTINGHQWLTGLRIEDGLLKKLGAEAVAQRVNEALHNAQAAASAYNDAAGEQLTAA
LSAMSRAMNEGMA"

CDS complement(4368212..4369594)
/codon_start=1
/note="gene_4018"
/transl_table=11
/translation="MTQSQTVTVDQQEILNRANEVEAPMADPPTDVPITPCELTAAKN
AAQQLVLSADNMREYLAAGAKERQRLATSLRNAAKAYGEVDEEAATALDNDGEGTVQA
ESAGAVGGDSSAELTDTPRVATAGEPNFMDLKEAARKLETGDQGASLAHFADGWNTFN
LTLQGDVKRFRGFDNWEGDAATACEASLDQQRQWILHMAKLSAAMAKQAQYVAQLHVW
ARREHPTYEDIVGLERLYAENPSARDQILPVYAEYQQRSEKVLTEYNNKAALEPVNPP
KPPPRHQDRPAPPPQEQGLIPGFLMPPSDGSGVTPGTGMPAAPMVPPTGSPGGGLPAD
TAAQLTSAGREAAALSGDVAVKAASLGGGGGGGVPSAPLGSAIGGAESVRPAGAGDIA
GLGQGRAGGGAALGGGGMGMPMGAAHQGQGGAKSKGSQQEDEALYTEDRAWTEAVIGN
RRRQDSKESK"

```
CDS             complement(4369701..4371005)
                /codon_start=1
                /note="gene_4019"
                /transl_table=11
                /translation="LETRYWWAGIALASLGVIVATVTFYGRRITGWVAAVYAWLRRRR
                RPPDSSSEPVVGATVKPGDHVAVRWQGEFLVAVIELIPRPFTPTVIVDGQAHTDDMLD
                TGLVEELLSVHCPDLEADIVSAGYRVGNTAAPDVVSLYQQVIGTDPAPANRRTWIVLR
                ADPERTRKSAQRRDEGVAGLARYLVASATRIADRLASHGVDAVCGRSFDDYDHATDIG
                FVREKWSMIKGRDAYTAAYAAPGGPDVWWSARADHTITRVRVAPGMAPQSTVLLTTAD
                KPKTPRGFARLFGGQRPALQGQHLVANRHCQLPIGSAGVLVGETVNRCPVYMPFDDVD
                IALNLGDAQTFTQFVVRAAAAGAMVTVGPQFEEFARLIGAHIGQEVKVAWPNATTYLG
                PHPGIDRVILRHNVIGTPRHRQLPIRRVSPPEESRYQMALPK"
CDS             complement(4371086..4372456)
                /codon_start=1
                /note="gene_4020"
                /transl_table=11
                /translation="MNLNEWSVLGVHRIFLITVALALLTASPASAITPPPIDPGALPP
                DVAGPDQPTEQRVLCASPTTLPGSGFHDPPWSNTYLGVADAHKFATGAGVTVAVIDTG
                VDASPRVPAEPGGDFVDQAGNGLSDCDAHGTLTASIIAGRPAPTDGFVGVAPDARLLS
                LRQTSEAFEPVGSQANPNDPNATPAAGSIRSLARAVVHAANLGVGVINISEAACYKVS
                RPIDETSLGASIDYAVNVKGVVVVVAAGNTGGDCVQNPAPDPSTPGDPRGWNNVQTVV
                TPAWYAPLVLSVGGIGQTGMPSSFSMHGPWVDVAAPAENIVALGDTGEPVNALQGREG
                PVPIAGTSFAAAYVSGLAALLRQRFPDLTPAQIIHRITATARHPGGGVDDLVGAGVID
                AVAALTWDIPPGPASAPYNVRRLPPPVVEPGPDRRPITAVALVAVGLTLALGLGALAR
                RALSRR"
CDS             complement(4372648..4374498)
                /codon_start=1
                /gene="eccA2"
                /note="gene_4021"
                /transl_table=11
                /translation="MVDTMGDLLTARRHFDRAMTIKNGQGCVAALPEFVAATEADPSM
                ADAWLGRIACGDRDLASLKQLNAHSEWLHRETTRIGRTLAAEVQLGPSIGITVTDASQ
                VGLALSSALTIAGEYAKADALLANRELLDSWRNYQWHQLARAFLMYVTQRWPDVLSTA
                AEDLPPQAIVMPAVTASICALAAHAAAHLGQGRVALDWLDRVDVIGHSRSSGRFGADV
                LTAAIGPADIPLLVADLAYVRGMVYRQLHEEDKAQIWLSKATINGVLTDAAKEALADP
                NLRLIVTDERTIASRSDRWDASTAKSRDQLDDDNAAQRRGELLAEGRELLAKQVGLAA
                VKQAVSALEDQLEVRMMRLEHGLPVEGQTNHMLLVGPPGTGKTTTAEALGKIYAGMGI
                VRHPEIREVRRSDFCGHYIGESGPKTNELIEKSLGRIIFMDEFYSLIERHQDGTPDMI
                GMEAVNQLLVQLETHRFDFCFIGAGYEDQVDEFLTVNPGLAGRFNRKLRFESYSPVEI
                VEIGHRYATPRASQLDDAAREVFLDAVTTIRNYTTPSGQHGIDAMQNGRFARNVIERA
                EGFRDTRVVAQKRAGQPVSVQDLQIITATDIDAAIRSVCSDNRDMAAIVW"
CDS             complement(4374577..4376190)
                /codon_start=1
                /note="gene_4022"
                /transl_table=11
                /translation="LTSKLTGFSPRSARRVAGVWTVFVLASAGWALGGQLGAVMAVVV
                GVALVFVQWWGQPAWSWAVLGLRGRRPVKWNDPITLANNRSGGGVRVQDGVAVVAVQL
                LGRAHRATTVTGSVTVESDNVIDVVELAPLPRHPLDLELDSISVVTFGSRTGTVGDYP
                RVYDAEIGTPPYAGRRETWLIMRLPVIGNTQALRWRTSVGAAAISVAQRVASSLRCQG
                LRAKLATATDLAELDRRLGSDAVAGSAQRWKAIRGEAGWMTTYAYPAEAISSRVLSQA
                WTLRADEVIQNVTVYPDATCTATITVRTPTPAPTPPSVILRRLNGEQAAAAAANMCGP
```

RPHLRGQRRCPLPAQLVTEIGPSGVLIGKLSNGDRLMIPVTDAGELSRVFVAADDTIA
KRIVIRVVGAGERVCVHTRDQERWASVRMPQLSIVGTPRPAPRTTVGVVEYVRRRKNG
DDGKSEGSGVDVAISPTPRPASVITIARPGTSLSESDRHGFEVTIEQIDRATVKVGAA
GQNWLVEMEMFRAENRYVSLEPVTMSIGR"

CDS complement(4376187..4377839)
/codon_start=1
/note="gene_4023"
/transl_table=11
/translation="MASPLNRPGLRAAAASAALTLVALSANVPAAQAIPPPSVDPAMV
PADARPGPDQPMRRSNSCSTPITVRNPDVAQLAPGFNLVNISKAWQYSTGNGVPVAVI
DTGVSPNPRLPVVPGGDYIMGEDGLSDCDAHGTVVSSIIAAAPLGILPMPRAMPATAA
FPPPAGPPPVTAAPAPPVEVPPPMPPPPPVTITQTVAPPPPPPEDAGAMAPSNGPPDP
QTEDEPAVPPPPPGAPDGVVGVAPHATIISIRQSSRAFEPVNPSSAGPNSDEKVKAGT
LDSVARAVVHAANMGAKVINISVTACLPAAAPGDQRVLGAALWYAATVKDAVIVAAAG
NDGEAGCGNNPMYDPLDPSDPRDWHQVTVVSSPSWFSDYVLSVGAVDAYGAALDKSMS
GPWVGVAAPGTHIMGLSPQGGGPVNAYPPSRPGEKNMPFWGTSFSAAYVSGVAALVRA
KFPELTAYQVINRIVQSAHNPPAGVDNKLGYGLVDPVAALTFNIPSGDRMAPGAQSRV
ITPAAPPPPPDHRARNIAIGFVGAVATGVLAMAIGARLRRAR"

CDS complement(4377824..4379353)
/codon_start=1
/note="gene_4024"
/transl_table=11
/translation="VTAPHKVAFPARCAVNICYDKHLCSQVFPAGIPVEGFFEGMVEL
FDADLKRKGFDGVALPAGSYELHKINGVRLDINKSLDELGVQDGDTLVLVPRVAGESF
EPQYESLSTGLAAMGKWLGRDGGDRMFAPVTSLTAAHTAMAIIAMAVGVVLALTLRTR
TITDSPVPAAMAGGIGVLLVIGALVVWWGWRERRDLFSGFGWLAVVLLAVAAACAPPG
ALGAAHALIGLVVVVLGAITIGVATRKRWQTAVVTAVVTVCGILAAVAAVRMFRPVSM
QVLAICVLVGLLVLIRMTPTVALWVARVRPPHFGSITGRDLFARRAGMPVDTVAPVSE
ADADDEDNELTDITARGTAIAASARLVNAVQVGMCVGVSLVLPAAVWGVLTPRQPWAW
LALLVAGLTVGLFITQGRGFAAKYQAVALVCGASAAVCAGVLKYALDTPKGVQTGLLW
PAIFVAAFAALGLAVALVVPATRFRPIIRLTVEWLEVLAMIALLPAAAALGGLFAWLR
H"

CDS complement(4379350..4380093)
/codon_start=1
/note="gene_4025"
/transl_table=11
/translation="VSGKGGVGVTTMAACIGGVFRECRPENVIAIDAVPSFGTLADRI
DESPPGDYAAIINDTDVQGYADIREHLGQNTVGLDVLAGNRTSDQPRPLVPAMFSAVL
SRLRRTHTVIVIDTSPDLEHDVMKAVLQSTDTLVFVSGITADRSRPVLRAVDYLRAQG
YHELVSRSTVILNHTDSITDKDALAYLTERFTKVGAIVEAMPFDPHLAKGGIIDTVHE
LNKKSRLRLFEITAGLADKYVPDAERAAQ"

CDS complement(4380468..4381298)
/codon_start=1
/note="gene_4026"
/transl_table=11
/translation="MLTTTVDGLWVLQAVTGVEQTCPELGLRPLLPRLDTAERALRHP
VAAELMAVGALDQAGNADPMVREWLTVLLRRDLGLLVTIGVPGGEPTRAAICRFATWW
VVLERHGNLVRLYPAGTASDEAGAGELVVGQVERLCGVAEAAPLRPVTVDADELLHAV
RDAGTLRSYLLSQRLDVDQLQMVTMAADPTRSAHATLVALQAGVGPEKSARILVGDST
VAIVDTAAGRICVESVTSGQRRYQVLSPGSRSDIGGAVQRLIRRLPAGDEWYSYRRVV
"

CDS complement(4381716..4382039)
/codon_start=1
/note="gene_4027"
/transl_table=11
/translation="VADTIQVTPQMLRSTANDIQANMEQAMGIAKGYLANQENVMNPA
TWSGAGVVASHMTATEITNELNKVLTGGTRLAEGLVQAAALMEGHEADSQTAFQALFG
ASHGS"
CDS complement(4382150..4383349)
/codon_start=1
/note="gene_4028"
/transl_table=11
/translation="MPDPGWAARTPEANDLLLKAGTGVGTHLANQTAWTTLGASHHAS
GVASAINTAATAASWLGVGSAASALNVTMLNATLHGLAGWVDVKPAVVSTAIAAFETA
NAAMRPAPECMENRDEWGVDNAINPSVLWTLTPRIVSLDVEYFGVMWPNNAAVGATYG
GVLAALAESLAIPPPVATMGASPAAPAQAAAAVGQAAAEAAAGDGIRSAYQGVQAGST
GAGQSTSAGENFGNQLSTFMQPMQAVMQAAPQALQAPSGLMQAPMSAMQPLQSMVGMF
ANPGALGMGGAAPGASAASAAGGISAAATEVGAGGGGAALGGGGMPATSFTRPVSAFE
SGTSGRPVGLRPSGALGADVVRAPTTTVGGTPIGGMPVGHAAGGHRGSHGKSEQAATV
RVVDDRR"
CDS complement(4383428..4383661)
/codon_start=1
/note="gene_4029"
/transl_table=11
/translation="MVWSVQPEAVLASAAAESAISAETEAAAAGAAPALLSTTPMGGD
PDSAMFSAALNACGASYLGVVAEHASQRGLFAG"
CDS complement(4383928..4385706)
/codon_start=1
/note="gene_4030"
/transl_table=11
/translation="VLRESLYNVPHRSPRRPWLAPLEDPEPVDRLVAAYRGKPWHVDY
GQNPGLMFPVGVMDIPEESQQVVHAVDALRSNIIVVGAKQRGKTTTLMALMCSAATMY
TPERVTFFCIGGATMAQIGSLPHVTDIVSPKDAEGIERILSTMDALIDAREEAFRRAK
IDMDGFRERRFGIGGDGVGGTDPTDAFGDVFVVLDDYDDLYAKDTLLGDRIISLSSRG
PEYGVHLMCSAGGWIHGQRQSLLQNVTARIQLRLADPGESQMGHLSIESREAARRTLN
RPGFGLTESLHELRIGVPALADPGTGELVGITDVGARIADVAGVTKHASLQRLPQRVE
LSAIVEHEAVHQGGDDLSIAFAIGERHELGPVPIKLRESPGLMILGRQGCGKTTALVA
IGEAVMNRFSPQQAQLTLIDPKTAPHGLRDLHAPGYVRAYAYDQDEIDEVITELAQQI
LLPRLPPKGLSQEELRALKPWEGPRHFVLIDDVQDLRPAQSYPQKPPVGAALWKLMER
ARQVGLHVFSTRNSANWATMPMDPWVKSQTSAKVAQLYMDNDPQNRINRSVRAQTLPP
GRGLLVGADGDVEGILVGYPSVPGEQ"
CDS complement(4385618..4388119)
/codon_start=1
/gene="eccC"
/note="gene_4031"
/transl_table=11
/translation="MSKKAFPINRVNIDPPKPVRVAPNPPIALPEREPRNIWVMIGVP
ALIVALIGTIVMLYVSGVRSLATGFFPLMGIGAFSMLAFSGRFGRARKITWGELEKGR
RRYLRDLDTNRDEIQTAVCAQREWQNAVHSDPPGLGAIIGGPRMWERGRGDVDFLEVR
VGTGVQHAPDSVLSVTWPDISSDEELEPVTGQALRDFILEQRKIRDIAKVVNLRSAPG
FSFVSEDLDRVRSLMRSVLCSLAVFHNPRDVKLMVVTRNPEVWAWMVWLPHNLHDELF
DACGWRRLIFATPEELEAALGAELHMKGKRGAWTPPTVASPTAMGSALETGQVGVDLG

PHLVIVDDNTGSPDAWESVVGQVGKAGLTVLRIASRVGTGVGFAEDQVFEMAQRHGAA
TAVKAGRDGADADDDQRPAPLLRARGTFFAHADQLSIHRAYRYARAMARWSPTSRSEV
TDSTSGAAELLRSLGISDPRELDVDRLWAERRGRGDDRWCEIPVGAKPNGELQNIILR
AKDFGGFGFHSVVIGTSGSGKSELFLSLVYGIALTHSPETFNVIFVDMKFESAAQDIL
GIPHVVAALSNLGKDERHLAERMRRVIDGEIKQRYELFKSVGARDANDYEEIRLAGRD
LPPVPVLLVIVDEYLELFANHKKWIDLIIHIGQEGRGANVFFMLGGQRLDLSSLQKVK
SNIAFRIALRAESGDDSREVIGSDAAYHLPSKENGFALLKVGPRDLEPFRCFYLSAPF
VVPKKKEVARTIDMTLTQPRLYDWQYQPLDARRRRGIGDRRGRRCGTRRIPLLRRRFQ
EEEDRRRAAGVAIQRAAPIAAPAVVGAAGRPRAGR"

CDS complement(4388120..4389607)
/codon_start=1
/gene="eccB2"
/note="gene_4032"
/transl_table=11
/translation="MPLSLSNRDQNSGHLFYNRRLRAATTRFSVRMKHDDRKQTAALA
LSMVLVAIAAGWMMLLNVLKPTGIVGDSAIIGDRDSGALYARIDGRLYPALNLTSARL
ATGTAGQPTWVKPAEIAKYPTGPLVGIPGAPAAMPVNRGAVSAWAVCDTAGRPRSADK
PVVTSIAGPITGGGRATHLRDDAGLLVTFDGSTYVIWGGKRSQIDPTNRAVTLSLGLD
PGVTSPIQISRALFDGLPATEPLRVPAVPEAGTPSTWVPGARVGSVLQAQTAGGGSQF
YVLLPDGVQKISSFVADLLRSANSYGAAAPRVVTPDVLVHTPQVTSLPVEYYPAGRLN
FVDTAADPTTCVSWEKASTGPQARVAVYNGRGLPVPPSMDSRIVRLVRDDRAPASVVA
TQVLVLPGAANFVTSTSGVITAESRESLFWVSGNGVRFGIANDEATLRALGLDPGAAV
QAPWPLLRTFAAGPALSRDAALLARDTVPTLGQVAIVTTTAKAGA"

CDS complement(4389651..4390478)
/codon_start=1
/note="gene_4033"
/transl_table=11
/translation="LTDPLTTQAIAALSRGHGLFAGGVSGADIDAPQIQQYANAISWV
ANAVPTAAAYRWRGAARALRRLANTGEALAQIMAAAQIDHAHARTATRALLEAAKTDA
MALTDTPLGRREAMARMAARLRAQHRHIARCRSRARLLGLRLRRLRYLRTAAARRPQV
TTPGGRAQVLAAVQKALDIKGVHDPAARARWTRGMDLVARRESNYNANAINHWDSNAA
RGTPSRGVWQFIAPTFAAITSRARRPTSTIWSPRRARSSTTREATTGWPPTHRIWPI"

CDS complement(4390667..4391656)
/codon_start=1
/note="gene_4034"
/transl_table=11
/translation="MTGDQNPAPGPAPGVPIKVTPEILLQVLTTPPASGPAPFPAVPV
DLPAPADIANGALFAAGNSGVPGDVESSGLEDLDRRAHAADAVQKFSANEADAAQQFQ
GVGAQAEAQGATQMMQQAVSGITGALGGAVGGVMGPLTQLPQQAMQAGQGAMQPLMSA
LQQTYGAEGLDVADGARLVDSIEGEPGLGGEPGAGDVGAGGGGGGTTPTGYLGPPPVP
TSSPPTTPAGAPAKSVTPDPVSGTPRASGPAGMTGMPMVPPGALGAGAEGANKDKPVE
KRVTAPAVPNGQPVKGRLTVPPSVPVKSADDKPVVTKSTRRILVVPNDDKVKE"

CDS complement(4391818..4392534)
/codon_start=1
/note="gene_4035"
/transl_table=11
/translation="MAGASALSATAGATAGAVSARAAEQQRLQRIVDAVARQEPRISW
AAGLRDDGTTTLLVTDLAGGWIPPHVRLPANVTLLEPTARRRDADVIDLLGAVVAVAA
HESNTYVAEPGPDAPALTGDRSARSAIPKVDEFGPTLVEAVRRRDSLPRIAQAIALPA
VRKTGVLENEAELLHGCITAVKESVLKAYPSHELTAVGDWMLLAAIEALIDEQDYLAN
YHLAWYAVTTRRGGSRGFAA"

```
CDS             complement(4392464..4392760)
                /codon_start=1
                /gene="TBEG_00400"
                /note="gene_4036"
                /transl_table=11
                /translation="VARLSPRDRCRLTALIYGPRRGSPRRALGSYGARIRRAGGALGV
                IGPIGGWGAGFSGGARSLESCGWTGWCELVDNGRRLGTVGHRRRDGGRGIGSGG"
CDS             4392859..4393155
                /codon_start=1
                /note="gene_4037"
                /transl_table=11
                /translation="VFAGVSTGPCVASMPAAMVGADTTGGVVIGGVACGGGTDPDCIA
                VIAPSDSECAPAAAGCPVTIPVANDCPIDVGDTPCPRAAGDSDPPGIATGGQWR"
CDS             complement(4393043..4393939)
                /codon_start=1
                /note="gene_4038"
                /transl_table=11
                /translation="VLVGAWWPAPSAALRAAAQHWATWAMQKQELARNLISQHDLLLR
                NQGRTAEDLIGRYLRGAKSEVTKAEKYEIKKGAFNTAADAIDYLRSRLTGIAGEGNKE
                IDDVLASKKPLPEQLAEIQAIQTRCNADAANASRDAVDKVMTAMQEILEAEDIGDDPR
                TWARANGFNVDDAPPPRLIRENDLAALTGPGARGGSFGSVEGAGDLASPQSVGAGGFS
                GSGVQAACSQPAPRAIGASSRHASAGPVPPAPVVTTPAAATPPVIATGPRWRCPAGRC
                RRRPSDRAYRLRRLGNRLRPGW"
CDS             complement(4394087..4394386)
                /codon_start=1
                /note="gene_4039"
                /transl_table=11
                /translation="VVAAIAAVVAVAALIVALTNARPAATPATTSVPTYTAAQTAAAQ
                RQLCDTYKLVAHAVPVDTNGSDKALARITLTNAAAILDNAAADPALDAKHRDAAR"
CDS             4394425..4394655
                /codon_start=1
                /gene="TMKG_01154"
                /note="gene_4040"
                /transl_table=11
                /translation="VLARSRRRWYPLTACLPLAPDHTNPDTPRRMRYVTGDHAVQVFQ
                LTSTVIDLTTKRKHTTVVYAATSMSGTPPLHR"
CDS             complement(4395033..4395563)
                /codon_start=1
                /note="gene_4041"
                /transl_table=11
                /translation="VTIGVDLSTDLQDWIRLSGMNMIQGSETNDGRTILWNKGGEVRY
                FIDRLAGWYVITSSDRMSREGYEFAAASMSVIEKYLYGYFGGSVRSERELPAIRAPFQ
                PEELMPEYSIGTMTFAGRQRDTLIDSSGTVVAITAADRLVELSHYLDVSVNVIKDSFL
                DSEGKPLFTLWKDYKG"
CDS             complement(4395560..4398100)
                /codon_start=1
                /note="gene_4042"
                /transl_table=11
                /translation="MAPLAVDPAALDSAGGAVVAAGAGLGAVISSLTAALAGCAGMAG
                DDPAGAVFGRSYDGSAAALVQAMSVARNGLCNLGDGVRMSAHNYSLAEAMSDVAGRAA
                PLPAPPPSGCVGVGAPPSAVGGGGGAPKGWGWVAPYIGMIWPNGDSTKLRAAAVAWRS
```

AGTQFALTEIQSTAGPMGVIRAQQLPEAGLIESAFADAYASTTAVVGQCHQLAAQLDA
YAARIDAVHAAVLDLLARICDPLTGIKEVWEFLTDQDEDEIQRIAHDIAVVVDQFSGE
VDALAAEITAVVSHAEAVITAMADHAGKQWDRFLHSNPVGVVIDGTGQQLKGFGEEAF
GMAKDSWDLGPLRASIDPFGWYRSWEEMLTGMAPLAGLGGENAPGVVESWKQFGKSLI
HWDEWTTNPNEALGKTVFDAATLALPGGPLSKLGSKGRDILAGVRGLKERLEPTTPHL
EPPATPPRPGPQPPRIEPPESGHPAPAPAAKPAPVPANGPLPHSPTESKPPPVDRPAE
PVAPSSASAGQPRVSAATTPGTHVPHGLPQPGEHVPAQAPPATTLLGGPPVESAPATA
HQPQWATTPAAPAAAPHSTPGGVHSTESGPHGRSLSAHGSEPTHDGASHGSGHGSGSE
PPGLHAPHREQQLAMHSNEPAGEGWHRLSDEAVDPQYGEPLSRHWDFTDNPADRSRIN
PVVAQLMEDPNAPFGRDPQGQPYTQERYQERFNSVGPWGQQYSNFPPNNGAVPGTRIA
YTNLEKFLSDYGPQLDRIGGDQGKYLAIMEHGRPASWEQRALHVTSLRDPYHAYTIDW
LPEGWFIEVSEVAPGCGQPGGSIQVRIFDHQNEMRKVEELIRRGVLRQ"

CDS complement(4398105..4398395)
/codon_start=1
/note="gene_4043"
/transl_table=11
/translation="VAEPFRVDPTVLADAVARMAEFGRHVEELVAEIESLVTRLHVTW
TGEGAAAHAEAQRHWAAGEAMMRQALAQLTAAGQSAHANYTGAVATNLGMWS"

CDS complement(4398526..4398699)
/codon_start=1
/note="gene_4044"
/transl_table=11
/translation="MGADDTLRVEPAVMQGFAASLDGAAEHLAVQLAELDAQVGQMLG
GWRGASGSAYGSA"

CDS complement(4398765..4399274)
/codon_start=1
/note="gene_4045"
/transl_table=11
/translation="VEYCIAGDDGSAGIWNRPFDVDLDGDGRLDAIGLDLDGDGLRDD
ALADFDGDDVADHAVFDVDNDGTPESYFIDDGSGTWAVAVDRGGQLRWYGLDGVEHTG
GPLVDFDGFGGLDDRLLDTDGDGLADRVLCAGEQRVTGYVDTDGDGRWDVRLTDTDGD
GTADGASSL"

CDS complement(4399299..4400741)
/codon_start=1
/note="gene_4046"
/transl_table=11
/translation="VPEAVQEADLLTAAAVALNRHAALLRELGSVFAAAGHELYLVGG
SVRDALLGRLSPDLDFTTDARPERVQEIVRPWADAVWDTGIEFGTVGVGKSDHRMEIT
TFRADSYDRVSRHPEVRFGDCLEGDLVRRDFTTNAMAVRVTATGPGEFLDPLGGLAAL
RAKVLDTPAAPSGSFGDDPLRMLRAARFVSQLGFAVAPRVRAAIEEMAPQLARISAER
VAAELDKLLVGEDPAAGIDLMVQSGMGAVVLPEIGGMRMAIDEHHQHKDVYQHSLTVL
RQAIALEDDGPDLVLRWAALLHDIGKPATRRHEPDGGVSFHHHEVVGAKMVRKRMRAL
KYSKQMIDDISQLVYLHLRFHGYGDGKWTDSAVRRYVTDAGALLPRLHKLVRADCTTR
NKRRAARLQASYDRLEERIAELAAQEDLDRVRPDLDGNQIMAVLDIPAGPQVGEAWRY
LKELRLERGPLSTEEATTELLSWWKSRGNR"

CDS 4401291..4401863
/codon_start=1
/note="gene_4047"
/transl_table=11
/translation="MRTVHETSAGGLVIDGIDGPRDAQVAALIGRVDRRGRLLWSLPK
GHIELGETAEQTAIREVAEETGIRGSVLAALGRIDYWFVTDGRRVHKTVHHYLMRFLG

GELSDEDLEVAEVAWVPIRELPSRLAYADERRLAEVADELIDKLQSDGPAALPPLPPS
SPRRRPQTHSRARHADDSAPGQHNGPGPGP"
CDS 4401860..4404268
/codon_start=1
/note="gene_4048"
/transl_table=11
/translation="VTALQLRWAALARVTSAIGVVAGLGMALTVPSAAPHALAGEPSP
TPFVQVRIDQVTPDVVTTSSEPHVTVSGTVTNTGDRPVRDVMVRLEHAAAVTSSTALR
TSLDGGTDQYQPAADFLTVAPELDRGQEAGFTLSAPLRSLTRPSLAVNQPGIYPVLVN
VNGTPDYGAPARLDNARFLLPVVGVPPDQATDFGSAVAPETTAPVWITMLWPLADRPR
LAPGAPGGTVPVRLVDDDLANSLANGGRLDILLSAAEFATNREVDPDGAVGRALCLAI
DPDLLITVNAMTGGYVVSDSPDGAAQLPGTPTHPGTGQAAASSWLDRLRTLVHRTCVT
PLPFAQADLDALQRVNDPRLSAIATISPADIVDRILDVSSTRGATVLPDGPLTGRAIN
LLSTHGSTVAVAAADFSPEEQQGSSQIGSALLPATAPRRLSPRVVAAPFDPAVGAALA
AAGTNPTVPTYLDPSLFVRIAHESITARRQDALGAMLWRSLEPNAAPRTQILVPPASW
SLASDDAQVILTALATAIRSGLAVPRPLPVVIADAAARTEPPEPPGAYSAARGRFNDD
ITTQIGGQVARLWKLTSALTIDDRTGLTGVQYTAPLREDMLRALSQSLPPDTRNGLAQ
QRLAVVGKTIDDLFGAVTIVNPGGSYTLATEHSPLPLALHNGLAVPIRVRLQVDAPPG
MTVADVGQIELPPGYLPLRVPIEVNFTQRVAVDVSLRTPDGVALGEPVRLSVHSNAYG
KVLFAITLSAAAVLVTLAGRRLWHRFRGQPDRADLDRPDLPTGKHAPQRRAVASRDDE
KHRV"
CDS 4404265..4407819
/codon_start=1
/note="gene_4049"
/transl_table=11
/translation="MRPSPGEVPTASQRQPELSDAALVSHSWAMAFATLISRITGFAR
IVLLAAILGAALASSFSVANQLPNLVAALVLEATFTAIFVPVLARAEQDDPDGGAAFV
RRLVTLATTLLLGATTLSVLAAPLLVRLMLGTNPQVNEPLTTAFAYLLLPQVLVYGLS
SVFMAILNTRNVFGPPAWAPVVNNVVAIATLAVYLAVPGELSVDPVRMGNAKLLVLGI
GTTAGVFAQTAVLLVAIRREHISLRPLWGIDQRLKRFGAMAAAMVLYVLISQLGLVVG
NRIASTAAASGPAIYNYTWLVLMLPFGMIGVTVLTVVMPRLSRNAAADDTPAVLADLS
LATRLTMITLIPTVAFMTVGGPAIGSALFAYGNFGDVDAGYLGAAIALSAFTLIPYAL
VLLQLRVFYAREQPWTPITIIVVITGVKILGSLLAPHITGDPQLVAAYLGLANGLGFL
AGTIVGYYILRRALRPDGGQLIGVGEARTALVTVAASLLAGLLAHVADRLLGLSELTA
HAGSVGSLLRLSVLALIMLPILAAVTLCARVPEARAALDAVRARIRSRRLKTGPQTQN
VLDQSSRPGPVTYPERRRLAPPRGKSVVHEPIRRRPPEQVARAGRAKGPEVIDRPSEN
ASFGAASGAELPRPVADELQLDAPAGRDPGPVSRPHPSDLQNGDLPADAARGPIAFDA
LREPDRESSAPPDDVQLVPGARIANGRYRLLIFHGGVPPLQFWQALDTALDRQVALTF
VDPQGVLPDDVLQETLSRTLRLSRIDKPGVARVLDVVHTRAGGLVVAEWIRGGSLQEV
ADTSPSPVGAIRAMQSLAAAADAAHRAGVALSIDHPSRVRVSIDGDVVLAYPATMPDA
NPQDDIRGIGASLYALLVNRWPLPEAGVRSGLAPAERDTAGQPIEPADIDRDIPFQIS
AVAARSVQGDGGIRSASTLLNLMQQATAVADRTEVLGPIDEAPVSAAPRTSAPNSETY
TRRRRNLLIGIGAGAAVLMVALLVLASVLSRIFGDVSGGLNKDELGLNAPTASTSAAS
SAPPGSVVKPTKVTVFSPDGGADNPGEADLAIDGNPATSWKTDIYTDPVPFPSFKNGV
GLMLQLPQATVVGTVAIDVASTGTKVEIRSASTPTPATLEDTAVLTSATALRPGHNTI
SVEAAAPTSNLLVWISTLGTTDGKSQADISEITIYAAS"
CDS 4407854..4408444
/codon_start=1
/note="gene_4050"
/transl_table=11
/translation="MPPPIGYCPAVGFGGRHERSDAELLAAHVAGDRYAFDQLFRRHH

RQLHRLARLTSRTSEDADDALQDAMLSAHRGAGSFRYDAAVSSWLHRIVVNACLDRLR
RAKAHPTAPLEDVYPVADRTAQVETAIAVQRALMRLPVEQRAAVVAVDMQGYSIADTA
RMLGVAEGTVKSRCARARARLARLLGYLNTGVNIRR"

CDS 4408537..4409301
/codon_start=1
/note="gene_4051"
/transl_table=11
/translation="MSAADKDPDKHSADADPPLTVELLADLQAGLLDDATAARIRSRV
RSDPQAQQILRALNRVRRDVAAMGADPAWGPAARPAVVDSISAALRSARPNSSPGAAH
AARPHVHPVRMIAGAAGLCAVATAIGVGAVVDAPPPAPSAPTTAQHITVSKPAPVIPL
SRPQVLDLLHHTPDYGPPGGPLGDPSRRTSCLSGLGYPASTPVLGAQPIDIDARPAVL
LVIPADTPDKLAVFAVAPHCSAADTGLLASTVVPRA"

CDS 4409395..4410402
/codon_start=1
/gene="trxB"
/note="gene_4052"
/transl_table=11
/translation="MTAPPVHDRAHHPVRDVIVIGSGPAGYTAALYAARAQLAPLVFE
GTSFGGALMTTTGVENYPGFRNGITGPELMDEMREQALRFGADLRMEDVESVSLHGPL
KSVVTADGQTHRARAVILAMGAAARYLQVPGEQELLGRGVSSCATCDGFFFRDQDIAV
IGGGDSAMEEATFLTRFARSVTLVHRRDEFRASKIMLDRARNNDKIRFLTNHTVVAVD
GDTTVTGLRVRDTNTGAETTLPVTGVFVAIGHEPRSGLVREAIDVDPDGYVLVQGRTT
STSLPGVFAAGDLVDRTYRQAVTAAGSGCAAAIDAERWLAEHAATGEADSTDALIGAQ
R"

CDS 4410399..4410749
/codon_start=1
/note="gene_4053"
/transl_table=11
/translation="MTDSEKSATIKVTDASFATDVLSSNKPVLVDFWATWCGPCKMVA
PVLEEIATERATDLTVAKLDVDTNPETARNFQVVSIPTLILFKDGQPVKRIVGAKGKA
ALLRELSDVVPNLN"

CDS 4410859..4412079
/codon_start=1
/note="gene_4054"
/transl_table=11
/translation="MPSPRREDGDALRCGDRSAAVTEIRAALTALGMLDHQEEDLTTG
RNVALELFDAQLDQAVRAFQQHRGLLVDGIVGEATYRALKEASYRLGARTLYHQFGAP
LYGDDVATLQARLQDLGFYTGLVDGHFGLQTHNALMSYQREYGLAADGICGPETLRSL
YFLSSRVSGGSPHAIREEELVRSSGPKLSGKRIIIDPGRGGVDHGLIAQGPAGPISEA
DLLWDLASRLEGRMAAIGMETHLSRPTNRSPSDAERAATANAVGADLMISLRCETQTS
LAANGVASFHFGNSHGSVSTIGRNLADFIQREVVARTGLRDCRVHGRTWDLLRLTRMP
TVQVDIGYITNPHDRGMLVSTQTRDAIAEGILAAVKRLYLLGKNDRPTGTFTFAELLA
HELSVERAGRLGGS"

CDS complement(4412100..4412834)
/codon_start=1
/note="gene_4055"
/transl_table=11
/translation="VSARITALRLEAFEQLPKHARRCVFWEVDPAILGKDDHLADPEF
EKEAWLSMVMLEWGSCGQVATAVPDERSHAEPPCLGYVLYAPPSAVPRAQRFPTAPVS
ADAVLLTSMGIERGQADDDLPHSLIARVIEELVRRGVRALEAFGRTPAATDLQNPGAV
TPDVRPVLEALGDCCVEHCIIDANFLMDVGFVVVAPHPYFPRLRLELDKGLGWKAEVE

AALERLLENARLQEPIAAGSTAGNTS"
CDS complement(4413124..4413993)
/codon_start=1
/note="gene_4056"
/transl_table=11
/translation="MGAIYREIPPSAIEANPRQPRQVFDEEALAELVHSIREFGLLQP
IVVRSLAGSQTGVRYQIVMGERRWRAAQEAGLATIPAIVRETGDDNLLRDALLENIHR
VQLNPLEEAAAYQQLLDEFGVTHDELAARIGRSRPLITNMIRLLKLPIPVQRRVAAGV
LSAGHARALLSLEAGPEAQEELASRIVAEGLSVRATEETVTLANHEANRQAHHSDATT
PAPPRRKPIQMPGLQDVAERLSTTFDTRVTVSLGKRKGKIVVEFGSVDDLARIVGLMT
TDGRDKGLHRDAL"
CDS complement(4414155..4414997)
/codon_start=1
/note="gene_4057"
/transl_table=11
/translation="MRVLHTTHEPLQRPGRRRVLTIANQKGGVGKTTTAVNIAAALAV
QGLKTLVIDLDPQGNASTALGITDRQSGTPSSYEMLIGEVSLHTALRRSPHSERLFCI
PATIDLAGAEIELVSMVARENRLRTALAALDNFDFDYVFVDCPPSLGLLTINALVAAP
EVMIPIQCEYYALEGVSQLMRNIEMVKAHLNPQLEVTTVILTMYDGRTKLADQVADEV
RQYFGSKVLRTVIPRSVKVSEAPGYSMTIIDYDPGSRGAMSYLDASRELAERDRPPSA
KGRP"
CDS complement(4415195..4415869)
/codon_start=1
/gene="gidB"
/note="gene_4058"
/transl_table=11
/translation="MSPIEPAASAIFGPRLGLARRYAEALAGPGVERGLVGPREVGRL
WDRHLLNCAVIGELLERGDRVVDIGSGAGLPGVPLAIARPDLQVVLLEPLLRRTEFLR
EMVTDLGVAVEIVRGRAEESWVQDQLGGSDAAVSRAVAALDKLTKWSMPLIRPNGRML
AIKGERAHDEVREHRRVMIASGAVDVRVVTCGANYLRPPATVVFARRGKQIARGSARM
ASGGTA"
CDS complement(4416001..4416564)
/codon_start=1
/gene="jag"
/note="gene_4059"
/transl_table=11
/translation="MADADTTDFDVDAEAPGGGVREDTATDADEADDQEERLVAEGEI
AGDYLEELLDVLDFDGDIDLDVEGNRAVVSIDGSDDLNKLVGRGGEVLDALQELTRLA
VHQKTGVWSRLMLDIARWRRRRREELAALADEVARRVAETGDREELVPMTPFERKIVH
DAVAAVPGVHSESEGVEPERRVVVLRD"
CDS complement(4416636..4417736)
/codon_start=1
/gene="oxaA"
/note="gene_4060"
/transl_table=11
/translation="VSLLFDFFSLDFIYYPVSWIMWVWYRLFAFVLGPSNFFAWALSV
MFLVFTLRALLYKPFVRQIRTTRQMQELQPQIKALQKKYGKDRQRMALEMQKLQREHG
FNPILGCLPMLAQIPVFLGLYHVLRSFNRTTGGFGQPHLSVIENRLTGNYVFSPVDVG
HFLDANLFGAPIGAYMTQRSGLDVFVDFSRPALIAVGVPVMILAGIATYFNSRASIAR
QSAEAAANPQTAMMNKLALYVFPLGVVVGGPFLPLAIILYWFSNNIWTFGQQHYVFGM
IEKEEEAKKQEAVRRRAANAPAPGAKPKRSPKTAPATNAAAPTEAGDTDDGAESDAST

ERPADTSNPARRNSGPSARTPRPGVRPKKRKR"
BASE COUNT   759165 a1454084 c1447269 g 758681 t
ORIGIN
```
        1 ttgaccgatg accccggttc aggcttcacc acagtgtgga acgcggtcgt ctccgaactt
       61 aacggcgacc ctaaggttga cgacggaccc agcagtgatg ctaatctcag cgctccgctg
      121 acccctcagc aaagggcttg gctcaatctc gtccagccat tgaccatcgt cgaggggttt
      181 gctctgttat ccgtgccgag cagctttgtc caaaacgaaa tcgagcgcca tctgcgggcc
      241 ccgattaccg acgctctcag ccgccgactc ggacatcaga tccaactcgg ggtccgcatc
      301 gctccgccgg cgaccgacga agccgacgac actaccgtgc cgccttccga aaatcctgct
      361 accacatcgc cagacaccac aaccgacaac gacgagattg atgacagcgc tgcggcacgg
      421 ggcgataacc agcacagttg gccaagttac ttcaccgagc gcccgcgcaa taccgattcc
      481 gctaccgctg gcgtaaccag ccttaaccgt cgctacacct ttgatacgtt cgttatcggc
      541 gcctccaacc ggttcgcgca cgccgccgcc ttggcgatcg cagaagcacc cgcccgcgct
      601 tacaaccccc tgttcatctg ggcgagtcc ggtctcggca agacacacct gctacacgcg
      661 gcaggcaact atgcccaacg gttgttcccg ggaatgcggg tcaaatatgt ctccaccgag
      721 gaattcacca acgacttcat taactcgctc cgcgatgacc gcaaggtcgc attcaaacgc
      781 agctaccgcg acgtagacgt gctgttggtc gacgacatcc aattcattga aggcaaagag
      841 ggtattcaag aggagttctt ccacaccttc aacaccttgc acaatgccaa caagcaaatc
      901 gtcatctcat ctgaccgccc acccaagcag ctcgccaccc tcgaggaccg gctgagaacc
      961 cgctttgagt gggggctgat cactgacgta caaccacccg agctggagac ccgcatcgcc
     1021 atcttgcgca agaaagcaca gatggaacgg ctcgcggtcc ccgacgatgt cctcgaactc
     1081 atcgccagca gtatcgaacg caatatccgt gaactcgagg gcgcgctgat ccgggtcacc
     1141 gcgttcgcct cattgaacaa aacaccaatc gacaaagcgc tggccgagat tgtgcttcgc
     1201 gatctgatcg ccgacgccaa caccatgcaa atcagcgcgg cgacgatcat ggctgccacc
     1261 gccgaatact tcgacactac cgtcgaagag cttcgcgggc ccggcaagac ccgagcactg
     1321 gcccagtcac gacagattgc gatgtacctg tgtcgtgagc tcaccgatct ttcgttgccc
     1381 aaaatcggcc aagcgttcgg ccgtgatcac acaaccgtca tgtacgccca acgcaagatc
     1441 ctgtccgaga tggccgagcg ccgtgaggtc tttgatcacg tcaaagaact caccactcgc
     1501 atccgtcagc gctccaagcg ctagcacggc gtgttcttcc gacaacgttc ttaaaaaaac
     1561 ttctctctcc caggtcacac cagtcacaga gattggctgt gagtgtcgct gtgcacaaac
     1621 cgcgcacaga ctcatacagt cccggcggtt ccgttcacaa cccacgcctc atccccaccg
     1681 acccaacaca caccccacag tcatcgccac cgtcatccac aactccgacc gacgtcgacc
     1741 tgcaccaaga ccagactgtc cccaaactgc acaccctcta atactgttac cgagatttct
     1801 tcgtcgtttg ttcttggaaa gacagcgctg gggatcgttc gctggatacc acccgcataa
     1861 ctggctcgtc gcggtgggtc agaggtcaat gatgaacttt caagttgacg tgagaagctc
     1921 tacggttgtt gttcgactgc tgttgcggcc gtcgtggcgg gtcacgcgtc atgggcgttc
     1981 gtcgttggca gtccccacgc tagcggggcg ctagccacgg gatcgaactc atcgtgaggt
     2041 gaaagggcgc aatggacgcg gctacgacaa gagttggcct caccgacttg acgtttcgtt
     2101 tgctacgaga gtctttcgcc gatgcggtgt cgtgggtggc taaaaatctg ccagccaggc
     2161 ccgcggtgcc ggtgctctcc ggcgtgttgt tgaccggctc ggacaacggt ctgacgattt
     2221 ccggattcga ctacgaggtt tccgccgagg cccaggttgg cgctgaaatt gtttctcctg
     2281 gaagcgtttt agtttctggc cgattgttgt ccgatattac ccgggcgttg cctaacaagc
     2341 ccgtaggcgt tcatgtcgaa ggtaaccggg tcgcattgac ctgcggtaac gccaggtttt
     2401 cgctaccgac gatgccagtc gaggattatc cgacgctgcc gacgctgccg gaagagaccg
     2461 gattgttgcc tgcggaatta ttcgccgagg caatcagtca ggtcgctatc gccgccggcc
     2521 gggacgacac gctgcctatg ttgaccggca tccgggtcga aatcctcggt gagacggtgg
     2581 ttttggccgc taccgacagg tttcgcctgg ctgttcgaga actgaagtgg tcggcgtcgt
     2641 cgccagatat cgaagcggct gtgctggtcc cggccaagac gctggccgag gccgccaaag
     2701 cgggcatcgg cggctctgac gttcgtttgt cgttgggtac tgggccgggg gtgggcaagg
     2761 atggcctgct cggtatcagt gggaacggca agcgcagcac cacgcgactt cttgatgccg
     2821 agttcccgaa gtttcggcag ttgctaccaa ccgaacacac cgcggtggcc accatggacg
```

2881 tggccgagtt gatcgaagcg atcaagctgg ttgcgttggt agctgatcgg ggcgcgcagg
2941 tgcgcatgga gttcgctgat ggcagcgtgc ggctttctgc gggtgccgat gatgttggac
3001 gagccgagga agatcttgtt gttgactatg ccggtgaacc attgacgatt gcgtttaacc
3061 caacctatct aacggacggt ttgagttcgt tgcgctcgga gcgagtgtct ttcgggttta
3121 cgactgcggg taagcctgcc ttgctacgtc cggtgtccgg ggacgatcgc cctgtggcgg
3181 gtctgaatgg caacggtccg ttcccggcgg tgtcgacgga ctatgtctat ctgttgatgc
3241 cggttcggtt gccgggctga gcacttggcg cccgggtagg tgtacgtccg tcatttgggg
3301 ctgcgtgact tccggtcctg ggcatgtgta gatctggaat tgcatccagg gcggacggtt
3361 tttgttgggc ctaacggtta tggtaagacg aatcttattg aggcactgtg gtattcgacg
3421 acgttaggtt cgcaccgcgt tagcgccgat ttgccgttga tccgggtagg taccgatcgt
3481 gcggtgatct ccacgatcgt ggtgaacgac ggtagagaat gtgccgtcga cctcgagatc
3541 gccacggggc gagtcaacaa agcgcgattg aatcgatcat cggtccgaag tacacgtgat
3601 gtggtcggag tgcttcgagc tgtgttgttt gcccctgagg atctggggtt ggttcgtggg
3661 gatcccgctg accggcggcg ctatctggat gatctggcga tcgtgcgtag gcctgcgatc
3721 gctgcggtac gagccgaata tgagagggtg gtgcgccagc ggacggcgtt attgaagtcc
3781 gtacctggag cacggtatcg ggtgaccgg ggtgtgtttg acactcttga ggtatgggac
3841 agtcgtttgg cggagcacgg ggctgaactg gtggccgccc gcatcgattt ggtcaaccag
3901 ttggcaccgg aagtgaagaa ggcataccag ctgttggcgc cggaatcgcg atcggcgtct
3961 atcggttatc gggccagcat ggatgtaacc ggtcccagcg agcagtcaga taccgatcgg
4021 caattgttag cagctcggct gttggcggcg ctggcggccc gtcgggatgc cgaactcgag
4081 cgtgggggttt gtctagttgg tccgcaccgt gacgacctaa tactgcgact aggcgatcaa
4141 cccgcgaaag gatttgctag ccatggggag gcgtggtcgt tggcggtggc actgcggttg
4201 gcggcctatc aactgttacg cgttgatggt ggtgagccgg tgttgttgct cgacgacgtg
4261 ttcgccgaac tggatgtcat gcgccgtcga gcgttggcga cggcggccga gtccgccgaa
4321 caggtgttgg tgactgccgc ggtgctcgag gatattcccg ccggctggga cgccaggcgg
4381 gtgcacatcg atgtgcgtgc cgatgacacc ggatcgatgt cggtggttct gccatgacgg
4441 gttctgttga ccggcccgac cagaatcgcg gtgagcgatc aatgaagtca ccagggttgg
4501 atttggtcag gcgcaccctg gacgaagctc gtgctgctgc ccgcgcgcgc ggacaagacg
4561 ccggtcgagg gcgggtcgct tccgttgcgt cgggtcgggt ggccggacgg cgacgaagct
4621 ggtcgggtcc ggggcccgac attcgtgatc cacaaccgct gggtaaggcc gctcgtgagc
4681 tggcaaagaa acgcggctgg tcggtgcggg tcgccgaggg tatggtgctc ggccagtggt
4741 ctgcggtggt cggccaccag atcgccgaac atgcacgccc gactgcgcta aacgacgggg
4801 tgttgagcgt gattgcggag tcgacggcgt gggcgacgca gttgaggatc atgcaggccc
4861 agcttctggc caagatcgcc gcagcggttg gcaacgatgt ggtgcgatcg ctaaagatca
4921 ccgggccggc ggcaccatcg tggcgcaagg ggcctcgcca tattgccggt aggggtccgc
4981 gcgacaccta cggataacac gtcgatcggc ccagaacaag gcgctccggt cccggcctga
5041 gagcctcgag gacgaagcgg atccgtatgc cggacgtcgg gacgcaccag gaagaaagat
5101 gtccgacgca cggcgcggtt agatgggtaa aaacgaggcc agaagatcgg ccctggcgcc
5161 cgatcacggt acagtggtgt gcgacccccct gcggcgactc aaccgcatgc acgcaacccc
5221 tgaggagagt attcggatcg tggctgccca gaaaaagaag gcccaagacg aatacggcgc
5281 tgcgtctatc accattctcg aagggctgga ggccgtccgc aaacgtcccg gcatgtacat
5341 tggctcgacc ggtgagcgcg gtttacacca tctcatttgg gaggtggtcg acaacgcggt
5401 cgacgaggcg atggccggtt atgcaaccac agtgaacgta gtgctgcttg aggatggcgg
5461 tgtcgaggtc gccgacgacg gccgcggcat tccggtcgcc acccacgcct ccggcatacc
5521 gaccgtcgac gtggtgatga cacaactaca tgccggcggc aagttcgact cggacgcgta
5581 tgcgatatct ggtggtctgc acggcgtcgg cgtgtcggtg gttaacgcgc tatccacccg
5641 gctcgaagtc gagatcaagc gcgacgggta cgagtggtct caggtttatg agaagtcgga
5701 acccctgggc ctcaagcaag ggcgccgac caagaagacg ggtcaacgg tacggttctg
5761 ggccgacccc gctgttttcg aaaccacgga atacgacttc gaaaccgtcg cccgccggct
5821 gcaagagatg gcgttcctca acaaggggct gaccatcaac ctgaccgacg agagggtgac
5881 ccaagacgag gtcgtcgacg aagtggtcag cgacgtcgcc gaggcgccga agtcggcaag

5941 tgaacgcgca gccgaatcca ctgcaccgca caaagttaag agccgcacct ttcactatcc
6001 gggtggcctg gtggacttcg tgaaacacat caaccgcacc aagaacgcga ttcatagcag
6061 catcgtggac ttttccggca agggcaccgg gcacgaggtg gagatcgcga tgcaatggaa
6121 cgccgggtat tcggagtcgg tgcacacctt cgccaacacc atcaacaccc acgagggcgg
6181 cacccacgaa gagggcttcc gcagcgcgct gacgtcggtg gtgaacaagt acgccaagga
6241 ccgcaagcta ctgaaggaca aggaccccaa cctcaccggt gacgatatcc gggaaggcct
6301 ggccgctgtg atctcggtga aggtcagcga accgcagttc gagggccaga ccaagaccaa
6361 gttgggcaac accgaggtca aatcgtttgt gcagaaggtc tgtaatgaac agctgaccca
6421 ctggtttgaa gccaacccca ccgactcgaa agtcgttgtg aacaaggctg tgtcctcggc
6481 gcaagcccgt atcgcggcac gtaaggcacg agagttggtg cggcgtaaga gcgccaccga
6541 catcggtgga ttgcccggca agctggccga ttgccgttcc acggatccgc gcaagtccga
6601 actgtatgtc gtagaaggtg actcggccgg cggttctgca aaaagcggtc gcgattcgat
6661 gttccaggcg atacttccgc tgcgcggcaa gatcatcaat gtggagaaag cgcgcatcga
6721 ccgggtgcta aagaacaccg aagttcaggc gatcatcacg gcgctgggca ccgggatcca
6781 cgacgagttc gatatcggca agctgcgcta ccacaagatc gtgctgatgg ccgacgccga
6841 tgttgacggc caacatattt ccacgctgtt gttgacgttg ttgttccggt tcatgcggcc
6901 gctcatcgag aacgggcatg tgtttttggc acaaccgccg ctgtacaaac tcaagtggca
6961 gcgcagtgac ccggaattcg catactccga ccgcgagcgc gacggtctgc tggaggcggg
7021 gctgaaggcc gggaagaaga tcaacaagga agacggcatt cagcggtaca agggtctagg
7081 tgaaatggac gctaaggagt tgtgggagac caccatggat ccctcggttc gtgtgttgcg
7141 tcaagtgacg ctggacgacg ccgccgccgc cgacgagttg ttctccatcc tgatgggcga
7201 ggacgtcgac gcgcggcgca gctttatcac ccgcaacgcc aaggatgttc ggttcctgga
7261 tgtctaacgc aaccctgcgt tcgattgcaa acgaggaata gatgacagac acgacgttgc
7321 cgcctgacga ctcgctcgac cggatcgaac cggttgacat ccagcaggag atgcagcgca
7381 gctacatcga ctatgcgatg agcgtgatcg tcggccgcgc gctgccggag gtgcgcgacg
7441 ggctcaagcc cgtgcatcgc cgggtgctct atgcaatgtt cgattccggc ttccgcccgg
7501 accgcagcca cgccaagtcg gcccggtcgg ttgccgagac catgggcaac taccacccgc
7561 acggcgacgc gtcgatctac gacaccctgg tgcgcatggc ccagccctgg tcgctgcgct
7621 acccgctggt ggacggccag ggcaacttcg gctcgccagg caatgaccca ccggcggcga
7681 tgaggtacac cgaagcccgg ctgaccccgt tggcgatgga gatgctgagg gaaatcgacg
7741 aggagacagt cgatttcatc cctaactacg acggccgggt gcaagagccg acggtgctac
7801 ccagccggtt ccccaacctg ctggccaacg ggtcaggcgg catcgcggtc ggcatggcaa
7861 ccaatatccc gccgcacaac ctgcgtgagc tggccgacgc ggtgttctgg gcgctggaga
7921 atcacgacgc cgacgaagag gagaccctgg ccgcggtcat ggggcgggtt aaaggcccgg
7981 acttcccgac cgccggactg atcgtcggat cccagggcac cgctgatgcc tacaaaactg
8041 gccgcggctc cattcgaatg cgcggagttg ttgaggtaga agaggattcc cgcggtcgta
8101 cctcgctggt gatcaccgag ttgccgtatc aggtcaacca cgacaacttc atcacttcga
8161 tcgccgaaca ggtccgagac ggcaagctgg ccggcatttc caacattgag gaccagtcta
8221 gcgatcgggt cggtttacgc atcgtcatcg agatcaagcg cgatgcggtg gccaaggtgg
8281 tgattaataa cctttacaag cacacccagc tgcagaccag ctttggcgcc aacatgctag
8341 cgatcgtcga cggggtgccg cgcacgctgc ggctggacca gctgatccgc tattacgttg
8401 accaccaact cgacgtcatt gtgcggcgca ccacctaccg gctgcgcaag gcaaacgagc
8461 gagcccacat tctgcgcggc ctggttaaag cgctcgacgc gctggacgag gtcattgcac
8521 tgatccgggc gtcggagacc gtcgatatcg cccgggccgg actgatcgag ctgctcgaca
8581 tcgacgagat ccaggcccag gcaatcctgg acatgcagtt gcgtcgcctg gccgcactgg
8641 aacgccagcg catcatcgac gacctggcca aaatcgaggc cgagatcgcc gatctggaag
8701 acatcctggc aaaacccgag cggcagcgtg ggatcgtgcg tgacgaactc gccgaaatcg
8761 tggacaggca cggcgacgac cggcgtaccc ggatcatcgc ggccgacgga gacgtcagcg
8821 acgaggattt gatcgcccgc gaggacgtcg ttgtcactat caccgaaacg ggatacgcca
8881 agcgcaccaa gaccgatctg tatcgcagcc agaaacgcgg cggcaagggc gtgcagggtg
8941 cggggttgaa gcaggacgac atcgtcgcgc acttcttcgt gtgctccacc cacgatttga

9001 tcctgttctt caccacccag ggacgggttt atcgggccaa ggcctacgac ttgcccgagg
9061 cctcccggac ggcgcgcggg cagcacgtgg ccaacctgtt agccttccag cccgaggaac
9121 gcatcgccca ggtcatccag atccgcggct acaccgacgc cccgtacctg gtgctggcca
9181 ctcgcaacgg gctggtgaaa aagtccaagc tgaccgactt cgactccaat cgctcgggcg
9241 gaatcgtggc ggtcaacctg cgcgacaacg acgagctggt cggtgcggtg ctgtgttcgg
9301 ccgacgacga cctgctgctg gtctcggcca acgggcagtc catcaggttc tcggcgaccg
9361 acgaggcgct gcggccaatg ggtcgtgcca cctcgggtgt gcagggcatg cggttcaata
9421 tcgacgaccg gctgctgtcg ctgaacgtcg tgcgtgaagg cacctatctg ctggtggcga
9481 cgtcaggggg ctatgcgaaa cgtaccgcga tcgaggaata cccggtacag ggccgcggcg
9541 gtaaaggtgt gctgacggtc atgtacgacc gccggcgcgg caggttggtt ggggcgttga
9601 ttgtcgacga cgacagcgag ctgtatgccg tcacttccgg cggtggcgtg atccgcaccg
9661 cggcacgcca ggttcgcaag gcgggacggc agaccaaggg tgttcggttg atgaatctgg
9721 gcgagggcga cacactgttg gccatcgcgc gcaacgccga agaaagtggc gacgataatg
9781 ccgtggacgc caacggcgca gaccagacgg gcaattaatc aggctcgccc gacgacgatg
9841 cggatcgcgt agcgatctga ggaggaatcg ggcagctagg ctcggcagcc gggtacgagt
9901 gttaggagtc ggggtgactg caccgaacga gccgggggcg ctcagcaagg gcgacggccc
9961 gaatgcggat ggcttggtcg accgtggggg cgcacatcgg gcagcgaccg ggccaggccg
10021 cataccagat gctggagacc cgccgccgtg gcagcgtgct gcgactcggc aatcccaagc
10081 ggggcatcgt cagccgccgc cggtatcaca ccctgagggg cgcccgacca acccgcccgc
10141 cgccgccgat gctcggctga atcgcttcat ctccggtgcg tctgccccgg tgaccggccc
10201 agccgccgcg gtcaggaccc cgcagccgga tcccgacgct tcgctggggt gtggcgacgg
10261 ttcccccgcc gaggcctatg ccagcgagct gcccgaccta tccggcccga ctccgcgggc
10321 cccgcaacgc aaccccgcgc cggcgcgtcc cgcggagggt ggcgcgggat cgagagggga
10381 ttcggccgcc ggttcgagcg gcggtcgttc gattaccgct gagagtagag acgcccgtgt
10441 ccagctgtcg gcgcggcgaa gccgcgggcc ggttcgagcc agcatgcaga tccgacggat
10501 tgatccatgg agcacgttga aggtgtcgct gttgttgtcg gtggcgctgt tcttcgtctg
10561 gatgatcacg gtcgcgttcc tctacctggt gctcggcggt atgggcgtat gggccaagct
10621 caacagcaac gtcggtgacc tgttgaacaa cgcgagcggc agcagcgcgg aacttgtctc
10681 cagcggcacc atcttcggcg gcgcattcct gatcggcttg gtcaacatcg tcctgatgac
10741 cgcgcttgcc accatcggtg cgttcgtcta caacctgatc accgatctga tcggcggcat
10801 cgaagtgacg ctggcagacc gggactaatg ttttgagagt cgggcgccgg ttgcggtaat
10861 ctcgtcgctc ggccgtacgc gagtacgggc ctatagctca ggcggttaga gcgcttcgct
10921 gataacgaag aggtcggagg ttcgagtcct cctaggccca cgaccatgtg cccgtcacga
10981 cgttcggtga ggttcgcatt gccactggcc gcgatcgctg tggcggccat cgtcgtgcgg
11041 ttccgacgcg gagccgatgt ctggcatgtg gccggcgatc cacctcctga tcacataacc
11101 ggtgacgaag aggggcctta gctcagttgg tagagcactg cctttgcaag gcaggggtca
11161 ggggttcgag tcccctaggc tccacaagtg aaaagcgtag ctcggatact tcgaatgacc
11221 acgtttgatc acaatcgcga gtgaagaggg cgttgatggc cactccgacg gcctcgacac
11281 ccgacccgta caggtggcgg tagcggtcca aggtcaaccc ggcggagtcg tgttcgagca
11341 tgttctgaag tgccttgaat tcgccccggc ctggatcgcc aacgacgccg ggtgtgccga
11401 gctcatgcag ttttgaactc ctacaccacc gccggcttcc cggtagcgtc catcacagtc
11461 tgagggaaca gctgcgccgc ggtcaccgcc tgcgaccacc accggcgccg cacatggctg
11521 ccgcgcatgt agccgcccgc cgagtccggg aacgctagaa gctcagcaac ccatcgaacg
11581 cggtcggccg gttgtcggcg tccacgagca cgcaccctag agcgaaagtc atggatccgc
11641 cgttggcggg gtctccggta ttgccggact cgtctatgta agcgaccagc acgcgacgat
11701 gctggcacga ttcttgggcg attgaccaca gttacagata actactgtta accgcagttg
11761 tgtcctttcg caggtggact gagttgtaac ccattgatct gcatcatgat tcgcctgtgc
11821 aaggcggggg tcaggggttc gaatccctag gccccaccgt gtgacgaccg gcctcagggg
11881 cgcggttgca cctcgacgct cggtggtcgg ggcgacggct ccggtcgcga cgagcgccgg
11941 acgatgctga aggcgacggc accgccggcg aggatggccg ccgcgatccc cgcgaagatc
12001 cagaggtggt gtttgctgcg acgttgggtc cgggcgtcct gtagggcctg cggtaggttg

12061 gccaccacgt cctgggcagc ggtcagctct tgagcgagcg tctcttgggc ggcagcgacc
12121 tcgcgggcca atcggccttc ccggtaacgg cggcgaagcc cggccgcggt cgaccgggcg
12181 gactgaagcc caagtccgac cccgagttcg aggaggcctc gggtcacgtc caccggaccc
12241 accgcagagt aggccagacc ccgggtcagc cgctcgcgtg gggtcaaccg ggtttccacc
12301 tgctcactca ttttgccgcc tttctgtgtc cgggccgagg cttgcgctca ataactcggt
12361 caagttcctt cacagactgc catcactggc ccgtcggcgg gctcgttgcg ggtgcgccgc
12421 gtgcgggttt gtgttccggg caccgggtgg gggcccgccc gggcgtaatg gcagactgtg
12481 attccgtgac taacagcccc cttgcgaccg ctaccgccac gctgcacact aaccgcggcg
12541 acatcaagat cgccctgttc ggaaaccatg cgcccaagac cgtcgccaat tttgtgggcc
12601 ttgcgcaggg caccaaggac tattcgaccc aaaacgcatc aggtggcccg tccggcccgt
12661 tctacgacgg cgcggtcttt caccgggtga tccagggctt catgatccag ggtggcgatc
12721 caaccgggac gggtcgcggc ggacccggct acaagttcgc cgacgagttc caccccgagc
12781 tgcaattcga caagccctat ctgctcgcga tggccaacgc cggtccgggc accaacggct
12841 cacagttttt catcaccgtc ggcaagactc cgcacctgaa ccggcgccac accattttcg
12901 gtgaagtgat cgacgcggag tcacagcggg ttgtggaggc gatctccaag acggccaccg
12961 acggcaacga tcggccgacg gacccggtgg tgatcgagtc gatcaccatc tcctgacccg
13021 aagctacgtc ggctcgtcgc tcgaatacac cttgtggacc cgccagggca cgtggcggta
13081 caccgacacg ccgttggggc cgttcaaccg gacgccctca cgccaagtcc gctcaccttt
13141 ggccgcgacc ggcgtaaccg gcagcggtaa gcgcatcgag cacctccact gggtcgctgc
13201 cgagatccca gcgggacaaa atcagcagcc cccgctgacc gtttcgatct cgagcaggcg
13261 caccaggcgg ccgtaacggc gaaactcgtc gattcggatg atcttgatat tggaatgtcg
13321 taatagctgc gtccggaacc aacctcggat cgccaggccg tcgggggtaa ttgccagcct
13381 tggacgtgcg cgccaagtgg cgctcgcaaa caagatcaga cccagcgcgg caactccggt
13441 caacacccgc ccgggcgtgt ctgtgactaa ggtcacagac gcaatagcca tcacgactcc
13501 cccggctccg caaccagcga ttcccgaggt gcgaggcgcc catgctgttt gctgcatgta
13561 ttccttagac cctctcacca ctgcagacaa agttatccac agacgctatc aacagtgggg
13621 atgaatcaca tgcgtgtgat tgagtgacca aaaggttgct ggcacagtaa cgacccgacc
13681 agaatatgaa ttcattctat cggcggcgtg gatcaatgcc agcgcatcgt gagcaacaaa
13741 ccggtgatca tgaaagcgaa cgcgatcgca tagttccagg gaccgagttg cgccatccaa
13801 ttgagcgctg tgggggcttg gctgccaatg gctgccaact gaaacaccat taaccagatg
13861 agtccgatca gcatcagacc gatgaacaac gagacgaacc atacgctcga cggtccgacc
13921 ttcaccttca tcggcgtgcg gctcaccgcg ctgacggtga agtcgttctt cttgcggacc
13981 ttggacttgg gcatcacttt cctcgggatc tggcgggact acctcgacaa gacgacgaat
14041 ggcccggggt gcaacgatag aagttgcagc tgcaggcata ccttgttatg agactaaccc
14101 acccaacacc ctgcccggaa aacggagaga ccatgattga tcggcgccga tcggcgtggc
14161 gtttcagtgt ccccttagtg tgcttgctgg cggggctgct gctggccgcc acgcatgggg
14221 tgtcgggcgg caccgagatc cgccgcagcg atgcgccgcg actggtcgac cttgtccgtc
14281 gggcgcaggc atcggtgaac cgtctcgcca ccgaacgcga agcgctgacc accagaatcg
14341 actcggtgca cggccgatct gtcgataccg cgttggcggc catgcagcgg cggtccgccg
14401 agctggccgg tgtggcggct atgaatccgg tccatgggcc gggcctggtg gttaccctgc
14461 aagacgcgca acgcgacgcc aacggccggt ttccgcgcga cgcgtccccg gacgatctgg
14521 ttgtgcatca gcaagacatc gaggctgtcc tcaacgcgtt gtggaatgcc ggtgctgagg
14581 cgatccagat gcaggaccag cgcatcatcg cgatgtcgat agctcgttgt gtcggaaaca
14641 cgttgctgct caacgggcgt acctatagcc cgccctacac gatcgccgcg atcggagacg
14701 ccgccgccat gcaggctgct ctggctgcgg ctcccctggt gacgctctac aagcagtacg
14761 tggtccggtt cggcctcggg taccgcgaag aagtccatcc tgacttgcag atagtcggct
14821 atgccgatcc cgtccggatg cacttcgcgc agcctgcagg ccccttggac tactgaacga
14881 ctgccggcag ggtcaggcgg tagcctgtca cgatgcggat cctggtcgtt gacaactacg
14941 acagcttcgt gttcaacctg gtgcagtacc tcggccagct cggcatcgag gccgaggtgt
15001 ggcgcaacga cgaccaccgg ctatccgatg aggccgccgt cgccggccaa ttcgacggtg
15061 tcctgctcag tcccggtccg ggtaccccgg agcgcgcggg cgcgtcggtg agtatggtgc

15121 acgcgtgtgc ggcagcacac accccttgc tgggggtctg ccttgggcac caagccatcg
15181 gcgttgcgtt cggcgccacc gtggaccgtg cgcccgagct attgcacggc aagaccagca
15241 gcgtattcca caccaatgtc ggtgtgctac aagggcttcc ggatcccttc acggccactc
15301 gataccattc gttgacaatt ctgcctaagt cgctgccagc ggtgctgagg gtcacggccc
15361 gcactagcag cggtgtgatc atggccgtgc agcacaccgg gctgccgatc cacggtgtcc
15421 agttccatcc ggagtcgatt ctcaccgagg gcgggcaccg catactggcc aactggctca
15481 cctgctgcgg atggacgcaa gacgacaccc tggtacgtcg gctggaaaac gaagtgctca
15541 ccgccatctc accgcacttc ccaacttcaa ccgctagcgc gggcgaagct actggccgaa
15601 cctcagcgtg atgatgccgt cccggttgac gccggtcccc gccggcgggt tttgatagac
15661 gacccggttg tgttgggagc caccggcgtc gacgtcggcc cctttgtcga gcatcccggt
15721 ccagcccagc gcgcgcaatc gtggttcggc gtcgacccag aacatgccgg ataggtcggg
15781 catgacgaat tggttgccct tggacacctg tagttcgatg actgaatcga ccggaactgt
15841 ggtgcctgcg ggtggattgg tgccggtcac ctcgccggcg ggacgggggc tgtccaccga
15901 ggcctgactg aatttggtga agccgtagac gttgaggttc ttctgcgcca cgtcgacggt
15961 ctggcccgcg acatcgggaa tgtctttggt cgccggacca gagccaacga tgatgatgac
16021 cacattggtg atggccgacg tctggttggc tggcgggttg gtcccgatga ccttgcccac
16081 cagttccggg gtggacggcg aattcgcttg cttgaagcgg ccgaatccgg cggcagtcag
16141 tttcttgacc gcttcggcgt atgtcagcgt ggagacgtcg ggtatttcgc gttgctcggg
16201 tccggtggac acgttgactg tgatctcgtc gcctgcactc accgacgtgt tggcggccgg
16261 gtcggtgccg ataacgtggt ccggtgggat tgtcgagtcc ggcttctgca aggtgcggat
16321 tttgaagccc cggttttgca gtgtggcgat ggcgtcggcg gaggattgac cccgaacgtc
16381 gggaacttga acgtcgcggg tgatgccgcc gaacgtgttg atggcgatgg ttaccacgac
16441 ggtcagcaca gcgagcacgg cgaccaccgc aacccaacgg cccaccgaac cgatgctgcg
16501 gtcacggtcg gtgtcgtcta agtcctggcg tggtagcgga tcggtgcgcg gaccgctaag
16561 gttgccggcc gcagacgaca gcagcgaggt ccgctcggca tcggtgagca ctttgggcgc
16621 ctcgggcggc tcaccgttgt gcacgcggac caggtcggcg cgcatctccg ccgctgtctg
16681 atagcggttt tccggatttt tggccagcgc cttgagaacg acggcgtcca ggtcggcgga
16741 gaggccttcg tgccgcgccg aaggtgggat cgggtcttcg cgcacatgtt ggtaggcaac
16801 cgagacgggt gagtcgccgg tgaaaggtgg ctccccggtg aggacttcat aaagaacaca
16861 gcccaaggaa tagacatcgg atcgggcgtc gacggaatca ccccgggcct gttcgggtga
16921 caggtactgc gccgtgccga tcactgctgc ggtctgggtc acgctgttgc cgctgtcggc
16981 aatggcgcgg gcgatgccga aatccatcac ctttactgca ttggtcgcgc tgatcatgat
17041 gttcgccggc ttgacgtcac ggtggatgat tccgttctga tgactgaagt tcagcgcttg
17101 gcaggcgtcg gcgatgacct cgatggcgcg tttgggcgtc atcggccctt cggtgtggac
17161 aatgtcgcgc agggtaacgc cgtcgacgta ttccatgacg atgtagggca atggcccggc
17221 gggcgtttcg gcttcaccgg tgtcgtagac cgcgacgatt gcagggtggt tcaatgccgc
17281 ggcgttttgc gcctcacgcc ggaagcgaag gtaaaaactg ggatcgcggg ctagatcagc
17341 gcgcagcacc ttgaccgcaa cgtcgcggtg caaccggagg tcgcgggcca ggtggacctc
17401 ggacatgccc ccaaatccaa ggatttcgcc aagttcgtag cggtcggaca ggtgggaagg
17461 ggtggtcatt gcgctatctc gtatcgggcc agcgacgcgc gcgaatgcgg tgtcggcggg
17521 acaacccagc tttgcagtcc agaatgacgt gtttccccgc gttccgtcca attgagtcgc
17581 gggctagcat cagtcccgcc agtgttgctg gccggagggt tcccggtggt ggtcacggtc
17641 ggcgtcggtg cctgctgcgg gctgttgtcc ccgggcgctt tgatgacgag cagcacggcg
17701 atgatgattg ccagcgcccc cagcaccccc gcggcccaga gcagcgcacg ctgaccggac
17761 gaaaacgtgc gccgcggcgg ccggtgacca cccgtggccg ggcgggatcg acgggatgcc
17821 gcagtccggc cagcagagtt ggccgcgacc ctggctgtcg tacccgacgg aatggccgcc
17881 ggggcggccc ggccaggggg gggtgtctgg ctgggccgcg ggggccggcg gccggcgcgc
17941 accgctgcca ccgcgtcggc gaacggtccc ccactgcgat agcgcatcgc ggggttcttc
18001 accagagtta tctcgatgag ttctcgcaca ttgggcggca ggtcgggagg cagcggcggc
18061 ggcggctcct tgatgtgctt cattgccacg gtcagggcac catcgccggc gaacggccgt
18121 ttacccgaaa ccgcttcata cccaacaact cccagtgaat agacgtcgct ggccgggctg

18181 gcgtcgtgac cgagggcctg ctccggcgcg atgtattggg cggtgcccat caccatgccg
18241 gtctgggtca cgggcgctgc atcgacggct ttggcgatgc cgaagtcggt gatcttcacc
18301 tgcccggtgg gggtgatcaa gatgttgccc ggtttgacgt cgcggtgcac caggccagcg
18361 gcatgcgcga tctgcagagc gcggccggtc tgctcgagca tgtccagtgc gtgccgcaac
18421 gacagccggc cggtgcgttt gagcaccgaa tttagtggct cgccgttgac cagctccatc
18481 accaggtagg ccgtgcgacc ctccccgttc atctggcttt cgccgtagtc gtgcacgctg
18541 gcgatgcccg gatggttcag catcgcggtg gtgcgcgctt cggcccggaa ccgttcgatg
18601 aactccggat cggaggagaa ctcgctcttg agcaccttca ccgcaacacg ccggcccaac
18661 cggttatcca cggcctccca gacttggccc ataccaccgg tggcgatgag gcgctgcagg
18721 cggtatctgc ccgacagcgt cacgccaact cgggggctca tggttccccc tgcagtgcgg
18781 cttcgatcac cgcccgcccg atcggtgccg cgagggcacc tccggtggcg gacagccgat
18841 cagccccgtt ctccaccagc acggcaacag ccaccttggg cgcttgtgcg ggcgcaaagg
18901 cgatgtacca agcgtgcggt ggagtgtgac gagggtcggt gccatgttcg gcggtgcccg
18961 tcttggatgc gatctgcacg ccggggattg cccctttctg ctgtgcgact ttctcggcgc
19021 cgaccatcag ctctgttagc ttagcggcga cctgcggtga caccgcgcgg cgctgctggt
19081 atccgacggt ggttgagata ttggctaggt ccggtccctt gaggctgccg actagataag
19141 gcctcatcgt aatgccgccg tttgcgatgg tcgcggctat ttctgcgttc gctagcgggg
[truncated: 623,047 more chars]
